# Identification of conserved miRNAs and their targets in *Jatropha curcas*: an in silico approach

**DOI:** 10.1186/s43141-023-00495-9

**Published:** 2023-04-07

**Authors:** Foeaz Ahmed, Md. Nazmul Islam Bappy, Md. Shariful Islam

**Affiliations:** 1 Faculty of Biotechnology and Genetic Engineering, Sylhet Agricultural University, Sylhet, 3100 Bangladesh; 2 Department of Molecular Biology and Genetic Engineering, Sylhet Agricultural University, Sylhet, 3100 Bangladesh; 3 Department of Animal and Fish Biotechnology, Sylhet Agricultural University, Sylhet, 3100 Bangladesh

**Keywords:** *Jatropha curcas*, Conserved miRNAs, Bioinformatics, Phylogenetic analysis

## Abstract

**Background:**

MicroRNAs (miRNAs) are small endogenous RNAs with an approximate length of 18–22 nucleotides and involved in the regulation of gene expression in transcriptional or post-transcriptional levels. They were found to be associated with leaf morphogenesis, flowering time, vegetative phase change, and response to environmental cues in plants, where they act as a critical regulatory factor. The nature of high conservancy of plant miRNAs within the plant species made it possible to detect the conserved miRNAs by computational approaches. Expressed Sequence Tags (EST) based comparative genomic approaches provide advantages over wet lab approaches as it is convenient, easy to carry out and less time consuming. EST-based in silico approach can unravel new conserved miRNAs in plants, even when the complete genome sequence is not available.

**Results:**

To identify the novel miRNAs, a total of 46,865 ESTs from *Jatropha curcas* were searched for homology to all available 6746 mature miRNAs of plant eudicotyledons. Finally, we ended up with 12 novel miRNAs in Jatropha that range from 18 to 19 nucleotides where their respective precursor miRNAs had 54.11–71.76% (A + U) content. The putative miRNAs belong to 12 individual miRNA family and most of them have higher (A + U) content ranging from 47.36 to 77.77% than their respective miRNA homologs. Many of the target genes by the newly identified miRNAs were associated with plant growth and development, stress response, defense and hormone signaling, and oil synthesis pathways.

**Conclusion:**

These findings have the potential to speed up miRNA identification and expand our understanding of miRNA functions in *J. curcas*.

**Supplementary Information:**

The online version contains supplementary material available at 10.1186/s43141-023-00495-9.

## Background

*Jatropha curcas* L. is a tough, perennial plant from the Euphorbiaceae family. It is a drought resistant plant that may thrive in poor or marginal soil, easy to cultivate, can be grown quickly and yields seeds up to 50 years [1]. The plant can be used to control erosion, as a fence (particularly to keep farm animals out) and as a commercial crop. In many parts of the world, the ancient systems of medicine have included Jatropha as a medicinal herb. It has numerous therapeutic potentials, for instance, leaves with some processing can be used as a medication in vaginal bleeding, wounds, jaundice, and malaria [2, 3]). Also, the stem barks have potentials as antimicrobial agents as well as strengthening the gums [4]. Furthermore, Jatropha after some processing can be a highly nutritious supplement in animal feeds with economic importance [5].

Due to the increasing expansion in agriculture, industry, transportation and other sectors, the need for using fossil fuels is increasing worldwide. Again, because of the increasing use of hydrocarbons from fossil fuel globally as energy source and its threat to global warming, researchers are searching for renewable sources. Finding an alternative of fossil fuel, even in a small percentage would significantly improve the environmental conditions and economy of the current world. In recent years, plant oils as a source of biodiesel in particular has got considerable attention because of the higher price of petroleum and the need to reduce CO_2_ emission and increase fuel security [6]. As edible oils have incredible demand as food and are expensive, the Jatropha oil made it noteworthy to be used as a fuel source at present because of its non-edibility. The seeds of Jatropha contain higher oil content than oil producing soybean, rapeseed, oil palm, and sugarcane [7]. Jatropha seeds contain about 30–50% oil where the kernel itself has 45–60%. Moreover, Jatropha is a prospective source of biodiesel and considered as recyclable, renewable and environment-friendly [8]. A certain percentage of Jatropha biodiesel (5%, 10%, 15%, and 20%) can be blended with petroleum derived diesel thus substituting fuel for transportations [9]. Nowadays, Jatropha biodiesel is being used widely as an alternative to the fossil fuels in Costa Rica, Ethiopia, Ghana, India, Mexico, the USA, and many other countries to achieve sustainable goal development [10].

MicroRNAs (miRNAs), which are generally 18–22 nucleotides in length, belong to a class of endogenous small regulatory RNAs [11]. These small non-coding RNAs derived from their precursor sequences, bind to their target mRNAs and negatively mediate the gene expressions in pre-transcriptional or post-transcriptional stages by impeding mRNA translation [12, 13]. Though miRNAs are mostly known to mediate post-transcriptional repression, they may also influence the pre-transcription phase. They may occasionally cause histone modification and DNA methylation of promoter sites, which affects the expression of target genes [14, 15] Moreover, transcriptional inhibition through microRNA-mediated chromatin reorganization is one of the nine mechanisms of miRNA action that described and assembled in a unified mathematical model [16]

Many earlier research has reported that miRNAs play vital functions in a variety of biological processes in both plants and animals [13, 17]. Plant’s miRNAs are found to be involved in signal transduction, hormonal biosynthesis, root and shoot formation, floral development and flowering, reproductive growth, as well as responses against pathogens, drought and salinity, and also in lipid metabolism [18–20]. Only a limited number of miRNAs have been detected despite their enormous importance in plant biological processes [21]. According to the publicly available database for miRNA, miRBase (http://www.mirbase.org; Release 22.1: October 2018) (accessed on May 31, 2021) [22], there are about 38,589 entries from 271 organisms containing sequences for precursor miRNA transcript (termed mir in the database) and mature miRNAs (termed miR) [23]. Among those entries, around 10,114 mature miRNA sequences have been currently recognized from 82 plant species which are available in the miRBase [22]. MicroRNAs are also evolutionary conserved among the plant species and this nature of conservancy renders an effective approach for their identification and characterization using comparative genomic analysis [24]. Based on this, several strategies have been established to detect miRNAs in plants by means of expressed sequence tags (ESTs) [25]. EST analysis is recognized as a prevailing tool for the searching of conserved miRNAs in plants when the complete genome sequences are not available, and this allows us to understand the conservancy and evolutionary relationships of miRNAs among different species [26]. Besides, EST-based techniques have several benefits than other computational methodologies for identifying plant miRNAs [27, 28]. Study revealed that miRNAs identified by EST analysis can be verified using a high-throughput sequencing technique [29]. EST-based approach to predict miRNA has also been reported in many earlier studies [30, 31].

However, despite the enormous significance of *J. curcas* in terms of biodiesel and feedstock, there have been no experimentally validated Jatropha miRNAs deposited in the miRBase database. Though Wang et al. [32] used a cloning approach for identifying 46 miRNAs in *J. curcas*, the use of cloning strategies has some disadvantages, and for instance, there is a possibility of degradation and skipping of loosely expressed miRNAs. Moreover, Galli et al. [33] performed deep sequencing of small RNAs only from mature seeds to identify miRNAs and also, predicted targets by in silico approach. Another similar research was also done to predict miRNA in seeds through the deep sequencing of sRNA [34]. But involvements of miRNAs in other developmental stages remained unclear. Thus, the future endeavor is needed to be the prediction of more new miRNAs in Jatropha. In present research, we carried out an EST-based homology search and a series of computational and bioinformatics steps to identify conserved miRNAs in *J. curcas* using publicly available 46,865 ESTs from the NCBI Genbank database and also their targets were predicted. As a result, we have identified 12 potential miRNAs which were found to be involved in different biological and metabolic processes as well as lipid and fatty acid biosynthesis.

## Methods

**Fig. 1:**
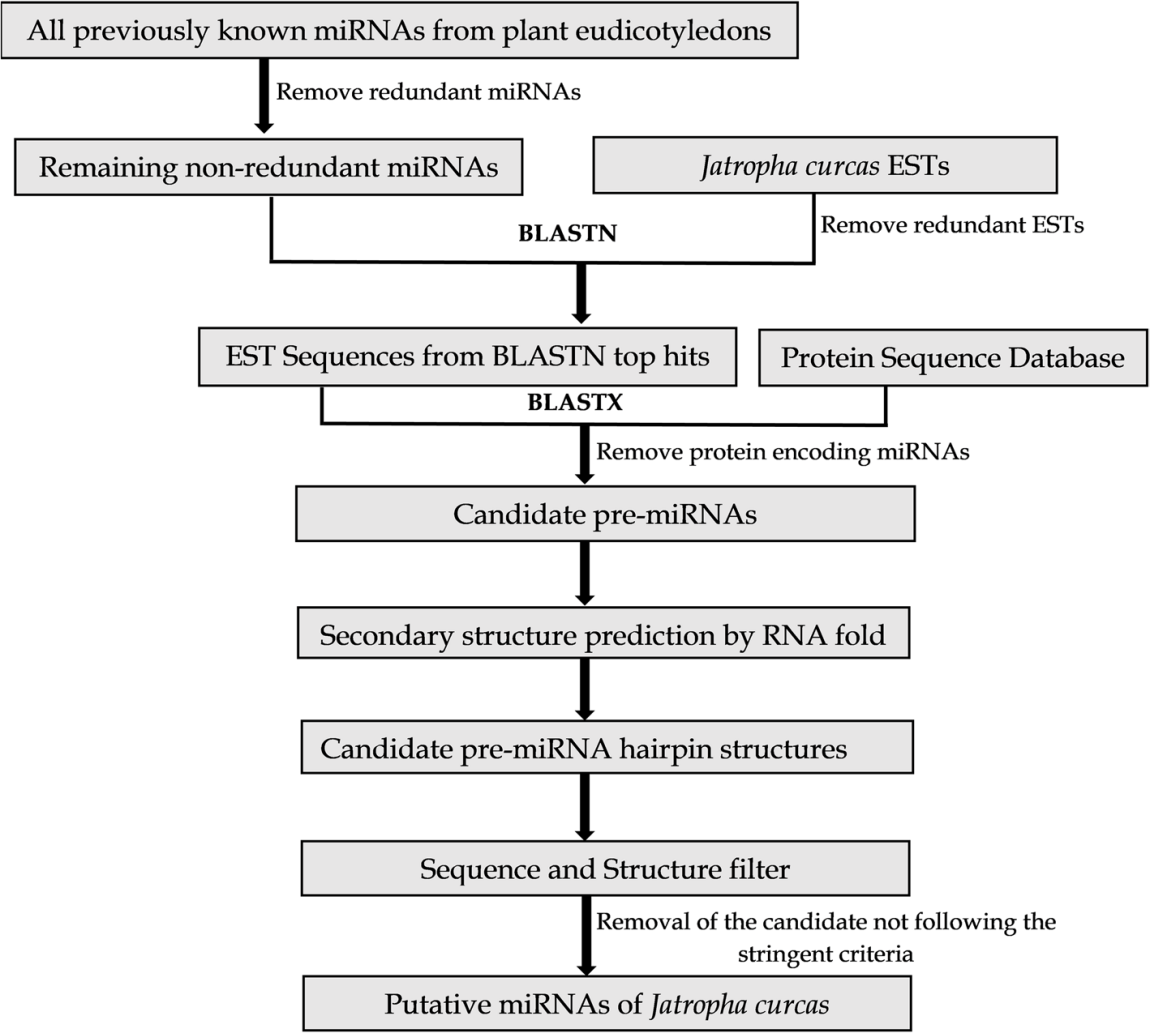
Schematic workflow for the identification of novel miRNAs in *J. curcas*

Here, we utilized several bioinformatics resources to detect conserved miRNAs in *J. curcas*. The schematic workflow for identifying miRNAs is depicted in Fig. 1.

### Sequence collection and software information

EST sequences were considered for the genome-based identifications of microRNAs. The suggested EST sequences of Jatropha (ID txid180498) were collected from the NCBI Genbank nucleotide database (http://www.ncbi.nlm.nih.gov/) (accessed on May 22, 2021). To find possible miRNAs in the *J. curcas*, completely available miRNAs of the group viridiplantae (Chlorophyta, Coniferophyta, Embryophyta, and Magnoliophyta) were retrieved from the mirBase (http://www.mirbase.org/) (accessed on May 31, 2021), a database for validated miRNA sequences. All the redundant or repeated sequences from both miRNAs and EST sequences were then eliminated by CD-HIT (http://weizhongli-lab.org/cd-hit/) (accessed on June 18, 2021) by keeping the value of sequence identity cut-off to 1.

### Search for non-coding miRNA candidates

The screened mature miRNAs of viridiplantae were used as query sequences for homology search against the ESTs of *J. curcas* by using BLASTn of NCBI database (https://blast.ncbi.nlm.nih.gov/Blast.cgi) (accessed on June 25, 2021) by setting up the parameters to default. Only the top result for each BLAST was selected for further analysis. Again, the ESTs from the top hit of BLASTn were subjected to redundancy check by using CD-HIT. The obtained ESTs were then aligned against nr database of BLASTx (https://blast.ncbi.nlm.nih.gov/Blast.cgi) (accessed on July 1, 2021) for eliminating protein coding sequences and only the non-protein coding sequences were selected to finalize the potential precursor miRNA (pre-miRNAs) by considering the miRNA precursor determinant properties.

### Identification of pre-miRNAs and putative miRNAs

The non-protein coding ESTs were then analyzed in mirEval 2.0 (http://mimirna.centenary.org.au/mireval/) (accessed on July 6, 2021) to determine the pre-miRNA candidates. All the candidates had a length of 85 nucleotides by default in mirEval. In the mirEval server, the genome was selected for others and the prediction of the strand sequence was set to original. Precursor miRNA was identified according to Zhang et al. [20]. The mismatches among the putative miRNAs and all the previously known miRNAs were determined by the local blast carried out in miRBase database (http://www.mirbase.org/search.shtml) (accessed on July 12, 2021).

### Prediction of hair-loop secondary structure and naming of pre-miRNAs and new miRNA

Precursor miRNA sequences were then tested for the secondary structure predictions by using publicly available server MFOLD 3.1 [35] (http://www.unafold.org/mfold/applications/rna-folding-form.php) (accessed on July 28, 2021). All the parameters were set to the default value. The candidate miRNAs were chosen based on the criteria suggested by Zhang et al. [20] that allow us to largely reduce false positive results for identifying miRNAs [36]. ΔG values (kcal/mol) were provided during the prediction of secondary structure in MFOLD which can be useful for calculating their negative minimal free energies (MFEs). Adjusted minimal folding energy (AMFE) and minimal folding free energy index (MFEI) were measured as the anticipated secondary structure should have higher minimal negative MFEI and MFE. AMFE is defined as the MFE of a 100-nucleotide length.

$$AMFE=\frac{MFE}{Length\;of\;precursor\;sequence}\ast100$$

MFEI for all single pre-miRNAs were calculated according to Zhang et al. [20]. The MFEI of secondary structure was calculated using the following equation:

$$MFEI=\frac{AMFE}{\left(G+C\right)\%}$$

To find the related miRNA families of identified miRNAs, the putative sequences were locally BLASTed in the miRBase database. Newly identified miRNAs were named following the nomenclature described by Griffiths-Jones et al. [37].

### Prediction and functional analysis of newly identified miRNAs targets

In this study, we applied a homology-based search method for determining the potential targets of identified miRNAs. Because of the limited gene availability of *J. curcas*, we used *Arabidopsis* as a reference organism for determining the targets of the candidate miRNAs. The newly identified mature miRNAs were used as query against the *A. thaliana* DFCI gene index (AGI) release 15 and *A. thaliana* TAIR10, cDNA, removed miRNA gene (release date 14^th^ December 2010) using a miRNA target prediction server psRNATarget (http://plantgrn.noble.org/psRNATarget/) [38] (accessed on August 6, 2021). All the parameters in psRNATarget for target prediction were kept in default except the following: (1) The (HSP) size was kept within 18, and (2) central mismatch for translational inhibition was 9–11 nucleotides. The target proteins, molecular functions and biological process in *J. curcas* were analyzed by searching the mRNA IDs in UniProt (http://www.uniprot.org) (accessed on August 3, 2021).

The identified targeted genes of jcu-miR11155c-3p, jcu-miR7805-3p and jcu-miR8786 were networked using GeneMANIA (http://genemania.org/) (accessed on January 12, 2023) based on automatically selected weighting method [39] as most of the target genes of those three miRNAs were found associated with diesel production, stress tolerance, and hormonal regulation. It provides a number of co-expressed genes relevant to target genes to make the regulatory networks more complete.

### Phylogenetic analysis of predicted miRNAs and validation of identified miRNAs

The related families of newly identified novel miRNAs were collected from miRBase by sequence search and collated with the putative miRNAs to carry out a phylogenetic analysis in Clustal Omega, which utilizes seeded guide trees and HMM profile-profile techniques to generate sequence alignments between three or more sequences (http://www.ebi.ac.uk/Tools/msa/clustalo/) (accessed on August 14, 2021). Then, the sequence similarities were viewed and phylogenetic tree was constructed in MEGA X software using the distance based method [40]. As the miRNAs need to be non-protein coding, all the putative miRNAs of Jatropha were analyzed for their non-protein coding properties in the BLASTx program.

## Results

### Acquisition of *J. curcas* ESTs and reference set of miRNAs

A total number of 46,865 ESTs of *J. curcas* were extracted from the NCBI nucleotide database (Supplementary file 1). For the reference set of miRNAs, a total of 6746 mature miRNAs of plant eudicotyledons, belonging to the 20 plant families were collected from the publicly available miRbase database (Supplementary file 2). Analysis in the CD-HIT server removed 4491 redundant ESTs, retaining 42,374 ESTs without repeated sequences for further study (Supplementary file 3). These ESTs will be the potential homologs for finding the target miRNAs. On the other hand, 3514 mature miRNA sequences were selected from 6746 eudicotyledons miRNAs after the removal of redundancy (Supplementary file 4).

### Screening for non-coding miRNA candidates

Homology-based search of the non-redundant eudicotyledons miRNAs were carried out in BLASTn against 42,374 ESTs of *J. curcas* by considering all the default parameters. Each miRNA query resulted in a top hit against the non-redundant ESTs in BLASTn analysis. Six miRNA queries did not find any homologs against the ESTs and thus the BLASTn results in 3508 sequences of Jatropha ESTs from homology search with the reference miRNAs. By this approach 3508 potential miRNA containing ESTs were obtained. Further redundancy checks by CD-HIT resulted in 2880 non-repeated ESTs which were kept for further analysis (Supplementary file 5). From the BLASTx analysis of ESTs, about 389 non-coding ESTs were obtained which are to be investigated as potential miRNA precursor sequences (Supplementary file 6).

### Identification of both pre-miRNAs and putative miRNAs in *J. curcas*

Different pre-defined criteria were followed to obtain pre-miRNAs from 389 non-coding ESTs in *J. curcas*. By careful evaluation based on mismatches, lengths etc. described earlier, these numbers were reduced to a total of 21 pre-miRNAs as candidates of Jatropha miRNAs. All the pre-miRNAs were 85 nucleotides in length as predicted by mirEval with default parameters.

### Determination of secondary structure of the pre-miRNAs and putative miRNAs

**Fig. 2:**
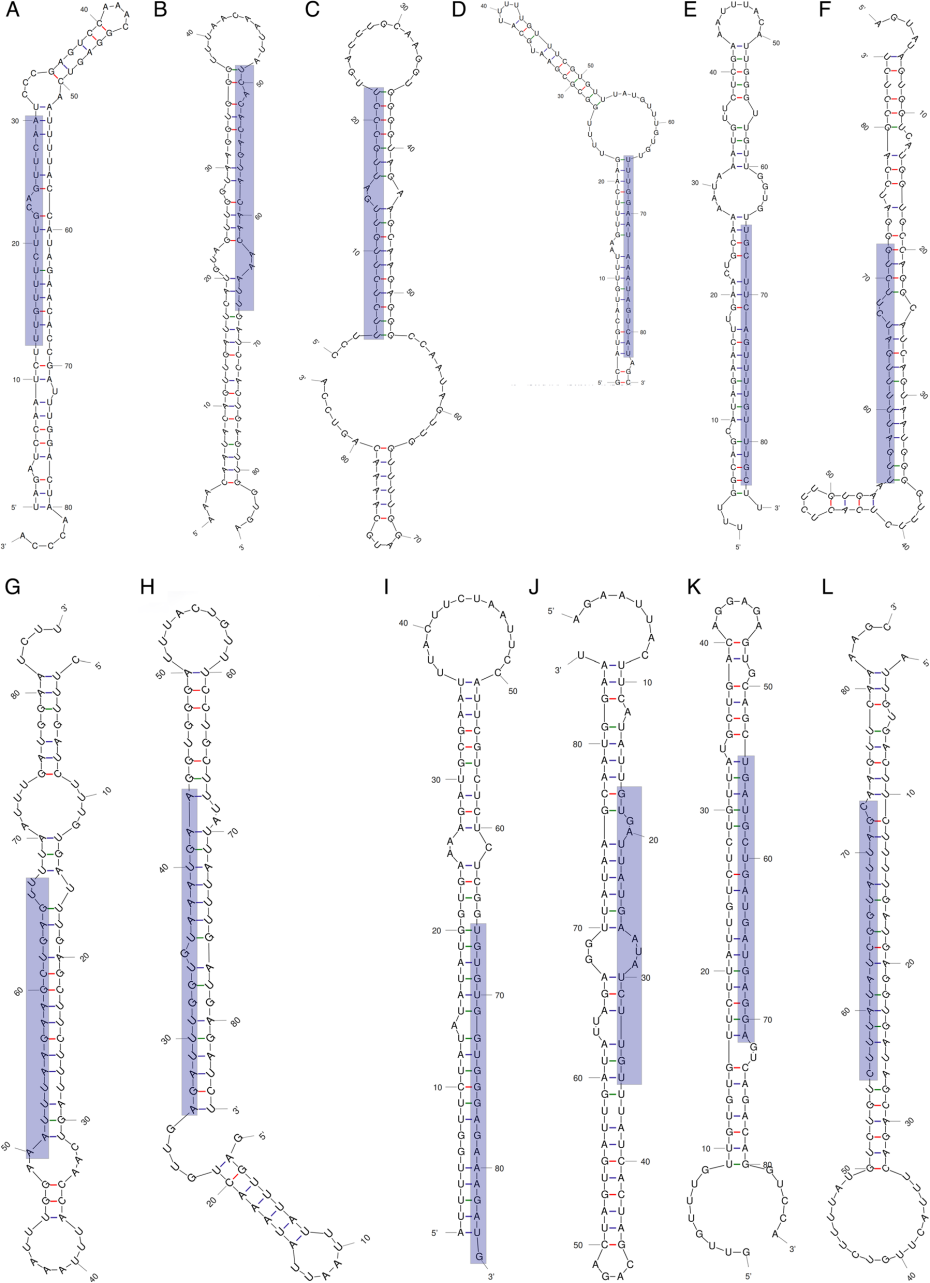
Newly identified mature miRNAs (highlighted) in the stem loop structures of pre-miRNAs. **A** jcu-miR5277, **B** jcu-miR9741, **C** jcu-miR7121, **D** jcu-miR1534, **E** jcu-miR6149-3p, **F** jcu-miR11155c-3p, **G** jcu-miR4249, **H** jcu-miR7805-3p, **I** jcu-miR8786, **J** jcu-miR3520-5p, **K** jcu-miR5658, **L** jcu-miR2112-3p

Following the analysis of the secondary structure formation capacity, 9 candidate pre-miRNAs were excluded as they failed to meet all the criteria mentioned earlier and only 12 pre-miRNAs that were able to form appropriate hair-loop secondary structures suggested by mirEval (Fig. 2, Supplementary file 7). By this investigation, 12 mature miRNAs were finally obtained and the identified candidate miRNAs have a maximal mismatch of 3 against its homolog (Table 1). There was nominal loop involvement in the secondary structures for all the miRNAs and all of them were incorporated in the single arm of the hairpin. Seven of the mature miRNAs were characterized by involving a single loop in their associated arms while the other 5 were located into a single arm and did not involve any loop. Size differences in the identified miRNAs suggest that they might be involved in various functions for regulating miRNA biogenesis or gene expression. Moreover, positioning of the miRNAs in various different locations of pre-miRNAs candidates suggests their diversity in the Jatropha plant. The number of mismatches between mature miRNAs and the reverse sequences (miRNA*) on the other side of the arms were not more than six. The MFE (ΔG in kcal/mol) values of the pre-miRNA secondary structures were calculated to be in between − 23.70 to − 15.80 (kcal/mol) in this study. The MFEI values ranged from 0.50 to 0.84 (− kcal/mol) for the pre-miRNAs. During the screening of putative miRNAs, the (A + U) percentages of pre-miRNAs was also calculated and the percentage was between 54.11 and 71.76, which was in accordance with suggested range (30–70%) for miRNAs identification (Supplementary file 8).

**Table 1:** Major properties of the newly identified Jatropha miRNAs

miRNA names	Predicted miRNAs of *Jatropha curcas*	EST Id	LoC	LM	NM	(G + C) %	MFEI
jcu-miR5277	UUGUUUCUUGCAGUUCAA	FM887831.1	5	18	2	33.33	− 0.664
jcu-miR9741	UCACACAGUACAACAAAUU	FM887543.1	3	19	3	31.57	− 0.633
jcu-miR7121	UUCUCUUGUUGAUUGCCCU	GW618852.1	5	19	3	42.10	− 0.607
jcu-miR1534	UUUGGAAUAAAUAGUCAU	GW879796.1	3	18	3	22.22	− 0.544
jcu-miR6149-3p	UGCUUCAGUUUUGUUUGC	JK317548.1	3	18	3	38.88	− 0.64
jcu-miR11155c-3p	UUGAUUUUUGAUCUUCUG	GW879253.1	3	18	3	27.77	− 0.502
jcu-miR4249	AAUUUAAGAAGCUGAGUU	GW875825.1	3	18	3	27.77	− 0.737
jcu-miR7805-3p	AGAUUUGGUGUAAAUGAA	FM890278.1	5	18	1	27.77	− 0.844
jcu-miR8786	GUGUGGUGGGAGAAAGAG	GW877957.1	3	19	3	52.63	− 0.540
jcu-miR3520-5p	GUGAUUAUGAAUAUCUUU	FM888667.1	5	19	3	26.31	− 0.800
jcu-miR5658	UGAUGCUGAUGAUGAGGA	GT971969.1	3	18	2	44.44	− 0.592
jcu-miR2112-3p	CUUUAUAUCGGUAUUAGC	GW611464.1	3	18	3	33.33	− 0.617

*LoC* Location of miRNAs, *LM* Length of miRNA, *NM* Number of mismatches, *MFEI* Minimal Folding Free Energy Index

### Nomenclature of predicted microRNAs

**Fig. 3:**
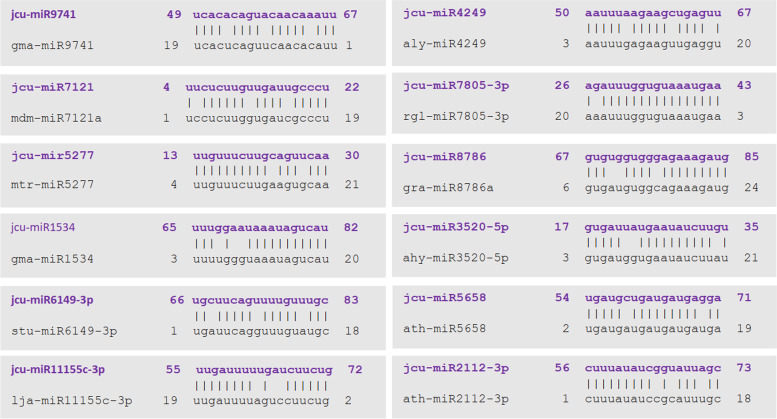
Alignments of the putative miRNAs of *J. curcas* and its homologs from respective microRNA family

The BLAST analysis of all unique miRNAs against all viridiplantae miRNAs in the miRBase database predicts which families the miRNAs are belong to, as well as the degree of similarity between them. It has been revealed that all the newly identified miRNAs belong to 12 different miRNA families (miR5277, miR9741, miR7121, miR1534, miR6149-3p, miR11155c-3p, miR4249, miR7805-3p, miR8786, miR3520-5p, miR5658, and miR2112-3p) and based on the nomenclature criteria, naming of the putative miRNAs was done (Table 1, Supplementary file 8). A sequence alignment has been displayed in MEGA X to have a better idea of the sequence relationships and distance covered between each putative miRNA and its homologous member of the mirBase. None of the identified miRNAs are aligned completely with respective full length miRbase homologs and have 1, 2, or 3 mismatches (Fig. 3).

**Fig. 4:**
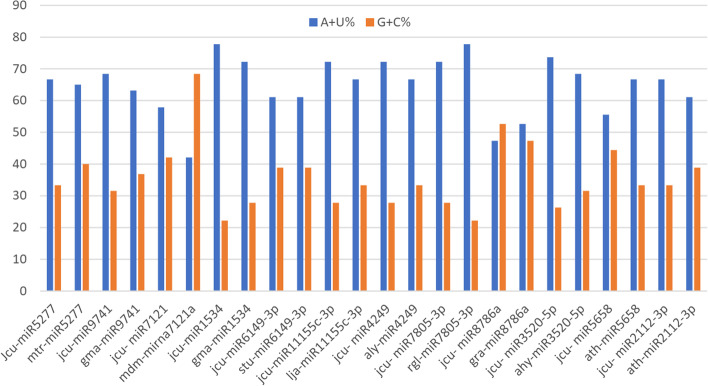
Overall nucleotide compositions (A + U% and G + C%) of putative miRNA from *J. curcas* and the most related miRNA homologs

In addition, the (G + C) compositions of maximum miRNA homologs were higher than the newly identified miRNAs except the homologs rgl-miR7805-3p, gra-miR8786, and ath-miR5658 which had lower (G + C) percentage (Fig. 4, Supplementary file 8).

### Target prediction and functional analysis of newly identified *J. curcas* miRNAs

Based on their perfect or nearly perfect complementarity with their target sequences in *Arabidopsis*, the 12 putative miRNAs were discovered to be engaged in targeting 893 genes (Supplementary file 9). Moreover, within our filtration technique, we discovered that 93 of the total 893 targets have unknown functions in the model plant *A. thaliana*. The miRNA family ‘miR5658' has the most individual target genes (211), while the family ‘miR9741' has only 16 targets (Supplementary file 9). On the other hand, the number of gene targets for the rest of the miRNA families - miR5277 (89), miR7121 (73), miR1534 (34), miR6149-3p (95), miR11155c-3p (134), miR4249 (58), miR7805-3p (60), miR8786 (56), miR3520-5p (43), and miR2112-3p (24) were also predicted. In all the gene targets, some targets such as transposable gene element and kinase protein family were found as common targets. It has been revealed that a single gene can be inhibited or degraded by an individual miRNA by their complementary binding on multiple sites of that gene. The predicted miRNAs target a number of gene families that have been linked to a variety of biological functions, including metabolism regulation, transcription factor activity, biosynthetic processes, growth and development, defense mechanisms, hormone biosynthesis, and biofuel synthesis (Table 2). The most vital functions of the gene targets are given on the Table 2 with their names, e-values and associated proteins.

**Table 2:** The selected gene targets of newly identified miRNAs

miRNA Acc.	Target Acc.	(E)	Target description	Target function
jcu-miR5277	AT5G24120.1	1.5	Sigma factor E	Female gametophyte development
	AT1G30860.1	1.5	RING/U-box superfamily protein	Protein modification
	AT4G13720.1	2.5	Inosine triphosphate pyrophosphatase family protein	Avoiding chromosomal lesions.
	AT5G61420.2	2.5	MYB28	Defense against herbivorous insects
	AT1G08970.3	2.5	Nuclear factor Y	Flowering, and gibberellic acid pathways
	AT1G06520.1	2.5	glycerol-3-phosphate acyltransferase 1	Biosynthesis of triglycerides, phosphatidic acids
jcu-miR9741	AT1G16070.2	2.5	AtTLP8	Stimulus from a fungus
	AT5G04870.1	3	Calcium dependent protein kinase 1	Signal transduction
	AT5G50950.3	3	FUMARASE 2	Photosynthesis to cold
	AT5G20240.1	3	K-box transcription factor family protein	Transcription factor
jcu-miR7121	AT3G15620.1	2	DNA photolyase family protein	Repair of UV radiation-induced DNA damage
	AT1G73960.2	2.5	TBP-associated factor 2	Transcription factor
	AT5G35750.1	3	Histidine kinase 2	Responses and abscisic acid (ABA) signaling in abiotic stresses
jcu-miR1534	AT5G59260.1	2	Concanavalin A-like lectin protein kinase family protein	Defense response to bacterium
	AT2G34640.1	3	Plastid transcriptionally active 12	Plastid gene expression
	AT3G20710.1	3	F-box family protein	Growth and development, auxin signaling
jcu-miR6149-3p	AT5G03180.1	1.5	RING/U-box superfamily protein	Protein modification.
	AT2G44950.1	2.5	Histone mono-ubiquitination 1	Defense, control of seed dormancy and germination
	AT4G16780.1	3	Homeobox protein 2	Metabolism and Lateral root formation
	AT1G21360.1	3	glycolipid transfer protein 2	Lipid metabolism
	AT5G03540.1	3	exocyst subunit exo70 family protein A1	Cell growth and organ morphogenesis
jcu-miR11155c-3p	AT4G34060.2	2.5	Demeter-like protein 3	DNA repair
	AT2G32510.1	2.5	Mitogen-activated protein kinase kinase kinase 17	ABA signal during abiotic stresses
	AT3G05630.1	2.5	Phospholipase D P2	Diacylglycerol synthesis
	AT5G22420.1	2.5	Fatty acid reductase 7	Lipid metabolism
	AT4G24230.5	3	acyl-CoA-binding domain 3	Phosphotidic acid biosynthesis
	AT4G36870.1	3	BEL1-like homeodomain 2	Lipid metabolism
	AT3G15730.1	3	phospholipase D alpha 1	Phosphatidic acids metabolism
jcu-miR4249	AT1G77020.1	2.5	DNAJ heat shock N-terminal domain-containing protein	Protein modification
	AT2G32430.1	3	Galactosyltransferase family protein	Protein modification
	AT1G72470.1	3	Exocyst subunit exo70 family protein D1	Exocytosis protein transport
	AT1G74910.3	3	ADP-glucose pyrophosphorylase family protein	Regulation of L-ascorbic acid biosynthetic process
jcu-miR7805-3p	AT3G51770.1	3	Tetratricopeptide repeat (TPR)-containing protein	Ethylene biosynthesis, post- embryonic root development
	AT2G21220.1	3	SAUR-like auxin-responsive protein family	Response to auxin
	AT3G50310.1	3	MAPKKK20	Abscisic acid (ABA) responses, abiotic stresses
jcu-miR8786	AT5G18410.3	3	PIR, KLK, PIR121, SRA1, PIRP, transcription activators	Development
	AT5G52830.1	3	WRKY27,	Disease resistance, hormonal responses
	AT4G12610.1	3	RAP74	Transcriptional regulation
	AT1G31550.2	3	GDSL-like Lipase/Acylhydrolase superfamily protein	Lipid metabolism
jcu-miR3520-5p	AT3G09560.1	2	Lipin family protein	Biosynthesis of diacylglycerol, lipid metabolism
	AT5G65800.1	2.5	ACC synthase 5	Ethylene biosynthesis, fruit ripening
	AT5G55490.1	3	GEX1	Gametophyte development, pollen maturation.
	AT1G02720.1	3	GATL5	Metabolism
jcu-miR5658	AT5G16560.1	2	Homeodomain-like superfamily protein	Transcription factor
	AT3G13930.1	2	Dihydrolipoamide acetyltransferase	Fatty acid biosynthesis
	AT5G59450.1	2	GRAS family transcription factor	Growth and development
	AT2G45880.1	2.5	Beta-amylase 7	Transcription factor
	AT4G34810.1	3	SAUR-like auxin-responsive protein family	Response to auxin
jcu-miR2112-3p	AT4G16860.1	2.5	TIR-NBS-LRR class family protein	Metabolism
	AT1G74030.1	3	Enolase 1	Metabolism
	AT1G73460.1	3	Protein kinase superfamily protein	Transcription factor
	AT1G51720.1	3	Amino acid dehydrogenase family protein	Metabolism

**Fig. 5:**
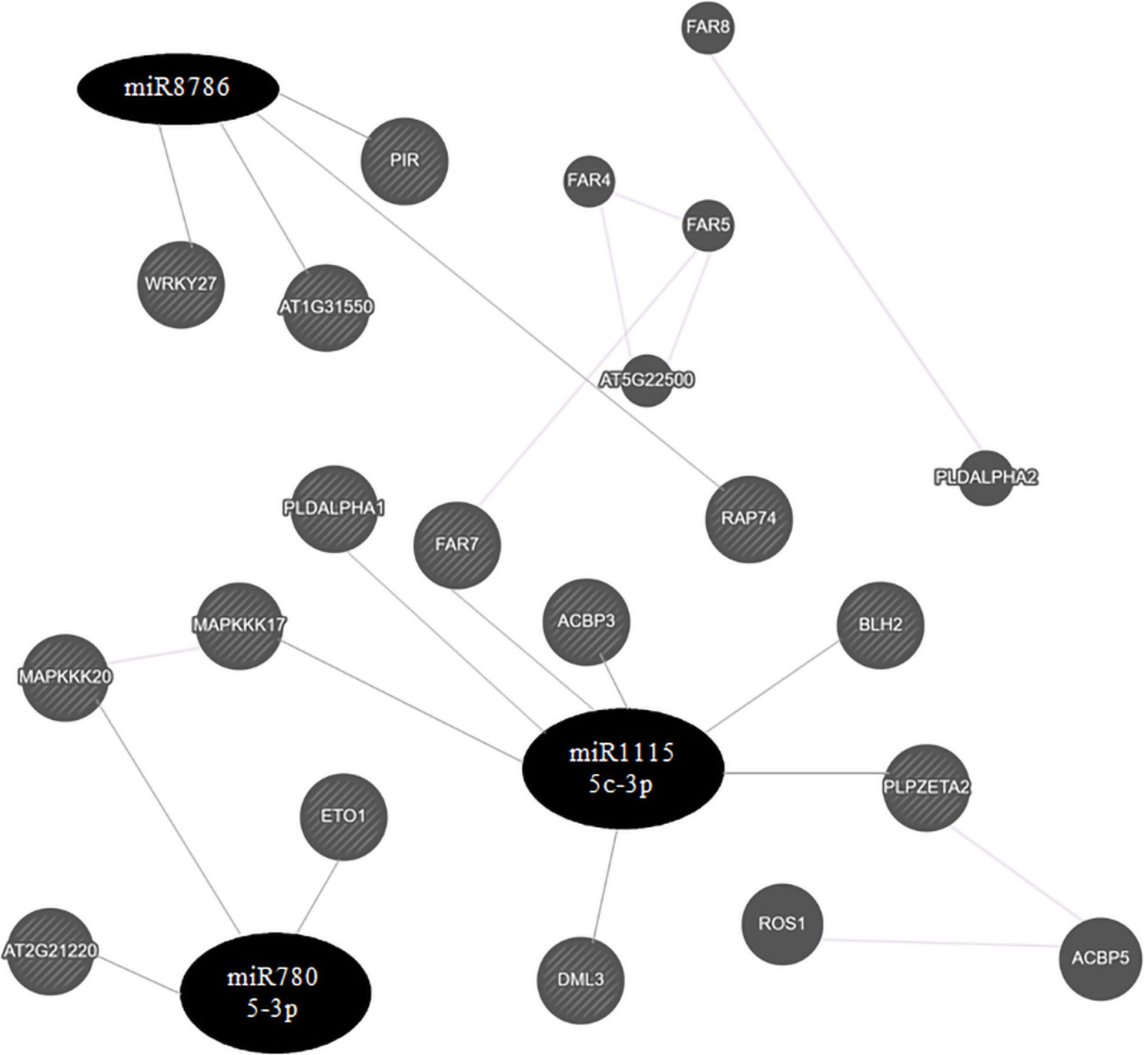
Co-expression network of potential target genes of the selected miRNAs

A study by Eisen and colleagues suggested that co-expressed genes were functionally related. Sets of co-expressed genes that may be associated with target genes can be elucidated by gene co-expression network building. Co-expression analyses can lead to characterize genes of unknown function [41]. The network analysis revealed that the target gene AT5G22420.1 (FAR7) of jcu-miR11155c-3p is co-expressed with the highest number of other genes (Fig. 5).

### Phylogenetic analysis

**Fig. 6:**
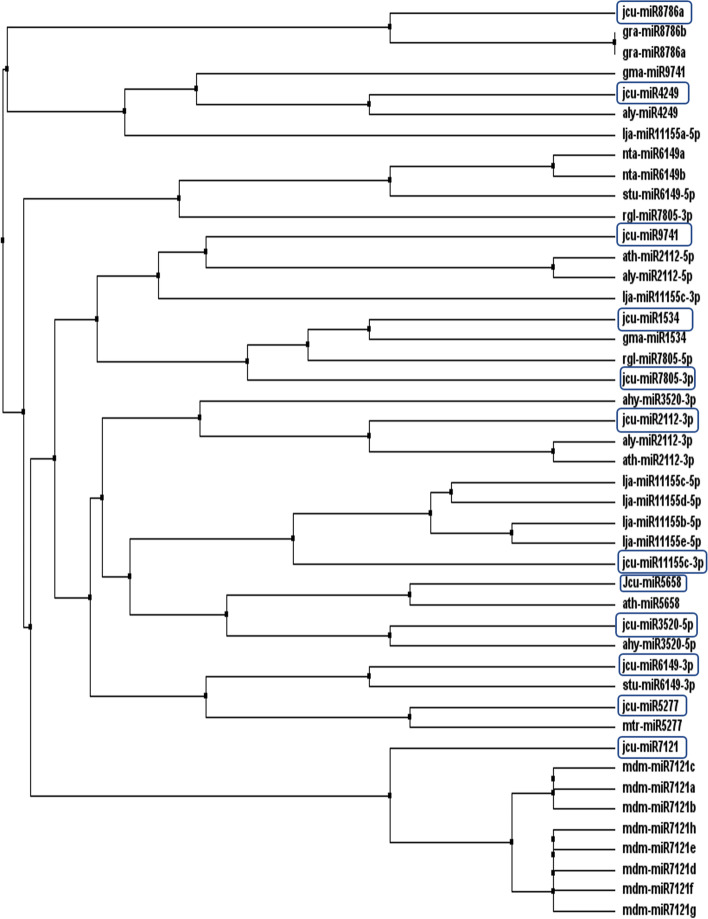
Phylogenetic analysis of 12 newly identified miRNAs of *Jatropha curcas*

When the newly identified mature miRNA sequences were matched to those of other members of the same family, it can be concluded that the majority of them shared a high amount of sequence identity. The phylogenetic tree illustrating the evolutionary relationship of Jatropha miRNAs with the other family members has been depicted in Fig. 5. It has been revealed that all miRNAs belong to their respective miRNA family except jcu-miR9741, which contained two related members ath-miR2112-5p and aly-miR2112-5p respectively (Fig. 6).

## Discussion

MicroRNAs have become vital candidates for research as they act as gene regulators in many plants [42]. Finding new miRNAs provides a novel insight to understand their regulatory roles and functions. The availability of EST sequences of *J. curcas* made the identification of conserved miRNAs relatively straight forward. In plants, it is believed that there is at least one miRNA per 10,000 ESTs, implying that the minimum frequency of discovering a miRNA from ESTs is 0.01% [25]. Searching of potential miRNAs using in silico approach in the EST sequences of *J. curcas* revealed 12 putative miRNAs belonging to 12 individual miRNA families (Table 1). Similar study was performed by Vishwakarma and Jadeja [26], where they identified different miRNAs than ours with a lower number (05) of miRNA families as well as lower number of targets (78). To predict new miRNAs from ESTs, we considered conservancy nature of sequences and ability to form hairpin secondary structure of the potential pre-miRNAs (Fig. 2). Because folding of pre-miRNAs into stem-loop hairpin structure is a vital stage in miRNA maturation. On the other hand, a stem-loop hairpin structure is not only a distinguishing feature of miRNAs but also other RNAs such as mRNA, rRNA, and tRNA can have comparable hairpin structures. Thus, criteria like MFE, AMFE, and MFEI for annotating new miRNAs were explored to avoid misleading categorization of other RNAs as miRNA candidates. The lower the MFE value, the secondary structure of the matching sequences is more thermodynamically stable [43]. Therefore, we have selected the pre-miRNA candidates having MFE values ranged between − 23.70 to − 15.80 kcal/mol and within the suggested value of other reported pre-miRNAs, and also have lower values than many of the tRNA and ribosomal RNA [44]. It was observed that the stem portion of the hairpin structures contain all of the putative mature miRNA sequences of *J. curcas* as required. The identified miRNA hairpin structures also revealed that the stem region contained at least 18–19 nucleotides engaged in Watson–Crick or G/U pairs between the miRNA/miRNA* and no extensive internal loops or bulges were found (Fig. 2), confirming the findings of Zhang et al. [20]. Furthermore, the (A + U) content of pre-miRNA was discovered to be between 54.11 and 71.76 percent, which was similarly reported in the miRNAs of higher plants [21]. The results showed that miRNAs derived from pre-miRNAs had only one respective family, which supports previous research in numerous plant species [26, 45, 46]. Interestingly, biasness of the uracil (U) content at the first position found to be one of the attributes of the miRNAs [31]. The uracil’s position at the 5′ end of miRNAs is critical since it is thought to have a role in recognizing and binding to their target genes [46, 47]. In our findings, among the twelve predicted miRNAs, uracil was the first base at the 5′ end in seven miRNAs (Table 1, Fig. 2).

The identification of mature miRNAs’ targets is a crucial step in determining their involvement in biological and metabolic processes, as well as post-transcriptional gene control [30, 48]. The ability of miRNAs to bind their target mRNAs with perfect or near-perfect sequence complementarity offers a powerful approach for identifying new miRNA targets by comparing miRNA sequences to mRNA sequences [48–50]. Our target prediction of the Jatropha’s miRNAs indicated that a single miRNA can effectively function in many genes, which is consistent with previous studies in other plant species [51]. Furthermore, it was observed that Jatropha microRNAs might have numerous target sites on a single mRNA, which is thought to improve the miRNA's recognition of the target gene [52]. Here, we have found that predicted targets of miRNAs includes transcription factors, enzymes, and protein families that play significant roles in metabolism, growth, bacterial and fungal defense, protection of DNA damage, hormone biosynthesis, and production of fatty acids and oils (Table 2). A number of transcription factors were found to be targeted by Jatropha miRNAs such as sigma factor E and nuclear factor Y (jcu-miR5277), K-box transcription factor family protein (jcu-miR9741), TBP-associated factor 2 (jcu-miR7121), plastid transcriptionally active 12 (jcu-miR1534), homeobox protein 2 (jcu-miR6149-3p), RAP74 transcription activators and WRKY DNA-binding protein 27 (jcu- miR8786), and GRAS family transcription factor and beta-amylase 7 (jcu-miR5658). Sigma factor E is an essential protein and act as an initiation factor for plastid encoded RNA polymerase, and also plays a crucial role in reproduction and female gametophyte development [53]. On the other hand, the nuclear factor Y stimulates various genes and maintains responses of flowering signals by directly regulating SOC1 transcription and also regulates the gibberellic acid biosynthesis pathways [54]. Another important gene targeted by miRNA was the homeobox protein 2 mainly involved in the negative regulation of the cell elongation and lateral root formation. In addition, the WRKY DNA-binding protein 27 was associated with a plethora of biological activities like cellular heat acclimation, bacterial and fungal defense, positive autophagy control, and gibberellin response [55]. Moreover, GRAS family transcription factor, which is a transcription regulator, plays a key role mainly in plant growth and development, gibberellic acid signaling and phytochrome A signal transduction (Vivek et al. 2018). Both of beta-amylase 7 and GRAS were targets of jcu-miR5658. Also, the genes encoding proteins which are directly or indirectly associated with the biosynthesis and regulatory pathways of plant growth hormones were the predicted targets of miRNAs. The F-box family proteins were targeted by both jcu-miR5277 and jcu-miR1534. This protein is involved in plant growth, development, and auxin signaling probably by the action of two F-box family proteins, F-box 2 and F-box 3 [56]. Furthermore, the protein histidine kinase 2 (HK2/AHK2) was the target of jcu-miR7121. It works by negatively regulating drought and salt stress responses, as well as abscisic acid (ABA) signaling. It also inhibits the ABA response in combination with AHK3 during cold stress condition, whereas it is believed to have a positive regulation in cytokinin signaling pathway and have vital roles in cell division, seed germination as well as seed size [57–59]. One of the most important targets of predicted miRNA was the ACC synthase 5 (ACS5) which plays vital role in ethylene biosynthesis. Ethylene functions in the process of fruit ripening [60], which is expected to be inhibited by the jcu-miR3520-5p. In this study, we have also identified the gene transcripts for the proteins such as myb domain protein 28 (MYB28), DNA photolyase family protein and demeter-like protein 3 (DML3), which were found to be the predicted targets of jcu-miR5277, jcu-miR7121, and jcu-miR11155c-3p respectively. MYB28 protects the DNA from free radical attack by producing detoxifying enzymes while DNA photolyase repairs UV radiation-induced DNA damage [61–64].

In silico identification of the miRNA in *J. curcas*, a biofuel producing plant may help us to understand the mechanism of genes related to oil production. Interestingly, our results showed that some gene targets (09) of predicted miRNAs (06) were related to oil and biofuel production. Among these six miRNAs, maximum number of genes were regulated by the jcu-miR11155c-3p, which included phospholipase D P2 (PLDP2), fatty acid reductase 7 (FAR7), acyl-CoA-binding domain 3 (ACBP3) and phospholipase D alpha 1 (PLDALPHA1). PLDP2 mediates the biosynthesis of phosphatidic acid and is involved in hydrolyzing phosphatidylcholine and phosphatidylethanolamine to produce diacylglycerol. Again, FAR7 and ACBP3, both are involved with fatty acyl-CoA, thereby having a significant role in lipid biosynthesis [65, 66]. Moreover, PLDALPHA1 has a function in fatty acid metabolic processes apart from its involvement in ABA signaling pathways and seed germination [67]. Similarly, jcu-miR5277 might prevent the enzyme glycerol-3-phosphate acyltransferase 1 from being transcribed which functions in an essential step during the synthesis of glycerolipids such as triglycerides, phosphatidic acids and lysophosphatidic acids [68]. Ceramide is a waxy lipid molecule found in plants to which ceramidase 1-phosphate tends to bind for the biosynthesis of sphingolipids and fatty acids. The expression of glycolipid transfer protein 2 (GLTP2) which functions to bind ceramide 1-phosphate with ceramide get blocked by one of our predicted miRNAs, jcu-miR6149-3p [69]. This miRNA can possibly show a key role in the sphingolipid biosynthesis pathway [70]. Production of PAH1 and thus phospholipid biosynthesis might be altered by the jcu-miR3520-5p. Other than these proteins, the enzyme dihydrolipoamide acetyltransferase was a possible target for the jcu-miR5658 which is involved in the fatty acid biosynthesis process in *Arabidopsis* [71].

## Conclusion

In conclusion, the findings of the present study provide new insights into the miRNAs of *J. curcas*. In silico identification of 12 putative miRNAs along with their possible targets will aid in future research on further understanding of their role in various aspects of biological processes such as defense mechanisms, hormone biosynthesis, signal transduction, lipid and fatty acid production in Jatropha. The identified miRNA in Jatropha showed maximum similarities with their respective miRNA homologs, and the functional analysis revealed that the miRNAs could potentially target various biological and metabolic processes with near-perfect complementarity. It is clearly evident that the identification of more miRNAs is yet to be done. However, experimental validation as well as expression analysis of computationally identified miRNAs needs to be performed to justify their predicted functions.

## Supplementary Information

**Additional file 1.** Retrieved 46865 EST sequences of *Jatropha curcas.* 

**Additional file 2.** A total of 6746 of plant eudicotyledones miRNA from miRBase database.

**Additional file 3.** Total of 42374 non-redundant EST sequences of *Jatropha curcas.*

**Additional file 4.** Total of 3514 non-redundant miRNA after redundancy screening.

**Additional file 5.** Potential 2880 ESTs of *Jatropha curcas* genome for predicting putative miRNA.

**Additional file 6.** Total of 389 putative non-coding EST sequences of *Jatropha curcas* genome.

**Additional file 7.** Non-coding EST sequence containing the predicted precursor miRNA.

**Additional file 8.** Major characteristics of miRNAs, pre-miRNAs and miRNA homologs. 

**Additional file 9. **Gene targets of the predicted miRNAs. 

## Data Availability

All data generated or analyzed during this study are included in this published article (and its supplementary information files).

## Abbreviations

miRNAs: MicroRNAs
EST: Expressed Sequence Tags
MFEs: Minimal free energies
AMFE: Adjusted minimal folding energy
MFEI: Minimal Folding Free Energy Index
SIGE: Sigma factor E
PEP: Plastid-encoded RNA polymerase
PLDALPHA1: Phospholipase D alpha 1

## References

[1] Openshaw K (2000). A review of Jatropha curcas: an oil plant of unfulfilled promise. Biomass Bioenergy.

[2] Singh YN, Ikahihifo T, Panuve M, Slatter C (1984). Folk medicine in Tonga. A study on the use of herbal medicines for obstetric and gynaecological conditions and disorders. J Ethnopharmacol.

[3] Staubmann Schubert-Zsilavecz M, Hiermann A, Kartnig T (1999). A complex of 5-hydroxypyrrolidin-2-one and pyrimidine-2, 4-dione isolated from Jatropha curcas. Phytochemistry.

[4] Igbinosa OO, Igbinosa EO, Aiyegoro OA (2009). Antimicrobial activity and phytochemical screening of stem bark extracts from Jatropha curcas (Linn). Afr J Pharm Pharmacol.

[5] Becker K, Makkar HP (1998). Effects of phorbol esters in carp (Cyprinus Carpio L). Vet Hum Toxicol.

[6] King AJ, He W, Cuevas JA, Freudenberger M, Ramiaramanana D, Graham IA (2009). Potential of Jatropha curcas as a source of renewable oil and animal feed. J Exp Bot.

[7] Achten WM, Verchot L, Franken YJ, Mathijs E, Singh VP, Aerts R, Muys B (2008). Jatropha bio-diesel production and use. Biomass Bioenergy.

[8] Pandey VC, Singh K, Singh JS, Kumar A, Singh B, Singh RP (2012). Jatropha curcas: a potential biofuel plant for sustainable environmental development. Renewable Sustain Energy Rev.

[9] Tiwari AK, Kumar A, Raheman H (2007). Biodiesel production from jatropha oil (Jatropha curcas) with high free fatty acids: an optimized process. Biomass Bioenergy.

[10] Rahman KM, Mashud M, Roknuzzaman M, Al Galib A (2010). Biodiesel from Jatropha oil as an alternative fuel for diesel engine. Int J Mech Mechatron (IJMME-IJENS).

[11] Zhang B, Pan X, Cannon CH, Cobb GP, Anderson TA (2006). Conservation and divergence of plant microRNA genes. Plant J.

[12] Zamore PD, Haley B (2005). Ribo-gnome: the big world of small RNAs. Science.

[13] Zhang B, Wang Q, Pan X (2007). MicroRNAs and their regulatory roles in animals and plants. J Cell Physiol.

[14] Hawkins PG, Morris KV (2008). RNA and transcriptional modulation of gene expression. Cell Cycle.

[15] Tan Y, Zhang B, Wu T, Skogerbø G, Zhu X, Guo X, He S, Chen R (2009). Transcriptional inhibiton of Hoxd4 expression by miRNA-10a in human breast cancer cells. BMC Mol Biol.

[16] Morozova N, Zinovyev A, Nonne N, Pritchard LL, Gorban AN, Harel-Bellan A (2012). Kinetic signatures of microRNA modes of action. RNA.

[17] Ambros V, Chen X (2007). The regulation of genes and genomes by small RNAs. Development.

[18] Chen X (2005). MicroRNA biogenesis and function in plants. FEBS Lett.

[19] Körbes AP, Machado RD, Guzman F, Almerão MP, de Oliveira LF, Loss-Morais G, Turchetto-Zolet AC, Cagliari A, dos Santos MF, Margis-Pinheiro M, Margis R (2012). Identifying conserved and novel microRNAs in developing seeds of Brassica napus using deep sequencing. PLoS ONE.

[20] Zhang B, Pan X, Cobb GP, Anderson TA (2006). Plant microRNA: a small regulatory molecule with big impact. Dev Biol.

[21] Ahmed M, Ahmed F, Ahmed J, Akhand MRN, Azim KF, Imran MAS, Hoque SF, Hasan M (2021). In silico identification of conserved miRNAs in the genome of fibre biogenesis crop Corchorus capsularis. Heliyon.

[22] Kozomara A, Birgaoanu M, Griffiths-Jones S (2019). miRBase: from microRNA sequences to function. Nucleic Acids Res.

[23] Griffiths-Jones S, Saini HK, Van Dongen S, Enright AJ (2007). miRBase: tools for microRNA genomics. Nucleic Acids Res.

[24] Qiu CX, Xie FL, Zhu YY, Guo K, Huang SQ, Nie L, Yang ZM (2007). Computational identification of microRNAs and their targets in Gossypium hirsutum expressed sequence tags. Gene.

[25] Zhang BH, Pan XP, Wang QL, Cobb GP, Anderson TA (2005). Identification and characterization of new plant microRNAs using EST analysis. Cell Res.

[26] Vishwakarma NP, Jadeja VJ (2013). Identification of miRNA encoded by Jatropha curcas from EST and GSS. Plant Signaling Behav.

[27] Frazier TP, Sun G, Burklew CE, Zhang B (2011). Salt and drought stresses induce the aberrant expression of microRNA genes in tobacco. Mol Biotechnol.

[28] Zhang BH, Pan X, Stellwag EJ (2008). Identification of soybean microRNAs and their targets. Planta.

[29] Kwak PB, Wang QQ, Chen XS, Qiu CX, Yang ZM (2009). Enrichment of a set of microRNAs during the cotton fiber development. BMC Genomics.

[30] Akter A, Islam MM, Mondal SI, Mahmud Z, Jewel NA, Ferdous S, Amin MR, Rahman MM (2014). Computational identification of miRNA and targets from expressed sequence tags of coffee (Coffea arabica). Saudi J Biol Sci.

[31] Vivek AT (2018). In silico identification and characterization of microRNAs based on EST and GSS in orphan legume crop, Lens culinaris medik(lentil). Agri Gene.

[32] Wang CM, Liu P, Sun F, Li L, Liu P, Ye J, Yue GH (2012). Isolation and Identification of miRNAs in Jatropha curcas. Int J Biol Sci.

[33] Galli V, Guzman F, de Oliveira LFV, Loss-Morais G, Körbes AP, Silva SDA, Margis-Pinheiro MMAN, Margis R (2014). Identifying microRNAs and transcript targets in Jatropha seeds. PLoS ONE.

[34] Yang M, Lu H, Xue F, Ma L (2019). Identifying high confidence microRNAs in the developing seeds of Jatropha curcas. Sci Rep.

[35] Zuker M (2003). Mfold web server for nucleic acid folding and hybridization prediction. Nucleic Acids Res.

[36] Ambros V, Bartel B, Bartel DP, Burge CB, Carrington JC, Chen X (2003). A uniform system for microRNA annotation. RNA.

[37] Griffiths-Jones S, Grocock RJ, van Dongen S, Bateman A, Enright AJ (2006). miRBase: microRNA sequences, targets and gene nomenclature. Nucleic Acids Res.

[38] Dai X, Zhao PX (2011). PsRNATarget: a plant small RNA target analysis server. Nucleic Acids Res.

[39] Warde-Farley D, Donaldson SL, Comes O, Zuberi K, Badrawi R, Chao P, Franz M, Grouios C, Kazi F, Lopes CT, Maitland A, Mostafavi S, Montojo J, Shao Q, Wright G, Bader GD, Morris Q (2010). The GeneMANIA prediction server: biological network integration for gene prioritization and predicting gene function. Nucleic Acids Res.

[40] Kumar S, Stecher G, Li M, Knyaz C, Tamura K (2018). MEGA X: molecular evolutionary genetics analysis across computing platforms. Mol Biol Evol.

[41] Eisen MB, Spellman PT, Brown PO, Botstein D (1998). Cluster analysis and display of genome-wide expression patterns. PNAS.

[42] Sunkar R, Jagadeeswaran G (2008). In silico identification of conserved microRNAs in large number of diverse plant species. BMC Plant Biol.

[43] Prabu GR, Mandal AKA (2010) Computational identification of miRNAs and their target genes from expressed sequence tags of tea (*Camellia sinensis*). Genomics Proteomics Bioinformatics 8(2):113–121 10.1016/S1672-0229(10)60012-5 PMC5054452 20691396

[44] Bonnet E, Wuyts J, Rouze P, de Peer YV (2004). Evidence that microRNA precursors, unlike other non-coding RNAs, have lower folding free energies than random sequences. Bioinformatics.

[45] Panda D, Dehury B, Sahu J, Barooah M, Sen P, Modi MK (2014). Computational identification and characterization of conserved miRNAs and their target genes in garlic (Allium sativum L.) expressed sequence tags. Gene.

[46] Subburaj S, Kim AY, Lee S, Kim KN, Suh MC, Kim GJ, Lee GJ (2016). Identification of novel stress-induced microRNAs and their targets in Camelina sativa. Plant Biotechnol Rep.

[47] Felice KM, Salzman DW, Shubert-Coleman J, Jensen KP, Furneaux HM (2009). The 5′ terminal uracil of let-7a is critical for the recruitment of mRNA to Argonaute2. Biochem J.

[48] Pani A, Mahapatra RK (2013). Computational identification of microRNAs and their targets in Catharanthus roseus expressed sequence tags. Genom Data.

[49] Schwab R, Palatnik JF, Riester M, Schommer C, Schmid M, Weigel D (2005). Specific effects of microRNAs on the plant transcriptome. Devolopmental Cell.

[50] Wang XJ, Reyes JL, Chua NH, Gaasterland T (2004). Prediction and identification of Arabidopsis thaliana microRNAs and their mRNA targets. Genome Biol.

[51] Jones-Rhoades WM, Bartel DP (2004). Computational identification of plant microRNAs and their targets, including a stress-induced miRNA. Mol Cell.

[52] Axtell MJ, Bartel DP (2005). Antiquity of microRNAs and their targets in land plants. Plant Cell.

[53] Yu F, Shi J, Zhou J, Gu J, Chen Q, Li J, Cheng W, Mao D, Tian L, Buchanan BB, Li L, Chen L, Li D, Luan S (2010). ANK6, a mitochondrial ankyrin repeat protein, is required for male-female gamete recognition in Arabidopsis thaliana. PNAS.

[54] Hou X, Zhou J, Liu C, Liu L, Shen L, Yu H (2014). Nuclear factor Y-mediated H3K27me3 demethylation of the SOC1 locus orchestrates flowering responses of Arabidopsis. Nat Commun.

[55] Mukhtar MS, Deslandes L, Auriac MC, Marco Y, Somssich IE (2008). The Arabidopsis transcription factor WRKY27 influences wilt disease symptom development caused by Ralstonia solanacearum. Plant J.

[56] Peng J, Yu D, Wang L, Xie M, Yuan C, Wang Y, Liu X (2012). Arabidopsis F-box gene FOA1 involved in ABA signaling. Sci China Life Sci.

[57] Higuchi M, Pischke M, Mähönen AP, Miyawaki K, Seki HY, M, Kakimoto T,  (2004). In planta functions of the Arabidopsis cytokinin receptor family. PNAS.

[58] Mähönen AP, Higuchi M, Törmäkangas K, Miyawaki K, Pischke MS, Sussman MR, Kakimoto T (2006). Cytokinins regulate a bidirectional phosphorelay network in Arabidopsis. Curr Biol.

[59] Nishimura C, Ohashi Y, Sato S, Kato T, Tabata S, Ueguchi C (2004). Histidine kinase homologs that act as cytokinin receptors possess overlapping functions in the regulation of shoot and root growth in Arabidopsis. Plant Cell.

[60] Vogel JP, Woeste KE, Theologis A, Kieber JJ (1998). Recessive and dominant mutations in the ethylene biosynthetic gene ACS5 of Arabidopsis confer cytokinin insensitivity and ethylene overproduction, respectively. PNAS.

[61] Hirai MY, Sugiyama K, Sawada Y, Tohge T, Obayashi T, Suzuki A, Saito K (2007). Omics-based identification of Arabidopsis Myb transcription factors regulating aliphatic glucosinolate biosynthesis. PNAS.

[62] Nakajima S, Sugiyama M, Iwai S, Hitomi K, Otoshi E, Kim ST, Yamamoto K (1998). Cloning and characterization of a gene (UVR3) required for photorepair of 6–4 photoproducts in Arabidopsis thaliana. Nucleic Acids Res.

[63] Ortega-Galisteo AP, Morales-Ruiz T, Ariza RR, Roldán-Arjona T (2008). Arabidopsis DEMETER-LIKE proteins DML2 and DML3 are required for appropriate distribution of DNA methylation marks. Plant Mol Biol.

[64] Schleicher E, Hitomi K, Kay CW, Getzoff ED, Todo T, Weber S (2007). Electron nuclear double resonance differentiates complementary roles for active site histidines in (6–4) photolyase. J Biol Chem.

[65] Gaudet P, Livstone MS, Lewis SE, Thomas PD (2011). Phylogenetic-based propagation of functional annotations within the Gene Ontology consortium. Brief Bioinform.

[66] Leung KC, Li HY, Xiao S, Tse MH, Chye ML (2006). Arabidopsis ACBP3 is an extracellularly targeted acyl-CoA-binding protein. Planta.

[67] Fan L, Zheng S, Wang X (1997). Antisense suppression of phospholipase D alpha retards abscisic acid-and ethylene-promoted senescence of postharvest Arabidopsis leaves. Plant Cell.

[68] Chen YQ, Kuo MS, Li S, Bui HH, Peake DA, Sanders PE, Cao G (2008). AGPAT6 is a novel microsomal glycerol-3-phosphate acyltransferase. J Biol Chem.

[69] Airenne TT, Kidron H, Nymalm Y, Nylund M, West G, Mattjus P, Salminen TA (2006). Structural evidence for adaptive ligand binding of glycolipid transfer protein. J Mol Biol.

[70] Nakamura Y, Koizumi R, Shui G, Shimojima M, Wenk MR, Ito T, Ohta H (2009). Arabidopsis lipins mediate eukaryotic pathway of lipid metabolism and cope critically with phosphate starvation. PNAS.

[71] Iida K, Fukami-Kobayashi K, Toyoda A, Sakaki Y, Kobayashi M, Seki M, Shinozaki K (2009). Analysis of multiple occurrences of alternative splicing events in Arabidopsis thaliana using novel sequenced full-length cDNAs. DNA Res.

