# Supplementary material for: Identification of conserved miRNAs and their targets in Jatropha curcas: an in silico approach
Source: J Genet Eng Biotechnol. 2023 Apr 7;21:43. doi: 10.1186/s43141-023-00495-9 (PMC10079790; doi:10.1186/s43141-023-00495-9)
Supplement: Supplementary file 5 — Additional file 5. Potential 2880 ESTs of Jatropha curcas genome for predicting putative miRNA. [file 43141_2023_495_MOESM5_ESM.docx]

**Supplementary File 5:** Potential 2880 ESTs of *Jatropha curcas* genome for predicting putative miRNA

>GW616807.1 Jc2-015-H07-M13F.H07.ab1 Jatropha curcas flower and seed Jatropha curcas cDNA, mRNA sequence

TATAACAACACAGCCCGTTATTTCTGGATATGCCTCTCCTATTCCATCAAGGCAAATCAATAACCTGATCCATGACCAGG

TGGAGAAGGGGAAAAAATATGAAAATTCTGTAGGCTGCCGTTTATTTGGAATTGATATAACAAGCAATTCCAATGCTGCT

GCTACTCCAGAGAAGGAGGCTTTGTGTCCAACTGTTGACTCCCATGGCACTGAAGGACCTGCTCCAGCTTCAAGTGATTC

TGACAAGGCTCAAAATATGGATGTTTCAAATCCCTTAAAAGATCAGAAGCAAGGTGCATCAGAAGTAGTGCCAAGAGAGA

CACACAGCAAGCTAGGTTCCACTTCTTCCACAAGAACTCGTACAAAGGTGCAAATGCAAGGAGTTGCTGTTGGTCGTGCT

GTTGATCTGACAGTGCTGAAAGGGTACGGAGATCTTATAAAAGAGCTGGAGGAAATGTTTGAAATCAAAGGAGAGCTTTC

CACTCGCGACAAATGGGCCGTTGTGTTTACTGATGATGAGGGTGATATGATGCTTGTGGGTGATGATCCGTGGCCGGAGT

TCTGTAAGATGGTTAGGAAGATCTTAATATATTCAAGTGAGGAAGTGAAGAAAATGAGTACTAGCAGCAAGTTTCTTGCA

TCATCTC

>GW615361.1 Jc1-049-G02-M13F.G02.ab1 Jatropha curcas flower and seed Jatropha curcas cDNA, mRNA sequence

CTCTGTATCTCTCTGAAAGATATCATTACCGATCGTTTATTGGTGAAAATGTCTCTTCTATCGGATCTTATCAACTTGAA

CCTTTCTGATACAACTGAGAAAATTATTGCAGAATATATATGGATCGGTGGATCTGGTCTGGACATTCGAAGCAAAGCTA

GAACTCTTAATGAACCTGTAAGTGATCCCCAAAACCTTCCAAAATGGAACTATGATGGTTCAAGCACAGGCCAAGCACCT

GGAGATGATAGTGAAGTCATCTTATACCCACAAGCAATATTCAGGGACCCATTTAGAAGGGGTAACAGCATCCTGGTGAT

GTGTGATGCTTATACACCAAGTGGGGAGCCAATTCCAACAAACAAGAGATATAATGCTGCAAAAATTTTTAGCCATCCAG

ATGTTGTTGCTGAGGAACCCTGGTATGGGATAGAACAGGAGTACACCTTGTTGCAAAAAGAAACCAGATGGCCACTTGGG

TGGCCAACAGGTGGTTACCCTGGACCTCAGGGCCCTTATTACTGTGGTGTTGGTGCTGATAAGGCCTTTGGCCGTAACAT

TGTTGATTCTCATTACAAAGCATGTCTTCATGCCGGGATTAACATTTCTGGGGTCAATGGAGAAGTTATGCCTGGCCAGT

GGGAATTCCAAGTGGGCCCTGCTGTTGGTATCTCTGCTG

>GT977051.1 JGCCJG2005A08.b Jatropha curcas L. germinating seeds (mixed stages) Jatropha curcas cDNA clone JGCCJG2005A08 similar to cysteine protease, mRNA sequence

TGTTATTTGATGAATTGCAAAGTGCAAGTACAGTTGGAATGAAAAACCAGATCATGAAAGCCCTATCTACAGTGGATAAT

TGAACCTGATTAATATAATTTGTTCTTCCAATGCAACATGATAAATAGGAAGCTATATACATAATCCGCATCTAAATAAA

TAATCTTTTGAGTAAACCATCCTTCTTTCAATATCCTAATGGGGGAAAATGGCTGCAGCTTCCCAATAACCAAGGAGGAG

ACAATCAACCCCTGGTCCGATTCTTCTGTTCCTTCCTGTTCTATTGGCCATGCCGCAGCGACCTAACTTTTGTCTAAGCA

CTGCTCTTCTTGCTTCCATTTGCAAGAACCCAATGAGGCAAGGCACGAGTGCGCCTCATTGCCTTCACTCCTAATGGATT

GGCCTTGCTTATTAAGCAAGTTCCCTGGTTAATGTTACAGACGGGATATTCTTTTGGGCAGCAACTATAATGGTCTTCAC

AGCAGTTCGCCCTCGCATCGATGATATCATAATTCACGTCGACAAATTGATCTCAAAGTTTT

>JK612335.1 JCF25-19 Jatropha curcas, immature Seed cDNA subtraction library Jatropha curcas cDNA, mRNA sequence

ACCACCTATGCCTGCAACAACCATTCCTGTCCCAGCTGCTCCTGCCTCAGCACTTTCTCCTATGCCATATGGCATGGCAC

CTGGTTCTGCCCTTGCAAAAAATGACATGAGGAATGCTGGAATTGATCGAAGAAAAAGAAAAAAGGAAGTTTAACCTGCT

ATCAGCTGACTATGAAAGCAGTGGGGCAAACATATCTGCTGAGGCATCAATCATCTGTTTCGCAACTGTTTGATTGCCAT

CTTCTCATATTATGCACAGTGAAAAATTGGCTTACCCTCAAAAGTTTTGCTGAAATACTTGTCAACTGAATGAATGTCAT

GAAAGCCTCAAGAAATTGTCAGCTCCAAAGTTTTTAGATGCGGTCAGCATCAATCTGGAAAAAAAAAAAAAAAAAAAAAA

AAAAAAAGCTTGTACA

>GW613829.1 Jc1-032-E11-M13F.E11.ab1 Jatropha curcas flower and seed Jatropha curcas cDNA, mRNA sequence

CTATTCCAGATAAACAGAAGGTAAACAGGAAATGAAACTCTCACTTATGTCAGCCAGTGATCCATCTTTTTCTCCACTTT

CTGACTTAAAAAAAAAGGGGGAAGAAAATTGGAAAGAGATAAACTTTAATCCAATCCCATCCAATCCTTAATTTCCTAAT

TATTTATAATTTTAATACTAAACTAAGCTTTCATTGCTGCCTTTTGGTGTTTGCCGTGCAAGGAGTCCAACAGCAACTTG

GTTCTCAAGGTATAACTTAACTGACACTTTCTGCTCAGGTCTGAATCTTGAAATGGTCATGGTGTCATTTTGCCTCTTGG

ATAAGGATTATTGTGGTGAAAATTTTGATATATAAGAACAAGAAATTAAAGGTGCAAAAATGGTTCTGGCTTCTTCAGTG

AAAGTAGTTTTAGGTTCAATTGCCTTTTCAATCTTCTGGATACTGGCAGTTTTCCCTTCTATCCCTTTCCTACCTGTTGG

GAGGACTGCAGGGTCCCTTCTAGGGGCTATGCTTATGGTCATCTTCCGTGTCATGACCCCAGATCAAGCATATGCCGCAA

TTGA

>GW879244.1 JC003802 Seed specific Normalized cDNA library from Jatropha curcas L. Jatropha curcas cDNA clone N07003 5' similar to Unknown protein, mRNA sequence

GGAGGATTATGCTATTTCCTTCTACGCTTGTGATTCACTCGAACACTTTTGCTCTCAATGCCAATGCTTTATAAAACCCA

AAGCCGCAGCCAATTCTTCTGCAATTCATCCTGTAAACCATCTCCTTCACCGTCCAATCAGATTTCTTCCCGCCGGAGGA

TTACGTGTCAGACGATGTCTTGCTCGTCTCAAACTGCTTTCCCTACTCTAAAGATTGTTACAATACCTTTTCCGACCTCA

AGGACAAAAACGTAGACTTGTCAACAAAAATTGAGGAAGGTTTTGGACCGAATGGACTGGGGATTCTATCAGTTAAAGAT

GTGCCTGAGTTTTCTTCATTGCGTCAAAATCTTCTGCAGCTTTCGCCAAGATTAGCCAGCCTTCCTGAAGAGAAGAAGAA

GCAGCTTGAAGATCCAAATAGCAGGTACAACTTTGGATGGAGCCATGGAAAAGAGAAACTTGAATCTGGGAAGCCTGGTA

ATGATTCATAATGAATTTATGAATTATATGATTGGTCTGCCACAACTATCATTATGTGATAATTAGGATATGGGGCTACA

ATCTTGTTCTTAATCTTATTTTTGCTCAGCTCTATATTGGAAGATTGTGCAGGTCATGACAAGAATGTGGCTAAAATATG

CAAACTTGAAAATGCAATGTTATGGGATGATTGTTTCTGTGTTTCTGCTTAAAATATTGTTTTTCCAAAAATGCAGTTTC

TGTTCACAA

>FM889404.1 FM889404 Jatropha curcas embryo 35-55 (DAF) Jatropha curcas cDNA clone rjcfea0_003065, mRNA sequence

CAAAAAACCAAAAAAAATTTCTTCTCTATACCTTCATCCGTCGTCTCGCGTCCGGTTCTGTACGGTTCTCGGACGATTCT

ATCCCTATTTTACCCTTCTCTCTCCTCTTCCTTCCTATCCCTCAAATTATCAAAAAAAGAAGAAAAAAAAAGGCTCTCAA

ATTCACACTATTAGGTCATAGATTATAGTTCACTTTTCAGCTTTAGGATCAGAGTTATTGCCATTATTATCGTATCTGTC

TGCGTTTTCTAGCTATCTAAGGTTTATCTTCAAATAAGAAGAGGAGAGAGGGGGTAATAGATTTTTATTTATTTTTTTAT

TTGGGAAAGAAGAGAGAAGGATCTAGAACGTCTGTGGTTTCGAGATGGCACGGATTCAGGTACCGCATCCAGGCGGCGCC

GGTACCGGGGCCGAACGGAGTTGCGGCGACTCAAGCCGGGATTCCATTCATGCCGACGTCGCTCTATGTTGGAGATCTTG

>FM895763.1 FM895763 Jatropha curcas embryo 71-95 (DAF) Jatropha curcas cDNA clone rjcpga0_002745, mRNA sequence

AATAACAAAAATTTTTTTATCAAAATATATTAATAAATACAATTAATGTTGTTAATGAAATTACATTGTAAACAAACCCT

AATTATACAGCAATTCGCCACTGCAAAATAAATACTATGAAAAGGAAAAATAACAATAATAGTAATGGTAAATATAGAAT

AGATATTAAATGCACAATTTCTTACAATGATATGTACATCAAATGTGAAAGAATGCATAAACAGTAACACACTACAAAAC

AAAGTTGTAATCAAGCTCCAACCTCTTCTTTTAAGTTGGATTGACATCCCGCGAAACTTGACCTTGCCATCTTGAGAGTA

GAAACCTTGCAAGAGGGCTTAAATTGGCAGTCGAATTTAGTTCCTTATTTTGTTGCTTCTTCTTGTTCTCAGCCTCAGCA

TTTGCATTTTGTGTTTCATCAGGAGCTCCCTTGCCCTTGAGGAACAGGCGAGCTGCTAGCTCCTGTAGATCTTTCATTGA

AGATTCCTTTGAACTTGCCAATCGCTGAAGCAAACTTCTAAGGAGGACATCAGCAGATCCAGGCTTTGTTCGCTCCATAT

T

>JK317406.1 JCST173 Jatropha curcas L. seed cDNA library Jatropha curcas cDNA 5', mRNA sequence

GAACGGAGCTGCGACGCGTTCATTCGGGTCCAAAAAACCCATTTGATGTTCTGAGAGTTGCAAAAAAGGGTAAAACGATG

GAGTGAGAAAGCAATTGGCAGTTTACCGAAAAACAAGCCCGCTATATGCATTGACAAGATATCCAGAAAGGACAGAAATC

CAAGTGGTCAAAGATGGGAAGCCACAAAATGGGTCCCTAGGAGTTATTTCTGGAAACCCCTCAACGTTTGCAACCTTTTC

ACTTGGTCAGTTCATCTCTTCTATGGTTTAATACTGAATTCTATGACTCTCCAGCTGGAATCCGAGATAAAACGCGTACG

CAACCACCATGATACAGCGGCTAGCCCTAGAAAAAGCTAAATCGAATCGTCAAACACGCCGTTCAGAGTATCTAAAGTGG

CGCAAAGACACGAAACTCTATGTCTTTCCTGTGTCCACAGCAATCAGGAGTTGAGACACCTGAAGCCTTGACAAACCCTT

GTTCTTTCTAATGAAGAACAATGCTAATACGCGGATTGCCAACCTGACCTACAACAATTTTAGCCTTTTCAGTCGTCAAC

TCGGTTAACCACCTCGAAAAAATAAATTACTTAACCCTAAATGAAAAGAACCTCCATGCATCATTCAAACATT

>JK613393.1 JCF18_103 Jatropha curcas, immature Seed cDNA subtraction library Jatropha curcas cDNA similar to transaldolase-like protein, mRNA sequence

ATTCAAACATATTTGACTTTTGTTTACAGCTTTGCTCAAGCTGCAGCTGCAGCACAAGCTGGTGCTTCTGTTATTCAGAT

TTTTGTTGGACGCCTCAGGGATTGGGCACGCAACCATTCTGGTGACCCTGAGATAGAAGCTGCTCTAAAAAGAGGAGAGG

ACCCTGGTTTGGCTTTGGTGACAAAAGCTTACAATTACATCCACAAATATGGACACAAATCAAAGTTGATGGCAGCAGCA

GTTCGTAACAAACAGGATCTCTTCAGTCTTTTGGGGGTTGACTACATCATTGCACCATTGAAGGTATTGCAGTCTCTCAA

AGAGTCTTTTCTTTCTTCCTGATGAAAAGTACCTCTTTCGTTACCATTTT

>GT972107.1 GJCCJC2028F11.b Jatropha curcas L. developing seeds (mixed stages) Jatropha curcas cDNA clone GJCCJC2028F11 similar to calmodulin-binding protein, mRNA sequence

GCCATACGTAATGCTACACAAGCTGCTAATCGTATACATCAAGTATTCAGAATGCAATCCTTCCAGCGGAAACAGTTGAC

TGAGTATGGCGATGATGAATCCAATATATTGGATGGGCGTGCTCTTGCACTTCTAGCAGCCAGGACCCACAGGCCTATGC

ATTCTGATGGAATGGTCAATGCTGCAGCCATACAAATCCAAAAGAAGTTCCGGGGGTGGACGAAGAGAAAAGAATTCCTG

ATAATCCGGCAAAGAATTGTTAAGATACAGGCCCACGTAAGGGGACACCAGGTAAGGAAGCAATACAGAACAATCATCTG

GTCAGTTGGAATTTTGGAAAAGGTTATACTGCGCTGGAGGCGCAAAGGAAGTGGTTTGCGGGGATTCCGCAGAGATGCAG

TTAGGGAGACCAATGTACAACCCTTGGCTTCACAACCCTCGTCTTCGCAGCCCTTATGTTCACAGCCCTTGTCTTCACAG

CCCTTGCCTTCACAGCCCTTGTCTTCACAGCCCTTGCCTTCACAGCCCTTGCCTTCAAAAGAGGATGATTATGATTTTCT

AAAGGAAGGAAGGAAGCAAAATGAGGAGAGGCAACAAAAAGCACTTACAAGGGTGAAATCTATGTATCATTCTGCAGAAG

GACAAGCTCAATACCGGAGGCTGCTGACTTACTTCGAAAAATTA

>GW876639.1 JC005565 Seed specific Normalized cDNA library from Jatropha curcas L. Jatropha curcas cDNA clone N09541 5' similar to Unknown protein, mRNA sequence

GATCTATCTCCACGCTTTCCCTTCATATTTTTCTCGCCAAAAAAAAAAAAAAAAAGAAAAGAAATTCCCCAAAGAGAAAA

CTGAACTACGATTTCTCAGTCCTAATCTCACGAGCATTCTTAGCAAGCATCGCAAATTTTCCTTCGATCTGCAAATCTAG

AAAACCATCTTATCTATTTCCATGGCCCTAAGCTCTAATTAATTGTTAAATTCCGAAAATGTCAAACGGGACCTCTACGA

TTACTATCAGGGTCCAAACCCCGACCCGATCCTCCGACGACATCGTCGACACGACGCCGTTGCTTTCATCTGGCTCAAAT

GTCGAATCCAATAGCAATAACGGTCGCCGACCCGTTCGCCGTCCAACCCTCCGCGACGCTGCGAGGTTCGTCCGTCGCGC

CAGCAGCCGTCGCATGATGCGGGAGCCATCCATGTTGGTTCGTGAAACTGCCGCGGAGCAGCTCGAGGAACGACAGAGTG

ACTGGGCTTATTCGAAGCCCGTGGTGATCCTTGACATCATATGGAACTTCGCCTTCGTGGCGGTGGCTGTCGCAGTCCTG

ATATTGAGCAGGAGGAGCATCCAAGTATGCCGTTGAGGCTGTGGATTATTGGGTATGGATTCCAGTGCGTGTTGCATATG

G

>FM889771.1 FM889771 Jatropha curcas embryo 35-55 (DAF) Jatropha curcas cDNA clone rjcfea0_003506, mRNA sequence

GTTGGCTTGTTAAAACCTAGGAGGATGAGCATGTTTGGAACACTTTTGGTAATATGGGGCCTTGTGAAGGAAGGGATCCT

ACGAAAGCCGGCAAATACAGATCCTGCTAAAGCTGTTTATGTCTATCCAACAATGTTGCTTGCCTTGATCTGTGCCTTCT

CTTCTGTGAAGTATGATGTGAAGAAGGCTGTGAGAAGTGCCCCTTCTAGACCCATTGCAAAGCCTCTGCACAGCTCTTCA

AAGTCTAAACTGAAATGAGTTGATTGCCTTTAATCTTTTTGTAATTTTTTTTAATCTTAAAAACTTCACGTTGGTTGATG

AGTAGTTGAACTTGAAGCTGTATGTTTTCCTGCCAAAATCAATTTTGAACAGAAAAACTGATATTAGTATTAACTCACGA

ATATGTTGATATTAATCAATCAGTTAATCTTCCTTTTG

>GW875281.1 JC001988 Seed specific Normalized cDNA library from Jatropha curcas L. Jatropha curcas cDNA clone N03585 5' similar to Unknown protein, mRNA sequence

GGGGATTTTATTCACCACGAAGAAGATGACGGAAGCTTGGATTCGACCGCTAGTTGAAGCCATGCATTCCAGTCCTACTC

AGGCCGTCCTTTATCTCTCCGGCGGCGCTTCTCAGGCGTTGGGCTGCTTGATGTCAGTTCCTGGAGCTACAAATACAGTT

CTTGAAGCAGTGGTACCCTACTCTAGAATGTCCATGATTCAATTACTTGGAAAGGTTCTCTTCATTTCTTTTCCTTTTTT

TTTTTTTTTC

>GW881664.1 JC004507 Seed specific Normalized cDNA library from Jatropha curcas L. Jatropha curcas cDNA clone N07983 5' similar to Unknown protein, mRNA sequence

GAAGTCCCACTGTTCGTACTCCTTTTTACCACTTCCCACTAAGCAGCAGCGAAGAGACTATCAGAGCTAAACAGCTTCTC

TCTCTAGTGGACGCTTTCTCTCTCTAGCAGACTAAAGAAGAAATTTCAGATGGGAGAAGGTAAAGGCTCAACGCTGGTTC

ACCTTCTGGTGGTGGTTCTGTGCCTTGTTGCCTTTGGATTCGCCATTGCTGCCGAGAGACGAAGAAGCATTGTATGCCTT

TCTCTGTCTTTTGCCTTTTCACTTCATAATTGCTGTTCAGCTGTGTCTGCAGTTTGCATTTATCTTAGTACTCATTCTTC

GCGCTTTGATCTCTGTTCCTTGCATTAGATGCTTTGAGTTGTGGAGATCTGTGTATTTATTTGTTAATTCGTCACTTCTT

GTACACTTTTGATTGGGGTCTTTACTTAGGATTGAGGTTAGTATTTGTTAGTTCAATCTTTGGATACTTAGTTTAAGTAA

AGGTTCGGCGTTGGTTTTGTTGATAGATTTGAAAACTTATAATAAGAAAAAGTTTGACTTCAGAATTTTGATTGCTTCTG

ATGGATTACTCATAAGAGGGAGTGAATAGGGAGTCCACAGTTACGTGGCTAAAGAAGAGCAAGTTCAGAGGAGCAAGTTC

AGATGGATTAATAGCATGATAATTATACGTTGAGGATATTATGTAAAATTAGTTGAATAAGCTCTAATGTCCTA

>FM894548.1 FM894548 Jatropha curcas embryo 71-95 (DAF) Jatropha curcas cDNA clone rjcpga0_000334, mRNA sequence

AACAATGGGTGTACCTTTATATTTTCAATTACCTAATCTCGTAATATAAACTAACCATGTACTAAAAAGCAATAAAGAAC

GAAGCTATCATCCCAATTAATACAAATATTATGAAAAGAATGTTTGCAGTGGTATTTTGCTGGGAACAAAAAACAGTATC

GTAACAAAATTTTTCTTGCGACTATGTTAAAGGTCTCATTCTACCCCAGTAAATTATGAAAAATCTTCCAAGGACCAAAA

TTTAAATGATCCCTAATGATTCTCAACTACTCAACAACACATTGCTAGTGATCCCCAACTCCTCTCTGACGGGGATAAAA

TTCTAAGTTACAAGAGAGAATCTTCAACGAGAATTCTGTGGAGGGCAACTCCAGTTCGAATTATCAAGCTGTTTTCACTT

TGCGAGAGGGAGGCCGTCGGAATAAGCTTATGAGATCATCAAGACCCCTAGTTGTAATGACCAGAAAGCTGAAAAGCAAT

ATTAAATTCGATCCCAGCACAAGAAGCAAGAGCCGGTCAAATGTTCCGCTAGCAACCTGATAT

>GW876113.1 JC005490 Seed specific Normalized cDNA library from Jatropha curcas L. Jatropha curcas cDNA clone N09434 5' similar to Disease resistance protein RGA2, mRNA sequence

GTTTCCATTTAAATTCAACTTGGCAGACTCCAGTCGGCTTATTTCAAGCAAAACAGTTGCGGACATTTCTTCTGCCCAGT

CAAGAAGTTTGGTTAAGCAATGAAGATGGAAGATGGAAAATGCCAAACCTTGAAGCCATTTTTTCAAACTTCAGGCAATT

ACGCGTGTTTGATTTGCATAATTCTGGAATTGAGGAAGTGCCAACTTCCATTGACAAGTTGAAACATCTGAGATATCCCG

ATGTTTCCAAAAATGATCGAAGCAAGGCACTTCCAGATTCTATTACCAAGCTAAAAAATCTGCAAGTGCTGAAACTTTCC

GATTGTGAAGAGCTCAAGGAACTGCCAAAAGACATAAAAAAGCTTGTTAATCTTAGGCATCTGGACTTAGAGAGATGTTG

GGATTTGAGGCACATGCCACGTGGCCTTGGACAATTAAGTTCCCTTCAATCATTAACGTGGTTTGTGGTGGCCAACCATA

ATTCTGTCTCTAACCATATCGGGAGTTTGAAGGAATTGGATGCTCTCCACAACTTGAGAGGGAGGATAGAGATAAGAAAT

CTGAAGCATGTGAAAACTGGCTTATCAGAATTTGAGGTAGCTAATTTAAGAAAGAAGCAGCATCTTCAGTCGCTGTCTTT

GTGTTGGAATAGGGATGATGATGACTG

>FM895625.1 FM895625 Jatropha curcas embryo 71-95 (DAF) Jatropha curcas cDNA clone rjcpga0_002577, mRNA sequence

TGAAAACATATATAGATTATAAAGCTAAAGACATTAATGTTTTGGGTATAAAAGAGTTCACAAAACCTTAATACCAACAT

ACATTGATTGATAAAATAAATAATAACCCCTAACTATCTTCACCACCATTCTCTCCAAACCAAGAGGACAAGGGAAAGAA

AAAAACTCCAAACCAATAATAGTTCATAAAGGACTAACGAAAATAAATAAATTAAAAAAAAATAAATGACTCCCAAAACT

TCAAAACCCCTCCCTTTCTGTACCTCTCCAAAGATATCAGCAAATTCTCCCTCCAAACTTCAATCGAACTCTACTGTTGC

TTGCACTCTGGAGGCCTCTCACCCTGCTGACCATCCCCAGCTTGTGTACTTCAAACCTTATTATACCCGGCTGACAGCTT

CTTTAAAGTCTTGCTTTCATCCTCGTACTCATCCTCATATTCATCCTCTTCATCCTAATCATCATCATCAATATCTTCAT

CGTCATCGTCGTCATCATCATCATCATCATCTATTCCAAAGTCATCCCACTGAAT

>FM895396.1 FM895396 Jatropha curcas embryo 71-95 (DAF) Jatropha curcas cDNA clone rjcpga0_002312, mRNA sequence

TCCGGCTCTCGATTCGATTCCGCTTCTGCTTGGTGGGGTTCACTTCTTTTGCAGATACACAAGTTTTCCGATTTCCCTTA

TCTCAGGTTAATTGCAACACTTTTGGTAGCACACTTGTAACAAGAAAATACCTTCCCTAGGTATGGAAACTGAGGATATG

ATTGGCCTCCCTGCTTCCAGCAATTCTAGAGATAGCAGTGAGAGCGGTGAAATTCACAAGTCTGGATTTGGGTCAGGTGA

AGCTGATTCTCAGCCTAGGAATTTTGAAGCTGAAGAAGATGTGGGTGATGGAGATAGTTTGAGGCTCAATAATGACGTTG

ATATTGATACAGAAAAAGGAATAAGAGGCCCTTGATTGTTTGGCGCTTGTCGAAGACGATACTGGAGCAGAAAAAATATT

AGGAGATTCTGAGGTTTTGGACAGCAATGAAGATGATATTGGAACAGAAGAACATGTGACAAGATTAGAGAACGAAATAT

TAAGTTATTGATGCATTGAAATATATAATTTTCAACTAACATTTTATCCATCAAAGCATAATGGATTGG

>GW615998.1 Jc2-006-H02-M13F.H02.ab1 Jatropha curcas flower and seed Jatropha curcas cDNA, mRNA sequence

TACTACTCTGCTCTTGTGAAGGCTGCCTCTCCCAAGTCCATTGATTCTTCAGAGCCAGTACAAAATTTGGATGAGGTTAT

TTACCGAATCAAACTTCCAGGACCAGCCATTTTGGGTGAGGGAAAACCTGAAAATCAAAATCATGCTATTATCTTCACAC

GCGGTGAAGGCTTGCAAACAATAGATATGAACCAGGATAACTACATGGAAGAGGCCTTAAAAATGAGGAATTTGCTCGAA

GAATTTCTGAAAAGGCATGATGGTGTGAGGCATCCTACTATACTTGGGCTTACGGAGCATATCTTTACAGGAAGTGTTTC

ATCTCTTGCATGGTTTATGTCAAATCAAGAGACCAGTTTTGTGACTATAGGTCAAAGACTGTTGGCCAACCCGCTGAAGG

TTCGGTTCCATTATGGCCATCCAGATGTGTTTGATAGGCTTTTTCACCT

>FM892316.1 FM892316 Jatropha curcas embryo 56-70 (DAF) Jatropha curcas cDNA clone rjcaeb0_002839, mRNA sequence

CAGAAACATAGCATAACGCCAATAAATAAACCATCAATATGTGAGAATTACATAAAGTGAGACTTTCAATTGTCTCCTAC

CACATAAAAGTCACAAGACACCTCTGTCCTAATTCTAACATCCTTTTTCTTCCTCTTTTCATACTTTTCCCCTTTAAAAA

TTCTAGAGTTTTGGGGTACTGGAGAAGAAAGGGGCATGGTGGTGATCTGTTATTTTGTTTCCAGCAACAAGTCGTCCAAC

TACATGTCTCTTGATTCCCTGAGAAAAAACTATGACACATCTCTACAGCTCAACTACTAATAAGGACGGTATCTTCTGCT

ACGATTGCCATCATCTCCACTACTTCCGGCTCGTCCAGGCCCACTAGGACCATTGCTCCGATCGACTCTCCTAGGCCGAG

GTTGTGGCATGTGCACTCGAGGCTGCTGAAGAACATAGCCAATTTGACCATCAGGTAGAACCATTGGCACCATATGCATC

CCTGCTGGCATTGGACCTCTTCCATATATAACAGGCTGTTGAAAACTGGCAGAAACACCAAATCCA

>FM896165.1 FM896165 Jatropha curcas embryo 71-95 (DAF) Jatropha curcas cDNA clone rjcpga0_003331, mRNA sequence

GTCTCGTCTAACCTCCCTAGCTGGATGAAAAATGAAACATATTCACAAGCCAAATCCTCAGTAATGCATCCAAATGTCTC

AGCTTTCTCATAAATCGTCAATAGATAAGAAATAATATCTTCAGCATGATTGGATAATTTAGAAAGTTGGTTTTCTTCTC

TTTTAGGAGCAACAGCATCCATAAAGAACTTAGCATATAGATTAAACAGAATGGGAGAAGGCACAAACTGCAAAGCCTGC

TCATAAACCTGAACTGCTTTTTGTAGCTGTGGTGACATGATACCTTCACTTGTATCCTTTGTAGTTCTAGAATAAGTCAT

TTCAAGTCTAGCAAGCCAGTCCCAGTATTCGGGATCTTTAGAAAAGTCTCTTTTCATATCACTTAGGATCTCCTTTCGAA

TTTCTTCCGAATGTGCCAACTCTGTTGCCTCCAATATCTCAAATAAACGTTTTCTTAGTCCTAAGCTAGAAGGAAGAGCT

TCAATTGCTCCACTATAGATAGTCTTAAGTATATTCAAACCTTGTTCTCTAAATAAATCCATTTTCTTTTCTGACTCCCC

ATTATTAACTTTTGATCCTTCAACGTTTCTCATTTCTTCATCAAGTAACATAAACGCACCATTGTTTTCCATCTCTCC

>GW877946.1 JC003548 Seed specific Normalized cDNA library from Jatropha curcas L. Jatropha curcas cDNA clone N06576 5' similar to Tonoplast intrinsic protein, mRNA sequence

GGACCTTCTCCTCCATCAAAGCTAGTTAGTCCAGTTCCCTTATTAAGCTAAATTTTTTTCGAAGATGGCCAAAATTGCCT

TTGGTCGTTTTGATGATTCTTTTAGTTTAGGCTCTTTTAAAGCTTATCTTGCAGAATTTATCTCAACTCTGCTCTTCGTT

TTTGCTGGCGTTGGTTCTGCCATAGCTTACAATAAATTGACAGGAAATGCAGCTCTTGATCCTGCTGGTTTAGTAGCAAT

TGCTATTTGCCATGGATTTGCTCTCTTTGTTGCTGTCGCCGTCGGTGCCAACATCTCCGGTGGCCATGTTAATCCTGCTG

TCACCTTTGGATTGGCTCTTGGTGGTCAGATCACCATTCTTACTGGCATCTTCTACTGGATTGCTCAGCCTCTTGGTTCC

ATTGTTGCTTGCCTCCTTCTCAAAGTTGTCACCGGAGGCTTGGAAACACCCACCCACAGCCTTGCAGCTGGAGTAGGAGC

CATTGAAGGAGTGGTTATGGAGATTATAGTCACATTTGCTTTGGTATACACAGTGTACGCAACAGCAGCTGATCCCAAGA

AAGGATCACTAGGCACCATTGCCCCCATAGCCATTGGCTTCATCGTTGGTGCCAACATCTTGGCTGCAGACCCATTTTCC

CGGTGGATCCATGAACCCAGCCC

>GT969604.1 GJCCJC1013A05.b Jatropha curcas L. developing seeds (mixed stages) Jatropha curcas cDNA clone GJCCJC1013A05 similar to putative genomic DNA, mRNA sequence

AGGTCACATGATACATAGCTAATGAGATTAATAATCTTTACTTAGCAAAAGTTATGAGGAACTTAATTCTTTGTGTGAAA

GGTGAGAATATTAGTGTCGAACCTGTGCCTTGGGTTATGAATGTTCTACTTGATTTGACTTGTGGATATAGTACAACTCA

AGTTTAAATTAACTATAGTGATAAGGTAGATATCTAACTGTTCATCAGAACAAGAATTCTCCTGAGTGTAAATGTCATAA

CTTTTAGCAGTTGCAGCATTATGTGAGAAGCACAACCACCATTGTAGTAAACAACTGTTGCATCCTAGTTTCTTGTTAGC

TTTAAAATTCGAGGACGAATTTTTATAAAGGGGGAAGACTGAAATAACCCGAATTTTTATTTTCAAATATTTTCATTTAA

TTTAATAAACTGACAGCGTGGAAAATGTATTA

>FM889747.1 FM889747 Jatropha curcas embryo 35-55 (DAF) Jatropha curcas cDNA clone rjcfea0_003473, mRNA sequence

GTAAAATTCTAAAATGGATTGCTACCTATGCCTAGATCCCGGACAACTGGAAATTTTATTGATAAGACCTTTTCAATTGT

AGCCAATATCTTATTACGAATAATTCCAACAACTTCGGGAGAAAAAGAGGCATTTACTTATTACAGAGATGGAATGTCTG

CTCAATCCGAAGGAAATTATGCAGAAGCTTTACAGAATTATTATGAAGCTTTGCGGCTAGAAATTGATCCCTATGATCGA

AGTTATATACTCTATAATATAGGCCTTATTCACACAAGTAATGGAGAACACACAAAAGCTTTGGAATATTATTTTCGGGC

ACTAGAACGAAACCCCTTCTTACCACAAGCTTTAAATAATATGGCCGTGATCTGTCATTACGTGCGACTATCTCCACTAT

AGAAAGAAAAAAGAAA

>GW618610.1 Jc2-037-B10-M13F.B10.ab1 Jatropha curcas flower and seed Jatropha curcas cDNA, mRNA sequence

TACCCTTCTTCATGACATATTGTCTCCTGAGGCACAAACAAATCTCACCCATTATTTCCAGGCTGCTGCCAGGAAAAGGT

CACGAAGGCACTTGACAGAGACAGATGAATTTGTTACTAGCAACAATGAAGCTACTTTGATGGACTCGGTTGCAATGTCC

ACAGCTTACCAGAAGATGACTTCCCTTTGTTTGAACATTAAAAATGAAATTAGTACTGATATTGAAATCCACAATCAACA

TATACTTCCCAGCTTTATAGACCTTCCAAATTTATCTTCATCCATATACAGCACAGAGCTTTGCAATAGATTGCGGGCTT

TCCTACTTGCATGCCCTCCAACTGGTCCTTCACCTCATGTAGCGGAACTCGTTATTGCAACAGCTGATTTTCAGAGGGAC

CTTGCCAGCTGGAACATTAGTCATGTGAAAGGAGGAGTTGATGCAAAAGAGTTATTCCATTTGTATATCATGCTATGGAT

TCAAGACAAGCGCCTTTCTTTGCTAGAGTCATGTAAACTAGACAAGGTAAAATGGTCAGGAGTCAGGACGCAACATTCTA

CTACTCCCTTTATCGATGAGATGTATGACCGTCTTAGAGAGACTCTAGACAACTATGAAGTCATCACTTGCCGATGGCCA

GAGTACATTTTTGTCCTAGAAAATGCTATTGCT

>GW876121.1 JC005498 Seed specific Normalized cDNA library from Jatropha curcas L. Jatropha curcas cDNA clone N09446 5' similar to Paramyosin protein, mRNA sequence

GAGGAGAGACTGGTTGGTTGGTAACTTTCGTTTTTTGTTGTGTTCTGTTCTGTTTTTCACTCTGTTTCGGCCATCATCTT

CCTCCTCAGCCTGCTTCCCATTCCCATTTATACTTACAAATACGTAAGTCATAACCTTCCTCTCTATCTCCATTTTGTTT

CCCCTTTTAAGCAACCTCAATTAGTAAACTAGCAAGCAGTGTCCTTTTCCCAACCTTGATTTTCCTTTTATTCGAAGCAA

TCGTTTACTTTGGTTATTGGCTAGTCCCAATTCTAGGGCTTATCAAAGACAAGGAATTGCAATATTTGGAATTTAATTAA

TTAATAAAATGGTATTTTTTTAATTCAATTGATCACCGCTTCTGAATTCAATCTGGAATGGCGAGCGGTGACGGCGATGA

GGACGTCGCCGTTTTGAGCGATGTTGAGGGAGACGATCCGGTGCCGATCGTTGTAAGAAGTCCGAGACTGGAAGATGTGT

CCGTGGAGAAATATCGCGAGCTACTCGCTGAGCTCGATCGGGGGAGAGCTGCTCGTGAGGCAGCCGAGACGTCTAAGTCG

GAGCTTCAGGTTTCGTTTAATCGGTTGAAAGCGCTGGCGCATGA

>JK317566.1 JCST335 Jatropha curcas L. seed cDNA library Jatropha curcas cDNA 5', mRNA sequence

AGTAAACTGTACATTTAGTTAGAAGAAGAAAGGTCAGCATTTCAAGTAATGGCAACGATTTCATTATTGCCTACAGAAGA

CCAATACCAGTTATTACAGTTACCACATCTCAAGTTCCTTTTCGCTAGCAGGTTTATTTCTTGGGAATTTCATGTTAGGT

TTGATATAAAGCATTTCCTCTTTGAGATACTCTAAGCCAAGTCCTTCAAGCAGGTTCAAATCCCGCGTATTCACATGATT

ATTAGCCTTCATAGGGAAATCGCAGTGAGAGATTACAGTCTTGAACGGAGTATGCATTTGATGGGATTAAATGGAGCATG

CCTTCGGGTAGAACTATATGGCTGTCTACTTAATGATGCTGATGACTTAATATCCCAATCTTCAACGGTCTTTGGAACAC

ATACGTCGAAAAGTACTTGACCAAGAACACCATGAGCGTTAATACGCGGAGGGTTATGCAAATGAATCAAATCTTCATTG

TGTTCATCTTGCAAGGAACCCATATCTCTACCCTCATAATAGCCCACTGAGCTTGGCATGAAGAAATTGTCACTTGGTTG

GATAGTAACTCCACAAAAGCTCTGGTTTGGACTTTCTACTGCTTTCCTCAGATCGCCAATTGGTTCCGAGATCTCATCAT

GCGTCCCCTCTACACCCAATCTTTCTCCTTCAAGTCGTCGAATTCTTTGTGAGTAAACTGCAGCTGCCTTACCGAGGAGT

TCAGTCGCAGCAGCCTTGTAGTCAAATGGATAAAGTCGAAAATGACCTACATGAGGAGAGCCACTCATTGTCACAAGTTT

ACATCAGCCCCAAGTTCTTGCAGTCTTTGACTGAAATTGCAAATAACTTG

>GT976070.1 GJCCJC2072G02.b Jatropha curcas L. developing seeds (mixed stages) Jatropha curcas cDNA clone GJCCJC2072G02 similar to PGK (PHOSPHOGLYCERATE KINASE), mRNA sequence

GAAGGAATAATTGGTTGAATCAAGACCAAACCAAACCACCTCCCAATCTTCGTCTGCCTATATATCAAACCTTCTGAATT

CTTCTCCACTATCCAACTTGTCTTTTCTTGATCTCAACCGCATTTCTCTTTTCGATTCTTCTTGGATTTTTGTTGCGTTG

AAATATGGCTACAAAGAAGAGCGTGAGCAGTCTAAAGGAAGCTGATTTGAAGGGGAAGAGGGTGTTTGTGAGAGTGGATC

TCAACGTGCCTTTGGATGATAACTTGAACATCACAGATGATACCAGGATCCGCGCTGCCGTCCCCACCATCAAGTACTTG

AGGGATCACGGTGCTAGAGTTATTCTTGCCACCCACTTGGGACGCCCAAAGGGTGTTACACCTAAGTACAGCTTGAAGCC

TCTTGTGCCTAGGCTATCTGAACTTCTTGGTATTGAGGTTAAGATGGCAAATGATTGTATCGGTGAGGAAGTTGAGAAAT

TGGTGGCAGAGATCCCAGAAGGGGGTGTTTTGCTCCTTGAGAATGTAAGGTTCCACAAGGAGGAGGAGAAGAATGACCCT

GAATTTGCTAAGAAGCTAGCTTCTCTTGCAGATGTCTATGTGAATGATGCATTTGGCACTGCTCATAGAGCCATGCTTCC

ACAGAAGGGGTAGCTAAATACTTGAAACCTTCTGGTGCTGGTTTTCTGATGC

>GW879990.1 JC006502 Seed specific Normalized cDNA library from Jatropha curcas L. Jatropha curcas cDNA clone N11010 5' similar to Unknown protein, mRNA sequence

GGCCATTACGGCCTAGTACGGGGGACCCCGCTCTTCTCCGCATCGACAAGACCACAGCCAGCTCCTCTTCCCCACACTCG

AGCCTTTTCTTTTCTCTCTCAATTCAACAATTGCTTGTGATTTCCCAAACCCTTTTTGTATGGTTCCAAGACTCCAAGAA

CTTGAAAGTTTCGGAAGGATAAAGCAAGAAATTTTGAAGAATTACAAGGGAAGGAAGTTTTAGTGTAGAGAAGGAAGCGG

TTACTTTGGATTCTTTGAATTCAGGTATCATTATAAGCAATCTTTGCAAGGAGTGGTTTTTACTCATGGAAGTAGATGAT

AATAATGGCAGGAAGGAATCAGCATGTGGCTCATGATATGGTTAATCTGCAAGATAATGTGCCTTTTGGAGATGCATTGG

GCGAAAATATGGTTGATGTCATGGACGAGGCGCAAAATGGAGATGGTGGGGTTGCTAATTACCCCAAAACAGCTGTTGCA

ATGTTTGAAGAGAGCACAGACTTTGAGCCGGGTAATGGAATTGAATTTGAATCACATGAGGCAGCATACTCATTTTATCA

AGAATATGCTAAATCAATGGGGTTCACCACTTCAATAAAAAACAGTAGACGTTCGAAGAA

>GW880703.1 JC007042 Seed specific Normalized cDNA library from Jatropha curcas L. Jatropha curcas cDNA clone N11862 5' similar to Unknown protein, mRNA sequence

GCCCAGTTACGGGGGTGCTTATGTGGTAGTTGATGTGGCATAAATAAGCAACTATTGTAAATGATGTCCAGAATTGACAT

TTCACATTCAATATCTTTTTTAAGTTCTCATTTCTCTCTCTGACCTTCTCTCTGCTCTTGGCGATTCCAGAATTGTCCAT

CTTCATTTCCTACCCTTTCTCTCATTTTCTTCATGTTTCGTAGCTATCAGACATCATCAGGCATTTTATGTTTGCAATTC

CTAACATGCTGATTCATATGGGACATTCTAAGTTTGTGGATCAAAACTTTGCTAAGGAGTGTTATAACAAAACATTTAAG

GAGTTTATGGTCCATATACATCTAGGCCTACAGGACATTATTTTTTCTTGATGGATTAAATTAAACATGCCAATGAGATT

TTTACTACCCTTGCCCGATTGATTTTGTTCTACATGTGATGATAATTGGTCATCTAATTTTGTATCAAATAACTGTGTAT

ACCATAGGCCTGTTCCTTTTTGCGTTGGAGTGCCATGCGTCAATGCTGTACTCTTGGCAGTCCAACAGCAAAGCCAAACA

GCCTCTATGTACTTAATGATGCATCAAAATTGATGAGTTGCTCTTAGTTATAATCATCCTTGAGTAACAATTTTTCAGAA

AATATATTACCAGTCATGTAATACAACCATTAGCATTTGTTGTGCTACGATGAAAAAAAAATTGTTTTTGGTCGAGTA

>JK611726.1 JCF4736 Jatropha curcas, immature Seed cDNA subtraction library Jatropha curcas cDNA, mRNA sequence

ACCCTGGATGATTTGATCTTCTTCCTCTCTTTTTCCTGATGGTCCAATTTCAACATAGGATTGGCTGTCATAATTCTTCC

TATCTGTCCAATCTTGACCAACTTGCTCCGGCTGTTTTCCTCCCCTTCTTCTTGAAATGCCCTCGATACTGCGAGTGAAG

AAGATTGTTTCATTTTCCCAATGGCCCTGATGATCTATGTTTGAGGATTAGCTTGCCAATTCCTACCATCTAATTCTTAA

TTCCAATGGAAATGGCACATCTTACACTTGATTTCTGTGCATTGCCGGCTCTTCCAGGTTCTGTTTTAATTATCTTCTTC

TTTGGCTCTTCTTCCCACAATTATACTTTCTTTTCTTCTCCCCGCCTGTGTTTGGCTTAGGATCTTCCCAATTTCATTTG

AATCCGATTGCGACTTCATTGGTCTCTTCTCACTTTCTGTCTCTCGAACCTCTTTGACACTGTCCACCTCTGCCGCGACA

ACTCTCTTCTATATGATCTCTTTATGCACCCAGCTCTGATTCTTGGCTCTCTGCTTCTCCCCTACCCGTCTGTGAAAACC

TTGTCGCTACTCACCTAAATCGCCGAGTAGGCAAATAGCACTTTCGAAAGCTGGCTTTGTAGGGTACATGATAGCACTGA

TACGCACTATCGCATCCCTGACGCTGGCTGCATATGGGGAAATGAAAATGACATCGGTCATATTTAGTAAATGTACGTCG

TGACAGTTTATCGCTGAAAACCGTATCATGCTTTCACGAGTAGCGAGAATCGAGTCACTGCCTGTACTGAATTCACAACG

ATCATGACCTGAGATAAAGCCTTGACGGTGTCCAACGATTAGTGACATGATCAAATTCACAGTATCGTCGGAACTGCGCA

C

>JK317678.1 JCST449 Jatropha curcas L. seed cDNA library Jatropha curcas cDNA 5', mRNA sequence

TTCATCTATGTCGGGTGGTCGAGAGAGGTAATCCTTCTGTTAGCAAGATCCTCGCTTTGGAGTCGGTCTGTCTAGTGGCA

GCCTGGGTGAGACATCCTGCTGAGGCCAAGTAGGCAGAATATTTGGGCAACGCTGTGGACCTTCGCGGGCGCAAGAGGGC

TAGAAAATCGAACTTTATGGTGGGCGTCCCAACTTTGGGGGGGGCATGATCCCGAAACGGGGGGGCGGGGAGGGGGCAAT

TGAGAGAAAGTCGGGCTGGAGAGTGGTACGGTTGTATGAAGGCAGTAAAATGGTTGCATAGCCACCCAAACCGCCCGAAT

GTTGTATAAGCATTTTAAAGGGCCAGGGTAAGAACACATTTGGGTGGCCTATCCAAGTAACTGAAGCAGGAGGAACCGAC

CCGGTTTGCATTGTGGTGGGGCGACGCCACCAGGTTCCGCACGCGGCCGCCGCGTTGACCCCAAATTTAAAACAGCCGTG

CTCGTAGTGAACCCCCAACTATCAACCCCAGTAAGAGGAAAACGATGGGAAAGAAAGGTACTCCACGAGCACAAGTTAAA

AAGAAGGGAGCAGTTGTTGTTGGCAAAAAAAATCCCGCCCAGCCGGAATGGTACCCAGCATCTTTCCTCCGTTAAACCAC

ACCCAATAGAGGAACATAGAAATGGGACAGAGCGAGGAAGGAGCCGCTAGACTCGTAAAGAAAGCAAACTAGATGTNNTA

GTGCATAAGGATTGTGTGGGGTATACGAACTAGTGCGCTAGCTCGTCAACTCTATATACTGATAACATCCCNGAACCCCC

GAACACGACGCAACGTATAGGACTGTNANCACGTAGGTGGTATTGGGTAGAGGGGGNCTGTATAGGTAATC

>GW876145.1 JC005522 Seed specific Normalized cDNA library from Jatropha curcas L. Jatropha curcas cDNA clone N09476 5' similar to Unknown protein, mRNA sequence

AAAAGAATTATATTAATGGAACTTTTTTGGAGGACATTGTCACTATACAAATTTGCATTTGTTCTTCTAATATTGTTATT

ACAGCTTAGAAATTAGATGGGTTTTATATTGAGTTATTGAGTTAAAGCAATGCTGTACTTTTGGAAAATATTTTCCCCCT

ATAGCGACAATAAGGAATGATATTTGAATTGCTTAAACTTATAATTAAGATACTTTGTATCAAGAACCCCAAAGCAGAAA

AGAAAACTGTATCTTCTCTGTTGAAATCTGGAGTTTTGTCGCATGTGCAATCACTTATGGGTATTCACATGACAGCACCA

TCCATTTAGCATTGATCAATATTTAATTGTTTTACAAATACTTGTGTTAGTCTTATTCACTAATGCATTGTCTGATGCAT

TGATATACGTGCAAATAGTCCTGCATTCTATTTCCTACAGAAATTTGGAATAAGAAGTTGACAGTGTTCTAGATTCTCTG

GGTCTTTCTTTCTTTGATTTTGGATGTGCGTGTGTGCACATTTAGGTTTCGAATTTTGTCATTTATGAACCAATCTTAGA

AAATTTTATTTTACATTTTCCCTCTTTAAATCTTGGAGAATTGCTTTTTAATTTTTAATGCACTGGCATTAATTTTGTGC

AG

>JK317696.1 JCST467 Jatropha curcas L. seed cDNA library Jatropha curcas cDNA 5', mRNA sequence

ATTTTTTTTTTTTTTTTTGTTTTCTCTTATATTATCAAAATCTATGGCGAAGCTAATTTGCCAGACAAGGTTCTTAAAAC

GTTCTACAAAATGCTTGCATTTAACTGCAAGCCTTTGCCCAAACAGTTAAACCTCATCCTTGAATTCCTTGTTTCTCATC

GGAACTACAGGAAACCTGCCTTGGATCTTTTAAATAATGCTCATAGATACGCTGTTTCGTCCAACACTAAATCCTACAAC

ATTTTGATGCGCGCCTTTTGTTTTAGTGGGGAACTCAGTGTTGCATACAAGCTGTTCAATCAAATGTTTAAGAGAGATGT

TTTACCAGATGCCGAGTCTTATCGAATTTTGATGCAGGGATTATGTAGAAAAAGTCAGGTTAATGGAGCAGTTGATTTGT

TGGAGGATATGTTGAATAAAGGGTTTGTGCCTGATTCTTTGAGTTATACTACTTTGTTGAATAGTTTGTGCAGGAAAAAG

AAGCTCAGGGAGGCATATAAGCTTCTCTGCAGAATGAAGTTTAAGGGTGCAATCCTGATATTGTCATTATAATACAGACA

TACTGGGATTTTGGAAAAAGGACGTGCAATGGATGCTTGCAAGGGTCGTGAAACATGGAGCCAAAGGGTGGTTAGGCTAA

TCTGGAATCGTATAAGGCTTAGGAAAGAGGGTATATAAAAAAAGAGTTACGGGGGAGACGGTGCGTGTCGCGGATGTGTG

ACATCTGACGGGCAACGACGAAAAGAGAGTGAAAATGTATGCGGAGGAGTGAAGGAGAGTGTCGTATATGAATATGATGA

ACGAGAAGATGAGTATATAACACGAGAAGAGCAGAGC

>GW618595.1 Jc2-037-A06-M13F.A06.ab1 Jatropha curcas flower and seed Jatropha curcas cDNA, mRNA sequence

TCTGCACCTTCCTTTTCCTACCTTCGCATAATCGCGAAATCAGATCGGTGAACCCCTGATCTCTTCATTCTGACCCATCA

GTTCAATTCACATAACCAGGGAGAAGCAGAAATCAGAAGGCAAAAAAGTGACAATGTCAGGTTCCACCATGGTGGGAGAT

CCAAGCTCGTGGTCACGCGCGTTGGTGAAGATCTCTCCTTATACATTCTCTGCCATTGGCATCGCCATCGCCATCGGCGT

GTCAGTCCTCGGTGCCGCTTGGGGGATTTACATAACTGGAAGCAGTTTGATTGGAGCTGCAATCAAGGCTCCTCGTATTA

CTTCTAAGAATCTCATCAGTGTAATCTTTTGTGAGGCTGTTGCTATATATGGTGTCATTGTGGCTATTATTCTACAAACC

AAATTGGAGAGTGTTCCCTCCTCACAGATTTATGCCCCAGAATCTCTTAGAGCTGGATATGCAATTTTTGCCTCTGGGAT

CATTGTGGGCTTTGCAAATCTTGTCTGTGGGCTGTGTGTAGGAATAATTGGAAGCAGCTGTGCATTATCTGATGCCCAGA

ATTCCTCTCTTTTCGTGAAAATTCTCGTGATTGAGATCTTCGGTAGTGCACTTGGGTTGTTTGGAGTGATTGTGGGAATA

ATCATGTCAGCTCAAGCTACATGGCCTGCAAAAGTG

>GT974398.1 GJCCJC2158G11.b1 Jatropha curcas L. developing seeds (mixed stages) Jatropha curcas cDNA clone GJCCJC2158G11, mRNA sequence

CTTTCCTTTAGTACACAAATTATGAGCTGCCAGCTAACATTTTGCATTCCCAATTAAAACTTCAAGACAAATACTTGCAC

AATCGCATCCAATAAATGAAGAGCGCCACTAGTTAAAAGAGAGCATAAGACTATAAATATGGAAACCAACAAAATGAACA

TTAGAAAATTGTTGGTCTGCCAAAAAAACCCATTAAGATGACGTATAAACTTACTTACATTACAATGTGGGAGAGGGAAA

AACAGTTAAATGTACAAAGTGGGATGGTTTGAAAAAAGCAAGGCTACCATTGCCTTGCTCAAGATAAGGGGTATATATCA

TTATTATATATTATATATTATATATTATTTATATATATTATGTATAACTGGACACCAATAATACTAACTCCATTAACATG

TCCAAAATTGGGTATATATCATTCTTGCCATCTATTTCTGAGATACCAACAAGCGTCCGTCTTAGAACAGGTTAAGACTC

GCACCCCATCTCAAATTTCGCAACTGAAAAGGCTATAGCAAACCTTGCTTTGCTGCCTGTCTCGAAAACCTGGCACATAA

GATTCACAAAAGTTAATTACAACGGTATATTCAAGAATAATTTTAAAGAGCATCAATAACCATCCTGTATTACTTTTTTA

ATTAAACAAAAAGATAAAGCATATAGCTCTTGCATTAGATATTCAGCATAGCTAATTTGTTCACAACCCTCAAAATTACC

TAGAATTTTCAAAGTAGCCCAACCTAAAATT

>GT981494.1 JGCCJG2056C06.b Jatropha curcas L. germinating seeds (mixed stages) Jatropha curcas cDNA clone JGCCJG2056C06 similar to unknown protein [Arabidopsis thaliana] (TAIR:AT1G78890.1); similar to hypothetical protein OsI_027317 [Oryza sativa (indica cultivar-group)] (GB:EAZ06085.1), mRNA sequence

GGTGGAAGGAAATGAAAGTCCACACCCATCTCCTCCTCCACCTCCACACTCACTCTCTCCTTTATCCAATAGGCAAATCA

ATTCGCTCTCTATATATTCTCACCCCCTCCTCTTCTCTCTTCTATAACAAAAAGAATGGCCCTTTCTCTCTCCAAAACCT

ACCTAACCAAAGCTCCATCAAGGTCCCTCCCTCGTCTGCTGGCCTTGCGCGCCCAATCTAAACTTCTTTCTCGCCCTAAC

AACGTTGATCTATGCGATGATTCCTCATCCTCCAACGACGCATTGCTTCAGAAGCTTGAGGATGCCATCTACCGGATAAT

CGTGCCGTCGCGCTAGCACCGATTGGCTCCCTTTTCATACCGGCTCCGCTTACTGGGTCTTAACTCCTAGATCCGATGCT

GAATCCCTTGGGATTGCCCTGTTGATTGAGAAGTTGGTCAATTGATTGATGAATGAGGAATCGCTCTCTACAACAACCGT

CAGAGGTTGG

>GW879526.1 JC004085 Seed specific Normalized cDNA library from Jatropha curcas L. Jatropha curcas cDNA clone N07398 5' similar to Unknown protein, mRNA sequence

GGTTGATATTTTTTATAATAACACGTTTATAGGTGGTGGAGCTAACCTCCTGGAATCAACCGGATATATATATTGTTCCT

TTTTCCTTTCGCTTTTGCAAGTTTGGGTTGGCTTCTTTGGCTTCATGTCGGTCTCAAAGCTCCTGGTTTTTTCGCTCCTT

CTTGCCCTAATTTTCTCCGCCGTTAGGGCGGAGGCGGATGTTTCAATCCAAACCGATAATCAAGTCATTGGATCAGACGC

CGCTGATTCCTCTGCTTTGAAGATCGAAATTGACCAACTTAAGTCTAAGATCCATGCACTTGAATCCCACATTGATGAGA

GAACTCGAGAACTGAAGAGCAAGGATAATATATTAGCACAAAAGGAGCAATGGTTACTGATAACAACTCATCTTGAACCA

CATGTTCAATCACTGAGTGCAAAAACAATCGAAGCTTTTGAAGCCTTAAAGACTGCAATAACTCTATACGTTGTCAGAGT

ACAAGAATTTGTCGATCCTCACTACCAGGAAGCTAAGAAGTTAAGTAAGCCATACATCGATCAGGTTGTAACTGTGACAA

GACCCCATGTTGATAAAGTTAGGGTGGCTCTGA

>GT229112.1 JC677 Jatropha seeds from fruits at three stages of maturation Jatropha curcas cDNA clone PL16SE.F07.scf 5', mRNA sequence

AATAATAATAATACATTCATTTTTAGAATATTTTGTTCACAACACCAAAACTTCCAAGAGGAAGTGCAAATAAAGCATCT

TTAAAGACTCAAAATAATGAGCAAAAGCTTAAAAGAGAGAGAGAGAGAGAGAGAAAAGTGAACATTAAATTGAAATTAGT

CTCCTAAACTTTAAACAGCTAAACAGCCTGCCTGGTTCTCTTCTTGACACCAGACTTGGCAACCTTAAGGCTCCTGTGAA

CTGCACTAAGCCTAGCAAGGGCTGCCTTCTTCAAATCTGGCCTGTAGTAATTATCTGCAACCTGATTCGAAACAGCCTTA

GCCATGCGTTGGAACTCCTTCCTCATGACCGACTTGTGTAACAAACTGGAAGGCTTGTTTTGCTTCTTGGTCTTCGTGGT

AGCGAGCACAACGGAGAGATCCTTACCACCAGGCTGAATGGTAACAGTCTTCTTGTTAGCCAAACCAGAGTGCTTGTAGG

AGTTGAGGTTGTAGAGATTGTTGCTTTCCTTGCTAAACTGAACACTCGCATTGCCTCTGCCAAACTCTTTCACCAGGAAA

GAATTGTTCTTCTTCACGATCTCCCAAATCAACTGGCCTGGAACTGTCGCCATTTCTAGTCACACGTGACGGAAGAGC

>FM888014.1 FM888014 Jatropha curcas embryo 35-55 (DAF) Jatropha curcas cDNA clone rjcfea0_001324, mRNA sequence

AAACAACTCATTTCTGGGGACCTGTTGCCAATTGGGGATTTGTCGTTGCTGGCTTGGTGGACATGCCAAAGCCTCCAGAA

ATGATTTCCGGAAATATGACAGGAGCAATGTGTGTTTATTCAGCATTATTCATGAGGTTTGCATGGATGGTACAACCTCG

CAACTATCTACTTCTAGCATGCCATGCTTCAAATGAGACTGTGCAACTCTATCAATTCTCTCGTTGGGCAAGGGCTCAAG

GGTACCTCTCATCAGAGAAAAAAGAGAAAGCCTCGTCAGAGTAATAGAATACTGCTTTGATTGTCCTGTGTTGCTGGGAT

TCATATTTTGATTTATGGAGGAGCTGATGGTAATTGGAATGTGAAAACACTTTCATTGTACTACTAGTAATAACCATGAA

GCAGATTATTGACTGAAGTTTGGTGTTCGTGATTTAATTCACCAATTCATTCAGAACAGTATATGAAATAATTGGCTTAA

TTTC

>GW614735.1 Jc1-042-G09-M13F.G09.ab1 Jatropha curcas flower and seed Jatropha curcas cDNA, mRNA sequence

GCTGTTGGAGGTCTACCAGAGAAGATTCTAACTGCATTTCAATCAGTAACTGATGAACATCTTGATGAAGAAGCTGCTTT

AAACGAGTCTACTGCTGCTCTTTCTCGAGTTGGTAAAATTAGGGAAGAAATGGAAAATGGTTCAACTCAAGGAAATCAGC

AAGGCAGTTCATTTGTGGAGGAGTTACAAGTGCAGGAAAATGTTCTCCAGCAGTGTGTTGGGAAACTTGAAAGTGCTGAA

GCAACCAGGGCTATGCTGATTTCCCAGCTCAAGGAAGCACTTCAGGACCAAGAATCAAAGCTGGACGTCATTCGTGCTAG

GTTACAAGCTGCTCGAGTCCAGATTGAGCAAGCAGTCAGTCTGAGAAATAGGTTGACATCTTCCATTGTTCCTGGCCCCT

TGACCACCATTACGATGCCCTCGGCGGATGCTGCCAAGGTTGTAGAACACAGCATAGCTCCAGTTCAGCCAACCAGTACT

CCACCTCAGCCTCAACTTACACAACCTGTGGTTTCTTTTGCGCTGATGAAGACTACTGATGAGGATAGCAAGAAAGCCGC

GGCTGCTGCTGTTGCTGCTAAGCTTGCTGCCTCTACATCCTCAGCACAGATGCTTACCTCTGTTCTTTCATCTCTTGTTG

CTGAGGAAGCTGCCTCCTTGAATGGTGGCTTAAAAT

>GT972927.1 GJCCJC2043A04.b Jatropha curcas L. developing seeds (mixed stages) Jatropha curcas cDNA clone GJCCJC2043A04 similar to Acyl-CoA-binding protein, mRNA sequence

GCATGGAAGGCTGTTGAAGGCAAATCTAAGGAAGAAGCAATGAGTGACTATATCACGAAGGTTAAACTGTTGCTGGAAGA

AGCTGCATCAACGGCTTCCGCTTATTGTGTTGGCCATGCATATGTAACTGTTGTGTTCATTATAGTTAAAAATCAGTTCG

ATACTGTATCTGGATTTGTAATGCGATGTTAACTTGTGCCCAATAACTTTCATAATTAATTTATGTACTTTGTAC

>GT978286.1 JGCCJG2018G12.b Jatropha curcas L. germinating seeds (mixed stages) Jatropha curcas cDNA clone JGCCJG2018G12 similar to hypothetical protein, mRNA sequence

GAAGCCATCTGGAATTGGCCGAAGCCCTCAACAACTAAAGCTTTGAGAGGGTTTTTTGGGCTATGCAGTTATTACAGGAA

ATTCATTCACATTTTGGGAAGATTGCTAGTCCCTTAACCAACATGTTGAAGAAAAATGGGTTCAGCCGGTCACCATTGGC

AGAAGATGCCGTCAATAAATTGAAGGAAGTAATGACTTCTGGTTCTGGCGTGGGAGCCGTATTGATGCAAGAATGCCCAA

TTGCCTTCTTTAGCCAAGCATTACATGGTAAGCATCTCCTCCTATCTACTTATGAGAAGGAAATGTTGGCATTGGTTTTA

GCTGTGCAGAAATGGAGATCATACCTCCTTGGGCAAAAATTTATTGTTCGAACTAACCATCAAAGCTTAAAGCATCTGTG

GAATCAAAAAATAACCTTCGCTGCCCAGCAGAATTGGTTGTTCAAATTAATGGGATTTGATTTTGTCATTCAATACAAGG

GAGGTAACGAAAACACCGTTGCTGATGCTTTGTCTATAAGAGAAGAACAAGGATAGGAAGAAGGTTCTCTTTTAGCAATC

TCTTGCCTAATTCCCAATTGGATTGAGGCAAATCAAGAAGAAACTAATTCATTGGCAAAGTTGAAAGAGAATGTGCGGAC

AGTTTGTGATGGTGAGGCATTGGGACCATGGGAATACAGGGATGGCATCTTATTCTTCAAAGGGAGAATT

>GT974856.1 GJCCJC2064C08.b Jatropha curcas L. developing seeds (mixed stages) Jatropha curcas cDNA clone GJCCJC2064C08, mRNA sequence

ATATATATATATATTCCAGAAGTAAAAAAATATTTAAAAAATAGCTAGACATGATGCTTTGTTTTTTCAGTATTCTATTT

TTTAAAAAACTATTTTTAGACTTTTTTTTTACTATGATTTTTTCTTTGTTTCTAAAATTTGATTGGTCATCAAAATATTT

TTAAAATTAATATTTTATGATAATAGTCAACATTTTTAAAACAGTTTCTACAATTTGATTGGCCTTTAAAATATTTTTTA

AATAATATTTTATGATAATAACCAACATTTTTTAAATTTAATCTTATCAGCAATATACTTTCCTTTTGTTGAATCTTAAT

TTTTATGTTTATTAAGTTTTGTATCTAAGCCTTCTACTTGCTCTCTCCTTCTATTGTTCTCGTATGATAGTTTTTATTTA

CTCCAAACTTCTATTTTTACAGGCATCATGTACAGAAAGATTCCTGTATTGTGCAACAAACCATGCTGACAAATCAAAGA

ATGTGCTTTAGTGTATTTCGCTCATTATTTTCATCCAGATTCAACTATGAAGAGTTAGGGAGAACTTAAGTTACACTTTA

AAGATTTTCAACATGAAAATGTTTTTTTTGGATACTTCTGTTATGAATTTGCAGTTAAAATAAGACTTTTATT

>GW877468.1 JC007272 Seed specific Normalized cDNA library from Jatropha curcas L. Jatropha curcas cDNA clone N12235 5' similar to Unknown protein, mRNA sequence

ATGCCTGTTAGGCTATTACCTTAGTTATACTTCTTCTCTCTTTCTCTCTCTAAAACATGCCAAGTTCTGTACTTTGTCTC

TCTTTCTTTCCTGTTTGCTTCTTGAAGCCTCATATCTAAGCCAGCCAAGATAAGAGAGAAAGAAAACAAAAAAGAAAGAA

AATCAAAAGTGATGATAGACACGCCTTACCACACCCACAAAACCATTCCTTCAATCCAACTCTACATGCCCACATTCACA

AATCTTGACCTTCTCTTAGAAAACCCAATAAAATAAAAATCAAGAAAACAAATTAAGTAATACCCAAATAAGACCATTTC

TTGTTTTCAATTCTTTGACACTAAAATTGAAAAAGAAATTTGTCTTTCTTTAATTTCCCCTAAAACTAACAATGACTGAA

GTACTTCAATCATCCCCATCTCACTTCCCTTCTTCTTCAAGCTCCTCTTCCACCCCATGTGTC

>GW615669.1 Jc2-003-B04-M13F.B04.ab1 Jatropha curcas flower and seed Jatropha curcas cDNA, mRNA sequence

GGATGGTTCATGGATGTGTTTAATGTTACTGACCAAGATGGTAACAAAATCATAGACGAGGAAGTCATCAGTTATATTCA

AAGAAGAATTGAAAGCAATGCAAGCTATGTTCCCTCGATGAGCGGTTCTGTTGGGGTGATGCCATCTGAAGAGCATACCT

CAATCGAACTTACTGGTCCTGACAGGCCAGGATTATTGTCTGAAGTATGTGCAGTTTTGACAGACCTCCACTGCAATGTG

GTAAATGCTGAGATATGGACGCATAATGCCAGGGCTGCAGCTGTAGTTAATGTCACGGATGATTCCACACGATGTGCAAT

TAAAGATCCAAAGCGACTATCAATAATTAGGGAATTGCTTTGCAATGTTCTCAAAGGAAATAATGATTTGAAGGCAGGAA

AAATGACACTCTCTCCTCCTGGGATAACTAGTAGGGAGAGAAGATTGCATCAGATCATGTTTGCTGATAGGGACTATGAA

AGGGTTGAAAGGGGTGGACTAGGGAGGCATGAGGATAAGAGCTCAAGACCCCATGTCACTGTATTGAACATCGAAAAAGA

TTACTCTGTGATTACAATGAGGTCCAAAGATCGTCCTAAACTGTTGTTTGACATCGTTTGCACTTTAACAGACATGGAAT

ATGTAGTCTTTCATGGAATGGT

>GW881040.1 JC006268 Seed specific Normalized cDNA library from Jatropha curcas L. Jatropha curcas cDNA clone N10708 5' similar to Calcium-binding protein, mRNA sequence

CGGGGATTGATGTAAGTAGTTACAGTAACGACCAACCGTTGGCATAAAATACAACACAGTACCGTCGGATCTCACGAAAT

ATTAATAAACAAATTTAAATTAAAAAAAAAAACAACAACAAAGCAACTACAAAACTCTAGTCTCTCTTTTCTCAAATCCA

GAACAAAAAACCAAGACCAAAAAAATCTAGAAAGTGCTCAACAAGAACAGTGGGGGTTTCTATTCAGAGTAAGGATAGGA

GCCTGCGTGTCGAAACCTGCGGCGGCTGCTGCTGCTGAGAGTGCCTCTTCGAGGGAGGTGGTGCAGAGGCTCTCGCAGCC

GCCGGCAGCAGCAGCGGCGCCGGTGGTAACAATTACAACAGCTACATCAGCTGTAGCCGGAGGGCCAGTGAACTTACCTC

ATTCGAAATCGGAAAGGATGAAGGGACTTTTCAAGTCTAAGCCTCGAACTCCGGTTGATATTGTTCGGCAAACGCGAGAT

CTCCTCATCTACGCTGATCGCAGCCCCTCATCCGATGCTCGCGATCCCCAGCGTGAAGAAAG

>GW881699.1 JC003136 Seed specific Normalized cDNA library from Jatropha curcas L. Jatropha curcas cDNA clone N05930 5' similar to Unknown protein, mRNA sequence

GGATATCCTGCCGCGGCTTTATCAATAGATCAAGGAACAGAGCATTTCTCCATACGCGTTGAGAGAGAGAGAGAGAGAGC

AGAGCTTAAACTAATCTCTCTTACACAAAATCCCAATCAGGGTCCTTCTCTTTTACCCTCTAATCTTTTTCCATTGATAT

TTTCTTCTTCATATCTTCTGCTACTTTCTTCCCTTCCCCATAAGTTTTTATCCATTAATCTGTTCTTTTCTTGGCAGAGC

TTGTTGGGGTGGGTGTGGAGTTGGCTGTTTCCTTTCTTCTTCTTTTAAATAATAATTCCTTTTTTTTAAAAAAATTATCC

ATCTGTTGGTTTGTGTCTCTAAAACATG

>FM891533.1 FM891533 Jatropha curcas embryo 56-70 (DAF) Jatropha curcas cDNA clone rjcaeb0_001843, mRNA sequence

ATTGAATAATGGAAATTTCAAGCGAGAAGAATGGCTACAGAGCAAGAAAGGGCTGAACTTGATGCTAGAGCAAGGCAAGG

AGAGACTGTTGTACCTGGTGGAACTGGTGGCAAGAGCCTTGAAGCCCAGGAACATCTAGCTGAAGGGCGGAGCCGTGGAG

GGCAGACAAGGAGGGAGCAGCTGGGAACTGAGGGGTACAAGGAACTTGGTCACCGTGGAGGTGAGACAAGAAGGGAGCAG

ATTGGGCATGAAGGGTATCAGGAGATGGGCCGAAAAGGCGGACTTAGCACCATGGACAAGTCTGGTGAAGAACGTGCTGC

TGAGGAGGGGATTGAAATTGATGAGTCCAAGTATAAAACCCGTTAGTTAGGTTCTTCATATCAGTGTTTTAACTAGTAGT

TCCTCTTTTTGAACTAGTTGCGAGTAGGTTTGTACATATGGTAGTAGTAACTGCGGTATGTTGGT

>FM888703.1 FM888703 Jatropha curcas embryo 35-55 (DAF) Jatropha curcas cDNA clone rjcfea0_002161, mRNA sequence

GGCTGCAGGAGAGATGAACCTTGCTTGTTTATCCCGACGAGGAAGCGGAGCTCTCAAACAAGCCTTTCTAGCCGCATCTT

GAATCGGCATCCCTATCCATATCTT

>GT978327.1 JGCCJG2019C07.b Jatropha curcas L. germinating seeds (mixed stages) Jatropha curcas cDNA clone JGCCJG2019C07 similar to zinc finger (DNL type) family protein, mRNA sequence

GAGACAAGTCATGTAAAAGATCGTCGTCACGTCTCTCTTGGTACATCGAAATAGCCATGGAAACTTTAAGCCCATCTGCT

TCCATCGTCTCTTCTTCGTCTTCTTCACTCTCCTTCTTCTCCCCGAAGAGATTGGATTCTCCTGCAAGATTCCTCCGATT

TCATACTTCTTCCAAAAAGAATGAAAGCGAGTCCGATCTCCGATCTGAATCGAACGATTCAAGCATCGTCCCTTTCTTTA

ACAACCCAACTCTTTCCAAGGACGCGGCGATGGGGTTGGTTTTGAGTGCTGCTTCCGTCAGGGGCTGGACTACTGGTTCC

GGCATGGAAGGGCCTCCGGTGCCTGCTGGCGCCGACGAAGGATCCAATACGGAGAAGGTTTCGACCTTACCGTGGTCTCT

CTTCACAAAATCTCCACGTAGGAGAATGCGCGTTGCTTTCACTTGCAATGTTTGTGGTCAAAGGACCACTCGCGCTATTA

ATCCCCATGCCTACACAGATGGCACGGTTTTCGTTCAGTGCTGTGAGTGCAATATTTTTCACAAGCTTGTGGATAATTTG

AACCTGTTTCACGAGATGAAGTGCTATGTGAATCCGAGCTTCAATTATAGAGATGCAAAATGGGATGGGTGGATTCAGTT

CTTTGATATGGATGATCATGATGGTGATGATGATCGAAATGATGTGGTTCCAATCTAAAGAAGTGAAATATTTGATTTTA

TAATGTAAAGTCAT

>GT982622.1 JGCCJG2070G01.b Jatropha curcas L. germinating seeds (mixed stages) Jatropha curcas cDNA clone JGCCJG2070G01 similar to heat shock protein 70, putative / HSP70, putative, mRNA sequence

TCCACACACCACTGGCCATCTTCACCATCAGTATATAACCATTCTTGTACCTCATCAAATCTTTCATTGAAAAGCTTGAT

GAGGTACAAGAATGGTTATATACTGATGGTGAAGATGCTACTGCCACTGAGTTTCACGATCGTCTCGATTCATTAAAAAC

TATTGGTGACCCTATATTTTTCATGTATAAAGAGCTTACTGCAAGGCCAGCAGCAGCTGAACTTGCTTTAAAGTATCTTG

GAGAGCTGCAACAGATTGTGCACGGCTGGGATAAAAACAAACCTTGGCTTCCAAAGGATAAAATACATGAAGTTCTAAGT

GATGCTGAGAAGCTAAAGAGTTGGCTGGATGACAAAGAGGCTGATCAGAAGAAGATTTCTTCATTCATCAAACCAGCATT

TACATCTGAACAAGTATACGAAAAGTTATTTAATCTTCAAAATAAGGTTGCTACTGCTAACTAAATTCCGAAGCCAAAAC

CTTAAGCTGAGAAGCTTATCAAAAATGAATCTGAGGAAACCAGTGAGAAATCAAGCACTTCTAATTCTACTTCTAAGAAA

AATGCTGAAACTGAAAAATCAACTGTAGATTCA

>GT973588.1 GJCCJC2050B08.b Jatropha curcas L. developing seeds (mixed stages) Jatropha curcas cDNA clone GJCCJC2050B08 similar to SUS2 (SUCROSE SYNTHASE 2); UDP-glycosyltransferase/ sucrose synthase/ transferase, transferring glycosyl groups, mRNA sequence

GATGATATCTAGAATAGAAAAACAAGGATTGGATGTTACCCCAAAAATCCTTATAGTCACACGACTAATACCCAATGCAA

AAGGCACAACATGCAACCAGAGACTTGAAAGAATCAGCGGTACGGAACACACATATATTTTGCGGGTGCCTTTTAGAAAT

GAGAATGGTATTCTTCATAAATGGATTTCAAGGTTTGATGTGTGGCCATTCTTGGAGACCTTTGCAGATGATGCATCAAA

TGAAATTGCTGCCGAATTACAAGGAGTTCCAGATCTTATTATTGGAAATTATAGTGATGGAAATCTTGTTGCTTCTTTGT

TGTCTTATAAGCTAGGAATTACGCAGTGCAACATTGCGCATGCATTGGAGAAAATAAAGTATCCGGATTCTGATATATAC

TGGAGAAAATATGACGACAAGTACCATTTTGCAAGTCAGTTTACAGCTGATCTTATTGCAATGAACAATGCAGATTTTAT

TATCACTAGTACGTACCAGGAGATTGCAGGAAGCAAGACTAATGTTGGACAGTATGAGAGCCATACTGCTTTTACTCTTC

CTGGGCTGTATCGAGTTGTTCATGGTATTGACGTTTTTGATCCCCAGTTTAATATAGTCTCTCCGGGGACGGATATGTGC

ATATATTTTCCATACTC

>FM895544.1 FM895544 Jatropha curcas embryo 71-95 (DAF) Jatropha curcas cDNA clone rjcpga0_002484, mRNA sequence

ATTCCAACGGGGTGAGTAAACCCGCAAGGCGCAAGGAAGCTGACTGGCGGGATCCCCCTGAGGGTTGCACCGCCGACCGA

CCTTGATCTTCTGAGAAGGGTTCGAGTGAGAGCATGCCTGTCGGGACCCGAAAGATGGTGAACTATGCCTGAGCGGGGCG

AAGCCAGAGGAAACTCTGGTGGAGGCCCGCAGCGATACTGACGTGCAAATCGTTCGTCTGACTTGGGTATAGGGGCGAAA

GACTAATCGAACCGTCTAGTAGCTGGTTCCCTCCGAAGTTTCCCTCAGGATAGCTGGAGCTCGGGACGAGTTCTATCGGG

TAAAGCCAATGATTAGAGGCATCGGGGGCGCAACGCCCTCGACCTATTCTCAAACTTTAAATAGGTAGGACGGCGCGGCT

GCTTCGCTGAGCCGCACCACGGAATCGAGAGCTCCAAGATGGGCCATAATTGGTAAGCAGAACTGGCGA

>GW615095.1 Jc1-046-G05-M13F.G05.ab1 Jatropha curcas flower and seed Jatropha curcas cDNA, mRNA sequence

ACCGGAACAACAACCCCTCTAACGCTCTTTTCAGATCTCTTCTCTTTTTGAAAACAACGGAAATCCTAACTGAAGCGGGG

GCCTCTCTCTTCTCGTTTTTTGAATCTATTGCCCATGTCTCAGATATTTCGGATTCTGTCTTTGAATTTGCTTTTACTTC

TTAGCTTTAAAGCGGCGTCGTTTCCTTCGGGATCCCGTTCGATCCTTCGAGATATAAGTAGCAGCGACAATGTCGTCAAG

CCTGATTACGCTGTCGATTTGAATATTACCAATTTTGATTCAGTTCTTAGAGACACTCCAGCTACTTTTGCTATTGTTGA

ATTCTTTGCTCACTGGTGCCCTGCTTGCAGAAATTATAAGCCACATTATGAAAAAGTTGCAAGACTTTTCAATGGACCTG

ATGCAGTGCATCCTGGTATTGTGTTGATGGCAAGGGTAGATTGTGCATCAAAGATAAATAATAAACTCTGCGATCGATTT

TCTATATCTCATTATCCCATGCTATTTTGGGGCCCACCTTCCAAATTTGTAGCTGGTGGTTGGGAGCCTAAAGAAGAGAA

AAGTGAAATACGTGTAATTGATGATGGACGGACAGCTGAACACTTGCTT

>GT229002.1 JC567 Jatropha seeds from fruits at three stages of maturation Jatropha curcas cDNA clone PL12SE.B08.scf 5', mRNA sequence

ACTCGCGAATGCATCTAGATCAGTGGTGTAAACGCAGAGTGGCCATTACGGCCGGGGGATAAAAACAGCTGAGATCAGGT

TGGTTGCTGCCAGAAAAATGAAGCAAGCTGCAAGAGCAGCAGAAGCAATTGCTTTTGCAGAGATCAAGGCTGTGTCAGGA

CATGAGAACTCATCGGAAGATCCTTTACAGAAAGCTGAAGGAGTAACTCTTACATTTGAAGAGTATTCAACTCTAACCTG

CAAAGCTCAGAAAGCCGAGGAATCATCAAAGGCTAAGGTAGTAGATGCTATGCTTCAAGTTGATGAAGCAAATGTCTCAA

GGACGGAAATCTTAAAGAAGGTAGAGGAAGCTACAGAAGAAGTCAAAACCAGTAAGAAGGCCTTGGAGGAAGCTTTGAAC

AGAGTAGAGGCAGCAAATAAGGGAAAGCTGGCCGTTGAAGAAGCTCTTCGCAAGTGGAGGTCTGAGCATGGGCAGAAAAG

ACGTTCGGTTCACAACTCTACCAAGTTTAAGCACTCATACCCTTCTCACCATCGAAGGGATTCTCGTTTGCTTGATGTAA

ACGGGCTGAACTTAGTAAATGATGAGTCAAGTCCTGTTTTGAAGCCGACCCTATCCATAGGCCAAATTCTTAGCAGGAAG

CTGGCCTTGCCTG

>FM887253.1 FM887253 Jatropha curcas embryo 35-55 (DAF) Jatropha curcas cDNA clone rjcfea0_000411, mRNA sequence

TGAGAGCGGCTTCTCTTTCAGACCCTCCTCCGCCTTCTTCCACNTACTTAAAAAAAACTAATGCTAATGGCGTCCAATTT

ATTCAATCACGAACTAAACTCTCTCCCCCCGCCTAAAGTGAAAGGAAAGGGTGATTGATGCTATCTCTCTATATCCCTGG

CAAAGGCCATCCATCAAAGGCTTCAGCTGAATACATGGGGCCACTAAGCTTTCATTATTAGCAGCAAGCGCCTTAGCAGG

AAAGTCTGCACGGTTACTCAGCTCCTTTAGGTCGTGGGCTTCTATAGGTCATTCATCGCGGTAAGTCTCCGCGTAAAGCA

TCCAACGCCGGTTAACACTCCCTCCCCTCGAAGTCCGTTGTCGCATCCATAAGCCGAGTCGGGCATTCCATCCAGCCTCC

TGTCCCGCACCATCGTCGTATGTGCATTTCGTCCCTCTTATCTCTTAGGCGAACTCTCGTAAACCATATTTGGATCTCTC

CACTTTCGACAACTCACTTTATATTCTACAACCAGCTCACTCTTCATATGATATCTCGAACTTCTCTTAAGATCTCTGAC

GATGATTGGCTAAATCCTGAGCTAAGCGACTGTAACTTATTCTCATATGCAATAATCCAAGGAGGCATCCCCATTAAATA

GCGTAAGAAGGGAGTGGTAAAGAGTCCTCCTTAATAG

>FM890225.1 FM890225 Jatropha curcas embryo 35-55 (DAF) Jatropha curcas cDNA clone rjcfea0_004041, mRNA sequence

TTTTTTTNTGGAACTGGGCCAATACTAACACAGTTCTATTCATAATAACAAAGATTACAAATAGCAAATCTCTGATCAAT

TAAATAACAGCACCATAACTCTAGTAGAATACCAACAGGCTAAAAATCCATAAAATCAGCTGGGCCTAAAACAAAGTACT

ACTACTTGATAACAAAGAAAAATAATACTCGGCAGTGCCAGAAAAGATGCATTCTACAAAGTCCATACAAACTCTGGTGC

ATCTTTCCGCTGCCCTACCAATGTCCGATACAAGTACATCTTATCTACTTTCTGGCTATCATCAACACCAAATTCTGCCT

GCTCATTCTCATTCATGATCTCTACATTGAGCCTTGGCATCTTCTTCGCAAGGGTCTTGCAGCCTCCAAGAGTCACTTCA

CAGGACGACATCCAAAGGGATCGCATTGTTTCATACTTTCCCACGTCCATTAGAAGTGCCGCATTACCAAAAGGGCTATC

CCTTATCTCTAACTTGCGAAGTCTTTTGCCACCATTTAACACGTACTGCATTCCCTTATCACTGTCTCCAGCAAATGCA

>GW618744.1 Jc2-038-F09-M13F.F09.ab1 Jatropha curcas flower and seed Jatropha curcas cDNA, mRNA sequence

TGGGTTCCTAAACCCTAACTTCCGTATCTAACCAGGAATGAAGCGAAAGCGTGGACGCCCAAAAAAATCTGTTGCAAAAA

AGCCAAAAACTTTAGCGAATAAGGGTGAAGAAGCTTTCTTGAACTTTGTTGGCCAAAATGTCCAAGAAAAATCTGCATCA

GAGCATGTCAATGATAATGGGTTTGATCAATTTAGTTCTGATAAGGTCTCTAGTTCGTCTTCCTCTTCTTCTTCCTCAGG

TGAAAGTGATGATGGGTTACAGTTTGGGAGAGTTAACAGTGATTCTGATGAACCACAAATTGATAGAAGGGTTAAGAGAA

CAGCTAACAGGCGTGGTAGGACTAAAAAGCTGGAAGCTTCAAAGAAAAGTTTAAATGCCAATGTTGTTTCTTCTAATGGT

TTGGGTTCATCAAGCAAGAAAGAATTACCACCATTGAAGATTCCAGCTCAGGATTCAATGTTAAATAAGAAAGATTTGAA

AGCTTCCTTAGCGGTCATTAAAAAGGTAATGGAAATGGAAGAAGCCGTACGTTTTAGTGCTCCTGTGGATCCTGTTTCTC

AGGGATTACCCGATTATTTCAACATCATAGATACACCAATGGATTTTGGAACGATATGTA

>GT971220.1 GJCCJC2018C01.b Jatropha curcas L. developing seeds (mixed stages) Jatropha curcas cDNA clone GJCCJC2018C01 similar to BIP1; ATP binding, mRNA sequence

GGGACATGAAGCTTGTTCCATATAAAATTGTCAATAAGGATGGAAAGCCCTATATCCAGGTTAAGATTAAGGATGGAGAG

ACCAAGGTATTCAGTCCTGAAGAGATCAGCGCTATGATTCTGACTAAGATGAAGGAAACAGCTGAAGCATTCCTTGGGAA

GAAAATCAAGGATGCAGTGGTCACTGTTCCTGCCTATTTCAATGACGCCCAGAGGCAAGCTACCAAAGATGCTGGTGTTA

TTGCTGGACTAAATGTGGCCAGAATTATTAATGAACCAACTGCTGCTGCCATTGCTTATGGTTTGGATAAGAAAGGTGGC

GAGAAGAACATTCTTGTATTTGATCTTGGTGGTGGAACATTTGATGTCAGTATCTTGACAATCGACAATGGTGTTTTTGA

GGTTCTCTCAACAAATGGAGACACACATTTGGGAGGTGAGGACTTCGATCAGAGGATTATGGAGTACTTCATTAAATTAA

TCAAGAAGAAGCATGGAAAGGATATCAGCAAAGACAACAGGGCTCTTGGAAAGCTCAGGAGGGAAGCTGAGCGAGCCAAG

AGAGCTCTTAGCAGCCAGCACCAAGTGCGTGTTGAGATTGAATCACTCTTTGATGGAGTGGACTTCTCT

>FM890840.1 FM890840 Jatropha curcas embryo 56-70 (DAF) Jatropha curcas cDNA clone rjcaeb0_000789, mRNA sequence

TTCACAAGAACAAGGACATATAGAAAGATTAAAAGGGTAGCAAGTATTACATGAAATATGACCAGTTTATCTCTTAAATT

CAATTTATAAAGCACAAACCCTTCTCAGGCTCTTATAATACCATTACCAACTCTAGAGAACTCTCTCTTCATGACTAAAC

ATAAAGTAGAAAGAAAGACAGTTCTCAAACTCTAGATCTTATCAATATCTTCATCTTCAAACTCAACATAATCATCACCA

GCACCATCATCTTCTTCATCAAGGCCTCCAGCAATACCCTCATTGAGACGTGTGTTCTCAGGAAGTTCACCATAGGCCTT

TAGAAGCCTAGCTTCATCTGGCATGTACTTCAAGATAACATCAGCCTTGTCATCCTGATAGTCACGCAACCCAACCAGTA

TTATATCACCAGCCGCAATCCAGACCTTCTTGTGCATCTTCCCGCGAATATGGCATAAACGCTTGGTACCATCGATACAC

ATGGCTTCACATCGGCCATTACCAAGCATACGAAGCACTTGGGCATACTCCTGTCCATCTTCTTTAAAGATGAGCTCACG

CTTCTCGTCGTCTGCTTCGTTCTTTCCTCTCTTCCTATTCTTTCCTCCCTTTCCCTTATTCTTCGGCATGGTTTCTTATT

CTTTGCGGTCTGTGGCGTAGAAGAAACATTCGAATTTAACAAAATATTACGCTTACAATTA

>FM889220.1 FM889220 Jatropha curcas embryo 35-55 (DAF) Jatropha curcas cDNA clone rjcfea0_002820, mRNA sequence

CGGCCGCTCTAATATGGATCCGGGCTGCGGCCGTATCACGGGTTGATCGACCGGACGGTGGCACTTGTCAACTCTTCGTC

GACAGTCTCAGAAGGATGTTNAAAGGAAAGAGGCGATTTCTTGCCTAAGATTACGTTGACGAAGATTGATAGACAATGGG

TTCATGTATTGAGCGAAGGTTGGGCAAGTCCACTTTCTGGATTCATGAGAGAATCCGAGTTCCTCCAAACTCTTCATTTT

AATGCCATCCGTCTCGAAGACGGGTCGGTTGTTAACATGTCGGTGCCGATTGTGCTCGCCATCGACGATTTGCAGAAGCA

ACGGATCGGCGAGTCCAAAAGGGTCGCTCTCGTCGATTCTGACGATAAGACGATTGCAATTTTGAAC

>JK613512.1 JCF19-112 Jatropha curcas, immature Seed cDNA subtraction library Jatropha curcas cDNA similar to ribosomal protein s12, mRNA sequence

ACATACGACTTTCGTCGAATACGGCTTTCCACAGAATTCTATATGTATCTATGAGATCGAGTATGGAATTCTGTTTACTC

ACTTTAAATTGAGTATCCCTTTCCTTCCTTTTCCTGCTAGGATTGAAAATCCTGTATTTTACATATCCATACGATTGAGT

CCTTGGGTTTCCGAAATAGTGTAAAAAGAAGTGCTTCGAATCATTGCTATTTGACTCGGACCTGTTCTAAAAAAGTCGAG

GCATTTCGAATTGTTTGTTGACTTTGTTGACGCGGACAAAGTCAAGGAAAACCTCTGAAATTATTCCAATATTGGACCTT

GGACATATAATAGTTCCGAATCGAATCTCTTTAGAAAGAAGATCTTTTGTTTCACGGTAGCCTGCTCCAGTCCCCTTACG

AAACTTTCGTTATTGGGTTAGCCATACACTTTACATGTTTCTAGCGATTCACATGGCATCATCAAATGATACAAGTCTTG

GATAAGAATCTACAACGCACTAGAACGCCCTTGTTGACGATCCTTTACTCCGACAGCATCTAGGGTTCCTCGAACAATGT

GATATCTCACACCGGGTAAATCCTTAACCCTTCCCCCTCTTACTAAGACTACAGAATGTTCTTGTGAATTATGGCCAATA

CCAGGTATATAAGCAGTGATTTCAAATCCAGAGGTTAATCGT

>FM889973.1 FM889973 Jatropha curcas embryo 35-55 (DAF) Jatropha curcas cDNA clone rjcfea0_003738, mRNA sequence

TGAGAGGAAGGTTAGGGGCAACCAATCCATGACAGCCAGTAAGAGACAGAAGAGAGAGAGCACAGATAGAAGGAGGGCAG

ACTAGAATGCCCTATGAAAGCATCGGTGCAAAAGGAAAGCACGTAGTCTGTCGGTCACCACTCAATTATCTTACTGATTA

TATATCCATCGATGCATTGAATATCCTATCTATCGTCGTAGAAGGAGTTAAGTTCCTAGGTGGAACATGAGCCTTCTCAA

TAATCCGGTTCTGCTTCCTGTTTTGGTTTATTGATCTATAGTTCATACATAATGAAGTACATGTTGTCCAGACCAATAAA

AGGGCGAGTAGGAAAGTGGACGTTGGTACTTATTTTCTATACAATAACAATACGTACCCCGAAAGGAAGTAAAGGGGAAA

GCACTGGCCTCTTTCATTGCTTTCTTTCCTCGTCACGGACATCAAGCTTTCGGTC

>GW614372.1 Jc1-038-G10-M13F.G10.ab1 Jatropha curcas flower and seed Jatropha curcas cDNA, mRNA sequence

AAAGAGAGAAAGTCATAACAAAAGCCATAAACCTGGAGTGGAGGAAAGGGAAAGTGAGCCCAATAGTGAGTAGTATAAAG

CAGAGGTTGAAGCTTAAACCGATGGCGGTTAATATAGTTCATGATGTTGATGGATTGCCCAGGATTATCTTGACTGACCC

TACTGGTTCATCAGCTGAGGTGCTACTGTATGGCGGGCAGGTTGTTTCTTGGAAGAATGAACGCAGAGAAGAATTACTCT

TTATGAGCAGCAAGGCTGTTTGGAAGCCGCTTAAAGCTAACAGGGGAGGTATACCAGTCTGTTTTCCACAGTTTGGAAAT

CTTGGTTCACTGGAAAAACATGGTTTTGCAAGGAACAGATTGTGGTCATTGGATAGTGATCCTTCACCTTTGCCTCCAGT

AAATAGCCAGTCATCAGTTGATCTGATATTGAAGTCCACAGAAGACGATTTGAAGACCTGGCCACGTAGCTTTGAGTTGC

GGCTTCGTATTTCTCTGGGTCCTGGCAAGCTCACTTTGATCCCTCGTGTGAGAAATACAGATAACAAGGCCTTCTCTTTT

ACATTTGCGCTGTGCAACTACTTATCTGTG

>GT978826.1 JGCCJG2025C11.b Jatropha curcas L. germinating seeds (mixed stages) Jatropha curcas cDNA clone JGCCJG2025C11 similar to ANAC100/ATNAC5 (Arabidopsis NAC domain containing protein 100); transcription factor, mRNA sequence

GTACTTTTTTTGTGTAAGAGACAGAAAGTACCCAACTGGTTTGAGGACAAACAGAGCTACTGAAGCTGGCTACTGGAAAG

CCACAGGAAAAGATAAAGAGATCTACAGAGGAAAATCACTTGTTGGAATGAAAAAGACTCTTGTTTTCTATAAGGGTAGA

GCCCCAAAAGGAGAGAAAACTAACTGGGTTATGCATGAATACAGATTAGAGGGTAAATTCTCTGTCCACAACCTGCCCAA

AACTGCAAAGAATGAATGGGTCATTTGCAGGGTATTTCAAAAGAGTTCTGGTGGGAAAAAGACCCATATTTCAGGATTAG

TGAGATTAGGCTCTTTTGGTAATGAATTGGGTAATTCTGGATTACCACCATTAATGGATTCTTCACCCTACAGTGTCAAG

ACTAGTAAACCAGTAGCTGAATCGGCTTACGTGCCCTGCTTCTCCAATACCATTGATGTTCAAAGAAACAACCAACAAGA

ATCCATTGATAGTTTCAACAATAATTATTTTGCTGTTTCTTCAAATCCTTCTGATGTTTTTGCAAGGATTCCAATTCAAA

ACTCATTCTACTCTGCTCAAAATATTCCAATACCAGGAAATTTGCAATATCCAAGTTCTGGTTTAATGCAAGACCACTCG

ATTTTAAGGGGCCTTAATTGAAATCAAGGGTCTAACTTGAAACAGATTTTCAAAACAGAAAAGGATCAAATGGTTACTGC

CT

>GT976459.1 GJCCJC2078D05.b Jatropha curcas L. developing seeds (mixed stages) Jatropha curcas cDNA clone GJCCJC2078D05, mRNA sequence

CACCTATCATATCAAAAACTATCCGTTACACTCTTTTTTTTTACACTTGAAAGTATTTTTTTATATAAATCAACTATTCA

TGTACCATTTTTTATATAAATTTAAGTCAAAAAATGTGTATTTTTTTCACAACTTTTTATAACTTATGTACCATTTTACT

TCTCTTCACCATAAATCAAATCAACTACAATCAAATCAATTGATATAAATTTTTTTATAAAAATTTTAAGCTACAATTTT

TTGTGTATCCGGTTCGAACCGGACCGGAACCGCCGGTTCCGTGTCATATTTTAACATCTACATGAACCTTAACCGGCCTT

TGAACCGCCGGTTCCGGTTCAAAATTATGAACCGCCGGTTTCGGTCCGGTTCCGGTTCGTCTACAGTATGGTTCGGTTCT

AGTTCGGTTCCGGTTAGAACCGAACCGGTGGCCATGCCTAGATTGTATAAACTAAAAGACTAAATAATTTACCTTTTTTA

TTTTTACTCCAAATTACCATCATAATTTCTTTTTTAGAAAAAAAGTAAAAACATAATTTTCTATTTTATTCATATTTAAT

ATGCAAATTTTATCAAGAAACATTACTATTATCATTGATAAAATCT

>GW618965.1 Jc2-041-B02-M13F.B02.ab1 Jatropha curcas flower and seed Jatropha curcas cDNA, mRNA sequence

CTCGTGGTCTATCGCCATGCTGTTGCCACCATTGTCATTGCTCCTTTTGCATTTTTCCTAGACAAGAAAGTCAGGCCAAA

GATGACTCGCTCAATCTTCATCAAGATGGGGGTGCTTAGCTTATTAGAACCAGTTATCGATCAAAACTTGTACTTCTTAG

GGATGAAGTATACAACGGCAACATTTGCTGCTGCTATAATCAATATTCTGCCTGCCATTACCTTTGTAATGGCTTGCATT

GTTAGGCTTGAGAAGGTGAAAATGACATCACTCCACAGCCAAGCAAAGGTGGTAGGGACAATAGCAACAGTTGCAGGAGC

CATGGTGATGACAGTAGTGAAAGGCCCACCTGTTGATTTGTTTAATAGCACAAAAGGAATGAGAATTTATCATCACAATC

AACAAACATCTGATGATGTAAATCTCCATAATTCAATTAAAGGTGCTCTGATGATTACAATTGGGTGCTTCGGTTGGGCT

TGTTTCGTCATCCTCCAAGCAATTACACTTAAAACATATCCTGCCGAGCTGTCTCTTACTGCTTGGATTTGCCTCTTTGG

TACAATTGAGGGCTCCATTGTGGCGTTGGTGATGGAAAG

>JK610214.1 JCF161 Jatropha curcas, immature Seed cDNA subtraction library Jatropha curcas cDNA similar to nucleotide binding protein, mRNA sequence

ACTTTGACTAATTCATACAAATTGTAATAATGACAATCCCCTGAAACTTCCTAGATTTCCTATACAAACCAAAAGTTTAT

CTTACATATTTGCACAGACGTAGGGACGAGGGAAGAATCATTTACAGCTCATTAGCACAGAATTTATAACATGCATAAGA

AGACGTATACTGGCAGATTCTGAAGCAGATGTAATAGGCAGGTTCCTTTGATGGCTCAATATCTGATAGACCTGATCAAA

TATTGGTCTCACGTGCACTGCGATCACAGGATCTGCAGGGTTTATAGCAACAACCACATCTGTCATCCATGCCAGCTTTT

TAGAAGTATCATTGCTGATATCGCAAGCCAATTGCTGTAGAAGAGCCAGTAGT

>GT971822.1 GJCCJC2025A10.b Jatropha curcas L. developing seeds (mixed stages) Jatropha curcas cDNA clone GJCCJC2025A10 similar to oxidoreductase, mRNA sequence

GGTAGCAACAATGGCGAAATATTTTAAGGAAATTGAGATCCCAAGACAAAAGTACTTCGATGCCCTTCCTTTCCCCTTCC

GTTGTTCCCCCCAAACACTTCCCTATCATTTCCTTCCTCGCTTTCTCTCCTCACAGACGCCATTAAAAATGAGAACCTTT

ACCTCGATTCTTTACTACACAAAACTGGAGCGATTCTCTTTAGAGGCTTTCCCGTAAACACCGCCTCGGACTTTAACGAC

GTCGTGGAAGCATTCGGTTTCGAGGAGTGACCTTACGTTGGTGGCGCCGCTCCTCGGACTAATGTCGT

>GW879969.1 JC006481 Seed specific Normalized cDNA library from Jatropha curcas L. Jatropha curcas cDNA clone N10987 5' similar to Unknown protein, mRNA sequence

GCCATTACGGCCTACTTACGGAGGACTGTCCCCATGATAATCTCATCGTGTACCTCTAGTAGTTGAAGCTGCCAGCATGA

TCTGAGCTTTCCTTAATACTCATCAATCCTACCCTATCCAGAGAGGAGGGATTATATCATGTGGCAGTTTCACCTGTTAA

TGCTAGCATGAAAATAAGAACCCTAATCGGGATAAACCATAATTTCCTGATTCTATCCAATTTTGTCAACCCATTACAGA

TTAATCATGGAAAAACTCTACACGATGAGCTATACTATTGTTTATATTGATTAATTGCTATTTTGTTTTCTTTCTTTTAT

TTCTTTTCTC

>GW881157.1 JC004421 Seed specific Normalized cDNA library from Jatropha curcas L. Jatropha curcas cDNA clone N07868 5' similar to Unknown protein, mRNA sequence

GGATATCTTATCGTATTTCTTTCCTTCTCTTTCTACAAGCTCTGCCTAACTTTTCTGGTTCAGATCTCCTCTTAATGGCT

TCTTCTCAAGGATTGTGGCTTTCGCTGTTGAATGGATCGTTCTCCGATCTATTTTCAGATGATGGTTTCAGGGCTTGACT

CAAATAGGGACGTGTAGATATCAGGTTTGTGTAATAATAGATCTCTCAGCTTCCCTTCATATGCTAGTTATATTTCCACA

ATACTTTTACTAGACATGTTGTGAATATGTATTTCTGAATAGATCTTGCAATGGTCGTGTAGCTTGCAACTTCGAACTGT

CCATTGAATTGATCTTCTGACTGGTTCGGGTCATGAAAAAGCCGTTGAAAGTGTCTATTCCCTTTTTCTTTGGTAGCTGT

TGCAGGTGGTGTAAAAGGACCTGTCACCTTAAGGATCCGCTCGGCCAAGATCAGTGCGTCTGCCTGTAATTAATGTGTGG

TTTTTGTAGCAGACGTTTTGATCAGATGTTAGGAGGCGGAAAAATATATATCTCCACATATATATATGTTTATATTCATG

TCTCTGGATGTAGAAGATATATCAGGACGAGAAGCCCATATATTGTTTGATTGATGGAGGGGGCTTTAATTCTTTGGTTG

ATATCTTTGTCATCGGACCTTTGCCATG

>GW880753.1 JC007092 Seed specific Normalized cDNA library from Jatropha curcas L. Jatropha curcas cDNA clone N11920 5' similar to Unknown protein, mRNA sequence

ACTGAATTTCACGCCACTGTCAGCTATATCCACAATTTCCACTAGCCACTTCTCACTTTCCAAGTCTTGCAAAATACACT

GCATATATATCTGAAATCATGGGAGTTGCAGAAACAAAATGTTCTTCAGGTTACGGCAAGCCTCCTTGGGTATTCAAAGT

GCCTTGTACCAGCTTCACCTTGTGAAAGCAGAAACAGCTCGAACATTCATTCCAAAAGAGTTTAGATTAGTTGAAGCTTT

TGGGTATACTCTTGGAGGGTTTTTTCTTGCCAACTACGAAGAAAGTCCGGCAGGGGCATTCGATGAGCTAGTGGTGATAG

CGGGAATCGTGTGGAACCCTCCAACATCCTGCGCATGGGCAGCCAGAGTGCTTGTGAGCAGCAATGATGCTTGTGATCAT

GGAAGAAAGGAAGTAGGGCTCCCCAGTCATGTTGCCAAGTTTTCAAAGAGAATCGAAGGGATTCCGAGGCAGCAAACAAG

TAAATTTAATCGCTTTCTCAGCATGATTGGCTTAGATACTGCATCCTCTACACCCAATGGTGGCATGGATGTTCAAGTAA

CTGAGATCAATGGTCCTCGTGCAATAAACATCTGCGAAATCAACCTTCCAACTTTTGGCCCAGGGTTCAACTGGACAAGG

GG

>FM891702.1 FM891702 Jatropha curcas embryo 56-70 (DAF) Jatropha curcas cDNA clone rjcaeb0_002088, mRNA sequence

TGAATTCATCATGAATCCACTAGTATTGTTGGTTCTGGCAACTGTGGCTTTGGCCAACCCTGAGGTGAAATTTCAGCAAC

CAGATGCAGATGTTAGTCCGTGTGAAGTATGCACTATGGCCTTGGATCAATTGAAAAAACTGTTGCAAATTGATGCTGTC

AAGACCGAGGTTGAAGTGTTGATGAAAGGCGTTTGTGCAGCGTTTGGTCCTGTCAAGGGTTTGTGCGAAGCGTTGGTTGT

CAAGGGAATCGATATTGTCTTCGACCTCATACAGAAGCAACAACCGCAGGATATATGCGCGAAAATCAGACTGTGTTAGA

AACGAGGAAGAATAAAAATGGTTGATTGAATGGTGATACCACATTCATCACATTACATTCGTTGCATGAATAAGACTGTT

TTGCGTTAAAGAAAAAAAGGGGGGGCCCGGTAC

>GW875881.1 JC005258 Seed specific Normalized cDNA library from Jatropha curcas L. Jatropha curcas cDNA clone N09095 5' similar to 4-alpha-glucanotransferase, mRNA sequence

AGCCAATGGGGTCGTTTTTCTCATTATTCCAAGGAAAGGATTGAGAAACTTGTCTCAAAAGATAGCTTGCACTATGACAT

AATTTGCTTCCATTACTATATCCAGTTCCATTTACATTTGCAATTGTCAGAAGCGACAGAGTATGCAAGAAAAAAAGGAG

TAGTGTTGAAAGGAGATTTGCCTATTGGTGCTGACAGAAACAGTGTGGATACTTGGGTCTATCCAAATCTATTTCGCATG

AACACATCTACTGGAGCACCTCCGGACTACTTTGCCAAAAATGGACAAAATTGGGGCTTTCCCACTTATAACTGGGAGGA

AATGTCAAAAGACAACTATGCCTGGTGGCGAGCTCGTTTGACACAGATGGCCAAATACTTTACAGCATACAGGATTGATC

ATATATTGGGTTTCTTTAGGATCTGGGAACTTCCAGAGCATTGTCTGACTGGTCTAGTTGGAAAATTCAGACCATCTATC

CCTCTAAGTCAGGAAGAGCTTGAAAGAGAGGGAATTTGGGACTTCGATCGCTTGAGCCGCCCATACATACGACAGGAATT

TTTGCAGGAAACTTTTGGAGCTTCATGGATTTTTATTGCCTCAAATTTTCTTAATGAACTTCAGAAGGGTCGGTATGAGT

TCAAAGAGGACTGTAATACAGAGGAAAAAATCGCTTCCAAGTTGA

>JK612313.1 JCF26-200 Jatropha curcas, immature Seed cDNA subtraction library Jatropha curcas cDNA similar to vacuolar type atpase subunit a, mRNA sequence

ACTCAACGGCATTGGAGTCTTTCTATGATCAATTTGATCCAGATTTTATCAACATCAGGACAAAGGCCCGGGAAGTGCTA

CAAAGAGAGGATGACTTGAATGAAATTGTCCAACTTGTGGGAAAGGATGCTTTAGCCGAAGGAGATAAGATTACACTAGA

GACCGCAAAGCTTTTGAGGGAAGACTATCTTGCTCAGAATGCCTTTACTCCATATGATAAGTTCTGTCCTTTCTACAAGT

CTGTTTGGATGATGCGCAACATAATTCATTTTTACAATTTGGCTAATCAGGCTGTGGAGAGAGCAGCAGGTATGGATGGC

CAGAAGATAACATACAGTCTCATAAAGCATCGGTTGGGAGATCTCTTCTACCGTTTAGTGTCTCAGAAATTTGAGGATCC

AGCAGAGGGAGAAGCAGCCCTGGTGGCAAAATTCAGCAAGTTGCATGATGATCTAACTGCAGGTTTCCGTGCTCTGGAGG

ATGAGACTCGATGAATTCCCAGAGTTTCAAATTTTGGTTTTTCACAAATCGT

>FM896460.1 FM896460 Jatropha curcas embryo 71-95 (DAF) Jatropha curcas cDNA clone rjcpga0_003679, mRNA sequence

TGCAGGCCAAGCTATTCCGCAGCAGCTGGACCAAGCTATCATCCACCACGCACTCCACCGCCGCCACCACCGCCTTCTAA

TGTTGGTTATATACCTACTTTTCTTCCAAATACTGATTACATAAACATGCCATCATCTGCAGCAGCCTCTAGAATGGGAG

CAAGACCTGGTGTGGCAATGGGACTTGGTGCTGGAGCTGTGGCTGCTGGTGCTGTGATTTTTGGTGATGACTTCTTGTCA

GGATTTGATATTCCTTCAAGCTTACCTGATCCTAGTCTAACAATATCAGTAGAGCCTCCTTTCTAAAGGAAGGGTGCTAC

AATTTCCTGTAACACGCTGAAATGCTATGAAAACATTGAGTTGGTTATCTCAAAATTTTCCACCTTCCTCAATAGTGAGA

ACTCTGTTCTGCAAATAACAGACACCATGATTCCGAGAGAAAAACATTTTTGAAGAAAAATTTTTGAAGAGGAATTTTTA

AATCATTTGATATATTAACAAATAGAACAAAGCATAAAATAGGCAACAATAA

>GT974864.1 GJCCJC2064D05.b Jatropha curcas L. developing seeds (mixed stages) Jatropha curcas cDNA clone GJCCJC2064D05, mRNA sequence

CCATTATAGTAAGACAGTTAAAAGTGTAAGCCTAATCCTATAAAGACTTGGGATGGAAAGGTCCATAAGATTCTTTATAA

ATAGTCTAAGTCCCCTCTCTTTCCTTAACCCTAATATTGGCTAGTCCCATTAAAAGCCGCATTGTGAATAAGAAAACGGT

GAGGAAGATCTAGTCCCTCTTCAATAAATACTAATTTCATATTTGAGTGTCTTCATTATGGATAAACACAAGACAGCAAG

GAATCATGAACTCTACTTTAAATCACAAATCTGGTACGCAAATTGCTTCCGCATTATTAATAGTTGGTGCTCTAAATTTA

TGTACTAGATCTATTAATTTCTTACAATTTGTTGCACAGTTTATGGAATTTGGATATTCCTGTTTAGGTAAAAGAACTTT

TGTGGAAGGCTTTTAATGAAAATTTTATGCATAAAGGTTAATCTTGTTTTATAAGGTATGTAGATAGATGTTTATTGAGC

TTTTTGTGGAGGCATGGAAGCTTTGACCATGTCCTTGTTGATTATAATAAGATGAAGCTTGTTTGGAGTATGGATGGTTT

TGAAGCTTATCTTACTGATGGATCATTTAAGACTCTTTTTTCCAGGTTAAGAAAATTTATCTCAGAGCATTTGAAATTTG

GTTATTTGAATAAGAGAGATTTGGTCATCACGTATTATGCTTATATGGCAAGCATTATACAATTAGTTACGTAGATGTAT

CATTCAATTCT

>GW611742.1 Jc1-009-B01-M13F.B01.ab1 Jatropha curcas flower and seed Jatropha curcas cDNA, mRNA sequence

TTGCTGTCTCTAGCATGTAGGTCTAGTTATCTGAAGTTTGGCAGGTAGAATTTTGGCCTGAGAGAATCCAGGATAAGATA

TCACCATGCATTTTTGTCTGCTTTGTTTGAAACAAGGGATGTGTATCCTTTCTTTATCCCATCAAAACTGCCACAAGGAT

GAGAACGTGAAAATGTAAAATTGTCCAAATCAATTACTGAATTACTTCTTAGCTACCAACTTTTTGAGTGGGAGCAGGAA

GTTTGCATGGCGGATCTCTCATCAGTTGCCCTTTTTTCTTCTACTTTTGAGCTGTGTGCTATATATTAGGCACGCTTGCT

TATAAGGTTTTGGTTTTGCAGTAGCTAGCAAGGAATTTGTACATTGTTTTGGATTATTTAAAAGAAAATGAAAGAGAGAA

GTTATTGC

>JK610320.1 JCF759 Jatropha curcas, immature Seed cDNA subtraction library Jatropha curcas cDNA similar to peroxidase, mRNA sequence

GAGCGGGCGCCCGGGGAGGTGTTTCTGCCTCTTCCCTGATATTATATCAGCTCAACTTAGACGAAATTATTATGCAAATA

TTTGCCCAAACGTGGAATCCATTGTTAGAAATGCAGGTCAAAAGAAATTTCAACAGACATTTGTCACTGTTCCTGGAACC

ATTCGTCTCTTTTTCCATGATTGCTTTGTCCAGGGATGTGATGCTTCAGTTATAATTCAATCCACTCCAAATAACAAAGC

AGAGAAGGACAATCCCGATAATTTATCGCTGGCCGGAGACGGATTTGACACCGTTATAAAGGCGAAAGCCGCCGTGGACG

CAGTCCCAAGTTGCAGAAATAAGGTCTCATGTGCAGATATTCTTGCTATGGCAACAAGAGATGCTATTGCTTTGTCTGGT

GGACCTTCATATGCTGTTGAATTGGGAAGATTAGATGGTCTAAGCTCAACTGCTTCTAGTGTCAATGGCAAACTTCCTCA

GCCCACTTTCAATTTGAATCAGCTCAATTCACTGTTTGCTTCTCATGGACTTTCCCAAACCGACATGATTGCTCTTTCAG

GGGCCCACACCGTCGGATTTTCCCATTGCAACAAGTTCGCTAACAGAATATACAATCTCACCCGTCAAAATCCGGTGGAC

CCTACACTCAACAAGGGTCTACGCAACTCAGCTACAAGACATGTGTCCTAGGAACGTGGACCCCAGGATAGCAATTAACA

TGGACCCCGCAACACCGCAATACTTCTGACAACGTGTACCTCGGGCGCGACCACGCTAATCTAG

>GW611493.1 Jc1-006-D04-M13F.D04.ab1 Jatropha curcas flower and seed Jatropha curcas cDNA, mRNA sequence

TAGAAACTTCTCTTGCAGAAGATATACCATTCAGAGAGGTTAGCTAATAGCTATCTAAAAGATAATAAATGGGTGCTGAG

AAAGAAGCTGCAAAGAGTGAAGGAGAGAAGAAACCCGCCGCCGACAAAGACGATGCCAAAGTCATCTCCGTTTATAAGAT

GGACATGCATTGCGAGGGCTGTGCTAAGAAAGTTAGACGTGCCGTTAAACATTTGGAAGGTGTGGAAACTGTGAAAACAG

ATTGTGAGGGCAACAAATTGACGGTGACCGGGAAAGTAGACCCGGAGAAAGTCAAGGCGAGGTTGGAGGAGAAAACAAAG

AAGAAAGTGGAGATTGTGTCTGCTCCACCGAAGAAAGACGGTGGCGGTGATAAGAAACCTGAAGAGAAATCGGACAAAAA

ACCGGAGGAGAAAAAGCCTGAAGAGAAGAAACCGTCTCCTCCTAAAGAGAGTACGGTCGTTTTGAAGATCAGAACCCACT

GTGATGGTTGCATTACAAAAATGAAGAAGATCATCTTGAAAATCAAAGGCGTTAAGAACGTGACGGTGGACGGAGCTAAG

GACTTGGTGACGGTAACAGGAACAATGGACGTGAAGGAATTGGCGCCTTATCTAACGGAGAAGCTGAAGAGGAACGTGGA

GGTTGTTCCGCCAAAGAAA

>GT981334.1 JGCCJG2054C10.b Jatropha curcas L. germinating seeds (mixed stages) Jatropha curcas cDNA clone JGCCJG2054C10 similar to ATAAH (ARABIDOPSIS THALIANA ALLANTOATE AMIDOHYDROLASE); allantoate deiminase/ metallopeptidase, mRNA sequence

GGTGGCAAAGAAGAGGCATTGGTCAGCCGCGGTGGCGAATTCAGTTTTGGCAAGCTATCGCGGTTGGCCATAGCGATTTC

TCTTGCAACTAAACGCATTTTCATTACAAGAAAACGCTCGCTCATGGCTAACCGGCTGATTTTCTTTCTCGTTATGGTGA

TTTTCCTCTTATCAACTTGTTATGCTTCTGCTTATACGTTCTCTGATGTTGACAGCAGAAGGAATGATTTGTATCGGGAA

ATCTTGAGAGACGAGGCAGTTGCAAGGCTCAATGACCTTGGAAAGGTGAGTGATGCTGATGGCTATCTTGAGAGGACATT

TATGAGTGTAGCTTCTGTGAAGGCTGGAAATCTTATTCGTAGTTGGATGGAGGATGCTGGTTTGATGACGTGGATGGACC

ACATGGGCAATGTACATGGTCGAGTTGAGGGATCAAATCCAAGTGCTGAGGCTCTCTTGATTGGTTCTCATTTGGATACT

GTTGTTGATGCTGGGATATTTGATGGGTCGTTAGGCATAATCTCAGCATTGTCTGCTTTAAAGGTGTTGAAAAGCAAAGG

AATGCTGAGCAAACTAAAACGGCCAGTTGAGGTTATTGCATTTAGTGATGAAGAAGGGGTGAGGTTTCAATCTACATTCT

TAGGCAGCGCCGCTGTAGCTGGTATTTTA

>GW878285.1 JC002661 Seed specific Normalized cDNA library from Jatropha curcas L. Jatropha curcas cDNA clone N05049 5' similar to Arginine decarboxylase, mRNA sequence

GGTAGCCGGGGCCTCGGCCTCGGCGGGTTTTAAAGCCCCCACCTGCACAAACTGAGAAGAAAATCATAATAATAACCCTA

ACTTCTTTTTAACCCAAAACAGATCGTCGCGGAAGAGGATGCCGGCCCTGGCTTGTTGCGTAGATGCTGCCCTTGCGCCT

CCCGGCTACGCTAATCATGCAGGGGATAGCTCCCTTCAGTCGTCGATCCTATTTTCCGGCGTACCTCCGGCACCTACCAC

CACCACCGCATCCGCCATTGATAACTCTCCTTTCTCTCATTGGTCACCTTCCCTCTCTGCCGCTCTCTATAAGATCGACG

GATGGGGTGCTCCTTACTTTTCTGTCAACTCCTCCGGCAATATTGCCGTCCATCCTTATGGGACTGATACGCTGGCTCAC

CAGGAAATTGATCTCATGAAAATCATGAGGAAAGTTTCGGATCCGAAGTCCATGGGCGGGCTTGGATTGCAGCTGTCTCT

TATTGTTCGCTTGCCTGATATCCTCAAAAACAGGATTGAGTCCCTGCAGTCAGCTTTCAATTATGCAATCCATTCTCAAG

GCTTTGAGGCTCATTACCAAGGTGTTTATCCTGTGAAATGTAACCAG

>GW879879.1 JC004447 Seed specific Normalized cDNA library from Jatropha curcas L. Jatropha curcas cDNA clone N07904 5' similar to Transferase, mRNA sequence

GGCCATTACGGCCTAGTTACGGGGAAGCGCTATTAATCTTGGGATCGATAACAATGGAGGCTAAAACCAAGGCACCTCCT

CTTCCCCTTCACACAGTTAGATCCATGCCTCAAACAACCTTCAACCGTGTATTTGCTTCAGTCTACGCTTGTGCCATTCT

TGCTCTACTCTATCACCATCTCATATCTCTTCTTTACTCCAAAACCATCGTCTCTTTCTCCATAACTCTCATTTTGTTAA

TCTCTGATTTAATTCTTGCCTTCATGTGGATCAACACGCAGTCCACTCGCATGTATCCAGTCTATCGACAACAATTCCCA

GAAAATTTGAAGAAAGTTTTGAAAAGAAACGATTATCCAGGTTTAGACGTGTTTATATGTACTGCAGATCCATATAAAGA

GCCGCCAATAAGTCTAGTGAATACAGCTTTATCAGTAATGGCTTATGATTATCCAGTGGAGAAGGTTTCTGTGTATGTAT

CTGATGATGGTGGCTCAGCCTTGACTCTCTTTGCTTTAATGGAGGCGGCTAGATTTGCCAGCCATTGGTTACCATTTTGC

CAGAAGAATCGCCCAATGGATAGAAGTCCTGAAGCTTATTTTAAATCATCAAATCAAATTTTGTCTCCAGATACTGAAAA

GATTAAGATAATGTACGAAAGATTGAAAACGAAAATAAAACGTGTTCTTGAGAGAGGGAAAGTTGACGAAAATTCATC

>JK611593.1 JCF4385 Jatropha curcas, immature Seed cDNA subtraction library Jatropha curcas cDNA similar to conserved hypothetical protein, mRNA sequence

GATAAAGTTGATGATCTCTGCCACGTGTATGAAACAGTGGAGAGAATACTACTCAGCCCTGACGAAGTCTTGAAGGCAGT

AAAGCAAGCGGAAGAAGCCATCACCCCGACTGTTGGTGAACCTAAACGAAGATTTACGAGGATTGGCCTGAACTTTGGCA

GTAGGGACAGAAACCAGAGACAAGAAGGGAAAAATCCGGAGATTTTGCCTCAACAGCCATTCTCTAATTTCTTTGATGGC

AAGTCATCCTTGTTTTCAAAAAGCCTCCAAAGCCTGAGAATGCATCTGCAGAAAAAAGCCCCTAGAACAGTTGAAAGTGA

TTGACAATTGTTTAATCAAATAAAAATCGAATTTTATTTATTTATTTTCCCATTAATTTTGGGTTAGTCGATTTGGGAGA

ATTGTAATTTTTGGAAATTCTTGCCATATAATGGAAATAAATACATTTGTTTTTAAAAAAAAAAAAAAAAAAAAACTCCC

TGCCCGAACCCCTCCAAGGGACATAAACAAGTTCGAGAGCGCAAATAGCGGTTTTACACGCGAGAGAACGGGTCCAATAT

CGTGCACCTCCTCTCGCGTGAAAAGCCCCCCCTCCTCCTGCCCGAGAGAGAGCTATTTACAGTTGTAAACTCCAGGGATC

TATAACAGTGGTTTGCAATAGACGACGAAAGCAGACACACAATATGGCACTCATCTTTCGAGGATGAGTTTGGGGATCCC

CGCCACTCGATTAGTCAATAGCATGCATCGTAGACTGACG

>JK612267.1 JCF26-140 Jatropha curcas, immature Seed cDNA subtraction library Jatropha curcas cDNA similar to conserved hypothetical protein, mRNA sequence

ACTGACATGGTGGAGGTTTGTTTGACGGTTAAGCGGCAACTGGATGTTTTACAAGCTGAGAATGGGCTATTGAAGAAAGC

TGTGGAGGACCTCAGGCGGGAAATTGCTGGAGATAAATTTAGTTTGCCAGTGACGGATTTCAATTCCAGCAAATACAGAG

AAATAGAGAGAAACGGAATGAAAAATAGTAGTAGAACTGAAAGGAGAAGCAATGATAAGAAATCAAGGGAGGGAGATGCG

AATGAAGAGTTGAAGAAGGCATTGAAGGGAGCTACTGGTGTTGGTGCTTGAATTTGTAGAAACAGCCCAAGGT

>JK610935.1 JCF3263 Jatropha curcas, immature Seed cDNA subtraction library Jatropha curcas cDNA, mRNA sequence

ACCTTTGATGTGTTGTGCGGCAGACATGTTCTCTGTGCCTCATCAGAAGCAAGTTGTCTTAGTTGGCCACAAGCCATCCT

CGGAGTTTGACAATATGTTGGCTGCTGCTCATGCATCATATGATCCCAACAGAAGAGTAATTCATATAGATCCCACCAAC

AATGAGGAAATGGAATTTTGGGATGATAATAATAGCAACGTAGCACTCATGGCAAGGAATAATTTTTCTGCAGATAACGT

GGTAGCTTTAGTCTGTCAAAACTTCACCTGCAGTCCTCCAGTCACTGACCCTAAATCTCTTGAAGCTTTGCTTTCCAAGA

AACCTGTTGCTGTATAGAACTTGGCTGGT

>GW876201.1 JC005756 Seed specific Normalized cDNA library from Jatropha curcas L. Jatropha curcas cDNA clone N09827 5' similar to Nucleic acid binding protein, mRNA sequence

GGCCATTACGGCCTAGTTACGGGGAAACACAAAAGAAATTGAAGCAGGAGGATTCTACCAGGTTTTGAGTCGTTCCTATT

TCACGTAACCACGCGAAAATTACAAATACGAATCCTAATTTCCCTGTAAACCCTAGATATCTAATCGCCGACAACTTTCG

GCTTGTCTTTCATATCAGGACATCAGATTTTCTTTATCAATTTCAATTTGAAATTTAGAGAAAGAAAGCCATGGACGATA

GTTGTGCGGTGTGCGCTGAAACCCTGGAATGGGTGGCCTATGGAGCATGTGGCCATCGAGAGGTCTGCTCCACCTGCGTG

GTTCGCCTTCGCTTTATTTGCAGCGATCGACGGTGCTGCATCTGTAAGACCGAATCTCACGTCATCTTTGTCACCAAGGT

TAGGGTTTTTCTACTCCTTGAACAATCAATCTATTTGGAATCATTCTTTTTTTCTCTCCATTCGTAATCTAGTAGTGTTT

TGCAATTAAATTATTGTCGGTTGATTTGGATTATGTTGTAGGCTTTAGGTGATTATACGAGGATGGTTAATGACTTCGCA

GTCTTTCCATCTGAACCCAAGGGAGGGTAGAGTTGGGTCATACTGGTATCATGA

>JK610473.1 JCF1343 Jatropha curcas, immature Seed cDNA subtraction library Jatropha curcas cDNA similar to o-linked n-acetylglucosamine, mRNA sequence

CCTTCCACGAATGGTATCACTAGCACTTGCTACGAAGGACCCCATTCGATCATCTGAATAATTTTGAACAATATCTTCCT

AATGCCACTATTCCTGTTCTCTAGATCTTATCTGCGGTGGAGAATATTCACACTCCTCTCATCTAACAAAAGGGAACTCT

GAATCATTCTCCGCAACTTTGAAATGCCAGGGCTGCTGAAGTCTCAAGAGTCTATTCCACAATTTGAAATATGCCCTCTC

CAATTCCGGACCCCTGCGCTATATCAATGAAGGACGCTCCCCTCGACTGCCTGCAGTTTATTGCTCAGAGCTTGCAGTTT

CGACGTATTCCGAGCGCGGGACACTGCTCTCTCTTCCAATTCCTTCGTGCCGCTCTCTATCTTCCCGTCCCCAAGTCCAC

TGCGACCTGACTGGGATCAAGCAACTCTAGCAGCCATTTTCTCAAGGTGGCAGTGATCCCCCTGGCAAAACTGCCCACAG

AATATCTGTTCCCCGCACATGTGCATTACATACAACGCGTGCCCAGAAATAGATCTGCTAAGGCATACCGCCTGATATGA

TCCTGTTTCATGTCACCATCAATTTAAATGATCTGCTCTCGTTGCTTCCCTTGTGCGACAACATATGATCAATGTCTCAT

TTCTCCAGCGACAGGAAACATATGGGGCCAAGTGCTGCTATTTGTCGCAACATTAGGACTATTGCAACAAACATTAACTT

CTTTCGTGAACCAGCTTGAAACCTGCTTTGAAGACGCAGACTGAACTGTCCCCACGAACAACATCAGCTTGTTCGCGTGC

GAGGACACGTTAGAATCCAAAAACATGAACACTTTTTACGGATCCCACTAATTTCAGACTTGTGCGAGATGAAGCCGGGT

GACACAGCGTTTTGTCTAGATGAGAGATCTA

>GW881693.1 JC002871 Seed specific Normalized cDNA library from Jatropha curcas L. Jatropha curcas cDNA clone N05381 5' similar to Signal recognition particle 19 kD protein, mRNA sequence

GGCGCGTTACACACCCCTTGTAAAAGAAGTCGCATCATTTTCCAGCTGAGCACCAGCCGAGATCGTCCCGTTTCTCAGCC

GAAGACTAACCTCTGTCGTCCCGTCCGTAATGATGGATGGAAATCTCCCCAATATAAAGAAATGGATAGTTTTGTATCCC

ATTTACATAAACTCGAAGAAGACCATAGCTGAAGGCAGGCGAATCAGTACTACCAAGGCTTGCGAAAATCCAACTTGTGT

TGAGATTGGTGGTTGCTGCAGCCATCTTAAACTCCCTTTTGCCATTGAGATTGACAAGGCATATCCGCGGGATTTTATGC

AAATAGGAAGGGTGAGGGTATTGCTGAAGAGGGAAGATGGGACTTTGTACAATCCAGCCATTCCTACTAGAAAACAGCTA

ATGCTCCATATTGCAGAGTTGGTGCCTAGGCATCCAGGGAGGACAAAGAAGCAGGAGCCTGCCTCCACATCTGGCGCTGG

ACCATCCAAGTCTGGGAAGGGTGGGAGAAAGAAGAGATAGGAGTGCATCAGATCATCATTTTTTTGTCAGCATAGCATAG

GCTTTCTTGTAGCAGGCTTAAACTCTCAGTGAGCTCAAATTTCACAGATACCTTTGTATTTTTCAATCCTTTTCGGAAAT

ATAGTTTGTGTTGACTTGAAACT

>GW879973.1 JC006485 Seed specific Normalized cDNA library from Jatropha curcas L. Jatropha curcas cDNA clone N10991 5' similar to GPI ethanolamine phosphate transferase, mRNA sequence

GGCCATTACGGCCTAGTTACGGGGAAAAAGCGAAATGGTTAGTAGTGATGGGATCTTGGGGATCAAAGAAGGAGAAAGAA

TAAAAGCAGAAACTCTTAAAAGAAAGAAACGGCTAAAGAGACGAGAGAGATGGTTGGTAATACTTGGCGTGATCCTTCAC

GCAATTTATATGCTAAGCATTTTCGATATCTACTTCAAAACCCCAATTGTCCATGGCATGGATCCTGTCGAACCTCGCTT

CAAGGCTCCAGCGAAACGTCTCGTGCTTCTAGTAGCGGATGGTTTACGCACTGATAAGTTTTTTGAGCCGGATTCGGAGG

GAAATTATAGAGCTCCATTTTTGAGAAGTATAATTAAAAGCCATGGTAGATGAGGAGTTTCACATGCTCGGCCCCCTACA

GAATCAAGGCCTGGGCATGTTGCTATAATTGCTGGTTTCTATGAGGATCCTAGTGCTGTCACAAAAGGATGGAAAGCTAA

TCCAGTTGAATTTGATTCCGTTTTTAACCGAAGTAGGCACACATTTTCTTATGGAAGCCCAGATATTGTCCCAATATTCT

GTGGTGCCTTGCCACACAGCACATGGAAGAGCTACCCTCATGAGTTTGAAGACTTTGCAACTGGTTTG

>GW877381.1 JC006904 Seed specific Normalized cDNA library from Jatropha curcas L. Jatropha curcas cDNA clone N11617 5' similar to WRKY transcription factor, mRNA sequence

GGCCATTACGGCCTAGTTACGGGGGACCCAGAGAAAGAACACACACACACTCTCTCTCTCTCTCAGCTACCTACCTACCA

TAAAAACCCGATCAACCCTACCCTACTATCCCTTTCTTTCTTCAAAGCTGTAGAAAAAGCCCTCTTTTTTTCTCGTTTTC

TCTATTTATCTCCTTGTGTATTCTGTAGGTATTGATGAAGAAATTGGTTGTCTGATATAGCTATGGAAGGGAAAGAAGCG

GTAAAGATAGAGAATATTGCTGGATCGACGACATTTCCTGATCATATTCAAAGTAGTTATTCTCTAAATGGTGTATCTGA

GTTTTGTGAAGGAGATAAGAGCTATTTAGGGTTTATGGAGCTACTGGGTATGCAGGATTTTAATCCTTCTATGTTTGATG

TTCTACCGTTACCATCTACGGTACCGCTGCCTACAGTTAGTATTAATCCAGTTAAAAAGATGGAGTCGCCACCGGAGGTT

TTGAGTCAGCCTGCAACGCCTAACTCTTCGTCTATTTCTTCAGCTTCTAGTGAGGCTTTAAATGATGAGCCGGTTAAACC

TGTGGATATTAATAATGAGGAAGAAGAGCAACAGAAGACTGCAAAAGAGTTAAAACCCAAGAAGACAAACCAGAAGAGAC

AGAGAGAGGCAAGATTCGCATTCATGACAAAGAGCGAGGTTGATCATTTAGAAAATGGGTACAGATGGAGAAAGTACGGT

CAAAAGCTGTGAAAAATAGCCCCTTTCCTAG

>GT971907.1 GJCCJC2026B03.b Jatropha curcas L. developing seeds (mixed stages) Jatropha curcas cDNA clone GJCCJC2026B03 similar to leucine-rich repeat transmembrane protein kinase, putative, mRNA sequence

AAAATTGAAAGTAACAGCATAGCAATGGATGTCAGTGGAGGTGCTTCAATTGTAATGTTTCATGGGGACTTGCCTTACTC

TTCAAAAGACATCATTAAAAAATTAGAGACCTTAAATGAGGAACACATCATAGGTTGTGGGGGTTTTGGAACGGTGTACA

AGCTTGCAATGGATGATGGCAATGTATTCGCTTTGAAAAGAATTTTAAAGATGGATGAGGGTTTTGATCGTTTCTTTGAG

AGGGAACTTGAGATTTTGGGAAGCATAAAACATCGATACTTGGTGAACTTGCGCGGTTATTGCAATTCTCCAACCTCAAA

ATTGTTAATTTATGATTTCCTCTCTGGTGGCAGTCTTGATGAAGCACTCCATGAAAGATCTGAGCAACTGGACTGGGATG

CACGTCTGAATCTTATCATGGGAGCAGCAAAAGGATTGGCCTACTTGCATCATGATTGTTCCCCCAGGATCATACATCGT

GACATAAAATCAAGCAATATTTTGCTTGATGGCAATTTGGATGCTCGAGTATCTGACTTTGGACTAGCCAAATTGTTAGA

GGATGAAGAATCACACATTACAACCATTGTTGCAGGGACATTTGGTTATCTAGCTCCTGAGTACATGCAGAGTGGTAGAG

CCACTGAAAAGACTGATGTTTATAGTTTTGGGGTCCTGGTGCTTGAAGTGTTAAGTGGAAAGCGACCCACAGATGCATCA

TTCATCGAGAAGGGTCTGAACATTGTTG

>FM892271.1 FM892271 Jatropha curcas embryo 56-70 (DAF) Jatropha curcas cDNA clone rjcaeb1_002775, mRNA sequence

GTTAAATCTAATTTAACTAATATTTTCAACAGCAATAACTGGATCATCTCACAAGCATGAGATCCATCCAGGTGATTTAT

GCTGCTTCCACTTTTGTATTGCAACCATAAGCATGAGATCCATCACATTCCAAGTAGCTTTCAGGAACTTGTCAACTATT

ATAAATGCCACTCCACAATGCACCAGTACTAATCATCATGTGCAAATGCATACAGAATTGCAAAAACACAACAGTATTCA

CATTAATTATTAGGAAGAAGATACTGCTAAAAGATTTGAAATCAAGTCCTATATGAAAACCAAGTGTCTATTTTACTCCA

ACAATAGACATCAGAAACCTAAGCTTATCATTCACTTCA

>GW874751.1 JC000141 Seed specific Normalized cDNA library from Jatropha curcas L. Jatropha curcas cDNA clone N00242 5' similar to Unknown protein, mRNA sequence

GGTCTCAGACTTCTACCTTTTATCGCCCGAGATCTCGAATTTCTCGCCGTAGCTTCGAGTTCGTCGGTCCTCCTAAGTTG

GCCTTTCTTTTTCTCGTCTGCAAAGACCTGCCTCTCGATTTCCCCTGGGGAAGCTTCTTCGAGAATGCCGACGTGGCTAA

TTTCTCCATTTTTATTCACTCGGCGCCTGGTTTTGAGTTCGACGAGTCAACAACACGGTCGCATTTCTTCTACGGTCGGC

AGTTAAAGAATAGTATCCAGGTAGTCTGGGGAGAGTCGAGTATGATTGAAGCAGAGAGGTTGTTGCTTGCGGCTGCTTTG

GAAGATCCGGCAAATCAAAGATTCGTGCTTCTCTCTGACAGCTGTGTTCCCCTGTACAACTTTAGCTACATATACGGCTA

CTTGATGGCTTCTCATAGGAGTTTTGTGGACAGCTTTATTGAAACAAAAGGAGAGCGCTACAACCCGAAAATGTCGCCTA

TCATACAAAAAAAGAAATGGCGAAAAGGATCCCAG

>GW876552.1 JC007186 Seed specific Normalized cDNA library from Jatropha curcas L. Jatropha curcas cDNA clone N12097 5' similar to Glucan endo-1,3-beta-glucosidase precursor, mRNA sequence

GAAGAGCCATTTATTGGTGTTAACATTGGAACTGAACTCTCTGACATGCCCCATCCCACTCAAATAGTGGCCCTCCTCAA

AGCCCAACAGATTAGGCATGTCCGGTTATATGATGCTGATCGTGGGATGTTAATTGCGCTTGCAAATAGTGGCATTCAGG

TTATGGTCTCTGTCCCTAATGAACAACTCCTTGGAATTGGTCAGTCAAATTCCACTGCAGCTAATTGGGTATCTCACAAT

GTTGTGGCTCATTATCCAGCCACCAATATCACGGCTATTTCAGTAGGTTCCGAGATTCTGACCACCCTTCCTAATGCAGC

ACCAGTCCTTGTCAATGCCCTTAAGTACATTCACTCGGCCCTTGTGGCATCGAATCTAGATCGTCAAATCAAAGTTTCAA

CACCTCTTTCTTCCTCTATTATTCTTGATTCTTTCCCACCCTCCCAAGCCTTCTTTAACCGCTCGTGGAACCCTGTTTTA

GTTCCAATGCTCAATTTTTTGCAGTCCACAGGCTCATACCTCACACTTAATGTATACCCTTATTATGACTACATGCAATC

AAATGGTGTAATTCCATTAGACTACGCACTCCTCAAGCCTCTTCCTCCAAACAAAGAAGCTGTAGATGCTAATACACTAG

TCCACTATTCCAATGTCTTTGATGCTATGGTTGATGCAGCA

>GW616712.1 Jc2-014-H07-M13F.H07.ab1 Jatropha curcas flower and seed Jatropha curcas cDNA, mRNA sequence

AGAACCAAGAATTGCGAAAGAAACAGACAGAAATCTTGCCGATGCAGAAAAATCAGGTATTAGAGATGATGAATCAAGAG

CAGGGAGCCGAGAAACGATGCTTAAGGAGGACTCAGACAGGTCCATGGTAAAGTGCAGGCATGGTTGTGATTATACAGAT

GAAAATTATGTATGGTAGGTGGCTGTACATATTTGAGAGAAGTGCTACTTTTTGTAGATTAAGAAAATGCAAGTTCACAA

TGGAGGAGATGATAATTTTACCGT

>FM894472.1 FM894472 Jatropha curcas embryo 71-95 (DAF) Jatropha curcas cDNA clone rjcpga0_000144, mRNA sequence

GACAAGTTGATACNTGTATTATTGGAAAGAATTATGCAAGAAATGGAAGGAAGAGCATCAAGTAGATCTTCTGTGTGCCT

ACCTGTGACTGATGATTATAGCTCCACCAATCAAACTTGTTTTGGTGCTTCCCATAATGCTGTACATGATTCAGAAATAC

AAGCTCAATGGTCAAGGGAAACTGAGCTGTTAGTGGAGTTGCTGGGTAAAATACTTCAGCAGATAATTAAAAGTGATACA

AGAGCGGATATTTGGGGCTTATATGCAAGATGGCACAAGATCAAAGGAGATCTTACAATGTGCTCTGAAGCGCTCCTGAA

GCAAGTCAGATCGTATCAGGGATCTGATTTATGGAAAGATAGAGAACGATTTAAAAAGTTTGCACATGCCTCATTGGAAC

TATGTAGAGTGTACATGGAAATATCTTCTTCTACCGGCAGTCGTCGTGAACTCTTTACAGCTGAGATGCACCTGAAAAAC

CCAGTGAAACAGGCTGAGAGCTT

>GT970721.1 GJCCJC2013D07.b Jatropha curcas L. developing seeds (mixed stages) Jatropha curcas cDNA clone GJCCJC2013D07 similar to ATFUC1 (ALPHA-L-FUCOSIDASE 1); alpha-L-fucosidase, mRNA sequence

GAGCAAGTCCCAACACCACCACTACCAGTCTTACCACTTCCATCATTTTCTCAGTTGAAATGGCAACAAAGGGAGCTTAT

CATGTTCCTTCACTTTGGAGTCAACACATTCACGGATTCTGAATGGGGTACCGGACATGAGAACCCGGCCATATTCAACC

CGGTTGGTCTCAACGCTAACCAATGGGTCAGTGCAGCAGCAGAGGCAGGAATCTCTCTGATGATACTCACTGCAAAACAC

CATGATGGGTTCTGTTTATGGCCTAGTAAATACACTGACCATTCTGTTGAAAGAAGTCCATGGAAGAATGGCCGTGGAGA

TGTTGTCCAAGAACTCGTCAGTGCAGCCAAGAATTATGATGGGATTGATGTCGGACTTTATCTATCGCCATGGGATCGAC

ACGGCAGAAGATATGGTCATAATTTGCAGTACAATGAGTATTACTTGGCTCAATTGCAAGAACT

>GW615996.1 Jc2-006-G12-M13F.G12.ab1 Jatropha curcas flower and seed Jatropha curcas cDNA, mRNA sequence

TCTGTTGACAGAAAACTATAGATAATCAAATCCCTAAATTCACCTTTACAAAACGTGTATTTTCTCAACAGACCTTCTTT

CGAGAATCCAGGCTTTTCTACTACCCTTTGAGATCCCTTATTCTTTACTGCTACAAGAGCTTCAAGTCTTACCAATTCAG

GTATATCTTGAAATACACTAGACAGAGCCATCTTCAGTGCAATTGTGGCTATCCCTTGTCCCCAATACTCGGTAGCTACT

GCATATCCAATGTTTGCTCTGCATCTATCATCACCGGAGTATTGCCAAATGGAGATACATCCAATTGAACGATGATCCAA

ACATATAGATCGGCGCCATGGATGGGATATGGCAACCTCTTTGAGGTGTTTTAGTGCTTCTTCTGTAGTGGTAATGGAGT

TCCATCTCAGGTTTGAGGTTACTCTATCATCACTTGCCCATTTTAGGAAATCGTCAACATCAGAAAGCTTGAAAGGACGG

ATGGAAATTCTTGATGGATCCATAAAGATTGGGAACGATCAGCTTAGCTTAGAACAAGGATATAAATACAGGCAAGGATA

TATGCAGAGGACTGATTCCATGGATGAGGTATAGCTACATTTTGAAGATGTTCTAGTGCTTCTTTCTTGGAGGTAATGGC

ATCCCATC

>GW881332.1 JC001450 Seed specific Normalized cDNA library from Jatropha curcas L. Jatropha curcas cDNA clone N02704 5' similar to Unknown protein, mRNA sequence

GGATTGGAGCTTCATTATGTAAATGCTCTCATTCAGGCTCAATCGAATGAGATCCAATGGCCCTTTGACTCACTCTCTTC

TTCAGTAGTCATGTTTGTCTTTGCAAAGGGAGCTCTCTTTAGTTCTCTTCTTCAAGTTTAGGCGCATCGTGCTTTTTGTT

TCTATGCATTAAGGATTTTTTTTTTATTAAACCGCTGCGTTTTAGTATTGTACAAGGAGCTTAGGTAAATTTAACTCAAC

CTAATTTGATGATACCCATTTTAATAATTTTTTATTCAAAACCCTAGTTAATTTTGATTTTAGGATGATAATATTACAAA

GTTTTTAAATTGGAGGGTTCCAAGAAACGAAGGTCAGATTAAAATGTTTTTACAACCAACTCCAAATCTCGTAGTTTTTT

TTTATAGGTTAAGTGCTGATTTACAATATAATATGATTTTTCTTTCGATTTAATAAAATTTAAATTTAAATTTAATTAGA

AAGTTAATATCATCGATTAAAATTAAATTAGCAATTTCGGACAATTCTAAAGTTCCCTATTCCCAGTTATAGAAACTCGT

CCAACTCTTCTTGCGTCTTGTTGACTAATGTCAAAATCTGCAAAATAGGAGTGATAGATTAA

>GW613120.1 Jc1-024-D10-M13F.D10.ab1 Jatropha curcas flower and seed Jatropha curcas cDNA, mRNA sequence

AATTATACAACAATGGGTTCTGTTGGGAACCAAAATCCTTGGTCGCCATATGATACTGCTTATAGAGATTGCTCCCAAGG

AATTTGTAGCATATATTGTCCACAATGGTGCTACATAATCTTCCCTCCTCCGCCTCCTTTTACTCTCGGCGATGATGATT

CCGGCACTGATTTTTCTCCTCTAATTATAGCCGTTATCGGTATCTTGGCTAGCGCTTTTATTTTAGTGAGTTACTATACT

ATCATTTCCAAATATTGCAGACGCCGAGGCAGCCACCCTGAGAATACGATGGAATTGGATGGGAATCGCGACCAAATCAG

TAGTGAAGCTTGGCAAAGCATCAATGGCGGATTGGATGAGTCTATTATTAAATCAATTACTGTTTGCAAATACAAAAAAG

GTGATGGGTTTGTTGATGGCTCTGATTGCTCTGTTTGCTTAAGTGAATTTCAAGAAAATGAAGACTTAAGATTGTTGCCT

AAATGTAGCCATGCATTTCATCTTCCTTGTATTGATACTTGGTTAAAGTCTCATGCCAGTTGTCCTCTTTGCCGTGCCAA

TATCGCTTCTACAAATTTTTTACCACCGCCGGAAACTCAAGAAGCTCCTCCTCCGCCAACTAGCACTACTACTACGGCTG

CCCATGAATATCAACATA

>FM891934.1 FM891934 Jatropha curcas embryo 56-70 (DAF) Jatropha curcas cDNA clone rjcaeb0_002351, mRNA sequence

ATTCCATTAATAAGCACATTTCCTAACCAGGTAATACAAAGTTACAATAGAAACACTTCATAGCAAGCTGCTTTTTAAGC

ACACTTAGACCCACTATTTTATTTATGGCTTTCCCTGTTTATAAGATGCTACATAAACTCTCATCTTAGAGAAAGCCCAA

GACCCCAATACACACATAAAATTTATTAGCCATAGGTAGCAGTACTAGTAGCTAATACCTAGTGTCTGTGTCCCTATAAG

TTGTGGTCTGCTGATGGCCAGAACGATCCTTAACATGATCTTCTTCTTCTTCATGGTGAGCCATACCAAAAGCGCTTTTG

ACAGTATCAGCAGTTTTTTGAGCTGCATGCTTCATTTTCTCCCCAGTTTCTTCCAGAACTCCCTTTGTCTTCTCCTTCCC

TTCATGAGCCTTCTCCTTGGTTGCCTCTGCATCCAGGTCTTGTGTTCATATTTGGTTTGGCTATGCAGTTGGTTGACTCC

TTGGAATGGTGTGGAATCTAGCTGTAGTTTGTTGGCATTTGTACATTGTGATCT

>GT977842.1 JGCCJG2013C10.b Jatropha curcas L. germinating seeds (mixed stages) Jatropha curcas cDNA clone JGCCJG2013C10 similar to EMB1923 (EMBRYO DEFECTIVE 1923), mRNA sequence

GGAAAAATGGCTAATTTTCTGTAGAACCATAGCCATGTCGATTCCAGTCGCCGCTCTCCCTTCGCCAACATTCAAAATTT

CATCACTTTCTCGTTTCTCCACTCGACTACCTAACCAGGTCAATTCATTTCGTTCTTTACCGATCAAAATCGAACCCTTC

AATCCAAAACTCACCCAGAAAAGATTTGGGCTTTTATACACTAGAAAGTTAGGGTTTTGTCTAAATGCAGCTGATAATGG

AGAAGTAGAGAGCAAGAGTAGCGTTGATGACGATGGTGCTGAAAAGATGGCTCGAGGGGAGAGTACTATGCCTGATAGGT

TCAGGTACCTGACTAAAGAAGCACCTGACCCTCCCGTTAGATGGCCTTGGTTTGTCGCACTAGGTTTCCTTGTTTATGCA

TGGAGGGCAGTCTTATTGGAACTAGCTAACTGGAAAAAGTCCGTACAAGCAGTTATTAGTTTTGTGGGATACCTCCTGAA

ACTTCTGTTAGCTGTTATCTTCCATTTCATCGGGGATCCAATCACTTCATTGATCAAATGCATAGAGACTTTGGTCTATG

CTATTCGGGCCTACTATTCTGGGATAGTGGCTTATGCTCCTGTTCCCGAATTGACTATGATTATTGTGCTTGCATCAGCA

GTTTTTGCTATTGCAGAAGCTGCTGGGTCAAATTCATAACCAGCCAACCATATCTTCTCACATTATCCGGCCTGGATGGC

TATGCAGCGGTGAAGAACTACATCTCT

>FM893856.1 FM893856 Jatropha curcas embryo 56-70 (DAF) Jatropha curcas cDNA clone rjcaeb0_004785, mRNA sequence

ATTGGACAACCGATAGCGTGATGGGACTTCCCAAAGCTCTTACTGAAAAACTTATGAAGGAGGCTCTCTAGCCTAGTGCA

GCTATTATCTTTTCCATTCTTTTGGTATTTCCCGACTGACATCCTTTGCTTTCCTTTACTGTTATGCTTCCTCTGAACAT

TGCTTGATTTGAAATGGTTAAACAAATTATTGGTTATCGTTCCCCATCAGCTGATAGAGTAGGCACTGATTTTCGTTTAT

CAGGGATGACTAGTTACTTAAATGTTGAGCTGTGAACAAAATGCTATTTGCCAAAGTTGCCCTTTTGTTAAAGCTACTTG

ATAGGAATATATAGTAGACTGCATATTTTTATGTTACTGGGCATGATATGAGTAGAGAAAATTTTATTT

>FM895288.1 FM895288 Jatropha curcas embryo 71-95 (DAF) Jatropha curcas cDNA clone rjcpga0_002088, mRNA sequence

ATTCCTCCTTTTTTAATTCTCGAAAAAAGAAAAGGAAATGACCGGAAGATAAATGTTCTTTGATTAGATAGAGCTGGAAT

CCCAGATATTGTGATGGAAAAAAGGAAATGGTTGTGGAAGAGGAAGTCATCGGAGAGAAGCCCGGGCGAAACTGAAAGTT

CAGGATCAATATCTTCACAATCTGAGAGATTCTCTGATGAACAGGACAACTTGAAGGCATCTCCTAATAATGAAACTCAA

TCTCCAGAGGTTACATCAAAAACTGTAGTGAGGGATGAAGATGTCAATGATAGTGTTAGGATTCTAACAGAGAAGTTATC

AGCTGCCCTTGTGAATGTTAGTGCCAAAGATGACTTGGTAAAGCAGCATTCCAAAGTTGCTGAAGAGGCTGTTGCAGGAT

GGGAAAAGGCTGAAAATGAAGTAGCAGCTTTGAAGAAACAACTTGAAGCTGCTATTCAGCAGAACTGTGCTCTGGAAGAT

CGGGTGAGTCATCTTGATGGGGCACTAAAGGAATGTGTAAGGCAGCTAAGACAAGCAAGAGAAGAGCATGAGGAAAAGGT

CTACGAGGCTGTGACAAAGAAGACAATTGAATGGGAATCCGTTAAATCTGAACTTGAAAATCAGCTTCTTGAACTGAAGA

CAAAAGCCGAAGCTACTAAGTCAGAATCT

>GW874798.1 JC000188 Seed specific Normalized cDNA library from Jatropha curcas L. Jatropha curcas cDNA clone N00331 5' similar to Unknown protein, mRNA sequence

GGATTACTCTGATTTGCTGCTATTGTCTTCTTCATCTGCTTCACACAATCGTAAAAATGTCTCGTTATCACTACCAGAAG

AAGAACTAGAAAGACTGAAAGCAATAAGGAAAGCTATCATTGAAAACTTAGGTCCCAAAGGTCCAGGTCTCCTTTCTATC

ATTGGTGTCCCCAGAGACGCTTCTCTTCTTCGCCGCAATCTCCTTCCTCTTGCTCGCAACTTAGCTCTTCTCGACCACGA

GCGCCGCAAACGCGTTCTAAAGGAGCACACATTAGGGAGTGACGTTCCCCTGAAGAATCCTGATAGAAATGTGTCCTCTT

TTGCAATGCAACTCAAATATGTAGATGCTATGGAATCTGCTTCTGGTAAACCAAGTCATGGCAGCTATGCACACTTCAAT

TCAGAACCAATGCATTTGCATGTTGATAAGGTAAAAGAATTTGAGGCTGATGAATTTAAAAATCTTGCTGATGCCTTTAG

AAAGTTGGGATATTTCATGATGGAGCTGGGGCTCCTTCTTGCTCAAATATGTGACGAGTTGCTCG

>FM893485.1 FM893485 Jatropha curcas embryo 56-70 (DAF) Jatropha curcas cDNA clone rjcaeb0_004319, mRNA sequence

ATTCTCCTTATTGAGCTTAGTGACGAGAAACAATCTCTCTCTCTCTCAAAACCCTTCAAGTAGCGTTTTCTGCAATCGAC

CTTGAAGAAATGGCTAACAGTAATCTCCCTCGAAGAATCATTAAGGAAACTCAACGTCTTCTGAGTGAACCAGCTCCCGG

AATAAGTGCTTCACCATCTGAGGATAATATGCGGTATTTTAATGTGATGATTCTTGGCCCAACACAGTCTCCATATGAAG

GTGGGGTTTTCAAACTGGAATTATTTCTACCAGAAGAATATCCAATGGCTGCTCCAAAGGTTCGATTTCTGACCAAAATC

TACCATCCTAACCTTGACAAGCTTGGAAGGATATGCCTTGATATTCTGAAAGACAAATGGAGTCCTGCTCTTCAGATCCG

AACTGTA

>GT981650.1 JGCCJG2058C01.b Jatropha curcas L. germinating seeds (mixed stages) Jatropha curcas cDNA clone JGCCJG2058C01, mRNA sequence

GGCCGAATAGAGTAACGAGACAAGAAAGGACTCGGCCCCTAACTCCCAAGTCATCTAGTCGCTCCCCATTACACTCAAAC

GAAAGCATTGATAAAATCTTGCTATATCCGCATTTGAACTTGGGTGATCAATCACAATGCAGTCTGGTGAAGCCTCTTGT

TCAATGGAAGTAGAAATGGTTATAGATGAAACATCTTAGCTGGCGAAGCCAAAGTTTGAGCCTTTTGAAAGGCTCATGAG

ATGTCTG

>GW611587.1 Jc1-007-D07-M13F.D07.ab1 Jatropha curcas flower and seed Jatropha curcas cDNA, mRNA sequence

TAGCAGTACAAAGCAAAATAGAATCTCATTCAAGCTTCGAAAACCCTAACTTTCGAAGCTAAAACATTTCTATCCCTCAT

AAAATTCTTGTTTATTTATTTACTTTATTCACATAAAGATTCTTCTACTTGTCATATATCTAAAAATCTTCGAATCTCTT

TTTTTGCTTTTTAAGCAAAGATTTACATGGCAACTGCAGTATCTGTAACTTCAAGCTCTTCACAGCATCGTTTTGGACAA

GCTTCTTTCTCAATTCCAAAAGCCAATATCTCCATAACTACACCGATCATTAGCTTTCGTAGAAAGGGATTTGTTTCGAG

TAAAAAACAGAGGTTCAAGACATGCTCAGTGATGAAGAAGAAAACAGAGGATGCTGTTGTTGAAGTTATTATCGACGAAG

ATGATAAAGCACAAACCTTTGATGATGACATCAAGAAAAATCGACCGTTGATTTCACGTGTAGAAGAGAAACTTGCAAGA

AAGAAATCGGAGAGGTATACGTATCTTGTAGCTGCTATCTTGTCAAGTATTGGCATCACTTCAATGGCTGCCATGGCTGT

TTATTATAGATTTTCATGGCAAATGGAGGGTGGAGAAATTCCTGGCTTGGAAATGTTTGGTACTTTCGCACTTTCTGTGG

GGGCTGCAGTGGGTATGGAGTTTTGGGCAAGGTGGGCCCACAGAGCGTTATGGCATGCTTCGTTATGGCATATGCACGAG

TCTC

>GW877882.1 JC003484 Seed specific Normalized cDNA library from Jatropha curcas L. Jatropha curcas cDNA clone N06497 5' similar to Unknown protein, mRNA sequence

GGATTCCACAGCCTTAAAGGCGAAGGAATCGCTTCATTTTCGTCTCTTTGACTCTCTCTCTAAGCTTCTCTTCTCTTTGT

AAAATTAATCAACCGAAAAAGAAGATAATTTAGAGAAAAATGGGACTTTTATCTAATAAGATCGGAAGAGATGAGCTGAA

GCCAGGAGATCACGTATACTCTTGGAGGCTCGCTTATGTATACGCCCATCACGGGATATATGTTGGGGATGGAAAGGTTA

TCCACTTCACTCGTGGAGCAGGTCAGGAAATTGGGACAGGCACTGTGTTAGACCGTATTATTTTCAGCTCTTCTCCCTCT

CATCCTTCAGACAATCCATGCCCGAACTGCGGTGATCAATCAAGACTTGATGGTGTTATCTCATCCTGTATAGACTGTTT

CCTTGCGGGTGGGAATTTGTACCTCTTTGAATATGGTGTCTCTCCAGCCTTCTTTCTTGCCAAAGCTAGAGGAGGGACCT

GCACCCTTGCTGCTTCTGATCCACCCGAAGATGTCCTGCATCGGGCTTTTCTCCTACTGGAAAAAGGTTTTGGTGTCTAT

CACATCTTCAAGAACAACTGTGAGGATTTTGCAATATACTGTAAGACAGGCCTACTCATATTCACAAGCATCAGCGTTGG

TCGGAGTGGGCAGGCAGCATCCTTTTTAGCTGCTGCCAATGCTATTATTTCTTCACCACTTCGATTTTTAACGACCAGTG

TTAGTGGGTTGGCAGCTG

>GW875045.1 JC000436 Seed specific Normalized cDNA library from Jatropha curcas L. Jatropha curcas cDNA clone N00829 5' similar to Farnesylated protein, mRNA sequence

GGATGCCTGTCACACACTTCGTAGCTGGCGGTATATTTTTCACTTCTTAAGTTCTAAACTCAAAACCTAAAAGCGGAAAG

CTCTTCTTCTTATCTGCTTTTTATACACAACTCTTGGTTCTTGTGCCTTTGATTTGTATTGTAGTCATTTACATCAAAAT

CAAAATCAAAGCTTGTATTGAAGTAGAGGTAATCAATGGGTGCTCTCGATCATTTCTCTCATATCTTCGACTGCTCTCAT

GGCAGATCCAAGCTCAAGAAACGCAAGCAATTGCAGGTATATATGTGCTTTTGATTAGCTTCTTTCATTTTCCTCTTCTT

TTATAAAATATATATTCCTCTTTTTGGAGCTCAGATTAATTTTCTGCTCTTGGCTTTGTTCGTTCTTACCAAAAAGAAAG

AAAGAAATTTATGGTATCTCTCTCTCTTTTTTTTTTCTCAAAAATGCTTTTTTAAATATTCAAGTTCATCTCAGATCTAA

CCTATTTGCAGTTTGTTACTATTATCAAA

>FM892828.1 FM892828 Jatropha curcas embryo 56-70 (DAF) Jatropha curcas cDNA clone rjcaeb0_003483, mRNA sequence

ATTCATTCATACATCTCTTAGTTGAAACCATGGATCGCCATTTTGGCAATGTGTGTGAGCTAGACATCATGTTCCATTTA

GAAAAGGCGCATTTCATGTTGGAGGAGATGGTCATGAACGGTTGCATTGTTGAGACAAGCAAGTCTAACATCCTGGCGCC

AATTCAGCTGATGGACAAATCATCATGAAAAGAGTTGATTAAGCAGACTCCTGGATTTTCGATGGTTTTGCATTTTTTAT

GTTTCTTGTTGTCTATTGTAAAATAAGCTTATAAATTCACAAAATTTCTTATATCTTCCCTTGGTAAGTGGAAAAACTTG

GTATTAATGTTGTTCTGTTTTTATCTTGGTGGTTTAATAATTTTGTTGGTGATATTAATATAGCATTCCCAAGAATTTTG

TATTCAGATATCTATTTAGTAGAAGGTTATAAATTTAAATTCCCTT

>GW613806.1 Jc1-032-C03-M13F.C03.ab1 Jatropha curcas flower and seed Jatropha curcas cDNA, mRNA sequence

AGCCTCTGATCCAAATCTATCTTTTAATATATACCATGGCTTTGCAAAATACTGCGGCTCTACTAATCTTCACGGGAATC

ACCTTCTGTGCAGCCTCCGTGGGCCTCCAAAACTGTGTACACGGTAGGTGATTCTGACGGTTGGCATCTTAACGTTGACT

ACGATAGGTGGACTGCTGATAAGCATTTCTTCGTTGGTGACATTCTAATTTTTAACTACGACCAAGATTACCACAACGTC

CTGCTGGTGAAGAACCAAAATTTCTTATCATGCGATGGATCGTCTCCTAATTCAAGTTATGCCTCTGGTCATGACTCTAT

CACTCTTACAAGTGAAGGCGATTTCTTCTTCATATGCGGTTTCCCTGGCCATTGCGAAGCTGGTCAGAAGCTTTTTATCC

AGGTTGAGGATGCTGAAACTGTTTTGCCAAATAGTGAACCGTATCCTGATGAATGGTAGGCTCAAGTTCTCGCCTTCTCC

GGTCGGTGAATTAATATGCTTGGGAAATTAATAGATCCTAGTAGTAGAAACGAAGAATATGTCGAGTTGGTTATTTCATG

TCAGTTGTCACTGTAGCCTGCATAGGAAGAAATAAAAATA

>GW880030.1 JC007259 Seed specific Normalized cDNA library from Jatropha curcas L. Jatropha curcas cDNA clone N12215 5' similar to Unknown protein, mRNA sequence

ATCTCTTCATCATCACCAATCTCTCTCCCCCTATCTCTCTCGCTCTCATCCCCCATGAAGATAGGCGAGACTCTTGGGGG

ATAGAAGATAAAGACTTTTACCCAAAGAAGAACACAATGATTTCCCTTTAACTCTCTCTACTGAAGAAGAAGCTGTGCAA

AGGGAGCTTTCTCTTTTGAGGGGAATGTTGTCTGGCTCGAGGACTCTCTTTTGATCCATCTTTGTTGATGATTTTTCTTT

AATTATATCCATAGATCTAATAGGTGTATGGATTTAGAATTAGATTGATCATGTGATAGTGTCGTCGGACCAGGCTTCAT

TCCCCCCAATTAATGCTTCCATGGAGATCTGATTTTTCTTTTCAAGGGTTGCATGCAAAGGTTTGGGTTCTCTCAACGTT

TCTCTAATTTGTTTATATTTGTCTTCTTTTTGGGAAAACGCAGGCACTTTTAGATCGTGGGTTTTCGTTTCTAGGCTAAT

CAAGGTTGTTGGTCTTGTGCAGATTGGTGTAATGATGAAGTATTTTGACCAATCTACTTTGAATAAAAGTTTAATCATTA

CTTTCTTTTTATTTTCATTCCTGTTCGTGCGATGATGGTGTTTTTTTTTTTTCTCTTTTG

>FM889946.1 FM889946 Jatropha curcas embryo 35-55 (DAF) Jatropha curcas cDNA clone rjcfea0_003707, mRNA sequence

TGAAAAGAACTTATTCCCCTTCATATCTTATAGCAACTGAATTGTAAAATGTGGATGACTTCCATGTGGTTAGGCGAGCT

GGTGCTTGGATTTTCAACCATATGTGTCTCCTCATATGCATATTATGCTGTGAAATGATAACTGCAATTTTACCTTTCGG

TTTTGTTTGTCATAGTTTGTTTTTTGTTGCTATATTTCTTTCTTTAATTGACATTTTGTTACTC

>GW615666.1 Jc2-003-B01-M13F.B01.ab1 Jatropha curcas flower and seed Jatropha curcas cDNA, mRNA sequence

AATCTCAATTCCTCTCAAGTCCGAGTGGAAACAGCGATTGCAGCTTTCTTACTAGTATTTCTTTTTGTTCCCACTACCCT

CCATCTCCGACACGCCGACTCTTGGCCTTGGTTTCGGTTTCGTTTTCAACCATATTTCAAAAAACGCTCTCTCTTTGTCT

CTCTGTGTGAATTTTTGATCCATTGGCAATGGCGAGAGCAAGCAACAAGAACATACAAGCCAAGCTGCTTTTATCAGGTA

CTTCTTGGGGACATGGGGACTGGAAAGACAAGTTTGGTATTGAGATTTGTCAAAGGCCAATTTTTTGATTTCCAGGAATC

AACAATTGGAGCAGCCTTTTTCACTCAGGTTCTATCATTAAACGAGGCCACCATAAAATTTGATATATGGGACACAGCAG

GGCAGGAAAGATACCATAGTTTGGCACCCATGTACTATCGCGGTGCAGCTGCAGCTGTGGTGGTGTATGATATCACAAGC

ACGGAGTCATTTCAACGAGCCAAAAAGTGGGTTCTAGAGTTGCAAAGGCAAGGAAATCCAAATTTGATAATGTTCTTGGC

GGCCAACAAAGCT

>FM896837.1 FM896837 Jatropha curcas embryo 71-95 (DAF) Jatropha curcas cDNA clone rjcpga0_004243, mRNA sequence

GCAGAATAAAAACTGTAATAAAATTCGTTTCCTGAATAAAGGAGCACATAGTGAACTTACAATAATAAAGCCAGAAATAT

CTAGATTTAGAAATCTAAATTGAGCATTAAAAAATTGACTCAGTTTGTAACAACAGAAACATGAAAAGGTTTCCTGGATG

CAAGCTCACCAACACATTATAAGTTCAGTCAGATTACAAGGGAAAAGACCCTGACAAAGCGCAGAAGAAAGATGCCGTAT

CTTGACAGCATCCTGAAATTTGAAACTACGTACGAACCCTGCTGCTTTCTGCATTTCTAATTGGATAGAAGTAAGGATGG

GCCATTGCTTCTTTTGCAGTAGGCCTTTCTTGGTGATCATATCGCAGCAACTTGTCAAGGAAGTCAACAGCCTCAGGAAC

TGCCAAATGCTGATTGTCAACATTAATAAACTTTGTCCATGGTTTCCGGCTATGCCTGCCAACTAAAGCTGCAAGATGTG

GATCCAACTCTATGCGGTACTTGTTCAAATAAGCATTTAATTCATCTGTTCCAAGTACCTTGGCTATTTTGACCAGTTGA

TCATAATTATCATGCCCATAGAAGAACGGCTCCTTACGGAATAT

>GW618901.1 Jc2-040-D08-M13F.D08.ab1 Jatropha curcas flower and seed Jatropha curcas cDNA, mRNA sequence

AAAGGTTACGACGGTGCTAAGGTGGATGTTTGGTCATGCGGTGTCATCTTATATGTGCTCATCGCCGGTTATTTACCGTT

CAATGATACGAATCTCATGGTCTTGTATCGGAAGATTTATAGAGGTCAGTTCAAATTTCCGAGATGGATATCTCCCGATT

TACGACGATTTTTGTCTCGGCTACTCGACCCAAATCCCGAGACTAGAATTACCGTCGATGAGATTCTTCGGGACCCATGG

TTTATTAAGGATTACAAAGAGGCAAAGTTTCATTTGGAGGATTTTGATTCCAAGGTGCAAGACAATCACAAGCGTTTGAA

CGCTTTCGACATAATTTCATTTTCGTCCGGCTTTGATTTATCTTGTTTGTTCAATGACAACGATATCTCGGCCTGCAGTG

AACGGTTCGTTTCATCCAAATCGCCTGCAAAGATAATAGAGAGAGTAGAAGAAGTTTTCAAGGGCGAAAACGTAAGAGTA

ACGAAAAAGAAAAAGTGGGGAGTGAAATTGGAAGGATTGAATGGTTGTTTCGTGATGGCTATTGATATTTTTCCATTAAC

AGAACAACTTGTTGTTGTAGAAGTGACAGGGAAGGAAGCAAATGTAAATCCAGGCCATGAAATTTGGAAAGATATACTAT

GGCCACAGCTTGGTGGCTTGATATAT

>GW880076.1 JC000590 Seed specific Normalized cDNA library from Jatropha curcas L. Jatropha curcas cDNA clone N01117 5' similar to Unknown protein, mRNA sequence

GAAATTCGAACGACATTCACTTTCCCTCTAAAACGCTCCGTTTGTATTTCAAATCAAAGATTCAGATCTCTTTTTGCCTA

ATTTTAGATCAACAATGGATTCTACCAAGGAGAAGAAGGGTGCAGGAGGAAGAAGAGGAGGAGACAGAAAGAAGGCTGTT

TCAAAGTCGATTAGAGCTGGGCTTCAATTTCCAGTGGGTCGTATTAGCCGGTTTCTCAAAAAGGGCCGATATGCGCAGAG

ATATGGTGCCGGAGCTCCGATCTATCTTGCTGCCGTTCTCGAGTATCTCGCCGCCGAGGTGCTTGAGTTGGCTGGGAATG

CAGCAAGAGATAACAAGAAGAACAGGATAAATCCTAGGCATGTGCTGCTAGCGGTGAGAAATGATGAGGAGTTAGGCAAA

TTGCTTCAAGGGGTTACTATTGCAAGTGGTGGAGTGCTACCAAACATCAACCCTGTTCTGT

>GW611736.1 Jc1-009-A07-M13F.A07.ab1 Jatropha curcas flower and seed Jatropha curcas cDNA, mRNA sequence

CTTTGATGTTGGCCAAAGCTCTCAAGAATCCTATTTCCACACTGGAGATAGAGGCTACCATAAGTTATGGGACAGCAGGC

TCTTTAATTATGCTAATTGGGAAGTTCTTCGCTTCCTGCTGTCGAACCTGAGATGGTGGCTTGAGGAGTACAAATTCGAT

GGCTTTCGGTTTGATGGAGTAACTTCAATGCTGTACCATCACCATGGAATCGACATGGCATTTACTGGGAATTATAACGA

GTATTTCAGTGAAGCAACTGATGTTGATGCTGTTGTTTATTTGATGTTGGCCAATTCTCTGATTCACAACACCTTGCCAG

ATGCTACTGTGATTGCCGAAGACGTTTCGGGCATGCCTGGACTTGGTCATGATGTCTCTGAGGGGGGAATTGGTTTTGAC

TATCGCTTGGCAATGGCTATCCCTGACAAATGGATAGATTACTTGAAAAACAAGAGTGATGAAGAGTGGTCAATGGAGGA

AATTGCATGGAGCTTAACTAATAGGAGGTACACTGAGAAATGTGTTGCTTATGCTGAGAGTCATGACCAAGCCATTGTTG

GTGACAAGACAGTTGCCTTTTTGCTAATGGATAAAGAAATGTATTCTGGAATGTCTTGCTTGACAGATGCTCCACCTATC

ATTGAGCGAGGGATAGCACTTCACAAGATGGTACATTTTTTAACTATGGCATTAG

>FM888723.1 FM888723 Jatropha curcas embryo 35-55 (DAF) Jatropha curcas cDNA clone rjcfea0_002185, mRNA sequence

GTATAATATATATATATAGAGGTCTGTGCGGTAAACAGAACTGCTCCTATTACAATTTGCAAATGATCCAAGGACATGCA

GATACTGGACTGAAAGACAATAACCCACAAAGGAATTTAGAGAAAAAAAAAAAAAAGAAGCAACCAAGGGAGAACACCAA

TGGTTTCCACCAGCAAAGTTCGAGATACCAAGATTACTATTAACAAAATGATGAAACCAAATCCATTTGGTTCTCTCTCC

CCAGATACATTTCTACAAAACCAGGGGGGGCCCGGTAC

>FM894150.1 FM894150 Jatropha curcas embryo 71-95 (DAF) Jatropha curcas cDNA clone rjcpga0_004338, mRNA sequence

AGGGATTTATCTTTGCGCCGGTCTTCCCTCTCATTTCCAACCTCCTTGGCTATCTGATGAAGGATATCGATAATTTCAGA

GAAGTTGGGTCTTTGAGCTGGATCATGCTGCCAGCATCTCTCGAGCAGTTCCGCAAGTTTTGGATGAGCATGCTTGGGGA

TTGTAGGCCTTAGACCCTTTTGCACTACACCTACAGCTGCTTGCAACGGGGTCAAGTACGA

>GT981795.1 JGCCJG2060B07.b Jatropha curcas L. germinating seeds (mixed stages) Jatropha curcas cDNA clone JGCCJG2060B07 similar to NAP57 (ARABIDOPSIS THALIANA HOMOLOGUE OF NAP57), mRNA sequence

GTTTGGGACCGAGGGCTTCGATGAAGAAGAAGTTGATTTCCGAGGGGAAATTGGATAAGCACGGCAAGCCAAATGATAGT

ACCCCTCAAGAGTGGATGAGGAATTTGGTTTTGCCTACTGGAGGAGATTCTATAGTTGCTAGCCTTGCAGCTGCAAATGA

GCCAGCAAAGGATGTTGCCGCAGAGAAGAAAGCGAGTGTAGAAGTGGAGATGGAGAAGGAGAAGAAAAAGAAGAAGAAGA

GCAAGGATGGTGAAGATGGGGAAGGGCGCAAGCGCAAGCTGGATGAAGGCACTGACAGTCCAGGTGTTCAAGTTCCCGCT

AAGAAAGTTAAAGTTGAACAAGTTGAAGAGAAAGAGGCAGTTGAGTTGAAGAAGGTTAAGGGAGATGCTGTTCAGGAGAG

CAAAAAA

>GW875507.1 JC004643 Seed specific Normalized cDNA library from Jatropha curcas L. Jatropha curcas cDNA clone N08181 5' similar to Common plant regulatory factor CPRF-1, mRNA sequence

GACGACACGTGTCCGGTGACGTGGATGTTGCCTTGGCGCTCCTTTCTCGTTCTTCTTCTTCTTCAACCCTTCCCCTCTCT

CTTTCTCTCTCCTCAATTTCTCTCTGCAAAAACGAAACCGAACTTTTTTATTTTCCTTCTGTTTATAGAGAGAGAAAAAT

GCAGGAGTTCTGAACTTACTACTCAGGTTTCTATCAAGTAATTTAAGTTTTACTTTTAGCGAACATTTGAAGATAGTTGA

AGCATTGAAGTTTCCCTCGCTTATCTAATTTCGTATTTTCTGAAAGCCGAAGAGAAGCAGATTTGGGATTGGATAATTGA

AGTTGACTAAGTTTACTCAACAAAGAAAAGTGATAATTGTGCTTACTGTCCATATTATTTGAGGATTTCCTCCTATTGCT

TTACCATGGGAAACAACGAAGAGGGAAAGTCCTCTAAGCCTGATAAATCAGCTTCACCTGCACCAATGGATCAGACCAAT

ATTCATGTATATCCTGATTGGGCGGCCATGCAGGCATATTATGGCCCTCGAGTTGCTCTTCCACCATATTACAACTCGGC

AATGGCATCTGGTCACGCTCCCCATCCATACATGAGGGGCCCACCGCAGCCTATGATGCCACCTTATGGGACTCCTTATG

CGGCAGTCTACTCACATGGAGGAGTTTACGCGCATCCAGCAGTTCCAATTGG

>JK613102.1 JCF22-103 Jatropha curcas, immature Seed cDNA subtraction library Jatropha curcas cDNA similar to small rubber particle, mRNA sequence

GTTTTTTTTTTTTTTTTTTTTTTTTTTTTTTTTAAAAAAACTTCAAAAAATTTGATAACAACTGCAGAGGCGATGCAACT

TACAAAACAATTAAAAAAAAACCTTATATCAAAATTCAAAAGTTCCGTTGCTCAATCAAATTTCTAGCCCTTATAACTTA

TACGGAGTCTTTTTTTTTTTTTTTATTATTAAAAAAAAAAGAAAAAAAAGACCGAAAAACAAATAGCTTTCACTGGGCCG

GGATCCTTTCTCCACTGATTGAGCCGTTGGGCCCATTCACGCTTTCATCAAAAAGCTTAGTAATCCTATCGACTGGGATC

AACGGCAAATACGTCGCCGCTGTATATCCTCTATTTGCTGTATGACAAACGGCTTGATTGTATTTTTCAAACCAGTAAGC

GGCAGTAGGGACTACAATCTGTGCTGCTTGAGGAAACAGAGGAAGCCGATTCAATGACCTCCAGGCCAACACCGCATAAT

GCTCAGCCACCGGCTCGT

>GW877324.1 JC006846 Seed specific Normalized cDNA library from Jatropha curcas L. Jatropha curcas cDNA clone N11530 5' similar to Unknown protein, mRNA sequence

GACTGCATGAGCGGCCTTCTTTGTGGCTTCATTCCTCAACATATCTCTGAGCTGCCATTCTCATATCGTCGAAGCAGCAG

GTTAGTGGGTTTTGTGATCTCTTTACAAATTAGCTTAATCGGTTCTTTTTTTTTTCTTTGAAATCTGATTAGGTTTTGTA

CCCTCTCTTCTGTTCATCAGTCTTATGCTGGAACGATGTGATTGCTAGCAGAAATTTGTTCATTTTTGTTTTTATTTGAA

GTTTTTATTCTTGAAGATTATTGTTTTATCCATCGCAATACACTAGTAAATAAGCGTATTTATTACTAGTTGTATTGTTT

TGCCGATGAATCACATAGGCAGCTGCCTTTTCCTTTTTCTTATTTCTTTTTCCTTCTTTTAGCAATTTTGTTATCTGTTC

AGGAGGGAAGTGTGCTTCACCTTTTATGTAGTTGTGATTCATTTGGTGGAGGCTCTGAAGATGTTTTCCTTTCAGTTTTG

GTTGAAGAAGCTTCCATAAATGTAGAGTGTTGTTAGCATGACACCTAAACAGTTATTGAGATCTTTAAGTCTG

>GT973541.1 GJCCJC2049F05.b Jatropha curcas L. developing seeds (mixed stages) Jatropha curcas cDNA clone GJCCJC2049F05 similar to unknown protein, mRNA sequence

ACTCCTCATACTATGAGCATAGTATGCTGGCGGTCGGGCAAATGTGCCCCCATCGTCTAGTGGTTCAGGACATCTCTCTT

TCAAGGAGGCAGCGGGGATTCGACTTCCCCTGGGGGTAGGGTATTACGAAAGGAAGTTGATCATGGATTATTAATAAGTC

TGGAATTGATTCTTCCTGGGTCGATGCCCGAGCGGTTAATGGGGACGGACTGTAAATTCGTTGGCAATATGTCTACGCTG

GTTCAAATCCAGCTCGGCCCAATAATTCACTGATCCGCCATGAAATGATATAACCCCCTTGTTCTCTCGAAATACCTGAT

ACAGAAAAAAATAGAAATTTCTGATATATTTCTACCTGTTCTTTATTTGA

>GW875486.1 JC004622 Seed specific Normalized cDNA library from Jatropha curcas L. Jatropha curcas cDNA clone N08148 5' similar to N6-DNA-methyltransferase, mRNA sequence

GGACTAAAGTCAAACGTATTTCTCCTCCCTCCCTTCCCCGCGCCGCTCGTTTTCTGCACTTCTACAGCCACAGTCTTTCT

TTTTACCGTCCCGTTGGTTTCGTTCTTGGCCATCACTGCCGCAGCACTCGTGCTCTTCAAGCGAAGGGAGAAGGCGAAAC

TTTACTTTCAAGTCTGTCCCAAGCGCTCTTCAGTCACAGAGGATAATAGAGACTTGAGAGAGGAAGGATTTGTGGGCATT

AGAAAAGCTAAGGCTTTGGTGGAGAGCAATTGTTGCCAGAGGTTGCATCAATCTGGCACATAAAAATGTCCTTTAGAGTA

GCACAAATTCGCCTTGTAAGTTCACATCCTGAGGTTTATGAGCCATGTGATGATTCATTTGCACTGGTTGACGCACTTCT

CGCTGATCGCACAAAATTGTTAGAGCATAATCCAAGAATATGTATGGAAGTGGGTTGCGGCAGTGGTTATGTTATTACGT

CTCTAGCTCTCATGCTTGGGCATCAGATTCCTGGGGTTTACTACATTGCCACTGACCTCAATCCTCATGCGGTCAGAGTG

ACTTATGAAACATTAGAAGCTCACAATGTTCATGCAGAGTTGATAGTGACTGACATTGCATCTGGTCTAGAAAAGCGTCT

GG

>FM888784.1 FM888784 Jatropha curcas embryo 35-55 (DAF) Jatropha curcas cDNA clone rjcfea0_002260, mRNA sequence

TTTTTAAATAAATGTTGAATTTATATATATCTTCCAAATTAATCTATATACATACAATATGTTAGAGGCTCTCTAAAAAC

AGAGCCATCTAATTGAAATTGAATGATACAACACATTTATACTTCATAGTTATGTTTGCTGTCAGAAATAACAAAGCAAA

TGTCAAAATCCTGAAAAAGGTGCGATGCTGTTCTAATATCAGCACAAGTCATCTCTGGATAATTAATGTTTCCAGAGCTG

AGAATGCTCTTATTTCTTTTTCTTGTTGTCCCATGCTTATTGCTGTTTCTAAAGGAGAAAACCAAGATCAGGCGCGATCC

TGTGATCTTGCAGCAAATCGCGAGCATTTCTTCCGATTAGTTTTGCTAAGTGCACAGACACAAGT

>GT971282.1 GJCCJC2018H06.b Jatropha curcas L. developing seeds (mixed stages) Jatropha curcas cDNA clone GJCCJC2018H06 similar to EMB1674 (EMBRYO DEFECTIVE 1674), mRNA sequence

GCTGTAAAGATAATGAAAATGGAGAAGATAGCTAGCAATTGTAGAAACCCAAAAGCCTCGTCGTTTCCAGTTCCATAGCT

GATAGAAAACCCCGTCATTCCTGCCTCTTTTTTGCCCCTCAAATCTGTATTACGGAAAGATTGGGGTGGGAATACGAACA

AAAACAAAGGGATTGCTGTTGCTGGGTTTGCTTTAAGCGACTTAGGAGCAAGACTCTTTTCCTCTGCAACAATATCCAGG

AGAATCAATGCCACCACTCTTGAAACCAGGGATGGAATCACAATTGCACTAAACGGCTTCATAAATAGGTCCCGAACACT

TCAAAGTGGCTTTCCAGTTCTGGTTTGCAATCATTTCCAGATTGGGTTTCCATTTGACTGGGAAGGGTGCCTTTCAAAAT

TTTATGTGGAAGAATCTGCTAAAAGAGATTCTACAAGTAGA

>GW612521.1 Jc1-017-F07-M13F.F07.ab1 Jatropha curcas flower and seed Jatropha curcas cDNA, mRNA sequence

CAAATTAAATTTTCGACTCTGCAAAACGGCCTCTGAAGCTCGACAATGTTCTATGATAAACATCCAAAGAAGGGATAAAT

GAAAGACAAAATACAAATTCTTTTATTTTTGGCAACAGAGGAACGATGAGAAAGAAAAACTAAACTGCGTATAACCATTA

TCAATTTTTTATTATCTAATAGGAGATTGGAATTTGTCATCTCTTTTCCTGGTAGAAAGAAGAATCTCGTGAATCTTTTT

CCTCCTACTGTAGAACCCCTTTTGAGTAAATCTTGACTCTCTCTTTAATCTCTCTCTACTTCGAGTTGTCTCAGTTCAAT

TCTGTGATCTCGAAAAAAGATACTTTTCTTGATCCAAACAATACGGAGATGTCTAGATATATGTCTGCTTTATATGAAAA

CAATATGAAGTCTACTGGTGAAGATTTGGTAACTGCTATAGTGCCTTTGATGAAACTTATCTGCCTTACAGTTATTGGAT

TGATTCTTGCACACCCAAAAACCCAAATCACCCCCAGAGCAACATTTAGACTTCTAAGCAAGCTAGTTTTTGCCCTATTC

TTGCCCTGCTTAATATTTACTGAGCTTGGTGAAAGCATTACGCTCCAGAACATTGCTCTTTGGTGGTTTATCCCTGTCAA

TGTATTGGTTAGTACATTGATTGGTTTCTTGCTTGGGCTGTTAGTTGTGGCTATATGTCGCCCCCCGCCTGAATTTAATA

GATTCACTATTATCATGACTGC

>FM895988.1 FM895988 Jatropha curcas embryo 71-95 (DAF) Jatropha curcas cDNA clone rjcpga0_003111, mRNA sequence

GTGTGAATGCTAGAACTCATTTCCCATTATTTGAAAATTACATATACATTTGGGGCACCACGGTAGTCATATTCAAACAA

TTGCCTGGCCTAGGAAAATGCAGTTTACATTAAGGGGAAAAAAATTAATGGGATAAAGAATAAAGGAAACAATTAACTTA

TGCCACTTTAACTCCACTTGGGCGTTTTCCTTCCATGGCGTCCTTCTTATTGCGGCAATCAGCACATAGGAAAGGCCCTT

GCCATGGTTTTGAGCTGGTGGAGAAGATTGAACCAGCATGAACAGAGATGCCCTCGCTTGGTGCAAGGTCAACTTGACAT

ACAGCACATGTGAAAAGACCAGATGTTGATTGTGTTGCGGTGTCATCGTTCCCCAGTCTTTCAGCAAGCATTGCTGAGCC

TTCTTCAAACAATTCTTCCACTATACCTACGGCAGAAAGCTTTTTTGATAGCTTATTAGTAGAATCATGTGATTTCTTCC

TGAATAATAAGGCTGTAAGTTTGTTCTGCTTCTTGGGAGGAGTTGATGGCTGTACTGAATTATCAGGAGGAATTATGTCA

ACAAT

>FM888886.1 FM888886 Jatropha curcas embryo 35-55 (DAF) Jatropha curcas cDNA clone rjcfea0_002396, mRNA sequence

CATATTTATTTGCAGATGGGACTGTGCACAACTTGAATGAATACATGGAAATCCACGCCTCCGATGAGTTGACAAATCTG

GGAGCTGATTCGATCAATTTAGTTTTCACTCAAAAACATGGTGTGGTGATAGGTGAAAGCCGACTCGAGGAATTCTTTTC

GCAAATATCATCTCAAATATCTTGACCTTCGGTTGCAGTCAATTACATCGATAGCCTAAGGATTGCATGTGTAAGCTGTC

ATTAGCTTCGAAAATCAGATCTGTTACTTTTGAGACAGAATAAGCTGTTTTTACCAAAGGTGCATGGAGTAAATATTTCT

GCTAAGTTTTGTATGTATGAGGC

>FM894094.1 FM894094 Jatropha curcas embryo 56-70 (DAF) Jatropha curcas cDNA clone rjcaeb0_005070, mRNA sequence

GGAAAAGTAAAATGTATAATATATATAAAAACAATCTGTTGTGAATCTATGAGAGCAACCTACATTGTGAACACATAACC

AGATGAACTGTCTACAATATAGTTAAATACCTTTTCAACTCAGCATTAAATGACAAAGATTCGAAAAACCAGCCTCTCTA

TAGCAAGTCTACTGATTCTTCTTTAGTGTAGAAGTTGAGGATCGACGATCTTGGTTAGAAGTTCTGATAATAAAAAGCAA

AGTTTCTACAAGCATTCCACAAACTAGACCAAGAATCCCTCCAGCAGCACTCATTCCAAGATTGTGGCCAAACAATGCCC

TAAATGTTGCATATCCCACCAAGTAGCCAGTAAACATGGTTAGAGCAACATGTAAACCAAAACCAAGCTGATCCTTGTAA

GAGGAGAAAGGCTCAGTCTCGTCGTTCCTTGGTGTAATATCCATAACAAGCTTCTGGTATGCATTCCTCTCTGCCAATTC

CTCAAGTTTCCTCAGTCTTGCCTTTAACGCTTCACTCTTTTCTCTAGGTTTAGGACTAGTAAAGAGGAATTCAGATCCTG

AGAAGAGGCTGGCCAAATCAGGTCGGTTGGAAGGCTGGCATGCTATCCATAAGCTACGTAGAGACTTGTAGTGTATATTA

TTTTGAGTGAGGAGAGTGGAAGCAAGGGCTCGAAGGTTCTG

>GW620035.1 Jc2-054-A04-M13F.A04.ab1 Jatropha curcas flower and seed Jatropha curcas cDNA, mRNA sequence

TATACTATCAAAAAAAGCCATGGATTGGTTCTCTTGGCTATCAAAAACTGGCCTAGAGCCTTCTCTTGTATATGAATATG

GTCTTGCCTTTTCTCATAATGAGCTTGAAGAAGAAGATATTGCTTATTTTAACCATGAATTTCTTCAAAGCATGGGGATA

TCAATAGCAAAACATAGGCTAGAGATTCTCAAACTTGCAAGAAAAGAAAAGGGAGCTACCCCTCATCCAATGGCAAGAGT

TCTTGTTGCCATTAAAAGGACAAAGATGCGTTTAGCTAAGTATATAAGAGCACTGGCTCACCGTGATGAATCTGCTCTTG

TTGTTGTTCCTAGGCCTGCAGGTTATGCTACTAGATGGAGAGGAGCAATGTTGAAAAGAAACAAGAAACTGATGATGGGT

AAGCAAGGGAGGTTATTGCTTACAAATGGAAGTCCTATGGTGGTTTCTGGTCCTAGACTTGATAGTTTTTCAAGTCCTGT

GGTATATGATCTTCATAAAGAAGAAAAGATTGATGAAGGTGATGATGATGGGTATTGGTCAACTGGTGTTGAAGAGATCA

GGTGGGATACTATGTTCCAGAATCTCAAACCAACTTGAATTGTTA

>JK611657.1 JCF4604 Jatropha curcas, immature Seed cDNA subtraction library Jatropha curcas cDNA similar to glutamyl-trna amidotransferase subunit, mRNA sequence

TTCGAGGGGCCGCCCGGGCGGGTACCTTCACATGGAGATTTTTAAAAAAGTTGATGTCATAGTCACGCCAACAACTGGCA

TGACAGCACCCAAAATACCTCCTAGCTCTCTTAAGTATGGGGAGACTGATATGCAGGTTACTGGTTACCTCATGCGATTT

GTTGTTGAACCGAATCTTCTTGGTCTTCCTGCCATTTCTGTTCCTGTTGGTTATGATAAACAAGGGCTTCCTATAGGTTT

GCAAATAATCGGTCGTCCATGGGCGGAAGCAACAATTTTGCGTTTAGCTTCTGCTCTTCAGGAATTATGTTCAAAGCCAA

TTAAGCACCCTGCATCATTTTATGATGTTTTGAAGAAAAAGTGAATCGTTCTATGTGGCTTACCTGAAACCCCTTCGGGA

AATCTGGCTTAACTTTTAATGTTTGAAACATAAGAATTTGTAATCAAGCTTCATAAATGGGT

>GW878560.1 JC002159 Seed specific Normalized cDNA library from Jatropha curcas L. Jatropha curcas cDNA clone N03886 5' similar to Unknown protein, mRNA sequence

GGTTTTGATTCTGCTTCGTTTAATACGGTGCGTTTCATTCCAAAAATTCCATTTCCGGCTCCTATAAAGCCACGTGTTGC

CACGTCAGATATTTCGCGACGGCTGTTCTTTTCTTTCCTTTTACCTTCCATTTGGAGAAAATCATTTCCCAAAAATGTGC

GGCGACTGTTAAAACGCATGACTCTCTGCCGTTTACGTATATATCCTTTCCTCCTCGTCCTCCAAAAAATCGTCGAATCG

CTTCTTATTCTCCACCTGTCGGAGAAACATTGGAGGGGAGAGATGGAAGGCGACGAGAAGGTGAAAAACGACGCGTTGCA

GATAATCGGGCAATGCGAGGACTTGCCTCGCTTGGTCGTCTTCGATCTCGATTACACTCTATGGCCTTTTTACTGTGACT

GCTACTATGAAGACGATACGCCATATCTGTATCCACAAGCTAAAGGTATATTAGAAGCGTTGAAGGAGAAAGGTATTGAG

GTTGCTATTGCTTCAAGATCGCCAACTTCTCAGATTGCCAAATCGTTTCTTGCCAAATTGGAATTAACTCCATGTTTGTA

GCTCAGGAAATTTTCTCCAGCTGGACTCATAAAACTGAACATTTTCAGAGGATCCATAGGAAGACTGGAGTGCCTTTCAG

TTCAATGCTCTTCTTTGATGAAGAGGAAAGGAACATCCAAGCTACATCAAAATGGGTGTAACTAGTATCTTAGTTGGCAA

TGGGGTGAGTCTGAGCTTTG

>GW878621.1 JC002386 Seed specific Normalized cDNA library from Jatropha curcas L. Jatropha curcas cDNA clone N04424 5' similar to Glycosyltransferase, mRNA sequence

GGCTCCCGCTAACTCCTCTTCTCCATATATATACTAGTTTTCTTTTGTTCTCTTTTCCATATCTCAATTAACTGTTCTTC

CAAACCCAGAATCCAGGTTTTCTATGATTCTATCGAATTTTGATGAAATAAAAAGTAAAATACCCTTTGTATCAGCTGGG

TAATTCAAGAATCTATCAAAGTAATATACCTTAGACACTGACTTTTTTTGCTTATCTGAACCACATTCTACTTTTTCCAA

AACGTTCATCATTAAACCACTCTTCTAGTTTGGATCTCTTCTTTGATTCAAGGTTTGAGTTTTCTCTTTGCCTTTTCTTT

GTTGTTGAATTCACTTCCAATCTACAGTCTCTACCCTTGCATTTACTAGCGAATTGGTTGTGCCATTAATGAGAAATTAA

TGGGTTTGAGATTGGGAGGGAAGATGACAGAGGGACTTGTTTGATTACTTGGGTTAGAAGAAAGAGAAACCTTGAAGGCC

TCCGGGGACAGCCTCCTATTGGATTATGAGTTTAATGTGAGAATATTCATTTCTCCTGTTGCCAGTGATGTGAAGAATGA

GGCAGAGGCAAAGTGCAGCTAAATCGCCGTCCTCTGAAGTCAAATCTCAGAGTACCAATCTCTCCAAACGAGAAGGATTA

AGCAATGAAAACATATTTCAAAAATCTAACACCTTCTTGTCTCCAAAGAACATTTTTGCATTCTGTCTCGCATTTCGATT

GGCAAATTCTCTGCTGAAACAAACGTATTTTAATCCGG

>JK610319.1 JCF755 Jatropha curcas, immature Seed cDNA subtraction library Jatropha curcas cDNA similar to map3k delta-1 protein, mRNA sequence

ACTAAGATGTTTCAGTTACTAATTGCTAGTCTCAACTATGTCTCCGAAAACAGCTCTCTATTACTCGAAGCATTCCATCC

ATATTATTACGTGGCTTGGTGGAAGCACTATTATTGTCCACCCATTTGTATCAACTGAATTGGAGACTTCTGCAGCTTCT

TCAATGTCTCTACTATCTTACCAAAAGTCGGGCGCTGAGCTGGATCATCAGCCCAGCAAGATTCCATAAGAGAAGTCAGC

GCTGGGGAGGTATTTGGTGGTATGGTAAGCCTTCTGTTCTGGAAAGCTACAGCTCCAACCACCTGTGCAGGACTAAGTCC

ATTCCAAGGCTGTTGCATGGTAACAAGCTCCCATAAGATGACTCCAAAACTATAGACATCAGATTTCTCATTTGAGGGCT

CTCCACGGAGAAACTCTGGAGCCATCCACTCAGGTGTTCCTGCTACTGATTTTGATGATATGAATGTGTTTGCTTTAAAA

CCTAGACAGCCCCAAAATCACACACCCTTCACTGGTCCAATTTTTATCCACCAAACAAATTTTGGGAGATTTAAAGGTCC

CCAATGAACCAACCGTGAGGGTTCAGACAATGAAAGGTAAATTTGA

>JK317866.1 JCST637 Jatropha curcas L. seed cDNA library Jatropha curcas cDNA 5', mRNA sequence

ATTTTTTTTTTTTCTGGTTAAATTGGGGACTTATAAACAATGAAGCGTTTTGTTGTTGTTTTGTTCTTCTGGTTATCAGC

TAGCTGTTTGATGACCTCAGCAAGTGTGGTTTTGATTGGAAGCAACGTTACTTTGTCTGTCGATGATGTTGAAGCTAACT

TCGCTCCAGCAGTGAAGAGCTCAGGTGAATGTGGGTCATTGTCTTTGGCAGAGCCCCTTGATGCGTGTTCAGAATTGTCG

AATAAAGTTGAAAAAACTTCAAATTTTAGTTCCCCCTATGTGTTAATTATAAGAGGAGGTTGTAGCTTTGAGGAGAAAGT

TAGAAGGGCACAGAAGGCTGGTTTCAAAGCTGCAATTGTCTATGACGATGAAGATGATGGCATCTTGGTTTCAATGGCAG

GGAATTCGGCTGGTATAAAAATACATGCCGTATTTATATCTAAATCTTCAGGTGAAAAGCTCAAGAATTATGCTGGGTTA

AGTGGCATGGAGGTGTGGTTAATCCCAAGTTTTGAAAATTCAGCATGGTCTATCATGGCAATCTCTTTTATTTCTCTTCT

GGCCATGTCTGCTGTGCTCGCTACCTGCTTCTTTGTTCGTAGGCATCGTATAAGAAGAGAGCGGCCTCATTCTTCTCACG

TTAGAGAGTTCCATGGGATGAGCAGCCGCTTGGTGAAAGCAATGCCAAGCGTAATATTCACTGCTGTCCTAGAGGATAAC

TGTACTTCAAGAACTTGTGCCATATGCCTTGAAGACCATAGTGTTGGAGAAAGCTCAGATCCTTCCATGTCGGCAACAAA

TTCCATGCTTTCTGCGTGGACTCTGCTTACTTCATGAGACTTT

>GW879509.1 JC004068 Seed specific Normalized cDNA library from Jatropha curcas L. Jatropha curcas cDNA clone N07373 5' similar to Beta-Mannosidase, mRNA sequence

GGATCTGACTCTTTGCATCGTCGTCTGTAGTGGACTTCTGTCTTACTGCTACAAATACTTCTAGTTACTCGGTCATTTTT

GTTTTCTAGTCGCTTTTTAGATTGAATTTGGAAAATTTCTGAGTTATGGCGGAGATAGGGAAAATTGTGCTTGACTCAGA

TTGGTTAGCGGTGAGATCGACGGAGGTGCAACTCAACGGGACGCAACTCACCACCACTCACCCTCCTACTGGTCCCAACT

CGCCATGGATACCAGCTGCTGTTCCCGGAACTGTTTGGGGAACTCTTGTAAAGAGCAATGTGGTGCCTGATCCTTTCTAC

GGTTTGAATAACGAAGCAATTTTGGATATAGCTGTTTCTGGAAGGGAGTACTATACATTTTGGTTCTTCACTACTTTTGA

GCATAAGCTGTTAAGGAATCAACACCTGGAGCTCAATTTTCGTGCAACCAATTACTCTGCTGAAGTGTATTTAAATGGAC

ACCAGATGGTGTTGCCAAAAGGGACGTTTCGAAGGCATTCTCTTGATGTTACTAGTCTTCTAAATCCTGGAGGGCGGAAT

TTACTG

>GW880457.1 JC000991 Seed specific Normalized cDNA library from Jatropha curcas L. Jatropha curcas cDNA clone N01923 5' similar to Unknown protein, mRNA sequence

CTGCCCCGATCTCCTCTTTCCTCCCACTTTCGAAACCTAATGTGCCGGTGCCATAATCATCTATTCCTTCTTTCACCACC

GCATCCTTAATCGCTTCTCCATTGACATAAACGAGCGATCGCCGACGACAGTACGCCGATCGGCGCAGGCATAATCGACC

ATTTCTACAAAAGCTCAGATCGAGAAACCAAAAGCCTACTGTTGGAATAAGCTTGATTCTGGTTAGGATGTGTGCTGTCA

GACTGGGTACTGGACTGGAGTAAAGCTGTTCTTGAAAACTGTTATTTGCGTAGGTATTCTCACAGTTTAAGACTTTTTCT

AAACATTATGGCTGTTCACTCTAGATTTCGGCTTATCTGTCATTCGCAAGAGCTTGAAGATGGACAGCCAGTATATGTTT

CTTCAAATTCCCTTCCAATTAAGGCTTTAAAGCTTGAGCCTGCTGGACATGCATTCCATGCTGCTGCTTTGAAACTCCTT

GGCTGTGAGGAGGAAGATGCAGAGAGTGCAGACCAGAAAGTTCCCAATGAAAAGGAGCAGTCATATATGCCATCATCTGA

TTCTTATAGCAGTAAAGGTAAAAAGAAATCTGGTACAGGAGACAGCAACAAGATCACTATGCCCTGTTAGGTTTGAGCCA

TTT

>FM895864.1 FM895864 Jatropha curcas embryo 71-95 (DAF) Jatropha curcas cDNA clone rjcpga0_002860, mRNA sequence

ATTCATTTTAATTCTTGGTGGGTTATCTTTCCCTAGATTAAACCCTTCACCTAGTGGTTTATACAATCAATTCTCTCCTT

TTGGTGATATTTCTGCTAGTTCTGCAACTGCTGGTAGTGTTACTAGTCCTTGTTTCAGTCTGGACCCATCTGGAAGCTCT

TCTGGTACTTTGATGGGGCTTAACTATCCGATTTCAAGTGGCTTTACCGGTGCAATTCAAGAAATGGGCGGTGGCGGTGT

TGGTGGCGGAAATGGCGGTTCGATGAATGTTCATACTAACCTTGCATCATCAATTGAGTCTTTGAGTTCTATAAATCAAG

ATTTGCACTGGAAATTGCAGCAACAACGATTGGCTATGCTTTTTGGAGGAGAAGACCAGAAAGATAATAATAGTGTTTCT

TCGGTTCCTATTGAAAACCAAGTGCAAAAGCCTCAACCCATTTTGTTTCAAAATCTTGAAATTTCAAAACCAGAAGTAAA

TGTTGCTGGAAATTCAAGAAGATTAGGTGATACAACCACCGAATGGT

>GW618834.1 Jc2-039-F08-M13F.F08.ab1 Jatropha curcas flower and seed Jatropha curcas cDNA, mRNA sequence

GCATCCGGATTAGGAAAGTCATATCTCCAGAGGTTTTTTGAATGTGAATTCTATCATAATGAAGAAAAAGGATTTGTCAC

AAAACTAGTAAGGAATTATAGATGCCACCCTGCAATTCTACACCTCCCTTCTAAGCTTTTCTACAAAGGAGAGTTGCTCG

CATGTAAAGAAGATACTTGTCCCTCGATAGGTTTTGACGTGGATTTTCTCCCTAGAAAAGAATTTCCTGTTCTATTCATT

GGTATTCAAGGCTGTGATGAGAGAGAAGGCAATAACCCTTCATGGTTTAACCGAATTGAGGCGAGCAAGGTTGTTGAGAT

TGTAAACAAGTTAATAGACAATACAGATCTAAATGAGACAGATATTGGGGTAATAACACCTTATAGGCAGCAGGTCCTCA

AGATCAAAAAAGCACTTGAAAGTTGGGAAATGTCAGATGTTAAAGTTGGGAGTGTAGAACAATTTCAGGGACAAGAAAGA

GAGGTCATTATTATATCTTCTGTGAGATCAACTGTTAAACACAATGACTTTGACAGAACCTACTCTCTGGGTTTCCTGAG

CAACCCAAAGAGATTTAACGTTGCAATTACCCGTGCCAGATCCTTGCTTATCATTGTTGGGAATCCACACATTATCAGTA

AGGATCCTTGCTGGGAGAAGCTTTTGTG

>GT970231.1 GJCCJC2009F12.b Jatropha curcas L. developing seeds (mixed stages) Jatropha curcas cDNA clone GJCCJC2009F12 similar to phosphoesterase family protein, mRNA sequence

GATAGCAACTGTCATAAAAACACAAACACCATCACCATCACCATCTTCATCATCATCCTCACAAATGGTAACCAGACAAT

CTATCATTGTTCTGCTTATAATTCTGCTTAATATCCATAGCCAAACCATCCATGCAAACCCAATAAAAACCGTAGTTGTG

CTTGTAATGGAAAATCGTTCATTTGATCACATGCTTGGCTGGATGAAGAAATTCAATCCAGAGATCAATGGTGTTGATGG

AAGTGAATCAAACCCATTAAACGCAAGTGATCCGAATTCTAATAAAGTCTTCTTCCAGAATCAAGCTCAGTATGTTGATC

CTGATCCTGGTCATTCTTTTCAGGCTATCAGGGAGCAAATTTTTGGGTCTAACGATACCTCAAAGCTTCCTCTAATGAAT

GGTTTTGCTCAACAAGCTCTTTCTATGGACCCTTCAGCTGCCATGTCTAGAGATGTCATGAATGGGTTTGATCCTGACAT

GGTCGCTGTTTACAAGACTCTTGTTTCAGAATTTGCTGTCTTTGACAGGTGGTTTGCCTCTGTACCATCATCAACACAGC

CCAACAGACTCTACGTACATTCAGGGACTTCAGCCGGAGCCACCAGCAACGTTCCGGCCTTACTCATAAAGGGTTACCCA

CAGAGAACCATTTTTGAGAATCTTGATGATGCCAAGATATCATGGGGAATATACTTCCAGAATATCCGGCCACCTTGTTT

TTACAGAATCTGAAGAAACTTAATACA

>GW880057.1 JC000571 Seed specific Normalized cDNA library from Jatropha curcas L. Jatropha curcas cDNA clone N01086 5' similar to Unknown protein, mRNA sequence

GGGGGTTCCGTCTTTGTAGTTATGAATACTTAGACTCCTGGTAGTGGTTTGGCCAATTCAGAGGAGGTCAAAACGGACCG

CAACACTGCAAGCCATATTCCTCCTTCAGAAATGCAACAATTTCAAAAGTCCCTCCTATTTTTCTGTATTAATTAGAAGT

CGTTCATATTTGTTCTTAGATGTGTGGCGTTTCAGTGCTTGCGTGTCTTTACCTCTACATTCCTAAGGAGTTTCCATAAG

TATTTTCTTGGATCCTTGTTTTTGCTTTGTCCTTTAACAGGAATATGAAAGAGAACATTACTGAAATGCAAAATAAAAAT

AAGAAGATGCTCTTTGTATCCAGGAAAAAGAAACACAGAAGATTCTTTAGATCTTGCCATCTGCGTTTTGCAATCTTTAG

CTATAAAGATAATTATGTGGCAACTGCCGACTTATTGATGATTATACTTTATATTCCCT

>GW613990.1 Jc1-034-D09-M13F.D09.ab1 Jatropha curcas flower and seed Jatropha curcas cDNA, mRNA sequence

AGGTTTCAACATGGGAACAAAATGCAATGCTCTTTTGATCACTCTTCTACTTTTAATTCCTGTGGCTCTTTCTGTACACA

ATGTTGGGTTAATCAGAATTGGATTAAAAAAGCGGAAAATGGATCAAGTCAATCGTCCTGCTGGAACCATTGACTCCAAA

GAAGGGGAATCAATGAGAACAGCAACCAAAAAGTACCATTTTCGTAATGATCTCAGGGACTCTGGGGATGCTGAATTTGT

TACACTAAAAAACTACTTAGATGCTCAGTATTTTGGTGAAATTGGTATTGGCACACCTTCCCAGACTTTCACTGTGATAT

TTGACACGGGAAGTTCTAATCTCTGGGTACCCTCTTCAAAATGTTATTTCTCGCTTGCTTGCTACTTCCATTCCAGGTAT

AAGTCCAGCGAATCAAGTACCTATGAGAAGAACGGTACATCTGCTGCTATCCGATATGGCACTGGTTCAATTGCCGGTTT

CTTTAGCCAGGACAATGTCCAAGTTGGTGATCTTGTTATTAGGGATCAGGATTTCATCGAGGCAACAAAAGAGCCAGGCA

TCATATTCATGGTTGCCAAGTTTGATGGTATATTTGGCCTTGGCTTCCAGGAAATCTCAGTTGGGAATGCTGTTCCTGTT

TGGTATAATATGGTTAATCAAGGTCTTGTTAAGGAACAAGTTTTTTCCTTTTGGC

>JK611284.1 JCF3851 Jatropha curcas, immature Seed cDNA subtraction library Jatropha curcas cDNA similar to alpha-expansin 6, mRNA sequence

ACTGAGCAATTCGTTGGAAGATAGGCTGAGAGAGATCGAAATGATGCTGGGGAGGGTTGCACCAGCCCCCAGCATTATTA

GGGAGAGCATTATTGGACACTCATATTTTAACCTAGTCTTGATCACCAACGTTGGTGGTGCAGGAGATGTTCAGGCAGTT

TCAGTCAAAGGTTCAAGAACTGATTGGCAACCCATGTCAAGAAACTGGGGTCAAAATTGGCAAAGCAATTCATATCTTAA

TGGTCAACGCCTGTCATTTAAGGTCACAACTAGTGATGGAAGGACTGTCGTCTCTAACAATGTTGTTCCTGCTGGATGGT

CCTTTGGTCAAACCTTCACTGGTTTACAATTCTGATTAAAAAATTAAAACTAATCTATAAATGATAAATAATTAGTTTAA

TTTAGGTAAAAAAAACAAAAAGGGAAATGAGCTCCGTTTTAAATTAGCACATTTTTCAAATTGATATTTTTAGATTTTGA

ACCTTGTTTGGAGGGTTAGGGTTCTGTTTATGTTTCTGCTAGCTTTTTGAAAAAGGGCAGAACTGGGAAATTTTGGGCGG

GAGGTGGGGTAATTTAATAGATTGGAATATATAAATTTTTAAAAAACCCAATTAATTTAATTTAGCAGAAATAAGTCCGA

AATAAAAATTTCCGCGGTCAAATATATCCAGAATTTTGGTAAATTTTTTACACCCCCAGCCGTCCTTTTTTTTTTCCTTT

CCAAGTAAATCCCGGGTAATTGGATTAAGTAGGAGAAAAAAAGCAAACTTTGCCTTAAGCCATGAAACTCTATGGATACA

CCACGCCTTTTAGCTCGGAGTATTGGGGCGACGCAAC

>JK610729.1 JCF2131 Jatropha curcas, immature Seed cDNA subtraction library Jatropha curcas cDNA similar to atp synthase subunit h family protein, mRNA sequence

ACTCTCAGTATCTGATAGAATGACTGCTCAATGTATTCAAATTGAATGAGAGAGCAATAAAACAGATAAAGTGAACCACA

ACCTAAATCAATTCAAAGTTCACATCGATAAATTTTACACACAGGCCCAAAATGAGAAGATACTGAATGATCCATGAGTC

CTAATCAGATATTCTATGGAGATCGACGCACAAGCCACATTGCTGGTTTACCAATGAGGTCCAATTATGCTTATCCAGGG

ACATCTTTACTCTCCTTCACTTAGAATTGGAACAATGAGAGGTTTCATCTGTGCAAGATATACAATAGCCCACATCATCC

AACAACAGACGGTCGCTGTTATAACCAATGTCAAATGGAACAAATTAGTGGAGGGGCCTCTATTGCAACAGATTCTGGT

>GW615328.1 Jc1-049-D03-M13F.D03.ab1 Jatropha curcas flower and seed Jatropha curcas cDNA, mRNA sequence

GGTGCTCCGGTATGGAACAACAACTCCTCCATGACCGTCGGACAAAGAGGTCCGATCCTCCTTGAAGACTATCACATGTT

AGAGAAACTCGCCAACTTTGACAGAGAGCGAATTCCAGAGCGGGTTGTCCATGCTAGAGGAATGAGCGCAAAGGGTTTCT

TTGAAGTCACTCATGAAATCAGTAATCTTACCTGCGCCGATTTCCTTCGAGCCCCCGGAGTTCAAACTCCGGTGATTGTC

CGTTTCTCCACTGTCATCCATGAGCGTGGCAGCCCTGAAACTCTCCGAGACCCTCGAGGTTTCGCTACCAAGTTTTATAC

TAGGGAGGGTAACTTCGATATTGTGGGAAACAATTTCCCTGTTTTCTTCATCCGGGATGGAATAAAATTTCCTGATGTGA

TCCATGCATTCAAGCCAAACCCAAAGTCTCATATCCAGGAGTACTGGAGGATCTTCGATTTCTTATCACACCACCCTGAA

AGTTTGAGCACGTTCGCTTGGTTCTTTGACGATGTTGGCATTCCACAAGATTACAGACACATGGAAGGCTTTGGAGTTCA

CACTTTCTGTTTCTTGAACAAAGCGGGCAAAGTGACCTATGTCAAATTTCACTGGAAACCCACTTGCGGCGTCAAGTGTT

TGACGGATGATGAAGCAATGAAAATTGGAGGAGCTAACCACAGTCACGCTA

>GW877062.1 JC006577 Seed specific Normalized cDNA library from Jatropha curcas L. Jatropha curcas cDNA clone N11112 5' similar to Unknown protein, mRNA sequence

CGTGTCGTGTTATTGTTTTTTTTTTTTTTTTTTTTCTTCCTTCATTTTGATTTTTTTTTTGGCTTAAACCCCACTCTGCT

TGTTCCCTTCCTTTGCTTTCATAAAGAGGAACCATACTTTTGAGAGAGACAAACAAGTATAGGGAGGGAAAGTAAAGAGA

GTGAAGCTGTCGGGTTTCTAGTCATGGGAGCTCTTGCTCCTGTGTCTCCTTGGATACCAAAAAATGATCTTTTGTTAAAG

AATGCTGTTGAGGCTGGTGCTTCCTTAGAATCACTTGCTAAAGGTGCAGTGCAGTTTTCTCGAAAATTCACTGTTCGAGA

AATACAAGAACGATGGCATTCTCTCCTTTGTGATCCAATTGTTTCTGCAGAGGCTGCTTTTCACATGATTGAGTTCGAGC

GATCTGCTTCAACTCTTCAATCAAAATTCAGTAAATCA

>GW617824.1 Jc2-027-D01-M13F.D01.ab1 Jatropha curcas flower and seed Jatropha curcas cDNA, mRNA sequence

GAGTTAGCTCTAGAGGCGGCCCGACAAGGCATAGTTCTGCTCGAGAATCGTGGGGGAGCCCTTCCATTGTCCGGTGCCCG

TCACCATACCGTAGCAGTCGTTGGACCGAATTCTGATGTTACCGTTACCATGATAGGGAATTATGCTGGTATTGCATGTG

GTTACACTAGTCCATTGCAAGGAATCGGCAGATATGCAAAGACAATTCACCAACCCGGGTGTGAAAATGTGGCCTGCACT

GGCAATCAAAATTTTGCAAGAGCTGAGGTTGCAGCTCGCCATGCTGATGCTACTGTCCTTGTAATGGGTCTTGACCAATC

CATAGAAGCAGAATTCAGGGATAGGGTAAGCCTTCTCTTGCCTGGAAACCAACAAGAACTTGTATCAAGGGTAGCTAAAG

CCTCTAGAGGTCCAACTATATTGGTGTTGATGAGTGGGAGTCCTATTGATGTCTCCTTTGCTAAGAATGATCCTCGCATT

GGTGCCATTTTATGGGTTGGGTATCCGGGACAAGCCGGAGGCGCTGCCATTGCTGATGTTTTGTTTGGCACTACAAATCC

AGGTGGGAAGCTGCCAATGACATGGTATCCACAAAGCTATATTGCAAAAGTACCAATGACAAATATGGGAATGAGGGCTG

ACCCTTCAACAGGCTATCCTG

>FM888164.1 FM888164 Jatropha curcas embryo 35-55 (DAF) Jatropha curcas cDNA clone rjcfea0_001510, mRNA sequence

ACTAGTGATCCCCGGGCTGCAGGATGACCCAAGAATTCGCTATTTCGATTTTGCTAGTCCTGTGGAACAGCGTAGCGACG

GATAATTCAGAATCAGAATAAATAGGTCCAGTCCAGGGACAAATCAATAGGAAATGCTATTTGCTTTGTAAGAATAAGAA

TTCACTTCTATGAAATGAAAGAGTTTCACGGGAATTCTCTTGATCGTCGGATATCATTGAGAAATAGGAAGAAGTGTTAC

CGAACGATTTCCTGCAATTCTTACCAGTATGGAATGAGTAAAGAATCAAGCTACAAGTCTCGATCTATACAAAACGCACT

GGGGTTTTGTCTTTCTTTCGGTTTCATTGATAGCAGAAGAGCCTTTCTCTATAGGGGCGCTAGCGCTTTACTAATAGAAT

ATCAAGTAAAGGGGACTGAAAAACGTAATAGGCTGCTACTCTAGGCTCGCTTCGCTTCTTCAAGCTCCGCCCCTTATTGA

AGACTAAAGTCTTGAAGAGCCTTCTATTTTTTAGTAAAGCAACCTTTCGCGCTAGGCGCTCACGTTTTTTGTCTGGGTGG

AAGCTGGCGGCAGGGCTTGCTTAACCGCATACACGGAATTGGGAT

>JK613473.1 JCF19-73 Jatropha curcas, immature Seed cDNA subtraction library Jatropha curcas cDNA similar to oligopeptidase a, mRNA sequence

ACTATCTGCAGATGCATTCTCAGCTTTTGAGGATGCTGGATTAGAAGACAGCAAGGCTGTCAAGGAGACCGGACACAGGT

TCCGTGAGACCATTCTTGCGCTTGGAGGTGGAAAAGCACCACTAGAGGTTTTTGTGCAATTCCGAGGGCGCGAACCTTCA

CCAGATGCACTGCTTCGGCATAATGGCTTATTATCAGGCGCAGCCTCAGCATGAGTCCTACAGTCTACTGGTCATCTGTA

TTTCTGTAGGCATGTAGTGTTCTCTAAATCATGCAGGACTTGTTCATTAGGCCTCGAGTTATCCACTGGTTATGGTTTAG

GTTCTGAGACAAAAGTGCCTCTGGTCCCCATAGGGATGCTGTGGCAATTACTAGTCCTTCAGTATACAAGAACTTGAAAA

TAACGTTATATGGAACAATGGCTTTCTTCTTTGTCATCTCCTTCTCTTGGTCTGT

>FM888811.1 FM888811 Jatropha curcas embryo 35-55 (DAF) Jatropha curcas cDNA clone rjcfea0_002304, mRNA sequence

TGAGAAACAAGTCCAAAAGAATGATTGGTTTGGTCACGATTATACACAGATAGCTGTACTTCGGGTTTCGAAAGATGATG

AAGATGGGTCTTTTATAACCATTATTGTTGATCAGAACAAAGAAAGAGAGCTTAGTCATACACCTCAGCAGTACCATTCA

AGCACTTCACAAGTACTACCTCTTGCATCTTCACCTTCGAGATTCACATCAGCAGCCAAGAAAAGTCCTGCCTTTCATTA

GTGCTTGCGTTTCATGATCAATTAAAATGTACTTT

>GW881568.1 JC000763 Seed specific Normalized cDNA library from Jatropha curcas L. Jatropha curcas cDNA clone N01448 5' similar to BADH, mRNA sequence

GGCTGTTGAAAATTATGAAAGCTTTATTACAGCAGCCAACTTACAACAGAAGAAAGAAAAGAAAAGAAAAACAAAGAGAA

CATAAACACAAAGCCATAACATAAACCCATTTCCACCATTACCGCCTTCCCTTTCAGAAGCTTCTCTCTCTCATGCTTCT

TCAAGCTTAATTAAACCTTCTCAATCATCTTTCTTAACCAAACAAAGGGAACTTTGAATCATGGGTTGCTGTGGATCTTC

ATTTCTAGCTGAAACTCATCCTGAGAAAGACCATACCATAACCAAACAAACCCAACCTAATCAAAGCCATCCATCACCTT

CTTCTCTTCTTGACCCTTCTACTGGAATCCCTTCTTTCTCCGAATTTTCCTTTTCTGACCTTAAAGCAGCCACCAATAAC

TTCAGTTCTGATAATATCGTCTCTGAAAGCGGCGAAAAGGCACCTAATCTTGTCTATAAGGGTCGTCTTCAAAACCGCCG

TTGGATCGCTGTCAAAAAGTTTACTAAGATGGCTTGGCCTGATCCTAAGCAGTTTGCGGAGGAGGCATGGGGTGTAGGAA

AGCTGATGCATAAAAGGCTTGCTAATTTGATTGGGTACTGCTGTGATGGGGATGAAAGGCTGCTTGTTGCTGAGTACATG

CCCAATGATACTTTAGCTAAGCATTTATTTCACTGGGAAAATCAAACAATTGAATGGGCAATGCGTTTAAGAGTAGCCTT

CTACATTGCTGAGCATTA

>GW611250.1 Jc1-003-F03-M13F.F03.ab1 Jatropha curcas flower and seed Jatropha curcas cDNA, mRNA sequence

GAATTTACAAAACAACAGCTTCAGCTATCAAATCCCGGAGCAGATTAGTCATTTGAGAAGACTGGAAGAACTCAGACTGA

ATAACAATTCGATAAGTGGAAAAATGCCCAGTAATATATCAAGTTGTTCAGATCTCATTGTTATTCGGTTAGGTAATAAT

AAGTTGAAGGGAGATATTCCTGAAAAATTGGGTTCGTTATCGAAACTTCAAGTTCTTTCCTTATTTGAAAACAATCTATC

AGGAACTATCCCTCATTCACTTGGAAATTTGTCACAGCTTCTGCTACTCTCCTTAGCTGTCAATGGCTTGGCAGGAACTG

TACCTTACTATTTAGGCCAGTTAACCAATCTTCAGTTCTTGAGTCTTCACACAAATAGGCTTTCAGGTATCTTCCCTTCA

TCAATCTTTAATATTTCCTCCATAACAGAATTTGACATAGGAGTGAATCATCTCCATGGAAATCTTCCTTCAGAATTAGG

CTCTTCACTTCCTAAGATTTGATTTTTTTCTATTGGTGCTAATGAGTTCACTGGACAAATCCCTTCTTCTTTTTCTAATG

CCACTAATTTGCAGAGACTTGCTTTGGCAAAAAACAAGCTTACTGGACAAGTTCCTTCTCTGGCAAAGTTGAATAAACTT

CGTGGTTTTTCTGTTTCTTTAAACTATCTGGGAAATGGGGATTCTGATGACTTGAGTTTCCTTTACTCTCT

>GT977387.1 JGCCJG2008A04.b Jatropha curcas L. germinating seeds (mixed stages) Jatropha curcas cDNA clone JGCCJG2008A04 similar to ATNUDT9 (Arabidopsis thaliana Nudix hydrolase homolog 9); hydrolase, mRNA sequence

CGATTCATAGCCATCCAGAGCCTGAGGAAGTTGGAATAACTTGTCATCAGAGCAGCAAAGATCTTACAGACTCGAAGATG

ATTAACAATAACGTTTCACAAAAAATGTTTGACAGTATTATTCGCGAAGTGGTCGAGGAAATTGGAGTACCAGCAACATC

TCTTTGCAATGTACGTTTTATTGGTATATCCTGTCGAGTGTTAAATGTTAGACCTGCTGCCTTTTTCTCCATCAGATCCC

ACCTTGGATCAGAAGAAATTCATCAACTGTATCACAATGCTCAAGATGGCTATGAGTCGACCCAACTTTTTACAGTTTCA

TTGATTGATTTAGAAAACATGGCATCTAAAATGCCTGGCTGCCATCAAGGTGGATTTGCTCTCTATATGCTGATGGGGGA

AACTCTGAAAGATACCTGAATACT

>GW619809.1 Jc2-051-D11-M13F.D11.ab1 Jatropha curcas flower and seed Jatropha curcas cDNA, mRNA sequence

CTAAAGAGATCAGAGAAAGAAGAAGTGTAAAGATGTTGCTAGACAAGATGTGGGACGACGTCGTTGCGGGGCCTCAGCCC

GACCGTGGCCTTGGCAAGCTCAGGAAGATAAGCACCAAGAAATTAACCGTAGTCACGGAGGGAGAAGGAGAGAGTAGCAA

GTTTCAGAGGTCGATGTCGATGCCAGCAACTCCAGGGACACCTTCCACTCCGGTGACACCCACAACTCCGGTTTCCGGTC

GCAAGGATAATGTATGGAGGAGCGTTTTCCACCCTGGAAGCAACCTTGCCACCAAGGGTCTGGGTGCTCAGCTCTTTGAC

AAGCCGCAGCAGCCTAATTCCCCCACCGTTTATGACTGGTAATTTCCGTAAACATATATATCATCTTTCTTGGCTCTATG

CATCTTTTTCAGGTGTTTTAGTGGCGCGTGGGAATAGATCTTGGTCTTCTTGCTTGCATGCATGCAAGTACACACGATAT

GGTATTTTATATGTCACGTTGGTTTGTGCTTTAGTTGAATCTTGCCGTCAGATCCGTTTACCTTTTGTATCAGATGGCTT

GGATATTTATTTATTTTTATTTTTAAAATTTTTGTTCTTTTATCT

>GW881227.1 JC006403 Seed specific Normalized cDNA library from Jatropha curcas L. Jatropha curcas cDNA clone N10889 5' similar to Unknown protein, mRNA sequence

GGGGTGGGTATTTTCTATATTGTCATGTCAAAACTTGCTAGATTCTCATCTGCTCCATTTTTACACTTGTCTTTCTCTTC

TCTTCAATCTTCGATCTTTATCTTCTTTCTCTTATTTTTTTGTCTGCCCCTGCCTCTTTATTGGCCACTCTGATCTTTCC

TTCCATTTCTATTTCTGTTTTGATCTCTTATATAATAAATATATTATTCCTCTATACAAACAGCAATGGCTGGCAAGGAA

ATCCTACACAAGATGAAGGAAAAAGTTGGATTAGGTTCATCTGCAGACTCTGGAAAGGGCAAGAGTAAGATATCAAAGAA

TGTAACACAAGGATATCACTTAGTGAAGGGAAAATCACATCATGCTATGGAAGATTATATCGTTGCACATTTTAAGCAAG

TCGATGACAATGAACTTGGTTTGTTTGCAATATTTGATGGTCATCTGAGCCATATTATTCCAGATTATTTGCGGTCAAAT

TTGTTTGACAACATCTTAAAAGAGCCAGACTTCTGGACAGAACCAAAAAATGCTATGAGGAGAGCATATCGTATAACTGA

TACTATGATTTTAGAGCAAGCAGCCGATTTGGGTAAAGGAGGTTCTACTGCTGTCACAGCAATACTGATCAACTGTCATA

CACTGGTAGTAGCCAATGTTGGTGATTCTCGAGCTGTTATCTGCA

>GW875667.1 JC004984 Seed specific Normalized cDNA library from Jatropha curcas L. Jatropha curcas cDNA clone N08705 5' similar to Unknown protein, mRNA sequence

GAGCGACAATTATAAGATCAAAGAAGAAGGAACAAACCTACTAGGCTTCTTCGATAATGGCGTTCCCTTCTCGTCTCTTA

TCCAGATCCAAGCAGCTCTATGGCAGTCAAGTCATTCTACAACAGGATCACGCTATTCCTGTCCGTTACTTTGCCAAAAA

AAGCTGATCCTCCAAGTCAAAAAGGAGATAGTCCTCCAAGGCTTAAGGGAGATGAGTTGTTAAAGGGCATATTTCTGGAA

GTCAAGAAGAAATATGAGACAGCTGTTGGAATACTTAAGAAGGAGAAGATCACCATTGCTCCCGAGGATCCTTCAGCTGT

TTCTCAATATGCGAAGATCATGAAGACAATTAGAGAGAAGGCTGGTTTGTTCTCTGAGTCTGAAAGAATTAAATTCACTA

TTGAGACACAAACCGTCGATATTCCAGATGCTCGGACATGTTTGTTGACATTGAAAGGAAATTCGAATCAAGAGAGGTCT

CATTGATGAACTTGGGTGCTGAAGCCATGATGATGGATGCTTTGGAAAAAGTTGAAAAGGAAATCAAGAAACCTCTA

>FM887498.1 FM887498 Jatropha curcas embryo 35-55 (DAF) Jatropha curcas cDNA clone rjcfea0_000714, mRNA sequence

CAAAAGGCTTGGAAATCTGCCTCTANGGTTCATGGTCATCAATATCTTAGTCCTCATTTCGTTTCTTAGAAGGTTGCTGA

GGAGGCTGGATCACCATTAACTATGACGGCAGTGTGAACCTTTAGTTCGGGCTTGCAGCATGTGGTGGGGTCTATAGGAA

TAGTGATGGCATTTGGCTAACTAGGTTTGCAGCTAGTCTAAGTGTTTGCGATATGGTTGAAGCTGAACTTTTGCGTATCT

TCCATGGCTTCTCAGTAGCTTAGGTTAGAGGGTGGAGATGTGTTCAAGTTCAGGTGGACCTACAGACTGTTGTTCACATG

CTGTCTTCTCAGCAGATGAAGATTTGTCACTTTCAAAATTTGGTTCAAGCTTGTGTGAGGCTCTTGGCCAGGCAATGGCA

GGTTAGTGTTATTTAGATTTTTTGTAAAGCGAACTTCGTGACAGACTCCATTGCTAAGGATGCGATAATGAATCCCCATG

GTCTATTGATGATCCATTT

>GW876006.1 JC005383 Seed specific Normalized cDNA library from Jatropha curcas L. Jatropha curcas cDNA clone N09277 5' similar to Transcription factor, mRNA sequence

CATTTTTAGTGAATATTTACAAAGTTTATCGATCTTCCTCTTCTCCTTTCTCTTGATTTCTTAGCTTTTCAGTTAAAATT

TAAAGAGATTGATTTGTGAAGTTTGTGATGTCTCCACCTCTGTTGGGTGTGGAGGAGGAAGGGGAGAACAATGTCTCTTT

AGTGGCCTCTTCGCCTTCCATTGATTGTATCTCACAAAATGGCTGCGGTTTAACAGAGCGGAACTATTTGGGTTTATCAG

ATTGTTCTTCAGTTGACAGTTCTGCTGTCCCAAGTTTATCAAAGGAGACTAAGAACAATCTAAATTTAAAGGCTACTGAG

TTGAGACTTGGCCTTCCTGGATCCCAATCTCCTGAAAGAGAAACAGATGTTTCGTTGCTAAGTACAGGGAAGCTTGATGA

GAAGCCTCTGTTTCCTTTGCTTCCTTTGAAAGATGGAATCTGTTCATCATCACAGAAGCATGTTGTTTCAGGCAACAAAA

GAGGATTTTCTGACACCATGGATGGATTCTCTGAGGTAAAGGGTTCTGTGTATTCAGAAAAAAACTGGTTATTTCACTCG

GCTGGGACAGATACTGATTCTCTACAATCTTTGGGACAAGGGAAGTTCCCCGGTAATTCTGGGATAAGTGCAATCTTATC

ATCTAGGCCATCTGTGGCCCAATCAGCCATGATGAAA

>GW620077.1 Jc2-054-E02-M13F.E02.ab1 Jatropha curcas flower and seed Jatropha curcas cDNA, mRNA sequence

TCGGTCCACCCCCATGGCAGAAGAGAAACAGCACCACCACCTCTTCCACCACCACAAGGAAGGCGAAGGAGGAGGAGAAG

TTGACCACAAGAAGGAGGAGAAACACCACAAGCATCTTGAGCACCTCGGCGAGCTAGGCGCGGCTGCTGCCGGCGCTTAC

GCTTTGCATGAGAAGCATGAGGCTAAAAAAGACCCAGAGCATGGCCACAGCCATAAATTAAAGGAGGAGATCGCGGCGAC

GGCTGCCGTGGGAGCCGGAGGATTTGTCTTGCATGAGCATCATGAGAAGAAAGAAGCCAAAAAGGAAGATGAGGAAGCTC

ATGGAAAGAAACACCACCATCTCTTTTAATTAAATATAATTATATATCTATTTTGTGTGCTGTGTGTAATGGTTTCACGA

TATCCATGTTGTTTAATTACCCTAAACTGGAATATTGAGAATCCTTTACTAAACCAGATCATATATACTGTCTGGTTCTC

TTTTTATGTATCTAATTATAAAATAATGTTAATTTTGGATATGTTTCTATGAGTTTAATTATTTTCAATAAATTATATTT

TGC

>GW618499.1 Jc2-035-H07-M13F.H07.ab1 Jatropha curcas flower and seed Jatropha curcas cDNA, mRNA sequence

ATAACAGACCCGTCTAACCAACCCTCGATCCTAATCCTTTCTCTCCTTTTCTCTTCGATTGGCTCGCCCCTTTAACTGAG

TTCACTCACCGTTTTACCTTTTCCAATCGATTCCATCTCTTTCAATTTTGATTCTTGATTGATTGGTTTATTGGTTGATT

GATTGGTTGATTCATCTTCAATTTGAGATATGACGAAGAGAGCTAGAACGAGCCCTGATAAGGCTGTGGTGAATGTTTGG

CAACGAGAGGTTGGTGAACTTTCTACACGGAATTTCGCTCACCGGCTTGCCGCTTCTGAGGATCTTGTATTGCGGCTTGA

TATATTCAAAAAGCTAGAGAAGCACAGAGGTTGTGTGAATACAGTAAGCTTCAATGCTGATGGTGACATTCTGGTGTCAG

GTTCTGATGACAGGCGAGTCATACTCTGGGACTGGGAAACTGGGCGTATCAAGCTTTCTTTCCATTCTGGTCACAATAAC

AATGTTTTTCAAGCCAAAATCATGCCTTACACTGATGATCGAAGCATTGTTACCTGTGCTGCTGATGGTCAGGTTCGACA

TGCTCAAATTCTGGAAAATGGAGCAGTGGAAACTGCATTGCTA

>FM888975.1 FM888975 Jatropha curcas embryo 35-55 (DAF) Jatropha curcas cDNA clone rjcfea0_002516, mRNA sequence

GAAGGATCATCATACCTCGGGCAACCATTGCCGTTCTCTATAACTACGTTGATCTGGATTGAGGTTTTGGTGATTGGATA

CATTGAGTTCCAAAGAAACGCAGAGCTAGACCCAGAGAAGAGGCTATACCCTNGGAGGCAGTTTCTTTGATCCATTAGGT

CTAGCAGCTGATCCAGAGAAGAAGGCGACCCTTCAATTGGCAGAGATAAAGCACGCTCGCCTTGCCATGGTTGCTTTCCT

TGGTTTTGCAGTTCAAGCTGCTGTTACTGGGAAAGGACCTCTCAACAACTGGGCTACTCACTTGAGTGACCCTCTCCACA

CGACCATTATTGATAAACTTGACCTCTTAAAACATTACCGCTGGTCATCCTTTTTTCTTGTGAGAAAGTTGAAAATGTT

>GT975153.1 GJCCJC2067H05.b Jatropha curcas L. developing seeds (mixed stages) Jatropha curcas cDNA clone GJCCJC2067H05 similar to CSD2 (COPPER/ZINC SUPEROXIDE DISMUTASE 2); copper, zinc superoxide dismutase, mRNA sequence

GCACATTATCATTTCTTTTCTCTCTCTCATTTTCTAAAGGGGAAAAAATGCAAGCAGCAGCTGCTGTAGCAGCCATGGCT

GCTCATACAATCCTTGCAGCCTCTCCATCCTCTCATCCTCTCCTCTATCCATTCCCAAACCCTATCCTTTCCCACTCTTC

ACCTCTGCATTCCTCATTCCAAGGTGTTTCTCTCAAACTTCCTCGTCAATCGCTACCTCTTTCTCTTACCACCGCCGCCG

CCCCAAAAAAACCCCTTGCTGTCGTTGCTGCTACCAAAAAAGCCGTCGCTGTCCTTAAGAGGTACCTCCAATGTTGAAGA

GCGTTGTCACTTTGACCCAGGAAGACGATGGTCCCACAACAGTGAATGTTCGTGTCGCTGGCCTTACTCCAGGGCCTCAT

GGATTCCACCTACATGAGTATGGTGACACAACAAATGGATGCATTTCTACAGGAGCTCATTTCAATCCGAACAACAAGAC

ACATGGTGCTCCTGAAGATGAAATCCGTCATGCGGGTGACCTGAGAAACATAGTTGCTAACGCTGATGGGGTGGCAGAAG

CAACAATTGTGGATAACCAGATACCATTGAGTGGTCCAAATGCAGTAGTCGGAAGAGCACTTGTTGTCCATGAGCTTGAA

GATGACCTTGGATAAGGTGAGCATGAACTTAGTTTGACCACTGGCAATG

>JK610364.1 JCF1138 Jatropha curcas, immature Seed cDNA subtraction library Jatropha curcas cDNA similar to wrky transcription, mRNA sequence

ACATTTGAAGTAGCTCCTAGGATTTGGATTTCCCTTAACCACTTTCTGCCCATATTTTCTCCATCTGTAACCATTATCAA

GAATGTCAATGTCACTTGTAGTTTGAACCACAACTCTAGGTTCTCTCACTGTTCTGCTTCCAGGTGCTGAAATTCCTTCA

TTTTCAGTCTCTGTTTTCCATCTTTTGGCTTCTGGTTCATCTTCATCAAACTCCTCACTTCTGGATTTACTCTTTTGTGA

ATCAAAATCATCATCTCCTACTGATATTGAGGAATTATCAGTTGTAGCAGCGGAGTCCATCTGTCCACTTCCATGGGTAA

CAAATGATTGATCTTCAAGCTCCTAATCATGTAACAAATGATCTTCAAGATCAATCATTTGTTACATGATTAGGAGCTTG

AATTGCCAGATTGGTAGCTGAAGATGAAGAAGATGATGATCTTCTTGTAGATTGAGGCTTAGGATGATTATGGCTACCTT

TGTAAACAATTTCAGTTATTTGTCCATCTAAGACTTCTCTACCTTCTTTTTTTGTGGGACAATTAGGGTAAAGTGCACTT

GTAATAACTTCTAAGATTTCACTTTCCCTTTAACTTGTTTTTGACCATATTTTTCTCCAATTATATCCCATCTTCTGAAC

TTTTTGGCCACTAAATTGGACTGAATACC

>GW614942.1 Jc1-045-A09-M13F.A09.ab1 Jatropha curcas flower and seed Jatropha curcas cDNA, mRNA sequence

GTTTTTTTAGTCTCTGTTTCTCTTATTTTTATTGTTTTTTCTTAATTTGAACTTCGATTGTGGGATTTACAGCTACTTTT

GGTGGTTGTAGTCTCACAGATATATGCGTACAGAGAGAGCTAGCGAAAGAAAAAGAGAGAAATGGGGGAAATCCGAATTT

GCTGACTCTGATTTCATTTCCTTTCGTGGGATTTCCCCCTTAACTTCTTCTCAGGTCCCTTTTGTTGACTGAAGGTTGTG

TTCTTGGTCACTTTTTCTTGAAATAAATATTTATACTAGTTTGTGATTATTACTGTCAAAATGGGTGGATGCGTCTCAAC

AGCCAATGCAAGGCCAAGAACACACAGGAAACACCACCACAAATCTTCTAAACGCCGTGGGAAGATTCCCACTGCTATTT

CTGATGTACCTATCAAAAGGTTTAGTGGCTCTGATATAGGAGATTTTGCTGTTAGTGAGTTTGTTCATCTTGATTTCGAA

AAGGGTGCTGCTACGACTTGCAGAAGATCTGAAACTACTAACAAGAACTTCCATGTGACTCAGCTTCAATGGAATCATAG

CCAAATTGATGCAAAAGGAATATGCCAACAGGAAGAAGTATGGTTTGATTCTGTGAGTATTATAGACTCAGATTCTGACG

ATGACTACATTAGTGTCCATGGAGGAGATGGATTTGGTTGTGTTGGG

>GW615232.1 Jc1-048-C11-M13F.C11.ab1 Jatropha curcas flower and seed Jatropha curcas cDNA, mRNA sequence

AGACCCTTCACTAATTGCTTCACATCGGAGCTTATTGGCTGCACAGAGAAACGCCCAACAGCAGATCCCTAATTGCAGTG

GGATGGTGGCCAGATCTCAGTGCTCACAGAACCCTAAGTGCCGCTGGTGCAAGAGCGAGGCTCTTGATGACATGTGCTTT

AGCAAGATCGAGGCTTGGAGGCTGCCTCAACAGGTCTTTGTTTGCGATTGATTTTGTACCGTTAAACATCTCTCTTCCTA

TACATAGTCGTATTGGAAATGTAATTTAGGGACGTGTATGAATGGAACTGGAAAGTTTACAATTGCTGTTGTTGTTGTTG

TTGTTGTTATAAATAGTTTTTAGATTAGAATGGTTGGAGAGTCCCCAACTTTTTTATGTTTTTCTTGTTCTTCTGTATTG

ACAAGAAACAAAGTTCTTATTTTGTTCTAAAACGTATATTGATCGGGTGACTGCTACTATTTATTCATTTTTATTAAATT

TTACTTTTTTGTGCAAGTAGGGAAAGAATGAGCATTAGAGTTTGGATCT

>FM890548.1 FM890548 Jatropha curcas embryo 56-70 (DAF) Jatropha curcas cDNA clone rjcaeb0_000433, mRNA sequence

ATTCCGGAGCACGACTTCCGCCAAAACCGAATGCCACCACCATCATCCGCCGCCACGCGTTCCGACCATGATGACGACGA

TCATGCTCCCTCGTCCTCCGATCCACCTGTCAAAATCTCTGATTCCAAGCTCGATAACGACAAACAACACGAGGTCGAAG

TTGAAGAAGTAGAGGAACAAGAAGAAGACGTTAATGATAATGACGATGGCAGCGAAGAAGAAGAAGCAGAAGAAGAAGAG

TACGAAGAGATTGAAGT

>GW878718.1 JC002984 Seed specific Normalized cDNA library from Jatropha curcas L. Jatropha curcas cDNA clone N05691 5' similar to Unknown protein, mRNA sequence

GGAACTAATTTTTGTTTGAAATGCTACCTCAAGAGAAGGGGGCCCCTGGTCCGGAAGATGCCTTATTTATAAATTTATTA

AATTAAATAAGGGTATATACTACTACGATTGTATCTATTCGCTGAGAGTCCCAATCTTCTTGAAAAAACGTGATACAGAG

AACCAACACATCTTGATGAGTGACTTTTCCAGTTTTCATTTCTACATTTAGTAGTCTCATTCGACAGCTAAAATTCGTGC

TTTTGACGTAGCATCGGGAGTTCAATTGGCTAACCTTTCTATAGTGCCCCCATGTTCCTATGATATCCCAACCCGAAATA

TTAAGCTTTTGCTGTGCCATTGAGAAAGCTAAGCGCACCTGTACATCTCACCCAATTAGTCACTTTCTTTCATATGATTC

AAAGATTCTGCTCGTGAGCTGGTATAAACCTGTCTGACTGTGCCGTAACAGAAGATTTATCTCAACCCTATCAGGAGACC

CAAGCAATGCCCAAGTACTCCCGTGCCTTTCCGGATCATTGGTCAACTACTTTCTCTCTATTCTAACCGAAGATATATCA

TATATAAAGAAAAGACTTTCTTCATTCTAGTTCGTTAGTTGACGATAGGCCACCGCACC

>FM891174.1 FM891174 Jatropha curcas embryo 56-70 (DAF) Jatropha curcas cDNA clone rjcaeb1_001171, mRNA sequence

ATTATAAACAGTGTTACAGGGAAAAACCATAAAGCTAGTTTATTTCAGGGTATCTACAGGCATTTTTTGCGAGAAAATGA

TGGTACAAGGGTTGCTGAAATTAACGGTTCTGAGGTAGGAGTTGCCTTTTTCCTTAATTTCTCTGTTCATGCAACAAAGC

CTATGGGCTATGACCACAAGGAAGGAGAAGACACTAGAATTCTCCCTGTTCATTACTCGGAGAACTATTTTTCGCTTGTG

CCAGGAGAGCAGATGTCAATCAAGATATCTTTTGAGGTTCCTCAAGGAGCCAGCCCACGGATAAAACTTAGTGGCTGGAA

TTACCATGGCTATCTTTCTGTTTTCTAAACCCAGCAAGTACTATCTTTTCCATCGGTCATCTGTCAAAATCTTGCATGAT

CAGCCTCCTAAATGGTTAGAAATTCGAGGTTACTTCATCCATGTTGGACATTAAACGGCAAGTGTTCTTATTATTTGGAT

TCAACTTCTGGACATTATCGTCTCAGTTGCTCCGGCGTGGAAGTTCTTCTAGTGATACTGAAAAATAAGGGCAATTGTCA

ATCT

>JK611594.1 JCF4386 Jatropha curcas, immature Seed cDNA subtraction library Jatropha curcas cDNA similar to secreted protein, mRNA sequence

ACAGTAGCATTCAGTAACGAATACATATTAATTAAACACGTATTTTACGTAATTAAGACCATTTTTAGAACCTGCCTATA

GAGGATTCTACTTCCAAAAACACCAAAACCAAAAAACAAGCAACCACCTATACATCCTGATATCCACACTGACACTATCC

ATATTGGTTTTTTACTTTATAAACACAAAATCTGCATATATATGATCATGGAAAAGGTGCCCTCAATTTCATTTCAGTCC

TTTCAATGTCAATGGCAAAACCTTTCCCATTCTGCTTCCAGTAATCTGCCTGAGGATTGATCCACTCTGGCTCTGCAATC

TCAATCCTCGAGTTCTTTATAGCATCTGTCATATATTATACTTGTATCCCTTTTCTCAATCAGGCTGGAAAAACCTGCCC

AATTCACTGCTGTGGGGACTCACCATGAGGGGTTCAATGGAGCCAATGGCTGAAAAAGTTCAATTCTTAAGGTGCAACCC

TTTAATTAGGTTATTGTTTTATCCTTACCTTTCCTGGCCCAAATGCCAATTTGGAAATTTCTTCGGTCCCCCTTTGGAGT

TGGGTTCCCACCTTTCCCCAAGGGGAGCAATTTTTTCTGGGGAAAAAAAATCACAAAAATTCCCCCCCGCGCCCACTATG

TTTGGGGGAAAACGGGTTTAAGTGGAGAAGTGTCATATCTCGCCCTTGGGGGAGAGACGCCCCCCCCCGCCGGAGAGAGA

TTTATTTTTATTTATTTTTTCGGCGCACACATATTGGATATATCATGACGCAGCTCGACTCTGTGTAGCGAGAGCAGTCG

CCTTTATTATTATTTA

>GW615376.1 Jc1-049-H05-M13F.H05.ab1 Jatropha curcas flower and seed Jatropha curcas cDNA, mRNA sequence

TCCAGTTCAAGAAAACACATAGGACTTGTTTAGAAATTGAAAACAGAGAAGATTTGTTCCCCTGTTCTTCCATTTTCATT

TCTCTCTTATATACAGAATTTCTTCCTCACACAGAGACAACCGGGGGTTCCATCGTCAGTAACTTCTTCGGCTCAGCGTC

GACGTCGTTGCTGGGCCACCGGCTTGTGGCGTTCATAAGTTTAAGATAGGAAGGGCGAAGATTGTATCCTTTTGGCAGTA

GTCGCGACCAGCACAAAAAATTTGGGATTTTTGTGGTTCTAATCCTTTTATAATTACAAAAATCAGACTGGATTGAAGAG

AGAGACGGACGGAAAGAAGAAGAAAGCTGAAGTAGCAACAAAAATGGTGGTCTCCAGTAGTAATTCCAACAAGGAGATAA

GTGTCAGGAGAAGGATTGCCAGCATATACAATAAACGACAGGAAGATTTTCCATCTTTGAAGGAATACAATGATTACTTG

GAGGACGTTGAGGACATGATTTTTGACTTGGTTGCTGGAGTG

>GW611685.1 Jc1-008-E01-M13F.E01.ab1 Jatropha curcas flower and seed Jatropha curcas cDNA, mRNA sequence

CGTTTTGGCTTGGAACTTGGCCTCTCTTTCCAGATTGGAGGCCTCTCTGCATACGAAATTCTCCGCAACAATTTGAAATG

TAAGAACGGTTGCCCTTTTCACCTTTTATTATCAGATGGACAATGCAATTATTTTAGCATTCTTTAGCTACGATTATGGT

CTATAAAGAGGGAAAGTTATAGCTACTTGTATTAATTCGGGTTTCATTGCTTGAATTCCACGAGTTATGACAACGGATTC

CCGTGATTCTTTTGCCTGGAGGATTTTTATAGTTTTGCTGTAATTGTAAAGGTTTAGCTGCTTGACAATAAAAGGCAAAG

GATAGAGATTGAGCATGCATGGATCATTTGGGAATAAGAAGTAATAGCTTAGTTGATTTGGAAATAGGTGGGAGTTCTAG

TGCAAATGATGAGGTAAGAGGACTGGGTTCAAGTGGTCAAAAACCCAAGACTGATAGGTTAAAAAGTGGATCTCTGAGTT

CCGCTCGGAAGATGAGGAGTGATGATGAAATAAGTTTAAGTGAAGGTGAATTGAGCTCTAGTGGAAATTTGGAGATATTG

GTTGGTGAGAGAGGGGAAAAGGCAAAAACTAGTGTGGAGAATGGAACAGTGAAGGACAAAAGGAAGAAGAGGAGTGCTAA

GCCACCATTGC

>GT978616.1 JGCCJG2022F02.b Jatropha curcas L. germinating seeds (mixed stages) Jatropha curcas cDNA clone JGCCJG2022F02 similar to NADH dehydrogenase, mRNA sequence

GGGGAGAAAGATTAGCAATGTTTCTTCGGGTGATCGCACGGCCATTGATGGCCAAGGTGAAGCAGACTACGGGGATCGTG

GGTCTGGATGTGGTCCCCAACGCGAGAGAGGTGCTGATCAATCTTTACAACAAAACCCTAAAGGAGATTAAGGCGGTCCC

TGAGGACGAAGGATACCGTAAGGCGGTGGAGAGCTTCACGAGGCATAGGCTGCAGGTGTGCCAAGAAGAAGAAGACTGGG

AAATGATCGAGAAAAAGCTTGGGTGCGGCCAGGTCGAGGAGCTTATCGAGGAGGCCCAAGACGAGCTCAAGCTCATCGAG

AAAATGATCGAGTGGGATCCTTGGGGTGTTCCTGATGACTATGAGTGCGAAGTTATTGAAAACGATGCTCCTGTACCAAA

GCATGTTCCTTTACATCGACCTGGTCCTCTTCCTGAGGAGTTCTACAAGACGCTTGAGGCTGTGCAGTCCAAAACTGATG

CACCTGCAGTCACATCTGGTCAGTCAGAGACAAAGGAGTAATGCACAACTGACACATCTTGAGGAACAACAAACTTCGGA

GTTGAATCCTTGTTCTTAGTTTAATATTTGTGTTTGTTCTCTTGTAATTCATGTTTACCAGATTGGATAGGGTTTTCATG

AGAACACAGCCAGAATGATTCAGTGAATAAGCCG

>GW613318.1 Jc1-026-F06-M13F.F06.ab1 Jatropha curcas flower and seed Jatropha curcas cDNA, mRNA sequence

CTCTCTCGACCCAAAGCACTCCATCGTCAGTTTCTCTTTCCTCTGATCCAAAACAGGTTTTAGTAACATGAATCAAGGTG

GGTATACTGTAGAAGTTACTGGCCTCTCTCCCAAAGCTACTGAGAAGGATCTCTATGAGTTCTTCTCCTTCTCAGGTGTG

ATTGAACATGTTGAAATAGTCAGGTCTGGAGAATATGCGTGTACTGCGTATGTGACTTTCAAAGATTCATATGGTCAAGA

GACTGCTGTCTTACTCAGTGGTGCTACAATTTTGGACCAACGTGTGTGCATAACACGCTGGGGGCACTATGTAGATGAAT

TTGATTTTTGGAACAGGGCTTCAACAGTGGTTGAAGATGGAACTGAATCAAATGTGCTGCCACAAAGAAGTCAATATGTC

CCTAGTGCTGGAGAAGCAATGACCATGGCTCAGGATGTTGTCAAAACTATGCTGGCAAAGGGATATATACTCGGAAAAGA

TGCATTACAGAAAGCAAAAACTTTTGATGAGTCTCATCAAGTTAGTGCAACTGCTGCAGCTAAGGTTGCCGAGCTAACAG

AGCGAGTTGGCCTTGCTGACAAGATTTTTGCGGGCATGGAAGCTGTTAAAGCTGTG

>FM891554.1 FM891554 Jatropha curcas embryo 56-70 (DAF) Jatropha curcas cDNA clone rjcaeb0_001864, mRNA sequence

ATTCTTCCTCTCTAGAAAAAAGCAATTTAGCTTTGACACATCAAAACACATATCACCACCTCTATATATAGAGAGAGAAA

AGGAGAAGGAGAAAGAGAAAGAGGTTTTGGTGATGGATGGCTCTTCCTCTATGAATAACTCATCAATGAATAGAGGTGTT

GTTTCTAATATTATTAATAATAATAATTCTACTGAAGAAGAGAGTGGATGGACAGCTTATTTTGAAGATTTCTCTAACCA

TAGAGAAGAAGAAGGAGAAGAAGATAGTTTCTGTAATGCTGGTTTTTAAGGTAGTACTTCTATGGTTTCTGATGCTGCTT

CTTATCCTGCATGGAAATCATCATCAACATCAAATAATCAACATCATCATCATAATTACAACAA

>FM893969.1 FM893969 Jatropha curcas embryo 56-70 (DAF) Jatropha curcas cDNA clone rjcaeb0_004924, mRNA sequence

TTTTTTTTTAGCTCCACAAACTTTCATTATCTCATAACATAAGGCATATACAGATCTACATTTTTATATTATTGCAAGGA

ACTAAAACTGCCTATAAAAATAGGCTTATTCTTCTCCAAATCTAAACTATGTAGAAACCAGTTTATTATACATCCTCTTG

GTATAATTGCATAGTTGCAATATTAATTACGAGATCTAGACCCAGGGCTAAACACAGTGACTTCCTGTCTATTCTCCTTA

AGCCTCCTGGCATCTTCTCTGGAAATCTGGAATGCGTTTGCAACCACTGCCTCTGGCAGAGCCCTAATTGCCGAGGTCCG

TCCTGCTAGTGGGCTAATTTGTGCATTTTCGTTAGTCTTGAAGGATACCCACTCTAGCCCTTCATTGCTTGCCCTCTTCA

AAACCACAAAGTTCTGTGGAGCTGTTAATATTTGACCTTGTTGAACTTGTCCATCGAACACTGCATCTCCATTCTCATCT

ACGATTTGGACGTGACCATTTCCTCTTGTAAAGTAATATATGCTATGCGCATTGATGTTCCAGTGTGGTGCAAATATAGC

ATTCCTAAAGAGGACACCTTTCTGAACGCTGAGTCGGAGGTGTCTGAGGATCGGGAAGTTGTGGCTATTGACATTAGTGA

CATGGCCACACGTGGATTATAGATTTCGGCACGTGATGGGGGGGCCCGGTACCAATTCGCCTAT

>GW877491.1 JC002024 Seed specific Normalized cDNA library from Jatropha curcas L. Jatropha curcas cDNA clone N03646 5' similar to Serine/threonine protein kinase, mRNA sequence

GGAGCTCTGATCGCTCGGGCTCTACAAATCACGAATTTGTTATCATTTCAGAGCTTCTAGAGAGAGAAGCGGTGCTCTCT

AATTCGCAGAGGAACAGTAAACCTCGAGCTCTCAAACTCTAAGCCACAAGCCCTAATATTCTCGTAAAGATACTCCGGTC

ATCAATGAAGCATAATTACATGTAATGTTACGTGGTTACTGGATATGGAGAGGGTTGCAGAATCAAAAGCTCTTCCTGTG

AAATTGCCCGTTGTAGGCCAGGTGTCAAATGCATCCATGGCCTATGGAAGAGAAGGGGGTCAGGTAACAAAGCACCAGAC

AGGATTGGTAAGAGAAGTAGTGGGACGATTTAATGCACAAGGCTCTGTAGTTATGGACTTACCTGTCCCTGTAAGACCGT

GGAAGGGAAAAGACTCTTTGCCTGCACGGGAAGAGCTAATGCTTGATGTAGTCACTTCTATGGGAAGTGGTGATTCATTT

GATGAAGGTGGCCCTAGTTCTTTTTCTGGGGCTAGTCATCCCACCAGAACCGATTGACACAGATCTAATGAAAACGG

>GT980491.1 JGCCJG2043H04.b1 Jatropha curcas L. germinating seeds (mixed stages) Jatropha curcas cDNA clone JGCCJG2043H04 similar to chromatin protein family, mRNA sequence

TCCACACACCACTGGCCATCTTCTCTAGCCTTCTGCTCTGCAACATAAAGTGCCTCTGATAGCTTGGCAAAATTATCATT

GATCTGAACATCCTGTGACTGTGAAAGATCAGCAGGATTGGAAAATCCCTCCTAGTATCTCAAATTGGAAGAAGCAAAAA

GG

>GT981301.1 JGCCJG2053H06.b Jatropha curcas L. germinating seeds (mixed stages) Jatropha curcas cDNA clone JGCCJG2053H06 similar to unknown protein [Arabidopsis thaliana] (TAIR:AT3G03160.1); similar to unnamed protein product [Vitis vinifera] (GB:CAO42347.1), mRNA sequence

GGAGTAAGGAAGGAAAAGAAAGTTCAAAACAAGGCTTCAAAGTTCAGACTTGGATCTAGGGTTTCCAATTTTCATATTCT

ATTGAAAATGGCACTTGAGTGGGTTGTCCTTGGCTACGCTGCCGGCGCAGAAGCGATCATGGTCCTTCTCCTTACGATCC

CAGGCCTCGATGGACTCCGTAGGGGACTGATCGCTGTCACTCGTAATCTCCTCAAGCCGTTCCTGTCGGTGGTGCCGTTT

TGTCTGTTCCTTCTTATGGATATTTACTGGAAATACGAGACGCGCCCGAGCTGCGAGGCAGATTCCTGCACGCCGTCTGA

GCACCTCCGCCACCAGAAATCCATCATGAAGAGTCAGAGGAACGCGCTTCTAATTGCAACGGCACTTGTCTTTTACTGGT

TGCTTTATTCAGTTACAAATCTAGTGGTCAAGATCGAGCAGTTGAATCAGCGTATCGAGAGGCTCAAGAACAAAGATTGA

TCATGTGACAATGAATTGTCAATTTGAGATAAGTGGATTTGTTGTTTGAATTTTGTGTTATTTATTATGATATTTCACGG

TTATCTGAAATCTTGACGGTACTAGTGTAGGGCACTAAGTTGTGTTTGGATGTTGATCTTAATAATGAAAGCCCTTTT

>FM895000.1 FM895000 Jatropha curcas embryo 71-95 (DAF) Jatropha curcas cDNA clone rjcpga0_001328, mRNA sequence

TTTATTTCCTTAAATGCATAAAAAATCGAAGGGGAAATGAAAAAGAGAACCATCTCTCCTGCGACGCATAGCTTCTTTGT

CTCTTAAATTTTGTGTTTCTGTAGCAGAAAAATTGTCATACATTCTTAATTTAGGTAATTTATATAGGAATAGCCATTGA

GGCAACCATGCGAATTTAAGTTATTCAAGATGCTTACAAACCCTAATTCTGGATTAGGGTTATAAGCCAATGGATAGAGT

GATAACTTACATATACATTGGCTAAATACAATGTAAAAGAGCCTGAGGAATAAGAGTTTTACTCCCCAAAAAAAAAGGCA

ATATTGTAGCGATCTGTTCACTGGTCCTGGCTCAGCAGATTGGTGAAGAAAGGCACTGAGAATGGAAAACAGGCATATAT

AGCAGGAAACAAGAAGAAAAGAGGATCCTATACTGCACCAGCCAGCTCAGATGTTAGTTTTTTTGCTCTGTTTTTACCCG

TTTATGGTTCGTTAGTTGTTCAAGCCCTGCTGTGTAACCCTATTAGTTTGGTGAA

>GW879348.1 JC003906 Seed specific Normalized cDNA library from Jatropha curcas L. Jatropha curcas cDNA clone N07143 5' similar to Ras-GTPase-activating protein-binding protein, mRNA sequence

GGTTCGTTCCTTTTTTATTCTTTCGCTGACTGTTTATCTCTTCACTTTCCTCACCTCTATATCTCTACTCTTCAAGGCAC

TGTATTCTCTCTTCTTCCCAAATCTCCTAATTGCAAAGCTTTAACATTTCCATTTCAGAATGGCTTTGCAAACTTCAACT

TCTCCCAGTGCCGAAGTAGTTGGAAATGCTTTTGTCGAGCAGTATTACCATATTCTTCACACCTCACCAGAAGTGGTCTA

CAGGTTTTATCAGGATTCAAGTGTGCTAAGCCGGCCAGATGCCAATGGCGTAATGACATCAGTCGCAACCATGCAAGGTA

TCAATGAGAAGATACTTTCATTGAACTTCAAGGATTATAAGGCAGAAATAAAAACGGCAGATGCTCAAAAATCTTATAAA

GAAGGGGTTACTGTGTTAGTAACTGGATGCTTAATGGGCAAGGACAACTTGAAAAGGAAATTTGCCCAGTCATTTTTTCT

TGCACCACAGGACAATGGATATTTTGTTCTGAATGATGTTTTCACATTTGTGAAGGATGATGAACCATTGGAGAACAATC

CAGTTAACAATATTGACAGCGCCCCAACAGTCCCCTCAAACCCAGATTCAGAGCCTTCTCATGTTCCTGATATAGAGCGA

GTTTATGAAAAAGAGGTGGTTGTGGAAACTCAATCTGATTCCAATTGGAATAATGATTCTGTCGTTGTTGAATC

>GW612432.1 Jc1-016-F12-M13F.F12.ab1 Jatropha curcas flower and seed Jatropha curcas cDNA, mRNA sequence

GAAAACTTCTTCCTCTATTCTGGTGTGTTGTTCTCAAAATTTCTGCTGGTTTTTCTTTTGTGCAGTCTGATATTCTAACA

CTTGCTCAGTCTGATACTCTAACAATCTTATCTTCTACGGACACTCTCTCCTTCTCTCTCCGCTTCCCGCTTTCAAGTTC

GTGCAATGGCTGACTCTGCTTCTGCTCCTTTCAAGAAAATCCAAATCCAGAGAGAAGATACTACATTTGATGCATATGTG

GTGGGCAAAGAAGATGCATCTGGGATTGTAGTGCTTCAAGAATGGTGGGTGTTGATTTCGAGATCAAGAACCATGCTGTA

AAAATCTCCCAATTGGAGCCTGGATATAAGGCGCTTATCCCTGACTTGTGCCGGGGAAAGGTAGGCTTGGATGTTGCTGA

AGCGCAACATTTGATGGAAGATCTTGACTGGCAAAGTGTTGTTAAGGATATACGTGCTTCAGTTAATTGGCTCAAGGCTA

ACGGTTCAAAGAAGGTTTGGCACATCTAGTTCTTTGCTGTTAGAATCATGGATTCTGGAATGCTTTTATGCCAATTGCCT

GATCATGCTCGTTAGATATCTACTGTTCTGTTTTGTCCTATTTCTACGCTATGCCCAACATCTATAATTCCATGTGG

>GW614095.1 Jc1-035-F04-M13F.F04.ab1 Jatropha curcas flower and seed Jatropha curcas cDNA, mRNA sequence

AGAACTCTCTTCAATGGCCGCCAGTTCGAGCCGAGGCGGTTCCTTCTATGGCGGCGCTGCTCCCTATAGATCGAAGGATG

GGCTCAGCACGAGACCGGCGGCGAGCTCCGATGAAATACAATTACGGATTGATCCGATGCACGCTGACTTCGATGACGAG

ATCTCTGGTCTCCGTAGCCAAGTTAAGCAATTGAGAAATGTGGCTCAAGAGATAGGATCAGAAGCAAAGTTCCAGAAGGA

TTTCTTGGATCAGCTGCAAATGACTGTGATAAAAGCTCAAGCAGGTGTGAAGAATAACATAAGGAAATTGAACAAGAGCA

TCATTAAGAATGGTGGAAACCATATCGTCCACGTGGTTCTTTTTGCACTCCTATGTTTCACTGTGGTCTACCTGTGGTCC

AAAGTATCCAGAAGATGAGTTATGTTTAGACTGAAAATATGTTCATATCAATAGCTATCCATGGATGGACCGGTATTTGC

GAGCTATATTGCAGGATCCGTTGTAAAATCTTCCCATTAGCTTCTTTATTGAACATTGGCTTTTGTTGGGTTATGCCTAA

AATCTAGATCTTTCCTATTTAAAAAAGAAAAAAGAGATTACTACTATGCTTGATCTCTA

>GW878467.1 JC002846 Seed specific Normalized cDNA library from Jatropha curcas L. Jatropha curcas cDNA clone N05347 5' similar to Lactoylglutathione lyase, mRNA sequence

GGCGGAACAGGTATCATTTCTGGATAGTGCGTCTAACTTGTCTTCACTGTCTGTTACTGTTCGCTAGTGTTGACAATGGC

TGAGGCTGCTTCAAATGCTGAGCTCCTGGAATGGCCAAAGAAGGATAAGCGCCGACTTCTCCATGTCGCGTATCGTGTTG

GTGATCTTGAGCGCACCATCAAGTTTTATACTGAATGTTTTGGGATGAAGCTGTTGAGGCAAAGAGATATTCCAGAGGAG

AAGTACTCAAATGCCTTTCTTGGTTTTGGACCTGAACAAACTAATTTTGTTGTGGAGTTGACTTACAATTATGGAGTAAC

TAAATATGATATTGGAGATGGCTTTGGGCATTTTGCGATTGCAACTCAGGATGTTTACAAGTTGGTCGAAGACATCCGTG

CCAAGGGTGGCGAGATCACTAGAGAGCCTGGTCCAGTCAAGGGCGGAACAACTGTCATTGCCTTTGTGAAGGATCCGGAT

GGTTATACTTTTGAACTCATTCAAAGAGGACCGACTCCTGAGCCTCTGTGCCAAGTAATGCTTCGTGTTGGTGATCTAGA

TCGCTCTATCAAGTTCTATGAAAAGGCCTTA

>GW611525.1 Jc1-006-G02-M13F.G02.ab1 Jatropha curcas flower and seed Jatropha curcas cDNA, mRNA sequence

AAGAAGCTAGGTCTTGTTGATGCCGTTGTGTCTTCTCAAGAACTGCTGAAAGTATCTCGGCAATGGGCTTTAGACATCAA

AGAAAGGCGAAAACCATGGTTGCGTTCTCTTCATAATGTAGACAAAATTGGTTCCCTTTCTGAAGCACGTGAATTATTGA

AAGCTGCCAGAGAACATGCCAAGAAGACTGCCCCAAATCTGCCTCAGCATCAAGCATGCCTTGATGTAATTGAGGAAGGC

ATTGTTCATGGAGGATATTATGGAGTTCTAAAGGTATTCCTGGGCTGGAGACATACAGTTTGTTAAACTAGATATATTAA

ATTGTTTACCTGTATTAAATTTTCAGTGATTGTGGAATCTTCCTCTTATGCTTGCTTTTGTTTTGAGGTGTGCATTTGTG

CAATAATTGACTTTTTCTTTCATGTCACAAGCTTATTTCCACAAGCATGGTGGTTATTTTGACTTAATTTTCTTAATGTG

CACGCCAATGGATAAATTTTATTGCAGGAAGAAAAAGTATTCAAGGAGTTAGTTTTGACAGACACTTCTAAAGGTCTTAT

TCATGCCTTCTTTGCCCAGCGTGCAACATCGAAGGTGCCCAATGTCACTGATCTTGGGCTCAAACCAAGGGAAATAAAGA

AAGTTGCTGTCATTGGTGGAGGTC

>GT974851.1 GJCCJC2064C01.b Jatropha curcas L. developing seeds (mixed stages) Jatropha curcas cDNA clone GJCCJC2064C01, mRNA sequence

GGTGAACTACGTAAATTTCTGTGTGTCACAATGGTGGTTATTTCGCTGTGTTAATTTTCTCTTAATTGGTTGAAAAGGCC

AACAAGTGGTATCAGAGCTGATGCCCAATAACTGGTATCAGAGCTGATTCCTAACAACTGGTATCAAAGCCGAAAGTTTC

GGTAGGTCCAGCCGATCAGATAGCGGTACCGGATCCGTGGATATACTTTGAAAATAAATATACCACGACGACGAGAAAAT

TACGACGAGTCTATTGGTGCTGTCCGCTCGGAAAGATATTATAGTATCGACTTAGTGGTGAATAGTAAAACAGTAACTCA

AAGTTGGTGGTGAACAGTATTATTGGCGTTACTGTTGCTGAGCCGTGACTCTTAAGCCCAGCACTATTCACGCATAGTAC

TATTGACGTGTACAGTTACTATTTACGTGTACTGATACTATTCACGTATACTGTTACTATTCACAGCTACTATTCACGTA

TACTAATAATCGAGGCGGAATCAGAGTAGAGTGAGCCAGTGTTTGTAATGGTGGACGATATCTGAGAAAGTCCAGTGGCC

AACGACT

>FM892214.1 FM892214 Jatropha curcas embryo 56-70 (DAF) Jatropha curcas cDNA clone rjcaeb1_002706, mRNA sequence

ATTCACTATTTAGAAGCAGTACAGGAGATCTTGGTTGTGCTCTCGCCGGTGCCGGAGATGGATGGTAAAGGGAAAGAGAA

AAGGAGGAGGATTGACAAAGAATCCGATGAAGCTAAGAGTAAAAAGGTTAGACATGGAGATAATAAGGAGGAGGCGACGG

ACGAGGAAGTTGAGGAATTCTTTGCGATTCTAAGGAGTATGCAAGTAGCTGTCAAGTACTTCGAGAAAGGAAAACGAGAA

GGCTGGCGTGCTGCCGTCGAAGCTGAAGTGGTTGCGGTGGTTGGTGGTTCCAAAGAAGATGAAGACGATGATCATCAGAT

TAAAAAGAAGAAAGGATCGCCAGCAAAGATAAATCCGATTGTGGAGGAGGAGGAGATGGCGGTTTTGGATTTGAACGCCT

TGCCGGCGGTGGAAAGTAGTGAGATATGTATAACTGAATCGCCTGATCAAACGTGGTTGAGATCAAGGAAGCTGCCGTCG

CGTGCGGTAGTGGAGGAGCAGCTCGTGAG

>GW877521.1 JC002244 Seed specific Normalized cDNA library from Jatropha curcas L. Jatropha curcas cDNA clone N04114 5' similar to Unknown protein, mRNA sequence

GGTTACACTGCAGGAACACTATTCGCCACAAACGAATAGCAGTCTCGCAATTTTCAAATTCCAAATCTATTTCTCTCTTT

CACAGATCGGAATGAGGTTAAGCGGAAGGACAAAACTGTCGAAAGAAACTTCATTGAATCTCATAGTAGTCGATCAACGA

TCAGAAATTATTTCTTCCCTTATTCTGAAGTTGCGATCATGGTCGAATACATGTATTCGAAAATCTGTTTCATTTTTCTC

ACCGATTTTGGTAAACGTCAACGTTTTTGTCACAAGAATCATACCGACCAAATTGTGGAGGAAAAAAAATCATCAGGTTA

ACGATGTAGCCGCCTGTGATCGAATAACATTCGAAGGGGATACAGATTTAGAAGACGATATAGAAAAAGAATTAATGTCA

CATGGTGTTGGTGGATTGGCTAAAAGTTTCGATTTTATCACGGACAAGGTGTTTGATACTTCATTGCGAAGCGCTGGGCA

ATTTACACGGTTAAAGATCAATAATGTTAAAGGGATGCTTCTCTATGGGCCACCTGGAACTGGGAAAACATTATTGGCAA

AAGCACTTGCAAAGATGCTACACGCTAAGGAATCTGATTTTCTCCCTCCCACTTTACAATTATACAAATGCACACAAAAT

TTTTACTTGGCAATATCTTGGATTTTAAACTGTAATAGCTATAATGAATAATGAAACGTTCTAAAAATCAAA

>FM887872.1 FM887872 Jatropha curcas embryo 35-55 (DAF) Jatropha curcas cDNA clone rjcfea0_001155, mRNA sequence

GGATAAATACAGCAGTAAGATTACATAAACACAGAGGACATATACCTACTTCAAAGTCAAAATATGATGATATCCCCTAA

AACATGATATACCTACAGAAGTATAAGCTGTATGATAAAAACCCAGCTAGAAACATCTGGGATCATTGACAATACTAAAC

AACATTGCCTTCGTATTTTTGGCTACCTCAAAACCCCCCATGTTTTACGTTCTCTGAGATCACTGGGGATGATAAAAACT

GAATCTACACAAAGCCCACCTTTAGAATGTGTGCAATCAATCTGTTTCATGGAAAATTGGACCTCAGTGGCAGGTTCTGA

GTTGGTGACAACAAATTCACCTACCTTGTACTCTATCCAGCATCCACGTTTATGGCTGCCATTTAATTCATCTTGTTCGG

TCTCATCCAAACAACATTCGGAAGATGCTTGCTGACCATCAGAAGTAGACAACGTTAATCGTACTGGCTTTATATCCCAA

CCATGGGTGTGCTCAGAATTGCACACACGCCGTTCCAACCTTTTGGCCAATCTGCCA

>GT971703.1 GJCCJC2023E08.b Jatropha curcas L. developing seeds (mixed stages) Jatropha curcas cDNA clone GJCCJC2023E08 similar to transport protein, putative, mRNA sequence

GGAGACAGAGGAAGGATTTGATGCCACTCGGTGGTTAGATAGGAACCTCATCCGGCTCTGTTCCAAATTTGGTGATTATC

GGAAAGATGATCCAGCATCTTTTACATTGAATCCTTCTTTTTCATTGTTCCCTCAGTTTATGTTTAATCTGCGACGATCT

CAGTTTGTACAGGTCTTCAACAACAGTCCTGATGAGACAGCTTATTTCCGCATGTTGTTAAATCGGGAAAACATCACAAA

TGCTGCTGTCATGATTCAACCGTCATTAATATCATATTCATTTAACTCTCTGCCTCAACCTGCATTGTTGGATGTGGCTT

CTATTGCGGCTGACCGTATTCTCTTATTGGATTCATATTTTAGCGTGGTCATTTTCCATGGTATGACAATAGCACAGTGG

CGTAACATGGGTTACCAGAATCAACCTGAACACCAGGCATTTGCACAGCTATTGCAAGCACCACAAGAGGATGCTGCATT

GATAATTAGGGATCGATTCCCCGTTCCTAGACTGGTGGTATGTGATCAGCATGGATCTC

>GW877932.1 JC003534 Seed specific Normalized cDNA library from Jatropha curcas L. Jatropha curcas cDNA clone N06559 5' similar to Unknown protein, mRNA sequence

GGATCTTCAAGGCGTGCCATTTTATGATTTTCTCTTTTACATTAAATTTTTATTTTTCTTTCTTTCTTGTGCTAATCAAG

GCGTGCCATTTTGGTTCTCATTAGATATTACTCCTCTTTTCCTCAAAACGCATCGATTGCTCTCTCTCTCTCTCTCTCTC

TCTCTCGTCCTCTTCCTCTTTCTCTTTCTCTCTGTTCATGTTTCTTTCTTCTCCTCCAGTCCTCCTTGTTTTCAATAATG

CCCATTTTCTTTTCACATGATTTGATATCACTTCTCTTATTTGCAGCCTTTTGATACTCTCTAAACAGACAACAAAAAAA

GAGGGAACACGTCAACGTACTCTCTTCTTGTTTCTTTTTCATCTCTGTTTTTCTCTCTATATCTTCTTCATTTTCTTGTA

TTTTTACAAATTATCTACAAAGAAACCATGCATAAGTGGGAGAAAGCACTAAAAGAAGACAGGTACAAGCAAGAAGGAAA

GAACCCATCTTTCTCTTCAAGTCTTCTTGATGAAATCTACCGTTCTATTTGTGAAGGTGACACAAAGCATGAAGATACGA

AATTTTTTAGAGAAACAATGCCCAAGAAGCAAAACAAAAGTGTTGGTGTAAAAAGTTGCAGA

>GW878139.1 JC002333 Seed specific Normalized cDNA library from Jatropha curcas L. Jatropha curcas cDNA clone N04319 5' similar to Unknown protein, mRNA sequence

GGAGCGCCATATCTCCAAGAAGCTTTTTCTTCTTCCTCCCTCTCTCTCTCTGTGTGATATATATATATTTATATATAGTA

TAATCATACATGTCTTGTATAAAAGAAGGTGGCGAGTGATAGGGAGAGCATGTGATTTGATGAATTGGATTCGATTTCTG

TGTAAAAGCTGAGGATTCTCTGCGCTTCTCACTTTGGTTTGTTTGCCATTTTTAATAATTCGAAAGGAAGATCTAAGAGT

ATGCAAATATAACCACAAAGACAGAGAGAGAGAATTCATGTCTGATTTTCCAGGAAAGGTACCCTGATTTTGCTGGAACA

TTTCTGTCTCTCGCATCCCGAGGAGCTTAATTTGGTTTTTTACTCTCTCTCTCTCTCTCTCTCTCTCTCTCTCTCTCTGT

ATCTCAGTTCTAGGGTTTGTTTGTAAGTTAAGAAAAACGCTCGTTTCTAAATGTTTCAAGCTCGTTTTTTACTTAGATCT

CCATTTTGTCTAGGGTTTGTTGTGTGATCTGTGTTAATTGATTGTTTTCTTTGTATCTCAATTACCATGGTCAATCTCTT

GTCTTGAAATATCATTTCTGTATTCTAGGGTCATTTCAACTATCCGTTTTTTCTTTTATTCGTTGCCTATTATAGCTTCC

AAGAATAGATACTGAATCTCATTTTATAGAGCTGAAAATTTCTTTGCCCTAAGAT

>JK317735.1 JCST506 Jatropha curcas L. seed cDNA library Jatropha curcas cDNA 5', mRNA sequence

TGTCATCTATGTCGGGTGGTCATCTATGTCGGGCGTCACCTATGTCGGGTGTCTTCTAAGAAAGGTGAACCCAAGTGCGA

TCTTTCATTATGCACCCACAGGATTGATTACCACTTCTTTTATTGGAGACGGCTCTCATTGTAAATGCTTTCTGAAAATG

ATGGACATCCTGCCCAGAAACATGAAACTTCTGTGTTTCTTCCTCCTCCTGGTGCGGGCTCCCCCATATGAGTGTCCCAT

GAATGCCGATTTCAGCATGGGATATGCTGCCTCAGATCAGAACTGTCAATAAGCATTTTTCTCTTCACACCGATCCTGCC

CCACGCGCAGCTTCATCACTGTGGCCCAGGACTGTTGAAACCTTCCATAAAACTGCCCCTCACCTACGATGTTGTTGGTG

TGTGGTGGGTCTAACGAATTCATGTGGATACTCCCTCCCCCGGGTTGGGGGTGACAATGCATGCGGATATCAAACAGAGT

GCAGCACAAGCTACCCCTGTCCTTCATGAGCCTAATTTGTATATCGGGAGATAGGTCCACGCTTCGGGTTCGCCCTGACT

TTCATCCCTGATGCAGGCCGAAGGATTCAGTTGTGTCTCGCCTTACGAGAGTTAGCACCAATAGTGATGCCGGGCACAGA

CGGGTGCGCTAGTTGTGTTGTGCTGCAGCCAACAGACCGCGATTGACGTCTCGCCNGTAGAAAGGACAACAAAAGACACT

AGAAGTTCTCTACAGGCTATGACGTACTCATCTGGTNATAGGGAGTCTAGTCTGGCTCCACAGNACTAAGCAGAGGTTCT

TTGAGGAATGGGTCGTGATACCATAACTTCTGGAGATGTTATGTATTTCTGATAGATGTGCTA

>GW618153.1 Jc2-031-H01-M13F.H01.ab1 Jatropha curcas flower and seed Jatropha curcas cDNA, mRNA sequence

GAATCTTTAATTCCGCTTGCTTCTTTGTTCTTTCCTACTTCAGTTATTATTATTATTATTATCATTATTAGTTATTAGAC

TTTTACTGCTTCAATAAAACATGGAAGCAGGCGAGTGTTGTTCCAGTACTTCTCCCTCTACTTCTACTCCCACAAGTGCC

GAGAAACGCAAGCACAGGCAACAACAAGAAAAGCCTTACAGAGGTATAAGGATGAGGAAGTGGGGAAAGTGGGTCGCTGA

AATTAGAGAACCCAACAAACGTTCTAGAATTTGGCTTGGCTCTTATTCTACCCCTATCGCCGCCGCGCGTGCATATGACA

CCGCCGTTTTCTACCTTAGAGGCCCTTCCGCTAGACTTAATTTCCCCGATTTGATATTCCGCGATGACGAACAGACAAGA

GACATGTCTGCTGCTTCTATACGCAAAAAAGCAACCGAAGTTGGGGCTCAGGTGGACGCCCTGCAACAAACGGCTCCCCA

CAGCCATGCATCGGATCAGAACTCGAACCGAGTAGTAGTTTCAGAGAAACCTGACTTGAACCAGTATCCGAACCCGGAAA

ATTCTGATGAAGAGTAGAAGAAATAGAATCGTTTGATTAATTAGTAATATTAAAACTCCTTGTTATCTTTTCTTTTTAAA

GAAAGCTAGCTATAGT

>FM888277.1 FM888277 Jatropha curcas embryo 35-55 (DAF) Jatropha curcas cDNA clone rjcfea0_001659, mRNA sequence

CTAGTGGATCCCGGGCTGCAGGCGCCTGACGCCTCTTTTCTCTGGTGCCTTTTCGCTCCCTCCCCGCGCTGGGAGGCCCA

AAAAAGCTCAGGAACTCGCTTCAGTCCCCTTCATACCTAATCCCTGATTATTTCACTGTCTGTAATCTTAGTTGCGCTCA

ATTTTGAATCTCTGATTTAGGTCAAAGTTTCAATTCCACCTTTACTTGAACGATTCGCTGATTTCTTTCTTAGAAATGGC

AGTTATTTGCTTCTAGGGTTTCCTAGATTTTCTTTCTCACTTTCCGAAGCCAATATTTACCTAATCCTTATCAGGATTAT

CAGAATTGCTGATATGGCTATTCGCGTCACCTTCACCTACTCTGGCTATGTCGCCCAAAACATCGCCGCCTCCGCCGGGA

ATCGGGTTNGAAATTGCCGCTCTCTCCACGAATGTTGGGTTCGCTCACGCATCTTCTCTTCGCCCACTGTTCAGAATACC

GATTACGAA

>FM890254.1 FM890254 Jatropha curcas embryo 35-55 (DAF) Jatropha curcas cDNA clone rjcfea0_004071, mRNA sequence

CACTGGAATGCTGTTGGAGATGAGCAAGTCTGAATAGTTACCTCTTGAATTCCCCAAATTCCTTCGCTTTACACTTGACA

GGGCAGCTCAAGTGCTGAAGGAGGCCAATGCTATGAAAGCTGCAGATACTGGTGCGGTTTTGCCCAAGTGTGCTTACTAC

TTGAGATACTAGATTTAGTGGTGATTCCGGTTTCTAACATCTTAGTACTATCTTCAAAAGTGGATGTTAAAACACAGCGT

TTTAACACGACACGATTTGTCAATTGGTGCACAAATTGCAT

>JK611563.1 JCF4348 Jatropha curcas, immature Seed cDNA subtraction library Jatropha curcas cDNA similar to polygalacturonase-like protein, mRNA sequence

ACAAACACTAAAATAGAAGACTGTTACATAGTCTCTGGAGATGATTGTGTGGCCGTTAAAAGCGGTTGGGATGAGTATGG

GCTCCCCATTTGGGATGCCAACAAAGCAACTAATCATCAGACGGCTCACGTGTATTTCCCCATACAGTGCTACAATTGCC

CTTAGGGAAGTGAAATGTCCGGTGGAATCCCAAGATGTTAGAGCCAAGGACATTACAGCCATTTCATACGGAATTCAGGG

TCAGGATCAAAATGGCCGGGGGCAAAGAGGATTGTTAAAACTTTTTTGTAAAGGAAGACCTGCTTCTTAAAATGGGTTTT

GGGAACGGAAATTTGGGCCCTGGGGAAAAAATTAACCCATTTTTGGGGTTTTTTGGGGTTTTTTGGGGAATTTTTTTTAT

AATACCCCCCCCCGGGGAACCCCCCCCCCCCCCAATTTTTTTTTTTTTCTAGGGGGGGGGGGGGGGGGCCCCGCGGGCCC

CCCCTTTTAAAAAAGAGAGAAAAAAAGGTTTTTTTTTGGGGGGGGGGGAAAAATTTTCACCTCTCCCCTCCGGGGGGGGG

CTTTCCTAGCCCGGGGGGGGGGGGGGGGCGTTTTTTGAGCCACACCCCCGAACACGAGAGGAGCAAAAAAAAAAAAAAGG

GGCGGGGTTCTATTTTCGTTTTTATTTTGTTCGCTAAGACGATCCTTGTCATAACAAACACATGGTGGAAACCCCCCGAG

GGCTGGGCTATCTATGTCGGGAAGTGCAGTGCTCGACTGCAGTAGCGTGCAGTACGCGTGTAGGTGCTCGTGCTGTAGAC

TAGATCGATCGTATCAGCGTCGGAGGTAGTCGTATAGAGGCGGTCG

>FM890967.1 FM890967 Jatropha curcas embryo 56-70 (DAF) Jatropha curcas cDNA clone rjcaeb0_000940, mRNA sequence

ATTCCTTTAGNTATTGGATTGATGAGGTTTTGAATTTGCTGAAAAACTTGTTTGTATCAAAATTTCCTTTAACAGAAACT

TTCTTGAGGCCTAAAGAATTACCTGATTTTGGCAAGAAAACTTTTCAAGCGAGTTCATAATTAAGCACCAACTTATTAGG

GAAAATGCACGAAAAGAAAATGTGAAGATTTGTGCAAGGCTATTTCTCAGCAAATCCAAGAAAAGGGTGTGCTATGCAGA

AGTTGGTGATGATTTTGTTGATTTACTCTTTAGTTTCCTTACCATTCCTCTTGGGTTTATAATGAAAGAAATGAATGGTG

CTCATTCAAAAGGGTGCATAGACTATTTATATAAAAGTATTATGGATCTTGATAGTGAGAAATACTTCCAAACAAATGTC

CATAAAGAGATTTTACTTAGTCCCAAGATTGCTCCTGATTTTGGCTATGAGAACCAGCTTCTAGGGGTGAAGGAAGTTGC

CCACCAGCTCTGCTACCTCAAATTGAGTAGTAATGGTTTAGGGTTTAATGTTGGGATTTCTTTGGATAAACCTACTAGCA

ATAGAAGCATTGCCTTAAGAGTAAAGGATCCAAAATCAACATACAAGAGCGGTAAAACTGGCAGAGGATTTATGAAGGG

>GW877536.1 JC002259 Seed specific Normalized cDNA library from Jatropha curcas L. Jatropha curcas cDNA clone N04138 5' similar to Unknown protein, mRNA sequence

GGGGCCTAGTTGGAGAGACTCGCTGCCTGAGTATTGGAACATCTGATATTGTCTTTGATGATCTATCTTTTGATGGATCA

ATGCCTCTAGTAGGACTAGAAAATAGTCAAACTTCGTATTTACATAATGATACATTGCCAGAAATTATAGAGAGCTATTG

CTTGTCTGAGTTGCGCCCATTATCAGGATTATCCATAGGCAGGCAATCACCAGTTGAGATTGTTAAATCCCGATCCAGGA

ATTTTGGAGATGGAGATATTCGAAGAGGAAATAATGGATGGTATAAGGATGCCCCTTTAAGCATTGTTGAAAATCACATT

TCTGAAGCAAGTGGAGAAGGTATTCATGTTTCTGAAGACAAGCTTCCATCTTTTGACTGTTCAAGATCTGACGACATAGG

AAAGCCCAATGGACGTTTACTTCTTAAGAACATTAATGTGACCTGGAGAATGTTTGCTGGTTCTGACTGGTGTGCACATG

AAAAGAATGGCGAGTCTTCTAGAAGTATTCATGGAAGGGATAGAACTGCTTGTCTGGAACTTGTGTTATCTGGAATGCAA

TTTGAATACGACTTTTTTTTCCTGTTGGTGGGATATGTGCTTCTAAGCTTTCTCTTTCAGTTCA

>GW615742.1 Jc2-004-A03-M13F.A03.ab1 Jatropha curcas flower and seed Jatropha curcas cDNA, mRNA sequence

GGCTTAATTCTTCTGTGGCCATGGCAACGAATGAGAATCTTCCCCCAAATGTCATAAAACAACTTGCCAAAGAACTGAAG

AACCTTGATGAATCTCCCCCTGAGGGTATTAAAGTAGGAGTAAATGATGATGATTTTTCAACCATATATGCTGACATTGA

AGGGCCAGCTGGAACACCGTATGAGAATGGTGTGTTCCGCATGAAGCTGATATTATCACGTGACTTCCCACATTCTCCTC

CCAAAGGGTACTTCCTTACAAAGATTTTCCATCCGAACATTGCAACCAATGGTGAAATTTGTGTCAATACGCTAAAAAAG

GACTGGAATCCAAGTCTTGGACTGCGCCACGTTCTCATTGTAGTTAGATGTTTATTGATTGAGCCGTTTCCAGAATCAGC

TTTAAACGAGCAGGCTGGCAAGATGCTGCTAGAAAATTATGAGGAGTATGCTAGGCATGCCAGGTTGTACACTGGAATTC

ATGCCAAACCAAAACCAAAGTTGAAGTCGGGAGCTATTTCAGAGTCTACTACAGCGCTGAATGTTGATCAAAGTAACACC

TCAGTTCTCAACGGTGACCAGAAGAACACAGCAGTTACTGTTGCAATAACATTGCCATCCCCATTGGCTCCTTGTACAAT

ACCTATGAAAGGA

>JK610915.1 JCF3190 Jatropha curcas, immature Seed cDNA subtraction library Jatropha curcas cDNA, mRNA sequence

CACCTATACGAGTTCTGGATACTCTTCCGTTTGATATTCTCAGGAGGACGGGTGGCCTTCCCTGCGAGGTAAGAACTATA

TAGATGGCAATAACAATTCAGTAATAACTAATCAAAGCTTTTCCATTAAAGGAGAAAATCCTATCTTTTATTTTCCTTTT

CCCTGGTGAGAAACCAACTACTGAATGCTGTTATCTTTTTGCAAATTAAAACCTCAGGCCATCTTGGAAATTAGTTTTCT

TCTTTTTTTTCCTTTAAATGCGTCAATTTTTGTCTCTAAGCAAGTGTATAGGATATGATACTCTATTTATCTTTTGCTTT

CCTTCTTTTTGAAGTTTAAGCATAGAATAATATTGCTGATATTTTCATGACATAGTGAGTGGTTTTCTAGGTGATATAAT

ATATTAGTGGTTATCTTCAAACCCCGACATTAATGAATTTTCATTCATACCCTTCTTTCCCAAAAGCGAGTTCTTCTTTT

TTCAATCATTATGAAATGAGTAATCTCACGTTAAGAGCAAAATTCTATTTTTAGGCATTTGTAATGGTATTACATAAAGA

GAAAATTCGTTTCCTAAAGTGAGACCATCTGCGCATGGACATTCACAAAATGAATATGCATAAATTATATGATGGCACTA

ATTAAGGTTCTGAACTGCATTATTTGGGTTATTTTGTGCATCAAGATACAATTTCTGT

>JK611492.1 JCF4183 Jatropha curcas, immature Seed cDNA subtraction library Jatropha curcas cDNA similar to predicted protein, mRNA sequence

ACCTTCCCCTGACCTTAGGCTATCCTAATACCGATCTCTTATATAAGTCATTCCATCCCTAAGATCCGAGTTTAGGAATC

AAAGAGACTTGATTCAAATTCAAGGGCACATCTTGAGGGGAAAGAAGGAAAGAGCCTCCTCAGAGTTCTCAGAGATAGCG

TTAACGGATCCATTTCAAAGTGCTACGCTTCGTTCGCATGAAGAAAGGAAGGTGCGAAGCGTCTAGTCTCTTTCCCTTAA

CAAGAGGGGCAGCACTCACAGGTTATGGGCCTTGCTCTTGGAGAGGTTGGGTATACTACATCACTTCTTCAGGAGCAAGG

TGGGCATTGATGAATATCGGGCGTGGTTCATCGTCCGTGCCAAAATGGATCTGCTTGACTTCTCTTCCGTGGGGAGGAGC

TTTACATGAATAGGGGGCTCTTCTTCTTTCTGACCCTGCAATGTTAAGCTTGGAAGTTTTTCTTCATCTTCCTCGCCGGA

TTCTCCTGCGAGCTCTTTAGTAATTAGGACGCCTGCGATTTATCTGCTCGGTAGAAAGGAATCTCCAGTTAACTCAGGTC

CCACTTTGGAGGAAAACTAATCTAAATTCGACCCTCTCTCGTCAATTGAGAGGCCCGCGCGCACACATATATATGCTTCG

AGAGACAGCTAATCCGGCGTTTAACCGCGCGGAACCGGGTTCCAATTATCTTGGAACTCTTTGCGGGGTAACAGAGGC

>JK317579.1 JCST350 Jatropha curcas L. seed cDNA library Jatropha curcas cDNA 5', mRNA sequence

AGCTGGAAACTTTACTGAATGCAGACTTGAAGCTTTGACATTATTGAAGAGTAAAGAAGAGAAGTGCTTACGTCCACCAT

GTAAAATTGCACCGTTTTTCTTCTCAGAGATACAAAGCAAGCTTGTTTCTCAAGAAACCTTGTTGTATACTTCTGAGTTT

TTTGGGCTGGTACCTATGTCGAGTTTGTCTGAAGTGGGGACTGATGGTTGTGTTTTGGAGTTTGGGCCGGCCTGGTGATT

CGACGACTAAACGATTAAATGATGTATTCGGCTAGGCGACGAGGGTGTGAGGAGGAGCGGCGTGGTACACGACCGACAAG

AGTTGTACACGGCCGCGGCGAAACATGGTTGTGGAGACTCGGAACGCCCCCGCTGGTAGGGGAGATGGACCCCCTGGGAA

AGGGTTGATGTTTTTGGAAATGGAAAAATGGCCGGCCGTGCAATACGATGGGCTGGTGGAGCAGAAGGATCTTCCGAGGG

AGCAGCGTCTAATGGGCATGAACCGAGATGGGGTTAGAAACTGGTCGTTGGAGAGTGATACGAAAACCCACGCCGGGGCC

AGCGTGCCTTGATAACTTGAACCAGACGTTCAGAGGATGAGCACTATATAATCCGATTGCCAAATGAATTCGATGGGTGG

ATCCTCTTGGTTGACAGAATTTGAGTGAAGAGCCCAGACCACATCAGTGACAAGGGATGTATGACGAGGGAAGAGCGCGA

CATAAGAGTACAGCTCGTCATTGTAATACAGAAGGATATATCGCCTTAAAAGAAAACAATATAGCGTGCAGGAAATGGTG

AGAACGGAGAGATAGCATGATACAGACAAATAAAATTGT

>GW880081.1 JC000595 Seed specific Normalized cDNA library from Jatropha curcas L. Jatropha curcas cDNA clone N01125 5' similar to Unknown protein, mRNA sequence

GATTTTCTCTAATTTAGAACAAGAAAAGTCTTTTAAAGGAGAAGGGTATCAATACTTTTTTGTTTATTATATTAAAAAAC

AAGAAACATTTCTGTCTTTAGTGTGTTAACACACAGTTTTGCTTTGTCAGTACTTTCTTGCTTCTATGTGCATCTATGGT

TATAATTTCTTTATGCACTAATTTTAAAACCCATCAATTTGTTTGTGATTGATATGAAAAGATGGAGGATCTCTGTCCTC

TATGGCTCAAATATCTTAATTCAATTTGAAA

>FM888481.1 FM888481 Jatropha curcas embryo 35-55 (DAF) Jatropha curcas cDNA clone rjcfea0_001906, mRNA sequence

CAGAAGAATGTGTACCTGGAATATCATTCGGCATGGCTAATGTCGAACTTCCACTTGCACACTTACTATATCATTTTGAT

TGGAAACTTCAATCTAGGAATAAACCCAGAACGCCTTGATATGACAGAATCATTCAGCGTTACAGTCAAAAGAAGTAGTG

CTCTAAATGTAATTCCCATCCCTTACATTCCTTCCCGTGTAATGTAAAATCTTTATTTTTAAATGCTTTCCTTTTTATGA

ATAAATATTCCCATATATAAATATGATTGATT

>GW881479.1 JC001366 Seed specific Normalized cDNA library from Jatropha curcas L. Jatropha curcas cDNA clone N02578 5' similar to Sucrose transporter 4, mRNA sequence

GGGTCCTCTGATTTCTAAAAGCTGAAAAACACTTGACAGAGAGAGAGTGTGTGTAAAGACTGAGATAAGCAGACGCGCAC

AAACTCAGAAACAGTTACTTCGATTGCCATAGATAGTGAGTGTTCGGGGCTTGTGCGTGTTCCTTTCGAGGAAAAGGGAT

GGCAATCCCACAAGCGGAGTCACATCGAGCCAGAGCTAGACCACCGGTAATCCGCAGAGTACCGTTGCGGCAACTACTAC

GTGTGACTTCAATCGCCGGTGGCATACAGTTCGGATGGGCTTTACAGCTCTCTCTCTTAACACCTTACGTGCAGGAGCTA

GGAATTCCGCATAAGTGGGCCAGTATTATATGGCTTTGCGGACCATTATCGGGCCTAGTCGTGCAGCCTCTAGTCGGCCA

CATGAGTGATCGCTCCACCAGCCGTTTTGGCCGCCGCAGGCCGTTTATTCTTGTGGGCTCGGGTTTTAATTTGTCTTGCA

ATTTT

>GW878716.1 JC002982 Seed specific Normalized cDNA library from Jatropha curcas L. Jatropha curcas cDNA clone N05685 5' similar to UDP-glucosyltransferase, mRNA sequence

GGACCCATTTAGTGCACCCTCCATTTCACTTCGCTCACCCCGTGCATCCCACGCGCTAAAGAACCACCATCGCCATGCTT

CAACCTCACTTTCTTCTCGTAACCTTCCCGACCCAAGGCCATATAAACCCAGCTCTCCAATTCGCTAAGCGATTAATACG

AATTGGTGTTGAAGTCACTCTAGCCACCTCTGCATATGCTAAACGTCGCATGACCAAGACCTCATTTCCTAAAGGCTTGT

CATTGACCACCTTTTCCGATGGGTATGACGACGGTTACAAGGGTAGCGACTCCGATGCCCATGACAAATACATGTCGGAG

ATAAGGCTTCGAGGCTCGGAAACTCTAAGTGATCTCATAATAACTAGTGCAAATGAAGGAAAACCAGTTACTTGTTTAGT

GTACACGTTACTTCTTCCTTGGGCAGCTGAGGTGGCGCGTGCACATCACCTCCCATGTGCGCTTCTTTGGATTCAACCTG

CAACAGTTCTTGATATCTACTATTATTACCTCAATGGTTATGAGGATATGTTTAGCAAGTGCAGTGATCCATTATACCGA

GTCGAGTTGCCTGGACTTGAACCCCTAACTAGTCGCGACCTTCCTTCATTTCTAGTTCCTTCTAACGCTTACGCTTTTGT

TCTTTCGTTGTTTGCAGAACAGTTAGAGATGCTTAACAAAGAAACCAATCCAATAATATTGGTAAACACTTTTGATGCTT

TAGA

>JK317419.1 JCST186 Jatropha curcas L. seed cDNA library Jatropha curcas cDNA 5', mRNA sequence

TCGTATCTGCNTANGATGGAATAAATCCTTCAGAGGGCTATGAGAACAAAAATGCATTCGAAACTGGCAGGCTAGAGTCT

TGTAGAGTGGACGGAAGAAATTCCAGGTGTAGCGGTGAAATGCGTAGAGATCTGGAGGAATACCGGTGGCGAAGGCGGCC

CCCTGGACAAAGACTGACGCTCAGGTGCGAAAGCGTGGGGAGCAAACAGGATTAGATACCCTGGTAGTCCACGCCGTAAA

CGATGTCGACTTGGAGGTTGTGCCCTTGAGGCGTGGCTTCCGGAGCTAACGCGTTAAGTCGACCGCCTGGGGAGTGCGGC

CGCAAGGTTAAAACTCAAATGAATTGACGGGGGCCCGCACAACCGGTGGAGCATGTGGTTTAATTCGATGCAACGCGAAT

AACCTTACCTACTCTTGACATCCAGAGAACTTTCCAGAGATGGATTGGTGCCTTCGGGAACTCTGAGACAGGTGCTGCAT

GGCTGTCGCTAGCTCGTGTTGTGAAATGTTGGGTTAAGTCCCGCAACGAGCGCAACCCCTATCCTTTGTTGTTAGCCGTT

CGGGCGGGGAATTTTAGGAGACTGCCAGTGATAAACTGGAGGAAGGTGGGGATGACGGCCAGTCATCATGGGCCCTTTAC

GAGTAGGGGTACCCTCGGGCTACGATGGGCGCATACTAAGAGAAGCGACCTCGCGAGAGACAAGCCGACCTCATAAAGTG

CGTCGTAATCCGGATTTGGAGTCTGCATCTCCACTCCCTGAGTCCGAATCGCTACTAATCTTAGATCAGAATGCTACGGT

GAATACGTTCCCGGGCTTGTACACCCGCCCGTCACCCATGCGTAGTGGGGTTGCAAAGAAATAGTAGTTAACCTTCTGGA

GGCGCTAACACTTTG

>GW616164.1 Jc2-008-G04-M13F.G04.ab1 Jatropha curcas flower and seed Jatropha curcas cDNA, mRNA sequence

CATCTCCCAAGTTTAAAAGAAACCTTATTTTCTTTCTTTTATTTCTTTTTTGGTTCTTGTTCTTGCATATGTTCAATGGC

TGTCACTTGTTTTTCTTCTCCTTACATCCTAGTGCAAAAGTCTAGGCCAGTTGAAAAGAATAGAACCAGAAGTATTATGC

TTAAACACAGTCAAATTCCACTCAAGAAAGCATCTACACTTTCAGTCAGATCATCTTTCCAAGACAAGGTGTTTGAGAAT

AAATCTGAGGGCATAATTTGTTATAGAGATGATAGTGGGGAGATAATTTGTGAAGGATTTGATGAAGGTCCTCGATTCCA

TCATCATCTTCCAAGAACAGCATATAATTCAAGGGATGCTGAGATTATCAATCTCCTTCAACAAAGATTGATTCATGTTG

TTAATGGCGGTGAGTTTGACAACAGTGACAATGGTGTTATTACTGTGAAAGAGGACTTCAACTTCAAATGGAATGAGTTT

AATAAATTCTGCTGATAAATGACTAATTAACCCTTGTTAATTTCATCAATGTATTTCAAATAATGTGAATCAATCCAAAA

CATTGTATTTCTTGCAATCTCAGAATGTAAATGAGCCTAAAAGTACATTATTTCTCTTGTATTCTCAAAATTTCAGTTCT

TTTGCTTGCAA

>GW615699.1 Jc2-003-E03-M13F.E03.ab1 Jatropha curcas flower and seed Jatropha curcas cDNA, mRNA sequence

AAGAGGCTAATGGTTACGTTTTTGTGTATATTTGCCGCTGTTTTTGCTTATAATATGGTTGCTGTACTTCTTTTTTTTGC

ATTGGCATTTTTAGTTTGGGGTACAAGTTTTGCGTTCATATCTCTGTTGATTTTCATGATTTTATACTTTGTGGGTATTG

TGTATATGAGCATAATATGGCAATTAGCCAGTGTTGTTTCTGTTTTGGAAGAGGCTTGTGGAATTCAGGCTATGGTGAAG

AGTAAAGCTTTGATCAAAGGGAAGATTTGGATGACTTCAATTATATTCTTTATGTTGAATGTGTCTCTTTTTATAATTCA

ATTTGCATTTGAGAGGTTGGTTGTTCATGGACATAGAAGATCCATTGGGATGGTTAACAGAGTTTCTTATGGGATAATAT

GTTTGCTGTTGTTATTGATGTTCTTTTTGTTCGGTCTTGTTATACAAACGGTGATCTATTTTGTCTGCAAATCGTTTCAC

CATGAAAATATTGACAAATCGGCTTTATCGGATCATCTTGAAGTTTATTTGGGAGAATATGTTCCTTTGAAGGCAAAGGA

CGTTCAGCTAGAGCAACTTGATCATGTTTGACTCAGTTTTTGAATTTTATATATATAGTATATTACAAAAATTAATGTAT

AACAATAATCTCTGCATTA

>GT971950.1 GJCCJC2026F02.b Jatropha curcas L. developing seeds (mixed stages) Jatropha curcas cDNA clone GJCCJC2026F02, mRNA sequence

AGGAAATCTGTGCGAAGGCTTTCTTTGGGTAAGGCAAAGGTAGATGTAATATTGCGATATGTGTCTTACCTGGGTAATTC

CTTTATTTACCTAGTGGGTCTAACTAGATTTTATGTCTAAGGAAGAGGAGAATCGCCCTAAAGAGCCTGCCTAGTCCTTC

TCGAATCCTGTGAGTGATGTTACATCCTCAGTTCTTTCTCAAACCAGTGACTTCGTTACTACAAGACAATGTGAGTCCGA

ATCCATACATTTCATTTGTAGGTACCACAGAAAGCTAATGGGATTAGAATGAAAATGGAGGAATAGCACGATTATTTATT

TAAATTATGTGTGTGGAAATGACTCTAATTAGAATGAGGGGTAGATCTATTTTAGCGAGGACTAGTTGGAGTGTAGTAGA

GGGCTGCAGAGATAGATTATCTGGCTGGGGGATAGCTATAGATAAGATTCCGATAAGATCTTCTGGGGCAGGTGAATTGA

TTGTTTTCTCTGACCGGTCCATAAATGGTTCTCTTTAGATAACCAGTGCCAGTTGGTGTGATAATGTGATAAGATAAGCG

CTTTCTTTAAAAGGCCTCTTCTTCTTATAAGTTATAAGCCTGAGAGTTCTGTACCATTTCGTAAGCGAGTGTCCAGTTGG

AATTTAGTTATGTTGTGCCAAGCTCTTATGGTCCTTTCTATGTGTGTCTGCAAGAGCAAAAGGAAGTTTCATTTATCTTT

ACATTTAGAATCACTACCTACCTCGGGTTAACTCAGTCACAGGTGGGAAAGATAAGGAGCTTGGTTCGACC

>GW880171.1 JC001030 Seed specific Normalized cDNA library from Jatropha curcas L. Jatropha curcas cDNA clone N0198891 5' similar to Unknown protein, mRNA sequence

CATGGCCAGTTAGGAAAATTATCAATATTACCAGGAGATTACAGTGACCTGGAGGCGAAGCCTGTTGGAACATTAGAAGT

GAAACTAATACAAGGCAAGGAGTTAACCAATAAAGATGTTATTGGGAAATCTGATCCTTTTGCAGTAGTTTTTATACGTC

CACTGCGCGACAGGATGAAAACCAGTAGAACAATTAATAACAATTTGAATCCCATTTGGAACGAACACTTCGAATTCATG

GTTGAAGATCCATTAACTCAACGCTTGACAGTAAGAGTTTTTGATGATGAAGGAGTTCAGGCTGCTGAACTCATTGGTTG

TGCTCAAGTGGCACTAAAGGACCTTGAACCAGGAAAAGTGAAGGAAGTATGGTTAAAACTAGTGAAGGATTTGGAGGTCC

AAAGAGATACTAAATATAGGGGTCAGGTGCACTTAGAGCTCCTGTACTGTCCATTTGGCACAGAGAGCAACTTGAAAAAT

CCTTTTAATCCTGTTTTCCAACTAACCACATTGGAAAAGGCTTGTAAAACTGAAGAAACAGAA

>GW877532.1 JC002255 Seed specific Normalized cDNA library from Jatropha curcas L. Jatropha curcas cDNA clone N04133 5' similar to Unknown protein, mRNA sequence

GGGAACATGGTGTTTTTCTTGTTGTTGTTTGTTGGGTTTTTAATGTCCGAATCTTGTGGGCAAAAAGTCGTATGCACGAA

CACACATACACCCATTTTCTTTTGTTTTTGATTTTGAAAAGTTATCCAATCAGATGTTAAACAAAAGCTGGAAAAACTTC

AATTTTTTATAATAATTATTAATAGAAATAATTTGTTTTCGGGTTATTTCAAAAGAAAAAGGAAAAGATGATATGAAATG

CGATTTTATATTTATGTGTTTAATTGTTTTTGTTTTGACCCGATTGGGTATAGAAACTGTAGTAAAAACTGACACTTTCT

CTGAACCAAAAAGGGGTCTTTGATTGATTGTCAATTACAAAACATTTACTAGTCGTATTTTCAATTTATTTATTGCGTGA

CTCTATTTTGTAGATGATTTTTTATTTATTTACTATTTTTTTCTTCTTTTGTTTTTGTTTTGGTTCGGTGCGTGAGGGGA

GATTTTTATTAGAGAAAGAAAGAGATGTCCTTTCGGGTTTTCTTGAGATTTCTTTATTGCTAGTCCTATTTAGGGTTCCC

TTAACTTTACTCTTATTATTCTCTTTTCGAGTTTTTGGGTTTCCGATTTTGTTTTTGGGTAGCTTCAGTTCTGGGTTTCC

TTTTGTTTAATTCCTAAATTTTGCCTCCTTTGACCC

>GT970120.1 GJCCJC2005D08.b Jatropha curcas L. developing seeds (mixed stages) Jatropha curcas cDNA clone GJCCJC2005D08, mRNA sequence

CTACTAATAACTCTGCAGGAAATCAGTTTAGCTGGGCTGACCTTGCGGTGTTAAGTTTATAAAGCTGAGTTTTGTAGATG

AATTGTTAGTAAATTTCCTGGTCAGTTTTGTTCTATCATATGTAGGAGCTCACAATGTCAGTTTTAACAGTTATATCAGC

TTACTTTAGATGACAATCATACAAAACACACAATTATTATGAAAAATAAAAAATACAAGATATTAATTGATAAAATTAAA

AGAATTGACCAAATTGTAAAGTGTCAGAATTGAGGATTTTTTTTGTCATTTGGCCTTGCCGTGGTTATCAAACTTTGCGT

TTAATTTCATTAAGGCTACATAATTTTAATTTGTCATTTTTTGTTTCAATAGAAGTCACCCACCAGAATCAAGTCATGGA

AAATACTAGCATAATACTAAAATTAAGTATTCAAAAAAACCTTATTTATTCTTAATAAACTAATATGGGACTCACTCATC

AATTAGCTAGCAAATGGTACCTGCCAAATGAAATAAGCTTAGAAAAATTTTCAACTCTCTATCTTTGTATTTTGTAATGT

ACTTTTTCCCTTCT

>GW875490.1 JC004626 Seed specific Normalized cDNA library from Jatropha curcas L. Jatropha curcas cDNA clone N08159 5' similar to Unknown protein, mRNA sequence

GACGTGAAAATGGAAGACATATGGAAGAGGGCAAAGCTCTTCGCAGAAGAAACGGCCAAAAAAATCACAAACCCTGACTT

CATCCAACAAAATTGCTGATCTAGTCGCCGAGACCGCTAAGAAATCCAAAGAGCTCGCATTTGGAAGCGTCCAAGAAAGC

CGATGAGCTGAAAACCGCCGCTCTTAAGCAAGCCGATCAGATCCAAATCAAGTTTATTTCAGATATCATTCCTCCTCAAC

TCTCCTCTCTCTCGATTGTGAATTCTTCTTCCTCTTCTTCGGGATCGGCGGATTCGGATACTGAGGAGCTTCGCAAGTTT

GGCGTCACTGATGATTTGAGAGATTTTGTTAAGGGACTGACTTCCAGTACTTTCCAGAACTTTCCAATTCAAGATGAAGC

TGAGGTGTCTGATGTGCCGACTACTGCATCAAATGTACGGAAAGATCTTAATGAGTGGCAGGAGAGACATGCCACACTCG

TGCTCACTAACGTTAAGCAAATCTCGAAGTTAAGATATGAATTATGTCCACGAGTGATGAAAGAAAGGAGATTCTGGAGG

A

>JK611037.1 JCF2499 Jatropha curcas, immature Seed cDNA subtraction library Jatropha curcas cDNA, mRNA sequence

CAGAAGTATTTTTTGCTGTGAAAATGGATCTTCTTAATCTTCCACAGTGTTGCATATCATTTGGGAGAGTTCATTTATAA

AATGGAGCCGAAAACACAACTTATTAATAATATTATAAAATTTACAGATTGAAGAAAAGACTAGGAAAAGTTTTGTGAAG

ATGAAAAAGTTTTCCTGTGGTCAAGTTTCCCTTAGCATATTAAAAATAAAGGAAAAGAATCATGTTGTTTTCTTAGTGGT

CTTTTCGTAATTTCAATAACCAATTGCTTATGCAAGATGTTAATTGATAAAAATGTAATAATAGAAGATGTTAAAAAAAT

GCATTCTGTAAATGGAGAACAATGTTTTTCCCAAAAAAAAAAAAAAAAAAAAAAAAAAAGCTTG

>FM890719.1 FM890719 Jatropha curcas embryo 56-70 (DAF) Jatropha curcas cDNA clone rjcaeb0_000672, mRNA sequence

ATTCCATTCGTCTTCAAAGTTTATAATTCACGCATTATCGTCGATTTTGGAGCAACGGAAGATGTAAAGATAGGTCATGC

AGTGTGCGCCATTGATCAAGTTTCTGCAAAAGGAACTCATTTGGAAGATGGAAGAGAAATAATGCAGGTACTCTCCGAGG

AGAAAAATTTTCCCTTGACTATCAAATTTGCCAAGCCGGTCCCCACTTCCAACGATCGTCTACATTTGGCCGGATTGTGG

CACGGTTTGCATCGTTTGTCTCGACACTTGAGTCCATTGTCTGAAACGTCAACTAACGACTTTTCCGATTCGCAAGAAAA

ACGAGCTGCTGCGGTTTTCAACTCGGGCATCCAGAGTCTAGAATGCGCATCCTGTCGTATTCACTGCTTGGAATCGCGTG

TTGGTGTGAAATTTTTACTGATTACTGATGCTAAATTGCCGTCAGCCTCGCGTGAAGCACTCAAGCGAATTTACGAAGCG

TATACAGATTATGTCCTGAAAAACCCCTTCTACGCGCCAAATCAACTGTTCAACTACGATT

>GT974509.1 GJCCJC2061D12.b Jatropha curcas L. developing seeds (mixed stages) Jatropha curcas cDNA clone GJCCJC2061D12, mRNA sequence

AGAGGGGGGCTCAAAGGAACAAGAACCACAACAATAGGCAATCCATTAGCACTTCCTAGTGGACCAATTACAAAGAGCCG

AGCAAAGAAGTATAGAGAGCAACCATGTCCTTACACATACAAGAACAAGTTACAAAAGAGCTCCAAGACCTTGCTTTCAA

CAAGTGCTATGAAGAACTTGAAGGAACACCTAAGTTTCTCACATTAATAGAAGCACAAGTTGAGTGGGAACATGAATTGG

TTAGCACGCCCGTGCTAGATGGCTCTCCCCAAGCCGTGCCCTCGTCTGACTTGCTGAAGAAAACAACAGCCTTGAAGCAC

CCCCGTGCCAAATAGCACACCCTGGCCGTGCTTCCCTCTGACTCTGAAGCACCCCCGTGCTAAATAGCACACCCTGGCCG

TGCTTCCCTCTGACTCTCAAGAAGGAACTCCTACATTGAAGCACGCCCGTGCCTAATAGCACACCCAGGCCGTGCTTGTC

ACTGCCTCTCATTAAGTTAGTTAGCTTATTTTGAGTTTTTGCT

>GW881832.1 JC001719 Seed specific Normalized cDNA library from Jatropha curcas L. Jatropha curcas cDNA clone N03157 5' similar to ATP-dependent helicase NAM7, mRNA sequence

GGATTTCATGCACTTGAACAGTTGAACTCTCCTGCCAATAGAAAAGCAAAAGCCTTTCGCATGATTTGTGCATTTAATAC

AAGAAAAATAGGAACCAGTGAAGTGCACTGAAGAGTCTGCTTTTCATTTTATTTCATTTCTCAAGGAGATTAGAACAGGG

AGGCATCTGGGTTCTGCTTCTTTTCCATTAAAGAGTCCAAATTTTGAATACCCGTTTCCCTTTTTTAGTTGTTCTGATCA

AAGATTGAACCTCAGGTAGAGTGATATTGCTGTTGATATTGAGCTGATGATGGATACAGTTAAAAAGGATGATTGGGCTG

ATGAATACTCTGTGATTGGGGAGAAGGGTGAGATTGGGTTCATTGATTTTGAGGACGATAAATCTGTCTGTAACTATGAT

TCAGATGAGGGGGGGACAGTTGTTATATCTGTCCCATTTCCTTTTGTAAGAGGGAAGCCTCAATCTATTCTTGTTGCTGA

AACTTCGAAATGCTCAATTACCATATCAAACACCATTAATGAACCTGTGGAGCTTTGGGGAGTTAGGATATTCTGCTCAA

ATCCTGCAGATTCATCCACTTTATCTCTTATAGAGCCTCCATCAGTCAATTCAAAGGCGGAAAACGTCTCTGGA

>JK611045.1 JCF2508 Jatropha curcas, immature Seed cDNA subtraction library Jatropha curcas cDNA similar to ATP-dependent RNA helicase, mRNA sequence

ACATGCAATGTGCCACTGCGGTGGAGCCTCAGTGGCTGGCTGAGCTGGGGCCAATGTTCTTTTCCGTGAAGGACTCAGAT

ACATCCATGTTGGAGCATAAGAAAAGGCTGAAAGAAGAAAAAACTGCAATGGAAGAGGAAATGGAAAATTTGAGGAAGGA

GCAAGCAGAGGTAGAGAGGGAAAGCAAGGAAAAAGAGAGGCAAAAGAGGGCAAAGCAACAGCAGCAAGTGTCAATGCCAG

GGATGCGACAAGGCACTTCCACTTACCTGAGACCCAAAAAGCTCGGTTTGTGAATGTTAACTAGGGCTGTTCACATCGCT

TATTCGGGTTACCAGTGTAAGAGATGCAAAAAGCTGACAGAGCACTAAACATGTTAGAGATGAATCTGTTGGTGACCTGG

ATAGAAAATTTAAAATGTTCCAACTGGTTCCAAAACAACCCAAATTGAACCGCACTAACCAGGATGTATTTATCTGTTGA

ATGTTATTAACATCTACCTACTATAACAGATGTTGTGCAAGGGGGAGGAAAATCATTAGCTGATATAAATTTCTTTTTTT

CTTTTAATGTAAATATGTAGCCATAATATTGAAGTGCTCGAGTGAGGGTCGGATAATTTATTGCCTTGT

>GW881671.1 JC006522 Seed specific Normalized cDNA library from Jatropha curcas L. Jatropha curcas cDNA clone N11035 5' similar to mRNA, mRNA sequence

GGTTCTTCTGCACTTTCAAAGGCTGAGGGTGCAGCTCGCCTCGCTGCCGCACACAACTCACCTTTTATGTTATCTTCTCT

CTTCAATTCCCTTCCTCCGCCACCACCCACTCTTCTCTATCTTGTACCGATGTGCGTATTACGAACATTAACATCCTTCT

AATCCTTGAAGCTCACAATTTTTGAAGCTAGAAACCAATATTGGCGCGAAAAAGTTAAGAAATACTCTGGTACTTTCAAA

GTTGTAATATTTTTCATGCAATCCACTTTATATTCACCTACCAATTCTCGCCAATGCCATTTCCCATGGAAATCCCAAGA

ATTTCATCTTGTATCCAGGAAACCTATTTCGAGGAAGCTCAAATTATTGTCACCGAAAACCATAACCCTTACCTGTTCAC

TTAGAACCTCCATTAATTATGGGCCCGAAGAAGGTCTAAATGAGGGACCTAAACAACTACCTATCTTGCCGGCGCGTCTA

CCGGTGGTACTTAGGCAGTCCGGTCGAGTCTCCAGGTATATGTGGGATGGTAGTAATTTGACGTTGGTGAGTGTTGATGG

TGGTGTGACGTCGTTTTCTCTTGATTTTGAA

>FM891240.1 FM891240 Jatropha curcas embryo 56-70 (DAF) Jatropha curcas cDNA clone rjcaeb0_001365, mRNA sequence

CTCCCTTCCAATAAAAACAAACAGTTCATTTATTTCCATAAACATCACAAACAAGACTATTTCAGACCAACATTGACTAT

TTGGATTCTTGACAATGTTTGATTACATAGCATTAGGTAATATAAGAGCTTTAAACACACAAACAACTAGAGAAATAGCT

ACTTGAACAAACTTAGAGAACCTTTAGGATTTCATCAACACCGGAGACAGTGAACTCTCCACCTTCTTCAAGATTTGCCG

CCTGCACCTTCAGATTATCAACTAAAAGAGCAAATCTCCTTGATCGAGTGCCAAGTCCCTTCTCTGTCAAGTCAAGCTGA

AGCCCAAGAGCATGAGTGTATGTTGCTGAGCCATCGGCCAAAAACTTCACATGCTTGTTTTCAGGGTAAGTCTTGGCCCA

TGCCTTCGGGGGGGCCCGGTAC

>JK612661.1 JCF20-99 Jatropha curcas, immature Seed cDNA subtraction library Jatropha curcas cDNA, mRNA sequence

ACGAAAGATTGCTATTCAAAGCATCGTTAAGAGTTCTGTTTTTCTAGGGAGTAGTCAAGATAAGATGAGTCTGATCTTTC

CTTCTATGGAGCCGGATGTGAGCTTCTCGCCTACGGATCTTACTCAAAAGAGTCTCAGGGAGCGGACACATCAATAGCAG

CAGACGCAGAGGCAAGATCTCTATATGTTGATAGATACCCGAGAGACTAAGTCCTTTCCTTAAGGCATGCCCCTACTCAA

CTTCCTATTGCAAATGCCAAAGCAACAAGAGCGGCTACTCCAAGTTCTTTTCTTTCTGAACCTCAGCTCAGGGAAAAGAG

CTAACAGCTGCGGGAGAGGCAACTTCTCTTTCACTTCATTCACGGGAGACCTACCAAATCTGCGCATTTCTATAAGTCAT

GGTCACATGTCTTGTGAACATTCAACCATCAAGAGGGAAGGTCAAAGAGAGCTGCGTCTTCGACGGTGCTGCTAAGAAAG

TGCAGCTTGTAGCTCTGAGAGACTTTATGAATTGAAAGTAAGCCTCAAGCGAAGTTTCCATCAAAGGAAGTGAAGGATGA

GGGAAGGGAGGGAGAATTCCTGCAACGAATAAAGCTCAGAATGCCGCTCAACGAAAGAGAATTGCAGCTAATGCGCTTTC

ATGGCAGTTAGCCGGTTTCAGTCCGGCTCTTCTCCGGCTGTCAAAGCAGTTCTTCTAGCTCTTAAGCCTGT

>GW614326.1 Jc1-038-C06-M13F.C06.ab1 Jatropha curcas flower and seed Jatropha curcas cDNA, mRNA sequence

TGCAACAAGTGAAATAACCAATGATGATGTAAATCAACTGTACATGATTGGGAAACCAGCTTGTCAAGGCTGTCGTGTGA

ATACTAAAGATAATCCCAACTGTTTTTGTGGCCTGATTCCACCACCCAATGGCAGTCGCAAATCTGGGTTATGGCAAAAA

TTGTCTGATATTGTTCAAGCCCTTGGCCCAGACCCATGCAAGGATCTTCGTGCTTCTGCTGGTTCACCTGCTGGCCTCAC

AAACCTGGGAGCAACATGCTATGCCAATAGCATACTTCAATGTCTTTACATGAATACATCTTTTCGAGAAGGGGTTTTCT

CTGTTGAATCTGAAGTATTGAAACAACAGCCTGTTTTGGATCAGCTTGCTCGGCTTTTTGCACAGTTGCATGCAAGTAAA

ATGGCTTTCATTGACTCAGCTCCATTTATAAAGACGCTGGAGTTAGATAATGGAGTTCAGCAGGATAGCCATGAGTTCTT

GACGTTACTTCTTTCCTTGCTTGAACGCTGTCTAAGCCATTCTAAAGTTTCCAAAGCAAGAACAATCGTTCAAGATCTCT

TCTGTGGAAGTGTGTCACATGTAACAAC

>GW877400.1 JC006923 Seed specific Normalized cDNA library from Jatropha curcas L. Jatropha curcas cDNA clone N11651 5' similar to Unknown protein, mRNA sequence

GGCCATTACGGCCTAGTTACGGGGGCCTCGCCACAGCACAAAAGCTTGAAAACCCTAATCAGACCAGTACTTTTTTTCTT

TTTTCTCTTTTTGTTCTCTCTTCTTTTCAATTTTACACCAAAACCCTTTTTTCTCTTTACTATTCTTCCTTGTCTTGAAT

GAATCTTCAGTCTCTCATTTTCAACAATTAGTCGTCGATTTCTTAACCATCCGATGGCCGAAGGCAAGCTCGACATGACC

GACGTTCTTGTTATCTCGAAACCGTCCGATCGATCTTAGACTCCGATAGTTAAGGATTCAGTATTATTGATCTTTGTTTT

TTCATTCTTGAAACCGATTTTTAATATGATAGATTGATTTGGGGTTTGTTTCAACGTTTATAATGTTGCAGGTTGTTTAC

AGCAAAAGATTTAAGAAGGAATAATGAAAAACGAGAAGAGTTATGTGCCACCACCTTATATTCCCATTGGACAATCGGAT

TGTGACTTAGAAGAAGCAGAGACTGTTCAACGTGTTGAAAATGCTGCTCCTTTTCATCAAACGCAATGGTCATCTGGAAT

CTGTGCTTGTTGCGATGATGTGCAAAGCTGTATGCTACTTTCTGTCTTGGGACCCTTCATTTATCAAATTCCAAAAAAAT

TTAATATGAGGTGCCTAAT

>GW874957.1 JC000348 Seed specific Normalized cDNA library from Jatropha curcas L. Jatropha curcas cDNA clone N00645 5' similar to Electron Transporter, mRNA sequence

GGGTTGGTAGGATAAATAGGCAAAATCCCGGCTTTATCCCTGAGTGAGTCAATGGGTGTCTTTTAGATTTTGACAATCTG

CCTCCCTATCTTTGCTGCTTCTGTTGTTTGTTGGTTTATTGAAGAGAAAAGAAGGGATTTTTTTTTTTTTTTTTTTTTTT

TCGTTTTTCCTTCTAAATGCTCCTTTTGGAACTGAAGAGATGCTTTGTCTAAAAGCAGAAGTCCTCGATGACCACGAATC

CAACAATACCACTAGTATTTCTACTAGTACCAACAATGGCGCTGCGGGGTTTGGGTTTATACTGCCGATGAATTTGGGAA

GTTCTATTCATAAATATTTATGGAGGAGTGGTAGGTTGTCTGCTCGATCTATTTCTTCTGGAGATGGGTCTCCTTCTTCA

ATTTCTGATTTTGAGGATATTAAACCCCTGGGCAAAATAAGTGGAAAAATGAATGGTGGGGAACACGTCTTTTTACAGAT

ACCAGCTTG

>JK613573.1 JCF19-183 Jatropha curcas, immature Seed cDNA subtraction library Jatropha curcas cDNA similar to cysteine proteinase, mRNA sequence

ACCTGGAGCGATTGAAGGAGCTAATTTTATAGCAACAGGGAAGCTTCTTAATCTTAGTGAGCAACAATTGGTGGACTGCG

ATAATAAGTGTGACATGGTGGACAAGACTGCATGCGATAGTGGTTGCGGGGGAGGTCTTATGACGAATGCTTATAGATAT

TTGATGGAGGCAGGAGGGTTAGAAGAAGAGAGTTCATATCCATATACAGGAAAACGCGGTGAATGCAAGTTTGATAGGGA

GAAAATAGCAGTCAGAGTTGCTAATTTCACCAATATCGCTATTAATGAGGATCAAATTGCAGCTCATTTAGTTCACTATG

GACCTTTGGCAGTTGGATTGAATGCGATTTTTATGCAAACTTACATTGGAGGAGTGTCATGTCCACTTATTTGCGGCAAG

AAATGGATCAACCATGGAGTTTTGCTTGTTGGGTATGGAGCTAAAGGGTTCTCCATTCTTAGATTAAGTAACAAGCCGTA

TTGGATCATCAAGAACTCTTGGGGT

>FM895439.1 FM895439 Jatropha curcas embryo 71-95 (DAF) Jatropha curcas cDNA clone rjcpga0_002361, mRNA sequence

TTAGAATTAGGGTTAGGGTTGGTATAGTGAAAGAGGGATATCAGGCCTACAGCTATGGCCTGTTCAAGAGGTGGAGAAAT

GATCACTCCACTCTCGTGGTCTACTCTTCCAGTAGATCTCTTAACTGATATAGCCAAACACCTCGATCCCTATTTTGATG

TCCTTCATTTCCGTTCTGTTTGCCATTCATGGCGAACTTCCGTTCCTGCTCCTAAAAAGTCTCCATCTTTGCCTTTGGAG

CTTAATTTCCTCACTGACGATCCTTTCCACCCTTTTGCTACTTTTTCTGTCTATCGAAGGACAGTCTTTCGTCTTGAACC

TGAAGGCTCTCAACATGGTAATAGCTGGTTGATTAAGGTTCAAGAAGAGAGTGATGCAGGCAAGCTGCTTGTAACAAGTC

CTTTCTCAAGATATCCCATTTATAATTTACCTCCCACATTTCCAAGGGAGTTGAACCTGCTCAGTTTCCGTGTCTGGCCT

ATTGCTAGTACTTTCAGCCTTGAACGACATGATGAGGATGAGGAGCTCGATAGCAACGATGTTACTTATCACCCCATGGA

GATCGTTCATGGAGGAGTAGAAGAAAATGATAACGATAGTTTTATTAGCAATGACGGGGTTACCATGTCTAGCAAGGTAA

CCGTTTCAGCTTCTGGAACGGATTCAG

>FM887550.1 FM887550 Jatropha curcas embryo 35-55 (DAF) Jatropha curcas cDNA clone rjcfea0_000774, mRNA sequence

GTATGTTTGTCATAGATAAGAGCCACCAAGAAATGTTGGCACAGCATGGGCAAGATACCAGATTTTCATATTTTAGTTTT

ACATATTCCTGTTACGGTACATATTGTTCTACCAATATTTGAATAATAGGAAGACGTATATATTAATTGTAGGCAAGCAA

AGATCCCCAAAAAATTAAAATAAATGGGAATTATGTCAAACAAAGAACTTAAAATTTTCCATCAAGTTTGAGAGGAATGT

TTTTAACTTTTTCTTAAATAACACACACTGACCTTTTCAATGCTGCATCCCAGTCTTGCTCCTCTTTATAATTGAAAGCA

GCATCGAAACCAAACTTGTTCGTCAATAAGTCAATCTTTATGTGAGACAAACCAGAACAAAATTAAATGAGTTCAGCATG

TTTAATCATCATTGAAACCAAACTTGTTCTTCATAAAAAACCATCCAATCACTACATTTAATAAAGAACAGACATAATAC

AAAATATATACTATATGCAAAATGCAGAAAGTGATCATACAATAGAACTTTGGCAAGTAAATAGGTTAAATGACGATTAG

GATGATAATTCTTGATTC

>GT976172.1 GJCCJC2073H08.b Jatropha curcas L. developing seeds (mixed stages) Jatropha curcas cDNA clone GJCCJC2073H08 similar to PP2A-2 (protein phosphatase 2a-2); protein serine/threonine phosphatase, mRNA sequence

GCAACAAAAACACAGAAACACCAATTTTAAAAAACAGAGAGAGAAGCGAAGGCGAGGAATGATAGACAGATCTCCAAAAC

GAATCGTTTTCCTCTACACAACCGGCGCCACAGATCGGTAGCTGTGGAATCTTTTACCAGTATCTGATTTGATTCTGATT

TAGAATCTACTGTTGACCGTACGATCTTTTGTGGACCGGAGAGGATGCCGTCACACGGGGATCTGGACCGTCAGATCGAG

CACCTGATGGAGTGTAAGCCCTTGCCGGAGGCGGAGGTGAAGACGCTCTGCGATCAAGCCAGAGCGATTCTTGTTGAGGA

ATGGAACGTTCAGCCGGTGAAATGTCCGGTCACGGTTTGCGGCGATATTCATGGCCAGTTCTACGATCTCATCGAGCTCT

TTAGGATAGGAGGGAATGCGCCTGATACTAATTATCTCTTTATGGGAGACTATGTAGATCGTGGGTACTATTCAGTGGAA

ACCGTCACACTCTTAGTGGCCCTGAAAGTTCGTTATAGAGATAGAATTACAATTCTTAGAGGAAACCATGAGAGCAGGCA

GATTACTCAAGTGTATGGTTTCTATGATGAGTGCTTGAGAAAATATGGAAATGCCAACGTGTGGAAGTATTTCACTGACC

TTTTTGATTACCTACCCCTTACGGCCCTCATTGAGAGTCAGGTTTTCTGGTTGCACGGTGGACTTTCACCATCTTTGGAT

ACGTTAGATATATCCGAGCTTTGGACGTATACAGAAGGTTCACATGAAGGACCATGTGTGATCTATTGTGGTCTGAT

>JK612521.1 JCF24-80 Jatropha curcas, immature Seed cDNA subtraction library Jatropha curcas cDNA similar to 26s protease regulatory subunit, mRNA sequence

ACCAGGAGGTCCATAAAGGAGGACACCCTTGGGAGGTTTAATCCCAACTCTAAGGAAAAGCTCAGGGTTCATAAGAGGTA

GTTCAATAGATTCCCTCAATTCTCTGATTTGATCTGATAAACCACCCACAGCTGAGTAGCTCACATTACCGGGATCTTCA

TGCAGCATATTATAAACAACTGGATCTACTTCACGTGGAAGAGACCGCATGATTGTGAGTGTTGTCATATCAAGAACCAC

CCTAGTTCCAGCAGTCAGCTTTTCCTTATCGACCTTGCTGCGGCAACCAACCACATACCTGGGGCCACTGCTTGCTTTAA

CAATCAACCGTTCATTGTCAAGGGGTCGAAGAACTTCTCCAATGATCTGCCCAACACTTTGAAGAGACTTCAAATCATCC

TCTGTTTTGTTAAACTCTTTCTTAGCAGATCTTAAATTCTCTCTAACAGCTCTCACTCTTGATTCCAACTCCTTGTGTTG

GAGTAACTTCTTGCGATAGTCTGAAACAGCATTACGACGTCTTGCTGCATCCTCTCCTTCGGTGCTCATTCTTCAAAATC

GAAATCGGCTCTTGGCACTAAGATATTTTCCCTGTTTCTTTATCGAAAGAAGGCACGCTTTATTTCGCTGCTG

>GW878012.1 JC003614 Seed specific Normalized cDNA library from Jatropha curcas L. Jatropha curcas cDNA clone N06658 5' similar to Serine/threonine protein phosphatase, mRNA sequence

GGCCGCATTGAAATTGCAGCCGTCTCCTTCTTCTTTATTGACACTTTCTCCTTTTTTTCCCCTATAAACAAATCTTTACC

TTATTGGAGTATTGTTTTCCTTCTCTTTCCCACTCTTTTTTTCCAAGAAAATAATTTTTTCTGCCCCACCAAGCAGGCTA

AATTTGAACTAACCTAGCGCAAAACCACGAAACTGAAAAGGAGAAACCCACAACGACTAGGAGACCAAATAATCTTCAAA

ACTCTCTACCCATTCTGATTTCTAGGGTTTTTTTCTTTGTTTGGAGGAATCAGGGTCTTTAAAAGGAAGGAGAAGAAGAG

ACGAAGAAAAACATCCGATGGCAACACAAGGGCAGGCATTGATGGACCCTGCCGTACTGGATGATATAATCAAACGGCTA

ACGGAGGTCCGATTAGCGAGGCCAGGCAAGCAAGTGCGACTCTCAGAGGCGGAGATCAAGCAACTTTGCGTGGCTTCACG

AGATATCTTCATGCAACAGCCTAATTTGCTTGAGCTTGAAGCCCCCGTTAAGATTTGCGGCGACATTCATGGGCAATATA

GTGATCTATTAAGGCTTTTTGAGTATGGAGGTTTTCCACCTGAAGCTAATTACTTGTTTTTGGGGGATTATGTTGATCGT

GGCAAACAGAGTTTGGAAACTATTTGCCTATTGCTTGCGTATAA

>GW615915.1 Jc2-005-H10-M13F.H10.ab1 Jatropha curcas flower and seed Jatropha curcas cDNA, mRNA sequence

TCTCATTTCTGTCATAAACCCCATATTCTAAAGTTAACGTCTTTTTCAACATTTTCGTTGCTATTTCATGATGGAAGCTG

CCAATCAACTCGGATCAATTGCTCAACCGAGCTCCGTACCGGCTTCTGTAGCAGGTAAAACCTGCAGTGGCACTTTGGGC

CGCCATTTGGCTCGGCGATTGGTGGAGATCGGCGTCAGTGATGTTTTCTCGGTCCCTGGAGACTTCAACTTGACGCTTTT

GGATCATTTGATAGCAGAGCCCGGGCTGAACTTGGTCGGCTGCTGCAACGAGCTGAACGCCGGATATGCTGCCGATGGGT

ATGCACGTGCCAGAGGCGTGGGGGCTTGTGTAGTGACTTTCACTGTTGGTGGTCTTAGTGTGCTTAACGCAATTGCTGGT

GCTTATAGTGAGAATTTGCCTGTGATTTGTATTGTGGGTGGTCCTAATTCTAATGATTATGGGACTAACAGAATTTTGCA

TCACACTATTGGTTTGCCTGATTTTAGTCAGGAGCTCAGGTGCTTCCAGACAGTCACCTGTTTTCAAGCAGTAGTGAACA

ACTTGGATGATGCGCATGAGCAGATTGACACTGCAATTTCTACTGCTTTGAAGGAAAGCAAGCCTGC

>JK612392.1 JCF25-91 Jatropha curcas, immature Seed cDNA subtraction library Jatropha curcas cDNA similar to seed maturation protein pm23, mRNA sequence

ACGCATAGCACCTTCAAGCAACTTGATCAACAGCCATCATTTTCCACATTGCTTGTTACAGACAGAAGTTCCATCAACTC

CATTCTATATTCTAGGCCTCGAAGGTAGTGTCCCAAAAGCATACACCAGAAGAGCAGACGTGCAAGATAGTCCCTTGTTA

AGGAAATGTGGGGTTGGAACTGAAGCGAGGTGTTTTCAACAAGTTCAGGACAGCCTTCTCTCCCAATTTTTCGTGTCCCC

ATTAATGTGCTGTTCTCTGATTGATGTGGAGCCTTGGAATGCATCTTAGGAGAAAGAGTTGCCAGGAGGCCATGGACTAC

AGAGTGGATTACTTCTTTCAATTCAGGGGATGTAGGCTCTGATAACTCAGCCACCTTCTCCGGTTTTAGTGATCTTAAAT

AGTCAAGTAAATCATTCTTTTCTTCGCCAACAAACTGTTGCATCTGAAGAGCAGCACTTTTCCTCTTGACTTCATGAAGC

TCCTTTTTCACAGAAGATAAACGAGACTGCAAATCAAGAATATATTGCTGAGCTTCAGGGGAGACTTCACCAAGGCCTTG

GATGCCAATATTATCACATAGGTCTTCAGTTGCTTTTTCAAGTTTAGCATTTCTTTCATAATATTGGTTCATTTTCTCAC

TATTTTCTTCTGTCCCTTGCAAATCAAGTTTAGAGTTTTCTGGGTTCTTGGCTTTCAGTATTGCCTTCATAATTCAAAGT

TTTTTTCAAAGGCAAAGGCCTAATACCTCAGCATTGCGCAATGTA

>GT970597.1 GJCCJC2011A07.b Jatropha curcas L. developing seeds (mixed stages) Jatropha curcas cDNA clone GJCCJC2011A07, mRNA sequence

CTGCAAATAAACCATCATCTGTTAGAACATCATCTTCACTGCCAATCAGAGCTCTTAGTGACTTATTGGGGCACTCGTGT

AATGGAGAATAGGGCTGTTTGAACGGAAACACGGTCCCTTGGCTCGAAGCCCTTGGCAATCATTATGGGAGTGGTGGCGA

ATGCCACGAAATCTGTTGGTAGTATTGGGTGGTGGAGGAGCAGGTAGGGGGAGAGGTTGATTTTGGTGGGTTAGGGTTTG

TGTTTGGTCGTTTGATCTAGGGGATGGGTGGGAATGATTTCTGTCCGGGTCGGGTAAGTAATTTGGTGTGGGGCACAGGT

TGTAATTGGGAAAACGGGTTTTGGGCCAAGGCCAAAACGTTGTGTAGTATGTAAGTCGCTAACGTTTTGACGATAGGTTT

GTTTAAAGAAGGCGGTGGTCGGATTAAGCAAGGCGACTTCCATTTCAACTTCTCAAGCTGTGCGTAGAGCCCTGTATAAT

TCGCCAGTATCAGAGGAACGGATTTTTACACGTATTCCTTCCTTGAGTCCATTGAGAAAGTAACCAATCAATTGAAGATC

AAAGAGAGCTGGAGCTTGAGTAGCTCTGA

>JK612152.1 JCF27-191 Jatropha curcas, immature Seed cDNA subtraction library Jatropha curcas cDNA similar to nucleolar protein, mRNA sequence

CCAGGTACTTTTCATTCCTAGGGAATTTATCAAAGTTGATCCAAAGGTATCTTCAAGAGGTGACACCTTTTAGATGGTGT

CAAAGTTTATAGGCTCGCGCAGCAACCTCTCCATCTCCTTCGCTCTAGTCCTAATGGACAAGTCAAAGACCTTCATATCA

AACTCTACCAAAAGCCCCCCCAAAATACTTGGATCAATCTTCTTTTCCAGCTTAACTTTCTTCCCTTGTCCAATTATCTC

TTGCAGTGTTTCCTTCAATTCTTCCTCCTCTTCTCCACCCAAGGGAATGACTGTTGTGACTATAGCTTTAACCTCTCCCC

TGTCTGCCATGGTCAACTCCACAAATTTCTTGGCTATGCTGTCTATGTTCTTTAACCTTCCATTCTCAGCCAGCAAAACC

AAAAAGTTCTTTGTGATATCTGAAAATTTAGCTTGATCAGAAATATCATTAATGGCCTTGACTCTTGTGTCGGCAGGCAC

TGACAAGTCCTTTGTGAACTGAGTAAACGTAGGACTTCTCTTTATAGCCTCCACAACATCAAGAACTTCAGCCTCAACCT

TGTCCAGTACATTAGACTTTTTGGCTGCAAGGTACAATGCAGAGGCATAGTTTCCAGCTCCTCCAAACAAAGCAAGCGGC

ACCTTCACCTTCTTCTCTTGCCCTGAAGAAGTGGCATAATTTTTTGAAGTCTCAGATGTAAAAGCAGGCATAAAACTCGG

CCGCGACACGCTA

>GT970576.1 GJCCJC2010G06.b Jatropha curcas L. developing seeds (mixed stages) Jatropha curcas cDNA clone GJCCJC2010G06, mRNA sequence

AGCCCTGTTAGAGCTGCGTGTCGCAGCTAAGGGTGCCGTCATGCAGGTCCTACCTGCGTGACGCAACCCTAACGCGGGTC

AGGCTCCTTTGGGCGGTTTGGTTCAAACACTTCTGGTTTATCGTGATACCTTATCCCTTGTCCTCATATCATCATCGGGT

TAAGTGGAGGCATAAAATCACGCATCAACAATACCTGTCTAAGCCTTAGATTACAACCTCTCCTACCAATATTTATTAAA

ACTCCTAGATTAGCTTACATCAATATTAGTTAGGAATTCTTATCTCAATATTAAATCTCCTAATTATAATTATGGTTCAT

GTAATTTTATTTGACAATGTTCTAAACTAATTTGCAATTACGTTATTCTAAAATCCTTAACGAGTTCACTAATCATATTC

GCTATTGCAATTACGACACACTAACCAATATAATTTATTCACAAAAGACTATAAATGTTCTAAATATATTTTAAGCTATT

CCAATAACCTCATTTCTTATTATTCAATTTAATCTATTTAATTACTCAATTAAATTCATTAATTAACTAATTCAGAATTT

ATATAAAAATTGAGAATTGAGTCTTAGGGCATACCGATTGAAATTTGGGCTACAATTCCACGATCCTCACGAAGAGAGGA

AAATTCTGTTCTTTTGGTGTCTCGCTAGAGGCTCGCCGGAACGCCGTCATCGCCGACATCCACGGTGGCCGAAAATCAAT

TTTTGCTTAAAAAT

>FM888148.1 FM888148 Jatropha curcas embryo 35-55 (DAF) Jatropha curcas cDNA clone rjcfea0_001489, mRNA sequence

TGTTTTCTGGGCATCAGTGTGTAACCTGTTGTATTCCCCACTGACAGGAAGTGGCATCCCATGGTTTTTGGGTCTTCTTT

TTTGGCTTTATGTTTAGTTGCCACTCCGTAGAATTGTAGTTGGTTTGTGACTTGCAAAATGAAAAAACGGTCACTTTTGG

TTTAAAAAAAAAAAAAAAAAAAAAAAAAAACCAACACCTAAGCCTGTTGATGAGGACTTGTACAAAATCCCACCAGAGCT

TCTTTATCAAAAACCCAAAAAGAAAAGAGGATTGTGCTTCTTTTCAGCTGCTTGCTTCCTACTTGTGTATTGTGAAGCTT

GTTGGATTATGGGTACATATATATATATATATGGAGAGATATGAGAAGAATATATATATGTAATTATACCTTCTTCTTTT

TTGGGTACTTAATATTAGGATATTCTCGGATCAGTTTACACGAATCATCCGATTGGATTCCAATGGAATGTGGAGAATAT

TGTTGAGTATTATTACTATTAGTTTCT

>HO004487.1 JC007303 Seed specific Normalized cDNA library from Jatropha curcas L. Jatropha curcas cDNA clone N10211 5' similar to Conserved hypothetical protein, mRNA sequence

ACATGAGAAGAGGAGCCAAGCTACTAGTGTATGCCAATGGAGAGTCCAGATTTTTCACCTTCCCACGTGGACGCATCACG

TCCGTCTCTTGGTTTCCCTCTCGGTACTGCCTTACTCTTGATCATCATCTTCACCTTGAGCGGTATTTTCTCTTGCTGTT

ACCATTGGGAGAAGCTCAGATCTCTCCGTCGATCTTTCTCCGATCAACCTGATCCTGAGGTTGACATCGAGGCTCCACTT

TCTAAACCTAAGCCTGACAACACAGATTTCAAGCAAAATCAAAGCCAGAGCTTGCCGGTGCTAATGCCTGGAGATCAGAT

TCCGAAATTTATAGCATTACCATGCCCATGTGAGCCGCTGCGAGCGGAGAAAGTGGTGGTGAAAGAGCAAAAGCTGCCGA

AACCGCTGCGATTTCCGGTGCCTCTGTATTAGCAATGGAAAAAAGATCTTACCATTTGATGTATATAAAGGGGAATTAAT

TCAGTTTTCTTCAAAATTTACAATCTTTTAGCTTATTTTCACCATTTGAGAAAAAAAAAATCGCAAAAAAAAAAAAAA

>FM892702.1 FM892702 Jatropha curcas embryo 56-70 (DAF) Jatropha curcas cDNA clone rjcaeb0_003319, mRNA sequence

ATTCGTCACTTTAACTTCTAGTGGAACAGCATCATCAGAGTTCAAACATGGCCATGTAGGTTTGGACACGAAAACAAGGA

CAAGATGAACTCAATGCCAGAAAAAAACCATCCTGTTAATACAGCTATTTCTATCAGCCAAAATAGTGAAGTTAAGGGAA

TGGAATGCCATGATACAGCAGATTCAATAAACACACGGAATTTGTCTGAGCAATCGTGCAGTGTGGGGAAGGTAGGTGTT

GATTCTTATATACTCAGAGAATAACTCAAGTAATGGTAATTGCCAGAGGACTAACAGGAAGGAATGCATTGGGCTGGATA

CATGCATTGGAATTGACAGAAGATCAACTCATGGCTGCTCAAAGATCAATGATAATGCACATGGTGGTGCTCTGCGTTAT

GCCCTGCATCTCCGTTTTCTATGCCCTTCCCCTAAAAAGTACTTCTAGGTCAGTTCAGAGATGGCAATCTGATCCCACTT

CTCTAAAACAAAATACACATTTAGATGCGGAAGGGGACCGCAGGTTCTATTTATACAATGACCTGAGAGTTGTGTTCCCT

CATCGCCATTCAGATGCTGATGAGGGCAAGT

>GT973069.1 GJCCJC2044F11.b Jatropha curcas L. developing seeds (mixed stages) Jatropha curcas cDNA clone GJCCJC2044F11 similar to Retrotransposable element Tf2 155 kDa protein type 3, mRNA sequence

CGGAGTACTGTTAAGATGCGTATCGATGAAGGTAGCTGAAGGGATTATGGAACAGATCCATGCAAGGGTTCGCGGCACCT

ACATGAATGAAAAAGTGCTTGCTAAGAAAATTCTCAAACAAGGATTCTTTGGTCGACAATGGAAAGGAATTGTATTTCTC

TCGTGAAAAGGTGCTTCGAATGTCAAATACACGGAGACCTGAGTCACATCCCACTTTCAGAATTACATCCGTATATTTCA

CCATGGCCATTTGCAGCTTGGGGAATAGATATCATTAGCAAGATTTCACCACCTGCTTTTAACGGACACAAATTTATAGT

GGTTGCAGTGGACTATTTCTTAAGATGGGTTGAAGGAAAATCTTTTAAAAACATCAATGCCAAGCAAATGGCTAAATTCA

TTGAAAAGAACTTGATCTATAGAAATAGAATTCCCCATCATAAAATGACAGATAATGGAGTTTATTACCAAGAAGAAGTA

AGGACATTGTTAGAGAAATATGGAAAAGAACAACATAAATCTTCACCTTACAGACCACAAGCTAATGGGGGCAGTGGAAG

CCGCAAATAAAAACATTAAGAAGATATTAAGGAAAATGGTAGAAAATGACCATGATTGGGCTAACGAGCTACAGTATGCT

TTGTGGGGATACCAGACCACAGTCAAATCTGCGAATGGGGCAACCCTTTACTCTCTGGTCTACGGAATGGAAGCTGTATT

A

>GW615346.1 Jc1-049-E11-M13F.E11.ab1 Jatropha curcas flower and seed Jatropha curcas cDNA, mRNA sequence

AGATATGCAAAGTCCTCAACATTGGCATTCTTTGTACCGGTCCCCTACCCATCAACCGACCATCGATGAGAAGGGTTGTA

AAAATGCTGCAAGAAATTGTGCCAGAGAACATGCTCAAAACTGTAAAAAAAGATGGGAAATTGACGCCATATTACTATGA

GGATGGCTCAGATCAGGGGAGTGTAGCTTGAGAATTACGATCAAGTTTTGTCGCAAAGTCTGATATTGGAGCAGCAGAAT

ATGAGCTTAGCTTCAAAACATTTTCTTTTTCTTGGGCCAACTTGCCCATTTCCACTTCGATTTGGTGTTTGCTTTACAAA

AATGAAGGAAAGATGAAGTTTTTCTGTCCAAATGATAGGGTAGTTTCTGAAGAGGGGGTT

>GW620183.1 Jc2-055-F07-M13F.F07.ab1 Jatropha curcas flower and seed Jatropha curcas cDNA, mRNA sequence

CCCCTCCAATTCCACGGCTCCGATCACTTCTTCAGCTGCGGATCTTTTTCGCTCCGCCTCAAGTAAAGCATCTTCGAAAG

AGATGGAGCGAATTGATAACCTATTTTATTCCTATGCAAATAGGTCTTCTGGTATGATTGATCCAGAAGGAATTGAAACC

CTATGTTCAGACATGGAGGTTGATCATACTGATGTTAGGATCTTGATGCTAGCTTGGAAAATGAAAGCTGAAAAACAGGG

ATACTTTACGCTGGAGGAGTGGCGCAGAGGTCTTAAAGCGCTGAGAGCTGATACTGTGAGTAAATTAAAAAAGGCGCTTC

CAGAGCTAGAGAAAGAGGTCAAGAGGCCGTCAAACTTTGTGGATTTCTATTCCTATGCATTCCGATATTGCCTGACAGAG

GAGAAACAAAAGAGTATTGACATAGAGAGCATCTGTCAGTTAGTGGATCTTGTTTTAGGATCTCACTTCCGTGCTCAAGT

TGATTATTTCATCGAGTATTTAAAGATTCAAAATGATTATAAGGTCATAAACATGGATCAGTGGATGGGCTTTTATCGTT

TTTGCAATGAGATAAGTTTTCCAGATCTTAGTAATTACGATCCAGAACTTGCTTGGCCATTGATTGGATAATTTTGTAGA

ATGGATGCGAGAAAAAAGGACTTAAAAAACCTACTGTCTGTGTCGACACGATTCTCTAC

>GT979247.1 JGCCJG2030B12.b Jatropha curcas L. germinating seeds (mixed stages) Jatropha curcas cDNA clone JGCCJG2030B12 similar to unknown protein [Arabidopsis thaliana] (TAIR:AT3G24506.1); similar to unknown [Populus trichocarpa] (GB:ABK93895.1), mRNA sequence

GGAAGATCGAAGAACAGCAACAATAAGGAGGAAAGAAGATATTGTATTAGGCTGAAGCTATGGCGTCTATTTGCCTCTCT

TTATGCTCGACTTCGAAACCCTTAGTGGTGCCTCCTAAATCGGCAGCCGGAGTTGCTTCTTCTTCTTCTTCTTCGTCTTC

ACCGAGCTGCCATGGTTGCCGTTGCTCTCTCGTTTTCTCCACGCCGATTTTTGGTTCCGGAAGAATGATTTCTTCTACTA

GCAGCAATACCAGTCGGAGCAGGCGACAAAAGCAAGTGGTTTGTATGGCACCAGATGAAGAAAAATTGACTCGCCGCAAT

CCTCTTGATTTTCCTATCGAGTGGGAAAGGCCCAAGCCTGGGCGCAGACCTGATATATTTCCTCAGTTTAGCCCCATGAA

AACACCTATACCACCCCCATTGCCATATGATCCTCCAGAAGAAGATGAAGAAGAAGAGGAAGAAAAAAAGAAAGAGGAGG

AGGAGGAGGATCCTGAAAAGGAGGAAGAACCAGACAAGCCTGAGAAGCAGTAGTGTCTATCATATTCAATTTCTTTTTGG

TTCTCTATGTAAACTCCACCGAGTTTTTGATTAGTTTACATCTAGCTTTAAGTGCTGATAATAGGAAGTGGACGAAATGC

AATAATCCTCATCCAGGAAGAATTTTTGTAATGGCTGTTGTTGCCCACTTTGAGCTGAAAGGATCTTGGCAACCTCCTTT

TGGACTTTATAAAGGCTAGAGTTT

>FM895292.1 FM895292 Jatropha curcas embryo 71-95 (DAF) Jatropha curcas cDNA clone rjcpga0_002092, mRNA sequence

ATTCGATTAAGCCATGCATGTGTAAGTATGAACTAATTCAGACTGTGAAACTGCGAATGGCTCATTAAATCAGTTATAGT

TTGTTTGATGGTACCTGCTACTCGGATAACCGTAGTAATTCTAGAGCTAATACGTGCACCAAACCCCGACTTCTGGAAGG

GATGCATTTATTAGATAAAAGGTCGACGCGGGCTCTGCCCGTTGCTCTGATGATTCATGATAACTCGACGGATCGCACGG

CCATCGTGCCGGCGACGCATCATTCAAATTTCTGCCCTATCAACTTTCGATGGTAGGATAGAGGCCTACCATGGTGGTGA

CGGGTGACGGAGAATTAGGGTTCGATTCCGGAGAGGGAGCCTGAGAAACGGCTACCACATCCAAGGAAGGCAGCAGGCGC

GCAAATTACCCAATCCTGACACGGGGAGGTAGTGACAATAAATAACAATACCGGGCTCAATGAGTCTGGTAATTGGAATG

AGTACAATCTAAATCCCTTAACGAGGATCCATTGGAGGGCAAGTCTGGTGCCAGCAGCCGCGGTAATTCCAGCTCCAATA

GCGTATATTTAAGTTGTTGCAGTTAAAAAGCTCGTAGTTGGACCTTGGGTTGGGTCGATCGG

>FM894141.1 FM894141 Jatropha curcas embryo 71-95 (DAF) Jatropha curcas cDNA clone rjcpga0_004328, mRNA sequence

GAAAAGAACGTTTGAGATAATATACTGACAGCGTCTAAATGTTTGACAAACTATACACACAACAGGCCTGTAAAAATGAC

ATGCTCTGACAGTAACTAAGCATGATATTTATACTTGCTTGAGTTTCAATGGACAAAATACATAGATAAAAAGTTCCAAG

TTAATCAACGATGATGAACTTCTTGTTTTCTCCCCTTCACATGCTGATCTGCTGCAACTGAAATTGTCGATTCCTTCAAA

TTGTGGTCAAACTCACTTGCCATTTACAGCTTTCTCATTCTGCTGCCTGGCAGTCAAACAGCATACAGCCTTATGATCTG

ATCAATCTTCGCACGACACACAGGGCAGCCCCATTTTTTTGCCTTGACCTCCTTTAAACAAGACATACATCCAGCCATAT

GGCCACATGGAACGCAAGCCCCCTCAACTGGAGCATCCAAACATATCACACAAGACGAAGAACTGCCATCTTCTTTCTTT

TCACCAGTACTAGCTGGCAAGTTCTCCATAGGGGGGGAAGATATATCAATAGGACTAGAATCAATTGATGGATAATGAAT

GGGACCATCTTCTACAATC

>GT969593.1 GJCCJC1004H02.b Jatropha curcas L. developing seeds (mixed stages) Jatropha curcas cDNA clone GJCCJC1004H02 similar to unknown protein [Arabidopsis thaliana] (TAIR:AT4G30710.1), mRNA sequence

CCATGGGACAAAGCCGCAAGTCTCCAAAACCTGGGTCCCGGTCGAATAAGAAACCAGCCAATAAGCAGAAAACCAGGGCT

CGCATCCTCAGCCTCATCCGGTCAGGGTCGTCCAAGCAAGTCTCTGAAAATTCATCAATCAGGGGCAGCAGAATCGCCTT

CTGATCATCCACAATCATAGCCCTAGCCTGTGGCCGGCTAAACCGAAACAAGTTCTCAAATGACTTAACATACCCAACTT

TCACCTCGTGCCTAACCAAGGGAGCATTGGGATCACACCCTATAATCGCACAAAATTCCTCAAAAAGAGGGCACATTTCC

TCCATCATAGCCCCAAACCGAAAAACGTGAGCCTCAGGATCCCAAAATCTAATACAGGCTCGCAAAAAGTCCCAGTCAAC

TACTACGCCCCTAAGAGTGATAACGGGAGAAATTCCGTACCTCCCCAAATTGATCTTTTCAACTTTGGAGAGACTCCTGG

TCCAGCTATGCATGCTATGGTTGTAAAAGGGATTCATGTTTTTTATTTATTTATTTATCCTGTTTTTATTAAT

>GW875596.1 JC004913 Seed specific Normalized cDNA library from Jatropha curcas L. Jatropha curcas cDNA clone N08592 5' similar to Unknown protein, mRNA sequence

GCAACCAGCAATCTCCATTGAAGCCTTATTCCCTGTTTTTGAGATCAACTTCTAGTAGCAACGATGTTGTTGAGAGCTGC

TTCAGCTTTTTCTCTGACAACCAAACAGGGGGATTCTGCTTCGTCTATGGCGGCCCTGACATTATCACCGTCGAGGCGGC

GAAGCAACAGTAACCTCGTGGTTTCTGCCATGGCGGATGGCAGTTTACAGCTTACTGGTGTAGTGTTCCAGCCATTCGAA

GAGGTCAAGAAGGATGAATATTTGGTTCCTATCGCTCCTCAAGTTTCTCTTGCTCGCCAATTTTATGAAGATGAGAGTGA

AGCTGCAGTTAACGAGCAGATCAATGTGGAATACAATAATTCGTATGTATACCACGCCATGTTTGCATATTTTGACAGGG

ACAACGTTGCCTTGAGGGGCTTAGCCAAATTTTTCAAGGAGTCTAGTGAAGAAGAAAGAGAGCACGCTGAAAAGCTAATG

AAGTATCAAAACGTACGTGGAGGGAGAGTAAAACTTCATCCTATCCTGAATCCTATTTCAGAATTTGATCATGCAGAAAG

GGGAGATGCATTGTATGCTATGGAATTGGCATTGTCTTTGGAGAAGTTGACAAATCATAAACTACTGGGCTTGCACAGTG

TGG

>GT973791.1 GJCCJC2052F11.b Jatropha curcas L. developing seeds (mixed stages) Jatropha curcas cDNA clone GJCCJC2052F11 similar to Retrotransposable element Tf2 155 kDa protein type 3, mRNA sequence

CTGCTTTAAGCTTTAAAAAGAGTCGTTAGCTGATCTGGGAGTCAGTGTCTCGATGCCGAAGTCACTGGTCAATAAGTATA

AGTAGGCAAAATCCTCTGGGGATGTAAGCGAAGCTATTGAAGTAAGAGTTACCGCCCCGACAAGTAAGTGGTAGTTGCTT

CCTCGCCTAGCCTAACGGTTTATATCCAATTCCTACTGCCAAAGTTAAGGCTAAGCTTAGTAGGATTCTAACCCTGTCGC

CCCAGGATTCCTTCAATAAAGTTAACAGGATGGGGATGGATGGGATATTCTTACAAGAGCTGAGGAAATGGGAGCTCCTA

TTCACCGGAGCTAATTCCTTTCTTTGCGGAACCATAGCTAATGGCTCAATTAACTATCTTCCCTTTTACCTTTTGTCCCT

CGCTTCCCCATTCATTGGGGGACAATATAGCTAGGCCTATTTGAACTAATAAGAATCTCTGCCCCGAGCGGAAATACACA

GTACACGAAAAGGAGATGGCCGCCATAGTACACTGTCTATGCACTTGGAGACACTATCTGCTCCATTTCCAACACAGAAG

AAGCTCAGCCCTAAGCAAGCAAGGTGGCAAGACTTCTTGGCTGAGTTCGATATGCGTATCGAGT

>JK613543.1 JCF19-151 Jatropha curcas, immature Seed cDNA subtraction library Jatropha curcas cDNA similar to senescence-associated protein, mRNA sequence

TCGAGCGGCCGCCCCGGAAGGGACGAATCCGAACCGTGGAAGCGTGGAATTGCGATCCTTGAAACCTTCGCCATTTGAAG

CTTTAGGTGTCCGAAAAGTTTAGTTCATGATAACTGGCTTGTGGCAACCAAGCGTTCAGGGCTGCGTTTCTTTTTGATCC

TTAGATGCCTGCTCTTCCTATCCTTGGGAAGCAGAATTCACCAAGTGTGGGATTGTTCACCCAACAATAGTGAACTTGAG

CTGGGCTTGTTTTTCCCTGGGAAAGGTTACTTTTACCCTACTAAACATGGTGTCACGATGGTACTTTTGACACATTCCCC

AGCCGCGACCAAGCTAATCTATATGCCGTCCCGATGGACCAAGATAACATTCACTGGGCGTCCTGGTTCAACTTCATGAC

TGGGATAACCCTGACGTTACCCATGTTAATACTCTTGCAGAACATGCCTCTTTCGCCAATTCGCGCAATCCCCTATATGC

TCGTGGCGTTCTCTCTTCCAGACAGTTGCGCAGCTCGAATGGCTAGATGCATTCGTAGGAATGAACTTTGATAAAATTCG

CGTTAATTTTTTGTTAAATGACCGGATTAACTATCCAGTAAGCCGATATCATCTTAATCCCTTAGAAATCAATATCATAA

ACCGACATAGGGTTGAGTGGTACTCCACTATGGACCATTACTCCGCTCTGAAATGACATGAGAGTCCATCGTCAATGACG

AAGTACCGCTCTATGCATGACGATCGCCCTACATCTTGAGATCAGTCGACCGTTAATCCATGCTATGCTAGAGTGCCAGA

AGTCGTATAGTTCTGTAATCGAATACGCTGACTGAGCTACGTACTATCGACTGTCGGCAACTTCGTACTACGATGCCAGG

TGTAAG

>FM889601.1 FM889601 Jatropha curcas embryo 35-55 (DAF) Jatropha curcas cDNA clone rjcfea0_003302, mRNA sequence

TGAAAATGGCGTTGCAGTGGATGATACTCACTTACGCCGTGGCAGTGGATGCGGTTCTAGCTGCAGTTTTAACCCTGCCC

TCGCCGAAGCTGTTGAAATATCGATTGGTGTCTCTCGTCTCCCTCCTTCTCCAACCTGCTCTCTTCATCGTCCCTTTCGC

TGGGTTTCAGCTCCTGGATATCTACTGGAAGAATGAACATCGGTTGATGTGCACCTCTGAGATCTGCACTGCCGCAGAAA

GGGATCGCTATGAGAAATCTATTTACAAATCCCAAAGGAATGTGATCTTATGTGCTTCAGCTTGCTTGCTCTACTGGTGT

ATCTATCGTATATGCAAATATCATAAAGAGATTCACAGTTTGGAGGATGTGGAGAAGAGGTACAAGGACCAGTAGTTCAG

TTCTCTGTCCCACTTCAGCATTGGGAGCACACAACAAATTATACTTCTTGTTGTCAGTGTTTAGTTATGAACTTGGAT

>JK610655.1 JCF1668 Jatropha curcas, immature Seed cDNA subtraction library Jatropha curcas cDNA similar to anaphase-promoting subunit 10 family apc10 family, mRNA sequence

CAACAATACCTCCGCCACTGGATCGGCCGCACATAAGTTATATTTGCAGATTGGAATAGAAAATGGCGACTGAATCGTCA

GAGGGCGAAGAAGAAGGGAAGATTACAGGAGGGACTCAACATCTGATTGTAGATGATGATCTTAGAGAAATGGGCAAAAA

GGCTGCTTGGAGTGTCAGCTCTTGCAAACCTGGCAATTGCGTATCTTCTCTTCGTGATGACAACCTTGATTCCTTTTGGC

AATCTGACGGTGCACAGCCTCATTTGGTGAATATTCAATTCCAGAAGAAAGTAAAGCTGCAACTGGTTATACTCTATGTG

GATTTCAAGCTTGATGAGAGCTATACCCCAAGTAAGATCTCTATTCGAGCTGGCGATGGATTTCATAACTTGAAGGAGAT

CAAGACTATGGAGCTTGTCAAGCCATCTGGCTGGGTTTATCTATCCTTATCTGGAAATGATCCTAGGGAAACTTTTGTCA

ATACATTTATGTTACAAATTGCGGTGCTATCAAATCATCTTAATGGAAGGGATACTCATGT

>JK613213.1 JCF22-223 Jatropha curcas, immature Seed cDNA subtraction library Jatropha curcas cDNA similar to senescence-related protein, mRNA sequence

ACAATTGTGAATTCTAAAGCGGGCAAGAAATTTTTTAGCTTGTTGCCTGGAGAAATTGTGCTTGCTTCATTGGATGGATT

TAACAAGGTGTGTGATGCTGTTGAGGTTGCAGGAAGGAATGTCATGTCAACATCATCAGTTGTTACAACAGAGCTTGTCT

CACAGAGGTATGGAGAACAAGCAGCAAAGGCGACAAATGAGGGGCTTGATGCTGCGGGACATGCCATTGGCACTGCTTGG

GCAGTTTTCAAGATAAGAAAGGCTCTTAACCCAAAGAGTGTCTTCAAACCTACAAAACTTGCTAAGGCTGCTGCGGAAGC

AACTTCTGCTGAAATGAAGGCTAAATACAAGAAATAACGGCAGCTAGTTTTCGGGTTGATTTTTGTAACATTTGGTGGCT

ATGGAAAAGGATAGCAAATTCTACAACATATAAATCTTATTTATTTAATTTTGTATAGACTTGTATCTTTGTAGTATGCT

AAGAATGTGAACTGGTCATTGTATTTGTTGAATTATTATCATTTATTTTTACCGAATGGTTTAACTCAAAAAAAAAAAAA

AAAAAA

>GW874847.1 JC000238 Seed specific Normalized cDNA library from Jatropha curcas L. Jatropha curcas cDNA clone N00433 5' similar to Unknown protein, mRNA sequence

GGAGAAGCTAGAACTAAAAACCATTCTTGAGGCATAGGCATTGATGTAAGAATTAGCAAGAAATAGAAGTTCTTTATCTG

AATCATCCTCATCCTCATCCTCGTAATTATAGTTATGATCTCCATCATCACCGCCAATCTTGAGGCTTTCTTGATCATCA

TCAGCACTATCCAACAACTTTTCTATCTCAGTAACCTTTTGATAAAAAGAGAATACCAATTCTTCATCCTCGTTGTTAAA

ACAATCATCAGCAGTATCGAGTAGCTCTTCAATCTCAATAACCTTCCTCCAAAACGATAATTC

>JK612656.1 JCF20-94 Jatropha curcas, immature Seed cDNA subtraction library Jatropha curcas cDNA similar to nucleic acid binding protein, mRNA sequence

TACCGTGGGCCCGGCCGACGTGCGCAATAGTAACAACCGAGCGTGGCGATTGGTTTTTCGTAGAGGACTAAATGAATGTT

GGCCTTTGTGTTCTGAAGTTTCCACCAAAATCAGGGGTTCCACCAATTATCTCACAAGGCATTGAATATATGGCTCCTTT

ACTCCACCGGAAAGCTATCTCTTCCACCTCCAGCATATGTTATAATTACTCCTACTATCACCAATGAATCTGCGCATGTA

CGGCGAACGCCTCTTGATGAACTCCTTGAACTTCCATCCACACCAGGAAAAGCTTTTGATATTAAATCGGTATTCTGGGC

ATCCCAAACTCGCTGCGGATATCATCATCCTTTCAATTTAATTTCCAAATCACCGCCTTGTGAACTTAGGAACGGGGATG

TCCCTCTGAGACCGGAAGAGAGCGTTTGCTCCTACTACGCAAAGCTATGCTTTTGCACATTTTCGGACATGCTTGAACAT

GATATCTTTCGACACATCCACTCTCGAAGACATGCCTTGCTATCGATAAAGAATCCTCGGAAAAAGAACACACTCATCTT

GATGCGCTATATTTCTCGAAAGCTCGAATTCGCTGCCCATCATGATACATCGTCCATGACATCCAATAACCCCCTCTCGA

CGACTTGCTATCGACAGTAGGAGAATATCCATCCATATCAGCGAGCTGATCCTATTATGGAGGATGCCGTCAACTAATTC

GACTTCCTACCAACAAGATGACGCAACCATGGAGGATGTATTTGGGAGTTGGGAATGCTGATTACTAATTGCAAGATCTC

GCATGTACTCATAGCTCGAGTCAGCTGCATCGTATATACTCCATGAGTCGAGATCGTAACGATGCTCGTATAACGGAACG

ACTGTATCCGACGATCGGTATAGGTGATCCTAGCTTGACGAGCAGTACTCCTCTGACGACACACCA

>GT975731.1 GJCCJC2062F10.b1 Jatropha curcas L. developing seeds (mixed stages) Jatropha curcas cDNA clone GJCCJC2062F10 similar to calmodulin-binding protein, mRNA sequence

GTTCGATCAAGAAGACAACTATAATAGTAACAATTCATCACAGAATGTAAAAGTGACTAAACCGTCCTACGCGTCAACTT

TCTTGTCTGCGATATCATTTTCGTCAAAAGGTTACAAGAAATGGAAGCTTAAAGATTTATTACTATTCAGAAGTGCATCA

GAAAGAAGAGCAACAGCAAATAATAAAGACCCATTAACAAAGTATGTTGTGTTGTCTAAAAAAGAGGCGGTGGTGGAGGA

TGCCAAGAATGCTAGTTTTCGGTCTGCTGATAGTAGTATCGGCGGCTCTTCACGGCGAAGAAGGCCGGTCTCAGCCCACG

AATTACATTATACGGCGAACCGGGCGGTTTCGGAAGAGTTGAAAAGAAAAACGTTTTTGCCTTACAAACAAAGACTATTG

GGTTGCTTAAGATTCAACCCTGGTGTGCATGAGATCTCTAGAGGTGTTGGGTCTTTGACACGTGTATGATCCAGATTGGC

TTGATCTGTTTCTCTTGGTTAAGCATATACTAGTTTCTTTTATTGATTTTTATTTTTAACTTTAATGGTTTATCTTCATA

TATTTGTTACTTACAATACATAATGGAGTGTCACCATCTGTATCAAATATACACAATTGTATGAGAATACACTACTATTG

ATT

>GW875924.1 JC005301 Seed specific Normalized cDNA library from Jatropha curcas L. Jatropha curcas cDNA clone N09162 5' similar to Polyadenylation factor subunit, mRNA sequence

GTACCGATACGGTTTTCAGTTGCAGAGCCTGCCCACCCCGTTTCTCTACCATAAAACCTCCACTTTCTCCGGTTGCTTCA

CATAATTTCTCTCTCCCCCCCTCTTTATCTCTCTTTCTGTAGCTGGACCGTGATATTGTCTACAGATTCGAATTCCGGTC

CACGAATTTGGGAGAATGACCTCTATTACCTCCGCCGAGTTAAATTACCTCGTTTTCCGATACCTTCAAGAGTCAGGTTT

TACACATTCAGCTTTCGCTTTAGGATATGAAGCAGGTATTAACAAATGTACCATTGATGGCAATATGGTTCCACCTGGTG

CTCTTATTACATTTGTGCAAAAGGGAATTCAGTATTTGGAGATGGAAGCAAATTTGAGTAATAGTGATGCAGATGTTGAT

GAAGATTTCTCATTCTTGCAACCTTTGGATCTTATAACAAAAGATGTATATGAATTGCGGCAGATGATAAAAGATAAGAA

AAAAAATCTGCACAAAGACAGAGATAAAGATAAAGATAAAGATAAAGATAAAGATAAAGATAAGGAGTGTGACAAGGAGT

GTGACAAGGAGCATGAAAGAGAACGTGCTCGAGTAAGAGAGAAGGAAAGGCATGAAAGGGAGAAGGAAAGTGAAAAGGAT

AGAGAGAGGGTGGAAAAGGAAAAAGAGCGAGAAA

>GW880093.1 JC000607 Seed specific Normalized cDNA library from Jatropha curcas L. Jatropha curcas cDNA clone N01148 5' similar to Unknown protein, mRNA sequence

GGATTCACTCTCTGTCTCCTTGCCGTACTCTTGTTCTTTAATGTTTCTTCACCCCGAGTTCGTTTATTTCAATCTTACTA

TAACATTTCCCTCTTCTCTCTTTTTTTAGTGTAACTTTCCTTGAAACTTCTCGTCCTTTAATGATACTTTTGAAAGAGAA

AAGGAAAAAAAGAGTATTCAATCATCTCCGTTTCAAGGCTTCTCTTTAACGTTGTGTTCTTTCTCTCAACTTTCTTTATA

TCCTCTGTCTCTCTTTCTTTATTCTCCCTCGTGTCCAGTCTCAAAGGAATCTACTCTCTTTTCTTTTTTTCATTCTCTCT

ATTTCACGCTCTCCTCCTCCCTCACTTCACTTTCAATGCGAATCTGAAAGAACCCAAAAATAGTAAGCAGAAAAGCAACA

GACAAAAACCCAAGCCCGCTTCTCGAAATGGGATCAAAATAGTCTTATGTCCATGAATCCAGTCAAGTTCAGCGGACATC

>GT978645.1 JGCCJG2022H09.b Jatropha curcas L. germinating seeds (mixed stages) Jatropha curcas cDNA clone JGCCJG2022H09 similar to nodulin MtN3 family protein, mRNA sequence

CACCATCAACGTATTTGGATGTGCAATTGAGACCATTTACATTTTCCTGTATATGTTTTACGCTTCCAAAGAAATTCGGG

TCTCAACTTTCAGACTTCTTGTGTCCATGAACATAGCTTTGTTCTCCTCCATTGTCCTCTTCACTCATTTTCTAATGAAG

GGTTCGATCCGAGTTCAGGTTCTTGGTTGGATTTGTGTTGCTGTTTCTGTCAGTGTTTTTGCATCTCCTTTAAGCATTGT

GGCTCAAGTTGTCAGAACTAGAAGCGTCGAGTTCATGCCATTCACTCTGTCGTTTTTCCTTACACTGAGTGCTGTTATGT

GGTTTGCTTATGGTTTTACTACAAAGAACATGTGTGTCGCTCTCCCAAACATCTTAGGATTCATCTTGGGGCTACTTCAG

ATGCTGTTATATGCAATCTACAGGAAAGCAAAGGTGGTGGTTATAGAGGAAAAGTTACCAGAAAAAAATTTGAAGAGCAT

TGTCATACTAAGCACATTAGGCAATTCTGAGGTTTATCCTGTGGATGTTCGACCCGAATCCGAACCCGATGAGAATAAAG

TTGCAAAAGAACAAGAACAAATAGAGGAAGGAAGCAAGAAGAGTAATGAGAGAAGCTTGGAAACATCAAATGATCTTAAT

CCAAATGAAATTGCAGTTTAATATGTTCTCATGTATTAATTATAAATGGCTGAATAATTAATGTTTGTATACTCTCTTTT

TTTTCCCTTTTTGTGTAATATTACCTCCCTTGGTTGATGAAGGAAAGTATGTGTATATGTAAGCATAA

>GW881129.1 JC004393 Seed specific Normalized cDNA library from Jatropha curcas L. Jatropha curcas cDNA clone N07828 5' similar to ADP-ribosylation factor, mRNA sequence

GGAACAAAAGCAAACGCATTTTCTCTTTCTTCTCACTCGTTATGCCTCTGAATAAAAATATATAAAACTCAAAAAAATCT

AAAAAAAAAAAAAAAGAAAAAAACACTGTCAAATCGATTTCGCTCTGAATCTCTGAATGTGAACTTCCATCGGAAAATCA

TAGCATAGCGTAGAAGGAGATCAAAGCCCAAATTGAAAGAAGGTAAAATTAAAAAAACCTTCATATTCAGCATCAAAGGG

GCGGCTACGCAATGGGTCAAGCCTTTCGCAAGCTCTTCGATACCTTCTTTGGCAACACTGAGATGCGGGTGGTGATGCTA

GGGCTGGATGCAGCTGGTAAAATAACCATACTTTACAAGCTACATATTGGAGAAGTCTTATCTACTGTTCCAACAATTGG

GTTCAATGTGGAGAAAGTTCAGTATAAGAATGTGATGTTCACTGTTTGGGATGTTGGTGGGCAAGAGAAATTACGGCCAC

TTTGGAGGCATTATTTTAATAATACAGATGGATTGATCTATGTTGTTGATTCCTTGGACCGTGAAAGAATTGGCAAGGCA

AGAGCAGAGTTTCAGGCCATCATCAAAGATCCTTTTATACTCA

>JK317670.1 JCST441 Jatropha curcas L. seed cDNA library Jatropha curcas cDNA 5', mRNA sequence

TTTTTTTTTTTTTTTGAGAAGTAAGATTTAACCACAGCTTGTTATTGCTCTCCACTAACCATTTGTGAGAAGAGATTTAT

TGTACATTAATGGATTGGGCCAGTCTCATCAAGCCCACAATCAATGATATTAGTCTTGTAGATTTTCTTGAACTACAATC

ATTTACACATTACAAGCAGGATTAGAAGAAAGGCTTGACACCCAAACTGAATCACTTATAACTGCTTCATCATCATCATT

TCTATGCCATCTCCAAAGTGCATGACTTGCATTCACCACCTCAAGCTGTCCATGCCCAAAGCTTGCCTCTCTAAACACTG

ATATTTCTGGCTTTGGATCTATATAGTTTTTGGCAAGGCCTTCACGGTTGCCGCCATCCCCGATGTTTATGTAAACAGGA

CCACAATTGTTTGCTTTCCCTTGGTAAACACGAGCAAATCGCTCATATGCATGCACATGGCCAGCAAAAACGACATCAAC

ACGAGCTTGATAAATTAACTTCTCCATAAAATCCCTCATATGAACAGACTCAGCTTCACCTTGATGAGCAGAATTAGAAT

TATACCAAGGTGCATGAATTAACACAAACACCCAAGGAGTTTTTGCCCTTTCAATCTTCCCTAAATCCTTCTGCAACCAC

TTGTACTGATCAGAACTTGGATTGAAATCTGCATATGACCCTAACATAATCACACGTACTGTACCACCGGCTGCATTGAA

CGAATAGTATAGGTTTGAACTGGACCCACTTTCTTGAAGTGGCATATGCCATCTTGAATTGTAGGCAGTGAATTTGGTGC

TATGTAGAAATGGATTTTCTCCACTTCATGGTTGCC

>GW879315.1 JC003873 Seed specific Normalized cDNA library from Jatropha curcas L. Jatropha curcas cDNA clone N07092 5' similar to Unknown protein, mRNA sequence

GGAAACAAAATCATACACGAAACTGCCCATGCAATTTGCAGCCTGGTAAGTGACTAGTGGAGTGCCAATCTTGGCCACCC

AAAACCTCTGCTAAAACTGAACCACAAAACACCAAGGACAACAAACACATGCAATCTGTTTCCAAGAAAGCAACATTCAC

CACTTTCCAGAAGCTTACATTACTATACTACGCCACACCCAACCGTCGCATCCCATATATATATATTTCTCTCTTGGGTT

GCTTTGTTTTTTTCCTTCTAAATAAGCAGCTAAAACAAGCAGCGTTTAATGGCGGCGAACGATAAGGAGAAAGAAACGTC

AGAGTTTTTCTTGGAAGACGGTGATGATGATTTTGGCAGAGACTTAGAAGTAAACAAGGTTGAGATTGGAGGCCGGAGTA

GCAGTAACAGTAGCAGTAGCGGTGGTGAAGAGGCTGATAGTGCTGAAGGATGTGGAACTTTCTCTTCTCAGCAATGGCCG

CGGAGTTTCAGAGAAACAGTGGATTCTTACTCAATCTCGATGTCACCTAACTTTGGGTTTCTTGGGCGTGGACAAA

>GW619117.1 Jc2-042-G02-M13F.G02.ab1 Jatropha curcas flower and seed Jatropha curcas cDNA, mRNA sequence

CTCTTCTCTTCTCTATCAAAGCTGCTTCACATGGTTTTTTGCTGGTTGATGGTGTTGAAGTCACTCATCTAAACAGACCA

AATGACAGATTGTTTGAATGGTTCTTTCACCCTTTGCTTGTTGTAAAGGAACAAATCAGAGTCATCAAATTGGAAGAAGG

GGAGGAAAGATTCTTGCAGAAAGTAATTCTGTTTGGAAATGATACACAGCGGATGGAGGCCTGGGATAATGGCAGTATAA

TACCTCAAGAGGCTCTCAGAGCTGCTCAAATACAAGGCCTTAGCAGAAGGATGTTAGGAATGGTGAGGAGTGCATCAAAG

CTTCCTACTTACAGAAGGAGATTTCGGCAAGTGGTGAAGGCATTGATAGCATATTCAACAGATAAGGAGAGTGATGTTAG

ATCCAATTCTGTAAAATCAGTTGGCAGCACTAAGGAGGGTGGTGTTAGATCCAGTTCTGTAAGATCAGTTGGCAGCACTA

AGGAGGGTGGTGTTAGATCCAGTTCTATAAGATCAATTGCCAGCACTGATCTTGCGGTGTGAATTCAGTTTTATCTTTTC

TTTCTTCCTGGTTTGTGTGGAAAGCTTATCATAGTTTGTGAATCACTTTTAAGGCTATTCTCACAT

>FM895543.1 FM895543 Jatropha curcas embryo 71-95 (DAF) Jatropha curcas cDNA clone rjcpga0_002482, mRNA sequence

ATCCTGTCCTGTCTCTCTCTCTCTCTCTCTCTCCCTTCTCTTTCTCTTTTCTCCAAAGCAAACCCTATAAATCCCATCTG

TCTCTTTCCCTCTCTCTCTCTCTCTCTCTTCTCTCTCTAACCCATTTCCTCAATTAAAACCATCAGTTTTAAGAGACAAT

ACCAGAGAGTTAGAGAGAGAAAGCTCAGGCACATGCACCTTCTCCGGATTACTATAAATTGGCGAAGACAAGTGCTGTAG

ACGATTTAGTTTTGAGGAGGAGAGACAAGATGCGCAGGTGGCTTTGTTGTACCTGTCAAGTAGAGGAGTCGTACCCATCA

CGTGAGAATGAGCACCTAAGAAGCCCAAAGCACTATCCAGATGGAAATCCTACGAAAGGCCCGAAGGTAGCAGCTCCAGT

GAAATCCGAAGTGCAGAAGGAAGCCCCTCCAATTGAAGTGCCTGCATTGTCTTTGGATGAACTGAAAGAGAAGACCGAGA

ATTTTGGTTCAAAAGCATTGATTGGTGAAGGATCCTATGGAAGAGTCTATTATGCTAATTTAGAGAATGGA

>GW878618.1 JC002383 Seed specific Normalized cDNA library from Jatropha curcas L. Jatropha curcas cDNA clone N04419 5' similar to Unknown protein, mRNA sequence

GGTAACTCATCCTCTCCTTCAACGCCGTCAGAGTCTCAGTCTCCTAATTACTCTCCTTCGCAGTCTCCTTCACGCTCTCG

TTCTCGCTCCCCTTCTCCAGCTCCTTCTCGAGCCCCGTATCGGCTACGATCGAGTGGGAAGAAACCGGCTAGGGCCGCTA

CCGGTGGTGAGAGTAGAAAGAGCGTGGGAATCCGTACTCTTTCTGATCTGAATCGGGAGCCTGAGGCGGGGTCGGGGAGT

GACGATGACGATGATGATTATGAACCACAGCAGTATTACACTGGTGGCGAGAAAAGCGGAATGCTAGTCCAAGACCCTAC

CAAGCATTATGATGCAGATGCTGTTTTCAATCAAGCTAGAAATTTAGGTGTGGAAAGACCTGTTGATCACCTTCGATCTT

CGTCGAGTTCAATAAGCTTCACTGGAACTGGGAGATTACTCTCAGGTGAGACAGTTCCTTCTGCTCCTCAACCGCCTGAG

CCTGTCCATCACACCGTCACTCTTTGGAGGAATAGTTTTACTGTGGATGATGGCCCTTTGCGAAGGTTTGATGATCCTGC

TAATGCATCCTTCTTGGAGAGCATCAAGAAGTCCGAGTGTCCCCTAGAGCTCGAGCCAGCAGATAGGAGGACTCAGTCCA

TCTTGATCTCATAAGGGGGAAGAAAATTATTCTGAACCAGAGAGACGCCAAACTCCATTTCAAGGGGTTGGAAGAACTAT

AGGTAGCAGCAGTGCCCCCCCTGCCCCAACTTCTCCCCTTCTTTGAAGGGAGCTCC

>FM890908.1 FM890908 Jatropha curcas embryo 56-70 (DAF) Jatropha curcas cDNA clone rjcaeb0_000868, mRNA sequence

ATTCCCTGNAATGAAATTAAGGACTCTGTTGTTGCTGGGTTCCAGTGGGCATCAAAGGAAGGAGCACTGGCTGAAGAAAA

CATGAGAGGTATCTGCTTTGAAGTCTGTGATGTGGTGCTCCACACTGATGCTATTCATAGAGGTGGTGGTCAGATTATTC

CAACTGCTAGGAGGGTTATCTACGCATCACAGCTTACAGCCAAGCCCAGGCTTATGGAACCTGTTTACTTGGTGGAGATC

CAAGCCCCAGAGCAGGCACTTGGAGGTATCTACAGTGTTCTTAACCAGAAACGTGGGCATGTCTTTGAGGAGTTACAGAG

GCCTGGTAACCCATTGTACAACATCAAGGCATACCTACCGGTTATTGAGTCCTTTGGATTCTCAAGCACTTTGAGGGCTG

CCACATCAGGACAGGCCTTCCCACAATCCGTGTTTGATCACTGGGAGATGATGTCTTCTGATCCAATGGAAGCTGGTACA

CAAGCATCAACTCTCGTGGCAGAGATTCGCGAGAGAAAGGGTTTGAAGGAGCAGATGACTCCTCTATCTGAGTACGAGGA

CAGGCTGTAATCATTTGTTGAGTG

>FM887434.1 FM887434 Jatropha curcas embryo 35-55 (DAF) Jatropha curcas cDNA clone rjcfea0_000634, mRNA sequence

CAACAACCTTGTCCTAAATGTTGATGGTGCAATTGGGTCCCTTTTCTTGGATCTTCTTGCTGGCAGTGGAATGTTTACCA

AACAGGAGATTGATGAGATCGTGGAGATCGGTTATTTGAATGGGCTCTTTGTGTTGGCACGTTCCATTGGTCTGATTGGG

CACACCTTCGACCAGAAGAGATTGAAACAACCACTATATCGCCACCCGTGGGAGGACGTTCTCTACACCAAGTGAGAGAA

TGACAAAGAGCAGGTGCAATGCTGATCTGCTTTCGACTTTCAGCAAGCTTTTCACTTCCCAAATTT

>GT970409.1 GJCCJC2007H05.b Jatropha curcas L. developing seeds (mixed stages) Jatropha curcas cDNA clone GJCCJC2007H05 similar to Transcription factor RF2a, putative, mRNA sequence

AGGGTATTTGAATCCTGGCCTGACCCAACCAAGGCCTGCAACTATTAGCTCAGATGGGCTTGTGATTTGAATATGTTAGG

GCCTGACCCTACCTGGCCTACTGGAAGAATAAAAAATTAATACGTTACGTATGTTGTTTGTAGTGCTGGGTCAGGTCAGG

CTTGTGCCCGACCCAAAAACAAGAACATGTTTCCACATTGTGTATGTATGTGTTTATCTTTTTGATCTTTGAATGATATA

ACGGATGCTCAACCTTTTGAGGGTGCTTTCAGTGGAGCATGAAGTCTTGGAGAGGGAGATTGGGAGACTACGAGCCTTGT

ATCAGCAGCAACAACAACAGCGACAGCCACAACAGCAGCAGCAGCAACCATCTTCCAGCCATCGCCGAACTAACAGCAGG

GACCTTGAATCCCAGTTTGCAAACCTCTCCTTGAAACAAAAGGATGCCAATCCTGGTCGTGATCCTGTGACAGGTCCACT

CCGCACTTAGATAAATAAATTAACCAGAGATTTGTTGTGCCACCATTTTGCTTAGTTGGGCTACCACATTAACCATCATT

ATTTTCTCCTCGAGTGGGGTAAAGCCGAGTTCCTCCAGTCTCCATTCCGCCTCTCTGTCTGATTTGCACAATTAATTTTT

ACTTTGTTTCTTG

>GT977949.1 JGCCJG2014E06.b Jatropha curcas L. germinating seeds (mixed stages) Jatropha curcas cDNA clone JGCCJG2014E06 similar to RNA-binding protein Nova-1, putative, mRNA sequence

CAGAAGACTCATGCTACAAGCAACACTTACGATCACAGCTACAGTCAGCAGGCACCAAGTTATGGGTCAGCTGCTGTGTC

ATCTCAACCAGATGGGGCAATTCCTTCCCAATCCACACAAGCAGCCCCTGCATACCCACCTCCTTATAATCAACCAGTGA

CCAACCCTCAAACATACTGGACTCAACAACCACCCCAAACAGGATATGATCAAACAGGGTACTCCCAAATTGCTTATGGA

GGTCTACAACCAGTCCAGTTCATGCCCTCATCAACTCAGCCAGTTTATGGACCAGGTGGTTATCCTCTTGAGCCATCTCC

TGCAACTGCAAATTCTGTTCAGGGGACACATCCTCTTACTTACGGGCAGGCACAGGTGGAGACACAACCCCAGTCACAGC

AGCCAAACAATGGCCATTCACAGTCATTGGCTTATGGTGCTGAAACTCAGGATGGGAATTCAAACTCTGCTGTGCAGGAA

CCAGTTGCTTCTCAAAGCTGAATTCCAACGCATTCAAAGGTGGCAGTTAGGTCCCTTTCTAAAGATTATTTGTTCCCAGC

ATGAAATTCCTCTTCGTTTGTATACTACGATGTTATCATGAGAGGGAACTGCATTTCTGGGATTATAATGGTGGAAAATT

TTGTCTGATGTCTTAATCATTTGTAACGTTAAACTCTGCAGTCGAACTAGCTATGTCAGTTTAAAGATAGAGCTTTTGGA

TAACTTTTGGCAAAATTGGGCCCTTAAGTGTGCCTGTATGG

>GT972355.1 GJCCJC2033D12.b Jatropha curcas L. developing seeds (mixed stages) Jatropha curcas cDNA clone GJCCJC2033D12 similar to TdcA1-ORF2 protein, mRNA sequence

CCTATTTCAACATAAGATCGGTTTTCATCATCCAATCTATCCATTAAATCTTCAACAACTTGCATCGGATGCGTATCAAT

GCTAACTTCTTGAAATGGCTCCGATACTGCGAGTGAAGTAGATTGTTTGGTTTCAAGTGCATGAACAACTGATCTAGCCT

TGATTTTACACACTGCCATCCAATCGCAACAATCTCGTTTAAACGATGGATATGGGACATAGTACACCTGTGCAGCTTGC

ATTGCCAACACAAAAGGTTCATACTTTGTATATCTTCTTCTTTGATTAATATCTACCAGATTATACTTAGGATGAACCTT

CGTGCCTGGCGTAGGATCAAACCATTCACATTTGAATAAGATTGAGCACTTCATTGGTAGGCCTGGATATTCTAACTCTA

AAACCTCAACCAATTTTCCATAAAAATCATTTGACACGTCACTGTAGTTTGATCCCTTTATGCAAACACCGCTGTTCATT

ATCTTTCTGCTTCTCCCATAACCCTCAGTGTGAAACTTGTAACCATACAAAGTAGCCAGAGTAGGCATTGGCAACCGGTA

GAGGTCCCTTTGCTAGATCCGTAATGAATCTGTTACCAATATCGATTCTCGAGCTTCGAGCCTATGGGAAATAGTAATGA

CAGGAGTGAGATTTAATAAATGTACATTCCATGTTGTGTACATTGAAAAGTACTTACATAATATTTGAACCATTCTGCAA

ATTCGGAGTCCAGCTTTGC

>GO246920.1 JcrME_RL0464 Expressed sequence tags from Jatropha curcas root cDNA library Jatropha curcas cDNA, mRNA sequence

TATCAACGCAGAGTACTGCGGGGAAGAGAGACCGGTATGGCACACCATTAACTGCGATGTTCTTTCCAAGGATTCAAAGG

GAACCAAGGCGGAGAAATTGAAAACTGAAGAGAAGCAGATTATTATGGTCACAGGCATTGAATATTCATCTAATTCTGAG

TCAAATGAATGATAGGTGGTGCACTGGACACGCGGGCCTCTGTTAATACTAACTGAATTAATGCAGTGAAAATTTATGTT

CTTGATGGTTTACTGACCGTTTTTCACCCCTTTGAAAAGTTGAAGCTATTGAATCTAATGACCAATACATATACATTTCG

TGATACA

>JK612225.1 JCF26-82 Jatropha curcas, immature Seed cDNA subtraction library Jatropha curcas cDNA similar to protein binding protein, mRNA sequence

ACTGCTCTCCTAAATATACCTTTTGTGTTGGAGAAGGGGGGGCAGCATCCACAGGAGAAAGAGTCATTGCTTTATTTGCC

AACTTGTAGAGAGCTACAGCTCCATCAAGTTGTTGTTTTAAGTTTGTAGAGCCAAGAAGCCCCAGAAGTAACTCCAATCC

ATTGTTGTCAACAAATATGGTTCTCTGATCATCTGGGGAACAAAGATGTGCAAGAGCTAAAGCAACACGCCGTTGGACAG

CCTTCTCTGCTACACGCATTAGATATAATAAATGGTTTAAAACTCGTCCATGAATTTTTTCCTCTAATCTTTTCAAAGTC

TTGGATACACAATCTTTTGTCGCTTGAACAATAAACTCTCCATCCTGAAGCTTCTGAACACCTCGTCCGCGACCACGCTA

>FM895130.1 FM895130 Jatropha curcas embryo 71-95 (DAF) Jatropha curcas cDNA clone rjcpga0_001907, mRNA sequence

AAAATCAAAACCCCCCTTATAGTATCAGAGTTTATTTCTTTATTGCACTACTGAAAAGATAAATTTTCTCTCTTGTAATT

TTACAATGATTACAGGAAATAAATCAGCCTCATTGGTGATTAAACGAAATACATTATCCTCCCCCCTATTGCGTCTGACA

ATAAATATGGGGGAACAGTCTTCTTCCCAAACCCAAAATATGAATACAAGACGTATTGGTTCAATGTGTTAGGAAATTAA

ATTAACTAACTCAGGTACATTCTCTTTTAACTTATAGAGTTTGTTCTTTCTAAACTAGCTCAGCTGCTCAAGACATTCTC

CTTTTCTGTATCTCTCCTACTTCAACAGATATTATATACTTTGGAGGTTAGACTTAGGCCTTACCAGGGTGGGTGGTCAA

GCTCTGGCGTCTCTGGTGGCATTGGTTTCCAATCGGGTTTCTCATCCCAGTACATGTCATAATATACGTGTCCTGGGGTG

TGATTTTCCACGCAGCACCATATACCAACATACCGCTTCACACGATTCCTAAATTTTTCTTCTCTTGCCTTGTCGGTGAA

ACCTAGGAATCCATCTTGCATTGAAG

>GT979872.1 JGCCJG2036G06.b1 Jatropha curcas L. germinating seeds (mixed stages) Jatropha curcas cDNA clone JGCCJG2036G06 similar to 40S ribosomal protein S10, mRNA sequence

GACGAGAGAAGGAAGTACTACGCCTCACTGAATAACACTCGTTGCGGCCTCATATCGCACTTCCTGCTCATCGCCACCGG

CAACCATGATCATTCCGGAGAAGAACCGCCGCGAGATCTCTAAGTACCTCTTCCAAGAGGGAGTATGCTATGCAAAGAAG

GACTTCAACCTTGCAAAGCACCCACAAATTGATGTGCCGAATCTACAGGTTATTAAACTGATGCAGAGCTTCAAGTCAAA

GGAGTATGTCCGCGAGACATTTGCATGGATGCACTACTACTGGTACCTCACTAATGATGGTATTGAGTTTCTGCGAACTT

ACCTCAATTTGCCATCTGAAATTGTTCCAGCTACATTGAAGAAACAAAGCAAACCCCTTGGTAGGCCCATGGGAGGCCCA

CCAGGAGATCGCCCACGTGGTCCTTCACGTTTTGATGGAGAGAGGAAGATTTGGTGGTGATCGAGATGGATACCGTGGAG

GTCCACGTAGACCTGGTGGTGAGTTTGGTGACAAGGGTGGAGCCCCTGCTGATTACAGACCTACCTACGGGGGTAGTGGT

GGGAAGCCTGGCTTTGGCCGTGGTGCTGGTGGCTTTGGGGCAAGGCCTGCCAGCTCGAATCTTTCTTGATTGATATCGGT

TTTGGCCGCTACAATTCCAACAGTGTTCAATATG

>JK612831.1 JCF21-46 Jatropha curcas, immature Seed cDNA subtraction library Jatropha curcas cDNA similar to vesicle-fusing atpase, mRNA sequence

ACGGCCAAGTGCATCGGAGAAGGCAGTATTAACCACACCAGCAAGATCAGCACCACTAAAGGCATCCGTCATGTTTGCTA

TCTCTCGAAGGTCCACATCTTGGAAGTAGCCCCTTCGCTCGTAAGGTTCTGTGTGAACCCTCAATATGTTCAAACGAGCT

TCCTCATCTGGGAGGCCCACTTCAATGGGGAGAAGCCTGCCAGGTCTCAAGAGTGCTTCATCAATTAATTCCTTCTTATT

TGTTGTTCCAATAATAAGTATGTTATTCAGGTCCTCCACACCATTAATCATAGTCAGCAATTGGCTAACAATCCTGTCAG

TCGCAGTCTCTCCAGGAGTTATCCCCCTTTTCCTTGCAATTGAATCTAATTCATCAAAGATGATGATATGAAGGCCACTT

TTCTCTCCATATCTTCTAGAATCTTC

>FM895877.1 FM895877 Jatropha curcas embryo 71-95 (DAF) Jatropha curcas cDNA clone rjcpga0_002877, mRNA sequence

ATTATTGAAATTATGATTGTAGATTATATAAGCAAATAAGAGTACATAAATATGAAATTGTTACCGCTTCTCAGTGCTCT

TCTAATTCTCAAATTCTAACGAAAAAGCACTCTACTGGTTTAGGATTTTCTGGGATTACAAAGTCTAAGCTATGGAAGAA

GAGATTCAGGTTGCCTTCTTCTTTCCGAACAATAAGCTCCATGCATATGTGTTGCCTTTCTCAGAAGCCGCTTCAACTCG

CTTTGCCCCGATCTGGTCCATAAGCCAGTAACCAATGGTTGGCATGTACTGTACTAGATACATAACGGCTAGTACAGGCT

GGTATGATATCCAAGCTTCCTTTAAACCATGGGTCATAGCAATAATTGTCAGTTCCGCACACCTCTCTGATGACACACGC

TTCTCAGAACCCTTTTTTGCTGTAGTGTAGCCATTTGATGTCTCTATTGGC

>JK317588.1 JCST359 Jatropha curcas L. seed cDNA library Jatropha curcas cDNA 5', mRNA sequence

ATTTTTTTTTTTTTTTTTTTTTTTGACTACTGTCGCACGGGTGTGTACCATATATGCAACCTTCCTTTAACTGGAGCATA

GCCCTTCGAATGAGGGGTAATACTCCATAACATAATGAAGAGGCATCACTTTATTATTAAACCGCAGGGGGTTTAAGATG

GGCTTGCGGTCTTTACCTAGTTGGTGAGGAATGGTCACCAATGTTACGATGATTAGGGGTTCTGAAGGACATACCCCCAA

CTGGTAGTGATCACGGACCAGACTCCTACCGGAGGCACCAGAAGGAATATTGTACAATGGGGGCAACACTGATGCTACCC

TGCCGCGTGCAGGATGACGGATCTATGGGTTGCAAACTGCTTATGTACGGGAAAAAACCCTCGTTAGTGACCGCCGCTGA

TGGTACCGTACGAATAAGGAACGCCTAACTCCGTGGCAGCAGCGCGGTAAAACGGAGGATCCAAGCATTCCCGTCTATAT

TGGGCTTGTTCTGATCTGGCCATTAGTATTTCTGTGGTGAAATCCTGCTACTTATTGTTATCTGACTTGAACTGTTAACT

AGCTATATTTGAATTGTGGCAATGTGCTTGAACATGAATCGCTTAAATATAGCACCGAACACCATTGCAAGTCAGGTTCT

CTAAACTATTACTGACGCTAGGCACGACGTCTGGGGATCATAATGGATTAGAACCCTGTTACCCTTACAGATAACGTCGA

TACTCGATGTCGCATACACTGAACGCGCATAGTCAAACCATAGGAACACCTGCTAGAAACTCTCAGATGAACTCAAGGAT

GACGAGGCCTCTAACAGAGGACATGTGGTTAATCCA

>FM889932.1 FM889932 Jatropha curcas embryo 35-55 (DAF) Jatropha curcas cDNA clone rjcfea0_003689, mRNA sequence

GACAGCAAGACTCTGAATTTGTGACATTATCTATAAAAAGAACTATTTAACAAAGCTAACCCAATGTACTCTACTGTCGC

TCTCGAATGACCTAAATATAAGCCAAAACTAACCCTACAACCACTGTAAACTAAGACTAGAACTGTTTAATATAGCCACA

AACCTACCAATTATGTCACATGATGACACCACATACACAAGCAATGGCTTACTGTGTCGATGGAGCATTCATATATCATC

AAAAAGCTGCAACAGAAGGCACTGGAGGTTCTGGGGGTCCATAATCCATAGAAAGCAATCTAGTTAAGATAAGACCAGAC

AAGTATAATTTAGACTCTTCGGCGATTAATTCCACTCTTCACCTCACTTCCCTGTCCATTAGACTTCCTCTTGATACT

>GT979274.1 JGCCJG2030E06.b Jatropha curcas L. germinating seeds (mixed stages) Jatropha curcas cDNA clone JGCCJG2030E06 similar to RNA polymerase Rpb7 N-terminal domain-containing protein, mRNA sequence

TCGAAAGCAAAACGTCTCATCCCTCTGCACACTCACTTGCCGCCACCTCCGTTTCTTGGCCGCGTTGTCGAGGATTTTTT

GGCCGCGTTAAGGATTCTGAGAAGCAAAGAAACATGTTTATCAAAGTGCAGTTGCCTTGGAATGTTATAATCCCTGCCGA

AAACCTGGATGCCAAAGGACTGATGCTGCAAAGGTCGATTATCATCCGCCTGCTGGAAGATTTTGCTATTAAAAAGGCTA

CCAAAGATCTGGGGTACTATCTTGCTGTAACCACTTTGGAGAAAATAGGGGAGGGCAAAGTGAGGCAGCATTCAGGGGAC

GTTCTATTTCCTGTTGTGTTCAATGGCATCACCTTTAAGATCTTCCGTGGAGAGATTCTAGAAGGGGTTGTTCACAAGGT

GCTCAAACATGGGGTTTTCTTGAGATGCGGACCTATTGAGCACATATATCTCTCTTGCATGAAAATGCCGGATTATCACT

ACGTGCCTGGAGAGAATCCAGTCTTCCTCAATGAAAAGACGTCGAAGATTGAAAAAGAGACAGTGGTCAGATTCATTGTT

CTTGGAACAAAGTGGCTAGAGGCCGAAAGGGAATTTCGGGCATTGGTCAGTTTGGAAGGTGATTACCTTGGACCTGTCTC

CTAGAGCCCTTCTGTCTACTTGGCTAGAGATGCTTGTAAATCCCTTTTATTTTCATTTGGGCAAAAACTTAACTTAAATG

CGGCATGCTGGGTGTTTCTCTTGAAGCTGAACATCTGCCTGCATGCCATAGCT

>JK610477.1 JCF1353 Jatropha curcas, immature Seed cDNA subtraction library Jatropha curcas cDNA similar to auxin-induced protein, mRNA sequence

GGGTACAGCAATATGCGTAGGAGGAGCCATGTTGTTGTCATTTTACCATGGAAGTAGAATCAATGTAGGAGAGTCCAGCA

TTCATTGGAAATATGCAGATGATATCGGTAGTCAAAATTCTAATGATGGTTCAAAATCAAACTTCATTTTAGGCCCTTTA

TTTATATTGGCTAGTGCTGTTTGTTGGGCAATATGGTTTACAATTCAGGCCAAAGTAAGTGAAAAATTTCCCGCTCCTTA

CACAAGCACATTTCTACTGTGTTTCATGGGCAGCATTCAGTGTGTTCTGATTGGCTTTGGTGCCAATCATGAAGCAGCAG

ATTGGTCATTGCGCGATCCGGGTCGCCTTGTTGCAGCTCTTTATGCAGCAATTGTGTGTTCTGCTCTAGCATTTTCCCTC

ACTTCATGGAGTATCCAAAAGAAAGGAGCACTTTTATGTCTCATTGTTCAGCCCCCTTGTTTGCTGGTTATTGTTGCTGT

TCTAAGTTGGGCAATTGCTTCGTGAGAAAATTATATGTCGGAACTGTTGTAGGTTCTGCGTTGATTTGTGGCCCGGGCTC

TTCCCAA

>GW878093.1 JC002220 Seed specific Normalized cDNA library from Jatropha curcas L. Jatropha curcas cDNA clone N04033 5' similar to Unknown protein, mRNA sequence

GGCCGTCACCTGAGAAAATCACTGCACAGAGGAATTTTGGTTTTGGTTATTGAGTACGTGCCCTATCTCGACAACGATTT

CATAGATTTTCTGTGAAATCACCGGTCACCTCACCTGAGAGACCTGATACGCCACCGAAACCGAATCCTTCCTTGTCCGC

GTTTCGCATCAAAAGCATTCTCTTTCTCGAGAAGAACCATACTTTTGAGAGAGACAAACAAGTATAGGGAGGGAAAGTAA

AGAGAGTGAAGCTGTCGGGTTTCTAGTCATGGGAGCTCTTGCTCCTGTGTCTCCTTGGATACCAGAAGATGATCTTTTGT

TAAAGAATGCTGTTGAGGCTGGTGCTTCCTTAGAATCACTTGCTAAAGGTGCAGTGCAGTTTTCTCGAAAATTCACTGTT

CGAGAAATACAAGAACGATGGCATTCTCTCCTTTATGATCCAATTGTTTCTGCAGAGGCTGCTTTTCACATGATTGAGTT

CGAGCGATCTGCTTCAACTCTTCAATCAAAATTCAGTAAATCAGGGAATCAAAAAGAGAACAAATTTATCTCGGGGAAAA

GAAAAGCTGAAAGTGTTCGCAGTTGTTACTATGCTC

>GW875557.1 JC005164 Seed specific Normalized cDNA library from Jatropha curcas L. Jatropha curcas cDNA clone N08948 5' similar to Unknown protein, mRNA sequence

ATTACGGCCTAGTTACGGGGATCGTCCTCTCACAAACGCCAAAATATACACACGAGTAACCTTCATCCCTCCTCCTCTTC

TCTGTTTCATTTTTTACTTCTTAATCGACGCCGGCTATTTCACAGCCGGAAATTTCAAGAAATTGACCGGAAAATGGAGG

CTTCGGCGATAGATTGTGCTTTAATTAACCAAAATGAAGGCGTGACGTTATCGTGCCGTCGCGTAGTTCGGGAACTTTAA

CCGTATGTAGAAATAAGCAAAAGCGACTTTACTTAGCAGAGTTTAGAAGTTATTCCATGCTTTGTCCGTCAAATCAGAGG

CAAGATACTATATAATAATATCGTCGGAGTAGAGTTAAGGAGTCTCGAATTTTGTTAACGCATTCATCTTTGGCGAAATT

CTGGATTTCCGTGTCGATTCGATCTGCTGATATCATCCTGTGGACTTATCAATGAGTCTGGTGCATCCTTTGTGATTGTT

TCGATCCATGTGTTTTTGGGATTTTCTTTTGCGAAATTACATTTCTTCCATCTCAATATTTGTACTGAAGGCAGGGAATG

AAGAAAAGTAAATAAAAGCTCTGAAGTTTGGTAGTTTTAGGAACTAATTAGTATCGCGATTAAGCTTTTTGAAAATGG

>JK317653.1 JCST424 Jatropha curcas L. seed cDNA library Jatropha curcas cDNA 5' similar to elongation factor 1-alpha, mRNA sequence

CTCTCCCAAATTTTATCATGGGTAAGGAGAAGGTTCACATTAACATTGTGGTCATTGGCCATGTCGACTCTGGCAAGTCA

ACAACAACGGGTCATTTAATCTACAAGCTTGGAGGTATTGACAAGCGTGTGATTGAGAGATTTGAGAAAGAAGCTGCTGA

AATGAACAAAAGGTCATTCAAGTATGCTTGGGTCTTGGACAAGCTTAAGGCTGAACGTGAGCGTGGTATCACCATTGATA

TTGCTCTTTGGAAGTTTGAGACCACTAAATATTACTGCACTGTCATTGATGCTCCTGGACATCGCGACTTTATCAAGAAC

ATGATCACTGGTACCTCACAGGCTGACTGTGCTGTGCTCATTATTGACTCTACCACTGGTGGTTTCGAAGCTGGTATTTC

CAAGGATGGTCAGACCCGTGAGCATGCTCTGCTTGCTTTCACTCTTGGTGTGAAGCAGATGATCTGTTGCTGCAACAAGA

TGGATGCCACAACCCCCAAGTACTCTAAGGCTAGATACGATGAAATTGTGAAGGAAGTTTCTTCCTACTTGAAGAAGGTG

GGTTACAACCCTGACAAGATCCCATTTGTGCCAATCTCTGGTTTTGAGGGTGAAAACATGATTGAGAGGTCAACAAACCT

CCATTGGTACAAGGGCCCCACCCTCCTTGAGGCCCTCGACTGGATGAATGAGCGAAAGAGGGCAACAGAGAGGGCACTCG

TCTCGCAATCAAGATGTCCACCGAATTAGTGCACGCCACAGTAGCACTTTATATGGAGACATGGGGTCTTAGGACTATTT

AGTCGGTCTCCTACACATTGACAGAAGCTTAATATGTAT

>GT973390.1 GJCCJC2048A02.b1 Jatropha curcas L. developing seeds (mixed stages) Jatropha curcas cDNA clone GJCCJC2048A02 similar to ATSLY1; protein transporter, mRNA sequence

GCAGTGGAAATTGAGACGCAATTGAATAAGTACAAGAAGGATGTTGATGAGGTAAACAGGAGGACTGGGGGAACAGATGG

AGCAGAATTTGACGGCACAGACTTGATTGGAAATACGAAACATTTGATGAATGCAGTGAACTCCCTGCCTGAGTTGACTG

AGCGGAAGCAGGTGATTGATAAGCATACAAACATCGCAACTGTATTATTGGGTGAGATCAAGGAGAGATCTCTTGATTCT

TATGCCAAAAAGGAGAATGACATGATGATAAGAGGAGGGATTGATAGAAATGAACTTTTGGCTGTGCTTAGAGGGAAAGG

GTCCAAAATTGATAAGCTGAGATTTGCAATCACTTACCTTATATCATCTGATAGTATTAACCAGTCAGAAGTTGAAGCAG

TGGAAGCAGCTCTTAGGGAGTCTGAGGTTGATACTTGTGCATTCCAGTATGTGAAGAAAATGAAGTCCCTCAATGTTTCA

TTGGCATCAACAAATTCTGCTAGTAGAAATAATATTGTTGATTGGGCTGAAAAGCTTTATGGACAGTCAATTAGTGCTGT

GACAGCAAGTGTGAAAATTTATTATCTAATGATAGGCAGCTTGCATTAACAAGGACTGTGGAAGCTTTGATGGAGGGGAA

ACCTAAT

>GW612226.1 Jc1-014-D06-M13F.D06.ab1 Jatropha curcas flower and seed Jatropha curcas cDNA, mRNA sequence

TCGTTTCAAACCCGCCGCCGCTCTCTCTTCCCCAATAATAAAACGGCAAAATGTCTCTCTGTTTCAATATCATTCTAAAA

CCTAATCTCTCTCTATTTCAAGTCTTAATTTATGTGGTCTCTACTTCGGTTTTCAAATCCATAGACAAAACCCTACTTCC

AATTTATTCTCTTCTCTTTTAGTTTCTTGTTTCTTTTTTGTTTTATTGCTTGAAACTCTCAGTGTCTGTCTCTGCAGAAG

TCTCATGAAAACCCCATGAATTGGTCTATAAAATACACAGCGTCATCTATCTGGTGCGGATCCATTCCCAAAATCAAACT

GTGTAGGTGGCAAAAGCAATTAGTTAAAAGCAAATTTGATTCATCCAGTAACATTGATGATGCTTTGGCTTCCTTTAATT

TGATCCTTCGTAGGAATCGGTCACCACCTTTGGTGGAATTTATGTAGATCATGAGACTGTCATTTGTTTGTCTATCTAAA

GGGATGGAATTGTTTGGTGTTTAACTCAATTTTTATTCTATTAAATATTTGACCTAATTGCTTTTTCTTCTTACATCGTG

CGGATTCTGGGTACTCTCCTATTCAGATTGATTCCATAGCTTCATCAAATTTATCCTCCCTACCTTTAATGGGCTTGGTA

GGGAGGATAATTTTGTTGAAGCTGTGTAATCGTTTGATGATATTGTTGCGAAAGGGTATCAC

>JK613096.1 JCF22-96 Jatropha curcas, immature Seed cDNA subtraction library Jatropha curcas cDNA similar to predicted protein, mRNA sequence

AATTTTACTACACTAATGTGAGACGCCAAGTTGGAAACATGACAATGGCGACATACGAGGCAAGTCTGGGCAGACAAACC

AGCACAAACTATCCTCTTGTTAATTTCTTGATGGCAACAGAAGCAGATTTTTTCATCGGAGCATTAGGTTCAACATGGTG

CTTTCTCATAGATGGAATGAGGAATACTGGAGGGAAAGTAATGGCTGGTTACCTGAGTGTTAACAAGGATCGGTTCTGGT

AGGGATCAACCGGTCTCAAAAATCCAAGTAAATACACATGTAAATTAGAATAAAATGATCTACGATTCCGGTTCTCATCT

GTAAATGAGTATTCAATTTTGT

>FM889279.1 FM889279 Jatropha curcas embryo 35-55 (DAF) Jatropha curcas cDNA clone rjcfea0_002903, mRNA sequence

TGGTGAACAGCCTATGTTGATATATAGTTTAATCTCTCCTTGCAACAATTTGACATTGGCCAGTGAGTTGGCAGGCATGG

TTTGCAACTTAGACTTCACACGATATTCAGTCGATATTTTTGGATTTTTTATTTTGGGTTGGTGTATGGGTGTCTAATAC

TTAGTAATTGAATATAATTACTTACAGGCTTGATCGTTTTTTGCAAAGTGCCCT

>GW875635.1 JC004952 Seed specific Normalized cDNA library from Jatropha curcas L. Jatropha curcas cDNA clone N08648 5' similar to Unknown protein, mRNA sequence

GGTCCAAAAAAACAAACAAGCAGAGCCAGAAAATGGCCCTCTCAATCTCCGCATATCAACTCAATAAACTCTCCTTCTTC

TTCTCTTTTAAATCTCACCATCCCGCCAGACTCTCTATCTCAAACCCTAAAATCAGCTTACCAGTGCTACCCATTAGCCA

AGCCACAAGGTCTCAGACTGGTCCACTTAAGAAGCGGTCCTCTGCTTCTACTCCTTCTTCAGCCTCCACCGGCAAGAAGA

AGAAGAAAAAGGGCAAAAGTGACGCCCCTAATCATTTGAGGGATGTTCAAATCATTAAAGCTGATGATAATATTGATAAT

GATTCATATTCATATTCAAATTCGAGGTCATTGCCTTATCATCCAAACACGCCACTGCCAAAGGCGCCTGCGGGATTCGT

GGTAGATGAAACTGGAAGGGTTCTCATGGCTTCTAACAAGCGGATTGCTACTCTTGTTGATTCCTCCAATAATTATCCAT

TAGAGTGCATTATAAAGGAGAGTATTTAGAAGTTCAAATGGGAATGAATGCATGCTGCTGTGCCCAGTAAACACGCCTGT

GCAGATACTAAAGAGTACGAATATTGATGGGTGGTCAGCTGTATGTTTCTCTGGCCCTTAAC

>FM896646.1 FM896646 Jatropha curcas embryo 71-95 (DAF) Jatropha curcas cDNA clone rjcpga0_003917, mRNA sequence

CGAATTCCTCAACCTTCAAACTCAAATTGGACAATAGCATTAGTTTGCTTTGTTGTTTCCTGGTTCACATTTGTGATAGC

TTTTCTTCTGTTATTAACCGGTGCTGCACTGAATGATCAACACGGTGAAGAGAGCATGTACTTTGGCAACTACTACTGCT

ATGTTGTAAAACCAGGAGTCTTCGCAGGTGGTGCTGTCTTGTCTTTTGCAAGTATCACTCTGGGAATTCTCTACTACCTA

ACCTTAAACTCATCAAAGAGCATTAACAGTACATGGGGCAACCCTCCTGTTTCTAGTTCAACTGGCATAGCCATGGGACA

ACCTCAAAACACATCACAAGGTGGCACTCAAGATCCTACTTTTGTACACGAAGATACTTATATGAGACGACAGTTCACTT

GATCGGTAAATTTCACGAACCAATTGAGTTGCTATGTTAAATTTGATGGAGAAATATGCATATTTTGGTTCCAAAGCTTG

TTACTGGAAGCAGAAGTCTACTGTGAAG

>GW614126.1 Jc1-035-H11-M13F.H11.ab1 Jatropha curcas flower and seed Jatropha curcas cDNA, mRNA sequence

ACTGACTGGATCAAGTGACAGTGAGGAAGCAGGTGACAGGGCAACAGGAGCAGAAGAAAGGGATAATGACGAACGGAGGC

CTAAAAGAAGACACACAGATGTCCAAACATCTGAGGTTGTTTTACCACACAAGACAGTCACAGAGCCAAAAATCATTGTT

CAAACTAGAAGTGAAGTTGATCTTTTAGATGATGGCTACAGGTGGCGCAAATATGGCCAGAAGGTAGTCAAAGGGAACCC

CCATCCAAGGAGCTATTACAAATGCACTAGCACAGGATGCAATGTCCGTAAGCATGTTGAGAGAGCTGCAGCAGATCCCA

AAGCTGTCATTACTACGTATGAAGGAAAACACAACCACGACGTTCCAGCAGCTAGAAACAGCAGCCATAACACAGCTAAC

AACAATGCTCTGCAATTGAAACCTCAAAAGTTGATGGCTGAGAAGCATTCTTTGCTCAAGGGGATGGACTTTGGGAACAG

TGACCAAAGACCGGTGCTTCTAAGACTAAAAGAAGAGGAAATCACTGTATAAATGCTCTCTCTGATGATAGCTGCTAGAG

TAACATAAGGATCCATCAAGGTAACGTG

>FM896015.1 FM896015 Jatropha curcas embryo 71-95 (DAF) Jatropha curcas cDNA clone rjcpga0_003144, mRNA sequence

TAGATCTTTATATAGATGCTTGATTTTCTTTGCTGTAGGGTTCTTTTTGGGTTTGTTCTTGTTTGGTCCTTTGGAAAATG

ATGTCCAGAATCACGACTTCTCCTTCGAGATCAAGCCGCCGCATGTCAATGTTCAATTGGATGACGGCGGCGGCGATAAT

CGCGGTATTAAGCGAGACGTTTTTGCCCTAAATTCTGTGAGTTTGGGGGTTGATACGCAAGTTAATTACATTTCGAGATT

TGATTATGTTCCGAGGAAGCAATTAATCGTGATTACGCCAACATATAATCGTGCTTTGCAGGCCTTCTTTCTGAATAGAT

TAGGGCAAGTTCTAAGGCTAGTGCAGCCTCCGTTACTGTGGATTGTGGTGGAAATGAAAACCGCCTCTGTGGAGACGGCA

GAAATTTTGAGGAAGACGGGAGCTATGTATAGGCATTTGGTTTGCGAGAGAAATTCGACT

>FM887290.1 FM887290 Jatropha curcas embryo 35-55 (DAF) Jatropha curcas cDNA clone rjcfea0_000453, mRNA sequence

TTGAAATCTCCTTGGAAACATTTTTTTTCAGTGCAGTTTCCATAAATTAGGTAATTTGGAAACACGTTTCCACTCTCGGA

AATACGTTTCTGTAAACTGGAAACTGTTGGAAACTCATTTCTTTAATTAAAATAAATACATAAATAAAAAAGAATTCACA

AAACATGTATTTTTTTATGGGTATGGAACTTGTAAACGACTTTTTTTTTTTTTTTTTTCCTTTGGATAACGATTAGATGT

TAATTGGACTTGACTTGTTGCTGAATATATCTTATGGTTTACAATAATTTATTTCTTTATATTTGTAAATATAACCCTAT

GTTTTTCATATTTATGTGTTTCCCCCTGTTCCCATTTCCTATATTTTAAAAAATACTGTTTTCCTGTGTCCATTTCCGCA

TTTCCACACTTCCATTTTTCTGTTCTGGTGCAACATAGCTCACTGCTCTATATCCTCTTTATACTTTTGCTTAGTGAATT

CTATTTTTAGGAGTAAATTGTAACCCAGCCAAAATTCTAGCTATGTAGTTAGATTCAAATCAAAATCTATGGTTCGTTGC

AACAGGCTATTTCCTGAGAATTCACATTTGATGTCTTATTTTCAATATTTTAATCATCATGCTTGCTTCGTACTGGTACT

ATTCTCTGCATAC

>GW875012.1 JC000403 Seed specific Normalized cDNA library from Jatropha curcas L. Jatropha curcas cDNA clone N00754 5' similar to Cytochrome B6, mRNA sequence

GGAAATTAATATTTGAAATTAATTGAATGAACAATTTCTAAAAAGAAAAGGAAAATTTTCTACAATATTCCTACAATATT

CCATCGATAATATTCCATCGATTCTTTAGTTCTTTACTGTGTTACAATTTTTGTATTGAGATTTATGAGCCATATAGATT

AATATTTAAGGATAGATATTACCTCTCTTTTTCCCTTTTTCAAATAAATTGAAATGATTGAAGTTCTTCTATTTGGAATC

GTCTTAGGTCTAATTCCTATTACTTCGGCCGGATTATTCGTAACTGCATATTTACAATACAGACGTGGCGATCAATTGGA

CCTTTGATTAATTAACACTTTGTTTTTTGACCTCCTCCTTTCTTTAGTCCGCAGGAGGTCAAATTCAGATTGCTCCTCTA

TTTTTTCCGTATAAATTTTGACCTTTTTGACCTAACAAAACAAGAACAGAATCGCGCTCTGTAGGATTTGAACCTACGAC

ATTGGGTTTTGGAGACCCACGTTCTACCTAACTGAACTAAGAGCGCTCTCTTATCACAGTCACAAAAAATGCGACTGTAA

GTAAAAAGATTCTTTTTTTT

>GT970836.1 GJCCJC2014H03.b Jatropha curcas L. developing seeds (mixed stages) Jatropha curcas cDNA clone GJCCJC2014H03 similar to calcineurin-like phosphoesterase family protein, mRNA sequence

TCTATATTCTGAAGCAGGTGAAAAATCATAGTAGGCATGATCATCCGGACTTGAAATATTCAATAATGGTAGTAATTTCT

CACGGGGAAGATTATAGAGGCAATGATTACCAATCATGTGATAGACAGGGCCATTGAACTTCTCAAATTCATAAGTTACT

TTCTTCACAGCAGTTAGAGATTTGTCCTGGGGGCATTTTCCATCAACAATGTCTCCGAAGTTAATCACAAACTTAAGTTT

CTGATGGTTGTTCCATTGTTGTACTGCCCTTTGCAATACGTGGATGCTATGCCGATAATACCTGGGGACGCCTACGAATG

AGTAACCGTCAGGAATATCAGCATATTGGACATCAGAGATGACTCCAAAAGAGAAAACAGGTTGCTTCCCTTGTGCACTT

ACTAATCCACTTGTGGACTCCATATAATAAATAAAACTGATATCCTACAAGACATCATGATGATTTATCATTAGCCAAGT

CATCAAATCATTAGCCAGATCACACAAGCCATGGTTAACAAGTTCATAAAATGAGCATAGAAGACAATACTATGCTGAAT

AGA

>GW875540.1 JC005147 Seed specific Normalized cDNA library from Jatropha curcas L. Jatropha curcas cDNA clone N08920 5' similar to tRNA-splicing endonuclease, mRNA sequence

ATTACGGCCTAGTTACGGGGGCAGCCAAGACTTGAAAAGTTAGTTGAAAGAGAACAGAACAAAACGAAGTTTAGAAAGGG

TGAAACGGAGCTGCCATTTTCCATTCCCCGCGATTCGCGAACCTTTTGTTGATCATTCTAAGAAAGCGGTGCTTATACAT

GTTCATACCGCTAAACTTATCCCCATCCTTGATTCAATCTGAAATATTGGGGATTGAAAGTCTGGGAAATAGCAAATTTG

AGAACTTCAGGTTTAGGAAGTTATCGAAACAATTGATATCTCTGATTTACTCCGCTGTCTCTGATTTTTTTTTCATCTTT

GCTTCCTCCTGATAATCGATGTGTTCCCTTGAGATGTTATAGACTTATATTTTAGGGACGACGTAAACAACCTGTTCGAC

GAAACGCCCACGTAATCTGATTACTCGTTGAACATAAGGAGCGGCATGAGAAAGCAAGAGAATTGAGGTGATCGTTTATT

TGGCGATGACAGTTGCATCCAATTACAAATCGCTTTGTTCGTTTGAAGGATAACTAAATGGGACCTAGATGGAAAGGAAA

GGGCTCAGAAGCCAAAGCTCTTGCAGATCCCATGTCAAAGATAGTTTCAGAGCTTTGTTCTTCTCTCATCCAAATGGCTT

CTCATGGGTTACTCTCAGGATGCAGCGTGCTTCTCGCAGT

>FM888666.1 FM888666 Jatropha curcas embryo 35-55 (DAF) Jatropha curcas cDNA clone rjcfea0_002121, mRNA sequence

TGGAAGTGTACAAGGTTGATGAGAGAGGAATGAGGTGGTTCTACACAGGTGACATTGGGCAATTTCATGCTGATGGTTGC

CTTGAGATAATTGACCGTAAAAAAGACATAGTCAAGCTTCAGCATGGGGAATATGTCTCTTTGGGAAAGGTTGAGGCTGC

TCTTATCGTAAGCCCCTATGTTGACAACATGATGCTGCATGCTGATCCATTTCACAGTTACTGTGTGGCCCTTGTGGTGC

CTGCACAACCTGCCCTGGAAGATTGGGCTTCAAAACAGGGAATCAGCTTTACAAGTAATACAGACTTGTGTGAGAGACAT

GAAGCGATAAAGGAAGTGCAAGCATCACTCGGCAAGGAAGCTAAGAGAGCACGTAAGGAAAAGT

>JK611113.1 JCF3516 Jatropha curcas, immature Seed cDNA subtraction library Jatropha curcas cDNA, mRNA sequence

ACTACATAACATAGTTAGCTTAGCTTAGCCCGCTAACTCCGATTTCATAGTTATTTATCTTACCCCGGATATCCCGATAT

TGATTAAATCCTCTTTCCGTAAAGTAAGGCAAGGAACTTCAATGCTGGAGGAAGGTTTGAAGGACAACTGCTATTGAATC

AAGTGCGGGTATTGATTACTTTTCCGAGACTAGGAAAATTGGCTTCTTTCTTCCCTATCTGTCTTAGGAATAGGACTGGA

CATAACCCGATGTTAGAGTTCCGTGCTTAAATCCCTAGTTTGAAAGGGATGGTAATTTAGTTCCGAAAGAAATGCTCAAT

GTGAGAAATTGTAGACAAAGTCTCATACATATGTGCACATCGTGAGAACACTTATTCATTTGTTTGTGCTCCTACACCGG

CTTGATAAACTTCTATTTTACAATGAGATCAGAGAAAGAAGGTGGCACTACTTCAATGTGGAATGTCAAGTGTAAAGCTT

TACCCAGACTGCTTTCGTTTTGAGTTGTCTACCGT

>GW879813.1 JC004773 Seed specific Normalized cDNA library from Jatropha curcas L. Jatropha curcas cDNA clone N08378 5' similar to Organic anion transporter, mRNA sequence

GGCCATTACGGCCTAGTTACGGGGGAAGAAAGGAGTTGGAGTTTGAGTTGAAGGAAGATTGGGGATAGATAAAAGATTTT

GAGAGCGAGACTCCCCTTTCCGGACACCATTTCTCCTCCTGCATTCTGCTGATACAGACTCCATTACCTATTTGCATTTT

CCTTTCCCATTTCTATCTATTCTCTGTAATATAATAGGAAACTCTTTCTTCTACCATCCATTACCTCATATCTCCACTTC

CAAATCTATCAATTTCATCTATTTGATCTCGCCAGAGACAATTGATGCACGTTTTGTGACTTGGGAAAATCAGCCGACCA

GAAAAGCCCAAAGCGCGTTTCCTCGTCTCTTCCCTTCTTTTTCTCTCCCTAGAGAATTGAAGGAAAGAGATCCGTTTCTA

TCTGCATAAATAAACAGATCCTTCGCAATTTTGAGATTGAAGGAAAAAGAAGCAGCATGGAGGGCGAGAACAAGAAGTCG

GCTGTATCGGATGTTGGAGCTTGGGCCATGAATGTTATCAGTTCCGTCGGAATCATCATGGCCAATAAGCAGCTTATGTC

TAGTAGTGGTTATGCTTTCAGTTTTGCCACGACATTAACGGGTTTCCACTTTGCGGTAACTGCGC

>JK612351.1 JCF25-37 Jatropha curcas, immature Seed cDNA subtraction library Jatropha curcas cDNA, mRNA sequence

ACCGCGAGGAAGTAGCTCCCATGAGAGAGGTCTCCAGTGCTCACAAGGCTTTTCCTTCCCATCTTGGAGTAGGGAATCGA

GGCCGTTAAGGTGAAGGTAGTCTTTCCTGTCTGCTTTCTTGTGAACGCCTTTCTATCACTCTGCCTAGTATCACTCGGTC

TGTAATGCCTTCGGTCTTTTCGCTCTTCTCTTTCTGCCTCTAGTAGTAGCCCTGTTGACTCTTCCAGGTCTTTTCTAAAG

GAAGGTGATCGAGAGTAGTCCACTCGGATCCCTAAGATGAAAGTTCTCATTGTCAATCGGTCATCTCAGTCACTCAATCT

TATCACGAAATCAATCTTCGAACGAAGTCTTCCACTTTACCCCTCTTCTCCAGTAGTATAGGAATAGGGTGGGACAGGGG

GAATCCAGAGGAATTCTAGTCAGGTTGTCTCGCTTATCCGGTCACCGGGGCATCCTTCTTCTTATCAACGCTTTGATGGG

TCTTATCAACGCTTGTCAATTCCTGGTGTAATACTCACTTGTAATGATTGGTGTCAGATTGCAAAAAGTCGAATAGTCGT

CTAGCCCGAAAGTATATCCAATAGTAACGCATGAAGCCTGCACCGGATCATTTCTGCTATTCCATCCGGTGATGGAAACT

TGCAACCCATGCCAAAGTTGCGAATGAAACTGTAAGGAAGACCAGATCTCTCATTA

>GT971795.1 GJCCJC2024F11.b Jatropha curcas L. developing seeds (mixed stages) Jatropha curcas cDNA clone GJCCJC2024F11 similar to putative Zinc finger, CCHC-type, mRNA sequence

CACTAACACGGATGCGCATATAATCATGCCATATTACAGAATAATTCTTAGGATCAGATGCAAGAAAAGTGCCAATGTAA

TCGCCAACATCACGGCCAACTCTCTCAAGTATAAAATCAATCGATAAATTATGAAATTGAATCCAAAAATCCACTGTTGT

TAGCTCCACCTCTCTTGGCTTATCTCCATGCTTTAACTCCTTAATCAACAACAAGTTATTAGAAAAATTTCTTGGACCTC

CTCCAATGACTGGTTCTACATCCTGTTCATGAAAAAATTGGAAAGTATAAGCATTATGTCCTAGCTCCTTAATCCCAAGA

CCCCTAACCAACCTCCATATATTTGCCATGCTCTGCTAGAATGCAATAAAATTAATATATTAGCCTCATAAGCCACATAC

TCAAGTCGCCCCTGCCGCTCTTCAAGATTCAGACGTGCTTGAATCTTAGCTACCAACTGCTTCTCCTCACCTCCTGTCGC

CATGAATCAGTTTGTGGGCATAGGGAAAAAAGAAAGTCGAAGGAACGTAAAAGAATAAACTTATTATCTAAACAAGAGAA

AAAGAAAACTTTTGTCATT

>GW611671.1 Jc1-008-C10-M13F.C10.ab1 Jatropha curcas flower and seed Jatropha curcas cDNA, mRNA sequence

TTCAAGCAAAAGTGAATCAGATGCTAGCCAAGTGGGAAATGGATGTGATTCAGAAGAGGTTGCTCCAGAGGAATCAGATG

AGAATAATGAATCCATGGATATTGACCCAGCAGTTGTCCCAGATGGAAATAATGGAAAAAATATTGGAAGTAATTCTTCA

CTAGAATCTAGCTTTTTTGTTAGTTCAACATCAGTTTCAAAGGACCCTGTCCATGAAACTTGGCCTCTTCATCGCAAGAA

GATACGTAAAGTAGTAATTAGGAGAGAAAAGAAGGTTCGAAAGAAAGAAGAAAACTTTGAGAGAGCTGTTCTGATTGTTG

ATACAAGAACAAGGGTTGATGTGGCATGGCAGGATGGAACCATAGGACGTGGGATGGAATCAACAACTTTGATACCAATT

GATAGTCCTGGCGATCATGAATTCGTTGCGGAACAGTATGTGGTGGAGAAGGCCTCGGATGATAACGATAATGCTTCTGA

AGCTAGACGTGTTGGGGTTGTGAAAAGTGTTAATGCAAAAGAGCGAACAGCTTGTGTGAGGTGGTTAAAGCCAGTTGCCA

GGGCAGAAGATCCTCGTGAGTTTGACAAAGAGGAAATTGTTAGCGTGTATGAGCTGGAGGGACATCCAGATTATGATTAT

TCCTATGGGGATGTAGTTGTGCGATTATCTCCAGCTGCTGTATCAACTTCTGATAGTGAGTCT

>FM894282.1 FM894282 Jatropha curcas embryo 71-95 (DAF) Jatropha curcas cDNA clone rjcpga0_004590, mRNA sequence

ATTCCTATCCCTGAAATTATCGTGACGGACACGCCCTCCCCTCTTCTCTCTCCGCATTTGAAATTTCTAGGGTTTTGATC

TGAAAATTGGAGATGGTACTGTCGCAGAAGCTTCACGAAGCTTTCAAAGGTACAGTAGAGAGAATCACAGGGCCGCGAAC

GGTGTCCGCCTTCAAAGAAAAAGGTGTTCTTAGCGTTAGTGAATTCATTCTTGCCGGCGATAATCTCGTCTCTAAATGCC

CCACTTGGTCCTGGGAGTCAGGTGAACCAAACAAGAGGAAGTCTTACTTGCCCCCAGAAAAACAGTTCTTGATTACTCGG

AATGTTCCTTGTCTAAGACGAGCTGCATCTGTTGAAGAAGAATATGAAGCTGCTGGAGGTGAAATTCTGCTTGATAATGA

AGATAATGATGGCTGGCTGGCAACTCATGGCAAACCAAAAGAAACTAAAGGTGACGAGGATGATAATGTACCTTCAATGG

AAGCCCTAGAAATCAGCAAAAAGAATCCTATTCAACCGATCCCCACATACTTTGGGGGTGAAGAGGAAGAAGATATACAG

ATATGGCAGACTATGAAGAACCTGACCATTTAATTGAAACAGATCCTGGCACACTCCCGTCTACTTATCTTGTAGCTCAT

GAACCTGATGATG

>GW874833.1 JC000224 Seed specific Normalized cDNA library from Jatropha curcas L. Jatropha curcas cDNA clone N00406 5' similar to Unknown protein, mRNA sequence

GGACTTTTGAGTTTTCTTTGTCCATTTCAGTTTCTTTAAGGTCCCTCTCCTCTCTCTCTATCTCTCTCTCTCTCTCTCTC

TATTTATTTTGCATCATAGTGTGGGGTTACTGCATCTTAGCCTATATTTTCCTTTTTTGTCTAAATGAAGCTGATTGATA

TTTCCTCTGTGAATTATGAAAAATGAACTGGGTTTCCTGTCATTACTCTCTTTTGTGGGTAATATGGGATGCAAGGCTCA

GTTCTAAAATCAATGCTTCAAGAAATTCAATACCATAAATTAGTTTTCACCAGCAAATGGATGCCAATTTTGTTTTTTTG

TTTTTTTCTTTCTGTTTTTAACTTTTCCAATTTCTGGAATACTTATGAGTACTTCCTTCAGCCTCTGGTGGGAGCATATA

ATGTGTTTTGATTGGTAAATTGAATGCAACCATTTTTTTGCTTTTCAAGTACTTTCTGTAGAAATGATTGGGTGTTGAAT

GTGAAAGTTCTTGAGTTTTGCCTCAAATTTATAGAGATGA

>GW619660.1 Jc2-049-G04-M13F.G04.ab1 Jatropha curcas flower and seed Jatropha curcas cDNA, mRNA sequence

GGTCATTGCCTTACCAATGATGTAGGTGGTGGTGGATATATGTTCTTAGGTGATGATTTTGTACCACGCTGGGGTATTGC

ATGGGTTCCCATGCTCCACAGTATTTCCATAGAATCTTATCATACTGAGATTTTGAAGTTGAATTATGGAAACAGCCCCC

TTAGTTTAGGTGGGCAGGACAGAAGTGTAAGACGAATAGTGTTCGATACTGGCAGTTCTTACACATACTTCACAAAAGAA

GCATACTCTGAATTGGTTGATTCTCTGAAGGAGGTTTCTGAAGAGGGACTTATACAAGATACATCTGATACAACATTGCC

TTTCTGCTGGCGAGCGAAGTTCCCTATCAGATCTGTTACAGATGTTAAGCAGTTCTTCAAGACTTTAACCCTTCAATTTG

GAAGTAAATGGTGGATTATCTCTACTAAGTTTCGGATTCCTCCAGAAGGCTACTTGGTCATCAGTAATAAAGGCAATGTG

TGCTTGGGCATTCTTGATGGAAGCAAAGTGCATGATGGATCCACCATTATACTTGGAGACATATCGCTGCGCGGACAACT

GGTGATATATGATAATGTGAACAAGAAAATCGGGTG

>GW881255.1 JC000819 Seed specific Normalized cDNA library from Jatropha curcas L. Jatropha curcas cDNA clone N01562 5' similar to Unknown protein, mRNA sequence

ATAACAAGCAAGAAAAGAACTACTAGTGAAATTTATAGCTATACATGTAGCTAGCTTGGGCTCTCTCAATAATAATCTAC

GACGAGACTGCCCATCTTCTTATCGTGTAACACTATCAGTTGAAGCTGCCAGCATGATCTTAACCTTCCTCCATAGTTGA

GGAAAGATCAGATCATGTGGCAGCTTCACCCGTTGTGGTAGCACGAGAAACCCTAGTTTCACTCGAAACCCTTTTCTTTA

AATGCTTAAATGGGCTGTTAGGGTTTTCTTTGTTCAATCCCTAGAAAAGGAGGGTTTTCAGTGACGGGGTTTTTGCGTCT

CGGTGCAGTTCAACGATTTCTAGATCTTCCCTGGCATTTTTGGTGATTGAGCCTTGACATTCCTTAATCTGCCGTACTGC

TTTTTG

>JK317882.1 JCST653 Jatropha curcas L. seed cDNA library Jatropha curcas cDNA 5', mRNA sequence

TTTTTTTTTTTTTTTTTTTTTTTTTTTTTTTTTTTTTTTTTTTTTAACAAGAGATTATTCATTTCTAATAAAGTTGTAAA

TTCAAATATACTCGGTCTAAAAAATTAAAAAAACAATTAGCTTCATTATCTAAGTATTGTACTAAAATAAGGACTCTCTA

AACAATTGGTGTTACAAAGTATTTTGTCACACTAAGCTCCTTCAACATACAAAAACCAAAATCCCTTTCCATCTCTCTCC

AAAGTTGTTTCTTTTCCTCCTTCTGATACATAGTTATCTAATACACATGTCTGCCAAATTTAAATCTTAATGTTGAAGTA

ATCTGGATCGAAATGATGTAACCTCGCGTACCCATCTTCACCTCCGCTTGAGGAGCTTTTCCCATCAGGGTTGAAAGCCA

AAGCATTTATAGGTCCAAAATGACCTTTCACGCCTCCAATTTCTTCTTGAAGAATCTTGTCATAGAATTTGGCTTCGAAT

TTCCCAGCACGGTGATCAGTGGTAGTCACAGCTGAAGCATCCTGACCACCTCCAAGCACAACATGATCAAGAAGTGGAGA

CATAGTAACAGCATTGACTGGACGTTCTGTCACATAGGTCTTGATAAGAGTCAACGTTCTGATGTCCCATAGCTTTGCAG

ATTTATCTAGAGAACCAGTAACAAAGTGTGAACCATCAGAAGATTTTGTAAGTGATGTTATCGTCTTCTTATGCCCAACT

TCTTTATCTGACTCTTTCAAAAGCTTTCCAGTCTCAGAATCCCATATGCGAATTACAGCATCCTCTCCAGCACTTATAAT

GGTCCTGTTCAAAGGTCCCCAAACAGCTCTGTTAATTCTTCC

>JK611544.1 JCF4326 Jatropha curcas, immature Seed cDNA subtraction library Jatropha curcas cDNA similar to conserved hypothetical protein, mRNA sequence

ACAATTTATCAAACCAATATGATGAAGTGCGAAATAGCACATGAAATTGATTATTTTAAGATAGAAGGACTATAAAAGGG

GATTGACTTCAATGTTTCAAAACATGGATGTGCTATAAAGAATAAACAGTTAAAACTTTATATTAAAACCCATTGGATTA

GAATCTTTGATTATCAACTGTAGGATAAAAAATCTTGGGACTAACAAAAGCAAGCCAGTTTACCTTTTGTTTTGAGAATT

CTTTTTGTTACCTAGCATAAGGTTCTCCAGAAGTATTGACCACAAATAACATAAATGCCCCAACTTCTCTCTCAACCCCA

AATCCACCATTGCATATCCAAAATTTTTCTGATACATTTTTTATGTAACTGAAAGGGATAGAATTTTGTATCACCATCTC

TGAAATTGGTATTTTATGT

>GT969945.1 GJCCJC2003D04.b Jatropha curcas L. developing seeds (mixed stages) Jatropha curcas cDNA clone GJCCJC2003D04 similar to VIP5 (VERNALIZATION INDEPENDENCE 5), mRNA sequence

GATGTTGGTGATGATCTTTACAAGGATGAGGATGATCGGAGGAAGCTTGCTCAAATGAGTGAACTTGAAAGGGAGATGAT

ATTATCAGAGCGGGCAGACAAGAAAGGTGATAAGAATTTAACTGAGAGGATTAGATCAAAACGGGACAGTGAGAGGGCAA

CCCGATCCAGAAAAGAGACTCCACCTCTGCCATCATCTCGCGGAGTTCGCACATCAGCTAGATCGGCAGACAGGGCAGCT

GCCAAAGATGATGCATTGAACGAACTCAGAGCTAAGCGTTTAAAGCAGCAGGACCCAGAGGCTCACCGTAAGTTGAGGGA

TGTCTCTAGAGGAACTTCTGGCAGTCGGGGTGTTTCACCTGTCAGGAGAAAACGTTTCACTTCGGCAAGCCTGAGTAGCT

CTAGCAGTGAGAGTGACAGTAGGTCTCATAGTGAAGATGAAGCGTCAACAGGTGACGGTGGAATGGCAGATAGTGATGAG

GACAGAGAGCCTGGATCCGAAGGTCTGACATATGATGATATACGGGAAGTCACTATCCGGAGGTCAAAACTTGCTAAATG

GCTTATGGAGCCATGGTTTGAAGAGTTGATTGT

>GW880393.1 JC001121 Seed specific Normalized cDNA library from Jatropha curcas L. Jatropha curcas cDNA clone N02152 5' similar to Fasciclin- like domain-containing protein, mRNA sequence

GAAACCAGCACATAACTCACCACCACCCCTTTTGTCCACATTAACTCACTCTCTTTCTCTTCTTCACACAACTCACAACC

AACACTCTCAACTTCTGATCTTTTTCACATTTTCTCGAGCACCATGGATCCTCACATCTATGGTGTCTCCAAATTATGCT

TCATTTCTTCTCTGTTTCTCTTCTCTTTAATTACTGTTTCTGCATTGCCTCATACCCCGTCTTCTAAATTCTCTTCTTCT

TCGTCTTCTAATAATACAGGAATCAACTCTAACTCCATTCTGGTTGCGCTTCTCGACTCGCATTATACTGAGTTGGCTGA

ACTCGTTGAGAAGGCTCTTCTTCTTCAAACTCTTGAAGAAGCTGTTGGCAAACACAATATTACCATTTTTGCTCCTAGAA

ATGAAGCCCTGGAACGCCAGCTTGACCCCGAATTCAAGCGCTTCTTGCTTGAACCCGGGAATCTCAAGTCTCTTCAAACT

CTTCTAATGTTCCACATTATTCCTAAACGGGTCGGATCTAGTCAATGGCCGAGTGAAAAATCTAAGCCACTCAAGCATTC

TACTCTCTGCAATGATCATCTTCGCTTAATTAGCAAAAGCTCCGGCAAAAAGGCTGTTGACTCTGCTGAAATTATCCGAC

CCGATGATGTCATCCGACCCGACGGCGTTATCCATGGAATCGAGAGGCTTCTAATCCCGCAATCAGTACAAGAAGA

>GW878345.1 JC002722 Seed specific Normalized cDNA library from Jatropha curcas L. Jatropha curcas cDNA clone N05142 5' similar to Unknown protein, mRNA sequence

GGCATCAAAAGAAATGCTGCAAATTGGTTCTCAGATACCAGTTTCAACTGTGCAAGATGAACATTCTCCAAGTTTGATGA

ATTTGCCTCAAGTTACCCAGGATGTCAGGTATAATGTTGATGCTGGAGCGTCTTCTTTCCAACTTCCTCATCAAATATTT

GGGAATATTAATAGTCAAAAGAGTTGGGATACTACTCTTCCTGAACAAATTAATGAAATCCATGAGGAGTCTTTGCTGGA

ACCATCGCTTGTAGAGATGTCTTCATCTCTAGGGTCAATGGACAAATCTTCACAGGAACCTTCCCATGCACATGAGCCTC

TCCTTGCTTCTGCTTGCCTTACTCCTCTTTCTGTGGAGCAGATATTAGAAGACACACGAACAACTGAAAAAGCTTTGAAT

GTTGCAATACCAGAGGCTACAACAGGTACTGCACAGCTGGAGTCTCCTGGAATCTCCTTTACTAATCCTCTATCAGGAAC

TTGTGAGGACGAAATTACAAAGCCTCAACTACCCTGCGTTATGAAAGTTCAGCTGGATGGTACTCTCAGCGAGCAGCAAG

TTGAAAAAGAAAGGAGCACTGATGATCCTGCTATAGTGGCTGAAGTGAAAAATATTGAAGTTCGAGAGGTAAGAAAAGCT

TCTGAGAAAAAATCCAGAAAGCAAAAATCTGCTAAATCCAGCTCTATTGACCAGGTAAAGGG

>GW880640.1 JC004754 Seed specific Normalized cDNA library from Jatropha curcas L. Jatropha curcas cDNA clone N08356 5' similar to Unknown protein, mRNA sequence

GAATTTCATTTCCGAATTCAACATTGTCATTCTACGATCATCTTATGGTCTCGTTGCCCAGCAGAACCAGGTTTCTGTAA

CGCTAGATTCAATCGACCATTGCCTGCTTAATCTCGCAAGGATGTCAGAACCACCTTTTAGACCCCGGGAGAAGCTCCTT

GAGAAGCAAAAATATTTCCAAAACATCCACAAGCACACATATTTGAAAGGACCTTATGACAAGATCACATCTGTTGCCAT

TCCTTTAGCTTTGGCAGCTACTTCATTGTACATGATTGGTCGAGGGATTTACAATATGTCCCATGGGATAGGGAAGAAGG

AATGAGGAGGCTGTCGGTTTAACGATGGGCTACATGTTTTCTGCTCATATTTTGAAGGATTATTTGCTCTTGAACATGGA

CCAATGTTGTTTCTCAAGGTTATTTTGTTTAGTGCCTTCAATAATTGCATAGTTGCAGACTTGTAGCTACCAATGGAAAT

TGACACTTCAATCTTGATGGTTTTATTTTCTGAATCCTTAATATTTCATCTTTGAACAAAAAAAAAAAAA

>GW879593.1 JC001768 Seed specific Normalized cDNA library from Jatropha curcas L. Jatropha curcas cDNA clone N03226 5' similar to Protein arginine n-methyltransferase, mRNA sequence

GGCCGTTGGAAGGTAAAAGAAGGTTCTTCTCTTCCTGTCAGCAGCGTCTGAGCCTGTGAATGAGTTGACAGTCTTCTCCA

GAAGCAAACCACATGTATTCGAGTTCTGATTACAGTAATGGCTATCACAACGAGCCTCATCGAGTTGGGGCTAGTCACCG

GGAGCGAGCAAGAAGAGGGGTGCGTGGCGGTCGCTCACGCGACTGTAGAGATGGGTTTCGAGTTTCTAAAGAGCAGCAGT

CGGAGGATGAGCAGAACGTAACTCCTTGTACGGATTTCGACATGGCATACTTTCATTCCTATGCTCATGTTGATATCCAC

GAAGAGATGATTAAGGTATACCTCTCCTCTCCTTTTCTCTCTTATGGTTGTGACTTTTTTTCCTGTTCATTGCAGTGGTG

TTATTGTTCCCTTCTTACTTTGGTTGTTGTTTTTGGGAAATCATCTCGCTTTATAGATGTTGACTAGCATCTTTTTTTTC

CCTTTCATTTTCTTTGTAGAGTCAGTCTCTTTGGCTATACTCGTTTGTTAATCCATGGGTGTGAACTATTAATGAACAGC

CAATAAATTAAGAAATGAATTTCATATAATAAGATTACTGCGGCATAAAGATTTTTGTTGCACGTCCCTAGGAAAAAATA

AGTCTTTC

>GW877644.1 JC002462 Seed specific Normalized cDNA library from Jatropha curcas L. Jatropha curcas cDNA clone N04624 5' similar to Electron transporter, mRNA sequence

GGCGAATTTACGCACGGTCTTGGGTCCTGAGATTTCTAGTTCGGCTGCCCTGTTGCTCGTCTTGGTTTTCTCCGGCTGCC

GTCACTTAAATTCGGACGTCTCAATTGGGTGTCTGATCTGCAGTCTTTCACTCTCTTACAGAGTCTTGCGCCGTTTGAAG

CTAATTGCAAGTACGGAAGATGAAGATTGATCCTTCTCCTAAACAACCTTTATTCTGCTTGAAATGGCCATGGGATATTG

AACATCATCCTAAAAATTCCAATGGTTGCAGCTTTGAGACTCCTTGGCTATTCAAATCATTGCATACTCTTGGATCAATT

GCTTTCAATTCCTTTAATTCAATTTCCAAATCCTCAAATTCTTTGATTCATACCTTTAATCCCATCAAATTGGATGTCGG

AACCAGCCAAAGTAAAAGCATAAAGTCCCGAAAGAAGGTCTTCACTCCTGAAGAGCAAGGAGAGGCAGTGCAAAGAGCAT

TTGCATCTGCACTAGCTAGTGGGAAAGAAGCTACTGTGCTTGAATTCTACTCACCCAAATGCAGGTTGTGTAATTCTTTG

CTTAATTTTGTCTTGGAGCTTGAGAATAGGAATTCAGATTGGCTAAATATTGTCATGGGAGATGCGGAGAGTGATAAATG

GCTGCCTGAGCTACTCCATTATGACATAAAATATGTTCCTT

>GW616292.1 Jc2-010-B10-M13F.B10.ab1 Jatropha curcas flower and seed Jatropha curcas cDNA, mRNA sequence

ATATACCCCCTAACAATACCCAACCCATGTCTGCAATCAGACCTTTTCATCAATGCCTCCATATATAGTATCATTGGTTC

CTGAAGCGAAAACTAGTTATTGGGTTTTGAGGCAAACAATAATTCCAGCTCCAATAGCCTATCTCCTATGGCAGAGAAAG

TTGTTGCACTGTTGGGTCAGAAGAGAAGTAGAAATAGAGATACTGTTAATCATGGCGGTGGAGGAGAATCAGAGCATGAG

GTTCACATCTTGACTGAGAGAGAGAGGAGAAAAAAGATGAGGAACATGTTCACAAGTCTTCATTCTTTGCTTCCTCAACT

CCCCAATAAGGCAGACAAGTCCACCATTGTTGATGAAGCAGTAAAATACATAAAGAGCCTCCAAGAAACTCTCCAAACGC

TAGAGAAACAAAGGCAAGAAAAGCTGCAAGGAGCAACAATTGTTGACAGCATTGGACAATCACTAATTACATCACCCACA

GAAGCACTTTTTGAATCCAGGGAAGCATTCTTGGCTATTCAAGGACCATCAAAGGGCTATCCAATGCATAATTCTTTTCC

GGTCACTCTTTCCCCAGCTTGCTATCAGACTTGGTTTTCTCCAAATGTTGTTATGAGCATGTGCGGCGATGATGCACAGA

TCAGTGTGTGTACACTG

>FM887773.1 FM887773 Jatropha curcas embryo 35-55 (DAF) Jatropha curcas cDNA clone rjcfea0_001037, mRNA sequence

GGAATCGAGTCAGGAAGAAGAGGTTATATATTTCCTTACCGAGGTAATCTTCCATTCGTTACTCACTCTCGGAGAGGAAA

GATTTTGAAGATCGAATTAAGTTTTTCATTCTCTTTTGTAACGATCGCTTCTAAGGCAAGTATCTATGGCATTCTACGCC

CCATACCTCTATCTACTAGCTCTTTGACCTTGATTTGATAGTTCCGATTCGATGAGCTCAAACAAATAAGTAGATACGTG

CCCAGACCCAATTTTTGAAGAAGAGCCTGATTCTATGATTGATTTTTGAGTGAAGTGACCACAATGGGGATTGGGCACGA

ACGGGATGCCAAGCGCTCCTTCTCTTTTACGCTTAACGCCCTTCTAGTAATAAAAAAATGCATAGCCGACATGTAATTTT

CTGACTTCTAATTCTGACTGGGGAAATGACGTAAGAGAAGAAATAGAGTGCTGAGTCTCTTTTACCAGGTTCTATACTCG

GATCGATTAGGCTACTTTAACTTTATTTAAGAAGCTAAAAGAGTACCATTTCTCTATATGCTTTCGTTGAGCTACGTAAC

CTATCTACCTTACTTAGCCCCTTTTCCTCTTCTTCTTCTTTTCCTAAGAAAAGAACCGATCTACTATTTAGCGGA

>FM890971.1 FM890971 Jatropha curcas embryo 56-70 (DAF) Jatropha curcas cDNA clone rjcaeb0_000945, mRNA sequence

ATTCCAAGCCAGCAACTAGTAGATTTGGTTCATGAACATCTGAGGATGGAAAGCAAGCTCTCTGTAGTGTGTGAGAGAAT

ACTCGATAGGTGTTTGGCACCGTCAACAGCAGGTGGCGAAGGATGCGACAACATGACCATGATCTTGGTGCAGTTTAAGA

AACCTATTCAGCCAACTGCCACTGCAAATGATCAGTCCTCACAATCTGAATTTGCAGATGCCGAAACTAAACCGGAGGAA

GATGAATTGAAGTAGCTGCAATGGGTGGTTGATCTCTTGTGCCATTGTGCAGCCCTCTGGCTGGAAGAATGTGGGAGGAT

CCCTCATTTTCTGGGAAAAGTTGGATCCTCATATATCGTTGTAATGGTTTANCATGAATGGGTGAAATATTAAGAAATTT

TTCATTACATGTACTGGTGTGTGCTTTATGTTTGAACCATTCTCACATTCAATAACACATGTACAGCTCTTTGAAAACAA

CATTTTACCATGCCCATCTCCAGTGTACTTTTCTGCACACTGAATATTTATACCTATT

>GT970780.1 GJCCJC2014B11.b Jatropha curcas L. developing seeds (mixed stages) Jatropha curcas cDNA clone GJCCJC2014B11 similar to AT3G62370.1 | Symbols: | carbohydrate binding / catalytic, mRNA sequence

GCGTTCACTTACATTGTGATAGGTGGCATGATCACCAATTGGAAACATGAGGGCCACAGATGGGCATTTTTCGTTATTGC

TGCAGTAAACAAAATACATTGAATCCAATACAATCCTTCTAAGTCTATAAGTCAAAGCACCAGTTTCAGTGATTGATTTT

GATTATGTGATCTAACTAAACATGTATCCCATGATTATTCCCAACTCCCAAAAGTCTAACCATGGCAAAATAAAATCTGT

CTGCAAAAATTTAAATAGTATAGGAATCAGGAAGCACGGGAAATAATTTCCCTCAAATCAAATCTTCATTGATATGCCTT

TTATGTAAATCAAAATTTTATTCATTTATAGACAGGATTAGATTGCAATACACTTCAAGGAGCATCCTGCTCCAGCTACA

TGCTTAATATTGGACAACAATGCACATGATTATTTATAATCTGCATCTTTCTTTCGAAAGGAGACTTACCCTTTCGTGTA

AGCATACTCCCCGTCAACTTGCAACAAGAAGAACACGTCCCTGCCATCGTGCAAAGCCTTTAAAACCACAGCCGCATATC

ATCAT

>GW881390.1 JC000918 Seed specific Normalized cDNA library from Jatropha curcas L. Jatropha curcas cDNA clone N01798 5' similar to Unknown protein, mRNA sequence

GGCAAAATATTCTGTAATCGTTTCATCATTTCGCATTTCCAGGATCTCAAATTCTCTTCTTAAGGCATTGAGAAGAGATT

TCTTCACCTTCTGATTTCCTCCAAACTTCCGTTTCAGTGAGTCCCAAACAATCTTAGATGTACGACGGTCTAAGATTTGT

TCGAAAACAGTTCGATCAATAGCCTGGAACAGGTAATGTTTTACCTGATAGTCCTTGGCCTTTGCATCATCCAGCTCTGC

CTGTTGAGCCTCAGTTAATTTCGTCCCTTCTGTTGGTTCCGTGAAGCCTTGCTCCACCAAATTCCACAAACCTTTTGCTC

TAAGCAAATTTTCCATCAACTCACTCCAATGATCATAGTGACCGTCAAAGTGAGGGAGCTTTGTTAAAGTTTTGTCATCT

GCCATTCTCTCAGTGTTTGACAAACGTTGAAGATTGCAACTTCTCGTTTCAGAAACCCAGTGGGGGCTCTGATACCAATT

GTTAAAGCTTAAGAGAAACTTGAACAAAGAACTTTAAACAACTGGAATTCTTATTTGCTGAGGATTAAACGGTTCAGACT

TGCTATATATAGCTTTACAATAAAGACAAAAACAAAACGTAAACAGCAAAACAAATAAATCCATAAGACTAGGACTCTCC

TAAGGAATAGCACTTCCCTAAAGAATAGGTCTTTATTCAACAGAATCCAAAAACTAATCAACTCCTAACACGGA

>GW619652.1 Jc2-049-F08-M13F.F08.ab1 Jatropha curcas flower and seed Jatropha curcas cDNA, mRNA sequence

AAATTCAACCTGAAATCGATCTCCGATATCTCTTGATTTTGACGATTATTCTTCGAATCAAAACTCAATTCGTTTAATTC

AAACTCTCCTTTTTCGATCGATATCTCTTTCGACTTTTGCCGTTAGTTCTGACATATTCTAGGGTTAGGGTTTCGCGTCG

TGCTCTCTTCGGCTCTCTAATCTCTCTATTTCGTATGGGTGTTGAGTCTCCTCTGTATATATCTTAATTTCCCCATTGAT

TTTGGCTCTATTTTTGTATATTATTGCTGTTCTGTTCGACCTTTAGGGTTTCGAAGTTTTCGATTCGTGTAGGATTTGTT

GCAATCTGTAAATTGGTTTAGGGGTTTGATTCTCTTTGTTATTTCGCCTCTTATCATCCTCTTTTCCGTCCGAATTTGGT

TGGATATTTTGTGAATTGGGAATTTAGGGTTAGGGTTTTTTTTATGAAAATTTTAGGTTCCCTTTTCATAAATTTGTTTA

GGGATACGCCTAACCAAATCAATTAGTATCTTGGCGATACAATTGGGGATCCTTTACGCCTAAATTAAGTGCCATACTAC

TCCTATTCTGTTTGGTATTTTGAATTCAA

>FM890084.1 FM890084 Jatropha curcas embryo 35-55 (DAF) Jatropha curcas cDNA clone rjcfea0_003876, mRNA sequence

AATAATAAATGAACTCAATCAATTATACTTGTTGACTCCAAGTCAATGCGGAAAACAAAAAGTGGTAATAATAATTTTCC

ATTGAAGTAGACTGCCTTTTCAAATTAAATTATCTAAATATATCAATTGGATGATAGTTTGATTGATCTTCTCACTTATA

TAACGAGTGTTCGAAGTCCTGCTATATTTTCAAATTTTGATATTTTACTGTTTCATAAAAGGGTAAAGGGCTGACTGCCT

CAATTTGAAAAATTATATTCTTTTTTTCATACTAGAAAAAAGAATTTTCACACCAATTTGTAGTGTTTTGATTGGTCTCC

TCCAAAGTCTCGACTTCGAGTCTTTTTCACCAAAAAAATTGCCATTAAATTTATCTCTTCACCAAAAAAATTGTTATCAA

ATTTAGTGAAGGTTATTGAAAAGAGAGAGTCATCATATTGTGTAGCTCACAGTGTTAGTTTGATTGATTGGATATTTCTT

AGTTACTAGTTCTAATAGTCCAAATTGTAATTTCAGGATTGGATATTCAAAACCTTTTAATATACAATTTTAAAAGTATA

TTGCAGTATTATTACCTTTTACTACTGGTG

>GT976593.1 GJCCJC2080A01.b Jatropha curcas L. developing seeds (mixed stages) Jatropha curcas cDNA clone GJCCJC2080A01 similar to ATP binding protein, putative, mRNA sequence

GATGACAGAAAGAGAGACAACACTTTGAATCCCATTCCACGTCACATGGTGAAACTGAACGAATGTTACCTTTGACAGGA

TTTGGGGATGGTAGTGCAGCCGCTGGCACCGAAATTCCAACGAATGAGCATACACAAGTGATTTGTATGCTAAATGGTTT

CCCATATGAGCATATTATAGCATACACTGAATCAAGTACACAGGATGATAGATGATCTTGTCAGCCCCGATGATAATGAA

TCATCTCCGAAATCCGTGGCAGTTGATCTGTCAAGTACACACGAACATGAATTTAGCTATGAGCTGGAGTTGCAGATAGA

ATCTCATGACAGACAGCACCACATCAAGTGGTTCTGAGATCAACTTTGCTGGCAGGACAGAAAGTGTCCGGACGAAGTAT

CCTCCGCTAGATTTTGATGCCAATGCTGCCAGATTATCCAGAACTGATTCTCACTCATCTC

>GW881247.1 JC006423 Seed specific Normalized cDNA library from Jatropha curcas L. Jatropha curcas cDNA clone N10914 5' similar to Unknown protein, mRNA sequence

GGGGTATATCAGAAGTCTCAGTGCTTCACTGCTTCTCTGATTTTGATGCAAGAGATAAATTAAAATTAAAATCAAAATTA

AAACTAAAAAATAAACTAACAAAATAAGAAATATACAGGAAGAGCTGTGCTTGTGCTTTGTCTATATCAGTACATGTCCC

TACTTCTCGCATTTGAGTCTCTCTCTGGCTCTTCTTTAAAGTTTTAAGAGGGGAACCCAGCAAGAATTTGCCGGTCTTGT

GCTTCAAGGATTGGAAGGTTCTTAATGATGCTCACTAGGAGAAACAGATCATGTTAAAAAGGGGCATGCTTTTTCAATTT

AATGGCTGATTCTGCTCCAGAGTCCCATTGCCAATCTGATGCATTGGGTCTGAGACATACAAGCAGTTCATTTTTCAATC

TTCCTGGTTTCTTTGTGGGATTTGGTTCTAGAGGTTCAACAGAATCTGACTCAGTTAGGAGCCCAACTTCTCCTCTTGAT

TTCAGTTTTTTCTCAAACCTTAGCAATCCATTTAGCCACAAGTCCCCAAGATCACCACCAAACCAAAATGGCTATCAAAA

GAAATGGGATAGCAGTAAAGTAGGCCTCAGCATCATAAATTTACTTGCTGATGAAACCAAACCAACTAGTGAAGTTCTCA

ATTCACCAAAGAGGAA

>JK611380.1 JCF4048 Jatropha curcas, immature Seed cDNA subtraction library Jatropha curcas cDNA, mRNA sequence

ACCTCGAATTACTGAGAAACTTACTTGGATTTCTTCTCTCGTAGCAATTGCTCTAGGCCTGGCCATTATCATTAGCCAGT

TTTCCTGCTATAGCTTGTATTGATCCATCTAACACGACATCAAGATTCACATTTGCAAGCCAGCATTTTAGTGTTAGAGA

AAGATCTTTAGAAACAAATAGTCAAATTTATTTCCCATAAAAAAACATCTGCGCAGCATCTTATCTCCGTAAGAGTCATC

TTTTACTTCCTTTTTTCCCCCCTCCCTCCTGAAGATTGAAATTTTTGAATAAAATTGAAACTGGCCTTTTTAAAATACAA

GCTTTCAAAAGCCCCACCCCAGGGGTGCCCCTTGAAAAGGGGAGGGGGAGGTAAGGGTAAGAAACCTACAAACCCAAAGG

GGAAAAAAAAAAATGGTTTGGACTTTTGCCCTGGGTTGCCCGGGGTCCCCCAAAGGTTTGGAAGGGTTTCCCGGTTTGAT

ACCCTCGGGGGGGGGGGGGATTTTTAAAAGCCCCCCAAAATTTTTCCCCGAAAATATTTTTTTTCCCTTTTTCCCGAAAA

ATGGGTCCCCCCCCACCCCTTAAAGTTTTCGGGACGGATCTTTCGGGCGTTTTAAACGGGGGAAACGCGACACTACAAAG

AGACCCCCTCCTGGGGAAAAAAAAACGCACCTTTTCCGCGGGGGGGGGGGGGGAATTTTTATCTATTTTGTGTTTTTCTT

TCGGCGCAGCTCTAAAAAAAAAAAAGGTGGTGTTGTGTGCTATACACAATCGACGAAACAACGATCTGTGCA

>FM896000.1 FM896000 Jatropha curcas embryo 71-95 (DAF) Jatropha curcas cDNA clone rjcpga0_003126, mRNA sequence

GGATGATAATCTGTCACTTTTACTCCCTCCTTTGCCACCAGAATGAACTGTTCGTGTCCTNTGGTGACTGTGAGCTGATT

TTGCTATTTGATGGAAGAGAATGGCGTCGGGCAGGGGTATTTTTCTCGGCCCCATCTTGGCTAAACCTTGGGGAACCTTG

TGCTCTCAACTTTGCCTTTGCAGATTCTGTTGCTGCCATATAGCTTGGAAGTGCTGGACTACTCTGTAATCCATTCTCTG

CACGTTCTTGCTTTACTAGATTCGAAGCTTTTTTGCTGATTTTATGATTCTCGTTGCTCGATGGATCTTCCTTATGGTTC

AACTCCCCATTTGTAACAAGAGTATTCTCATCTTTATCACCACTCTCGACTGAAGGCTTCGATTCAACCACTGTTAGATC

ACAATTTAATTCTGGTGTTTCATTCATTTCCAATGTATTCAGAGTGGTTTCTACATCAGGCACTTTCAACGCTGTCAAGG

TTGGCTCAGTCTTCACCA

>GW881258.1 JC000822 Seed specific Normalized cDNA library from Jatropha curcas L. Jatropha curcas cDNA clone N01565 5' similar to Unknown protein, mRNA sequence

GGGATTTTGAAGTGAGGAAGGCAGCACATAGGGAGAAGAGTGATGATAACCTTCTCTACTGTCCAAATGCTCTTCGGCTG

GGAAACAATGAGATTGAGCATTTTCAAATGCACTGGACAAGGGGTGAACCTGTTATTGTTAGAAATGTGCTTGAAAAAAC

ATCTGGTCTTAGTTGGGAACCAATGGTTATGTGGAGAGCCCTTAGAGGTGCACAAAAGATATTAAAAGCCGAAGGACAAA

GGGTTAAAGCCATTGATTGCCTTGACTGGTGTGAGGTTGAAATTAATATTTTCAAGTTCTTCAGAGGCTACTTAGAGGGT

CGT

>GW878120.1 JC002314 Seed specific Normalized cDNA library from Jatropha curcas L. Jatropha curcas cDNA clone N04283 5' similar to Unknown protein, mRNA sequence

GGAGAGAGCTACCTCAAAATACGGGACAGCTTATTTTTACATTATAGCTTCTCTCCGTATGTTAATGTTATTACATCTAA

CCGAATCTTTTGCCAAGTTTTTCTCAGTTTTTCAAAGTTATTTTATTTTATTTTTCTTTTCTTTTCTCTCCTTCCTTCTG

CCACCCTAAATAATCTCTCTTTGTCGTTCTAATCTTTCTCACACCACAATAATCAAAACCCATTTCTTCAACTCGACTCA

GTCGCAAGATAGCGAGTCATGGACCAGTGACACAATGTAAATCCCTTTGCTTTTCCGTTTCAGCTGTAGAAGTGATATTG

AGATTGCTTAACTGAGAAAAAAGTAGAGACCTTTTTCAACATTTTTGATTAATGGAGAACGATCAAACCTCGGCAACCCT

TGAAGTTGAACTGCCAGATGGAAAAACCCTAGAAGCTTTAAACCCCGAGTATTGTAATAATCTAGAAAAAGCTTCTGGGT

CGTGTGAGCTATTTGGCTTAAGTGAGAATTGTACTGATGTAGAACCAAGTTTGGAGATTGCCGTACAAGAAAATGGGAGT

GGAGTTGTTGGTGTCGGTAATGTGGAATTAATGAAAGGAATTGATGGCATTGAAGAAGAGAATTTGGATGCTAGTGCAAA

TACAATGGATGCTTTTGATGTGAATTATATCGGAAATAAAGGAGGGTTCTTTGGTGAATAATAGGG

>FM896592.1 FM896592 Jatropha curcas embryo 71-95 (DAF) Jatropha curcas cDNA clone rjcpga0_003844, mRNA sequence

AATTCAAACGAACATACATGGATAGGAGTAAAGAAGTAGGCGGAGGAGGAGGAGGAGGCCAGGGGAAGGCGAGAAAGCTA

GAAAAAATCAAAATCAAAGCGAAGCTAGAGGCGGAGTTGCTCCCAAAGGAAAATCATGCATAGGATATCTCTATCACTCT

TCCTCTCTTAAATCCAATGGCATCAATCCTCGTTGCATCGGCATCCCTCGTACTCTTCAACAAGTTCCCAGTAACCTTGT

TGGAGTATCTAAGGCTGAAGCTTCTAAAAAGGGCGGGATTCTTACGGATATGTATTATGGTTGTGCTGGGTACTCTATCT

ATGTCACTAATGACCACTCTACAGATAAGCTACTGACAAAGCCAGCATGTTCGGGCCTTGAGTAGATAGTGGAAGGAGTT

GCTAATGCTGATAGTGCATCTGCTCCGGCTCATGTTCATAGTAAAGAAGATGGCAGAGAATTTCCTCAGCCTCGAAACAA

TAAACCAGGGCATTCTGCAGGAGATGATTTTCTGAGCAGGTATACAAGGAATGCCAGCTTGGTTGCATCAGGAGTGGCGA

GA

>GW613231.1 Jc1-025-F06-M13F.F06.ab1 Jatropha curcas flower and seed Jatropha curcas cDNA, mRNA sequence

GAACTTCCTGATGAATTTGGAGCTGAGGTGAACCAGCTTATGAGCCTGAAGCTTGAAGGATCAGTTGGTTAAGCTGCAAA

TGAAAATCATGTTTTTTTTCGTCAGTGATTTGATTATTGGAGACGTTTCATGAAGTTTGATCTGTAAATGCATCACTCAA

GAAAGCTGTATACTTGGTGGTTTCAGAGTAGGGCAAGAATCATCTGTGACCCTTTGTTCTGCCAAAAAGGAAAAAACAGA

AAATAAAAACACGAACTCATATCTAACGTTGTAATAGTATGAGATGTAGTATGTAACGTGTGGCATGGTAATAATACTGA

TCTGTAACATAATTTGTGTTCACATGTTGTT

>GT975358.1 GJCCJC2069D01.b1 Jatropha curcas L. developing seeds (mixed stages) Jatropha curcas cDNA clone GJCCJC2069D01, mRNA sequence

ATGGAAGCTAAATAAGAATGAATACCACAGACGTCCTCAAATTCAGATTACCGCCTATTAGGAAAGCACCATCAACTTTG

TGTAGATGACCTTTCGCTTGTAGAATAGGATGCTCCAACAAACACTAGCTGTGTCAAGAACTGGAGACCTCTTTGCTGCA

GACAGGACAAACCTGCAGTTTTAGAGGGCAGGAGTCATACAACACATTCTTCACATCATATACCAATGGTGATCTTTAAA

CAAGTGATTTACTTGCCATTTGTTCTTTAAAAATTTAATTGGTAGAAAAGAGAGCAAGAGCAGTAATGCAAAATCAAGAA

GAGAGGCAAAAACATGGAATAATGACATTCTCGCCAAACCCAGGACGTTTATCATATAACAAGTAGTTAGGGGAATCTGT

GAATTCAATTGAATTATTTCTGCATTTTATGCTGTTATTTTTTCAACAGAAAAGAGCTGTTCCATTGTTTGGTTGGGAAG

ATG

>GW877117.1 JC006632 Seed specific Normalized cDNA library from Jatropha curcas L. Jatropha curcas cDNA clone N11186 5' similar to Unknown protein, mRNA sequence

ATCTTGAACTGTAACTTGCTAAGTCATCAAGGATGGCAGCCTCAGTTCCTCTTCAGACTTGTTTTGCTGGAAATATTGGA

AAGAGCAATGGGTCTTGTATTATCCGGCCAAAACCAGAAGGTTCAAAATCAAGACTGGTTTCCTGGAACTTGATGCAAAA

GAACAAGAAGAGGAAATCAATTGCAGTTGTTGCAGCTGTTGGAGATGTATCACCTGACGGCATAACCTACCTATTTGCAG

GCGCAGCCACCGTCGCCCTGCTTGGAACTGCCTTCCCCATCCTTTTCTTTCGCAAGGACCTGTGTCCTGAATGCGATGGA

GCGGGTTTCATCAGGAAGGCAGGTGGGGCTTTGAGGGCGAATGCTGCTCGCAAAGATCAAGCTCAGATTGTATGTCCTAA

TTGCAATGGTCTTGGCAAGCTTAACCAAATTGACAAATAAACCCGAAGTTATTCTGGCTTTTCAAATGGACAGGTCCTGT

TTATTGAATCTATATACCAGAGTTCAAAAGTGATTATTGGAATTTTATCACCCATATCTTTGTATTTCTAATCAAAAGCT

TCCGTTAATTTTGATTTGGTTGTTTTAGATTTGTGTAATACGACCAATCATGTTGTAAGAACCATGCTAATATCCGTACT

GAATACACCCTTCTTGTAATTGTTTGAAAAAAAAAAAAA

>FM888818.1 FM888818 Jatropha curcas embryo 35-55 (DAF) Jatropha curcas cDNA clone rjcfea0_002313, mRNA sequence

CGGAGCTCCGAGAGAGATACAGAAAGAGAGAATCCTGAAGAAATTTCGGCTAAACCTTCATCGTGTAGCCCTTATTCTCT

CTTTTCGTTTCTCTCTTTAGCGCTACCTCCNGATTTCTTCAACATTCAGCTTTCTTCGTCGGCCAGATTGGTGCATATCT

GCCATTACTCCCTCGCCAGTTCTTAAACTCGGTCTTCGATCTTCTTGAGTTATACCAAAATGGTGTTCTGGGTTTTTGGC

TATGGTTCGTTGGTGTGGAACCCGGGATTTGAATATGATGAGAAAGTGATAGGCTTTATCAAGGATTACAGACGAGTATT

TGATCTTGCATGCATTGATCACAGAGGTACACCTGAACGTCCTGCGAGAACTTGCACATTGGAAAATGTTGAAGGATCTG

TTTGCTGGGGTGCTGCTTACTGTGTTCGTGGAGGACCTGAAAGGGAAAGACTCACCAATGGAGTATTTGGAACGGAGAGA

ATGTG

>GW615372.1 Jc1-049-H01-M13F.H01.ab1 Jatropha curcas flower and seed Jatropha curcas cDNA, mRNA sequence

AACCCTCCTTAATTCTCAATTCCATCTCGTCTCTCTATAAAACACACAAATGAAAGATTAAAAAACAAATTCCCACTTAT

TCATTGCTGTATTTTGTATATAAAATAAAAGAAAATTTCGATTGCCTTTTTCAATTTTTATGGGAAAATCGTTTGCAGCT

ACTACAGCAAAGATACACAATCTAGCGAAGCATCTAAAACCTTCAAAATACGCAAAACCCACGTCCCGAGTTCAGACTAA

GAAGATGGAAAGCAACGCGGATACAGACAATCTCAACAATATGAACACGAACAGTAATAGCAGTAACAACAGCAATATCA

ACATCAACGCGCGCTTGCCCTTATCGGAGGTTGTATCGGATTGCGTAAAGAGATGGTTTAAAGACACCCTCAAAGATGCT

AAGTCTGGAGATATTAATATGCAGGTCTTGGTCAGTCAAATGTATTATAGTGGATATGGAGTGCCTAGAGATGCCCAAAA

GGGAAGGATTTGGATGACAAGAGCATCAAGGACTAGGTCTTCAGTTTGGAAAGTCAGCGATAAGCATCCAGGTTATAATG

CCAGTGATTCAGACTCTGACGAATTGAAGGGTGATTCTTAATAAGAGCTGCCGGAATTTTATGCCGAAACAATCAATGGA

TGATGCCGTTTCTGCTCTGCCTCAGGTTTTATGTGCT

>GT971882.1 GJCCJC2025G12.b Jatropha curcas L. developing seeds (mixed stages) Jatropha curcas cDNA clone GJCCJC2025G12, mRNA sequence

CATGGATCTGTCTCCATGGCTCAGGGGACAAAGCCGTGAGGGTGACCTCGTACGGAAGGGCCCAAAGCGAACAACACTAA

CCAGGGCGGAGCCATGACATTTGGTATCAGAGCTTAGGTTTAGACAGTTCTCGGACTATCTGCGTATATGTGTAGGAATT

TATCTATTTTGAGGTGAGAGTATGAGTAATTTAAGTTAAATACATGCCATATTAGTTTGAATTGGAAACAACCAGCCATT

GGAGCATGGTGTTGTGGTAAATGGATTATGTTATTGAGGTTCCTACTGATGATTAATGGTTATATCCTTCTAGACCATTT

GAATGAATTATTCTTGGAGGAAATTCTTAGAGAATAATGGATAGGGATTCGATGTAGTGGTTATTGGAGATGAGATATTA

TTGAGGCATTAAGGATTTGAGCTCTTTTTCTCGAGTTGATATGATTTCTAAGTGGTATAAATATATTTGAGATTTGACTT

TTTAACATATGAATTTTGTTTATTTATTTTGAATCCATAAGAAGCATATTGTTTTTATGTTGCTTATGGATAAATGATGT

TGCTATGAAGATATTTTTTTTCCTTCTATTGAATCCTTTGG

>GW875545.1 JC005152 Seed specific Normalized cDNA library from Jatropha curcas L. Jatropha curcas cDNA clone N08931 5' similar to Protein brittle-1, chloroplast precursor, mRNA sequence

ATTACGGCCTAGTTACGGGGGAAACAGGAAACCAACAAATCTCCGATGAGAGCAATGTGACAACGGCGGTGCAAGCCAAG

TGACCGCTGCATCTCCTACAGCATCTTCCAAATTCCCGCTGTCACTTTCATTTCTCCAACTACACCACCACCACCACCAC

GTCCATACTATTCTCTCTTCTTCTTCAAGCCTCTCTACTATATCAATTTTCCATTTCTTTATTATAATACTCTCATGTTG

TTACTCTTCTTCTCTGCTTTCTACCTTTCTTTCAGTTTCACCAACTCTCGCAATGTACGGACACGATCTCTTCTTCTCCA

CTCTAATCAAGCTCAAGCTTGATTCTGAACCGTCCAATCAGCACTTCCTTCATGGTGGCGGTTTGTTCCTCGATCAAGAA

ACTGTTTGTCGCTCATTTGTTTCGTTAATATCGTCCAAGACTGAACCGCGTTCTTCTTGTTTTTCTCGGAAAACGTTGAG

GTTCGTTGATCGGCGGCGAGGAAAGAAGGGTCGCAAAGCAGGATTGTTTTTATCGGTGAGTTTGTCAATAAAGGGAAGCG

AAGAAGAAGAAGGATATGTTGGGGAATCGCGAGAGAGTTTGGGGCAAAATGGAGAGGAAAAGAATTTGGAGGAA

>FM891421.1 FM891421 Jatropha curcas embryo 56-70 (DAF) Jatropha curcas cDNA clone rjcaeb0_001704, mRNA sequence

ATTCCTCTTCTTCTATCTCTATTTCCATATATTTATGTAATAATTATTTCATATATATATATATATATATATATATTTTA

TTTGGATTAGGTTATCGATTTGGGGATTTTTTTTTTGGGTCGTTTGGGACTAGGTTTAGGGTTCTGAGCAAAGCAAGGAA

TACAAATATGCAACAACCGCCGCAGATGATCCCAGTGATGCCTTCCTATCCACCTACAAACATCACCACTGAGCAGATCC

AGAAGTACCTTGATGAAAATAAAAAATTGATTTTGGCGATCTTGGACAATCAAAACCTTGGAAAACTTGCTGAATGTGCC

CAGTATCAAGCTCAGCTGCAAAAGAAT

>FM892258.1 FM892258 Jatropha curcas embryo 56-70 (DAF) Jatropha curcas cDNA clone rjcaeb1_002759, mRNA sequence

GAGAAGGGTACAACTTTTATTTATCCAAACAAAGACGACATTTAACACCTATATAAGCTATAGAAGGAGACACCATTAAA

CCAGCAACACTCTAGATATATAACAAAAAGACAAAAAGAACTTTTGACTTTCTTCACAGCTATCTCCATCAACCATTCAC

AATAACGCCTCCATTGGGGTGAAGAACTTGGCCAGTGATATGGGAGGAGCAATGGTTACAAGCAAGAAACACATAAGAGG

GTGCAACCTCACTTGGCTGGCCAGCTCTCTGCATAGGCGTCTGTATCCCAAACTTAGCCACCTCCTCTTCACTGAAGAGA

AGAGGGTATCCATAGGAGTCCACATAGGTCCAGGAG

>FM896521.1 FM896521 Jatropha curcas embryo 71-95 (DAF) Jatropha curcas cDNA clone rjcpga0_003753, mRNA sequence

CTGGCAATCCCATAAAAATCAAATTCCCAGATGTGAAATCAGCTCCAGATGGGTATGTTGCTAAATGTGTGTGGTTTGAT

TTACAAGGCTCACCAAATTTTAGCAATGTAGCATCTGGAAATGAATGTTCAACAATTGACCAAGGGCATTTTTCTATAGT

TGTTGAATATAATGCCCCTTCTCCAGCACCAGTTTTTCCTACTCCATCTGGTGAAGCTCCAAATGTTCCAGGACGAGAAG

GACATAAAAAGAATAATTCTAAAGTGTGGATAATTGTTGGTTCTGTGCTGGGTGGCCTATTGTTGTTGGTGCTATTGTCA

TTTCTAGTGCTGTGGGTGCATAAGTTGAAGGAAAGGAAGAAACTGCAAGAGATGGAGAGGGCTGCAGATGTGGGAGAAGC

CCTGCAAATGACATCAGTTGGGGAAACAAAAGCACCTGCAGCAATGGTTACTCGAACGCCACCAACCCTTGAGAATGACT

ATGTGCCATAACTCTCACCATTTGTACAAGCTTCCTTTACCTTG

>GW877940.1 JC003542 Seed specific Normalized cDNA library from Jatropha curcas L. Jatropha curcas cDNA clone N06569 5' similar to Glutathione peroxidase, mRNA sequence

GGATGCTATAAAGCAATCCTCATTTCCCCAAATCCAGTGGAGAGTTTCAGTAAACAAAATCGACATGGCTTCTGTTCCTT

TTCAGAGTCTAAAGCAATTCCAAATAAACCCAAAGTCTTGTTCTTTCTCGCCTGCAATGGCTTTTTGCATTCCATCCATG

AAGACCTCGTTTGGTCCCTCTAAATCGGCTTTTCTGCAACATGGTTTCTCTTGCAATTGCCAAATTTTCCTGGGTTTCTC

TCAAAAACTCATTCTTTTGCTGTTTATGCAAGAGCGGCCACTGAGAAAACCATACACGACTACACTGTCAAGGACATTGA

TGGGAACGGCATTCCTCTCAGCAAATTTAAGGGGAAAGTTCTATTGATTGTCAATGTTGCTTCAAAATGTGGTTTGACGG

CATCAAATTACACAGAGCTCTCACACATATATGAGAAGTACAAGACTCAAGGATTTGAGATTCTAGCATTCCCTTGCAAT

CAATTTGGAGGACAAGAACCTGGGTCAAATCCTGAGATCAAACAATTTGCTTGTACCAGGTACAAAGCAGAATTCCCAAT

ATTTGATAAGGTGGATGTGAATGGACCAAATACAGCTCCAGTTTACCAATTCCTGAAGTCAAGTGCTGGAGGATTTTTAG

GTGACCTGATCAAGTGGAATTTTGAGAAGTTCTTA

>GT972569.1 GJCCJC2037H05.b Jatropha curcas L. developing seeds (mixed stages) Jatropha curcas cDNA clone GJCCJC2037H05 similar to adenosylmethionine decarboxylase family protein, mRNA sequence

GAAGAAAAGGTAGCGTTCTAACATACGAGTCTCTGTTTCGAGAAATTATTGGAGATTTAAGGAGCTTCTCGTTGCTTATT

TTTATCGCTCTTCACCGTTTCTTCTGATCGGGTTTGATTTGAGGTTCACAAAAGGAAGGTTTTCAAGAGGAATTCAAGTG

TTCAGATTGGATTATATTGTATTTATTGGAAAGACTGAATGAACTAATGGAGTCTAAAGGTGGCAAGAAGAAGTCTAGCA

GTAGTAAATCCTTATTCTACGAAGCTCCTCTCGGTTACAGCATTGAAGACGTGCGACCCCACGGTGGAATCAAGAAATTC

AGATCTGCTGCTTACTCCAACTGCGTGAGGAAGCCATCCTGAGTTCGCCCAACTGTGAACATATATCCCCGTCTTAGCTC

TCATACTTACAATATTGTTTTTTATTCTGCATTTCTTTTCTACTTACCTCGTTCTCTCATACTCCTTCACAAGTGTTATT

TGTTCCTCTGAGGCAAGATGGCCTTGCCTACCTCTGCTATTGGATTTGAAGGCTATGAAAAGAGGCTTGAAGTGTCCTTT

TCTGAGCCTGGCTTCTTTGCTGACCCCGGAGGGATGGGCCTCCGTTCTCTGTCCAAAGCTCAAATAGATGAGATTCTAAA

GCCAGCTGAATGCACCATTGTTGATTCTCTTTCAAATGATCATGTTGACTCTTATGTTCTTTCAGAATCTAGTCTCTTTA

TTTA

>JK612970.1 JCF21-196 Jatropha curcas, immature Seed cDNA subtraction library Jatropha curcas cDNA similar to protein with unknown function, mRNA sequence

ACATCTCTGTCATTTCCGGTGTTCCTTCGACTCTTCACCCAGGGGCTTGTATACTGGTTGTTGGAAAGTAAAGCCCTCCA

GTTATTGCCTTGTACTGGGATAAGAAACTGCAGTTCTCCCTTCAGGGAAGGACGTCATTGTGGGATTCCATCTTGCTTTT

ATGCCACAGGATCAATGAAGGATATGGAGGCATAGTTCGGGGAATGCATCTTGGTTCATCAGCCTTAAGCATCCTCCCAG

TTCTCGAGTCCGAAACTGATGACTGACTTGATTAGGCATAAGCAACAAAGGATATGTCTACATCTCATCTAAATATGTTA

ACTGCATAACTTGCTAGACCAATTGAGATCTTGCTTAATTTAAAAGGCTGTATAAAATGTGGGCAATGAACTTTCTGACA

GTCCGCTTATATCAACCTCAGTCAGCGGGAGCTTTCACCAATGATGTTTGTCTTAAGTTTTCTCCTGAGATGAACAGAAG

AGTTGGCAGAGAAATTAGGGACTTCTGTTTGTATTTTTCTTCTTTGCAGTTGTTTTATTTTTGTTTTTCAATTTTCTCTT

TTTAACCTTAATGTTTCTGAGGTGGAAAGAATTAGT

>GW877922.1 JC003524 Seed specific Normalized cDNA library from Jatropha curcas L. Jatropha curcas cDNA clone N06547 5' similar to Acid phosphatase 1 precursor, mRNA sequence

GGATTCTCCTTCTCTCTCTCTGTCTCTCTCGTTTCTGCAAAAAATGGGAAGAATGTGTGTGTTCACACTAGCATTGGCAA

GCCTCTTCACTGGGCTAGTGGCAGCTGCAGATTGGAACATTTTGAACCTGAAAAGGAGAAACTATGGGATTGAATCTAAT

AATTTGAAGAATTATTGTGAGAGTTGGAGAATTAATGTTGAAGTAAGCAACATTAGAGGTTTTGAGGTTGTGCCTCAAGA

GTGTATAGCTTATATCAAACACTATATGACATCTTCTCAATATGAGGCAGACATAGAAAGAGCCATTGAAGAGGTCAAGC

TCTATCTCAGTAGCTGTAGTTGTTGTACTTTGGAAGGTGATGGTAAAGATGCTTGGATTTTTGATGTTGATGATACTCTT

TTGTCTACCATACCTTTCTACAAGAAACATAATTTTGGGGGAGAGAAGTTGAATAAAACATTATTGGAAGGTTGGATGAA

AGAAACAAAAGCACCAGCTTTAGAGCAAACCCTAAAGCTCTTCCATGAGATGAAAGACACAGGAATCAAAATCTTCGTTG

TCTCTTCAAGAAGTGAAACCCTAAGATCTTCTACCGTTGATAATCTCATCAATGTTGGCTATCATGGATGGTCTAGTCTC

TTATTAAGGGGTCTTGAAGATGAAACCATGAAAGTGCAAGAATACAAATCCCAAGCAAGAAGAAATTGATGGATGAGGGT

ATCGCATTTGGGGA

>JK610506.1 JCF1393 Jatropha curcas, immature Seed cDNA subtraction library Jatropha curcas cDNA similar to senescence-associated protein, mRNA sequence

ACGATTCCCATCCGGGAAAGGATGGACTCTCTTTCCTATAAACCTTAGGAATTTGAAGCTGAATGTGTCAGAAAAGTGAC

CACAAGGATAATCGGCTTGTGGCAATAAAGCGTTCATATCGGCCGCGATTTTTGATCCTTCGATGCATTCTCTTCCTATC

GATGTCAAATTCAATTCACCTCGTGTTGGATTGTTCACCCTGGAATAAGGAACGTGTGCTGGGATTAATCCCCTTGCAGA

ACATCCCCCTTTAGCCTACTGATGAAGGAGCCAAGATGGCCATTCCGATTACCCTTCCCGCCGCTACCACACTCAGCTTG

ATGAATGCACAATGTACCCAT

>GW879362.1 JC003920 Seed specific Normalized cDNA library from Jatropha curcas L. Jatropha curcas cDNA clone N07159 5' similar to Unknown protein, mRNA sequence

GGCGCCAGATGGCTCCAACTTTCTCCTTCTCTGCAACTTTCTTCCTTTCCTCTGCAATGCATTTCTAGGGTTTAACCTTT

TCTCAAATCTTTCTCTCCTAAAATTCTTTTACTGGATATTTCTTCCAAAAAACCTTTCAAATTGATAAATCTTCTACTTT

CTGGATGATTTTCCCCAACATCGTTGTTTTTTTATCTTCTTTCCAAATTTAGGGTTTTCACATTGTTTCCTTGTTCAATT

CGCATTATTCCCTCATTAGCATTTTCTTGAAGATATATCAGTTTTGGCAATTGCAGTCCTCTTTGATGAATTGTTAGGGT

TTAAAAGATCAAAACTTTGTTATTTGCTGGGTCATTTGTTATTAGTTTTCAAACTTCTTTGTCCAATTCAGTTTTCAAGA

GATTTTAGGGTTTTTATTATTGATTTAGTTGCTGGTTGTTTGGTTGGGGCATTGTTAAGGGGGATGAATAGTGTATTCAG

TGAACAGATACTTGCAGACAAGCTCTCTAAGCTCAACAGCACTCAACAATGTATTGAAACTTTGTCACATTGGTGTATAT

TTCATCGAAGCAAAGCAGAACAAGTCGTTGCAACATGGGATAAACAATTCCAAAGTTCAGAGATGGTTCAGAAAGTTCCT

CTGTTGTATCTTGCAAATGATATCTTGCAGAACAGTAAACGTAAAGGAAATGAATTTGTCTCA

>FM887902.1 FM887902 Jatropha curcas embryo 35-55 (DAF) Jatropha curcas cDNA clone rjcfea0_001186, mRNA sequence

ACGCGTGGCGGCGCTCTAAACTAGTAGATCCCGGGCTGCAGGCAGATATTATGGGGCAGTTTCAAAACTCACCCTCTTAG

AGCTATCCAATAACCAATTATCAGGGCAGCTACCCACTTCTTTATTCAACTTGCCAAACTTCAAGACTTGGCCTTGGACC

ACAACCAGCTCATTGGAAAAATCCCAAACCAAATTGGTAGCCTGAAATCCCTTACTCATCTTACATTGAGCTCTAACAGG

TTTACAGGTCAAGTCCCTGAATCCATAGCAAGGTTACAGAACCTTTGGTACCTCAACTTATCCAGAAATGCATTCTCAGA

CCCTTTACCAATGATACAAAGTAGGGGCCTTCCTTCTCTATTGTCTGTGGACCTGTCTTATAATAATCTCAGTCTAGGGA

CAATTCCTAATTGGATATTTGAAAAAGAGCTTTCTGATGTCCATTTAGCTGCATGTAAACTTAGAGGTAACCTTCCAAAG

TTTACCAGACCTGCTTCTTTGAACTCAATAGACCTGTCCAATAACTTTCTCTCAGGTGGCATTTCTGGCTTCTTCACCAA

CATGTCCAGTGTGCAAAGGCTCCAGCTTTCAAATAATCCATTGAAGTATGATATTTTAGAGATTGAACTGCCAG

>GW878616.1 JC002381 Seed specific Normalized cDNA library from Jatropha curcas L. Jatropha curcas cDNA clone N04417 5' similar to Unknown protein, mRNA sequence

GGAAGCCGGTGGCAATGGCGAAGAGAGAGAGAGTTAAAGAAGGGGAAAAAGAGAAGAAAGGAAGGTGAAAGAGTAATTGA

CGCGGCAAGTATAGAAAAAAAAAAGGGAGCAGAGCATGTAGATATCGAAAGTACCTGAGGGGATCATGATGTTCTTCTTC

CATTAAATATAGTTTTCAATCTGAATCGAAGCTCCATTTTTTTTTTTTTTTTAGCTGCTCCTCTTTTTCTTCTTCTTCTT

CTTCCTTGGCACCCTCTGTCTTCTCTCTCTATACCTGCCAACCAGTCGCTTCCCTTTGGGCCTCGTGATGAATTTACAAA

TCTGAAGGTTCAATTTTTTTTTTTTTTTTTTTGCGTTGCCATGATTATAACTTAGAGGATTTTCAAATTATGTATTAGTC

AAATTTTGGGTAATCTTACTCATTAATTTTTTAAAAACCTAATATATGTTTTTAATATGATTAATTTATTATTGAAAAAA

AAAATTAATTTTTCATAAAAAAATTAAGCTTGGCCGAAACTCTTTCCCTGCAGTCTATGTGAGCTTACAGTATATGCCGG

ATCGTATATATATATGGGATTTACATTGGAATTTTAAATTATTTTAAATTTATAATATTCCAAAAAATACTTTATCAAAT

TGCATTATTTTTCCCATTGGGAAATTTAAAAAGAAAAAAAAAATAAAA

>FM890111.1 FM890111 Jatropha curcas embryo 35-55 (DAF) Jatropha curcas cDNA clone rjcfea0_003911, mRNA sequence

TCCAAATCTTCAAAGCTATGACTATAGCTGCTAACTCCAAATCATGAGTATGGTAGTTCTTCTCGTGTTGTTTAAGCTGT

CTAGAAGCATAAGCAACTACCTTACCCTCCTGCATAAGCACACAACCCAATCCATTATGTGAAGCATCACTATACACATC

AAACGGTCTACCCGAAACTGGCTGTGTCAACACTGGAGTGTTCAGTAAGCATCGCCTTAAGCTTCTCAAAACTATTCTGA

CATTTCTCCTTCCACTCGAATCTGACATCTTTTTGAAGAAGTCTAGTCAAAGGAGCCGCAATGAGAGAAAATCCCTTCAC

AAATCTTCTATAATAACCAAACAATCG

>GW875328.1 JC002064 Seed specific Normalized cDNA library from Jatropha curcas L. Jatropha curcas cDNA clone N03727 5' similar to CXE carboxylesterase, mRNA sequence

GGAATCTCTTCCCTACATTCCCATTTAGTTGATAGCTTATTCATATACCCTTTGATGGCCTCCATTAATAAGTAAGTAGC

TGGAGAGCTTCTCCCTTTCCTTAGAGTTTTCAAAGATGGATCAGTAGAGCGATTAATGGGAGCTCCAATAGTCCCTCCAT

CACCTGATCAAGACCCGGAAACTGAAGTCTCATCAAAAGACATCAGCATCTCGCAACACCCACCAATCTCTGCAAGACTT

TATCTCCCAAAACTCTCTCAATCTGATGACCAAAAAAAACTCCCCATTTTAGTATACTTCCATGGCGGAGCGTTCTGCAT

TGAGTCTGCTTTCTCTTTAACCGAAACCAAATACATGGACAACTTAGTTTCTTTAGCCAAAGTCATTGCCATCTCAGTAG

AGTACAGATTAGCCCCTGAACACCCTCTTCCTACTGCTTATGAAGATTGCTGGTTTGCTCTTCAATGGGTTACATCGCAT

GACAATAAAGAGCCATGGATATCTAACCATGGCGATTTTGACAGATTCTTTATTGGAGGAGATAGTGCAGGAGCTAATAT

TGCTCATAATATGGCAATGCGAATTGGGACTGA

>JK612806.1 JCF21-20 Jatropha curcas, immature Seed cDNA subtraction library Jatropha curcas cDNA similar to rna polymerase beta subunit, mRNA sequence

ACCTATCATGAGATTTATGAACACTATCTAATAGTAAGAAATATAAAAAAAGAAATTATTTGTATATACATTCGAACTAC

TGTTGGTCATATTTCTTTTTATCGAGAAATCGAAGAAGCTATACAAGGGTTTTGTCAAGCCTGCTCAGATGGTATCTAAT

CGGATCTAAGTTAAGTAATTCTATGACATCGGCATGAATTCCGATCTCTTTGGTTCAACTAGGACCGTAGCTCCTGAATT

TCTACGCGAATCAAGATCAAGAAAAGGAAATTTCCAATCAGTGACTCAAATCCATTGTCGAACCCTACTCAGCAGAATAT

GGAGGTGCTTATGGTCGAACGGGCCAGTCTGGTCTTTCACAATAAAGTGATAGATGGAACTGCCATTAAACGACTTATTA

GCAGATTAATAGATCATTTTGGAATGGCATATACATCACACATCCTGGATCAAGTAAAGACTCTGGGTTTCCAGCAAGCC

ACTGCTACATCCATTTCATTAGGAATTGATGATCTTTTAACAATACCTTCTAAGGGATGGCTAGTCCAAGATGCTGAACA

ACAAAGTTTGATTTTGGAAAAACATTATCATTATGGAAATGTACACGTGGTAGAAAAATTACGCCAATCTATTGAGGTAT

GGTATGCTACAAGTGAATATTTGCGACAAGAAATGAATCTTAATTTTAGGATGACGGAACCCTTTAATCCAGTTCATATA

ATGTCTTTTTCGGAGCTAGGGGAAATGCATCTCAAGT

>GW875206.1 JC001874 Seed specific Normalized cDNA library from Jatropha curcas L. Jatropha curcas cDNA clone N03393 5' similar to Unknown protein, mRNA sequence

CTCCTCTCGTTTCATTTCTCTCGTCGTCACCACTGCTCTCTCTCTCTCTCTCTCTCTCTCTCTAATTATCTACAGTCACT

GTTTCTGCACACAGAAATCTAGCGTGTCTCTTTCCAGCAACGATAACTTGGCGGGGACGACGATGATGGTGAAGGACGAG

TGGGTAAGAGCGGCGATGACGGATGATACAGTGGTTGTGGAGCTGCTTGTACGGCTTAAGCAAGCACAAGCGG

>GW880370.1 JC000955 Seed specific Normalized cDNA library from Jatropha curcas L. Jatropha curcas cDNA clone N01871 5' similar to Unknown protein, mRNA sequence

GGGAGTTATTTTGATTATGGTGATTTGTGGCTGAGCATGAGAACTAAGAACGAAAGCATAAAGTTTGTTTCTTGGTTTCA

TTTTATATGAATCTGGATTCAGAAAGAGGCTTTCTGGATATTGAGTTATCTAGTAAGATAAATATTTTGAGAGAAATGAT

TAAAGAGAGCAGAGAACCATTGATGCCTTTTCTTTTTCCTCTGGGTTATTTTTGTTATTTTGGTTCAGTTGCATAAAATT

GGGAAACTTGGAGCATACAAATTAGCTTGCTGAATCTGGTTAAGGGTAGAAAAATAACTTTCGTAAAGGATTGAAGCTCT

ATTTAGAACCCATTTTTCAATTTTTATTTTTTTGTTTATGTCTTTCCTGCATATATCATTGATAGATTTTTAGTTTTTGA

ACAATTCAACGAACATTTAGAAAGAGCAAGTTTTGAGTTCCTAATATTTCAAACAACAGATCTGTACTATTGGTTTCTTA

GACTCAAATCACCATTTGACGTCTAGTATGAAGTTTTGCTCTAACCTCATATTCTCTTCTCAAAGACATATCACTAATTC

ATACTCTTTGATTTTCTCAACTCGTAGACTGC

>GW878509.1 JC003262 Seed specific Normalized cDNA library from Jatropha curcas L. Jatropha curcas cDNA clone N06112 5' similar to RNA binding protein, mRNA sequence

GGACTGTCATTTCTCTCTCTCTCTCCCCCTCACACACATCGAACGAACCCGTTTTCATTTCGCTAGTGAGCGAGTACGAA

TACAAAGAGGAAAAATCCTCTTTCTCTTTCTTTCTCTCTCTCTTACTTACTTGACCTATCGGTTTTCAATTAACCGGAAC

CCTAGCTCGATCCACCGGCAAATTAAAGATTCCGATGAGCGTACGATTGGGGAAGACTTGAGGATCGACCGGCCTGGATT

TCGTCACCGGATGGAGGGAGGTAGCGTCTCTAGACTTACTTGTTCTGGTATACACCCATGGATTATGATGACAATGATTT

TCAAAGCCAGAATCTCCATTTAGCTGGTGAAGGAAGCAACAAATTTCCTCCTGTTCTAAGGCCGTATGCTCTTCCAAAAT

TTGATTTTGATGACAGTCTTCATGGACCTTTAAGGTTTGATAGTTTGGTTGAAACTGAGGTTTTTCTTGGTATTGAAAGT

AACGAAGATAGCCAGTGGATTGAAGAGTTCTCTCGGGGTAGTAGTGGGATACAGTTTAGTACAAGTGCAGCAGAGTCTTG

CTCAATTTCGAGGCGTAACAATGTCTGGTCTGAGGCGACTTCCTCAGAATCTGTTGAAATG

>GW876460.1 JC007094 Seed specific Normalized cDNA library from Jatropha curcas L. Jatropha curcas cDNA clone N11923 5' similar to Phox (PX) domain-containing protein, mRNA sequence

GCGCACGCCAACAGCCTAAACCTAACGGTCCTCGCATTGAATTCTCCTTCTTCATTTATGAGTTACCCGATTCTCTACTT

TTTCTCTCTATCCAAGCACCGTTTTCCCTTTCAAATCTGTCCAATTTTCCTTTGCCTTTTCTTAGCTTCACATCCTTTCT

GACTTGGCTCGGTCTTTTAAACAAATGTCGGTTTTTTTAGAACAATTGTCGCTGCGATACAAGAGTGAAAAGTTCAAATT

GAGTTTATGATTTCTTGATTTTTCTTTAGTCGGAAAAATCGAAGAGATTTTTTGCTTTAGCTCGGCGAATTCGAAAATGA

ACACGCAAAGGCAAGTGACCGTGCGGGACCTAGTGGAAGAAGCGAAGAAGAGGATCGTCTTTTTGGTTATTTCCGTTGTT

GGATTATCTTATCTCATGTCTTTGTCAAGCTCCTCAGTTTGGGTCAACTTGCCGGCTGCTGCCTCCTTAATAGTTTTCCT

TCGTTATTTCTCTTTGGATTATGAAATGCGGAGGAAAGCAGCTGCTTACAACAGCAAACCTTCCTCAGCAAATACAGTGT

CTCAAAACAACACTTCTGATCCTACTAGGGCTCTTGAAAGGCCTGACTGGAGAAGAAAAGTAAATTCGCCAGTTGTTGAG

GATGCAATTGATCACTTCACAAGACATTTAATTTCTGA

>GT973487.1 GJCCJC2049A07.b Jatropha curcas L. developing seeds (mixed stages) Jatropha curcas cDNA clone GJCCJC2049A07, mRNA sequence

CCTTTATGTGTAGAACTTCCACCTTGTGCTTAGCCGATGTGGGATTTTGCCTAAGGTGCAGTCCCTGACATACACCCCCC

CTTAGGGACTCAGCGTCCTCGCTGAGGTTTGCCCCACCACCAGCCATAGGGACAAGGCTTTGGCTCTGATACCAAATTGT

AACGCCCCGGCCCATGGTAGATATTGTCCGCTTTGGGCCCAGCCCGCACGGTTTTGTTCTTGGGCCAGCAACACATGCTG

TAACCCAAAACGCGTCTACCATGTTCTAGAGTGCCACCCTTTATGTATAGGACTTCCACCTTGTGCTTAGCCGATGTGGG

ATTTTGCCTAAGGTGCAGTCCCTGACAGGTATTTACTTTCATGATTCACTTAAATTTAATAATATTTAATTAGAACATCA

AATTAA

>FM890261.1 FM890261 Jatropha curcas embryo 35-55 (DAF) Jatropha curcas cDNA clone rjcfea0_004078, mRNA sequence

GATCCAAAGAAAAGGCCTCAATGCCATATATAATATCCAAATTAAACAAAAAAACAAAGATCCAAAACACTTTCAACAAA

ATCTTAAATAGTTTTTTAAGCTTCAACTACTACAAAGAAGATAATTTGTTTCGAATCCAAAACCAAAAAGCAATCTTCAC

TACTACTAATTTCTCCTTAGTAGCGTCCAATCCCCCAGACTCTAACAACACCATCTGTATAACCACTGAACAAGGTACTT

CCATCTGCACTCCAGTTCAAGCTTGTGCAGTAGATGTTCTTCTTCTTGATACTTGTACCGGTGGCAGTGCTGCCTTCGGA

TTTCTCAGCCTCAGCTTTGAGGTCCACCTTCAAGTCCTCCACTATGCTCTTGCTCTCCAGATCCCAGATCTTGATGCTAT

GTTCTGTAGCTGCACATAGCCAATACCTGTTAGGACTGAAACACAGAGCATGGATGACTGCAC

>FM889282.1 FM889282 Jatropha curcas embryo 35-55 (DAF) Jatropha curcas cDNA clone rjcfea0_002907, mRNA sequence

CAATTATTGAAACGAAGTTTCACCGCTAGTTTCGCATTGAGGAGCATAATTTGATGCATTTTTTTCTGGTCTGACCAGAA

GGAAATGAAGGCATAGATACAAGTTTGTATAGAGCAGACCTTTTGACTATTTCCAATTTTTGGCTGTTGGCAGACAAGTG

AATTAGTTTTGTACAGCATTAAAAAAGACGATCTATATTCAAAAATTAGTTTTTAGTTCAGTATATATCTTTTTTTCCCT

TTCAAAAAAGCAAAAAA

>FM888576.1 FM888576 Jatropha curcas embryo 35-55 (DAF) Jatropha curcas cDNA clone rjcfea0_002015, mRNA sequence

TTAATGGCATAATTCGAGCCTTGAAGGAAAAAGAAGCAAAGCTTAAAGCAGCAAGGATGAGCATATATGTGAGGAGAGGG

GGTCCAAATTACCAGAGAGGCCTTGCAAGAATGAGGGCACTTGGCGAAGAACTTGGCATTCCTATTGAGGTTTACGGACC

TGAAGCAACAATGACCGGTATATGCAAGCAGGCAATTGAGTGCATCGCGGCAGCTGCTTAAGCCACCAACTCCTGGACCA

ATTGTCTCTTCTTTCTTTCTTTCTTTTTGTT

>GW878532.1 JC001968 Seed specific Normalized cDNA library from Jatropha curcas L. Jatropha curcas cDNA clone N03546 5' similar to Unknown protein, mRNA sequence

GGCGGCGTCATATTGCTAGAAGAGATGGTCAAAGGAATAGATCATCAGCTCAGGGTCGTCCACAGTTCTTAGTATTTTCT

AGTAATGCCAATTCACCTCATGCTGGACCAATTTCTTCCTCTCCAACTCAGAGAGGAGAAGGTGAGCCTGCTGGTGCAAT

TGCAATAACCCCTCCATCTTCCACTCCCATGAATGTCGTGGAAGAATCTTTACAACCTGTTATACCACAATCTTCTAATG

TCCAAGCTGAATCGGCCTCTGCTTTAGCTTCTGGATCTAGTCCTCTTGCTTCCACTCAACATGACAATACCTTGAATAAT

AGGAGATCTCCTAATCAGTCTTCTCCAAATAGTCAAGACAGAGACGACGATC

>FM888660.1 FM888660 Jatropha curcas embryo 35-55 (DAF) Jatropha curcas cDNA clone rjcfea0_002113, mRNA sequence

AATTTCATAGGAATATTCATATTTTAAAATTCATACCTCCATTGGAGACTGGGAATTCTAACTTAGTGTTACTATCCCAA

TCCATTTTAAGAGTTTTGAGTGTACAATAGAAAGTTCAGTTCATTAATAGAAGACTTAGTTCCTTGAAACTTATTTGCCC

CAGGGTTCAAAGTGCACTTTTTATTGAACATTAATTTTATTATTTGTATCTAAAATTTTGATTTCTTGATCAAATTATCA

TTTTGCACATGTATGCTTTCATGGATTTAGTTCCATAATTAATTGTCATACTTGAAAATTATATTGTATTGCAGCAGTAT

TAATTGTAAAACTAGAATTTTTTTTATTAATTAATAGATTAAATAAATCCGAGTGAACTGCACTAATATAAACCAATATA

AATTTATGAATGAAGTTTATTATTAAACCTTCCATATTT

>GW878515.1 JC003269 Seed specific Normalized cDNA library from Jatropha curcas L. Jatropha curcas cDNA clone N06120 5' similar to Unknown protein, mRNA sequence

GGTAAATTTTTTCCCTTGTTCCCCTTCTTTCATCATCATCATCATCATCATCATTATTGTTATCATTCAAATTTTATGTG

ATTTATCACAGCATTTATCAAGCTTAGTGTGGAGTCATTGACTGGATTGATGTTTGTTTGTTATCATGTTATCCCTTACA

ATTATTTTGATGGCAGTGAAGGGGGATTCTTTAATGCCACTATGAGTTTTCCTTCCAATTATCCCAATAGTCCTCCAACA

GTGAAGTTTACTTCAGAGATATGGCACCCGAATGGTTGGTCAATTTTCTGCCCATTGTAATTTGAATTTGCTCTTCTCAT

TCTGAATTGGCAAGGTTTGATTTTAGTTGCTGCTGGAATTGCAGTTTATCCTGATGGGCGTGTTTGCATATCAATTCTTC

ATCCACCGGGTGATGATCCAAATGGTTACGAGCTTGCAAGTGAGCGCTGGATGCCTGTGCATACTGTATGTCATAATTAC

TTTGTCTCTTGGAAATTGAAACATGCATTTAGAAACTGAAAGTTTTCAACTGAGTCCAATCATACTTGTCAAATAGTGAT

CTGAATCAAGGAAATGAAATCCTGAATTTGCTTTTTAGAACAGA

>GO247639.1 JcrME_RL1183 Expressed sequence tags from Jatropha curcas root cDNA library Jatropha curcas cDNA, mRNA sequence

GGAGAAATTAGGGTTTTTACTAATCGGAAGATGTTGGAGGTGGTGTCGAACGATCGGTTGGGGAAGAAGGTGAGAGTGAA

GTGTAATGATGACGACACCATTGGCGATCTGAAAAAACTGGTGGCGGCGCAGACCGGTACCAGAGCTGAGAAGATAAGGA

TACAGAAGTGGTACACCATTTACAAGGACCATATAACTCTGAGGGACTATGAGATCCATGATGGCATGGGTCTTGAGCTC

TATTACAGCTAAAAGGTTTTTTACTCTTGTAAATGCATTGGGAATTCTGGAGCATACCAGAAGATGCCTGCTGTTATATT

TTACTTTCCACCGGGCAATAGATGCCAATTCAGTATGAAAGGATTTTATTTATGTACGTGTGAAGACTGTTATGTTTGGT

AATTACCTAATTTGCCAATTACACTCCTTCAGTATATGTAATTGCTTGATAAAGTCTGTAATTGAAATGTTGGCATTTGT

GTAATGAAATTAAATTATCCCAATA

>FM890695.1 FM890695 Jatropha curcas embryo 56-70 (DAF) Jatropha curcas cDNA clone rjcaeb0_000620, mRNA sequence

ATTCGGATGCAAGATTTAAGAAGACTGAGATTTGCCAAACGTGTAGTAAGTTGAAAAATGTTTGTCAAGTCTGTCTTTTG

GATCTTGAATATGGATTGCCGGTTCAGGTTCGAGATACTGCTCTCCATATCAGTTCCAATGATTCCATTCCAAAGAGTGA

TGTCAACAGGGAGTACTTTGCTGAGGAGCATGACCGAAGGGCTAGAGCTGGTATAGATTATGAATCTTCATATGGGAAGG

TGCGGCCAAATGACACTATTCTGAAGCTTCAAAGGACGACACCGTACTATAAAAGAAATCGAGCACATGTTTGTAGTTTC

TATGTTCGAGGTGAATGCACACGAGGTGCTGAGTGCCCTTATAGGCATGAGATGCCAATAACTGGGGAGTTGTCACAACA

GAATATAAAGGATCGCTATTATGGAGTAAATGATCCAGTGGCTTTGAAGCTGCTTAACAAGGCTGGTGAAATGCC

>GW874700.1 JC000090 Seed specific Normalized cDNA library from Jatropha curcas L. Jatropha curcas cDNA clone N00149 5' similar to Unknown protein, mRNA sequence

GCCAAATTCCTTTTCCTTTCTTGCAGCCTTCTCTTCATCCTAACTCAACTCTTGTTCCTAGAGAGAAAGACGAAATCTTT

CATCGAGTGAGAATGAGGGCGGGCAAAAAAGCAGTCCCTTCAGAATGAATTCAGCGAAGCAGGAGAACAAGAATAATAAA

AACAAAGAAAAGAGTATAAAAAACCAATCAAAGGAAGACAAACTTAATAATAGTGAAGAGCCTGAACACTATCTGGAAAA

AAAAGAAATTGCTACTGATTCCGAGTCTGAGTTTGAGGAACTTTCTGATTCTGAGGGCGAGTATTTTGAATCATCTGGGG

ATTCAGAAGCTGGAGAAAGCAATTCATCTGGAGATGAGGATTCTATTGACACTGACAATAATGATAGCGTGGACAATGAA

GATCAAAGTGAAGGTTCAGAATCTGATAAAGTTGCTGAAGAAAGTGATTCCTCTGAGGATGAGGTGGCTCCTCGAAATAC

GGTTGGAGATGTTCCCTTGAAGTGGTACGAGGATGAAAAACATATTGGATATGATATAGCTGGG

>GT971427.1 GJCCJC2020D11.b Jatropha curcas L. developing seeds (mixed stages) Jatropha curcas cDNA clone GJCCJC2020D11, mRNA sequence

GACGACATGAACACATGTATAAACACAAAATGCAAGCATGCAAATGATGTGAAACTAACATGACACAAGTTGCCCGCTTG

GCAAGGCTATCAGCATACAGGTAAGCAGTAAGGTTTATGAAATTATGAAGCAGTTAATTTCATGACACAAGCATGCAAAT

TATGACATAAGCATGTGAAAATAACATGACACAAGCATGCAAATTATGACACAGTTACTATGCTTTTGTTTTATCTATTG

ACATACCATTAATATGCTCCATTGAGGTTAGGTAGAAACCACGTAGATAAGGCTATGTCGTGACATAGGATAGCAGATCT

GCACCCCATACAGTTTGGTACTCCGGCTACAGGCCGTTGAGTGGATATGCTTATATATTGGCCATAGCAGCCCAGTCATA

CAGTATTCATACAGTTTCATACAGTTTTTCTTATCACTTTGGTTTCACTATAACCTTATGGAGCTATACAGTTATATTGA

CTACAAATTGCATAATCATATAGTACAATGAGCAGCAAGTATGATAATGAAAGTATCATGATATGTGTGCTTACATTCCA

TATGTGAGTACATGTTTATTTCTACATATGTATGTGCTTATATGTTTCTTTAATCGTTGCGATATTCACTGAGTTTTAAT

CAAGTTGCAGAATTCATACAGCATAAAGAACATCGGGTAAGATCATGAAAGTATAATGCTATATATGCTTA

>GW617522.1 Jc2-023-G11-M13F.G11.ab1 Jatropha curcas flower and seed Jatropha curcas cDNA, mRNA sequence

CAAGCCTAACCCTAAGGATGAACCAGACAAACAAAGGAAGGATGCAGAGTTTGCTTGATCAACTCGAGGTGATTGCTTAT

CTGCTGTAGCTTGACAAGGCAAGGAAGGACAAGGCAGAAGGGGCAGGAATCACAGCCTTCAGGGTTATCCCTGTGAAGGT

AAGGCCATATAATTCCATCAGGTACAATCCTTTTGCAGCATTCCACTTGTTTAAGGGGGCTACCACAGATAAGCAATCAC

CTCGAGTTGATCAAGCCTAACCCTAAGGATGAACCAGACAAACAAAGGAAGGATGCAGAGTTTGCTTCTGCTGTCTTTGG

TCCTGACATAGATTTGGTAGGGGGGAATGCCCAAACTGAGAGTTTCATTGGCCTGAGTGATGTTAAACATTGTGGAAAAA

TGCGGGCACTGGAAAAGTTAATGTTCTCTTGGGCTTCACGAGGTGACAAGCTTCTTCTATTCAGCTACTCTGTCAGGATG

CTGGACATACTGGAAAAGTTTCTCATACGTAAAGGGTATAGTTTTTCAAGACTTGATGGTTCCACTCCAACTAATTTGCG

TCAATCTATGGTTGATGAATTCAATTCAAGCCCAAGCAAACAGGTGTTCCTCATA

>GW879500.1 JC004059 Seed specific Normalized cDNA library from Jatropha curcas L. Jatropha curcas cDNA clone N07357 5' similar to Unknown protein, mRNA sequence

GGAGCAGAAAAATGAAAACTCCATTAGCAGCAGCAGTGTTCCTTCTCTTCTCCTCTTTTTTTCTCTCATTCTCCAATGTA

AATTCTGTCTCTGCCGATAATGACTTCTCCTCCACCAACCAAAAACAGAAACAGAACCCATTCACTCCCAAAGCATCTTT

AACGCGTTATTGGAGCAACCAAATAACTACAAAATTACCCATTTCTCCATTCATTCTTTCAAAAGCTTCTCCATTGGATG

CTCTCACCTCTGTTTCTTTTTCCAAACTCGCCTCTCAAAACGCTCTCTCTGCTCACCTCCCTGCCTTCTGCTCAGCCGCT

AAACTCTTTTGTTATCCCCATTCATCGCCTAGTCTTCAAGAACAACGTAAAGACTCAGACTTTGCAACCTATCAAAACCA

AAACTTTACTAATTACGGCACGGGTCAAGTCGGCTCAGCTGCATCATTCAAGGAGTACTCAGAAAGTGAGAACGAGGCAA

ACGATGCGTTTCTCCGGTACAGCCGAAGCGCAGAGGATAATAAAGAAAAATTTTCTGAATATGCTCATGCCGCAAATCTT

GCTGTTGGCAACTTCAAAACTTACGGCACCGGAGCTACCGGCGGTTCTGGCGATTCCAAAGGTTACCATAACGGAGTAAA

TTTTGAAAATCTCGAATTCACTTCTTAC

>GW880299.1 JC000869 Seed specific Normalized cDNA library from Jatropha curcas L. Jatropha curcas cDNA clone N01697 5' similar to Unknown protein, mRNA sequence

CTCTCACTCAAAAGTTGAGTTTCTGCCTGGGTTTAACGGTCCACTTCCTTTTGAACTTGAAACTGGATATATCGGTGTTG

GCGAATCAGAGGATGTGCAGCTTTTCTACTACTTCGTTAGATCACAAGGGAATGCAGAAAAAGACCCTCTTTTGCTTTGG

CTAACAGGTGGCCCTGGCTGCTCTGCCTTGTCTGGTTTCATTTATGAAATCGGCCCTCTATATTTTGAGGTAGTGGAGTA

CAATGGGAGCTTACCTACACTGTTCTTGAATCCAAACTCATGGACGAAGGTGGCTAGCATAATATTTATAGATTCACCTG

TTGGCACTGGATTCTCTTATCCAAGAACCCCACTTGCTTCTGTGTC

>GW876191.1 JC005746 Seed specific Normalized cDNA library from Jatropha curcas L. Jatropha curcas cDNA clone N09815 5' similar to rps8, mRNA sequence

GGCCATTACGGCCTAGTTACGGGGAGTCTACGAATCTATCCTAACTATCAAAAAATTCCTAGAATTTTGGGCGGGATGGG

GATTGTAATTCTTTCTACTTCTCGGGGTATAATGACAGACCGAGAAGCTCGACTAGAAAGAATCGGTGGAGAAATCTTGT

GTTATATATGGTAATCTTTCTGATATCTGAATTGTATCCAGACTTCCTATTTGTTTTGTGAAAAAAAAGAGAAAGAACAG

ATTTCTAATATCATTCCTCTCGCATTAGTTAATACTTCGATTAATACTTCGAGAGGATTTTGGATGGAATGAAAGAACAA

AAATGGATTCGGGAAAGTTTAATTACTGAATCACTTTCAATAGTATGTTCCAAGCCCATTTAAGATCTGGTTTTAGGTTA

TGTTTCAGAAAAGATCCAACGTAGTCTTGTCTTGTTTTATACATATACTACTACCAAATATAGAAGTAAATCCACGACCA

TAGCGCATCTAATTTAGATACTCCGTAAGAAAGATTTGAATGCTGAATGATTCGGCAGTTTTTTTTCAACTTTCAAATTC

CTTTCACTTTCAAAAAAACTTATTTTCTTCAAAAAAGTATGTATATTCAAGGACGGAAAATATGAAAATAAGAGCTTCTG

>GT974140.1 GJCCJC2057G11.b Jatropha curcas L. developing seeds (mixed stages) Jatropha curcas cDNA clone GJCCJC2057G11, mRNA sequence

CTGGAAATCACACAAAAATACAAAGTCAATATACAAATCTCATATGAATCCTTTTTTAAGAAGGATTTACCAGTCATTAC

CTCATTAATGTCATCAAGAAGAAGAGCCAGGTGATTCACACGACCCAAGGATACCTCCCTAAGAGACGAAGTACCCTCAA

GAGTGTAATAAGAAATGCTGTATGGAAAGTGCAAATTGGCCAAGCCCGGATGGACAGGCCTGGTAGCCTCTGAAATAGC

>JK317716.1 JCST487 Jatropha curcas L. seed cDNA library Jatropha curcas cDNA 5', mRNA sequence

AGCAAAAGAAAGGACTCTCTGGTCCTTCACTCAGCTGTATAGGATTGCACAAACACATCTCATTTCCTCATCCATACATA

TAAAAAAGAAGATTCCTTCTTTTTGTCATAATGGATAGCTGCTTGTCTTCATCTCTAAGAGTTATTTAATAATTCCCTGC

AAAAGTTGAGCACTCCTAAGTTGAAGTCATGGTAGTGGATGGTCGGTTAGCTTTTTGCCATTTCTAACTTTTCTCTGTTC

TGAATATTTCCCTGAAATTCTCATTACATGAAGACTATTTTTCGAAGAAAGGGCCCTCACTGCCAATAAATTATGTTTAT

CTGAATTTCCATTAATCTGAATTTCTATCTCACATGCCCAGTATCAATCAAGAGCGGTGTCATCTGCACTGAACTTGCCC

ATGGAAAGATGAAGTATATCAGCAATATTGTTCACATGCTTTAAGGGTGGCTAAAAGAGGAGAATTCTTTGGGATTAGTC

GTTATGAAAACGGAGGAGGTTGGAGCTCTCTCAGGGGCCGAAGTAACGGCAATACATGACAGCACCCAAGTTCTCACGGT

GCGGAGAAACTCACCAGAGTAAAAAAAAAGTAGGGATGATGGTCATGTCGCGTGGGCAACGAGCGGCTGCAAGGACGACT

AGCAGACAGCAGAGGGAACTCGGGTCCTTAATAAATGCACCTTCGGGACCATCCTTAGGTTCATCAGGCGGTCGGGGGGA

GACTTTGAGACAAAGATTGGAAGATTACAGGCTATGCCTCACTCCTGCTTTTTTTTTGGAGCATTTGTCGCGATTGTGGG

GACTTCATANGGAGCGCTGGATCTTCGCCAGACCGTGGG

>GW878614.1 JC002379 Seed specific Normalized cDNA library from Jatropha curcas L. Jatropha curcas cDNA clone N04415 5' similar to Unknown protein, mRNA sequence

GGTTAAACGAAGAATACAGAATATGGAAAAGGAACACTCCTTTTCTTTACGATCTGGTTATAACTCATGCCCTCGAATGG

CCTTCCCTTACCGTCGAGTGGTTACCCGACCGCGAAGAGCCTCCTGGCAAGGACTACTCTGTCCAGAAGATGATTCTCGG

CACTCACACCTCTGAGAATGAGCCCAACTACCTTATGCTCGCCCAGGTCCAGCTTCCCCTCGATGATGCCGAGAATGATG

CCCGCCACTACGACGATGATCGCTCTGAATTTGGTGGCTTTGGAGCCGCCAACGGCAAGGTGCAAATAATTCAGCAAATC

AATCACGATGGAGAGGTTAACAGGGCTCGTTATATGCCTCAAAACCCTTTTATAATTGCCACCAAGACTGTTAGTGCTGA

AGTTTATGTTTTTGACTACAGCAAGCACCCATCTAAGCCGCCTCTTGACGGTGCTTGTTCTCCCGATTTGAGATTGAGGG

GCCACAGCACTGAAGGTTATGGTTTGTCTTGGAGTAAGTTCAAACAAGGCCACTTGCTTAGCGGTTCTGATGATGCTCAA

ATTTGCTTGTGGGACATCAATTCCACTCCCAAGAACAAGGCCCTGGATGCTATGCTAACTTTTAAGGTTCATGAAGGCGT

TGTAAAAGAAGTAGCATGGCATCTTAAACATGAATACTTATTTGGCTCTGTCGGGGAAGATCAATACCTGCTTATATGGG

A

>GW611106.1 Jc1-002-A03-M13F.A03.ab1 Jatropha curcas flower and seed Jatropha curcas cDNA, mRNA sequence

TAAAAACTGCACAGGTAGAATGATTGATCCTTCTAAATCCTCAACTTTCAATTATGTATTTTGTAAGTCCAATGAGTGTC

AAAAAAATAACGGAGTCGCTTGCATATCAAACAACTGTCATTTCTCATGTGATTCTGCAGACCAAGTTTCTTGTCCAAAT

GGAGACCTTGCTACAGACACAATCACCTTGAGCTCCATTAATACTACACTTTCCCTTCCCAATATTAATTTTATCTGCGG

AAATAACATTTCCAGTGACTTTCCAGGAATTGGTTTTGTCGGTCTTGGACATGGGTCACTTTCTTTAATATCCCAAATGG

ATCATCTGATAGATGGAAAATTTGCTTACTGTCTAGTACCTTACTCTTCTAACCAGTCTAGCAAAATCGATTTTGGGTGG

AAAGGTGTGGTTGAAGGTGATGGTGCAGTTTCTACACCATTGATCCAAAACATCGGTATAGGCAGTTACAGTGTTATTCT

CGAAGGAATTACTGTAGGAAACAAGAGTACTGTAACTATTGGTGAAATAAATGGAGCCTATATTGATACAGGGACAATGT

TAACATACTTGCCAAGCGATGCCTATGATTTGCTACAACAAGAGGTTATAAAAGCTATTAATCAGAAGCCTATGGAAGAA

GATGATACTCCAAGACTGCCTCTTTGCTATCTGTACACACCTG

>JK317510.1 JCST277 Jatropha curcas L. seed cDNA library Jatropha curcas cDNA 5', mRNA sequence

ATTTTTTTTTTTTTTTTCAGATTTTGTTCTTTATTTTTATGTGTTTGGATAATAGGATCCCAAGGAATGGCCCATAATAG

AGCCATTGCCTTCTTATGGAAGAGGTAGAGAGAGATTAGGAGGAAGACATATTAGCCTTATTCATGGAAATGGACTTACT

AATGTTGTCATTACAGGAAACAATGGAACAGTTGATGGTCAAGGGAAGATGTGGTGGGAATTATGGTGGAATAGAACACT

GGATTACACGAGAGGCCACCTTGTAGAAGTAATGAACTCTGAGAACATTCTCATCTCCAACCTTACCTTCCGCAATTCTC

CCTTTTGGACCATTCATCCCGTTTATTGCAGTAATGTTGTGGTTAAAGACATGACAATTTTGGCTCCCCTTAATGCACCA

AACACTGATGGTGTAGACCCAGACTCAAGCACCAATGTCTGTGTTGAAGACTGTTACATTGAGAGTGGGGATGATCTTGT

AGCAGTGAAGAGTGGGTGGGATCAGTATGGGATCAAAATGGCTCGTCCTAGCTCAAACATCATAGTGAGGAGAGTTTCAG

GCACAACCCCAACTTGCTCTGGGGGTTGGGATAGGTAGTGAGATGTCTGGAGGGATATTTAATGTAATGATTGAAGCTGT

TATATCTGGGATTCACCAACATGTGTGAGAATAAACCTGAGAAGGGTAACGAGATAATACCAATATAACTAAAATCTATA

ACAATGGAGAAATTAGATATAACTGGTTCTTACAGGTCGATGTCACGCTGAGGAAAGTGGATACGAAGTAACCACATGCG

ACGTGACGTAGACTAATGGTTCCATTCACTTGCTCG

>FM889140.1 FM889140 Jatropha curcas embryo 35-55 (DAF) Jatropha curcas cDNA clone rjcfea0_002724, mRNA sequence

CAAGAAACAATTCTGTTGTGTCATGACATTTTCTTGAACAAGGCAAATTAGGCAGCAAAAAGTTTACACTCAGAAATTTA

CAAAAACCACAAGTTCAATAAAGATCACAGTAGTTGATCGAGATATGGATCCCGTTGAGCTTTTCTTCCTCCTTGATTTC

CTGAACCACTAAACAAGGAAGCACCCCATGCCAACCATCCAGAGTTGATCGTCCAGGCGAACCTCTTTTCTCAACTAGCA

GAGGCGAGGAAGTTTTCATACATCGGCGTCACCGTACGTACGAACCATGATCGGAGAACCCAATCAGTTGTGCTCCTTCC

TTTTCTTAGATTAGATCGTGTCTTCATTGACTGAGTTGCCGGTGAACTGAATCGTCTATCTAAATGAAAGCTAGAAGGGA

ACAAACATTGGCACGAGTTCGAACAGAAGACTCCCGAAGAAGACTTTCGGACAAAAAGCATAATCCAAAGACGAGACATT

TAGTAACTCTACAGGTACCACCAGTCATTTCATTTCT

>JK612446.1 JCF25-153 Jatropha curcas, immature Seed cDNA subtraction library Jatropha curcas cDNA, mRNA sequence

TAGCGTGGGCGCGGCCGAGGTACTAGTGGGAGGTGTGAGAGTCACTTTACTCGCTTAAGCCGAATCACTTTTTCACTCAC

AGCTTCCCATGAGCGACTTTCCTTTTATTCTGTCCTTTCTTTCTAGCTACTTTCATAAGGGGCATGAGCTACTCTCTTCT

AGCCTTTGCTTTGAAAGCAGTTCCCAGTCGAAGGTCAAATGAACAGGAGCCAGGAGCTAGCCTTATTCCGAAAACACCGA

GTCTTGCTCCTTCTTTTTATGCGGAATCCTACTAATATAAGTTACATCATTTCCTTTCTAAAGCCCATACTAAAGCAAAA

GCACCAAGAGCGACAACGGAGCCATTCTCTTCCCTACTTTGTTCACTCGGAGTTCTGTCAGTCTAGTCTGGGTCTGCTTT

TCCAATGCTTGAACTGATTCACGCTCAAGCTGCTAGTCGAACTTCAGCAGAAGGGAGCAGCGTATAAAGGAAGTTCATAC

AAGGAGTCAAGCAGGTCATTTGCTTTTGCTTTCAATGCCAGCTAGATTGTTAAAAATAAAGAGTAAGTCGTCTTCGCCTT

TTCATCTCTCGTTCCTTACCTCGGTCCCGACGACTTTGCTTCAAAATGAAAAGGAGCCCTTTCTCTAGAATACGAGAGGA

ATAAATCCAATGAAAGAGATGAAACTAAAGCAAGAACCCTCTAAGTCACCTTTCCCCTCAACTTTATATATAGCATCCCT

CCTTATAGATTCTCATCAAAAGAAAAAGGATAAGGAACGTAAGCTTGACGGTAAAATAGTTGAAAGTGATTGAGTGCGCA

TATGAGCTATCAGTCATCAAGCAATCTAGTCATTCTTGCTTGCGATTGACTATCCTTTTTGGGTGGTCTGGAAGTTCCTT

AAAAGACG

>FM892569.1 FM892569 Jatropha curcas embryo 56-70 (DAF) Jatropha curcas cDNA clone rjcaeb0_003158, mRNA sequence

CTTCTCTCGGCATCGGCCACCAAAGATGCAGATCTTCGTGAAAACCCTAACGGGGTAAGACCATTACCCTCGAGGTTGAG

TCTTCCGATACCATCGACAATGTGAAAGCCAAGATCCAAGACAAGGAAGGAATTCCACCGGACCAGCAGCGTCTAATCTT

CGCT

>GT976553.1 GJCCJC2079E02.b Jatropha curcas L. developing seeds (mixed stages) Jatropha curcas cDNA clone GJCCJC2079E02 similar to UBQ3 (POLYUBIQUITIN 3); protein binding, mRNA sequence

AACACAACCAATATAATAAAATTGGATTTCAGGCCAAGCCATGTGAAGACATTCAAGATGCAAATCTTTGTGAAAACCCT

TACTGGCAAGACAATCACCCTTGAGGTGGAAAGCTCCGACACCATTGACAATGTCAAAGCAAAGATCCAGGACAAAGAGG

GAATTCCCCCACACCAGCAGAGGCTTATCTTTGCTGGTAAGCAACTCGAGGACGGGCGTACACTTGCAGACTACACTATC

CAAAATGAATCCACACTCCATTTGATGCTGCGATTGAGGGGTGGTATGCAGATATTTGTCAAGACTCTCACTGGCAAGAC

TATCACTTTGGAAGTTGAGAGTTCGGATACCATCGATAATGTCAAGGCCAAGATACAAGACAAGGAGGGTATTCCACCAG

ATCAGCAAAGATTGATTTGTGCTGGGAGACAGCTAGAGGATGGTCGCACACTTGCTGACTATAATATTCAGAAGGAGACT

ACGCTTCACTTGGAGCTCCGACTCCGTGATGATATGCAAATCTTTGTGATAACCTTAACTGGCAAGACCATCACCTT

>GW881862.1 JC001749 Seed specific Normalized cDNA library from Jatropha curcas L. Jatropha curcas cDNA clone N03201 5' similar to Unknown protein, mRNA sequence

GGAATTTCCTCAACATCTATTTCTTGAAATCTTCTTTAAGTTCAAAAAAATCTGCTACCAGAACCATCTTGAAAGAAAAG

AGCAAGTTCAATTAGCCTCTTACCTGTACTATCGATTAAATGCTTTGAAGTAGTCCCAATATCAGTTCTTTTCAGGAGTT

CAAGAGCCCATTCACTTTGAGAACCTAAAGCTTCCATCAAGATGATCCATTGCATTCCATTTCTGATTTCAAGATCAACT

ATATGTATTTTTTTAACCTGCCACGTGCTCTAGAATAGCCTGAATTACTGAAAAAATGGGCAATTCGACAGAAGGGAATT

GCTTGGTGATATGCAATGGCGGGTGAGCTTAGGGTTGTTATTGCTTCATGCATATCTAACGACTGCTTTTTCACCACGAT

TCCTAATTGTGCATCTATTCTCTCTTGAAGAGCTTCAGGGAAGTAATAAACGACTCGTTGAACTGGATTTAACGGAAAAA

GGCCATGAATGTTTTTTAGCAATCTCCCCCCACTGTAAAACTGTAGATTGCCCACTTTCTCAGCAGAAGCCAGGAGGCAT

TGCACGAGTTCTATGTCTCTGATTGCTTCATCA

>GT980212.1 JGCCJG2040B07.b1 Jatropha curcas L. germinating seeds (mixed stages) Jatropha curcas cDNA clone JGCCJG2040B07 similar to 60S ribosomal protein L13A, mRNA sequence

GAAAGAGAGTGAGGGAGAAAGTGTTTGCAAAAATGGTGTCCGGATCGGGGATCTGCGCGAAGAAGGTGGTGGTGGATGCG

CGGCACCACATGTTGGGGCGGCTTGCGTCGGTGGTGGCGAAGGAGTTGCTTAACGGACAAAAAGTGGTGGTGGTGAGGTG

CGAGGAGATATGCATGTCCGGTGGATTGGTAAGGCAGAAGATGAAATACATGAGATTTCTTCGCAAACGCATAACACTAA

GCCCTCTCATGGACCCATTCACTTTCGCAATCCTGCTAAGATCTTCTGGCGTACTGTCCGTGGAATGATTCCACACAAGA

CTAAGCGAGGGGAAGCTGCACTTGCACGATTGAAGGCCTATGAGGGTGTCCCACCTCCATATGACAAAACGAAAAGGATG

GTTATCCCTGATGCTCTCAAGGTCTTGAGACTTCAGAAGGGACACAAGTATTGCCTGTTGGGCCGCCTGTCATCTGATGT

TGGATGGAACCACTATGACACCATCAGGGAGTTGGAGAAGAAAAAAAAGGGAAGGTCTCCAATCCTGTATGAGAGGAAGA

AGCAATTGAACA

>GW879586.1 JC001761 Seed specific Normalized cDNA library from Jatropha curcas L. Jatropha curcas cDNA clone N03217 5' similar to Unknown protein, mRNA sequence

GGATTGCACTGTCCTTTCAGTTGTTTACGAAGACATCAAGAGTAAAAGGCTCGAGCCTCTGCCTCTGCCTCTCTCCCCCT

CCCTATTCTCTCTGGCCATCCTTCGTTGACCTACAATTATTTTTTCAACAAGACATAATTTACTGATACTCAGTTCAAGA

TCTATTCACTACAAGCCTTGATTTCTCAAATCCAAGATCTATACCAAATAAGAAACGACGCCACGTGGGACATCGAGCTC

ACAACTGGACTAGTTCAGGTTTTGGGTGATTATGCTTATTTTTTTCAGATCTGGGGTTTATAGTATTAGCTACTCTGTTG

CTTTTTGAATTCGTTTGTTGGAAATGTCAATTATACCTAAGAATGACGACTCCATTCAAGTCCGAGAGGTGTGGAATGAC

AATCTCGAGGAAGAATTTGCTTTGATTCGTGAAATTGTTGATCAATTCAATTATGTAGCTATGGATACTGAGTTCCCGGG

CGTGGTTCTGCGTCCGGTGGGAAATTTTAAGAACATAAATGACTATAACTATCAGACTTTGAAAGATAACGTTGATATGT

TGAAATTGATCCAATTGGGTCTCACTTTCTCTGACGAGAATGGAATTTGCCCACTTGTGGAACCGATAAGTTTTGCATTT

GGCAATTCAATTTTCGTGAATTCAATATCAGCGAGGACATATTTTGCTAGTGACTCTATTGAGCTGTT

>FM890898.1 FM890898 Jatropha curcas embryo 56-70 (DAF) Jatropha curcas cDNA clone rjcaeb0_000857, mRNA sequence

ATTCCTATGACAAGAGAGGTTTGCTGAATCAAAAAAGGAATTGGATGCTCTGCTTTCGGATGAAGCTCTTGCCAATGTTC

CATTTCTCATCTTGGGGAACAAAATTGACATACCTTATGCTGCTTCAGAGGATGAGTTGCGATACCACCTTGGCCTAACA

AACTTCACCACCGGCAAGGGGAAGGTAAACCTAGCTGACACAAATGTACGCCCCCTAGAGGTGTTTATGTGCAGTATCGT

ACGCAAAATGGGATACGGTGATGGTTTCAAGTGGCTCTCTCAGTACATTAACTGAGGTGATTGTCAGGAAGATGTTTGGC

AGAACCATGAAGACCTCACCCAAGCACGCCATCTCCTACCTGAAAAGTTGTATTTACCCCCCTGTTTAAATTGGATGTAT

TGTGATCTGCATCTGTGTGGAAGGAGGTATTAATATCACTGCTCTGTAAAATTTACTTGTGGCAGCAAGTTGTTCTTTGC

TCTTGTAGTTTGTCAATTGACTTTTGCCATCTCTTGCTGTAATCGTAATGTGTAGAGCC

>GW879245.1 JC003803 Seed specific Normalized cDNA library from Jatropha curcas L. Jatropha curcas cDNA clone N07004 5' similar to Respiratory burst oxidase, mRNA sequence

GGATATATAACAAACAAACACTTCCCCCTGCAAAAGAACAAGAAACAAAACAAAACACAAAACAAAATTATAGTTTTCAT

TTGCTTTACAAATCTAGTCTGCAAGAATTCAGCTTTTAAAGGGCCGTCTTCAACTTAGATTCAAGAAATGGGAGAAGAAG

ATCCCCATCACCAGCATCATCATTCTGATACAGAGCTTTTGTCAGGCGAAAAGGTACCTTACAGTGGACCTTTAAGTGGA

CCTCTTAACAAAAGAGGAACAAGAAAGAGTGCAAGGTTTAACATTCCAGAATCTAGTTCATCTCAAGATGAACAATATGT

GGAGGTTACACTCGATGTTCGTGATGATTCTGTAGCAGTCCACAGTGTTAAAGCAGCAAATGGTGTTGCAGAAGATCCCG

AAGTGACATTACTAGCTAAAGGACTAAAAAAAAGATCAGCATCTAAATTGTTAGAAATGCTTCTGCAAAAATCAGACAGG

AAATAAAGCGTTTGGCTTCATTTTCAAAAAGGCAGCCTCCTAGTCGTCTTGACAGAACAAAAACTGCGGCTGCACATGCT

TTGAAAGGACTTGAGTTCATTAGCAAGACTGATGGAGGTGCA

>JK613094.1 JCF22-94 Jatropha curcas, immature Seed cDNA subtraction library Jatropha curcas cDNA, mRNA sequence

TTCCAGGGGCCCCCCCGACGGGCATGTTCTCGCTTTATGGGGGGGATTGCAACTTCAATGTGGTCTGGGGAAAAGAACAT

CTGCCTTGCTGGTCCAAGCCGGGATTGTGTACAATGATAATATAAAAATCCATGGTTTCTGACTTCCACCCCTAAATTCT

CTGCCTGGGGAAACCCCCTCCTCCTGCGGAGGTTAAATTGGTTGATCTTTAAAATCATGATCCACTGCTCATTCCCGGAT

CTTGTGAGTTTGATATGAATATATTGAAATACATGCCTTGATTGATTTTCAATGGGCAAGGTCTGTGTGGGATGAAAAAG

AAGGTTAACGTTTCTGTAATCTCCTCTCTTCTGAATGGAGACTGTGTGTGAAGGTGAACTGTGAGAATATATTTCTTTTC

CTTTGGGATGCATGTTTCTGGGAAAAAAAAAAAAAAAAAGCTTGTACCTGCCCGGGCGGCCCCTCATCATCCATTCTCAA

GGTACCTTTCTCGCTTACACTGGTAATCAAAGACTTGAGGTACCTCTGCCGCAACCACTGCTAATCTCCATGTTATCCCG

AGTGCAGCAGATCCCCCTTTCTGCAGCTCGCTTAACAACGAAGAGGCCCGCAACGATCTCCCTTCCCCCCACTTGCTCGC

CCTGCATGGCATATGCATTTCGTCAGCTGGAATATTTTGTTAAATTCCCGCTTCAGATTTTCCTTACCCACCTCAGTTTT

TTACTCATAGGGCATAGTAGTCGTAATCCCTTATATTCATAGATACACGTAGATTTGTTGTAGAGTTAGCTTCATTTTTT

ACCAGTAGTCCACGTATCAGGCAACGTGCACTCACGTCAAGAGATCGACGACGATCTGTCTAGACCGATGTCCAGATTCG

TACCGATGCTCCTATTCAGGTATCTCGTTG

>JK611457.1 JCF4139 Jatropha curcas, immature Seed cDNA subtraction library Jatropha curcas cDNA similar to protein binding / ubiquitin-protein ligase, mRNA sequence

TACCGGGGGCGCCGGGGAAGGTGTTAAATTTGCTCATAAATGGAGACTTTTTAAATGTCCCTTTCAACTGGGTATTGGTT

TTTTTTTTTTTTTTTTTTAATGAAGTGTATCTGTATGTTAAAAAACTTTATCTATGTATATGTTGCCCTTTTTTTTTTTG

CAAAAAAAACCAAAAACCAACCCTGTAATGTGGCTTTCCCTCCCTGTGGGAAATAAACTTGGTATATACATACAATGAAG

CTCGAACATAATTGCCAAGGGGCAAAATTGCCATGAGAATTCCGAGTAAAGCCAGGAATTGACCATACTCTGATTACTGG

ACAGCTTCCCCCCTTCCGATACTTGATATACAAAATGACAGGCCGACTACTTGGACCACACTGAACCATTTGACTACCTT

ACTGGTTTCAGATTTGGAGAAGATTGTTGACAGTGACAGTTACAGAATTTGTTCGCTGCAAGTCCCGGAGTATCTTTTCA

TCTAGGATATGAGCCAGAACCTCGCGTACCTCGGCCGCGACCACGCTAATCTAGGTGCGTGCGTTATGTCCCGAGCTCGA

ATTAACTGGCCGTCGTTAACGACGACTTGACTGGCAAAGCCCTGAAATTACCCAACTAGTCGACGTGCACTACATCCACC

TTTCGCCAGATGGAGTATATCGGAGTCGACCTGCACGATGCCCTTACCCAGCAGCTGCACAGGCATGCACCGCAATATGA

ATGCAGGATAACAGTCTGACTAGACTCGCAGTGATGTTAGATAGTCAGCTCTGTCTACGATACGTCGAATCGGACAGATA

CCGTTTGAGAGCATAACAGCAATAGACG

>FM891664.1 FM891664 Jatropha curcas embryo 56-70 (DAF) Jatropha curcas cDNA clone rjcaeb0_002022, mRNA sequence

AAAGAATATGTAAGGATGTAATAATATGAACTTAGCTGATATCTGTAAAGTTCTAGAACTCAGCATTGATCACAACAAAG

AAGATGAATCCATAACAAACATTTTCTGATTGCTAAATAACCTCATCATTGTCAAAATTTAAAGAGCGCACTACAAGTAA

TTCAGACGAAAAACATTCAACAAAAGATGTTATAGCTCTCAGATACTTTTATGCCTCGGGTTACTGGAAACTCA

>GO246852.1 JcrME_RL0396 Expressed sequence tags from Jatropha curcas root cDNA library Jatropha curcas cDNA, mRNA sequence

GGGCACCACTTCAGGCGAAAAAACTCTCTCTCTCTCTCTCTCTCTCTCTCACTCTGTGTCACCATCTCCGATCAACCGAA

AATGAAGAAATTTTCTTACTCAGCCCTATCGCTCGTGGTGGTAGTGGTGGTGGTGATGGCATTTGAGGTGCGCTTGTCAA

ATGCTGTGACCTGTAGCCCTCTGGCGCTGAGTCCATGCCTGCCGGCGATTAACTCATCGTCCCCGCCGTCGAATGATTGC

TGCGTCAAGTTAAGAGAGCAAAAGCCTTGTCTTTGCGGATACTTGAAGAATCCAAGCCTGAAACAGTATGTGTCTTCTCC

TGGTGCAAGAAGAGTTGCTAACAGCTGTGGTGTTCCTATCCCAACCTGTTAATTCATTAAATTAAACTAATGAATAATTA

AGCAGAGCCAAAATAAATAATTATAAATATGTATTGGAAAATAAAATCTTTGAGGGACAAATCACATATAGTGATTGGAG

GACTTTATGTAGTTGAATCTCTACTGTTATATTAATAAATTATAGCGGAAATATTTGGCTA

>FM889134.1 FM889134 Jatropha curcas embryo 35-55 (DAF) Jatropha curcas cDNA clone rjcfea0_002716, mRNA sequence

CACACTCACGCTCACTCTTTCTCTGTCTCTCTCTTTTTCTCTCTCTAGAAAATGCAGGAGGGCGGATGGCAGTTGAAGAG

CCTGTTAGGCTCTCCTCAGTTCTATCTTCTTCAAAACCTAGCTATTTACCTTCACTAACAAAAATAGTTGGAACACTTGG

ACCTAATTCGCAGTCAGTAGAAACCATTGAGGCATGCCTTAATGCAGGAATGTCAGTTGCAATGTTTGATTTTGCATGGT

TGGATGTTGACTACCATCAACAAACTCTTCAAAATCTCAGGATAGCTATGAAAAATACTAAGAAATTATGTGCTGTTATG

TTGGACACTGTTGGCCCTGAACTTCAAATCTGTAATCAGACTGGAAAACCAATCGAGCTGAAGGCTGATGACCGTT

>GW881059.1 JC006294 Seed specific Normalized cDNA library from Jatropha curcas L. Jatropha curcas cDNA clone N10732 5' similar to Unknown protein, mRNA sequence

GGGGGACCCCTGCAAAAAAAGAGGAGCGAGAGTGCCGGTAACTGCCTTCAGAAAATTAATTAGAATGTCTAGCGAGAGTG

AAGCTGCGCACGCTATTTCACCTGTTGCGGACTCCAACACGACCACCGGCGACGATGACATGCAACTCCTGAATGAAGCT

GTTCGTTCTTTGCCTTCTCACGCCGTGAAGGACCTTCTGTCCATTGGGGTATGCACCAGATGCATCTTTCGGCTATTTGG

AATCCGTGGACGTATTTATTCTTCCTCTTCTCTCTTGCCATCAATAATTTGTAGTATTCTTGGCGAGCCAACATGTACAG

TTAAGCATGCAGTGAACTCCAGTTTGAAAGTCGATGAAGATGTTAAGGATCCTTCAATATTTAAGGAATCAGAAGAAACT

GAGCCAGAATTATGCCGCGTTTGCTTAGGCATCTTGCAGTTCATTTACTGTGATGATAAAGGAATAACAGTAAAAAAGAA

CAGTGCTATTAATTTGGCTTTATCAATTGCTGAGCTGATAAAGCGAGAGGGTCATCAGATTGATAGCTTTTCTCTTGAAG

TCTCCATACCACACATTATCCTTGAAAACGAACAAATTGTTCATTTGTACATGAAAAAGAAGTATAGATCAGAACTTTGG

TTCCAAGAAAGACTTTCTAAATGCATTTCTGCAAAGGA

>FM888353.1 FM888353 Jatropha curcas embryo 35-55 (DAF) Jatropha curcas cDNA clone rjcfea0_001751, mRNA sequence

TCGAAGAAACTTTTGTGCTCATTCAATTCAATCGTTGAAGAGCACATGGACATTGATATCGAACACATGATGGAATCCTC

AACGCTGCCAGGGAGCTCAGATGGGTCTAACCCAAACAGCTCAAGTCTTGTAT

>GW878288.1 JC002664 Seed specific Normalized cDNA library from Jatropha curcas L. Jatropha curcas cDNA clone N05053 5' similar to Unknown protein, mRNA sequence

GGAGTGCAACGAGTAACTTCGTTCCAAGAACTCTTTTTGGATTTTCTGTTGAGTTCAGAGTATATGGTAATTCTGGGTTT

TGGGTGGTTCTTGAATTTCTTCCTTTGGTTTTTTCATAGTTTGCTTTCTTGTACTGTTATTTTCTTTTCTGGTTGGACTC

TTCAGGTGAGAATTCTGTGTCTTCTTTCTTTGAATAGAATTTCAGTTCAGCATAAAATAAACTTTTTTTCCCTGTTTCTA

TCACTCTAAAGTCATTTCTTGTGTTTCTGGTATTGAATTTTATTGGGTTTGAGCAAAAATAGTCATTTGCATCTAAAAAG

GTTTCTGGGTATCAGTTAGTTGTTAATTCTGATTAGCAAATGTTTGTGTTCTTGCATAACAGAGTGGAAGCTTAAGGGCT

AAAGTTTTTACCTTTATTTTTTGGGAATTAATAGCAATTCTTTACAATGCTTTCCACAAAATCTGAATCTGATATTACAA

GTTTAGCCCCATCATCACCTTCAAGGTCTCCAAAGCGCCCTGTATATTATGTGCAAAGCCCTTCAAGGGATTCACATGAT

GGGGACAAGTCCTCTTCAATGCAACC

>GW617233.1 Jc2-020-E12-M13F.E12.ab1 Jatropha curcas flower and seed Jatropha curcas cDNA, mRNA sequence

TTTCTTCTTCACGATTAAATGTGGAAAATTAATGAGATTCCTAACTGCGCGCTTTCATTTGGTTAATCTTTATAGCAGTG

GATCAATAGAAATTTAGAAACAAATGAAACGATGGCTGTCGGTGTCGGATACTGCCCCCACAGCCACAGGCTCTCGAATC

TAATAGAGTCAGGGTGATGAAGCGGATGGTTCTGCGAGGCTGAGACTAGTCTAACCACTGGTTTGTTTGTCTGCTGGTAT

TGGCCCATTTTTTCCAATTTCTTATCTTCAGTCTTCTCACTTGCCTTCTATGCGGCTTCCCTGGAGCCCAACACCTACCA

TTTTCATCCCCCTTAGCTACTATTAGTCGTTCTACTACCCATCAAATCCCACCAAAAAAAAAAAAATCTCCTTGATTCGT

TTAAATATCATTCCCACTCGAACCACAGATAGCTCTTATCAGTGTACGTTCTGTCTGGCCAGTGAGCTTTTGCTATTGCT

TGATTCGCTGCTTGTCATGGGCTGCCTATCCAATAAGTTCTTCAGACTTAAATTCCGGCACAGATCTACAGTAAGTGGTG

TTGTGGCTAATGGTGATCATATAAAGGAAAAGGATGATAATGATGATGATGATTA

>GW877857.1 JC003459 Seed specific Normalized cDNA library from Jatropha curcas L. Jatropha curcas cDNA clone N06468 5' similar to Unknown protein, mRNA sequence

GGCGTTTTGCAGAGCAGTCACCGAGCATACAATTTCTTCCATTTCCCTTTGTTTTTCTTCTTTCGCCAGGTTGATTGGCA

TTGACCAACTGATGCTTTTCCGGCTTGTGTAAGGGAATTCCTGCTATTTGTTATTTATTCATGAATCCGAGATTCCTACA

TGTTCTTGTATTTTGAACATTGGACTTAGTTCCAAAGGAATTGCAATGTTGCATTAATTGGGTATGTTGGACTGATTTGT

TACGGATAATCTCCTGTTATAAGCTTTATAGAGGTTTAAACCAACACAATTGATTTGGTTTAGGGTCATAGAAAATAGCT

AAACGGGGTCGCTTTGTTGCGTGGCTGCAAGGCCACATGGGTCAAATACGACCCGTGGGGACTGGCCTATGGGTCAGCCT

GAGCCTTACTGGAGAACTAATATAAGCTTCTCACCACCCCCATCGAGATGGGATTTTAGTTTCTCATCTGAAGAGCTACT

GAATGATTCGCAAGATGGTAACCTGTTGCTTGGGTCCTCAACATCATCAAACAGTAAAGAAAGTGGATGGATGAGAGGAA

ATCATCTTTACAATCATCACTGTTCTGCATCTGATGGTGTGGGCTTGTTTCTTAGTAGTCCTCATCTTTCTCCAGGTCCT

CAATGGACACCTCCAGCAATACAGGAAATTAATGTTGATGATTATGACACTGCAACAAGGAGAGGTCAAGTTCTGCAGCA

TCACCCTCCACACCTAT

>FM891475.1 FM891475 Jatropha curcas embryo 56-70 (DAF) Jatropha curcas cDNA clone rjcaeb0_001775, mRNA sequence

GGGAGAGAAAAGATTACTTCGTCTCATTGTTAAAGAGAATGCTATAACATGCAAAACAGGTTACAGGTACATGTAGACTG

AACATAAAACTCGGTGCCATTTCTATGTCCGCTTCGAGAAGACACTTGTTATAAGTACATACGCAAATACACTCCTACAC

ACAAGGGGAAGTAACAGAACAAAACAAAGCAAACATCATATACAAACAAACACCATTCATATTAGTAACTCTTCATTAGT

GTATCAGTAGTTAATGGTCTCATTAAGCCTAGCAGCTGTCGTCATTGACACCGCAACACCACCTGGATGCGTGTTTAAAT

TCGGATTGTTCCTCAACTCTGCACTAACCACCCCTTCAGCATCTGCCCTTGTCACGACCTTATCCGCCGCCAATTTCGAA

GTTGCACCCGATAGAACGTCGGCGAGTTTATCTTTTACTCAGCGCGATTCGTAACG

>JK613005.1 JCF22-3 Jatropha curcas, immature Seed cDNA subtraction library Jatropha curcas cDNA similar to thioredoxin h, mRNA sequence

GCCGCCGCTGCCGAAGACAACTCGGCCGATCACTCTGGAGTCACTACTTTCCACTCACCTGAGCGATGGCAGCTCCACTT

CAATTCCGTCAAAGATTCATCGCAGCTAATGGTGATAGATTTTGCCGCTTCTTGGTGCGGGCCTTGTAAGCTAATGGAAC

CAGAAGTCAAAGGCATGGCTGTTAAGTTTACAGATGTGCAGTTCGTGAAGATTGATGTGGATGAATTGTCTGATGTGGCT

CAGGAATTTGGAGT

>JK317822.1 JCST593 Jatropha curcas L. seed cDNA library Jatropha curcas cDNA 5', mRNA sequence

TGTCATCTATGTCTGGTGATAGGACTAATGCGAGTGCGGAAGCGTATTATATCTGGTCTGCCTGGGGTGGACGCCATCAA

GATGACCTCCGATTTAGGGAGAGCGCCACTGGCGAGTCGGGGCGCTCGTGTTACCTCGACACTGACTGAGTGCGTCCAAA

ATGAGGGCAGGTGATGTACGACGCGGGTGTTTGCGGCGGTGGGGTAACACTATAAGGGGGCCAGGGCTGAACAGCTATAT

CATTGCTGGGCCCTCTGCCGTCGCGCGATACCTGAAGTCGAAGTCGCTGGGCAACTTAATGATAAACGGAAAGCGACCGC

AAGTTGGATTAGAGCAATGGAGGTTGCAAAGGCCAGGCAACTTGCATGGGTCTTAGTACGGTTGTGGGAGGCCCCCCGAT

CGACGAGACACCAGCCAAACTTTTACGTAGAAGAGGAGCATGAGAGGAAAGACGATGGAAATGGTTAGCGCAGGATGTGG

GCCGTGCAGCGGTAACGGAATTGGCACCAGGACCATCAAGGGCGAACCAATGGTGGCCAGGAGACCCAAATAATATCGTT

TATCTAGGTACAAGTACTGCGTAGTGTCACGGCAGCAGCCGCGGACGACGCGCGTTCAGTAGAGGCGATCTCGGAGAGGG

TAGTAGTGAAAGAAGAGGAAGCTCTCGAGGTCATAGCGAGTATAGCACTGCGAGACGACAATCATGGGGCGAAGGCAATT

TTTAGCTGACTGGTATGTCTACTCTTTAGTAGTCGCTATCGACTGATCAATTCGNCTGCTGTCCGTTTTAGAATGACAGT

GGGTGCCTGTCTTACGTTAGGTANCTGGCATGCAGTGTGTAGATAGGGAGGAGGAGGATGGCNATTGNNAGGTAGGT

>FM889952.1 FM889952 Jatropha curcas embryo 35-55 (DAF) Jatropha curcas cDNA clone rjcfea0_003714, mRNA sequence

TGCACCGTTTGGGCTCAGAGCTCTACCTTTCCTCAGTTTACAGCTGAAGAAGTAACTGGAATTATGAATGATTTTGCTGA

ACCTGGAACTCTTGCACCAACTGGCCTCTTTCTTGGTGGCACAAAGTACATGGTGATTCAGGGTGAACCAGGGGCTGTTA

TACGAGGAAAGAAGGGCTCTGGCGGAGTAACTATCGAGAAGACCAATCAGGCTTTGATAATTGGCGTATATGATGAGCCT

TTAACTCCTGGTCAATGCAATATGATTGTGGAAAGGCTTGGGGATTATCTAATTGATCAGGGTCTTTAGTTTCTTAGGTA

TGCCTTTCTATTTCTTTTATTCTTTGAGAAAAATGCTTTTTAGGTTTGGAGGCTTGCATCTACCTACATGGGTTGGATAT

TTTTACAGTTGAAGATGTTGGGATCTACAAGCACTACAAAACTTGTAAACTTGGTTTCTGCTGTATTGAGTATTTGTATT

TGTGA

>GT979069.1 JGCCJG2028B02.b Jatropha curcas L. germinating seeds (mixed stages) Jatropha curcas cDNA clone JGCCJG2028B02 similar to mitochondrial substrate carrier family protein, mRNA sequence

GAGAAAATGGGAACTTTTTGTGCTGGTGAAAATGGATGTATGAGGTGAGAGAGCAAGAAAGTGGGTTTTCTTCTTATGTT

AACAGAAGGGATAAGCAACTGAGCAAAGAAGACGGCCGCGGTTATGGTCGAAGCATCATTGAGAAATGGAGAAGAGTATA

AAGCGGCTGCTGGATATTATGGGCTTTGCGCTGTTAGTGGAATGCTCAGTGCTGGCACAACCCATCTTGCAATCACTCCT

CTTGATGTCTTGAAAGTCAATATGCAGGTGAATCCGGTCAAGTACAACAGCATATATGCTTGCTTTACCACTCTATTGAG

AGAGCAAGGTCCTTCAGTGTTTTGGAGAGGATGGGCAGGCAAGTTATTTGGTTATGGTGTTCAAGGTGGTTGCAGATTTG

GTTTGTATGAATACTTCAAGAATCTTTACTCAAATGTCTTGGTTGACCGCAACAGGAGTTTTGTCTTCTTTATGAGTAGT

GCTTCAGCTGAAATGTTTGC

>GW611196.1 Jc1-003-A03-M13F.A03.ab1 Jatropha curcas flower and seed Jatropha curcas cDNA, mRNA sequence

AAAAGAAGAAAAAGGAAAAGGAAAAGGAAAGAGCTTGCTGACCGTATCTACTGGCTATGTTTTGATCATTTATGGAAGTG

AAGCCTTCAGTTTTAGATTTTTGCTGCATGCTGCTTAATTCATGGAATTTGGGATGGGAACCTCACTTCTGCACTTGGAT

GATTGGTACATGTATTATATTAATGTATCATTAGGCTTCATTTCATTCGTCCAGTAAATAATCACATGGAATTATAAAGA

GATTGTGGTCAAAATGGCTGCAAAAGAGACTTCACTTGATAGAGTACGGAAGAATTTGCAGGGCTTTATGACCATTTTAA

AATATGCAGCTTGTGAATTGCTTTTGACTTTTTTGCTGCTTATTGATGCTGTGTTCTCATATCTGCTGACAACATTTGCG

CGTCATTGCAAACTACAAATACCTTGCATTTTGTGTTCTAGGCTTGATCATATATTGGGTAATGAAAAACCTGGATTTTA

TTGTAATCTACTGTGCAGAAACCATAGATCAGAAATATCATACTTATTTTCTTGTTGCATACATGGCAAGCTTGCTGATG

GCCGTGGCATGTGTGAGGAATGTCTCTTGTCATTTACCATGAAAACCAAATCAAACACAGATATGAACAG

>JK613442.1 JCF19-39 Jatropha curcas, immature Seed cDNA subtraction library Jatropha curcas cDNA, mRNA sequence

ACTGATCTAGATTTAGATCTAGTAAGAGGTTCACCCTTTACTTTTTAGTTCTCAACTTTTTCAGCTTGAGCATGGATCTC

ATGATTCTGATTTTCCTCAACCGGAATGAGGGTGCCCCCCTTACTAGCTAGAAAGAGACAACCAAGCTGCTTCAGCCCTC

CCGATTATTAGTCGGGGCTCGCTCAAAATATGTTCCTTATCTGATAACAAAGCAGATTGGTCGAGCCCTTTAAGTAAGCA

AAAGTAAGCAATTTCTTACCATCTGAAGGAAGTAAGATCAAAAGAAGAAGAAGCTTTTCGTCTCCTTGATAGTATCAGTT

TTAACATTTTCTGACCCAAAATAACAATCTCTATCGCTTCTGATATTCAAGAGATTCATTCTTTGCTGATCTGCTCACCT

ACAATGCTTTGGGCCCAGTCTTCTCTATGCATGGCATTTAAAGAGAGAAAACTGCAAGAAATCTCTTTGAATTTCTATGA

ACAGAATCGGATTTCTTCGAAGAGATATCTGCGAATGTCTCTTCTTTTGGAACGATATCAGAATGATTGTTTCAGAGATA

TGGCAATGAGACTGCTGTAAAGGAGGATTACTAGCATTGATATCATCATGAGTGGATTGATGAATCTATGTATTGAGAGC

AGATGGAGCACGTGATCGTAGATCAAATCAGTGCTCGAAATCTCATACAATTATTCTGCTGGGAGTGCTTATTCACTCAT

TGATTGCCTTTTTCGTGAGAATCTGAGCCTTGAGTTTGTATTACACAATAGGCAGCCTATTGAATGTGTACGTCTTTGTG

ACGCTTTTCTGAACCCTTTTTTTTTTTGATGG

>GW611225.1 Jc1-003-C12-M13F.C12.ab1 Jatropha curcas flower and seed Jatropha curcas cDNA, mRNA sequence

GAGCTTATCTTCTGTAAAGAAAATTCAGGAGAATGGGGATATATTCCAACATGGAAAACCCATGGCAAGAAAAGGTAAGG

AGAAGGTAAAGGAAGAGAAATCCGAGGTGGGAAAGCAGGTGTGTGGAATATGCCTATCAGAAGAGGATAAAAGAAGATTG

AGGGGAACACTGAATTGCTGCAGTCACTATTTCTGCTTCACTTGCATCATGGAGTGGTCAAAAGTGGAGTCACGTTGCCC

TCTGTGCAAGCAGAGGTTTAATACAATAACCAAGAATGGAAGAGCAGCTGTGGGAGTGGATCTTAGAAATGTGGTGATAC

AAGTTCCCAAGCGCGATCAGGTCTATCAACCATCAGAGGAAGAAATCCGGAGCTTTATTGATCCATATGAAAATGTGATT

TGTACAGAATGCCATGAAGGTGGGGATGATGGCCTCATGTTACTATGCGATCTCTGTGATTCACCCGCACATACCTACTG

TGTTGGTCTTGGGCGGCAGGTACCTGAAGGCAATTGGTATTGTGATGGCTGTCGTCCTGTTGCTCTTGGATCCTCGAGCT

CCCTACCTCAGGATACTTTGCCTGATCAAAGGACAGCAAACAACATGTTTAATAGACCATCTCCTGTTGTGAATAATGGT

GAAGGCATAGACCTGACTTTAGAGCCTTCACCTCGGCTAGCAT

>GT971586.1 GJCCJC2022C01.b Jatropha curcas L. developing seeds (mixed stages) Jatropha curcas cDNA clone GJCCJC2022C01 similar to 11S globulin seed storage protein 2, mRNA sequence

CATCGGAGGATCAGTTTCAGTGTGCCGGTGTTGCCGCTTATAGAGATAGAATCCAGCCCAACTCTCTCTCCTTGCCCAAG

TTTTTACCTGCTCCTCACTTGGTTTATGTCATCCAAGGTAGAGGAGTGCTTGGACTTAACTACCCTAACGGATGCCCCCA

AACATTCCACGCAGAGCAACAATCGAGAGGAGGAGAAAGCCAAAAAGGAGCAAGACACGACCCACACCAGAAAAATTCTC

AGGATTCGTCGTGGAGACATCATTGCCATACCACATGGCGCAATTGATTGGTGCTACAATGATGGTAACGAGGAGCTTGT

AACCGTCACTGTTGTCCATCTTAACAACAGGCACAACCAGCTCGACCAGTACTTGAAGGCATTTTTGCTCGGAAGCGGCC

AAT

>FM895466.1 FM895466 Jatropha curcas embryo 71-95 (DAF) Jatropha curcas cDNA clone rjcpga0_002390, mRNA sequence

TTTTTTTTTTTAATGAGAAAAAAGAAAGTAAATTTTATTCACAAACTAAGAGAAACTCATTAACGACTCTGAGGATAACT

AGATAAACGAAAAGGGTAAAAAAAAGCTACTACCAACAGCTATTACAGTCTGTGTTTGCAACCACGTTTTATTCTTTACA

TTACTCTTCTCTAATCAAAACCCATTATCATATCATTATCAAAGCTCTTTATCAATTTATGCACCCTTTCAGAATTAGCC

GCCTCCTTCAACTTGTCTGGTGGTGCTGCAGATGCTGAAGGCAATATAACTGACTCGCGTTGTGAATTAACAAAGTGCTC

CAATCCTTCCTCGCTTAAGCCAAATGCAGTTGCAAGCTCAGGGCCTCTTAAGGTAGTAAGAACTGAGTTTGAACCAGCTA

AGAATTGGGGTCTATTCTTTTGAGCTGAGGTTGTGAATCCGAAGAACTCACAAGGAGCTGTTCTTGATGCAATTTGACAG

AATGGGTGATATCTTGGCACCCAAAACACATCATTTACTGCTACTTACGCTTACATTGCTTGTGTACC

>JK317650.1 JCST421 Jatropha curcas L. seed cDNA library Jatropha curcas cDNA 5', mRNA sequence

TTTATTGTTAAACCAATTTTGAACTGTTTCAGATTCACATATTTCAAAGAATACAAGATTGATATCATACACATTATACT

ATATCACAAAAAATCCAAACAAAGATACATCAGGGGATACAAAATATCAGTTTTTTAATGCTTGTCTGAGTCCATTAGGT

CTCTCCCAACTGTCTTCACAAATATTATCCACCGCATTGTTTCCTATGCCCCATCCCCTTCCTCAAATATGCACTATCTT

TTAACTTCCAAGGGGATCTATGTTCCAAGATCTGCATCTGCAGCAAAATTATCTAACTGTAAAGTCACACAAGATGTACT

TTAACTCAAAAACGGGGACAGCTTATGGTTTCCAGGGTGCTGGAGGTGGTGGTGAGAAGTAACAAACCCAGATCTTCAAA

CAAGCTTGGCAGCTTCCACGAGACCACTCCAGATAGAAACCCTTCTCCCAGAGATGAATATCTCTCTCACGGTCACAGAA

ATTATAAGGGAGCTCTGCAGAACTGCATCGTAACTCAAAGGGCACATGTGAGATGCGGCTGACGAAGGAAAATGAGGTAT

GAACTTGGGGGGACCAGAGAGCAACTACGAAACTAGATGATCCATAAGAGGAACATGATTAGGAGAGGAAGAAGTGAGAG

GGAAAAGGATTAACTGCCACAGGGGGACGTTGCCCAAGTCCGGCAGTCATCCAATTCCTTCATTGCATCCTCAAATCATC

ACTTTGCTTGGATTGGGATCTACGCTGATGAACACAACNNAATTCCTTGTAGTTCCAAATATACTGTTAGATAACAGTCC

TTTCTGACCGTAGGAACTGCATTCCAGGTTCCATTG

>FM890964.1 FM890964 Jatropha curcas embryo 56-70 (DAF) Jatropha curcas cDNA clone rjcaeb0_000937, mRNA sequence

ATTCCTCNAGGTTGATCTTGCTCATATGTTAGCTGCTCGTGAACAAGAATTACGGACTCTTTCTGCTGAGATGAATCAAC

TACAGACTGAACTAAGGCTTGCTCGATCCTTGATAGCTGAGAGGGACGCTGAGGTACAGCGAGTACGCACTACAAACAAT

CAGGTGTCTCCAATAGCTATAACTGGCATTCTCAGTTTAAATTATTTTATTCTTGTTACATATTATTTCTGCTGTATTAA

ATAAATGCTGCTAAATGATTGGTGCTTTCTTTATGTTTAGCTGTTGTCTGCAAAAATATTTATGTGTCTACGTTGCTGCA

GTATTTGTCAGGTTATAGTTTTTAGATGTTTCCCAGTGTTCTCTCTCTCTCTCTCTCTCTCTCTCTAATACTGCTAATTC

TGCACACGCTTTAAGAAAAGGTTGTATGTGATTTGCATATTCTACAAAATACAAATCACTGTGGTCATGTTGAGCTTTTC

CTAATTTCATTAACGAATTGTCGGAACAGTATGTGGAAGAGAATGACAGACTCAGAGCTGTTTTAGGGGAATGGAGCACA

CGAGCAGCAAAGCTTGAGCGAGCA

>GW619663.1 Jc2-049-G07-M13F.G07.ab1 Jatropha curcas flower and seed Jatropha curcas cDNA, mRNA sequence

TCATAGATAGATGGGAAATGTAAATGGTAGAGAAGGTACAGATGAAGAAGGATCTAACGGTGAATCAAACATCCAAGCCG

GCGGCTCCTCCTCCTCTTATTCGCATCGGCAACCACCTGCTGTTCCTTCTGATTCCATGCCCAATACATTTACTACTACA

GCTATTGTTAATAATAATACTAACACGCCGCCTCATAGCCCTGCTCGCTCCGTTTCCCCTCTTTTGTTCGCTCCTCAGGT

CCCTGTAGCTCCTTTGCAGAGGGGTGATGGCCCATCTTTCCTAAACCAAATTTGGCGAACTGAGTCTTCTGAAGTTGATC

ATCCTCCTGAACAAGGAATCCCCACGATCATATCCTGGAACTACGGTGGCAATGAAGTGTTTGTGGAAGGATCTTGGGAC

AACTGGATGTCCAGGAAGCAACTACAAAGATCTGGTAAGGACTATTCTATCCTTCTGGTCCTCCCATCAGGAATATACCA

TTATAAGTTCATTGTAGATGGGGAATGGAGATATATTCCAGAACTTCCTTGTGTAGCTGATGAAATGGGCCGTGTTTGTA

ATCTTCTCGATGTT

>GW614407.1 Jc1-039-C01-M13F.C01.ab1 Jatropha curcas flower and seed Jatropha curcas cDNA, mRNA sequence

CAGAGCAGCCGGTTGCTCAAGTTTGTACGAACTGTGGTGTCAATATGGGGGAATATTTCTGCGGACTCTGCAAATTCTAT

GACGATGATACCGACAAAGGACAGGTGCCGTTGTGATGATTGTGGGATCTGTAGAATTGGTGGTCGCGAGAACTATTTTC

ACTGCAAGAAGTGTGGTTCTTGTTATGCAATCAGCCTACGTGGTAATCATTCATGTGTGGAGAACTCTATGCGCCACCAC

TGCCCAATATGTTATGAGTACTTATTTGACTCACTAAAAGACACTTCTGTGATGAAATGTGGGCACACAATGCATTTCGA

ATGTTATGTTGAGATGATAAACCGTGACAAATATTGTTGTCCCATATGCTCCAAATCAGTGATTGACATGTCTAAAACAT

GGAAGAGAATAGACGAAGAGATAGAAGCAACCATCATGCCTGAGGATTATCGCTACAAGAAGGTTTGGATCCTATGTAAT

GACTGTAATGACACAACTGAAGTCTACTTCCACATAATTGGGCAGAAATGTACCCACTGCAAATCATACAATACTCGCAC

GATTGCACCTCCTGTTCTTCCTCAATGAGAGAGTTATGGTTCTGGGACAATATCCAGCCATATGAGAATATTGAAAGCTT

GGCATCCTGAGTATAAAACAGAGAGGGTACATTTTATT

>FM887260.1 FM887260 Jatropha curcas embryo 35-55 (DAF) Jatropha curcas cDNA clone rjcfea0_000418, mRNA sequence

CAAATGACTGATCATCTAAATGGACCCTCCATTTATTTACCAATACTCACTGCAGCATTCTTCTCACTCCTTAAACTAGT

CTTCAATCATATACTAAAGTATATGAATAGGTTGAAGCTAGCTGATGAGTAAGAATTGCATTTAACGTGCTACCATAAAA

CATTTACAAAAGGAAATGTAAAAGCTAGATCGTTTCTAGCATAATGGTTTAGTAGAGGCTTCTTGAATGTAAGCAATGGC

TTCCTCCTCTGTTAACCGTCCTCCTTCCACAAGGCCTTTTATTAAGGTGTTGGCATATTCTTTGCAAGGTGCACGCAAAG

GGACTTCCCCTGATTTAAACCTTTCGATTGCCGAAAGTGGGCAGGTCATAGTCAGTATAGGGATGTCCTGCTCCTTTCCC

AGGTAACATACATTGTGGTACCAACCCGTCTTAAAACCTCCAAGAAATGGAT

>FM889961.1 FM889961 Jatropha curcas embryo 35-55 (DAF) Jatropha curcas cDNA clone rjcfea0_003723, mRNA sequence

CTTTCTCAAAATAGTTAAAACTATTTAAATTATGAAACAGTTCAACTGGTCTTAGTGCCCAGACCAGAGGAAACATGTCG

GTTAAAAACTAATTACCTTTTTGTCTCTTTTGTTTGAGACATGAACAGAAAAATTAAGTCTCTCATTTTCTGAACCTAGA

TATTGCTAGTTCTCATACTCAAAAAAGTCATAATCAGATGTCTGAATGACTTTCAAGTAGCTATGGCTGAGTTCATCTCA

TTGAGAATACGGTAATATTTGTGGTGATCTGATGTGTTTCCAAATCCAGGAATGAAGTCTGAAAAGAATCTCAGGACAGC

TGCAAGAAGCTGAGAATCATTGGAAGTCAAAGCAGCTTCGAGAAACAATGAAGGCCGGACAGTACTGACCTTATGCTTCC

TTGCATAACGCAAGGCTTCAAGATAAT

>GT228617.1 JC182 Jatropha seeds from fruits at three stages of maturation Jatropha curcas cDNA clone PL02SE.A09.scf 5', mRNA sequence

GCAATAATAAAATCAAACAAGAGGTCAGAAAGTTCAATAAGGTAATTGTTTCTTTCATGGCCTACCTTCTTTGCATTGGA

GTATGTATCTCAATTACATTTGAAAAATAAGGCAACAGTATAACAGTTCTTTTTTTGGTGGGGGTGGGGGGGTGAGAAAA

AAAATAAAGCATTGACAATTACGACTAAACGGTTTACTTCCTATAATCATTGATAAGCACGCTAAAACTCATTTAGCACC

TGGTTCCTCGGCTGTTCTCTTGTATTCTGGCTTCAGTTCGTATGATCCTTGATTAGTACCCTTATTGTTGTAGACACAAA

GTTCTTTAAGGATATCCTTCAGAAATTGTTCAGGTTGGTCAGTCTCTTGGATGAGTTGTCTTAATGTACAATTTGATTGC

CTTACAAAGAGCTTAAACATGATATCTTCCCCGGCCGTAATGGCCACTCTGCGTTGATACCACTGATA

>JK610574.1 JCF1478 Jatropha curcas, immature Seed cDNA subtraction library Jatropha curcas cDNA similar to senescence-associated protein, mRNA sequence

GTGGGGAGTTTGGCTGGGGCGGCACATCTGTTAAAAGATAATGCAGGTGTCCTAAGATGAGCTCAACTAGAACGGAAATC

TCGTGTGGAACAAAAAGGTAAAAGCTCGTTTGATTCTGATTTCCAGTACGAATACAAACCGTGAAAGCGTGGCCTATCAA

TCCTTTAGACCTTCGGAATTTGAAGCTAGAGGTGTCAGAAAAGTTACCACAGGGATAACTGGCTTGTGGCATCCAAGCGT

TCATAGCGACGTTGCTTTTTGATCCTTCGATGTCGGCTCTTCCTATCATTGTGAAGCATAATTCACTAAGTGTGGGATTG

TTCACCCACCAATAGGGAACGTGAGCTGGGATTAGACCGTCGTGAAACATGGTAGTTTCACCCTACTGATGATGGTGTCG

CGATGGTAATTCAACCTACT

>GW879174.1 JC003732 Seed specific Normalized cDNA library from Jatropha curcas L. Jatropha curcas cDNA clone N06877 5' similar to Unknown protein, mRNA sequence

GGACCCAATCCCATCCAACAAACCGCCGCAGCCATCCGCTATTACTTCACCAGGTGGCGCTCTATTACCTCCGCCGCAGT

ACACTCTGCCCATCAGTCATCACCGATGACCGCCCTCCCACGCTAATATTACTAGAACGCCCTTACATTTTGTCCAAAAA

ACAAATGCAAAGCCCAGTATTTAAGACCATCTGTTGGCCCAAAACACCGTCATTTTGCTTCCAAGTTGATGCTCTTTCTT

CCTCTCTACTCATCAGACCGAAAAAGTTTAACAGCAAAAGGGGAGGAGAGATAAAGGAGAGACAGTGGAGTAGTACTGGT

AGTAGGAGGAGGAGGAGCAACTATAAGAGCAATAGAAGTGCCATGCCTTGTTTCTCTGCTAGCACTGTATCTGGTGGTGT

TGGTGGTGGAGGAGGCGGAGATGATAATGGTTCGCTTC

>GW881713.1 JC000691 Seed specific Normalized cDNA library from Jatropha curcas L. Jatropha curcas cDNA clone N01299 5' similar to Embryo sac development arrest 35, mRNA sequence

GACTCACTGATATAATTCGATAGCTACAAGGTGAAAACTGTTTCTACACTCTCTCTCTCTCTCTCTCTCTCTCTCTCTCC

CCGCTGCAGAAACACTAAAAGGACGAACAAGAACACCTCGCGTCCAAGACCAAAGAGGTGACGGCTAAGGCCACTTGTTG

CATAAATAGGAGGTTATTAGGAGGTTCTCTATTGACTTACATCTTAGCAATGAAGGAAGAAGAAGTGAATCGATGCCAGA

TTCAGGAATGGTACCCAAAATTCAATCTGTATCCATCAAAACTATAACTCATGAGCTTCCAGAATCTTTTGTTGAGTACC

TTCTTGATGATTCAGTCCTTTTATCCTCCCCGTTTCTGTCTCTAATGAAGATGCCTTACCCAATAGAATTCATAATCCTA

TAGATGAAGAGGATTATC

>GT229102.1 JC667 Jatropha seeds from fruits at three stages of maturation Jatropha curcas cDNA clone PL15SE.D08.scf 5', mRNA sequence

GTGTATCAGCGCAGAGTGGCCATTACGGCCGGGGGATTTGGAGGATGAAAATGGTGGTGCAGGTGTATACGCTGCTAGTT

TGAGGAAGAATTACATCTTAGCCAATGATGAATGGAAAGAAGATGTATTGCCAGAAATTCTTGATGGTCACAATGTTTAC

GACTTCATTGATCCTGATATCTTACAGAGACTTGAGGAATTAGAACGAGAAGAAGGTATTCGGCAAGCGGAGGAAGAAAA

TGGGGATTTTGAGATGGATGGTGAGGAATTAACTGAGGAAGAGAAAAAAGCTTTGGCTGAGATTAGGAAAAAGAAAAGCT

TACTCATTCAGGAGCATAGGATTAAGAAGAGTACTGCAGAGAGTCGGCCTATGGTACCTAGAAAATTTGACAAAGACAAA

AAGTTCACAACAGAGAGAATGGGCAGACAACTCTCTGCTTTGGGGATTGATCCTTCTTTGGCAATTAATCGTGCCCGCAG

CAGGTCTCTCTCTAGGAGAGGCCGCAAAAGGGAGAGGTCAGTTGTTAGGGGAGACAACAATGATGGTGATGCTATGGATA

TGGATGTTGACCATTCAAATAAGAAGCTGCGCATTAGATCTAGCTCTCGATCAAGGTCAGTGTCAAGGCCTCCAGGTGAG

GTTGTCCCTGGAGAGGTGCTTCAAGACTCTTCTCAAAGTTGAAAGCTCTTAAGCTC

>JK611363.1 JCF4018 Jatropha curcas, immature Seed cDNA subtraction library Jatropha curcas cDNA, mRNA sequence

ACATACGCTTAGACAAAATTAATTAAAACATCTATGGACTCATAAATTATCTAACCTATTTTTAATACCACGACTCGCCA

CAATACATAAATTCCCTATAACTGGCGGGAGTATACTAAACAAGATAAAATTACAAAATAAAATCCCTCCGTAACATTAC

CAAATTAAATTTAATGAAATTAAATTTTCCTGAAAATTTAGAAAAAATTTCTCCTCCCTAACTTTTATGGGGGAAAGGAA

ACAAAACCTTTTTACCCAGGAAATTAAAAAAAAACCTACTTCCCTGAAAATTTTAAACAAAAAAAAAAATGGGAATTTTT

TTTAATGGGCCATTTTTTTTTTGGGCCAAACCCCTTGTTTTTCCCCCCAAAACCTTTAATTTTTTCAACCCAGCCCCCCG

AAAAAAAAAAAAGAACAGGGGGGGGGAACCCCCCAAATTTCCCAAACCCCCCCCTCCAATTTTAACCCCCCCCCAAATTT

CCCCCCCGGGGAAATTGGTTTGGGCCCTTTAAACCCTGGGGAGGGTTTTTCCCCCGGGCCCCCCCCTTTAAGGGGGCCCC

AAGAGGGAATCCCCCTATATGGGGGGGGGGGGATTTTTTTTTTGGGGGGCCCCCCCGTGTTTTTTTTCTTTTTTTTTTTA

AAAGGAGGCCCCGATGTGTATACAATATAATATAAAAGAGAGGGGGGGGGGGGGCTCTTTTTTTCTAGGGGGAAAAAAAA

AAACACACACAGAGGGGGGGGGGGTTCTCTAGCGGCGCAACATGATATATATTAGACTGAGAGAGCGTACTATCTTCGTC

GGCGACGTTGTTCTTCTGCTATCAGAGAGAGGAAACGCTCTTCATTCCGTCCTATCTTG

>FM887047.1 FM887047 Jatropha curcas embryo 35-55 (DAF) Jatropha curcas cDNA clone rjcfea0_000116, mRNA sequence

CTTTGGTATAAGCAAGAGATATGAGAAGAAGAAGAAGCGCATTCGTGTTTACATGGATGGTTGCTTTGATCTTATGCATT

ATGGACATGCCAATGCTTTAAGACAGGCTAAAGCTTTGGGTGATGAATTAGTGGTAGGTGTTGTCAGTGATGAAGAGATA

ATTGCCAATAAGGGTCCCCCTGTTTTGCCCATGGAGGAGAGACTGGCTCTTGTCAGTGGATTGAAGTGGGTGGATGAAGT

CATAGCCAATTCTCCTTATGCCATTACAGAGCAATTCATGAACACTCTCTTCAATGAGCATAAAATTGACTATATCATAC

ATGGCGATGATCCTTGCCTGCTTCCTGATGGAACTGACGCTTATGCATTGGCCAAGAGGGCTGGACGCTATAAGCAGATT

AAACGCACTGAAGGGGTCTCCAGCACTGATATTGTAGGAAGGATACTTTCTGTGGATGATCCANAAGTTTGTGAAGCTCA

TGATGATAAGTTGCCTTTACCTAGAGATGCCTGTAAAGCAAATCAATCCAGTGGTGCCTATATATCTCAATTTCTACCAA

>GT972247.1 GJCCJC2032C03.b Jatropha curcas L. developing seeds (mixed stages) Jatropha curcas cDNA clone GJCCJC2032C03 similar to heat shock protein binding, mRNA sequence

GAAGTAAGGGATGGACAAACTGCAAAAGCAAGCAAATATAAAGAAGCTGAAGATGCTGCAAAGAAGAATCAGCAAAAGAG

TGCAGATGATATTGAATCCTTTTTTAGCATGGGTTCTCGATCCACAAGTGCACCAAAGTCAAGGACTGCAACTTTGGACC

CTTTATTTGATGCAAAGTCAAATGGCAGAGGAGAGCCTGTAGTAACCCAGAAGAAGTCTAGCTTTGCCTCATCTGGGATA

AGGAAGGCTTCTTCTACAACAAATATAATTGATGATTTTATGTTTGGAGATGCCTCATTATTTGGAGAATTTGAGGAAGT

AGAAGGGGAAAGTGAAGAACGACGAAGAGCCAGATTGGGACGTCACCAGAGGACTCAGGATCGTGTGGTGTGTGTTTTTC

CCTGTTCATTTTCTGTTTCCTTAGATGGTATTGGTGGATGTTTTTATATTTTTGCTAATTGGCTATTTGAATGCTGAAAT

TCTTGGATTGAAATTTGTAAAAAAGTTTGCAAGTGACCATTTCCCGCTCAGTTGCTGAAAGATAAAGAGATAAGCTTTCT

AAATGAGATTGAGTTCCACGAATATGCAGGCTAGAAAGATGTGATTTGCTGCTATAACATGGTAAAAGATGTTTAGTTAT

TTCCTGTTAGATCCTATACCTAACAAATTAAGTTGTCTATGCATTATTAGTTTGATGTTCTTCATCTCTATAAGTTCGAT

CTATGTAGCACTTCAGCGTGCTGCAACTGTAAGAATGCCCTTTTGGGCTG

>FM893183.1 FM893183 Jatropha curcas embryo 56-70 (DAF) Jatropha curcas cDNA clone rjcaeb1_003915, mRNA sequence

TGAAGGCAAGGGCAATGAGAAATCCCCCAATATTCAAGGGAATAGGAAAGGAAACCGAAGATGAATGCCCTGAACATAAG

CTTGATAATGTGTTAGTATAAAGCTCCAGTTCAAAATGGGCAGCTCTCATATCTAGGCAGTGTCTGACAAATTTCTCTAA

TGCTGGATTTTGTTTGCCCTCAGTCAGCCTATGCTTATTGACATGGAAAAGAGAAAGAAAGGGTTGTGTGCATTAAATTG

TTTGTACAATTACTCTTATGTCAAACTTAATAGCATTTCGAATCAAGGAATTTTCTTGATTTGATATAAAGGAGATCTTA

ACCTCCCTTTTGAATAAAAAAAAAAAAAAAAAAAAAAAAAAAAACTCAAGACTAGTTCTCTCTCAGAAAATGTCGAGCTG

CGGTACTGCCTGCAGATGCGGCTCTGGCTGCAGCTGCGGCAGCGGCAACGGATGTGGCATGTACCCTGACATTGCAGAGA

GCACCAGAACTGAGACCCTCATTGCAGGAG

>FM896484.1 FM896484 Jatropha curcas embryo 71-95 (DAF) Jatropha curcas cDNA clone rjcpga0_003707, mRNA sequence

AAATAAATTATTCCAAGAGAAAAGTTGGAGTTTTAAAGAAGGCAAAGGAACTTTCAACACTATGTGATATTGATCTTGCA

ATTATCATGTTTTCTCCCACTGAAAAGCCAACCCTCTATGTTGCTCATGACAAACAATTAGTTACTGTTTTGGAGAGGCT

CTCAAGTTTATCTGTTGAAGAGAGAGAACACAGGAGGGCCTACAGCATGAGGAAAGTGAACAAGATGGCCAACCCAGAAG

TGGCTAAAAAAAACTCTTCTCTCAACAAAGATGAAGCAGTCAAACCTGGCCAAGCTCTGCAGCTGCAGAAGAATCATTTG

GGAGAGCTGAAACAAAAACTCATTGAGAAGTCAAGAATTCTCAGGGACTGGAAGAATCCATACACTGTTGACAACTTAGA

TCAGATCAAAGTGATGGAGGAACATCTCATTGCAAGTCTTGAGAGGATCACCGAAAGGAAGATTCAGTTGTGGGAGCAGC

AAAAAGGACACAAAGCCTCTAAGGCCAAACGCACAAATTGACCTCTGTTTCTTCATCTGGGTCCTCAGTGTCA

>JK317562.1 JCST331 Jatropha curcas L. seed cDNA library Jatropha curcas cDNA 5', mRNA sequence

CTTAGCAAATTTCAAGAGAACCCTAACAATCATGGGTTCTAAACTTGAAGCTTTGTTATTCATTTTTATGATTTTCATGG

CAATATCATTGCCACCAATTTATGCTTGTGCACCTTGTACTCAGCCACATCCACCAGGCCACCGTCCAACCCACCCAAAA

GTTCCTCATCCTAACCCACCAAGCACCAAGCACCCACCACACCACGGCGGCCACCCAAAACCACCTTCAACAAAAAACCC

ACCAGTGGTTTTGCCACCAATAGTCATTAATCCTCCTCTTGTAGTAAACCCTCCTGTAATAATAACACCTCCTCCTGTAA

TAACACCGCCGCCTTCTTCAGGTTACCCACCATACACGCCTAGCCCTCCATCTGGTGGTGGCGGAGGCGGCGGCGGTGGC

GGAGGCGGTGGAGGAGGAGGAGGAGGAGGTGGTGTAGTCTTTTTTCGAAAAAAAATAATTCAACCGCTTGAACACAAAAA

AAATTGAATTTTTCACCCTCAAAAAACACCAAAAGGAAATATTTTTCGGTTTGAAATGAAAACAACCTAAAGGATATAGT

CAAATTTAATTTAATCAATAAAGCAACAAAAATAACCCGGGTCGCTCTTCCCGCTACCATACTTTTTGGGGAGAAACACG

GTGCAAAGGGAGAACCACTCACCAGAGTTGGGTCTTTTCTCAGTTTTTTACTTGAAGCAGAGAGGAGAAAGTGGTATTAA

AAAACATAACCTTTATTTTGATTAAAAGGAGTGTTAACCTGACAAAACATAATGAGTCAGTAAAATTTTAATATGTACTA

TCAATATACAGACCCCCAAAAAAAAAAAGACACA

>GW877674.1 JC002492 Seed specific Normalized cDNA library from Jatropha curcas L. Jatropha curcas cDNA clone N04662 5' similar to Unknown protein, mRNA sequence

GGATTGTGTTCACTGTTATTTGCTCTCCCTCCAAAAATAGTAAAAAAATGTCGGATTACTTTCTTCCTGAAATTTGGCGG

AATATTCTTCAAAGGCTTCCTGTGGAGTTCCTCGTGCGATGCACGTGCGTCTGCAAGCGATGGTATTCTCTAATCACTAG

CTCTCATTTCATCTCCACCCACTTATCTTTCTCTCTGTCTCAACCTCCTCTCTCCCGTCCTCTCTCCTCCGACGCTTCAT

CGAGTCGTCAAGAACTCAGCTTTTCTCTTTCCATCTCGATTCGTCTCCCCATTCCCTCACTAATTACAAGAATGCCTTCA

CTTCACCATTTAAGGCAAAAGACGGGCTCTTCATTCGTATCATCGGTTGTCTCCATGGCCTTCCTTACCGCTCCGACGAT

ATCTTCGGGATGACCGGAGTTGTCATCCTCTGGAACCCTCTCATTTAAACGCTATGTTTTACTCTCTCGACCCCGTATCG

TTTTTGACGAGGTCGGTCCCTACATGTTTGTTCTAGGATTTGGTTTCGATTCGGTGAGAAATGATTATAAGG

>FM895455.1 FM895455 Jatropha curcas embryo 71-95 (DAF) Jatropha curcas cDNA clone rjcpga0_002377, mRNA sequence

TTTTTCCTGAATTCTCTTTTTCTGTAGCCCCTACCCTATAATGCGCGATGGTGTGTTTTACGCACCCCTCAATCCACCGC

TTCTTCTCTCTCCCTCTCCGACACCAACCTCCGTGAACAATTCCATTGCTGCCTCCAACCAAAACATGCTCGTCTCCGTT

TTTTTGGCCCTTTTCTTGCCTTGTATCGGCATGAGCGCAGTTTTTCTCGTCTATATTTGTCTCCTGTGGTACGCTGCAAG

TCACCAAGCCGAAATCCCACCGCCAGTAAAGGCGGCACCGGAGAAGGGATTATCGGCTTCTCAGCTCGAGAAATTGCCTA

AATTGACTGGGAAGGAGTTGATATTGGGGACGGAATGCGCGGTGTGTCTAGATGAGATCGAGAGCGAACAGCCGGCTAGA

CTGGTTCCCGGTTGCAATCACGGTTTCCATTTGGAATGCGCTGACACTTGGCTTTGCAAGCATTCTGTGTGTCCTGTTTG

TAGGGCCAAGCTTGATGCTCTGTTCTTCGTTGCTTCTAATGAGAATCCTTGTTAATTTTAATTTCTGAACAAGCATGATC

CATCAAACAAGAAGTTCTTCCAGCTCTGTTCTGCTATGTTGCCAGAAGATTGCCGATTGCGACTCATAATTACTAGG

>FM891179.1 FM891179 Jatropha curcas embryo 56-70 (DAF) Jatropha curcas cDNA clone rjcaeb1_001180, mRNA sequence

TTTTTTTTTAAGACAAAAAAAACCAACTAGCATAACAAAAGATAAATTACAATAGCATTTCCCAGAAAAAGAGAAATTCT

TTAGGGTATATTAAAGCTTAAGCAACTCCATAAATCAAAATTCTAGTCTTGATATGACAATGGCTGGGAAAATGTAAACC

GCACAGCAAAAGCTCATAACAGCACTTTTAATCACTCTAGCTATAGCATAACAAAAGAGTCTTGATACTAATACAAAATT

AAACCATAAAAAAAGAAGTGGGTAGAGATGCACATATTAACGAAGCCTCCTGGCTTCTCTTTCAGACTCAAGAAGGGTGA

GCACATCACCTTCTCTAACAGGTCCCTTGACATTCCTCATGATGAATCGATTCTGATCATCAAGAAACTTGACTCTGACT

TGAGTCACCTGCCCCCTCGATCCTGTACGACCCATAACTTTCACCAACACGGCGTGCTTGATTTGTGAATCCATCATGAG

AACGATTCCGATAAGGATCGCCTGAAGCAAGATAGGGTCAGAGAAAGAAGCTCGGATGGGGGG

>GW875148.1 JC000539 Seed specific Normalized cDNA library from Jatropha curcas L. Jatropha curcas cDNA clone N01032 5' similar to GATA transcription factor, mRNA sequence

GGATCTCACAGTTTCCTCTCTCAAACACAATCCCCTTTTACAAACCTCAACTCTCTCTCCTCTTTTCCCCCTTCTTTCCC

CCGTTTTTTATTTTTTTCACTTATTCCTAACTCGATTAGGGTTGGGGTTTAATCAGAATTCCCTAATTTATTCTTGTTTT

TTCTTTGTAATGCTTCAAAATTTTCTGATCTCGCTTCAGATTTACCGATGTATACACATTCCCATTCTCAGTCTATGAAC

GTTCACCACCAGATCGCTGCCACTGCTGGCCCTGCTGGCGATGACGACGACGGGGCTGCTGTCGACTCCATTGATCATCA

TCACATTCGTTATGAAGATGGCAATGCTACTGGCGTTGTAGAGGAAGTATCTCCTGACTCTGTCTATGTTCCTAGTAGTG

GCGCTGGCTCAGAACTCGCTATACAGCGAGGTGATGTATCAAGCCAACTCACATTGACGTTTCGTGGTCAAGTTTATGTG

TTTGATGCTGTCACTCCTGATA

>GO247607.1 JcrME_RL1151 Expressed sequence tags from Jatropha curcas root cDNA library Jatropha curcas cDNA, mRNA sequence

ATTTCTCCTTCCTTTCTTTTAGTTGCTCCAAAGCATTTCATTCCCAGCAGAGCCTAGGGTTAGGGGGTGTGGATTCAAGC

TTTCCTTTAATCCAAGATTCAGCTTTTCCCACCTGTGTCAAATGCCGCCATTTCTTTACTCTCTGTATAGAACACAGACA

CGCTTCTCTTTTTAATCAATTCCTCTTCTCCAAAATTTCAGATCATAAGAAATCTTCATTTTACAAAAGATTTTAGGTTA

TTATATTTCTTTTTGACAAAAAGAAAAAAGAAAATCTGATGGATAAGAAAAGGGTGGCGGTTCCACTTGTGTGCCATGGT

CATTCTCGCCCAGTCGTCGATCTGTTTTACAGTCCAATCACTCCTGATGGTTTCTTCCTCATAAGTGCTAGCAAGGATTC

TAGTCCAATGCTTAGAAATGGAGAGACCGGAGATTGGATTGGAACTTTTGAAGGACATAAGGTGCGGTGTGGGAGTTGCT

GCTTGGATACTAATGCTTTACGTGCTGCTTCTGGTCTGCTGACTTTACTGGGAAAGTCTGGGATGCATTAACAGGGATGA

CTGCATCATTTGAACCAAGGCATTGGGCGGCTTTGCATTCCAAGGAATCCATTTTTTGCTTCTGGGGGAATAGAAGGTCT

GATTTGAATGAATGTCAATGTCCCTCAGAAAGTGAAGTTCTGTCAACGAACTTGCGGTTCCAGGAACAAAAATTGATCGC

>JK613383.1 JCF18-88 Jatropha curcas, immature Seed cDNA subtraction library Jatropha curcas cDNA similar to atp binding protein, mRNA sequence

ACCCCTGATGAATTTGTATATTTCCATCCATTAACATAACCAAAATGCTTCACTGCATGGAATTTCTACTTTGAAAGTTC

ACATCCTCAATATCTCCTTTTTCATATGAAAAAAGGGAAACCAACTCATTATAGCAAGTCCAAAAGAGGCGGCTGCCCTG

CCGCTGGTTTGGCATTGCTAGACCTCGAAGTAGAAGTAGCACCTCTTCCTCTTCCTCTTCCTCTACCAACAGCAGTTGAT

GGAGGTGGAGCCAATTTAGGTGCAGGCTGGTCATTTTTGCTGGAACTAAATATTGATTCAAGATCTGTTGATTTCTTTTC

GTTATAAGAACCCAAAGTAGACATACCTTGATTCTGCTTTAAGAAACCAAGAGAATTCTGAGGATTAACGCCACTGCTCA

AACTACCTGTATTCATTGTAGAAGTCCCTTGGTTTGGTTTCATTGGCTCAAATGAATTTTGGCCATTGAAGGCCCAGCTG

TTATTTCCATTGCTCTGCAAGTTCATATTGTTGAGAGTGTTCATGTTCAAACTTGTAGTGTAATTGTTTGTCAATGATCC

CATAGAGCCATTTTTGGAAATTCCTGATGGACTAGAAGCATTGCTTGGTCGTGGAGGCCAATCAGCAAAAGGATCTAGAT

CATCAAAACTTGAAGAGGATGATGTTCCTGTGTTCATTTGCTTCTCAATATTGCTTGACTGAGTGTAACACCTGAGATGA

TGCTCTAGAGCCATTCTATGTCAACCGGAGGGCATGATTCAGCTGTTTGTGACAGATACAGCAGAAATCACGAGGATCTG

ATCTAAGAAGCCACTGAATGGCCTGACTGTTCAGAACAGGAGCTGACGTGCTTGCCTGTGAAGCTGCTTGTAGTGGTAGC

TTCTTTTTTTGGTAACAC

>GT982150.1 JGCCJG2064E08.b Jatropha curcas L. germinating seeds (mixed stages) Jatropha curcas cDNA clone JGCCJG2064E08 similar to armadillo/beta-catenin repeat family protein / U-box domain-containing protein, mRNA sequence

GGATGGGCATGGCGGAGAAGGCAATGGTGGTTTTGAGTAGCTTGGCTGCTATAGAGGAAGGGAGAGAAGCAATAGTGGAG

GAAGGAGGGATTGCTGCACTTGTGGAGGCAATAGAAGATGGGTCTGTTAAAGGAAAGGAATTTGCAGTGCTTACATTATT

GCAGTTGTGTACTGATAGTGTGAGGAACCGTGGGTTGCTTGTTCGGGAAGGCGGGATTCCTCCCCTTGTTGCCCTTTCAC

AGACGGGGAGTGTTCGTGCTAAGCATAAGGCTGAGACACTACTTGGGTACTTGAGAGAGCCAAGACAGGAGGCTTCATCT

TCTAGTCCTTAAAGAAGTGCCCTTTTCCAGAACTCTTTTATCAGAGGTAATTACTAATTAGACTGAGTGGTTGATTGTTA

TTGAGTTTGTATATAGGGAGTGTTTTTTGGTGGTTTGTACAGTGTGGTTATTAAATTTGAGGAAAAATGTGGAGTCTACT

GCAATTAGGGGAAGCTAGTGAGCAAGAGACTGACTTGTTAGATGTATGCTCATGCGTTTAACTAGGTTTTTGGCGTTTTT

TACTATCATGGTTTTCTAGAGGAGGAAGTATAGTTTTTCTCCTATGTTGGTTGTTGTCCGAGTCGATGAAGATCTGGGTT

TTTTCCTAACGTTTTGTTTGGATGACCCAAATTTCTTCTACTGATTATTGTTGTCAATGTTTAAACTGGTGAAGAAGATA

GAGAGATTATGCTAAAAAAAGAAAGAGAAG

>GT972864.1 GJCCJC2042B11.b Jatropha curcas L. developing seeds (mixed stages) Jatropha curcas cDNA clone GJCCJC2042B11, mRNA sequence

GGTGTGCGAATCGCAAATTTCTGGGCAAGATTTGATTTTCCGGCCACCATGCATGGCGGTTGGCGGCGGCGGTCCGATGA

CCGGCCGGCGACACTCCAGCGAGGCCCCGAGAGACAGAGTCCTCTTCTCTTCGCGCTGATCATGGAATCGTCCCCCGGTT

CTCAATTGGTATGCCCTAAGACTCAATTCTCAATTTTTGTATATAAAGAATTAATTGATTAATGAATTTAATTGAGTTAT

TAAATAGATTAAATTGAATAACAAGAAAATAGGTTATTGGGTTAGCTTAAAAATATATGAACATTTATAGTCTTTGTAAA

TAAATTAGATTGATTAGTGTGCGTATTTGGATAGCGAACATGATTTGTTAACTAGTTTAGGATTTTAGAATAATGTAATT

GCAAACTAGTTTAGAACATTGTCAAATAAATTACATGAACCATAATTATAATTAGAAGGTTTGATATTGAGATAAAAATA

CCTAACTAAATTTTATGTAAGTTAATATAGGAATTTTAATAAATATTGTTAGGAGAAGTTGTAAGTTAAAGTAAATAGTT

ATAGGGCAATTAACACTATTAATGAATCACGCTTGGAGGTTAATTTGTTGAAATTATTAAGTATATATGTAAGTGTGATG

AATGATATATGTAGCTTTGAGTTAATGGGATGTCTGAATTTGATTGATCTATGATTGAAAAATAAGATATGGCGGACGGG

TTGAGTTGTTTTGAAGGGAGATGGGGATATAGTTGATTTTATGCCCGATGAATGGGCTGTGTGCATTGATTG

>GT973095.1 GJCCJC2041A12.b1 Jatropha curcas L. developing seeds (mixed stages) Jatropha curcas cDNA clone GJCCJC2041A12 similar to PFN3/PRF3 (PROFILIN 3), mRNA sequence

CTGGCTTAAACTGCATACAATCCGATAAAACCAATATGAGTGTCAATGAAATCATTTAGATCACAATAATGACAGACCAA

ACACATAATAAAGAGTTGATAATTTGCTAATGTAAGAGGTAGATTGTCAAAGACAGATACTTTCGCAGGAATTACATTAG

GGAAATGAAGATTATTATGTTTATATTTAATAAAACCAGATCGAATTCAAATCTAGAAAAAATAAAGCAATTAACGAACA

AAAAACAACAATCCAAACAAACAGATCAGGACAAAAAATAGAGATACAAAGATTGAGAAGCAACCTGAGGGAAGCTGGTA

CTCTGGGCCCATACACTGCCATCGTGACCGATAATGGCAGCGGCGGTGAGACGTTGGCCATCGATATCGCACATGAGGTG

CTCATCAACGTACGTTTGCCACGACATTTTCGATCGAGAAAATCCTAACCGTTGATTCTAATTACTAAGTTTTTTCTTCT

TTTATAGCTTTGGCTTTTCTTTTACGTTCTCTGCTTTGAAATCTTCTAGTTCAAAGAAGAGAGGTATATATAGTGCTCTC

AAAGCAGAGAGAGTGCGTGTACGTGTTTAGTGGGTCCCGTGGCGCTATGATGGATCATGGTGGTTTTCTGATTTTTGATC

CGAT

>FM895018.1 FM895018 Jatropha curcas embryo 71-95 (DAF) Jatropha curcas cDNA clone rjcpga0_001351, mRNA sequence

TATTTCCAGTGTATATCCATGTAATACAACAAGGTCAGAGTCAATCAATTATTGGAGCTTCTTTTGTGTAACAGAAAAAA

AAAACATGCATCATCATGATTGTCATTTGTTTGAATTCAATCATACAACGCATCCACATTACACTCCCCAAGGAAAACTT

TGATACTGAGCGGTGAACCACAACTACCTTCAGCTATAATTGCTCTGTCATTGGCGGCCATGTGTGCAATAATCTTGTAT

ATCATTTCAACATCTTCTTCTGCATGGCAACGTTCAGCTACTTCTTGAAGTGTCAATGGTTCAACAGGCTCTTTACAGCT

TGCCTCATTAAGTACTGCCAAAACCCGCTTCTGAAGGGCTAAAACTTCCCCTGCTGCCTTTTTCCCAGCTTCCACACCAG

GTTGATGATAAGCATTGATATTAACTAGTGAGGCATATATCCCAACTGCTCGTTCATAAAGTGCAACAAGAGCCCCCACG

GTCCT

>GT981005.1 JGCCJG2050A09.b Jatropha curcas L. germinating seeds (mixed stages) Jatropha curcas cDNA clone JGCCJG2050A09 similar to 29763.m000195 conserved hypothetical protein, mRNA sequence

GTGTCATGCAAGCACGCTCGTGCTGACGAGACTACGAAGAATCAAGTTTTGGCACGCCCATGACATGCCAAGCACACCCC

ATGCTAGCCCCTGAGAATCTACGGCACGCCCGTGCCGCCTCCCTCACTAAGGTTTAGTTTATCTACGAATTTTGTGCTAT

TTTTAGGGTTTTATAACTTTTAGCTTAAACCTATAAATAAGTTATTTGTATTATTGGCAAGGATTACCTTTGATTATTAT

TCATATCAATAAACTTCTTGAGAGTTTTAATTCTCTATTTTATTTGTCAGATCATACGTTATTGATCAAAACTCCTAACA

GAGTATAATCATTATTCCCAATCACGTAACCGTCCCATGTCAGTTGGTATCAGAGCCGAAAGGTGAAGTATCTCCATGGC

CGACAATCATGAGACACGACTCATAGTCTTAGAGGCGAGGTTCGGAGAGTTAACCGAACAAATTAGATAGTTGACTCTCA

CAATTCAACAAGGCAACCCACAACCTGCAACTCCAAATCAGCGGCAACCAGATTATGAAGGCACTAAAATCGTAACAGGC

CGAGATTATAAAAACAATGGTGAAAACTTTCATAATGGATACAAAATGAAGGTAGGATCTTCACCTTTCTGAAGATCACT

AGATATTGGAAGCACTCTTGATTGGCTAGCGGAGGTCAAATGATTCTTTAAGGTTATGAATGTTGAGGATGAACGTAAAG

TCTCAATTGTGGGCTATAAACTGA

>GT971771.1 GJCCJC2024D01.b Jatropha curcas L. developing seeds (mixed stages) Jatropha curcas cDNA clone GJCCJC2024D01 similar to MAPKKK5 (Mitogen-activated protein kinase kinase kinase 5); kinase, mRNA sequence

GCATAATATCAAGAACAAAGGCTAAGAAGAAATGCTTGACCACTTGATGAGTTTAAGAAGAAAGAGGATGAGAAGAAATA

ATCTGCATATGAATTCAAAACAGCTGCGGTGACTAACCTGTTCTAATTGCTTTATACACTCTGCGGATTTTGGGTCATCT

GGAAATATGTCGACTTCCTTCATTGCACATAATGCCCCAGTTTCCCTGTTGGAAATGAGAAACTGCGATTAGCGTTATTG

CTGAAAGGAAGCGAGCCTTGAGAAATACAGAGTTTCCATACCTGTTGCTGGCAACGTAAACACTACCAAATGTGCCCCGT

CCAATAAGCTTTCCCTTCTGCCACCGACTTTTCAAGGGCATGGAATCTGCTTTGGCAATAACTTGAGGGATAGGAGTTGA

TGTTGAAAGCCTGGCTGCTCCAGGAGGAAGAGGTAATGGGTGGACCTCTACATTAGCACTACTTTCCCGCCCTGGAGTTA

AGGTTTCAATTGACAACCTCCCAGGTAATGGTGATGC

>JK611774.1 JCF29-40 Jatropha curcas, immature Seed cDNA subtraction library Jatropha curcas cDNA similar to phosphomannomutase, mRNA sequence

ACTGCGGGAGAAGTTCTCTCACCTTAACCTTACATTTTCAATAGGTGGACAGATAAACTTTGATGTTTTTCCTCAAGGTT

GGGACAAGACATACTGCTTGAGATACCTCAATGAGTTTAATGAAATCCACTTCTTTGGAGACAAAACTTACAAGGGAGGA

AATGACTATGAAATATATGAATCAGATCGAACTGTGGGTCACACAGTTACCACCCCTGATGATACAGTGGTGCAGTGTAA

AGCTCTCTTCCTAGCAAATCCTTGAACTATATGGCAATTATTTTTTGGTTCAATTGTATTCCCCATTGATAGCAAATGAT

TATCTGTTGACTGGGGGAAATCCTATTTAAATTAAATTTACATTAAAAGGCGAAAATTATGCCCTTACTCATTTGTATAT

TACACTTTTGAGAATAAGTGTCAAAGTAGAATTTTTGCCCAAGTAATTTTTCTAACAGCTATTAACAAACATGTATATCT

TACAAGCTTGTACACCTCGGGCGCGACCACGCGAATCTAC

>GW876750.1 JC005676 Seed specific Normalized cDNA library from Jatropha curcas L. Jatropha curcas cDNA clone N09713 5' similar to DNA binding protein, mRNA sequence

GAAATGTATTCGAATTTATTTTCTTAAAATTTGAAAAGCCAAAATCTCCCGCTTCTCTCAACAATGGAACCTCCTCTTCT

GAAGCTAGTAATGGTACAGGGCCCTAGAGAAGGCGAGAAGTTGGAGTTTCGAACCGGATCCACCATTCGAATAGGTCGGC

TAGCCCGCGGCAACAACATCAGTATCAAAGATGCAGGCATCTCCTCCAAACATCTCTTTATCGGATCCGAATCGGGTAAG

TGGATTGTCCAAGACCTCGATTCGTCCAATGGCACTACTCCCAACTCCACCAAGCTCCCTCCCTTCACGCCGTTCGATCT

TCAAGACGGCGACAGTATCAAGCTCGGTGAAGTCACCTCCATCCTCGTTCGGTTTGTCGGCGGGGATGAGCCGTCTCAGT

TAAGACGCAATCCGAGGAGAAAAGTCAATGAATTGGATAAAAAAAGGATCGGTTGCAGAGGCCCCGAGCCAGAGGGCAGA

GGCTGTGATAATAACTGAAGAAAATAGCGGATTAGAGAGTGAGAATGTCGAGAATCGTAGAAGAGGAAGGCAGAGGAAAG

CTAGGGTTTTGGATAAAGTTACAGAGATTGATAAGGAATCAGAAAAATTGGACTTTGTGGCCGAGAAACCGGAGACGCGG

AGGGTGAATGTTAGAGTTACTAGAAATAGAAAGAATGAGGATTGCCTGATTTTGGAAAATCTGGGTGGAAAATGG

>GT973539.1 GJCCJC2049F03.b Jatropha curcas L. developing seeds (mixed stages) Jatropha curcas cDNA clone GJCCJC2049F03, mRNA sequence

GACTGTTCGCAGTCCGGAAGAAAATAGGCTTTTGAGCCTCAAAATTTGAGTGTTCACAGGATCCAGCAAAGGGCACGCAA

GACTTCAAATAGTACTGAGGCAAAGCCGATTGAAGGGGTTAGGGCCAGAATGGTTTCTCCGAAAACCCAGGAATGGAAGA

TGCTCCGACAGAGAGGTGTCTGAATTTTGAGCTTAAAAGCGACAGAATCAAGAAGTTATGTTTGGAATTGGGTTGGGTCT

GCACGAAGTCGCAATGCTATCCATACACTACTCAAATCATCTCAGAATCAAGTTGCCTCGAAATTCTTTATGGAGAGGCT

GCTTCTAAGCAAGCAAAAGAAAAAAACGGGTCAAAAGGAACCTCTGCACAAAAGAAAGAAATTAAGGACAAAGCTACAGG

AAATCAAGTTGTCAAAAACGGGTCAACTGCAAGCCCTCCGTTAAATTCAAGCCCATCAAGGGTAGATGACAAATCACTTT

AGCCATAAGGACAAAGACCAAGAAGCTGCTCACATTTTGAAAAAGCAAGTTAAAAGAAGCGGTCATTGCCACAAATTGGA

TGACTACAAACACCTTGAAAATCAAGCGCCAGGCTGGCACAAGTACTAGTGTCCTTATAAA

>FM887603.1 FM887603 Jatropha curcas embryo 35-55 (DAF) Jatropha curcas cDNA clone rjcfea0_000837, mRNA sequence

GCGGTGGCGGCGCCTAAACAGTGGATCCCCGGGCTGCAGGTTTTTTTAAGAAAAATAGGAGCCAATCAGCCGAGGGTGCA

TTTTCAGATCGAGAAACTGCAAAAGGAATTACATGGATCAACACGTTCAGCTTTCAAGTTCCAACAGTTTAGGTACATTA

CAAATACAGGGCCTGTCGCTTGCGGTAATACTATGCAATAAAGGAAGCTAAGCTATATCCACATATGTCAATATCAGTGC

CACTCCTTCGTAGATTAGCAAAAGCCCATCTCATGGAAGACAACTGCCGCAAATATGTTCCTTGCCCGATCGCTAAATGC

ATAGCGTTTAGCAAACAAATTCAATTTGGCGTAGTTGAAATCNATCTGGTGCCATATGGCCATCAACAAAAGCTTTTAGG

TTGTTGAAATAAATTCTTAACTGCGAAATGGAGGTTGGTTCTCAAAAGAAATTCGATGGGCGTTGCATTGCCATAAATTC

TTCCGAGTTGTAGGAAGCTCTCTGCTCAGGAAGAAATATGTGGATCACTACATCACCAAAATCCAGCCGGGTCCAAGATT

TAGGTTTTGTGTCCCCTGACGGAAATTTTCCGTACTTCTTCTCAGCAAGATCCCTTATTTTCGACGCCAAAGCATCAGTC

TGAGGGCGTGAAAACGCTGTTGCAATGATAAAA

>GT971927.1 GJCCJC2026D01.b Jatropha curcas L. developing seeds (mixed stages) Jatropha curcas cDNA clone GJCCJC2026D01, mRNA sequence

GCTCGTGAAATTGGGTTCTTCATTGTGCTATCCATCCTTCTATCGTTTCCTTTGATGCTCTGTATCGTTTCTTTCAATTT

CGGTTTTGCTTTTATCTCGCAATTTAGAAATAGAAGACTTTATCTTTACATGATTTGATTGGATTGATTTCGGTTAATAC

CACGCATTTTCTTACTTCGATCTTTAAATTCTTCGAATCAGCTTTGCTTCTCTGTCTTTTTCTTTCCTTCTCATTGCGAG

ATGCATATTACGATAACCTATTGAAGTGGTAATTTCTTTTTTGGCATTTCTTTCTAATTATAATTGCTTGATAATACGTG

AGATTATTATGATTTTGAATGATCAAAACAGACAAAAATAGATGATGATGATGACTACTACACTCTTTACTGGAAGTACA

CTCTCTACGGGAGTGACGACGATTAGGGAACGCTTTGGAGTAACCATAGGACTCATACGATGTCTTTGGCATACCGACCT

GGATTCCTGAACGATTATGATGGAGGGATGTGTCCAGCTGATGCTTGTCAATGAAATACCGACAATGTTTATCACCACTT

CCGAACCG

>FM889186.1 FM889186 Jatropha curcas embryo 35-55 (DAF) Jatropha curcas cDNA clone rjcfea0_002777, mRNA sequence

GTGAATCTGTGAGACGACAGACAGTCGACAGATAATATGCTTTTTTCCCTATTTGCAAATGGTGGTATTTTAGCATTTAG

ATAATTAATTTCCACGTTTTTTCCTTATATTTTTTAGATTGACTTATCTAGACCTGTCTATATTCTATTCGTAATCAGAA

CTAATCAATCTACTTAGTCTCCCAATGACTTTTTGTTTAATCTGAGATATTATGTATGTCTTTTTCTGTTTTATTATTTT

TGTTTTTTTTTTTTTTTTTTGAGTTTAGTAGTACTTCTAATATGTTCAATGTCCTATCACACATTCTTTTTTTGTGGGGT

CTTTAGTTTTATTTTCTTCGAGATTCTCAAATCCACCTTTGAAATGCGAGAAACATAATAACTATAGTAATATCTCAGGC

CTTTCTATAATATCTTCTCACCTATTATTTCTTCCAATAGTCTTTTTGCCTTAGTCAAGTTAGGCTTGGCATCAAAACAT

GGATGCAGGAGAACAAAAACTAAGTCTTGAATCACCATTGATATCTCAGCCAAGAATATCAGAATC

>GT976179.1 GJCCJC2074A05.b Jatropha curcas L. developing seeds (mixed stages) Jatropha curcas cDNA clone GJCCJC2074A05, mRNA sequence

CCTCAAGAGTTTTACATGATGGAAGGACAAAATGGTAAATGGAAAAAGTTAAAAGTGCCGGATGAATTTTCGGGGATTGT

TCAATCTGGTTGCTTTTGGGAGATATGAACAAGAGGCAAGAGCCGCTCGTGAACATCTTCTCTTGTGTATATAGAACTCT

TCCATTGGCTTTGTGAAACTACAAAGAAGGAACCTTTTCTTCAAGTACTTCCAAATGTCGTTAAATATATTAATTTTGAA

TGCTTTCAAAATTTTTTGAAAATATCATATATCATAAAAATACATCAAATATGTTTTCAACACATATACATTAAAAAGTG

TATTTTTTATAACAATAAAAGTATAAGGTTCATAGTTCCATTGCATCAATTAAGTGACATCCGATTGATAGAAGTTATTA

TAATGATTATGCGACATTTGCGAGCTTATCAAACATATATTAGCCTAATTCAGAAACTTCTATTGGATTTACCTCAAGAT

AATCAGTGTTCATCTTCGCGCATCAACCGGCTACTCGTGCGCGCACACACAAAAAGAATTCATGGGGTTCCAAGTACAGA

AATTAGATCTACCATTTAATACTCCACATTAGCACAATTAAACAAATTCACAACAAAACACAATCAACAGAATCTCATAC

ACAAGCTAGTCTAATTAC

>GT971577.1 GJCCJC2022B04.b Jatropha curcas L. developing seeds (mixed stages) Jatropha curcas cDNA clone GJCCJC2022B04 similar to WD-40 repeat family protein, mRNA sequence

GGAAGACAAAAGATAAGAAAAATCAGCGGCAGATTTGCAAGGTAGAATGTCTTACTAGCATTTAGGGGAATTCGATTTCA

TGGCGGAGGAAGGAGAAGGGAGATGAAATCGACCAAATGGAAGATGGAAGTAATTATGGAGGACAAGACAGTGTAGAAAT

GGAAGCAGATGAATATGACATGCTGACCAAGGTCACTGATACATCTTCTGCCCAAGCTAGGGATGCGAAAGACATACAAG

AAATTCCATGGGAGAGATTGAATACAACTAGGGATAAGTACAGATTGACAAGGCTTGAACAGTACAAGAATTATGAGAAC

ATTCCTTTATCTGGTCAAGCTGTGGATAAGGAGTGCAAACAAATGGAGAAGGGAGGGAACTACTACGAATTCTTCCTTAA

TACTGGATTGGTGAAGCCCACAATTCTTCATTTTCAGCTTAGGAACTTGGTTTGGGCTACTACAAAACATGATGTCTATC

TCATGTCTAAATATTCAGTCATGCACTGGTCATCATTATCTAGCAATTTGTCCGAGGTTATCAATTTTGCACGACATGTA

GCACCTAATGAGAAACATGCATGGAGTTTGTTAGAAGGTTTTACCCAAACTCAGATTAGCACATTAGCAGTCAAGGATAA

TTTTCTTGTAGCAGGCGGCTTCCAAGGAGAGCTTACTTGCAAACGTTTGGATAAAGAAGGCATTAGCTTTTGTACCCGCA

CACATATGATGATAATGCCATCACAATGCTATTGAGATATATGACAGCTTGAGGG

>FM890409.1 FM890409 Jatropha curcas embryo 35-55 (DAF) Jatropha curcas cDNA clone rjcfea0_004262, mRNA sequence

TCTATACATCAACCAGATTATCTGAGTATCGAGGATGGGGTTCGAGACCAGCCAACCCTAACCATCGCCATTTTACATGG

GGTGTAAGATCATCAGAGAGGCATGTGGTTGACTTCTACATCTCTGATTTCCAATCAGGGCTCAGGGCATTGGTTAAGAC

TGGCAATGGCTCAAGGGTAACTCCATTTGTTGATGATTCTTTTAATATTGATATTGATCCAAAGAAGAAAGACTTGTCTC

CACAGTTTGCCAGGTGGTTGGGACAGAAAAACTCTTTCACGTGATGATCGTACAATGCCCATTGAAAGAAGGGTTCATCA

AAGAAGGTAGCACTGTTAGCTGTGATGGGAATTGTTCCCACAACTGACAATGTACTTATGATTGCTCCGCCACCCGAGCC

ATTGGCGACTGGATGGAAGTGGCCTAAATGCATCTTTCCAGTTAGCCTTGACGGTATTG

>GW880508.1 JC001134 Seed specific Normalized cDNA library from Jatropha curcas L. Jatropha curcas cDNA clone N02175 5' similar to Unknown protein, mRNA sequence

GCCAGAATTATAATAAACAAACAAATTTGTAAAAAAAAAAAAAAAAAAAAGCAGCAGCAAGCAATGGCCATAGGCAAGGT

AGAGGAGAAGGAGCAGCTTATCCTAAAGAAAAGAAACGAAGAGCTAGAAACAGCGCTCAAGGAAAGCAAGCAGAGGGAAG

AGCAGATGAGAGAAGAGCTGCAGAAGACATGGCGGAGACTCCAAGTGGCGGAGGAAGCGGAGGAGCGGCTTTGCACACAG

TTAGGTGAGCTAGAAGCTGAGTCTGTTAATGAGGCGCGTGCGTACAACGCTCGAATCCTTTCTCTCCTGGACCAGCTCTC

TCAAGCTCACCATCTCATCCATAATCAATAAATTAACAGTTGAAGTATATAAATATATAATTTGTGTGTATTAATTAAGT

AGTTTAGATCTCTTGGACTCTTCTTATTTGTTCTTAAAAGAGTCTATTTTCATCTCGAAAAAAAAAAAAAAA

>GW876782.1 JC006028 Seed specific Normalized cDNA library from Jatropha curcas L. Jatropha curcas cDNA clone N10278 5' similar to Unknown protein, mRNA sequence

GAACTCTGGAGTTGCAGCCAGAAGTTAATGAAGTGGTTGACTCTATGATTGAGCGGTTGAGAACTTTGAGCCGCAAGTCC

GATGGACGGTTTATTGCCGTGGACTTGAGAGTTGATATATTGGAAAAAAAGAGTTGCCACGGTAGTGATGCTGGTGCAGC

CAAAAATTGTTATAGTGCTCAGGAAATTGCCTTGTTTTTAAGAAAGATTGGATTTGATAAGGACACCACAATCTACTTAA

CTCAGTCCAGATGGGATGAAAGCCTTGATGTTCTTAAGGACATATTTCCAAAAACTTATACAAAGGAAAGCATCATGCCT

GAAGATAAAAAGACCAAGTTCCTGGAATCCGAAGATTCGGAGTTTGAAAAAGTTATAGACTCCTACTTATGTTCTCAGAG

TGATGTGTTCGTACCAGCCATATCTGGCCTATTCTATGCCAATGTAGCTGGTAAGAGAATAGCTTCTGGTAAGACACAAA

TTCTTGTTCCAGCCAACATTCCGGGAACCTCTGCGTCGGTCACCAATCATTTCTCCCCATATATCTCAA

>FM892155.1 FM892155 Jatropha curcas embryo 56-70 (DAF) Jatropha curcas cDNA clone rjcaeb0_002650, mRNA sequence

ATTGGCNTACAAAAGAACTCTTTTGCTCCTCTGCTTTGAGAAACTGCAATGAGAATCCCATCTTGATCTTGACACACGGC

GACTTATTAACAAGTGAGGAGAGAATTGAAGGCAGGCTAAAGATATGTGAAAGTTTAGGCATATCAGAGACAAATGGGAT

ATACGATGTCGTTTGCCTTACAGAATATGGATTTTTAGCAGAAGAATCTGACCCTGTTAGTGCTTACAATGTAGCTGAAG

CAGTATATAGGGCATTGCTCATTTCTGACAGGGAACACTTGCCAAAGAAAACGTTGCAAGACTGGACGCTCTTCATATTA

TCATGGCTAATGTCTCTCATGGCTATTTTTTTCGCTTTTCTTGCTCAAATTTGCTCTGCACTTGGACATAGCAACAAGCT

GAAGCGTTACAATTTAAAGAGAAGGAGATTAGGAAATACATACATATATATTTTCTGTATATTATATTGTTATGTGTAGT

TTAGCTTATAATGACAATTTCAGTAAGGCTAACTGAATATCTGTTTAATCT

>FM893547.1 FM893547 Jatropha curcas embryo 56-70 (DAF) Jatropha curcas cDNA clone rjcaeb0_004405, mRNA sequence

ATTCCCTTGAGAGAAAGAGAGGAACCTTAAAATATGGCGACACTGCAGAAATTCAAGCTGCTCTGCTACGCAATGCGGCG

TGGCGGAGAGTCCAACACGAAGTCCCAGAACAAGTCCTATCGTCCACCTCCGCAGCCGCAAGAAAACCACTCTCCGCATG

CTTCTCACCCGAAGCTCCGGCTCTGGCCGACGGTCACCTCGGCGACATGAGCCATCGCCGATTCCTCACCAGCAACGTCT

CCTTCCACCGCTGCCGGAGAAAAAGAGAGACACCTTAAAGGACTTGTATGTGTCGTCTCCGCTCATTTGAAGAAGAAGAA

GAAAACAAAGATAAGGTTAGAGAAATGATTGGAGGCAAGTGTGAAGTGTCTCGGATGAGAGTGAACGGTTTAGGAGCTGA

ACTGGCCGGTTCACCTCGACCGGGATGGATCGGTTTAAGACATAGGTATCTGATTAGAAGAGCTTGGCGACCTATGCTAG

TTGGTATTCCAGAAGAAGAAGAGTAAAATTAACTTAACCGAA

>GW879157.1 JC003715 Seed specific Normalized cDNA library from Jatropha curcas L. Jatropha curcas cDNA clone N06838 5' similar to Unknown protein, mRNA sequence

GGATTTTTATTTTTATTATTTTCTTTTCTCCAAAGTAAAAATAAGAAAAGGGAAAAACAGAAAAAAAGAAAAAAAGGAAA

ATTCCTTCATTCTCTTCCCTTTCTGGCCAACTGCAGACTGTAGATTGTTTCCGGTTTCAATTGCTTCATCGTCAAAGTTC

TCCCCGCTTGTACGTCCGGTTCTGGATTTTGCTAGATTCTTCCAATTTTATCAGCTCTCAATTTGTCAGGATTGTTGACA

TTAACTTTCCACCAAGTTGGAGGTCTGAGGGAATATTGCCTTGAAACTTTTTGACGCTGCTGTGCATGAGCATACTGTGA

TGCCTTAGCATGGAGATGGAGTCACAGGTCTCCTTGGACTCTAAGGTACAGTTTCATGCTGAGAATACTTCAATTCAACA

TGGGAACCCTGGTATAGTATCATCAATACCTGACCACGTTGAACCACAACAGCCAGCAAAAAAGCCAACGCGACAGTGGG

CTGCTTGGACACGTGAAGAGGAGGAAAGTTTCTTCACTGCATTACGACGGGTTGGCAAGAACTTTGAGAAGATAACTCGT

CGTGTCCAAAGCAAAAACAAGGATCAGGTCAGGCATTATTACTATCGCCTTGTAAGGCGTATGAACAAGTTGTTGGGTCC

AGGATTATGTCTTGATGCCAAAAAACTCTAAAGATACTAATGCTGCAATGCTTCGATGGTGGTTTTTATTGGAAAAGTAC

AGCTGCAAAGCCTCAAAGCTTCACTTGAAGCCACGAAGGTTTAAA

>JK613615.1 JCF1338 Jatropha curcas, immature Seed cDNA subtraction library Jatropha curcas cDNA similar to conserved hypothetical protein, mRNA sequence

ACTAGCCCATTGTCCCGAAACCTGAACCTCCTGCCAACTCTTTGGGTGAAATCTCTTCACTGTCTCAGAAAATTATGCAA

TCTCCTTACCTTAGGAATCTCTGCCTCAGTTGCATGCCAGGCCCACTAGAACAAGATAGAGATGGAGAGCTGCTATTTCT

CCTCCTACCATGCAAAGACCAATAACAACTCATTATCTTGGTCTTTCCTCTCCTGAATGTTTGATAACCTCTGCTACATA

TCTCTCCTTGCGGGGTTTTGCTCATGGGATTTCTCAGCTTCCTGTTGCCAATCTTGCTGGGGTTAACACCTCTTGCTTGG

AGAATCCAGTATGCACCTCGTCAGATTTGGTCACACTTTCTCTTCTTCCGTGTCAACCTTGGGATCCTGCCATTGCTCCC

AAAATCAGATGCATGGGGCGACGCGGAGAGAAAAAGGTAATTGATTTGAATATTGGAGCTGAAATCCCATAAGATATAGA

CCTCTTGCGGCTCTGCGAGAACGTCCTGCTAACTTAACAAATGTGATAGTACCTCAGCCAAGTAGGCCAGTTGGCTCCAG

GATCAGCGTCGTGAGCATAACATGAAGACGCAAGTTAGCCTCTTCACTGGAACTGCCATAGGATGCCAGGGGACGTTGAT

GAAGACATGCATCTGATGCCTTGCCAGCTGGGATATGCGATCTACTACGAACGTGAGCTAGCAATCTGCTAGGACACTGG

TGGGTCACCAGAATGTTATCGCTCGACTCGATGATAAGGGATGTCGTCTGGTGGGTCAGCATACGAGTATTGGCAGTATG

CCGAATCTTGTTGTCAGAGGCATACTCCAATGCGTTCTCTAACTCGGGGAAATACAGTGGACTCAAG

>JK317690.1 JCST461 Jatropha curcas L. seed cDNA library Jatropha curcas cDNA 5', mRNA sequence

CATTTTTTTTTTTTTCGTCAGAATCTCAGTTCTCTTCCGGTATTCTTCCGTTTCGATTTTTGTATTATAATCGGAGAATA

TGCGTTCAAGTCGCGTGCTTCTCAAGGAGCCGATAAGGGTGGATTCCGGTCTGAAGCCGTGAAAGGGGAGTTGCGTTCCA

GGACTGGGGGGGATTGCGGGAAGCTAAACGGCCGTCCGGGCTACCCTACACGAGCTTTGGTGGTGGGTAATATGAAGGTT

GCTTGGCTTAAATCTTCAGGAACCGCATCCGGTGGGACACAGGGAGGTGGGGGTGAGACAGCAATGGGGGGGTGTTGTGA

AACGCGTTAATTTATTTAGTGTGACTTGTGTTAGCAGAAGCCCCGAAACGCAATGGTGTGGGAACGCCCCTAATAGTGTC

GGGGCCTTAAGATTTTCGTGATAAGTGGTGGATAGCGCAATGGTGGGCGCTTTATTTGCCTCCCATATGTGAGCGCTATA

TGAAGTGGTGGCGAGATGGGAGGATTCGCAAATATAGGCGGCGCGATGGAGGATCTGCATGGATAATCCTCGAGCGTAGG

TAGTGTTGTTAAGCGTACCCAGTGTAAATGAGCTTGACAGAGTAGTTCAGGATTAAGAGGATGNACATGACGTGAGNNTG

CTGGACCACAGTTGGATTACTATTCGGGACTACGAGCTGCGATGCNGATGCTGTTAACTTACCGCTACGGTTTGTCGGGC

CAGTGGTAGACGAGTCTGTCCAGTTCATCAGTTAGAGACTGAGGTACCATTAGGGACANACGGTGCTATCTTGATTTAGG

CACCACGCACACCCTCGTTACCTAGGGAATTTTNNTC

>FM895785.1 FM895785 Jatropha curcas embryo 71-95 (DAF) Jatropha curcas cDNA clone rjcpga0_002771, mRNA sequence

TTCGCATTTGCCCTCTAAGAAGCGTACGCATAGCATCTTTATCTGCTTCTCTTTTAATTGCCAAGGAATCATCTTTCAAT

TCCAAGCAAACTCTTTTGCGATCTCTTCATAAGTCACCTCTTCCTTTGGTTCCTCGCTCGATCAGAACAGGGAATTCAAG

GCCTGTGCTCTATTCTTTGAGATTCGATCATACAATGGCTACCCAATCTGAGCCTAAATCAGTCCATGACTTCACTGTTA

AGGATGCTAGGGGGAACGATGTTGATCTCAGCACATACAAGGGAAAGGTTCTCATTATTGTTAATGTTGCTTCACAATGT

GGCTTGACCAATTCCAACTACACTGAACTGACTCAGTTATACCAGAAGTACAAGGATCAAGGTTTGGAGATTCTGGCTTT

TCCATGCAATCAATTTGGATCTCAGGAGCCAGGGACCAATGAACAGATTGTGGAGTTTGCCTGCACTCGCTTCAAGGCTG

AGTATCCAATATTCGATAAGATCGATGTCAATGGAAACAATGCTGCTGCTTCTTTACCAAGTTCTTTGAAGTCCAGCAAA

GGAGGAATTTTTGGGGATAGCATCAAGTGGAATTTCCCCAGTTTCTGGCTGATAAAGATGGCAATGTTGTGAACCGAATG

CTCCACTACT

>GW876735.1 JC005661 Seed specific Normalized cDNA library from Jatropha curcas L. Jatropha curcas cDNA clone N09684 5' similar to Multicopper oxidase, mRNA sequence

GCTTCTCCCACTCCAAGAACCTAAGCCTTACGCTTCCCGCTTTCAAAATTTCTCTCTGTCCCCTTCTCTCTGCCTTTCTG

ATCGGATTTGCTTCCATAAATAAAAAAGAAAATGCCGCTAAGATTAGCGGGAAAAGGTGCTGTTTGCACCTTGTTTTTCG

CTCTTTTTGCCATTGTTGGGGCTGAAGATCCGTACAGGTTCTTCAACTGGAGTGTTACTTATGGGGATATATACCCACTT

GGAGTTCGTCAAAGGGGTATTCTTATCAATGGACAATTTCCAGGACCTGATATTCACTCTGTTACAAACGACAATCTTAT

TATCAATGTGTTTAACAGCTTAGATGAGCCTTTTCTTCTCTCCTGGAATGGAATTCAACAGAGGAGAAATTCATATGAGG

ATGGTGTTTTCGGAACCACACGCCCAATTCCTCCAGGGAAGAACTTCACATACATTCTTCAAGTGAAGATCAAATAGGAA

GTTTCTATTATTTCCCATCTCTTGCATTCCACAAGGCTGCCGGTGGTTTTGGAGGAATTAGAATCCTCAGTCGCCCAAGA

ATTCCCGTTCCCTTTGCTGATCCAGATGGCGATTACACCGTTCTTATTGGAGATTGGTACAAAGCTAATCACACGGAGCT

GA

>JK317918.1 JCST689 Jatropha curcas L. seed cDNA library Jatropha curcas cDNA 5', mRNA sequence

TTTTTTTTTTTTAAGAATTTTGCAAGTCTCTGAGATAAACTTCTCAATTGTGTGAGGGGGGGGGGCTGTGGCCTTGAATA

TCCCTACATCCAAGACTAATTCATAGTATTTGTCAGCAATTATTATTTTCCGGAAGGCGCCCGTCTACATCGACACGCTG

CTTTGAGAGAGCGCCCGCCGGGGAAAGAAGAGTCGTATGGAGCGTTACCCACTAAAAGGTTATGTTTTAGGGAAGAAATG

GGATGGGACCTTCCGGGATAATTTGGTCACAAGAACTCTATGCAGCAATGCTATCGGGATGGGAAAGATTAAAATGAGAA

CCGTAATACCATGATGCCTATTGGTTCAGGGGGTGCATAGGCGGGAAAGTGACTAGTGGACGTGGGACGCGTGCGGTTCT

GAGTGTGGGGGCCTGAGCTAGCAGGATCACGACTACGGGCTTATGAGGGAAGTTTAGTAAGGGGAAAGGGAAAGCCGTAC

AAAAGGGGAACGGGGCCAGGGGCAGTGGACGACAAGATTCGAATAAAGGGGGAAGTTAAGGGGGCGGCTCTGATGGTAGG

CCGTTCGTAAGCAAAATCCACGTTTTGAGCAATGCTACCAGATAATTCCATAAAAAAGCATAAGCGAGGTAATGGGGAGC

TCACAGCGGAGATAAGACAAGCCAAATGAGTAGTAGAACATTATCGTCTCCCCAAACTAGGAGTTTTTAGAGGGACGCAG

AACGGGAAAGAAGGAAACACGTAGCGAGCGGTCGAGGTCGATGGATGAGCTATAAGCGATTCTGGCTCGAGTACGTCTAC

TCGCCGCGACACTATGAATCAG

>GW876126.1 JC005503 Seed specific Normalized cDNA library from Jatropha curcas L. Jatropha curcas cDNA clone N09451 5' similar to Unknown protein, mRNA sequence

ACAGTAGGAAACGCATTACCAATTCTCTGTCGTATCTTTGTTTCAGCACTAAACAGAAACAGGACATTACTCACTCCTTA

GATCTTCCCTTTAATACAAAAAGATTTTGCAAACGCCATTCGTGAAACCTCTTGCGTCTGTTGATAGATATATTCGAAGC

TTTTATAGAGAGATTTTAATTGGAAAGAGCGAGATAGAGAATAGGCTGATGGGTTATAAAAACAGAGCAGAAACAGAAAC

AGAGAATTGTTTAAGCGACGCTTTGTTGCTTGCTACCATGTGCATAATTGGACTCCCCGTTGATGTCCATGTTAGGGATG

GCTCTGTTTATTCTGGAATCTTCCATACTGCCTCTGTTGACAAAGACTACGGTATTGTGTTGAAGGAAGCTAAACTGACT

AAGAAGGGGAAATATGATGCAAATGTGCCTAATGGGAGTGTGATAGAAACGCTTGTAATTCTTTCAGGTGATCTTGTTCA

AGTTGTTGCCAAGGAAATTCTATTCGCAGCTGACGGTATCACGGGAAATGGAGCTAGCGATGATGTAGAAGCTTCTGTGG

CCAATGTTTCCTCTTGTGAGGTTGTAATGTGTGAGGCAAAGGAATCTAATAGGTCTGATGTGGACAAAAAGAAAATTCAC

CTGAACAGGATTTC

>FM894214.1 FM894214 Jatropha curcas embryo 71-95 (DAF) Jatropha curcas cDNA clone rjcpga0_004514, mRNA sequence

GTTTCATGTAAATCACAGTTGGTTTCAATGGAAAAATATTCTCTTTGATTTATGTCTGAATATAAGAATCTTTANGGTTT

AAAACCCTAGACCTTTTTCAACCAATAAAGCTAGCTCCTTGGCTGGGCATTTTGTTGAAGCAGAGATGATTCAGTTGCTA

TTCTTAGTTCTCTTTGCTGAGGGAGTTGTGGCATTCTTATTACTGGTGAAAATTGGTCCCTTGAGAGAGCTGGTGTTAAA

AAGTTTGGATCAGGTAAAGATGGGAAAGGGTCCAGCTACTGTGAAAACTATTGCTGGTACCATGTCAGTGATTCTTCTAT

CAAGCCTTATGAGCATTGTTAAGATCCAAAACAAGGGTGCAAAGCTTGGCACCATGTCGCCCATGGATCAGGTCCTATGG

AGAACCCACTTGCTTGAGGCATCACTGATGGGTTTCACTCTCTTTCTTGGGTTCATAATTGATCGTATGCACCATTATCT

TACAAAGCTTATTAGTCTACGAAGTAACATTGGATCTTCTAAAGAGGAGGTTGAACGGCTTCAGAAAGAGAAAATGCAGC

TTAAAGGGAA

>FM892335.1 FM892335 Jatropha curcas embryo 56-70 (DAF) Jatropha curcas cDNA clone rjcaeb0_002862, mRNA sequence

TGAGCCCCGTGGCTACTTTGGTGACTTCTTTGATGGAGTGCTGCCGCTGCCCCAAAACAGTAGAGTCTAAGAGGTATCAG

CAATGCAGGTTTGGTCACTCCCCTAATGAATGACCATATTTTCAAATGATATCTGCTAATCTTCTTTGGATTGTCTGATT

GCGAGTAAATTTGGTTGCTGTAACATAGCCGAGTGCCATGTAATCAGTTTTCCTCGTCCTTTGATGGTTTTCCTTCATCT

GTATATCTTGATGTAACCACAAACTGCACATCAACTTCCATATCAAGTGCAAATTCTTAATCCTGTCATTGTTAATTTGA

ATGCTGAATTTGCAAGGTGAAAATAGCGAGTTATTAATATATAGATTTTGTCTCTCTAGTTGTTTTTAACCTTGTTCTTA

CGCTTTGAAAGAAAATAAAGGGTTATGAATCTGATCATGCGGTC

>GW616975.1 Jc2-017-G06-M13F.G06.ab1 Jatropha curcas flower and seed Jatropha curcas cDNA, mRNA sequence

TGAATCTTCCTTGCTAAAATTCAATTCTCAATTTAACTTGTCACATTGACTCTTCTTCTCTTTCTCACTACAATCCCTTC

TCTGCATTTCTCAAGGATTATCCTATTACCAGTTGGAAGATAGCGTAGTTGCAGAGCTGTAAAAGTTTGTAGAGTTGACA

ATCTATTGGCTGTTCGTTCTTCCATGGAAGAGGAGAATGCTGCTATGCTCCTGCAGAGATACCGGAGGGACCGGAGAATA

CTTTTAGACTTTATACTTTCCGGCACCTTAATTAAGAAAGTCGTAATGCCTCCCGGTGCAGTTACATTAGATGATGTGGA

TCTAGACCAAGTCAGTGTAGATTACGTTCTTAACTGTGCTAAAAAAGGTGGAATGCTTGAGCTCTCAGAAGCAATAAGAG

ATTACCATGATAACACCGACTTACCCCATATGAATAATGGGGGTTCTGCAGATGAATTTTTCTTAGTTACAAATCCTGAA

TCTTCAGGATCACCTCCAAGAAGGGCCCCACCACCCATTCCAGTTTCAGCACCACTATCCATTCCAGTTTCAACAACAGC

TCCTATTTTTGCATCATCCCCTGATGTGTCATTGTCAAGTGTAGGAAAGTCAGTGTCCTTTAACTCCACTGAAGATCGAG

AACTGACTGTT

>GT974341.1 GJCCJC2158B09.b1 Jatropha curcas L. developing seeds (mixed stages) Jatropha curcas cDNA clone GJCCJC2158B09, mRNA sequence

GGGCCCATCACCTTCTTTCAACGCCCAATAAGCCCGTGCGGCCCGTCACCTCTTTCCGAGACAGCAGTCCAACAGCCATC

TTTTCAAAGGGCTCGCAATGGTCCGCTTCGGCAATTTAACGTCGATGCGCGGCACGTACACTTCTGCGATTTTTCTCTAC

TCACCGCACAGGGGGCAGAAACACCAAATTGCTAAATTACCCTCAGGTAAGTTTTCTTTCGACCACTATATCATAAAAGG

TGTATTTGAAAATGCAGATAACAAATCCTCTGTTAAATCATTTATGCCAAAATTTCTGCATGGGAACATGTGATGTTCCA

ATATGTTTC

>GT972030.1 GJCCJC2027F09.b Jatropha curcas L. developing seeds (mixed stages) Jatropha curcas cDNA clone GJCCJC2027F09, mRNA sequence

GATATTGATACGCCAATCCCCGGCAACGGCGCCAAAAACTTGACATGCGTTTTTCGCAAGTGTACGAAATCGCAACAAGT

AGTATAGAGTAAGAAAGTATCGTATCCACAGGGATTGGTTATCAATTACCAAAATTGTGTTAACCTATTATTATTTAAAC

GATGATAAAGGGAATTTGCTATAAAGTAAATAAAATTCAAAGTAAAAGAGATCAAACAAAATTATTGTGAAAAGAAATCA

GGAGATTAAGCTACTAGGGAATTTGATTTCATTTAATTCTATCCAGCTTATTCTTATTAGTTCATTAATCAAGTTTAATT

GTTCAAATAGGAGAAAACAACCCTAAAGTCACTAACAAGCTCTCCCGATTTATTGTTAGCGATTCTAATCCAAATTAATC

ACCTGCCTTAATAGAGAAATTAAACATGAATCAGATCATTAGGTTTTATGGTTGTTTTATGAATCCACAACTTAATACTG

TATTAACTTATGAAAACCCCCCTTGAGTCAAACCATATAGTTTCCACATCAAGTTATTATTCTCAAGTAATTTATTACCT

TGTTCATATCAATAACCCTTCTCCCGAATGTAATTATTGAAATAAATCAATCCAGGGGAATTATTCTGCCTGGAAGCCCT

TAAGCACATAATAAATTATAACAAGAAACAAGAAAAAATAATCCTATATTATAAGCTGGAATCGAATTAAATTACATCAA

ATCCCTAGTATAAAAGTTAGCTAGACATAGTAATTAATTTAACTCAATCACACTTGAATCATT

>GT975483.1 GJCCJC2070G01.b Jatropha curcas L. developing seeds (mixed stages) Jatropha curcas cDNA clone GJCCJC2070G01, mRNA sequence

GCTGGGGACATATGTAATCATTTTGCATAAAGTGGACTATAATTGCTGCATCAACTAACCTGCCCTCCTTCAAGGTATTA

TTTTTGTTGTGCTTTGTTTTGCAGGGTACCCTAACACGAATCAAGTGTTCTTTCTTTAAGAATACTGGATAAGGAAATTT

GGACTTAATTGTAAAATTTAATAGAAAACATTTAAATTTGTTGATTTATTTGCACGATTGAGAATCTTTTTCTCCATTGC

ATAAAAAACGAATTTCAAATGAATTTCTTCTCTTCTGTTCAAACTTCTAAGTCTGGATAGCGAGTAGCACTGATCGGCAA

GTCTCCTTGATTATGTCGACAGACAGCCAAAGCGTCCTCCCTGTTACCGCAAACCATCCCTAATCCTTTTGGGGGTGGCC

CATGAGTATGCGTACTGTTAGACCCACGCGAAACTCTTTGTTTCACATGTGGTTAGGTATATCCTTACTCAAAGTTCAAA

GGTCCCTTAATAAGCCAATTGTCTATTTGTGTCATAGCCTATTAGTCCTATCCTTTAAGGCTGTATGCACCCAATGATGT

GTATCATATCA

>GT969967.1 GJCCJC2003F03.b Jatropha curcas L. developing seeds (mixed stages) Jatropha curcas cDNA clone GJCCJC2003F03, mRNA sequence

GGTGCTTTCATAGGCCACGATTGTGTGATGGGGGGTGGTGCTTTCATAGACCACAGTGGTTCTTTCACAGGCCACGATTG

TGTGATGGGGGTGGCGCTAACATAGGCCACGATCAAATCTGCTTATGTTCATTCTGTATTAGTACGAGCCTTAACTAAAG

AATTAACATATGAAGAGATTGATTATTTCCTTTGAAGAATTAGCTTATGAAGAATTAACATCTTTGTATTTTTAATCGTC

TTATTACTTTCAAAGTATTTGAATTAAACGAATGAGATCATATTATTTTAGTAAACCTTACTTTGGAAATTAATGATTAT

GTTATCAAATGAATTTTCAAAGCATGATATTCCGGTACATGGTTTTAAACATAAATAGTATATGTATTGGTATAATAAAT

GTGCAAGTAATGAATTTATGAGTTAAAAGAATTAAATTCTTTATGTTATTACTTAATAGTTTATTTGATATTTCTTTTGC

TACATGTATGTTGAAATTCAATGATTTTATGTCATTTGTGTTTTAAGTAAGTTGTCATCACAACTTTAGTAAGTATGTGA

ATTATGAGCACCACTGAGTTTTATACTCAGCGCGTTGATTTTTATCACGCGCAGGTTAAAGACTGTGGTTGCTGCTTTGA

GACTAGAAGAGTAGCATCAGGGCAGAAGTGACTATTGTGGTAGTAGT

>GW881292.1 JC001223 Seed specific Normalized cDNA library from Jatropha curcas L. Jatropha curcas cDNA clone N02319 5' similar to Unknown protein, mRNA sequence

GAGCCTGAGCTAGTTTGATGTATTTCCAGGGGTTCTGCGAGGAGAAGATTGAGTTCTGGGGTTATTTGCCTTGTCATAAA

TTTTTCAATATTTTATTGATCTGTTACTATGTCCATCTCTCTCTTTTTTCCTTCTCACCTTGGTGTGGCTATAATTTCAA

TCGATATGTGGATTCTATTATTGTCGTCCCAAGCTTTTTGTTTTTGCAGTGATCAACACTCCCTCAATCTTGCAGAACTC

AATGTGAGTTAGTTTTAAGCTTTGATCACTTCACTTGACTTTTCCATTTCATTTTCTGCGTTCTCCTGTGGTTTTCCTTG

CTGAATATGATAAAAACTGTTTTATCTTTTTTTTTTTCAAGAAGATTATACTGTTTTATCTTATTCAATATCTGCAGCTA

TCTGCAATTCTGTTCTGTCTATTCAAGTTAATGCAGCTATCAGTAATATGCGTTGTTTTGTTATTTGGTATCTCCCGCTA

GTGTTGGCTAAAAATTGGACAGCATTTTGAGTTACTGGCTTATGCACTTGGGATTTTCCCAATGTTGTGTAATGGTCGAA

TCACCATGAGGAAAGACAATTTACATTTTTGGAAAGTCAGA

>GT976435.1 GJCCJC2078B03.b Jatropha curcas L. developing seeds (mixed stages) Jatropha curcas cDNA clone GJCCJC2078B03, mRNA sequence

GGCATCTTAAGAAGCACCATCAAAGGGGAAGTTTTGAAGGGTAATCATGGAAAGGATGCAGCAAAGGGTACGCAAGACTT

CAAACAGTACCGAGACAAAGCCGATTGAAGGGGTTAGGGTCAGAATGGTTTCTCCGAAAACCCAAGAATGGAAGATGCTC

CGATAGAGAGGTGTCTGAATTTTGAGCTTAAAAGCAACAGAATAAAGAAGTTATGCTTGGAATTGGGTTGGGTCTGCACG

AAGTCGCAATGCTATCCATACACTACTCAAGTCATCTCAGAATCAAGTTGCCTCGAAATTCTTTATGGAGAGGCTCCTTC

TAAGCAAGCAAAAGGAAAAATGGGACAAAAGGAACCCCTGGTCAAAAGGAACTTCTGCACAAAAGAAAGAAATCAAGGAC

AAAGCTACAGGAAATCAAGTTGTCAAAAACGGGTCAACTGCAAGCTCTCCGTTAAATTCAAGCCCATCAAGGGTAGATGA

CAGATCATTTCAGCCATAAGGACAAAGACCAAGCGGCTGCTCACATTTTGAAAAAGCAAGTTAAAAGAAGCAGTCATTTC

CACGAATTGGATGACAACAAACACCTTGGAAAACAAGCGCCAAACATGGCACAAAT

>JK317682.1 JCST453 Jatropha curcas L. seed cDNA library Jatropha curcas cDNA 5', mRNA sequence

TTTTTTTTTTTTTTTTTTTACATTTTAACTTTAAGGACATTTTATTTTTCATTTCTACAGAAGTAAAATTGCTCATCTCC

AAATGCATATTGTGCGCTCAACAAGATAGAAAGTCCTTAAAATTTTTCATTTTATATCTGTGGAGAAGTTGAGAATGATA

TCAACAAAGTAACAGAACCCAGAGTGTCAGAACATACAGAACCCCAAAAACTATAGACGTGAGACTGTTGGACCATGCTT

AGAAAAGGGTGGCATGTGTGAATGAGACTGTCACTCGGGATAAATCTCATCTTCAATTTTTGTCGGAGCAAGCTTCCCTT

GCATCCAGCTTTTCAGCATCTGTAATGCAGCTTTTGGCTGATCCATTGGAACCATGTGCCCAGCCTCATGAACCTTGAGA

AAAGTGAGAGGGCCATGGGATTTGAGTTGTCCTGCTTCTTCACCTTCGACAATAAATGGAACATCTGGAGATGCTCCAAA

TTCTTTCTGGCCAGACCATTCCATTGCCTGAACCCACCTCGAATTTCCTAGCCAGTTGCATATGAGATCTTTCTCGCCAG

CATAGATAAGTACTTTGATACCATCCTCAAGGAGAGCAGGGATTCCCACTTCAAGATTCCTCATCCAGTCCGTTACCATA

GCATCATAAACCTCACTGCTGCATGAAACAAATTCAAGCTCTCCAACCCCTAGAGCATCCCTAACTGTTTTCTTGTTAAG

GAAAGTCTCCAGGTTTGAGAAGTCATAGCATAATTGCCCATCACATGTCTTTCTGATGTCATAGTAATTTATGTCTCCAG

CAACTTGCAAGATCGCATTGAATATGTCGTGCACAC

>FM887831.1 FM887831 Jatropha curcas embryo 35-55 (DAF) Jatropha curcas cDNA clone rjcfea0_001107, mRNA sequence

GCGGTGGCGGCGCTCTAGAATAGTGGATCCCGGGCTGCAGGCTCCTTTTGGTAAAACAAATTAAGAACCGTTTCCTCTCT

CTCTCTCTCTCTCTCTCTCTCTCTCTCCTCTTCCAATCTAAGCAATATCACAGCTGTATTCACCTCTCTTTCTGTCTCTC

TTGTGCTCTCTCTTTCTTTGTCTTTCTTTCTTCTTTCCTTTCTTTAAGCGCCGACACAATTCTTCAATTTCTTCCATTCT

GATACTCTCTTATTCTCTTTTAGATCCAATCTTTGTTTCTTGCAGTTCAATCCCGAGTCCAAACGGAGTCAATTTTACCA

TAGAACACCGATTTGGACTAACCCACCTGAAAAAGCTTTGAGCTTAGTCTGGAAGTTAGTTCTAACCGAGGTGGGTGTGC

CTGCAAAATCTGTACAAAGCCAGCTACAAAATACAATACGCCTTGGAATTTTCTGTCTTTGACATTTTGGTTAAGTTGAG

TCTATTGAAGTTGGTGCTACGTAGGAATTCGTAAAGAAATGGTGGTTTCGTGATAAAGGGTGATCAGTGGAGAATTTGTT

GGTTTCTTTTATTTAGGTACAGCCATGGAGACGGCAGGGGCAACGACCACTCAGAGCTTGACGGACTCCTACAAAGGCAT

GTCCTCTGATAATATCCAAGGTTTGGTTTTGGCG

>GT971354.1 GJCCJC2019F08.b Jatropha curcas L. developing seeds (mixed stages) Jatropha curcas cDNA clone GJCCJC2019F08 similar to encodes a chloroplast ribosomal protein L2, a constituent of the large subunit of the ribosomal complex, mRNA sequence

CTGGGTCCAATGGAATTCATAGGTATAGGAAGAAGCCCTGTCAAATAGAGATTTTTTCTTTCGACCATCTTTCGATTGTT

AATACGATATATAAGAACCACTACTACAAATAGTACTACACCCTTGATCGTGAAATATCGATTGCTTGTTGAACCCTGTG

AATTGCGTGAAAGTAAGATACTCCGAATTCGGGAGTCCAAGAGTTTTATAAAACGTTCTTGATGGAAAAAAATGTGAATG

AAAGATCCCACTGAATTGAATTGGGTCCATGAATCTAAGAAATAGTGAGAATTCTTGATCTCTCTCAATATCTCTCTCAA

TTCGAAAATCCAGGATTTGAATTGATGTCCTTTCATTGATTCCTCCTAAATTGCATTGATTTATCCTAAAGATTTCATTT

CAATTGGAATTTGGTTATTCACCATGTACGAGGATCCCCGCTAAGCATCCATGGCTGAATGGTTAAAGCGCCCAACTCAT

AATTGGCGAATTCGTAGGTTCAATTCCTACTGGATGCACGCCAATGGGACCCTCCAATAAGTCTATTGGAATTGGCTCTG

TATCAATGGAATCTCATCATCCATACATAACGAATTGGTGTGGTATATTCATATCATAACATATGAACAGTAAGAACTAG

CATTCTTATTGAGACTAGAACTCATAGGGAAGAAAATAGATTTATGGACGGAATCAAATATGCAGTATTTACAGACAAAA

GTATTCGGTTTATTGGGGAAAAATCATATACTTTTAATGTCGAATCAGGATCAACTAGGACAGAAATAAAGCATTGGGTC

>JK317572.1 JCST341 Jatropha curcas L. seed cDNA library Jatropha curcas cDNA 5', mRNA sequence

ATTTTTTTTTTTTTTTTTTTTTCAGACAACGCAAATATGGAATTATTATCAAATCTGCAAATCAGCCAACATGCCAAAAG

CTCGACGAAAAATATATCTTGTTCTACCAAATATGTTCAAAAACCTATCAAACACACTACAAGTTCCAACTTCCAAGTTC

CAACAACCTACTTAAGCCTAGAATAACCAAATATTGGATGGTCTAAATCTGCTTAACAAGAAATAAAATGGTGTCTAGAG

CTGCTTAACAATAAATATAACTAACTCTTGAAATTGCAAAACCACTTGATGGGCATGTCTTAGTTCACCAAAAACATTCT

TTAGGAAACCAGGCCATCATTCAGGGATAGTGATGCAAGCTGATCGGCAGGGTTGGCTGCCTGCTGCTGAACAGCAACAT

TTCTAAGGACTTCCATGGCCTCTGCAACTTTTGCTTTTAAAGCCTCTGGTGATTCAAGCAGGCGTAGAACTTCAGTTTGA

TCCGTCTCCAGAAGCATGCCTGTAACCTTGGCTGCTGAATCATGCTCCAGTTGATCCACCAATGGGTACAGACTTTCACC

CAACAGCGTCCTTTGCTGCTCAGGGGTAGCATTTGCAAGGGCTGTAGCCAAAGCAGTGATTGGCATGGGCTGCCCTGTAG

CAGCATCCCTTATCGGCATGCCACCCATGTCATAGGGAACAGAAAGCGGCATTCCAGCAACACCTGCCATTGGAACATCT

GGCATGTTTCGACCAGTCGGGTAACGATAGACACGTCCCCTAGAAGCATCTGCTGTGCATTAGTGAACAGGCTGCTGGCT

CTGTGCACAGGACCAGCTCCACGTATCCAAAAGCGCTGCT

>FM894762.1 FM894762 Jatropha curcas embryo 71-95 (DAF) Jatropha curcas cDNA clone rjcpga0_000628, mRNA sequence

CTAGTGGATCCCCCGCGGCTGCAGGTTTTTTTTTACACGAAAATAGATCCACTTTCTAAAATAGAAATTTAAGGAAAACT

GAAATAAACAGTAAAGAAAGATGGGCGAAATTGAAGAAAAATACTACAAAATAAGTTCCTAACTTTCATCATCACATTTA

AGAATGTGAGATCTANCTCCGCTAATTCTTCAAGGACCTCCTCTTCATCCCTGTAAACAAAATCAATCATCCTCCATATC

TGAAGAGTACCACCTCCTGAAGTGCTTTCACAATCATCAGAAACACTGACAATTGTCCAGGGATCAGAGGCATTCCAATG

GAAGTCAACAACCTTATCCCTATGCCCAGCATGCCGGAAACATAATCCTGCAGGAGCATTTGGCAATTTCAACCCAACAG

AATCTTGCTTCTTACCAACCTTCTCATAATCCCATATATATAGAATAGCCCTC

>FM890588.1 FM890588 Jatropha curcas embryo 56-70 (DAF) Jatropha curcas cDNA clone rjcaeb0_000480, mRNA sequence

ATTATTCACTCTACATATTCACATCCAAGTGAACCAAGCACAAAAACAACGTCGCCGTACTTATAGGGCACATGGAAGGA

TAAATCCTTACATGTCCTCACCATGCCACATTGAGTTGATTTTGTCAGAAAAAGAAGAGCCTGTTAAGAAAGAGCCTGAG

ACTCATCTGGCTCCTAGGAAACCAAAGGCCCAAGCTCTACGCAGTGGGGCTTCCTCCTGAAGGCAAGCAGTAAACAGGTC

CTGAAAATTGGCTTTATCTGTTTCTTCTCTGCTGGAATGCTGTCTCATCAGTGACCTCTGCGTTTGTTTTCTGAGATGTT

TGTCCTAGATCTTATACTCATTTAGACTCTTACTGTAAACTGATCCCCCTACTCTGCAGTGGATGGGCCAAAGATTGGGA

TATGAAAGCTTTCTCTAAAATTTTGAT

>GT228516.1 JC081 Jatropha seeds from fruits at three stages of maturation Jatropha curcas cDNA clone Contig82 5', mRNA sequence

GCGAGAAATGGAGAAGTCTTTTGAATAAAACACTGAAAAGCTTCTAGACAACTTGTTTCTTGGAAGTTGAGTCCCCAGTA

GATTTCAATTTTGTCCGATACATGAATGAGTACAAGACCGAACTATAAACTATCCCTTAGGAATACGATATATTCAACAT

AATCAAAGCAAATGACAATTTTCATAACCAAAAACTTCAGACTAGGGGCCGTGAGTGTCAAACAGTATTCACAGTATCTG

CAAGGGCACGCAATATGCAAACCAGGACAGCTTTCTCTGTCAGCTTTTCATGTATTCGTAGGCATAGGTCGCATGTGGGA

CGATCTCCAGGTTGACAACCCCCTATACAAGTACTTCATGAGAATATCATAGTACTCAGGATCCAAAGAAGAGAACATCC

CTTCCACATCTCTTATCGCCATGATCGCTCTATGTACGACTATCCAATTTGCAGACTTGCATCGCTCGTCTCGGGTCTTG

GGAGGCGAGCCTTCAAGAGCCGTTTTGAGAGCTTCGACGGGTTTGAACTGTTTAAGTAAGCTTTCGATCTTGCGAGATTT

GTGTTCAATTCTGGTGATTATGGCCTCTGCATTGTCTGCTTCTACGAATTCCTCCTTTCCTGCCATTCGCTTCTGCTGCT

GCTCTCTGTCTCCCTCTTGTACAACCCGATTTCAAACATTTGTTATAATTCTACCCCCCCCGGCCGTAATGGT

>GW619122.1 Jc2-042-G07-M13F.G07.ab1 Jatropha curcas flower and seed Jatropha curcas cDNA, mRNA sequence

TGGGACCAAACACAATTCACATTCCCACAGGAGAAAAACTCTATGTGTTTGATGCAGCAATGAAGTACAAGACTGCTGGA

CATGACACCATTGTCTTGGCTGGAGCCGAGTATGGAAGTGGAAGCTCCAGAGATTGGGCTGCCAAGGGTCCAATGCTATT

GGGAGTTAAAGCGGTGATTGCTAAAAGTTTTGAGAGAATTCACCGCAGTAATTTGGTGGGAATGGGAATTATTCCACTTT

GTTTCAAGGCTGGTCAGGATGCAGATACACTAGGGTTGACTGGTCATGAGCGGTACACCATTGACCTTCCAAGCAATATC

AGTGACATAAGGCCTGGCCAAGATGTAACCGTCACAACAGATAATGGAAAATCTTTCACCTGCACAGTTCGCTTTGATAC

TGAGGTGGAATTGGCTTATTTCAACCATGGAGGCATTCTTCCATATGTTATCCGCAACCTGATGAAAGAGTGAAGATCTT

TCTTTCAATCGTAAAATGTGAGAAGGGAATAAAGTTTTCAATAATAGGAGAGAAGAAAATCCCTTTTTGATGGGGCAGGC

ATTTTTTTTAGCACTTGAGAAAGAATTACCACTATGAATTCTTTTTTTTACTCTGAT

>GT978045.1 JGCCJG2015F05.b Jatropha curcas L. germinating seeds (mixed stages) Jatropha curcas cDNA clone JGCCJG2015F05 similar to unknown protein [Arabidopsis thaliana] (TAIR:AT2G35585.1); similar to hypothetical protein [Vitis vinifera] (GB:CAN79460.1), mRNA sequence

GGTTGATCTATTATTCGACACGCAAAACTGAGATTTGAATATAATACCCTTTGGTTTCCAAGCAGTTTTTTGCTGTGTAG

AAACGAGAGATGGTGGGTATTTTCTCAAGATTTTCTGTTGGCAGAGTTGGCCATAGGAGAGCCCAAAGTGCGCTTGATGA

GAGGGAAGTATTGCCCCCAATTACCGAAGTGAACAGGGCGTCTACCGCTGCTGCTCCACATGGAATTGAAGTAGCGGTAG

AGTTTAAGCCAGTTGAGCACCCAATTGAGCCTCCCAACAGTGATCAACCAATTCAATGCCCATTGCCAGAACATTCGATT

CTTAATGATGGAAGAATATGGAAAGAGAGGGTATCTGCAACCGTGCGGAGGAGATCTGACTTGCCTGTCATGAAAGAAGG

TGGGTCTCTTGAATCTGAATCTGCTGGGACAAGGCCACGACAATCCAATAGAGTGATTCTACCATCCATCAGTGCTCCTG

AACATAATTTACTAAAACTACTTGAAGAATGTGACGCATCGGGGCCCTAAAACGCTGTTTCAGGTTCTGATATGAGTGCT

GCGTTACATAATTCCCTTACATAATTTCCTGTCCCTTTCACTACCGGATTATCGACTTTTTTCAGACATATTTACTTGAA

TACATGTATTACTCTGTC

>JK613198.1 JCF22-207 Jatropha curcas, immature Seed cDNA subtraction library Jatropha curcas cDNA similar to senescence-associated protein, mRNA sequence

GCTGGAAATCAGAATCAAATTAGCTTTTACCCTTTTGTTCCACTGAGATTTCTGTTGTCGTTGGGCTCTGCTTACGGCAC

CTGCTATATCTTTTAGCAGATGGGCCGCCCCATCCGAACTCCCCACCTGACAATGTCTTCCGCCCGGATCGGCCGCCGAA

ACGACCTTGGGTCCAAAAAGAGGGGCATAACCCCGCCTCCGATTCTGGGAATAAGTAAAATAACGTTCAACTTCCTGGTA

TTTCACCGTCTCCTTTCGATTCCCACTTATCCTACACCTCTCAAGTCATTTCACAAATACCTCCGCCGCGACCACGCTA

>GW881526.1 JC000744 Seed specific Normalized cDNA library from Jatropha curcas L. Jatropha curcas cDNA clone N01410 5' similar to Unknown protein, mRNA sequence

GGTGCTTTCTACTACTCAAACCCACCCCAACCAACTATATAAACTGAATCGAAAAACACGCCTCTCAGCTTCCACCAACA

CACCCCCCTCCTACTTGTCGACATGGCCATCGCCGCCTGCTTCTTCACCGCCTCCCTTCCCAACTCCGACCCCAAACTCA

ATCTCCACCGTCCATTCCTTTCCTCCTCCTCATCACCCCACCTTCTTCTCCGTCTATCCGTCCCTTCCTCCTCCCCACTC

TCTCTCCGTCACCAACACAGTAACCTTCTCCCTCTCTTCGTCCCTTCTGCTTCTTACTCCGGTGGAGATGACGGCTTCAA

CAACCGCAATCATGCCGGCGGCGGCGGCGGCGGTGGAGGTAATGACAACAATGGAAAATAATGGTCACGGTGGTGACGAG

GATGGGGATATGCCGCGAATAGGAATAGGAAAGAGGCGATTATGGTATT

>JK611907.1 JCF28-A32 Jatropha curcas, immature Seed cDNA subtraction library Jatropha curcas cDNA, mRNA sequence

ACTGTCGATGGAAAAGACCCTAAGGATTTGCAGCAAGAGATTACTGATGGTGATGTTGAGGTGCCGCTTGATTGAGATCG

CAAGCTTTAGTGAGCTATTTCCTTTTTCTTTTTCTTCTACTTCTTTTTTTTGTTAAATTTTATAAAACAGAGAAACCTGT

GCTTTTATCTAATTTGTGTTTTTTGACAAAAAAAAAAAAAAAAAAAA

>GW614457.1 Jc1-039-G05-M13F.G05.ab1 Jatropha curcas flower and seed Jatropha curcas cDNA, mRNA sequence

AACTTTCACCAACACGAGGCCCGAGTAAGGCTGGTACAGAGGCATTGGAAACTGAAGACAAATCAAAAAACAGCTAATGG

GCATATGACAGAACTGCAAAATTTCCGGCTATCAATTACCAACATGCAGCCAGAGAAATCCTCAACAATGGAAAATTGGA

ACTTAGCATCTGCGATTGCTATCTAAGACTAAAAATAGCTAGGATAAGCTCTTCTTCTGTTATCTAACAATGATATTATT

GTCAATTTATAGGTAGAGACGTACAAAAAAAAGGCAACTAAGCTGGAGGCAGCGGTGAGACGCATGATTACGGATGAAAA

TGCTGAGCTGCTAGTTGTTCTTGACATTATCGATAATGTTCAAAGGTTAGGATTGGGATACCGGTTCGAGAATGAGATAA

GGAGAGCCCTTGACAGATTTGTGTCTTCTGGGATATTTGATGCCGCGGTGAATAATGGCCTCCACGCTACTGCTCTTAGC

TTCAGACTTCTTAGAGAACATGGCTATAAGATGTGTTAATGAAATTCAAGGGTGAAAATGGAAATTTCAAGGAATGCCTT

GCGGAGGATGTAAAGGCAATTTTGGGTCTATATGAAGCCTCATTTCTTGCTTTTGAAGGAGAAAATCTGTTGGATGAGGC

TAATAAATTTGCAA

>GW880378.1 JC000963 Seed specific Normalized cDNA library from Jatropha curcas L. Jatropha curcas cDNA clone N01883 5' similar to Beta-fructofuranosidase, mRNA sequence

GGAGTCACCACTTACCACCACAAATACATACTCATGATTTGCCTATCTGTACCCCCACAACGGCATCAGCAACCGTCCGT

ACAACAAACATATATCCAGGTGACAAAAGAAAAAAATTCACCCTTCAGTCTCCCTCTATCTCTGTCTAACCGTTAGTGTG

ACGATGATGATGATGATGAACACCCATCTGGGTCCACCTTCATTCTTCACTTTGTTTTAGTTATAAAAGCCATATAGTTG

AAAAGCTAAGAGATGGCAGGTTTTAGTGTAGATTTGTCTAGAAATGGTAACCCAACTGAAATTGAAGAATGGGATTTCTC

AAAGTTATTAGAAAGACCAAGGCCATTGAATATTGATAGGCAAAGATCACTTGATGAAAGATCTATCAATGACCTGTCAA

TTGGTGTTTCTCCTCGCTTAACAACAAGAATTGATAGCACTGCTAGGTTAGTAGACCATGTTGATTCTTCGTATTCCCCT

GGTAGAAGGTCAGGGTTTAATTCTCCTAGGTCTGATGCTGGGTTTGAGACCCATCCTACTGTGGCTGAAGCTTGGGAAGC

TTTAAGAAGATCATTGGTTTATTTTCGAGGTCAACCGGTTGGGACTATTGCTGCTTTGGACAATTCTGAAGAGAAACTTA

ATTATGATCAGTGTTTGTGAGAGACTTTATCCCAAGTGCAATGGCATTTTTGATGAATGGAGAACCTGAAATAGTCAGAT

TTTC

>GT974616.1 GJCCJC2059A04.b Jatropha curcas L. developing seeds (mixed stages) Jatropha curcas cDNA clone GJCCJC2059A04, mRNA sequence

ACATCATTAAGAATAAAGTAGCAAGTGGACTCAACCCGTTCTTCGTGGGATCGATCTTTTATTACTACGTTGCGATCCGTATACTTGCAAGAACTCAAAAGAAACGCAACACCTAGCATACCAACATTACCCTACCACACAGGGTCAAACCGTCCCATCT

CACAGGGTCAAGTCCAAATACCTGTACCCATACACTACACTACCTCACACTATCTCACATACCCTATGAAAACACCTTAT

ATAGGATAGTCTAATCGTTTTGCCTATGTCCTTTCACAGGTACCCCTCCAAAGACCTCCTGTACCCGAAACACCGACTGA

GCTCTCACCCACAAGTCCAACAACTCCAACTGCACCAATGGCATCACTTACTGACATCCAAAACCTCATTGAACAACTGG

AGGCCAGAATGGAGGTTAGAATGAATGAGGCATTGGCTGCCCAAAGGACTGCCCTAATAGCTGAACTAAGTGGTGGTAAT

GGAAATGGGGCTTCCGCCGGGGAAAGTGGTCCTGCTACCTCCTATCAGGGACCCGGTGCTGCACCGACGTCCTCTGACCC

TGCCCTGGACGACATGCCCTCCA

>JK317921.1 JCST692 Jatropha curcas L. seed cDNA library Jatropha curcas cDNA 5', mRNA sequence

TTTTTCTATTTCTGATTTTTTCTTTCCCTGTGGAAACCGCACATTTCTGACAGTATGACAAATGGAGGCATTGATGTCCC

TACCAGGGAAAGCTTTCGACACTTGATATCGATTATTTTTTGAGTCATTGGTATCTTCCAGTAAAAATTATTGTGAAAGA

ACTAATGTTTATCTTACAAGGAACAAACGAAACAACGGTGGAAGGATGATCTTCGAGCTGATTTATACATTATCTATTTA

GCACGATCTGGACGTCCACACTAAACAAACTTTTGTTTAGTCGCCATGGCACGGTTACCAAAAGCATTAATTTTCGACGA

AGGCCCAACCCCATCGAATGCTTATCGTAGACAAAATATGAGCTCTTGCAAAGGCCACCGCGAAATGTTATAAAGAGCTA

CCAAACGCACCCCCGTATATTCAAAAACCTCGTAATCTTTGATCCAATACTAGCGATAATAAACACAACCGGAATGCTTT

GATCATTTTTCGAGGCACGTAACCCCTTAATGACCAAAGACAGATAACGATATGTTGACTTCATAAGTCACGTAAGTCTT

CAGCAGCACGAT

>GW614612.1 Jc1-041-D11-M13F.D11.ab1 Jatropha curcas flower and seed Jatropha curcas cDNA, mRNA sequence

GTTGTTGGAGGGATTACGCCTACACCACTTGGGGAAGGCAAATCAACTACTACGGTTGGGCTTTGTCAAGCTTTGGGAGC

ATTTCTTGATAAAAAGGTTGTCACCTGCCTTCGCCAACCATCACAAGGACCTACTTTTGGAATTAAAGGGGGTGCAGCAG

GTGGTGGTTATAGTCAGGTGATTCCAATGGACGAATTCAATCTTCACCTAACGGGGGATATCCATGCAATAACAGCTGCA

AACAATCTCCTTGCAGCTGCTATTGATACTCGAATTTTCCATGAGTCATCTCAATCTGATAAAGCTCTTTTCAACCGATT

ATGCCTACCTAACAAAGAAGGAAAAAGAACCTTTAGTGATATAATGTTTAGGCGTTTGAAGAAGCTTGGTATCTCCAAGA

CTAAACCAGAGGAACTCACTGCACAAGAAATTAAGAAGTTTGCTAGACTTGATATAGATCCAAATTCAATTACATGGAGG

AGAGTTATGGATGTCAATGACCGATTCTTGAGGAAGATTACTGTTGGTCAAGGTCCTGAAGAAAAGGGAATGCTGAGAGA

AACAGGATTTGATATTTCTGTTGCTAGTGAGATAATGGCAGTTCTGGCCCTTACTACATCTCTTGCTGATATGCGGGAAA

GGCTTGGGAATATGGTTGTTGG

>FM890208.1 FM890208 Jatropha curcas embryo 35-55 (DAF) Jatropha curcas cDNA clone rjcfea0_004022, mRNA sequence

CAAATTTAACTATATGTTGTTATTTTAAGCTCTAATTTTGTAATTAGAAAAGTTGGAGCATTTTTAAAGGTCTTTTTTTT

CTCCTAAATTTAACCATTCTATTGATTACCAATTTTTAACTAGTATAACCTCTAAGAAAAGATAGATGAATGCTAATAGG

GCTTATTTTTATAGTATTTTTAATATAGTTTTTATTAGAACATTTAGTGTTTTTAATTAGTTTTTGTTTAGATTTATTTA

TATTTTTAATTAAGTTTTTTTAATTGTTGATAAGCTTGATTAGAAGGGTTTTTAGCAAGAAAATAGCTAAAATTTCCATA

AAAAAAGGTTAGAGGTAAAATTGGAATTCAAAATGGCCAAGTCAATAAAATACACGGCTTGGCCGTGTCAAATGCTATGG

TCGTGTTACCAAGAAAAGCTGCAGATTCAGAGTCAGTCGAATTTAATCATTTAGCCCGTGTAGATCTGAAGTCTAGGAGT

GGCATGTGCATGGAGCTGCATGG

>GW881421.1 JC001173 Seed specific Normalized cDNA library from Jatropha curcas L. Jatropha curcas cDNA clone N02235 5' similar to Acyl-CoA dehydrogenase, mRNA sequence

GGATTTGTTCGAAGCCAGACGATAATATCACCACCGTCCAAGACCCATTTCCTTACACGTCCCTAGTTCCCGGTTCCTCC

CTTCGGCCTTTTGCTTCCGTGTTCATGTCATATGGTTGAAACGGCCTAATCTCTAATCTTATAATCTTCTTCCTTTTCTC

TAATTTTTGTTACCAAACAAAACGAAATTGAAAATGGCGATCCATACATCTGAGTTATTAAGGCAAGTTCAGCAAGCTCA

CGAGTTTGACCGTGACGCTTTGTTTCGCTACGCCTCTGCTAATGTTGCTGGTTTCCCCGTCTCTCCCTCCACCTTCATCG

TCAAACAGTTCGGGCACGGACAATCGAATCCTACGTTTCTGTTGGAAGTGGGAACAGGAGCCTCCGTTAAACGCTACGTT

TTGAGGAAGAAGCCTCCCTGGCGAATTGCTCCAGTCTGCTCATGCTGTAGACAGAGAATATCTGGTTTTGCGGGGCGCTG

GGGGAACACACGCGAGTTCC

>GW612019.1 Jc1-012-B08-M13F.B08.ab1 Jatropha curcas flower and seed Jatropha curcas cDNA, mRNA sequence

ATCAGGAGTGGAGAGAGCATACCAATTGTCTTTCAGCTTTGATAAGCAATGAAAGCTCATCTGAGGAGTTTCTTCCTTCA

ACAGCTCTTAGATCTGGTGGAAGCTTTCTGCTCAAAACAAACACCGGTGGAACTGATTCTACTTCATCATCAGTTCCAAA

TACTACAGATGGTCCGCAACCAGAATGCAATGGTGAAAGAATTGATTTGCTGGTGAGGGTTGTATTACTAAAGTTAGTAA

GTGGGGTTTCTGGTCTGACACAAGAAACTTTGCCTGAAACCTTCATGCTTAACCTGCCTCGTTTGAGAGCTGCCCAAGCT

CAAATGCAGAAGATCATAGTAATATGTACCAGCCTTCTTGTTTGCCGGCAAACACTCCTGATGGAGCGAATTGTAGCCAG

TGGTGCAGACTTGGAAACCATAGTGTCAAAGTGCACCAAGCAACTGCTGGACCTCCTAGACAGTGTAGATGATGTGGGCA

TTGAAGAGATTGTCGAAATAATAAGTGGATTCTCACAAGAAGGTGACAAAGCTTTAGACCTTGAGAAACTTCAATCAAGG

AAGTTAGTGATGGCTCGGATGTTAGCAAGGAGCCTGCAGGCTGGGGACCCTGTTTTTGAAAAGGTCTCTCATGCTGTTTA

TTTGGCTGCAAGGGGAATTGTACTAGGCGGCAGTGGGCCTCGAGGAAGAAAATTAGCAGAAATGGCCCTCCGACAGGTAG

GGGCTGCAATGCTGACT

>FM887543.1 FM887543 Jatropha curcas embryo 35-55 (DAF) Jatropha curcas cDNA clone rjcfea0_000766, mRNA sequence

TCACGCGGTGCGGCCGCTCTAAACTATGGATCCCGGCTGCAGTTTAATTAATGATCAAAATTTTACTCGAATTGACCGAA

AATTTTACTCAAATTGACCGAATGTTCGCCCCTATCCAAATTTAATGATCAAAATTTTACTCGGATCGACCAAATGTTGA

CCCTTATCCTGTGGTTTATGTACACACAAAAACTTTCATTTTGGGTATTCTTTTCCATATTAGCATGGTATAACAGAAAC

TTTTATGCTTAATATTTCCAAAATGCTAGTAACTTTTTTTAGTCCTTTTCTTCTCTCCTATATTAAAAAAACAAAATTCA

AGTTTTCCATTATTAAAACATAAAATCACCCTTGCCAATAACATTATTTTATAGTTTGAGAAAGAGGAAGGCCAGAGCCT

GTCTTTTTGTTTTAGTTAGAAAGCATAGAGTTCTTTCATCTTGGATGCATCTTTTGTAAAATCTGCACGCTCTTTGTTTC

ATGACATAAGCCTCGACTTTCAGTCAAGTTTACATTGTTAAGTATTGAACAGTTGAACTTCCAAGAATATAGTAAACGTA

AAACAATACAGTTGATTCATGTAGTTGGTAAGGTGGGTTTAACAATTATCACACAGTACAACAAATTGATCCACTGAGTT

GGTGAGGTGGGTTTACAATTTCTCCT

>GW611426.1 Jc1-005-F06-M13F.F06.ab1 Jatropha curcas flower and seed Jatropha curcas cDNA, mRNA sequence

CATTTGTGAGATCGAGATCGAGCCTCCTATTGCTAAACAAATCAAAACTACCCAGCGAGAAGATGTCGTACTCCAGAAGG

TCAAGATATTCCCGATCTCCTTCCCCGTACAAGCGATACAGCAGGTCTGTCTCGAGATCATTGTCCAGGTCAAGGTCGAG

GTCGACGGATAGGTTAAGATCAAGGAGTCCTTCAAGTGATGTGGACAATCCTGGTAACAATTTGTATGTGACAGGGCTGT

CACCTCGAATCACCAAGAGAGACCTTGAGAAGCATTTTGCTAGTGAAGGAACGGTGGTTGATGTTCATCTTGTTGTTGAT

CCATGGACTAGAGAATCTCGTGGTTTTGGCTTTGTTACTATGTCCAGTGTTGGGGAAGCTGATCGCTGCATTAAGTATTT

GAACCGCTCTGTTCTTGAAGGTCGTGTCATTACGGTGGAGAAGGTATAACATTCAATCATTTTTCTACAGGGAGCCTACA

AAAATTTATTGTTTCTTGGTTACTTTGCTACAATTAAATTGTGTTAAGAATTCTGGATGGTTTGCTATTAGTTCTTATGA

GTTTGTAAGTTGTTTGCTTCTTTCCACTTGAAGTTTCTGTTGCAGCAGGTTAGCTGTGTGTAGCAGCTCTTCGTCAAACT

CTCAAGTTCTTACCACAACTAAACAAACAAATCAAACATCAACAAGTA

>GW616599.1 Jc2-013-F08-M13F.F08.ab1 Jatropha curcas flower and seed Jatropha curcas cDNA, mRNA sequence

CCGTTTCAGGCAAGCCTAGGACACAAGATTTGAATTTCCGGCCACCGTGCACGGTGGCGACGGCGGTGGTCCGGCGACTG

GCCGGCGACACTCCGGTGAGGCACCAAAAGGCAGAATCTTCCTCTTCTCATGTTTATAAAATACAAAATGCGAGGACAAA

AGCTGGAAGCATGTGGCTAAAAAGGCAGTCCTTTTAAAGGACAATAAAGAACTGTAATAGAAAGAAAGAAAAGCCTTGTG

GAGTTGAGACACTTATTGTAGAAGACTGTTAATGATAGAAATAACGATGTAAATACAAAGGTAGAGTTCCGCAATTGTAT

TCCAGTTGGGTTGTAATTAAATAATGAAAGTCCATTTTAAAT

>GW880197.1 JC001320 Seed specific Normalized cDNA library from Jatropha curcas L. Jatropha curcas cDNA clone N02479 5' similar to Nudix hydrolase 20, mRNA sequence

GACTGCCGCTTAACGGACTTTCAACGCTTCAACTATCCTCTCCCTCCGTCCAAGTTCCGTCTCTAATGGCTGCCTCCGGT

ATGTCCCTCCGGCACCACCTTTTTTCTAACAAAATCCGTCTCGCATTTCCTTCTCTCTGCGTCTCAAAATCCTTCATATC

TCATCCAACGACTCAAACATTTCCACTATGCTCCATTTCTAACTATTCTGCTTCAATACGATCCAGTTCTCGCTCTCCAC

CAGTTATCTCTCATATCAGTATTAGCAGCAACAGCAGCTTCACTTGGGACGACGTCGAAAAAGTTTCGCAACCTGAATAT

GTCCCTAACGATTCCTCGGATCTCTCTGGCTTCTTCGAAAAGATCCAATTCTGCAATCGGGGCTTTGAAATGCAATCTGA

GTTTATTCCATTCATTATTGAAGATGAAATTGTTGGTTATGTACATTATGGTTTTCTTGACCAGTTGAGGAGGTTCAAGG

ATGTATTTCTTTTCCCTCAAAGTAATTCTCATGAAGGCCGGTTTGAGAGCTATGTAACTTTAAATGAAA

>GW611555.1 Jc1-007-A09-M13F.A09.ab1 Jatropha curcas flower and seed Jatropha curcas cDNA, mRNA sequence

AAACAGTTGGTGACTTGACAAGTAGGCTTGGGTCAGATTGTCAGCAAGTGTCTCGAGTTATTGGAAATGATCTTAATTTG

ATAGCACGCAATGCTGTACAGGGTACAGGTGCCTTGATCTACTTGCTAATTTTGTCCTGGCCACTTGGTTTATGTACGCT

TGTGATATGCTCAACTATAGCTGCTTTAATGATGATATATGGCATGTACCAGAAGAAGGCAGCAAAGTTAACTCAGGAGT

TCACTGCTTCTGCTAATCAAGTGGCACAAGAGACATTCTCTTTGATGAGAATCGTTCGCATTTATGGAACGGAAAAACTA

GAACTTGAAAGGTATAAGCTGTGGCTGGAAAAATTGGCTGATATAAGCTTGCGGCAAAGTGCAGCATATGGATTTTGGAA

TATGAGCTTCAACACACTTTATCACTCCACGCAGATCATTGTTGTGCTAGTAGGAGGAATGTCTATTCTGGCTGGTCATA

TTACAGCGGAGAAACTGACAAAGTTCATATTGTATAGCGAAACGCTGATATATGCTACATGGTGGGTTGGGGATAATTTA

TCATCTCTGATGCAGTCAGTTGGGGCAAGTGAAAAGGTCTTCCAATTGATGGATCTCTTGCCAAGTGACCAATTCACATC

GAAAGGATTGAAGTTGCAGAGACTGATGGGAAATATAGAGTTTGTAAATTTATCTTTTCATTATCCAGCAAGGGCAGAGG

TTCCTGTTCTGCAACATGTA

>GW876395.1 JC005958 Seed specific Normalized cDNA library from Jatropha curcas L. Jatropha curcas cDNA clone N10133 5' similar to Unknown protein, mRNA sequence

ATTTTCAGAGAAAGAGCTTTTTGGATAGGTGTTAGCAGATGGCATGAGTTTTGCGTTTCTGGGTTAGATTTCCTATTGGT

AGATAAAGTTGGAATTTTTATGTATGCAAGGTATCTGATAAAAAGCAGCAATGAGTCAATTTTTATGATCTTGGTTCTAG

AGCTAGAGCTTGGAATTGAGACTAGCCATTATCTCTCTCTGTAAGCTTGTTTCTCCCTTTCTTGGCTTCTTGTTTTGCAG

TTCTGGAGAGTGATTTTCAAGTTATTTATGGTAACTTTGTTGGTTTGTACCTATGCTTTTTGATATTTCTATTGTTTCAG

TATTTTAAGCAGCTGTGATGTGATTAGAGTGAAAAGATGAAATCTTTGAGTAGTGTAGGACTTGGTTTGAGTATAGTTTT

TGGGTGCCTTTTATTAGCTCTTATAGCTGAGCTTTACTATTTG

>GW879979.1 JC006491 Seed specific Normalized cDNA library from Jatropha curcas L. Jatropha curcas cDNA clone N10997 5' similar to Secretory carrier membrane protein, mRNA sequence

GGCCATTACGGCCTAGTTACGGGGGGAAATTCGCTTCTTCAATTTTGTATATTCGAAAGTTGTAACACAACACATTTTAA

ATATTAATTATTAAAATTTTGTAATATAAATTCCGAACTTTTTTACGTCCAAATTTTTATTTTTTATCACATGGTAAATA

AATAAGAGCTCAAAGAAAATTAAAGTTAAATAAAAACTTAATATTCCAGTGTTATGCTTGTAAGAGATCAAATTAAAGCT

TCAAGAAAATAATATGGGATAGTTTCAATAGGTGGAAACGCTTAATCACATGATAGTTTCAATAGGTGGAAATGCTTAAT

CGCATATTTTATGTTTAATGTAGAACATATCATTTGTTTTTTTTGCTTCTATTTAGAGGAGTTTTTAGCTCCAAATAATT

TTGGTGTGTTATCATGCAGATTTTCTACTTGGTGGGGTTTGGCTTGTTTTGCTTAGAGTCCCTCCTCAGCTTGTGGGTTC

TCCAGGTTAGATGTGAAACATTTCATTTACTCTTCAATTGTTTGTTGTTATCTTTGCAACCTATGCACACGAGCACAAGC

ACAAGCACGAAAAAGGTTGCACTATTCTTATTAACTTGGTTATGATTTCTTTATATCTTTAAGAGTAACAAGTTGGCCCA

TTTTTTGGGAGTGGTAAATAGGTATATCATTTCTTTAACTAGAAAGGAAAATTTTACCATCCTTCCCTTCTCA

>GT982581.1 JGCCJG2070C01.b Jatropha curcas L. germinating seeds (mixed stages) Jatropha curcas cDNA clone JGCCJG2070C01 similar to zinc finger (C3HC4-type RING finger) family protein, mRNA sequence

CCGGAAGTGAATGGGACTGGCCTGAGTTAAGCTAGCCCAACTTTTAGGGCTTCCTCAAGCAGCATGCGGTTGGGGCATGT

TGCACCTTCAGATGATGGCATGCCATTGGTTGCTGAAAACTTCTCCTCCAGACAATCAAGGCTATTATCCTCTTTAGCTT

GGAGGAATGGTGATAGGAATGGGAGATCAAGGATTTCTTATGAGAGACACCGATCAGTGTCCAATGAACCAGGTCTTCAT

GTTCGATTCTCATCTGAGGGTTTCATGGTTGTGGACCGCTCAGCCTTTTATGGGTCCAGAAACATATTGGATCAACATCG

AGAGATGAGGCTAGACATAGATAACATGAGCTATGAGGAACTACTTGCACTAGGTGAAAGGATAGGCAGTGTCAGCACCG

GTTTGTCTGAAGATCTGGTTACGAAGTGTTTAACACAAACAGTATATTGTTCTTCAGGCCAAAGCCAGGATGAAGGAAAT

TGTGTAATTTGCCTGGAAGAGTACAAAACCATGGATGATGTTGGGTCACTGAAAATTTGCGGCCATGATTAACATGTGAG

CTGCATCAAAAAATGGCTATCGATGAAGAACTTGTGTCCAATTTGCAAAACTTCTGTT

>GT970424.1 GJCCJC2008A11.b Jatropha curcas L. developing seeds (mixed stages) Jatropha curcas cDNA clone GJCCJC2008A11 similar to prenylated rab acceptor (PRA1) family protein, mRNA sequence

CCGCAGCACATGCAATTATCATCTCCGTCTCCATCTTCCAATTTGTTCTTGGTGCGCGACCAGTACACCTATATTCTCTA

TTTTTCTCGCTGTTGAGATCTGAAAGAGAGAGACATCTTCCTTCTCCACTTCCTTACACGCGCCACCATTATGGCCGCTC

CGACGATCCCAATCTCAAACCCTCAGTCCCAACCACGAACGCAACCACCAATCGCCACACCTGCCTTCCGCGCGTTCCTT

TCACGCCTCTCCTCCTCGATCCGCCAGAGCTTCTCTCAACGCCGCCCGTGGTCAGAACTGGTTGACCGTAACTCCATTTC

CCGACCCGATTCCCTCTCTGAAGCGGCTTCCCGGATCCGTAAAAATCTGTCTTATTTCAAAGTCAATTATATTACCTTAC

TGGCAATTGTACTCGCCCTCTCTCTTTTGTCTCACCCTTTCTCTCTGCTAGTCCTTCTCTGTCTTCTTGGCGGATGGATC

TTTCTCTACCTATTTCGACCGGCGGATCAGCCTGTGGTTGTTCTCGGTCGAACTTTCTCAGATCGAGAAACCCTAGGTGT

TTTGGTCGTATTGACTATAGTCGTGGTATTTTTGACTAGCGTTGGCTCGCTTTTGATCTCTGCTTTGATGATTGGATTGG

CAATTTGTTGTGCTCACGGTGCGTTTAGAGTGCCTGAAGATCTGTTTTCTGATGATCAAGAGCCCTGCAGTGCTGGGTTC

CTCTCT

>JK613631.1 JCF4589 Jatropha curcas, immature Seed cDNA subtraction library Jatropha curcas cDNA similar to cysteine proteinase, mRNA sequence

ACTGACTAACCAGATTATAGGAAATTTCCAAGCAATTTCAATCTGACAATCCAACAGTCGGTTGTTCTGACAACCCCAAG

AAGAGGAATTTCCAGGAAATTCCAATCCAATGCTTTGGGTGGACATATGACAGATAAACAGTTCGAATTATCAAAGCTGA

CATAATATTATAACATACTTATATACAGAAGATCCAGCATACATAACATCGCTGGATGCACCAGCCTACTGTGAGGAGGT

TTGAACTGCAGCAACAGTTGACACCATGGAGTCTACTCCACAAACATTGCGACCCCTGCAGATTTTGTAATATCCGCTCT

CTCCCCAAGTTTCTCCCCAGGAGTTCTTGATAATCCAGTATGGCTTCTCCTTCAATCGGATAGGAGCATAGCCAGCCGAA

CCATATCCAAC

>GW615316.1 Jc1-049-C02-M13F.C02.ab1 Jatropha curcas flower and seed Jatropha curcas cDNA, mRNA sequence

CAAACAATGGCGGCGATTTCGTGTCACTCTGCGGTTCCATCAAACAAAGTCTCTCTCTCCTCGTCTTGCTCTGCTTTCTC

CAGTAATCTTCTGTCGCGGTCTTCCCGATTTACCAGTGCATCGGTGCGATCTCAGGCAATTCATGGAAGAATCTCATGCC

AAGCTTCTTCCGCTACATCTTCATCTTCCTCTTCCGTCAATGGCCAAGCAACGCCAGAGAAGACGGATTTTGTACACATT

AGTGATTTTGACAAATCCACTATTATGCGGATATTGGATCGAGCTGCAGAGGTCAAGGCTTTGTTGAAATCTGGAGACCG

GGCATTTCTTCCATTTAAAGGAAAGACAATGGCTATGATCTTTGCAAAACCTTCAATGAGAACACGGGTTTCATTTGAGA

CTGGATTCTTCTTGCTTGGAGGCCATGCTATATATTTAGGACCTGATGATATCCAGATGGGAAAACGAGAGGAAACTCGT

GATGTTGCTCGTGTTTTATCTCGTTATAATGATATAATCATGGCACGTGTTTTTGCTCATCAGGATATTCTTGATCTGGC

AAAGTATGCATCTGTACCTGTCATTAATGGCCTGACTGACTACAACCATCCTTGCCAAATAATGGCTGATGCCCTCACAA

TGATTGAACACATTGGTCAGTTGGAAGGAACCAAGGTTGTCTATGTCGGGGATGGGAATAACATTGTGCACTCT

>JK611371.1 JCF4033 Jatropha curcas, immature Seed cDNA subtraction library Jatropha curcas cDNA similar to nadh dehydrogenase subunit i, mRNA sequence

ACATTGGTCAAGGTTTCATGATTACCTTATCCCATGCAAACCGTTTACCTGTAACGATTCAATATCCTTATGAAAAATTA

ATCACATCGGACCGTTTCCGCGGTCGAATCCATTTTGAATTTGATAAATGCATTGCTTGTGAAGTATGTGTTCGGGTATG

CCCTATAGATCTACCTGTTGTTGATTGGCAATTGGAAACTGACATTCGAAAGAAACGGTTGCTTATTACAGTATCGATTT

TGGAATCTGTATTTTTGGGCACTGTGTTAGTATGTCAACAAATGTTTTCATGCTGAGAAAAGACTTTCATTATGATGTCC

GATGATTATACAATGCTTAGGCGTTACATGGCGTAGTGCGATTCATTCACATTTGATCATCACAATTCCCAAATAGAACC

TTATGTAAATCCGCGGGGCATTAGTTCGGACATCTTCGGCGTTAACTGGGAACCGGTCAATCTGGATCTTCGGGAAGAGC

GCGTCTCACGCGCGAGGAGAGCATTAACTTTAACTTCACGCACTAAACGAGTGTTGTTACCCAATCGTCTCTGTGCTACA

TCTGAATAAAGTGACCTCACAACCCGCCTAATACTATTTTATCCTGATTAATGGAC

>GW877609.1 JC002426 Seed specific Normalized cDNA library from Jatropha curcas L. Jatropha curcas cDNA clone N04552 5' similar to Transcription factor, mRNA sequence

GGACCTCCCGTCTTACCTAGCGTCGTCGTTCTACAACCGCTAACCACCCTAACTGTAACGTCCTTTCCATGGCTTCTTGT

GACGACGATTTCTCTCTCCTCGGTGAGGATAACCACCACCAAACAAACCCTAACCACCACCACCCCCACCACCATCACAT

CCTCCACTCAGCTTACGCACCTCACCGACTCCCAGCAAAATCAGCTCCTATCCACGCTCCACCTCAACCGATATTACCTT

CTTCCGTCGCCGGATCCGGTAACGGTGCCAAGAATATCGGAGGTGGAATCGAAGAGGCAGAAGAACAAGTAGAAGAAGAA

GAAGAACATAACGAGACTGATTCAGCTTTCCCTGAGGTGAATCCTTTTAGTGACAATTCAAATCCTTTCAATAATGAGAC

TAATTTGCGAACTGACAAGAGAAGAGAAGGAGAAGATCACCGCGATGGAGGTAACAGCAATCCGTACAGTTACAAAAAAC

CAAGGCCGTCCAGTAACGCAGGGAATTCTGGTGGAGAGTACAGGAAGGATCGAGAAGAGTGGAGCGATGCTGCGATTGAA

TGCTTGCTGGACGCTTATACGGACAAGTTTACACAGCTCAACAGAGGTAACCTACGAGGGAGGGGCTGGGAGGAGGTGGC

GGCGATAGTGAGTGAGCGGTGCCAGAAGCA

>GO247005.1 JcrME_RL0549 Expressed sequence tags from Jatropha curcas root cDNA library Jatropha curcas cDNA, mRNA sequence

GGATGGCGGCCAATTTTTTAAAGGACCACCCCTCTCTTTCTCCACCTTCTCCCATTTCTTCTTCTGCTTCTCAAATCACC

ACCAACCTAACCCTTCCCCCCTCTCTCTCTCTCTCTCTCAAATATCTCTCCTTTCTTATTCTTTTTCATCAACATCTCGC

CGGCCGGCAACGGCAGTCAATTACATAGATATATACGTAGTGATCGATCAATATAAGTATTAATTGTTATCAGCTTTGGT

AAATAATGGACGGCAGGTTCAACAACAGAATAATCACTGACCAAAAAGAGAACGACAATACAGAAAGTGGTCCTGATTCA

CCTCCTTCTTCTAGCTTGCTTAACGACATGAAGATGACTTCCACTTCTTCTTCATCTCCAAAAAGAAGCAAACGGGCAAC

GCAAAAAAGGGTTGTCTCAGTTTCACTCAAGGACGTAGATGGCTCGAGGCTGAAAGGCGAAACCGCACCTCCACCGCCCG

ATTCTTGGGCTTGGAGAAAGTACGGCCAGAAACCCATCAAGGGTTCCCCTTATCCCAGAGGATACTACAGATGTAGTAGT

>FM892953.1 FM892953 Jatropha curcas embryo 56-70 (DAF) Jatropha curcas cDNA clone rjcaeb1_003618, mRNA sequence

ATCCGTATAGTATTCTTACAGTAAGAGTTTATTTGATATTAGTAAAATCAGACCCTTACACAAAAAGCAGAAGGGGATTT

TTGATTAGTTAATATCTCACATGATATCTGATCTCCAAAATTTAAAAAGAGGAAAAAAAAAAAAATTAAGACCAAAATTT

TATGGCTCATCCTCAATAACTGAAGCCAAGCCAGTCACGATCTTAACCCCAATCGGAAAACTCTTGGGCTTAATTTCAGG

CGGTTTCCATTCTTTCACCATCACGTCGTTCTTTTCATCCACAGAACTTTCAGATGACAAAGCAAATACAGGGTCGCCCA

ATTCAGTATCCTCCACTGAATTATTCTCTTCATCAACGGTTCCAAACTCGCTGACGGCTCTTGGAACCGAAGGTTGCTTC

TTTTCAGGTTTGGCTCGCGTATAAGGAGAAAGCCATTTGGCTTTGACGTACTCGGCTTTTCTCCATGGTGGAATGTGCAT

GTCCTTCTTTTTCAGGCTTTGTTCGCTGCATCCGATGGGGGGGCCCGGTAC

>GT972276.1 GJCCJC2032E09.b Jatropha curcas L. developing seeds (mixed stages) Jatropha curcas cDNA clone GJCCJC2032E09 similar to AAP2 (AMINO ACID PERMEASE 2); amino acid transmembrane transporter, mRNA sequence

GATCTCTGAATTTTCATTTCACTTCAGAACATATTCACTGACATTTGCTTTCTTTTTCTTCTGATAATCAAAGATGGTGG

AGAACACATCAGGAAGATCTCATTCTCACCACCAAGTCTTTGATGTCTCCATTAACATGCACACCCAGATGAGCAATGGC

TCCAAATTGCTCGACGACGATGGTCGTCTTAAGCGAACTGGGACTGTTTGGACTGCGAGTGCACATATCATAACGGCTGT

GATTGGGTCTGGAGTTCTGTTTTTGGCTTGGGCAATAGCGCAGCTTGGATGGATTGCAGGCCCTGCTGTTGTGTTCTTGT

TCTCTTTTGTTACTTACTATACTTCAACTCTTCTCTCTGCCTGTTACCGTTCCGGCGATTCTGTCACCGGAAAAAGAAAC

TACACCTACATGGATGTTATTCGGTCTAATCTTGGTGGTGCTAAGGTCAAAATATGTGGAATTGTTCAGTATCTGAACCT

TTTTGGGGTTGCCATTGGTTATACAATTGCATCATCCATAAGCATGATGGCAGTCAAGAGGTCTAATTGTTTTCACAAGA

GTGGAGGCAAGAATCCATGCAAAATGAATGCAAATCCTTACATGATTGCATTTGGCATTGCAGAAATTATTTTTTCTCAA

ATTCCTGATTTTGATCAGCTATGGTGGCTTTCCATTCTTGCTGCTGTTATGTCCTTCACTTACTCGACTATTGGCCTTGG

ACTTGGAATTTGC

>JK317412.1 JCST179 Jatropha curcas L. seed cDNA library Jatropha curcas cDNA 5', mRNA sequence

CTATGTNCAGGTGGGTGTGACAAAAACCTATCCCTAAACCTGTTCACTGCATTTGAGCTGGTCGGCCAGCGTCTTGTTCA

GCGGGGTAGAATTCAAGGTGTTCGGTGATATGTTTACAGATCTGGAGGAATACCGGTGGCGAACGCGGCCCCCTGGACAA

AAACTGACGCTCAGGTGCGAAAGCGTGGGGAGCAAACCGGATTAGATACCCTGGTCGTCCACGCCGCAAACGATGTCGAC

TTGGAGGTTGTGCCCTTGACGCGTGGCTTCCGGAGCTAACGCCTTCATTCGACCTTCTGGGGAGTACGGCCGCCAGGTTA

ATACTCTTATGAATTGACGGGGCCCCGCACAACCGGTCGAGCATGTGATTTAATTCTATGCATCCTCTAACCTTACCAAC

TCTTGACATCCATAGAACTTTCTAGAGATTGCTTGATGCCTTTTGGAACTCTGATACAGGTGCTGCATGGCTGTCGCCAG

CTCCTGTTGTGAAATGTTGGGTC

>GW616303.1 Jc2-010-C11-M13F.C11.ab1 Jatropha curcas flower and seed Jatropha curcas cDNA, mRNA sequence

CATCCGTCTGTTCGTTTTCTCTCTGTGTAAGCAAAGCAAAACTTCCTTTTCTCTCAAAGATCCAAATCGATCTGAAATTA

TGAGCGATGTCTTCGAAGGATACGAGCGCCAGTACTGCGAGCTCTCTGCAAATCTCTCCCGTAAATGCAACTCCGCTTCT

CTTCTCTCAGATGGAGAGGAGAAGAAGGAGAAGATCTCCGAAATAAAATCTGGACTGGATGATTGTGATGTGTTGATTCG

GAAGATGGACCTTGAAGCGAGAAGTTTGCAGCCAAGTGTGAAGGCTATGCTTCTTGCAAAATTAAGAGAGTATAAATCTG

ATCTTAATAAGTTGAAGAGGGAATTTAAGAGAGCAACTTCAGGTAATGCTGGTCAGGTTGCCCATGAAGAGTTGTTGGAG

GCTGGAATGGCTGATGCTTCTGCGGATCAAAGGGAAAGATTGTCTATGTCAGTAGAGAGACTAAACCAGTCAGGTGATAG

GATTAAGGAGAGTAGAAGAACAATGCTGGAGACAGAGGAGCTTGGTGTTTCAATTCTTGAAGACTTGCATCAGCAGCGAC

AAACACTTCTACATGCCCATAACAAGCTTCATGGAGTAGATGATGCCATTGACAAGAGTAAGAAAATCTTGACTTCTATG

TC

>GT971209.1 GJCCJC2018B01.b Jatropha curcas L. developing seeds (mixed stages) Jatropha curcas cDNA clone GJCCJC2018B01 similar to retrotransposon, mRNA sequence

CTCCATCCTTCTTATCATTTTTATCCTAATTTGTTTTTCACATATACTAAAAAAAAATTTAATGTGATTAACATAAAAAT

TTTTACTTTGTTTTTTTAAATATATTATTATTTTAATTATTAGATTATCGTATTATTTTATTACCCTAAATTTATTCTAT

TTTAATTTTTGGAATATAAAGATCAAGGAATAAAAGTTAAGCTAATATTAGTGACCATGATAATATTTTTATTAACACAA

ATGCATAAATAGGGATAATATAATAAACTTAAAAAATTATCTTATTCTAAACTAGATAATAGGATTATAATTTATGACGA

ATAAAAAAATAAGTTAAGATTTGTCATAAGTTTCCCATGAAACAGAAGCCATAAGAAACACCGTACTCGTTGAGAAACTT

TCAGGCTCCAAATCAGAGACCATAACTGACTCCTTTGTACCTCCCCTCCGCCTCGAACCATGCTATAAGCAGAGGAAACA

GAGAACTACCAATAGGCGTACCCGATCAAGCAATGGTGTCTTGTATACTGGGATCTACCTCCAAAAAAATAGACTCCAAA

ATACCACAAATCGTAGTTGGTAAATAAGGCTGAATTAGCTGCCAGTTCCACGTATCCCCATTTCAATAACTAGCCACCGG

CGTCTGCTCAT

>JK317395.1 JCST162 Jatropha curcas L. seed cDNA library Jatropha curcas cDNA 5', mRNA sequence

GGTAGGAGATCTTCTAGAATGATGTCTTCTATGTCGGGTGGGGGAGCTCAGGGTTGGGAAAAAGGGGGTAATGCTGATGC

TTGGGACTGAGCTTCTATAAAAAGCCAGGTAGGAAATCTACAATTTTCTTTATTCTGACCCTGACTGATTTTTTTTCCTT

ATTAACTTGGGAAGTATATGTATAAAAAAGTGACCTCAATGACCCCCTGTCCCTCACACATGCGGTAATCCCAAAGAATG

AATTTGATTTTGCTGAAAGTTACCTCATCTCCTCTTCCCTTGAGACAGAAGGCCAAAATCTGCTCTTTTTTTATTTTTTT

TTTTTGAA

>FM889324.1 FM889324 Jatropha curcas embryo 35-55 (DAF) Jatropha curcas cDNA clone rjcfea0_002957, mRNA sequence

TGGATCCCCCGGGCTGCAGATGGCATGCTCAATTATCTCTTAACCTAGCTATGTTAAGTTCTTTAACCAATTGTTGTAGC

TCACCATATGTATTCCATGCCCCCTTATCCATATTTAGCTACTGACTATGGTACACAACTGTCATTGTTCACACATCACA

TGTGGATTGGTGGATTTCTCATAGTTGGTGCTGCTGCACATGCAGCCATTTTTATGGTAAGAGACTATGATCCAACTACT

CGATACAACGATCTATTAGATCGTGTTCTTAGGCATCGCGATGCAATCATATCACATCTCAACTGGGTATGTATTTTTTT

AGGTTTTCATAGTTTTGGTTTATATATTCATAATGATACCATGAGCGCTTTAGGGCGCCCTCAAGATATGTTTTCCGATA

CTGCTATACAATTACAACCCGTCTTTGCTCAATGGATACAAAAC

>GW877307.1 JC006829 Seed specific Normalized cDNA library from Jatropha curcas L. Jatropha curcas cDNA clone N11507 5' similar to Unknown protein, mRNA sequence

GGATAAAATTACGCGGTCTCGCTTTCTTTATTTCTTTTCTTTCCTCTCTCCGACTGTTCTCTCTCTCTCTGTTTTGTTGT

TTTTCTTCCATGGCTATCAGAGAGAAATACATTTGATTTAAGTTAGAACATGTACATTAAGTTAGCGAGAGAATTGTGTC

GCTCAAACTTAGGGTTTCCTTTTTGAGTTTCGTTGAAGTAGCGAGGAAGAGAAGGAGGCGGAGGAAGGAAGGGAAAGGAA

GACAAAGGCAGGCAAACAGAGGG

>GW618852.1 Jc2-039-H03-M13F.H03.ab1 Jatropha curcas flower and seed Jatropha curcas cDNA, mRNA sequence

AAAAACCTCTTCTCTCTTCTATTGTGATGAAGATGTCTATGCTCAAGTTTACTCTCGTTGCCTTTCTCTTGTTGATTGCC

CTTGATTTGCAAGGTGGGGTAGAAGCAAGAGGGCCAATAGTTGGTTTTGGATGCAAAACAGTCCAAGACTGTGTTGCAAA

TAATCCACTATGTTCTGCAGAAGTTTGCAAACCACCCTACTGGTGTTTCTGTATAAATGGACAGTGTGCATGTCAACCAG

ATTCATTAAGTGCTACAACCCTGATTGGAAGCTAAGGAAAAGGAAGTGCAAAAACATTTGAATAAAGCAAAAATAGAGAA

GTTTGAGATACCAATATCCAGCTCTCTGATAATGATACTTGTAAACCTCTAATTACAAGTTATAATAAGGATTATATGTT

TGTATCCTCGTTATAAAAATCATTATAAAGAAAGAGAAATATATGCAGTTATCTTATTATTTTCCTTT

>GW877743.1 JC003345 Seed specific Normalized cDNA library from Jatropha curcas L. Jatropha curcas cDNA clone N06282 5' similar to Beclin-1, mRNA sequence

GGAGGATCCGGCAAGGGCAGAAACCGGATCCGGACTCTTCACATATTACGCAAAATCGCTCGGGGAAGGGAATTTTCAGA

AGCTAACAAAAGCCCAAAACTGTTGTGATCTTCTCTCTGAGCTCAGCAAAGAAGCAACAAAGAAGAGAATATATGCTGTT

CGTTGTTTCGCCTTGTTGCCATTTCCCTTACATATAGAATGAAACGATAGCTAGGAAGAAGGAAGATATGATGGGAGATA

AAGGTCGGACCTTGCCGGTAGATCCGAATCTTCCTCGATGGGTGTGCCAGAACTGTCGCCACTCCCTTTGCATCACCGGC

GTCGATTCCTACGCCGACAAGTTTTTAAACGATTCCTCTTCTCGCTCTGCAATGCATGGCTTTTCAATGCATGGAGCCAA

CAGTGTGTTAGGCTCAACACGTATGGACAATTCGTTTGTTGTTTTGCCAAGACAAAGACCACAACTGCAGGGAACCCCTC

CATGTCCTCATGGTGGAGCTGCTCAGCCTGATCCAGGCCAGCCTGGAAAGGCAATGGAAGAATCTTTTGTAGTGGTGTAT

AAGTCTGAGCCTACATCTGATGGGGGTGCAACACAATTACCATCAATAGATGGAGGATCTAATGTCCAGTCGCAGCCCAA

CAATGCAGGTTTCATTCTACCATAACTGTCCTGAAACGTGCATTTGAGATTGCTACAACCCAGACACAGGTTGAACAACC

TTTATGCCTTGAGTGCATGAGAGTGTTATCTGATAAACTCGAT

>GT976453.1 GJCCJC2078C10.b Jatropha curcas L. developing seeds (mixed stages) Jatropha curcas cDNA clone GJCCJC2078C10 similar to PAC1 (20S proteasome alpha subunit C1); peptidase, mRNA sequence

TCCCCGTAATTGAGCTGTTGTTTAATGCCCTAGTACCACTGTTGTCATCCATGTCTCATTATCATCAATCAAAAAATAAC

TTAATTTCACGTTGGAGCGCATCTATAAGAACACTCAATCATGAATAGCGATAAATTAAAATCATTGGATACAAGCATGA

TAGTGATCAAGAAAGCATATATGTGAAAGTGAATGCACAACCAAGGCTTTCCAAAGAGAATGCATAAGGACACCAAAATA

ACATTTGTTCACAGAAATTCAATCAGATCTTAGATATCATCAGCCTTATACCCCAGATCAAACATGAGAGTTCAATATTG

AATTTGTCCAACCAAAGCAAGATCGTCTTTTGAGAAAAAGTTGCATAAGTACTCAAAACGACATACACCTACCAAATTAC

TGCTGAAACTTACCTAAGCAGCCTCAGCAGGAGGTTGGGTAACTCCAGACTTCACTAACAACCTAGTCAGAGAGTCTGGT

GAACGAACCTGGTACTTAACTTCTCCAGTGGGCAGGAGAAAGACCTCAGCTAGTTCGAGCTTATCAGAAGTGAGACTTGT

ACTGTCCATTGTCTTGCTCAGCACCTTAAGTGCAAGCTGAACTGCCTCCTCTCTTGTGATATCATCTTTGTAGTCCTGCT

TCAACATTGACTGTGCTGCCCTGGTGTTTGCTCAACAGCTGCAGCCTTCCATCACCGTAGTTTC

>GT977259.1 JGCCJG2001E02.b Jatropha curcas L. germinating seeds (mixed stages) Jatropha curcas cDNA clone JGCCJG2001E02 similar to unnamed protein product [Vitis vinifera] (GB:CAO40945.1), mRNA sequence

GAGAGAAACAAAAACAGGCATCAAGATATGGCTTCTTGGAGTAGAACTCTTGTTTCTTTAACGATTGATTCGCGCTCTAT

CTTCTCTTCTTCCACTTCTTCTCCCATTATCACTCCACCTGTTGTGGGTTCACGTTTCTGTGCAGCTGATTCCATTACTA

GTATCAAACTTGCACAGCCTTGTTTATACTCTTCAATCAGCAAAATCTGCAAGAACAAGGAAAGAAAACAAATTTGCAGA

GCAGGAGAATACAAGTTTCCAGACCCAATTCCAGAATTCGCTGATGCAGAGGCAGAGAAATTTAGGACCCATCTTCATAA

AAAGCTTGTAAAGAAAGATATATGCGGAGATTCAGTTGATGACGTCGTCAGAATCTGCACTGAGATATTCAGTACTTTCT

TGCACACAGAGTATGGCGGTCCTGGAACACTCTTAGTCACACCTTTCATTGACATGGCTGATACTATAAATGAACGAGGT

TTGCCTGGAGGACCTCAAGCTGCACGTGCAGCTGTCAAATGGGGCTCAGATCATGTTG

>JK610630.1 JCF1613 Jatropha curcas, immature Seed cDNA subtraction library Jatropha curcas cDNA similar to hypothetical protein, mRNA sequence

GCTTTTTTTTTTTTTTTTTTTTTTTTAATAGAAAAGCCAATTTTATATATTTCACATAGTTGTTGCAATTTTCAGGGTGA

ATCTCACAAAATTATGTTCTATTCCTATCCTCACAACAAGCTCCAAACTGACTTTCACATGCCTACTTTTACATTAACAC

CTTGATTAAAATGTAGCACCCAAATCAATAATCAGCTAAAAAAGGCGTCTAATCATTTGTTTTCGTCACTGTATTCTTCT

GATTCTTCGGTTTCACCATCATAGTAATAGTAGTCGTCATCGTCATCGTCGTCCTCCTTAGCATCATCTTTGTCATAACC

TATCAACTGCTGTTTCCTTAGATGCTCCTGAATTCGATCTCTGATTGCTGTTCCCATTTTATGGCAACGTTCTTCAAACA

TCTCGAGAAACCCAGCAACCAGACGATCAGCATTTTCCACCCATTCATTTCGATGT

>GW874836.1 JC000227 Seed specific Normalized cDNA library from Jatropha curcas L. Jatropha curcas cDNA clone N00412 5' similar to Brefeldin A-Sensitive Golgi Protein, mRNA sequence

GGATCAAACAAACTAACATAAACAACCAAGAACCAGAGTCCAAATGGTGGATTCCATGTCAACTCCACCGCCAAGATCCG

CTACGGACCTCTTCTCGGATCCACTCGACTCCCACCCGCTTTGGTTCAAACCCAATCTCTTCCTTTCCCCGACCTTCGAC

TCCGAATCCTACATCTCGGAGCTCCGAACATTCGTTCCTTTCGATACGCTCCGTTCGGAGCTCCAAGCATACCTTGCCTC

CCTTAATCACGACCTCATTGACCTAATCAACCGTGATTATGCTGATTTCGTCAATCTAAGTACTAAACTCGTCGATGTAG

ATGCTGCCGTTGTTCGCATGCGGGCCCCGCTATTGGAGCTTCGAGAGAAGATCGAAGGATTTAGAAGAAGCGTTGAAGGT

TTACTTGTCGCTTTGAGGAATGGGTTGCAGCAGAGATCGGAGGCTGCTGCTGCAAGAGAGGTTTTGGAGCTGTTGTTGGA

TACA

>FM892858.1 FM892858 Jatropha curcas embryo 56-70 (DAF) Jatropha curcas cDNA clone rjcaeb0_003528, mRNA sequence

ATTCATTTCATTTATGCAACAGCCGATTCGGTCAGTCAAAATAGCAAAAAACATGTCTATAATTTCTCTTCCACCTCTTA

CTCTTACGGATGTGCTGCAGCCGGCCAGATCGGTAACGCCGTGCAAAATCTAAACCGGCAGTTTGCAGAAAGAAGATACT

CGTCGAATTGCCGCCGATATTGCCAAGAATACCACCGTTTTTGCCTGCCGTTATGGTCTTAAAACTGTCTTACCAGAGGA

ACTTGTGAATACTGTTGTGCCGTGATCTTTTTATGCTAACAACAAATTGAAGAATATGGAAAAA

>GT977409.1 JGCCJG2008C05.b Jatropha curcas L. germinating seeds (mixed stages) Jatropha curcas cDNA clone JGCCJG2008C05 similar to 30024.m001697 conserved hypothetical protein, mRNA sequence

CTGGGTGCACTCTGAGTCTGGTCTCTCCTCTGCTGAGACCGCTGGCGTCTGGTACGCTGACTGGCCTGAATTCGCTCTAT

CTCCTGAGCTGCCTCTACCAAAGCTGAGAACTCTCTGAATCTGGAAGCTACCACTCCCATCTGAATCTCATCATTCAGGC

CTTCCTGAAACTTTTTACACCTTTCGGCCTCTGTAGCCACTATCCCCTTACCATACCTGCTGAGCCTGGTGAATTCTCTT

TCATATTCTGAAACTGACATTATGTTCTGCCTCAGGTATAAGAATTCTTTCCTTCTCGCATCTAGGTAAGCCTCTGTGAT

GAACTTTTTCTTGAATTCATCCAAGAAGAATTGTCATGTACGCTGCTCTTCTGATACTCGCTGCTGAACAGTAGTCCACC

ATGTATATGCTTCATCTATGAGCAATGATGTGGCACAAACCAACTTATCCTCTGGTGTACATTGTAATTGTTCCAGAACT

CTCGATGTCCCATCTAACCAATACTCAGCTCTCTCTGGACTATCATCCTTGTTAGCACTGAATTCTTTCGCCCCATATTT

CCGAAGCTTCTCTAGTGGGGGCTTTCTTTCTCTTTCTACTACTATTGGTGCTTGAGGCATCACCCCTGCAATCTGTCAAA

AATATCTGCAAGTTGTTTTACAAAACCTCTTGA

>GW615586.1 Jc2-002-C01-M13F.C01.ab1 Jatropha curcas flower and seed Jatropha curcas cDNA, mRNA sequence

CACAGAAACAAGAAACTCTCTTTTAACAATTCCCAGAAATAGCGGTTAAAGCGGTGGTGTGGTTGATTATGAAAATGGTG

GGAGAAGCAAAACTGAGGAGTGAGGACTTGAACCAGTGCTTTGAGAGGTTGATGACGGTTGGTAGTGGAAATAGTGGGAT

TGATAGTGGTATTAGTCAGGGAGGAGTTAAAATGGAAGGAATTGTTATCACTGAGTGGAAAGATATTCCTATGGAACTTT

TGCTGCGGATAGTTTCTCTTGTTGATGATCGGACCATAATTATGGCATCTGGGGTTTGTAGTGGTTGGAGGGATGCTATC

TGCTTGGGCCTCACCCATCTCTGTCTCTCTTGGTGCAAAAACAACATGAACAACTTGGTTCTGTCACTGGCTCCTAAATT

CACAAAACTGGAAACTCTGGTCCTGCGCCAAGACAAACCACAACTTGAGGATAATTCTGTCGAGGCTATTGCAAGTTACT

GCCATGATCTTCAGGACCTGGACCTAAGCAAAAGCTTTAAGCTTAGTGATCGTTCTCTCTATGCATTAGCTCATGGTTGT

CCTAACCTGACCAAACTGAACATTAGCGGTTGTACGTCATTTAGTGATGCTGCTCTTGAATATCTGACTGGATTTTGC

>JK612344.1 JCF25-29 Jatropha curcas, immature Seed cDNA subtraction library Jatropha curcas cDNA similar to o-linked n-acetylglucosamine, mRNA sequence

ACAATCACATTGACAATCACTAACAGTAAGTAAACACATCACCATTCCATCATCTGAATAATTTTGAACAATATCTTCCT

AAATACACTATTCATTTTCTCTTGCTCTTATCTACAATGCCGGATATTCACTCTTCTCTCATCTATCACAAGGGAACTCT

GAATCATTCTCAGCAACTTTGAAGTGCCGGGGCTGCTGGCCCAAGCAATGTATATTCCACATTTTGAAATATGCCCTCTC

CAGATTCCGGACCCAGCGTGCTGTATCAAACAAAGGACAACTCATTCGGACTGCCTTCAGTTTATTGGTAAGAGCTTGGA

GTTTTGACCTATTCAAAGCCAGGGACACTGCTCTCTCTTCGTATTCCTTCATGCTGCTAACTATCATCTCGTCCCCAAGT

CCAGTGGCAACACAAAGCGATCCAGCAACTCTAGTAGCCATCTTCTCAAGGGGCAGTGTTACCATTGGCAAACCTGCCCA

CAGAATATCTGTTCCAGTGGGTATGTGCATTACATAACGGCGTGTCCAGAAATAGATCTGCTAAGGCAGACCGCCTGATA

TGTTCCTGTTTCATGGCAACATCAGTAAAAATGATCTGCTCAGGTTGCACCCCTTGTGCGACAGCATATGATCGAATTCT

CATTTCGCCTGCAGCAAGCAACCTAACGGAGCTAAAGTACACTATATTGGCCACACGTTTAAGAATATTGCACCTAGTAT

TAGGTTATTTGCAGGATCCATCTTAGCATTACTTGGTTGAAGCATTCATATGATGACTGTGTGCTCAGAGACCATAATTC

TGATCGCTAGGGGTTGACCCTGTATGTATCAAGTACCTCCGAAATGTCG

>GT980287.1 JGCCJG2041C01.b1 Jatropha curcas L. germinating seeds (mixed stages) Jatropha curcas cDNA clone JGCCJG2041C01 similar to unknown protein, mRNA sequence

GGAGGCAGAAGCAGCTGGCAAAACCCTGAAATCGGAGAGGATTGTACAAACCTCGCAACCATCAAAAATTGAAGAAGAAG

AAGAGAAAGCTATTGGATATGTTTTTTGGAAGAAATTTCTGTCCTTCCCCATGGTAATTGGAGCCGTAGTGATCGGGGTT

GTTTCAAGAAAAGCATTATTTGGGCCCCCTCTTGATGAGTACTGGAAAAAGAAGCTCCAGGATGAGGCAGCTACCTATGA

TACCGACTCAAGCTCTAATCAATAGATACTGTACTGAATTATCTTGATACTGGGAACTGGGAGGAGGGAGGTTGTCTTTT

CCAGATGCTGACTGATAAAGCCTAATATACATTCATATTCTCATTGCACGCTGTTACAATATAGACCTTGGATTTGGACA

ATGCAC

>GW877934.1 JC003536 Seed specific Normalized cDNA library from Jatropha curcas L. Jatropha curcas cDNA clone N06561 5' similar to Nuclear transcription factor Y subunit A-1, mRNA sequence

GGAACCCCTAAGTTTATCAGCAAGTATATTCAATTTCTTTCTTTTCTCTTCCTTCAAATGCTTGCTCTGCGTACACTCTT

CCTACCAAATTGTCCTGTCAAAGATGAAAGTCATAACTCATAAATCAAAGAAGAAGAATTAGAAGGTCTCTCTTCACTTG

GTAGGCGGCAGATCTGGAAATTTTCAATTTCATATAGTAAGAGGCATGCAAATGAACTCTGATGACAATAAAAATTCAAA

ATTATATTTTAATAGCAATAGTATTTGTACACCTACTTCCAAATCTTGGTGGCATAGCATCGGGCATAATGTTGTCTTCT

CAGACATGGTAGAGGAAAGCGCAGCAAAATTTTCTCTGTCAAAGTACAAGGATGGTTTTGTGGGGAAATCTAGCAAATCA

CAAGCTAACTATCTATTGGAAAAGGGAACTGATGCTAGTAAAGAAATGTTCACTATTTTGCCACAATCAGATGGGAAATA

TGGAGAAGAACATCCTAACTCACAGCATTCTGTATCCATTATGTCTCCAACAATGGTTGAATGCCTTGCGCCACCTACCC

AACCAGATCTTGATGGATACTACTCATTTGTTCATTCTCATCTCGGAGCCTATAACACTAGAATGG

>GW878749.1 JC003015 Seed specific Normalized cDNA library from Jatropha curcas L. Jatropha curcas cDNA clone N05744 5' similar to 60S ribosomal protein L24, mRNA sequence

GGTTACCTTACGTCCGAAAACCTACTTGAACTCCATTCCATTTCTCAATCCATTCTTCAAACTCGACCGGCCGACCCATC

GTCCCTATTTTCCTCCTTTCCGCACTCTCTCTGCCCCACCTCGCCGTCGCTGACTACTGCACCTGCCGTTTCTCTGGCGT

TCGCCTCCGTTCTATACCTTGCACTTCCTCCTCCCAGATTCAAGAAGAATTTGAAGTAGCTAAATAAGAACCTTGAGTGA

GCAAAAACTACTGAAAATGGCCTTTAGAGGAAAGGAGATAATGAAGAAAGTACTGAAAAAGGTAGGAGAGAACAATTTAA

CATCTGGAGTTAAGGAATCTCTCAAGAAATCCATCCCTGATAGCAAGGTTGTTATGGGCCGAGCCAAGCGTGGCCTCTTT

GCTGGCCGGCATATCCAGTTTGGCAATCGAGTCAGTGAAGATGGTGGTAACAAGTCTAGGAGGACTTGGAAGCCGAATGT

CCAGGAGAAGAGGCTTTTTAGTTACATCTTAGACCGACACATTAAAGTCAAAGTCACAACTCATGCTCCTCGTTGTATTG

ACAAAGCTGGAGGGATTGATGAGTACCTGCTGAAGACTCCTTACCAGAAAATGGACACAGAAATGGGCCTCTTATGGAAA

GCTAAGATAGAAAAATTGTATGAAAACCTTGGGAAGATGGAGG

>GT975068.1 GJCCJC2066H06.b Jatropha curcas L. developing seeds (mixed stages) Jatropha curcas cDNA clone GJCCJC2066H06 similar to Putative ribonuclease H protein At1g65750, mRNA sequence

ACCACGTAAAATAAAGAGAAGAATTTGAGAATTTGTATTCACAATTTGATATCTACAAACATTAGCAGAAAGCTATATAT

ATAGTCTATACAGGAAGCTAATATAGAAAGAAAACAAATTACATTAATTTAGGCTATAATTTGCAATCAGATTAGGTAAC

TAGAATATTTCTATCCTACAATTGCTCATTGACAGCTCACGTTAATACAGATTTATTGAGGTAAGGAATAGAATAGAATT

TGGAGCTGAAGATAATCGTTAACAAAGAGGAGTGGCTATCACACGAGACAATAATCAACCCCTATCTACATACTTAAATT

TTTGTGTTTGAATGGAGAAAATAAAAGAAAAATTTAATGAAGAAAGTATATATATATATGCGAATGTATATGCATACAAA

TATGGGATCCATCGTTGACTGGTTAAGTTCGGATAGCCAGCCTGCTAACAGTTACAGGTCTGTTGTCAGAGCTATTAAAA

TGTTGCTTCAATGGAGTTAGGTTTTCCAGGCTTGTCCTGTCTATAGGGAAGCTAATCAATGTGCAGACTGGTTGGCTTCT

CATGCTTTCTCTCCTGGCCTTGGTGTCCATCCTCTTGATTCTCCTCCTATTGATATTTTTTTCTTTGATTTTTAATGAAA

GTTGCTGTGTTGCGTCTAGGCGTTCTATCGCCTCTTGTAGCTCTTCTTGAGCCTTAGAGCTCCCTTTAATTCGAGCT

>FM889959.1 FM889959 Jatropha curcas embryo 35-55 (DAF) Jatropha curcas cDNA clone rjcfea0_003721, mRNA sequence

CAAAAGATGGTCTTTTCTCTGTTGGCGAAATGGAATGCATGGGATGCTGTGTAAATGCTCCCATGATTACTGTTGCTGAT

TATTCCAATGGATCTGAAGGATATACTTACAATTATTATGAAGATGTACTCCAAGCGAGTTGTTGAGATAGTTGAGATGT

TAAGAAGGGGAGAGAAGCTACCGCCTGGCACACAAAACCCAGAACGCATCAAGTGTGGGCCTGAAGGAGGCAATACTACA

CTGCATGGTGAGCCAAAACCACCTCCATGCCGGGATCTCGATGCCTGTTAATGTTATGGTAAATGTTGTTACCAATAATG

CAAAGCTTGGATTTTTTTCAGGCTTTCCTGAGTCATCTTTGTATATAAAGCTCAACCATTTATAGTATTTGCCTGCCAGC

ATGGTTTCTGGCAGATACTCAGTTCCATGCATCTTCGTATTCAAGAAATGGCTTACTAGTTCCTTGGTGCAAAA

>FM889561.1 FM889561 Jatropha curcas embryo 35-55 (DAF) Jatropha curcas cDNA clone rjcfea0_003249, mRNA sequence

AACAAAAGCTTCCCTCTTTTATCTTTTGTTTAAATGCTGATTTTCAAATAACAAATCTTAAGTTACGTTAGATCACAAGA

TTAGAAAAAACAGAGCATTTCTATGGGTATCACGGTTGCATATCCTTCAACTTTGTGATGACTTCTGCTCTTTCTTCTGA

ACTACCTGACAGATTCTCCGGCTTGGCATCGCACTCCTGAAGTTCCCGTACAGTAGTNTAAAAAAAAA

>GT975942.1 GJCCJC2071B12.b Jatropha curcas L. developing seeds (mixed stages) Jatropha curcas cDNA clone GJCCJC2071B12, mRNA sequence

ATTATAGCATTTGGAGAAATCACGGCCGTAAATCTCGATTCTTTCCTGGGGCATATCTCCACCATTATGACTTCATGCCA

AACTGATATGATCAGCCAATCCTCGAAATGGGTTGCCTTCATGGAGAAGTGATAAGTTTGATGACGATGATGAACTGTAA

TTGAATGCTTAAATAGAAGCTAGAAGTTTGTATCATGGGTATAGTTGGGTGGGTCAGGTTTGTGTGTGGAGCCCCAATTT

CAATAAATACTTTTGGTGAAAGAAAATGAAATTTGTTCTCTATGTATTCCTGGATTTTGTAAATATGTTCTTAACTTGAT

AAATGAAGAGTTGTTTTTAATCCATTTGCTGTCCGAATGAAATTGGATTATTATGTTTCTTCGAAGCGAAATAAA

>GW881252.1 JC000816 Seed specific Normalized cDNA library from Jatropha curcas L. Jatropha curcas cDNA clone N01547 5' similar to Mevalonate kinase, mRNA sequence

GGAGGAAAAAGAGAATGTATCTTTGGTTTTCTCCGCCAAAAATTGAGTTCCAGTTCCTCCCATGTCACCCTCTTCTTCCA

CTTTTGTCAATTAATGGATCCGTAAATCTCACAAACTACTAAAAGATTCCAACTTTATTTTTTTCTGGTAACGGAATTCG

AGAAACGGAAAAAAACAGATGGAAGTTAAAGCAAGAGCTCCAGGAAAAATCATTCTTTCCGGTGAACACGCAGTGGTGCA

CGGATCGACTGCAGTCGCTTCATCCATTAATCTCTACACCAATGTCACCCTCTCTTTTCCCACTTCTGAGAATGATGGTA

CACTGAAACTCCAGCTCAAGGATATGGCAATAGAATTTTCATGGCCAATTGGCAAAATCAAAGAAGCATTACCTAACTTA

GGTGCTCCTTCCGCTTCGACACCCACCTCTTGCTCAATAGAATCAGTCAAGTCAATTTCAGCTTTGG

>GT976566.1 GJCCJC2079F05.b Jatropha curcas L. developing seeds (mixed stages) Jatropha curcas cDNA clone GJCCJC2079F05 similar to unknown protein [Arabidopsis thaliana] (TAIR:AT4G29400.1); similar to unnamed protein product [Vitis vinifera] (GB:CAO65989.1), mRNA sequence

GAGGACGGAGATTAGCAAGGATTCGGGAGGAGAAGAGGAAACGCGAACTCGATCGTCTTCATAACTATCCTTCCTGGGCC

AAAGTTCTTGAAGATGCTTGCAAAAACGACGAAGAGCTTCGAGCTGCTCTTGGAGATAGCATTGGCAACCCCGAACTCAT

GAGGAAAAGAGTTGAAGATAGAGTAAGGAAGAAAGGTCGCGACTTCTACAAGTCAAAAACGGGTTCAGTCCTTGCCTTCA

AAGTCAGCTTTAGAGAATTTATTGTAAGCATCTTGGAGATGGAAATTACTGGAAAGGGTATGCTTTATTGCTTCAGAATC

TATAGTTCTTGACCATTGGAGATCTATGCAGACTATTCAGTCATGGTATGTTATGGGGAGCTTGGGTGCCTTCAATTCTT

CAAATTTGCAGCTGGCAAATCAATCAATGGAGTACAATCCCCTCTATGATGCAGATAAGGGCTTTAAAGTGATGCCATCA

TCTTTCCATGATATCAGTGATGTTGAGTTTCAAGATAGCTGGGGCCGTTTTTGGGTAGATCTTGGGACATCTGATTTTTT

TGCTATAGATGTGCTTATCAACTGCTTAACTGTATTGAGTTCGGAATATTTAGGCATTCAGCAAGTAGTTTTTGGAGGCC

GCCGAATAAGTGATTGGGAGGAGGGAATGACCCACCCTAATGA

>GT977188.1 JGCCJG2006F10.b Jatropha curcas L. germinating seeds (mixed stages) Jatropha curcas cDNA clone JGCCJG2006F10, mRNA sequence

GACGCTCTGTACACCGATGTAGTCCGACTGGAGCCTTCATGTTAATGGAACCTTGGACGTGACCAGATGACCGCTGACGC

TAAAAGCCCCATGTATTCCGACTGGAGCCTTCCCGTTAATGGAACCTTGGACGTGACCAGATGACCGCTGACACTAGAAA

CACCGATGTATTCCGACTGGAGCCTTCATGTTAGTGGAACCTTGGACGTGACCATATGACTGCTGACTCCCATCACGCCC

ATGTGGTCCGACTGGAGCCTTCAGGTTAATGGAACCTTGGACGTGACCAGACGACCGCTGACGCTCAGTACACCGATGTA

TTCCGACTGGAGCCTTCAAGTTAATGGAACCTTGGACGTGACCAGATGACCACTGACGCTAAGCACACCGATGTATTCCG

ACTGGAGCCTTCAGGTTAGTGGAACCTTGGACGTGACCTTATGACGGCTGACGCCCAAAACGCCGATGTATTCCGACTGG

ATCCTTCAGGTTAATGGAACCTTGGACGTGACCCGAGGACTACTGACGCCC

>FM888963.1 FM888963 Jatropha curcas embryo 35-55 (DAF) Jatropha curcas cDNA clone rjcfea0_002502, mRNA sequence

ACGTCAAAATGGATTACTATAGGAGAAGCGCTGGAAGCCTCAGCACAAACAGCTGGTGACAAGGCAGTGGACCAGAGCGA

TGCTGCCGCAATTCAAGAGGCAGAAGTAAGAGCAACTGGCAGCAATGTCATTGTACCAGGTGGAATCGCAGCCACAGCTC

AATCTGCCGCATCTTACAATGCCGCTACGAATCGCGCTGAGGAAAAGATTAAACTCGCCGACGTTCTATCGGTAACCGGA

ATTTGTTACTAATTTTGTCATTACTAATGCACTTAATTAAGTACAAGGTAGCATGGATGTGACATTTCGGGTGGGTGCAG

GGTGCAACTTCGAAATTGGCGGCGGATAAGGTCGTGACAAGGGCAGATGCTGAAGGGGTGGTTAGTGCAGAGTTGAGGAA

CAATCCGAATTTAAACACGCATCCAGGTGGTGTTGCGGTGTCAATGACGACAGCTGCTAGGCTTAATGAGACCATTAACT

ACTGATACACTAATG

>GW618245.1 Jc2-032-H05-M13F.H05.ab1 Jatropha curcas flower and seed Jatropha curcas cDNA, mRNA sequence

TGCTTGTTACCAAAGGGGAGGCAGTGGTGATATCTGTGAAGAATTTCTCAGGATTTGTATCAATCAGTGGTGATGGTAGC

TGGGTTTGTGAAGGTAGCAGTAAACACAAGGCCCAGCGTTATCCTGATCCTGTGGAGGAGGCAAAGAAACAATCTTCAGT

TCTTGAATCATATCTTGAACAAAGAGGAGTTTCTCTTCCAGAAGGATATTTGTCGTACAAAGTTGTTCTTCCCAATCCAA

AGTTCTGGACTGTTCACTCAAGCTATTTCCCATCTGAGGTCATTACTTATGACCAATGGTTACAGTTGAAACCAGAACCC

AAAAGTATGTTTTCTGGTTGGATTAAGGGTGCCTTTCGTGGTGGAAAGAAAGAGATGCAGGAATCTATACATGAGAAGCT

TAATTTCATTCTGAGCACTGCTCCAATGTGGGATAGGTTGGAGCTTAAAGGTAACAAATATGTCTTAGGAGAATTTCTGG

AATTTAAAGGAAAGGAAGAAGATATCACAGCTTTGAGAAACATCAAGAGATCAAAAGTGGGTCGTCTTGTTGTCCAAAAG

ACAAGCATGTTGGGACTGGCTAAATCAAAGCTTCAAGTTTTGTACTCTC

>FM887721.1 FM887721 Jatropha curcas embryo 35-55 (DAF) Jatropha curcas cDNA clone rjcfea0_000973, mRNA sequence

TTGTGAGTGGAAAGAAGAGATTAGCAGAGATGGATATGTCTTTCTTCACTTCACTTGCGCAAGATAAGCCAACTCGTTCG

AAGAGCAACCTTCAATTAGCTTCTCAGGTTGCTAACGATGTTGACAGCCCCATTCTTCCTGGGCTGCCTGATGATGTGGC

AAAGTATTGCCTTGCACTCGTTCCTCGTCCGAATTTTCCAGCTATGGGAGCTGTCTGCAAGAAATGGAGATCATTTATTA

GAAGCAAAGAATTCCTTGTTGTGAGAAAATTGGCTGGGTTGCTTGAGGAATGGCTATATGTCCTAACTATGGATTCTGAA

GGAAAAGAAAGCCATTGGGAGGTTTTGGATTGCTTGGGACACAGACATCAGCTTCTTCCATCAATGCCTGGTTCTATGAA

GGCTGGGTTCGGGGTGGTGGTTCTCAATGGAAAGCTTGTTGTCATGGCTGGCTATTCAGCTCTTGACGGGACTGTTTCTG

CCTCATCTGATGTTTATGAATATGATTCTTGCCTCAACAGTTGGAGCAAATTGCCAAACATGAATGTTGCACGCCATGAT

TTTGCATGTGC

>GW611009.1 JC2-048-G11-M13F.G09.ab1 Jatropha curcas flower and seed Jatropha curcas cDNA, mRNA sequence

TCCCCAAAAAAAAAAAAGAAAAAAGAAAAAAAAAACACTTTCCCCCAAATCATGAAACCCTAAATTCACAAATCACCAAA

TTCCCAATTATACTTTAACTTCCCATCTCCGATCCATCTGTTTTGTTTTTATTTCTGTAGGTTTATTAATTGTACAAACA

AATGGATGGGAACAAAGACGACGCGTTGAAATGCTTGAAAATCGGCAAAGATGCACTGGATTCCGGCGATCGAAGTCGTG

CTTTAAAGTTCATCAATAAAGCTCGTCGCCTTGATCCCAATCTCCCCGTTGACGATCTCTTGTCGGAAATTGAAAAGGAT

TCGTCTTCCTCTGATCAAACGGCTGAGAGTACTAATGGGCCCACCAGCACCACGACAACCGTCGCCGATGAATCTAAAGT

TCGCAACAGGGCTCCCTCAACTGGGTCTTCTTCATCTGCCTCTGCCTCGGCAACATATACTGAGGAGCAAATCACGATCG

TGAGGCAAATCAAGAAGAAGAAAGATTATTATGACATTTTGGGATTGGAGAAAACTTGCTCTATTGAAGATGTTCGGAAA

GCTTACAGGAAATTATCTCTGAAAGTTCATCCTGATAAAAATAAAGCT

>GT973802.1 GJCCJC2052H05.b Jatropha curcas L. developing seeds (mixed stages) Jatropha curcas cDNA clone GJCCJC2052H05 similar to Jatropha curcas microsatellite ssr 308 containing mitochondrial DNA, mRNA sequence

CGGTTGAAAACATGTGTGAGGCCTTTTGGTGGTGACATGGCTGGAGAGTTGGAAGAACTCCAAGGTTAAGCGTGCTCGCT

TAACGGCAATCCTAGGATGGGTGACCTCCTGGGAAGTTTCCCATTTCCCCCAAGAACCAAACCATGCGGGCCGTAGGGCC

CAGAGTGGACAATATCACACATGGGCTGGGCCGTTACAATTGGTATCAGAGCCGAAGCCTCGCTCCTCTGACCGGTGGTG

GGGCAAACCTCAGCGAGGACGCTGAGTCCCCAAGGGGGGGTGAGCGGTGGCTGTGAGCGGTGGCTGTAGTGACAGTGCAC

TCACAGTGCACTCACCTTAGGCGAATCCCAGATCGGTTAAGTGCGAGAGGGAAGTGATGTACTTAAGGTGGGCGGTTGAA

AACATGTGTGAGGCCTTTTGGTGGTAAAATGGCTGGAGAGTTGGAAGAACTCCAAGGTTAAGCGTGCTCGCTTAACGGAA

ATCCTAGGATGGGTGACCTCCTGGGAAGTTTCTCATTTCCCCCAAGAACA

>GW874708.1 JC000098 Seed specific Normalized cDNA library from Jatropha curcas L. Jatropha curcas cDNA clone N00165 5' similar to Unknown protein, mRNA sequence

GGAGCCCACTCCCAACAATCTCCAAACATAGCCTCTGCCATGAGAGGGTTTTTGGTTAGGGTTTTTCGATCTAAGCTATC

AAACACTAACAAATGGCGTCTCTAATGGCGATTCGGCGTTCAAGGAATTGGAAATTGGTTTCTTCTTCATCTTCCCCTTC

GGTTTTCAAGGTACGGTCTATTCTGCATTCTGATTTCTTTTACAATAGAACTTTAATCCTCTCCAAAACCCTAAGCTCTT

CCTCTGCTGTTCCTGATAACTATCAAAGACCTCTTCCTTCTAGTTATCAAGATGAGAGAAACCTTAATCGCCAATGGAAT

CAGTGGAATGCACAGCAACAACCTCCTCAATATGACAAAAATCAGTTCAATTACCAAAATAGAGGGTACTCTAATTCATT

TCCTCAGCAACAACCACACCAGAACCCTAATCAATGGAATTCTCAGGGTCAGAATTCTCCCAAGTACCCAAACCGTAATC

AAATGAACCCTAACCAGTGGAGTTCTTCAACTCACAATTTTCCTCAACACCAACCGCAACAAATCAAAATCAGTGGATCT

CTTCAGCTCACAATTTTCCTCA

>GW615604.1 Jc2-002-D08-M13F.D08.ab1 Jatropha curcas flower and seed Jatropha curcas cDNA, mRNA sequence

CTGGAGAGACAGCAAGTACAAGACATTAGCTTATAGCAACTCAACTCAACTCATTTTAACTAACTTCATTTGCATATTCA

ACTTTTCAAGTCGGACCTAAATCTCATCACAAAGAATGGGTGAAGAGAAGAAAGAAGAAGAAAAGAAAGAAGAAGTGAAG

GAAGAAGAGAAGAAGGAAGAAGAGAAGAAAGAAGAAGAGCCTCCAGAAATTGTACTTAAAGTTGATATGCATTGTGAAGC

TTGTGCTAGAAAAGTTGCCAGAGCTTTGAAAGGATTTGAAGGAGTGGAGGAAGTAACTGCAGATAGCAAAGCCAGTAAGG

TGGTGGTGAAAGGCAAAGCAGCAGACCCCCAAAAGGTATGTGAGAGGCTGCAAAAGAAAAGCGGCAGAAAAGTGGAATTA

ATCTCTCCGTTGCCAAAACCACCCGAAGAGAAAAAAAGAGGAAGAAAAGAAAGAAGAACCTAAGGAAGAGAAAAAGGATG

AGCCTCCTCCTGTCATAACGGTTGTATTGAACGTTCGAATGCACTGCGAAGCATGTGCTCAAGTTTTACAAAGACGAGTT

CGAAAATTCCAAGGAGTTGAATCGGTCGAAACAGACTTAGCTAATAGTCAAGTAATAGTAAAGGGCATAGTCGATCCAGC

AAAGCTAGTC

>FM894441.1 FM894441 Jatropha curcas embryo 71-95 (DAF) Jatropha curcas cDNA clone rjcpga0_004793, mRNA sequence

GTTTCCAAGTGCTCAAGAAGAACAAGCGGGGACAAGTTAGGAAACCCTATTTGGTTTTTGAGATGGCAGACTTGGAGAAA

CAAGTGGTGGAGGCGGAAGAAGAGGAAAGGCTGATTGAAGGAATGGCAGTGTTGGATTTTGATATGTTGTGTCCACGGTG

GCTTTGCAGACGCAAGGGAAATGGAGGAAACTGGAAACTGAAGATTTTGATGCTATGGATGCTAGTGGTGGGGAATTTGG

AGGTGTTTGAACGATGTGGGAAGGTGAAGTCCTTGGTTGCTTCGACGACCGCCGCATCGCTATTGAGTCCTTTTGTTGTC

CATGCTACAGATTTGGGAAAAACATGACACGAGCTGGTTTTGGTTCTTGTTTTCTTCAGGGAACTGTATATTACGTTCTT

GCTCTTACTGCCCTTCTTCACTTCATTGCATTCATAGTCACCAAGCGGCAATGCTTTCTTTATTTGGCAGCAGCATTTAC

TATTTCAATAGGAATGTATTTGGGTTTCTTCCGCACGCAGATGAGAAAGAAATTCAATATTACGGATGGTGATAGTTCCT

TGGATGATTTCATCTGTCACCTTTCCTGACCCTGCTGTG

>GT972332.1 GJCCJC2033B11.b Jatropha curcas L. developing seeds (mixed stages) Jatropha curcas cDNA clone GJCCJC2033B11, mRNA sequence

ATCCATGCATTTGATCATTTCATTTGAGACGGCAAGGTTTCCTTCTCTGCCCATCCTCAAAATTTGCTCCTTCAAACAAC

CACTGACTCTCATAAATCGTTTTTATGATAGCCAGTGGGGGCCAACTGTGAATACTCAAATTTTGACATTTGCTACAGTA

AATTTGACTTTGCTACAGTTGCTACATTTGAAATAATCCGGACTACAAACTGTCCGACCGGACCGAAGGCCATTTTGGAA

GTTCACCGAAAGTATGCCAATATGGTACTTAAAACGTCCGGAATGTCGCGAGGATCGCGAAACAGGTCCCGAAATATTTT

TCTGAGGTTCGGAACTCCCGAAAATGCCTCCGGAATGAAAAGCCCGAGGG

>GT975852.1 GJCCJC2030B02.b1 Jatropha curcas L. developing seeds (mixed stages) Jatropha curcas cDNA clone GJCCJC2030B02 similar to transporter, mRNA sequence

GGATGAGAGGCGAACCAGAATCGGTTCATTGAAGAAGAAAGCCCTTAATGCTTCCACTAAATTCAAGCATTCTCTGAAGA

AAAAGAGCAGTAGAAGAAAAAGTGATGGTCGAGTCAGCTCTGTATCAATTGAGGATGTTCGAGACGTTGGGGAACTTCAG

GCTGTTGATGAATTTCGACAGACATTGATCATGGATGAATTACTTCCAGACAAGTTTGATGATTATCATATGATGTTGAG

GTTTTTAAAAGCAAGGAAGTTTGACATTGAAAAAGCAAAACACATGTGGTCCGATATGCTTCAATGGAGGAAGGAATTTG

GTACTGATGCTATAGTGGAGGATTTTGAATTTAAAGAGTTTAATGAAGTATTGAAGTATTATCCCCATGGTAATCATGGT

GTGGATAAAGAAGGGAGACCTGTTTACATTGAAAGGTTGGGAAAAGTTGATCCGCATAACTTATGCAAGTTACAACTATG

GATCGGTATATAAAATACCATGTGCGGGAGTTTGAGAAAAGCTTCGCGATAAAGTTTCCAGCTTGTACCATTGCTGCAAA

GAGGCATATAGATTCAAGCACAACCATTTTAG

>GW611108.1 Jc1-002-A05-M13F.A05.ab1 Jatropha curcas flower and seed Jatropha curcas cDNA, mRNA sequence

ATTTGTGGGCAAAGGTTGAATGAGGAAACTGGTGTCACATTGGCCTACATACACAAGGGGTTAAGGCTTGGCAATGACGA

GATTGTGCGATTACGCAACTCAGATACGAAAAATTTATTACGCCATAAAAAGCTTTACTTGGTTCTTGATCTAGACCATA

CATTGCTGAACTCTACTCAGCTTATGCATATGACAGCAGAAGAGGAATATCTGAAGAGTCAACTAGATTCTCTGCAAGAT

GTTTCTAATGGTAGCCTCTTCAAGTTGGACTTCATGCACATGATGACCAAGTTAAGGCCCTATGTGCACACATTTCTGAA

AGAAGCAAGTCAAATGTTTGAGATGTACATATACACTATGGGTGATAGGGCATATGCTTTGGAAATGGCGAAATTACTTG

ATCCTAGAAGGGAGTACTTCAATGCTAGAGTTATTTCACGTGATGATGGAACTCAAAGACATCAAAAAGGTCTTGATATT

GTTCTGGGCCAAGAAAGTGCTGTTCTGATACTTGATGATACAGAAACTGCATGGACAAAGCATAAAGATAATTTGATCTT

GATGGAAAGATATCACTTTTTCGCTTCAAGTTGTCACCAATTTGGCTTCAGCTGCAAATCCCTTTCTGAGTTGAAAAGTG

ATGAGAGTGACTCTGACGGAGCACTTGCATCTGTTCTTAAAGT

>FM893826.1 FM893826 Jatropha curcas embryo 56-70 (DAF) Jatropha curcas cDNA clone rjcaeb0_004754, mRNA sequence

GCTATCAAAGATAAAAGCATTCATAAAAATCATAAAAGCACTATATATAACAACTTCATAATACAATCACCACCCGAGGC

TCAAAATATCAAGAAGCAAAACTTTAAGTTATCTGGCCCCATTAGATAATTCTATGCATACCCAGCCATCCCGACATTCG

AACTAGTACGATTTGCCTTGCTGGCTGCCTGAGGGGCAGTAGCCTTGGAGAAATACATGTAGTAGTATACATTAAGAATC

ATTGTTGCAACTACAAACACAGCTCCAGCAACAAAAACACCCTTACGCAATGTTTCACATGAGAAATTTTGAGCATATAT

CATTCCCCTGTACTTGGTGTGGTATGCATTTTTTGTTGCACCTGCAAGTAGACATGCTTCTGCAACCAGAAAAGTCAACC

ATGATGAGACAAAATATATTATAGACCATGCTCGATTTCCACCTGGAGCTAATGGTCTTCCAAAACACATGCACTTGGTT

ACACCCATTAGCAGTGATTCACTCGAGAGA

>FM895170.1 FM895170 Jatropha curcas embryo 71-95 (DAF) Jatropha curcas cDNA clone rjcpga0_001951, mRNA sequence
[truncated: 1,708,963 more chars]
