# Supplementary material for: Identification of conserved miRNAs and their targets in Jatropha curcas: an in silico approach
Source: J Genet Eng Biotechnol. 2023 Apr 7;21:43. doi: 10.1186/s43141-023-00495-9 (PMC10079790; doi:10.1186/s43141-023-00495-9)
Supplement: Supplementary file 6 — Additional file 6. Total of 389 putative non-coding EST sequences of Jatropha curcas genome. [file 43141_2023_495_MOESM6_ESM.docx]

**Supplementary File 6:** Total of 389 putative non-coding EST sequences of *Jatropha curcas* genome

>GW615669.FM889404.1 FM889404 Jatropha curcas embryo 35-55 (DAF) Jatropha curcas cDNA clone rjcfea0_003065, mRNA sequence

CAAAAAACCAAAAAAAATTTCTTCTCTATACCTTCATCCGTCGTCTCGCGTCCGGTTCTGTACGGTTCTCGGACGATTCT

ATCCCTATTTTACCCTTCTCTCTCCTCTTCCTTCCTATCCCTCAAATTATCAAAAAAAGAAGAAAAAAAAAGGCTCTCAA

ATTCACACTATTAGGTCATAGATTATAGTTCACTTTTCAGCTTTAGGATCAGAGTTATTGCCATTATTATCGTATCTGTC

TGCGTTTTCTAGCTATCTAAGGTTTATCTTCAAATAAGAAGAGGAGAGAGGGGGTAATAGATTTTTATTTATTTTTTTAT

TTGGGAAAGAAGAGAGAAGGATCTAGAACGTCTGTGGTTTCGAGATGGCACGGATTCAGGTACCGCATCCAGGCGGCGCC

GGTACCGGGGCCGAACGGAGTTGCGGCGACTCAAGCCGGGATTCCATTCATGCCGACGTCGCTCTATGTTGGAGATCTTG

>GT969604.1 GJCCJC1013A05.b Jatropha curcas L. developing seeds (mixed stages) Jatropha curcas cDNA clone GJCCJC1013A05 similar to putative genomic DNA, mRNA sequence

AGGTCACATGATACATAGCTAATGAGATTAATAATCTTTACTTAGCAAAAGTTATGAGGAACTTAATTCTTTGTGTGAAA

GGTGAGAATATTAGTGTCGAACCTGTGCCTTGGGTTATGAATGTTCTACTTGATTTGACTTGTGGATATAGTACAACTCA

AGTTTAAATTAACTATAGTGATAAGGTAGATATCTAACTGTTCATCAGAACAAGAATTCTCCTGAGTGTAAATGTCATAA

CTTTTAGCAGTTGCAGCATTATGTGAGAAGCACAACCACCATTGTAGTAAACAACTGTTGCATCCTAGTTTCTTGTTAGC

TTTAAAATTCGAGGACGAATTTTTATAAAGGGGGAAGACTGAAATAACCCGAATTTTTATTTTCAAATATTTTCATTTAA

TTTAATAAACTGACAGCGTGGAAAATGTATTA

>GW880703.1 JC007042 Seed specific Normalized cDNA library from Jatropha curcas L. Jatropha curcas cDNA clone N11862 5' similar to Unknown protein, mRNA sequence

GCCCAGTTACGGGGGTGCTTATGTGGTAGTTGATGTGGCATAAATAAGCAACTATTGTAAATGATGTCCAGAATTGACAT

TTCACATTCAATATCTTTTTTAAGTTCTCATTTCTCTCTCTGACCTTCTCTCTGCTCTTGGCGATTCCAGAATTGTCCAT

CTTCATTTCCTACCCTTTCTCTCATTTTCTTCATGTTTCGTAGCTATCAGACATCATCAGGCATTTTATGTTTGCAATTC

CTAACATGCTGATTCATATGGGACATTCTAAGTTTGTGGATCAAAACTTTGCTAAGGAGTGTTATAACAAAACATTTAAG

GAGTTTATGGTCCATATACATCTAGGCCTACAGGACATTATTTTTTCTTGATGGATTAAATTAAACATGCCAATGAGATT

TTTACTACCCTTGCCCGATTGATTTTGTTCTACATGTGATGATAATTGGTCATCTAATTTTGTATCAAATAACTGTGTAT

ACCATAGGCCTGTTCCTTTTTGCGTTGGAGTGCCATGCGTCAATGCTGTACTCTTGGCAGTCCAACAGCAAAGCCAAACA

GCCTCTATGTACTTAATGATGCATCAAAATTGATGAGTTGCTCTTAGTTATAATCATCCTTGAGTAACAATTTTTCAGAA

AATATATTACCAGTCATGTAATACAACCATTAGCATTTGTTGTGCTACGATGAAAAAAAAATTGTTTTTGGTCGAGTA

>JK317678.1 JCST449 Jatropha curcas L. seed cDNA library Jatropha curcas cDNA 5', mRNA sequence

TTCATCTATGTCGGGTGGTCGAGAGAGGTAATCCTTCTGTTAGCAAGATCCTCGCTTTGGAGTCGGTCTGTCTAGTGGCA

GCCTGGGTGAGACATCCTGCTGAGGCCAAGTAGGCAGAATATTTGGGCAACGCTGTGGACCTTCGCGGGCGCAAGAGGGC

TAGAAAATCGAACTTTATGGTGGGCGTCCCAACTTTGGGGGGGGCATGATCCCGAAACGGGGGGGCGGGGAGGGGGCAAT

TGAGAGAAAGTCGGGCTGGAGAGTGGTACGGTTGTATGAAGGCAGTAAAATGGTTGCATAGCCACCCAAACCGCCCGAAT

GTTGTATAAGCATTTTAAAGGGCCAGGGTAAGAACACATTTGGGTGGCCTATCCAAGTAACTGAAGCAGGAGGAACCGAC

CCGGTTTGCATTGTGGTGGGGCGACGCCACCAGGTTCCGCACGCGGCCGCCGCGTTGACCCCAAATTTAAAACAGCCGTG

CTCGTAGTGAACCCCCAACTATCAACCCCAGTAAGAGGAAAACGATGGGAAAGAAAGGTACTCCACGAGCACAAGTTAAA

AAGAAGGGAGCAGTTGTTGTTGGCAAAAAAAATCCCGCCCAGCCGGAATGGTACCCAGCATCTTTCCTCCGTTAAACCAC

ACCCAATAGAGGAACATAGAAATGGGACAGAGCGAGGAAGGAGCCGCTAGACTCGTAAAGAAAGCAAACTAGATGTNNTA

GTGCATAAGGATTGTGTGGGGTATACGAACTAGTGCGCTAGCTCGTCAACTCTATATACTGATAACATCCCNGAACCCCC

GAACACGACGCAACGTATAGGACTGTNANCACGTAGGTGGTATTGGGTAGAGGGGGNCTGTATAGGTAATC

>GW876145.1 JC005522 Seed specific Normalized cDNA library from Jatropha curcas L. Jatropha curcas cDNA clone N09476 5' similar to Unknown protein, mRNA sequence

AAAAGAATTATATTAATGGAACTTTTTTGGAGGACATTGTCACTATACAAATTTGCATTTGTTCTTCTAATATTGTTATT

ACAGCTTAGAAATTAGATGGGTTTTATATTGAGTTATTGAGTTAAAGCAATGCTGTACTTTTGGAAAATATTTTCCCCCT

ATAGCGACAATAAGGAATGATATTTGAATTGCTTAAACTTATAATTAAGATACTTTGTATCAAGAACCCCAAAGCAGAAA

AGAAAACTGTATCTTCTCTGTTGAAATCTGGAGTTTTGTCGCATGTGCAATCACTTATGGGTATTCACATGACAGCACCA

TCCATTTAGCATTGATCAATATTTAATTGTTTTACAAATACTTGTGTTAGTCTTATTCACTAATGCATTGTCTGATGCAT

TGATATACGTGCAAATAGTCCTGCATTCTATTTCCTACAGAAATTTGGAATAAGAAGTTGACAGTGTTCTAGATTCTCTG

GGTCTTTCTTTCTTTGATTTTGGATGTGCGTGTGTGCACATTTAGGTTTCGAATTTTGTCATTTATGAACCAATCTTAGA

AAATTTTATTTTACATTTTCCCTCTTTAAATCTTGGAGAATTGCTTTTTAATTTTTAATGCACTGGCATTAATTTTGTGC

AG

>GT974398.1 GJCCJC2158G11.b1 Jatropha curcas L. developing seeds (mixed stages) Jatropha curcas cDNA clone GJCCJC2158G11, mRNA sequence

CTTTCCTTTAGTACACAAATTATGAGCTGCCAGCTAACATTTTGCATTCCCAATTAAAACTTCAAGACAAATACTTGCAC

AATCGCATCCAATAAATGAAGAGCGCCACTAGTTAAAAGAGAGCATAAGACTATAAATATGGAAACCAACAAAATGAACA

TTAGAAAATTGTTGGTCTGCCAAAAAAACCCATTAAGATGACGTATAAACTTACTTACATTACAATGTGGGAGAGGGAAA

AACAGTTAAATGTACAAAGTGGGATGGTTTGAAAAAAGCAAGGCTACCATTGCCTTGCTCAAGATAAGGGGTATATATCA

TTATTATATATTATATATTATATATTATTTATATATATTATGTATAACTGGACACCAATAATACTAACTCCATTAACATG

TCCAAAATTGGGTATATATCATTCTTGCCATCTATTTCTGAGATACCAACAAGCGTCCGTCTTAGAACAGGTTAAGACTC

GCACCCCATCTCAAATTTCGCAACTGAAAAGGCTATAGCAAACCTTGCTTTGCTGCCTGTCTCGAAAACCTGGCACATAA

GATTCACAAAAGTTAATTACAACGGTATATTCAAGAATAATTTTAAAGAGCATCAATAACCATCCTGTATTACTTTTTTA

ATTAAACAAAAAGATAAAGCATATAGCTCTTGCATTAGATATTCAGCATAGCTAATTTGTTCACAACCCTCAAAATTACC

TAGAATTTTCAAAGTAGCCCAACCTAAAATT

>GT974856.1 GJCCJC2064C08.b Jatropha curcas L. developing seeds (mixed stages) Jatropha curcas cDNA clone GJCCJC2064C08, mRNA sequence

ATATATATATATATTCCAGAAGTAAAAAAATATTTAAAAAATAGCTAGACATGATGCTTTGTTTTTTCAGTATTCTATTT

TTTAAAAAACTATTTTTAGACTTTTTTTTTACTATGATTTTTTCTTTGTTTCTAAAATTTGATTGGTCATCAAAATATTT

TTAAAATTAATATTTTATGATAATAGTCAACATTTTTAAAACAGTTTCTACAATTTGATTGGCCTTTAAAATATTTTTTA

AATAATATTTTATGATAATAACCAACATTTTTTAAATTTAATCTTATCAGCAATATACTTTCCTTTTGTTGAATCTTAAT

TTTTATGTTTATTAAGTTTTGTATCTAAGCCTTCTACTTGCTCTCTCCTTCTATTGTTCTCGTATGATAGTTTTTATTTA

CTCCAAACTTCTATTTTTACAGGCATCATGTACAGAAAGATTCCTGTATTGTGCAACAAACCATGCTGACAAATCAAAGA

ATGTGCTTTAGTGTATTTCGCTCATTATTTTCATCCAGATTCAACTATGAAGAGTTAGGGAGAACTTAAGTTACACTTTA

AAGATTTTCAACATGAAAATGTTTTTTTTGGATACTTCTGTTATGAATTTGCAGTTAAAATAAGACTTTTATT

>GW877468.1 JC007272 Seed specific Normalized cDNA library from Jatropha curcas L. Jatropha curcas cDNA clone N12235 5' similar to Unknown protein, mRNA sequence

ATGCCTGTTAGGCTATTACCTTAGTTATACTTCTTCTCTCTTTCTCTCTCTAAAACATGCCAAGTTCTGTACTTTGTCTC

TCTTTCTTTCCTGTTTGCTTCTTGAAGCCTCATATCTAAGCCAGCCAAGATAAGAGAGAAAGAAAACAAAAAAGAAAGAA

AATCAAAAGTGATGATAGACACGCCTTACCACACCCACAAAACCATTCCTTCAATCCAACTCTACATGCCCACATTCACA

AATCTTGACCTTCTCTTAGAAAACCCAATAAAATAAAAATCAAGAAAACAAATTAAGTAATACCCAAATAAGACCATTTC

TTGTTTTCAATTCTTTGACACTAAAATTGAAAAAGAAATTTGTCTTTCTTTAATTTCCCCTAAAACTAACAATGACTGAA

GTACTTCAATCATCCCCATCTCACTTCCCTTCTTCTTCAAGCTCCTCTTCCACCCCATGTGTC

>GW881699.1 JC003136 Seed specific Normalized cDNA library from Jatropha curcas L. Jatropha curcas cDNA clone N05930 5' similar to Unknown protein, mRNA sequence

GGATATCCTGCCGCGGCTTTATCAATAGATCAAGGAACAGAGCATTTCTCCATACGCGTTGAGAGAGAGAGAGAGAGAGC

AGAGCTTAAACTAATCTCTCTTACACAAAATCCCAATCAGGGTCCTTCTCTTTTACCCTCTAATCTTTTTCCATTGATAT

TTTCTTCTTCATATCTTCTGCTACTTTCTTCCCTTCCCCATAAGTTTTTATCCATTAATCTGTTCTTTTCTTGGCAGAGC

TTGTTGGGGTGGGTGTGGAGTTGGCTGTTTCCTTTCTTCTTCTTTTAAATAATAATTCCTTTTTTTTAAAAAAATTATCC

ATCTGTTGGTTTGTGTCTCTAAAACATG

>FM888703.1 FM888703 Jatropha curcas embryo 35-55 (DAF) Jatropha curcas cDNA clone rjcfea0_002161, mRNA sequence

GGCTGCAGGAGAGATGAACCTTGCTTGTTTATCCCGACGAGGAAGCGGAGCTCTCAAACAAGCCTTTCTAGCCGCATCTT

GAATCGGCATCCCTATCCATATCTT

>FM887253.1 FM887253 Jatropha curcas embryo 35-55 (DAF) Jatropha curcas cDNA clone rjcfea0_000411, mRNA sequence

TGAGAGCGGCTTCTCTTTCAGACCCTCCTCCGCCTTCTTCCACNTACTTAAAAAAAACTAATGCTAATGGCGTCCAATTT

ATTCAATCACGAACTAAACTCTCTCCCCCCGCCTAAAGTGAAAGGAAAGGGTGATTGATGCTATCTCTCTATATCCCTGG

CAAAGGCCATCCATCAAAGGCTTCAGCTGAATACATGGGGCCACTAAGCTTTCATTATTAGCAGCAAGCGCCTTAGCAGG

AAAGTCTGCACGGTTACTCAGCTCCTTTAGGTCGTGGGCTTCTATAGGTCATTCATCGCGGTAAGTCTCCGCGTAAAGCA

TCCAACGCCGGTTAACACTCCCTCCCCTCGAAGTCCGTTGTCGCATCCATAAGCCGAGTCGGGCATTCCATCCAGCCTCC

TGTCCCGCACCATCGTCGTATGTGCATTTCGTCCCTCTTATCTCTTAGGCGAACTCTCGTAAACCATATTTGGATCTCTC

CACTTTCGACAACTCACTTTATATTCTACAACCAGCTCACTCTTCATATGATATCTCGAACTTCTCTTAAGATCTCTGAC

GATGATTGGCTAAATCCTGAGCTAAGCGACTGTAACTTATTCTCATATGCAATAATCCAAGGAGGCATCCCCATTAAATA

GCGTAAGAAGGGAGTGGTAAAGAGTCCTCCTTAATAG

>GW881157.1 JC004421 Seed specific Normalized cDNA library from Jatropha curcas L. Jatropha curcas cDNA clone N07868 5' similar to Unknown protein, mRNA sequence

GGATATCTTATCGTATTTCTTTCCTTCTCTTTCTACAAGCTCTGCCTAACTTTTCTGGTTCAGATCTCCTCTTAATGGCT

TCTTCTCAAGGATTGTGGCTTTCGCTGTTGAATGGATCGTTCTCCGATCTATTTTCAGATGATGGTTTCAGGGCTTGACT

CAAATAGGGACGTGTAGATATCAGGTTTGTGTAATAATAGATCTCTCAGCTTCCCTTCATATGCTAGTTATATTTCCACA

ATACTTTTACTAGACATGTTGTGAATATGTATTTCTGAATAGATCTTGCAATGGTCGTGTAGCTTGCAACTTCGAACTGT

CCATTGAATTGATCTTCTGACTGGTTCGGGTCATGAAAAAGCCGTTGAAAGTGTCTATTCCCTTTTTCTTTGGTAGCTGT

TGCAGGTGGTGTAAAAGGACCTGTCACCTTAAGGATCCGCTCGGCCAAGATCAGTGCGTCTGCCTGTAATTAATGTGTGG

TTTTTGTAGCAGACGTTTTGATCAGATGTTAGGAGGCGGAAAAATATATATCTCCACATATATATATGTTTATATTCATG

TCTCTGGATGTAGAAGATATATCAGGACGAGAAGCCCATATATTGTTTGATTGATGGAGGGGGCTTTAATTCTTTGGTTG

ATATCTTTGTCATCGGACCTTTGCCATG

>GT974864.1 GJCCJC2064D05.b Jatropha curcas L. developing seeds (mixed stages) Jatropha curcas cDNA clone GJCCJC2064D05, mRNA sequence

CCATTATAGTAAGACAGTTAAAAGTGTAAGCCTAATCCTATAAAGACTTGGGATGGAAAGGTCCATAAGATTCTTTATAA

ATAGTCTAAGTCCCCTCTCTTTCCTTAACCCTAATATTGGCTAGTCCCATTAAAAGCCGCATTGTGAATAAGAAAACGGT

GAGGAAGATCTAGTCCCTCTTCAATAAATACTAATTTCATATTTGAGTGTCTTCATTATGGATAAACACAAGACAGCAAG

GAATCATGAACTCTACTTTAAATCACAAATCTGGTACGCAAATTGCTTCCGCATTATTAATAGTTGGTGCTCTAAATTTA

TGTACTAGATCTATTAATTTCTTACAATTTGTTGCACAGTTTATGGAATTTGGATATTCCTGTTTAGGTAAAAGAACTTT

TGTGGAAGGCTTTTAATGAAAATTTTATGCATAAAGGTTAATCTTGTTTTATAAGGTATGTAGATAGATGTTTATTGAGC

TTTTTGTGGAGGCATGGAAGCTTTGACCATGTCCTTGTTGATTATAATAAGATGAAGCTTGTTTGGAGTATGGATGGTTT

TGAAGCTTATCTTACTGATGGATCATTTAAGACTCTTTTTTCCAGGTTAAGAAAATTTATCTCAGAGCATTTGAAATTTG

GTTATTTGAATAAGAGAGATTTGGTCATCACGTATTATGCTTATATGGCAAGCATTATACAATTAGTTACGTAGATGTAT

CATTCAATTCT

>GW611742.1 Jc1-009-B01-M13F.B01.ab1 Jatropha curcas flower and seed Jatropha curcas cDNA, mRNA sequence

TTGCTGTCTCTAGCATGTAGGTCTAGTTATCTGAAGTTTGGCAGGTAGAATTTTGGCCTGAGAGAATCCAGGATAAGATA

TCACCATGCATTTTTGTCTGCTTTGTTTGAAACAAGGGATGTGTATCCTTTCTTTATCCCATCAAAACTGCCACAAGGAT

GAGAACGTGAAAATGTAAAATTGTCCAAATCAATTACTGAATTACTTCTTAGCTACCAACTTTTTGAGTGGGAGCAGGAA

GTTTGCATGGCGGATCTCTCATCAGTTGCCCTTTTTTCTTCTACTTTTGAGCTGTGTGCTATATATTAGGCACGCTTGCT

TATAAGGTTTTGGTTTTGCAGTAGCTAGCAAGGAATTTGTACATTGTTTTGGATTATTTAAAAGAAAATGAAAGAGAGAA

GTTATTGC

>FM892271.1 FM892271 Jatropha curcas embryo 56-70 (DAF) Jatropha curcas cDNA clone rjcaeb1_002775, mRNA sequence

GTTAAATCTAATTTAACTAATATTTTCAACAGCAATAACTGGATCATCTCACAAGCATGAGATCCATCCAGGTGATTTAT

GCTGCTTCCACTTTTGTATTGCAACCATAAGCATGAGATCCATCACATTCCAAGTAGCTTTCAGGAACTTGTCAACTATT

ATAAATGCCACTCCACAATGCACCAGTACTAATCATCATGTGCAAATGCATACAGAATTGCAAAAACACAACAGTATTCA

CATTAATTATTAGGAAGAAGATACTGCTAAAAGATTTGAAATCAAGTCCTATATGAAAACCAAGTGTCTATTTTACTCCA

ACAATAGACATCAGAAACCTAAGCTTATCATTCACTTCA

>GW881332.1 JC001450 Seed specific Normalized cDNA library from Jatropha curcas L. Jatropha curcas cDNA clone N02704 5' similar to Unknown protein, mRNA sequence

GGATTGGAGCTTCATTATGTAAATGCTCTCATTCAGGCTCAATCGAATGAGATCCAATGGCCCTTTGACTCACTCTCTTC

TTCAGTAGTCATGTTTGTCTTTGCAAAGGGAGCTCTCTTTAGTTCTCTTCTTCAAGTTTAGGCGCATCGTGCTTTTTGTT

TCTATGCATTAAGGATTTTTTTTTTATTAAACCGCTGCGTTTTAGTATTGTACAAGGAGCTTAGGTAAATTTAACTCAAC

CTAATTTGATGATACCCATTTTAATAATTTTTTATTCAAAACCCTAGTTAATTTTGATTTTAGGATGATAATATTACAAA

GTTTTTAAATTGGAGGGTTCCAAGAAACGAAGGTCAGATTAAAATGTTTTTACAACCAACTCCAAATCTCGTAGTTTTTT

TTTATAGGTTAAGTGCTGATTTACAATATAATATGATTTTTCTTTCGATTTAATAAAATTTAAATTTAAATTTAATTAGA

AAGTTAATATCATCGATTAAAATTAAATTAGCAATTTCGGACAATTCTAAAGTTCCCTATTCCCAGTTATAGAAACTCGT

CCAACTCTTCTTGCGTCTTGTTGACTAATGTCAAAATCTGCAAAATAGGAGTGATAGATTAA

>FM893856.1 FM893856 Jatropha curcas embryo 56-70 (DAF) Jatropha curcas cDNA clone rjcaeb0_004785, mRNA sequence

ATTGGACAACCGATAGCGTGATGGGACTTCCCAAAGCTCTTACTGAAAAACTTATGAAGGAGGCTCTCTAGCCTAGTGCA

GCTATTATCTTTTCCATTCTTTTGGTATTTCCCGACTGACATCCTTTGCTTTCCTTTACTGTTATGCTTCCTCTGAACAT

TGCTTGATTTGAAATGGTTAAACAAATTATTGGTTATCGTTCCCCATCAGCTGATAGAGTAGGCACTGATTTTCGTTTAT

CAGGGATGACTAGTTACTTAAATGTTGAGCTGTGAACAAAATGCTATTTGCCAAAGTTGCCCTTTTGTTAAAGCTACTTG

ATAGGAATATATAGTAGACTGCATATTTTTATGTTACTGGGCATGATATGAGTAGAGAAAATTTTATTT

>FM889946.1 FM889946 Jatropha curcas embryo 35-55 (DAF) Jatropha curcas cDNA clone rjcfea0_003707, mRNA sequence

TGAAAAGAACTTATTCCCCTTCATATCTTATAGCAACTGAATTGTAAAATGTGGATGACTTCCATGTGGTTAGGCGAGCT

GGTGCTTGGATTTTCAACCATATGTGTCTCCTCATATGCATATTATGCTGTGAAATGATAACTGCAATTTTACCTTTCGG

TTTTGTTTGTCATAGTTTGTTTTTTGTTGCTATATTTCTTTCTTTAATTGACATTTTGTTACTC

>FM888723.1 FM888723 Jatropha curcas embryo 35-55 (DAF) Jatropha curcas cDNA clone rjcfea0_002185, mRNA sequence

GTATAATATATATATATAGAGGTCTGTGCGGTAAACAGAACTGCTCCTATTACAATTTGCAAATGATCCAAGGACATGCA

GATACTGGACTGAAAGACAATAACCCACAAAGGAATTTAGAGAAAAAAAAAAAAAAGAAGCAACCAAGGGAGAACACCAA

TGGTTTCCACCAGCAAAGTTCGAGATACCAAGATTACTATTAACAAAATGATGAAACCAAATCCATTTGGTTCTCTCTCC

CCAGATACATTTCTACAAAACCAGGGGGGGCCCGGTAC

>GW880057.1 JC000571 Seed specific Normalized cDNA library from Jatropha curcas L. Jatropha curcas cDNA clone N01086 5' similar to Unknown protein, mRNA sequence

GGGGGTTCCGTCTTTGTAGTTATGAATACTTAGACTCCTGGTAGTGGTTTGGCCAATTCAGAGGAGGTCAAAACGGACCG

CAACACTGCAAGCCATATTCCTCCTTCAGAAATGCAACAATTTCAAAAGTCCCTCCTATTTTTCTGTATTAATTAGAAGT

CGTTCATATTTGTTCTTAGATGTGTGGCGTTTCAGTGCTTGCGTGTCTTTACCTCTACATTCCTAAGGAGTTTCCATAAG

TATTTTCTTGGATCCTTGTTTTTGCTTTGTCCTTTAACAGGAATATGAAAGAGAACATTACTGAAATGCAAAATAAAAAT

AAGAAGATGCTCTTTGTATCCAGGAAAAAGAAACACAGAAGATTCTTTAGATCTTGCCATCTGCGTTTTGCAATCTTTAG

CTATAAAGATAATTATGTGGCAACTGCCGACTTATTGATGATTATACTTTATATTCCCT

>FM890548.1 FM890548 Jatropha curcas embryo 56-70 (DAF) Jatropha curcas cDNA clone rjcaeb0_000433, mRNA sequence

ATTCCGGAGCACGACTTCCGCCAAAACCGAATGCCACCACCATCATCCGCCGCCACGCGTTCCGACCATGATGACGACGA

TCATGCTCCCTCGTCCTCCGATCCACCTGTCAAAATCTCTGATTCCAAGCTCGATAACGACAAACAACACGAGGTCGAAG

TTGAAGAAGTAGAGGAACAAGAAGAAGACGTTAATGATAATGACGATGGCAGCGAAGAAGAAGAAGCAGAAGAAGAAGAG

TACGAAGAGATTGAAGT

>FM895000.1 FM895000 Jatropha curcas embryo 71-95 (DAF) Jatropha curcas cDNA clone rjcpga0_001328, mRNA sequence

TTTATTTCCTTAAATGCATAAAAAATCGAAGGGGAAATGAAAAAGAGAACCATCTCTCCTGCGACGCATAGCTTCTTTGT

CTCTTAAATTTTGTGTTTCTGTAGCAGAAAAATTGTCATACATTCTTAATTTAGGTAATTTATATAGGAATAGCCATTGA

GGCAACCATGCGAATTTAAGTTATTCAAGATGCTTACAAACCCTAATTCTGGATTAGGGTTATAAGCCAATGGATAGAGT

GATAACTTACATATACATTGGCTAAATACAATGTAAAAGAGCCTGAGGAATAAGAGTTTTACTCCCCAAAAAAAAAGGCA

ATATTGTAGCGATCTGTTCACTGGTCCTGGCTCAGCAGATTGGTGAAGAAAGGCACTGAGAATGGAAAACAGGCATATAT

AGCAGGAAACAAGAAGAAAAGAGGATCCTATACTGCACCAGCCAGCTCAGATGTTAGTTTTTTTGCTCTGTTTTTACCCG

TTTATGGTTCGTTAGTTGTTCAAGCCCTGCTGTGTAACCCTATTAGTTTGGTGAA

>GT974851.1 GJCCJC2064C01.b Jatropha curcas L. developing seeds (mixed stages) Jatropha curcas cDNA clone GJCCJC2064C01, mRNA sequence

GGTGAACTACGTAAATTTCTGTGTGTCACAATGGTGGTTATTTCGCTGTGTTAATTTTCTCTTAATTGGTTGAAAAGGCC

AACAAGTGGTATCAGAGCTGATGCCCAATAACTGGTATCAGAGCTGATTCCTAACAACTGGTATCAAAGCCGAAAGTTTC

GGTAGGTCCAGCCGATCAGATAGCGGTACCGGATCCGTGGATATACTTTGAAAATAAATATACCACGACGACGAGAAAAT

TACGACGAGTCTATTGGTGCTGTCCGCTCGGAAAGATATTATAGTATCGACTTAGTGGTGAATAGTAAAACAGTAACTCA

AAGTTGGTGGTGAACAGTATTATTGGCGTTACTGTTGCTGAGCCGTGACTCTTAAGCCCAGCACTATTCACGCATAGTAC

TATTGACGTGTACAGTTACTATTTACGTGTACTGATACTATTCACGTATACTGTTACTATTCACAGCTACTATTCACGTA

TACTAATAATCGAGGCGGAATCAGAGTAGAGTGAGCCAGTGTTTGTAATGGTGGACGATATCTGAGAAAGTCCAGTGGCC

AACGACT

>GW878139.1 JC002333 Seed specific Normalized cDNA library from Jatropha curcas L. Jatropha curcas cDNA clone N04319 5' similar to Unknown protein, mRNA sequence

GGAGCGCCATATCTCCAAGAAGCTTTTTCTTCTTCCTCCCTCTCTCTCTCTGTGTGATATATATATATTTATATATAGTA

TAATCATACATGTCTTGTATAAAAGAAGGTGGCGAGTGATAGGGAGAGCATGTGATTTGATGAATTGGATTCGATTTCTG

TGTAAAAGCTGAGGATTCTCTGCGCTTCTCACTTTGGTTTGTTTGCCATTTTTAATAATTCGAAAGGAAGATCTAAGAGT

ATGCAAATATAACCACAAAGACAGAGAGAGAGAATTCATGTCTGATTTTCCAGGAAAGGTACCCTGATTTTGCTGGAACA

TTTCTGTCTCTCGCATCCCGAGGAGCTTAATTTGGTTTTTTACTCTCTCTCTCTCTCTCTCTCTCTCTCTCTCTCTCTGT

ATCTCAGTTCTAGGGTTTGTTTGTAAGTTAAGAAAAACGCTCGTTTCTAAATGTTTCAAGCTCGTTTTTTACTTAGATCT

CCATTTTGTCTAGGGTTTGTTGTGTGATCTGTGTTAATTGATTGTTTTCTTTGTATCTCAATTACCATGGTCAATCTCTT

GTCTTGAAATATCATTTCTGTATTCTAGGGTCATTTCAACTATCCGTTTTTTCTTTTATTCGTTGCCTATTATAGCTTCC

AAGAATAGATACTGAATCTCATTTTATAGAGCTGAAAATTTCTTTGCCCTAAGAT

>JK611492.1 JCF4183 Jatropha curcas, immature Seed cDNA subtraction library Jatropha curcas cDNA similar to predicted protein, mRNA sequence

ACCTTCCCCTGACCTTAGGCTATCCTAATACCGATCTCTTATATAAGTCATTCCATCCCTAAGATCCGAGTTTAGGAATC

AAAGAGACTTGATTCAAATTCAAGGGCACATCTTGAGGGGAAAGAAGGAAAGAGCCTCCTCAGAGTTCTCAGAGATAGCG

TTAACGGATCCATTTCAAAGTGCTACGCTTCGTTCGCATGAAGAAAGGAAGGTGCGAAGCGTCTAGTCTCTTTCCCTTAA

CAAGAGGGGCAGCACTCACAGGTTATGGGCCTTGCTCTTGGAGAGGTTGGGTATACTACATCACTTCTTCAGGAGCAAGG

TGGGCATTGATGAATATCGGGCGTGGTTCATCGTCCGTGCCAAAATGGATCTGCTTGACTTCTCTTCCGTGGGGAGGAGC

TTTACATGAATAGGGGGCTCTTCTTCTTTCTGACCCTGCAATGTTAAGCTTGGAAGTTTTTCTTCATCTTCCTCGCCGGA

TTCTCCTGCGAGCTCTTTAGTAATTAGGACGCCTGCGATTTATCTGCTCGGTAGAAAGGAATCTCCAGTTAACTCAGGTC

CCACTTTGGAGGAAAACTAATCTAAATTCGACCCTCTCTCGTCAATTGAGAGGCCCGCGCGCACACATATATATGCTTCG

AGAGACAGCTAATCCGGCGTTTAACCGCGCGGAACCGGGTTCCAATTATCTTGGAACTCTTTGCGGGGTAACAGAGGC

>GW880081.1 JC000595 Seed specific Normalized cDNA library from Jatropha curcas L. Jatropha curcas cDNA clone N01125 5' similar to Unknown protein, mRNA sequence

GATTTTCTCTAATTTAGAACAAGAAAAGTCTTTTAAAGGAGAAGGGTATCAATACTTTTTTGTTTATTATATTAAAAAAC

AAGAAACATTTCTGTCTTTAGTGTGTTAACACACAGTTTTGCTTTGTCAGTACTTTCTTGCTTCTATGTGCATCTATGGT

TATAATTTCTTTATGCACTAATTTTAAAACCCATCAATTTGTTTGTGATTGATATGAAAAGATGGAGGATCTCTGTCCTC

TATGGCTCAAATATCTTAATTCAATTTGAAA

>GT971950.1 GJCCJC2026F02.b Jatropha curcas L. developing seeds (mixed stages) Jatropha curcas cDNA clone GJCCJC2026F02, mRNA sequence

AGGAAATCTGTGCGAAGGCTTTCTTTGGGTAAGGCAAAGGTAGATGTAATATTGCGATATGTGTCTTACCTGGGTAATTC

CTTTATTTACCTAGTGGGTCTAACTAGATTTTATGTCTAAGGAAGAGGAGAATCGCCCTAAAGAGCCTGCCTAGTCCTTC

TCGAATCCTGTGAGTGATGTTACATCCTCAGTTCTTTCTCAAACCAGTGACTTCGTTACTACAAGACAATGTGAGTCCGA

ATCCATACATTTCATTTGTAGGTACCACAGAAAGCTAATGGGATTAGAATGAAAATGGAGGAATAGCACGATTATTTATT

TAAATTATGTGTGTGGAAATGACTCTAATTAGAATGAGGGGTAGATCTATTTTAGCGAGGACTAGTTGGAGTGTAGTAGA

GGGCTGCAGAGATAGATTATCTGGCTGGGGGATAGCTATAGATAAGATTCCGATAAGATCTTCTGGGGCAGGTGAATTGA

TTGTTTTCTCTGACCGGTCCATAAATGGTTCTCTTTAGATAACCAGTGCCAGTTGGTGTGATAATGTGATAAGATAAGCG

CTTTCTTTAAAAGGCCTCTTCTTCTTATAAGTTATAAGCCTGAGAGTTCTGTACCATTTCGTAAGCGAGTGTCCAGTTGG

AATTTAGTTATGTTGTGCCAAGCTCTTATGGTCCTTTCTATGTGTGTCTGCAAGAGCAAAAGGAAGTTTCATTTATCTTT

ACATTTAGAATCACTACCTACCTCGGGTTAACTCAGTCACAGGTGGGAAAGATAAGGAGCTTGGTTCGACC

>GW877532.1 JC002255 Seed specific Normalized cDNA library from Jatropha curcas L. Jatropha curcas cDNA clone N04133 5' similar to Unknown protein, mRNA sequence

GGGAACATGGTGTTTTTCTTGTTGTTGTTTGTTGGGTTTTTAATGTCCGAATCTTGTGGGCAAAAAGTCGTATGCACGAA

CACACATACACCCATTTTCTTTTGTTTTTGATTTTGAAAAGTTATCCAATCAGATGTTAAACAAAAGCTGGAAAAACTTC

AATTTTTTATAATAATTATTAATAGAAATAATTTGTTTTCGGGTTATTTCAAAAGAAAAAGGAAAAGATGATATGAAATG

CGATTTTATATTTATGTGTTTAATTGTTTTTGTTTTGACCCGATTGGGTATAGAAACTGTAGTAAAAACTGACACTTTCT

CTGAACCAAAAAGGGGTCTTTGATTGATTGTCAATTACAAAACATTTACTAGTCGTATTTTCAATTTATTTATTGCGTGA

CTCTATTTTGTAGATGATTTTTTATTTATTTACTATTTTTTTCTTCTTTTGTTTTTGTTTTGGTTCGGTGCGTGAGGGGA

GATTTTTATTAGAGAAAGAAAGAGATGTCCTTTCGGGTTTTCTTGAGATTTCTTTATTGCTAGTCCTATTTAGGGTTCCC

TTAACTTTACTCTTATTATTCTCTTTTCGAGTTTTTGGGTTTCCGATTTTGTTTTTGGGTAGCTTCAGTTCTGGGTTTCC

TTTTGTTTAATTCCTAAATTTTGCCTCCTTTGACCC

>GT970120.1 GJCCJC2005D08.b Jatropha curcas L. developing seeds (mixed stages) Jatropha curcas cDNA clone GJCCJC2005D08, mRNA sequence

CTACTAATAACTCTGCAGGAAATCAGTTTAGCTGGGCTGACCTTGCGGTGTTAAGTTTATAAAGCTGAGTTTTGTAGATG

AATTGTTAGTAAATTTCCTGGTCAGTTTTGTTCTATCATATGTAGGAGCTCACAATGTCAGTTTTAACAGTTATATCAGC

TTACTTTAGATGACAATCATACAAAACACACAATTATTATGAAAAATAAAAAATACAAGATATTAATTGATAAAATTAAA

AGAATTGACCAAATTGTAAAGTGTCAGAATTGAGGATTTTTTTTGTCATTTGGCCTTGCCGTGGTTATCAAACTTTGCGT

TTAATTTCATTAAGGCTACATAATTTTAATTTGTCATTTTTTGTTTCAATAGAAGTCACCCACCAGAATCAAGTCATGGA

AAATACTAGCATAATACTAAAATTAAGTATTCAAAAAAACCTTATTTATTCTTAATAAACTAATATGGGACTCACTCATC

AATTAGCTAGCAAATGGTACCTGCCAAATGAAATAAGCTTAGAAAAATTTTCAACTCTCTATCTTTGTATTTTGTAATGT

ACTTTTTCCCTTCT

>JK611037.1 JCF2499 Jatropha curcas, immature Seed cDNA subtraction library Jatropha curcas cDNA, mRNA sequence

CAGAAGTATTTTTTGCTGTGAAAATGGATCTTCTTAATCTTCCACAGTGTTGCATATCATTTGGGAGAGTTCATTTATAA

AATGGAGCCGAAAACACAACTTATTAATAATATTATAAAATTTACAGATTGAAGAAAAGACTAGGAAAAGTTTTGTGAAG

ATGAAAAAGTTTTCCTGTGGTCAAGTTTCCCTTAGCATATTAAAAATAAAGGAAAAGAATCATGTTGTTTTCTTAGTGGT

CTTTTCGTAATTTCAATAACCAATTGCTTATGCAAGATGTTAATTGATAAAAATGTAATAATAGAAGATGTTAAAAAAAT

GCATTCTGTAAATGGAGAACAATGTTTTTCCCAAAAAAAAAAAAAAAAAAAAAAAAAAAGCTTG

>GW880093.1 JC000607 Seed specific Normalized cDNA library from Jatropha curcas L. Jatropha curcas cDNA clone N01148 5' similar to Unknown protein, mRNA sequence

GGATTCACTCTCTGTCTCCTTGCCGTACTCTTGTTCTTTAATGTTTCTTCACCCCGAGTTCGTTTATTTCAATCTTACTA

TAACATTTCCCTCTTCTCTCTTTTTTTAGTGTAACTTTCCTTGAAACTTCTCGTCCTTTAATGATACTTTTGAAAGAGAA

AAGGAAAAAAAGAGTATTCAATCATCTCCGTTTCAAGGCTTCTCTTTAACGTTGTGTTCTTTCTCTCAACTTTCTTTATA

TCCTCTGTCTCTCTTTCTTTATTCTCCCTCGTGTCCAGTCTCAAAGGAATCTACTCTCTTTTCTTTTTTTCATTCTCTCT

ATTTCACGCTCTCCTCCTCCCTCACTTCACTTTCAATGCGAATCTGAAAGAACCCAAAAATAGTAAGCAGAAAAGCAACA

GACAAAAACCCAAGCCCGCTTCTCGAAATGGGATCAAAATAGTCTTATGTCCATGAATCCAGTCAAGTTCAGCGGACATC

>FM889932.1 FM889932 Jatropha curcas embryo 35-55 (DAF) Jatropha curcas cDNA clone rjcfea0_003689, mRNA sequence

GACAGCAAGACTCTGAATTTGTGACATTATCTATAAAAAGAACTATTTAACAAAGCTAACCCAATGTACTCTACTGTCGC

TCTCGAATGACCTAAATATAAGCCAAAACTAACCCTACAACCACTGTAAACTAAGACTAGAACTGTTTAATATAGCCACA

AACCTACCAATTATGTCACATGATGACACCACATACACAAGCAATGGCTTACTGTGTCGATGGAGCATTCATATATCATC

AAAAAGCTGCAACAGAAGGCACTGGAGGTTCTGGGGGTCCATAATCCATAGAAAGCAATCTAGTTAAGATAAGACCAGAC

AAGTATAATTTAGACTCTTCGGCGATTAATTCCACTCTTCACCTCACTTCCCTGTCCATTAGACTTCCTCTTGATACT

>GW875557.1 JC005164 Seed specific Normalized cDNA library from Jatropha curcas L. Jatropha curcas cDNA clone N08948 5' similar to Unknown protein, mRNA sequence

ATTACGGCCTAGTTACGGGGATCGTCCTCTCACAAACGCCAAAATATACACACGAGTAACCTTCATCCCTCCTCCTCTTC

TCTGTTTCATTTTTTACTTCTTAATCGACGCCGGCTATTTCACAGCCGGAAATTTCAAGAAATTGACCGGAAAATGGAGG

CTTCGGCGATAGATTGTGCTTTAATTAACCAAAATGAAGGCGTGACGTTATCGTGCCGTCGCGTAGTTCGGGAACTTTAA

CCGTATGTAGAAATAAGCAAAAGCGACTTTACTTAGCAGAGTTTAGAAGTTATTCCATGCTTTGTCCGTCAAATCAGAGG

CAAGATACTATATAATAATATCGTCGGAGTAGAGTTAAGGAGTCTCGAATTTTGTTAACGCATTCATCTTTGGCGAAATT

CTGGATTTCCGTGTCGATTCGATCTGCTGATATCATCCTGTGGACTTATCAATGAGTCTGGTGCATCCTTTGTGATTGTT

TCGATCCATGTGTTTTTGGGATTTTCTTTTGCGAAATTACATTTCTTCCATCTCAATATTTGTACTGAAGGCAGGGAATG

AAGAAAAGTAAATAAAAGCTCTGAAGTTTGGTAGTTTTAGGAACTAATTAGTATCGCGATTAAGCTTTTTGAAAATGG

>FM889279.1 FM889279 Jatropha curcas embryo 35-55 (DAF) Jatropha curcas cDNA clone rjcfea0_002903, mRNA sequence

TGGTGAACAGCCTATGTTGATATATAGTTTAATCTCTCCTTGCAACAATTTGACATTGGCCAGTGAGTTGGCAGGCATGG

TTTGCAACTTAGACTTCACACGATATTCAGTCGATATTTTTGGATTTTTTATTTTGGGTTGGTGTATGGGTGTCTAATAC

TTAGTAATTGAATATAATTACTTACAGGCTTGATCGTTTTTTGCAAAGTGCCCT

>FM887290.1 FM887290 Jatropha curcas embryo 35-55 (DAF) Jatropha curcas cDNA clone rjcfea0_000453, mRNA sequence

TTGAAATCTCCTTGGAAACATTTTTTTTCAGTGCAGTTTCCATAAATTAGGTAATTTGGAAACACGTTTCCACTCTCGGA

AATACGTTTCTGTAAACTGGAAACTGTTGGAAACTCATTTCTTTAATTAAAATAAATACATAAATAAAAAAGAATTCACA

AAACATGTATTTTTTTATGGGTATGGAACTTGTAAACGACTTTTTTTTTTTTTTTTTTCCTTTGGATAACGATTAGATGT

TAATTGGACTTGACTTGTTGCTGAATATATCTTATGGTTTACAATAATTTATTTCTTTATATTTGTAAATATAACCCTAT

GTTTTTCATATTTATGTGTTTCCCCCTGTTCCCATTTCCTATATTTTAAAAAATACTGTTTTCCTGTGTCCATTTCCGCA

TTTCCACACTTCCATTTTTCTGTTCTGGTGCAACATAGCTCACTGCTCTATATCCTCTTTATACTTTTGCTTAGTGAATT

CTATTTTTAGGAGTAAATTGTAACCCAGCCAAAATTCTAGCTATGTAGTTAGATTCAAATCAAAATCTATGGTTCGTTGC

AACAGGCTATTTCCTGAGAATTCACATTTGATGTCTTATTTTCAATATTTTAATCATCATGCTTGCTTCGTACTGGTACT

ATTCTCTGCATAC

>JK611113.1 JCF3516 Jatropha curcas, immature Seed cDNA subtraction library Jatropha curcas cDNA, mRNA sequence

ACTACATAACATAGTTAGCTTAGCTTAGCCCGCTAACTCCGATTTCATAGTTATTTATCTTACCCCGGATATCCCGATAT

TGATTAAATCCTCTTTCCGTAAAGTAAGGCAAGGAACTTCAATGCTGGAGGAAGGTTTGAAGGACAACTGCTATTGAATC

AAGTGCGGGTATTGATTACTTTTCCGAGACTAGGAAAATTGGCTTCTTTCTTCCCTATCTGTCTTAGGAATAGGACTGGA

CATAACCCGATGTTAGAGTTCCGTGCTTAAATCCCTAGTTTGAAAGGGATGGTAATTTAGTTCCGAAAGAAATGCTCAAT

GTGAGAAATTGTAGACAAAGTCTCATACATATGTGCACATCGTGAGAACACTTATTCATTTGTTTGTGCTCCTACACCGG

CTTGATAAACTTCTATTTTACAATGAGATCAGAGAAAGAAGGTGGCACTACTTCAATGTGGAATGTCAAGTGTAAAGCTT

TACCCAGACTGCTTTCGTTTTGAGTTGTCTACCGT

>GW874833.1 JC000224 Seed specific Normalized cDNA library from Jatropha curcas L. Jatropha curcas cDNA clone N00406 5' similar to Unknown protein, mRNA sequence

GGACTTTTGAGTTTTCTTTGTCCATTTCAGTTTCTTTAAGGTCCCTCTCCTCTCTCTCTATCTCTCTCTCTCTCTCTCTC

TATTTATTTTGCATCATAGTGTGGGGTTACTGCATCTTAGCCTATATTTTCCTTTTTTGTCTAAATGAAGCTGATTGATA

TTTCCTCTGTGAATTATGAAAAATGAACTGGGTTTCCTGTCATTACTCTCTTTTGTGGGTAATATGGGATGCAAGGCTCA

GTTCTAAAATCAATGCTTCAAGAAATTCAATACCATAAATTAGTTTTCACCAGCAAATGGATGCCAATTTTGTTTTTTTG

TTTTTTTCTTTCTGTTTTTAACTTTTCCAATTTCTGGAATACTTATGAGTACTTCCTTCAGCCTCTGGTGGGAGCATATA

ATGTGTTTTGATTGGTAAATTGAATGCAACCATTTTTTTGCTTTTCAAGTACTTTCTGTAGAAATGATTGGGTGTTGAAT

GTGAAAGTTCTTGAGTTTTGCCTCAAATTTATAGAGATGA

>JK611544.1 JCF4326 Jatropha curcas, immature Seed cDNA subtraction library Jatropha curcas cDNA similar to conserved hypothetical protein, mRNA sequence

ACAATTTATCAAACCAATATGATGAAGTGCGAAATAGCACATGAAATTGATTATTTTAAGATAGAAGGACTATAAAAGGG

GATTGACTTCAATGTTTCAAAACATGGATGTGCTATAAAGAATAAACAGTTAAAACTTTATATTAAAACCCATTGGATTA

GAATCTTTGATTATCAACTGTAGGATAAAAAATCTTGGGACTAACAAAAGCAAGCCAGTTTACCTTTTGTTTTGAGAATT

CTTTTTGTTACCTAGCATAAGGTTCTCCAGAAGTATTGACCACAAATAACATAAATGCCCCAACTTCTCTCTCAACCCCA

AATCCACCATTGCATATCCAAAATTTTTCTGATACATTTTTTATGTAACTGAAAGGGATAGAATTTTGTATCACCATCTC

TGAAATTGGTATTTTATGT

>FM887773.1 FM887773 Jatropha curcas embryo 35-55 (DAF) Jatropha curcas cDNA clone rjcfea0_001037, mRNA sequence

GGAATCGAGTCAGGAAGAAGAGGTTATATATTTCCTTACCGAGGTAATCTTCCATTCGTTACTCACTCTCGGAGAGGAAA

GATTTTGAAGATCGAATTAAGTTTTTCATTCTCTTTTGTAACGATCGCTTCTAAGGCAAGTATCTATGGCATTCTACGCC

CCATACCTCTATCTACTAGCTCTTTGACCTTGATTTGATAGTTCCGATTCGATGAGCTCAAACAAATAAGTAGATACGTG

CCCAGACCCAATTTTTGAAGAAGAGCCTGATTCTATGATTGATTTTTGAGTGAAGTGACCACAATGGGGATTGGGCACGA

ACGGGATGCCAAGCGCTCCTTCTCTTTTACGCTTAACGCCCTTCTAGTAATAAAAAAATGCATAGCCGACATGTAATTTT

CTGACTTCTAATTCTGACTGGGGAAATGACGTAAGAGAAGAAATAGAGTGCTGAGTCTCTTTTACCAGGTTCTATACTCG

GATCGATTAGGCTACTTTAACTTTATTTAAGAAGCTAAAAGAGTACCATTTCTCTATATGCTTTCGTTGAGCTACGTAAC

CTATCTACCTTACTTAGCCCCTTTTCCTCTTCTTCTTCTTTTCCTAAGAAAAGAACCGATCTACTATTTAGCGGA

>GW619652.1 Jc2-049-F08-M13F.F08.ab1 Jatropha curcas flower and seed Jatropha curcas cDNA, mRNA sequence

AAATTCAACCTGAAATCGATCTCCGATATCTCTTGATTTTGACGATTATTCTTCGAATCAAAACTCAATTCGTTTAATTC

AAACTCTCCTTTTTCGATCGATATCTCTTTCGACTTTTGCCGTTAGTTCTGACATATTCTAGGGTTAGGGTTTCGCGTCG

TGCTCTCTTCGGCTCTCTAATCTCTCTATTTCGTATGGGTGTTGAGTCTCCTCTGTATATATCTTAATTTCCCCATTGAT

TTTGGCTCTATTTTTGTATATTATTGCTGTTCTGTTCGACCTTTAGGGTTTCGAAGTTTTCGATTCGTGTAGGATTTGTT

GCAATCTGTAAATTGGTTTAGGGGTTTGATTCTCTTTGTTATTTCGCCTCTTATCATCCTCTTTTCCGTCCGAATTTGGT

TGGATATTTTGTGAATTGGGAATTTAGGGTTAGGGTTTTTTTTATGAAAATTTTAGGTTCCCTTTTCATAAATTTGTTTA

GGGATACGCCTAACCAAATCAATTAGTATCTTGGCGATACAATTGGGGATCCTTTACGCCTAAATTAAGTGCCATACTAC

TCCTATTCTGTTTGGTATTTTGAATTCAA

>FM890084.1 FM890084 Jatropha curcas embryo 35-55 (DAF) Jatropha curcas cDNA clone rjcfea0_003876, mRNA sequence

AATAATAAATGAACTCAATCAATTATACTTGTTGACTCCAAGTCAATGCGGAAAACAAAAAGTGGTAATAATAATTTTCC

ATTGAAGTAGACTGCCTTTTCAAATTAAATTATCTAAATATATCAATTGGATGATAGTTTGATTGATCTTCTCACTTATA

TAACGAGTGTTCGAAGTCCTGCTATATTTTCAAATTTTGATATTTTACTGTTTCATAAAAGGGTAAAGGGCTGACTGCCT

CAATTTGAAAAATTATATTCTTTTTTTCATACTAGAAAAAAGAATTTTCACACCAATTTGTAGTGTTTTGATTGGTCTCC

TCCAAAGTCTCGACTTCGAGTCTTTTTCACCAAAAAAATTGCCATTAAATTTATCTCTTCACCAAAAAAATTGTTATCAA

ATTTAGTGAAGGTTATTGAAAAGAGAGAGTCATCATATTGTGTAGCTCACAGTGTTAGTTTGATTGATTGGATATTTCTT

AGTTACTAGTTCTAATAGTCCAAATTGTAATTTCAGGATTGGATATTCAAAACCTTTTAATATACAATTTTAAAAGTATA

TTGCAGTATTATTACCTTTTACTACTGGTG

>JK611380.1 JCF4048 Jatropha curcas, immature Seed cDNA subtraction library Jatropha curcas cDNA, mRNA sequence

ACCTCGAATTACTGAGAAACTTACTTGGATTTCTTCTCTCGTAGCAATTGCTCTAGGCCTGGCCATTATCATTAGCCAGT

TTTCCTGCTATAGCTTGTATTGATCCATCTAACACGACATCAAGATTCACATTTGCAAGCCAGCATTTTAGTGTTAGAGA

AAGATCTTTAGAAACAAATAGTCAAATTTATTTCCCATAAAAAAACATCTGCGCAGCATCTTATCTCCGTAAGAGTCATC

TTTTACTTCCTTTTTTCCCCCCTCCCTCCTGAAGATTGAAATTTTTGAATAAAATTGAAACTGGCCTTTTTAAAATACAA

GCTTTCAAAAGCCCCACCCCAGGGGTGCCCCTTGAAAAGGGGAGGGGGAGGTAAGGGTAAGAAACCTACAAACCCAAAGG

GGAAAAAAAAAAATGGTTTGGACTTTTGCCCTGGGTTGCCCGGGGTCCCCCAAAGGTTTGGAAGGGTTTCCCGGTTTGAT

ACCCTCGGGGGGGGGGGGGATTTTTAAAAGCCCCCCAAAATTTTTCCCCGAAAATATTTTTTTTCCCTTTTTCCCGAAAA

ATGGGTCCCCCCCCACCCCTTAAAGTTTTCGGGACGGATCTTTCGGGCGTTTTAAACGGGGGAAACGCGACACTACAAAG

AGACCCCCTCCTGGGGAAAAAAAAACGCACCTTTTCCGCGGGGGGGGGGGGGGAATTTTTATCTATTTTGTGTTTTTCTT

TCGGCGCAGCTCTAAAAAAAAAAAAGGTGGTGTTGTGTGCTATACACAATCGACGAAACAACGATCTGTGCA

>GT975358.1 GJCCJC2069D01.b1 Jatropha curcas L. developing seeds (mixed stages) Jatropha curcas cDNA clone GJCCJC2069D01, mRNA sequence

ATGGAAGCTAAATAAGAATGAATACCACAGACGTCCTCAAATTCAGATTACCGCCTATTAGGAAAGCACCATCAACTTTG

TGTAGATGACCTTTCGCTTGTAGAATAGGATGCTCCAACAAACACTAGCTGTGTCAAGAACTGGAGACCTCTTTGCTGCA

GACAGGACAAACCTGCAGTTTTAGAGGGCAGGAGTCATACAACACATTCTTCACATCATATACCAATGGTGATCTTTAAA

CAAGTGATTTACTTGCCATTTGTTCTTTAAAAATTTAATTGGTAGAAAAGAGAGCAAGAGCAGTAATGCAAAATCAAGAA

GAGAGGCAAAAACATGGAATAATGACATTCTCGCCAAACCCAGGACGTTTATCATATAACAAGTAGTTAGGGGAATCTGT

GAATTCAATTGAATTATTTCTGCATTTTATGCTGTTATTTTTTCAACAGAAAAGAGCTGTTCCATTGTTTGGTTGGGAAG

ATG

>GT971882.1 GJCCJC2025G12.b Jatropha curcas L. developing seeds (mixed stages) Jatropha curcas cDNA clone GJCCJC2025G12, mRNA sequence

CATGGATCTGTCTCCATGGCTCAGGGGACAAAGCCGTGAGGGTGACCTCGTACGGAAGGGCCCAAAGCGAACAACACTAA

CCAGGGCGGAGCCATGACATTTGGTATCAGAGCTTAGGTTTAGACAGTTCTCGGACTATCTGCGTATATGTGTAGGAATT

TATCTATTTTGAGGTGAGAGTATGAGTAATTTAAGTTAAATACATGCCATATTAGTTTGAATTGGAAACAACCAGCCATT

GGAGCATGGTGTTGTGGTAAATGGATTATGTTATTGAGGTTCCTACTGATGATTAATGGTTATATCCTTCTAGACCATTT

GAATGAATTATTCTTGGAGGAAATTCTTAGAGAATAATGGATAGGGATTCGATGTAGTGGTTATTGGAGATGAGATATTA

TTGAGGCATTAAGGATTTGAGCTCTTTTTCTCGAGTTGATATGATTTCTAAGTGGTATAAATATATTTGAGATTTGACTT

TTTAACATATGAATTTTGTTTATTTATTTTGAATCCATAAGAAGCATATTGTTTTTATGTTGCTTATGGATAAATGATGT

TGCTATGAAGATATTTTTTTTCCTTCTATTGAATCCTTTGG

>GW878616.1 JC002381 Seed specific Normalized cDNA library from Jatropha curcas L. Jatropha curcas cDNA clone N04417 5' similar to Unknown protein, mRNA sequence

GGAAGCCGGTGGCAATGGCGAAGAGAGAGAGAGTTAAAGAAGGGGAAAAAGAGAAGAAAGGAAGGTGAAAGAGTAATTGA

CGCGGCAAGTATAGAAAAAAAAAAGGGAGCAGAGCATGTAGATATCGAAAGTACCTGAGGGGATCATGATGTTCTTCTTC

CATTAAATATAGTTTTCAATCTGAATCGAAGCTCCATTTTTTTTTTTTTTTTAGCTGCTCCTCTTTTTCTTCTTCTTCTT

CTTCCTTGGCACCCTCTGTCTTCTCTCTCTATACCTGCCAACCAGTCGCTTCCCTTTGGGCCTCGTGATGAATTTACAAA

TCTGAAGGTTCAATTTTTTTTTTTTTTTTTTTGCGTTGCCATGATTATAACTTAGAGGATTTTCAAATTATGTATTAGTC

AAATTTTGGGTAATCTTACTCATTAATTTTTTAAAAACCTAATATATGTTTTTAATATGATTAATTTATTATTGAAAAAA

AAAATTAATTTTTCATAAAAAAATTAAGCTTGGCCGAAACTCTTTCCCTGCAGTCTATGTGAGCTTACAGTATATGCCGG

ATCGTATATATATATGGGATTTACATTGGAATTTTAAATTATTTTAAATTTATAATATTCCAAAAAATACTTTATCAAAT

TGCATTATTTTTCCCATTGGGAAATTTAAAAAGAAAAAAAAAATAAAA

>GW880370.1 JC000955 Seed specific Normalized cDNA library from Jatropha curcas L. Jatropha curcas cDNA clone N01871 5' similar to Unknown protein, mRNA sequence

GGGAGTTATTTTGATTATGGTGATTTGTGGCTGAGCATGAGAACTAAGAACGAAAGCATAAAGTTTGTTTCTTGGTTTCA

TTTTATATGAATCTGGATTCAGAAAGAGGCTTTCTGGATATTGAGTTATCTAGTAAGATAAATATTTTGAGAGAAATGAT

TAAAGAGAGCAGAGAACCATTGATGCCTTTTCTTTTTCCTCTGGGTTATTTTTGTTATTTTGGTTCAGTTGCATAAAATT

GGGAAACTTGGAGCATACAAATTAGCTTGCTGAATCTGGTTAAGGGTAGAAAAATAACTTTCGTAAAGGATTGAAGCTCT

ATTTAGAACCCATTTTTCAATTTTTATTTTTTTGTTTATGTCTTTCCTGCATATATCATTGATAGATTTTTAGTTTTTGA

ACAATTCAACGAACATTTAGAAAGAGCAAGTTTTGAGTTCCTAATATTTCAAACAACAGATCTGTACTATTGGTTTCTTA

GACTCAAATCACCATTTGACGTCTAGTATGAAGTTTTGCTCTAACCTCATATTCTCTTCTCAAAGACATATCACTAATTC

ATACTCTTTGATTTTCTCAACTCGTAGACTGC

>FM889282.1 FM889282 Jatropha curcas embryo 35-55 (DAF) Jatropha curcas cDNA clone rjcfea0_002907, mRNA sequence

CAATTATTGAAACGAAGTTTCACCGCTAGTTTCGCATTGAGGAGCATAATTTGATGCATTTTTTTCTGGTCTGACCAGAA

GGAAATGAAGGCATAGATACAAGTTTGTATAGAGCAGACCTTTTGACTATTTCCAATTTTTGGCTGTTGGCAGACAAGTG

AATTAGTTTTGTACAGCATTAAAAAAGACGATCTATATTCAAAAATTAGTTTTTAGTTCAGTATATATCTTTTTTTCCCT

TTCAAAAAAGCAAAAAA

>FM888660.1 FM888660 Jatropha curcas embryo 35-55 (DAF) Jatropha curcas cDNA clone rjcfea0_002113, mRNA sequence

AATTTCATAGGAATATTCATATTTTAAAATTCATACCTCCATTGGAGACTGGGAATTCTAACTTAGTGTTACTATCCCAA

TCCATTTTAAGAGTTTTGAGTGTACAATAGAAAGTTCAGTTCATTAATAGAAGACTTAGTTCCTTGAAACTTATTTGCCC

CAGGGTTCAAAGTGCACTTTTTATTGAACATTAATTTTATTATTTGTATCTAAAATTTTGATTTCTTGATCAAATTATCA

TTTTGCACATGTATGCTTTCATGGATTTAGTTCCATAATTAATTGTCATACTTGAAAATTATATTGTATTGCAGCAGTAT

TAATTGTAAAACTAGAATTTTTTTTATTAATTAATAGATTAAATAAATCCGAGTGAACTGCACTAATATAAACCAATATA

AATTTATGAATGAAGTTTATTATTAAACCTTCCATATTT

>JK317716.1 JCST487 Jatropha curcas L. seed cDNA library Jatropha curcas cDNA 5', mRNA sequence

AGCAAAAGAAAGGACTCTCTGGTCCTTCACTCAGCTGTATAGGATTGCACAAACACATCTCATTTCCTCATCCATACATA

TAAAAAAGAAGATTCCTTCTTTTTGTCATAATGGATAGCTGCTTGTCTTCATCTCTAAGAGTTATTTAATAATTCCCTGC

AAAAGTTGAGCACTCCTAAGTTGAAGTCATGGTAGTGGATGGTCGGTTAGCTTTTTGCCATTTCTAACTTTTCTCTGTTC

TGAATATTTCCCTGAAATTCTCATTACATGAAGACTATTTTTCGAAGAAAGGGCCCTCACTGCCAATAAATTATGTTTAT

CTGAATTTCCATTAATCTGAATTTCTATCTCACATGCCCAGTATCAATCAAGAGCGGTGTCATCTGCACTGAACTTGCCC

ATGGAAAGATGAAGTATATCAGCAATATTGTTCACATGCTTTAAGGGTGGCTAAAAGAGGAGAATTCTTTGGGATTAGTC

GTTATGAAAACGGAGGAGGTTGGAGCTCTCTCAGGGGCCGAAGTAACGGCAATACATGACAGCACCCAAGTTCTCACGGT

GCGGAGAAACTCACCAGAGTAAAAAAAAAGTAGGGATGATGGTCATGTCGCGTGGGCAACGAGCGGCTGCAAGGACGACT

AGCAGACAGCAGAGGGAACTCGGGTCCTTAATAAATGCACCTTCGGGACCATCCTTAGGTTCATCAGGCGGTCGGGGGGA

GACTTTGAGACAAAGATTGGAAGATTACAGGCTATGCCTCACTCCTGCTTTTTTTTTGGAGCATTTGTCGCGATTGTGGG

GACTTCATANGGAGCGCTGGATCTTCGCCAGACCGTGGG

>JK612446.1 JCF25-153 Jatropha curcas, immature Seed cDNA subtraction library Jatropha curcas cDNA, mRNA sequence

TAGCGTGGGCGCGGCCGAGGTACTAGTGGGAGGTGTGAGAGTCACTTTACTCGCTTAAGCCGAATCACTTTTTCACTCAC

AGCTTCCCATGAGCGACTTTCCTTTTATTCTGTCCTTTCTTTCTAGCTACTTTCATAAGGGGCATGAGCTACTCTCTTCT

AGCCTTTGCTTTGAAAGCAGTTCCCAGTCGAAGGTCAAATGAACAGGAGCCAGGAGCTAGCCTTATTCCGAAAACACCGA

GTCTTGCTCCTTCTTTTTATGCGGAATCCTACTAATATAAGTTACATCATTTCCTTTCTAAAGCCCATACTAAAGCAAAA

GCACCAAGAGCGACAACGGAGCCATTCTCTTCCCTACTTTGTTCACTCGGAGTTCTGTCAGTCTAGTCTGGGTCTGCTTT

TCCAATGCTTGAACTGATTCACGCTCAAGCTGCTAGTCGAACTTCAGCAGAAGGGAGCAGCGTATAAAGGAAGTTCATAC

AAGGAGTCAAGCAGGTCATTTGCTTTTGCTTTCAATGCCAGCTAGATTGTTAAAAATAAAGAGTAAGTCGTCTTCGCCTT

TTCATCTCTCGTTCCTTACCTCGGTCCCGACGACTTTGCTTCAAAATGAAAAGGAGCCCTTTCTCTAGAATACGAGAGGA

ATAAATCCAATGAAAGAGATGAAACTAAAGCAAGAACCCTCTAAGTCACCTTTCCCCTCAACTTTATATATAGCATCCCT

CCTTATAGATTCTCATCAAAAGAAAAAGGATAAGGAACGTAAGCTTGACGGTAAAATAGTTGAAAGTGATTGAGTGCGCA

TATGAGCTATCAGTCATCAAGCAATCTAGTCATTCTTGCTTGCGATTGACTATCCTTTTTGGGTGGTCTGGAAGTTCCTT

AAAAGACG

>JK613094.1 JCF22-94 Jatropha curcas, immature Seed cDNA subtraction library Jatropha curcas cDNA, mRNA sequence

TTCCAGGGGCCCCCCCGACGGGCATGTTCTCGCTTTATGGGGGGGATTGCAACTTCAATGTGGTCTGGGGAAAAGAACAT

CTGCCTTGCTGGTCCAAGCCGGGATTGTGTACAATGATAATATAAAAATCCATGGTTTCTGACTTCCACCCCTAAATTCT

CTGCCTGGGGAAACCCCCTCCTCCTGCGGAGGTTAAATTGGTTGATCTTTAAAATCATGATCCACTGCTCATTCCCGGAT

CTTGTGAGTTTGATATGAATATATTGAAATACATGCCTTGATTGATTTTCAATGGGCAAGGTCTGTGTGGGATGAAAAAG

AAGGTTAACGTTTCTGTAATCTCCTCTCTTCTGAATGGAGACTGTGTGTGAAGGTGAACTGTGAGAATATATTTCTTTTC

CTTTGGGATGCATGTTTCTGGGAAAAAAAAAAAAAAAAAGCTTGTACCTGCCCGGGCGGCCCCTCATCATCCATTCTCAA

GGTACCTTTCTCGCTTACACTGGTAATCAAAGACTTGAGGTACCTCTGCCGCAACCACTGCTAATCTCCATGTTATCCCG

AGTGCAGCAGATCCCCCTTTCTGCAGCTCGCTTAACAACGAAGAGGCCCGCAACGATCTCCCTTCCCCCCACTTGCTCGC

CCTGCATGGCATATGCATTTCGTCAGCTGGAATATTTTGTTAAATTCCCGCTTCAGATTTTCCTTACCCACCTCAGTTTT

TTACTCATAGGGCATAGTAGTCGTAATCCCTTATATTCATAGATACACGTAGATTTGTTGTAGAGTTAGCTTCATTTTTT

ACCAGTAGTCCACGTATCAGGCAACGTGCACTCACGTCAAGAGATCGACGACGATCTGTCTAGACCGATGTCCAGATTCG

TACCGATGCTCCTATTCAGGTATCTCGTTG

>FM891664.1 FM891664 Jatropha curcas embryo 56-70 (DAF) Jatropha curcas cDNA clone rjcaeb0_002022, mRNA sequence

AAAGAATATGTAAGGATGTAATAATATGAACTTAGCTGATATCTGTAAAGTTCTAGAACTCAGCATTGATCACAACAAAG

AAGATGAATCCATAACAAACATTTTCTGATTGCTAAATAACCTCATCATTGTCAAAATTTAAAGAGCGCACTACAAGTAA

TTCAGACGAAAAACATTCAACAAAAGATGTTATAGCTCTCAGATACTTTTATGCCTCGGGTTACTGGAAACTCA

>FM888353.1 FM888353 Jatropha curcas embryo 35-55 (DAF) Jatropha curcas cDNA clone rjcfea0_001751, mRNA sequence

TCGAAGAAACTTTTGTGCTCATTCAATTCAATCGTTGAAGAGCACATGGACATTGATATCGAACACATGATGGAATCCTC

AACGCTGCCAGGGAGCTCAGATGGGTCTAACCCAAACAGCTCAAGTCTTGTAT

>JK317822.1 JCST593 Jatropha curcas L. seed cDNA library Jatropha curcas cDNA 5', mRNA sequence

TGTCATCTATGTCTGGTGATAGGACTAATGCGAGTGCGGAAGCGTATTATATCTGGTCTGCCTGGGGTGGACGCCATCAA

GATGACCTCCGATTTAGGGAGAGCGCCACTGGCGAGTCGGGGCGCTCGTGTTACCTCGACACTGACTGAGTGCGTCCAAA

ATGAGGGCAGGTGATGTACGACGCGGGTGTTTGCGGCGGTGGGGTAACACTATAAGGGGGCCAGGGCTGAACAGCTATAT

CATTGCTGGGCCCTCTGCCGTCGCGCGATACCTGAAGTCGAAGTCGCTGGGCAACTTAATGATAAACGGAAAGCGACCGC

AAGTTGGATTAGAGCAATGGAGGTTGCAAAGGCCAGGCAACTTGCATGGGTCTTAGTACGGTTGTGGGAGGCCCCCCGAT

CGACGAGACACCAGCCAAACTTTTACGTAGAAGAGGAGCATGAGAGGAAAGACGATGGAAATGGTTAGCGCAGGATGTGG

GCCGTGCAGCGGTAACGGAATTGGCACCAGGACCATCAAGGGCGAACCAATGGTGGCCAGGAGACCCAAATAATATCGTT

TATCTAGGTACAAGTACTGCGTAGTGTCACGGCAGCAGCCGCGGACGACGCGCGTTCAGTAGAGGCGATCTCGGAGAGGG

TAGTAGTGAAAGAAGAGGAAGCTCTCGAGGTCATAGCGAGTATAGCACTGCGAGACGACAATCATGGGGCGAAGGCAATT

TTTAGCTGACTGGTATGTCTACTCTTTAGTAGTCGCTATCGACTGATCAATTCGNCTGCTGTCCGTTTTAGAATGACAGT

GGGTGCCTGTCTTACGTTAGGTANCTGGCATGCAGTGTGTAGATAGGGAGGAGGAGGATGGCNATTGNNAGGTAGGT

>JK317650.1 JCST421 Jatropha curcas L. seed cDNA library Jatropha curcas cDNA 5', mRNA sequence

TTTATTGTTAAACCAATTTTGAACTGTTTCAGATTCACATATTTCAAAGAATACAAGATTGATATCATACACATTATACT

ATATCACAAAAAATCCAAACAAAGATACATCAGGGGATACAAAATATCAGTTTTTTAATGCTTGTCTGAGTCCATTAGGT

CTCTCCCAACTGTCTTCACAAATATTATCCACCGCATTGTTTCCTATGCCCCATCCCCTTCCTCAAATATGCACTATCTT

TTAACTTCCAAGGGGATCTATGTTCCAAGATCTGCATCTGCAGCAAAATTATCTAACTGTAAAGTCACACAAGATGTACT

TTAACTCAAAAACGGGGACAGCTTATGGTTTCCAGGGTGCTGGAGGTGGTGGTGAGAAGTAACAAACCCAGATCTTCAAA

CAAGCTTGGCAGCTTCCACGAGACCACTCCAGATAGAAACCCTTCTCCCAGAGATGAATATCTCTCTCACGGTCACAGAA

ATTATAAGGGAGCTCTGCAGAACTGCATCGTAACTCAAAGGGCACATGTGAGATGCGGCTGACGAAGGAAAATGAGGTAT

GAACTTGGGGGGACCAGAGAGCAACTACGAAACTAGATGATCCATAAGAGGAACATGATTAGGAGAGGAAGAAGTGAGAG

GGAAAAGGATTAACTGCCACAGGGGGACGTTGCCCAAGTCCGGCAGTCATCCAATTCCTTCATTGCATCCTCAAATCATC

ACTTTGCTTGGATTGGGATCTACGCTGATGAACACAACNNAATTCCTTGTAGTTCCAAATATACTGTTAGATAACAGTCC

TTTCTGACCGTAGGAACTGCATTCCAGGTTCCATTG

>JK611363.1 JCF4018 Jatropha curcas, immature Seed cDNA subtraction library Jatropha curcas cDNA, mRNA sequence

ACATACGCTTAGACAAAATTAATTAAAACATCTATGGACTCATAAATTATCTAACCTATTTTTAATACCACGACTCGCCA

CAATACATAAATTCCCTATAACTGGCGGGAGTATACTAAACAAGATAAAATTACAAAATAAAATCCCTCCGTAACATTAC

CAAATTAAATTTAATGAAATTAAATTTTCCTGAAAATTTAGAAAAAATTTCTCCTCCCTAACTTTTATGGGGGAAAGGAA

ACAAAACCTTTTTACCCAGGAAATTAAAAAAAAACCTACTTCCCTGAAAATTTTAAACAAAAAAAAAAATGGGAATTTTT

TTTAATGGGCCATTTTTTTTTTGGGCCAAACCCCTTGTTTTTCCCCCCAAAACCTTTAATTTTTTCAACCCAGCCCCCCG

AAAAAAAAAAAAGAACAGGGGGGGGGAACCCCCCAAATTTCCCAAACCCCCCCCTCCAATTTTAACCCCCCCCCAAATTT

CCCCCCCGGGGAAATTGGTTTGGGCCCTTTAAACCCTGGGGAGGGTTTTTCCCCCGGGCCCCCCCCTTTAAGGGGGCCCC

AAGAGGGAATCCCCCTATATGGGGGGGGGGGGATTTTTTTTTTGGGGGGCCCCCCCGTGTTTTTTTTCTTTTTTTTTTTA

AAAGGAGGCCCCGATGTGTATACAATATAATATAAAAGAGAGGGGGGGGGGGGGCTCTTTTTTTCTAGGGGGAAAAAAAA

AAACACACACAGAGGGGGGGGGGGTTCTCTAGCGGCGCAACATGATATATATTAGACTGAGAGAGCGTACTATCTTCGTC

GGCGACGTTGTTCTTCTGCTATCAGAGAGAGGAAACGCTCTTCATTCCGTCCTATCTTG

>GT971927.1 GJCCJC2026D01.b Jatropha curcas L. developing seeds (mixed stages) Jatropha curcas cDNA clone GJCCJC2026D01, mRNA sequence

GCTCGTGAAATTGGGTTCTTCATTGTGCTATCCATCCTTCTATCGTTTCCTTTGATGCTCTGTATCGTTTCTTTCAATTT

CGGTTTTGCTTTTATCTCGCAATTTAGAAATAGAAGACTTTATCTTTACATGATTTGATTGGATTGATTTCGGTTAATAC

CACGCATTTTCTTACTTCGATCTTTAAATTCTTCGAATCAGCTTTGCTTCTCTGTCTTTTTCTTTCCTTCTCATTGCGAG

ATGCATATTACGATAACCTATTGAAGTGGTAATTTCTTTTTTGGCATTTCTTTCTAATTATAATTGCTTGATAATACGTG

AGATTATTATGATTTTGAATGATCAAAACAGACAAAAATAGATGATGATGATGACTACTACACTCTTTACTGGAAGTACA

CTCTCTACGGGAGTGACGACGATTAGGGAACGCTTTGGAGTAACCATAGGACTCATACGATGTCTTTGGCATACCGACCT

GGATTCCTGAACGATTATGATGGAGGGATGTGTCCAGCTGATGCTTGTCAATGAAATACCGACAATGTTTATCACCACTT

CCGAACCG

>JK317690.1 JCST461 Jatropha curcas L. seed cDNA library Jatropha curcas cDNA 5', mRNA sequence

CATTTTTTTTTTTTTCGTCAGAATCTCAGTTCTCTTCCGGTATTCTTCCGTTTCGATTTTTGTATTATAATCGGAGAATA

TGCGTTCAAGTCGCGTGCTTCTCAAGGAGCCGATAAGGGTGGATTCCGGTCTGAAGCCGTGAAAGGGGAGTTGCGTTCCA

GGACTGGGGGGGATTGCGGGAAGCTAAACGGCCGTCCGGGCTACCCTACACGAGCTTTGGTGGTGGGTAATATGAAGGTT

GCTTGGCTTAAATCTTCAGGAACCGCATCCGGTGGGACACAGGGAGGTGGGGGTGAGACAGCAATGGGGGGGTGTTGTGA

AACGCGTTAATTTATTTAGTGTGACTTGTGTTAGCAGAAGCCCCGAAACGCAATGGTGTGGGAACGCCCCTAATAGTGTC

GGGGCCTTAAGATTTTCGTGATAAGTGGTGGATAGCGCAATGGTGGGCGCTTTATTTGCCTCCCATATGTGAGCGCTATA

TGAAGTGGTGGCGAGATGGGAGGATTCGCAAATATAGGCGGCGCGATGGAGGATCTGCATGGATAATCCTCGAGCGTAGG

TAGTGTTGTTAAGCGTACCCAGTGTAAATGAGCTTGACAGAGTAGTTCAGGATTAAGAGGATGNACATGACGTGAGNNTG

CTGGACCACAGTTGGATTACTATTCGGGACTACGAGCTGCGATGCNGATGCTGTTAACTTACCGCTACGGTTTGTCGGGC

CAGTGGTAGACGAGTCTGTCCAGTTCATCAGTTAGAGACTGAGGTACCATTAGGGACANACGGTGCTATCTTGATTTAGG

CACCACGCACACCCTCGTTACCTAGGGAATTTTNNTC

>JK317918.1 JCST689 Jatropha curcas L. seed cDNA library Jatropha curcas cDNA 5', mRNA sequence

TTTTTTTTTTTTAAGAATTTTGCAAGTCTCTGAGATAAACTTCTCAATTGTGTGAGGGGGGGGGGCTGTGGCCTTGAATA

TCCCTACATCCAAGACTAATTCATAGTATTTGTCAGCAATTATTATTTTCCGGAAGGCGCCCGTCTACATCGACACGCTG

CTTTGAGAGAGCGCCCGCCGGGGAAAGAAGAGTCGTATGGAGCGTTACCCACTAAAAGGTTATGTTTTAGGGAAGAAATG

GGATGGGACCTTCCGGGATAATTTGGTCACAAGAACTCTATGCAGCAATGCTATCGGGATGGGAAAGATTAAAATGAGAA

CCGTAATACCATGATGCCTATTGGTTCAGGGGGTGCATAGGCGGGAAAGTGACTAGTGGACGTGGGACGCGTGCGGTTCT

GAGTGTGGGGGCCTGAGCTAGCAGGATCACGACTACGGGCTTATGAGGGAAGTTTAGTAAGGGGAAAGGGAAAGCCGTAC

AAAAGGGGAACGGGGCCAGGGGCAGTGGACGACAAGATTCGAATAAAGGGGGAAGTTAAGGGGGCGGCTCTGATGGTAGG

CCGTTCGTAAGCAAAATCCACGTTTTGAGCAATGCTACCAGATAATTCCATAAAAAAGCATAAGCGAGGTAATGGGGAGC

TCACAGCGGAGATAAGACAAGCCAAATGAGTAGTAGAACATTATCGTCTCCCCAAACTAGGAGTTTTTAGAGGGACGCAG

AACGGGAAAGAAGGAAACACGTAGCGAGCGGTCGAGGTCGATGGATGAGCTATAAGCGATTCTGGCTCGAGTACGTCTAC

TCGCCGCGACACTATGAATCAG

>GT972030.1 GJCCJC2027F09.b Jatropha curcas L. developing seeds (mixed stages) Jatropha curcas cDNA clone GJCCJC2027F09, mRNA sequence

GATATTGATACGCCAATCCCCGGCAACGGCGCCAAAAACTTGACATGCGTTTTTCGCAAGTGTACGAAATCGCAACAAGT

AGTATAGAGTAAGAAAGTATCGTATCCACAGGGATTGGTTATCAATTACCAAAATTGTGTTAACCTATTATTATTTAAAC

GATGATAAAGGGAATTTGCTATAAAGTAAATAAAATTCAAAGTAAAAGAGATCAAACAAAATTATTGTGAAAAGAAATCA

GGAGATTAAGCTACTAGGGAATTTGATTTCATTTAATTCTATCCAGCTTATTCTTATTAGTTCATTAATCAAGTTTAATT

GTTCAAATAGGAGAAAACAACCCTAAAGTCACTAACAAGCTCTCCCGATTTATTGTTAGCGATTCTAATCCAAATTAATC

ACCTGCCTTAATAGAGAAATTAAACATGAATCAGATCATTAGGTTTTATGGTTGTTTTATGAATCCACAACTTAATACTG

TATTAACTTATGAAAACCCCCCTTGAGTCAAACCATATAGTTTCCACATCAAGTTATTATTCTCAAGTAATTTATTACCT

TGTTCATATCAATAACCCTTCTCCCGAATGTAATTATTGAAATAAATCAATCCAGGGGAATTATTCTGCCTGGAAGCCCT

TAAGCACATAATAAATTATAACAAGAAACAAGAAAAAATAATCCTATATTATAAGCTGGAATCGAATTAAATTACATCAA

ATCCCTAGTATAAAAGTTAGCTAGACATAGTAATTAATTTAACTCAATCACACTTGAATCATT

>GW881292.1 JC001223 Seed specific Normalized cDNA library from Jatropha curcas L. Jatropha curcas cDNA clone N02319 5' similar to Unknown protein, mRNA sequence

GAGCCTGAGCTAGTTTGATGTATTTCCAGGGGTTCTGCGAGGAGAAGATTGAGTTCTGGGGTTATTTGCCTTGTCATAAA

TTTTTCAATATTTTATTGATCTGTTACTATGTCCATCTCTCTCTTTTTTCCTTCTCACCTTGGTGTGGCTATAATTTCAA

TCGATATGTGGATTCTATTATTGTCGTCCCAAGCTTTTTGTTTTTGCAGTGATCAACACTCCCTCAATCTTGCAGAACTC

AATGTGAGTTAGTTTTAAGCTTTGATCACTTCACTTGACTTTTCCATTTCATTTTCTGCGTTCTCCTGTGGTTTTCCTTG

CTGAATATGATAAAAACTGTTTTATCTTTTTTTTTTTCAAGAAGATTATACTGTTTTATCTTATTCAATATCTGCAGCTA

TCTGCAATTCTGTTCTGTCTATTCAAGTTAATGCAGCTATCAGTAATATGCGTTGTTTTGTTATTTGGTATCTCCCGCTA

GTGTTGGCTAAAAATTGGACAGCATTTTGAGTTACTGGCTTATGCACTTGGGATTTTCCCAATGTTGTGTAATGGTCGAA

TCACCATGAGGAAAGACAATTTACATTTTTGGAAAGTCAGA

>FM887831.1 FM887831 Jatropha curcas embryo 35-55 (DAF) Jatropha curcas cDNA clone rjcfea0_001107, mRNA sequence

GCGGTGGCGGCGCTCTAGAATAGTGGATCCCGGGCTGCAGGCTCCTTTTGGTAAAACAAATTAAGAACCGTTTCCTCTCT

CTCTCTCTCTCTCTCTCTCTCTCTCTCCTCTTCCAATCTAAGCAATATCACAGCTGTATTCACCTCTCTTTCTGTCTCTC

TTGTGCTCTCTCTTTCTTTGTCTTTCTTTCTTCTTTCCTTTCTTTAAGCGCCGACACAATTCTTCAATTTCTTCCATTCT

GATACTCTCTTATTCTCTTTTAGATCCAATCTTTGTTTCTTGCAGTTCAATCCCGAGTCCAAACGGAGTCAATTTTACCA

TAGAACACCGATTTGGACTAACCCACCTGAAAAAGCTTTGAGCTTAGTCTGGAAGTTAGTTCTAACCGAGGTGGGTGTGC

CTGCAAAATCTGTACAAAGCCAGCTACAAAATACAATACGCCTTGGAATTTTCTGTCTTTGACATTTTGGTTAAGTTGAG

TCTATTGAAGTTGGTGCTACGTAGGAATTCGTAAAGAAATGGTGGTTTCGTGATAAAGGGTGATCAGTGGAGAATTTGTT

GGTTTCTTTTATTTAGGTACAGCCATGGAGACGGCAGGGGCAACGACCACTCAGAGCTTGACGGACTCCTACAAAGGCAT

GTCCTCTGATAATATCCAAGGTTTGGTTTTGGCG

>JK317921.1 JCST692 Jatropha curcas L. seed cDNA library Jatropha curcas cDNA 5', mRNA sequence

TTTTTCTATTTCTGATTTTTTCTTTCCCTGTGGAAACCGCACATTTCTGACAGTATGACAAATGGAGGCATTGATGTCCC

TACCAGGGAAAGCTTTCGACACTTGATATCGATTATTTTTTGAGTCATTGGTATCTTCCAGTAAAAATTATTGTGAAAGA

ACTAATGTTTATCTTACAAGGAACAAACGAAACAACGGTGGAAGGATGATCTTCGAGCTGATTTATACATTATCTATTTA

GCACGATCTGGACGTCCACACTAAACAAACTTTTGTTTAGTCGCCATGGCACGGTTACCAAAAGCATTAATTTTCGACGA

AGGCCCAACCCCATCGAATGCTTATCGTAGACAAAATATGAGCTCTTGCAAAGGCCACCGCGAAATGTTATAAAGAGCTA

CCAAACGCACCCCCGTATATTCAAAAACCTCGTAATCTTTGATCCAATACTAGCGATAATAAACACAACCGGAATGCTTT

GATCATTTTTCGAGGCACGTAACCCCTTAATGACCAAAGACAGATAACGATATGTTGACTTCATAAGTCACGTAAGTCTT

CAGCAGCACGAT

>FM890208.1 FM890208 Jatropha curcas embryo 35-55 (DAF) Jatropha curcas cDNA clone rjcfea0_004022, mRNA sequence

CAAATTTAACTATATGTTGTTATTTTAAGCTCTAATTTTGTAATTAGAAAAGTTGGAGCATTTTTAAAGGTCTTTTTTTT

CTCCTAAATTTAACCATTCTATTGATTACCAATTTTTAACTAGTATAACCTCTAAGAAAAGATAGATGAATGCTAATAGG

GCTTATTTTTATAGTATTTTTAATATAGTTTTTATTAGAACATTTAGTGTTTTTAATTAGTTTTTGTTTAGATTTATTTA

TATTTTTAATTAAGTTTTTTTAATTGTTGATAAGCTTGATTAGAAGGGTTTTTAGCAAGAAAATAGCTAAAATTTCCATA

AAAAAAGGTTAGAGGTAAAATTGGAATTCAAAATGGCCAAGTCAATAAAATACACGGCTTGGCCGTGTCAAATGCTATGG

TCGTGTTACCAAGAAAAGCTGCAGATTCAGAGTCAGTCGAATTTAATCATTTAGCCCGTGTAGATCTGAAGTCTAGGAGT

GGCATGTGCATGGAGCTGCATGG

>FM887543.1 FM887543 Jatropha curcas embryo 35-55 (DAF) Jatropha curcas cDNA clone rjcfea0_000766, mRNA sequence

TCACGCGGTGCGGCCGCTCTAAACTATGGATCCCGGCTGCAGTTTAATTAATGATCAAAATTTTACTCGAATTGACCGAA

AATTTTACTCAAATTGACCGAATGTTCGCCCCTATCCAAATTTAATGATCAAAATTTTACTCGGATCGACCAAATGTTGA

CCCTTATCCTGTGGTTTATGTACACACAAAAACTTTCATTTTGGGTATTCTTTTCCATATTAGCATGGTATAACAGAAAC

TTTTATGCTTAATATTTCCAAAATGCTAGTAACTTTTTTTAGTCCTTTTCTTCTCTCCTATATTAAAAAAACAAAATTCA

AGTTTTCCATTATTAAAACATAAAATCACCCTTGCCAATAACATTATTTTATAGTTTGAGAAAGAGGAAGGCCAGAGCCT

GTCTTTTTGTTTTAGTTAGAAAGCATAGAGTTCTTTCATCTTGGATGCATCTTTTGTAAAATCTGCACGCTCTTTGTTTC

ATGACATAAGCCTCGACTTTCAGTCAAGTTTACATTGTTAAGTATTGAACAGTTGAACTTCCAAGAATATAGTAAACGTA

AAACAATACAGTTGATTCATGTAGTTGGTAAGGTGGGTTTAACAATTATCACACAGTACAACAAATTGATCCACTGAGTT

GGTGAGGTGGGTTTACAATTTCTCCT

>GW876395.1 JC005958 Seed specific Normalized cDNA library from Jatropha curcas L. Jatropha curcas cDNA clone N10133 5' similar to Unknown protein, mRNA sequence

ATTTTCAGAGAAAGAGCTTTTTGGATAGGTGTTAGCAGATGGCATGAGTTTTGCGTTTCTGGGTTAGATTTCCTATTGGT

AGATAAAGTTGGAATTTTTATGTATGCAAGGTATCTGATAAAAAGCAGCAATGAGTCAATTTTTATGATCTTGGTTCTAG

AGCTAGAGCTTGGAATTGAGACTAGCCATTATCTCTCTCTGTAAGCTTGTTTCTCCCTTTCTTGGCTTCTTGTTTTGCAG

TTCTGGAGAGTGATTTTCAAGTTATTTATGGTAACTTTGTTGGTTTGTACCTATGCTTTTTGATATTTCTATTGTTTCAG

TATTTTAAGCAGCTGTGATGTGATTAGAGTGAAAAGATGAAATCTTTGAGTAGTGTAGGACTTGGTTTGAGTATAGTTTT

TGGGTGCCTTTTATTAGCTCTTATAGCTGAGCTTTACTATTTG

>JK317395.1 JCST162 Jatropha curcas L. seed cDNA library Jatropha curcas cDNA 5', mRNA sequence

GGTAGGAGATCTTCTAGAATGATGTCTTCTATGTCGGGTGGGGGAGCTCAGGGTTGGGAAAAAGGGGGTAATGCTGATGC

TTGGGACTGAGCTTCTATAAAAAGCCAGGTAGGAAATCTACAATTTTCTTTATTCTGACCCTGACTGATTTTTTTTCCTT

ATTAACTTGGGAAGTATATGTATAAAAAAGTGACCTCAATGACCCCCTGTCCCTCACACATGCGGTAATCCCAAAGAATG

AATTTGATTTTGCTGAAAGTTACCTCATCTCCTCTTCCCTTGAGACAGAAGGCCAAAATCTGCTCTTTTTTTATTTTTTT

TTTTTGAA

>GW877307.1 JC006829 Seed specific Normalized cDNA library from Jatropha curcas L. Jatropha curcas cDNA clone N11507 5' similar to Unknown protein, mRNA sequence

GGATAAAATTACGCGGTCTCGCTTTCTTTATTTCTTTTCTTTCCTCTCTCCGACTGTTCTCTCTCTCTCTGTTTTGTTGT

TTTTCTTCCATGGCTATCAGAGAGAAATACATTTGATTTAAGTTAGAACATGTACATTAAGTTAGCGAGAGAATTGTGTC

GCTCAAACTTAGGGTTTCCTTTTTGAGTTTCGTTGAAGTAGCGAGGAAGAGAAGGAGGCGGAGGAAGGAAGGGAAAGGAA

GACAAAGGCAGGCAAACAGAGGG

>GW618852.1 Jc2-039-H03-M13F.H03.ab1 Jatropha curcas flower and seed Jatropha curcas cDNA, mRNA sequence

AAAAACCTCTTCTCTCTTCTATTGTGATGAAGATGTCTATGCTCAAGTTTACTCTCGTTGCCTTTCTCTTGTTGATTGCC

CTTGATTTGCAAGGTGGGGTAGAAGCAAGAGGGCCAATAGTTGGTTTTGGATGCAAAACAGTCCAAGACTGTGTTGCAAA

TAATCCACTATGTTCTGCAGAAGTTTGCAAACCACCCTACTGGTGTTTCTGTATAAATGGACAGTGTGCATGTCAACCAG

ATTCATTAAGTGCTACAACCCTGATTGGAAGCTAAGGAAAAGGAAGTGCAAAAACATTTGAATAAAGCAAAAATAGAGAA

GTTTGAGATACCAATATCCAGCTCTCTGATAATGATACTTGTAAACCTCTAATTACAAGTTATAATAAGGATTATATGTT

TGTATCCTCGTTATAAAAATCATTATAAAGAAAGAGAAATATATGCAGTTATCTTATTATTTTCCTTT

>GT975942.1 GJCCJC2071B12.b Jatropha curcas L. developing seeds (mixed stages) Jatropha curcas cDNA clone GJCCJC2071B12, mRNA sequence

ATTATAGCATTTGGAGAAATCACGGCCGTAAATCTCGATTCTTTCCTGGGGCATATCTCCACCATTATGACTTCATGCCA

AACTGATATGATCAGCCAATCCTCGAAATGGGTTGCCTTCATGGAGAAGTGATAAGTTTGATGACGATGATGAACTGTAA

TTGAATGCTTAAATAGAAGCTAGAAGTTTGTATCATGGGTATAGTTGGGTGGGTCAGGTTTGTGTGTGGAGCCCCAATTT

CAATAAATACTTTTGGTGAAAGAAAATGAAATTTGTTCTCTATGTATTCCTGGATTTTGTAAATATGTTCTTAACTTGAT

AAATGAAGAGTTGTTTTTAATCCATTTGCTGTCCGAATGAAATTGGATTATTATGTTTCTTCGAAGCGAAATAAA

>FM888362.1 FM888362 Jatropha curcas embryo 35-55 (DAF) Jatropha curcas cDNA clone rjcfea0_001763, mRNA sequence

ATTGAAAATAGACAACCTATTCTTCTTATCCGAAAGGATAATAAGTTGCAAGAGTCTTCTTCATCCGAATTAGGTAACAA

GCCGTGGAACATGAAAGACTCCAGTCAACGAAAGACTAAGAGAAGAACATGAAGAAAACATATTACCAACAGAGGAAAGA

GATAAGGCAGTCCCATCAGCAGTAAATATAGAAGAGGAGGGGCGAAGAATTATAGTTCTAGTTC

>FM892219.1 FM892219 Jatropha curcas embryo 56-70 (DAF) Jatropha curcas cDNA clone rjcaeb1_002711, mRNA sequence

ATTGTTTACTTTTAGTATTGATCTAAGCGGACCTGAAAATTTTATTTTCGGGTTTGTCTTCTCTATTTTGTTGCAAAGTT

CGGTTAATCACTGCTAACTTGGGGTAAGTTTATGAATCCCCGAGTCAATTTCCAAGACAGAATATATGCACTATAACCTC

AAGAATACCATGGGAAAAAAAAAAAAAAAAGAGGTAGGCTAAACTTGGATGTTCTGACCATTGCAATTATCTTGGGTGTA

TCATACAAAGAGGAGAATATTATGAGGAAATCCATGTGAATGGAGAAGGGGGATAATACTTTAATGTACAGAAGTCCTTG

AAGGACTGAAAGAATACAATTCCCCCCTTAAAACTTGTCTAATTATGAAAGTGAACACAGAAAAGCAA

>JK317809.1 JCST580 Jatropha curcas L. seed cDNA library Jatropha curcas cDNA 5', mRNA sequence

TGGATCAAAACGGGTGTCTTCTATGCGGGGTGGAAGAGGGGCATGTGCTTCGGAGGCGGTCGTGTTACGTTGTACGACGG

GGAAACTTAAAACGAGGAGCCGGAAACGGCATTGTCTCCTGTCCTTATTAGTTCTAGTGAAGAATATAGCTGAAGAACAT

TGGCGGCCGCTTGTATCCTCCTCCTACCTTCCTGCCAATTGCACTTCTTGGCGTACAACAGGGAACGGAACTAACCAAAT

TATTTTTCCTTCCTACAGCAGGCTCGTAAGACGGGTGCTGTTATACTCGTTTTCACTTATGGTGGTTCTCATTAATGTTG

GCTGAGTAACCTTGCAGGCCATTTATCCGTTTTTTTTCATTCCCTATATAAAACGCACCTTCTTGAGAATGACGGAACCA

CTTATATTTGGGATCGTATAATAGCTCTTACTACGGCTATAATCTTTCGATAATACATTAGGGGGATAGAGTTAAACAGT

TAACGACAAGAAAACCACTATTCTAGCCGTGGGGCCACCCATATAAGTTCAGAAAGCTACGCTGGTATTTCGCTTATATA

AAGAG

>FM890137.1 FM890137 Jatropha curcas embryo 35-55 (DAF) Jatropha curcas cDNA clone rjcfea0_003938, mRNA sequence

TTTGTTAGTTTTTATTTGAGTAGTTTGTCATAAAATGTTAAAAACTGAACGTTTATAAAACACTATTTTAACTTTTAAAA

ACTAGTTCATATATTATATTACTAAATATTATAAAAAATAACGTTTAGTATTTTGATCGGTCAAAATTCTAAAAGCTATT

TAAAAATATCACTAACTCTTTGAGTCTTCATTTCCCACTATGGCATTGGCAAAAGGTAGATCTCAAATTAATTGCAGAGA

AATTTTCTCAAATTTTTCAATGAAGCTAAACTCAACATAACACAAGAACCAGATTGTAGGGTCATTTTATATCAGATGCA

TAATGGGTGACATCAANGTATGTCAGAACATATTTATCTACTTATATATTGATTTTAATCAAATCTAAGTAAAAAAATTT

TAAAAAAAATCAAATTTAGTTTGCCTTTCAGTTCTAGATATTGCCATTGGGACCCAAAATAGGCTCATCTCAGTCTTTTA

AAACATGAGCTGACATAAAACAGGATTCTTGAACATGGTTTCTGCTCCTCTGGATTTCCTC

>GW881652.1 JC001233 Seed specific Normalized cDNA library from Jatropha curcas L. Jatropha curcas cDNA clone N02344 5' similar to Unknown protein, mRNA sequence

AGCGACCGGACATAGGCAAGAATTGTGGTTTACCATTCGTCTTCCTCTCTTTCTCTTCCTCTTCCTCTGCGAGTCTTTGC

TGCGATTTCCTCGCTGCCCGTGCCTGCTCCGCCTCCGTCCCTCCTTCACTCGCCCCCTCCCTTGCGTGGGCCACCATTTC

CACCTTTGTTTTCCTCACTAATTCAAAAAAAATAAAAGGAAAAATTTCTCTTTCCTTTCTTCTTCTTCTTCTTCTTCTTC

TTCGTCGTCCGCAGACCTAGCTTTCTGCAAATCACATGAAACAGTAACCAAAAAGCCGATCCGTCTCATCATCGGATATT

AATTAAAATATAAAAAGGACACTTGGCCGGTGGTGAGGTGAGAGATCTGATCTGCATCTGATTCACTGTTTCGCTCTGCC

ATTTCACCATGATTTCGTTTTCAGTGCATTGATATATTTCAGATATCAGTCATCTTCGTTTCTCTAATCCCGAGGCGATT

GCCTGTCTGATTGCTTCCTTATTCACTCTGGTACGTTTCTACTTTTTCTTTTTTCACTTATTTTATTTTGGGGAAGA

>GT974022.1 GJCCJC2056B04.b Jatropha curcas L. developing seeds (mixed stages) Jatropha curcas cDNA clone GJCCJC2056B04 similar to porin, putative / voltage-dependent anion-selective channel protein, putative, mRNA sequence

GAAAACAGAACAAAAATACTCTCACCGATAAAAAAGCTGAAATTATGGTTTTTTCGGTTCCTATAAATCACAACCCCAAA

TCGTGTTCCATTTTCCCATTTTCAAGCTCGACAACAGCTCTCTCTCTTTCTCTGGTATTTTTATTTTTGGCGATTCTTTA

TTAATTAATCAATCATGGGTAAAGGTCCTGGTCTCTACACTGA

>GR209260.1 JCST39 Jatropha curcas L. seed cDNA library Jatropha curcas cDNA 5' similar to unknown, mRNA sequence

ATGGCGCCCGCCGGGGGACGGTCTTTTAGGTCCCATGTCCTTTGGGAACAAAGGAAAGGAGCAGACCAAAAGGCTAGCCA

TGCAGCCTCCCTGTTGGGGAACGGTGGAGGGGAGAGAGAGGCGAGGAGAGACCGTCGGACAGGGCTCACATTGCACAAGT

GCTTTCCTCCCATACATTCCCATGGTGCCAAACGGGGTTTCCAGCTTAGAAAACCCCAGGATGCCCGATCTCCGACCCCG

AGCCCCCGCAACCCCCTACGGAATTTTCACGTTGCCGGCAAGTAAGGGGCTGGTTTTAAAGCGCCCAAGAACCAAAACAT

TTAATATTCCAACCCAGTTAGATAACAGTGGAGAGCCAGAGGCAGGGAAACGCGAATTTGAGACGGCGGGAGATGTCGAT

TGAAAAAATTTGGGGAAGAGGGGAGGTGGGCCAAGGGCCCCCTTCCTAGCCCCTAAACTATTATAAGGAGCCGAGGGGGG

GGGAAGTCCCGGGGGCGGGTACCGAGAACAAAAAAAATTTCCTATCACGCAAAGTACATTAGCTTTTAGAATTCTCACAT

TAATAGCGCAGCGCACATAGAACCAGAGGACAAAAGTAAAAACAACCACCAATCGAATCTCCAGAAAGCACGACACATAC

AAACCGATCTAACCATGGAGGAGAGAAGAGTAAGAAGAAAGACAAAGACACACAGATTAGCAAAGAACATCATCGGGCCC

GAGTACTAGTGATCGAGACGTGGAGTTGTTCTATGTTAATATAACCAGCCGACTTAACAGAGCAGTCGCTCGAATGGACA

GGTGCGTCCGTGTGACGAGGCAAGATCATA

>FM891348.1 FM891348 Jatropha curcas embryo 56-70 (DAF) Jatropha curcas cDNA clone rjcaeb0_001612, mRNA sequence

ATTCCTTATAGGGACACAGACACTAGGTATTAGCTACTAGTACTGCTACCTATGGCTAATAAATTTTATGTGTGTATTGG

GGTCTTGGGCTTTCTCTAAGATGAGAGTTTATGTAGCATCTTATAAACAGGGAAAGCCATAAATAAAATAGTGGGTCTAA

GTGTGCTTAAAAAGCAGCTTGCTATGAAGTGTTTCTATTGTAACTTTGTATTACCTGGTTAGGAAATGTGCTTATTAATG

AAAAATGAAACAGGTGTCCTTTTTTACTTCTAAAAAAAAAAAAAGAGAGAGAGAGAGAGTAGTCTCGAGTTTTTTTTTTT

TTTTTTTAAAACCTTAATCCTCCTTTTATTGCCCAAGACAGGTTAACGTGTTGAAAAAGCAAGGGGAAATAGAGAATTGG

GGTAAATAACAAAAAACTTTAACAGGCTCTTCCAAAAATCATGGTGTAAATATGGGGTACGAGCTACGTTACAAAAGAAA

TTTGAGGCAAGCTAACAAATCTCCAAGATACTGAGTTAGTAATGTGAATATCAGATTTTGATACAAAAATGGTTGTCTAT

AACAGCCATCTTCCTGATATCGATCAACGTTTGACCCATTGGAAAG

>GW880332.1 JC001418 Seed specific Normalized cDNA library from Jatropha curcas L. Jatropha curcas cDNA clone N02644 5' similar to Unknown protein, mRNA sequence

ATTAAAATCCGATATCTAGTTATCGTTTTGATGGATACCCACTTATTTATATAGTTCAAATGTTATGTAAATAATGAAGG

ATGAGTTTTGATTAAAGTTAATGCTCGATGGATGGGCTGTATATTGAATTGTGCCTTCGTATACGTGATTTGATAGCTAT

CCCGATTTACAAGGTAGATGCAACTAGATTTTCGGTAAAATGTATAATAACGAATTAAAATAATTATATCATTAAGAGAA

ATGACTAATAAAATTACCAGTACTCGTATTACCAATATAATAATAATAATAATAATATTATTATTATTATTACTGCTACT

ACTTACCAAAGGACTACAAAATTTATTAAAGTTTATATATCAGAGTAAATAAGTAAATAAAGTAATAGTAGATATACGAT

TTGATAAGTTAAGACTAACTATTACATTAGCCAAAAGATAGTCCTATTAAGGACGATAAAGAACTGTGAGTAGAGTTAGA

TAATCTGTATCACTTATGCATTATACAATCATGCATACATGTATAATATGATATCTACGTGAAACATGCATACATGTAGA

AGTGCCATATAAGCGTGTATGCCATAAGCCGCATCAAACACATATAGTTTTATATTGGAATATATAGAGATGTTGGATAT

CATACACTGTGTGAGTGATATGAAATTTTGTGA

>JK611903.1 JCF28-A28 Jatropha curcas, immature Seed cDNA subtraction library Jatropha curcas cDNA, mRNA sequence

ACTATCAACAGTAAGGTTATCTTTAAACCCGGCAGGAGTTAAAATTTCTTACTTTTTTTTTTTTTTTAAGTTTTAATTTT

TGAAAGTTTTTTCGAAGTTTCGTCTTGGATTACTGCGTGCCCTTTAATTTTATCATTCTTGTTTTAATTCTTGATTAATT

TAATAATCAATTCACTTGACTTCGGGATTTTTTTGTTTGTTTGTTGGGTTCGAAGCGGAGTTCATGTTAATTGCATTTCG

CTCAACTTTAGCGATCTTTTATTCTTTGCATATTAAAAAAACTATTTTGTTCTTTGTTTGGTTGCTGAGAAAATGTGGGA

GAGGAAAGGAAAGCGGAGACAACTCGAAGAGTGCTTAAGCATCCAATAATTTCCGGTTTCGAAAATAATGTTATCACGTT

CGCATTCTGTTCATTTCCATATTAAAACTTCATATCTTCGCAGGGATTTTCTTTCCCTTTGTGTAAAACTCGTAGCTTTC

TTTCCCTATAAATACACCCTCCAAAAATCCCCCAATCCTCATTGACTTCTCTCTTTTCAAATCTCTTGCGAGTCTCCTCA

AAGCCTTCTTCCCAACCAGGCTACCATTTGTTATTCTAAATCCCTTTGAGATGATATTCACTTTTTTTTTTCAAAATTTC

TTTTATGCGTTGCATATTGGGAATCCGGCTCTTTTTGTTTACTGCTATTTTTGAGTCGGTGGAGGCTTAGTGCAAAAAGC

ATCTTTACTATTCTGATTTGTATTTGAATGCTTGAT

>GT969466.1 GJCCJC1002C10.b Jatropha curcas L. developing seeds (mixed stages) Jatropha curcas cDNA clone GJCCJC1002C10, mRNA sequence

ATATATATATATAAAGTTGTAATGCTTGACCCCTAATTAGCGACCAAAACCAACTGGTGGCAACAAACAGCAATTGGTAA

TAGCTGGTAGACTGCGCAAATAAAGTTCTTCGAGGGCATAATTCCATTCATCGTCCTGGAATTTATCACTAAGCTCAACA

GAATCATTATATTTTAAAATTTACACTAGCGCTAAAATCACTCAAAAAATGCACCGATTCTACATTTTGTTAGAATTGCA

AATATTATATCCAAGTAAAAATTATTCTAATACATTAAT

>GW616626.1 Jc2-014-A02-M13F.A02.ab1 Jatropha curcas flower and seed Jatropha curcas cDNA, mRNA sequence

GGGATTTGCAAGTTCAAATTAAAACGAAGCTTGAAATCTTCATTTCATGGCTTCTCAGTTTTATCAAGAAGTGTAAAAGG

CGTTTTGAGCTCAAGCTTATGCTATCAGAGATTGATGGCATCGATCCGGTTTATGTTATTCCATCCGAAATGTATCCACT

TAAGGCTAAAACGAAAACTATCTAATTCCATGTTTTCTTGTATCTATTTTTGTTTTTATTAATCTATTACGTTTGCTTTC

TTGATGTCGTATTAAATTACAGAATTTGTGCAATTAATCGGAGTTATT

>GW880352.1 JC000706 Seed specific Normalized cDNA library from Jatropha curcas L. Jatropha curcas cDNA clone N01324 5' similar to Unknown protein, mRNA sequence

GGAAGCCCGGTGGTTTGCTCGAATGGGTTAAAGATAATGATTGATAGTATTACATTGAAGTACTTCACTGCATTCATTAG

AAGAAATAATAATTTTGTTCTGACGACGAAATTTCAGTTCAGTACAGGGACTGATCCCTCAAGCTAATTAATCTTCTCCA

GCAAGGGCCACAGATCCGTCAATTTTGGATGCTTGTAAACAAAAGATCTGCGAGTAACCAATTGGTGAAAAAAGCGCAAA

CAGGAACAGAAGAAGCAAGCAAAGGAAAGAAGAGGGACAAAAAAAGGAGAAAAATAAAATCTTTTAAGAGCTTGACGCCT

GTAACTATACCCAACATTGTTTCAGTGGCTGAGGCTGCCATTGCAGGCTTTGCTTTTTGAGAGTCTTCTGTGCAAACTGC

AAAAAGCTTAGCCGAAAGAGAAGCTGAAAAGGTTTTCTCTTTCTATCTCCTCTCTCCTGACTTGGACCGTGAT

>JK317843.1 JCST614 Jatropha curcas L. seed cDNA library Jatropha curcas cDNA 5', mRNA sequence

TGTCATCTATGTCGGGTGGTCGACAAGAGGTAATCCGTACTTTAAGAATTAATTAAAGAAACCCCACACCTACATCACAT

TGATAACCATTTAAAAATCGAATTCTCCACATCGGTCTCCCGTTCGGAGGAGCAACCGACACTTTCCTTCATAAGAGTTA

GTGCGCGACCCAGAATATGGAGGTAAGCTCGGCCTGGTGGAAGGTCTGGTTTAAAAAAGAAAGAAAACGGTAATGCGGGG

TATAGGGGATAAGGATGGGGACCTGGGAAACGGGGAGGGTGGGAGAGAAAAGACAGGAGAAAGAATGAAGGCGGGGGGGT

AAAGAGCAAAGTATTTATAAGAAAAAAGATAAGAAAAAGACGCGGAGAATCGGGGGAAGAGTACCTAGAAAGTGCGGGAA

TGGCGGGTTGGGAATAGGGGGGGGTAGTAGGTAAGGAGGAGAGGAGGGAAAGGAGAAACGAGAAGGCGAAGGGTAAGTTG

GTGGAAGGGGCGCGGTGGGACCTAATGCGAGCTAGGCCCAAGGGGAGGAAGCGCAGAAGGCGGGATGAAAAGGACGGACG

TTGACCAGACTAAACGACAAGGATACAAATGAGAAATAAAAGGGGGAGGTGAACATGGAAACAGAGAGAGAAAATGGGAA

AACGGAAGACAAGAAGGAGGGATCTGGGTACGGAGGTGTACACGAGGGCACGAACAAGGAATGACACTAGCGTCGGTACT

AACCGCGGGAAATGGCTCGGCGGCGCTAGACGACCTAAGAATCAGGCTAAGAGGAGGGGAGCGAGGAGACATGGGAAATA

ACGATCGGAGAAGAAAAGACGAAAATCAGGGAGAGAAACGAAGAAATAATGCAAGAATGCATTTGGGGACAAGACTCTCC

CAATCCAGCAACCGCCGATACAAAACAGCCCAC

>JK613435.1 JCF19-32 Jatropha curcas, immature Seed cDNA subtraction library Jatropha curcas cDNA, mRNA sequence

ACTTTATACAGCAAAGCATATTTTCACCCGCTTTCACATATTCAATCATGCAAAAGCAGCAGAAGACACCTTACAATAGG

TAAAGACGCATTGCAAAAACTTATGACAACACCATCTTGTAAACAAACACAGCTATCTTTGCCGAGAGAGACAACCAAGC

CAAAAAAAGAAAGACTAGCTGCAGAAGTGACTGAGGCCTCCTAGACAACAACTGCGGATTGTGAGACTAGGGATCATTTG

TGTAACAAGCTTGTCTCGATCGTAGGCCACAATAATTGTTAAAAATACTTAGACGCACCACTAAACCGACAATTAGCACA

TAATTGCAATACAGCGAAATTAGTATATAATTGCAATCCAGCATGGCAACTTGGAAGCAACTAAGACGTTGAAGCAGATG

AGGAACTGCAACAGGCAGGAAAGTAGCTCAGTAGCACAGCCGCCGATTTGGCACATAGTAGAAGAGAGGACAAAATAAGA

AGTCTGAGTAACAGTTGGATACACATCCCTCTCTCAGAAACATGGAGCAGTTAAATCAACCCAATCACAAGAAACCACAG

GTATCATCTGTAAGGCTGCCATCCCTTTGTATAAACAATTGACGCTTGATCCAAAATCCTGCTGAGAAGCCAACCTTCAC

ATCGTGAGCAG

>JK612440.1 JCF25-144 Jatropha curcas, immature Seed cDNA subtraction library Jatropha curcas cDNA, mRNA sequence

ACTGATTCTGATGACAAATATGAGGGAGAGAGGGACAAGGACAGAGACAAGCATAGAGACGGGGACAAGGATAGAAGGAG

CCGGGACGATGAAAGAGAAAGAACCAAGGATAAGAGCAGGAAGAGAGACAGAGACAGTGAGAGGGATGAGAAACGGGAGA

GGGCAAGAGACAGAGACAAAGAGAGAGAGAGGGACAGGGAGAGGGAAAGGGAAAGAGATAGAGAAAAAAGGGAGCGTGAA

AGAGAAGAGCGTGAGAGGGAGAGAGATAAAGAGAGGGAGAGGAGAGAGCGTGAGAGGGAGAGAAGAGAACGTGAGAGGGA

AGAACAACGAGAGAGAGAGAGAGACAGGGAAAGGGAGAGAAGAGATAGAGAAAGGGAAAAAGAAAGAGAAAGGGAGAGGA

GGACAAGGGAAAAGGAGAAGCATCGAGAAGTTAATAGCGATGATAATAGTGACGATGATAGCAGGGAGCATGATAAGAAG

CGTCGTAGAAGAGATGATGACGACTACAAGGACAGACAAATTGAACTGAGCAATAGCAGATCAAACAGGCACAGAGATGA

CACTGAAGGAGCCCAAGGAAAAAGAGCAGTGAGGATGACTCAGATAGAAGAGAGATACACGAGAGAGATAGAGACGACAA

AAAAAAAAAAAAAAAAAAAGCTTGTACACTGCCCGCCGCGCTCGA

>JK611540.1 JCF4320 Jatropha curcas, immature Seed cDNA subtraction library Jatropha curcas cDNA similar to hypothetical protein, mRNA sequence

TCTGCCACTTTCCCGGGTTTCCCGTCAAGCTTAACGGGGGGTCCTTTGGGTCCGATTAGGTTACGGCCCGACCCAAAACT

GAAAGGGGAGGTCCGGTGGGCCTCCCCTAAACGTTTCCCTTGAGGGGGCCCGTTTTAAAGGGATTTGCAAGGAAACACAC

CTCCGTATTTTATAAAGATCGCTCCCCGGAAAGTTAAAATGGTTAAATATATTGGTTGGGGGGCCTTTTTTTTTACCTCA

CACCAGAGTAAAGAGATTTGGCTCTTTTTTCTGCTCAGGAATAGAGCGGCTCAGCAACTCTCCTATACTGGTGGTGCCAG

AACCCCCTAATATCTATGAGTACGCCTCAACAGAGGATAGTCAGTATCGCAGCGACCATTATACATTGTGGTGTG

>JK317845.1 JCST616 Jatropha curcas L. seed cDNA library Jatropha curcas cDNA 5', mRNA sequence

TGTCATCTATGTCGGGTGGGCTCCAAGAAATCAGACCACTGTCGCCGTTTTCGGTAGTTGCGCCGGGACCCCCCAGTAGG

GAGACCGGCACTTGTTCAGCAAACAACCTTGGATTGCTTGAGTCTTGGAGGCCAACAATTCATAAAAGTTGAGGGGAATT

ACGAAGCACATCACCAGGCTATTGTGATACAAGCGATAGGGAGGCAGTCTTAGAGAATGTGCCGCTTCCAGGAATAGGAA

TCGGGTAATCCAGCAAGGAAGCTGGACGAGTAACGGAACGACGCTTCCTCAAAGGGATGAAGGAGCCATCCACCATGTCC

TGTTGCGCGTTGGGGAAAATTAGGCTTGGTCGGTAAAGTAGGATGGCTCACTGTTCGTTCTCTCGTAGGGTGAGGGGGAG

TCGGCGTCTTGGGGGAGGAGTTTTATTCAGTGGGAACAACAAGTTGGAGAGAACAAATAAGTTTTTAAACATTGTAGTGT

AAAACAAAGATACAACCTGTGTGACGTCTTCATGAACTGCACCGGGTTTCATTGCAACAAGGATAAAGTAATAAAGAATT

GCGGGACGGCTACCGTATAGTCGAAAACACTGCGCACGAAGCCTCGAAGACCCCTCTGACTTGGCGTCGAGGGACCAGAC

GATGTCGTGGTTAGACGGGGTGCTCGGGTGTGAGCGAGGGTGCTGATGTGAGTTCCACTCAGCTTATCAGATTGGAGCGC

GAAAGCAGGATGCGAGGGTTATGTCCCCACAGACTAGCGATGCCACCTATCTTCAGATATAGCGAGAGTCGCCGCCTGTG

CGGGCGACCTGGTCAGGGATGACAGCTTATGACCGACCTACCCGTCGCCGGACTGACCAGCCGAGCACGCA

>FM888018.1 FM888018 Jatropha curcas embryo 35-55 (DAF) Jatropha curcas cDNA clone rjcfea0_001328, mRNA sequence

CACTTCAGTGTAAGATTCATAACCTAAAAGAGTAGTTATGAAGTGCTTTGCCCATCCTTAACAAATGCGGTCTTCTATTT

GAATATGGAGATATTGGAGATGCAGTTTATGTTACTTGCGTCCAGCTCTGCCTAGCCATCCCTCCTATGTATTTTGTGAG

TGAATCTGGGGGGAAA

>FM888414.1 FM888414 Jatropha curcas embryo 35-55 (DAF) Jatropha curcas cDNA clone rjcfea0_001828, mRNA sequence

TTCAATATACGATAGATCGATCTTTTCAAAATTTCAAGATGAAATAGAGATAAAATCCTAAAATTCAGAGGTAATTCCAT

ACATTAAACCGATTTTATCTTGATGTGCAAGATTTCTCTTGCCATTTTTTAATCAAAATAAATATTTTTGGTCTCTACAT

ATCTTTTATTTTTTTGTTTTCAATACCACTTGAAGTACTTCAAAGGTTCATAAATAATATTAATATAATTCACTAAGTAG

TCCACAATCACATTGAATCTTGGAGGTTTACATCCTTATCAACTAAATTATCGAGTTTTGATACATTAAAATATAGCCCA

GTGGTAAGTGTACGCTGTGTAGTAAAAAAATTTTCAACCTCTAGGTACTAGCATAAGCAGGGCCATAGTCTGCCAAATGT

AATGCGATGTCGTTTGCTTCAGTTTGTTGCTTGTAATGGTCGGTCGTTTTATTTTAACCGCGGGTGTCAAATTTTTTTTA

ACCGCGCACAAATTGGTGGGCCCCAATTTCGGGTCAGCGGCTTGCACAATGTCCTTCGCCGGCCATCGAACCTAGTCAAA

ATATAGACATTATAAGAAAAAAGCACAATATATATAGTGGAACATTCACAAATTCTCATTTTGTTTTCCCTTTTTGTCTC

AGATTTTGC

>GT973543.1 GJCCJC2049F08.b Jatropha curcas L. developing seeds (mixed stages) Jatropha curcas cDNA clone GJCCJC2049F08, mRNA sequence

GAATGGCATATGTTAAGGTGGTAATAATTAATTAATTCATCCTTAAAGAAAGTAAATCATGAGTTTTGCAGCTTTTGCTT

TGTTTATGGCCCATTGCATCCTATTCTAAAAGAGGTGTGTGTTTGCCTTGGATTCTAGGTGAAGTAACAAGATTGTTAAT

AATTTGACAAGCCTCATTAGAGATAAGGTAAAGAAAGAACAGACAAAGAAATCATTAGATACAAAGATTGTTAAGGTAAA

GAAGAGACCATGAAATCATGAACTAGCAAAGATTTTAGGTTGTCTGGTGAACTTTCTAATGGTTAGAAACATTTAGGGGG

CCATTGTGTGTTGTAAAGTTCTAAGCTTTACAATGAAATCATTTTTATTCTTGAAAGCTAAGTTTTGTAATTTTTAAAGA

AATTATTACAATTTCTGTATAATCTTTAACAAATACATGAAATGATTCAAGCAAATGT

>GT976488.1 GJCCJC2078G01.b Jatropha curcas L. developing seeds (mixed stages) Jatropha curcas cDNA clone GJCCJC2078G01, mRNA sequence

GCGGCGGTGGGGGTGAGGAATATGAATACTCATTAGGTGGAGGAGGATTTTGATAATGAGAAGGTGATGGTGGGTAATGC

CAATGATGCTGTGGGGGTGGGTGGTGGTAGCTTGGTGTGGATGTTGGCGGCGGTTCTAATGGTGGTGAGTGGTGATAGCT

TGGTGTAGATGTTGGTGGCGATGATGGTGGTGGGTGGTGATAGTTTGGTGAGGAGGATGGCGGCGGTGATTCTGGAGTGG

TGCATCCAGGCCCATGTGGAGGAGGTGGACTTGCCGTTGAATGCTCATGAGTTGGAGGAGGGGAGTGTGTATATCCATTC

TTTGGTGGAGGAGGAGGCGGACTTACCGGTGAATGCTCATGAGTTGGAGGAGGAGAGTGTGTATAGCCATTCGTTGGTGG

AGGAAGCGGCGGTGGTGATGGATGGTATTGATAGGGAAGGGCATGATAATTAACAGTGGTGGTGGAGGTGCTTCATGTTG

ATAGGGAGGTGGGT

>GW875289.1 JC001996 Seed specific Normalized cDNA library from Jatropha curcas L. Jatropha curcas cDNA clone N03602 5' similar to Unknown protein, mRNA sequence

GGGGCTCTTTCTTTCTCTCTTTTCTTTCGTCCTCCTCTGCTCAACAAGACCATCGTCTGCTTCTCTCCTTCATTCTCTAT

CTTCCTCTAGGGTTTTTGTCTCTTTAACATTTTTAAGCTCCACTTAAAAGTCTGCACATATCTGTACATGTATATATAGG

TTCTTATTTTAATGGCAACAGCTCCTGTTAAGCCGCAGCCGCTGCATAACTTCCCACTCTCCCTAAAATGGGGCCAGACC

ACTACACTCGCTGCCTCCACCAACCACCACCACCATCGTAGCACGTCTACACTTGCACCTGACTCGGAAACTGAATCAGA

CCCCGAACAAGCAACAATCCGGCATCTTCCTCCTCCTCGCGTCGGATCCCGCTCTGCTCGTTTACATCGGTACTCTTTTA

CCTCTTGCTCTACTCTTTTGCCAAAGCCTAAAAATCCATCGACAGAAGACCTGCAGAAACAGACGGCTTTATCGGAAACG

GAAGTCGCAGAAAAGCAGCAGAAAAAAGGCTTGGTTTTGGAAAACCACCACGAAGTTGACGCTGAAGAAGAAGAAGAGGA

AGAGGAGAAACGAAAACAAGAAGAGGAAGAAGG

>GH296344.1 JCL794 Jatropha curcas total leaf library Jatropha curcas cDNA, mRNA sequence

GATTACTCAGTCCTGTCCTTCGGTTGAAATGCTCTTGTTGTCGACTCCAACATTACTCGTGTTATAGTAGTGGAAACTGA

TTGTCTGGATTCTGGAAGTATTGTATCATTAATACATCAGACTCCTTTGTGCGTGAACCCATCTATATTGTTGGAATGTG

CCATAAAATTTAATAATTTAGCCACACCAGTAATATCAAATGTTTTCAGTCTTTCATACAGTTGCAACAACAGCTCCATT

ACTCCCTAAAA

>GW876410.1 JC005974 Seed specific Normalized cDNA library from Jatropha curcas L. Jatropha curcas cDNA clone N10169 5' similar to Unknown protein, mRNA sequence

TACTATATTTTTTTCCATTCTCTGTCAATTTTACGTTTTTCTTTTTCTTTTTCCTTTATTTTTAGTTTCTAATCAGCGAA

TTTTATCATCCTTCAATCTATTTTCCTTCTCGTTACTGTCCAAAAGCCACATCAAAATTAAGGCTGTTGAACAATTTTCT

AAATCACTGGAATAATTTTTAAGATGCTGCGGCGGCGTCACGCGAGCGAGAGATCTGCCTCTTCTTGGCAAAGTAGAAAA

TCGCGACACTTTATTCCTGTGAAGCCAGAAGAAGTTGTAGCACTACTTGGCAAGGCCATTTGCCCATATGAGTTAAATAA

TGTTCCTGCATCTGTTATTCCAACTTCAGTTTAACCCAAAATGGCAATGCCGTTGGTATATAGGTGTTCGTGTAGCGGTT

CGGTTCGGTTCGGTTCGTTTGGTTGGTTTTGACTCTAAAACCGTAAACAAACTAAAGTTCAGTTTTTTTTAAGTTTTACA

AACTTAACCAAACCAACAATTTTTTAAACCACAGCCCCGCTTCCGCTGCCATCATCCCACCCACCGAAAACTGCTGCAAC

CAATTCTAATTTGGATCCAATAGAAAACGAAATTATGGCTATTAATCTACAAGGAAAGGATATGTCTCTCTTATATCATT

GCTAAACAATCACTACTTCGCTCCCTCTCTTCCAAAGGACTCCACTATATCCTA

>GT974516.1 GJCCJC2061E11.b Jatropha curcas L. developing seeds (mixed stages) Jatropha curcas cDNA clone GJCCJC2061E11, mRNA sequence

AGGTCCAAGGATGAAAGCTCCAATTGATAGATCACCATGAAGATCAGCAAAGGTGCCTATCAAGAATAAGAAGTACTTGG

ACTAAGTCTTTGCTTTAGCTTATATCTCTGCTGCTGTTGGAATTATTTTCCGCTGTAATTATTTTTTAAGTTGGTTTAAT

AAAGTTAATTTGCTGTTGTGATTGTGTCAGCAAATCTGGGCTATTTTAACTCATAAAGTGCCGTTTGAGGCCTTTAGAAT

AGGACTAAGTAGTTTTCATTCTTTTCCTATTTATTTCCCTTGTATCTGTAGCAGAGTTCATCAATGATAAACACTTGAAA

CTTCTTACTCTCTTGTTCAAGTCTCTTGGCTTTGACAAGGAACCATTAGTGGTATCAGAGCCAGTTTATCTTATTCTCTT

CTTTTCTTTTCCTCTTTTCATGGCTACTAACAAAGAGCATATCGAAAATTTAGAGAGTTGGGCTAGGCCAGCTCCAAGAT

AATTTATCTA

>GT229269.1 JC834 Jatropha seeds from fruits at three stages of maturation Jatropha curcas cDNA clone PL20SE.F02.scf 5', mRNA sequence

CCGATAGCGTGGTATCAACGCAGAGTAGCATTACGGCCGGGGCTTGGTGCTTTTTTCCCAGCTCTGACATTGTTGTCTAA

ATCACCAATATACCAAAATCTATGGAAGACAATTAAAACTTGATGAATTTGCATTGAACAGAAAATTCAGGTCTTATCAG

TTGATCCTTGATCAAGAATTATTCGCTCTCATGAAGTGCGAGTAGGTTCAGAACACTGCGGTATTCCATCCGCAGCAACC

TCTTTCTTTATTCACCGAGTGTTCTATTGAATTCATAACAAGTCCCGCGTTTCTGAATGGGATGAAAAGGGAAAGATTGA

CGTCAGCTGTCTTTCATGTGCGTACAGATTTTCGTCATGCTTCATTTTCTGTAGTCGTCAGCATTGTATCATATTTTGAG

GTGGTTATTGTATTTCCCATGATGTTAAATTGTCTATTTTATTCTATTGTTCTTAGTGTATATTTTCCTCCCCTTGATTA

TTTTCAATGTACATTTGTGTATTTTTCCTGTTGAAAGGTAGTCGATGTAAATTACTAAACTCAGCATTTTTTTTAGGGTA

ATGACTTTGCAGAGTT

>JK613118.1 JCF22-121 Jatropha curcas, immature Seed cDNA subtraction library Jatropha curcas cDNA, mRNA sequence

ACTAGTCGGTTAACGAGTTACCACTGTTAACTGAGTTCCTTCTCCAGCATCCTTAGTAGCACCATTAGCAACATCTTGAG

TTCCGGCACCAGTAGACAGTGGAGCGGTTGGTTCCGCTTTACGCGAGATAAGAAGGCTAGAGGAGATGTTTACTGCTGCA

ATGGTAGAACCCAGGGTGACAAACGAAAGACTGTAAAAAAGAAATGACTTCTTCTAACTACTGACTTTTAGCTAACACTC

GAAACTGTGAAAAAACCCGGGAATTTTGGCATAACTGCAATTTCCTTCCGTCGATACCTTATCTTCTTTCTATTTACACA

TGTTATTGCCAATTTTTATCATCTTTCTATCAATCATGCATCTATTAGTGGATAGCACTACAGACTTGGTTACGCAGTGA

TATACGAGAATCGAATGCTTTCAGTTTCAGGGGAATCGTCTTAGCAAGATAACCTGGTAAATGTAAATCCTGCTCTCAAG

AGATAAGATAGAATATAACCATATCCTTTACGCGAGGGGTTGTTAGAGTAGCAGTTCAAGCAGTTCCCGCATATAGGGGA

AGATCGTTAACAGGGATAAGGGAT

>JK317892.1 JCST663 Jatropha curcas L. seed cDNA library Jatropha curcas cDNA 5', mRNA sequence

TTCAATTGAACGGGGGGGGGCAAGAGGGAACCAATTAAGTAGAAGGCAAACCCGAGAAGCACCGAAGACATGGAGGCTTG

CCGGATTCAGCACGGCTGCCGTATCACCAAACGTTTCAAGCAGCTAGATGCTTACTTCGCGGATCAAGCTATAAGGCGAA

CGGTAAATGAACTTTCTGGACTAAATTTTTACATGCCCAGACTCATCCATTTTCAACGCGTTATATGGATCTCCAACTGA

CCTGCTGACCTCGTTTTAGCAGGGTGCGACAGTTAACATTCCGCTTGATGTGGTTGAAGATGTCCCGAAGAACGTTATGA

ACTCCAAGGTTTTGAGTCTTAATTTTCGCCCTAGAGCAGCATGTACAACCTGGCCCAGATGCAGGTGGGCTCTGTGCAAT

TGCCGCTGAAGGAGGAGGATTGCTCGCCATTTTTCCCAACAGGTCGCACAACATACCAGGAAATTAACGCAGCCGTTTCG

TCACCGAATATCTCCCAAACGTGCGCTAATCCCACCCTAGTTTTAAGGATGTTTTCTTTTTTCATTTTGCTAGCAACATA

GAACAACCCAATTCCACTTGGCGTTGTCCTTGAGGCGGG

>GW880102.1 JC000616 Seed specific Normalized cDNA library from Jatropha curcas L. Jatropha curcas cDNA clone N01170 5' similar to Unknown protein, mRNA sequence

GGAAGGAAATTTCTCGCAACATTTGTCATCTAGCTAAAACGCTTCAAACATTTCCCAAATCAGTTCCTTTTCTTCTGAAT

TTTCTTGGCAACCAAACAGAAACTCCTTTTGTCCTTTTACCAGTTACACCACCGAACACAAAGAGATTCCTTCGCGGTAA

GTCAATCAAATTCATTCTATTTCGAATACACAATCCTATTTATTTTCACTCATATCTTGTACGGTAACCTCAAGAATAAA

AACCCATCAAAACCATGTTATGTTTCAATCTAACCATGTCGGTCTCTCCAAATTTCTTCAAAATCTAACTCTTTTGTCAA

TTCTTTT

>FM890570.1 FM890570 Jatropha curcas embryo 56-70 (DAF) Jatropha curcas cDNA clone rjcaeb0_000458, mRNA sequence

ATTNCGAATTCATTTGATGACGTGTTTGAGACTTGGATTGCAGATAGTAAGTTTAAACTATGGCGTTGTTGCCTCTGTAA

CAAATTACCTAAAATAGTGCAGAAGAAAAACCCTAGCAATTGGACCAGTGAAGGAGCTCCGATCAAGAATACTATTGTGC

ACTCATCTTTCAACTTAAGGCTTGAGAGGCAAACAATGGTAAACGCGCAGCAAACAATGGTAAATGCGCAGTGAACTGTG

GGGATATATATAGGGAGAGACGAGAGAGAATTTATTTAATTTTTTTTTTCCAAATTTTACAGCAAAATAGGGATTATGCA

AAGTTTATTTATCTTTCAGTTTTGAGAAAAATAAAAGTCTCCCTTACTTTCACCGTTTTGATGCTTTATCTATATTTTTG

TTCTTTTTCATATTTGAGTTCTGGAAATTATTATAAGTTTTGGGGCTGGTCTGAATTTCTGGAAGATCTCTAGCCTATTA

ACATCAACTTCCTTACAGATTATCATGTTGTACTTCATGTGTTGAAGAGCTTTG

>GT975335.1 GJCCJC2069B01.b1 Jatropha curcas L. developing seeds (mixed stages) Jatropha curcas cDNA clone GJCCJC2069B01, mRNA sequence

GAGATAAGGGTTATAGAAAAGAAATAAATATCTCCCACATAAAAAATAAGTTACACCATTGATGTATACGAAAACAACAT

ACTAAGGACTTAGTGGTAATTTAGGCGCAATTCTCCAACAATTATAAGGACTAATATGAATATTCAACATAGAATCTTCA

TTAGAAATATATATATTTTCTTAATTAAATACTGACTTAGGGTTCTTGAACCCTGCATATACAACATTTTTTCTCAGTAG

AGGATATCGCTCTCAAAGCAATAAAACTCTAAATGTTATTAATCTTTTTCTAAAATTGTCTCTAATATTTTCGAAGCTAT

CAATTGGTGCGGTGGACATGGAACGCACATTCCACAACAATATGTAAATATTGAGTTTCCTTCAAAAATA

>GW875653.1 JC004970 Seed specific Normalized cDNA library from Jatropha curcas L. Jatropha curcas cDNA clone N08680 5' similar to Unknown protein, mRNA sequence

ATATCCTCCACTCTCCCAACCATACCAGTCATTAATTTCTAGTAGATTTTGGCCATCGCTACAGCTGTGCTCCTTTGCCA

AACAATTGAATTCACAACACAACGCGTCCAAACTCCCTAAATCTACAGCTTTTTAGTCACCATAGCTGACCGATTAATTT

TACACAGCATATAGAAATATGTAGTTAGATAACAATTGATTTGAGACAAAATTAAATTCGTCCATTTCTCTTTCTTTCTT

CTCTATATAAACAGGTTTCGACAAGTCTCATTTTAGTTGAGTCTTGGAGAGCAAAACACAGAGAGAGAAAAGAGAAATAC

TAAAAAGTAATATAACAAGCTGAACATCTCTCTCTCTCTCTCTTTTGAAACACTTTGCATCTTCCCCTTGGCTTCTTCGA

TTACTAATCTACTATATTAGGGCTGTTGCTTTTAATCTTTAAACTCTCTACAAGTCTAGTTTGCTATAACAAATATTACT

TTTTGCTTTTCACGACAAAGAACGCTTATTCTACTGGCACAAAGGTATGCATAAAGCTTAAAACCCAAGAATACCCAAGA

AAATCAAGAAACGGACAAGCAAGAAAATCAAGAAACAGAAAAGC

>FM895200.1 FM895200 Jatropha curcas embryo 71-95 (DAF) Jatropha curcas cDNA clone rjcpga0_001982, mRNA sequence

ATTCCAAATAGGTCCAATATATTAAAAAATGCACAAACGCATCCTTACATCTAAAATTACTCAAATTTTCGTCAAATGGC

AAATAAAATGACCATACTAACCTTTATAATATACAAAATTTTCTTTACAATTAATTACTTCTATTCCTCCATCTCTTTCC

TTGAATCCTTCACAGAAACAGAAAACGGTCATCTCTCTCTTTAATGCCGTGATCTGCACTTTCTGAACTCTCTCATTTTC

ACAAATCTGAACCATAAATTACGCATGGAGCAATCTATGTGAGAGCAAGGCCTTCAAAAATGAAATTACGAACAAATAAA

TGAATTTAAGCATAGGCTAATTTCCTTTCCCTTTCTCATGGTAGAGATGCCATTATTGCTATTTCATTATTTTTTTTTCC

TTTTCCTATCACCGGCTCATGTACGGCTAGCAGCGACCTCCTCCCTTTGCCTCACCGCCGTTCTCTCTCTCCATTTCTTT

TCCGATTGGAGCAATGACAGGGTGTGAGCTGCCTTCGTTTTCCGAATGGAGTGGCTCCACCTCAGTCTCCTTCAATA

>FM889918.1 FM889918 Jatropha curcas embryo 35-55 (DAF) Jatropha curcas cDNA clone rjcfea0_003673, mRNA sequence

GGATCCCCGGGCTGCAGGCACGAATCCCATTATCTCTTATAAATAGTTTTTAATTATAAAATTAAATTAAATAACTTAAA

TATATGACTTATAAAACCTCTCACTTTTATAAAATCTAATTATATAAATTGAACTATATAAATAATATCTTGTGACTTCT

AATTAATTATAGTTTTTACCTTAAATTTATTTATATTTTTTTAAAATAACAGATATTACATAATCATATTATAATTAATA

CAAAATTTTAAATTTGTGAAAATTTT

>GT971762.1 GJCCJC2024C02.b Jatropha curcas L. developing seeds (mixed stages) Jatropha curcas cDNA clone GJCCJC2024C02, mRNA sequence

ATTGTTATTGTCGTTCTTGTTGACGATGGATGATGATCGATGATCGATGATAAGCTTTATAAACTTCAAGACAATTATTG

TTCACTCTCCCTACTGACCTCCGACCAGCGAAGCTTGCCATTTCTGGCAATATTTCCGCCGGAAGCCCTTGAGGGAGAGT

GAGCAATGAAAATGTCTTGTTATACTCTTGATTGATGATGAAGCTATTCTTGATCGATCGATTGCCATGAGCAAGCCACT

GCCTGTTGTACTTCCCACAGGGGCTCCTGCCCTAACCAAACCCTAGTCATGTTTGCTCAAAAGTTAAGTTCTTATTAATT

ATATT

>GW876024.1 JC005401 Seed specific Normalized cDNA library from Jatropha curcas L. Jatropha curcas cDNA clone N09304 5' similar to Unknown protein, mRNA sequence

GAACCGAGTCATTAGAGGAAACGGAGTCATATTGTGAGGTCGCCATTAGAGGAAACGGAGAGGGAGTTCAGGATTTTCGG

AAGGTTCTGGGATGGGTGAGCGGGGAAGATAAATTAATTATGTAAAATTTAATTATTTTTTAATTGTTGTTGTTATTATC

TTCCCTACTCCACCCATGCCATAGGTTTCCGACCATTCGTCCCTCTCCTTAAACCTCTGTTTCTTGATCATCTCCGTCGT

CAGCTGTTTTCTTCCGATCCGGTTTGTATTTCTTGATCTTTGATTGTCTGGTACTGGCTTATTTTTGTTTTAGTTGATTT

GAATCTGAAATTTACAGGTCAATTTCGTTGGTTTGTTTATAACCGGATGATCTTACTTAACTAGCACCACAATAAGCGAC

CCGAATGTTAGTCTTCACGTTGTCGTTCGACGCCCACAAAAAAAAAAAAAAAAAAAGAAAAAAAAAAAAAATTCAAAAAT

TCAAAAATTAGGGAATATAAAAGCAAACATTAATGAAAATTTATTATTATATATGA

>FM896723.1 FM896723 Jatropha curcas embryo 71-95 (DAF) Jatropha curcas cDNA clone rjcpga0_004010, mRNA sequence

CTGGAAAACTCATTTCTGCATTTAGTAAAGACGGCAGACAACTGTCATTAACGGCATTTTTCTCACTGGCCATAGACTTC

TAAGTTCTAACTGCTAATACACGGACAAAAAAAAAAACGTAAACATTGGCGTCTTATTTTTCATGTATGGGGAAAACTGT

GCTGAGAAAGTAAAAATGAGAAAATGATTGCCTCTTGAAAAAAGATTAAAATTTCTGGACACTCATCTTCTCCAACATTC

CCGCACAATCAGCACAAAAGCTTAGGCAAACACATCAAACCTTGTTCCAGTCATCCAACAAATGATCTGTTGCAATGATT

CTCTGGTATACCAGCCCCACAGTAAACAAATAAACGATCTAGAAAA

>JK317834.1 JCST605 Jatropha curcas L. seed cDNA library Jatropha curcas cDNA 5', mRNA sequence

ATTTTTTTTTTTTTTTTCCTCTCTTGTAAATCGATCTTGTAGAAACTGAAACTACAAATCTAAGCTACCAATTGATTATT

TTAGTGACATATTTAGAAGAGAGAGAGACTATTGGCTGCTGGTTTTACTTACAGCTCTCAGACTTTCGCAGGAATGGCTG

CATCTTTGAGGGTAGGTAATGCAGAGGTTGCTGCCTCGCAATTGCCGAAATATGGTTCGAAGTTGCTGAAGGGAACCCGG

TCGGGAGCGGGCCGGTTGGGTGCAACTGGGTTTACGCAGCGTGGTGCGGTGCTTGAATAGTTACGGGGCGGAACGTCGTG

GAGTACGAACGCTTTGAGAATAGGAGGGCCGCAAGGAGGTACCGAGCACGGGAATGAGGGGGAACAGCGGAGAGGGATAA

GGCCGCATGGGTCCAGGACGAACTGGGGAAGGGGCGCTGAGGGGCGGAAAGTGGGGAAAGGGGGCGGGGACGATAGTGGA

GAGCTGCCCGGTTGAAACAATGGGGGAAACAAGGGAGGGGGGTTTAATAGCGAGATTGGGAGGGAACGCACCGCCCGCCT

GCGGGGCAGGTGTTTCGAGAGTGCCTATATGACCAACCCCGGACGGCAGGACGCACCGGCAAGCGTATGTGTCATGGGAT

ATGCGTGGTCTGAGGAGGGGGAGCGACCCTCGCCCCACCACTGCCCATGGAGACAGGCGGCGGGACGCGGCAACGCCAGA

GGATAGGCATGCCGAAACAACACAGATAAGCAGTGAGAGCTTAAGGGGCTGTATTGACAGACTAGAAGNNACGAGTCCGG

CAGTCTGGTTGTGGCTTTCCTGGGATGGATGAACCCGCCCTATTTCC

>JK610134.1 JCF55 Jatropha curcas, immature Seed cDNA subtraction library Jatropha curcas cDNA similar to function, mRNA sequence

ACTAATGTTTCTTAGGGCCATTACAGAAATCAATGTTGGTTGAACTTTAATTACTATGGCATCAAGTGTGCATCTCTTCA

ACATTTCTCTTTAGTTTCTATACCTGTCTTGTTTTCTTTTTTGAGAAAAGTACCTTTCTAATTTTGGAAGGGGCAAGTAA

TGTGTAAACTTGTGTTGTTCGAAATCATTCTAAAATTGGCCTGTTATTTGGGTTTGTCTAGTCCACTTCGGAATTGGTAT

TAAATGGTAATTATTGTCTAAATTAATAATCTTTATTC

>GW617862.1 Jc2-027-G05-M13F.G05.ab1 Jatropha curcas flower and seed Jatropha curcas cDNA, mRNA sequence

AGTTAGCAATAAACAAAACCCTAGCCCACAAACCCTTCAGAGAATTCCTAACCCACTTTTGATGGAGTAGGATATTTGGG

TCACCAAGATTGGGAATTTCCTCAAGGGTTTTCTTGGATTATTCCGGAATTTTAAAGCCCAGTTGTTAATTTGAACTCAC

TGCTGATTTCAACAGTAATTCATAGGCGGATCCAGTGGACTGTTTCTGGTAATTTCCCTGTCTCTGTTTCGTGATTTGAT

TCCGCAAGTTTTTGAATTTTCTTGAGAAGATGGAAAAGGAATTCCTTTCTGGACTTGCTTTTTGAAAAACTCGTGCTTTG

TTTATTTTTTGCATGAAAGCTTATTTTTTTGTTTTGTTTATCCTGTAGTTTGGGCTGATGCTCTGTTTGGTTACAGAGAA

AATGTTGGAAAATAATGGAAGTGAAGTTTGCGAATTGAATGTTGAGAAACACACTGCCTTGAGATTCTTTTTGGCGTTTG

AATTATCTTTCTTTGTGCAAGTCGTGTTCATTCTCTCTAGGGGAATGAGAAACAGTGTCCTCTTTTTCTTCACTCTTTTC

TGTCCCTGTGCAACCAAACATCAAGCCTTTTTTTTTTTTTTTCT

>JK612516.1 JCF24-75 Jatropha curcas, immature Seed cDNA subtraction library Jatropha curcas cDNA, mRNA sequence

CATGAATGGAGATTGGAACAACTTTCTCACGAAAAACATATTTTTCTTTCTTCATACCTCTTGGCTTCATCATACGCAGG

ATTATTTGACACCATGCATTAAGAAGGGTGTTATTACATGTAGAAAGATCACCTAAAGCTCAAACTTAATGAATTCCTAG

ATGAGACCGGGGTAGGGTTGAGGAATTTGGTAAGAAGAAGAAGGAGCAAGCTCGGTCGTTAAGCTTTGTTGCATAACTGT

ATTAACGGCTTTTGCAAATGTAGAAACAAACTCTATAGCCAATTTGCAAAACCCTATGCAAATCTCCACCTTCTTTATCA

TCATAGCTCCCATAATTTTTACGTGATATCTTTGGTATTTTTCTCTCTCACTAAGCAGAAGAAATTCTTGTGGGTTTCGA

GAAGCCGCGTAAATGTGATTTTACCTCGGCCGCGACCCCCTAATCTAGATGCATTCGGGAGTACGAGTCGATTCACTGGC

GTCGTTTACACGTCTGACTGGGAAACCTGCGTACCAATTATCGCTGCACAATCCCTTTCGCAGTGGGAATACGAGAGGCC

GACGTGCCTTCCAAATTCGAGCTGATGCGAGGATTGAGCTTAATTTGTAATCCGTAATTTTGTAATCGCCATTTAACAAG

GCGAACGGAATCCT

>GO247604.1 JcrME_RL1148 Expressed sequence tags from Jatropha curcas root cDNA library Jatropha curcas cDNA, mRNA sequence

AGGTAGAAACCCCGGATCGGACTACTAGCAGCTGTATACGACTCACTATAGGGAATATTAAGCTCGCCCTTAAGCAGTGG

TATCAACGCAGAGTACGCGGGGGGAGTGCCATTTGAATATAGACGTGTACACAACATTTAATGAGTTTCTGTTTCTGGGT

TGGTTTTTGTTAACGCAGGGGAGTAATTTCTGAGTGACAGCCCTTTAAGACCTTTTCAATCGCCACGAGTCACCGGTTTT

TAAAGATTATCTCCTCTGAAAGCAGGAAATTCAACTTATTTAAATAATGGAGCTCAATGAGACAGGATGCCAAGTTCCAC

TTGGGGCTCCTAAACTTGTGCCATGGTTGTGGATTCTTTGGAACTGCACCACCATGAACTTGGCTCTAGGGCCCCAAACT

TCTTATTGAGCAGGACACACTTGGTTCATCGTCTTGGAGATTGGGACGGGACTCAAGAACTCGGAATACCTGGTTTCCTT

TGTTGCAATTTTTCCAAAACTTGGCGATTCTCCCCGCCTTATTCCCCCCAAAATGGAACGGTTGAAAAACCCATAAGGGC

CCTTCTGGAAAAGTGGTACAGAATTTTTCTCCGGAAATTTTTTTTTTCCCTCACACAAAATGGTCTTTATTACGGCGAAA

ATTTCGCGCCCCCTTGGAGAAGAATTACCCCCTCCCATCTGGGAAACTTTTGGACGCCGCTTTTCTTGTAATTGTTCAAA

GATAACG

>JK317550.1 JCST319 Jatropha curcas L. seed cDNA library Jatropha curcas cDNA 5', mRNA sequence

TTTTTTTTCGAGCAAAATTAACAATATAGTAACATTGCAAACATTATTACACCCTTCCAAGCAGTAAACTAATGACCCAA

AATGATATACGATCCCATGACCATGAATATTCAGAAATGAAATTCAGCCTAGTTTTTAAGCAGATGCGAAAGAACAGATT

ACTGATAGACAAATGTAACTCCCATGTGTAAATACACCCGGAAATCCTTTGTGCTACTGGATCTGGTTTCTTTCGGGTTG

CGTTGCCGAGTCAATTACAGGCACCGTCACGCGGTCCCCCCTTCACCCAGCTCAAAATTGAAAAAGGCACAGAAGCGGAC

ACAAGGAAGAAAATAACAAAAAGAACTAAAAAGACGTTGGGCGCGGCCTCGCCCAGTGAAGTCCGGGGGCTTTGGTAATT

TGAGAGTTAATTACACGCAGGGTGATTATGAATATTCTCGTGCGGACGTATTTTGTGACAGAATATACTAAGAGGGGGGC

GGACAAATAGGTGCGGGAGAGATGTGAAATGTTCGGAAAAATATGGGAACAAAAGTTTACGAAGCAGAGAAAATAATCCA

GCTCAAGGGTCAAGCTTCTGACGGGACTAGCTCGAAGATTAAAGGGTAGAATGCGAATTTCAGAGCATAGGAGCTACGAC

GTGACTCCCTTCATAGACGAACCAGAGACGATACACAGTAGGACGAGGAGTGACTTGTCTTCAGGAGAACCTAACAGCTC

GTCGGATATATTAATGAGAGTATGACGGAGAAAGATAATGAACAGATGTACATATGCGAGGTTGCAGGNGGAGCGGGGAG

>JK317393.1 JCST160 Jatropha curcas L. seed cDNA library Jatropha curcas cDNA 5', mRNA sequence

TCTTCTAGAAAGTGTCTCTATGTCGGGTGGATCGATGGCGGGGGGTGCGGGGGGGGGGGGAGGGGGAAGGAGGGAGGGGG

GGGTGGAGCCTCTTTGAGGGTACGGATTTTTTTAAGGTGATTATTGAATTCTTCAGACCCAGGCCACCAGGGCGAAGTGG

ATACCCCATTGTGTTTGTGCAGAGAATGAGGGTACCACTTACTGACCAGTTGAACTAAATAACACTTGAATCCCAAACCA

ACGCACTTCAGTTGTTCAAGGTTCCCCACAATTGCAGACTGGATGCCTGCATGCAACTCTGAGGGCTAATTTCAGTTTTT

TCACCTTGTTTTCTTAGGGTTGACAATCTTATCTTTCTACATGAATAACAAGTATTCAGTCTGAAAACGGTTGATGTTAT

CACTTCCGTCTTCAAATGATTTTTGATGCATTGTCTTTACTGGAGTGATTATCCGTTATTTACACCCTCTCTGGAGATAA

TGAGAAATAGCTGAACGCTAAGTTCCTGGTGGAGAAGCAACTAGAGTCTCAGGAAGCCAACCACTCACCCTCCTCTCTGG

GATCAACCCGTCTAAGTCTGCAAAAATACATTGTTTGCAGTCTCCGGATGACTGATGTTGTGCGCACAAACAATTGTGCA

ATGTTCGACTCTTTTGACCAATAAATACACTTCTGGTAATTCCATCAGAGAGTCTAGGACAAGATATGTATCTGCTGGCA

AATATTTCTGCCCAGACTTAAACCCATCAGGATTGAGCTCCTGAGCTGCGCCCGGACGACCTGCTGAGGTATGGATTGCC

ATGAAAAGGTAATCAACAGACTTTCATGCTCCTGTTTCCCGTGTGTAGCTGACTGGACCTAGTTGGCCTCTGTCCAGATG

GTCGAGCAGTGGGACAGGTCTGTGACACTGTCTGTAATCTGGAATGATCTAGA

>FM889403.1 FM889403 Jatropha curcas embryo 35-55 (DAF) Jatropha curcas cDNA clone rjcfea0_003064, mRNA sequence

GGCGAATTCGTGGTGAAAGGGCCTAAATATCAATTGCTGTTTGGTCATTTAGCTCGCTGTTCTTGTGCTTCTACTTTTGT

CTCTATTTTGCTATGCTAATATCAATTACTGAATGCTTGTAGTTGACTTTCTAAATGTTGGTAGTTTGTATAATTCACTG

CTTGTTAAGATGACAACTAATAGTATTATGCCCTTCTATATCATTTTTCTTGCTTAGTCAAAAAAAAAAAAAA

>GW881786.1 JC001673 Seed specific Normalized cDNA library from Jatropha curcas L. Jatropha curcas cDNA clone N03080 5' similar to Unknown protein, mRNA sequence

AATACAGTAATACTTAAAACCAAAGAGCCTCCGTTTGAGAGAGGGCGCCAAACAAAGTCCAAAAGGCGGCACATACAATT

ACAAAAAAACAACCCCTGTACTGTCCTGCTCTGCCCTTGACCCTGGCCGGCCATGTGACTCACTTTCACTTCATTCCTCC

GTCTCTCTAACCTACAACAACTTCTCTCCCTCTCTTGTTTTCTTCAGCTTCCTCCATGTTTTCTACTTCCAGTAGGTCCC

ACAGATCTTCGGCCATCTCGCCCTTCCGTTCCCGCAAGTCGCCTGCCCAGCCTCCGCCGTCGAAATCCACCGGAGGGTCG

GTAACTCATTCCTCGACGACGTCATCTAGGCCTCCCTCCAGGCTCTCAGCCACTCC

>GW615424.1 Jc1-050-D08-M13F.D08.ab1 Jatropha curcas flower and seed Jatropha curcas cDNA, mRNA sequence

CCCCTCGAATTTCAAGTACTGTACTTTTCTTTTCCCTTTCTTTTATTTTTTAATATAAATTCTTTTTATCTTCTTAGTCA

GGAGGCAATTTGCATTCTCCCTCTTCTTCTGTGTTCTTTTTTAGGGTTTTAGCGTTTGGAAAAGATCAAGAATCGACCTC

GACGGGACAGGTATTTATTGTGTTGTGTTTGTATATAAAGCTAATTCTTTGCTGGTTTTAATTTTGCTTTGCTCGAATAG

AGTTGTAGAAATTAATTAGCCGAATAGGAACCCGAAATCCGTGCCTTTTCTTGTTGTTATTGGAGATTTTTTTAAAAAAT

TTTTGTATTTGTTTTCCAAAGCGTCGAAAAAGGACAGTTTTAGAAATTATCTGTCGTTTATCTTAATTTAGTGTTGTGTT

TGTCTTGCGGATCTCACACGCATGTTCCGTAGAATAAACTCCTGTGACGTTACTATTTAGGATCTAGACAGGGTGAATTT

CCAAGGTTTTGTTTGTTTCTTTTACTAGGTATAAGGAATATGATTTCTGTCGTCGTTGGTAATGAATATCACATGAAAAA

TAAGGACTTGATTGGAAATTTTAATTCCAGAATATAGCATATTTGCGGAGGAATTATGTTTTGAAAGATTTTCTTCATCG

ACTTTCTTTGTTATTACTACTGT

>FM889414.1 FM889414 Jatropha curcas embryo 35-55 (DAF) Jatropha curcas cDNA clone rjcfea0_003075, mRNA sequence

TCTAAAACTAGTTGGCTAAGAAACGTAAAACGAGTTTTAGTCGGTTAACAGTTAGATGCGAAATTTTGATAAATTAAGGT

TTGGTAGGCCTAACCACTTTCAATATTGATATAATAATCGAATTTAAATAGGTAGTAGATATAATTAGTTATTGCTAAAG

TAAGTTTTAACAGCTTAACAAATTAGTTACTATAAAGCTATTAATTTAGTTAACTTATGATTTATTAAATAATATTATAT

GGATATTATTGGAATTGGTTTGTAAATTTATGGTTGAAGTTTAAGGAGGATGGGTACTTAAATTAATTGATTACCCGATA

GATGGGCTGTGTGAATTGAGTGAATGAGTAGATGAAAGAATACTAGTTATGGCGAACGGGGTTAGTTATTTTGGATTTGC

CCGAAGGATGGGCTTTGAAAACGTACTGTTGAATTGAGGTTTAATCAAATTTTAGGAAAGGTGCTGTCGAAATTTCGACA

GA

>FM888961.1 FM888961 Jatropha curcas embryo 35-55 (DAF) Jatropha curcas cDNA clone rjcfea0_002494, mRNA sequence

ATAGGATTTGAGGAACGAAGTAACTCGACTGTAAGGAAAGGACAATTATTCAGCAAAATAGAGTGGAGGGGGTTCCATAT

TCATGAAAAGCCGGGGTTGAACCGGAACTAAGACAACAATTATTACTAATAATAAGAAAGAGTTAAGCGCCTCCAAGGCC

TATAAATAGAAGCGGATTTCAGTCTATATTTTCAAAGAGAGACGTAGAAGTCAGAAAGAAAGTGAGTAGACATAGCCATG

GATCCCGCTGGAAGACTGGCGCTTATTCCGCACGAAATCTCTCAAGTAGAAGAGGAGAAGAAAAAAGGAAAAAGCCACTA

CGGGGAATAGGACATGATTAATCACTCGCTTTCAAGGAAGACCGCTAAAAGAAAATAGAGAATACGTAAGATAGGCTACG

GAAATACAGATAGACAGACCAGTGCCTGCAATGCTTACTCCTTGAATAAGTGGAGCTTTAATCGGATTCTCATCCGGTAC

GAAACGAAGGCGGCTTGTCCTA

>GO246830.1 JcrME_RL0374 Expressed sequence tags from Jatropha curcas root cDNA library Jatropha curcas cDNA, mRNA sequence

ATAGAAAGCAGTTAAAGCTTTTATTGTTAAATAATCCTTATTATTACAGCATAATATTTGGATTTACATTCACTCCCTGC

CTACAAGGATAGCACACATCACAACTATTAACCAAAATATCAAACTTCATAAAGACAACTTTGCCCTTTTTTTTCTTTCT

TTTTTTTTTTATTACTTTTTCCTCCATTTCTGTAGATAGGGCACGAACAGAAAAACTTCAGTACACCTAGATAATTAGGT

ATATAATGAGAGGATCACCATTCATTAATTAATCGCTATCGCTATCACTGCTGCTGCTATGACCATGTTTATGGCCGTCC

TCATGCTTCTTCTTTTCCCCCGCGTACTCTGCGTTGATACCACTGCTTAAGGGCGAGCTTCCAGGTCACCCATTCAAGGG

AAGCCTATCCTAACCCTCTCCCCGGCTCGATTCTACCCGAACGGGCATCATCACCATCACCATTGAGGTTCTAGAGGGGC

GCCTCTG

>GT972245.1 GJCCJC2032C01.b Jatropha curcas L. developing seeds (mixed stages) Jatropha curcas cDNA clone GJCCJC2032C01, mRNA sequence

CCTATATAGATCATTGACGCTTCAAGTAATTTTCCATTAAAAAAAACACTATATCGAAAACTATAGAATATGTATATATT

GCTTATGTTATACGGTCCAATATTTCAGCTAGCAAGAGAAACTAATAAATGGAGCAGGTATTTTCTTTAGGCATATTCCT

CGTATGATACGAGATATTTCCTCCTCCTTTTGGAGAGAAGAACTAATAGAATTGCAAAGAAAAGTTGACAACCTAGTAAA

GTGGAAGAAGAAAATGAAAAAAAAGAGAAGGGACTTCTTAGCTATTTAGGTTGCAAAAGAGGAGAACAAAGAGACCCCAT

ATGTACCAAATCGAGGGGACAAATGGATGTAAGTGCATCGCAAGAAGATCATGCGGGACATGCGTGTGGTGTGCTTCTAA

TACGGCCACTGCTTACCTCTTGACCTTTACCTCCAGCATCTCACATGAGAGAAAATATTGCTTACTATAGTCTATTATTG

TTCTTGTAGTATACAATATGCCATTCTAAAATCATCAGTCCTCTGCTACATATATAATTTCTTTCTTGTAATAGGAAGAC

ATGTACATGACCACCGGCCAAATTCTTTCAAATCTTGGAAAGAGTCCAATATTATTTGCTTTAAATAAAGTATTTCTTCA

ATCA

>JK610928.1 JCF3204 Jatropha curcas, immature Seed cDNA subtraction library Jatropha curcas cDNA, mRNA sequence

AGCTTTTTTTTTTTTTTTTTTTTTTTTTTTTTGAGGGGAAACACAAACACCTTAACAAATAATGAAATGTGCCCAAATCG

TGAACCCAAAAATGGAAAACACGAACATCTTACCAAAGAATGAAAAGTAGCAAAATCAAGACCCAAAATGACATTGGTAA

AAACGAGACATATCCTACAAATCAAAAAAATAGAAAAATTTTAAAAAAATTTTTTCCCCCCCCCCACCTTGGGGGGGAAT

GGAAAAGGAACCCCACCCCCCCCTCCCAAAAAAAAAAAACGGGCCCCCGGGGAAGAAAAAAATTTTATTTTTTTAAAAAA

AAAAAAGGGTTTTGGTTTAAAATAAATTTGCCTTCCCCCATTTCTTCAACATCCCGTATGCCTTCCTCCCTCATGGCAGC

CCATCAAAGATTCATCTGAAGGTCAATTCTTACCCTTATGAAGCAATTACCAATTTCACTGTAAAAATTTGCAGACCCGG

TGTTCCGGACTTATCAGTCTCCCTTGGGTTCTCCACCTCGGCCGCGAACACGCTAATCAGATGCTTTCGCGAGGTACCGA

GGCTCGATTCAGGGCCGTCTTTTTAAACGCGAGATGGCAAACCCTGGGCGTAACCCACTAATCCCGTCCCACCATCCTCA

CTTTCGCAACTGCGAATATCCGACAACCCC

>GO247235.1 JcrME_RL0779 Expressed sequence tags from Jatropha curcas root cDNA library Jatropha curcas cDNA, mRNA sequence

GGAATAAAAGAGCAATAAGCTTCTTGAAAATTCAATATGAAAATAAGTTACTATAGAAAATGCCTACCTAAATGTAACTT

TTTTATTTTATAACTTGCATCTCACTCAATAACATCCTTTACGTAAAGTTCAAGAACCAGAAAATAAATCTATGAGAAAA

AAAAAAGAAAAAAAATTATAATAGCTGGGATTTTGATCTCCCAGGTGTAAAGAGATCCCTGAAATCAGCTGATCTAAAAT

GCTCAATTCTGTCTTTTTCCTCTCCCGCGTACTCTGCGTTGATACCACTGCTTATGGGCGAGCTTAATATTCCCTATAGT

GAGTCGTATTACAGCTGCTAGTAGTCCGATCCGGGGTTTTTTCTCCTTGACGTTAAAGTATAGAGGTATATTAACATTTT

TGTTGATACTTTTATTACATTTGAATAAGAAGTATACAACCGAAATGTTGAAGGATTAGTTAAGTGGTTATGCAGTTTTG

CATTTTATATCTGTATAGATCAAATCATCGCTTCGCTGATTAATTACCCAAAATAAGGCTAAAAACTATCGCATATCCTC

TATGGTGGTATTGATTCGTCATTGAGGTTGGGGGGCAGGTACTGCAATTTTTCCCTCTAACCAAAACTAGTTGGAAATCT

TTTTTCGGACAGGCGGCGGGGCACCTGCTTTCGGACCGACGGGAAAAAAACCCCGGGGGAAG

>FM888124.1 FM888124 Jatropha curcas embryo 35-55 (DAF) Jatropha curcas cDNA clone rjcfea0_001458, mRNA sequence

TCACTGCCGTATATCAAAGATCCGGTGCGCTCCCGGTGGCGGGTCAGCTGAAGATGAAGAAGATTCACATGATGAGCTGT

AAGAATATTTGAAGAAATGAGGATATAGGCTTAGGCGAAGAGGTTGATTGAGATACTGTAATTTTTCTGATGGTAGAAGC

AAAAGGGAGAGGAAATTCCCCTTTCTTCTCATGTTTTTTTGAAGGTTAAAAATACGTTGAGGAGGTCCTTTTATGTTTTT

ATTACTAATTTGTAATTTCTTTGTTTAAAAACTTAATTAAATTCCTTCTATCAAAAAAAAAAAAAAAAAAAAAAAAAAA

>FM887645.1 FM887645 Jatropha curcas embryo 35-55 (DAF) Jatropha curcas cDNA clone rjcfea0_000881, mRNA sequence

GACTTTCTAACCTTTGAAGTTCATTTTGCGTGAGGTCTGCATATCTCCAAATAAATGAAGACCTCATATAATGTAAATTG

CTTATTTATTCCATTGATGCTGACTTAAGCTCACATAACCGGTATGGAAAAACAGCTTTTGGGATCTATTGAAATGATTT

CTTCTTCCTCTCTTTTCTGTTTGATGCTAGGTGCCATACAGATTTATTGTCAACCTATAGTTTATAATATTAACCAATCA

GAAGTGGTAAAGCCGCATATTTGTGGGCGGCATCCACATAATGTTTCCTTAAGCACGTGCTTACCAAAGCCAAAATTTGG

CCAACTCAGTTTGGACCTTCGTCCCTTTCTTCATCAGTTTATGCCGCAGGCTTCCCTTTAATCAAGGTACATGGGGGCAA

TCTCTCTGCACCTTTCTTTAATTTTCTTCCCCCCTATATACGCAAGTTTATTTGAACAATGGTTTGTGAAAATGTGCAAA

TTCAGTCTATAGAAAGAAACAATTGATAAATCTATTATTGCATAGAGGACATAGGTCATCTTTTATATTGTAGTTTATGC

ATAAGCTTTTACTTTGAAGATGTATGATTTCTG

>GW616386.1 Jc2-011-C03-M13F.C03.ab1 Jatropha curcas flower and seed Jatropha curcas cDNA, mRNA sequence

CAATAAAAATAATGATTTGTACTGATAATAAATCCATATTAAATAACATAATATCCAAAACAAGGAATTTAAGCAGGAAA

AAAAAAAAGCCACTTCCACGCACCCAAGCCCGATAAAGCCCTAAACAAACCTCAAATTGCCAACCTCATTAACAGATCAA

TGGAAAAAATCAACACCAATCATGAACTCGATTTTGATCCACAGCATTAAAACAAAAGAAAATAACTCGACTCAGCCTCA

TCACACCAATGGATTACGTTTGCTTGCCATACATCATTCTTTTAAGGAAACAAAACAATATTCCTAGAAGGAAACCTTCC

AAGCTCATATTGATCAACAAGCCACCAAAGTGAATTTTCTATACCTAAGTAAAACACTGACCAAATTTTAACGGAACCAA

CCAGACAACAAAAGAAGAAAAACAGACACCATTCTATTTTGACACCATATCAGCAAAATCAGCACAGCTCCTTTTGAAAG

AAACAAGTTAACAGTCGTCTCTAACAATCTGAC

>JK613243.1 JCF23-108 Jatropha curcas, immature Seed cDNA subtraction library Jatropha curcas cDNA, mRNA sequence

ACCTTATATATGATATGTGATTAAGAAAAAAACAATGCAGTTTTGTGACACATACTGAAAAGAGTCTTTGTAATAAAGAT

GTTCTAATTATAATGTGCTGTATGTGGATCTATGTGATGGTAATAAGTTTCACGTGATATATTATTGGATGGTTATTCGG

AAAAAAAAAAAATAAAAAAAAAAAAAAAAAAAAAAAAAAAAAAAAAAAGCTTGTAC

>JK317570.1 JCST339 Jatropha curcas L. seed cDNA library Jatropha curcas cDNA 5', mRNA sequence

GCCACCAATTTCAATGTCCTCTATGTCCCGGGGGCGACCAGAGGGAATCCATTTTTTTTTTTTTTTTTTTTTTTTTTTTT

TTTAATATTTGTTTATTATTTTTTATTGGGTTGTTAGATGACATGACTTTGATTTGCTTCCTTTTGCTAAAAATGAATTG

AGGCTTTTCTTGATTCCTCCTATTAACGAATGATCATCGCCACCATCGTCTCCTCCTCTTCCTCTTGGCTCTTTTTTCTC

TTTTGCTTTCTGGGTTTTCTTCAGAATTTTGATTTAATTCTTATGTTTTGAACGAATTTGGAATTAAAAAACAAACATTG

TTTGTTGAGGATTTCAAGTGCCTGGGATTCAAAGCACTAAAATTTTTATATGTAATGGGAAGAATCATAAGAAAATGATT

TCTATCATTGCAAGGCTTTGGGGCAATTACCCTGATTCAACTAAGAATGTTACCGAAGAGAAAATTTTGACCTGTAATTT

CCTGAACCTTAATGGACTTGCTTCCAAATATAAACAACCATATAATTTGAAAGCAATTCGAATTCTGTAGCCGCAATGGG

TTGATTAGGTTGACCTTCCTTTTTCACCGCCCTTTATCATGCCTTCCTTCACTCAAATGGTAAATTGCGCCAACAAACCA

GACCAATTCATACGGGTTCCTTGTCATAGAATCCGATTCGTATCTCGGAGGACCACTGTTTGTAGGTCCGCCGATACCAC

GCGGAAGACATATTTAGACAATATCCTTTCCGAGTTTGTCATACGCATGTTGCTATCCCCGCTACCTCGCTCTCTTTATG

GTTGCTACATCAGTAATCGCCGTCGTCCGCGCGCCGCTAAAACGTCGAATTTTAGCCGATAATTATTAGACGCTCGCTGT

C

>GT975066.1 GJCCJC2066H04.b Jatropha curcas L. developing seeds (mixed stages) Jatropha curcas cDNA clone GJCCJC2066H04, mRNA sequence

GATGGAAAGAAAGTTAAGTGGAAAGGGCAAAGATGTGAAAATGCAGCAAGCAAGTTCATTAATCTTTGAATTGCATATTT

TACTTATGTTTATGAAAGCAATACGTATACTTTTTTGAGGAAATTTGCTTTTCTCAAGAGGCATGGATGGTGTGCCAGGA

CCTATTAAGTAAGATTATTTTTTGCTATAAACATGAAATTATATTACTCCTATGCCCATGAATTCCTTCCCATATAAGAT

GGATGGTGATCATACTAAGACTATCTGTTTTTAATGCCTCATTGACTGCTTTAGACAGGTTGGTACATGCTTGCCTGTAA

AAGTAATTTGCTTTTGGCAGATGATGTATCTGTCTGCACCTGGCATTTTCTTCAGTGCCTTGAGGCATTACACAGAATAA

GGCTTAGTACAAAGTTTCTGCCTGAATATCTGAGCTCTTATCCTGAGATTGACATGACAAGCTTATATAAACTTGTAAAT

TTACCAGGCTAATATTCTGTGGTCCTTGTTTGCCTTAGAATTTCTTCATTAATTTGGAATACAACTGTAAACTATCTTGC

TTTTGATTGGTTACCAATTTTTTCTCTGTAATTAGTTCTGTATTTATTGCTTAAAATTTATGTGTGCACTTTTAGTTTGA

GCTTCATAAGCTCATATTTAATCTGCTATAAGTCATTCCGAACATTTTAGTGTGTTAGTC

>FM888391.1 FM888391 Jatropha curcas embryo 35-55 (DAF) Jatropha curcas cDNA clone rjcfea0_001796, mRNA sequence

TCAACTTTTTCTTTGCTCAACAACCCACACTTTCTAGAAATAATGGAAAAAGTAATAAGATTTTTTATGCTTATTCATTG

TTATGGCAATCATTGCCACCATCTCTTCTTCTTGAACGGAAAGTGTTTCTTCACTATCGTGGCAATCACTGCCCCCACCG

GTGAACCCGTCTTGATGAAGGATCTATGGGACCAAAACGAGGTTTGATTTTCTGAAAATCTAATTCTTGAAAATTTGCAG

GGAATGGCTATTGTTGCGCTTTTGATTTCGGTGTATGGAAGTTTGAAGTCTGAGGGAGACGGTGCTTGGTCAAATTTCGG

TTAGCTAATTCCAGTGGGCATTAACAACATCTATGCCATGAGAAAGAGAAAGGGGAAATAGGATTTGAGTTTCAAAGGAG

ACAGGTAGAGGAGAAGCAATTAATTGTCAATTTTGTACATTATCAAGGTTAATACGGTCATTTGATTTGTCATTTAACGG

>GT969774.1 GJCCJC2002B07.b Jatropha curcas L. developing seeds (mixed stages) Jatropha curcas cDNA clone GJCCJC2002B07, mRNA sequence

GAATCATACAGTATACTATACAGCAAGAAAGATCATCAAGTGAAACATGACAGTATCATGATATGTGTGCTTACATTCCA

TATGCGAGTAAATGTGTATTTATTTCCACATATGTATGTGCTTATATGTTTCTTCATTCATTATGATATTCACTGAGTTT

AATCAAAGTTGCAGAGTTCATACAGCATAAAGAACATCAGGTAAGATCATGAAAGTATAATGTTATATATGCTCACAAGT

TTATTTATTTAATTCTTAAGAAGCCATACAGTCATGAGTTGTTATTAATCATAATTTATATGATGTGACTGAAAGGGCAT

AAGTCATACAGCATAATGAATAGCAAGTAAGACAATGAGCATATACTGTTATGTATTCTTACATATTTATTGATTTCATG

CAGACAACACAATGAACAGCAGGTAAGACCATGATAGTATCATAATGTGTAATATACGGTTCATGTAAAGGAGTTATGAG

AGAAGTAGATTAATTATGTTTATGAGTTAGAAGTTTATTCGTTGCTACAAAGAGACATTCATGTTAAAATTTACTGATTC

TATGAGTATCAATGCTTTATGAAATTGGTATTTGAAGTTAGTTGCCATCGTACTTTGTTTAGTATGTGAATTATGAGCAC

CACTGAGTTTTATACTCAGCGCGTCGATTTTCATCACGCGTAAG

>GR209314.1 JCST93 Jatropha curcas L. seed cDNA library Jatropha curcas cDNA 3' similar to :Arabidopsis thaliana phytochrome E (PHYE) identical to SP|P42498, mRNA sequence

TTCTTTAGCTTAATAACACACACACACCCAACCACCAAATGATAACAAGCGATTTTTAAGCTTCGGTTGAAATATGAGAA

TGAAGTCATACAGTGTTTTTTCAAAGCTATAACTCATAGCTAAAAGAATAAAGTGATTCTGGTAATGAATACACATGCAA

TAAGATTGTATCTCAAGTAGGATAAATTGTAGAGCAATTTTTTAGACAACTTGCAAGATGAAAAAAAGCAAAGGCACAAT

AAATTTATGAAAGCAATAGGTTGAGAAACTGAACTAACACAAAAGAAACATCCATGATATAATAGCAACATAATGCAACT

GGCAAAAAAAGAAAAAGAGATCACCTATTAATCTTGTTATTATGTGACCATTAATCAAATATGTTTGTTTTCTCCATATT

TTTTATTATTATTTCATGGCCTTTTTTATCACGAAGGATGGTAGGGATAGACAGAAAAAGTTTAGTACTTCACATTGTCC

ACATACAAGGTACATGAAATCCTACAATAATAAGCAGGTTTTTGGTAAATGTGCCACCCGGGAAACCAAACACACAATTT

AAGTGAAGGAGAGATGTGGGGAGATAACATTGTTAATATCAAGTTGCTATATAAAAAAAATACAAATGCTACATAAGCTT

CAAATACTTGTTTGTAGGCTTGCACCATCCTCAAGCACTAACCAGGAAAATTTGTCAAAGTAACACAGATAAAAGAGGTT

AGAAAAAGAGCGCATAATTAGGAGAAATCAAAAAATTAACTAAAAATCTTTAATCTGGGGATTCAAAAAAAAAAAAAAAA

AAAAAAAAAAAAAAAAATGGATT

>FM895832.1 FM895832 Jatropha curcas embryo 71-95 (DAF) Jatropha curcas cDNA clone rjcpga0_002828, mRNA sequence

AAGATATGCAGGAAGGTGGGGAATGGGAATGGGAAGGGGAGAGGCTGTGATCTCGCATATGTAGTGTGGTATTATGAATT

GATGAGCTCCTCTCCTTTGCTTTACCACCTAAGTGAAAGAGAGAGAGAGAGAGAGGGAGGGTACCTCCTTGCTTTTAGAG

ATTGGTGGTTTACCATTATACAACTCCTCCAGTGTAATTTAGCAAAGTACATAACAGGATTTTGACTAGGGGATGTATAC

CGGGTATAGTTGGTTGCATTTGACAATTTCAATTCTTGTCCTTATTTTTTTCTTGTCGCCAGTTTTCTCGGGGATCGGCT

TGTAAAATTATTGGTTGAATTTACGCTTCTGGGCTTTAGGATTCATGTAAAAGAATTGTAATG

>FM888934.1 FM888934 Jatropha curcas embryo 35-55 (DAF) Jatropha curcas cDNA clone rjcfea0_002457, mRNA sequence

TTCTAAGACAGTCCCCCATCTTGCAGATGCTATACACGCTGCTGTCACGCGCTTTGCTTAAGATCAATAATCCCTGGCCA

TGGGCATTACTTTTGCATTTTTGACTGACTTTTGCATCTTCAAGTGACTGTAATTTAACGTACTCCGTCCAAAATGTCTT

TTTCGGATTTTGATGGTTTTGTTCATTTTATTATAATGCTGCATCCTCGGCTTTGACTGAGGAACATA

>FM888406.1 FM888406 Jatropha curcas embryo 35-55 (DAF) Jatropha curcas cDNA clone rjcfea0_001817, mRNA sequence

GAGAGAGAGAGAGAGAGANACTAGTCTCTCTTTAAAAAAAAAAAAAAAAAAAAAAAAAAAAAAAAAAAAAAAAAAAAAAA

AAAAAAAAAAAAAAAAAAAAAAAAAACTCCAGGGGGGGGCCCGGTACCCAATTCCGCCCCTTATAGTTAAGTTCGTTATT

ACCCGCCGCTCACGTGGCCGTTCGTTTTTACAACGTTCGTTAACTGGGAAAAACC

>FM889167.1 FM889167 Jatropha curcas embryo 35-55 (DAF) Jatropha curcas cDNA clone rjcfea0_002756, mRNA sequence

TCGAGCTCACAGCTCGTTGTTAGATGCGGTGTACGTACCTGCAGCTCATGCTGATGAAATTTAGGGGGGCTCTGGAGATA

GTTTGGCATAGAGATATTAGACATGCCAGTATCCCTTCTCACGTAATTCTTACAGCAGAATCTCGTTGTTGTATTGTATG

ATGGCATTCCTCTGAAACGCCGTCTCCCCACCATTTCTTTGGCTTCTAAAATGCCCTCCCCTCGCTCACTTGTTTGTATT

CTTATAGTTAGCAGTACTGTGGCTGAAATCAGTCTCTCATGTATATAGTATTGTATTAAAGGGGTGACTAAATGTAAATA

TATTGCCAATTTAAGAATGTATTTTTTTTCTT

>GT975336.1 GJCCJC2069B02.b1 Jatropha curcas L. developing seeds (mixed stages) Jatropha curcas cDNA clone GJCCJC2069B02 similar to putative microsatellite, mRNA sequence

CTCTATCATAATCTCTATGTCTGTGAATATCAGTAAGCAATAAATGATGAATGCTTTGTTCAATAAAAGAAAAATATGAA

AGATGGAACAGTATTTAAATCATCTGAAATGGCTCGATTTTTATTGCCTGTCTCGACTTCGGTAACGATCCTCATCATCT

CTACCTCTGCCCCGTTCTCTCTTCTCACTGCGATCTCTGCGGCGATCACGATGTCCATGGTGAGAGTCACTGTCCTTGTC

TCGGTCTCTTTCCCTATCCCTATCCCTGTCCCTACTTCTCCCCTTATCATTGTCCTTCTCACGATTCCGATCACTGTCTC

TCTCACCATCTCGATCCCTCTCCTTATCAAAAACCTTTTCTCTTTCCTTTTCCCTACTTTTTGAAGACTCTCTTGTATAG

TCATGAGACCCATGCTGCATTGACAAATTGTAGAAGCCCAATTCCAGAAAATAAGTGATGTTTAATAATAACAAAACATA

AAGATATAGGACACTAAGCTTACCAGCAGATAAAATCCACATATTATCAAGAATTAATATAATCTAGTGAATAATTCATT

TCA

>FM890814.1 FM890814 Jatropha curcas embryo 56-70 (DAF) Jatropha curcas cDNA clone rjcaeb0_000757, mRNA sequence

CCCAAAACAAGCTTGGTACTTATAATTTGAAGCTTACAAAAACACTACCATANAGGGCATCAAAACAAATCTTCCGCTTG

CACTGCTACATAAGTAACACATAAATGGCAAAAATGCATGAAGACCTTTAAGCAAATCCAGAAGAACTAGTTTGAGCTGC

CCCACCGAGAAATATGAGGATGATTACGCGAGGGAGACTCCATATGACAAATACAGTTACTAAACTCACAAGGTGAACCA

CTCTCCAACACTGGTAAATATTGATTTTATTACGATAAATATATCAAAACAAGTTCCAAAAAAAAGGGGGGGCCCGGTAC

>GO247671.1 JcrME_RL1215 Expressed sequence tags from Jatropha curcas root cDNA library Jatropha curcas cDNA, mRNA sequence

GACTCTTTGCATATTTTAAAAACCAGTGATAAACACAAGAGATTCCATTTTCTTGGTTTGACCTATAGCAAGAAAACAGA

GCTCCATGGAGAGTGACCAAAAACCCAAACCCAAATAACCAGCCCACCTAAAATCTTCTTCTTCTTCTTTCTTCTTCTCA

CTTCACACCTATCCTTTTCAAAAACCTTATCTTCTTTGAAAAGAGAGAGGGGGAGTTGTCTTCGAGGCTGCGTTACAGTT

GGTGCATGATGGAATTGAGTGCAGCGTAGGGCCTGATATAAATTTAAAGGGTGCAGAGATAAAAGAATTAAAGATAGAGA

CTTTTCTTTCTTTTTCTTTTTTTTTTTTTGGGTTAATATTCGTAATTTTAATTGGTGGAGACATCATTCCAGAATATCTT

TAAGATTAATTCATGCCACAAAAGTAAGAGTGCTATTAATGCATTAGCCACTTCTTTTTTG

>JK317728.1 JCST499 Jatropha curcas L. seed cDNA library Jatropha curcas cDNA 5', mRNA sequence

CATCCTAGGCTCTGCCTCCCTGACTAAAACTACCGATCTCAGCGCTCGAGGAGCAGTGGGGAGGGGGACTGAGCCACTGC

GGAGCTAGAATTTGCGACTGGCACAAGCCTGGCATCGTGACTCGGCCACACCGAGCGCGGGAGGACGAAAGAAGAAAACA

AACCACGAGGCGCGAGAGCGGACGCTGGACTGTAGACTACGCGCTGGAGGGGAGAGGGGGGGGGCGCGAGGGGAGCTGGA

ACCTATCAAAAGCGAAGGCGCGATAAGCGCAAAGGAGGAGTACGGGCGATCGAAAAGAAAATCTGAAGAAAGTGAGAGAG

AGACAAAAGGAAGAAAAGACAACTAATACGAACAGACAAATAACAAGACGACAACAATGCGAAACAGGTTAGGGGCAGCA

GCGCGCAGCGAGGAAGCTGGGCTAGACGGCACCCGGGTCGTCACGCGCGCATCCCTGGGTGTACAATATCCCAGCCCACC

G

>JK317923.1 JCST694 Jatropha curcas L. seed cDNA library Jatropha curcas cDNA 5', mRNA sequence

TGTCATCTATGTCCGGTGATCGACGAAGGGTTGGCAGCGCTAGTAGCAGGAGGACGGATTGACTTTATAGACAAAGGAGG

AAAATTCGGGGGGTAGTTCTTGAACGGAAGTTTTGATCGACGGAGAGGGAGTCACCTTCCGAGATGAAAAAGAAGATGAA

TCTGTAGGCTAGTGAGGACTTGCGGGGGTATTAGACTGAAAGGAGGTAACAAGGGGCCAAGCAATTGAAGACTCGAACGG

GGGAGGGTAGAGCGGGGATTTATCAAGTTCGGACAAAGAGGGAGAAACGGGGGAATGGCGAACACATTAGGCGGTGACAC

GGGTGAAGCTTCGAGGGTAATAATATAACGTAGTGAACGTATGACAAGAAAAATGGATATAAAGGAACGCAGGGAGCACT

GATAAAGAAAACACTTGCCGTAGGAATCAAAGGAGTGCTCGAAGGCTGTGAATGGTAGGAAAGCGCCGGAGGTTTAAGCG

AACATAGTGGTGGGACTGGTAGATTGCAGGGGTGAAGACGGAGGGCGGGAAAGCCGATTGGGGGTTTAATGAGTAGGCGA

CGCTGGAGAGTTACGGGCACGTTCGGCTACGAAAGACAGTGTCTGGGCCGGGGGATGCCGGATGGCCGGTGAGGGTGGGG

TATGTGTAGAAAAAATCTATGAAATTCCACGTCACCCGAGTTACGAGAGGGACACAGNTGCGNCACCGAAGCTATATTAT

ACACGCGCTGTGGTGTGGGGGGAGGGCGGCTTGGGGGTCGGGGTGAGGNAGCAATTGACCC

>FM889774.1 FM889774 Jatropha curcas embryo 35-55 (DAF) Jatropha curcas cDNA clone rjcfea0_003509, mRNA sequence

TTTATATAATTTCTCTCCATATGTGTGTTCCATTCAACAACACTCAAACTAAAATCTGTGTCAGAAGAACTAACGGTAAC

TGGCTGCTTAATATATATACACACACACACACCATTAGCGTTACAATATTCCTAGCAGTAGTACTGGGAGTGAAAGAACA

TAACGGTGATTTTCTACATCGATCAGGTGGATTGTCAAAATGGTGGGACAGCCGTAATCTAACATTTCAATGATTTCAGC

TGCACTGTCAGTCTATACATGCATCAACCGCAATAACCACCGATAACCTCTGCATCTTCGTAAAGAAAATTATCAACACA

TGCAAATAACACGAGAGAATTTTCCCCAATGGTGGTTGGTGAAAAGACAATTCTTACTGATTTTCCTCANAGAGCGTTCA

ACCTTGTGAATAGAAAAACTTCTTGATTGGAACACTTGACCCGGCGGCCATCAAGATTAATTTAACCTAATGGTATATAA

AAAAAAATCTTAAATGTTTGAACTTTTACGTTATTTAATTATGAT

>GW879024.1 JC002300 Seed specific Normalized cDNA library from Jatropha curcas L. Jatropha curcas cDNA clone N04239 5' similar to Unknown protein, mRNA sequence

GGGACCTGTTGTTCGTATTTTTATCTCTTAAACCATTTAATTTAATTTCTCTCTCAGGTGCTCTCGTTCTTTCTTTTTTC

TGTGCCTTGATTACTATAAAAATTGCATCTTTCACTGAAATTCTTAATTACAATGGTCATTCTTTAGCTTCAATTTTTTG

TTTCACTGTTTGACTTTGCAGCTTTTTTTTTTTTTTTTTTTTTTCTAATGCTAAAATCGTAGTCCTTTTTCTCTTCACAC

TGTTAGTGTTCAGTTACAGTCACTGGTTATATTCTTTTTCTATATTTTGTTTTTTCTTTTCATAATATGGGTTGATTCTC

GTAAATGAATCGAACTTATTAGCATATCGCTTGAACAGTGTAAATGGATTGATCAGTGAAAGATTTGTCGACCCAGTTCG

TTTTTCTGCTTGTTTGGGTATAAAATTGGCTGATCGGTTGGTGATAAGATTGTGAAACTTGATCGAATTCTTTGTTTTAA

ATTTATAAGCAGAAACAACAAGACCTAAATGGGTAACTTTATTAAAGAGCTTACTCTTTTGTTGCAAAATGGAATATTTA

GATCTTGGTAAAATTTATGATTTAATTCCTCCTGAAAGAATATAA

>GT980382.1 JGCCJG2042D07.b1 Jatropha curcas L. germinating seeds (mixed stages) Jatropha curcas cDNA clone JGCCJG2042D07, mRNA sequence

GAGAATATAATGTTTGGTGCTAAGGAGAATACGAGGGAATTTTTTCCATTTTATCCATTAAGCCCCACTATTTTTTTTCT

AGTTCTCGAACTCTTCCAGTTGGATCTAAGAACAATGGGGGAAAAGTTTCCCTTGCTAATGCTAGGTACCGTTGCTGTCT

CTGGGCTTTGATTACATTTATTAGATGCGAAAAATTTGCGAGCTGGAGCTGTCCCAATTCGGCCTGACCCAACCCAAAGG

TTGAATTAAAATGGTATGGACTGTTTTATTTTCATTGTTTGGGCCAGACATCTGCTAGGCCCGCATTCAGTCAAATGAGC

CTACCACCTTGTTTTTTGGCCTTTGTATTTTTCTCTGTATTTCCCTATTTCACATACATATATCATAATTTCTTCTACAT

AAAATGGAAGCCAAAAACTATACTGTATTATTCTCGCCAAAGAAGACTGAATTGATCACTTACATACATCTAAATGGATT

CCATGCACTGGCCGAAGCTTGGTACTCCCACTCCCACACACAAACACATTATTGTTTGATAAGATGTAATCATTTCTTGA

GAACTAACAGTTCATACTCGGTTTGGTGTATAAGCCAATTAATTATAGCTCTGCACACACATTATATCT

>FM889330.1 FM889330 Jatropha curcas embryo 35-55 (DAF) Jatropha curcas cDNA clone rjcfea0_002964, mRNA sequence

AAACGTAAAACATACAGTTGATTCATGTAGTTGGTAAGGTGGGTTTAACAATTATCACACAGTACAACAAATTGATCCAC

TGAGTTGGTGAGGTGGGTTTAACAATTTCTCCTCCTAGTACAAATAATGTCTTTTGAATTCCTCAACAAAATCTCTAACC

TAACACAGTAAAATCAATCAGAACATTGATGGTCAAAGGAGAAAAAGAAAAGAAA

>JK611424.1 JCF4098 Jatropha curcas, immature Seed cDNA subtraction library Jatropha curcas cDNA, mRNA sequence

ACCTCCATACCCTTACTCTGAGCACTTTACCAATCTATAACTTTGATATCATCAAAAGAATGAAAAATAAAAAGAGCCCT

TTTTCCGGCGGCAAGACGAATATAAAATAATGTAGGATCTTTATGAGATCAAGCACGCACATATTCAAAAGGAGAAGATC

TTCCCTATATGGGGTTCTGAGATCAAGGAGAGATGCGAGAATTAGACGAGACTAACTTGATGGTCTTTATGCGAGCACAG

CATCCCTTCAGAGTATAGTAGTGTGTAGAACTGAGCATACCATACGCGTAATCAACTCGCATAAGGCTGTTCTCGGTTTT

AGTTCGAGCCCCTTCCGGTAAATCCAGTAAAGCTAGCTAATGAGGAAAGAGTATAGAGTTACCTCACCTGGAGGTGGAGT

CCCGCCGTTAGTTTCGAGACATCATTCTGGGTTACGGGTGAAACGGTCATACCGCACCTCCGGGAAAGCCCACCCCCAGC

GCGAGAGATGCATTAAACTTTAACTTCCGGCATAGACAGAGTGTGACTAAGACGAGGGATCTTTTTGTAAAAAACTTAAA

AAAAAAGGGGCCCACGCGCGAGATCTATATATCATATTTTTTGGAA

>GW879796.1 JC004293 Seed specific Normalized cDNA library from Jatropha curcas L. Jatropha curcas cDNA clone N07668 5' similar to Unknown protein, mRNA sequence

GGCCATTACGGCCTAGTTACGGGGGAAATACTCTTTTTAATCGTTGTTGAGCATGCATGTTTAAGTTTCAAGTTTTGGCG

CGAATGCATTTTTGTTTCGTGTTTATGTTTGTGTTTTGGAATAAATAGTCATAGCGACTTATATGTAATTAGTCGTTGTG

ATGTCGAAAAAAAAACAGGAAAAGAAAATTAGAAAAGAAATAAAAAAAAAAAAGAAAAAAGAAAAAAAAAGAAAGGAAGA

AAGAATAAATAAACAGCAGCAATAAAATAAATAAAAGGAAAGAAAAATAGAGGGAAAGGCTACAATGTTATGTTTTGTTT

TGTGAAGTTGCATTTGTTGCCATCTAAACATTAAAGTTAATAACGTGCATTGCTCTTTGAACTTAATGTGAAAGACTTAT

TTTCTTGTTACTCTCTAGCCTTACATTATCCTTTTCTTTTGTAACCATTACCCTAGCCTAACCCATATAAAAGACCTGAT

GATCCTTAGTTGAGACTTGTCCTACATTAGTGGAGAGGGAACCGCGAGGAGAGCTTATGGTTGTTCATTATCTTGTTTTC

TTGAAGCTACAAAAATGTATTTTATATGCTTTGATTATTGCTCGATAAAACATTTGTTGATTCAAGCTTCGTATGTGTGC

GTGAGTGTTTTC

>JK317426.1 JCST193 Jatropha curcas L. seed cDNA library Jatropha curcas cDNA 5', mRNA sequence

GGTCCTAGGGGGTNNNCAAGAGGAAATCCCGAGCCCCTCTGCGGGTTTGTTCATCCTGATCCTCCCGCTCCNNGAGGAGG

GGGGGGCCATACANGTGGGTTGTTCGAGGCTTGTAGATCTTTTCGGAAACCGGAGGCTACCGAGGGCCCCTGGATGGGAT

GCCGCTAGGAGTGAACGTTTGGGGTGCATCAGGATTTGATACCCTGGTGGCCACTTCGTCCCGATGTTTACTTGGAATTC

GCGCCCTTGCCCTGTGGCTTACCCTGGATAACGCCTTTAGTCTAGCGGCTGGGGAGTGCGGTCGGCGGGGTATTAGTTTT

TTCTTTAGTGGGTGCCCCCAACCCGCTGAAGCATGAATTATNACAAAGAACGTCTAAGAACCGCACAAATTTTTATTCCT

AATAAATTGTGGCAGGCCGTAGTTCGGCTGACACATTTTGACACACGGGGAAGGCAAACACGGTAGTGTCGGACNGGACG

GGGCATATAGCGAAAAGACGACTGTATTCATGCTTTTCATTTTTTAGCTGGAATAAAAAAAGTCCCTGATATATCCAAAC

GGTCTGAACGAAGGAAATNTNACCTGAGTAAAAGGTCAAGNGCAGCCTTAATAGGCAGAAAGCCCCACATAGCCACCATC

CAAAATACATGTATCCGTCTTCTGTAGTCTGGCTCTCATACCGAGCCCATCCCATAAATAGATGCGACATGATNCAGTAC

CGACATGTGACGAGCCATACAAACGAAAAGACATACGACGNCAGTGATCTNNTGTAATTAGCGTNNGTGCTCTGTCTTGT

CATACATTTGCGACNGNGCANCAGCTTGCGCATGCAGAANNCACNCCCACAAANGTNNTACCNAGTANNNNCACNCANAC

TAGAAAAGATNTANNACTAANNNNACGCGTNNTCATACANAGCA

>GO246606.1 JcrME_RL0150 Expressed sequence tags from Jatropha curcas root cDNA library Jatropha curcas cDNA, mRNA sequence

GGAAACTCCAAACTATTAATAACCATCATTTTTTTTAAAAAATCTAGTATATCACTATGAAACATTAGTTATAGTTTCTT

TTGAAATCCAGTAATAGTTTCATAGTAATAGCGTTATATTTTACTATTTTATCGAGCAGCAACAATTATTTTTAAATGCA

ATTAGTAGTAACATTCTTAATATTATTTTAGTTTCCAAATTAATTACTTAAATCAATAAGACTTTTTACATCAATGAAAA

AATAATTATTATGATGAATATTACTTCTTATATAATTAATTAATTAGCGGGGATATTACTGATTTATTATATCAAATTTA

T

>JK611614.1 JCF4411 Jatropha curcas, immature Seed cDNA subtraction library Jatropha curcas cDNA, mRNA sequence

ACATATACAATTTTTGCATAAACTAAAGCTGATGATTGCTACAATCGGAGGGAAAAAGACGAAACAAAAAGGAAAAAGGT

TGACCAAGTCCTAAAATGCCGAAACACAACACACTTCCTCGCACTACTAATTTAAGAGAATACAACCAACACAAGTATAT

TGGATTCTTTTTCCATCAGGCATGGCCTCAGAGCTCTCCAGTCAAGGTGGCCGTTCTTATTCTACAATGAAGCAGGCTGC

AAAAACGAGGTCTACCTGAAGCATTATACATACAAATCAAATACTTAATATTACCCCATCCCCTAGTACCTGCCCGGCGG

CCGCTCGAATCTAATGAATTGCAAGTACCGACTTGAATTCTTGGCGCGTTTACACCTCCTGACGGAAAACCCGGTGTCCC

ATTTATCCTTGAGACATCCCTTTCGCGGGGGTATCGAAAGGCCCCAAGCCTCTCAATTGCCCGTGGAGAGAGATATGCAT

ATTTTCCCCTTTTTAATTTTTGAGGCGACCTCTTATAATAGGGGGGGTGTGCCCCCTCATTTTAAAAACGACGCACTACC

ACTATTTTTTTTAAAAAGATACGACGTCACTGATAAAGGTTTGTAATACCCCACGTTAACGCATTCCCACAAATTTCAAA

GCGCCAGATATGCGTATGATCAGCTATATCTACTTTAC

>JK317598.1 JCST369 Jatropha curcas L. seed cDNA library Jatropha curcas cDNA 5', mRNA sequence

ATTTTTTTTTTTTTTTTTTTTTTTTACTAAGCAACTATATTACTTTAAATACATTAATACGTCATGAGTAAATATTTCGC

AGGAAATCGAACATTGTTATGCCAGATACCCGCAACAAAGAGATACCGAGATCTTTTGCAGGCAAGGCTTCAGGTTAAAC

TGGCGGAATGAACGAGGAGAGGGCGTATTTCTCCGTGGGGTGCATTGGGGGAGTTTCGAACAAGAGAAACCTGGCGGCCT

GAGACGCGGCAGACCACCCCGGCAGGGTCAGAGGCTGGGGTGGAACACCAGTCCATACCGGCGTATCTGTTTTTTCAAAA

GTCGCTGGTCCGAGTGTTGCATCGATTGCGTTTCACATCGAAAGCATAGATAATTCGGGTGTTATCGACTGTTGCAAACA

GCATACCGAAAACTATCGTCGTCCTC

>JK612777.1 JCF20-222 Jatropha curcas, immature Seed cDNA subtraction library Jatropha curcas cDNA, mRNA sequence

CCAGCATAGTTGTTCTTGGCTCTTGTATTCTGACCATCCTAATTTTCCACGAAAAAAAGGTTGGATAGTTTCTTTAATTT

GGGAAAGTTACAACTAAAGGGAGAGGATATGCTGAAACATATCCAGTTTTGTTACCTTAAAATCTGTAAATTTGTATGTC

TACCATTGTAGAGAATCAAGAGAGGTTAGTTGAGTTCATTGAATTTAGTTGCAGATAGGTAGTTCTTTCTTTTTTTGTTT

TTGGAGTGTTGCTGTCTCCTGTATAAGGTCCTTGCTCATGTATAGAAGGAACTCTGTATTCATGT

>GW612927.1 Jc1-022-C03-M13F.C03.ab1 Jatropha curcas flower and seed Jatropha curcas cDNA, mRNA sequence

TCCTTCTGATGCAGTCAATGAAAACCAAACAGAAGTGTTATTTTTTAGGAATTTTTTGTTTCTGTTAGAAGATTTAGAAA

GTCGTTTTTTCTTCCTATTTTAGAATTGGGTTGAAAAATTTTCTTCCTAGTTTGAGATACTTAATTTTAGTAGATAACAA

GTCTTCCACTAATGGTTGTCAATATTTTATTCGAAAGTCTTGGTGGTTACAGTGTCTCCTTTCCTCTTCTTTTCTTTCAT

TCAAATATAGTAGCACTGATAGGACCAGCAGTTGTTCACATCCTAAAAAAAAGAAATTAATTTTCCTCTTCTTGCACTTG

ATTCTGCTACACCTTCAAACAACCTTTGTTTTTTTTACTTGCTTAAATCATCCATAATTCTGGTCCTTAACCTTTTTGTT

CTTTAAATCTGAATCTAAATCATTCAGTTAAATTTTCTAAAGAATAGTATATTTTCTAAATAGAAAATATACAAGATGAT

AACTAAATAATTAAGAGAATCCTTAAATTTATATTTTCCTGATTGCAACGCTTCCTCTTCCTCTTCCATCTCTATCAAAG

GACACATATC

>GW877437.1 JC006961 Seed specific Normalized cDNA library from Jatropha curcas L. Jatropha curcas cDNA clone N11727 5' similar to Unknown protein, mRNA sequence

GGAACCTTATCTTCTCCGTCCCTTTCCAACAACAACAATAATCATGGAATAAACAAAATCAAAATTCATGAAAAAGAAAG

CAAAATCAGGTGGTGGGTGTAGGATTGAAGAGTTAACCAAAAGGAGTGCTAATCCACCGTGTCATTTCGCCGGTTTTGAT

CTTCATCCCCTGGTTTTTTAGGTCACTGTGTAATTCCTTGCATTCTTTTTCCCTTTAAGCATTCTCTCCTTTTCATCATT

TCTGGATCTTCTCTCGATTCTCTAACAATTAGCCAGCTCATCTTCCCTCTGTTCTTTGACATTATTTTTAATCTTCACAC

CATTTTATCTCTTATTATCAGTTTCCCCGTTCTGTCACTTTCATTACACTTTTGATTCTTACAGTATAATCTTATTCTTC

TGTCTGTTTCATTTTTTCCCCATCAATTAACTTAATGGATCTGGTTTTGCTTTTCAAATTCGTGGACTTTTTTTGTGATT

AGATCTAATTGTACGATTCTTTGATGACAGTTTATTCGTTTGTGTACTCGAATTTATTGGTTTCTTTTAAATTTCTTGAA

TTTAATTTCATTTTGTCTTCAAAATAGTCATGTACTTTGTACATTAACTCCTGGGTTTCTTTGAAATTAATTCAGTTCGA

TCCTTTTTTGGTGTTTTGAGTTTCTGGGTGTCACAAAAAAA

>FM892094.1 FM892094 Jatropha curcas embryo 56-70 (DAF) Jatropha curcas cDNA clone rjcaeb0_002571, mRNA sequence

CATTCCGACCAAGGGGTTTCGTTTTTGGCTATGGACTGCATTTGCTGGAAAACTACCCNCTTGGAGAATGTGAAACATAA

TAAGAGCTTATTCATGAGGTTTCAAGGAGTTAATGCCGGAGCGAGATGATCTCACCGGATGGTGCAGACGAGATTGCGAA

CCATTGAAGATGTATTTCCAACACCAGTTGGACAAATTGATTAATCTAGCAATGCTTACTGAAAGATCTCCATTCTATTT

TAAAAATGAGTTTGCCTGTATTTTAAAATGTAATTCTCATTATAGCTTATGTATTTCTCCATTTTGTTTTGAAGTTACTA

CTATTTATTTCAATTATATCTATCTAACTAGGATTTATTCTTGTGGTAACTTAACAACAATGGAATTCATAATAAAAACG

AAAATAACTTATAGTTTCATAAAAC

>JK611567.1 JCF4352 Jatropha curcas, immature Seed cDNA subtraction library Jatropha curcas cDNA, mRNA sequence

ACTACATTTGCAGTGGATAATTAACTTCGGGAATCTGAAGTCAATCATTTTAAAGGCTTGAAATTTGCTAGAGTTGTCAC

AAATCCATTTAAATGAGCATGCAATCTTCTCTTACTATCTGGCTGCCATGACCTTACCACATTTTTTGATCCTCTGTCAA

TTATTAATATAGAATATTCTCATGATCTACGCTGATATTTGAGCCTCTTTTATCTCTTGTTGGTGCACTAAAATTTCTTA

CCTTCATGTTCCCAGAAAGTGCATATCCAGTTGTGGGAGAGCATTGTGCAGGCATGTTTCTGGTCTTTTGGGCTGTTTGC

TGCGTCTACACATTATATGATAAGCTCAAGCATGTAAAAGTCAAAAGGTAAGGGGCCCTTGAAATCTGTAAGAATTGTTG

TATATTACCTGAGGTTTCATGTGCCATCTTTAGATCACATTGTGGAAACGCGGGGACCAGAGAGTCGCGCGCCCCAATAA

GTCGGACACACTCTGCTTAACGGGAACGTTATTTTCTTTCTTCTTGAAACACCTTCGGGAGAGTATATTTTTTCCACAAC

GAGAACTCACAGACACGCGGCGTGACTTCTTCCGTGGCTTACTGAGTAGTGCGGTT

>FM888476.1 FM888476 Jatropha curcas embryo 35-55 (DAF) Jatropha curcas cDNA clone rjcfea0_001901, mRNA sequence

AATATTGTATTTTTCTATGATTTGTTATGCTGAGTTGAATTGTTAATGAATTGCTAATATTGATGCAAGCAGTGAATCTG

AAGATATTGAAATTGAGCAAAATTTAGGAGATCTTAGAGATTATTACGATTAAGAATGGATATTTTATTTTATCATGTTG

AACAATGTTTAGGCTTATTTTATGGATGTTAAATAATATTGATGAGTTTGATTATATTTTTAGTACTTGTAGATTGGGAT

TCGAATTGTTGAGTTTGATTGGATGATATCGAATTGTAGTATTCCTTTGAATTATTTAGTTTAATTGAACGGTGAACAAT

ATTATATAATTGTAGTGTAATATTTGAACAGTTGAGTCTGATTGGATACTGAACAATTTTACCCTTTTTATATTATTTTT

TCATTATTAATAAAATTTATATTTTTAATTATATTTAGAAATATTATATATACCAAATTCGATTGAACCGTATTGAACC

>GT973976.1 GJCCJC2055E05.b Jatropha curcas L. developing seeds (mixed stages) Jatropha curcas cDNA clone GJCCJC2055E05, mRNA sequence

GAGGATTCTAGGTCAGATTTGTTGGCTGCAATGTATTCCAATTGTCATTGTTAACATGTTATATCCAGTTGTATAATGCA

GTCTACAATAACTTTTTACATTTGTGTCCATTTTACATGCCTTTGCTTCTGTGTTCGGCAATTACCTTTATAGAACTTAG

CTGACGCTTGTAAATGTGATTTTGATTTTAGGAAATGTAATGAAGATGGGGAAAGCTTGTAGATGTAAGTTATTAATGAT

CCTGGGGAGAAGGCTGTAAAGCTCTAGAGATGTGACCTGGCACTTGCCTTCTTCTTGAGTTGGACACTTGGCCATTCACC

ATATGCAACGCTAGTGCAGGATTGGTGCAAACGGTCTTCAACGTCCTTATCATGCCATGAGATTTTTTATACCTATAAAT

TGTTGAGTGCGCCTACGAGGGTGAGGATTCAATGAATTAAAAAACCAATATTGTATGAATTATTTTTCTTGGTTTCAAAG

AGTTGTTTCCAGT

>GW876377.1 JC005940 Seed specific Normalized cDNA library from Jatropha curcas L. Jatropha curcas cDNA clone N10096 5' similar to Toprim domain protein, mRNA sequence

ATCCGTCTTCTTTTTCTTAAAAAGTTAAATCTGGACCGCGAACCAAGCTTCCCTCTCTGGTTTCTTGCCGCTCTGCACGT

TCCCGCTGTATTTTTAACAGAAATTAAACCCTAACCCTAATTAGATTTTCTTCGCTTTTTCTCAATTTATCGAATTCATT

CAATTTCGGAACTTGTTTTTGTTCGTCAATCTTAGATCTACAAGATACTCCATTAAAGCCCATTTCGTGCATTGATTGAT

TGATAATTGGAACTTCTGTTTGGTTTCAATCTAGAGGCTCTCTCTTGATTCTGTTTCTGAGATTTTGAATTGATTTTGCA

GGCTATATAGAGAGGTAGGCGTTGATAAATTTTCACGTGTAGAAGGCTTTGGTTCTTTGAATTGAATTGATTGAGCGGTT

TAGCGGTTGTTGACAGTTTGAGAAGTTCTTGGATCGATTTTAATGCTACTTATGTGAATATTTTGCTTCTTTCTGGTTGC

TAATTAATAATTCTATCTAATGTTAACTATCTGGAGAGGACTAATTGAGTTCAATCGCCATTAAAATTTAGGGTTTG

>GT969760.1 GJCCJC2002A05.b Jatropha curcas L. developing seeds (mixed stages) Jatropha curcas cDNA clone GJCCJC2002A05, mRNA sequence

CCGGTTCCTTGTTTTTTATAAATGAAACCAATGGCTGTTGTTCGAAACTATAAAAGAAAAGAATAAAAATTATTTAAATT

AAAAACTATCAATGAAACTTTCAATTTATTTTCTATTTTGTTATAATTTTTTTAAATAAATGTTAAGATTTTTTACTAAA

ATCATTTAAAATTTTCATTACTATTATTAGCATCACCATTATTATTTATTTATTTATTTATTCATGATACAAAACTAAAA

GAATCAAAAATTTTAAGAAAAAATTGAAAAAAGTTATTTAACTTAATCATTAAATTGAAATCGAACTAAAATCAAACGGC

TCTAAAACTGCCACATATTAAAAATTAAAAAATAACAATTAAAAAAATAATATTCATTTATTTATTCATTTAGTAGTGAC

AGAGAAACAGTATTATTGATAAGAGAAATAACAATCACGTACTTGTATAACAATAATAGAAAATTCAAACATTACAAACA

ATTTTTGTTCTCTTAATTAACATCAACCATTAATTCAAATATTTTTGAGATTTCGATGAATATAAGTTTGGCAATATTAA

AAATGAAAAATATATTAATAATTAATACGCTTTAATTTGTAAAATTCATCTATTCAATGTACGAACTCGTAAGATCACAA

CTATTAAATAAAAATTCTATTTATTCAATTTGCTCGTATGGCCCATAAACAAAAGTATTAAAATT

>GW611103.1 Jc1-001-H12-M13F.H12.ab1 Jatropha curcas flower and seed Jatropha curcas cDNA, mRNA sequence

GAAAGGCTAATTGGAATTCTGAAATTGTCGGGGCTTAGTTGCTTGTGGTCTCGGTTGGGTTATTTGCTTCACTAGATTTA

CTCTGCATGGTTTTACAATTATCTGCAATATTTATTCTTTTGTGCACTATTCCTTTGAACATGAAATTAGTTAGTTATTT

CATTGGTTAAATAACTCAACTCATGTTATGTAATTTATTAATCTTTTCCCTGCTTGTGTCTTGTAACACCTATAAATTGC

ATTGGAATATTTGCTTTGATAAAAATATCAGGAGATAAATGCATTTTAAG

>GW878387.1 JC002765 Seed specific Normalized cDNA library from Jatropha curcas L. Jatropha curcas cDNA clone N05216 5' similar to Unknown protein, mRNA sequence

GGTAGGAGCCGGAGCAAATCTCCCAAAACTAAGTCTACGCGTCGCTTGCCTGCGAGGTCAAGATCAAGGTCTGCTTCTCG

TTCTCGTTCTGGATCAAAGCCACATTCTCTGTCAAGGTATTCTTTCATGTCTATTTAATTTCCACATTTGTATGAAGAAA

TTACATTTTAATATTGATATCAGTGATATGTTTCCTATTCCTTTTATGTATATGTATGCCATCTTCTTGTATCCCGGAAT

TGCCAACTGTAAATAAAAAAAAATATTTTATAATACACTGAACAACTTATATAAAAGATTATCTCGTAAAATCAATTATA

TTATATGCACACTAAAAGCATATTATTGGAGCTGGGAAAACTAATTACGAGTTGGAGATTACCTGTCTGGTTTTGTCAGC

AATAAGCAGGCCGTTGCTGATATGTGAGAAAGTCATGTTATAAATTGCTTAGAGTTAGTTTACCAGATGGTTTGAATTGG

AAGACAAACACTTCTGCTACTGGTCTAACATTGATATCTTCTGATCCTCTTTCTGTTATCAATTGATTTTGTTAGTCATA

AAAGTTTATTCACCTCTACTGTAACTGGAATACACCCAGTGCTTGCTGGCAGTAATGCTATCAAGTTAATTTACATTATG

ATATTTATAGAGGTAATGTATTAATAATTTGATTTTTGAATTATGTTGGAGC

>JK610784.1 JCF2299 Jatropha curcas, immature Seed cDNA subtraction library Jatropha curcas cDNA similar to 40S ribosomal protein, mRNA sequence

CTTCGAGTGGTCAACGGGACCTTGATCAAGTGGCCGGACGGATTGTGGTTGCCCCTTGATAAACTTAAAGTTGGGTATGC

TGCAGAATACCAATTTATTTTGGTAGTTTTAGCTTTCTGTTTTAGTCTATTGGAGAGATTTTACTGCTTGATGCTTAAAC

CATATTACATGTTGTGAAAAGTTTTGGGTTCAATTATTACTGTCTTTGAGATGAACATGGTTCCTCCTGCTGTTGCTTTT

TATTTATAAAATTTATGGTTTAACTCGTTATGTTCAAAAA

>FM893880.1 FM893880 Jatropha curcas embryo 56-70 (DAF) Jatropha curcas cDNA clone rjcaeb0_004817, mRNA sequence

ATTCCGATAACATAAAAACAACTCAATTTTAATCTGCAAACCCTCTTCCCACACAAACACTCACCTTCCCATTATGTGAC

TCTCTTGGCTCTCTCCGGCCACCCCTTTAGGGTGGCTGGAGGCCGGCTGCTTGGCTTGGTGTTTGTGAAGTTATTTGTGT

GTGTCTGTGTGTGTGCATGTACGGTCTGTCCCAGTAATTTTCTGTGATTCTTCGTAGAAGTATTGTAGAAAATTGTAATG

GGAGGGTGTGGTTGGACCTGAATCGCCTTATTTAAGGGCGGTTTGTCTTCTTATAGGGCAACTTCGATCCTTTGGCATTT

TCATTTCCTTTTGAAATCTCTCGTTGCTAGCTTTATATGCAAGAGTGAAGCTTGGAGGAAACTTTCACAAGGGCGAGTCA

GATTATCAATTGTCTGTCATTTATATATGTACGTTTGTTTTCTTTTTTCTTATGAATCTAATTATGGTATATGCATGTTT

AGCTGGGAAGTATATACATATTGTATGGCCAAGTGAACAGTTTGGTAAATAAATTATATATGTAGTA

>FM887625.1 FM887625 Jatropha curcas embryo 35-55 (DAF) Jatropha curcas cDNA clone rjcfea0_000859, mRNA sequence

GGCTGCAGGCAACTTCAGTGAGTGTAGCCAGAACTTTAGTAGCAATAAGTAATTTTTTAATAAAAAAAGTACAGATTGTC

AACCAATCAGAATGGCAGCAGTATTTACGTAGCAGCCATTCGTAATCTCTTGCCAAGAGTATGATCTCGTGCCAAGCTGT

GGTCTAATCTCTCTGTCCTTCCGTCCCCCCAATTAGCTAGTTGTCCCGTCCGTCGTCCAATCCCTCACTTCGTTTGTGAT

AGTCCATTAGTAGTCCCTACTGAGCAATCTAGCTGCAATCGAGCTACCATTGAAATGGAAAGAGTAGAGTGAAAGTAGGA

CGAGGTCTGCCTTTACCCTTCCTGTTAGTAGGAGTGGATTAGTTAGAGTCGGTCCGTTCTCTTGTTTCAGAAGCTAGGGC

TAGGGTCAAGCAAGCCTGCCTCCATCCGTTTAATTCCATAGCCTATCGAGCCAAGCCAGATATGCAGCCAGTGACGATAA

CTAAGTAGTTAGTAGTTGTAGTACTTAACTGGGACGATTCACTAGATCCATAAGCCCCGTAACGGAAAAAGCCTTTCTTT

TCCCAAACCTCGTTTCGATCCCGAACTGCATTC

>JK317548.1 JCST317 Jatropha curcas L. seed cDNA library Jatropha curcas cDNA 5', mRNA sequence

CTAGCATTGTTCATGGTCTACCACTTGCAGTACTGCTAGGGTGCTTATTCTTTGGCAGCATAGAACTTGAACTGCAAAAT

AAATGTTCTCGAAATTTACATTGGGTTGTTGGTGTTGCTTCAGTTTTGTTTGCTTATAATAGCCTGCGTTATCTTTGTTT

ATTTTTTTTAATTGTTATCTCAAAGGAATTAGAATGTTCACGTGTTGCTTGAGAGTGGTATGTTTAGAGCTTTTCGTCAT

TTCCCTCCTATTCATTTCTTGGAGTTATGTTCACGTATTTCAATACAATTCTTTTGGTTTCTGTTAACTTCTGGAAACAT

TTACAAATATATAGGCTACATAGTTGAAAACCCCATACCATTAAAGCATGGCCACTTTTGGTAGATATTCTCATAATAAC

GCCACTTTCTGTTTTCCTCCTATGACTATTTAACTAATTTTCCCCAAA

>FM895706.1 FM895706 Jatropha curcas embryo 71-95 (DAF) Jatropha curcas cDNA clone rjcpga0_002674, mRNA sequence

ATTCCTTTCTCTTCAGTATATTTTCTGAACTTGGGTTCACTGTTTTGGAATATGATTAAGAACCCATGGGTATGTAACTT

ATTCCATTAATTAAGTTGTGGCAC

>FM887720.1 FM887720 Jatropha curcas embryo 35-55 (DAF) Jatropha curcas cDNA clone rjcfea0_000972, mRNA sequence

CTGAATTTTGGTTACACTTTAGAAATCGATAAAATTTCCGTTGCTGATACGTTATTAAGAATCTGAGGCCTCAGCAACGG

AAGAAAAAATCTATTTGTGTTATAACTTAGAAACGGAAAAAAATTCCGTTTCGAAGACCTTTATAGAACCCTTCGTCGAC

CCTTAGTAATGGAAAAGTAATGAAACAAGATTTGCTAATTAATAAAAATAATATGATTAAGAAAACTACTATAATCAATC

TAATTAAAATTAAAATTTTCTCTCTTTCAAAAAAAAAAAAAAAAATTTCCTAATTAAATGGTCCATTAAGAAAATTACTA

TAATCAATCTAATTAAAATTAAGATTTTCCTAATTACATGGTACTATCTCTTTTAATATATTTCATGATAATACTATTCA

GGAGGGAAAACTTATAAGCTTATAATTATTCATGTATACTGAACAA

>FM887903.1 FM887903 Jatropha curcas embryo 35-55 (DAF) Jatropha curcas cDNA clone rjcfea0_001187, mRNA sequence

ACTAGTAGATCCCGGGCTGCAGGAAAACTACTATACAAAAACCTAAAAAGAGAAGGGATCAACTTCCTTAGGATGCAACT

TATTCATATTTACTTTAACTGTATTCTACTTAATAATTTCTAACCCAGTTGGACAACATACGCTTTGTGCATGTATGATT

AAAATAAAGAAAGGAAGAAAGAAAACTTTGATTCCTGAGATATATGGATATAGCTGGGAGGAGGAAAAATATGAAACATC

TCTTTTAGAACTCTTCCATCCACCTAATAATTTTAAAGGATTTCTTTTTCCCCCTCCTTCCTAATATTTTTCAACAAGAA

AGTGAATCCTACGTCAAAAGGTCACTTGATCGTCTTCTTTGGGTTACAGCTACCGTGCATACATTATTAATAATAATAAA

CTGAATCTAATTCACTACTTAATTTAAAAAACTATTCCATCAATCTGATTTTCCCAAACCCTATTCACATTTTTAATTAT

AAGTCTTTTAATCAGCTATTGCATTCAATACTTTCAGATGAATAGCAAGTTTTAAAAAAAAAAAGGTTTTATTCTCTAAA

ATTTGAACTCAATTTTTCAAGCAAAAGGTTAAAGATTGTGTGTGGTA

>FM889904.1 FM889904 Jatropha curcas embryo 35-55 (DAF) Jatropha curcas cDNA clone rjcfea0_003657, mRNA sequence

CAAGCTAACAGGGTCGGTTCAAACCGGAACTGTATCATAAAGGGGATTGAAATCAACTTAAAAATGGCTCAGATTTGAAT

TAGTTTCAATTTCAATATTTGAAAGTACAATCACTTGGTTTTGAATCAAAATCAAAATTGAACCAACTAGTTTGAAACTG

ATATCAGTTTTATTTCTAAAATTTTCGAACAATTAATTTATGAATGGGTCGATCATGCTTTTTTTTTTTTTAAAGATAAA

GAATAATTTCATTTCATATCATAGAAAGAAACTAAATAGCTTGAAATTATATTCCAAACTTGAAGACAATCATATTTATA

GGAAGCTCGTATTAATGAATGAGTTCGAGTTAAATTGCCTTCTGATTCATCTAATGGGTCTTTTAGGATTAAAACTCAAT

TCAACTCTTTTCTTTTNTATTTTTAGCCCAATTACCATTTTTAAATCATCAGATTCAACTCTCAAATCAAAAAATTAAAA

GCTTTATTCTAGATAATATTTAGGCGAAATTTTTATAATATCAAAAAATAACTATACCAAACTCTTAATATTTTC

>GT228726.1 JC291 Jatropha seeds from fruits at three stages of maturation Jatropha curcas cDNA clone PL06SE.B03.scf 5', mRNA sequence

GCAGTCTACGGGCCCGGGATCCGATTAGCAGTGGTATCAACGCAGAGTGGCCATTACGGCCGGGAGATCTGCTTTGATTA

TTACTCCAAACCAAACTAAACCGATCCCCAACAGCTCTACCTAGAACTCAAATTCTCTCGCCTCCCTCAGCCCAGCGCCA

CTCTCCGCCGCCACAAACCACCACTCCCTTGCCGCCAACCATAATCTCCTATCACCAAACCACCACGGAAGCTAAAACCC

CACACCGAAACCCGATCTAACCATCGTCAACCTTCACCTATATCTCTCTCTCCTTCATTCTTTGTCGCCTCTTTTCTCAA

AATTCCTAAAACGAAACCTCCACTCCTCTATCATCTATTCATCCTGCAGCCACCACCAACCTCCACTTCCATAAGAGTCA

CCTCTCACCTCCTTTAATAGAATACCGACTCTCAACAAGCTTAAATCCAGTAGAAACTTGAATTTTGGCTAAGAAGGTTC

AATTCCCAGACCTCTTTTCATTTTTTTTCCTAATTCTTTCCTTAAAATTTTCAATTATAGTTCATTCTTTCAACTATG

>GW881439.1 JC001542 Seed specific Normalized cDNA library from Jatropha curcas L. Jatropha curcas cDNA clone N02875 5' similar to Unknown protein, mRNA sequence

AAAAAAATGGTGCGTATCTCGTTTCTTTATACCATTAAAGTGAAAAGAAACAAACAAAAGCAGAGCTAAAGTAGTCGTTT

TTACGAAAATCCAATTTCAAATTTGCAGAGAAACAGAGCAAAGCAAGAGAAAGGGATCTGACTTCAAAATTCTCAACTTG

TCTCTATCTCCGCCTCTGCTGCCTCTGAGTATCAGCAGAATCCCACTATCTTTGATTCCTTTTCTCTTTATCAACAATAA

AAATTCACATTCCTTTCCTGTTCTGTTTTGTCTTCAAAGACTATACGGTCTAATTATGGGCTTTGTTGATAGTATTCTCT

TTCATTTGTCAACATTACGATTTTCTTTGCGTAGAATTCAATTCCCCTTTTTTATTTTTTTGAATAATTAGTCATTTCAA

ATTATTCATCATTACCTCGTATTCATTACCTTTTTGATTACTTACGAAGCTTTGTTTCCCGCCTATTAATCTTTTTTTTT

TTCTTTTTCTTTTGTAATGCGCAAGTCATGGAATTTTCATTCTCTGCTTTGAAGGCCCTTCCCTTTGTTGGTTATAGCTT

TTGGTCATTGGTTATTGCAA

>FM894530.1 FM894530 Jatropha curcas embryo 71-95 (DAF) Jatropha curcas cDNA clone rjcpga0_000313, mRNA sequence

TTTTTTTTTTTTGACAAATTTCTAGAGAGATCTTAAAATTAAATAGGAATTCGAGCTAGTTTACATAAACTCAGTCTAGT

AATGTAATGAACTGCAAAAATTTTGATGTATGAATAAATACAGTTACAAGCAAATTATACAATGGATCAGAGTCGAGCAC

GAATCGCTCGGGTAATTTCCTATACCATCTTATTACAATGGTGCCTAGGTGATTCAGAAAACTGGCACATGAAATACTAC

TATACACTAAATAATTTATCTCATTTTGAGGAAAATTAATCCTCTATTATTTGAACAGCCTAAAATGAGAAGTTTCTAGC

TGATTGATGTCCACTTAAGCTGGCATTCTGTGACTGAATAAAAATTTAGTTCTATTTGCATAAATTAGAAGGTTTTGGTA

ATTATATGAAGTCAGGTTGAGCCTGTTGAAGTCAAGCAACGATGCCACATGCGGCTGTTAGGATCTACTGC

>GW876930.1 JC006178 Seed specific Normalized cDNA library from Jatropha curcas L. Jatropha curcas cDNA clone N10580 5' similar to Unknown protein, mRNA sequence

GTTGGAATTGCACTGGCATTTGTGTGAGAAGTATTGTAGTAGCTGAATGGATTGTTGTGTGTGAATTGTGAAATTTCTAG

CAGTAGGGTGTTTACTTAGAAAATTTGTATCACAATTAAATGGTTAAGTAATTTCTTCTTATTCAGTTTTACTTATCAGA

CCATGATATATGATCTGTGACTTTCTACAATCTAAAAATACATTCATATGGCTTTATGCTGCATATTCTTTTTTCCACTT

ATTTTGTAGCCTCCCTGAGGGCCATGCACCATGTAGTTCGAAAAATCCCCTATGGAGATACAGATTGCTTTGAGTTATTT

GATGGATTTAATGCTCACAATAAGTTATTTATTCATATTGCAGCCGACCCCAACTATTTTTTATTAAGGCTCAGTTGTTG

TTGTTGTATCACCCCATCAGTTAACAAGGAGCTGTCAATTTTCCCATCTGTTAAATCTAGCAATTTATGTATCATACTTG

TAATTTGAGCAGATGGACAATTACAATTTTTAGAAGATCATGTCATCTGGCTATCTTAGCTGTTCTGGCCCTTTGACCTT

TGAGAGTG

>FM889252.1 FM889252 Jatropha curcas embryo 35-55 (DAF) Jatropha curcas cDNA clone rjcfea0_002861, mRNA sequence

AAGACGACCCTTCTGTTGAGGGTGAAAGAGAAGCTGAAAGCAAAGGGAAAACCCTGGAGGAGGATGAGCTTACCTAAGTT

CGAATTTTACATCTTTTGTTCTTTTGGTTTTTCTGCAATGATTGCATTGTATTATCTGTCAGTTTACAATCACTCTAATT

TACACTGTCTCTGTTTTAAATGTTTTCTTGGCTTGGATTTTAATCAGAGAGTTGGTTGCTTAAAAAAAAAAA

>FM895088.1 FM895088 Jatropha curcas embryo 71-95 (DAF) Jatropha curcas cDNA clone rjcpga0_001654, mRNA sequence

GTAGAAACTATCCCCTTTCTATTAGGTAAATCTCCATACTGATGATTCGTAACACCAGAAGCATAGTAAAACAGTATGCA

GTGTTCTGGATTGATTTTATGTCTTTCTCCTACTCATATAACAATAATGAAAAGAAAAAGAAATTAAAAGAAGCAAAGAT

TAAAATTAGTTCTTAAGATTCCCATGCCTATAAAGATTTAAGCAGTATGCCTATAAGATCCATTTTTTACCTATTTGAAC

AACACAACTTCATAGGCCTACAGATAAATGGTGAGAGAGTAATCCCCAGCAAAATCCTCAACTGAAACTTGGTAAATACT

GCAGAAGTTGAAGTGCCTAATGGAAAAAAAAAAATATGACAAGATTTAAGAACATTCCTGGAAGTTACCAGATATACTTC

CGAATGGCTTTGACTTATTCCATTACAACTTTGGCCCCGACTGCTTTCATCTTCTCAATTATTTTCTCAGCTTCTTCCTT

TGACACTCCTTGCTTTCAG

>JK610927.1 JCF3203 Jatropha curcas, immature Seed cDNA subtraction library Jatropha curcas cDNA, mRNA sequence

GATCTTCAGTTTTGAATGTTTGGGGTCCCAGCTAACTACATGTCTCTGCTTCTGTTTGATTGAACTCTCCGGCTGATATG

CCTCTCTCACTCTGTAGTGGATTTCTTTTATTTAAGTTTAAAGGATAGAGAGCTGCTTCAAACGCTTCTGGCAGCATTGT

CCAGTGCATGCAAGCCTTTAGCTGCAGACATTTCTTGATGGCCTCTTGAGTTTCTTATCTTTATCACCGACAAGTTGACA

GTGATTTTCTACCTCTGGCAACTTTGACCCTTGGTGAAAGGGTTTGGCTCTGTTTTTTAGAATGAGCCGGAAGATTTGAC

CAGCAGAAATTCTACTGCCTTCTCTTATTTGCCGTATCTCTACCGAGGAAGATTGTATGCATTCATCATGGGCTGAGGTT

TATTTGTAGTGAATCAGGTGATTAAATACTTCTGGTTCCACAACTTGAAAGCTAATATCTGCAAGTTGTCTGCAAATTCA

GATTAGTTTTTTGGAGTGGCTCATTTGGACAGCTCACCCTCCTAATTCTGGTGCCTTATCTTCTGTTTAAATTCTTAGAA

ATTATGTCATCTCAGAAGTTTTATTTGGTCTGAAGTATGAAATATCCCATCAGATTATGTTAATAACATTTTATTTGCTT

TTATCATCTGTCTACTAGAAAATTCATTTGATTTTTGGAA

>GT971250.1 GJCCJC2018E08.b Jatropha curcas L. developing seeds (mixed stages) Jatropha curcas cDNA clone GJCCJC2018E08, mRNA sequence

CTCTACTGAAAGCACTTTTCTTTATGAGTCTGATGCGGTGTTAGAAATATTAGACATCTTATCTTTTTATTAAAATAAAT

TTAGTACGATATATAAAAAACTTAGTTATGATACTGTATTAAAATTAAATGAGAATCTAAGAAAAAAAAACTTCACATTT

TATTTTGTATTATAGAAAAATGGTGCCAGCAACTAATCCCTTTAGAAGATTGAATGTATAAGTGGAGACATTTCAAAGAG

ATCTGTTATACCAAATCATTGATTAATCAATTAATTGCCTAACCACTTCTTTCTTCTTATAGTAAATAATATTTTTTTTG

GCAGTTCATCAAACAGACACCTAACCTATTTCTTTCATTTGCATATCATTCTCATACTTCTTAATCTTACAATTTCAATA

TTTTCAAGAGAAGAGAGGAAAATAAAACAGTAATTTCTAATTTAAGTTATAGAGTGAACAAATTTGCAATCAACAAACAT

CCTGTGTCTGCATTAAATATATTCATCACGTTGGCCCATCGATTTGGTTATTATAAGACTAATGACCAAACTTTTTTTAT

TATTTATAATTTTCTTTAATCGAGTATTTCAAAACACTCGATTAAAGAGTGAATTATAACGCTCGATTTAAATACTGTAG

GATATTTCTATATTTATCTCCAATGTATTTATTTATTTATCGAGAAAAACATCATTTTTCTCTACACATGGCTAACGCTT

TTTAATATTTGATATAATTTATAAAATTTGAATATAGCTATTTAATTTTTCATATTTACATAAGATAC

>JK317363.1 JCST127 Jatropha curcas L. seed cDNA library Jatropha curcas cDNA 5', mRNA sequence

AACTTTATTGAATTATTACATGTGTAGACACAAGCAGCAAATTCTAAACAACACTAACTGTCAAAACGAATTATACACTG

TCTAAGAAAATAGTGTAAATAACCTGCAATAGTAATCCATGATCTCAGGAATTAAAAATACGGGGTGAATTACTGCGGAG

GACTGTTAGGAGGCGTTTCTATGAGGTCGGTCGAGGGGCGGGGGTGTAGCCTAAACAAATATGTGCGCTAGGTTCCACCC

GAGGAGGTACGCTCGAGGTGGGGGGTTTTTACTTCGCCCTTCCTCCTTATAGCACCACATCACTCCTCGCTGCCTTCGTA

ATAAAAGGTAGGTGTGAGTACGTTTACGGTGTATGGCAGCTTTCCTTAGCTAGGTCCATCGGGCTTCAGATTGTACGTTG

ATCGCGCCCGTTTATACAAGATTTTGTTTTTGATGTTAACAGAACGCGGGTGGTTCATAGAAATCCGAGTAAGTCCCAAT

GACTCACTGTACAGACCCGATATAATTACTTTATTGGGTCACGACTTCTTCAGTTTCTTGTTATGCGCTGTTCAGCTATC

TGGTTCATGTCCATAGTTTGACGATTACAATGCCAGAGATGTAAAAGAAAACAACGCAAAAGCCACCAAATCCGGCAATG

>GT973911.1 GJCCJC2054F01.b Jatropha curcas L. developing seeds (mixed stages) Jatropha curcas cDNA clone GJCCJC2054F01, mRNA sequence

AGGAATAACCATCGGGTATGTACTCTCAAGTGTAATATTGCAATAACTTTTAATTGTCTCTCCAAAGAGGTATTTCATTG

CTCCTCTTTAATTACTGTGATTATCTTTTCTCAACATTTTGTAATTTCATTATTGAGAAGAGAGATGACTAGAAATTTTG

TAAATTTTATTAGTGAGAAGAGAGATTAGAATTTAATTCAGGAGGAGAGAAACAAAAGAATAATGACATCATTCTCACTT

GTAAATGGTAATAATAATGTGAGTAATTGAGAAATGAATGCCCGTGTGTGAATTTATTGTATCCACAACACTAAAATGTA

TGTTTATGAGCAAGAGAGTGCACATACCCTTGATCCATTAATTTGGTTTGATTAAAAATTTGATTCAAATCCAACGAGTA

CTCAACCAGACAACCACCCAAAAATTGAAGTCCAAACATGCTACAACCCGACCCGATATTTTTTGCTGGCCGGCTTCAAC

GGCATTTTCGGCCGTTTTGTTGGGATTTTGGCGC

>FM887493.1 FM887493 Jatropha curcas embryo 35-55 (DAF) Jatropha curcas cDNA clone rjcfea0_000708, mRNA sequence

GTAATCACTATAATTAATGGGCTATTGGATTGCTGGGCTCACTCTTTAATGGAAAAATCTTATATTACATATATAAACAA

TTAATATTTATATTGCTTTCAAAAAAAAAAAAACAATTAATATTTATATTTAAAAATTTCAATCCAAACTAAATTAAATT

AAAAAAATTCATAATCACATACATAATATTACATGTGTGTGTATATATATATATATTTTATTGGTTGGATTGGGTCTGCA

AGTTTATCTAATGAACCCGTGACTTAACCCAATCCGTGAATCATAAAGATAATCTGCCCAACCCAACCCATTATATTTTT

AAACCCGTCGGGTTGGGTTGGATCGAGTTACGACGGGTCATGTGCAGCCCTACTTGTATCCGTGAAATTGCCTAAACTAA

ACCTAAAGTGTATGATTGAATCATTTAATCACTATAATTAATAGGCTCACTCTTTAATGGAAAAATCTTATCTTACATAT

ATAAACAATTTAATATTTATATTTA

>FM890636.1 FM890636 Jatropha curcas embryo 56-70 (DAF) Jatropha curcas cDNA clone rjcaeb0_000541, mRNA sequence

ATTCAAACAGGTATATTTATGATCATTTATTTGTTGCCCACACATTTGAATGTTTGTTGTATTAACTGCCGTATCAATAC

AAAACCTGAAAGGTAAGATAAATATCTAAACATAGGGTTACAGCTCTGGTAACTGAATTGCGAAACTTTCTGAACTAATG

TAAATGTTTGTTTCACTAATTTTTTGGACTGAACTGGATAACAAAATTAACTGACATTTTTTCAGTTAACCGGTGGTTTC

AGTTCAGTCTGCCAGTTTATTGTCGTCATTTTTCAGCCAAATGACACCTTTTGAATTTTCTGTTCACCTCTTACTGTGTT

AAAGCATGTCAGAGTCAAGCACAGCTAGCACTCAAATCATTCAGAGACAAAATTAAACCACAAATGTCCAACTAGGCTAT

AAATGAATGGAGGAGGTGAATGCATCTGAGGAAAAAAAAGGGGGGGCCCGGTAC

>JK610476.1 JCF1349 Jatropha curcas, immature Seed cDNA subtraction library Jatropha curcas cDNA similar to function, mRNA sequence

ACTGGAATATGCTTTTAAGGAGCGCATTCACGAGCACTAGCTTTCCTGGCTTCCTTTCAAGCTTAGAATATCACGTCTTC

CTTAGAAAGCTGTTAGCCTAGGTCGGATCCTAGAATTACTCGTAGCTCGTTACTCGGATTAGCTCGGGGTTTTTTTTCCC

TTCATTTCATTCTTTCTTTCCGGTTTAGCTAAAGCTAAGGTCGAAATCAATCCCTCTTTTTCCTGTGCTTTACTAGTAGG

GCTAATGAACGACCCTTTGATCTATGTCGTTCCCAGTTCAGCCAGGTCTGATTAGAAATGCTAAAATGAGTGGAGCGAAG

GGCTTTAGAAGGATAAAGGGGATATGGGCGAGCTTTAATTGAAGCAGGGAGTCAATTAACAACGAGGCCGTAGTTTACTA

ATAAGGACTATTGCGACTAATATAGCAACCCCGAAGAGAAGGAAGGCCACAAACAAGAGCATATTTTTTAGATATCCCCA

CAATTAGAGCTATTAGCTTAGCTGGAGTTTTGCTTGGTCGGCTGAGTAC

>JK610286.1 JCF681 Jatropha curcas, immature Seed cDNA subtraction library Jatropha curcas cDNA similar to function, mRNA sequence

AGCGTGGCCGCGGCCGAGGTTTTAAATGCGAGGTGTGAACATCACAAAATGATCCCTTTGATTTATTAGCTTCATGGGGC

ACTTTGGATAAAATTGATATTTTGCCTCATTTGTTGCTTACAGGTTTTCTTGTGGCTTGGGTTTTTAATGAAGCTTGAGA

AAAATTATATGCGGGCTTCTCCAACAACTATTATACTAAAAAAAATTTGAAAAAGAACAAGAACTATTGTGCAAAATCAG

TTTTCTCGGTGAAGTTGAGTGTTCCTATTTATCTAAGTGCATGGTGTTTGAGTTTTCCAGTTGGACTTTGATGACCTTAA

CTTTGTGGTTGAGTCTGGCTTCGAGAGTAACCACTTTATGCGGAACCCGTATTTAGACGACTGACTTGACCTGCTGGCCG

GCTTTCCGTTCGAAGACTAGAATGCATACCTT

>FM889153.1 FM889153 Jatropha curcas embryo 35-55 (DAF) Jatropha curcas cDNA clone rjcfea0_002741, mRNA sequence

CAAAGCTAGTGCTCACCATTGATAACCAATCCTCCAAGAAGAAGAAGCTTCTCTATATGTCCAAGGAGCGGATCACTTTC

TGAATAAAATTCAAGAATCCAATGCCATTTGTGGAGCATTGCAGAAATAATGAACAGGGTCTTTTGTTTTTTGTTTAAAT

TTAAATTTTTGTTCTATAAAGTATGGGGAAATTTATTTATATTTTTTATTGGGGGGTTTATGTTATTTGGTGTTTGGATT

GGATGCAAATCACTGGTAAAGAATGCACTGGACAAGACACATCTGAGGTTGTGTTGTGGGAGGTTACAATGTTTGTGTCT

CCATTGTGGACAGTAACTCTGTTATGATTCTTACTTTTACCTGTAAAATTTGTGTAAATGTGATGGTGTAAAACTTCTCT

TCTATTACCA

>GT980138.1 JGCCJG2039B09.b1 Jatropha curcas L. germinating seeds (mixed stages) Jatropha curcas cDNA clone JGCCJG2039B09 similar to dehydration-responsive protein-related, mRNA sequence

ACATAAGGTGGTGGCTTTACAGTAGGAGGTTTTGGTATATGGGGTGGATATACAATTGGAGGCTTGACAACCGGTGGTTT

GGGTACGGTTGGTGGTTTGACAACGGGTGGTTTGGGTACATAAGGTGGCGGCTTTACAGTAGGAGGTTTTGGTACATGGG

GT

>GW881495.1 JC001382 Seed specific Normalized cDNA library from Jatropha curcas L. Jatropha curcas cDNA clone N02597 5' similar to Unknown protein, mRNA sequence

GAGGTACTTGTCTCTCCCTCCCACATTCAGCTTTTTCAAATGCAGACCGTAATAAACGCCATTTTTGAGTACCCATATGC

TTTATGGAGCTTCAAATCCCAGAAAAAGTTTGAAACTCGAAAACAGTGGCTTTTAGATCAGAGACACAGGGGAGGGCTTA

AACGGTGAGAAACCATTTGCTTACTTTGTGGGTTTATTTTGGTTTGTGATGGTAAAAGGCGTATTCAGTTTGAAGCTTGC

GTTTTTGTTTTTTTAAGGCTTAGCCAGTTGGTAGGTTTGGTGGGTTTTGAACCACTTTGATTGATATGGGAAATTTAATT

GGCTTAAAGGTAGCTTGTTTAATTACTTGGAATATCTTCACCCTCTTTTAGTCACAAATCTTAGATGGTAATATTTATTT

AATAAACTTTTTCATGACATAATTAACTCCAATACCCATTTTGATTATTTCTTTAATCTTAGTTGTTTGTAGCCATTGCC

AGGGTTTAAGGCGTATGACCCGTGATCTTGTTTCCTCTTTAGTGCTTGCTATGAATTTTATATTATGATTTCTTTCTTTT

CTTTTTTTTTTTTTCTTTTTGGTCTTTATTTGCTCCAATTTACTGTTCGTGAAAGCAAAGATTCCTTTAATCACATAATC

ATTAGATCCGTCTTATTTAAGGTTTTTGATCTTTTTCAAAGATGGAGTTTGCGCCTCTAACCCTCTACCCTTCTTCAAAC

ATACCAAAATGGTCTCTAAATTTGCTAAAGTTTGAAAACTG

>FM895496.1 FM895496 Jatropha curcas embryo 71-95 (DAF) Jatropha curcas cDNA clone rjcpga0_002427, mRNA sequence

AAGAAGATGAATGGAACAATTCAGAATTGATGAGATTTTTACAAGAATAAAAAGTTAAAAAAAACTTCAAAAAGCAATCC

TTAAAACTATTGCGAGAAACCCTAAAACCCCCACCCTACGATAATCAAGTTGATAAAATCAAAATCTGCCTCCCTTTTCC

TTCTCTTTCTCTCCTGTTTCTAAGCAAACAAACAAGCTAATCACGAAACCCCTTAAGAAAACACTAAAAAATATTGCAAG

CCAAAACCCAAAAATACCGAAACTATAATTACACCCTCAATTTACCGACAACAAAAGGCTAACAAGACCCAAAAGGAGAG

AATAAAAGAAAACCTCAATCTTCCTTTTCCCACCAGGGTAAACAGAAACAGAGAGAAAATCAAAACCCTAAAATAAAGTC

CCAGAAGAAGACCCAGACTAAGTAAAAGGATTACTTAAAAGAACACCCACCTTCTCTCTACCTTCAATGTCCGAAACATT

TCCCCAAACGAAGCCTCAATGCCCAAATCCCTACTCTCA

>GT978255.1 JGCCJG2018E02.b Jatropha curcas L. germinating seeds (mixed stages) Jatropha curcas cDNA clone JGCCJG2018E02, mRNA sequence

GGGGGGAGACTCAAAATCAAAATGTCTGATTGAAATCTCACTTATTAAGTTAAGGGGCGTACCGAACAATATCATAGTTC

CTTTTTTGGATTCCCTTTTTTAAACAACTCTTTCTTGTGCTTAGGAAAAGGGGAAGGGAAGCGGGAAATCCTCCGTGCTG

TGGACTAATAAGGACCCCCCCCTAAGCCGGGAGTGAGATGAAACTAGTAAAAAAAGCGAGTCTTGCCCGGGCTTCTCAGG

ATCTAGGGTAAACAAACCTAGGGGAAAGAGAGTCTTAGTTAGTCTTTTTTTGGAGCTAGTGCGAAACTCTTCTCGAAAAT

GGGCGTAGCGTTTCAGATTTCACACCAAGAGCGGCTTTCTCGACATTTTGTTGAAGAGACTAAAGGGATTCCAAACCAGC

GTTTAGAGTCCAACGAAGGAGGCCCTAGGTTGAGTTCAGATCTCATGATTTATTCCCTGGCCCATCTCTTATCTTTGACT

CCAGAGCCGACTTCCTGGCCTTAGTAAGAAAGCATTCCAGAGAATGCCCAGATAGATTCCTTACACCCTTTCTCAGGGGT

TGCA

>GW879874.1 JC004442 Seed specific Normalized cDNA library from Jatropha curcas L. Jatropha curcas cDNA clone N07897 5' similar to Unknown protein, mRNA sequence

ACATTTGTCAGATTTTTATTAGGGTTTGATCTAAGGTTAACCTTAAGTGCGATTGCTCTGGTACATGGAATGGAAATGAG

AATGTGAAATTTCTGTCTAAACATGTAGTGTGATGCCTCTTTTTAGATTTTTGGATTGCTAGGTAGGAATTTGATATGTA

ATAACATCTATGCAAGATCTCATATACTGAATTATAGATAGTAAGAACAACTGCAAAGATTTGATAAGATCAATTAACAA

CTAATTCTATACTACAAAATAAAGGGGAAAATACAAAAAACTATCATGTCATTTGCAATTCTATTAATAGGGGTTTGTTG

TTTTTATAGACACGTAGGTCCCTGTGGTTTGCTTCAATAGACGCTCAATTGCCAACTGCACTAACACCATTTGTGTTTGA

TGATATTATATATTAATATCAATTAAAAATATTTTTGCGGATGTAGAGAAATTTCTTTTATAATTTGGGATGGGGATTTA

GGATTTTCGGATTAAATGAAACTTATAAAAATTATTTTGCTTTTCTTTTATGAAAAGTATTTTTCATTTTAGTAATTAGT

TAAAATGTTTATTTTTAGTTGAGCTAGCTCCATAGTACCTAGTGGAAATTCTATGTTAGCCAAATATATTTT

>FM887563.1 FM887563 Jatropha curcas embryo 35-55 (DAF) Jatropha curcas cDNA clone rjcfea0_000790, mRNA sequence

AAGAAGAAGACACATCACAGGCTCATGGAATACATTAAATGCTTCTAGCATGTTAAGACTCAATTGCGCCAGAAGTTTCT

GTACAGGGAGAAACTTGGCTGAAAGATCACAACCTTCTACAAGAACTCTATAAACACAACTGTTCTTGCTAATTGCCAAA

AGTCAAATTCATGCTTTAGCAGCGATTGCTGCAGCTTCAGCTCTGGGGGGGGCCCGGTAC

>GW876231.1 JC005788 Seed specific Normalized cDNA library from Jatropha curcas L. Jatropha curcas cDNA clone N09867 5' similar to NADH dehydrogenase subunit 4, mRNA sequence

GGCCATTACGGCCTAGTTACGGGGGAGGAAAGACAGTCATTTTCGTTAAAAAAAACAAAAAAAAAAAAAAAAAAAACGGA

GCATTCCGATGGACGCAGCTCTAGCAGAGTAGTAACAGAGAAATCAAGAAAAAGAGGCACCAAATCTAGCCCATGATTGC

GAACCCACAAAACTTTCAGGTCTTCAACGTCTTGTATGCTTCCATTGCATTTCTCGTAATTCAAGAAGAATACATTCTTC

CAATTCACTGCAATGTCTAAAATTAAGAAAAGCACAGCGGATAGGCAAAACAGGAAGAAGTTAGACCCAATTGA

>FM889943.1 FM889943 Jatropha curcas embryo 35-55 (DAF) Jatropha curcas cDNA clone rjcfea0_003704, mRNA sequence

GTGGATTGCCCCGGGCTGCAGGCTCATTGTTCCAATTCTAAGTGAAGGAGAGTAAGATGTCCCTTGGATAAGCATAATTG

GACCTCATTGGTAAACCAGCAATGTGGCTTGTGCGTCGATCTCCATAGAATATCTGATTAGGACTCATGGATCATTCAGT

ATCTTCTCATTTTGGGCCTGTGTGTAAAATTTATCGATGTGAACTTTGAATTGATTTAGGTTGTGGTTCACTTTATCTGT

TTTATTGCTCTCTCATTCAATTTGAATACATTGAGCAGTCATTCTATCAG

>FM889418.1 FM889418 Jatropha curcas embryo 35-55 (DAF) Jatropha curcas cDNA clone rjcfea0_003079, mRNA sequence

AATTGGGGATCCATCCCAACCAGCGACCGTTTGTATAGCTCATCTGTGTGAGAAATTTACAAAAAATCAAATTTTGATCT

TTGCATGGGACATGGAAGTAAAATGAGCAATTTACGATGTTTCCTTATCAAATAGCTAACTCTGATTTCTTTTTTTCCTT

CATAAGTATCTTATAAAAGAACAAAAGTATACCATGCTACACTACTACATGCCCTAAATACTGTGTCCTAAGCAAAATGT

ATGTATCACTACTCCCCTTTTTATTAGACAAGGGACATGTTGACACCTCAGCTTAAATTATTCGAGAGAGCGAATGTTTC

CGTACTTGTAGTCTTATCAGAGTGCATCAACATTTAATAAATATTCGATTTTTTCTATACTTCCCAAAAGAACAATGTCT

ACATCATGCAGCATCTATGCCACTCGCATCCCTCATTAGACCGGTCATTCTGTGTATGGCTGCATCCTCGCTCGGTTTCC

AAAAGACATGCAATACTTGGCACCATAATTTATGAACCTGAGAGGAATGGAACCTCTGCTAGTGTT

>GW881608.1 JC000669 Seed specific Normalized cDNA library from Jatropha curcas L. Jatropha curcas cDNA clone N01263 5' similar to Unknown protein, mRNA sequence

GGAACCCTTTTTTTTTTTAACTTAACCTTTTGCCTTTTATTTTAAGTTTTATATATTTTTTTGGGTAAATCTCAACACAAAATTTTGTTAATATTAAGAAATTTACAGGATATTTTCATATGTATAGAGTTGTTATGTTTTTTGGTTTTTGTAGCGTGTTGACTTGGTCTTTGAAATTTAAGCAAGAAACTTCTTGCCCCCTGTTGGTATTATAGGGGATATGAAATAAAGTCATTAGCAAAAACTTGACTTTTCAATTTCAAGGTTACTAGTTTTAGTGCTTTGATGGATATGATAATGGATTTCAATAGAAGAGCATGTTTGGTAGCAATTTTTCAACTTTGGTTATGTTTGTATCTCCAATACCCATTTTGAAATTCAGAAAAAATGATTTCTTTTTATTTAATTTTCTGGTTTCTGAATGTCATAAACCCTTATCGTGTAGCTTCTGTATGTTTTGCAATTTTTTTTTCTCTTAGATAGATACTTCGATGCATCCGCCATAGATTTTTTTC

>JK611451.1 JCF4133 Jatropha curcas, immature Seed cDNA subtraction library Jatropha curcas cDNA, mRNA sequence

ACAAAAACCAATCTCCCTTCCAGCTTTCATCCTTAACACATGAGAGCCATTGGAGCGACCACCAAACAATTGCCCACCAG

AACCAACCACAACACCCTGACTACCCTCATATGGATCATAGCATGGTGAATACCGCACAAGACAACCCGAGAGATCCACT

TCGTAAGACTGGTTGGGAAGACCTCACATACAGAAGAAAACCTTTCCACAGAATGGACATGCAAGCTATTACAACTCAAT

AAGACAAAAAGAGAGATTTTTTTCCACGCACTCCATTCACTCATCTGCACTATGCCCATATTATAGCAAC

>FM887482.1 FM887482 Jatropha curcas embryo 35-55 (DAF) Jatropha curcas cDNA clone rjcfea0_000692, mRNA sequence

CGAGGAGAAGGATATCATTTCGGGACCGGCCCGCGATGAAGAACTCAAGGGAACCCCCCACTGATTGTAGAACTTCGTCC

TTTTCCAACTCTGACTTCTTCCACTTTTGGTTGGCATGATCGGGCGGGGGTTCGAACCCGCGAACTTCTGNGCGTGACAG

ACCAGCACTCTAACCGACTGAGCTATTTCCCCC

>GW879253.1 JC003811 Seed specific Normalized cDNA library from Jatropha curcas L. Jatropha curcas cDNA clone N07014 5' similar to Unknown protein, mRNA sequence

GGTCAGTGGGAGTATAGTGGTCATGGTGCCAGGCATCAGTAATGGGGTTTCTCACTCTTGTGAATTGATTTTTGATCTTC

TGGGATCCAGCCTCTTTGATATATATGGCTAAAGAGGTTAGTCTACTCATGCTTATTTTCAAAATGTATTTGATTATATC

AATTATATATTTCATTACAGTTCACTAAACATCTTAGTTTATTTTGGAGGTTCTCTTTTGTATGCATCTTGCCCTTATCA

CTGATTCAAACTCATGGCCTTCCCAAGTTGCAGCTCACCCATAAAAAACAACAATTCTATGCATAAGAAGATAGAACTTG

TAGAAAACTGCCACTTGTATAAAGAAACATGCGCTTCAATATTTATTCCATTTCCCTTCCCTGAAGTCTTCGTCGGCTTT

ATTCAAATACAAGTCTCAATTCCAGATGAAACCGGGAACTGCAAGTCTCTCGAGTTTCTTGAACTACCAAATTCATCCAT

TTTTTTGTAGTTTTCTGGTTCTTGACTGTTAAGCAAAGCAAACTACCAGCGAGATCTCCTGAACGCCGATTTGACAGGAA

TTCATTGATTTGCAGAAATTCGAATCAGATTTTGCTAAAAATTGCGTCTGCAGCCAAAAAGTTCGCTCCA

>GW881331.1 JC001449 Seed specific Normalized cDNA library from Jatropha curcas L. Jatropha curcas cDNA clone N02703 5' similar to Protease, mRNA sequence

GGAGAAATAGAATTCGAGCACTGTAATCAGATAAGATGTCGACGAGACTACTTTCGTTTTAACTCTAGAAGCCGTTGATT

GTTGTTCCGGCAAACATCTTTGCTCTCGAGCTTCTTCGCCAATCCACATGAAATCGCTGAGCTGCTACAAAGCTTGCTAA

TTACAAAAACTTCGGTTGCAAAATCAAGTAACTCCAGGTAAGGGTTTCTACTTTCTGCCATTTGCTTTTCCACTTCATTC

ACTCATGAGCGAGAGTGGTGAAAAGATTCAGGACCTTATATATCATATGAACCCAATAAATTCCAATTTGAAAGATTATT

TGTGGGAAATTGGCCTCTACAACAGAATTTTTCATCTATTGCTAGTAGGGAGTAATCATGTCCAAAGGTTGTGTATTTAT

TGCAAGTGATTTGAAGTTAATAGCATAAGGGGTTTAACTATTCGTTGATGTTCAGTTTGG

>FM890039.1 FM890039 Jatropha curcas embryo 35-55 (DAF) Jatropha curcas cDNA clone rjcfea0_003827, mRNA sequence

TCGAATAGACTGTACAAACTAATTATTTTAGCAGATTATAAATTATTGATCAAAGATCATTTTGACTCTCTCTCGTAGAG

AAAATATGAAAAAAATTATGTTGAAAAATAATTGTAGATTGGTTCAATTGGTTCAGCCAAAGCTGTGGAGACATGACCAA

ATCACTTAGATTTAATGAATCAATTAACCTCTTTCCTTGAGCCATTAAATACCCTAAAAATGAAATTCAAGGAAAGAATT

ACAGAAGGGTATTTAGAGCCCATGAAGACAGCAGATCTCCACAGTGGAGAATGTTAATATTAAAAGATCACAACCATCAA

TTTAGGGTGGTTTCAGTCTTTCAGAGCCCAAGTGGTCCAATAACATGAGAAATCTCTAATCCATACACCCTAACAAATAA

TTAGACCCAAAGTAGAATATATGGGCCTCCAAAATGGGCCTTTTGAGGTCCAATGGACCATAATTTTGGTGCACAGTCCT

CTACAATAGGTTTCCTTCTCATTTTGATTCCATCAAACAAACACTCCTTAATTTGCAT

>JK317420.1 JCST187 Jatropha curcas L. seed cDNA library Jatropha curcas cDNA 5', mRNA sequence

GGATGCTCGAGTTTTTCAGCAGATTCATCTATGTCGGGTGGTGTCATCTATGTCGGGTGTCATTTAAGTCGGGTGTCATC

TATGTCGGGTGTATCTATGTCGGCCGTTAACTAAGTCGGGTGTCATCTATGTCGGGTGTCATCTATGAAGGATGTAATCT

AGGTCGGATGTCCTCCTTGATGAGTGACAACATAATGCGGTATCACCTATGTCGTAACCATATTATATACCCTGGTTTTC

CTAATCCCCTACTATTTATTTTAATTACGTTCACATGCCTATTTTCTAAACTACTAACATCATCA

>GW880797.1 JC004698 Seed specific Normalized cDNA library from Jatropha curcas L. Jatropha curcas cDNA clone N08258 5' similar to Unknown protein, mRNA sequence

AGGTCTTTAAAGCTTCAAGCTTTCTGTTTTTTTCAGAAGAAAGATTTTATATTTTGAACCCAGAAAGTAAAAATCTTTAC

ACAAGGAGGGATAGATAGATCTCTCCCCCTTACATATCAAGATGGATTTGACAAAGTGTTCTGCCATTTTCATGTTCCTT

CTGTTTGTGTTTCTTTTTGCACCTCCTTCTTCTGCTTCCAGTTTCATTTCAGATTCTGTTTATGAATCCCATGCTTCTAC

TGCAAGGAATCTACTTC

>JK611604.1 JCF4400 Jatropha curcas, immature Seed cDNA subtraction library Jatropha curcas cDNA, mRNA sequence

ACTTGGTCAAGAATAGAGAAATTAAAGGAAAAACATGAATACTACATTCTCATGCTTAAATTTGCATCCTTATGGTAATA

TGTGTAGGTCTAATATGTAGTATGGAGTTAATATAAATATTAATTTCATGCTACAGCTTCTAAGAGGAGATAATTAGAAA

GAGAAGTTAATAGGTAAAGGAGGGGAGCTAGGGGAGTCTTCTAGCTCATGTTAGTGCATTATATCTATAATTTATACTGT

TTAAGTGATAAGTAAAATAGTTTTAAAAAATATTTTATGAAAAGTTACAAGGTAAAGAAAGCAAATGTTTATATCAAATG

AACTTTTACAAGAGAAAAAGTTGACTTATTTATCAAATGTTTATATCAAATGAAATTGAATTCAAATGTGATTATTGCAA

TTGTGAATTGATTATAAAGAAAGCAAATGCTAAAGTTATAGTTAAATGTTTACACAAAATACTAAAGAGCTAATGTTAAA

TGTTTTATAATAGTTGGAAATGAATTTCAAAGT

>JK611026.1 JCF2484 Jatropha curcas, immature Seed cDNA subtraction library Jatropha curcas cDNA, mRNA sequence

ATCTACCTAGTTGGCCATGGTTCTTAGATGCTCCTACTCCAACTGTGAGTTCTGTTTCTTGTGACCTGTTTCAAGTTGAT

CATGAAGGCTCTGCTCACGATGTGAAGGTTGGCTTCTCAGCAGGATTTTGGATCAAGCGTCAATTGTTTATACAAAGGGA

TGGCAGCCTTACAGATGATACCTGTGGTTTCTTGTGATTGGGTTGATTTAACTGCTCCATGTTTCTGAGAGAGGGATGTG

TATCCAACTGTTACTCAGACTTCTTATTTTGTCCTCTCTTCTACTATGTGCCAAATCGGCGGCTGTGCTACTGAGCTACT

TTCCTGCCTGTTGCAGTTCCTCATCTGCTTCAACGTCTTAGTTGCTTCCAAGTTGCCATGCTGGATTGCAATTATATACT

AATTTCGCTGTATTGCAATTATGTGCTAATTGTCGGTTTAGTGGTGCGTCTAAGTATTTTTAACAATTATTGTGGCCTAC

GATCGAGACAAGCTTGTTACACAAATGATCCCTAGTCTCACAATCCGCAGTTGTTGTCTAGGAGGCCTCAGTCACTTCTG

CAGCTAGTCTTTCTTTTTTTGGCTTGGTTTGTCTCTCTCGGCAAAGATAGCTGTGTTGTTTACAAGATGGTGTTGTCATA

AGTTTTTGCAATGCGTCTTTACCTATTGTAAGTGTCTTCTGCTGCTTTGCATGATGAATATGTGAAGCGGTGAAAATATG

CTTTGCTGTATAAAGTACCTGGCCAGCGCCGCCTCGAATTCTAGAATGCATCGCGAGTTACGAGCTCGATTCCATTGCGT

CGTTACACGCTGGACTTGAACTTGGCGTACAACTATGCCTTGACCATCCTTTGCCACGTGGCGTAATGGCGCA

>FM887653.1 FM887653 Jatropha curcas embryo 35-55 (DAF) Jatropha curcas cDNA clone rjcfea0_000891, mRNA sequence

TGGTTATATCCTTCTGGACCATGTGAATGAATGATTCTTGGAGGAATTCTTAGAGAATAAGGGATAGAGATTCGATGTGG

TGGTTATTGGAGATAAGATAGTATTAAGGCGTTAAGGATTGATGTTCTTTTTCTTGAATTGATATGAGTTCCAAGTGTTA

GCAGTATATTTGAGATTTGACTTGTGTAAATCATATACAAGGTAGGGATTTCTACCACATATTTCATACTCATCATACAT

CATCTTAAGAGCATAAACCAACAAAGAGAACAAGAAATCTTAAGGCCTTTTTCATCAATAGGTTCTGCCACTTTTGGAAG

ATGAAGAAGAAAATGTTTTCCTCATCAATTCTACATATAAACATGCTTAGAAATCTCCCAAGAACACATTTATACCACCA

AAATTCAATAATCAAATCATTTCATATAAAATTCCCAATTTTCACAAAATAGTAGAAACTTCACAAAATCAAAAATTTAA

ACAAGGTAGGAGGATTTATGAACACTTACTTTAAAACAAGCCTTAGCAAGCTTAGATTAAAGCATTCTAAGCTCCAACTT

TGCCTTTACTCCTTGGTAGAGGTTTCCTAAGCCTTTGGCTTTCCAAGTTTCTTGAAAATAATCACCACAAAT

>JK317920.1 JCST691 Jatropha curcas L. seed cDNA library Jatropha curcas cDNA 5', mRNA sequence

CAAAACATAATGTCGTTGAAGGAGGAGGGGGAGAGAGGGGTCCAGAAGAAGGAGGAGCTTAAATTGAGAGTTCAGTTCGG

CCTTGCCTGAAATATCTCGGGCATGTCAGGATTTAGCGTTGGTTGTGAATACCTCCTTAGGTGATTGAAGCATTAGATAC

GGAAATTTTCTTGCCTCTATTTGAAATCTTCCAGTAAACATTATTTTGCCAGGTCATGTGTATGCACTTACAAGCTTCCT

TCAGTTACAAACGCCTGGAAGACGATCTTTCTGCGTTCTGCTTGCA

>JK611054.1 JCF3343 Jatropha curcas, immature Seed cDNA subtraction library Jatropha curcas cDNA, mRNA sequence

ACCAAAGAATTTATGGAAAGCAGACTTCAGTGCGCAAGCTACCCAAACACTAACATCAGAACTACAACTTGCTTGTTATC

ATATTGTTCCTATTACGCGCTGACTTTTTTCCCTACTTCGATTTTCCATTGCAGCAAATACCACAACAAAAAACAATGAG

CCAAAAGTCCCTTCCTATATAGTAATTATTAAATATGAAATCAGACCTACCTTTGCGATCTCAAGCACCCCTATTCGTAT

GAAGTTTGCTTCAGTTTAAATCCAACATCTAATACCCAAACATTGATGTTTCACCAAGACTTTCTTGCCACTACATTACC

AGATATTAAAAGCAGCTGCAGCACTTAGCATCCTCAAATGAACAGAAGCTTCTCACTTTGT

>FM887272.1 FM887272 Jatropha curcas embryo 35-55 (DAF) Jatropha curcas cDNA clone rjcfea0_000433, mRNA sequence

TACTGCTTTTGCTTAGTAATTTTTCATTCCTGGTGCTTCCTTAGTATCGAGCTTAAAACGGTTGGTAGTTAAATTTCTGA

TTAATGAATTATATATTTTCCTCG

>GW881310.1 JC001264 Seed specific Normalized cDNA library from Jatropha curcas L. Jatropha curcas cDNA clone N02392 5' similar to Unknown protein, mRNA sequence

GATAAAAGAATTTCACTGTTTTTTCTCTCTCTTTCCCTTCTTTTCTCTCTAAGACTCTCTGTCTTTCTATCTCTCTCTAA

ACCCTTCTCTCTGTCTCTTTTTATATCATCTCCAATCCTGTCTCTATTCATCCGTTAGTCTGTTTTGTTGTTTATTATAA

ATTACTTTGTCTTTATATTCAAAATTAATAAATTATAGAAAATTTTGCTTCTTGTATTATCTTCTCCTAAATCTTCTACT

AATTTTGATCTCATTTCTTTCTTTCTTTTTTGTTTTCAATCTTGGTTTTTGTCTATTATAGTAGGCCTCTGTGTATTCCA

TCAAAACCCTGAATCTTGTTTATTTTCATGGATCAATTAATGCGATTTTGTGGTTTTTACTTCATATAAGTGAATGAAGA

GATATGGGATTAGAATCTGTAGGTGGATTGTTGTAGTTCTTTGATGATCAACCCCAATTGGGTAATCGAACCAGACTGTT

CAATTGCTGATCAATTGGATTTTGTCTGCTATTTGATTTATAGCAATGGA

>FM888437.1 FM888437 Jatropha curcas embryo 35-55 (DAF) Jatropha curcas cDNA clone rjcfea0_001853, mRNA sequence

CTCTTAGAACCTATAGTCGTATCCCTCTTACTAGTGCTATTTGACTCTCAACTCTCATGGAAATCAATCTATCTATGACC

TATATGGATAACTCCCACTCAAAGAAATCTATGTCTTATCCTTCTGCTTGCCTAAAATCCAAGACAAGAGACTGCCACGA

AGGCGAATGCGGGTCATTTTGAATGCTAGGATAGAAACTACTGTAATGACTCCTACAATGCCAATATTCATAAGTGAATA

GTAGCAATGGCTACTGGGACAATGCCAAGAGGGAAGAGGGGAAACTACCTTTATCACTGGCTTGTGCTTCCATATCCGAA

GCTATCCCTACTTGCTTACCTGATTACCTACGAGTGTAAACTATGCCCGCATATTACTTCTATGGGTAATTCTATGGGAA

AGTCTTACCGCCTATGAAACCTAGTCGCTTGCTGCCTTATACCTGTGGGTGCCTTGGAAAATTTCACTAGGACTAGGAGT

AGGGGGGTCCGAAGGAGATCGCTAGAAACTGGTTCTGCCATTGAAAGACTTGAAAACTCACTTTGGAAAAGAAGGAGAAC

ATATTCTTT

>GW881607.1 JC000667 Seed specific Normalized cDNA library from Jatropha curcas L. Jatropha curcas cDNA clone N01257 5' similar to Unknown protein, mRNA sequence

GGTCACATAAAATCAAAGAAAAAGAAGCTCTTTTCATTACTGTTTCTCTCTCTCTCTGTGTACAAAATCCAACCTTTTTT

GACTCTTATTTGCTTTCAATTTTAGGGTTAATTTCTGTAAATTCTCTCTAGTTCACTTCTCTTTCTGTGTTCATTCCTAA

AGTCAAAAGCAAGAATCTTTATAAATTTGCGTATACAGTAGCTCTTGTTCTTATTCTTCTTCTTTATATCTCCAGAACAA

GAAACTTCCTCTCCAATTTTTCATTGTTTTGTTGCTTGTCTAGATATTAAACTATAAAACAGTGTTGCTTTTCCTGGGTT

TTCACCCATCAAGATCCAATTTGATGTATAGAAAAAATAGTGGCTCCTTATTAAAAAGAGAGAATCTTTGATAAAATTGT

ATGTTTCGGACGAAAATAGTGGGTCTTTGTTAAAATTCGATTTAAGTGAACAATTTGCTTGATTTGGGTTCTTTTGGGTT

TGTGATCTAAACCAAAACCATGGCCCGTTTCAACAATAGCTTTTATATGCGA

>GT975893.1 GJCCJC2030F03.b1 Jatropha curcas L. developing seeds (mixed stages) Jatropha curcas cDNA clone GJCCJC2030F03, mRNA sequence

GAACACAAGGTAGCGTTCTATCATACGACTCTCTGCTTCAAGATATTATTGAAAAGTGCCTGATCTTCTCGTGGCTGATA

TTATATCACTCTTCCCCGGTTCATCTGAGCTCGCTTGATTTAAAGTTCACGATGGGAGAGCTCTCTGGAAGAATTCCAGT

TTTTCCTAGTTGGATTATATTGTAGTTATTAGACACACGTGAGTGAACTACTGCAGTCTACTAGTGGCAAGAAGACATCT

ACCTTGATGAAAACCTTATTCGACTAAGATCCTCTCGGTTACTGCAATTGATAAGAGCGACCCCTGCGATGAATCACAGA

ATGTCAGATCTGCTGCTTACTCACTCTGCTTGACGAACCCATGCTGAAATCGCCCAGCTGTGAACATATATCCCCGTCAT

AGATCGCATACTCTACTCTACTGCTGTTTATTCTGCATTTCTTTTCCACTTACCTCGTCTCTCTCATACTCCTTC

>FM892521.1 FM892521 Jatropha curcas embryo 56-70 (DAF) Jatropha curcas cDNA clone rjcaeb0_003090, mRNA sequence

ATTCCATTGTTCACTTGGGCACTGATCTGAGGCTTGTAATCTCCTTTGGGCTCGATCTTTTGTTAAGTTGTTGATCTTTG

TTAAGTAGTTGTTGTTTATGGGGGACCTTGGAAGGATATTTATCAGTGCATGACAGTTAATGCAATGCACTTGCTAACAA

AATACATGGTATAATTGAAAACATATTTAAAGGCTAGGATTGTTATGTGTTTT

>JK611362.1 JCF4017 Jatropha curcas, immature Seed cDNA subtraction library Jatropha curcas cDNA, mRNA sequence

GGCCTAAAGGCCACAATTTTAATAAAGGGGCTTTCCTTCCATTTTTCAAGAAAGATGAGTTTTTGACGTGTTTTACCTCT

ACAAAAAAAAAAAAATGCATTAAACTTATTCAAAACAAAACCTTCCTCCATTTGAATGTATTTCTTTTCATTCGAGAATT

CCAACCTCAAATTCAACCGAATGGGCATTTTTCCTTTGTTCCCTGAAATGGGGCTTCCTTCCAATTTTTTTTAAGGTTGG

TGTTCATCCTTCCGAATAAAGGATTTGCCCCGATTTTTATTTTGCACCATTAGGGCAAAAATCGCCCAAATACCGCCTCC

TTTCCCTTTGGGAAAAACTTCTTTTTTTATATTAATTCCAAATTGAATTTACCTTTTCGCCCAAAATTTTAACGATTCTT

TTTCATTTTTTTTGGGGGAAATGATAAACATTTGGAATTTTCCCATTTTTAAAAAAAAGAAGAAGACAGGGGAACCATAT

ATAATATCCATGGGGTTTTTAAAGAGAGCCGGACATCTATGTGTGTCTCACACCCCATATATCTATATGAAAATCGGGAC

GCGGGGGGGGCACATATATAGTCGAGACACACATATCCGCGCGTTTAACGCGGGAGACGCGACATCTTTTGGGCCTCTCT

CCGGAAGAGAGACCAGCTCTCTCCCTCGCTGGGAGATCATATAATAATACTATATAACTACTTATAATAC

>GW875825.1 JC005202 Seed specific Normalized cDNA library from Jatropha curcas L. Jatropha curcas cDNA clone N09000 5' similar to DNA repair protein RadA domain protein, mRNA sequence

GAATTTAATCTGACCGGCAACGTTTCGTCAGCTCTCTCAACCGCTCTTTCTTGTTCTTCTTCCTTTTGCTCCACCTGAAA

ACTCCACTGCAGATCCAAAGCCTCAATCTTTCACTTTGTAACTGCGATTTCACTACTCGGCATCGAACCTCCAAGTTTGG

TAATTTCTTCTGTTTTCTTCTATTAAATACGATTATAATATGTTAATTTTTCTTTTTAATCTCTAGATCATGGCCTTCTA

ATCTTTTTGTTTCGATCAGTTGTTTTATATGATATGGATTCATTGGTATACTTAACATCTATTACCTAATAATACCCATA

TAATTAAGATAATAACTTTACTTTGATCTTTGTGATTTGAGCTTCTTTAGTCAACCATTTAAATTTGGAAATTTAAGAAG

CTGAGTTTTAATTTGATGGAATCTTTGTTTGCATAAAATTCAATTACTCATGATATATCGATTAGAAGGTGAAAGAATGA

TGTATTATGAGTAAATGGATTCTAAGTTTTTGGAAAATGCTGCTTTCCTTGTCCAGTTAAATGATAGTTATTAGTAAAAG

GGGGAAAAAACTGTTAGTTGCATGATATATATAGTGAGTAAATAAATAATGTGACTGCATTTATCTGCTGCTTTCTCATT

CTAATCCAGGTATTACTCTTTTTTAATAAAATCTGTTAAATCATATCACCTTACAGCTAAATATTTTACCTC

>JK611239.1 JCF3765 Jatropha curcas, immature Seed cDNA subtraction library Jatropha curcas cDNA, mRNA sequence

ACTTGCTTTATCTACCTAGTTGGCCATGGATCTTAAATGCTCCTGCTCCAACTGAGAGTTCTGTTTCTTGTGACCTGTTT

CAAGTTGATCATGAAAGCTCTGCTCACGATGGGAAGGTTGGCTTCTCAGCAGGATTTTGGATCAAGCGACAATTGTTTAT

ACTAAGGGATGGCAGCCTTACTGATGATACCTGTGGTTTCTTGTGATTGGGTTGATTTAACTGCTCCAACTTTCTGACAG

AGGGATGTGTATCCAACTGTTACTCTGACTTCTTATTTTGTCCTCTCTTCTACTATGTGCCAAATCGGCGGCTGGGCTAC

TGAACTACTTTCCTGCCTGTTGTAGTTCCTCATCTGCTTCAAATTCTTATAAGCTTCCAAGCAGCCATGCTGGATTGTAA

TTAAAAACTAAAATCCCTGTAATGCACTTATAAGCTAAATGAATAGTTTCGTGGTGCGTCTGAGTGTTGTTACCAATTAT

AACGGCCTACAATATAAACAAGCTTGTTACACAAAGGATCCCTACTCTCAAACAACCGATCATGATGACTACCAAGTCTC

TGACAATTCTCCATCTAGACTTTCTTGGTCTGGCTTGGTTGATCTATCTCGAAAACGATACCCTGAAATGGATCCCCACG

ATTGGAGATGTCGATCAGTTAAAACCAGTGGGACCTTCACGTAAATATAAAGGAGTTCATCCTGCGTACGTAACGCAACA

TAAAATATGCTCGAAGCCGTATGAATATATGGTTCTGGCTGACAAACGTACCCTGCCTCGGGCAGAAGCTACGAAAATCG

GATTGCATTTTGGCGAGGATATCTGAGCGCAAATATGACTGGGACACCCTTTCTAG

>GW880578.1 JC001512 Seed specific Normalized cDNA library from Jatropha curcas L. Jatropha curcas cDNA clone N02822 5' similar to Unknown protein, mRNA sequence

GGGTTTGAATTGCAAACAGTGTATCATACGAGTAAACTTGCCTATTATAACACATACCTTCACATTTCTAGTTGTGATTA

GAGTTAGCTTTACAAGAATCAATTAATTTTGAAATAAAAATATAGTAACAGAGAGAGGAACCAAAGAATACAACGCATTT

TGAGTTCTACTTAAGATGGCAGTAGCTACTAATTAAGTTCTTTTAGATATGAACTATTTCTTTTTCATTTATCACTAGGT

TTTACACTTCCACTTCAAAAATATAAATATTAGTCATATTGAAAAAAAAAATTCATTCATTAACATGTCTATTATTAATTTT

>JK610970.1 JCF3333 Jatropha curcas, immature Seed cDNA subtraction library Jatropha curcas cDNA, mRNA sequence

AAAATACACATTTACGCGGCTTCTCGAAACCCACAAGAATTTCTTCTGCTTAGTGAGAGAGAAAAATACCAAAGATATCA

CGTAAAAATTATGGGAGCTATGATGATAAAGAAGGTGGAGATTTGCATAGGGTTTTGCAAATTGGCTATAGAGTTTGTTT

CTACATTTGCAGTTCCGGTAATACAACAATGCAACAAAGCTTAACGACCGAGCTTGCTCCTTCTTCTTCTTACCCATTCC

TCAACCCTACCCCGGTCTCATCTAGGAATTCATTAAGTTTGAGCTTTAGGTGATCTTTCTACATGTAATAACACCCTTCT

CAATGCATGGTGGCAAATAATCCTGCGTATGATGAAGCCAAGAGGCATGAAGAAAGATAAATATGTTGTTCGCGAGAAGT

TGTTTCCCATCTCCATTCATGACCTGCC

>GT976121.1 GJCCJC2073D01.b Jatropha curcas L. developing seeds (mixed stages) Jatropha curcas cDNA clone GJCCJC2073D01, mRNA sequence

CTCCCAATACTGTAAAAACTTGCTGTAGCTAATTATAATTTTCTCTTTAGTCTTTTATTGTATGTATTATTTATTTCTGT

TGAATTTTAATTCTTTTGTTCTATTCTAAGATTACTGGTACTGTTGTTCTAGAACATTTCAGGTTAAATTGTTTTATTAT

TTCAGGTTGAATTTTAATGTATTATATATGTATATATTTTTTTGCATTTTCAGTTTCTGTTTTTCAGCGGATTCGGTAGT

ATGCCGTTAATTCTAAACGGATTCGGGGTACTTAAATTGATTGTTGGGTTCAGATTCGGGTAGTTGATTTCTAGCGGGTA

CCTGCCCTGCTGCCATCCCTAAACATTTATACCAGGAAGCGGTGAGGGCATACATTCTGAACTATGGCAGCTCAAACTTC

TGGAACCAAGTCCCTAGAAGAATATTTGGGAAGGTATGAAAGCATCAATGAGGAAGAGAAGAAAAAGAAAAGAAGAAGAA

GAAAAGTGAATCTGATGGGCCTGGTGTTTTTGTTGTGGATG

>GW881682.1 JC006924 Seed specific Normalized cDNA library from Jatropha curcas L. Jatropha curcas cDNA clone N11652 5' similar to Unknown protein, mRNA sequence

GACCACCACCTCTACCACCATCTTCTCTTCTTCGCTCCAACTTAAAGCCCCTCCGGTTTTATCTGTGCACATCTCACACG

ATTCTCGTTTCTGTTTCTCTCTCTGTGACCTCGTTTTCATAACACGAGCATGTATTGTCTCGGGAACCAAACATTGTGTG

AAACAACCTGCTAGCTGTTTCATTGACCTTATGGTAGTTTCGTTTTCTTTGCACTATGGCCACTTCAGGTGTTGGTAGTT

ATAGAAATGGCAATC

>FM888757.1 FM888757 Jatropha curcas embryo 35-55 (DAF) Jatropha curcas cDNA clone rjcfea0_002226, mRNA sequence

TCTAAATCGACGGTTATGAATGATTTTAAGAGTTGCGACACGGATTGTGCTTTTGGAGAGGTGGCGCTTTCGAAGTTCTA

GCGGGGGTTGATTTGATCGTTTGATCTATTCTCATCGACGGCTAAAAAAGATCAATCCGCGTGAAAGGGGAAAATTGGAT

ATGAACCGTGGATGAGGTCTTAATCTACGATCAAGAACAGTATTTGAGGTTGTGACACGGATTATGCTTTTTGGTATTTT

GCATATTCGGTGTTCAGCGAGTTGATTATGGTCGTTGGATTACATGTCCACCAACGGCTGGGAGAGTCGATCCGCGTGAA

GGTACATCTTTGATGTGGACCGTCGAGAACTTTCAAATCGACGCTGATGAACTGGTTTCGAGGATCAGTTGCACGGATTG

TGTTTTTTTTTGCAGATGATGCTTGTGCAGTGAAAACGGTTGAAAATTTTCTTCTTAAAACCACGGTGTCAGACTCTATT

ACATGTTATCTGCCTCTTCGTGTAAATATAT

>GT971256.1 GJCCJC2018F03.b Jatropha curcas L. developing seeds (mixed stages) Jatropha curcas cDNA clone GJCCJC2018F03 similar to putative microsatellite, mRNA sequence

GGAAGAAGGAAAGATAAATGAAAAAAAACAAAGGGCAGTTGAGAATATTAGATAGGCTTATCGCATGTTTGCTTCACGCA

AGCAGCTAAATGGGGGAGGGCTTTGCTCTATCGCGTGGCTTTAAAGACTTCTTCTAGCGTTTCAAATCCTCCATTAAATA

ATAACAATAATTACAAAAACCCCAAATACCAGAAACCAACACCCACCAACCAAACCCTAGACCATCCCTCTTCTTCTTCT

CCTTCTCCTTCTTTCCATCCTGCATATATACAAACCCTAATTATGTAACACAGTGGTTTTCC

>FM887877.1 FM887877 Jatropha curcas embryo 35-55 (DAF) Jatropha curcas cDNA clone rjcfea0_001160, mRNA sequence

AAATTCGAATTCTAAAATGAATAATTCTAAATCTATCTAACAATTAAGACAGAGTAGATTCATATATCTTATTCACTTTA

CTTTAACTTTAGGAAAAATTTTAGAAAGACTCAAGTCAAGTCAAATAAACTACTCATAAAATGAGGAAATGCTTATATGT

ATGTATTCTTAGCTTATTAACACTTTTGTTTCCTTTTTTGTAATGTAAAAAAAAAATGGTGATTCTTTACTTATTATATT

ACTTAATTTTATTACTTAATTTTAATATTTACTTAATTTACTTAATTTAAATATTTAACATAGAATATCCATAATTAGTT

TCAGTGACAATTCTTTAGTACTATCTGATAAATAAGATGACTTTCTAGTAATTTCGATTCTGATTTTAGCATTCAGTAGG

AAAGAATAATAACTTCCAATTTTCCTTACATTAGTCTTTTTATCCACCACTACACTAAAAAAAAAATGGGAATACCCATT

TATTTGTTTTACATCGTTGATGGTTTAATCAATAAGGGGGGTATTTTCTCTTATGATG

>FM889822.1 FM889822 Jatropha curcas embryo 35-55 (DAF) Jatropha curcas cDNA clone rjcfea0_003559, mRNA sequence

TTCAAATCAACCTTATCAAGAATCAAAGAAAATTAAAAATATAAATAAATTTAAACAAAAACTAATTAAAGTCATTAAAA

ACACTAAAAATTCTAATAAAAATTATATTAAAAATACTATAAAAATAAGACCTATCAGAATTCATGTTGAAGCTATTCCT

TGTGATAATTAAATTAAGTGGTTTGTTGCTATGCCAGAAGAATGGAATCTCTCCATAAGAATGGTACTACCTCTTAGAGA

CAGACAATAAGGTTATAGGTTTTAAACGGGGAAAGGAAGCTATTCCAGCTGTTCAATATTGTACATACAATAGTCAAGTT

TATGGAATTGACTTTAATACTAATTTTTTTTTTTTTTTTATTATTTTTACCTATTACTAAACTGAGCTTTATTTGTATCT

TACTCCTTTCCGTTTCCAAATACACATCGCATTCGTATATTTAATAAATAATAAATTAGTTTTTCCATTTTTATCTTTTG

GATTTTATAAATTAAAGATAATATAATAAATTCA

>JK612753.1 JCF20-197 Jatropha curcas, immature Seed cDNA subtraction library Jatropha curcas cDNA, mRNA sequence

TCCCAGGGGCCGGCCCGGGTGCTTATAGGGCTCGATCGGGTCCCCTTGTCATATAAATAAAAGGTCCCACCGGTGTCCTA

TCGATCCTTCTTACCTTCTCAATTTGATTTGGATTCTGTGCCATGTCTACCATCAGGATAACTGGCTTGTGGCACCCAAA

ACTGGATCTAGAATTGCTTTTTGATCCTTCGCTGCTTCTTGTACCTATCATTGCTCATCAGAATTGACCATTGGTTGGAT

TGTACTTCCGCCATTATGAAAGTTGACCTGGGCTTATAACTTAACACCTGCTGTTAGTCCTTCCCTTCTGAAGATAGTGT

CGCGACGGTAGTAATCCCGGTCACCTCGGCCGCGACCACGCTAATCTTTTGCCTTGAAAGTACCTCCGAGCTCAAATTCC

CTGGCTAGATGCATTCAACAGGTACCGAGGTCGAACCCTGGCGTTACCCATTTACAATCGCCTTGACTGGCATCCCCCTT

TCGTTACCTGGCTTAATCGCCTTGCAGGCACATCACCCCATCTCGCCTTCCGGCGTAATGCGCGAAGAGGAATGGCACCA

TGGAAATCTGTCCCAACTTAATATTTTGCTAAAATGGCGAATGGAAATTGTGATAAATCAATATCATTGTTTTAACCAAT

CGGCCAATTATCGTTAAATCCCCTCATAAATCAAAAGAATAGACTGAAGATCAGCGTTGACTCTTGTACCTCATTAGAAC

AGAACTACACTAGTGATGAAACGTGTACTCCAATCGTCAACATGACTCAACATCTTACAGATACAGGGCGACTCGTACCG

TTACGTGAAGCAATCAGCCCTCATATCCAGGTCTATGAGCCCGTAGGTGCAGTCATGCACCTTAATTCAGAATCTCTAGA

GGGTAGACGCCGCCCATTATAAGCACCTTGAACGCC

>GR716939.1 JcrRL_ASE0008 Salinity tolerance responsive genes from Jatropha curcas root cDNA Jatropha curcas cDNA, mRNA sequence

GGGGGAAGGTTGATGTATGCAGCCTTCCCCAATTCTTTTGCACCAAGCCAAAAATTGTTACTCTCCATCCAATCAAACCC

ACCAAATAAAAAAGACAGCATAATGTCTTTCTCATAATTAAAAGAATTGTAAGTTATTCAACTCTAAGAAAATAAAATGA

>FM895532.1 FM895532 Jatropha curcas embryo 71-95 (DAF) Jatropha curcas cDNA clone rjcpga0_002470, mRNA sequence

AGAAAGCAACTGTCAACTGCCATCAAATTTGCGGGGGAAAAATAAAAAAAATCAAATACAACAGTGGCTGATTGGTCAGT

GCATGGTTTTGTCACAACGTACAAAGCTTCTAACTCAGAGTTACAAACATCACTTTATCTATTTCATTGTATCTATTGTG

ACAATCACAAAAATGGCCATAACCTGGCCCATCTCCTAGTAACCTTTCCATTCTCACTTGTGGAGGTTAACAGTACCATA

AACTGAGCTAAATCTCTAATCTCACCGTCCTCTTGCTTGGAAAATTCTAAGTATCCAAGTAAACCAATGGGAACTACAAC

TCAATTTCCAGAAGCATGTCTTCGTTGCACCAACAAATCATCAGCAGAGTAATAATCGGCAATG

>JK611477.1 JCF4164 Jatropha curcas, immature Seed cDNA subtraction library Jatropha curcas cDNA similar to Vacuolar-processing enzyme precursor, mRNA sequence

CACTCGTTGTTTATGCAATACGTCAAGGTCGGTGCAAGCCCTTATGAATCCTTCTAATATACCGAGATGAGGTTGAGCGC

TGTTGCAGACTGTAACAAGAGTACACATGTGTAGAACGTGGACTCGAGTTACAGATCGCGAGAAAGCGTCTATCACGGCG

ATGGCCCACTACAAGAACCATCACTCTTCTACTGTGGTTAGGGGTCGATGTCTAGTAGATCAAAAATCGGAAAACTATTT

GGAGGCCCCGATTTTAAGGTTGACATCGATACTATGCTATCGTCCAAATGACTGCAGTGCATGAATTGTCATCTGTAATG

TGCTGTAGGATCTGGCATTTCTATGCGGACTGCTGATCGATCCTTCATCACATTCGCCGCATAATGAGCAGCTCAGCGTC

CCTCTGGGTGACCACTTTCAAGAAGGCTTTGGAGGTATGCCATTATCTGGGGAGCTATCTGAACTTGCGAGGTTTCCTCC

GCCCGGACAACTTACCTGATGATGCTCAGGACTGATTAGTCAGTGGCGGTCTTAACATCGCGGGACGCGGTTAACCTGGG

TGTGACGCA

>GT975189.1 GJCCJC2068D02.b Jatropha curcas L. developing seeds (mixed stages) Jatropha curcas cDNA clone GJCCJC2068D02, mRNA sequence

CCATAAACTTTGAAATTTGATTGGCTCGAAAGATTTGAATTTACCTGAAGACTCCAATCGGACTACATTAGCATTCTAAG

CATTGGCAGTCATTTGGTCACGTTCAAGATGATTTTGAAAATAGGCAATTTCTGTATACAAATAGATCTCGCCTTGGTAT

GTCTAAAGAGCAATTTCTATATACAAACAGATTTCGCTTGGTGGGCTCAAATAGCAATTTCTGTATACAAACAGATTTTG

CTTGGTGCAATTTCTGTATACAAACAGATTTTGCTAAATTTCTGTATACAAACAGGAGGGACCAAATAGCAATTTCTGTG

TACAAACAGATTTCGCTTTATGGAGTCAAAGAGGAATTTCTGTATACAAACAGATCTCGCTTAGTGGGACCAAGTCTCAA

TTTCTGTGTACAAACAGATCTTGCCTTGTGGGCCCAAAGAGCGATTTCTGTATACAAACAGATCTCGCTTGATGCAATTT

TCTGTATACAAACAGATTTTACTTTGTCCTAAGCTTGATTTAACCGGCTTTGTTGTTCATCTCCTCAGC

>JK610798.1 JCF2318 Jatropha curcas, immature Seed cDNA subtraction library Jatropha curcas cDNA similar to function, mRNA sequence

ACCCAGCAATAATGAGCGGTGCAGTGAGCCATAGATGATTGACATGTTGAAGAGCCAGGTAAAGGATGTTGTCCAAAAAA

AAAAAAAAGCAAATTGAAAGAGAAGATGAAAGTAACATCGGGTGACAATTTTTTTAAAAGAAGGGCCATGTCACCTTAAT

TTATAGGCTACAACATAAGGAGTTGGAATATGGTGCTGCAAAGCACTGTATTTGCAACTGTTATTAATGTAGAAGTGGAA

CATATTTTTCTGGGTGGCTATAATATATGTTGAATAAGTTCACTCAAAAAAAAAAAAAAAAAAAAAAAAAAAAAAAGCTT

>JK317361.1 JCST125 Jatropha curcas L. seed cDNA library Jatropha curcas cDNA 5' similar to A.thaliana mRNA for Kap alpha protein, mRNA sequence

ATTTTTTTTTTTTTTTTTTTTTTTTTTTTTTTTTTTTTTTTTTTTTTTAAAAAAGGAACCACCGAAAATAAGGTTAAAAA

GTTCTTATCACATAACTTGCTTAATCACCTTCCGCCGTCGTGGTTCCTTCTGAATCGAGCTCATCATCTGCAAGTGCAAA

CTTGTGGACATCACAAGTAACAGCATCAATATCCAGACGCGGTCCAAGCTTCAACCCCAGCCGCGTCCGATTGGGACGTG

GGACTTAGGCATAGCGCTGAATCAGAAAAAAAAAGATCAAGGGATTAGGAAAAAGGGGATAAGGCCACCGGGTATTGAAA

ATTGGAAACGAAAATGGCTTGGGGGTATGAAGAAAGAGTCATGGCGAGGGAACGAGCGGAAAAAATATCTGGGAATTGGT

CAGTAAAAAATCACATATTTAAAACACCGCAAGGGAGACAAGTAAAAGAGTAAAGGCTCCTCCGGCTGCAAGCTAGAAAG

GACAGGGCGGGGTTATAGTACCTTACTAGAAAAAGTTGGAAGCTAAATAAACAAATGCAACTTTTGGGATACCCGACAGG

AAGGGCTGCACCCACTATGGGTGAAATAGCCTACACAGAAAAGGACGTCAATGGACGACATCTT

>GW880266.1 JC000756 Seed specific Normalized cDNA library from Jatropha curcas L. Jatropha curcas cDNA clone N01439 5' similar to Unknown protein, mRNA sequence

GGAGACTGAAAAAAAAAAAAAAAAAAAAAAGCAACTAACCCTTACACACACAACAACTGTAACCTTCTCTTCGTCGTCGT

ATGTTCTCGGAGCTCCGGCTCCCTTAACGGAGCCCAGCTCAGTAATCAAAACCAGTAACTCTGCATTCTTAAATTATAAA

AGCTAAGGATCTTTCATTTCCTGATTTTCCGTTTGCAGATCTTTAATTTCGTCTTACTTTTTTTCCTTGTAAGCAATGTC

GAAAGCTTGGGGTGCCATCGGCGATTGGGCCGCCGATGTAAAGCGCGAGGAGCGGGAGCGGCTG

>FM887739.1 FM887739 Jatropha curcas embryo 35-55 (DAF) Jatropha curcas cDNA clone rjcfea0_000996, mRNA sequence

AGGTGGACCACAAGATCTAGCAAGAAATCATGTTACATCCAGTCAGTGTTTCACATTTCATTTCCTTGTACTCAATTACC

AAGGATAATGCCAAAAATATTTTTACATGTGATTGCAAACATATCATTAAAACCCATTCCACAAAATATTTTCCATTTCA

CCACTGCTAATCTCACTTAACCCTCATGTCAACCATCTTCAAAATAATTCCCTTGCCAATCAACTATGCTTGAACTCACG

CAGTCCCTCAAGTTTCTACAAACTATGCGAGAATTATCCTTGTCCATCATATAGCCTTCAGTCAAAGTTGCAACTAATGA

ACCTCAATGCTAACATGGTTATCAGTGCATGCATACCTGAGGTCATAGACCATTATGCTCACTTGGTTTTCTCCAGCTCA

TAATCAATTTGAACTACATGTTCCTTCACTACAACTAAACTTCAGAACACTCACGACTAGCCTAGTTGCTCCCGTGGACC

ATAAAATACACACCTCTTGCAGAACATCAATGTTGAAGAACAATGAATTGTGAAGTTAATGGG

>FM890278.1 FM890278 Jatropha curcas embryo 35-55 (DAF) Jatropha curcas cDNA clone rjcfea0_004101, mRNA sequence

GAGTTTATTTAATTATAAACTGTTGAGATTTGGTGTAAATGAAGGTGGGATTTACTGTTTCCTGCTTTATTATTTGATGA

GATCTTTAACTTTAACCACAGTTTCTTTATTTATTTTCTCTCTTTTTTCTTTTTTTTTCTCTCTCTTTTTTTTTTTTTTT

TGTTCTTAATGAGCAATTTTAGGTTTAAATGAAATGAAATGATATAGCCGTTGCTTGTAATTCCTTTTATCGCTGAATTA

GCATATTGGCAGAGAATTTCT

>FM888826.1 FM888826 Jatropha curcas embryo 35-55 (DAF) Jatropha curcas cDNA clone rjcfea0_002321, mRNA sequence

TGTGGAAATTTAATTTTTTAATAGGTATAGTTTTATAAATTAAAGTATTTGATGGGTTAAAAGTAAAAATTGAGAGTACG

CGGAATGAAAGTAAAAAGTTTAAAGGATGAAATCCAACTTATTCATTCGATTAAAGTAACAATTTGATTTATTTATATTA

CAGAAATTCGATTCAATTTGAAATCCAACTGACTATTGGTGTCTAATTGCAAGAAATTACCAAGTTTAGGCACTTTTTCA

ATTAGAAAAATAAGATAAAATAAGTTCAAATTAAGGGAGAGATATTGGGATTTTGTTTAGAGTTTATTTCTTTATTTGGC

AATGAAGTGTTTCTTGGGTGTGAAATGTTAAGTGTGAACTACAATCCAAAAACCCTTCACGAGACATTACAAGACAAATC

TCAACCTCCTTTGTTTCTTCCCTACATCCTAAGTTTTTCTTTACATAGCCATAAAAAACTAGATTACCTCTATAAAATCC

GAG

>GW615908.1 Jc2-005-H03-M13F.H03.ab1 Jatropha curcas flower and seed Jatropha curcas cDNA, mRNA sequence

AAAAAAAAAAAAAAAAATCGTTAGGTGCTCTCTCTCGGTCTGTTTAATTTTCTCAATTAGTTATTTTTCTTCCGAATATA

TGGATAGTACAGCTAAAAGTATGTTATGATCATATACAGTTTCTTTCTTGTTCTCTCTGTTATTATTATCTTTTTTTTCT

TGATATGTGTTGATTAATATTTGGAACATGCTAATGGAAGATTGAAGACAGTAAGGATATTGTATTTATAAGTTATAAGA

TCAGATGATTTCTTCTGAGTTTCTAGTTGTGTGGATCAATTAAGCCATGGTTTGAGCCTTTTGTTGTAGTTTCTGGAGTG

GAATCTGTTGCGAAATTTAACCTTTGATTGCTAAGTTGCTTCTTTTTCGATTCTGTAATTGCTAATCTGATCAATTGGAG

CAGTTGCGGCATACAACATTTTAAACTTTGAGCTAGGTATTGGAGCAATTATTTTGGCTATCTAATTAGTTTATTGGCAA

ATATCAGATGCCCGGTTGGTGAAAAGAAGAAGAAGAAAAAGTTAGAGATATCATTGAGCTAG

>JK317422.1 JCST189 Jatropha curcas L. seed cDNA library Jatropha curcas cDNA 5', mRNA sequence

TCGGNATNTCGGAGGTCGAGTTTTTCGCAGTTCATCTATGTCGGGTGCATCTATGTCGGTTGAAGCCTGAAGGGCACTCG

CCGGACTCGTGACCCGGCATAATTACACCCGCCATGTGGCCGTAATTGTGACTGCAGAGATGGATGAATCCGAATAAAGA

GGCCCGAGGATAAGACTGACGCTCAGGTGCAACGGTGGGAGCAAACCGGATTGATACCCCGCTATTCCACGCCGCGCCGA

TGTCGACTTGGAGGTGGTGCCCATGACTGTGGCTTTCGGAGCTACCACCTTATTTCTACCGACTGGGGCTGCGGCCCCCA

GGTTTTTCATTAACGCATTGACGGGGGCCCGCACACCCGCAGGAGCATGGGGTTTCATGTTTACAACCCTAAAATCTGAC

CTACCTTACTGATCCATATAATTTCGAGATCTGGATCGATGCCTTTGGAATTTTGATACAGGTGCTGCATGGCTGTCGCC

TGCTCCCTCTTCC

>GW878689.1 JC002955 Seed specific Normalized cDNA library from Jatropha curcas L. Jatropha curcas cDNA clone N05615 5' similar to Unknown protein, mRNA sequence

GGTGGCTCGCAGTGTCAATAATTAATTCGTGAAGTGCGCGTTTTTGCGCTCTGAAATTCAAATCACACATTTTCCTTCAC

CACGAAAGAATTTCTCGAGAACCAAACAGAATTTCGCGGTTATGTTCCCAAAAAGGTGAAATTTAGGGGTTATTTTTTAT

ATTGATGGGTGGATTAATTACCTTAAGAGATGACGATCCAGTCGGACTTTTGAGGTTTGATCCTTAAAAACCCTCAAACA

CTCTGGTTTGCTTCAATCCACAGCTTTATATATATAGTCTAGCATTTCTGCATGTGTGCTTCCCTAAATTTATCTCCTCC

ATGATATGATAATTTTTTTGGATTTGAAATGATATAAAATTGTCAAATCTACTATAGGCATGAACTTTAGAGATGAAAAT

AATTTGGAAAATTTAGGTTAATTTTGTTTTACCATGTGTCACAACTAGTAACTTCCCAAGGTGGGAAGCGTCACGCCTGC

AATAAGTTTTATCGTCATTACAATGAATCTCAAATTGGTGGCTTAATTATTAGTTACAAGTAATATATTGCCTGGTTTGA

AGGGGGAATAACCTTTTGATTTTCGGCTAGCCTATATGCCTGCACTTGTTTGGAGAGATCACTATAAATGATTAAAGTGT

GTCGTTTTCAATCCATAAATATTCAGCCAGAGCTGCTCCTTGTATTTTTTAG

>FM890773.1 FM890773 Jatropha curcas embryo 56-70 (DAF) Jatropha curcas cDNA clone rjcaeb0_000712, mRNA sequence

AATGATCAAGGTTAGAACTTTATTTTCTGCGAGATCTCTGGTTCAAATCCTGCTTAGGATTGCGAAGAGAGCTATAGAAA

GATGCAGTCCTATTGGTCATCTGACTTGTTCTATCTTTCAGAAAAAGAAAAGAAAACATCTCCATTGGCGATTGCGAGCA

CTAGTTAAGAGAGACTCATTTAACATGCCTTTCAAAAAGATGAAGTTTCTGAACTAATTTTGGCGATAATAAACAACCAA

TCAATCCAGTTCTTGGTTACGCATAATCGCTCCAGATTCTGTAGTTGTTGGAGTACATTTGGATAGAACAAACCTTGAAA

TGAATAGGATGAAGAGCAAAACCCCTTGCTTTTTTTTTCTCAAAAATAAAAATTATCATTGTTTATAGCGGGCGGCCGTT

GGCCAGTTACATCTCTTTTGCTCAAACACTATGGTTAATGTTAGAGACACAGCAATGCTATGAGCCTAA

>GW877466.1 JC007270 Seed specific Normalized cDNA library from Jatropha curcas L. Jatropha curcas cDNA clone N12231 5' similar to Unknown protein, mRNA sequence

TATTCGCTTTCATAAACAATGTCAGACAACTTCCTTCTTTTCCCTTCATTTCTTTTTATTTCTTCCCGGCAAATAAGGAA

GAGAGAAAATAATTCTCCTATGAAAATTTCCCCATATTTTCTGTAACTTCACTTTTGTTTTTCTTTTTCCTTATTTTATT

ACATGTTATCATTTTTTTTTTTTTTTTTTTTTGTTGCAAAATAATAGACTTGAACCTTACAGAAAATTTTATTCTACAAG

GTGATAATTTCATATGCATTAGTTAGTCAATCACAAAAATGTTATGTTTACCATTCCATTTCAAGGTTATCTTGTAGCGA

ACTGTTTAAAATTTTGAACTTTTGAACTGGAAGCTTTCTAATAACATTTCTTAAAAAATTAAGTTTGGGATTCCGAACCA

AGGAGAAAAGTAAAAGCACTATTACTAAATTTGCAATTTTACTCTATGCTTTTGTTATTTACAAATCTGACCAAATCCAA

AAATACATTTCTAGATGA

>JK317925.1 JCST696 Jatropha curcas L. seed cDNA library Jatropha curcas cDNA 5', mRNA sequence

TTATGCTTCGTTTCGGTTCGATTTCTTGGTTCATTGTTAAAAAATTTCGAAACAGACCCAATGAGGGCAGTAATGTCAGG

TCTTTATAGAGCAGTAGGAAATTTGACGAAGTGTCTTTACTTTTCAACTTTAAATGCACATTGCTTATTTTATATATCAG

CCCTCGTCCCGAGTACAATAGTAAGAACACTAAAGAACAATCTTGAAGCATAACACACTAGAACCGAAATCATTTTTAGT

GCGATGAGTGGGCATAAATAACCACACCTCCAACATACGGTATAACTAGCCCTGAAATAATATCCCGCTTGTGGGAGATA

GGAACAATGCGACCGCGTCGAGGGAGCAAATAACATGCAGTCCTCTTAGTCTAGGAGTGCGGATGGTGTTGCGTACGTGT

CCTCTGGGGGGGTCGTGGCAGGGGGAGCGGTCTTGCACTGATCCAGGCCTCCGGACAAAAAAAAAAAAACTGAAAAGAAC

AATATGAAACGCCTATTTGCAGCCCTCTCCAAGTCCCGATCAGATTATTCGCTGTTTGTGATAAAAGCGTGCATCTTAAG

ATAACCAACAATACGACTTATGAGCCCACTGCATCACGCTATAAAAAAGTTCAATTAGTATCAAATGGAGAAAAAAAGGA

ATACATTATACCAATGCCCACAAACATAAAACCACAGGGATAAAAATAGGAGACATCTAAACGACGATCAACGCGTTGCC

TCTAGTCGTCGCACGAACGCTCAAGTCTATTACTACTAAACTCATATGTATAGAAAATAGATAGTAGTTCTATATATAAT

ATAACTTATGTAGAATCAGGTA

>GT969702.1 GJCCJC2001D03.b Jatropha curcas L. developing seeds (mixed stages) Jatropha curcas cDNA clone GJCCJC2001D03, mRNA sequence

ACCCTATGCCAACTATAATTTACTCTTTCTTTTCTTTTCTTTATCATTATATAACTAAGATCCCGAATACGACTTAATAA

TATGTGAGTCAGCATTCTCGCCAAGCAAAATTTCAGGTAACGAACCCATTTTATTTGTCTCTCAAATCATAAATAAGCAT

TTATACATCAATCATCATGAAATCTTGAAAGTACGCTCATTTACATATATTTATTCTTCTAAAGCAACACAAAAAACATC

AAGGTAATTGATGTCAATTCGCAAAAAGAATTAAGTTTTTCTATCTTAAAATCATGGATCACACCAACTAATTAATTCCA

CGCAAGAATTAGATTTACAACCTTAATTGTACCATGTCAAACCAAAAATCAATAGAAATAATTGAAGATAGCTGCTGCCA

ACCTAAGCAAATCAAGGTTTCTCGATCAAAATCCATATTATAAACACTAGTTAACTCATTTACCAATAAAACTTAACTTA

TCATGTCAAATGCCCCATACCAAAGTAGAAATTTTAAGCAAATAGCTAGGTTGGCTGCTACCATATCACAATCATTCTAA

TTTCTTTCATAGGACCTACAATAAACACCCTAACTTACACATTTCCTTGAAAAATGCAGTTATAGTGTCAATTAATAGTC

AAAATCAGATTTTTCCCAACTTCAGTCGTGTGAATAGTGTTTCAGGAACCTAGCATTTCTCATTCATGATT

>GW878244.1 JC002620 Seed specific Normalized cDNA library from Jatropha curcas L. Jatropha curcas cDNA clone N04982 5' similar to Unknown protein, mRNA sequence

GGTGTAAGTGTTGGTGTCTTTTTTTTCCTTTGTCAAATTCTGTGATGAATAGTGCAGTCAATCTTTTGGAATTTTAGAAT

GGCAGCAAGGAAATTACTAGGGTGGATTCTGTGTAATGATTAAGTTAATTCACTTGTAACTTGAAGATTATGGGTTCATT

TTTTCGCAAAAAATAAAGGGAAGCCTGCATATATCAACCTTTTCTAAGGCTTACAAAGGTAGGATAAGTAATGTATTGGC

TCATCCCTTAAAAATGCAATAAGAAACAAGAATAGTAAAATCTGTTTAATTTAATTAAAACTTAAGATACTAATATGGTT

TAGACAAAAAAACAAAAGGTAAGTACTTTATTGATGTGCTTTGCCTTGCACTAATTATGCCTATAATTTGATTTTTCAAG

TTGGCAAGAGGAAGATTAACTCGAAAAACTAAGACATATTGATTTGGTAGGCGTAACATACATAGCAACATTGAAGGATT

AAAGGAAATTGAATTAAGTTTAATATGCCTTGTATGAAGATATAGAGTAAGTACTGTACTCATCCTGCAATCTTTTGTGT

GTTATTTTTGTATGATAATGGAAGAGATTCATCATTTCCCAATTAAAGGGATCAAATCTATTAATTAAATCTCAAAGTTT

TTTCATAAAATTTGCAGTGCATTGATCATTTTTTGATTGATTTTAT

>JK610920.1 JCF3196 Jatropha curcas, immature Seed cDNA subtraction library Jatropha curcas cDNA, mRNA sequence

ACATTCCATGCTGATACAAATTCCAATCACCAATCTTACAGCATACACATTAGATTATGGGGGAAAGCAACCATTCCCCA

CCGCTACACTAACATTGCTTAACATGTAAGCAAGGACAAGTAACATCATCTAACCAAAAAAAGAAAACCCCAACCCCAAG

CCACAGAACAAATATTTTTGGTCCATAAAAATATTGGAACAAAAATTAATAATTGAAAAACTAAATTGTTAAGTGCAAGA

AAATGGTAAGCTGCAGAAGGTAAACCTCAAATTTATGCAAACAGCAAAATAGAATTAGAGTGCCTACTCAACTTCTATCA

ATTTGGCGAACCATCTTACAGCGTGTCATCGACAGCAGTTGCAGTCTTCAAATTACCGCATTCTGTTACCAAATTCTATG

ATGTTCATTATTTTGATTGGATGATTTTAAAGAGTTAGTTAAAACCGATGTAGGCGCAAGAAGAAAAAATATAGCCACCC

TATGCTCCAATTGGCAAAAAGGTTGACCGGGACTGGAGCACGAGCATTCCATTGAAAACAAGACTTGTATATTAGATAGC

TACAGTGAAATAGTGCATGTGAAACACGTGACAAAGGCACGATAGTAGAACATTACTAGCTTAAGCATCAGCTGCTGCTG

AACCTCGGGCCGGGAACCACCCCTTATTCTTAG

>FM893376.1 FM893376 Jatropha curcas embryo 56-70 (DAF) Jatropha curcas cDNA clone rjcaeb0_004183, mRNA sequence

ATCCAAAGAAAGAGTTAGCAGCAAGCATTCATAAGGGAATTTCATTTGTCAGGAAATGAGTCATGGCTAATAACAAGGGT

TCATTTCTAATTTTCTGTCCCTGTTTTATTCCTTTGATGCATTTCCTCGATATCTGGGTTCCTTGAAATAAGTAGCGTCA

TCTCGTTTTAGCTGTCCATAAAAGGCAAGCAAACAATTGTAAGAATATGGTGATGGGGCTTTCTTCAACAGGGCTTTGGT

GATAATTATGTAATAAATACAGAAGCACTTCAAAGCTAGGAACTATTCTCGTCTCTTCTCTTCATAATTTTTGTTTCCAA

GATAATCTCACCAACTCTGTACTCAAAAAAAAAAGGGGGGGCCGGTACCAATCGCCTATGTGAGTGTAT

>JK317618.1 JCST389 Jatropha curcas L. seed cDNA library Jatropha curcas cDNA 5', mRNA sequence

ATTTTTTTTTTTTTTTTTTTTCTTTACATATTTTCTAGCAGAAGAGCGCCCCTGGGGTGGCAGGGGTTGTTCGTTTTCTC

TGCGTGCTGGGGTGGGTATAAGGCCGGAAAAGCCCTCCGTGACATCTCCAGACCTAGCCAAAAAAGTGGAAAGGGGTCTG

CTCTATTAAAGGACGGAAGAAAAGAGTGAAATTCCCGGTGTCGCGGTGAAGTGCCTAAAGTTAGGGAGGACGGCCGGTGG

CGAAGGCGGCGGTCTGAAACATTTCTGACGCTGACGCTCGGAAGCGTGAGAAGGAACAGGATTCGAACCGTGCAGCCCAC

GCCTTAAACTATGGGCCTAAGTGTCGGACTTTACGGTCTGTGCCACGCTAACGCACTGTATTTGCGCCTGGGAAGGGCGG

GCACAAGGCTGATGATCAAAGGAATTGACTAGGGCCCGCACATGAGGGGAACATGTGATTAAATTCAACACAAACCAAGA

ATCTTACCCATACTGGACATGCAAATCGTAGAATGGTGAAAGCCTGACGACATCGACATGCCTCCTGATAAGTGCTGCTA

TGGTGATATTAGTTGAGACGTGAGGATTGGTTTAGTCTACATGAGCACAAGCCTGTCTTTGATACTAACAGTACGCGACA

TTCGTACTGCTGACAGCAGAACGGCGCGGAACGTAGAGAGACGGAAGTCACATGGGTGATGCTGCGCACTCACTACTACA

TGCATGGCCATTCATCTAACTGCAAGACTAATCCATTGATCATCATGATTTGAGCTGAGAATCATGGCATCATGTTCGTG

AGACAATTGTATCGCCCTAATGCGAAGTAATGCGCTCGGAGCATA

>GO247676.1 JcrME_RL1220 Expressed sequence tags from Jatropha curcas root cDNA library Jatropha curcas cDNA, mRNA sequence

TAAAATAGAAAGCTCATATATTTCTAGTCACTAAAAGTACCAGGAAACTAAAAGGTCCACGCTTTCTCGCATCTGCAAAT

TTAGAATCACTTACATCCAATAACTACAAGTAGCCATATTAGCCAAAATACAGCGCTCGAAGCTATGTCATTAGCACTAA

TAAAAAGCCATAATCACTGGGTTTTGTTTACAGGTTCTAGCGCTTC

>GT228781.1 JC346 Jatropha seeds from fruits at three stages of maturation Jatropha curcas cDNA clone PL07SE.H08.scf 5', mRNA sequence

ACCGGGATCCGATTAGCAGCGGTATCTACGCAGAGTGACCATTACGGCCGGGCACTGAGCTCATCCTATGGCGCCTTATT

GCCATGCACTCTAGCCAGTGAGCAAGTGTATCACCAATTGCAACCTTTTTACCACCTAATTGTAGTTAAATGACTTGGGG

TAAATTTATGTATTAAAAGCTTGGGCAAAATTTAG

>GW616463.1 Jc2-012-B10-M13F.B10.ab1 Jatropha curcas flower and seed Jatropha curcas cDNA, mRNA sequence

GGGAACTCCTTTTGATCTAGGGTTTTCAGATTCAATCCTACACTTCCTCTGTTCTCTCTTCATTTCAGTTTCGGATCGGA

CGCCCCGTTTTGGAGATCCGATTGGGTCTCTTGTTCTTGGCTCGAGATCCGGGCCAAGCACAGGAGTGTCGGATTCCGGT

TTTTCGATTGAATCCTCCTCAACTATACTTGCGTAAGCGTACAGGTTCTTTCTCTTTCTCTACCATATGATTTTTTTTTT

TTTTACTGGTGGTATGTTTGGTTACTGGGAAAAGGAGGGAAAGGAAAGGAAAGTGAGCTTTTGAATCTTAGGTTTTCGCA

TTTTTTTATATTTCAAAAAACACAGCAACTTTACCTAAACAATTTACTGTCAGAGAGTGACTTTTATGCTTAGAAGATGA

TACTATTGTTTGAATACCATAGCTTCTGCTTTAAGTTTTAGCATCTTAATCAGTTCTATCTTGTTTTCGTAATTCATGGT

TTAAAATTCATTGTTTCCTTCTGTACTGTGAGTTATGCTTGTCCAAGTCTTATTTTTCTTTTTAGTTGAAGTTTTTACTT

AACTATCCGTGCTGGCATTCTTTACATTTCTAATTAATTGTCAAGACTTTTCTATGGTCAGATTATTCTGATAAT

>GT970096.1 GJCCJC2005B07.b Jatropha curcas L. developing seeds (mixed stages) Jatropha curcas cDNA clone GJCCJC2005B07 similar to putative microsatellite, mRNA sequence

CCGGCCACAAGCAGTTGCTGCCCTTCAACCCGGCAACTCCCATTCTAGCAATTCGACAACAACTATATTTGGGGTATAAT

TAATTTAAATACATTTTAGTTAATCGATTTAATTTTATTCATGATAGTTAATTAAAGTTAGTTATTTTAGTTAGTTTAAT

TTTAACAATTTTAGTTAATCTAGTTAATGTTAGTAATATTAGTTAATTAAATTAATTTTATTATTTGTAGTTAATTAATT

TAGTAATGTTGGTTAATGAAATTAATTTTTATTAATTGTAATTAATGAGTTAGTTAAGTTTAGTTAAGTTAATGTTAGTA

ATGTTAGTTAATTAAATTAAACTTATTACTTTGTAGTGAATTAATGTAATTAATTTAATTTGGTAAATGTGGATAGTTAA

TAGCTTTGAAATTTTAGTGTTGATTTAATTAAGATAGTTTAAACATAATGTTATTGTGTGGTGATGTTGAGTTTGATGGA

GGATGGGAAATTTGATTGATTGATTGCCCGATGGATGGGCTGTGTGGTTTGGAAATATTAGATATATTGTTGATATTTTA

ACAGGTGGATAGTCCAATTTTTAGGGGGAGATGCTGCCGAAATTTCGGTAGAAAATAAAATAATTTTTATAGAAAATAAA

ATAGGAAATAAAA

>JK317937.1 JCST309 Jatropha curcas L. seed cDNA library Jatropha curcas cDNA 5', mRNA sequence

TTTTTTTTTAATCAACAAAGCAACCATTATTATATAGATCAGTTCCCCTCTTAAAACCGAAGGAAAAATTAAAGTATTAG

TTAAAAGGAGAGAAACATAAAATCAATACCTAAAAAATATCAATCAATCATCGCAGATAGAACTGGGCAGAGAGTAACTT

TAAATTCACTAAAAACCTATTGAAATGCAACAGGGTGCATCGTTTCCTCACAAATACTTATTGCCATAGCATATATGAAT

AAGAAATCTAAAAAGCTAAGAAGGGTTTAGATAGTATGAAGGGAGGAATATTATCCTGGGGCTACACCTTGGCTTCCAAC

TTTCTTTCGCTAAATGGAGAAGAGGTAAAAAAAAATAAATTCTCATTCACTCAAGGGCTTTTCTGTCGGTAGACTCCCTA

ATGTTCGGTAGTTAATTTCCTTTAAAACTATGTTTGTTCTTTTACAGGATCTTTAAAACGTAGCCCGGTTTTAATACGAC

TCTACGCAGGAATCGTCAAGCAAAGGTGTTAAAAGCACAGGGTTCGCCAAGCCGCTTTGGACGTTCCTTACCCTTATTGG

AGCACAACGGGGCGTCTAAGATGAGAACTTTAATTTATTAACAGTTCATTACTTCTTTATCCACTTGCACTCTGGCAAAA

TCGTCAGCAAACACGAGATGAGTAATATACGCTAACCGCAGGCTTCTCGTTGGAGCACTGCCGCGTTACTCGGCCTTTAT

GAGACGCTCAGGCGCGTAAGTGAGTCCGACTTACTCGCCACGTGCTCGTCTTTTATTAGCTATTGACGTTTTGCTACGAC

ATGCGCGCGGCACGAGGAGCACAATCAGACAGACA

>GT972427.1 GJCCJC2034C03.b Jatropha curcas L. developing seeds (mixed stages) Jatropha curcas cDNA clone GJCCJC2034C03 similar to putative genomic DNA, mRNA sequence

CGCTCCTTAGGCTATTAGTGAAAGCGGAATCACACATCCGGAGTTAAAGTTAAGCTTAAACAGCAACTTAAACAGCCTTC

CTCATCTCTCTCTCAATCTCATGAGTTCAAACCCGGGAGAGGGACGAAGTGACTCGACTGTAAGGAGAGGAATTTTTAAT

AGCAGTCGGGAGAGAGCGGCGTGGAAGTTTGCAGTCGTTAGGTATTTATTCGTGAATCTATGCAGGCCATGGAAGAGAAA

GACTTCATATATGCACAAAAGGCACTCGATTAGCTGGCTACGACCCTTATTCCAGTAGAGATGAATCCCAAAAGAAAGGG

TTGGTGCTAACTCTGCATCGATTCCGCCCATTGCAAGAAGACGGGGTTAGCCTTTCTTTGACTGTCCAATCTCGGGCAAA

GGAACTGACATATGTTTTCAAATGTCCATTCCCGTATTTCCATAAACTGTATCGACGGACGTCTATTAAGGGAAATCAAA

TCGGATTGATGCCAAAATTCCCACTGTTCTGGAGGTTTCTGCAAGTGAATCAGAATAGGACAAGGAAGTTTCATCCATTT

CTTACTTTCCTGAGCGAAGAGGGGAATGCGCTCTTGCTGCAAGAAGGGCTTTTCTCGAATCAACTGTGGCACTTGAGCTA

GTGGCATATCTTATTGAATGTCCATCTTTAGAAGAAATATCCGACATTCCACTTTCGGAATAAAGGCTATCAGATAGTGC

ACATTCCCCTTGTTGGGATACCTTTCATATGAAATTTAAAAGAAAGAATTCCTAGATGGGCTTGGTCTCGAATGCGCTCT

TACTGC

>GW877957.1 JC003559 Seed specific Normalized cDNA library from Jatropha curcas L. Jatropha curcas cDNA clone N06587 5' similar to Unknown protein, mRNA sequence

GGACCTCTCGTGAAGTAGAAGGCTACAAGGTGTGCACAGACGTCCTTCATTATTATGATTTTTTTCATTTCTCGTTTTAT

TTTCAAAATTTTCTTGGTGATGTTACTTGCCGTTTGACATCCAAGAAAATCTCTTCCTTTTTTTTTTTTTATTTGGGAAA

TCGGCATTTTGGGTGTTAGATTTCAGAGTCTGGTGGCTTGATTTTGTTGGGTTCTTTCCATTTTTTATGGGTTTAAGTAT

TTGCTTTACTTTCTCCGAAATAATTTTCTTTCCCTCGCTTTCTTTCTCTGTTCTTTTTTTTTTTTTTTTTTTTAACATTT

CGCTTGATTATTATCTTGTTGAATTTTTCTTTGATTATTTGTTTAATTTGTGCCTTGAAAACAATTTATTTTAGATTGCA

TTTTTTTATGGTTGCCTTCTAGATTGATTGTCTTAATGGGAAAAGAAATGTTATGGTTCCAATCAATTGGATTTTGGTTC

TATATATATGGTGAAAAGATGCGAATTTACTTCTAATTCCATTCGTCTCTCTCGGTGTGTGGTGGGAGAAAGATGGAGAG

CTTTGGGGAAAATGAATTTCAACACCGTCAAAGTTCCTAAGG

>GT971710.1 GJCCJC2023F03.b Jatropha curcas L. developing seeds (mixed stages) Jatropha curcas cDNA clone GJCCJC2023F03, mRNA sequence

CTCCGTGTCTTGGACTCCCACTGGCACCACGTTCGCTTGCATCAAAAGTTGGCTCATGCTTACAAAGGACTTAATAAGGG

ATTTCGTGGGCATAGGGTTATCCAGCAATTACATAGTAGCCCCGAGTTAATATTTGTAGTTTCCTATAAAAAAACAGATT

TCTCAGAGTATAAATTCACTATATTTATAATTGTGAGAACATATTAGAGATTTTATAAACTTTATTAATTAGCATGTATT

TTTTTAGTTGATGCGACTCCACTTTCTTCATTAATAATTAATCCTATTACAGGTTTGAATTGTAGTAATATTGTTAATCA

GCATGCTTTATGTGTGTTTGAGTTTTCGGCACTGTGACTCATAACACATTTGTAAGTTTCTGTTTACCAGATTGTTGCAT

ATTAATGGTTGTTTAACTGGCTAGTTGCTTTACTAATTCAAATTGGATGTGCTCATAAATTTTTGAACGGATCAAGAATA

AAAGCTAGCAGCAGATTAATGAGTGCCTTATATGGAATAGGAAGATAAAACTCATAATCTAAGTACCTAACTTATTTTTT

AGAGGGTATTATTATATTAGTGAAATACGAAGATTACCCTACACTTATAAACTTAAGAGGCTTGAGAGTTTACACAGGT

>FM891553.1 FM891553 Jatropha curcas embryo 56-70 (DAF) Jatropha curcas cDNA clone rjcaeb0_001863, mRNA sequence

ACCAAATCAAAAAACATTTTTATATTTATACACCGATACAAATATTTACCGTATTTAACATCCCAAGAATTGGAAAGTTA

TAAAAAAAAAAAAAAAAAGAAAGAATAGAGCCACTCACAATCCTCAACCTCAGCCACACCAACTTAGGCCTCCAAATTTC

TAAAAATACCCTCACACTCAAAACAATGAATAGCACTCGGGAGTAGTAATAATATACACATTCCTAAACAAAATTTTTCC

TATTTTTCACTTTCATATCTGATGCAGAAGAAAAACTCTACAGAAATTTTTATATAAGCAGTTTATGGAAAAAGAAATGG

AAGATATCAACACATAATAATCTGCAACGAGTACAGGCAAACACATTTCCAAACTCCATGACATGCCCACCAATGCACAA

GGAACTCAATTGACTGGATACAGCTTCTTCCAAAATTAAG

>GW880199.1 JC001322 Seed specific Normalized cDNA library from Jatropha curcas L. Jatropha curcas cDNA clone N02481 5' similar to Unknown protein, mRNA sequence

GTCAGCTAACGGCCTCCTGACCTGTCGACCTGTCTTGCTTCGTCATGTTGCCAAATGACAAATTCATGTGCCCATTACTG

AGTCTACTCACGATTATTAGCCTTTTTTTTATTTATGTTTTAGATGCAAAAATATCTTCCTTCATCCATTTCTAGTATTA

GTGTAATGGTGTTTTTACTAATTTTCTGCAAGTATTTGCAAGAGGATGTCAGAAACTGATTTTTCATATTCTTGCTATTT

ACAGGTTGCTTCACTGGAGAAGCTTTATAAGATTTTCGAGAGATGCTAACTGAAAATGGTTCTCTTCAAAACCAATCATA

TAATCTTAGATTAGGTCTTTTTTTTTTTGGTGGTCTCCTGCCCGAGTTTTTGCAGTCCCTTTCGCGGTCAAACAGGAGCT

TATATATATATACAAATTAGTTGTGTATATTTTCCGTAAAAATAGATGTCCAATTGCTTTTAGTTCACGGATGCTTCATC

AAACCAAATCTCTCCCTTGCATGTGTAATTGCGTTTTTTTTTTTTTTATTT

>GT975095.1 GJCCJC2067C02.b Jatropha curcas L. developing seeds (mixed stages) Jatropha curcas cDNA clone GJCCJC2067C02, mRNA sequence

AACTTCTTCCTTCCAAGGATCAGATCAGAGTATCTCTCTCTGGGTTTTGCTTTGTATTTTGACAAATGCAACGTATGTAC

CCCACTTTTGAATTTTAGCCAAAAAGACCCCATGTACCAATGAATAGCTTGAACTTGAAGAAAAGTTTTTTCCTAAAATT

ATTTTTGCGTTTCTTTTACTACTTGATTTGGTTTATCTAATAGTGGGTTTTTTTTATGCATTTCATGTCAATCTCAAATC

TCAAATTAAGCTAAAACTGTGCTGGGAACAATTTTTTAAATCAGAAAATATTATGGATTATATAATAGAATAATTTTCTC

GTTTATTCATCAAATTCATTTGACAACCGAGAAGACTCTCTCCCTCAAACCCGAGACCTCCCACAATAAACAAATTCATT

TGACAACCGAGAAGACTCTCTTCTCGAATCCGAACTCGAGACCTCCTATTAATATTTTTACACATTTGTCTTGTTACTTT

ATAACTTCTAGCTCAGCCACAGTATAAAGATATGTTAATATATATTTTCAAAATCCATGTTGAAAAGAATAACAAAACTG

ACCCAGCA

>GW878534.1 JC001970 Seed specific Normalized cDNA library from Jatropha curcas L. Jatropha curcas cDNA clone N03548 5' similar to Unknown protein, mRNA sequence

GGACAAACGGATCCCATTTCACAAATCCGACACACACACGCACAGAAGTAAGAAAAAGAAAGAGAGAAAGTTGAAAAGAA

AGAAAGGAAGAAATTAGGGAGTTGGGACTAACATTCAAAGCAGAGAAGCCAGAAAAAGATTAAGAAAACGGAGGAAAATA

TTGCTTGGCGGCGTGTTAACTCTATACAAAAGTGGGTGTAGGTTAAGGCTAGTCCGAGTAAACAACGGCTGAGTCCTGAA

CTTTGAGAGAGAGAGAGTCCAGTGGAGTAACGAACAGAGACACACCTCTCTCTCGTCTCCGACACAAGAGACGTGTCCTA

AACCAGAACCCACGAAAGAGAAAGAGATGTAAACGCACCGTTTTGTGCAAACCATTTACTTGTGTTAGCTAACTGAAGCT

GCAGGTGTGGGGTTCATTACGTAATTTTGGCCGTTGGAGTGCAATTGTTGAAATTATGGTGGTTTACCCGTGTGCAGCCT

AACGAAAGGAAAATCTCGTTGTGGTTCGCCTTTCGCTTGGCCTCTCCTGCGGTAGGATTTCTTTTGCTGCATTTTTGAGG

>GT982346.1 JGCCJG2066B09.b Jatropha curcas L. germinating seeds (mixed stages) Jatropha curcas cDNA clone JGCCJG2066B09, mRNA sequence

GTGGTATCCATCGATAAGAAAGGCTCGACGAAGTGAGAAGACGTCGGATGAAAGGAGAAGGCAGCGGCCCAGAGACGAGG

TTCTGTAGGGACGTAGCGAGTTGATCTTAATAAACAATATTATTTCATAATCGGGGAAGCGCCCAACGGGATAAGAGCTG

AAGGCTGACACGGGGTCGATCTAATTTCAACTAATCATTTTCGCCGGCAAGGAAGACTGATCGGGCAGGCATAGGTGCAA

CTAGTCCATATACGATTCAATATGTCTCACTAGCACACTTTCTTAGCCATAAGCATAAGGAGGAGTATAAAGACTAGTAT

CTCGTCCGGATACATACCTATGACCATGAGCGGGAGAAGCAGGGGCGGGGGCACCAGTAGATCGGACCCATAGCCTACAG

CAGCATAGGTAGTGGCATCAGTACCAGCAGCATCATAGGCATCATCTTAGCCGGATCGATAGGCAGATTCCTTAGTGATC

TAGAGCCATACAAGCATCCTTTCGCCCAACTCCATACGTAGATTCACTAGTGACCTTAGCTTGGTTCACAAGTCCAGGTG

CAGATTTTCTAGTGACAGAAGCAAGGTTATTAGATACCTAAGGGATTTTTTGGTCTTAGCATGTTAAGACCGGTTATTCC

TATCCTATTATATATTTCTCCTATTTCTATTAC

>FM888294.1 FM888294 Jatropha curcas embryo 35-55 (DAF) Jatropha curcas cDNA clone rjcfea0_001680, mRNA sequence

AAATCCTGAATGTGACTGTCCCCAAGTCTTAGAAGAGAAAGAAACCCTTCCGTTGGTGAATCTGTCTTGATATTGTCAGA

GTTTACACTGCCATGTTAGATGAACAGAAAGGAAGATTGCCATATTCTTTCCATAGTCTGACATTGTGCCTTCCAAGAGG

TTTTGCGGTTCTTGGGATTGTTACAGTTACCAGGTTGTAATGATGTATGAGTAGTTAAATGCAGTCCTTTATGAATTGTG

GTCAATTTTACAGGCACGTGCTTCTTTGTGGTATTTTCACACATTTCATTGCTCTGAATTGAATCACCTTTCACCAATGT

ATGTGGTCGTATAGTGTGTAGTTCCTAATCCTAGATCATACTAGTTCATTGATATCCTTTAACACCACCTATATTGGCAT

ATGTTATTATTATTATTATTATTTTTTTTTTGGTGTAATGAAAAGGTGTAAGCTTCTTGAGTATAAAGTTTCTGGCTCTT

CAAGCAAAGTGCACTTTCTAGGAATTGAACCGGTGTTCTCACCCTTATGGGAATTGAGATTTCTTA

>FM888700.1 FM888700 Jatropha curcas embryo 35-55 (DAF) Jatropha curcas cDNA clone rjcfea0_002158, mRNA sequence

CAATTAAGCGGAACAAGCAACGGACTTTGATGGCCCACTCTGATCATTTAAGATTACTAAAGATGGAATTGTTCATGTGA

AAACTATCATTTATATAAACCTCTACCGAGGTAATGGGGACCCCCTACTCTTGTACAAAGTCCTGCGAATCACATATCCC

TATTAATAGTACTCACCACTTGCTAGTTGTTCTAAACTAACTACTACTTTATGCAAGTTCAAGACCCAAAAAATGTGTCC

CTATAATGACACCATTATTGGTATGAAAAGAATAGGTGATATATGTATATATTGATAT

>GW612582.1 Jc1-018-C12-M13F.C12.ab1 Jatropha curcas flower and seed Jatropha curcas cDNA, mRNA sequence

AATGCTCGAAATAATTTGGTTTGTTTACATGTTATGGGAGGCCGCATAATTAGTGTTAGTTTTGGCTTTGATTCGGCATT

TGGTTAACCGGTTCTAAACCATGAAGGCTGTCCTAGTTCAATTCTGAGTTTCAACTTTATTTAAATTGCATTGGAATGGA

ATTTGTAATGTGAAGAATCTAGATAGACCAGGAAAAGAAGAAAAATGGAATCTTTGTGTTGCTTCTTTCTGGAATCTCTT

CTTGGGACTCGTTGATGGCCTTCATTGCTATACTATACATGAACTTGCTC

>GT972167.1 GJCCJC2029D04.b Jatropha curcas L. developing seeds (mixed stages) Jatropha curcas cDNA clone GJCCJC2029D04, mRNA sequence

ATACCCATCATTGATTTTCACCTTTCAAATTTAATCACAATCAAATAAATGGTTTGTTTGACTGCACCTATTTTATATTT

CATGACCTATAACCAATTATATACAACACTAAATCCTAGATTTCCTTTAATAAGCCAAGTATAACTTACAAATTTACGTG

TCTAACAACCCTGCTGGTTATTATATCCTTACGATGAATCTGAGTCCAACATACAAGTATGTGCTAAGTAAACTCATATC

TTGGTTTCCTAAGCATACTTTAATTCATGTTTTATGAATTCAGCTTCGTCTTGGCGTAGAAAACTAAATATTTTTACCCG

TTACTAATTGTAAACACGTGTCTATTTATCCTTATATCATAATCTGTATATGAAATCAATAACACTAAAAGTTTCCTTAT

TATAAAACTATAACTTCGATTTAATCTCAAGTCATGCTAACAAAATAAGCTAACAACTAATTTATTCATGTGATTAGAAT

ACCCAATAACTTAGCTACTGCATTTCTACCAACCCCTCCTTAATTTAACTTTCTGTGATATTTACGGCTTAGAAGCCTAA

TTTTCGATTTCTGTCGTCTAATGATTCGAACACTATACCTTATCAAAGTGCAAACTAATTGCAAAACTAGATTCTATGTA

AAAATTGGAATATACTGTCTATGAGGAATTCCGGTCACCGAATCCACCGCTGTCGCCGCCGTCTATGGTGGCCGGAAGTA

CCTTGGTTCCTAAATCTTGC

>GO247236.1 JcrME_RL0780 Expressed sequence tags from Jatropha curcas root cDNA library Jatropha curcas cDNA, mRNA sequence

TGGCTTACCTTCGATGGGTGACCTCGAAGCTCGCCCTTAAGCAGTGGTATCAACGCAGAGTACGCGGGGAGATAATTGGA

TCTTATCTGTGGATCTATTTATCCCTGTTCCATAAATTTCTCTTTCCATAGATTTCTGTTCCATAGATTTGGTTTTTTTC

TTTATTCAAGTAATGGGTTTCTTGGATTTTTTGTGATACAATCGCTTCCCTGATTAATTACCCCAGAAATAAGGGTAAAA

AAACTAATCCCCATATCCCTCCTATGGGTTGTTAAATTTAATTCCTTCCATTTAAAGGGTTTGGGGGGGCCAGGTTACCG

GCCCATTTTTTCCTCTTTCTAAACCATAAAAACTAATATTTGAAAAATTCTTATTTGTTCGGAAACA

>FM896644.1 FM896644 Jatropha curcas embryo 71-95 (DAF) Jatropha curcas cDNA clone rjcpga0_003915, mRNA sequence

ACTTTAATCGCTTATATCAGTTCATGGCGACTGAGTGAAATTAAACCATCCAAAAGAGCTCCTTCATGTCCAAGATAATC

TACTTACCAGAGTTCCTAAGAACAGACATATTAAGGTCACACATTCCAAGCCATTAATCTTCAATGCAAGTAACTGTGTG

ACTACTTCCTGACAAAACCCCAAGCCTCAAACTAATTATTACTGCATTGAACTT

>GH295843.1 JCL292 Jatropha curcas total leaf library Jatropha curcas cDNA, mRNA sequence

ATTGCCTACCTTACCCTTAGTCCCTACACTTCCAATTCCCTCCCTCCCTATCATTTCTCCACCACCAGCTTGCAACTAGT

CTGTTCACCAGGAGGACCGAGTTTGAATAATGATTAAATTGTTTCATTCTGGTTTAGTCCATATTTGTTTGTGCATTATT

GTTATTTATAATTCTGTTAGGTTTGTTTTTGTATGATTTTGTAATTCTGTGATTACCAGTTCCCATTGATTGTACTTTGA

ATGCAACTGTTTATCTTTGCTTGTTCTAAAAAAAA

>GW875698.1 JC005015 Seed specific Normalized cDNA library from Jatropha curcas L. Jatropha curcas cDNA clone N08753 5' similar to Unknown protein, mRNA sequence

GTCTGTGTGAAATCTGATTACAGCATTGGCTTTCAATCTTCAGGTTTCTGTTAATGGCTTAGATTTTTTTTGACTTCTCA

ACCGCTCTTTGCTTGTTCGTGTTACCATCTTATTGTGAAAACAACTACTCCCTTCTCCGCCTTTTCACTTTCGGCTCTGT

TTTTCCCCTGAAAGAAAAGAAAAGATAAACCATATCATTTTTTCTACTTCATTTTCGGTTTGTACTGTCTTACTCTTGTC

TCAATCCTGTAATCAGAGCTTTCTTCAAGTGGGTCTCTCGTTTCTTGTACAACAACACAACGCATTTGCTTCTGTTGCTG

TAGAAAAGCCCGCTCTGCTCTAAACCAAAGTTGGGTTTTTAAGAAGAAAGGGTTTTGCCTTTTTTCCTTCGTATCATTCC

TTCATTTTCATTACCATCGTGAATTTCTTCAGTTGCATTTATAATTGTTTCCAGAGTTTTTATGGTCTTTCTAGGCAGAA

TTCTAAAGAGCGAAGTTAGTATACTAAAAACGGGTGTCGTTCAA

>FM888358.1 FM888358 Jatropha curcas embryo 35-55 (DAF) Jatropha curcas cDNA clone rjcfea0_001757, mRNA sequence

AAGAGAAAGGAAAAGGAGGCTAACATCATTGGGAAAAGAAAGCAAAGTTTCTTGTTAGACTAAGATCTTTGACCAAGTCC

GAGTCTGGTCGCAGGGGTGTGTGACATGTAGTAGGTGATGACCTGTTGGTCTGTTTTCTCAATGGACCCGCTAACTCCTC

AGTCCTAGCGGAATTTTGGTTCATTTTTCGTTTAGTTGTAAAAAAATCCTGGCAACTAATTAATATAATAACTGTTTTTT

>GT975995.1 GJCCJC2071H01.b Jatropha curcas L. developing seeds (mixed stages) Jatropha curcas cDNA clone GJCCJC2071H01, mRNA sequence

CCCTGTGTCTTGAGAATTCTCTCGTTCTGGATTGGTTTCATTTGAGTGTTTGGTGAAGTAGGAATTCAAGAAACATTGGA

CTTGGTTCTGCTGAATCAGGTCTGATCTGTTGGTCTATGTTCTGAGCTTTTGTTTGCTATCTTACTCTTTGATCTGGGCT

GTGGTCCCTTCTGAACATTTGTAGCATTTTGGCTTAGCTTTCTAATGAATCTAATTTCATTCAATTTGGAGACCCATAAC

TCAAATTATGTGTTAAAAACCCAGCAAAGGTCAAGTTTATAACAATGCTGAAAAGTTCTTGATTTATGTACTTTTATATT

ATATTAAGTATATATACATTTATAGTGTTTTGAGTAAAATATTTGAACTGATGAGTACTAAATGAAGAGGTTTTGATTAA

GAATTATTGCTTTTGTGAAAGAATGCTTTTGATTTAGGCTCTACTATGCATGATTGAGACGTAGTATGATGAGATGGATT

TTCTTATTGTGAAGCTATGATCATATGCTAGGGTTATCAACCCTTATAAATAGGCGGACGCGCTTATTTGTGTGATGGGG

TGGTGCTTTCAT

>GT981219.1 JGCCJG2052H03.b Jatropha curcas L. germinating seeds (mixed stages) Jatropha curcas cDNA clone JGCCJG2052H03, mRNA sequence

GAGTGCACCTTAATTCGCGAGGAAGTGACACGTGGCCCACCAAGATGACAAATGGCTTTTACGAAATTTAAAATTTCAAGAATCCGTGGGTCCATTTTATAAAGCTTGCTGTGATCACAGGTAGAAAAACTTTTGGGTAGGGGCCAATTGGATGGACGCAACTATTAAAAAAGTCCAACATGACGTCAGTGGTATCAACCGCAAACCTTCTAATCATAATATAGTAGTAATTGACTAATCATTTATGGAGGTAATGTATCGTAATATTTCAAATTTTATAAATTTTATAATTTTACTATTTAAAATTTTAATCAATTTTATTTCAATTTTCAATTTTATAGTAATATACAATTAAGGAAGAATACATCAAATAAAAAAGAATTTTTTTCAGATATACCTTTTTGTTTTTCTTTAACAATTAGGTGTTCAAAATTTACTGGCCTGATTAATTCGGAGTTGCGCCGAATAGGCCCACAAAGGGGAGCAAAGCGCTCCCTTCCGAGCATTTCAAGGGATTTTAAATTCATGGCTCAAACTCGAGACCTCCGGGTTAAAAGAGAAGAGATTTTTGTCATTCCATTTCACCCTTCGGAGATTTTAGATATAATATATCTTAATGGTGAATAAATA

>GO246470.1 JcrME_RL0014 Expressed sequence tags from Jatropha curcas root cDNA library Jatropha curcas cDNA, mRNA sequence

ACGCAGAGTACGCGGGATTCATCATGGTGCAGATAGCATTTGTTTAACTTCTGAAAAATTATAATTCCGCAACCCAAAGT

TACTGTGGCCAGAAATTTAGGCTTGCGACTTCACTCTGTTAATATGTATTCGTACTGATGGGTAGTTGACTCATTCCTCT

TTCTACTGATTAGTGTAAACTAGTAGGTCAGTAACCAATGACAGAAGCCTGCACTTAATGGCAATGGCATATTTTGAAAA

ATTAAAAACTGGACTCTGTTCGAAGTAATTTAATGATGTTGTTGTGGAATTTGTTTAGAGATTTGATTCCAAGAACTTCA

TTGCAATATGTAGTCTTGATTCTTGAAAAGATGCAAGCAGGGAAATATAGTTGGTTGCTTGGGAGGCTCTTTT

>GT972650.1 GJCCJC2038H03.b1 Jatropha curcas L. developing seeds (mixed stages) Jatropha curcas cDNA clone GJCCJC2038H03, mRNA sequence

AGCAGACTTCAGACTGAGACATCGAGGTACACATACACCGAGGGATTTTGTTGCGAGATTCCTGTTTGGCTAATTTTTTC

TGTAATTTTTTTATCTCAAAAATCCTTCTCTCTCACTCTGTTGGAACCGGAGGAGAGAGAAGGTTGAGTATCTCTCCTCT

CTGCCTGAACTGAGTGTTTTTTTGGGGGACTTTTTGAGTTTT

>GW875008.1 JC000399 Seed specific Normalized cDNA library from Jatropha curcas L. Jatropha curcas cDNA clone N00740 5' similar to Unknown protein, mRNA sequence

GGGTTACAAACACAACTGACAAAACGAATCAACACATTCAACGGTCAGGATCCACGAACCTCCATTGCCGTTAGTTGCTC

CTTTCTCTCTCCTACTCTCTGTGTTATCCCGCACAACACGGCTCTTCTCAACCTTTTTCTCTCGTCTTTCCGTTCTCTCT

CTTCCCAGAACTTTCACCTCTTGTCTCTTTTTCTACCTGATTCAAAATCGTTTCAAAATTTACTTCGAAGACGTGTTTCT

TTTCTCTCTCACTTAAAAATTCC

>JK612757.1 JCF20-201 Jatropha curcas, immature Seed cDNA subtraction library Jatropha curcas cDNA, mRNA sequence

ACTACGAAAACAAAGGGCTAATGATCCAAACCCACCTTCCCTTAACGTTTCAAATTTATCCAACTATCTTAATATTATCA

GACCTATTTCATAGTTCATAATTGGTAAAAATTGAATCCAATACCATCCTAAGCTAGCATTGAATTGCCATGGTCAGGCA

ACTAGAATGACATTTCAACTATTGACAACATTTTAGGAAGTAGACAGTTTCAAGTGGGCCCCAGTCAAATAGTTAACATT

CTTGTGTGTGTCTCTATGTGGTTCATGATGTAACTCTTATCCTTAGATACAAAGCTACCTATAAAGTATGAGAAAAAGTT

AAAAGTAAAATAAAGAATCAAACAACATTTCAACTAGCCCAAAGGTTAGGTTATATGGAATCGTTACACCCTTAAAAGCA

TGAAGGGTCATCAACATTCTAGCATCAGAATGGGTCATTGCTATAGTTGCATTCTGCATAAAACAATAGTTGT

>FM887704.1 FM887704 Jatropha curcas embryo 35-55 (DAF) Jatropha curcas cDNA clone rjcfea0_000954, mRNA sequence

GGGGATTTGCTTCAGAGTGGGATGCTGCAGCTCCACCACCACAATATGCTGGTGCAGCTCCACCCGTTGCTGCACCTGGT

GCTACTGCTACTGGCTGGGAAATGGACACTTAATACTATGTTTTAATGCTTCTTCCTNGGATTAGTTATGAACATCAAAG

TGGGAAATGGACACTTATTTTATTGTCAATTTGCGCATAGTTTTGTTTTAGAGATTGTATTTTAAAAATTTTGGCTATAT

CTTAATCAAGTAATGTTATTTTTTCGT

>JK612741.1 JCF20-185 Jatropha curcas, immature Seed cDNA subtraction library Jatropha curcas cDNA, mRNA sequence

ACGAGGCCAAAGAAATTGAAGTTGAAGAGTTGTTGGATGTGTCAGAAGAGACACTTTCGTTTGAATTTGGACAGGAGTAA

GGACTATTAGTTTTTATATATTCCCAGAGTTCATACGTTCAATGCAACCAACTTGGATGCGTGAAGTTGTGCAGATACGC

AAAAAGCTTGGCTACGGGTTGGATATTTGTAGTTTCAGTGGTTGAATTTGTTTTAGGAACTTATGGTTGATGCTTTTCCT

GATCTGACTCCTCTGTTGATCTGTATCAATTTCTTAAATTTTGTTTGCCGGATAAGTATATCGCCTTTCTTATAGTTCTT

CTGTTACTTATTGTGGCTCCCTTCATTCTATTCAATTTATGCAAGTTTAACTGTAAGCTCAAGCAGGGGTTAGCTTGTTT

ATTTTTCCAATAGGCAATAGCCTGGTAGATTATTTTGGCATTTTGAGTTTGT

>FM888864.1 FM888864 Jatropha curcas embryo 35-55 (DAF) Jatropha curcas cDNA clone rjcfea0_002367, mRNA sequence

CGTCCACTAATCCACTAGATCTCATACTGCCGGGAATGAAAGAAGATGAAAGTAGGAGTCAAAATCTTACCCGGAAAAGA

GCACTGGATGTAAAGAAACGAACCTTCAAAGGCCAAGTAACAAATTAACTAAGCTGGCTTTCCATGTCCCATTCCTT

>FM888667.1 FM888667 Jatropha curcas embryo 35-55 (DAF) Jatropha curcas cDNA clone rjcfea0_002122, mRNA sequence

TTTGGAAATGGAATGAAGACAATTTTATGAAACATGTAAAAGAATTACTTCATATTGTGATTATGAATATCTTGTTTATC

ACTAGCAGACTAGTGATTGATATTAGAGGTTATAAGCAATGGAATCAGATTTTTGAATTAACTCATCATTTGGTGGGTTT

TGACCAACTTTGAAATTGAAATGAAAAAATCAGATTGAATTCTAATATAAGAAATTGAAATGAAAACAGCAAGTAGCAAT

TAAATTATAATCTTAATCTGAAATGATTGACATAATTGAGGAATTGATGTTCTATACAAGAAAATATATAAGCTTAATAT

ATCATTAAAAGGC

>FM894811.1 FM894811 Jatropha curcas embryo 71-95 (DAF) Jatropha curcas cDNA clone rjcpga0_000684, mRNA sequence

GAAAACAGAATTTCTGTTGCCTCTTTACCACAACTTCTGAACCAAACATGTCCTAGATGTCCACAAGTCCTCATAATGCC

CTACGCTACTTGGGCCAAAAACGGAAAGAAATTTCTTGATCTGCTAATAAACCATTGATTCAGCAAGTTGCGTTGCATAA

TTTGTGTTGGAGAAAGCTTACAATTCGTTGCACAAACATGCACTAAGCAGACAAGTATAAATTTTCTACCAAGTTTGTTT

GACAACATTACATGACGACTCCCATAAAGAGCATCAAAAACTGATCTGACTAGAGCAGAGTGTTTAGACAACAATCAAGA

AGCCTAAACAATAAACCTTTACCAGAGAACCTGCTGCAAATAAGCTGATGTACACTTCAATGGCTGAAGGAGGAAAACAG

AATGAGCCCAGAACCCAAAAACAGGATTAACCTGTTCCCTCAGAAACCTGCCTGTGCCTGCCCTGTTGATCAGATAGATA

ATACAAGAGACCCAGGGCCAACAGGCCAAACAATTTCCTATATCATAGCAATCAGGTCCCAACTCTTT

>FM890140.1 FM890140 Jatropha curcas embryo 35-55 (DAF) Jatropha curcas cDNA clone rjcfea0_003941, mRNA sequence

CCGAAATTGACCCACCAGAGCAAGAGAGAGAGACACACACAAAAGAATAGCTCTTCGTTATCTCACTCCTTCGAAGCCCC

TCCCCCAAAAACCTACCCTTATCTATATCATCATTTCCTTGGACTCCAATTCCCCATTTCTCTAATAGCACAAGGAAAAT

TCAAGATCACACCTTTATCAATTGATCAAATCATCACTGTTACTGTTCATTTCCGGTTTCGATTGGTTTCTGACAAGGAT

CGGGTGGGATTTTTGGAGCTAAAATTTTAATTATTTCATTAATTAAAGAAGCCACTATGGATGCTGATTCGTGGAGCGCT

CGTCTCTCCTTCAGCTT

>FM888907.1 FM888907 Jatropha curcas embryo 35-55 (DAF) Jatropha curcas cDNA clone rjcfea0_002425, mRNA sequence

AGAACATTTATTATGTTAGGCAGATAAATGAATAAATGTATTAATAAGTAGCCCAGATGCTAAGACATCTACCACGAAAA

TCAGCGTAAAAATTTAATACAATTTAATTTAGTAGTTGAAATGCATTATGGTGATTATATGATCGAGTCCTAAAATCAAT

ATGTTATAAAAAATTTTTAATGTAATTGCACGATAGTCGTAAAATCAATATATTATGAAGGATTTTTTAATATAGTCGCT

TCCTAGTTCCAAAGCGAATAATCTCCCGTTTGATCGTTTGAGATAGAAAGAGTATAAAGTGTTACAATAAAGTACTTTTT

>FM887596.1 FM887596 Jatropha curcas embryo 35-55 (DAF) Jatropha curcas cDNA clone rjcfea0_000830, mRNA sequence

ACAAAATCACTCGGTTGTCCATTCTCATTGCCAATTGCCAGGCATCTACCCAGCTAATCGAGATAGAATCAATTTGTGAA

CTAAATTAAAAAAAAATACAGCATTTGCATTCCAATCGTCTAAACACGAACATCAGCTCTTCATAGTTGGGCCAGCTTTT

GTCATTGAGAATGGACAACCGAGTGATTTTGT

>GW616335.1 Jc2-010-F08-M13F.F08.ab1 Jatropha curcas flower and seed Jatropha curcas cDNA, mRNA sequence

CCCCGGGGGAAAATGGAACACTTCACAAGATGGACTTCTATAGTCTCTTTCTATGAGAAGATTTCTATCCTCAATTATAT

GGACAAAGTAACTTGCAAGGGTCTCAAAATGAGGTTTATTAATCATGGGAGATGCTGGTACTTGAAGTCTGCAGCTCAGA

TTGTGGACCATTTGCAAATATCTATGTACATTTATTATAGGTTATAGGCTATGGAGCAGGTATGCTGTGTAATACACTGT

GGATCATGTGAATATTAAATTCCTATCATGGACTAGGGGTACCTGGAGAACTATTGGCTTAGTGCAGTAATGGAATAATA

TTGTCCAGTTCAGAGCTCAATTGGCATTAGATGATTCTGTTTATTAGTTTGTAATTTTTCTTAACCCCTGTATTAGACAT

TCACTCGTGTTGTCAATGATATTGGCATGTTATCTGTTCCAGTTAGAGAGAATCTCCAGGTCATAGGACTTAAAACAGAG

GACACTTGTCAGTTCTACTTTAAGCCATGAACTTCAAAGAGAATGTGTGTCAGGCATAGACCTTTCATGACTAATGGAAC

AACAGGAAATGTGCATGAGAAATGCTCTTGATATTCTCTTGAATTTTTTCTCTATAAATAGAATCTACCATTGCAAGC

>GT971860.1 GJCCJC2025E07.b Jatropha curcas L. developing seeds (mixed stages) Jatropha curcas cDNA clone GJCCJC2025E07, mRNA sequence

GTAGCACTAAAGATATTCTAAAATATTTACTTTTCTTAGAGTGCATTCCTCAAACTCCTATAAATTGACGGTAATATTGT

TATAAATACACGAAATTCAAGCTATGCAACTTGAATGCATTAGTATTTACTTTATCTCTCACCTCCACTCTCCTTGTCTC

TCTCTCCTGTAACATCCCTTCCCGGTCTACTCATGAACCCGAATGAACCGAGCCAAGATGCTATAATCTGGTTGAACCTA

TGTCCTATCATGCTGATATCATCCTATATGCTAAATATTATGTCATGCTGTTGCTTTATACATCATTTATATACAGACAT

TTATTTAATATATACAAGACATTACACTATTTTCCAAAATGAAATGGGTCCAACTATGTGCCATGTACATGCCACAGGAC

CAAAATATCAAAATGAGTAGTTACCCTGAAGACTTAGTCTTCAAAAGATGAACTGCAGCAGTTGTAAAGATGACTCTCTA

CAGTCCAGATCCAAAACCTGCGAGTTGAAACAGGTAACTCGCTGAGTAAATAAAGCTCAGTGGATATCTCAACTTAATAA

GAATTAAATAAATAAGCATGTAAGCACACATATTATTATACTTTCATGGTCTTACCTCCTGTTCATTATGTTGTATGAAT

TATGCAATCGTATAATTTATAAATAACACAATTTCATAACTGTATGGCTCCATAAGTTTACTGGTGAACCGAAGTG

>GW879376.1 JC003934 Seed specific Normalized cDNA library from Jatropha curcas L. Jatropha curcas cDNA clone N07177 5' similar to Unknown protein, mRNA sequence

GGCTCTACAAATCACGAATTTGTTATCATTTCAGAGCTTCTAGAGAGAGAAGCGGTGCTCTCTAATTCGCAGAGGAACAG

TAAACCTCGAGCTCTCAAACTCTAAGCCACAAGCCCTAATATTCTCGTAAAGATACTCCGTACATATTCATATTCTTCGT

TTCTCTGGTTCCTTTTCTTTTTGGAGCGTTTCTTTGTTCTCTGTTAGAATGTTCCTTTCCTAGTCCTTCCTCTTTATGTT

GTGATTTTTTTGGGTTCCGAGATCTCTTCCTGTTTCACTGCCTCAGAATTTTGGATTCTCTGTTTTGTTCTCTTTTTTGC

CTTTTGTTTTGCGTAGAATTGAAGAGTTTTGTTGTAAGGATGGGATGCTAGTGGGAATACAACAAGTTCGGGGAATTTCG

CATTTCTTGCTTTCTTGAACTTGCGGGTTTACTTTTTTATATCTTCTTTTGCTGATTGAGCAAGTTTCTCTGTTAGTTTT

CCTTTGATTGTGAGAGAAGTTAAGGATTTGGTTGAGTTGCTTGTGGAAGTTGCTTGATCCACTGAGCTAGGAGCCCTGCC

AATTAGGGTTTCCTACCCAATTATTTTTGCTCTTCAAGGCACACACAGTAGACGGGTCATCAATGAAGCATAATTACATG

TAATG

>GT971681.1 GJCCJC2023C08.b Jatropha curcas L. developing seeds (mixed stages) Jatropha curcas cDNA clone GJCCJC2023C08, mRNA sequence

ACCAAATTCTCTAGCTAAATAATTTATCCATTTCTATATGGAGTTTCAATTTATACCTCCATTATAAACAATATCAAGTC

CCAAATTAATGGCGCTTTACCATCCAAATATCACATCGAATCAATCTCATTAACTCCTTAGAGTCTGATTTAAATATGCT

ATTAACTTCTTTGCTCTTTTGTCCATATTCTCCTAGATTACAATTTCAAACCATAATGCCAACATTTTAATTAGTCTCAT

TAACCAAGTATTAGGTACCATATGTGGAACTATTAACTTAGCATCTATCTATTGCTAACTTATATCAAATTCAAATTCAA

CTCACCTGTCTCACCAATCTTTAACTTCATGTTTGACTTCAGAATTTTCAACCAATCTAGTAAGGGATTCATAAATTCCT

CTCATTTTTATCTACCACTTACTCCTCATATATATTACAACTCGTCATATATTATTCCTTATACCCTTAAACTATTCAAG

TTCATTCTTAATGTTATCTTTCTCTCTTTACACATTAACAGTCCTATACTCCAAATTTAGTAGCAACTTAACTCCTATTC

CAAAATTGTCTACTCGACCTTTCTAACGTTCGTGTTTTTGTCTTGACTATGAACTATCTACTTACATTTATCATTGTTAT

CATTATCATTACCATCCCATTATCTTATTCTACTAGCCCCGTGATATAAATAGACAACTCTCAAAAAAA

>GW881639.1 JC000905 Seed specific Normalized cDNA library from Jatropha curcas L. Jatropha curcas cDNA clone N01775 5' similar to Unknown protein, mRNA sequence

TAGATTTTTTTTCCCTACTGACTAATTTCACTCTCATCTTCTTCATGTACCTTTCCAGATGATACTTCAATGTAATTTTC

CTTTTTATTTGCCTTTATTCTCTTCTTAATTCCTATTTTGTTGTTTTTTCTATCAGAAACTTAAAAATACTCATTCAGGT

TATAGATATTGGCCTCTTCTTCTTTTCACAATGAGTTTTGAAACAAGAGAAATCAATTTCTTATTCTTTTGTTTGGCATG

AAACCTATACAGAAAATTTTCCAGGAAAGGAGAAGATAATAGGAAAATTGTGGGTATTATATATGGAGACAGAGTAGAAT

CAGAGTAGAAGAGAAAATGAATATTTTTGGAATTTGAATATTCCAAAGGGGAAGGAACCTTTGGGATGGCTCAGAAGTGA

GATTGAACCAAGGACAAAGACTTCAACCCTCAAAAGATATGAAAAGTCTGGAAAGCTCATTACTTGGGGTTCAACAGACG

A

>FM893496.1 FM893496 Jatropha curcas embryo 56-70 (DAF) Jatropha curcas cDNA clone rjcaeb0_004334, mRNA sequence

CCTTCAGTCAGGTTTCATGACAGACAAGAAAAGAGAGAACTGTCCACAAAGCCAAACTTATCCAATTAATTTGATAAAAT

CTCAGACGATTTCTACGATCAAAGCAACTCCCTTCATAAACTACAATACCTAGCGTATATCTGCTAGCGTTAAATTCCGA

ATCCTAACTTAATATAGCAGATTAAAGAAGCCAGCATCAAATTTAAATATCCAAAACTTTTTTCCCGGTTAAATTACCGA

TCCGTTTCCTTTTTCAGCTGTTGTGTATTTGCATCACCTATAACTTGGACAAAACACACAGTAGGGATTACTACATGGGG

AGATACAGATAGATAGGCAAAAAGATGCGTTTATATTTATTTCTAATTGTTTTCAAAGATATGTTAAATCCAAAATATAG

TTTGCGCTTTTCTTCTCCCCAATAAAGACAGCAGAATACAAGGAATATTTACGAAGGAGCTAAAACTACAATCTTTACGA

AGCAGAACATAGACGCAATCTTCTAACACGG

>FM896081.1 FM896081 Jatropha curcas embryo 71-95 (DAF) Jatropha curcas cDNA clone rjcpga0_003224, mRNA sequence

GAATAACAATTACTCAACCAGCATCCCAGCTGGAAGGCAGAGTCCATTGATCAAATGATTTTTTTAATACGCAACTGAAC

CAGAGTTAGCATCACTGCCGCAAGCTGTGGCCCAATTTTTGCAGTCAATAATCAAAGCGTTAGAGCTAAGCCAAAAAAAA

GAAATTCCCCCAAAAAGTTTCGACGAAAATACTTAAATTAGCTGAACTACAAAACGAGGGTGTCTTCGTATGGTGCATGC

ATCAACAGTAACCCACATTCATAACAAGTTTTTCATGCAAAACAACCAAAAATAAAATTTTTGACCACCCACCTACAAAA

CTAGCAACACAGACACCTAACAGACGCCCCCCACGCCCCCAGTGAGTAATGATGTCATGAAGGTCATGAAGGGACCCCAA

CGTCCGCTGGGGCATGACCACCATGAACTCCTGAACCATGATTGTTATAAGGGTGCCCTCCGCCCCTGCCACCCCTTCCC

CGATTGTTATAGTAATTTCTTGGATAATAGTTTCCCGACTGGTCATAATATTGGCTGCG

>GW879341.1 JC003899 Seed specific Normalized cDNA library from Jatropha curcas L. Jatropha curcas cDNA clone N07133 5' similar to Unknown protein, mRNA sequence

GGTGCAAACAAACACTGCCTCCAAATCTTTCCTTCTTCTCCAACGGGAGAAAAGTGGGGGAGAGAGAAAGACAAAAGCAA

GAAAGAAAAAAGAAAAAAGAAAAAAACTTTCCCTTTTACCAAACAAAAGAAAGTTAACAAAAGTTAGAATTCAATTTTAT

TCTTTGGATTCCGTCGCTTTGATTTCGCGTGCTTCTGCAAGCTCCAGCCCGTAATCTGTGATTTACTGATTTCCATCTTC

AAATTCGAAGTCACGACTCTACTGGGTCTCTCGTCTTTGTCTCTGTCAGTCTATAATCTTTATCTATTGATTGTTTATGG

TGCCTTTGGGGTTTTGTTATGAGTCGATTGGGTGGGTTACAATGTGGATTTGATTGCCCTTTTAGTAGAGATCTTACGTA

CTGGGTGTTTGGTTTTTTCTGGTTGTATTTGCTATATGTTTTGTGGAAGGAGACTGATGATGCATTTTATTATGTTAGAA

AATATCACTTTTGGATATTCGTTTGTGTGAATTAGAACTAAATCAAGACATCTGCAAGGCCAGTGGTGATTGCCACAGAA

AAGAAATCAGCTTTTTTAATTATGAGTAATAATTACCTGTAAAGGGGGTTCAATTTGATTTGTGTGGAACTTACGGAGTG

ATATTG

>JK611411.1 JCF4085 Jatropha curcas, immature Seed cDNA subtraction library Jatropha curcas cDNA, mRNA sequence

TACCAGGGGCCGGGCCGAGGTGTCAGAGATCCTATGGCAATGTATTGAAGAATCTCTTGTGCTGATGGGTTGTTCGGTTC

TTCAAACGCTGAAAAGGTGAGAGGTCCTACTTTAGTGACCAATTCCAGGCTAAACGTGCTTCGAACACGGATAACTGACA

GAGGAAGAGCCCCCTACTAGAGGAGCGCTTGGCTTACGGCTGCGCATAGAATCACCCACACTACCTGTCGACAAGAAACA

GATTGAGCGAAAAGGTCCAGAAGGAATGGAGAAGGATGTATGCTTGGGTTACATCTGATTCCGTCATGCTGCTGGAAGAA

CTAACTGTAAGAACAAGGTTTCCGTTCCGGACTTCTTAAACTTAAGCTTAATCCAACCTGAACTTAATAAAGAAGGGCTT

TATGACCAGCCGGCCCATGGGACTCCGGGCCGGCCTTTTTTATTTATTAGGAAAACGGGGCTTCACTGGTCCTCCCGTAT

AGAGTCACCCCAACGCCAAAGATTCCCCTTCGGGTATTCCCTTCAACCCGCAAAATATATGTGTGTTGTGTTAAACCACA

AAGAGGGGGGGCTCGTGGGGCAGGAACGTGAGTGTGCGACGCCTTATGTGCCACGAAGATGCTGCTGTCGGGCGAGTGCG

TACCAAAATAATTTCCGTGGAAGCAGCATGATGAAAGGGGCGCTGATCGGCGGCTAGTAAGCCCTG

>GT970928.1 GJCCJC2016A02.b Jatropha curcas L. developing seeds (mixed stages) Jatropha curcas cDNA clone GJCCJC2016A02, mRNA sequence

GGAAAAAAGTATTTTAAAAGAAATCAATATCGTCATTATAACGATAAAGATTAAGGACATGGCGTAAATAAGAAGAAAGA

ACAAGTCCTTATGCTCGGAAATGTCATTGCCTATGAGTTTGGGTGCGTTCTGCTTGCCATCTCCCCGATTGTATAGATAG

ATCTTACAATGTTCGCTTCACTCTTTAAGTAGTCCGATAGTTCAACCAACGCTGTAGAAGGTTCTTTCTAAGCAGCGTAA

GAGAAAGCAATAGTAAAGAAAAGAAAGCAGGCAAGTAGTAAGCTGGCCAAGTCAAACTAAGCAGAAGTTGAAGGCAGATC

TGTCTTGGCCTGCAGCCTATGATGATGAATGTCCAGCCTTGATCTTATTATACCGCTCGCCACAACGGATTAGGGAGGCT

GTCCCGAGAGTGAAGGGGAGCTTGAGCTCTGA

>JK612000.1 JCF28-A147 Jatropha curcas, immature Seed cDNA subtraction library Jatropha curcas cDNA, mRNA sequence

ACCACGCATAAGAAAGCAGAAACAAACAATTGGAAGGTGAGATGGAACCGTAGAGTTGTGCCGCTATTTTCTCTGTGGCT

GAGAGAGGCTTCAGATTCAGAATGGCTCGCACTCAATTTCATACCGGTCTTGTGATACGGTATCTTCGCGCACCATGTGA

TCACGCTACGCTAGCAATGTCTTATGCTTTGAGTTTCGGACACGTAATAATTTTCAATGGACCTTCCAACCGTTGACAAG

CAATGCATTGTGCATTATGCATTATGCAAATGCTTTGTTTATTTAGCTTCCTCCTTCCCTCCTTTGTATTTCTTATAAAT

ATTTATTTCTTAATTTACCACCTCAAACTTCTCCATTCGTGAAACATAGTCCTTTTTTCACTCTTCTTTTATTTAATCAT

GGCAAATACTTTTAGTTATTTCACTTCTCTAGATTGTTCATTTGTTATCTGTTGGGAACATAATGTGCCAGCTGTCTCCA

ATATTTGAAATGGCCCACTTCTCTATTTCCTTTTATTTTCAATTCAAGTTCAGATTATTTAAATTATTTTCAAATTAACA

GGCCAAATTATAGTCCGACGTCATGTTTTGGAATTTCGAGCTTTCCACCTTCATCAGCTTTATCTCCGCAATAAAGTCCC

TATACCAAGAAAAAAAAAAAAGAGCTCGTAC

>GW612095.1 Jc1-013-A02-M13F.A02.ab1 Jatropha curcas flower and seed Jatropha curcas cDNA, mRNA sequence

ACAATCCTCACGTCTCCTCACCATCCCTCCCATTCCTCTTTTCCCTTCTCAGGGTATTCTAAAAGATGAGAAGAATGGAG

CTATTAGAGAGCAAATGTTGAATTCGTGCTTTATTTTGGTTTCTATGGTTTCAAATTTAATTTTTCTTTTAATTTATTTG

GTGGGAGGATGGCATATCCTTAATTGTCGATTGCAGTGCCATGATGAACTGTATTCTCATATTTGCATATTTTTACTTGT

TTGTTTCAAAAAGGACGACGAGCAGACTGAAATGGTCATATGTTAGAAAGGGATGTCAAACTATTATGCAAAAGAGGGAT

GTGGGCCTCAACTTGTCTGCAATGTTTATTTAGACAAATCATCTGCTCCACTGGAAAGTTCTTTGTGCCATGTTATCCTC

TCTATTGGTTTTTGTGTGCCAAGGTTTTTAGATGTCTTTAATTTCCTTAAAAACTCCCCATGGAAGTTCAAAACTGAATC

TTGAGCCTTCTTGGCAGACCATAACCACCCTTAACCTTAGAGGAGTTCTTGTATTATGTAAGAAAATGTTTGTAACTTAG

GCTGTTATATTGGCATGCATTGTGATATATTTGCACGCGTTGTGTTCATGGTACAACTAAATTCTGGAGATAAATTTTAT

TCTATTATTTTGCCCTGCATGAAATGGAAAAATGTTTGTAACTTTTACATCTGAAGATATGCCAATT

>GW618938.1 Jc2-040-G10-M13F.G10.ab1 Jatropha curcas flower and seed Jatropha curcas cDNA, mRNA sequence

TTTCAAAAGCTCTCTAATATTAAGCGATGGCTGTGTCTCCAATCCAGTCGGTGCTTGGGATGTTGGGTGTTATGTCCACG

GCACAGCGTCAAACTGGGTTGAATATCAGGTCATTTTACCCACCTGTTGCACAACTTCTTGGCACCGTATTGTTGGGTTA

AGTCCTTTACCGCTTCTGGATGAAGACTGAAGCTTGTGTTTGTGTTATCCTTTTAAGTAATGTTAATAAGCATGGCTTTG

ACTGATCCACCATCTCCACTGGAGTGTGTGTGTGAATCTCAATGCTATGAGCAAATAAATTCTGTCTTTATGTACTTCTA

TTTTATGTGTTTTTAATAAATCAGTCTTTCCAGTGCGGTC

>GW877229.1 JC006747 Seed specific Normalized cDNA library from Jatropha curcas L. Jatropha curcas cDNA clone N11370 5' similar to Unknown protein, mRNA sequence

GGCCATTACGGCCTAGTTACGGGGGAGTGTAGCCAGAACTTTTAGTTGCAATAAGTCATTTCTTAATAAAAAAAGTCAAG

ATTGTCAACCAATCAGAATGGCAGCAGTTTTTACGTGCAGCCATTCGTAATCTCTTGCCAAGAGTTTGATCTCGTGCCAA

GCTGTGGTCTAATCTCTCTGTCCTTCCGTCCCCCCAATTAGCTAGTTGTCCCGTCCGTCGTCCAATCCCTCACTTCGTTT

GTGATAGTCCATTAGTAGTCCCTACTGAGCAATCTAGCTGCAATCGAGCTACCATTGAAATGGAAAGAGTAGAGTGAAAG

TTGGACGAGGTCTGCCTTTACCCTTCCTGTTAGTAGGAGTGGATTAGTTAGAGTCGGTCCGTTCTCTTGGTTCAGAAGCT

AGGGCTAGGGTCAAGCAAGCCTGCCTCCATCCGTTTATTCCATAGCCTATCGAGCCAAGCCAGATCTGCAGCCAGTGACG

ATAACTAAGTAGTTAGTAGTTGTAGTACTTTACTGGGACGATCCACTAGATCCATAAGCCCGTAACGGAAATAGCCTTTC

TTTTCCCAACCTCGTTCGATCCCGAACTGCATTCAAAATGAAGCAAGACCTGGTTCTCTGAGCA

>GW881631.1 JC000896 Seed specific Normalized cDNA library from Jatropha curcas L. Jatropha curcas cDNA clone N01759 5' similar to Unknown protein, mRNA sequence

GTCAGATTTGTCCAATTAATGACTGAGACATGTACATCTGAGGCCTTAATCCTTAATTAATATTACCATGATTTTGTATC

TAAAATTGTCATTTGGTAACTTATCATAAGGTGGCCTGGTATGATATTAAGATTTGCATATGTTGTGGTATTGTTCAGAC

TTTAGTCCATGACTAATATGTTAAGCAGCTACTGCTTATTCTTTTTATTTGCATGGCTAACCTGATGATATAATGATTAG

GAAAAGTTAATGTGGCCTATGATGATTAATTATTTGCCTACATTGTCAGATTGTCTAATTCATGACTGAGACATACAGCT

GAGGCCTTAATCCTTAATTAATATTCTCATTATTATTACACATGATATTGATTTGGGGAAGTGCTATTAATCTAATTGTT

GCAGTTTTCTGTTATTGCCTGATAATGCATCTAAAATGGCCATTTTTCTGAATCTTGTCTAA

>JK611618.1 JCF4476 Jatropha curcas, immature Seed cDNA subtraction library Jatropha curcas cDNA, mRNA sequence

ACCCACTAATTTATCGTCACCTTTCACCTAAGCCCCCACGTGCTCCGTCTCTCTCTCTCTACTTCTCTTTGCAGCTCGAA

TTTCTTTCTTTTGCTTTTCCAATTCTTTTTTTACCATTTTTGTTCATCCAATCCTTTCTTCTTCATGGCCCCCATCTCTT

CAAAATTTTCACTTTTCAACAGTGGAGATCAAAAGTAAAGAGCTTTCAAATATATAGATACTCTTCACTTCCTATTTTAC

AAAATCCTTGGAGCATCTCCCCCCCCCCCCCCCTTTCTTTCTTTCTTTAAAAACCATCTCACCCCTTCTTATTATTTCCA

TTTTTTTTTAAAATTTCATTCATGGTCAGAAGTACTTGGCCGAACCCGAATTAAAGCATCCGGGTCCAGTGATTCTGGGG

GGTTTAACCCGGGGGGAAACCGGGTCCCATTAACCCTTGAAACCCCTTCCCGGGGGAAAAAAAGGGACACTTCTAAAGGC

CGGGGGGGAAAGGGATTTTTAACCTTTTTTCTTTTTCCAGGAGGACACAAAAAAAGGGGGGGGGGGTATTAAAAAAAAAA

CAAGCAACCACTGTTTTGGGAAGTGTGTAGAGAGGGGGGGGGGGACTCCGTGGGGTAGACATATATTATTCTTATATATA

ACTCGATATAGCACGCGGACTATA

>GT971837.1 GJCCJC2025C04.b Jatropha curcas L. developing seeds (mixed stages) Jatropha curcas cDNA clone GJCCJC2025C04, mRNA sequence

TCTTACAGATCCATCCTTTTACGTGGACCGACCTCACTGTCATCTCCAACACCAAACTATCAACCACACCTACCCCATTC

ATCATAACCATTTACTATGTATTTCCCTTATTCAATGGATGAAATCCAATTATTAACGCCAAAAATAAATTCATTTTGCT

TATTCATTCTGTGCCCTGATTTGTTAGCTCCTTTATTCTCTCTATCACATATATTCTTGCAAATAGCATTAAAGAAAATG

AAAATGTTGTTGTTAAGGTTTCTCGAATTCTATTTGAGAAAATTATCGGTCTCCAACATAAATTATGTAATTAAAATTTA

AAATTATATGCATATACATTATGTATATGTCTTTTGATTCTTTTGCATTGCTGTATTGTATACACCAAAACCCAACTCTT

ACAACAACTTAAGCTATTCAAGGGATAATTGAAAAAAGAAAAAGGGAAAAGAAAAGCTTCCTCTTCATTTTCCTTTTCTT

TTACCT

>JK317367.1 JCST131 Jatropha curcas L. seed cDNA library Jatropha curcas cDNA 5' similar to unknown, mRNA sequence

ATGGGGGGGCGACTTCTTCAAAGAATTGGTACGTTGGGTGGAATGGGTAAATCATGGGACCCATAAAGAGCAAAAAAAAT

AACTAAATCCCGGTAAAAAAAAATCATGTCTTTTATAACAAGGTTATAGAGGGACATAATTACGCCATCGTGCCGGTGTG

AATAATGTCACTAATGCCTCATCTGATAGTAGTATTTAAGTTGGTGCGAGGGACTAATGTGTCAGCCTAATCATGAGTTA

AACAGAATTCGCCTGTTGGCTTCTTTATGCTGTGTAGCCTTGCCCCTACCC

>GW878518.1 JC003272 Seed specific Normalized cDNA library from Jatropha curcas L. Jatropha curcas cDNA clone N06123 5' similar to Unknown protein, mRNA sequence

GGACATAAGTAAGACCCTAGTATATGTAGGATGTTTAATAAGTCAGACTAAGTTTTTGGATCTAGTATAATAAAATGAAA

GTAAAATATAATAGAGGAAACTAGTGGACTTGGGATTGGAACGCCATTATGTTGGGTGATAATTATATTAGAAAATGATA

ATTAGAATATAATTTGAAGGAGAATAATATTTGTGTAATTGTAAAGTTTCTTGTTGTATTGATGAAAAAAAAAAAAAAAA

TAATAATAAAATAAAATAAATAGAGATAAGGTGGATATTAAGAATTTAAATGATCATAAATAGTAATTTTATGCTGTAAA

TAAATTTAAAATTTATGAAAATTTAGAAAGTTAACAGATGTTTTAGAATAAGTAGTTAAGACTATGTAGTTAAACAAAAA

AGGAGCGAAATATAATTAAAGATAGAATAATGGAACATGTATACTATTGAAGGTAGGAAAAATGGCTTTGAGGTTTAAAA

TAATTATCACTGTTGATGATAATTCTAATAGGATATTAAATAAATAATTGATAATGTTACAACCAAACTTTTGAGTTGAG

TTAATCGGATCTTTGCGAGGTGAGTTTCGAGAATTTAGTGGTTCATAAATAGTAAATTTAAATCTA

>GW879952.1 JC006464 Seed specific Normalized cDNA library from Jatropha curcas L. Jatropha curcas cDNA clone N10967 5' similar to Unknown protein, mRNA sequence

GGCCATTACGGCCTAGTTACGGGGAGACCCAGCACCAGCAGAGCGGCGACGCCATCATCATCGTCATCTTCCTTTCGCCA

TGGTTCTCAAGTACGCTTTCTCTCTATCTCTTACTCGCAGCAATTTCTCTTTGTATTTTAATGCACATTAACTGAATATC

TCATAGATTGGATGCTGAATCCTTCGTGTTCATTTCTCCAATTATGTAAATGCTGAATCTCTGTATTTTAGTTGTTTTAG

GCATTAGTATCAGATAATGTAGTTAAAAATTACGAAGATTTCTTATTTAATTTTGTATTTCAGTGAGCTTCACATTATTA

TTTGTTTGATTCGTGTTTTTTTTTCTCATGTGGATTCGAACTTTAAATCTTTTTTTTAAAGGATCTTTAAGCCCCAAAAT

CCACGGACTTGAGCGGAAAAAGTTATCAACTTTAGAAATAAACTGAACTTTACCCTCAGTTGTAGATGTTGTTGGTTCTT

CTATATTTATACAGGGTACATTACAAGTTTGTTGCTTATTATATTTATATATAGCAAGCTGTGTCTTCTTCAGCATCCTT

CTCTTGCTAAAGGCATCCTCAGTTATGG

>FM889002.1 FM889002 Jatropha curcas embryo 35-55 (DAF) Jatropha curcas cDNA clone rjcfea0_002551, mRNA sequence

CAGCAAAGGGCCTTGATGCAGAAACTCAAAAGGAAGCTTCAAGACAAATCTCCCACAATATCAATAAAGGAAAATTTTGG

TTCCCAGAAAATCTTAACATTCTTTCTGAGTCAAAATCCTCCAGATCAATGCCAACCTTGCTTCATGTCAAAAACCTCTT

TGCTTGTCAAACTTTTGGTTCCATTTCCCTTCACAACCTCTAAATTCATCCTCCAAAATATATCTCCTTAAAACGCCTAG

AAGCACACTCTAAAATAGACTGATAACCAATGCAGTGAAAATTCTTCCACCTTCTCAATCCCCCCCATTCAAGATAGCCA

C

>GT970290.1 GJCCJC2006D10.b Jatropha curcas L. developing seeds (mixed stages) Jatropha curcas cDNA clone GJCCJC2006D10, mRNA sequence

GAAACATTAGTTGCAATGGGGAAGATTCATATGGCAAAAATTACCAATATAAAACAAGCTCCTCCACTGCAACCTTAAAA

TCAGAGGTCAAGGAGCTAATGCTACAATATACTGCCAGTCGATATCCCAAAATAGAACATCTGCTTAGTTGAAATGTCAG

ATTTCACATAATTGCCGTCTAAGGTGGGGTAGTAGCTAACACATATCCCAAAATAGAACAACTCCACTACAGAGTCTCTC

ATATTATTGGAATTTCATACACAAATGAATATCCAATTCACAGAACTTGCAACAAAACAGCACCAAAATCTATTGAAAAC

AAAACAAAACAATCATTAATGACAACTATTTCTGTCCTAGAGTACGAAAAGTATTAAACCGGTTTTCGCAAGCAATTGCA

ACG

>GT977221.1 JGCCJG2001A08.b Jatropha curcas L. germinating seeds (mixed stages) Jatropha curcas cDNA clone JGCCJG2001A08, mRNA sequence

ATCTTCATTTGAAGCAGAAGTCTCATTGCCAAAAACAAGCCCAACCATCCCCTGCCACCATCAATTTGCGAATTAGACCC

ACAAACGTAAATGCAAAACACTGGTAGAATTGAATCATATACAGATGGGAAAACTTTACAAGAAAATCATCAAAATTTAA

GATAAACTTATCATATCAAAATGCGCATTAGATCACAATCTAAAAACAAAAGGACAAACAGCACTATACACCATAACCTA

ATCGAGTAGTGATTCTTAACTATATACAATGAAATCCGATTCCAGAAGCGATTAATATATCAAGTTGCATAAATCAACTA

ACATTGAGAACCAAAACCAGAAAGAGAAAGAGAAACGATCACCTTGTAACGAGAAGCCAAATCCATGTTGCTAGCTCAGC

TTAATACCTAGAACCAGAGTGAATCTAAGATTTTGGATGATTAGGGCTTTAGTTCATCTAAACAATCTGAAGGACAAATT

TAGGATCCCTAACGATGAAGATGAAGTTCAAAAGGAGAAAAGAACAATAATTGCAAATAATAAGTCAATAAATATATTTT

CATGAGTAGAGAGAGAGAGAAACAGATTTTGACCG

>GW880833.1 JC006311 Seed specific Normalized cDNA library from Jatropha curcas L. Jatropha curcas cDNA clone N10751 5' similar to Unknown protein, mRNA sequence

GAAGTTATATAATAAGATGGCCAAGAGTCTTCGAAATGTTTTCTCTGTCTTCTTTATCCTCCTCGTCGTTTTGCTTCTCG

GGATGACACCGCTGTTATCTGCTCGTCAACTTCTACGAGGTAATGATATTTTTTGTAAATAGATACATACGGTAATATTT

TTAATTTAATTTAATCAATCACAAATTACAAAAATTAAATCTCTTTTTATTTCACAAAATATTTTTTTAAGATATTGTTG

GCAATTTAATGGAAAATCTTTTACAAATTAAGGAAATTGAAGATAGCTTTTTTAGGAGCCAAATTAAAATTATATATTCA

TTAAAATATTATAAAACTTTGTATAAAATAAATTGTAATACGTTTGTTTGTGACAATTTGAGCCATTTGCTCGCTTTCAA

TTGTAATTAATGATGAAATTGCAAATAAAAAAGTATTTTCATTCTTTATTTAATAACGATAAAATAAATTTTCTCAAAAT

AAAAGATAAAATAAAATTTAAATATCAAATTATATTTATAATTTAGTTGGCACATTTATAGATTCAGAGACATAATATTT

GACGCATGTGCAATTAAAATAATTAAGAAATCACTTATTTATTTAAAAAAAAAGAATAATTTCACTATATTATCGATTCA

AAAATTACTACAATTACAATTTTTAATCCTTCAATATTTTTTAAAATATTGTTGGCAATTTAATGGAAAATCTTTTTCAA

ACTAAGAA

>FM890270.1 FM890270 Jatropha curcas embryo 35-55 (DAF) Jatropha curcas cDNA clone rjcfea0_004088, mRNA sequence

GGCTGCGGAAAATATGAATGAGTTAATATTCTGTTCTAAGTAAGATTTTTGCTGTTTCTGATGGAACTGCAGAAAGTCTG

TNCAGGAGTTTTGGCAGTGGAAGGAAAACTAACAACCTCCTTTTAATATTAGTTGTAAATAAAATTACTCTTAAATCATT

GTTAATTCTGGCTGATCACCAGATTTACCATAGCTATGTTGATGCTTTTATTTATTTTACGGGGCTAAGGTTTCAATTGT

CATCAAGAAGTCCAATTGCCTAACGGGAAAAGAAAAGAAAAGAAATATATAAGCCTGTGGTTGTTTAATTTTGAGTGATT

CCCAGAGACTTGGAACTCTGCAAAACGTGAGTCTCGGACTATTATTTTCTTGCTTTAAGCCTCGAGGGGGGGCGCGGTGC

>JK317864.1 JCST635 Jatropha curcas L. seed cDNA library Jatropha curcas cDNA 5', mRNA sequence

TTTTTTTTTAGAAAAAAGAAAGTAAATTTTATTCACAAACTAAGAGAAACTCACACACAAGATTGTATGGGAACGCAGCA

TAAAAAAAAAAGGGGGAAAAAAACAACCTCTCCCCCCACCCGTTGGTTGGGTGGGGTGTGCCACCTTGTTATCTGGTTTT

TTTTTTTTTCAAAAAAACCTTTTTTTTTTTTTAACCAAAAAACAAAAAAAACGGGATTCTAACTTTCTTTTTTCCTCTCG

TACTATGTATGAAATCCATATGAATAGATGTCATGAAACCTTACTGCTGAAATGTCCCTTACGACTAGGATCTCCTATCA

CATCATACACTCACCACATGATTCTGTTCCTATTAAACTATCCGATAGACCAGGGGGCAAAAAAATTTTTTAAAATAAAG

GGGGTTTTTTTGGGGGGAATAAAAAATGATTTTGAAACCCCCCAAAAAAGGGGGGCCTTTTCTTTTTAAACTCAAATTTT

TAAACCCCCCGGTTTTTTAGGGGGGGGGTGTCTTGATGCGGTTTTTTAAAAAGGGGGTGATATTTGGTGCCCCAAAAAAA

AAACATTTTTGGGGGAATTTGGGTTATAAAAAATTTTACCACCTTAAAAAAAAAATTTTATTTTTCACCTTTCCTCCTCT

AAAAAAAAACCCAATATTTTGGGGGGGGGGGGGGTACAAAAGGGGGGGGGCCCTTTTTTTTCCCCCCCGGGGGGGGTGAG

GGGAAAAAAAATTTTTTTTTAGTGGAAAAAGTGTGTGGGGGGGGTGTCTTTCGTTGGGTGCCTCTCCCCCAAAAACCCCC

CACACACCACCAAAGGGGTTTTTTATAAAAAGCGAGGC

>FM887200.1 FM887200 Jatropha curcas embryo 35-55 (DAF) Jatropha curcas cDNA clone rjcfea0_000328, mRNA sequence

TTTCTTCTCCTCATGACAGAGAGAAGTTACTAGATGATGTGCATACTGAAAGATGAATATTGGTTTTTGGAAGATGGCAT

GACTTATGTATTGATATCAGGTCTCGTGTAGCAATGGAAATTCTGCTGTTTGTGTGAACTGAACTTTGAGAACAAGGAAA

CACGGGTTTTGTTTTTTTTTTTTCTCTCTTTTTTCTTTTTTCTTTTCTTTCAAAAGGTTTGTACTTTCTGTGAAACAAGT

TTCTAGTACTACTGTTATTTATGTTGTATTTCTTGTTTATTGTTATTATTATTATTATTATTATTATTATTATTATATTT

GTACTTAAACCTC

>GT971969.1 GJCCJC2026H02.b Jatropha curcas L. developing seeds (mixed stages) Jatropha curcas cDNA clone GJCCJC2026H02, mRNA sequence

GGAGAGAAGTGGAAATCCATGTCTCCTGCTGTAAGTACTGGAATTTTTTCTAATATGTGATTTTTTGTTTGTGAGGTTGT

GTATCTCTTGGTTTAAACTTGCTATATGAATCTATATTCTTTGTTTCTTGATTACGTGGGATTTGTTTGCAAGCTTCAGA

ACACATTAACAACTCATCACATTCTTTTCGATGCAAGCACACTAACCATTTTATTGGGATTTACTTGAATTAACTGTTTT

TGTTTCCTTTTAGCATCTTTTTTATTCTTGTATTTGGAAACAGTTGTTGAAAGAACTTCTTATTCTGTGAAATGGTTGTA

AATAATTTTTAAGTTTCATTTCTTGGTTTGGCAGGAAAAAGCACCATACGAAGCTAAAGCTGCCAAAAAGAAGGATGACT

ATGGAAAGCTTATGAATGCATACAGCAAGAAGCAGGTGAGCATAATGCATCAACCATTCGCCTGTCAAAGGAGCGTTCTA

GTTGTTTGTTGTGTGTTCTTATTGTCTCTGTTATGCTGACAGGAGAGTGCAGCTGATGCTGATGATGAGGAGTCAGACAG

GTCCAAATCTGAGGTAAATGATGAAGATGATGAGGCTACTGGAGAGGTTGGTCAACCTGTTTATCACTGGGTTGAGTTGC

CGCATTATAGCTTATTTTTCACAATATA

>GT971853.1 GJCCJC2025D12.b Jatropha curcas L. developing seeds (mixed stages) Jatropha curcas cDNA clone GJCCJC2025D12, mRNA sequence

GGGAAAAACGCGTATTTGTTTTTTCAAAGGTTGGCAGCCAACCATTCAAAGGTTGGCATGCCATTTGACAAAGGAGTGCC

GTGCATTGGTAATGCACGGCTCTCTCAAATTCTCCCATTCCCTCTCCTATAAATAGGAGAGGATGTCCCTCAGTTTATGC

ACACAGCAGAAAGAGAAAATGTAGCGAGAGAGTGTTAGCGTTTTAGGATGGGTATCCTAAACCGTGGGGAGAGAAAAATA

GTGAGGTTATTTTGGGGTGTTTTTGGGAAACACTTGTGTGCCACTATTTTTGTATTACTCTCTTTGTGTACCTACTATGT

GTTTAATAGTGGAAGAATGATTCGGAGTTTGTCCCGTGGACGTAGCCCTAAATAGTTGAGGGTGAACCACGTAAATTTCT

GTGCGTCACAAGTTTATTATTCCGCTGCGTTAATTTTCTCTTAATTGGTTGAAAGGGCCCAATGAGTGGTATCAGAGCTG

ATGCCCAACAAGTGGTATCAGAGCCGAAAGTTTCGGTAGGGCCAGCCGGTCAAATAACGGTACCGGATCCGTGGATATAT

TTTGAAAA

>GT969498.1 GJCCJC1003C05.b Jatropha curcas L. developing seeds (mixed stages) Jatropha curcas cDNA clone GJCCJC1003C05, mRNA sequence

GAGAGAGAGCACACACCAAGAATGGCAAAATATGGAGAAGCTTAGTAAGGTGGTGTTTTGTTTGAAACTTTGAATGGAGA

GGGCTTAGGGTTGGGCAATCCAAAGATAGAGCAAAGAAAAGCATAAAGGAACCCAATTAGCACAAGGAACCAAGACTCTC

TCTCTCTCTCTCGTTGGTCATTTTTGTTGCATGGAGAGAAGAGCTTGGAATTCTAAAGTTACTTCATTTATTTATTGCAT

AAAGCAACTTCTTG

>JK317577.1 JCST346 Jatropha curcas L. seed cDNA library Jatropha curcas cDNA 5' similar to Jatropha curcas chloroplast, complete genome, mRNA sequence

ATTTTTTTTTCTTTTTTAGGATATGCCACACAGCTGATAAATATAGCTTACAGCCCATTTTCCACTGTGCAGGTATGATG

TACAGGTTCACAGTTTAATTCTTCTTTTTCACTAGATGGAATTTCAATGAATTAGATTTATGAGAACCGCTTGTCATATG

GAATGTGAAAATGTCTTTTGCATCTCTTACCTGCATTCATAGGGGTTGACTGTGAGATCAAATGAATAATGACCTCTTTA

ATGTGGTTCAGAAAGGTTTTGTTCGCTGGGATTCAGAACTTAACCAGGGCCAGGAGACGGAAGGAATTCAACGGTATATT

GTCATTGGGGGACTCTTTTTGGAAACTAGGGAGACGCAAAGCTATAGGAACCAACGTGTGGACACACTACAAATTATCAA

GTAGGGACAAGTAGCCAAGGAGAGAATACCGAATGAGTTTTAGACACGAATACGATTTACTGCAAATGAAGAATAGGAGA

AGCCCTTGCAGGGAAGGATTTGGTAGTGGAAAGGTCATGGATGAGAGCTCGAAGATCAAGACCATTGAACATGACAGATA

CGATCACCGTAGCATGAATATTTCAAATGACTAAAGCAACATATCGAATCAAAACTGAGCGTGGCGGCTGGAGTTGAGCG

CCCAAACGAGGAAGACGGAACAAGAAGACACGCGTGACTAGACGGAAGATATCTAAGGATCTTAGTGAGAGCGACGATCT

GCACGAATAAAAATGACAAAGAATATGTTCACCGTTGTCTCCTACTAGAGAGACATCACTATCTNTACAGATTAATTATG

CAGCCTCGCAGACGCTAGAGATTTCTACAGAATAGC

>GW880486.1 JC000688 Seed specific Normalized cDNA library from Jatropha curcas L. Jatropha curcas cDNA clone N01293 5' similar to Unknown protein, mRNA sequence

GGCTAAATACAATGCTTAAAACATCTTCTTTGAAACAACTATCTGCCGCTCAAAAAGATCTGATTCCCTTTTTCTACTTT

GCTACCTTTAATGCTTCCCTTTCTCCATACCCACCCTGGTAGTGGTTTTCAGTTTTTCAAATTTCAAATTCAATTTCTTT

GTTTTTCATCTGGTTTTGTTTTAACCCATTAAATTTCTGCAGATCACCCATTTCATTTAGGTTTTTTCAATTTCTTGCTT

TTCTCTATTCTTCTGTTCTTAGTCCATTTTGAGTCTAAAAGGAAAAGGGAGAATTTTTTTTAGTTCCCTGTTTTCCTTCG

CATTCGTTTTCTTTTTATATCCTCTGTTTCCTTCTGAACAGAGTTGCCATGACCATGGAAAGACTGCATG

>GR209294.1 JCST74 Jatropha curcas L. seed cDNA library Jatropha curcas cDNA 3' similar to Unknown, mRNA sequence

CTTTTTAATTTACCCCTACCTAATTACTTCATTAGACAATGGTAGAGAGGTGCCAGTGCTGTAAAGAAAACCCTATCGCC

GGCGTGGCGCATCATCAAGCGAGGTGTAATATAGCAACAAGTGTCGAATGCTTTAACCTTTTTTTTTTTCAGTGGCGAGG

TGTTCGGTGAGGTAGTGACTCATCGCGGGACGTCTTGGTATCCTTCATTACATGGGCAGCCAAAATTGGGTAAGCGTGAT

TTAACCAGGGGTGCAATGGACATTTTTTCCATGGGGGCTGATTTTCCCTCACCCTGCCCCTCCTTACCCATTCATATTTT

TTTCGTCTAATGTCGTCCATTCCTTTATTCCTATCAAATATCTAGACGGAAAATGTTGATGTTCATATGGCGAACTGTGA

ACATAAACTTTCGGGTTGGCTGACTTTTTTTTAGCAGCTGCCCCCCAGTTCTCGATCCGGCCCTGATTCAAGTCAGGAGG

CCCGGTAAGGGACCTGGTGGAAGTGTAGAAGGTAGAGTGGTTTGGCCATCTCCCCCAAGCAGCGGGCACTACCCACAAAG

CGTCTCCTGACTGTCGCCCCGTTATCAGCTACCCAATGATTAAGTTATAGTCCCTTACTCAAATTATCTAATCCGGCGAA

ACCAGAAAAAAAGACAAGGAGCAAAAGCGGAAACCTTTCGTCGACCCCTGGTCNATTGNATGCCAA

>FM888003.1 FM888003 Jatropha curcas embryo 35-55 (DAF) Jatropha curcas cDNA clone rjcfea0_001310, mRNA sequence

CACCCCAAGATCCTCCTAAAGGCAAAGGAGAGAAGCCACCACGGAAGTTGAAGTCACCCTACAAACCTCCATCAGCCAAC

TGAGTGCTTCACTACTATGCATGGTTATATATATACATAGCTAGATAGCAATAAACTGTCTCTTACTTGGATATGTTTAG

TCAGTTGCTTTTATGTATTGGGTTGTGGCTTATGAGCATTACTCTATATTAGTATGCTCTTCCAATGTATGTTTACCTTA

CGTTGCCAGTATTATAAACCATGCATGTTTTCTAGTTT

>JK317402.1 JCST169 Jatropha curcas L. seed cDNA library Jatropha curcas cDNA 5', mRNA sequence

GGGTTGNACACCGTTTCNAACNNNNNNNAAAAACCCACATTATCGTTATTTTATTTTGGTTTTTACTTGGGAATGAAGAA

AAGTTAAAAGCCAAAAAGGTTTGGAGAAGTGCAAAGCGGGATATCAGAACGGGTAAACGAAGTTGGTTATAGCAGGGCGA

AGGAACGAAGTGAAGGAATAATAGGGATGAAGGGACGCCGCGAAAGAGAGGTATAGGGAATCAAAGAAACCATAAGGGTG

CGGCAGACCAAAACGGGGATGGATCTAGAAAAGTAAATGATCCGGTTAAATGTGTGACTTAGATGATGCCCTTAGGGCAG

GATCATGACGAAGCTAGAAGGGGTATTTATCCGTGGCGGGTCTTTGTGTCCTTCCCCGGGAAGGTGC

>FM894526.1 FM894526 Jatropha curcas embryo 71-95 (DAF) Jatropha curcas cDNA clone rjcpga0_000309, mRNA sequence

TTTACCAAGGAACAAGCTATCATCTTACTGTATTCATTACTTTAATTTATGGTGGTCTTGAATTCAAATACACTCTCCCA

ACAATCAAAAATAGGAAACCAAAATCCAAGGAAAATGACTTCCGTTACTCTCTTGCAATTCTGATAGTGTGAAACTTCAC

TACCATAAATAAAATATGAACATTCTCTTCTTACCAAGAAAAGAGGGAAAAAAGGGGAGATGGGAGTTAAATTTTGCGCT

CTAATAAAGATGGTTGAC

>FM887894.1 FM887894 Jatropha curcas embryo 35-55 (DAF) Jatropha curcas cDNA clone rjcfea0_001177, mRNA sequence

ACATAAATCCCTCAAACTACAACCAACTTAATTAACCATGCATAAAAATAGAGTTCTTCATAAACCCTGGCTATAAACCA

GAAATGAGATTAATATTTTCATGGATAAGGAAGAACGCCAGTTCCACTGCCAACGCCTCCGCCAACGCCACCGCCAACAC

CGCCGCCAACGCCACCGCCACCGCCAACGCCACCCAAACCACGGGGGGGCCCGGTAC

>FM887809.1 FM887809 Jatropha curcas embryo 35-55 (DAF) Jatropha curcas cDNA clone rjcfea0_001079, mRNA sequence

GGTTTATTTTAATTTTATTTTTACTTATTTGATGCCAAGTAAGAGCATACTGGACGGGAAAGTGTTAGTTCATTCAAAGT

GGGGACTCAGCACAAACCCCCGTTGGTTCAACCCAGAGTGGAGATCCTCCTAATTGTTAATCTATCTGCATAAGTGCAGT

GGAGGAGGATGCCGTTGGGAAGTTGCTTTAAGCAGCATAAATAGTTATTGTCAATCTTACATGTAAGAAAATCAGAGT

>GW876525.1 JC007159 Seed specific Normalized cDNA library from Jatropha curcas L. Jatropha curcas cDNA clone N12053 5' similar to Aminotransferase class IV, mRNA sequence

GAACGTTAGGTTCTATAATAAAGCTTAAAAAGCTTCACATTTCCACATTGATTCCCCTCTTGCCCCTACCCTCCATACGC

TCCTCCATGAAAAATCAATACTCCACAGTTCATTTTTTTTATATATAGATCTGAATGAAGTGAACTGGACCAACCATAGA

ATAATATATCTCACTGCATTTTCACTTTTTTATTGGCCAATTCATTTTGGACTTGAATAAAGACTGTACAAAAAACACTT

TCACGCCTCTTAATCAGACTCCAAAAATTGTTCAATCATTGCAAATAAGTTCTTGGGCTTCTCAAATTTCTGGGGTTTTT

GAGAAATTTATGCTCAGCTTTTGACTTCTTTTTGTTTTTCTTGCTTCAAGTCCTAGGGTTTCCTTATTTCTTGGGGTTCT

TTTGAAAATTAAGTTCACTGTCTTTCTGTCTTTATGATAAATGGGATTTTATTAAATTTCAGGTTTTTTATTGTTTTTGA

GTTC

>FM889578.1 FM889578 Jatropha curcas embryo 35-55 (DAF) Jatropha curcas cDNA clone rjcfea0_003270, mRNA sequence

TAGAACTGGGTGCTGGAGGTAAATATTCTTATGTTTCCAGTAGCCCTTTGAGTTGCTAGTTCTATTCAGAAATTGATTGC

CATTTGTAGCTTGGGCGAGTTCTTTGTGGCTTCTGATTCGATTTAGTGCAAAAGATTCATTTGGCCAAGGAACAAAGCTA

TCTATGTATTTACATTTTACACCCAACTTAAATGAAATTTATTAGAATTTTTCCATTCATAAGGTTGTTGCTTCTGTATA

TACTTGTTGGGCCCATGAATACTTCTCTTAGAATCAAATTGATCCAGCAGATAACTGTTTAACAGATTATTCTGTAGGCT

ATTTATTACCAAAGATTACGCTAATTCACGTAATTCTGCATTTACTATCGCAAAAATGATTTCTTCTGGGAGATGGGTAT

ATAGAATGAATTAGAACAGAATGATCTGATACGCTTAAAACATCCACATAGATGTGCTTCTGGCAGTTGGCAATTGGTAT

TGTGGAAGGTGGATAAATCATCCGTGATCATGAACATAGAGAATAACTATGAACCTTCTTCAAACTGA

>FM894152.1 FM894152 Jatropha curcas embryo 71-95 (DAF) Jatropha curcas cDNA clone rjcpga0_004340, mRNA sequence

CTCTCTCTCTCTCTCTCTCTCTCTCTCTCTCTCTCTCTCTCTCTCTGCTCTCTCTCTCTCTCTCTCACTCTCATCTCTCT

CTCTCTCTCTCTCTCTCTCTCTCTTCTCTTTCTCTCTCT

>FM893206.1 FM893206 Jatropha curcas embryo 56-70 (DAF) Jatropha curcas cDNA clone rjcaeb1_003971, mRNA sequence

ATTCATCGAATGAACTGGAAAAGAAAAATCAACCCAGCAACATTCTCTCTCCACCTCTTTTACTCTGCTAAATCATCCTC

TTAATCGGACAGGGCACGAGACCTGCCATGGCTTCGAGCTGTTGAAGAGATCGAATAGTGATTGCTCTTCTCTTTCGTTG

ATTTTCGTTTTCAGGGACTTGGGGATTGCAAGGATTTGATTTTGGAGATTTAGGTGTTTAGGGATTTCATCCCCCAATCT

CTTCTTATCGTTATATATTCTAAAAATAAAAATAAATTTAATTATTTTCTTCTTTAATTTGACCGGTTGAGAAACACGCG

TGCCTGGTCAGCTAAGAAATAGGTCTTTTGTAGATTTGTGGCCAAGTTCGCGATGGATTTATCAAAAAAAGGTGAAATAT

GGGGTTTTTAATGTTGAACCCAAGTTCAGTGGTCAATTTGAACCTTCTTTAAATAACAGGTCCGTGTCCTCTCCTCTGCA

CAATTCGAAGCGATCAAACATTGCAATTGCAGATTTATATTTTTGTAGAGTAGAGCTGTTGTGCATATTCCTCTGTCTTA

TATAAGGTTTATGAAGGATGGACATAATCT

>GW614984.1 Jc1-045-E06-M13F.E06.ab1 Jatropha curcas flower and seed Jatropha curcas cDNA, mRNA sequence

CCCCATTCACTTCATCTCCTACTTCCACAATCTTGCCTCCTCCTCCCCTTGCCTTTGCTTCATTTTGCATTTTAGTGTCT

TTTTATTTTTATTTCTCATTTCAAAAATGTTACTATTTTTTTTTTTTGCTTTTGCACTTCCAATTTAAGCTATAAGTTAA

GTAATTTATTGTGGTTGCTTCATGGGTATATATATATATATATATATATATATATACAAAATATTTTTTAATGTCAAGTT

TTGAACTCTTTTATTCAATTAGTGAGGCCTTAAAAAAGTGTTTGTATCTTGAAATTTATGGCCGTTCGTTGATTTTCCTT

TCTGGGTTTTGATTGTTACAAAGTGGGATTTGTCATAGTTTCACTCATAACGTAATTAACAGTCATTATTATAGATATAC

TCCAGAATAGTGAGTCTTGGGTGGGGATATTATCTGGTTTTGGATCCTGTAGTTATGAATTAGATTTTTCTTTTTCATCT

TTAGTGTTCATTGATAGGGAAACTTGAAGAGAATATTTCATGAAAGTTTTGATTTTTAATTAATGGTCTTATCGTCGTAT

TGTCTTCTTCTGATGTAAACGTGACAAGTTTCAGGTGATTTCTTTATCAATTGTAGCTAGGTGTTATCCTATGCACTTGC

CAAATGTTTCATTTTCT

>FM889369.1 FM889369 Jatropha curcas embryo 35-55 (DAF) Jatropha curcas cDNA clone rjcfea0_003020, mRNA sequence

TTTTGGATGCATAGAAGACGATGAGGAAACTTATGTGGCTGATCCTCCGCTTGATTACTGCCCGGCTAATTGCTGCTGGC

GGTTCCATGGCTGGATTTGATTTTTGCCTTACTCATCTGAGTCTTTCACTGCTCTAATTTTGATTTCTCAAGATAGCTAA

TGGCTATTCAAGGTATGCTTCTTCTTCCTCTTCTATTTTTAGGGTTACCTGTTAAGTTCATGTACATAACATGATCCAGT

TTTAAGTTTTATATACGCAATGATATGTATATAAATGTGTCTGTATGATTAAACAAATTACTTGCTGCTGACTGATTCCT

TTTTCTTGCCC

>JK612410.1 JCF25-112 Jatropha curcas, immature Seed cDNA subtraction library Jatropha curcas cDNA, mRNA sequence

ACTAACTGATCACCCAAATTCTTATGGCTCTTTATGCCTACTGAAGAATTTCTGAAGGAATAACTAGCATACAAGTTCGC

CGCTATGATGCTATCAAAGAAACATGCCAATTGTTTTAAATTTACAAATCTAACATATTGTAAGGGAGCAGCAAATTCAA

GTTCATCCATAAAAAACTTCTAGGGAAACAAATTAACGAAATCAGTTCAATTTGACAATTTATGGTAATCATCACTGCAG

TCACAGGGTAAGGATGAATCCAAAATCATGTTCGTCTTTCCTTCCTTTACAATGATGAATGATAGCTTTTTCCAAAAAAC

ATATCACGAGCAATGAACAAGGTGATTAATTGTCTGCAAAATCTAACCACGTTTGCTCTTGTAACTAGT

>GT228916.1 JC481 Jatropha seeds from fruits at three stages of maturation Jatropha curcas cDNA clone PL10SE.C02.scf 5', mRNA sequence

GTATCGGTCAAATTGATTTTTGTAGCGATCAACAATAAGCGGCTGGTACATATTGGTCCGGCTAGGGTATGCGATACGGC

ATTCCTTGGTTCAAAGTATTAATATTCTCGATACGGGTTCGGTACATAATACAACATTCCTTGGTTTAAAGTATTAATAT

TCTTCTATTAGACAGTCCCTGATGTTCAA

>FM889916.1 FM889916 Jatropha curcas embryo 35-55 (DAF) Jatropha curcas cDNA clone rjcfea0_003671, mRNA sequence

GAACAAACTCTTAATTTAATAGGCTAGTGATAAGTTGTGATCCCTTACTGCCTGCATATTAATAACAACAGGTTGAAACA

AACCAGCTATTCTTTGAAAGATGGAGTACGATCTTTCCACCAAAAAGTAATTATAATTTTAAAATGTTAACTACACCCCA

ACAAGTAACAGCAGCTTCTTAAATGCTTTCAGAGCTCACTGATAAAGTTTTTTCACAATGAATGTGGATCAGATCTTGAG

TGGATATGATCTAGAATTCCATTCTGTTAACGAGCAATATGGAATATGAAATCNGTACATAGCTTATTATTTTTCTTAAA

TTACTTACCACCAAACCAGGATATGCACATGTACACAGAATAAGGTCCTTAAAACTCTAAAAGTTATTATTACCACAT

>FM887294.1 FM887294 Jatropha curcas embryo 35-55 (DAF) Jatropha curcas cDNA clone rjcfea0_000457, mRNA sequence

TTTTCCTCAATAAATAGGCTTGTACGTTCAAAAAAAACGAGCATCCTTGGTTTAGCATGAACTGCAGAATAGATTTTCAA

AGTAGTCTAATTAGGTACAGAATACAAATTGACGAAGTTGTCAGTGGATCAGTATTCTTGTACTTGTTTTTGCTTGATTT

TGTTATAGTTGTTACATCCTTTGATCTGTTCACCGTTTTAAGTTAGACAACCTTCTCGATGGTGGCTGGTACTGTTACCT

TTGTTTTGTAATGGAATCGTTCACCGATTTTCAATATCAGATCATTTTTCAGTTTTGGTCTGTTTC

>JK612427.1 JCF25-130 Jatropha curcas, immature Seed cDNA subtraction library Jatropha curcas cDNA, mRNA sequence

TATGCCCCGTTCTGTAATTCTTTCCCTTCTTAGCTAGAAAGGAATAGTTTACTTCCGCTTTATACCCATCGTCTCCAAAG

CTGCTCTGATCAACTGAATCGATCCGTTGGTCTCTCTTTTGACTCTATCGACCGCTCGCACGGGTAACCTCAAACTATAA

GTAATAATAAGGGCCTGTCTCGT

>GW880330.1 JC001416 Seed specific Normalized cDNA library from Jatropha curcas L. Jatropha curcas cDNA clone N02637 5' similar to Unknown protein, mRNA sequence

AGAATAGTGAGTCTTGGGTGGGGATATTATCTGGTTTTGGATCCTGTAGTTATGAATTAGATTTTTCTTTTTCATCTTTA

GTGTTCATTGATAGGGAAACTTGAAGAGAATATTTCATGAAAGTTTTGATTTTTAATTAATGGTCTTATCGTCGTATTGT

CTTCTTCTGATGTAAGCGTGACAAGTTTCAGGTGATTTCTTTATCAATTGTAGCTAGGTGTTATCCTATACACTTGCCAA

ATGTTTCATTTTCTGGTGTCTCTTCGTTTGAGCCTTTGGTTTTTTCCTCAGTAACAACATTTTCTCTTGCTGTCTTGCAT

ATTAATATACGAGGGTAATCTGTATTGGGGTTCTCTAGATCGAAGATATAGTAGGAGGCTGTGCTTTGAATTCATTAGTA

TGGTCTTCTTGCCATTGTTGGTGCTCTAAAAAGCCTGATTAGAGATTAGAATTCCCTTGTGTTTAGATTACAAATTCTTT

TTTCTTCTCCTCAATTTTTCTTTTT

>FM894403.1 FM894403 Jatropha curcas embryo 71-95 (DAF) Jatropha curcas cDNA clone rjcpga0_004748, mRNA sequence

AGTTATTTTATCTGGCATTCTGCACACAAAAGGTTTCAGATGCCGTCACTATGGCTTAAAAAGGTTGTTTTCTCCAAGAT

TAACAAGTGAAGTGAAAAGTTCAGAACAACTGAAATCTTTGCAAAGATGTAACAAGAGGCTGTAAAAATATTGTTTTCCA

ACTCATGTTGCAGAATGCATAATAGACATAAGTACTAGAACTTCCATCAAACAACAGAATCCTGGACCAGGCACAAGTCC

AAGTCACAGCCAATAAAGCAGCTTATAAAGATTCCCTGTGTCAATAATTTGATGGCAGAATACAAGACTGGAACTTACTG

GTTTGGACTACACATGCCAAAGGATGGACCTTTAGATGGGAATGA

>FM890197.1 FM890197 Jatropha curcas embryo 35-55 (DAF) Jatropha curcas cDNA clone rjcfea0_004007, mRNA sequence

GAAAAAATTTCCCCGCCCCCGGGCTCGGTTCCGGCCAATGATACGGCCGTTCCCCATGCTCTCCCATAAATACTCTAACA

ACAATTGCAATCAATATATCTGTCATAAAAAGTCATAAAAAATAATATATGGGGTGCCAAAAAGTTATAAAAATAAGTTG

ACAGTTTAGCAACTTCACCAACAATCGGCATAAAAAGTTATATGTGGCATAAAAAAAAGCCTCAAAAAAAGTTGATGTTA

TATGACGTAAGAAGAAAGGCATAAAAAATAGATCATGTTTTTACAAGGAAAAAGGCATAAAAAAGTTGATGTTTTAGCGG

TTAGAACCTGCGACCAAAACATAAAATATACATCCTAATACCATTTCAACTACAATAATTTACAACTTAAAATTGGATAT

ATCTCTTTTTATATTGTTTTACATCACACCTTAATAATGTCTAGTTGGT

>JK612457.1 JCF25-166 Jatropha curcas, immature Seed cDNA subtraction library Jatropha curcas cDNA, mRNA sequence

ACTGTCGCACCAAGACTCAACCTGAGCCAACCTTTTGCAATGCAGCTTTTAATTTCTTGTTTTTGAAGTCTTATGTTGTA

GTAATAATTGTGGGTTCAAAATAGGTTGTTTGTATTGTGATTAGCCATGAGTTCATTTTAGTCTTGAACTGAAAACCTTG

ATGGGATTGGGATTGTTGTGTAATGGTAAATTCTGAATGTTTTAAGTGTTAAATTTGCTGAAACAACAACATATGTAATC

TTTTACTGCTACCCATTTGTTTTTTCTTCTCACTAGATTAGATTGGCTCGTATGT

>FM889576.1 FM889576 Jatropha curcas embryo 35-55 (DAF) Jatropha curcas cDNA clone rjcfea0_003268, mRNA sequence

TGCTAAAGATACTGGCATGTGACACGAATCCATTTCTTGTTTTGAAGTAGAAACAACGACTGAATACACATCTCTACCCT

TACAAGTACAGTATTATAACGAGTAATGATCATTTTTTTTTTTTTTTTTTTTGTAAGCTACCACAATCACAAACTTTCCT

TGGTTTCGATCTAACCCTTTTTAATAGAAATTGAACCCAACGTGTGATCCAATTAGCAAGGAGATGGAGACAAAGAGTTA

TGAAATCTTATCCTCATCTTTTTCTCTAGCCGGTTAGGATTGGAGTGTTAGCCAGATACATAATGATAACTAATAGAAAA

GGTCGTATGATATGTCTATATTGTAATTCAACGTATTATCTAGATTAAACCATGTAAAAAGATTTAAAAAATTATTAATT

TCATTGATTTGATTCATAGATGAAGCTATAAATCTAGTCTAGACAAGTATTTACTGTTTTGCTTTACTTCTTTAAAGATC

ATTAGTCACACCAAAAATAAAGTATACCCTTCATCATTATTTTGACTTTTACATTTAATAAGAAAAAAATAGTTACTAGG

ATTACT

>GW612755.1 Jc1-020-C12-M13F.C12.ab1 Jatropha curcas flower and seed Jatropha curcas cDNA, mRNA sequence

TCTTATTCTTAATTATGTAATTGTGTTTTGACAGCAATTGCTGAACTACTGGATAATGCTGTTGATGAGGTACCGCTCAA

CTATTCAAGTCAAGTTGCATTTATTTTTTACTCATTAATTAATAATAAACAGTATTTTTATAATATGCCATACAATTATG

CGTTTTTGTTTACCCTTTTCCTTGCTGCACATCAGTTATCTAAGTTCTGTATCACAATATGGATAGCTGAAACAGAAATT

TAAGGCCTACCTTTCAGTTGATATTGGTATATGTGGAAAGAATGTAGGGCCATTAAACCAAAAGTTTAGAGATTTTGCAT

ATGTTAGCTGATAATCAAATTTTGGAACTGTTAGTAAGGAAAGCGCTTGGGTTAGAGAAGAACTGTTGTGCTGCAATGAT

CAACATCTTGCAATTGCATAACTGAAGGTTCTGTTTTTCTGTTCTTCCCCTTCTCTTTCTCTTGTAGTTTATATATTGCA

CTGGCATTTCACAATTGTAGAAAGAAGGCTGATTTCCCTCCCTCTCTTAGACCAGCAGACTTTACTATTTCTTAATCCAA

GCTTGAGATGTTTGATATGTGTTCGTTTTCCAATCCTGACTTCTGATTATGTTCACCTTAGTTGCAAGTTGCAATGTCAT

TCTTGTTGCTTTCTTAGTTGTTTGTTGTTCATTTTCTTTTTAGTCTTAGATTCATCTA

>JK613114.1 JCF22-117 Jatropha curcas, immature Seed cDNA subtraction library Jatropha curcas cDNA, mRNA sequence

ACTACCCTAGTTTGACCATTATTTTTTAAACTTACATTACATTCTTCTCATCACCTATATATTTGTATTCTTCGTAACTT

AAACTTCTATAAATATATTCTTAATCAAGCCTAATACAAGACTTAATTTCTCCCTCAAAGGCAGCTTGCGGGTTGAACAA

AGCCAGAAGAAGTTCCACCAGCATTGGT

>GW876027.1 JC005404 Seed specific Normalized cDNA library from Jatropha curcas L. Jatropha curcas cDNA clone N09310 5' similar to Unknown protein, mRNA sequence

GGTTCTCTCTTTATTTTCTTTTTGGAATCCATTTTGAGTTCAAATGGGTTCTCTAAGGATTTGAATTTGCTTGAAGCTTT

TCTTGTGAATTTTTTTGGTTTATATCTATTTTCTTTTCTCAATCTGCCACAACATTTTACTTTGACAAGATAGTTCTTCA

TTAAACATATTTGTATAGTGCAAAGCTTGAAAGATGGATTCTTTTGTGTAATTTTTCTTTCAGTTTATTGTTTGTCACTT

TTATATTTGTTAAAAAAACAGGTGCTCTGTCTAGTTGCTTTACTCAACCAGTTCTCTTTAAAAAAATTGTGACTTCTCTC

CATATGGGTATTAATAATTGATGTTTCTTGAAAATGCTCTTTGAGAATGAAGCATTATTTTAAACTAAATCTCATACTCG

AGAGATTATGAGTTACACTTGCGGATACAGTTTTAATGAAAAGTTGCCTATGCCAGAGTGCCAGCAGCAATATTTGAGGG

ATTTTGGGTTAGGGGA

>JK317661.1 JCST432 Jatropha curcas L. seed cDNA library Jatropha curcas cDNA 5', mRNA sequence

TAGANNNGNAGGANCTGGGACCACACGTGTGGTCTAGAGCTAGCCTAGGCTCGAGAAGCTTGTCGACGAATTCAGATTGT

CATCTATGTCGGGTGGTCGACAAGAGGTAATCCCATTTTTTTTTTTTATTTGTTTTGATTAAGAGGAAGCGCGACAGAGG

TCGCGAAGGCATGAGGTTGCTCTTTTAATGGAATGCGTGTGGAAATGGGTCTGAATAGGAATCTAACTTTGACATATCGT

TTATAGCGACGGGTGGCAAGGGCCGCTTAACTCACATGCGGTGAGCCGGGGACCCCAGGTGTTTTAACCTTGAGTTCCAA

TTACTGTGGGTGATAAGGCGTGGATGCCTGGGAGCTTGGATGAACTTTGTGGGGAAACAACCCCTCAGCCAACGGCGCTG

AAGCGGTAGTGGCCCTACAGTAGCGGGAGATTGCAAGACCCGCACACCCCACAACCGACAGACAATTGATCATTCGGGTG

TTTTGGAACGACACTGGGTGGAAGCTTTGTCCGCATCTGCGAAACCCGGAACACCTGGGCGTCCGATTAGATAATATCGT

ATATACAAGTCTGGAAATGGGAAGACAGTGAGGAGACCAATACGAAGCAGACCAAGGCCCAAAATAAAGACCCTGCGCCA

GGGTGACTTGCCCTGAAGGTAAGTTTATGCGGCACCCTAGTGCAAGATTCCCGAAGAAACATTGGGTTCTGAGAGAGGGT

GGAAAAAAAACTTTAGTTTGCGACACGTTGGTGAGTGAGAGAGAGTGAGTAACGAGATGAGCAGAGTTATATCGGGGGTG

AGGAGCATGAAGAGTAAGGAATATATACAGAGACAGAATCGCAACAAANCACGACAAGAGACGATCAGTGTGGGTCTGTG

TCGGCCGGTCAGAGATAGACATGCCTGTACGCCAGTGTAGATAGCGTGAGATGGTTGAGGGGTGAGTGTC

>GT969785.1 GJCCJC2002C07.b Jatropha curcas L. developing seeds (mixed stages) Jatropha curcas cDNA clone GJCCJC2002C07, mRNA sequence

AAAGTGTGGTTTTTGAAAGAGTAAGAATCAATTGATTAAAGTTTCAGATCTCTCAAAGCTCTCTCTTTGACCCCCCCCTC

TTATTTTTTTCATCTATTTGCTTTAAGAGATAATTTTTTCAAAGAGAAAAGAAAATTTAATAACAACATTCTCTGAAGAA

TAATTTTGAACTTTCTTTCTATTTTCAAAATCTGTTTTCTATTGTTCATGTGCTTATAACTCTGTCTACATAGATTTTTG

TAATCTGTTGTAGACTAGAGGCTTTTAAATGAAAAACTTTTGTGTAACAGCTTAGGTGAAAATGTGTGTAGTAAAAAATT

ATCAAGCAACCTTCTCCTTGTGTGAGATTTAATGTCTTGATGTGAGTCTTCAGTCCTCTGCAAAATAGAAAGGTAGAAGT

TGATGATAGGTGTGATTCTT

>FM889581.1 FM889581 Jatropha curcas embryo 35-55 (DAF) Jatropha curcas cDNA clone rjcfea0_003274, mRNA sequence

TGTTACTTGATTTAAGTTGCATCAAGGATTTCTTGATTTTTCGTTTCATTTTTTTTATAAAAAAGAAAAGAAAATAGTGT

TCCAACTGTTAAATGATCCATCAAGGATTATTATTATTATTTAGTAGTTTGTAATAATAAGTAGATGATCTCTAAAACAA

CTGGTTTGAAGGAATCTGAGCCATTTGATGATTGACATTCGAGATGGATGCTTTTCTTGGCTTTATTGTTGATTCTCCTA

CAAGCTACCTGAGGTATAATAATAAGATACAAAACCTCATCCATGCTCGCTCGTGGCTTTTCATGTCAACATAAAACCTC

TTTAATCTCCTTCTTTTTTGGCCCTTTAGCTTGCTTTGTTTTCTTTCTTTCCCCTTTAACCAATTTTTCAGGCCTAAAAA

ACGAAAAGCTCAGCAAAAATGTGTGCTAAGAGCGCATTCACTCGCTTTCTTGAAAGAAAATTTGCACTAAATTTTAGCAA

AAGAGGTGTAAATGAAACTTTGTTTAAAAGCAACAGTGGCATAAAGTTGCCAAATCTGCAAAAAA

>JK610435.1 JCF1260 Jatropha curcas, immature Seed cDNA subtraction library Jatropha curcas cDNA similar to DNA / pantothenate metabolism flavoprotein, mRNA sequence

ACACTCACCCATTCTTAGGATAGATTAAGTGAAATTCTGTATTTTGGAGAGTCTGGAGACCGTTTGCTACTTTTAGTATG

CAGTTCTACTAATTCATCACCGTTAAAATCACAATTTATGTTTGCGAGATGGCTAGGATTATTCTGAATGGTCAATGAAG

TATTATATCAATGTTCATTGTATAAGTGCAGCATTTCCTCGTTTGGTTTGGGATCATCAGTCAGTTTGTTATAGAGGATG

CTGAGGAGGAAGAGGCAATGGCTGTTTTATATGTGGATGACACAATCCTGGCCTATGATCCCTCAAGTGGGAAAGAAAGG

TAAAAATACTCAAATGGGAAAGAAGGGTTAGGAAAAGAAAAACGAAGAAGAAAAAAATGAAATCCTTTTTGAGTCCTGAA

CATGGAACCCATCTAATGCACATTGAGTTGCTTGCCTTCTTCTGTATTATGAGATGGGCCAGAAGCTCGAAACTAATAAA

TAATGTGAATACGGCATATGATTGAATGTATGTGCACGTCAAATTCAAGCAAAGTGCCCCCACTTGTGCCCTGTAAATTG

CAATTATGGAAGGACACATGGGCCTGAAAAAGTTTAAGACAACAAAAGCTACATTCCGAAGGTATTCCGATTCATGTGGT

CGCGTTATTCCTGCAGACTGGCTGGGTGTGTGTGGTGGTTTGCACGAGTGTATTATTGTGATAATATTAGTAGTATACTT

TTATTTATTTGTTGAATTGATATTGTAAATCTACTTATAGGCACTATCCTTATCTTTCAAAAAAAAAAAAAAAAAAAAGT

TTGT

>FM895656.1 FM895656 Jatropha curcas embryo 71-95 (DAF) Jatropha curcas cDNA clone rjcpga0_002616, mRNA sequence

AACTTGAAACCTAAATCGAATTTTCTGTTTGCAACAGTTTCTCTGCCGATGACGGTTCTAGTCTACAACTAGGCCCAATT

TAATCCAATCACCAGTACGCCTGACGAAAAATTATAGCACAGGCACTACTAACATGAACCCAAAAAAGAAACCAGAAAAA

ATGAACCCAACACTCAAAACTCAACCTGCAATCAATGCGATAGACCAAAATGACAAATCACGAAAAGCCCAGTTGCTAGA

TGGTCTTTCGTGTCTTTGCCGGTTTATAGTATACTCTCGCTTCATCAATAAGCATCCGCTCGGAGGAGTTAATTTTTAGT

TTGCTATAAAAGGCGGCAGTAGCATACCTACAACGTTACAATCAAGGGAATAACAAAGAACCAACCCAAAACCAAACTCT

TCAACATTATCAACACCAAAAGCCACATATCCGTCCCCTTCTTCCGTTGTTAATAGAGAACAAAAGATCTGCTTCTTCAG

ATATACTGAACC

>FM894461.1 FM894461 Jatropha curcas embryo 71-95 (DAF) Jatropha curcas cDNA clone rjcpga0_000131, mRNA sequence

ATTCCTCAAGAAATCTGAGAATCTAACAAGCAAGCAAGAGAAAGGCAACCCATAATGGGAAACAGAACTACGCTTTTCTT

CTTACTGCTAGTTATTTGCTA

>GW880776.1 JC004677 Seed specific Normalized cDNA library from Jatropha curcas L. Jatropha curcas cDNA clone N08223 5' similar to Protein disulfide isomerase, mRNA sequence

GCCGGAGGTATGTGATAACTTTTAAATCCTAGCTTGGCTCCTGGCCGGAGATGTTATTCTCACCGTATGCAAAAGAAACA

GAGATGACATGGTAGTTTGAGTTTAAAGACTTCCATACTCCTCTATCCTTCTAACAAAGAAGGTCAATTCACCAGATCAG

CACTACCCGACAAGCTTCCTTCAGCGATGACTTCTACACCTGAAGATGCTTCAGAAAAAGCTTCTTTTTATACATGTGGA

AACTAGGAGTAGAGGTTCAATTGCAAGAAGACTGTGCTTATAAATTTGGCATCCCTACGAACCATTATTCTTTCCGCAGC

TGCATGCAGATTGGAGCCTCTTACACTGAAATGGCAATGCCAAGACACTTATATCACGGTGAATTTACCTAAATTGGTAT

TAAGGTAACTTGGTTATTTTCTCCTCTTATTAATTTGCTTCTTACTATAATTTTTACTTCGCAAATTTATGGAATTACTG

AATGAATAATGCATTAAAAAATAATTCAACCTGCATTCATAAATACATCTGAATTTAAAAAAGTGAATTTGAGTCATCTC

TATAATAAAAATTATAATTAATTTAATGAATGCTATTTAAAAAGAGATGGTTACTTTGTGTTGTGTAGCTTACATATTCT

CTCGGAAAAACATGTTAATGTAGTGAATTTGGAAAAAAAAAAAAAAAGGCCGCCTCGGCCCTCGAGGGGGGGCCCGGTAC

CCA

>JK612804.1 JCF21-18 Jatropha curcas, immature Seed cDNA subtraction library Jatropha curcas cDNA, mRNA sequence

ACTAAGTTGAATTAACATCAATGCACCATCCTTTAAAAGGTAAAACTAACCTGTCTCAAATCCGTCTGACTACAGCTCGT

AAAACCTTTTGGTGGGGGAACCCTTCAACACTTGTGGAATTCTGGTTCACATCTGCTGAAGAGCCGACATCATATGATCC

TGATGGACCCTCTTGTCAACGCTTGGCTGATTGCCCCCCGTGCTCCCTATGCTTTCTTTCTTGACCCCTCTGACTTCATT

GAAGGAATGTCTAAAGGATCCTTGGCCACCTGCCCGGGCGGCCGCTCTAAATCTCTTGATTTAACGAGGTACCGAGCTCA

CCTTCTCCTTTTGCTTTTTCTTGCTCCCTTGCCTGGTAAAACCCTGCCCTCCCCCAGATTAATCGCCTTTAGAGGATCGA

CGATGAGCCTTCTGGTTCATGAGCGCAAATGCCCTCAACCATTCACCTACCTTTCTCTGGCGCTATCTGAATGACGAATG

CGGGCGGCCGCTCTAATATTTTATGCAAATTCGAGGTACATAGCTGTTATTTCCTGTCATTTTTTAACCAATATCCCGAC

TGGGAAAAATCCCTTATAAATCAACTTAATCGACCGAGATAACATTGAGTGTTGTCCCAGTGGGGAACAAGAGAACACGC

TTACAGAGATCGCACTCCAACACCGATAGGGCGAACTGAATGGTATAAGGACGATGGACCCTACATATACCGTCACACTA

ATCATGTTATTTTGGGGTAAATGTGCTCATATAGCACTACAATCAGGAACGCATAACGGCAGAATCCGATTATAGAGCAT

AGAAGAGGAGAACGCAGGACAAAGGTGACCTGGTAATGCAAGGTTAGAAAAGAGAAATGCACTAGTAACTTAAGGCTGCA

AGTCAAATCGTAACAAGTCGACAATGACGCTATACAGGACAATGGGCGCAATAACTGGAGAGCACACGCATTTACATACA

CCACGGCGAT

>FM888265.1 FM888265 Jatropha curcas embryo 35-55 (DAF) Jatropha curcas cDNA clone rjcfea0_001646, mRNA sequence

CTGACATGCAATATGATTTATATTTTGTGTATTGCAGGTTTTAATGTACAGTACAACTTGGTTTAATGTTCATGCAACAG

CAACGAAAGATTGTGCTAATGCAACATACAAATTGGCATTCAAAATATCAGCAAAATGCAAGTTCTGCTATCATCTGAGA

ATATGGTATTTTTAAGTTTGACCGATTGCAAGTTCTTTCTAAATCCTAGCTTTAACCCTGTTCAGCTCAAGCACAACAAT

ACACTCCTAGAAATAGCCTAGCAGTGGAATCTCCCTCGAACCCACATAATCCACTTGGTATTCCTATCTGTATGACTTGC

AAGAGGACTCAAGTTTCCATCTATTCAGACTACGAGAGCAGATGCATTGATCGCATGTCNACCTTAGCAGCACTCCTCGT

CTCATTATAATTGTGAGACCTGGAAACATGTAGCACTGGGGTTGAAGGTGGAGGTCGATTTGTTT

>FM887998.1 FM887998 Jatropha curcas embryo 35-55 (DAF) Jatropha curcas cDNA clone rjcfea0_001304, mRNA sequence

CTTTTGGGTTCTAAAATTTGCCTAAAGAAATTCCATAGATTATAAGCATTTAAAGAATGCCTTTAGAGCTGAATTACTAA

TAGCTAGTTAAACTTCATCCTTCTCAAAGTATTGTTATGGACAGAAAAACCCTAAAGTGCAGGTTCTCTTTTGTTTTCTG

CTTGTTATAAAGTTTGTCCCAGATGAAGCTAATCTTGCGTCATGATATTCTTTTCTTTTTCTTCTTTTTTCATCTGTGAC

AGGAGAAGGCAATAGAAGCGCAAAATAGTATGCTAGCAAAGCAGGTAACTTATCTTTATTATTCAAAAGCATACAAATCA

CTCTTTCCAGTACTACATAGAGAAAGGTTCATATAAGTACATAGATTGTGAAAATTGCCAATTGCCAAGTTCGATTACTT

TTTTGACAATGCCAAATCAAAAGGCATTCTGCTTGAAGATAACAGGAAATTACGATTTTCAAACTGGTACGGCGCNATTC

TTGCAGTTTTATGAACTTTGTTTCTTAGAACAGTAGAATTACTGAGTA

>FM894293.1 FM894293 Jatropha curcas embryo 71-95 (DAF) Jatropha curcas cDNA clone rjcpga0_004606, mRNA sequence

TTTCCCGGTTTTCTTAGAGAAAGCTATTCTGTTGATCTTATATCAATATGGATGTTTATATTGAATGTTTCACAATACGT

AACGGAGCTCGCAAACAAGAGCATCCATTGCCACACAACTACGGATAAGCTATGATACAGTTCATATATGTTACAATTCT

GCTTTCTCGAAACCGAAATACTGCAATAATATTCACAAAGTCTACCACAAATAACTTCCCATTACACAGACCAAATTCTA

ATTTTGTGAAAACTAACTTG

>GT971784.1 GJCCJC2024E08.b Jatropha curcas L. developing seeds (mixed stages) Jatropha curcas cDNA clone GJCCJC2024E08, mRNA sequence

CCATACTTGACCTCTTTTCACCGCAACGTTAAGCCGACTCCAGAGAGCCAGACCTAATCTCCTCCATTCGTCGGGAGAGT

CCCAGGGTTGATAGTAAGCCAGGGCACTTTCTTTACTACCAAGCCCAATGATGACATCGTCAGCGTAGCGAAGAAGCCTC

ACACCTGCGCTTGGGAAAATAAGGACTGGAAAATATTCATCAAAATCAGCCTTGAAGTGAAGTATGCCCACCTCAACGCA

CGTATAGTCCAAATGAAATGAGTGAGTGATCCGGATCACGAACGAATGGATTGAATTCTCTATCTGTCTTGCCCGCCTAT

CCCCTACAAACCTAAGCTGACTAAGGAGCCCCACCGAAAGAGCGTAGTCTACGAAGCGAAAAGGAGAAATGTCGATAACT

GTAAGGTTGGACTGAAACTTAAACAATTCTTTCAAGCCAGTACGGTACCAATGCTTTCTCTTTATGGTTTATAGATAGTA

AGTAGTGAGCCAGTCAAGTAAGGCAGTATATTTCCCTGGTTGTCAACGGGAAAGATTGGCGGAATTCCCCTTCTCGCACC

GGTAATTCACCCCAGAGTGCTTAGGCTTTGAGCTCGTACTCAGACTCTCATATCGGATACGAAAGCAGCGAAGTCAATAG

CAACTGGCACCTTTACAAGAAAGATGCTCACGAAAGACCAGCAGCAAAATAAAGGCCTTAACAACCAGAGAATAGTTTTA

GTTAACC

>JK317715.1 JCST486 Jatropha curcas L. seed cDNA library Jatropha curcas cDNA 5', mRNA sequence

TTTTTTTTTTTTTTTTTTTTTATGTGATTAGCTGAATATTCACTTGCTTGGAAAATGAGCTTCCTGTGTGGATTAAACAT

CCTTGGGGGAGGGGCGCTCTGTGCGAGTGACAAAACCAGTCCCCCTCCCCCCAAGACAACCCCCCGATTAGGAGGTTGGT

GATCTTGTCTAGCCGTCTTCACAAATCTCCCGGCTTGAACTGGCTTGGGTTAAAGTGGTTCCTGTGCCTAAATCCGGACC

TCAACGTCGCTTTTTTGGTGTTTCCCCCGGAGAACTTTGCCCCCACACCCTTGAACCCTCCCCTGCCCAAGCTTCCCTTC

CAAATTACTGGAAAAGGAAAAGGTTCGCTTTTCCCACTGATATTAAGCCCCCGACCGCAACATTTGCGATGATTCCTAAA

CTCTTCTCTGACAGGGCAAGGAGTTTTAGACCGAAAACCAGTAACCATTGACGAGTGGAGTAAAAAGGAATAACTAAAGT

GTTGTCACGGTGGGCGTTCACTCAGCTAATCATCAATCACAACGTGGGCCACCGAGGACCATGGGTCAAGGGGCATCCCC

CAACAAGACCATTAGGACGGAAGCCGGGTGCGGAATGAGACCAGATCCGCTCGCAATATTCATTTCAATTTATCTGGTTA

AATTGGTTGTGAAAACTTGGGGGGATATTGATAAACCCTCCCACATAATAATCCGCCACGAGTCCTACCCCCTATCCGGG

CTCAGATTGGCGTGAAACTCCATATATGATCTCCTTTATCCGGAGTCATTTGTTAGTGTATAAGTGTATGGTGCCAAGAT

GACAGATGAGGCAATCATGAAGNGAGTAGGCCCTGAT

>GW877281.1 JC006800 Seed specific Normalized cDNA library from Jatropha curcas L. Jatropha curcas cDNA clone N11455 5' similar to Unknown protein, mRNA sequence

AGTGTTAAGTTGGTGAGAGTTCGAAAAAGAAAGAATTTAGCTTGAGTTTTAACAAACTAATCAAAATGACTCTTCGAATG

TGCTTTCCGTGCTGATTTTATTTCCTGGAATCCTCTTAGAAACTCCAGATCAAAGTCTGCCTTTCCCAACTGTTTTTTAG

CCTGCCAAAGATATACAAGTTTAGTTCTAATATATTTTATTCTTCATTAGAAAACTTATTTCATCCTATTGAACAAGTTA

CGTCTTTGCATACCTTTATGTCATCTCAGATGCAGCATGCACTTCAGCTCCATTAAGTACTGTTTGCTGGGCATTCTCTG

TATTAAGAGTCTGCAGTCATCTTTTGTCTTTTACATGTGCTGTTGATGACCAAAAGAATGAACATTTTGTTCACAGGGGT

TAAAAAGAGCTTAAAGTCTGGCATTGATTTAGTGCCACAAATTTAGATATTGCAATTTTCCTTTATGCACCTAAATTTAT

GATCCACCCCTGTAAAAATATTCTCGTTCTGTTAAACCTTCGTTTG

>GT976673.1 GJCCJC2080H07.b Jatropha curcas L. developing seeds (mixed stages) Jatropha curcas cDNA clone GJCCJC2080H07, mRNA sequence

ACCATCCTGTATGCATCAAAAGATATGCTAAAATACCAAATATCTCCAACGGGAGTTTCCAACAAACTGTGGCACTCTGA

GGTAACTAAGTTACAAATATGAACTGCTATGCAGAGGCCAACAAAGCTGCAGTAAGATACTTCAAGATATAATATAGATT

GCTGGTTTCTTTGCTTACATGAAACCACCAGTCCTTCCGCTCCATAATTGGCTCAGTTACTACATTTGCCCAATTTTTTC

AACCTTAAATCCATCACATTCCACAGCTCATAAAGACCACAGTTGTATAGATCAATGCAAGTATCGCATTATATATTTGC

TAAACATCATTTCACCTTTTCAAGGAAAAATCAAACCCTAGTACAATAATGTTCAAAATTTTTCCACACATCATTCAATA

AAACCGATAACTCAAAATCTTTCTCCTCTACAAAGAAGCACAGAGTGATAGGTCAATTTAGATTACAAAGTTGATAAGCT

GAATCTCAAGATTCACTCGTCCAATTTCTTAATCACAGCAACAGATACCAAATTTTGAAACAGAGAGCAATCTAGCTTGC

CACTCAACTAAGAGAGTCATGCATAAACTATAGCATTTCACTAGACTCTGTCAGAAAATTGATATAGCTATACTCAACCA

ATTGAAAA

>GW876874.1 JC006122 Seed specific Normalized cDNA library from Jatropha curcas L. Jatropha curcas cDNA clone N10464 5' similar to 80 KD MCM3-associated protein, mRNA sequence

GAAATGAATAATGGTGGCGCATTTTAAGCGCTGAAATCGAGCCAATGCTTTTCCCTGCAGCATTAATGCACCTCTTTCCC

TCTAGTTCTGACTCTCTCTACTCGTTGCTCTTTTTCGTTCGTTTTCTCTCTCTCTCTAAGCTTGACTTTGTCAGTTTCTC

TATTGCACTGTGTAGTGTGTATTATTATATGTTGGGTAAGTAACTATTGGCGGTGTTAGTAGTGGTGGTGCTGTTGGTGG

AGGAGGAGGTGGAACTAACGGAATCACATTGTATACAATCGTCAGTATTTAGAACTCTCTTTTTTTCCCTTGCCATTGGC

GTGCCTTCCTTTTTCCTCCATTCTGCTCTGCCAGATCTTTCTTTCTCAAGATTTTCACTGTTAAGCTTGTTGCTTTGTTT

TTGTGCTCTCTAGTCTAATCTCTCGATTCTCTCTGCTTATTTTTGAAGAGTTCTCATACTGTCTCTCTTTCTAGTTTCTT

TTCTGTTCTGTTTCTCTCTCTAGCCAGGATTTCTATTTTCTCTCTCTAGGGTTTGAGGCCTTTAGCTGCTTACGTTTTTA

>GT973971.1 GJCCJC2055D11.b Jatropha curcas L. developing seeds (mixed stages) Jatropha curcas cDNA clone GJCCJC2055D11, mRNA sequence

CATCCTTCTAATACAACACAAGAACTCAAATGGTTGACGCCAACTTGCAGAAATAAAAGTTTTCTAAGTCTAAATCATGG

ATTAAACATTAACTAACTATTCCTATCGAAATCAAATTTATAACTTCAATGTACTATACTAGGCTACAATTTTCCAAAAA

TAAATAAATTGAAATAATTGCTCGCCAAATCTAAAAGTCTAGACTTCATGATTAAATTTTCATATTTTACACACTAGCTA

ACTTACTACCCTACAAGAACTTCATTTTACAACTTCAAATACCCATCCCAAACATTATGATTCAAAGAACCAACTAATTA

ATTGCTGCTAACTCAAGGAATTCAAAATTCTCTATTTAATACCCAAAATATATCCACCATTCTTTCATCATTACTAAAAT

TCTAAATTAAGCATTACAAACCAATGTTTCCATTTAAGTTTGAATTTAGTAATCTCATCTCTCAAG

>GW879240.1 JC003798 Seed specific Normalized cDNA library from Jatropha curcas L. Jatropha curcas cDNA clone N06997 5' similar to Unknown protein, mRNA sequence

GGATCTACATATTTTTGCTATAGTGCGCTTTCCTTCTTATCCTTCGCTCCCTCCTCCACCCATTTCTACGGTGTCTTTCT

TTCTTAAAGTTTTATTGGAATTAAATTTTCCCTTTTTTTGTGATCGTTGGTGATTTTTTTTGTAAAACCCCCCCCCCTTC

CAAAAAATGGAAGAGGTTATAATTTTTAAGGTTCCCCCCCAATTTTTGGGGTTTATATTTTATTTCATCTTGAGGGGGGA

ATGATTTTAAA

>GW879539.1 JC001574 Seed specific Normalized cDNA library from Jatropha curcas L. Jatropha curcas cDNA clone N02921 5' similar to Unknown protein, mRNA sequence

GGCTGCATACCAAATTTCTCTTCTCTTAGTTTAGCTCGATGAGAAGCAGAGTGATTGTATCGAAGAATCGAAGCAGAATC

TTACGAAATACAGGCACTAACACAGTATCTTCTTCTGGGTCAGCTTTTCTTCACTGTCTTTACTATTCCGTGGTTCATTC

TTTGAACTCTTACAATTTGTAAACTTTATTGCTCGAATTTTATGCAAGCAGAGTCTTTTTATAGAATAATAGAATTGGGT

TAATATCAAAATATGGAAACAAACCATGCTCTTTGTCTGGGTCTTATTCTATTGCTACTTGCCGTTGACTGCTCTCTTCT

TGATTGGAAGGTAACTGATGAGGAATTAAACTTAATTAAGTGTTTTGTATTGTCAAATATGTCATTATGTGATCTTTTAT

TATGGATTA

>FM889614.1 FM889614 Jatropha curcas embryo 35-55 (DAF) Jatropha curcas cDNA clone rjcfea0_003316, mRNA sequence

CTCAGACAACGCAAATATGGAATTATTATCAAATCTGCAAATCAGCCAACATGCCAAAAGCTCGACGAAAAATATATCTT

GTTCTACCAAATATGTTCAAAAACCTATCAAACACACTACAAGTTCCAACTTCCAAGTTCCAACAACCTACTTAAGCCTA

GAATAACCAAATATTGGATGGTCTAAATCTGCTTAACAAGAAATAAAATGGTGTCTAGAGCTGCTTAACAATAAATATAA

CTAACTCTTGAAATTGCAAAACCACTTGATGGGCATGTCTTAGTTCACCAAAAACATTCTTTAGGAAACCAGGCCATCAT

TCAGGGATAGTGATGCAAGCTGATCGGCAGGGTTGGCTGCCTGCTGCTGAACAGCAACATTTCTAAGGACTTCCATGGCC

TCTG

>GT971064.1 GJCCJC2031E03.b1 Jatropha curcas L. developing seeds (mixed stages) Jatropha curcas cDNA clone GJCCJC2031E03, mRNA sequence

GAAAGGTGATTTCTGTATGAACGTATATAGCTCATGTTTTGAAGTCAAATATGCAATCAGTAGAATTGGTTTTTTCAAGT

ATTTATTTAACAGTCTTTTGGGTGTCATTCTTTGTCTAATTTAGGAGTGGTCATTGTGGATGCAGGATCGGTTGGACAAG

GCAAGGGTCGTTTTTCTGTTGCCATAGGCTCGGTAATTTTGCAACCCATGGTCCTTGTTTGATATATGATACAATGGCAC

TAGGATAATGCCGTCATTGTGAAGTTTAAAACTTTAAACGAGGTGCAAGCAAAATGGCTGGTGTGGTAAGATGCATATGC

TTAACTTCTTGTTGAATTCTGCTCTGGATGTGTAGTCACTTTCCTTATTCTTGAAGGAATATGAACAAATAGATTATAGC

ATCAATTCTCTGCAGGAGTTTTGTGACTTTCCCGGTGTTATGGTGTTATGTATGGAAACTTCTTTTCGTGTACTGCACAT

GTCCCAAGAATGAGAGTTTTTGGTAGATATGAATTATCCATGTGAGTGCATATTTTCTGGAGGTAGTGGTTGGAAGGTTC

CTTTCTGTGCCAACACGCGACAACAGACTTTTATGAGATTAGAAATTTGCAGAAGAAGAAAGGCAAAGTTTGATTT

>GW614466.1 Jc1-039-H02-M13F.H02.ab1 Jatropha curcas flower and seed Jatropha curcas cDNA, mRNA sequence

ACATTGACATAAATCAAATTAGTCTCGTACCTAAAAATAGTAAAGGAAACCTAAAAAAAAACAAACCGTTTCATATCAGA

CTGCTGCTGCTATTTACTTTTGTATGTGGGGTTATTTATTGTATGTATTTTACTTAACTTTTCAAGATCGGATAGGGTTT

ATTGGAAAAAGCCTAATGGAGTGGGTGTAGTAAACTAGCTAGTTGAATTATTTTTCCTGAAAATAATTTTTTTATTTCTA

TTTAATTTTTAAATAAAAATTTTCTTTGCTGATAAAAAAAAAAAGAAGAAAAAGTAAACTATATTGTAAAAAATGCACAG

ATAATTCAATATATATATATATATATATATATATACACACACACACCACTAAGGGCTAAAGTTTTGAAAGAGAAACAAAA

CAAATAGGGAAGTTTGTTAGATAAAACACAGTTACTTATGATACATAGGCATGTAGAAGACAACTTCATACTTGATGAGC

ACCCTTTATAAGGCATACCCTTTGAGGCTCTTACATTACCAAATCCTCTCCTTCTCTAATTTTTGAGCTAAGAAATCCTG

AAATGGCCAACATCAAAACAACATTTGTCTTGGCAATACTCTGT

>GT976636.1 GJCCJC2080D12.b Jatropha curcas L. developing seeds (mixed stages) Jatropha curcas cDNA clone GJCCJC2080D12, mRNA sequence

ATGCCCTTAAATTGAATGTTGTTTATTGCTTTTTTTTATATAAAACTTTATTTATACCCTTGAATTATAATGAATTTGCT

TATTGATATTCCAAAATATTTCACTTATATGAATACTATCTTTTAAAAAGAACTTGTTAGGAACACAAATTATTATAGAT

TGAAAAATAATTCTTACTATTTTCAAATATATTAAAGCCAATTATACGATCATTTGAAAGAAAAAAATAATTAATGTGTA

TTGTAATCCTCCAATCATATGATACATATTAAATATAGAAATAGGAATTATAGTTTCTTTTTCTTTTCCGTTCAATACAT

ACAATTTCACCATTACAGAAATTGATCATCAAATGTTC

>GT972912.1 GJCCJC2042G10.b Jatropha curcas L. developing seeds (mixed stages) Jatropha curcas cDNA clone GJCCJC2042G10, mRNA sequence

AGGGGAGCTAGAGCTTCTCACTTAAAAAGTTGTTAGACAAGTGAAACAACTTAGTAGATTCAAGTTTAATAATTAACCTA

CAATTCTAAAAGTCCAAAAGATTCATGAAGCAGACCCTAGTTAGTTGTCGTTGCCTGTTTTGTGCTTTGTTCTACTTATT

AATATATCAAATGATTTTAGAATTCCCCTTCAAAAGTTTTCTTCTACAAAAATTATTTTAAAATTTATCAGAGCGAATAT

TGGAAACAAAGAATTCCTGAGTCAAACTCTGGTATGTAAGGGAGAAGTGCAACTCCTCTTAAAACTCCATTAATGATAGA

AGAAACAGCAACTTCCATTTATATAGTCAACCCCAACTAATTCGGGACTAAGCTTAGTTGTTGTTGTTGTAATCTACAAT

TATGTCTTGATTTTGATAAGAGTTCTGTTTTAACTTGTTATAATTTACCATTGAGTTGGTCGTCCACGAATTTCCCAATG

GAGACAGCTCCAAGCATCACTCTATTGGCATTACTATGGTTGTGTCTATATGATACTGTATGCATGTGAAAGAATGCTTA

CATGTTTGTAAGCTATAATCAAGTCCTATGGTAATGTGAAAGGATAAAAAACAATGTTAGACAACATGTTTGGTGGGGAT

CACATGCACTAAATTTGGGTGAAGTGATGAGCTAGATTTAAGGAGAACATTCCCCCCAAAGACACACATCACTAAGATGT

GGCACATCCA

>FM891637.1 FM891637 Jatropha curcas embryo 56-70 (DAF) Jatropha curcas cDNA clone rjcaeb0_001975, mRNA sequence

ATTCGAAGAACTCCTTGATTCTATTGAGATTGGAGTAGAACTTTCACTTATTGATGGAAGAAGTGGGGTGGGATAGCTTA

GCAAGGTTTAGAGTTAAGGTGGTGC

>GW874828.1 JC000219 Seed specific Normalized cDNA library from Jatropha curcas L. Jatropha curcas cDNA clone N00398 5' similar to Merozoite Surface Protein, mRNA sequence

GGAGATGCAGTCAGAGAGAGCGTTAAGAAAATAAAATTACAATGCAGAGAGATGGAGCGATAAGAAGAAACAGAAAATGA

AAGATAGAGACAATCCATCGGTTGTTGCGGCTCCTTCAAGAGAGAGACGGTGACTTACGCGCGGATGCGGGTGCGGATGG

GATCTACCACTGCTTCTACTTGTTGCTGCTGTTGCTGCCACTACTTCTACTTCGCCAACTGTTTTTGATTCAAATCCACA

TTCCGTTTCCTTTTCCATTTTTCAGCAGGACTTTGTGAATCATTTCACCAAATTGCCAGTTCATTTTGTGGTTTCTTTTG

GATTTCTTTAAGAAGCTTGATGGGTTCCAAATACCCATCTCATAAACTCAGTAATGGCCTCTATGTGTCGGG

>FM888116.1 FM888116 Jatropha curcas embryo 35-55 (DAF) Jatropha curcas cDNA clone rjcfea0_001450, mRNA sequence

AAGTATGTATCTATTCAGTAAAGTAAGTACTTTAAGTATGTATAATTCCCAATAAATTTTTGGTAATTTGGCATTGGGGC

TAAGCCTTTTATATGAACCTTTTTTTTTTTTAAATATAAAAAATAAAAAACGTAGCTAATGCTAAATTTTTCAGCATAGA

TGTGCATTTCTAATTCTGGAGTAAAAATGTATTAATCACATATCAAGCTTTAGAATTTTCTTGGAGATTCC

>GW879330.1 JC003888 Seed specific Normalized cDNA library from Jatropha curcas L. Jatropha curcas cDNA clone N07115 5' similar to Unknown protein, mRNA sequence

GGTGAATACAACCATCCTCTTCTTTGTTTATTTTTTATTTTTTAACGTTTTTTCAGTCAATAAATGGTAGCTTATAAAAA

AAGATGTGGTTCACCACTGTGTAGATTGGATTTGATCATTGTGTCACTATCTCTATGACACTCTCAATGGCATGTTGAAT

TGATGCAACATAGTGCATGAAGTCAATGTTCATTTTAGAACATTCTTTTTGTTGCTCAGGTATTTTGGATATAGTGGAGT

AACTTGGTTTAAAATTTATAAAAAAAAAAAAAAAAAGGAAGAAAGTTTCCTGAATGTAACTTAGTTCATTTACATAATGA

TTTCTGGACTGGTTTAAACATTCCCCCTTGTTTACACACTAGAAACACTCAAATCTCTTACAAAATTGTGAGACTTTTAA

AGAGCCAACTTGATTGTGCTTTCTAAAAGTAATAGGTAAACTCTAGCTTACAGATTGCCAGGGCTTCATTTTCCTTGGCA

AATTCTTTTACTCGCATTGATTTCCATTTTACATTCTTCTTGCTCAGGATTTGAATGATTAACATTGTTATTCGGTGCAT

TGTCTGGTCATCAATTAGCTAGATTTAGTGGATATATATATATATATATATATATGAAGAGA

>FM890762.1 FM890762 Jatropha curcas embryo 56-70 (DAF) Jatropha curcas cDNA clone rjcaeb1_000691, mRNA sequence

GATCCCCGGCTGCAGGAATTCGACATCCTCAGAAGCTCGCGAAACTGTTAAGGATTTGGTTGATAGAAGTTTGTAATAGC

TCATATTAGAATCTCGTCGAAAAATTTTCCCCAAGAATAATGAGCAGGAAACCTAGATAGACAACAATTATAAACAACAC

AAAAAGTGATAGTGTTATCATTGCTAGGCAGCCTAGTTTACCATGTTCTCGCTGCATTTTCTCTTCTGTTAATTTTTTCA

TGTGTGAATAGACAGGATGTTGTATTTATTTCATTTTGCATTAGTTCAGGCGGATGCATGACCTTGAAGCTCCTAGTTTT

AGTATGAGATCAATGTTTTCTTGATTATGTATGATTTTGTGGTGCTTAAGTTGATCTTTTGTGATTCACAAACAC

>FM889375.1 FM889375 Jatropha curcas embryo 35-55 (DAF) Jatropha curcas cDNA clone rjcfea0_003030, mRNA sequence

GATAGAGGAGCATCGTAAAGATCAATTAGCGAGATTTTGGCCGATACAATAAGAACTGCTTACTTATGTCATAATATGAG

ACAAAAGTAGGAATCAACTTATGTAACAGAGTTGATCTCCTAAAGTATTGAGCAGCGGTGTAGCATCAGATCCAAAGAGA

GTAAGTCTTTCTTTTGAGGGAAGGTCTTTTTCAAAGATTCTATATATATTTATATATAAAAATAGAAAAATAAAAATAGA

AAACCGGGATAGTTACCTTTCAGAAAATTCTAACGATAGTAGAACGCATACGCTTTATTCTTCTGAAGGTGGGAGAAAAG

ATAAAACTGATTATT

>GW611464.1 Jc1-006-A10-M13F.A10.ab1 Jatropha curcas flower and seed Jatropha curcas cDNA, mRNA sequence

CCTTATACAAATTGTGACTTCTTTTGATGAGGTGATAGCAGACTTTACTTGTCTTTTATGTCTGTCTTTATATCGGTATT

AGCAAGTTCAAAAGCACAGGCATATATGCCCACATGAAAGAACCCATGTTCAGTGTGGACAACTACTTGGGGTTTATAAT

TTTCAATAGATATATTAAGAAAATTTTAAAACGTTACTTGTATGTCCTCTGTTGATTGGATATGGTAGTATTTTAAGTGG

TGGTCATGAACTAATTTAAATATTTAGCAAAAGAGCCGGGATCAAATCTATAATTTTAAAAATGAGATATTGGTAAACCT

GCTCTTTTGCTCAATTCTCTTTATCAATTTTTTTTTCTCTCTCTCTCTCTCTCCTCCTCTACTAGGATTTAAAGCATATG

CCAAAAACTAGAAGGGAATTTCTCTTTCTAATACCTTTCTATCAGGACTAATAAAAACTATTGGCTTACTTTTGAAGCTT

TATTAAGTTCCTATTTTCTTTTTCTTGCATGTAGGTAATTTTAATTTGCTCATGAAAAGGTGGTAGTGAGAATTAAAAAT

TATCAGCTTGTTATCATTTCACGATCAGAACTTCCTGCCTCTTTCAGGATTTTCTGGCTTCACACGTGACAAAACCCCCA

TCACCTTTTTAGCTAGCAAGA

>FM887271.1 FM887271 Jatropha curcas embryo 35-55 (DAF) Jatropha curcas cDNA clone rjcfea0_000432, mRNA sequence

GTCAAATGTTAGTTTAGTTTTCAGTAAGATTTTTTTTTTTAGCTAATTTTCAAAAACCGTTGTTTGATATAATTGCATTT

TAAGAATAGAATTTATTTACAAGTGATAAAATTTTGTTCAGTTTTGTTAAATTTCATAAATTTTAATTTTAATGAGTGAA

ATGAACCTTTTCATTTATACCAAATATGAAAAATGAAATTTCAGACAAATGTCACCAAATTTTATGAAATGAAATTCATT

TACACCCATCAGTCTTAGAAAATTCATGTGGAAGTTAATTGGATTCATCTAGTTAAACAAGTTTCGTTCTGTTTTTTCAT

GGTGCACACCACAGCTATAGGGTGATTAATGGTCCATAGTTATAAATTTGCTGTTCTTATTTCCTCATAAAGCTTGGACT

GGATTAAATAGTGTCTGCACTCGATTTTGCTCTTTGAATTGCAGTTTCTAAATTTTACCTAAAACATAGTCATGGTTATG

CGTTGGGTTTCCAATTGGTGACTAGTATTGGATTTTGAAATAAG

>GT980132.1 JGCCJG2039B03.b1 Jatropha curcas L. germinating seeds (mixed stages) Jatropha curcas cDNA clone JGCCJG2039B03, mRNA sequence

GTACCTTTTTTTTCTTTTTATCATTTGCAGATTCAAATTCTGATGAGCAGAAGCAAAGTCACCAGACTGTTTATGAGCAG

GTTGAGCTCCATTTATTTAGTTGATAACAAACCAATGGTATTATTAGGACTATTGAGGATATTAGAAAGCAGTGTAGCGT

TGATTTTGTTTGATTTATTGATTATATTGTACCTTTTCTTTATTAAAATGCTTTAGCTATATGCTAAAGTTAGCTCAGAC

TACTTCTACTCTTC

>FM887163.1 FM887163 Jatropha curcas embryo 35-55 (DAF) Jatropha curcas cDNA clone rjcfea0_000271, mRNA sequence

CTGTTCGGCTTAACAATCCCTACTGGTTCCATTTGATGTATTTGGCTGAGTTTCATTTGAGTTTGAATCCGCTCAGCCGA

AAAATGAAAAGTCCCTTTGTTATAATTTGACCTCTTAAGGATCTACTCAGTGCGAAATTGTAAATTTGATCGGTGTTCTA

CCCACAACTGAATGTCTGAAATAGCTTGGGTTCAGCATTGTAGTATATCCACCAGATTGTCGACTGAGTTTGACATCTTT

TGAATTTCTTGTCGAAGCTCCTATTACATTTGTTGATAATCCAGAACGGAGAGTGTCATAATTATTGCAATCTAGTTGAC

CATACCAAAC

>GT970262.1 GJCCJC2006B01.b Jatropha curcas L. developing seeds (mixed stages) Jatropha curcas cDNA clone GJCCJC2006B01, mRNA sequence

GGGGTGGGGGCTTGAGGGCAACCAGTCACCTCCATAAGACCCACAGGGCCCAGCTCAGCTTCCCTTGTAAATGTTGTTTT

ATTATCTTAATCAGAGCTTTTAATCTAAACCGTCGAATACGCGAAACCGTCTGATTTGAACCGTTGATCAAGGATAAAGC

GCTCCCAGACGCACGATATTCTTGCTTTATTAGCGTTTCATTTTGGGATTGTTAAAACAGGATAGAATATGATTCCTTAT

TTTAGCTAAAAATATGTTAAAACTCATCATGGCCGTTGATATTGTAACAGATGCTGATGGAATAGTCAGAGGCACAAGTC

TCGTGACGCGGGATTTTTTAAATGATGAGCTAACGTGTCAACAAAGTATGCAATGTGGTGTGCCTTAGCACGCGACGCTA

TATGACATGACCCCTCCGCCCTCTCTTCTCTTATGAGCCAAGTGAAGAAGGGGTATGTAATAATAGAGGAGGCGTGAGTA

TGATGCAACAAACTGCATAAAGGAAGGTGCTTGCCTAGAAGCAAGTTGTGCTGTGTTGTGCCGCATCTCATTAAAATGAT

GTCAAAGCAAAGCAGCTATGATTGGTTGTTGAGTACAAGGAACATATGATTCATTATCATTTACTCACACACACAGGTAA

CGCTAAAAATGGTATATACATAAAGTAAGCTT

>GO247117.1 JcrME_RL0661 Expressed sequence tags from Jatropha curcas root cDNA library Jatropha curcas cDNA, mRNA sequence

GGAGATCACTAATTAATAGCTCAAAAAATGAGTCTCAGTCTCAAGCACTTGCTAGTATTGTTCCTCGTTGGAGCTTTGAT

TCTTTCTTTAACCCCTTCGCTTGATGCTCAACTCTTACCACCAAGAAGGCCACCAAGAATTCCACCAAGAAGGCCACCGC

GTGGAAGGCCTCCCTCTAATTCTGGAAGAAAAACGTTATCTTTTGGTAACCAGTTATTATTTCCGTTGCAGAAACAGCCA

TGTTCTGGTGTTGTAAAGTTTCCGAGGGATAGAAAGTGTTAATTGCATGTCGAAGCTATCCAACACCTATCTCTGCTCCA

CCATTTATGTATTGATAACAAATAATCATGTCGGTCCCTGTATTTATATCTTATCATGAAGATTATAGTCTCATGGTACG

TGTGTGAAAATAAGGGCTTAGAGTATATAATGCCACCACTTAAATAAAATTTGTGGATTGTGTTGTTTGTGCTGCTTTTG

CTTAGCTGAATATGTTGCTTGAACTGAATAATATTAAATAAAAAAGTATATTTCCTGTGA

>GW876209.1 JC005764 Seed specific Normalized cDNA library from Jatropha curcas L. Jatropha curcas cDNA clone N09837 5' similar to Histone h2a, mRNA sequence

GGCCATTACGGCCTAGTTACGGGGAAAGCTCAAATCCAAAAATCACATCTCTCATTTTCAATCTCCAGTCTCCTCTCTTC

ACACCTACTTTGACCTCGCATCTTCAACGCATACCTCAGGTCTCTCTCTCTGTCTCTGTCCCTTATAGTTTTCGTTCTGT

AGAATTTTGATTCCTTTTCTTACAAGTTTCCTTTTTAGATCTGATATTTGTTCTGTACTGAATGCTTAGATCTGCAAAAT

AAAGTTCTGCCCTTCCTTTTTTTTTTTTTTTTTGGTTATTTAGAATTATGATGATGTCGTTTATGCTTGGTTTGATTTTG

TATGTAATTTCGAACTTCATTCTGCTAACTGTGTTCTAAGGAATTATGCTCTTCATGCGTCGAAATTTTGATTTCATGAG

GTTTGTTTGGCTCTAAAATTGTAAATTTGGAAAAAAAAGAGTAGGTGAAAAATGGCTGGTAAAGGAGGGAAAGGGCTTTT

GGCAGCTAAAACCACTGCAGCAAACAAGGATAAGGAAAAAGATAAGGACAAGAAAAGGCCTATTTCCCGGTCTTCTCGTG

CCGGTATCCAGGTAACCTTCACCAGCAATTTTCTGCTTA

>JK317599.1 JCST370 Jatropha curcas L. seed cDNA library Jatropha curcas cDNA 5', mRNA sequence

CATTTTTTTTTTTATTTTTGTTTCCGGTACCAATGTGCTCTACTACCGGTGGTTTTGACTGGTATTGTTCTGTAAGTTAC

ATTATTATGACTTTGGGTATCAAGAATAACGTCGACAAACATTTGCACAGTACTTGGGAGCAACAAATTCAGGGTAGAGC

AGGGAGGGACTGTGTCTGTCTGCTTCTTCCTGTTCTATGATATGTTCTCAAATGCTCTAACACGCTTGCAGCCTTGTGCC

CAACCTCTACCCAAGGCTTATTGCTGGAGTTGGGACTGCGTTCGGGCCTCGGGTAAAGGGAGTTCTAGAGTTTAGACATA

GATGGAGCTCGTACAAGTGGCACGTGTTTAGCGCAATTCTATGCTCCCCTGACCACCGCGTTGGATAAGAATTACTAAAG

AAATGAGACGAGCAGACGCGGGGCACGAGGCGCGCAGTACCACGGCATGCCCCAAATCTTCTGACCCTTTGTAACTTGAC

AGGAGTATAAATCCTCGATTCGTTAGCACGACAGGATTGTTCGTAAGCGGAGATGGTAGACCTAGTTTCGTAGGTTGAAA

GATAAGGCAGAATAGG

>FM888968.1 FM888968 Jatropha curcas embryo 35-55 (DAF) Jatropha curcas cDNA clone rjcfea0_002508, mRNA sequence

TATGCATATATTCTATTGCACCAATAAATTTAGTTAAATTTAGAAAATTCTATTTAATTACTGATTTTGATTCAATTTCT

TTTAATTTTATTAAGAGGTATGCGAATGAGGTTATTAATTTATTAGTTAAGGTAACTTATTCTATGTTAGATCCTAAAAG

TGGTTTAACGCCTCTTAATTTTATTTTAGCTGCTTTAATCAATAAAACTTAATATAATTAAATATCATATTTTCCTATCT

AAGAAACTATACATTGTAACTCTTTTTCACAATTATTTGAGTAAATGGTGTGACTCAATTTTAAATAATTTCATTAAATA

AAAATCAAACTAAACTATTTTGAAAATGGTTTGCTAAATTTTGAACTAAATTAAATTATTTAAAAATAATTTAACCTCCA

TGCAAGTCTTTCCTTTAAATTTATTATAG

>FM892575.1 FM892575 Jatropha curcas embryo 56-70 (DAF) Jatropha curcas cDNA clone rjcaeb0_003167, mRNA sequence

ATTCCAGAGAATCCAATCCTATTATCCATTAAAAGAACAAAAAGCACGAAAAGTGGAATTTTTGATTGAGGGTTTAAAAA

AGGGTTCAGTGTTTGAGACAAAAAGAAGCCATTTTTGGAACGAGTACAGATTGCAGAGCGAAAAAAAAAAAAAAAACAGA

GACAAGAGACCTCTGAAATCAATCACTATCAAGAACGTTAAAACAGAGTATTATATGCAATTCCTGATAAAAGGCAATCA

ATTTAGGGAAGATGACGGCCATGGACTGACTAAAGTCGATATGCAAATATCTCAGTTCGCAACAACTTATAACACAAGCG

ACGAAGAGTGCGAACAAACAGGGAGACTAGACCAGACCAGAGAAGTACAAAAGAGACAGAGCTGTTTTTCATGGGCTTGT

CATTATTGAGTAATTGATTCCTGAATTTCGACCGTAAAAGAGGATCAACCCATGTACTCTTTCCATTTCTTCTTCAGTTT

CTCTAGAACTTGAGGTTTCTGCTGCAGTATCTTTTGCTTACTGGT

>FM889493.1 FM889493 Jatropha curcas embryo 35-55 (DAF) Jatropha curcas cDNA clone rjcfea0_003169, mRNA sequence

TCAGTAAGACTGATAACATTCTGATATGGCATTATGACTATTAATGTTTCAGTATAAGTATATATTCTGATAATATCTTG

ATATAGTACATATGCTCTGAATCCATCTAATAAAAAGGATATACTCTAAATTTATTCTGATGTGGTGTACATTTTGTCGA

CATTCTGAAGTAGTGTGTGTTTGAAATATAAAGAAATATGATCATTGATACCTGAGTTGTTTGTATTCTATTACATTGAG

AATTGAAACGAGCCTCATGAGATATGAATTACTAATGTTAATACTGTTATATGATTACATGTGACACAAGTTACTTAAAG

TCATACAGTTATGTTGTTGCTTGTAATGCAACTTTCTTATATTGCAACTAATGTTTAATTACTTGGAATTCTGGTTACTA

TTGTTATAACTTTGCCGTGTATTTAAGCTTATTAAAATGATGTATAATCATTTGTGTTAGAGTTATAAGGTGAAACTCCT

TGCTTGATAACCAATG

>JK613030.1 JCF22-30 Jatropha curcas, immature Seed cDNA subtraction library Jatropha curcas cDNA, mRNA sequence

AGGTACAGGGACACCACTACCATTAACCTTAACGGGATTTTTCATGACCATCCTGTGCATCCAATAGTTGAAAAGAATGC

GAACAAGGATGGATAAGGCACACCAAACAATAAAATCCCACCTCAATAAATTTTAAGTATCAGGTCCCAATCTTACATGC

AATACTGTTAGAATCGATTGACACAGCATAGAACACGTGATCCCGGGCCATGATATCTTTATCATATGCAAAACGACAAA

AATGAGTCATGGGCTTCTGTTAGAATGTCAAACTGTGAAAACCCTGAGGTCCTCCTTAGGATTGCTACACATATTTGTAG

GTATTCTTCCAGGTTAACCATGTTTGTGGCCTCATATTTTTTTATATCGATCAGTTTCTTCTAAATTTAAAATTCCGTGA

CGTAAGGCGTGTATTTCAT

>GT972603.1 GJCCJC2038C10.b1 Jatropha curcas L. developing seeds (mixed stages) Jatropha curcas cDNA clone GJCCJC2038C10, mRNA sequence

CCGAAGATATCCCAGAGCCTTTATTGGACTCAAATCCCAAAAAGATTCTTTAATGACATCATCTAATGACGTCATTTTCC

AGTCTGAAACCAAAAATCAACACACGTCACATGTCAGCTATACAATGTCATCAACTTAATGCCACGTCATCTTTCATGCC

ATGTCAGCATATATATCCCGTTGCCATCTCATTGATAGCTATACACATCATCGCTTATGTACACGTTATCGATGAAACTG

AGGATGTTGCCAACACGTCTTCCAATTGCAACACATCACCAATGTGCCATGTCATACCACCTTTCTGTCGGCAGCCAAAT

AATCGTAAGAACCTAACACTTCTGCATGGGAGCCACATCTTCTGCTACATGGTTAATAATATTAGACCGATTGACATGTT

AGCAGAGTTCGGACACATCTTTAATGCCACGTTATCTACACAACTCAACATATTTAGCAAGTCAAGTCAAACCAAGATCG

TTAAGTTGTGACATGCATCAACATGATGATACACCAGCTGCACTACTTACAAGTCATATTATACGGCAACCAGACAATGG

CTCATGATATGCCACATAATTTGTTTCTTCACACGTCATAGCACACACCAACGGTCATGACACATCAAGCCTGCACACGT

CACCTTTTCAGTACACATTAGCAAATTGAGCCACATCATCTGCTATGTTGTTAAAAAATCTTGG

>FM891360.1 FM891360 Jatropha curcas embryo 56-70 (DAF) Jatropha curcas cDNA clone rjcaeb0_001631, mRNA sequence

ATTCAGCACCTTCGTTTCGTTTCCATCGTTTTCTTTTATCTTCTTCTTCGCATGTCTTAATATCTGCGGCCCTCATCTGC

GATTAAAGCGCCTCCGTATATCTTCGATCTGTCTTTTTCCTTCCGGTAAGGCCATTCAGTTCTTGTGTCTTGTCGAGGAC

AGACGGCCTAGAGCACAGAATTCATGGTCTAGGGTTTTCAATTTTGATTTTTGCGTTGAATCGATTGCTTTTTTCCTCTC

TCTGGTTATCGCTGAATCTGGTTGAGTTTACTCTTTGTTAATATTTTTGTTTGTTTGCTAAAACAAATCTGTAATATCTT

GCTAATTTCTAGGGTTTGATTGGGATTACTTTTG

>GW874808.1 JC000199 Seed specific Normalized cDNA library from Jatropha curcas L. Jatropha curcas cDNA clone N00356 5' similar to Unknown protein, mRNA sequence

GGCCCTTTGCGGGGGGTTTCTTTGAGGACTGAGATAACCATCGAGAGCGAGATAGAAATAGCCCCTTCTTAACAAACAAC

GAAAACCCCCAATACCAATTCTTGAGATTTGACATCAAATGATCTGATAATACACAAAGGAAAAAAGGGAACCCATTTAT

AGATACAAAGACAAGGAAACAGGTAGAGAGAGATTGAGATAGAGAAAGGATCACTTTGAAAGCTTGTTTCTTTGCCATTT

CCTTTTTCCTTGTTTGAGATCTTGCCAATCTTCTTAC

>FM889987.1 FM889987 Jatropha curcas embryo 35-55 (DAF) Jatropha curcas cDNA clone rjcfea0_003755, mRNA sequence

CTGCAGGAAAAAACATGGCAAGAGCTCTATATACACAGGTTTTCCCAAAATTTGAAATCATAGTGAAAGAGATGAAGTTC

CTGCTTCAATTGCCTTGTTGTTCGTGTTTCTGTTTCATCAAGTCCAAAAAAGGAAAACACAAAGACAAGGAAGGAAAAAG

TGACTGAAATTAGCAATTATGCTTGCCAAGATTTCCTCATGCAAGGATGACTGGGATCAATGGTGTTCCTTTGCTGTTCT

TTTAATTTCTTCTTTTCTTTATGGATGAATGTATCCAATATTGCAATCAGCTTTTCAGTTTAATTGTCATGAAATAAAGT

TGCTCTTTCCTCC

>GH295575.1 JCL24 Jatropha curcas total leaf library Jatropha curcas cDNA, mRNA sequence

GTGGATCANGGGCAACTGTTTCAAAAAAGGGGGGTTGGAGATGGTTTTGTGGGGGGTGTTAGATGGGAAGGGGGGGGAGG

TAGAGAAGTAGGGATTCAGAGAGGAAGATAGAATGGAAGGAGTAGGAAATGGTAGGGATTGGTAGATTTGGGGGGGAGGT

ATTGAAGATGGTGANATTGTAGAAGGTAAAGGAGAAAGGAGTAGAGAAATTGTAATTGAGGGAGATTGTGAGAGTGATGA

TTATATTAGAATGAATGAGTGGATTGAGGATAGATAGATGGTAAGGGATAGTAATGTGTATATTGATGTTATGGTGGGAT

TATTTAATGAATAAAGAGTGAAATGTGTAAGGGAAAAAGTTGTTATGAGAATTAATTGAAAGTGAATAGAATAAATTTTA

ATGATTATTGTGTAGGGAATATTTTATTTGTTTAAAGAATATTGTTATGAATTGGTTTGGGAAAGGGAAAAAAGATAATT

TTATATTTGTTTTGAATAGATTTAATTTTGTTTAGGAATTGAGAATAATTATTTTAATTTATATTAGTAATGTTGTTTGA

AAATGTTTATGTATGTAATATGGTTTAGTAGTTAGGGTTAGTTTTTATATGTTAGGATATGATAAA

>JK610383.1 JCF1157 Jatropha curcas, immature Seed cDNA subtraction library Jatropha curcas cDNA similar to function, mRNA sequence

CGGATGCATTTACTGTAAGTAGCTGTGTTTTTGTTTTAGTCATGTTGCTTACACAATTTTGTTGCGGCGAGTAGAATAGA

CATTGTAGTGTCATGTGCATAATTATGTATTGTGTTTCAACTTAAATCTAACTCCTTGCTGACTTTCGGTGTCAGTAGTA

TTACATTCTACCATAGCTTTCGGCTTTTACTCCTGTCTAGTGCAAGTTCAATAATCCAGCTCCCTCTCCCTCACTCCAGT

ATTGGATCAGTTTGAATTGTGTATCATTTGGAGTTAATTACTATGAGATTTCTCATAGCAATGAATATATTAAAGCAGCT

GCCTATAAACTTCCATGTGTATATTGTAATATGT

>FM888292.1 FM888292 Jatropha curcas embryo 35-55 (DAF) Jatropha curcas cDNA clone rjcfea0_001678, mRNA sequence

ATGATTTCCCATAAATAAAATTAGTAACACGACAGTAGTCATCTCCAAACTAGGCAAAATTTGACAAATAAGTCCAAACT

ACTGTTTAATTTCATGGGAAAAGAGAGGGGGGAGTCCGAATTGAGAGGCCTCATACATTTTAAAAGGAAAGCTGATATTG

AAAGAGAAATGTATTTCTACTCAGTGATCACCNAGGAGGCTCTTGATGATATCAGAATCGGGGGGGCCCGGTAC
