# Supplementary material for: Identification of conserved miRNAs and their targets in Jatropha curcas: an in silico approach
Source: J Genet Eng Biotechnol. 2023 Apr 7;21:43. doi: 10.1186/s43141-023-00495-9 (PMC10079790; doi:10.1186/s43141-023-00495-9)
Supplement: Supplementary file 7 — Additional file 7. Non-coding EST sequence containing the predicted precursor miRNA. [file 43141_2023_495_MOESM7_ESM.docx]

**Supplementary File 7:** Non-coding EST sequence containing the predicted precursor miRNA

| ***EST Accession No.*** | ***Non-coding EST sequence containing the predicted precursor miRNA (highlighted)*** |
| --- | --- |
| **>FM887831.1** | GCGGTGGCGGCGCTCTAGAATAGTGGATCCCGGGCTGCAGGCTCCTTTTGGTAA  AACAAATTAAGAACCGTTTCCTCTCTCTCTCTCTCTCTCTCTCTCTCTCTCTCCTC  TTCCAATCTAAGCAATATCACAGCTGTATTCACCTCTCTTTCTGTCTCTCTTGTGC  TCTCTCTTTCTTTGTCTTTCTTTCTTCTTTCCTTTCTTTAAGCGCCGACACAATTCT  TCAATTTCTTCCATTCTGATACTCTCTTATTCTCTTTTAGATCCAATCTTTGTTTCT  TGCAGTTCAATCCCGAGTCCAAACGGAGTCAATTTTACCATAGAACACCGATTT  GGACTAACCCACCTGAAAAAGCTTTGAGCTTAGTCTGGAAGTTAGTTCTAACCG  AGGTGGGTGTGCCTGCAAAATCTGTACAAAGCCAGCTACAAAATACAATACGCC  TTGGAATTTTCTGTCTTTGACATTTTGGTTAAGTTGAGTCTATTGAAGTTGGTGCT  ACGTAGGAATTCGTAAAGAAATGGTGGTTTCGTGATAAAGGGTGATCAGTGGAG  AATTTGTTGGTTTCTTTTATTTAGGTACAGCCATGGAGACGGCAGGGGCAACGAC  CACTCAGAGCTTGACGGACTCCTACAAAGGCATGTCCTCTGATAATATCCAAGGT  TTGGTTTTGGCG |
| **>FM887543.1** | TCACGCGGTGCGGCCGCTCTAAACTATGGATCCCGGCTGCAGTTTAATTAATGAT  CAAAATTTTACTCGAATTGACCGAAAATTTTACTCAAATTGACCGAATGTTCGCC  CCTATCCAAATTTAATGATCAAAATTTTACTCGGATCGACCAAATGTTGACCCTT  ATCCTGTGGTTTATGTACACACAAAAACTTTCATTTTGGGTATTCTTTTCCATATT  AGCATGGTATAACAGAAACTTTTATGCTTAATATTTCCAAAATGCTAGTAACTTT  TTTTAGTCCTTTTCTTCTCTCCTATATTAAAAAAACAAAATTCAAGTTTTCCATTA  TTAAAACATAAAATCACCCTTGCCAATAACATTATTTTATAGTTTGAGAAAGAGG  AAGGCCAGAGCCTGTCTTTTTGTTTTAGTTAGAAAGCATAGAGTTCTTTCATCTTG  GATGCATCTTTTGTAAAATCTGCACGCTCTTTGTTTCATGACATAAGCCTCGACTT  TCAGTCAAGTTTACATTGTTAAGTATTGAACAGTTGAACTTCCAAGAATATAGTA  AACGTAAAACAATACAGTTGATTCATGTAGTTGGTAAGGTGGGTTTAACAATTAT  CACACAGTACAACAAATTGATCCACTGAGTTGGTGAGGTGGGTTTACAATTTCTCCT |
| **>GW618852.1** | AAAAACCTCTTCTCTCTTCTATTGTGATGAAGATGTCTATGCTCAAGTTTACTCTCG  TTGCCTTTCTCTTGTTGATTGCCCTTGATTTGCAAGGTGGGGTAGAAGCAAGAGGG  CCAATAGTTGGTTTTGGATGCAAAACAGTCCAAGACTGTGTTGCAAATAATCCACT  ATGTTCTGCAGAAGTTTGCAAACCACCCTACTGGTGTTTCTGTATAAATGGACAGT  GTGCATGTCAACCAGATTCATTAAGTGCTACAACCCTGATTGGAAGCTAAGGAAA  AGGAAGTGCAAAAACATTTGAATAAAGCAAAAATAGAGAAGTTTGAGATACCAA  TATCCAGCTCTCTGATAATGATACTTGTAAACCTCTAATTACAAGTTATAATAAGG  ATTATATGTTTGTATCCTCGTTATAAAAATCATTATAAAGAAAGAGAAATATATGC  AGTTATCTTATTATTTTCCTTT |
| **>GW879796.1** | GGCCATTACGGCCTAGTTACGGGGGAAATACTCTTTTTAATCGTTGTTGAGCATGCA  TGTTTAAGTTTCAAGTTTTGGCGCGAATGCATTTTTGTTTCGTGTTTATGTTTGTGTTT  TGGAATAAATAGTCATAGCGACTTATATGTAATTAGTCGTTGTGATGTCGAAAAAAA  AACAGGAAAAGAAAATTAGAAAAGAAATAAAAAAAAAAAAGAAAAAAGAAAAAA  AAAGAAAGGAAGAAAGAATAAATAAACAGCAGCAATAAAATAAATAAAAGGAAAGA  AAAATAGAGGGAAAGGCTACAATGTTATGTTTTGTTTTGTGAAGTTGCATTTGTTGC  CATCTAAACATTAAAGTTAATAACGTGCATTGCTCTTTGAACTTAATGTGAAAGACT  TATTTTCTTGTTACTCTCTAGCCTTACATTATCCTTTTCTTTTGTAACCATTACCCTAG  CCTAACCCATATAAAAGACCTGATGATCCTTAGTTGAGACTTGTCCTACATTAGTGG  AGAGGGAACCGCGAGGAGAGCTTATGGTTGTTCATTATCTTGTTTTCTTGAAGCTAC  AAAAATGTATTTTATATGCTTTGATTATTGCTCGATAAAACATTTGTTGATTCAAGC  TTCGTATGTGTGCGTGAGTGTTTTC |
| **>JK317548.1** | CTAGCATTGTTCATGGTCTACCACTTGCAGTACTGCTAGGGTGCTTATTCTTTGGCA  GCATAGAACTTGAACTGCAAAATAAATGTTCTCGAAATTTACATTGGGTTGTTGGT  GTTGCTTCAGTTTTGTTTGCTTATAATAGCCTGCGTTATCTTTGTTTATTTTTTTTAA  TTGTTATCTCAAAGGAATTAGAATGTTCACGTGTTGCTTGAGAGTGGTATGTTTAG  AGCTTTTCGTCATTTCCCTCCTATTCATTTCTTGGAGTTATGTTCACGTATTTCAATA  CAATTCTTTTGGTTTCTGTTAACTTCTGGAAACATTTACAAATATATAGGCTACATA  GTTGAAAACCCCATACCATTAAAGCATGGCCACTTTTGGTAGATATTCTCATAATA  ACGCCACTTTCTGTTTTCCTCCTATGACTATTTAACTAATTTTCCCCAAA |
| **>GW879253.1** | GGTCAGTGGGAGTATAGTGGTCATGGTGCCAGGCATCAGTAATGGGGTTTCTCACT  CTTGTGAATTGATTTTTGATCTTCTGGGATCCAGCCTCTTTGATATATATGGCTAAA  GAGGTTAGTCTACTCATGCTTATTTTCAAAATGTATTTGATTATATCAATTATATAT  TTCATTACAGTTCACTAAACATCTTAGTTTATTTTGGAGGTTCTCTTTTGTATGCAT  CTTGCCCTTATCACTGATTCAAACTCATGGCCTTCCCAAGTTGCAGCTCACCCATA  AAAAACAACAATTCTATGCATAAGAAGATAGAACTTGTAGAAAACTGCCACTTGT  ATAAAGAAACATGCGCTTCAATATTTATTCCATTTCCCTTCCCTGAAGTCTTCGTCG  GCTTTATTCAAATACAAGTCTCAATTCCAGATGAAACCGGGAACTGCAAGTCTCTC  GAGTTTCTTGAACTACCAAATTCATCCATTTTTTTGTAGTTTTCTGGTTCTTGACTGT  TAAGCAAAGCAAACTACCAGCGAGATCTCCTGAACGCCGATTTGACAGGAATTCA  TTGATTTGCAGAAATTCGAATCAGATTTTGCTAAAAATTGCGTCTGCAGCCAAAAA  GTTCGCTCCA |
| **>GW875825.1** | GAATTTAATCTGACCGGCAACGTTTCGTCAGCTCTCTCAACCGCTCTTTCTTGTTCTT  CTTCCTTTTGCTCCACCTGAAAACTCCACTGCAGATCCAAAGCCTCAATCTTTCACTT  TGTAACTGCGATTTCACTACTCGGCATCGAACCTCCAAGTTTGGTAATTTCTTCTGTT  TTCTTCTATTAAATACGATTATAATATGTTAATTTTTCTTTTTAATCTCTAGATCATGG  CCTTCTAATCTTTTTGTTTCGATCAGTTGTTTTATATGATATGGATTCATTGGTATACT  TAACATCTATTACCTAATAATACCCATATAATTAAGATAATAACTTTACTTTGATCTT  TGTGATTTGAGCTTCTTTAGTCAACCATTTAAATTTGGAAATTTAAGAAGCTGAGTTT  TAATTTGATGGAATCTTTGTTTGCATAAAATTCAATTACTCATGATATATCGATTAGA  AGGTGAAAGAATGATGTATTATGAGTAAATGGATTCTAAGTTTTTGGAAAATGCTGC  TTTCCTTGTCCAGTTAAATGATAGTTATTAGTAAAAGGGGGAAAAAACTGTTAGTTG  CATGATATATATAGTGAGTAAATAAATAATGTGACTGCATTTATCTGCTGCTTTCTCA  TTCTAATCCAGGTATTACTCTTTTTTAATAAAATCTGTTAAATCATATCACCTTACAGC  TAAATATTTTACCTC |
| **>FM890278.1** | GAGTTTATTTAATTATAAACTGTTGAGATTTGGTGTAAATGAAGGTGGGATTTACTGT  TTCCTGCTTTATTATTTGATGAGATCTTTAACTTTAACCACAGTTTCTTTATTTATTTTC  TCTCTTTTTTCTTTTTTTTTCTCTCTCTTTTTTTTTTTTTTTTGTTCTTAATGAGCAATTTT  AGGTTTAAATGAAATGAAATGATATAGCCGTTGCTTGTAATTCCTTTTATCGCTGAATTA |
| **>GW877957.1** | GGACCTCTCGTGAAGTAGAAGGCTACAAGGTGTGCACAGACGTCCTTCATTATTATGATTTTTTTCATTTCTCGTTTTATTTTCAAAATTTTCTTGGTGATGTTACTTGCCGTTTGACATCCAAGAAAATCTCTTCCTTTTTTTTTTTTTATTTGGGAAATCGGCATTTTGGGTGTTAGATTTCAGAGTCTGGTGGCTTGATTTTGTTGGGTTCTTTCCATTTTTTATGGGTTTAAGTATTTGCTTTACTTTCTCCGAAATAATTTTCTTTCCCTCGCTTTCTTTCTCTGTTCTTTTTTTTTTTTTTTTTTTTAACATTTCGCTTGATTATTATCTTGTTGAATTTTTCTTTGATTATTTGTTTAATTTGTGCCTTGAAAACAATTTATTTTAGATTGCATTTTTTTATGGTTGCCTTCTAGATTGATTGTCTTAATGGGAAAAGAAATGTTATGGTTCCAATCAATTGGATTTTGGTTCTATATATATGGTGAAAAGATGCGAATTTACTTCTAATTCCATTCGTCTCTCTCGGTGTGTGGTGGGAGAAAGATGGAGAGCTTTGGGGAAAATGAATTTCAACACCGTCAAAGTTCCTAAGG |
| **>FM888667.1** | TTTGGAAATGGAATGAAGACAATTTTATGAAACATGTAAAAGAATTACTTCATATTGTGATTATGAATATCTTGTTTATCACTAGCAGACTAGTGATTGATATTAGAGGTTATAAGCAATGGAATCAGATTTTTGAATTAACTCATCATTTGGTGGGTTTTGACCAACTTTGAAATTGAAATGAAAAAATCAGATTGAATTCTAATATAAGAAATTGAAATGAAAACAGCAAGTAGCAATTAAATTATAATCTTAATCTGAAATGATTGACATAATTGAGGAATTGATGTTCTATACAAGAAAATATATAAGCTTAATAT |
| **>GT971969.1** | GGAGAGAAGTGGAAATCCATGTCTCCTGCTGTAAGTACTGGAATTTTTTCTAATATGT  GATTTTTTGTTTGTGAGGTTGTGTATCTCTTGGTTTAAACTTGCTATATGAATCTATAT  TCTTTGTTTCTTGATTACGTGGGATTTGTTTGCAAGCTTCAGAACACATTAACAACTC  ATCACATTCTTTTCGATGCAAGCACACTAACCATTTTATTGGGATTTACTTGAATTAA  CTGTTTTTGTTTCCTTTTAGCATCTTTTTTATTCTTGTATTTGGAAACAGTTGTTGAAAG  AACTTCTTATTCTGTGAAATGGTTGTAAATAATTTTTAAGTTTCATTTCTTGGTTTGGC  AGGAAAAAGCACCATACGAAGCTAAAGCTGCCAAAAAGAAGGATGACTATGGAAA  GCTTATGAATGCATACAGCAAGAAGCAGGTGAGCATAATGCATCAACCATTCGCCT  GTCAAAGGAGCGTTCTAGTTGTTTGTTGTGTGTTCTTATTGTCTCTGTTATGCTGACA  GGAGAGTGCAGCTGATGCTGATGATGAGGAGTCAGACAGGTCCAAATCTGAGGTAA  ATGATGAAGATGATGAGGCTACTGGAGAGGTTGGTCAACCTGTTTATCACTGGGTTG  AGTTGCCGCATTATAGCTTATTTTTCACAATATA |
| **>GW611464.1** | CCTTATACAAATTGTGACTTCTTTTGATGAGGTGATAGCAGACTTTACTTGTCTTTTAT  GTCTGTCTTTATATCGGTATTAGCAAGTTCAAAAGCACAGGCATATATGCCCACATGA  AAGAACCCATGTTCAGTGTGGACAACTACTTGGGGTTTATAATTTTCAATAGATATAT  TAAGAAAATTTTAAAACGTTACTTGTATGTCCTCTGTTGATTGGATATGGTAGTATTTT  AAGTGGTGGTCATGAACTAATTTAAATATTTAGCAAAAGAGCCGGGATCAAATCTAT  AATTTTAAAAATGAGATATTGGTAAACCTGCTCTTTTGCTCAATTCTCTTTATCAATTT  TTTTTTCTCTCTCTCTCTCTCTCCTCCTCTACTAGGATTTAAAGCATATGCCAAAAACT  AGAAGGGAATTTCTCTTTCTAATACCTTTCTATCAGGACTAATAAAAACTATTGGCTT  ACTTTTGAAGCTTTATTAAGTTCCTATTTTCTTTTTCTTGCATGTAGGTAATTTTAATT  TGCTCATGAAAAGGTGGTAGTGAGAATTAAAAATTATCAGCTTGTTATCATTTCACG  ATCAGAACTTCCTGCCTCTTTCAGGATTTTCTGGCTTCACACGTGACAAAACCCCCAT  CACCTTTTTAGCTAGCAAGA |
