# Supplementary material for: Identification of conserved miRNAs and their targets in Jatropha curcas: an in silico approach
Source: J Genet Eng Biotechnol. 2023 Apr 7;21:43. doi: 10.1186/s43141-023-00495-9 (PMC10079790; doi:10.1186/s43141-023-00495-9)
Supplement: Supplementary file 8 — Additional file 8. Major characteristics of miRNAs, pre-miRNAs and miRNA homologs. [file 43141_2023_495_MOESM8_ESM.docx]

**Supplementary File 8:** Major characteristics of miRNAs, pre-miRNAs and miRNA homologs

| **miRNA names** | **pre-miRNAs** | **EST id** | **Best miRNA homolog** | **A+U%**  **of (miRNA)** | **G+C % of**  **miRNA** | **A+U%**  **Of**  **homologs** | **G+C %**  **Of homologs** | **A+U%**  **of**  **pre-miRNA** | **G+C% of**  **Pre-miRNA** | **MFE** | **MFEI** |
| --- | --- | --- | --- | --- | --- | --- | --- | --- | --- | --- | --- |
| jcu-miR5277 | UAGAUCCAAUCUUUGUUUCUUGCAGUUCAAUCCCGAGUCCAAACGGAGUCAAUUUUACCAUAGAACACCGAUUUGGACUAACCCA | FM887831.1 | mtr-miR5277 | 66.66 | 33.33 | 65 | 40 | 58.8 | 29.41 | -16.60 | -.664 |
| jcu-miR9741 | AAACAAUACAGUUG AUU  CAUGUAGUUGGUAAGGUGGGUUUAACAAUUAUCACACAGUACAACAAAUUGAUCCACUGAGUUGGUGA | FM887543.1 | gma-miR9741 | 68.42 | 31.57 | 63.15 | 36.84 | 64.70 | 35.29 | -19.00 | -.633 |
| jcu-miR7121 | CCUUUCUCUUGUUGAUUGCCCUUGAUUUGCAAGGUGGGGUAGAAGCAAGAGGGCCAAUAGUUGGUUUUGGAUGCAAAACAGUCCA | GW618852.1 | mdm-mirna7121a | 57.89 | 42.10 | 42.10 | 68.42 | 54.11 | 45.88 | -23.70 | -.607 |
| jcu-miR1534 | GCAUGCAUGUUUAAGUUUCAAGUUUUGGCGCGAAUGCAUUUUUGUUUCGUGUUUAUGUUUGUGUUUUGGAAUAAAUAGUCAUAGC | GW879796.1 | gma-miR1534 | 77.77 | 22.22 | 72.22 | 27.77 | 65.88 | 34.11 | -15.80 | -.544 |
| jcumiR6149-3p | UUUGGCAGCAUAGAACUUGAACUGCAAAAUAAAUGUUCUCGAAAUUUACAUUGGGUUGUUGGUGUUGCUUCAGUUUUGUUUGCUU | JK317548.1 | stu-miR6149-3p | 61.11 | 38.88 | 61.11 | 38.88 | 64.70 | 35.29 | -19.20 | -.64 |
| Jcu-miR11155c-3p | AGUAUAGUGGUCAUGGUGCCAGGCAUCAGUAAUGGGGUUUCUCACUCUUGUGAAUUGAUUUUUGAUCUUCUGGGAUCCAGCCUCU | GW879253.1 | lja-miR11155c-3p | 72.22 | 27.77 | 66.66 | 33.33 | 55.29 | 44.70 | -19.10 | -.502 |

| **miRNA names** | **pre-miRNAs** | **EST id** | **Best miRNA homolog** | **A+U%**  **of (miRNA)** | **G+C % of**  **miRNA** | **A+U%**  **Of**  **homologs** | **G+C %**  **Of homologs** | **A+U% of (pre-miRNA)** | **G+C % of Pre-miRNA** | **MFE** | **MFEI** |
| --- | --- | --- | --- | --- | --- | --- | --- | --- | --- | --- | --- |
| jcu-miR4249 | CUUUGAUCUUUGUGAUUUG  AGCUUCUUUAGUCAACCAU  UUAAAUUUGGAAAUUUAA  GAAGCUGAGUUUUAAUUU  GAUGGAAUCUU | GW875825.1 | aly-miR4249 | 72.22 | 27.77 | 66.66 | 33.33 | 71.76 | 28.23 | -17.70 | -.737 |
| jcu-miR7805-3p | GAGUUUAUUUAAUUAUAA  ACUGUUGAGAUUUGGUGU  AAAUGAAGGUGGGAUUUA  CUGUUUCCUGCUUUAUUA  UUUGAUGAGAUCU | FM890278.1 | rgl-miR7805-3p | 72.22 | 27.77 | 77.77 | 22.22 | 70.58 | 29.41 | -21.10 | -.844 |
| jcu- miR8786 | AUUUUGGUUCUAUAUAUA  UGGUGAAAAGAUGCGAAU  UUACUUCUAAUUCCAUUC  GUCUCUCUCGGUGUGUGG  UGGGAGAAAGAUG | GW877957.1 | gra-miR8786a | 47.36 | 52.63 | 52.63 | 47.36 | 62.35 | 37.64 | -17.30 | -.540 |
| jcu- miR3520-5p | CAUAUUGUGAUUAUGAAUA  UCUUGUUUAUCACUAGCAG  ACUAGUGAUUGAUAUUAGA  GGUUAUAAGCAAUGGAAUC  AGAUUUUUG | FM888667.1 | ahy-miR3520-5p | 73.68 | 26.31 | 68.42 | 31.57 | 70.58 | 29.41 | -20.00 | -.800 |
| jcu- miR5658 | GUUGUUUGUUGUGUGUUCU  UAUUGUCUCUGUUAUGCUG  ACAGGAGAGUGCAGCUGAU  GCUGAUGAUGAGGAGUCAG  ACAGGUCCA | GT971969.1 | ath-miR5658 | 55.55 | 44.44 | 66.66 | 33.33 | 54.11 | 45.88 | -23.10 | -.592 |
| jcu- miR2112-3p | AUUGUGACUUCUUUUGAUG  AGGUGAUAGCAGACUUUAC  UUGUCUUUUAUGUCUGUCU  UUAUAUCGGUAUUAGCAAG  UUCAAAAGC | GW611464.1 | ath-miR2112-3p | 66.66 | 33.33 | 61.11 | 38.88 | 65.88 | 34.11 | -17.90 | -.617 |

Highlighted sequences are mature miRNAs in pre-miRNA sequences
