# Supplementary material for: Identification of conserved miRNAs and their targets in Jatropha curcas: an in silico approach
Source: J Genet Eng Biotechnol. 2023 Apr 7;21:43. doi: 10.1186/s43141-023-00495-9 (PMC10079790; doi:10.1186/s43141-023-00495-9)
Supplement: Supplementary file 1 — Additional file 1. Retrieved 46865 EST sequences of Jatropha curcas. [file 43141_2023_495_MOESM1_ESM.docx]

**Supplementary File 1:** Retrieved 46865 EST sequences of *Jatropha curcas*

>FM895301.1 FM895301 Jatropha curcas embryo 71-95 (DAF) Jatropha curcas cDNA clone rjcpga0_002106, mRNA sequence

CGGGAGAGCAGAAAGAGAAAACATGGTATCGCTAAAACTACAGAAGCGGCTCGCCGCTAGCGTTCTCAAG

TGCGGGAAAGGCAAGGTCTGGCTCGATCCTAATGAGGTCAATGAAATTTCCATGGCCAATTCCAGGCAGA

ACGTTAGAAAGCTAGTGAAGGATGGTTTCATCATTAGGAAGCCAACAAAGATTCACTCTCGTTCTCGTGC

ACGCAGAATGAAGGAGGCCAAGAGAAAGGGTCGTCACTCTGGATATGGTAAGCGCAAGGGTACCAGGGAG

GCTAGGCTGCCAACCAAGATCCTTTGGATGAGGAGAATGCGTGTCCTGAGGCGTCTGCTGCGCAAATACA

GGGAATCCAAAAAAATTGACAAGCACATGTATCATGACATGTACATGAAGGTAAAGGGTAATGTATTCAA

GAATAAACGTGTGCTGATGGAAAGTATCCATAAATCAAAGGCTGAAAAGGCAAGAGAGAAGACTTTATCT

GACCAATTTTG

>FM895300.1 FM895300 Jatropha curcas embryo 71-95 (DAF) Jatropha curcas cDNA clone rjcpga0_002105, mRNA sequence

TTTTCTAAAACTCTTTCTCTCTCTCTCTCTTGGCGATTTCCATACACAGACAATAATAAAAGCCTCAATA

ACATACATAGACATGGCCAAGATTGGAAAGCTAACAAAGCTCAAGTCAGCAATAAAGAGATGGCCATCTT

TCACTAAGCTGGGTCGTACAAACAGCTCCATTGCTGCTAGTACTGACGTGATCGAAGACAACATTTCAAG

GGGAACTCCAAGCAGTTTACGTCGGTAAATCAAGACGGCGATATCTTTTAAGCTCTGATGTCATTTGCCA

TCCTCTTTTTCAAGAATTGATAGAAAGATCAGGTGGGTTTGATGACGGACAAGTAGTCATTTCTTGTGAA

GTTGTGTTGTTTGAGCATTTGTTATGGATGCTTGAAAGCGGTGGCTCTCAGTTAGGCTCCATGGAAGAGC

TTGCTGACTTTTACTACACTCGCTAATAACTACTTAGCTAGTATTACTGCGAGTATCCCTA

>FM895299.1 FM895299 Jatropha curcas embryo 71-95 (DAF) Jatropha curcas cDNA clone rjcpga0_002104, mRNA sequence

TGCAGGTTAGTCTGGTTTCACTTTCATTGGTTTAAATCGCTTTTCGGAGTTTATGGTTCACAAGCCTGAA

GAAGGATTCTTCTCTTGGATCTTGGCAATCTTGCTTTCACCTCTGCTTTGGATATTTTCAACATTAATTG

AGTTCTATTTGAAGTGGACACAACCATTGAAGAAATATAATATGGTCCCATCTCATAGTTTCATCAAACA

GATTTCTTCGTGCTCGCTCTCATCTCTACCAGCTAATTTTTATGGCAATGTCAAAGAAGGAAGCCTTATC

CCAAAGAAATCAGAAAACCTCAGCTTCTGCAAAAATGGTTTGATCATAGAAGGTGAGGAGAATCCAATGG

AAACTGAAATTGTTATCTTTGCAACGGGATACAAAAGTGAAGAAAAGCTCAAGAACATTTTCAAATCCAG

CTACTTTCAAAAATGCATTATTGGGTCATCAGCTCCTTTATACCGAGATTGCATTCATCCTCGAATTCCA

CAGCTAGCTATACTTGGATATGCATACAGTCATTCAGATTTATATGCTGCGGAAATGA

>FM895298.1 FM895298 Jatropha curcas embryo 71-95 (DAF) Jatropha curcas cDNA clone rjcpga0_002103, mRNA sequence

ACGCGGTGGCGGCGCTCTAGAACAGTGGATCCCCGGCTGCAGGAAAGGATTCTATCCGTCGCCCGATGGG

AATTGTACTTTAAGTGCGGCCCTCGCGGCTCGTCCGCCGCGGGGGCTTGGCCAACGGCACGTGCCTCTGG

GGGCCGGATGGCCCCTACTGCGGGTCGGCAAACGGGCGACGGACACGCGCGTCGCTTCTGGCCCGGATTC

TGACTTAGAGGCGTTCAGTCATAATCCAGCGCACGGTAGCTTCGCGCCACTGGCTTTTCAACCAAGCGCG

ATGACCAATTGTGCGAATCAACGGTTCCTCTCGTACTAGGTTGAATTACCATCGCGACACCATCATCAGT

AGGGTAAAACTAACCTGTCTCACGACGGTCTAATCCCAGCTCACGTTCCCTATTGGTGGGTGAACAATCC

AACACTTGGTGAATTCTGCTTCACAATGATAGGAAGAGCCGACATCGAAAGGATCCAAAAGCAACGTCGC

TATGAACGCTTGGCTGCCACAAGCCAGTTATCCCTGTGGTAACTTTTCTGACACCTCTAGCTT

>FM895297.1 FM895297 Jatropha curcas embryo 71-95 (DAF) Jatropha curcas cDNA clone rjcpga0_002102, mRNA sequence

TTCATTTTCAATTAATCAACTGGGCGTTTTCTTCAACGCTTTCTTCCCTCGTTTCCTCCGCCTCTTTTTC

TCCTCTTAAACTCCTCTTCCTTTCCCTCTCTTCCCCTCATTCTCTCTTTCTCTTGCTTCTGCAGCTTTCT

GTTTACTACAGAAACAGAAAAGAGAGAGAGATAAGTTAGCGCAACTCCAATGGCGACTGCTTTCTCTGCT

ACCAAGTTTACCCAGCCTTTGCCCCTCAATACCACCTCCTCCAGATCTCATGAAAAGCACACTCTTTTTG

ATCCTCTTAAAACTACTGCTTCTTTTCTCGGATCCACCCGCAAACTTCGTTTCAATTCTGTTCCTAAATT

CAATCAAATCAATTCCCACCGTCGATCTGCTATTGTTGCTGTATCTGAAGCCGTTAAGGAGAAGAAGCTC

AAATCAACCTCCAATCTGTTGATCACAAAAGAAGAAGGCTTAGAGCTATATGAGGATATGGTGTTGGGCA

GATACTTTGAGGACATGTGTTGCCAGATGTATTAAAGAGGAAAAATGTTATGGTTTGTTCATCTCTATAA

TGGCCAGG

>FM895296.1 FM895296 Jatropha curcas embryo 71-95 (DAF) Jatropha curcas cDNA clone rjcpga0_002096, mRNA sequence

CACAAAGGGTGTTGGTCGATTAAGACAGCAGGACGGTGGTCATGGAAGTCGAAATCCGCTAAGGAGTGTG

TAACAACTCACCTGCCGAATCAACTAGCCCCGAAAATGGATGGCGCTTAAGCGCGCGACCTATACCCGGC

CGTCGGGGCAAGAGCCAGGCCCCGATGAGTAGGAGGGCGCGGCGGTCGCTGCAAAACCCGGGGCGCGAGC

CCGGGCGGAGCGGCCGTCGGTGCAGATCTTGGTGGTAGTAGCAAATATTCAAATGAGAACTTTGAAGGCC

GAAGAGGGGAAAGGTTCCATGTGAACGGCACTTGCACATGGGTTAGTCGATCCTAAGAGACGGGGGAAGC

CCGTCCGACAGCGCGTCCTCGCGCGAGCTTCGAAAGGGAATCGGGTTAAAATTCCCGAACCGGGACGTGA

AGGCTGACGGCGACGTTAGGGAGTCCGGAGACGTCGGCGGGGGCCTCGGGAAGAGTTATCTTTTCTGTTT

AACAGCCCGCCCACCCTGGAAACGACTCAGTCGGAGGTAGGGTCCAGCGGCTGGAAGAGCACCGCACGTC

GCGCGGTGTCCGGTGCGCCCCCGGC

>FM895295.1 FM895295 Jatropha curcas embryo 71-95 (DAF) Jatropha curcas cDNA clone rjcpga0_002095, mRNA sequence

GGATCTATCCGTCGCCCGATGGGAATTGTACTTTAAGGCGGCCCTCGCGGCTCGTCCGCCGCGGGGGCTT

GGCCAACGGCACGTGCCTCTGGGGGCCGGATGGCCCCTACTGCGGGTCGGCAAACGGGCGACGGACACGC

GCGTCGCTTCTGGCCCGGATTCTGACTTAGAGGCGTTCAGTCATAATCCAGCGCACGGTAGCTTCGCGCC

ACTGGCTTTTCAACCAAGCGCGATGACCAATTGTGCGAATCAACGGTTCCTCTCGTACTAGGTTGAATTA

CCATCGCGACACCATCATCAGTAGGGTAAAACTAACCTGTCTCACGACGGTCTAACCCAGCTCACGTTCC

CTATTGGTGGGTGAACAATCCAACACTTGGTGAATTCTGCTTCACAATGATAGGAAGAGCCGACATCGAA

GGATNCAAAAGCAACGTCGCTATGAACGCTTGGCTGCCACAAGCCAGTTATCCCTGTGGTAACTTTTCTG

ACACCTCTAGCTTCAAATTCCGAAGGTCTAAAGGATCGATAAGCCACGCTTTCACGGTTCGTATTCGTAC

TGGAAATCAGAATCAAACGAGCTTTTACCCTTTTGTTCCACACGAGAATTCTGTTCTCGTTGAGCTCA

>FM895294.1 FM895294 Jatropha curcas embryo 71-95 (DAF) Jatropha curcas cDNA clone rjcpga0_002094, mRNA sequence

GTTCCACACGAGATTTCTGTTCTCGTTGAGCTCATCTTAGGACACCTGCGTTATCTTTTAACAGATGTGC

CGCCCCAGCCAAACTCCCCACCTGACAATGTCTTCCGCCCGGATCGGCCGCCGAAACGACCTTGGGTCCA

AAAAGAGGGGCATAGCCCCGCCTCCGATTCACGGAATAAGTAAAATAACGTTAAAAGTAGTGGTATTTCA

CCGTCGCCTTTCGGCTCCCACTTATCCTACACCTCTCAAGTCATTTCACAAAGTCGGACTAGAGTCAAGC

TCAACAGGGTCTTCTTTCCCCGCTGATTCCGCCAAGCCCGTTCCCTTGGCTGTGGTTTCGCTGGATAGTA

GACAGGGACAGTGGGAATCTCGTTAATCCATTCATGCGCGTCACTAATTAGATGACGAGGCATTTGGCTA

CCTTAAGAGAGTCATAGTTACTCCCGCCGTTTACCCGCGCTTGGTTGAATTTCTTCACTTTGACATTCAG

AGCACTGGGCAGAAATCACATTGCGTGAGCATCCTCAGGGACCATCGCAATGCTTTGTTTTAATTAAACA

GTCGGATTCCCCTTGTCCGTACCAGTTCTGAGTCGACTGTTCGACGCCCGGGGAAGGCCCCGGAAGGGCC

>FM895293.1 FM895293 Jatropha curcas embryo 71-95 (DAF) Jatropha curcas cDNA clone rjcpga0_002093, mRNA sequence

TCTTTTTGTTTTTTACAGAAAAATCTAATCCAATTGTCTTCAAAATAGTTATTCAAACCGTCCACCCGCA

TTTACCAAATTAAACATATAATCCTAAAAAGTACTGATAATTAATCATACAGGATTCAAAAACATAATCC

ATAAGCATATCCTATCCTTCTTTCTACAAAGATGAGATGGGTTTAAGAGAAAGGGATAGAGAGATCGACC

TATATGTTAATGGTCATTCCTCAACCCAAGAAGGGTCCCAGCCCTATCGCTTGGATGGTCTCTCATCATC

CTCTCCACCACCACCATCATCATCGTCATCTGAGTCGTCTTTATTCGACCTCTTCCTCTTTGCTGGAGCC

TCCACCTTCCCATCTTCTCCATCATCTTCACCTTCCTCATCAACATCCTCATCCTCTCCATTCTCTTCTG

GTTCAAAATCACTAGCATCCTCCTCATCTTCAGCACGACCTAATGGTCGAACAAGGTATTCGGTTACCAT

GTCCTCCTCCTCTTCAGCATCTTCATCCTCCTCGTCTTCCTCATCATCGTCGTCATC

>FM895292.1 FM895292 Jatropha curcas embryo 71-95 (DAF) Jatropha curcas cDNA clone rjcpga0_002092, mRNA sequence

ATTCGATTAAGCCATGCATGTGTAAGTATGAACTAATTCAGACTGTGAAACTGCGAATGGCTCATTAAAT

CAGTTATAGTTTGTTTGATGGTACCTGCTACTCGGATAACCGTAGTAATTCTAGAGCTAATACGTGCACC

AAACCCCGACTTCTGGAAGGGATGCATTTATTAGATAAAAGGTCGACGCGGGCTCTGCCCGTTGCTCTGA

TGATTCATGATAACTCGACGGATCGCACGGCCATCGTGCCGGCGACGCATCATTCAAATTTCTGCCCTAT

CAACTTTCGATGGTAGGATAGAGGCCTACCATGGTGGTGACGGGTGACGGAGAATTAGGGTTCGATTCCG

GAGAGGGAGCCTGAGAAACGGCTACCACATCCAAGGAAGGCAGCAGGCGCGCAAATTACCCAATCCTGAC

ACGGGGAGGTAGTGACAATAAATAACAATACCGGGCTCAATGAGTCTGGTAATTGGAATGAGTACAATCT

AAATCCCTTAACGAGGATCCATTGGAGGGCAAGTCTGGTGCCAGCAGCCGCGGTAATTCCAGCTCCAATA

GCGTATATTTAAGTTGTTGCAGTTAAAAAGCTCGTAGTTGGACCTTGGGTTGGGTCGATCGG

>FM895291.1 FM895291 Jatropha curcas embryo 71-95 (DAF) Jatropha curcas cDNA clone rjcpga0_002091, mRNA sequence

ATTGGATTCTATCCGTCGCCCGGTGGGAATTGTACTTTAAGGCGGCCCTCGCGGCTCGTCCGCCGCGGGG

GCTTAGCCAACGGCACGTGCCTCTGGGGGCCGGATGGCCCCTACTGCGGGTCGGCAAACGGGCGACGGAC

ACGCGCGTCGCTTCTGGCCCGGATTCTGACTTAGAGGCGTTCAGTCATAATCCAGCGCACGGTAGCTTCG

CGCCACTGGCTTTTCAACCAAGCGCGATGACCAATTGTGCGAATCAACGGTTCCTCTCGTACTAGGTTGA

ATTACCATCGCGACACCATCATCAGTAGGGTAAAACTAACCTGTCTCACGACGGTCTAAACCCAGCTCAC

GTTCCCTATTGGTGGGTGAACAATCCAACACTTGGTGAATTCTGCTTCACAATGATAGGAAGAGCCGACA

TCGAAGGATCAAAAAGCAACGTCGCTATGAACGCTTGGCTGCCACAAGCCAGTTATCCCTGTGGTAACTT

TTCTGACACCTCTAGCTTCAAATTCCGAAGGTCTAAAGGATCGATAGGCCACGCTTTCACGGTTCGTATA

CGTACTGGAAA

>FM895290.1 FM895290 Jatropha curcas embryo 71-95 (DAF) Jatropha curcas cDNA clone rjcpga0_002090, mRNA sequence

ATTCAACTTAAATAGCCAAAATTATCATTCCAACTAGTCAACAATTAAGACCATAAATTGTAAGCTCAAC

AAGAAGAAACTTAAAATCAAAAGCTTGGTCTACCCTCTGAGAAACAATTTCAAAATCATTCTCCACCAGC

CTTCTGGTAAACATAGGTGAGGCCGCACTTACCACAGTAGTGCCTATCAAAATGATTAGCCATAAAAGTG

CCAGCTCCACACTCCGCATTGGGACACTCCTTCCTTAACCTCTGCACTTTACCACTGTCATCGACCTTGT

AGAACTGAAGAACAGCGAGCTTGACCTTCTTCTTCTTGTGCTTGATCTTTTTTGGCTTGGTGTAGGTCTT

CTTCTTTCTCTTCTTGGCACCACCTCTCAAGCGAAGCACCAGGTGAAGAGTCGACTCCTTCTGAATGTTG

TAGTCTGCGAGGGTCCTGCCGTCTTCCAGCTGTTTTCCAGCGAAGATTAGACGCTGCTGGTCCGGTGGAA

TTCCTTCCTTGTCTTGGATCTTGGCTTTCACATTGTCGATGGTATCGGAAGACTCAACCTCGAGGGTAAT

GGTCTTACCCGTTAGGGT

>FM895289.1 FM895289 Jatropha curcas embryo 71-95 (DAF) Jatropha curcas cDNA clone rjcpga0_002089, mRNA sequence

TTTTTTTTTTAGATAAATAAACACCGAGTACATATAGACATTGTATAATTTAAGATGCAGGGACACAGAG

CCCTTCTTGGCCACCAAGCAGCGCAGGAAATCAATAACAAGTTTGCTCTAGGGCCCCAAGTAAGAATGCA

TAATCGCAATTTTGTAAGTCACAAGAGACAAGATTTCTGCCCAATAAAATGTATCAAGCAATTCAAGAAG

TAGCCATGGACTCAATCAGGAGTTGGTAACCTTCTGGTACTGTCGTCTGTGTTTGCACACACTCTGCCAA

TGCTGGAACATCACTAATATCCAGAAACTTGGCTAGAAACAACAGCAGATGAAAATCAGAAATTCTTTTC

ACGAATGGAAGATTCTTTGTTCGATCCAAATGAGTCTTCAGTGCCCTCACTGTCACTTGGGTGATCCTGT

TCTCAATGGGAAAGGAAGATGAAAGAGGACCCTGGTGATCCAAAATCTTAACCACGACCAAGAAAAAATC

ATTATCAACCTCTCTAACATCCTTTCCTCCAACAACAACATCCTTCTTCATTTTGGACAACTTAGGATCA

ATATCCTCCCCAATATCTGTCTCAAACCAATTTTCCTTAAACAGTCTAATGCACATATCACTTATCTGAA

ACGCCTCAA

>FM895288.1 FM895288 Jatropha curcas embryo 71-95 (DAF) Jatropha curcas cDNA clone rjcpga0_002088, mRNA sequence

ATTCCTCCTTTTTTAATTCTCGAAAAAAGAAAAGGAAATGACCGGAAGATAAATGTTCTTTGATTAGATA

GAGCTGGAATCCCAGATATTGTGATGGAAAAAAGGAAATGGTTGTGGAAGAGGAAGTCATCGGAGAGAAG

CCCGGGCGAAACTGAAAGTTCAGGATCAATATCTTCACAATCTGAGAGATTCTCTGATGAACAGGACAAC

TTGAAGGCATCTCCTAATAATGAAACTCAATCTCCAGAGGTTACATCAAAAACTGTAGTGAGGGATGAAG

ATGTCAATGATAGTGTTAGGATTCTAACAGAGAAGTTATCAGCTGCCCTTGTGAATGTTAGTGCCAAAGA

TGACTTGGTAAAGCAGCATTCCAAAGTTGCTGAAGAGGCTGTTGCAGGATGGGAAAAGGCTGAAAATGAA

GTAGCAGCTTTGAAGAAACAACTTGAAGCTGCTATTCAGCAGAACTGTGCTCTGGAAGATCGGGTGAGTC

ATCTTGATGGGGCACTAAAGGAATGTGTAAGGCAGCTAAGACAAGCAAGAGAAGAGCATGAGGAAAAGGT

CTACGAGGCTGTGACAAAGAAGACAATTGAATGGGAATCCGTTAAATCTGAACTTGAAAATCAGCTTCTT

GAACTGAAGACAAAAGCCGAAGCTACTAAGTCAGAATCT

>FM895287.1 FM895287 Jatropha curcas embryo 71-95 (DAF) Jatropha curcas cDNA clone rjcpga0_002087, mRNA sequence

TACATGAGGCAACAAGCCATGTACCGTGGTGATGTTGTGCCCAAGGATGTCAATGCTGCAGTTGCCACCA

TCAAGACCAAGCGCACAATTCAGTTTGTTGATTGGTGCCCAACTGGATTCAAGTGTGGTATCAACTACCA

GCCACCCACTGTTGTTCCTGGTGGTGATCTTGCCAAGGTGCAAAGAGCTGTTTGCATGATCTCCAACTCA

ACCAGTGTTGCTGAGGTGTTCTCACGCATTGACCATAAGTTTGATCTGATGTATGCCAAGCGTGCTTTTG

TCCACTGGTATGTGGGTGAGGGTATGGAGGAAGGTGAATTCTCTGAGGCTCGTGAGGATCTTGCTGCCCT

TGAGAAGGATTATGAAGAGGTTGGGGCTGAATCAGCTGAGGGCGAGGAAGGTGATGACGATGAATATTAA

GCAGTTTCTGGGAATCTATATATGCTTTTGTCAATGGCGTATGTTTTTACTTTTTTGGATTGTCTTGTTA

TGAACCATTCTCTTAATTGAAGCCATCTTGCTTCAAGAATATGCTTCAAACTATTCTAGTAATGAATGCT

ATTTACTGTTTAATAAACTATTCTGTCGGGATTGCGTTCTGACTACTTTTGTT

>FM895286.1 FM895286 Jatropha curcas embryo 71-95 (DAF) Jatropha curcas cDNA clone rjcpga0_002086, mRNA sequence

ATTCGAATTCCTTATCGCTCCCAGTTTAAGCGCCGTCTAGCCAGGATAAGGCGCTAATATTTTTCCAACT

TGAAAACGTATAATCAATTTTGCTTTGAACGAGCCAAGTGAGGCAATAATCGGCGAAGAATGGCGCATTC

ATTAGTATCCTCATCGGCCTCTTCCCTCAGATTCAGCTCTCTCGTCTTTTCTCCAAACCCTAGCACTACT

TCCGATTGCCAGAAGCTTTCTTTACCTTTTGAGCCTCTTCGTTCCAGGAAAGTGGGAAAGTTGAGTAGTT

TTAGAAAGAATTTTCAGCCATCTCCAACAAAGGCTGTTTATTCTGGCGAGTTTTGGGCACCTCAGAGAAC

TTCTCGAAAGGGGATTTGGTCTATAAGGGATGATTTGGAAATCCCATCTTCACCTTATTTCCCTGCATAT

GCCAATGGACAAGGTCAAGGACAAGGGCCACCTCCAATGGTGCATGAGAGGTTCCAGAGTGTTGTTAGTC

AACTTTTTCAACATAGAATTATACGCTGTGGTGGAGCTGTTGATGATGACATGGCAAATATTA

>FM895285.1 FM895285 Jatropha curcas embryo 71-95 (DAF) Jatropha curcas cDNA clone rjcpga0_002085, mRNA sequence

ATTCCGGAGGCGGGGCTATGCCCCTCTTTTTGGACCCAAGGTCGTTTCGGCGGCCGATCCGGGCGGAAGA

CATTGTCAGGTGGGGAGTTTGGCTGGGGCGGCACATCTGTTAAAAGATAACGCAGGTGTCCTAAGATGAG

CTCAACGAGAACAGAAATCTCGTGTGGAACAAAAGGGTAAAAGCTCGTTTGATTCTGATTTCCAGTACGA

ATACGAACCGTGAAAGCGTGGCCTATCGATCCTTTAGACCTTCGGAATTTGAAGCTAGAGGTGTCAGAAA

AGTTACCACAGGGATAACTGGCTTGTGGCAGCCAAGCGTTCATAGCGACGTTGCTTTTTGATCCTTCGAT

GTCGGCTCTTCCTATCATTGTGAAGCAGAATTCACCAAGTGTTGGATTGTTCACCCACCAATAGGGAACG

TGAGCTGGGATTAGACCGTCGTGAGACAGGTTAGTTTTACCCTACTGATGATGGTGTCGCGATGGTAATT

CAACCTAGTACGAGAGGAACCGTTGATTCGCACAATTGGTCATCGCGCTTGGTTGAAAAGCCAGTGGCGC

GAAGCTACCGTGCGCTGGATTATGACTGAACGCCTCTAAGTCAGAATCCGGGCCAAAAGCGACGCGCGT

>FM895284.1 FM895284 Jatropha curcas embryo 71-95 (DAF) Jatropha curcas cDNA clone rjcpga0_002084, mRNA sequence

TTTTTTTTGAGAAAAAAATAAAATAATATTAAATACAACAAGCCAGGCTGCAGGAGAGAAAAGGACCAAC

TAGAACAAGTTATACAAAAGCTGTCCTGAAAATTGTCGGCAAAAGATTGATTTTGCATACATTAGCTGAA

GCTGAACATTGAACTGTACAGTCGTCGGAAAAATAATCTTGAACGCGCTGACGTGCTTGCGTTGTATTGA

GCTATAAAGTGATGCAGTTTTAGTCAAGTTCCTTTATTCCATTTTCAAACATACAGAGGGAAGGCCGAGG

GACGAGGATAAGCGGCTGCAGTTCTTCCACCACTAGCATCATCTGCACTGTGTCGACGCATCCTTTGTGC

AGAGCCTGAATGGGTGTTAACATTTCCAATTTCCTTGGAATCCTGAAGCTGTTCCAATGATGTCACCACC

TCGTCCATGTTGGGCCGGAACTTTGATTCTATGGATAGACAACGTAGTGCAAGGGTAGCTGCCTTATTGG

CAATGTCCATCGAGTATTGGCCTTCAAGACGATTGTCCAGGATGCGAAATATCTTTCGCTTGTTTCCCAG

GTAGGGCTTAGCCCATTCCACTAGAACATGCTCTCCAGTTGGCCGGTTTTTATCTATTGCTCTTCTGCCA

GATAACATCTCT

>FM895283.1 FM895283 Jatropha curcas embryo 71-95 (DAF) Jatropha curcas cDNA clone rjcpga0_002083, mRNA sequence

CTTCTACGGCCATAGCCACACAGAGAGACGCAGGAGGCGGATAGCTTTCGATTCTTTCAGGTTGTGCAAC

AATGGGTGCCGGTGGCAGAATGTCTGTTCCTCCTTCCCCCAAGAAGTTGGAAGCTGAGGTCTTGAAACGA

GTTCCATACTCGAAGCCACCATTCACACTTGGTCAGGTCAAGAAAGCTATCCCACCTCATTGTTTCCAGC

GTTCTGTTCTCCGCTCATTCTCGTATGTTGTTTATGACCTGACCCTTGCCTTTATCTTTTATTATGTTGC

CACCAATTACTTCCACCTCCTTCCTCAACCCCTCTCTTATGTGGCCTGGCCAATTTACTGGTCCCTCCAA

GGCTGTGTCCTCACTGGCATTTGGGTTATAGCACATGAGTGTGGGCATCATGCCTTTAGTGACTATCAAT

GGCTTGATGACATAGTTGGCCTTCTCCTCCATTCCTGTCTCCTTGTCCCTTACTTTTCATGGAAACATAG

CCACCGCCGTCATC

>FM895282.1 FM895282 Jatropha curcas embryo 71-95 (DAF) Jatropha curcas cDNA clone rjcpga0_002082, mRNA sequence

CCACACGAGATTTCTGTTCTCGTTGAGCTCATCTTAGGACACCTGCGTTATCTTTTAACAGATGTGCCGC

CCCAGCCAAACTCCCCACCTGACAATGTCTTCCGCCCGGATCGGCCGCCGAAACGACCTTGGGTCCAAAA

AGAGGGGCATAGCCCCGCCTCCGATTCACGGAATAAGTAAAATAACGTTAAAAGTAGTGGTATTTCACCG

TCGCCTTTCGGCTCCCACTTATCCTACACCTCTCAAGTCATTTCACAAAGTCGGACTAGAGTCAAGCTCA

ACAGGGTCTTCTTTCCCCGCTGATTCCGCCAAGCCCGTTCCCTTGGCTGTGGTTTCGCTGGATAGTAGAC

AGGGACAGTGGGAATCTCGTTAATCCATTCATGCGCGTCACTAATTAGATGACGAGGCATTTGGCTACCT

TAAGAGAGTCATAGTTACTCCCGCCGTTTACCCGCGCTTGGTTGAATTTCTTCACTTTGACATTCAGAGC

ACTGGGCAGAAATCACATTGCGTGAGCATCCTCAGGGACCATCGCAATGCTTTGTTTTAATTAACCAGTC

GGATTCCCCTTGTCCGTACCAGTTCTGAGTCGACTGTTCGAC

>FM895281.1 FM895281 Jatropha curcas embryo 71-95 (DAF) Jatropha curcas cDNA clone rjcpga0_002081, mRNA sequence

ATTCCTGAGATCTTGACAGTGATTGGGAAGATCCCATATCTTTCTGAGTTTCTAAATTCTTTATATGAGT

GCCAGTACAAGTCATTTTTCTCAGCATTTGCTGGCCTCACAGAGCAAATTAGACTTGACCGCTATTTGCA

TCCACACTTTCGATATTACATGAGGGAGGTCAGAACTGTTGTTTATTCTCAGTTTTTGGAATCCTATAAG

AGTGTTACTATTGAATCGATGGCAAAGGCTTTTGGTGTGACAGCAGACTTCATTGATTTGGAAGTATCGC

GTTTTATAGCAGCAGGGAAGCTTCATTGCAAGATTGACAAGGTTGCTGGTGTTCTTGAAACCAACCGTCC

TGA

>FM895280.1 FM895280 Jatropha curcas embryo 71-95 (DAF) Jatropha curcas cDNA clone rjcpga0_002080, mRNA sequence

ATCCAATCTCTCACCGATCGGATATTATAATATTTAATTACAGAGAAGCAACAACATCACCAAATGGAAT

CCACTACTGAAGCTCTTTCAAAGCTTAACATCAATGGCGATTTTGCATCCTCGTTCCCTAATTTACATAA

AAACCTTCACCTTCTCCCCCCTGAGCAGATTGAACTGGCGAAAATATTACTAGAAATGGGGCAGAGTCAT

CTGTTTGAGCATTGGCCATAACCTGGCGTTGATGATGAAGAGAAGAAAGCTTTGTTCGATCAGGTGGCTC

GCCTCTATTCCAGCTACCCTGGAGGTTTGGCATCATATATTAGAACAGCAAGAGAACTGTTGGCCGATTC

ATATGCAGGAGAGAACCCCTTTGATGGCTTTACACCGTCTGTTCCAGCAGGAGAAAATTTAACTTTTGGT

GACGAAAACTTTGTCAAATTTGAGGAGTGCAGGAGTTAGAGAAGCCCATAATGCTGCATTTGTGCTTGTT

GCAGGTGGTCTTGGCGA

>FM895279.1 FM895279 Jatropha curcas embryo 71-95 (DAF) Jatropha curcas cDNA clone rjcpga0_002079, mRNA sequence

ATTCCTCCTTTCTATAGCAATGGCTAACTCTTCTTTTCTCTCTCTCGGTCTCTGCTTTCTCGTTCTTTTC

CAAACCTGCTTTGGTCAATTAGAACAATTAACAACTGAGAATCGCCGGCCCTGGCAATTCCGTGACCAGC

GCTCTCAACAGGATGCGTGTAGACTCGAACGCATTAACGCTGTGGAGCCTTCCCGTAGAATTCAATCAGA

GGCCGGTGTAACTGACATCTGGGACGAAAACGACGAGCAGTTCCGATGCGCTGGTGTCGTAGCCATGCGT

CATACAATTCAAGAGAGAGGCCTCTTGTTGCCCCTATATGCCAATGGTCCTAAGCTCATTTACGTGCTCC

AAGGTAGAGGTATCCACGGAGCCGTATTTCCAGGTTGTCCTGAGACATTCCAGTCACCATCAGAACCACA

GGAACGGTACGGATTCGGTGCAGGAGAAAGAGAGAGTCGGAGATTTGATCAACACCAG

>FM895278.1 FM895278 Jatropha curcas embryo 71-95 (DAF) Jatropha curcas cDNA clone rjcpga0_002078, mRNA sequence

CAAACTCAAAACTCACTTCATCTTCTTCATTTGGATTAAGCGATGGATACTGATTCTGTGTTTAGCAATA

TCGTTCGAGCTCCTGAAGATCCTATTTTAGGGGTTACTGTTGCTTATAACAAAGATACAAGCCCTAACAA

GTTGAATTTGGGTGTTGGTGCTTATCGAACTGAGGAAGGGAAGCCTCTCGTTTTGAATGTGGTGAGGAAA

GCGGAGCAGATACTAGTAAATGACCAGTCTCGGGTCAAAGAGTATCTTCCTATTGTTGGACTTGCTGATT

TTAATAAACTGAGTGCCAAGCTTATCTTAGGAGCTGATAGCCCCGCTATACAAGAGAACAGAATAACTAC

TGTGCAAGGCTTGTCTGGTACCGGTTCTTTGAGAGTTGGAGGGGAGTTTCTTGCAAGGCATTACCATCAA

CTGACAATATACATTCCACAGCCAACATGGGGAAATCATACCAAAGTCTTCACTCTTGCAGGGTTGTCTG

TTAAAGACGTACCCGGTACTATGAT

>FM895277.1 FM895277 Jatropha curcas embryo 71-95 (DAF) Jatropha curcas cDNA clone rjcpga0_002077, mRNA sequence

AGGATTCTATCCGTCGCCCGGTGGGAATTGTACTTTAAGGCGGCCCTCGCGGCTCGTCCGCCGCGGGGGC

TTAGCCAACGGCACGTGCCTCTGGGGGCCGGATGGCCCCTACTGCGGGTCGGCAAACGGGCGACGGACAC

GCGCGTCGCTTCTGGCCCGGATTCTGACTTAGAGGCGTTCAGTCATAATCCAGCGCACGGTAGCTTCGCG

CCACTGGCTTTTCAACCAAGCGCGATGACCAATTGTGCGAATCAACGGTTCCTCTCGTACTAGGTTGAAT

TACCATCGCGACACCATCATCAGTAGGGTAAAACTAACCTGTCTCACGACGGTCTAACCCCGGCTCACGT

TCCCTATTGGTGGGTGAACAATCCAACACTCTGGTGAATTCTGCTTCACAATGATAGGAAGAGCCGACAT

CGAAGGATCAAAAAGCAACGTCGCTATGAACGCTTGGCTGCCACAAGCCAGTTATCCCTGTGGTAACTTT

TCTGACACCTCTAGCTTCAAATTCCGAAGGTCTAAAGGATCGATAGGCCACGCTTTCACGGTTCGTATTC

GTACTGGAAATCAGAATCAAACGAGCTTTT

>FM895276.1 FM895276 Jatropha curcas embryo 71-95 (DAF) Jatropha curcas cDNA clone rjcpga0_002076, mRNA sequence

TTTTAAAGTTAAACTACTCGCTAACAAGGACAAACCAGCTATCATCAACGTTAATATAGGCACAACTGTC

AAAGGAGCAGTTGATGACCTTGATCTTGTAATACAAACGCTTGAAGAAAGTGGATTTACGCATGATCGGT

TCTACATCCACTGTGATGGAGCGTTATTTGGACTTATGATGCCTTTCGTCAAGCGTGCACCAAAGGTTTC

TTTCAAGAAGCCTATCGGAAGTGTGAGTGTTTCTGGTCACAAGTTTGTTGGCTGTCCAATGCCATGCGGT

GTTCAGCTAACAAGGTTGGAACACATTAATGTTCTCTCAAGGAACGTCGAGTACCTTGCTTCAAGGGATG

CCACAATCATGGGGAGCCGCAATGGTCACGCTCCTATCTTCCTTTGGTACACCCTCAACAGAAAAGGTTA

CAGAGGATTCCAGAAAGAAGTACAGAAATGTCTCAGAAATGCTCATTACTTGAAGGATCGCCTACGTGAT

GCTGGGATTAGTGCAATGTTGAATGAGTTGAGCAGTACTGTTG

>FM895275.1 FM895275 Jatropha curcas embryo 71-95 (DAF) Jatropha curcas cDNA clone rjcpga0_002074, mRNA sequence

GCCCGAGCAGACAGAGAGACGGATACCTAGTCGACCCCTTCAAACCCTAGAGGACTGTTACTGTCCGACA

ATTGATCGAAACCCCTATCTCCACCGTTACTTAATTCTTGTTCTCGACGAACAGCACTCGTGGTTGACAG

TTTTGATCTTGCAACGGAGAAGCTAGCAGATGGAGGATTGGGAGGATGAACAAATTCCACCTCTCCTTTT

AAAGGAGCAACCTAAAAGCAAATGGGACGATGAAGACGTGGATGACAATGATGTGAAAGAATCATGGGAG

GATGAAGATGAACCAGCTCCGGCACCTGTAACACAGCCTCCTCCTGAAAAACCCTCTAAGAAACCCGTGA

CAAAATCTACAGAGAAGAAAGGGAAAACTGTTGAAGTAGCAAAGGAAGAGCCTGAACTTCTAGATCCTGT

TGCTCAGAAACTTCGCCAACAACGGCTAGAAGAAGAAGCTGATTACAAATCTACTACAGAACTGTTTGCT

AAGAGAGATGATGAGAAGACGCTTGATAATTTCATTCCCAAATCAGAGAGTGATTTTATGGAATATGCAG

AAC

>FM895274.1 FM895274 Jatropha curcas embryo 71-95 (DAF) Jatropha curcas cDNA clone rjcpga0_002073, mRNA sequence

ATTCCTACCTTTCAGCTTTCGATCTCTGCCGAGATTCTTCTTTTCCAATTCCTCTCTAAAGATCATGAAG

CTCGATACCAGTGGTCTTGAATCAGCGGCACCGCTCTTTGGAGCTACTGGCGAGCTGTTTAATGGGTTTT

CTACTGCGCCTTCATTTGAACTTCCTGTTACTACTGATTTTGATGGATTCCAGAAGGAAGCTATCCAAAT

GGTGAAGCCTGCCAAGGGGACAACAACACTTGCTTTCATTTTTAAAGAGGGTGTCATGGTCGCTGCTGAT

T

>FM895273.1 FM895273 Jatropha curcas embryo 71-95 (DAF) Jatropha curcas cDNA clone rjcpga0_002071, mRNA sequence

AAAACAATGGACTTTCCATATTCGTATATAAGATGGAAAAAAATTAATAATTAATAAAGAAAAAAAAATG

AAAACCATGCAATTAGATTATCTTCATCAAATAATCTAAATTTGTTTCTCACATTTGGTACAAAAAAAAA

AAAAAAAAAAGAAATAGCTGAAAAATGACATGAAAATATCAATGTAGAGGGTGCCTATGGCAAGCCATGT

TTAATGGAATTGGTGGGTCTGAAAATGAACCATCTTTAATATTTTCAAACATCCATTTGTTTGCTGCTTG

AGTGAAATGAACTCCATCCCAATTAATCCAAACTGAAGGATCTTCACATGCTTTCCCCACCAAAACTTGT

CTTCCACCTTTTGTAATATATCCTCCACATCCAATATTCTATGTTGTAGTTATACTTCCCACCATGTCCA

CAAAAAGCTCTCAATGACTCATTAAATCCGAAACTTTCTGGCTTGACTGAAAAGACTATATTTTAGAGAG

TAAATATCAACATATGTGATTGCTGCTTCTGGGCAA

>FM895272.1 FM895272 Jatropha curcas embryo 71-95 (DAF) Jatropha curcas cDNA clone rjcpga0_002070, mRNA sequence

ACTAGTGGATCCCCGGGCTGCGGAAAGGATTCTATCCGTCGCCCGATGGGAATTGTACTTTAAGGCGGCC

CTCGCGGCTCGTCCGCCGCGGGGGCTTGGCCAACGGCACGTGCCTCTGGGGGCCGGATGGCCCCTACTGC

GGGTCGGCAAACGGGCGACGGACACGCGCGTCGCTTCTGGCCCGGATTCTGACTTAGAGGCGTTCAGTCA

TAATCCAGCGCACGGTAGCTTCGCGCCACTGGCTTTTCAACCAAGCGCGATGACCAATTGTGCGAATCAA

CGGTTCCTCTCGTACTAGGTTGAATTACCATCGCGACACCATCATCAGTAGGGTAAAACTAACCTGTCTC

ACGACGGTCTAATCCCAGCTCACGTTCCCTATTGGTGGGTGAACAATCCAACACTTGGTGAATTCTGCTT

CACAATGATAGGAAGAGCCGACATCGAAGGATCAAAAAGCAACGTCGCTATGAACGCTTGGCTGCCACAA

GCCGGTTATCCCTGTGGTAACTTTTCTGACACCTCTAGCTTCAAATTCCGAAGGTCTAGAGGATCGATAG

GCCACGCTTTCACGGTTCGGATTCGTACTGGAAA

>FM895271.1 FM895271 Jatropha curcas embryo 71-95 (DAF) Jatropha curcas cDNA clone rjcpga0_002069, mRNA sequence

GGATTCTATCCGTCGCCCGATGGGAATTGTACTTTAAGTGCGGCCCTCGCGGCTCGTCCGCCGCGGGGGC

TTGGCCAACGGCACGTGCCTCTGGGGGCCGGATGGCCCCTACTGCGGGTCGGCAAACGGGCGACGGACAC

GCGCGTCGCTTCTGGCCCGGATTCTGACTTAGAGGCGTTCAGTCATAATCCAGCGCACGGTAGCTTCGCG

CCACTGGCTTTTCAACCAAGCGCGATGACCAATTGTGCGAATCAACGGTTCCTCTCGTACTAGGTTGAAT

TACCATCGCGACACCATCATCAGTAGGGTAAAACTAACCTGTCTCACGACGGTCTAAACCCAGCTCACGT

TCCCTATTGGTGGGTGAACAATCCAACACTTGGTGAATTCTGCTTCACAATGATAGGAAGAGCCGACATC

GAAGGATTCAAAAGCAACGTCGCTATGAACGCTTGGCTGCCACAAGCCAGTTAT

>FM895270.1 FM895270 Jatropha curcas embryo 71-95 (DAF) Jatropha curcas cDNA clone rjcpga0_002068, mRNA sequence

GTTAAGAAAGGATGGGCTTCTCTTCAGGGTATTCCGAGGACAGTTGCTTTTGACACAAAGAGTGGCAGCA

ATTTGCTTCAGTGGCCAGTCGAAGAAGTAGAGAATTTGAGATTAAGAAGCAAAGAATTCAAGAACGTAGA

GGTCAAGCCAGGGTCAGTTGTGCCTCTAGATCTTGAAGCAGCTACACAGCTTGATATCTTCGCTGAGTTT

GAGATAGACAAGAATGTTTTGGAGAACATAGCAGAAACCGATGAAGAATTTAGCTGCAGCACTAGCAATG

GAGCTGCTCACCACAGTGCATTAGGACCTTTTGGTCTCCTGGTTCTCGCAGACGAGAGTCTTGCAGAGCA

AACTCCTGTTTACTTTTATGTTACGAAAGGATTTAATGGCACTCTCAAAACATTCTTCTGCACAGACCAA

ACAAGATCCTCTGTTGCAAATGATGTTAATAAAC

>FM895269.1 FM895269 Jatropha curcas embryo 71-95 (DAF) Jatropha curcas cDNA clone rjcpga0_002067, mRNA sequence

TTGAATTTTCTTATTCCAAACCCATGGAAGCAAGTGATTTAGTTAAGATACTAGGCGTTCACCGAGTCAC

ACCATTAGCTAGCTCACCCAAATCCACTACCGAGTCATCTCTTGCTCTTACACATTTTGATATGATTTGC

CTCAAATTTTATCCAGTTGAGCAAATCTTTTTCTATGAGTTCAACAATTTAACTTTGTCATTCTTCAACT

CAGTGATCATCCCTAAACTCAAATCATCACTCTCACTTGCTCTCCTCCATTTCCTTCCTCTAGCAGGAAA

CATCAAATGGCCTTCTAATGCTACCAAACCATGCATCTTATACACCGTAAATGATGGCATTTTGGTTAGG

ATTGCAGAATCAGCTGCAAACTTCACCCATCTTTCGAGCAACCAAATGAAGGAAGCAATTGAGTCACGTC

TTTATATACCCGAATTGAATATATCTGACTCAACAG

>FM895268.1 FM895268 Jatropha curcas embryo 71-95 (DAF) Jatropha curcas cDNA clone rjcpga0_002065, mRNA sequence

GTCATGGAAGTCGAAATCCGCTAAGGAGTGTGTAACAACTCACCTGCCGAATCAACTAGCCCCGAAAATG

GATGGCGCTTAAGCGCGCGACCTATACCCGGCCGTCGGGGCAAGAGCCAGGCCCCGATGAGTAGGAGGGC

GCGGCGGTCGCTGCAAAACCCGGGGCGCGAGCCCGGGCGGAGCGGCCGTCGGTGCAGATCTTGGTGGTAG

TAGCAAATATTCAAATGAGAACTTTGAAGGCCGAAGAGGGGAAAGGTTCCATGTGAACGGCACTTGCACA

TGGGTTAGTCGATCCTAAGAGACGGGGGAAGCCCGTCCGACAGCGCGTCCTCGCGCGAGCTTCGAAAGGG

AATCGGGTTAAAATTCCCGAACCGGGACGTGAAGGCTGACGGCGACGTTAGGGAGTCCGGAGACGTCGGC

GGGGGCCTCGGGAAGAGTTATCTTTTCTGTTTANCAGCCCGCCCACCCTGGAAACGACTCAGTCGGAGGT

AGGGTCCANCGGCTGGAAGAGCACCGCACGTCGCGCGGTGTCCGGTGCGCCCCGGCGGCCCTGAAAATCC

GGAGGACCGAGTGCCT

>FM895267.1 FM895267 Jatropha curcas embryo 71-95 (DAF) Jatropha curcas cDNA clone rjcpga0_002064, mRNA sequence

ATTCCTCATGGCAAAAGATGGTAGTTTGAAGAAATTATCCAATGAGTGGACTTTAGTTGGTTCTCTCACT

GCATCAGGACTTCCCGCAGCTATTTATGCACTGCAAAACAGCTTGCTGCAGATATCTTACAGAAATCTTG

ACTCACTCACGTTTTCTATGCTGAACCAGACAAAAATTTTTTTCACTGCATTCTTTACATTTATAATATT

GAGGCAGAAGCAGTCGATTCAACAAATTGGTGCTCTGTTCTTGTTGATCATGGCAGCTGTTCTTCTAAGT

GTTGGTGAAGGCTCTAGCAAGGGAACCAGTAGTAGTAACCCTGATCAAATTTTATTTTATGGGATTGTCC

CCGTGCTGGCTGCTTCTGTTCTTTCTGGCCTTGCTTCAGCTTTGTGTCAATGGGCTTCTCAGGTTAAGAA

GCACTCATCGTACTTGATGACTGTAGAAATGTCCATCGTTGGAAGCCTGTGCTTGTTGGCCAGTACCACC

AAGTCTCCAGATGGTGAATCAATCAGACGATATGGATTCTTT

>FM895266.1 FM895266 Jatropha curcas embryo 71-95 (DAF) Jatropha curcas cDNA clone rjcpga0_002063, mRNA sequence

CAGAGTTTCTTCTTGGCACCTCAAGACAAAGGCTATTATGTTTTGAATGATGTCTTCCGTTATGTTGATG

ATGCCAAGAATCAAAATGGAACTCTTGGTTTTTTTAACACTGTTGAAGCTCCAGTTAATCCAGGCCAGGA

TCTCCCTTCTGTACAAGAAAATCACATTT

>FM895265.1 FM895265 Jatropha curcas embryo 71-95 (DAF) Jatropha curcas cDNA clone rjcpga0_002062, mRNA sequence

GTCTGACATGTGTGCGAGTCAACNGGGTGAGTAAACCCGCAAGGCGCAAGGAAGCTGACTGGCGGGATCC

CCCCGAGGGTTGCACCGCCGACCGACCTTGATCTTCTGAGAAGGGTTCGAGTGAGAGCATGCCTGTCGGG

ACCCGAAAGATGGTGAACTATGCCTGAGCGGGGCGAAGCCAGAGGAAACTCTGGTGGAGGCCCGCAGCGA

TACTGACGTGCAAATCGTTCGTCTGACTTGGGTATAGGGGCGAAAGACTAATCGAACCGTCTAGTAGCTG

GTTCCCTCCGAAGTTTCCCTCAGGATAGCTGGAGCTCGGGACGAGTTCTATCGGGTAAAGCCAATGATTA

GAGGCATCGGGGGCGCAACGCCCTCGACCTATTCTCAAACTTTAAATAGGTAGGACGGGCGCGGCTGCTT

CGCTGAGCCGCGCCACGGAATCGAGAGCTCCAAGTGGGCCATTTTTGGTAAGCAGAACTGGCGATGCGGG

ATGAACCGGAAGCCGGGTTACGGTGCCCAACTGCGCGCT

>FM895264.1 FM895264 Jatropha curcas embryo 71-95 (DAF) Jatropha curcas cDNA clone rjcpga0_002061, mRNA sequence

TTGCTACTTTCTTAGTTCTTTCTTATTGTTTGCTTCTCAATTTTATTTTTTGGTTCTTATTAGGTACTTT

TCTCCTTGGATTCAAATGGCTACTGTTGAGGTAGTATCGGCACAAACTGCACTTTCAGAGGAAAAAGCAG

AAGAAGTGAAGGCTTCAGAGACTGTCACTCAAGTAACAGATCAGTCAGTTACTGATGAAAAACCAGACAC

AGGAGAAAAGAAGGAAGAACAAGAAGAAGTTGCAGGAACTAAAGAAGAAGCTGTAGAACCAGAACCAGAA

GTTGCAACTGAAGTAGTGGTGGAAACTAAAGAAGTAGTAGAAGATGAGACCAAAACTGTAACAGAAGAGC

CCCCATCGGAGAAAACTGAAGAAGAAACTCCTAAGGAAACAGAACCAGAAAAAGAAGAAACTCCTAAGGA

AACAGAACCCGAACAAGAAGAAAAGGAGTCCCCGAAAGAAACAGTAACTGAGGAGACTGTTGCTGAGGAG

ACTACTAAAGAGACCACTGAGAGTACTGAGACAACAACTC

>FM895263.1 FM895263 Jatropha curcas embryo 71-95 (DAF) Jatropha curcas cDNA clone rjcpga0_002060, mRNA sequence

CTCCTCTGTATACATATGTCGGAAACGATTTGAATATTTTTCTGCCCCTAATGTACACGTAACTTTAGCA

CCCTTGAGGAAGACTTTTCGTTCTTTTTTTTAAAAAAAAAAAAAAAATTAACAAAAAAGAAACTACAAAA

AGAGGTATACTGCAGCAACTGTGGCTTGCTTCTCATGGCTGCAATGAAACTGATGGCCGATCTTCTGGCT

TCTCGACAAGTTTACACCTTGAAGGTGGACCAAAAATGCTCAAAACTACTGGCAGAAATACTAGCCCATG

CAAGAAACCAAGAAGGACCAACGCCAAGTACATTTGGAAGTAATATATCACAAAAACTTCCGTCCTTGAG

AAACAAAGAACGAGCACGCCGACTAGCTTTGTTAGTGTGATACCGCTGAAGACTGAAGCCCCCATTGTGC

CTAGAGCCTCTTTCATTCTTTCTTCTCTATCTCCAGTGCTTACCGTGAAAGCATGTG

>FM895262.1 FM895262 Jatropha curcas embryo 71-95 (DAF) Jatropha curcas cDNA clone rjcpga0_002059, mRNA sequence

GTTTTAAGAACCACTTCATAGTGTAGCATAGAAATGGTGTTTCTTGTTGAAACAATCCAGCACATATAGA

AGTTAAAACGCTAATGAAAAACAATAAACAAAAAAAAGCTCAAAAATAAAAAGTCCCAAAAACTGCCAAA

CTGAAAAACTGAAACTGCAGATAAAACAGAGGTTACATAGTACCAAAAACAACAAACATGTAGCAGTAAT

TTAGCTCGAAACATCAATACTTCTGCGTCTGAGAATCTCAACCCCCAAATAATCGAGAGGACCATGAACA

GCAACATGAGGCTTCCCAACCTTTACGCGAACAGCAGATATTTGGGGGTATTTTGTCAGGGTCGTAGAAG

CAATCTTGTGAGCCACTGACTCGAGAAGATTATGAGGGGCTCCTTCCACAACTTCCTTGACTATGCGATA

AATTTCAGTATAGCTAATCGTATCTGACAGCCGATCAGTTTTACCAGCTTCCCTAAGATCCATCCAGGCA

TCTACATCAATCAAGAACTTTTGTCCCAATGTCCTTTCTTCTGGTTTCACTCCATGAAAGCCATGGAACT

TCAATCCTCTTAATATAAGTTTATCT

>FM895261.1 FM895261 Jatropha curcas embryo 71-95 (DAF) Jatropha curcas cDNA clone rjcpga0_002058, mRNA sequence

ATTCATGCAGTCCTGGTTGTATTCTCAGTTCGAACACGCTTCTCACAGGAAGAGGAATCTGCACTTCGGA

GCTTGCAGACTTTGTTTGGAAGCAAGATATTTGATTACATGATTGTAGTCTTCACTGGAGGAGATGAGCT

TGAAGATAACGATGAGACATTACAAGATTATCTAGGTCGTGAATGTCCTCAGCCTTTAAAGGAAATTCTT

AATCTCTGCAAGAATAGACTAGTGCTTTTTGATAACAAAACCAAAGATGAACTTAAGAGAGCTGAACAGG

TTCAACAGCTTCTTTTCCTTGTAAATATGGTCATGGCACAAAATGGTGGACAGCCATATACAGATGAGAT

ATTTGTCGAGTTGAAGAAAGGAGCGATGAAACTTCGTGATCAACAAGAAGAGGTTGATTCCCTGGAAGGA

TATACTAAACGAGAAATATTGGAGCTAAAGGAGCAGATGCACAAGTCATATGAAGAACAGCTGAAACGAA

TAACTGAGATGGTTGAGTCAAAACTTAGAGAGACAACAAATAGGCTTGAGCAGCAGTTGG

>FM895260.1 FM895260 Jatropha curcas embryo 71-95 (DAF) Jatropha curcas cDNA clone rjcpga0_002057, mRNA sequence

CAAAGGGTGTTGGTCGATTAAGACAGCAGGACGGTGGTCATGGAAGTCGAAATCCGCTAAGGAGTGTGTA

ACAACTCACCTGCCGAATCAACTAGCCCCGAAAATGGATGGCGCTTAAGCGCGCGACCTATACCCGGCCG

TCGGGGCAAGAGCCAGGCCCCGATGAGTAGGAGGGCGCGGCGGTCGCTGCAAAACCCGGGGCGCGAGCCC

GGGCGGAGCGGCCGTCGGTGCAGATCTTGGTGGTAGTAGCAAATATTCAAATGAGAACTTTGAAGGCCGA

AGAGGGGAAAGGTTCCATGTGAACGGCACTTGCACATGGGTTAGTCGATCCTAAGAGACGGGGGAAGCCC

GTCCGACAGCGCGTCCTCGCGCGAGCTTCGAAAGGGAATCGGGTTAAAATTCCCGAACCGGGACGTGAAG

GCTGACGGCGACGTTAGGGAGTCCGGAGACGTCGGCGGGGGCCTCGGGAAGAGTTATCTTTTCTGTTTAA

CAGCCCGCCCACCCTGGAAACGACTCAGTCGGAGGTAGGGTCCAGCGGCTGGAAGAGCACCGCACGTCGC

GCGGTG

>FM895259.1 FM895259 Jatropha curcas embryo 71-95 (DAF) Jatropha curcas cDNA clone rjcpga0_002056, mRNA sequence

AGGATTCTATCCGTCGCCCGGTGGGAATTGTACTTTAAGGCGGCCCTCGCGGCTCGTCCGCCGCGGGGGC

TTAGCCAACGGCACGTGCCTCTGGGGGCCGGATGGCCCCTACTGCGGGTCGGCAAACGGGCGACGGACAC

GCGCGTCGCTTCTGGCCCGGATTCTGACTTAGAGGCGTTCAGTCATAATCCAGCGCACGGTAGCTTCGCG

CCACTGGCTTTTCAACCAAGCGCGATGACCAATTGTGCGAATCAACGGTTCCTCTCGTACTAGGTTGAAT

TACCATCGCGACACCATCATCAGTAGGGTAAAACTAACCTGTCTCACGACGGTCTAAACCCAGCTCACGT

TCCCTATTGGTGGGTGAACAATCCAACACTTGGTGAATTCTGCTTCACAATGATAGGAAGAGCCGACATC

GAAGGATCAAAAAGCAACGTCGCTATGAACGCTTGGCTGCCACAAGCCAGTTATCCCTGTGGTAACTTTT

CTGACACCTCTAGCTTCAAATTCCGAAGGTCTAAAGGATCGATAGGCCACGCTTTCACGGTTCGTATTCG

TACTGGAAATCAGAATCAAACGAGCTTTTA

>FM895258.1 FM895258 Jatropha curcas embryo 71-95 (DAF) Jatropha curcas cDNA clone rjcpga0_002055, mRNA sequence

TTTTTTTTTTGGATCATATGTTTTAGACTTTTTTAGCTCAATGAATGTTTGTAAAAAAGCATTCTTATAA

TTATCTAACAGTTCAACAGATTTCCCATTAACAAAGTGAAAGCCGGTCAGGTGAAGTTTCAGGAGTAGAG

GCTTTTGCCCTTGTTGATACATCAAGGGCTTGTTGTAATTTTACAACAACTTGCTCCATTGTTGGTCTTT

GTTGCTTAAGTGCTGTGCATGACAATGCTAGCTGAAATACTAGAACAAAAGCTTCTGCTGAATATTCTCC

ATTCAGCTTAGGATCTGCAAATTCTATTATGCTGCCTCCTCTGGTTATGGATTTTGCCATTTTATCCAAT

GGCATTGGATTCTTCAGATTCATATTAATAACCCTCTTTCCTGATAGAATCTGCAGAAGTACTATTCCAA

AGCTATAAACATCGCCTGATGAATTTACATGGTGATTATTTCGGTACTCAG

>FM895257.1 FM895257 Jatropha curcas embryo 71-95 (DAF) Jatropha curcas cDNA clone rjcpga0_002053, mRNA sequence

GTCTGCTATTCTAGCACGCTTCCTTTGCAAAGTCAAAGGAGTCACCAACCTCTGAATTTTGGGAGCTTTG

CTAACCTTCTTACCAGATTTGGTTGTAAATGTTCGGCGGTAAGTGTTGACATACTTCCGGACATCATCCT

CCTTGGAGAGGTTAAACAGTTTCCTGATCTTTGATGCCCTCTTAGGACCTCTCATTCTAGGCTTCTCAGT

GTCTGTCAGTCCTGGCAGATCCTGTTCACCTTTCTTAACAATAACCAAGTTCAAAACTGAGAGATCCTGA

CTCACAATGCATCCACGAACAGACTTCCTTCTTCGTTCTCCATTACGCCTCCCATATCCACGGAAGCAAG

GGGTACCTCGGTGAAGCAAAAGTCGAACACGGCCTGGAGTTAGAACTCCCTGCTTCATTGGAAAACCTTG

TTTGTCACAGCCTCCCATAATCTTAAATACATAACCTTTGAACTCCTCACCCAGGGCATCTCCACTGACT

TCTTGTGATATCCTCTTGTCAAAAAATGCACGGAGTTTCTGGTCATCGTCTATCTCGAGGGGGGGCCCGG

TA

>FM895256.1 FM895256 Jatropha curcas embryo 71-95 (DAF) Jatropha curcas cDNA clone rjcpga0_002052, mRNA sequence

GTTCCGATTGATCAGACGATCAAAGTACTAAAAAATACAATCTCTTATACACAATACAAATACTAAAAAT

TAACATTCAAATGCACACACAGTACATACATAGTTTAATAATGTCCAAAAATATTCACTACTTGTCGATT

TCCTTTGGTGGCTTCACAATAAGGATGGGGCATTTTGCATGATGGGCACAATAGTAACTTACACTGCCCA

ACAATGCCCTCTTAATTTTCCCAAGACCGCGGCTGCCAACCACAACGAGATCAACCTGCATTTGCTCTGT

AGCTTTACAAATCATGTCCTTCGCATCCCCTTCAAGAATTAGACTTTCTGCTTTCACCATTTTTTCATCG

CACATCTTTAATGCTCGGGAGACTATTGCTGCAGAATTTTCTTGCTGAGCTTTCTTCACAGAATCCGCTA

TTGAAGACGGTGTGTAAAGAGCTGCTCCAGCAGGACCAAGAGGGACGATACTAGTCTGGAAAGAATGTTG

GACATGAACTAGTGTAACCATATCTGAATCTTGACTAACACCTTTGATTATAAG

>FM895255.1 FM895255 Jatropha curcas embryo 71-95 (DAF) Jatropha curcas cDNA clone rjcpga0_002051, mRNA sequence

ATTCAAAGGATTCTATCCGTCGCCCGATGGGAATTGTACTTTAAGGCGGCCCTCGCGGCTCGTCCGCCGC

GGGGGCTTGGCCAACGGCACGTGCCTCTGGGGGCCGGATGGCCCCTACTGCGGGTCGGCAAACGGGCGAC

GGACACGCGCGTCGCTTCTGGCCCGGATTCTGACTTAGAGGCGTTCAGTCATAATCCAGCGCACGGTAGC

TTCGCGCCACTGGCTTTTCAACCAAGCGCGATGACCAATTGTGCGAATCAACGGTTCCTCTCGTACTAGG

TTGAATTACCATCGCGACACCATCATCAGTAGGGTAAAACTAACCTGTCTCACGACGGTCTAACCCCAGC

TCACGTTCCCTATTGGTGGGTGAACAATCCAACACTTGGTGAATTCTGCTTCACAATGATAGGAAGAGCC

GACATCGAAGGATCAAAAAGCAACGTCGCTATGAACGCTTGGCTGCCACAAGCCAGTTATCCCTGTGGTA

ACTTTTCTGACACCTCTAGCTTCAAATTCCGAAGGTCTAAAGGATCGATAGGCCACGCTTTCACGGTTCG

TATTCGTACTGGAAATCAGAATCAAACGAGCTTTTACCCT

>FM895254.1 FM895254 Jatropha curcas embryo 71-95 (DAF) Jatropha curcas cDNA clone rjcpga0_002050, mRNA sequence

TTTTTTTTTTTGAAAATCAACAATGGAACCCAAAAGACCAAAAAGAGAAATACAAATGCAACAAAGAAGA

TCCCCCATAAACCAACTCAACTACCTCAAAACAACACCCATACAACAAAAGTAGTCTCGTACCAAAACCC

AGCTCTCTGACTTCAACCCTTAATAACCAACAGCATTTCTCTCTACTTATTTCCCTTCATGTGCTTTCCT

CTGGATTTCTTGGCCTACTTCCTTGGTCTTCTGTCCCACAAAACCAGCCATGTCTTGCATACGCTTCTTA

GCTATATCCAGCTGCTCCGGCATTCGCCGAGTGACCTCCTGGAGGTATTTCAGAACCCACGAGAGTGATG

ACAACCCTGTGAGCCCCATAGCCCCGGAAGCCAAAAATGCCATGACCGAAAGGCCAATAACTAATGCAGC

TGGGACAAGAACAGGGCTAAAGATGACAAAAAGAGGGGTAGTAATAGCTAGGCCGATTAGGGTACCTACT

AAGGTTATACCAGCTAGTGCAAGAAGCCCACCGCCTACCGGCAGGAGGGTGATCACAGCTAACACTTTCT

GAGCTGATGGACCTTTTTGTTGACCTTTGAAGGCAGCTTCATAGCGGTGTTGTG

>FM895253.1 FM895253 Jatropha curcas embryo 71-95 (DAF) Jatropha curcas cDNA clone rjcpga0_002049, mRNA sequence

TTTTTTCTTTTTCTACACTTATTACCTGCTTCAACATCTTTAACTATATCTGTACTTACTTCAATATCAC

AAACATTATCAACCTTGGCAGTGAGTTCTTTATATATATCCTTGTCTTCTTCTTTTTCTTCCTCTTCATC

TTCTTCTTCTTCTTCTTCTTCTTCTTCTTCTCCTTCTTCTCTGATAACTATCTGATTCTTTTCTTCAGCT

TGCCGAGAAAGAGACAAATTCCCACCTAACAGGTTCCGTTCAAAAGCACGAATTCTCTGAGTGCATCCAT

TTCTATACAGCTCAGGCTCTTTGCTTCTCATCACTATGGAGGCAAAATCAGGACTGCAAACAAAACAATT

GTCTTTATGACAATCATGTCCACTATAGCATTTGCTGCCATCGAAAGTCCCGTTGAAAGTTGAATTCTTC

ATCTCATGAGTTATTACTGGATAACATTGTGAGGCTGGCAGAA

>FM895252.1 FM895252 Jatropha curcas embryo 71-95 (DAF) Jatropha curcas cDNA clone rjcpga0_002048, mRNA sequence

GCACAATATCAAAATCTTGATAAACATTGCAAATAGGTTTATTCTGCATCTATAAACAAATTTTCATGTC

AATGTTTCAGGATCCTCACTCTTCACAGGAGTCCAGCTGCCTTTTGCGGTGTTTTGCTTCCTGTAGTATG

AAATACACCCAAGAACGTAGCCAATAAACGCCCCAAGCACCAGGACACTCGCAGCCTTCATATACTTTGA

AAATGGAGATTCAGCCACTACCTCAATCCTAGTTCCGGTAGCATCACTAAAGTCATCACTACCACTGCTA

ACCTGGCCGGATGCCCAAATCTCCACCATATCATGCACCTCCCCGTCATGGTTCCCTACATAAGAAAGTG

TCAGCTTCTCCCTTGTGGCAAACAATCTAGTATACCCAAATTCACCCCCGCGGTACATCGATCGCTCAGG

CTGTGGAAAGACAGGAACATCTGGGTGGTCAGGTCTGGGTTCCCAGATAGGCTGCCAGTCTTGTCCTGCC

ATCCCAATTACAACATG

>FM895251.1 FM895251 Jatropha curcas embryo 71-95 (DAF) Jatropha curcas cDNA clone rjcpga0_002047, mRNA sequence

CGAAGCAGAATGACTAATTTCGTTTAATTGAGATGAATTTGTAATGTTCAGCAGCTCAGCTTCTGCAATA

AGTTTACAGTCCCATCCATGAGTATTATAACATCAACTAGCTTAAAGATTAAAGTTTCACAGTCTACAAG

TGCCACCAAACCCTTTTGGTACCATTCATAGTTAATAACACCATAATTTTAACA

>FM895250.1 FM895250 Jatropha curcas embryo 71-95 (DAF) Jatropha curcas cDNA clone rjcpga0_002045, mRNA sequence

CTTTGTTTATGGGATTCCTNGAGAAAGGAAAGCCAGTGACTGGGGCATCAGATAATCTTCGAGAGTGGTG

GAAGGATAAAGTAAGGTTTGATCGAAATGGTCCGGCTGCCATTGCCAAGTATCAAGCGGATAATTCGATC

CCAGGCAAGAATGAGGGTTGTAATTCAATTGGTCCAACTCCACATACCTTGCAGGAGCTTCAAGATACTA

CTTTGGGTTCGCTTTTGTCAGCTTTGATGCAGCACTGTGATCCTCCTCAGAGGCGGTTTCCATTGGAGAA

AGGTATTCCTCCACCATGGTGGCCCAATGGAAATGAAGAGTGGTGGCCTCAATTAGGCTTACCTAAGGAA

CAGGGTCCTCCACCTTATAAGAAGCCTCATGACTTGAAAAAGGCATGGAAGGTTGGGGTTCTTACTGCAG

TGATCAAACATATGTCTCCAGACATTGCCAAAATTCGCAAGCTTGTGAGACAGTCAAAGTGTTTACAGGA

CAAAAATTGACAGCTAAGGAG

>FM895249.1 FM895249 Jatropha curcas embryo 71-95 (DAF) Jatropha curcas cDNA clone rjcpga0_002044, mRNA sequence

GCAAAGGATTCTATCCGTCGCCCGATGGGAATTGTACTTTAAGGCGGCCCTCGCGGCTCGTCCGCCGCGG

GGGCTTGGCCAACGGCACGTGCCTCTGGGGGCCGGATGGCCCCTACTGCGGGTCGGCAAACGGGCGACCG

GACACGCGCGTCGCTTCTGGCCCGGATTCTGACTTAGAGGCGTTCAGTCATAATCCAGCGCACGGTAGCT

TCGCGCCACTGGCTTTTCAACCAAGCGCGATGACCAATTGTGCGAATCAACGGTTCCTCTCGTACTAGGT

TGAATTACCATCGCGACACCATCATCAGTAGGGTAAAACTAACCTGTCTCACGACGGTCTAATCCCAGCT

CACGTTCCCTATTGGTGGGTGAACAATCCAACACTTGGTGAATTCTGCTTCACAATGATAGGAAGAGCCG

ACATCNAAGCATCAAAAAGCAACGTCGCTATGAACGCTTGGCTGCCACAAGCCAGTTATCCCTGTGGTAA

CTTTTCTGACACCTCTAGCTTCAAATTCCGAAGGTCTAAA

>FM895248.1 FM895248 Jatropha curcas embryo 71-95 (DAF) Jatropha curcas cDNA clone rjcpga0_002043, mRNA sequence

AATCAAGAAAAATTCACTCTTATTCAAATCTGTATCTGGGACAGTTACAAATTTATTGTCTCCAGAAATT

ACAACGTAACTTAACAGAACATACCCCAAACATCCAACTTTTTGTTTATATTCCATCTAATGGAAATATT

AAACAGGTGCTTACTAAGTAGATAAACAAAGCTAAGCAAAATGTACTAAAGCTGAAAAAGGGTGATAGCT

ATTAAGGAAGTCTTATTGCGTTTACAGAAGACTAAACTATTCAAAGTTTAGAGGTAGCAAGCTGTATCAG

ATCCTTCCTGAGGATCTTGCCTGATGCATTCTTAGGGATGGCAGAAATGAATGCAACTCTTCGGATTCTC

TTGTATGGAGCCACCTGTCTTGCTACAAAATCCATTACAGCAGTCTCTGATAAGCTACTTCCAGCCTTTC

TTACCACATAGGCCATGGGAATCTGTCCAGCCTCCTTGTCAGGAAGTGGTATCACGGCAGCATCAGAAAT

GTCCACATGAGTCAGCAACAAGGCTTCTAGTTCCGCTGGGGGAACCTGATAT

>FM895247.1 FM895247 Jatropha curcas embryo 71-95 (DAF) Jatropha curcas cDNA clone rjcpga0_002042, mRNA sequence

TTTTCTCTCTCTCTCTCTTAAACGGGTCGGACTTTCTGCGGACATGGACGCCGGAGCTAAAGTCAAAAAG

GGTGCCGGTGGTAGGAAGGGTGGAGGCCCGAAGAAGAAGCCTACTTCTAGGTCCGTTAAGGCTGGCCTTC

AGTTTCCCGTTGGGAGGATCGGTCGTTACCTGAAGAAGGGAAGGTATGCGCAGCGGGTCGGATCCGGAGC

TCCTGTTTACTTGGCTGCTGTGCTCGAGTACCTAGCTGCTGAAGTACTTGAGTTGGCCGGTAATGCTGCC

AGAGATAATAAGAAGAATAGAATAATACCAAGGCATGTATTGCTTGCCGTTAGGAATGATGAAGAGCTTG

GAAAGTTGCTTGCAGGAGTGACAATTGCTCATGGAGGTGTTCTTCCCAATATTAACCCTGTTCTATTGCC

CAAGAAGTCAGAGAAGGCAGCAACTAAGGAACCCAAGTCTCCATCCAAGGGAACAAAGTCTCCAAAGAAA

GCTTAGGCCATTCCAGTTATTATTCTCTTATGCTCTTGTAGTATGTAG

>FM895246.1 FM895246 Jatropha curcas embryo 71-95 (DAF) Jatropha curcas cDNA clone rjcpga0_002041, mRNA sequence

ATTCCTAAAATTAGAGGCAAAGTCTGGGTCTACGGAGGAGAAAAAGAAGTCGCTCTTTCTTTAATTCCCG

TCTCCACCTCCAAAACATCCATAGCGCAATAATCATTAATCAATGGCTATCTCCCCTGTTTTCATCACCA

AAGAATCACTGCGCTCCATCCTCTCACACAGCTCCTTAATCCAACACTTCCATACATCTTTACCCACCGT

CTCGCCCACACTCCAAACTCCAATCCGCCAAAGCTACGCCGTTTCACCATCCTCTTCTCTTCTCCTAATG

CCTTCTTGGTCCTCCTCCCCTTCCCTTCCTTATATAGGCGTAAAGCTTGTCACTCACTTCCCGGAAAATT

CCATCATAAACTTACCCGGAATCCATGCAAGTTATGTGCTTTTTAGCTCCACAACTGGACAAACTTTGGC

ATCTATGGATGGCACTGAGTTAACCCTATATAGAACAGCTTGTGTTTCTGGCTTGGCCTCAAAACTCTTG

GCTAATGATAGTAGCAAAGTACTGGTGATGATTGGTGCAGGCTCTTTGGCTCCCCATTTGATCAAGGCCC

ATATGGCTGCAAACCCCAGTTT

>FM891647.1 FM891647 Jatropha curcas embryo 56-70 (DAF) Jatropha curcas cDNA clone rjcaeb0_001990, mRNA sequence

CAAGAAATAAAATTTATTTCAGTAAACAGAAACATACAGCCAACCAAAGCTATGGGCAAACAAACAAGTC

TTATTACATATTTATGAAATCCATCATCAAACTCAAACTATATTAGCTTATATTATTTACCAAACAACAA

CTACTGCTAGACAACCTGATCATGTCTTTGCAACTTCCTGGGCCTTCTGGCCCACCTCCCTAGCCTTCTG

GCCCAATTGACCCGTCGTTTCTTGCGCGCGCCGCTTTGCCATTTCCATCTGCATCGTCCAAGATCCTCTC

ATCCTGCGAATATAATTAACCATCCACGACAGTGAAGAAAGCGCAGTGATTC

>FM891646.1 FM891646 Jatropha curcas embryo 56-70 (DAF) Jatropha curcas cDNA clone rjcaeb0_001988, mRNA sequence

ATTCCACACTAAACCATAATACGTTCTCTCACCGATCTTTGATCTTCTCCAGAAACCAATCAATGAGAAT

GTCTCTGATTCCAAGCACCCTCTTCGGTGGCCAGGAGAACCGGCATTTTCGATCCCTTCTCTCTCGATAT

CTGGGATCCATTTCAGGACTTCCCTTTCACAAGCACTGCTCTTTCTGCTCCTCGTTCTGAGTTATTGAAC

GAAACGGCTGCGTTTGCCAACACCCGCATAGACTGGAAAAAAACACCAAAAGCTCATGTATTCAAGGCTG

ATCTTCCAGGGCTTATAGAAAAAGGAATTGAAAGTGGAGATAGAGGAAGGTAGGGTCTTGCAGATAAGCG

GATAGAGAAGCATAGAGAAAGAAGATAAGAATGATAAGTGGCATAAATTTGAAAGGTCAAGTGGTAAGTT

CT

>FM891645.1 FM891645 Jatropha curcas embryo 56-70 (DAF) Jatropha curcas cDNA clone rjcaeb0_001986, mRNA sequence

ATTCGTTCCAACACATTTTGCTGAGTTCATCACGGGCAATGAGGCTTTGAGGAAGTCCATTGAGGGAAAA

ACCTTACTGAACAGGCTTGGTACCACAGGAGACATGGCCTCCGCAGCTGCATTTTTGGCATCTGATGATG

CTTCTTACATAACCGGAGAAACTTTGATTGTGGCTGGAGGAATACCCTCTAGACTTTAGTTTTCTTCAAT

GGATTAGCTTTAGTCCACAGTAATAAACAAATAAATATCCTAGCAGAGAGAATTTTTCTTGCTGCTTTTG

ATGGATTAGCTTTCATTTTTCATGCGTTACATGAATTATGTTACAAACAAAATAGGTACGACTTTTTATT

TTCCATGAGAAAAGTAAAAAATTTCTTTTATTGTCTGAAAAAAAAAAAGGGGGGGCCCGGTAC

>FM891644.1 FM891644 Jatropha curcas embryo 56-70 (DAF) Jatropha curcas cDNA clone rjcaeb0_001985, mRNA sequence

AAACTCAAAATCATTAGTTAATTTAACTCTATAAGAAAATTTTAGTCGAGATTACATACTAAATAAGAAC

TACCCAATAATTCAAAACGGAAACAGGCAACACCAAAATAAAATAAGCATCACTAAATGCTGCAAAGAAA

CTCCATGAAGAGATCTGCAATCTGGCTGTAGCACTCATTTCTTTTTCTCTGCAGCTCCACCAGCTCTCAT

TTTCCGAAGAACAGAAGACATTTCCTCACGCTTCTTCTTGGCTCTCTTATGAGTACCCAACTTTCTCTTT

GCCACCTTCAGTGCACGCTTATCTTTTCCAACCTTTAGAAGCTCAGTGATCCTCTTCTCATAAGGAGCAA

AACCAGCCACCTCCCTGATATGTCCTC

>FM891643.1 FM891643 Jatropha curcas embryo 56-70 (DAF) Jatropha curcas cDNA clone rjcaeb0_001984, mRNA sequence

TCCAATTCATTTGAATAGCCAAGTGAGTGAAAATGGAGTCGCTGAAGGAAACCGCAGCTAACGTTGGAGC

CTCTGCTAAGGGCTGGCATGGAAAAAACCAGAGCAGAGGCGCAAGAGAAGGTGGAGAGAATGTCAACACG

TGATCCAGTGCAGAAGGAAATGGCGAGAGAG

>FM891642.1 FM891642 Jatropha curcas embryo 56-70 (DAF) Jatropha curcas cDNA clone rjcaeb0_001982, mRNA sequence

AATCTTAAAAGGAACTAATATTATTATCGTTATATAACAGAAAATACCTGACATAAAAGATTCAAACACA

ACCTCATAGATTTAATACAAACAGTCATACCGAGCAAAATTACAGAACAAACGACATTAATTCTTTCTTC

ACCATCTCTGTTAACATATTTTTTGCTAATATGTGTAAGTTT

>FM891641.1 FM891641 Jatropha curcas embryo 56-70 (DAF) Jatropha curcas cDNA clone rjcaeb0_001979, mRNA sequence

ATGGCTTCTCTCAAACTCAGAATCTCACTATTTCTGATGCTTCTTGCCACTGCTTTGCTTTCTTCTCCAG

TGCTCTCTGATGGTACAGATGAAGATAAGATTCTTCAGGGCATAAACAGTTACAGGACATCCTTACGACT

TCCATCGCTTGCCCAGAATGATAAAGCATCCTGTCTGGCCGATAAAATTGCAGACAAACTAGAGGACCGA

CCATGTCCTCCAACTCCCACCACAGTCCAACAACAAGATTACCCTGAATTACTAGAAAAATGTGGCATTG

ACAGTAACCATACTACAGACGGTGTCGTTTTGCCTGTTTGTGTGCCTAAATTGGTTCAAACTCTTGTTCT

TACCAATTATACACGCACGGATCATGCCAAGTACTTAAATGACTCCAGGTTTACTGGAGCAGGACTTGGG

TCCGAGGATGATTGGATGGTCGTGGTTTTGACCACCAACACTCCCGGAGGTAACTTCGCCGGAGCTATGA

GTTTGTTTTCTAAGGTTGGTTTTGGTCATTGCTTGATGTGTTTGTTGCTTGGAATGCTTGTTTATGTAGT

AGTGTAGTAGTAATTGAAGTGCTTCCTATATGATTTGTATGTGATCATGTATTTTGTGTGATCTCGTAGC

CGAAA

>FM891640.1 FM891640 Jatropha curcas embryo 56-70 (DAF) Jatropha curcas cDNA clone rjcaeb0_001978, mRNA sequence

ACATACATAAACTCAGCATTCTATTTAATTATTATTAACTACGCACCATAATGTGCTTATAATTTTTTAT

TTTCACTAATAACAAAATCACTCAAATCTCTAGCAAAACCACCAGAGCTGTGACTTTTATTCTTCTACCA

GTACAGCATTTATCCTACGCCTTGCCACCTTACGTCCACC

>FM891639.1 FM891639 Jatropha curcas embryo 56-70 (DAF) Jatropha curcas cDNA clone rjcaeb0_001977, mRNA sequence

CGAATTCCTTCTTGGTTCTATTGTTGCTTCTCTCTTGTTGAGGCTTGTCACTAATGGGATGAGACCGGAA

GGATTTCATGTTACAGCAGGAGTTGGAGAGGTTCATGGGCTTATAATGGAGATTGTAATGACATTTGGAC

TGGTTTACACAGTTTATGCAACAGCAATTGATCCCAAAAGAGGAAGTTTAGGAATCATTGCCCCTCTAGC

CATCGGTTTAATAGTTGGGGCTAACATTTTAGTGGGAGGTCCTTTTGATGGTGCGGCAATGAATCCAGCA

AGAGCATTTGGGCCTGCGCTGGTCGGATGGAGATGGAGTAACCACTGGATTTACTGGGTTGGTCCTTTCA

TCTGGAGGAGCCCTGGCTGGTCTCATTTACGAGTACATGGTCATCCCAACAGAGCCGCCACTACATCATA

CCCATCAGCCTCTGGCTCCTGAAGACTACTAAATCCTTTGGTTACAAGTTAATTAATTCTGCTTAGACAA

ACGA

>FM891638.1 FM891638 Jatropha curcas embryo 56-70 (DAF) Jatropha curcas cDNA clone rjcaeb0_001976, mRNA sequence

ATTCATACAACAAATGGGCTGAACGTTCACAGCCACACCAAGTTCAAGTGCACCCACAACACCGCTATGA

AGCTGCCTTCAAAGGTCAACAAAAAGGTCCATCAGCTCAGAAAGTGTTAGCTGTGATCACCCTCCTGCCG

GTAGGCGGTGGGCTTCTTGCACTAGCTGGTATAACCTTAGTAGGTACCCTAATCGGCCTAGCTATTACTA

CCCCTCTTTTTGTCATCTTTAGCCCTGTTCTTGTCCCAGCTGCATTAGTTATTGGCCTTTCGGTCATGGC

ATTTTTGGCTTCCGGGGCTATGGGGCTCACAGGGTTGTCATCACTCTCGTGGGTTCTGAAATACCTCCAG

GAGGTCACTCGGCGAATGCCGGAGCAGCTGGATATAGCTAAGAAGCGTATGCNAGACATGGCTGGTTTTG

TGGGACAGAAGACCAAGGAAGTAGGCCAAGAAATCCAGAGGAAAGCACATGAAGGGAAATAAGTAAAGAG

AAATGCTGTTGG

>FM891637.1 FM891637 Jatropha curcas embryo 56-70 (DAF) Jatropha curcas cDNA clone rjcaeb0_001975, mRNA sequence

ATTCGAAGAACTCCTTGATTCTATTGAGATTGGAGTAGAACTTTCACTTATTGATGGAAGAAGTGGGGTG

GGATAGCTTAGCAAGGTTTAGAGTTAAGGTGGTGC

>FM891636.1 FM891636 Jatropha curcas embryo 56-70 (DAF) Jatropha curcas cDNA clone rjcaeb0_001974, mRNA sequence

TCCGCCTGCTCATTGGAATCTCATTGGCGATCTTTCTCCCTTTCCGATTTACAGTCTGCTGCTGCTGCCA

GCAACTTTTGATAACTGATTTGACGTATTCTAATTGAAGATATTGACAACATACGACATTGACATATAAC

TTCTACTCGACACTGAATCGCCAAGGAAACCATGGTGTTGAAAGGCGTTATAGCCGGCTGTCTACTCTTT

CTAA

>FM891635.1 FM891635 Jatropha curcas embryo 56-70 (DAF) Jatropha curcas cDNA clone rjcaeb0_001972, mRNA sequence

ATTCTCAAAAACCTGGATTACAGAAGCCAAAATAGCATAAGGTGGAACTTCTGGCCCATTTCTCTCCTTT

TTTCATAGCAGCTCATCAAAAGGTTTGAATTTCATAGTTACATGATGCACACTCTTCTCTCTGTACAAAC

TAAACATCAAACATTATACAAGAGATAACAAACTGCACAAAACATTATCAGATGTAAATATGGCTCACTA

GACCAATTTATTGAGATGGGACGAAGAACACCAAGGAATTGAATGCTTAGCCAGGTTTTCTAGCAGAAAA

CATCACAAATTGCCTATTCCTTGTAGCTGTAGATCCAACAAGCCCACAGGCTCTGCATATATACTTCTAG

TTCACGCTCACGAAGGAAGATTTGACTGCCTGATACTTGTGCAATATTCTGGCGCAGTGGACCCAAAAGT

GGGACAACATTGAAGGGCCGATCAAGTATATAGGTGCTAGCAACAAGAACACCTCCAGGTCTCAGAACTC

GGCTTATTTCAGCTACAGCCACTGATGGTGAAGGCCGGCAATGTATAGCAGCACCAGCATGGACAGCATC

GA

>FM891634.1 FM891634 Jatropha curcas embryo 56-70 (DAF) Jatropha curcas cDNA clone rjcaeb0_001971, mRNA sequence

ATTCCACCCTTGTTGTTGTGGTTTCTAAAACTTTTACTACTGCTGAAACTATGCTGAATGCACGAACACT

AACGGCATGGATTTCAAAAGAACTTGGACCTTCAGCAGTTGCAAAGCATATGGTTGCAGTTAGCACAAAT

CTTACACTTGTAGAAAAGTTTGGTATTGACCCTAATAATGCTTTTGCATTCTGGGACTGGGTTGGTGGGA

GGTATAGTGTTTGCAGTGCTGTTGGTGTGTTGCCTTTGTCTCTCCAATACGGTTTCTCAATTGTTGAGAA

GTTCTTAAAAGGAGCATCAAGCATTGATCAACACTTCTCTTCAGCGCCATTTGAGAAAAATGTTCCTGTA

CTTCTAGGTTTATTGAGTGTATGG

>FM891633.1 FM891633 Jatropha curcas embryo 56-70 (DAF) Jatropha curcas cDNA clone rjcaeb0_001970, mRNA sequence

ATTCTGCAGATATGCTCGTTTACGATTTCGACTTTGTCACCGTGGATAATTGCCGGAAAAGCAAGATCTT

CTTCATTGCATGGTCTCCAACAGCATCAAGAATTAGAGCAAAAATGTTATATGCAACCTCCAAAGATGGA

TTGCGGAGAGTGCTTGAAGGGATTCACTATGAAGTCCAAGCAACTGACCCAACTGAAATGGGATTTGATG

TTATAAAAGATAGAGCTAAATGAATCTAATTAATTAGCATAATTATTATATTATTAGCTAAATTACATAT

AATAATAATAATAATAACAACTGTGTTCTACCATTTAATATATGATCTTACTTTCTATTT

>FM891632.1 FM891632 Jatropha curcas embryo 56-70 (DAF) Jatropha curcas cDNA clone rjcaeb0_001969, mRNA sequence

ATTCCTCGTACACAACATCAATGACCCGTCACGTGCCGATGTATTCAACCCACGTGCTGGCCGTCTCACC

AACATCAACGGCCAAGACCTCCCCATCCTCAGTTATATCCAGCTAAGCGTTCAGAAAGGTGTCCTCTACA

GAAATGCTCTAATGTCACCACACT

>FM891631.1 FM891631 Jatropha curcas embryo 56-70 (DAF) Jatropha curcas cDNA clone rjcaeb0_001968, mRNA sequence

GCATTAAACTTGTCTTTTTTCTTATAATTATTATTATTATTATTACTTATCATTATAAACTTTACTATAC

ACAATCTCAGGCTCTATGCCACATCACATCTTCATTTTGCATATGCAACCTTAAATTAAAATCAACCGCA

AACCAAAAAAAAACAAGGAAAGAGGTTACAATCTTTACTGTATATCTACATCGATAACCTTGCGTTCTAC

TTTAGTCTTTGGAATAGAAATAAAGAGAACTCCATTCTTCAACTCGGCCTTGATTTTATCCTTTTCGCAG

TTATCTGGTAGCTTANGACGGGTGTCGTAAGAGCTTAGACTCCGGCCAAACCAGAATCATCGCGGGATTC

TTCCTCCTTGTGCTCTCCCTT

>FM891630.1 FM891630 Jatropha curcas embryo 56-70 (DAF) Jatropha curcas cDNA clone rjcaeb0_001966, mRNA sequence

AAAAGACAATATTAGGTTTCTAATTGGGAATTCAACGATAATAATAGTTGCTGCTACAAGTTTAATCTCT

CATCTCATAAAAAATACAATCTCCAGACACAAGGAAAAGAAAGAGGGGGGAGACAAGTCTGATCTGAATA

GAAAGTTTGGATTGCAAAATTTTAGGCAGCCCCAGCTCCCTTGCCCTTCTTCCTCTGGATCTTCTTCATG

TAGAACTCCAGCTCCTTACCCTCCAGAATATACCCATCAGCACGGCCAGACTGACCAGGGCGTGAGTGAG

ATACAAGCGATTAGAGGACCACCAGCAAATTGTTCTTCAATATGAGGATCAAGCTTTAGAGTCTGCTGTC

TCTTCTCCAGCTTTCTCAAAACATGATTACTCTTCTTCACTTCCTCAGTAGCACCTTCACCTTCCTCTCC

TTCCTTCTTGGTAGCC

>FM891629.1 FM891629 Jatropha curcas embryo 56-70 (DAF) Jatropha curcas cDNA clone rjcaeb0_001963, mRNA sequence

ATTGTTGCCATTCCTTGGCAGAAAGGTGATTTTCTGTTGATAGATAACTGGGCTGTTCTTCATGCCCGGA

AACCCTTCAATCTGCCTCGTCGAGTACTAGCTTCCCTTTGCAAGTAGTTTCCTTATCACCATGCTAATAG

CAAGGAAGCAACNTAGTGGCAAAAAATAGAGCTTGTACCACCATCATATTAAAAATACCAAGATGTTGAA

TTCAGATGCCTTAGTTCTTCAACTAAAGGTAATTAATTAGGTAAAAAAA

>FM891628.1 FM891628 Jatropha curcas embryo 56-70 (DAF) Jatropha curcas cDNA clone rjcaeb0_001962, mRNA sequence

ATTCTCTACATTACATATCTTTCTCACACTTATCATCAACAAATGGCTGAACGTTCACAGCCACACCAAG

TTCAAGTGCACCCACAACACCGCTATGAAGCTGCCTTCAAAGGTCAACAAAAAGGTCCATCAGCTCAGAA

AGTGTTAGCTGTGATCACCCTCCTGCCGGTAGGCGGTGGGCTTCTGCACTAGCTGGTATAACCTTA

>FM891627.1 FM891627 Jatropha curcas embryo 56-70 (DAF) Jatropha curcas cDNA clone rjcaeb0_001961, mRNA sequence

ATTCCATATATTCAGGCAAAAGCAAGGAGCTATTGTATAAGCCTCCAGGGAGCAAATTCAAGCTATGAGT

CATCGTGAAGAAGGTGGCGGCGTTTGGCCTTTCGGAACTGAATCCATCGGTCCAATTAGTATCCTGAAGA

ACCCTGTTTTTAGGAATAACTATGGTCAACTCGTATGAAGTGGCTCTAGAGATACAAGCAACGGCTCCGA

GA

>FM891626.1 FM891626 Jatropha curcas embryo 56-70 (DAF) Jatropha curcas cDNA clone rjcaeb0_001958, mRNA sequence

TGAGAGCCATGCCTCTTGATTCTGATGAACTCTTACAGAATGTCTCCAAGCGAAGCTCATCAAGTGAAGA

GGAACAGTGGATCCACAGTCCATGTTTGTTTACTCCACACCAGACAGTGAGACACATAAGTAATGTTACA

TACATAAAATATGCATTGTGATTGGTGCTTCTACTAGCCAAGGCCGCAACTAAGAGATCCTTCTTGTAAA

TTTAGAGAAGGGATCCGTCGTATATTGTAGTATAGAGAGTTGTAGAGTG

>FM891625.1 FM891625 Jatropha curcas embryo 56-70 (DAF) Jatropha curcas cDNA clone rjcaeb0_001955, mRNA sequence

TTAATTCTTATAATTAAACAGCAGACACTTGATATGCAATGTCAATGAAACTACATATTTACAGGCACAG

AAACATCTTATTGTTCCCTATTAACAGTTTATACCACTTAATCAACAAATTCTTTTATCAATCAAATTCA

AATACCA

>FM891624.1 FM891624 Jatropha curcas embryo 56-70 (DAF) Jatropha curcas cDNA clone rjcaeb0_001954, mRNA sequence

GGCTGCAGGAATTCGAATTCCTCTCTGCAGAGCAAGGAGAAGAAGGTGGCGGCTGCTAAGGAGACTAAGG

CGAGGTTCGAGGAGCGGTTCAAGACTGGAAAGAATAGGTGGTTCTTTACCAAGCTCAGGTTTTGAGGATT

CATTTCTTAAGAAGTTTTGTTTGGTTTCTTTATCTTTTATAA

>FM891623.1 FM891623 Jatropha curcas embryo 56-70 (DAF) Jatropha curcas cDNA clone rjcaeb0_001953, mRNA sequence

AGAGAGTAGAGCACCAAATTCTAGCTACTGTTCAACCAAAATATCAGGACACAATCTGTAGAGCATACAA

AAAGGTGAAAGAATGTGTGAAAAACACGGTTGAGATACAAAAACCAATACATACATCTAAACTAGCATAC

AAAGTAGCCATAATGTATACTTCTCCCCTGTTAGTAAAACATAAAATTAGACAATGTGAACTGCCACAAA

GTGACATTCAAGGAAGCTTCACTGCAATCAAA

>FM891622.1 FM891622 Jatropha curcas embryo 56-70 (DAF) Jatropha curcas cDNA clone rjcaeb0_001949, mRNA sequence

ATTCGGTTGATTGAGCTTTCTCCAGGATTGAGATTGCTCATGTAGCTGACCTAATTGAATTGCCTATAGA

TCATGTAGAGAAGAAACTGTCTCAGATGATTCTGGATAAGAAGTTTGCCGGAACTTTGGACCAGGGTGCT

GGATGCCTCATCATTTTTGATGATCCCAAGACAGATGCAATCTACCCAGCAACATTGGAGACTATTTCCA

ATATTGGCAAGGTTGTCGATAGCCTGTATGTGAGGTCGCCAGATAATGGCATGAATTTCTGATGTGGGAC

GCTCGAATAGCTGTTGTGCT

>FM891621.1 FM891621 Jatropha curcas embryo 56-70 (DAF) Jatropha curcas cDNA clone rjcaeb0_001947, mRNA sequence

ATTCGGCAAGATTGCTGATTCTGAGACTGTTGATCTCTTCTCTAGTGCTCATAATATTCCCAAATCATCG

GACTCCCGCCACTTCCACTGATCAATCTCAGAGCCACCGCCCCTCGAATGCCGCCGGAGGAAATGCTACC

AATAACAATCCCACCACTACCAATGATTGAGAGTTTTATTTTGTTTCATGTAATCTGAGAGCGTGTCGTA

GAAAAAAGAAGTGTTAGAATGTTTCTTCTGCTGCTCTCCAGAAACTATATGTGTAATAATAATTTAGTTT

AAGAAATACCTAAGCATAGCAATATATAACAAATTCATTAGATAGTTTTGTAATAATTCCTG

>FM891620.1 FM891620 Jatropha curcas embryo 56-70 (DAF) Jatropha curcas cDNA clone rjcaeb0_001946, mRNA sequence

ATTCCTTCATTCTGCTTGGAGGCGCCTCTCTTTCTCTGCTTTCTGCATCCGCAACCATGGTGAGAGTTAG

TGTTTTGAATGATGCTTTGAAGAGCATGTACAATGCTGAAAAGCGTGGGAAGCGACAGGTTATGATCAGG

CCTTCCTCAAAAGTGATCATCAAATTTCTGCTGGTGATGCAAAAGCATGGATACATTGGCGAGTTTGAGT

ATGTTGATGATCATAGAGCTGGTAAAATTGTGGTTGAATTGAATGGGAGGTTGAACAAGTGTGGAGTTAT

CAGCCCTCGTTTTGATATCGGTGTC

>FM891619.1 FM891619 Jatropha curcas embryo 56-70 (DAF) Jatropha curcas cDNA clone rjcaeb0_001945, mRNA sequence

AACTTAGAATTTATCTCTTTATTAATTATTAAACCAGAAGCCACTATAGTCCGCAGCAGTGATCTTTCTC

TTTTCTTAAAGACTAATTTACACATGTACCTTCACCTTTCATTATTTATTCACCATACACAGCACTATTT

ACATTAGTAAACCCTCAAGGTCGATGTCACAGTCACCCCAAAAAGCCGAAACACTTGCCATCACGTTATT

AGCTTCTCATGCCACAGCATGACCCTCGAAGGGACCTCTCCTTGGCCCTGGAAAGAACCACTCCTCGTTT

TGTCTTCCAAAAGCCTGATCCACTTCTCTTTCACTTACACCGAAGGCTAACTCTTTGGCTTGCTTCTCCA

TCTTATTTACAATATTATTTTTCCCTGCAAGTAGATGCCTAGTATTGCCTTCTTCATTAATCTCAAAGCA

GACTACTTCCAGATT

>FM891618.1 FM891618 Jatropha curcas embryo 56-70 (DAF) Jatropha curcas cDNA clone rjcaeb0_001944, mRNA sequence

GCCATAAAGCATAAGTGAAGTAATGTCATAATGCACTCTCATGGATCAAAAATTTTCAAGATAACATATA

TAATCCTTAATGTCTAACTTAAAAAGATTGAGACAGCAAATTTCATTCCTTAGAAAATTTAACGAGCAAC

TTCAAAACAAAACATAGAACAGAAAGAATTCGACACTTAGGTCCTAGCAGCAACACCGGCACCTGCAGGA

CGAGCTACTTGACCGGTCTGACCTGGCTGTCCAGGCTTTGGCAGGTCATCCCTGCGCCTGAGGAAGCGCT

GTGGTGGTTCACGGTTCCTGCGCTCAGTGCG

>FM891617.1 FM891617 Jatropha curcas embryo 56-70 (DAF) Jatropha curcas cDNA clone rjcaeb0_001942, mRNA sequence

ATTCCANGATACTCACAAAATGAGTCCTGAAGAAGTGAAGGCAGCAGGAGTAGAGGGCTCCAAAAGGCCA

CCAGGCCATAACCCAGGAGGGGTCTTGCACCAGAGGCGCAATTTGCCTTTCAGCAAAACCACCATTACAG

TAGCTGGCTTCTTTATCACTGCTGCTATTGGATACATGGTCTTGTATGCCAAGAAAAAGCCTGAAGCAAG

CGCTAGAGATGTTGCCAAAGTTGCCACTGGTACTGCTGACCCTAGAGATACTCATCCTAGGAAGTAAATA

TAACTGTAATTTTGTGTTTTTTCTGTTCTGTTCGTTATCTAATATATGCATTACTTTTAGTGTTTAAAAA

AAAAAAAAAAAAAAAAAACTCGAGGTTTTTTTTTTAATTTTTTTGACAGACAATGGAAATTTATTTCCAG

TGTATATCCATGTAATACAACAAGGTCAGAGTCAATCAATTATTGGAGC

>FM891616.1 FM891616 Jatropha curcas embryo 56-70 (DAF) Jatropha curcas cDNA clone rjcaeb0_001941, mRNA sequence

ATTCCTACTCTTTTTCTCCCTTCCAAGAAAAAGCTTATTACGATATCTATTTTAAGCTTCCATGGCCGAG

TATGAGCGACGACCAGGCGGAGAAGGGCACAAAGGAGCCCTAAAGGAGAAGGGCGGCTTCTCGACCTCAC

AGATTTTAGCGGTTGTCACTCTTTTGCCGGTGAGTGGGACGCTTCTTTTCCTTGCTGGAATAACGCTTAC

TGGAACACTCATTGCGCTAG

>FM891615.1 FM891615 Jatropha curcas embryo 56-70 (DAF) Jatropha curcas cDNA clone rjcaeb0_001940, mRNA sequence

CCTTGCTCTCTGCAGCAATTGCAAAAACAAGGGTTTTCCTAGCCAACGGCTATCAATCATGATTATTTCA

GAGAAGAACAGGAGGGAGATCTCCAAGTATCTCTTTCAAGAGGGAGTTTGCTACGCAAAGAAGGATTTCA

ACCTTGCAAAACACCCAAATATCGATGTCCCAAATCTGCAGGTTATTAAGCTTATGCAGAGTTTCAAGTC

CAAGGAGTATGTTCGTGAGACATTTGCATGGATGCACTACTACTGGTACCTCACAAATGATGGGATTGAG

TTTTTGAGGACTTACCTTAATCTTCCATCAGAAATTGTTCCTGCTACATTGAAGAAATCTGCAAGGCCCA

CTGGTCGGCCAATGGGTGGCCCACCTGGTGATCGCCCCCGAGGCCCACCTCGCTTTGATGGAGACCGCCC

AAGATTTGGTGATCGTGATGGGTCCGTGGAGCCTCAGGGTGTTTGGTGGTGAAGGGAGTGCTCACGACTT

CAGCCATCAATAGGGCTCAGTGTAGCTGCTTGGTCGTGTGGTGTGTGGT

>FM891614.1 FM891614 Jatropha curcas embryo 56-70 (DAF) Jatropha curcas cDNA clone rjcaeb0_001939, mRNA sequence

TTTTTTATTTTTTGGAGAAACTAAGATAGTTTAAAACAGTTTACCCAAGATTCTACAGATAACTTCCATA

CAAACAATTAAAAGCAGCAAATTAGACCTCTCCCTTTTGGTCTGCTCATAAGAGGCAGAGATAAACACTT

TCATCATTCATACATATATATATATATTTTAACTTAAAGACCAAAGCCACAGGGAAAGAAGCCCTCCCTA

TCACACATACAAAGCAGCATAGTTAACCCATAGCATACTTCTGGGTCCAGCTCCTTGCTGTTGCCTCGCT

ATTTGCTCCTATCAGTCTTATACATGTGGGCAATTTCTGGCACCAACGGATCGTCAGGATTTGGGTCCGA

CAATAGAGAACAAATTGAAAGCAAC

>FM891613.1 FM891613 Jatropha curcas embryo 56-70 (DAF) Jatropha curcas cDNA clone rjcaeb0_001938, mRNA sequence

ATTCCTAAGTGAGGAAAGAGAGCGGGCCGCGAGTAGCTGCAGAAGGATTGATCCATTCGAGAATCTATTT

CTCAGGGGATCGAATAGCTTCTGCAGTATCTGCTTACTCTGTACATATATCTACCAATAAAATGACTCGC

TCTCTTTCCACCGCTAAATTTCTGGTTGCTTCAATTGTTGAGGATCTACCACTTTCTATCTTCCGGAGAG

GCTATGCGGGGTGCAGCACAT

>FM891612.1 FM891612 Jatropha curcas embryo 56-70 (DAF) Jatropha curcas cDNA clone rjcaeb0_001936, mRNA sequence

ATTCAGAAGACCAAGGAAAAAGCAATTGAAACAAAGGATTACACTGCTGAGAAAGCTAAAGAAGGGAAAG

AAGGTGCAGCTAGTAAGCTTGGTGAACTCGCTGAATCAGCTAAAGGAGCAGCAAGGAAAGCTATGGACCT

CTTCGGTAAGAAAGATGAAGCAAAAGAAAAAACATCAGAAACTAAAGAAGCAGCTAAGGAGAAACTTGGC

GAAGCAGAGGAGGAAACTAGGCGGAAGATGGAGGAGTTGAAAATGGAAAAAGATGAGGCAGAGCAAAAAG

ACATTGAAGCTGAAAGGGGAAGTGCAGCAAGGGACAATATTTTTGAGAAGGCAGGATTAGGGAGCATCAA

AGACTCCATTAAAGGGAAGCTTACAATGCCACGGACTCTGGGAAACGTG

>FM891611.1 FM891611 Jatropha curcas embryo 56-70 (DAF) Jatropha curcas cDNA clone rjcaeb0_001935, mRNA sequence

TTTTTTTTTAAAACTAATAGATAAAGATAACTCTTAAAAACGAAAAGCCATTCGGAGTACTAAGCGTTTA

ACTTACAATGTCACCAAATAGTTTATTTAGGAACCCTGTTACCCAAAAAATTCTTCAGAACATCCATGAC

AGCCCCAAATTCTGCCTTAACTTGCACAGGAGGGTTAGCAAACCTGCAGGCCATATCTGGTATTGCTTTG

GAGTGGTAATCAATCTTAGATTGAGTAAATTTCAATCCATGAGCTGTGCTCACCACCACTGTCCTATCCA

TAGGCCCTATAACCCCACTATTCCTAAGCTTATTCAATGCGGTCAATGCAACTCCCGTATGTGGGCAGAT

AAACATCCCTGTAGAATCAGCTTGTGCCATTGCATCCATCAACTACTCCTCTGTGGCTTCTCAACAACCA

TTTGAATCTCAAGCTATACAGCTCTGTCTATAAACCGGGTCCCATTTGTATAGCCGAGCAAATGTGGAAT

TCGCTTTACAGGCTTAAAATCTTCCATCCGGACTTGTAAT

>FM891610.1 FM891610 Jatropha curcas embryo 56-70 (DAF) Jatropha curcas cDNA clone rjcaeb0_001934, mRNA sequence

ATTCCTCTAGAAGAGGGAATGTCTGTTGATTCAGATGATGAGGATGAGGATGAGTCTGAGAGTGAAGATG

AGGAGACCCCAAAGAAGGCTGAAAAAGGCAAAAAGAGGCCCAATGACTCTGCAACAAAGACACCTGTCCC

CTCGAAGAAGATAAAATCAGTTACTCCTCAGAAGACTGACGGTAAGAAGGGCGGGCCATACTGCAACTCC

CCATCCA

>FM891609.1 FM891609 Jatropha curcas embryo 56-70 (DAF) Jatropha curcas cDNA clone rjcaeb0_001933, mRNA sequence

TTTTTTTAACTACAAAAATTCTTATAATTCATTTAATTTGATTCCCAAGTCAGTCCGCTACATTTAAACG

AGAAAAAAAAAAAAAGCAGAATCCTTAACTCAGATAAGGAATCCTTCCATACTGTTGAGGGCTTTCTTTG

CAACCTTGAATTTGTTAGCAGCTGCATTGTTAGTTTTGATAACATACTCTCTGATGAAGAATTCAATGCC

ATGGCGAAGACTTTCCAGCTCCGGCTCTTTAGCTATACATGTAAACATGTTCCATACATCCTTGTCAGGA

TACTCAAATATGGCCTCGAATAGTATCCGAAAATGCATGATCCTCTTTGATGTCAGCTGTCCAGCATCAC

TCAAATCACTCTTTAAAATTGAAAGAGAGAGGGTCCCAAGCGACATTT

>FM891608.1 FM891608 Jatropha curcas embryo 56-70 (DAF) Jatropha curcas cDNA clone rjcaeb0_001932, mRNA sequence

ACTAAAAGATACCTTTTATTCAACTGGAATTGCTTAACAAGAAAAAGACACCAAACAGAAAACATAGATA

TATTACATAACAGGAGCTAAAGCACATGCCATGCCCTACACACGTGGGTTATGGCCAGTGGTGTCTCCGG

GCAACCCAGATGGGCGAGTCCTGGTCAAACCCGTTGGGTCCACCTCTCCACCGTATGGCTGCCCTGTGCC

GTGTCCAGGCATGGCCGACATTTGCTGGGTCCCTGTAGGGTAACCGGTTGTCCCAGTTGTTGAATGGGTA

TAGGTTTCGGTTGTGCCTTGAGCGCCCGGACCAGCAGTTGTGTACTGTACATGGCCACCAGCCTTGGCTG

CCTGCCTCGCCGCTGCGTTATCCTCGCGCGCTTCTAGTTTCCGCAATTCTGCCTCCGTTTTCCTCTCTTC

TTTCTTCTCTCTCGCCATTTCCTTCTGC

>FM891607.1 FM891607 Jatropha curcas embryo 56-70 (DAF) Jatropha curcas cDNA clone rjcaeb0_001931, mRNA sequence

GGACATGTTGAAGAATTTTATTTATTAGCAGCAACTCTACATGTAAAAAAAAAAATCAGTTAGTATCATG

CTAGCAGTTTAGCAAGTTCATCTATCCCTAGGCTTCCTAATGGTGCTGAATCCATGGATTGTTCATGTGA

TCTTTCATTTCTTGGAAGTTTAAAACTTTTCTTCTTCTTGCTTTTTTCTGTATGAGAAATGGTTGTTACT

TCTTTAGCTTCCAAATCCTTGCTTTCTTGCTCCTCATCAGACTTTCGAATCCATACCTTCCTGGGTTTTT

GAATTCTAAAAACAGGACCACGTGGAGCAGACTGGGCAAAGGCAACTGCTTTTTCATAAACATTATCAGC

ATCCAAGTTGGCATCCTCTAATATGACTGCACTTC

>FM891606.1 FM891606 Jatropha curcas embryo 56-70 (DAF) Jatropha curcas cDNA clone rjcaeb0_001930, mRNA sequence

GCAAAAGATAATTATATTGGTGGAGAAATTTGTTAGCAAAAAAGGGCAAAAATGTCTTCAAATCAATCAA

GACAAGAGCTTGATGAAAGAGCAAGGCGTGGAGAGACTGTTGTCCCTGGAGGTACTGGGGGCAAGAGCCT

TGAAGCTCAAGAGCATCTTGCTGAAGGGAGAAGCAGAGGAGGA

>FM891605.1 FM891605 Jatropha curcas embryo 56-70 (DAF) Jatropha curcas cDNA clone rjcaeb0_001928, mRNA sequence

ATTCGGTTGATAAAACCAATCCTTCATATCATTTAAAAGAGTGAATATACAGAAGTAATTGAGATAACCA

GAGTCGACAGGTAGAATGTCTCTTTATAGCCTATGACTGACCTTCAACTCCTTGGTCTGCAGTTGAATTG

CCATTAGCTGCCAGACCGCGAATCTTAACATCATAATGCACTTCCTCGACTCTTCTTAAGTCATATTTCA

TACCGTCAAACTTCTTGCGCAGAAAATCATTCCGGAAATTAAGCATGCGGAAGGCTTCATGAAGATCTGT

TAAGAACTTCAGCACCTTTCTTGGGCAATCATAGTCTCCAGCAGTTACTCGGTTCACCACATATCTAGGC

ATTTCATTTGACATGAAGCAAACACCAATAAGATAGTCCTCAATGTCCAAACCAAACTCTGAACTATTCA

ACC

>FM891604.1 FM891604 Jatropha curcas embryo 56-70 (DAF) Jatropha curcas cDNA clone rjcaeb0_001927, mRNA sequence

NTAAAAGAAAAACTTACATTTTATTAACGAAAACTCAGATTACATAATAAAAACAGCAACTCCATAAGGA

AACAAGCTCACAACAACAAACCCATTACACACATCTATTTATTTTATTGGATGGATGGGTACCATTTAGA

CTTAGACACACTCAAACATAACTAAAAGTTTCAACCAGAAATCTCAATAGCCTTAACGTCCGGTTTCTGN

ACTTCCACNTTTAGGCACTGGGTCACAGTCAAGACTCCATTTTCCATGCTAGCCTTAACCTCATTAACCT

TTGCATTCTCTGGTAGCCTAAACCTTCTCAAGAACTTACCACTTGACCTTTCAACTCTATGCCACTTATC

ATTCTTTTCTTCTTTCTCTTTGCTTCTCTCTCCGCTTATCTGCAAGACCCTACCTTCCTCTATCTCCACT

TTCACTTCCTCTTTCTTTAGCCCTGGAAGATCAGCCTTGAATACATGAGCTTCTGGTGTTTCTTTCCAGT

CTATGCGGGTGTTGGCAAACGCT

>FM891603.1 FM891603 Jatropha curcas embryo 56-70 (DAF) Jatropha curcas cDNA clone rjcaeb0_001926, mRNA sequence

GATAATGGATAGTTTTCTTCATTGATATGATAAGTCATAACCAAGACTACAGGAGTCTTTAAGACAATAA

GAGTGGTATGACGCAACTGGCAACATTTAAAAACTTCTAGTACAGTACTACTATATGCTGGTGTAAGAAA

CACACTAAAAAGTGAACGCCACCACTTCAGCATCTGAATCTCAGACTTCTACCGTGGCCTAACCCGGTTG

TGCAATAGCATTTCCCGGAGCTCAGCCGGGTCGATCTCAGCCGCAGAATTAGCTGGCCTGTAGTACCCCG

TGATGGGGTCTGGTGCCCACGCAGTAGATGATCCTGAATCATCTTCTTTCGTCGCAGCTCTTTCTTCTAC

TTTCCCCACAATACTAGTCCTTGACCCTCGCCTACCAAAGCTAGCCGACTC

>FM891602.1 FM891602 Jatropha curcas embryo 56-70 (DAF) Jatropha curcas cDNA clone rjcaeb0_001924, mRNA sequence

ATTCCACAAAATGCTGTTTCCAGAAATGTCCACTCCTCTCTTTACATGTCACCAATTCCTTCTGTGTTTC

TCCACCGATCAAGTAAAAGCTTGGAGTTCATTCCTTTAGAAAAATGTCTCAAGCTCTTTCCAATTTAACC

TTTTCTCTTCCTTTATCATCAAAAAGCACTTCTGCTCGATCAAGAAACCTTCGTTTCATTGTCAAAGCAA

AGGCAGCAGAAAATAGAGACAATCTTGATCATTTACAGAAAGCCACCAAGCAGCAGCACCATCAACACCC

CCAAACCAAGAAAAGAACGGCTCCTGTTGCTCCAGTTGGGCTATGGGACAGATTTCCTACAGCTAGAACA

GTACAGCAGATGATGGAGACGATGGAGAGAATGATGGAAGCACCCTTTTGTCTATACGGCTACTTGGCCA

TCAGAGCCACCTTCACCAGTTGAACGAAGTGGGTATAGCAGAGGAAGGACTCCATGGGAGATAAAAGAAG

GAGAAACAGAGTACAAGATGAGATTTGACATGCCTGGAATGACTAAAGAAGATGTGAAGGTGTGGGTTGA

AGAGAAGATGTAGGTTTTGAAAGCAGAGAAGGTGCCCGAGAAAAATAATGTCAATGGTGCACAAAATGGA

>FM891601.1 FM891601 Jatropha curcas embryo 56-70 (DAF) Jatropha curcas cDNA clone rjcaeb0_001923, mRNA sequence

ATTCAACAGAGCAAAAGATTAATTATATTGGTGGAGAAATTTGTTAGCAAAAAAGGGCAAAAATGTCTTC

AAATCAATCAAGACAAGAGCTTGATGAAAGAGCAAGGCGTGGAGAGACTGTTGTCCCTGGAGGTACTGGG

GGCAAGAGCCTTGAAGCTCAAGAGCATCTTGCTGAAGGGAGAAGCAGAGGAGGACAGACAAGGAAGGAGC

AGCTGGGGACAGAAGGATATCAGGAGATGGGCCGGAAAGGTGGGCTGAGCACGACGGACGAGTCTGGTGG

AGAAAGAGCTGAGAAGGAGGGCATCACTATCGATGAGTCGAAGTTCAGGACTGGGAGCTAGTGTGATGAT

TAAAACTATAAATGGTATTTTGTAGTAATAATAATAATAATGTTGGTAATAAAGTATTAGCTTAGCAGTG

TACTTTAGTTTAGTAATGTAGTTTACATGTGGGTTAAAATGACTAGGGTTTAGCTAGTATGTGTGTTGTA

ACTTTGAGAGGGGTTTATGGTCGTTCCCTTTCTGGGAGGACTGCTATACTTCTCCCTATTGTAAATACCT

GAAT

>FM891600.1 FM891600 Jatropha curcas embryo 56-70 (DAF) Jatropha curcas cDNA clone rjcaeb0_001921, mRNA sequence

ATGTGTAACAGAGCAAAAGATTAATTATATTGGTGGAGAAATTTGTTAGCAAAAAAGGGCAAAAATGTCT

TCAAATCAATCAAGACAAGAGCTTGATGAAAGAGCAAGGCGTGGAGAGACTGTTGTCCCTGGAGGTACTG

GGGGCAAGAGCCTTGAAGCTCAAGAGCATCTTGCTGAAGGGAGAAGCAGAGGAGGACAGACAAGGAAGGA

GCAGCTGGGGACAGAAGGATATCAGGAGATGGGCCGGAAAGGTGGGCTGAGCACGACGGACGAGTCTGGT

GGAGAAAGAGCTGAGAAGGAGGGCATCACTATCGATGAGTCGAAGTTCAGGACTGGGAGCTAGTGTGATG

ATTAAAACTATAAATGGTATTTTGTAGTAATAATAATAATAATGTTGGTAATAAAGTATTAGCTTAGCAG

TGTACT

>FM891599.1 FM891599 Jatropha curcas embryo 56-70 (DAF) Jatropha curcas cDNA clone rjcaeb0_001920, mRNA sequence

ATTCGGTGGAGAAATTGTTAGCAAAAAAGGGCAAAAATGTCTTCAAATCAATCAAGACAAGAGCTTGATG

AAAGAGCAAAGCGTGGAGAGACTGTTGTCCCTGGAGGTACTGGGGGCAAGAGCCTTGAAGCTCAAGAGCA

TCTTGCTGAAGGGAGAAGCAGAGGAGGACAGACAACGAAGGAGCAGCTGGGGACAGAAGGATATCAGGAG

ATGGGCCGGAAAGGTGGGCTGAGCACGACGGACGAGTCTGGTGGAGAAAGAGCTGAGAAGGAGGGCATCA

CTATCGATGAGTCGAAGTTCAGGACTGGGAGCTAGTGTGATGATTAAAACTATAAATGGTATTTTGTAGT

AATA

>FM891598.1 FM891598 Jatropha curcas embryo 56-70 (DAF) Jatropha curcas cDNA clone rjcaeb0_001919, mRNA sequence

ATTCCTCCTACGCAAGCTCTGAAGCCTGGACAGAGAAACTCTCGATATGTTGATGCAGTGATGACTGTTC

CTGCTAGGGCCATGATGCCCATTAGTGGAATCAACATTGCTTTTGATCGCGAGCTGGTGGGCCCTGCTTT

GTTGCCAGCTTTGAAGTTAGCTGGGGAAGGAAAGTTGAGGTGGGAAACAATGGAAGATATCTGGAGTGGA

ATGTGTGTTAAGGTTGTATGTGACCACCTTGGGCTTGGCGTGAAAAGTGGGCTCCCCTATGTGTGGAGAA

AGGATCGAGGTAATGCGATTGACAGCTTGAAAAAAGAGTGGGAAGGCATGAAGCTCATGGAGGAAGTTGT

CCCTTTCTTTCAGTCAGTGAGGTTGCCACGGACAGCAGCCACAGCAGAAGATTGTGTGATTGAGATTGCA

ACAGCAGTGAAGCAACAATTGGGACCATTAGATCCTGTTTTTACTCGTGCTGGTGAAGCCATGTTCGAAT

GGATCAAGATTTGGA

>FM891597.1 FM891597 Jatropha curcas embryo 56-70 (DAF) Jatropha curcas cDNA clone rjcaeb0_001918, mRNA sequence

AACTAGAATTTATCTCTTTATTAATTATTAAACCAGAAGCCACTATAGTCCGCAGCAGTGATCTTTCTCT

TTTCTTAAAGACTAATTTACACATGTACCTTCACCTTTCATTATTTATTCACCATACACAGCACTATTTA

CATTAGTAAACCCTCAAGGTCGATGTCACAGTCACCCCAAAAAGCCGAAACACTTGCCATCACGTTATTA

GCTTCTCATGCCACAGCATGACCCTCGAAGGAACCTCTCCTTGGCCCTGGAAAGAACCACTCCTCGTTTT

GTCTTCCAAAAGCCTGATCCACTTCTCTTTCACTTACACCGAAGGCTAACTCTTTGGCTTGCTTCTCCAT

CTTATTTACAATATTATTTTTCCCTGCAAGTAGATGCCTAGTATTGCCTTCTTCATTAATCTCAAAGCAG

ACTACTTCCAGAT

>FM891596.1 FM891596 Jatropha curcas embryo 56-70 (DAF) Jatropha curcas cDNA clone rjcaeb0_001917, mRNA sequence

ATTCCGACGACCAGGCGGAGAAGGGCACAAAGGAGCCCTAAAGGAGAAGGGCGGCTTCTCGACCTCACAG

ATTTTAGCGGTTGTCACTCTTTTGCCGGTGAGTGGGACGCTTCTTTTCCTTGCTGGAATAACGCTTACTG

GAACACTCATTGCGCTAGCTGTTGCGACTCCGCTGTTCGTGATTTGTAGTCCGGTGCTGGTACCAGCGGC

GTTGGTCATTGGGTTGTCGGTGTTAGGGTTTTTGACGTCCGGTGCTTTTGGAATCACTGCGCTTTCTTCC

A

>FM891595.1 FM891595 Jatropha curcas embryo 56-70 (DAF) Jatropha curcas cDNA clone rjcaeb0_001915, mRNA sequence

ATTCTTTTTTTAAACTCAACCTGCAATCAATGCGATAGACCAAAATGACAAATCACGAAAAGCCCAGTTG

CTAGATGGTCTTTCGTGTCTTTGCCGGTTTATAGTATACTCTCGCTTCATCAATAAGCATCCGCTCGGAG

GAGTTAATTTTTAGTTTGCTATAAAAGGCGGCAGTAGCATACCTACAACGTTACAATCAAGGGAATAACA

AAGAACCAACCCAAAACCAAACTCTTCAACATTATCAACACCAAAAGCCACATATCCGTCCCCTTCTTCC

GTTGTTAATAGAGAACAAAAGATCTGCTTCTTCAGATATACTGAACCTATTCCTCATCATTTTGACTTTC

ATCTTTGACAAGCTTGATCATGTCATCAATTCGAAGGATTGTTATGGCAGCTTCAGTGGCAAACTGGATT

ATCTTCACTTTGCTCATTGCAGGCTCAATGACTCCAGCTTCCAAGTTGTTCCGAACAATGCCCTTTGAAA

GGTCTAATCCCATGCTGGATAAATGTTTCTTCTCAGCCCTTGTCTGTGCTGTGTGGTGGTAAGCCCGTAG

CTTTGCTACTAACTCAGTGGCATCCTT

>FM891594.1 FM891594 Jatropha curcas embryo 56-70 (DAF) Jatropha curcas cDNA clone rjcaeb0_001914, mRNA sequence

ACTCAGACATCCAGATAATTATATTAAGAAACTAAACAAAAAAAAACCAGTATCAATATCCATCATACTC

AACTAATCAAAATTATGCAAAAAGTAGCAAACTTAGAATTACCAAATCCATATCACGAAACAAAAACGCA

TTAACAATAACCTAATCACCGCCAGCCTTATTGTAAACATAAGTCAAGCCGCACTTGCCGCAGTAGTGCC

TATCAAAATGGTTGGCCATGAAAGTTCCAGCTCCACACTCAGCATTGGGGCACTCCTTCCTCAGCCTCTG

CACCTTTCCGGAGTCATCGACCTTGTAGAACTGAAGCACGGCAAGTTTGACCTTCTTCTTCTTGTGCTTG

ATCTTCTTTGGCTTGGTGTAAGTCTTCTTCTTTCTCTTCTTAAGCACCACCAACGAAGACGAAG

>FM891593.1 FM891593 Jatropha curcas embryo 56-70 (DAF) Jatropha curcas cDNA clone rjcaeb0_001913, mRNA sequence

AATTAAAAATTATAATAATATTATTAATTTTCATAAAAAAAATTATATTAATGATTACAGAAATATCAAA

AAAAAAAAAAAAAAATGAGGTTCACTTGCTTGAGCTTAAGCATCACCACCACTGTCACTATTGCACTACT

CGTCTGCACCTCTTCTTCTTTTGGTCTTATTCTCCTCCGAAAGCAACGGAATCATATCTCATACTACCAC

AGGTTTCATCATCCAAACCTTAACTACTCTCCTTCTTGACTTAGTCTTCACTACTCATCTAAACGCCACC

ATTTCAGCTTCTTTCCCGTTTTTCCCTTTTAAATCTAAAAAACTTCTATTTCAAGCTTGCTATGGATTCT

CAACTTCTCTTTTATTTTGTTTCTATCTACAAACATAATTGAGTAAAAAGGAAAATACCCAAAAGCTATG

GAGTTTCAAGAAGCACTTATGAGACTAGTTATACATAAGCATCCTGTAGAGAAACGACTTGTCATTGATG

ACTCACAGAAAGAAACAGAGAAGCAGAAGGGGGGGCCCGGTAC

>FM891592.1 FM891592 Jatropha curcas embryo 56-70 (DAF) Jatropha curcas cDNA clone rjcaeb0_001912, mRNA sequence

AAGCAGTTATTATCATTTTATCACTATCATACATAAAAAGTGGCAGAGTTGCCTACAACTCAGAACAGAA

GAAATAGGGGGTGAAAGAACTGAAACCAGTACTCTGGAGAAAATTCACACCATTATAATTTCTAAAGTAA

ATAGAAAACAAAGACATTATTAGATACATCGACCGTTATTGCACTTATTTAGTCAACTTCCTCCATCTTG

CTCTCCTCATTTCCTCCTTCCTCCAAAGCTGGCATGTCTATGTCCTCCTGTTCACCACTCTCATCCTCAT

CAATGCCCAGACCCAACTTGAGCATCCTGTGAATTCTTGCTGCAAATGTGTTTGGATCATCAAGACTGAA

ACCAGAAGTCAAAAGTGCCGTCTCAAATAGCAGTAGCACCAAGTCCTTAACTGATTTGTCATTCTTATCA

GCCTCAGCCCTCTTTCTCAGCTCATCCATTATAGGATTGTC

>FM891591.1 FM891591 Jatropha curcas embryo 56-70 (DAF) Jatropha curcas cDNA clone rjcaeb0_001911, mRNA sequence

AATCGAATCACCGGCTGTTCAACCTTTATCTTGGCAAAAAGAATAAGAGCAATGTTGCGGAAATTGTTAA

CAGACGGCTCTGTGCGGCAACCATTATGGTGGATGATGTCAAATCGACGCTCCACCACCAACATATCCGC

CGCCGTGGATTCCATGTTGCTCCGCTCCCTTAAAGAACACTATCACGAAGTCTCCAGAATGAACCCTCCT

CCTAAGGTGAGCCCCCCATCAGCCTTTACAGTATTAAAAGGGGCTTTAGATGGGAATGGACCCGTTCTCA

CTAGAACCTTTGGCAATGAGAATATTAATATCTCTGTAATGCGCTTGGCCAATATCATTCCGGGAAGCGG

AGGGGAGGATGATGATGATGACATAAACCAGTTGTTCCTTCACGTGGATGTGTCAAAGCCTGGACAGGAA

AAATCTTTGCAGTTCCTATGCGGCCTATATCCAGACGCATTGGGAATTCATTCTGTTTCATTGAGACAGA

AGGCTGATGACCCAGAGTTTCCATCTGCATATAACGGCCCTCTTTTTGAGGAACTTGA

>FM891590.1 FM891590 Jatropha curcas embryo 56-70 (DAF) Jatropha curcas cDNA clone rjcaeb0_001910, mRNA sequence

ATCCATAAACAAGCAAAGGATAAACCATATCTCAAAATAAACAAAAGGAAAGAGTAGCCTCACCAATAAC

ATGCATGTCAAAAGGGGGAATCAAGCCAACCCCAAATCAGCATGTCCTGCTCCTACACATAAAAAGGATA

GAAGATGGGAAAGACAATCAACCCTCAAATACACAAAACATTATCCACATAATTACTTGGTACACATACA

CACACACAGCGTTCTACAAGGGTTAACAATTATCTTAGAATAACATTAAGACAGAATGCCAATGGGAAAG

GAATGTGCCATCATCACTAGACCAAATTCAGCACCGTACTCCTGCTAACGAAAGACCATAGGTTTGGCCA

CGAACTGAATACCAGACTGTCGCTCCTTAAACTCCATGCCCAGATGAAGAGGTTTTCGGAACAGCTGAGC

GGAGAGAATGAGCTAGAAAGCAGTGAAGGAGTTCTCTCTCCATTTTGTGATTTTGAACTTTTCTTTCTCT

CTCTATTTTGTGATTTTGGTCTTTTCTTAAACTCCTGAGTTCTTTCTCCATTTTCTCCATCACAACTCGC

AATTCTTTTACCTCCTGTTTATGATCTGGTAAGACAGTTTTAAGACCATAAACGGCAGGCAAAAACGGTG

GTATACTTGGGCATATCGGCGGCATTCGAC

>FM891589.1 FM891589 Jatropha curcas embryo 56-70 (DAF) Jatropha curcas cDNA clone rjcaeb0_001909, mRNA sequence

ATTCTGTACATGTAATGCTAAAAGAATTATCTTGTGTCATTTACATATCCCCTTTTGGCGGGGATGTATC

TGGGTCTGGTTTGGACTTTGGACTTTGGACTTTGATCGTCCTTTATGTTTAATTAATCCACTTGTGTTCA

TGTACGTAT

>FM891588.1 FM891588 Jatropha curcas embryo 56-70 (DAF) Jatropha curcas cDNA clone rjcaeb0_001908, mRNA sequence

AAGCAAAAATCGAGAAGCTCAACATTTTCACTCAGATTGTTGGGATTCCAAAACAGCAGTCTTTCCAATA

TCCAAAATTTCAAACTCAAAGTAATAACAATTTTGTTCTCCAGACAAAACCCATAAACTAACACTTCCAA

GAACATCAATGAAATTAACGGTAACGGCGAAGTGAGAGAGTGTTATTTCTCTTCCATGTTGCTCTGCGAG

AAGGACGATTCTTGTGGTGGAGATGTCCCCTTCCACGTAGACCCCTATATTTCTTGCCAGCAGAGGTGAG

ACCTCGGAACTCCCTGTGCTTATGAACAGGGTCACATAGCCAGTTGATCCTTGGGTCATTTCGAATGGCA

TTATGTGCTACATCAACTAAGATTACCTCAAAAATACTTGTAAGTAGAATCCTCATTATTCCCAGTAAGA

ATTGAG

>FM891587.1 FM891587 Jatropha curcas embryo 56-70 (DAF) Jatropha curcas cDNA clone rjcaeb0_001907, mRNA sequence

TATCAATCGGCGTTGAAAGATCAGTTCTCCTTTTTGAGTATCTAAAAATGTCTTCTTGCTGTGGTGGAAA

TTGCGGCTGCGGCTCCGGCTGCAAGTGTGGCAGCGGTTGTGGCGGATGCAAGATGTATCCTGACATGAGT

TTCTCCGAGAAAAACACCGCCGAGACTCAGGTTCTCGGAGTTGCACCGGAGAAGGCACGCTTTGTCGATG

GAGGTGAGGTCGGCGTTGGAGCTGAGAACGGATGCAAGTGCGGTGACAACTGCACCTGCAATCCTTGCAA

CTGTAAATGAGATCGGCGGCGATCAGTCTGTTTTTTAGAAGTAAAAGCAGAGATGACTCTGCTTCTCTCT

TTCTAAAAAAGGAGAAATAATTAATAGAGTCTAGATATCGTATGAGTTTGTTCGTTTGTTTCTGGTTTTG

AGCTTCAATAAAAGTAGTGGTGGCTTGTGAGCTTGTGTTTAGCAATGGCTGCCTGTTGCTTTAACGTTAT

ATATTGTATAAATTAAAAACTAGCATATGAATATGTAAATGAGTACGAGGGGTGAAATAGCTTTGAAAGC

TTAATGAGAAACTCCTTTTC

>FM891586.1 FM891586 Jatropha curcas embryo 56-70 (DAF) Jatropha curcas cDNA clone rjcaeb0_001906, mRNA sequence

ATTCGATCCATTCTTAAAGTTTACATTTACTTCAGATGTGGAACTGTCAGTGAGAAAAGCAATATCAGAT

TTGGATAGTTGGTTAGCCAAAGCAGATTCAGGGCTTGTAGATGACTTGGAGAAGCTTCCTTATGTTTGTG

CTGCAATGGAGCAACTAGAACAGAGGAAGAAATATTGGAATGAGCAGCAGGTGGGCTATAATAATATAAA

TTGTAAATGTGCTAAGAGTCAGAAACAATCTCAAGAGCTTTTGTCAAATCAAAATTTGAACTGTGCTTAT

CAAAACTCTAGTTCTAGATGTTCAGTGCAGTCTATTGATGCCAGTGATGGAAATAAGTACTTGCGCAATG

TGAAGTTGGACATGATTGAGTCATTAGAAGGTCACGATTCAGTTTTACCATCTTCATTTGATGAGAGCAA

GAGTTTGCAGAAATCCTTTAATGGATCAAGAGATGTTAAGTACAATGACTCTTACAATTTCTACCAGGCA

GTTGACGGTCAGCACCTTATTCTTCATCCTTTAAACATGGGGGGGCCCGGTAC

>FM891585.1 FM891585 Jatropha curcas embryo 56-70 (DAF) Jatropha curcas cDNA clone rjcaeb0_001905, mRNA sequence

ATTCCTAAGTGCTATTTGTCGATGTGAATCAATACATTATCTGTTAGAGAAGCAGCTGGAGGAAGGAGAA

GTTGGAAGCGAAGATGAGGCAAGGCGCAGGACTAAAAATATAATTGATGTTTGCTTTGGGTCTATATGCC

CTCGTTAAAATGCTAGCTGCAGGTGAATAAAACTTCATTCACTTGTATGTGAGTGAGAGTTATATATAAT

GATGTATAAGAAGTAAGAGCATCAGAATAAGTTTGTTCGAAACTGCTTCTTGTAACGTTGCTTCAAGCTT

TCCTAATGTCTTGTAATAAAAACTG

>FM891584.1 FM891584 Jatropha curcas embryo 56-70 (DAF) Jatropha curcas cDNA clone rjcaeb0_001904, mRNA sequence

TGAATTCCTTCGTGATCCTTTGAGGGACTATGAACCAGTAGGCACACCGATGCTTGCTCTTATTCAAGTA

GAGAGAGTCTTTGTCCCACCAAAGCCAAAAATATACTGTAAGGTTTCGGAGCTAAGCAATGATAAAGAGG

ATGACGATGAGTCAGAGTTGGTGGTGAAGGAAAAGGTTAAGGAAGAGAAAATTGGGGATACAGCGTCTGA

AGAGGAAAGAATTCCTCAGTTCAGCATCACGGAAGTACGTGTTGCTGGTCTCAAGACTGAGCCTGAGCCT

GGAAAGAAGAAACTCTGGGGTACTGCAGCTCAGGAACAATCTGGTTCTCGTTGGCTGCTTGCTAATGGAA

TGGGGAAGAACAATAAGCAGCTTTTTACAAAGTCAAAAGCTGCTTCTAATAGAACTGCAACCTCTTTGAC

AACAAAGCTGCAACGTGGTGATAAATTATGGAGCATCTCATCTCGGGCAAAATAGGAATCAGATTGCATC

GAATCGACACACAGAATCCAAGTACAAAATTCCTTAAATGAAACTATTAGGTTGAGGTAAATTGCACA

>FM891583.1 FM891583 Jatropha curcas embryo 56-70 (DAF) Jatropha curcas cDNA clone rjcaeb0_001903, mRNA sequence

ATTCGTTTCGAGATGATTAGCAAACTTTTTCTGGATTAGCAGCAATGGCACCAAAAGATAGTTCAAAAAA

GCCTGATGCAAAGGCACAAGCTGTCAAGGCTGCAAAAGCTGTGAAGTCTGGACCAACCTTTAAGAAGAAA

GCTAAGAAGATCAGAACCAAGNTCACATTCCATCGCCCAAAGACTCTAAAGAAGGAGAGGAATCCCAAAT

ACCCCCGCATTAGTGCTCCACCAAGGAACAAGCTTGATCATTATCAGATTTTGAAATATCCTCTTACTAC

TGAGTCAGCTATGAAGAAGATTGAAGATAACAATACCTTAGTTTTCATTGTTGATCTTCGTGCTGACAAG

AAAAAAATCAAGGATGCAGTCAAGAAGATGTATGATATTCAAGCCAAGAAAGTGAATACCTTGATAAGGC

CTGATGGAACAAAGAAAGCATATGTAAGGTTGACACCAGACTACGATGCATTGGATGTGGCATACAAAAT

TGGCATCATCTAAATTAGTACCTGGCTTTTTTCTAATT

>FM891582.1 FM891582 Jatropha curcas embryo 56-70 (DAF) Jatropha curcas cDNA clone rjcaeb0_001896, mRNA sequence

ATTGGTATGCCTCTTATAATCTTCACCTTCGAAGCTCAAGAAACCCTCGTCGCCGTCGAAGAAACTCGTC

TCTGCAACCATGGGGAAGACACGTGGTATGGGAGCTGCCCGCAAACTGAAGTCCCACAGAAGAAGGCAAA

GGTGGGCTGACAAGGCATACAAGAAATCCCATCTTGGTAATGAATGGAAGAAGCCATTTGCTGGCTCTTC

CCATGCCAAAGGCATTGTTCTTGAGAAGATCGGTATTGAGGCCAAGCAGCCTAACTCTGCTATCAGAAAA

TGTGCTAGAGTTCAACTGATTAGAACTGGAAAGAAGATTGCTGCTTTTGTACCAAACGATGGTTGCTTAA

ACTATATTGAGGAGAATGATGAGGTTTTGATTGCT

>FM891581.1 FM891581 Jatropha curcas embryo 56-70 (DAF) Jatropha curcas cDNA clone rjcaeb0_001894, mRNA sequence

ATTCCTGAGCTCCGAGACCGAGAAGGAACGGAAAAATGCCAAAGCAAATACATGAAATCAAGGATTTCCT

TCTTACAGCAAGAAGAAAGGATGCTCGTTCTGTCAAGATCAAGAGAAGCAAAGATGTGGTGAAGTTCAAG

GTTCGCTGCTCCAAATATCTTTACACACTTTGTGTGTTCGACTCTGAGAAGGCTGACAAGTTGAAGCAAT

CTCTTACCCCAGGTTAGACTGTACAAGACCTGTGAAGAAACATAAAGACTAAAGTTTTTGGCCTTATGAG

AAATGAAGGGATCCAAGT

>FM891580.1 FM891580 Jatropha curcas embryo 56-70 (DAF) Jatropha curcas cDNA clone rjcaeb0_001893, mRNA sequence

ATTGTTCCTACAACTTGAAAATGGGGTGGCTTTGATTAGCTGATTCCATGAAAACTTGATGAAGCTCTTC

TTGTATGCAAATGGTCCTATTGGTCCTGTTATTATTTTTAGTGGTCTTTTAAATTTTATAATCCAAACCA

AGTCACCTACTTATGAACATCCCTAATTATTGTTATTATTATTTTGAAATATAATTTTTGTTAGAGAGAT

C

>FM891579.1 FM891579 Jatropha curcas embryo 56-70 (DAF) Jatropha curcas cDNA clone rjcaeb0_001892, mRNA sequence

ATTCCTTCCTCTTCATCGAGGTCTAATGGTGGGTCAGGTGCAGGTTCTGAAGCAGGTTCTTCTGCTGGGT

CCTATGCTGGTTCAAGGGCTGGGTCAGGCTCAAATGGCCATGGAAAATGAGTTCACGTACGTCTGTCTTT

ATGATGATTTGCAAAGTTAAAGCAAAAAATAATAGTAATAAGTGCATACACCACTACAAGCATAGATAAT

TATTTAGACGTCCTCATGTTGTGTGTTATCCTAAATCAATGTATTGTTGTTGCCTTTGTATTTCAAGTTG

CTTGCTGGATCTAAATTTTACAATTGTTGTTTAAAAGGGGGGGCCCGGTAC

>FM891578.1 FM891578 Jatropha curcas embryo 56-70 (DAF) Jatropha curcas cDNA clone rjcaeb0_001890, mRNA sequence

NAACAACTCAACTGTCGATTAGCTTTTATTACAGGACATCAAGAAAACTTGAAGCAACACTACTACCTTA

TAAGGTTCATGCTCTTAGTTTTTTATAGATTATTACACATAACTCTGTCTCACTCACACTCAAGCGAGTG

AAATTTTATTTACCTGCAGCATTTTAACGAGGGCAAACCGAGCCAAAGCAAACATCAATTATATTGAAAG

CCCTACGTTCTGCCTCATCATATTCTTGGCTGCAATATTTTCAGTTTTTGCT

>FM891577.1 FM891577 Jatropha curcas embryo 56-70 (DAF) Jatropha curcas cDNA clone rjcaeb0_001889, mRNA sequence

ATTCATTTACTCTGTGCTTCGATCGCGTCAATGGCGGAGCACCCACAGTCGCAGCATGTTGGCCAACAGC

CACGATCCCACCAAGTAGTGAAGGCAGCCACTGCAGTCACTGCCGGTGGATCTCTTCTAGTTCTCTCCGG

TTTGACCCTTGCTGGTACGGTCATTGCACTGACCGTAGCCACTCCATTGCTGGTCATATTCAGTCCGGTT

CTTGTTCCTGCGGTTATCACAGTTTGTTTGATAATCACAGGGTTTTTAGCCTCAGGCGGATTTGGCGTCG

CTGCCATTTCTGTCTTGTCCTGGATTTATAGGTATGTTACTGGAAAGCATCCACCAGGAGCAGAAAGTCT

GGAACAGGCACGCTTAAAGTTGGCGGGAAAGGCAAGAGAGATGAAGGACAGAGCTGAGCAGTTTGGACAG

CATGTAACAG

>FM891576.1 FM891576 Jatropha curcas embryo 56-70 (DAF) Jatropha curcas cDNA clone rjcaeb0_001888, mRNA sequence

ATTGTGAATCAATACATTATCTGTTAGAGAAGCAGCTGGAGGAAGGAGAAGTTGGAAGCGAAGATGAGGC

AAGGCGCAGGACTAAAAATATAATTGATGTTTGCTTTGGGTCTATATGCCCTCGTTAAAATGCTAGCTGC

AGGTGAATAAAACTTCATTCACTTGTATGTGAGTGAGAGTTATATATAATGATGTATAAGAAGTAAGAGC

ATCAGAATAAGTTTGTTCGAAACTGCTTCTTGTAACGTTGCTTCAAGCTTTCCTAATGTCTTGTAATAAA

AACTG

>FM891575.1 FM891575 Jatropha curcas embryo 56-70 (DAF) Jatropha curcas cDNA clone rjcaeb0_001887, mRNA sequence

CTACAACTATTAATTTCTTTTTAAGAAAGGAGAAGGACAAAATAAGAGGGAGCAGAGACATCCTTCTCTT

ACAAGACAAAAGCAAGAAAAAAATTAACAAGCAAATATCCCCATTAACATGAGTTGTTCAAACATTTGTC

ACAGCCTAAATGACTATGCTACTCCTCTCCCCCTCTTCCTACTCATCATCGTCCTCCTCTCCACTTCCCT

CCTCATCATCTTCTTCCTCATCATTCACCTCAGACATTGACTTCTCAGATTCCTCTTCTTCCTTGGGGCC

TTCAGCCTGTCCCTTGTTGTAGGCTTTCAATTTCTTCTCATATTCTATCTTCCTCTTATCTGCCTTAGAT

ACAAAAGGTGCTTTCTCAGCATCTGACATTGACTTCCATTTATCTCCACCAGCTTTGCCAACAGCAGCAA

CGGATTTGTTTTTAGGATGTTCCTTCTTGTACTGCTCTCTGAACTCTTCCATGAAAACGAAGAAAGCACT

GGCGGGCCTCTTTGGCTTATTGGGATCCTTCGCTGCCTTACCCGCCTTCTTACTGGCCTTGGCGGG

>FM891574.1 FM891574 Jatropha curcas embryo 56-70 (DAF) Jatropha curcas cDNA clone rjcaeb0_001886, mRNA sequence

ATTCCAGAGCAAAAGATTAATTATATTGGTGGAGAAATTTGTTAGCAAAAAAGGGCAAAAATGTCTTCAA

ATCAATCAAGACAAGAGCTTGATGAAAGAGCAAGGCGTGGAGAGACTGTTGTCCCTGGAGGTACTGGGGG

CAAGAGCCTTGAAGCTCAAGAGCATCTTGCTGAAGGGAGAAGCAGAGGAGGACAGACAAGGAAGGAGCAG

CTGGGGACAGAAGGATATCAGGAGATGGGCCGGAAAGGTGGGCTGAGCACGACGGACGAGTCTGGTGGAG

AAAGAGCTGAGAAGGAGGGCATCACTATCGATGAGTCGAAGTTCAGGACTGGGAGCTAGTGTGATGATTA

AAACTATAAATGGTATTTAGTAGTAATAATAATAATAATGTTGGTAATAAAGTATTAGCTTAGCAGTGTA

CTTTAGTTTAGTAATGTAGTTTACATGTGGGTTAAAATGACTAGGGTTTAGCTAGTATGTGTGTTGTAAC

TTTGAGAGGGGTTTATGGTCGTTCCCTTTCTGGGAGGACTGCTAATACTTTCTCCCTATTTGTAAAAT

>FM891573.1 FM891573 Jatropha curcas embryo 56-70 (DAF) Jatropha curcas cDNA clone rjcaeb0_001885, mRNA sequence

ATTCCGATCGTGCCTGATGAACGAGAAGAAGAAGAGTTCACGTACAGCAGACCCGGCCAGAGCGGGAGGT

GGATGAATGGAATGGAAGAAGCCATGTGCACAATGAAAACTATACATAACCTTGAGTCTCGTAGGGAAGC

TGATTTTTACACAAAGGAAGCTGGTAGAATCCACCTTGCAAATCAACGAAAGCTCCCCATTCTCAGTTTC

ATGGACATGAGCGCTGAGCGAGGCGTTCTGCTGCCGAGGGCAATGCACAGCCCACACTGGTCCATAAACG

ATCAGAGAATAGTGTGTGCTCTTCGCGGCGACATGCACATACAGATTGTGGATGACAGTGGAAACACAGT

AATGGACGAAAGGGTGAAAGAAGGAGACATGTTCGTGATCCCTCAATTCTATGCAGCACTTGGAAGGGCA

GGAGAAAATGGGTTCGAGTACGTAACATTTAAAACAAGTAGCCAGCCTATCAAGAGCCCATTGGCGGGTT

ATACATCAGTGATGAGAGCCATGCCTCTTGATGTTCTGATGAACTCTTA

>FM891572.1 FM891572 Jatropha curcas embryo 56-70 (DAF) Jatropha curcas cDNA clone rjcaeb0_001884, mRNA sequence

ATTAACAACTTTTAGCAGACGCTTTCAACGTCAATGTTGAGGTTGCAAGAAAGATAAAAGGACAAAACGA

TAACAGAGGATTAATTGTAAGCGTAGAACGTGATTTTGAGTTGCTATTCCCACCAAGAAGCGCAGAAGAA

GAAAGACGAGAAAGAGAGGAAGAGACCCAGAAACAATTAGAGGGAACAGGTTACAGACCAGGTGGCGGCC

AAGGAAGATTCGTTAATGGTGTGGAAGAAACTTTCTGCACTGCTAGACTCGTACACAACATCAATGACCC

GTCACGTGCCGATGTATTCAACCCACGTGCTGGCCGTCTCACCAACATCAACGGCCAAGACCTCCCCATC

CTCAGTTATATCCAGCTAAGCGTTCAGAAAGGTGTCCTCTACAGAAATGCTCTAATGTCACCACACTGGA

ACGTCGATGCCCATAGCATAAACTACTTCACAAGAGGAAATGGCCATATCCAGATCGTAGATGATAATGG

AGAGACAGTATTTGATGGAGAAGTTCAAGCAGGACAAATATTAACATCTCCACAAAACTTTGTGGTAGTA

AAGAGGGCAGGAAGCGAAGGTCTTGAATGGGTATCCTTCAAGACTAATGACAATGCCAAGATTGACCAAC

TAGCAGGACGTGTCTCGGCAATCAGATCAATT

>FM891571.1 FM891571 Jatropha curcas embryo 56-70 (DAF) Jatropha curcas cDNA clone rjcaeb0_001883, mRNA sequence

ATTCGAATAGCCAAGTGAGTGATTGAATTTGAATAGCCAAGTGAGTGAAAATGGAGTCGCTGAAGGAAAC

CGCAGCTAACGTTGGAGCCTCTGCTAAGGCTGGCATGGAAAAAACCAGAGCAGAGGCGCAAGAGAAGGTG

GAGAGAATGTCAACACGTGATCCAGTGCAGAAGGAAATGGCGAGAGAGAAGAAAGAAGAGAGGAAAACGG

AGGCAGAATTGCGGAAACTAGAAGCGCGCGAGGATAACGCAGCGGCGAGGCAGGCAGCCAAGGCTGGTGG

CCATGTACAGTACACAACTGCTGGTCCGGGCGCTCAAGGCACAACCGAAACCTATACCCATTCAACAACT

GGGACAACCGGTTACCCTACAGGGACCCAGCAAATGTCGGCCATGCCTGGACACGGCACAGGGCAGCCAT

ACGGTGGAGAGGTGGACCCAACGGGTTTGACCAGGAC

>FM891570.1 FM891570 Jatropha curcas embryo 56-70 (DAF) Jatropha curcas cDNA clone rjcaeb0_001882, mRNA sequence

ATTCTTCGCTGCAGCAGAGATTTTGAATTCTTTTGATAGCTCTTAATAGAACAGACAAACAAGATAATAT

AATATGATTCATTTTGTCCTTCTTATAAGTCGACAAGGAAAAGTGAGATTGACAAAATGGTATTCGCCTT

ATTCACAGAAGGAAAGAACTAAGGTTGTTCGTGAGCTCAGTGGGGTAATTCTGACTCGAGGGCCCAAGCT

TTGTAATTTTGTTGAATGGAGGGGATATAAAGTTGTTTATAAAAGGTATGCCAGTCTATATTTCTGCATG

TGCATTGATCAGGACGACAATGAGTTAGAGGTCCTCGAAATAATCCATCATTTTGTTGAGATTCTGGACC

GATACTTTGGCAGTGTGTGTGAGCTAGACTTGATCTTCAACTTCCATAAGGCCTATTATGTACTGGATGA

GCTTTTGATCGCTGGTGAGCTCCAAGAGTCAAGCAAGAAAACAGTTGCCAGGCTAATAGCTGCACAGGAT

TCATTGGTGGAGACTGCAAAAGAGCAGGCCAGTTCCATAAGTAATATTATTGCACAGGCCACCAAGTA

>FM891569.1 FM891569 Jatropha curcas embryo 56-70 (DAF) Jatropha curcas cDNA clone rjcaeb0_001881, mRNA sequence

ATTCATTTACTCTGTGCTTCGATCGCGTCAATGGCGGAGCACCCACAGTCGCAGCATGTTGGCCAACAGC

CACGATCCCACCAAGTAGTGAAGGCAGCCACTGCAGTCACTGCCGGTGGATCTCTTCTAGTTCTCTCCGG

TTTGACCCTTGCTGGTACGGTCATTGCACTGACCGTAGCCACTCCATTGCTGGTCATATTCAGTCCGGTT

CTTGTTCCTGCGGTTATCACAGTTTGTTTGATAATCACAGGGTTTTTAGCCTCAGGCGGTTTTGGC

>FM891568.1 FM891568 Jatropha curcas embryo 56-70 (DAF) Jatropha curcas cDNA clone rjcaeb0_001880, mRNA sequence

CCATAAGCCTTCATTCTTTAAGGGTTTCATTTTCGAAGATCTGAATATCATATACAGATACAGACAATTA

GAATGCACTTATACATGCCATTAAGGATCAGCTTTGCCAGCAACTTGGTCATCCTCAGAGGCATAATCAT

CATCGCTCAGCTCTTCATCGCTCGATTTATCTTCCGGTTATGGCTCCAATGTCCTCTGCCTTAGCATATT

TCTCACAGTATTCTTTTAATCTCTGTTCATAAGCAGAGCGATCACGCATCATTAGAGCAGCAGCTTCTCC

ATTCAATGGATCTGATGGATTGGGATACAAAAGAAGCTGAGGAAGAAACACTTCAAACACATTTACCAGA

TCAAACATGGGGCTCCAAGTCTGGTTGATAACATCTACACAAACTGAACCT

>FM891567.1 FM891567 Jatropha curcas embryo 56-70 (DAF) Jatropha curcas cDNA clone rjcaeb0_001879, mRNA sequence

ATTGTTAGTTTTACCCTACTGATGATGGTGTCGCGATGGTAATTCAACCTAGTACGAGAGGAACCGTTGA

TTCGCACAATTGGTCATCGCGCTTGGTTGAAAAGCCAGTGGCGCGAAGCTACCGTGCGCTGGATTATGAC

TGAACGCCTCTAAGTCAGAATCCGGGCCAGAAGCGACGCGCGTGTCCGTCGCCCGTTTGCCGACCCGCAG

TAGGGGC

>FM891566.1 FM891566 Jatropha curcas embryo 56-70 (DAF) Jatropha curcas cDNA clone rjcaeb0_001878, mRNA sequence

GCAAATAAAGGGAAAGGATGTTAAAATTCACATAGAATAAAATTTACAGTTCAGTTTTCCAAGGGGCACC

CGTTCACCTAATAACAAGAGGTTGAAGGAATAAAATTTACAGTTCACTTTTTCAAGAGGCACCCACTTAC

CTAATAACGCCCAACTTGAGAAAAAAAAAACTATCTTAAATGAAAAACCTACTCCGAAGTCAATTATTTC

AAATAAGAAGATGATGAACAACAACACACAACAACAACAATGCTTGGTGTATAGAATATAACCTGAATTT

AGTAACTTATGGCCTGCTAGTACAAACGCCCCCGACATTCACTTG

>FM891565.1 FM891565 Jatropha curcas embryo 56-70 (DAF) Jatropha curcas cDNA clone rjcaeb0_001877, mRNA sequence

TGTGGGGCATAAACCAGAGCTCCACTAACTTGATTACACTCCTGGAGAACCTCTCTGTTTTTTCGTTTTT

ACACCCTGCCAAAAGATTATTATTATTATTATTATTTTTTTTTCGTATGTTCCCGTCATTGCTTCTTAGT

TATTAGCATCCACTTGTCTGTTTAGGCTCATAGCATTGCTGTGTCTCTAACATTAACCATAGTGTTTGAG

CAAAAGAGATGTAACTGGCCAACGGCCGCCCGCTATAAACAATGATAATTTTTAAAAAAAAAAAAAAAAA

AAAAAAAAAAA

>FM891564.1 FM891564 Jatropha curcas embryo 56-70 (DAF) Jatropha curcas cDNA clone rjcaeb0_001876, mRNA sequence

NTGCTAAATAAAAATGAGATGGATATAGTAACATAAATAGCAAAAGTTTGTGGCTCCAAAATCCGCAAAA

CACAATACAACAATTCAGAACGACACAAAAGACAGTAACTTGAGAACTTAGGTCACAAAACTGCCAGATA

ACTGGAACTGAAACCAAAAACAACAGGAAGACATTACTTAAAACAAAACATAACTAAACTAATAACTGTA

ACATCATTCACCCAATGCTTTACTTTTGGCCAAAGTGATACACTTTTTGTCGTGTCCTTTCGAGAATGTA

TAAACTTTGAATATCTATGTAAATAGACTTTTACTTGGTTTCCTGC

>FM891563.1 FM891563 Jatropha curcas embryo 56-70 (DAF) Jatropha curcas cDNA clone rjcaeb0_001875, mRNA sequence

ATTCCACTGATCGTAAACTCCATTGAATTTACTCTGTGCTTCGATCGCGTCAATGGCGGAGCACCCACAG

TCGCAGCATGTTGGCCAACAGCCACGATCCCACCAAGTAGTGAAGGCAGCCACTGCAGTCACTGCCGGTG

GATCTCTTCTAGTTCTCTCCGGTTTGACCCTTGCTGGTACGGTCATTGCACTGACCGTAGCCACTCCATT

GCTGGTCATATTCAGTCCGGTTCTTGTTCCTGCGGTTATCACAGTTTGTTTGATAATCACAGGGTTTTTA

GCCTCAGGCGGTTTTGGCGTCGCTGCCATTTCTGTCTTGTCCTGGATTTATAGGTATGTTACTGGAAAGC

ATCCACCAGGAGCAGAAAGTCTGGACCAGGCACGCTTAAAGTTGGCGGGAAAGGCAAGAGAGATGAAGGA

CAGAGCTGAGCAGTTTGGACAGCATGTAACAGGTCAACAGACTTAAAAGAGAAGTGGGTTTTGGTGGTTG

ATGCAGAGAACATAAAATGGCTTTGATATATATATAAAAATATATGAGTCCTTTAATTAACATGGA

>FM891562.1 FM891562 Jatropha curcas embryo 56-70 (DAF) Jatropha curcas cDNA clone rjcaeb0_001874, mRNA sequence

CATACTCGTACTGGTACGTAGTTTATGATCGTTGCATGATAATGGCTTCTGAAAAAGCATACCGGGTGTT

GCTCTGCTTGGGGTTCGCGATGCTGTTGATAGTTGTTGTTGTCGGTAGCCTAGAACATATGCCTTCCACG

AGCGAAAAAAGCGAGGACTCGGAAGCAATTAAGGACAGGGCAAAAGATGCCAAACGAACATCGGAGTCGT

GGACAGAATGGGCGAAGGACAAAATCTCCGAGACTGTCGGACGAAAACAAGATGAAGCCAAGGAGGCTGC

TAAGAAAGCGTCTGACTCTACCGCTGAAACTGCTCAGAGGACCAAACACAAAGCTTCTGAAAAAGCTGAA

GAGCTGAAGGATGCTGCAACGCAGAAGGCCAACGAAATGAAAGATAAGGCCTCTGAAATGACGAATGCGG

CTAAGGAGAAGCTCAGTGCGGCTGAAGGCC

>FM891561.1 FM891561 Jatropha curcas embryo 56-70 (DAF) Jatropha curcas cDNA clone rjcaeb0_001873, mRNA sequence

GAACACACAAAAAGCTCTGATGTGAAACTTATTGTACTCTTTCCAACTATGAGCAAAGGACCCATTAAAT

AATATTTCCATTCCATATGGACATAACCCGCGTCACATGATCAGCAGGCTAAACTATTTTAGACCAACAG

CATCCAAATTACAATCCAAATCCAGTCGAGAAATGAACAAAAAGAAAAAAATTAGCATCGCCAGAGGTGA

GAAAGAGGTCTCCCGTTTTTACATGCCTTGACATGTTCCAAAACAATCTCTTGGAGCTGTTTTTGAACCT

CATCTATGGGTTGACAGGCATCTATTATCTTCCAGGAGGAATCATGGAGGGTCTGATAGCATTGTGCTAC

TTTTCTCTGAAATTCAAGTTTCTCGTATCTTTCACCTCCATAGCCTCCTCTTTCTGCAGCTTTCTAATGA

TGCCAACATGGGGGGGCCCGGTAC

>FM891560.1 FM891560 Jatropha curcas embryo 56-70 (DAF) Jatropha curcas cDNA clone rjcaeb0_001872, mRNA sequence

ATTCATTATACGCACGCAATGCCCTTATTTGTTATGTTGTGTGGCTGATTTGCTTTTTATCGATCAGTTT

GTAATAGGGTGATGTGTGGCTGGTTGAGCTTGCATCCCCAAGTGTGTTGGAGCTTTGGTTTGTGTGTTTA

AGAAAGGACTTCTGTCTTCTGTTTGGAGAAATGTAATAACAAAATTTGGGATTTACTGTAATACTTTTCT

GGAAACATAGCAGCCATAGAAGCAAATATGTAGTTATGTAGTGTTGTGAACCTGGAGTGTGATGTAGCGA

ACGCGGGAAATTCATTACATTTGCAAGTTGCTTTCATTATAAGTTATTAAAAAAAAAGGGGGG

>FM891559.1 FM891559 Jatropha curcas embryo 56-70 (DAF) Jatropha curcas cDNA clone rjcaeb0_001871, mRNA sequence

TGGACTCCCCCGGGTTAGCAGGTTTTTTTTGAAAATGAAACGCTATTTAAATTACTCAAAACAGTCAACA

TATAGAGAAGCTACAACATCAACATACGTCCAATATAGAAAAACTACACGCAAATCTTTTTTC

>FM891558.1 FM891558 Jatropha curcas embryo 56-70 (DAF) Jatropha curcas cDNA clone rjcaeb0_001870, mRNA sequence

GAAATGCAAACAAAATATAAGTAGAATTCTAACAAAATCATGCAATACAAAAAGATAGCGTATCAAAAAT

AAGGCATTCGATATCCCAACTACGAACACAAATCCTAAACATAAAAAACAAAAACTCATTGATACCACAA

CAGACAATTTGTCCAGAAGAAGATAAAAACAAGACATGGAGTTCTAGAAAACAAGGCTCCTTAAGCACCC

TTGTCTGCTTTGGCAGCTGTTGCTGCAGCTTGTCCTCTGAGACGACCAGTCCTCCTTGCAGCAATGAGAC

CAACCTTTTGGCCAGGAGGAGCATCGCGGCGAACAGTACTAGCATGTCCAATGTGTTGGTGGTTACCTCC

ACCATGGGGATGCTCTACAGGATTCATAGCAACACCACGAACCTTAGGCCAGCAGTTCCTCTTGACTCTG

TATTTGTGATATGCATTTCCTGCCTTTAACATGGGTTTCTCAGTCCTTCCACCGCCTGCAACTTGACCAA

CCATTGCACGGCAACCAC

>FM891557.1 FM891557 Jatropha curcas embryo 56-70 (DAF) Jatropha curcas cDNA clone rjcaeb0_001867, mRNA sequence

ATTCAGAAGAAGTTATTAACTGGACATCTCTTGCAATATAGCAAGGACATCTCAGTTCCTCAAAAAATAA

TTGTGCCATCAGTTGGATCTCAGAGGCCTTCTTACCCATCTTCAAAAACTAGAGCAAAAGACTGGGATAA

GTTGGAAGCCCAAGTAAAGAAGGAGGAAAAAGATGAGAAGCTAGATGGCGATGCTGCTTTGAACCAAGCT

ATTTAAGGACATTTACCCAAAATGCAAATGAAGATATGAGGAGGGCTATGACCAAGTCTTTTGTCGAATA

AAATGGGACAGTGCTATACA

>FM891556.1 FM891556 Jatropha curcas embryo 56-70 (DAF) Jatropha curcas cDNA clone rjcaeb0_001866, mRNA sequence

TCGCAATCCCATCATTGCCAAGATGTATCAAGGTGCTGGTGCTGACATGGGTGGCGGCATGGAAGATGAT

GTTCCTCCAGCAGGTGGCAGTGGTGCTGGTCCCAAGATCGAGGAAGTTGACTAAGTGGCTTGTTTGATGC

TTGAGTAAAGATGGTGATGAATATTTTATTTTCTTTATTGGTCAGTACAATTATTATTATTATTATTTTA

ATATTCTACTTAGACATTATGTAAAAAAAAGGGGGGGCCCGGTAC

>FM891555.1 FM891555 Jatropha curcas embryo 56-70 (DAF) Jatropha curcas cDNA clone rjcaeb0_001865, mRNA sequence

CGAAGACCGAGTGGCTGGACGGAAAGCATGTCGTGTTCGGTCAGGTCGTGGAAGGTATGGATGTTGTGAA

GGCCATCGAAAAGGTCGGGTCTAGCTCCGGAAGGACCTCAAAGCCTGTTGTGATTGCAGATTGCGGTCAA

CTTTCTTAAGTGACCTAAGATCTACAGTCTTATAAGTTGCTGAGGCGGCGGAGGTGTCTTTTATATGTGA

TGATGTGTGTTAGTAATTTAGGGTCTGGTGTTTAGGGTTTGGGTCATGAGTCGATTTCCGGGTCTTAATG

TTTGGGGTTCCTAAGACTTTGTTTTATGTGTTGATGAATTGAGAACTACCATAAATAAGATCGGCTATTT

TAAC

>FM891554.1 FM891554 Jatropha curcas embryo 56-70 (DAF) Jatropha curcas cDNA clone rjcaeb0_001864, mRNA sequence

ATTCTTCCTCTCTAGAAAAAAGCAATTTAGCTTTGACACATCAAAACACATATCACCACCTCTATATATA

GAGAGAGAAAAGGAGAAGGAGAAAGAGAAAGAGGTTTTGGTGATGGATGGCTCTTCCTCTATGAATAACT

CATCAATGAATAGAGGTGTTGTTTCTAATATTATTAATAATAATAATTCTACTGAAGAAGAGAGTGGATG

GACAGCTTATTTTGAAGATTTCTCTAACCATAGAGAAGAAGAAGGAGAAGAAGATAGTTTCTGTAATGCT

GGTTTTTAAGGTAGTACTTCTATGGTTTCTGATGCTGCTTCTTATCCTGCATGGAAATCATCATCAACAT

CAAATAATCAACATCATCATCATAATTACAACAA

>FM891553.1 FM891553 Jatropha curcas embryo 56-70 (DAF) Jatropha curcas cDNA clone rjcaeb0_001863, mRNA sequence

ACCAAATCAAAAAACATTTTTATATTTATACACCGATACAAATATTTACCGTATTTAACATCCCAAGAAT

TGGAAAGTTATAAAAAAAAAAAAAAAAAGAAAGAATAGAGCCACTCACAATCCTCAACCTCAGCCACACC

AACTTAGGCCTCCAAATTTCTAAAAATACCCTCACACTCAAAACAATGAATAGCACTCGGGAGTAGTAAT

AATATACACATTCCTAAACAAAATTTTTCCTATTTTTCACTTTCATATCTGATGCAGAAGAAAAACTCTA

CAGAAATTTTTATATAAGCAGTTTATGGAAAAAGAAATGGAAGATATCAACACATAATAATCTGCAACGA

GTACAGGCAAACACATTTCCAAACTCCATGACATGCCCACCAATGCACAAGGAACTCAATTGACTGGATA

CAGCTTCTTCCAAAATTAAG

>FM891552.1 FM891552 Jatropha curcas embryo 56-70 (DAF) Jatropha curcas cDNA clone rjcaeb0_001862, mRNA sequence

ATTCCTTTTCCCTTGNAAGAAATATGTCGACTATGAGGGTGCCCACATCTTGCTTAATCAGAACCACACC

CCCAACCAAAGCTGTGAGCCCAACCAAATCTTGTGCCTTGATCAAGGGTTCGTTTTCTCTGGGTTCTGCG

AAAAATTTATCTAAAGCATTTGGTTTGAAATCTTCATCCTTCAAGGTTTCAGCAATGGCTAATGTATACA

AGGTGAAACTAATCACGCCAGAGGGTGTAGAGCATGAATTTGATGCCCCTGATGATACTTACATCCTTGA

CTCTGCTGAAAATGCAGGAATAGAACTGCCTTACTCATGTAGGGCAGGGGCCTGCTCTACTTGTGCTGGG

CAGCTGGTTTCTGGATCTGTAGACCAATCAGATGGGTCATTCCTGAGCGAAGAGCAAATGGAGAAAGGAT

ATGTACTTACCTGTGTCTCATATCCAACTTCAGACTCTGTTATTCGTACTCACAAGGAGAGTGATCTCTA

CTAAGTCATGTAAGAAGCAAGAATGAACTTGCATTAAATCGTAGATTCGTACAAT

>FM891551.1 FM891551 Jatropha curcas embryo 56-70 (DAF) Jatropha curcas cDNA clone rjcaeb0_001861, mRNA sequence

ATTCCTCTGTGCTTCGATCGCGTCAATGGCGGAGCACCCACAGTCGCAGCATGTTGGCCAACAGCCACGA

TCCCACCAAGTAGTGAAGGCAGCCACTGCAGTCACTGCCGGTGGATCTCTTCTAGTTCTCTCCGGTTTGA

CCCTTGCTGGTACGGTCATTGCACTGACCGTAGCCACTCCATTGCTGGTCATATTCAGTCCGGTTCTTGT

TCCTGCGGATATCACAGTTTGTTTGATAATCACAGGGATTTTTAGCCTCAGGCGGTATAGGCGTCGCTGC

CATTACTGTCTAGTCCTGGATATATAGGTATGTTACTGGAAAGCATCCACCAGGAGCAGAAAGTCTGGAC

CAGGCACGCTTAAAGTTGGCGGGAAAGGCAAG

>FM891550.1 FM891550 Jatropha curcas embryo 56-70 (DAF) Jatropha curcas cDNA clone rjcaeb0_001860, mRNA sequence

ATGCAGAATCAATATCCATGATTTATTCATTGTTTATTCCGACATTTCCACAATTTTATAAACAGACGTT

TCTAGCACAGTTTGATTAAACGGCAGATTTCCGTTGTGTTGTGTTGATTCACGTGATCGATAAAATAATC

GACTTCCTGTTGAACGAATTCAATGCATGCATCCTGAGAGCGAGCTCGTTTGCACAAGTATTTCACCAAG

TATCCAATGTGCTCCTGGACGGTCTGATCTTCGAGCATTTTCTTTATTACATCCATCGTGTTCGTGCATA

AATTACATAGGTCAATGTCGAATTTTTTTTGCTTTGATGGCACATCAAAGCTTGCGAAAGTTACTGCCGC

CAGCATTAACACGAACAGTGGGTTCATGATTTGCGGATGGGTGTTCTCTCTGTCTACAAGGATCTCCTTC

AAACAACAAAAATGGCTTCGGAGAAGAAGCTCTCAAACCCAATGAGAGAGATTAAGGTACAGAAGCTTGT

ACTAAATATATCCGTCGGAGAAAGCGGTGATAGG

>FM891549.1 FM891549 Jatropha curcas embryo 56-70 (DAF) Jatropha curcas cDNA clone rjcaeb0_001859, mRNA sequence

CTCTCTCGCCATTTCCTTCTGCACTGGATCACGTGTTGACATTCTCTCCACCTTCTCTTGCGCCTCTGCT

CTGGTTTTTTCCATGCCAGCCTTAGCAGAGGCTCCAACGTTAGCTGCGGTTTCCTTCAGCGACTCCATTT

TCACTCACTTGGCTATTCAAATTGGGGGGGCCCGGTAC

>FM891548.1 FM891548 Jatropha curcas embryo 56-70 (DAF) Jatropha curcas cDNA clone rjcaeb0_001858, mRNA sequence

ATTCGAATTGTCCGAATACTGTGTATCTCAAGTGGTTATTGCTGGCTTAAGACGCTTTCTGGTATATTGT

TGATCGGTTGAAAGTTCCAACACTTTTGGCTTTATCACGTAGAGGTAGAGGCGAGGAGCGGGAAGAAGAA

GGAATCATCGAAGAGACCTTCATTAGTTGGTTTGATTGTCCTCTCTTGAGTACTTGAAGAAAGATAGAGA

AAAAAAAAAGTTCATCTTTGTATTGTTATATTTTTGTTTGAATTCAGTTGAATATCAGGCCACTTTTTTT

TTTTTTACGGGCC

>FM891547.1 FM891547 Jatropha curcas embryo 56-70 (DAF) Jatropha curcas cDNA clone rjcaeb0_001857, mRNA sequence

ATTCGGCTCTTCCAGCCGCTGGACCCTACCTCCGACTGAGTCGTTTCCAGGGTGGGCGGGCTGTTAAACA

GAAAAGATAACTCTTCCCGAGGCCCCCGCCGACGTCTCCGGACTCCCTAACGTCGCCGTCAGCCTTCACG

TCCCGGTTCGGGAATTTTAACCCGATTCCCTTTCGAAGCTCGCGCGAGGACGCGCTGTCGGACGGGCTTC

CCCCGTCTCTTAGGATCGACTAACCCATGTGCAAGTGCCGTTCACATGGAACCTTTCCCCTCTTCGGCCT

TCAAAGTTCTCATTTGAATATTTGCTACTACCACCAAGATCTGCACCGACGGCCGCTCCGCCCGGGCTCG

CGCCCCGGGTTTTGCAGCGACCGCCGCGCCCTCCTACTCATCGGGGCCTGGCTCTTGCCCCGACGGCCGG

GTATAGGTCGCGCGCTTAAGCGCCATCCATTTTCGGGGCTAGTTGATTCGGCAGGTGAGTTGTTACACAC

TCCTTACCGGATTTCGACTTCCATGACCACCGT

>FM891546.1 FM891546 Jatropha curcas embryo 56-70 (DAF) Jatropha curcas cDNA clone rjcaeb0_001856, mRNA sequence

ATTCCTGATGAACTCTTACAGAATGTCTCCAAGCGAAGCTCATCAAGTGAAGAGGAACAGGGATCCACAG

TCCATGTTGTTTACTCCCACCAGCAGGAGACCATAAGTAAGTTACTACTAAAATAGCATTGTGATTGGTG

CTTCTACTAGCCAAGGCCGCAACTAAGAGATCCTTCTTGTAAATTTAGAGAAGGGATCCGTCGTATATTG

TAGTATAGAGAGTTGTAGAGTGTTAAAAATGTAGAACTTCAACTTCTGCAGAAATCAATAAAATGTGGCC

TTCTTTTGTTTCTAAAAAAAAGGGGGGGCCCGGTAC

>FM891545.1 FM891545 Jatropha curcas embryo 56-70 (DAF) Jatropha curcas cDNA clone rjcaeb0_001855, mRNA sequence

GGCACATACAAAGATAATACATTGAAAATCAACAATGGAACCCAAAAGACCAAAAAGAGAAATACAAATG

CAACAAAGAAGATCCCCCATAAACCAACTCAACTACCTCAAAACAACACCCATACAACAAAAGTAGTCTC

GTACCAAAACCCAGCTCTCTGACTTCAACCCTTAATAACCAACAGCATTTCTCTCTACTTATTTCCCTTC

ATGTGCTTTCCTCTGGATTTCTTGGCCTACTTCCTTGGTCTTCTGTCCCACAAAACCAGCCATGTCTTGC

ATACGCTTCTTAGCTATATCCAGCTGCTCCGGCATTCGCCGAGTGACCTCCTGGAGGTATTTCAGAACCC

ACGAGAGTGATGACAACCCTGTGAGCCCCATAGCCCCGGAAGCCAAAAATGCCATGACCGAAAGGCCAAT

AACTAATGCAGCTGGGACAAGAACAGGGCTAAAGATGACAAAAAGAGGGGTAGTAATAGCTAGGCCGATT

AGGGTACCTACTAAGGTTATACCAGCTAGTGCAAGAAGCCCA

>FM891544.1 FM891544 Jatropha curcas embryo 56-70 (DAF) Jatropha curcas cDNA clone rjcaeb0_001854, mRNA sequence

ATTCCCAGACTTCTGGGACCTAGAAGGCTTCTCCCCTTTACTAGATCCATTTCAAAACTTTCCTTTCCCT

TCTTTACTTTCCGCTCACTTCCCCTCAGTTTTCTCTTCCCTTGAAACCCAGGTGGATTGGAGAGAAACCC

CAAGAGCCCATGTGTTTAGGGGTGTGTTTGGAGGGTCCAACAATGAGGATGTGTTGGTTTACATTGACGA

TAACAATATTCTTCAAATAAGTACAGAGAGTGGTAAGTTCATGAGCAAGTTTAAGCTGCCTGATAATGCT

CAAAGGGATAAGGTTAGTGCTTCTATGGTGAACGGAGTTCTTATTGTTACTGTTCCTAAGGAAGGTGGTG

ATAGGATCTCTAATGTAAGGGCCATTGAAATTTCTGGTTCTGATTAGAATACATGTATAGTTAGATCTTG

TGCTTCTATGTCTGTAGGAGAGGATCTTGTGCTTCTGTGTCTGTAGGAGAGAGGGTTGGGTTTTCCCGTG

TGGTGTCTGTACGAGAGGATCTTCTGCTTCTGTGTCTGTAGGAA

>FM891543.1 FM891543 Jatropha curcas embryo 56-70 (DAF) Jatropha curcas cDNA clone rjcaeb0_001853, mRNA sequence

ATTCCTAGTAATAGGTGTATAAAATATAGCAGAAACTATTACCGGCTACCGGTTACCACAATCAATCATC

TAACAATAAGCAGTACACTTTTTCTTACAGTCGATGGTGCAGCCACCCCCACCACCGCCAGCTCCATATC

CCTTCCCTGACCGGCTATAGGAGGTGAAACACTTTGCAGGACAAGTCAGCTTCTTCTTGTAACAAGGACC

TCTCTCCTTGCACACCACAGTAGGCCTTATAATACCACCTTTTGAATACCCTCCATTTGGACCTCCATAA

CCAGCTCCATACCCGCCACCAACAATCCCATTTCCCCAACCTTTTCCAAATCCCGGTATTCCAAACCCGG

CTCCGGGCCCAAAGTATCCGCCCATTCCAGGATTATCATTTCCACGGGGGGGCCCGGTAC

>FM891542.1 FM891542 Jatropha curcas embryo 56-70 (DAF) Jatropha curcas cDNA clone rjcaeb0_001852, mRNA sequence

ATTCGAGAGAGAGCAGGAAAAGAAAGAGAAATTAGAGGGTCTTCCAGTTGAGGACAGCCCTTATCTGCAA

TACAAGGACTTGGAAGATTACAAGCTAAAGGGCTATGGAACTCAAGGCCATCTCCAGCCAAAGCCTGGCC

GCGGAGCCGGCGCCACCGATGCACCCACTCTTTCTGGTGGCTCTGTCCCTCGAGATGCTGATGTTAGTAC

CATTGATGCTATAACTAGTCGTCCCAAGAAGCCTTAAATTTTATTCCTCAAAATTAAAAAAGAAAACCAC

CTTAGTTTTTTTTAGTTTTTTTTTTTTTTTTGGATGAATATATAGATGGGGATTAAATAAAGTTGGGTAA

TTAAACTTTCAGCTTTCTTTTTTCAAATTAAAAAAAGATAATAAATAACAGTGATTAAGGAAAAAAAAGG

GGGGGCCCGGTAC

>FM891541.1 FM891541 Jatropha curcas embryo 56-70 (DAF) Jatropha curcas cDNA clone rjcaeb0_001851, mRNA sequence

ATTCGAATTCCTGAGCTCCGAGACCGAGAAGGAACGGAAAAATGCCAAAGCAAATACATGAAATCAAGGA

TTTCCTTCTTACAGCAAGAAGAAAGGATGCTCGTTCTGTCAAGATCAAGAGAAGCAAAGATGTGGTGAAG

TTCAAGGTTCGCTGCTCCAAATATCTTTACACACTTTGTGTGTTCGACTCTGAGAAGGCTGACAAGTTGA

AGCAATCTCTTCCCCCAGGTTTGACTGTACAAGACCTGTGAAGAAACATAAAGACTAAAGTTTTTGGCCT

TATGAGAAATGAAGGGATCCAAGTCTTTTGCCTTTTTTCTATTGTCTTTTGGGTAATGTTATCAATTTCG

TTGACTGTTGAAGTAGTTATGCTGTTTGCCCAACGTTTGCTTATGGACTCCATATGAACTGGTAGACATG

AGCTGTTGAGTATGTTATGAATTTTGATAATACTTTCAACTTGAGCACTTGGTATTAC

>FM891540.1 FM891540 Jatropha curcas embryo 56-70 (DAF) Jatropha curcas cDNA clone rjcaeb0_001850, mRNA sequence

ACAGGAACATTTTCTTCGTTGACTAATCCATCCATGTAGAAAATATACATATGGGCTCATACAGGCCTCA

CATGGGCTCTGTCGTCACTCATCCCAACTATACTTCATTTCTACTTCATTCAAGCCCAACAAAATACAAG

ACAGGACACCAGATATGTAAAATAGACGCCAAAACGACAACAGAACAGCCAAAGAAACAAATCAGAGCTC

TTCGTCCGGCAATTCACCACCGGCACCAGCCACGTTATCCTTGAGATGCTGTGATGTCTTCTTTGCCTTA

TCTTTT

>FM891539.1 FM891539 Jatropha curcas embryo 56-70 (DAF) Jatropha curcas cDNA clone rjcaeb0_001849, mRNA sequence

ATTCGGCAGAGGTCAGAGGAACGAGCGTATTTGCCTGCACTGACCGGTGCGGATGCCCAGTTCCATGCCC

CGTGCGGCACAGCATGCAGGTGCAGAACAGTAAGTGAGACGGCAAGCGGTGGTGGCGCTATGGGAACGGG

ATATGAACACAGCCGGTGCTCGTGCGGCGAGCATTGCGGTTGCAATCCGTGCACATGTCCGAAAGGTAGA

GAGACTATTGCGGCTGGTACGGCGAATTGCAAGTGTGGCGTTGGTTGCACGTGTCCTACCTGTAGCTCTT

AAAGACTGAAAATGAAGAACAGGCACTTACAAGTCGACTCTATAATACTCCTACTAAGTATTTTGTGAGT

TATATATAATATATATATATATATAGGAGAGAGAGAGAGAGAGAGAGAGAGAAAGAGA

>FM891538.1 FM891538 Jatropha curcas embryo 56-70 (DAF) Jatropha curcas cDNA clone rjcaeb0_001848, mRNA sequence

ATTGTTTCTTCTTTGCATTCAATCCAAAACCATCTGAGAACTTACCGGAAAAATCATTTTCCGGCAATTT

TTTTTGCTAGTCAAAGACCCACTACATTACTGTTTGTAAGAATGGCTTCCGGCGAGCAGCAGTTTCCTCC

TCAAAGGCAAGAGAAGCAGCCTGGGAAAGAACATGTCATGGACCCTATCCCACACTATGCCATGTCCGAT

AGCAAGCCCTCTGATAATCTCAGGGGAAAGGTGGCACTAGTCACTGGTGTGGACTCGGGAATAGGACGTG

>FM891537.1 FM891537 Jatropha curcas embryo 56-70 (DAF) Jatropha curcas cDNA clone rjcaeb0_001847, mRNA sequence

GGGAGAGAAAAGATTACTTCGTCTCATTGTTAAAGAGAATGCTATAACATGCCAAACAGGTTACAGGTAC

ATGTAGACTGAACATAAAACTCGGTGCCATTTCTATGTCCGCTTCGAGAAGACACTTGTTATAAGTACAT

ACGCAAATACACTCCTACACACAAGGGGGAAGTAACAGAACAAAACAAAGCAAACATCATATACAACCAA

ACACCATTCATATTAGTAACTCTTCATTAGTGTATCAGTACGTTAATGGTCTCATTAAGCCTAACAGCTG

TCGTCATTGACACCGCAACACCACCTGGATGCGTGTTTAAATTCGGATTGTTCCTCA

>FM891536.1 FM891536 Jatropha curcas embryo 56-70 (DAF) Jatropha curcas cDNA clone rjcaeb0_001846, mRNA sequence

GATTAATGAAAACATCCTTGGCAAATGCTTTCGCAGTTGTTCGTCTTTCATAAATCCAAGAATTTCACCT

CTGACTATGAAATACGAATGCCCCCGACTGTCCCTGTTAATCATTACTCCGATCCCGAAGGCCAACACAA

TAGGACCGAAATCCTATGATGTTATCCCATGCTAATGTATACAGAGCGTAGGCTTGCTTTGAGCACTCTA

ATTTCTTCAAAGTAACAGCGCCGGAGGCACGACCCGGCCAGTTAAGGCCAGGAGCGCATCGGCAGAAGGG

ACGAGCCGACAGGTGCACACCGCGAGGCGGACCGATCGAGCCAACCCAAGGTCCAACTACGAGCTTTTTA

ACTGCAACAACTTAAATATACGCTATTGGAGCTGGATTACGGGCTGTGGCACCAGATTGCCTCCATGGAT

CTC

>FM891535.1 FM891535 Jatropha curcas embryo 56-70 (DAF) Jatropha curcas cDNA clone rjcaeb0_001845, mRNA sequence

ATTCCCGACTTTCCCTCTGAACAGTGGCCATCCCATATATAAACATTCATATTTGTGAGATTTATCATCT

ATACTTTATAAAAAATGGCTGATTTACGAGATGAATATGG

>FM891534.1 FM891534 Jatropha curcas embryo 56-70 (DAF) Jatropha curcas cDNA clone rjcaeb0_001844, mRNA sequence

ATTCGCTAACTTCATTTAAAATTAAACAGGTAACCACATCTTACTGTGCTTATTACAGATGATGCCCATA

ATCTTGGCTGACAAGCTAATCAAATCCTGAAAGTTAAATAAGGACCAAGACAAGAATCATCGTACATTGT

TTTAAACTGCACAATGGTCAGGATTTTAATGACACTCAGCATCTAAGCACACCAAGGTTGTCAACCAGTG

CGGATCCCCCCCATAAATGTCCTAGGATCAGGGCAGTTTCCCGAGAATTGAGCCTGTGAGAAGTCAAAAC

CAGGATTCTCTTCTTGAAATCTCTGAAGCATGAGACGCTTCTGTTCGAGATCAGTGGAATAAGGATCCAA

CTGACCCTGACCCAATATAGGAGAAGACCATGTCTGACCCTTATCTCTTTTTTGCAGGGTCAAGTGCATT

ATATCTCCTCTAATCCAAGGGGGGCCGGTACCAT

>FM891533.1 FM891533 Jatropha curcas embryo 56-70 (DAF) Jatropha curcas cDNA clone rjcaeb0_001843, mRNA sequence

ATTGAATAATGGAAATTTCAAGCGAGAAGAATGGCTACAGAGCAAGAAAGGGCTGAACTTGATGCTAGAG

CAAGGCAAGGAGAGACTGTTGTACCTGGTGGAACTGGTGGCAAGAGCCTTGAAGCCCAGGAACATCTAGC

TGAAGGGCGGAGCCGTGGAGGGCAGACAAGGAGGGAGCAGCTGGGAACTGAGGGGTACAAGGAACTTGGT

CACCGTGGAGGTGAGACAAGAAGGGAGCAGATTGGGCATGAAGGGTATCAGGAGATGGGCCGAAAAGGCG

GACTTAGCACCATGGACAAGTCTGGTGAAGAACGTGCTGCTGAGGAGGGGATTGAAATTGATGAGTCCAA

GTATAAAACCCGTTAGTTAGGTTCTTCATATCAGTGTTTTAACTAGTAGTTCCTCTTTTTGAACTAGTTG

CGAGTAGGTTTGTACATATGGTAGTAGTAACTGCGGTATGTTGGT

>FM891532.1 FM891532 Jatropha curcas embryo 56-70 (DAF) Jatropha curcas cDNA clone rjcaeb0_001842, mRNA sequence

ATTGGCAAGAGCCTTGAAGCTCAAGAGCATCTTGCTGAAGGGAGAAGCAGAGGAGGACAGACAAGGAAGG

AGCAGCTGGGGACAGAAGGATATCAGGAGATGGGCCGGAAAGGTGGGCTGAGCACGACGGACGAGTCTGG

TGGAGAAAGAGCTGAGAAGGAGGGCATCACTATCGATGAGTCGAAGTTCAGGACTGGGAGCTAGTGTGAT

GATTAAAACTATAAATGGTATTTTGTAGTAATAATAATAATAATGTTGGTAATAAAGTATTAGACTTAGC

AGTGTACTTTAGTTTAGTAATGTA

>FM891531.1 FM891531 Jatropha curcas embryo 56-70 (DAF) Jatropha curcas cDNA clone rjcaeb0_001841, mRNA sequence

ATTATCTTTCTCACACTTATCATCAACAAATGGCTGAACGTTCACAGCCACACCAAGTTCAAGTGCACCC

ACAACACCGCTATGAAGCTGCCTTCAAAGGTCAACAAAAAGGTCCATCAGCTCAGAAAGTGTTAGCTGTG

ATCACCCTCCTGCCGGTAGGCGGTGGGCTTCTTGCACTAGCTGGTATAACCTTAGTAGGTACCCTAATCG

GCCTAGCTATTACTACCCCTCTTTTTGTCATCTTTAGCCCTGTTCTTGTCCCAGCTGCATTAGTTATTGG

CCTTTCGGTCATGGCATTATTGGCTTCCGGGGCTATGGGGCTCACAGGGTTGTCATCACTCTCCGTGGGT

TCTGAAATACATCCCAGGAGGTCACTC

>FM891530.1 FM891530 Jatropha curcas embryo 56-70 (DAF) Jatropha curcas cDNA clone rjcaeb0_001840, mRNA sequence

ATTATACACTCTCTCTTATACCTAAATTTAAAGTTAGAGTTTTCAGAAATATGGCTACGAAGAACACATC

AACAGTTGCTCTCTTTCTTGTATTTAACCTTTTGTTCTTTACCCTAGCCAACGCATGTGGTGGGGGCTGC

CCTACTCCTAGGCCACCAAGACCAAAGCCAACTCCAAAGCCAACACCAACTCCGTCACCTTCAGGCGGAA

AATGTCCTAGAGATGCACTTAAACTAGGCGTATGTGCTAAGGTGCTCGGTTCATTGCTTAATATCACCAT

TGGCGATCCTCCTGTCAAACCTTGCTGCTCTCTTCTAGAGGGTCTTGTTGATCTTGAAGCTGCTGTTTGC

CTTTGCACTGCCATTAAAGCTAATATTTTGGGTATCAACCTCAATGTTCCACTTTCTTTGA

>FM891529.1 FM891529 Jatropha curcas embryo 56-70 (DAF) Jatropha curcas cDNA clone rjcaeb0_001839, mRNA sequence

ATTCGTGACAAAAGTGGCACCGTTGATATTGAAGAGTTTAGAACTGAAGTCAGGAAAATATTGCTTGCGA

TTGCTGATGGGTTAGGATCCAGTCCCATCCAGATGGCTCTTGAAGATGATGATCAGAGTTTTCTCAAGCA

GGCTGCCGATCTCGAAGCCTCTAAGTTTCAGTCTTCTGTTTCTTAACTAATTTAGATCCGTTTTCTATCT

TGCCTGTCAAATCTTTTTATCTTTACATCTTATAAGGCTTTTGTTTGTTCCTACCTTCTTTATCTGTTTT

TCAATATTTCAATAAATTTTGGCAATTTAATCT

>FM891528.1 FM891528 Jatropha curcas embryo 56-70 (DAF) Jatropha curcas cDNA clone rjcaeb0_001838, mRNA sequence

CGAATCGTGAAATGGCACGGATAAATCAGGATTCAAGCCGGTCTATCTTGGGTTCACGACCGTGACGGCA

ATGTACCCTCCAACGCAATCGACGCCGGCGACGGTATATACGTCGGCCGTTTGATGCACGATGGAGACAT

GCTACCCGCGAAGGTAGTGCCTAGACTGGGAAAAGCATACGTATGTCACGGGGGCCGAGAACATGAATAT

CACTCTTATGAAGTACTGTGTGATACAAAGGCTCCTGGAACACAAAAATGTTACGTCTGGGAACACGCAC

GGGGTGGCCATGTTCCAAAATACGCTGTGCTAGCCGGTCTCTCGGATTCCGGTGATCCTATTTACGTTTC

TCGTTCGGAAATCGATGGTGAACGCGTCGTGGGCAAGGTTCACAGTGGCCATGACTGTGCCTACTTCCCC

TACGGTGGTCGTGAACATCAGAAAAGTAGCTACGAAGTTCTGGTTATGAAAAATG

>FM891527.1 FM891527 Jatropha curcas embryo 56-70 (DAF) Jatropha curcas cDNA clone rjcaeb0_001836, mRNA sequence

ATTCCTTCTTTCATGGCGCATGTTTCCTGCAGTATTAGTATTGGACCTTGTAACTACTTTTATCATAACA

TCAACTATAAGTACTTCAGTTTTAGTACTCAAATTATCAATCATAAGCAGACTTTGTTCGCTTGCATTAG

TAATAACAGAATACACAGTATTGTTCACTCTAAGAAGCAGCAGGCTTCCTCCATTACTTGTTCTGCCTCT

AACAGACCCTCTTCTCCTACTGATGTTAGGTTCTTTTTACAAGTGAGGCTTCCTTGTAATTTTTCTTTTA

TTTGTTCTTCAGTTCTACAGCCAAGATAAGAAGTGAAGTTCTCTCTCCATTTCGGTCTGTGCGGATGTTT

TTCTATCTAGCCTTTATTGCTAGTGGTGCTCTGGG

>FM891526.1 FM891526 Jatropha curcas embryo 56-70 (DAF) Jatropha curcas cDNA clone rjcaeb0_001835, mRNA sequence

ATTCATCAGCTCATTTCAAAGGATAGTTATTCAGAATTGTCCCATTCAATACCGGTTCAGTTTCCCCATT

ACACGTCCCTCCTGAGTCCCAATCCCCACCTCTGAAATGAGCTGATGAATATCCACGATAAAAGATCAAC

TGCTTCGCAGGATTCACATTCTCGTCAATCCACTTTCCCCAGGTTCTCAGAGCCCTTCTGTAAGCCTCAA

CTGCATCAAACTGAGGATAGAGATAATCACCTTCTTTATAGTAATTTTTCCCACGTGCAGTTTTTCCATG

AGTCCACCAGTGCCCTGTATTGAAAACAAGAATGTCTGCTCGCTTCCAGCGGCCTGATGTCTTATCAATT

CGATCAATGGACAAAGTTGGGTTTGAGTTTCTTTGTTCATTTATTCGAACCCCTTCCCTTACAAGGAAAT

GTGACCTTACAAACTCCACGTACAGTTATAATCATAATTGAAG

>FM891525.1 FM891525 Jatropha curcas embryo 56-70 (DAF) Jatropha curcas cDNA clone rjcaeb0_001834, mRNA sequence

ATTCATTCAACCCACGTGCTGGCCGTCTCACCAACATCAACGGCCAAGACCTCCCCATCCTCAGTTATAT

CCAGCTAAGCGTTCAGAAAGGTGTCCTCTACAGAAATGCTCTAATGTCACCACACTGGAACGTCGATGCC

CATAGCATAAACTACTTCACAAGAGGAAATGGCCATATCCAGATCGTAGATGATAA

>FM891524.1 FM891524 Jatropha curcas embryo 56-70 (DAF) Jatropha curcas cDNA clone rjcaeb0_001833, mRNA sequence

TGCTGAGGAGGGGATTGAAATTGATGAGTCCAAGTATAAAACCCGTTAGTTAGGTTCTTCATATCAGTGT

TTTAACTAGTAGTTCCTCTTTTTGAACTAGTTGCGAGTAGGTTTGTACATATGGTAGTAGGAACTGCGGT

ATG

>FM891523.1 FM891523 Jatropha curcas embryo 56-70 (DAF) Jatropha curcas cDNA clone rjcaeb0_001831, mRNA sequence

ATTCNCGAAAACACTAGGGAAAAATGGATGTCAGGTTCGTAGGTCTCGATTCACCACTTCTCCACACTCT

CCAGCAAATGATGGACATTGGCGACGATGCTGACCACAAATCCTTCAACGCGCCAACACGCACTTATGTG

AGGGACGCGAAGGCTATGGCTTCTACTCCGGCCGATGTCAAAGAGTATCCGAATTCTTATGTGTTCATTA

TCGACATGCCTGGATTGAAGTCTGGAGATATCAAAGTGCAGGTGGAAGATGATAATGTGCTGTTGATTAG

TGGAGAGAGAAAGCGTGAGGAGGAGAAGGAAGGAGCTAAGTATGTGAGGATGGAAAGGAGAGTTGGAAAA

TTGATGAGGAAGTTTGCTCTGCCTGAGAATGCCAATACTGATGCCATTTCTGCTGTGTGTCAAGATGGGG

TTTTGACTGTCACTGTCCAGAAATTGCCACCGCCTGAGCCTAAAAAGCCAAAGACTATTGAGGTTNAAAT

TGCTTGAGCTCAGAGGATCGTTTTGTCACGTTTGACTGCGTGCTGGAAATAATTTGGGCTATGTTGAAAT

CGTAATCTGCTTGTGTTTTTCTT

>FM891522.1 FM891522 Jatropha curcas embryo 56-70 (DAF) Jatropha curcas cDNA clone rjcaeb0_001830, mRNA sequence

ATTCCTTCATGCTCTTTCTATTTCTCACTTCACTACATGTACAATACCAACTTTTAGTGCAAGTAAGCAA

AGCCAAGCTATGGACGAAGAATTCTCACTGGAGGATCCTACTCCTTTACTTGAATCCGCCTCTGATTTCG

CTTATTTTCCCGGTGTTCAAAATGATGCCGTTGTTAAGGACTTTCTTGATCGTTTTCCCCTTCCTCTCAT

TATCAGTTCTTTACAAACAAAAACAGATGTGCCTGGTTTGGAGAAGACTCTGGTTGCTTGTCTAGAAAGA

ATATTTAAGACAAAGTATGGTGCATCTCTGATTCCGCAGTATATGTCCTTCGTGCAGCTTGGTTTGAAGG

CAGATTCACAATTAGTAAGATGTTTAGCTTGCAAAACGGTACTTGTATTGGTCTATTGAGCTACCTGGAA

AGTAATTGATGATGGGACCAGCTCCAGTTAGATGCTGTTTTGGTTGGAAGAGAAGACGTTTTTTATGATC

TAGAATCAATTTTAGTTTGAAAGGATTTTCAGAAATGAAAGTTGATATACATCATGGATTTTTGGAATAC

CATAACTGGGCCAGACTCCAGATGTCTGACTGTGCTGTTGAAAAA

>FM891521.1 FM891521 Jatropha curcas embryo 56-70 (DAF) Jatropha curcas cDNA clone rjcaeb0_001829, mRNA sequence

ATTCCCACCACCAATCTCTCCCTCGCCGCCTACCAGCAGTCATCAATGATGACAAAAGATGCCCTAAACC

CTAAAACGACATTGAGAGAAGAGGGTTTCGGTTCTCCTTTCTCTTAATTTCGTTTTGTTTTTGTTCCTTT

TTTTTTTCTATTTCTATATTTATATAAATTATATATACCTATAATCTTCATCATGAAGTTTTTTTCTGGA

ATTCATGTCTTGTTGCGGTCCCACCGGTGGTGAAGTCAACCAGAAGGATATGCCGGAGCTGTACGGGCGG

CGTTCGGAGGAGACGCGGGCGCTGGTGTCGTCACTGACGCTGCGGTCGTTGACGACATCGAGAAGTAGAA

GAAGGAAAAGAGGTACTACATCTCCATCGTCGGCGACTGTAGTGGCTGTGTGGAAGCCCACGCTATGCTC

GATATCAGAGGATACCGTGGTGATGGTGGTGGAGGAAGGAACGGAAAGGGTGGTGAAGAGGAAAAGTAGC

GGTGGTGGTGGGTCCCGTAATATAGCTAGTGTTGCCAGCTTCAATGAAGATTACAGGCATAACAATCACT

TCACAGCAATTCCTACATTCTCAGCCACTCCCTTCATGTTTT

>FM891520.1 FM891520 Jatropha curcas embryo 56-70 (DAF) Jatropha curcas cDNA clone rjcaeb0_001828, mRNA sequence

AGAGAGAGTTGATGGAGAAAGAATGGCAAAGAGTAAGGATGATATCAAGTATGGGACAGCACAGGCAAAG

CTATCAGAAGATGAAAGTCTAAGGATGAGGTATAAGCATGGGACTCCGCTTGAAGGAGGCAAGATTGCTG

ATTCTGAGACTGTTGATCTCTTCTCTAGTGCTCATAATATTCCCAAATCATCGGACTCCGCCACTTCCAC

TGATCAATCTCAGAGCCACCGCCCCTCGAATGCCGCCGGAGGAAATGCTACCAATAACAATCCCACCACT

ACCAATGATTGAGAGTTTTATTTTGTTTCATGTAATCTGAGAGCGTGTCGTAGAAAAAAGAAGTGTTAGA

ATGTTTCTTCTGCTGCTCTCCAGAAACTATATGTGTAATAATAATTTAGTTTTTTTTTTCCTAAGATAGC

AATATATAAAAATTATTAGATAGTTTTGTAAT

>FM891519.1 FM891519 Jatropha curcas embryo 56-70 (DAF) Jatropha curcas cDNA clone rjcaeb0_001827, mRNA sequence

ATTCTTTCAACACTGGGCTATTTAAACAGCCATATATTCAGACTTGCAATATTGAGAATGACACTGGATA

CCATCAGATCAGAACACATACAATGTATTCAATCTGATCGGTTTAAAGTTTTACCAACATAAAATAAACA

CAAACATAGTTATTTTTTATTAACAAGGTAGTCCAACTCCTTTATAGCTTAAGCCTTGGCAACAGCAGCA

GCAAGAGCTCCAGCTTCAGTAAGCAGAGGAACCAGCTTTCCTTCACCGTGCAAGCCTGTAGTTGCATCAC

AGCCACCAATGTGCTTGCCCCCAATGAAAACGTTAGGCACAGTGCGTTGTCCAGTCCACTCTGCTAAAGC

TGATTGTATCTCTTTACCATCACTCTCAGTATCCAATTCGATGGCCTTGAAAGCTGCTCCCAATTGATTA

AAAAGCTTCTTCACGGTTGTGCAAAAAGGGCAAGTAGTTTTTGCTTAAAAACGACGACGGAATTGGAAG

>FM891518.1 FM891518 Jatropha curcas embryo 56-70 (DAF) Jatropha curcas cDNA clone rjcaeb0_001825, mRNA sequence

ATTCTTTTTTTTTGACAAAAGTACAGACAAACTGTATTTCTGTAAACTGTAATCAAAAGGAACAAGCTTT

AGAAATATTAATAATTACAGAAATTAAACCAGATAAAGCATTAACAATCCACGCATATAGATATTATCAT

TTCAGTATATCAAGGAGTTAGACGTGTGCCTCCATCTGATTGAGAAGGAAGTTGCCTTCATTGTCATAGT

GCACCTCAACCTTGTACTTCTCTACATTGATTCCCCTCTTCACCTTAAGGAGCATATCAAACTTTGCAAA

ATCATCCACTACCTCAGCCTTCGCATGAACAATCTCTTGAAGCTCATAAGGGAACAATGAATTGGACCTC

TGCTGAATGGTCTACACAGCATGATTTGCAACATCTTGCACTGCAGGATCATGTGCTGGCA

>FM891517.1 FM891517 Jatropha curcas embryo 56-70 (DAF) Jatropha curcas cDNA clone rjcaeb0_001824, mRNA sequence

ATTCTGCTAGACTCGTACACAACATCAATGACCCGTCACGTGCCGATGTATTCAACCCACGTGCTGGCCG

TCTCACCAACATCAACGGCCAAGACCTCCCCATCCTCAGTTATATCCAGCTAAGCGTTCAGAAAGGTGTC

CTCTACAGAAATGCTCTAATGTCACCACACTGGAACGTCGATGCCCATAGCATAAACTACTTCACAAGAG

GAAATGGCCATATCCAGATCGTAGATGATAATGGAGAGACAGTATTTGATGGAGAAGTTCAAGCAGGACA

AATATTAACATCTCCACAAAACTTTGTGGTAGT

>FM891516.1 FM891516 Jatropha curcas embryo 56-70 (DAF) Jatropha curcas cDNA clone rjcaeb0_001823, mRNA sequence

ACTAGTTGATCCCCCGTGTGCTGCAGTGAATTCGAATTCGTGCAGAAGCAAAATCAAAGTGATAAATGTA

GTGTGATGTGAGTTTTGCTTTGAGTGCGTCATGGAAGTCTGCACTGTAAATGTGTTTGTATCTGGGCTTC

ATTTTGTTTATGACATTAATAAAGTTAGGGAAAAATACATGTTTATTTAGATAAAAAGGTTTTTCGAATG

TAAAAAGTATATAGCACTAATATTGCTAGACCTTAACGCCCCAAAATTTTCTTCTTTTCCTC

>FM891515.1 FM891515 Jatropha curcas embryo 56-70 (DAF) Jatropha curcas cDNA clone rjcaeb0_001822, mRNA sequence

TGTAACAGAGCAAAAGATTAATTATATTGGTGGAGAAATTTGTTAGCAAAAAAGGGCAAAAATGTCTTCA

AATCAATCAAGACAAGAGCTTGATGAAAGAGCAAGGCGTGGACAGACTGTTGTCCCTGGAGGTACTGGGG

GCAAGAGCCTTGAAGCTCAAGAGCATCTTGCTGAAGGGAGAAGCAGAGGAGGACAGACAAGGAAGGAGCA

GCTGGGGACAGAAGGATATCAGGAGATGGGCCGGAAAGGTGGGCTGAGCACGACGGACGAGTCTGGTGGA

GAAAGAGCTGAGAAGGAGGGCATCACTATCGATGAGTCGAAGTTCAGGACTGTAAGCTAGTGTGATGATT

AAAACTATAAATGGTATTTTGTAGTAATAATAATAATAATGTTGGTAATAAAGTATTAGCTTAGCAGTGT

ACTTTAGTTAAGTAATGTAGTTTACATGTGGGTTAAAATGACTAGGGTTTAGCT

>FM891514.1 FM891514 Jatropha curcas embryo 56-70 (DAF) Jatropha curcas cDNA clone rjcaeb0_001821, mRNA sequence

ATTCCCAAGTTCAAGTGCACCCACAACACCGCTATGAAGCTGCCTTCAAAGGTCAACAAAAAGGTCCATC

AGCTCAGAAAGTGTTAGCTGTGATCACCCTCCTGCCGGTAGGCGGTGGGCTTCTTGCACTAGCTGGTATA

ACCTTAGTAGGTACCCTAATCGGCCTAGCTATTACTACCCCTCTTTTTGTCATCTTTAGCCCTGTTCTTG

TCCCAGCTGCATTAGTTATTGGCCTTTCGGTCATGGCATTTTTGGCTTCCGGGGCTATGGGGCTCACAGG

GTTGTCATCACTCTCGTGGGTTCTGAAATACCTCCAGGAGGTCACTCGGCGAATGCCGGAGCAGCTGGAT

ATAGCTAAGAAGCGTATGCAAGACATGGCTGGTTTTGTGGGACAGAAGACCAAGGAAGTAGGCCAAGAAA

TCCAGAGGAAAGCACATGAAGGGAAATAAGTAGAGAGAAATGCTGTTGGTTATTAAGGGTTGAAGTCAGA

GAGCTGGGTTTTGGTACGAGACTACTTTTGTTGTATGGGTGTTGTTTTGAGGTAGTTGAGTTGGTTTATG

GGGGATCTTCTTTGTTGCAT

>FM891513.1 FM891513 Jatropha curcas embryo 56-70 (DAF) Jatropha curcas cDNA clone rjcaeb0_001820, mRNA sequence

ATTCCTCGCCTGTATTTAGCCTTGGACGGAATTTACCGCCCGATTTGGGCTGCATTCCCAAACAACCCGA

CTCGCAGACAGCGCCTCGTGGTGCGACAGGGTCCGGGCACGACGGGGCTCTCACCCTCTCCGGCGCCCCC

TTCCAGTGGACTTGGGCCCGGTCCGCCGCTGAGGACGCTTCTCCAGACTACAATTCGGACGCCGGGGGCA

TCCGATTCTCAAGCTGGGCATTTCCCGGTTCGCTCGCCGTTACTAGGGGAATCCTGGTAAGTTTCTTTTC

CTCCGCTTATTGATATGCTTAAACTCAGCGGGTAGTCCCGCCTTTTTTTTTAAACAGAACATTCCTTTAG

ATTAAATAACAACAACACAAAATATAAATATAAATATAAATAAATTCCTCCCTTAGAACAAAATGGAACT

CTATTAACCAATAATAATACTAGTAATTGTAAATCTAATACTTCAAATTTTACCAGTATTTCAGTAACCT

CAAATTGTTGCTATAATCTGATATGGAACAGATCAANGCTGTTGCTGTGCATCCATGTAGCCAGCATCAA

CAAGCACCTTCTCTCTTTTCGCTATTTCAATATCTTCATCCACCATCATCTTAAACAGTTGCTCAAACCC

AA

>FM891512.1 FM891512 Jatropha curcas embryo 56-70 (DAF) Jatropha curcas cDNA clone rjcaeb0_001819, mRNA sequence

AACCAACAGGTAATTATATATACTGCAGGTTATTTTACAAATAGGGAGAAAGTATTAGCAGTCCTCCCAG

AAAGGGAACGACCATAAACCCCTCTCAAAGTTACAACACACATACTAGCTAAACCCTAGTCATTTTAACC

CACATGTAAACTACATTACTAAACTAAAGTACACTGCTAAGCTAATACTTTATTACCAACATTATTATTA

TTATTACTACAAAATACCATTTATAGTTTTAATCATCACACTAGCTCCCAGTCCTGAACTTCGACTCATC

GATAGTGATGCCCTCCTTCTCAGCTCTTTCTCCACCAGACTCGTCCGTCGTGCTCAGCCCACCTTTCCGG

CCCATCTCCTGATATCCTTCTGTCCCCAGCTGCTCCTTCCTTGTCTGTCCTCCTCTGCTTCTCCCTTCAG

CCAGATGCTCTTGAGCTTCAAGGCT

>FM891511.1 FM891511 Jatropha curcas embryo 56-70 (DAF) Jatropha curcas cDNA clone rjcaeb0_001817, mRNA sequence

CGAATTCTTCTTCTCTCGCAATCGATTCACCGATCCAATTCTCCAAAATTTCTAATGGCAACTGGTTTTA

GCGGCGATGAAACCGCTCCTTTCTTCGGCTTCCTTGGCGCCGCGGCGGCCCTCGTTTTCTCATGTATGGG

AGCTGCCTATGGGACAGCCAAGAGCGGTGTTGGTGTAGCATCAATGGGCGTTATGAGACCAGAGCTCGTG

ATGAAATCTATAGTTCCTGTTGTTATGGCTGGAGTGCTTGGTATTTATGGTTTGATTATTGCTGTTATTA

TTAGTACTGGAATTAACCCTAAAGCCAAATCATATTACCTCTTCGATGGTTATGCTCATCTTTCTTCCGG

TCTGGCCTGTGGTCTTGCTGGCCTTTCTGCTGGTATGGCTATAGGTATTGTTGGAGATGCTGGCGTCAGA

GCTAATGCACAGCAACCAAAGCTTTTAGTTGGCATGATTCTCATTCTCAT

>FM891510.1 FM891510 Jatropha curcas embryo 56-70 (DAF) Jatropha curcas cDNA clone rjcaeb0_001816, mRNA sequence

AGTTGTGAAACTTTCTGTGCATATTTTTAATATTTTTCATGAGAGCTTCTGATAATCTCATAAGAATTAA

CTATGGACAGAAAGCTCCCATCTTACATGTAATATAGATGATGCACAAAATAATATAGATAACAGAAAAC

AAACTTAGCTTGATGTGCTCATTCTTTTGTGCATTATTATACATAACTCTGATCACTCCCCCACCAAGTG

AGTGAAATTGTTATTTTACTGAAGCACATCCACGTTATAGCTTAGCGATTAGGAACG

>FM891509.1 FM891509 Jatropha curcas embryo 56-70 (DAF) Jatropha curcas cDNA clone rjcaeb0_001815, mRNA sequence

ATTCGACAGACCGGTAAAACAGCAGTAGCCACAGATACAATTCTCAATCAACAAGGACAAAATGTAATAT

GTGTTTATGTAGCTATTGGGCAAAAAGCGTCTTCTGTGGCTCAGGTAGTGACTACTTTACAGGAAAGAGG

AGCAATGGAGTACACTATTGTGGTAGCCGAAACAGCGGATTCCCCGGCTACATTACAATATCTCGCTCCT

TATACAGGAGCAGCTCTGGCTGAATATTTTATGTACCGGGAACGACACACTTTAATCATTTATGATGATC

TCTCCAAACAAGCGCAGGCTTATCGCCAAATGTCTCTTCTATTACGAAGACCACCGGGTCGTGAAGCTTA

TCCAGGAGATGTCTTTTATTTGCATTCACGCCTTTTGGAAAGAGCCGCTAAATTAAGTTCTCGTTTAGGT

GAAGGAAGTATGACTGCTTTACCAATAGTTGAGACCCAATCAGGAGACGTTTCGGCTTATATTCCTACTA

ATGTAATTTCCATTACAGATGGACAAATATTCTTATCCGCCGATCTATTCAATGCTGGAATCAGACCCGC

TATTAATGTGGGTATTTCCGTTTCCAGAGTAGGATCTGCGGCTCAAATTAAAGCTATGAAACAGGTAGCT

>FM891508.1 FM891508 Jatropha curcas embryo 56-70 (DAF) Jatropha curcas cDNA clone rjcaeb0_001814, mRNA sequence

ATTCGACTGTAGTAACCTAAAAAGAGGTGGTATGGTAAAAGTGAAAGTTGCTAGTTACTAGGCCAGGCTG

CTCTGCAGTAGAAAGTGATTTCGATTAAAGTAGGCTTCTAAGGCGGAGTTTTGTCTGATTTTCAACAACT

GTTTTTGACTGCTAGTGCTAGGACGTGTTTGAAGGAAGTGTGAGTTAAGATTTAGAATAAGAAAGAAAAT

AATAAGTCCATTGATTCTTGCAATTTTATGGCAGTTTCTGTATTCTAGGTTGGTGGGGACTCTCTCTGGA

TCTTATCTTCTCCCCATTTTTCATTTCTTCTTCTCTCCTTTCCTTTACAGCCTGTAAATGAGTCCTCTTC

CTGTAATTTTTTTTTCTTTGTCGTTTCTGTCTCGCTGTGTTCCAGTGTTTGTGTGGGAAAATGTAGAGGT

GGTGTGGCATTTTTGGCCTTGATGTGAGATTG

>FM891507.1 FM891507 Jatropha curcas embryo 56-70 (DAF) Jatropha curcas cDNA clone rjcaeb0_001813, mRNA sequence

GTAAAAGCCACCGCTTTGACAGAAAAGAGAGAACCCAATGCAATCCATGTAAAACACCTCCATCCCCTTT

TTCCGCTGTCACAAACGCCGTCTCCACCACCACCATTACCGCGTATCAATTAAACCCAGAATCAAAAAGT

TTTGGTTGCTAATATTTTTTTATCCAATATGGCCTCACTCCTTGATACTACTCCACCTTCTACACCAGGC

AAATTCAAACCGGACAAATCGTCAGCGTACCTTCATCCGCACCATCGCTCTCTCCGGTTACACTCTTCTC

TCTCTAAACTCACTTTCTATTCCTTTCTCTTTCTAATTCTTTTTCTCTCTCTCTCTCTCGTTAAAAGGTT

AATTGGGAAGTTTTGCAAATCTTTGAGTGCTTTCAGACTGATTTTAGTCTATTATTATCATCATCATTCA

GATAATTTCTTTGCTGTTAAGATTTTCAGACAGTTGTTTACCATGTGGTTGTAGTATGCCGTTATTATCA

ATGCTTGGCCTCTTTTTTGGGACGT

>FM891506.1 FM891506 Jatropha curcas embryo 56-70 (DAF) Jatropha curcas cDNA clone rjcaeb0_001812, mRNA sequence

ATTCCAAAAACATCTCCCACAATAATATCATCTTTATCCTTTCATTTGCACAAGAAGAAGAACAAGAACA

AGAAGAGATGGGAGTGGTGGTAATAGATGGATCCACGGTCCGTAACTTTGTGGAGAATGAAGCCCACTTC

AAGAAGAGCGTGGAAGATACGTTCGCCAAGCTGGACCTTAACAACGACGGGGTGTTGTCCCGCTCTGAGC

TCCGACGAGCCTTCGAGTCGCTGCGTTTGGTGGAAACCCACTTCGGAGTGGACGTCACCGCCACACCAGA

GCAGCTGACTCAGTTGTACGACTCTATCTTTGAGTCTTTTGATTGTGACAAAAGTGGCACCGTTGATATT

GAAGAGTTTAGAACTGAAGTCAGGAAAATATTGCTTGCGATTGCTGATGGGTTAGGATCCAGTCCCATCC

AGATGGCTCTTGAAGATGATGATCAGAGTTTTCTCAAGCAGGCTGCCGATCTCGAAGCCTCTAAGCTTCA

GGTCTTCTGTTACTTAACTAATTAAGATCCGGTAACTATCTTGCCTGTCACATCTTTATATCTTTACATC

>FM891505.1 FM891505 Jatropha curcas embryo 56-70 (DAF) Jatropha curcas cDNA clone rjcaeb0_001811, mRNA sequence

ATTCCGGTTACCCTACAGGGACCCAGCAAATGTCGGCCATGCCTGGACACGGCACAGGGCAGCCATACGG

TGGAGAGGTGGACCCAACGGGTTTGACCAGGACTCGCCCATCTGGGTTGCCCGGAGACACCACTGGCCAT

AACCCACGTGTGTAGGGCATGGCATGTGCTTTAGCTCCTGTTATGTAATATATCTATGTTTTCTGTTTGG

TGTCTTTTTCTTGTTAAGCAATTCCAGTTGAATAAAAGGTATCTTTTAGTNAAAAAAAAAAAAAAAAAAA

AAAAAAAAAAAA

>FM891504.1 FM891504 Jatropha curcas embryo 56-70 (DAF) Jatropha curcas cDNA clone rjcaeb0_001810, mRNA sequence

ATTCAACAGAGCAAAAGATTAATTATATTGGTGGAGAAATTTGTTAGCAAAAAAGGGCAAAAATGTCTTC

AAATCAATCAAGACAAGAGCTTGATGAAAGAGCAATGCGTGGAGAGACTGTTGTCCCTGGAGGTACTGGG

GGCAAGAGCCTTGAAGCTCAAGAGCATCTTGCTGAAGGGAGAAGCAGAGGAGGACAGACAAGGAAGGAGC

AGCTGGGGACAGAAGGATATCAGGAGATGGGCCGGAAAGGTGGGCTGAGCACGACGGACGAGTCTGGTGG

AGAAAGAGCTGAGAAGGAGGGCATCACTATCGATGAGTCGAAGTTCAGGACTGGGAGCTAGTGTGATGAT

TAAAACTATAAATGGTATTTTGTAGTAATAATAATAATAATGTTGGTAATAAAGTATTAGCTTAGCAGTG

TACTTTAGTTTAGTAATGTAGTTTACATGTGGGTTAAAATGACTAGGGTTTAGCTAGTATGTGTGTTGTA

ACTTTGAGAGGGGTTTATGGTCGTTCCCTTTCTGGGAGGACTGCTAATACTTTCTCCCTATTTGTAAAAT

A

>FM891503.1 FM891503 Jatropha curcas embryo 56-70 (DAF) Jatropha curcas cDNA clone rjcaeb0_001809, mRNA sequence

AATAAAATTCAAACGGATATTACATAATTATTCCAATTGGTAACTCCTTGTAATCCTACAACATGCCTAT

GCACAAGTACATATTGATTATTCATATCAGAAAATCTCATAATCTTTAAAGCTACAACTTTTAGGCACAC

AATCAGGAACTCGTGGCTTCACATTAAAGTAAAGTAAAGCCCAAAATTACAAACACAAACCATATTCATT

ATTTAAATCAGAAAATCTTATTTTGCTCACTGCTGCTACACTTGCTGAGCCCATATCTTACACGGATAAA

ATCACAAAATTAGCGGTAGCTTCTATATACAGGACTGTACATGCAACTCTCAGCATATTTCACAAGGTTC

TCCGGACGAGGTAGACGTGTAGCCAACCCAAGTTCATATGCCTTTGCGGCTACATTAGCAGCAATATGTG

CTGAAATTTAACTGATGTTAGAGAAAGGTGGGTAAATCAGCCCCTTACTATAGTTCTCTTCCGTAACTTT

GGCCAGCCAACGCTTCCGATGCTGCCAGAAGCATTTCATCATGAA

>FM891502.1 FM891502 Jatropha curcas embryo 56-70 (DAF) Jatropha curcas cDNA clone rjcaeb0_001808, mRNA sequence

ATTCCCAATATTTACTGAGTGAGACCATTGGAGAAAATGGAGAGGGTGAAGCTGTGCAACCACCAGAAAG

GTCTTTCTGGGCTAAATATTGGATGTACTTGATTCCTCTTGGACTCATAGTGATGAATGCCGTAACTCAA

GCGATGAATCTGCCTGAGGAACAAGCCATGGGTCAGGCAGGTGCCCCTTCGGCAGCAGCAGTTCAGCGAG

GGCCGGGTTCTGCTGTTAGAAGAAGATGAGTTGTCTGAACGTTCTGTTTCTACAAATGCGAAAGGATCAG

ACTTTATATTCTTGTATTAAAGCAATAGAGTCCTCTGTATATTCATCAAGTGATTGGAGATTGCGCTGGT

AGCTAGCACAGCTGGGATGGTTGTAGTTAGGGCTATGA

>FM891501.1 FM891501 Jatropha curcas embryo 56-70 (DAF) Jatropha curcas cDNA clone rjcaeb0_001807, mRNA sequence

ATTCCACTCTAGCTCTGTTCAAACTCTAGAAATGTGCATGACTGGCTTGGACAGAGACCGAGCATCTGTG

TTTTACAAGACACCTGCAAGTTCAGCTGCTGGGATGACCGAGGAATCTGGTATCAGGAAAATCCTTCCAC

AATCTGAGATATGTGATTTTGATTTTGAGCCTTGTGGCTACTCTATGAATTCCATCGAAGGGGGTGCAAT

TTCTACAATCCATGTTACTCCGGAAGACGGATTTAGCTATGCAAGCTTTGAAGCTGTTGGTTACAATCTT

GAACAAGTGAAATTAACTCTGCTGCTCGAAAGGGTGTTGGCTTGCTTTGAACCGGCCGAGTTCTCCATTG

CATTGCATTCTGACTTTGTGCATGATGAACTTGGACTTATGTCGTCTCTGGATGTGAAGGGATATTCCTG

CGGAGGCAGTAACTATGAAGTTCTTGGGAAGGGTGGATCGATCATCTATCAGAGTTTTGTGAAAACCCAA

AGGCTGTTCATCTCCAAGGTCAATCCTGAAATGTTGCTGGAGTGAGGATGAGAAAGACGAGGAAGTTGAA

TAGTTATTAATTTTATTATATGTTGCTTTCCTTTTCTGCCTTTTAAATTGAATAAAGCGGTGTGAGATGG

GGTTATA

>FM891500.1 FM891500 Jatropha curcas embryo 56-70 (DAF) Jatropha curcas cDNA clone rjcaeb0_001806, mRNA sequence

TTCGAATTCTGTTGGTCTACCAGGATTTGCTTACCGGTGATGAGCTTCTCTCGGATTCGTTCCCTTACAA

GGAAATTGAGAATGGCATGCTGTGGGAAGTTGAGGGAAAGTGGGTTGTTCAAGGAGCCGTTGATGTGAAC

ATTGGTGCAAATCCCTCTGCTGAAGGGGCGGATGAAGATGAGGGTGTTGATGACCAGACTGTTAAAGTTG

TTGACGTTGTGGACACTTTTAGGCTCCAGGAGCAACCTGCTTTTGACAAGAAGCAGTTTGTCACATACAT

GAAGAGGTACATCAAGTTGTTGACACCCAAATTGGATGAGGAGAAGCAACAGGCTTTCAAGAAAAACATT

GAGGGAGCAACCAAGTTCCTGCTCTCGAAGCTCAGTGACCTCCAATTTTTTGTGGGGGAGAGCATGCATG

ATGATGGGAGTTTGGTGTTTGCTTACTACAAGGATGGCGCCACTGACCCAACATTTTTGTACTTCGCTTA

TGCTTTGA

>FM891499.1 FM891499 Jatropha curcas embryo 56-70 (DAF) Jatropha curcas cDNA clone rjcaeb0_001805, mRNA sequence

ATTCTAAAAAATGGCTACTGCTACTGTACTCTTCTCTACCATTTCCACCGCTCTCTTTATCCTCTTCTCC

TTTGGTCAAGCTGCGCCTCTTATTTTAACCCTAGTCAACAACTGCCCTTTCACCGTTTACCCTGCCATCC

AACCCAACGCTGGCCACCCGGTCCTCGAAAAAGGTGGCTTCCCACTCTCCACCCTAACACACCGCTCTTT

TCCCGTCCCTAACCAGCACTGGACTGGCCGGATCTGGGCTAGAACCGGATGCACTCACACCAACGGCAAG

TTCCACTGCGCCACCGGTGATTGCAACCACCAACTAGAATGCAACGGCCTCGGTGGTGCCCCTCCAGCAA

CTCTCGCTCAGTTCAGCCTTCACCATGGCCATAACGACTTCTCCTCTTACGGCGTCTCTCTCGTCGACGG

ATTTAACCTTCCGATGACCATAACTCCACATGAAGGCCAAGGTG

>FM891498.1 FM891498 Jatropha curcas embryo 56-70 (DAF) Jatropha curcas cDNA clone rjcaeb0_001804, mRNA sequence

TTTTTTATTATTTTATTCTCTTTTTGTTCTTTATCTCTTCAAGGTTATAATCATTAGATTTGCAGTATGT

GTCAACCTTACTGAGGCAGCGTCACCGCATTTCAGAAGAATTACATTTCTATTAAACGATTGCTTATAAG

AACTGGAATAAAAGGCAGCTGCCTTAATGAAACTTTCATTTCCTCTTCAACACCTACTATACACAGCAAT

ATATATATATACCTCTGAATGGCAACAAGTTACAGGGGTGCTTGACTATAAAACCTCTGTAGTCTCAAAA

GACTGGTTGTGAAAAATGAATTTGGTACCTAGGTGGTACAAGGACCGAAAAATTTCTCGGCACAAACAGG

AATGATGACAGGACATCCGTTGGGAAGGAATCTACACTCTTAGAAAGATACATGAGGATCGTTCATGCGC

AAAGAAATCACGTCCTTATGACCAGTTTATTTACCCAGGATACTTGCTC

>FM891497.1 FM891497 Jatropha curcas embryo 56-70 (DAF) Jatropha curcas cDNA clone rjcaeb0_001802, mRNA sequence

ATTCCAGGTTTCTTCAGGGTTTACGTCTCAAAGTAACAAAAATGGCTTTCTGTAACAAACTTGGGAGCCT

GGTGAGACAAAGTATCTCTCAGAATGGGCAAGTCCAGTGAATTCAATGCTGAATTCTATTCGGTGCATGT

CGTCTTCCAAACTTTTCATAGGAGGCCTTTCATATGGAACTGATGACCAGTCTCTGAGAGATGCCTTTTC

TAGTTTCGGTGAAGTCGTTACTGCAAGTTGTCACTGATAGAGATACTGGGAGGTCAAGGGGATTTGGGTT

TGTTGACTTCTCCAGCGAGGAGTCTGCTAGCTCGGCCTTATCAGCCATGGATGGCCAGGAATTACAACGA

CGAAGTGTACGCGTCAGTTATGCTCAGGACAGAACTGGTCCTCCAAGAAATAATTATACGTAATTTACGT

GGGAATGG

>FM891496.1 FM891496 Jatropha curcas embryo 56-70 (DAF) Jatropha curcas cDNA clone rjcaeb0_001801, mRNA sequence

GCTGCAGGAATTCGAATTCGATTGAATTTGAATAGCCAAGTGAGTGAAAATGGAGTCGCTGAAGGAAACC

GCAGCTAACGTTGGAGCCTCTGCTAAGGCTGGCATGGAAAAAAAACCCCCCAGNAAAAGGCCAGAGGCGC

AAGAGAAGGTGGAGAGAATGTCAACACGTGATCCAGTGCAGAAGGAAATGGCGAGAGAGAAGAAAGAAGA

GAGGAAAACGGAGGCAGAATTGCGGAAACTAGAAGCGCGCGAGGATAACGCAGCGGCGAGGCAGGCAGCC

AAGGCTGGTGGCCATGTACAGTACACAACTGCTGGTCCGGGCGCTCAAGGCACAAACCGAAACCTATACC

CATTCAACAACTGGGACAACCGGTTACCCTACAGGGACCCCAGCAAATGTCGGCCATGCCTGAACACGGC

ACAGGGCAGCCATACCGTGGAGAGGTGGACCCAACGGGTTT

>FM891495.1 FM891495 Jatropha curcas embryo 56-70 (DAF) Jatropha curcas cDNA clone rjcaeb0_001796, mRNA sequence

TGCGTTGAAAGATCAGTTCTCCTTTTTGAGTATCTAAAAATGTCTTCTTGCTGTGGTGGAAATTGCGGCT

GCGGCTCCGGCTGCAAGTGTGGCAGCGGTTGTGGCGGATGCAAGATGTATCCTGACATGAGTTTCTCCGA

GAAAAACACCGCCGAGACTCAGGTTCTCGGAGTTGCACCGGAGAAGGCACGCTTTGTCGATGGAGGTGAG

GTCGGCGTTGGAGCTGAGAACGGATGCAAGTGCGGTGACAACTGCACCTGCAATCCTTGCAACTGTAAAT

GAGATCGGCGGCGATCAGTCTGTTTTTTAGAAGTAAAAGCAGAGATGACTCTGCTTCTCTCTTTCTAAAA

AAGGAGAAATAATTAATAGAAGTCTAGATATCG

>FM891494.1 FM891494 Jatropha curcas embryo 56-70 (DAF) Jatropha curcas cDNA clone rjcaeb0_001794, mRNA sequence

CTAATAAAAAATACCTTTATTCACTGGAATTGCTTACAGAAAAAACCCAAACAGAAAACATGATATTTAC

ATAACGGAGCTAAGCAATTCCTGCCCTACCACGTGGGTTATGGCCAGTGGTGTCTCCGGCAACCCAAATG

GGCGAGTCCTGGTCAAACCCGTTGGGTCCACCTCTCCACCGTATGGCTGCCCTGTGCCGTGTCCAGGCAT

GGCCGACATTTGCTGGGTCCCTGTAGGGTAACCGGTTGTCCCAGTTGTTGAATGGGTATAGGTTTCGGTT

GTGCCTTGAGCGCCCGGACCANCAGTTGTGTACTGTACATGGCCACCAGCCTTGGCTGCCTGCCTCGCCG

CTGCGTTTATCCTCGCGCGCTTCTAGTTCCCGCAATTCTGCCTCCGTTTTCCTCTCTTCTTA

>FM891493.1 FM891493 Jatropha curcas embryo 56-70 (DAF) Jatropha curcas cDNA clone rjcaeb0_001793, mRNA sequence

AGACGTAACGATATATATTGAACACTGACTTTTTATACGTACATGAACACAAGTGGATTAATTAAACATA

AAGGACGATCAAAGTCCAAAGTCCAAAGTCCAAACCAGACCCAGATACATCCCCGCCAAAAGGGGATATG

TAAATGACACAAGATAATTCTTTTAGCATTACATGTACATATATTATTACATTGTTGTTGCTTAAACCCG

AGTCCCTGTCACTGCTGTGCCACCCTTTGGAATTGGTTCCCCACCAGCTCCTACAGCGTATTCGGTGTCC

>FM891492.1 FM891492 Jatropha curcas embryo 56-70 (DAF) Jatropha curcas cDNA clone rjcaeb0_001792, mRNA sequence

AACTAGAATTTATCTCTTTATTAATTATTAAACCAGAAGCCACTATAGTCCGCAGCAGTGATCTTTCTCT

TTTCTTAAAGACTAATTTACACATGTACCTTCACCTTTCATTATTTATTCACCATACACAGCACTATTTA

CATTAGTAAACCCTCAAGGTCGATGTCACAGTCACCCCAAAAAGCCGAAACACTTGCCATCACGTTATTA

GCTTCTCATGCCACAGCATGACCCTCGAAGGAACCTCTCCTTGGCCCTGGAAAGAACCACTCCTCGTTTT

GTCTTCCAAAAGCCTGATCCACTTCTCTTTCACTTACACCGAAGGCTAACTCTTTGGCTTGCTTCTCCAT

CTTATTTACAAGTATTATTTTTCCCTGCAAGTAG

>FM891491.1 FM891491 Jatropha curcas embryo 56-70 (DAF) Jatropha curcas cDNA clone rjcaeb0_001791, mRNA sequence

GAATTCGATTCAAAACCCTAGCAAGTGCTTGTTAACTCAAAAACAGCAGATCCTCCATTCTCTTTGCCGA

TCCATAATCAAAACAATGAAGGTCATCGCTGCATATTTGCTCGCCGTTTTGGGAGGTAACACCTCACCCT

CCGCCGAGGACCTGAAGGAAATCCTCG

>FM891490.1 FM891490 Jatropha curcas embryo 56-70 (DAF) Jatropha curcas cDNA clone rjcaeb0_001790, mRNA sequence

ATTCCTGGATTTATAGGTATGTTACTGGAAAGCATCCACCAGGAGCAGAAAGTCTGGACCAGGCACGCTT

AAAGTTGGCGGGAAAGGCAAGAGAGATGAAGGACAGAGCTGAGCAGTTTGGACAGCATGTAACAGGTCAA

CAGACTTAAAAGAGAAGTGGGTTTTGGTGGTTGATGCAGAGAACATAAAATGGCTTTGATATATATATAA

AAATATATGAGTCCTTTAATTAACATGGAAGGCCATTGCTTGTCTGGTGTATTTCTTTTTTCGCTTAAAT

CTTTTTGTTTCTTCTATGTAATTTGTTAACGGGTCTTCCCTCTTTTGAGAGCTGTCTAAAATAATTTTCT

TGTCATGTGTTGAAGGTCTCTGTAATCAAGTCTTACTTTGTTTGTTGATGAATTTGATGTTTCCATACT

>FM891489.1 FM891489 Jatropha curcas embryo 56-70 (DAF) Jatropha curcas cDNA clone rjcaeb0_001789, mRNA sequence

ATTCCTTGCTTTACTGACTTCCTCCAGTGTTCGAATGGGTGAAGCAGTCCAACCATTAACTGATGCTGCG

AAGCAATACCTTGCATCTCGTTATAGGGATATGACCAAATTCCAAGAAGAGGTGATGTCCTTGCCCCTGG

CTGGAGTTGAGGCAATTTTATCCAGTGATGACCTACAGGTTGCCTCTGAAGATGCTGTATATGACTTTGT

GCTGAAATGGACAAGAGCTCAGTACCCAAGCATCGAGGAGCGTCGTGAAATCCTGGGTGCACGCCTAGCA

CATTATATTCGCTTCCCATACATGACTTGTCGGAAGCTTAAGAAGGTTTTAACATGTACTGACTTTGATC

ATGATGTTGCATCCAAGCTTGTCCTTGAGGCCCTATTTTTCAAGGCTGATGCCCCGCATCGGCAACGAAC

CCT

>FM891488.1 FM891488 Jatropha curcas embryo 56-70 (DAF) Jatropha curcas cDNA clone rjcaeb0_001788, mRNA sequence

AAATATAACGAAAGCATAAACATATAAACGAAAATTGATGCTACATAGTGCCAATATTTTAAACTTCTTC

CATCTAAGATATGAAGTAACATAGTCTAAATCAACAAAAGACCACACCAAATCAACAAAACTATTCTGCT

CAAACAACAGAATGTCAGAACCTCTGGATCTCATTTCTTGGATTTCTTAGACCCCTCAGTTGGTTGAGAT

GCAGCAGCAGGCGGAGCAGCTACAAGTTTCTCTCCCCCAGGACCCTGGGCCAATCGTTCCTCCCTCCTCG

CAAATTTCCTTTCCCTGCTTGCCTTGTTCTTTGCCCTCTTAGCCTCAAACTGATCAGACAGAGTCTTCTC

TCTAGCTTTCTCAGCCTTTGACTTGTGAATACTTTCCATAAGAACACGCTTGTTCTTAAAGACATTACCC

TTCACCTTCATGTACATGTCATGGTACATGTGCTTGTCAATCTT

>FM891487.1 FM891487 Jatropha curcas embryo 56-70 (DAF) Jatropha curcas cDNA clone rjcaeb0_001787, mRNA sequence

CCGGTTACCCTACAGGGACCCACAAATGTCGGCCATGCCTGGACACGGCACAGGGCAGCCATACGGTGGA

GAGGTGGACCCAACGGGTTTGACCAGGACTCGCCCATCTGGGTTGCCCGGAGACACCACTGGCCATAACC

CACGTGTGTAGGGCATGGCATGTGCTTTAGCTCCTGTTATGTAATATATCTATGTTTTCTGTTTGGTGTC

TTTTTCTTGTTAAGCAATTCCAGTTGAATAAAAGGTATCTTTTAGT

>FM891486.1 FM891486 Jatropha curcas embryo 56-70 (DAF) Jatropha curcas cDNA clone rjcaeb0_001786, mRNA sequence

ATTCCATCGATTTTGGTATTGCAGAGGGTGTTGATTTTATTGCAATATCATTTGTCAAGTCTGCTGAAGT

GCTTAATCATCTCAAGAGCTACATAGCTGCTAGGTCTCGTGATAGTGACATAGCTGTCATTGCAAAAATA

GAGAGTATTGACTCATTGAAGAACTTAGAAGAGATCATTCTAGCATCAGATGGAGCTATGGTTGCAAGAG

GAGATCTGGGTGCTCAGATACCATTGGAACAGGTTCCATCAGCCCAGCAAAAGATTGTTCAGCTCTGCAG

GCAGCTAGATAAGCCAGTCATTGTTGCTTCTCAATTACTTGAATCTATGATTGAATACCCTACACCCACC

AGAGCAGAAGTAGCTGATGTTTCTGAAGCAGTTAGGCAACGGGCTGATGCTTTGATGCTCTCTGGTGAGT

CAGCCATGGGCCAGTACCCAGAGAAGGCACTGGCTGTTCTGAGAAGTGTCAGTGTGAGAATCGAGAAATG

GTGGAGAGAAGAGAAGCGTCATGAAGTTATGGAACTCCCAGCCATAGATC

>FM891485.1 FM891485 Jatropha curcas embryo 56-70 (DAF) Jatropha curcas cDNA clone rjcaeb0_001785, mRNA sequence

ATTCCTCACACTTATCATCAACAAATGGCTGAACGTTCACAGCCACACCAAGTTCAGAGTGCACCCACAA

CACCGCTATGAAGCTGCCTTCAAAGGTCAACAAAAAGGTCCATCAGCTCAGAAAGTGTTAGCTGTGATCA

CCCTCCTGCCGGTAGGCGGTGGGCTTCTTGCACTAGCTGGTATAACCTTAGTAGGTACCCTAATCGGCCT

AGCTATTACTACCCCTCTTTTTGTCATCTTTAGCCCTGTTCTTGTCCCAGCTGCATTAGTTATTGGCCTT

TCGGTCATGGCATTTTTGGCTTCCGGGGCTATGGGGCTCACAGGGTTGTCATCACTCTCGTGGGTTCTGA

AATACCTCCAGGAGGTCACTCGGCGAATGCCGGAGCAGCTGGATATAGCTAAGAAGCGTATGCAAGACAT

GGCTGGTTTTG

>FM891484.1 FM891484 Jatropha curcas embryo 56-70 (DAF) Jatropha curcas cDNA clone rjcaeb0_001784, mRNA sequence

GGGTGCGGATTCGAATTATTGAAAATCTCACACAGCAGATGAAGCGAGCTGGATATGTTCCAGATGATGG

TGATCATGAGGCTTATGAGGAACAGAGCGACTTAGATGTAGGCTTCTGTTGCCAAACGGAAATAACTGCT

GATGTTGCTGTCATGTAATTTGCTCGGAATGGTTTATTCTATCGTTTTATTCTTTATACTTGTGACCAAA

TTGTTCTCTTCCCGCTAGGAAATAAAAGCTTCTCTGATAATCTTCTT

>FM891483.1 FM891483 Jatropha curcas embryo 56-70 (DAF) Jatropha curcas cDNA clone rjcaeb0_001783, mRNA sequence

ATTCCGTACTTGTGCTCTCTCTACTTCTTCTCTTTGTGGTTCGATCCGGGAGAAACTCAGAATCATGTTG

GTCTACCAGGATTTGCTTACCGGTGATGAGCTTCTCTCGGATTCGTTCCCTTACAAGGAAATTGAGAATG

GCATGCTGTGGGAAGTTGAGGGAAAGTGGGTTGTTCAAGGAGCCGTTGATGTGAACATTGGTGCAAATCC

CTCTGCTGAAGGGGCGGATGAACATGAGGGTGTTGATGACCAGACTGTTAAAGTTGTTGACGTTGTGGAC

ACTTTTAGGCTCCAGGAGCAACCTGCTTTTGACAAGAAAGCAGTTTGTCACATACATGAAGAGGTACATC

CAGGTTGTTGACACCCAAATTGGA

>FM891482.1 FM891482 Jatropha curcas embryo 56-70 (DAF) Jatropha curcas cDNA clone rjcaeb0_001782, mRNA sequence

CGAATTCCTCTCATTTTTCGCTGTCTCTAGAATCTTCTCTTGTACAATTTTACACAGCGAGATCCTGTCG

CCGTAGCTGATCGCCGGAATCCATGGACGACGATTTCGAGTTCGCTGATCACTCTCCACCGTCGTTCCAG

CAGCGCATGGGGAATGTGGTCAATGATGTTGAGGCTAAAGGATTCAATCCTGGACTGATCGTGTTGCTGG

TTGTTGGGGGGCTAGTTCTAGCATTCCTCATTGGAAATTATGTTCTCTATGTGTATGCACAGAAGACCCT

ACCTCCCAAAAAGAAGAAGCCAATCTCTAAGAAGAAGATGAAGAGGGAAAGATTGAAGCAAGGCGTCTCT

GCACCAGGAGAGTAAATTTGGGGGTCGGTTTCCTATTCAATTACAGTGTTGAACTCGTGTCGAATATCTT

GTCTTCGTGTCAGAGAG

>FM891481.1 FM891481 Jatropha curcas embryo 56-70 (DAF) Jatropha curcas cDNA clone rjcaeb0_001781, mRNA sequence

ATTCGCGGCCTAATCGTTCGAGCTTATTTCTCCGGTTAGGGTTTTCTGATTCCAATCAACTACCAGCAAC

CATGGCGGATGTGGAGACAGAGGTGGCTGCAGGACAGCCAAAGAAGAGAACGTTCAAGAAGTTCAGCTTT

AGGGGAGTGGATTTGGATGCTCTCCTTGACATGACCACTGATGAGCTTGTCAAACTCTTTCCTGCCCGTG

CTCGCAGAAGGTTTCAGAGAGGTCTGAAGAGGAAGCCAATGGCTCTTATCAAGAAGCTGCGCAAGGCGAA

AAGGGAAGCACCACCTGGTGAGAAACCAGAACCAGTTCGAACCCACCTCCGCAATATGATTATTGTTCCC

GAAATGATTGGAAGCATTATTGGTGTGTACAATGGGAAGACCTTTAACCAAGTTGAAATCCAGCCTGAGA

TGATCGGGCATTATCTTGCTGAGTTCTCA

>FM891480.1 FM891480 Jatropha curcas embryo 56-70 (DAF) Jatropha curcas cDNA clone rjcaeb0_001780, mRNA sequence

TCCTTTTCCTTCTTTTCTCTCTCCCTTACAAGCCCTAACCCTAACTCAGACCCAGATTCGCCATGAAAGG

AGGTAAATCAAAGTCAGACACCAAGAACGCCAAGCTTTCCGTGAACAAGAAACCCGCCAAGGCAAGTAAG

AAGGCGGGTAAGGCAGCGAAGGATCCAAATAAGCCAAAGAGGCCCGCCAGTGCTTTCTTCGTTTTCATGG

AAGAGTTCAGAAGAGCAGTACAAGAAGGAACATCC

>FM891479.1 FM891479 Jatropha curcas embryo 56-70 (DAF) Jatropha curcas cDNA clone rjcaeb0_001779, mRNA sequence

ATTCCCTCAGATCACTGTGTGTTTTGATATTGATGCTAATGGTATCTTAAATGTGTCTGCCGAGGATAAA

ACCACTGGCCAGAAGAACAAGATAACTATCACAAATGATAAGGGGAGGTTGTCCAAGGAAGAGATTGAAA

AGATGGTACAAGAGGCAGAGAAATACAAAGCAGAGGACGAGGAACACAAAAAGAAGGTTGACGCCAAGAA

TGCTTTGGAGAATTATGCCTACAACATGAGGAACACAGTGAAAGATGAGAAGATCAGTTCTAAACTTGCT

GCAGACGACAAGAAAAAGATTGAGGATGCCATTGAACAAGCTATCCACTGGTTAGATGCCAACCAGCTGG

CTGAAGCAGATGAATTCGAGGACAAGATGAAGGAGCTTGAAAGCATCTGCAACCCTATCATTGCCAAGAT

GTACCAGGGTTCCGGTCCTGACATGGCAGGTGGCATGGACGAGGATGCTCCTCCAGCTGGTGGCAGTAGT

GCTGGCCCTAAGATCGAAGAAGTAGATTAAGCTGCTTTTTAGAATGTTTTTTGGTGGTGGTGTTTTGTGG

A

>FM891478.1 FM891478 Jatropha curcas embryo 56-70 (DAF) Jatropha curcas cDNA clone rjcaeb0_001778, mRNA sequence

ATTCGCCCNCAAATAGCAGCTGCCGGCCCGCCCGCCCGCCTCTTTCGTTACGCCAATTTTGTTTATATGT

ATTTTACCTTTGGGCTCTGTGTGTATTATCGTTGCGTTTCATTTTCTAAATTGTGGTTAAAGTCGGCTGG

CAGCCTGTGTGTGTAATCTACAAGATTCAAATGCGAAGGGAGCAAGTTTACACCCTGGGGCTTGTTTTGC

TTCAATCTTGGAATTGGAATGAAAATGAAATAGATAATTAGTTT

>FM891477.1 FM891477 Jatropha curcas embryo 56-70 (DAF) Jatropha curcas cDNA clone rjcaeb0_001777, mRNA sequence

CGAATTCGATGGATTTTTCTAATTTTGGTAATATGGGCGGCATGGGGGGCATGGGCGGCATGGGCTTGGG

TGATGATGGTATGGGTGATTTTGAGGACAGTGATGATGAAGAGCAAGAGGTGGCAAAGCCTGACACAGCA

GAAGGAGCTTCAAAGCCAGATGAACACGGTACAGATAATGTAGAGGAGAAAAAGTAAGCTTCGCCAAGCC

CAAAGCACTGAAGGACAAACAGAATTTGCTTTAAAAGCCAACAAATGGGGGTGTTGGTTTAAGATGTTGA

TTTACTGAAGGGATAGTTGTGCTTTCTACATTTTTTCGGTATCATGACTAGTTGAACGAGTGTCTTTTTT

TTTTCGTTAGTTGAGTGGGAGTTAAAAATTTGCCTAGTCTTTAGATGCTTGCAAACATTCGTTTCTGCCA

TGTAGACATCCGAAATTGCTCCATAGCTTTAATGTTGTACTTTTTTAGTCATTCCTTCTTGAATGATTAT

TTTGAACTT

>FM891476.1 FM891476 Jatropha curcas embryo 56-70 (DAF) Jatropha curcas cDNA clone rjcaeb0_001776, mRNA sequence

ATTCGGAAAGAGAGCGGGCGCGAGTAGCTGCAGAAGGATTGATCCATTCGAGAATCTATTTCTCAGGGGA

TCGAATAGCTTCTGCAGTATCTGCTTACTCTGTACATATATCTACCAATAAAATGACTCGCTCTCTTTCC

ACCGCTAAATTTCTGGTTGCTTCAATTGTTGAGGATCTACCACTTTCTATCTTCCGGAGAGGCTATGCGG

GTGCACACA

>FM891475.1 FM891475 Jatropha curcas embryo 56-70 (DAF) Jatropha curcas cDNA clone rjcaeb0_001775, mRNA sequence

GGGAGAGAAAAGATTACTTCGTCTCATTGTTAAAGAGAATGCTATAACATGCAAAACAGGTTACAGGTAC

ATGTAGACTGAACATAAAACTCGGTGCCATTTCTATGTCCGCTTCGAGAAGACACTTGTTATAAGTACAT

ACGCAAATACACTCCTACACACAAGGGGAAGTAACAGAACAAAACAAAGCAAACATCATATACAAACAAA

CACCATTCATATTAGTAACTCTTCATTAGTGTATCAGTAGTTAATGGTCTCATTAAGCCTAGCAGCTGTC

GTCATTGACACCGCAACACCACCTGGATGCGTGTTTAAATTCGGATTGTTCCTCAACTCTGCACTAACCA

CCCCTTCAGCATCTGCCCTTGTCACGACCTTATCCGCCGCCAATTTCGAAGTTGCACCCGATAGAACGTC

GGCGAGTTTATCTTTTACTCAGCGCGATTCGTAACG

>FM891474.1 FM891474 Jatropha curcas embryo 56-70 (DAF) Jatropha curcas cDNA clone rjcaeb0_001773, mRNA sequence

ATTCACGTTCTTTTGGACGTCACGATCGTACCACTTTTTCGGCCCTCGATCTAATGGTATTCCTGGACCC

CGTGGACCGATAACACGCATTTTTACACGTTCATCCTCGAGAGATTCGACTAGGATGGACTCACGCTACG

ATGAACTGGGTCAAGATGGAGATAATTATCCCATAACTAGTCCACGACTGGCTCGTTCGCAATACTCCTC

GCATCAGCCCCCCTTGCTACCATTGTTGCCAATGCATCAGAATTTATTCTTGCCTCGTAGATCACCAAAT

ATGCGCCAGGCCATGTCTTACCGTGGAGCCTATTTCGGTCCACCTGTTCTGTTTGGATATCGACGTGGTG

CGAACCTGTTGCGTAGACGATTTGCCAATACGTATGAGCCTGCTCTGTTTGGTACTGGGGGACCACACAC

AATTCGCCCCAGCTCGGGAACGAATGGTAA

>FM891473.1 FM891473 Jatropha curcas embryo 56-70 (DAF) Jatropha curcas cDNA clone rjcaeb0_001772, mRNA sequence

CAAAACCAAATTTAATAGAATTGTTTACAATGTACCAATGAAATATGTTACATACAAAAGTTCCAGCTGA

AACTGATATTTTAAGATGAAAGGCCATTATGCCACACCTTGCAATGCAATCAGTGTCACAGATGCATTTC

TATATGTTGATGAAAGCTGGAGGACAAAGTTAACCTGCAAATTAGCTGGGTGGCAGGAGACAAATAATTT

CTCCACCCAAATCACCACAAAGTCCTGCTAAAAAAAGTATGAATGATCGTCTGAGAAACAATTTTCGCAA

CTTCCTTCCAATGGATATCCCTCACCTCAACTTCATCCTGTGTCAGTCATGTGGCAACTAGAGTACAGAG

ATTAACAGCCAAGGCCTGTTAACCAAACAAACCGCCGGTACCTAGCTCCCCTTGTTGCAGCTCCTTTCTG

CGCTGCTCTTCTAGAGGATCTGGGCTCTTCAATATTCTTCGCAAACGCCTGTCTTGAGCATGCTGAGCAG

GAAAAGTAGGCATAAAATCTTTGGATTCGGGAAGAGGAACTTCACGAAACCACTCATGGTTTAGAGCATC

TTCGGCAGTAATTC

>FM891472.1 FM891472 Jatropha curcas embryo 56-70 (DAF) Jatropha curcas cDNA clone rjcaeb0_001771, mRNA sequence

ATTCAGCAAGCCACAAGAGTAGCCTTCGGTGTAAGTGAAAGAGAAGTGGATCAGGCTTTTGGAAGACAAA

ACGAGGAGTGGTTCTTTCCAGGACCAAGGAGAGGTTCCTTCGAGGGTCATGCTGTGGCATGAGAAGCTAA

TAACGTGATGGCAAGTGTTTCGGCTTTTTGGGGTGACTGTGACATCGACCTTGAGGGTTTACTAATGTAA

ATAGTGCTGTGTATGGTGAATAAATAATGAAAGGTGAAGGTACATGTGTAAATTAGTCTTTAAGAAAAGA

GAAAGATCACTGCTGCGGACTATAGTGGCTTCTGGTTTAATAATTAATAAAGAGATA

>FM891471.1 FM891471 Jatropha curcas embryo 56-70 (DAF) Jatropha curcas cDNA clone rjcaeb0_001770, mRNA sequence

GACTTCAAAACAATTTGAATGATATTCATTGTCATTCGCGCATTACCCAAACAAAATTGTTAATGATAAT

TATTGCGTTTACACAACCAAAGATATAAATAGAGAATGATTACAGAAAAAACCTCCAATTTATCACTATG

AATTAATAAATGCATGGTACTGTAAATCTGATTAGCCGCTGCAATATACAGACCGGTACAGGCAACTGAA

CTCCTAACCAAAATTTTCAGCCTCTTCTGCACATGAGACTCACTCTCTTTCCATCTACACAGAACTTCTG

ACTGCCTTCCAACTTTTGTTTCAGGTGAGTAAGAGCATCTCTTTTTGAAGAGAAATTGGAAAAAACAGTA

GATGAATGCTCTTCAATAAGAACTTTCAGCTTTTTTAGCTTTAAGGACTCCCCTGGTACCTGACGTAGAA

TTCGTTTGCACAGTTTTTTCAACTTTACTTTTTGTTCATCAACATTTTTTCCTGGAGCGTCGCGTTTTCT

TTTTCCTAAGGAGCCAAA

>FM891470.1 FM891470 Jatropha curcas embryo 56-70 (DAF) Jatropha curcas cDNA clone rjcaeb0_001769, mRNA sequence

CGAATCCTCTCTTCTATATATATATATATATATATATTTAACTTTGTTAATTTTGATTTTAATTTTGGAT

GCTGAGAGGATCTAGGGTTTGTGGCGTTGTGTAGGGATTTTATCTTTTGTTTTTGGTGATGGCATCGAAG

CGGATCTTGAAGGAGCTCAAGGATCTCCAGAAGGATCCTCCTACCTCTTGCAGCGCAGGCCCTGTTGCGG

AAGACATGTTCCATTGGCAAGCAACAATTATGGGTCCTCCTGATAGTCCATATGCAGGTGGAGTGTTTCT

AGTGACGATTCATTTTCCTCCAGACTATCCATTTAAACCACCGAAGGTTGCATTCAGGACAAAGGTCTTT

CACCCCAATATCAACAGCAATGGTAGCATTTGCCTTGATATTTTGAAGGAGCAGTGGAGCCCTGCCCTTA

CCATTTCCAAGGTGTTGCTTTCAATTTGTTCTCTATTGACGGACCCAAATCCTGACGATCCGTTGGTGCC

AGAAATTGCCCACATGTATAAGACTGATAGGAGCAAATACGA

>FM891469.1 FM891469 Jatropha curcas embryo 56-70 (DAF) Jatropha curcas cDNA clone rjcaeb0_001768, mRNA sequence

GAATTCAACTTCGCTACCGCCACGAGTACCAACTCCACAGCTTCGGCGCCTTCAAGTAATACCTCAAGCA

CTTCCCAACCTCCACCTTCATCGGCATCTCCCAATGGCTCTACGCCGGCAGTTGTAGTGGATTCTAGTCC

GCCGAACGGGCTGCCTCCTGCCGCTGTGACGGGCCTCATAGTGGGAGTAGCTTTAGGAGCAATAATTGTG

CTGATTGGCGTCGGCGCTTTCTTCTTTTTTTACCGGAAGAAGAAGAGAAAGGAAGCAGAGTGGGGGGGGC

CCGGTAC

>FM891468.1 FM891468 Jatropha curcas embryo 56-70 (DAF) Jatropha curcas cDNA clone rjcaeb0_001767, mRNA sequence

ATTCTGTTGGCCAACAGCCACGATCCCACCAAGTAGTGAAGGCAGCCACTGCAGTCACTGCCGGTGGATC

TCTTCTAGTTCTCTCCGGTTTGACCCTTGCTGGTACGGTCATTGCACTGACCGTAGCCACTCCATTGCTG

GTCATATTCAGTCCGGTTCTTGTTCCTGCGGTTATCACAGTTTGTTTGATAATCACAGGGTTTTTAGCCT

CAGGCGGTTTTGGCGTCGCTGCCATTTCTGTCTTGTCCTGGATTTATAGGTATGTTACTGGAAAGCATCC

ACCAGGAGCAGAAAGTCTGGACCAGGCACGCTTAAAGTTGGCGGGAAAGGCAAGAGAGATGAAGGACAGA

GCTGAGCAGTTTGGACAGCATGTAACAGGTCAACAGACTTAAAAGAGAAGTGGGTTTTGGTGGTTGATGC

AGAGAACATAAAATGGCTTTGATATATATATAAAAATATATGAGTCCTTTAATTAACATGGAAGGCCATT

GCTTGTCTGGTGTATTTCTTTTTTCGCTTAAATCTTTTTGTTTCTTCTATGTAATTTGTTAACGGGTCTT

CCCTC

>FM891467.1 FM891467 Jatropha curcas embryo 56-70 (DAF) Jatropha curcas cDNA clone rjcaeb0_001766, mRNA sequence

ATTCCAAAACTTTGTTAATTAATGCTCTCTCTGCCCTAGAATTTGTTTCTTATGAGCACAAGCTTGTTCA

CGGGCTTCAGTTTTATCAAAGCATTGGAAATCATTCACCAAATAAGCCAAAGATCATATAAATAATGGCT

TAGGTAGTTGCTCATTCTGTCGTGGACACACCTTTGCATGCAGTTGTTTCCAACTTTGATTCACCCACAG

TTTTTCTCTAAATGCAACCACCAAATGCATTTTGGATTTTTTCTTTATTGTTTTTTTCATATTCTTTGGT

AATTAATTAGTGTAGGTTTGTTATTTTCACTTAGATTAGATTAAGGTTGCTTAACTCTAAGCATTGTCTG

TGGTACAAATAAATTAATTTATTATAAGTATTTGTTATGAAAATAAACTTACACCAATAATATGAAAATA

AG

>FM891466.1 FM891466 Jatropha curcas embryo 56-70 (DAF) Jatropha curcas cDNA clone rjcaeb0_001765, mRNA sequence

ATTCCTTTCTCTCACCGATCTTTGATCTTCTCCAAAAACAATCAAGAGAATGTCTCTGATTCCAAGCACC

CTCTTCGGTGGCCGGAGAACCAACATTTTCGATCCCTTCTCTCTCGATATCTGGGATCCATTTCAGGACT

TCCCTTTCACAAGCACTGCTCTTTCTGCTCCTCGTTCTGAGTTATTGAACGAAACGGCAGCGTTTGCCAA

CACCCGCATAGACTGGAAAGAAACACCAGAAGCTCATGTATTCAAGGCTGATCTTCAGGGCTAAAGAAAG

AGGAAGTGAAAGTGGAGATAGAGGAAGGTAGGGTCTTGCAGATAAGCGGAGAGAGAAGCAAAGAGAAAGA

AGAAAAGAATGATAAGTGGCATAGAGTTGAAAGGTCAAGTGGTAAGTTCTTGAGAAGGTTTAGGCTACCA

GAGAATGCAAAGGTTAATGA

>FM891465.1 FM891465 Jatropha curcas embryo 56-70 (DAF) Jatropha curcas cDNA clone rjcaeb0_001764, mRNA sequence

CGAATCCTGTAGGAGATGGCCCTAATGGAAGGAAAGTAGAGGGGTTTCTTTACAAGTATAGCGAAGGTCA

AGTGAACATAGTATGCATTTGTCATGGCAGCTTCTTCTCTCCGGAAGAATTCGTAAGGCATGCAGGCTGT

GAAGATGTAGCCAACCCCATGAAGCGTATCACAGTCTC

>FM891464.1 FM891464 Jatropha curcas embryo 56-70 (DAF) Jatropha curcas cDNA clone rjcaeb0_001762, mRNA sequence

TCGTTATGAGTCTGATACTTGGAAGTTGATTTGAGTAAAGAGCTTACACAAACTCAAAAGCCGATATCAG

AATGTGTTGCTGAGGCTGGAAACAAAGACAGCATTGGCATTTATAAAAAATGGCGGTTGGTACCAACACG

CCGTGAGCAGGAGAAGTGGGATAAAGCAACTAAGGCTGCAACTGGGGGCAGTGATGTGATGTTGAGGGAA

GTAGAAGGCCTCAAGGAGATCCGGAAATATTGGCTGCTCAATCAAGGGAGCAATATTTTAAGTTGAAAAC

AAAAATGCAGATTCTCACATTGGGTATTGGTGGCGTCGGGCTGGTCTCAGCTTATGTTTCTTATTCTCCG

GAAAT

>FM891463.1 FM891463 Jatropha curcas embryo 56-70 (DAF) Jatropha curcas cDNA clone rjcaeb0_001760, mRNA sequence

ATTCGATTTACCCAAATATTCGCCGAGTTATCAACAAAGCAAAGGAGAGATCAAAACATCTGATTTCTTT

CGGTTGTGATGGCTTCTTCAGCTATGGTTTTGGATCCGAAACCCGTATCTGAACCTCCGCCATCATTTCT

GCGGCGAAACGGACGTACAACATCCTTCGCCGACCTATCAAACTCCGACGAAGACGACCTTTACAGCCGC

CTAAAGTCTCTCCAGCGTCAGCTCGAGTTCATCGACATCCAGGAGGAGTATGTCAAAGACGAGCAAAAGA

ATTTGAAGCGCGAGCTTCTCCGTGCTCAGGAAGAGGTCAAGCGGATCCAATCGGTGCCACTTGTAATTGG

ACAGTTCATGGAGATGGTCGACCAGAATAATGGTATTG

>FM891462.1 FM891462 Jatropha curcas embryo 56-70 (DAF) Jatropha curcas cDNA clone rjcaeb0_001759, mRNA sequence

ATTCCTTCATATCTCAGATCTCTCGCGAAAACACTAGGAAAAATGGATGTCAGGTTCGTAGGTCTCGATT

CACCACTTCTCCACACTCTCCAGCAAATGATGGACATTGGCGACGATGCTGACCACAAATCCTTCAACGC

GCCAACACGCACTTATGTGAGGGACGCGAAGGCTATGGCTTCTACT

>FM891461.1 FM891461 Jatropha curcas embryo 56-70 (DAF) Jatropha curcas cDNA clone rjcaeb0_001758, mRNA sequence

ATTCGTTTTTTTTTCTTAACCAACAGGTAATTATATATACTGCAGGTTATTTACAAATAGGGAGAAAGTA

TTAGCAGTCCTCCCAGAAAGGGAACGACCATAAACCCCTCTCAAAGTTACAACACACATACTAGCTAAAC

CCTAGTCATTTTAACCCACATGTAAACTACATTACTAAACTAAAGTACACTGCTAAGCTAATACTTTATT

ACCAACATTATTATTATTATTACTACAAAATACCATTTATAGTTTAATCATCACACTAGCTCCAGTCCTG

AACTTCGACTCATCGATAGTGATGCCCTCCTTCTCACTCTTTCTCCAAAGACTCGTCGTCGTGCGGGGGG

GCCCG

>FM891460.1 FM891460 Jatropha curcas embryo 56-70 (DAF) Jatropha curcas cDNA clone rjcaeb0_001757, mRNA sequence

CGAATTCCTTTCGATGCTATAAAGCTTTTGTTCCCAACAGCAGATGCTGCACGACCATTTTCCATGCTTG

GGCTTGGTGACATTGTTATCCCTGGTATTTTTGTAGCGCTGGCATTGCGATTTGATGTGTCTAAGGAAAA

CACAGTCAATATTTTAAAAGCNNGCCTTTCTTGGATATACATTTGGTTTGGTCCTTACAATTATTGTAAT

GAACTGGTTCCAAGCTGCACAGCCTGCACTTTTATACATTGTAC

>FM891459.1 FM891459 Jatropha curcas embryo 56-70 (DAF) Jatropha curcas cDNA clone rjcaeb0_001752, mRNA sequence

ATTCAATACTCTTTTTGTCTTCTTTTGTGATCGGAGAGTCAGGTTTTTGCGGCGACAAAATGGCCAGACT

CGGTGGAGTTCGCGAAGTAAGCGCCAACAGCGTCGAGATCGACGGTCTGGCTCGTTTCGCCGTCGAAGAA

CACAANNCAAGAAAAGAGAAATGCGTTGTTGGAGTTTGCGAGAGTGGTGAAGGCGAAAGAGCAAGTGGTG

GCTGGGACTCTGCATCACCTCACTATCGAAGCGATTGAGGCTGGAAAGAAAAAGCTTTATGAAGCGAAGA

TTTGGGTCAAGCCTTGGCTAAACTTCAAAGAATTACAGGAGTTCAAGCATGCTGGGGATGTTGATGGACC

TGATGGTGGTGCTTCATCCTTCAACTCTTCAGATCTTGGTGTCAAGAGAGATGGGCATGGGCCTGGATGG

AGAGAAGTGCCAGCACATGATCCTGC

>FM891458.1 FM891458 Jatropha curcas embryo 56-70 (DAF) Jatropha curcas cDNA clone rjcaeb0_001751, mRNA sequence

ATTCCAAGTATCTTAAAGGATGAGCCAATTTCCTTGGCATTGCTGCGGAAGCACTGTCTGCAACAATGCC

AAGGAAATTGGCTTCATCAAGTATCGTTAAAGGATGAGCACCTGTTCCACAAGAGCGATGGACAAGTATG

ATTGGACTTGTGGTTTCTTTTTGCATGAACACTAATATAATGTTATATTTAGTTAATGTCGCGTTGAACT

TCTATTGATTATGGAAGTATATTTTGCCGTTACAATGTTTGGAATTAGTCATTGAGGATAATTAATTTCC

TAAGTCTAC

>FM891457.1 FM891457 Jatropha curcas embryo 56-70 (DAF) Jatropha curcas cDNA clone rjcaeb0_001750, mRNA sequence

ATTCAGCTAACCCTGCAGAGTGGTAATTAGTTGCCCAGAAAGGTTAGGAATTGCAGGGAAGCTTGTTCTA

CTACCAAAGACCTTGCAGGAGTTGCTGGAGATTGGTGCTAAGAAATTTGAGCTCAATCACCCTAAAATTC

TCAGCAATGATAGAGCTGAAATCGAAGATATTGAGGTGATTAGAGATGGTGATCATATTTTA

>FM891456.1 FM891456 Jatropha curcas embryo 56-70 (DAF) Jatropha curcas cDNA clone rjcaeb0_001749, mRNA sequence

ATTCCTCTTCTAGTTCCCGGTTTGACCCTTGCTGGTACGGTCATTGCACTGACCGTAGCCACTCCATTGC

TGGTCATATTCAGTCCGGTTCTTGTTCCTGCGGTTATCACAGTTTGTTTGATAATCACA

>FM891455.1 FM891455 Jatropha curcas embryo 56-70 (DAF) Jatropha curcas cDNA clone rjcaeb0_001747, mRNA sequence

CGAATCTCATCTGATAAGTATTTATTCAGCTCTATAACGATGTAGCTGCTAAGAACCCAACGGTATTGGT

AGAGATTTCAGATTCATCAGAATCAAAATCTAGCTTAATCTGTATTGATAATTTAAAGGCTGTCTCTGAA

TTCTCCTTGGATTTCTTGAAGGATAGAGTGAAAATTAGGGCATCTTGTAATGGCCTTTTGTGTTGCTCTA

GTATTCCTGATAAGGGTGTTTACTATGTTTGCAATCCGATGACGAGAGAATTTAAGTTGCTTCCTAGGAG

TAGAGAAAGGCATGTGACTAGGTTTTATCCTGATGGTGAGGCCACTCTAGTTGGTTTGGCTTGCAATTTA

TCAATGCAGAAGTTTAATGTTGTTTTAGCAGGTTACCATCGTACCTTTGGTCATAGGCCAGATGGGACAT

TTATATGCTTGGTATTTGATTCGGATTCAAATAAATGGAGGAAATTTGTTTCATTTCGAGATGATCAATT

TACGCATATGAATAGGAACCAGGTTGTATTTGTAAGTGGTGCACTCCATTGGCTAACTGGT

>FM891454.1 FM891454 Jatropha curcas embryo 56-70 (DAF) Jatropha curcas cDNA clone rjcaeb0_001746, mRNA sequence

ATTCTTTTTAATCAAATAGCATGCTTCAAATTGTGAAGTTTACGTTTCTAATAACTATAGTTAAACAACA

AACCAAAATAACAATATGTTGAGAAATAAAACAAAAGCAAAGTACAAGATCGGATCCGACTAGATGGAAG

GCTAGAAAGAAATCTATGGGACCAAAAGGATAATTACCAAAATTTGTCTGGCCTACTACTAGGAGGAAAA

ACTTTGGGCATCTTTATAAGAATAAATAGAAAACAGCATCAGCTATTCTATACAACAAAAGCAAAAAAAT

CACCATCCATACTTGGCTTGCGTTTGAGCTTTCGTGAGAAACCCTTTGGCACGCCTTTCTGTCAGTTCCA

TCTCTTTCTGCTTCATATCGAAGTACAACGAGCCAATCTGATGCTTCCTCTTGTGAAGCTTTGATGGCTT

CCCCTTGGTAGAAGTAGGCTCATAGGAAGGCCCAAAAGCAATTCCAGTTAACTTGACCTGGTCCTCCGGG

GGGGCCCGGTAC

>FM891453.1 FM891453 Jatropha curcas embryo 56-70 (DAF) Jatropha curcas cDNA clone rjcaeb0_001745, mRNA sequence

ATTCCACAACTACCGTTTGGCGGGTGTTGTTTCTCCATCTCTAAACATGGCGGATGTTGAGACCGAAGTC

GCTGCTGCAGGCCAGCCAAAGAAGAGGACGTTTAAGAAGTTCAGTTTTAGAGGAGTTGATTTGGATGCTC

TATTGGACATGACTACTGATGATCTCGTCAAGCTCTTTCCTGCTCGCGCTCGCAGAAGGTTTCAGCGAGG

TTTGAAGAGGAAGCCAATGGCACTTATTAAGAAGCTTCGCTGAAACCTTCTGCGAGGGGGGGGCCCGGTA

C

>FM891452.1 FM891452 Jatropha curcas embryo 56-70 (DAF) Jatropha curcas cDNA clone rjcaeb0_001744, mRNA sequence

ATTCCTTCTGTCAAGGGAGCAAAAGTTGTCACTGATCTAAATACTGGCCGCTCAAAAGGTTATGGCTTTG

TTAGGTTTGGTGATGAAAATGAGAGGTCAAGGGCCATGTCAGAGATGAATGGTGTTTATTGCTCTAGTAG

GCCAATGCGCATAGGTGTTGCAACTCCCAAGAAGTCATCTGCATATCAACAACAGTACTCTTCACAAGCT

TTGGTGTTAGCTGGTGGGCATGCTCCAAATGGTGCTATTGCCCAAGGATCCCAATCTGATGGAGACTCTA

ATAATACAACTATATTTGTCGGAGGAATCGACTCTGATATCAGTGATGAGGATCTCAGGCAGCCTTTTTC

CCAGTTTGGTGAAGTTGTGTCTGTGAAAATTCCAGTTGGCAAAGGATGCGGTTTCGTGCAA

>FM891451.1 FM891451 Jatropha curcas embryo 56-70 (DAF) Jatropha curcas cDNA clone rjcaeb0_001743, mRNA sequence

ATTCCATAAAACAGCTTCCAGAATCATACTCAAGCTGTAAACAGATATGTTTCCATCATCACGCAACAGC

CTCTTCCTCGTGCAGAAGCATCTGGTCGAAATGCCATACTCCATGTCCTTTCCCCACTCTTCTAAGTTGA

GCAACATATTCAGAGATTTTTTTGATGGCATCCACCTGTTCAGCTAAGAACTCGCTTTCAACAAAATCCG

ATAATTGCACATCATTGTTTTTTGAAGCCACACTGTGTAAGTTCAGGAGCTTTTCATTTGTTAATTTTTC

CAGAGATAAAGCAAGCTCCATCGCATACAAGGCATCCCCCTTTTCGACGTGATCATACTCAGTAAGAGGC

ATCACTATAGACTGCAGCTTCACTTTTCCACCCCGTTTGTTCTGGTATTCCATCAATTTCTCAGCATGTT

CTCTTTCTTCAAGGCTTGATTCCTTGAAAAACTTGGCAAGACCCTTGAGGGCAACATTATCTCTG

>FM891450.1 FM891450 Jatropha curcas embryo 56-70 (DAF) Jatropha curcas cDNA clone rjcaeb0_001742, mRNA sequence

AATTCTNAAGAAATCATCATCTCAGCCCCAAGCAAGGATGCTCCCATGTTTGTTGTTGGTGTCAATGAGC

ACGACTACAAGCCAGATATTCACATTGTATCAAATGCCAGCTGCACCACTAACTGCCTTGCTCCTCTTGC

AAAGGTTATCAATGATCGCTTCGGCATTGTTGAGGGTCTTATGACAACTGTCCACTCCATTACTGCTACC

CAAAAGACTGTTGATGGGCCATCAAGCAAGGATTGGAGAGGTGGAAGAGCTGCTTCCTTCAACATCATTC

CTAGCAGCACAGGAGCTGCTAAGGCTGTTGGGAAGGTGCTACCTGCTCTGAACGGCAAGCTTACTGGAAT

GGCTTTCCGTGTTCCAACTGTTGATGTCTCTGTGGTTGATCTCACTGTTAGGCTAGAGAAGGCTGCTACA

TATGAACAAATTAAAGCTGC

>FM891449.1 FM891449 Jatropha curcas embryo 56-70 (DAF) Jatropha curcas cDNA clone rjcaeb0_001741, mRNA sequence

ATTCCTCACTCACTGCGACAGAGCCGCTTCTATCTCCTTTGCTCTCAGAGATGGCCAAGTCAAAGAACCA

TACAGCTCACAACCAGTCCTACAAGGCTCACAAGAATGGCATCAAGAAACCAAAGAGGCATCGTCACACC

TCCACCAAGGGGATGGATCCCAAGTTCTTGAGGAACCAGAGGTATGCCAGGAAGCATAACAACAAGAGCG

GGGATTCAGCCTCCGAACAAGAGTAGAACTACCTTCTTATTGAGTTATGGTTTCCTATTTCCATGTTAGG

GATTTACTTTAATAGTGTATTGGCTTTATTTCAAGTTGCAATTTTTGAATAGTGGCTGTACTGTACTGTT

AAGCCATTTATGTTTTGGATAATTGTTACTTAAATATTTTGCTTAAGATTTTCT

>FM891448.1 FM891448 Jatropha curcas embryo 56-70 (DAF) Jatropha curcas cDNA clone rjcaeb0_001740, mRNA sequence

ATTGTATGTTCCAAGATTTGTAGTTATGCACAAGGGATGAATTTGCTGAGGGCTAAGAGTGTTGAGAAAG

GTTGGAACTTGAATTTGGGAGAGTTGGCAAGGATTTGGAAAGGTGGGTGTATTATAAGGGCAGTGTTTTT

GGATAGGATTAGAAGGCTTTCAAGGAATCCCAATTTGGCTAGCTTGTTGTGGATCCTGAATTTGCTGGGA

GATGGTACAGAGGCAGGCAGCTTGGAGAAGAGTTGTGGGTTTGGCTATTTCAGCAGGCATTAGCACTCCA

GGCATGTGCGCAAGTCTTGCTTACTTTGACACTTATAGGCGTGCTAGGCTACCGG

>FM891447.1 FM891447 Jatropha curcas embryo 56-70 (DAF) Jatropha curcas cDNA clone rjcaeb0_001738, mRNA sequence

ATTCGTCACTGGATCCGATGGAGTCAAAGCAAAATCAGGACGATATCAATAAAGAGCCTCTGCTTAGGAA

GAGTTTGCCCATTAGCATTGCAGCTGGTACTACAGAAGATAGTGAACCTAGTGTCATGGAGGAAGAAGAA

TCTGAAACAGCTGCTAGTAGGAAAATTGGTTCACAGAGACTGGGCCGCAAGTATCGGACAAAGAACAGTT

TAGAAATAATGGATGAGTCTGTTTAAGAGGGCTGCCATCTTCAGTGAGCTGAGCTACTATATCCTGACTG

CTACTTTATTGAACCCATCTGACTGTGTAATTACTAATTTATGCATTTGTATTTCATAGGTATCTGTTGA

TTTTAGTTCTCGCTTGACTTTCTTTAGGTGGGAGCTTGGTTGGCTTCATCTTCTCAGAAATGATATACTG

ATGCGATATACAT

>FM891446.1 FM891446 Jatropha curcas embryo 56-70 (DAF) Jatropha curcas cDNA clone rjcaeb0_001737, mRNA sequence

ATCTCGATCTTCAACTCCGGCCGATCCCTCAATTATATGACCTGATTCTGAAATGGCTTTAGGAGGAACA

GCTCCCCCAAGAGGCAGTGCAGCGGCTGCTGCAAGTATGCGTAGGAGGAGGACAACCAGTGGTGGGGCCT

CAGGAGGAGCATCAGGGACTATGCTTCAATTTTACACTGATGACGCACCAGGACTCAAGATCTCCCCCAA

TGTTGTACTGATCATGAGCATCGGATTCATAGCTTTTGTCGCAATCCTTCATGTTGTGGGTAAACTCTAC

TTGGTTCGTAGAGAGGCTTAGAAATATACTGATGGTAGGGAATCAGTTTGGGAAAGTTGAATTGTGGATT

TTGAGTCAGTTTGGAAATTGTACTAATGGATATCGTTTTTGAATTACGTGGATATGATTAAACATTTACA

GTATAAAAGAAAAAACAA

>FM891445.1 FM891445 Jatropha curcas embryo 56-70 (DAF) Jatropha curcas cDNA clone rjcaeb0_001736, mRNA sequence

CGAATTCCTCACCTGCTCCTGACATGAAACGGATTGATAAATAAGCTGCATACGTTGAAAGTTTTCTTGG

AGTTGCTTCCGTGCATAGCTGATATGAGTATATCCCGTGGATGTATGAGATTTATGATCTGATTGTTTCC

GCCCCAGTTATTTTCCCCAAATTAGAACTTGGTCCGTGTAATGGGAAGTTATTTCTGCTAGACTTTGTGA

ATATTATTGCAGTATTTCGGTTTCGAGAAAGCAGAATTGTAACCTATATGAACTGTATCATAGCTTATCC

GTAGTTGTGTGGCCATGGATGCTCTTTTTCGAGCTCCTTACTATTGTAAACTTCAATAAAAACATCCATA

TTGATATAAGATCAACAGAATAGCTTTCTCT

>FM891444.1 FM891444 Jatropha curcas embryo 56-70 (DAF) Jatropha curcas cDNA clone rjcaeb0_001735, mRNA sequence

CTTCGTGCTCTCTCTCTTTCTTGCGTGCTGCTTCTGTTTTTGTTGAACCACACGCCATGGCTTCGATTAC

CGGAACTTCAATCTCCATGACCTCTCTTTCGAGGTCTCTTAGCACCCAGGCACCAAGAAATGGTATCTCT

AGTTTGAGGATTTCACTTCCCATTAGTGGAAGAAGCTTTCCTTCTCTTAGATTGCAAAAGGGTGCATCTC

GCTTCCGTGTCTCCTGTGCGGCCAAACCAGAGACGCTAAATAAAGTGTGTGAAATTGTAAGGAAGCAGTT

GGCACTAGCACCTGAAACCGCTGTTAACGGAGAGTCGAAGTTCGTTGCATTGGGAGCTGATTCTCTTGAC

ACGGTTGAGATTGTAATGGGACTTGAGGAGGAATTTGGAATCAGTGTAGAAGAAGAAACGGCCAGAGTTA

CTCACTGTCAGATGCGCGATCTGATTGAAGCTTATCGAG

>FM891443.1 FM891443 Jatropha curcas embryo 56-70 (DAF) Jatropha curcas cDNA clone rjcaeb0_001734, mRNA sequence

AATGCATTAAATGAATAAAATTGAATTCTTCCATTTAAATTGTTCAAATTAAAAATTAAAGAACAGGGAG

CCTAAAACTGGAACTTAGTTTGACCTTGCAAGAGTCGTATCTTGAACAAAGGTTAGCAGGTCGTATCAGG

TTCTCTTCAAACTTTTGACAATTTAGGAATTTACATTTAAAACACCTAAAAAAAATTAGGAACTTTCACA

TACTTAGCAAATAAGCAAATTCAAACTCATAAGGAAATTGCTCAACAATTTGCTAGCTATGTTTACCTAC

AGCGCGTTTAGTTGCCCAAACTACTTGGAGCGCAAACCAGGGGATATTCCTTGAAGGCCCATCTTGCCAG

AACAAGCTGGGTATGGCGGAGGTTGTCAGTCCATACTTCG

>FM891442.1 FM891442 Jatropha curcas embryo 56-70 (DAF) Jatropha curcas cDNA clone rjcaeb0_001733, mRNA sequence

GCTCTAAATCTTTATTTATAATGCTCCCAACATTTTCTTTCAAGCGAAACAGAAAGAGAAAGAACTGCCT

TACAACATAAACGAGTTTAAAATTGCTCTTAGAGAAGATCAAGCTGCAAAAGAAACCTCTGATATGGAGC

TCTGGATATAATCAAACTTCTCCAAATCCCTTCACTAGCAAGTGCTCACTCAATCTCCTCATGATGGTTC

TTGCTCCTCTTCTCAGACCGCTTTGCAGCCCTCCCAACCCGTGGTTCATTTGAGACGCACACATTAGAAT

TAGATGTCAATGTTGGCTTCCTGCTTCTCTGGCTGTGCCTTAAGCCAGAGTCATCTGATCCATCTGAATC

AGACTCATTAGAATATCTATGACTTCTTCTACCCCTCTTCTCTTTCCTACTCTTCCTCGTATGTTGCTGC

TGCTCGCTCTTTTCCTCAGAAGACTCATCCCTCTTTCCCCTAGTCTCCCTCCTTTTCCTTTCACCTTCAT

CCTCAGAATCATTT

>FM891441.1 FM891441 Jatropha curcas embryo 56-70 (DAF) Jatropha curcas cDNA clone rjcaeb0_001732, mRNA sequence

ATTCCCTAGCAGCACGTCGCTTGTTAACTCAAAAACAGCAGATCCTCCATTCTCTTTGCCGATCCATAAT

CAAAACAATGAAGGTCATCGCTGCATATTTGCTCGCCGTTTTGGGAGTTGGAGCTGATGCTGATGATGAT

AAGATTCAGTTATTGTTGTCCCAAGTTGAAGGAAAGGATATCACAGAATTGGTTGCAGCAGGAAGGGAGA

AATTGGCATCTGTGCCTTCTGGTGGTGGTGTAGCTGTTGCTGCTGCTGCAGCACCTGCTGCTGGAGGTGG

TGGTGCTGCTCCTGCTGCTGAGGCCAAAAAGGAGGAGAAGGTTGAGGAGAAAGAGGAGTCAGATGATGAT

ATGGGTTTCAGCCTCTTCGACTAGGAGATCTATTTGCTGGTTCCATTTGTATCAACGCTTTCTTTTATCT

TCTAGTCTTTTAGTTAAAAAATTTACAAGTTTTTGGTCTGTAGTTGGTTATGATATTTAGAACTTGTTTG

CGTTCTTGAATTTAGAGTTAGGTAATGGAGATTAAACCATTTCACATAT

>FM891440.1 FM891440 Jatropha curcas embryo 56-70 (DAF) Jatropha curcas cDNA clone rjcaeb0_001729, mRNA sequence

ATTCGTTAAGTCTGTTGAATGCACCACGAAGCTCTCCAGGAGGCCCTTCCAGGTGACAATGTTGGCTTCA

ATGTGAAGAATGTTGCTGTCAAGGATTTGAAACGTGGTTTTGTTGCCTCTAACTCCAAGGATGATCCTGC

GAAGGAAGCTGCCAACTTCACCTCTCAGGTCATCATCATGAACCACCCTGGTCAGATTGGAAATGGATAT

GCTCCAGTCCTTGACTGCCACACCTCCCACATTGCTGTTAAGTTTGCTGAGATCCTAACCAAGATTGATA

GGCGATCTGGTAAAGAGCTTGAGAAGGAGCCAAAATTCTTGAAGAATGGTGATGCTGGATTTGTTAAGAT

GATTCCCACCAAGCCCATGGTTGTGGAGACCTTTTCTGAGTACCCTCCTCTGGGTC

>FM891439.1 FM891439 Jatropha curcas embryo 56-70 (DAF) Jatropha curcas cDNA clone rjcaeb0_001728, mRNA sequence

ATTCGTGGAAAGAATGAGGAGGAGGAGTTCAACACTGGACCCCTTTCGGTACTCATGATGAGTGTAAAAA

ATAACACACAGGTACTCATAAATTGTCGGAACAACAAAAAACTTCTGGGTCGTGTTAGAGCCTTTGATCG

ACACTGCAACATGGTTCTTGAAAATGTCAGAGAGATGTGGACTGAGGTACCAAAGACAGGGAAAGGCAAG

AAAAAGGCCATGCCAGTCAACAAGGATAGATTCATTAGTAAGATGTTCCTCCGCGGGGATTCTGTAATTA

TTGTTCTTAGGAACCCTAAGTGAGTGAAAGCTGCTTTTCAAGTTGATGGATACTCTTTTGAGTGGCTATT

AATGTACTGAAATGTGTAATTGGGTGCTATTATTGTGTCTTT

>FM891438.1 FM891438 Jatropha curcas embryo 56-70 (DAF) Jatropha curcas cDNA clone rjcaeb0_001727, mRNA sequence

AAATCAAAATATAACGAAAGCATAAACATATAAACGAAAATTGATGCTACATAGTGCCAATATTTTAAAC

TTCTTCCATCTAAGATATGAAGTAACATAGTCTAAATCAACAAAAGACCACACCAAATCAACAAAACTAT

TCTGCTCAAACAACAGAATGTCAGAACCTCTGGATCTCATTTCTTGGATTTCTTAGACCCCTCAGTTGGT

TGAGATGCAGCAGCAGGCGGAGCAGCTACAGGTTTCTCTCCCCCAGGACCCTGGGCCAATCGTTCCTCCC

TCCTCGCAAATTTCCTTTCCCTGCTTGCCTTGTTCTTTGCCCTCTTAGCCTCAAACTGATCAGACAGAGT

CTTCTCTCTAGCTTTCTCAGCCTTTGACTTGTGAATACTTTCCATAAGAACACGCTTGTTCTTAAAGGGG

GGGCCCGGTAC

>FM891437.1 FM891437 Jatropha curcas embryo 56-70 (DAF) Jatropha curcas cDNA clone rjcaeb0_001726, mRNA sequence

ATTCCAGATCTTTCCTGGTAAGATAGAAGACGATAGAGATTTCTACTTCTGGTGCTCAGAGAAATCAAAG

CTTTTAGGGATGGAATTGCTGTCAAAGATAAAGGATATACTCAAGAGGAGACCATCAATAGCAGATTTAA

CTGGTATTAGTAAGTCACGTCTTGATTGCTTTGCTACACACCTCCGCACCTATCTTGTTGGAAGCATCCG

AGAAAATGCTGTATGCTTTCCTGCCACTTCATTGGGCACCAATCTTGACCTCGCTTGGATGGTACATCTT

CTATATCATCTAAGTCTCTGCGTTCCCGACACGTTGGCAGTCAGGCTGTTAAGGGCAAATTCATCTTAT

>FM891436.1 FM891436 Jatropha curcas embryo 56-70 (DAF) Jatropha curcas cDNA clone rjcaeb0_001725, mRNA sequence

ATTCCAAAAGATTAATTATATTGGTGGAGAAATTTGTTAGCAAAAAAGGGCAAAAATGTCTTCAAATCAA

TCAAGACAAGAGCTTGATGAAAGAGCAAGGCGTGGAGAGACTGTTGTCCCTGGAGGTACTGGGGGCAAGA

GCCTTGAAGCTCAAGAGCATCTTGCTGAAGGGAGAAGCAGAGGAGGACAGACAAGGAAGGAGCAGCTGGG

GACAGAAGGATATCAGGAGATGGGCCGGAAAGGTGGGCTGAGCACGACGGACGAGTCTGGTGGAGAAAGA

GCTGAGAAGGAGGGCATCACTATCGATGAGTCGAAGTTCAGGACTGGGAGCTAGTGTGATGATTAAAACT

ATAAATGGTATTTTGTAGTAATAATAATAATAATGTTGGTAATAAAGTATTAGCTTAGCAGTGTACTTTA

GTTTAGTAATGTAGTTTACATGTGGGTTAAAATGACTAGGGTTTAGCTAGTATGTGTGTAGTAACTTTGA

>FM891435.1 FM891435 Jatropha curcas embryo 56-70 (DAF) Jatropha curcas cDNA clone rjcaeb0_001724, mRNA sequence

ATCTAAAATCCTTCGATTTGACGATCCGCGAGAAGGAGATCTATACTCTAAAGAGGAGTAAAAGAAAGAA

TATGATACTAGTGGCCATAGTTGCAGAGCTGCTGGAGGAGTACACGGCGGTACTGGCAAGGATGCTGGAG

CACATGTTTAATGAAGCCCCTTTCCCAAGGCGGATTCGTTTCCTCATTCTGCGCAGCCTGCCTTTCGCTT

CTTCCTCACACCCTCCTCCTTTAATTCGAGGTCCTGCTTCAGCTTAATTACAATGTTTTGAATTCCGGTT

CTGTGTACATATTTGGAGGATTTTGTTTCTCTGTACTAGTTTGTTTGATTTGATTAGATTAATGTTCTTC

AATCTTCAAT

>FM891434.1 FM891434 Jatropha curcas embryo 56-70 (DAF) Jatropha curcas cDNA clone rjcaeb0_001723, mRNA sequence

ATTCGTCAATAATGTATCTTAATAATATCATAAAACTGCAAACTAAACAAGAGAGCAAATCTTTGACACA

AATAACAACCAACCGAGCAACATCAGTATGTTCAAATCTTTAAAACATAACGCTAAAAAAAAATTCTTAA

GAGAGACAAAATTCTTCCTCTGCCATACAGTATTGATCTCTTAACGATTGGCCTTAGCAACTCTTTCAAT

CTCATCCTTCTTCTTGATGGCATAGCTGTTGGAGGAGCCCTTTGCTGCATTGATAAGCTCATCAGCCAAA

CATTCTGCAATGGTCTTGATATTTCTGAAAGCAGCTTCCCGAGCACCAGTTGTAAGCAGATAAATAGCTT

GGTTTACACGCCTCAAAGGTGAAATATCAACAGCCTGACGCCTNGACACACCAGCAGAACCAATACGGGT

GGCATCTTCCCTGGGACCACTGTTAATCACAGCATCAACAATAACTTGGATTGGGTTTGATCGGTCGAAC

ATGAATGATCTCAAGCAT

>FM891433.1 FM891433 Jatropha curcas embryo 56-70 (DAF) Jatropha curcas cDNA clone rjcaeb0_001722, mRNA sequence

ATTCATTCGGTAAGAAAAAATACGACAAAATTAAGAACTACCCAGAAAAATTCAAGACTTAAACCTACCC

ATTAGTCTTATAAATACATATATATTGTTAATTTATCACGCCTTAGACCTTTTTTCCTTGTAAGACATAT

AATTAGTAACAGCTTTAGACCCTTCAGCAATAGCATGTCTTCCAAGTTCTCCAGGCAAAACCAATTTAAC

TGCATCCTGAATTTCCCTGGACGACAGTGTCGTTCGGTGGATATAATCTGAAAGCTTTGCTGCTTCATAA

ACAATTCTTTCAAACATGTCCTTCATTAAATTGTTTATAATACTCATTGCCATTGATGATATCTTCAGCT

CAGGATGCACTTGCTTTAGTACTTTAAACACATACCTTTTATAACCCTCTCTTTCTCTCCCGACTCCTCT

TCCTTTCCTCTTCTTTTTCCTTTCTTGTCCTTTCTCTTGTT

>FM891432.1 FM891432 Jatropha curcas embryo 56-70 (DAF) Jatropha curcas cDNA clone rjcaeb0_001718, mRNA sequence

ATTCTTTTTATATTTATATATTATATAGACAACAATGAAAGGAGATATATTAACCATTTAATAGCAATAT

CATAATTGAAGTTCCCCTTCCAAAATAACACAATAGGCTCCTTAAAGAACAACAAAGCAAAAGTTCAAGA

CAATACATACAACAAGAAACAACAGGAAATCAAATCCCACATGACAGGGTTTGAGAAGGTACCCATGGCT

TCTTTTATTTACACCAAACATGGATTATTACACATAATTCAACTGATTAACCATAAAAGGCCAAGACTAA

TAACTGCTTTGTTGGAGCCTTTTCCCTTCTGGTTTCTCATTAGCAGTTGCATGGGTCACAGTTGCAGTTT

GATCCACACTTGCAGCCATGACCACCCTCAGCTCCAAAGCTCATCTCAGAACTCTCATTCAACTTCTTGG

AAGGTGCAACTCCTGCAATGAGGGTCTCAGTTCTGGTGCTCTCTGAAATG

>FM891431.1 FM891431 Jatropha curcas embryo 56-70 (DAF) Jatropha curcas cDNA clone rjcaeb0_001717, mRNA sequence

ATTCCAACAAATGGCTGAACGTCACAGCCACACCAAGTTCAAGTGCACCCACAACACCGCTATGAAGCTG

CCTTCAAAGGTCAACAAAAAGGTCCATCAGCTCAGAAAGTGTTAGCTGTGATCACCCTCCTGCCGGTAGG

CGGTGGGCTTCTTGCACTAGCTGGTATAACCTTAGTAGGTACCCTAATCGGCCTAGCTATTACTACCCCT

CTTTTTGTCATCTTTAGCCCTGTTCTTGTCCCAGCTGCATTAGTTATTGGCCTTTCGGTCATGGCATTTT

TGGCTTCCGGGGCTATGGGGCTCACAGGGTTGTCATCACTCTCGTGGGTTCTGAAATACCTCCAGGAGGT

CACTCGGCGAATGCCGGAGCAGCTGGATATAGCTAAGAAGCGTATGCAAGACATGGCTGGTTTTGTGGGA

CAGAAGACCAAGGAAGTAGGCCAGA

>FM891430.1 FM891430 Jatropha curcas embryo 56-70 (DAF) Jatropha curcas cDNA clone rjcaeb0_001716, mRNA sequence

ATTCGGCATTTTTGGCTTCCGGGGCTATGGGGCTCACAGGGTTGTCATCACTCTCGTGGGTTCTGAAATA

CCTCCAGGAGGTCACTCGGCGAATGCCGGAGCAGCTGGATATAGCTAAGAAGCGTATGCAAGACATGGCT

GGTTTTGTGGGACAGAAGACCAGGAAGTAGGCCAAGAAATCCGAGGAAAGCACATGAAGGGAAATAAGTA

GAGAGAAATGCTGTTGGTTATTAAGGGTTGAAGTCAGAGAGCTGGGTTTTGGTACGAGACTACTTTTGTT

GTATGGGTGTTGTTTTGAGGTAGTTGAGTTGGTTTATGGGGGATCTTCTTTGTTGCATTTGTATTTCTCT

TTTTGGTCTTTTGGGTTCCATGTTGATAACATGTATTATCT

>FM891429.1 FM891429 Jatropha curcas embryo 56-70 (DAF) Jatropha curcas cDNA clone rjcaeb0_001715, mRNA sequence

ATTGATCANAGAGAGAAGCTTGATTGATATTAAAAATAGTGCAGACACCACTATTTACAGCATTGAGAAG

AGTTTGAATGAATATAGGGACAAGATCCCTTCCGAAATTGCCAAGGAGATTGAGGATGCAGTTTCAGACT

TGAGGAAGGCAATAGGTGGAGAAAATATTGAAGAGATTAAATCTAAGCTTGATGCTGCAAACAAAGCTGT

GTCAAAGATTGGAGAACACATGTCTAAGGGAAGTGGTGATGGTGGTGCATCTGGTGGTTCACAAGGCGGC

GACCAGACTCCCGAGGCAGAATATGAGGAAGTGAAGAAGTGAGTGGTTAGCTGATAATAACATTGTTAGT

TTTGCTCGATTGGACTTTGAACAACATCTGAACATGAATTTTCTGAATTTTTACGTTCTCGTTAGGTTAT

ATATTTCTAGCGGTTTGTATCTAACAGTGGAGGCTGAATGTACTA

>FM891428.1 FM891428 Jatropha curcas embryo 56-70 (DAF) Jatropha curcas cDNA clone rjcaeb0_001714, mRNA sequence

ATTCCCCNTTGGTCTTCGCCTCTCCAGTTGAATCTCTGTTCTTCTCTTCTCTTCAAAACAGTCCCTTTGT

AGCCCAAAACCCAGAAGAAGCTCATCTCTTCTTCGTGCCCTTCGCTTCTGGTATCTCCACGCGCTCGATT

GCTCATGTAATTAGAGATCTTCGGATGGATTTCCCCTATTGGAATCGTACTCTCGGTGCCGATCATTTCT

ATGTTTCATGTTCTGGTCTCGGCTACGAATCGGACCGAAATCTTGTGGAGTTGAAGAAAAACTCGGTTCA

GATTTCCTGCTTTCCGGCACCGGAAGGTAAGTTTGTTCCCCACAAGGACATAACATTGCCTCCGCCGGTA

TATTCTCTTTCTGCGCACCCGCCCAGAAACAAAACTGCGAGATATCGAGGTTTTGTGAAACACAATGGGG

TCAAAGAGTCAGCTTTAATCAACGACTTGAGGAATGCTTCAG

>FM891427.1 FM891427 Jatropha curcas embryo 56-70 (DAF) Jatropha curcas cDNA clone rjcaeb0_001713, mRNA sequence

ACATAAAGCACCATTTCTATAATTGTCTTGATTAGAAATGATATCCTCACAATAGAGATGCTGTCGAGAC

TAAAGAGCATATTAGCTCAGACTAGAGAAATTGGAAGTAAATACAAGATAATGTGCACTTTAACTGCATA

CTGCCATATTTGATAACTAAGAAACGAGAAATAAATAGTTGCAACTAGAGTGAAATCGAATTGAGATGCA

TTTACATGTAAGCAGCCTCATTTGTCTGCTGCATCCACAATGATATGGCTGATATAATTCGTCGTGATAA

GGTTGACATTTTTTTCTCCTTGTAGCAAACTGGAACGATCGGTCTTGATTTACCCCTTTGGATGGCATAA

CGTGATACTTCACTGCAAGCTTTGGGTGCAGAGTCGTTGTGTGTATAGTGCTTGTATGCCTGCATATGTA

ACAATAAATTATCAT

>FM891426.1 FM891426 Jatropha curcas embryo 56-70 (DAF) Jatropha curcas cDNA clone rjcaeb0_001712, mRNA sequence

ATTCGGAGAGGTGATGAAGAAGGGTTAAAGAGCCTCAACATCAGTCAGTGCACAGCACTCACTCCTCCAG

CTGTGCAGGCACTATGCGACTCATTTCCTGCACTTCACACCTGCTCAGGGAGGCATTCCCTCGTCATGAG

TGGCTGTTTGAACTTGACATCTGTGCATTGTGCCTGTGCAGTCCAGGCACACCGCACATTGAGTTATATT

CCCAATCATGCTCATTAAGCAGCGCAATGAGCATGAAATTTTGAGTTCATTTGGAAGCTCACTGAAAGGA

GTATGCCTCTGTACATAAATGCTTGTGTTAATAACCGGACTGTTTTGGCTGGAAACGAGCAGCCAGCTCT

TTTGTTGTTGGAATCACTGTGTGTATAGTTGTAGACTTCTTGTGCTGGCTCTTGCTTTCTAAATTAGCAG

CATTAGGCTAATTGAAGCATTGAAGCCAAGTAGTGTCATGTATGGGATCGGAAGGA

>FM891425.1 FM891425 Jatropha curcas embryo 56-70 (DAF) Jatropha curcas cDNA clone rjcaeb0_001711, mRNA sequence

ATTCCANTTGCTTTGCTGCCGGAGTTGCCCATTGGTGTTACAACGACGGCAATGAGCCTGTTGTCGCCGT

TTCTGTTCTTGACACCGCCAACAGCGCCAACCAGCTTGACATGAACCCTAGACATTTCATCTAGCTGGTA

ACCCAGAAGATGAATTCCCGCAACTGCATGAGGGTCGCCGTGAGGGTGGCAGCCAGGAAAAATATCCAGG

GGGCCGCCGTGCCTCGAGAGAACGTGGCTCTTGTAACAATCTTTTTTGCGGAATGGACTCGAGATTTGTT

GCTGAAGCTTTCAATATCGACGAATCGTTGGCTAGGAAACTCCAAAGCGAGAATGACA

>FM891424.1 FM891424 Jatropha curcas embryo 56-70 (DAF) Jatropha curcas cDNA clone rjcaeb0_001710, mRNA sequence

ATTCATCAAATATATGATCGTAAACCTCCATTGAATTTACTCTGTGCTTCGATCGCGTCAATGGCGGAGC

ACCCACAGTCGCAGCATGTTGGCCAACAGCCACGATCCCACCAAGTAGTGAAGGCAGCCACTGCAGTCAC

TGCCGGTGGATCTCTTCTAGTTCTCTCCGGTTTGACCCTTGCTGGTACGGTCATTGCACTGACCGTAGCC

ACTCCATTGCTGGTCATATTCAGTCCGGTTCTTGTTCCTGCGGTTATCACAGTTTGTTTGATAATCACAG

GGTTTTTAGCCTCAGGCGGTTTTGGCGTCGCTGCCATTTCTGTCTTGTCCTGGATTTATAGGTATGTTAC

TGGAAAGCATCCACCAGGAGCAGAAAGTCTGGACCAGGCACGCTTAAAGTTGGCGGGAAAGGCAGAGAGA

TGAAGGACAGACTGACATTTGG

>FM891423.1 FM891423 Jatropha curcas embryo 56-70 (DAF) Jatropha curcas cDNA clone rjcaeb0_001708, mRNA sequence

AGCAAGAAAAAGTATTTTCATTAAAAATATCAATCCTACAAGCAAAATTACTTCCTCAATACAAATGGAA

AGCCAGGCATAATGTTCTCTAGATAGGCTTGATCTTGAAATGGAGTGAGATAAGGGAGAAAACAAAGCAC

TTCAAGTCCTGAACAAGGTAATAGAAGATACGAAGCCCTTCAGGATCCTTACTGCTCTGGACATCAACAA

GTGACCCAATCTTTGAGGTAGTGAAGGAGATATGCTCGTTTCCCATTACAATCTCGAGCTCTTGTCTCCC

AACCCTGTCTGGTTCTGGCCAGTTGGCATCATCTTCCTTCATGATCTCGCTCTCGGCGATGATGCGGCGG

CATTCCTTAAGAACGGCGGGGGTGAGGAAGACTTCCTTCCGGATCATGGTGTCGTTCTTGTAGTG

>FM891422.1 FM891422 Jatropha curcas embryo 56-70 (DAF) Jatropha curcas cDNA clone rjcaeb0_001706, mRNA sequence

AATTCCNTTTTTGTTTCACACACATATGAACATGTATTACTCGTTCATATAACATAGCTCGCACAGGCTC

TGGCTTCATAAAAGACAAACTTTCTAGTGTACTGTCATGGACTATAGAAAGTCCCCCCCCCCCCCCCCCC

TAAGGCGCTTTGGATG

>FM891421.1 FM891421 Jatropha curcas embryo 56-70 (DAF) Jatropha curcas cDNA clone rjcaeb0_001704, mRNA sequence

ATTCCTCTTCTTCTATCTCTATTTCCATATATTTATGTAATAATTATTTCATATATATATATATATATAT

ATATATTTTATTTGGATTAGGTTATCGATTTGGGGATTTTTTTTTTGGGTCGTTTGGGACTAGGTTTAGG

GTTCTGAGCAAAGCAAGGAATACAAATATGCAACAACCGCCGCAGATGATCCCAGTGATGCCTTCCTATC

CACCTACAAACATCACCACTGAGCAGATCCAGAAGTACCTTGATGAAAATAAAAAATTGATTTTGGCGAT

CTTGGACAATCAAAACCTTGGAAAACTTGCTGAATGTGCCCAGTATCAAGCTCAGCTGCAAAAGAAT

>FM891420.1 FM891420 Jatropha curcas embryo 56-70 (DAF) Jatropha curcas cDNA clone rjcaeb0_001703, mRNA sequence

CTGCTCCTCTCCTCTGCAAAAATGCAGATTTCGTGAAAACCCTAACCGGGAAGACGATAACCCTAGAGGT

TGAGTCTTCTGATACTATCGATAATGTCAAGGCCAAGATCCAGGACAAGGAAGGCATCCCTCCAGACCAG

CAGCGACTGATCTTCGCCGGAAAGCAGCTAGAGGATGGCCGCACTCTTGCCGACTACAATATCCAGAAGG

AATCCACTCTCCACCTGGTGCTTCGTCTTCGTGGTGGTGCTAAGAAGAGAAAGAAGAAGACTTACACCAA

GCCAAAGAAGATCAAGCACAAGAAGAAGAAGGTCAAACTTGCCGTGCTTCAGTTCTACAAGGTCGATGAC

TCCGGAAAGGTGCAGAGGCTGAGGAAGGAGTGCCCCAATGCTGAGTGTGGAGCTGGAACTTTCATGGCCA

ACCATTTTGATAGGCACTACTGCGGCAAGTGCGGCTTGACTTATGTTA

>FM891419.1 FM891419 Jatropha curcas embryo 56-70 (DAF) Jatropha curcas cDNA clone rjcaeb0_001702, mRNA sequence

ATTCCAAAACCACGAGTTTGAGCAAAAATGGCAAAGCTAATTACCTCAACTGCTCTAATCTGCATTCTTC

TAGCTGATCATAGCCAATGCAGTCTCCGTGCCATATGACAACCACCACCAGCCACCACCACCGATTGAAA

TGGACGATGCTTATGAAGTTCGGGAAATGTGCAAGAGAGAAGCTGAAAGAACAAATTTGAGCTCTTGTGA

GAGGTATATAAGACAGTCGAGAACAAGGTCAGAAGCTCTGTTAGCTATGAAAGGAATCGAAAACCGGCAC

CATAACGTTCCTAGACAATGTTGTGACCAAGCAAGGAAGCTACGTGCCATTTGTCGATGTGAATCCATAT

TGTATCTACCAGAGAAGCAAGGATTTGTTGGAAGCGAAGAATATGAAGAGGCAGGAGCAGTAGGTCTTAC

AATATAATCGAT

>FM891418.1 FM891418 Jatropha curcas embryo 56-70 (DAF) Jatropha curcas cDNA clone rjcaeb0_001701, mRNA sequence

ATTCGATGGTGTCCGATGGTAATTCAACCTAGTACGAGAGGAACCGTTGATTCGCACAATTGGTCATCGC

GCTTGGTTGAAAAGCCAGTGGCGCGAAGCTACCGTGCGCTGGATTATGACTGAACGCCTCTAAGTCAGAA

TCCGGGCCAGAAGCGACGCGCGTGTCCGTCGCCCGTTTGCCGACCCGCAGTAGGGGCCATCCGGCCCCCA

GAGGCACGTGCCGTTGGCCAAGCCCCCGCGGCGGACGAGC

>FM891417.1 FM891417 Jatropha curcas embryo 56-70 (DAF) Jatropha curcas cDNA clone rjcaeb0_001696, mRNA sequence

ATTGGTTGCTGTTGTGAACATTATCTTCCTTATCTTCCTCATCATTGTCTACTCTGTTGGATGCTGTGCT

TTCAGGAACAACAGAAGGGACAATGCTTATTCTGGTGGTGGATGGAATAAGCACCCTTGAGACTTTCAGC

TTGGAGGGTGTTATTAGTTTTTAGGTTCTCTTTATTATTATGAGTAGATGGTGTTCTTCTTTTGTTTTTG

TATAGCATCTTATATGCTTGATTTTATGTTCATTAGAACAAATATCGGATATATATAAATATATTTTTCT

GTTATTAAAAAAAAAGGGGGGGCCCGGTAC

>FM891416.1 FM891416 Jatropha curcas embryo 56-70 (DAF) Jatropha curcas cDNA clone rjcaeb0_001695, mRNA sequence

ATTCCTGAGTTCCTGTTCTAATCTTTAGAGCTAGCTAGCTAGCTATGGCTCGCTCTCTTGAACCACTGGT

GGTTGGGAAAGTGATCGGTGAAGTTCTTGACATGTACAATCCAGTAGCTGAGTTCACTGTGCATTATGGT

TCTAAGCAAGTCGCTAATGGCTGTGAAATCAAGCCTTCTGTTGCTGCTCAGAAACCACATGTCCATATTT

TGGGTTCTCGTCTATCCTCCGATCTCTATACCCTGGTTATGGTTGATCCTGATGCTCCAAGTCCTAGCGA

ACCAAAATGGAGAGAGTGGCTTCTTTGGATTGTTGTAGATATTCCAGAAGGATCCGATGCTACCAAAGGG

CATGAGTTGGTGTCGTACATGGGACCTCAGCCTCCAACCGGCATTCACCGTTATGTTTTTGCGCTGTTCA

AGCAGAAAGGTGCATTGATGGGAAGGATTCAACCTCCAGATGGTCGTGGCAACTTCAACACCCGCCACTT

TGCTGCCCAAAGTGGACTTGGGCTTCCGGTTGCTGCTGTGTATTTTAACTCGCAGAAGGAACCGGCAGTT

AAAAAACGTTAATTCTGTTTAGTTCTTTTTTTTTTTTTTTAACACGGGATAGCAACATTGGGATTACGGT

TATATGTTTACGCTGCGTCCTGTA

>FM891415.1 FM891415 Jatropha curcas embryo 56-70 (DAF) Jatropha curcas cDNA clone rjcaeb0_001694, mRNA sequence

ATTAGAATGGTTTTGGTTTCATGGNCACCAATGACCATGGAGAAAGAAAGAGACAGATCATGTCCAATAC

CAGATGGATTGCCAAAATCGAAGGAGGAGCTTGAAGAGGAAGAGAGAGCAAGAATGCCAGATTCACAGTA

TACTAGATTACTTCGAACAAAAGGAAGATTTCCAGCTTGGTACTCACCTGCTCCTGACCATGAAACGGAT

TGATAAATAAGCTGCAATACGTTGAAAGTTTTTCTTGAGTTGCTTCCGTGCATAGCTGATATGAGTATAT

CCGGTGGATGTATGAGATTTATGATCTGATTGTTTCCGCCCAAGTTATTTTCACAAAATTAGACTTTGGT

CTGTGTAATGGGAAGTTATTTCTGCTAGACTTTGTGAATATTATTGCAGTATTTCGGTTTCGAGAAAGCA

GAATTGTAACATATATGAACTGTATCATAGCTTATCCGTAGTTGTGTGGCAATGGATGCTCTTTTTCGAG

CTCCTTACTATTGTAAACTTCAATAAAAACATCCATATTGATATAAGATCAACAGAATAGCTTTCTCT

>FM891414.1 FM891414 Jatropha curcas embryo 56-70 (DAF) Jatropha curcas cDNA clone rjcaeb0_001693, mRNA sequence

TCATCAAATCGGCGTTGAAAGATCAGTTCTCCTTTTTGAGTATCTAAAAATGTCTTCTTGCTGTGGTGGA

AATTGCGGCTGCGGCTCCGGCTGCAAGTGTGGCAGCGGTTGTGGCGGATGCAAGATGTATCCTGACATGA

GTTTCTCCGAGAAAAACACCGCCGAGACTCAGGTTCTCGGAGTTGCACCGGAGAAGGCACGCTTTGTCGA

TGGAGGTGAGGTCGGCGTTGGAGCTGAGAACGGATGCAAGTGCGGTGACAACTGCACCTGCAATCCTTGC

AACTGTAAATGAGATCGGCGGCGATCAGTCTGTTTTTTAGAAGTAAAAGCAGAGATGACTCTGCTTCTCT

CTTTCTAAAAAAGGAGAAATAATTAATAGAGTCTAGATATCGTATGAGTTTGTTCGTTTGTTTCTGGTTT

TGAGCTTCAATAAAAGTAGTGGTGGCTTGTGAGCTTGTGTTTAGCAATGGCTGCCTGTTGCTTTAACGTT

ATATATTGTATAAATTAAAAACTAGCATATGAATATGTAAATGAGTACGAGGGGTGAAATAGCTTTGAAA

GCTTAATGAGAAACTCCTTTTCT

>FM891413.1 FM891413 Jatropha curcas embryo 56-70 (DAF) Jatropha curcas cDNA clone rjcaeb0_001692, mRNA sequence

ATTCCAGAGAACTATAAAAGCTAAACCCTTAAAACACAAAAGCTTCATGCCCCGACAGTGGCACCTAGGA

AATGAGTTTCGGCCAAACCAGTGACATGCAAACGCAGAAACGATCCAATCTCACCTGGTATGCTTATCGT

TCCTATGCCAAATCCTCAGAAAACATTCGACAGACCAGAGAGAGCAAGGATTATCGTTCCATCTAGAAGA

ATTAATGAAGAACTCCTTGATTCTATTGAGATTGGAGTAGAACTTTCACTTATTGATGGAAGAAGTGGGG

TGGGATAGCTTAGCAAGGTTTAGAGTTAGGGTGGTGCTACAGGTTTTTATTTAGCGGCGGATTTTGGAAA

TCCTCCGACGATGGTTTTTCCCCCCAGACTTCCACCTAACCCCCTGTGAGAGTTGCCCATTCTCGTCTAC

AGTTAGTGCTTTTTGGTGAAGATGGGAAGAAAAAAATTTTGCTTTTTGGTTGTAAAATAGAAAAAGAAAA

AA

>FM891412.1 FM891412 Jatropha curcas embryo 56-70 (DAF) Jatropha curcas cDNA clone rjcaeb0_001691, mRNA sequence

ATTCCCATTTTATACCATGAGCGCAGCTTGACAAGAAACTAACCAGCCACCATGCTCAAGACACTCAGTC

CTTACTCTACAACAGCAAAAACTGCTGAAATCATGTCTAGGTACAGGCCCATTGCACCAAAGCCTGAGCC

TCCTGCCAACTCTTTGGGTGAAAGCTCTTCAATGTCTCAGAAAATTAGGCAATCTCCTTACCTTAGGAAT

CTCTGGCCTCAGTTGCAAGCCAGGCCCACTAGAACAAGAAAGAGAGGGAGAGCTGCTATTTCTCCTCCTA

CCATCAAAAGACCAAGAACAACTCATTTTCTTGGTCTTTCCTCTCCTGGTCATTTAATAACCTCTGCTAG

AGATCTCTCCTTGCGGGGTTTTGCTCATGGGATTTCTCAGCTTCCTGTTGCCAATCTTGCTGGGGTTAAC

AGCACTTGCTTGGAAAATCCAGTCACCACCTCTTCAGATTTGGTTACGCTTTCTCTTCTTCCGTGTCAAC

CTTCGGTTCCTGCCGTTGCACCCGAAATCAGTTGCATGGAACGACGCGGAGAGAAAAAGGTAATTGATTT

GAATATTGTAGCTGAAATCCCAGAAGATAAAGACCTCTTGCAGCAACTGCAAGAACCACCTGCTAGCTTA

ACAAATGTTATAGCACCTCAGCCAATTAGGCCAGTTGGCTCCAGGATAAGCGTTGGGTGCATAACAG

>FM891411.1 FM891411 Jatropha curcas embryo 56-70 (DAF) Jatropha curcas cDNA clone rjcaeb0_001690, mRNA sequence

ATTGAATACAATTATATTTATAGAATATATTGATTAGGGAATACTTAATTACTAATTAGGATGATTACAT

TACTTTCATCACTTATTTTCATAATATATTACATACTTCATTAGCGTGAGATCATAACTCCCCTCGTAAT

ACTACTTACTTTCACTATCTACACACCCAAAAAGGCTACTTCATTATTTACTCCTCGGCATATCTTCCAG

ACTCGGTCCTGGAAATAGTCAAGGTGGTCTCTTGTCTTCCAAACTTAATCCTTCTTGCCTCCTCTGCTGA

TACCCTAAATGCATTTGCAATAACCGCCAACGGCATTGCTCTAACGGCGGAGGAGCGACCTGCAAGATCA

TTGGTCATCGCGTTATCGCTTGTCTTGAAGGTTACATATTCGAATCTTTCACTTTCTGCTCGTTTCACCA

CTACGAAGTTTTGTGGCACCGTCAAAAGGGGGGGCCCGGTAC

>FM891410.1 FM891410 Jatropha curcas embryo 56-70 (DAF) Jatropha curcas cDNA clone rjcaeb0_001689, mRNA sequence

ATTGGCATTAACAATGGCCATAACTAACTCTTTCCTTCTTTCACTGTGTCTAATTGTTATCTTCACTGGC

TCTATAGCAAGTCAGCAACTTCAGCAGCAAAACCAGTGTCAACTCGATAGGCTCGACGCCCTCGAGCCAG

ATAATAGTATTCAAAGCGAAGCTGGTGTGGTCGAGTCTTGGAACCCTAACCAAGACCAGTTCCAATGTGC

TGGTGTGGCTATTGTTAGGCTCACCATTCAACCCAAGGGCCTTCTGTTGCCATCTTATAGCAACGCTCCT

CAACTCGTTTACATTGTCCAAGGTACGGGTATGACAGGAGCCCTGTTTCCTGGATGCGCTGAAACATATC

AGGAGTCTCAAGGATCAGGTGGTAGCAGTAGGGTTGAAGCCCAGCACCAGAAGATTCGTCGTTTCCGTCC

AGGTGATATCATTGCTTTGCCTGCCGGAGTTGCCCATTGGTGTTACAACGACGGCAATGAGCCTGTTGTC

GCCGTTTCTGTTCTTGACACCGCCAACAGCGCCAACCAGCTTGACATGAACCCTAGACATTTCAATCTAG

CTGGTAACCCAGAAGATGAATTCCCGCAACTGCATGAAGGTCGCCGTGAGGGT

>FM891409.1 FM891409 Jatropha curcas embryo 56-70 (DAF) Jatropha curcas cDNA clone rjcaeb0_001688, mRNA sequence

ATTCAGCAATTGTACCTTGTGGTCTTATAGCATGGAGTTTGTTTAATGACACCTATAGCTTCACTCGCAA

CAATCAGCAGTTGCAGGTGAATAAGAAGGGGATCTCATGGAAAAGTGACAGGGACCACAAATTTGGCGAT

GATGTTTTTCCTAAAAACTTCCAGAGCGGAGGTCTCAAAGGTGGTGCAAGTCTGAATACATCAATACCAT

TGGATGAGCAGGAGGACCTCATTGTTTGGATGCGGACTGCAGCTCTGCCAACTTTTAGGAAGTTGTATGG

GAAGATAGAGCAGGATCTACAAGTCAATGATGAGATACATGTGATATTGGAGAACAACTATAATACGTAC

AGTTTCAATGGCAAGAAAAAGTTAGTGCTTTCAACCACAAGCTGGCTTGGTGGAAAGAATGACTTTCTTG

GCATTGCTTATCTCACCGTCGGGGGCCTCTGCT

>FM891408.1 FM891408 Jatropha curcas embryo 56-70 (DAF) Jatropha curcas cDNA clone rjcaeb0_001687, mRNA sequence

ATTCGAGGAACTATGATCAAAGTAAAGACTCTCACTGGTAAAGAGATTGAAATTGACATTGAACCTACCG

ATACCATTGACCGAATCAAGGAACGGGTTGAAGAAAAAGAGGGCATTCCTCCTGTGCAGCAAAGGCTTAT

TTATGCAGGTAAGCAGCTAGGAGATGACAAAACAGCTCGTGACTACAATATTGAGGGTGGCTCTGTGCTT

CATCTTGTGCTTGCACTGAGGGGTGGTTGTTATTAAACGGTGTTTATATCATTCACAAGCTCTAGTTATC

TAAGTCATTATTGCCTGTGATGTACGGATAAACATTGAGGATACTAAATGAAAAATGTTCAGCTCATTTT

GTGGGCATGAAATTGTTCAAAAAAAAAAAAAAAAAAAAAACACTATTTAACTTCTATTTCTTCGGCTCTA

GTAATGGATATGGGATCGCATCAAAACCCTCATGGTTTCCTCCTATATGGGCTCTACATTTCTTCTCCTT

CGTTTCGACTTTCTTGATGGGTCTGTCCGCTTGGCTCGTTTGGGCTGAAGGAGGGTTTCATAAAAAACCA

AGGGCTATTTCTCTTTACTTGGCTCAGTTAGGTTTGGGTTTGGCTTGGGACCCGATTGTGTTCCGGATGG

GAGCTACTTGGGTCGGGTTGCTTGCTTGCTTGGCTATGTTTTGGAGCCATTGTTGGGTG

>FM891407.1 FM891407 Jatropha curcas embryo 56-70 (DAF) Jatropha curcas cDNA clone rjcaeb0_001685, mRNA sequence

ATTCGAAAAACATTTGATTTGAGTCTTTCTTCACAAAATCAAAAGATCAAAAGAAGATGAATAGATGCTT

ATCAGATCTAGAGGCTGGAAGCCTGGGAGAAAGATGTATGACTTATGATATGAGCTATACATAGCAAGCC

AAAATTATAACCCATCAGTCTGGCACCTGGTCAGTTCTCAAGCAAAGCTGAGCTCATGGATTGGCAAATT

TACTCCCTAATATCAAGAATGGAGGCAAGAGTTACCTCAAGTTTGTCTTCTCTAGCCTTGTGATTCTTTG

GAAGCTGCCGAAACTCCTCAGTTAAGCGAAAGCACTCATGGAGATTTTCAGGAGAGACAAAATCTACTGC

AACTTTCGTACATGACTGTTTGTTTTGCAAAAAGAAAGGCCTTATTATAAGTAACTTACCTATTATATAA

TGCTAGATTGCTTATTTATTTTCTTAAAAGAGAAAAGCAAAATGTAGTAGTTTTCATT

>FM891406.1 FM891406 Jatropha curcas embryo 56-70 (DAF) Jatropha curcas cDNA clone rjcaeb0_001684, mRNA sequence

ATTCGACACACCGCCGTGGAATTTTGATCAGACGACAAAGATGCCGATCAGAAACATTGCCATAGGCCAT

CCTCAGGAGGCAACTCATCCAGACGCCTTGAAAGCGGCGTTGGCTGAGTTCATCTCCACTCTTATTTTTG

TCTTCGCCGGTGAAGGCTCCGGGATGGCCTTTAATAAGCTCACTAACAACGGCGCCACCACTCCTGCCGG

TCTAGTCGCCGCATCTTTAGCCCATGGTTTCGGTCTTTTTGTTGCTGTCTCAGTTGGAGCCAACATCTCC

GGCGGTCATGTAAATCCAGCTGTTACCTTCGGTGCTTTCGTCGGTGGAAACATCACTCTCCTCCGCGGTA

TTCTCTACTGGATTGCTCAGCTTCTCGGATCCACCGTAGCTTGCTTGCTTCTCAAGTTCAGCACCGGTGG

CTTGACTACGTCCGCCTTTGCTCTCTCCTCTGGTGTTGGTGTGTGGAACGCTTTCGTTTTTGAGATTGTG

ATGACCTTCGGGCTTGTATACACCGTGATATGCCACCGCAATTGACCNCAAAAAGGGCAGTCTGGGAACG

ATTGCACCAATCGCCATTGGTTTCATTGTTGGAGCTAACATTTTGGCGGGAGGAGCCTTTGATGGAGCTT

CAATGACCCAGCGGTGT

>FM891405.1 FM891405 Jatropha curcas embryo 56-70 (DAF) Jatropha curcas cDNA clone rjcaeb0_001683, mRNA sequence

ATTCCCAAGAAACTTTCTCTCACCGATCTTTGATCTTCTCCAAAAACCAATCAAGAGAATGTCTCTGATT

CCAAGCACCCTCTTCGGTGGCCGGAGAACCAACATTTTCGATCCCTTCTCTCTCGATATCTGGGATCCAT

TTCAGGACTTCCCTTTCACAAGCACTGCTCTTTCTGCTCCTCGTTCTGAGTTATTGAACGAAACGGCAGC

GTTTGCCAACACCCGCATAGACTGGAAAGAAACACCAGAAGCTCATGTATTCAAGGCTGATCTTCCAGGG

CTAAAGAAAGAGGAAGTGAAAGTGGAGATAGAGGAAGGTAGGGTCTTGCAGATAAGCGGAGAGAGAAGCA

AAGAGAAAGAAGAAAAGAATGATAAGTGGCATAGAGTTGAAAGGTCAAGTGGTAAGTTCTTGAGAAGGTT

TAGGCTACCAGAGAATGCAAAGGTTAATGAGGTTAAGGCTAGCATGGAAAATGGAGTCTTGACTGTGACA

GTGCCTAAAGTGGAAGTCAAGAAACCGGACGTTAAGGCTATTGAGATTTCTGGTTGAAACTTTTAGTTAT

GTTTGAGTGTGTCTAAGTCTAAATGGTACCCATCCATCCAATAAAATAAATAGATGTGTGT

>FM891404.1 FM891404 Jatropha curcas embryo 56-70 (DAF) Jatropha curcas cDNA clone rjcaeb0_001682, mRNA sequence

ATTCTAGCTATGGCTCGCTCTCTTGAACCACTGGTGGTTGGGAAAGTGATCGGTGAAGTTCTTGACATGT

ACAATCCAGTAGCTGAGTTCACTGTGCATTATGGTTCTAAGCAAGTCGCTAATGGCTGTGAAATCAAGCC

TTCTGTTGCTGCTCAGAAACCACATGTCCATATTTTGGGTTCTCGTCTATCCTCCGATCTCTATACCCTG

GTTATGGTTGATCCTGATGCTCCAAGTCCTAGCGAACCAAAATGGAGAGAGTGGCTTCTTTGGATTGTTG

TAGATATTCCAGAAGGATCCGATGCTACCAAAGGGCATGAGTTGGTGTCGTACATGGGACCTCAGCCTCC

AACCGGCATTCACCGTTATGTTTTTGCGCTGTTCAAGCAGAAAGGTGCATTGATGGGAAGGATTCAACCT

CCAGATGGTCGTGGCAACTTCAACACCCGCCACTTTGCTGCCCAAAGTGGACTTGGGCTTCCGGTTGCTG

CTGTGTATTTTAACTCGCAGAAGGAACCGGCAGTTAAAAAACGTTAATTCTGTTTAGTTCTTTTTTTTAT

TATTTTTAACACGGGATAGCAACATTGGGATTACGGTTATATGTTTACGCTGCG

>FM891403.1 FM891403 Jatropha curcas embryo 56-70 (DAF) Jatropha curcas cDNA clone rjcaeb0_001681, mRNA sequence

CGAATTCACTAAACCCTTCTTTTCTCTATCTCTATCTCCATCTCATTCTCATCTCGTCGCAGCCATGTCG

AAAAGAAAGACTAGAGAGCCTAAGGAAGAAAATGTGACACTTGGACCTGCTGTCAGGGAGGGTGAGCATG

TTTTTGGAGTTGCCCACATCTTTGCATCTTTCAATGATACGTTCATTCACGTGACTGATCTTTCTGGAAG

GGAAACCCTGGTTCGTATAACTGGTGGGATGAAGGTGAAAGCTGACAGAGATGAGTCTTCCCCATATGCT

GCCATGCTTGCAGCTCAGGATGTTGCACAGAGATGCAAGGAACTTGGTGTTGGTGCTCTTCATGTTAAGC

TGCGTGCTACCGGAGGGAACAAAACAAAAACACCTGGCCCCGGTGCACAGTCGGCCTTGCGTGCCCTTGC

TCGATCTGGGATGAAAATTGGTCGTATAGAGGACGTTACACCAATTCCAACTGACAGCACTCGCAGAAAG

AGTGGTAGAAGAGGGAGAAGGCTGTGAGCTTCTTTCCTTAGTGGTCGAGGATCTTGTTTCTTATGTGGGA

ATTATCTGGAATGGATATTATATTGATCTCAGCTAAAGTCAGTCTTATTTCCT

>FM891402.1 FM891402 Jatropha curcas embryo 56-70 (DAF) Jatropha curcas cDNA clone rjcaeb0_001680, mRNA sequence

GGGCTGCAGGATTCGAATTCCTCTATCCCCCTCTTCTTTATCTTATCTTCCTGAATACACGGCACCGTTT

CGTTAATCCCTGTCGCCATTTTCTCGTTCTCCTGCAATTCAACTGCAGCTACATTCTCATCTCGCCTGCA

GCAGAACCGCCACCACCAAAACTCTCCACGGCAAAGGTGCAGAAAACTGTGAGTTTATTAGTATACTTTG

TAATCAAAGAACAATTCAGATGATGGTCTATCCAGTTTGCTCAGTTCTCTTCACAAAATTGTTTAAAGGA

CTTCACTCGCTTGACATTCTGGAGATTGTCTATCACATATCCACGAAGTAATCGTGGTTCAAACACTTTT

CTATTGCCAACTTTGGGTTATGTGCGTGGAATACACAAATTTCCAAGCAAGCATGGTACATTCAAATTAT

CATACATCGGTTTTGAAGAGTGAATCCGGATTTGGCAAGCATTTCCAGTTAAAACTTGGTCAGGATTTAG

CTGAAACTCTATCTTCGAAGGAAGATAGTCTACTGGACAGTTGCCAGGACTCATCTA

>FM891401.1 FM891401 Jatropha curcas embryo 56-70 (DAF) Jatropha curcas cDNA clone rjcaeb0_001679, mRNA sequence

GATAGAATTCTTTTAGACTATTATTTTACACACAAGTAAGTAATCCACATGGGAGGATTGCTGAAGGCAG

TTTATTTCTTCAACAATTTTACTTAACAAATGAAAATGAGCTTATTATGACAGAATTTTCTTCCTCTAGT

TGCCATTTTCAGCTTGCACCCCTGTCAATATTTGCTGAAAGCACGACAAAGTGTACTGCACATCACTTGC

TGAAATTTGATGGTGAAGAACAATCCGAAAGCTGAATGGACCCTCTAGCATCACAAGTACACCATGTTGT

TCCAAGCTCTTACATAGTTTTTCTGCTGTAAATTTTGAGCTCTCTACTACATCCACATATATGATATTGG

TTTCTACTGCAGCAGCATCCACTCTTAGTTCCTTAAACTGATGCAGTCCCTCTGCTATTGTCTTGGCTTT

CTTGTGATCGTACTCGAGCTTTCCGACATTCTCTTGTACAGCAACTAAACCAGCAGCACAAAGCATACCA

ACCTGCCTCATCCCAACTCCTAATG

>FM891400.1 FM891400 Jatropha curcas embryo 56-70 (DAF) Jatropha curcas cDNA clone rjcaeb0_001678, mRNA sequence

CTGCGAAGCAGCAACCAGTTACTTCTCTCTTCTCTCCCACTGCTGCCGGAGCTTTGGTAAGCGATGCACT

GCTTCAACCACCAATTCGATTTGCAAAGATGTACACTACTTGGAAGAAGATCCGAAAAGACAAGGATGCT

GAACCAACTGAATTTGAGGAGTCAGTTGCTCAGGCCCTGTTTGATCTCGAAAATACCAACCCAGAACTGA

AAAGTGATTTAAAAGATCTCTTCATAAATCAGGCAGTTCAAATGGATGTTTCTGGAAATCGGAAAGCTGT

TGTTATTTATGTTCCCTACAGATTGAGGAAGGCCTACCGCAAGATTCATCTTCGTCTTGTTAGAGAGTTG

GAGAAAAAGTTCAGCGGAAAGGACGTTGTCCTCATTGCCACCCGGAGGATCGTGCGCCCACCGAAGAAGG

GTTCTGCTGTTCAGCGCCCTCGTTCCCGAACCCTTACATCTGTGCATGATGCCATGCTTGAGGATGTAGT

GTATCCCGCTGAGATTGTTGGAAAACGCATCAGATACAGAATCGATGGGTCCAAAATTATCAAGATTTTC

TTGGATCCCAAAGAGCGCACCAACACTGAGTA

>FM891399.1 FM891399 Jatropha curcas embryo 56-70 (DAF) Jatropha curcas cDNA clone rjcaeb0_001676, mRNA sequence

ATTCACCCATTCTGTCAAATTGCATCAAGAACAGCTCCTTTTGAGTTCTTTGGATTCACAACCTCAGCTC

AAAAGAATAGACCCCAATTCTTAGCTGGTTCAAACTCAGTTCTTACTACCTTAAGAGGCCCTGAGCTTGC

AACTGCATTTGGCTTAAGCGAGGAAGGATTGGAGCACTTTGTTAATTCACAACGCGAGTCAGTTATATTG

CCTTCAGCATCTGCAGCACCACCAGACAAGTTGAAGGAGGCGGCTAATTCTGAAAGGGTGCAAAAATTGA

TAAAGAGCTTTGATAATGATATGATAATGGGTTTTGATTAGAGAAGAGTAATGTAAAGAATAAAACGTGG

TTGCAAACACAGACTGTAATAGCTGTTGGTAGTAGCTTTTTTTTACCCTTTTCGTTTATCTAGTTATCCT

CAGAGTCGTTAATGAGTTTCTCTTAGTTTGTGAATAAAATTTACTTTCTTTATTCTCATT

>FM891398.1 FM891398 Jatropha curcas embryo 56-70 (DAF) Jatropha curcas cDNA clone rjcaeb0_001675, mRNA sequence

AAGAAACTAAACCAATAAAACCAGTATATGAATATCCATCAGAAGCATACTCAACTAAACAAAATTAAGC

CAAAAGTAGCAAACTTAGAATTACCAAATCCATATAACCAAACAAAAACGCATTAAAAATAACCTAATCA

CCGCCAGCCTTATTGTAAACATAAGTCAAGCCACACTTGCCGCAGTAGTGCCTATCAAAATGGTTGGCCA

TGAAAGTTCCAGCTCCACACTCAGCATTCGGGCACTCCTTCCTCAGCCTCTGCACCTTTCCGGAGTCATC

GACCTTGTAGAACTGAAGCACGGCAAGCTTGACCTTCTTCTTCTTGTGCTTGATCTTCTTTGGCTTGGTG

TAAGTCTTCTTCTTTCTCTTCTTAGCACCACCACGAAGACGAAGCACCAGGTGGAGGGTGGATTCCTTCT

GGATGTTGTAGTCGGCAAGAGTGCGGCCACCTCCGCTGCTTTCCGGCGAAATCAGTCGCTGCTG

>FM891397.1 FM891397 Jatropha curcas embryo 56-70 (DAF) Jatropha curcas cDNA clone rjcaeb0_001674, mRNA sequence

ATTCAGCACATTGAGACCGTCTAGCGAAACATGTGGGCTGTAACTCTACCACAAAGACCATTCTTTCCTC

TCATTCCCAAACCCAACAAACCACTCGCCGTATCAGCCGCCTCTCAGCCCCCTCGCCACCGCGGCTCTTC

CCTGACCAGAAAAGATCGCCAACAGATACAAAATCAAGAATCCAAAAACCAAAAACAAAGTGGAGCAGTA

ACTAATCAGCTCAGTGGACTAGATGTGCTATGGGCTATGCAGAGAGCGGCTGCTGAGAAAAACAGAGCGA

GCGGCGGCGCTACTAAAAAGAATCGGAAAACAAAGGGGTTGCTCTCTGCCGGCGGTGGTGGCCAAAGGGA

GGAGGATGATGTGGACGATAGCAATGTCAGGCCTTGTGTATAAGAGCGAATGGA

>FM891396.1 FM891396 Jatropha curcas embryo 56-70 (DAF) Jatropha curcas cDNA clone rjcaeb0_001673, mRNA sequence

ATTCCTTTAAACTCTTAAAACCCTTTTGCTCCAATTATGTTGCCAGAAACATCCAACCTAAATCCTACAC

TCGTTACTCCAATTCCAGTTTCACTCAACAGACAAAACCCGGAAACCCCAATTCTAATTTTACAGCTTTG

AATGCCAGACTCCAACACAACAAAAAACCACAAATTTCAGTCACAGCTTTCTCAATTTCGCAGCAAAATC

CAAAAATCATGACTTTATTAGGTCAAGAGCAGAAGAGGCCAGATTGTTTTCATCATCCCTAATTTCTGAT

TTGAAGCTAAAGGAGATAACTTTAGCAAATACCCTTTTAAAGACTCAAGAACCCAGACATTTTTCAAGCT

CAGATTCATCTTCTGATCCTGAAAAACCCCAAAATCCATGTGAAAATCCAAGCAAGATCTCTAATTTCAT

AGAGGAATCCACTGCAGAAAGAGAGAGAGACAGAGACGTATCAGCTTTGGCCAATGAGACCATGTTGGAA

TTAAGAAAGATAGTGAAGAAGACACTTCGTGAATCCATGGTTGATGCTT

>FM891395.1 FM891395 Jatropha curcas embryo 56-70 (DAF) Jatropha curcas cDNA clone rjcaeb0_001672, mRNA sequence

ATTCGGGGCTCACAGGGTTGTCATCACTCTCGTGGGTTCTGAAATACCTCCAGGAGGTCACTCGGCGAAT

GCCGGAGCAGCTGGATATAGCTAAGAAGCGTATGCAAGACATGGCTGGTTTTGTGGGACAGAAGACCAAG

GAAGTAGGCCAAGAAATCCAGAGGAAAGCACATGAAGGGAAATAAGTAGAGAGAAATGCTGTTGGTTATT

AAGGGTTGAAGTCAGAGAGCTGGGTTTTGGTACGAGACTACTTTTGTTGTATGGGTGTTGTTTTGAGGTA

GTTGAGTTGGTTTATGGGGGATCTTCTTTGTTGCATTTGTATTTCTCTTTTTGGTCTTTTGGGTTCCATT

GTTGATTTTCAATGTATTATCTTTGTATGTGCCATCTGTGATTCTTCTCACACATCTCATCCTCGCATGC

TTGTCTTATATTAGGAGTCTACGGTCCAGTTTCTTTCACTTCAGATGA

>FM891394.1 FM891394 Jatropha curcas embryo 56-70 (DAF) Jatropha curcas cDNA clone rjcaeb0_001671, mRNA sequence

ATTCGTCCAAAGGTGTAAATGGCTGATGATAATGAACATGAGAAACAATATCAAGCTGGTCTCCTGCATA

CGAGGGACAATCAAGGCAAAGCTCAAAATGGTTCGACAAGGAGTTGTACTAAGGTTCAAAAGCAGGGGAT

TGCACTTGGTAGGTCTGTTGACCTTACTAAGTTCGGCAATTATGATGAGTTGATTGCTGAATTGGATAGG

TTGTTTGAGTTTGACGGTGAACTCCTGGCTCCCAAAAGGAATTGGTTGATTGTGTATACGGATGATGAAG

GCGATATGATGCTTGTTGGAGATGATCCCTGGCAGGAATTTGTTGGCATGGCTCGGAAGATTTTCATCTN

CNCTAGAGAGGAGGTACANAAGATGAATCCAGGGACCCTGAATTCAAAGGGGGATGAGAATTTGTTGGAG

GTGGACGGTGTGGATGCAAAAGAAGTGAAACGTTTGCCACTTCCTTCAGCACATAGCACCGAGAATTGTT

AGGTTGGGCTACCTTGAAAATTCTAGGTTTTCG

>FM891393.1 FM891393 Jatropha curcas embryo 56-70 (DAF) Jatropha curcas cDNA clone rjcaeb0_001669, mRNA sequence

AGTGGATCCCCGGGCTGCAGGATTCGAATTCCAAGAAATAAATTTATTTCAGTAAACAGAAACATACAGC

CAACCAAAGCTATGGGCAAACAAACAAGTCTTATTACATATTTATGAAATCCATCATCAAACTCAAACTA

TATTAGCTTATATTATTTACCAAACAACAACTACTGCTAGACAACCTGATCATGTCTTTGCAACTTCCTG

GGCCTTCTGGCCCACCTCCCTAGCCTTCTGGCCCAATTGACCCGTCGTTTCTTGCGCGCGCCGCTTTGCC

ATTTCCATCTGCATCGTCCAAGATCCTCTCATCCTGCGAATATAATTAACCATCCACGACAGTGAAGAAA

GCGCAGTGATTCCAAAAGCACCGGACGTCAAAAACCCTAAAACCGACAACCCAATGACCAACGCCGCTGG

TACCAG

>FM891392.1 FM891392 Jatropha curcas embryo 56-70 (DAF) Jatropha curcas cDNA clone rjcaeb0_001667, mRNA sequence

ATTCCGATTCGTGACTTGACTGTACTCGTTCATCACTCTTCCCTTAAATACCTTACGCGATTCCCTCTAC

TATTCTCATCCATCCGCTCGCTTACTGCTCGACTCTATCCCTCTCTTTCACACTCTATCAAATTCCCTGA

CAAAAGCACATAGGTGTGCGCAGAAATGCTGCAGGTT

>FM891391.1 FM891391 Jatropha curcas embryo 56-70 (DAF) Jatropha curcas cDNA clone rjcaeb0_001666, mRNA sequence

CTAAAAGTATAAATTCGATCAAAACATTAAACAACCATTCTCTGTCGCTAAACATCAGATAAAGCATGAA

CCTATAATTGGAAAAACAACTTAGAAAGAACATAGGAAGTATATAAAATTGCACAAAATCACTTTATATC

TTCCTTAGGAGCAGCATTACCCTCCTCCGCAGCAGCTTCAAAAGTCTCCCCAGCAACAAGCTCTGGGACC

TCATCTTCATCATCCTCTTGGGGTGCAGTTGCAGCAGAGCCTGCATTAGGTGCCTGCTTCTGTATCTGCT

CAGCTAGCTTCTTCAGATTGTCCAAATTATCAGGCCCCAAATGGCCAAGAACTTGAGGAAGAATATCCTG

CAATTTCTTTGTTTGTGGAGCACCACTAACAACCCATGTATTGGCAGCAATTGATGCTTGAACTTTGGGG

TTCAAAAACTGGATAACTATGTCATCCTTAAATATGTTAACTTCCTCAATTGCAGGAATAGCATTCACTC

CTATTCTTTTCAGG

>FM891390.1 FM891390 Jatropha curcas embryo 56-70 (DAF) Jatropha curcas cDNA clone rjcaeb0_001664, mRNA sequence

ATTCGCATTGCTATCATCAGTAGATCGGGACTGTCTGAGCGGTTGGGGAGGACACGTCTATGTTGTTACT

CCCACGGAAGTTACAGAAAGAATCTTAAAGGGAAGGATGGATTGAACTTTGTTCATAGATGCCAAGCCCT

TCTATACACAGATCAGGCCACCCGTTGTTCAATATTGTTAACCAAACTCCAATTTGAATGTGATCTGAGC

CATCTGGTTTCGTCAAGACGGTTGTTTAGTAACTTGAAATCAAAGTATTCTTGGGCTTGATAACTTGACA

TTACTGTTATGATATTTGAATGCAGCTTGTAGTGCCAAAGTTTTATTTGTCGGTTGTTTTTCAGAGCAAT

GCAAACATTATGGATGAATTTAGTTCTCTTAAAAAAAAAA

>FM891389.1 FM891389 Jatropha curcas embryo 56-70 (DAF) Jatropha curcas cDNA clone rjcaeb0_001663, mRNA sequence

ATCGAATCCTGTTTTCTCTACATTAACATATCTTTCTCACACTTATCATCAACAAATGGCTGAACGTTCA

CAGCCACACCAAGTTCAAGTGCACCCACAACACCGCTATGAAGCTGCCTTCAAAGGTCAACAAAAAGGTC

CATCAGCTCAGAAAGTGTTAGCTGTGATCACCCTCCTGCCGGTAGGCGGTGGGCTTCTTGCACTAGCTGG

TATAACCTTAGTAGGTACCCTAATCGGCCTAGCTATTACTACCCCTCTTTTTGTCATCTTTAGCCCTGTT

CTTGTCCCAGCTGCATTAGTTATTGGCCTTTCGGTCATGGCATTTTTGGCTTCCGGGGCTATGGGGCTCA

CAGGGTTGTCATCACTCTCGTGGGTTCTGAAAT

>FM891388.1 FM891388 Jatropha curcas embryo 56-70 (DAF) Jatropha curcas cDNA clone rjcaeb0_001662, mRNA sequence

AGAAAAAGTTATACTTATGACATTTCTAAAGATCTTTTTACAATAACAACAAAATCCTTTCTAACACAGA

TTGACAGATACCATATATAGCATGTACATGGTTAAGACCCAGAAAAGGAAAAACAAAAAAAAAAGATGAA

AAGAAAAACTTAATATAACCCGAAAAAAAAAGAAAGAAAAACCACAAGGCAAAACATTTTACAGGTCTAT

CTTTCTGTTGGATTCGCGGATATGGTTACAACACCGTCAAGTATCAACAGCATATCCCCTTTCCCTTGTA

GCCTCGATTCAAGAAATACAGCTTAGATTTACAATGGCTAAAAATGCAGCCCAGGCCAAACAAGGCTTAA

CCAAAT

>FM891387.1 FM891387 Jatropha curcas embryo 56-70 (DAF) Jatropha curcas cDNA clone rjcaeb0_001661, mRNA sequence

ATTCCAACAAATGGCTGAACGTTCACAGCCACACCAAGTTCAAGTGCACCCACAACACCGCTATGAAGCT

GCCTTCAAAGGTCAACAAAAAGGTCCATCAGCTCAGAAAGTGTTAGCTGTGATCACCCTCCTGCCGGTAG

GCGGTGGGCTTCTTGCACTAGCTGGTATAACCTTAGTAGGTACCCTAATCGGCCTAGCTATTACTACCCC

TCTTTTTGTCATCTTTAGCCCTGTTCTTGTCCCAGCTGCATTAGTTATTGGCCTTTCGGTCATGGCATTT

TTGGCTTCCGGGGCTATGGGGCTCACAGGGTTGTCATCACTCTCGTGGGTTCTGAAATACCTCCAGGAGG

TCACTCGGCGAATGCCGGAGCAGCTGGATATA

>FM891386.1 FM891386 Jatropha curcas embryo 56-70 (DAF) Jatropha curcas cDNA clone rjcaeb0_001660, mRNA sequence

CGAATTCGAATTCCCAATTTGTCCTCGTCGGTTATAAATACAAGATTATATCCCCTCCACAGCCTTACGC

AAATATTTCTTTCTAGCTACTAACAGTACTAGCTCACAGTAATGGCAGAGATGGCTAACGGCGATTCTCA

TGATTTTCGCTCGCTTCTCTCTTCGTCAGATTGTGATTACCTTATTCGCAAC

>FM891385.1 FM891385 Jatropha curcas embryo 56-70 (DAF) Jatropha curcas cDNA clone rjcaeb0_001659, mRNA sequence

ATTCGCTTNGCTCGTGGGTTGCATTATACAATGTTCCTGTCATGTGCAATGATGTCTCTATTGTTCGCCG

GATTGCAAGGATGGTGAGTGCTCGTGGCGGTGGCCTTCCAACAGTGCAAGCATTGGCTCTGGTTCACGGT

GAGTATTCAGTTGAGGTAGCTTGTATGCTTTTGGAGCCTAGTAGGATTGGAGCAGAGAAGGTTCAGGCCC

GGATTGAGATGCTAGCTGCTGAAGAAGGGTTAGATGTTGAGCAAGGATATTTCACTCACTTTTCACCCGG

AAATGATTGTTGAGAAGTACATGAATCTAATCTCTGCTACCAGAAGCTGATTAAACACTTGTTAAAGTTT

AG

>FM891384.1 FM891384 Jatropha curcas embryo 56-70 (DAF) Jatropha curcas cDNA clone rjcaeb0_001658, mRNA sequence

ATTCCCCAAATTGGATGAGGAGAAGCAACAGGCTTTCAAGAAAAACATTGAGGGAGCACCAAGTTCCTGC

TCTCGAAGCTCAGTGACCTCCAATTTTTTGTGGGGGAGAGCATGCATGATGATGGGAGTTTGGTGTTTGC

TTACTACAATGATGGCGCCACTGACCCAACATTTTTGTACTTCGCTTATGCTTTGAAGGAGATCAAGTGC

TAAGAATGGAGAATAGCTGGCTTCGAAGTGGAAGTTCTTGCCATATTTCTAGCTTTATGTGTCCTTTTCC

ATCTCTGTACTGTTATATTTGGTGCTTCATGCCTTATATTGGTTAATTATTGACTAGACATGTTTTCCTC

CTTCTTTGGTTATTAGTTTCTTGTTTGACCAATAATTCCATCTTCTATTGGGCGGTGTATTCTAAAATTT

ACCCAGCCTAAGTTGGTAATTGTTTGATGTTA

>FM891383.1 FM891383 Jatropha curcas embryo 56-70 (DAF) Jatropha curcas cDNA clone rjcaeb0_001657, mRNA sequence

ATTCCTTAAAACACAAAAGCTTCATGCCCCGACAGTGGCACCTAGGAAATGAGTTTCAGCCAAACCAGTG

ACATGCAAACGCAGAAACGATCCAATCTCACCTGGTATGCTTATCGTTCCTATGCCAAATCCTCAGAAAA

CATTCGACAGACCAGAGAGAGCAAGGATTATCGTTCCATCTAGAAGAATTAATGAAGAACTCCTTGATTC

TATTGAGATTGGAGTAGAACTTTCACTTATTGATGGAAGAAGTGGGGTGGGATAGCTTAGCAAGGTTTAG

AGTTAGGGTGGTGCTACAGGTTTTTATTTAGCGGCGGATTTTGGAAATCCTCCGACGATGGTTTTTCCCC

CCAGACTTCCACCTAACCCCCTGTGAGAGTTGCCCATTCTCGTCTACAGTTAGTGCTTTTTGGTGAAATG

GAAAAAAAAATTTGCTTTTGGTT

>FM891382.1 FM891382 Jatropha curcas embryo 56-70 (DAF) Jatropha curcas cDNA clone rjcaeb0_001656, mRNA sequence

CGAAATTCCGCATTCTTTCCCTAATGGTACCAAGAGCACAAAAATGGGAGAAGCATCTTCCCATGAAAAA

GTTAACAAGCTCTTCAGTAAAATACTAAACGGTAGCAAAACTAATTCAGAACCAAAGAAGAACAAGTCTT

ATGGAAAGCAAAAGATGTTTCTAGATGAGACAACAGAGTTACTGAGGAACGCTGAATCCGCCTCCACAGA

CTCCGAGATGAAATGTCAAGACATAACACTTTCACATCCTTGGATCCAGAGATGGTGTCATAAACCAGCT

GTGTCTTCCAAAAAGAAGCCAGAAGCTATGGTGGTGGTTTTTGAGCCACAGAATTCAAAGGGGGCCCTAG

ACGAATTACAGGAGAAGCAGTTTCCAAGCATTGCTGCTATGGCTTTGATGGGGAAGGCCATGACAGGTTT

TGGTCCATGTGATTGAAGAAATGGGAGCATCTGTAGTTGGAATACCAAACGA

>FM891381.1 FM891381 Jatropha curcas embryo 56-70 (DAF) Jatropha curcas cDNA clone rjcaeb0_001655, mRNA sequence

GGAAATGAAGTTCTCATCTCTCTAATAAAAAACTCTAATCAAGTCGAGCATTTCAATTTTACTAGCAATT

CCAACAAAACAAAAATAACAGCAATCCAAGAAGAAAAGGTGATCTTTCAAGTAAAGACACTTCTATAGGT

AACTGGAATGCTACATCTTGTAAAAATCTCAAAACTCCAAATAAGCTGCTCCTTAAAAGACGCGGACTGG

GTTACCCATTGTAGTCTTGATGTTTAGGCACTTCACATTTTGCCAGTTCTTCTTCAACAAGGAAACAAGG

AAATTGACGCTCATTTGCACATTTTGGAAGACTTGCT

>FM891380.1 FM891380 Jatropha curcas embryo 56-70 (DAF) Jatropha curcas cDNA clone rjcaeb0_001654, mRNA sequence

ATTCAATCAACCATAGGCTAAGCTCACCATCCTCTTAGCCTCCTTCATAGCCCTCCTCTTCCTCGTCGAC

GCCTCCATCTACCGAACCACCCTCATCACCGACGAAAACGACGATAACCAATTCCCGCCATACGACCGCC

CAACAAGCTGTCAACAGCAAATCCAATCACAGCAAAGGTTAAGAGCATGCCAGCAGTATCTCCGGCAACA

AACTGGCTCCCCCTGGGGCCGTCGTTTCGACAACGAGGAAAATCAGATGGAGCGAGAAGAGTGCTGCAGA

CAGCTTCAAAGAATGGAGCTCGGTGCCGGTGCGACGGTCTCCGACGGC

>FM891379.1 FM891379 Jatropha curcas embryo 56-70 (DAF) Jatropha curcas cDNA clone rjcaeb0_001652, mRNA sequence

ATTCGGAGGATTCTGCAACAAAGGAAATTAAATAAACAAAGAAAGAAAGGAAGAAGAAAAAGGAAATGGC

AGCAATATTTTTGTCAAAGAGAACCACCATTAACATCTCTAAAGCATCACTACTAAAACAAAAGCCTCCT

TATGTCTGCTTTTCCACTGCCTTTGAACGTGCTGAGACAAGAGGAGAAGGAACAGGACCAGATGTGAGAC

CAGAAATATCAAGTAACGATCCAGCAGGTACTCAAAGGCATGTACGAGGCAAAGCAACAATCATTAGACA

TGGATGAGAGCGCAACCGCTGGAGCAACTCAGTTTGTAGCTGACTCGGCCAAAGAAGGTGTTCGAAAAGC

TACAGAAATTGCAGATACTGTTGGAGATACAGCTAAAAAGACCATGGATGGTGCTTGGAATGTAAGTAAG

AGATTAATCAAAAGATTAAGGAATCGTACGATATGAAATAATTACAGTGATACTGTGAATATAGAAATTA

CATGGAGAGAGGA

>FM891378.1 FM891378 Jatropha curcas embryo 56-70 (DAF) Jatropha curcas cDNA clone rjcaeb0_001651, mRNA sequence

GCTGCAGGAATTCGAATTCGCCTCTCTCTCTCTCATGCTGCTTCCCCTTTCTTTCCCCCCCTCTCTCTCT

CTCTCTCTCTCTCCCTATTTATATATATTTTGAAGACTAGAGGGAATTAATTCTGTTGAGACTCCATCTC

CCAGCGACACAAAGTTGTCATCTGCACATTGTCTCTAATCTATTCCTGCAGCACTGATCACTCCTGTATA

AAAATAAAAAGAAAAAGAAGATCGATCGAAGACAATTAATGGCTTCTTGGAAAAAAACCATTGCTTCCCC

TTTCAAGAAAGCTTGCACTTTCTTCAATCAGCAACCAAGAGATAAGAAATCTCAGCCAGAGCCAGGAAAT

GAGAACCGAGTGATGGTCATGGATCTCCTGGGGAGGTGATGGCTGCGGTCACGAGGATGTTCAAGTGATG

TGGTCATTCTTGATAAGTCAAGTCCCCACTGCACTTAACTTCTTCTGATCTGTTCTGCTTCAGCTCTTGT

AATACAACAGATCCGTTGCTTGTGCCCTACATCTCCAATTTCAGATTGACG

>FM891377.1 FM891377 Jatropha curcas embryo 56-70 (DAF) Jatropha curcas cDNA clone rjcaeb0_001650, mRNA sequence

GAAAAGTTGAAGCTTGTTAGAAATTAAGAAGCAAGGAAATCCTCCATTAATTACAAATCACACTGAACAG

AAATTTATAAACACAAAACTTAGGTCCCTGATACATATGTATAAATAAAAAGAAAAACCTTAAACCTTAA

ACTATCCAACCAATACAAATATTGCATATACCATTCCTGGTATATATCCCAATATGGTCAGCAATAGATC

TATCCAAAACTCCACCCCACAGCCATATCGCAGGAAGACACCAACAGGAGGTACAAGAATGGCCAATATC

ACCTCTAAGAAAGTCTCTGAACCCATTTTCTTCTTCTCTTTTCTTCTTCTTTCTTTTTTTGTTCTTCTTC

TTCCTCCTGTCGATGAAATTTGATTATTTGGGGGGGGGCCGGTA

>FM891376.1 FM891376 Jatropha curcas embryo 56-70 (DAF) Jatropha curcas cDNA clone rjcaeb0_001649, mRNA sequence

CTTTAAATACTAAATATTACGTTTTGCACAACATAAAGAAAAATATGAAAGTTGAACATTAGTGTTGAGA

AACAAAATTTAGCATGTCAGAATAATTTAATATGTTGAAAAACTGTGGATAAAATTGACACGGGATCTAA

CAATCTAGTAACTTCTGCCCCAATAGGTCCATTTCTTAGCTGTCTTTCCAGATGCAAAGCATTTAGTACC

TTCAGGCAGTTCTGGCTGGTCAAATGGAGTACAAAGGCTCTTGGCAGCTCCCATCTCACCTCTTGTTCGC

TCTTTCACATCTTGTTCCACCTCCGCCTCATCACACCAGGGAGCTAAGATCATTTCCTTTGGCTCAAGCC

TCCTTGAATCATCCCAAGTCTTCACAATTTGAACGCAACATTCGCTTTGTTCGCAGCATCAAACAACTTG

TTGAACATCATCCAAAATATTTTCACAGACTCAA

>FM891375.1 FM891375 Jatropha curcas embryo 56-70 (DAF) Jatropha curcas cDNA clone rjcaeb0_001648, mRNA sequence

GAAAAGCCTTACAGAGGTATAAGGATGAGGAAGTGGGGAAAGTGGGTCGCTGAAATTAGAGAACCCAACA

AACGTTCTAGAATTTGGCTTGGCTCTTATTCTACCCCTATCGCCGCCGCGCGTGCATATGACACCGCCGT

TTTCTAC

>FM891374.1 FM891374 Jatropha curcas embryo 56-70 (DAF) Jatropha curcas cDNA clone rjcaeb0_001647, mRNA sequence

CAAGAAAGCCATAACATCAGCATATTTTTATATTCAGTTTTTAAATACTCATGTGACCAGAAACAGAACT

CCAAAGTCTTTATTCATCTACTTATTCTTTTCTCTCATGTTCTTCATATCCCGCACTAGGTTTGTCTTGT

CCAATTATAAGCTCTTTAGTTGTATGAGCAATCTCTGCTATAGTTTCGCCAATGGCGCCGAGCAGGCTGC

TGCTCCCTTTGCTGCTGCCATTGATGATGCTCTTCTGGCTTTCTTCTGAGCCTTGAGTGGTTTTCCGGTG

AATCTCCCCGCAGTTTCCCATTCTTCTCCTCCTCCTGTTGAATGAACCTG

>FM891373.1 FM891373 Jatropha curcas embryo 56-70 (DAF) Jatropha curcas cDNA clone rjcaeb0_001646, mRNA sequence

CGAATTCCCCTAATCGGCCTAGCTATTACTACCCCTCTTTTTGTCATCTTTAGCCCTGTTCTTGTCCCAG

CTGCATTAGTTATTGGCCTTTCGGTCATGGCATTTTTGGCTTCCGGGGCTATGGGGCTCACAGGGTTGTC

ATCACTCTCGTGGGTTCTGAAATACCTCCAGGAGGTCACTCGGCGAATGCCGGAGCAGCTGGATATAGCT

AAGAAGCGTATGCAAGACATGGCTGGTTTTGTGGGACAGAAGACCAAGGAAGTAGGCCAAGAAATCCAGA

GGAAAGCACATGAAGGGAAATAAGTAAGAGAATGCTGTTGGTTATTAGGGTTGAAGTCGAGAGCTGGGTT

GGTACGAGACTACTTTGTTGTATGGGTG

>FM891372.1 FM891372 Jatropha curcas embryo 56-70 (DAF) Jatropha curcas cDNA clone rjcaeb0_001645, mRNA sequence

CAGTTGATAGAAAAAATGGCTGCAGCTTATGATCTTTCCAAGAACTATCATCAAGGGAGAGAGGGAGCAA

AGATTCATCAATAACAGTGAATAACATAAAGGTCAACATTTGTGTATTTGTCTTTGATATCATGTTTGCT

AATGGAGAACAGTTGCTGGAGCTTCCTCTCCGCCAAAGACGGAAATACCTAAAGGATTTGTTTTGTGATG

AAAGGATGGGCTTTTTGAATATG

>FM891371.1 FM891371 Jatropha curcas embryo 56-70 (DAF) Jatropha curcas cDNA clone rjcaeb0_001644, mRNA sequence

ATTCAGCCGCTTCTATCTCCTTTGCTCTCAGAGATGGCCAAGTCAAAGAACCATACAGCTCACAACCAGT

CCTACAAGGCTCACAAGAATGGCATCAAGAAACCAAAGAGGCATCGTCACACCTCCACCAAGGGGATGGA

TCCCAAGTTCTTGAGGAACCAGAGGTATGCCAGGAAGCATAACAACAAGAGCGGGGATTCAGCCTCCGAA

CAAGAGTAGAACTACCTTCTTATTGAGTTATGGTTTCCTATTTCCATGTTAGGGATTTACTTTAATAGTG

TATTGGCTTTATTTCAAGTTGCAATTTTGAATAGTGGCTGTACTGTACTGTTAAGCCATTTATGTTTGGA

TAATGTTACTTAATATTTGCTTAGA

>FM891370.1 FM891370 Jatropha curcas embryo 56-70 (DAF) Jatropha curcas cDNA clone rjcaeb0_001643, mRNA sequence

ATTCCAACTGCACAGGAAGTTGAAGCAGCTGCTCAGCAGTTCAATGGCTATAGAACTCGAGGGGAGTGGC

ATTGAGGGTGAATGCCGGACCTCCTCCACAAAA

>FM891369.1 FM891369 Jatropha curcas embryo 56-70 (DAF) Jatropha curcas cDNA clone rjcaeb0_001642, mRNA sequence

ATTCCCTTGATCTTCTGAGAAGGGTTCGAGTGAGAGCATGCCTGTCGGGACCCGAAAGATGGTGAACTAT

GCCTGAGCGGGGCGAAGCCAGAGGAAACTCTGGTGGAGGCCCGCAGCGATACTGACGTGCAAATCGTTCG

TCTGACTTGGGTATAGGGGCGAAAGACTAATCGAACCGTCTAGTAGCTGGTTCCCTCCGAAGTTTCCCTC

AGGATAGCTGGAGCTCGGGACGAGTTCTATCGGGTAAAGCCAATGATTAGAGGCATCGGGG

>FM891368.1 FM891368 Jatropha curcas embryo 56-70 (DAF) Jatropha curcas cDNA clone rjcaeb0_001640, mRNA sequence

GATTCAAATTACAGGAACATTTTCTTCGTTGACTAATCCATCCATGTAGAAAATATACATATGGGCTCAT

ACAGGCCTCACATGGGCTCTGTCGTCACTCATCCCAACTATACTTCATTTCTACTTCATTCAAGCCCAAC

AAAATACAAGACAGGACACCAGATATGTAAAATAGACGCCAAAACGACAACAGAACAGCCAAAGAAACAA

TCAGAGCTCTTGTCCGGCAATTCACACGGCACCAGCCAGTTATCCTTGAGATGCTGTGATGTCTTCTTTG

CCTTATCTTTTGCCTCTTCGGACTTGTGGTATGCTCTCTCTTCCGCAGCTCC

>FM891367.1 FM891367 Jatropha curcas embryo 56-70 (DAF) Jatropha curcas cDNA clone rjcaeb0_001639, mRNA sequence

TGAAGCAGCTGATGGAGATGAAGGATGTGATCCTTCAACTTCTGCTACGACAACCAAGGATGATGGTCTG

CTTAGAGTCCCTAGAGAACTTGCAGATATTGAACTCATGGAGAGTTTATATATGAAAAGCAGTATATCAG

CATTGCGGATTTTGAAGGAAATTCGAAGTGGAAGCTCAACTGTTAGTGTTTTTTCATTGCCACCTCTTCA

GTCGAGTGCGTTGGAAGATACTTGGAAGAAGGTTCCAATTCTGGAACAGGCCGCTAAGTAATTAATTGGG

TTAGAGGAGAATTGAGTATACTTTTGTTTTGTATTAATACTCTTTTATTTATACCTTTATATTTCTTTTT

TTGAGTTACCTTTCATGCATATTTTATGCGTGGTCTAGATACTAACACAATGTAAAATGTATAATTAAAG

CTGGATCTAATATTATATAT

>FM891366.1 FM891366 Jatropha curcas embryo 56-70 (DAF) Jatropha curcas cDNA clone rjcaeb0_001638, mRNA sequence

ATTCCGTCGTTTCAGAAATACAGTATGGTGGAGAAATGTTAAGCTCACGGTTGGTCTAGATAATCCTCCT

CCTAGTGATTGTCTATGTTGTATTGGCCTTTGTATGCCATGGACTAGCACTACCTTCGTGCCTGAAG

>FM891365.1 FM891365 Jatropha curcas embryo 56-70 (DAF) Jatropha curcas cDNA clone rjcaeb0_001637, mRNA sequence

TGCAGGAATTCGAATTCTGTGCTTCGATCGCGTAAATGGCGGAGCACCCACAGTCGCAGCATGTTGGCCA

ACAGCCACGATCCCAGCAAGTAGTGAAGGCAGCCACTGCAGTCACTGCCGGTGGATCTCTTCT

>FM891364.1 FM891364 Jatropha curcas embryo 56-70 (DAF) Jatropha curcas cDNA clone rjcaeb0_001636, mRNA sequence

ATTCGAGAGAGAGGAGAGAGAACTAGTCTCGAAGTTTTTTGTCTATTGGTTATTTTACTAAAAGATACCT

TTTATTCAACTGGAATTGCTTAACAAGAAAAAGACACCAAACAGAAAACATAGATATATTACATAACAGG

AGCTAAAGCACATGCCATGCCCTACACACGTGGGTTATGGCCATTGGTGTCTCCGGGCAACCCAAATGGG

CAATTCCTGTTCAAACCCGTTGGGTCCA

>FM891363.1 FM891363 Jatropha curcas embryo 56-70 (DAF) Jatropha curcas cDNA clone rjcaeb0_001635, mRNA sequence

ATTCGGCAAACTTTATCTTCTACCTCCAACGAACTGCGAACTTTCTTTCTGTAGAGAGTGGAGAGTAGTA

TTCAAGACCCTGAAAATTCACTCAGTTTATGACTAGAAGGAGCTCA

>FM891362.1 FM891362 Jatropha curcas embryo 56-70 (DAF) Jatropha curcas cDNA clone rjcaeb0_001634, mRNA sequence

ATTCGAGGAGGGCAACATAGAAGTGGTCTATACACATAACGAGGAACATGGTGCTATTGCTCTTTGCGGG

ATACACGACACAGAAGTCAAACCAATTTTGTTATGCTCTTGCAATGACAATTCGGTTCATCTGTATGACC

TGCCATCATTCAATGACAGAGGCAAATTTTCTCAAAACGAGAAATCGGAGCATTGCACGTGGGTCCATAC

GGCTTATTTTCTCTGGCGACAGCACAGGAATGCTCACTGTCTGGAAGCTGGTGGAATCACATAA

>FM891361.1 FM891361 Jatropha curcas embryo 56-70 (DAF) Jatropha curcas cDNA clone rjcaeb0_001633, mRNA sequence

AAAGAAAACTATGCATCACTTGATGTCATACTGTACATTACTTGATGTCTTAATATGTATACAAGGTAGA

AAATAAGGAGCTTTTCTAGTGCACAGTTGAATATTCAGACATTGGTGGCGGTGGTGGTCCTGCCGGTTTG

AGAACCTCCCTGCTTTGAGTGTGGAACCTCCAAATTGTAGAGATAATCCTTCGAACTCGCAGGAACAATT

CTGATAACTTCCCAAAAAAATTGGATGGCTTACTTCTTCCCTTCATGTCCCC

>FM891360.1 FM891360 Jatropha curcas embryo 56-70 (DAF) Jatropha curcas cDNA clone rjcaeb0_001631, mRNA sequence

ATTCAGCACCTTCGTTTCGTTTCCATCGTTTTCTTTTATCTTCTTCTTCGCATGTCTTAATATCTGCGGC

CCTCATCTGCGATTAAAGCGCCTCCGTATATCTTCGATCTGTCTTTTTCCTTCCGGTAAGGCCATTCAGT

TCTTGTGTCTTGTCGAGGACAGACGGCCTAGAGCACAGAATTCATGGTCTAGGGTTTTCAATTTTGATTT

TTGCGTTGAATCGATTGCTTTTTTCCTCTCTCTGGTTATCGCTGAATCTGGTTGAGTTTACTCTTTGTTA

ATATTTTTGTTTGTTTGCTAAAACAAATCTGTAATATCTTGCTAATTTCTAGGGTTTGATTGGGATTACT

TTTG

>FM891359.1 FM891359 Jatropha curcas embryo 56-70 (DAF) Jatropha curcas cDNA clone rjcaeb0_001629, mRNA sequence

ATTCCACCAATTTAACCTCTCAAACAAAAATCTTCCACTTCTCCCCTTCCTTCCTTCACCTTCTCTCCTT

CTGCTCCCTATCCGATCTGCCACAAAATGGGTCCGCTCGTCTTGACTCAGCTCGCTACAGGGCTTAGTGT

GCTTGCCGGAGCTGTTATCGTCAAATCGGTGATGGACCAGAAGCCTATGGCCGGTCAGTTCCAACGGTGT

CCAAGTTGCAACGGTACAGGTCGTGTCACTTGCCTCTGCTCACGTTGGTCAGATGGAGATGTCGGGTGCC

GGACTTGTGCAGGTTCAGGTCGCATGGTTTGTAGTAGTTGTGGAGGGACGGGTACGGGTCGACCTCTTCC

GGTACGAATATCTGCTCGGCCACCTAACGGCCGGCTCTAACCGGTTTGTGCCTCGAGATTCTATAGCTCC

TTTCTCACTTATATGTTTGTGTAATTTTTTGTCCCAATTACAGAATTAAAT

>FM891358.1 FM891358 Jatropha curcas embryo 56-70 (DAF) Jatropha curcas cDNA clone rjcaeb0_001628, mRNA sequence

ATTCCAGCAGTCCAAATGCGTAGATTGAGAATGGAGATGCAATGTTCGATGGACAAGTTCAAACAAGGTC

AAATATTAACATGCTCCACAGAACTTAGTGGTTTTGAAGAAGGCATGCAATGAAGGGCTACAGTGGGTAT

CCTTCGAGACTAACGAAAATGCACAATTAGCCCACTAGCAAGACGGACCTCGGCAATTACGGCTCTGCTA

TAGGCAGTGGTTGCGGACGCATTCCGCATTTCCATAGAAGATGCCAGGAGGCTTAAGGAGAATAGACAGG

AAGTCACTGTGTTTAGCCCTGGGTCTAGATCTCGTAATTACTATTGCAACTATGCTATTATACCACGAGG

ATGTATAATAGACTGGTCTCTACATAGTTTAGATTTGGAGAAGAATAAGCCTATTTTTATAGGCGGTTTT

AGTTCCTTGCCATAATATAAAAATGTAGATCTGTATATGCCTTATGTTATGAGATAATGAAAGTTTGTGG

AGCTAAAA

>FM891357.1 FM891357 Jatropha curcas embryo 56-70 (DAF) Jatropha curcas cDNA clone rjcaeb0_001625, mRNA sequence

GGAAGCAAATATTATTTATCAAGCATTTATTGAGCTCATAGATGGATACTATCATTTTTCCCGCAAGATA

TAATTTACATTAATGATAGTGCAAAGAACCAAACAATGTCTTTATCATGCCCCACTTAT

>FM891356.1 FM891356 Jatropha curcas embryo 56-70 (DAF) Jatropha curcas cDNA clone rjcaeb0_001624, mRNA sequence

ATTCAGAAACACACTAAACCAAGAAACTTTCTCTCACCGATCTTTGATCTTCTCCAAAAACCAATCAAGA

GAATGTCTCTGATTCCAAGCACCCTCTTCGGTGGCCGGAGAACCAACATTTTCGATCCCTTCTCTCTCGA

TATCTGGGATCCATTTCAGGACTTCCCTTTCACAAGCACTGCTCTTTCTGCTCCTCGTTCTGAGTTATTG

AACGAAACGGCAGCGTTTGCCAACACCCGCATAGACTGGAAAGAAACACCAGAAGCTCATGTATTCAAGG

CTGATCTTCCAGGGCTAAAGAAAGAGGAAGTGAAAGTGGAGATAGAGGAAGGTAGGGTCTTGCAGATAAG

CGGAGAGAGAAGCAAAGAGAAAGAAGAAAAGAATGATAAGTGGCATAGAGTTGAAAGGTCAAGTGGTAAG

TTCTTGAGAAGGTTTAGGCTACCAGAGAATGCAAAGGTTAATGAGGTTAAGGCTAGCATGGAAAATGGAG

TCTT

>FM891355.1 FM891355 Jatropha curcas embryo 56-70 (DAF) Jatropha curcas cDNA clone rjcaeb0_001623, mRNA sequence

CAGATGGTGAGTTCATGGTGGTGGCTGAACCTGCAGATTTTGTTCATGTGTATAGTACCAGAGCCGATTA

CAAAAGGCGGCAAGAGATAGATTTCTTTGTGTGAGATATCAGGAGTGGCTCTAAGTCCAGACGACGAATC

CTTATACATCGGAATTTGGGATCGGACTTATGGAAGTTTGCTACAATATAGCAAAAGGCATACATAATGA

TATCTTGATTCATACCTATGATATATTTTTACCCGAACGATTTTCTTTGGAAGTGCGCTTTTTGTATCAT

CTTCAGGTGAACTAATTAGTTGAGTTGAGATAAAAGACAATGATAAAATAAATATGTCAGTAAGAAACTT

AGATTGTGTACAGTTGATATTTTGATCTTCATATAAAGTGAGAAAAAACTGGTCTTTGTAAGCCC

>FM891354.1 FM891354 Jatropha curcas embryo 56-70 (DAF) Jatropha curcas cDNA clone rjcaeb0_001622, mRNA sequence

AACTAGAATTTATCTCTTTATTAATTATTAAACCAGAAGCCACTATAGTCCGCAGCAGTGATCTTTCTCT

TTTCTTAAAGACTAATTTACACATGTACCTTCACCTTTCATTATTTATTCACCATACACAGCACTATTTA

CATTAGTAAACCCTCAAGGTCGATGTCACAGTCACCCCAAAAAGCCGAAACACTTGCCATCACGTTATTA

GCTTCTCATGCCACAGCATGACCCTCGAAGGAACCTCTCCTTGGCCCTGGAAAGAACCACTCCTCGTTTT

GTCTTCCAAAAGCCTGATCCACTTCTCTTTCACTTACACCGAAGGCTAACTCTTTGGCTTGCTTCTCCAT

CTTATTTACAATATTATTTTTCCCTGCAAGTAGATGCCTAGTATTGCCTTCTTCAT

>FM891353.1 FM891353 Jatropha curcas embryo 56-70 (DAF) Jatropha curcas cDNA clone rjcaeb0_001620, mRNA sequence

ATTCAGCTTTTGAGATGGCAATGTCATAACTGCAAAGTATGAATACAATGACAAAACATCTGTATGTAAC

ATTACTCTATTTAATCCAAAATAATGCTCAGTACCATATACCACTATTCATGTACAAGTGTAAAAGGCAA

TCAAAACAAATGAACAAATGAAAATAAATAGAAATCAAAGAAAAGGTAACAG

>FM891352.1 FM891352 Jatropha curcas embryo 56-70 (DAF) Jatropha curcas cDNA clone rjcaeb0_001619, mRNA sequence

GCTCTAAACTAGTGATCCCCGGGCTGCAGGGCAGAGGTAGAGGAACGAGCGTATTTGCCTGCACTGACCG

GTGCGGATCCCAGTTCCATGCCCCGGCGGCACAGCATGCAGTGCAGAACAGT

>FM891351.1 FM891351 Jatropha curcas embryo 56-70 (DAF) Jatropha curcas cDNA clone rjcaeb0_001616, mRNA sequence

GCCAGNTAAAACATATCTATTTTGACAAGCCTTCCACAAATACAAAGGGAACAAAGAAGTTGTAACATCA

AACAATTACCAACTTAGGCTGGTAAATTTTAGAATACACGCCCAATAGAAGATTGAATTATTTGTCAAAC

AAGAAACTAATAACCAAAGAAGGAGGAAAACATGTCTAGTCAATAATTAACCAAAATAAGGCATGAAGCA

CCAAATATAACAGTACAGAGATGGAAAAGGACACATAAAGCTAGAAATATGGCAAGAACTTCCACTTCGA

AGCCAGCTATTCTCCATTCTTAGCACTTGATCTCCTTCAAAGCATAAGCGAAGTACAAAAATGTTGGGTC

AGTGGCGCCCATCCTTGGTAGTAGCAAACACCAAAATCCCATCATCATGCATGCTCTCCCCCCACAAAAA

TTGG

>FM891350.1 FM891350 Jatropha curcas embryo 56-70 (DAF) Jatropha curcas cDNA clone rjcaeb0_001615, mRNA sequence

ATAAAATTGTATGATTTCATTGTTACTAACAAGGAGTAACATTTTATTACTACTCAAGTAAAAAGCTCTC

TCAACTTACATAACTCTAACTAGAGACAATCAAATTATCAAGTCTCTGTTTTATTTCTCCATGCCTTGTA

TTTACAGAAAAACAAAGGCTCTCCTCATTTTAATTAAGCAACAGCTGTCCCTTCCTGCAGGTCGAGACCT

TGAAGTACCGAGAGTCGTCTCTTCTCTTCCGAACTTGATTCTCCTTGCGTCCTCTCTGGACACCCTAAAT

GCATTGGCGATCACTTCCAAGGGGGGGCCCGGTACCCA

>FM891349.1 FM891349 Jatropha curcas embryo 56-70 (DAF) Jatropha curcas cDNA clone rjcaeb0_001614, mRNA sequence

AATCACACACACACACACAACGTTAAAAATCAAGATCTTACACATCCATTTCTTTTAAAAACCAAAAAGA

GAAAGAAAACAGGGTTTGATAGTGTAGGCTCACCGGAAGATAGAAAGTGGTAGATCCTCAACAATTGAAG

CAACCAGAAATTTAGCGGTGGAAAGAGAGCGAGTCATTTTATTGGTAGATATATGTACAGAGTAAGCAGA

TACTGCAGAAGCTATTCGATCCCCTGAGAAATAGATTCTCGAATGGATCAATCCTTCTGCAGCTACTCGC

GGCCCGCTCTCTTTCCTCACTTGGGGGGGCCCGGTAC

>FM891348.1 FM891348 Jatropha curcas embryo 56-70 (DAF) Jatropha curcas cDNA clone rjcaeb0_001612, mRNA sequence

ATTCCTTATAGGGACACAGACACTAGGTATTAGCTACTAGTACTGCTACCTATGGCTAATAAATTTTATG

TGTGTATTGGGGTCTTGGGCTTTCTCTAAGATGAGAGTTTATGTAGCATCTTATAAACAGGGAAAGCCAT

AAATAAAATAGTGGGTCTAAGTGTGCTTAAAAAGCAGCTTGCTATGAAGTGTTTCTATTGTAACTTTGTA

TTACCTGGTTAGGAAATGTGCTTATTAATGAAAAATGAAACAGGTGTCCTTTTTTACTTCTAAAAAAAAA

AAAAGAGAGAGAGAGAGAGTAGTCTCGAGTTTTTTTTTTTTTTTTTTAAAACCTTAATCCTCCTTTTATT

GCCCAAGACAGGTTAACGTGTTGAAAAAGCAAGGGGAAATAGAGAATTGGGGTAAATAACAAAAAACTTT

AACAGGCTCTTCCAAAAATCATGGTGTAAATATGGGGTACGAGCTACGTTACAAAAGAAATTTGAGGCAA

GCTAACAAATCTCCAAGATACTGAGTTAGTAATGTGAATATCAGATTTTGATACAAAAATGGTTGTCTAT

AACAGCCATCTTCCTGATATCGATCAACGTTTGACCCATTGGAAAG

>FM891347.1 FM891347 Jatropha curcas embryo 56-70 (DAF) Jatropha curcas cDNA clone rjcaeb0_001611, mRNA sequence

AACTAGAATTTATCTCTTTATTAATTATTAAACCAGAAGCCACTATAGTCCGCAGCAGTGATCTTTCTCT

TTTCTTAAAGACTAATTTACACATGTACCTTCACCTTTCATTATTTATTCACCATACACAGCACTATTTA

CATTAGTAAACCCTCAAGGTCGATGTCACAGTCACCCCAAAAAGCCGAAACACTTGCCATCACGTTATTA

GCTTCTCATGCCACAGCATGACCCTCGAAGGAACCTCTCCTTGGCCCTGGAAAGAACCACTCCTCGTTTT

GTCTTCCAAAAGCCTGATCCACTTCTCTTTCACTTACACCGAAGGCTAACTCTTTGGCTTGCTTCTCCAT

CTTATTTACAATATTATTTTTCCCTGCAAGTAGATGCCTAGTATTGCCTTCTTCATTAATCTCAAAGCAG

ACTACTTCCAGATTATTGTTTCTTGAAGCTACAAAAGCCGCCGGATGGCCTGCAGGGACGACCACTGCTG

TTCCACGTTTCAACCGTGAACTTACTTTCTGGTAAGTCTTCTGACTTTCACCCGTCGTG

>FM891346.1 FM891346 Jatropha curcas embryo 56-70 (DAF) Jatropha curcas cDNA clone rjcaeb0_001610, mRNA sequence

AATTAAAACCAAAAAGTATGGAAACATCAAATTCATCAACAAACAAAGTAAGACTTGATTACAGAGACCT

TCAACACATGACAAGAAAATTATTTTAGACAGCTCTCAAAAGAGGGAAGACCCGTTAACAAATTACATAG

AAGAAACAAAAAGATTTAAGCGAAAAAAGAAATACACCAGACAAGCAATGGCCTTCCATGTTAATTAAAG

GACTCATATATTTTTATATATATATCAAAGCCATTTTATGTTCTCTGCATCAACCACCAAAACCCACTTC

TCTTTTAAGTCTGTTGACCTGTTACATGCTGTCCAAACTGCTCAGCTCTGTCCTTCATCTCTCTTGCCTT

TCCCGCCAACTTTAAGCGTGCCTGGTCCAGACTTTCTGCTCCTGGTGGATGCTTTCCAGTAACATACCTA

TAAATCCAGGACAAGACAGAAATGGCAGCGACGCCAAAACCGCCTGAGGCTAAAAACCCTGTGATTATCA

AACAAACTGTGATAACCGCAGGAACAAGAACCGGACTGAATATGACCNGCAATGGAGTGGCTACGGTCAG

TGCAATGACCGTACCAGCA

>FM891345.1 FM891345 Jatropha curcas embryo 56-70 (DAF) Jatropha curcas cDNA clone rjcaeb0_001608, mRNA sequence

GAAAATAAACTGAATTTTATTGCAATTTGATATTCAAATTTAATAGTCCATCTGATTTAACAATAACAGA

GAAATAAGTAACTATTTACATGATCTACAAGTTAAGGACTAAAAACTAGTGAAAGAAATACTTGTTTGAA

TGCAGAGGTTGCATTCAAAGCTGCAAATGCACTGCTATTTTCTTGGATCAGCATTCATCCAGAATGTCTA

AAGAAATCTAGTCAAGGTTTAGTTAAGAGGATAAATGAACAAAGACAAATGGATAAGTAATCTAATTTCA

GTACATTGTTCAAAGCTGGCTCCTTGCCTAACAAAATAAATTTGAGTATAGCAACTACAACATCACTCCA

CTAAATTTCAAAAGCAAAATCAATTTTCAGGCTATGCTTTCTTCCAGCCATTGATACTGTAGACGAGTAA

GTACGTAAGTTCTATGATTCAGAAACATTGTCCATAAAATTCAGAAATATAGAAAGTGAACTTTGTACAC

AGCCAATATCAGTATGCTTGAAGATTAAACTCTATGCTTGAAAAGCTCAGGTT

>FM891344.1 FM891344 Jatropha curcas embryo 56-70 (DAF) Jatropha curcas cDNA clone rjcaeb0_001607, mRNA sequence

GTAGGGTCTTATGTACATGATCCAGATTCTGAGACCTATAATAAATTAGTGACTGCTATTCAAAAGTGTA

AATCAGTCAGGAGTTTGTCTGGGTTTTTGGAAGTTGCTCCCCACTGCCTACCAGCTTTTCACTTGATTTG

CCCGACCCTGACCTCCTTGAACCTGAGTTATGCTCCAGGAATTCAAGGTAGTGAGCTTACAAAGCTAATT

CGCCACTGTAGGAAACTAAATT

>FM891343.1 FM891343 Jatropha curcas embryo 56-70 (DAF) Jatropha curcas cDNA clone rjcaeb0_001606, mRNA sequence

AAAAAGAGCAATACAACATTTAAGAATATAAGAGATGCCAAATATCCAAAAATTTATAATCCAGTGATTC

CTATGAAACAACCAAAAATATATCTCCATGTTCGGGTTAAAAGCAGAGACAACAAAAACTATTAACCCCT

CAATATGGAAACAACAGAGATAAGTATAAATGCTGCTCTTATTCTTCTCCAACTACCGTGCCCTTCTCAC

TGACATAGATGCCGTCCAGGAACTTCCGGATATCTTTGTTCTTAACATGACATTTCTGGGGGGGGCCCGG

TAC

>FM891342.1 FM891342 Jatropha curcas embryo 56-70 (DAF) Jatropha curcas cDNA clone rjcaeb0_001605, mRNA sequence

ATCGAATTCCTCGGCAATTAGGGCTCTGCCAGAGGCAGTGGTTGCAAACGCATTCCAGATTTCCAGAGAA

GATGCCAGGAGGCTTAAGGAGAATAGACAGGAAGTCACTGTGTTTAGCCCTGGGTCTAGATCTCGTAATT

AATATTGCAACTATGCAATTATACCAAGAGGATGTATAATAAACTGGTTTCTACATAGTTTAGATTTGGA

GAAGAATAAGCCTATTTTTATAGGCAGTGTTAGTTCCTTGCAATAATATAAAAATGTAGATCTGTATATG

CCTTATGTTATGAGATAATGAAAGTTTGTGGAGCTATAAGTTATAGTCCAAAGGGGGGGCCCGGTAC

>FM891341.1 FM891341 Jatropha curcas embryo 56-70 (DAF) Jatropha curcas cDNA clone rjcaeb0_001604, mRNA sequence

CATTCGTTGATGGGCGAGCATCTATTTCTTTGTCTAGTTATAGAACAAAAAAAAAAACAAACAAACAAAC

CAACCATCGCTGACTTTATTATTTCTTGCCTACTCGTACCATTGCGTCTACTAACTGGGCCAGGGCAAAA

CGAGAAGACCCTCCTTCGCTCGCTGCCTCCGCCGCAGCCTCTTTCATGGCAATAACTCGCTTTTGAATCG

TTTGCCGGGGGGGCCCGGTAC

>FM891340.1 FM891340 Jatropha curcas embryo 56-70 (DAF) Jatropha curcas cDNA clone rjcaeb0_001603, mRNA sequence

ATGGCAAGTTCTTTTCCACAACTCTATAACTTGTAAAGGATGAGTAACACAGACAAAATATATATATATA

TATATATATATATATATATATATATATATATATATATATATAAAATAAAAAACAATAAATAGAACCATCT

CATCAGAGAGAAACTTCTACGTAACAAACAATCACAGTTGGGAAGGATAAGTCACAGAAGGAAAAAAGAA

CATAGTAACTGCAGGAACTACTACTCTATCATGCTAAATGAAAGTTCTCTCCAACGACCCACTTGCTTCT

GCAAAAAAGTTCAAGCACGTCTTCCTTGCTTTCCTTTCAGCTCAGGGTCCCTCTGAATATGGTCAAGAAT

AAGGAGCCTTGGTCCAGGTGGGATCCTGACATCTCTAAGAGCACTATCGGTTAGCAATGGCAAAGTGCTG

TCTGTTAAGAAGCCTTTCTTAAAGTTTTTCGGGGGGGCCCGGTAC

>FM891339.1 FM891339 Jatropha curcas embryo 56-70 (DAF) Jatropha curcas cDNA clone rjcaeb0_001602, mRNA sequence

ATTCGAAACTAATTAATAACATCTTAGTAATAATTCATACATAATTGCATTACATCATATTTTTCCATAC

TAGCACCCAAATTTAAAATTAAAAATTAAGCAACTAAACTTTACAATTTATATTTCTTGTAGATAATAAA

ATCCCTAAAAGCATCTAAAGAACTAATTGAACTAATTGGAAACCCATTTTCAAATTGAGCACTAAGCAAT

CTTGCTTGGTTCTTGATTTGTTCATCTCCTATCAACCTCTCGATTCCTTTAATTATATCATCTTTACTAA

TCAGTCTTGAAAAATCATTAGAAATCATAAATCCAATTTTGAGATGTTTAACTATCAATTGGGCATTATA

ATATTGATCACCTCTAATTGGCCATCCCAAGATTGCTTTTCCACCTCCAATTGCTTCCACACTAGAATTC

CATCCACAATGTGATAAAAATCCACCTATTGATGGGTGGCTCAGTATCAACAATTGGGGTGCCCATCCAT

GTATTATCAAACCTCTTTTT

>FM891338.1 FM891338 Jatropha curcas embryo 56-70 (DAF) Jatropha curcas cDNA clone rjcaeb0_001496, mRNA sequence

ACATAATTCAATGCATCATCCTTACTTAACTCTCATACACGAATCCAATATATATGTCCCATAAGACAAT

ATTCATGTACTCACTAAGGAAATACTAGCGTGTAAAACAGGCACACCTATTTTCCATGTACTACATCTCT

AATTCTGACGTAGACGGTCCTGATTACTACCCCGAATGTGGATTCCAAACTGCAAATCCGCAACAGAAAC

CAAGAACTCTGCAGCTTGGTATGGGGTCAACATATCACCAATCATCCTGACTG

>FM891337.1 FM891337 Jatropha curcas embryo 56-70 (DAF) Jatropha curcas cDNA clone rjcaeb0_001494, mRNA sequence

ATTCCACACGAGTACCTTCATCCCTCCTCCTCTTCTCTGTTTCATTTTTTACTTCTTAATCGACGCCGGC

TATTTCACAGCCGGAAATTTCAAGAAATTGACCGGAAAATGGAGGCTTCGGCGATAGATTGTGCTTTAAT

TAACCAAAATGAAGGCGTGACGTTATCGTGCCGTCGCGTAGTTCGGGAACTTTAACCGTATGTAGAAATA

AGCAAAAGCGACTTTACTTAGCAGAGTTTAGAAGTTATTCCATGCTTTGTCCGTCAAATCAGAGGCAAGA

TACTATATAATAATATCGTCGGAGTAGAGTTAAGGAGTCTCGAATTTTGTTAACGCATTCATCTTTGGCG

AAATTCTGGATTTCCGTGTCGATTCGATCTGCTGATATCATCCTGTGGACTTATCAATGAGTCTGGTGCA

TCCTTTGTGATTGTTTCGATCCATGTGTCTTGGGATTTTCTTTTGCGAAATTACATTTCTTCCATC

>FM891336.1 FM891336 Jatropha curcas embryo 56-70 (DAF) Jatropha curcas cDNA clone rjcaeb0_001493, mRNA sequence

GAACTGAATGGTATTTCTGTTGCCATAATTTAAGAGAGACTTTCAACCATTTCAGCACTATAAAGTTCAA

AACAACCACTTACATAAAAACATCTAGAATGTAAAATTAGTACTCAATGGGATCATGCTATGATAAATTC

TACAACCAACAAGGACCAACAAAATTAATGCGTGTTCTTGAAAGCCTGAACTCTCAGTCATCTTCACGTT

CAGGGGGTAGACCGCATGGCAAAAGCATTTTCTGCATAAAGTTATAACGCTCTCTTCCAATTGACTCATT

TTCAGCAACAGCAAAATAAGCCAATATAGGGTAATGCCCAATATATGATGCATAACTATCACGCTGGATA

TTCATCGCCCACTCGAATCTGTTTAAATCGGCGTGACCAGTTCCAACATACTTGGCCTGAAGATGCTCAA

GCTGGGAATTGATGTTAAACCTATCACTGGCCTGCGGGGGGGCCCGGTA

>FM891335.1 FM891335 Jatropha curcas embryo 56-70 (DAF) Jatropha curcas cDNA clone rjcaeb0_001492, mRNA sequence

GCTGCATGAATTCGAATCGACGAACAACAGCCAGTCGCTCACTCAGAAGTATTCTTGGACGAAGCATCAA

TGTCTACTGGCATGAAAATCACTTTGAAAAGCGCCGACTGTGAGACCTTCGATGTAGACGATGCTGTTGC

TCTTGAATCGCAGACGATTAAACACATGATAGATGACGACTGCTCCGATAATTGGAATCCCTCTGGCCAA

TGTCACCAGCAAGATCCTATCTAATGTAATATGAAGTACTTGTAAGAAGCATGTTGAGACCCCCTAAGTT

CGACGATCGCCCCTCTTCTGCCGACGAAGAACTTAAGACCTGTGACTCGGAGTTTGTCAAAGGTTGATCA

GGCTACCCTCTTTGATCTAATCTTGGCTGACAATTATCTGAACATCAA

>FM891334.1 FM891334 Jatropha curcas embryo 56-70 (DAF) Jatropha curcas cDNA clone rjcaeb0_001491, mRNA sequence

ATTCCANAGAAAGCCATAACATCAGCATATTTTTATATCAGTTTTTAAATACTCATGTGACCAGAAACAG

AACTCCAAAGTCTTTATTCATCTACTTATTCTTTTCTCTCATGTTCTTCATATCCCGCACTAGGTTTGTC

TTGTCCAATTATAAGCTCTTTAGTTGTTTGAGCAATCTCTGCTATAGTTTCGCCAATGGCGCCGAGCAGG

CTGCTGCCTCCCTTTTGCTGCTGCCATTGATGATGCTCTTCTGGCTTTTCTTCTGAGCCTTGAGTGGCTT

TTCCGGTGAATCTCCCCGCAGTTTCCCATTCTTCTCCTCCTCCTGTTGAATGAACCTGTTCTTGTTCTGC

TTTCCTGGCTTCCACCTCTCTCTCTGCCTCCTGTTTCTTCCTAGCAGTATACTCCTTCATAGTCTCACCA

GTAGACGCAACAGCATCCTTAGCAGCAATGAATGGCTTTGCAGCAAGCTCTGCTGCTTTCTCTGTTGCTC

CTTGAACGGGCCTTGCTGCTGCTTTAGTCCCCTCCACTGTTTTCTCAGTGGTGTAATGCGCAGCGGTCCA

ACCAG

>FM891333.1 FM891333 Jatropha curcas embryo 56-70 (DAF) Jatropha curcas cDNA clone rjcaeb0_001489, mRNA sequence

ATTCAGAAATCAAACAACTGGTGCTGGCACCATTCATTAAGCTAGCGTAACAACTGCTGCTGCTGCCATA

CTTAGACCAAACACATTTCGACATTTAACATTTAGAACAAAGGAGACAAGCTAGTAACTGGAAAGATGCC

AGTTCACATGTTATTTCAACCTTTTGAGCAACACGACATCTAAATAACAAGAACAACGAGATAAAAGCCT

TGCATGTGGCATCCCAGAACATAGATGTGATACCTTCAGCAGAAGTTTGATGAGGGTCAGATGTTATCAA

GCTTTTTAGCTTTCACAATGGGGTTGGCCTTGGCAATAGCATTTTGTCCAGCAGTTCGATAATCATAGGT

GCAACCGGGGGGGCCCGGTAC

>FM891332.1 FM891332 Jatropha curcas embryo 56-70 (DAF) Jatropha curcas cDNA clone rjcaeb0_001487, mRNA sequence

ATTCCCCGCTCTGAGCTCCGACGAGCCTTCGAGTCGCTGCGTTTGGTGTAAACCCACTTCGGAGTGGACG

TCACCGCCACACCAGAGCAGCTGACTCAGTTGTACGACTCTATCTTTGAGTCTTTTGATTGTGACAAAAG

TGGCACCGTTGATATTGAAGAGTTTAGAACTGAAGTCAGGAAAATATTGCTTGCGATTGCTGATGGGTTA

GGATCCAGTCCCATCCAGATGGCTCTTGAAGATGATGATCAGAGTTTTCTCAAGCAGGCTGCCGATCTCG

AAGCCTCTAAGCTTCAGTCTTCTGTTTCTTAACTAATTTAGATCCGTTTTCTATCTTGCCCTGTCAAATC

TTTTTATCTTTACATCTTATAAGGCTTTTGTTTGTTCCTACCTTCTTTATCTGTTTTTCAATATTTCAAT

AAATTTTGGCAATTTAATCTC

>FM891331.1 FM891331 Jatropha curcas embryo 56-70 (DAF) Jatropha curcas cDNA clone rjcaeb0_001485, mRNA sequence

ATTCGCAATCACCGCTTGAGTTTTGAGAAGAAGTAAAAAATGAGCCAGGGGCAACCACGAAGAACTCAAT

ACGACCAGGAGCCAATCAAATACGGCGATGTGTTTAATGTCGGAGGCGATGTCGCCTCCCAGCCGATTGC

ACCAGTAGACGCTGCTAACATGCAGTCTGCTGAGAGCCAAGTCCTTGGAGAGCCTCAGAGAGGCGGGCCT

GCCTCAGTCATGCAATCTGCGGCAAACGTCAACGTTAGGACTGGTGCCGTCGAGCGGGATGATGTGAGTG

ATGTTGTTAGAGAACAGGGCATTAACGTTGCTGAAATAGATATCGGCGGCACTCGCGTTATCACAGAGAA

AGTCGGTGGAGAGGTTGTGGGACAATATGTTCAGCCAAGAGTTCCAGCGACATATCCAATGCCAGGTATG

GATATTACAATGGGTGAAGCTTTAAAGGCAACTGCTTATTCTGCCGCNAGAGACAAACCAATTGATCAAA

GCGACGCCGCTGCTATAAAAGCTGCAAAGGTGAGAGCTCTTCGCAGTACTCAAACCCGGCGGGTGGAAT

>FM891330.1 FM891330 Jatropha curcas embryo 56-70 (DAF) Jatropha curcas cDNA clone rjcaeb0_001484, mRNA sequence

GGAATTCGAATTCCCCACAGTCGCAGCATGTTGGCCAACAGCCACGATCCCACCAAGTAGTGAAGGCAGC

CACTGCAGTCACTGCCGGTGGATCTCTTCTAGTTCTCTCCGGTTTGACCCTTGCTGGTACGGTCATTGCA

CTGACCGTAGCCACTCCATTGCTGGTCATATTCAGTCCGGTTCTTGTTCCTGCGGTTATCACAGTTTGTT

TGATAATCACAGGGTTTTTAGCCTCAGGCGGTTTTGGCGTCGCTGCCATTTCTGTCTTGTCCTGGATTTA

TAGGTATGTTACTGGAAAGCATCCACCAGGAGCAGAAAGTCTGGACCAGGCACGCTTAAAGTTGGCGGGA

AAGGCAAGAGAGATGAAGGACAGAGCTGAGCAGTTTGGACAGCATGTAACAGGTCAACAGACTTAAAAGA

GAAGTGGGTTTTGGTGGTTGAT

>FM891329.1 FM891329 Jatropha curcas embryo 56-70 (DAF) Jatropha curcas cDNA clone rjcaeb0_001483, mRNA sequence

AAGTAAAACAGAACAATACAAGCATTAACAGCACAAAACTAGCAAATACAGGACGCAGCGTAAACATATA

ACCGTAATCCCAATGTTGCTATCCCGTGTTAAAAAAAAAAAAAAAGAACTAAACAGAATTAACGTTTTTT

AACTGCCGGTTCCTTCTGCGAGTTAAAATACACAGCAGCAACCGGAAGCCCAAGTCCACTTTGGGCAGCA

AAGTGGCGGGTGTTGAAGTTGCCACGACCATCTGGAGGTTGAATCCTTCCCATCAATGCACCTTTCTGCT

TGAACAGCGCAAAAACATAACGGTGAATGCCGGTTGGAGGCTGAGGTCCCATGTACGACACCAACTCATG

CCCTTTGG

>FM891328.1 FM891328 Jatropha curcas embryo 56-70 (DAF) Jatropha curcas cDNA clone rjcaeb0_001481, mRNA sequence

AAAAAAAAAGTAGAAGAGAATAATTCTCACAATTGGTTATTTATTTACAAATGATCAATATGATGAAAAT

TACAAAAAATGCCTCAAAATGCTGGCATGCCCTCTCCACTCTTCACGATGACAAGAAATACATCAATGAT

GGC

>FM891327.1 FM891327 Jatropha curcas embryo 56-70 (DAF) Jatropha curcas cDNA clone rjcaeb0_001480, mRNA sequence

ATTCACTAAAACCTCTTCAAAATCCTAAAGCCCAAAACACTAAAACAGAAGAAAATGTCTTGCTGCGGAG

GAAACTGTGGCTGCGGTTCTGGCTGCATGTGCAGCGGCTGCGGCGGGGGATATGGCATGTACCCTGACAT

TGCAGAGAGCACCAGAACTGAGACCCTCATTGCAGGAGTTGCACCCTCCAAGAAGTTGAATGAGAGTTCT

GAGATGAGCATTGGAGCTGAGGGTGGTCATGGCTGCAAGTGTGGATCAAACTGCAGCTGTGACCCATGCA

ACTGCTAATGAGAAACCAGAAGGGAAAGGCTCCAACAAAGTAGTTATTAGTCTTGGGCTTTTATGGTTAA

TCAGTTGAATTATGTGTAATAATCCATGTTTGGTGTAAATAAAAGAAGCCATGGGTACCTTCTCAAACCC

TGTCGTGTGGGATTTGATTTCCTGTTGTTTCTTGTNTTATGTATTGTCTTGAACTTTTGCTTTGTTGGTC

TTTAAGGAGCCTTTTGTGTT

>FM891326.1 FM891326 Jatropha curcas embryo 56-70 (DAF) Jatropha curcas cDNA clone rjcaeb0_001479, mRNA sequence

TTGAATAGCCAAGTGAGTGAAAATGGAGTCGCTGAAGGAAACCTGCAGCTAACGTTGGAGCCTCTGCTAA

GGCTGGCATGGAAAAAACCAGAGCAGAGGCGCAAGAGAAGGTGGAGAGAATGTCAACACAGTGATCCAGT

GCAGAAGGAAATGGCGAGAGAGAAGACAGAAGAGAGGAAAACGGAGGCAGAATTGCGGGAAACTAGAAGC

GCGCGAGGATAGCGCAGCGGCGAGGCAGGCAGCCAATGCTGGTGGACATGTACAGTACAGGGCACAGGGC

AGCCATACGGATGGAGAGTGTGGAACCAACGGGTTTGAGCAGGACTCGCCCATCTGGGTTGCCCGGAGAC

ACCACTGGCCATAACCCACGATGTGTAGGGCATGGCATGTGCT

>FM891325.1 FM891325 Jatropha curcas embryo 56-70 (DAF) Jatropha curcas cDNA clone rjcaeb0_001478, mRNA sequence

AGATTAATTGCCAAAATTTATTGAAATATTGAAAAACAGATAAAGAAGGTAGGAACAAACAAAAGCCTTA

TAAGATGTAAAGATAAAAAGATTTGACAGGCAAGATAGAAAACGGATCTAAATTAGTTAAGAAACAGAAG

ACTGAAGCTTAGAGGCTTCGAGATCGGCAGCCTGCTTGAGAAAACTCTGATCATCATCTTCAAGAGCCAT

CTGGATGGGACTGGATCCTAACCCATCAGCAATCGCAAGCAATATTTTCCTGACTTCAGTTCTAAGGGGG

GGCCCGGTAC

>FM891324.1 FM891324 Jatropha curcas embryo 56-70 (DAF) Jatropha curcas cDNA clone rjcaeb0_001477, mRNA sequence

ATTCCACGAAAGCTCTCCAGGAGGCCCTTCCAGGTGACAATGTTGGCTTCAATGTGAAGAATGTTGCTGT

CAAGGATTTGAAACGTGGTTTTGTTGCCTCTAACTCCAAGGATGATCCTGCGAAGGAAGCTGCCAACTTC

ACCTCTCAGGTCATCATCATGAACCACCCTGGTCAGATTGGAAATGGATATGCTCCAGTCCTTGACTGCC

ACACCTCCCACATTGCTGTTAAGTTTGCTGAGATCCTAACCAAGATTGATAGGCGATCTGGTAAAGAGCT

TGAGAAGGAGCCAAAATTCTTGAAGAATGGTGATGCTGGATTTGTTAAGATGATTCCCACCAAGCCCATG

GTTGTGGAGACCTTTTCTGAGTACCCTCCTCTGGGTCGTTTTGCTGTGAGGGACATGCGCCAGACTGTTG

CTGTTGGTGTCATCAAGAGTGTTGAGAAGAAGGATCCATCTGGTGCAAAGGTCACCAAATCCGCAGCAAA

GAAAAAGTAAAGCATTGGGTGATTGAAGTTACAGTTAGTTT

>FM891323.1 FM891323 Jatropha curcas embryo 56-70 (DAF) Jatropha curcas cDNA clone rjcaeb0_001476, mRNA sequence

CAGATGAAACCCTTCAATGCATTGATATTAAATTTCAACATTTCCAAAATACATGATGTTGAGGAGTTTT

TCCCTCTTTAACTTCAAAGCAATGAAGATTTTATAAAATTAGATAACTAGTATACAAATTTTAAATTAGG

AGTCATCTTGTGGCAGCTACTGACAGTAGATATTCATATTTTTCTTCCCATCCCATGCCACATTAACCAA

CAATATTGCTTAATTAAAATTGCTTCTCACTTTCTTCCTACTATGCACAATCTTGATGATCTAGAACAGT

GGAAAATGTAAAAGTATTTACATTAAAATAATTAACTTACATGAATAATAATTAGTGTGGATAGATGATC

AGCAAAACCAAAAGCTCACTCCGACTCTGATAAACTAAGTATCGATACTGCCCAGAACCCTCTTAAGATC

AGATTTCTGTTCTGCTGTAAGGGGGGGCCCGGTAC

>FM891322.1 FM891322 Jatropha curcas embryo 56-70 (DAF) Jatropha curcas cDNA clone rjcaeb0_001475, mRNA sequence

ATTCCACANAATTCCAATTAGAGATGATAAAGAAGCAAATGAAGCATCGCTAGAATTGGTAAGAAAGGAC

AAGCTAAGAGAGGTTAAAGCAGGGCATGATGGAACATGGGCAGCACACCCAGGACTTATCCCAGCATGCA

TGGAAGTATTCACAAACAACATGGGAAATGCTCCAAACCAAATTCACGCCAAGAAAAGAGAAGATGCTGC

GAATATAACAGAGGAAGACCTATTACAAAGGCCTCGAGGAGTTCGAACAATGGAAGGTCTTCGCCTAAAC

CCTAGAGTTGGAATCCAGTACTTAGCAGCCTGACTTACTGGAACGGGCTCAGTGCCTCTTTACAATCTTA

TGGAAGATGATGACACTGCTGAAATTAGCAGGGTCCAAAATTGGCAATGGATTAAGTATAGAATGGAGTT

GGATGGAAATGGACTGGGAGTGAAAGTGA

>FM891321.1 FM891321 Jatropha curcas embryo 56-70 (DAF) Jatropha curcas cDNA clone rjcaeb0_001473, mRNA sequence

ATATCACTAATATAATATTTTATTAAATACAACGATAAAAAATACATATGCTTTTTTTCTTTTTTTCTCT

CTTGAATAACCAAATGCACACGAGAACATAAATAACACGAAAAGACTGTTTTTTGGGCAACAGATTAAGG

AGAAGCACGTTTGGATCTGATATAATCTTCAAGGGCTTCCATTTGTTCTTTAGACGGCGGGGTTTCGTTG

TTCTTTATAGATCTATGCACGTCATACGTTGTTGAAGCCAAGAACACAACCCCAGAAGCCATCAGCATTA

TTTGCCCTGGAGATACGTATTTTCCGAAGATCTTCATCGCCTCTCTCTCTCTCTCTCTCTGTCTCTCTCT

CTCCGTGAAGCTTCTTGAGAACTAGT

>FM891320.1 FM891320 Jatropha curcas embryo 56-70 (DAF) Jatropha curcas cDNA clone rjcaeb0_001472, mRNA sequence

GAAGCTTAAGAACTAGGTTTATTAGTAAACTCAAGATAACATTTGAAAATTAGATACAATTCCAAATTTT

ACATTTCCACTGTCCGGTTCCAAAATGGTCTAAACTGTAATGGTTCCTCCATAGCTTTCTATCAATGAAA

TCCACTGATGAAGTTGGGGAAGCCACTTAGACCTTGTCAAGCCTTGACATTTATCCTGCATTGATGATTG

TCTCTACCCGTCCGAAATTCATGGTCCTTCAATCCACATCTTTGCAAAGGGATGCAAACCTAACTAAATA

CGACAACTTGCACACAGCCAAATCATTCTTGCAGATATCTCGTAACTCATCGTAAACCATAAACAAGTGT

ATTCTCACTTCCATAATTCGCTGGATGTATCAAAATTCCTTCCCAACTTTGAGATAATTCAAGCCCTGTT

TTTCTTCTTGCCTCTCTTTTTTGAACTAGCTTCAGGCTCTGCTT

>FM891319.1 FM891319 Jatropha curcas embryo 56-70 (DAF) Jatropha curcas cDNA clone rjcaeb0_001471, mRNA sequence

ATTCAAATGGCCAGACTCGGTGGAGTTCGCGAAGTAAGCGCCAACAGCGTCGAGATCGACGGTCTGGCTC

GTTTCGCCGTCGAAGAACACAACAAGAAAGAGAATGCGTTGTTGGAGTTTGCGAGAGTGGTGAAGGCGAA

AGAGCAAGTGGTGGCTGGGACTCTGCATCACCTCACTATCGAAGCGATTGAGGCTGGAAAGAAAAAGCTT

TATGAAGCGAAGATTTGGGTCAAGCCTTGGCTAAACTTCAAAGAATTACAGGAGTTCAAGCATGCTGGGG

ATGTTGATGGACCTGATGGTGGTGCTTCATCCTTCACCTCTTCAGATCTTGGTGTCAAAAGAGATGGGCA

TGGGCCTGGATGGAGAGAAGTGCCAGCACATGATCCTGCAGTGCAAGATGCTGCAAATCATGCTGTGAAG

ACCATTCAGCAGAGGT

>FM891318.1 FM891318 Jatropha curcas embryo 56-70 (DAF) Jatropha curcas cDNA clone rjcaeb0_001469, mRNA sequence

ATTCCNNCTAAAGGCAGTGAAAATGAGCCAAGAACAACCAAGACAAACCCAGACAGAGCAAGATCGAGAT

CCCATCACGTACGGCGACATTTTCAATGTCCATGGAAATCTTGCTTCCACACCCGTTGCCCCTGTAGACG

CAGCCACCATGCAGGCTGCTGAAAACCGTGTACTCGGAGAGACCCGGAGGGGTGGTCCTGCCTCCGCCAT

GCAATCTGCGGCGAATGTCAACTTGAGAGAAGGCTTCGTTGACCCTGATGAAGCCAGCAATGTCGCTAGA

GACGACCAAATCGAAGTATCAGAAAGTGATGGTCCCGGCGGTGATCGCGTTATCACTGAAACAATAGCAG

GACAGGTGGTGGCGCAATATATTGAGCCAAGGGCGGAAAGAACAACTCCGGGATCAATTGCTAACCGCGA

CGCAGTCA

>FM891317.1 FM891317 Jatropha curcas embryo 56-70 (DAF) Jatropha curcas cDNA clone rjcaeb0_001468, mRNA sequence

ATTCCGAAGCAACATCTTCGATCCTTTCTCTGCTCTCGACGTTTGGGATCCATTCAGAGACTTTTCTTTC

CCTTCATCTTCTTCCCTCGTCTCTCGTGAAAATTCTGCTTTTATTAACACTCGCATTGACTGGAGAGAGA

CACCAGAGGCTCATATATTCAAGGCTGATCTCCCTGGGCTCAAAAAAGAGGAAGTGAAAGTAGAAATTGA

AGATGACAGAGTGCTTCAAATCAGCGGGGAGAGAAATGTGGAGAAGGAAGACAAGAACGACACGTGGCAT

CGCGTGGAGCGTAGCAGTGGCAAGTTCATGAGGAGGTTCAGGCTGCCGGAGAATGCAAAGATGGATCAAG

TAAAGGCTTCAATGGAAAACGGAGTACTTACTGTCACTGTTCCTAAAGAGGAAGTCAAGAAACCTGATGT

TAAGGCTATTGAGATCTCTGGTTAAGTTTCAGCTTATAATGATGTTATCTCAATATATATATATATATAT

ATATATCTAT

>FM891316.1 FM891316 Jatropha curcas embryo 56-70 (DAF) Jatropha curcas cDNA clone rjcaeb0_001467, mRNA sequence

ATTCCTTTCTATAGCGGAACAGTGGCCAATGTTCCGTCCACTCAACACGAAACAGCCGCCATTGCCAACG

CACGAGTGGACTGGAGGGAGACTCCGGAGGCTCACATATTTGCTGTTGATCTTCCTGGGTTGAAAAAAGA

AGAGGTAAAAGTGGAGGTAGAAGATGGCAGAGTTTTGCAGATAAGTGGGGAGAGAAGCAAAGAACAAGAA

GAGAAGAACGATAAGTGGCATCGCGTAGAAAGAAGCACTGGTAAGTTCTTGAGGAGGTTTAGGTTGCCGG

AGAACACAATGATGGATCAAGTCAAGGCCACTATGGAGAACGGTGTGCTTAAAGTGGTTGTGCCTAAAGA

ACAAGAGAAGCAGCCTCAGATTAAGCCTATTGAGATCACTGGCTAGAAGTGCAAATATGGTCTTTTACTA

CTAATAGATAAAGCTTTTAACGCTGCAGCTTCTGGAGTTATGGGTATGACTAAAATACAGTACTAGGGGT

GAGCGTATTGCGAAATTTGGATGTATGTAGCG

>FM891315.1 FM891315 Jatropha curcas embryo 56-70 (DAF) Jatropha curcas cDNA clone rjcaeb0_001464, mRNA sequence

TCGAATTCTGATTAGGGAATACTATACTAATTAGATGATTACATTACTTTCATCACTTATTTACATAATA

TATTACATACTTCATTAGCGTGAGATCATAACTCCCCTCATAATACTA

>FM891314.1 FM891314 Jatropha curcas embryo 56-70 (DAF) Jatropha curcas cDNA clone rjcaeb0_001463, mRNA sequence

ACTAAAAGATACCTTTTATTCAACTGGAATTGCTTAACAAGAAAAAGACACCAAACAGAAAACATAGATA

TATTACATAACAGGAGCTAAAGCACATGCCATGCCCTACACACGTGGGTTATGGCCAGTGGTGTCTCCGG

GCAACCCAGATGGGCGAGTCCTGGTCAAACCCGTTGGGTCCACCTCTCCACCGTATGGCTGCCCTGTGCC

GTGTCCAGGCATGGCCGACATTTGCTGGGTCCCTGTAGGGTAACCGGTTGTCCCAGTTGTTGAATGGGTA

TAGGTTTCGGTTGTGCCTTGAGCGCCCGGACCAGCAGTTGTGTACTGTACTGGCCACCAGCTTGGCTGCT

GCCTCACCACGCTTTATCCTCGCGCGCT

>FM891313.1 FM891313 Jatropha curcas embryo 56-70 (DAF) Jatropha curcas cDNA clone rjcaeb0_001462, mRNA sequence

ATTCCCTGTTTTAGGAATAACTATGGTCAACTCTTTGAAGTTGGCTCTAGAGATTTCAAGCAACAGCTCC

GAGACCTTGACCTTTCAGTCTCCGTTGCCAACATCTCTCGTGGAGCAATGTCTGGGCCCTATTACAACTC

CAGAGTAACCAAGATATCAGTGGTTTTGGATGGCGAAGGCTACTTTGAAATGGCTTGTCCTCACCTTAGC

GGCTCGCGGGGAGACACGACGGGTGAAAGTCAGAAGACTTACCAGAAAGTAAGTTCACGGTTGAAACGTG

GAACAGCAGTGGTCGTCCCTGCAGGCCATCCGGCGGGCTTTTGGGTAGCTTCAAGAANACAATTTAATCT

GGGGGAAGTAGTCTGCTTTGAGATTAATGAAGAAGGCAATACTAGGCATCTACTTGCAGGGAAAAATA

>FM891312.1 FM891312 Jatropha curcas embryo 56-70 (DAF) Jatropha curcas cDNA clone rjcaeb0_001461, mRNA sequence

CGAATTGTCCAGGTGCGTATCATGAGCACTTTCAGCGAGATGATTGGTACTGGAAGGCTGATGCATCCTT

CAGAAGATCAGAGAACTAACTTCAGGACTCCAAGAAGAGAGTGCAAGCTACCCATTATCACATCATTATT

CAGTTTTGGGCCTTGAAGGTGCAGGGCAACACCATACACGGAAGCTGAGATTAAGAAAGCATTCAGGGCC

AAGCAAAAGAGTTCCACCCAGATCAGAACCAAGATAATAACGAAGTGGCTGAGGCAAAGTTCAAAGAGGT

AATGG

>FM891311.1 FM891311 Jatropha curcas embryo 56-70 (DAF) Jatropha curcas cDNA clone rjcaeb0_001460, mRNA sequence

ACTCCAAATGACAAATAATATACAAAAAAGGGCAAAGCTGTTCTCAAATTTACATGCTTGTTTTAATTCC

TCCCGAAACTCAAACAAAGGACAACAGCTTGAGCTATTATCATCGTAGTTGTCATCATCATCACTAGTTA

ATAGTAAAATTTTAACAGGAGAGAGACAGAGACACAGAAAGAGCTAGACTACCTTCCCTGAGCCTCAACA

CATTCACAATACATTACCTCATCATTAAGCTCCTTTAATCCCACAGGAAAGGCAGTGTCTGCATTAGGAT

TGTGATCTAGCTGATGAAGATTCACCTAATGAAACAGAAGCCAGGGAGGAAGCCCAAACGGGGGGGCCCG

GTAC

>FM891310.1 FM891310 Jatropha curcas embryo 56-70 (DAF) Jatropha curcas cDNA clone rjcaeb0_001459, mRNA sequence

GGATCCCCGGGCTGCAAGAATTCGAATTCCAAAAACGACCCAGCAGCAAGCAAAGGAACCGACTCTCTCC

TCTTCTCTCGCAGCCTCAGGTTTCCGCAGAAGATTGTAGAGTCTTATTGCCCAGAAAGAAAGACGTTTTG

TGGGAGGTGTTTATACGTAATGGAGTCGGCTGAAATTTCACATGATGCAGATGAAAACAGCACAAAGAAG

GAAGCAACTGAATCCAAGCATGATGGTGCAAAAATGTCTAAGACTGGTCCAGTCCCCCTACAATTATGAA

CACATAAACAT

>FM891309.1 FM891309 Jatropha curcas embryo 56-70 (DAF) Jatropha curcas cDNA clone rjcaeb0_001457, mRNA sequence

GAAAAAATGGAGAAGCTTACAACCTCAGCCGCTCTACTCTGCATTCTGCTAGTGCACATAGCCAATGCAG

TCTCCGGCTATGTGACAACCACCATCACCACCGTTGAAATCGACGATGAT

>FM891308.1 FM891308 Jatropha curcas embryo 56-70 (DAF) Jatropha curcas cDNA clone rjcaeb0_001456, mRNA sequence

ATTCCACAGCTTCGGTCTTTGACAACGGAACAGCGGACAAAGCTGAAATGCAGCTTTATTCATATTTGAT

GATCCTAGTTTCTGTGAATGGATGCGAGGCTTGAAAGTAGTG

>FM891307.1 FM891307 Jatropha curcas embryo 56-70 (DAF) Jatropha curcas cDNA clone rjcaeb0_001454, mRNA sequence

TTTTTTTTTAAGGGCAAAAAACATGCTCATATTATCTCTTTGTTTATAACAGAGAACTATGAAACGTTGA

ACTTACAAAAAGCCTTGATGAACACTGAATATTTACAATGCTGCACCCTGCACAAGTTGTATTCTACATA

ATGTACAATAAAACTAC

>FM891306.1 FM891306 Jatropha curcas embryo 56-70 (DAF) Jatropha curcas cDNA clone rjcaeb0_001453, mRNA sequence

ATTCGCAAAGAAGGATTTCAACCTTGCAAAACACCCAAATATCGATGTCCCAAATCTGCAGGTTATTAAG

CTTATGCAGAGTTTCAAGTCCAAGGAGTATGTTCGTGAGACATTTGCATGGATGCACTACTACTGGTACC

TCACAAATGATGGGATTGAGTTTTTGAGGACTTACCTTAATCTTCCATCAGAAATTGTTCCTGCTACATT

GGAAGAAATCTGCAAGGCCCACTGGTCGGCCAATGGGTGGCCCACCTGGTGATCGCCCCCGAGGCCCACC

TCGCTTTGATGGAGACCGCCCAAGATTTGGTGATCGTGATGGGTACNCGTGGAGGCCCNTCGAGGGGGGT

GATTTTGGTGGTGAAAAGGGAGGTGCTCCAGCGGACTTCCAGCCATCGTTTAGGGGCTCAGGTGGTAGGC

CTGGCTTTGGTCGTGGTGGTGGTGGTGGTTATGGTGGGGCAGCTCAAACTAGTTCTTCTGGAT

>FM891305.1 FM891305 Jatropha curcas embryo 56-70 (DAF) Jatropha curcas cDNA clone rjcaeb0_001451, mRNA sequence

GCACATTTAACTTACCCAGCAAATATATTANTAGGTAATAACCAGCACATGAAGTTGAAACAAAATACAA

AAGTTGTTTAGTATCGATCATAATCAAATAAATGCCCATAGGTTCCAAACAACTAACAACTAGCTATAGA

AACAAGCCCATAGATTGGGATAACCGCTTAGAAGCCTTCATTGTTAAAATTTCCATTCCCACGAAAATTA

CTATAATTATTTCTTGGAGGACCAGTTCTTTCCTGAGCATAACTGACGCGAACACTTCGTCCTTGTAATT

CCTGGCCATCCATGGCTGATAAGGCCGAGCTAGCAGACTCCTCGCTGGA

>FM891304.1 FM891304 Jatropha curcas embryo 56-70 (DAF) Jatropha curcas cDNA clone rjcaeb0_001450, mRNA sequence

TGATGATGGTGTCGCGATGGTAATTCAACCTAGTACGAGAGGAACCGTTGATTCGCACAATTGGTCATCG

CGCTTGTGTTGAAAAGCCAGTGGCGCGAAGCTACCGTGCGCTGGATTATGACTGAACGCCTCTAAGTCAG

AATCCGGGCCAGAAGCGACGCGCGTGTCCGTCGCCCGTTTGCCGACCCGCAGTAGGGGCCATCCGGCCCC

CAGAGGCACGTGCCGTTGGCCAAGCCCCCGCGGCGGACGAGCCGCGAGGCCGCCTTAAGTACAATTCCCA

TCGGGCGACGGATAGAATCCTTTGCGGGGGGGCCCGGTAC

>FM891303.1 FM891303 Jatropha curcas embryo 56-70 (DAF) Jatropha curcas cDNA clone rjcaeb0_001449, mRNA sequence

ATTCGATTCCAGAGCAAAAGATTAATTATATTGGTGGAGAAATTTGTTAGCAAAAAAGGGCAAAAATGTC

TTCAAATCAATCAAGACAAGAGCTTGATGAAAGAGCAAGGCGTGGAGAGACTGTTGTCCCTGGAGGTACT

GGGGGCAAGAGCCTTGAAGCTCAAGAGCATCTTGCTGAAGGGAGAAGCAGAGGAGGACAGACAAGGAAGG

AGCAGCTGGGGACAGAAGGATATCAGGAGATGGGCCGGAAAGGTGGGCTGAGCACGACGGACGAGTCTGG

TGGAGAAAGAGCTGAGAAGGAGGGCATCACTATCGATGAGTCGAAGTTCAGGACTGGGAGCTAGTGTGAT

GATTAAAACTATAAATGGTATTTTGTAGTAATAATAATAATAATGTTGGTAATAAAGTATTAGCTTAGCA

GTG

>FM891302.1 FM891302 Jatropha curcas embryo 56-70 (DAF) Jatropha curcas cDNA clone rjcaeb0_001448, mRNA sequence

TTTGAATTCCTCGCTTCTGCTTATGTTTGGTGCTTTTTTAGAATGTAATGCAAAGAAATTAAATTCTATA

AACCATTTCACTGCTTGCAGATCTCAAAGGAAGTATGTCACCGAAACGGCCATCCATTGATTCTATATTC

AGTATTAGATCTTACTTATTTTTCC

>FM891301.1 FM891301 Jatropha curcas embryo 56-70 (DAF) Jatropha curcas cDNA clone rjcaeb0_001447, mRNA sequence

ATTCGAATTCTAATTCCTTTTGTTTATTTTGCATCTCTGCTTAAGATGACTACCGAAAAAGAAGCTGAAG

TTGCTTCCACCGTTGAACAGCCACCGCCTGCGACAGAGGAGGCTAAGCCGGCGGAGACTAAAACTGTGAA

GGAGAAGAAAACCAGAGCTCCAAGAGAGAAGAAGCCTAAACAGCCTAAAACTGCTTCTCATCCTCCATAT

TTTCAGATGATCAAAGAAGCTCTGCTGGCTTTAAACGAAAAGAGTGGATCGAGCACATACGCCATAGCTA

AATACATGGAAGAAAAGCACAAAGCAGTATTACCAGCAAATTTCAAGAAAATCTTAGCTTTACAGTTAAA

AAACTCGGCTGCTCGAGGAAAGCTGACAAAGATCAGAGCTTCTTATAAATTATCAGCAGGCAAGAAAGAG

AAAGACACAACCACCAAAGAAGTTTCAAAGAGTACTAAAACTACTAACAAAGAAAACAACAAACCCAAAA

CAGCTACGACAAGAAAGACAAGGTCTGTTAACAAAACTGTCGAAGCTGCAAAGAAGCCTGCAAAGAGAGC

TGTT

>FM891300.1 FM891300 Jatropha curcas embryo 56-70 (DAF) Jatropha curcas cDNA clone rjcaeb0_001446, mRNA sequence

ATCCACGTGCCGATGTATTCAACCCACGTGCTGGCCGTCTCACCAACATCAACGGCCAAGACCTCCCCAT

CCTCAGTTATATCCAGCTAAGCGTTCAGAAAGGTGTCCTCTACAGAAATGCTCTAATGTCACCACACTGG

AACGTCGATGCCCATAGCATAAACTACTTCACAAGAGGAAATGGCCATATCCAGATCGTAGATGATAATG

GAGAGACAGTATTTGATGGAGAAGTTCAAGCAGGACAAATATTAACATCTCCACAAAACTTTGTGGTAGT

AAAGAGGGCAGGAAGCGAAGGTCTTGAATGGGTATCCTTCAAGACTAATGACAATGCCAAGATTGACCAA

CTAGCAGGACGTGTCTCGGCAATCAGATCAATTCCAGAAGAGGTGTTGGCAAACGTATTCCAGGTTTCAA

GGGAGGACGCGAGGAGACTGAAAGAGAATAGACAAGAAGTGACTCTGTTCAGCCCTGGGACTAGGCCTTC

TTCTCGCAATTGAAGGGACTAAAAAGGCAGCTATGTAATTGTAATAAGGAGAGAAAAAACGCTTA

>FM891299.1 FM891299 Jatropha curcas embryo 56-70 (DAF) Jatropha curcas cDNA clone rjcaeb0_001445, mRNA sequence

TCGAATTCGTTGCCTCTTATAATCTTCACCTTCGAAGCTCAAGAAACCCTCGTCGCCGTCGAAGAAACTC

GTCTCTGCAACCATGGGGAAGACACGTGGTATGGGAGCTGCCCGCAAACTGAAGTCCCACAGAAGAAGGC

AAAGGTGGGCTGACAATGCATACAAGAAATCCCATCTTGGTAATGAATGGAAGAAGCCATTTGCTGGCTC

TTCCCATGCCAAAGGCATTGTTCTTGAGAAGATCGGTATTGAGGCCAAGCAGCCTAACTCTGCTATCAGA

AAATGTGCTAGAGTTCAACTGATTAAAAATGGAAAGAAGATTGCTGCTTTTGTACCAAACG

>FM891298.1 FM891298 Jatropha curcas embryo 56-70 (DAF) Jatropha curcas cDNA clone rjcaeb0_001444, mRNA sequence

CTTTTCATCGAAAATTTCACTTTAAATAAGAACTGCTCAAGTTAAGATCCCTGCTGCCGCTAACTAAAAC

AAAAGAGCCACTAATTTAGGAAAAATAAAACATTATACATCCACAATAATTTGTGACAATATCACACCTT

GGTACTGAAACAGAGGTTCATTCAGAACACGTATGGTAAATTCTACTCTATGCATACTGACAATCTGAAT

CCTTCCCCCCTACTAAAAGCTACCTACCCAACTGGGAACACAAGACCTACAGTTACTCTCACTTTCTCTT

CTTAGCAGGTGGCTGGGGAACTTCTTCTTCCTCCTCTTCTTCCTCTTCTTCATCCTCTCCATCTTCTTCT

TCATCATCTTCATCACCATCATCGTCGTCGTCGTCATCATCGTCATCATCACTTCCTCCTTCTCCATTGG

CTTCAGGATCATCCTCAGGATCTCCTTCCTCCTCCCCCTCATCCCCTGAGAA

>FM891297.1 FM891297 Jatropha curcas embryo 56-70 (DAF) Jatropha curcas cDNA clone rjcaeb0_001443, mRNA sequence

CGAATTCGCGAGGCGTTCTGCTGCCGAGGGCAATGCACAGCCCACACTGGTCCATAAACGATCAGAGAAT

AGTGTGTGCTCTTCGCGGCGACATGCACATACAGATTGTGGATGACAGTGGAAACACAGTAATGGACGAA

AGGGTGAAAGAAGGAGACATGTTCGTGATCCCTCAATTCTATGCAGCACTTGGAAGGGCAGGAGAAAATG

GGTTCGAGTACGTAACATTTAAAACAAGTAGCCAGCCTATCAAGAGCCCATTGGCGGGTTATACATCAGT

GATGAGAGCCATGCCTCTTGATGTTCTGATGAACTCTTACAGAATGTCTCCAAGCGAAGCTCATCAAGTG

AAGAGGAACAGGGATCCACAGTCCATGTTGTTTACTCCCACCAGCAGGAGACCATAAGTAAGTTACTACT

AAAATAGCATTGTGATTGGTGCTTCTACTAGCCAAGGCCGCAACTAAGAGATCCTTCTTGTAAATTTAGA

GAAGGGATCCGTCGTATATTGTAGTATAAAGAGTTGTAGAGTGTTAAAAATGTAGAACTT

>FM891296.1 FM891296 Jatropha curcas embryo 56-70 (DAF) Jatropha curcas cDNA clone rjcaeb0_001442, mRNA sequence

ATATTGAACACTGACTTTTTATACGTACATGAACACAAGTGGATTAATTAAACATAAAGGACGATCAAAG

TCCAAAGTCCAAAGTCCAAACCAGACCCAGATACATCCCCGCCAAAAGGGGATATGTAAATGACACAAGA

TAATTCTTTTAGCATTACATGTACATATATTATTACATTGTTGTTGCTTAAACCCGAGTCCCTGTCACTG

CTGTGCCACCCTTTGGAATTGGTTCCCCACCAGCTCCTACAGCGTATTCGGTGTCCCTGTAACCAGGGCC

ATACCTGCTGCTAAATACCCCAGCATGAAGCAACAGGACATACAAGAGTTGGGTAAATGTCAAGATTATC

ATGAATGCCTCTAATACCCTCAGTCTCCATCCTCTGTGCCCTCCTAGGTTTATTTCCTTGCATGCCAACC

CAAAAGCTAGGGCAGTGATGGCCCAAGTCACAAGTGAAGAAGAGCCTGCAGAGGCTAGACTGTCATTTCT

CCAGCTCCTGAATATGAAGGCCACCTGCCAACTTGGATACTATACCAAACATAGAAGCTAATATAGCCAA

AGTCAAAAAGAAAGCTGTTGCTCCATTCCCTCCAAAACTTGGATGGTAGGTTTGTCCATTGATATAC

>FM891295.1 FM891295 Jatropha curcas embryo 56-70 (DAF) Jatropha curcas cDNA clone rjcaeb0_001441, mRNA sequence

ATTCCCCTAATTGGGGACGACTTGACGAATTCTTGGAACAAAAGTTTGGAACTGAAGCTGGTTAAACAAT

TGTTGTCGACTACTGAATTGATTGCCCTTCTGATTAACGCTGTAACAAGAATTTCAGGGAACCAAGTTTT

TTGAAGTTATTTTTGTTTATTGCTTCGTGCATTTTAGCTGTGTGAACTATATTACCTATTAGATGACAAG

ACTTTGGATATTTAGCCTGGTATGAAATGCAATTTCAGTTTTGACAAAAAAAAGGGGGGGCCCGGTAC

>FM891294.1 FM891294 Jatropha curcas embryo 56-70 (DAF) Jatropha curcas cDNA clone rjcaeb0_001439, mRNA sequence

ATTCCTCAGTGGCTCCATGGATGTTCATTTAACCGTGCTTGAACTCCTTTACACCGACGATTTTACCACC

GTAATAAACTCTGCATCCACGGCTCTTGATATTCTACCTGTGGCGTTAGAGTTGTTATTCGAAGATTGCG

TGAAGAATTGTGTTAAATTCCTGGAAGCGGTCCCGTGGAGCGAAGAAGAGGAGAAACGTGTGCTCAGCTT

GATTCCTTTTTTGCGTGAAGAGGAATCTCAAGAGCTTTTAGCTAGGACTTCACCGGGGAAGGATGATTCG

TGCGAGGAAATGCTTCACGGATTGATATTAGCTGCAATTCATAACCATCCAAACATGGCTTTCGTGAAGG

CATTTGTGGCTAAATTGTTGAGGGAATTTTCGTCGAGAGAGTCCGCTAGGAGAGTTCTGGAGAGAGCTTT

TGAGGAGAGTTTGAAGATTGTGAAGGAATCGCTTGAGGAGTACTCAAGTCCTGATTTTAGAGGGGATCAC

AATGAGACGGAGGCAATACCGAGATTGAATCTGCACACGGCCTTGACTAATGGGAAGCCATTGCTGTGGC

TGGTGGAGAGGATGATTGA

>FM891293.1 FM891293 Jatropha curcas embryo 56-70 (DAF) Jatropha curcas cDNA clone rjcaeb0_001438, mRNA sequence

TCAGCTTTAGAAGAGCTACGAAGCTTCCTTTAGCTGGCTCTCTATCTCGAGATGCGTCGCTCTGCAGCCA

GGAAAACGGGTCAATCCAATTCCACGGCTCCGATCACTTCTTCAGCTGCGGATCTTTTTCGCTCCGCCTC

AAGTAAAGCATCTTCGAAAGAGATGGAGCGAATTGATAACCTATTTTATTCCTATGCAAATAGGTCTTCT

GGTATGATTGATCCAGAAGGAATTGAAACCCTATGTTCAGACATGGAGGTTGATCATACTGATGTTAGGA

TCTTGATGCTAGCTTGGAAAATGAAAGCTGAAAAACAGGGATACTTTACGCTGGAGGAGTGGCGCAGAGG

TCTTAAAGCGCTGAGAGCTGATACTGTGAGTAAATTAAAAAAGGCGCTTCCAGAGCTAGAGAAAGAGGTC

AAGAGGCCGTCAAACTTTGTGGATTTCTATTCCTATGCATTCCGATATTGCCTGACAGAGGAGAAACAAA

AGAGTATTGACATAGAGAGCATCTGTCAGTTAGTGGATC

>FM891292.1 FM891292 Jatropha curcas embryo 56-70 (DAF) Jatropha curcas cDNA clone rjcaeb0_001435, mRNA sequence

ATTCGTACTTGCTGCTCTCTCTACTTCTTCTCTTTGTGGTTCGATCCGGGAGAAACTCAGAATCATGTTG

GTCTACCAGGATTTGCTTACCGGTGATGAGCTTCTCTCGGATTCGTTCCCTTACAAGGAAATTGAGAATG

GCATGCTGTGGGAAGTTGAGGGAAAGTGGGTTGTTCAAGGAGCCGTTGATGTGAACATTGGTGCAAATCC

CTCTGCTGAAGGGGCGGATGAAGATGAGGGTGTTGATGACCAGACTGTTAAAGTTGTTGACGTTGTGGAC

ACTTTTAGGCTCCAGGAGCAACCTGCTTTTGACAAGAAGCAGTTTGTCACATACATGAAGAGGTACATCA

AGTTGTTGACACCCAAATTGGATGAGGAGAAGCAACAGGCTTTCAAGAAAAACATTGAGGGAGCAACCAA

GTTCCTGCTCTCGAAGCTCAGTGACCTCCAATTTTTTGTGGGGGAGAGCATGCATGATGATGGGAGTTTG

GTGTTTGCTTACTACAAGGATGGCGCCACTGACCCAACATTTTTGTACTTCGCTTATGCTTTGAAGGAGA

TCAAGTGCTAAGAATGGA

>FM891291.1 FM891291 Jatropha curcas embryo 56-70 (DAF) Jatropha curcas cDNA clone rjcaeb0_001434, mRNA sequence

TCGAGAGAGAGAACTAGTATTGTGGGGAAAGTAGAAGAAAGAGCTGCGACGAAAGAAGATGATTCAGGAT

CATCTACTGCGTGGGCACCAGACCCCATCACGGGGTACTACAGGCCAGCTAATTCTGCGGCTGAGATCGA

CCCGGCTGAGCTCCGGGAAATGCTATTGCACAACCGGGTTAGGCCACGGTAGAAGTCTGAGATTCAGATG

CTGAAGTGGTGGCGTTCACTTTTTAGTGTGTTTCTTACACCAGCATATAGTAGTACTGTACTAGAAGTTT

TTAAATGTTGCCAGTTGCGTCATACCACTCTTATTGTCTTAAAGACTCCTGTAGTCTTGGTTATGACTTA

TCATATCAATGAAGAAAACTATCCATTATC

>FM891290.1 FM891290 Jatropha curcas embryo 56-70 (DAF) Jatropha curcas cDNA clone rjcaeb0_001433, mRNA sequence

ATTCCCTAGCAGCAGCGTCGCTTGTTAACTCAAAAACAGCAGATCCTCCATTCTCTTTGCCGATCCATAA

TCAAAACAATGAAGGTCATCGCTGCATATTTGCTCGCCGTTTTGGGAGGTAACACCTCACCCTCCGCCGA

GGACCTGAAGGAAATCCTCGGATCCGTTGGAGCTGATGCTGATGATGATAAGATTCAGTTATTGTTGTCC

CAAGTTGAAGGAAAGGATATCACAGAATTGGTTGCAGCAGGAAGGGAGAAATTGGCATCTGTGCCTTCTG

GTGGTGGTGTAGCTGTTGCTGCTGCTGCAGCACCTGCTGCTGGAGGTGGTGGTGCTGCTCCTGCTGCTGA

GGCCAAAAAGGAGGAGAAGGTTGAGGAGAAAGAGGAGTCAGATGATGATATGGGTTTCAGCCTCTTCGAC

TAGGAGATCTATTTGCTGGTTCCATTTGTATCAAGGCTTTCTTTTATCTTCTAGTCTTTTAGTTAAAAAA

TTTACAAGTTTTTGGTCTGTAGTTGGTTATGATATTTAGAACTTGTTTGCGTTCTTGAATTTTGAG

>FM891289.1 FM891289 Jatropha curcas embryo 56-70 (DAF) Jatropha curcas cDNA clone rjcaeb0_001432, mRNA sequence

ATTCCAGTGGGTGAGTCTTGTTGATGGAAGCTTTAGGCGGTGGCAATGGCGGGTTTTTTTGGGGGTGGAA

CAGGGAAATAAGAAAACGTAAGCCATATAGTTGTAAAAGTAATGCGTCGAAATCCTATGATGGAGTGGCA

GGAGGCCGTCACCAGTTCCCTTTAAAGCAAGCTGTGACGGCGGGTTCTCTCGCACTTGCTGGAGACACAA

TCGCCCAAGTTAGAGACCGCTGGCTAAACAATAAAGCCTCCATGCAAAACTCTGTTCTCCCTTCTAATGA

TTATAGCGAGCGACAAGGATCTTATGGGGGCCATCCTTTTGGACCATGATTGGCTTCGTGCCTTGCGGAT

GACTTCTTATGGGTTTCTTTTTTATGGCCCTGGTTCTTATGCATGGTATCAATATCTGATTCTTGTATGC

TAAG

>FM891288.1 FM891288 Jatropha curcas embryo 56-70 (DAF) Jatropha curcas cDNA clone rjcaeb0_001430, mRNA sequence

ACGAAAAAAAAATAATAATAATAATAATAATCTTTTGGCAGGGTGTAAAAAACGAAAAAACAGAGAGGAT

TCTCCAGGAGTGTAATCAAGTTAGTGGAGCTCTGGTTTATGCCCCCCTTTCCTTCAGTTATCTGTCTTCA

ATATTAAGAATCCAGCGCCACAGCTTCCGTGAGTGGACGTAGTTTAAGGGCTGGAACATAGGAATTAAGC

GCAACAAGTACCTTCCTCACTTCATTTGCCAATTCAATCACCATGTCATCTTCATGACCAATATCAAACA

TGGCTTTTTCTAACAGGAATAGGTAGTCTCTGTTATTCCCACAAGGTCCATGGGCCG

>FM891287.1 FM891287 Jatropha curcas embryo 56-70 (DAF) Jatropha curcas cDNA clone rjcaeb0_001428, mRNA sequence

AAATAAAATATCTACTAAAAGATACCTTTTATTCAACTGGAATTGCTTAACAAGAAAAAGACACCAAACA

GAAAACATAGATATATTACATAACAGGAGCTAAAGCACATGCCATGCCCTACACACGTGGGTTATGGCCA

GTGGTGTCTCCGGGCAACCCAGATGGGCGAGTCCTGGTCAAACCCGTTGGGTCCACCTCTCCACCGTATG

GCTGCCCTGTGCCGTGTCCAGGCATGGCCGACATTTGCTGGGTCCCTGTAGGGTAACCGGTTGTCCCAGT

TGTTGAATGGGTATAGGTTTCGGTTGTGCCTTGAGCGCCCGGACCAGCAGTTGTGTACTGTACATGGCCA

CCAGCCTTGGCTGCCTGCCTCGCCGCTGCGTTATCCTCCGCGCGCTTC

>FM891286.1 FM891286 Jatropha curcas embryo 56-70 (DAF) Jatropha curcas cDNA clone rjcaeb0_001427, mRNA sequence

CGAATTCCATCTCAAAATCAACTCTTTCTTTCTCTTTAACCTCTCTCGCTTTATCCTCAAGCTTAAAAAT

TTCCATTAAAGGTTCATGTTGTCTCCTTTTCTCGGTTAGATCAGCACCGTCGCAACATCTCCATGGAACA

GCAAAAACCTCAATATCAAAATTCTCCTTTTGCATGTGTCTCATTTTCTTAATCTCCTAATCAATTTTAT

GAAATATAGGTATGTGTATATTATCCAACTCTCTTCGCCAGTCAGATCAGCGTCGTCGCCGCTAACATCG

TCAAATTGTCAAACTTTTGATTTATGGTTCTATTTCTCTTGTTTCATAATCATCATATTTTGTTATTTGT

GTGTTTTTTTTTTTTTTTTTAATTATTGAAGAATTTGTTTCAAGAT

>FM891285.1 FM891285 Jatropha curcas embryo 56-70 (DAF) Jatropha curcas cDNA clone rjcaeb0_001425, mRNA sequence

ATTCAGATTTCAAGCAACAGCTCCGAGACCTTGACCTTTCAGTCTCCGTTGCCAACATCTCTCGTGGAGC

AATGTCTGGGCCCTATTACAACTCCAGAGTAACCAAGATATCAGTGGTTTTGGATGGCGAAGGCTACTTT

GAAATGGCTTGTCCTCACCTTAGCGGCTCGCGGGGAGACACGACGGGTGAAAGTCAGAAGACTTACCAAA

GTAGTTCACGGTTGAACGTGAACAGCAGTGGTCGTCCCTCAGCCATCCGGCGGCTTTTGTAGCTTCAAGA

AACAATAATCTGGAAGTAGTCTGCTTTGAGATAATGAAGA

>FM891284.1 FM891284 Jatropha curcas embryo 56-70 (DAF) Jatropha curcas cDNA clone rjcaeb0_001424, mRNA sequence

ATTCATCAAATCGGCGTTGAAAGATCAGTTCTCCTTTTTGAGTATCTAAAAATGTCTTCTTGCTGTGGTG

GAAATTGCGGCTGCGGCTCCGGCTGCAAGTGTGGCAGCGGTTGTGGCGGATGCAAGATGTATCCTGACAT

GAGTTTCTCCGAGAAAAACACCGCCGAGACTCAGGTTCTCGGAGTTGCACCGGAGAAGGCACGCTTTGTC

GATGGAGGTGAGGTCGGCGTTGGAGCTGAGAACGGATGCAAGTGCGGTGACAACTGCACCTGCAATCCTT

GCAACTGTAAATGAGATCGGCGGCGATCAGTCTGTTTTTTAGAAGTAAAAGCAGAGATGACTCTGCTTCT

CTCTTTCTAAAAAAGGAGAAATAATTAATAGAGTCTAGATATCGTATGAGT

>FM891283.1 FM891283 Jatropha curcas embryo 56-70 (DAF) Jatropha curcas cDNA clone rjcaeb0_001423, mRNA sequence

ATTCCTCCTTGCACACACACTCACACACCCAAACCTAACAGAGCGCTCTCTGATTCAGGAAAATGGGTCG

CGTACGTACAAAGACAGTGAAGAAATCCTCTCGCCAAGTGATAGAGCGCTACTACTCGCGCATGACACTT

GATTTTCATACCAACAAGAAGATTCTTGAAGAGGTGGCTATCATCCCTTCAAAACGTCTCCGAAATAAGA

TTGCAGGGTTTTCTACCCATCTAATGAAACGGATCCAAAAGGGTCCGGTTCGCGGGATCTCACTCAAGCT

TCAAGAGGAAGAGCGCGAGCGGCGCATGGACTTTGTACCTGATGTCTCTGCCATCAAGACTGATCAGATT

GAGGTCGATAAGGAGACTATTGACATGCTTGCTGCTTTGGGTATGACTGATATTCCTGGGATTCTCCAAG

TCGAACCGGCTGCAGCGCCTGCGACCCAGTTCGTGTTTGGGAGAGGTGGTGCTGGTGGCAGACC

>FM891282.1 FM891282 Jatropha curcas embryo 56-70 (DAF) Jatropha curcas cDNA clone rjcaeb0_001422, mRNA sequence

AACTAGAATTTATCTCTTTATTAATTATTAAACCAGAAGCCACTATAGTCCGCAGCAGTGATCTTTCTCT

TTTCTTAAAGACTAATTTACACATGTACCTTCACCTTTCATTATTTATTCACCATACACAGCACTATTTA

CATTAGTAAACCCTCAAGGTCGATGTCACAGTCACCCCAAAAAGCCGAAACACTTGCCATCACGTTATTA

GCTTCTCATGCCACAGCATGACCCTCGAAGGAACCTCTCCTTGGCCCTGGAAAGAACCACTCCTCGATTT

TGTCTTCCAAAAGCCTGATCCACTTCTCTTTCACTTACACCGAAGGCTAACTCTTTGGGCTTGACTTCTC

CATCTTATTTACAATATTATTTTTCCCTGCAA

>FM891281.1 FM891281 Jatropha curcas embryo 56-70 (DAF) Jatropha curcas cDNA clone rjcaeb0_001420, mRNA sequence

ATTCCTCAGTATCGAGCGACAGGTCTGCTCTTCGTATTGAGTCCTACTTTACCTTCTCTTCTACTGTACT

CTGGATTATTAGCAATCTAAAATGGAGGACGAGAAAGATGCTTTTTACGTTGTGCGGAAAGGAGATATTG

TTGGCATTTATAAAAGCTTAAGTGATTGTCAAGCTCAAGCTGGATCATCAGTATGTGATCCTTCTGTAAC

TGTGTTCAAGGGGTATGGTTTGCCTAAGGAAGCTGAGGAGCACCTACTCTCNGCATGGGCTCAAGAATGC

TACCTATTCTATCCATGCTGTTGATGTTCCAAAAGGATCTTTTTGGTAAAATTGTTCCCTGCCCTTTTCA

GCAACCGAATTCTTCTGGAGGAAAAGCATTAGATTATTATTCTCCGCCACAGAGATTGCAAGATGTGCTT

GGTTTGGCAGTAGC

>FM891280.1 FM891280 Jatropha curcas embryo 56-70 (DAF) Jatropha curcas cDNA clone rjcaeb0_001419, mRNA sequence

TCGAATTCCAACAGGAAAAACGAATTGAAACAAAGGATTACACTGCTGAGAAAGCTAAAGAAGGGAAAGA

AGGTGCAGCTAGTAAGCTTGGTGAACTCGCTGAATTAGCTAAAGGAGCAGCAAGGAAAGCTATGGACTCT

GTCGGTAAGAAAGATGAAGCAAAAGAAAAAACATCAGAAACTAAAGAAGCAGCTAAGGAGAAACTTGGCG

AACAAGGAGGAAACTAGCGGAAGATGGAGAGTTGAAATGGA

>FM891279.1 FM891279 Jatropha curcas embryo 56-70 (DAF) Jatropha curcas cDNA clone rjcaeb0_001417, mRNA sequence

CGAATTCGATCGATAAAGTGTAACAGAGCAAAAGATTAATTATATTGGTGGAGAAATTTGTTAGCAAAAA

AGGGCAAAAATGTCTTCAAATCAATCAAGACAAGAGCTTGATGAAAGAGCAAGGCGTGGAGAGACTGTTG

TCCCTGGAGGTACTGGGGGCAAGAGCCTTGAAGCTCAAGAGCATCTTGCTGAAGGGAGAAGCAGAGGAGG

ACAGACAAGGAAGGAGCAGCTGGGGACAGAAGGATATCAGGAGATGGGCCGGAAAGGTGGGCTGAGCACG

ACGGACGAGTCTGGTGGAGAAAGAGCTGAGAAGGAGGGCATCACTATCGATGAGTCGAAGTTCAGGACTG

GGAGCTAGTGTGATGATTAAAACTATAAATGGTATTTTGTAGTAATAATAATAATAATGTTGGTAATAAA

GTATTA

>FM891278.1 FM891278 Jatropha curcas embryo 56-70 (DAF) Jatropha curcas cDNA clone rjcaeb0_001415, mRNA sequence

GCTGCAGGAATTCGAATTCGTGATCAATTTGGACTGGGTGAGCCTTGTTTCGCCGAAAATGACCCTGACA

GTGGCTTCGCCACCGCCGTTCATGGCGTTTTCGTCAGGCAAGCTTTGTCGCCGAAACTGATGGGAGAAAG

AGACTCGCTGGGAAGAGAGGAGTCGATTGAAGCATGTCCCTCGCCTTGAAGCTGCCGGAAATGGTGTGGA

TCGCCGGAATTTGGTGAGCACGAGCTGTCGAGGGCAACAACTCCGTTTCGCTCCCATTGACGCTCTTTCC

GCGCG

>FM891277.1 FM891277 Jatropha curcas embryo 56-70 (DAF) Jatropha curcas cDNA clone rjcaeb0_001413, mRNA sequence

TTTATTTATTTTTTGAATCTCCGTCGACCTCATCGGCGAATCTCCGATCAGTGATGAAACATTGAGATGC

CAAATCTAATTACGAGGGTGTGAAGTCTTGAAATCAGCCACTTCATGCAAAATGCTGAAGATTAGGACTG

TATCCAGAACACCCTGCCAGAAGCCTGCTTATATGGAACCTGAACCAAGCGATGGCTTTCTATTTTGTAT

AAATATGTATAGGACGGTTATTTATTATCTTCTAAATGTCTAATTGTCTATGCTTTTCTTTTCCC

>FM891276.1 FM891276 Jatropha curcas embryo 56-70 (DAF) Jatropha curcas cDNA clone rjcaeb0_001411, mRNA sequence

ATTCGCTCCTTGGTGTGGGCCCTGCCGAATGATCCATCCTTTTGTAGCTGAACTATCAGAGCAATATGCT

GGAAAAGTGAAGTTCTTTAAATTGAACACTGATGAAAGTCCTTCTACTGCAAGCAAATATGGAATTCGAA

GCATTCCAACAATAATGATCTTTGAAAATGGAGAGAAGAAAGATGCTGTCATTGGTGCTGTGCCCAAAAC

AACACTAACTACCACCATTGAAAAATTCTTGTAGACGGAAGGTAATAGAATCTTGTCAAGATCTCCTGTT

TTATACTTTTGGAGCCTTTCTTTTCTGCCTTATTCTGTGAGTTCGAAAAATTCATCTCCCTGTATAATTA

TGGATGCTGCCATCTTTTATTTTTCATGACAATTTTGCTTAAATGACAGCAATAAGTGTTCGTTTTGCTT

AAAAAAAAAGGGGGGGCCCGGTAC

>FM891275.1 FM891275 Jatropha curcas embryo 56-70 (DAF) Jatropha curcas cDNA clone rjcaeb0_001410, mRNA sequence

ATTCCTTCTACCAGCTCCGTTTCTTCTACGCCACAGACCGCAAAGAATAAGAAACCATGCCGAAGAATAA

GGGAAAGGGAGGAAAGAATAGGAAGAGAGGAAAGAACGAAGCAGACGACGAGAAGCGTGAGCTCATCTTT

AAAGAAGATGGACAGGAGTATGCCCAAGTGCTTCGTATGCTTGGTAATGGCCGATGTGAAGCCATGTGTA

TCGATGGTACCAAGCGTTTATGCCATATTCGCGGGAAGATGCACAAGAAGGTCTGGATTGCGGCTGGTGA

TATAATACTGGTTGGGTTGCGTGACTATCAGGATGACAAGGCTGATGTTATCTTGAAGTACATGCCAGAT

GAAGCTAGGCTTCTAAAGGCCTATGGTGAACTTCCTGAGAACACACGTCTCAATGAGGGTATTGCTGGAG

GCCTTGATGAAGAAGATGATGGTGCTGGTGATGATTATGTTGAGTTTGAAGATGAAGATATTGATAAGAT

CTAGAGTTTGAGAACTGTCTTTCTTTCTACTTTATGTATAGTCATGAAGAGAGAGTTCTCTAGAG

>FM891274.1 FM891274 Jatropha curcas embryo 56-70 (DAF) Jatropha curcas cDNA clone rjcaeb0_001409, mRNA sequence

AAGATGATAAAATTCTCGTATCTATATATAGTACTTCGTATTGATTTACAATATTAGAGCTAGCTGATTA

CAGAAAATGAACACTGTGATCAGTTGTTGGGACCCCAACAGCTCAAGAACATGACTGTCCTCAAAGATTC

TTCTGCTTGTTTTGATTCCTTCACTTTATTGCTAGAGACAGATGAGGAAGAAGAGAGATTCCTGGCCTGA

GATAGAGACCTGAGGTTATTCTTGGTATGTTGATGCAATGATCTTAAGGTGTAGTTCCATCTGCAAAAAC

CTAGCTGGTCCTTTAATGCTTCCACAGTTCCAATGCTGGCTGCAACCGCCCAACCTTTTCCTGCTGAACC

CATTTCTCTCTATTCTTAGTTTTCTTGGGGGGGCCCGGTAC

>FM891273.1 FM891273 Jatropha curcas embryo 56-70 (DAF) Jatropha curcas cDNA clone rjcaeb0_001408, mRNA sequence

TTGAATTTGAATAGCCAAGTGAGTGAAAATGGAGTCGCTGAAGGAAACCGCAGCTAACGTTGGAGCCTCT

GCTAAGGCTGGCATGGAAAAAACCAGAGCAGAGGCGCAAGAGAAGGTGGAGAGAATGTCAACACGTGATC

CAGTGCAGAAGGAAATGGCGAGAGAGAAGAAAGAAGAGAGGAAAACTCGAGACTAGTTCTCTCTCTCTCT

CTCTGGGGGGGCCCGGTAC

>FM891272.1 FM891272 Jatropha curcas embryo 56-70 (DAF) Jatropha curcas cDNA clone rjcaeb0_001407, mRNA sequence

CGAATTCCTGAGCTCCGAGACCGAGAAGGAACGGAAAAATGCCAAAGCAAATACATGAAATCAAGGATTT

CCTTCTTACAGCAAGAAGAAAGGATGCTCGTTCTGTCAAGATCAAGAGAAGCAAAGATGTGGTGAAGTTC

AAGGTTCGCTGCTCCAAATATCTTTACACACTTTGTGTGTTCGACTCTGAGAAGGCTGACAAGTTGAAGC

AATCTCTTCCCCCAGGTTTGACTGTACAAGACCTGTGAAGAAACATAAAGACTAAAGTTTTTGGCCTTAT

GAGAAATGAAGGGATCCAAGTCTTTTGCCTTTTTTCTATTGTCTTTTGGGTAATGTTATCAATTTCGTTG

ACTGTTGAAGTAGTTATGCTGTTTGCCCAACGTTTGCTTATGGACTCCATATGAACTGGTAGACATGAGC

TGTTGAGTTTGTTATGAATTTTGATAATACTTTCAACTTG

>FM891271.1 FM891271 Jatropha curcas embryo 56-70 (DAF) Jatropha curcas cDNA clone rjcaeb0_001406, mRNA sequence

ATTCAACTGGCCTAGCTACCTCTAAGCCTGCAACCATTGCAAATGTTCTGCAGATCGATGTGCCGGATAC

TGGCCTTTTGGCTAGTACGGAGGAGCCACCAAAGGGCCACACTGGCGAGCATGTAGGTGGTGCTGAACCT

GAGCCCGTGAAAGCGGAGCAACCGCAACAACAACAACAAGAACTTGAAAGATCTAGTAGTTCAAGCTCTA

GCTCGTCAGAGGATGATGAGCAAGGAGGGAGGAGAAAGAAAAAGGGATTGAAGGAGAAAATAAAGGAGAA

GCTGACAGGTGGGAAGCACAAGGAAGAGCATAAACAGACAGCAATCACTGAAACAACCCCAGGGGATCCT

CAACACCATCAACATGAGAAGAAGAGTGTAATGGAGAAGATAAAAGAGAAGTTGCCTGGCCACCAC

>FM891270.1 FM891270 Jatropha curcas embryo 56-70 (DAF) Jatropha curcas cDNA clone rjcaeb0_001405, mRNA sequence

CCTTGCTTCTCTGCAGCAATTGCAAAAACAAGGGTTTTCCTAGCCAACGGCTATCAATCATGATTATTTC

AGAGAAGAACAGGAGGGAGATCTCCAAGTATCTCTTTCAAGAGGGAGTTTGCTACGCAAAGAAGGATTTC

AACCTTGCAAAACACCCAAATATCGATGTCCCAAATCTGCAGGTTATTAAGCTTATGCAGAGTTTCAAGT

CCAAGGAGTATGTTCGTGAGACATTTGCATGGATGCACTACTACTGGTACCTCACAAATGATGGGATTGA

GTTTTTGAGGACTTACCTTAATCTTCCATCAGAAATTGTTCCTGCTACATTGAAGAAATCTGCAAGGCCC

ACTGGTCGGCCAATGGGTGGCCCACCTGGTGATCGCCCCCGAGGCCCACCTCGCTTTGATGGAGACCGCC

CAAGATTTGGTGATCGTGATGGGTACCGTGGAGGCCCTCGAGGGGGTGATTTTGGTGGTGAAAAGGGAGG

TGCTCCAGCGGACTTCCAGCCATCGTTTAGGGGCTCAGGTGGTAGGCCTGGCTTTGGTCGTGGTGGTGGT

GGTGGT

>FM891269.1 FM891269 Jatropha curcas embryo 56-70 (DAF) Jatropha curcas cDNA clone rjcaeb0_001404, mRNA sequence

ATTCATCGGAAGATGGGAGACACTGCTCTTATTTCAGAAGGGTCCCTGCACGAGTTTACCAGAAGGTGCT

GCTGAATTGTGTGGCATACAACCTTCAGAAGTGGCATGGTTAGAAGAAACTCCGCCTCCAGAAAACTTTG

TGGTGCAGAGGGGTCTATATAAAGGCCCAAATATTAGGCTTTAATAAAGGGTTTTTCCCCCCTCCCCTTT

TGGATAGGATGTAAAGTACCATTAATCAGATCAGATGAACAAATGGATAACCCTGGAATTGATTGCTGGA

AAGTTAGCTGTCAACTGACATGGCAAGTAATAATTAGTCCTTGGCTTTGT

>FM891268.1 FM891268 Jatropha curcas embryo 56-70 (DAF) Jatropha curcas cDNA clone rjcaeb0_001403, mRNA sequence

ATTCAATGCTTTTACTTCTTTTGCATTTTTAACCCCTGATCAAGATGAGGCCAAGGAGTCTTTTGACAAA

GCAGCTGTCTACTTACAACAAGCTGTTGATGAGGATCCAGAGAATGAAATTTATCGCAAGTCTTTGGAAG

TGGCTTCTAAGGCTCCAGAATTACATGCGGAGATTCATAAGCACGGTTTAGCTCAACAGACGATGGGGGC

TGCACCTGCTTCTGGGCCTTCTACTTCATCCAG

>FM891267.1 FM891267 Jatropha curcas embryo 56-70 (DAF) Jatropha curcas cDNA clone rjcaeb0_001402, mRNA sequence

ATTCGAAAGAACAAGCGTATATCCAAGGGAAAGAAGGGAGGCAAGAAGAAGGCAGCTGATCCGTTTGCCA

AGAAGGACTGGTATGACATTAAGGCACCTTCTGTTTTCAATGTCAGAAATGTTGGCAAAACCCTTGTCAC

CAGAACTCAGGGAACCAAGATTGCTTCTGAGGGGCTGAAACATAGAGTTTTTGAAGTATCTTTGGCTGAC

CTTCAAAAAGATGAAGATAATGCCTATAGGAAAATCCGTCTCAGAGCAGAGGATGTGCAAGGAAGGAATG

TGCTGACAAACTTCTGGGGAATGGACTTCACTACAGATAAGCTGAGATCACTGGTGCGGAAATGGCAGAC

ACTGATCGAAGCCCATGTTGATGTGAAAACAACCGATAATTACACTCTGAGGTTGTTCTGCATTGCTTTC

ACTAAGAGACGTGCAAACCAGGTCAAGCGGACCTGCTATGCTCAGTCCAGTCAGATCCGCCAGATTCGCC

GGAAGATGAGGGAAATTATGATTAATCAGGCCTCATCATGTGATTTGAAAGAACTGGTGGCAAAGTTCAT

TCCTGAGATGATTGGTAGAGAGATTGAGAAGGACACTTCCAGCATAATACCATTGCAAAATGTC

>FM891266.1 FM891266 Jatropha curcas embryo 56-70 (DAF) Jatropha curcas cDNA clone rjcaeb0_001396, mRNA sequence

ATTCAGAGAGAACTAGTTCTCTCTCTCTCTCTCGGCAAATCTAATTACCTCAACAGCCCTACTCTGCGTT

CTTCTAGTGATCATAGCCAATGCAGTCACTGGCTACAGAACTATCATCACCACCGTTGAAGTCGACGATT

CTTCCAATGTTCGGGATATATGCAAGAAGGAAGCTGAAAGAAGAGATTTGAGTTCCTGTGAGAATTATAT

AACACAGAGAAGAGGAAGGTCAGAAGATATGTTAGCTATGCCAGGAATTGAAAACCCTCGACAGCAGGTT

CCTAGACAATGCTGTAACCAAGCAAAGGAACTAAGTGCTATTTGTCGATGTGAATCAATACATTATCTGT

TAGAGAAGCAGCTGGAGGAAGGAGAAGTTGGAAGCGAAGATGAGGCAAGGCGCAGGACTAAAAATATAAT

TGATGTTTGCTTTGGGTCTATATGCCCTCGTTAAAATGCTAGCTGCAGGTGAATAAAACTTCATTCACTT

GTATGTGAGTGAGAGTTATATATAATGATGTATAAGAAGTAAGAGCATCAGAATAAGTTTGTTCGAAACT

GCTTCTTGTAACGTTGCTTCAAGCTTTCCTAATGTCTTGTAATAAAAACTG

>FM891265.1 FM891265 Jatropha curcas embryo 56-70 (DAF) Jatropha curcas cDNA clone rjcaeb0_001395, mRNA sequence

ATTCCTCTTTTTCTCCCTTCCAAGAAAAAGCTTATTACGATATCTATTTTAAGCTTCCATGGCCGAGTAT

GAGCGACGACCAGGCGGAGAAGGGCACAAAGGAGCCCTAAAGGAGAAGGGCGGCTTCTCGACCTCACAGA

TTTTAGCGGTTGTCACTCTTTTGCCGGTGAGTGGGACGCTTCTTTTCCTTGCTGGAATAACGCTTACTGG

AACACTCATTGCGCTAGCTGTTGCGACTCCGCTGTTCGTGATTTGTAGTCCGGTGCTGGTACCAGCGGCG

TTGGTCATTGGGTTGTCGGTGTTAGGGTTTTTGACGTCCGGTGCTTTTGGAATCACTGCGCTTTCTTCAC

TGTCGTGGATGGTTAATTATATTCGCAGGATGAGAGGATCTTGGACGATGCAGATGGAAATGGCAAAGCG

GCGCGCGCAAGAAACGACGGGTCAATTGGGCCAGAAGGCTAGGGAGGTGGGCCAGAAGGCCCAGGAAGTT

GCAAAGACATGATCAGGTTGTCTAGCAGTAGTTGTTGTTTGGTAAATAATATAAGCTAATATAGTTTGAG

TTTGATGATGGATTTCATAAAT

>FM891264.1 FM891264 Jatropha curcas embryo 56-70 (DAF) Jatropha curcas cDNA clone rjcaeb0_001394, mRNA sequence

CGATAGCTTAGACTATGCACGCAACCAACAAGAAAAATACAAACAAAAAGCACCAAGAACGCAACATACA

TCCAAATTTCGCAAAACGCTCACCCCTAGTACTGTATTTTAGTCATACCCATAACTCCAGAAGCTGCAGC

GTTAAAAGCTTTATCTATTAGTAGTAAAAGACCATATTTGCACTTCTAGCCAGTGATCTCAATAGGCTTA

ATCTGAGGCTGCTTCTCTTGTTCTTTAGGCACAACCACTTTAAGCACACCGTTCTCCATAGTGGCCTTGA

CTTGATCCATCATTGTGTTCTCCGGCAACCTAAACCTCCTCAAGAACTTACCAGTGCTTCTTTCTACGCG

ATGCCACTTATCGTTCTTCTCTTCTTGTTCTTTGCTTCTCTCCCCACTTATCTGAAAAACTCGAAGGGGG

GGCCCGGTAC

>FM891263.1 FM891263 Jatropha curcas embryo 56-70 (DAF) Jatropha curcas cDNA clone rjcaeb0_001393, mRNA sequence

ACAGTAAATCCCAAATTTGGTTATTACATTTCTCCAAACAGAAGACAGAAGTCCTTCTTAAACACACAAA

CCAAAGCTCCAACACACTTGGGGATGCAAGCTCAACCAGCCACACATCACCCTATTACAAACTGATCGAT

AAAAAGCAAATCAGCCACACAACATAACAAATAAGGGCATTGCGTGCGTATAATACATTATTAAGTAGAG

TATTAAAGAACACCTAGTGGTGCTCGTGGTGGTGGGAGCCAGGAAGCTTCTCCTTAATCTTATCCATTAT

TCCTTTCTTCTCATGATCATGGCCATACTCAGCTGAATGATAACCACCTGGAGTTGTAGCTGAGGTTGCT

TGAGTTCTTTCTTCCTTATGATGACCTGGTAGCTTCTCTTTTATCTTTTCTTTTAGCCCTTTCTTCCTCC

TTCCACCATGTCCATCATCTTCAGAGGAGCTAGAGCTGGAGGAGCCAGAACGGGAGAGAGAGAATAGACA

AGAAGTGACTCTGTTCAGCCCTGGGACTAGGC

>FM891262.1 FM891262 Jatropha curcas embryo 56-70 (DAF) Jatropha curcas cDNA clone rjcaeb0_001392, mRNA sequence

ATTGAAAAGCAAATTTACAAAGTCCAAAACAAAAGTCTCCAAAAACGAAGGTATTGAAATCCATAAATGC

ATATGATTATTACAGTAGACAAAAAAAGGACATTCAAAGACATCCCTATACAACTATACAATAGCTTCCA

TGGATTGAAATGTGCAACAGCATAACCAAACTGTTTTCCTATGTCTTATCACCACAGGACCTCCAGATGA

AAACCTACCAAACTTAATAACCCTGTAGTTGTGCATCTCGACTCGTTAGAGGAACGTAGCGAACAGAAGT

CTCGCTCCGGATACTAATGGAACCATCTTGGTTCTTGTCGATCACCTTCAAATCCTGGAATATGTTTCCT

ACTGGAATTACCATTCTGCCACCTGTTTTCAACTGATCAATGAGCGCCTGTGGGATTTCTGGTGCAGCTG

CTCCCACATGTATTGCATCATAAGGTGCAAACTCTGGCCAGCCTTGTCTTCCATCGCCAACATGCACTGA

GAGGGAACCTTCTTTCAATAATGGGAATTCGAAGGGGGGGCCCGGTAC

>FM891261.1 FM891261 Jatropha curcas embryo 56-70 (DAF) Jatropha curcas cDNA clone rjcaeb0_001391, mRNA sequence

ATTCCCCAAATTCATTTCACTCTTCTTATGTAAAATCAACCATGGCTAAGCTCACCATCCTCTTAGCCTC

CTTCATAGCCCTCCTCTTCCTCGTCGACGCCTCCATCTACCGAACCACCCTCATCACCGACGAAAACGAC

GATAACCAATTCCCGCCATACGACCGCCCAACAAGCTGTCAACAGCAAATCCAATCACAGCAAAGGTTAA

GAGCATGCCAGCAGTATCTCCGGCAACAAACTGGCTCCCCCTGGGGCCGTCGTTTCGACAACGAGGAAAA

TCAGATGGAGCGAGAAGAGTGCTGCAGACAGCTTCAAAGAATGGAAGCTCGGTGCCGGTGCGACGGTCTC

CGACAGGCTATTGATCAGCTACAGAGGCGTGGAATCCTTGGGGGCCAGGATGTCCGTGAGGCGTACATGT

TAGCTAGGCAATTGCCGTCGGAATGCGGCGTTTCACCAAGGCAATGCCATTTCGGGACACGGTGGTGGTT

CTAAAAGAGTGGTGGACGTAAGGTGGCAAGGCGTAGGATAAATGCTGTACTGGTAGAAGAATAAAAGTCA

CAGCTCTGGTGGTTTTGCTAGAGATTTGAGTGATTTTGTTATTAGTGAAAATAAAAAATTATAAGCACAT

TATGGTGCGT

>FM891260.1 FM891260 Jatropha curcas embryo 56-70 (DAF) Jatropha curcas cDNA clone rjcaeb0_001390, mRNA sequence

ACACAAAGCCACACTATTATTTTATTATAGCATATATAAGAAACACAGTGGCCATTACATAAAAGGAACA

CAAAGAACCTGAAAACAAAACAAAAGCATCAAATCTATTAATTAAACGGGAATTGAATTGGTGACTGATG

GGTAAACGACAGTCGCTGGATGTGCCATGATCCTGCGACCTTTCCTGTGTTTAAAGAGGTAAACAAAAGC

TCCCACCGGCCACTCTACCACCGGGGGGGGCCCGGTAC

>FM891259.1 FM891259 Jatropha curcas embryo 56-70 (DAF) Jatropha curcas cDNA clone rjcaeb0_001389, mRNA sequence

NTGATAACCTTCCAATCTTATCCATATAAGATTAGGCACATAAGATCCAGAAGGGCAAACAAGCCCACAA

AAGCTTTGTTTATAGTTACTCAAAACAAAAAAAAAGCACAACAAGAAGGCCCAAAAAGCTACTAATGAAC

ATGGTGGTGTAAAAGTGAAAAAGTAAAAGAAGTAGGAAGTACACCGTGAATTCGGTGGACGTTATCACTT

ATTGTCTGAACGGTGGACGACCTTTAGATGAGTAACACCTTCATCATCTAACCGTCCAACATCGTTGAAA

GTTTGAGCACTAATCTGATCAGACTCTTTCAGGGTGGATGCCACGGTTCCTGCTGGTGTTTCTTCAAGAA

CCACCACCTCTTCCTTTCTTCCTGTGCCTCCATGCTCACGTGCAGCACACGTTTCTCCCACGACGTCCCG

TGGCATTGTAAGCTTCCCTTTAATGGAGTCTTTGATGCTCCCTAATCCTGCCTTCTCAAAAATATTGTCC

CTTGCTGCACTTCCCCTTTCAGCTTCAATGTCTTTTTGCTCTGCCTCATCTTTTTCCATTTTCAACTCCT

CCATCTTCCGCCTAGTTTCCTCCTCTGCTTCGCCAAGTTTCTCCTTAGCTGCTTCTTTAGTTCTGATGTT

TTTCTTTTGCTTCATCTTTCTTACCGAAGAG

>FM891258.1 FM891258 Jatropha curcas embryo 56-70 (DAF) Jatropha curcas cDNA clone rjcaeb0_001388, mRNA sequence

AAATATTAACTTTATTGTCCATATATTATAATAATAGAGTAAAAGGCGGCGACAGGATGAACCCATCACA

GCAATTTCAAACACTGACATCTAAAGTGATGAAACTTGGACACAACAAGAGAATAAAAGAAGTTCAGCTT

ATTTATGAAAAATGAAACAGGTGTCCTTTTTT

>FM891257.1 FM891257 Jatropha curcas embryo 56-70 (DAF) Jatropha curcas cDNA clone rjcaeb0_001387, mRNA sequence

ATTTCGCCATTTGGAAGTGATCGCCAATGCATTTAGGGTGTCCAGCAGAGGACGCAAGGAGAATCAAGTT

CGGAAGAGAAGAGACGACTCTCNGGTACTTCAAGGTCTCGACCTGCAGGAAGGACAGCTGTTGCTTAATT

AAAATGAGGAGAGCCTTTGTTTTTCTGTAAATACAAGGCATGGAGAAATAAAACAGAGACTTGATAATTT

GATTGTCTCTAGCTAGAGTTATGTAAGTTGAGAGAGCTTTCTACTTGAGTAGTAATAAAATGTTACTCCT

TGTTAGTAACAAAAAAA

>FM891256.1 FM891256 Jatropha curcas embryo 56-70 (DAF) Jatropha curcas cDNA clone rjcaeb0_001386, mRNA sequence

GTGCAATTTTCTTTCCTTTTTATTTATATAATAATAGAAGTAAGACTAACCTAGGCCTATACATAAAAGA

TAAAACTAGCCTTTGACTTTAATGAAAAATAAGACAAAAGCAGATGATCCATCCTTCTCTACTTTACATC

TGGCATCTAAGAAAATGACTCAACTGTAATTGAATCCACCGTGGATTCCACAGATCCGATGAACTTTGCT

TATTTTACTCTTCCAAACTGCGGTGTGACAACCGCCGTTCGCCGCCAGTGTCTTGTTACAATTTCCCACT

TCTTTGCCTACGCATCCTAGCTCCCCAAGACATGTTCATCTCTGTTAAATCTTCGTCTTCACTACCCCTT

TCCCTCTCTAACTCTGTTAGCTGAGACGTCCATTGCTCCCTCCACTCACTTCTTTCTTATCTTTCCTTGG

CATAGCGGGATTGCTTTCCAGCAAAGAGAGCCATATGCAAGCCTCTGCTCATATCTCTGTCATAAATAGC

TCTTCTTTTAGGGTCAGACAAAGTCTCATAAGCTTCCTGCACTTCACTAAACATCTTCGGAGAGAGAGAG

AGAGAGAACTAGT

>FM891255.1 FM891255 Jatropha curcas embryo 56-70 (DAF) Jatropha curcas cDNA clone rjcaeb0_001385, mRNA sequence

ATTCCACGAAAGCGTGACTAAAGGGCAGAGAAAACAAGCGTTGGAGAAACCTCCCTCGCTCCTTCCTTTG

GAGAACGGGATCAGCTGCCAACGCCTCTCTGCTGAATGCAACCCGTTCTCCGCCACCCTAATCTAATAAA

TCCATCTTTCTGATTTGGTCGATGGGTCATCCACTCGCAGACAGATCTCATACATTGATACTATGCCTGT

TTATGTTAAGGAGCTCATTGCTGGTGGAGCCGCTGGAGGATTCGCCAAAACTGCTATTGCGCCTCTTGAA

AGGATTAAAATTCTTTTGCAGACAAGAACTGTAGGATTTCAATCTCTCGGGGTGTTTCAGTCACTGAAGA

AATTATTAAAGCATGAGGGAGTTCCCGGATTTTATAAGGGAAATGGCGCTAGTGTTATTCGGATTGTTCC

CTATGCAGCCTTGCATTTCATGACATATGAGCAGTATCGTTGTTGGATTTTGAATAACTGTCCTGCTCTA

GGTTCAGGACCTGTTGTAGATTTGTTAGCTGGTTCAGTGGCTGGAGGAACAGCAGTGATGTGCACATATC

CCTTAGATTTGGCTCGCACTAAGCTTGCTTATCAGGTCTTGGACAAAAGAGGAAGTTTCAGTAGTGGCAT

GAAAAGTATGTGTTCTCAACCCTCTTATGGTGGCCTAAAAGATGTTCTTACAAGCGT

>FM891254.1 FM891254 Jatropha curcas embryo 56-70 (DAF) Jatropha curcas cDNA clone rjcaeb0_001383, mRNA sequence

ATTCCTCTTTCCTCTGATCAAGGGCAACGTTAATGGCGACACTTGGCTCCTTCGAGCCCGTTCCGGCTGA

CTAGAGGACTGATCTCACCTAACCAACGCCAAAAATGGACTCTTCAATGAAATCCGAGCTTGATGGTATG

AAGAGCGCTCGATTTTGGGCATCTACGACTTTCCGGCCATCGTGGGCGGCGGCGCAGCAGCATTCCGGCG

ACTGGTCGGGGAGACCCGGGAGGCAGAAACTTAAGACTTGTACTGTAATTTAAAATTTAGTTTATTAAAT

TTAATGTAAATTTCAATTTAGAAATTATTGCTACTGGGTT

>FM891253.1 FM891253 Jatropha curcas embryo 56-70 (DAF) Jatropha curcas cDNA clone rjcaeb0_001381, mRNA sequence

TCATTGGCAGCTGGAGGATCGATGGTCCATCCACAGGTTCCAGGCATCCTCTTTACACCAATTTGTCCAC

ATTCATTGTCTTTTCGGCCTCTGATATTACCTGAACATGTAACAGTAAGGGTACAAGTTCCTTTCAACAG

TAGAAGCTCTGCTTGGGTATCATTTGATGGCAAGGACAGGAAACAGTTAGCAGCCGGCGATGCACTTGTG

TGCAGCATGGCGCCTTGGCCTGTGCCTACAGCATGCCAAGTTGATTCCACCAATGACTTCTTGCGCAGCA

TTCACGAGGGCCTTCACTGGAACCTTAGAAAGACCCAATCTTTCGATGGCCCTGTGATAAATAATAATAT

TTAGTTTAATTTTTCAAGCAAATCTAGCTAGTATGCATTATAGAAGCGGAAATACATAAGAGTTTGAAAT

TTAGCAAAATTAGTCAACAATTATCTGCAGATGATGATGAAATCTGTTAGCAAGCCTTTGCAGATTTACA

AGAGGTTCAGTCCTGGCTGAAAGAGAAGGACGCTTGGAGATGAA

>FM891252.1 FM891252 Jatropha curcas embryo 56-70 (DAF) Jatropha curcas cDNA clone rjcaeb0_001380, mRNA sequence

ACAGTAAATCCCAAATTTTGTTATTACATTTCTCCAAACAGAAGACAGAAGTCCTTCTTAAACACACAAA

CCAAAGCTCCAACACACTTGGGGATGCAAGCTCAACCAGCCACACATCACCCTATTACAAACTGATCGAT

AAAAAGCAAATCAGCCACACAACATAACAAATAAGGGCATTGCGTGCGTATAATACATTATTAAGTAGAG

TATTAAAGAACACCTAGTGGTGCTCGTGGTGGTGGGAGCCAGGAAGCTTCTCCTTAATCTTATCCATTAT

TCCTTTCTTCTCATGATCATGGCCATACTCAGCTGAATGATAACCACCTGGAGTTGTAGCTGAGGTTGCT

TGAGTTCTTTCTTCCTTATGATGACCTGGTAGCTTCTCTTTTATCTTTTCTTTTAGCCCTTTCTTCCTCC

TTCCACCATGTCCATCATCTTCAGAGGAGCTAGAGCTGGAGGAGCCAGAACGGTGAAGCTGACCAGACTC

TCCGTAGTGTTCTTGGCGGTGTCCCATGC

>FM891251.1 FM891251 Jatropha curcas embryo 56-70 (DAF) Jatropha curcas cDNA clone rjcaeb0_001379, mRNA sequence

GGAGAGCCACAGGAATGCACTGGTTGTGAACTAAGTCAGTCCGGCAGATTCTACAGAGTTGACTCTTCCT

ACGCCACAAACTTGGAAGCCTCCGAACTCGGAAATTTGTCATGGATTTAAATACGGAGGCTATGGCAGCG

GCGATCGGAGTATCGATCCCGGTGCTTCGGTTCTTACTTTGTTTCGTGGCGACAATTCCGGTGAGTTTCA

TCCACCGACTCGCCCCTGGTCGACTCGGCAAGCACCTATACGCGGCCGGTTCTGGGATTATTCTTTCGTA

TCTTTCATTTGGGTTCTCTTCAAATCTTCACTTCTTGGTGCCTATGTTGTTGGGTTATGCTTCCATGGTT

CTCTTTCGCGCCTATTGTGGGATTCTTACGTTCATTTTGGGATTCGGTTACCTCATTGGCTGCCATGTGT

ATTACATGAGTGGAGATGCCTGGAAGGAGGGCGGAATTGATGCCACTGGGGCCTTAATGGTGTTAACTCT

GAAAGTCATATCATGTGCAATAAATTACAATGATGGATTGTTAAAGGAGGAGGAATTACGGGAGGCTCAG

AAGAAATACCGACTCATTAAACTACCTTCATTGATTGAGTACTTTGGTAACTGCCTCTGTTGTGGC

>FM891250.1 FM891250 Jatropha curcas embryo 56-70 (DAF) Jatropha curcas cDNA clone rjcaeb0_001378, mRNA sequence

ATTAATTAGAAACAAAAGAAGGCCACATTTTATTGATTTCTGCAGAAGTTGAAGTTCTACATTTTTAACA

CTCTACAACTCTCTATACTACAATATACGACGGATCCCTTCTCTAAATTTACAAGAAGGATCTCTTAGTT

GCGGCCTTGGCTAGTAGAAGCACCAATCACAATGCTATTTTAGTAGTAACTTACTTATGGTCTCCTGCTG

GTGGGAGTAAACAACATGGACTGTGGATCCCTGTTCCTCTTCACTTGATGAGCTTCGCTTGGAGACATTC

TGTAAGAGTTCATCAGAACATCAAGAGGCATGGCTCTCATCACTGATGTATAACCCGCCAATGGGCTCTT

GATAGGCTGGCTACTTGTTTTAAATGTTACGTACTCGAACCCATTTTCTCCTGCCCTTCCAAGTGCTGCA

TAGAATTGAGGGATCACGAACATGTCTCCTTCTTTCACCCTTTCGTCCATTACTGTGTTTCCACTGTCAT

CCACAATCTGTATGTGCATGTCGCCGCGAAGAGCACACACTATTCTCTGATCGTTTATGGACCAGTGTGG

GCTGTGCATTGCCCTCGGCAGCAGAACGCCTCGCTCAGCGCTCATGTCCATG

>FM891249.1 FM891249 Jatropha curcas embryo 56-70 (DAF) Jatropha curcas cDNA clone rjcaeb0_001377, mRNA sequence

ATTCGAATTCAATAAACCTCAATCCTCAGAACCCTTTCCCAGATTTCCTTCCTCTCTATCTCTCTCTGCA

GCTTCTAGCATGGCCTCCTCTTGTCACCGATTCATCAGCAGATCATCTCTATCGTCCATTAAATCCGCTA

TCAGATCTAACGCTCCAAAATCTCCGATTACTAGATCTCCCACTTCTCCCTCTCTCTCGGCCTCTCCTCG

TTTACCTTCCTCTTCCCTTCCTCGCCGCTTTTCCTTCTCATCCAGGTCTCCGTCTGAGCTAGGATGCGTG

CAGTCGTTGCTGCCGCTACACAGTGTCGTGGCGGTGGCGAGGATGACTTCATGCTTGAGTACGACATCGA

GGAGTTGCCGAGCGCTCTCACAGGGCACATAATTTGAAAGAAATAAATTCAGGACCCGTCAATTATGGCC

AACTGCATCTTTATCAAAGCTTGGCCTGTCAGTTCCAAGGTGACTTGTTTGGAAAGAATCAATATGATAT

GGGAGAACATCTTCACATGAATGGAGAAGCAAAGCCTAGTAGCTTTTCATTTTTTGAAGAGTTTTTGTCA

ATCTATAAAATGCTGCTGAACCTTCAAAGCAGTAGAGCTGATTTTGATTCTCCATAACACACAGGATAAT

TTTCTTCTGAGATGTAGCATTAAATGCTATTGTTTAACACCATCAGTGTATGCTCGATTGGA

>FM891248.1 FM891248 Jatropha curcas embryo 56-70 (DAF) Jatropha curcas cDNA clone rjcaeb0_001375, mRNA sequence

ATTCGAATTCAAGGGCTATCGCTATAAGATGCGATTTGTCTATGCTCACTTCCCTATCAACGCCTCCATT

ACCAATTCCAACACAGCTATTGAGATCCGCAACTTCCTTGGCGAGAAGAAGGTCAGGAAAGTTGACATGC

TGGAAGGTGTCACTGTTGTTCGGTCAGAGAAGGTTAAGGATGAGTTGGTATTGGATGGCAATGACATAGA

GCTCGTGTCACGTTCTTGTGCCCTCATAAATCAGAAATGCCATGTGAAGAAGAAAGATATTAGGAAATTC

CTTGATGGCATCTACGTCAGCGAGAAGGGCACTGTAGTTGAAGAAAATTGAGAGCAGTAGTTCTACTTAT

CTTTATTATTGCTTTATTCAGGGGTTTATAGTTTTTGTTATCTGAATGTCAAGGATATATTTTTGGTTGT

TTTCTAGGAATTCTTGAATTACCAATTTTTGATAATTGCAACCTGTTATGTTTT

>FM891247.1 FM891247 Jatropha curcas embryo 56-70 (DAF) Jatropha curcas cDNA clone rjcaeb0_001374, mRNA sequence

CATTAATAAGCACATTTCCTAACCAGGTAATACAAAGTTACAATAGAAACACTTCATAGCAAGCTGCTTT

TTAAGCACACTTAGACCCACTATTTTATTTATGGCTTTCCCTGTTTATAAGATGCTACATAAACTCTCAT

CTTAGAGAAAGCCCAAGACCCCAATACACACATAAAATTTATTAGCCATAGGTAGCAGTACTAGTAGCTA

ATACCTAGTGTCTGTGTCCCTATAAGTTGTGGTCTGCTGATGGCCAGAACGATCCTTAACATGATCTTCT

TCTTCTTCATGGTGAGCCATACCAAAAGCGCTTTTGACAGTATCAGCAGTTTTTTGAGCTGCATGCTTCA

TTTTCTCCCCAGTTTCTTCCAGAACTCCCTTTGTCTTCGGGGGGGCCCGGTAC

>FM891246.1 FM891246 Jatropha curcas embryo 56-70 (DAF) Jatropha curcas cDNA clone rjcaeb0_001373, mRNA sequence

AGACAAAAAAAAAATTTACAAGAATTCTCAAGTTGCAAACCAGTCATTTGAGTTTCAATTTGAAGACACG

GGTGAAAAACAAATGAACCAAGGAAACACTGGCGACCATCACAAATATGATTTTCAATTCCCAGGGGAAA

AAAAGTACAATGCTGGGGAAATTATTGGAGTTGCTCAACAAAACGACCTTAAAGAGGTAGCTACAAAGTT

TCAAGAAAGCCTTTGCCAAGATAATTATTTCGTCACTGTCGCATAGTCTACCCACAATATTTCTTCAATT

AACCCTCCAGTGCTGATGCTCCCGATATAATCTCAATCAACTCGGTGGTGATGGATGCTTGACGAGTTCT

GTTATAAGTAAGTGTCAAGCGGGGGGGGGCCCGGTAC

>FM891245.1 FM891245 Jatropha curcas embryo 56-70 (DAF) Jatropha curcas cDNA clone rjcaeb0_001372, mRNA sequence

ATTCGTACAATTCTTGTCAAGGACTACTATTATCCAACTTCTTTCTTCTATGATCAGCAACACAATGACA

GATGTAATATTAAGATCAAGGGCGATGATGATCAGTGGAAGGCTCCTAGCCAAGGAAAACACATATGTAT

TCATTGATGAATCTTGTGCTAAAACTGTTATGTCAGCCATTGATGGAAGGCTTAGTTTTGAGAGCCAAGA

TTTGAATGAATGTGAATGTGCACTTGAAGCATTGGGCCAAATTGGATTGTCGGTCCCAGGAGCAACATTG

CTGCTCTCGATGTCGCCTTCTCCAGCAAGACATGTTATCGATGCTGCTTTTGATAGACAAGCACGTGGCA

AACAGTTGGCTGCATTGCATTCTCTTGGAAACATCTCAGGTGAATCTCAGTCCGAGAGCAACAAAGTACT

TAATGCTGATGCGGAAGAAAATCTTCGGCGCTTGATATATGAAACGGCATCCAAGACTTCAAAGCTTACA

CCATCAGGTCTTTTCCTATCAGTCCTTCAACAGGATTCAGAAATTCGTTTGGCGGCTTACAGGATGATTA

CTGGGTTGGTGGCTAGGCCATGGTGCCTGATGGAGATTTGCTCAAAACAGGAGATTGTAAATATTATAAC

TGATCCAATCACTGAAACAACAAAAATAGGCATGGAAGCT

>FM891244.1 FM891244 Jatropha curcas embryo 56-70 (DAF) Jatropha curcas cDNA clone rjcaeb0_001370, mRNA sequence

AACCAACAGGTAATTATATATACTGCAGGTTATTTACAAATAGGGAGAAAGTATTAGCAGTCCTCCCAGA

AAGGGAACGACCATAAACCCCTCTCAAAGTTACAACACACATACTAGCTAAACCCTAGTCATTTTAACGG

GGGGGCCCGGTAC

>FM891243.1 FM891243 Jatropha curcas embryo 56-70 (DAF) Jatropha curcas cDNA clone rjcaeb0_001368, mRNA sequence

ATTCCTTCGTGGACTAATCCATCCATGTAGAAAATATACATATGGGCTCATACAGGCCTCACATGGGCTC

TGTCGTCACTCATCCCAACTATACTTCATTTCTACTTCATTCAAGCCCAACAAAATACAAGACAGGACAC

CAGATATGTAAAATAGACGCCAAAACGACAACAGAACAGCCAAAGAAACAAATCAGAGCTCTTCGTCCGG

CAATTCACCACCGGCACCAGCCACGTTATCCTTGAGATGCTGTGATGTCTTCTTTGCCTTATCTTTTGCC

TCTTCGGACTTGTGGTATGCTCTCTCTTTCGCAGCTCCAACGGGGGGGCCCGGTAC

>FM891242.1 FM891242 Jatropha curcas embryo 56-70 (DAF) Jatropha curcas cDNA clone rjcaeb0_001367, mRNA sequence

ATTCACCTAATCCAGCTTCTAAAATTTCACCAATTTCTTCCCTTCTTTCGNTGGTTGAAGTTTAACCATG

GGCAGAGGTTCTTCTCAGTCTCAAACAACCACCTCAACCACCTCCCGTCCTGGCGGCACGGCTCCTCGCG

GCTCTGCTGCTGCAACAGCTGGTATGCGTCGACGCCGTTTNGGATCTTCCGTTTCCGCTTCTGCATCCAA

CGCCGGAGCTATCGGAAGTGGCAGCAGTAATATGCTTCGGTTTTACACCGATGATGCTCCAGGCCTCAAG

ATCTCCCCTACCGTCGTTCTCGTTATGAGTCTCTGCTTCATCGGCTTCGTCACCGCTCTGCACGTGTTTG

GCAAGCTTTATCGTACTAGTGTAACTCCTGGAGCATAATCTATACTATCCGAAACCATATTCCCAACCCG

AATCCAACCGATGATTCGGATCCTTGATTTGTAACTTAAACTTCTTTGTTTCTCTTAAGCCCTTCCCTTG

TATCAGGCTATGTACAAATAGTAGTTGTGTTGTTG

>FM891241.1 FM891241 Jatropha curcas embryo 56-70 (DAF) Jatropha curcas cDNA clone rjcaeb0_001366, mRNA sequence

AAACAAACAGCAAAAGGCAAAACATAAAATAGGAGGAAAATACATAAGGGGGAAAACAGATACTAAATCT

GATTGGGAATATCAAAATAAACAGAAGTAATAAACAACAATCTCATTAAAAGGCCATCTAAATAACTCCT

CTAGAAGTTCCAACCAAAAGGTAAAACTGGTTTTAAGTCTCGCAAAAAAAAAAAAAAAAAAAAATCAAAA

CACTTAAACAGGGAGCTCAAACTTGAACAATCTCTTCCACCTTAAAACCCTAGCTTAGTACGGGGCCAGT

GCCTGCGCTTAGCATGGTACCTGATAGTGTGGTCGGTTCTCATGCGAATCCAGTGAGGGATGGGCCTGTT

CTGCCTCATCTTCTTCGCCAATTTCTTCTGGATCATGAAAGTCTTGTGTGAAGGGATTTACGCGGCTTGG

GAGCTCGGAGGCTAAATGCTGCAGCTGCAAATGGGGGGGCCCGGTAC

>FM891240.1 FM891240 Jatropha curcas embryo 56-70 (DAF) Jatropha curcas cDNA clone rjcaeb0_001365, mRNA sequence

CTCCCTTCCAATAAAAACAAACAGTTCATTTATTTCCATAAACATCACAAACAAGACTATTTCAGACCAA

CATTGACTATTTGGATTCTTGACAATGTTTGATTACATAGCATTAGGTAATATAAGAGCTTTAAACACAC

AAACAACTAGAGAAATAGCTACTTGAACAAACTTAGAGAACCTTTAGGATTTCATCAACACCGGAGACAG

TGAACTCTCCACCTTCTTCAAGATTTGCCGCCTGCACCTTCAGATTATCAACTAAAAGAGCAAATCTCCT

TGATCGAGTGCCAAGTCCCTTCTCTGTCAAGTCAAGCTGAAGCCCAAGAGCATGAGTGTATGTTGCTGAG

CCATCGGCCAAAAACTTCACATGCTTGTTTTCAGGGTAAGTCTTGGCCCATGCCTTCGGGGGGGCCCGGT

AC

>FM891239.1 FM891239 Jatropha curcas embryo 56-70 (DAF) Jatropha curcas cDNA clone rjcaeb0_001364, mRNA sequence

CAGGAATTCGAATGTTTTTCTCTACATTAACATATCTTTCTCACACTTATCATCAACAAATGGCTGAACA

GTACACAGCCACACCAAGTTCAAGTGCACCACAACACCGCTATGAAGCTGCCTTCAAAGGTCAACAAAAA

GGTCCATCAGCTCAGAAAGTGTTAGCTGTGATCACCCTCCTGCCGGTAGGCGGTGGGCTTCTTGCACTAG

CTGGTATAACCTTAGTAGGTACCCTAATCGGCCTAGCTATTACTACCCCTCTTTTAGTCATCTTTAGCCC

TGATCTTGTCCCAGCTGCATTAGTTATTGGCCTTTCGGTCATGGCATTATTGGCTTCCGGGGCTATGGGG

CTCACAGGATTGTCATCACTCTCGTGGGTTCTGAAATACCTCCAGGAGGTCACTCGGCGAATGCCGGAGC

AGCTGGATATA

>FM891238.1 FM891238 Jatropha curcas embryo 56-70 (DAF) Jatropha curcas cDNA clone rjcaeb0_001363, mRNA sequence

ATTCGAGAGAGAGAGAGAACTATAGTATTCCACATAATATTGTTCTTCTCCAAAAGAAAATGGTTCCATT

CAAGTCTTTAGCTCTCATTGGTTTGGTTCTTGCACTATCAGCCATTGAATCCGAAAGCAGAGCAGCAAGA

AAGGACTTGGGTATTGATCTTGGTGGTGTAGGCCTTGGGGTTGGAGCAGGAGTAGGTTTAGGCATAGGAG

GTAGCGGAAGTGGCTCAGGAGCTGGAGCTGGCTCCGGTTCAGGATCTGGTTCAGGTTCTGAATCAAGTTC

ATCTTCTTCTTCCTCATCCAGGTCTAGCAGTTCAGGCTCTGGTGGAGGTTCTGAAGCAGGTTCATCAGCC

GGTTCCTATGCAGGGTCTCACGCTGGATCGGGTTCAGGCGGTGCAGGTTCTGAAGCAGGTTCTGAAGCGG

GTTCTTCCGCTGGGTCTCGAGCAGGGTCAGGTTCAGGTCGTCATGGAGGAGGTTCCGGGTCTGGTGGAGG

ATATGGTGGAGGTTATGGCACAGGAAGCGGCTCCGGAGATGGTGAAGGATACGGTGAGGGCGGTGGCCAT

GGTGAAGGGTATGGACGTGGCGGTGGCAA

>FM891237.1 FM891237 Jatropha curcas embryo 56-70 (DAF) Jatropha curcas cDNA clone rjcaeb0_001361, mRNA sequence

GGTGGCTACAACTCAGCGCTTCCTATGGCGTTCTCCGCAATGATGCCATGAGATTACCCCACTGGCACAT

AAACTCTCACAGCGTAATTTACGCACTCAAAGGACAAGCGCGGGTTCAGGTGACGGATGAAAACGGCAAC

ACCGTCTTCGACGGAAGTGTAAGCAAAGGACAGGTTTTGACGGTGCCACAAAACTTCGTAGTGGTGAAAC

GAGCAGAAAGTGAAAGATTCGAATATGTAACCTTCAAGACAAGCGATAACGCGATGACCAATGATCTTGC

AGGTCGCTCCTCCGCCGTTAGAGCAATGCCGTTGGCGGTTATTGCAAATGCATTTAGGGTATCAGCAGAG

GAGGCAAGAAGGATTAAGTTTGGAAGACAAGAGACCACCTTGACTATTTCCAGGACCGAGTCTGGAAGAT

ATGCCGAGGAGTAAATAATGAAGTAGCCTTTTTGGGTGTGTAGATAGTGAAAGTAAGTAGTATTACGAGG

GGAGTTATGATCTCACGCTAATGAAGTATGTAATATATTATGAAAATAAGTGATGAAAGTAATGTAATCA

T

>FM891236.1 FM891236 Jatropha curcas embryo 56-70 (DAF) Jatropha curcas cDNA clone rjcaeb0_001360, mRNA sequence

ATTCAATAGCAGCAGCTCTGAAATCATGGCCGTCGGAAAGAACAAGCGTATATCCAAGGGAAAGAAGGGA

GGCAAGAAGAAGGCAGCTGATCCGTTTGCCAAGAAGGACTGGTATGACATTAAGGCACCTTCTGTTTTCA

ATGTCAGAAATGTTGGCAAAACCCTTGTCACCAGAACTCAGGGAACCAAGATTGCTTCTGAGGGGCTGAA

ACATAGAGTTTTTGAAGTATCTTTGGCTGACCTTCAAAAAGATGAAGATAATGCCTATAGGAAAATCCGT

CTCAGAGCAGAGGATGTGCAAGGAAGGAATGTGCTGACAAACTTCTGGGGAATGGACTTCACTACAGATA

AGCTGAGATCACTGGTGCGGAAATGGCAGACACTGATCGAAGCCCATGTTGATGTGAAAACAACCGATAA

TTACACTCTGAGGTTGTTCTGCATTGCTTTCACTAAGAGACGTGCAAACCAGGTCAAGCGGACCTGCTAT

GCTCAGTCCAGTCAGATCCGCCAGATTC

>FM891235.1 FM891235 Jatropha curcas embryo 56-70 (DAF) Jatropha curcas cDNA clone rjcaeb0_001359, mRNA sequence

GAAATAGATTAAACTTTCATTGCATTTCCCAATAGCAAGTAATTCATTAAACATAACTAATACAACACTT

ATTTGTTTCGATTAACCCCCCAATAAACAGAAATACAAACATTTAACAAGACTATTAAACAAAACAAACG

ATTACAACCTTAAAATACAAACATAAGTTTAGCGAACTCCTAAACTTGATCGAACAACACGTAAAACTCA

AAACCCATATATAGATATATAGTTAAATGTCTATATATGTTTAGGCGCATTGGAAATCAGAAGGAACCTT

TTTGCTACAAACATTTAGAAGCAAACTCAAAGAAAGTGGAACATTGAGGTTGATACCCAAAATATTAAGC

TTTAATGGCAGTGCAAAGGCAAACAGCAGCTTCAAGATCAACAAGACCCTC

>FM891234.1 FM891234 Jatropha curcas embryo 56-70 (DAF) Jatropha curcas cDNA clone rjcaeb0_001358, mRNA sequence

CGAATCGTTTTACTGTAAGAAATACTAAAAGCTCTATCTTTCTTTCGTGTTAGGCTTTGAATTAAAAGGA

CAAAAGATGGCAACTGTGGGCAGGAACATAGCAGCTCCATTGCTGTTTCTTAACTTAATCATGTATGCTA

TTGCTTTAGGTTTTGCCAGTTGGTGCCTTAATAAGTATATCAATGGACAAACCTACCATCCAAGTTTTGG

AGGGAATGGAGCAACAGCTTTCTTTTTGACTTTTGCTATATTAGCTTCTATTGTTGGTATAGTATCCAAG

TTGGCAGGTGGCCTTCATATCAGGAGCTGGAGAAATGACAGTCTAGCCTCTGCAGGCTCTTCTTCACTTG

TGACTTGGGCCATCACTGCCCTAGCTTTTGGGTTGGCATGCAAGGAAATAAACCTAGGAGGGCACAGAGG

ATGGAGACTGAGGGTATTAGAGGCATTCATGATAATCTTGACATTTACCCAACTCTTGTATGTCCTGTTG

CTTCATGCTGGGGTATTTAGCAGCAGGTATGGCCCTGGTTACAGGAACACCGAATACGCTGTAGGAGCTG

GTGGGGAACC

>FM891233.1 FM891233 Jatropha curcas embryo 56-70 (DAF) Jatropha curcas cDNA clone rjcaeb0_001357, mRNA sequence

GAGTAGCAAGAACTAATTAGTAATTACAAAAGCTTACAACTTCCTTTTTTTCAAGAAATCAGAAAAATAT

ATATAATAAATAAATATAATATATATAAGAAGGAACATCTATTAATTCCAAGAACTGAACAAAGGAAGAT

TCTACACAGGCGTTGTGTGACATAAAGAGACTTAATGCCACATCAGTCGACAAAGAACAGAGGGGTATTG

GATCCTTAGGCACTTTCAACTGGAATCTGACCCATAATTCCAAAGTTGCTGCGATACTTTGGCCTCCACG

CAGTCCTTCCCCTTACTATCTCAGCACCTTCCTCCAGTCGAAGCAAGTGCTGACACTCAATTTCACCAGC

ATTTCCTAAATTTCCTAAGCCGGTGCCAGAGACAGCAGTCAGCGACTGAAGCACACTATCCCTTCCACAC

TCCCTCCTATATTCCATAATAATGGATGATAGCTCATGACTCTCCAGGATCGGCAGCGGAGCACTCTCAA

GGATCCAGCCAATGTACTTCACATTGTTAACATGTTGGTTGACATCT

>FM891232.1 FM891232 Jatropha curcas embryo 56-70 (DAF) Jatropha curcas cDNA clone rjcaeb0_001356, mRNA sequence

TTTTTTATTTTTTCGGAAAGAGAGATCATCACATAGACATTTCATATCACTCAATTCTGAGATTAAACAC

CCATATTTATTTTGATCAACCAAGTACAATCTAGGACTCTAGGTTCAGCGTGAGAGCTAAAAGCATCCTT

TCTCATATTGGACATATCTGCAATTCTTCATAAAGTCAATGAGTAAAGCATTAGAGGCAATTTCCTGAAA

CCCACTCCACCATTTCATAAAAGTGCTTTTTTGCAAGAAAATCTCTGGAGATACTGCATAGTTCTTGATT

TGTTCCATTAGATAAGCCTCAAAGTTAATTAGGTACTCTCTTGATGACTGTTTGTCTATTACACTGGATG

TTGCATCTTTCAACAATTCACTTGAAATAAGAGCTTCCTCCACCTTTGCCCAGAAGCAAGAATCCTCTGT

CAATATACCAGCCACATTTTGTTTCTTTTTGTTCGTTGGAGCACTTGCAGGTTTCTCTGCACCCTCTTTT

GCCCACTTCTCCAATAGAATGTAATGTTTAGACCTTCCTTGACTCTCGTAGTTTCTTTTGCCAGCTCTAT

AGTACTCTGCTATATCAAGAGGTTC

>FM891231.1 FM891231 Jatropha curcas embryo 56-70 (DAF) Jatropha curcas cDNA clone rjcaeb0_001355, mRNA sequence

AACCAACAGGTAATTATATATNACTGCAGGGTTATTTTACAAATAGGGAGAAAGTATTAGCAGTCCTCCC

AGAAAGGGAACGACCATAAACCCCTCTCAAAGTTACAACACACATACTAGCTAAACCCTAGTCATTTTAA

CCCACATGTAAACTACATTACTAAACTAAAGTACACTGCTAAGCTAATACTTTATTACCAACATTATTAT

TATTATTACTACAAAATACCATTTATAGTTTTAATCATCACACTAGCTCCCAGTCCTGAACTTCGACTCA

TCGATAGTGATGCCCTCCTTCTCAGCTCTTTCTCCACCAGACTCGTCCGTCGTGCTCAGCCCACCTTTCC

GGCCCATCTCCTGATATCCTTCTGTCCCCAGCTGCTCCTTCCTTGTCTGTCCTCCTCTGCTTCTCCCTTC

AGCAAGATGCTCTTGAGCTTCAAGGCTCTTGCCCCCAGTACCTCCAGGGACAACAGTCTCTCCACGCCTT

GCTCTTTCATCAAGCTCTTGTCTTGATTGATTTGAAGGGGGGGCCCGGTACCCAATTCGCCCTATAGTG

>FM891230.1 FM891230 Jatropha curcas embryo 56-70 (DAF) Jatropha curcas cDNA clone rjcaeb0_001354, mRNA sequence

ATTCCACACAACTACCGTTTGGCGGGTGTTGTTTCTCCATCTCTAAACATGGCGGATGTTGAGACCGAAG

TCGCTGCTGCAGGCCAGCCAAAGAAGAGGACGTTTAAGAAGTTCAGTTTTAGAGGAGTTGATTTGGATGC

TCTATTGGACATGACTACTGATGATCTCGTCAAGCTCTTTCCTGCTCGCGCTCGCAGAAGGTTTCAGCGA

GGTTTGAAGAGGAAGCCAATGGCACTTATTAAGAAGCTTCGCAAGGCGAAAAGGGAGGCCCCAGCTGGTG

AGAAACCTGAGCCTGTTAGGACTCACCTGCGCAACATGATTATAGTACCTGAGATGATTGGTAGCATAAT

TGGAGTTTACAATGGCAAGACCTTTAATCAAGTTGAAATCAAGCCTGAAATGATAGGGCATTATTTAGCC

GAGTTCTCAATCTCCTACAAGCCTGTTAAGCATGGAAGACCTGGTATTGGTGCTACTCACTCATCCAGGT

TTATCCCTCTCAAATGAAGATTTTGCAGATGTCTTAACTTCAATGGGAAGTGCTTTCTTATGTTTGTCGT

TAAATGACCTTTCTGTTAACTGCTTTTCATCTCT

>FM891229.1 FM891229 Jatropha curcas embryo 56-70 (DAF) Jatropha curcas cDNA clone rjcaeb0_001353, mRNA sequence

ATTCCAATCTCAGAGCCACCGCCCCTCGAATGCCGCCGGAGGAAATGCTACCAATAACAATCCCACCACT

ACCAATGATTGAGAGTTTTATTTTGTTTCATGTAATCTGAGAGCGTGTCGTAGAAAAAAGAAGTGTTAGA

ATGTTTCTTCTGCTGCTCTCCAGAAACTATATGTGTAATAATAATTTAGTTTTTTTTT

>FM891228.1 FM891228 Jatropha curcas embryo 56-70 (DAF) Jatropha curcas cDNA clone rjcaeb0_001352, mRNA sequence

ATTCATTCAAATTTCTGCCCTATCAACTTTCGATGGTAGGATAGAGGCCTACCATGGTGGTGACGGGTGA

CGGAGAATTAGGGTTCGATTCCGGAGAGGGAGCCTGAGAAACGGCTACCACATCCAAGGAAGGCAGCAGG

CGCGCAAATTACCCAATCCTGACACGGGGAGGTAGTGACAATAAATAACAATACCGGGCTCAATGAGTCT

GGTAATTGGAATGAGTACAATCTAAATCCCTTAACGAGGATCCATTGGAGGGCAAGTCTGGTGCCAGCAG

CCGCGGTGGGGGGGCCCGGTAC

>FM891227.1 FM891227 Jatropha curcas embryo 56-70 (DAF) Jatropha curcas cDNA clone rjcaeb0_001351, mRNA sequence

ATTCGTCAATTAGAACAATTAACAACTGAGAATCGCCGGCCCTGGCAATTCCGTGACCAGCGCTCTCAAC

AGGATGCGTGTAGACTCGAACGCATTAACGCTGTGGAGCCTTCCCGTAGAATTCAATCAGAGGCCGGTGT

AACTGACATCTGGGACGAAAACGACGAGCAGTTCCGATGCGCTGGTGTCGTAGCCATGCGTCATACAATT

CAAGAGAGAGGCCTCTTGTTGCCCCTATATGCCAATGGTCCTAAACTCATTTACGTGCTCCAAGGTAGAG

GTATCCACGGAGCCGTATTTCCAGGTTGTCCTGAGACATTCCAGTCACCATCAGAACCACAGGAACGGTA

CGGATTCGGTGCAGGAGAAAGAGAGAGTCGGAGATTTGATCAACACCAGAAAGTTCGTTCACTCCGACAA

GGTGATGTTATTGCTTTGCCAGCTGGAGTAGCTCAGTGGATTTACAACAGTGGCCGGACTCCTCTTGTTC

TTGTCCAAATTATTGATACTGGCAATGCTGCCAACCAGCTCGATGAAAATCACAGAGCGTTCTTTCTTGC

CGGTAATCCACAAC

>FM891226.1 FM891226 Jatropha curcas embryo 56-70 (DAF) Jatropha curcas cDNA clone rjcaeb0_001350, mRNA sequence

CAAAATCAAAAGATCATCTTATGCTGCTGTTAAATAAAAGACAATATTAGGTTTCTAATTGGGAATTCAA

CGATAATAATAGTTGCTGCTACAAGTTTAATCTCTCATCTCATAAAAAATACAATCTCCAGACACAAGGA

AAAGAAAGAGGGGGGAGACAAGTCTGATCTGAATAGAAAGTTTGGATTGCAAAATTTTAGGCAGCCCCAG

CTCCCTTGCCCTTCTTCCTCTGGATCTTCTTCATGTAGAACTCCAGCTCCTTACCCTCCAGAATATACCC

ATCAGCACGGCCACACTGACCAGGGCGTGATGAGATACAAGCCATTAGACGACCACCAGCAAATTGTTCT

TCAATATGAGGATCAAGCTTGCGAGTCTGCTGTCTCTTCTCCAGCTTTCTCAAAACATGATTACTCTTCT

TCACTTCCTCAGTAGCACCTTCACCTTCCTCTCCTTCCTTCTTGGTAGCCGCAACTTTCTTCTTGCGGCC

AATATCAACTCCATAGTGCTGAAGATACCACTGTTTAAATGGTGCAGCATCAACCTGGACAATGGCACTC

TTCACCAAGGTTTGAGTACGAACCAGCTCATTGTTAGATGCATTGTACACCACATCAAGAATTCTGGTCT

TTCTAGTA

>FM891225.1 FM891225 Jatropha curcas embryo 56-70 (DAF) Jatropha curcas cDNA clone rjcaeb0_001349, mRNA sequence

ATTCGTCATCGTGAAGAAGGTGGCGGCGTTTGGCCTTTCGGAACTGAATCCATCGGTCCAATTAGTATCC

TGAAGAACCCTGTTTTTAGGAATAACTATGGTCAACTCTTTGAAGTTGGCTCTAGAGATTTCAAGCAACA

GCTCCGAGACCTTGACCTTTCAGTCTCCGTTGCCAACATCTCTCGTGGAGCAATGTCTGGGCCCTATTAC

AACTCCAGAGTAACCAAGATATCAGTGGTTTTGGATGGCGAAGGCTACTTTGAAATGGCTTGTCCTCACC

TTAGCGGCTCGCGGGGAGACACGACGGGTGAAAGTCAGAAGACTTACCAGAAAGTAAGTTCACGGTTG

>FM891224.1 FM891224 Jatropha curcas embryo 56-70 (DAF) Jatropha curcas cDNA clone rjcaeb0_001348, mRNA sequence

TAGAATGGCTTTAAGCTTAGGAAGGAGGAGCAACATCTTTGATCCGTTCTCCTAGATATTTGGGACCCAT

TTGAGGGCTTACCTTTCTATAGCGGAACAGTGGCCAATGTTCCGTCCACTCAACACGAAACAGCCGCCAT

TGCCAACGCACGAGTGGACTGGAGGGAGACTCCGGAGGCTCACA

>FM891223.1 FM891223 Jatropha curcas embryo 56-70 (DAF) Jatropha curcas cDNA clone rjcaeb0_001347, mRNA sequence

TTTGCAGCTTGCAATTCGCGGTGATGAAGAACTTGATACTCTGATTAAAGGTACCATTGCTGGTGGTGGA

GTCATCCCTCACATCCACAAATCACTTATCAACAAATCATCCAAGGAGTAGACTCTCAAGGATTTTACCG

TGTCAATTTGCCAAAGGGAAAACAGTTCCTACTTTCTGTTACTGTTGATCTGGTCAAAACCTACTGTTTA

AATTGTTAGCTCTTACTTATTGTTTTTTCATTTCGGTTGGCCTTAGTAGGTTTCTCATACACTATGGATA

TTTGGATAATGACCTTATTTGGCAACCTTTTTCTTTTT

>FM891222.1 FM891222 Jatropha curcas embryo 56-70 (DAF) Jatropha curcas cDNA clone rjcaeb0_001345, mRNA sequence

TTTTTTATTTTTTAATCAGGAATTATTATATTTTACATTATTAAAATGAAACAGTAATAATAAACATTCT

TTCAGTACATACTTTCTCACACACATATCATTATATATATCATATATATATATATATATATATATATATA

TATATATATATATTGAGATAACATCATTATAAGCTGAAACTTAACCAGAGATCTCAATAGCCTTAACATC

AGGTTTCTTGACTTCCTCTTTAGGAACAGTGACAGTAAGTACTCCGNTTTTCCATTGAAGCCTTTACTTG

ATCCATCTTTGCATTCTCCGGCAGCCTGAACCTCCTCATGAACTTGCCACTGCTACGCTCCACGCGATGC

CACGTGTCGTTCTTGTCTTCCTTCTCCACATTTCGGGGGGGCCCGGTAC

>FM891221.1 FM891221 Jatropha curcas embryo 56-70 (DAF) Jatropha curcas cDNA clone rjcaeb0_001344, mRNA sequence

TCGAATCCTTGGATTGGTGTCGCAGACATGGGCANAGAAATTGGGAGGCCATGCTCAGAGACCCCTCACT

TTCTTTCTCCAAGCATAAAACCATAGAAGATCTATCGCGACGGTGGGAGAAAGAAAGGCTTAAAATCTTG

TATCNCTGGGAACTTACCAATTCATGGATCAGGTAACTTACCTTCCGACATGTTCAACAACTCANGTTGA

CTATAGGAAGAAGCGTCTTAAAGCAGCTGATAGTACTTTACCATTTNGTTGAGGATCAGCTGCCTTCATN

GTGGGGAAAAGAAAGTCAGCAAACCTTCTTCTGGTGGATTATTAACATTTCCATGTTGAGAAGAGACCGA

AGAGAGTGAGAAATCGAACCAGACTCACAGCTTAAAGCAAAGAATTTCTTCAGAAGACACCATTTCTGAT

TAGAATTGCATATCATTATTATCTAATATTGTCCATAC

>FM891220.1 FM891220 Jatropha curcas embryo 56-70 (DAF) Jatropha curcas cDNA clone rjcaeb0_001342, mRNA sequence

CGAATCCGGAGCTCCGAGAGAGATACAGAAAGAGAGAATCCTGAAGAAATTTCGGCTAAACCTTCATCGT

TTAGCCCTTATTCTCTCTTTTCGTTTCTCTCTTTAGCGCTACCTCCGATTTCTTCAACATTCAGCTTTCT

TCGTCGGCCAGATTGGTGCATATCTGCCATTACTCCCTCGCCAGTTCTTAAACTCGGTCTTCGATCTTCT

TGAGTTATACCAAAATGGTGTTCTGGGTTTTTGGCTATGGTTCGTTGGTGTGGAACCCGGGATTTGAATA

TGATGAGAAAGTGATAGGCTTTATCAAGGATTACAGACGAGTATTTGATCTTGCATGCATTGATCACAG

>FM891219.1 FM891219 Jatropha curcas embryo 56-70 (DAF) Jatropha curcas cDNA clone rjcaeb0_001341, mRNA sequence

TTTTTTGGAAAAGATATGGTCATCACACATACCTTTATTTATNCTGAAATCCACAGAAGCCGGTAAAATC

AAAATATTTAAATATATATTCCATCCTGCCGACACACAAGTTTGCTCGATCGAGAAATGAGCAGCTCCTG

TTCCCTTCCCATCTCCTTCATTACAAGCCTTATGAGTCTTGATTACAAAGCTAAAATACCATCTTAATAA

AAGTTTGTTGTACATATTCATAGTAGATAAAATTCTCTATCAGCAAACATAAACTAGAATTGCTTTCACC

ATCAGCTATGGCTAATGCTATAGCATCATTTTTAATTCTTAGGACCAATATCCTTAAGAGCACAGATCTG

CTCCTCTCCCATTGCAGACATAACAGTCACCACAACGTCCTTCCCCTCAGCAAACCCATCTTTAATCTGG

CTAAGCAGATTTTCAT

>FM891218.1 FM891218 Jatropha curcas embryo 56-70 (DAF) Jatropha curcas cDNA clone rjcaeb0_001340, mRNA sequence

CGAACAAACTTATTCTGATGCTCTTACTTCTTATACATCATTATATATAACTCTCACTCACATACAAGTG

AATGAAGTTTTATTCACCTGCAGCTAGCATTTTAACGAGGGCATATAGACCCAAAGCAAACATCAATTAT

ATTTTTAGTCCTGCGCCTTGCCTCATCTTCGCTTCCAACTTCTCCTTCCTCCAGCTGCTTCTCTAACAGA

TAATGTATTGATTCACATCGACAAATAGCACTTAGTTCCTTTGCTTGGTTACAGCATTGTCTAGGAACCT

GCTGTCGAGGGTTTTCAATTCCTGGCATAGCTAACATATCTTCTGACCTTCCTCTTCTCTGTGTTATATA

ATTCTCACAGGAACT

>FM891217.1 FM891217 Jatropha curcas embryo 56-70 (DAF) Jatropha curcas cDNA clone rjcaeb0_001339, mRNA sequence

ATTCCCGTCTGCAATAGAAGAGGAGGAAAGAAGGAAGCAATTCAAGCGGAACCCTAAAACAGAATAGATT

CTTCTAGTCGAGTATGGCAACCAATTCTAATTCCAGTGTTGGGCTTGAAGTTAGTAACACTCTTCAGTCT

ATCTGCTACAAACGCGGCTCGCTTCATCTCCTCGACCAGAGAAAGCTTCCTCTCGAGACGGAATATCTGG

TTATCGGAGATGCATCAGATGGATGGTCTGCTATAAGAGAGATGGTGGTCCGTGGGGCACCTGCTATTGC

TATTGCAGCAGCTCTCTCACTCGCAGTTGAAGTTTTTAACTTGCAGGATTTAATGGTACATCTGATGATG

CTGGCTCGTTCCTCTTCCAGAAATGGACTATCTCGTTTCAAGCCGGCAACTGCAGTGAATCTTTCAGATG

CTGA

>FM891216.1 FM891216 Jatropha curcas embryo 56-70 (DAF) Jatropha curcas cDNA clone rjcaeb0_001338, mRNA sequence

ATCAAAACAAGGCAAAACATTTAGTTGACAAATAAGTACTTGGCTCCACTCTCAAATTAACATAATAAAA

TATTCTAAAACTTATCAACCATGATAAGATCTCAAAAGAGTACAACATCTACTCTGGATATCCCAAAACT

TTGCTGCACAAAGTACTCCTAGCCTAGGCCACAAGTGTGCTAGCAGTGGTTGACTCGTATTTCCAAACAG

GAGGTAGCTTCTTCGTTTTCTTGTAGTATCGAGCAGCCGTGAATCCGGCTCTCTACAGGATCAACCTGAA

CTTGGAATCCTTATCTTTTCTGTTCCTCTCCAATGCTTCCTAATTGCAACAGCCTTCTTGATGAGATGAC

CAAATCCTCAGGAATTTCAGGAGCAAGACCATGAGCCTTAAGGATGCGCAAAATCTTGTATCCAGTGACA

CTCCTCAACTGAGCAATACCATGAGAATCACGACGAATGACACCAATCTGAG

>FM891215.1 FM891215 Jatropha curcas embryo 56-70 (DAF) Jatropha curcas cDNA clone rjcaeb0_001337, mRNA sequence

ATTCAGAACTATCATCACCACCGTTGAAGTCGACGATTCTTCCAATGTTCGGGATATATGCAAGAAGGAA

GCTGAAAGAAGAGATTTGAGTTCCTGTGAGAATTATATAACACAGAGAAGAGGAAGGTCAGAAGATATGT

TAGCTATGCCAGGAATTGAAAACCCTCGACAGCAGGTTCCTAGACAATGCTGTAACCAAGCAAAGGAACT

AAGTGCTATTTGTCGATGTGAATCAATACATTATCTGTTAGAGAAGCAGCTGGAGGAAGGAGAAGTTGGA

AGCGAAGATGAGGCAAGGCGCAGGACTAAAAATATAATTGATGTTTGCTTTGGGTCTATATGCCCTCGTT

AAAATGCTAGCTGCAGGTGAATAAAACTTCATTCACTTGTATGTGAGTGAGAGTTATATATAATGATGTA

TAAGAAGTAAGAGCATCAGAATAGTTTGTTCG

>FM891214.1 FM891214 Jatropha curcas embryo 56-70 (DAF) Jatropha curcas cDNA clone rjcaeb0_001336, mRNA sequence

CGAATTCCAAGAGAATAGCTGGGGAACTTATTGTCCTGGAATGGACCCTTCTCCACCTCCAGAATTCATT

ACTTGTTTAGGGGATTTGTATAGTGTTGCTTGGATGGAGGACAGCGAGACTCATAATCTGAAGAGGGAAA

CCATAAAGCAACAATATAGGACGGTAAAGGAGAGGACTTCTAATTATAACACATATACTGCTGGGTCTCA

TGTGATGGAATATGGAAATTCAAGCCTTAAAACAGAGAAACTCTACTTATATCAAGGTTTCGACCCCGCA

AATGTCAATTTGCCCCCAGTTAATGCACATGTCAATGCCCCTATGGAAGTTGTTAACCAGAGAGATGCAG

AGCTTCTTTTCATGTGGCAAATGTACAAAAGATCAGATGAGCAGTCAGGAACGAAAGCACAAATCCTACA

GCAG

>FM891213.1 FM891213 Jatropha curcas embryo 56-70 (DAF) Jatropha curcas cDNA clone rjcaeb0_001335, mRNA sequence

ATTCGGCTTTTCAATCGCCACAACGTTCTTCTGCAATCCTTCCGTCATTGGTGCATGTCCGTCATGTTTC

GTCAAGGGAGCGACGGAAGAGGAGGAAGCCAATGACACCAGTAACCTCCAAAGTCAAGAAAATCAAAATG

AAGTCTTATTCGTCCTACAAGTCCAGATTTCGGACATTGAGCGACGGGAGCATTCGTCGTTGGAGAGAGG

GCAAGAATCATAATGCGCACTTGAAGTCAAAGAAATCAAGGCGCAGACTTAGAAAACCTTCTACTGTGCC

TGTAGCTTATGCCAAAGTTATGAATAAACTTAATTTCTGTGGTTAAATCTGTGACATGCATTCTGCCAGT

CATCTTAAGGAGATAGGGAATACGTTTTCAGAGCAGAGTTTTGTGGCTAACAAAATTTTGCTGCAGATGT

TCAGAGCAGTATTTTTGAACATTTTTGCCTTTCAATCTCGGTCAATCTTACCTCCAGACGGCGGCTTATG

TTAGTCTTTTAGCACATCTGTTTGCTTATGGAATAACAAGAAATTGTACTTTGTAATAATTTTACAATTC

AGTTATCAAATTACCATAGTTTTGGATTTTACCCTTGGTTTTGAGATTAATATT

>FM891212.1 FM891212 Jatropha curcas embryo 56-70 (DAF) Jatropha curcas cDNA clone rjcaeb0_001333, mRNA sequence

ATTCCAACCGGTTACCCTACAGGGACCCAGCAAATGTCGGCCATGCCTGGACACGGCACAGGGCAGCCAT

ACGGTGGAGAGGTGGACCCAACGGGTTTGACCAGGACTCGCCCATCTGGGTTGCCCGGAGACACCACTGG

CCATAACCCACGTGTGTAGGGCATGGCATGTGCTTTAGCTCCTGTTATGTAATATATCTATGTTTTCTGT

TTGGTGTCTTTTTCTTGTT

>FM891211.1 FM891211 Jatropha curcas embryo 56-70 (DAF) Jatropha curcas cDNA clone rjcaeb0_001332, mRNA sequence

ATTCGTTTCTGTCTAGACACACAGAAGTCGAATTCGTCCGAGAGGACTGATTTCTGCTGACTATGTACCG

AGTTGTTTCGTTTGCATTCGCCCTCTTCGTTCACACGTCTCATGTCCTGTGAACCTATCAAAGACGCACA

GTGACTTACCCATTATTCATGGACTGCACACATTCGTGCCTTAAAGTGTTGTCGATGCTCACCTGTCAGA

GCAATCAGCAGTCCTATGCGAGTACGTTTTTTTCGTCAATGGGATGTTGATTGCACTGAACCATGCTGCC

TTCGTCGACAACCGTTTACATGGCTGTGAAATGAGTATATATTTTGGTTTTGAAGATTGTTTTTCCCTTC

CCTTTGTTTCTGTTTCTGTCTAGACACACAGAAGTCGAATTCGTCCGATAGCAATAAGTCTGATTTATTA

TTACTCATTTGCAGTGCCGGTCCATCCATCCTTGTGCCTGTCAGCGTTTGCCTACCAAAATACTGATCAT

CAGTGTGATTCAACTGGTCTGATAGAATGTACCGAGT

>FM891210.1 FM891210 Jatropha curcas embryo 56-70 (DAF) Jatropha curcas cDNA clone rjcaeb0_001331, mRNA sequence

CGAATTCATCTTGCAAAAAGTGTCACGACACAGTAAGGAAGGTGAAGCTTACAATTCTCATGTGCCTCCG

ATGCTTACCCGTCGTTTAGGAATCGGAAGCTTGCCTTCGTCTGGGGGTTTTTTGACTACCCAACTCTAAA

TCCTTATTGCGGTGACTTCTTGAGTTGAGGTTGTAAAATTGGTGCCATTGTAAATAGAGAGATAGGCTCA

TTACTGATTTTGTTGACATCCTGACCACCTCCATCACCCGTTGTCCTGCTTGTTCGAGTTTGTATGCATG

ATGTATTACAAATCAAATTCTCTCCAACTCGTTTGTACTGTGTAAGATGATTCTTGGAAGGCTATATAAA

GCTCTAAAATATAATAAC

>FM891209.1 FM891209 Jatropha curcas embryo 56-70 (DAF) Jatropha curcas cDNA clone rjcaeb0_001330, mRNA sequence

ATTCGGAATAGTTTACCCATGGGAGACTTATACNTGGATGCGTGCAGTTGGTTTTAACCCANATGTCTCT

CTTGTTATGGCCCTGGTCATCAATGGAGGCCTGAGCTATCCTACTTTGCCCGGGTGGTTTCCGTCGCCCT

TTTTACCCATATACATACGCAACATACACAAAGCCAAGCATGGTAGCGATTCAGGAACTTACGACACGGA

AGGACGATATATGCCCGTGAATCTTGAGAACATTTTCAGCAAGTATGCTAATACAGCTCCTGATAAGTTA

ACATTTGGCGAGCTGTGGGACATGACACAGGGACAGCGAGTTGCCTTCGATTTATTTGGATGGGTTGCAG

CCAAGTTAGAGTGGGGTATACTCTATGTTCTCGC

>FM891208.1 FM891208 Jatropha curcas embryo 56-70 (DAF) Jatropha curcas cDNA clone rjcaeb0_001327, mRNA sequence

ATTCCTCAGTGATGGCGTCGTTAATAGCGAGAGCTCTACCGATTATTCCTCAGTCACTGAACTCATCTTC

TCGCTTATCGTCGTCTCAATCCGAAAACCTAGGCATTTCTCTTTCCTATTCAACACCAACTCTGTCCCTT

TCTCTCTCTACCGCTTCCTCTTCTTCAGTCCCATTTGTGTATTGCGGAAGAGGAGACAGGAAGACCGCGA

AGGGGAAGCGATTCAACCATTCGTTTGGGAACGCGAGGCCTCGGAACAAGAAGAAAGGTAGAGGACCGCC

GAGAATACCAGTTCCTTCTTCTCCGCCTAAACCTGATCGATTTGAAGAGGACCAAGTTGTTAAGATCGAT

ATCGATGAGTCTCTCACTTAACGTTTTGTAATTCCTAAAACCTCTAATTTGCTTCTGTTATGATTAGCAT

TGTATGTGTTGTGTTGTTGCCTTGCCAATTAGAATTCTATTGAGCCTTATGAATTTCCAGCAACTTATCG

TATGATTCTTTGATTTAAAATTCTTATAGTTGCTTATTGTG

>FM891207.1 FM891207 Jatropha curcas embryo 56-70 (DAF) Jatropha curcas cDNA clone rjcaeb0_001326, mRNA sequence

ATTCATCCAACTTCTGTTCTCTCTGGTTTTGAACCAGAAACACTTTCAACTGCCTTTAATGTCACAATGG

ATGAACTTAGGGATATCATGACAAGGCAAAATGAGGGTCCAATCGTATATTTGGACGATTCTCGAGCACC

GAAAGCAAGCGTATGGAAAAAGTTCATTCAAATGAAGGAGCAAGACAGACTTCAGCACCTAAAGAGAATG

ATGAAGTTCCAACAAGAACCAATGGAAGAAGAGGAAGAAGAGGAGGAAACAACATGGTCATGGAAGAAAC

TCATGAGTTCTGTTTTCGGGCAGGAAAATGAGAAAAAAAAGCGTGAGAGGACCGGTAAATCACCAAAATC

TTATAACATGTACAAGACCCCCGACTTCAAAAACAACTATGGATCCAGCATTGCCCTGGATGACACTAGT

TATGATCCTCTCAAACACTCTGGCATTGGAGTATACTTCGTCAATCTCACTGCGGGATCAATGATGGCAC

CGCATGTGAATCCAACAGCAACAGAATACGGAATTGTTCTGAGAGGAACTGGCGTAATACAGATTGTGTA

TCCAAACGGAACACAAGCA

>FM891206.1 FM891206 Jatropha curcas embryo 56-70 (DAF) Jatropha curcas cDNA clone rjcaeb0_001325, mRNA sequence

GTTCTTCTAGTGATCATAGCCAATGCAGTCACTGGCTACAGAACTATCATCACCACCGTTGAAGTCGACG

ATTCTTCCAATGTTCGGGATATATGCAAGAAGGAAGCTGAAAGAAGAGATTTGAGTTCCTGGTGAGAATT

ATATAACACAGAGAAGAGGAAGGTCAGAAGATATGTTAGCTATGCCAGGAATTGAAAACCCTCGACAGCA

GGTTCCTAGACAATGCTGTAACCAAGCAAAGGAACTAAGTGCTATTTGTCGATGTGAATCAATACATTAT

CTGTTAGAGAAGCAGCTGGAGGAAGGAGAAGTTGGAAGCGAAGATGAGGCAAGGCGCAGGACTAAAAATA

TAATTGATGTTTGCTTTGGGTCTATATGCCCTCGTTAAAATGCTAGCTGCAGGTGAATAAAACTTCATTC

AC

>FM891205.1 FM891205 Jatropha curcas embryo 56-70 (DAF) Jatropha curcas cDNA clone rjcaeb0_001324, mRNA sequence

NTCGAATTCCAAAAACATCTCCCACAATAATATCATCTTTATCCTTTCATTTGCACAAGAAGAAGAACAA

GAACAAGAAGAGATGGGAGTGGTGGTAATAGATGGATCCACGGTCCGTAACTTTGTGGAGAATGAAGCCC

ACTTCAAGAAGAGCGTGGAAGATACGTTCGCCAAGCTGGACCTTAACAACGACGGGGTGTTGTCCCGCTC

TGAGCTCCGACGAGCCTTCGAGTCGCTGCGTTTGGTGGAAACCCACTTCGGAGTGGACGTCACCGCCACA

CCAGAGCAGCTGACTCAGTTGTACGACTCTATCTTTGAGTCTTTTGATTGTGACAAAAGTGGCACCGTTG

ATATTGAAGAGTTTAGAACTGAAGTCAGGAAAATATTGCTTGCGATTGCTGATGGGTTAGGATCCAGTCC

CATCCAGATGGCTCTTGAACGATGATGATCAGAGTTTTCTCAAGCAGGCTGCCGATCTCGAAGCCTCTAA

GCTTCAGTCTTCTG

>FM891204.1 FM891204 Jatropha curcas embryo 56-70 (DAF) Jatropha curcas cDNA clone rjcaeb0_001323, mRNA sequence

ATTCCATGACTCTGTAAAGATCACAAGTTTGATATCGCTTTTGAATTTTTTGATGAAATGGTGTCGAGGG

GTCTTGAGCCTCGGCTTTCAACACATGCTGTATTTATCAAGGGATTCTTCAACTCACAGCAGTACGGGGA

GGCATACAAGTATGTTGTGGGCTCAGATGATAAGTATTCAGCCAGTATGAATTACACCTTGCTTGCAAGC

CTTCATCAGAAGAGGGGAAATCTGGTTGCTGCTGAAAATATTCTTTCTGAAATGATTAAGAAGGGTCTTA

GACCTCATTTTAAAGTGTATATGAAAGTCTTAAAGAACCTCGGGAAGTCAGGCGACGAAAAATTGGCTTT

AGATTTGCAACAGAAGTTTTTCCAGCTTGAACATAGAGCTTAGTACAAGTATCACATGGGTTTTTTTTAT

ATTTTTAATTGTAGCAATTAGTTTGCCCTGTGACTGACTAAGTTGTTTAATTGTAATTGACACATGTAAA

GGGTAGTCAANGTTTAAATTTTGATTAGGAGTTTGTCTTAACCAGATCTATAAGCTAA

>FM891203.1 FM891203 Jatropha curcas embryo 56-70 (DAF) Jatropha curcas cDNA clone rjcaeb0_001322, mRNA sequence

AGAAACAAAAGAAGGCCACATTTTATTGGATTTCTGCAGAAGTTGAAGTTCTACATTTTTAACACTCTAC

AACTCTCTATACTACAATATACGACGGATCCCTTCTCTAAATTTACAAGAAGGATCTCTTAGTTGCGGCC

TTGGCTAGTAGAAGCACCAATCACAATGCTATTTTAGTAGTAACTTACTTATGGTCTCCTGCTGGTGGGA

GTAAACAACATGGACTGTGGATCCCTGTTCCTCTTCACTTGATGAGCTTCGCTTGGAGACATTCTGTAAG

AGTTCATCAGAACATCAAGAGGCATGGCTCTCATCACTGATGTATAACCCGCCAATGGGCTCTTGATAGG

CTGGCTACTTGTTTTAAATGTTACGTACTCGAACCCATTTTCTCCTGCCCTTCCAAGTGCTGCATAGAAT

TGAGGGATCACGAACATGTCTCCTTCTTTCACCCTTTCGTCCATTACTGTGTTTCGGGGGGGCCCGGTAC

>FM891202.1 FM891202 Jatropha curcas embryo 56-70 (DAF) Jatropha curcas cDNA clone rjcaeb0_001321, mRNA sequence

ATTGAACAAGTATTGGGAAGAATGGAAGCGCCAAAATCAATTGCTCATGAAATCGGGGGCATCCAAAACG

ATGCTCTTCGATTCGGCCTTCATGGCGTCAAGAGCGACCTCGTCGGAGCTCACCCTCTCCAAACAGCCTA

CCAATCGTCCAGGAAAACCCAAGAGGAGATGAATAGAAAAATCCTTGCAAACACGTATGGGTCAGCCTTC

CCGTTGAAGATGGATCTTGATAGGCAAATTCTTTCCCGTTTTCAGAGGCCACCAGGACCAATTCCATCTT

CAATGCTAGGACTTGAAGCTCATACAGGAAGCTTGGATGATTTNGGTTTTGAGGATTATTTGAACGATCC

TCGCGAATCTGAAACTCACCGGCCTCTCGAC

>FM891201.1 FM891201 Jatropha curcas embryo 56-70 (DAF) Jatropha curcas cDNA clone rjcaeb0_001320, mRNA sequence

ATTCGGAGGACGCGAGNGAGACTGAAAGAGAATAGACAAGAAGTGACTCTGTTCAGCCCTGGGACTAGGC

CTTCTTCTCGCAATTGAAGGGACTAAAAAGGCAGCTATGTAATTGTAATAAGGAGAGAAAAAACGCTTAG

CTAGTGGCTATGTAGGAGAGTAATAGAGAGGTGTAAAAATAAAGCCTTTATAAGTGGGCATGATAAAGAC

CTTGTTTGGTTCTTTGCACTATCATTAATGTAAATTATATCTTGCGGGAAAAAAAAAAAGGGGGGGCCCG

GTAC

>FM891200.1 FM891200 Jatropha curcas embryo 56-70 (DAF) Jatropha curcas cDNA clone rjcaeb0_001319, mRNA sequence

GGTGTTTACATATCACAATTCATTTAAATTTCAACCTCGAAAAACAAAATAAGTGGAGAAGAATGAAATA

AGAGGATTTACAGGNCTACAATGAGGAAAAGTTTTCAAGTTCATCCTAAAATCTAAATTCTGCACGGTTT

TTTCAACTCTTCCTTCGCTTTCTTTTCAAGAAAGGCATTGCACTGAGACGTCCAATAGTTTCACTTCCAG

TGGTTTTAGCCAGAGATGAATTGGCTTTGTTAATTCACAACGCGAGTCAGTTATATTGCCTTCAGCATCT

GCAGCACCACCAGACAAGTTGAAGGAGGCGGCTAATTCTGAAAGGGTGCAAAAATTGATAAAGAGCTTTG

ATAATGATATGATAATGGGTTTTGATTAGAGAAGAGTAATGTAAAGAATAAAACGTGGTTGCAAACACAG

ACTGTAATAGCTGTTGGTAGTAGCTTTTTTTTACCCTTTTCGTTTATCTAGTTATCCTCAGAGTCGTTAA

TGAGTTTCTCTTAGTTTGTGAATAAAATTACTTTCTTTTTCCTC

>FM891199.1 FM891199 Jatropha curcas embryo 56-70 (DAF) Jatropha curcas cDNA clone rjcaeb0_001318, mRNA sequence

ATGTAAAATTTAAATGGTATTATTCTGTATTCTGTATTCATCAATGATTATTCTACAAGNGAAAACTTCC

ACAATAAAAAAATGAGATGCAAAATGCTCTGGATGATGCAAATGTAGCTGCAACTCATCACAGTTTCTGT

GGTATTGGATCTGAAGTAGGAAGCTGAGCATACATATTTGATATGGCTTTGGCAAAGTATTCCTTTGCTT

GAGGCCTCTTGATCTTGAAAGTTGGAGTAAGGAGGCCATTCTCCATAGAAAATGGTTCAAGCACAAGTGT

TACAGCTTTTGCAAATTCGAAACCTCTCAACTGGGCTTCCTTTCCAACCTCATCCATCTCAGCAAGCACT

GCAGCTCTTGCTCTTGGATCATTGCACAACTGTCCTAAATCATCATACTTAATCCCCTCATATGCAGCCC

AATCTCTCAACACATCTGGCTCCACAACAACTACAGCTACTAAAAAGGAGTTAAAGCTGTCACCATATAC

AAAACACTGGGAAACAAATCTGCATTTGGCATATACATTCTCAATCTTTTCTGGTGCT

>FM891198.1 FM891198 Jatropha curcas embryo 56-70 (DAF) Jatropha curcas cDNA clone rjcaeb0_001316, mRNA sequence

ATTGTGCGTTCTCCTTATGGTGTCGTGCATGGGAAAAGTCTCCTGTGTATTATGATGAGAAAGGGGAAGA

TGGCTTGTTTTCTGGGTTAGAAAACCCTTTCACTGCTGGGAGATATCATAGCCTTGTGATAGAAAAGGAC

AGTTTCCCGAGCGAAGAACTCGAGATTACAGCATGGACAGAAGATGGACTAATAATGGCTGCTCGACACA

AAAAATATAAGCATCTTCAGGGGGTGCAGTTCCATCCAGAAAGCATTATAACATCGGAAGGCAAGAGTAT

CGTCCGCAATTTCGTGAAAATGATAGAGAGAAAAGAGGCTGAATCTCAGAACTAGTATAATGTCATCCAA

TAAATAGAAAAATAGGGAGGAAAGTTTAATCTTTGTCCTGATACTTCTCTTTACCCTGTGTGTCAATGTT

TTGCAACTTTTAAGTCTCATATAATTTCTATAATTTTCAGTTTTCAGTTG

>FM891197.1 FM891197 Jatropha curcas embryo 56-70 (DAF) Jatropha curcas cDNA clone rjcaeb0_001315, mRNA sequence

ATTCGGCGAGGTCGAGGAGCGGTTCAAGACTGGAAAGAATAGGTGGTTCTTTACCAAGCTCAGGTTTTGA

GGATTCATTTCTTAAGAGTTTTGTTTGGTTTCTTTATCTTTTATAAGAAAGGAGTATGATCTTGTTATTG

TAAGACTTGTTAAGTAGTAATCGTTAATTTTGGATTTTAAGATTTGTGTTTTGATGAACTTACTCAGATC

TGATCGGATGAGTTGTTTCTAAGTTTATACAATGCATTTTGAATCTTT

>FM891196.1 FM891196 Jatropha curcas embryo 56-70 (DAF) Jatropha curcas cDNA clone rjcaeb0_001313, mRNA sequence

ATTCCGTTTCATAACACACAGCCACCACTAAACCCTTCTTTTCTCTATCTCTATCTCCATCTCATTCTCA

TCTCGTCGCAGCCATGTCGAAAAGAAAGACTAGAGAGCCTAAGGAAGAAAATGTGACACTTGGACCTGCT

GTCAGGGAGGGTGAGCATGTTTTTGGAGTTGCCCACATCTTTGCATCTTTCAATGATACGTTCATTCACG

TGACTGATCTTTCTGGAAGGGAAACCCTGGTTCGTATAACTGGTGGGATGAAGGTGAAAGCTGACAGAGA

TGAGTCTTCCCCATATGCTGCCATGCTTGCAGCTCAGGATGTTGCACAGAGATGCAAGGAACTTGGTGTT

GGTGCTCTTCATGTTAAGCTGCGTGCTACCGGAGGGAACAAAACAAAAACACCTGGCCCCGGTGCACAGT

CGGCCTTGCGTGCCCTTGCTCGATCTGGGATGAAAATTGGTCGTATAGAGGACGTTACACCAATTCCAAC

TGACAGCACTCGCAGAAAGAGTGGTAGAAGAGGGAGAAGGCTGTGAGCTTCTTTCCTTAGTGGTCGAGGA

TCTTGTTTCTTATGTGGGAATTATCTGGAATGGATATTATATTGATCTCAGCTAAAGTCAGTCTT

>FM891195.1 FM891195 Jatropha curcas embryo 56-70 (DAF) Jatropha curcas cDNA clone rjcaeb0_001312, mRNA sequence

CGAATCCTTTGCTCTCATCAAAGACTGAGGAACGAAAGGTTGATGTGGAGAAAGAGTTTGGCTCCATGCT

TCAGCTTTCAAACAAGAAGGGCAATGATGACATCTTTATCAAACTGGGTTCTGAAAAGGACAAGAGAAAG

GATGCTGCTGACAAAGAAGACCGAGCCAAAAAGTCTGTCAGCATAACTGAGTTCCTGAAGCCTGCTGATG

GAGAGAAATACTACTCTGGTGGCCGTGGTCGAGGCCGTGGCCGTGGTTCAAGAGGTGGATATGTTGGAGG

CAATGCACGTGATGTTGCAGCCCCCTCAATTGAAGATCGTGGGCAGTTCCCCTCCTTGGGTGGCAAGTGA

GATGTTACTTCATCTTTCATGGTCGCAGAGGGCATCTACAGTCTGAAATTCCTTTGCTATGTGCTTGGGA

GCTTGATTCAGAGGTTGTCAACAGGAAATGTTATTTCATGCATTATAAACCTTTTTTTTTTTTTTAACAT

TTAGTCGTTTGGGTGTAAATTTTAGATATTCCTCCATGGGTATCTGCCTGGGATTCCTTCTCGACTTTGA

TATGCATGCATACTCTTTTTCCCCCCACCCC

>FM891194.1 FM891194 Jatropha curcas embryo 56-70 (DAF) Jatropha curcas cDNA clone rjcaeb0_001311, mRNA sequence

AAGATGATACATTACTTTCATCACTTATTTTCATAATATATTACATACTTCATTAGCGTGAGATCATAAC

TCCCCTCGGTAATACTACTTACTTTCACTATCTACACACCCAAAAAGGCTACTTCATTATTTACTCCTTG

ACTATTTCCAGGACCGACGGGGGGC

>FM891193.1 FM891193 Jatropha curcas embryo 56-70 (DAF) Jatropha curcas cDNA clone rjcaeb0_001310, mRNA sequence

ATTCTTTTTTTTCCCGCAAGATATAATTACATTAATGATAGTGCAAAGAACCAAACAAGGTCTTTATCAT

GCCCACTTATAAAGGCTTTATTTTTACACCTCTCTATTACTCTCCTACATAGCCACTAGCTAAGCGTTTT

TTCTCTCCTTATTACAATTACATAGCTGCCTTTTTAGTCCCTTCAATTGCGAGAAGAAGGCCTAGTCCCA

GGGCTGAACAGAGTCACTTCTTGTCTATTCTCTTTCAGTCTCCTCGCGTCCTCCCTTGAAACCTGGAATA

CGTTTGCCAACACCTCTTCTGGAATTGATCTGATTGCCGAGACACGTCCTGCTAGTTGGTCAATCTTGGC

ATTGTCATTAGTCTTGAAGGATACCCATTCAAGACCTTCGCTTCCTGCCCTCTTTACTACCACAAAGTTT

TGTGGAGATGTTAATATTTGTCCTGCTTGAACTTCTCCATCAAATACTGTCTCTCCATTATCATCTACGA

TCTGGATATGGCCATTTCCTCTTGTGAAGTAGTTTATGCTATGGGCATCGACGTTCCAGTGTGGTGACAT

TAGAGCATTTCTGTAGAGGACACCTTTCTGAACGCTTAGCTGGATATAACTGAGGATGGGGAGGTCTTGG

CCGTTGATGTTGGTGAGACGGCCAGCACGTGGGTTG

>FM891192.1 FM891192 Jatropha curcas embryo 56-70 (DAF) Jatropha curcas cDNA clone rjcaeb0_001309, mRNA sequence

AAACCAACAGGTAATATATATACTGCAGGTTATTTACAAATAGGGAGAAAGTATTAGCAGTCCTCCCAGA

AAGGGAACGACCATAAACCCCTCTCAAAGTTACAACACACATACTAGCTAAACCCTAGTCATTTTAACCC

ACATGTAAACTACATATACTAAACTAAANGTACACTGCTAAGCTAATACTTTATTACCAACATTATTATT

ATTATTACTACAAAATAGCATTTATAGTTTTAATCATCACACTAGCTCCCAGTCCTGAACTTCGACTCAT

CGATAGTGATGCCCTCCTTCTCAGCTCTTTCTCCAGCAGACTCGTCCGTCGTGCTCGGCCCACCTTTCCG

GCCCATCTCCTGATATCCTTCTGTCCCCAGCTGCTCCTTCCTTGTCTGTCCTCCTCTGCTTCTCCCTTCA

GCAAGATGCTCTTGAGCTTCAAGGCTCTTGCCCCCAGTACCTCCAGGGACAACAGTCTCTCCACGCCTTG

CTCTTTCATCAAGCTCTTGTCTTGATTGATTTGAAGACATTTTTGCCCTTTTTTGCTAACAAATTTCTCC

ACCAATATAATTAATCTTTTGCTCTGTTACACTTTATGGGGGGGCCCGGTAC

>FM891191.1 FM891191 Jatropha curcas embryo 56-70 (DAF) Jatropha curcas cDNA clone rjcaeb0_001308, mRNA sequence

ATTCTCAGCCTCCGAACAAGAGTAGAACTACCTTCTTATTGAGTTATGGTTTCCTATTTCCATGTTAGGG

ATTTACTTTAATAGTGTATTGGCTTTATTTCAAGTTGCAATTTTTGAATAGTGGCTGTACTGTACTGTTA

AGCCATTTATGTTTTGGATAATTGTTACTTAAATATTTTGCTTAAGATTTTCT

>FM891190.1 FM891190 Jatropha curcas embryo 56-70 (DAF) Jatropha curcas cDNA clone rjcaeb0_001307, mRNA sequence

CCAATTGATCAAAGCGACGCCGCTGCTATAAAAGCTGCAGAGGTGAGAGCTCTTCGCAGTACTCAAACCC

CGGCGGGTGGAATAGGCGCAGAAGCACAGTCGGCAGCTGATCGTAATACCAGAGTTATGCTCGACGAAGA

TAAGACTACCCTCTCTGATGTTTTAGCGGACGCGACTGCCAAGTTGCCTAGAGACAAGACGGTAACTCGG

GATGATGCTGAAGGTGTGATTGGAGCGGAAATAAGAAACAAACCTAATATGAGGACTACGCCTGGTGGAG

TTGCTGCTTCTGTGGCTGCAGCTGCTAGGCTTAATCAGAACCCATAATACCATTCACGTTCTGTTTTCTT

TTTCTTTCTTTTTATAGAGAAGTTGCCTTCGTTTTATATAATTGCTGCACTGTCGCTTGCTATATGAACC

GTTCCCCTGTTTTGCTAGTTACGAATGATGGTGGCCAAAGTGATACACTTTTTGTCGTGTCCTTTCGAGA

ATGTATAAACTTTGAATCTCTATGTAAATAGACTTTTACTTGGTTTCCTGC

>FM891189.1 FM891189 Jatropha curcas embryo 56-70 (DAF) Jatropha curcas cDNA clone rjcaeb0_001306, mRNA sequence

AGTCATATAATAAGTTCATAGCACAACTTATCTCAAGGTTCGGCAAATTATCGCGAGTAGTCACAATACT

TAAACAAAACTCAAAACTAAAAATCTTAAATGTCATTGTAAAAGCTTTTGACTCTAATGATACAACTTCA

TCAGTCAAAGCCTCCCATGGACCAGCTGCTAACAATCCAAGAGGCCCATCACACCGAATTGCAATCCGTA

ATATTTCATATCAAGAGTTTTTCAATAGCATCCTTCAAGTGTTGTATTTGAGTCTTCAATTGGCTACCCT

CATTTGTTGTCACAGAACAGGCAATAACAGGTCTTGTGACACCACATGCTCGGCCAAGTGCTTGCTTTGA

AGGGACAAATACATAGGGAACGTTCTTATCCTCAGCAAGCAAAGGAAGATGGAGAAGAATCTCGAGCGGC

TCAGTATCCGCGGCCATCACGATAAACTCAGAGATACCCCTATTAAGGGTCTTAGTGGCTTCATTTGCAC

CTTTTTTGAGTTGCTTGTAGTTGGCAGCTTGTTGAACAAGATCCAGAATTGTTATGGTGAGTTGTGCATC

GGCAAGTGGGTATGCCTTAGGGTTCACCGCTTCAACTCCTCCTGTCATTCTTCTTGTTTTGCTTTGAATG

CAAATA

>FM891188.1 FM891188 Jatropha curcas embryo 56-70 (DAF) Jatropha curcas cDNA clone rjcaeb0_001305, mRNA sequence

GAAAATACTCTTTCCAACCCTTCAACTTCAAATCAAGTATCCAGCTAGAATGGCTTTAAGCTTAGGAAGG

AGGAGCAACATCTTTGATCCGTTCTCACTAGATATTTGGGACCCATTTGAGGGCTTACCTTTCTATAGCG

GAACAGTGGCCAATGTTCCGTCCACTCAACACGAAACAGCCGCCATTGCCAACGCACGAGTGGACTGGAG

GGAGACTCCGGAGGCTCACATATTTGCTGTTGATCTTCCTGGGTTGAAAAAAGAAGAGGTAAAAGTGGAG

GTAGAAGATGGCAGAGTTTTGCAGATAAGTGGGGAGAGAAGCAAAGAACAAGAAGAGAAGAACGATAAGT

GGCATCGCGTAGAAAGAAGCACTGGTAAGTTCTTGAGGAGGTTTAGGTTGCCGGAGAACACAATGATGGA

TCAAGTCAAGGCCACTATGGAGAACGGTGTGCTTAAAGTGGTTGTGCCTAAAGAACAAGAGAAGCAGCCT

CAGATTAAGCCTATTGAGATCACTGGCTAGAAGTGCAAATATGGTCTTTTACTACTAATAGATAAAGCTT

TTAACGCTGCAGCTTCTGGAGTTATGGGTATGACTAAAATACAGTACTAGGGGTGAGCGTTTTGCGAAAT

TTGGATGTATGTTGCGTTTTGGTGCTTTTTGTTTGTATTTTTTTGTTGGTTG

>FM891187.1 FM891187 Jatropha curcas embryo 56-70 (DAF) Jatropha curcas cDNA clone rjcaeb0_001304, mRNA sequence

ATTCTCTCATCCTCCNGAGCAGCTAAAATGCCGCCCAAATTTGACCCTTCTCAGGTCGTAGATGTATTCG

TTCGGGTCACCGGTGGTGAAGTAGGTGCGGCCAGTTCTCTTGCTCCGAAAATCGGGCCTCTAGGTCTTTC

CCCTAAAAAGATCGGAGAAGACATTGCTAAGGAGACCGCCAAGGATTGGAAGGGACTTCGAGTTACTGTC

AAGCTTACGGTGCAGAATCGTCAGGCCAAAGTAACGGTGGTGCCATCGGCGGCCGCTTTGGTGATCAAGG

CACTCAAGGAACCGGAGAGAGACAGGAAAAAGACTAAGAATATCAAGCACAGTGGCAACATCTCGCTCGA

TGATGTTATCGAGATCGCGAGGGTGATGAGACCAAGATCCATGGCGAAGGATCTGAGCGGAACTGTTAAG

GAGATATTGGGGACTTGCGTTTCGGTTGGATGTACTGTCGATGGAAAAGACCCTAAGGATTTGCAGCAAG

AGATTACTGATGGTGATGTTGAGGTGCCGCTTGATTGAGATCGCAAGCTTTAGTGAGCTATTTCCTTTTT

CTTTTTCTTCTACTTCTTTTTTTTGTTAAATTTTATAAAACAGAGAAACCT

>FM891186.1 FM891186 Jatropha curcas embryo 56-70 (DAF) Jatropha curcas cDNA clone rjcaeb0_001303, mRNA sequence

ATTGATCGATAAAGTGTAACAGAGCAAAAGATTAATTATATTGGTGGAGAAATTTGTTAGCAAAAAAGGG

CAAAAATGTCTTCAAATCAATCAAGACAAGAGCTTGATGAAAGAGCAAGGCGTGGAGAGACTGTTGTCCC

TGGAGGTACTGGGGGCAAGAGCCTTGAAGCTCAAGAGCATCTTGCTGAAGGGAGAAGCAGAGGAGGACAG

ACAAGGAAGGAGCAGCTGGGGACAGAAGGATATCAGGAGATGGGCCGGAAAGGTGGGCTGAGCACGACGG

ACGAGTCTGGTGGAGAAAGAGCTGAGAAGGAGGGCATCACTATCGATGAGTCGAAGTTCAGGACTGGGAG

CTAGTGTGATGATTAAAACTATAAATGGTATTTTGTAGTAATAATAATAATAATGTTGGTAATAAAGTAT

TAGCTTAGCAGTGTACTTTAGTTTAGTAATGTAGTTTACATGTGGGTTAAAATGACTAGGGTTTAGCTAG

TATGTGTGTTGTAACTTTGAGAGGGGTTTATGGTCGTTCCCTTTCTGGGAGGACTGCTAATACTTTCTCC

CTATTTGTAAAATAACCTGCAGTATATATAATTACCTGTTGGTT

>FM891185.1 FM891185 Jatropha curcas embryo 56-70 (DAF) Jatropha curcas cDNA clone rjcaeb0_001302, mRNA sequence

ATTCTAAGAGTAGTCAAGAATCATGGCAAGTGTAAGCTATTATGATTGTAGCCTGCGTGATATGATGTTG

GCCCATGGGCACAAAGCGCATTCTGTGTGGATATGTTATGAGAGCTCTGACTCCGTGCGTGGGCTATTTG

CAGAGTGGTGGGCAAGTTCCTGGCAATTGCTGCAACGGCATTAGAACACTTTATAATGCTGCTCAGACCA

CACCTGATCGCCAAGCCGTTTGCCAGTGCTTGGAATCTGCTGTTAGTAATTTCCGCTACTCCAGCTATAA

CCTTGGCCTCGCTCAGAGTCTTCCTGCTAAATGCAATGTCAATATTCCTTACAAGATCGACCCCTCCACC

AACTGCAAGAACGTTAAGTGAAGATGATATAGATGATCTTGCAGCATCGAAGCTACTGGTGTATGTAGAC

GGAGAATAATGAAGGCTTCCATAGTCTATCTTGAGGAAGTCTCAGTGTTTGTCTGTAGTGGGCTATAGTC

TACTATTTTGTACGACATTATCGACTTGATGTGTAGGATATGAGACGATGAAGTTGTATAGTGTGCTATC

TAACTT

>FM891184.1 FM891184 Jatropha curcas embryo 56-70 (DAF) Jatropha curcas cDNA clone rjcaeb0_001301, mRNA sequence

GAACCAACAGGTAATTATATATACTGCAGGTTATTTTACAAATAGGGAGAAAGTATTAGCAGTCCTCCCA

GAAAGGGAACGACCATAAACCCCTCTCAAAGTTACAACACACATACTAGCTAAACCCTAGTCATTTTAAC

CCACATGTAAACTACATTACTAAACTAAAGTACACTGCTAAGCTAATACTTTATTACCAACATTATTATT

ATTATTACTACAAAATACCATTTATAGTTTTAATCATCACACTAGCTCCCAGTCCTGAACTTCGACTCAT

CGATAGTGATGCCCTCCTTCTCAGCTCTTTCTCCACCAGACTCGTCCGTCGTGCTCAGCCCACCTTTCCG

GCCCATCTCCTGATATCCTTCTGTCCCCAGCTGCTCCTTCCTTGTCTGTCCTCCTCTGCTTCTCCCTTCA

GCAAGATGCTCTTGAGCTTCAAGGCTCTTGCCCCCAGTACCTCCAGGGACAACAGTCTCTCCACGCCTTG

CTCTTTCATCAAGCTCTTGTCTTGATTGATTTGAAGACATTTTTGGGGGGGGCCCGGTACCCAATTCGCC

CTATAGT

>FM891183.1 FM891183 Jatropha curcas embryo 56-70 (DAF) Jatropha curcas cDNA clone rjcaeb1_001196, mRNA sequence

CGAATTCCAAAGTAGGAAAGCCTTTCTGTTCCATCTCTTCGCGTTGTCTCTAATCTCTACGATTAATAAC

AGATACTTGTATCTCTCTCTGCTTGTTTGTTGTTGGGATAAGCTGAGATTATTTTCAATAATGGCAACTA

GGCTTATGTTTGAGAACTCATGTGAGGTTGGAGTATTTTCCAAGCTCACCAATGCTTATTGCTTGGTTGC

TATTGGAGGCTCTGAAAGCTTCTACAGCACATTTGAGGCTGAGTTAGCAGATGTTATCCCAGTGGTTAAA

ACTTCAATTGCAGGCACTAGGATTGTTGGCCGTCTTTGTGCTGGTAACAAGAATGGGCTTCTCCTGCCCC

ACACCACCACAGACCAAGAACTTCAACACTTGAGAAACAGCCTACCTGACAATGTTGTGGTTCAGCGCAT

TGAAGAGAAATTATCTGCATTGGGAAATTGTATAGCCTGCAATGACCATGTTGCTCTAACACATACAGAT

CTTGACCGAGAAACCAGAGGAAATGATTGCGGATGTTCTTGGTGTTGACGTTTTAAGACCGACAGTTGCT

GGTAATATTCTTGTGGGAAGTTATTGTGCATTCTCCAATAGAGGTGGCTTGGTT

>FM891182.1 FM891182 Jatropha curcas embryo 56-70 (DAF) Jatropha curcas cDNA clone rjcaeb1_001195, mRNA sequence

ATTCCCAATTGCGAGAGACAAAAGGGAGAAGAGAGAGCGCTTTGTCGCTTCCGGTGCCAACAGAAGTTTG

GAAGGGACGAAGGGGAACACAGTTGGGGAGAAGAAAAGAACCCTTATGTGTTTGAAGAAGAGCATTTTAC

TTCAATAATTAAAACAGAACATGGAAGAGCCCGCGTTCTACAGAAATTCACTAAAAAGATCGGAGCTTCT

ACGTGGAATTAAGAATTATCGTGTAGGAATTCTTGAAGCTAATCCTCAAACATTTGTATCTCAGGGCCAT

TGGGATGCTGATGGAGTTCTCTATGTGGCTAGAGGACGAGGAACAATATCTATGATTATTGAGGAAAAGA

GACAGAGCTTTAACGTCCAGCGTGGAGATGTGCTAAGAATTCGTGCAGGCACTCCTCTCTATTTGATTAA

CAGAGACGAAAAAGAGAAACTTTACGTTGTCAGCGTCATGGTACCCGTCAATATTCCGGGCGAGTTCGAG

ATATTCAGTGCGTCTGGTACTGAAGATTCAGAGTCCTTTTACGAGGCATTCAGCTGGGAGTTGCTTGAGG

CTGCTCTTAAAACCCAAA

>FM891181.1 FM891181 Jatropha curcas embryo 56-70 (DAF) Jatropha curcas cDNA clone rjcaeb1_001190, mRNA sequence

ATTCCCCAGATTCGCCATGAAAGGAGGTAAATCAAAGTCAGACACCAAGAACGCCAAGCTTTCCGTGAAC

AAGAAACCCGCCAAGGCAAGTAAGAAGGCGGGTAAGGCAGCGAAGGATCCAAATAAGCCAAAGAGGCCCG

CCAGTGCTTTCTTCGTTTTCATGGAAGAGTTCAGAGAGCAGTACAAGAAGGAACATCCTAAAAACAAATC

CGTTGCTGCTGTTGGCAAAGCTGGTGGAGATAAATGGAAGTCAATGTCAGATGCTGAGAAAGCACCTTTT

GTATCTAAGGCAGAAAAGAGGAAGATAGAATATGAGAAGAAATTGAAAGCCTACAACAAGGGACAGGCTG

AAGGCCCCAAGGAAGAAGAGGAATCTGAGACGTCAATGTCTGAGGTGAATGATGAGGAAGAAGATGATGA

GGAGGGAACTGGAGAGGAGGACGATGATG

>FM891180.1 FM891180 Jatropha curcas embryo 56-70 (DAF) Jatropha curcas cDNA clone rjcaeb1_001186, mRNA sequence

GGCTGCAGGATTCGAATTCTTGACTATTTCCAGGACCGAGTCTGGAAGATATGCCGAGGAGTAAATAATG

AAGTAGCCTTTTTGGGTGTGTAGATAGTGAAAGTAAGTAGTATTACGAGGGGAGTTATGATCTCACGCTA

ATGAAGTATGTAATATATTATGAAAATAAGTGATGAAAGTAATGTAATCATCCTAATTAGTAATTAAGTA

TTCCCTAATAAATATATTCTATAAAAAAGGGGGGGCCCGGTAC

>FM891179.1 FM891179 Jatropha curcas embryo 56-70 (DAF) Jatropha curcas cDNA clone rjcaeb1_001180, mRNA sequence

TTTTTTTTTAAGACAAAAAAAACCAACTAGCATAACAAAAGATAAATTACAATAGCATTTCCCAGAAAAA

GAGAAATTCTTTAGGGTATATTAAAGCTTAAGCAACTCCATAAATCAAAATTCTAGTCTTGATATGACAA

TGGCTGGGAAAATGTAAACCGCACAGCAAAAGCTCATAACAGCACTTTTAATCACTCTAGCTATAGCATA

ACAAAAGAGTCTTGATACTAATACAAAATTAAACCATAAAAAAAGAAGTGGGTAGAGATGCACATATTAA

CGAAGCCTCCTGGCTTCTCTTTCAGACTCAAGAAGGGTGAGCACATCACCTTCTCTAACAGGTCCCTTGA

CATTCCTCATGATGAATCGATTCTGATCATCAAGAAACTTGACTCTGACTTGAGTCACCTGCCCCCTCGA

TCCTGTACGACCCATAACTTTCACCAACACGGCGTGCTTGATTTGTGAATCCATCATGAGAACGATTCCG

ATAAGGATCGCCTGAAGCAAGATAGGGTCAGAGAAAGAAGCTCGGATGGGGGG

>FM891178.1 FM891178 Jatropha curcas embryo 56-70 (DAF) Jatropha curcas cDNA clone rjcaeb1_001179, mRNA sequence

ATTCCGTGTGACCCACGATACACGCTCGATCAAAAGCCCACGACAGGCATCATCTGAGTTCCTGTTCTAA

TCTTTAGAGCTAGCTAGCTAGCTATGGCTCGCTCTCTTGAACCACTGGTGGTTGGGAAAGTGATCGGTGA

AGTTCTTGACATGTACAATCCAGTAGCTGAATTCACTGTGCATTATGGTTCTAAGCAAGTCGCTAATGGC

TGTGAGATCAAGCCTTCTGTTGCTGCTCAAAAACCACATGTCCATATTTTGGGTTCTCGTCTATCCTCCG

ATCTCTATACCCTGGTTATGGTTGATCCTGATGCTCCAAGTCCTAGCGAACCAAAATGGAGAGAGTGGCT

TCTTTGGATTGTTGTAGATATTCCAAAAGGATCCGATGCTAACCAAGGGCATGAGTTGGTGTCGTACATG

GGAC

>FM891177.1 FM891177 Jatropha curcas embryo 56-70 (DAF) Jatropha curcas cDNA clone rjcaeb1_001177, mRNA sequence

ATTCGCTATGGCTGCAGTGGGTTCGATCCAGAAGACGGAGGAAGAGTGGCGGGCTATTCTCTCTCCGGAG

CAGTTTCACATCCTTCGGGAGAAAGGAACTGAGTTGAAATTCACAGGAGAATATGATAAATTCTTTGGTG

AAGGATTTTACAATTGTGCTGGTTGTGGAACACCACTTTACAAATCAGGTACCAAGTTTAATTCTGGCTG

TGGTTGGCCCGCGTTCTTTGAAGGTCTTCCTGGAGCCATAAATCGGAGTCCTGATCCTGATGGGAGGAGA

ACTGAGATTACATGTGCAGCTTGTGGAGGTCACTTGGGGCATGTTTTTAAGGGTGAGGGATTCAAAACTC

CAACAGACGAACGCCACTGTGTTAATAGCATTTCAATCAAGTTTGTTCCTGGAAATTAGTCTTCACATGT

GAAGATGATAAATATTGTCGTGAAAAGACTCTTACTTGATGTGCTTCTGTCTTTCTATTGTTAAATTTGT

TGAGCCTATATCAATAATATATGGGACAGAGTTGGGTGAAAA

>FM891176.1 FM891176 Jatropha curcas embryo 56-70 (DAF) Jatropha curcas cDNA clone rjcaeb1_001174, mRNA sequence

ATTCGTGTTAAACATGTTGGGAATAAAACTCAAGTCCTTTTCAACGAGATAGATAAACCGAAAAAAGAGC

AAGAGTATACGATCATGGATGTCAAATTTATTGGAGAAAATCATATAGTGTGTCACAAGCAGAAATATCC

ATATGCTGTGTATTATTGCCACGCACTCAATGGCACTAAAGTTTACACCGCTTCGGTGGTGGGTGCTGAT

GGTACTAAGGCTAAAGCAGTAGCTGTATGCCATTTTGATACCTCTTCTTGGAATCCAGGACACTTGGCCT

TCTTGCTCCTCAACATGAAGCCAGGGGAAGGAACTGTCTGTCACTTTATCAGAAGTGATACACTTGTTGT

GGTCTCTAATAACTAAATTAGAGTTTAAGCATACTCTGTAGTTGTACTTGTAACTGTGCTACTTTTCTCA

ATAATGCACTCTTTTTAAGTGGC

>FM891175.1 FM891175 Jatropha curcas embryo 56-70 (DAF) Jatropha curcas cDNA clone rjcaeb1_001172, mRNA sequence

ATTCCTATAATCAACACGACATCAGTTAATGCATACAAGGGAAATAAGAAACTGCTTGATTACACAGCAA

CTAAAGGGGCAATAGTGGCTTTCACTAGAGGACTAGCACTTCAGCTAGTGAAAAAAGGCATACGAGTCAA

TGGTGTTGCTCCTGGACCTATTTGGACTCCATTGATACCCTCTTCTTTCAGTGAAGAGGAGGTGGCTAAC

TTTGGGAAACAAACGCCTATGCAGAGAGCTGGCCAGCCAAGTGAGGTTGCACCCTCTTATGTGTTTCTTG

CTTGTAACCATTGCTCCTCTTATATCACTGGCCAAGTTCTTCACCCCAATGGAGGCGTTATTGTGAATGG

TTGATGGAGATAGCTGTGAAGAAAGTCAAAAGTTCTTTTTGTCTTTTTGTTATATATCTAGAGTGTTGCT

GGTTTAATGGTGTCTCCTTCTATAGCTTATATAGGTGTTACATGTCGTGTAAGTATGGATAAATAAAAGT

TGTAACCTTCT

>FM891174.1 FM891174 Jatropha curcas embryo 56-70 (DAF) Jatropha curcas cDNA clone rjcaeb1_001171, mRNA sequence

ATTATAAACAGTGTTACAGGGAAAAACCATAAAGCTAGTTTATTTCAGGGTATCTACAGGCATTTTTTGC

GAGAAAATGATGGTACAAGGGTTGCTGAAATTAACGGTTCTGAGGTAGGAGTTGCCTTTTTCCTTAATTT

CTCTGTTCATGCAACAAAGCCTATGGGCTATGACCACAAGGAAGGAGAAGACACTAGAATTCTCCCTGTT

CATTACTCGGAGAACTATTTTTCGCTTGTGCCAGGAGAGCAGATGTCAATCAAGATATCTTTTGAGGTTC

CTCAAGGAGCCAGCCCACGGATAAAACTTAGTGGCTGGAATTACCATGGCTATCTTTCTGTTTTCTAAAC

CCAGCAAGTACTATCTTTTCCATCGGTCATCTGTCAAAATCTTGCATGATCAGCCTCCTAAATGGTTAGA

AATTCGAGGTTACTTCATCCATGTTGGACATTAAACGGCAAGTGTTCTTATTATTTGGATTCAACTTCTG

GACATTATCGTCTCAGTTGCTCCGGCGTGGAAGTTCTTCTAGTGATACTGAAAAATAAGGGCAATTGTCA

ATCT

>FM891173.1 FM891173 Jatropha curcas embryo 56-70 (DAF) Jatropha curcas cDNA clone rjcaeb1_001170, mRNA sequence

GTTAACAGGTATCTGACAGTTTTCACCATTAATAAATATATTCTATTTCCACAAGCAAAAGAATTGAGTT

GCAGAAAGTCGCTATTCAAATTAACTGCAATTACACGGCAAATCTAATTACCTCAACAGCCCTACTCTGC

GTTCTTCTAGTGATCATAGCCAATGCAGTCACTGGCTACAGAACTATCATCACCACCGTTGAAGTCGACG

ATTCTTCCAATGTTCGGGATATATGCAAGAAGGAAGCTGAAAGAAGAGATTTGAGTTCCTGTGAGAATTA

TATAACCCAGAGAAGAGGAAGGTCAGAAGATATGTTAGCTATGCCAGGAATTGAAAACCCTCCGACAGCG

GTTCCTAGACAATGCTGTAACCAAGCAAAGGAACTAAGTGCTATTTGTCGATGTGAATCAATACCTTATC

TGTTAGAGAAGCAGCTGGAGGAAGGAGAAGTTGGAAGCGAAGATGAGGCAAGGCGCAGGACT

>FM891172.1 FM891172 Jatropha curcas embryo 56-70 (DAF) Jatropha curcas cDNA clone rjcaeb1_001168, mRNA sequence

ATTCCAAATATCACTGATCGTAAACCTCCATTGAATTTACTCTGTGCTTCGATCGCGTCAATGGCGGAGC

ACCCACAGTCGCAGCATGTTGGCCAACAGCCACGATCCCACCAAGTAGTGAAGGCAGCCACTGCAGTCAC

TGCCGGTGGATCTCTTCTAGTTCTCTCCGGTTTGACCCTTGCTGGTACGGTCATTGCACTGACCGTAGCC

ACTCCATTGCTGGTCATATTCAGTCCGGTTCTTGTTCCTGCGGTTATCACAGTTTGTTTGATAATCACAG

GGTTTTTAGCCTCAGGCGGTTTTGGCGTCGCTGCCATTTCTGTCTTGTCCTGGATTTATAGGTATGTTAC

TGGAAAGCATCCACCAGGAGCAGAAAGTCTGGACCAGGCACGCTTAAAGTTGGCGGGAAAGGCAAGAGAG

ATGAAGGACAGAGCTTGAGCAGTTTGGACAGCATGTAACAGGTCAACAGACTTAAAAGAGAAGTGGGTTT

TGGTGGTTGATGCAGAGAAACATAAATGGCTTTGATATATATATAAAAA

>FM891171.1 FM891171 Jatropha curcas embryo 56-70 (DAF) Jatropha curcas cDNA clone rjcaeb1_001167, mRNA sequence

GAAGAAATCAAACAACTGGTGCTGACACCATTCATTAAGCTAGCGTAACAACTGCTGCTGCTGCCATACT

TAGACCAAACACATTTCGACATTTAACATTTAGAACAAAGGAGACAAGCTAGTAACTGGAAAGATGCCAG

TTCACATGTTATTTCAACCTTTTGAGCAACACGACATCTAAATAACAAGAACAACGAGATAAAAGCCTTG

CATGTGGCATCCCAGAACATAGATGTGATACCTTCAGCAGAAGTTTGATGAGGGTCAGATGTTATCAAGC

TTTTTAGCTTTCACAATGGGGTTGGCCTTGGCAATAGCATTTTGTCCAGCAGTTCAGATAATCATAGGTG

CAACCATGTATGTCTGAGTAGCGATGAACTGAACAGAAAAGATTCCCGCAACGA

>FM891170.1 FM891170 Jatropha curcas embryo 56-70 (DAF) Jatropha curcas cDNA clone rjcaeb1_001166, mRNA sequence

GGAATTCCTTCTTTCTGATTTGAAGAAAGAGGGTTATGTTCCTCAATCTTACCTTCCAAATGCACCCACA

AACTTATCGGNATGATGATCATTGGGAAAATTTTGTTGTGTTTTGCAATCTTGGAAGCCAGAAAGAGGGA

CGATTTAGGATATGAATTATGAATGATGGT

>FM891169.1 FM891169 Jatropha curcas embryo 56-70 (DAF) Jatropha curcas cDNA clone rjcaeb1_001164, mRNA sequence

ATGGAAACATCAAATTCATCAACAAACAAAGTAAGACTTGATTACAGAGACCTTCAACACATGACAAGAA

AATTATTTTAGACAGCTCTCAAAAGAGGGAAGACCCGTTAACAAATTACATAGAAGAAAAAAAAAGATTT

AAGCGAAAAAAGAAATACACCAGACAAGCAATGGCCTTCCATGTTAATTAAAGGACTCATATATTTTTAT

ATATATATCAAAGCCATTTTATGTTCTCTGCATCAACCACCAAAACCCACTTCTCTTTTAAGTCTGTTGA

CCTGTTACATGCTGTCCAAACTGCTCAGCTCTGTCCTTCATCTCTCTTGCCTTTCCCGCCAACTTTAAGC

GTGCCTGGTCCAGACTTTCTGCTCCTGGTGGATGCTTTCCAGTAACATACCTATAAATCCAGGGGGGGCC

CGGTAC

>FM891168.1 FM891168 Jatropha curcas embryo 56-70 (DAF) Jatropha curcas cDNA clone rjcaeb1_001163, mRNA sequence

ATTCTTTCCTTTCGTTTCCGGGATCATTTCCAACTTCCAACTTAGGACAACAAGAGCCATCGAGTACTCG

TAGTATAAATATCAATATTTTGTCAGCTGGTGGGACACAAAACGCTTCTGAATCTGAGAGACAGAACAGC

ATTTTGCAGCTCCTGAGGAACCTCCTTCCAGTTGGGGAAATCCATGTGGAGGATGGAGGTTTGCAGGGAA

CAGCTAATGGTTCTAACCCTGAGAATACAGGGGCGTCTACTGTTCCTGTTGAAGCACAATCAGGAGCTAC

TGATGAAGGAATATTCTTGTCTCATTTGCTTCATGAAATTATGCCATTCATATCTCAACGTGGTGGTGCA

GAGCCAAATGTTATTCCCCAGGGAGATTTAGGTGCCTCTGGCAATCAGGGAGCTCAAGATTCATCCACCC

AGGCTGAAAGCTCTGAAGGTGGGGCATCTCGTCGCCGTAATGATACTGAACACAGCCCCCCAAATGCAAA

ACGCCAAAAGACAGAGTGAGATATAACATGAAAGAGTATTGACGTGGTGTTACGGTTGTGATTGATCAGT

GCGGAGGGTTGTGAATGATGTAGCGGTTCTAATGGCCT

>FM891167.1 FM891167 Jatropha curcas embryo 56-70 (DAF) Jatropha curcas cDNA clone rjcaeb1_001161, mRNA sequence

ATTCGAATTCCAAGGTCGTTTCGGCGGCCGATCCGGGCGGAAGACATTGTCAGGTGGGGAGTTTGGCTGG

GGCGGCACATCTGTTAAAAGATAACGCAGGTGTCCTAAGATGAGCTCAACGAGAACAGAAATCTCGTGTG

GAACAAAAGGGTAAAAGCTCGTTTGATTCTGATTTCCAGTACGAATACGAACCGTGAAAGCGTGGCCTAT

CGATCCTTTAGACCTTCGGAATTTGAAGCTAGAGGTGTCAGAAAAGTTACCACAGGGATAACTGGCTTGT

GGCAGCCAAGCGTTCATAGCGACGTTGCTTTTTGATCCTTCGATGTCGGCTCTTCCTATCATTGTGAAGC

AGAATTCACCAAGTGTAGGATTGTTCACCCACCAATAGGGAACGTGAGCTGGGATTAGACCGTCGTGAGA

CAGGTTAGTATTACCCTACTGATGATGGTGTCGCGATGGTAATTCAACCTAGTACGAGAGGA

>FM891166.1 FM891166 Jatropha curcas embryo 56-70 (DAF) Jatropha curcas cDNA clone rjcaeb1_001158, mRNA sequence

TAAACTATGGATCCCGGGCTGCAGGAATCGAATCGCTCTGCTGCTGCTGTTGCCAAGCTTAAGCTATAAA

GGAGTTGGACTACCTTGTTAATAAAAAATAACTATGTTTGTGTTTATTTTATGTTGGTAAAACTTTAAAC

CGATCAGATTGAATACATTGTATGTGTTCTGATCTGATGGTATCCAGTGTCATTCTCAATATTGCAAGTC

TGAATATATGGCTGTTTAAATAGCCCAGTGTTGAAAGAATATTAACATGAC

>FM891165.1 FM891165 Jatropha curcas embryo 56-70 (DAF) Jatropha curcas cDNA clone rjcaeb1_001157, mRNA sequence

AATCCTCTATAACTGAAACAAGTCACTCAAATAGCTAGCAATGGCAGTCGCATTCTATGATCTCGGCTCT

GAGTCTGGCCTTAAGAAACTGGATGACTATCTCCTTACCCGCAGTTACATCACTGGTTATCAAGCTTCAA

AAGATGATGTTACAGTGTATGCTGCTTTGCCAAAGGCTCCATCATCCCAATATAGTGAATGTGTCCCGGT

GGTACAAGCACATTGATGCACTCCTAAGAATTTCTGGTGTATCTGGAGAAGGGTCAGGAGTTACTGTTCA

GGCTTTTGCACCCGTCACANAGGATGCAATTGCAACTCCTCCAGCTGCTGATTCAAAGGCTGCTGACGAG

GATGATGATGATGATGTGGATCTGTTTGGT

>FM891164.1 FM891164 Jatropha curcas embryo 56-70 (DAF) Jatropha curcas cDNA clone rjcaeb1_001155, mRNA sequence

TTTTTAACATTGAGCCAATTGCATATATTATTGCAGTAGAGATAATGATCAATAAACCAAAATCAATACT

GTTTCCTTCAAGACATTAAATGCCAGTAGAATTGTAACTCGAGACAAAGTATTGTTTAACTTTGGTAAAC

ATCAAAGTGAACAGTAAAGAGTAAAGAGTAGGTACGGTAAAACAAGGTCCAAAACTTCTCATCTGCACAG

CTCACCTTTGAAAGGTTCATGTCCCAGTATATTTCTACTGGTAACATATGCAACAAACTTAGCTTTAGAA

GACTCAAGTCCCTGCATTTCTGCGGCCAACACGAGCAACAAAACCAGCTTTGATGGGGGCAACTGATCGT

CCAGAACCACACTTTTCACATCTGAGAAAAAATAGACGATTCTCCTTCGAAAGTATAGTGTCTGGACTTT

TGCAACCAAGGCAAATGACATATTCATTAATATATCTACGCAGGATCCCCTCAAAATTCTTGGGAGCAAA

TCTGCCCTTCACAACTAATCTTTGCTGTCCATCAAGAGATCCACTTGTTCCCAACTCAGCAAGTAAGAAG

GTCATGACATGGTCTGGCTGCCGATGCATTGTCTTGCAGAGATCCATGAAATTCACAAAAACAGTCTTCT

TAGTACCCTCAC

>FM891163.1 FM891163 Jatropha curcas embryo 56-70 (DAF) Jatropha curcas cDNA clone rjcaeb1_001154, mRNA sequence

AGCCAATCCCAATTTTATAACAATATTATATATAATAAAATATTTACTTTTTATTACTTTTTTACCCTCC

TTCCTTTTTGAAAATCTATTTTACACGTCACGCAACACGACAAACCCCCCGTCCTCCGCCTATGGACAAT

ACCAGTACAAAATATTAAAC

>FM891162.1 FM891162 Jatropha curcas embryo 56-70 (DAF) Jatropha curcas cDNA clone rjcaeb1_001153, mRNA sequence

TGGATCCCCGGGCTGCAGGCAAAGGTGGAAACAAAGAGGCGTCGAAGGTGATAAAGTAAATAAACAAACT

ATGTTGATATAGAATGATAATGCAGGAATTGGAGTATCAATCTTTTTAACTTGGTTAGGCTTCAAGCAAA

CACGGAGGCGAAATATTTGCACAACAAGCTGTAGTACTTATGTGTCAGATGGCAGAGGGAAACTCGTTTG

TTATATTTATATGGTAAGGGGTAAGTGACCTATGTTTTCCTTTGAAAGTCGAGGACGTGTTTGGCTGTTT

ATAGTTGTGTTATGTAGTTGTAATCATACTCTGCTTTCTTGTATATATAACTTAGGGTTAGTGTATAATG

GTAACCACTTCAAGCTTATTCAAAGAAAATAGATACAAGAGTCTCGTTATGCCTCTTCTTGTG

>FM891161.1 FM891161 Jatropha curcas embryo 56-70 (DAF) Jatropha curcas cDNA clone rjcaeb1_001152, mRNA sequence

ATTCCTCTGNATTCAGGGAAAATGGGTCGCGTACGTACAAAGACAGTGAAGAAATCCTCTCGCCAAGTGA

TAGAGCGCTACTACTCGCGCATGACACTTGATTTTCATACCAACAAGAAGATTCTTGAAGAGGTGGCTAT

CATCCCTTCAAAACGTCTCCGAAATAAGATTGCAGGGTTTTCTACCCATCTAATGAAACGGATCCAAAAG

GGTCCGGTTCGCGGGATCTCACTCAAGCTTCAAGAGGAAGAGCGCGAGCGGCGCATGGACTTTGTACCTG

ATGTCTCTGCCATCAAGACTGATCAGATTGAGGTCGATAAGGAGACTATTGACATGCTTGCTGCTTTGGG

TATGACTGATATTCCTGGGATTCTCCAAGTCGAACCGGCTGCAGCGCCTGCGACCCAGTTCGTGTTTGGG

AGAGGTGGTGCTGGTGGCAGACCTGGTGGAAGGTTTTAAAAACTTATTAGAAAAATTTTTGTTTTTATTT

CGTTTTATGTTGTTTTTGTTTGACTGGATTTTTATCATGTTGGAAATATTTGTTGCTATGCAATCTATAA

GTACTTGTGATGATTTAGTTAAAAGGGGGGGGCCCGTAC

>FM891160.1 FM891160 Jatropha curcas embryo 56-70 (DAF) Jatropha curcas cDNA clone rjcaeb1_001150, mRNA sequence

GCTGCAGGTTTTTTTTACTCAAACTAATCTACTTCAATAACAATAATTTCCAACTTCCAATTACACTGAA

ATTTAGCGAACTGAATTTAAACAACTGAATCGTAAATATATACCCTTTGCAATTTTGGGTTTCGGAATCG

GGTTCAGTGCGAGAAGCCGGTATTTCTGCCCCCCCGAGGGTCTCGCCGGAGACTCGTCGGCAAATCGCCG

GGTTGTCGCCGGCGCCGCCTTGCTCGGTGGCCGGATATTTCAAACAACCCAGAAATGCACGTATTTATAC

ATCTTTTTGCTCCGTTTCATTAGGAGATCTCCGATCTCATGTCCGACTAACGAGATGGGTATCTCAGAGG

CCGGAAATGGTGATACATCACCGGAGAAAGGATTTGCTACAGTGACAGAAGCTTCAAGCT

>FM891159.1 FM891159 Jatropha curcas embryo 56-70 (DAF) Jatropha curcas cDNA clone rjcaeb1_001149, mRNA sequence

CGAATTCCAACTAAACTTTAAAAAAAATGGCTGATTTAAGAGATGAATATGGCAATCCAATTCGACTTAC

CGATGAACATGGAAATCCAGTTGAATTGACCGATGAACATGGCAACCCAGTGCATATAACTGGCCTAGCT

ACCTCTAAGCCTGCAACCATTGCAAATGTTCTGCAGATCGATGTGCCGGATACTGGCCTTTTGGCTAGTA

CGGAGGAGCCACCAAAGGGCCACACTGGCGAGCATGTAGGTGGTGCTGAACCTGAGCCCGTGAAAGCGGA

GCAACCGCAACAACAACAACAAGAACTTGAAAGATCTAGTAGTTCAAGCTCTAGCTCGTCAGAGGATGAT

GAGCAAGGAGGGAGTAGAAAGAAAAAGGGATTGAAGGAGAAAATAAAGGAGAAGCTGACAGGTGGGAAGC

ACAAGGAAGAGCATAAACAGACAGCAATCACTGAAACAAACCCAGAGGATCCTCAACACCATCAACATGA

GAAGAAGAGTGTAATGGAGAAGATAAAA

>FM891158.1 FM891158 Jatropha curcas embryo 56-70 (DAF) Jatropha curcas cDNA clone rjcaeb1_001148, mRNA sequence

ATTCGAATTCGAGGCAAGAAGGATTAAGTTTGGAAGACAAGAGACCACCTTGACTATTTCCAGGACCGAG

TCTGGAAGATATGCCGAGGAGTAAATAATGAAGTAGCCTTTTTGGGTGTGTAGATAGTGAAAGTAAGTAG

TATTACGAGGGGAGTTATGATCTCACGCTAATGAAGTATGTAATATATTATGAAAATAAGTGATGAAAGT

AATGTAATCATCCTAATTAGTAATTAAGTATTCCCTAATCAATATATTCTATAAATATAATTGTATTCAA

AAC

>FM891157.1 FM891157 Jatropha curcas embryo 56-70 (DAF) Jatropha curcas cDNA clone rjcaeb1_001147, mRNA sequence

ATTTCAATAACCAAAACCGTCATGTTGAGTCTCTCAAAAACCTTCCACTACTCCCGTCCTATCTTTCTCG

GATCCTCCAGTAAAGTCTCCCGGGTCTGCTTCACTTCTGCCTCAAAACATATCCAGAGTCGAAATGCAGA

GGAGGAGAATAGAGACAGAGCATCGGATTTTACTGATATAGCCAGAGAAGCTACAAAAGAAGGCGTTGAA

AGAGCCAGAGAGCGCGCAGAGCGGGCAAAAGAACAGTCTAAGGAAATGAAAGAAAAGGCAAAAGACCGCG

CAGAGCAAATGAAAGAAAACGCAAAAGGTTATGCTCATGAAACCAAAGAAAGTGCCAAAGGCATGGCTCA

ATCGGCTGCAGAGAAGACCAAGGAAGGAGCTCATAAAGCAACAGAGACTGCAGAGAAAACAAAAGAGAAA

GCAGAAGATTATGCATATGATACTAAGGAAAAGGCAAAAGAAGGGACGGGATAAGTAGCGGACACTGCGC

ATGATGTGA

>FM891156.1 FM891156 Jatropha curcas embryo 56-70 (DAF) Jatropha curcas cDNA clone rjcaeb1_001146, mRNA sequence

ATTCATTTGAATAGCCAAGTGAGTGAAAATGGAGTCGCTGAAGGAAACCGCAGCTAACGTTGGAGCCTCT

GCTAAGTGCTGGCATGGAAAAAACCAGAGCAGAGGCGCAAGAGAAGGTGGAGAGAATGTCAACACGTGAT

CCAGTGCAGAAGGAAATGGCGAGAGAGAAGAAAGAAGAGAGGAAAACGGAGGCAGAATTGCGGAAACTAG

AAGCGCGCGAGGATAACGCAGCGGCGAGGCAGGCAGCCAATGCTGGTGGCCATGTACAGTACACAACTGC

TGGTCCGGGCGCTCAAGGCACAACCGAAACCTATACCCATTCAACAACTGGGACAACCGGTTACCCTACA

GGGACCCAGCAAATGTCGGCCATGCCTGGACACGGCACAGGGCAGCCATACGGTGGAGAGGTGGACCCAA

CGGGTTTGACCAGGACTCGCCCATCTGGGTTGCCCGGAGACACCACTGGCCATAA

>FM891155.1 FM891155 Jatropha curcas embryo 56-70 (DAF) Jatropha curcas cDNA clone rjcaeb1_001145, mRNA sequence

ATTCCTTATTTATGTGTGGTTTTGATTTTGTATATGCCCTCAGCCACGTAAATTTGGGAATAGAGAGAGA

TTGCTAGGCGCTATACTAGCATTTGTTGAACAAATTTCATTACTCTGTAGTTTTGTTTGCTTGCTCGAAT

TTCATTGTGTTTGTAAATGCTGTTTAACTTTTGTATAATTTGAGTTATTGAGCCTTAGCCATTTCCTTTA

TTGGGAATAATTTCTGCCCCCTTTTGTTCTTGCCTAGCGGGCTAGTTTGAGTACACAGGAGTGGCTACTT

TGACATCGGCCATGTTATCTGGGGTGGCAGCACGCATTATGACTCATGATAGCTCTATGGAGGCACGTGT

AGGCTGTAGCTGATTGTGTTATATGGATTACTATGTACTTGAATTAAATGAAGACATTAAGTTGTCTAGC

TAACATATAAATGTTAGA

>FM891154.1 FM891154 Jatropha curcas embryo 56-70 (DAF) Jatropha curcas cDNA clone rjcaeb1_001144, mRNA sequence

ATTCGGAAGCTCCAACCGATGCTCTTAAACAGGTTCTCCAGCATTGTACTGGATGGCAGCATGTTCCTAG

ACCAGTTGATCAGAATCAGATATTGCCTGCACAATGTTAGTGAAAATGTATTTATATGGAAAATACTCGT

CTAGTCCATCGGGTACCATTATACCATCTAAGATGATAGATTATGTGTAAATCCCACCTTATATGAGATG

GTATATATATGGTGAGCTAAAGTCTATATAAGAATTGCTGTATTGATTTATTTATTTTTATTTTTATTTT

TATTTTTGATTTTTTGAGAATCTGTATTAATACTTGGTTATTCCTTTCTTTACTT

>FM891153.1 FM891153 Jatropha curcas embryo 56-70 (DAF) Jatropha curcas cDNA clone rjcaeb1_001143, mRNA sequence

ATTCCACAAACCCTCTCTACTTCTCAGACCTACAAACCCTCTCTTTTTCCTCTCCACTCCAAAAGCCTCC

ACAGACAATGGTGCTGGACTTTCCGCTGCTGTAGAGGAACCAAAAGCTGAGCAAAAGGCTGCAGAGCCTT

CTGGCTCGGTGCCGGAGGCTAAAGAGAGCTCTTTGGAGTCTAATGGAGCGGTGGCGGATGGAGAGGTGAA

GCTGGAAAGTAAATTTGTGGACCCCAGGTGGATTGGAGGGACTTGGGACTTGAAGCAGTTTCAAAGGGAT

GGAAAAACTGATTGGGATGCTGTAATTGATGCTGACGTTAGAAGAAGGAAATGGCTTGAGGGTAATCCAG

AATCATCCAGCAATGATGATCCTGTAGTTTTTGACACCTCGATCATACCATGGTGGGCATGGAT

>FM891152.1 FM891152 Jatropha curcas embryo 56-70 (DAF) Jatropha curcas cDNA clone rjcaeb1_001142, mRNA sequence

ATTCGAATTCCACAAGTTTGCGAGGAACCAAAAGAAAATCTAAGGCATGGCTTCTCTAGCAACCTTTGGT

TCTGTGCAGCCAGCCACCGTTAAGGGCCTTGGTGGCAGCTCCCTCTCAGGAACAAAGCTCCATGTCAAGC

CTTCTCGCCAAAGCTTGAGACCCAAACATAAGGAATGGTGCTGTTGTGGCTAAGTATGGTGATAAGAGTG

TATACTTTGATTTGGAGGATTTGGGTAACACTACTGGCCAATGGGACTTGTACGGATCAGATGCTCCATC

TCCATACAACCCACTGCAGAGCAAGTTCTTTGAGACATTTGCAGCTCCATTCACCAAGAGGGGTTTGTTA

CTCAAGTACTTGATCTTAGGAGGTGGCTCCACCTTAGCTTACCTGAGTGCTACAGCTTCTGGTGACGTAA

ACCAATCAAGAAGGGCCC

>FM891151.1 FM891151 Jatropha curcas embryo 56-70 (DAF) Jatropha curcas cDNA clone rjcaeb1_001141, mRNA sequence

CTAGAAAGTTATCAAAATCGACAACTTCGCTCAGCATTACTGTTTAGCAAGTTATGGAACAAATCATCTA

AATGACAAATATAGCTTTAAATAAAAGATTACCAACATGAATTCACATCTCTCAAAATTGAGGTCTTCAA

CTAACGCTATGCCCCCAAAAAACATAAAACCAAAAGAGAAAAACAAGTTTCGTCACGCAACACATGTTGA

TGATCCTTCAAAACTAACTCATGTTTATAGTAAATGTCCAGTACATAGCGTAACATTAAAAAACCTTACT

CCCGGTTACCACC

>FM891150.1 FM891150 Jatropha curcas embryo 56-70 (DAF) Jatropha curcas cDNA clone rjcaeb1_001140, mRNA sequence

AGAGGAAAATACTTAATAGACTTTATAATATTTCTAGTAAACATCAGCCTCATCAGAAACTAATGTCAGA

CGAAAACACCCAAAAAGAAAAACACAAGCAGATTACGATTTCAACATAGACCAAATTATTTCCAGCACGC

AGTCAAACGTGACAAAACGATCCTCTGAGCTCAAGCAATTTTAACCTCAATAGTCTTTGGCTTTTTAGGC

TCAGGCGGTGGCAATTTCTGGACAGTGACAGTCAAAACCCCATCTTGACACACAGCAGAAATGGCATCAG

TATTGGCATTCTCAGGCAGAGCAAACTTCCTCATCAATTTTCCAACTCTCCTTTCCATCCTCACATACTT

AGCTCCTTCCTTCTCCTCCTCACGCTTTCTCTCTCCACTAATCAACAGCACATTATCATCTTCCACCTGC

ACTTTGATATCTCCAGACTTCAATCCAGGCATGTCGATAATGAACACATAAGAATT

>FM891149.1 FM891149 Jatropha curcas embryo 56-70 (DAF) Jatropha curcas cDNA clone rjcaeb1_001139, mRNA sequence

ATTCGATTGAATTTGAATAGCCAAGTGAGTGAAAATGGAGTCGCTGAAGGAAACCGCAGCTAACGTTGGA

GCCTCTGCTAAGGCTGGCATGGAAAAAACCAGAGCAGAGGCGCAAGAGAAGGTGGAGAGAATGTCACACG

TGATCCAGTGCAGAAGGAAATGGCGAGAGAGAAGAAAGAAGAGAGGAAAACGGAGGCAGAATTGCGGAAA

CTAGAAGCGCGCGAGGATAACGCAGCGGCGAGGCAGGCAGCCAAGGCTGGTGGCCATGTACAGTACACAA

CTGCTGGTCCGGGCGCTCAATGCACAACCGAAACCTATACCCATTCAACAACTGGGACAACCGGTTACCC

TACAGGGACCCAGCAAATGTCGGCCATGCCTGGACACGGCACAGGGCANCCATACGGTGGAGAGGTGGAC

CCAACGGGTTTGACCAAACTCGCCCATCTGGGTTGCCCGGAGACACCACTGGCCATACCCACGTGTGTAG

GGCATGGCATGTGCTTTAGCTCCTGGGTATGTATATATCTAT

>FM891148.1 FM891148 Jatropha curcas embryo 56-70 (DAF) Jatropha curcas cDNA clone rjcaeb1_001138, mRNA sequence

ATTCGTTCGTCCGCGTGGGCGAGATGCAGTTTGCCCCTACTGCAGTGGAGGATTTGTTCAAGAACTTGAT

GAATTGGAGCCATTCAGCCCATTTGAGTTCTTTGGATTGGACAGTGATCATGATCGAGACCAAAGATTTG

GCCTCATGGAAGCTTTCTCAGCCATAATGAGGCAGCGATTAATTGATAGAAGCCACGACATTAGGAGAAG

CCATGACATTAGAATGAGATCAGATCCTATATCTATTCCTGGACGAAATCCAGGTTTTGGTCCTCTGTTG

ATTTTTGGTGGTGAAGTTCCCATTAGATTGTCCGGAAATGGTGGGTTTGAAGCTCTTTTCCAAGGTACGC

CTGGAATTGCTTTTACACGAGGTAATGCTGGTGATTATTTTATAGGCCCAGGGCTGGAAGAATTATTTGA

GCAACTTTCAGCTAATGATAGGCGGGGTCCTCCACCAGCTTCAAGATCATCTATTGATGCAATGCCCATC

ATTAAGATTAGCCAGAGGCATCTTCGTTCTGACTCACACTGTCCAGTCTGCAAAGACAAATTTGAGTTGG

GAAGTG

>FM891147.1 FM891147 Jatropha curcas embryo 56-70 (DAF) Jatropha curcas cDNA clone rjcaeb1_001137, mRNA sequence

ATTCAATGCCAAGCTTCGCATTTTTTGGCCCTGGTCTTGTAACTGAGCAAGGGAGTTGTAAATTGGATCT

CACAAATGACATTTCCAATTTGAATGCAATTGATGGAGAGGTCGGCTTGGAGAAGATGCTCGTGTTGCCA

GAGTCTTCTCTATCCTGTGGACGTGAATCCAGAGAAATGGAACACAAGCCAGTTGAAGTTAAAAGGTCAG

GAGGAGCCATTGGTTCTAGAATGGGTATGCACAGTAAGAGGAACCGTACAATTGGGCTTGGTGAGCATGA

GCAACTTGATCTGATGGTGACCGAGTCAGTTATCCGATGGGCATCATGCACACATCTTCCAACTGCTCCG

TCCCTTAGGTCTTCTCTTGTCTGTTGAACCTTGAAAGACAGGACTGTCCCATGACCTGATGCCTAGCAAT

AAAAGACGCTCTGATGGATGACTACAGTGAAGAAAGTGGTTAATCCAGACTAGTTCTATCCGATGTATTT

ATGATGTGTATGGTGCTGTCCTATGTGCTAAATCATT

>FM891146.1 FM891146 Jatropha curcas embryo 56-70 (DAF) Jatropha curcas cDNA clone rjcaeb1_001136, mRNA sequence

ACATTGAAGCTGAAAGGGGAAGTGCAGCAAGGGACAATATTTTTGAGAAGGCAGGATTAGGGAGCATCAA

AGACTCCATTAAAGGGAAGCTTACAATGCCACGGGACGTCGTGGGAGAAACGTGTGCTGCACGTGAGCAT

GGAGGCACAGGAAGAAAGGAAGAGGTGGTGGTTCTTGAAGAAACACCAGCAGGAACCGTGGCATCCACCC

TGAAAGAGTCTGATCAGATTAGTGCTCAAACTTTCAACGATGTTGGACGGTTAGATGATGAAGGTGTTAC

TCATCTAAAGGTCGTCCACCGTTCAGACAATAAGTGATAACGTCCACCGAATTCACGGTGTACTTCCTAC

TTCTTTTACTTTTTCACTTTTACACCACCATGTTCATTAGTAGCTTTTTGGGCCTTCTTGTTGTGCTTTT

TATTTGTTTTGAGTAACTATAAACAAAGCTTTTGTGGGCTTGTTTGCCCTTCTGGATCTTATGTGCCTAA

TCTTATATGGATAAGATTGGAAGGTTATCATCTCAAAAAAAAAAAGGGGGGGCCCGGTAC

>FM891145.1 FM891145 Jatropha curcas embryo 56-70 (DAF) Jatropha curcas cDNA clone rjcaeb1_001135, mRNA sequence

ATTCCTTGATGCTAGAGCAAGGCAAGGAGAGACTGTTGTACCTGGTGGAACTGGTGGCAAGAGCCTTGAA

GCCCAGGAACATCTAGCTGAAGGGCGGAGCCGTGGAGGGCAGACAAGGAGGGAGCAGCTGGGAACTGAGG

GGTACAAGGAACTTGGTCACCGTGGAGGTGAGACAAGAAGGGAGCAGATTGGGCATGAAGGGTATCAGGA

GATGGGCCGAAAAGGCGGACTTAGCACCATGGACAAGTCTGGTGAAGAACGTGCTGCTGAGGAGGGGATT

GAAATTGATGAGTCCAAGTATAAAACCCGTTAGTTAGGTTCTTCATATCAGTGTTTTAACTAGTAGTTCC

TCTTTTTGAACTAGTTGCGAGTAGGTTTGTACATATGGTAGTAGTAACTGCGGTATGTTGGTTGTTTTTG

GTTGGTGTCGGGGTTTGTTTTCTGGGCATCAGTGTGTAACCTGTTGTATTCCCCACTGACAGGAAGTGGC

ATCCCATGGTTTTT

>FM891144.1 FM891144 Jatropha curcas embryo 56-70 (DAF) Jatropha curcas cDNA clone rjcaeb1_001134, mRNA sequence

ATTCGACCTATTTTGCTTCATGTACAGTTTCACTTCCACTCCGGATAAAATATCAAACAAGTACTACTTG

GGACCTGCTCCCTTGGAGGAAATGGCTAGCCAAATTGCAACGGCCCATGGACCTTGTGGGAATAACAGAG

ACTACCTATTCCTGTTAGAAAAAGCCATGTTTGATATTGGACATGAAGATGACATGGTGATTGAATTGGC

AAATGAAGTGAGGAAGGTACTTGTTGCGCTTAATTCCTATGTTCCAGCCCTTAAACTACGTCCACTCACG

GAAGCTGTGGCGCTGGATTCTTAATATTGAAGACAGATAACTGAAGGAAAGGGGGGCATAAACCAGAGCT

CCACTAACTTGATTACACTCCTGGAGAATCCTCTCTGTTTTTTCGTTTTTTACACCCTGCCAAAAAATTA

TTATTATTATTATTATTTTTTATTCGTATGTTCCCGTCATTGCTTCTTAGTTATTAGCATCCACTTGTCT

GTTTAGGCTCATAGCATTGCTGTGTCTCTAACATTAACCATAGTGTTTGAGCAAAAGAGATGTAACTGGG

CAACGGGCGCCCGCTATAAACACTGATAAATT

>FM891143.1 FM891143 Jatropha curcas embryo 56-70 (DAF) Jatropha curcas cDNA clone rjcaeb1_001133, mRNA sequence

GACAACAATGAAAGGAGATACATTCACCATTTAATAGCAATATCATAATTGAAGTTCCCCTTCCAAAATA

ACACAAAAGGCTCCTTAAAGACCAACAAAGCAAAAGTTCAAGACAATACATAAAACAAGAAACAACAGGA

AATCAAATCCCACACGACAGGGTTTGAGAAGGTACCATGGCTTCTTTTATTTACACCAAACATGGATTAT

TACACATAATTCAACTGATTAACCATAAAAGCCCAAGACTAATAACTACTTTGTTGGAGCCTTTCCCTTC

TGGTTTCTCATTAGCAGTTGCATGGGTCACAGCTGCAGTTTGATCCACACTTGCAGCCATGACCACCCTC

AGCTCCAATGCTCATCTCAGAAATCATCATTCAACTTCTTGGATGGTGCAAATCCTGAAATGAGGGTCTC

AGTTCTGGTG

>FM891142.1 FM891142 Jatropha curcas embryo 56-70 (DAF) Jatropha curcas cDNA clone rjcaeb1_001132, mRNA sequence

ATTCCCCAACTGCAAGAATTCATTGAGAGTGAGTTTTTAGGAGAACAGGTTGAAGCAATTAAGAAGATAT

CAGGGTATGTCGCTCAATTAAGGAGGGTTGGTAAAGGACATGGAGTCTGGCACTTTGATCAGATGCTCCT

CCATGAGGGTGATGCTGCGTAATGAACCATGCAGTTGTATTGTTCTTGGTTTAAGTATTTCCTTTACTAT

TGAAACTTTTAGGAGCTTTATTATTGTATAAATGCTTATGAACTTCCATTGCTGTACGTAGGGTGACTAT

AAATGTTGTAATAAAAAGCTTGTAGCTTTTTTGTTAAAAAAAAAGGGGGGGCCCGGTAC

>FM891141.1 FM891141 Jatropha curcas embryo 56-70 (DAF) Jatropha curcas cDNA clone rjcaeb1_001131, mRNA sequence

ATTCCTTGTCTTCACTGTCTGTTACTGTTCGCTAGTGTTGACAATGGCTGAGGCTGCTTCAAATGCTGAG

CTCCTGGAATGGCCAAAGAAGGATAAGCGCCGACTTCTCCATGTCGTGTATCGTGTTGGTGATCTTGAGC

GCACCATCAAGTTTTATACTGAATGTTTTGGGATGAAGCTGTTGAGGCAAAGAGATATTCCAGAGGAGAA

GTACTCAAATGCCTTTCTTGGTTTTGGACCTGAACAAACTAATTTTGTTGTGGAGTTGACTTACAATTAT

GGAGTAACTAAATATGATATTGGAGATGGCTTTGGGCATTTTGCGATTGCAACTCAGGATGTTTACAAGT

TGGTCGAAGACATCCGTGCCAAGGGTGGCGAGATCACTAGAGAGCCTGGTCCAGTCAAGGGCGGAACAAC

TGTCATTGCCTTTGTGAAGGATCCGGATGGTTATACTTTTGAACTCATTCAAAGAGGACCGACTCCTGAG

CCTCTGTGCCAAGTAATGCTTCGTGTTGGTGATCTAAATCGCTCTATCAAGTTCTAT

>FM891140.1 FM891140 Jatropha curcas embryo 56-70 (DAF) Jatropha curcas cDNA clone rjcaeb1_001130, mRNA sequence

ACTAAAAGATACCTTTTATTCAACTGGAATTGCTTAACAAGAAAAAGACACCAAACAGAAAACATAGATA

TATTACATAACAGGAGCTAAAGCACATGCCATGCCCTACACACGTGGGTTATGGCCAGTGGTGTCTCCGG

GCAACCCAGATGGGCGAGTCCTGGTCAAACCCGTTGGGTCCACCTCTCCACCGTATGGCTGCCCTGTGCC

GTGTCCAGGCATGGCCGACATTTGCTGGGTCCCTGTAGGGTAACCGGTTGTCCCAGTTGTTGAATGGGTA

TAGGTTTCGGTTGTGCCTTGAGCGCCCGGACCAGCAGTTGTGTACTGTACATGGCCACCAGCCTTGGCTG

CCTGCCTCGCCGCTGCGTTATCCTCGCGCGCTTCTAGTTTCCGCAATTCTGCCTCCGTTTTCCTCTCTTC

TTTCTTCTCTCTCGCCATTTCCTTCTGCACTGGATCACGTGTTGACATTCTCTCCACCTTCTCTTGCGCC

TCTGCTCTGGTTTGTTCCATGCCAGCCTTAGCAGAGGCTCCAACGTTAGCTGCGGTTACCTT

>FM891139.1 FM891139 Jatropha curcas embryo 56-70 (DAF) Jatropha curcas cDNA clone rjcaeb1_001129, mRNA sequence

ATTCCTCTCTCTCTCTCTCTCTCTCTCTATTTATCTTTTCCTTCTTTTCTCTCTCCCTTACAAGCCCTAA

CCCTAACTCAGACCCAGATTCGCCATGAAAGGAGGTAAATCAAAGTCAGACACCAAGAACGCCAAGCTTT

CCGTGAACAAGAAACCCGCCAAGGCAAGTAAGAAGGCGGGTAAGGCAGCGAAGGATCCAAATAAGCCAAA

GAGGCCCGCCAGTGCTTTCTTCGTTTTCATGGAAGAGTTCAGAGAGCAGTACAAGAAGGAACATCCTAAA

AACAAATCCGTTGCTGCTGTTGGCAAAGCTGGTGGAGATAAATGGAAGTCAATGTCAGATGCTGAGAAAG

CACCTTTTGTATCTAAGGCAGAAAAGAGGAAGATAGAATATGAGAAGAAATTGAAAGCCTACAACAAGGG

ACAGGCTGAAGGCCCCAAGGAAGAAGAGGAATCTGAGAAGTCAATGTCTGAGGTGAATGATGAGGAAGAA

GATGATGAGGAGGGAAGTGGAGAGGAGGACGATGATGAGTAAGAAGAGGGGGAGAGGAGTAACATAG

>FM891138.1 FM891138 Jatropha curcas embryo 56-70 (DAF) Jatropha curcas cDNA clone rjcaeb1_001125, mRNA sequence

AAGTAATATTTGAAATAATTGCATTGATTGTGGCTTCAGTTTAAGAACTGCTCAGACCAAGCACATTGTT

AACCATACAAGAACAGGGGTAGACAAGAATTGCAGGAGGAAGCTAATTGTACATGCATGAAGTTCGCCAA

ATATAAATCTAATCCGCTCAAATTCCGACGAGGGAAATTCAAAAGAAACGCAGAGACATAACATAGTGAC

AACCATCTCCAAAGAAATTTCCAGCTCTACAAAGCTCTATTTGCTAATCAGCATCCATTTCTTCAAGTGC

GGTCCTGGCAGCCGTCTTGGCCTCCTCTAAAAGTTCATAGGGAACCTTCTGATGCAGTCTCTTTG

>FM891137.1 FM891137 Jatropha curcas embryo 56-70 (DAF) Jatropha curcas cDNA clone rjcaeb1_001123, mRNA sequence

ATTCCTTTCTCTCACCGATCTTTGATCTTCTCCAAAAACCAATCAAGAGAATGTCTCTGATTCCAAGCAC

CCTCTTCGGTGGCCGGAGAACCAACATTTTCGATCCCTTCTCTCTCGATATCTGGGATCCATTTCAGGAC

TTACCTTTCACAAGCACTGCTCTTTCTGCTCCTCGTTCTGAGTTATTGAACGAAACGGCAGCGTTTGCCA

ACACCCGCATAGACTGGAAAGAAACACCAGAAGCTCATGTATTCAAGGCTGATCTTCCAGGGCTAAAGAA

AGAGGAAGTGAAAGTGGAGATAGAGGAAGGTAGGGTCTTGCAGATAAGCGGAGAGAGAAGCAAAGAGAAA

GAAGAAAAGAATGATAAGTGGCATAGAGTTGAAAGGTCAAGTGGTAAGTTCTTGAGAAGGTTTAGGCTAC

CAGAGAATGCAAAGGTTAATGAGGTTAAGGCTAGCATGGAAAATGGAGTCTTGACTGTGACAGTGCCTAA

AGTGGAAGTCAAGAAACCGGACGTTAAGGCTATTGAGATTTCTGGTTGAAACTTTTAGTTA

>FM891136.1 FM891136 Jatropha curcas embryo 56-70 (DAF) Jatropha curcas cDNA clone rjcaeb1_001121, mRNA sequence

ACGCCTGTGAAAGGTATCCAGATTTATTACTCAAGCCATAGAACTACATGATATAGATAGATGAAGGATT

TCAGATCACAAAGCCACAGGGAATATAATTGCAGTCAGTATATGTCAACCCTCAGCCCTCAGGGTTTTGT

CCATGATAGGCAACCCAAAAAAAAAAAAAGAAAAGAAAAATAAAAAAGCTCTGCATCACTGGTAAAGCAT

AAGTTACATAGGAGTTTAATCATCATCCTCCAGAACACTCCGCCGCCTCTGGATTGTGACTTTTGTCTGT

TTAGTTGTTTGGTTGTTAATCTGTTCCAGTAATGAAATGAGCTTCTCTTCAGAAACCTTCTCAACTATCT

GACCCATTTGAGCAGCTCTCAGTATAACGTCTTCAACACCCCTTGCTTTCTCAGGTTTAACTAAAGCAAT

TAGGGCCATTCTTTCACGTGCCTCAGCAGATAGAATCTG

>FM891135.1 FM891135 Jatropha curcas embryo 56-70 (DAF) Jatropha curcas cDNA clone rjcaeb1_001119, mRNA sequence

GTTTTTAGGGTGGGAAATGTGTTCTTGCTTCTGATATGATATCAACGCGTTTAGTTGAGCTTATGGGTGT

TAGAGAAGCCCTTGGTTGAAAAACCATGTTGGATGTGTAGCGGTTGAGGTTGAAGGTGACTGCCAGTTAG

TTATGCAAGCTTTACAAGGTTCTGTCGTTGATAATTCTTATTTTGGTGTTTTATTTGATGGTTGTAGAAC

TCTTCTGTCTGAGTTCGCTTCATTTCTATTCTTTTTTTTTTTTTTGAAAATCAGTGAATCAGTGTGCCGA

TTCCTTGGCAAGGGCTGCTAATAGGATGTTTTGAATTTTGATC

>FM891134.1 FM891134 Jatropha curcas embryo 56-70 (DAF) Jatropha curcas cDNA clone rjcaeb1_001118, mRNA sequence

AAACAATACTTAATTACTTATAGTTTTTATTAATCACACTGTTACAGAACATTAACATAATCACATAACC

AGGAGTTGTATTAATGTTTATCTGCAACTCCTTTATTCTCCCTCAAAAATATACACAACACAAGATGACA

TTTCATAGACAACATCCTTCAAGCAAAATAAGCAAACAAGACAGGCATAAAAGCACTCCTTCAGTCTTCA

AACACATTTTGATACGATACAGATTTTATACATATGGTACAAATGTATTGATCTGCACATTTTAATGTGC

TAACTCTGCAAATTGGCTGCAATCAATTCCGTGGGCAGAAAATGCCTCCACCAATTTTGGGAAGATCTTA

GCAAGCATTAGTTTGAAACCAGTAGAACTGCCCCTTTCACATCTATGCTCCATCTGTCCACTTCCAGTTA

GGCATGCTACTGGAGCTCCTTCCATATTTGCTTTACAAGCCAATAGAATATTTTGAGATCGTTGCTGGCA

GTGTTCCTCCATGAGTGCTTCAAGTGCTTTGGCGGGGCGGGAAGCAGTAAAGCA

>FM891133.1 FM891133 Jatropha curcas embryo 56-70 (DAF) Jatropha curcas cDNA clone rjcaeb1_001115, mRNA sequence

ATTCCCGGCCGGCGCTGAGAACGACGGCAAGTGCAAGTGTTTGTCCAAGCTGCACCTGTNGTTGACTGCG

TGTTGTGTGTCATTGAAGCATATAATAAAAATGAAAGAGTGGTGTGTGAGAGTTTACTAATAATGTAATT

GTGCTAAATAATTTGTATTTCCTGTTAAAATACATGTGGGTGTGAAGTGAAATGTGTGAAAGAGCCATGT

CTCTGTTTCCTTGCATACCTTGTGTAATGGCCATATCTATGGCTTCTTGTGTGGTTTAATGAATTACTCT

TAGTAT

>FM891132.1 FM891132 Jatropha curcas embryo 56-70 (DAF) Jatropha curcas cDNA clone rjcaeb1_001114, mRNA sequence

TTTTTTTTTTTGGAGAGATTTATAACCAACAATAAACTATAGATGAATAACACAACCACAAAGATCTGTA

TTTGATCTGATTACAAAAATATTAACAAACCTGAACCGACAACAATCACACTAGCTGCAGAATACAATAT

AAACTGCAAAATGGTAGACAACAGAAAACCCATAACCTCTGCTACCTATACAACCACACAACCACTGCCC

ACTCAAAAGAAAAACCTGACAAAAGGTTAGCCCAAAGTCAATTTCTTCCCTTTTCTTTTTTAGTAGTATT

TTTTTTCTGCTGATCTCTAAGCAGAGGAAAAACATTCTATGTCAGAAGCATGCCAATTTCCTTCATCCTC

TGATACTGTTCTCTTCCCTGAGTTTTTACTTCTCTTTGCCAATTACAATAGTCACACTGGGTCATTGGCA

TGCGAGTTACCATCTTATAATCAATCACAGTAGAAGGGGAAAATAAAAGAAAA

>FM891131.1 FM891131 Jatropha curcas embryo 56-70 (DAF) Jatropha curcas cDNA clone rjcaeb1_001109, mRNA sequence

CTGCAGGAATTCGAATTCCGTCTTCTTCGTGTGCTCTGCAATAAGACAGCGGAGGAGAGCACCGTCAGCT

AACAAGAATGGTACACGTCTCATTTTATCGCAACTATGGGAAAACCTTTAAGAAACCAAGACGTCCCTAT

GAGAAGGAGCGTTTGGATGCTGAGCTGAAGCTTGTTGGAGAATATGGTCTCCGCTGCAAAAGAGAGCTTT

GGAGGGTTCAGTATGCATTGAGCCGCATTCGTAATGCAGCAAGAATGCTTCTCACCCTTGACGAGAAAAA

CCCACGTCGTATTTTTGAGGGTGAAGCACTTCTGAGGAGGATGAATAGATATGGTCTTTTGGATGAGAGC

CAGAACAAACTCGATTATGTGTTGGCCTTGACTGTGGAGAACTTTCTTGAGCGCCGTCTTCAAACTCTTG

TGTTCAAGTCGGGTATGGCCAAGTCAATCCACCATGCCAGAGTGCTCATTAGGCAAAGGCATATTCAGGT

AGGGAGACAGGTGGTTAATATTCCCTCATTTATGGTGAGGGTGGACT

>FM891130.1 FM891130 Jatropha curcas embryo 56-70 (DAF) Jatropha curcas cDNA clone rjcaeb1_001105, mRNA sequence

ACTCAGACATCCAGATAATTATATTAAGAAACTAAACAAAAAAAAACCAGTATCAATATCCATCATACTC

AACTAATCAAAATTATGCAAAAAGTAGCAAACTTAGAATTACCAAATCCATATCACGAAACAAAAACGCA

TTAACAATAACCTAATCACCGCCAGCCTTATTGTAAACATAAGTCAAGCCGCACTTGCCGCAGTAGTGCC

TATCAAAATGGTTGGCCATGAAAGTTCCAGCTCCACACTCAGCATTGGGGCACTCCTTCCTCAGCCTCTG

CACCTTTCCGGAGTCATCGACCTTGTAGAACTGAAGCACGGCAAGTTTGACCTTCTTCTTCTTGTGCTTG

ATCTTCTTTGGCTTGGTGTAAGTCTTCTTCTTTCTCTTCTTAGCACCACCACGAAGACGAAGCACCAGGT

GGAGAGTGGATTCCTTCTGGATATTGTAGTCGGCAAGAGTGCGGCCATCCTCTAGCTGCTTTCCGGCGAA

GATCAGTCGCTGCTGGTCTGGAGGGATGCCTTCCTTGTCCTGGATCTTGGGCCTGACCTTATCGATAGTA

TCAGAAGACTCAACCTCTAGGGTTATCGTCTTCCCGGTTAGGTTTT

>FM891129.1 FM891129 Jatropha curcas embryo 56-70 (DAF) Jatropha curcas cDNA clone rjcaeb1_001104, mRNA sequence

CGAATTCCGCTTTTAAGCCGTATATAGGTATTAAAAATTACTGTCTCTTCCATTCCTTAATATCGAAGTT

TTTCTTTCTGGTACTGTATACACATATATACATACATTTCGTGCTTGAAGCGTATGTTTGACGAATCGTC

TGCAATAAGTCATTCCGAGATTTGGTTTAACTTTAATTCATGTCGGCAAGAACGGTAAGGAGGAGACTTC

ATCACGGGGATGTTGGTGGGAAAAGGCAAGAGCATTTGGACTCATCAGGGTATGATGGTCTCAGTGAACC

TCTACTTGGGACTCAAGAAAATAATGATATGCAAACGTCGGGAATCACACTAGAAGACATTTGGGATGCT

GAACAGAGAAGGGAACGGCTCCATTGGACTTTGTTACTATCGCATTTGATTGCTGAGTGGGCACAATGGT

TAGCAAATATTGTCCTAGGTACTGGATCACTCATTGGACGGTTTGTGCCTTTTGCTGGTCCAAATGGATC

AGACAGGAAGCTTCTTCAACCTTACCTTAATCCTTTACAGGAAGCAAGAATCAGAAATCTTACAGCA

>FM891128.1 FM891128 Jatropha curcas embryo 56-70 (DAF) Jatropha curcas cDNA clone rjcaeb0_001194, mRNA sequence

TTTTTTTATAAAAAGCTCAAGCTCCCATCTTTAAAACTTCAAAAATATAGCAATAACTATATGACGGTTT

GCATCCCTCCAGCCTACTCTTTTGTGATTTTAAATAAATAATGTATAACATCAATGGTTGAACCAGATTG

TGTGCCCTGAATCCAGTTACAACCCCATCATCATTGTTAACCAAAAAAGATACAACTACCAAATCATCAA

TTTTGCTGCATTGCTCCTAACCCAGCTGAATCGTAATTGGGCAAACAGATTTCGCGTCCATCAAAAGCAG

TCAACCACTAAAAATAAGAATCTGCATTTTCAAGCTTCTTTCCTAGTTATCCTGAAACAATTAATGCCCA

AGGCCGTTGTGATTTCATGAATGACTGACGTAGCAAAATGTAAATATAGTCCCACTGTGAATAAACAGTA

ACCAAGAAGGACCCAGAACTCATCTACCAAAGGAACTCCATCATTCAGTCTGGCTGTGAGTGCATTTGCG

GGGGGGGCCCGGTAC

>FM891127.1 FM891127 Jatropha curcas embryo 56-70 (DAF) Jatropha curcas cDNA clone rjcaeb0_001193, mRNA sequence

AATCTACTCTTTTTCTCCCTTCCAAGAAAAAGCTTATTACGATATCTATTTTAAGCTTCCATGGCCGAGT

ATGAGCGACGACCAGGCGGAGAAGGGCACAAAGGAGCCCTAAAGGAGAAGGGCGGCTTCTCGACCTCACA

GATTTTAGCGGTTGTCACTCTTTTGCCGGTGAGTGGGACGCTTCTTTTCCTTGCTGGAATAACGCTTACT

GGAACACTCATTGCGCTAGCTGTTGCGACTCCGCTGTTCGTGATTTGTAGTCCGGTGCTGGTACCAGCGG

CGTTGGTCATTGGGTTGTCGGTGTTAGGGTTTTTGACGTCCGGTGCTTTTGGAATCACTGCGCTTTCTTC

ACTGTCGTGGATGGTTAATTATATTCGCAGGATGAGAGGATCTTGGACGATGCAGATGGAAATGGCAAAG

CGGCGCGCGCAAGAAACGACGGGTCAATTGGGCCAGAAGGCTAGGGAGGTGGGCCAGAAGGCC

>FM891126.1 FM891126 Jatropha curcas embryo 56-70 (DAF) Jatropha curcas cDNA clone rjcaeb0_001192, mRNA sequence

ATTCCGAAGCTACTCTCAGCTACGATCGACCAAATCTAAGGCTTTCAGATCGAAAAATGGCATTGATTCC

AAGCTTTTTCGGTAACTCACGAAGCAACATCTTCGATCCTTTCTCTGCTCTCGACGTTTGGGATCCATTC

AGAGACTTTTCTTTCCCTTCATCTTCTTCCCTCGTCTCTCGTGAAAATTCTGCTTTTATTAACACTCGCA

TTGACTGGAGAGAGACACCAGAGGCTCATATATTCAAGGCTGATCTCCCTGGGCTCAAAAAAGAGGAAGT

GAAAGTAGAAATTGAAGATGACAGAGTGCTTCAAATCAGCGGGGAGAGAAATGTGGAGAAGGAAGACAAG

AACGACACGTGGCATCGCGTGGAGCGTAACAGTGGCAAGTTCATGAGGA

>FM891125.1 FM891125 Jatropha curcas embryo 56-70 (DAF) Jatropha curcas cDNA clone rjcaeb0_001191, mRNA sequence

ATTCGAAAAATGCGATTGTTCATATTAGCCGTCCTCACGGTCGGAGTGCTTGGTTCGAATGATGATTTGT

GGCATCAATGGAAGCGAATGTACAATAAAGAATACAATGGGGATGACGATCAGCACAGACGAAATATTTG

GGAAAAGAATGTGAAACATATCCAAGAACATAACCTACGTCACGATCTCGGCCTCGTCACCTACACATTG

GGATTGAACCAATTCACGGATATGACATTCGAGGAATTCAAGGCCACATATCTAACAGAAATGCCACGCG

CGTCCGATATACTCTCACTCGGTATCCCG

>FM891124.1 FM891124 Jatropha curcas embryo 56-70 (DAF) Jatropha curcas cDNA clone rjcaeb0_001189, mRNA sequence

ATTCCTTCAAATCAAGTATCCAGCTAGAATGGCTTTAAGCTTAGGAAGGAGGAGCAACATCTATGATCCG

TTCTCACTAGATATATGGGACCCATTTGAGGGCTTAC

>FM891123.1 FM891123 Jatropha curcas embryo 56-70 (DAF) Jatropha curcas cDNA clone rjcaeb0_001187, mRNA sequence

GAAGATTAACCAAACCCTTTTCATATATCCCGTTACAGGTGGACCACGATTACTCAAAACGCATTCTACC

AGGCTACAAGACAAAATTTCAGAGACTGCCTCTTTTTTTTTTTTTTGTTCTCTTCCTACACAAAAAGGGC

AATTCTGACTTGTACTCACTCTACTACAGTACATAATATCAGCAACAATAATTAATAAAAACAAAGAAGA

GATTACATGGGACCCTTATAAGGCAACGGTAAAAATAACGGTGATCATTTCATAACTTGGATGGATTAAG

ATTAGGAGACTAAAATTACTCTAGAAAACCTGTTTTTGTCAAAATGGCATTCCTCACTTTGCTAAGATCT

CTACCTTTGAGCGTCCTTCCAATCCCTTCACATACAGAGAAAGAAGAAATCTGACTTCTTGCAAGCTTCT

TAGCTATCCACTCATGTGGAGGGACCATTCCTTCTCCGTCATCATTCTCATTATCAGCATCATTATAAGG

TCCATTAACAGGGTCATGGTCTGTCCTGTCCTCAGCCCAGGAGCCCATTTTTGTCGTTTTACCATAAATC

TTTGACCAATCAGGGATTTTAACAGGAGCTGATGATTGTTCAACTGCCTGAGCTTCATGGAGGATGGTCG

CTTCGGCTGCTGGAATCATCTTGG

>FM891122.1 FM891122 Jatropha curcas embryo 56-70 (DAF) Jatropha curcas cDNA clone rjcaeb0_001185, mRNA sequence

CGAATCATTTATTTGCGGGTGTCAAGATTTGGGGGAAGCGTTAAGAAGGGTAAGGGAAGGTGCGGCAATG

ATAAGGACACAAGGAGAGGTATTAGGCCATGGCAATATCGCCCTGACTGTTAAGAATGTGAGAAATGTAA

TGAAAGAGATTCGAGTTTTGAATAACATGGACGAAGATGAGGTTTTTGCATTTGCTAAAAAGATAGCTGC

TCCTTATGATCTTGTAGCCCAGACTAAGCAAATGGGAAGGTTGCCTGTTGTGCAGTTTGCTGCAGGAGGG

ATTATGAGTCCTGCAGACGCAGCTTTAATGATGCAATTGGGTTGTGATGGGATTTTTGTAGGGAATGAGG

TATATGATTGCGCTGATCCAAATAAGCGAGTAAGGGCTATCGTGCAAGGCTGTTAGGCATTATAATGATC

CTCACGTGATTGGTCGAGAGTAGCTGTGAATTGGAGGATGC

>FM891121.1 FM891121 Jatropha curcas embryo 56-70 (DAF) Jatropha curcas cDNA clone rjcaeb0_001183, mRNA sequence

AACCAACAGGTAATTATATATACTGCAGGTTATTTACAAATAGGGAGAAAGTATTAGCAGTCCTCCCAGA

AAGGGAACGACCATAAACCCCTCTCAAAGTTACAACACACATACTAGCTAAACCCTAGTCATTTTAACCC

ACATGTAAACTACATTACTAAACTAAAGTACACTGCTAAGCTAATACTTTATTACCAACATTATTATTAT

TATTACTACAAAATACCATTTATAGTTTTAATCATCACACTAGCTCCCAGTCCTGAACTTCGACTCATCG

ATAGTGATGCCCTCCTTCTCAGCTCTTTCTCCACCAGACTCGTCCGTCGTGCTCAGCCCACCTTTCCGGC

CCATCTCCTGATATCCTTCTGTCCCCAGCTGCTCCTTCCTTGTCTGTCCTCCTCTGCTTCTCCCTTCAGC

AAGATGCTCTTGAGCTTCAAGGCTCTTGCCCCCAGTACCTCCAGGGACAACAGTCTCTCCACGCCTTGCT

CTTTCATCAAGCTCTTGTCTT

>FM891120.1 FM891120 Jatropha curcas embryo 56-70 (DAF) Jatropha curcas cDNA clone rjcaeb0_001178, mRNA sequence

ATTCAGTTGAGTGGCTATGAGAGGACATGAGGCGATGGACTGCCCGGAAAAGACAATGGGGTCGACGGCA

GTGGTGGTTATGGTGGGGAAGGAAGAGTTGGTGGTAGCAAATTGTGGGGATTCTAGAGCAGTTTTATATC

GTGGAGGTATGGCTATGGCCTTGTCTAATGATCATAAGCCTGACAGGCCCGATGAACGTGAAAGAGTGGA

AGCAGCAGGCGGAAGAGTCATAGACTGGAACG

>FM891119.1 FM891119 Jatropha curcas embryo 56-70 (DAF) Jatropha curcas cDNA clone rjcaeb0_001173, mRNA sequence

ATTCNGGTTTGTCCAGTTTGTGCAATGAGGGTGGGGGTTGACATGGTTGCGCACATAACCATACAACATG

GAAATATATTCAAGATGCAGCGTAAGAGGAAATCACGTAAAGGCGGACATCATTCAACGCTTACTTTATT

GAGGAAAGAGCTACGAGAAGGAAATCTACAATCTCTTTTTGGGGGTTCTTCCTGTATAGTGTCATCATCC

AATGCAGCGCCTGATCCATTGTTGTCTTCGTTCATTTTGCCTATGGCTGATGATTTCACAAGTGCTCAGC

CTTCTTTCTCAAATGAAACAAGTTCAGCAAAGAAAAGCTCAGATGAGAATGTTTCAGAGCGAAATGTCAA

GTCAACCCCCCTATCAATCAAGGATAAGGAAGAGAAGGCCAAAAGGAGTGAGTTTGTTAAAGGGCTGTTG

TACTCTGCTATTCTTGATGACATCTTATGAAAAGGGACTCATGCTGGCAGCTAGAGCTATG

>FM891118.1 FM891118 Jatropha curcas embryo 56-70 (DAF) Jatropha curcas cDNA clone rjcaeb0_001169, mRNA sequence

ATTCGAATTCGAACACAATGATGGATCAAGTCAAGGCCACTATGGAAAACGGTGTGCTTAAAGTGGTTGT

GCCTAAAAAACAAGAGAAGCAGCCTCAGATTAAGCCTATTGAGATCACTGG

>FM891117.1 FM891117 Jatropha curcas embryo 56-70 (DAF) Jatropha curcas cDNA clone rjcaeb0_001165, mRNA sequence

GCTAGTTTTTAATTTATACAATATATAACGTTAAAGCAACAGGCAGCCATTGCTAAACACAAGCTCACAA

GCCACCACTACTTTTATTGAAGCTCAAAACCAGAAACAAACGAACAAACTCATACGATATCTAGACTCTA

TTAATTATTTCTCCTTTTTTAGAAAGAGAGAAGCAGAGTCATCTCTGCTTTTACTTCTAAAAAACAGACT

GATCGCCGCCGATCTCATTTACAGTTGCAAGGATTGCAGGTGCAGTTGTCACCGCACTTGCATCCGTTCT

CAGCTCCAACGCCGACCTCACCTCCATCGACAAAGCGTGCCTTCTCCGGTGCAACTCCGAGAACCTGAGT

CTCGGCGGTGTTTTTCTCGGAGAAACTCATGTCAGGATACATCTTGCATCCGCCACAACCGATGCCACAC

TTGCAGCCGGAGCCGCAGCCGCAACTTCCACCACAGCAAGAAGACA

>FM891116.1 FM891116 Jatropha curcas embryo 56-70 (DAF) Jatropha curcas cDNA clone rjcaeb0_001159, mRNA sequence

AATTCCCTGCATTGATGATGTCACCACAGGCAGTGAACTTGGGCATGGTTTCTCCAGCCCTGATTGAGAA

AAGGGATTACAAGTATAATATATTGACAGATGCTATAAAGAACTCGAGGATTGAGATTGCAGAGCCAGAG

TGGATCAATCCTCAGGCAGATTACTGGAAGCAGAATGGGAAAGGTTTTGCCATTGACATTGAAAGGACTG

AAATGAAATTGAGGGCACCTTTTCCATGATCATATATATGCAGATTTAGTGTATATAAAGTAAAAAAACA

ATATGGATAGTGTAAGTGTGGATATCAGGATGTATAGGTGGTTG

>FM891115.1 FM891115 Jatropha curcas embryo 56-70 (DAF) Jatropha curcas cDNA clone rjcaeb0_001156, mRNA sequence

ATTCCATGATTGTCCATGTGAAGGAATAAAGTATGTATTAGAGCAGCAGCTGGAGCAAGGACAGCGAGAG

CGTTTGGAGGCAGTGCGTAGGGCTAGAAGTATACCTTCTGCTTGTGGCCTGCGTACAAGCTGCGATATTC

GTTCCTAATCTGCTAGCTATAACGTGGATGTGCTTCAGTTAAATAAAAATTTCACTCACTTGGTGTGTGA

GTGATCAGAGTTATGTATAATAATGCACAAAAGAATGAGCACATCAAGCTAAGTTTGTTTTCTGTTATCT

>FM891114.1 FM891114 Jatropha curcas embryo 56-70 (DAF) Jatropha curcas cDNA clone rjcaeb0_001128, mRNA sequence

ACTAAAAGATACCTTTTATTCAACTGGAATTGCTTAACAAGAAAAAGACACCAAACAGAAAACATAGATA

TATTACATAACAGGAGCTAAAGCACATGCCATGCCCTACACACGTGGGTTATGGCCAGTGGTGTCTCCGG

GCAACCCAGATGGGCGAGTCCTGGTCAAACCCGTTGGGTCCACCTCTCCACCGTATGGCTGCCCTGTGCC

GTGTCCAGGCATGGCCGACATTTGCTGGGTCCCTGTAGGGTAACCGGTTGTCCCAGTTGTTGAATGGGTA

TAGGTTTCGGTTGTGCCTTGAGCGCCCGGACCAGCAGTTGTGTACTGTACATGGCCACCAGCCTTGGCTG

CCTGCCTCGCCGCTGCGTTATCCTCGCGCGCTTCTAGTTTCCGCAATTCTGCCTCCGTTTTCCTCTCTTC

TTTCTTCTCTCTCGCCATTTCCTTCTGCACTGGATCACGTGTAGACATTCTCTCCACCTTCTCTTGCGCC

TCTGCTCTGG

>FM891113.1 FM891113 Jatropha curcas embryo 56-70 (DAF) Jatropha curcas cDNA clone rjcaeb0_001126, mRNA sequence

ATTTAAATAATTAATTTAGATTTAATATAAACATTACGAAAACAAAATATTGAATACAATTATATTTATA

GAATATATTGATTAGGGAATACTTAATTACTAATTAGGATGATTACATTACTTTCATCACTTATTTTCAT

AATATATTACATACTTCATTAGCGTGAGATCATAACTCCCCTCGTAATACTACTTACTTTCACTATCTAC

ACACCCAAAAAGGCTACTTCATTATTTACTCCTCGGCATATCTTCCAGACTCGGTCCTGGAAATAGATCA

GGTGGT

>FM891112.1 FM891112 Jatropha curcas embryo 56-70 (DAF) Jatropha curcas cDNA clone rjcaeb0_001124, mRNA sequence

AGAAAATAGAAAAGGAGTTTCTCATTAAGCTTTCAAAGCTATTTCACCCCTCGTACTCATTTACATATTC

ATATGCTAGTTTTTAATTTATACAATATATAACGTTAAAGCAACAGGCAGCCATTGCTAAACACAAGCTC

ACAAGCCACCACTACTTTTATTGAAGCTCAAAACCAGAAACAAACGAACAAACTCATACGATATCTAGAC

TCTATTAATTATTTCTCCTTTTTTAGAAAGAGAGAAGCAGAGTCATCTCTGCTTTTACTTCTAAAAAACA

GACTGATCGCCGCCGATCTCATTTACAGTTGCAAGGATTGCAGGTGCAGTTGTCACCGCACTTGCATCCG

TTCTCAGCTCCAACGCCGGGGGGGGCCCGGTAC

>FM891111.1 FM891111 Jatropha curcas embryo 56-70 (DAF) Jatropha curcas cDNA clone rjcaeb0_001122, mRNA sequence

ATTCGCCAGATCTAAAAAGCTCACAGGTAACAGTAAAGGGAGTTTTTGATCCTCCGATACTTGTCGATTA

TGTGTACAAAAGAACAGGCAAGCATGCAGTAATAGTGAAGGCAGAACCGGAGAAAAAAGAAGAGAAAGGA

AAGGAATCCAAAGAAGAGAAAAAAGAGGAGGAAGGTGACAAAGAAAAGAAAGGCGGTGAACAGGAGGAGA

ATAAAGAAAATAAAGAAAGTAAAGAAGGTGGCGGCGGGGGTGAAGGGGAAGCAAAACCAGAGGCGGCAGC

GGAAGCGCCACCCGCAGAAGAAAGTACAGTTGTAGAGCTGAAGAAAAATGAATATTACTATTTCCCACCA

AGACCGTATGCCATGGAATTGTATGCATACCCTCCTCAGATCTTTAGCGACGAGAACCCCAATGCATGTT

CTGTTATGTAAAAAGTTAATGAAATCAGGGGCAAATATGAGATTTTAGGGGAAAATATGCTCATAACCCG

CCCATTCTGGACTCTCTGATGTGCTGCTCCCTC

>FM891110.1 FM891110 Jatropha curcas embryo 56-70 (DAF) Jatropha curcas cDNA clone rjcaeb0_001120, mRNA sequence

GTGAAAAATAAAGATGGATTCTGGACAAACTTGCAATAATTACATTAAGTTATAGACATGATCCATAAGT

TGAAGTACAAATCTAAGTGCATATCCACATTGGCTAGTGCACTTCAAATGACTTTTTGTACACTATATAA

GGAGAGATTAGAAGCACAAAACAAAGTAGAGTCAAGGAAGCTTGCCTATGGTTTAATAAAATAAAAAGGC

CCTAAGGTTAGCTCTCTAAACTTCGGTCAGTCTCTGGTTGTTGCTTTGGGGCAATAGCACCTCCATTCAT

TCTTGCACGTGCCTCAGCAAACATCCTTTGCTGCTCCGCTACTGCTTCTTCCTCGGTCATCTCCGCTCCA

TTGCTCCATTTACCACCTTTTAATGAGTCCTGCATAGTCTCAAGCTTGTGTTGCTCGTATGCAGCATAAA

CCTCTTCGATGTACTCTCCAAAACCAAGAACCTCTAACGCCTTGAGTACATGCTCAGGCGCAATCGTCCG

CTTCTCCTCTTTACTGCATACTTCATTGGACTCTGATGATACAAGGTTTA

>FM891109.1 FM891109 Jatropha curcas embryo 56-70 (DAF) Jatropha curcas cDNA clone rjcaeb0_001117, mRNA sequence

ATTCATTGAATTTGAATAGCCAAGTGAGTGAAAATGGAGTCGCTGAAGGAAACCGCAGCTAACGTTGGAG

CCTCTGCTAAGGCTGGCATGGAAAAAACCAGAGCAGAGGCGCAAGAGAAGGTGGAGAGAATGTCAACACG

TGATCCAGTGCAGAAGGAAATGGCGAGAGAGAAGAAAGAAGAGAGGAAAACGGAGGCAGAATTGCGGAAA

CTAGAAGCGCGCGAGGATAACGCAGCGGCGAGGCAGGCAGCCAAGGCTGGTGGCCATGTACAGTACACAA

CTGCTGGTCCGGGCGCTCAAGGCACAACCGAAACCTATACCCATTCAACAACTGGGACAACCAGGTTACC

CTACAGGGACCCAGCAAATGTCGGCCATGCCTGGAC

>FM891108.1 FM891108 Jatropha curcas embryo 56-70 (DAF) Jatropha curcas cDNA clone rjcaeb0_001116, mRNA sequence

ATTCGACCATCTGACCAAGGAACAGGTCATCGATAGAGTCCTCGATGTGGTTAAGAGTTTCCCCAAGGTC

GATCCATCCAATTGTGAGTCCTGATGTACATTTCCAGAAGGATCTGGGTTTGGATAGCTTGGACAATGTA

GAGATTATAATGGCTATAGAAGACGAGTTCAAGCTGGAAATTCCAGACACCGAAGCAGTTAAGATTGACT

CCTGTAATCTTGCTATT

>FM891107.1 FM891107 Jatropha curcas embryo 56-70 (DAF) Jatropha curcas cDNA clone rjcaeb0_001113, mRNA sequence

CTCTTCGCCATTTCCTTCTGCACTGGATCACGTGTTGACACCCTCTCTCTCTCACACCCTCTCTCTCTCC

CTCCATATTCGAAAATGGTGAAGGGACGCCAAGGAGAGCGAGTCAGACTCTACGTCAGAGGGACCGTCCT

TGGATACAAGAGGTCCAAGTCAAACCAGTACCCAAATACATCATTAATCCAGATTGAGGGAGTGAACACC

AAGGAGGAGGTTGCCTGGTATGCTGGTAAGCGCATGGCTTATATCTACAAGGCCAAGGTAAAGAAGAATG

GATCACACTATCGCTGCATTTGGGGAAAGGTCACTAGGCCTCATGGTAACAGTGGTGTTGTACGAGCTAA

GTTCAAGTCCAATCTGCCTCCGAAATCAATGGGTGCAAGGGTTAGAGTCTTCATGTACCCTAGCAACATA

TAAGTATCACAAGACCGGTAGATAGCAGATTGGGCTTATTGATAGCTTGGAGGATCTGCTTTTTAGTTGT

TCTATATAAGCTAAGTTTAAATGTGTTGAATGCTTTCGAGTTTTGAGGAGTTACTATTGTTTTTTAGAAT

TGTGGATGAACAATATTTGGTTACCTTATTTAATCTCAATCGGG

>FM891106.1 FM891106 Jatropha curcas embryo 56-70 (DAF) Jatropha curcas cDNA clone rjcaeb0_001112, mRNA sequence

ATTCCTCTTTCTTTTGTTTTCTTTAGCTAAGAGAAGCTAAGCTCTTAGCAACTTAGAAATCCTCACAGGA

GGTTCACTTGCAGCGATTGAGCTAAGTTAATCAGCAGATACTGAAGAAGAAGAAGAAGTAGAAGAAGAAG

AGAGCAAG

>FM891105.1 FM891105 Jatropha curcas embryo 56-70 (DAF) Jatropha curcas cDNA clone rjcaeb0_001111, mRNA sequence

ATTCTTCTCTTTGTGGTCGATCCGGGAGAAACTCAGAATCATGTTGGTCTACCGGGATTTGCTTACCGGT

GATGAGCTTCTCTCGGATTCGTTCCCTTACAAGGAAATTGAGAATGGCATGCTGTGGGAAGTTGAGGGAA

AGTGGGTTGTTCAGGGAGCCGTTGATGTGAACATTGGTGCAAATCCCTCTGCTGAAGGGGCGGATGAAGA

TGAGGGTGTTGATGACCAGACTGTTACAAGTTGTGGACGTCGTGGACACTTTCAGGCTCCAGGAGCAACC

TTGCCTT

>FM891104.1 FM891104 Jatropha curcas embryo 56-70 (DAF) Jatropha curcas cDNA clone rjcaeb0_001110, mRNA sequence

ATTGCCACTTTGTCGTGCTCGTTGTCAGCTGTAACAAAGATGAGCAGTAATTTGGAGACAAGGTCATTGA

TGGATGAATTCCGTAACTTGGAAAAAGGTTGGTCCTTTGATCTTGGCCATCCTCTCCTCAACCGCATCGC

TGAGAGCTTTGTCAAAGCTGCTGGGATTGGAGCAATTCAGGCGGTGTCACGGGAGGCTTATTTGACAGCC

AGTCAAGGTAATGGACTTGATTCAAGTAGTGGTCTGCCGCCGGAGCTCTCTGGTGCTGCAAAGAAACGAC

GTTTCCCTAACCTCAAAGGAGAAACCAGCGTCAAATCTCTTGAAGCATTGGTGAAGAGAACAGGAGAAGA

ATCCTTACAGTGGGGACTGGCTGCAGGGGTTTATTCAGGTCTTACTTATGGACTGAGAGAGGCTCGTGGA

GCACATGATTGGAAAAATAGCGCAGATGCTGGGGCTTTAACAGGGTTGGCATTGGGG

>FM891103.1 FM891103 Jatropha curcas embryo 56-70 (DAF) Jatropha curcas cDNA clone rjcaeb0_001108, mRNA sequence

ATTCGGAGAAGGCACGCTTTGTCGATGGAGGTGAGGTCGGCGTTGGAGCTGAGAACGGATGCAAGTGCGG

TGACAACTGCACCTGCAATCCTTGCAACTGTAAATGAGATCGGCGGCGATCAGTCTGTTTTTTAGAAGTA

AAAGCAGAGATGACTCTGCTTCTCTCTTTCTAAAAAAGGAGAAATAATTAATAGAGTCTAGATATCGTAT

GAGTTTGTTCGTTTGTTTCTGGTTTTGAGCTTCAATAAAAGTAGTGGTGGCTTGTGAGCTTGTGTTTAGC

AATGGCTGCCTGTTGCTTTAACGTTATATATTGTATAAATTAAAAACTAGCATATGAATATGTAAATGAG

TACGAGGGGTGAAATAGCTTTGAAAGCTTAATGAGAAACTCCTTTTCT

>FM891102.1 FM891102 Jatropha curcas embryo 56-70 (DAF) Jatropha curcas cDNA clone rjcaeb0_001107, mRNA sequence

ATAGAATATATTGATTAGGGAATACTTAATTACTAATTAGGATGATTACATTACTTTCATCACTTATTTT

CATAATATATTACATACTTCATTAGCGTGAGATCATAACTCCCCTCGTAATACTACTTACTTTCACTATC

TACACACCCAAAAAGGCTACTTCATTATTTACTCCTCGGCATATCTTCCAGACTCGGTCCTGGAAATAGT

CAAGGTGGTCTCTTGTCTTCCAAACTTAATCCTTCTTGCCTCCTCTGCTGATACCCTAAATGCATTTGCA

ATAACCGCCAACGGCATTGCTCTAACGGCGGAGGAGCGACCTGCAAGATCATTGGTCATCGCGTTATCGC

TTGTCTTGAAGGTTACATATTCGAATCTTTCACTTTCTGCTCGTTTCACCACTACGAAGTTTTGTGGCAC

CGTCAAAACCTGTCCTTTGCTTACACTTCCGTCGAAGACGGTGTTGCCGTTTTCATCCGTCACCTGAACC

CGCGCTTGTCCTTTGAGTGCGTAAATTACGCTGTGAGAGTTTATGTGCCAGTGGGGTAATCT

>FM891101.1 FM891101 Jatropha curcas embryo 56-70 (DAF) Jatropha curcas cDNA clone rjcaeb0_001106, mRNA sequence

ATTCCACNGAAGTAGCGTCACTTGCAAGCGGCGAAACAAAGAAAAACAAAGCCGAGAGCCAAACAAGTGT

AAAGAAATCCAGAATCCGATTTCTAGATGGATCCAGACGCGGTAGCGAAGGCGTTTGTCGAGCACTACTA

CTCCACGTTCGATGCGAACCGTGCTGGCTTGGCTAACCTCTACCAAGACGGATCGATGCTCACCTTTGAA

GGCGAGAAGATCCAAGGCTCTCCGAACATCGTCGCTAAGCTCACTAGCCTTCCTTTCAAACAGTGCCAGC

ATAGAATCACAACCGTCGATTGTCAGCCATCTGGTCCTGCCGGTGGCATGCTTGTCTTTGTCTCTGGTAA

CCTCCAGCTCGCCGGCGAACAACACGCTCTCAAGTTCAGTCAGATGTTCCATTTGATGCCAACACCACAG

GGGAGCTTTTACGTGTTGAATGATATTTTCCGGTTGAACTATGCCTGAAGACCATATCAAACTCAAATAG

TCACTGTGGCCCAAAATGTTCAGGATTAAAATCACTTTTTCTTGCCTCTGCTTTTAGACGTTTGAAATGA

AAACTGTGAT

>FM891100.1 FM891100 Jatropha curcas embryo 56-70 (DAF) Jatropha curcas cDNA clone rjcaeb0_001103, mRNA sequence

CTACTAAAAGATACCTTTTATTCAACTGGAATTGCTTAACAAGAAAAAGACACCAAACAGAAAACATAGA

TATATTACATAACAGGAGCTAAAGCACATGCCATGCCCTACACACGTGGGTTATGGCCAGTGGTGTCTCC

GGGCAACCCAGATGGGCGAGTCCTGGTCAAACCCGTTGGGTCCACCTCTCCACCGTATGGCTGCCCTGTG

CCGTGTCCAGGCATGGCCGACATTTGCTGGGTCCCTGTAGGGTAACCGGTTGTCCCAGTTGTTGAATGGG

TATAGGTTTCGGTTGTGCCTTGAGCGCCCGGACCAGCAGTTGTGTACTGTACATGGCCACCAGCCTTGGC

TGCCTGCCTCGCCGCTGCGTTATCCTCGCGCGCTTCTAGTTTCCGCAATTCTGCCTCCGTTTTCCTCTCT

TCTTTCTTCTCTCTCGCCATTTCCTTCTGCACTGGATCACGTGTTGACATTCTCTCCACCTTCTCTTGCG

CCTCTGCTCTGGTTTATTCCATGCCAGCCTTAGCAGAGGCTCCAACGTTAGCTGCGGTTTCCTTCAGCGA

CTCCATTTTCACTCACTTGGCTATTCAAATTCGGGGGGGCCCGGTAC

>FM891099.1 FM891099 Jatropha curcas embryo 56-70 (DAF) Jatropha curcas cDNA clone rjcaeb0_001102, mRNA sequence

ATTANGGGTTTTTAGCCTCAGGCGGTTTTGGCGTCGCTGCCATTTCTGTCTTGTCCTGGATTTATAGGTA

TGTTACTGGAAAGCATCCACCAGGAGCAGAAAGTCTGGACCAGGCACGCTTAAAGTTGGCGGGAAAGGCA

AGAGAGATGAAGGACAGAGCTGAGCAGTTTGGGACAGNCATGTAACAGGTNCAACAGNACTTAAAAGCAG

AAGTGGGTTGTGGTGGTTGATGCAAGAACATAAAATGGCTTTGATATATATATAAAAATATATGAGTCCT

TTAATTAACATGGAAGGCCATTGCTTGTCTGGTGTATTTCTTTTTTCGCTTAAATCTTTTTGTTTCTTCT

ATGTAATTTGTTAACGGGTCTTCCCTCTTTTGAGAGCTGTCTAAAATAATTNTCTTGTCATGTGTTGAAG

GTCTCTGTAATCAAGTCTTACTTTGTTTGTT

>FM891098.1 FM891098 Jatropha curcas embryo 56-70 (DAF) Jatropha curcas cDNA clone rjcaeb0_001101, mRNA sequence

CGAATTCTTTTAATCAGGAATTATTATATTTTACATTATTAAAATGAAACAGTAATAATAAACATTCTTT

CAGTACATACTTTCTCACACACATATCATTATATATATATATATATATATATATATATATATATATATAT

ATATATATATTGAGATAACATCATTATAAGCTGAAACTTAACCAGAGATCTCAATAGCCTTAACATCAGG

TTTCTTGACTTCCTCTTTAGGAACAGTGACAGTAAGTACTCCGTTTTCCATAGAAGCCTTTACTTGATCC

ATC

>FM891097.1 FM891097 Jatropha curcas embryo 56-70 (DAF) Jatropha curcas cDNA clone rjcaeb0_001096, mRNA sequence

GAGAAGGGTACAACTTTTATTTATCCAAACAAACACGACATTTAACACCTATATAAGCTATAGAAGGAGA

CACCATTAAACCAGCAACACTCTAGATATATAACAAAAAGACAAAAAGAACTTTTGACTTTCTTCACAGC

TATCTCCATCAACCATTCACAATAACGCCTCCATTGGGGTGAAGAACTTGGCCAGTGATATAAGAGGAGC

AATGGTTACAAGCAAGAAACACATAAGAGGGTGCAACCTCACTTGGCTGGCCAGCTCTCTGCATAGGCGT

TTGTTTCCCAAAGTTAGCCACCTCCTCTTCACTGAAAGAAGAGGGTATCAATGGAGTCCAAATAGGTCCA

GGAGCAACACCATTGACTCGTATGCCTTTTTTCACTAGCTGAAGTGCTAGTCCTCTAGTGAAAGGGGGGG

CCCGGTAC

>FM891096.1 FM891096 Jatropha curcas embryo 56-70 (DAF) Jatropha curcas cDNA clone rjcaeb0_001095, mRNA sequence

CGAATTCGTCAATGGCGGAGCACCCACAGTCGCAGCATGTTGGCCAACAGCCACGATCCCACCAAGTAGT

GAAGGCAGCCACTGCAGTCACTGCCGGTGGATCTCTTCTAGTTCTCTCCGGTTTGACCCTTGCTGGTACG

GTCATTGCACTGACCGTAGCCACTCCATTGCTGGTCATATTCAGTCCGGTTCTTGTTCCTGCGGTTATCA

CAGTTTGTTTGATAATCACAGGGTTTTTAGCCTCAGGCGGTTTTGGCGTCGCTGCCATTTCTGTCTTGTC

CTGGATTTATAGGTATGTTACTGGAAAGCATCCACCAGGAGCAGAAAGTCTGGACCAGGCACGCTTAAAG

TTGGCGGGAAAGGCAAGAGAGATGAAGGACAGAGCTGAGCAGTTTGGACAGCATGTAACAGGTCAACAGA

CTTAAAAGAGAAGTGGGTTTTGGTGGTTGATGCAGAGAACATAAAATGGCTTTGATATATATATAAAAAT

ATATGAGTCCTTTAATTAACATGGAAGGGCATTGCTTGTTG

>FM891095.1 FM891095 Jatropha curcas embryo 56-70 (DAF) Jatropha curcas cDNA clone rjcaeb0_001094, mRNA sequence

ATTCGGAAACATCAAATTCATCAACAAACAAAGTAAGACTTGATTACAGAGACCTTCAACACATGACAAG

AAAATTATTTTAGACAGCTCTCAAAAGAGGGAAGACCCGTTAACAAATTACATAGAAGAAACAAAAAGAT

TTAAGCGAAAAAAGAAATACACCAGACAAGCAATGGCCTTCCATGTTAATTAAAGGACTCATATATTTTT

ATATATATATCAAAGCCATTTTATGTTCTCTGCATCAACCACCAAAACCCACTTCTCTTTTAAGTCTGTT

GACCTGTTACATGCTGTCCAAACTGCTCAGCTCTGTCCTTCATCTCTCTTGCCTTTCCCGCCAACTTTAA

GCGTGCCTGGTCCAGACTTTCTGCTCCTGGTGGATGCTTTCCAGTAACATACCTATAAATCCAGGACAAG

ACAGAAATGGCAGCGACGCCAAAACCGCCTGAGGCTAAAAACCCTGTGATTATCAAACAAACTG

>FM891094.1 FM891094 Jatropha curcas embryo 56-70 (DAF) Jatropha curcas cDNA clone rjcaeb0_001093, mRNA sequence

ATTCCAACTCTCTCTTTTTCCCTCTCTCTTCTTTCTCTCTCTTTTGCCATCCTTTTGTTCTACTCTCTTA

AAGTGCTGTTAATTTCTCTTCTTTTGCTGCTCAATCTTGCATAATCATGGCTGTTCTAGTGTATTTAGTG

CTTTTCTTAGCACTTACTGGTTATTCAAGTGCTACTTACTGTGTTTGTAAAGATGGATTGAGCGACGCAG

CACTACAAAAGTCTCTAGATTATGCTTGCGGTGCTGGAGCTGACTGTACTCCGATCCTTCAAAATGGTGC

TTGTTTCCAACCCAACACCGTAAAAGACCACTGCAGCTACGCTGTGAACAGCTATTTCCAAAGGAAGGGT

CAAGTAACTGGAAGCTGTGATTTTGCAGGAACTGCTACTACTAGTGCCAGTCCCCCTTCAGGTGTTCCTT

CTACTTGTGTTTATCCTGCATCTTCAAGCACTGCCGGCACAGGCACCACAACCCCAAGCACAGGCACCGG

CACAGGCACAGGCACCGGCACA

>FM891093.1 FM891093 Jatropha curcas embryo 56-70 (DAF) Jatropha curcas cDNA clone rjcaeb0_001092, mRNA sequence

GGGAGAGAAAAGATTACTTCGTCTCATTGTTAAAGAGAATGCTATAACATGCAAAACAGGTTACAGGTAC

ATGTAGACTGAACATAAAACTCGGTGCCATTTCTATGTCCGCTTCGAGAAGAGACTTCTTATAAGTACAT

ACCCAAATACACCCCTACACACAAGGGGAAGTAACAGAACAAAACAAAGCAAACATCATATACAAACAAA

CACCATTCATATTAGTAACTCTTCATTAGTGTATCAGTAGTTAATGGTCTCATTAAGCCTAGCAGCTGTC

GTCATTGACACCGCAACACCACCTGGATGCGTGTTAAATTCGGATTGTTCCTCAACTCTGCACTAGCCTC

CNCCTTCGCATCTGCCCTTGTCACGACCTTATCCGCCGCCGGGGGGGCCCGGTAC

>FM891092.1 FM891092 Jatropha curcas embryo 56-70 (DAF) Jatropha curcas cDNA clone rjcaeb0_001091, mRNA sequence

AAGAAACAAAAGAAGGCCACATTTTATTGATTTCTGCAGAAGTTGAAGTTCTACATTTTTAACACTCTAC

AACTCTCTATACTACAATATACGACGGATCCCTTCTCTAAATTTACAAGAAGGATCTCTTAGTTGCGGCC

TTGGCTAGTAGAAGCACCAATCACAATGCTATTTTAGTAGTAACTTACTTATGGTCTCCTGCTGGTGGGA

GTAAACAACATGGACTGTGGATCCCTGTTCCTCTTCACTTGATGAGCTTCGCTTGGAGACATTCTGTAAG

AGTTCATCAGAACATCAAGAGGCATGGCTCTCATCACTGATGTATAACCCGCCAATGGGCTCTTGATAGG

CTGGCTACTTGTTTTAAATGTTACGTACTCGAACCCATTTTCTCCTGCCCTTCCAAGTGCTGCATAGAAT

TGAGGGATCACGAACATGTCTCCTTCTTTCACCCTTTCGTCCATTACTGTGTTTCCACTGTCATCCACAA

TCTGTATGTGCATGTCGCCGCGAAGAGCACACACTATTCTCTGATCGTTTATGG

>FM891091.1 FM891091 Jatropha curcas embryo 56-70 (DAF) Jatropha curcas cDNA clone rjcaeb0_001090, mRNA sequence

ATTATTCTCTTAAAGATTACTTCCCAACCATGGAAATGAGACCAAATCCTCAATGTTCAAATGCTGCTTG

TTTGGAGCGTCAGAAAGAGTACATCCTTNCAAAGCCAGCTAGGGATGCTGCAGCTAAAGCAAAGATGGAG

GCAGAAGCACTATTAGTTACGGAAATCACACTTCATGCAGATAATGAATGGAACATAAGTGTTGTGGATG

ATAGCGAGCTGGAAGGGACAGACGCCACAAGTTCAGATGCTCTTCCAGAAGGTCTTGCACGTGAGCTCCC

AAGTGCGGATGAGTCCCCGGCTGCTGAAACAGCTACAACTCCAATTGACGACCTCGAAGAACTTCGGAAG

CAACTTGAAGCCCTTAATGCACCATAAAGCATATGTAGCATTGAGAAGAAACTAGGTTTCAAGAAGATAA

GGCAATCGATTTCTTT

>FM891090.1 FM891090 Jatropha curcas embryo 56-70 (DAF) Jatropha curcas cDNA clone rjcaeb0_001089, mRNA sequence

ATTTATATATATATATATATTAACCCTTGTAACCGAAGCTTGAGTCCTGCATTCTGGAAATTGCCTTCTC

AAATTGTGACCATTTCTGCTCCAAATTGCCAATATACCCAATAGACATCCTAATTAACCCAGGCGAAATC

CCAGCTAGTTTCTTTTCCTCAGGACTCATTTCACTGCTGGTGCTACTCCCAGAAACTGACATTAACGTCT

CGTAGTATCCCAAACTAACTGCCATAAGCCCAAACTGGGTGTAGTTCTGCAAGTGGTTCATCAACCGGTT

AGCCCTTTCTTCAGTTTCCATGTCAATGCAAACAAGTGCACTGAAACCATAATCTTTATTAGCCATGGAT

TTCAAGAGCTGATGTTGTGGATGCTCTTCAAGCACTGGGTATATGACTTTGAGTCCCAGTTTCTTCATTC

TGGTGGCATATTCCATTGCTCTGTGACAATGTTCTCTCATTCTCAATCCCAAGTGAGGAAG

>FM891088.1 FM891088 Jatropha curcas embryo 56-70 (DAF) Jatropha curcas cDNA clone rjcaeb0_001087, mRNA sequence

GGGCTGCAGGAATTCGAATTCCATACTCGTACTGGTACGTAGTTTATGATCGTTGCATGATAATGGCTTC

TGAAAAAGCATACCGGGTGTTGCTCTGCTTGGGGTTCGCGATGCTGTTGATAGTTGTTGTTGTCGGTAGC

CTAGAACATATGCCTTCCACGAGCGAAAAAAGCGAGGACTCGGAAGCAATTAAGGACAGGGCAAAAGATG

CCAAACGAACATCGGAGTCGTGGACAGAATGGGCGAAGGACAAAATCTCCGAGACTGTCGGACGAAAACA

AGATGAAGCCAAGGAGGCTGCTAAGAAAGCGTCTGACTCTACCGCTGAAACTGCTCAGAGGACCAAACAC

AAAGCTTCTGAAAAAGCTGAAGAGCTGAAGGATGCTGCAACGCAGAAGGCCAACGAAATGAAAGATAAGG

CCTCTGAAATGACGAATGCGGCTAAGGAGAAGCTCAGTGCGGCTGAAGGCCAGACCACCGATGCTACAGA

AGCAGCAAAGGAGAAAGGTGAGGAGGCAAAGAACACGGCCTTTGAGAAGG

>FM891087.1 FM891087 Jatropha curcas embryo 56-70 (DAF) Jatropha curcas cDNA clone rjcaeb0_001086, mRNA sequence

ATCCGGAATCGTTCTCAGGATGGATTCACAAATCAAGCACGCCGTGGTGGTGAAAGTTATGGGTCGGACA

GGATCGAGGGGGCAGGTGACTCAAGTCAGAGTCAAGTTTCTTGATGATCAGAATCGATTCATCATGAGGA

ATGTCAAGGGACCTGTTAGAGAAGGTGATGTGCTCACCCTTCTTGAGTCTGAAAGAGAAGCCAGGAGGCT

TCGTTAATATGTGCATCTCTACCCACTTCTTTTTTTATGGTTTAATTTTGTATTAGTATCAAGACTCTTT

TGTTATGCTATAGCTAGAGTGATTAAAAGTGCTGTTATGAGCTTTTGCTGTGCGGTTTACATTTTCCCAG

CCATTGTCATATCAAGACTAGAATTTTGATTTATGGAGTTGCTTAAGCTTTAATATACCCTAAAGAATTT

CTCTTTTTCTGGG

>FM891086.1 FM891086 Jatropha curcas embryo 56-70 (DAF) Jatropha curcas cDNA clone rjcaeb0_001084, mRNA sequence

AACTAGAATTATCTCTTTATTAATTATTAAACCAGAAGCCACTATAGTCCGCAGCAGTGATCTTTCTCTT

TTCTTAAAGACTAATTTACACATGTACCTTCACCTTTCATTATTTATTCACCATACACAGCACTATTTAC

ATTAGTAAACCCTCAAGGTCGATGTCACAGTCACCCCAAAAAGCCGAAACACTTGCCATCACGTTATTAG

CTTCTCATGCCACAGCATGACCTCGAAGGAACCTCTCCTTGGCCCTGGAAAGAACATCCTCGTTTTGTCT

TCCAAAAGCCTGATCCACTTCTCTTTCACTTTACACCGAAGGCTAACTCTTTGGCTTGCTCCTCCATCTT

ATTTACAATATTATTTTTCCCTGCAAGTAGATGCCTAGTATTGCCTTCTTCATTAATCTCA

>FM891085.1 FM891085 Jatropha curcas embryo 56-70 (DAF) Jatropha curcas cDNA clone rjcaeb0_001083, mRNA sequence

AATTCGCCCTCGACTTGATCAATGAGCCAAAGAGGCCAACAGACAAGCCACTCCGTCTCCCACTTCAGGA

TGTCTACAAGATTGGTGGTATTGGAACTGTCCCTNTCGGTCGTGTTGAGACTGGTGTCTTAAAACCTGGT

ATGGTTGTCACCTTTGGACCATCTGGACTGACCACTGAAGTTAAGTCTGTTGAGATGCACCATGAAGCTC

TCCAGGAGGCCCTTCCAGGTGACAATGTTGGCTTCAATGTGAAGAATGTTGCTGTCAAGGATTTGAAACG

TGGTTTTGTTGCCTCTAACTCCAAGGATGATCCTGCGAAGGAAGCTGCCAACTTCACCTCTCAGGTCATC

ATCATGAACCACCCTGGTCAGATTGGAAATGGATATGCTCCAGTCCTTGACTGCCACACCTCCCACATTG

CTGTTAAGTTTGCTGAGATCCTAACCAAGATTGATAGGCGATCTGGTAAAGAGCTTGAGAAGGAGCCAAA

ATTCTTGAAGAATGGTGATGCTGGATTTGTTAAGATGATTCCCACCAAGCCCATGGTTGTGGAGACCTTT

TC

>FM891084.1 FM891084 Jatropha curcas embryo 56-70 (DAF) Jatropha curcas cDNA clone rjcaeb0_001082, mRNA sequence

TCGGGAAAAATAATATTGTAAATAAGATGGAGAAGCAAGCCAAAGAGTTAGCCTTCGGTGTAAGTGAAAG

AGAAGTGGATCAGGCTTTTGGAAGACAAAACGAGGAGTGGTTCTTTCCAGGGCCAAGGAGAGGTTCCTTC

GAGGGTCATGCTGTGGCATGAGAAGCTAATAACGTGATGGCAAGTGTTTCGGCTTTTTGGGGTGACTGTG

ACATCGACCTTGAGGGTTTACTAATGTAAATAGTGCTGTGTATGGTGAATAAATAATGAAAGGTGAAGGT

ACATGTGTAAATTAGTCTTTAAGAAAAGAGAAAGATCACTGCTGCGGACTATAGTGGCTTCTGGTTTAAT

AATTAATAAAGAGATAAATTCTAGTT

>FM891083.1 FM891083 Jatropha curcas embryo 56-70 (DAF) Jatropha curcas cDNA clone rjcaeb0_001081, mRNA sequence

ATCGATTCGAGAGAACTAGTGTTCGATGGACAAGTTCAACAAGGTCAAATATTAACAGCTCCACAGAACT

TTGTGGTTTTGAAGAGGGCAAGCAATGAAGGGCTAGAGTGGGTATCCTTCAAGACTAACGAAAATGCACA

AATTAGCCCACTAGCAGGACGGACCTCGGCAATTAGGGCTCTGCCAGAGGCAGTGGTTGCAAACGCATTC

CAGATTTCCAGAGAAGATGCCAGGAGGCTTAAGGAGAATAGACAGGAAGTCACTGTGTTTAGCCCTGGGT

CTAGATCTCGTAATTAATATTGCAACTATGCAATTATACCAAGAGGATGTATAATAAACTGGTTTCTACA

TAGTTTAGATTTGGAGAAGAATAAGCCTATTTTTATAGGCAGTTTTAGTTCCTTNCAATAATATAAAAAT

GTAGATCTGTATATGCCTTATGTTATGAGATAATGAAAGTTTGTGGAGCTATAAGTTA

>FM891082.1 FM891082 Jatropha curcas embryo 56-70 (DAF) Jatropha curcas cDNA clone rjcaeb0_001080, mRNA sequence

CGAATCCAAAAACAATCAAGAGAATGTCTCAAATTCCAAGCACCCTCTTCGGTGGCCGGAGAACCAACAT

TTTCGATCCCTTCTCTCTCGATATCTGGGATCCATTTCAGGACTTCCCTTTCACAAGCACTGCTCTTTCT

GCTCCTCGTTCTGAGTTATTGAACGAAACGGCAGCGTTTGCCAACACCCGCATAGACTGGAAAGAAACAC

CAGAAGCTCATGTATTCAAGGCTGATCTTCCAGTGCCTAAAGAAAGAGGAAGTGAAAGTGGAGATAGAGG

AAGGTAGGGTCTTGCAGATAAGCGGAGAGAGAAGCAAAGAGAAAGAAGAAAAGAATGATAAGTGGCATAG

AGTTGAAAGGTCAAGTGGTAAGTTCTTGAGAAGGTTTAGGCTACCAGAGAATGCAAAGGTTAATGAGGTT

AAGGCTAGCATGGAAAATGGAGTCTTGACTGTGACAGTGCCTAAAGTGGAAGTCAAGAAACTGGACGTTA

CGGCTATTGACATTTCTGGTTGAAACT

>FM891081.1 FM891081 Jatropha curcas embryo 56-70 (DAF) Jatropha curcas cDNA clone rjcaeb0_001079, mRNA sequence

ATTCAGAAAATGCAGGCCTTGGGAAACTGTTACTGCTAAGAGATAATGTCTCTATTTAGTGGGCTGCTTA

AAATAGGATAGAGCAACTCGACTGCACAAAATATAGTGGAAAAGTGTGAGCCCCACCCAACAGGAAAGTG

CAGCATTTCTGCACCACCAGGGAGAAACTATGATATATAGAGCACCTAACAATAAAATTGGTGGTCAGCT

GAAACCCCTTTTCTGGGATTTGACCATGCAAAATGCTTGATGCAGATTGGTATAGTAGAATCACTTGAGA

TGAAGCAGAAAAAAGGGTAAATACAAATTTTCCAGCACTTGAAGCCTAATTGGAATAATTTTGACTATTC

TTGAATATATGAACCAGCCAAAAGAAAGAAAAGTGATTGATCATATTGGTGATTAGGAAAATAGTATATA

TAAGAAAAAGGTGAAAAGAAAAGGCATGTATTACTTGCTTTGTAATGATAAATATAGTTGAA

>FM891080.1 FM891080 Jatropha curcas embryo 56-70 (DAF) Jatropha curcas cDNA clone rjcaeb0_001078, mRNA sequence

ATTCCTGAGCTCCGAGACCGAGAAGGAACGGAAAAATGCCAAAGCAAATACATGAAATCAAGGATTTCCT

TCTTACAGCAAGAAGAAAGGATGCTCGTTCTGTCAAGATCAAGAGAAGCAAAGATGTGGTGAAGTTCAAG

GTTCGCTGCTCCAAATATCTTTACACACTTTGTGTGTTCGACTCTGAGAAGGCTGACAAGTTGAAGCAAT

CTCTTCCCCCAGGTTTGACTGTACAAGACCTGTGAAGAAACATAAAGACTAAAGTTTTTGGCCTTATGAG

AAATGAAGGGATCCAAGTCTTTTGCCTTTTTTCTATTGTCTTTTGGGTAATGTTATCAATTTCGTTGACT

GTTGAAGTAGTTATGCTGTTTGCCCAACGTTTGCTTATGGACTCCATATTAACTGGTAGACATGAGCTGT

TGAGTTTGTTATGAATTTTGATAATACTTTCAACTTGAGCACTTGGTATTATGATCGTA

>FM891079.1 FM891079 Jatropha curcas embryo 56-70 (DAF) Jatropha curcas cDNA clone rjcaeb0_001076, mRNA sequence

GCTGCAGGAATCGAATTCCCTGGTTCAGCAGCTGCTAGTCAATCTCCTAGTGGGACTGCTGGGTACGGGA

ATCAAGTTATGGGTATGGTAATTATGGTGGAAATGATGGATCTTATGTGAATCCTGCTGGATATGGTGCT

GTTGGCGGGCGTTCTGGGGGTAACCCAAACAGCAATGCTGCTGGTGCAGGTGGAGAACTACAGGAGACTG

GCAGTGGCTACATGGGAAGTGGTTATGGCGATACAAATGGAAATTCAGGATATGGAAATGCAGGATGGAG

ATCTGAACCATCACAAGCTTCTGGAAACTATGGAACTCCTCAGGCAAATGGACCTCATGGTGGGCAAGTT

GGTTACGTAGGGGGATATGGTGGTGCTCAGACTCGACAAAGCCAACAACAGTGATCATGGACAGATTGAT

GCCAGCTGGATCTTCTTCATTGTTTAATTGGTACCCCTGCTTTTCTCATGAGGTCCTTTGAAGATTTCTC

ATTCCCAGCAGCA

>FM891078.1 FM891078 Jatropha curcas embryo 56-70 (DAF) Jatropha curcas cDNA clone rjcaeb0_001075, mRNA sequence

CGAATCCACAAACCTAATATTTAATTAAGAGAGAGATAAATTTAGTTATAAAATGGATGGAGAGTACTGG

AAATAAGAGCAAAATGGAGACAACGTGATACCAGGGTCGGTTGGGGGCAAAAAGGAATGAAGGGTGTCTC

GTTTACTTTTGCTCATTCGGTAATGTTGTTTTATAGGTTTATGTTTGTTATGCTAATTATGAATGTTAGT

AGGAATGTCAGGGATTTGAAATTCCTTTCACAAATGTCC

>FM891077.1 FM891077 Jatropha curcas embryo 56-70 (DAF) Jatropha curcas cDNA clone rjcaeb0_001072, mRNA sequence

ATTCAACGTTATGGCTCTGCCAGATGCAGTGGTTGCAAACGCATTCCAGATTTCCAGAGAAGATGCCAGG

AGGCTTAAGGAGAATAGACAGGAAGTCACTGTGTTTAGCCCTGTGTCTAGATCTCGTAATTAATATTGCA

ACTATGCAATTGTACCAAGAGGATGTATAATAAACTGGTTTCTACATAGTTTAGATTTGGAGAAGAATAA

GCCTATTTTTATAGG

>FM891076.1 FM891076 Jatropha curcas embryo 56-70 (DAF) Jatropha curcas cDNA clone rjcaeb0_001069, mRNA sequence

TCCCATTCTTATAGTCTGGTTAATTTACCGACCCTACAAACCTCAGTTCACTGTAGTTGGTGCTGCCATT

TATGACCTTAACACATCATCTCCACCTTTCATCTCCACTTCCATGCAGTTCACTCTTGTGACTAGAAATC

CTAACAAAAGAGTCTCCATAATCTACGATAAACTCTCTGCTTACGTTTCTTACAGGGACCAAGCAATAAC

GCCTGCAATAGCGTTGCCTCCGCTTTATCATGACACGAAGAGCACTGTGGCGCTGTCGCCAGTGCTGGGT

GGCTCTAAAGTGCCTGTGTCGGCGGACGTGGCGAATGGGTTGGTGATGGATGAGAGTTATGGGGTGGTGG

CATTAAGGGTTGTGTTGTTGGGGAGATTGAGATGGAAAGCAGGGGCAATAAGGACAGGAAGATATGGGGT

TTATGTCAAGTGTGATATTTGGGTGGGTTTGAAGAAGGGTTTGATAGGGCAAGTTCCTTTGCTTGGATCT

CCAAAGTGCA

>FM891075.1 FM891075 Jatropha curcas embryo 56-70 (DAF) Jatropha curcas cDNA clone rjcaeb0_001068, mRNA sequence

GGATAAATCTCACATTGTCTAGGATTGCACCTGTTCCACACCTTCACTGACCTACACGAGACTTGCCTAT

ATGACAAGGAATTAGTGATTTCGTACAAGATTGTCATTGTTAGGTTGGGGTACATCTCATGCCATAAATA

TAAATGAGTCCAGACTTTAATTAATTTTTCAGCTATACAAGTAAATTTTCTTGTCAAATAGGGGTATCCT

GTTTAATCGAAAAAAGAACCTCTCTTTCAAGCAATTCAAATATCTCCTTCAATCTATGTGTGAATTTCTC

AGTTATGTTTCGAGTGATCCTCTGCTGGAACTTGGTGCTCTTCAACCAGTTTGTCCCCACATAAACGTCA

CACTTGCATGCACTATGAGCAACGGTACATTGCTCAATCTGGTACCTAAAATTTACGCGGAAGTGATCAC

CAAATGGGACATTATGC

>FM891074.1 FM891074 Jatropha curcas embryo 56-70 (DAF) Jatropha curcas cDNA clone rjcaeb0_001067, mRNA sequence

ATTCCAAGAACCAGAAGATCAGNGATTATGGCGGCACCAGCAATTTCTGTAAAAGCAGAGGCCTTGACTA

TAGAGAAATCTGGAATCAAGATCGTTAGGAACCTTCCCGAGTCCAAACTCACTGAACTAGGAGTTCGAGC

TTGGCCTAAGTGGGGTTGCCCTCCAAGCAAATTCCCATGGACATATTCGGCCAAAGAGACATGCTATCTT

CTGGAGGGTAAAGTTAAGGTACACCCTGNATGGATCAGATGAGGCCGTCGAAATTGGTGCTGGTGACTTG

GTTGTGTTCCCTAAAGGAATGAGTTGCACTTGGGATGTTTCTGTAGGAGTTGACAAGCACTACAAATTCG

AATGAATAAAATATATTTGCTGGTTGTGTACCCTCCATCCCTCTGTCTCTCTCGTATGAATTAGTAAATC

TTCATATACTGATACTGAAACCATTTGCTAGCTGTATGAATTTGATATTAATCGGACCTGGCAATTAGCT

TACAATTTTCTCGTTTCCGTCGCCCTTTTTACCCATATACATACGCACAACCAAGCAAGCATGTAGCGAT

>FM891073.1 FM891073 Jatropha curcas embryo 56-70 (DAF) Jatropha curcas cDNA clone rjcaeb0_001066, mRNA sequence

ACTGAGCTCACGCGGGGCGGCGCTCTAAACAGTGGATCCCCCGGCTGCAGAATCGAATCCACACTAAACC

AAGAAACTTTCTCTCACCGATCTTTGATCTTCTCCAAAAACCAATCAAGAGAATGTCTCTGATTCCAAGC

ACCCTCTTCGGTGGCCGGAGAACCAACATTTTCGATCCCTTCTCTCTCGATATCTGGGATCCATTTCAGG

ACTTCCCTTTCACAAGCACTGCTCTTTCTGCTCCTCGTTCTGAGTTATTGAACGAAACGGCAGCGTTTGC

CAACACCCGCATAGACTGGAAAGAAACACCAGAAGCTCATGTATTCAAGGCTGATCTTCCAGGNCTAAAG

AAAGAGGAAGTGAAAGTGGAGATAGAGGAAGGTAGGGTCTTGCAGATAAGCGGAGAGAGAAGCAAAGAGA

AAGAAGAAAAGAATGATAAGTGGCATAGAGTTGAAAGGTCAAGTGGTAAGTTCTTGAGAAGGTTTAGGCT

ACCAGAGAATGCAAAGGTTAATGAGGTTAATGCTAGCATGGAAAATGGAGTCTTGACTGTG

>FM891072.1 FM891072 Jatropha curcas embryo 56-70 (DAF) Jatropha curcas cDNA clone rjcaeb0_001065, mRNA sequence

ATTGGAATACAATTATATTTATAGAATATATTGATTAGGGAATACTTAATTACTAATTAGGATGATTACA

TTACTTTCATCACTTATTTTCATAATATATTACATACTTCATTAGCGTGAGATCATAACTCCCCTCGTAA

TACTACTTACTTTCACTATCTACACACCCAAAAAGGCTACTTCATTATTTACTCCTCGGCATATCTTCCA

GACTCGGTCCTGGAAATAGTCAAGGTGGTCTCTTGTCTTCCAAACTTAATCCTTCTTGCCTCCTCTGCTG

ATACCCTAAATGCATTTGCAATAACCGCCAACGGCATTGCTCTAACGGCGGAGGAGCGACCTGCAAGATC

ATTGGTCATCGCGTTATCGCTTGTCTTGAAGGTTACATATTCGAATCTTTCACTTTCTGCTCGTTTCACC

ACTACGAAGTTTTGTGGCACCGTCAAANACTGTCCTTTGCTTACACTTCCGTCGAAGACGGTGTTGCG

>FM891071.1 FM891071 Jatropha curcas embryo 56-70 (DAF) Jatropha curcas cDNA clone rjcaeb0_001063, mRNA sequence

GCGCTCTAAACACTGGATCCCCGGCTGCAGAATTCGAATTCCTTCTATCTCCACAAATGCCAAATTTGAA

GTGCGAAGCCCCAAGCGGGTGCAGATCAAGTTTGAAGAAGGTGTCATTGGGACTCCCCAGCTCACGGACT

CCATAGTAATACCGGAAAATGTGGAAATTTCTGGGACAAAAGATTGACCTCACACCCCTTAAGGGCATAT

TAACCTCTGTCCAAGACACAGCATCATCAGTTGCAAAGACCATTTCAAGCCAGCCGCCATTGAAGTTTCC

AATCTCAAACACTAACGCCGAATCATGGCTGTTGACCACATACCTCGACGAAGACCTTCGAATCTCAAGG

GGTGATGGTGGCAGCGTGTTTGTTCTCATCAAAGAAGGCAGACCCCTCTTGAAACCTTGAACCTCGTGTA

AAACTAGTACTGTTTTATCAATCACAAATAAGTAAAGAAAAGTAGAAAGTGCTTTATAATGTATTACTGT

ATATAAACT

>FM891070.1 FM891070 Jatropha curcas embryo 56-70 (DAF) Jatropha curcas cDNA clone rjcaeb0_001062, mRNA sequence

CGAACAAACTTATTCTGATGCTCTTACTTCTTATACATCATTATATATAACTCTCACTCACATACAAGTG

AATGAAGTTTTATTCACCTGCAGCTAGCATTTTAACGAGGGCATATAGACCCAAAGCAAACATCAATTAT

ATTTTTAGTCCTGCGCCTTGCCTCATCTTCGCTTCCAACTTCTCCTTCCTCCAGCTGCTTCTCTAACAGA

TAATGTATTGATTCACATCGACAAATAGCACTTAGTTCCTTTGCTTGGTTACAGCATTGTCTAGGAACCT

GCTGTCGAGGGTTTTCAATTCCTGGCATAGCTAACATATCTTCTGACCTTCCTCTTCTCTGTGTTATATA

ATTCTCACAGGAACTCAAATCTCTTCTTTCAGCTTCCTTCTTGCATATATCCCGAACATTGGAAGAATCG

TCGACTTCAACGGTGGTGATGATAGTTCTGGGGGGGGC

>FM891069.1 FM891069 Jatropha curcas embryo 56-70 (DAF) Jatropha curcas cDNA clone rjcaeb0_001061, mRNA sequence

ATTCCCAAGAATAAGGAGGGGATCCCACCAGACCAGCAGAGGTTGATCTTTGCTGGGAAGCAGCTTGAAG

ATGGAAGGACTCTTGCAGATTATAATATTCAGAAAGAATCTACTCTGCATTTGGTGCTTCGTCTGCGTGG

TGGAATGCAGATTTTTGTCAAAACCCTGACAGGGAAAACCATTACCCTCGAGGTTGAGAGCTCTGACACA

ATTGACAATGTGAAGGCTAAAATCCAGGACAAGGAGGGTATTCCCCCGGAACCAACAACGATTGATTTTT

TGCTGGTAAACAACTTGAAGATGGACGAACCCTTNCAGACTATAATATCCAGAAAGAGTCCACTCTTCAC

CTTGTCCTCCGACTCCGTGGGTGGTTTCTAATCTGTTTGCTGTTTGGTTTTTAAGCAGTTACTTATATCT

ATAATATC

>FM891068.1 FM891068 Jatropha curcas embryo 56-70 (DAF) Jatropha curcas cDNA clone rjcaeb0_001060, mRNA sequence

GAATTCTCAGTGCGGCTGAAGGCCAGACCAGCGATGCTACAGAAGCAGCAAAGGAGAAAGGTGAGGAGGC

AAAGAACACGGCCTTTGAGAAGGCTGAGGAAGCAAAGGACAACGCCTTTGAGAAGGCTGAGGAAGCAAAG

GACAACGCCAGAGAGACAGTTGGAGCTGCGAAAGAGAGAGCATACCACAAGTCCGAAGAGGCAAAAGATA

AGGCAAAGAAGACATCACAGCATCTCAAGGATAACGTGGCTGGTGCCGGTGGTGAATTGCCGGACGAAGA

GCTCTGATTTGTTTCTTTGGCTGTTCTGTTGTCGTTTTGGCGTCTATTTTACATATCTGGTGTCCTGTCT

TGTATTTTGTTGGGCTTGAATGAAGTAGAAATGAAGTATAGTTGGGATGAGTGACGACAGAGCCCATGTG

AGGCCTGTATGAGCCCATATGTATATTTTCTACATGGATG

>FM891067.1 FM891067 Jatropha curcas embryo 56-70 (DAF) Jatropha curcas cDNA clone rjcaeb0_001059, mRNA sequence

ATTCGGAATTGAAAACCAGCAACAACGGGATCTTCGCCAATGTTGTAACGAATTAAGGCAGCTAAGTCAT

GATTGTCCATGTGAAGGAATAAAGTATGTATTAGAGCAGCAGCTGGAGCAAGGACAGCGAGAGCGTTTGG

AGGCAGTGCGTAGGGCTAGAAGTATACCTTCTGCTTGTGGCCTGCGTACAAGCTGCGATATTCGTTCCTA

ATCTGCTAGCTATAACGTGGATGTGCTTCAGTTAAATAAAAATTTCACTCACTTGGTGTGTGAGTGATCA

GAGTTATGTATAATAATGCACAAAAGAATGAGCACATCAAGCTAAGTTTGTTTTCTGTTAAAAAAAA

>FM891066.1 FM891066 Jatropha curcas embryo 56-70 (DAF) Jatropha curcas cDNA clone rjcaeb0_001057, mRNA sequence

CCCAAAAAAGTAAAAATACTTCTTCTGGTCTGGCCTTATCATTCTAGTTGACGGATACAGCTCATTTCAT

TTATACTAAACGACAATCATTCGAGTAAACGACCATTCTCACTGTATGAACTTACAACTTACAATCTCAC

ACTATACAAATGCAAATGCAAAAACAAATTTGAACTGTCAGCCACATTTTATTTTTTCATCTTTATATAA

TTAATTTCCAAATGTACTATATATCTGCCTTTTCGGGGGGGCCCGGTAC

>FM891065.1 FM891065 Jatropha curcas embryo 56-70 (DAF) Jatropha curcas cDNA clone rjcaeb0_001056, mRNA sequence

CGAATTCATAAAAACCAAAGAATATTTTGCTCAAAAGTTTCTCAGTTGATTATGGTGATGATGATAATCA

GCTGAAATTCCAAATTTAACATGGAAACCGCCGCTCTCCGTCACGCAATCGCAATCGGCGTGACCGACTT

CCCCTTGAGTCGCTCCAGACGGAGGCGAGTCAACTCGGGTCAATCTTATCTTTCACCGNAACTTCTCTTT

CTTCCCGNTTTTCACCTCAAATCCATATATCCTCCTCGCAAATCCTTCAATCCCCGTTACCAAACGCTCC

GTTGTTTGGCGGAAGCTAAAAATCACCGAGATCATGACCTCCACCAGCACGACGATCACCC

>FM891064.1 FM891064 Jatropha curcas embryo 56-70 (DAF) Jatropha curcas cDNA clone rjcaeb0_001054, mRNA sequence

ATTCGAATTCCAGCTCTTGAAATCCATGGCTAATAAAGATTATGGTTTCAGTGCACTTGTTTGCTCTTGA

CATGGAAACTGAAGAAAGGGCTAACCGGTTGATGAACCACTTGCAGAACTACACCCAGTTTGGGCTTATG

GCAGTTAGTTTGGGATACTACGAGACGTTAATGTCAGTTTCTGGGAGTAGCACCAGCAGTGAAATGAGTC

CTGAGGAAAAGAAACTAGCTGGGATTTCGCCTGGGTTAATTAGGATGTCTATTGGGTATATTGGCAATTT

GGAGCAGAAATGGTCACAATTTGAGAAGGCAATTTCCAGAATGCAGGACTCAAGCTTCGGTTACAAGGGT

TAATATATATATATATATAAATAAAAAATAAA

>FM891063.1 FM891063 Jatropha curcas embryo 56-70 (DAF) Jatropha curcas cDNA clone rjcaeb0_001053, mRNA sequence

CGAATCAATAAGACCTGCAATTGAGGAGCATGAGAGGGATAACCAGGATAGTGTAGCTACAAGTGTAGCT

GAGAAGTGGATAGAAGTATGCTGCCAAAAATTGAAGGCAGAATTTGACCTCTACTCTTCAATTATAAAGA

ATGTCGTCTGCACTCCTCGGAGAGCAACTGATCAACCACAACCTTCTGAAACAGGAGGAATCAACGATAA

TGAGGTGAAGTACTTGGGATTTTGAACATACAGTCATTTTCTTCAAAGTAACGATTTAGGACTTGTTTAC

TTTCATTTTGTTTCATTTTTAGAAAACTAGAGAATACATCCATCCACCTCTACTTATTACTCAACTTAGG

TTGTATTAGTATTATCTTCTGGAAGAGATGGAAGTTTTCTTTT

>FM891062.1 FM891062 Jatropha curcas embryo 56-70 (DAF) Jatropha curcas cDNA clone rjcaeb0_001052, mRNA sequence

ATTCGAAAACTATACATAACCTTGAGTCTCGTAGGGAAGCTGATTTTTACACAAAGGAAGCTGGTAGAAT

CCACCTTGCAAATCAACGAAAGCTCCCCATTCTCAGTTTCATGGACATGAGCGCTGAGCGAGGCGTTCTG

CTGCCGAGGGCAATGCACAGCCCACACTGGTCCATAAACGATCAGAGAATAGTGTGTGCTCTTCGCGGCG

ACATGCACATACAGATTGTGGATGACAGTGGAAACACAGTAATGGACGAAAGGGTGAAAGAAGGAGACAT

GTTCGTGATCCCTCAATTCTATGCAGCACTTGGAAGGGCAGGAGAAAATGGGTTCGAGTACGTAACATTT

AAAACAAGTAGCCAGCCTATCAAGAGCCCATTGGCGGGTTATACATCAGTGATGAGAGCCATGCCTCTTG

ATGTTCTGATGAACTCTTACAGAATGTCTCCAAGCGAAGCTCATCAAGTGAAGAGGAACA

>FM891061.1 FM891061 Jatropha curcas embryo 56-70 (DAF) Jatropha curcas cDNA clone rjcaeb0_001051, mRNA sequence

GCTGCAGGAATTCGAATTCGAATTCGCAACATCAACCTGCATAATTAGTGATTATCAATCAGTAATCTGT

AAAATTCCTACATCTATATACAAATATAATTTTGGTTCTTCTTCGAGATATCTGTCCAACACTGTCCTCT

AATAAAGTTATTATTGGTTCCTCAACAATCAAATATCCTCATAGAACTCCGGATTGCCTGCCGACAGAGA

GGACACTTTGGTGATATCTCCCGTTCAACTGATATCGCACAGAGTTGGCAACAAACAAGATGCCCGCATG

GAACGAATGCAGATCGTCTTCTCCTCATCAAGCAAATCACACATAATTGTCCATCCGGTACATCTCCTGT

CTCCACATCTGCATCAATCTGAGGGGCATCCCGCGATCACTAAGAGCATGTGCTTTGTTGCTGGAGACTG

TCTCTGATTGCCTTCCACTCTTTCCATTTATTCCAGTTCCTCACAACAGCATAGCCAAGAATACAATTGA

CAGCGAACCAAGAACAATACCACTCCACAACA

>FM891060.1 FM891060 Jatropha curcas embryo 56-70 (DAF) Jatropha curcas cDNA clone rjcaeb0_001050, mRNA sequence

ATTCCACACNTTATCATCAACAAATGGCTGAACGTTCACAGCCACACCAAGTTCAAGTGCACCCACAACA

CCGCTATGAAGCTGCCTTCAAAGGTCAACAAAAAGGTCCATCAGCTCAGAAAGTGTTAGCTGTGATCACC

CTCCTGCCGGTAGGCGGTGGGCTTCTTGCACTAGCTGGTATAACCTTAGTAGGTACCCTAATCGGCCTAG

CTATTACTACCCCTCTTTTTGTCATCTTTANCCCTGTTCTTGTCCCAGCTGCATTAGTTATTGGCCTTTC

GGTCATGGCATTTTTGGCTTCCGGGGCTATGGGGCTCACAGGGTTGTCATCACTCTCGTGGGTTCTGAAA

TACCTCCAGGAGGTCACTCGGCGAATGCCGGAGCAGCTGGATATAGCTAAGAAGCGTATGCAAGACATGG

CTGGTTTTGTGGGACAGAAGACCAA

>FM891059.1 FM891059 Jatropha curcas embryo 56-70 (DAF) Jatropha curcas cDNA clone rjcaeb0_001049, mRNA sequence

GTCTGGTAAACCAACTTTCCTCCGGGAGTTTTGACGACCCTGATGCTGGTTCGACTTCGTGGCATAGCTG

TGCCGTTTCCGATATGTGAGCCGCTAAACCATTTTATGTATTCTCCCTCTCCCACTCTCTCACCTAGGGT

TTAGAGTGTGGATGCTGCGTTGGCAACGGGGGGGGCCCG

>FM891058.1 FM891058 Jatropha curcas embryo 56-70 (DAF) Jatropha curcas cDNA clone rjcaeb0_001048, mRNA sequence

GAGAGAGAACTAGTCTCGAGTTTTTTTTCCAGAGGATTTGAGGGACAGGGTCGGAGGGGGGGACAAGTAG

AGTGTGATCATAAAACTGCTCTTGTTAGCAAATCAATCAATATAACTAACTTGTTTAGCCAGATAATTCT

TTACGGTTGATGCATTTTGGCTTCTCTTTTTCCCTAACACAACAATGTTCCCTTCTTTAACTTCTTTAAG

TTAGCAGTTGAAATTCGCCGTGTTTTAGTGTTTTGTACGTGTCGAGTTGAGTTGACGAAGGGAAGAACCT

GGGAAAGGAAAATAGCATTCAAAGATTTTCTGGGGGGGCCCGGTACCCAATTCGCCCTTTAGTGAGTCG

>FM891057.1 FM891057 Jatropha curcas embryo 56-70 (DAF) Jatropha curcas cDNA clone rjcaeb0_001047, mRNA sequence

ATTCGCCATGGCTGCCCTAAATCAAGAAGTAATGCAGCTTGGCCAGTCTCTTTACAACCAACCAGGTGCA

GGTGGTGCTGGCCCTGCTCCTGGCAGTGAAGCTGGGCCTGGACCTTCATATTCATCAAGCAAAGGACCTG

ATGGCGATGTTATTGACGCAGATTTCACTGACAGCAAGTGAGATGGCAAGATAGTTGGCTAGTTTTTGTG

CCCCTTGCTGCTGATGTGTATAAGAAATTTGATTTTGCCTGGTCTGAGGGTTACTGCATTTCTAGACGAC

TTTTGCTCTTTGTGGAGTGATAAGGAAATCGTTTTTAGGAATATAGAGAACCAAGCAGCAAACAGCAAGC

AACCCACAATAGTTAAAGAGAAGAACTTTGGGTGTTAAAGCCAGTGCACTTATTTATAGTGGTGTTTAGA

TTTAGTATGGCGGATTG

>FM891056.1 FM891056 Jatropha curcas embryo 56-70 (DAF) Jatropha curcas cDNA clone rjcaeb0_001046, mRNA sequence

CTTCGNTTGACTAATCCATCCATGTAGAAAATATACATATGGGNCTCATACAGGCCTCACATGGGNCTCT

GTCGTCACTCATCCCAACTATACTTCATTTCTACTTCATTCAAGCCCAACAAAATACAAAACAGGACACC

AGATATGTAAAATAAACGCCAAAACGACAACAGAACAGCCAAAGAAACAAATCAGAGCTCTTCGTCCGGC

AATTCACCACCGGCACCAGCCACGTTATCCTTGAGATGCTGTGATGTCTTTCTGGGGGGGGCCCGNGTAC

CCAAT

>FM891055.1 FM891055 Jatropha curcas embryo 56-70 (DAF) Jatropha curcas cDNA clone rjcaeb0_001045, mRNA sequence

GCAAACACAGAAAACTACTACTAGTTACTCATTTACATGTACTTGTTACCTTTACATAGGCTTTCGATCG

ATAGCTTAGACTCTTGCACGCAACCAACAAAAAAATACAAACAAAAAGCACCAAAACGCAACATACATCC

AAATTTCGCAAAACGCTCACCCCTAGTACTGTATTTTAGTCATACCCATAACTCCAGAAGCTGCAGCGTT

AAAAGCTTTATCTATTAGTAGTAAAAGACCATATTTGCACTTCTAGCCAGTGATCTCAATAGGCTTAATC

TGAGGCTGCTTCTCTTGTTCTTTAGGCACAACCACTTTAAGCACACCGTTCTCCATAGTGGCCTTGACTT

GATCCATCATTGTGTTCTCCGGCAACCTAAACCTCCTCAAGAACTTACCAGTGCTTCTTTCTACGCGATG

CCACTTATCGGTTCTTCTCTTCTTGTTCTTTGCTTCTCTCCCCACTTATCTGCAAAACTCTGCCATCTTC

TACCTCCACTTTTACCTCTTCTTTTT

>FM891054.1 FM891054 Jatropha curcas embryo 56-70 (DAF) Jatropha curcas cDNA clone rjcaeb0_001044, mRNA sequence

ATTCCAACAGCATCTGCCTTTATAATGCTTTGACTGACATGCTGTGGCACTTTGGCCAGAAACGAGGTGC

CCAACTGGTTGTGCTTGAAGGGAAACGTCGACAAGTATGGGAGAACGTGTGGTCTAATTCTTGCTTAGAT

TTGCATCTGATGTCTTCTGGTGCTGCTCGGGCAATGGTTCATGCATGGTTGCTGAACATTCGCTCTATTG

TGTTTGAGGGCCATGAATTGCCAAAATTATTAAGCATTTTAACTGGATGGGGCAAACACAGCAAGGTAGT

TGGTGATAGCACACTAAGGCGAGCTATCGAGGCGCTTCTTACTAGCATGGGTGCACCCTTTCGATTAGCT

GAATGCAACTTAGGTCGGTTTACATCAACAGGATCAGTTGTCGCTGCCTGGTTAAAAGAATCTGGCACAC

TAAAATTGCTTGTACTTCACGACGATAGAACTCATCCCGAAACTATGAGGTTTGATCAAATCTCTAATGT

ACAAATGCTTCCTTTGTAGTTATTTGTATGAGGTTGCAAATGTTTTGTTGCCCGCTTGTAACATGTTCGC

GTTAATTCC

>FM891053.1 FM891053 Jatropha curcas embryo 56-70 (DAF) Jatropha curcas cDNA clone rjcaeb0_001042, mRNA sequence

GGACTATAACTTATAGCTCCACAAACTTTCATTATCTCATAACATAAGGCATATACAGATCTACATTTTT

ATATGATTGCAAGGAACTAAAACTGCCTATAAAAATAGGCTTATTCTTCTCCAAATCTAAACTATGTAGA

AACCAGTTTATTATACATCCTCTTGGTATAATTGCATAGTTGCAATATTAATTACGAGATCTAGACCCAG

GGCTAAACACAGTGACTTCCTGTCTATTCTCCTTAAGCCTCCTGGCATCTTCTCTGGAAATCTGGAATGC

GTTTGCAACCACTGCCTCTGGCAGAGCCCTAATTGCCGAGGTCCGTCCTGCTAGTGGGCTAATTTGTGCA

TTTTCGTTAGTCTTGAAGGATACCCACTCTAGCCCTTCATTGCTTGCCCTCTTCAAAACCACAAAGTTCT

GTGGAGCTGTTAATATTTGACCTTGTTGAACTTGTCCATCGAACACTGCATCTCCCATTCT

>FM891052.1 FM891052 Jatropha curcas embryo 56-70 (DAF) Jatropha curcas cDNA clone rjcaeb0_001041, mRNA sequence

CGAAGCTAGCTGGCGGCGAGAGTTCTGAGGAGGACGAGAAGGAAGAAGCTTAAAAATGGCAAGAGGGTTG

AAGAAACACTTGAAGAGGCTTAATGCCCCGAAACATTGGATGCTTGACAAACTTGGTGGTGCATTTGCGC

CCAAACCATCATCTGGACCTCATAAGTCCAGGGAGTGCTTGCCTTTGATCCTTATTCTGCGCAACAGGCT

GAAGTATGCTCTCACATATCGTGAAGTCATCGCTATTCTGATGCAACGACATGTTCTTGTTGATGGGAAG

GTTAGGACGGATAAAACATACCCATCAACTAGAACATGTCG

>FM891051.1 FM891051 Jatropha curcas embryo 56-70 (DAF) Jatropha curcas cDNA clone rjcaeb0_001040, mRNA sequence

ATTCCAACCTGNCCACCAAGGTCTGGGTGCTCAGCTCTTTGACAAGCCGCAGCAGCCTAATTCCCCCACC

GTTTATGACTGGCTTTACAGCGGAGACACCAGGAGCAAGCATCGCTGAAGTGCTGAAGTGGCAGTCAAGT

AGGGTTGCCATGTAAATACCGTCGTTTTGTTTCCCGGTCTTCACTGCGTTGCTGTCGTTTTAATTAATGC

TTACTATGCTTAGTATTTGCTACGATCTGACTCAAAAGTGTTTAAGCTATGTGTGAGTAAAATGTTATCA

CGGTTTGTTGACCTCTGTAATTCGAGATCTGCCGTGGACGGTGGTGATGAAGCTACTTTATTGTTGTGGT

TAAATAAAGAAATTGCATTTGTAAGTTTTTTAATTTGAATGGGGGGCCCGGTTAC

>FM891050.1 FM891050 Jatropha curcas embryo 56-70 (DAF) Jatropha curcas cDNA clone rjcaeb0_001039, mRNA sequence

ATTGGAAGTTATTACTCCATACCTGTAAAGATGAAATTAGTTGCCTTTTTGTTCCTCTTGTTAACCGCCA

TCGCCTATGCACATCACTGGGATGGTTACGGCTACGGAAGCGGCTACGGAAGCGGCTACGGAAGCAGCTA

CGGACACGGCTACGGACACGGCCATGGACACGGCCACGGCTACGGTTATCGTCCACATCATGGTCACTGG

GATGGTCATGGTCATGGCTACGGTTACGGTTACGGTTACTAAATTTCTAGCCGCATCTAACATGATATTA

GTAATTTTGTTTCTAATCATAATAATAATGCATAAACATTA

>FM891049.1 FM891049 Jatropha curcas embryo 56-70 (DAF) Jatropha curcas cDNA clone rjcaeb0_001038, mRNA sequence

CTGCAGGAATTCGAATGTGAAAAGGATATATCTCTTGTATCCTTTCAGTCAGTCTCTAAAGGGTACTATG

GAGAGTGTGGAAAGAGAGGAGGTTACATGGAAGTCACTGGTTTTAGTCCTGAAATAATGGAACAAATATA

CAAAGTAGCATCTGTGAATCTTTGTTCCAATATTTCTGGTCAAATTCTTGCTAGTCTTGTCATGAGTCCA

CCCAAAGTTGGAGATGAATCATATGAATCATATTCTGCTGAGAAAGATGGAATCTTGTCATCCCTTGCAA

GGCGTGCAAAGACGTTGGAAGATGCACTCAACAATTTGGAGGGTGTAACATGCAACAAAGCGGAGGGAGC

AATGTATCTCTTCCCCCGCATTGTTCTGCCTCAAAAGGCAATTAAAGCTGCAGAGGCTGCAAAAAAAGCT

CCAGATGCATTCTATTGCCAGCGTCTCCTCAATGCCA

>FM891048.1 FM891048 Jatropha curcas embryo 56-70 (DAF) Jatropha curcas cDNA clone rjcaeb0_001037, mRNA sequence

ATTCGGAATTCCGGACTTATGTCACACTAGGATCTTTCGCTGGGATCAGGACAATGACAAAGTCAAGATT

TATGTCTCTATAGAGGGTGTCGAGCAGGAAAAGATTGAGACAGAATTCAAGCCAATGTCATTTGATGTCA

AATTTCATGATGTCCAAGGGAAAGAATTATCGATGTGCCATACCAAAATTGAACAAGGAGATTGTACCAG

AGAAATGTAAAGTGGTAGTTAAGCCTACCAGGGTTATCATCACTTTGTTTAAAGCTTCAAAAGGGAACTG

GTTAGACTTGCACTTCAAAGAGGACAAGCTGAAGCCCAATTTGGATTAAGAAACGGGATCCCATGGCTTG

GAATTATGGACTTAATGAAG

>FM891047.1 FM891047 Jatropha curcas embryo 56-70 (DAF) Jatropha curcas cDNA clone rjcaeb0_001036, mRNA sequence

CCCCCGGGCTGCAGGGCAAACTGAAGTCCCACAGAAAGAAGGCAAAGGTGGGCTGACAAGGCATACAAGA

AATCCCATCTTGGTAATGAATGGAAGAAGCCATTTGCTGGCTCTTCCCATGCCAAAGGCATTGTTCTTGA

GAAGATCGGTATTGAGGCCAAGCAGCCTAACTCTGCTATCAGAAAATGTGCTAGAGTTCAACTGATTAAA

AATGGAAAGAAGATTGCTGCTTTTGTACCAAACGATGGTTGCTTAAACTATATTGAGGAGAATGATGAGG

TTTTGATTGCTGGATTTGGACGAAAGGGTCATGCAGTCGGAGATATTCCTGGAGTTAGGTTTAAGGTTGT

GAAGGTCTCTGGTGTCTCTCTTCTCGCACTCTTCAAGGAGAAGAAGGAAAAACCAAGATCTTAAGTTTGC

TATGGAGAGTTTATCTACTGTTATAGTTTATTGCTAGTTCTTTTTGCGGTACTGGGAATGTTTCTCTATG

TNTTTAAGTGGATCTGTTGAAAACGATTTTTATCTTTCTAGTTATTGTACTGAATCTCTCTGATTTCAAT

TTTTGTT

>FM891046.1 FM891046 Jatropha curcas embryo 56-70 (DAF) Jatropha curcas cDNA clone rjcaeb0_001035, mRNA sequence

GGGCTGCAGGAATTCGAATTCTGTGTTTAGCCCTGGGTCTAGATCTCGTAATTAATATTGCAACTATGCA

ATTATACCANAGAGGATGTATAATAAACTGGTTTCTACATAGTTTAGATTTGGAGAAGAATAAGCCTATT

TTTATAGGCAGTTTTAGTTCCTTGCAATAATATAAAAATGTAGATCTGTATATGCCTTATGTTATGAGAT

AATGAAAGTTTGTGGAGCTAAA

>FM891045.1 FM891045 Jatropha curcas embryo 56-70 (DAF) Jatropha curcas cDNA clone rjcaeb0_001034, mRNA sequence

AAATAAAATATCTACTAAAAGATACCTTTTATTCANACTGGAATTGCTTAACAAGAAAAAGACACCAAAC

AGAAAACATAGATATATTACATAACAGGAGCTAAAGCACATGCCATGCCCTACACACGTGGGTTATGGCC

AGTGGTGTCTCCGGGCAACCCAGAATGGGCGAGTCCTGGTCAAACCCGTTGGGTCCACCTCTCCACCGTA

TGGCTGCCCTGTGCCGTGTCCAGGCATGGCCGACATTTGCTGGGTCCCTCATAGGTAACCGGTTGTCCCA

GTTGTTGAATGGGTATAGGTTTCGGTTGTGCCTTGAGCGCCCGGACCAGCAGTTGTGTACTGTACATGGC

CACCAGCCTTGGCTGCCTGCCTCGCCGCTGCGTTATCCTCGCGCGCTTCTAGTTTCCGCAATTCTGCCTC

CGTTTTTCCTCTCTTCTTTCTTCTCT

>FM891044.1 FM891044 Jatropha curcas embryo 56-70 (DAF) Jatropha curcas cDNA clone rjcaeb0_001033, mRNA sequence

AAAATAAACTAAGATGCCATTTATGTAGCAAAAACTGAAAATATTGCAGCCAAGATATTGAAAGTAAAAA

TCGGACAGATGGTTTACATAATGTCTAAGTAGAATATTAAAATAATAATAATAATAATTGTACTGACCAA

TAAAGAAAATAAAATATTCATCACCATCTTTACTCAAGCATCAAACAAGCCACTTAGTCAACTTCCTCGA

TCTTGGGACCAGCACCACTGCCACCTGCTGGAGGAACATCATCTTCCATGCCGCCACCCATGTCAGCACC

AGCACCTTGATACATCTTGGCAATGATGGGATTGCAGATGCTCTCAAGCTCTTTCATCTTGTCCTCGAAC

TCATCTGCCTCTGCAAGCTGGGGGGGCCCGGTAC

>FM891043.1 FM891043 Jatropha curcas embryo 56-70 (DAF) Jatropha curcas cDNA clone rjcaeb0_001031, mRNA sequence

ATTCCACCCACAGTCGCAGCATGTTGGCCAACAGCCACGATCCCACCAAGTAGTGAAGGCAGCCACTGCA

GTCACTGCCGGTGGATCTCTTCTAGTTCTCTCCGAGTTTGACCCTGACTGGTACGGTCATTGCACTGACC

GTAGCCACTCCATTGCTGGTCATATTCAGTCCGGTTCTTGTTCCTGCGGTTATCACAGTTTGTTTGATAA

TCACAGGATTTTTAGCCTCAGGCGGTTTTGGCGTCGCTGCCATTTCTGTCTTGTCCTGGATTTATAGGTA

TGTTACTGGAAAGCATCCACCAGGAGCAGAAAGTCTGGACCAGGCACGCTTAAAGTTGGCGGGAAAGGCA

AGAGAGATCAGGACAGACTGAGCAGTTGGACAGCATGTAACAGGTCAACAGACTTAAAAGAG

>FM891042.1 FM891042 Jatropha curcas embryo 56-70 (DAF) Jatropha curcas cDNA clone rjcaeb0_001030, mRNA sequence

CGAATCGATCGATAAAGTGTACAGAGCAAAAGATTAATTATATTGGTGGAGAAATTTGTTANCAAAAAAG

GGCAAAAATGTTTCAAATCAATCAAGACAAGAGCTTATGAAAGAGCAAGGCGTGAGAGACTGTTGTCCCT

GGAGGTACTGGGGGCAAGAGCCTTGAAGCTCAAGAGCATCTTGCTGAAGGGAGAAGCAGAGGAGGACAGA

CAAGGAAGGAGCAGCTGGGGACAGAAGGATATCAGGAGATGGGCCGGAAAGGTGGTCTGAGCACGACGGA

CGAGTCTGGTGGAGAAAGAGCTGAGAAGGAGGCTCATATCGATGAGTCGAAGTCAGGACTGGGAGCTAGT

GTGATGATTAAAACTATAAA

>FM891041.1 FM891041 Jatropha curcas embryo 56-70 (DAF) Jatropha curcas cDNA clone rjcaeb0_001028, mRNA sequence

ACTAGTCTCTCTCTCTCTCATTTTTGTCCTCGCAGAAATGGACCTGGCGTTTACTACCTCCTTCTCCCCT

GCAAGAGATAGAGCCATTTTCAAGATTAAGCCTGTTAGCTTAAACCTTAATAATGTCACTAGAAAACTGC

CCGTTCAGAAAAAGTTTCGCTGCTCAGCAACTGTAGCTGCTGACCTTACTCGGGTTTCTGAGTTTGAGAT

AGAGAAGAAAAAGCATGACCTTTTAAGTGCTACCCAAGACACACAACGAGGGCTTGTTACAACTGCTGAT

CAACGCTCTAGTATTGAGGAGGCTTTGGTGGACTTGGAAGGATACAACAAGGGTGCGCCAATTGACTTGG

TCCAGTTGGATGGGACATGGCGCCTGCAATATACTTCTGCCCCTGATGTGCTTGTTCTTCTGGAATCTTC

TGCTAGACTTCCTTTCTTGCAGGTTGGGCAGATCTTCCAAAAGTTTGAATGTCGGGATCCGTCTAATGGA

GGTATTGTCCGTAATG

>FM891040.1 FM891040 Jatropha curcas embryo 56-70 (DAF) Jatropha curcas cDNA clone rjcaeb0_001026, mRNA sequence

ATTCACCTGTTTATAGAGAAAGGATGGGCATCACAATCTCTAGTCGCTCTTGGATTAACTCATCGGTTTG

AATCCAAATTTGTTACTGTCGTCCTCTATAAGCCTCTCAAAAGGTAGCAGCATCTTTCATTTTCAGGCTT

AGTTTCATAAATGGAAGATAATCAATCACTTGCACTATGTGACTTTGTGAATGTATCTTTGTTTATAGGA

TACTCAGCTTTTCTTAACATGCTCTCTTATGTTTGATCTGTTGTAGTATTAACCTTTCATTTTCAAGCTT

TTTGCACCCTTTGTTGTTCTCCAAGGAACAGCGCAGGAAAGATAATTGAGTCCCTTTTTGGTTAGTACAT

CTCCTTTATCTCTT

>FM891039.1 FM891039 Jatropha curcas embryo 56-70 (DAF) Jatropha curcas cDNA clone rjcaeb0_001025, mRNA sequence

GCTGCAGGAATTCGAATTCCCAAGCTTGTACCGGTTGGATATGGTATAAAGAAACTGCAGATTATGCTTA

CTATTGTGGATGACCTAGTGTCTGTCGACAGTCTAATTGAGGAACATCTGACTGTTGAACCAGTGAATGA

ATATGTCCAGAGCTGTGACATTGTGGCCTTCAACAAGATATAATTTGAGGATCCTTCTCACTTGGACTAG

GTGCAGGAGAGGTTTCACTTTATGAAGATTGCGGAGAAATGATATTATGCTTCTTTGATCTTCATATTCC

TATTTTTGTTACACAAAAACCTTATTTAAATGTTTCTTGTGGATTTTTGTCCTGGTTGAATGGTTGTTAA

TGTAATGTATCTATCGAATGTGA

>FM891038.1 FM891038 Jatropha curcas embryo 56-70 (DAF) Jatropha curcas cDNA clone rjcaeb0_001023, mRNA sequence

GATGGAAGAGCAAACATGCAATTAAAGTTTAATAGTTTAATTAAATACACATTAAAATAAGTTTGGTGCA

GAAATAAAAGATGAAAACACACAACACAAACATTAAAACACCCAAATGTTACTATAAACCCGACAACAAT

GAAGCCCTTATTACTCTGATAATGTCTGTTAGAGCAACAAGTCCAACAAGAACACCTTGTTCATCCACAA

CCCAAACTCGATGCACATGTTTCGTAATAACCTTGTCGATGACTTCCTCGAGAGGGG

>FM891037.1 FM891037 Jatropha curcas embryo 56-70 (DAF) Jatropha curcas cDNA clone rjcaeb0_001022, mRNA sequence

GCTGCAGGAATCGAATTCGACGAAGCTACTCTCGCTACGATCGACCAAATCTAAGGCTTTCAGATCGAAA

AATGGCATTGATTCCAAGCTTTTTCGGTCACTCACGAAGCAACATCTTCGATCCTTTCTCTGCTCTCGAC

GTTTGGGATCCATTCAGAGACTTTTCTTTCCCTTCATCTTCTTCCCTCGTCTCT

>FM891036.1 FM891036 Jatropha curcas embryo 56-70 (DAF) Jatropha curcas cDNA clone rjcaeb0_001021, mRNA sequence

GACGAAGAGTGGAGGCAAGCAGCAGATACTCACAAAATGAGTCCTGAAGAAGTGAAGGCAGCAGGAGTAG

AGGGCTCCAAAAGGCCACCAGGCCATAACCCAGGAGGGGTCTTGCACCAGAGGCGCAATTTGCCTTTCAG

CAAAACCACCATTACAGTAGCTGGCTTCTTTATCACTGCTGCTATTGGATACATGGTCTTGTATGCCAAG

AAAAAGCCTGAAGCAAGCGCTAGAGATGTTGCCAAAGTTGCCACTGGTACTGCTGACCCTAGAGATACTC

ATCCTAGGAAGTAAATATAACTGTAATTTTGTGTTTTTTCTGTTCTGTTCGTTATCTAATATATGCATTA

CTTTAGTGTTAAAAAAAAGGGGGGGCCCGTACCAATCGCCCTATA

>FM891035.1 FM891035 Jatropha curcas embryo 56-70 (DAF) Jatropha curcas cDNA clone rjcaeb0_001020, mRNA sequence

TAAACTATGGATCCCCGGCTGCAGGAATCGAATTCTGAAGAGGTACATCAAGTTGTTGACACCCAAATTG

GATGAGGAGAAGCAACAGGCTTTCAAGAAAAACATTGAGGGAGCAACCAAGTTCCTGCTCTCGAAGCTCA

GTGACCTCCAATTTTTTGTGGGGGAGAGCATGCATGATGATGGGAGTTTGGTGTTTGCTTACTACAAGGA

TGGCGCCACTGACCCAACATTTTTGTACTTCGCTTATGCTTTGAAGGAGATCAAGTGCTAAGAATGGAGA

ATAGCTGGCTTCGAAGTGGAAGTTCTTGACCATATTTCTAGCTTTATGTGTCCTTTTCCATCTCTGTACT

GTTATATTTGGTGCTTCATGCCCTTATTTTGGTTAATTATTGACTAGACATGTTTCCTCCTTCTTGGTTA

TTAGTTTCTTGTTGACAAATAATTCATCTTCTATTGGGCGTGTATTCTAAAAT

>FM891034.1 FM891034 Jatropha curcas embryo 56-70 (DAF) Jatropha curcas cDNA clone rjcaeb0_001019, mRNA sequence

GGTCAACAAACCTCGATTGGTACAAGGGCCCTACCCTCCTTGAGGCCCTCGACTTGATCAATGAGCCAAA

GAGGCCAACAGACAAGCCACTCCGTCTCCCACTTCAGGGATGTCTACAAGATTGGTGGTATCGGAACTGT

CCCTGTCGGTCGTGTTGAGACTGGTGTCTTAAAACCTGGTATGGTTGTCACCTTCGGACCATCTGGACTG

ACCACTGAAGTTAAGTCTGTTGAGATGCACCACGAAGCTCTCCAGGAGGCCCTTCCAGGTGACAATGTTG

GCTTCAATGTGAAGAATGTTGCTGTCAAGGATTTGAAACGTGGTTTTGTTGCCTCTAACTCCAAGGATGA

TCCTGCGAAGGAAGCTGCCACTTCACCTCTCAGGTCATCATCATGAACACCCTGGTCAATTGGAAAGGAT

TGCCCTGTCCTTACTGCCCACTCCACATTGCTGTTAATTTGCTAATCTACCAAATTATAGCATCTGTAAA

ACTGAAAG

>FM891033.1 FM891033 Jatropha curcas embryo 56-70 (DAF) Jatropha curcas cDNA clone rjcaeb0_001018, mRNA sequence

GATTGTCTCAAACTATAACCGGTTGATGCGCAGCCATGACGAGCAAACCAATCACTGTCATGTTTAAGCT

ACAGCAGGCATGTCCTTGAGCCATGCATCAAGTGGCTTACCAATTGAGTATACAATGAAACCAATCTCCC

TCAGCTGATCCGCATTCACAACGTTCCTTCCGTCAAACACGAAGGCTGGCTTCTGCATACTTTCATAAAT

CCTCTTGTCATTTTTTCTTGC

>FM891032.1 FM891032 Jatropha curcas embryo 56-70 (DAF) Jatropha curcas cDNA clone rjcaeb0_001017, mRNA sequence

ATTCCAACAACTCCCTCATTACCGACCCTACCTGCGCCGCCAGCTCCTCCATTGTCGAGAATCTTATCGC

CTATCATGTCTACTCCTTTANTTCCTTCCTCTTCTCTAGCTTGCTCGCACTCGCGCTCAAACAAGCTCTC

CGAATAGCTAAAACCTCTCATTTCTCGGCTCATTTCAATCACTTGCCTGAGTTTTTCGCGCACATTAACA

AAGATGTGATACGGGTCGGTATGCTGGTGTCCGGTGTCGGTTCTGTTTGTGGGTGCGTGTTTTTGATGCT

GGCTTGATTAATGTGGATCAGTCAAGCTTGGAACTTGGCTGTGGAAGTGGACATAGCTTTGCAGCCGTAG

TGCCTCTGTGA

>FM891031.1 FM891031 Jatropha curcas embryo 56-70 (DAF) Jatropha curcas cDNA clone rjcaeb0_001016, mRNA sequence

ATTCCAACTCTACTTTTGTATTCTTCTACTTAGGACGTGAGATGGAGAAATTAGGAGGCAAAGGAGGAGT

TGCTAATGAAGAAACGACGAGCACCATGCGAACTGCAGAGACCATTCTGCGTCTTATACCAATGGCTTTA

TGCCTCTCTGCTCTCGTTCTTATGCTTAAGGACTCTCAGTCAAATGATTTCGGCTCTCTCTCTTACTCTG

ATCTTGGGGCTTCAGGTACTTGGTGCATGCGAATGGCATATGTGCTGGATATTCCCTTCTCTCAGCCATC

ATTGTAGCCATGCCTCGACCATCCACCATGCCCCAGGCCTGGACATTTCTTCTTCCTCGACCAGGTGTTG

ACATATGCGATTCTGG

>FM891030.1 FM891030 Jatropha curcas embryo 56-70 (DAF) Jatropha curcas cDNA clone rjcaeb0_001015, mRNA sequence

GGGACTATAACTTATAGNCTCCACCAAACTTTCATTATCTCATAACATAAGGGCATATACAGATCTACAN

TTTTTATATTATTGCAAGGAAACTAAAACTGCCTATAAAAATAGGCTTATTCTTCTCCAAATCTAAACTA

TGTAAAAACCAGTTTATTATACATCCTCTTGGTATAATTGCATAGTTGCAATATTAATTACGAAATCTAA

ACCCAGGGCTAAACACAGTGACTTCCTGTCTATTCTCCTTAAGCCTCCTGGCATCTTCTCTGGAAATCTG

GAATGCGTTTGCAACCACTGCCTCTGGCAGAGCCCTAATTGCCGAGGTCCGTCCTGCTAGTGGGCTAATT

TGTGCATTTTCGTTAGTCTTGAAGGATACCCACTCTAGCCCTTCATTGCTTGCCCTCTTCAAAACCCCAA

AGTTCTGTGGAG

>FM891029.1 FM891029 Jatropha curcas embryo 56-70 (DAF) Jatropha curcas cDNA clone rjcaeb0_001014, mRNA sequence

AAGGACCTAATAATAGAACTTGTATTTAGAAAGATTAAACATGCAAGAACCACAATATCTGGATACAGCG

ACATACAGTTGATAGATTTTATATCTGTGATCACATAAGTTCAATTACTGTCCAAAAATAGATAGGTAGC

AAGCAAACCAAACCAAGTTTCCAAAGGCAGCTGCTTTAACTCTTATCAATTTTTACAGCCATCACCATCT

GTTTGGAAGTCCAAGCAAAGGGTTCTGCTGGATCTGCTGCGAAGGTAAAAGCAACTTGTCCAGTACTAGT

TTGCAGATTTTTTCTTCCTAAAAGTGAACTTTCACAGGCATTTTGGACAAAAGATGTCCTGTAACAGTAA

TTTGCTTTGCTGGAGATGTTTTTATTCTCAGATCACCATCTAATA

>FM891028.1 FM891028 Jatropha curcas embryo 56-70 (DAF) Jatropha curcas cDNA clone rjcaeb0_001013, mRNA sequence

CTTTTCCAATACACAGAGAACTATCAAAGCTAAACCCTTAAAACACAAAAGCTTCATGCCCCGACAGTGG

CAGTAGGAAATGAGTTTCAGCCAAACCAGTGACATGCAAACGCAGAAACGATCCAATCTCACCTGGTATG

CTTATCGTTCCTATGCCAAATCCTCAGAAAACATTCGACAGACCAGAGAGAGCAAGGATTATCGTTCCAT

CTAGAAGAATTAATGAAGAACTCCTTGATTCTATTGAGATTGGAGTAGAACTTTCACTTATTGATGGAAG

AAGTGGGGTGGGATAGCTTAGCAAGGTTTAGAGTTAGGGTGGTGCTACAGGTTTTTATTTAGCGGCGGAT

TTTGGAAATCCTCCGACGATGGTTTTTCCCCCCAGACTTCCACCTAACCC

>FM891027.1 FM891027 Jatropha curcas embryo 56-70 (DAF) Jatropha curcas cDNA clone rjcaeb0_001012, mRNA sequence

ATTCCCTCAATCACAGCAGTTCATCATGTCTGATCAGCCATGGCCGATATCTCAAATGGTCCGCGATCCG

GCGCCACAGTCTTATCCGAGGGTCAAGTTCGCAACGGCGGTGACGCTGGGAGCAGCGTTCTTGATATTGT

CTGGGCTAACTCTAACCGGGACAATAATTGCTCTAATCATGGCGACTCCGTTTCTGGTGCTATTTAGTCC

GATTTTGGTTCCGGCGGCGATAGCGGTAGTTTTGGTAGCCAGTGGTTTCTTTTTCTCGGGTGGATGTGGT

TTGGCGGCAATAATGGTGTTAACTTGGATGTATAACTACTTAGCAGGGAAGCATCCGCCTGGTGCAGATA

AGATTGATTATGCGAGAGGACAACTGGCGAGAAAGGCTCATGATATTAAAGAAAAAGCTAAAGAGTATGG

ACAGT

>FM891026.1 FM891026 Jatropha curcas embryo 56-70 (DAF) Jatropha curcas cDNA clone rjcaeb0_001011, mRNA sequence

AAATAAAAAAACACTAAATAAAATTATTTACATACTGTTAAAAGTTATAAAACTTAAAAGATTAAAAAAA

AAAAAAAAAAGAGAATAAAGAAAAGGTAAAGAAACAACACACTACTCCTTTGTTTTAATTTTCTTTTGTC

CTCCTTTAACCACTTTCTTAACAAATCCTTTTAGACCTTTCTTCTCCTTTTTCTCAATCTTCTTCTGTTC

CTCCTTCCTCTCAGTCACAAGCTCTTCCTCCAACTTAAGTAAACGTTCCTCTGCCTGATCAAGCCTCTCA

ATGAGAGTGCCTTTGATTTCAGTCTCAGTCATCACACGGTTGATCGGACGGCAGTGCTTCTCTAGACATT

TTGGAGAAAAATCAATGGATGATACTTGACCTTCACTTGTAAGTGTCCCACTTGATGGAGTAGATGCAAT

CGAGCTCTTTGGAGACCTG

>FM891025.1 FM891025 Jatropha curcas embryo 56-70 (DAF) Jatropha curcas cDNA clone rjcaeb0_001010, mRNA sequence

CGAATTCGTAAGATCTCCAAATCACCCAGTGTATATGTAAGCAAATCTACACATAGCCCTTCAAAGGCCA

ATGAAAACAATGCTGGAGCTGGAGTTTACGTTATGCTCTTCATCTCCGTTTTTTATGTCCTTTCCCCAAG

AAATCTCCAAGGTCAGTCCAGAGGTGCAAATCTGATCCTTCATCGGCACCAGCAGGAAACAAGATGTACA

TGCGAAGGGGATCGTCGGTTCTACTTGTATAGCAATATGAAGGTGGTGTTCCCACAGCGCCACTCAGATG

CTGATGAGGGCAAGCTACATGTAGAATATGATTATCCCTCAGATCCCAAGTACTTCGACATTTAAATGCC

CGGTAAGCTATCCTCTTGAAAGCTGGTTATA

>FM891024.1 FM891024 Jatropha curcas embryo 56-70 (DAF) Jatropha curcas cDNA clone rjcaeb0_001008, mRNA sequence

ATTCCCTTAACTCACCTGACCGTGCTTGCAATCTTGCAAAGCAGGCTTTTGATGAGGCTATTTCTGAGCT

TGACACATTGGGTGAGGAATCTTACAAGGACAGTACATTGATCATGCAACTTCTCCGGGACAATCTGACA

CTATGGACTTCTGATATCACGGATGAGGCTGGGGATGAGATCAAGGAGGCATCAAAACGTGAATCAGGCG

AAGGACAACCACAGCAGTAATGAATTAAAATTTGTAGGATATGTTTACTCTGTACTTCTTTCTTGTGACC

TCCGCATAGATGCGATGCTGGTTGTTGTAGCTGGATTTGCATAATGAAATTGGTAAATCCTGAGTGCTGA

TCTGATGCTACTTGGGATTGAGGTAATTCCATAGATTATCAAGCTTCTGCTGTTGATGTTAAGAATTCAT

GTCTGTATTCTTATTGCT

>FM891023.1 FM891023 Jatropha curcas embryo 56-70 (DAF) Jatropha curcas cDNA clone rjcaeb0_001007, mRNA sequence

ATCCATCCATATCTGAAGGTGTTTCTGGATCACCTCCTAATCGTCAAGTGGGCATGAAGTCCAAACGTGA

GGAGAATGAAGGGGAAGATGATATTGAGTGGGAAGAGGCGCCAACTGGAGGCAATACAACTGAAAGTTAC

AGGATCAATGACTTGAATGTTGAAGCAGAAGCTTCAGCAGAGGATGAGGATGATGTAGATTGGGAAGAAG

GTTGAATGGCGCTTATTAACCTGTTATGGAATTGCGCTTACAATGTTTAGAAATTCTATATGAAAGGTAC

TCAAAATTTTATCTAAATATATTTTTCCTTTTCTAGATACACATC

>FM891022.1 FM891022 Jatropha curcas embryo 56-70 (DAF) Jatropha curcas cDNA clone rjcaeb0_001006, mRNA sequence

CTAGTTCTCTCTCTCTCTCCCGGCAACAACAGGTTCCTGGGCAATGTTGTAACCAAGCATGGAAACTAAG

TGGCTTTTGTCGATGTCAATCAATAAGGTATCTACCAGAGAAGCAAGGATACGTTGGAAGCCAAGAATAT

GATGAGGCAATGCGTAGGGCTGACGATATAATTAGTGTTTGCTTTGACAATATCTGCCCTCGTTAAAATG

CTATTGCTACCGAAAATAAAATTTCACTCGCTTGAATGTGAGCGAGAGTTACGTTTAATAATAATCCATA

AACAATATGAGTTTCGGAGTAAGTTTGTAATGTTGCTTCAAGTTTTCTTCATGTTCCTGTAATAAAAGCT

AATCAACTGTTGAGTTATTGAACTGAAAATCCAACATTTGTTTCAAAAACTAAACGAACCCATTTAGGTG

TTCTTTTCACATTAAC

>FM891021.1 FM891021 Jatropha curcas embryo 56-70 (DAF) Jatropha curcas cDNA clone rjcaeb0_001005, mRNA sequence

TCGAATTCGATTGTTTGTGTGCCCCCTACAAACTGTTACATTTTTTCCCAAGTTGTTGTTATTATTATTA

TTATTATTATTATTATTTTTAGGCTTACATTTTTATGAATTTGAACTTAATGAGTTTGTTTTAGCTCAAG

CTCCCTTCATTTTTAATTGGGATAGTTTTTAGACTAGGCATGTTGTTTTAAACATGTTATTTTTTTTTAT

TATTTCCGAGTCAGAATC

>FM891020.1 FM891020 Jatropha curcas embryo 56-70 (DAF) Jatropha curcas cDNA clone rjcaeb0_001004, mRNA sequence

ATTCAAAATGAATCCACTGATTTTGTTGGTTCTGGCAACTGTGGCTTTGGCCAACGCTGAGGTGCAATTC

CAGGAACCAGATGCAGATGTTAGTCCGTGTGAAGTATGCACTGTAGCCTTGGATCAATTGAAAAAACTGT

TGCAAATTGACGCTGTCAGGAACGAGGTTGAAGTTTTGATGAAAGGCGTTTGTGCAGCGTTTGGTCCTGT

CAAGGGTTTGTGCGAAGCGTTGGTTGGCAAGGGAATCGATATTGTCTTCGATTTCATACAGAAGCAAAAA

TCGCAGGATATATGCGCGAAAATCAGACTGTGCTAGAAACGTGCGTTTGAGGAAGAATAAAAATCGTTGA

TTGAATGGTGATACCACATTCATCACATTACATTCGTTGCATGAATAAAACTGTTTTGCGTTAAAAAAAA

GGGGGGGCCCGGTAC

>FM891019.1 FM891019 Jatropha curcas embryo 56-70 (DAF) Jatropha curcas cDNA clone rjcaeb0_001003, mRNA sequence

GCTGCAGGAATTCGAATTCACGAAAATACTCTTTCCAACCCTTCAACTTCAAATCAAGTATCCAGCTAGA

ATGGCTTTAAGCTTAGGAAGGAGGAGCAACATCTTTGATCCGTTCTCACTAGATATTTGGGACCCATTTG

AGGGCTTACCTTTCTATAGCGGAACAGTGGCCAATGTTCCGTCCACTCAACACGAAACAGCCGCCATTGC

CAACGCACGAGTGGACTGGAGGGAGACTCCCGAGGCTCACATATTTGCTGTTGATCTTCCTGGGTTGAAC

CAAGAAC

>FM891018.1 FM891018 Jatropha curcas embryo 56-70 (DAF) Jatropha curcas cDNA clone rjcaeb0_001002, mRNA sequence

ACTTAAAATATCATATCCTGATGTATGATAAACATTACTACTTTATCTTCCTTTACCAAAATGACAAATT

TATTACAGCAGCAATCAACAACACCATCCTAAATTGGGTATTTGTTCAATTCAAAGAACTTGCCTATCCG

CAGCCTCAGCAACAATGGGCAACCTAGGTACTTGTCCCAGCAAACAAGCACTAGACCCATAAATCAAACA

AACCAAAAGATATAAAAACACAGTACTATCCAAACTCATCATCAAATCCAACCCTAACCCATCTCTTGGG

TTAAAACTCCGGGGGGGCCCGGTAC

>FM891017.1 FM891017 Jatropha curcas embryo 56-70 (DAF) Jatropha curcas cDNA clone rjcaeb0_001001, mRNA sequence

ATTCCTGTTATGCTTTCCTTGTAATAGTAAGAAAAAACTCACCTGGGCATCCTAGATCTCCAAGAATTTC

AGAGGATATAAGAATTTCCAACTATATATACGTATAATTGGTATTTGACCTGGTACCTGGCAATTGACTA

TAACTCCCACTTTACCTGATGGATTTACTGAAAGTCGGTATGCAGAATTGATGAAGTGATGATATGTGTA

GTCAATATTTTCCCGCTGTGCCGGTCCTCTGGTCTGTGGTCCTGATTTTGGTTGGACACTGTTGTCTGCT

GGACAATGCAGCTGAAGAGAAGTCTTCTTCAGATCTTTTGCAAGAAGTGACGTCTCGAAGTCTTTGCCAC

AGCAAGTCCTTCTCAACACTGTAAGAATCCTCCATTGTGTTCATGTTAATAATTGAAAGGTATTCTTACC

ACCGATTTTAAGAGTCTAAATGCCAAATTCAAACAGCTTTAATCATTCCC

>FM891016.1 FM891016 Jatropha curcas embryo 56-70 (DAF) Jatropha curcas cDNA clone rjcaeb0_000996, mRNA sequence

ATTCTTTTATATAAACGTAACGATATATATTGAACACTGACTTTTTATACGTACATGAACACAACGTAGA

TTAATTACACATAAAGGACGACTCAAAAGATCCGAGAGTCCAGAGTCCAAACCAGACCCAAATACATCCC

CGCCAAAAGGGGATATGTAAATGACACAAGATAATTCTTTTAGCATTACATGTACATATATTATTGCATT

GTTGTTGCT

>FM891015.1 FM891015 Jatropha curcas embryo 56-70 (DAF) Jatropha curcas cDNA clone rjcaeb0_000995, mRNA sequence

ATTCATCAGTTCAAGACCGTCCGTTCTTGCCGTGATAAGGTTTCTGGGTCTTGTGGCAGAGTCTGGAATC

GGATTCTTGAGCTTCCAGCTCCAAGTGATGATATTCCGATGCTAATTACCGAGTAGAGGCAGCCAACTGT

TCTGTTAGCATGGGAAGTAGCTGAGGAAACTTGATACACTAGCAATTAGTAAAGTAATCTACTATTAGTG

TTGCTTTCAGACTCACAAACTCAGAGTCTGTCAGTCTTTATATACTCCTCTTATTTGAGCTTAATAATGT

CAATGAGCTGGGGAAGTTGTTTGTATATATATATATATATATATATTAGACATGTTTCAGCTTTACATGT

ACTAGTATCTGGTGTGTATGAACTCTCTGTTGAACAATTGTATTGAGGAAGAACTTTCAGCTAA

>FM891014.1 FM891014 Jatropha curcas embryo 56-70 (DAF) Jatropha curcas cDNA clone rjcaeb0_000994, mRNA sequence

ATTCGATTTTGGTGACTCATCACCAAGAAAAGCATACGAAGAAGACGATAAAAGGAGAGTATGTGCCTGT

TTATGTGGCGATAGGACTGATTGCTTTATCGGTCTCTCTGGGTCTGTACACTGCAAAGCAGCAGATATTG

CACTCGCCGAATGTGCGCGTGAAGAAGAAGAGAAGGGAGACGATACCGGAGGTGGAAGACCCTGATAGTG

TGGTGGATGAAGCTGATAAATTTCTGAAGAAGTCTTTCTTTAGGAAAGTAGCGCATGTTCAAGAGTTTGA

TAGTGGACTTCAAAGCTTGCCTGATGCTAATATTCATAGAGATGCATTTGCTTACAAGCCACACGCCGAG

ACTCTCAAGTCTGTTGGGATTGATCCTAAGCGGCATTGAGCTACGCGACATGTATACATGTTACTTTATT

TCTCTTTTAAGTTAGGGTTAGCCCTTAATTACCTTTTGCAGAAGAACGAGGAAGATGTGTATTTGAACTT

>FM891013.1 FM891013 Jatropha curcas embryo 56-70 (DAF) Jatropha curcas cDNA clone rjcaeb0_000993, mRNA sequence

ATTCCGTCAATTGAAGGTTTTGCCCAAAAGGACCAATGTTCCTGGGATGAAACAATATCGCCCTTAGCGG

TTCAATCCTTACATTGGTTACCGGTTTAGGAGGCCATATGCTCCCCCTTATTTCTACTCCCCTTATGGAT

ATGGGAAGGTTCCTAGGTTTAGAAGGCCAGTGCGATACATGCCTTACTACTAGATGACTGTTAACATTGG

CGAAGTATATCACGTGATTGTTTCCCTTTGGGATCTTTANCCTGCCTTGATTTTATTATCCGTTGAGAGG

GCAAAACAGATGCTTTATTGGCATAGGTATTATCGTTGCTGTTATATATAACTCTAGACATAAAAGAGC

>FM891012.1 FM891012 Jatropha curcas embryo 56-70 (DAF) Jatropha curcas cDNA clone rjcaeb0_000992, mRNA sequence

ATTCCTCAAAACGACAATACGAGCAATCTCTCAACTCTCTCTGCAACGATCACACGTCTATTCCCTCCCA

CTTATGAAGATCTGCCTGTCACTTCTTTCCTTGTGAGCCTGGCCCCCTATTCCCTTGTTAAACTGATGGA

TCTTGAAGGCAAGACATTTGGAAGGGGGCAAAGGGAGCTTGGTGGTGCTGTTGATCTCGTAAGTCAGTAT

AAGCTTTGGCCTCATCATGAATTCTTTTGCAAGAGGTCACTCCCTTTGTCAATATCTGAAACTCGCTATC

TCCAGAATGTTGTGGGTGACACAGAAATTAGGAAAGGGGAAGGAATGGAATTGGATCAATTGTTTCATAA

CACCCCCAATCTGAGAGAGAGAAATGTTCATGCTCGTATACTTCCTTTTGATTTAGGTGTACTC

>FM891011.1 FM891011 Jatropha curcas embryo 56-70 (DAF) Jatropha curcas cDNA clone rjcaeb0_000990, mRNA sequence

TTTTTTTTTATAGCTCCACAAACTTTCATTATCTCATAACATAAGGCATATACAGATCTACATTTTTATA

TTATTGCAAGGAACTAAAACTGCCTATAAAAATAGGCTTATTCTTCTCCAAATCTAAACTATGTAGAAAC

CAGTTTATTATACATCCTCTTGGTATAATTGCATAGTTGCAATATTAATTACGAGATCTAGACCCAGGGC

TAAACACAGTGACTTCCTGTCTATTCTCCTTAAGCCTCCTGGCATCTTCTCTGGAAATCTGGAATGCGTT

TGCAACCACTGCCTCTGGCAGAGCCCTAATTGCCGAGGTCCGTCCTGCTAGTGGGCTAATTTGTGCATTT

TCGTTAGTCTTGAAGGATACCCACTCTAGCCCTTCATTGCTTGCCCTCTTCAAAACCACAAAGTTCTGTG

GAGCTGTTAATATTTGACCTTGTTGAACTTGTCCATCGAACACTGCATCTCCATTCTCATCTACGATTTG

GACGTGACCATTTCCTCTTGTAAAGTAATATATGCTATGCGCATTGATGTTCCAGTGTGGTGCAAATATA

GCATTCCT

>FM891010.1 FM891010 Jatropha curcas embryo 56-70 (DAF) Jatropha curcas cDNA clone rjcaeb0_000989, mRNA sequence

AATAAATTCTAATTAACTTCTAAAGTGTAAATAATTAGACCCTACAAAAAGAAATAAAAAAAAACTAATT

AACAGGCTAAAAAAAACAACAAAAACCTAAATGAAAAATAAAATACTAGTACAAACAAAACCCTTCCCTC

CTAATTAGAAAATTACCTAAACCCTATAATTAAATCCGTTACAAAGCCTGCAATCTGCAAACAGACCCTT

GACCAACTAAGCCCTTCAAAAACTATGAACCATCTGGTTCAACCCGGAATCCTGTCCGGTCATTTCCTTT

CTCTTCCTAATTTCTAGCACTTTCCGGTGACTATTGGAGTGAACCTCCTCTGAAAATGTCGGG

>FM891009.1 FM891009 Jatropha curcas embryo 56-70 (DAF) Jatropha curcas cDNA clone rjcaeb0_000988, mRNA sequence

GGACTATAACTTATAGCTCCACAAACTTTCATTATCTCATAACATAAGGCATATACAGATCTACATTTTT

ATATTATTGCAAGGAACTAAAACTGCCTATAAAAATAGGCTTATTCTTCTCCAAATCTAAACTATGTAGA

AACCAGTTTATTATACATCCTCTTGGTATAATTGCATAGTTGCAATATTAATTACGAGATCTAGACCCAG

TGCTAAACACAGTGACTTCCTGTCTATTCTCCTGGAAGCCTCCTGGCATCTTCTCTGGAAATCTGGAATG

CGTTTGCAACCACTGCCTCTGGCAGAGCCCTAATTGCCGAGGTCCGTCCTGCTAGTGGGCTAATTTGTGC

ATTTTCGTTAGTCTTGAAGGATACCCACTCTAGCCCTTCATTGCTTGCCCTCTTCAAAACCACAAAGTTC

TGTGGAGCTGTTAATATTTGACCTTGTTGAACTTGTCCATCGAACACTGCATCTCCATTCTCATCTACGA

TTTGGACGTGACCATTTCCTCTTGTAAAGTAATATATGCTAGGCGCATTGATGTTCCAGTGTGGTGCAAA

TATAGCATTCCTAA

>FM891008.1 FM891008 Jatropha curcas embryo 56-70 (DAF) Jatropha curcas cDNA clone rjcaeb0_000987, mRNA sequence

ATTCCAGAGTTGGAGAAGCTACTCGATCGCCGAAGATGGATCCCGGGATATGGAGCCGACTGCCGGAGGA

GCTACTTGATCATGTATTGTCTCTATTGCCTCTAAAAGCATTCTTGAATCTCAGATCAACTTGTAAGCGT

TTCAGGTCTCTTATATTTTCTCCTTGTTTTATGTCTAAACACTGTTCTTCTGGTTCACCTTTCTCTTCTT

TTCTTTTACTCTCTCACCCTCAGTTTTATCATCGTTACTGTTTATATGATTCTATGATTGGTACTTGGCG

CAACTTAAACTTATCACTCTCTTTTTTGCTACGCTGCACCGCTGGTGTGGGTGGTAGTTCACAATCTTGC

ACTCTTGTGTCATGCTCTAATGGGTTATTTTGTTTCTCTCTCCCCAACTCATGTTCTTTTGTTGTTTGCA

ATTTCTTAGCCAAATCTTCGAGGGTTATTGAATTTCCTCTTTACCCTTTTGGTTTTGAGTCACTTACTTT

TGTTTCAACACCATTTGGGTCCAAAATCTTTGTGCTA

>FM891007.1 FM891007 Jatropha curcas embryo 56-70 (DAF) Jatropha curcas cDNA clone rjcaeb0_000985, mRNA sequence

ACACACTCAAAAATTACTGGGCAAAAGAACACCAGAAATAGTGCCACAGTAAATAGTCCATATACATATT

TAGTTATATACGTATCGTATATTACCATAATACACGTCGAGTCATCAAAATTACACAACCAAAATCCTGC

AACCTATAACATATTATATTTTACATGGCGTATCATATTATATGGTAAATTACACAAGAACCCTCT

>FM891006.1 FM891006 Jatropha curcas embryo 56-70 (DAF) Jatropha curcas cDNA clone rjcaeb0_000984, mRNA sequence

TTTTTTTTTTTAGACAAAACTCATTGATTGTTTCTCCATTCTCACCAAGAATTCTTCGAGTTCTCAGCTA

AAACCACAATGAAAAGCCGCCCCTCACGGATATATCCATATTACATTTGACCAGAAAGAGACAGTGACGG

CTAACAGGCATTTCACCGCACATATAAAATGAACTATAAAATAAATCTAATTCATTTAACATACATCATG

AATCATGACAAACGTTAGCTGTGATCCAAAGAAGCTGGCTAATTCAAAATGCAGTCATCCAACCAAGTTC

CATTTGAGATTCCATTTACTAAGCCAACAAGATGCGGCAACAACCAGGCCAACCAGATATTTTATCAACT

AAATTTACCAATTTCCATCTTCTTCTTGATTGCCTTTTGACCGTGATATATAACTGAATCCAGTATTCCC

GAAACAGTGAAAACACCTCCAACAATAGCGCACACATTTGTTAGGAAGTGCAAGAATGATACATGCTGCT

CTGTGAAAGTCACCTTTATTGGAGAAAGGTCATA

>FM891005.1 FM891005 Jatropha curcas embryo 56-70 (DAF) Jatropha curcas cDNA clone rjcaeb0_000983, mRNA sequence

ATTCTATCATATCAAATCGGCGTTGAAAGATCAGTTCTCCTTTTTGAGTATCTAAAAATGTCTTCTTGCT

GTGGTGGAAATTGCGGCTGCGGCTCCGGCTGCAAGTGTGGCAGCGGTTGTGGCGGATGCAAGATGTATCC

TGACATGAGTTTCTCCGAGAAAAACACCGCCGAGACTCAGGTTCTCGGAGTTGCACCGGAGAAGGCACGC

TTTGTCGATGGAGGTGAGGTCGGCGTTGGAGCTGAGAACGGATGCAAGTGCGGTGACAACTGCACCTGCA

ATCCTTGCAACTGTAAATGAGATCGGCGGCGATCAGTCTGTTTTTTAGAAGTAAAAGCAGAGATGACTCT

GCTTCTCTCTTTCTAAAAAAGGAGAAATAATTAATAGAGTCTAGATATCGTATGAGTTTGTTCGTTTGTT

TCTGGTTTTGAGCTTCAATAAAAGTAGTGGTGGCTTGTGAGCTTGTGTTTAGCAATGGCTGCCTGTTGCT

TTAACGTTATATATTGTATAAATTAAAAACTAGCATATGAATATGTAAATGAGTACGAGGGGTGAAATAG

CTTTGAAAGCTTAATGAGAAACTCCTTTTCTATTTTCTAAAAAAAAA

>FM891004.1 FM891004 Jatropha curcas embryo 56-70 (DAF) Jatropha curcas cDNA clone rjcaeb0_000982, mRNA sequence

AACCAACCAGGTAATTATATATACTGCAGGTTATTTTACAAATAGGGAGAAAGTATTAGCAGTCCTCCCA

GAAAGGGAACGACCATAAACCCCTCTCAAAGTTACAACACACATACTAGCTAAACCCTAGTCATTTTAAC

CCACATGTAAACTACATTACTAAACTAAAGTACACTGCTAAGCTAATACTTTATTACCAACATTATTATT

ATTATTACTACAAAATACCATTTATAGTTTTAATCATCACACTAGCTCCCAGTCCTGAACTTCGACTCAT

CGATAGTGATGCCCTCCTTCTCAGCTCTTTCTCCACCAGACTCGTCCGTCGTGCTCAGCCCACCTTTCCG

GCCCATCTCCTGATATCCTTCTGTCCCCAGCTGCTCCTTCCTTGTCTGTCCTCCTCTGCTTCTCCCTTCA

GCAAGATGCTCTTGAGCTTCAAGGCTCTTGCCCCCAGTACCTCCAGGGACAACAGTCTCTCCACGCCTTG

CTCTTTCATCAAGCGGGGGGGCCCAGGTACCAAG

>FM891003.1 FM891003 Jatropha curcas embryo 56-70 (DAF) Jatropha curcas cDNA clone rjcaeb0_000981, mRNA sequence

CTATTATTTGATAATGATAGACAAGGGAGAAAGAAAACACCTACCGGTACTACACTGCCAAAATTGATCG

TACGACAAAACGTTTTAATTCCCTTTTTCTATTTTCTAACCCTCAATTTTACATCAGGCTCAAATGAAAT

CAACTCCTTGGATTCCAGCCAAGCCTTAACAACAACAACAGAATCAAAAACCTTCATTTCTCCATTAACT

TTTGTTGCGGCCTCAATTTTCAGATAATATTTTATTCCAGAAACTACCTGCCTCTGCGCCTCCACTACTT

CCGAGAATATTAGTTCTTCTCCGCCGTTGCCGTGGCGGTGAATCTGCTCGTTGAACTCCTCGACGGAGAA

TTTGCCCAGATCTTGCACCTCTTGATTAGTCTCTACGTCCTTCACTTCCGTTCTGCCTCCAACCATTCCT

CTGTAACCATTTGCTAAGGAAACAAAGAGAAATGCAGAGGCCAGAAGAAGAAGAAGCTCAACAATTTTTG

CCATTTCAGAGAGAGAGAGAGAGAGAACTAGTCTCGAGGGGGGGGCCC

>FM891002.1 FM891002 Jatropha curcas embryo 56-70 (DAF) Jatropha curcas cDNA clone rjcaeb0_000980, mRNA sequence

ATTCACAGAGCGTTCTTTCTTGCCGGTAATCCACAACGAGACGTACAGAGCAAGAGGTGTGCATTTAGGG

GTAGCCGGATGCCCCTCGCAGGAAGGGAATCTCGAAGGCCCTCCGAGGAAAGATCAGGCAATATCTTTTC

AGGCCTTGACGAACAACTTTTAGCAGACGCTTTCAACGTCAATGTTGAGGTTGCAAGAAAGATAAAAGGA

CAAAACGACAACAGAGGATTAATTGTAAGCGTAGAACGTGATTTTGAGTTGCTATTCCCACCAAGAAGCG

CAGAAGAAGAAAGACGAGAAAGAGAGGAAGAGACCCAGAAACAATTAGAGGGAACAGGTTACAGACCAGG

TGGCGGCCAAGGAAGATTCGTTAATGGTGTGGAAGAAACTTTCTGCACTGCTAGACTCGTACACAACATC

AATGACCCGTCACGTGCCGATGTATTCAACCCACGTGCTGGCCGTCTCACCAACATCAACGGCCAAGACC

TCCCCATCCTCAGTTATATCCAGCTAAGCGTTCAGAAAGGTGTCCTCTACAGAAATGCTCTACTGTCACC

ACACTGGAACGTCGATGCCCATAGCATTAACTACTTCACAGGAGGAAA

>FM891001.1 FM891001 Jatropha curcas embryo 56-70 (DAF) Jatropha curcas cDNA clone rjcaeb0_000979, mRNA sequence

AACCAACAGGTAATTATATATACTGCAGGTTATTTTACAAATAGGGAGAAAGTATTAGCAGTCCTCCCAG

AAAGGGAACGACCATAAACCCCTCTCAAAGTTACAACACACATACTAGCTAAACCCTAGTCATTTTAACC

CACATGTAAACTACATTACTAAACTAAAGTACACTGCTAAGCTAATACTTTATTACCAACATTATTATTA

TTATTACTACAAAATACCATTTATAGTTTTAATCATCACACTAGCTCCCAGTCCTGAACTTCGACTCATC

GATAGTGATGCCCTCCTTCTCAGCTCTTTCTCCACCAGACTCGTCCGTCGTGCTCAGCCCACCTTTCCGG

CCCATCTCCTGATATCCTTCTGTCCCCAGCTGCTCCTTCCTTGTCTGTCCTCCTCTGCTTCTCCCTTCAG

CAAGATGCTCTTGAACTTCAAGGCTCTTGCCCCCAGTACCTCCAGGGACAACAGTCTCTCCACGCCTTGC

TCTTTCATCAAGCTCTTGTCTTGATTGATTTGAAGACATTTTTGCCCTTTTTTGCTAACAAATTTCTCCA

CCAATATAATTAATCTTTTGCGGGGGGGGCCGGTAC

>FM891000.1 FM891000 Jatropha curcas embryo 56-70 (DAF) Jatropha curcas cDNA clone rjcaeb0_000978, mRNA sequence

ATGAAGGGGGGGAAAAAAATAAAAAACATTCTCATTCCTCAGCTATATATTAGTTGTATTGTAATAGGTT

TTAAGAATTACTGCCATTGGAATATTAAGAACATGACCCTTATTTCCAAAAAGTCCAATCCTTCCATAGC

CAGCAACAGTGCTGTTCATAGTCGCATTCAACATTATATTCAATACCAGCTTTTCTCCACTGGCAATAGT

GAAGTGAGTTGGAGCCACCTTAACTGCCACCCCATATGGTGCGCTCCAACCGACATTATACGACTCGTTG

CCAGCAATGTTGGTTACTGTTCTTTGGACCATCCGATACTGATCTAGCTTAGAAATTGTGATTGAGGGCA

AGTTCAAATCACTACCGTTGATAGTTGAATTATATGTCCAACAATTTTGGCCTGTGTAATTGAAGACCAC

AGGACCAGAGCCATTGATTCCACAGAGAAATGACATATAATCATCATAACTGGAATCGAAAATTAGGCCT

GAATCCAGAGCCGCTGTTGCATTCACA

>FM890999.1 FM890999 Jatropha curcas embryo 56-70 (DAF) Jatropha curcas cDNA clone rjcaeb0_000977, mRNA sequence

ATTCCAGAATCGACGAGCAATCATAAGGTATTATGCAGAGAAGTACAAGTCGCAGGGAACTGAATTACTG

GGCAAGACCATAGAAGAAAGGGGATTGGTGGAACAATGGCTAGAGGTGGAAGCACAGAACTATCATCCTC

CAATTTACAACTTGACCCTTCACATTTTGTTTGCCCCAGCCTTGGGGTTTCCTCCGGATGAAAAGGTTAT

AAAAGAGAGTGAAGAAAAGCTTGCAAAAGTATTGGATATCTACGAGGACAGACTTTCAAAGAGTACATAC

TTAGCTGGGGAATTATTCAGCCTTGCAGACCTT

>FM890998.1 FM890998 Jatropha curcas embryo 56-70 (DAF) Jatropha curcas cDNA clone rjcaeb0_000976, mRNA sequence

ATTCGAGAGAACTAGTCTCGAGTTTTTTTGATGCCAAAGCTGGAATTGCTTTGAATGATAAGTTTGTGAA

GCTTGTTTCTTGGTACGACAATGAGGTTGGTTACAGCACCCGGGTGGTTGACTTGATCTGCCATATGGCA

TCTCATCATTAAGGCTGCAGCAATATGTTATGAGGCTTCCTCCAACATGTCTTGGTGGTGTTTATGTTAG

TACTGAGGCCAAATGAGAATAAATTCGTTCGAAGGCGGGATTGACCGATCAATTTCATGCATGCTTGTAT

TTGGTTTCTTAGATCTGGTATTTACTGTGTGTTGGTATTGAGAGGGAGTTTAAATTCCTCCTACTGGATT

TGAATATTTTGTCTTGCTAGTGTGTTGAGATATTTTGAAACAAAACTATCGGCTCTTGTGCATA

>FM890997.1 FM890997 Jatropha curcas embryo 56-70 (DAF) Jatropha curcas cDNA clone rjcaeb0_000975, mRNA sequence

ATTCTTTTTTTTTAAAAACTTCAAAATTTTATTCCTTTCAGCTGGCCATAAAATAAGAAAAGAAGTGAAA

CAAAGGATCATTGAACCCAAAACTAAAGAGGCATTCATTCGCATGCCACTAACCACTTGCTATGCAGCGA

GAGCTTAAAGTACAGGACAATGACATACACTAAGCCAGCGGGCATTTTCTATCAGCAAAGAAAAAGAAAA

TTGATTGAATAAGGCATAACATAACTTTTCTATGAGAAAAGGTGGCATGATACAAAGCAGATCATAGCAA

GGATCCCACTATGAGATACCTGGAAAATAACACATGGAACTCGATCCGCCATTCTTACAGTTACTTAATT

CCTTTCATTTCCTCCAGCCATTGATTTCAGCATCTCCAGACCTGGATTGTCAACGTCTTGGTCTAGGCTT

GCAACGAAATTCAGCTAAAAGTGCTATATGATCAGATGACCACTCTGGAGAAGGAAGGGCTGTATCTTTC

CTTAAGCTTTCCTCATCCAAGAGTTCCAACAAGGACTCCACTGTCAATGAGTCCGCTGTGTAAAATATAT

AATCT

>FM890996.1 FM890996 Jatropha curcas embryo 56-70 (DAF) Jatropha curcas cDNA clone rjcaeb0_000974, mRNA sequence

CACACCTCCTTCGCCGTGAAATTCTGGTGGTAAAGTCTCAATTTCAAGTACCATTGGAATTTACGAGAAT

AAAACGTTTAACCTAATTATTTAACTCGTCAAATGGGAAAAAAAAGTTTTATTGAAAATTCAAATAAATA

AATAATAGTGTACTGTAAAATGAAGCATGTCCTCTTTATTTCTGTGCATGTTCTAGATCATAATAAGTTG

TGAAATGGCAAGAGATCGATATTGTAAACTGATCTTTGTTCAATAATAGTTTTTTTTTTTTTTTTTTCCT

TTC

>FM890995.1 FM890995 Jatropha curcas embryo 56-70 (DAF) Jatropha curcas cDNA clone rjcaeb0_000973, mRNA sequence

CCAGTCCAAACGTACTTCCTTGATGTCTCCATCGAAGCATGCCAAAAATAACTAGTACAAATCCAGATTA

CACAAATATTACACCAAGGTAAGGTTAAAAAAATATATATAATAAAATAAAGGAAAGGAAAAAGAAACAT

CAAGTTCAAGATTCTACATCTTTCCATACATCATCGTTATGCAAATATAAGTGAAATCAATCCCATCCCA

CTGACAGAAAACTAAAATGGTCTAAAATCCTATAGCACAAGGCAAAGAGCGTTTGAGAATCTTATACTCA

AATACTATGAGTCTTGATTTCTCAGCCTTGATTCAGATAATCTCCTTAACGTTAATTTATTGCTGAGCAC

ATTGTACCCTCTGGGAACCACCATGCATATCTTCATCTTCATCATATGCCTCTGCAGCATGTTGTTGTTT

CCTCCGCATCTCCTCTTCAAAGTTAACATCATGTAAAGTTGTCTCCTCACATTCATCCAGCTCCATGTCA

GAGAGCTGCACCGACGATCTTGAGGGAAGCACTGCCTCTAGGGCTTTGCACTGATCTGGAGGTAGAGAGT

CAGGGAAATCTACAGTGAAATGAATGTACAACTTCCCCC

>FM890994.1 FM890994 Jatropha curcas embryo 56-70 (DAF) Jatropha curcas cDNA clone rjcaeb0_000971, mRNA sequence

ATTCAAAGAAACACACTAAACCAAGAAACTTTCTCTCACCGATCTTTGATCTTCTCCAAAAACCAATCAA

GAGAATGTCTCTGATTCCAAGCACCCTCTTCGGTGGCCGGAGAACCAACATTTTCGATCCCTTCTCTCTC

GATATCTGGGATCCATTTCAGGACTTCCCTTTCACAAGCACTGCTCTTTCTGCTCCTCGTTCTGAGTTAT

TGAACGAAACGGCAGCGTTTGCCAACACCCGCATAGACTGGAAAGAAACACCAGAAGCTCATGTATTCAA

GGCTGATCTTCCAGGGCTAAAGAAAGAGGAAGTGAAAGTGGAGATAGAGGAAGGTAGGGTCTTGCAGATA

AGCGGAGAGAGAAGCAAAGAGAAAGAAGAAAAGAATGATAAGTGGCATAGAGTTGAAAGGTCAAGTGGTA

AGTTCTTGAGAAGGTTTAGGCTACCAGAGAATGCAAAGGTTAATGAGGTTAAGGCTAGCATGGAAAATGG

AGTCTTGACTGTGACAGTGCCTAAAGTGGAAGTC

>FM890993.1 FM890993 Jatropha curcas embryo 56-70 (DAF) Jatropha curcas cDNA clone rjcaeb0_000970, mRNA sequence

CGAATCCACTGCTGATGGTTTCGTGGCCTTTGCCGATAATATTGAATTGCTTAAAGGTGCTTGTGGCAAG

TCCCTTGCCTCTTTCCCTGAAGGGTGCTTCTACTCCACAGCAGTTGGAGAACTGAGAAGCTTCGAAAATC

CTAAAAGCAAGATCACTGCTGTTCCAGCTAAGGAGGAAGAGATATGGGGTGCTACTTTCAAGGTTGAAGG

GCCAGCAGTTCTTGCAGCCAGAGAGTAGAAGAAAGAGCTTTTCAATATAAATGTATGAATTTAGCTGTGC

AATCGGCTTGAGGGGCCATGTGAATTATCGTGTATGAATGAGTACAAATTGTACTAGTGGAGTTAGTAGT

TGGTCTTTGTGGAAAAATTTAGGTTGCTAGTTTTCTCTTAAGCGCATCATTTTCCTCTTATGTACAGCTA

TGTTTAAATTAGTTTAAATAAAATAAGTTTGCTCAAGCTTTACGTCT

>FM890992.1 FM890992 Jatropha curcas embryo 56-70 (DAF) Jatropha curcas cDNA clone rjcaeb0_000969, mRNA sequence

ATTCCTTTCTGCACTGCTAGACTCGTACACAACATCAATGACCCGTCACGTGCCGATGTATTCAACCCAC

GTGCTGGCCGTCTCACCAACATCAACGGCCAAGACCTCCCCATCCTCAGTTATATCCAGCTAAGCGTTCA

GAAAGGTGTCCTCTACAGAAATGCTCTAATGTCACCACACTGGAACGTCGATGCCCATAGCATAAACTAC

TTCACAAGAGGAAATGGCCATATCCAGATCGTAGATGATAATGGAGAGACAGTATTTGATGGAGAAGTTC

AAGCAGGACAAATATTAACATCTCCACAAAACTTTGTGGTAGTAAAGAGGGCAGGAAGCGAAGGTCTTGA

ATGGGTATCCTTCAAGACTAATGACAATGCCAAGATTGACCAACTAGCAGGACGTGTCTCGGCAATCAGA

TCAATTCCAGAAGAGGTGTTGGCAAACGTATTCCAGGTTTCAAGGGAGGACGCGAGGAGACTGAAAGAGA

ATAGACAAGAAGTGACTCTGTTCAGCCCTGGGACTAGGCCTTCTTCTCGCAATTGAAGGGACTAAAACGG

CAGCTATGTAATTGTAATAAGGAGAG

>FM890991.1 FM890991 Jatropha curcas embryo 56-70 (DAF) Jatropha curcas cDNA clone rjcaeb0_000968, mRNA sequence

TCCTTACTAGAGTTAGGCATGATTAATATTCCAAGATTTTGGAGCTTCAAATCATCACCATTAGTGAAGT

CAAAATTTAAGTGTGGTCTACTTTTTTTTCGTATCCCTGGTTGGTGAGTTTGCCTTCTTCAAGGCTAAGA

ACTTCCAATCTACAATCTTCAAGAAGATGATATCTCCAACTACTACTAAATGAGCTTGCATGGAAAATGA

TTGATAGCTTTACTTGTGCTCTTCTTCGACTTCTTATTATCTTCTTGTTTAGGACGTGCGTTTATATTTC

TTTTTAATTATTCGAAATGTAAATAGAAATTAAAGAGCAATATAATGTTTAATTCTTAAT

>FM890990.1 FM890990 Jatropha curcas embryo 56-70 (DAF) Jatropha curcas cDNA clone rjcaeb0_000967, mRNA sequence

ATTCGGTCAGCAGAATTACTTTTCAACCTTCCACAAGACACCAAAGCGAAAGAAGTTCACAAAGAAAGAA

TGGGAGGATTCACGAGGGAATCGACCCAGCAAGCTGTAGCAGAATGGGCAGCTTCACCAGAAGTTACTAA

TTGGCTCATTGAGAATGCTGACCGAATTCAACTCCTTCCTAGTGATTGCAGTTCAGAAGAGACGGTGAGA

AGTGAATCGGACTCCGCAAACGAAACTGTTGGCAGTAGTGGCGAACGATTTAACTTGTTTAATTGGTAGC

TTGTTGATCAATAGCAATC

>FM890989.1 FM890989 Jatropha curcas embryo 56-70 (DAF) Jatropha curcas cDNA clone rjcaeb0_000966, mRNA sequence

ATTCCTTTTGTTCATATTAGCATGGGAAGCTTAGATGAACATGGTGTTTATAGTGGTCAATTTATTACAT

TTACTCTCTGTGGTTATGTTCGTGTGCAGGTTACTGTTCTATCCTTCTAAGAGATTTATTATGCACCCGC

AATCTGAGTTTATGCTAATTTAATTTTGTGCTGACTTTGTTAATTGCTCTATAGAAAAATTCTGCATGTG

TTGCTAATGAGATCTCTTTTCTCATTATTTTTTGTTCTTGACTCGATAAACATTACTAAGATTTTGTCAA

AAACTGAACTTTGTTTGAAGAAATTGAGGGCTCTGCTTTAAGATCTTTTTTGATAGCAACAATGCTTTTA

TCTATTACCATAATTTTGTGTTTTGCTACTAGCTTAGGTGCTTTTGACAAATTGGGGTTCTGTCTTATCT

TAAATTGGAGAATCTG

>FM890988.1 FM890988 Jatropha curcas embryo 56-70 (DAF) Jatropha curcas cDNA clone rjcaeb0_000965, mRNA sequence

ATTCCAAATATCACTGATCGTAAACCTCCATTGAATTTACTCTGTGCTTCGATCGCGTCAATGGCGGAGC

ACCCACAGTCGCAGCATGTTGGCCAACAGCCACGATCCCACCAAGTAGTGAAGGCAGCCACTGCAGTCAC

TGCCGGTGGATCTCTTCTAGTTCTCTCCGGTTTGACCCTTGCTGGTACGGTCATTGCACTGACCGTAGCC

ACTCCATTGCTGGTCATATTCAGTCCGGTTCTTGTTCCTGCGGTTATCACAGTTTGTTTGATAATCACAG

GGTTTTTAGCCTCAGGCGGTTTTGGCGTCGCTGCCATTTCTGTCTTGTCCTGGATTTATAGGTATGTTAC

TGGAAAGCATCCACCAGGAGCAGAAAGTCTGGACCAGGCACGCTTAAAGTTGGCGGGAAAGGCAAGAGAG

ATGAAGGACAGAGCTGAGCAGTTTGGACAGCATGTAACAGGTCAACAGACTTAAAAGAGAAGTGGGTTTT

GGTGGTTGATGCAGAGAACATAAAATGGCTTTGATATATATATAAAAATATATGAGTCCTTTAATTAACA

TGGAAGGCCATTGCTTGTCTGGTGTATTTCTTTTTTCGCTTAAATCTTTTTGGTTCTTCTATGTAATTTG

TAACCGGTCTTCCCTCTTTG

>FM890987.1 FM890987 Jatropha curcas embryo 56-70 (DAF) Jatropha curcas cDNA clone rjcaeb0_000964, mRNA sequence

AGCAAATCAAAATCTTTAGTTTTCTTTTATTTTCTAGCCTGTAAAACATCCATTTCTTCATCAAGGTTTA

CAGTACAAAACATACCCATTCTTTCTCCCAACATGCCCCATTGGGAAATTTGNCCAACTATTCCTACTGG

GTAAAAAATTTAACCCACCTGGGTACAAAAATTAATTAAAAAACATTATTTTGGAAGGAGAAGCATCTCT

GTGGATCTTGTTTGGATCCGTGACATAGCAGCACTAGCTGCTATTGTCATGGCAGTCGCGGCAGCCCCCA

GCTTTGCACCACCAGCAGCCTTCATTACACCAGCTACTTTTGGCGAATACAGCTAGACCCCCAACAAGCA

TAGCCCTTAAAACGACTGTGGGTTTTACGGTCATTTCTTAAAAGACCTAAGTTTCGAAATTGATGATGTC

GTTTCGCTGAAAA

>FM890986.1 FM890986 Jatropha curcas embryo 56-70 (DAF) Jatropha curcas cDNA clone rjcaeb0_000963, mRNA sequence

TCTTTTTTTGTTTAAAAAGAAAATTCCAAATTCATTAAGAAAATAAGAAAGAAATACAATCAGAAATATA

TTAGTGTCGAGACATTAAACGGGAAACATAAATTTTGATAATGGATAGTTTTCTTCATTGATATGATAAG

TCATAACCAAGACTACAGGAGTCTTTAAGACAATAAGAGTGGTATGACGCAACTGGCAACATTTAAAAAC

TTCTAGTACAGTACTACTATATGCTGGTGTAAGAAACACACTAAAAAGTGAACGCCACCACTTCAGCATC

TGAATCTCAGACTTCTACCGTGGCCTAACCCGGTTGTGCAATAGCATTTCCCGGAGCTCAGCCGGGTCGA

TCTCAGCCGCAGAATTAGCTGGCCTGTAGTACCCCGTGATGGGGTCTGGTGCCCACGCAGTAGATGATCC

TGAATCATCTTCTTTCGTCGCAGCTCTTTCTTCTACTTTCCCCACAATACTAGTCCTTGACCCTCGCCTA

CCAAAGCTAGCCGACTCAGACCCATGTGCTGCACCCGCATAGCCTCTCCGGAAGATAGAAAGTGGTAG

>FM890985.1 FM890985 Jatropha curcas embryo 56-70 (DAF) Jatropha curcas cDNA clone rjcaeb0_000962, mRNA sequence

ATTCCTGGTGGTTGGGAAGAGACGAGCAGAGAATCATGTCATCGTTCGCCGGTTCATTTGGTGACGAGTC

TCGGCTCACCGGCTCCACTAGTCCCTTCGACGATGATGGATACATCGGCTATGAGCCTCGCTTAAATTCT

CAGCGGTTCGAGTCTTACAGCAAATTCGATACTGACTCTGTCAAGGACTCGGCCGTTGAGTCGTCGCCGA

TCTTCACCTCTCATGCATATACCGGTGGCGATGACATGTACTCATCTCATCCAGTGAGAGAGAGTCCTCC

GTCGATCTATTCTGGTGGTGCTGGTGGAGGATTCTCTATTGCTTTCTCTCCGGAGCAGAATGGCGGCCAA

GGATTTAATGGTCCGATCTTGCCACCACCAGCAGGGATGCAGGCAGAGGAAGGATTTGCATTGAGAGAGT

GGCATAGGCTGAATGCGATTAGACTAGAAGAGAAAGAGAAGGAAGAGAAACAGATGTTGCAGCAGATAAT

AGAGGAAGCGGAGGAGTATAAACGGGAATTCTATCGGA

>FM890984.1 FM890984 Jatropha curcas embryo 56-70 (DAF) Jatropha curcas cDNA clone rjcaeb0_000961, mRNA sequence

ACTCATAACAAAAATTCAAATATGCTTTTGTTTTTGCATGAAAAAGATCAAATCATCTAGTATGCATAAA

ACAATTTCCTAACACAAAAAGAATAAAAATGGATACATGGAAATTCTGTCCTTACAATATCTCTGCAGCC

AGGAAACTATTTTGGAGAAGGAAAAAACTGAAAACCAACTTTCTCAATGTTTTTTTATTTAAAGGTGGAC

AGCCTGGGAAGCTTGTCTCTAAGATANTACAACCTTGCCCTTCTGACCTTCCTGTGGCTGACTACTTTTA

TTTCCTTAATATTTGGTGAGTAAATGGGGAATACAAACTCAAACCCTATGCCTGCGATGGGGGGGCCCGG

TACCCC

>FM890983.1 FM890983 Jatropha curcas embryo 56-70 (DAF) Jatropha curcas cDNA clone rjcaeb0_000960, mRNA sequence

ATTCTATTTGAAGTGGGGACCAACCCCTTAAAGACATGGTTGATCCTACTCTTGATTCCTTTGAAGAGGA

GAAACTAGAAAGAATTGGTCAAGTGATAAAATCTTGATGTGCACCCAGATCCTAAACAAAGACCGGAAAT

GAGAGAAGTTACTGCCACATTAAGAGAGATTACAGGAATAACACCTGAGGGAGCAACCCCAAAACTCTCT

CCTCTTTGGTGGGCAGAGCTTGAAATTTTGTCAACAGATCAAGATGCTACCTAAGTATATAAAAGACCAG

ACTGTAAGTATGAGCTCAATATCTCATTGTTAGTAGTGTTGGCGAGGCACAAAACAAGTTGCCTGTAAGT

TTAATATCTTTGTATCTATTATCATAGGCAATCAAGAGTTGCTTGTAAATAACTTAGGAAGGGTGAAAAG

ATATGG

>FM890982.1 FM890982 Jatropha curcas embryo 56-70 (DAF) Jatropha curcas cDNA clone rjcaeb0_000959, mRNA sequence

AGCTCAAAAGGAATGCCACAAATTCTTCGATATTAATGTTTTGGCAATTAAAGGAAATTTGAAGAACTGG

TGTAGCAGCTCAGACATGGGGCAATTACTAGTACATATACAATTTTTGCATAAACTAAAGCTGATGATTG

CTACAATCGGAGGGAAAAAGACGAAACAAAAAGGAAAAAGGTTGACCAAGTCCTAAAATGCCGAAACACA

ACACACTTCCTCGCACTACTAATTTAAGAGAATACAACCAACACAAGTATATTGGATTCTTTTTCCATCA

GGCATGGCCTCAGAGCTCTCCAGTCAAGGTGGCCGTTCTTATTCTACAATGAAGCAGGCTGCAAAAACGA

GGTCTACCTGAAGCATTATACATACAAATCAAATACATAATATTACACCATCACCTAGTACACCAGAAAA

TTTCCCTCTTATCCTTAGAAGAGGGTAAAGGAGCTTGCAGGAATTTGTATTCTTATAAGAAATAAGACAA

TTGCTCAGTTTTCCTTGAACCCCCTTCTCCATACAAATCATTCCACTGCTTCAACTCGCTCATTATAGAT

CCCTGAGATGCAAAGCTAGCA

>FM890981.1 FM890981 Jatropha curcas embryo 56-70 (DAF) Jatropha curcas cDNA clone rjcaeb0_000958, mRNA sequence

CTTTTTCAAAAATCAAACAAACAGTATTTAATAGATATTAACTTTATTGTCCATATATTATAATAATAGA

GTAAAAGCTGCGACAGGATGAACCCATCACAGCAATTTCAAACACTGACATCTAGAGTGATGAAACTTGG

ACACAACAAGAGAATAAAAGAAGTTCAGCTTATTCATGGCCACTCATTTACGACCATTGGACGCACACAG

ACATCAACAAATTTAAATAAACAATCACCCAAGATATTATAGATATAAGTAACTGCTTATAAACCAAACA

GCAAACAGATTAGAAACCACCACGGAGTCGGAGGACAAGGTGAAGAGTGGACTCTTTCTGGATATTATAG

TCTGCAAGGGTTCGTCCATCTTCAAGTTGTTTACCAGCAAAAATCAATCGTTGTTGGTCCGGGGGAATAC

CCTCCTTGTCCTGGATTTTAGCCTTCACATTGTCAATTGTGTCAGAGCTCTCAACCTCGAGGGTAATGGT

TTTCCCTGTCAGGGTTTTGACAAAAATCTGCATTCCACCACGCAGACGAAGCACCAAATGCAGAGTAGAT

TCTTTCTGAATATTATAATCTGCAAGAGTCCTTCCATCT

>FM890980.1 FM890980 Jatropha curcas embryo 56-70 (DAF) Jatropha curcas cDNA clone rjcaeb0_000957, mRNA sequence

ATTCTGAAGGACAGAGCTGAGCAGTTTGGACAGCATGTAACAGGTCAACAGACTTAAAAGAGAAGTGGGT

TTTGGTGGTTGATGCAGAGAACATAAAATGGCTTTGATATATATATAAAAATATATGAGTCCTTTAATTA

ACATGGAAGGCCATTGCTTGTCTGGTGTATTTCTTTTTTCGCTTAAATCTTTTTGTTTCTTCTATGTAAT

TTGTTAACGGGTCTTCCCTCTTTTGAGAGCTGTCTAAAATAATTTTCTTGTCATGTGTTGAAGGTCTCTG

TAATCAAGTCTTACTTTGTTTGTTGATGAATTTGATGTTTCCAT

>FM890979.1 FM890979 Jatropha curcas embryo 56-70 (DAF) Jatropha curcas cDNA clone rjcaeb0_000956, mRNA sequence

GTTCTGATGAACTCTTACAGAATGTCTCCAAGCGAAGCTCATCAAGTGAAGAGGAACAGGGATCCACAGT

CCATGTTGTTTACTCCCACCAGCAGGAGACCATAAGTAAGTTACTACTAAAATAGCATTGTGATTGGTGC

TTCTACTAGCCAAGGCCGCAACTAAGAGATCCTTCTTGTAAATTTAGAGAAGGGATCCGTCGTATATTGT

AGTATAGAGAGTTGTAGAGTGTTAAAAATGTAGAACTTCAACTTCTGCAGAAATCAATAAAATGTGGCCT

TCTTTTGTTTCTAATTAATAAAATATAAATTCCATCGGCAAAAAAAAGGGGGGGCCCGGTAC

>FM890978.1 FM890978 Jatropha curcas embryo 56-70 (DAF) Jatropha curcas cDNA clone rjcaeb0_000954, mRNA sequence

ATTCCACCGATCTTTGATCTTCTCCAAAAACCAATCAAGAGAATGTCTCTGATTCCAAGCACCCTCTTCG

GTGGCCGGAGAACCAACATTTTCGATCCCTTCTCTCTCGATATCTGGGATCCATTTCAGGACTTCCCTTT

CACAAGCACTGCTCTTTCTGCTCCTCGTTCTGAGTTATTGAACGAAACGGCAGCGTTTGCCAACACCCGC

ATAGACTGGAAAGAAACACCAGAAGCTCATGTATTCAAGGCTGATCTTCCAGGGCTAAAGAAAGAGGAAG

TGAAAGTGGAGATAGAGGAAGGTAGGGTCTTGCAGATAAGCGGAGAGAGAAGCAAAGAGAAAGAAGAAAA

GAATGATAAGTGGCATAGAGTTGAAAGGTCAAGTGGTAAGTTCTTGAGAAGGTTTAGGCTACCAGAGAAT

GCAAAGGTTAATGAGGTTAAGGCTAGCATGGAAAATGGAGTCTTCACTGTGACAGTGCCTAAAGTGGAAG

TCAAGAAACCGGACGTTAAGGCTATTGAGATTTCTGGTTGAAACTTTTAGTTATGTTTGAGTGTGTCTAA

GTCTAAATGGTACCCATCCATCC

>FM890977.1 FM890977 Jatropha curcas embryo 56-70 (DAF) Jatropha curcas cDNA clone rjcaeb0_000951, mRNA sequence

ATTCTACAGATTGTGGATGACAGTGGAAACACAGTAATGGACGAAAGGGTGAAAGAAGGAGACATGTTCG

TGATCCCTCAATTCTATGCAGCACTTGGAAGGGCAGGAGAAAATGGGTTCGAGTACGTAACATTTAAAAC

AAGTAGCCAGCCTATCAAGAGCCCATTGGCGGGTTATACATCAGTGATGAGAGCCATGCCTCTTGATGTT

CTGATGAACTCTTACAGAATGTCTCCAAGCGAAGCTCATCAAGTGAAGAGGAACAGGGATCCACAGTCCA

TGTTGTTTACTCCCACCAGCAGGAGACCATAAGTAAGTTACTACTAAAATAGCATTGTGATTGGTGCTTC

TACTAGCCAAGGCCGCAACTAAGAGATCCTTCTTGTAAATTTAGAGAAGGGATCCGTCGTATATTGTAGT

ATAGAGAGTTGTAGAGTGTTAAAAATGTACAACTTCAACTTCTGCAAAAATCAATAAAATGTGGCCTTCT

TTTGTTTCTAATTAATAAAATATAAATTCCATCGGCTTTACAGTGGAAA

>FM890976.1 FM890976 Jatropha curcas embryo 56-70 (DAF) Jatropha curcas cDNA clone rjcaeb0_000950, mRNA sequence

ATTGGGGGCCAACCTTGCTCAATCTCCTCTTGCCGTCGCCTCTCAATCTGATGTCGTTTTCTCTATCGTC

GGCTTTCCCTCCGACGTCCGTCAAGTCCTCCTGGATTCCACCACTGGAGCTCTCCAAGGCCTCCGCCCGG

GTGGAATCCTCGTTGACATGACCACATCCGATCCCTCCCTCGCCTCCGAGATCTCCGCAGCGGCATCCGC

CAAAAATTGCCACTCTATTGATGCTCCAGTCTCCGGAGGTGACCGCGGTGCTAAGAATGGAACCCTTGCA

ATATTCGCTGGAGGGGATAAGGCAGTTATCGATCGCTTGAATCCCCTGTTTGCTCTCATGGGAAAGGTTA

ATTACATGGGTGCTCCTGGCAAAGGCCAATTTGCGAAGCTGGCAAATCAAATTACCATAGCTTCTACCAT

GGTTGGCCTGGTGGAGGGGATTATNTATGCATATAAAGCTGGACTCAACGTGGAACACTACTTAAACGCC

ATATCTACAGGCGCTGCCGGGTCAAAGTCCCTTGATTTATACGGCGCTAGAATATTAAGAGGGA

>FM890975.1 FM890975 Jatropha curcas embryo 56-70 (DAF) Jatropha curcas cDNA clone rjcaeb0_000949, mRNA sequence

AATCAAAAGGAACAAGCTTTAGAAATATTAATAATTACAGAAATTAAACCAGATAAAGCATTAACAATCC

ACGCATATAGATATTATCATTTCAGTATATCAAGGAGTTAGACGTGTGCCTCCATCTGATTGAGAAGGAA

GTTGCCTTCATTGTTTTTGTGCACCTCAACCTTGTACTTCTCTTCATTGCTTCCCCTCTTCACCTTAAGG

AGCATATCAAACTTTGCAAAATCATCCACTACCTCAGCCTTAGCATGAACAATCTCTTGAAGCTCATAAG

GGAACAATGAATTGGACCTCTGCTGAATGGTCTTCACAGCATGATTTGCAGCATCTTGCACTGCAGGATC

ATGTGCTGGCAC

>FM890974.1 FM890974 Jatropha curcas embryo 56-70 (DAF) Jatropha curcas cDNA clone rjcaeb0_000948, mRNA sequence

CAAAAATAACAAAGATGAATTTAGATAAATAGCACAAAGCGTACCTCTTAAACATACAAGTCAAGAAAAT

CTATGAAGCAGTTGGTACGCCCACCTTAGCACATAATATCAAACTCCACTAAAAAAATATTAGAAGACAT

ACATAAAATCATTTTTTACTCCACAAGTTCATTAACCTAGGCAGAAAAAACACAAATTTCATACCAAAAT

ACTAAACAGCAACAGCCCTAGTACCGATCGTAGCGCCTGTGACGATCATAATCCCTGGAACGTTCTCTAG

ACCGAGATCGCGACCTGTGTCGACTTCTTGAATCATAACTACGAGAACGATCTGCATCTCTATCACGTTC

CCGGTCATGACCACGATCTCGATCATAATGCCTGTCACGATCTCTGCTCCTACGATCATGATCCCTCTCC

CGGTCACGGCTATCTGCCCGATCTTGATCCCTGCTGGATTCTCTGTCACGGTCTCTACTTCGCTCTTTTG

ATCTCCGATCATCATGCCGATCACCTCTGCGAAGCTTGTTTCTCTCTTCCTGAAGTTCTGCTAGTTTCTC

ACGGATTTGCATATATCCTAGATGAAGCTTCCCTCCAAAATGATCTGCTAAACGTCGATCACTGTCATAT

A

>FM890973.1 FM890973 Jatropha curcas embryo 56-70 (DAF) Jatropha curcas cDNA clone rjcaeb0_000947, mRNA sequence

ATTCAATCGGCGTTGAAAGATCAGTTCTCCTTTTTGAGTATCTAAAAATGTCTTCTTGCTGTGGTGGAAA

TTGCGGCTGCGGCTCCGGCTGCAAGTGTGGCAGCGGTTGTGGCGGATGCAAGATGTATCCTGACATGAGT

TTCTCCGAGAAAAACACCGCCGAGACTCAGGTTCTCGGAGTTGCACCGGAGAAGGCACGCTTTGTCGATG

GAGGTGAGGTCGGCGTTGGAGCTGAGAACGGATGCAAGTGCGGTGACAACTGCACCTGCAATCCTTGCAA

CTGTAAATGAGATCGGCGGCGATCAGTCTGTTTTTTAGAAGTAAAAGCAGAGATGACTCTGCTTCTCTCT

TTCTAAAAAAGGAGAAATAATTAATAGAGTCTAGATATCGTATGAGTTTGTTCGTTTGTATCTGGTTTTG

AGCTTCAATAAAAGTAGTGGTGGCTTGTGAGCTTGTGTTTAGCAATGGCTGCCTGTTGCTTTAACGTTAT

ATATTGTATAAATTAGAAACTAGCATATGAATATGTAAATGAGTACGAGGGGTGAAATAGCTTG

>FM890972.1 FM890972 Jatropha curcas embryo 56-70 (DAF) Jatropha curcas cDNA clone rjcaeb0_000946, mRNA sequence

ATTCCATCTCTCGTGGAGCAATGTCTGGGCCCTATTACAACTCCAGAGTAACCAAGATATCAGTGGTTTT

GGATGGCGAAGGCTACTTTGAAATGGCTTGTCCTCACCTTAGCGGCTCGCGGGGAGACACGACGGGTGAA

AGTCAGAAGACTTACCAGAAAGTAAGTTCACGGTTGAAACGTGGAACAGCAGTGGTCGTCCCTGCAGGCC

ATCCGGCGGCTTTTGTAGCTTCAAGAAACAATAATCTGGAAGTAGTCTGCTTTGAGATTAATGAAGAAGG

CAATACTAGGCATCTACTTGCAGGGAAAAATAATATTGTAAATAAGATGGAGAAGCAAGCCAAAGAGTTA

GCCTTCGGTGTAAGTGAAAGAGAAGTGGATCAGGCTTTTGGAAGACAAAACGAGGAGTGGTTCTTTCCAG

GGCCAAGGAGAGGTTCCTTCGAGGGTCATGCTGTGGCATGAGAAGCTAATAACGTGATGGCAAGTGTTTC

GGCTTTTTGGGGTGACTGTGACATCGACCTTGAGGGTTTACTAATGTACATAGTGCTGTGTATGGTGAAT

AAATAATGAAAGGTG

>FM890971.1 FM890971 Jatropha curcas embryo 56-70 (DAF) Jatropha curcas cDNA clone rjcaeb0_000945, mRNA sequence

ATTCCAAGCCAGCAACTAGTAGATTTGGTTCATGAACATCTGAGGATGGAAAGCAAGCTCTCTGTAGTGT

GTGAGAGAATACTCGATAGGTGTTTGGCACCGTCAACAGCAGGTGGCGAAGGATGCGACAACATGACCAT

GATCTTGGTGCAGTTTAAGAAACCTATTCAGCCAACTGCCACTGCAAATGATCAGTCCTCACAATCTGAA

TTTGCAGATGCCGAAACTAAACCGGAGGAAGATGAATTGAAGTAGCTGCAATGGGTGGTTGATCTCTTGT

GCCATTGTGCAGCCCTCTGGCTGGAAGAATGTGGGAGGATCCCTCATTTTCTGGGAAAAGTTGGATCCTC

ATATATCGTTGTAATGGTTTANCATGAATGGGTGAAATATTAAGAAATTTTTCATTACATGTACTGGTGT

GTGCTTTATGTTTGAACCATTCTCACATTCAATAACACATGTACAGCTCTTTGAAAACAACATTTTACCA

TGCCCATCTCCAGTGTACTTTTCTGCACACTGAATATTTATACCTATT

>FM890970.1 FM890970 Jatropha curcas embryo 56-70 (DAF) Jatropha curcas cDNA clone rjcaeb0_000944, mRNA sequence

ATTCGAAACATCCTCGTGATCAGTCATTGGCCACATACAGAGGTCATTCGGTCCTGCGCACTCTGATTCG

CTGTTACTTCTCTCCAGAATATAGCACTGGTCAGAAGTATATCTACAGCGGATCAAGTGATTGTTCTGTT

TATATTTATGATCTGGTGAGTGGTGCTGAAGTTGCAAGACTTGATCATCACGAAGGCCCTGTAAGAGATT

GTAGTTGGCATCCTCTCTATCCAATGATCGTTAGCTCTTCTTGGGATGGAGTAATCGCCAAATGGGAATT

TCCTGGGTATGGAAAAGCACTTGTGCCATTGAGACGAAGGTCAAATCGGAGGCGGGTTTACTACACTGAT

GTTGACTTATAGTTGTACAGATGCATTTTGGTTTTTTTGGATTCAATGGTTAGTAAAATCCAGGTCATGT

CGATCTCTGGCTGGAGTGTGCATCTCTGTAGAAAGACATACAGATTTGTACATCTAAGGGAGGTTATATT

TTGCTATCACTGCTGTGGAAAGTCCAGCAGTTGGTCTAGATAATAGTAATATTACAGATTCCTTTTGTTT

GAAATTTGATATTGCTGTATGTAAAAACTCCCATTTTATATC

>FM890969.1 FM890969 Jatropha curcas embryo 56-70 (DAF) Jatropha curcas cDNA clone rjcaeb0_000942, mRNA sequence

ATTCCTCCTTTCCCTCCCTTCGCAAGCCTAACAGCTCCACCAAACCAAAAAGAAACAATGCTCGCTCACC

GTTTCGCGGCGGCCGCCGCCTTCTCTCTCATCGCGGCTATCATTTTTTCCACACCATTTTCTACAGTGTC

AGCTCAAGCGCCTTCTCCTGCTCCGTTTTTATCACCTGAACCTTTGAAGCCGTTAGGGCCATTGGCGCCG

ACACCAGCAACGACAACAAACGACTGTATTACGCCGTTGCTTAACATGTCGGATTGCTTGAGTTATGTGC

AGGAACCGAGCACGGCGAAAGTTCCGGACAAGAATTGCTGCCCGGAATTGGCTGGATTGGTGGATAGTAA

TCCTATATGTTTGTGTCAGTTGCTAGGGAATAGTAGCTTGACGGAGTCTTTTGGTATAAAGATTAATATT

AACAGAGCTCTGAAGCTTCCTTCTGTTTGTGGTGTTGAAACTCCTCCGGTTAGCACGTGCGCAGCCGTCG

GATATCCGGTACCAGGTCCAACAGCAAGTGCGCCAGGTGTTCAGCCACCAGAAGGCATTGCCGGCATTCC

ATCAGCTGGAAACAATGACAATAGAGCTACAAATATTCCCGGTGTTCAGCCACCAAAAGGCATAGCCAGC

ATTCCATCAGTTGGAAACCAATGAC

>FM890968.1 FM890968 Jatropha curcas embryo 56-70 (DAF) Jatropha curcas cDNA clone rjcaeb0_000941, mRNA sequence

ATTCCATCAATCAAATCTTCTTAAGTGATATACAAATTAGAAATGGCTCTGATTCCAAGTTTCTTCGGTA

ACTCGCGAAGCAGCATTTTCGATCCTTTCTCTGCTTTTGACGTTTGGGATCCATTCAAAGATTTTCCTTT

TCCTTCATCTTCTTCAATCGTCTCTCGTGAAAATTCTGCTTTTGTGAACACTCGCATAGACTGGAAAGAG

ACACCGGAAGCTCATGTCTTCAAGGCTGATCTTCCTGGGCTCAAGAAAGACGAAGTGAAGGTAGAGGTTG

AAGATGACAGAGTTCTCCAAATCAGCGGAGAGAGGAATGTGGAGAAAGAAGACAAGAACGATACTTGGCA

TCGGATAGAGCGTAGCAGTGGCAAGTTCTTGAGGAGGTTCAGGCTGCCGGAGAATGCAAATATGGATGAA

GTGAAGGCTTCAATGGAAAATGGAGTTCTTACTGTGACTGTTCCTAAGGAGGAAATCACGAAACGTGATG

TGAAGGCTATTGAGATTTCTGGCTGAGTTTTACAAATTTGATCATTAAAATGAATGTACTAGGAGAGATT

CAGAAGTTGTTTGATGTCATAAACTAATGAACAAATATGGAGTCTTGAGTTGTAAAATCTATTTTCCAAT

TTTCTAAATTTCTAACAGCTGAATT

>FM890967.1 FM890967 Jatropha curcas embryo 56-70 (DAF) Jatropha curcas cDNA clone rjcaeb0_000940, mRNA sequence

ATTCCTTTAGNTATTGGATTGATGAGGTTTTGAATTTGCTGAAAAACTTGTTTGTATCAAAATTTCCTTT

AACAGAAACTTTCTTGAGGCCTAAAGAATTACCTGATTTTGGCAAGAAAACTTTTCAAGCGAGTTCATAA

TTAAGCACCAACTTATTAGGGAAAATGCACGAAAAGAAAATGTGAAGATTTGTGCAAGGCTATTTCTCAG

CAAATCCAAGAAAAGGGTGTGCTATGCAGAAGTTGGTGATGATTTTGTTGATTTACTCTTTAGTTTCCTT

ACCATTCCTCTTGGGTTTATAATGAAAGAAATGAATGGTGCTCATTCAAAAGGGTGCATAGACTATTTAT

ATAAAAGTATTATGGATCTTGATAGTGAGAAATACTTCCAAACAAATGTCCATAAAGAGATTTTACTTAG

TCCCAAGATTGCTCCTGATTTTGGCTATGAGAACCAGCTTCTAGGGGTGAAGGAAGTTGCCCACCAGCTC

TGCTACCTCAAATTGAGTAGTAATGGTTTAGGGTTTAATGTTGGGATTTCTTTGGATAAACCTACTAGCA

ATAGAAGCATTGCCTTAAGAGTAAAGGATCCAAAATCAACATACAAGAGCGGTAAAACTGGCAGAGGATT

TATGAAGGG

>FM890966.1 FM890966 Jatropha curcas embryo 56-70 (DAF) Jatropha curcas cDNA clone rjcaeb0_000939, mRNA sequence

ATTAGTTTTGATGCAATGGGATTGCAAGAGAACCTTCTGAGAGGCATCTATGCTTATGGTTTTGAGAAGC

CTTCTGCTATCCAACAAAGGGGAATTGTCCATTTTGTAAGGGTCTTGATGTTATTCAGCAGGCCCAATCT

GGAACTGGAAAAACGGCAACATTTTGCTCCGGCATTCTGCAGCAACTTGATTATGCTACAGTTCAATGTC

AAGCGTTGGTGTTGGCTCCGACTAGAGAACTGGCACAGCAGATTGAGAAAGTTATGCGTGCCCTTGGTGA

TTATCTTGGTGTGAAGGTTCATGCTTGCGTTGGTGGAACAAGTGTTCGTGAGGACCAGCGCATTCTTCAA

GCAGGTGTTCATGTTGTTGTGGGCACTCCTGGTCGTGTGTTTGACATGTTGAGAAGGCAGTCTCTTCGCC

CAGATTACATTAAGATGTTTGTACTGGATGAGGCTGATGAGATGCTTTCACGTGGTTTCAAGGATCAGAT

TTATGATATTTTCCAGTTGCTGCCAGCAAAAGTTCAGGTAGGAGTGTTCTCTGCTACCATGCCACCAGAA

GCTCTTGAGATCACAAGGAAGTTCATGAACAAGCCTGTTAGGATCTGGGTTAAGCGTGATGAGCTTACCC

TTGAGGGTATCAAGCAGTTCTATGTCAATGTTGAGAAAGA

>FM890965.1 FM890965 Jatropha curcas embryo 56-70 (DAF) Jatropha curcas cDNA clone rjcaeb0_000938, mRNA sequence

ATTCCGACGAAGCTACTCTCGCTACGATCGACCAAATCTAAGGCTTTCAGATCGAAAAATGGCATTGATT

CCAAGCTTTTTCGGTAACTCACGAAGCAACATCTTCGATCCTTTCTCTGCTCTCGACGTTTGGGATCCAT

TCAGAGACTTTTCTTTCCCTTCATCTTCTTCCCTCGTCTCTCGTGAAAATTCTGCTTTTATTAACACTCG

CATTGACTGGAGAGAGACACCAGAGGCTCATATATTCAAGGCTGATCTCCCTGGGCTCAAAAAAGAGGAA

GTGAAAGTAGAAATTGAAGATGACAGAGTGCTTCAAATCAGCGGGGAGAGAAATGTGGAGAAGGAAGACA

AGAACGACACGTGGCATCGCGTGGAGCGTAGCAGTGGCAAGTTCATGAGGAGGTTCAGTATGCCGGAGAA

TGCAAAGATGGATCAAGTAAAGGCTTCAATGGAAAACGGAGTACTTACTGTCACTGTTCCTAAAGAGGAA

GTCAAGAAACCTGATGTTAAGGCTATTGAGATCTCTGGTTAAGTTTCAGCTTATAATGATGTTATCTCAA

TATATATATATATATATATATATATATATA

>FM890964.1 FM890964 Jatropha curcas embryo 56-70 (DAF) Jatropha curcas cDNA clone rjcaeb0_000937, mRNA sequence

ATTCCTCNAGGTTGATCTTGCTCATATGTTAGCTGCTCGTGAACAAGAATTACGGACTCTTTCTGCTGAG

ATGAATCAACTACAGACTGAACTAAGGCTTGCTCGATCCTTGATAGCTGAGAGGGACGCTGAGGTACAGC

GAGTACGCACTACAAACAATCAGGTGTCTCCAATAGCTATAACTGGCATTCTCAGTTTAAATTATTTTAT

TCTTGTTACATATTATTTCTGCTGTATTAAATAAATGCTGCTAAATGATTGGTGCTTTCTTTATGTTTAG

CTGTTGTCTGCAAAAATATTTATGTGTCTACGTTGCTGCAGTATTTGTCAGGTTATAGTTTTTAGATGTT

TCCCAGTGTTCTCTCTCTCTCTCTCTCTCTCTCTCTAATACTGCTAATTCTGCACACGCTTTAAGAAAAG

GTTGTATGTGATTTGCATATTCTACAAAATACAAATCACTGTGGTCATGTTGAGCTTTTCCTAATTTCAT

TAACGAATTGTCGGAACAGTATGTGGAAGAGAATGACAGACTCAGAGCTGTTTTAGGGGAATGGAGCACA

CGAGCAGCAAAGCTTGAGCGAGCA

>FM890963.1 FM890963 Jatropha curcas embryo 56-70 (DAF) Jatropha curcas cDNA clone rjcaeb0_000936, mRNA sequence

ATTCGCAAGAAGAGAGAGAAGGATGCCCCTCCGGACGCGACAATGGTCTTGAAGAGACCATTTGCAGCAT

GAGAATTGGAGAGAACATTGCTGATCCCTCGCGTGCTGACGTTTATGTACCAGAAGCTGGTCGTGTTAGC

ACCGTTAATAGCCATAACCTCCCTATTCTTCGGTGGCTACAACTCAGCGCTTCCTATGGCGTTCTCCGCA

ATGATGCCATGAGATTACCCCACTGGCACATAAACTCTCACAGCGTAATTTACGCACTCAAAGGACAAGC

GCGGGTTCAGGTGACGGATGAAAACGGCAACACCGTCTTCGACGGAAGTGTAAGCAAAGGACAGGTTTTG

ACGGTGCCACAAAACTTCGTAGTGGTGAAACGAGCAGAAAGTGAAAGATTCGAATATGTAACCTTCAAGA

CAAGCGATAACGCGATGACCAATGATCTTGCAGGTCGCTCCTCCGCCGTTAGAGCAATGCCGTTGGCGGT

TATTGCAAATGCATTTAGGGTATCAGCAGAGGAGGCAAGAAGGATTAAGTTTGGAAGACAAGAGACCACC

TTGACTATTTCCAGGAACGAGTCTGGAAGATATGCCGAGGAGTAAATAATGAAGTAGCCTTTTTGGGTGT

GTAGATAGTGA

>FM890962.1 FM890962 Jatropha curcas embryo 56-70 (DAF) Jatropha curcas cDNA clone rjcaeb0_000935, mRNA sequence

AATGGAGAAAAAAGAAAGTAAATTTTATTCACAAACTAAGAGAAACTCATTAACGACTCTGAGGATAACT

AGATAAACGAAAAGGGTAAAAAAAAGCTACTACCAACAGCTATTACAGTCTGTGTTTGCAACCACGTTTT

ATTCTTTACATTACTCTTCTCTAATCAAAACCCATTATCATATCATTATCAAAGCTCTTTATCAATTTTT

GCACCCTTTCAGAATTAGCCGCCTCCTTCAACTTGTCTGGTGGTGCTGCAGATGCTGAAGGCAATATAAC

TGACTCGCGTTGTGAATTAACAAAGTGCTCCAATCCTTCCTCGCTTAAGCCAAATGCAGTTGCAAGCTCA

GGGCCTCTTAAGGTAGTAAGAACTGAGTTTGAACCAGCTAAGAATTGGGGTCTATTCTTTTGAGCTGAGG

TTGTGAATCCAAAGAACTCAAAAGGAGCTGTTCTTGATGCAATTTGACAGAATGGGTGATATCTTGGCAC

CCAAAACACATCATTTTCTGCTACTTTCGCTTTCATTGCTTGTGTTCCGTTTGGATACACAATCTGTATT

ACGCCAGTTCCTCGGGGGGGCCCGGTACCCAAATCCG

>FM890961.1 FM890961 Jatropha curcas embryo 56-70 (DAF) Jatropha curcas cDNA clone rjcaeb0_000934, mRNA sequence

CTAGTGGATCCCCCGGGGCTGCAGGTTTTTTTGCTAATAACATAATCACTGGTATAATATGTAATCCAAA

ATGTGCAATTCAGAAGCAAGAATGCAAAACAAAGTTGCACTATGTAATCAAATGTAACTAAAATCACAAA

AAGCTAGACTTTAATTTGTCTGAAACGAGAGATACAAACTCACTAACAAGGAAAACAAAATTAAGACGCA

GAGAAAAACTTAAATTAAAAGATTAAATAGTTGTTTCACTTAAAGCACTTGGCATCCAAAAGAGGCTCTC

CCTCAAAAGGCTCAAAATCTTCAATCATCTGGTTGACGCGCTCCTTTTGCTCTTCATCTGAGATGTCAAC

CTTCTTCCAGTCATATAGCTCCATGTCATAGCACTCATCAATAACAAATTGAGGAATTTCTGATCCCCGG

AAAAGCCACAGCCCCTTAACCTTGTATGGAGGGTTTGCGCCAATTACTAGCATCTTCCCAAATGCATACT

TGCGAGCCAAGGGGGGGCCCGGTAC

>FM890960.1 FM890960 Jatropha curcas embryo 56-70 (DAF) Jatropha curcas cDNA clone rjcaeb0_000933, mRNA sequence

CAGTTTTTATTACAAGACATTAGGAAAGCTTGAAGCAACGTTACAAGAAGCAGTTTCGAACAAACTTATT

CTGATGCTCTTACTTCTTATACATCATTATATATAACTCTCACTCACATACAAGTGAATGAAGTTTTATT

CACCTGCAGCTAGCATTTTAACGAGGGCATATAGACCCAAAGCAAACATCAATTATATTTTTAGTCCTGC

GCCTTGCCTCATCTTCGCTTCCAACTTCTCCTTCCTCCAGCTGCTTCTCTAACAGATAATGTATTGATTC

ACATCGACAAATAGCACTTAGTTCCTTTGCTTGGTTACAGCATTGTCTAGGAACCTGCTGTCGAGGGTTT

TCAATTCCTGGCATAGCTAACATATCTTCTGACCTTCCTCTTCTCTGTGTTATATAATTCTCACAGGAAC

TCAAATCTCTTCTTTCAGCTTCCTTCTTGCATATATCCCGAACATTGGAAGAATCGTCGACTTCAACGGT

GGTGATGATAGTTCTGTAGCCAGTGACTGCATTGGCTATGATCCCTAGAAGAACGCAAAGTAGGGCTGTT

>FM890959.1 FM890959 Jatropha curcas embryo 56-70 (DAF) Jatropha curcas cDNA clone rjcaeb0_000931, mRNA sequence

ATTCCCTATTAGTTCCAGTTTTTCAAAAAAAAAAAAAAAAAAAAAAAAAAACAATTTCCAATTCAATTTC

ATCATCTAAGGCCTAACTTTATCAAAGTCCCAAATTTCTATTCAAATTGAAACCTAAATTCTAAACCCCC

TTCAATCCTCATTCAATGACCAAATCAATCCAGAATAAGTTAATCGAAAGCATTGATAACTGATTTTCGA

CCAACCTGAGTAAAGGGAATCGCCAGAAAGTCGAAGAAGAGAAGCTGTGGAAGGAAACAGTAACGTCGCA

AGGAGAGAGAGAGAGCCTTGTTTCGTGTGGAACAGGCTTCGCTCGTCGAAGAGGTGGCCGAAGTTGAGGT

CGTCGTCGACGGAGGAGTTGGTGGCGAGAGATATTGAGAGGTTTGGTCCAATTGTCAAATTAAAATCCGT

TTTCGATTCGATTTGATAGAAGCTTCTAGATTGTTCAGGTTTTTGGATGGTTTAGGTCTGATTTATGGGT

TCTCTCTTGGGTTCCGGTGGTTCGAATTTTCCTTATGTATTTTTGTTTATAATCCCATATTTG

>FM890958.1 FM890958 Jatropha curcas embryo 56-70 (DAF) Jatropha curcas cDNA clone rjcaeb0_000929, mRNA sequence

ATTCCCTTTCGATCTCTCTCTAAACTGGCCTCTCTTTTTAATTGTCTTACACATTTCTTTACCTCGTTTT

AGCTTTTAATTACTTCAATTTCAACTGGGTGTCTTATATTTTGTGCTGATCCCATTTGCAATTTCTAGGG

TTTTCTGCTATTTTCTCTTTCTGGATCGTTCCAATTTCCAACTGAGGTTTTTGCTTCTCTCGTGTTTGTT

GTTAGGTTTTAGTTTAATTTCACATATTAATTACTTGTTTCTATATCTGTGTAGTCTTTTGGATCTTAAA

ATTCGTTTCTCTTGTTAACAGTTCTTCTATACTTGAAATTATTCTGTTTGAAGTAGTTCTTAATTGTTCA

TTCAATGTCACAAGGCTATGCAATTCAGCTCTATTTCGATCCGGCCCTTGAAAACCAGGTCTTGAAGGCC

TGGAACGTGCTTGCTCGACGCCAAATCAGTACCCAACTCATCGAAATCGAGTCTCGCCCTCATATAACCC

TCTATTCAAGCTTATTTGTTGATCCCACAAAGCTTGAAGCAATTGTCAAGACC

>FM890957.1 FM890957 Jatropha curcas embryo 56-70 (DAF) Jatropha curcas cDNA clone rjcaeb0_000928, mRNA sequence

ATTCGAATTGAGGCATTATGCACCTGGAGTACCAATTATCCTTGTTGGGACTAAGCTTGATCTCCGAGAT

GATAAGCAATTCTTCATAGATCACCCTGGTGCAGTGCCCATCACCACAGCTCAGGGGGAGGAACTGAGAA

AGCTAATTGGAGCTCCTGTTTACATTGAGTGTAGTTCTAAAACTCAGCAGAATGTGAAAGCAGTCTTTGA

TGCAGCCATTAAAGTGGTTCTCCAGCCACCAAAACAGAAGAAGAAAAAGAAAAGGAAGGCACAAAAGACA

TGCTCCATATTGTGATCGTGAAACAAAATTGTGGAAAAGAAAATATGACCAACTGTAGGCATTGATCCCG

TTAGTCATTCACCATCTCTCTTACATTTTCCGACCTTGACTCCGCAACGGGATTTGGTCGTAGTATTTAC

TGACAGAAATAGGAAGCACATTATAAGCATCAAGTTGTGAGTTTGTTGCGTTATGGTTGCGTT

>FM890956.1 FM890956 Jatropha curcas embryo 56-70 (DAF) Jatropha curcas cDNA clone rjcaeb0_000927, mRNA sequence

CGAATTCTGCAAATCAACGAAAGCTCCCCATTCTCAGTTTCATGGACATGAGCGCTGAGCGAGGCGTTCT

GCTGCCGAGGGCAATGCACAGCCCACACTGGTCCATAAACGATCAGAGAATAGTGTGTGCTCTTCGCGGC

GACATGCACATACAGATTGTGGATGACAGTGGAAACACAGTAATGGACGAGAGGGTGAAAGAAGGAGACA

TGTTCGTGATCCCTCAATTCTATGCAGCACTTGGAAGGGCAGGAGAAAATGGGTTCGAGTACGTAACATT

TAAAACAAGTAGCCAGCCTATCAAGAGCCCATTGGCGGGTTATACATCAGTGATGAGAGCCATGCCTCTT

GATGTTCTGATGAACTCTTACAGAATGTCTC

>FM890955.1 FM890955 Jatropha curcas embryo 56-70 (DAF) Jatropha curcas cDNA clone rjcaeb0_000926, mRNA sequence

CACCAAATTAATAAGTATTTTATTTATAGAGACCAACATACAAATATTACTATAAACTTAAATTACAATT

ACTCTACAACATAAAAAGAAAAAAAAAATCCTTGACATAACTAGCTGGCAAGCCCGTTTTCATGACAAAT

TTATAGATTCAAAACCCGATCCGATTTGACTTGAATCAAAGATCAGAACGTTTCACGAACTTTTCGGTCT

CACATTACATACATTAATTATTCATGAAACAAACGGAAAAACGACGGCGATCGAGAAATTGTTAACTCAT

CAAACCCTTGACGACGATAACGGCGTTGAAGAAGTTGTAGAAGAACCTTGATCACCATCATCAACCTCGT

CTAAGATCGTTGCACAATCCTAACTGCGTCGACGACCTTGCGATTTGCAAAGACGGTGAAGAAGACGCTG

AGGAATGGCAGGAAGCGGCTGCAACTAAAGCCAAGCAACCTTCGCTCCATGACCTACGAATCGGATACGA

A

>FM890954.1 FM890954 Jatropha curcas embryo 56-70 (DAF) Jatropha curcas cDNA clone rjcaeb0_000925, mRNA sequence

ATCCGTTTCTTCANCACCGAAACCCAGGTCACAAACATCGGAGGTGATGATCTTGATACCGTCGACGTGG

ATCGCCGCTCCACAGGTCGCAGCGTCTCTCGCCGCCGCGATATGTCTCCTGGTTTCTTCCCAGATTTGGT

GGACCCATTTTCGCCAACAAGAACTCTAAGCCAAGTCTTCAATTTAATGGACCAGTTAATGGATTTTCCC

CTGTCACGCGGCATAGGAGCTGGCGGTGTTCCACGGCGAGGGTGGGATGTGAAAGAGGATAATGATGCTC

TTTTACTACGATTCGACATGCCTGGACTAGGCAAAGAAGATGTGAAAGTTTGCGTAGAGCAAAACACTTT

GATTATAAAAGGCGAAGGACCTAAAGAAAATGAAGAAGAAGAAGAAGAAGAAGGAGAAAGTGGAAGGAGA

TATTCTAGCAGACTGGACTTGCCACCAAATCTGTATAAACTTCAGGACATTAAAGCTGACATGAAGAATG

GTGTTTCGAAGGTTGTGGTGCCTAAAGTGAAGG

>FM890953.1 FM890953 Jatropha curcas embryo 56-70 (DAF) Jatropha curcas cDNA clone rjcaeb0_000924, mRNA sequence

GACTGGGAGCTAGTGTGATGATTAAAACTATAAATGGTATTTTGTAGTAATAATAATAATAATGTTGGTA

ATAAAGTATTAGCTTAGCAGTGTACTTTANTTTAGTAATGTAGTTTACATGTGGGTTAAAATGACTAGGG

TTTAGCTAGTATGTGTGTTGTAACTTTGAGAGGGGTTTATGGTCGTTCCCTTTCTGGGAGGACTGCTAAT

ACTTTCTCCCTATTTGTAAAATAACCTGCAGTATATATAATTACCTGTTGGTTAAAAAAAAAGGGGGGGC

CCGGTAC

>FM890952.1 FM890952 Jatropha curcas embryo 56-70 (DAF) Jatropha curcas cDNA clone rjcaeb0_000923, mRNA sequence

ATCCTCCTTTTGGAAAGGGGAAAAGGGCGCGTGGAATCTCAGAGATTAGTGTTTTTCTTCTTTTTTTTTT

TTTCGAAGAGTAGCGATGGAAAATGGAGTGGAAAAGGCGATGGCCGCGAGGAAGCGAGGCGGAGGAGGAG

GAGGCGGCGGAGGAGTGGAAGGAGAGAGGACGTACAAAGGGATAAGGATGA

>FM890951.1 FM890951 Jatropha curcas embryo 56-70 (DAF) Jatropha curcas cDNA clone rjcaeb0_000920, mRNA sequence

AATTAGTGAACAGCAACCAAACCTACCATACTCGATTCCAATTGGATTGCAAATTTCAATTCTCTTCACG

ATTTCAATACAAAGCAAATCCTAGCATTTATTTTTGCAATTTGGGCTTAATGGGTCCATCCCAAATCCAA

GTCTAACTCAGTACCTAATACAATAACCATCCATATGTATATTAAGTATACAGGGTCCAGTAATCACATT

AGGCTACGTTTTAAAGATTAGAAACAGTACAAAGAAGAAGCAGCACCAGCACAAAAACACTATACATGAA

CGCGAACTCGGTCACTGAATGTAAGATTGACCAGTGATCATCAGATCTCGATCACCGGTAGAAGGTGGCG

GTTTGTGCTTGGGTTCGGCGGTGGACTTCTCCGCCGGTCCGTCAGCACTCTGTGTGTCGCTAGCGGGTTT

CTTGTTCTGTTTTTGCGGTTGTTGATGAAAGCGGCGGGCTAAAGAATAATCCGGTGGCTGCAATTGTG

>FM890950.1 FM890950 Jatropha curcas embryo 56-70 (DAF) Jatropha curcas cDNA clone rjcaeb0_000918, mRNA sequence

CCTTTCAAGAAAAAACATTGAGGGAGCAACCAAGTTCCTGCTCTCGAAGCTCAGTGACCTCCAATTTTTT

GTGGGGGAGAGCATGCATGATGATGGGAGTTTGGTGTTTGCTTACTACAAGGATGGCGCCACTGACCCAA

CATTTTTGTACTTCGCTTATGCTTTGAAGGAGATCAAGTGCTAAGAATGGAGAATAGCTGGCTTCGAAGT

GGAAGTTCTTGCCATATTTCTAGCTTTATGTGTCCTTTTCCATCTCTGTACTGTTATATTTGGTGCTTCA

TGCCTTATTTTGGTTAATTATTGACTAGACATGTTTTCCTCCTTCTTTGGTTATTAGTTTCTTGTTTGAC

AAATAATTCAATCTTCTATTGGGCGTGTATTCTAAAATTTACCAGCCTAAGTTGGTAATTGTTTGATGTT

ACAACTTCTTTGTTCCCTTTGTATTTGTGGAAGGCTTGTCAAAATAGATATGTTTTACTGGCTCT

>FM890949.1 FM890949 Jatropha curcas embryo 56-70 (DAF) Jatropha curcas cDNA clone rjcaeb0_000917, mRNA sequence

ATTCCTTCTCTTCTTTGTTTGTATAACCAACAATGTCGTCAGCTGTGGTTGCAGGAAGCTCTGCCACTTT

CACCAGAAACCACTCCATCAAAACCTCTCTAAACCCAGCAAAACCAGCAATATACTCTCAGTGCCAACAG

AAAACTGCCTTTCAAGGCTTATCACTTCAAGATGCCAAAAGGGTTTCTTCAGAAATATTTATAGCTGAAA

AGAAAACTACTTTCACTGATTCAAGAAAAGGGCTTCAAGTTACTGCAAGAACAGCTGGTGCTTCAAAGAC

TATCGAAGTTGAGGTTGACAAGCCACTAGGCCTTACTTTGGGTCAAAAGTCTGGTGGTGGTGTGGTCATA

ACTGCAGTAGAAGGAGGTGGAAATGCGGCTAAGGCAGGGCTAAAATCAGGGGATCAAGTGCTATACACTA

GTAGCTTCTTTGGTGATGAACTTTGGCCTGCTGATAAGTTGGGTTTTACTAAAACTGCAATTCAAGCAAA

ACCAGACTCTGTCTACTTTGTTGTTAGCAGAGGAGCAGAAGTAGATGTTACAAGACTACCAAAGCGTCCT

GCTCCGCCCGCTTTGGAAGGAAATTAACAGAGGCTCCAAAAGCTAGAGCTACTCACATAATGT

>FM890948.1 FM890948 Jatropha curcas embryo 56-70 (DAF) Jatropha curcas cDNA clone rjcaeb0_000916, mRNA sequence

TGCAGGAATTCGAATTCCGCAGATAACATACATGTCTCCCTTATATTCCCTCCGGACACTGATACTCCTG

CCCTTGCTGAGGAAAATAAAATGCGGCCACAACTCACAAGTATCATTGCAGCCTCTTCTTGGTGCCATGA

AAGCAGATGAAGTTGCCAAAAAAGCTTTTAAATGGCCTCAAATCTGGTAGTTTTGTCGTCCC

>FM890947.1 FM890947 Jatropha curcas embryo 56-70 (DAF) Jatropha curcas cDNA clone rjcaeb0_000915, mRNA sequence

ATTCCGTAACGATATATATTGAACACTGACTTTTTATACGTACATGAACACAAGTGGATTAATTAAACAT

AAAGGACGATCAAAGTCCAAAGTCCAAAGTCCAAACCAGACCCAGATACATCCCCGCCAAAAGGGGATAT

GTAAATGACACAAGATAATTCTTTTAGCATTACATGTACATATATTATTACATTGTTGTTGCTTAAACCC

GAGTCCCTGTCACTGCTGTGCCACCCTTTGGAATTGGTTCCCCACCAGCTCCTACAGCGTATTCGGTGTC

CCTGTAACCAGAGCCATACCTGCTGCTAAATACCCCAGCATGAAGCAACAGGACATACAAGAGTTGGGTA

AATGTCAAGATTATCATGAATGCCTCGGGGGGGCCCGGTAC

>FM890946.1 FM890946 Jatropha curcas embryo 56-70 (DAF) Jatropha curcas cDNA clone rjcaeb0_000914, mRNA sequence

ATTCCTCTGGAAAGAGGGCAATAAGAATGGAGCCCGTCAATTATTGGATTTGATGTTGGAGAAGGGTTGG

ATCCCTGATGCTACTACTCATAGATTGTTGATTGGATCTGTTGATAGGGTGGAAAGTGGAGAGAAAATGT

TGACATATGAAAACTTTACCATTAAAGATAATGTTCTTGACATACTTGCTGAGGGCCTGGGAAAAACGTA

AAACTGGCATTTTGATTATACCGACCGCTCAATTTCTTCTTTCAAGGAATTGAAATAGTTTTATCAATTC

TTTTTTATGGCTGTCATGAGTAGTATGTGCATAGTGTAGATGTATTAATTCTATTAGTGATTTTTCTGCT

GTATAAAATCTGTATTATGTACAATGATCTCAGACATTCCAAAGGCAACTGACTCAACCTCATTTTCAGG

AATGGCAAGGGCTTTTGAAT

>FM890945.1 FM890945 Jatropha curcas embryo 56-70 (DAF) Jatropha curcas cDNA clone rjcaeb0_000912, mRNA sequence

ATACCTTTCTTTTGGTTCTAAAGCTCCAAATCCTCTGTACTTTTCAATAATCCATATGGGTAATTTGGGT

CATCAACAACTGCACATTTCTTTCTTCCCCTTCATGGCTCATGGCCACGTGATACCAACAGTAGACATGG

CAAAGCTATTTGCTTTTCGAGGCCATAAAACAACAATAATCACTACTCCTGCAAATGAACCTCTCATCTC

CAAAACAATCCAACGAACAAAAGATTTGGGTTTCGATATCAATATTCAAATCCTCAAATTCCCTTCAGTT

GAAGCCGGACTACCAGAAGGTTGTGACAATTTAGATTCTGTTATTTCATATGATAATAGTCAAGACATTA

TGGACAGATTTTGGTTAGCCCACT

>FM890944.1 FM890944 Jatropha curcas embryo 56-70 (DAF) Jatropha curcas cDNA clone rjcaeb0_000911, mRNA sequence

ATTCGCCAAGAAGTGTAGGGTTTCGGCGGCACCACTCTCGCGGAGAAAATGGTTTCTGGCAAGAAAACTA

AGAAGACCCATGAGAGCATCAATAACAGATTAGCTCTCGTGATGAAGAGTGGAAGGTACACTCTCGGTTA

CAAAACCGTGCTTCGGTCTCTGAGGTCTTCCAAAGGCAAGCTTATCATAATTTCAAACAATTGCCCACCG

CTGAGAAAATCTGAGATCGAGTACTATGCCATGCTTTCTAAGGTTGGAGTACACCATTATAATGGAAACA

ATGTCGACTTGGGAACTGCTTGTGGGAAGTATTTCCGTGTTTCTTGCCTTAGCATTATCGATCCAGGTGA

TTCTGATATCATCAAGAGCCTCCCTGGTGATCACTGAGTAGAAATACATTTCTCTTTCAATATCAGCTTT

CCTTTTAAAATGTATGAGGCCTCTCAATTTGGACTCCCCCCTCTCTTTTCCCATGAAATTAAACAGTAGT

TTGGACTTATTTGTCAAATTTTGCCTAGTTTGGAGATGACTACTGTCGTGTTACTAATTTTATTTATGGG

AAATCATTTACAATCAAAAGGGGGGGCCCGGTAC

>FM890943.1 FM890943 Jatropha curcas embryo 56-70 (DAF) Jatropha curcas cDNA clone rjcaeb0_000910, mRNA sequence

CCTAACCAGGTAATACAAAGTTACAATAGAAACACTTCATAGCAAGCTGCTTTTTAAGCACACTTAGACC

CACTATTTTATTTATGGCTTTCCCTGTTTATAAGATGCTACATAAACTCTCATCTTAGAGAAAGCCCAAG

ACCCCAATACACACATAAAATTTATTAGCCATAGGTAGCAGTACTAGTAGCTAATACCTAGTGTCTGTGT

CCCTATAAGTTGTGGTCTGCTGATGGCCAGAACGATCCTTAACATGATCTTCTTCTTCTTCATGGTGAGC

CATACCAAAAGCGCTTTTGACAGTATCAGCAGTTTTTTGAGCTGCATGCTTCATTTTCTCCCCAGTTTCT

TCCAGAACTCCCTTTGTCTTCTCCTTCCCTTCATGAGCCTTCTCCTTGGTTGCCTCTGCAGTCTCAGATG

TCTTTTCTCTTGTCTCATGGGCTTTTTCTCCTGTCCAGTCTTTCGCTTCTCCAGCCTTCTCTTTCACCGT

ATCGCCCATTCTGTTCCCCTTCTCCTCGGCTCGGCCTGTAGCCTCACCGGCTCTGTAACTATAAGACTGA

TCATTAGATGCCATTTTCCTTTGCAATTGATATTTGAAAGCTTCTTTAAGAATGGATAAAGAAAGGGGGG

GCCCGGTAC

>FM890942.1 FM890942 Jatropha curcas embryo 56-70 (DAF) Jatropha curcas cDNA clone rjcaeb0_000909, mRNA sequence

ATTCTGAAATATTGAGATATGCACCTCAACACAACTGGTCTGCATACGAGGAAGCTCTAAAGACAAATCC

AGTTTTAGCAAAAATGATGATTAGTGGAGTGGTCTATTCTATAGGCGATTGGATTGCTCAGTGCTTTGAA

GGTAAACCACTCTTTGAGTTTGACCGAGCACGAATGTTTAGATCAGGCCTTGTTGGCTTTATGTTACATG

GCTCCCTTTCACATTACTATTACCAGTTCTGTGAGGAGCTTTTTCCATTTCAAGACTGGTGGGTGGTTCC

TGTCAAAGTGGCCTTCGACCAAACTGCATGGGCAGCAGTTTGGAACAGCATCTATTATATAGTGGTGGGA

TTCCTGCGTCTGGATTCCCCTGCCACCATTTTTAGTGAATTGAGGGCTACATTCTGGCCCATGCTAACAG

CAGGGTGGAAACTTTGGCCATTTGCACATCTTGTTACATATGGTGTGATCCCAGTAGAACAAAGGCTTCT

TTGGGTGGACTGCGTAGAACTCATTTGGGTTACAATACTCTCGACATATTCAAATGAGAAATCAGAAGCA

AGAATACCCGAGACACAAACAGAAGCTGGTTCCAATTCTTTGTCGAAAGGTTCTGCTGAGGAAT

>FM890941.1 FM890941 Jatropha curcas embryo 56-70 (DAF) Jatropha curcas cDNA clone rjcaeb0_000908, mRNA sequence

CGAATGCAAAGACATCGCGGATAGATTGCTGCCTGCATGGAATACGCAGACTGGGATCCCTTATAACATC

ATTAACTTGGTGCACGGCAATGCACATAACCCTGGATGGACTGGTGGCGATAGTATCCTTGCAGATTCTG

GCACAGAACAACTTGAATTTATTGCTCTTTCCCAAAGGACGGGAGACCCAAAGTATCAGCTGAAGGTGGA

GAAGGTTATAACTGAGATCAATAAAACCTTTCCTGCTGATGGTTTGCTTCCAATCTATATTAGTCCTGAT

ACTGGAACTGGGTCATACTCAACCATAACTTTTGGTGCCATGGGTGACAGCTTTTATGAGTA

>FM890940.1 FM890940 Jatropha curcas embryo 56-70 (DAF) Jatropha curcas cDNA clone rjcaeb0_000907, mRNA sequence

ATTCCTATTGATACAATTTTTCAACATTCTAAGTTGATGGTTCAAGAATTTTAAATTGAGTTTTTGCAGG

GATTTATACCATTTTTGTTTTGAAGAATGTGTTTTCACGTCACTCAGAATTTGAATCGTCCTTTTCAATG

CATGCCTTGCTCTGATGTACAGTGGAATTGTACTGCGTCCCCAGAGGCCTAAGAGACTGGTATTTTTTAT

GGAGGAGTACTTTTTGCTCTTAAATTTTTAGTTAATGGGAATAATCTGCTGAATTTCAACTGTACTCTGT

TATATGCTCTACTGGTTTTCTGGGGGTCATTTTGTTTTTGTTATCCCTTTAAGTACAGATCTATATCTAA

TATGGTGAAGTCTCGTCAAGGAGATCATCGAGTGCTAAATATAACATTGAGGCTTTACATGGACCAATAC

TAAAGTGTGGTTTTTGCGTGGGAAGCTTAGGATATTAGTTAATTGATGTGTGTTCACTTTGGGACCTTTG

GATCGGCTTTAGGGTTGAGAAGGGAGTTCCTTTTGCATTCTTTCTTGTCAAGTTGTATATAGTGGGTCTT

GATCTGAATCGTGTACTCTAT

>FM890939.1 FM890939 Jatropha curcas embryo 56-70 (DAF) Jatropha curcas cDNA clone rjcaeb0_000905, mRNA sequence

ATTCCATCATCGAAAAACTAGAAACACGACGAAGCTACTCTCGCTACGATCGACCAAATCTAAGGCTTTC

AGATCGAAAAATGGCATTGATTCCAAGCTTTTTCGGTAACTCACGAAGCAACATCTTCGATCCTTTCTCT

GCTCTCGACGTTTGGGATCCATTCAGAGACTTTTCTTTCCCTTCATCTTCTTCCCTCGTCTCTCGTGAAA

ATTCTGCTTTTATTAACACTCGCATTGACTGGAGAGAGACACCAGAGGCTCATATATTCAAGGCTGATCT

CCCTGGGCTCAAAAAAGAGGAAGTGAAAGTAGAAATTGAAGATGACAGAGTGCTTCAAATCAGCGGGGAG

AGAAATGTGGAGAAGGAAGACAAGAACGACACGTGGCATCGCGTGGAGCGTAGCAGTGGCAAGTTCATGA

GGAGGTTCAGGCTGCCGGAAAATGCAAAGATGGATCAAGTAAAGGCTTCAATGGAAAACGGAGTACTTAC

TGTCACTGTTCCTAAAGAGGAAGTCAAGAAACCTGATGT

>FM890938.1 FM890938 Jatropha curcas embryo 56-70 (DAF) Jatropha curcas cDNA clone rjcaeb0_000904, mRNA sequence

AATATGTAATCCAAAATGTGCAATTCAGAAGCAAGAATGCAAAACAAAGTTGCACTATGTAATCAAATGT

AACTAAAATCACAAAAAGCTAGACTTTAATTTGTCTGAAACGAGAGATACAAACTCACTAACAAGGAAAA

CAAAATTAAGACGCAGAGAAAAACTTAAATTAAAAGATTAAATAGTTGTTTCACTTAAAGCACTTGGCAT

CCAAAAGAGGCTCTCCCTCAAAAGGCTCAAAATCTTCAATCATCTGGTTGACGCGCTCCTTTTGCTCTTC

ATCTGAGATGTCAACCTTCTTCCAGTCATATAGCTCCATGTCATAGCACTCATCAATAACAAATTGAGGA

ATTTCTGATCCCCGGAAAAGCCACAGCCCCTTAACCTTGTATGGAGGGTTTGCGCCAATTACTAGCATCT

TCCCAAATGCATACTTGCGAGCCAAATCCATCCTCTGAAGGAATCCACCGACCTTGTTCAAAGTGACAAA

TGACACTGTATTCTCACGCATTGTACTTGTACTCACAGAACCACAGTGAGTATCCTTCGGGATCATACTT

GTCCCAGAAGCCTTTAATTGCAACCTCACGGAAGTTGGTC

>FM890937.1 FM890937 Jatropha curcas embryo 56-70 (DAF) Jatropha curcas cDNA clone rjcaeb0_000903, mRNA sequence

ATTCCTCTGTTCAAGCTTCAAAAAAAAGGAATGGTTTATGGTCTGATGGATCTTAGCGAAAGAAAATCAT

GGTGTGAAGAAAACGGGAGTGTTGAGATCAAGAAGTACTGTACTGATTGCAAAACCACCAAGACACCATT

ATGGAGAGGTGGACCTGCTGGGCCCAAGTCACTGTGCAACGCTTGTGGGATCAGATACAGAAAGAAGAGA

AGAGCTATGCTGGGTTTGGAAAAAGATTCAGTGAAGAAAAAAAGAACCCATATCACCAGCCTCACAACCA

TCACTACTAGCTCTGCAAATACCAGCAATAACACCTCAGATTCGAATTGTAACGCTGATGAATTGAAGGA

ATCTTTAAAGATGAGACTAATCGTTGTGGATGGGAAAATGATGTTTCAAAGATCATCAATAGTAAAGAAA

CAGAGGTGTCAGAGGAGAAGAAAGCTACGAGAGGAAGAGCAGGCTGCTTTTAGTCTGATGGCTTTGTCCT

GTGGCTCTATTTTTGCATG

>FM890936.1 FM890936 Jatropha curcas embryo 56-70 (DAF) Jatropha curcas cDNA clone rjcaeb0_000902, mRNA sequence

CATCCATAATGTTTGCATTGCTCTGAAAAACAACCGACAAATAAAACTTTGGCACTACAAGCTGCATTCA

AATATCATAACAGTAATGTCAAGTTATCAAGCCCAAGAATACTTTGATTTCAAGTTACTAAACAACCGTC

TTGACGAAACCAGATGGCTCAGATCACATTCAAATTGGAGTTTGGTTAACAATATTGAACAACGGGTGGC

CTGATCTGTGTATAGAAGGGCTTGGCATCTATGAACAAAGTTCAATCCATCCTTCCCTTTAAGATTCTTT

CTGTAACTTCCGTGGGAGTAACAACATAGACGTGTCCTCCCCAACCGCTCAGACAGTCCCGATCTACTGA

TGATAGCAATGCTTGAGACACGGTTTCAAACAATTCTTCAGCTTCCATGTCAGGCTTGAACAATGATTCA

CATGCACCATATAGTGACTCTGATGCTGTGCCTGCAACA

>FM890935.1 FM890935 Jatropha curcas embryo 56-70 (DAF) Jatropha curcas cDNA clone rjcaeb0_000901, mRNA sequence

ATTCTCCTTAATGAGGATAGTGGGTGGAGACCAGCCATCACAGTGAAGCAAATTCTTGTGGGTATCCAAG

ACCTGCTGGACCAGCCAAATCCAGCTGACCCTNCACAAACTGAAGGTTATCATCTCTTCTTCCGGGAATT

GGTGCCAAAGTTCCAAGAAAGGGGTTGCCAACAGCACATGCCTACAGCAATCCCCTCCTCTTGTTTTTGT

GTGAATAACTACCACTCTCTACGAACGTGCTGGTTCTTGGTAGCTGTTGGGTTGCCTGTGCAACTGTGTA

CTATTAAACTAGCTTTCTCTGAAATTATTTGCACTTGGTAATTTTGAGTGGCCTTTATGTGTGACTGACT

GCATGGTTGAACTGGCATTTCAGCTCTACAGTTTAACTTTTGTTGATATTGTTATAGTATACCAGTCAGC

CAGTGTTACTCATTCGTGATTATAACTTATATTGGTTAAAAATCTAACATAGAACTGTTGCAGTTAAAAA

AAAAGGGGGGGCCCGGTAC

>FM890934.1 FM890934 Jatropha curcas embryo 56-70 (DAF) Jatropha curcas cDNA clone rjcaeb0_000896, mRNA sequence

GTCCAAGATAATCTAATTACCAGAGTTCATAAGAACAGAATAATTAAGGTCACACATTCCAAGCCATTAA

TCTTCAATGCAAGTAACTGTGTGACTACTTCCTGGCAAAACCCCAAGCCTCAAACTAATTATTACTGCAT

TGAACTTCTATGACTTTATTTCAAGCTCCACCGCGGTGCTCTATGCTGTTTGAGATCGAGACGGTTGATC

TTTAATTTCAAAATCTACTATTGATTTAGCTGGTGATAGATGGAGGAGCATCTGGTTGTTTTTGTGGCAT

AGCTTTCTGGAATGCTGGTAGCTCATTGTATGCCTCATACAACCTCAATAGAAGAGGGAACTGAGTCATG

TCAACTTTGAACCTTTCTGCTGCATGAATCTGTGGTGCTAGAAACAAATCTGCCAGGAAAACTTCGTCTC

CAGTCGCATATTTTCCAGCATGATCTTTCAACAGTTTCTCAAGTGCTGCAAATCCTTTTTGAACATGATA

CTGAACCCAAGGAATTTTCTCATCTGGACTGATCTTTTCCCCTATAAATTTCAGCACATCTAGATTCTGA

AGGGGCTGTATGCTTGAGGAAACAATATTTGCAGCCTGGTAATTGATGGCTCTTCTCTGAACATCACGGG

GGGGCCCGGTACC

>FM890933.1 FM890933 Jatropha curcas embryo 56-70 (DAF) Jatropha curcas cDNA clone rjcaeb0_000895, mRNA sequence

ATTCCGATTGATGGGCACTTCCGTTCCCTCATTTATATGAGACCACTAGCAATTCGGAGCATGCAATGGG

CATTATCACTTCGTAAGGCAATTCTTGAGGCTCCAAAGATCAACATAATGGACAGACTTCTCCTGTCTCC

TAGCACAAGATTCTCTCTTCATGAAATGGGGGTCAGAAAGATTGCAACTAAAGCCAAGTGCTTTGGAAAG

TCTGTCTTCAATTGCGCATGCTAAGCATGCCATGTAAGTTCCTTCATCTTTGTAAAAAAAAAAAAAATGT

TTCACTAAAATCTTTGATACCTTTTCCCAATTACCCTTCCAGAAGAAGAAACTGGGAGAGGTTTTGGGAG

AGGTGTCTCTTTCTTAAGACGGGCAATAAAAGCTAATACCTTGGTAGGCATTTAGTTACATGGTATAGGA

TGAGTCTTTCTTTGTCATCGTGTAATATTCTTTTTTATGAAAATCTAATCTTTATTCCTT

>FM890932.1 FM890932 Jatropha curcas embryo 56-70 (DAF) Jatropha curcas cDNA clone rjcaeb0_000894, mRNA sequence

ATTCCAAACTTTAAATAGGTAGGACGGCGCGGCTGCTTCGCTGAGCCGCGCCACGGAATGCGAGAGCTCC

AAGTGGGCCATTTTTGGTAAGCAGAACTGGCGATGCGGGATGAACCGGAAGCCGGGTTACGGTGCCCAAC

TGCGCGCTAACCTAGAACCCACAAAGGGTGTTGGTCGATTAAGACAGCAGGACGGTGGTCATGGAAGTCG

AAATCCGCTAAGGAGTGTGTAACAACTCACCTGCCGAATCAACTAGCCCCGAAAATGGATGGCGCTTAAG

CGCGCGACCTATACCCGGCCGTCGGGGCAAGAGCCAGGCCCCGATGAGTAGGAGGGCGCGGCGGTCGCTG

CAAAACCCGGGGCGCGAGCCCGGGCGGAGCGGCCGTCGGTGCAGATCTTGGTGGTAGTAGCAAATATTCA

AATGAGAACTTTGAAGGCCGAAGAGGGGAAAGGTTCCATGTGAACGGCACTTGCACATGGGTTAGTCGAT

CCTAAGAGAACGGGGAAGCCCGTCCGACAGCGCGTCCTCGCGCGAGCTTCGAAAGGGAATCGGGTTAAAA

TTCCCTAACCGGGACGTGAAGGCTGACGGCCACCAACGACGACTATTCTCTCTCTCTCGGGGGGGCCCGG

TACCAATTCGCCCTATAG

>FM890931.1 FM890931 Jatropha curcas embryo 56-70 (DAF) Jatropha curcas cDNA clone rjcaeb0_000893, mRNA sequence

CAACACCAGAAAGTTCGTTCACTCCGACAAGGTGATGTTATTGCTTTGCCAGCTGGAGTAGCTCAGTGGA

TTTACAACAGTGGCCGGACTCCTCTTGTTCTTGTCCAAATTATTGATACTGGCAATGCTGCCAACCAGCT

CGATGAAAATCACAGAGCGTTCTTTCTTGCCGGTAATCCACAACGAGACGTACAGAGCAAGAGGTGTGCA

TTTAGGGGTAGCCGGATGCCCCTCGCAGGAAGGGAATCTCGAAGGCCCTCCGAGGAAAGATCAGGCAATA

TCTTTTCAGGCCTTGACGAACAACTTTTAGCAGACGCTTTCAACGTCAATGTTGAGGTTGCAAGAAAGAT

AAAAGGACAAAACGATAACAGAGGATTAATTGTAAGCGTAGAACGTGATTTTGAGTTGCTATTCCCACCA

AGAAGCGCAGAAGAAGAAAGACGAGAAAGAGAGGAAGAGACCCAGAAACAATTAGAGGGAACAGGTTACA

GACCAGGTGGCGGCCAAGGAAGATTCGTTAATGGTGTGGAAGAAACTTTCTGCACTGCCAGACTCGTACA

CAACATCAATGACCCGTCACGTGCCGATGTATTCAACCCACGTTCTGGCCGTCTCACCAACATCAACGGC

CAAGACCTCCCC

>FM890930.1 FM890930 Jatropha curcas embryo 56-70 (DAF) Jatropha curcas cDNA clone rjcaeb0_000892, mRNA sequence

ATTCCCATTCTCAGTTTCATGGACATGAGCGCTGAGCGAGGCGTTCTGCTGCCGAGGGCAATGCACAGCC

CACACTGGTCCATAAACGATCAGAGAATAGTGTGTGCTCTTCGCGGCGACATGCACATACAGATTGTGGA

TGACAGTGGAAACACAGTAATGGACGAAAGGGTGAAAGAAGGAGACATGTTCGTGATCCCTCAATTCTAT

GCAGCACTTGGAAGGGCAGGAGAAAATGGGTTCGAGTACGTAACATTTAAAACAAGTAGCCAGCCTATCA

AGAGCCCATTGGCGGGTTATACATCAGTGATGAGAGCCATGCCTCTTGATGTTCTGATGAACTCTTACAG

AATGTCTCCAAGCGAAGCTCATCAAGTGAAGAGGAACAGGGATCCACAGTCCATGTTGTTTACTCCCACC

AGCAGGAGACCATAAGTAAGTTACTACTAAAATAGCATTGTGATTGGTGCTTCTACTAGCCAAGGCCGCA

ACTAAGAGATCCTTCTTGGTAAATTTAGAGAAGGGATCCGTCGTATATTGTAGTAT

>FM890929.1 FM890929 Jatropha curcas embryo 56-70 (DAF) Jatropha curcas cDNA clone rjcaeb0_000891, mRNA sequence

ATTCCTTATCATCAACAAATGGCTGAACGTTCACAGCCACACCAAGTTCAAGTGCACCCACAACACCGCT

ATGAAGCTGCCTTCAAAGGTCAACAAAAAGGTCCATCAGCTCAGAAAGTGTTAGCTGTGATCACCCTCCT

GCCGGTAGGCGGTGGGCTTCTTGCACTAGCTGGTATAACCTTAGTAGGTACCCTAATCGGCCTAGCTATT

ACTACCCCTCTTTTTGTCATCTTTAGCCCTGTTCTTGTCCCAGCTGCATTAGTTATTGGCCTTTCGGTCA

TGGCATTTTTGGCTTCCGGGGCTATGGGGCTCACAGGGTTGTCATCACTCTCGTGGGTTCTGAAATACCT

CCAGGAGGTCACTCGGCGAATGCCGGAGCAGCTGGATATAGCTAAGAAGCGTATGCAAGACATGGCTGGT

TTTGTGGGACAGAAGACCAAGGAAGTAGGCCAAGAAATCCAGAGGAAAGCACATGAAGGGAAATAAGTAG

AGAGAAATGCTGTTGGTTATTAAGGGTTGAAGTCAGAGAGCTGGGTTTTGGTACGAGACTACTTTTGTTG

TATGGGTG

>FM890928.1 FM890928 Jatropha curcas embryo 56-70 (DAF) Jatropha curcas cDNA clone rjcaeb0_000890, mRNA sequence

TTTTTCTACAAAAGAAAACCACTTAGCTAAATCATTAATTTTGGTGCAAGGTACGCAAAAGCAATCTCCT

TTCTACAACTATTAATTTCTTTTTTAAGAAAGGAGAAGGACAAAATAAGAGGGAGCAGAGACATCCTTCT

CTTACAAGACAAAAGCAAGAAAAAAATTAACAAGCAAATATCCCCATTAACATGAGTTGTTCAAACATTT

GTCACAGCCTAAATGACTATGCTACTCCTCTCCCCCTCTTCCTACTCATCATCGTCCTCCTCTCCACTTC

CCTCCTCATCATCTTCTTCCTCATCATTCACCTCAGACATTGACTTCTCAGATTCCTCTTCTTCCTTGGG

GCCTTCAGCCTGTCCCTTGTTGTAGGCTTTCAATTTCTTCTCATATTCTATCTTCCTCTTTTCTGCCTTA

GATACAAAAGGTGCTTTCTCAGCATCTGACATTGACTTCCATTTATCTCCACCAGCTTTGCCAACAGCAG

CAACGGATTTGTTTTTAGGATGTTCCTTCTTGTACTGCTCTCTGAACTCTTCCATGAAAACGAAGAAAGC

ACTGGCGGGCCTCTTTGGCTTATTTGGATCCTTCGCTGCCTTACCCGCCTTCTTACTTGCCTTGGCGGGT

TTCTTGTTCACGGAAAGCTTGGCGTTCTTGGTGTCTGACTTTGATTTACCTCCTTTCATGGCGAA

>FM890927.1 FM890927 Jatropha curcas embryo 56-70 (DAF) Jatropha curcas cDNA clone rjcaeb0_000889, mRNA sequence

ATTCCAAAAGGTACTAAGCTATAATTGAATAAGTTTTGAGAATGCTAATTTTGCATATATACCTCATTAG

CTGCTGTGCATAGGGTTCATGCATCAATGTTCATAGAAATAAGTGGCTTTTTAGGGCCTGTTAGTGATGT

CAAATCAATTTTCTGAAGACCAAGAAGAGCAGGTTCAAGTGTGTTGGACGTGACAGTTAGATAGGATTAG

CTTACATTCTTGTACAATGCAATGGTTAGGAACTGAAGAATAATTAATATTAATGTAAGAGAACCTGTCT

CCAGTTTATCTCTTAATGACACTAGTTATAACAAATATTTGGTTAATCTAAAAAAAAAAA

>FM890926.1 FM890926 Jatropha curcas embryo 56-70 (DAF) Jatropha curcas cDNA clone rjcaeb0_000888, mRNA sequence

ACGCACTAAAAGGAGGAGGTAATAAAATCAAACGGATATTACATAATTATTCCAATTGGTAACTCCTTGT

AATCCTACAACATGCCTATGCACAAGTACATATTGATTATTCATATCAGAAAATCTCATAATCTTTAGAG

CTACAACTTTTAGGCACACAATCAGGAACTCGTGGCTTCACATTAAAGTAAAGTAAAGCCCAAAATTACA

AACACAAACCATATTCATTATTTAAATCAGAAAATCTTATTTTGCTCACTGCTGCTACACTTGCTGAGCC

CATATCTTACACGGATAAAATCACAAAATTAGCGGTAGCTTCTATATACAGGACTGTACATGCAACTCTC

AGCATATTTCACAAGGTTCTCCGGACGAGGTAGACGTGTAGCCAACCCAAGTTCATATGCCTTTGCGGCT

ACATTAGCAGCAATATGTGCTGAAATTTTTCTGATGTTAGAGAAAGGTGGGTCAATCAGCCCCTTACTAT

AGTTCTCTTCCGTAACTTGGCCAGCCAACGCTTCCGGTGGGGGCCCGGTAC

>FM890925.1 FM890925 Jatropha curcas embryo 56-70 (DAF) Jatropha curcas cDNA clone rjcaeb0_000887, mRNA sequence

ATTCCAGAGATGGCCAAGTCAAAGAACCATACAGCTCACAACCAGTCCTACAAGGCTCACAAGAATGTCA

TCAAGAAACCAAAGAGGCATCGTCACACCTCCACCAAGGGGATGGATCCCAAGTTCTTGAGGAACCAGAG

GTATGCCAGGAAGCATAACAACAAGAGCGGGGATTCAGCCTCCGAACAAGAGTAGAACTACCTTCTTATT

GAGTTATGGTTTCCTATTTCCATGTTAGGGATTTACTTTAATAGTGTATTGGCTTTATTTCAAGTTGCAA

TTTTTGAATAGTGGCTGTACTGTACTGTTAAGCCATTTATGTTTTGGATAATTGTTACTTAAATATTTTG

CTTAAGATTTTCTAAAAAAAAGGGGGGGCCCGGTAC

>FM890924.1 FM890924 Jatropha curcas embryo 56-70 (DAF) Jatropha curcas cDNA clone rjcaeb0_000886, mRNA sequence

ATTCCACANAAAGGAAAAAAGATGTTTGAAGAGAGACCGCAGCAGGGCGGTGCACCCCATGGAATCATAC

TAGCTGTAGTTGTCTGCACGGTGGTTTTAGCTCCATTTCTCCTCGGCGACCAAGGCGAGGCCATAACTGA

AGCTATTACTGAGCTCTTAAGCCCAGTCGGTCTTCTTCTTCTTCCCATTATCCTTCTCTTGACTATTCAG

TTCCTTTCCTCCGATCGCGGCTCATTTGTCTCCACTATCTTCTCTACTGGCGAACCAGATACCATTCACC

GAGTCAGTGGCTCACCTTTCGGCGTGGCTTTATTTCTAGTTCTGATCTTGTTCTTGCTTTATAATCGCAT

GTCCATCTTCGGCGGCGACGATGATTCCGGTGATTAACTTCTGGTGAGTTGGTGACTTTGCTTGCATGGA

TCTGAGAGACTTGGACTCGTCTATTATTTTTTTTTTTTGGTTTTTTAATTTAATTTCCTGTATTTGTATA

CAGTACAGTGGGGGGTACGTAGATTTGCTAGTACATGGGATGGTATTTTTGTATTTAGAATTTTAGTTCG

AGGGTATTATGGTAATAGAAAATATTGGA

>FM890923.1 FM890923 Jatropha curcas embryo 56-70 (DAF) Jatropha curcas cDNA clone rjcaeb0_000885, mRNA sequence

GGAAAAGAAAAATGTTAGCCANTATTTCATAAAGCTTTTGCTAACCCGCCAGATGAGCTAAACAGTCCAG

CTTCTAATAATGGCAGTAAAAAGCCAAAGCTTCCTCAAGAAACTCTTGATGAATTCCTTTCTCATCATCC

AAACAACACTTGCTCCATGACCTTTGGTCAAGCTGCAGTTCTTGCCTATGTTCGCCCTGAAAACCCTTTT

TCAAAACATCAAAGATTGTTCTGCGGGTTTGAGGACATATTTTGCCTTTTCTTGGGGAGCTTGAACAACT

TGTGTCTACTGAATAGGCAATATGGATTAACGAAAGGGAGTAACGAGGTCATGTTTGTAATAGAGGCTTA

TAGGACCCTTCGAGATAGGGGTCCATACCCTGCAGATCAGGTTGTTAAGGATCTTGAAGGAAGCTTTGCT

TTTGTTATTTATGACAGTAAGGCTGGAACTGTCTTTGTTGCTCTGGGCTCTGACGGTGGAGTGAAGCTTT

TCTGGGGCATTGCAGCTGACGGCTCTGTTGTGATATCAGATGATTTGGAGGTCATAACAGCTGGATGTGC

CACATCATTTGCTCCCTTACCAACAGGTTTTATGTTCCACAGCG

>FM890922.1 FM890922 Jatropha curcas embryo 56-70 (DAF) Jatropha curcas cDNA clone rjcaeb0_000884, mRNA sequence

ATTGTTAAAAAACCTCACAAATACATTAACATATACCTATACATTGGAAGGAAACAGAAACCAGAGCGAG

CAAACCACACATAATCTCATTTCATACACATCAACGACAAAGCAATTTATTGTTTTTATATCTTAAAGAA

GATTGAAACTCATGCTCATGCACCGCTAATCTCAATAGACTTGACTTGAGGCTTCTTCTCCTCTGCCTTT

GGAATAGTAACAGTAAGTACTCCATTCTCCATTGATGCCTTAACTTCACCAACCTTTGCATTTTCCGGTA

ACCAAAACCTTCTCAAGAACTTACCTCTGCTTCTCTCAACGCGGTGCCATTTCTCGTTCTTCTCTTCCTT

ATCAACATTTCTCTCTCCGCTAATCTGAAGAACTCTTCCTTCTTCAATTTCAACTTTCACTTCCTCTTTC

TTTAATCCTGGAAGATCAGCTTTGAATATATGGGCTGGGGGGGCCCGGTAC

>FM890921.1 FM890921 Jatropha curcas embryo 56-70 (DAF) Jatropha curcas cDNA clone rjcaeb0_000883, mRNA sequence

TCCTGCAAATGATATCCCGAGTGATACCTTTGATGTCAAGGTGATATCCCACAGTATACTTCAGTGTCTT

CAAGTGGGAATTTGGTGCAGTATGATGGGGATAGGACGAAAGAAGATATTATTGATTTCATCGAAAAGAA

TCGGGACCAAGATAAAGTTCCCCAGCAACAAGATAAAGAAACCCTGCAAGAATCAGAAGAGACAAAGGAT

GAGCTCTAAATCTTATCAATTTTGAGCTTAGTGTACTACTAAAAGTTATGTCAGAAATCAGAATAATCAG

TTAGTTTACTTAGCCCCTTTATGGTTAGTAAAAAAATAACTTCACCGTGTAAGCTGAGTTTTTCCGTTTA

ATATATTACTGGATTCTTC

>FM890920.1 FM890920 Jatropha curcas embryo 56-70 (DAF) Jatropha curcas cDNA clone rjcaeb0_000882, mRNA sequence

ATTCCAAGGAAGTACACAGGCGAAGGTCAAGTGCTCAAAAGAATATATCGCCGCCATTAGAATGGTACAA

CGTACCAGAAGGAACAAAGAGCCTGGCTTTGGTGGTGCAAGACATAGATGCACCTGACCCAGATGGTCCC

ATTGTTCCTTGGACCCATTGGGTTGTGGTTAATATACCACCTACGTTGAATAGAATACCAGAAGGGTTTT

CAGGGAAAGAAGAGGAAATAGGTGGTGAATATGCTGGAATCAAAGAAGGAAATAATGACTGGAAAGTTCC

AGGTTGGCGCGGCCCCAAGCTTCCTAATCATGGACATAGGTTTGAGTTCAAGCTTTTTGCCTTGGATGAG

GAATTGAATCTGGGAAATAAGGTAACAAAAGAGAAGCTGTTGGAAGCAATTGAAGGGCATGTTCTAGGAG

AAGCAGTGCTGATGTGCAAGTTTTGAACTTTGATTGTTTCTTTTCTTTTCTTTTCTTTTCTTTTTTCTTT

GTTTAGAGTTTACTTTTAGGTATAAAGTTTTGAAGTTCGATTTGATACATCGAGTGCTG

>FM890919.1 FM890919 Jatropha curcas embryo 56-70 (DAF) Jatropha curcas cDNA clone rjcaeb0_000881, mRNA sequence

CCGGTTACCCTACAGGGACCCAGCAAATGTCGGCCATGCCTGGACACGGCACAGGGCAGCCATACGGTGG

AGAGGTGGACCCAACGGGTTTGACCAGGACTCGCCCATCTGGGTTGCCCGGAGACACCACTGGCCATAAC

CCACGTGTGTAGGGCATGGCATGTGCTTTAGCTCCTGTTATGTAATATATCTATGTTTTCTGTTTGGTGT

CTTTTTCTTGTTAAGCAATTCCAGTTGAATAAAAGGTATCTTTTAGTAGATATTTTATTT

>FM890918.1 FM890918 Jatropha curcas embryo 56-70 (DAF) Jatropha curcas cDNA clone rjcaeb0_000880, mRNA sequence

TCCTCAGAGAGCGTTTACAATGGGTTTCTCAGAATTTCAGCAAGCATGCAAAGAGCACCCATATCATAAT

CACAAGCAAGGAGTTTGTCCATCTTGTCTAAGAGAGAAATTATCTAAACTATATGTCGCTCCTTTAAATG

ACAAAGAAGCAGCAGCAATAGCTCTTTCATCTTCTTCATTTATGTCTCTCTCTTCTGCTTCTTCTTCAAA

TCACATGTCTCCGGATCATCACCGTCCTCGCCGCCTTAATCATCATCATCGGAATGTTTCTGATATTATG

CATTCGATATCTTTTAGGGTGAGTATTGCAGGAAATGGATTAAAGAAAAGTAGATCACTTGCTTTTGTTA

CAGAAGGAAAGAATGGAAAGAAGAAGAATAAGAAAGGGTTTTGGTCAAAATTGCTTCATTTGAAAGGGAA

GAAAGAAGTTAATTTTGATGGAAAGGTTGCTTTAAATATATAATTTACTGATTTTATCTAAAAAAACAAA

TTTTTTTTGGTGTTAGATATTAATTAGTTTTGTATAGCTTTAGTTTTCCAATCTCTCTTGTGAATGACA

>FM890917.1 FM890917 Jatropha curcas embryo 56-70 (DAF) Jatropha curcas cDNA clone rjcaeb0_000879, mRNA sequence

ATTCATCTAATGTCAGAGATATTGCTAGATGATGATACGGTTCCTGAGGTTTTAGTTGATGAAGACTTAG

ATGTGAATGACTCTAAGTTTTACCTTGAATTGGAGGATTTGACAGGAAAGCCAAGAAGTTGGGATGGATA

TGTAACTGAGTTGGTACAGCAAGCGGGTTGTATTTAGTTCTTGATTGCACTGGATGTGAATTGTCAGGCA

GGTGGCCCTTCCATTTGAGCTGCTCCTAGCCCGCATTGGAAATGCTAGTGTTTGACAATGGTCATACATG

TTCATATTTCCTGAGTCGTTTTTTTCTTTTTCTTTTTCTTTTTATTCTGTCACCAATGATGAATCATCTA

GACTGTAAATATTCCCACACGCAGTAAATACCATAGTTTTATCATCCTGCATCAAAAGTTTTAAACTTTG

TAATATCAGTAGTCAGACTTTGGGTATCAAACTACATAATAGTATGGAATAGCACTTGAGGTTTAATTGG

TTTACAACAAAAAACAAAA

>FM890916.1 FM890916 Jatropha curcas embryo 56-70 (DAF) Jatropha curcas cDNA clone rjcaeb0_000878, mRNA sequence

ATTCTACAGTTTGGGTACTCTGTTTTGGTTTTAAGATCTGTTTCAGGATTTTGTTGTAACAAAAGAAGAA

AAAATGTTGGCAATATTCAACAAACAGTTAGTGAATCCACCACAGGAGCTGCATAGCCCAGCTTCTTTAG

CATCATCAAGAAAGCCAAAGTTTCCTCAGCAAATTCTTCAAGATTTTGTTTCTTCTGATCCTTCTAATGC

GTTCTGTATTAGCTTTGGTGGTGCTGCTTCTCTCGCCTATATTCCACCTAAAAATCATCACTTTGTTAAC

CAGAGGTTATTCTGTGGATTGAATGACATGTACTGCATTTTCTTGGGGAGCTTAAACAACTTGTGTAGCC

TTATTAGACAGTATGGGCTATCAAAAGGAACCAATGAGGCTATGTTTGTAATTGAAGCATATAGGACCTT

ACGTGACCGTGGGCCATACCCGGCCCATCAAGTCCTCAAGGATTTGGAGGGCACTTTCGGATTTGTGGTT

TATGACACTAAAGCTGGACATGTCTTTGCTGCTCTGGGTGCAGAAGAACGGGTGGACATGTACTGGGGAA

TTGCACGTGATGGTTCTGTTGTAATATCTG

>FM890915.1 FM890915 Jatropha curcas embryo 56-70 (DAF) Jatropha curcas cDNA clone rjcaeb0_000877, mRNA sequence

AATGGAAGGCCTAAAGGCCACAATTTTATTGCCGACACTATCAGTATGGGATAAAATACAGTGAAACAGA

GTCAGACGTACATTTTATTTACTACTATATCCATGACCAAAGCAGCACTTGACAAGACAAGAAACAGAAG

AAAGGTAGAAGAAAGCAGAAACACAAGTAAGTTACTACTAAAATAGCATTGTGATTGGTGCTTCTACTAG

CCAAGGCCGCAACTAAGAGATCCTTCTTGTAAATTTAGAGAAGGGATCCGTCGTATATTGTAGTATAGAG

AGTTGTAGAGTGTTAAAAATGTAGAACTTCAACTTCTGCAGAAATCAATAAAATGTGGCCTTCTTTTGTT

TCTAATTAATAAAATATAAATTCCATCGGCTTTACAGTGGAAATTCACTAC

>FM890914.1 FM890914 Jatropha curcas embryo 56-70 (DAF) Jatropha curcas cDNA clone rjcaeb0_000875, mRNA sequence

ATTGGAAACATCAAATTCATCAACAAACAAAGTAAGACTTGGATTACAGAGACCTTCAACACATGACAAG

AAAATTATTTTAGAACAGCTCTCCAAAGAGGGAAGAACCCGTTAACAAATTACATAGAAGAAACAAAAAG

ATTTAAGCGAAAAAAGAAATACACCAAACAAGCAATGGGCCTTCCCTGGTTAATTAAA

>FM890913.1 FM890913 Jatropha curcas embryo 56-70 (DAF) Jatropha curcas cDNA clone rjcaeb0_000874, mRNA sequence

ATTCCAACAAGGTCAAATATTAACAGCTCCACAGAACTTTGTGGTTTTGAAGAGGGCAAGCAATGAAGGG

CTAGAGTGGGTATCCTTCAAGACTAACGAAAATGCACAAATTAGCCCACTAGCAGGACGGACCTCGGCAA

TTAGGGCTCTGCCAGAGGCAGTGGTTGCAAACGCATTCCAGATTTCCAGAGAAGATGCCAGGAGGCTTAA

GGAGAATAGACAGGAAGTCACTGTGTTTAGCCCTGGGTCTAGATCTCGTAATTAATATTGCAACTATGCA

ATTATACCAAGAGGATGTATAATAAACTGGTTTCTACATAGTTTAGATTTGGAGAAGAATAAGCCTATTT

TTATAGGCAGTTTTAGTTCCTTGCAATAATATAAAAATGTAGATCTGTATATGCCTTATGTTATGAGATA

ATGAAAGTTTGTGGAGCT

>FM890912.1 FM890912 Jatropha curcas embryo 56-70 (DAF) Jatropha curcas cDNA clone rjcaeb0_000873, mRNA sequence

CGAATCGTCTAATTACCTCAACAGCCCTACTCTGCGTTCTTCTAGTGATCATAGCCAATGCAGTCACTGG

CTACAGAACTATCATCACCACCGTTGAAGTCGACGATTCTTCCAATGTTCGGGATATATGCCAGAAGGAA

GCTGACAGAAGAGATTTGAGTTCCTGTGAGAATTATATAACACAGAGAAGAGGAAGGTCATAAGATATGT

TAGCTATGCCAGGAATTGAAAACCCTCTCACAGCAGGTTCCTATACAATGCTGTAAGCGAGCGAAGGAAC

TGAGTGCTATTTGTCGATGTGAATCATTACATTATCTGTTAG

>FM890911.1 FM890911 Jatropha curcas embryo 56-70 (DAF) Jatropha curcas cDNA clone rjcaeb0_000871, mRNA sequence

CAGTTTTTATTACAAGACATTAGGAAAGCTTGAAGCAACGTTACAAGAAGCAGTTTCGAACAAACTTATT

CTGATGCTCTTACTTCTTATACATCATTATATATAACTCTCACTCACATACAAGTGAATGAAGTTTTATT

CACCTGCAGCTAGCATTTTAACGAGGGCATATAGACCCAAAGCAAACATCAATTATATTTTTAGTCCTGC

GCCTTGCCTCATCTTCGCTTCCAACTTCTCCTTCCTCCAGCTGCTTCTCTAACAGATAATGTATTGATTC

ACATCGACAAATAGCACTTAGTTCCTTTGCTTGGTTACAGCATTGTCTAGGAACCTGCTGTCGAGGGTTT

TCAATTCCTGGCATAGCTAACATATCTTCTGACCTTCCTCTTCTCTGTGTTATATAATTCTCACAGGAAC

TCAAATCTCTTCTTTCAGCTTCCTTCTTGCATATATCCCGAACATTGGAAGAATCGTCGACTTCAACGGT

GGTGATGATAGTTCTGTAGCCAGTGACTGCATTGGCTATGATCACTAGA

>FM890910.1 FM890910 Jatropha curcas embryo 56-70 (DAF) Jatropha curcas cDNA clone rjcaeb0_000870, mRNA sequence

ATTCCAAAAATGGCAAAACTTGCAATCTCAACATCTCTAATCTGCGTTCGTCTAGCGATCATAGCCAATG

CAGTCTCCGGCTATCTGACAGCCACCGCCATCACAGTTGAAATGGAAGATGCTTATCGAGTTCAGGGAAT

ATGCAAGAAGGAAGCTGAAAAAAAAGATTTGAGCTCTTGTGAGAATTACGTAAGACTGTCAAGAACAAGG

TCCGAAGAGACGCCAGCTACTAAGAAGAATTGGAAACCGGCAACAACAGGTTCCTGGGCAATGTTGTAAC

CAAGCATGGAAACTAAGTGGCTTTTGTCGATGTCAATCAATAAGGTATCTACCAGAGAAGCAAGGATACG

TTGGAAGCCAAGAATATGATGAGGCAATGCGTAGGGCTGACGATATAATTAGTGTTTGCTTTGACAATAT

CTGCCCTCGTTAAAATGCTATTGCTACCGAAAATAAAATTTCACTCGCTTGAATGTGAGCGAGAGTTACG

TTTAATAATAATCCATAAACAATATGAGTTTCGGAGTAAGTCTGTAATGTTGCTTCAAGTTTTCTTCATG

TCCTGTAATAAAAGCTAATCAACTGTTGAGTTAACAAAG

>FM890909.1 FM890909 Jatropha curcas embryo 56-70 (DAF) Jatropha curcas cDNA clone rjcaeb0_000869, mRNA sequence

ATTCTTTTTAATAAAATATCTACTAAAAGATACCTTTTATTCAACTGGAATTGCTTAACAAGAAAAAGAC

ACCAAACAGAAAACATAGATATATTACATAACAGGAGCTAAAGCACATGCCATGCCCTACACACGTGGGT

TATGGCCAGTGGTGTCTCCGGGCAACCCAGATGGGCGAGTCCTGGTCAAACCCGTTGGGTCCACCTCTCC

ACCGTATGGCTGCCCTGTGCCGTGTCCAGGCATGGCCGACATTTGCTGGGTCCCTGTAGGGTAACCGGTT

GTCCCAGTTGTTGAATGGGTATAGGTTTCGGTTGTGCCTTGAGCGCCCGGACCAGCAGTTGTGTACTGTA

CATGGCCACCAGCCTTGGCTGCCTGCCTCGCCGCTGCGTTATCCTCGCGCGCTTCTAGTTTCCGCAATTC

TGCCTCCGTATTCCTCTCTTCTTTCTTCTCTCTCGCCATTTCCTTCTGCACTGGATCACGTGTTGACA

>FM890908.1 FM890908 Jatropha curcas embryo 56-70 (DAF) Jatropha curcas cDNA clone rjcaeb0_000868, mRNA sequence

ATTCCCTGNAATGAAATTAAGGACTCTGTTGTTGCTGGGTTCCAGTGGGCATCAAAGGAAGGAGCACTGG

CTGAAGAAAACATGAGAGGTATCTGCTTTGAAGTCTGTGATGTGGTGCTCCACACTGATGCTATTCATAG

AGGTGGTGGTCAGATTATTCCAACTGCTAGGAGGGTTATCTACGCATCACAGCTTACAGCCAAGCCCAGG

CTTATGGAACCTGTTTACTTGGTGGAGATCCAAGCCCCAGAGCAGGCACTTGGAGGTATCTACAGTGTTC

TTAACCAGAAACGTGGGCATGTCTTTGAGGAGTTACAGAGGCCTGGTAACCCATTGTACAACATCAAGGC

ATACCTACCGGTTATTGAGTCCTTTGGATTCTCAAGCACTTTGAGGGCTGCCACATCAGGACAGGCCTTC

CCACAATCCGTGTTTGATCACTGGGAGATGATGTCTTCTGATCCAATGGAAGCTGGTACACAAGCATCAA

CTCTCGTGGCAGAGATTCGCGAGAGAAAGGGTTTGAAGGAGCAGATGACTCCTCTATCTGAGTACGAGGA

CAGGCTGTAATCATTTGTTGAGTG

>FM890907.1 FM890907 Jatropha curcas embryo 56-70 (DAF) Jatropha curcas cDNA clone rjcaeb0_000867, mRNA sequence

TTTTTTACAAAAATTAGCTCTTATTATTTATAGGAGATTATTATACATAACTCTCACTCACATTCAAGTG

AGTGAAATTTTATTTTTCTGCAGAATATATATATTAATATATCCATGTTGTAGGTAGCATTTTAACGAGG

GCAGGCCGAGCCAAAGCAAACATCGATTATATTGTAAGACCTACTCTCTGCCTCTTCATATTCTTCGCTT

CCAACAAATCCTTGCTTCTCTGGTAGATACAATATGGATTCACATCGACAAATGGCACGTAGCTTCCTTG

CTTGGTCACAACATTGTCTAGGAACGTTATGGTGCCGGTTTTCGATTCCTTTCATAGCTAACAGAGCTTC

TGACCTTGTTCTCGACTGTCTTATATACCTCTCACAAGAGCTCAAATTTGTTCTTTCAGCTTCTCTCTTG

CACATTTCCCGAACTTCATAAGCATCGTCCATTTCAACGGTGGTGGTGGTGGTGGTGGTTGTCATATGGC

CGGAGACTGCATTGGCTATGATCAATAAAAGAATGCAGATTAGAGCAGTTGAGGTAATTAGCTTTGGCAT

TTTTGCTCAAGGGGGGGCCCGGTACCAATTTCGCCTATAGTGAGTCGT

>FM890906.1 FM890906 Jatropha curcas embryo 56-70 (DAF) Jatropha curcas cDNA clone rjcaeb0_000866, mRNA sequence

AGTTCAATTTGTTTCTACGTGATGATGTTATTTCTATATAGCAACAAATGTTTCATCAACAACACAGGCT

ACTAATATAATAGGAAAGGATTTCCCGTTACATATATGAGAAAAAATAGTTTGCTTGTTTTATTGATCAT

CTGAAGACCTTAAAAGAGTGATTTGCAGCAGTGAAGCCCCCGTCGACCATTAGATTAGCCCCACTAATGT

ACCTTGCTTCTTCACTTGCCAAGAAGAGTACAACATTAGCCACATCATTAGGCGTCAATTCCACCCCTTG

CAAATTAGCATTCTTTGCAGCAAAGGCTCGGAAACCTGCCATTGCATCTTCTGTTCTCTCGTCCTCGGGC

AAGTGAGCTAAGGCCAGGTCGGTTAGAACTGCATAAGGAGAAACACAGTTGACACGTATCCCATATTTTC

CTAGTTCAGCTGCAACATTTCTGGTCAGCCCCAAAAGAGCATGCTTGGACCCTGTATATGCATGGGGACC

CAGGCCTCCTATTACACCGCTGACACTGCAGATAGAAACAATCGAACCCTTATTTAGCGGGATCATAATC

CGTGCTGCATGCTTCATTCCAATAAATGCACCCTTC

>FM890905.1 FM890905 Jatropha curcas embryo 56-70 (DAF) Jatropha curcas cDNA clone rjcaeb0_000864, mRNA sequence

ATTCGAATTCTGAACGTCCTGCGAGAACTTGCACATTGGAAAATGTTGAAGGATCTGTTTGCTGGGGTGC

TGCTTACTGTGTTCGTGGAGGACCTGAAAGGGAAAGACTAACAATGGAGTATTTGGAACGGAGAGAATGT

GAATATGATAAGAAAACCCTTGTAGAATTTTACAAGGAAGGGGATCACTCGCGACCTGCTTTAACAGGAG

TCATAGTTTTCACTTCCACTCCGGATAAAATATCAAACAAGTACTACTTGGGACCTGCTCCCTTGGAGGA

AATGGCTAGCCAAATTGCAACGGCCCATGGACCTTGTGGGAATAACAGAGACTACCTATTCCTGTTAGAA

AAAGCCATGTTTGATATTGGTCATGAAGATGACATGGTGATTGAATTGGCAAATGAAGTGAGGAAGGTAC

TTGTTGCGCTTAATTCCTATGTTCCAGCCCTTAAACTACGTCCACTCACGGAAGCTGTGGCGCTGGATTC

TTAATATTGAAGACAGATAACTGAAGGAAAGGGGGGCATAAACCAGAGCTCCACTAACTTGATTACACTC

CTGGAGAATCCTCT

>FM890904.1 FM890904 Jatropha curcas embryo 56-70 (DAF) Jatropha curcas cDNA clone rjcaeb0_000863, mRNA sequence

ATAATCACAGGGAACTTGCCTAATTAGGCTTTGGAATTATGATCATTTCAAGCCCCTTGAATAAAATCTA

GTGCCCAAATTGTTGTTATTAGAAGATCCATATAACAGGACATCAAGAGCAATATGTTCCATGATAGGAC

TGACAAAGAAGAAATGAACAAAAGCACACACTAGTGCAGACAACCTGTGAACAGCTCGGTGGAGAGAAGG

GGCCCTTAATATTTTATAATACATTAACACATCATTTTATTTCAATCATAAGAGTGGTGAGCGCTCTAAG

CTGAAGGGCATTCATTTGCATACACTTCACTTGCATACTTCCAATTTATTACCTTCCATATGTTCTTTAG

ATAATCAGGTCTGACATTCTTGTACTGTAAGTAGTATGCATGCTCCCAAACATCAATGCCAAGCAAAGGA

ACTAAAGTTGGTCCTTTAGTTACCAATGGGTCCTGGTTTGCAGTTGTCTCAACTACAAGTTTCTTTGCTT

CTTTGTCCAAACCAAGCCACGGGGGGGCCCGGTAC

>FM890903.1 FM890903 Jatropha curcas embryo 56-70 (DAF) Jatropha curcas cDNA clone rjcaeb0_000862, mRNA sequence

ATTCCTCCCTTCACATATGGCATCTCAAGCCAGCCTCCTCCTTCAAAAACAGCTTAAAGATCTTTGTAAA

AACCCGGTGGATGGGTTTTCTGCGGGTTTGGTTGATGAGAATAATATCTTTGAATGGAGCGTTACGATTA

TTGGACCGCCTGATACCCTTTATGAAGGGGGATTCTTTAATGCCATTATGAGTTTTCCTTCCAATTATCC

CAATAGTCCTCCAACAGTGAAGTTTACTTCAGAGATATGGCATCCGAATGTTTATCCTGATGGGCGTGTT

TGCATATCAATTCTTCATCCACCGGGTGATGATCCAAATGGTTACGAGCTTGCAAGTGAGCGCTGGATGC

CTGTGCATACTGTTGAGAGCATAGTTTTGAGTATCATATCAATGCTTTCCAGTCCCAACGACGAATCTCC

AGCAAATGTTGAAGCTGCAAAGGAATGGAGAGACAGGAGAGACGAATTCAAGAAAAAAGTGAGCCGCTGC

GTGAGAAGATCACAAGAAATGTTATGATCTTTCTTCCGAATGTTTCTCCTCTGTCTGCATGTACCAAGAA

ATGTCTTAATTTATTATGGTGGGGTATCGTGGGGGGTAATTTGTTATACTTGTAGAACAC

>FM890902.1 FM890902 Jatropha curcas embryo 56-70 (DAF) Jatropha curcas cDNA clone rjcaeb0_000861, mRNA sequence

CGAATTCCCACAACACCGCTATGAAGCTGCCTTCAAAGGTCAACAAAAAGGTCCATCAGCTCAGAAAGTG

TTAGCTGTGATCACCCTCCTGCCGGTAGGCGGTGGGCTTCTTGCACTAGCTGGTATAACCTTAGTAGGTA

CCCTAATCGGCCTAGCTATTACTACCCCTCTTTTTGTCATCTTTAGCCCTGTTCTTGTCCCAGCTGCATT

AGTTATTGGCCTTTCGGTCATGGCATTTTTGGCTTCCGGGGCTATGGGGCTCACAGGGTTGTCATCACTC

TCGTGGGTTCTGAAATACCTCCAGGAGGTCACTCGGCGAATGCCGGAGCAGCTGGATATAGCTAAGAAGC

GTATGCAAGACATGGCTGGTTTTGTGGGACAGAAGACCAAGGAAGTAGGCCAAGAAATCCAGAGGAAAGC

ACATGAAGGGAAATAAGTAGAGAGAAATGCTGTTGGTTATTAAGGGTTGAAGTCAGAGAGCTGGGTTTTG

GTACGAGACTACTTTTGTTGTATGGGTGTTGTTTTGAGGTAGTTGAGTTGGTTTATGGGGGATCTTCTTT

GTTGCATTTGTATTTCTCTTTTTGGTCTTTTGGGTTCCATTGTTGATTTTCAATGTATTATCTTTGTATG

TGCC

>FM890901.1 FM890901 Jatropha curcas embryo 56-70 (DAF) Jatropha curcas cDNA clone rjcaeb0_000860, mRNA sequence

ATTCACTTACCAGAAAGTAAGTTCACGGTTGAAACGTGGAACAGCAGTGGTCGTCCCTGCAGGCCATCCG

GCGGCTTTTGTAGCTTCAAGAAACAATAATCTGGAAGTAGTCTGCTTTGAGATTAATGAAGAAGGCAATA

CTAGGCATCTACTTGCAGGGAAAAATAATAGTGTAAATAAGATGGAGAAGCAAGCCAAAGAGTTAGCCTT

CGGTGTAAGTGAAAGAGAAGTGGATCAGGCTTTTGGAAGACAAAACGAGGAGTGGTTCTTTCCAGCGCCA

AGGAGAGGTTCCTTCGAGGGTCATGCTGTGGCATGAGAAGCTAATAACGTGATGGCAAGTGTTTCGGCTT

TTTGGGGTGACTGTGACATCGACCTTGAGGGTTTACTAATGTAAATAGTGCTGTGTATGGTGAATAAATA

ATGAAAGGTGAAGGTACATGTGTAAATTAGTCTTTAAGAAAAGAGAAAGATCACTGCTGCGGACTATAGT

GGCTTCTGGTTTTATAATTAATAAAGAGATAAATTCTAGTTAAAAA

>FM890900.1 FM890900 Jatropha curcas embryo 56-70 (DAF) Jatropha curcas cDNA clone rjcaeb0_000859, mRNA sequence

GGGCTGCAGGAATTCGAATTCCACCTCCTCCTCCTCCTCCCTCTCCTTCGATCTCCGTCCTCCGGCCGGC

AAATTCGGCAAATGAACACTCTAAAAACATCTACTCAACGCAAGGAAGAAGATTATAGCTTAGATTTGAA

CTGAAATTGCGTCTATTTTGAAGGGGAAGAGCTTGAAGTTTCGGCCACCGTGGTTACACTTTTTTCCGGC

GATTCACGGCGTTTCAGGCAAGCCTAGAGCGCAAGATTTGACTTTTTCGGCAACCGTGCACGGCGGCGGT

CCGGCGACCGGCCGGCGAGGCTCCGGCGAGGACTTCAAAGGCAGAATGTTCTCCTCTTTGCGCGGATCCC

GGAAATCAAATCCGATTATCGATCGGTTTATAAAATACAACATGCGAGGACAACAGCTGAAAGCATGTGG

CTAAAAAGGC

>FM890899.1 FM890899 Jatropha curcas embryo 56-70 (DAF) Jatropha curcas cDNA clone rjcaeb0_000858, mRNA sequence

GCACAATATCAACGACCCATCACGTGCCGACTATCTATAATCCACGTGGTGGCCATGTCACTAATGTCAA

TAGCCACAACTTCCCGATCCTCAGACACCTCCGACTCAGCGTTCAGAAAGGTGTCCTCTTTAGGAATGCT

ATATTTGCACCACACTGGAACATCAATGCGCATAGCATATATTACTTTACAAGAGGAAATGGTCACGTCC

AAATCGTAGATGAGAATGGAGATGCAGTGTTCGATGGACAAGTTCAACAAGGTCAAATATTAACAGCTCC

ACAGAACTTTGTGGTTTTGAAGAGGGCAAGCAATGAAGGGCTAGAGTGGGTATCCTTCAAGACTAACGAA

AATGCACAAATTAGCCCACTAGCAGGACGGACCTCGGCAATTAGGGCTCTGCCAGAGGCAGTGGTTGCAA

ACGCATTCCAGATTTCCAGAGAAGATGCCAGGAGGCTTAAGGAGAATAGACAGGAAGTCACTGTGTTTAG

CCCTGGGTCTAGATCTCGTAATTAATATTGCAACTATGCAATTATACCAAGAGGATGTATAATAAACTGG

TTTCTACATAGTTTAGATTTGGAGAAGAATAAGCCTATTTTTATAGGCAGGTTTAGTTCCTTGCAATAAT

ATAAAAATGTAGATCTGTATATGCCTTAT

>FM890898.1 FM890898 Jatropha curcas embryo 56-70 (DAF) Jatropha curcas cDNA clone rjcaeb0_000857, mRNA sequence

ATTCCTATGACAAGAGAGGTTTGCTGAATCAAAAAAGGAATTGGATGCTCTGCTTTCGGATGAAGCTCTT

GCCAATGTTCCATTTCTCATCTTGGGGAACAAAATTGACATACCTTATGCTGCTTCAGAGGATGAGTTGC

GATACCACCTTGGCCTAACAAACTTCACCACCGGCAAGGGGAAGGTAAACCTAGCTGACACAAATGTACG

CCCCCTAGAGGTGTTTATGTGCAGTATCGTACGCAAAATGGGATACGGTGATGGTTTCAAGTGGCTCTCT

CAGTACATTAACTGAGGTGATTGTCAGGAAGATGTTTGGCAGAACCATGAAGACCTCACCCAAGCACGCC

ATCTCCTACCTGAAAAGTTGTATTTACCCCCCTGTTTAAATTGGATGTATTGTGATCTGCATCTGTGTGG

AAGGAGGTATTAATATCACTGCTCTGTAAAATTTACTTGTGGCAGCAAGTTGTTCTTTGCTCTTGTAGTT

TGTCAATTGACTTTTGCCATCTCTTGCTGTAATCGTAATGTGTAGAGCC

>FM890897.1 FM890897 Jatropha curcas embryo 56-70 (DAF) Jatropha curcas cDNA clone rjcaeb0_000856, mRNA sequence

ATTCGAACATCTCTCCTTCTTTGGACGGCCTAACCGAGAAGGAAATTGGTGTCCTTAATGACTGGGAGAA

GAAATTCGAAGTTAAGTATCCCATTGTTGGCCGTGTCGTCTCTTGAATTTTCTTATGTTTCTATCTATCT

GTTGTATTTGTGTTAATCTCGAAGATCAATTACCCGTTGTGAGTATCAATTTACATCATATTGTTGTCTC

TGTTGGTAATATGGATGGGGATTGTTCCTCTGTCTTTGTAATCTCCGATTGAATAAATCTGGGTTATTGT

GAAGTTAGAAGCTTTTCAATTCTCCATTTTTCTTTGTGAAATGACCTTATGTTAATATTAATAAGGCATA

TTCTCCTGTTGAAATGTTCTGGAT

>FM890896.1 FM890896 Jatropha curcas embryo 56-70 (DAF) Jatropha curcas cDNA clone rjcaeb0_000855, mRNA sequence

ATTCCCTTAATGAAGGGTGTTGCCTCCACNCTTTGGCTGGTTGTTCCTCCACCTAAGCTGGTCATGGCCC

TTCCCCACTCTTAAAAACCATACTCCTCCCCACCCTTTGGCCCCCTCTGCCCTTTTAAAGAGAGCTGGCT

CCGCCCCTGGTTGCTCCTGCTGCTGCATTTTGTTATAGGTTCAAATTATTTTCTGGAGTGATACTTGTTC

TGCTGATACTCTTTAGGATTGTTTTGTACAGTGATTTGAGTATGGGTGGGCGTTATTAGCTTCCAGAGAG

AGAGAGATAATAGAGCTTGTTCCCGGTCTTTTAAATGGGACAGCTTTATTGTAATCTGCACTTGTACCCA

ACTTTCTGATGTTTTTAGTGCTTTCATAGCTGTACTGCAAAAC

>FM890895.1 FM890895 Jatropha curcas embryo 56-70 (DAF) Jatropha curcas cDNA clone rjcaeb0_000854, mRNA sequence

NCGATTCGGCTAGAGCTGAGTTAGACGAAATTATTGGTCAAGACCGATTAGTCGACGAGTCCGATTTCTC

CAAGTTGCCATACCTGCAAAGCATTATAAATGAGACACTGCGACTATATCCAGTAGCACCATTTCTGGTG

CCACATGAATCGTCGGATGACTGTACCATCGGAGGCTACTATGTACCGCGAGGCACAATGTTGCTGGTCA

ATGCTTGGGCTATTCATAGGGATCCAACGGTGTGGAGTGACCCAACAAAGTTCAAGCCTGAGAGGTTTGA

AGGTTTAGGTAATGAAGCATATAAATTGATTCCATTTGGGGTGGGAAGAAGGGCTTGCCCTGGAGCTGGT

CTTGCAAATAGAGTAATGGGTTTAGCATTGGCAGAATTAATTCATTGCTTTGAATGGGGAAGACCCAGTG

AAGAGCTAATAGATATGACTGAAGGTACGGGCGGAACTATGCCTAAATCACAACCTTTGGGAAGCCATGT

GCAGAGCACGTGAGTCTATGATTAGCA

>FM890894.1 FM890894 Jatropha curcas embryo 56-70 (DAF) Jatropha curcas cDNA clone rjcaeb0_000853, mRNA sequence

CGAAGTCTCCTCTTTTATGATGCTGTTTAATTCACCTGTTCAAATAATGTAAAACAATGTTAAAATCCTG

TTTATTTGTGCTTCTATTTCAAAACTGCAATCAGAAAGGAATATAAACTGTAATATTCTATCTGCTCATG

TTTGTAAGGACCAAGGAATATTGACTTTGAACAGTATTTGTACTTGGACAAAGCAATGACTTTCTTGGTT

AGACTTTTCTAATTTTCACAGAAAGTTCTGTCCTTC

>FM890893.1 FM890893 Jatropha curcas embryo 56-70 (DAF) Jatropha curcas cDNA clone rjcaeb0_000851, mRNA sequence

ATTCGGTATTTGCTGTTGCCAAAGACAAATCCCAGGTAGATGTGCTATCTCTGACTCAAAGCCCAGGAGG

GAGGGAGCGGATGCAACATGAAATCATTTTTTTGGCAACGGAAGCTGGATTTAAGGGGGTCGGATTCACA

TCTTATGTCTATCACTATTGGGTTTTGGAGTTCCTGAAGACTTCATCCGTGCTGTCTTGAAGTTGAGCTT

TGTCAGGTTATTGGGTGTTTGATTAGGTTGATGTAAGTATGTTAGGAAAACTAAATGCTTTCTTCCTGGT

TGCTTGATCTTTTGATACATTGAATCCCTCAAGCTGAAAAGAAGGCCCGGTATCTCTCAACAGGATTACT

ATTACCTATTGGATATCTTCTTCTGTGAACTCCAAGGAAAGAGCTTGCAATCCTTGTTTTTAGGGTG

>FM890892.1 FM890892 Jatropha curcas embryo 56-70 (DAF) Jatropha curcas cDNA clone rjcaeb0_000849, mRNA sequence

ATTCCTCTATGAGAGATAATGCGCAACAGCTGCAGCCACCTCCACCTCCAAAGGAGCCAACCTTCAACTG

TCCAATATGCATGGGTCCATTTGTTGAAGAAACATCAACAAAATGTGGGCACATTTTCTGTAAGGCTTGC

ATTAGGGCTGCAATTGGAGTACAGTCGAAATGTCCTACTTGTAGGAAAAAAGTCACTGTTAAAGAACTTA

TTAGAGTGTTCCTTCCAGCAACCAGTTGATCATGTTTCTCACAACCAAATATTATATATGGTTGATAGAT

GCATCTTTGAGATAGAGGTATGGAGATGGAAGAATTGCAGTATCATGTTGGTTGATTCTTATTGTTTACT

TCCTAAATTTGAATTTCATCTAACATGTGCAGTAATTCAGTCAAAATTTGATATGCCTGAAGGCAATAGT

GGACTTTCGTTTTTGGGTAAAAAAATTTATGTCAAATGGTTGATTGGATGGA

>FM890891.1 FM890891 Jatropha curcas embryo 56-70 (DAF) Jatropha curcas cDNA clone rjcaeb0_000848, mRNA sequence

ATTCTCTAATTCCAGATCTAAGCGCTCAAGATCAGATGATGAATGGTCTGGTTTGGATAAGAGAGTTAAA

GCTTCTCCAGCAACACCAGGGGGCAGTCTTGATTCCGGTTTTGGCAGCGTTCACTTGCCTGGAATGAGTA

ATTCTGCTTACAACATGATTGGTTATACATCTCCACAAAGTCATGGGATCTTTGATAGCCGAGCTGCAAG

TGAGCCAACAGGGGCAAATTCAAGTAATTCTTCAGCACCCCCATGTCCAACACTTTTTGTGGCTAATTTG

GGGCCCAATTGTATGGAGCAAGAACTAATTCAGGTATTTTCAAGATGCCCTGGGTTTTTGAAACTGAAGA

TGCAGAGCACTTATGGGGCTCCAGTTGCATTTGTTGATTTCCAGGATACTTCTTGTTCCACTGGAGCACT

AAATCATCT

>FM890890.1 FM890890 Jatropha curcas embryo 56-70 (DAF) Jatropha curcas cDNA clone rjcaeb0_000847, mRNA sequence

GAAGAGGACAAGAAGAAAAAGAGGATCAAAGTAGTAGCGAGCACAAAATCACCAAAACAAGAGAAAAATA

AGGACAAAAAGAAGAAGAAGAAGCAAAATAACACTGCCAATGGCACAACAAGAAGAAGCGAATCTCTCGA

TGTGATGGTAATATCGTCGTCAAGTTGCGGCTCGCAAGAATCAGAAGAAGAAACTGTTAAGAGAAGCGAA

ACAAAGAAACGAAGCATTACAAGTACCAATAGGACCAAGAAAAGCAACAGCAAGAGGAAAGAACAGGCAG

AAGAGGATAATGAGGAAGGAGAAGCTGAGATTACGTTTAGGTTTCCAATGGCTAGAATTAAGAGGATAAT

TACGCGTGAAGAATCTGGATTGCAACTTAATCAAGATGTGGTTTTCCTTGTCAATAAAGCCACGGAGAAG

TTCATTCAACAATTTTGTGAGGA

>FM890889.1 FM890889 Jatropha curcas embryo 56-70 (DAF) Jatropha curcas cDNA clone rjcaeb0_000846, mRNA sequence

TCGTGTATGGCTGGAATTCCAATATGCGTCCAATGCGGCACCACTACCAACCCTTGCCGGTGCAAGGTGG

TTGGTCCAACTCTGGGGTTCTTGGCGTTTGCCGTGGCGGCGGTGGTAGAGTGGCCGGTGGGAGCTTTTGT

TTACCTCTTTAAACACAGGAAAGGTCGCAGGATCATGGCACATCCAGCGACTGTCGTTTACCCATCAGTC

ACCAATTCAATTCCCGTTTAATTAATAGATTTGATGCT

>FM890888.1 FM890888 Jatropha curcas embryo 56-70 (DAF) Jatropha curcas cDNA clone rjcaeb0_000845, mRNA sequence

ATTCGACGAAGCTACTCTCGCTACGATCGACCAAATCTAAGGCTTTCAGATCGAAAAATGGCATTGATTC

CAAGCTTTTTCGGTAACTCACGAAGCAACATCTTCGATCCTTTCTCTGCTCTCGACGTTTGGGATCCATT

CAGAGACTTTTCTTTCCCTTCATCTTCTTCCCTCGTCTCTCGTGAAAATTCTGCTTTTATTAACACTCGC

ATTGACTGGAGAGAGACACCAGAGGCTCATATATTCAAGGCTGATCTCCCTGGGCTCAAAAAAGAGGAAG

TGAAAGTAGAAATTGAAGATGACAGAGTGCTTCAAATCAGCGGGGAGAGAAATGTGGAGAAGGAAGACAA

GAACGACACGTGGCATCGCGTGGAGCGTAGCAGTGGCAAGTTCATGAGGAGGTTCAGGCTGCCGGAGAAT

GCAAAGATGGATCAAGTAAAGGCTTCAATGGAAAACGGAGTACTTACTGTCACTGTTCCTAAAGAGGAAG

TCAAGAAACCTGATGTTAAGGCTATTGAGATCTCTGGTTAAGTTTCAGCTTATAATGATGTTATCTCAAT

ATATATATATATATATATATATATATATATATATATATATATATACT

>FM890887.1 FM890887 Jatropha curcas embryo 56-70 (DAF) Jatropha curcas cDNA clone rjcaeb0_000844, mRNA sequence

AAGGATTGAATTGCTTTTGCCTGTGTCATATATTTAATACTTGTTAATGAAGAATCAGTCCTTCATAGAT

CATCCAATCTATCGTTTCTCACCAACTTATGCAGAAGTCTAGACATGAAAACCCAAGAAGTCTCTGGAAC

TTTATGGTTAAAATGAACTACCAAGGCTGACATAATATCAAGAATAAAACTATGAAATTCAGGCTTCAGT

GCCATGCGTATAAGCATTGCAGTCCACAACTCTGGGGCTAACTTTACCGCTTGACGTAGATATTGAAACC

CATTAACTACCCTCATGAATGTTGAATGGCCCTCAGACTTAGATGCCTCGTTTCCTTCACGTTTCTGGTC

TAAAATGCAACCAACGATTTTATCTCGTAAAAAGACATCAGCACTTAATTATATCCCGTACAAGAAAACA

CGCACCTGCTAAGGGGGGCCGGT

>FM890886.1 FM890886 Jatropha curcas embryo 56-70 (DAF) Jatropha curcas cDNA clone rjcaeb0_000843, mRNA sequence

ATTCCTTCGAATCCAAAAATTACCATAACAAACCAAAACCCAGACCAATAAAAAAGGAAAAAGAGAAAAT

TTTGAAGACAAATCCAAGGTTGAAGAGCGAAAAATCAAAGCTTTTGTTTGCAAGGGTAATATATGCAGGA

TGGAAAATCAAGAAGAAGAAGAAGAAGCAGAAGCTATACCTGCACAAACTGCGTCGCCAAGGCCATCATC

GACGACATTCAATTTATTTCTGACAATTATGAGCAAAAGAAGAACATGGGTATGTTTTTTTGTGCTTGTA

TATGCAATCCTTTTATCATCTTCATGGAATTTACTCAAATATATACTTTCTTGGTATAAAGAACAAGCCC

AAGTCACTTCTGCCTCTTCTGGTTGGCCTGCTTTGTATGCATCAGTGCTTTTAGGGGCAGTTTTTGGTCT

GCTTTTCATGATCGCAGCTCTTGCTGTGGCTGTGCCAGCCACTTTGGGTTATA

>FM890885.1 FM890885 Jatropha curcas embryo 56-70 (DAF) Jatropha curcas cDNA clone rjcaeb0_000842, mRNA sequence

GATAAAATATAATAATTTAATTTCTCAAATAAGGACCTACTCACACCCAAATGTGCAGAACAGTCATAAA

AAATAATGATAGGAAGACAATACAAGCCCAATCACATAGTTGTAACAAAATTAGTTCCATCGCGAAATGT

CTACCAATCCCAGTCTTCAAGATGGCTTCTTTAGACCACACAAGCTGACTTTTGGGCCTTGATTGGCCTC

CTGCCTGATCTGCTTGGTTGATTTTGATTGTCTGTGGCTCACTTGAGTCAGTATCCNTGCAAGCCTTTGC

TTGATATCCCTAGCTATTGAAAAGAAAACCTCCTCCACATTTAAATTTGTCTTTGCACTAGTTTCAAAAA

ACTTGATGCCATATTCATCTGCAAGAGCCTGG

>FM890884.1 FM890884 Jatropha curcas embryo 56-70 (DAF) Jatropha curcas cDNA clone rjcaeb0_000841, mRNA sequence

ATTGTTCGAGCTTATTTCTCCGGTTAGGGTTTTCTGATTCCAATCAACTACCAGCAACCATGGCGGATGT

GGAGACAGAGGTGGCTGCAGGACAGCCAAAGAAGAGAACGTTCAAGAAGTTCAGCTTTAGGGGAGTGGAT

TTGGATGCTCTCCTTGACATGACCACTGATGAGCTTGTCAAACTCTTTCCTGCCCGTGCTCGCAGAAGGT

TTCAGAGAGGTCTGAAGAGGAAGCCAATGGCTCTTATCAAGAAGCTGCGCAAGGCGAAAAGGGAAGCACC

ACCTGGTGAGAAACCAGAACCAGTTCGAACCCACCTCCGCAATATGATTATTGTTCCCGAAATGATTGGA

AGCATTATTGGTGTGTACAATGGGAAGACCTTTAACCAAGTTGAAATCAAGCCTGAGATGATCGGGCATT

ATCTTGCTGAGTTCTCAATCTCAT

>FM890883.1 FM890883 Jatropha curcas embryo 56-70 (DAF) Jatropha curcas cDNA clone rjcaeb0_000839, mRNA sequence

TTTTTTTTTANAGAAATTAAATTCTTTTTCAGTATCCTCAAACTTTGCAAGAAGGTAAACTGTTACACTA

GGAAAGATCAAAATAGTCTATTAAACAAACAAATAAGGACAGGCACTACGGCTTAGATGCGACTAGGGTA

TTATTTTCAGCAGCATTCTCTTCCTTTCCTAGTGCAAGGACCACCTTCTTCAGGATAGCTTCAGTCTGTT

GGTGGGCATTATCTACAGCCTTTGAAATGGCTTGCCATTTCTCGCCATGCTTTGGTTCTGCCGCGACACA

CCTCTTTAACACATCCTTCTGGGTTTCCTCAGTGCCATGCTGAAGTTCAAATTTGTAATATAATGCCCAG

AAATCCCCAGTGTCCGGAGCAAGAGTAACTGCTCTGTTCAACCAGGTTCTGGCTTTATCTACCTTCCTAT

CGTGCCAGAACAACTTGGCCACAGCAGCAATAACATGAGGATCGTGATCACATTTCTTGAGGGCATCCAT

ACTCTTGCTTATGCGCTGTGGACGTGGAACCATCTCGATAGATGCT

>FM890882.1 FM890882 Jatropha curcas embryo 56-70 (DAF) Jatropha curcas cDNA clone rjcaeb0_000838, mRNA sequence

ATTCCAAATATCACTGATCGTAAACCTCCATTGAATTTACTCTGTGCTTCGATCGCGTCAATGGCGGAGC

ACCCACAGTCGCAGCATGTTGGCCAACAGCCACGATCCCACCAAGTAGTGAAGGCAGCCACTGCAGTCAC

TGCCGGTGGATCTCTTCTAGTTCTCTCCGGTTTGACCCTTGCTGGTACGGTCATTGCACTGACCGTAGCC

ACTCCATTGCTGGTCATATTCAGTCCGGTTCTTGTTCCTGCGGTTATCACAGTTTGTTTGATAATCACAG

GGTTTTTAGCCTCAGGCGGTTTTGGCGTCGCTGCCATTTCTGTCTTGTCCTGGATTTATAGGTATGTTAC

TGGAAAGCATCCACCAGGAGCAGAAAGTCTGGACCAGGCACGCTTAAAGTTGGCGGGAAAGGCAAGAGAG

ATGAAGGACAGAGCTGAGCAGTTTGGACAGCATGTAACAGGTCAACAGACTTAAAAGAGAAGTGGGTTTT

GGTGGTTGATGCAGAGAACATAAAATGGCTTTGATATATATATAAAAATATATGAGTCCTTTAATTAACA

TGGA

>FM890881.1 FM890881 Jatropha curcas embryo 56-70 (DAF) Jatropha curcas cDNA clone rjcaeb0_000837, mRNA sequence

ACCTCCATTGAATTTACTCTGTGCTTCGATCGCGTCAATGGCGGAGCACCCACAGTCGCAGCATGTTGGC

CAACAGCCACGATCCCACCAAGTAGTGAAGGCAGCCACTGCAGTCACTGCCGGTGGATCTCTTCTAGTTC

TCTCCGGTTTGACCCTTGCTGGTACGGTCATTGCACTGACCGTAGCCACTCCATTGCTGGTCATATTCAG

TCCGGTTCTTGTTCCTGCGGTTATCACAGTTTGTTTGATAATCACAGGGTTTTTAGCCTCAGGCGGTTTT

GGCGTCGCTGCCATTTCTGTCTTGTCCTGGATTTATAGGTATGTTACTGGAAAGCATCCACCAGGAGCAG

AAAGTCTGGACCAGGCACGCTTAAAGTTGGCGGGAAAGGCAAGAGAGATGAAGGACAGAGCTGAGCAGTT

TGGACAGCATGTAACAGGTCAACAGACTTAAAAGAGAAGTGGGTTTTGGTGGTTGATGCAGAGAACATAA

AATGGCTTTGATATATATATCAAAATATATGAGTCCTTTAATTAACATGGAAGGCCATTGCTTGTCTGGT

GT

>FM890880.1 FM890880 Jatropha curcas embryo 56-70 (DAF) Jatropha curcas cDNA clone rjcaeb0_000836, mRNA sequence

CGGACACGCGCGTCGCTTCTGGCCCGGATTCTGACTTAGAGGCGTTCAGTCATAATCCAGCGCACGGTAG

CTTCGCGCCACTGGCTTTTCAACCAAGCGCGATGACCAATTGTGCGAATCAACGGTTCCTCTCGTACTAG

GTTGAATTACCATCGCGACACCATCATCAGTAGGGTAAAACTAACCTGTCTCACGACGGTCTAAACCCAG

CTCACGTTCCCTATTGGTGGGTGAACAATCCAACACTTGGTGAATTCTGCTTCACAATGATAGGAAGAGC

CGACATCGAAGGATCAAAAAGCAACGTCGCTATGAACGCTTGGCTGCCACAAGCCAGTTATCCCTGTGGT

AACTTTTCTGACACCTCTAGCTTCAAATTCCGAAGGTCTAAAGGATCGATAGGCCACGCTTTCACGGTTC

GTATTCGTACTGGAAATCAGAATCAAACGAGCTTTTACCCT

>FM890879.1 FM890879 Jatropha curcas embryo 56-70 (DAF) Jatropha curcas cDNA clone rjcaeb0_000835, mRNA sequence

ATTCGCCTATCTTTACACAATTAGCTCTGCAATCAGAAGGCCTCTAGCTGCGACTGAAAAAATGGCGACT

GCTAGAACTGTGAAGGACGTGTCTCCCCACGAGTTCGTCAAGGCTTATGCTTCCCATCTCAAGAGATCCG

GCAAGATTGAGTTGCCTCCCTGGACTGATATTGTGAAAACTGGAACACTCAAGGAGCTTGCACCTTATGA

TCCAGACTGGTACTACATTAGAGCCGCCTCCATGGCAAGGAAGATTTACTTGAGGGGTGGCCTTGGTGTG

GGTGCTTTCCGTAGAATCTATGGTGGGAGCAAGAGAAATGGAAGTCGTCCACCTCATTTCGGCAAGAGCA

GTGGTTCAATTGCTCGTCACATTCTTCAACAATTGCAGACAATGAACATTATCGATGTTGACCCAAAGGG

TGGGAGGAGAATCACTTCTAGCGGTCAACGGGATCTTGACCAAGTGGCTGGACGGATTGTGGTTGCTGCA

TGATGAACCAATGGACCGTATAGAATACCAGTTAATTTTGGTAGTTTTAGCTTTCTGATTTAGTCTGTTG

GAGAAATTTTATTGCAGAAACCAGATTATCTGTCGGATTTTTTTTTTGGTTTCAATCTCGTGAAAATGGT

TAACCACCAGCTTG

>FM890878.1 FM890878 Jatropha curcas embryo 56-70 (DAF) Jatropha curcas cDNA clone rjcaeb0_000834, mRNA sequence

ATTCCAGTCTTCCTCTCTAACTTGAGAAAGATCGTATATTCTAGATGGATTGGCAAGGACAAAAGTTAGC

GGAGCAGCTGATGCAGATCTTGCTGCTTGTATTCGCGGTCGTGGCTTTTGTTGCTGGCTATGTTACAGGA

TCATTTCAGCTGATGATTCAAATTTACGGTGCCGGTGTGATACTCACTACACTGGTTACCGTACCCAATT

GGCCTTGGTTCAATCGCCATCCTCTCCAGTGGCTGGAATCCAGCGAAGCCGAGAAGCATCCCAAGCCAGA

GCCAGTGAATTCTAAGAAGAAATCCGCTAAAAAGTAGGTCTTTAATTTTAAATTTGAAGTCTTGGGATTT

TTAGAGTTTATTTTAAAATTAACAGTGGAATGGAATGTTATCCGGTGAATTGGATGTTGTAATGAAGTTT

TTTTGCTATAAATTTAGACAATAGCAAGAGTTTTATCAAAAAAAAAAGGGGGGGCCCGGTAC

>FM890877.1 FM890877 Jatropha curcas embryo 56-70 (DAF) Jatropha curcas cDNA clone rjcaeb0_000833, mRNA sequence

ATTCGTTCTTCTCTTCAATGTCTCTTTCGCTAGTGAGCAGGTGAAGCAGGAAAACGAATGCCCTCAGCTT

CAGCAGCAACACCAATGCCGACTCGACAGGATCGAAGCCCTCGAACCAGATACTCGCATTCAAAGCGAAG

CTGGTACGATTGAGTCATGGAATCCTAGCCGTGACCAGTTCCAATGCGCTGGTGTGGCTATTGTTAGGCG

CGTAATTCAACCTAACGGCCTTCTATTGCCAGCTTATAGCAATGCTCACCAGCTCGCTTTCATTGTCCAA

GGTAGAGGTATCACTGGAGAAATGTTTCCTGGATGCCCTGAAACATTCCAGGAATATCAAGGAGCGGGTG

GTAGCAGTAGGGTGGAAGACCAGCACCAGAAGGTTCGCCACTTCCGTCCAGGCGATATCATTGCTTTGCC

TGCCGGGGTTGCTCACTGGTGCTACAATGACGGAAATGAGCCTGTTGTTGCTATTTATCTCCTTGACACT

GCCAACAGTGACAATCAGCTTGACATGAACCCTAGACATTTCTATCTAGCT

>FM890876.1 FM890876 Jatropha curcas embryo 56-70 (DAF) Jatropha curcas cDNA clone rjcaeb0_000832, mRNA sequence

ATTCCTTCTTCTCTTTGTGGTTCGATCCGGGAGAAACTCAGAATCATGTTGGTCTACCAGGATTTGCTTA

CCGGTGATGAGCTTCTCTCGGATTCGTTCCCTTACAAGGAAATTGAGAATGGCATGCTGTGGGAAGTTGA

GGGAAAGTGGGTTGTTCAAGGAGCCGTTGATGTGAACATTGGTGCAAATCCCTCTGCTGAAGGGGCGGAT

GAAGATGAGGGTGTTGATGACCAGACTGTTAAAGTTGTTGACGTTGTGGACACTTTTAGGCTCCAGGAGC

AACCTGCTTTTGACAAGAAGCAGTTTGTCACATACATGAAGAGGTACATCAAGTTGTTGACACCCAAATT

GGATGAGGAGAAGCAACAGGCTTTCAAGAAAAACATTGAGGGAGCAACCAAGTTCCTGCTCTCGAAGCTC

AGTGACCTCCAATTTTTTGTGGGGGAGAGCATGCATGATGATGGGAGTTTGGTGTTTGCTTACTACAAGG

ATGGCGCCACTGACCCAACATTTTTGTACTTCGCTTATGCTTTGAAGGAGATCAAGTGCTAAGAATGGAG

AATAGCTGGCTTCGAAGTGGAAGTTC

>FM890875.1 FM890875 Jatropha curcas embryo 56-70 (DAF) Jatropha curcas cDNA clone rjcaeb0_000831, mRNA sequence

CGAATTCCAATAAAGTGTAACANGAGCAAAAGATTAATTATATTGGTGGAGAAATTTGTTAGCAAAAAAG

GGCAAAAATGTCTTCAAATCAATCAAGACAAGAGCTTGATGAAAGAGCAAGGCGTGGAGAGACTGTTGTC

CCTGGAGGTACTGGGGGCAAGAGCCTTGAAGCTCAAGAGCATCTTGCTGAAGGGAGAAGCAGAGGAGGAC

AGACAAGGAAGGAGCAGCTGGGGACAGAAGGATATCAGGAGATGGGCCGGAAAGGTGGGCTGAGCACGAC

GGACGAGTCTGGTGGAGAAAGAGCTGAGAAGGAGGGCATCACTATCGATGAGTCGAAGTTCAGGACTGGG

AGCTAGTGTGATGATTAAAACTATAAATGGTATTTTGTAGTAATAATAATAATAATGTTGGTAATAAAGT

ATTAGCTTAGCAGTGTACTTTAGTTTAGTAATGTAGTTTACATGTGGGTTAAAATGACTAGGGTTTAGCT

AGTATGTGTGTTGTAACTTTGAGAGGGGTTTATGGTCGTTCNCTTTCTGGGAGGACTGCTAATACTTTCT

CCCTATTTGTAAAATAACCTGCAGTATATATAATTACCTGTTGGTT

>FM890874.1 FM890874 Jatropha curcas embryo 56-70 (DAF) Jatropha curcas cDNA clone rjcaeb0_000830, mRNA sequence

ATTCTGCAAGCCCAGTTATGAGCCCCTTCTGTGCTGTGGGATCAAATCAAACATCAGGAGATGCTGCATA

TGTGCAATGGCCTAGCGCTTCGATGATGTATGCACATTCATATGAGCAATTTAGACATGCTGTTTTCCAG

GTGCCGTTCTGTCAGCAACCTCTAAGTTTTGATTACTCTCAAAACCATTAAGTCTCAGCTAGAAGGGAAT

TGTATTACTCATTTTTATCTGCAGATTTCTTTATACTTCTAGGAAATGTCTTGTTGGTAGGATTTTTCCA

GACACTTGTGTTGCAAACCATTTGTTCCAGTTATAGTTATGGTTTCCGCTTGCTTTTCAAGGGGGGGCCC

GGTAC

>FM890873.1 FM890873 Jatropha curcas embryo 56-70 (DAF) Jatropha curcas cDNA clone rjcaeb0_000828, mRNA sequence

TTTTTCTTTTTTCTCTTCAAGGATTTTCAGTCTAGAAATTATTTATCAAACAGGTCCATAAGGAGTGAAG

GGAAAAGAAAAAATGAAAGCAAAAACGTAAAAACCAAAAACAAAAAAAAAAAGAAGGGAAGAAATACAAA

CGATTCTACTTAGCTCCTCAGTCCTCACTTAGCTAATGCCACGACTATTTACAGTGGTTGCTCCTACTTA

CTGTCCATCCCAACTACAACACCTTCCAGTGAGAAAACTCTTTTACTGATATCTAATCCTAGTCATTGAT

AACTCCCACAAACCCTAGAAGTCTTATTTTCTTCATCTCAAATTCCAAAACTGGCACG

>FM890872.1 FM890872 Jatropha curcas embryo 56-70 (DAF) Jatropha curcas cDNA clone rjcaeb0_000827, mRNA sequence

GGAAACTCATACTCAAAAGTAATTCAACTGAAATGAAACAGTTCAAACTCTTTAACACTTCAAATATCCG

ATCTTAAAACAGAAAATAATCATCATAAGCAAAAATATCAGGGGCATAACAATCTTAATAGACTAATTTA

CTAGTACTGACACAAAATTTGATACTAAAAAAACATAATTATGCCTTCAGACGTCCAAAGAACTTCTGTT

TCTCCTGTGTTGTCTGGAAGCGGCCATGTCCAAACTTTGAAGATGTATCAATAAACTTGAGCTTAATCTC

CTCCAGAGCCAACCGAGATGTCTGGGTAAGCAGAGACTGGCGGAGAGTAACAACCCTCTTTTTAGTTCCA

ACACAGCATCCCTTGATCATTATGTAGTCATCCTTCACCTCACCATAGTGAGGGAAACCACCAATTGGAT

TGATACCTTTCTCAGTCCTGTCATACTCAGTTATAGCAGTGTGGGACTCCTGTCCAGCTTTGCCAAGTTT

GTAAACTTTCTTGTTCATTTCGGTACGGTGATGATATCCATTCTGACCAGCCCTGGCAACAGTAAATGAA

ACTCTAGCTGGATGCCAGGCACCAATACAAGCCAACTTGCGGAGACCCCTGTGGGTTTTACGAGGCAGAC

GGGTG

>FM890871.1 FM890871 Jatropha curcas embryo 56-70 (DAF) Jatropha curcas cDNA clone rjcaeb0_000826, mRNA sequence

ACCAACTAAACTTCGAAAGGAAGTCCTACTTGCAGGGGAAGATTGGCCATATGATCCGGAGAGGAAAGAG

ATGAGAACCAAGATAAAAGGGCATAAATGCGATAAGATATCTGCAGAGAAACGTGAGGACACTGCCAAAT

TGATGCAGCGAATGCCGGATATGTTGTTGGCCTACAAGAAGCGGAAGTGGGAGAAGAAAATGAAGGAGGA

AGACCAAATATATACAGTGAGGGACGAGGAAAAGAAGTCCAAGTAAATACTCCTTTTTTTGGTTCCAGAG

TTGCTTCCTATAGTGCAGCTTTTTCTTTTT

>FM890870.1 FM890870 Jatropha curcas embryo 56-70 (DAF) Jatropha curcas cDNA clone rjcaeb0_000825, mRNA sequence

ATTCCAATAATATCATCTTTATCCTTTCATTTGCACAAGAAGAAGAACAAGAACAAGAAGAGATGGGAGT

GGTGGTAATAGATGGATCCACGGTCCGTAACTTTGTGGAGAATGAAGCCCACTTCAAGAAGAGCGTGGAA

GATACGTTCGCCAAGCTGGACCTTAACAACGACGGGGTGTTGTCCCGCTCTGAGCTCCGACGAGCCTTCG

AGTCGCTGCGTTTGGTGGAAACCCACTTCGGAGTGGACGTCACCGCCACACCAGAGCAGCTGACTCAGTT

GTACGACTCTATCTTTGAGTCTTTTGATTGTGACAAAAGTGGCACCGTTGATATTGAAGAGTTTAGAACT

GAAGTCAGGAAAATATTGCTTGCGATTGCTGATGGGTTAGGATCCAGTCCCATCCAGATGGCTCTTGAAG

ATGATGATCAGAGTTTTCTCAAGCAGGCTGCCGATCTCGAAGCCTCTAAGCTTCAGTCTTCTGTTTCTTA

ACTAATTTAGATCCGTTTTCTATCTTGCCTGTCAAATCTTTTTATCTTTACATCTTATAAGGCTTTTGTT

TGTTCCTACCTTCTTTATCTGTTTTTCAATA

>FM890869.1 FM890869 Jatropha curcas embryo 56-70 (DAF) Jatropha curcas cDNA clone rjcaeb0_000824, mRNA sequence

ATTCCTGGACCTTAACAACGACGGGGTGTTGTCCCGCTCTGAGCTCCGACGAGCCTTCGAGTCGCTGCGT

TTGGTGGAAACCCACTTCGGAGTGGACGTCACCGCCACACCAGAGCAGCTGACTCAGTTGTACGACTCTA

TCTTTGAGTCTTTTGATTGTGACAAAAGTGGCACCGTTGATATTGAAGAGTTTAGAACTGAAGTCAGGAA

AATATTGCTTGCGATTGCTGATGGGTTAGGATCCAGTCCCATCCAGATGGCTCTTGAAGATGATGATCAG

AGTTTTCTCAAGCAGGCTGCCGATCTCGAAGCCTCTAAGCTTCAGTCTTCTGTTTCTTAACTAATTTAGA

TCCGTTTTCTATCTTGCCTGTCAAATCTTTTTATCTTTACATCTTATAAGGCTTTTGTTTGTTCCTACCT

TCTTTATCTGTTTTTCAATATTTCAATAAATTTTGGCAATTTAATCTCTTCTC

>FM890868.1 FM890868 Jatropha curcas embryo 56-70 (DAF) Jatropha curcas cDNA clone rjcaeb0_000823, mRNA sequence

ATTCCAGTAATGGACGAAAGGGTGAAAGAAGGAGACATGTTCGTGATCCCTCAATTCTATGCAGCACTTG

GAAGGGCAGGAGAAAATGGGTTCGAGTACGTAACATTTAAAACAAGTAGCCAGCCTATCAAGAGCCCATT

GGCGGGTTATACATCAGTGATGAGAGCCATGCCTCTTGATGTTCTGATGAACTCTTACAGAATGTCTCCA

AGCGAAGCTCATCAAGTGAAGAGGAACAGGGATCCACAGTCCATGTTGTTTACTCCCACCAGCAGGAGAC

CATAAGTAAGTTACTACTAAAATAGCATTGTGATTGGTGCTTCTACTAGCCAATGCCGCTACTAAGAGAT

CCTTCTTGTAAATTTAGAGAAGGGATCCGTCGTATATTGTAGTATAGAGAGTTGTAGAGTGTTAAAAATG

TAGAACTTCAACTTCTGCAGAAATCAATAAAATGTGGCCTTCTTTTGTTTCT

>FM890867.1 FM890867 Jatropha curcas embryo 56-70 (DAF) Jatropha curcas cDNA clone rjcaeb0_000822, mRNA sequence

AAAGAAAAGAAAAAGTTTATTTATCTTATAGGGATTTTTTTCTTTTTCTATTTTACAACCAAAAAGCAAA

ATTTTTTTTCTTCCCATCTTCACCAAAAAGCACTAACTGTAGACGAGAATGGGCAACTCTCACAGGGGGT

TAGGTGGAAGTCTGGGGGGAAAAACCATCGTCGGAGGATTTCCAAAATCCGCCGCTAAATAAAAACCTGT

AGCACCACCCTAACTCTAAACCTTGCTAAGCTATCCCACCCCACTTCTTCCATCAATAAGTGAAAGTTCT

ACTCCAATCTCAATAGAATCAAGGAGTTCTTCATTAATTCTTCTAGATGGAACGATAATCCTTGCTCTCT

CTGGTCTGTCGAAATGTTTTCTGAGGATTTGGCATAGGAACGATAAGCATACCCGGT

>FM890866.1 FM890866 Jatropha curcas embryo 56-70 (DAF) Jatropha curcas cDNA clone rjcaeb0_000821, mRNA sequence

ATTCAATTTACTCTGTGCTTCGATCGCGTCAATGGCGGAGCACCCACAGTCGCAGCATGTTGGCCAACAG

CCACGATCCCACCAAGTAGTGAAGGCAGCCACTGCAGTCACTGCCGGTGGATCTCTTCTAGTTCTCTCCG

GTTTGACCCTTGCTGGTACGGTCATTGCACTGACCGTAGCCACTCCATTGCTGGTCATATTCAGTCCGGT

TCTTGTTCCTGCGGTTATCACAGTTTGTTTGATAATCACAGGGTTTTTAGCCTCAGGCGGTTTTGGCGTC

GCTGCCATTTCTGTCTTGTCCTGGATTTATAGGTATGTTACTGGAAAGCATCCACCAGGAGCAGAAAGTC

TGGACCAGGCACGCTTAAAGTTGGCGGGAAAGGCAAGAGAGATGAAGGACAGAGCTGAGCAGTTTGGACA

GCATGTAACAGGTCAACAGACTTAAAAGAGAAGTGGGTTTTGGTGGTTGATGCAGAGAACATAAAATGGC

TTTGATATATATATAAAAATATATGAGTCCTTTAATTAACATGGAAGGCCATTGCTTGTCTGGTGTATTT

CTTTTTTCGCTTAAATCTTTTTGTTTCTTCTATGTAATTTGTTAACGGGTCTTCCCTCTTTTGAGAGCTG

TCTAAAATAATTTCTTGTCATGTGTTGAAGGTCTCCTGT

>FM890865.1 FM890865 Jatropha curcas embryo 56-70 (DAF) Jatropha curcas cDNA clone rjcaeb0_000820, mRNA sequence

ATTCCAGAGCCTATCTTTACCCTTTGCTCTGTAGAGAACGCCATCCGTGGAAAAATGGCGACCGCCAAGA

CTGTAAAGGACGTGTCTCCTCATGAGTTCGTCAAAGCTTACTCTGCCCATCTTAAGCGATCCGGCAAGAT

CGAGTTGCCTCCATGGACTGATATTGTGAAGACAGGAACACTCAAGGAGCTTGCACCTTATGACCCAGAT

TGGTACTTTGTTAGAGCTGCCTCCATGGCGAGGAAGATTTATTTGAGGGGAGGCCTTGGTGTGGGTGCTT

TCCGCAGGATCTATGGTGGAAGCAAGAGAAATGGAAGTCGTCCGCCTCATTTTGGAAAGAGCAGTGGTGC

TATTGCTCGTCACATCCTGCAGCAATTGCAGAAAATGAACATTGTTGATGTTGACCCCAANGGTGGGAGG

AGAATTACTTCGAGTGGTCAACGGGACCTTGATCAAGTGGCCGGACGGATTGTGGTTGCCCCTTGATAAA

CTTAAAGTTGGGTATGCTGCAGAATACCAATTTATTTTGGTAGTTTTAGCTTTCTGTTTTAGTCTATTGG

AGAGATTTTACTGCTTGATGCTTAAACCATATTACATGTTGTGAAAAGT

>FM890864.1 FM890864 Jatropha curcas embryo 56-70 (DAF) Jatropha curcas cDNA clone rjcaeb0_000819, mRNA sequence

ATTCAAACAACATCTGTTCTTTCAGTCTTTTAACAATTAGAGCACTTGCCCTTTCTCGCGCACAGTCTTC

GTGCTCTCTCTCTTTCTTTGCGTGCTGCTTCTTGTTTTTGTTGAACCACACGCCATGGCTTCGATTACCG

GAACTTCAATCTCCATGACCTCTCTTTCGAGGTCTCTTAGCACCCAGGCACCAAGAAATGGTATCTCTAG

TTTGAGGATTTCACTTCCCATTAGTGGAAGAAGCTTTCCTTCTCTTAGATTGCAAAAGGGTGCATCTCGC

TTCCGTGTCTCCTGTGCGGCCCAAACCAGAGACGCTAAATAAAGTGTGTGAAATTGTAAGGAAGCAGTTG

GCACTAGCACCTGAA

>FM890863.1 FM890863 Jatropha curcas embryo 56-70 (DAF) Jatropha curcas cDNA clone rjcaeb0_000818, mRNA sequence

ATTCCAAACACTTTCACCTATCCTTGCAAAACCTTTCCTAAAAAGAAACACCCCATAAGAGAAGAGATAG

GAAACTTCAAAAATGAAACAGGCGTTGGTATCCTTCTCTTTCAGCATCCTCTTGCTCTTGCTCTGCTTTC

CTGTGTCCTCTAATGGATCAATTCTGCCCTTTGTGGATAGTCCAGGAAGTCGGCTGACTGATTTCTGGGC

AGACCGCTTCTCCGACCCATTTAGAATTTTGGAGCAGATACCATTTGAGATTGAGAGAGATGATAGTGTG

GCACTCTCTCCGGCCAGAGTAGACTGGAAAGAAACACCTGAGGGTCATCTTATCATGTTAGATGTTCCTG

GTTTGAAGAAGGAAGAGCTGAAGATTGAAGTGGAAGAGAATAGGGTACTCAGAGTGAGTGGAGAAAGGAA

AAGAGAGGAAGAGAAGAAAGGGGATCATTGGCATAGGGTAGAAAGATCATATGGCAAGTTTTGGAGACAG

TTTAAGTTGCCAGACAATGTGGATTTGGACTCTGTTAAGGCTAAGCTTGAAAATGGTGTGCTTACTTTAT

>FM890862.1 FM890862 Jatropha curcas embryo 56-70 (DAF) Jatropha curcas cDNA clone rjcaeb0_000817, mRNA sequence

ATTCCCNGGAATTTANCAAAGATGAAGACTATATTGTCATCGGAGACTATGGATATACCCGATGGCGTTA

AAATCAAAGTTAACGCCAAGGTTATAGAGGTAGAAGGGCCTAGAGGCAAGCTCACTCGCAACTTCAAGCA

CTTGAATCTTGATTTTCAGCTCATCAAAGATGAGGCCACTGGGCAACGGAAACTCAAGATCGAAGCTTGG

TTTGGATCCAGGAAGACCAGCGCCTCCATTCGCACCGCCCTCAGCCATGTTGAGAATTTGATCACAGGTG

TCACCAAGGGCTATCGCTATAAGATGCGATTTGTCTATGCTCACTTCCCTATCAACGCCTCCATTACCAA

TTCCAACACAGCTATTGAGATCCGCAACTTCCTTGGCGAGAAGAAGGTCAGGAAAGTTGACATGCTGGAA

GGTGTCACTGTTGTTCGGTCAGAGAAGGTTAAGGATGAGTTGGTATTGGATGGCAATGACATAGAGCTCG

TGTCACGTTCTTGTGCCCTCATAAATCAGAAATGCCATGTGAAGAAGAAAGATATTAGGAAATTCCTTGA

TGGCATCTACGTCAGCGAGAAGGGCACTGTAGTTGAAGAAAATTGAAAGCAGTAGTTCTACTTATCTTTA

TTATTGCTTTATTCCGGGGTTTATAGTTTTTGTTATCTGAA

>FM890861.1 FM890861 Jatropha curcas embryo 56-70 (DAF) Jatropha curcas cDNA clone rjcaeb0_000816, mRNA sequence

AAAAGAAGTGAAAAGGATTGTATAGTAGTCTAAATAACAACGATAATATGTCAAAATATCTGGACTACCG

GACCAAAGACAAACTTCATAAAAACAAACCATTCAATAAGAAAGAAAATGCAAAATTCATCATAAAATTC

CTCTGTAATTATAACTTCTCTCCACTAAAACACTAGAAGATTAGGACATTCTTTGTGCAGCCTCCTTCGC

TAAGACTCTCTCCTTAGCCTTGGTCTTGGCTTGAGATTTCGAGCCCATAATACCACCACCCCACCTCTTG

CGGTGCTCATCGAACTTGTCATTAAAGTTGGCCTTAACTGCCTCCAAAATCTTGCTAAATTCCAGCTTAT

CTTCATTCTTCACTGATGTAAGGCATAAAGCTGCAGCTGTTTTCTTGTGCACAATCGCTCCAAGTCGTGA

CTTGCCCTTCACAATTGCATAAGGGACCTCCATTTTGCGGCACAGGGCAGGCAACCAAACAACAAGCTCG

ATTGGATCAACATCA

>FM890860.1 FM890860 Jatropha curcas embryo 56-70 (DAF) Jatropha curcas cDNA clone rjcaeb0_000815, mRNA sequence

GGCAGCACAAGTAAAATTTATTAATTCCTAAATGTCTCTGCCCTACGGGAGACGGTGTTCTTAACTGACA

CTAAACAGTCATATTCATATCAAAATTGAACAATACAGTAAAACAAAAATACATAAAAACAATACAAACA

CGAACATCCTAATTTTCCAGCTAGCAAGTCCCATGGAGCCACTGGCCTAAGTACACCAAGTGCCAAGTGA

GAGCCATCAAAAGGGGGGGCCCGGTAC

>FM890859.1 FM890859 Jatropha curcas embryo 56-70 (DAF) Jatropha curcas cDNA clone rjcaeb0_000814, mRNA sequence

ATTGATTTTTGGCAGCTTACGAAAGTAGGACACATTCTTTGAATTCTTTGTGTAGTGTTTGGGTGGATGT

GATGCTGGTCACAAGAGGGCTGCGCTTTGCTCTCCAATTCTTCAAGCATCACTGGTTAATTTTGTGCAGA

TTGGAAGGAGTCAAGTTCATTTTGGAAGATCAGGTGTAGCTCTCAAGATAGCCAGGTCTCTAAACCTGAA

TGCTTCCGCACTGTAAGCATTTTGTTTGTTTTATTAGCCTTGTTACCACATTCTAGATTTTTGTTCCATT

TGTTCATATTGGAATTGTTTTTCTCATGTGTGATTTCCTTAATATCTATGATATTGATATAATGAAGCAT

ATTTTTGTTTTT

>FM890858.1 FM890858 Jatropha curcas embryo 56-70 (DAF) Jatropha curcas cDNA clone rjcaeb0_000813, mRNA sequence

ATTCGGAAGAGAGAGCAGCCGCCGTTGCTGAAGCTCAGAGGAAGAAAGCGAAATGGCGAGAATCAAGGTC

CACGAGCTTAGACAGAAATCCAAGACGGAGCTTTTGAATCAATTGAAGGATCTCAAAGCTGAGCTCGCTC

TCCTACGCGTCGCTAAGGTCACCGGCGGTGCTCCTAACAAGCTTTCCAAGATCAAGGTGGTGAGGTTGTC

AATTGCACAGGTCTTGACTGTGATTTCACAGAAGCAGAAGGCTGCATTGAGGGACGCATACAAGAACAAG

AAGTTCTTGCCACTTGATCTGCGTCCGAAGAAGACAAGAGCCATCCGCAGGAGGCTTACCAAACACCAGC

AATCGTTGAAGACAGAACGTGAGAAGAAGAGAGAAATGTACTTCCCCATGAGAAAGTATGCTATCAAGGT

GTAGGGCAATTTTTGCTAAATAGATTGGTTTTGGTATCTTGCAATTATAAACCCATTTGGTTGTCAAGTT

TTGTCTTGGATTATCTTTTTGATGTGCTTGTGAACTAGAGTTGGCTTTGTTGAATCTTATGTCTGTAATC

TTGGAAGGGTGTATCTCACTCTAACCGGGTGCTAGATTTACTTGGAATTATAGCTGCAGAAACTTCTTTT

CATTTTTCTTCAC

>FM890857.1 FM890857 Jatropha curcas embryo 56-70 (DAF) Jatropha curcas cDNA clone rjcaeb0_000812, mRNA sequence

ATTCCGATAAGATCGATGTCAATGGAAACAATGCTGCTGCTCTTTACAAGTTCTTGAAGTCCAGCAAAGG

AGGAATTTTTGGGGATAGCATCAAGTGGAATTTCACCAAGTTTCTGGCTGATAAAGATGGCAATGTTGTG

AACCGATATGCTCCCACTACTTCTCCTCTTAGCATCGAGAAAGATGTGAAGAAACTGCTGGGGATTGCAT

GAATGCTAAGGCGGGCTATTGAATAAGTCAGGACATCAGTCTGTCTCTGTGATGAATAAGCAGCATGAAG

AGATGTAACTATATTTGTATGGCGTATTTCCCTTGTAGAATGCTTAAGACACATGAATGTTGATGATAGT

GTATTTTCTTGTTGTATGCTCCATGAGTTCTTTTCTAGCAGAAATCTCAGCACTTTGATGCTTATATTTA

AAAGATTGAGCAGGCAGTCCATTTCATTTCCTACACTGAATGGAGGCCTTGAAAAATAAAATACTTTCTT

GAAAAATAAAATGGCGATTAGCT

>FM890856.1 FM890856 Jatropha curcas embryo 56-70 (DAF) Jatropha curcas cDNA clone rjcaeb0_000811, mRNA sequence

ATTCCAATATGGTGACAATCATGAGGATACAACTGTAATGGGATTTGAAGTTCCAAAATCTCCTGATTCG

AGTTACAACAATACCTACCCTGGTAATGAAGATGAGGCACGGGACCCACCTGTAGTTCCTCCACACCTGC

AACAAACCTTGCTTAGCTATCCAGCAAGTGCAAACACTTTTGAAACTATCCCCTCGCCACAGAATGTAAT

TCTCAACCACCTTTACATCGAGAATCGAGAAGTCCCACGTTCTGTGGTGGCGCTTGGTTTTACTCATCGT

TTCTGTTCAAAATATGTCACCACTGTGCTATACAAACCTGTTCAGAGGAGGGGGAGTACCTGCACTTAAT

AATATGCATATAGTAGAAAATTTAATTAGAGTTAGTCTCATTTCACCGATTGTGATGGAATGACTTGAGA

TTGACTGAAATACAGAAATTGGCTAGACAGAATATTTATTGTCTCAAGCAGTTAATGGAATTAGGTTTGT

TTTATT

>FM890855.1 FM890855 Jatropha curcas embryo 56-70 (DAF) Jatropha curcas cDNA clone rjcaeb0_000810, mRNA sequence

ATTCCGTGCCTCGAGAGAACGTGGCTCTTGTACAATCTTTTTTGCGGAATGGACTCGAGATTTGTTGCTG

AAGCTTTCAATATCGACGAATCGTTGGCTAGGAAACTCCAAAGCGAGAATGACAACAGGGGCAATATTAT

CAGTGTTGAAGATGGTGAGCTTCAAGTGGTTAGACCACCAAGAACTCAACAAGAGGCGCAACAGCAAGAA

GAGAGAGAAGGATGCCCCTCCGGACGCGACAATGGTCTTGAAGAGACCATTTGCAGCATGAGAATTGGAG

AGAACATTGCTGATCCCTCGCGTGCTGACGTTTATGTACCAGAAGCTGGTCGTGTTAGCACCGTTAATAG

CCATAACCTCCCTATTCTTCGGTGGCTACAACTCAGCGCTTCCTATGGCGTTCTCCGCAATGATGCCATG

AAATTACCCCACTGGCACATTAACTCTCACAGCGTAATTTACGCACTCCAAGGACCAGCGCGGGTTCAGG

TGACGGATGAAAACGGCAACACCGTCTTCGACGGAAG

>FM890854.1 FM890854 Jatropha curcas embryo 56-70 (DAF) Jatropha curcas cDNA clone rjcaeb0_000809, mRNA sequence

ATTCGTTTGTGCTGCTGATTATCTTTCGAAGCGAAGCTTAATGGCTATGCCTGCAAAAAAGACATAGTAG

TTGATGATTTCGTCTCTGACGTTATAGCTAAGCCGGGATCCCCAGCGAACAGCTATGGCTCCGTCGCCAC

TGCGGCAAATGTTCTAAAAGTTCCCGGTCTTAACACTCTCGGTATCTCCATGTCTCGCATTGACTACGCT

CCTGGCGGCCTTAATCCACCGCACCTCCACCCACGCGCTTCCGAGATCATTTTTGTCCTCGAAGGTGAGC

TTGACGTCGGTTTCCTCACCACCAACAACACTTTGATCGCAAAAACTGTTAAAAGGGGAGAGATATTTAT

TTTTCCAAAAGCTTTAGTGCATTACCAGAAAAACAATGCCAATGCACCAGCCTCCGTTCTCGCCGCCTTC

AACAGCCAGCTTCCGGGGACTCAGGTCATGGCCACCACATTATTCGCCGCCAAACCGGAAGTTCCCGATC

GTATATTGAGCGAGACATTCCACGTGGGTTCTAAGGAAATTCAGAAGATAAAGTCTAGATTTGCACCAAA

GCAATAAATCAAATCATTCAATTTTATCTTTTAATATGTAATTGTTATTTTATTGGGTATGTTTTTCATA

ATATTTGACA

>FM890853.1 FM890853 Jatropha curcas embryo 56-70 (DAF) Jatropha curcas cDNA clone rjcaeb0_000808, mRNA sequence

ATTCCAGGTTTTAACGACCAGAGGCGTAGACAGGGAGATAGAGTGTAGGGAGAGAGCGAGCGAGCGTGAA

AAGAGTTTCGTATTAAAATGCCGAAGAACAAGGGAAAGGGAGGCAAGAACCGCAAGAGAGGAAAAAATGA

GGCAGACGACGAGAAGCGAGAGCTAGTTTTCAAGGAAGACGGTCAGGAATACGCTCAGGTCCTCCGTATG

CTAGGAAATGGCCGGTGCGAAGCCATGTGTATTGATGGTTCCAAGCGGCTCTGCCACATCCGAGGGAAGA

TGCACAAAAAGGTCTGGATCGCCGCCGGGGATATTATCCTTGTTGGCCTTAGGGATTACCAGGATGATAA

GGCTGACGTCATCCTCAAATACATGGCTGATGAGGCCAGACTATTGAAAGCTTACAGTGAATTACCTGAT

AATATGAGATTGAACGAAGTGATGGCTGGTGGTATTGACGACGAGGATGATATTGCTGGCGATGATTACG

TTGAATTTGAGGACGAAGATATTGATAAGATATAAATTCCCGTACTGTATTTCAATATAAAAGGTTTTTT

TTTTCCTTTGAAATTCCGGTTTGAGCTTTTTACTATCGTGTGTAGTATTAGCCATTAGGATGTGAATTGT

TAAGGATAGCCATTCTTGTGCTGATAGTGAGCTAATAACAAAATCAAGAATTACTTGATAAACAGAGAGA

GAGAGAGAGAG

>FM890852.1 FM890852 Jatropha curcas embryo 56-70 (DAF) Jatropha curcas cDNA clone rjcaeb0_000807, mRNA sequence

AAATAAAAATGAGATGGATATAGTAACATAAATAGCAAAAGTTTGTGGCTCCAAAATCCGCAAAACACAA

TACAACAATTCAGAACGACACAAAAGACAGTAACTTGAGAACTTAGGTCACAAAACTGCCAGATAACTGG

AACTGAAACCAAAAACAACAGGAAGACATTACTTAAAACAAAACATAACTAAACTAATAACTGTAACATC

ATTCACCCAATGCTTTACTTTTTCTTTGCTGCGGATTTGGTGACCTTAGCACCACTTGGGTCCTTCTTCT

CAACACTCTTGATGACACCAACAGCAACTGTCTGGCGCATGTCCCTCACAGCAAAACGACCCAGGGGAGG

GTACTCAGAAAAGGTCTCCACAACCATGGGCTTGGTGGGAATCATCTTCACAAATCCAGCATCACCATTC

TTCAAGAATTTTGGCTCCTTCTCAAGCTCTTTACCAGATCGCCTATCAATCTTGGTTAGGATCTCAGCAA

ACTTAACAGCAATGTGGGAGGTGTGG

>FM890851.1 FM890851 Jatropha curcas embryo 56-70 (DAF) Jatropha curcas cDNA clone rjcaeb0_000805, mRNA sequence

GTTTGGTGTTTGCTTACTACAAGGGATGGCGCCACTGACCCAACATTTTTGTACTTCGCTTATGCTTTGA

AGGAGATCAAGTGCTAAGAATGGAGAATAGCTGGCTTCGAAGTGGAAGTTCTTGCCATATTTCTAGCTTT

ATGTGTCCTTTGCCATCTCTGTACTGTTATATTTGGTGCTTCATGCCTTATTTTGGTTAATTATTGACTA

GACATGTTTTCCTCCTTCTTTGGTTATTAGTTTCTTGTTTGACAAATAATTCAATCTTCTATTGGGCGTG

TATTCT

>FM890850.1 FM890850 Jatropha curcas embryo 56-70 (DAF) Jatropha curcas cDNA clone rjcaeb0_000804, mRNA sequence

GTGGATCCCCGGGCTGCAGGATTCGAATTCCGCAAAACCGGCACTTAAACTATACCAAAAGGGAATTAGA

GAAAGAGTAAGACAAAAGATAAACGGTGGAAAGGTGATCAAGGTGGAAGCGCATCCAATACCGGAGCACC

CACGTCCTGTGAGAGTACGAGGACGGGTGGCACTGGTGGGAGACGCGGCGGGGTACGTGACGAAGTGCTC

CGTGCGAAGGGATATATTTTGCGGCGAAATCAGGAAGAATGTGCGGCGAGGCGATAGTGAAAGCATCAGA

AAGAGGAGAGAAAAGGGTGAAAGATGAAGATTTGAAAAGAGGGTATTTGAAAGAGTGGGATAAGAAATAT

TACAGTACTTTTAGGTTTTTGGATTTGTTGCANAGGGTTTTCTATGGCTCTAATATGGCTAAAGAAGCCT

TGGTTGAGCTTTGTGGAGATGAGTATGTGCAGAGAATGACATTTGATCGCTATTTGTATAA

>FM890849.1 FM890849 Jatropha curcas embryo 56-70 (DAF) Jatropha curcas cDNA clone rjcaeb0_000803, mRNA sequence

ATTCCAATTCTCACCCTTTCTCTCTCTCTCTCTCTCTCTCTCTCTCTCTATTTATCTTTTCCTTCTTTTC

TCTCTCCCTTACAAGCCCTAACCCTAACTCAGACCCAGATTCGCCATGAAAGGAGGTAAATCAAAGTCAG

ACACCAAGAACGCCAAGCTTTCCGTGAACAAGAAACCCGCCAAGGCAAGTAAGAAGGCGGGTAAGGCAGC

GAAGGATCCAAATAAGCCAAAGAGGCCCGCCAGTGCTTTCTTCGTTTTCATGGAAGAGTTCAGAGAGCAG

TACAAGAAGGAACATCCTAAAAACAAATCCGTTGCTGCTGTTGGCAAAGCTGGTGGAGATAAATGGAAGT

CAATGTCAGATGCTGAGAAAGCACCTTTTGTATCTAAGGCAGAAAAGAGGAAGATAGAATATGAGAAGAA

ATTGAAAGCCTACAACAAGGGACAGGCTGAAGGCCCCAAGGAAGAAGAGGAATCTGAGAAGTCAATGTCT

GAGGTGAATGATGAGGAAGAAGATGATGAGGAGGGAAGTGGAGAGGAGGACGATGATGAGTAGGAAGAGG

GGGAGAGGAGTAGCATAGTCATTTAGGCTGTGAC

>FM890848.1 FM890848 Jatropha curcas embryo 56-70 (DAF) Jatropha curcas cDNA clone rjcaeb0_000802, mRNA sequence

ATTTTTGACACACGCATGCTTTAGGAGTTGATATGCTGGATATTCAACATGCAATTTGATACAAGAAAGC

ATGAGTTATATTAACACGCTATCTGATATAAGAAGGCATACGATGAAATCTACAAGCATAGTGACAGCAA

CTAGAATGAGACCCGGATACCTAGCAATGCCATCAATTGAGAGATGAACTGTGGATGCCCTGGCCATGCT

GCTCCAGTAACCAAATTTCCATCAGTGTAGCATCGATCTATCGGATCAGGTTCTAGCCATGTTGCCCCGG

ACAAGACAACATTAAGCTTCACCCTAGGATATGCAGTACATTTTCTTCCCTTCAGAACACCAGCAGCAGC

TAAGATCTGCTGTCCATGGGCAATGGATGCCACAGGCTTCTTAGATCCCCTGAATTCTTTC

>FM890847.1 FM890847 Jatropha curcas embryo 56-70 (DAF) Jatropha curcas cDNA clone rjcaeb0_000796, mRNA sequence

CGAATTCCTTCAACCGATCTGTCATTTGTTTCATATTTTCTCGCACTTCCTCGGTCGTATTCTCGTTCTC

TCCTTCTCTAATCAATGGACGCGTTCGCGTCGTTCTTCGACTCCCAATCGGCTTCAAGGAGTAGCTGGAC

CTACGATTCGCTGAAGAACTTCCGCCAGATCTCTCCTGTCGTCCAAACTCACCTTAAGCAGGTCTATTTG

ACCTTATGTTGCGCACTGGTTGCATCGGCTGCTGGGGCTTACTTGCACATTCTGTGGAATATTGGTGGTC

TTCTGACAACTTTTGCGTGCTTGGGATGTATGGCTTGGCTACTTTCTGTACCTCCTTATGAAGAGCAAAA

GAGAGTCTCTCTTTTGATGGCAGCTGCACTTCTTGAAGGGGCTTCAGTTGGTCCTTTGATTGATTTGGCC

ATTGAGATCGATCCAAGTGTTCTGATCACAGCATTTGTGGGAACTTCGGTGGCCTTTGGTTGTTTCTCAG

CGGCAGCCACGTTGGCAAGGCGTAGAGAATACCTATATCTTGGTGGCTTGCTTTCATCCTGGCTTGTCCA

TCCTTCTCTGGTTGCAG

>FM890846.1 FM890846 Jatropha curcas embryo 56-70 (DAF) Jatropha curcas cDNA clone rjcaeb0_000795, mRNA sequence

CATCCTATTCCCACCAAGAAGCGCAGAAGAAGAAAGACGAGAAAGAGAGGAAGAGACCCAGAAACAATTA

GAGGGAACAGGTTACAGACCAGGTGGCGGCCAAGGAAGATTCGTTAATGGTGTGGAAGAAACTTTCTGCA

CTGCTAGACTCGTACACAACATCAATGACCCGTCACGTGCCGATGTATTCAACCCACGTGCTGGCCGTCT

CACCAACATCAACGGCCAAGACCTCCCCATCCTCAGTTATATCCAGCTAAGCGTTCAGAAAGGTGTCCTC

TACAGAAATGCTCTAATGTCACCACACTGGAACGTCGATGCCCATAGCATAAACTACTTCACAAGAGGAA

ATGGCCATATCCAGATCGTAGATGATAATGGAGAGACAGTATTTGATGGAGAAGTTCAAGCAGGACAAAT

ATTAACATCTCCACAAAACTTTGTGGTAGTAAAGAGGGCAGGAAGCGAAGGTCTTGAATGGGTATCCTTC

AAGACTAATGACAATGCCAAGATTGACCAACTAGCAGGACGTGTCTCGGCAATCAGATCAATTCCAGAAG

AGGTGTTGGCAAACGTATTCCAGGTTTCAAGGGAGGACGCGAGGAGACTGAAA

>FM890845.1 FM890845 Jatropha curcas embryo 56-70 (DAF) Jatropha curcas cDNA clone rjcaeb0_000794, mRNA sequence

GATAACTAAATTTCCAGCACTGAATTCCAAAAGAGTCGATACAGGGCAACACTAAAATCCTAAACGAGTG

CAAGCCGCAACTCAGATAACTGTGAAACGATAAGAAAGAATAAACACAGCAAAACCCCCCGCACAAAATG

ATCAATAGAAAACTCAGGACAAAGGGCAACAATACAACCCAGAAATAATTCAGTTCATACTCATACAGTC

ATGGACATAAAATCACATCCTCAAAAACCATATAAATAAAGCCCCTTCCATTACAATCCAACATCTATTG

TTTACTCAAATGTGTCATCATCGTCATCTGGAAGAGGTTGACTGGCAGCAGCAGCAAGCTCAGCCTCATG

CTGTTGCTGAGCTGCAAGGTCAATTTGAACTTCCGGAGGTGCCAAGGCAGGAGACTCGACAAAATGCAAG

TTAGGATCCCCAGCAAGTATCCTAGCAAGATAAAGAAAAGGCTTCTCGAAATTATAATTGCTCTTTGCTG

AAATTTCATAGTATTGCAGGTTCTTTTTCCTGTGACATGTAACCTGCTTTGCCTTCACTTGCCTATTTTT

CACATCAACCTTGTTTCCAC

>FM890844.1 FM890844 Jatropha curcas embryo 56-70 (DAF) Jatropha curcas cDNA clone rjcaeb0_000793, mRNA sequence

ATTCCTTCTAACACTAGTCAGCGAAAATGCAGGAGGGAAGCTGAAAGGGCAGATTTGAGCTCCTGTGAGA

GTTATATGAGATCGTCAAGAAGGCCGTCAGAAGAGATGCTAGCTTTGCGAGGAATTGAAAACCAGCAACA

ACGGGATCTTCGCCAATGTTGTAACGAATTAAGGCAGCTAAGTCATGATTGTCCATGTGAAGGAATAAAG

TATGTATTAGAGCAGCAGCTGGAGCAAGGACAGCGAGAGCGTTTGGAGGCAGTGCGTAGGGCTAGAAGTA

TACCTTCTGCTTGTGGCCTGCGTACAAGCTGCGATATTCGTTCCTAATCTGCTAGCTATAACGTGGATGT

GCTTCAGTTAAATAAAAATTTCACTCACTTGGTGTGTGAGTGATCAGAGTTATGTATAATAATGCACAAA

AGAATGAGCACATCAAGCTAAGTTTGTTTTCTGTTAAAAAAAAAAAAAAA

>FM890843.1 FM890843 Jatropha curcas embryo 56-70 (DAF) Jatropha curcas cDNA clone rjcaeb0_000792, mRNA sequence

ATTCCACCAATCGAGATGGCATTGACTTCTCAGATGTCCAAAGTATGCAAGCTGTTCAGGAGTGGGATTT

GGTTGAAAATTTTCAAGGAGTTTTAGAATACCAAACAAGATATGCCAAATTTCAAAGTGTGGCAAGTATC

ACATTGCATTTCCCTGATAATTTCGGTGGAGACACAAGTCGGATACATTATATTGGCTTTAAAGGTGAAG

CCACCCAGTTGAAGAGGGATGTTGTTGCAACAATAGTTTATGAACTTATGCCCAATCCTTCAGACCACAA

GACAAAGGCGGAAACGGGTGGAGGTTTTTCACAGGTGGAATGAGAAGCAAGTGATTTAGGATGATTCTTC

ACTAGGATTGGATCAAACCTTTTGTACATTGATAGTGTTTCAAACCCCTTATTCCCCTCCTCTCCTTTTC

CCCTGTTCTCGTAATGCACATTTTGATCTGATTGGGCGATCAAATCTAGCAGTACATTCTCTGGGGGGGC

CCGGTAC

>FM890842.1 FM890842 Jatropha curcas embryo 56-70 (DAF) Jatropha curcas cDNA clone rjcaeb0_000791, mRNA sequence

GATGGAGTTAAAACTATTCTATCGTCAAGCAAAACCACCCATAAAACACTGATTCTCCACCCAATATTAT

ACAAACTCATTAGAAGCATGGAGTATACACTCCACATTCTCAATCACACAACTTTCAAACACCATCATCC

TGGCAAAACAATTACAAAGTGTGTCTTTCTAGATCAGATCAGCAACATTCATTGGCATTTCGTCGATCTG

GGTACTATAATATTGTTCTATATCTCGTAAAATCTTGATATCATCACTTTTGACGAAGTTTATTGCAACA

CCCTTCCGCCCAAATCGACCCGAACGACCAATCCGATGAATGTACAGCTCTCGATTGTTTGGTAAGTCAT

AATTGATCACCAAAGAAACCTGTTGAACATCAAGCCCACGTGCCCAAACATCAGTGGTGATCAGAACACG

GGTAGTACCGGACCGGAACTCTCCCATAATTGCATCTCTCTCTCTCGGGGGGGCCCGGTAC

>FM890841.1 FM890841 Jatropha curcas embryo 56-70 (DAF) Jatropha curcas cDNA clone rjcaeb0_000790, mRNA sequence

ATTCACACGGTTTTGTACTCCAAGTACGCAGGGAATGACTTCAAGGGAAGTGATGGTACCAACTACATTG

CACTGAGAGCTTCTGATGTGATGGCAGTGCTCTCTTAGTTGTCTGTTCTTGATGTTTTTTGCTTTTATCA

CTGAATTTTGTGGTTGAGTTGCCTCAAGGCTTATGGATTATAAATCTAGCATTCTAGTATAAAAAAAAAA

AAAAAAAAAAACTCGGGGAGAGAGAACTAGTCTCGAGTTTTTTTTTTTTTTTTTTCTACTAAAAGATACC

TTTTATTCAACTGGAATTGCTTAACAAGAAAAAGACACCAAACAGAAAACATAGATATATTACATAACAG

GAGCTAAAGCACATGCCATGCCCTACACACGTGGGTTATGGCCAGTGGTGTCTCCGGGCAACCCAGATGG

GCGAGTCCTGGTCAAACCCGTTGGGTCCACCTCTCCACCGTATGGCTGCCCTGTGCCGTGTACAGGCATG

GCCGACATTTGCTGGGTCCCTGTAGGGTAACCGGTTGTCCCAGTTGTTGAATGGGTATAGGTTACGGTTG

TGCCTTGAAGCGCCCGGAC

>FM890840.1 FM890840 Jatropha curcas embryo 56-70 (DAF) Jatropha curcas cDNA clone rjcaeb0_000789, mRNA sequence

TTCACAAGAACAAGGACATATAGAAAGATTAAAAGGGTAGCAAGTATTACATGAAATATGACCAGTTTAT

CTCTTAAATTCAATTTATAAAGCACAAACCCTTCTCAGGCTCTTATAATACCATTACCAACTCTAGAGAA

CTCTCTCTTCATGACTAAACATAAAGTAGAAAGAAAGACAGTTCTCAAACTCTAGATCTTATCAATATCT

TCATCTTCAAACTCAACATAATCATCACCAGCACCATCATCTTCTTCATCAAGGCCTCCAGCAATACCCT

CATTGAGACGTGTGTTCTCAGGAAGTTCACCATAGGCCTTTAGAAGCCTAGCTTCATCTGGCATGTACTT

CAAGATAACATCAGCCTTGTCATCCTGATAGTCACGCAACCCAACCAGTATTATATCACCAGCCGCAATC

CAGACCTTCTTGTGCATCTTCCCGCGAATATGGCATAAACGCTTGGTACCATCGATACACATGGCTTCAC

ATCGGCCATTACCAAGCATACGAAGCACTTGGGCATACTCCTGTCCATCTTCTTTAAAGATGAGCTCACG

CTTCTCGTCGTCTGCTTCGTTCTTTCCTCTCTTCCTATTCTTTCCTCCCTTTCCCTTATTCTTCGGCATG

GTTTCTTATTCTTTGCGGTCTGTGGCGTAGAAGAAACATTCGAATTTAACAAAATATTACGCTTACAATT

A

>FM890839.1 FM890839 Jatropha curcas embryo 56-70 (DAF) Jatropha curcas cDNA clone rjcaeb0_000786, mRNA sequence

GAATTCCTCCATAATTTTTCTTGTGTTACTAGAAAATAAGCTGAGTGCTTGGCTGTTAGATACAGTTGCA

GTGTGGAAAACTTAGTGACTATTGTTTATGGATCGTTCTGGTTTTTAGCCAATGTTGAAAATTTCAATTT

GTCCTTGGCACTGGCTTGTTGTCTGAACTGCGAAATGTTGAACAAAGCTGGTGTTGAACTCTAAAAAAAA

AAAAAAAAAAAA

>FM890838.1 FM890838 Jatropha curcas embryo 56-70 (DAF) Jatropha curcas cDNA clone rjcaeb0_000785, mRNA sequence

TCTTTCTCACACTTATCATCAACAAATGGCTGAACGTTCACAGCCACACCAAGTTCAAGTGCACCCACAA

CACCGCTATGAAGCTGCCTTCAAAGGTCAACAAAAAGGTCCATCAGCTCAGAAAGTGTTAGCTGTGATCA

CCCTCCTGCCGGTAGGCGGTGGGCTTCTTGCACTAGCTGGTATAACCTTAGTAGGTACCCTAATCGGCCT

AGCTATTACTACCCCTCTTTTTGTCATCTTTAGCCCTGTTCTTGTCCCAGCTGCATTAGTTATTGGCCTT

TCGGTCATGGCATTTTTGGCTTCCGGGGCTATGGGGCTCACAGGGTTGTCATCACTCTCGTGGGTTCTGA

AATACCTCCAGGAGGTCACTCGGCGAATGCCGGAGCAGCTGGATATAGCTAAGAAGCGTATGCAAGACAT

GGCTGGTTTTGTGGGACAGAAGACCAAGGAAGTAGGCCAAGAAATCCAGAGGAAAGCACATGAAGGGAAA

TAAGTAGAGAGAAATGCTGTCGGTTATTAGGGGTTGAAGTCAGAGAGCTGGGTTTTGGTACGAGACTACT

TTTGTTGTATGGGTGTTGTTTTGAGGTAGTTGAGTTGGTTTATGGGGGATGCTCTTTGTTGCATTTGTAT

TTCTCTTTTTGGTCTTT

>FM890837.1 FM890837 Jatropha curcas embryo 56-70 (DAF) Jatropha curcas cDNA clone rjcaeb0_000784, mRNA sequence

ATTCCTTTANGCCTCCATAGATTCCGGCCATATTTTCAAGCCGATCAACTTCCACAGCTGCTCATGAAAT

CCTCTCTTCGCTGCCAAACTAAGCCTAGCCGGTTACCAACCATTATAGCTTGCCAAACAGACTCCAAACC

CACCGAAACCTCCACAAAGGAGAAGGTGCTTGCTGAGTCTGCTTCAGTAACCGATACTAAAACCGAAGGT

TCAACTTCGTCCCCCACCTCTGGATTGCCCGTTCCCAATAAAGATTTAAATAGGCAAATAGCGGCGGTTT

CTGTGCTTGCGGCGCTGGGGCTTTTCTTATCCGCGCGGCTGGATTTCGGTGTTTCTTTAAAGGACCTGTC

TGCTTATGCTGTACCATACGAAGAGGCTCTCTCCAATGGAAAGCCCACTGTTGTGGAGTTCTATGCGGAT

TGGTGTGAAGTGTGTAGAGAGTTAGCTCCAGATGTTTACAAAGTTGAACAGCAGTTCAAGGATCGTGTTA

ATTTTGTTATGCTGAATGTTGACAATACAAAATGGGAACAAGAACTTGATGAGTTTGGTGTTGAGGGTAT

TCCACATTTTGCATTCCTGGACAAAGATGGGAATGAAGAG

>FM890836.1 FM890836 Jatropha curcas embryo 56-70 (DAF) Jatropha curcas cDNA clone rjcaeb0_000783, mRNA sequence

ATTCAGAAAATACTCTTTCCAACCCTTCAACTTCAAATCAAGTATCCAGCTAGAATGGCTTTAAGCTTAG

GAAGGAGGAGCAACATCTTTGATCCGTTCTCACTAGATATTTGGGACCCATTTGAGGGCTTACCTTTCTA

TAGCGGAACAGTGGCCAATGTTCCGTCCACTCAACACGAAACAGCCGCCATTGCCAACGCACGAGTGGAC

TGGAGGGAGACTCCGGAGGCTCACATATTTGCTGTTGATCTTCCTGGGTTGAAAAAAGAAGAGGTAAAAG

TGGAGGTAGAAGATGGCAGAGTTTTGCAGATAAGTGGGGAGAGAAGCAAAGAACAAGAAGAGAAGAACGA

TAAGTGGCATCGCGTAGAAAGAAGCACTGGTAAGTTCTTGAGGAGGTTTAGGTTGCCGGAGAACACAATG

ATGGATCAAGTCAAGGCCACTATGGAGAACGGTGTGCTTANAGTGGTTGTGCCTAAAGAACAAGAGAAGC

AGCCTCAGATTAAGCCTATTGAGATCACTGGCTAGAAGTGCAAATATGGTCTTTTACTACTAATAGATAA

AGCTTTTA

>FM890835.1 FM890835 Jatropha curcas embryo 56-70 (DAF) Jatropha curcas cDNA clone rjcaeb0_000781, mRNA sequence

GGAGGAAGAAACCCTCAGTGATAGAATTAAACAGTTAATGTCAATGTCCTACTGAAGCAAAATACATGCC

AAGAAAACAGAGTATTACAACAGAAGTTCTCATCCAGGCAAAAGGTCCAACTGATTTCACATTCTATCTT

CTCTCCTCCGTGGGGCACAGGTAATAATTTCATCCACTCTGAGAATCATCTCAGCAGCCTCAGTTGCCGA

CAGTAGTATGGCCTGCTTAACTTTGTAAGCTTCAGAAATCCGGGGGGGCCCGGTAC

>FM890834.1 FM890834 Jatropha curcas embryo 56-70 (DAF) Jatropha curcas cDNA clone rjcaeb0_000780, mRNA sequence

TGCGAATGCACTAGGCTACAATCATTTGATTTCACTTTATGCAAGTTTGGGCAATAAAGATGAGATAATA

AGGTTATGGGGACTTTCAAAAGCCAAGTGTCAGAAGCAATTCAACAGAGATTATATAACCATGTTAGGTT

CTCTAGTCAAGCTTGGGGAGTTTGAAGAAGCAGAGAAATTGCTGCAAGAGTGGGAGTCATCTTGCGAAAC

TTATGATTCCCGAGTTCCAAATGTTCTCCTTATTGGGTATTGTCAAAAAGGATTAATTGAAAAGGCTGAG

GCAATGCTTCGAGATATAGGGAAAAGACAGAAGACTGTAACACCAAATAGTTGGTCTATTATGGCAGAAG

GATATGTGAATAAGCAAAATATGGAAAAAGCTTTTCAATGCATGAAGGAAGCTGTGAATGTAGCGTCAGA

AAATAAGGTGTGGAGACCTAAACCTAATTTGATTTCTGTCATATTGAGTTGGCTTGGTGATAACGGTGAT

GTTGAAGAGGTAGAAGCATTTGTGAGTTCATTGGAAACTGTGGTCCAGAAAAACAGGGAAATGTATCGTA

CGTTGATCAAAGCTTATATCAGAGGTGGGAAGGAGGTGGATGGGCTTTTGGAGAGCATGAAGGCTTGATA

AATTGATATAGAATGA

>FM890833.1 FM890833 Jatropha curcas embryo 56-70 (DAF) Jatropha curcas cDNA clone rjcaeb0_000779, mRNA sequence

ATTCGAAAAAGCTTATTACGATATCTATTTTAAGCTTCCATGGCCGAGTATGAGCGACGACCAGGCGGAG

AAGGGCACAAAGGAGCCCTAAAGGAGAAGGGCGGCTTCTCGACCTCACAGATTTTAGCGGTTGTCACTCT

TTTGCCGGTGAGTGGGACGCTTCTTTTCCTTGCTGGAATAACGCTTACTGGAACACTCATTGCGCTAGCT

GTTGCGACTCCGCTGTTCGTGATTTGTAGTCCGGTGCTGGTACCAGCGGCGTTGGTCATTGGGTTGTCGG

TGTTAGGGTTTTTGAC

>FM890832.1 FM890832 Jatropha curcas embryo 56-70 (DAF) Jatropha curcas cDNA clone rjcaeb0_000778, mRNA sequence

ATTCCCAAATCTAAGGCTTTCAGATCGAAAAATGGCATTGATTCCAAGCTTTTTCGGTAACTCACGAAGC

AACATCTTCGATCCTTTCTCTGCTCTCGACGTTTGGGATCCATTCAGAGACTTTTCTTTCCCTTCATCTT

CTTCCCTCGTCTCTCGTGAAAATTCTGCTTTTATTAACACTCGCATTGACTGGAGAGAGACACCAGAGGC

TCATATATTCAAGGCTGATCTCCCTGGGCTCAAAAAAGAGGAAGTGAAAGTAGAAATTGAAGATGACAGA

GTGCTTCAAATCAGCGGGGAGAGAAATGTGGAGAAGGAAGACAAGAACGACACGTGGCATCGCGTGGAGC

GTAGCAGTGGCAAGTTCATGAGGAGGTTCAGGCTGCCGGAGAATGCAAAGATGGATCAAGTAAAGGCTTC

AATGGAAAACGGAGTACTTACTGTCACTGTTCCTANAGAGGAAGTTCAGAAACCTGATGTTAAGGCTATT

GAGATCTCTGGTTAAGTTTCAGCTTATAATGATGTTATCTCAATATATATATATATATATATATATATAT

ATATATATATATATATATATATAAAGCTATGTGTGTG

>FM890831.1 FM890831 Jatropha curcas embryo 56-70 (DAF) Jatropha curcas cDNA clone rjcaeb0_000777, mRNA sequence

TTTTTATTATTTTTATATTTAGATAAATAGATGGATACATCTTATAAATAACACGGAAAGTACAAAGCAT

GGACTAAAAACACACAGACACAAAAACAGTAATAAGAAGAAAATTACAGAGAAGACGAGTAACTGAAGAA

GAAAGTAGTTTCTAAAAGTGGAAACAGAAACACCATCCTCTTCTCTCTTATTCTTATTCTCCTTAAGCTA

CCCTAACACCGGGCCGGAAAATCCATTGCTGCTCGATATGTAAACCTTACCTGACATTGTTGCAATTAGT

GGAGAGGCTGATCTTGTAAGGAATGTTAACTTTGCAAGCACCAGGCAGAGCCTCAGCGTTCCCGGGGATG

ATGCCAGAGACGCTTCCTGCTGCTTGTTTCAAGCACTGACATGCTTGTTGGCGGTCAGGGGTGGATTTAG

CGGCGGAGTTGATGCTTCTCACTCCGTTGCAGCAATAAGGCGGTGGAGTTAAGGCACCTCTTGTACTCAG

GTAAGTAATGCATGGAGCC

>FM890830.1 FM890830 Jatropha curcas embryo 56-70 (DAF) Jatropha curcas cDNA clone rjcaeb0_000776, mRNA sequence

ATTCCTTTTTCTCCCTTCCAAGAAAAAGCTTATTACGATATCTATTTTAAGCTTCCATGGCCGAGTATGA

GCGACGACCAGGCGGAGAAGGGCACAAAGGAGCCCTAAAGGAGAAGGGCGGCTTCTCGACCTCACAGATT

TTAGCGGTTGTCACTCTTTTGCCGGTGAGTGGGACGCTTCTTTTCCTTGCTGGAATAACGCTTACTGGAA

CACTCATTGCGCTAGCTGTTGCGACTCCGCTGTTCGTGATTTGTAGTCCGGTGCTGGTACCAGCGGCGTT

GGTCATTGGGTTGTCGGTGTTAGGGTTTTTGACGTCCGGTGCTTTTGGAATCACTGCGCTTTCTTCACTG

TCGTGGATGGTTAATTATATTCGCAGGATGAGAGGATCTTGGACGATGCAGATGGAAATGGCAAAGCGGC

GCGCGCAAGAAACGACGGGTCAATTGGGCCAGAAGGCTAGGGAGGTG

>FM890829.1 FM890829 Jatropha curcas embryo 56-70 (DAF) Jatropha curcas cDNA clone rjcaeb0_000775, mRNA sequence

ATTCCTCTTCAAAATCCTAAAGCCCAAAACACTAAAACAGAAGAAAATGTCTTGCTGCGGAGGAAACTGT

GGCTGCGGTTCTGGCTGCATGTGCAGCGGCTGCGGCGGGGGATATGGCATGTACCCTGACATTGCAGAGA

GCACCAGAACTGAGACCCTCATTGCAGGAGTTGCACCCTCCAAGAAGTTGAATGAGAGTTCTGAGATGAG

CATTGGAGCTGAGGGTGGTCATGGCTGCAAGTGTGGATCAAACTGCAGCTGTGACCCATGCAACTGCTAA

TGAGAAACCAGAAGGGAAAGGCTCCAACAAAGTAGTTATTAGTCTTGGGCTTTTATGGTTAATCAGTTGA

ATTATGTGTAATAATCCATGTTTGGTGTAAATAAAAGAAGCCATGGGTACCTTCTCAAACCCTGTCGTGT

GGGATTTGATTTCCTGTTGTTTCTTGTTTTATGTATTGTCTTGAACTTTTGCTTTGTTGGTCTTTAAGGA

GCCTTTTGTGTTATTTTGGAAGGGGAACTTCAATTATGATATTGCTATTAAATGGTGAATGTATCTCCTT

TCATT

>FM890828.1 FM890828 Jatropha curcas embryo 56-70 (DAF) Jatropha curcas cDNA clone rjcaeb0_000774, mRNA sequence

ATTCCGGCTATCGCGACGTGTGGTACCAATACTCAGGAATCTGTTTATCATCTAATGTCAGAGATATTGC

TAGATGATGATACGGTTCCTGAGGTTTTAGTTGATGAAGACTTAGATGTGAATGACTCTAAGTTTTACCT

TGAATTGGAGGATTTGACAGGAAAGCCAAGAAGTTGGGATGGATATGTAACTGAGTTGGTACAGCAAGCG

GGTTGTATTTAGTTCTTGATTGCACTGGATGTGAATTGTCAGGCAGGTGGCCCTTCCATTTGAGCTGCTC

CTAGCCCGCATTGGAAATGCTAGTGTTTGACAATGGTCGTACATGTTCATATTTCCTGAGTCGTTTTTTT

CTTTTTCTTTTTCTTTTTATTCTGTCACCAATGATGAATCATCTAGACTGTAAATATTCCCACACGCAGT

AAATACCATAGTTTTATCATCCTGCATCAAAAGGGGGGGCCCGGTAC

>FM890827.1 FM890827 Jatropha curcas embryo 56-70 (DAF) Jatropha curcas cDNA clone rjcaeb0_000773, mRNA sequence

ATTCATGGAAGGATCTGCCTAACAAGATCCATCACAAGGTGTCAGAGAACTGGATCAGCGCCACCCTCCT

TCTCACCCCTCTCATCGGCACCTACACGTATGTGCAGAACTACCAGGAGAAGGAAAAGTTGGAACACAGG

TTCTGAGGTCATCTTCCTTGTCGAAGGAGTGTCTTATTATTATTTTTTTTTTTTTGGTGGAAGACTCCAT

AATTTTTCTTGTGTTACTAGAAAATAAGCTGAGTGCTTGGCTGTTAGATACAGTTGCAGTGTGGAAAACT

TAGTGACTATTGTTTATGGATCGTTCTGGTTTTTAGCCAATGTTGAAAATTTCAATTTGTCCTTGGCAAA

AAAAAAAAAAAAAAAAAGAGAGAAAGCGAGCAAGGGGAAACCTACTATGTCATTTGCAACATCTAGGGTT

TTCAAGGGTTGCAGAGCCCTCTTAGCCCCAGCTAAGTCTGCTGCCGCTGCAAAAACTTCTAGTGCAAAAA

CCGCCGCTACCAAGGCGGCACCAGCTAAGCCCAAGACAAAACCGGAAAAACCACTTCAGCCCGCTGGAAT

ATTGAAGGTCACTCCTGTCTCCCCAGCTCTCCGCGACTTTCTGGGT

>FM890826.1 FM890826 Jatropha curcas embryo 56-70 (DAF) Jatropha curcas cDNA clone rjcaeb0_000772, mRNA sequence

ATTCTGATTACATTACTTTCATCACTTATTTTCATAATATATTACATACTTCATTAGCGTGAGATCATAA

CTCCCCTACGTAATACTACTTACTTTCACTATCTACACACCCAAAAAGGCTACTTCATTATTTACTCCTC

GGCATATCTTCCAGACTCGGTCCTGGAAATAGTCAAGGTGGTCTCTTGTCTTCCAAACTTAATCCTTCGG

GGGGGCCCGGTAC

>FM890825.1 FM890825 Jatropha curcas embryo 56-70 (DAF) Jatropha curcas cDNA clone rjcaeb0_000771, mRNA sequence

ATTCGGAAAGGCAAGAGAGATGAAGGACAGAGCTGAGCAGTTTGGACAGCATGTAACAGGTCAACAGACT

TAAAAGAGAAGTGGGTTTTGGTGGTTGATGCAGAGAACATAAAATGGCTTTGATATATATATAAAAATAT

ATGAGTCCTTTAATTAACATGGAAGGCCATTGCTTGTCTGGTGTATTTCTTTTTTCGCTTAAATCTTTTT

GTTTCTTCTATGTAATTTGTTAACGGGTCTTCCCTCTTTTGAGAGCTGTCTAAAATAATTTTCTCGTC

>FM890824.1 FM890824 Jatropha curcas embryo 56-70 (DAF) Jatropha curcas cDNA clone rjcaeb0_000770, mRNA sequence

ATTGGTTGATATTGCTAAGGCCTTGGCTAGACCTCCTTCCTACACCACTAAGTACTTTGGAAATGAGCTT

GGTGCTCAATCGAAATTTGATGAGAAGACTGGAATTTCTCTTGTTAATGGAGCTCATGACACTCCAAAGC

TTGCTGGTCTACTTGAGAACTTCATAAAGAAATATGTTCAATGCTATGGCTGTGGGAACCCTGAAACTGA

GATAGTTATCACTAAGACCCAGATGATCACCCTAAAATGTGCTGCATGTGGGTTTATTTCTGATGTTGAT

ATGAGAGATAAGCTTACAACATTTATCCTCAAGAACCCTCCTGAACTGAAAAAAGGATCCAAAGACAAGA

AGGCAATGAGGAGGGCTGAGAAGGAAAGACTCAAGGAGGGTGAGACTGCTGATGAGGAGCTGAAGAAGCA

TAAAAAGGAGGCAGTAAAGAAGAAAGGCTCCTCTACTGGTTCCAAGGAAGGTTCAAAAGTTACATCATCT

AAGAAGAGAGGCAATGGCTCAGATGAGGATCACTCCCCCACTTTAAGTGCGGCTGATGAAAATGATCAAG

TGAATGCTGATGATGAGGATGACGACGATGATGTGCAGTGGCAAACAGATACTTCTTTGGAGGCAGCCAA

ACAGCGCATAC

>FM890823.1 FM890823 Jatropha curcas embryo 56-70 (DAF) Jatropha curcas cDNA clone rjcaeb0_000769, mRNA sequence

GGAAGAAACAGGGGCTGGTCCCCAAACATCCGAATAAATTAGTTGAAGAGCCTCAGTGCTGTGCATTGTA

GACTCAGCAAAAGGTTGTTTGTGCATTTTATTGCACTTACAAGAGTCACAAAAATACTTATTATCAATAG

CAAAAGGCAATTTAATGGATGAACTAAGCAACTGTAAAATTGGAGTGGCAGGGTGTCCAAGCCTCTTGTG

CCAAACGGAGTGTGTAAGAGCCGTAGTGTTCGAAACTTCCGGTCGACACATGGAGGATGCATTTGAGGGC

CACATATACACACCATCACTTGGCTTTCCTTCCACCAGAATTTTCCCCGTGCATAAATCCGCTGCGCTAT

CTGGCTAT

>FM890822.1 FM890822 Jatropha curcas embryo 56-70 (DAF) Jatropha curcas cDNA clone rjcaeb0_000768, mRNA sequence

ATTCTTCCGCAATCGCTAATGTACAAGTTGATTGGAAGGAAACACCAGAAGCCCATATATTCAAAGCTGA

TCTTCCAGGATTAAAGAAAGAGGAAGTGAAAGTTGAAATTGAAGAAGGAAGAGTTCTTCAGATTAGCGGA

GAGAGAAATGTTGATAAGGAAGAGAAGAACGAGAAATGGCACCGCGTTGAGAGAAGCAGAGGTAAGTTCT

TGAGAAGGTTTTGGTTACCGGAAAATGCAAAGGTTGGTGAAGTTAAGGCATCAATGGAGAATGGAGTACT

TACTGTTACTATTCCAAAGGCAGAGGAGAAGAAGCCTCAAGTCAAGTCTATTGAGATTAGCGGTGCATGA

GCATGAGTTTCAATCTTCTTTAAGATATAAAAACAATAAATTGCTTTGTCGTTGATGTGTATGAAATGAG

ATTATGTGTGGTTTGCTCGCTCTGGTTTCTGTTTCCTTCCAATGTATAGGTATATGTTAATGTATTTGT

>FM890821.1 FM890821 Jatropha curcas embryo 56-70 (DAF) Jatropha curcas cDNA clone rjcaeb0_000767, mRNA sequence

ACTAAAAGATACCTTTTATTCAACTGGAATTGCTTAACAAGAAAAAGACACCAAACAGAAAACATAGATA

TATTACATAACAGGAGCTAAAGCACATGCCATGCCCTACACACGTGGGTTATGGCCAGTGGTGTCTCCGG

GCAACCCAGATGGGCGAGTCCTGGTCAAACCCGTTGGGTCCACCTCTCCACCGTATGGCTGCCCTGTGCC

GTGTCCAGGCATGGCCGACATTTGCTGGGTCCCTGTAGGGTAACCGGTTGTCCCAGTTGTTGAATGGGTA

TAGGTTTCGGTTGTGCCTTGAGCGCCCGGACCAGCAGTTGTGTACTGTACATGGCCACCAGCCTTGGCTG

CCTGCCTCGCCGCTGCGTTATCCTCGCGCGCTTCTAGTTTCCGCAATTCTGCCTCCGTTTTCCTCTCTTC

TTTCTTCTCTCTCGCCATTTCCTTCTGCACTG

>FM890820.1 FM890820 Jatropha curcas embryo 56-70 (DAF) Jatropha curcas cDNA clone rjcaeb0_000766, mRNA sequence

CCACATCAGCACTCGGTAGTATGCTATTAGTGTCAACCATAGAAATAGTAGAATTCACAAGAACAAGGAC

ATATAGAAAGATTAAAAGGGTAGCAAGTATTACATGAAATATGACCAGTTTATCTCTTAAATTCAATTTA

TAAAGCACAAACCCTTCTCAGGCTCTTATAATACCATTACCAACTCTAGAGAACTCTCTCTTCATGACTA

AACATAAAGTAGAAAGAAAGACAGTTCTCAAACTCTAGATCTTATCAATATCTTCATCTTCAAACTCAAC

ATAATCATCACCAGCACCATCATCTTCTTCATCAAGGCCTCCAGCAATACCCTCATTGAGACGTGTGTTC

TCAGGAAGTTCACCATAGGCCTTTAGAAGCCTAGCTTCATCTGGCATGTACTTCAAGATAACATCAGCCT

TGTCATCCTGATAGTCACGCAACCCAACCAGTATTATATCACCAGCCGCAATCCAGACCTTCTTGTGCAT

CTTCCCGCGAATATGGCATAAACGCTTGGTACCATCGATACACATGGCTTCACATCGGCCATTACCAAGC

ATACGAAGCACTTGGGCATACTCCTGTCCATCTTCTTTAAAGA

>FM890819.1 FM890819 Jatropha curcas embryo 56-70 (DAF) Jatropha curcas cDNA clone rjcaeb0_000765, mRNA sequence

ATTCCACAGAAGTATGCCATGGGTTGATACTGGTTGTCACTAATCGGGTGATGTTTATGGTGGAATGTGT

TGAAAACTGTTTTATTAACTTATTTACTTGGTTTGGTTCGATAGAGGCTAGGGATGGACTTGAGTTCTTC

AGTGGAATCTATATCTTGGGTTTGGCATGAAATAACTGTATGATCAAGATGCTTTGATGTAGACAATGTC

TGTTGTTTGGAGCTCTTACTCTTACC

>FM890818.1 FM890818 Jatropha curcas embryo 56-70 (DAF) Jatropha curcas cDNA clone rjcaeb0_000764, mRNA sequence

ATTCCGAAGTAGCGTCACTTGCAAGCGGCGAAACAAAGAAAAACAAAGCCGAGAGCCAAACAAGTGTAAA

GAAATCCAGAATCCGATTTCTAGATGGATCCAGACGCGGTAGCGAAGGCGTTTGTCGAGCACTACTACTC

CACGTTCGATGCGAACCGTGCTGGCTTGGCTAACCTCTACCAAGACGGATCGATGCTCACCTTTGAAGGC

GAGAAGATCCAAGGCTCTCCGAACATCGTCGCTAAGCTCACTAGCCTTCCTTTCAAACAGTGCCAGCATA

GAATCACAACCGTCGATTGTCAGCCATCTGGTCCTGCCGGTGGCATGCTTGTCTTTGTCTCTGGTAACCT

CCAGCTCGCCGGCGAACAACACGCTCTCAAGTTCAGTCAGATGTTCCATTTGATGCCAACACCACAGGGG

AGCTTTTACGTGTTGAATGATATTTTCCGGTTGAACTATGCCTGAAGACCATATCAAACTCAAATAGTCA

CTGTGGCCC

>FM890817.1 FM890817 Jatropha curcas embryo 56-70 (DAF) Jatropha curcas cDNA clone rjcaeb0_000763, mRNA sequence

ATTCGGCAGTTAAAGCCTCATCTTCTTCCTGTGATAGGATTTTTCTGTTTTGGGAGAAGGATATTATCAG

AAACCCTAGAACCATGGGAGTATTCACATTTGTTTGCCGTCAATCCGGTGGCGAGTGGTCCGCTAAGCAG

ATCGCTGGTGGTGATCTCGAGGCTTCCGCTTCTTCCACCTATGATCTGCAGAGAAAGCTTGTACAGACCG

CTCTCTCTTCCGACTCCTCCGGCGGTGTCCAGTCTTCTTTCTCTACAATCACCCCTTCTTCGGCCGTTTT

TCAGGTGATAATTGGTGGTGGCGGCGGCGGAGCTTTTATTGGTGGAGGAGCTGCTGCTGCAGCTCCAGCT

GGAGGCGCAGCACCAGCAGCTGAAGCACCTGCTGCTGAGGAAAAGAAGAAGGAAGAACCAGCGGAAGAGA

GTGATGATGATATGGGATTCTCTCTCTTTGATTAGAGAATATGCTAAGTTACTCTTTTTTTATGTGCTTT

ATTTTGTCTCTCTGTTTTG

>FM890816.1 FM890816 Jatropha curcas embryo 56-70 (DAF) Jatropha curcas cDNA clone rjcaeb0_000761, mRNA sequence

AGCACAAATCAATTTCATATTTGTATAAACTCCTAATACAATGTTGCATTTGCATACAAGAGGTAATTTC

CTAAAATTGAATAAGGTAGTAGATGCTAATACTTTCGGTTTCGCAAAATAGTCGTAACGTCAATTTCTAA

TTTCTAACTCAATAATAGTCCAGGTCTGTCAACATTTCACATTTGTGACAGACTGAACAGTATAAAGTAT

ACAACTCCAAGATTCCAGTGGCTGCATTGGGTGTACAAGTACAAGGAACCAGTAGGCAATCTTCCTTTCA

CGTAAAACTCATCTAGTGCTAGAATTTTTCTTCTGTGCTTCTTTGACGGGGGGGCCCGGTAC

>FM890815.1 FM890815 Jatropha curcas embryo 56-70 (DAF) Jatropha curcas cDNA clone rjcaeb0_000760, mRNA sequence

ATTCCAAGAGGAAATGGTCACGTCCAAATCGTAGATGAGAATGGAGATGCAGTGTTCGATGGACAAGTTC

AACAAGGTCAAATATTAACAGCTCCACAGAACTTTGTGGTTTTGAAGAGGGCAAGCAATGAAGGGCTAGA

GTGGGTATCCTTCAAGACTAACGAAAATGCACAAATTAGCCCACTAGCAGGACGGACCTCGGCAATTAGG

GCTCTGCCAGAGGCAGTGGTTGCAAACGCATTCCAGATTTCCAGAGAAGATGCCAGGAGGCTTAAGGAGA

ATAGACAGGAAGTCACTGTGTTTAGCCCTGGGTCTAGATCTCGTAATTAATATTGCAACTATGCAATTAT

ACCAAGAGGATGTATAATAAACTGGTTTCTACATAGTTTAGATTTGGAGAAGAATAAGCCTATTTTTATA

GGCAGTTTTAGTTCCTTGCAATAATATAAAAATGTAGATCTGTATATGCCTTATGTTATGAGATAATGAA

AGTTAGTGGAGCTATAAAAAAAAAAA

>FM890814.1 FM890814 Jatropha curcas embryo 56-70 (DAF) Jatropha curcas cDNA clone rjcaeb0_000757, mRNA sequence

CCCAAAACAAGCTTGGTACTTATAATTTGAAGCTTACAAAAACACTACCATANAGGGCATCAAAACAAAT

CTTCCGCTTGCACTGCTACATAAGTAACACATAAATGGCAAAAATGCATGAAGACCTTTAAGCAAATCCA

GAAGAACTAGTTTGAGCTGCCCCACCGAGAAATATGAGGATGATTACGCGAGGGAGACTCCATATGACAA

ATACAGTTACTAAACTCACAAGGTGAACCACTCTCCAACACTGGTAAATATTGATTTTATTACGATAAAT

ATATCAAAACAAGTTCCAAAAAAAAGGGGGGGCCCGGTAC

>FM890813.1 FM890813 Jatropha curcas embryo 56-70 (DAF) Jatropha curcas cDNA clone rjcaeb0_000756, mRNA sequence

ATTCATTGGCTCGTTAAGCGCAGGAAATAGAAACGCGAGGTTTTAAAAATGGCGTCGGCGATGATGCGAT

CTGGTCTTCGTAATGCTCTTCGCGGTGGCTCTAAGGTCTCTGCTCCTCCCAAGAGACGTTTTTCTTCCTC

TGCTCATCATGACGAAGCTTATGAGGCGGCAGAGGCATCAAAGTGGGAGAAGATAACCTATTTGGGCATT

GCTACATGCACCATCTTAGCTATTTACAACCTATCAAAGGGCCATCCCCACCATGAAGAGCCTCCCGCAT

ACCCATATTTGCACATTCGCAATAAGGAGTTTCCATGGGGACCGGATGGGCTGTTTGAGACAAAGAAACA

TTAAGTATTTGATGGCCGTTACATGAAGATATCTGAAGTTACCCTCTTTTAGTGCATTCCTTGTCTCGTG

TCAGTTATTAGACTATCTCGGATGTTCTTTGAATTTGATGCTAGCATTTGCATTCCAATAATGTGGAATT

TGTGATTTTG

>FM890812.1 FM890812 Jatropha curcas embryo 56-70 (DAF) Jatropha curcas cDNA clone rjcaeb0_000755, mRNA sequence

ATTCTTCAAATCAATCAAGACAAGAGCTTGATGAAAGAGCAAGGCGTGGAGAGACTGTTGTCCCTGGAGG

TACTGGGGGCAAGAGCCTTGAAGCTCAAGAGCATCTTGCTGAAGGGAGAAGCAGAGGAGGACAGACAAGG

AAGGAGCAGCTGGGGACAGAAGGATATCAGGAGATGGGCCGGAAAGGTGGGCTGAGCACGACGGACGAGT

CTGGTGGAGAAAGAGCTGAGAAGGAGGGCATCACTATCGATGAGTCGAAGTTCAGGACTGGGAGCTAGTG

TGATGATTAAAACTATAAATGGTATTTAGTAGTAATAATAATAATAATGTTGGTAATAAAGTATTAGCTT

AGCAGTGTACTTTAGTTTAGTAATGTAGTTTACATGTGGGTTAAAATGACTAGGGTTTAGCTAGTATGTG

TGTTGTAACTTTGAGAGGGGTTTATGGTCGTTCCCTTTCTGGGAGGACTGCTAATACTTACTCCCTATTT

GTAAAATAACCTGCAGTATATATAATTACCTGTTGGTT

>FM890811.1 FM890811 Jatropha curcas embryo 56-70 (DAF) Jatropha curcas cDNA clone rjcaeb0_000754, mRNA sequence

ATTCGAATTCGTTAATCGCAAATGAGAATGGTGAATTATGCGCTTATTTGTCGAATACAGGAAAGCGAAA

GTTTCAGTTCGATCATGTGTTTCAGGTGGATGCCTCACAAGAAGACGTCTTTGGTGAAATTTGTGACATT

ATCGCCTCCTCAGTGGATGGTTATAACGTGTGCATTATGGCTTACGGACAAACAGGAAGTGGAAAGACGT

ATACAATGGAAGGACCACCAGACAAACCTGGGGTAAACATTCTTTCAATAAGGGAATTGCTGAGAATCAT

CAACGAGAGACAAAAAGTCAGCTTCGAACTTTCTATGAGTATTGTGGAAATATACAATGAGAATGTTGTG

GATCTATTAAGTCCAACAAACACGTGTGATGCGGTGGAAATTCGTCACACTGGTCCTTCGGTGAGTATTG

TGGGAGCCACATGGGTCCGAGTGAGAAATGAAGCAGACATGCAACAAG

>FM890810.1 FM890810 Jatropha curcas embryo 56-70 (DAF) Jatropha curcas cDNA clone rjcaeb0_000752, mRNA sequence

ATTCATCAAATCCTCTTCTTTGTTGTTCAATCTTCATCATCCCACTTCAATCATGGCTGTCATCAAAGTC

CATGGAAACCCTATTTCAACCGCTACACAGAGAGTTCTAGCTACCCTCTACGAGAAAGATTTAGAATTTG

AGTTTGTTCATATCGATCTTGCCACGGGAGAGCACAAGAGGGAGCCTTTCATTTCCCTAAATCCATTTGG

TCAAGTCCCAGCTCTAGAACACGGAGATCTCAAGCTCTTTGAATCAAGGGCAATTACCCAATACATTACC

CATGAGAATCCTGACAAGGGAACCCAGCTTCTTTGCCCAGGCAAGGTCAAGATGCCAATATTAACAGTTT

GGATGGAAGTGGAGGCTCAAAAATTTGAACCAGCAGCCTCAAAGCTCAACTGGGAAGTGTTTTTGAAGCC

AATTTTTGGTTTGACAACGGATTCAGCTGCAGTAGAGGCGAATGAAGCTGAG

>FM890809.1 FM890809 Jatropha curcas embryo 56-70 (DAF) Jatropha curcas cDNA clone rjcaeb0_000751, mRNA sequence

CGAATTCGCTAAGGCAGGTTCATGGCAAGGAAACAACAGATAGAAGCAACTGTCGGATGCCGGGTCCCTG

AGAATGACGGGAGGAGACATGCTCTGACTTTGACTGTTAGCGACTACAAGAGAAGAAGAGGACTCCTCTG

AAATGTGATAATGCACCATCAACTTCCTTTCTGGTGACTACCTCAGATCGCACAGCCTTCAAGCATGTCT

CTGAATTGTGTTGATCTTGTAATTGCAAACCTGGTTACTGGGAAAGTATTGTGTCACAACATCTTTGCTG

TCTGAAGTTTTAATATTATGGGTATAGTGTTTGGCTTTGCCTTCGAATTATTCATGGAAAGTTTGACAGT

GATTTTTATTTAGAGAAAAAAAAAAAAAAAAAAAAAAAAAAA

>FM890808.1 FM890808 Jatropha curcas embryo 56-70 (DAF) Jatropha curcas cDNA clone rjcaeb0_000750, mRNA sequence

ATTCGATGATGTCACCACAGGCAGTGAACTTGGGCATGGTTTCTCCAGCCCTGATTGAGAAAAGGGATTA

CAAGTATAATATATTGACAGATGCTATAAAGAACTCGAGGATTGAGATTGCAGAGCCAGAGTGGATCAAT

CCTCAGGCAGATTACTGGAAGCAGAATGGGAAAGGTTTTGCCATTGACATTGAAAGGACTGAAATGAAAT

TGAGGGCACCTTTTCCATGATCATATATATGCAGATTTTGTGTTTATAAAGTAAAAAACCAATATGGATA

GTGTCAGTGTGGATATCAGGATGTATAGGTGGTTGCTTGTTTTTTGGTTTTGGTGTTTTTGGAAGTAGAA

TCCTCTATATACATGTTCT

>FM890807.1 FM890807 Jatropha curcas embryo 56-70 (DAF) Jatropha curcas cDNA clone rjcaeb0_000749, mRNA sequence

CGAATTGATCGATAAAGTGTACAGAGCAAAAGATTAATTATATTGGTGGAGAAATTTGTTAGCAAAAAAG

GGCAAAAATGTCTTCAAATCAATCAAGACAAGAGCTTGATGAAAGAGCAAGGCGTGGAGAGACTGTTGTC

CCTGGAGGTACTGGGGGCAAGAGCCTTGAAGCTCAAGAGCATCTTGCTGAAGGGAGAAGCAGAGGAGGAC

AGACAAGGAAGGAGCAGCTGGGGACAGAAGGATATCAGGAGATGGGCCGGAAAGGTGGGCTGAGCACGAC

GGACGAGTCTGGTGGAGAAAGAGCTGAGAAGGAGGGCATCACTATCGATGAGTCGAAGTTCAGGACTGGG

AGCTAGTGTGATGATTAAAACTATAAATGGTATTTTGTAGTAATAATAATAATAATGTTGGTAATAAAGT

ATTAGCTTAGCAGTGTACTTTAGTTTAGTAATGTAGTTTACATGTGGGTTAAAATGACTAGGGTTTAGCT

AGTATGTGTGTTGTAACTTTGAGA

>FM890806.1 FM890806 Jatropha curcas embryo 56-70 (DAF) Jatropha curcas cDNA clone rjcaeb0_000748, mRNA sequence

ATTCACAGGTAATTATATATACTGCAGGTTATTTTACAAATAGGGAGAAAGTATTAGCAGTCCTCCCAGA

AAGGGAACGACCATAAACCCCTCTCAAAGTTACAACACACATACTAGCTAAACCCTAGTCATTTTAACCC

ACATGTAAACTACATTACTAAACTAAAGTACACTGCTAAGCTAATACTTTATTACCAACATTATTATTAT

TATTACTACAAAATACCATTTATAGTTTTAATCATCACACTAGCTCCCAGTCCTGAACTTCGACTCATCG

ATAGTGATGCCCTCCTTCTCAGCTCTTTCTCCACCAGACTCGTCCGTCGTGCTCAGCCCACCTTTCCGGC

CCATCTCCTGATATCCTTCTGTCCCCAGCTGCTCCTTCCTTGTCTGTCCTCCTCTGCTTCTCCCTTCAGC

AAGATGCTCTTGAGCTTCAAGGCTCTTGCCCCCAGTACCTCCAGGGACAACAGTCTCTCCACGC

>FM890805.1 FM890805 Jatropha curcas embryo 56-70 (DAF) Jatropha curcas cDNA clone rjcaeb0_000747, mRNA sequence

CTCTCTCGCCATTTCCTTCTGCACTGGATCACGTGTTGACATTCTCTCCACCTTCTCTTGCGCCTCTGCT

CTGGTTTTTTCCATGCCAGCCTTAGCAGAGGCTCCAACGTTAGCTGCGGTTTCCTTCAGCGACTCCATTT

TCACTCACTTGGCTATTCAAATTCAGGGGGGGCCCGGTAC

>FM890804.1 FM890804 Jatropha curcas embryo 56-70 (DAF) Jatropha curcas cDNA clone rjcaeb0_000745, mRNA sequence

GGACTATAACTTATAGCTCCACAAACTTTCATTATCTCATAACATAAGGCATATACAGATCTACATTTTT

ATATTATTGCAAGGAACTAAAACTGCCTATAAAAATAGGCTTATTCTTCTCCAAATCTAAACTATGTAGA

AACCAGTTTATTATACATCCTCTTGGTATAATTGCATAGTTGCAATATTAATTACGAGATCTAGACCCAG

GGAAAATATCATTCGATTTTATTTTATTTTATTTTATTTTTCTATCGAGATGTTGGGAGTGTTTAGCAGC

TCTATAGTATCTCCGCCCGACGAGCTGGTGGCGGCAGGAAGCCGGACGCCATCTCCGAAGACAACGGCGG

ATGCGCTGGTGAAGCGATTCATCGACACCAATCCCTCCGCCGTATCCTTACAGATCGGAGACAACGCGCA

GTTGGCTTACACCCATCACAGCGAGTCCTTGCTGCATCCCAGATCATTCGCTGTTAAGGATG

>FM890803.1 FM890803 Jatropha curcas embryo 56-70 (DAF) Jatropha curcas cDNA clone rjcaeb0_000744, mRNA sequence

CGACTATGAGTGGTGCCCACATCTTGCTTAATCAGAACCACACCCCCAACCAAAGCTGTGAGCCCAACCA

AATCTTGTGCCTTGATCAAGGGTTCGTTTTCTCTGGGTTCTGCGAAAAATTTATCTAAAGCATTTGGTTT

GAAATCTTCATCCTTCAAGGTTTCAGCAATGGCTAATGTATACAAGGTGAAACTAATCACGCCAGAGGGT

GTAGAGCATGAATTTGATGCCCCTGATGATACTTACATCCTTGACTCTGCTGAAAATGCAGGAATAGAAC

TGCCTTACTCATGTAGGGCAGGGGCCTGCTCTACTTGTGCTGGGCAGCTGGTTTCTGGATCTGTAGACCA

ATCAGATGGGTCATTCCTGAGCGAAGAGCAAATGGAGAAAGGATATGTACTTACCTGTGTCTCATATCCA

ACTTCAGACTCTGTTATTCGTACTCACAAGGAGAGTGATCTCTACTAAGTCATGTAAGAAGCAAGC

>FM890802.1 FM890802 Jatropha curcas embryo 56-70 (DAF) Jatropha curcas cDNA clone rjcaeb0_000743, mRNA sequence

ATTCCTTTGAATTAAAAGGACAAAAGATGGCAACTGTGGGCAGGAACATAGCAGCTCCATTGCTGTTTCT

TAACTTAATCATGTATGCTATTGCTTTAGGTTTTGCCAGTTGGTGCCTTAATAAGTATATCAATGGACAA

ACCTACCATCCAAGTTTTGGAGGGAATGGAGCAACAGCTTTCTTTTTGACTTTTGCTATATTAGCTTCTA

TTGTTGGTATAGTATCCAAGTTGGCAGGTGGCCTTCATATCAGGAGCTGGAGAAATGACAGTCTAGCCTC

TGCAGGCTCTTCTTCACTTGTGACTTGGGCCATCACTGCCCTAGCTTTTGGGTTGGCATGCAAGGAAATA

AACCTAG

>FM890801.1 FM890801 Jatropha curcas embryo 56-70 (DAF) Jatropha curcas cDNA clone rjcaeb0_000742, mRNA sequence

CGAATTCGAATTCCTGTTTTTCTCTACATTAACATATCTTTCTCACACTTATCATCAACAAATGGCTGAA

CGTTCACAGCCACACCAAGTTCAAGTGCACCCACAACACCGCTATGAAGCTGCCTTCAAAGGTCAACAAA

AAGGTCCATCAGCTCAGAAAGTGTTAGCTGTGATCACCCTCCTGCCGGTAGGCGGTGGGCTTCTTGCACT

AGCTGGTATAACCTTAGTAGGTACCCTAATCGGCCTAGCTATTACTACCCCTCTTTTTGTCATCTTTAGC

CCTGTTCTTGTCCCAGCTGCATTAGTTATTGGCCTTTCGGTCATGGCATTTTTGGCTTCCGGGGCTATGG

GGCTCACAGGGTTGTCATCACTCTCGTGGGTTCTGAAATACCTCCAGGAGGTCACTCGGCGAATGCCGGA

GCAGCTGGATATAGCTAAGAAGCGTATGCAAGACATGGCTGGTTTTGTGG

>FM890800.1 FM890800 Jatropha curcas embryo 56-70 (DAF) Jatropha curcas cDNA clone rjcaeb0_000741, mRNA sequence

CGAATTCATGCCACGGGACGTCGTGGGAGAAACGTGTGCTGCACGTGAGCATGGAGGCACAGGAAGAAAG

GAAGAGGTGGTGGTTCTTGAAGAAACACCAGCAGGAACCGTGGCATCCACCCTGAAAGAGTCTGATCAGA

TTAGTGCTCAAACTTTCAACGATGTTGGACGGTTAGATGATGAAGGTGTTACTCATCTAAAGGTCGTCCA

CCGTTCAGACAATAAGTGATAACGTCCACCGAATTCACGGTGTACTTCCTACTTCTTTTACTTTTTCACT

TTTACACCACCATGTTCATTAGTAGCTTTTTGGGCCTTCTTGTTGTGCTTTTTTTTTGTTTTGAGTAACT

ATAAACAAAGCTTTTGTGGGCTTGTTTGCCCTTCTGGATCTTATGTGCCTAATCTTATATGGATAAGATT

GGAAGGTTATCATCTCTGTGAGTTTGTATTGTAATTTAATGGTTGTGATGTGAGGTTTAACAAGCGGACG

TTGAAC

>FM890799.1 FM890799 Jatropha curcas embryo 56-70 (DAF) Jatropha curcas cDNA clone rjcaeb0_000740, mRNA sequence

ATTCCACTCTATCTCCAGTTTCCATTTCACAGCCGAATTTTCCCGGAAAATGGGATTCCAGGAGAACAGT

TTTCAGGTTGAAAAGTTTTAGTGCTTTGAGTTCAAGAAAACACATCCTGAGACCGAAATTGAGGGTTTTA

TGTTCAGTTCAAGAAGGAGAAAACCAAAGCAATGGTGAGGAACCGCCAGAATCGTTGTTCATGAAGGAAT

TGAAGAGGAGAGGAATGACTCCAACTTCATTGCTCGAGGAAACTAGCAGAGGGAGTTATGGAGTGCAAGA

TGAAATAAAGATTGGAGAAGAAGATAGAGGTTTTCCTAAAAGAAACGCCGTTTCTACTGAATTTGATAAA

GGAATTTCTAATCAAAGAGAGCGATCCATGGCATTGAATAGTGAAGGCCTTGAGGGTTTAATTCCTCGTG

CTAAACTTTTGCTCACACTCGGAGGGACAT

>FM890798.1 FM890798 Jatropha curcas embryo 56-70 (DAF) Jatropha curcas cDNA clone rjcaeb0_000739, mRNA sequence

TTGGCCAACAGCCACGATCCCACCAAGTAGTGAAGGCAGCCACTGCAGTCACTGCCGGTGGATCTCTTCT

AGCTCTCTCCGGTTTGACCCTTGCTGGTACGGTCATTGCACTGACCGTAGCCACTCCATTGCTGGTCATA

TTCAGTCCGGTTCTTGTTCCTGCGGTTATCACAGTTTGTTTGATAATCACAGGGTTTTTAGCCTCAGGCG

GTTTTGGCGTCGCTGCCATTTCTGTCTTGTCCTGGATTTATACGTATGTTACTGGAAAGCATCCACCAGG

AGCAGAAAGTCTGGACCAGGCACGCTTAAAGTTGGCGGGAAAGGCAAGAGAGATGAAGGACAGAGCTGAG

CAGTTTGGACAGCATGTAACAGGTCAACAGACTTAAAAGAGAAGTGGGTTTTGGTGGTTGATGCAGAGAA

CATAAAATGGCTTTGATATATATATAAAAATATATGAGTCCTTTAATTAACATGGAAGGCCATTGCTTGT

CTGGTGTATTTCTTTTTTC

>FM890797.1 FM890797 Jatropha curcas embryo 56-70 (DAF) Jatropha curcas cDNA clone rjcaeb0_000738, mRNA sequence

CGAATTCCAACTTATAGGGACACAGACACTAGGTATTAGCTACTAGTACTGCTACCTATGGCTAATAAAT

TTTATGTGTGTATTGGGGTCTTGGGCTTTCTCTAAGATGAGAGTTTATGTAGCATCTTATAAACAGGGAA

AGCCATAAATAAAATAGTGGGTCTAAGTGTGCTTCAAAAGCAGCTTGCTATGAAGTGTTTCTATTGTAAC

TTTGTATTACCTGGTTAGGAAATGTGCTTATTAATGAAAAATGAAACAGGTGTCCTTTATCACTTAAGAA

AAAAGAAAA

>FM890796.1 FM890796 Jatropha curcas embryo 56-70 (DAF) Jatropha curcas cDNA clone rjcaeb0_000737, mRNA sequence

ATTCTCCAATTTTCCTGCAGAACTGAGTCTGAACTAATTATTAAAAAAATGAGTGGCCGTTCCTTCCTTT

TCTGCAACCTCTCTCGGCCATCAATACTTGTGAGGCCTTGGACGCCATATTTTCTGTACAACCCCATCAA

GGTAATAACTTATTGAATAATGTTTACTACGTTGAGAGTTTGCTGGTTTGAATCATTGAAACGCTATCGT

TTTAATCGACAGAGAAACAAGGGAAGTAGCGCGGAGAAAGAGAATGAAGGGCTTGCAATAGATCAGGAGA

GAGCTTCTTCAACAGCTGATGAGTTCATAAGAGTTG

>FM890795.1 FM890795 Jatropha curcas embryo 56-70 (DAF) Jatropha curcas cDNA clone rjcaeb0_000734, mRNA sequence

CCCGCAAGATATAATTTACATTAATGATAGTGCAAAGAACCAAACAAGGTCTTTATCATGCCCACTTATA

AAGGCTTTATTTTTACACCTCTCTATTACTCTCCTACATAGCCACTAGCTAAGCGTTTTTTCTCTCCTTA

TTACAATTACATAGCTGCCTTTTTAGTCCCTTCAATTGCGAGAAGAAGGCCTAGTCCCAGGGCTGAACAG

AGTCACTTCTTGTCTATTCTCTTTCAGTCTCCTCGCGTCCTCCCTTGAAACCTGGAATACGTTTGCCAAC

ACCTCTTCTGGAATTGATCTGATTGCCGAGACACGTCCTGCTAGGGGGGGGCCCGGTAC

>FM890794.1 FM890794 Jatropha curcas embryo 56-70 (DAF) Jatropha curcas cDNA clone rjcaeb0_000733, mRNA sequence

ATTCAATTACGCCACATGGACAACTGTGGAGCATTACTACCAGGTACTCGCATTTCAAGCATGAATTATT

TTCTCTTGCAAAAAGTGTCGAAATTGTATCACAATTTTGTGAATTATAGGCACACAAGTTTCTGGGTGTG

AATGATCCTGTAGCAAAAGATTGCATTGAGAAGATAAAATCTGCAAAAAGTCCAGAGGAAGCAGCACGAA

TGGGAAGGTCAATGCAGAGACGGCACCCTGATCTGGTGTGTTCCTCTATAGTCTATACCTTTTTTATGAC

CTAAAGATTGGACCTTTTACTGATACCAGAGTTTACAGATTTGTATTGCAGTCTAATAGTACTAGAAGTC

CAATACTAGATAAAATAACTGTATAATATATGATTGAAGGCGAAGTATTGCCATTCTCCAATTCTCATAA

ATTGCTGACATCAATTTATTGTTGGAAGGATGGTCAACAGGTAGTGCATGAGTGGGTGGTTTATTTTTA

>FM890793.1 FM890793 Jatropha curcas embryo 56-70 (DAF) Jatropha curcas cDNA clone rjcaeb0_000732, mRNA sequence

ATCTTATTCAGCAGAGACTATGGTCAGTTCTGCTTCAGCGTCCTCTGATTCAGCTCTCAGCACAAATGGT

GACACGGTGGTGAAAGCAAGTCAAAATAGGTGCGCCACTTGCAGGAAACGCGTTGGTCTAACAGGATTCA

GTTGTCGTTGCGGGAATCTTTTCTGTTCAGTTCATCGCTACTCAGACAAACATGGTTGCACCTATGATTA

TCGAACTGCTGGACAAAATGCTATTGCCAAGGCCAACCCCATTGTGAAAGCTAAAAAGCTTGATAACATC

TGACCCTCATCAAACTTCTGCTGAAGGTATCACATCTATGTTCTGGGATGCCACATGCAAGGCTTTTATC

TCGTTGTTCTTGTTATTTAGATGTCGTGTTGCTCAAAAGGTTGAAATAACATGTGAACTGGCATCTTTCC

AGTTACTAGCTTGTCTCCTTTGTTCTAAATGTTAAATGTCGAAATGTGTTT

>FM890792.1 FM890792 Jatropha curcas embryo 56-70 (DAF) Jatropha curcas cDNA clone rjcaeb0_000731, mRNA sequence

ATTCCACAAAGGATGAACCATTGTGGTTTACTCCATTCAGATGTGAACCTTGAACAAATAGCTTCCATGA

CTGTTGGTTGGACCGGGGCTGAGCTTGCCAGTGCTGTTAACTCTGCTCTCTCCAATGCTATTGTCCGTGC

AGATCCTAATATGATAGGGCAAGTAAATAAGAAGCAGATTAGAGTAAAACAAGCAGACTTTCTTCATGGT

GTTCGAGTTAGTATCCATGATAAATGATGGAAGAAATTTCAGTATAATATATATAAATATTTATCTGATG

TATAATAAATATATGTTGATAACATATAATAGTTTGCCTACGCATGAATCCAACGCTAATTTCTGTAA

>FM890791.1 FM890791 Jatropha curcas embryo 56-70 (DAF) Jatropha curcas cDNA clone rjcaeb0_000730, mRNA sequence

CATCAACAAACAAAGTAAGACTTGATTACAGAGACCTTCAACACATGACAAGAAAATTATTTTAGACAGC

TCTCAAAAGAGGGAAGACCCGTTAACAAATTACATAGAAGAAACAAAAAGATTTAAGCGAAAAAAGAAAT

ACACCAGACAAGCAATGGCCTTCCATGTTAATTAAAGGACTCATATATTTTTATATATATATCAAAGCCA

TTTTATGTTCTCTGCATCAACCACCAAAACCCACTTCTCTTTTAAGTCTGTTGACCTGTTACATGCTGTC

CAAACTGCTCAGCTCTGTCCTTCATCTCTCTTGCCTTTCCCGCCAACTTTAAGCGTGCCTGGTCCAGACT

TTCTGCTCCTGGTGGATGCTTTCCAGTAACATACCTATAAATCCAGGACAAGACAGAAATGGCAGCGACG

CCAAAACCGCCTGAGGCTAAAAACCCTGTGATTATCAAACAAACTGTGATAACC

>FM890790.1 FM890790 Jatropha curcas embryo 56-70 (DAF) Jatropha curcas cDNA clone rjcaeb0_000729, mRNA sequence

CATGTCAAAACTGAAAATTGCATTTCATACCAGGCTAAATATCCAAAGTCTTGTCATCAAAAAGGTAATA

TAGTTCCACCACAGCTAAAATGCACGAAGCAATAAACAAAAATAACTTCAAAAAACTTGGTTCCCAAATA

GGACTTTTAAGTAGCATGTGTACTAGGAAAAATTTTGACATATTTAAGCTCAATTGCTGATCTAGTATGT

TAACTCATCATCATCGTCTTCATCAGCAGCAGAAATTGAGAAGCTTGCAATCGTTCGATCAACTTGGCTT

TCCTTTCCTCATACGTCAACTTCCTCAAATTGTACCTCTTGTGTTCCTTTGGTGGTTGCTTCTCTATTTT

CTTCAGAGTAGGATCAGCACGAATTGCAGCATGAACCTTCTTGTATAATGCCTCCAAGTTGTCTGGCTCA

ATTCCCCTCTTGACATATTCACTGAAGTGAGTCTGGTATTTCTCTGGTTCATCCTCCATTAAGGTCTCCA

TATATGCAGCAACATGACCACCATAAATATACTTGCGGTGAACTTCAGCATCTAGTTGC

>FM890789.1 FM890789 Jatropha curcas embryo 56-70 (DAF) Jatropha curcas cDNA clone rjcaeb0_000728, mRNA sequence

ATTCCCAGCATCCAATTTCTTGGTTTCTTGCTTGTTATCGCTGTTTCTCTCTCAATTTACCCGACTTTTA

CTTACTTAGAGGGGTCCCTTCTTTTCTCTATGTTCTTGCTCAGGAACCGAAATTTGGGGATTTCTAATTG

AAACAAAGAACCAAGAAAAGAAAGGAAAACAAAAATGAGTGCTTCTAGGTTCATTAAATGCGTCACAGTT

GGTGATGGTGCTGTTGGCAAGACTTGCATGCTCATCTCCTACACCAGCAACACTTTTCCTACTGACTATG

TGCCTACTGTGTTTGACAACTTCAGTGCGAATGTGGTTGTGGATGGGAGCACAGTCAATCTAGGGTTGTG

GGATACTGCTGGCCAGGAAGATTATAATAGATTAAGACCTTTGAGCTATCGTGGGGCTGATGTTTTCTTG

CTTGCTTTCTCTCTCATTAGCAAGGCCAGTTATGAAAATGTTGCAAAGAAGTGGATTCCTGAATTGAGGC

ATTATGCACCTGGAGTACCAATTATCCTTGTTGGGACTAAGCTTGATCTCCGAGAT

>FM890788.1 FM890788 Jatropha curcas embryo 56-70 (DAF) Jatropha curcas cDNA clone rjcaeb0_000727, mRNA sequence

ATTCGCACTTCCTTTCACTCGCTCTGAACTTTGCCGCCGCATAGAGAAGAGAACTTCTCTCCGCATCTGA

AACCCTAGCCATGTCGCTGGTTGCCAATGAAGAGTTTCAACATATTCTTCGTGTCTTGAACACGAACGTC

GATGGGAAACAGAAGATTATGTTTGCACTGACCTCCATCAAAGGTATCGGTCGCCGTTTTGCCAACATCG

TCTGCAAGAAAGCCGATGTCGACATGAACAAGAGAGCTGGTGAACTATCAGCTGAAGAGCTAGATAAACT

TATGGTGATTGTTGCTAATCCTCGCCAATTCAAGATTCCAGACTGGTTCCTTAACAGGCAGAAGGATTAC

AAGGATGGGAAGTACTCTCAGGTTGTATCCAATGCATTGGACATGAAACTCAGAGATGATCTTGAGAGGT

TGAAGAAGATCAGAAACCACCGTGGTCTTCGTCACTACTGGGGTCTTCGAGTACGCGGACAGCACACCAA

GACGACTGGACGCAG

>FM890787.1 FM890787 Jatropha curcas embryo 56-70 (DAF) Jatropha curcas cDNA clone rjcaeb0_000726, mRNA sequence

CGAATCGTCAGAGTTGCTAATTTCACCAATATCGCTATTAATGAGGATCAAATTGCAGCTCATTTAGTTC

ACTATGGACCTTTGGCAGTTGGATTGAATGCGATTTTTATGCAAACTTACATTGGAGGAGTGTCATGTCC

ACTTATTTGCGGCAAGAAATGGATCAACCATGGAGTTTTGCTTGTTGGGTATGGAGCTAAAGGGTTCTCC

ATTCTTAGATTAAGTAACAAGCCGTATTGGATCATCAAGAACTCTTGGGGTACGCAATGGGGGGAGGAAG

TATACTATCGGGTTTGTAGGGGACATGGCATGTGTGGAATGAACAGGATGGTCTCTGCAGTGGTAACTCA

AGTTTCCTAAAATCAGCTCCAAACTAAAAAGGGTTGAGTAAGAAATAATTAGGAGAAACTGTTTTTGGGT

TTTTTGCTTAATATATATATATATATATATAATGGGATTTGATGATCTTTGGAAAAACTTGTCGAGGCAT

GTATGTTTTTCCCCTTCTGTTTATTTGGTTTTTGTCCA

>FM890786.1 FM890786 Jatropha curcas embryo 56-70 (DAF) Jatropha curcas cDNA clone rjcaeb0_000725, mRNA sequence

AATTAATTTGCTCTGATATTACATGCCTAATTAAGGCTAATCTTAAAAAGGTAATGCAAGATCATTCAAG

AAAGATCTTTCATGAGATTGCAAAACCTAGTCTGATATGAGCTTG

>FM890785.1 FM890785 Jatropha curcas embryo 56-70 (DAF) Jatropha curcas cDNA clone rjcaeb0_000724, mRNA sequence

ATTCCTCTTCCCGACAATGTGATTCTACATACCACAATGGGAGACATTCACATGAGACTGTACCCCGAAG

AATGTCCAAAAACTGTAGAGAATTTTACAACACACTGCCGAAATGGCTATTATGATAATCTTATCTTTCA

TCGTGTCATCAAAGGTTTCATGATACAGACTGGAGATCCTTTGGGGGATGGCACTGGTGGGCAGTCTATT

TGGGGAAGGGAGTTTGAGGATGAGTTTCATAAAAGTTTGCGGCATGACAGGCCCTTCACAGTGTCAATGG

CAAATGCAGGCCCAAACACAAATGGCTCTCAGTTCTTTATCACTACAGTTGCTACACCATGGCTGGACAA

TAAGCATACTGTTTTCGGTAGAGTTGTCAAGGGAATGGATGTTGTCCAGGCAATTGAGAAGGTGAAGACG

GACAGGGCAGACAAGCCATACCAAGATGTAAAAATCCTGAATGTGACTGTCCCCAAGTCTTAGAAGAGAA

AGAAACCCTT

>FM890784.1 FM890784 Jatropha curcas embryo 56-70 (DAF) Jatropha curcas cDNA clone rjcaeb0_000723, mRNA sequence

GAATACAATTATATTTATAGAATATATTGATTAGGGAATACTTAATTACTAATTAGGATGATTACATTAC

TTTCATCACTTATTTTCATAATATATTACATACTTCATTAGCGTGAGATCATAACTCCCCTCGTAATACT

ACTTACTTTCACTATCTACACACCCAAAAAGGCTACTTCATTATTTACTCCTCGGCATATCTTCCAGACT

CGGTCCTGGAAATAGTCAAGGTGGTCTCTTGTCTTCCAAACTTAATCCTTCTTGCCTCCTCTGCTGATAC

CCTAAATGCATTTGCAATAACCGCCAACGGCATTGCTCTAACGGCGGAGGAGCGACCTGCAAGATCATTG

GTCATCGCGTTATCGCTTGTCTTGAAGGTTACATATTCGAATCTTTCACTTTCTGCTCGTTTCACCACTA

CGAAGTTTTGTGGCACCGTCAAAACCTGTCCTTTGCTTACACTTCCGTCGAAGACGGTGTTGCCGTTTTC

ATCCGTCACCTGAACCCGCGCTTGTCCTTTGAGTGCGTAAATTACGCTGTGAGAGTTTATGGGGGGGCCC

GGTACC

>FM890783.1 FM890783 Jatropha curcas embryo 56-70 (DAF) Jatropha curcas cDNA clone rjcaeb0_000722, mRNA sequence

ATTCAAACCCGAAAGGGCGGTCTAGGCAAAAGTTAGGGCAAAGAACCTGCTAGAGTCAAAAACTCGAAAG

GGCGGCTACTAGGCAAAAGTTAAGGCCAAAA

>FM890782.1 FM890782 Jatropha curcas embryo 56-70 (DAF) Jatropha curcas cDNA clone rjcaeb0_000721, mRNA sequence

ATTCCCAATATTCTGGGTTGGTTAGTACCTAGATTTGGAAGAAGCCCATATCTTCCTCCTGCTGAGGAGC

ATGTAATTTCTACACTGAAGAGAGCTCTCAATGCTCTAAACACTCATCTTGCCTCTAACACCTACTTGGT

TGGGCATTCTGTGACATTGGCTGACATTATCATGACAATGAATCTCTATAGTGGATTCACTCGCCTCCTG

ACTAAGAGCTTTACCTCAGAGTTCCCACATGTTGAGAGATACTTTTGGACCTTGGTTAATCAACCAAATT

TCAAAAAGATATTGGGTGAAGTGAAGCAAACTGAATCACTTCCTCCTGTTGCATCTAAGAAGCCTGCCCA

GCCAAAAGAATCTGCTAAATCAAAGGCCAAGGATGAACCGAAGAAAGAAGCCAAAAAGGAAAAGGAGCCA

CCGAAGCCTACACCTGCTGCTGAGGAAGAGGAAGAGGCACCAAAACCCAAACCAAAGAATCCTCTTGATT

TACTGCCTCCAAGTAAGATGATACTGGATGAGTGGAAGAGGCTCTACTCGAACACCAAGACCA

>FM890781.1 FM890781 Jatropha curcas embryo 56-70 (DAF) Jatropha curcas cDNA clone rjcaeb0_000720, mRNA sequence

GATAGAAAACATTTATTTTTCTCTGTTTCAATTATTTAATTAATGGAACAGCAAAGTAATAACCAGATTA

CAAAAGCATCAAAATTTCAAGACATAAAAAGATCCCAATATGCTCCGAACATCACTTCATATGTCGTATC

TCGACTACCAAAGCCTCTCTTGAAGTTCTATTGTCACTCCAAATGGCATTTCCGTTTGATACAGATAGGA

TCATCATCATCATTCTGTTCCTTATCTAACAAATTATTGTCGTTGTCCACCTCCAATTCTCAGGCGTAGG

TTATCGTAAGTACGGGAAACTAGTAACAGCCGAGGATCATCTGAAGGTATCTCGCGTTCTTTTAGCCCAT

TATAATATGCTTCCACGGCTGGAAATGAAGGGGTTATTTTACGAGCTTGCATCAAGTCCCATATATAATT

AGCTGTCGTATATCCACCAGTCTTTTCACCAGCTGCTACAAGGAGTAGATCACTTCCTAGTTTAGGACTC

AAGAAAAAGTTTCTGGAATTCATATTTACCAGTGTGTCTTGAGCAATTTGCATTCCCTTGGGAGTATAGC

CTATCAGGGGGGGCCCGGT

>FM890780.1 FM890780 Jatropha curcas embryo 56-70 (DAF) Jatropha curcas cDNA clone rjcaeb0_000719, mRNA sequence

GAAAAGCAAACAAATCCAGTTCCACCTTTATCAAAAGAAAATGAGAGCTTGTCTACTACATCCAGAAATA

TCAAACAAAACCGCAAAACAAAAAGATAGCATGATTAAAGTCT

>FM890779.1 FM890779 Jatropha curcas embryo 56-70 (DAF) Jatropha curcas cDNA clone rjcaeb0_000718, mRNA sequence

TTTTTTTCAATAATATTCCCATGAAATTATAGAAAACTGTCGAAATGACATTAAGAATTCAATGAACTAA

GAAGCGTCAAGGCACTTTTCATAATCATCAAACGAAACACCTCAATGTATGAAAATTCAAATAAAAACCT

TAAACATTTCATCTTGCTAACAAAACTCCATGATTTTAAGTAACATGGACCTAAGCTGCAACAGATGGCT

TAGATGCAGCAGAAGAGCC

>FM890778.1 FM890778 Jatropha curcas embryo 56-70 (DAF) Jatropha curcas cDNA clone rjcaeb0_000717, mRNA sequence

ATTCGAATTCGAGCAGAAAGTCTGGACCAGGCACGCTTAAAGTTGGCGGGAAAGGCAAGAGAGATGAAGG

ACAGAGCTGAGCAGTTTGGACAGCATGTAACAGGTCAACAGACTTAAAAGAGAAGTGGGTTTTGGTGGTT

GATGCAGAGAACATAAAATGGCTTTGATATATATATAAAAATATATGAGTCCTTTAATTAACATGGAAGG

CCATTGCTTGTCTGGTGTATTTCTTTTTTCGCTTAAATCTTTTTGTTTCTTCTATGTAATTTGTTAACGG

GTCTTCCCTCTTTTGAGAGCTGTCTAAAATAATTTTCTTGTCATGTGTTGAAGGTCTCTGTAATCAAGTC

TTACTTTGTGGGGGGGCCCGGTAC

>FM890777.1 FM890777 Jatropha curcas embryo 56-70 (DAF) Jatropha curcas cDNA clone rjcaeb0_000716, mRNA sequence

ATTCCAAATATCACTGATCGTAAACCTCCATTGAATTTACTCTGTGCTTCGATCGCGTCAATGGCGGAGC

ACCCACAGTCGCAGCATGTTGGCCAACAGCCACGATCCCACCAAGTAGTGAAGGCAGCCACTGCAGTCAC

TGCCGGTGGATCTCTTCTAGTTCTCTCCGGTTTGACCCTTGCTGGTACGGTCATTGCACTGACCGTAGCC

ACTCCATTGCTGGTCATATTCAGTCCGGTTCTTGTTCCTGCGGTTATCACAGTTTGTTTGATAATCA

>FM890776.1 FM890776 Jatropha curcas embryo 56-70 (DAF) Jatropha curcas cDNA clone rjcaeb0_000715, mRNA sequence

ATTGCAAAACTCAGAGCGAAAATATGACCAATAAGTGGCATGTATACATGACAAAGGATGGAAGGATATC

CTTGGCTGGATTGTCTTTGGCCAAATGTGAATATCTTGCAGATGCAATTATTGATTCATACCATAATGTT

AGTTGAAATGATAGAGCATAACATACTCGTAATAGAAGCAAAGCTTGAAAAATTTTGGGCCAGTTCAATG

TTTACATCTTGTAATAAATTTAAGTTCAGTCAGAAATATCTGTAATAATAGTTCTTACTTAGACAGTTTT

ATAGACTCTGCAGTGGAAAGGACTTTGATGTATTTGCATATATTGCTCAAATAAAATTGTTCTTTTAATT

CGAAC

>FM890775.1 FM890775 Jatropha curcas embryo 56-70 (DAF) Jatropha curcas cDNA clone rjcaeb0_000714, mRNA sequence

ATTCATCAACAGCCACACTCCCTATCCTAAGGTTTCTCCAACTCAGCGCTTCTCATGTGGTTCTCCGGAA

TAATGCTGTGAGATTACCCCACTGGCACCTGAATGCTCATAGCATAATCTATGCTTTGAGGGGACAAGGA

CGAGTTCAGATAACGGATGAATTCGGAGAAATCGTCTTCGACGGCAGTGTAGGAGCAGGCCAGGTGTTGA

CGGTGCCGCAGAACTTCGTAGTGGTGAAACAGGCAGAGACAGAGAGATTTGAATATATAACCTTCAAGAC

TAGCGACAATGCCATGACCAATGATCTGGTGGGGCGAACCTCAGCCGTCCGGGCCTTGCCATTGGAAGTG

ATCGCCAATGCATTTAGGGTGTCCAGAGAGGACGCAAGGAGAATCAAGTTCGGAAGAGAAGAGACGACTC

TCGGTACTTCAAGGTCTCGACCTGCAGGAAGGACAGCTGTTGCTTAATTAAAATGAGGAGAGCC

>FM890774.1 FM890774 Jatropha curcas embryo 56-70 (DAF) Jatropha curcas cDNA clone rjcaeb0_000713, mRNA sequence

GGCTGCAGGAATTCGATTCGAGAGGAGCTAATGAGTGTGGTATCGAAGAGGATGTGGTTGCTGGTATGCC

TTCATCAACTAAATCTTAGTCTTGTTAGACAGGTAGCTGGCACACATATAGTTGGGGATGCTTCTGCATG

GAGTATCAGATTATTATACCACAAGTAGTGTAATTTATACGCCAGTTTATAGGGGCCAGACATTACATCT

GTGCCAAAAAGTAGAAGAAAAACTGGGTTTGTAAAAGTATCTTGTATTGTGGTTTAGTATCAACTAGATA

TTTGTTGCTTATGTCTTGATAACTGAATAGTCATTCAAGTGTTATATATAACATCTTCATAGTCATATGT

GATATGAGAT

>FM890773.1 FM890773 Jatropha curcas embryo 56-70 (DAF) Jatropha curcas cDNA clone rjcaeb0_000712, mRNA sequence

AATGATCAAGGTTAGAACTTTATTTTCTGCGAGATCTCTGGTTCAAATCCTGCTTAGGATTGCGAAGAGA

GCTATAGAAAGATGCAGTCCTATTGGTCATCTGACTTGTTCTATCTTTCAGAAAAAGAAAAGAAAACATC

TCCATTGGCGATTGCGAGCACTAGTTAAGAGAGACTCATTTAACATGCCTTTCAAAAAGATGAAGTTTCT

GAACTAATTTTGGCGATAATAAACAACCAATCAATCCAGTTCTTGGTTACGCATAATCGCTCCAGATTCT

GTAGTTGTTGGAGTACATTTGGATAGAACAAACCTTGAAATGAATAGGATGAAGAGCAAAACCCCTTGCT

TTTTTTTTCTCAAAAATAAAAATTATCATTGTTTATAGCGGGCGGCCGTTGGCCAGTTACATCTCTTTTG

CTCAAACACTATGGTTAATGTTAGAGACACAGCAATGCTATGAGCCTAA

>FM890772.1 FM890772 Jatropha curcas embryo 56-70 (DAF) Jatropha curcas cDNA clone rjcaeb0_000711, mRNA sequence

ATTCATTTGTCCCTTCAAAGCAAGCACTTGGCCGAGCATGTGGTGTCACAAGACCTGTTATTGCCTGTTC

TGTGACAACAAATGAGGGTAGCCAATTGAAGACTCAAATACAACACTTGAAGGATGCTATTGAAAAACTC

TTGATATGAAATATTACGGATTGCAATTCGGTGTGATGGGCCTCTTGGATTGTTAGCAGCTGGTCCATGG

GAGGCTTTGACTGATGAAGTTGTATCATTAGAGTCAAAAGCTTTTACAGTGACATTTAAGATTTTTAGTT

TTGAGTTTTGTTTAAGTATTGTGACTACTCGCGATAATTTGCCGAACCTTGAGATAAGTTGTGCTATGAA

CTTATTATATGACTTGTTTAGTGTGAGTTGCTGGCATGTTGCTGCATAAAGATCTAGTTTTTTGTTTGCC

AAATGTAATACCAGTTTCTAAACCTGAGTTCCCTTATATTACAATGGCATCTATTAT

>FM890771.1 FM890771 Jatropha curcas embryo 56-70 (DAF) Jatropha curcas cDNA clone rjcaeb0_000710, mRNA sequence

GCCACGAATAGTCCATCCATATATAAGAAAAATTTTACATACCAAACATACATTATTATCTCCGCTACTA

ACAAAGAGAGGAAGTGCTTGACATCTACGCAAAAACTATACAATAGTATTTCTTAGATAAGAGTGTATCA

TATCGTCCGGCTGAGATATATTCTAGTCGACATAGGTGAAACGTAGATATTCCTTCTTGGAAGGAAGGTC

AACAGTCTTGTATCCCTGTGGAAACATCTTCTTGGCCTCTTCATTCGACACATTAGAGGATATTACCACC

GGATCTCCTGGTTTCCAATCCACAGGAGTAGCAATCTTGTGCTTTTCTGCCCTCTGCAGGGAATCCAGCA

CCCGGATCACTTCATCCATGTTCCTTCCAGTACTTGCTGGATAAAGGAAGCTCAGCTTGATCTTCTTATC

AGCGCCCACAATATGCAGAGCTCGGGAGGGAAGAC

>FM890770.1 FM890770 Jatropha curcas embryo 56-70 (DAF) Jatropha curcas cDNA clone rjcaeb0_000708, mRNA sequence

ATTCAGAAATTTCAAGCGAGAAGAATGGCTACAGAGCAAGAAAGGGCTGAACTTGATGCTAGAGCAAGGC

AAGGAGAGACTGTTGTACCTGGTGGAACTGGTGGCAAGAGCCTTGAAGCCCAGGAACATCTAGCTGAAGG

GCGGAGCCGTGGAGGGCAGACAAGGAGGGAGCAGCTGGGAACTGAGGGGTA

>FM890769.1 FM890769 Jatropha curcas embryo 56-70 (DAF) Jatropha curcas cDNA clone rjcaeb0_000706, mRNA sequence

TTTTTATTTTATTTCAATAATACTCGCTATTATTATTCTTATTTTTATTCTTAATTTCTTGACACAGCAA

TACAAAACGCATCACTTAAACATAAAAAAAACAACACCACCACCGCAAAAAAAACCCAAAATCACGCTGT

CACGGGCTTAAACGACGTCAGATTCTTGAAATTCAGAATAAAAATAAGAAAATAAAGCGAGTATTATTG

>FM890768.1 FM890768 Jatropha curcas embryo 56-70 (DAF) Jatropha curcas cDNA clone rjcaeb0_000705, mRNA sequence

ATTCCTGCATTCTGTATGCACATAGCCAATGCAGTCTCCGGCTATGTGACAACCACCATCACCACCGTTG

AAATCGACGATGATTCTTCTAACACTAGTCAGCGAAAATGCAGGAGGGAAGCTGAAAGGGCAGATTTGAG

CTCCTGTGAGAGTTATATGAGATCGTCAAGAAGGCCGTCAGAAGAGATGCTAGCTTTGCGAGGAATTGAA

AACCAGCAACAACGGGATCTTCGCCAATGTTGTAACGAATTAAGGCAGCTAAGTCATGATTGTCCATGTG

AAGGAATAAAGTATGTATTAGAGCAGCAGCTGGAGCAAGGACAGCGAGAGCGTTTGGAGGCAGTGCGTAG

GGCTAGAAGTATACCTTCTGCTTGTGGCCTGCGTACAAGCTGCGATATTCGTTCCTAATCTGCTAGCTAT

AACGTGGATGTGCTTCAGTTAAATAAAAATTTCACTCACTTGGTGTGTGAGTGATCAGAGTTATGTATAA

TAATGCACAAAAGAATGAGCACATCAAGCTAAGTTTGT

>FM890767.1 FM890767 Jatropha curcas embryo 56-70 (DAF) Jatropha curcas cDNA clone rjcaeb0_000704, mRNA sequence

ATTCCACAAATCAAAGTTGATGGCAGCAGCAGTTCGTAACAAACAGGATCTCTTCAGTCTTTTGGGGGTT

GACTACATCATTGCACCATTGAAGGTATTGCAGTCTCTCAAAGAGTCTATCACTACTCCTGATGAAAAGT

ACTCTTTTGTTCGGAGGTTATCACCACAATCTGCTGCTGCCTACAGTTTCAGTGAAGGAGAGGTTGCAAA

ATGGGACCAGTTAAGTGTTGCATCAGCAATGGGACCAGCAGCAGTCGATCTTCTGGCTGCCGGTCTGGAT

GGTTATGTTGATCAAGCAAGGCGAGTTGAGGAACTATTTGCAAAGATTTGGCCACCACCAAATGTGTAAA

ATTTTCTAGTTCATTTCTTGGAGATATGGGCTTCATGAAATAAAAGATGGAACAGTTTTTCAATTAGCCC

TTGTTCATTCTATATCCTCTTCCAGTCTTCTGCTCTTCATTTTACTAAAATGATAATCAAAATTTTATCT

TTAGTC

>FM890766.1 FM890766 Jatropha curcas embryo 56-70 (DAF) Jatropha curcas cDNA clone rjcaeb0_000703, mRNA sequence

ATTCCTCGNTTGCTGACTAGCGGAAAACCAAAAAATGCGAATTATGAAAAATTCTGCCGGTATTATTCGC

TTGTGTTTTCGGCTTTGGCCATTGCAATTCTTGTGCGGGCTGCGATAAGGTTGACTTATCGATGGGCGCC

GATTGTGGATGACTAAGGACAGATCCGAGTATTAAAGAATCAGGTGACTTTGTAAGAGATAGCTATGGCG

ATTGCTGTGCGTTGCTTTCTCTGATCTGCCTAGGCAATGTTAAAGATGACAAACTCAATCCATGTAAGAA

ATAGCTATGGCTATCGCTGTGTGTTTCTTTATCTGATCTGCTTGGGCATTGGTGATCAAATCAATCAATA

AACTTGCGCTTTTCGTAAAATAGGGCTTAATTTGATTTTTTTTTTTAATTACTCCTATATTTTGTTATTT

TCAATATAACTCAATTGGAGTTAATTTTAAGAGTTTTGTTTATAATCAAGTATTTATCTTCC

>FM890765.1 FM890765 Jatropha curcas embryo 56-70 (DAF) Jatropha curcas cDNA clone rjcaeb0_000702, mRNA sequence

ATTCATCAAATATCACTGATCGTAAACCTCCATTGAATTTACTCTGTGCTTCGATCGCGTCAATGGCGGA

GCACCCACAGTCGCAGCATGTTGGCCAACAGCCACGATCCCACCAAGTAGTGAAGGCAGCCACTGCAGTC

ACTGCCGGTGGATCTCTTCTAGTTCTCTCCGGTTTGACCCTTGCTGGTACGGTCATTGCACTGACCGTAG

CCACTCCATTGCTGGTCATATTCAGTCCGGTTCTTGTTCCTGCGGTTATCACAGTTTGTTTGATAATCAC

AGGGTTTTTAGCCTCAGGCGGTTTTGGCGTCGCTGCCATTTCTGTCTTGTCCTGGATTTATAGGTATGTT

ACTGGAAAGCATCCACCAGGAGCAGAAAGTCTGGACCAGGCACGCTTAAAGTTGGCGGGAAAGGCAAGAG

AGATGAAGGACAGAGCTGAGCAGTTTGGACAGCATGTAACAGGTCAACAGACTTAAAAGAGAAGTGGGTT

TT

>FM890764.1 FM890764 Jatropha curcas embryo 56-70 (DAF) Jatropha curcas cDNA clone rjcaeb0_000701, mRNA sequence

ATTCGTGAGTGAAAAAACTGCTTATGTCTTTTCTTTTTCCCCCCCTTGAACCCTTGCCTCCTTTTTGTCA

AATCTAAAATTCCAACAGTATGTTTTCTTTGATTATAGAGACAGTAGTTTATTTGTTAAAATATTTTCAT

GGTGGTTTTTGTTCCCATTGAGCCACTGTAACCAGTGTGCGCCTCACAGCGTTCTATGGGTGTAGAAATT

GGTTTATATGACAATGTAAATTTAGTT

>FM890763.1 FM890763 Jatropha curcas embryo 56-70 (DAF) Jatropha curcas cDNA clone rjcaeb1_000696, mRNA sequence

CACAATGCTATTATAGTAGTAACTTACTTATGGTCTCCTGACTAGGTCGGAGTAAACACCCTTGACTGTG

GATCCCTGTGCCTCTTCACTTGATGAGCTTCGCTTGGAGA

>FM890762.1 FM890762 Jatropha curcas embryo 56-70 (DAF) Jatropha curcas cDNA clone rjcaeb1_000691, mRNA sequence

GATCCCCGGCTGCAGGAATTCGACATCCTCAGAAGCTCGCGAAACTGTTAAGGATTTGGTTGATAGAAGT

TTGTAATAGCTCATATTAGAATCTCGTCGAAAAATTTTCCCCAAGAATAATGAGCAGGAAACCTAGATAG

ACAACAATTATAAACAACACAAAAAGTGATAGTGTTATCATTGCTAGGCAGCCTAGTTTACCATGTTCTC

GCTGCATTTTCTCTTCTGTTAATTTTTTCATGTGTGAATAGACAGGATGTTGTATTTATTTCATTTTGCA

TTAGTTCAGGCGGATGCATGACCTTGAAGCTCCTAGTTTTAGTATGAGATCAATGTTTTCTTGATTATGT

ATGATTTTGTGGTGCTTAAGTTGATCTTTTGTGATTCACAAACAC

>FM890761.1 FM890761 Jatropha curcas embryo 56-70 (DAF) Jatropha curcas cDNA clone rjcaeb1_000690, mRNA sequence

GGATCCCCGGCTGCAGGAATTCGAATTCCCGTAAAAAATTGCTGCCATTGAAATTTTCACTCACATATAA

CCAGGTAGAAATTCCTCCAGTTCAGACCAAATAATGTCAACAACAGTTAAAAACAACAGGTTTACAACGA

CATATACTACAAATCAGATACATTTATATCCACAAAATTGTTTCATGACACAAAATACCTTATATCAATC

CACACGCTCTATGTATGTCTCAGACAGCGATATTACCATTTGTGATATTTGTCGTTCCTATTATCAAATC

CTATGCCATTCCTTCCTGAGCACTAAGATATCGGAGAGCAGAAATTGCTGTCAAATGTAACCCCTCCATT

GGGGGCCCGGTACCCT

>FM890760.1 FM890760 Jatropha curcas embryo 56-70 (DAF) Jatropha curcas cDNA clone rjcaeb1_000684, mRNA sequence

ATTCGAACAAAATCAGCAAAAGCACGCTCTGGAGGTAAATCCTTGAATTCCTTGCTTTTCTTTGTTCACC

TGGATACGGAGACAAACAGCAAGGACTGCTGTGCCTACACAAGAAAGTACTCCTGAAAGAAAAGAATTGA

ATGGAAATGATCCAACAATGGCCATGTAACCCACCTGAACTAGAGCAGTAAAAATCGCAAAGCCAACATA

AAGATCGATGATCTTGAGACCCGTAGGAGTGGCTGCATAAGCCGAGCGAAGGGACTGAAAGAGAGCTTGG

GCGTCCTTGCTCGA

>FM890759.1 FM890759 Jatropha curcas embryo 56-70 (DAF) Jatropha curcas cDNA clone rjcaeb1_000683, mRNA sequence

ATTCGAATTCTGCTTTTTCGTTCTAATAGACATGGCGTCCAAGTCATGCCTTCGAAGTTGCAGCCGAGGA

TTAAAAACCGTGTGGGAAGGAAGGTCAAAATGGTTGTACGAAAAGATGCGACAGGTTGACGAGAGCATCG

ATATAGAGGGAAAACCGGGCGGAATAATGCCGTTCACTTACACCACTCGGAACGCTCTCGCCTTCAAGTT

CATTATGTTTATGTCCTCAGCGTTTATCCCACCTTTCTTTCTTGTGTATTATCATATGAAGCCACCTGGA

TAACGGAATTATCGATTGCGTAGTACTAAAAAATTAGTCGTGGCTTAGAATAAATTTCCTGATGTC

>FM890758.1 FM890758 Jatropha curcas embryo 56-70 (DAF) Jatropha curcas cDNA clone rjcaeb1_000682, mRNA sequence

ATTCATTTGAGTTAAGTGTTTTCGGGGAACGAAACAATCTTCCAAGTATGCAGAACGACGAGGGTCAGAA

CATGGATCTTTATATCCCAAGGAAATGCTCTGCCACAAACAGGCTAATCACTTCCAAAGATCATGCTTCT

GTTCAGATCAACATTGGTCACTTAGATGCCAATGGTAACTACACCGGTCAATTTGACCACTTTTGCTCTC

TGTGGTTTCGTCCGAGCCCAGGATGACGCTGACAGTGCCCTTGACAGGCTTTGGCTGAAGAAGAAGGTTG

AACTTCGCCAACCATAGATTTAGTTGGAGTATGTTATTTTTCTTTGGACAATGTTTGTCAAATGAGAGAA

TGTTAACATCTTGATACCTCAGTTGCTGCTTTGGTTGC

>FM890757.1 FM890757 Jatropha curcas embryo 56-70 (DAF) Jatropha curcas cDNA clone rjcaeb1_000681, mRNA sequence

TTTTTTCTTTTTATACTTACAGGAACATTTTCTTCGGTTGACTAATCCATCCATGTACAAAATATACATA

TGGGCTCATACAGGCCTCACATGGGCTCTGTCGTCACTCATCCCACCTATACTTC

>FM890756.1 FM890756 Jatropha curcas embryo 56-70 (DAF) Jatropha curcas cDNA clone rjcaeb1_000677, mRNA sequence

GCTGCAGTGAATTCGAATCTTCCGCTGTCGCTTGAGTCACCACACTGTAATGCAGCTCAATCACTAATCT

GGTATCCAAATAATCCAACAGATAAACCAAAACTATTAGTTCCACCGAAACAGATTATTGTGCCTTGGGA

AACATGGACCAATGATATGGCTCCGGGGCGCAGTTTGAGTGTGAAAAAACCAGATCAGTGCAAATTGGTC

GGGGAGTGGGCTCACGATACAGAGATTGGGATCGATGTTAATCCGACCATCAAAACCGGTTCGGCCACAC

GGACATTGAAAGCAGCTCCGCCGGTCGATACGACCACTATATCCACTTCTCGCTCCGTACGAACTCCAAG

TC

>FM890755.1 FM890755 Jatropha curcas embryo 56-70 (DAF) Jatropha curcas cDNA clone rjcaeb1_000673, mRNA sequence

TGCAGGAATTCGAATTCTGATCTTCTCCAAAAACCAATCAAGAGAATGTCTCTGATTCCAAGCACCCTCT

TCGGTGGCCGGAGAACCAACATTTTCGATCCCTTCTCTCTCGATATCTGGGATCCATTTCAGGACTTCCC

TTTCACAAGCACTGCTCTTTCTGCTCCTCGTTCTGAGTTATTGAACGAAACGGCAGCGTTTGCCAACACC

CGCATAGACTGGAAAGAAACACCAGAAGCTCATGTATTCAAGGCTGATCTTCCAGGGCTAAAGAAAGAGG

AAGTGAAAGTGGAGATAGAGGAAGGTAGGGTCTTGCAGATAAGCGGAGAGAGAAGCAAAGAGAAAGAAGA

AAAGAATGATAAGTGGCATAGAGTTGAAAGGTCAAGTGGTAAGTTCTTGAGAAGGTTTAGGCTACCAGAG

AATGCAAAGGTTAATGAGGTTAAGGCTAGCATGGAAAATGGAGTCTTGACTGTGACAGTGCCTAAAGTGG

AAGTCAAGAAACCGGGACGTTAAGGCTATTGAGATTCTGGTTGAAACTTTAGTTATGTTTGAGTGTGTCT

AAGTCTAAATGGTACCCATCATCCATTAAA

>FM890754.1 FM890754 Jatropha curcas embryo 56-70 (DAF) Jatropha curcas cDNA clone rjcaeb1_000671, mRNA sequence

AATACAAACATGATTCTTTTACATTCCTTACAGATGTTTCATTATAGCAAGTAACATTTAACAAACCTGA

AGATATCGTCATCATCCTCGTCATCTTTCTATCCTAAGAATATAATAACCAAATCCATTCGGAAAATTTA

CCATAATAAGGTGTAAAAGACTATATATATAGAGAGAGAAAGAGAGAGAGCAGCATCTTTATATAAAAGG

AAAAGGATCACATTCCAAGGTTTCAAGTCTAATGTATTCCTGAACATCATCGCCTCAGGCTCTCCACTAT

GTAGTCACAATACAGATTCATGGAATGCTGAATTGCTTTGTAGGCATCAGAAGCTGGAAGAAAGTCATTA

ACGGCAACTTCCTTGTTTTCATTTTACTTGTAATCTTCAAAGAAGCGGCGGATCTCAGCCAAACGATGTG

GTGGGAGGTCCTTGATATCATCATAATGGCGATACTCAGGATCATCAGCGCAAACAGC

>FM890753.1 FM890753 Jatropha curcas embryo 56-70 (DAF) Jatropha curcas cDNA clone rjcaeb1_000666, mRNA sequence

TTTTTCTTCTTTTTCTATTTTACAACCAAAAAGCCAAAATATTTCTTCCCACCTTTACCAAAAAGCACTA

ACTGTAGACAGGAACGGGCAACTCTCATAGGGGGTTAGGTGGAGGTCTAGGGGGAAAAACCATCATCGGC

AGGATTTCAAAATCCGCCGCTAATCATGTAACAAAA

>FM890752.1 FM890752 Jatropha curcas embryo 56-70 (DAF) Jatropha curcas cDNA clone rjcaeb1_000662, mRNA sequence

AACCAACAGGTAATTATATATACTGCAGGTTATTTAGCAATAGGGAGAAAGTATTAGCAGTCCTCCCAGA

AAGGGAACGACCATAAACCCCTCTCAAAGTTACAACACACATACTAGCTAAACCCTAGTCATTTTAACCC

ACATGTAAACTACATTACTAAACTAAAGTACACTGCTAAGCTAATACTTTATTACCAACATTATTATTAT

TATTACTACAAAATACCATTTATAGTTTTAATCATCACACTAGCTCCCAGTCCTGAACTTCGACTCATCG

ATAGTGATGCCCTCCTTCTCAGCTCTTTCTCCACCAGACTCGTCCGTCGTGCTCAGCCCACCTTTCCGGC

CCATCTCCTGATATCCTTCTGTCCCCAGCTGCTCCTTCCTTGTCTGTCCTCCTCTGCTTCTCCCTTCAGC

AAGATGCTCTTGAGCTTCAAGGCTCTTGCCCCCAGTACCTCCAGGGACAACAGTCTCTCCACGCCTTGCT

CTTTCATCAAGCTCTTGTCTTGATTGATTTGAAGACATTTTTGACTTTTTTGCTACCAATTTCT

>FM890751.1 FM890751 Jatropha curcas embryo 56-70 (DAF) Jatropha curcas cDNA clone rjcaeb1_000661, mRNA sequence

TGCAGGATTCGAATTCTTTCAGAATTCGAAAAATGGCATTGATTCCAAGCTTTTTCGGTAACTCACGAAG

CAACATCTTCGATCCTTTCTCTGCTCTCGACGTTTGGGATCCATTCAGAGACTTTTCTTTCCCTTCATCT

TCTTCCCTCGTCTCTCGTGAAAATTCTGCTTTTATTAACACTCGCATTGACTGGAGAGAGACACCAGAGG

CTCATATATTCAAGGCTGATCTCCCTGGGGCTCAAAAAAGAGGAAGTGAAAGTAGAAATTGAAGATGACA

GAGTGCTTCAAATCAGCGGGGAGAGAAATGTGGAGAAGGAAGACAAGAACGACACGTGGCATCGCGTGGA

GCGTAGCAGTGGCAAGTTCATGAGGAGGTTCAGGCTGCCGGAGAATGCAAAGATGGATCAAGTAAAGGCT

TCAATGGAAAACGGAGTACTTACTGTCCCTGTTCCTA

>FM890750.1 FM890750 Jatropha curcas embryo 56-70 (DAF) Jatropha curcas cDNA clone rjcaeb1_000659, mRNA sequence

ATTCCTCATTTTCGTTACTAATCTCGCAGCTGGTCCTATGTTGAACGACCGGGTCTGCTCCTAATGTCTG

CTTTCTTAATTGCACAACTGGTTGCAACTTTGATTGCTGTATATGCCGACTGGGGTTTCGCAAGGATTAA

GGGAATTGGCTGGGGATGGGCTGGTGTAATCTGGATTTACAGTAT

>FM890749.1 FM890749 Jatropha curcas embryo 56-70 (DAF) Jatropha curcas cDNA clone rjcaeb1_000652, mRNA sequence

TTTTTTTTTTACAATAAATTAGATTTAGATTAGTTGCTGAAACGTCACAAATTCAGTCTTGTAAAGATTC

ACAACCACCAAAGAAGAAAAGCTAAGTATGAAACAACTCGAAAAAGAAGAAACACATATTTATAAAGCAT

ATTCACATATTTCTTAATTTGGTCCTCTCTTCTTCTGATTAAATCTCCATTAATAAGCCGTGCTCTCCTT

TCTTTGGAGTTATGAATTGAACAAACTTGCTCTTGCTCTTGTTCTTGCCATTCCCACTGCCACCATTAGG

GGAGCTATGATGATGGTGATCTCTAACGCCTGTATATTTCTTTATATTTTCCCTCACATATCTTTCATAA

ATAAAAGCTGCCCCTCTAAATTGTGGCAAGACAAGCCAAGCCACGAACGCTAGCTTCACTGTATACCAGA

TTGGAATCCATTCTAAGATGGATTGGAGAATCATTTCTGTGAGAGTTAAAAACGAGTAGAGAATCCAATA

AGCAAGCCACTGCTGATCATCCAGTTTGGAGGGGGCTCTCTATG

>FM890748.1 FM890748 Jatropha curcas embryo 56-70 (DAF) Jatropha curcas cDNA clone rjcaeb1_000650, mRNA sequence

GGCTGCAGGAATTGAATTCCTCATGCAACTGAAGAGTCTTCAAAGTTACTGGAGCTGATGAACAGGTGAA

ACCTGATGCAAACATTCCCTCAGCTTCTGCTGCCAGTCAGGAGATGATACAAGAGGGAAAGGTTGAAACT

GTTGAAGAATGTGCTTCTGTTCCTTCGGTAAAAACTGAAACAAATGATGAAATACTTTTCAATCCTAACG

TTTTTACCGAGTTTAAGCTAGATGGGAGTCCAGAGGAGATAGCAAAGGATGAAGAGAATGTGAGGAAAGC

TAGTTCATATCTAGCTGATACTGTGCTTCCTAAGTTCATCCAAGATCTTTGCACCCTTGAAGTGTCACCT

ATGGATGGGCAGACTCTTACTGAAGCTCTACATGCTCATGGAATTAACGTGCGCTACATTGGAAGGGTTG

CTGAAGGGACGAAACATTTACCTCATTTATGGGATCTTTGTTCTAACGAAATCGTAATCAGATCTGCTAA

>FM890747.1 FM890747 Jatropha curcas embryo 56-70 (DAF) Jatropha curcas cDNA clone rjcaeb1_000649, mRNA sequence

CAGGAAAAAAAAAAAAGAAAAAGAAGAGATAAATGAGGCTCAGATTCGCCTTTAATCAAACAATAAAAGG

AACAGAGTGACTGTGGCTGCAAAAATGGAGCTCTCTACAAAGAAAACATCCACATTGCCCCGCCAAACCA

TCAAATTGTTCTTTATTGTTAACTTCTTGCACTCTAGAAAAATGAAAATCATCATAGCCTCAGCAACAAC

AGAACCTCCACAGAGAAGATTATGTACTGCTGTAATGTAGATCAAACCTTAGGGACGAAATAATAATTTA

CCCAAAAATATATATACACATACATATACATATACGCAAGAGCGATTGATTCATAACACGACGGTGAAAT

CATCGGCTCCGCAGTCGTTCAGAGAGCAGAGCAACCGCCAAACTCAACAGTCTCTCTGAGAGCAAAGCCT

CATTCAGAAGAGGGCTTGAGGGGGGGCCCGGTACCCAATTC

>FM890746.1 FM890746 Jatropha curcas embryo 56-70 (DAF) Jatropha curcas cDNA clone rjcaeb1_000639, mRNA sequence

ATTCCAGGGAATCCACAGTCCATGTTGTTTACTCCCACCAGCAGGAGACCATAAGGTAAGTTACTACTAA

AATAGCATTGTGATTGGTGCTTCTACTAGCCAAGGCCGCAACTAAGAGATCCTTCTTGTAAATTTAGAGA

AGGGATCCGTCGTATATTGTAGTATAGAGAGTTGTAGAGTGTAAAAATGTAGAACTTCAACTTCTGCAGA

AATCAATAAAATGTGGCCTTCGTTATGTTTCT

>FM890745.1 FM890745 Jatropha curcas embryo 56-70 (DAF) Jatropha curcas cDNA clone rjcaeb1_000636, mRNA sequence

ATTCCCACTTTCCTGTGTGGCTCTGATCCCAGGGAAGTACCTTGAAGTTGCAGAGAAGGCAGCAACATTC

ATCAGAAAACATCTTTACAATGAGCAAACACGCAGGCTTCAGCACAGCTTTAGGAATGGTCCTTCCAAAG

CACCTGGGTTTTTAGATGATTATGCTTTCCTTATTTCTGGGCTGCTCGACCTTTATGAGTTTGGTGGTGG

AATATACTGGTTAGTGTGGGCAACCGAACTGCAGAATACCCAGGATGAATTATTTCTTGATAAAGAGGGA

GGAGGCTATTTTAATACCCCAGGTGAAGACCCTTCAGTCCTTCTCCGAGTAAAGGAGGATCATGATGGAG

CAGAGCCCTCGGGAAATTCAGTTTCTGCAATTAATCTCGTTAGATTGTCCTCTATGGTGACTGGTAGTAA

ATCTGATTATTATAGGCAGACTGCAGAGCATCTTCTGGCAGTTTTTGAGAGAAGACTAAATGAAATGGCT

ATGGC

>FM890744.1 FM890744 Jatropha curcas embryo 56-70 (DAF) Jatropha curcas cDNA clone rjcaeb1_000635, mRNA sequence

ATTCGGGAAGACGAANCCTAGAGGTTGAGTCTTCTGATACTATCGATAATGTCAAGGCCAAGATCCAGGA

CAAGGAAGGCATCCCTCCAGACCAGCAGCGACTGATCTTCGCCGGAAAGCAGCTAGAGGATGGCCGCACT

CTTGCCGACTACAATATCCAGAAGGAATCCACTCTCCACCTGGTGCTTCGTCTTCGTGGTGGTGCTAAGA

AGAGAAAGAAGAAGACTTACACCAAGCCAAAGAAGATCAAGCACAAGAAGAAGAAGGTCAAACTTGCCGT

GCTTCAGTTCTACAAGGTCGATGACTCCGGAAAGGTGCAGAGGCTGAGGAAGGAGTGCCCCAATGCTGAG

TGTGGAGCTGGAACTTTCATGGCCAACCATTTTGATAGGCACTACTGCGGCAAGTGCGGCTTGACTTATG

TTTACAATAAGGCTGGCGGTGATTAGGTTATTGTTAATGC

>FM890743.1 FM890743 Jatropha curcas embryo 56-70 (DAF) Jatropha curcas cDNA clone rjcaeb1_000634, mRNA sequence

GCAGGAATTCAATTCGTTGCTTGAGGTCTCTTAAAACCGAAAGGAGGAGGTTAGAGCATATATTCAGGCA

AAAGCAATGAGCTATTGTAAAAGCCTCCAGGGCAGCAAATTCAAGCTATAGAGTCATCGTAGAAGAAGGT

GGCGGCGTTTGGCCTTTCGGAACTGAATCCATCGGTCCAATTAGTATCCTGAAGAACCCTGTTTTTAGGA

ATAACTATGGTCAACTCTTTGAAGTTGGCTCTAGAGATTTCAAGCAACAGCTCCGAGACCTTGACCTTTC

AGTCTCCGTTGCCAACATCTCTCGTGGAGCAATGTCTGGGCCCTATTACAACTCCAGAGTAACCAAGATT

CAGTGGTTTGGATGGCGAAGTACTTTGAAATGGCTTGTCTCACT

>FM896129.1 FM896129 Jatropha curcas embryo 71-95 (DAF) Jatropha curcas cDNA clone rjcpga0_003286, mRNA sequence

ATTCGTTGCTATTCTGGAGGTCGGGTTAGGTGGAAGGTTTGATGCAACTAATGTGGTTCAAACACCTATT

GTGTGTGGTATAGCTTCCCTTGGGTACGACCACATGGAGATTCTTGGAAATACTCTTGGTGAAATTGCTG

GGGAGAAGGCTGGTATCTTTAAGCATGGGATTCCAGCCTTTACAGTGCCTCAACCTGATGAAGCAATGCG

TGTACTTGAGGAGAAGGCTTCTAAGTTAGATGTACCTCTTCAAGTGGCACCACCATTAGATTCCAACTTG

CTAAACGGTTTACAACTTGGGCTTGAATGTGAGCACCAATATATATATGCCGGCCTTGCCATTGCACTAT

CTTCTAAATGGCTTCAAAGA

>FM896128.1 FM896128 Jatropha curcas embryo 71-95 (DAF) Jatropha curcas cDNA clone rjcpga0_003282, mRNA sequence

GACAATGGGGGTTCNTGGATGGAATCCTTTTGAGAAACTCACGGAAAGTTTCAAGGGACAGTCAATAGAA

GATGTGTTACGACAGCAGCTTGAAAAGGGAGAATTTTATGATGGAGGCAGTGGGAAAAATCCACCTCGTG

GTGGCGGTGGAAGCGGTGGCAGTGGGGATGGGTATGGAGAATCTGAGGATGAGGGTATTGCAGGAATCAT

TGATGAGACTCTGCAAGTGGTTTTAGCTACCATTGGCTTTATCTTTCTGTATGTTTACATCATCAGTGGT

GAGGAGTTGACTCGTCTAGCAAAAGACTATATCAAGTATCTTTTAAGAGGAAGTAAGAGTGTGCGTTTAA

AAAGAGCCATGTATAAGTGGAAAAGGTTCTACCAGAAACTCACAGAAAAGAAGGAGATTGACAAGATGTG

GCTTGAAAGGGCAATTATCAATACCCCAACTATCTATGAC

>FM896127.1 FM896127 Jatropha curcas embryo 71-95 (DAF) Jatropha curcas cDNA clone rjcpga0_003281, mRNA sequence

CGAATTCCCTACTTCTCATCATGATGCTTCTGCTCTGCATGGACTTGTTCCACGAATTCATTACAATCTT

TACAACCCAATTGACCCATCTTCGGCTGCGCGTGAAATTCCACGCGCTCAGCAAGGACTGTCTTTAAGCC

TTTCATCTCAGCAACAGCAACAGCAGCCGCCACCGCCACCAGGTTATGGGTCGCAGGCTCAAGCCGTGTC

AGGTGAAGATATGCGAGTTTCAGGCGGGTCTGTATCGTCCGGTTCAGGTGTTACAAATGGGGTGTCAGGT

ATGCAAGGGGTTTTGTTGAGCTCTAAGTACTTAAAGGCTGCTCAAGAACTTCTTGATGAAGTTGTCAACG

TTAACAGTAATGAAATCAAGAATGAATTGTCCAAGAAAGCTAATGGGATTAGCAGTAACAATAGCAACAA

AATGATTGGAGAATCATCAGGTGGTGATGGGTCAGCCGGTGGAGACGCCGGCGGTGGTAAGCGCGCAGCA

GAGCTTACCACGGCAGAGAGG

>FM896126.1 FM896126 Jatropha curcas embryo 71-95 (DAF) Jatropha curcas cDNA clone rjcpga0_003280, mRNA sequence

GTGCCTGTTGTACTCCAGCCCATTGATTTGTCAAATGGGGTTGCAGGTGAACTGTCTTCAGTTGCAACTC

AGCCTGCTCATGCAGTGGGCTCTAAGGGCAAGCAGGCACTAGATGCTGCTCAGCCATCAAATGGATCACC

ACCTGCCAACCAATTGCAAGCAATTGAAACCCAGTTGCTTCACTTTCAGTCTCAAAATAACCACCAGGTA

ATGTCTACAGGCATACAGAAGGAAGCTGGTCTTAATCAAAATTCAGCTGAGGCAGCTGGTATGCCTTTTG

AGAAACTGTTAACTGAGGTCATTAAGAGAACTCAAGTACCATCTCCATCAAAAGAGAATAATACTAGCTC

AACTGTTAAATCATGTGCAGAAGATGATGACAGTAGCGCAGATCTGCCTCTTTCAGTTGAGCCCCTGCAA

TCTGTGCAGCCAGTTCTGAATAATCACCCTGCAGTTCTGTCAAGACCCTTGGAGAACTATAGAACTGGAA

AAATGACTGAGCTGCTACAGGTTTTGCAGGAAAACATGCCCCAAAACCAGGTCACCGTGGACCCCAAAAT

GAAGGAAGATGAACTAGGCCCANAAACCGAACATGGAGATGAAGATGGTGGCAATTCAATGAAACATTCA

CAGGCATCAAGTGTCTGACGTGATAAATGTTACCTTGGGGAGCAATC

>FM896125.1 FM896125 Jatropha curcas embryo 71-95 (DAF) Jatropha curcas cDNA clone rjcpga0_003278, mRNA sequence

CTCGTCGCAGATCTCTTTCGCTTGGCAATCTGAAGGTATAAGGTCTGGAGGATGGCAGATAATGAAGGAG

GGGAGAATGTTCCTCGCGGGAATGAGTGGGAAGTTGTTTCACTTACGGCATCAACATATGCTGCTGCTCC

TGGTCCTAATGAAGTGGAAATGAAAGATGAAAACAAAGATAATGCATATGTGGGGGATGAAGCAGAAACT

TCCCGTGCCTTGTTCATGTCTGGGCACTTTGTCTTTCCACCAAGCCAGCATGAGAATTTACCACTGGAAC

GAGATAAAAGCGAGATTTTAAATGAGCAAGTTGGGAAGAATGCTGCCTCTGAACTTGACGTGCAAGAAGG

GGATAAATCTGGTGGGAAGGATGACGAAATTTGGGCACCGAAGCATGAATCTGAAGAGTTCCCTGGGATA

CAGATTTTTGATGAAAAGGGTAACAGACTACCCATCTCTGGGGCTGAATTTGAAGAAAGCATGACTCTTC

CAGAGTTAAATCTAAATGAAAAGGAACAGAGTTTATATAGTGCTGCCACATTTAGTTCCTTCCATAGTGA

AACTGAGCTTGGTGGTTCAGCCACATATGAGGATAACCTGGGTATTCATGAAGCAAGTGAACAATCTGAG

CAGGAATTGGATTCTCTACAGATAGTTTCACTCTCA

>FM896124.1 FM896124 Jatropha curcas embryo 71-95 (DAF) Jatropha curcas cDNA clone rjcpga0_003277, mRNA sequence

CGAATTCTCTTTATCTATACCCNATCTGGGTAAATCCCAACCCTAAACGGAATTGCATCAGGGAACCCAA

AAAGCCTGAACCTCAGAGTTAGAGCGTCAAGTTTTCCTCTAGCTAGCAGAATCGTTGTTAAAAATTTAGG

ATATTCAGTCAGTGAAACTAGTTTGCGAAAGGAATTCTCAAATTTTGGCGAAATAGCTGAAGTTAAGCTT

GTAAAGGATATTGATATCACTAAGAGGCCTAAAGCATATGCATTTATTCAATATACTTGTCAAGATGATG

CCGTTTTAGCTCTCGAGAATATGGACCGCGAGAGATTTGATGGCAGATTGATTTACATTGAAATTGCCAA

ACCTGGGAAACACAGTTTTGGAGAATATCCAAAAGCCTCTGGACCTCCAAAGAAGGAGAATTTGCAGGAG

CAAGACGAGGTTTCAGATTGCTGGTACTGATCAAAGATGGTTTGAAACCAGAGAAGCAGGTTGTAAATTA

GGGAATGAGGTAGAATGAAGAGCTACTTCTCAAGTGAAAATCATTTCAAGATGACCATAACCGATAACGT

ATGACATTGAAATGGCAAGTGCTGGTCAGTTTCATTAAAGTGAGTATATTACATGAACTACGTACCCTAC

AGTTTCTTGAAAGCACCTTATGTATTGCATCATTATAACTTACAATCCTT

>FM896123.1 FM896123 Jatropha curcas embryo 71-95 (DAF) Jatropha curcas cDNA clone rjcpga0_003275, mRNA sequence

GGTCCAAAATAGAATCAACTCTTTATATATAGCACGATATGTTGTGAGATAACTTACAAAAACATCATAT

ACAATTCACATTGACACAAAACACTTGGAAAAAGTCATTGCGGCTGAAAAAAGATGCGGGCTATTCCCTC

TTGAAACATCCATTATGATAACTAAGGAAATTCAAGATGTTTTCCCCCACTTAAAAAGTTAGTCAAAACT

AGCTTTTTATGACTCAACTGATATGTCTGCATTCATCTGCTCGGTTATTTTGCCTGGAACTGCTTTTGTT

GCAATGTGTTCCTGTTTTGATCTGTCCTTGGTTTTGCGGGTGCCATTAGTTGTCTTAACCTTGGTTTTGG

CCATGATGGGGGACGTTGTGTCTTCCTTGTGATTACCAATGCTATGCCGATGTCGAGCACAATGTTTTCC

AACTTCATCCTTAGATGACTGGATTGCATCACCACAGCTCTTTCTCCGATTCAGTTTTGGCGACACTGGT

CGAGTCAATGGCAGCTTCTTGGGTTCAACTTTAGGAGGAGGTGGCGCATAGTAGAAACTAGGAACAGGAT

TTGCTCTAAACATCATGCTCTTTCTG

>FM896122.1 FM896122 Jatropha curcas embryo 71-95 (DAF) Jatropha curcas cDNA clone rjcpga0_003274, mRNA sequence

ATTCTCTAAACCCTAGGAGAGAGAGTGGGAGAGGGAGAATAAAAAAAATGGTTCAGCGGCTCACATATCG

GAAACGGCACAGCTATGCCACGAAGTCGAACCAGCATCGGGTCGTCAAAACTCCCGGAGGAAAGTTGGTT

TACCAGACCACAAAGAAGAGAGCTAGTGGCCCTAAATGCCCTGTTACTGGCAAGAGGATTCAAGGGGTTC

CTCACTTGAGACCTGCTGAATATAAGAGGTCAAGATTGTCAAGAAACAGGAGGACTGTGAATCGGGCTTA

TGGTGGTGTATTGTCTGGTGGTGCTGTCAGAGAAAGGATTATTCGTGCCTTTCTGGTTGAAGAGCAAAAG

ATTGTGAAAAAGGTCTTGAAGATTCAAAAGGCAAAGGAAAAGCAAGCCTCAAGAAGTTGAGATAGAAATG

TCCAGTGATTTGGAGGATTTGTCGAAACAGCCAATGTTAGTGATTCATGGAAAATATTAAGGGGATCCCA

ATTCGAATATGTATTTTGTTTTGGCTCTTGTGAACCATGGTTTCTTAGCTTTGTCCACAAATTATGCTGC

AATTTTGTTCCGCTTTATCATTTCTCTGTTGTGCTGTCTTGAACTATTTGCTTGGATGACTGTG

>FM896121.1 FM896121 Jatropha curcas embryo 71-95 (DAF) Jatropha curcas cDNA clone rjcpga0_003273, mRNA sequence

ATCCAAGAACAGCAACAACTCGAGCCATTTATTTCCGTTTTCACGAAGCCTCGGAGTCACTCAGATCGCG

GAACAGGGTGTATCCCGGTGACTGATTAATAATAATGGACGAACTCAGATCGGCTTTGGAAGACCACATG

GTGCAAATGGCGGATCTCGTCCAGAAACTTTCTGCAGAGCTCCGATCCGGCCTTCGACCCGCCTATGAGA

ATTTCATGGGATTCTTCCATGCCATAGACTGGAAGGAGCCTTGGTTGATGGGTTTGTTGACATTTCATGC

TGTGTTGCTGATAATAGCCATCATTTCAAGGAAACATGTGAACTTTCAGATGTTCTTGTTTCTCCTTGCT

TTGGGTGGTGTATACTTCGCCGAGAGGCTTAATAGAGTTCTTGGTGACAATTGGAGAAGCTTTGCAAGCC

AGAATTATTTTGATCCACATGGAATTTTTCTTTCAGCACTCTGGTCTGGGCCTCTTCTCATCATTGCAAC

CATTATTTTGATAAACACTCTCTTTTCATTATGTTACATGATTGTTAGGTGGAAAAGAGCAGAACT

>FM896120.1 FM896120 Jatropha curcas embryo 71-95 (DAF) Jatropha curcas cDNA clone rjcpga0_003272, mRNA sequence

ATTGTAACTATGACTCTCTTAAGTGTAGCCAAATGCCTCGTCATCTAATTAGTGACGCGCATGAATGGAT

TAACGAGATTCCCACTGTCCCTGTCTACTATCCAGCGAAACCACAGCCAATGGAACGGGCTTGGCGGAAT

CAGCGGGGAAAGAAGACCCTGTTGAGCTTGACTCTAGTCCGACTTTGTGAAATGACTTGACAGGTGTAGG

ATAAGTGGGAGCCGACAGGCGACGGTGAAATACCACTAC

>FM896119.1 FM896119 Jatropha curcas embryo 71-95 (DAF) Jatropha curcas cDNA clone rjcpga0_003271, mRNA sequence

AGGATTCTATCCGTCGCCCGATGGGAATTGTACTTTAAGGCGGCCCTCGCGGCTCGTCCGCCGCGGGGGC

TTGGCCAACGGCACGTGCCTCTGGGGGCCGGATGGCCCCTACTGCGGGTCGGCAAACGGGCGACGGACAC

GCGCGTCGCTTCTGGCCCGGATTCTGACTTAAGGCGTTCAGTCATAATCCAGCGCACGGTAGCTTCGCGC

CACTGGCTTTTCAACCAAGCGCGATGACCAATTGTGCGAATCAACGGTTCCTCTCGTACTAGGTTGAATT

ACCATCGCGACACCATCATCAGTAGGGTAAAACTAACCTGTCTCACGACGGTCTAATCCCAGCTCACGTT

CCCTATTGGTGGGTGAACAATCCAACACTTGGTGAATTCTGCTTCACAATGATAGGAAGAGCCGACATCG

AAGGATCAAAAAGCAACGTCGCTATGAACGCTTGGCTGCCACAAGCCAGTTATCCCTGTGGTAACTTTTC

TGACACCTCTAGCTTCAAATTCCGAAGGTCTAAAGGATCGATAGGCCACGCTTTCACGGTTCGTATTCGT

ACTGGAAATCAGAATCAA

>FM896118.1 FM896118 Jatropha curcas embryo 71-95 (DAF) Jatropha curcas cDNA clone rjcpga0_003270, mRNA sequence

GCAAAGGATTCTATCCGTCGCCCGATGGGAATTGTACTTTAAGGCGGCCCTCGCGGCTCGTCCGCCGCGG

GGGCTTGGCCAACGGCACGTGCCTCTGGGGGCCGGATGGCCCCTACTGCGGGTCGGCAAACGGGCGACGG

ACACGCGCGTCGCTTCTGGCCCGGATTCTGACTTAGAGGCGTTCAGTCATAATCCAGCGCACGGTAGCTT

CGCGCCACTGGCTTTTCAACCAAGCGCGATGACCAATTGTGCGAATCAACGGTTCCTCTCGTACTAGGTT

GAATTACCATCGCGACACCATCATCAGTAGGGTAAAACTAACCTGTCTCACGACGGTCTAACCCCAGCTC

ACGTTCCCTATTGGTGGGTGAACAATCCAACACTTGGTGAATTCTGCTTCACAATGATAGGAAGAGCCGA

CATCGAAGGATCAAAAAGCAACGTCGCTATGAACGCTTGGCTGCCACAAGCCAGTTATCCCTGTGGTAAC

TTTTCTGACACCTCTAGCTTCAAATTCCGAAGGTCTANAGGATCGATAGGCCACGCTTTCACGGTTCGTA

TTCGTACTGGAAATCAGAATCAAACGAGCTTTTACCCTTTTGTTCCACACGAGATTTCTGTTCTCGTTGA

GCTCATCTTAGGACACCTGC

>FM896117.1 FM896117 Jatropha curcas embryo 71-95 (DAF) Jatropha curcas cDNA clone rjcpga0_003269, mRNA sequence

CGCAGGTTTTTTTTATTGAGGATGTTTCTCAGTGTTGATGCATTCATTTTAAATGTTCAGAATTGCTGAA

CTGAGAAATATGATAGTCAAGTGGTGAATATGAAACACATAATCATCTTCTTGTTGAGCTGACTGAAGTA

TTTTTCCTTCCTAACCAGGTATTGGGTATTTACATGGGAATAGAGGAAATAAACCTGCTCTTTTTCACCA

GAATATATCAGCTGAGAAAGTGTTCATTGATAGACGATATAATCCATTACTCTCATATTCAGGCCTGCAC

AAAATTCTTGCAGATGACATTATCTTCTCTATGCTAAAAGCTAGTGCTGCCATGGGATACCTGGCTCCTG

AGTACACAACTACTGGCCGTTTCACTGAAAAGAGTGATGTTTATGCATTTGGTGTGATTATACTACAACT

CCTCAGTGGAAAACATAACGTTACACCATTGACTTGCCACGCAGGGGAATCATGCAATGTAGAAGACTTT

ATTGATGCAAATCTTGAAGGAAAGTACTCAGAATTAGATGCAGCCACACTTGGGAGATTAGCTCTTCTTT

GC

>FM896116.1 FM896116 Jatropha curcas embryo 71-95 (DAF) Jatropha curcas cDNA clone rjcpga0_003267, mRNA sequence

TAGCCGACCGATCACAATAATGTCCATGGATTCAACTCATAAGTTGGAGCTCGTCGATAAGGCAATTCAA

AAACTCTTAGCAGAGAAGAAAAACAAACAAGATGATACTGATCAGCTTCTCCTCTCCAGATTACTTTCTG

AGTTAGAAATGCTGAAAGAGGGCATTTCAGTTAACCAATCTGATGTATCGATTGATCAAGAAAAGGCAAA

TTCTCCTTCAGCTGATGAAGAAAGGTTCAAAGAACATAAAAATGGTAGCAGCAGTGAAATTATAGCAGAA

GAGATTGTGAAGGAGCTAAAAATCGTGAAGAGACAGAATATGGTGACTCACTGTCTTCTTTCAGCTGTTA

TTGTTCTAACTTTAGCCTGGCAAATATCTGAGGTTTCCCTCATTTTGAAATTTAAAGATGGATTGAGTCA

CCCATTTAAGTCCTTTGGAAGTATGGTTTTTGGAATGTTCAGATTTCCTGGAAGAAAAGCACAAGATGGA

GACAAACAACAGCAGGAAGAAGGATCTTCTTCTAGACCTTCTGTTCATATGCCTGAGCTTCCCCATCCGC

ATATGCCAGAGTTACCGCATGTTGATTTAGGCTNTAATGGTCATGACAGGAAATGATAATGATAGGATTT

GTATTGGGCTTCAAAATG

>FM896115.1 FM896115 Jatropha curcas embryo 71-95 (DAF) Jatropha curcas cDNA clone rjcpga0_003266, mRNA sequence

CTGCTCGTTTCATTTTTGTATTCAGCATGCCTTCTGCTGCATATAACTTCTCATTTTGCTTGCTAGTTGT

AGATTTTGTCTGAGTTTTGTTTGCATCATCTTCTTCAAGACCCATTGGTTGGTCTTCACCATCATTCATT

TCTTCAGAGTATTCATCACCCTGGGTTGATGACCGAGTCTCCATATCTCCCTGTATCATACTTTCATCAA

AATTGATAGGACAACTAGGAGGTACTTCAACAGGATTGTAGTCATTAACGGACTTGAGACTACCAATAAA

TGAGGATTCACCACTGTAGACCTCATCAACATAAAATTCCTTCCCAAGCTCTGAAACAATCCTAGCTTCT

GAAGGTTCCTCTCGATTCCTAATTGGAGGCATAGTATACTACGGGATTTTACCCTCATTCCAATCATGCA

GAACAATACGAGCAGAAGCTTCAACATCCACAAT

>FM896114.1 FM896114 Jatropha curcas embryo 71-95 (DAF) Jatropha curcas cDNA clone rjcpga0_003265, mRNA sequence

GCAATATCAGGGAAGCCCTAATTTTGCTTTGCCTTATTTTTATTTCTCAGCCTCCATTCTTTCAAATAAA

TTCCTCATTTTCTGATTTGTGATAATGGCCGGAAAAGGAGAGGGACCGGCGATCGGAATCGATCTAGGCA

CAACCTATTCGTGTGTCGGCGTTTGGCAACATGATAGAGTAGAAATCATAGCCAATGATCAAGGGAACAG

AACGACGCCGTCTTATGTTGCCTTTACCGATACAGAGCGTTTGATTGGCGATGCTGCCAAGAATCAGGTT

GCCATGAACCCCATTAACACCGTCTTCGATGCTAAGCGTTTGATTGGTAGGAGATTTACTGATGCATCGG

TTCAAAGTGACATCAAGCTCTGGCCTTTCAAGGTTATTCCAGGACCTGGTGATAAGCCAATGATTGTTGT

CAATTACAAGGGTGAGGAGAAACAATTTGCTGCAGAGGAGATCTCTTCCATGGTCCTTATAAAAATGCGT

GAGATTGCTGAGGCTTATCTTGGCATAACTGTGAAGAATGCAGTTGTCACTGTTCCTGCCTACTTCAACG

ACTCTCAGAGGCAGGCCACAAAGGATGCTGGTGTTATTGCCGGTCTTAATGTCATGCGTATCATCAATGA

GCCCACTGCTGCTGCCATTGCTTATGGTCTTGATAAGAAGGCAACTAGTGTTGGTGAGA

>FM896113.1 FM896113 Jatropha curcas embryo 71-95 (DAF) Jatropha curcas cDNA clone rjcpga0_003264, mRNA sequence

ATTGGATTCTATCCGTCGCCCGATGGGAATTGTACTTTAAGTGCGGCCCTCGCGGCTCGTCCGCCGCGGG

GGCTTGGCCAACGGCACGTGCCTCTGGGGGCCGGATGGCCCCTACTGCGGGTCGGCAAACGGGCGACGGA

CACGCGCGTCGCTTCTGGCCCGGATTCTGACTTAGAGGCGTTCAGTCATAATCCAGCGCACGGTAGCTTC

GCGCCACTGGCTTTTCAACCAAGCGCGATGACCAATTGTGCGAATCAACGGTTCCTCTCGTACTAGGTTG

AATTACCATCGCGACACCATCATCAGTACGGTAGAACTAACCTGTCTCACGACGGTCTAAACCCAGCT

>FM896112.1 FM896112 Jatropha curcas embryo 71-95 (DAF) Jatropha curcas cDNA clone rjcpga0_003263, mRNA sequence

CAAATGCCTCATTTAAGGCTTGCTTTGTTAAATGCAATGACAGTTTGCTACAAGGCGAAGAACCTTGCCA

CTGCAGCTAACTTTGCTAGACGGCTATTGGAGACAAATCCCACAATTGAGAACCAGGCAAAGACGGCCAG

ACAAGTTTTGCAGGCTGCAGAAAGGAATATGACAGATGCCTCGGAGCTAAATTATGATTTCAGAAACCCA

TTTGTGACGTGTGGCGCAACATATGTTCCAATTTACCGAGGACAAAAGGATGTCTCTTGCCCATATTGTA

GTTCACGGTTTGTGCCTAGCCAGGAAAGTCAGCTGTGTACTGTTTGTGATCTAGCCGTTGTGGGTGCAGA

TGCTTCAGGATTGCTCTGCTCTCCTTCCCAGATACGATAATTGAAACGCTGAACTTAGAAGATTTTCCCG

CTCATCTTATGGGCAGCAG

>FM896111.1 FM896111 Jatropha curcas embryo 71-95 (DAF) Jatropha curcas cDNA clone rjcpga0_003262, mRNA sequence

GGTTTATGAAGATGGAATAATGGATGGGGGATTTGGCTGAGAAGGGGGTAATGGAGAAGAAGTGAGATTA

GGTGAGAGAGAATTATAATCTGGGTAATCAGAATTTGGAAGAGAAGTGTCTACAGGCTGTGAAAAAGAAA

AATCAGAAGTAACATGAGAAGAGAATAGGAAAATGTGTTTTGAGAATTGGACATCTCTAGAAAGAAAAAT

CTCTCTAGTAGCTAGATCCATGAGAATATAAACCTTGACCCACGATTATAGCCTAAAAAGATGCCTTTGT

GGGGCCTTAGTGCTAACTTGGTTCTATGAGCTGCTAAAG

>FM896110.1 FM896110 Jatropha curcas embryo 71-95 (DAF) Jatropha curcas cDNA clone rjcpga0_003261, mRNA sequence

ATTCCAATGATATGGACCCGTATCTAAAGGACAAAAATGAACCATGTGTGTCTCACTAGGGACTCTTCGG

ATAAAATTTAACATTGAAATGAAAATGAACCAAGCCCATTCAATTAACGAATGGGTTAAATAAAGAACAC

CGATTTTATTAAATAAAATCGGTTAACGAAGCCTGTTCGATCGATTCTTTCCTTATTTTTTTGAAAAAAA

ATCTTGCGTCCTCAGGACTGGTGACTCCATTGGAGATCGAACTCAGAATCTTTTGGGTTCAAGTAGAGAG

CTGACTCCAAAAGTTGTCCACTTGTTCACAAATCTTTAATAAATGCTATTAACTTTTAAATTGAGTTT

>FM896109.1 FM896109 Jatropha curcas embryo 71-95 (DAF) Jatropha curcas cDNA clone rjcpga0_003259, mRNA sequence

CATATGCAAGTTTCCTGCTTGCAAAGGGCTTGAGAATTTGGTCCGTCATTTCTCCAATTGTAAGACCCGG

GTTCCTGGTGGATGCGTTCATTGCAAGCGCATGTGGCAGCTGCTTGAACTGCACTCCCGTATGTGCAATG

AGCCAGATTCTTGCAAGGTTCCACTTTGTAGACATTTCAAGGAAAAAATGCAGCAGCAGACTAAGAAAGA

TGAAGCTAGGTGGAAAATACTAGTTGACAAAGTAACAGCAGCAAAGGTTACACTTGGGCCATTCTCATCT

CGGAGTAGAGGTTCGTTATCAGCAAGCTTTTGAATCTTTACCTCGTGTCTTCATTGTTGCGTATTTTAAG

AAATAATCTCAACTGTAAAATAGAAAATATTATCATAGAAGGGCCAGAAAATGGTTTTTATGACCAAACA

TGGTCTTAATCTTTCTTCTCCCGCCCCTTATTTTATATATGCCAAGCATCTATGATTCACTTGGCTACCG

CTTGCTGGTTGGATTTTCACTGGTGTATTTGTTTGTTCAGCTTTTTAT

>FM896108.1 FM896108 Jatropha curcas embryo 71-95 (DAF) Jatropha curcas cDNA clone rjcpga0_003258, mRNA sequence

TGCCGAATCAACTAGCCCCNGAAAATGGATGGCGCTTAAGCGCGCGACCTATACCCGGCCGTCGGGGCAA

GAGCCAGGCCCCGATGAGTAGGAGGGCGCGGCGGTCGCCGCAAAACCCGGGGCGCGAGCCCGGGCGGAGC

GGCCGTCGGTGCAGATCTTGGTGGTAGTAGCAAATATTCAAATGAGAACTTTGAAGGCCGAAGAGGGGAA

AGGTTCCATGTGAACGGCACTTGCACATGGGTTAGTCGATCCTAAGAGACGGGGGAAGCCCGTCCGACAG

CGCGTCCTCGCGCGAGCTTCGAAAGGGAATCGGGTTAAAATTCCCGAACCGGGACGTGAAGGCTGACGGC

GACGTTAGGGAGTCCGGAGACGTCGGCGGGGGCCTCGGGAAGAGTTATCTTTTCTGTTTAACAGCCCGCC

CACCCTGGAAACGTCTCAGTCGGAGGTAGGGTCCAGCGGCTGGAAGAGCACCGCACGTCGCGCGGTGTCC

GATGCGCCCCCGGCGGCCCATGAAAATCCGGAGGACCGAGTGCCTCCCAC

>FM896107.1 FM896107 Jatropha curcas embryo 71-95 (DAF) Jatropha curcas cDNA clone rjcpga0_003257, mRNA sequence

ATTCCATCGTTCTGTCTCTGTTGACACCCTCAATCGAAAACCTAATTTTGCAAAAGATATCATGGGCGTG

AGAGTTGGGCTCTTATTTCTTCTTATGTTGGGTGCTGGCTGGGCTTGTGCTGCTAGACAAATGGCTGACA

CTGAGCTTTCTCGTAGAAGCATGGCGATCTCTGAGTTACCAGCTACACAAATGAGAGACCAGGTACAAGA

AAAGGAAAATCAAGCTTCAGATGGAGTTATCAAAAATAACCAAGTATGCACATTGTGTGAGGAATTTGCT

ACCCAAGCACTTGATTACCTAACTGAAAACAAGACTCAGACTGAAATTGTTGACATCCTTCATGGGGCCT

GCTCTAAGGTCCATTCTTTCAAGGAACAGTGCATCACTTTGGTGGACTATTATGCTCCTCTCTTCTTTCT

GGAGATTTCCTCCGTACAACCTGCAGATTTCTGTAAAAAAGTCAATCTCTGTCAGGAAATAGTATTGATT

TTCTCAAAACTCCAAGAAGACAA

>FM896106.1 FM896106 Jatropha curcas embryo 71-95 (DAF) Jatropha curcas cDNA clone rjcpga0_003255, mRNA sequence

CTTTCAGTCTTGGTGTTTGCAATGGGTGTCAACTCATGGCTCTATTGGGTTGGGTCCCAGGTCCCCAAGT

TGGGGGTGCACATGGTGCTGGTGGGGACCCGTCACAACCTAGGTTTATTCACAATGAGTCTGGTCGATTT

GAATGCCGCTTTACAAGTGTGAGGATAAATGACTCACCTGCTATAATGTTCAAAGGTATGGAAGGTAGTA

CGCTTGGTGTGTGGGCTGCCCATGGTGAGGGAAGAGCATATTTCCCTGATGATGGTGTTTTTGATCGCAT

GATTCATTCAAACTTGGCCCCAGTTAGTTATTGTGATGACGATGGGAATCCTACAGAAGTTTACCCTTTC

AATTTGAACGGGTCTCCCCTAGGAGTAGCAGCAGTCTGTTCTGCTGATGGGAGACATCTCGCCATGATGC

CTCATCCAGAACGTTGCTTCTTGATGTGGCAGTTTCCATGGTACCCAAAGCACTGGAATTTGGACAAGAA

AGGTCCTAGCCCATGGTT

>FM896105.1 FM896105 Jatropha curcas embryo 71-95 (DAF) Jatropha curcas cDNA clone rjcpga0_003254, mRNA sequence

CTCTCAGCGAATCGTTGTAAAGAGTTAATATTTAGATTGATATAGAAAAAAATGTCGCCTACTGAAACAT

CCCGTGAGGAAAATGTCTACATGGCCAAATTGGCTGAACAGGCTGAACGCTATGAGGAAATGGTGGAGTT

TATGGAAAAGGTAGCAAAGACCGTGGATGTTGAGGAGCTAACCGTGGAGGAAAGGAATCTTCTATCCGTA

GCTTACAAGAATGTAATAGGGGCTAGGAGGGCTTCATGGAGGATAATCTCTTCAATTGAGCAGAAGGAAG

AGAGCAGGGGAAATGAGGATCATGTCACCATCATTAAGGAATATAGGGGTAAGATCGAATCAGAGCTGAG

CAAGATTTGTGATGGGATATTGAGCCTCCTTGAGTCACATCTCATTCCCTCTGCCTCATCTGCCGAGTCT

AAGGTGTTTTATCTTAAGATGAAGGGAGATTACCACAGGTATCTTGCTGAGTTTAAGACTGGGGCAGAGA

GGAAGGAAGCTGCCGAGAGCACTTTGTTGG

>FM896104.1 FM896104 Jatropha curcas embryo 71-95 (DAF) Jatropha curcas cDNA clone rjcpga0_003253, mRNA sequence

CTAATTTTCAATCTTTGTTTCAATGGCGAACCCTAAGGTGTTCTTCGACATGGCCGTTGGCGGCCAGCCA

GTCGGCAGAATCGTGATGGAGCTCTACCAGGACGTCGTTCCTCGCACCGCCGAGAACTTCCGTGCTCTCT

GCACTGGCGAGAAGGGGATCGGCAAAAGCGGCAAGCCTCTCCACTACAAGGGGTCCAGCTTCCACCGTGT

GATCCCCGGATTCATGTGTCAGGGAGGTGACTTCACAGCCGGAAATGGTACCGGAGGTGAATCGATCTAC

GGAGCAAAGTTCGCTGATGAGAACTTCGTCAAGAAGCACACTGGCCCTGGCATCCTTTCCATGGCTAATG

CTGGTCCCGGAACTAACGGATCTCAGTTCTTTATTTGCGTTGCGAAGACCGAGTGGCTGGACGGAAAGCA

TGTCGTGTTCGGTCAGGTCGTGGAAGGTATGGATGTTGTGAAGGCCATCGAAAAGGTCGGGTCTAGCTCC

GGGAAGGACCTCAAAGCCTGTTGTGATTGCAGATTGCGGTCAACTTTCTTAAGTGACCTAAGATCTACAG

TCTTATAGGTTGCTGAAGC

>FM896103.1 FM896103 Jatropha curcas embryo 71-95 (DAF) Jatropha curcas cDNA clone rjcpga0_003251, mRNA sequence

TTTTTTTATCAAATTGCAACCAACTAGCATTGAAATACAGTTGCAAGATTCAAAAAGAATATCAGGTACA

AATCATCATCAATTCAATTACACGTTTCTTCCCAAATAATCAGCACTGGATAGCATTTCGCCTTCAACCA

AAATACAGCATTTGATATATCAATTGCTCCAGTATTCTCATCTCGCTTCTTGATTTATTCAGAAAATGAC

CGTCTCCTCAAGAAAGAGGCAACATTCCACGTTGTCCAATCTTTTGTTACCCNGTTTGCTTAAAAGCTGC

AAACCCACCTTATTGATAATTCAACTATAAGATATCAATGTTTTGCATGAATCAGGTTCCTAGATGACAT

ATTGCAAGATACCTCCATGATCAAAATATGCCAGTTCCACCTCTGTATCAAAACGTAAGGTGCATGTAAA

TGACTTGCCATTATCTGTCAACAC

>FM896102.1 FM896102 Jatropha curcas embryo 71-95 (DAF) Jatropha curcas cDNA clone rjcpga0_003249, mRNA sequence

TTTTTTTTTGCACAAAAGAAACTAAGCCTTTCATTACATGAGTTAGTACAATAAGAACAACAACAAATAA

ACAACATTAATTCATAGTAGAATTTCATTAACTACAAGCTCTAATGCCAAATAAATTCACAACAAAAGTA

GATACACAGCATGCCTAATTCTTAAGTACCTTCTCATCTTTCTCCACAACTACACAGACCTTTTGATACC

TGAAATCCACTGGTTGCACATCACCGTCAATTGGAAAAACCGTGCGCCTACTGACAAAGATCGCCCATGC

AAATAGCACCGCAAATGCCAGTAATGCTCCACACCCAGTTGCAAAAATCATCCCAGCAAGAATTGTCAAA

GGACAAAACTTCCTTCTTTGCACTTTTACAATCTCATTACTATTGTCCTTAGAAGCATTTGGATTGACCG

GCAATGAATGCATCCAATAAGGAATCTTCCTTAACCTAAACCTCCATGAATACACATAACTTTTATCTGA

TAAGTTGCCATTGTGCTTTGAACCTAAGCCCACATAAACCTCATTATCTTCCCACATTTAGTACAGATCA

ATGGAATATGCCATTATCGGATTATATGGTCTTAGATCACTCAATTTACTTAGCCTA

>FM896101.1 FM896101 Jatropha curcas embryo 71-95 (DAF) Jatropha curcas cDNA clone rjcpga0_003248, mRNA sequence

GCCGAACAGAACCGAGAAAGCATTTTTGAAACAGCCCAAGGTGTTCCTGAGCTCAAAGAAGTTAGGGAAA

GGAAAGAGGCCTGGAAAGGGAGGCAATCGGTTTTGGAAGAGCATTGGCTTAGGTTTCAAGACACCCAGAG

AGGCAATTGAAGGAACTTATATTGATAAGAAGTGCCCATTTACTGGTACTGTTTCTGTCAGAGGACGAAT

ACTTGCTGGAACTTGCCACAGCGCAAAGATGATGCGGACCATTATTGTCCGACGCAATTATCTTCATTTT

ATCAAAAAATATCAGAGATATGAGAAGCGGCACTCTAATATTCCAGCACACATTTCTCCATGCTTCCGTG

TGAAAGAAGGGGACCATGTTATTATTGGTCAATGCAGACCTCTGTCTAAGACAGTGAGGTGCAATGTTCT

AAAATTGATTCCAGCTGGTTCATCTGGTGGTGGAAAGAAGGCATTCACAGGGTTGTGAGTTTCTTAGGGC

TTAAAGTTCTTTTGATTAGTTTCTGT

>FM896100.1 FM896100 Jatropha curcas embryo 71-95 (DAF) Jatropha curcas cDNA clone rjcpga0_003246, mRNA sequence

GTTTTGATTTGCATAGTGATGGTGATGGCCAGAGACATTTGAATGCTGGCATTACAGAAAGTGAAGTGGA

TCATGCATTGGGATCTAATGGTTCAGTTTTAGGCTTCCCTCCCAGGCGTGCCTCATATTCCTGCATTCAG

ATGAACGGAAAAGAGGTCTTCCGCTTTGCTGTTCGATGTGTCCCACAATCTATAGAGTCTGCTCTTGAGA

AGGCCGGTCTCACAGGATCTAGCATTGATTGGTTACTGCTCCATCAGGCAAACCAGAGGATCCTTGATGC

AGTTGCGACACGATTAGAAGTTCCACCAGAAAGGGTCATATCAAACTTAGCAAATTATGGGAACACGAGT

GCTGCTTCGATTCCATTGGCATTGGATGAAGCGGTTCGAAGTGGGAAAGTGAAGGCGGGCCACACTATTG

CAACTGCAGGTTTTGGAGCTGGTCTAACTTGGGGTTCTGCAATTATTAGATGGGGCTGAAGGTTTCAATG

ACTAGTATAAAAGAAGAATGAAAATTACTTTGTAAC

>FM896099.1 FM896099 Jatropha curcas embryo 71-95 (DAF) Jatropha curcas cDNA clone rjcpga0_003245, mRNA sequence

GAAATTGTTCAAAATCTAACTCAACCCAACCGTATATCCAACAATGTTTCAAATAGAGAAACAGTACATG

AAAACTGATCCAAATGATGTCTTGGCAAAAACACAAAAAGGATCAGTCTCCTGTGCAAAAGGAACAAACT

CGGTTTACAATGAAACGACAAACTCCGCACAAAGTTTACATGATGATTCAAGTAAAGGTGAAAGAGATGA

GAAATCTACATATATCCTCCACAAATCACACTGTAATGCAAGTTATCGAGATAGAACGAATTTATCTTCA

GTTACCATCGGCCTTATGATCAGCTGTTCGTTAGTCTCATCACGACTTCAGATGGTGGTCGTAAAGTGCA

AACCATCTCTTTAGTCTTCCCAAAATAAACCTGATCTAATGAGTTCCTCAGCTTACCCTCCATTTCTTCT

ATCATCCTTCCCATGTTACAAAGATGACCTTCTGCAGTTGAGAGGTCCATG

>FM896098.1 FM896098 Jatropha curcas embryo 71-95 (DAF) Jatropha curcas cDNA clone rjcpga0_003243, mRNA sequence

GAAACCTAATGGCTGCAAATAGCAGACCTGTAACTTTTATATATAGGTATGACAAACATCAAAGCTGTTA

AAAAAGAGAAGCTATATAAGAGAAAACAATTCAAATAATTATTTCCCCCCTCCCTCAGCAATGCAATTTC

ACAAAGATGATTAAAGACCATGTGAGACCTGCTCAATCCATGAATTTAAACTGATGAGTGTCATCTTTTC

AAGCAACCTTGCATCAATATCCCCATTCAGAGTTCAGAAGCATTTGAAATTTTTTCAAAAGCTACAGCAT

AAAGCAAATGAGCCTAGGCCTTTGCCTTACAACATCAGGGTCCTAGGCCTTTGCCTTACAACATCAAGGT

TCCTGCCACCATGCTTTCCTTCATAGCCCCACCAATTTACCAGGAATCCATTAACAGCCAGTGCCAGCAA

CACATCACTTTGGGTAGAGAATATCATAGTGACCCGGACGATAAAGCAAGTTAATGAAGGGATTTTTGGT

CACAGAGCCATCAGTAGCAGGATTTGGGACATCACTGGCCATAGGAATGAAATCATGATGATTAACACTA

ACACCACCTGTATCACAAG

>FM896097.1 FM896097 Jatropha curcas embryo 71-95 (DAF) Jatropha curcas cDNA clone rjcpga0_003241, mRNA sequence

TTTTTTGGAAAAGGAAAGGTTTTATAACCACTGCACTCTACAATGTGTAGCACGAAGTGCAAACAGGTAC

ATAAAAACTTTGACTCTAAGTGGCATCTGTTTCACATAATTTACAGGAAAAGAAAAAGAAAAAGGAAAAA

AAAAAAGAGAGAGAGAATCCAAGAGAGGTCAGGAAAGAGAGTTAAATGAATGCATCAAGAAAATTTTCCT

AAAGAAAGAACCTAAATGTCTTCTTTGCATTATAGCTCTCTTTTTCCCTTGATCCCTTCCTTTTCCTTTA

AACTCAGGAACTAAATAAAATACATATTTCAAGTTAAAATTTTACATTGTACATTAGAAAATTAATCAAA

GGGCAGGCACCTTTTCTTCTCTTGCTCTTCTCTTAGCAGTAGCCACCAGCTTGTACATGGAGTTCTAAGA

AGGATCCTGCACATGGGAAAACATTATCAAATACCA

>FM896096.1 FM896096 Jatropha curcas embryo 71-95 (DAF) Jatropha curcas cDNA clone rjcpga0_003240, mRNA sequence

CCCGGGCTGCAGGATCGCATTTGCCTTGATAATGGAGGAGGGCTTTGCTTTTGCTCACTGCACAGAGTGC

AGGGCATTGTTCATATTACGTGCTAATGTCCCACCTGATCGCTGGTGGTTGAGATTGAAATTTCAGTTCC

TTGTTGCAAGAGACCACGCATTCATTTTTGTAGTTGTTCAGCTGATTGTTGCATTCTTGGGGATGCTTGT

GTACAAGTTTTATGGGGATGAGCTCAGGGAAATGTTTGGCTATGAAGAGCATCCATATGGATTTTATACT

ATGGCTGTTTTAGCTATTGTTTTGGTTGGTTTGCTATATGGTTTCTTCATTGCCATAATTTGTGGACAAA

GGATAAATGAACGCCATTATCATGTTCTTGCCAAACAAGAACTAACAAAGGAATATGTGGTAGAAGACCG

AGATGCTAGTAAAAATGTCCCTGAACTTGATGCCAGTCATGTCACAGAGCTAAGAATGTTGGGCCTTTAT

TAATTTGGATTTCCCTCGGTGTAGTACTCTTGGTATTTCTTTTCTGAGGATGGATCCT

>FM896095.1 FM896095 Jatropha curcas embryo 71-95 (DAF) Jatropha curcas cDNA clone rjcpga0_003239, mRNA sequence

AAATGAAAAGTTCATCTATTTCATTTCCATGGCGAATGCCATTATCCACAGAATCTAAAACATACAATCA

GACTACGTTCAATTTAAAAGAATGGGTGTGACACAAACAACAACATAAATTACCTATTAGCTTTTCTAAT

TTGTACAAACAGATTGGTAACCATTTGCCCAAATGCAAGCAAAGAACAGACCATGTCGCAGTTCTATATT

CATCTGGAACAAGTTTCTAATTGCAAGAGAATGAAAACAGATTGATGATCTTACATGTCATGCATAAGCC

AACAGAAGTCTTCTTTTTTCACCTAAGAGGAGCAACAGCCACTCTTCTGAGCAACAGGCTGTCCTCGTAT

CTGCACCGTTGGAGGTCTCGCATTGTCCATAGCTGGTTGGCTTGCCATTCTATCCTTGATTGCAGCGGCC

ATGGCCATGAAAGCTTGTTCCACATTAGTGGCATCTTTTGCACTGGTTTCCATGAAAGGAATACCGATCT

CATCTGCAAATGCCTTTGCTGTTTCATAGCATACAACTTTATTT

>FM896094.1 FM896094 Jatropha curcas embryo 71-95 (DAF) Jatropha curcas cDNA clone rjcpga0_003238, mRNA sequence

GATTAATGAAAACATCCTTGGCAAATGCTTTCGCAGTTGTTCGTCTTTCATAAATCCAAGAATTTCACCT

CTGACTATGAAATACGAATGCCCCCGACTGTCCCTGTTAATCATTACTCCGATCCCGAAGGCCAACACAA

TAGGACCGAAATCCTATGATGTTATCCCATGCTAATGTATACAGAGCGTAGGCTTGCTTTGAGCACTCTA

ATTTCTTCAAAGTAACAGCGCCGGAGGCACGACCCGGCCAGTTAAGGCCAGGAGCGCATCGCCGGCAGAA

GGGACGAGCCGACAGGTGCACACCGCGAGGCGGACCGATCGAACCAACCCAAGGTCCAACTACGAGCTTT

TTAACTGCAACAACTTAAATATACGCTATTGGAGCTGGAATTACCGCGGCTGCTGGCACCAGACTTGCCC

TCCAATGGATCCTCGTTAAGGGATT

>FM896093.1 FM896093 Jatropha curcas embryo 71-95 (DAF) Jatropha curcas cDNA clone rjcpga0_003237, mRNA sequence

ATTCCCACCATCCATCTACTAATATGTTAAGATTAGGCGGAGCTGGGGCCGCGAAATTCGAACACATAAT

CTCTCCTCCTCCTCCCAATTCGTCTACATTTTTCATGTCGGATTCAAACCAAACTTCATTTCCAAGCAAA

CCCTTGCATGGCCTTATGCAACTCCCTGATCTTCAAAATTCCACCAATAATAATAACTCTACCAATCTTT

TTAATCTCAGCTTCTTTTCGAATAATACTACCCCTAATCGAATGAGTGGTAATGACAATGGCGCAAATTC

CAGTACTGGTACAGCTACCAATTTGGTGAACTCAAGCTTCTTGAATCCAAATCCAAACCCAAATCTTTAT

AATATGGATGATCATGTTAGCTCCACAGCCGGAATACACTCTCTTTATAGTAACTCAATGCAGCAAGAAA

ATATCACCCCACACATGTCTGCAACTGCATTGCTTCAAAAAGCTGCTCAAATGGGTTCAACAAGTAGCCC

AAA

>FM896092.1 FM896092 Jatropha curcas embryo 71-95 (DAF) Jatropha curcas cDNA clone rjcpga0_003236, mRNA sequence

ATTCGAGAGAAACAGCCCAGATCGTACGCTAAGGTCCCTAAGCAATCACTTAGTGGAAAAGGAAGTGATC

GAGCGATGACAACCAGGAGGTGGGCTTGGAAGCAGCCATCCTTTGAAGAAAGCGTAATAGCTCACTGGTC

TAGCTCCATGGCACCGAAAATGTATCAGGGCTCAAGTGATTCACCGAAGCGACGAGACCTTGAAAGCTGC

TTTTTCAAGTGTCAGTAGCGGGACGTTCTGTCAATCGGGGAAGGTTTTTGGTGACAAGACCTGGAGATAT

CAGAAGTGAGAATGCTGACATGAGTAACGAGAAATCCTGTGAAAAACACGATCGCCTGCCAGTGGAAGGC

TTTCTGCGTTCAGTCAATCTACGCAGAGTGAATCGGTCCCTAAGGAACCCCCGAAAGGGCTGCCGTCCGA

TGGGTACACGAAAGTGACGAAGTTGCTTTGACTACAGAAC

>FM896091.1 FM896091 Jatropha curcas embryo 71-95 (DAF) Jatropha curcas cDNA clone rjcpga0_003235, mRNA sequence

ATTCGGAATTTGTGCACTTTTCATGCTTTACATTAAGTAGTGAAGCTATTCAAAAAGTAGTACAAATAAT

TTAGTATTTTAAAAAAGCCTTTAATGAATTTCTCATGAACTATACATTTTTCCACCAAAAAAGTTCTCAA

TAGAAAAATAAAACAAAATATATACAAGTTTGAGTGGTTTATGTAGAGCAATCAAAGCTGCCTTAAAAAG

CAAAATGCCACAAGATGTGTTTTTCCTAAGCCCTTTCATCTTACAGGATCAAACATGAGAAAATACTGGA

CAATTCATGAAACTTGTGAATAATGCAGTAGAAAAGCTCAAATTTTAAGGTTATCATAAAGTAATTGGTG

GAAGTGATGCATAGCTTTCTCAACTGTGGGAGCATATCTACCAACCGAATATGCCGGCGAGTGGAGTCCC

CTCTGAACGCCTTCGCATAGCAAAATATCTTCCATCTGCACTCTTTCACTGTCTACAAGACTTTGCTCAA

TGAAAGCTTTGTCATCCTTATGATAAGCTTCAATAAAATAGTCAAATATCACTTGGCATTTGCTTGTTCC

TAAATGG

>FM896090.1 FM896090 Jatropha curcas embryo 71-95 (DAF) Jatropha curcas cDNA clone rjcpga0_003234, mRNA sequence

CTAAAAAATGGCTACTGCTACTGTACTCTTCTCTACCATTTCCACCGCTCTCTTTATCCTCTTCTCCTTT

GGTCAAGCTGCGCCTCTTATTTTAACCCTAGTCAACAACTGCCCTTTCACCGTTTACCCTGCCATCCAAC

CCAACGCTGGCCACCCGGTCCTCGAAAAAGGTGGCTTCCCACTCTCCACCCTAACACACCGCTCTTTTCC

CGTCCCTACACCAGCACTGGACTGGCCGGATCTGGGCTAGAACCGGATGCACTCACACCAACGGCAAGTT

CCACTGCGCCACCGGTGATTGCAACCACCAACTAGAATGCAACGGCCTCGGTGGTGCCCCTCCAGCAACT

CTCGCTCAGTTCAGCCTTCACCATGGCCATAACGACTTCTCCTCTTACGGCGTCTCTCTCGTCGACGGAT

TTAACCTTCCGATGACCATAACTCCACATGAA

>FM896089.1 FM896089 Jatropha curcas embryo 71-95 (DAF) Jatropha curcas cDNA clone rjcpga0_003233, mRNA sequence

ATCCAACTGATTATGAAAGTGGAGCAATTAAAGGGATGGCATACTTGGAATTCAAGGATTCCGAAGCTTT

CAACAAAGCTTTAGAGCTTAGCGGTAGTCAGCTTGGAGACCAGTACCTAACAGTTGAAGAAGCAAAACCA

AGGGGTGGTGATAACCGCGAGGGTTGGGGCAGTGGTAGAGGTGGAGGCAGAAGCGGTGGCGGGCGAAGTG

GTGGTAGAGATAGTGGTGGTCGTTTCGGTGGTAGAGATGGAGGTGGTCGTTTCGGTGGTAGAGGTGGAGG

TGGAAGAGGTGGTGGTGGTCGTGGCAGAGGACGCGGAACACCTAACAAACCTGCTGTCTCAACAGGGAAG

AAGACAAAATTCAACGACGATGATGAGTAAAACGAATATTATATTTTTGTTATCGTCTAATTAAAGTACT

TATTTTGCTAGTTATAGCACAAGATTTTGTTTGCCTTGGTTGACTTGCGTCATTTAATTT

>FM896088.1 FM896088 Jatropha curcas embryo 71-95 (DAF) Jatropha curcas cDNA clone rjcpga0_003232, mRNA sequence

ATTCTGACTACTCTTTCAAGGTCCTACGTTTCATTCACTCATCCTCAAACCCACTTCTTATCATCAGGTT

CCTTGAATTCAAGTAACCAGAGTCTTTTACCCGTTGGCCATTGTAATCCCTCTAAAAAAGGAAGACTTGT

TGCATATAGAAGGCCATTTACTCTTCAAGCAAGCGGAGATCGGAGTGGGTCAAATAGTGCAGGTATCTTC

ATTGGTGGTTTTGTATTGGGAGGAATAGTTGTTGGCGCATTAGGCTGTGTCTATGCTCCACAGATCAGCA

AGGCACTAGCTGGAACTGACAGAAAAGGTTTGATGAGGAAACTGCCGAAGTTCATATATGACGAGGAAAA

GGCATTGGAGAAGACACGCAAAGTACTGGCCGAGAAGATTGCACAGTTGAATTCTGCAATAGATGAGGTT

TCTGCTCAGTTGCATGCAGAGGATACCCCAAATGGAACTGCAGTGAATTCTGATGAGATCCAAGCTTCTA

CATAAGAAACATATTTGTTGAGATTTCAAAAACAAACTGAAAAATGACAAAAAAAAGAAGGGTTTTACTT

GTGATCAGCACTAGAATATAATGTCTCAAGAATTT

>FM896087.1 FM896087 Jatropha curcas embryo 71-95 (DAF) Jatropha curcas cDNA clone rjcpga0_003231, mRNA sequence

TTTTTTTCAAGAACTAAAATTATTATTGATAAACTAACTGCAATAATGTACAAGGACACCAAACTAAAAA

CCAGTTCAGGTTTCAGTCACAAATAAGAAAATGAAAGAAGTCACCACTAGAACAACAATCTTTTTTGCCA

AAGTATACAAGAGAATTGAAAATCTCACACTCAGGTAGTCTACAAATATTCATCATCTTTTGCCAATTAT

TTCTTTTCAATTAGTATTTTGTCCATTCTCAACACCGAGACCATATAAAACTCCTGCGTACTTCTCTGCA

GAACCGTTGAAAAAGTTATCCCTCAGCTTCCTAAATGTGCAGGAAGTGAGCAAACTATCGGAGCCCGCTT

GATGGCATACACCAACTCTTTCCACCTCCAATAGCTCTGCAAGCTTGTTCAACCCCCCATGAAGGCTATT

GCAAAACTTCATCAAATGCTTGATATCATACACCACAGGAAAGTACATATTGATCAAGTCAAAAAATCCT

GCTTGTGTATCAGGCAAACTCCTGCAAGTCAAAAGCTTAAGCAAGTACCCAAAATCATA

>FM896086.1 FM896086 Jatropha curcas embryo 71-95 (DAF) Jatropha curcas cDNA clone rjcpga0_003230, mRNA sequence

ATTAGCAGGAAATGCAAAATATAAGATTAACGATTGACAAAATCCGAGTTCACATGAAGTAAAAGCCTTA

ATATCCTTAGATCCAAAACATACCACTAACAATAATATCAAAAAAGCTCTTAACACATAAAGCATCCTAT

CATTGAATATTTAGAAATGACAAAACAAACTTCACCGCTCAAGATTTCAAATCAATCAACGCTCTGCATG

ATATCCTCAGCTGTGAACTTGGTGCCCTTATCCTTATCAGCGGCGGCCCTACCCTTAGCCTTGCGGTCAA

GCAGAGACTTCCTGTCCTTGTCGAGACGGAGCTTGGTGATGACAACCTTGGAAGGGTTGATTCCAACGTT

GACGGTAGAGCCGTTAACCTTCTCCCTGGTAATGCGCTCAATGTGAATGACCCATTTGCGTCGGTAAACC

TGAACCACCTTACCTTCCCGGCCCTTGTAGGTTCCACGAACC

>FM896085.1 FM896085 Jatropha curcas embryo 71-95 (DAF) Jatropha curcas cDNA clone rjcpga0_003229, mRNA sequence

GACATTCAGAGCACTGGGCAGAAATCACATTGCGTGAGCATCCTCAGGGACCATCGCAATGCTTTGTTTT

AATTAAACAGTCGGATCTCCCCTTGTCCGTACCAGTTCTGAGTCGACTGTTCGACGCCCGGGGAAGGCCC

CCGGAAGGGCCGTTCCCAGTCCGTCCCCCGGCCGGCACGCGGCGACCCGCTCTCGCCGCGGGAGCAGCTC

GAGCAGTCCGCCGACAGCCGACGGGTTCGGGACTGGGACCCCCGGGCCCAGCCCTCAGAGCCAATCCTTT

TCCCGAGGTTACGGATCCATTTTGCCGACTTCCCTTGCCTACATTGTTCCATCGACCAGAGGCTGTTCAC

CTTGGAGACCTGATGCGGTTATGAGTACGACCGGGCGTGGGAGGCACTCGGTCCTCCGGATTTTCATGGG

CCGCCGGGGG

>FM896084.1 FM896084 Jatropha curcas embryo 71-95 (DAF) Jatropha curcas cDNA clone rjcpga0_003227, mRNA sequence

CTCAAGACAACCATCTCTATAAGTGGTCTCTCTCTCTCTCTCTCTCTCTCTCTCTCTCTCTCTGTATAGT

TGTAAAGTAGCAATTGAACTCAGAATACAAGGGAAGTGGACAAGCAGAGNAAGAAAAAGGCTCCCAATGT

GAAAATGGTAACAAGAACAGTCGATCTCCGCTCTGACACTGTCACTAAACCAACCGAAGCGATGAGAGCA

GCTATGGCAAACGCTGAGGTTGATGATGATGTTCTGGGAGATGACCCAAACGCCTTACGATTACAGACCG

AAATGGCCAGAATCATGGGCAAGGAAGCAGCTCTATTTGTTCCATCAGGCACTATGGGTAACCTTATTAG

TGTGCTTGTGCATTGTGACATTAGAGGAAGTGAAGTCATTCTTGGACACGATTCTCATATCCACATATAT

GAGAATGGAGGCATTTCAACACTCGGTGGTGTGCATCCAAGGACAGTGAAGAATAACAAGGATGGAACAA

TGGATATCGATTTAATTGAAGCTGCCATCAGAGATCCGAAATGGCAGCTTGTGTATCCAACTA

>FM896083.1 FM896083 Jatropha curcas embryo 71-95 (DAF) Jatropha curcas cDNA clone rjcpga0_003226, mRNA sequence

NTGTAAAAACCATCTAATGTTTGTATTGAACTGATGAAATTCTTTCTCCAACAACATCCTTTAAAAACCC

CATCTTTGACCAACCAAAATCAAAAATTACAAAAGAGCGGACACAAGTTTTATTAAACAAAGGGAAGCCT

GCATCTAAACATTTGACTCAATACACAGCAGTCAAAAATCACCATCAATGTCTTCAGTGGAGGGTGGAGG

CAATGTGAGCAATCAAGTCTAGCACACGTGAACTGTAGCCCCATTCGTTGTCATACCAAGAGACAAGTTT

CACAAAGTTGTCATTCAAAGCAATTCCTGCCTTGGCATCAAAAATACTTGATCTGCTATCGCCCACAAAG

TCAGAAGACACTACATCATCTTCAGTATAGCCCAAGATTCCCTTAAGCTTGCCCTCAGACTCCTCCTTGA

TTGCAGCTTTGATGTCCTCATATGTTGCCTTCTTTTCAAGCCTGACAGTAAGG

>FM896082.1 FM896082 Jatropha curcas embryo 71-95 (DAF) Jatropha curcas cDNA clone rjcpga0_003225, mRNA sequence

TTTTTTTTTTGATTAACTCATGCAAAATTCAATAATATTCCCATGAAATTATAGAAAACTGTCGAAATGA

CATTAAGAATTCAATGAACTAAGAAGCGTCAAGGCACTTTTCATAATCATCAAACGAAACACCTCAAGGT

ATGAAAATTCAAATAAAAACCTTAAACATTTCATCTTGCTAACAAAACTCCATGATTTTAAGTAACATGG

ACCTAAGCTGCAACAGATGGCTTAGATGCAGCAGAGAGCCTGGATCTTTTCTTGGCTAAGCTCTCACTTC

GTCGTTCTCTCTGCTCCTTCAATCTTGTGGCAAGAAGCTTCTGGTACTCAGCGGCCTCTGACTTGGCCTT

AGCAATTCTTTTCTTCTTGTCAGCAATTCTTGCTCGCTTTCTTTGCAAGGTCAATGGAGTAACCAACCTC

TGAATTTTGGGAGCTTTACTAACCTTCTTACCAGATTTAGTTGTGAATGATCTGCGGTAAGTATTTACGT

ACTTCCGAACATCATCCTCTTTTGAGAGATTAAAGAGCTTGCGGATCTTTGAAGCCCTCTTAGG

>FM896081.1 FM896081 Jatropha curcas embryo 71-95 (DAF) Jatropha curcas cDNA clone rjcpga0_003224, mRNA sequence

GAATAACAATTACTCAACCAGCATCCCAGCTGGAAGGCAGAGTCCATTGATCAAATGATTTTTTTAATAC

GCAACTGAACCAGAGTTAGCATCACTGCCGCAAGCTGTGGCCCAATTTTTGCAGTCAATAATCAAAGCGT

TAGAGCTAAGCCAAAAAAAAGAAATTCCCCCAAAAAGTTTCGACGAAAATACTTAAATTAGCTGAACTAC

AAAACGAGGGTGTCTTCGTATGGTGCATGCATCAACAGTAACCCACATTCATAACAAGTTTTTCATGCAA

AACAACCAAAAATAAAATTTTTGACCACCCACCTACAAAACTAGCAACACAGACACCTAACAGACGCCCC

CCACGCCCCCAGTGAGTAATGATGTCATGAAGGTCATGAAGGGACCCCAACGTCCGCTGGGGCATGACCA

CCATGAACTCCTGAACCATGATTGTTATAAGGGTGCCCTCCGCCCCTGCCACCCCTTCCCCGATTGTTAT

AGTAATTTCTTGGATAATAGTTTCCCGACTGGTCATAATATTGGCTGCG

>FM896080.1 FM896080 Jatropha curcas embryo 71-95 (DAF) Jatropha curcas cDNA clone rjcpga0_003223, mRNA sequence

CTAAGATACCAAAAACAACAATGAGTAGAGTTGTTTTTTGCCTTTTATTTTCTTTCTCCTTCTTTCTGCT

CGGTTTTGTCCAATGCAGTCCCAATTACAAGGATGCTTTACTAAAATCCATATTGTTCTTTCAAGGTCAG

AGGTCAGGGAGACTTCCCACTTCACAAAAAATCACTTGGAGGTCCAATTCTGGCCTCTCCGATGGTTCAC

TTGCTCAAGTGGATTTAACAGGAGGGTACTACGATGCTGGAGATAACGTAAAATTTAATTTCCCAATGGC

TTTCACTACTACAATCCTATCATGGGGGACTCTCGAATATGGTAATGGAATGGGCTCTGAACTACAAAAT

GCGAAGGCAGCAATCCGATGGGCGACAGAGTATCTACTAAAATGTGCTAGAGCCACTCCAGGCAAGCTCT

ATGTTGGTGTAGGTGACCCTAATGTTGATCACAAGTGCTGGGAAAGACCTGAAGATATGGACACTGTCCG

TTCTGTCTATTCTGTCTCTGCCC

>FM896079.1 FM896079 Jatropha curcas embryo 71-95 (DAF) Jatropha curcas cDNA clone rjcpga0_003222, mRNA sequence

GGAAGAAACTTGGAGAGCAGTATACATAAATCTGTAAAATGTTCCCTAACTAACGAAACAGGGAAGGAAA

GAGAAGACAAAAGACAAGTGTCTCCAATGAAGTTAGCATCAGATGCTCTGAGCAACCCTAATATACACAC

GCCAAAGAGCAAGAAGACAACAGTGCCGCCACCACCACTACAACAAGCATAATTACTATTATCATAACAA

GAAGAATAGAAAACACTGTCTAATGGCATGCTGATTGCTGTGCATTGAAAAACTCTTGAGAGCTGGAACC

ATATGCAAAGTCTGTGGACTTGCAACTTTATTCTCAATCCTTTTACACTTCCTCCCTGTCTCGGCTGCTA

CTCTTCAAGATATTGTACTTGCAAAGTGGGCAGGTAGCATTAATATACAGCCACTTATCTACACAGGCAC

AATGGAAGTGGTGACCACAAGGAAGTTCCCTTAGCTCAACTCCATCATCATAGG

>FM896078.1 FM896078 Jatropha curcas embryo 71-95 (DAF) Jatropha curcas cDNA clone rjcpga0_003221, mRNA sequence

GTGCGAGTCAACGGGTGAGTAAACCCGCAATGCGCAAGGAAGCTGACTGGCGGGATCCCCCCGAGGGTTG

CACCGCCGACCGACCTTGATCTTCTGAGAAGGGTTCGAGTGAGAGCATGCCTGTCGGGACCCGAAAGATG

GTGAACTAT

>FM896077.1 FM896077 Jatropha curcas embryo 71-95 (DAF) Jatropha curcas cDNA clone rjcpga0_003218, mRNA sequence

ATTCTCCCTTTCTTAACGGTTTCCCTCTGTATCGTATGCAAAGGGAGAAACTTCCTTGTCTCATCACTGT

CAGATCCTTATCTTCATTTTCTCTAAGCTTTTTCCAGAATTTTCTCTCCTTCTTATCCAAACAAACCTGA

GAGAAAATTGCGATGGCGGATACAGAGAATGGAGAATTCAGCTTTAAAAGCAATGCAAGCGGACGCCATG

GCTTGCCTAAAATCACGACACAGAAACCGGCGAACGACGTTTGTCACGACGATAGTGGACCGACGGTGAA

AGCACAAACAATCGATGAGCTTCATTCTCTTCAAAGGAAGAAATCGGCGCCTACGACGCCGATCAAAGGT

GCACAGGTTCCTTTCTCTCCTAGGTCAGAAGAGGAACGGCAGAAACAGCAACTCGAATCCATCAGTGCAT

CGTTGGCATCTCTGACGAGAGAAACAGGTCCTAAACTGGTGAAA

>FM896076.1 FM896076 Jatropha curcas embryo 71-95 (DAF) Jatropha curcas cDNA clone rjcpga0_003216, mRNA sequence

GACAAAAGGACCACATTGTTCTTATTGTAAGGGGACAAAAAAAATAGTCGTTACATGCCTTACAAACAAA

AATCCAAGATCGTTTATCTTGAATAATCCAGTCCCCAACAACTACTACCTACAACCTGCGAACCTCATTA

TGAATACATGGATGCCTTTGGACCTTCACAATAATTACTCCCTAAAAAATGGACAAAAAGCCCGGTACTC

TTGTTTATGTGTAGATTTGGGTGAATGTTCACGTGGATTAGTGTTTTCTCGAGGAACATCAGTAATCCAT

GCCCATGCCAGGAGGAATTGCTGCAGCATCTTTCTCATCCTTTGGAAGTTCAGATACAACAGCTTCAGTT

GTTGTCATCAATGATGAGACACTTGCAGCATCAACTAAAGCAGTTCTAATTACTTTCAATG

>FM896075.1 FM896075 Jatropha curcas embryo 71-95 (DAF) Jatropha curcas cDNA clone rjcpga0_003215, mRNA sequence

ATTCCAAAGGATTCTATCCGTCGCCCGATGGGAATTGTACTTTAAGGCGGCCCTCGCGGCTCGTCCGCCG

CGGGGGCTTGGCCAACGGCACGTGCCTCTGGGGGCCGGATGGCCCCTACTGCGGGTCGGCAAACGGGCGA

CGGACACGCGCGTCGCTTCTGGCCCGGATTCTGACTTAGAGGCGTTCAGTCATAATCCAGCGCACGGTAG

CTTCGCGCCACTGGCTTTTCAACCAAGCGCGATGACCAATTGTGCGAATCAACGGTTCCTCTCGTACTAG

GTTGAATTACCATCGCGACACCATCATCAGTAGGGTAAAACTAACCTGTCTCACGACGGTCTAATCCCAG

CTCACGTTCCCTATTGGTGGGTGAACAATCCAACACTTGGTGAATTCTGCTTCACAATGATAGGAAGAGC

CGACATCGAA

>FM896074.1 FM896074 Jatropha curcas embryo 71-95 (DAF) Jatropha curcas cDNA clone rjcpga0_003214, mRNA sequence

AGGATTCTATCCGTCGCCCGATGGGAATTGTACTTTAANGGCGGCCCTCGCGGCTCGTCCGCCGCGGGGG

CTTAGCCAACGGCACGTGCCTCTGGGGGCCGGATGGCCCCTACTGCGGGTCGGCAAACGGGCGACGGACA

CGCGCGTCGCTTCTGGCCCGGATTCTGACTTAGAGGCGTTCAGTCATAATCCAGCGCACGGTAGCTTCGC

GCCACTGGCTTTTCAACCAAGCGCGATGACCAATTGTGCGAATCAACGGTTCCTCTCGTACTAGGTTGAA

TTACCATCGCGACACCATCATCAGTAGGGTAAAACTAACCTGTCTCACGACGGTCTAACCCCAGCTCACG

TTCCCTATTGGTGGGTGAACAATCCAACACTTGGTGAATTCTGCTTCACAATGATAGGAAGAGCCGACAT

CGAAGGATCAAAAAGCAACGTCGCTATGAACGCTTGGC

>FM896073.1 FM896073 Jatropha curcas embryo 71-95 (DAF) Jatropha curcas cDNA clone rjcpga0_003213, mRNA sequence

GCAAAGGATTCTATCCGTCGCCCGATGGGAATTGTACTTTAAGGCGGCCCTCGCGGCTCGTCCGCCGCGG

GGGCTTGGCCAACGGCACGTGCCTCTGGGGGCCGGATGGCCCCTACTGCGGGTCGGCAAACGGGCGACGG

ACACGCGCGTCGCTTCTGGCCCGGATTCTGACTTAGAGGCGTTCAG

>FM896072.1 FM896072 Jatropha curcas embryo 71-95 (DAF) Jatropha curcas cDNA clone rjcpga0_003211, mRNA sequence

ATTCCGCAAATGTCAATTTGCCCCCAGTTAATGCACATGTCAATGCCCCTATGGAAGTTGTTAACCAGAG

AGATGCAGAGCTTCTTTTCATGTGGCAAATGTACAAAAGATCAGATGAGCAGTCAGGAAAGAAAGCACAA

ATCCTACAGCAGATTAAAGAGACAATTAAGCACAGAAGTCATTTGGATAATAGCATAGAATTGATTGGAA

CCTTATTATTTGGACCCCAAAAAGGTTCTACTGTTCTTCAATCTGTTAGAGAACCTGGTTTACCCTTAGT

AGATGATTGGACTTGCTTAAAATCAATGGTTCGAGTGTTCGAGACATACTGCGGATCATTAACCCAGTAT

GGCATGAAACACATGCGTGCATTTGCCAATGTTTGCAATGGAGGGGTCTCTCATGCCTCCATGGAAGAAG

CTTGCATTGCCGCATGCAGCGGCCATGATGCTGGCCAATGGCAGCCTTTGAACCGAGGTTATAGTGCCTA

AATTAGTCA

>FM896071.1 FM896071 Jatropha curcas embryo 71-95 (DAF) Jatropha curcas cDNA clone rjcpga0_003210, mRNA sequence

TCGTCTTAATCTTCGCATCTGCAGTTATCCATCTAAATCCATAATGGCGACCCAATGAGCAAGAAGCGAA

AGTTTGTTGCAACGGTGTTTCTTCGCCGAGCTTAACGAAGTTTTGACCAGAGAGCTCGCCGAGGATGGAT

ACTCTGGTGTTGAGGTTAGGGTCACTCCTATGCGCACCGAGATCATCATCAGGGCCACTCGCACCCAAAA

CGTTCTAGGTGATAAGGGTAGGAGGATCAGAGAGCTGACCTCCGTGGTGCAAAAGAGGTTTAAATTCCCA

GAAAACAGTGTGGAGCTCTATGCTGAGAAAGTTAACAGCAGAGGCCTTTGCGCCATTGCTCAGGCTGAGT

CTCTTCGTTACA

>FM896070.1 FM896070 Jatropha curcas embryo 71-95 (DAF) Jatropha curcas cDNA clone rjcpga0_003209, mRNA sequence

ATTCCATTTTTATCCATCTTTACTTCAAAGAAAGTCATGAAGATGCTGGATCTGCGGGTATGAAGTCCTT

GAGAACTTTTGCACTCCTTGGAGCTGGTCTTTCAGGGATTCTGAGTTTTGCAACGGTAGCATCTGCCGAC

GAGGCTGAACATGGATTAGAGTGTCCAAACTATCCTTGGCCTCACAAAGGCATCCTTAGTTCATATGACC

ATGCTTCGATTCGTCGTGGCCACCAAGTTTACACACAAGTGTGTGCATCTTGCCACTCTATGTCTTTGAT

ATCATATCGTGATTTAGTGGGTGTTGCATACACAGAAGATGAGGTGAAAGCTATGGCAGCTGAGATTGAG

GTGGTTGATGGGCCTAATGATGAGGGCGAGATGTTTACCCGTCCTGGTAAACTCAGTGATCGGTTTCCTC

AGCCATATGCAAATGAACAAGCAGCTAGATTTGCTAATGGAGGGGCCTATCCTCCTNGATCTAAGTCTTA

TTACGAAAGCTCGGTCACATGGTCAGAACTATGTT

>FM896069.1 FM896069 Jatropha curcas embryo 71-95 (DAF) Jatropha curcas cDNA clone rjcpga0_003208, mRNA sequence

TCCTCACACCTCCGTCCCGCTGTATTTAGGTCACTGCTATATGAGGCTTGTGGAAGGATAGTGAATCCCG

TGTATGGTTCAGTTGGGTTGCTCTGGTCTGGAAATTGGGCTCAGTGTCAAGCAGCCGTTGATGCAGTTCT

TAGAGGAACACCGATAATGCAAATGCCTTCCGCTCCTGATGCACCGCCACCACACTTGATTGATCCTTTC

AAAACCTACGATATTCGTCATGTGTCCAAGGACTCTAGCTCTGTTGAACTCAACAAGGTTAAGAACAGAA

CCCGATTCAAACGGCCAATTAATAAGCCAACTTCTCCTTCTGAGTCAATTGGTCGAGTTAACCTCGGTGA

CTTGAGTTTCGAACAGTTTCGTGAACCCTGGTCGAGCCAGATGGCGAATGGGGATAGTAGCAAAGATGAT

GAAAGCATGTTCTCCATTGAAACAGTAGAGGATGTG

>FM896068.1 FM896068 Jatropha curcas embryo 71-95 (DAF) Jatropha curcas cDNA clone rjcpga0_003206, mRNA sequence

TAAAGTTTGGGTGTTGGATGGAGGCTTACCTAGATGGCGTGCATCAGGATATGATGTTGAATCCAGTGCT

TCTGGTGATACCATCCTGAAAGCTAGCGCTGCCAGTGAGGCAATAGAGAAAGTATATCAGGGCCAGACAG

TTGGACCAATCACATTCCAGACAAAATTTCAGCCGCATCTTGTTTGGACCCTTGAGCAGGTTAAGAAAAA

CATTGAGGACAAGATGCACCAACATATTGATGCCCGTTCAAAGGCCAGGTTTGATGGAGCTGCACCAGAA

CCTCGAAAGGGAATAAAAAGTGGTCACATACCTGGGAGCAAGTGCATTCCTTTTCCCCAAATGTTGGATG

CTTCACAGACACTCTTACCAGCGGATGAGCTTAAGAAACGGTTTGATCAAGAAGGCATCTCCTTGGAAAG

CTCTATTGTTACTTCTTGTGGGACTGGCGTAACTGCTTGCATTCTTGCTTTGGGTCTGCATCGACTTGGG

AAGCCAGATGTTGCAGTTTATGATGGGATCTGGACTGAATGGGGTG

>FM896067.1 FM896067 Jatropha curcas embryo 71-95 (DAF) Jatropha curcas cDNA clone rjcpga0_003205, mRNA sequence

CAAAGGATTCTATCCGTCGCCCGATGGGAATTGTACTTTAAGGCGGCCCTCGCGGCTCGTCCGCCGCGGG

GGCTTGGCCAACGGCACGTGCCTCTGGGGGCCGGATGGCCCCTACTGCGGGTCGGCAAACGGGCGACGGA

CACGCGCGTCGCTTCTGGCCCGGATTCTGACTTAGAGGCGTTCAGTCATAATCCAGCGCACGGTAGCTTC

GCGCCACTGGCTTTTCAACCAAGCGCGATGACCAATTGTGCGAATCAACGGTTCCTCTCGTACTAGGTTG

AATTACCATCGCGACACCATCATCAGTAGGGTAAAACTAACCTGTCTCACGACGGTCTAACCCAGCTCAC

GTTCCCTATTGGTGGGTGAACAATCCAACACTTGGTGAATTCTGCTTCACAATGATAGGAAGAGCCGACA

TCGAAGGATCAAAAAGCAACGTCGCTATGAACG

>FM896066.1 FM896066 Jatropha curcas embryo 71-95 (DAF) Jatropha curcas cDNA clone rjcpga0_003204, mRNA sequence

CCACACGAGATTTCTGTTCTCGTTGAGCTCATCTTAGGACACCTGCGTTATCTTTTAACAGATGTGCCGC

CCCAGCCAAACTCCCCACCTGACAATGTCTTCCGCCCGGATCGGCCGCCGAAACGACCTTGGGTCCAAAA

AGAGGGGCATAGCCCCGCCTCCGATTCACGGAATAAGTAAAATAACGTTAAAAGTAGTGGTATTTCACCG

TCGCCTTTCGGCTCCCACTTATCCTACACCTCTCAAGTCATTTCACAAAGTCGGACTAGAGTCAAGCTCA

ACAGGGTCTTCTTTCCCCGCTGATTCCGCCAAGCCCGTTCCCTTGGCTGTGGTTTCGCTGGATAGTAGAC

AGGGCACCAGTTGGGAATCTCGTTAATCCATTCATGCGCGTCACTAATTAGATGACGAGGCATTTGGCTA

CCCTTAAGACGAGTTCATAGGTTAACTCCCCGGCCGGTTATACCCCGCGACTTGAGTTGAATTTCTTCAC

TTTGACATTCAGAGCACTGGGCAGAAATCACATTGCGTGAGCATCCGCAGGAACCATCGCAATGCTTAGT

TTCAATTAAACAGTCGGATTCCCCTTGTCCGTACCAGTTCTGAGTCGACTGTTCGACGCCC

>FM896065.1 FM896065 Jatropha curcas embryo 71-95 (DAF) Jatropha curcas cDNA clone rjcpga0_003203, mRNA sequence

ATTCGAAAGGCGACGGTGAAATAACACTACTTTTAACGTTATTTTACTTATTCCGTGAATCGGAGGCGGG

GCTATGCCCCTCTTTTTGGACCCAAGGTCGTTTCGGCGGCCGATCCGGGCGGAAGACATTGTCAGGTGGG

GAGTTTGGCTGGGGCGGCACATCTGTTAAAAGATAACGCAGGTGTCCTAAGATGAGCTCAACGAGAACAG

AAATCTCGTGTGGAACAAAAGGGTAAAAGCTCGTTTGATTCTGATTTCCAGTACGAATACGAACCGTGAA

AGCGTGGCCTATCGATCCTTTAGACCTTCGGAATTTGAAGCTAGAGGTGTCAGAAAAGTTACCACAGGGA

TAACTGGCTTGTGGCAGCCAAGCGTTCATAGCGACGTTGCTTTTTGATCCTTCGATGTC

>FM896064.1 FM896064 Jatropha curcas embryo 71-95 (DAF) Jatropha curcas cDNA clone rjcpga0_003196, mRNA sequence

ATTCCAAATTATCTCTCTAAATGGCTGATACTGCAATTGCCTTATCCATGGATGCTGAGCCAGTCCCAAC

AAAGAAACCGAGAAACAACAGGAGAGCTCTGAAACAGAAAAACCCAACCACAAATGAGGCTAATATCATG

GCACAGAAGCTCTCCGCGACCTCTCCCGTGGCTATTCCAGCATTAGCCACCGATCCTTCAAAAGAGAACC

ATGAGAGTCTCTCGCAACCTCGCTCTTCGTCCAAGAAAGCGAAGGCCAAGGTCTCTAAGGCTAAGCAAAC

CAAGCAATCATCTTCTTCATTTGAAACGGACATGCAAGAAATGCAAGAAATGCTTCAAAAGTTGAGGCTA

GAGAAGGAGAAGACTGAGGAGCTACTGAAGGAGAAAGATGAGATGTTGAAAGCAAAAGAGGAGGAGCTCG

AAACCAAAGGCAAAGAACAAGAGAAGTTACATTTGGAGTTGAAGAAATTGCAGAAATTGAAGGAATTTAA

GCCCAACATGACATTCCCCCTCGTTCAATCATTGAAGGAGGACCTAGACAAGAAAAAGAAGAAGGACGGC

CAAGAAAAGAAAAGGCCATCTCAACCTTACATCTTGTGGTGCAAGGACCAGTGGAATGAGGTGAAGAAAC

>FM896063.1 FM896063 Jatropha curcas embryo 71-95 (DAF) Jatropha curcas cDNA clone rjcpga0_003195, mRNA sequence

ATTCCTGCAAAACCCGGGGCGCGAGCCCGGGCGGAGCGGCCGTCGGTGCAGATCTTGGTGGTAGTAGCAA

GTATTCAAATGAGAACTTTGAAGGCCGAAGAGGGGAAAGGTTCCATGTGAACGGCACTTGCACATGGGTT

AGTCGATCCTAAGAGACGGGGGAAGCCCGTCCGACAGCGCGTCCTCGCGCGAGCTTCGAAAGGGAATCGG

GTTAAAATTCCCGAAACGGGACGTGAAAGCTGACGGCGACGTTAGGGAGTCCGGAGACGTCGGCGGGGGC

CTCGGGAAGAGT

>FM896062.1 FM896062 Jatropha curcas embryo 71-95 (DAF) Jatropha curcas cDNA clone rjcpga0_003194, mRNA sequence

CAGAGGTTATCTCGACGAACAACAGCCAGTCGCTCACTCAGAAGTATTCAATTAAGAAGCATCAATGTCT

ACGGGCAGGAAAATCACTTTGAAAAGCGCCGACGGGGAGACCTTCGAGGTAGACGAGGCTGTGGCTCTTG

AATCGCAGACGATTAAACACATGATAGAGGACGACTGCGCCGATAATGGAATCCCTCTGCCCAATGTCAC

CAGCAAGATCCTATCTAAGGTAATTGAGTACTGTAAGAAGCATGTGGAGACCCCTAAGTCCGACGATCGC

CCCTCTTCTGCCGACGAAGAACTTAAGACCTGGGACGCGGACTTTGTCAAAGTTGATCAGGCTACCCTCT

TTGATCTAATCTTGGCTGCCAATTATCTGAACATCAAGAGTTTACTGGATCTCACTTGTCAGACAGTGGC

TGACATGATCAATGGCAAAACTCCAGAGGAGATACGCAAAACGTTCAACATCAAGAATGATTTCACACCA

GAGGAAGAAGAGGAAGTTCGGAGAGAGAACCAGTGGGCCTTCGAGTGAAAAGACCAT

>FM896061.1 FM896061 Jatropha curcas embryo 71-95 (DAF) Jatropha curcas cDNA clone rjcpga0_003193, mRNA sequence

TGCAGGCAGTGAAGACGAGGCCAGATACTTTTTCCAACAACTTATTTCAGGAGTCAGCTACTGCCATTCC

CTGCAAATTTGCCATAGAGATTTGAAGCTAGAAAATACCTTGCTGGATGGCAGCCCAGCACCACGTCTGA

AAATCTGTGATTTTGGTTATTCTAAGTCATCCTTGCTGCATTCAAGACCCAAATCCACAGTTGGCACTCC

GGCTTATATTGCCCCAGAGGTTCTTTCTAGGAGAGAATATGATGGCAAGATGGCAGATGTATGGTCCTGT

GGAGTGACTCTGTATGTTATGCTGGTAGGAGCATACCCATTTGAAGATCAAGAGGACCCAAAGAATTTCA

GGAAGACAATCAATAGAATAATGGCTGTCCAGTACAAGATCCCTGATTATGTTCACATATCTCAAGATTG

CAGACATCTCCTTTCTCGCATATTTGTTGCCAACCCATCCAGGAGGATCACAATCAAAGAAATCAGGAGC

CATCCATGGTTTTTGAAGAACTTGCCAAGGGAACTGACAGAGGCAGCTCAAGCCATGTACTACAGGAAAG

AAAATCCTGGCTTTGCCTTGCAAACTGAATCAGAAATCATGAAGATTGTGGAAGAGGGCCAAAGTCCTCC

TCCAGTATCTCGATCTATTGGAGGCTT

>FM896060.1 FM896060 Jatropha curcas embryo 71-95 (DAF) Jatropha curcas cDNA clone rjcpga0_003192, mRNA sequence

TTGATGGTTTCAGAGAGGACATGATTATGTTGAAGTTAAATGTGGATGTACAAGCAAGAAGTATGGTGAT

GCCATCGGACTCCTCAGAGTTTTTGCGAGTGGCCAATTTCAGATAACCTGTGATTGTTCTCCTGTCTGCA

AAAGGGGAAAGAGATTCACACCATATGAATTTGAGAAGCATACAGGGAAGGGAGGGAGAAACAGATGGAA

AAGCCATATATGGGTAGTAATGAAAGATAAAAAGGTGCCATTGTGGAAAACTCCATTGCTGAAATATTAC

AAACATATTGCTAATGGGGCAAGTGGATTTATGAAACGAGTCTTTCATCGCGACGAGTTCATCAAGTGCT

ATAAATGCAAGAAAGAACGCAGGTTTCGTCTTAGGACTAAGGAACAGTGTAGGATTTATCATGATGCTTC

GCTCAATGAAAGATGGAAGTGTGCTGATAGCTTGCATTCCAAAATAACCTGCAAGGATGACGAAGAGCGG

GTCAGTCGCNAGAACTGCAGAGGCTGTCCTGACTGGCCGTCATGCAGTGGCTGCACTTCCTGTGTCTGCT

TTGGGTGCCTCCAGTGCAGGTTTCTCGACTGCAACTGCCGCACCTGTGTTGATTTCATGCCAAAC

>FM896059.1 FM896059 Jatropha curcas embryo 71-95 (DAF) Jatropha curcas cDNA clone rjcpga0_003190, mRNA sequence

ATAAGTAATAGCTTACTTTTATCTTCCAAAGACTGAAAAAGAAAAGATAAAGGACTTAGTAAATCAACCA

ATCCCTTTGCCTTTCCTCATTCATGATATCATACATTCCTTGTATCTTGGTTATTGAGCTAAATCAGAGA

GCTATATATGGTCATGAATGGTAAGCACCAAGGAACCTGTTCAACTAAATTAGCGATATGAAATACAACT

TGCTGCATAACATCCTCCAATAGAAGTCACAGACCTTAGATCTTCTTATGCCTTTGAGCCCGTAGAAAAC

CCCACTAAATTACAGCAGTAGGAACATATGAATTTCATTTAGGTGGNCAGATCAATCTCATGAAATGTGC

TCCAGCGAATGCCACCATTTGGTTACAGGCAGCCAAGCATATAAGTCTGTCACATCATCAGTACCAGTGA

TTCCCAGGCCCACCTTGAGCAGC

>FM896058.1 FM896058 Jatropha curcas embryo 71-95 (DAF) Jatropha curcas cDNA clone rjcpga0_003189, mRNA sequence

AACAAAACAAGGAAAGGAAATTGCTGATGAATTCTACAAGTAAGCTTTGATTAAATGTTGCTAAGATAAT

CACCACATCTTCTCGAATTGATCATTTCCCGCAGCTCACAGTTCTACAGTACTCTAATCATGCAAACGAA

AATAAAATAATTATGCATAACATGTTTGTCCTAAGAAGGCTACCTCATAATCTAAGTAGTTCCATAAAAA

GTTAGAGAACAAGTCACAGGAAATAACGTATTTGAGGAGCCTCTACTATTTTGTCTATCTCACTTCTTCC

TCTTCTTTGGGGGCTGGAGGGCTTCCTCGTCTTCATCCTCCTCATCTTCATTCTCTACCTCCTCAACTTC

CTCCTCATCTTCTTCCCCACCATCGTCGTCGTCGTCATCATCATCGTCGTCATCGTCATCATCTAGATCC

TCACCATCTTCTTCATCATCATCATCCTCTTCCTCTTCTCCATTCTCTTCTGCTCCACCCCCAGGACCCT

TTTTAGACTTGCTCTTGTTGTTGTTAGGGTTGTTGCCGATGTCTCCTCCATCTTCAGATGATATTTCCTC

TTCGCCTTCTCCAAATCCACCATCTCCATCGTCGTCGTCATCCCATCGTCT

>FM896057.1 FM896057 Jatropha curcas embryo 71-95 (DAF) Jatropha curcas cDNA clone rjcpga0_003188, mRNA sequence

ATTCCTTCTTCTTTACTTCCCCGTTTGCTCAGTTGCTTTCTTTGTGAAATCAATATGAAAGGTGGAAAGA

TGAATCTCTCCATTATGGTGGCTGCATGGTTTTGCTGGAGTAGTATTATATTCGGATGGGCATCGGCTAG

GGAAATAGTTTGTCCATTCTCATCAAACCAAAACTACAAAGCTGGTTCCCCTCCAACTTTAACCATTACT

TATGACGCTACTACTGATAAGAAAAACTACGCCCAGTTCATTAAAGATCTAAGAGAAGCATTTGGCTTCA

GTTATTCAAGCCATGAAATACCAGTCTTACGGGCCACAGTTGCTCCAAATCAGAAATTTATTGTAGCCAA

AGTCATAAATGTAGCGAATTTAGAAGTATCATTAGGATTAAACGTCGTTAATGCGTATTTAGTGGGTTAT

AAGGTAGGAGGTACTTCCTATTTCTTTAACGATCCGGAATCTTTGGCTGATGCAAAAACATATCTTTTCA

CAGACACAAAGCAACAAACGCTATCATTTACTGGTAGCTATGCAGATTTTCTATCTAGGGCAAACGTACA

CAGAGAGG

>FM896056.1 FM896056 Jatropha curcas embryo 71-95 (DAF) Jatropha curcas cDNA clone rjcpga0_003186, mRNA sequence

ATTCCTGACATGTGTGCGAGTCAACGGGTGAGTAAACCCGCAAGGCGCAAGGAAGCTGACTGGCGGGATC

CCCCTGAGGGTTGCACCGCCGACCGACCTTGATCTTCTGAGAAGGGTTCGAGTGAGAGCATGCCTGTCGG

GACCCGAAAGATGGTGAACTATGCCTGAGCGGGGCGAAGCCAGAGGAAACTCTGGTGGAGGCCCGCAGCG

ATACTGACGTGCAAATCGTTCGTCTGACTTGGGTATAGGGGCGAAAGACTAATCGAACCGTCTAGTAGCT

GGTTCCCTCCGAAGTTTCCCTCAGGATAGCTGGAGCTCGGGACGAGTTCTATCGGGTAAAGCCAATGATT

AGAGGCATCGGGGGCGCAACGCCCTCGACCTATTCTCAAACTTTAAATAGGTAGGACGGGCGCGGCTGCT

TCGCTGAGCCGCGCCACGGAATCGAGAGCTCCAAGTGGGCCATTTATGGTAAGCAGAACTGGCGATGCGG

GATGAACCGGAAGGCCGGGTTACGGTGCCCA

>FM896055.1 FM896055 Jatropha curcas embryo 71-95 (DAF) Jatropha curcas cDNA clone rjcpga0_003185, mRNA sequence

GGATTCTATCCGTCGCCCGATGGGAATTGTACTTTAAGGCGGCCCTCGCGGCTCGTCCGCCGCGGGGGCT

TGGCCAACGGCACGTGCCTCTGGGGGCCGGATGGCCCCTACTCCGGGTCGGCAAACGGGCGACGGACACG

CGCGTCGCTTCTTGGCCCGGATTCTGACTTAGAGGCGTTCAGTCATAATCCAGCGCACGGTAGCTTCGCG

CCACTGGCTTTTCAACCAAGCGCGATGACCAATTGTGCGAATCAACGGTTCCTCTCGTACTAGGTTGAAT

TACCATCGCGACACCATCATCAGTAGGGTAAAACTAACCTGTCTCACGACGGTCTAAACCCAGCTCACGT

TCCCTATTGGTGGGTGAACAATCCAACACTTGGTGAATTCTGCTTCACAATGATAGGAAGAGCCGACATC

GAAGGATCAAAAAGCAACGTCGCTATGAACGCTTGGCTGCCACAAGCCAGTTATCCCTGTGGTAACTTTT

CTGACACCTCTAGCTTCAAATTCCGAAGGTCTAAAGGATCGATAGGCCACGCTTTCACGGTTCGT

>FM896054.1 FM896054 Jatropha curcas embryo 71-95 (DAF) Jatropha curcas cDNA clone rjcpga0_003184, mRNA sequence

ATTCCTACATTAACATATCTTTCTCACACTTATCATCAACAAATGGCTGAACGTTCACAGCCACACCAAG

TTCAAGTGCACCCACAACACCGCTATGAAGCTGCCTTCAAAGGTCAACAAAAAGGTCCATCAGCTCAGAA

AGTGTTAGCTGTGATCACCCTCCTGCCGGTAGGCGGTGGGCTTCTTGCACTAGCTGGTATAACCTTAGTA

GGTACCCTAATCGGCCTAGCTATTACTACCCCTCTTTTTGTCATCTTTAGCCCTGTTCTTGTCCCAGCTG

CATTAGTTATTGGCCTTTCGGTCATGGCATTTTTGGCTTCCGGGGCTATGGGGCTCACAGGGTTGTCATC

ACTCTCGTGGGTTCTGAAATACCTCCAGGAGGTCACTCGGCGAATGCCGGAGCAGCTGGATATAGCTAAG

AAGCGTATGCAAGACATGGCTGGTTTTGTGGGACAGAAGACCAAGGAAGTAGGCCAAGAAATCCAGAGGA

AAGCACATGAAGGGAAATAAGTAGAGAGAAATGCTGTTGGTTATTAAGGGTTGAAGTCAGAG

>FM896053.1 FM896053 Jatropha curcas embryo 71-95 (DAF) Jatropha curcas cDNA clone rjcpga0_003183, mRNA sequence

CCACACGAGATTTCTAGTTCTCGTTGAGCTCATCTTAGGACACCTGCGTTATCTTTTAACAGATGTGCCG

CCCCAGCCAAACTCCCCACCTGACAATGTCTTCCGCCCGGATCGGCCGCCGAAACGACCTTGGGTCCAAA

AAGAGGGGCATAGCCCCGCCTCCGATTCACGGAATAAGTAAAATAACGTTAAAAGTAGTGGTATTTCACC

GTCGCCTTTCGGCTCCCACTTATCCTACACCTCTCAAGTCATTTCACAAAGTCGGACTAGAGTCAAGCTC

AACAGGGTCTTCTTTCCCCGCTGATTCCGCCAAGCCCGTTCCCTTGGCTGTGGTTTCGCTGGATAGTAGA

CAGGGACAGTGGGAATCTCGTTAATCCATTCATGCGCGTCACTAATTAGATGACGAGGCATTTGGCTACC

TTAAGAGAGTCATAGTTACTCCCGCCGTTTACCCGCGCTTGGTTGAATTTCTTCACTTTGACATTCAGAG

CACTGGGCAGAAATCACATTGCGTGAGCATCCTCAGGGACCATCGCAATGCTTTGTTTTAATTAAACAGT

CGGATTCCCCTTGTCCGTACCAGTTCTGAGTCGACTGTTCGA

>FM896052.1 FM896052 Jatropha curcas embryo 71-95 (DAF) Jatropha curcas cDNA clone rjcpga0_003182, mRNA sequence

CTGATATTCCCANAAGCCGATCTTCTTCTATCAGAATCTTGTTGACCCTTTCTCTCTGAAACCTTTAACT

TTCTCTTCCATAATAATTAAACCTCAACCCTAGATATTCAATCACCCTACCCAACCACCAAAGTTGCTCT

CCAACTGAGCACCTCATTTGTGGCGTCAACCATGGCGGCTCCGGCACCGACCTGGCCGTGGACTTTCTAC

AGCTTCTCTAACTGTAACCCTAGTTTTAACCTTAGTTTCCGTAGTTTATGGCAACTCCGAAGGAGACGCT

TTGTACACCTTGAGAAGGAGCTTGTCGGATCCGGATAATGTTCTCCAGAGCTGGGATCCCACCCTCGTCA

ATCCGTGCACTTGGTTCCACATCACCTGTAACCAGGATAATCGCGTGACCCGAGTTGACTTGGGGAACTC

AAACTTATCTGGACATCTAGTTCCCGAGCTTGGAAAGCTAGAGCACCTGCAATACCTGGAACTCTATAAA

AACAATATTCAAGGAAGCATACCTAATGAGCTTGGTAACTTGAAGAGCCTCATCAGCTTGGATTTGTACA

ACAACAACATCTCAGGGACCATTCCTCCTTCACTGGG

>FM896051.1 FM896051 Jatropha curcas embryo 71-95 (DAF) Jatropha curcas cDNA clone rjcpga0_003181, mRNA sequence

CTTTCAGGCCTTTCACGCTTCCGATCGTAACGTGCCGGGTTCTTCTTCTCCCCTTTATTTAAGAAAATAA

ACAACAGTAGAAAGTGTGGAATTCGGAAGCAGGGGATTATCATTCCCCGTGCTTTCATCACAAGAAGTGT

TTTGCCAATTACTTTGAGGCTTTTCACGCTTCTGATCATAACCCAATTTCTCAATTCATGAGATCCGATA

ATCAGTCCCTCTAAAGTTTCTGTCCTCTGTCCTGTCGTTTACGGGTAAACATGTTCTCCAAGAAGATGGA

ACATGAGGAAGCCTCCATTTTGCAATTGCAAAACCAGAAGGATGTTGAGATGATAGTATCATCTGAAGAA

AATGCCATTCTTCCTGCTACAGCACATTTATCTTGCAATTCTGTTTGTGCCTTCTCCAAACAAGAAAATG

ATACTCTTGAATCCGAAGAACGATCAAAGTCAGCTGTAAAACTTGTGGGGCTTATAATTTTCTATTTACT

AGTCATGACAGTAGAAATCGTCGGTGGCTTGAAAGCCAACAGCCTTGCAGTTATTACAGATGCAGCCCAT

TTGCTGACTGATGTTGCTGGATTCTGTGTCTCT

>FM896050.1 FM896050 Jatropha curcas embryo 71-95 (DAF) Jatropha curcas cDNA clone rjcpga0_003180, mRNA sequence

ATTCCTCTCTTTATCCTCTTCTCCTTTGGTCAAGCTGCGCCTCTTATTTTAACCCTAGTCAACAACTGCC

CTTTCACCGTTTACCCTGCCATCCAACCCAACGCTGGCCACCCGGTCCTCGAAAAAGGTGGCTTCCCACT

CTCCACCCTAACACACCGCTCTTTTCCCGTCCCTAACCAGCACTGGACTGGCCGGATCTGGGCTAGAACC

GGATGCACTCACACCAACGGCAAGTTCCACTGCGCCACCGGTGATTGCAACCACCAACTAGAATGCAACG

GCCTCGGTGGTGCCCCTCCAGCAACTCTCGCTCAGTTCAGCCTTCACCATGGCCATAACGACTTCTCCTC

TTACGGCGTCTCTCTCGTCGACGGATTTAACCTTCCGATGACCATAACTCCACATGAAGGCAAAGGTGTT

TGTCCGGTGGTGGGTTGTCGAGCAAACTTACTGACAACATGCCCTCAAAAGCTTCAGGTGAGGTATCCGG

TGGGTCATGGTCCGGTGGTGGCCTGTAAAAGTGGATGCTTGGCGTTCGGAACCGACGAGCTTTGTTG

>FM896049.1 FM896049 Jatropha curcas embryo 71-95 (DAF) Jatropha curcas cDNA clone rjcpga0_003178, mRNA sequence

TCTGACATGTGTGCGAGTCACGGGTGAGTAACCCGCAAGGCGCAAGGAAGCTGACTGGCGGGATCCCCCC

GAGGGTTGCACCGCCGACCGACCTTGATCTTCTGAGAAGGGTTCGAGTGAGAGCATGCCTGTCGGGACCC

GAAAGATGGTGAACTATGCCTGAGCGGGGCGAAGCCAGAGGAAACTCTGGTGGAGGCCCGCAGCGATACT

GACGTGCAAATCGTTCGTCTGACTTGGGTATAGGGGCGAAAGACTAATCGAACCGTCTAGTAGCTGGTTC

CCTCCGAAGTTTCCCTCAGGATAGCTGGAGCTCGGGACGAGTTCTATCGGGTAAAGCCAATGATTAGAGG

CATCGGGGGCGCAACGCCCTCGACCTATTCTCAAACTTTAAATAGGT

>FM896048.1 FM896048 Jatropha curcas embryo 71-95 (DAF) Jatropha curcas cDNA clone rjcpga0_003177, mRNA sequence

ACTGGAGCTCACGGGTGGCGGCGCTCTAAATATGATCCCGGGCTGCAGGTCATTCTTTGTTTGTGAAGCA

AAGGTGGGGTGGGGGGGATAAGAACAGCTGGCCTACAAAATGAAAATGCCAAAACATACAAAAAAAATAC

AAAAAATGGAGGAAACTGGAACCAAATTATGACAATGATGAATAAATTAAAAACGGCTAAAGGAGAACTA

AGCTATCTCTCATGGTTCTTCTGGCAATGGCCGCCAATGGGGATTATTATGTAGCTCTTGATTTATGTGA

CAAGTATCTGGTAATCCTTACATGGTGGAGAGGAGGAAAGACTGGAGGGCAGTGAAGAGTTCCCATCAAG

TAGGGTGTTTTGCTTGGCAAAGAAGGAAACATGAAAACTTGAAGAATAGTGATACAAGTGAAGCCATGAG

TTCAAAAGCTGTAATGCCTCACCGAATG

>FM896047.1 FM896047 Jatropha curcas embryo 71-95 (DAF) Jatropha curcas cDNA clone rjcpga0_003176, mRNA sequence

GAAGTGATAGAAAGGGAGAATGGCTCTGTTGTGCGCACAGTCTCCTGCTATGCTATTATCCTCCGCATCT

TCTTCTTCTTCGACTACTCCAAGTGCACCAAAATCTTATTTTTCTTGTGCAAGAAAAGATGCCATTTGTT

GCAGTCCGTTTACTGTGGGATTCTCATTGGTAAAAAAACCATGTGTACGGACAGAATTTCCTTTTAGTAG

TTGGAGCAGCAGCAATTTTGTGGTTCGGTCTCAGAAAAAGAAGGAAGAGACCGCCATTGCTCGTGTACCT

CTTGATCAACGCTGGATGTTCGAGGAGAATGAGATCAATGGCCCTGACATTTGGAATAAGACTTGGTATC

CTAAAGCTGCAGACCATATCAACACAGATAAACCTTGGTACGTTGTTGATGCCACTGACAAAATTCTTGG

GAGATTGGCATCCACCATTGCCATTTATATCCGTGGGAAGAATTTGGCCACCTATACTCCTAGTGTGGAC

ATGGGGGCGTTTGTAATTGTGGTGAATGCTGAGAAGGTTGCCGTATCTGGGAAGAAAAGGACCCAAAAGC

TATA

>FM896046.1 FM896046 Jatropha curcas embryo 71-95 (DAF) Jatropha curcas cDNA clone rjcpga0_003175, mRNA sequence

ATTAGATGATGTTTTTGTTACACTGGGGATGCACACAAGCTATTGAAATCACATTATCAGTTCTTGGACG

ACCTGGGGCTAACATTTTGCTACCAAAACCAGGATTCCCATACTATGAAGCTATTGCTGGACATAATAAT

ATTGAGGTTCGCCATTTTGATCTTTTACCTGATAAGGGCTGGGAGGTTGATCTCGAGGCTGTTGAGTCTC

TGGCAGATGAGAATACTGCTGCTATGCTTATAATAAACCCTGGAAATCCTTGTGGAAATGTCTACAGATA

TGAACACTTGAAGAAAATTGCAGAAACAGCTAGAGAGCTTGGAATTATGGTGATTGCTGACGAAGTATAT

GGGCATCTTACATTTGGGAGTACTCCATTTGTTCCAATGGGAGTTTTTGGATCAATCGTGCCTGTTCTTT

C

>FM896045.1 FM896045 Jatropha curcas embryo 71-95 (DAF) Jatropha curcas cDNA clone rjcpga0_003174, mRNA sequence

GCGCAAGGAAGCTGACTGGCGGGATCCCCCCGAGGGTTGCACCGCCGACCGACCTTGATCTTCTGAGAAG

GGTTCGAGTGAGAGCATGCCTGTCGGGACCCGAAAGATGGTGAACTATGCCTGAGCGGGGCGAAGCCAGA

GGAAACTCTGGTGGAGGCCCGCAGCGATACTGACGTGCAAATCGTTCGTCTGACTTGGGTATAGGGGCGA

AAGACTAATCGAACCGTCTAGTAGCTGGTTCCCTCCGAAGTTTCCCTCAGGATAGCTGGAGCTCGGGACG

AGTTCTATCGGGTAAAGCCAATGATTAGAGGCATCGGGGGCGCAACGCCCTCGACCTATTCTCAAACTTT

AAATAGGTAGGACGGCGCG

>FM896044.1 FM896044 Jatropha curcas embryo 71-95 (DAF) Jatropha curcas cDNA clone rjcpga0_003173, mRNA sequence

CTGACATGTGTGCGAGTCAACGGGTGAGTAAACCCGCAAGGCGCAAGGAAGCTGACTGGCGGGATCCCCC

CGAGGGTTGCACCGCCGACCGACCTTGATCTTCTGAGAAGGGTTCGAGTGAGAGCATGCCTGTCGGGACC

CGAAAGATGGTGAACTATGCCTGAGCGGGGCGAAGCCAGAGGAAACTCTGGTGGAGGCCCGCAGCGATAC

TGACGTGCAAATCGTTCGTCTGACTTGGGTATAGGGGCGAAAGACTAATCGAACCGTCTAGTAGCTGGTT

CCCTCCGAAGTTTCCCTCAGGATAGCTGGAGCTCGGGACGAGTTCTATCGGGTAAAGCCAATGATTAGAG

GCATCGGGGGCGCAACGCCCTCGACCTATTCTCAAACTTTAAATAGGTAGGACGGGCGCGGCTGCTTCGC

TGAGCCGCGCCACGGAATCGAGAGCTCCAAGTGGGCCATTTTTGGTAAGCAGAACTGGCGATGCGGGATG

AACCGGAAGCCGGGTTACGGTGCCCAACTGCGCGCTAACCTA

>FM896043.1 FM896043 Jatropha curcas embryo 71-95 (DAF) Jatropha curcas cDNA clone rjcpga0_003172, mRNA sequence

TTAAATAGTTTAATTTACATTGAAATTTGCAGCTGAGTTTCATTCTCCATACTATAACTGGAAATGTATA

TGTTTTTGGTATAGATGAATAAAGGTTAACTAATTATTT

>FM896042.1 FM896042 Jatropha curcas embryo 71-95 (DAF) Jatropha curcas cDNA clone rjcpga0_003171, mRNA sequence

CTTCTTTCCTGGGAACCCTAAATGGCCAGCTTCTAAATATGCACTCACTTACGAGCTTCGTTATGACACC

CCGCCTCACCTAATACCTCCTCTCCAGAATGCTTTTCAAGCATGGGCTAACGTTACGCCCTTCACATTTT

CTCCCTTCCATGGTCGTGGAAATCCAGATATAACGATTGGTCTTTACAAAGGTGATCATGGAGATGGGTT

CCCTTTTGATGCATTTGGTGCCATTGCTGCTCATTCTTGGCCTCCACAGGATGGAAGGCTACATTTTAAT

TCGTATGGGAGATTTTGTATCGGTGCATTTCCAAATTTATATGATTTTGGGACAGTTGCCTCGCATGAAA

TAGGGCACCTTCTTGGACTTGGACATAGCCGTGACGAAGAGGCTATTATGTATCCAGTTGCTCCTTTCGG

ACGGGTCAAGGGTATACATCAATATGATATTGAAGGCGTTCGAGCTTTATATGAATTTTAATACTATTAA

CTGTTTCTATTA

>FM896041.1 FM896041 Jatropha curcas embryo 71-95 (DAF) Jatropha curcas cDNA clone rjcpga0_003170, mRNA sequence

AAGAATATTAAACTGAAAATTTTGAATAGCTGAATCAAGAAATCTGTACACAGCATCAATCAAAGGTTTC

AACCTCTAAATAAACAGAAACGAACTTCGATCCTATCAGTACCAGAACATTTTATAGTAATGAAACAAAG

CAAATAACATCTAATACAAACTGTGCAACATATATCTCTACCATAACCAATATTTGCTGCAGGGTACCAA

ACTAGTGGTTTCATGAACCACCATTTATAAAATTTTGAAGGTAGAGCATTGCTTGCCTTCTCTTTCATAG

TTCCCTTCCCCATCCTTATCAAAAGCTAGTTTCTTATCTTCACTTGATAATCTCTTATGAATCTAACTAG

TAGCAGCAGTTGCTTTCTTCAGATTTTTCTCATCTTCAATATCCGAATTGCCAATTTTGTCAAAATCATC

ATCAAAGCTACCATCGCCATCCGCATCATCTGGACCTCCATCACTGTCTTCAAGATCTCCCANAAGAAAA

GAGTCCAAGTCCTGCTCAGCTGATGATGCAGTTGATGTCCTGCTTATCAGGTTCTTTTCTATCACCTCAG

ATGTAGAATTATCACCAGATGTAGCACTTTGTTTAAATTGTCATCTTTTATCTGCTCTTCTG

>FM896040.1 FM896040 Jatropha curcas embryo 71-95 (DAF) Jatropha curcas cDNA clone rjcpga0_003169, mRNA sequence

ATTCTGAAAATATAAATATATTCGTGTGTTTCTTACAGAACCTCACTCCTGTCTCTGGCATCAAACCCTC

TTCAAAACCCTTCTCTTCTCGTATCTCTGTCTATCTGCTGAATAATAATGGAATCTATTTCGAGTTCAAT

GTTCGTCTCTAGATTCCCTGAAACAGTCCCAATTATCAACACCTCATCTTCTTCATATGCTTCTTCTCCT

TCTTCATCTTCTTCTTCTTCTTCTTCTTCTTCTTCTGACATAATCTACCTATCAGATAAGCGAATTGGGT

CTCTCAAATTTCCTGAAACTGCTCGTTTTCCAGGCCTTCGGACTCGGGCTCTTGCACGTGAGCTATCAGG

TGGGGAAGGAGAAGATCACAGGCCTTTGGATAATGGGTTCAGCTTTCTTTCTGATGATGAGTCTCTTTTT

TTCTCTCAGGATAATAATGAAAAGGATGCAGAGAAAATAGTGAAGTTTGAGACACCTTTGGCGGT

>FM896039.1 FM896039 Jatropha curcas embryo 71-95 (DAF) Jatropha curcas cDNA clone rjcpga0_003168, mRNA sequence

ATTCAAAGGATTCTATCCGTCGCCCGATGGGAATTGTACTTTAAGGCGGCCCTCGCGGCTCGTCCGCCGC

GGGGGCTTGGCCAACGGCACGTGCCTCTGGGGGCCGGATGGCCCCTACTGCGGGTCGGCAAACGGGCGAC

GGACACGCGCGTCGCTTCTGGCCCGGATTCTGACTTAGAGGCGTTCAGTCATAATCCAGCGCACGGTAGC

TTCGCGCCACTGGCTTTTCAACCAAGCGCGATGACCAATTGTGCGAATCAACGGTTCCTCTCGTACTAGG

TTGAATTACCATCGCGACACCATCATCAGTAGGGTAAAACTAACCTGTCTCACGACGGTCTAATCCCAGC

TCACGTTCCCTATTGGTGGGTGAACAATCCAACACTTGGTGAATTCTGCTTCACAATGATAGGAAGAGCC

GACATCGAAGGATCAAAAAGCAACGTCGCTATGAACGCTTGGCTGCCACAAGCCAGTTATCCCTGTGGTA

ACTTTTCTGACA

>FM896038.1 FM896038 Jatropha curcas embryo 71-95 (DAF) Jatropha curcas cDNA clone rjcpga0_003167, mRNA sequence

CTGGTTTTCAAAAATCTCTGCAACATTGCTACACTATTTCATGTTCCTATAGAGTCAATTTCAAATCTGT

CAGAAACATATTTTCAAGTCTTTTTTTTTTAATTATTCTTTAATTATTATTATTATTATTGTTGTTGTTG

TTGTTGTTGTTCGACTTCGAGATTTTTCTCTTCTGATTTCCTCTTCCTTTTCAGTTGGCTGCCCAAATTG

CTGGTTTCAGTTAGGAACTGGAAACATTTATAGGGTTTTGTTTTGGATCCCATGGATACTTCAATTGCTG

AAGTCTGTTTCACAACTTAATTTTACTTCCTCTTAAATTTTTGATAAGGCCAGGCCTGCATAGGAAGAAT

TTGGTTTTTGTTGTGCTGGTGGAAAAACCATGGTAGGGAATTCATTAGCTCAGGTTTCAAAATGCTCTGC

TACCATGTTATCATCATCGTTGATTGTTGTACCCAATGGTTCTTCGAGCTTCTTGCCCTATCTTCCATCA

CGACAGAAAAAACAAGTCAGGCTGGTCAGAGCTATGTCAGCGGACGCAGGACCTAGCCAAGCATCCTCAT

CTTCATCAGAGAAAAAGAATCCACTTGCAGTTGTTTTGGAGG

>FM896037.1 FM896037 Jatropha curcas embryo 71-95 (DAF) Jatropha curcas cDNA clone rjcpga0_003166, mRNA sequence

CGATTCTATCCGTCGCCCGATGGGAATGTACTTTAAGGCGGCCTCGCGGCTCGTCCGCCCCGGGGCTTAG

CCAACGGCCGTGCCTCTGGGGGCCGGATGGCCCCTACTGCGGGTCGGCAAACGGGCGACGGACACGCGCG

TCGCTTCTGGCCCGGATTCTGACTTAGAGGCGTTCAGTCATAATCCAGCGCACGGTAGCTTCGCGCCACT

GGC

>FM896036.1 FM896036 Jatropha curcas embryo 71-95 (DAF) Jatropha curcas cDNA clone rjcpga0_003165, mRNA sequence

AAATCGTATGTTCATGGTCTTAACTAATCATATACTGCAATTCATGTATCAAAACTTGCTGCATATTTTC

AAAAATAAATGAAATACATAAAACTATGATAAAGGAAGCAGCTGGGACAAGATTCAAATAATCATACCAA

TAGTAAGTTGGTGGTTTTATCTTCTCCATAACTGTTTCATGTTTGAAGTACAGGGCAGGCATCATATTGT

ATTATGGAGGACAATGATTCTTATTGTCATCAATTAACCTTCTTCAAATTGAGAAATAGTTCACCAAGTT

GAGATTCACAAGCAGCTTGGGTCTCAGGAGTAAGATCTTTAAGAAAAGACTTGGTGATGTCATGGGATGA

CACGATCTGGAAGAGCTTGCCACAAAATTTCCGCACCTCAGCCCGCCTTGACTTGTTCTCTGGCATATTC

ATCACCAATTCCTTTACCAGATGACTGAGCTTTTGCAAATGGCTTTTCAAAATTTTCTTTGATTCATTGT

GACTGCTTTCATCTGTACCAGAAGAAACCATTGACTTTAATATTTCCATCACTAAATCAAGCGCATCAAC

TCGTCGAAACTCTGACTTTGCACCGCCACATTTCTCCAAAAG

>FM896035.1 FM896035 Jatropha curcas embryo 71-95 (DAF) Jatropha curcas cDNA clone rjcpga0_003164, mRNA sequence

ATTGCAAAGGATTCTATCCGTCGCCCGGTGGGAATTGTACTTTAAGGCGGCCCTCGCGGCTCGTCCGCCG

CGGGGGCTTAGCCAACGGCACGTGCCTCTGGGGGCCGGATGGCCCCTACTGCGGGTCGGCAAACGGGCGA

CGGACACGCGCGTCGCTTCTGGCCCGGATTCTGACTTAGAGGCGTTCAGTCATAATCCAGCGCACGGTAG

CTTCGCGCCACTGGCTTTTCAACCAAGCGCGATGACCAATTGTGCGAATCAACGGTTCCTCTCGTACTAG

GTTGAATTACCATCGCGACACCATCATCAGTAGGGTAAAACTAACCTGTCTCACGACGGTCTAAACCCAG

CTCACGTTCCCTATTGGTGGGTGAACAATCCAACACTTGGTGAATTCTGCTTCACAATGATAGGAAGA

>FM896034.1 FM896034 Jatropha curcas embryo 71-95 (DAF) Jatropha curcas cDNA clone rjcpga0_003163, mRNA sequence

ATTCCCTNGCTTCGGCTACTGCTCCTCTCCTCTGCAAAAATGCAGATCTTCGTGAAAACCCTAACCGGGA

AGACGATAACCCTAGAGGTTGAGTCTTCTGATACTATCGATAATGTCAAGGCCAAGATCCAGGACAAGGA

AGGCATCCCTCCAGACCAGCAGCGACTGATCTTCGCCGGAAAGCAGCTAGAGGATGGCCGCACTCTTGCC

GACTACAATATCCAGAAGGAATCCACTCTCCACCTGGTGCTTCGTCTTCGTGGTGGTGCTAAGAAGAGAA

AGAAGAAGACTTACACCAAGCCAAAGAAGATCAAGCACAAGAAGAAGAAGGTCAAACTTGCCGTGCTTCA

GTTCTACAAGGTCGATGACTCCGGAAAGGTGCAGAGGCTGAGGAAGGAGTGCCCCAATGCTGAGTGTGGA

GCTGGAACTTTCATGGCCAACCAATTTTGATAGGCACTACTGCGGCAAGTGCGGCTTGACTTATGTTTAC

AATTAAGGCTGGCGGTGATT

>FM896033.1 FM896033 Jatropha curcas embryo 71-95 (DAF) Jatropha curcas cDNA clone rjcpga0_003162, mRNA sequence

ATTCACGAGTTCTATCGGGTAAAGCCAATGATTAGAGGCATCGGGGGCGCAACGCCCTCGACCTATTCTC

AAACTTTAAATAGGTAGGACGGCGCGGCTGCTTCGCTGAGCCGCGCCACGGAATCGAGAGCTCCAAGTGG

GCCATTTTTGGTAAGCAGAACTGGCGATGCGGGATGAACCGGAAGCCGGGTTACGGTGCCCAACTGCGCG

CTAACCTAGAACCCACAAAGGGTGTTGGTCGATTAAGACAGCAGGACGGTGGTCATGGAAGTCGAAATCC

GCTAAGGAGTGTGTAACAACTCACCTGCCGAATCAACTAGCCCCGAAAATGGATGGCGCTTAAGCGCGCG

ACCTATACCCGGCCGTCGGGGCAAGAGCCAGGCCCCGATGAGTAGGAGGGCGCGGCGGTCGCTGCAAAAC

CCGGGGCGCGAGCCCGGGCGGAGCGGCCGTCGGTGCAGATCTTGGTGGTAGTAGCAAATATTCAAATGAG

AACTTTGA

>FM896032.1 FM896032 Jatropha curcas embryo 71-95 (DAF) Jatropha curcas cDNA clone rjcpga0_003161, mRNA sequence

CTTGCATTGTGCTTTATTTCTCTGCATTTCTCTCCTTCAATTTCATCTACTACAACCATCTCAAACGACA

TTCTAAAGCACAATTCCCCTCTTCAAAATCTCCTCTATAACAAAGCAAAACAAGAACTAATTTCAAGAAA

ATGGAGGCAATGAAGATGAACCTCTTTGTCGTTTTGATGGTTGTGTTGATGGCTTCCTCCAACATGCAAA

TGGCCACAGCAGTTGGAGCACCATCTCTAGCACCAGCACCAAGCCCTGCATCTGATGCTAATGTCTTTGT

TCCCACATTCTTTGCCTCTTTGGTTGCTCTTGCTTGTGGGTTCTTCTTTTGAACCATATTACATATTATT

TATGTTATGTACAATATTTTCCTTATTTCTCTCTCTACCCATTTCTCTTTCTCTCTTTAGAAACACACTT

CTATATCTAGTTTTCTTCTCTGTTTTTATTCTTTTTAATCCATCTTTCATGGAGTGGGAGAGAGAGAGAG

AGATAGACCAGAAATGGGTAGGGATTAGTTTGATGTGGTTATTATTTAGTTTAAATTTCTAATGATGA

>FM896031.1 FM896031 Jatropha curcas embryo 71-95 (DAF) Jatropha curcas cDNA clone rjcpga0_003160, mRNA sequence

ATTCCTTCTTTTTTGCAGACTGCTCAGCCTGTTCAAGAAGATGAGCATCACGAATCCTGTCAAGGCCAAA

GGAAGTTTGCAACCTTGCAAGCCCAGCTTCAAGAAGAACTGTTGCCATGTTAGTCTTTGCTTCCCACAGG

GTTCCCAAGAACGTTCCAGTTCTATCAACAGTTTCTATTTCAATCTCAACATCCCTCTGCATTATCCTTC

GTCTCATTAGTGCAATTGCCTCATCTGAATATGGCTCATCACGACCGGGACACCTAACACCAGAAAATGA

AAACGCAATGCTGCATGTCTCCTTGGGACATAAAACCTTAAAACGGTGACCACTGAGCACATATTCCACT

ACAGCAGCAATTTTTCTGCCCCGGTGCAGGAAAGGCAACTATTCTTTTGCTTTCTTGGATGATTGCGTGG

TGAGATCTAGTATATGCGATACTGGAGACTCCTTGCCAGAATGCATTCCT

>FM896030.1 FM896030 Jatropha curcas embryo 71-95 (DAF) Jatropha curcas cDNA clone rjcpga0_003159, mRNA sequence

AGCAAAAACAAACTCAGTTTCATATGAAAATTGAAAGTTCATATAAGGAGGATGCTCCTTATGGATTATT

GAATACAACAAAGGAAAATGCAAAATAAAAAGTCTAATTTCCCAGAGGAAACAAACTCAGGCAAAAGGGT

ACTAATGTAAAAAACACACAGTTTCCTGGGAATTTATTTTACTGCAACTTAAAACATAGAAATGGTGGAT

TAATTTGCAGATAGAGCAATTGTTGGCCACCTTTTTTTTTTTTTGTTTTCATATTGCCTACTTCTTTACA

TAAACATGCCAGACCATCTCACAAACAGAAGTACCATTGATTACAGTGATCACATAAAACTGGTTTAAGT

TCTAATGTAAGGCTAGCAGTTGTATCAATCCATCTTAGATTTATTACCTAGTCAGATAGTATACAATAAG

AAATATGACAACAAAGGATGCCACAAGCGTA

>FM896029.1 FM896029 Jatropha curcas embryo 71-95 (DAF) Jatropha curcas cDNA clone rjcpga0_003158, mRNA sequence

TTTTTAACACTTCAGATTCACTGCCCTTTCAATGTACTTATCATCCTCTAAGCACCATCACCAAATATTC

AGTCGGAAAAGGCAAAAACAAACTTTACAGTGATACACAACGAATGACTGTAATTGTCTATTGCATGTTT

CGACAGGTACAACTAGCAACACTGCTTTCACAAAAGGCTTTCTTCATACATCATGCCGTAAATTCTGCTG

CGATGAGAGTTCAGGTTCCCGTTGTAGAAAATTCTGCCTTGATGGTAAATCTGATTGGTCATGTTGCTGC

CGAAGCAGCACATCTTCATCCTGATGATCTACGTCTGTACTACTAGATGGTTTGGCACCAGAAGTAATAA

GGTTTTTAAACCAAGCTGCTTGGAATCTAGGGGTCCCTCTGTTTGAATTGCCAGGTGAATCAAGACCAGA

CCCATTATTCGAAGCAACACCTATAAGGAAACGGTATGCCACTTTGCTAGCTGATACAATCTCTGCCTTG

GCTTGTCTTAACCGTAAAGCTGTATTTCCCGCTACTTCCAAGGCTGTGTCACCAGCATTGGCAAAAACGA

TTAACCCA

>FM896028.1 FM896028 Jatropha curcas embryo 71-95 (DAF) Jatropha curcas cDNA clone rjcpga0_003157, mRNA sequence

GAAGAGTTTGATCCTGGCTCAGAAGGAACGCTAGCTATATGCTTAACACATGCAAGTCGAACGTTGTTTT

CGGGGAGCTGGGCAGAAGGAAAAAGAGGCTCCTAGCTAAAGGTAGCTTGTCTCGCCCAGGAGGTGGGAAC

AGTTGAGAACAAAGTGGCGAACGGGTGCGTAACGCGTGGGAATCTGCCGAACAGTTCAGGCCAAATCCTG

AAGAAAGCTAAAAAGCGCTGTTTGATGAGCCTGCGTAGTATTAGGTAGTTGGTCAGGTAAAGGCTGACCA

AGCCAATGATGCTTAGCTGGTCTTTTCGGATGATCAGCCACACTGGGACTGAGACACGGCCCGGACTCCC

ACGGGGGGCAGCAGTGGGGAATCTTGGACAATGGGCGAAAGCCCGATCCAGCAATATCGCGTGAGTGAAG

AAGGGCAATGCCGCTTGTAAAGCTCTTTCGTCGAGTGCGCGATCATGACAGGACTCGAGGAAGAAGCCCC

GGCTAACTCCGTGCCA

>FM896027.1 FM896027 Jatropha curcas embryo 71-95 (DAF) Jatropha curcas cDNA clone rjcpga0_003156, mRNA sequence

CCCTGTCCTTAATTTCTCTCTATCACATCACTCACCAACATGAGGATGAGCTGCAATGGCTGCAGGGTCT

TACGCAAAGGTTGCAGCGATGACTGCACTATCAGACCTTGCCTTCAATGGATCAAATCCCCTGATTCTCA

AGCCAATGCTACTCTTTTCTTAGCCAAGTTCTATGGCCGTGCCGGCCTCATCAACCTTATCGATGCCGGT

CCTCAGCACCTCCGTCCCGCTGTATTTAGGTCACTGCTATATGAGGCTTGTGGAAGGATAGTGAATCCCG

TGTATGGTTCAGTTGGGTTGCTCTGGTCTGGAAATTGGGCTCAGTGTCAAGCAGCCGTTGATGCAGTTCT

TAGAGGAACACCGATAATGCAAATGCCTTCCGCTCCTGATGCACCGCCACCACACTTGATTGATCCTTTC

AAAACCTACGATATTCGTCATGTGTCCAAGGACTCTAGCTCTGTTGAACTCAACAAGGTTAAGAACAGAA

CCCGATTCAAACGGCCAATTAATAAGCCAACTTCTCCTTCTGAGTCAATTGGTCGAGTTAACCTCGGTGA

CTTGAGTTTCGAACA

>FM896026.1 FM896026 Jatropha curcas embryo 71-95 (DAF) Jatropha curcas cDNA clone rjcpga0_003155, mRNA sequence

TTGAATCTTATCATTCGAGTGGATCTCAAGCCTAATGATTCTCATGCCCAACATTACAAATGGAAAAGTC

AATCTAAAGGAAGAAGAGAGAGTATATGGATTTGGATAGGATATTTTAATGGCTTGATAGCTTTAATCTG

TTCTTGTTTTCATTTTTTTTTTTGACATAACAGTTTAATATTTTGGACTTGTCAACTGCACAAATCATTA

GGTTCTTTTTTCACATTTAGTACGTGATTGGGAATTGAAATTTACATAAATACATTATTAGCAATTATTT

TTGTAGTATCTTATTGATAAAAGAGAAATATATCCTTAAATGTAAAAGTATAATTTTTAATTAATTTTGA

AAAGTTACATTGAATCTTATGATTCGATTCTCGATTCTACAAATAAGAATTTTGATTCTTGATTTAACAA

CTATGATTTAGGTATAGG

>FM896025.1 FM896025 Jatropha curcas embryo 71-95 (DAF) Jatropha curcas cDNA clone rjcpga0_003154, mRNA sequence

GGAGAGGTTTGAAATTAGCAATTCTTATGGTGGTTCAACAAGTCAAAAAGTTAATTCATCACACTCCCAA

AACTCGGTGGATGCAGAAGAGAAGATGCAAAAGGTATCACCGCCTCGTAGAAAAGTATCTAGGGAGGAAA

AATCAGAAAAGTTCGGTGACTGGTTGAAAAAGGATAGTAGTGGATCAGACATTTCCAACCCAAGGCTGCT

GAGTACAGGAAACTATACTGCGAATAATACTGGATCGCGACAACATGAGCCTGATCCTCCTTCAGATGGG

AATATCAATGCAATACTTGAGGAAGAAGAGGCCTTAATTGCAGCTCATAGAAAAGAAATAGAGGACACAA

TGGAGATTGTTCGCGAGGAAATGAAACTGCTGGCAGAAGTTGACCAACCAGGCAGTCTAATAGATAACTA

CGTGACCCAGTTGAGCTTTGTGCTCTCACGGAAGGCAGCTGGTCTGGTTAGTCTTCAAGCTCGCCTTGCT

AGGTTCCAACATCGACTAGAAGAACAAGAGATACTTAGTCGGAAGAGAGTTCCT

>FM896024.1 FM896024 Jatropha curcas embryo 71-95 (DAF) Jatropha curcas cDNA clone rjcpga0_003153, mRNA sequence

GGAAGTCGTCCGCCTCATTTTGGAAAGAGCAGTGGTGCTATTGCTCGTCACATCCTGCAGCAATTGCAGA

AAATGAACATTGTTGATGTTGACCCCAAGGGTGGGAGGAGAATTACTTCGAGTGGTCAACGGGACCTTGA

TCAAGTGGCCGGACGGATTGTGGTTGCCCCTTGATAAACTTAAAGTTGGGTATGCTGCAGAATACCAATT

TATTTTGGTAGTTTTAGCTTTCTGTTTCAGTCTATTGGAGAGATTTTACTGCTTGATGCTTAAACCATAT

TACATGTTGTGAAAAGTTTTGGGTTCAATTATTACTGTCTTTGAGATGAACATGGTTCCTCCTGCTGTTG

CTTTTTATTTATAAAATTTATGGTTTAACTCGTTATGTTC

>FM896023.1 FM896023 Jatropha curcas embryo 71-95 (DAF) Jatropha curcas cDNA clone rjcpga0_003152, mRNA sequence

AGTTCCACACGGTGGAAATCATCCGAATGAATAAGAAGAAAGTCTCAATACAAATTAGTTCACTACACCA

AGCTGAACGTTTTTTCCAATCAATGCACTGTATCCCTAGCCTTACACTCTCAGAACCAACATGTTTAATG

TCAAGTACACAGCTGCTCCTTACAGCAAATTTGGTCCAGAAAAAAGGATTTTGCAATATGAAAAGAAAGC

AAGCAAAACTGGCTGAACTGCCAAAGCGACTTACACTAGTCTCTGTTTTGCTGCCAACGAGTTTTTCCCC

TTCGAATGAGAAATTCTTACAATGAGCAGAGAGGAATATAGAACACGGCAAAAGAGCAAAGTGATAGAAA

ATCACAATGCAACTAGATCAGGTGTTTCAGCTTCAATGGATAACAAAGCAGTGTGTACTTTATCCAGCCA

ATTATCCAGTCGGTCGCGCAAGGATTTGATTTGTGGAATGCCCAGCACTCTCGGCTGCACCCAGGATACA

TGAACCGTTCCCTCAACTTGAATCATTATACCCTCAATAAGATGGACAGAAAGACTCTTCATGAGAA

>FM896022.1 FM896022 Jatropha curcas embryo 71-95 (DAF) Jatropha curcas cDNA clone rjcpga0_003151, mRNA sequence

TGAACACNCGGTTTTTACTGTACAACAATGATGCTACACAGCACATTTTTGAAGTGTCTGCAGGTCTTGA

CTCTCTTGTTCTAGGAGAAGGCCAAATTCTTGCTCAGGTTAAACAAGTTGTCAAAGTTGGCCAAGGAGTT

GTTGGTTTTGGGCGGAACATTAGTGGGCTGTTTAAGCATGCAATATCTGTTGGCAAGCGGGTTAGGACTG

AGACAAATATTTGTGCTGGAGCTGTTTCTGTAAGTTCTGCTGCTGTTGAACTGGCTTGGATGAAGCTTCC

TGAATCTTCCCATGGTACCGCCAGAATGTTGGTGATTGGAGCAGGCAAGATGGGGAAGCTTGTAATCAAA

CACTTGGTAGCAAAGGGTTGCACGAAAATGGTGATCGTCAACAGAAGTGAGGAGAGAGTTGCAGCTATAC

GTGATGAGCTGAAGGATGTTGAGATCATTTACAAGCCCCTTGAAGAAATGCTAAACTGTGCTGCTGAAGC

TGATGTTATATTCACCAGTACAGCATCAGAAACCCCATTATTTTTGGAAGAACATGTTAA

>FM896021.1 FM896021 Jatropha curcas embryo 71-95 (DAF) Jatropha curcas cDNA clone rjcpga0_003150, mRNA sequence

GATGATAAATTGACAATTGCCAGTAATATCATGAACGAACTGGGCAGAGTACTATGCAAGGAAAGCAACT

TTCTCTAAGGCGATTAGGAAAATTACAATTTCTTACTTAATGAGTACAAACTAAGATAATACAGATAAAT

AATCAAAAGCTTGTTTTATTATCCTCCTGTGAATTATGATGTGTTTGCACACTCATCAGCCTGATATCTT

CAATCAAACGGTTCAGGTTACAAAAAGAGGATCCACCTTCACTAATACTTTTCCTTGCCAACTCTGCCAT

CCTAGCAGCTGATCTAACAAACTCTTCCCTCTTATTAACCATCAGATCATTCACCATCTTCTCAACAACC

TTTCTGTTACAAACATCTTTCATGTCCAGTCCTAATTTCCACACTTCACTCACAAATCTACTATTGGTTT

GCTGGTCAGCAAAATAAGGCCAACAAATCATAGGCACACCTGCTACTATACTCTCCAATGTCGAGTTCCA

TCCACTGTGTGTCAAAAACCCACCAATTGCTTTATGTGCTAAAACCTCTTCTTGCGGGGCCCATTCACGA

>FM896020.1 FM896020 Jatropha curcas embryo 71-95 (DAF) Jatropha curcas cDNA clone rjcpga0_003149, mRNA sequence

TCAGAACTGGTACGGACAAGGGGAATCCGACTGTTTAATTAAAACAAAGCATTGCGATGGTCCCTGAGGA

TGCTCACGCAATGTGATTTCTGCCCAGTGCTCTGAATGTCAAAGTGAAGAAATTCAACCAAGCGCGGGTA

AACGGCGGGAGTAACTATGACTCTCTTAAGGTAGCCAAATGCCTCGTCATCTAATTAGTGACGCGCATGA

ATGGATTAACGAGATTCCCACTGTCCCTGTCTACTATCCAGCGAAACCACAGCCAAGGGAACGGGCTTGG

CGGAATCAGCGGGGAAAGAAGACCCTGTTGAGCTTGACTCTAGTCCGACTTTGTGAAATGACTTGATAGG

TGTAGGATAAGTGGGAGCCGAAAGGCGACGGTGAAATACCACTGCTTTTAACGTTATTTTACTTATTCCG

TGAATCGGAGGCGGGGCTATGCCCCTCTTTTTGGACCCAAGGTCGTTTCGGCGGCCGATCCGGGCGGAAG

ACATTGTCAGGTGGGGAGTTTGGCTGGGGCGGCACATCTGTTAAAAGATAACGCAGGTGTCCTAAGATGA

GCTC

>FM896019.1 FM896019 Jatropha curcas embryo 71-95 (DAF) Jatropha curcas cDNA clone rjcpga0_003148, mRNA sequence

ATTCCTCTCATATAATCGCAGCCGATACAGAGCTTCTTGCTGCTCTTTAGGGTTTTCGCGTTTCATAACA

CACAGCCACNCACTAAACCCTTCTTTTCTCTATCTCTATCTCCATCTCATTCTCATCTCGTCGCAGCCAT

GTCGAAAAGAAAGACTAGAGAGCCTAAGGAAGAAAATGTGACACTTGGACCTGCTGTCAGGGAGGGTGAG

CATGTTTTTGGAGTTGCCCACATCTTTGCATCTTTCAATGATACGTTCATTCACGTGACTGATCTTTCTG

GAAGGGAAACCCTGGTTCGTATAACTGGTGGGATGAAGGTGAAAGCTGACAGAGATGAGTCTTCCCCATA

TGCTGCCATGCTTGCAGCTCAGGATGTTGCACAGA

>FM896018.1 FM896018 Jatropha curcas embryo 71-95 (DAF) Jatropha curcas cDNA clone rjcpga0_003147, mRNA sequence

GTTTAGCAAATTTCAAGAGAACCCTAACAATCATGGGTTCTAAACTTGAAGCTTTGTTATTCATTTTTAT

GATTTTCATGGCAATATCATTGCCACCAATTTATGCTTGTGCACCTTGTACTCAGCCACATCCACCAGGC

CACCGTCCAACCCACCCAAAAGTTCCTCATCCTAACCCACCAAGCACCAAGCACCCACCACACCACGGCG

GCCACCCAAAACCACCTTCAACAAAAAACCCACCAGTGGTTTTGCCACCAATAGTCATTAATCCTCCTCC

TGTAGTAAACCCTCCTGTAATAATAACACCTCCTCCTGTAATAACACCGCCGCCTTCTTCAGGTTACCCA

CCATACACGCCTAGCCCTCCATCTGGTGGTGGCGGAGGCGGCGGCGGTGGCGGAGGCGGTGGAGGAGGAG

GAGGAGGAGGTGGTGGTGGTGGTGTTCCGGGGCT

>FM896017.1 FM896017 Jatropha curcas embryo 71-95 (DAF) Jatropha curcas cDNA clone rjcpga0_003146, mRNA sequence

ATTACAGATAAAGTCAGGGTGCTATTATTGCCTGAATAAGAGATTTTTATCATACAAACATACATAAAAA

GGTTCAACAAACAATGCACTTTACAAACTACAAACTCTTCAGTGTTCCTGTAAGGAGAGTTCGAAAACAG

GAACTTGTCCAGACTTAGACAGACTTGTCCAGATTTTTACCATACAAATACTTGTCTTTGGAGTTCCCCA

ATTTTTTATAAACTTACAGAGCTTAGACAACAAAAACTTAAACACAGTAAAGATAATAAACACACAGATA

CCCATGATATCATATCTGGTCCAGCACCCTATCATTGCTGTCCTAGTGGAAATGTGAATTCATCAGCTTC

TGTACCATTTTCCAAATTTTCAACCGTCAATTTTGCTGGACAGACAATATTAATCACATGTGCAGCTGGC

TAGTTCCGGTTCTTACATTTCTCTACAGCCCTTGGGGCTAGGCCAATTGCATCAGAGCTCTTCATAATCC

GTAGCCTCTTGCATGTATCAATGAACATCTCCCATGGAACATCACCAACAAGCATCCAATCAACATCTTT

ATCCTCATA

>FM896016.1 FM896016 Jatropha curcas embryo 71-95 (DAF) Jatropha curcas cDNA clone rjcpga0_003145, mRNA sequence

CGAATTCCTTTAATATAGCATTTGATGGAGGCATCAACCAGAAAAACCCTAAAGCTTCTTTATCTTGTTT

GAAAATTAGACTAGTTGGTATCTATTTCTAGGTAATCGAGTACGAAAAGGATGAGTAAAGGTGCATTTCT

GTTAGTTGAATTTTAGTGATGTATAAATATTGATTATTGAATTTTGAGAGATTGAGCCGGTAATGGATGG

CGGAGGAGGAAGGAAGATAACAGTGAGCCCGAGACCATGCAGTGGAAGGAGAGTCTTAGCAAAGAAGCGA

GGTAAGGGTTTAGGAGGCATAGATGGGTTCGTCAACAGCGTTAAGAAACTTCAGAGAAGGGAAATCTGCT

ACCAAGCGCGAGCGTGCTTTCACCATGACGGACGCGCAGGAACGCTACAGAAATATCCGCTTGCAGGAGG

AGTATGATACCCATG

>FM896015.1 FM896015 Jatropha curcas embryo 71-95 (DAF) Jatropha curcas cDNA clone rjcpga0_003144, mRNA sequence

TAGATCTTTATATAGATGCTTGATTTTCTTTGCTGTAGGGTTCTTTTTGGGTTTGTTCTTGTTTGGTCCT

TTGGAAAATGATGTCCAGAATCACGACTTCTCCTTCGAGATCAAGCCGCCGCATGTCAATGTTCAATTGG

ATGACGGCGGCGGCGATAATCGCGGTATTAAGCGAGACGTTTTTGCCCTAAATTCTGTGAGTTTGGGGGT

TGATACGCAAGTTAATTACATTTCGAGATTTGATTATGTTCCGAGGAAGCAATTAATCGTGATTACGCCA

ACATATAATCGTGCTTTGCAGGCCTTCTTTCTGAATAGATTAGGGCAAGTTCTAAGGCTAGTGCAGCCTC

CGTTACTGTGGATTGTGGTGGAAATGAAAACCGCCTCTGTGGAGACGGCAGAAATTTTGAGGAAGACGGG

AGCTATGTATAGGCATTTGGTTTGCGAGAGAAATTCGACT

>FM896014.1 FM896014 Jatropha curcas embryo 71-95 (DAF) Jatropha curcas cDNA clone rjcpga0_003143, mRNA sequence

ATTCCAATCGTACACGTCGGTCGCTTAAAGATAACTCAATAATTGGTCGAGAGTTTGTATGCTCAAAAGA

GGGTTTTTGTGCAGGAAAATGTACAAAGAGAGAAAACAGAAATTTTTCAACACGATCTGCTACTAGAGAG

GGATGCAATGCAATGTTAAGGATAGCTGCAAAAGATGGAGGAAAATGGGTCATTTATGGTTTCATAAAAG

AACACAGCCATGAACTGAATCCTAGCAAAATACCACCTCGACGATCACATAGGATTGCATTCAACGAGGA

TGAGAAAGATCTGAAAATCAGGGAGCTATCCACAGAACTGCATCGCGAGAAAAAGAAATCAGCTGCTTAT

CAAGAGCAACTACAAATGGTGTTGAAATATGTCGAGGAGCATACACAACGGCTAGCATTAAAGATTGAAT

CAGTAGCTAATAAAGTAAGAGAGTTTGAGTTTGAAGAACTTGATGGTTCAGATTCTGAATAGAATGGCAG

TCAAAAATCTTGTTTGCTTCATAGATCGGATAATCTAATTTTGAAGCTTTATATTTGCTAATGGTAACCC

TTGTGCA

>FM896013.1 FM896013 Jatropha curcas embryo 71-95 (DAF) Jatropha curcas cDNA clone rjcpga0_003142, mRNA sequence

ATTCCTCCTTTCGCTCTCTTCTCTCTCGATCTTGCTCCTCGACGTCTCTCTGAGATAATAAGATGGGGCT

TTCTTTCACCAAGCTCTTCAGTCGGCTGTTTGCCAAGAAGGAGATGCGAATCCTTATGGTGGGTCTTGAC

GCAGCTGGTAAGACAACAATCTTGTACAAGCTCAAGCTTGGAGAAATTGTCACGACCATCCCCACAATTG

GATTTAATGTTGAGACTGTGGAGTACAAGAACATAAGCTTCACTGTCTGGGACGTTGGTGGTCAGGACAA

GATTCGACCATTATGGAGACATTACTTCCAAAACACTCAAGGGCTGATATTTGTGGTTGATAGCAATGAC

CGTGACCGTATTGTTGAGGCTCGAGATGAGCTGCATCGCATGTTGAATGAGGATGAGTTGAGAGATGCTG

TGCTGCTTGTATTTGCTAACAAGCAAGATCTTCCAAATGCTATGAATGCTGCTGAGATTACTGATAAGCT

TGGCCTCCACTCTCTTCGCCAACGCCATTGGTATATCCAGAGCACATGTGCCCATTCAGGTGAAGGGCTT

TATGAAGGACTTGACTGGCTCTCCAATAACATTGCTAACAAGGCA

>FM896012.1 FM896012 Jatropha curcas embryo 71-95 (DAF) Jatropha curcas cDNA clone rjcpga0_003141, mRNA sequence

TGCAAAGGATTCTATCCGTCGCCCGATGGGAATTGTACTTTAAGGCGGCCCTCGCGGCTCGTCCGCCGCG

GGGGCTTGGCCAACGGCACGTGCCTCTGGGGGCCGGATGGCCCCTACTGCGGGTCGGCAAACGGGCGACG

GACACGCGCGTCGCTTCTGGCCCGGATTCTGACTTAGAGGCGTTCAGTCATAATCCAGCGCACGGTAGCT

TCGCGCCACTGGCTTTTCAACCAAGCGCGATGACCAATTGTGCGAATCAACGGTTCCTCTCGTACTAGGT

TGAATTACCATCGCGACACCATCATCAGTAGGGTAAAACTAACCTGTCTCACGACGGTCTAAACCCAGCT

CACGTTCCCTATTGGTGGGTGAACAATCCAACACTTGGTGAATTCTGCTTCACAATGATAGGAAGAGCCG

ACATCGAAGGATCAAAAAGCAACGTCGCTATGAACGCTTGGCTGCCACAAGCCAGTTATCCCTGTGGTAA

CTTTTCTGACACCTCTAGCTTCAAATTCCGAAGGTCTAAAGGATCGATAGGCCACACTTTCACGGTTCGT

ATTCGTACTGGAAATCAGAATCAAACGAAC

>FM896011.1 FM896011 Jatropha curcas embryo 71-95 (DAF) Jatropha curcas cDNA clone rjcpga0_003140, mRNA sequence

GCAAAGGATTCTATCCGTCGCCCGGTGGGAATTGTACTTTAAGTGCGGCCCTCGCGGCTCGTCCGCCGCG

GGGGCTTAGCCAACGGCACGTGCCTCTGGGGGCCGGATGGCCCCTACTGCGGGTCGGCAAACGGGCGACG

GACACGCGCGTCGCTTCTGGCCCGGATTCTGACTTAGAGGCGTTCAGTCATAATCCAGCGCACGGTAGCT

TCGCGCCACTGGCTTTTCAACCAAGCGCGATGACCAATTGTGCGAATCAACGGTTCCTCTCGTACTAGGT

TGAATTACCATCGCGACACCATCATCAGTAGGGTAAAACTAACCTGTCTCACGACGGTCTAAACCCAGCT

CACGTTCCCTATTGGTGGGTGAACAATCCAACACTTGGTGAATTCTGCTTCACAATGATAGGAAGAGCCG

ACATCGAAGGATCAAAAAGCAACGTCGCTATGAACGCTTGGCTGCCACAAGCCAGTTATCCCTGTGGTAA

CTTTTCTGACACCTCT

>FM896010.1 FM896010 Jatropha curcas embryo 71-95 (DAF) Jatropha curcas cDNA clone rjcpga0_003139, mRNA sequence

AATTAGTTCTTTCTCCTTAACCTCATATATTTTCATTCATTCTATATCTCCTTATTTGCTTTCCATATGG

CCAGTGTTGAGGTTCAATCAGCTAAAACAGCATTGCCAGAGAATGAGACGCAAGAAGTGACCAAGACTGA

GGAGACAACCACTGAACCTGCTGCACCATCCGCACCAGAACCAGTAGCAGAACTACCGAAGGAAGTAGTC

GCAGAGGAACCAGCGGCCAAAGAACCAGAGGTTGAAACCAAGAAAGAAGAAGAAGAGAAGAAAGAGGAAC

CAGAGGCAAAAGCTGAGGATCAGCCTGAGGCTGCACCAGCAGCAGAAGAGACTCCAGATGTGAAGCCAGC

AGAGCCTGTCACTGAGGAGGAGGCCAAAGATGAGAGTACTGAGAAAGTGACAGAGGCGCCACCAGCAGCA

GACGAAGAGAAAACAGCTACGGAAGAGAAGCCAGTTGAAACAGAGGAGGAAGCAAGCAACACAGAGGTTC

CAGTGGAGAAGACTGAAGAATGAATAATGGA

>FM896009.1 FM896009 Jatropha curcas embryo 71-95 (DAF) Jatropha curcas cDNA clone rjcpga0_003138, mRNA sequence

AACAAAACAATTCAATTATTAATTGAAAGCTTGTCATATTTACATTGATAGAAACTGTCAATAGGAAGTG

TAGGAATAAAATGGTTTTGATTTGCACACAGGATGAAACACCGGAAAGCCCCATAGATTTCACAAAGTAA

AATTACAAATTTACAATTCATTTTGTTTGTTCCAGTGACGTGGCCTTCTTCAAAGTGCCTTTAGAACGAC

TTTGCTGGTTTTGTGAACGCCCTTTGCAGCAAGTTTCAGAATAGATCGAGTCCGATACAGCCCCCACAGG

GCAAGTCCCCAGGTAGCCAGAGTGTAAAATAGCTGTCCTGCATGCCAAATATTTAATATCTTCTCCCATT

TGCTCACACCTTCCAATGCTTTTTCCAGGGACTTCTGCAGCTCCTCTGGAGTCATTTTCTGAGCCTTAGG

ATTTGATAGAATGCATTTAGCCATAAAATTGGCAACTGCATCCACCACATG

>FM896008.1 FM896008 Jatropha curcas embryo 71-95 (DAF) Jatropha curcas cDNA clone rjcpga0_003137, mRNA sequence

ATTCGTCGTCTGCAAAGGATTCTATCCGTCGCCCGATGGGAATTGTACTTTAAGGCGGCCCTCGCGGCTC

GTCCGCCGCGGGGGCTTGGCCAACGGCACGTGCCTCTGGGGGCCGGATGGCCCCTACTGCGGGTCGGCAA

ACGGGCGACGGACACGCGCGTCGCTTCTGGCCCGGATTCTGACTTAGAGGCGTTCAGTCATAATCCAGCG

CACGGTAGCTTCGCGCCACTGGCTTTTCAACCAAGCGCGATGACCAATTGTGCGAATCAACGGTTCCTCT

CGTACTAGGTTGAATTACCATCGCGACACCATCATCAGTAGGGTAAAACTAACCTGTCTCACGACGGTCT

AAACCCAGCTCACGTTCCCTATTGGTGGGTGAACAATCCAACACTTGGTGAATTCTGCTTCACAATGATA

GGAAGAGCCGACATCGAAGGATCAAAAAGCAACGTCGCTATGAACGCTTGGCTGCCACAAGCCAGTTATC

CCTGTGGTAACTTTTCTGACACCTCTAGCTTCAGATTCCGAAGGGTCTAAGGATCGATAGGCCACGCTTT

CACGGTTCG

>FM896007.1 FM896007 Jatropha curcas embryo 71-95 (DAF) Jatropha curcas cDNA clone rjcpga0_003136, mRNA sequence

GGAAATCAGAATCAAACGAGCTTTTACCTTTTGTTCCACACGAGATTTCTGTTCTCGTTGAGCTCATCTT

AGGACACCTGCGTTATCTTTTAACAGATGTGCCGCCCCAGCCAAACTCCCCACCTGACAATGTCTTCCGC

CCGGATCGGCCGCCGAAACGACCTTGGGTCCAAAAAGAGGGGCATAGCCCCGCCTCCGATTCACGGAATA

AGTAAAATAACGTTAAAAGTAGTGGTATTTCACCGTCGCCTTTCGGCTCCCACTTATCCTACACCTCTCA

AGTCATTTCACAAAGTCGGACTAGAGTCAAGCTCAACAGGGTCTTCTTTCCCCGCTGATTCCGCCAAGCC

CGTTCCCTTGGCTGTGGTTTCGCTGGATAGTAGACAGGGACAGTGGGAATCTCGTTAATCCATTCATGCG

CGTCACTAATTAGATGACGAGGCATTTGGCTACCTTAAGAGAGTCATAGTTACTCCCGCCGTTTACCCGC

GCTTGGTTGAATTTCTTCACTTTTGACATTCAGAGCACTGGGCAGA

>FM896006.1 FM896006 Jatropha curcas embryo 71-95 (DAF) Jatropha curcas cDNA clone rjcpga0_003135, mRNA sequence

TGGAAAAAAAAAAAAACATGATGGAAGGAGACGGAAGCTCAGAGATGGAATACACAGAGATCAACACAAA

CGCAGAATCTCTGGACAATTCGGTGATCTTCCATGTCATTAAAGACGTTCTAGGCTTCGTTCTCTACATG

CACCAGCAGATCCCTTCCGTTTTGCAAGATATTGGACTCGAATTTGATTCTTTGAAAGAGGAATATCAGG

AGCTGGAGATGGCTCTTGCACAAGATGAAGCAAAGGCATCTATTCGAAGAAAACATAATAGCAGAAAGAG

AGAAGTCAAAAGTGGAATTAAGAGATTAGAGAAGTTGATGAATACTCTTAATGGCCTTGAAAGTGCATTG

CAACTGATAATTAGTGAGATTCCTAGTCTCGAGAGTTTGATTTTGATTCTTGGAGCTAGTCCATTACGGC

CACGGCATGTATATGAATTGTGGTTTTCACATGGAAAAGTTGTCCTCAGAGGAGCAAATGATTTTACTAA

AAGCAAAGCATCCGAAGGGCTCTCAAAAAAGGCTATTCGTACAC

>FM896005.1 FM896005 Jatropha curcas embryo 71-95 (DAF) Jatropha curcas cDNA clone rjcpga0_003133, mRNA sequence

ATTCCAAAGGATTCTATCCGTCGCCCGGTGGGAATTGTACTTTAAGTGCGGCCCTCGCGGCTCGTCCGCC

GCGGGGGCTTAGCCAACGGCACGTGCCTCTGGGGGCCGGATGGCCCCTACTGCGGGTCGGCAAACGGGCG

ACGGACACGCGCGTCGCTTCTGGCCCGGATTCTGACTTAGAGGCGTTCAGTCATAATCCAGCGCACGGTA

GCTTCGCGCCACTGGCTTTTCAACCAAGCGCGATGACCAATTGTGCGAATCAACGGTTCCTCTCGTACTA

GGTTGAATTACCATCGCGACACCATCATCAGTAGGGTAAAACTAACCTGTCTCACGACGGTCTAACCCCA

GCTCACGTTCCCTATTGGTGGGTGAACAATCCAACACTTGGTGAATTCTGCTTCACAATGATAGGAAGAG

CCGACATCGAAGGATCAAAAAGCAACGTCGCTATGAACGCTTGGCTGCCACAAGCCAGTTATCCCTGTGG

TAACTTTTCTGACACCTCTAGCT

>FM896004.1 FM896004 Jatropha curcas embryo 71-95 (DAF) Jatropha curcas cDNA clone rjcpga0_003132, mRNA sequence

AGGATTCTATCCGTCGCCCGGTGGGAATTGTACTTTAAGGCGGCCCTCGCGGCTCGTCCGCCGCGGGGGC

TTAGCCAACGGCACGTGCCTCTGGGGGCCGGATGGCCCCTACTGCGGGTCGGCAAACGGGCGACGGACAC

GCGCGTCGCTTCTGGCCCGGATTCTGACTTAGAGGCGTTCAGTCATAATCCAGCGCACGGTAGCTTCGCG

CCACTGGCTTTTCAACCAAGCGCGATGACCAATTGTGCGAATCAACGGTTCCTCTCGTACTAGGTTGAAT

TACCATCGCGACACCATCATCAGTAGGGTAAAACTAACCTGTCTCACGACGGTCTAAACCCAGCTCACGT

TCCCTATTGGTGGGTGAACAATCCAACACTTGGTGAATTCTGCTTCACAATGATAGGAAGAGCCGACATC

GAAGGATCAAAAAGCAACGTCGCTATGAACGCTTGGCTGCCACAAGCCAGTTATCCCTGTGGTAACTTTT

CTGACACCTCTAGCTTCAAATTCCGAAGGTCTAAAGGATCGATAGGCCACACTTTCACGGTTCGTATTCG

TACTGGAAATCAGAATCAAACCAACTTTTACCCTTTTGTTCCA

>FM896003.1 FM896003 Jatropha curcas embryo 71-95 (DAF) Jatropha curcas cDNA clone rjcpga0_003130, mRNA sequence

CCAAAATTACCAATATATAGAATCCAATTCCACCGATTCCAAGCACAGAACGCCACAAATTTTTTCCCTT

CTTCTTCTTCCTCCACATTTGTGTCCAAACCCATAAGGCTCTACTACTTCTATCTCCATTTTTAAGTTAT

TTGTTTTTTTATCAAAGAATTCGAAGATGAGGCTGTTGAAGGTAGCTACTTGTAACTTGAACCAGTGGGC

TATGGACTTCGACTGTAACTTGAAGAACATAAAGGAGTCAATATCTAAGGCCAAACAAGCTGGTGCGGTT

ATTAGACTCGGCCCTGAACTTGAAATCACTGGCTATGGATGTGAAGATCATTTCTTGGAGCTTGACACCG

TCACTCATGCATGGGAGTGTCTGAAGGAAATTTTGCTCGGCGATTGGACTGATGGGTTATTGTGCAGTTT

TGGGATGCCGGTTATTAAAGGATCAGAGCGTTACAATTGTCAAGTTTTGTGCTTTAACAGGAAAATTTTA

ATGATACGTCCAAAAA

>FM896002.1 FM896002 Jatropha curcas embryo 71-95 (DAF) Jatropha curcas cDNA clone rjcpga0_003129, mRNA sequence

TCCGTCGCCCGATGGGAATTGTACTTTAAGGCGGCCCTCGCGGCTCGTCCGCCGCGGGGGCTTGGCCAAC

GGCACGTGCCTCTGGGGGCCGGATGGCCCCTACTGCGGGTCGGCAAACGGGCGACGGACACGCGCGTCGC

TTCTGGCCCGGATTCTGACTTAGAGGCGTTCAGTCATAATCCAGCGCACGGTAGCTTCGCGCCACTGGCT

TTTCAACCAAGCGCGATGACCAATTGTGCGAATCAACGGTTCCTCTCGTACTAGGTTGAATTACCATCGC

GACACCATCATCAGTAGGGTAAAACTAACCTGTCTCACGACGGTCTAACCCCAGCTCACGTTCCCTATTG

GTGGGTGAACAATCCAACACTTGGTGAATTCTGCTTCACAATGATAGGAAGAGCCGACATCGAAGGATCA

AAAAGCAACGTCGCTATGAACGCTTGGCTGCCACAAGCCAGTTATCCCT

>FM896001.1 FM896001 Jatropha curcas embryo 71-95 (DAF) Jatropha curcas cDNA clone rjcpga0_003128, mRNA sequence

TGGAAATCAGAATCAAACGAGCTTTTACCCTTTTGTTCCACACGAGATTTCTGTTCTCGTTGAGCTCATC

TTAGGACACCTGCGTTATCTTTTAACAGATGTGCCGCCCCAGCCAAACTCCCCACCTGACAATGTCTTCC

GCCCGGATCGGCCGCCGAAACGACCTTGGGTCCAAAAAGAGGGGCATAGCCCCGCCTCCGATTCACGGAA

TAAGTAAAATAACGTTAAAAGTAGTGGTATTTCACCGTCGCCTTTCGGCTCCCACTTATCCTACACCTCT

CAAGTCATTTCACAAAGTCGGACTAGAGTCAAGCTCAACAGGGTCTTCTTTCCCCGCTGATTCCGCCAAG

CCCGTTCCCTTGGCTGTGGTTTCGCTGGATAGTAGACAGGGACAGTGGGAATCTCGTTAATCCATTCATG

CGCGTCACTAATTAGATGACGAGGCATTTGGCTACCTTAAGAGAGTCATAGTTACTCCCGCCGTTTACCC

GCGCTTGGTTGAATTTCTTCACTTTGACATTCAGAGCACTGGGCAGAAATCACATTGCGTGAGCATCCGC

AGGGACCATCGCAATGCTTTGTTTTAGTTAAACAGTCGGATTCCC

>FM896000.1 FM896000 Jatropha curcas embryo 71-95 (DAF) Jatropha curcas cDNA clone rjcpga0_003126, mRNA sequence

GGATGATAATCTGTCACTTTTACTCCCTCCTTTGCCACCAGAATGAACTGTTCGTGTCCTNTGGTGACTG

TGAGCTGATTTTGCTATTTGATGGAAGAGAATGGCGTCGGGCAGGGGTATTTTTCTCGGCCCCATCTTGG

CTAAACCTTGGGGAACCTTGTGCTCTCAACTTTGCCTTTGCAGATTCTGTTGCTGCCATATAGCTTGGAA

GTGCTGGACTACTCTGTAATCCATTCTCTGCACGTTCTTGCTTTACTAGATTCGAAGCTTTTTTGCTGAT

TTTATGATTCTCGTTGCTCGATGGATCTTCCTTATGGTTCAACTCCCCATTTGTAACAAGAGTATTCTCA

TCTTTATCACCACTCTCGACTGAAGGCTTCGATTCAACCACTGTTAGATCACAATTTAATTCTGGTGTTT

CATTCATTTCCAATGTATTCAGAGTGGTTTCTACATCAGGCACTTTCAACGCTGTCAAGGTTGGCTCAGT

CTTCACCA

>FM895999.1 FM895999 Jatropha curcas embryo 71-95 (DAF) Jatropha curcas cDNA clone rjcpga0_003125, mRNA sequence

CACGAAATTGATGAAATCGGTTCCCTTACATAGACTATCCGATTTCCGAAACTGATGAAATCGTTTCCCN

TCGTCGATCTTTCTCGCTTAATTGAGTCTTCATTTCATTTCCACATTCATCAATAATTTCTCAAAAATGG

GAGATCAGATAGCTCGAGCAGAGGATTTCGA

>FM895998.1 FM895998 Jatropha curcas embryo 71-95 (DAF) Jatropha curcas cDNA clone rjcpga0_003124, mRNA sequence

CTCTTTTTGGACCCAAGGTCGTTTCGGCGGCCGATCCGGGCGGAAGACATTGTCAGGTGGGGAGTTTGGC

TGGGGCGGCACATCTGTTAAAAGATAACGCAGGTGTCCTAAGATGAGCTCAACGAGAACAGAAATCTCGT

GTGGAACAAAAGGGTAAAAGCTCGTTTGATTCTGATTTCCAGTACGAATACGAACCGTGAAAGCGTGGCC

TATCGATCCTTTAGACCTTCGGAATTTGAAGCTAGAGGTGTCAGAAAAGTTACCACAGGGATAACTGGCT

TGTGGCAGCCAAGCGTTCATAGCGACGTTGCTTTTTGATCCTTCGATGTCGGCTCTTCCTATCATTGTGA

AGCAGAATTCACCAAGTGTTGGATTGTTCACCCACCAATAGGGAACGTGAGCTGGGTTTAGACCGTCGTG

AGACAGGTTAGTTTTACCCTACTGATGATGGTGTCGCGATGGTAATTCAA

>FM895997.1 FM895997 Jatropha curcas embryo 71-95 (DAF) Jatropha curcas cDNA clone rjcpga0_003123, mRNA sequence

TTTTTTTCCAATTCGGTTCTGGTGCCGTTCTTTATAAAACCAAAACCTGAAACATAAATTGCGTTAGGTG

TGGTACTGGTTCTTATAAGACCTATCCTCTTTTGTTCCGATTGGGTTTAATAGATTTTTTCGGTTTGGTT

GGATTCTATGGAATACTGTGCGCAGCCTATTACAAACAAACCATAAACCACAAAGAAATCAAATTTAAAA

ACCAATACGACAGAGAGTTTGGAAACATAGGATAGAAGATGGAATCATTTCCAACACCCTTCTCTTCGTT

ATAATTCAAGACAAAACCTCATCAACGAAAGCCTTAAGATTCTTATCCGAAGACCCACTTTCCTTGACAG

CTTCTCTCGCCAAATCCTTCCATTTTTTAGCATTTTTTCTTACATCTTTTCCTTTCTCTCCATCTCCCAT

AACCAAGTCTAAACACCTTGTTATCTCCTCACCTTCTACTATTCCTTCTTCATTTGGCACAACCCTTACC

CCTATCTCCCAATCATC

>FM895996.1 FM895996 Jatropha curcas embryo 71-95 (DAF) Jatropha curcas cDNA clone rjcpga0_003122, mRNA sequence

CAAATGCACAGTAATCCATGGCCAAGTAGGCATATAAGTCTGTAAAACGCTACAAATGAAAAAACTAAGA

ACTACAAGATAAAGGGGAAAAAGGAAAGTTTTTTTTCATATGTAAACTCAATGTAATAGAAGTGAAGCAT

TTTGGTTACCATTCTTAATTTTGAAACTTACATTAAGTTTTTACCTGACAAATTAATTCATACCAGTATA

GTTCTTACGGATATTTGGCCTACCAGACCTTCTGACTGTGCCAGAACTTGCTTTCAAGTTTGGATTGAAA

GCT

>FM895995.1 FM895995 Jatropha curcas embryo 71-95 (DAF) Jatropha curcas cDNA clone rjcpga0_003121, mRNA sequence

ATTCCTGACATGTGTGCGAGTCAACGGGTGAGTAAACCCGCAAGGCGCAAGGAAGCTGACTGGCGGGATC

CCCCCGAGGGTTGCACCGCCGACCGACCTTGATCTTCTGAGAAGGGTTCGAGTGAGAGCATGCCTGTCGG

GACCCGAAAGATGGTGAACTATGCCTGAGCGGGGCGAAGCCAGAGGAAACTCTGGTGGAGGCCCGCAGCG

ATACTGACGTGCAAATCGTTCGTCTGACTTGGGTATAGGGGCGAAAGACTAATCGAACCGTCTAGTAGCT

GGTTCCCTCCGAAGTTTCCCTCAGGATAGCTGGAGCTCGGGACGAGTTCTATCGGGTAAAGCCAATGATT

AGAGGCATCGGGGGCGCAACGCCCTCGACCTATTCTCAAACTTTAAATAGGTAGGACGGCGCGGAT

>FM895994.1 FM895994 Jatropha curcas embryo 71-95 (DAF) Jatropha curcas cDNA clone rjcpga0_003120, mRNA sequence

GGATTCTATCCGTCGCCCGATGGGAATTGTACTTTAAGGCGGCCCTCGCGGCTCGTCCGCCGCGGGGGCT

TGGCCAACGGCACGTGCCTCTGGGGGCCGGATGGCCCCTACTGCGGGTCGGCAAACGGGCGACGGACACG

CGCGTCGCTTCTGGCCCGGATTCTGACTTAGAGGCGTTCAGTCATAATCCAGCGCACGGTAGCTTCGCGC

CACTGGCTTTTCAACCAAGCGCGATGACCAATTGTGCG

>FM895993.1 FM895993 Jatropha curcas embryo 71-95 (DAF) Jatropha curcas cDNA clone rjcpga0_003118, mRNA sequence

CTATAAGCAGCTGCAGCCTCTTCTGCAGTCGTGTAAGTACCAAGCCATACTCGAACCTTTCGAAATGGAT

CTCGAATCTCTGCGGAATATTTCCCCCATGGCCTTCTTCGAACCCCTCTGTAACTACAGGATGATATTCT

AGTCTTAGTTTTAAATTTGTCTCCATTACTAGTTTCTCCTGGACAATTCTCACTTCTTGATTCATTCGGC

AACATTGAGAGATTGATTTCTTTTACAAAACGCTTAGCACCGATTGGATTAATGTGTGCTTCATCTTCAT

CGCTTGAAGAGTCTGTGGCATCAGGATCATGGCAAATTATGCGAACTTTTCTTGTGGCAATCATTTCCTT

TGGTTTTTACTTCTTCTCTTTGTCGAAACTCAAACTTTGATCCAAAATTGGCCTTCTAAGGCCTGGCATA

TTATGAAATTGAAATTTATAGAGAGATTTCTCACTCTAATCTCAAAAAGGAAATACATAAT

>FM895992.1 FM895992 Jatropha curcas embryo 71-95 (DAF) Jatropha curcas cDNA clone rjcpga0_003117, mRNA sequence

TTTTTGATGGAAACTACAAGTTACTTTCAACATTGAAGTGTAGTACATTCAAGATAAAAGAATCAGATTC

TCATCTTCAGATCAATCAGAAACAAATGAAATTCTATCAAATAGTCTTGAAGTCGAAAGAATTTGAAAAC

AGGGGAAAGAAACTACGATGTAATTGAAAATCCCGCCATAAATGTATATGTACGATAACATATGTACATA

TACACATATATACCAATGTTATCTACCAAATTTAGAAATCCGTCAACGCAGAGACTCCAGCATCTCATCA

AGGCGACGGTCCATGATGTGTGATACTGTCTCAAGGAACGCAACTTCGCTTGTGCCAGGAAGTACAGAAT

CCTCTGCTTCTTGGACAATCTCCTCAATAACAGATGCCTTCTTCAAGGTCTCTCTGCACATTATGCTTCC

AATAGCAAAGGGGGTGAAACCCCTCTCCACCCCCTTCTTCAGAAGCATGGTGGAGATGCGAAGTGTCCGC

GCACTTTCAACCGGCATGTCCCATCCATGAAACTTCAGAAGAGGCATATCCTCTTCTGGGTCCAGTGATT

TA

>FM895991.1 FM895991 Jatropha curcas embryo 71-95 (DAF) Jatropha curcas cDNA clone rjcpga0_003115, mRNA sequence

CAAAGGATTCTATCCGTCGCCCGGTGGGAATTGTACTTTAAGGCGGCCCTCGCGGCTCGTCCGCCGCGGG

GGCTTAGCCAACGGCACGTGCCTCTGGGGGCCGGATGGCCCCTACTGCGGGTCGGCAAACGGGCGACGGA

CACGCGCGTCGCTTCTGGCCCGGATTCTGACTTAGAGGCGTTCAGTCATAATCCAGCGCACGGTAGCTTC

GCGCCACTGGCTTTTCAACCAAGCGCGATGACCAATTGTGCGAATCAACGGTTCCTCTCGTACTAGGTTG

AATTACCATCGCGACACCATCATCAGTAGGGTAAAACTAACCTGTCTCACGACGGTCTAATCCCAGCTCA

CGTTCCCTATTGGTGGGTGAACAATCCAACACTTGGTGAATTCTGCTTCACAATGATAGGAAGAGCCGAC

ATCGAAGGATCAAAAAGCAACGTCGCTATGAACGCTTGGCTGCCACAAGCCAGTTATCCCTGTGGTAACT

TTTCTGACACCTCTAGCTTCAAAT

>FM895990.1 FM895990 Jatropha curcas embryo 71-95 (DAF) Jatropha curcas cDNA clone rjcpga0_003113, mRNA sequence

TTGAATGGAGAGTTGATCATCCCCTTGGCTGGTGGTGCTAATGTGAATCTTGACATGTCAAAGAAATTAG

ATAAGGAATTTATAGCAAGTCTCTTGGCTTTGTTCCACAATAGTAGAAGAGCAATAGAATTGCATGAAGA

CCTGTCACAAGTTGCTCAAGGACCTGCAGAGTTAATAATGGGTAGTTTTGATGGCATTAAGGCTTTGGAA

GGGCAATATGGACAAGGAGTTGTTGTTCAGCAAAGGATGGAGCTATTGCTTGCTACATTGTCTAAAATGC

TTGATTCCTTCCAAGTAGCATACAAAGGTCAAATTGTTGGAATTATCATCTGTAATGATGCATCCTCTTC

AGAATCAGAAATGATGTTGAATGTCATTTTGAATTCTCGGCCATCTGCACGTTGGTTGGCTGAGGTAAAA

GGATCAAATGCAACTAATGCAACCCTTGTGGAAGTGGCCTTGGTAAGGAAGACCCTAGCTTGGTTGACGG

GGATTATTCTCATCATTTCTACTCTA

>FM895989.1 FM895989 Jatropha curcas embryo 71-95 (DAF) Jatropha curcas cDNA clone rjcpga0_003112, mRNA sequence

AGGATTCTATCCGTCGCCCGATGGGAATTGTACTTTAAGGCGGCCCTCGCGGCTCGTCCGCCGCGGGGGC

TTGGCCAACGGCACGTGCCTCTGGGGGCCGGATGGCCCCTACTGCG

>FM895988.1 FM895988 Jatropha curcas embryo 71-95 (DAF) Jatropha curcas cDNA clone rjcpga0_003111, mRNA sequence

GTGTGAATGCTAGAACTCATTTCCCATTATTTGAAAATTACATATACATTTGGGGCACCACGGTAGTCAT

ATTCAAACAATTGCCTGGCCTAGGAAAATGCAGTTTACATTAAGGGGAAAAAAATTAATGGGATAAAGAA

TAAAGGAAACAATTAACTTATGCCACTTTAACTCCACTTGGGCGTTTTCCTTCCATGGCGTCCTTCTTAT

TGCGGCAATCAGCACATAGGAAAGGCCCTTGCCATGGTTTTGAGCTGGTGGAGAAGATTGAACCAGCATG

AACAGAGATGCCCTCGCTTGGTGCAAGGTCAACTTGACATACAGCACATGTGAAAAGACCAGATGTTGAT

TGTGTTGCGGTGTCATCGTTCCCCAGTCTTTCAGCAAGCATTGCTGAGCCTTCTTCAAACAATTCTTCCA

CTATACCTACGGCAGAAAGCTTTTTTGATAGCTTATTAGTAGAATCATGTGATTTCTTCCTGAATAATAA

GGCTGTAAGTTTGTTCTGCTTCTTGGGAGGAGTTGATGGCTGTACTGAATTATCAGGAGGAATTATGTCA

ACAAT

>FM895987.1 FM895987 Jatropha curcas embryo 71-95 (DAF) Jatropha curcas cDNA clone rjcpga0_003110, mRNA sequence

GCGAGTTTCAACCTAAATAGTGAAAAATTAGAAAAAGAATTTCTAGTTTAGGACAATTAGAAAAGAAGCC

AAGAAAAGATAGACCAATCTGAAACATAAAATGAAGACCTTTTGTCACCTCTTTGAATCTTTGCTTAAAA

CACCTGCGAACAAGTCCTTAACTAAAAAGAAATAACAACTGACATTGGAAAACCGAGACCAGCTTTATGC

AGGAGCAACTTCAGGCAAGTAACCAGAAGCAAATACTTGACTATGCCACGAGGGTGCTGGCAGTAGTTGA

TTCATATTTCCATACTGGTGGAAGCTTCTTTGTCTTCTTGTAATAGCGAGCTAGGCGGTGGATCCTGCTC

TCAACCAAAATCAACCTAAATTTGGAATCTTTATCCTTCCTGTTCCTCTCCAAATGTTTCCTAATGGCAA

CTGCTTTCTTGATGAGGTGGTACAAATCTTCAGGTATTTCAGGAGCAAGCCCATGTGCCTTCAATATCCG

CAAAATCTTGTTACCAGTAACACTCCTCACTTGGGCAATACCATGAGAATCACGAAGGATGACACCAATT

TTGAGATGGAGTCAAGGCTTTCTTTGCAAAT

>FM895986.1 FM895986 Jatropha curcas embryo 71-95 (DAF) Jatropha curcas cDNA clone rjcpga0_003109, mRNA sequence

AGCCACCAGTCATTTGAGTTTCAATTTGAAGACACGTGGTGAAAAACAAATGAACCAAGGAAACACTGGC

GACCATCACAAATATGATTTTCAATTCCCAGGGAAAAAAAAGTACAATGCTGGGGAAATTATTGGAGTTG

CTCAACAAAACGACCTTAAAGAGGTAGCTACGAAGTTTCAAGAAAGCCTTTGCCAAGATAATTATTTCGT

CGCTGTCGCATAGTCTACCCACAATATTTCTTCAATTAACCCTCCAGTGCTGATGCTCCCGATATAGTCT

CAATCAACTCGGTGGTGATGGATGCTTGACGAGTTCTGTTATAAGTAAGTGTCAAGCGGACAAGCATATC

TCCAGCATTTCTGCTTGAGCTGTCCATAGCAGACATCCGTGCTCCTTGCTCACTGCAAGCATTCTCCAAT

ACAGCATTGAACATGACACAAGAAAACTGGAACTCTGTAAGATTTTGAAGTATTTCACCCTTTGTTTCAC

CACCTTCAATCTCGTAGGAGTCGAGATCTCCAAGCTTCCCCCAGATTCAGCCTCTCTTTCGACAACCTCA

GGGGACAACACAGTTGACATGGTTGGAATGAATAAAACGACTGAATGGAACTTG

>FM895985.1 FM895985 Jatropha curcas embryo 71-95 (DAF) Jatropha curcas cDNA clone rjcpga0_003108, mRNA sequence

ATTCCAACAAAACCTGTCCTTAACGGGATGGTACTGACTTTCATACAGGTGCTGCATGGCTGTCGTCAGC

TCGTGTCGTGAGATGTTTGGTCAAGTCCTATAACGAGCGAAACCCTCGTTTTGTGTTGCTGAGACATGCG

CCTAAGGAGAAAGTCTTTGCAACCGAAGTGAGCCGAGGAGCCGAGTGACGTGCCAGCGCTACTAATTGAG

TGCCAGCACGTAGCTGTGCTGTCAGTAAGAGGGGAGCCGGCGCCTTTCGAAGCACTTTCTAGTGTGCGCT

TTAGAAAGATTGCAGCTAGCGCGCTTTACTTTACTAATAACAGAGAAAGGGGCTTTTCT

>FM895984.1 FM895984 Jatropha curcas embryo 71-95 (DAF) Jatropha curcas cDNA clone rjcpga0_003107, mRNA sequence

ATTCCTTACCTTCTCCTCCATCAAAGCTAGTTAGTCCAGTTCCCTTATTAAGCTAAATTTTTTTTCGAAG

ATGGCCAAAATTGCCTTTGGTCGTTTTGATGATTCTTTTAGTTTAGGCTCTTTTAAAGCTTATCTTGCAG

AATTTATCTCAACTCTGCTCTTCGTTTTTGCTGGTGTTGGTTCTGCCATAGCTTACAATAAATTGACAGG

AAATGCAGCTCTTGATCCTGCTGGTTTAGTAGCAATTGCTATTTGCCATGGATTTGCTCTCTTTGTTGCT

GTAGCCGTCGGTGCCAACATCTCCGGTGGCCATGTTAATCCTGCTGTCACCTTTGGATTGGCTCTTGGTG

GTCAAATCACCATTCTTACTGGCATCTTCTACTGGATTGCTCAGCTTCTTGGTTCCATTGTTGCTTGCCT

CCTTCTCAAAGTTGTCACCGGAGGCTTGGAAACACCCACCCACAGCCTTGCAGCTGGAGTAGGAGCCATT

GAAGGAGTGGTTATGGAGATTATAGTCACATTTGCTTTGGTATACACAGTGTACGCACCAGCAGCTGATC

CCAAGAAAGGATCACTAGGCACCATTGCCCCCATAGCCATTGGCTTCATCGTTGGTGCCAACA

>FM895983.1 FM895983 Jatropha curcas embryo 71-95 (DAF) Jatropha curcas cDNA clone rjcpga0_003106, mRNA sequence

ATCAACTTAAAATTTATTCATGATCAGCGGCGAAATGTAAACAAAAGATTCATACAGCTGATACTAAATG

ATTTAGGATTATCGACTCTAACGAATGTAATGGCATAGGATTCATATAGAGCCGATTTTAACTAGTTTAA

TTAGACATATCTATAAGGCCATTAACAAATAGTGAATTGAAAATCTCCATTTCACGCTTCTCCAGAACTA

TACCAACCTCAACACCGCCATTTGAACCTTTGCTCTTCAACAAAGAAATGCCTCCATTCCAGTCTATAGA

TGTAAAATCAACCTTCTTTGGTCTTCCCCACCCAAAATCCGTCTCATAAACTTCAAATTTTGGTGAAGCT

GAAACTGCGATCACATCTTCATGACCTACTTCAATACTCTTCAGAACGAGTCCAAGCTTCTCCTTTTCTC

CTGCAAGAATTCCTTTCTCTAATCCTTTTATCATATCGCTAAGCTTCATTGCAACAAAGCTAAACCCATT

CTCGTCAATGAGAGTTTCTGCTTCTGTAAATATAGGATTTGTAGTAACACAACAACCAATATAATTCTTA

GGCAATGGAGGGTCTACACGATCTCTCCAGTCTGCGGCAAATGCTATGATAATCTTT

>FM895982.1 FM895982 Jatropha curcas embryo 71-95 (DAF) Jatropha curcas cDNA clone rjcpga0_003105, mRNA sequence

TGAGCTCACGCGGTGGCGGCGCTCTAGAATAGTAGGATCCCCCGGGTGCAGGAATTCATTCAAAGGATTC

TATCCGTCGCCCGATGGGAATTGTACTTTAAGGCGGCCCTCGCGGCTCGTCCGCCGCGGGGGCTTGGCCA

ACGGCACGTGCCTCTGGGGGCCGGATGGCCCCTACTGCGGGTCGGCAAACGGGCGACGGACACGCGCGTC

GCTTCTGGCCCGGATTCTGACTTAGAGGCGTTCAGTCATAATCCAGCGCACGGTAGCTTCGCGCCACTGG

CTTTTCAACCAAGCGCGATGACCAATTGTGCGAATCAACGGTTCCTCTCGTACTAGGTTGAATTACCATC

GCGACACCATCATCAGTAGGGTAAAACTAACCTGTCTCACGACGGTCTAAACCCAGCTCACGTTCCCTAT

TGGTGGGTGAACAATCCAACACTTGGTGAATTC

>FM895981.1 FM895981 Jatropha curcas embryo 71-95 (DAF) Jatropha curcas cDNA clone rjcpga0_003104, mRNA sequence

AGGATTCTATCCGTCGCCCGATGGGAATTGTACTTTAAGGCGGCCCTCGCGGCTCGTCCGCCGCGGGGGC

TTGGCCAACGGCACGTGCCTCTGGGGGCCGGATGGCCCCTACTGCGGGTCGGCAAACGGGCGACGGACAC

GCGCGTCGCTTCTGGCCCGGATTCTGACTTAGAGGCGTTCAGTCATAATCCAGCGCACGGTAGCTTCGCG

CCACTGGCTTTTCAACCAAGCGCGATGACCAATTGTGCGAATCAACGGTTCCTCTCGTACTAGGTTGAAT

TACCATCGCGACACCATCATCAGTAGGGTAAAACTAACCTGTCTCACGACGGTCTAATCCCAGCTCACGT

TCCCTATTGGTGGGTGAACAATCCAACACTTGGTGAATTCTGCTTCACAATGATAGGAAGAGCCGACATC

GAAGGATCAAAAAGCAACGTCGCTATGAACGCTTGGCTGCCAAAAGCCAGTTATCCCTGTGGTAAC

>FM895980.1 FM895980 Jatropha curcas embryo 71-95 (DAF) Jatropha curcas cDNA clone rjcpga0_003102, mRNA sequence

GGAAAAGAACAACAGTTCTCTAACAATTTATATACAGACATCGATGAAGGCCTTCAACATTTCCATATAA

GCAAACATAGAAAGATCTAATATATAGAAAGTATTAAACACAAACGGTGGTGGCCTCGCATGACCACATA

ATAAGATAAACATATTGGTTCCAGATAATACAAATATTTGTAAGACAGTTAACTAAGCTGCCATTAGTGG

ACATGGAAGTCCTAGTGTTCTTCATCCATTAAGCTGCTGCAACCTCTGTGGCATGCTTTACAATTGTCAT

CTGCAGTTCCTCTTTCTTGGCACCAACAACCTTGTCCACGATTTTCCCTTCTTTCAAGAACATAAAAGTA

GGCATTGCCTCAACAGCCCAATCTTCAGCAACAGTCCTCAATTCATCCACGTCCACCTTCAAGAACGTGA

CATGAGGCATCTTCTTAGCCAAATCTTGCAGAATTGGAGTGATGAAACGACAAGGCCCACACCAGGTAGC

AGTAAAATCAACCACAATCAGGGTCTTGCTTTCCTTTCCCTT

>FM895979.1 FM895979 Jatropha curcas embryo 71-95 (DAF) Jatropha curcas cDNA clone rjcpga0_003101, mRNA sequence

TGAGCCCTTTGCTGCATTGATAAGCTCATCAGCCAAACATTCTGCAATGGTCTTGATATTTCTGAGAGCA

GCTTCCCGAGACACCAGTTGTAAGCAGATAAATAGCTTGGTTTACACGCCTCAAAGGTGAAATATCAACA

GCTATGCCATCAAGAAGAAGGATGAGATTGAAAGAGTTGCTAAGGCCAATCGTTAAGAGATCAATACTGT

ATGGCAGAGGAAGAATTTCGTCTCTCTTAAGAATTTCTTCTTAGCGTTATGTGTTAAAGATTTGAACATA

CTGATGTTGCTCGGTTGGTTGTTATTTGTGTCAAAGATTTGCTCTCTTGTTTAGTTTAGCAGTTTCATGA

TATTATAAAGAATACATTATTGACCT

>FM895978.1 FM895978 Jatropha curcas embryo 71-95 (DAF) Jatropha curcas cDNA clone rjcpga0_002995, mRNA sequence

TTTTAACATGAAGGACCAATAACTATTAGAAAGCTAAATAAAAATGGAATGAAGATAGTATGATAAATTG

AAGTTTGTGGCTCTAAAATCCAAAAACAACATATAACAATCAGAAGAGGATGCAAAAGACAATAACTTAA

GAACCTAAGTCACAAAACTGCCAGATAACTGAAACTGAAACCAAAAACAACAGCAAGACATAACTTAAAA

CAAAACATAACTAAACTAACTGTAACTTCATTCACCCAATGCTTTACTTTTTCTTTGCTGCGGATTTGGT

GACCTTTGCACCAGATGGATCCTTCTTCTCAACACTCTTGATGACACCAACAGCAACTGTCTGGCGCATG

TCCCTCACAGCAAAACGACCCAGAGGAGGGTACTCAGAAAAGGTCTCCACAACCATGGGCTTGGTGGGAA

TCATCTCCAATCCAGCATCACATTCTTCAG

>FM895977.1 FM895977 Jatropha curcas embryo 71-95 (DAF) Jatropha curcas cDNA clone rjcpga0_002994, mRNA sequence

CTGCAAGGATTCTATCCGTCGCCCGATGGGAATTGTACTTTAAGGCGGCCCTCGCGGCTCGTCCGCCGCG

GGGGCTTGGCCAACGGCACGTGCCTCTGGGGGCCGGATGGCCCCTACTGCGGGTCGGCAAACGGGCGACG

GACACGCGCGTCGCTTCTGGCCCGGATTCTGACTTAGAGGCGTTCAGTCATAATCCAGCGCACGGTAGCT

TCGCGCCACTGGCTTTTCAACCAAGCGCGATGACCAATTGTGCGAATCAACGGTTCCTCTCGTACTAGGT

T

>FM895976.1 FM895976 Jatropha curcas embryo 71-95 (DAF) Jatropha curcas cDNA clone rjcpga0_002992, mRNA sequence

CGAATTCCATAGATTACTAGATAAATTTATTAAAATATACATATCAGTACCATCAACAATTCATAGAAAA

TCCGTATTGCAACATACTACAAGTTACCCACCAAATCCAGTTAGAAGATTTCAGTAACAACTAGTACAGA

CAAGACAATGACAAAATGAATACATTTGTTTCTATGTTGGAGGTGCAAATGCAAGCAGATTTGCACAGTA

GGTTGCTAACTTAAGCAACGGGTGGACGGAGCACATGATCTTGAGAAGTATCCAGTGACAACAGGTGGCA

TAAACTGCCACATAGCAACTCCTGGGTAACCGATGAAAGGCACTAACTTGTTTCCAGATGCTTGGCCTTG

GGCAAGCAAATGCAGCAGGGATTGCAGGAGGAGGAGGC

>FM895975.1 FM895975 Jatropha curcas embryo 71-95 (DAF) Jatropha curcas cDNA clone rjcpga0_002991, mRNA sequence

TTTTTTTTTTTTAAGTTGTCAAACCAGAATTCTCTTTGGCATGGATTAAGAGTTTGATAGTTAACAAACA

AGCATTAGGGTTCAGAAACTTCTCAACACATTTGTATTCTAATACAAATCCACATGATTTACTTCCATGC

CTCCATCACAATATCAAGTTCCCGATAGAAAATCATGTTACCACCTGCTGTATGGAAACTAGCAAACAAA

ATGACAGACATCCTCCTGGCATCTATCTGCTACGCCTGGGTGACAAGTCAGCAGATCTAGACCGAGATCC

AGAAGGTGACCTTGATGCTCTACCAGGTGATTGCCTCCAAGCTCGTGCTTCCTCAGAATCATAGGCAGGT

TTCTCGCCATAACCATTGCCATTTCCATTTAGATCATCTACACCATATCCAGGAGAATATGACCTGCGAA

TGTGGCCACCATCTCGATCTGGTGGAGGGCCTCCAGGAGACCTTAGATGCTCTGCATGCCTTCTACCTGG

GGAAGCAGAGTAGTCTCTCCGTCTTGGGGCAGGAGAGTA

>FM895974.1 FM895974 Jatropha curcas embryo 71-95 (DAF) Jatropha curcas cDNA clone rjcpga0_002990, mRNA sequence

GGATCCCCCGGGGCTGCAGGCTGACATGTGTGCGAGTCAACGGGTGAGTAAACCCGCAAGGCGCAAGGAA

GCTGACTGGCGGGATCCCCCCGAGGGTTGCACCGCCGACCGACCTTGATCTTCTGAGAAGGGTTCGAGTG

AGAGCATGCCTGTCGGGACCCGAAAGATGGTGAACTATGCCTGAGCGGGGCGAAGCCAGAGGAAACTCTG

GTGGAGGCCCGCAGCGATACTGACGTGCAAATCGTTCGTCTGACTTGGGTATAGGGGCGAAAGACTAATC

GAACCGTCTAGTAGCTGGTTCCCTCCGAAGTTTCCCTCAGGATAGCTGGAGCTCGGGACGAGTTCTATCG

GGTAAAGCCAATGATTAGAGGCATCGGGGGCGCAACGCCCTCGACCTATTCTCAAACTTTAAATAGGTAG

GACGGCGCGGCTGCTTC

>FM895973.1 FM895973 Jatropha curcas embryo 71-95 (DAF) Jatropha curcas cDNA clone rjcpga0_002989, mRNA sequence

ATTCCTTTAAATAGGTAGGACGGCGCGGCTGCTTCGCTGAGCCGCGCCACGGAATCGAGAGCTCCAAGTG

GGCCATTTTTGGTAAGCAGAACTGGCGATGCGGGATGAACCGGAAGCCGGGTTACGGTGCCCAACTGCGC

GCTAACCTAGAACCCACAAAGGGTGTTGGTCGATTAAGACAGCAGGACGGTGGTCATGGAAGTCGAAATC

CGCTAAGGAGTGTGTAACAACTCACCTGCCGAATCAACTAGCCCCGAAAATGGATGGCGCTTAAGCGCGC

GACCTATACCCGGCCGTCGGGGGCAAGAGCC

>FM895972.1 FM895972 Jatropha curcas embryo 71-95 (DAF) Jatropha curcas cDNA clone rjcpga0_002987, mRNA sequence

GTTTAGCAACTATCGTTCGAGCTCCTGAAGATCCTATTTTAGGGGTTACCTGTTGCTTATAACAAAGATA

CAAGCCCTAACAAGTTGAATTTGGGTGTTGGTGCTTATCGAACTGAGGAA

>FM895971.1 FM895971 Jatropha curcas embryo 71-95 (DAF) Jatropha curcas cDNA clone rjcpga0_002985, mRNA sequence

ATTCCTCACAGAAGTACTACGTGACTCCTTTGTTGGCAACTCTAGGACTGTTATGATCTCTTGCATTTCT

CCAAATGCAGGCTCCTGTGAACATACACTCAATACTTTGAGATATGCAGACAGGGTTAAAAGTCTTTCAA

AAAGTGGAAATTCAAAAAAGGATCAGACTTTGAATTCAATACCCCCAACTACGAAGGATGTGTCATCAGC

ATCATCATTGCCTGTTTATTCTGAGGTAGATGATGTTTATGAACAACAAGAGGCAAAAGCAGTTGATACA

GTTAGGAGGGCTGTAGAAAAAGAAGCTATTTCTTACATACCCACTCCTGATTATGACAAACCGCCCCCTA

GTTTTACTTCAAGTTATTCCTTGAATGGACGTGAAGAAAATGGGTCTTCTGGTTCAACAGATAGGGAGAG

GTTTGAAATTAGCAATTCTTATGGTGGTTCAACAAGTCAAAAAGTTAATTCATCACACTCCCAAAACTCG

TGGATGCAA

>FM895970.1 FM895970 Jatropha curcas embryo 71-95 (DAF) Jatropha curcas cDNA clone rjcpga0_002984, mRNA sequence

ATTCCTCTTCAAGCTGTTTGAAAGAAAAGAAAAGAGAGGAAAGGAAAATGGGGAATTGTCAAGCCATAGA

TGCAGCAACGCTAGTGATACAACACCCAAGTGGTAAAGTTGACAAATTTTAT

>FM895969.1 FM895969 Jatropha curcas embryo 71-95 (DAF) Jatropha curcas cDNA clone rjcpga0_002982, mRNA sequence

GCTGCAGGAATTCGAATTCTGCAAAGGATTCTATCCGTCGCCCGATGGGAATTGTACTTTAAGGCGGCCC

TCGCGGCTCGTCCGCCGCGGGGGCTTGGCCAACGGCACGTGCCTCTGGGGGCCGGATGGCCCCTACTGCG

GGTCGGCAAACGGGCGACGGACACGCGCGTCGCTTCTGGCCCGGATTCTGACTTAGAGGCGTTCAGTCAT

AATCCAGCGCACGGTAGCTTCGCGCCACTGGCTTTTCAACCAAGCGCGATGACCAATTGTGCGAATCAAC

GGTTCCTCTCGTACTAGGTTGAATTACCATCGCGACACCATCATCAGTAGGGTAAAACTAACCTGTCTCA

CGACGGTCTAAACCCAGCTCACGTTCCCTATTGGTGGGTGAACAATCCAACACTTGGTGAATTCTGCTTC

ACAATGATAGGAAGAGCCGACATCGAAGGATCAAAAAGCAACGTCGCTATGAACGCTTGGCTGCCACAAG

CCAGTTATCCCTGTGGTAACTTTTCTGACACCTCTAGCTTCAAATTCCGAAGGTCTAAAGGATCGATAGG

CCAGGCTTTCACG

>FM895968.1 FM895968 Jatropha curcas embryo 71-95 (DAF) Jatropha curcas cDNA clone rjcpga0_002981, mRNA sequence

ATTCCCAAAGAAGTCATTGTTTTATCTTCAGGGTATTTTGTGTTTGATCTTCTAGGAATTTAATTAATCA

GCCTATCGAGATCTCGGAAGGAAGGAAAAGAAAGGAAGGAGAGGAATGGAGGAGGAGAAGCAGCAGCAGC

CGCCGCAGGGTGCATCATACACATATTGGGTGAGGGAAGCGACGGCAGATGCGGCTCCTCTTCCTCTTCC

TAAGAAACTCTCTACTAATGATTTAATTACCTCACAATCTCAGCCACCAACATTGGGTTCCGTCTGGAAT

CGGGCTGGAACTTGGGAGGAGAAAAACCTTAATAAATGGGCAACTGATAGGATAAAGGAACTTCTTGTGT

CAGTTGGCTCCTTGGAGTTCTCCTGTGGCAAAGCTGAAATAACAGAAGTGTCAAAATGTTTGGGTGACGC

ATTCTTGGTGACTGTGAGAAACAAGAAACGAGTTGGCTATACTTATGAGTTGACATTGAAAATCAAAGGG

GAATGGACAGTCAAAGAGGAGAAAAAGAATGTGAAGGCCCATATAGATATCCCAGAGTT

>FM895967.1 FM895967 Jatropha curcas embryo 71-95 (DAF) Jatropha curcas cDNA clone rjcpga0_002980, mRNA sequence

TCGAGTCAAAATTGAGAAAGCTTAAACCTTTACCATGTCTACTGCTTCTGCCTCTTCTCTTGTTCTTCCT

TCTCTCAATCCTAAAACCCTTTTTCTCTGCAACCCAAATAAACCCACTTCACTTTTTTTTGTTTCTCCCA

ATTCTTCTTCCCTAACCTTCCATGCGAAGCCCGTTTCTCTTTCCCACTCCTTAGGACAACAACAACCCTT

CTCTTCTCGCTTTGTTGCTAACGTTGCTATTTCTTCTGAGTTTGGACAAGAAGAGGAGGTATCTAGCGAT

GGAGATGAGTCCGGTTTCTCTTCTGAAGAGCCCAGTTTCTCTCCTGACCTTAAGTTGTTTGTGGGTAATC

TTCCTTTTAGCGTTGACAGTGCTCAGCTCGCCGGCTTGTTTGAAAGCGCTGGAAATGTTGAGATGGTTGA

GGTAATATATGACAAAGTAACTGGAAGAAGCAGAGGATTCGGTTTTGTGACCATGTCAACTGCACAGGAA

GTTGAAGCAGCTGCTCAGCAGTTCATGGCTATGACTCGAGGGGAGGC

>FM895966.1 FM895966 Jatropha curcas embryo 71-95 (DAF) Jatropha curcas cDNA clone rjcpga0_002978, mRNA sequence

CTGACATGTGTGCGAGTCAACGGGTGAGTAAACCCGCAAGGCGCAAGGAAGCTGACTGGCGGGATCCCCC

TGAGGGTTGCACCGCCGACCGACCTTGATCTTCTGAGAAGGGTTCGAGTGAGAGCATGCCTGTCGGGACC

CGAAAGATGGTGAACTATGCCTGAGCGGGGCGAAGCCAGAGGAAACTCTGGTGGAGGCCCGCAGCGATAC

TGACGTGCAAATCGTTCGTCTGACTTGGGTATAGGGGCGAAAGACTAATCGAACCGTCTAGTAGCTGGTT

CCCTCCGAAGTTTCCCTCAGGATAGCTGGAGCTCGGGACGAGTTCTATCGGGTAAAGCCAATGATTAGAG

GCATCGGGGGCGCAACGCCCTCGACCTATTCTCAAACTTTAAATAGGTAGGACGGCGCGGCTGCTTCGCT

GAGCCGCGCCACGGAATCGAGAGCTCCAAGTGGGCCATTTTTGGTAAGCAGAACTGGCGATGCGGGATGA

ACCGGAAGCCGGGTTACGGTGCCCAACTGCGCGCTAACCTAGAA

>FM895965.1 FM895965 Jatropha curcas embryo 71-95 (DAF) Jatropha curcas cDNA clone rjcpga0_002977, mRNA sequence

CGAATTCCGTTATTTTACTTATTCCGTGAATCGGAGGCGGGGCTATGCCCCTCTTTTTGGACCCAAGGGT

CGTTTCGGCGGCCGATCCGGGCGGAAGACATTGTCAGGTGGGGAGTTTGGCTGGGGCGGCACATCTGTTA

AAAGATAACGCAGGTGTCCTAAGATGAGCTCAACGAGAACAGAAATCTCGTGTGGAACAAAAGGGTAAAA

GCTCGTTTGATTCTGATTTCCAGTACGAATACGAACCGTGAAAGCGTGGCCTATCGATCCTTTAGACCTT

CGGAATTTGAAGCTAGAGGTGTCAGAAAAGTTACCACAGGGATAACTGGCTTGTGGCAGCCAAGCGTTCA

TAGCGACGTTGCTTTTTGATCCTTCGATGTCGGCTCTTCCTATCATTGTGAAGCAGAATTCACCAAGTGT

TGGATTGTTCACCCACCAATAGGGAACGTGAGCTGGGGTTAGACCGTCGTGAGACAGGTTAGTTTTACCC

TACTGATGATGGTGTCGCGATGGTATTCAACCAGTACGAGAGGAACGTTGATTCGCACAA

>FM895964.1 FM895964 Jatropha curcas embryo 71-95 (DAF) Jatropha curcas cDNA clone rjcpga0_002976, mRNA sequence

AGACTCTGTCGTTTTAATAATGTGTTTGTTAGGAACATGCTACATGGTCCTTGGAAAGAAGCTAGTTCTC

CTGTTGTGAACTTGCATGTGGATGATAAGAATGTTAATGGGGAGGCAATAACTATGGCTTTGGCTTATCT

TTATGGGCATCACCCCAAGTTTAATGATAACAACGCGTTTCGTGTCTTGGCTGCTGCTTCCTTTCTTGAC

CTTCAGGATTTGTGTGCTATATGCACAGACTTCATTATATCTGAACTGTGGACTTCTAACTTCTTGGCAT

ATCAGGTATTTGCAGAGAGCCAAGATTATGGTTTACATGGAGAGCGTGTAAGAAATGCTTGCTGGGGCTA

CCTTTGTCAGAGTGGTGCCATGGAATTGAAAGAGGTGCTTCCCAAGCTTTCTTCTCAAACTCTGCATGCA

TTACTGACCTCAGATGAATTATGGGTACCTAGTGAAGAGAAACGGTAAGAGGTGTAGGTTGGGTTGCTGC

AGAATTGGGTTTACTGGACACTGGTTGAAGTCTTGATTAGGTTGGATAATCCATGTTTTCTTTTCTGTT

>FM895963.1 FM895963 Jatropha curcas embryo 71-95 (DAF) Jatropha curcas cDNA clone rjcpga0_002975, mRNA sequence

TTTTTGGTAAGCAGAACTGGCGATGCGGGATGAACCGGAAGCCGGGTTACGGTGCCCAACTGCGCGCTAA

CCTAGAACCCACAAAGGGTGTTGGTCGATTAAGACAGCAGGACGGTGGTCATGGAAGTCGAAATCCGCTA

AGGAGTGTGTAACAACTCACCTGCCGAATCAACTAGCCCCGAAAATGGATGGCGCTTAAGCGCGCGACCT

ATACCCGGCCGTCGGGGCAAGAGCCAGGCCCCGATGAGTAGGAGGGCGCGGCGGTCGCTGCAAAACCCGG

GGCGCGAGCCCGGGCGGAGCGGCCGTCGGTGCAGATCTTGGTGGTAGTAGCAAATATTCAAATGAGAACT

TTGAAGGCCGAAGAGGGGAAAGGTTCCATGTGAACGGCACTTGCACATGGGTTAGTCGATCCTAAGAGAC

GGGGGAAGCCCGTCCGACAGCGCGT

>FM895962.1 FM895962 Jatropha curcas embryo 71-95 (DAF) Jatropha curcas cDNA clone rjcpga0_002974, mRNA sequence

TCTGACATGTGTGCGAGTCAACGGGTGAGTAAACCCGCAAGGCGCAAGGAAGCTGACTGGCGGGATCCCC

CCGAGGTTGCACCGCCGACCGACCTTGATCTTCTGAGAAGGGTTCGAGTGAGAGCATGCCTGTCGGGACC

CGAAAGATGGTGAACTATGCCTGAGCGGGGCGAAGCCAGAGGAAACTCTGGTGGAGGCCCGCAGCGATAC

TGACGTGCAAATCGTTCGTCTGACTTGGGTATAGGGGCGAAAGACTAATCGAACCGTCTAGTAGCTGGTT

CCCTCCGAAGTTTCCCTCAGGATAGCTGGAGCTCGGGACGAGTTCTATCGGGTAAAGCCAATGATTAGAG

GCATCGGGGGCGCAACGCCCTCGACCTATTCTCAAACTTTAAATAGGTAGGACGGCGCGGCTGCTTCGCT

GAGCCGCGCCACGGAATCGAGAGCTCCAAGTGGGCCATTCATGGTAAGCAGAACTGGCGATGCGGGATGA

ACCGGAAG

>FM895961.1 FM895961 Jatropha curcas embryo 71-95 (DAF) Jatropha curcas cDNA clone rjcpga0_002973, mRNA sequence

TGAACCACAGGTTGATTTAAGGGAGCTAGAAGTTGGTACTAATTTTCTAGCAACCTCAAGAAATGAAAGA

ACTACTCCATTACAGGAGCCATTACGTGTAATGGATTCAAAGGTTTCACAGATCTTAGGCGTATTAAGGA

GGCACTTCTCGTGTATTCCTGATTTTAGGCACATGACAGGGCTCAAGGTTAGCATATCCTGCAGTTTGAA

GTTTGAATCTGACTCTTTCAATCAGTTATGCGGGAATAGCTCTCCAACCAGTCGGTTAGATGGAATAGAT

GCGCTTCCTGCTTTATATGCAACTGTGCTAAAATTTTCATCATCAGCACCATATGGGTCCATCCCACCAT

ATCACATACCTTTCCTTCTTGGTGAAGCCACCAAGAAAGA

>FM895960.1 FM895960 Jatropha curcas embryo 71-95 (DAF) Jatropha curcas cDNA clone rjcpga0_002972, mRNA sequence

GGAGCTGACATGTGTGCGAGTCAACGGGTGAGTAAACCCGCAAGGCGCAAGGAAGCTGACTGGCGGGATC

CCCCTGAGGGTTGCACCGCCGACCGACCTTGATCTTCTGAGAAGGGTTCGAGTGAGAGCATGCCTGTCGG

GACCCGAAAGATGGTGAACTATGCCTGAGCGGGGCGAAGCCAGAGGAAACTCTGGTGGAGGCCCGCAGCG

ATACTGACGTGCAAATCGTTCGTCTGACTTGGGTATAGGGGCGAAAGACTAATCGAACCGTCTAGTAGCT

GGTTCCCTCCGAAGTTTCCCTCAGGATAGCTGGAGCTCGGGACGAGTTCTATCGGGTAAAGCCAATGATT

AGAGGCATCGGGGGCGCAACGCCCTCGACCTATTCTCAAACTTTAAATAGGTAGGACGGCGCGGCTGCTT

CGCTGAGCCGCGCCACGGAATCGAGAGCTCCAAGTGGGCCATTTTTGGTAAGCAGAACTGGCGATGCGGG

ATGAA

>FM895959.1 FM895959 Jatropha curcas embryo 71-95 (DAF) Jatropha curcas cDNA clone rjcpga0_002971, mRNA sequence

ATTCCGCAAGGCGCAAGGAAGCTGACTGGCGGGATCCCCCCGAGGGTTGCACCGCCGACCGACCTTGATC

TTCTGAGAAGGGTTCGAGTGAGAGCATGCCTGTCGGGACCCGAAAGATGGTGAACTATGCCTGAGCGGGG

CGAAGCCAGAGGAAACTCTGGTGGAGGCCCGCAGCGATACTGACGTGCAAATCGTTCGTCTGACTTGGGT

ATAGGGGCGAAAGACTAATCGAACCGTCTAGTAGCTGGTTCCCTCCGAAGTTTCCCTCAGGATAGCTGGA

GCTCGGGACGAGTTCTATCGGGTAAAGCCAATGATTAGAGGCATCGGGGGCGCAACGCCCTCGACCTATT

CTCAAACTTTAAATAGGTAGGACGGCGCGGCTGCTTCGCTGAGCCGCGCCACGGAATCGAGAGCTCCAAG

TGGGCCATTTTTGGTAAGCAGAACTGGCGATGCGGGATGAACCGGAAGCCGGGT

>FM895958.1 FM895958 Jatropha curcas embryo 71-95 (DAF) Jatropha curcas cDNA clone rjcpga0_002970, mRNA sequence

ATTCCGAATAGAAAGGGATGTATCCCTAAAGCAGATTGTGGACTTGGATACTGTAATTGGATTTTGTATT

GAGGCAGGGCAAGCACTTGCTTTTGATCAGCTTCTTGCTGAAGCTGCTGGAATTGTGCTCGAAGAAGGTG

CACCCTTGATATATTCGCTACTTGCTGGGATAGTTAAGCATATACCTGAAGAAATGCCAGAAAAGAGAGA

AATTAAGAGGATAAGAGGCGTAGCAAATAGTGTTGGTCTGGTTGTGGATCATGATTCTGAGTGGGTAAGA

TCAATTCTGGAAGAGGTGGGTGGTGCAAATGATGGTTCTTGGACCTTGTTGCCTTATTTATTTGCAACAT

TCATGACATCAAGCATCTGGAACACCACTGGGTTCAATGTTGACACAGGAGGATTCAACAACAATATTCA

TTGCTTGGCAAGGTGCATGAGTGCTGTAATTGCAGGAAGTGAGCTTGTTAGATTGGAACGTGAACATCAG

CAGAGGCAATCTCTCTCTAATGGCCATGTTGGTGA

>FM895957.1 FM895957 Jatropha curcas embryo 71-95 (DAF) Jatropha curcas cDNA clone rjcpga0_002967, mRNA sequence

GATAAATCGATCACTCATTCACCAACCTCAGATAAAAACATACAGAGATGAAAATAAAAAGTTGATTAAG

CACTATATAAGAAATATATATTATATATAGGAATAAGCTAAAAAAAAAAAAACATGCACTACCAGATCTT

CCTCGAAATAACAACAAAATCCTAGAGCTTTGAATGTCTTATAACAAAGACACCAAATTAGGCAAAAGAT

GAAACAAGATAAGAACATAAAACAAACAGAAAGATACTAAGGCAGTCATTTTTGGACCCTTTTAACTTCT

CATTCACACAACCAACATCCCTTCCCTTCTTCCTCTTGCTCCTGCTCCTGCTGCTTGTCAAGTCAATACC

ATTTCTTCTCTCTGTTTCTTTTCCTTTTTCTAACAAAGACCACCTTGTAATAGAAAGAAGCACGAAAAAA

ACCTTTCCATCAAAGGCGTACTAATTAATTAATTAATAATTCATTAATTATTCAGACCATTAGATAATTA

CTACACCAGTGGATTGGTCGTCGTGGACGATCCTAGCGTGACATCCATCCATGATCCAGAT

>FM895956.1 FM895956 Jatropha curcas embryo 71-95 (DAF) Jatropha curcas cDNA clone rjcpga0_002965, mRNA sequence

CTAGTGGATCCCCCGGGGCTGCAGGTTTTTTTAAACAAAATAATGAAATGCAAGTTTCTCAATAAATAAT

AAGAAAAAGAGCAAGTCTCTGAGAGAGAGATCTCAACTGCTTGAACATCATACAACACTTGACTTAATTA

CTCCCTCAAGTATAACCCTAGAATGGAATTTAATTCACTGTTTGGCACTGCGAATCAAACCCTAAATGAT

CAAGTCACAGACACTAACACTAACCAAAAAGAAAGAAACTAACACAAAAACTAAGGACAAATGAATTTAC

CCCATAAGATTAACCTTATTGGTGGAGTAGTACAATGTTTCAAAGAGGAGGACACACAAAACCAGGAGGT

GGATTCTTTCCACAAGTTATTAAAACTTGAAGAGCAAGAGGAATGAAAATGTTGATATTAAGAAGCTTAA

GCCTTATAGTTGTGCATAGACAAACTGCTGCTTCAAGCTCTAGCAATCCTTTAATTACGGGGCAGCAAAC

ATTCTCAACTGGATTCCCTAATCCAACATGCACTAATCCTCCTAATACATCAACACAAGCCCTACTTAGG

GCGTGTTGGACAAGGGGTGAGTA

>FM895955.1 FM895955 Jatropha curcas embryo 71-95 (DAF) Jatropha curcas cDNA clone rjcpga0_002964, mRNA sequence

CTAGCGGAGTTATACTCTAAGCTTTCGCATTGGAAGGATGTAGAGGTATGTCTAGGAAAGGCCAGAGAAT

TGAAACACTACTCTGCAGGTTTGCTTCATACAGAAGGCATTATGTCTCAAGGACGCGGCCAACTCCAAGA

AGCCATGTGTAATTTCACAAATGCTCTTTTGTTTGAACCATCTTATGTGCCTTGCAAGATACTGATTGGT

GCCACGTTATCCAAGATGGGCCCCAATGCGTTGCCTGTTGCACGAAGCATACTCTCAGATGCATTGAGGA

TAGAACCTACTAGCCGAATGGCTTGGTATTACTTAGGGATGGTTCACAGAGATGATGGGCGAATGGCTGA

TGCTACAGACTGCTTTCTCGCAGCTGCCATGCTTGAAGAATCTGATCCCATCGAAAGCTTCAGCTATGTC

GGTTAATTGGATATGCGGTTCATTGAAGGCCTCAGCAACTGATGATCACTACAGAAAGGCCAAAGAGAGG

AAATTTGAGGAAGAAGTTTAACCCAATTCAGGAAATTAGAGGAAAAGAGCAGAGGTATTTGATTGTAGCA

TAGTCACCTGTTAGGTGGTATTTAATAATTAATTTGGATTA

>FM895954.1 FM895954 Jatropha curcas embryo 71-95 (DAF) Jatropha curcas cDNA clone rjcpga0_002963, mRNA sequence

CAGTTTGTTTGGCAACAATTTCACTAATTTCCAGCGACCATTACTTCCCAGTTCATCGTCATATCCTCTT

TATCAGCCAAATCGCTCAAACAATGCCGGAACGACGCTGTCGTTTGATAGAGCTTCTACCTATGGTCTTA

CGAGTAATGCCACAAATGGCCTCATTAGTGGTCCTCAACATAATCAAGCAATTAGAAGATCAAATCCTTT

CAACAGTGACAACCCTTTCAAATTTGGCTCTTCCAATCATGCTTCAGGCTCAGGAACTGTCAACAACTCT

TATCCTTTTCTTAATCCCAACAGTAATTATGCTGGAATCCGATTGACTAGTGACGGAGAGCTTATCGGAT

CAGGACCAATCGGATTCTCAGGGAAAAACCTTTCAAGTGGTTCCAGTTTGATGAATTGGACTCGTAATAA

CAATGCATCATCGACGGACACGCTGAATGGGCTATTTGGTTATCAAGGAGCGTCTTCTTCTGCTGCTGCT

GCTGCTGCTCCTCCTCCTGCTCCTGCTGCTACTGCTGCTGCTCCTCCTCCT

>FM895953.1 FM895953 Jatropha curcas embryo 71-95 (DAF) Jatropha curcas cDNA clone rjcpga0_002962, mRNA sequence

CTTTCTCTCTCTCTCTCTCTCTCTCTCTCTCTCTATTTATCTTTTCCTTCTTTTCTCTCTCCCTTACAAG

CCCTAACCCTAACTCAGACCCAGATTCGCCATGAAAGGAGGTAAATCAAAGTCAGACACCAAGAACGCCA

AGCTTTCCGTGAACAAGAAACCCGCCAAGGCAAGTAAGAAGGCGGGTAAGGCAGCGAAGGATCCAAATAA

GCCAAAGAGGCCCGCCAGTGCTTTCTTCGTTTTCATGGAAGAGTTCAGAGAGCAGTACAAGAAGGAACAT

CCTAAAAACAAATCCGTTGCTGCTGTTGGCAAAGCTGGTGGAGATAAATGGAAGTCAATGTCAGATGCTG

AGAAAGCACCTTTTGTATCTAAGGCAGAAAAGAGGAAGATAGAATATGAGAAGAAATTGAAAGCCTACAA

CAAGGGACAGGCTGAAGGCCCCAAGGAAGAAGAGGAATCTGAGAAGTCAATGTCTGAGGTGAATGATGAG

GAAGAAGATGATGAGGAGGGAAGTGGAGAGGAGGACGATGATGAGTAGGAAGAGGGGGAGAGGAGTAGCA

TAGTCATTTAGGCTGTGAC

>FM895952.1 FM895952 Jatropha curcas embryo 71-95 (DAF) Jatropha curcas cDNA clone rjcpga0_002961, mRNA sequence

TTTTTTTTTTACACTCGCACATAAATTACACGTTAAAACAATCTAAATTGATCCACATTTTAAGCATTTA

AACGACAGCCACGTCTCAAACTTCCAGATGCTGCGTTTTTCTGAGTTGGAAAAAAATTCTGAACAAGAAA

ATTTATATTTGACCCAAGTTAATGATACTACCAGAACCATTCTTGTCTTGCATCTGCAGCTTTTACGTTA

GTTGAAACAAGAGGCACAGCTCCATATAGGGAAATCAAGTCGTTGAGACATTTATTTCTCCTGTTAGTAT

TACCTTTCAAACTACTCATGCCTGAGGATCCAATTTCATGTATCTTCCCTTTCACCCATATTGGAGGAGC

GCCTGTAGCATTGGCAACTAGTAAAGACATTGCAATATCTTCACAATCCCTTTCTCTTGAAACATAGTCT

TGTATGGACGGCGACATTGAGTGAGTATACAAGTCCAAGTACTTCTTGTGAAAGAAAGCAGCTTTTGAGA

GAACTATACTGTAAGCACCTGTCCACCAAACTGACCACCATCCTCCATATTTGTAATATGGGACACCATT

CTTCTGGCCATCCAGCAAATGGATACGAGGT

>FM895951.1 FM895951 Jatropha curcas embryo 71-95 (DAF) Jatropha curcas cDNA clone rjcpga0_002959, mRNA sequence

CTGCACTTCTGCAGTTTATCNTGGCCACACAAACCATGACCAGCACCGGATGAAACCCTTTTAACATTTA

TGGCGGCTTGGCGTGCTATGCTTCGCCAATTTGATTTGCAAAATGACCCATTTAGGGTCATCTCTTTGTT

CAAAGCCATGCAAATAGGGAAAGAGAACGACCCAGTTACTGATCCTTTTGTGTATGCTTCTTTGATAAAA

GCTTGCAATAAGGTTTTGGGTATTAATGAAGGGAAATCAATACATAGCCGCGTAGTTAAGCTTGCGTTGG

ATTATAACTTGAATGTTTTGAATAGTTTGATTCATTATTATGCGAGTGTAAATGGGTCAGTGAAGTATGC

TCGTGTCTTGTTCGATAAAATGCCTGACAGAACGGTTGTTACTGTTAATAGTATGGTTTCTGGGTTTCTG

AAGAATAACCAATTTGATTTTGGTTTGAGCTTATTCAAGAAAGTCCTTACTGGTGCTTTTGGTCTGAGTA

TGAAGCCAAACTTTGTTACTCTAATGGTTTAGATTTCTGGGTGTGTTGAAAACGGGGGATACAGCATTGG

GAGATTGC

>FM895950.1 FM895950 Jatropha curcas embryo 71-95 (DAF) Jatropha curcas cDNA clone rjcpga0_002958, mRNA sequence

GATAAAACCAACTCCAAAAATTCCAGAATTTCTCACCTTCCATTATCCGTTTCTCAATGTTTTCCTCTGC

TCTCCTTTCTCCCTTATCTAATCCAATCTCTCAGTTTCAACACACAACAAACAAGCTGCTCTCGCACTAG

CTGACAGAACGTGTATTCTCCCTCCTTTCTCTTTCAATTTTAGGATGAGAAGAATAAATTCAGCCAGGAA

TTTCAAATTGTAGAGCGAGAGAGAAGTTTTGTTGGATAGAGTTGGCGAATGGGGACGGGTTCATCAAAGG

ACGCTGATGGTAACTCTCATGGAGGCGAAGAGCGAGAAGAAAATCTTGATCAAGCTGGAGGTCAGCTTTA

TGTCTCTCTTAAAATGGAGAACTATAAGCAAAAGCGTGACCTTATTCCTCACGTCTATGGCTCTGTGCCT

CTTATTGGCTCCTGGGATTCTTCTAAAGCTCTTTCAATGGAACGCGAATCGGCGTCAATGTGGGAATTGA

GCTTTGTTGTCCCTCCTAATCATGAAACTTTGGATTTCAAGTTCCTGTTGAAGCCGAAGTACAGCAATAT

TCCTTGTGTGGTTGAGGATGGGCCAA

>FM895949.1 FM895949 Jatropha curcas embryo 71-95 (DAF) Jatropha curcas cDNA clone rjcpga0_002957, mRNA sequence

ATTCGTTGTTTATTTCAAGTGATAAGTTCAATTATGGCTGATCAGGAGCACCATCAGAAGAGTTATGAGC

CCGAGACCACTGCAGTTGAGGCCACGGATCGTGGGTTGTTTGATTTCGGGAAGAAGAAAGAGGAAGAGAA

GCCTCAAGAGGAGGTGATCGTTACAGAGTTTGAAGAGAAGGTTCAGGTCTCTGAACCAGAGGAGAAGAAG

CATGGCATCTTGGAGAAGCTTCACCGATCAGGAAGCAGCTCTAGCTCTTCTAGCGATGAAGAGGAAGGAG

AGGGAGAGGAAAAGAAGAAGAAAAAGAAA

>FM895948.1 FM895948 Jatropha curcas embryo 71-95 (DAF) Jatropha curcas cDNA clone rjcpga0_002956, mRNA sequence

ATTCCATTGTGAAGCAGAATTCACCAAGTGTTGGATTGTTCACCCACCAATAGGGAACGTGAGCTGGGTT

TAGACCGTCGTGAGACAGGTTAGTTTTACCCTACTGATGATGGTGTCGCGATGGTAATTCAACCTAGTAC

GAGAGGAACCGTTGATTCGCACAATTGGTCATCGCGCTTGGTTGAAAAGCCAGTGGCGCGAAGCTACCGT

GCGCTGGATTATGACTGAACGCCTCTAAGTCAGAATCCGGGCCAGAAGCGACGCGCGTGTCCGTCGCCCG

TTTGCCGACCCGCAGTAGGGGCCATCCGGCCCCCAGAGGCACGTGCCGTTGGCCAAGCCCCCGCGGCGGA

CGAGCCGCGAGGGCCGCCTTAAAGTACAATTCCCATCGGGCGACGGATAGAATCCTTTGCGAATTGCTGC

GGAGAAACCACAAGAGAGACAACAAAAGAGTGGACCTAGCTCAGAAGTAGCTACAGTTTCATCAACAAAT

TTGGTATCTTATTATCAGGCCCCTGAAGCACTTAAAGTGGTAAAACCATCACCGAAATGGGATGTTACTC

TTATGGAGTGATGTTACTAGAAATGATA

>FM895947.1 FM895947 Jatropha curcas embryo 71-95 (DAF) Jatropha curcas cDNA clone rjcpga0_002955, mRNA sequence

GCAACAGCTGCCTTCCTTTCCTTCACCGTTTGCTGCTTCTGGAGTTAGGGCACAACATGATTCTCAGGAA

CTTGGTTATGCTCAATTACCTGCAGCCACTAGTTCAGTGAACGATCCAACACGCTTCCCGCAGGGACTTC

AGGCTTCCCAGGAGTGGATGTGGAAACACAAGGCAGCTGGTTCTGGATCATGAAGTGGAGAAGTGGATTC

CCAGGCGGATGAGTGAAATATTGCTGCTGTTGAAATGCCTAGGAAGAGAACGGTGGTGCTCTCAGCGACA

ACGCAAAGAGAAGGCAGCAGCAGCAGCAATTGCTATGAGAGCTTAAAATTCTAAATGAGATGCAACTGTA

ATGGTTTTAACCAACCTTTAGTTTTGTTAGTTATTTTGGCGAATTTACTGTTGTATTAATTTAATTAGCA

AATTAGCCTATGTACCATTAGTATTGGAGCAAGTTGTGTCGCAACTGTGTATTGTTGAAAGTAAAAAATT

TATGTAACATGTTCACTAAGTAATG

>FM895946.1 FM895946 Jatropha curcas embryo 71-95 (DAF) Jatropha curcas cDNA clone rjcpga0_002954, mRNA sequence

GGAAAACCCATTTGGGCTAGTTGATCTTCTTTTCCGTTAACATCTCTCTCTATAAATGGCCATCACAACC

TATTTTTCTTCTACACAACATCACTACTACATTTATATTACTAAACATGACCAAGTCAAGCGACTTAATC

CCGGGTCTTCCAGAAGAAATAGCTCTCGAGTGCTTAACTCGACTCCATTACTCAACGTACCGAGTTGCTT

CCCGAGTTTCCAAACGCTGGCGTCATCTTCTTCGAAGCAGAGAGTTTTATTACCAGAGAAAACTATCTGC

TCGTACCTATAAAGCTGCTTGCTTTATCCAATCTATTCCTCTTCAATCTGACTCTAAATCAACTGGGTCA

CCCAGTTATGGAGTTTCTATGCTTGATCCAATCAGTGGAGCTTGGGAACGAGTTGACCCGGTTCCTGACT

ATCCCGATGGTCTTCCTTTGTTTTGTCGAGTTACTAGCTCAGAAGGGAAGATTGTAATCCTGGGAGGGTG

GGATCCAGTGAGTTACGAACCACTGAGTCACGTGTTCATTTATGATTTTACGACTCGGAAATGGAGGAAG

GGTAAGAATATGCCCGAATATCGTTCTTTCTTCGCTGTAGGCGAGTTAAACGGTCGAGTTATAATCGCCG

>FM895945.1 FM895945 Jatropha curcas embryo 71-95 (DAF) Jatropha curcas cDNA clone rjcpga0_002953, mRNA sequence

TCGTACAACTGGTATCGTGCTGGATTCTGGTGATGGTGTGAGCCACACTGTGCCAATCTATGAAGGATAC

GCTCTTCCACATGCTATCCTCCGTCTTGACCTTGCCGGTCGTGATCTCACTGATGCTTTGATGAAGATTC

TCACTGAGAGAGGGTACATGTTCACCACCACTGCTGAACGGGAAATTGTCCGTGACATGAAAGAGAAACT

TGCATATGTTGCACTTGACTATGAGCAGGAACTTGAGACTGCCAAGAGCAGCTCCTCAGTTGAAAAGAAC

TATGAGCTTCCTGATGGCCAAGTCATCACGATTGGAGCTGAGAGATTCCGATGCCCAGAGGTCCTTTTCC

AGCCATCTCTCATCGGAATGGAAGCTGCAGGCATCCACGAGACTACTTATAACTCAATTATGAAGTGTGA

TGTGGATATAAGGAAGGATCTTTATGGTAACATTGTGCTCAGTGGTGGTTCCACTATGTTCCCTGGTATT

GCTGACCGTATGAGCAAGGAGATCACCGCCCTTGCTCCAAGCAGCATGAAGATTAAGGTGGTTGCACCAC

CCGAAAGAAAATACAGCGTCTGGATTGGAGGATCTATCCTTGCATCCCTTAGCACTTTCCAGCAGATGTG

>FM895944.1 FM895944 Jatropha curcas embryo 71-95 (DAF) Jatropha curcas cDNA clone rjcpga0_002952, mRNA sequence

ATTCCATGAATCCTTTCTTTGAGCAACTAGCCATCACCTATCAAGATTTTCTGTTTCTCACAGTGGATGT

GGATGAGCTTAAGGGTGTGGCTACAAAGATGGAGGTGAAGGCCATGCCAACATTTGTGTTGATGAGTAAA

GGAGCAAAGGTTCATAAACTTGTGGGTGCTAACCCACATGAGATCAAGAAAAGGGTCCATGCCTTTATTC

TCTCTACTTAATTATAATTATTTAATTAATTATGTGGCTTCACATTTTATTTTAAACATCTTTTTTTAAG

TTAAGGTCTTTAAGGGTTGTAATTTTTCTTTTTATTAGAATTCCTGTTAGTAAAAAACAAGTCATCTATC

AAATTTATATATATCCATGGTGAATATTTGCATTGCTTGAAAA

>FM895943.1 FM895943 Jatropha curcas embryo 71-95 (DAF) Jatropha curcas cDNA clone rjcpga0_002951, mRNA sequence

CAGGATATTGCTCTTGCTGAACTGGCTCCTACTCACCCAATAAGGCTTGGGCTTGCCCTTAACTTCTCCG

TATTCTACTATGAGATCCTTAACTCACCTAGACCGTGCTTGCAATCTTGCAAAGCAGGCTTTTGATGAGG

CTATTTCTGAGCTTGACACATTGGGTGAGGAATCTTACAAGGACAGTACATTGATCATGCAACTTCTCCG

GGACAATCTGACACTATGGACTTCTGATATCACGGATGAGGCTGGGGATGAGATCAAGGAGGCATCAAAA

CGTGAATCAGGCGAAGGACAACCACAGCAGTAATGAATTAAAATTTGTAGGATATGTTTACTCTGTACTT

CTTTCTTGTGACCTCCGCATAGATGCGATGCTGGTTGTTGTAGCTGGATTTGCATAATGAAATTGGTAAA

TCCTGAGTGCTGATCTGATGCTACTTGGGATTGAGGTAATTCCATAGATTATCAAGCTTCTGCTGTTGAT

GTTAAGAATTCATGTCTGTATTCTTATTGCTTGACCATGTAATAGTGTTGGAACTAGGGGTTCGAACCTT

CCCACTTACATATAACATCAAGTGCAAGAAAATTGTTGGGTGTTAAAAAAAAAAA

>FM895942.1 FM895942 Jatropha curcas embryo 71-95 (DAF) Jatropha curcas cDNA clone rjcpga0_002950, mRNA sequence

GGGTTAATTTTTCCTTTATTATTTAAGAAACAAAAAGTTGCAAGATTACAAGATGCTACACATAAGATGA

AATGAAACCCAGAAAAACAAACTAGCAAATGAAAGTTTGGGTCTTATGTACAACAGTCAACAACTACAAA

TTAAGTAAATAATCTGAGTTATTCTCAAACTCACTGACTCACATACTCTACCTCGGCTGCTGAATCGTTC

GTGGACTCACTCTCACACTAACAATCCTTGGTGCTTTTTCTACATACTTGGGTACCTGCGCCACCTGAGC

TCGACCCTTTTGCCACTTCATTACTCCTATTGAAACCAAATCCTTGCTATGCTCCTTCTCTTCCATCTCC

ACCGCCTGTTTGGTATCAACGCAGTCGCAAAAGAGGGAATCGACATCGACTTCGTCGAAAAAATTGAAAT

GGAGAGGATCAATTTGAGGATCAGTGAAGCATTCTTCAAGCCAGTAATCGCGGAAGGAAGGAGAAGAGTG

GACGAGGGACCACCCTTGATCGG

>FM895941.1 FM895941 Jatropha curcas embryo 71-95 (DAF) Jatropha curcas cDNA clone rjcpga0_002949, mRNA sequence

CAAAGGATCTATCCGTCGCCCGGTGGGAATTGTACTTTAAGGCGGCCCTCGCGGCTCGTCCGCCGCGGGG

GCTTAGCCAACGGCACGTGCCTCTGGGGGCCGGATGGCCCCTACTGCGGGTCGGCAAACGGGCGACGGAC

ACGCGCGTCGCTTCTGGCCCGGATTCTGACTTAGAGGCGTTCAGTCATAATCCAGCGCACGGTAGCTTCG

CGCCACTGGCTTTTCAACCAAGCGCGATGACCAATTGTGCGAATCAACGGTTCCTCTCGTACTAGGTTGA

ATTACCATCGCGACACCATCATCAGTAGGGTAAAACTAACCTGTCTCACGACGGTCTAACCCCAGCTCAC

GTTCCCTATTGGTGGGTGAACAATCCAACACTTGGTGAATTCTGCTTCACAATGATAGGAAGAGCCGACA

TCGAAGGATCAAAAAGCAACGTCGCTATGAACGCTTGGCTGCCACAAGCCAGTTATCCCTGTTGGTACTT

TTCTGACACCTCTAGCTTCAAATTCCGAAGGTCTATAGGATCGATAGGCCACGCTTTCA

>FM895940.1 FM895940 Jatropha curcas embryo 71-95 (DAF) Jatropha curcas cDNA clone rjcpga0_002948, mRNA sequence

TTCTATCTGCTTCCTCTCCGCCGGTGCAGAAGGCGGCTGTTGATATTGTTCGGGACTTAACTGGGTCTGA

AGATGGGTTACTGTCCCTTTCTAAGAGGTATCTGAGCCTGCAGCAGAAGCTCTTGTGAATCTGTCACAGA

ACTCAAATTTAGCTACTAAGATGGTTGAGACTGGGATGATTCAAATGGCCATGGATCTATTATATAAGCC

AGACTCTAGCATTACCCGATTACTTGTAATGCTCCTAGTTAATCTCACTCAGTTGGATGCTGGTATTCAA

TCCTTGCTTCAGACTGATGATGAGAAGATGCAAGGACTGTTTGTTATGAAGCTTGTGAGATCATTCTGTA

GAACCTCCAGTGAAACTAGAGACAATCCATTTGATCATGTTGGTTCCATTCTTGTAAATATATCAAAGAA

AGAAGCTGGTAGGAAAATGTTATTGGACCCTAAGCGAGGGTTGCTGAAACAGATCCTCAGACAGTCTGAC

TCCACAAGTCCATTGCGGAGGAAAGGGGTTTCTGGAACGATTCGC

>FM895939.1 FM895939 Jatropha curcas embryo 71-95 (DAF) Jatropha curcas cDNA clone rjcpga0_002947, mRNA sequence

GGCTGCAGGCCACAAGCTCATCGATCCCAAGCTAGCTGAGAAGCTCGAAGAAGATCTACAAAGAGCCAGA

TGTATGATGCTTGATGGAGACGCAGCTGCTTTTCTTCCCACCAAAACCCAGGGCAAATTCCTCAAAATGT

TTCTGGGACCCATTAATGTCCGTGCTTCTCGCAAAGATGTCAATTGAAAGTTAAAGAGGAATACAACAGT

TATAGGGACAGAACCGCCCTTTTGTTTCTTCTTTTTCCATCAATTCTACTGACCCTAAGATCTTGGCTCT

GGAATGGATGTTTGCCGGCATTTCCTGTTCAACTCTACCAGGCATGGCTGTTGTTCCTCTATACTGGTTT

GGCTTTGCGAGAAAATATTTTGAGAGCCAATGGAAGTGATATCCGTCCATGGTGGATTTACCATCATTAC

TGTGCTATGATTATGGCCCTTGTTAGTCTCACATGGGAGGTTAAAGGACAACCAAATTGTGCTCAGAAGC

AGAGAGGGGTGCAACTTTTCTTGCAATGGGCAATGATGCAAGGTGTTGCAATGCTTCTACAGAACAGATA

TCAACGTCAAAGACTTTATACACGTATTGCCTTAGGAAAGGCCAAGAGAATGGATGTTGTCTGGGGTGAA

ACAGCTGGCGTGCATGGCCAACTGTG

>FM895938.1 FM895938 Jatropha curcas embryo 71-95 (DAF) Jatropha curcas cDNA clone rjcpga0_002946, mRNA sequence

AGGATTCTATCCGTCGCCCGATGGGAATTGTACTTTAAGGCGGCCCTCGCGGCTCGTCCGCCGCGGGGGC

TTGGCCAACGGCACGTGCCTCTGGGGGCCGGATGGCCCCTACTGCGGGTCGGCAAACGGGCGACGGACAC

GCGCGTCGCTTCTGGCCCGGATTCTGACTTAGAGGCGTTCAGTCATAATCCAGCGCACGGTAGCTTCGCG

CCACTGGCTTTTCAACCAAGCGCGATGACCAATTGTGCGAATCAACGGTTCCTCTCGTACTAGGTTGAAT

TACCATCGCGACACCATCATCAGTAGGGTAAAACTAACCTGTCTCACGACGGTCTAAACCCAGCTCACGT

TCCCTATTGGTGGGTGAACAATCCAACACTTGGTGAATTCTGCTTCACAATGATAGGAAGAGCCGACATC

GAAGGATCAAAAAGCAACGTCGCTATGAACGCTTGGCTGCCACAAGCCAGTTATCCCTGTGGTAACTTTT

CTGACACCTCTAGCTTCAAATTCCGAAGGTCTAAAGGATCGATAGGCCACGCTTTCA

>FM895937.1 FM895937 Jatropha curcas embryo 71-95 (DAF) Jatropha curcas cDNA clone rjcpga0_002945, mRNA sequence

TAAAGTTGCTGCATGGGCTGCTGCTTCTGCTGCTAAAAGTCCTGCCTTGTGCCGTGCCCTTGCTCAAATC

AAGAGACAGGTCTCTTTGAAACCCAGTAGAAAACTTGAAGCCCAAGAAAGTCCGATCACAGAGGGGGGCA

CTACCCATTTACAGTCATCACCAGCAGAAGAGTACATAGAAACCAGTGCCATGCCATGGACAAATGCAAA

TGGAAAGTCAATGGATACTTCCGAAACCAAACTCGAAAATCCGCCAAAAAAGATGAAACTTCTGAACGAT

GGGTTCGAAGATCATGGAATGGATGTGATGAAACAGATGCCTACTGTGACAACTGTAGGAGATGGCCCTA

ATGGAAGGAAAGTAGAGGGGTTTCTTTACAAGTATAGCGAAGGTCAAGTGAACATAGTATGCATTTGTCA

TGGCAGCTTCTTCTCTCCGGAAGAATTCGTAAGGCATGCAGGCTGTGAAGATGTCGCCAACCCCATGAAG

CGTATCACAGTCTCCTCTTCTTTTTCCTTTTAGTAGCCCCAATTGGATCGGGAACAATGACATAC

>FM895936.1 FM895936 Jatropha curcas embryo 71-95 (DAF) Jatropha curcas cDNA clone rjcpga0_002944, mRNA sequence

TTACCCGCGCTTGGTTGAATTTCTTCACTTTGACATTCAGAGCACTGGGCAGAAATCACATTGCGTGAGC

ATCCTCAGGGACCATCGCAATGCTTTGTTTTAATTAAACAGTCGGATTCCCCTTGTCCGTACCAGTTCTG

AGTCGACTGTTCGACGCCCGGGGAAGGCCCCCGGAAGGGCCGTTCCCAGTCCGTCCCCCGGCCGGCACGC

GGCGACCCGCTCTCGCCGCGGGAGCAG

>FM895935.1 FM895935 Jatropha curcas embryo 71-95 (DAF) Jatropha curcas cDNA clone rjcpga0_002943, mRNA sequence

AGGATTCTATCCGTCGCCCGATGGGAATTGTACTTTAAGGCGGCCCTCGCGGCTCGTCCGCCGCGGGGGC

TTGGCCAACGGCACGTGCCTCTGGGGGCCGGATGGCCCCTACTGCGGGTCGGCAAACGGGCGACGGACAC

GCGCGTCGCTTCTGGCCCGGATTCTGACTTAGAGGCGTTCAGTCATAATCCAGCGCACGGTAGCTTCGCG

CCACTGGCTTTTCAACCAAGCGCGATGACCAATTGTGCGAATCAACGGTTCCTCTCGTACTAGGTTGAAT

TACCATCGCGACACCATCATCAGTAGGGTAAAACTAACCTGTCTCACGACGGTCTAAACCCAGCTCACGT

TCCCTATTGGTGGGTGAACAATCCAACACTTGGTGAATTCTGCTTCACAATGATAGGAAGAGCCGACATC

GAAGGATCAAAAAGCAACGTCGCTATGAACGCTTGGCTGCCACAAGCCAGTTATCCCTGTGGTAACTTTT

CTGACACCTCTAGCTTCAAATTCCGAAGGTCTAAA

>FM895934.1 FM895934 Jatropha curcas embryo 71-95 (DAF) Jatropha curcas cDNA clone rjcpga0_002942, mRNA sequence

CTGTAGGAACTTCGAAAAAATGATCTGTCCGTTTTGCAATTTCTTCAAAAAGCCAAAGCCCTTGCTGATG

AACTTTCTACTGCTGGCCGACCTATCTCCATTGCTGAATTCAATGCGATTATCTATTGCACTATTGGTCC

CGACTTTCATTCTGTCATAACCACTCTAAGTACTCGTCCTGAACCAGTTAAGTTTTATGAACTCCATGGC

CATTTGATTGCACATGAACTCCTTCCAAAAGCCCTTATCTCCCTATTGCAAATCTTTCTCTTAAAACCAC

AAATACTGCCCCTCTTTTGCCTACTGCAACCAATACCTCTTATCATCCAAATACCCAACCTTCTTCAAGA

CCACAAAATCAATTTCTCTCAAACAATTTCACTTTTAATCGTGGTTGTTCAGGACCCTGTCAGATTTATG

GTTATAAAATTATCCTGCCTTAACCTGTCGTAGATGCTACCAAAGAGATCAAGTAGCCCTAAAGTTTCCT

CAACATATTATAGCACCTCTAAGGTCTTCTTATAATCACTCCATTATGGCAAACTATTCCAACGTTGCAC

CTCACCATAAC

>FM895933.1 FM895933 Jatropha curcas embryo 71-95 (DAF) Jatropha curcas cDNA clone rjcpga0_002941, mRNA sequence

TTCCTATCATTTTCCTTCTAAATTATCACCCAATAGAACTCTTATGTTTCTCTAATTACGCTTTTCCAAT

AAAAAGAATCAAGAAAATGAGGAAAAGAGATCTTCAAGAAAGTTCCGATGTGGGTGGCGGCGGCGACGGC

AAAGGTGAATCTTTGTCAATGGGGAAAGGAAAATTATGGAATGAAGAGCAAGATAGTGGGGCTGTAGATG

AATTACTTGCTGTATTAGGTTACAAAGTCCGGTCATCGGACATGGCCGACGTTGCTCAAAAGCTTGAACA

ACTTGAGATGGTAATGGGTACAGCTCAAGAAGATGGGATTTCTCATCTTTCTACTGATACTGTACATTAT

AATCCTTCAGATCTCTCCGGTTGGGTTCAAAGCATGCTTTCTGAGTTTAATAATTCCCCTTTCGGTCTTG

ATTCCTCTGATATCCTTCAAAATCAAGATTCTACAATTTCTTTCTCTAGGAATCCACAACCAGGAATTTT

CAGTGACGACTCCGAATACGATCTTCGTGCAATTCCTGGTGTGATTGCTTATCCACCG

>FM895932.1 FM895932 Jatropha curcas embryo 71-95 (DAF) Jatropha curcas cDNA clone rjcpga0_002940, mRNA sequence

CTCAGTGATAAAGGAGGTAGGGGTGCAGAGACAGCCAGGAGGTTTGCCTAGAAGCAGCCACCCTTGAAAG

AGTGCGTAATAGCTCACTGATCGAGCGCTCTTGCGCCGAAGATGAACGGGGCTAAGCGATCTGCCGAAGC

TGTGGGATGTAAAAATGCATCGGTAGGGGAGCGTTCCGCCTTAGAGGGAAGCACCCGCGCGAGCAGGGGT

GGACGAAGCGGAAGCGAGAATGTCGGCTTGAGTAACGCAAACATTGGTGAGAATCCAATGCCCCGAAAAC

CTAAGGGTTCCTCCGCAAGGTTCGTCCGCGGAGGGTGAGTCAGGGCCTAAGATCAGGCCGAAAGGCGTAG

TCGATGGACAACAGGTGAATATTCCTGTACTACCCCTTGTTGGTCCCGAGGGACGGAGGAGGCTAGGTTA

GCCGAAAGATGGTTATCGGT

>FM895931.1 FM895931 Jatropha curcas embryo 71-95 (DAF) Jatropha curcas cDNA clone rjcpga0_002939, mRNA sequence

CATTTGGAACAAAATATATAAATTCATAAATGAATTCCTGTCATGTCAATTAATATTATCTAATGATGTA

CAATTTACCACTTAAAGCAATGCAAGTTTCTCATCCAAGAATTACAACATGAGACCTTAAAGATCATCCA

GGTTAATCATCCATTCACTGCACCAATAAGTGTCTAAAGACTTCGTTTTAGAATCAGCTTCTGAAGAAGA

GTCACGATCTGCCAAGGCTTTGTCTAATGAATCTGCTAAATTAGCATCCCATAAAGCCAATCTGCAACCT

TTGTAGTAGGTATCATAACTTGCAGCTCTTGCACCTTCTGAGATCATCTCCCTTAAAGGTCCAAAAATCT

CTGGACTATTATTCAGGACATCTAGAAAAGTTTTAGACATACCCCGTTGCCAGAAAACCACAATGGCACA

GCTCTTGTCTACTAATTTAACATCAAATTCCTCGGAAAAAATTTTATGAGACTCTTGAAGCAGGCTCTTC

AGTAGCCCTGCTGACATCCCATCCCTAAATCCCCACAAGAAAACCAAGTCCTC

>FM895930.1 FM895930 Jatropha curcas embryo 71-95 (DAF) Jatropha curcas cDNA clone rjcpga0_002938, mRNA sequence

ATTCCAAAGGATTCTATCCGTCGCCCGATGGGAATTGTACTTTAAGGCGGCCCTCGCGGCTCGTCCGCCG

CGGGGGCTTGGCCAACGGCACGTGCCTCTGGGGGCCGGATGGCCCCTACTGCGGGTCGGCAAACGGGCGA

CGGACACGCGCGTCGCTTCTGGCCCGGATTCTGACTTAGAGGCGTTCAGTCATAATCCAGCGCACGGTAG

CTTCGCGCCACTGGCTTTTCAACCAAGCGCGATGACCAATTGTGCGAATCAACGGTTCCTCTCGTACTAG

GTTGAATTACCATCGCGACACCATCATCAGTAGGGTAAAACTAACCTGTCTCACGACGGTCTAATCCCAG

CTCACGTTCCCTATTGGTGGGTGAACAATCCAACACTTGGTGAATTCTGCTTCACAATGATAGGAAGAGC

CGACATCGAAGGATCAAAAAGCAACGTCGCTATGAACGCTTGGCTGCCACAAGCCAGTTATCCCTGTGGT

AACTTTTCTGACACCTCTAGCTTCAAATTCCGAAGGTCTAAAGGATCGATAGGC

>FM895929.1 FM895929 Jatropha curcas embryo 71-95 (DAF) Jatropha curcas cDNA clone rjcpga0_002937, mRNA sequence

ATTCGCATAGTCTTTCTGTTGGGAGATGTAGATGCTAGTCAAGGTGCCTTCCAGTATTTTGGACTCAAAG

AGGATCAAGTGCCTCTCATAATCCTACAAACAAATGAAGGACAAAAATATCTGAAGCAACACTTGGAACC

TGATCATATTGCTCCTTGGGTGAAGGAATACAAGGAAGGCAAGGTGCTACCATTCAAGAAATCTGAACCT

ATCCCGGAAGTTAACAATGAGCCTGTGAAAGTTGTGGTGGCTGATAGTCTCCAGGACATTGTTTTCAACT

CTGGAAAAAACGTTCTTCTAGAGTTTTATGCTCCATGGTGTGGACACTGTAAAAAATTGGCTCCAATCTT

GGATGAAGTTGCCGTCTCATACCAAAATGATCCTGATGTAGTCATTGCAAAGCTTGATGCAACTGCAAAT

GATATCCCGAGTGATACCTTTGATGTCAAGGGATATCCCACAGTATACTTCAGGTCTTCAAGTGGGAATT

TGGTGCAGTATGATGGGGATAGGACGAAAGAAGATATTATTGATTTCATCGAAAAGAA

>FM895928.1 FM895928 Jatropha curcas embryo 71-95 (DAF) Jatropha curcas cDNA clone rjcpga0_002936, mRNA sequence

TGCGTGTCTGGCTAGGAACATTCAATACTGCAGAAGAAGCTGCAAGAGCATATGACGCGGAGGCACGTAG

AATTCGTGGCAAGAAAGCTAAAGTTAACTTCCCCGAGGAAGCTCCGCATGCTTCACCAAAACGTCCATCT

AAGGCAAACTCTCAGAAATCACTTGGCAAAACAAACTTGGCTGAGAATTTGAATTACTTGGACAATCCAG

AACAGGACTACTTCAATTCTATGGGCTTTGTTGAAGAGAAACCGCCAGTGAGCCAGTTTGGTATAATGGA

CTCTCTTCCTGTTAATGGAGATGCTGTAGTAAACTCCATTACTCCAAGTGACAATGTTCCCATGTATTTC

AATTCTGATCAAGGGAGCAACTCTTTTGAGTGTTCTGACATTGGATGGGCAGAGCAGGCTGCAAAGACTC

CTGCAAATCTCATCTGTTCTCTCAGCTACTCCTGAAATTGACGAATCTCTATTTATAGAGGATGCTAACC

CCAAAAAGAAGCTGAAATCTGACTCTGGCAATGAGGCGCCTGCAGAAGAAAACAATGGAAAATCTCT

>FM895927.1 FM895927 Jatropha curcas embryo 71-95 (DAF) Jatropha curcas cDNA clone rjcpga0_002935, mRNA sequence

GATTTTTCAATGATTTCCATTTTCCATTTTTCCCCATAATTAACAGCAAAAAGATTCAAAAACCCAAAAA

CAAAATTTACATTGATAAGTCTTCTTAGTTCTTAGCCTAGGCCTATGAGACAAGTAAAGAAAAAAAAAAA

AACATTTCTTTGCCATTCTCTGCTAAAAGCTTTCTCTGCTCTTAGTAGACAACCGTTTCAAGAATTCATA

AGTAGTGATCATAGTCGTTGCAGACATAGACATTGACACCCATCTTGGTCCTAATCCTCTATAACAAGCT

GCAAATCCACCTTCCTTGACGAGATTCTTAACTGTTTGCATAACCGTTAATGGTTGTCTTCTTCCATTTT

GTTCTCCATCCAAAACCTGCATTCTTGTCTTAATCGTGTCAAGTGGCATTGTAATCATAACCGACATTCC

ACTAGCCATAGCTGCACATAACCCTTGCACTGCCAACATTGCCTTTGAATCAGGCCTATAAGCACACCTA

CCACCACCAGCATTAACAAA

>FM895926.1 FM895926 Jatropha curcas embryo 71-95 (DAF) Jatropha curcas cDNA clone rjcpga0_002934, mRNA sequence

GATTAATGAAAACATCCTTGGCAAATGCTTTCGCAGTTGTTCGTCTTTCATAAATCCAAGAATTTCACCT

ACTGACTATGAAATACGAATGCCCCCGNACTGTCCCTGTTTAATCATTACTCCNGATCCCNGAAGGCCAA

CACAATAGGACCGACAATCCTATGATGTTATCCCAGTGCTAATGTATACAGAGCTGTAGGCTTGCTTTGA

GCACTCTAATTTCTTCAAAGTAACAGCGCCGGAGGCACGACCCGGCCAGTTAAGGCCAGGAGCGCATCGC

CGGCAGAAGGGACGAGCCGACAGGTGCACACCGCGAGGCGGACCGATCGACCCAACCCAAGGTCCAACTA

CGAGCTTTTTAACTGCAACAACTTAAATATACGCTATTGGAGCTGGAATTACCGCGGCTGCTGGCACCAG

ACTTGCCCTCCAATGGATCCTCGTTAAGGGATTTAGATTGTACTCATTCCAATTACCAGACTCATTGAGC

CCGGTATTGTTATTTATTGTCACTACCTCCCCGTGTCAGGATTGGGTAATTTGCGCGCCTGCTGCCTTCC

TTGGATGTGGTAGCC

>FM895925.1 FM895925 Jatropha curcas embryo 71-95 (DAF) Jatropha curcas cDNA clone rjcpga0_002933, mRNA sequence

ATTCTCATCTTCTTCATTTGGATTAAGCGATGGATACTGATTCTGTGTTTAGCAATATCGTTCGAGCTCC

TGAAGATCCTATTTTAGGGGTTACTGTTGCTTATAACAAAGATACAAGCCCTAACAAGTTGAATTTGGGT

GTTGGTGCTTATCGAACTGAGGAAGGGAAGCCTCTCGTTTTGAATGTGGTGAGGAAAGCGGAGCAGATAC

TAGTAAATGACCAGTCTCGGGTCAAAGAGTATCTTCCTATTGTTGGACTTGCTGATTTTAATAAACTGAG

TGCCAAGCTTATCTTAGGAGCTGATAGCCCCGCTATACAAGAGAACAGAATAACTACTGTGCAAGGCTTG

TCTGGTACCGGTTCTTTGAGAGTTGGAGGGGAGTTTCTTGCAAGGCATTACCATCAACTGACAATATACA

TTCCACAGCCAACATGGGGAAATCATACCAAAGTCTTCACTCTTGCAGGGTTGTCTGTAAAGACGTACCG

GTACTATGATCCAGCAACACGTGGGCTGGATTCCCAAGGCTTGCTAGAGGATCTG

>FM895924.1 FM895924 Jatropha curcas embryo 71-95 (DAF) Jatropha curcas cDNA clone rjcpga0_002932, mRNA sequence

ATTCTCATCTCTGCAAGGACATATCTGTTGACTCTGTTGCTAGCATCTTGGCCTTGGCCGACAGTCATCA

TGCTACAGAATTGAAAGCTGTTTGCCTAAAATTTGCTGCTGAAAACCTTGCAGCTGTAATGCGGTCAGAT

TCCTTCGAGCATCTCAGGGAGAACTGCCCATCTCTGCAATCAGAGCTACTGAAGGCAGTGGCTGGTTGCG

AGGAAGATTGCAGCAGTGGAGGTGGAAAGTCTCGCAGTGTGTGGGCTCAACTCTCAGACGGCGGTGATAC

CAATGGCAGGCGGGTCAGACAAAGAACCTAATATCTAATCATTAGTCTTGTAAATGCAGATGATGATGGT

ATAAGAAAAAAAAAAAAGAAAAGAAAAAGGAAAGCAGAACTCAAAAACAGAAACCTTTTGTAATGTGGCA

GGAGTTTCTGCCAGTTTTCCGTTTATCATAAAAGGTAGCTGGCATA

>FM895923.1 FM895923 Jatropha curcas embryo 71-95 (DAF) Jatropha curcas cDNA clone rjcpga0_002931, mRNA sequence

GCAAAGGATCTATCCGTCGCCCGATGGGAATTGTACTTTAAGGCGGCCCTCGCGGCTCGTCCGCCGCGGG

GGCTTGGCCAACGGCACGTGCCTCTGGGGGCCGGATGGCCCCTACTGCGGGTCGGCAAACGGGCGACGGA

CACGCGCGTCGCTTCTGGCCCGGATTCTGACTTAGAGGCGTTCAGTCATAATCCAGCGCACGGTAGCTTC

GCGCCACTGGCTTTTCAACCAAGCGCGATGACCAATTGTGGGAATCAACGGTTCCTCTCGTACTAGGTTG

AATTACCATCGCGACACCATCATCAGTAGGGTAAAACTAACCTGTCTCACGACGGTCTAAACCCAGCTCA

CGTTCCCTATTGGTGGGTGAACAATCCAACACTTGGTGAATTCTGCTTCACAATGATAGGAAGAGCCGAC

ATCGAAGGATCAAAAAGCAACGTCGCTATGAACGCTTGGCTGCCACAAGCCAGTTATCCCTGTGGTAACT

TTTCTGACACCTCTAGCTTCAAATTCCGAAGGTCTAAAGGATCGATAGGCCACGCTTTCACGGTTCGTAT

TCGTACTGGAAATCAGAATCAAACGAGC

>FM895922.1 FM895922 Jatropha curcas embryo 71-95 (DAF) Jatropha curcas cDNA clone rjcpga0_002930, mRNA sequence

GTCAAACCAGAATTCTCTTTGGCATGGATTAAGAGTTTGATAGTTAACAAACAAGCATTAGGGTTCAGAA

ACTTCTCAACACATTTGTATTCTAATACAAATCCACATGATTTACTTCCATGCCTCCATCACAATATCAA

GTTCCCGATAGAAAATCATGTTACCACCTGCTGTATGGAAACTAGCAAACAAAATGACAGACATCCTCCT

GGCATCTATCTGCTACGCCTGGGTGACAAGTCAGCAGATCTAGACCGAGATCCAGAAGGTGACCTTGATG

CTCTACCAGGTGATTGCCTCCAAGCTCGTGCTTCCTCAGAATCATAGGCAGGTTTCTCGCCATAACCATT

GCCATTTCCATTTAGATCATCTACACCATATCCAGGAGAATATGACCTGCGAATGTGGCCACCATCTCGA

TCTGGTGGAGGGCCTCCAGGAGACCTTAGATGCTCTGCATGCCTTCTACCTGGGGAAGCAGAGTAGTCTC

TCCGTCTTGGGGCAGGAGAGTATGACCTTGAGCGGTATCGAGACCTAGAACTCATATGATGTCGAGGGGA

TCTTGATCGGGATAGTGAACGTGACCG

>FM895921.1 FM895921 Jatropha curcas embryo 71-95 (DAF) Jatropha curcas cDNA clone rjcpga0_002929, mRNA sequence

CTTAGCAGTCGCTTTGATCTTTATCTCACAACCACCTGCCAAGCTTTTGCCCTACACACGGCGATAATGA

AGAATCTTAGTGTTTTCTTCTTCGCTCTCCTCCTCCTCGCTTCCCCTCTCCTTCAAGTCGCTAGGTGTCA

GTCAGATTCTGATGTGGAAGTCACAGACGCTGCTGAAGAAGTCAGTGATCTTGGGATTGTTGGTGAGGAT

GCTCAGGATTTTGGTGATGGGACTTTTAGCCCGGCTCCTGGAGTAGAGACTGTTTGTGTTTTCCCCAAAA

ACAGTGCCCGTGTGGTGCCAGCTGGGGAAGAGGCTGAACTACTTGTTGGAATTAAGAATGATGGGGAGTC

GAGCATAAATATCATTGCAGTTAAGGCCAGTGTTCACCTTCCTTTTGATCATCGTCTGCTGGTTCAGAAT

CTCACTGCTCAGGCCTTCAACAATGCAACTGTTCCTCCCTCAACACAGGCGACTTTCCCATACGTTTTTG

CTGTCAGCAAGTACTTGCAGCCTGGCAACTTTGATCTTGTAGGATCCATTATTTATGAGATAG

>FM895920.1 FM895920 Jatropha curcas embryo 71-95 (DAF) Jatropha curcas cDNA clone rjcpga0_002927, mRNA sequence

ATTCGATGCCCGGGCATTGAGAAGGAAGGACGCTTTCAAAGGCGAAAGGCCATGGGGAGATACCGTCTGT

GATCCATGGATCTCCGATCGGGAAACCGTATCCAAGCTCCGTGGCTAGTCTGCGCTCTTTGGACTTTTCA

AACTTAGCGAACTGAAACATCTGAGTAGCTAAAGGAAGGGAAATCAACCGAGACCCCGTTAGTAGCGGCG

AGCGAGAGCGGATTGGGGGTTTGAAGAAAAACAAAGACGTCTTGTCAGAGCCTCAGCAAAGTGTTCACTT

CTTTTTCGCCAGGTTTCATTCGATTTGTTGTGGATTGGATGATGGAAAAACCAAGCAAGCCGCCGCCCTT

GATTTGATACAAGGAGCGGGCGCAGTGAACTGTAATTGTGAAAAGGTTGGAAGATCTGGCCAAAGAAGGT

GATAGCCCTGTAGATTCGTTCCCATGGTTCGATCCTTCCCAGTAAAACGCGGCGTGTTCGAATTCTGATC

GCTTTTACGCGAGAAAGGGGGACCACCCTCTAAGCCTAAGTATTCCTCAATGACCGATAGCGTACAAGTA

CCGTGAGGGAAAGGTGAAAAGAACCCTATTTAGGGAGTGCAATAGAG

>FM895919.1 FM895919 Jatropha curcas embryo 71-95 (DAF) Jatropha curcas cDNA clone rjcpga0_002926, mRNA sequence

ATTAAAACCATTACTACTTCAACTTGTTTTGCTTGTCATTGTTTCATCAGGTACTTCAAATCAAGTTATC

GACAAATTTCTCCAATGTCTTCCTCATCAAATTTACTGCTCCAAACCCATAAATAAAGCCATTTATACCC

CAGACAATCCTTCTTTTGAATCTGTTTTACAAGCCTACATTAAAAACCTAAGGTTTTTAAACAATACCAC

CCCAAAACCACTTGCTATCATCACCGCCTTGCATGAAGCCCATATCCAATCAACTATTATCTGCGCTAAG

TCCAACGGCTTGCAACTCCGGATCAGGAGCGGCGGCCATGATTTCGAGGGTCTTTCTTACAGATCTGACG

TCCCGTTTGTAGTTCTTGACTTGTTCAATCTCCGAGACATAGACATAGATATGGCTACAGAGACTGCATG

GGTTCNAGCCGGGGCAACAACTGGTGAACTTTACTATAACATCGCCAAGAAAAGCAAAGTCCACGCCTTT

TCGGCAGGAGTTTGCATTGCACTTGGTTTAGGCGGTCATTTTAGCGGAGGAGGGTATGGAAATATGATGA

GAAAG

>FM895918.1 FM895918 Jatropha curcas embryo 71-95 (DAF) Jatropha curcas cDNA clone rjcpga0_002925, mRNA sequence

TTTTGACTAAGGAAAACATATATAGATTATAAAGCTAAAGACATTAATGTTTTGGGTATAAAAGAGTTCA

CAAAACCTTAATACCAACATACATTGATTGATAAAATAAATAATAACCCCTAACTATCTTCACCACCATT

CTCTCCAAACCAAGAGGACAAGGGAAAGAAAAAAACTCCAAACCAATAATAGTTCATAAAGGACTAACGA

AAATAAATAAATTAAAAAAAAATAAATGACTCCCAAAACTTCAAAACCCCTCCCTTTCTGTTCCTCTCCA

AAGATATCAGCAAATTCTCCCTCCAAACTTCAATCGAACTCTACTGTTGCTTGCACTCTGGAGGCCTCTC

ACCCTGCTGACCATCCCCAGCTTGTGTACTTCCACCCTTCTTATGCCCGGCTGACAGCTTCTTTAAGGTC

TTGCATTCATCCTCGTCCTCATCCTCATCTTCATCCTCTTCATCCTCATCATCATCATCAATATCTTCAT

CGTCGTCGTCGTCATCATCATCATCATCATCTATTCCA

>FM895917.1 FM895917 Jatropha curcas embryo 71-95 (DAF) Jatropha curcas cDNA clone rjcpga0_002924, mRNA sequence

CGAATCCATGTGTGCGAGTCAACGGGTGAGTAAACCCGCAAGGCGCAAGGAAGCTGACTGGCGGGATCCC

CCTGAGGGTTGCACCGCCGACCGACCTTGATCTTCTGAGAAGGGTTCGAGTGAGAGCATGCCTGTCGGGA

CCCGAAAGATGGTGAACTATGCCTGAGCGGGGCGAAGCCAGAGGAAACTCTGGTGGAGGCCCGCAGCGAT

ACTGACGTGCAAATCGTTCGTCTGACTTGGGTATAGGGGCGAAAGACTAATCGAACCGTCTAGTAGCTGG

TTCCCTCCGAAGTTTCCCTCAGGATAGCTGGAGCTCGGGACGAGTTCTATCGGGTAAAGCCAATGATTAG

AGGCATCGGGGGCGCAACGCCCTCGACCTATTCTCAAACTTTAAATAGGTAGGACGGCGCGGCTGCTTCG

CTGAGCCGCGCCACGGAATCGAGAGCTCCAAGTGGGCCATTTTTGGTAAGCAGAACTGGCGATGCGGGAT

GAACCGGAAGCCGGGTTACGGTGCCCAACTGCGCGCTAACCTAGAACCCACAAAGGGTGTTGGTCGATTA

AGACAGCAGGACGGTGGTCATGGAAG

>FM895916.1 FM895916 Jatropha curcas embryo 71-95 (DAF) Jatropha curcas cDNA clone rjcpga0_002922, mRNA sequence

GGATTCTATCCGTCGCCCGATGGGAATTGTACTTTAAGGCGGCCCTCGCGGCTCGTCCGCCGCGGGGTGC

TTGGCCAACGGCACGTGCCTCTGGGGGCCGGATGGCCCCTACTGCGGGTCGGCAAACGGGCGACGGACAC

GCGCGTCGCTTCTGGCCCGGATTCTGACTTAGAGGCGTTCAGTCATAATCCAGCGCACGGTAGCTTCGCG

CCACTGGCTTTTCAACCAAGCGCGATGACCAATTGTGCGAATCAACGGTTCCTCTCGTACTAGGTTGAAT

TACCATCGCGACACCATCATCAGTAGGGTAAAACTAACCTGTCTCACGACGGTCTAAACCCAGCTCACGT

TCCCTATTGGTGGGTGAACAATCCAACACTTGGTGAATTCTGCTTCACAATGATAGGAAGAGCCGACATC

GAAGGATCAAAAAGCAACGTCGCTATGAACGCTTGGCTGCCACAAGCCAGTTATCCCTGTGGTAACTTTT

CTGACACCTCTAGCTTCAAATTCCGAAGGTCTAAAGGATCGATAGGCCACGCTTTCACGGTTCGTATTCG

TACTGGAAATCAGAATCAAACGAGCTTTTACCCTTTTGTACCACACGAGATTTCT

>FM895915.1 FM895915 Jatropha curcas embryo 71-95 (DAF) Jatropha curcas cDNA clone rjcpga0_002921, mRNA sequence

CTATCCTTCAGTGTTTTTTTGCGTTTTCCTCTAGTTTTTTCCATCAGAACTATCATCTGAAGAAGGAAGC

TTTCTTGATTTCTTCTTCTTTCCTGCATCCTTTCCAACAACATCCTGAATTTTGTTTTCAAAAGTTTCAC

CACAATCTACAATTTTTACAGGGCGAATGGGTTTGCCATCACCTGTTCCCACCTGTTCAATTTTTTTCAC

GACATCTAATCCCTTTATGACCTTTCCAAAAACCACATGTTTCCCATCAAGATGAGGCTGACGCTTAAAA

GTTACAAAGAATTGGGAACCATTTGTGTTTGGACCACTATTTGCCATGGAGAGAAGACCTGGTTCATCAT

GCTTCAATATAAAATTCTCATCTGCAAACTTCCCTCCATATATACTTTCTCCACCAGTGCCATTTCCCTT

TGAAAAGTCACCACCTTGGGCCATGAATCCTCTGATAATTCGATGGAAGAAAGATCCTTTATAGTGAAGA

GGTTTCCCAGTTGTTTTCCCAATGCCCTTCTCACCTGTACAAAGAGCCCGGAAATTCTCCGCAGTCTTGG

GG

>FM895914.1 FM895914 Jatropha curcas embryo 71-95 (DAF) Jatropha curcas cDNA clone rjcpga0_002920, mRNA sequence

TTTTTTGTTTGTTTCGGAGCCGTTTTGGCGGGGCGTACTAAACCCACTACGTACCACACCACCGGGCGGC

TCGCCTGAATGCCGAGTCTTTCTCCGCCGCCAACTCGACGTCGTCGTCACCTGCAAGATAAGGCCAAAAA

CTGGACTTTACTAAACAAGCGAGAAAAGCCCCTTTCTCTGTTATTAGTAAAGTAAAGCGCGCTAGCTGCA

ATCTTTCTAAAGCGCACACTAGAAAGTGCTTCGAAAGGCGCCGGCTCCCCTCTTACTGACAGCACAGCTA

CGTGCTGGCACTCAATTAGTAGCGCTGGCACGTCACTCGGCTCCTCGGCTCACTTCGGTTGCAAAGACTT

TCTCCTTAGGCGCATGTCTCAGCAACACAAAACGAGGGTTTCGCTCGTTATAGGACTTGACCAAACATCT

CACGACACGAGCTGACGACAGCCATGCAGCACCTGTATGAAAGTCAGTACCATCCCGTTAAGGACAGGTT

TTGTTGTTCATATGTCAAGGGCTGGTAAGGTTATGCGCGTAGTATCGAATTAAAACACATGCTCCAACGA

TTGTGCAAGCCCCCGTCATTCCTTTGAGTTTCGGTCTTGC

>FM895913.1 FM895913 Jatropha curcas embryo 71-95 (DAF) Jatropha curcas cDNA clone rjcpga0_002919, mRNA sequence

GTGATCAACCGGATTCATTTCACTCAGAAAAAGATGAGCACGTATTTCAAGGTGCAAAGNCAAAAAACCA

AAATGAAAAAGTATTTTAGCAACTTTAGCCATCCCTAAATCAAACCCCCAGAAAACTAGACAAAAAAAGA

TTAGAAGTCTAGAATTCCTGAGAAGCAGATCCAATTTCGCCGTTTGCCCTTGCCCATCTTCTTAGGAAGG

AGTGTCTGGTGAATATTTGGCAATACACCACCATTGGCAATGGTAACTGAACCCAGAAGTTTACTCAGCT

CCTCGTCATTCCTCACAGCAAGCTGAATATGCCTTGGCACAATACGATTCTTCTTATTATCTCTCGCTG

>FM895912.1 FM895912 Jatropha curcas embryo 71-95 (DAF) Jatropha curcas cDNA clone rjcpga0_002918, mRNA sequence

CTGTCCAGCTTCTTCGCGTCATCTCCGCTGCCTTTGCCATTGTCAGTCGGACCCGGCCACTTCCGGCGCG

CGTAGCTCCGATCCAGCGCGAGCAAACTCCCGATCTAGCAACAAATTCGAGAAAAGATTATATTTTGGGG

TAACAAAATTAATTTTAATAATTTTAGTTACTTGAATTAATTTTATTATTGTAGTTAATTAATTTAGTTA

ATTTAATTTAGTAATTGTGAGTAGTTAAATTAAGTTAATTTAACTACTCACAATTACTA

>FM895911.1 FM895911 Jatropha curcas embryo 71-95 (DAF) Jatropha curcas cDNA clone rjcpga0_002917, mRNA sequence

CAAAGGATCTATCCGTCGCCCGATGGGAATTGTACTTTAAGTGCGGCCCTCGCGGCTCGTCCGCCGCGGG

GGCTTGGCCAACGGCACGTGCCTCTGGGGGCCGGATGGCCCCTACTGCGGGTCGGCAAACGGGCGACGGA

CACGCGCGTCGCTTCTGGCCCGGATTCTGACTTAGAGGCGTTCAGTCATAATCCAGCGCACGGTAGCTTC

GCGCCACTGGCTTTTCAACCAAGCGCGATGACCAATTGTGCGAATCAACGGTTCCTCTCGTACTAGGTTG

AATTACCATCGCGACACCATCATCAGTAGGGTAAAACTAACCTGTCTCACGACGGTCTAAACCCAGCTCA

CGTTCCCTATTGGTGGGTGAACAATCCAACACTTGGTGAATTCTGCTTCACAATGATAGGAAGAGCCGAC

ATCGAAGGATCAAAAAGCAACGTCGCTATGAACGCTTGGCTGCCACAAGCCAGTTATCCCTGTGGTAACT

TTTCTGACACCTCTAGCTTCAAATTCCGAAGGTCTAAAGGATCGATAGGCCACGCTTTCACGGTTCGTAT

TCGTACTGGAAATCAGAATCAAACGAGCTTTTACCC

>FM895910.1 FM895910 Jatropha curcas embryo 71-95 (DAF) Jatropha curcas cDNA clone rjcpga0_002916, mRNA sequence

ATTCGAGAGAACTAGTCTCTCTTTCAATGGCAGCCGCTGCCGGTTCATCAATCTCCATGCAGTCTCGTCC

TAGACTGGCTGCAACCAGAATCTCTAGCCTCAAATCTTTTTCGTTTCCAAGCCAGAGAAGTTTCCTGTCT

TTCAGAGTGCAGCCAGTTCCAGCATCTCGCCTTCGGATTTCTTGTGCTGCCAAACCAGAAACAGTAGAAA

AGGTGTGTCAAATAGTGAAGAAGCAGCTGGCATTAGCCCCAGAAGCTGTTGTTACCGGCGAGTCAAAATT

TACAACACTAGGAGCAGATTCTCTTGATACAGTTGAGATTGTGATGGGACTGGAGGAGGAATTCGATATC

AGTGTGGAAGAGGAGAGTGCCCAGAGCATCACAACCGTTCAGGAAGCTGCTGATCTTATTGAAAAACTTG

TTGAGAAGAAGGACGCTTAGAAGATCAGTCTAGGAAATCCTTCAATTCCTTTCCCCCTGGCGCCCCCCCC

CCCCCCCTCAAACATAATTGCAGTTATGCTTAGTGTTAGCCCGCCATATGATTGTGCCAGGCAAT

>FM895909.1 FM895909 Jatropha curcas embryo 71-95 (DAF) Jatropha curcas cDNA clone rjcpga0_002915, mRNA sequence

ATTCCTCTTTCTTCTCTCTTGCGTTTCTTTCTTAACTTTTCAAGGGTTGTTACTGTTTTTGTTTCTTTAA

GGCAGCCATGAATGCTCTTGCAGCTACCAACAGGAACTTCCGCAATGCAGCTCGCATTCTTGGTCTTGAT

TCTAAGCTTGAAAAGAGCCTTTTGATCCCTTTTAGAGAAATCAAAGTGGAATGCACCATCCCGAAAGATG

ATGGTAGTCTGGTTTCTTATGTTGGATTTAGAATCCAGCATGACAATGCTCGTGGCCCGATGAAAGGCGG

CATCAGATACCACCCTGAGGTTGATCCTGATGAGGTGAATGCTTTGGCTCAACTAATGACATGGAAGACA

GCAGTAGCAGACATTCCATATGGTGGAGCTAAGGGCGGTATCGGCTGTGATCCTAGGGAGTTGAGTTTCA

GTGAATTAGAGCGCCTTACTCGGGTTTTTACTCAGAAAATCCATGACCTTATAGGAGCTCACAGGGATGT

TCCTGCTCCTGATATGGGAACTAATGCACAGACAATGGCCTGGATGTTGGATGAATACTCAAAGTTTCAT

GGTCATTCACCTGCTGTTGTTACTGGAAAAACCATTGATCTTGGGGG

>FM895908.1 FM895908 Jatropha curcas embryo 71-95 (DAF) Jatropha curcas cDNA clone rjcpga0_002914, mRNA sequence

ATTCCTTTCTGTTTCTGAGATGGATCTTTCTTAGATTTCTTCAGATCCCTTAAATTTCACGAAAGCAGAA

AGAGAGAAACAGAAAGAAACCAAAAAAGGAAGATGACAACTCCAGCTAGGAAGAGGCTGATGAGGGATTT

CAAGAGGCTTCAGCAAGATCCACCTGCCGGCATCAGTGGCGCTCCTCAAGAGAACAATATCATGCTCTGG

AACGCTGTCATTTTTGGGCCTGATGATACTCCTTGGGATGGAGGTACGTTTAAATTGACACTGCAATTCA

CAGAGGATTATCCCAACAAACCTCCAACAGTCCGTTTTGTCTCAAGGATGTTTCATCCAAATATATATGC

AGATGGAAGCATTTGTTTAGATATTTTACAAAATCAATGGAGTCCAATATATGACGTTGCTGCTATACTT

ACTTCTATCCAGTCCTTGCTTTGTGATCCAAACCCAAACTCGCCTGCAAACTCTGAAGCTGCTCGCATGT

TCAGTGAAAATAAGCGGGAGTACAACCGAAGAGTGCGGGAAATAGTAGAACAGAGCTGGACTGCTGACTG

AAAACCCAATGAAAGCAGCAGCAGCCC

>FM895907.1 FM895907 Jatropha curcas embryo 71-95 (DAF) Jatropha curcas cDNA clone rjcpga0_002913, mRNA sequence

TGCCAAAAACCTGTAAATCAAACATCGATTACCAAACCAGCAATAAAATCTTATCAACTCAAGGCCGTAG

TATTGCTGCAACGACCGACAGGCATTGATGGATCCCGAGAAATGTTCAAAGGCAATCAAACACCAGTAAT

TGAAGCTTACACCCGTCAATTATTATTGAATTAATATTTCACAAGTTGAAGGGTTCGTGTGATTTTGAAC

ACGTCCCAACTAATTGGACACATTGGGTGCTTATTTACAAAGTCAGCATGGCTCCATTTCAGGTAAAACT

TTAAACCTTTCAATTGCTGATATCGAAATATCAAGGATCGTCCGGGAAGTG

>FM895906.1 FM895906 Jatropha curcas embryo 71-95 (DAF) Jatropha curcas cDNA clone rjcpga0_002911, mRNA sequence

TCACGGGTGAGTAAACCCGCAAGCGCAAGGAAGCTGACTGGCGGGATCCCCCTGAGGGTTGCACCGCCGA

CCGACCTTGATCTTCTGAGAAGGGTTCGAGTGAGAGCATGCCTGTCGGGACCCGAAAGATGGTGAACTAT

GCCTGAGCGGGGCGAAGCCAGAGGAAACTCTGGTGGAGGCCCGCAGCGATACTGACGTGCAAATCGTTCG

TCTGACTTGGGTATAGGGGCGAAAGACTAATCGAACCGTCTAGTAGCTGGTTCCCTCCGAAGTTTCCCTC

AGGATAGCTGGAGCTCGGGACGAGTTCTATCGGGTAAAGCCAATGATTAGAGGCATCGGGGGCGCAACGC

CCTCGACCTATTCTCAAACTTTAAATAGGTAGGACGGCGCGGCTGCTTCGCTGAGCCGCGCCACGGAATC

GAGAGCTCCAAGTGGGCCATTTTTGGTAAGCAGAACTGGCGATGCGGGATGAACCGGAAGCCGGGTTACG

GTGCCCAACTGCGCGCTAACCTAGAACCCACAAAGGGTGTTGGTCGATTAAGACAGCAGGACGGTGGTCA

TGGAAGTCGAAATCCGCTA

>FM895905.1 FM895905 Jatropha curcas embryo 71-95 (DAF) Jatropha curcas cDNA clone rjcpga0_002910, mRNA sequence

ACCAAATCTTGAACATCTGATTTTTAATAATTAGGCGTCTTTTACCAATCCAACCAATCACAACAAAGCA

TCTCCAGAATTTTGAGACAACAGGGCACAATGCAACACTAAACATAATCATAACCAACCAAAAATCACAG

TTTTCATTTCAAACGTCTAAAAGCAGAGGCAAGAAAAAGTGATTTTAATCCTGAACATTTTGGGCCACAG

TGACTATTTGAGTTTGATATGGTCTTCAGGCATAGTTCAACCGGAAAATATCATTCAACACGTAAAAGCT

CCCCTGTGGTGTTGGCATCAAATGGAACATCTGACTGAACTTGAGAGCGTGTTGTTCGCCGGCGAGCTGG

AGGTTACCAGAGACAAAGACAAGCATGCCACCGGCAGGACCAGATGGCTGACAATCGACGGTTGTGATTC

TATGCTGGCACTGTTTGAAAGGAAGGCTAGTGAGCTTAGCGACGATGTTCGGAGAGCCTTGGATCTTCTC

GCCTTCAAAGGTGAGCATCGATCCGTCTTGGTAGAGGTTAGCCAAGCCAGCAC

>FM895904.1 FM895904 Jatropha curcas embryo 71-95 (DAF) Jatropha curcas cDNA clone rjcpga0_002909, mRNA sequence

GGATTCTGCTTGTTTTTTCTGCTGTTCTTTGAGCCTAGCTCTCCTATTCAACTCAACTCGGGTGACCCTC

TTTGTTTTATTAGACTTTTTTTCTAGTGCAGCATCCTCATTCCCATTTAAATCCTCTTCATTATCATCAC

CATTATCCGCCTCAAGGAAGTACATATCTTCTTCATCAATCACTCTCTGGTCCCCAGGAACAGTTAATGG

AACAGGCTGAGGTCCCAACTCGCTTTGATACACTTTCTGCATTTCCTCTGCGACAGCTTGAGCTAAAGAA

TCCTGATGAGCCTCAAATGAAGGATTATACGAGCAACCTGGAGGCTCAACTTCCACTGCTGGAATAACTG

AAGGTATGGATACCTTTCTAGTTTTGTTATTGCATTCACCTTCATTTTCCCACAAATCAACCATGCCACC

ATCATCCTTAGGATCATCTCGAGTCACATCAGAATTTTTCTGGGTTTTCTAAGATTTCTT

>FM895903.1 FM895903 Jatropha curcas embryo 71-95 (DAF) Jatropha curcas cDNA clone rjcpga0_002908, mRNA sequence

CAAGGATTCTATCCGTCGCCCGATGGGAATTGTACTTTAAGGCGGCCCTCGCGGCTCGTCCGCCGCGGGG

GCTTGGCCAACGGCACGTGCCTCTGGGGGCCGGATGGCCCCTACTGCGGGTCGGCAAACGGGCGACGGAC

ACGCGCGTCGCTTCTGGCCCGGATTCTGACTTAGAGGCGTTCAGTCATAATCCAGCGCACGGTAGCTTCG

CGCCACTGGCTTTTCAACCAAGCGCGATGACCAATTGTGCGAATCAACGGTTCCTCTCGTACTAGGTTGA

ATTACCATCGCGACACCATCATCAGTAGGGTAAAACTAACCTGTCTCACGACGGTCTAAACCCCAGCTCA

CGTTCCCTATTGGTGGGTGAACAATCCAACACTTGGTGAATTCTGCTTCACAATGATAGGAAGAGCCGAC

ATCGAAGGATCAAAAAGCAACGTCGCTATGAACGCTTGGCTGCCACAAGCCAGTTATCCCTGTGGTAACT

TTTCTGACACCTCTAGCTTCAAATTCCGAAGGTCTAAAGGATCGATAGGCCACGCTTTCACGGTTCGTAT

TCGTACTGGAAATCAGAATCAAACGAGCTTTTACCCT

>FM895902.1 FM895902 Jatropha curcas embryo 71-95 (DAF) Jatropha curcas cDNA clone rjcpga0_002907, mRNA sequence

AAATCAAACATTAAGGCTTTTAAGGAACTTGCCATTTTGAAGATTCTAAACTTAGTGCTCGTGTTTTTAC

AATACAAAGAAATCCCAAACACAATCTTTAAAAATATTCTCAATAACAGTCTTAGGTACCGAGGTTTGGT

ATACACACACCAGCATCTGTATTTTGCTGTTCCTTCCATATCCTCCCAGTAACTCTAAGCTTCAGTTCTT

TCCTATCCCCTCCAGAAAAGAAAAGTGCCATGTTTCGTTGAATAATGAGACCGTCATGACTGTCCCTGAC

CTTCCTGTGGCTATCTCGTATACTTCGTGGGGTACCCTCCCAAATGAGTTTCCTACCATTTCCTCCAACC

TCAAGGCTGTAGTTATAGTTACGAGCCTCAGTCTCATCACCCATGAATCGAAGGAATGCCATGTAAACAG

AGGCCATACCGAGTTGGAAGGCCTCAAAGTGAAGGCAAAAGTATTGACCATAGCAATGGAAGACCGTGAG

CATCCAAGTTGCATTGTCCACTTCACGGGGATTTGACTTCACATAACGATGGTTGAAAGTGCATCCTGAG

TGCATGTCCACCTTGTGATCATCC

>FM895901.1 FM895901 Jatropha curcas embryo 71-95 (DAF) Jatropha curcas cDNA clone rjcpga0_002906, mRNA sequence

CTATAACGGTGGCGTGGTACTCGGTGCTGACTCTCGCACCAGCACTGGAATGTATGTTGCTAATAGGGCA

TCTGACAAAATTACTCAGCTAACTGACAATGTTTACATTTGCCGTTCTGGATCGGCTGCAGATTCCCAAA

TTGTATCTGATTATGTGCGTTACTTTCTTCATCAACACACAATACAGCTAGGGCAGCCTGCAACTGTCAA

AGTTGCTGCGAATCTTGTCAGGCTACTATCTTATAACAATAAGAACATGCTGGAAACTGGCATAATAGTT

GGTGGGTGGGACAAGTACGAGGGTGGTAAAATTTATGGAATTCCTCTTGGTGGGACAATAATAGAGCAGC

CTTTTGCTATAGGAGGATCTGGCTCCAGTTATTTGTATGGATTCTTTGATCAAGCATGGAAAGAAGGAAT

GACAAAAGATGAAGCTGAGCAATTAGTTGTGAAGGCAGTTTCCCTGGCAATCGCAAGAGATGGCGCTAGC

GGTGGTGTTGTGCGCACTGTCATTATTAATTCAAGGGAGTGACAAGAAACTACTATCCTGGCGACACACT

TCCGCTGTGGCATGAAGAGTTAGAGCCTCAGAATTCACTATTGGATATGC

>FM895900.1 FM895900 Jatropha curcas embryo 71-95 (DAF) Jatropha curcas cDNA clone rjcpga0_002905, mRNA sequence

ATTAAAGGATTCTATCCGTCGCCCGATGGGAATTGTACTTTAAGGCGGCCCTCGCGGCTCGTCCGCCGCG

GGGGCTTGGCCAACGGCACGTGCCTCTGGGGGCCGGATGGCCCCTACTGCGGGTCGGCAAACGGGCGACG

GACACGCGCGTCGCTTCTGGCCCGGATTCTGACTTAGAGGCGTTCAGTCATAATCCAGCGCACGGTAGCT

TCGCGCCACTGGCTTTTCAACCAAGCGCGATGACCAATTGTGCGAATCAACGGTTCCTCTCGTACTAGGT

TGAATTACCATCGCGACACCATCATCAGTAGGGTAAAACTAACCTGTCTCACGACGGTCTAAACCCAGCT

CACGTTCCCTATTGGTGGGTGAACAATCCAACACTTGGTGAATTCTGCTTCACAATGATAGGAAGAGCCG

ACATCGAAGGATCAAAAAGCAACGTCGCTATGAACGCTTCGCTGCCACAAGCCAGTTATCCCTGTGGTAA

CTTTTCTGACACCTCTAGCTTCAAATTCCGAAGGTCTAAAGGATCGAT

>FM895899.1 FM895899 Jatropha curcas embryo 71-95 (DAF) Jatropha curcas cDNA clone rjcpga0_002904, mRNA sequence

TGAAATTGTTGTTTCAGACATGGAACCTAAGGTTTTCAAGGCTTTGCTGCACTTCATATACAAAGATACT

CTTGTTGAAGATGAGGAGCTTTCTATGTCAAGTTCGTCTTGCATATCATCTGTATCCGATACTTTGGTTT

CAAAGTTGTTGGCTGCAGCAGACAAGTATGACTTGCCTCGACTCAGATTAATGTGCGAGTCTGTACTTTG

CAAGGATATATCTGTGAATTCTGTTGCAAAGATTCTTGCCCTGGCTGACCGTTATCATGCTATGGATTTG

AAATCTGTTTGCCTAAAATTTACTGCAGAAAATCTTGTAGCTGTCATGCGCTCGGACGGTTTTGAGTATC

TCAAAGAGAACTGTCCACTGCTGCAGTCAGAACTCCTAAAGACAGTAGCAGGATGTGAAGAAGATTGCAG

TGGAGGGGGAAAGAGCCGAAGTGTATGGGGACAGTTTTCGGACGGGGATGACACAAATGACAGGAGTGTT

CGGCAACAGCAATGGGAAAGTGGAGTAGAAAGAAGCCGAAGCTTGTGGGTCCAACTCGATGATGGCGATG

C

>FM895898.1 FM895898 Jatropha curcas embryo 71-95 (DAF) Jatropha curcas cDNA clone rjcpga0_002903, mRNA sequence

CTGGTGTTGAGCATATCCTTTTGGTGGGGAACTCGGGACGGAAGTTTGATATAATTCTAGATTTTCTTGG

ACTCGTTGAGAAATTTTACTATCTATCTGGCAGATATGACATTCAGTTGACTGTTGGTGATGCAGTCATG

GAGAACTCTTTCTTAAAGGTTATTGGTCATGTAGACCTAGACCTACCAGAACCACCTGAAAAGGCACCCC

GTCCTCCCCTGCAGCCAGTTGAACTATACTCAAGATACGGCCCAAAGGCTGAGATAACCCACATCTTTAG

GGCACCTGAAAAGCGTCCGCCTGAGGAGCTCTCCCTTGCTTTCTTGGGTCTTACACTTGTGCCATTCATT

GGATTCTTAATTGGGCTTTTACGTCTAGGGATAAACCTGAAGAATTTCCCTTCTTCACCTATACCTGCTA

CATTTGCCATCCTCTTCCATTTTGGAATCGCAGCAGTTCTGTCGCTCTATGTGCTTTTCTGGCTAAAGTT

GGATCTTTTTACAACATTGAAAGCGCTTGGTTTCTTGGGAGCTTTCCTGATGTTTGTTGGACACAGAACC

CTTTCCTACCTGGCATCAACGTCA

>FM895897.1 FM895897 Jatropha curcas embryo 71-95 (DAF) Jatropha curcas cDNA clone rjcpga0_002902, mRNA sequence

TCCATTTTTGACCTCAACCTTCTTCTCCTCCTCCTTCAACCTCTAGCTCTCTTTTTGCTTGGCAGCAGCT

TCCCTTTCTCTATCTTCCGCCATCTTCTACTTAACCTTATCCTTAGCTGCCATAACGACCGCCACGATCT

GCTTCTCCGCTGTGTCTTGAGCCATAAAGTCGGTCTCTATCACCAGAAAATCAAACAGTTTCTCCACAAT

CCCTATCGGATTGCTCGGATCGAATGTTGCGTTAAACCGGCAATGTTTTGGGTGATGACGATG

>FM895896.1 FM895896 Jatropha curcas embryo 71-95 (DAF) Jatropha curcas cDNA clone rjcpga0_002901, mRNA sequence

ATTCGCAAAGGATTCTATCCGTCGCCCGATGGGAATTGTACTTTAAGGCGGCCCTCGCGGCTCGTCCGCC

GCGGGGGCTTGGCCAACGGCACGTGCCTCTGGGGGCCGGATGGCCCCTACTGCGGGTCGGCAAACGGGCG

ACGGACACGCGCGTCGCTTCTGGCCCGGATTCTGACTTAGAGGCGTTCAGTCATAATCCAGCGCACGGTA

GCTTCGCGCCACTGGCTTTTCAACCAAGCGCGATGACCAATTGTGCGAATCAACGGTTCCTCTCGTACTA

GGTTGAATTACCATCGCGACACCATCATCAGTAGGGTAAAACTAACCTGTCTCACGACGGTCTAAACCCA

GCTCACGTTCCCTATTGGTGGGTGAACAATCCAACACTTGGTGAATTCTGCTTCACAATGATAGGAAGAG

CCGACATCGAAGGATCAAAAAGCAACGTCGCTATGAACGCTTGGCTGCCACAAGCCAGTTATCCCTGTGG

TAACTTTTCTGACACCTCTAGCTTCAAATTCCGAAGGTCTAAAGGATCGATAGGCCACGCTTTCACGGTT

CGTATTCGTACTGGAAATCAGAATCAAACGAGCT

>FM895895.1 FM895895 Jatropha curcas embryo 71-95 (DAF) Jatropha curcas cDNA clone rjcpga0_002896, mRNA sequence

ATTTTAAACTTTATTTTTAAATATTTATAAAAATTATAATGCGATAAAAAATAAAAATAGTTTCAAAATT

AGCGCAAAGCGCAAGACAGATGTAGAATAACTCTCACATAACTTCATAAAAGAAATGGGGAAATCATCAT

AAGTACAGAAAAAAAGAAAAAAAAAAAATTACAGTGATGAACCTTTTTCGTCAAGGGATTCTCGAACTGC

CTTTAAAAGAGATGGCATCAACTTAGGATTGGTGACAATGATGCCAGTGTCAAGATCCAGATATCTTCCT

TTANAAAAATCTAAAGGGTTTCCAGCAGACATCTGTGGCCACTCCACCAGCTTCTGTCACAACTATATAT

CCAGCAGCATGATCCCATATCTTCTCCCGGTAACCTTTACGAGGGAAACGCAGATATATAGTCCCGTCTC

CTCTGGC

>FM895894.1 FM895894 Jatropha curcas embryo 71-95 (DAF) Jatropha curcas cDNA clone rjcpga0_002895, mRNA sequence

ATTCCAATTTATCATATTTCCGTTTCCTGCGTGGGAAACCTATTCATTCTCGATTTAATTCACTCTTATT

TAAGATGGGATTGTTTAGCTAAAGCTGACACTATTTGATTTTTCTTTTCTTTGTAATATTTTATAATTTC

TAGATATGTTTCACCCTCACACGAATACCCTCAAACCAACTGGTTCTCTACCTCCTCTCAAGATCAGAGA

TCATGCTTTGATTTCAGACAGAGTTGGAGAAGCTACTCGATCGCCGAAGATGGATCCCGGGATATGGAGC

CGACTGCCGGAGGAGCTACTTGATCATGTATTGTCTCTATTGCCTCTAAAAGCATTCTTGAATCTCAGAT

CAACTTGTAAGCGTTTCAGGTCTCTTATATTTTCTCCTTGTTTTATGTCTAAACACTG

>FM895893.1 FM895893 Jatropha curcas embryo 71-95 (DAF) Jatropha curcas cDNA clone rjcpga0_002894, mRNA sequence

CGAATTCCTGAAAGTTGCTGTTGCATCTAGTGCTGATCGTATCAAAGTTGATGCAAACTTAGCTGCTGCC

GGTTTGCCATTGTCGATGTTTGATGCTATTGTATCAGCAGATGCTTTTGAAAATTTGAAACCAGCTCCTG

ACATTTTCTTGGCTGCATCAAAGATCTTAAATGTGCCGACTAGTGAGTGTATTGTTATTGAAGATGCACT

AGCTGGAGTACAAGCTGCAAAAGCTGCAGAGATGAGATGCATAGCTGTGAAAACTACATTGTCAGAAGAG

ACTCTAAGGAATGCTGCTCCATCCCTTATCAGGGATGATATTGGAAATGTTTCACTTGAGGATATTCTCA

GTGGTGGATCTAATGGCTATAATGAGATGATGCAGAGGCCTCAGGTTCTGCATAGTTCTGAACAAACTTT

GGCCTCAATGCTCGAAGAAAAAAAAGATAATGGATCACTTCTTAAC

>FM895892.1 FM895892 Jatropha curcas embryo 71-95 (DAF) Jatropha curcas cDNA clone rjcpga0_002892, mRNA sequence

ATTCCTAGCCCCGAAAATGGATGGCGCTTAAGCGCGCGACCTATACCCGGCCGTCGGGGCAAGAGCCAGG

CCCCGATGAGTAGGAGGGCGCGGCGGTCGCTGCAAAACCCGGGGCGCGAGCCCGGGCGGAGCGGCCGTCG

GTGCAGATCTTGGTGGTAGTAGCAAATATTCAAATGAGAACTTTGAAGGCCGAAGAGGGGAAAGGTTCCA

TGTGAACGGCACTTGCACATGGGTTAGTCGATCCTAAGAGACGGGGGAAGCCCGTCCGACAGCGCGTCCT

CGCGCGAGCTTCGAAAGGGAATCGGGTTAAAATTCCCGAACCGGGACGTGAAGGCTGACGGCGACGTTAG

GGAGTCCGGAGACGTCGGCGGGGGCCTCGGGAAGAGTTATCTTTTCTGTTTAACAGCCCGCCCACCCTGG

AAACGACTCAGTCGGAGGTAGGGTCCAGCGGCTGGAAGAGCACCGCACGTCGCGC

>FM895891.1 FM895891 Jatropha curcas embryo 71-95 (DAF) Jatropha curcas cDNA clone rjcpga0_002891, mRNA sequence

ATTGAGAGACAGCAGACTCGCAAGCTTGATCCTCATATTGAAGAACAATTTGCTGGTGGTCGTCTAATGG

CTTGTATCTCATCACGCCCTGGTCAGTGTGGCCGTGCTGATGGGTATATTCTGGAGGGTAAGGAGCTGGA

GTTCTACATGAAGAAGATCCAGAGGAAGAAGGGCAAGGGAGCTGGGGCTGCCTAAAATTTTGCAATCCAA

ACTTTCTATTCAGATCAGACTTGTCTCCCCCCTCTTTCTTTTCCTTGTGTCTGGAGATTGTATTTTTTAT

GAGATGAGAGATTAAACTTGTAGCAGCAACTATTATTATCGTTGAATTCCCAATTAGAAACCTAATATTG

TCTTTTATTTAACAGCAGCATAAGATGATCTTTTGATTTAAAAGGGGGGGCCCGGTAC

>FM895890.1 FM895890 Jatropha curcas embryo 71-95 (DAF) Jatropha curcas cDNA clone rjcpga0_002890, mRNA sequence

GCTGCAGGCATATACAAAGATACTCTTGTTGAAGATGAGGAGCTTTCTATGTCAAGTTCGTCTTGCATAT

CATCTGTATCCGATACTTTGGTTTCAAAGTTGTTGGCTGCAGCAGACAAGTATGACTTGCCTCGACTCAG

ATTAATGTGCGAGTCTGTACTTTGCAAGGATATATCTGTGAATTCTGTTGCAAAGATTCTTGCCCTGGCT

GACCGTTATCATGCTATGGATTTGAAATCTGTTTGCCTAAAATTTACTGCAGAAAATCTTGTAGCTGTCA

TGCGCTCGGACGGTTTTGAGTATCTCAAAGAGAACTGTCCACTGCTGCAGTCAGAACTCCTAAAGACAGT

AGCAGGATGTGAAGAAGATTGCAGTGGAGGGGGAAAGAGCCGAAGTGTATGGGGACAGTTTTCGGACGGG

GATGACACAAATGACAGGAGTGTTCGGCAACAGCAATGGGAAAGTGGAGTAGAAAGAAGCCGAAGC

>FM895889.1 FM895889 Jatropha curcas embryo 71-95 (DAF) Jatropha curcas cDNA clone rjcpga0_002889, mRNA sequence

ACTATGGATCCCCCGGCTGCAGGTTTTTGGTAAGCAGAACTGGCGATGCGGGATGAACCGGAAGCCGGGT

TACGGTGCCCAACTGCGCGCTAACCTAGAACCCACAAAGGGTGTTGGTCGATTAAGACAGCAGGACGGTG

GTCATGGAAGTCGAAATCCGCTAAGGAGTGTGTAACAACTCACCTGCCGAATCAACTAGCCCCGAAAATG

GATGGCGCTTAAGCGCGCGACCTATACCCGGCCGTCGGGGCAAGAGCCAGGCCCCGATGAGTAGGAGGGC

GCGGCGGTCGCTGCAAAACCCGGGGCGCGAGCCCGGGCGGAGCGGCCGTCGGTGCAGATCTTGGTGGTAG

TAGCAAATATTCAAATGAGAACTTTGAAGGCCGAAGAGGGGAAAGGTTCCATGTGAACGGCACTTGCACA

TGGGTTAGTCGATCCTAAGAGACGGGGGAAGCCCGTCCGACAGCGCGTCCT

>FM895888.1 FM895888 Jatropha curcas embryo 71-95 (DAF) Jatropha curcas cDNA clone rjcpga0_002888, mRNA sequence

CTGGCTGAATGGAAAACATAGATCTGCCTGCCATGGCATTCATAATGGGCAAAAATTAATTGCCAGCGTA

CATTCTTAATACATTCCAAAACACAAGTCTGTCTAAAAAACCTGGAGAGAGATAGAAACCTCGCAGGTTA

CAGTAGAATCCACCTCCTACTGTCAGAAGTGTAGAAAACATGCCCCTATATGGCTTTACAAGAGAATGAT

TTTAATCTTCAAGCTCAATAATTTTAACCACAGATGCATCTCCAACTTTCTGGCAAACAACTACCCTGTC

ATGAGACTTAATAACTCCTGAGGCCTTTCCATGATCAAGAGCAACCTTCAGGACAGATTCATTTGTTGCA

CTCGTAGATTCCGCAGGATGTCGTGGATCAGCAAGCATAGGGAAGAGACCTCTGACAATGAGTGATTGCC

TTGCTTCAAAGGCCCCACTGAAGCTCCATCTCAGTTGATT

>FM895887.1 FM895887 Jatropha curcas embryo 71-95 (DAF) Jatropha curcas cDNA clone rjcpga0_002887, mRNA sequence

CCAGGTGACAATGTTGGCTTCAATGTGAAGAATGTTGCNTGTCAAGGGATTTGAAACGTGGTTTTGTTGC

CTCTAACTCCAAGGATGATCCTGCGAAGGAAGCTGCCAACTTCACCTCTCAGGTCATCATCATGAACCAC

CCTGGTCAAATTGGAAACGGATATGCTCCTGTCCTTGACTGCCACACCTCCCACATTGCTGTTAAGTTTG

CTGAGATCCTAACCAAGATTGATAGGCGATCTGGTAAAGAGCTGGAGAAGGAGCCAAAATTCTTGAAGAA

TGGTGATGCTGGATTTGTGAAGATGATTCCCACCAAGCCCATGGTTGTGGAGACCTTTTCTGAGTACCCT

CCTCTGGGTCGTTTTGCTGTGAGGGACATGCGCCAGACAGTTGCTGTTGGTGTCATCAAGAGTGTTGAGA

AGAAGGATCCATCTGGTGCAAAGGTCACCAAATCCGCAGCAAAGAAAAAGTAAAGCAT

>FM895886.1 FM895886 Jatropha curcas embryo 71-95 (DAF) Jatropha curcas cDNA clone rjcpga0_002886, mRNA sequence

ATCCCTGNCCAGTAGTCATATGCTTGTCTCAAAGATTAAGCCATGCATGTGTAAGTATGAACTAATTCAG

ACTGTGAAACTGCGAATGGCTCATTAAATCAGTTATAGTTTGTTTGATGGTACCTGCTACTCGGATAACC

GTAGTAATTCTAGAGCTAATACGTGCACCAAACCCCGACTTCTGGAAGGGATGCATTTATTAGATAAAAG

GTCGACGCGGGCTCTGCCCGTTGCTCTGATGATTCATGATAACTCGACGGATCGCACGGCCATCGTGCCG

GCGACGCATCATTCAAATTTCTGCCCTATCAACTTTCGATGGTAGGATAGAGGCCTACCATGGTGGTGAC

GGGTGACGGAGAATTAGGGTTCGATTCCGGAGAGGGAGCCTGA

>FM895885.1 FM895885 Jatropha curcas embryo 71-95 (DAF) Jatropha curcas cDNA clone rjcpga0_002885, mRNA sequence

TCTGACATGTGTGCGAGTCAACGGGTGAGTAAACCCGCAAGGCGCAAGGGAAGCTGACTGGCGGGATCCC

CCCGAGGGTTGCACCGCCGACCGACCTTGATCTTCTGAGAAGGGTTCGAGTGAGAGCATGCCTGTCGGGA

CCCGAAAGATGGTGAACTATGCCTGAGCGGGGCGAAGCCAGAGGAAACTCTGGTGGAGGCCCGCAGCGAT

ACTGACGTGCAAATCGTTCGTCTGACTTGGGTATAGGGGCGAAAGACTAATCGAACCGTCTAGTAGCTGG

TTCCCTCCGAAGTTTCCCTCAGGATAGCTGGAGCTCGGGACGAGTTCTATCGGGTAAAGCCAATGATTAG

AGGCATCGGGGGCGCAACGCCCTCGACCTATTCTCAAACTTTAAATAGGTAGGACGGCGCGGCTGCTTCG

CTGAGCCGCGCCACGGAATCGAGAGCTCCAAGTGGGCCATTTTTGGTAAGCAGAACTGGCGATGCG

>FM895884.1 FM895884 Jatropha curcas embryo 71-95 (DAF) Jatropha curcas cDNA clone rjcpga0_002884, mRNA sequence

ATTCCAAAGGATTCTATCCGTCGCCCGATGGGAATTGTACTTTAAGGCGGCCCTCGCGGCTCGTCCGCCG

CGGGGGCTTGGCCAACGGCACGTGCCTCTGGGGGCCGGATGGCCCCTACTGCGGGTCGGCAAACGGGCGA

CGGACACGCGCGTCGCTTCTGGCCCGGATTCTGACTTAGAGGCGTTCAGTCATAATCCAGCGCACGGTAG

CTTCGCGCCACTGGCTTTTCAACCAAGCGCGATGACCAATTGTGGGAATCAACGGTTCCTCTCGTACTAG

GTTGAATTACCATCGCGACACCATCATCAGTAGGGTAAAACTAACCTGTCTCACGACGGTCTAACCCCAG

CTCACGTTCCCTATTGGTGGGTGAACAATCCAACACTTGGTGAATTCTGCTTCACAATGATAGGAAGAGC

CGACATCGAAGGATCAAAAAGCAACGTCGCTATGAACGCTTGGC

>FM895883.1 FM895883 Jatropha curcas embryo 71-95 (DAF) Jatropha curcas cDNA clone rjcpga0_002883, mRNA sequence

ATTCCCTCGCTTCAGGTCTATTTGTCCCTTTTATGCATCAAGTTCCAGCAAACAAATTCAGGAATATCAA

TGCTGGTCCTTCATTTATCTCTCAATCAGCTGCTGATGAAGGGTCCAGAACTGGAATTAAAGGTCCTGGA

ATTTTGAGTTCCATCAATACTGGCACAGGCATTACCGAGAAAAATTCATCAGGCGCACTGGCAAGTGGCA

GTAAACCTAAATCTGGGACTCATAATTCAGAACCGGAATTTCAACTCCTTCAAGTCGGCACGGTTTAACA

TCTGCCAGTCGTCAGATGACTATATTTTATGGTGGCCAAGCTCATGTTTTTGATGATGTCCACCCAAACA

AGGCAGATGTTATAATGGCCTTAGCAGGATCAAATGGAGCATCATGGTCAACCACCTACTCGCCTAAACC

TACCGTGAGGCCGGGTGGTGAAAATTACATG

>FM895882.1 FM895882 Jatropha curcas embryo 71-95 (DAF) Jatropha curcas cDNA clone rjcpga0_002882, mRNA sequence

GCATTTAAAATGATCCTCGTGTTTTATTGTGGCTATGCATGAATATAGAAATTAAGTACATAATTCATTA

GCGAACACAATCCAATCCAACAATTCTCGGTGGAATGCATCCAGATGGAGCAAGCATAAAAATTACCCTG

AAAAATTATGGTCCTAGCTTCTGATTCATTGGTTACAGATTTAACATACCTCTCGTGCCGCCAAACTCAG

CTTCCATTTCTTCCACGGGAACAACCCTTATCAATAACTTCCCTTCATCTCTTGCGCTTTANTGTCTTGA

TATTCTTCACAATATAACCAATCACCTCCTTCAACGTGGCTTTCCTTCCTTCTTTGAAGTTCCAAAGCTC

AGGAGTAACATATCTCCCACTCGGATTATACTCGTTTATCTCTTTTAAAGCAGTAACAACTGCCATGTAA

ATGTCAC

>FM895881.1 FM895881 Jatropha curcas embryo 71-95 (DAF) Jatropha curcas cDNA clone rjcpga0_002881, mRNA sequence

GCTGCAGGCTAGCTTCACATCCAGTGTAATTAAGACACACCGCCGTGGAATTTTGATCAGACGACAAAGA

TGCCGATCAGAAACATTGCCATAGGCCATCCTCAGGAGGCAACTCATCCAGACGCCTTNAAAGCGGCGTT

GGCTGAGTTCATCTCCACTCTTATTTTTGTCTTCGCCGGTGAAGGCTCCGGGATGGCCTTTAATAAGCTC

ACTAACAACGGCGCCACCACTCCTGCCGGTCTAGTCGCCGCATCTTTANCCCATGGTTTCGGTCTTTTTG

TTGCTGTCTCAGTTGGAGCCAACATCTCCGGCGGTCATGTAAATCCAGCTGTTACCTTCGGTGCTTTCGT

CGGTGGAAACATCACTCTCCTCCGCGGTATTCTCTACTGGATTGCTCAGCTTCTCGGATCCACCGTAGCT

TGCTTGCTTCTCAAGTTCAGCACCGGTGGCTT

>FM895880.1 FM895880 Jatropha curcas embryo 71-95 (DAF) Jatropha curcas cDNA clone rjcpga0_002880, mRNA sequence

CTGGTATTAGCGAATTTGTGAAGTCACATGGCAGATAAATGGAAACAAACTGCTCTCTTAGTCATTGACA

TGCAGAATGATTTTATATTAGAAGATGGATTGATGAGAGTCAATGGAGGGGAAGCCATCGTCCCGAATGT

GATTAAGGCAGTTGAAATTGCTAGGCAGCGCGGCATCCTTGTTGTTTGGGCTGTTCGCGAACATGACCCA

CTAGGAAGAGATGTCGAACTTTTCCGCCGGCATTATTACTCTGCGGGTAAAGTGGGTCCCACCTCTAAGG

GAAGTGAGGGTGCAGAACTGGTTGATGGCCTTGTGATCAAAGAGGGGGATTATAAGGTGGTGAAGACATG

CTTCAGTGCATTTTTTAACACCCATCTCCATTCATTTCTTCAAACTGAAGGAATTAAGAATTTGGTGATT

GTTGGTGTTCAAACTCCAAATTGCATTAGGCAGACTGTGTTTGATGCAGTAGCTCTGGAT

>FM895879.1 FM895879 Jatropha curcas embryo 71-95 (DAF) Jatropha curcas cDNA clone rjcpga0_002879, mRNA sequence

ATTCCAATGATTAGAGGCATCGGGGTGCGCAACGCCCTCGACCTATTCTCAACTTAAATAGGTATGACTG

CGCGGCTGCTTCGCTGAGCCGCGCCACGGAATCGAGAGCTCCAAGTGGGCCATTTTTGGTAAGCAGAACT

GGCGATGCGGGATAACCGGAAGCCGGGTTACTGTGCCCAACTGCGCGCTAACCTAGAACCCACAAAGGGT

GTTGGTCGATTAAGACAGCATGACGGTGGTCATGGAAGTCGAAATCCGCTAAGGAGTGTGTAACAACTCA

CCTGCCGAATCAACTAGCCCCGAAAATGGATGGCGCTTAAGCGCGCGACCTATACCCGGCCGTCGGGGCA

AGAGCCAGGCCCCGATGAGTAGGAGGGCGCGGCGGTCGCTGCAAAACCCGGGGCGCGAGCCCGGGCGGAG

CGTGCGTCGGTGCAGA

>FM895878.1 FM895878 Jatropha curcas embryo 71-95 (DAF) Jatropha curcas cDNA clone rjcpga0_002878, mRNA sequence

ATTCCACCCTTTCTCTCTCTCTCTCTCTCTCTCTCTCTCTCTATTTATCTTTTCCTTCTTTTCTCTCTCC

CTTACAAGCCCTAACCCTAACTCAGACCCAGATTCGCCATGAAAGGAGGTAAATCAAAGTCAGACACCAA

GAACGCCAAGCTTTCCGTGAACAAGAAACCCGCCAATGCAAGTAAGAAGGCGGGTAAGGCAGCGAAGGAT

CCAAATAAGCCAAAGAGGCCCGCCAGTGCTTTCTTCGTTTTCATGGAAGAGTTCAGAGAGCAGTACAAGA

AGGAACATCCTAAAAACAAATCCGTTGCTGCTGTTGGCAAAGCTGGTGGAGATAAATGGAAGTCAATGTC

AGATGCTGAGAAAGCACCTTTTGTATCTAAGGCAGAAAAGAGGAAGATAGAATATGAGAAGAAATTGAAA

GCCTACAACAAGGGACAGGCTGAAG

>FM895877.1 FM895877 Jatropha curcas embryo 71-95 (DAF) Jatropha curcas cDNA clone rjcpga0_002877, mRNA sequence

ATTATTGAAATTATGATTGTAGATTATATAAGCAAATAAGAGTACATAAATATGAAATTGTTACCGCTTC

TCAGTGCTCTTCTAATTCTCAAATTCTAACGAAAAAGCACTCTACTGGTTTAGGATTTTCTGGGATTACA

AAGTCTAAGCTATGGAAGAAGAGATTCAGGTTGCCTTCTTCTTTCCGAACAATAAGCTCCATGCATATGT

GTTGCCTTTCTCAGAAGCCGCTTCAACTCGCTTTGCCCCGATCTGGTCCATAAGCCAGTAACCAATGGTT

GGCATGTACTGTACTAGATACATAACGGCTAGTACAGGCTGGTATGATATCCAAGCTTCCTTTAAACCAT

GGGTCATAGCAATAATTGTCAGTTCCGCACACCTCTCTGATGACACACGCTTCTCAGAACCCTTTTTTGC

TGTAGTGTAGCCATTTGATGTCTCTATTGGC

>FM895876.1 FM895876 Jatropha curcas embryo 71-95 (DAF) Jatropha curcas cDNA clone rjcpga0_002876, mRNA sequence

TTTTTTTTTTGAAAAATTACTGAAATACTGAACTATTATGCTTGTTAAATGGTACATCTTATGGAAAATA

TACGGTGGTCCTGGTATTAAATGGCGCCACTTATAACTTAAAATTTACATACAAGTGTGAGATACTAGCT

GATAAAACAAACCAAGAACAAGCCCTATGATGATGCTGATGAAGCTCCTCAGATGTCCCCACGTGGAGCA

TGGTGGACAGGAATCTCTCCTTGATTATCCAATGGCATGTATTGCGCCATGATAGCCCGTATCTCCGAGT

CCATGTATCTCCGAATTCTGTACTTGTAAATTGCATATCCAGCAATTCCAGCAGCAGCCAAGCCTAGAAT

AATAGT

>FM895875.1 FM895875 Jatropha curcas embryo 71-95 (DAF) Jatropha curcas cDNA clone rjcpga0_002875, mRNA sequence

CTACAAGTAAGCTTTGATTAAATGTTGCTAAGATAATCACCACATCTTCTCGAATTGATCATTTCCCGCA

GCTCACAGTTCTACAGTACTCTAATCATGCAAACGAAAATAAAATAATTATGCATAACATGTTTGTCCTA

AGAAGGCTACCTCATAATCTAAGTAGTTCCATAAAAAGTTAGAGAACAAGTCACAGGAAATAACGTATTT

GAGGAGCCTCTACTATTTTGTCTATCTCACTTCTTCCTCTTCTTTGGCGGCTGGAGGGCTTCCTCGTCTT

CATCCTCCTCATCTTCATTCTCTACCTCCTCAACTTCCTCCTCATCTTCTTCCCCACCATCGTCGTCGTC

GTCATCATCATCGTCGTCATCGTCATCATCTTGATCCTCACCATCTTCTT

>FM895874.1 FM895874 Jatropha curcas embryo 71-95 (DAF) Jatropha curcas cDNA clone rjcpga0_002872, mRNA sequence

CTTACGAATCGGAGCGACAAAGGGCTGAATCTCAGTGGATCGTGGCAGCAAGGCCACTCTGCCACTTACA

ATACCCCGTCGCGTATTTAAGTCGTCTGCAAAGGATTCTATCCGTCGCCCGATGGGAATTGTACTTTAAG

TGCGGCCCTCGCGGCTCGTCCGCCGCGGGGGCTTGGCCAACGGCACGTGCCTCTGGGGGTCGGATGGCCC

CTACTGCGGGTCGGCAAACGGGCGACGGACACGCGCGTCGCTTCTGGCCCGGATTCTGACTTAGAGGCGT

TCAGTCATAATCCAGCGCACGGTAGCTTCGCGCCACTGGCTTTTCAACCAAGCGCGATGACCAATTGTGC

GAATCAACGGTTCCTCTCGTACTAGGTTGAATTACCATCGCGACACCATCATCAGTAGGGTAAAACTAAC

CTGTCTCACGACGGTCTAACCCCAGCTCACGTTCCCTATTGGTGGGTGAACAATCCAACACTTGGTGAAT

TCTGCTT

>FM895873.1 FM895873 Jatropha curcas embryo 71-95 (DAF) Jatropha curcas cDNA clone rjcpga0_002871, mRNA sequence

GACTAAGGAAAACATATATAGATTATAAAGCTAAAGACATTAATGTTTTGGGTATAAAAGAGTTCACAAA

ACCTTAATACCAACATACATTGATTGATAAAATAAATAATAACCCCTAACTATCTTCACCACCATTCTCT

CCAAACCAAGAGGACAATGGAAAGAAAAAAACTCCAAACCAATAATAGTTCATAAAGGACTAACGAAAAT

AAATAAATTAAAAAAAAATAAATGACTCCCAAAACTTCAAAACCCCTCCCTTTCTGTTCCTCTCCAAAGA

TATCAGCAAATTCTCCCTCCAAACTTCAATCGAACTCTACTGTTGCTTGCACTCTGGAGGCCTCTCACCC

TGCTGACCATCCCCAGCTTGTGTTCTTCCACCCTTCTTATGCCCGGCTGACAGCTTCTTTTTGGTCTTGC

TTTCATCCTCGTCCTCATCCTCATCTTCATCCTCTTCAT

>FM895872.1 FM895872 Jatropha curcas embryo 71-95 (DAF) Jatropha curcas cDNA clone rjcpga0_002869, mRNA sequence

TGAGCTCACGCGGGGCGGCGCTCTAAACTATGGATCCCCGGCTGCAGGAAAGGATTCTATCCGTCGCCCG

ATGGGAATTGTACTTTAAGGCGGCCCTCGCGGCTCGTCCGCCGCGGGGGCTTGGCCAACGGCACGTGCCT

CTGGGGGCCGGATGGCCCCTACTGCGGGTCGGCAAACGGGCGACGGACACGCGCGTCGCTTCTGGCCCGG

ATTCTGACTTAGAGGCGTTCAGTCATAATCCAGCGCACGGTAGCTTCGCGCCACTGGCTTTTCAACCAAG

CGCGATGACCAATTGTGCGAATCAACGGTTCCTCTCGTACTAGGTTGAATTACCATCGCGACACCATCAT

CAGTAGGGTAAAACTAACCTGTCTCACGACGGTCTAATCCCAGCTCACGTTCCCTATTGGTGGGTGAACA

ATCCAACACTTGGTGAATTCTGCTTCACAATGATAGGAAGAGCCGACATCGAAGGATCAAAAAGCAACGT

CGCTATGAACGCTTGGCTGCCACAAGCCAGTTAT

>FM895871.1 FM895871 Jatropha curcas embryo 71-95 (DAF) Jatropha curcas cDNA clone rjcpga0_002868, mRNA sequence

TTTTTTTTTTACCCCGCACAAACCATCATATAAATTGTACGTAAATATCACACTTCAAGGGGTATACAAC

CCCACGAATCCACAAATATATAACGACACGACTTAGAGAAGCAAATTCTAGGTTCAAATTTTTATAAACA

TTACATTTAATGGACCAGATAATATGGAATCTCCATCACATTGTAGGTATCCTACATTCAATTTGATCGG

CTTCTAATAACATCAAGACCCATTTCTGTAGGATCAGTTGCTTGCAACTCTACCTGAATACCGTCCAACT

CTCTTTTGAACCTGTCCTTTGAGCTAGCATAAATCATCTTGCTTCTCACCTTAGATGTATCAGGAGACCA

TGCAATAAAAACAATCCTACTCTTCTGGACATTCTCCTCTGTTACAAAATCAAAATCATAGACAGCATAA

CGGCACTCATCAGCAGGAAGGCTTGCTGTGAAATCCTCATAACTGTTAGCTGGTTCGCCAAGCTTCTCCA

CAACCA

>FM895870.1 FM895870 Jatropha curcas embryo 71-95 (DAF) Jatropha curcas cDNA clone rjcpga0_002867, mRNA sequence

TGCAGGCAAAGGGTGTTGGTCGATTAAGACAGCAGGACGGTGGTCATGGAAGTCGAAATCCGCTAAGGAG

TGTGTAACAACTCACCTGCCGAATCAACTAGCCCCGAAAATGGATGGCGCTTAAGCGCGCGACCTATACC

CGGCCGTCGGGGCAAGAGCCAGGCCCCGATGAGTAGGAGGGCGCGGCGGTCGCTGCAAAACCCGGGGCGC

GAGCCCGGGCGGAGCGGCCGTCGGTGCAGATCTTGGTGGTAGTAGCAAATATTCAAATGAGAACTTTGAA

GGCCGAAGAGGGGAAAGGTTCCATGTGAACGGCACTTGCACATGGGTTAGTCGATCCTAAGAGACGGGGG

AAGCCCGTCCGACAGCGCGTCCTCGCGCGAGCTTCGAAAGGGAATCGGGTTAAAATTCCCGAACCGGGAC

GTGAAGGCTGACGGCGACGTTAGGGAGTCCGGAGACGT

>FM895869.1 FM895869 Jatropha curcas embryo 71-95 (DAF) Jatropha curcas cDNA clone rjcpga0_002866, mRNA sequence

GCCATTTTTGGGTAAGCAGAACTGGCGATGCGGGATGAACCGGAAGCCGGGTTACGGTGCCCAACTGCGC

GCTAACCTAGAACCCACAAAGGGTGTTGGTCGATTAAGACAGCAGGACGGTGGTCATGGAAGTCGAAATC

CGCTAAGGAGTGTGTAACAACTCACCTGCCGAATCAACTAGCCCCGAAAATGGATGGCGCTTAAGCGCGC

GACCTATACCCGGCCGTCGGGGCAAGAGCCAGGCCCCGATGAGTAGGAGGGCGCGGCGGTCGCTGCAAAA

CCCGGGGCGCGAGCCCGGGCGGAGCGGCCGTCGGTGCAGATCTTGGTGGTAGTAGCAAATATTCAAATGA

GAACTTTGAAGGCCGA

>FM895868.1 FM895868 Jatropha curcas embryo 71-95 (DAF) Jatropha curcas cDNA clone rjcpga0_002865, mRNA sequence

ACACACTAAACCAAGAAACTTTCTCTCACCGATCTTTGATCTTCTCCAAAAACCAATCAAGAGAATGTCT

CTGATTCCAAGCACCCTCTTCGGTGGCCGGAGAACCAACATTTTCGATCCCTTCTCTCTCGATATCTGGG

ATCCATTTCAGGACTTCCCTTTCACAAGCACTGCTCTTTCTGCTCCTCGTTCTGAGTTATTGAACGAAAC

GGCAGCGTTTGCCAACACCCGCATAGACTGGAAAGAAACACCAGAAGCTCATGTATTCAANGCTGATCTT

CCAGGNCTAAAGAAAGAGGAAGTGAAAGTGGAGATAGAGGAAGGTAGGGTCTTGCAGATAAGCGGAGAGA

GAAGCAAAGAGAAAGAAGAAAAGAATGATAAGTGGCATAGAGTTGAAAGGTCAAGTGGTAAGTTCTTGAG

AAGGTTTAGGCTACCAGAGAATGCAAAGGTTAATGAGGTTAAGGCTAGCATGGAAAATGGAGTCTTGACT

GTGACAGTGCCT

>FM895867.1 FM895867 Jatropha curcas embryo 71-95 (DAF) Jatropha curcas cDNA clone rjcpga0_002864, mRNA sequence

CGGCGCTCTAAACATGGATCCCCGGGCTGAGGAATTCGAATTCCACTTGCTTGTTGCGGTAATCCGTAAA

ACTGTTAGAGCGTTTCTTGGAACCTGCCAATAATGAGTAAGCTTCAAAGTGAGACATTGAGAGAGGCTAT

CTCTACTATTATGGCTGCTTCTGTGGAGAAGAAGCGTAACTTTACTGAGACAATTGAGCTCCAGATTGGA

CTGAAGAACTATGATCCTCAAAAGGATAAGCGTTTCAGTGGCTCTGTGAAGTTGCCTCACATCCCTCGTC

CTAAGATGAAGATTTGCATGCTTGGAGATGCTCAGCATGTTGAGGAGGCTGAGGCAATAGGACTACAATG

GATGGATGTGGAAGCATTGAAGAAGCTAAACAAGAACAAAAAATTGGTTAAGAAACTTGCAAAGCAGTAC

CATGCCTTCTTGGC

>FM895866.1 FM895866 Jatropha curcas embryo 71-95 (DAF) Jatropha curcas cDNA clone rjcpga0_002863, mRNA sequence

CAAGTTTCAAGCAGATCTCAGAAGATGGAAGCTTAGATTTCGAAGCAACCTAACTCAATAAAATGCCGAC

CTATCCCCTACCGAGTCGACGACTCGTTCTCATTCTGCTTCTATTTTTCCTTGCAGTTTCAAAAGCATCA

GCTTCAAGACCTGGTTTTCTCTTCACAAGAACCAGAGGAAGATGCACTCCGCAGTAAGAGTAACCTTCGG

TCGAGTTTCCCGAGTTGAGTTACGAGTTAATATTGAACTGTTGAGTTTGTGTGTTACAGGTATTGGAGTA

GCAGGCGAGAGGCGTGGCCGAGGATGGTACCGCAGACATCAACGGTGTCAAACGTGTTTGGGTCGATAAT

CTTTGAACGGTACAGATCTGGATTGACTTTANTAGAATCAACGGCTAGGAATGACGATGAGAATGCGTTC

GGTAGGTTGTTGAAGCAGGGGAGTACAGCTCTTTTGAACTCTTATGCTAGAAAGGGGTTCAAGTACTCTG

CTTGGGAAGTGAAGACTCTGTTTATTCAAGCTTTGGTATCGGA

>FM895865.1 FM895865 Jatropha curcas embryo 71-95 (DAF) Jatropha curcas cDNA clone rjcpga0_002862, mRNA sequence

GGATTTCTTTCCTCCTCTTCTTCTTCGATTATAGCTGTGTTGCTCGAAAATGGCCATGAAACGTGCTGCT

ACTTCTGTGATTCGCGCTTTCACTTCCTCATCTGCAATTTCCGGCTCTTCAATTCTCGCTAGAGACCTCC

ATGCTTCTGCAGGGAGCAAAAAAATTGTCGGGGTTTTCTACAAGGCCAATGAATATGCTTCAAAGAATCC

CAATTTTGTGGGATGTGTAGAAGGAGCTTTGGGCATACGTGACTGGCTGGAATCACAAGGCCATCAATAC

ATTGTCACTGATGACAAAGAAGGGCCTGATTGTGAACTTGAAAAGCATATTCCTGATCTCCATGTCCTCA

TAAGTACCCCATTCCACCCTGCTTATGTTACGGCAGAAAGGATCAAAAAAGCAAAAACTTGCAATTGCTC

CTCGCAGCTGGAATTGGCTCAGATCACATCGATTTGAAGGCTGCTGCAGCTGCAGGGCTAACTGTTGCAG

AGGTCACA

>FM895864.1 FM895864 Jatropha curcas embryo 71-95 (DAF) Jatropha curcas cDNA clone rjcpga0_002860, mRNA sequence

ATTCATTTTAATTCTTGGTGGGTTATCTTTCCCTAGATTAAACCCTTCACCTAGTGGTTTATACAATCAA

TTCTCTCCTTTTGGTGATATTTCTGCTAGTTCTGCAACTGCTGGTAGTGTTACTAGTCCTTGTTTCAGTC

TGGACCCATCTGGAAGCTCTTCTGGTACTTTGATGGGGCTTAACTATCCGATTTCAAGTGGCTTTACCGG

TGCAATTCAAGAAATGGGCGGTGGCGGTGTTGGTGGCGGAAATGGCGGTTCGATGAATGTTCATACTAAC

CTTGCATCATCAATTGAGTCTTTGAGTTCTATAAATCAAGATTTGCACTGGAAATTGCAGCAACAACGAT

TGGCTATGCTTTTTGGAGGAGAAGACCAGAAAGATAATAATAGTGTTTCTTCGGTTCCTATTGAAAACCA

AGTGCAAAAGCCTCAACCCATTTTGTTTCAAAATCTTGAAATTTCAAAACCAGAAGTAAATGTTGCTGGA

AATTCAAGAAGATTAGGTGATACAACCACCGAATGGT

>FM895863.1 FM895863 Jatropha curcas embryo 71-95 (DAF) Jatropha curcas cDNA clone rjcpga0_002859, mRNA sequence

CTCTTATTGAACTCTCGCTACTCTCTTTCTCGACGATCAACTCCATCTCTCTTTCAATGGCAGCCGCTGC

CGGTTCATCAATCTCCATGCAGTCTCGTCCTAGACTGGCTGCAACCAGAATCTCTAGCCTCAAATCTTTT

TCGTTTCCAAGCCAGAGAAGTTTCCTGTCTTTCAGAGTGCAGCCAGTTCCAGCATCTCGCCTTCGGATTT

CTTGTGCTGCCAAACCAGAAACAGTAGAAAAGGTGTGTCAAATAGTGAAGAAGCAGCTGGCATTAGCCCC

AGAAGCTGTTGTTACCGGCGAGTCAAAATTTACAACACTAGGAGCAGATTCTCTTGATACAGTTGAGATT

GTGATGGGACTGGAGGAGGAATTCGATATCAGTGTGGAAGAGGAGAGTGCCCAGAGCATCACAACCGTTC

AGGAAGCTGCTGATCTTATTGAAAAACTTGTTGAGAAGAAGGACGCTTAGAAGATCAGTCTAGGAAATCC

TTCAATTCCTTTCCCC

>FM895862.1 FM895862 Jatropha curcas embryo 71-95 (DAF) Jatropha curcas cDNA clone rjcpga0_002858, mRNA sequence

ATTCCTATTCTCAAACTTTAAATAGGTAGGACGGCGCGGCTGCTTCGCTGAGCCGCGCCACGGAATCGAG

AGCTCCAAGTGGGCCATTTTTGGTAAGCAGAACTGGCGATGCGGGATGAACCGGAAGCCGGGTTACGGTG

CCCAACTGCGCGCTAACCTAGAACCCACAAAGGGTGTTGGTCGATTAAGACAGCAGGACGGTGGTCATGG

AAGTCGAAATCCGCTAAGGAGTGTGTAACAACTCACCTGCCGAATCAACTAGCCCCGAAAATGGATGGCG

CTTAAGCGCGCGACCTATACCCGGCCGTCGGGGCAAGAGCCAGGCCCCGATGAGTAGGAGGGCGCGGCGG

TCGCTGCAAAACCCGGGGCGCGAGCCCGGGCGGAGCGGCCGTCGGTGCAGATCTTGGTGGTAGTAGCAAA

TATTCAAATGAGAACTTTGAAGGCCGAAGAGGGGAAAGGTTCCAT

>FM895861.1 FM895861 Jatropha curcas embryo 71-95 (DAF) Jatropha curcas cDNA clone rjcpga0_002857, mRNA sequence

ATTCGCAAAGGATTCTATCCGTCGCCCGATGGGAATTGTACTTTAAGGCGGCCCTCGCGGCTCGTCCGCC

GCGGGGGCTTGGCCAACGGCACGTGCCTCTGGGGGCCGGATGGCCCCTACTGCGGGTCGGCAAACGGGCG

ACGGACACGCGCGTCGCTTCTGGCCCGGATTCTGACTTAGAGGCGTTCAGTCATAATCCAGCGCACGGTA

GCTTCGCGCCACTGGCTTTTCAACCAAGCGCGATGACCAATTGTGCGAATCAACGGTTCCTCTCGTACTA

GGTTGAATTACCATCGCGACACCATCATCAGTAGGGTAAAACTAACCTGTCTCACGACGGTCTAAACCCA

GCTCACGTTCCCTATTGGTGGGTGAACAATCCAACACTTGGTGAATTCTGCTTCACAATGATAGGAAGAG

CCGACATCGAAGGATCAAAAAGCAACGTCGCTATGAACGCTTGGCTGCCACAAGCCAGTTATCCCTGTG

>FM895860.1 FM895860 Jatropha curcas embryo 71-95 (DAF) Jatropha curcas cDNA clone rjcpga0_002856, mRNA sequence

CTTACGAATCGGAGCGACAAAGGGCTGAATCTCAGTGGATCGTGGCAGCAAGGCCACTCTGCCACTTACA

ATACCCCGTCGCGTATTTAAGTCGTCTGCAAAGGATTCTATCCGTCGCCCGATGGGAATTGTACTTTAAG

GCGGCCCTCGCGGCTCGTCCGCCGCGGGGGCTTGGCCAACGGCACGTGCCTCTGGGGGCCGGATGGCCCC

TACTGCGGGTCGGCAAACGGGCGACGGACACGCGCGTCGCTTCTGGCCCGGATTCTGACTTAGAGGCGTT

CAGTCATAATCCAGCGCACGGTAGCTTCGCGCCACTGGCTTTTCAACCAAGCGCGATGACCAATTGTGCG

AATCAACGGTTCCTCTCGTACTAGGTTGAATTACCATCGCGACACCATCATCAGTAGGGTAAAACTAACC

TGTCTCACGACGGTCTAACCCCAGCTCACGTTCCCTATTGGTGGGTGAACAATCCAACACTTGGTGAATT

C

>FM895859.1 FM895859 Jatropha curcas embryo 71-95 (DAF) Jatropha curcas cDNA clone rjcpga0_002855, mRNA sequence

AATGGATGAAAGTCTAACAAAAGTCCTTTCTTCCTTACTGTTGTCTTTTGCTTCCTTCCCTTGTATGTTC

AGTCGCAGTGCAAAAAAAATCCGGTAGTGTTNCACCTTTGGAGACTCCAATACCGACACCGGTGCATATT

TTTCCGGCTTGGGTATGCTTTTTGGAGCTCCCAACGGACGTACATACTTCAATAAGCCATCTGGCCGGTT

GTCGGACGGACGTTTAGTCATTGATTTCCTATGTGAAAGTTTGAACATTAATTACTTAACACCATATCTT

CAACCCCTGGGGCCTAATTTCAAGTATGGAGTTAATTTTGCCTTTATTGGTGCAGCCACTTCTCCTAGAT

TTAAACCTTTTAGCTTGGACGTTCAAATTCTTCAGTTCATTCACTTCCGAACTCGCTCTCCTGACCTATT

ATCACAAGGATACAAAGACTTGATCAGTGAGGAAGACTTCGAAGATGCACTGTACATATTTGACATTGGA

CAGAACGATTTGGCTGCTTCATTTGAA

>FM895858.1 FM895858 Jatropha curcas embryo 71-95 (DAF) Jatropha curcas cDNA clone rjcpga0_002854, mRNA sequence

TGCAAAGGATTCTATCCGTCGCCCGATGGGAATTGTACTTTAAGTGCGGCCCTCGCGGCTCGTCCGCCGC

GGGGGCTTGGCCAACGGCACGTGCCTCTGGGGGCCGGATGGCCCCTACTGCGGGTCGGCAAACGGGCGAC

GGACACGCGCGTCGCTTCTGGCCCGGATTCTGACTTAGAGGCGTTCAGTCATAATCCAGCGCACGGTAGC

TTCGCGCCACTGGCTTTTCAACCAAGCGCGATGACCAATTGTGCGAATCAACGGTTCCTCTCGTACTAGG

TTGAATTACCATCGCGACACCATCATCAGTAGGGTAAAACTAACCTGTCTCACGACGGTCTAATCCCAGC

TCACGTTCCCTATTGGTGGGTGAACAATCCAACACTTGGTGAATTCTGCTTCACAATGATAGGAAGAGCC

GACATCGAAGGATCAAAAAGCAA

>FM895857.1 FM895857 Jatropha curcas embryo 71-95 (DAF) Jatropha curcas cDNA clone rjcpga0_002853, mRNA sequence

GCTGCAGGAATCGAATTCGGAGTCTGACATGTGTGCGAGTCAACGGGTGAGTAAACCCGCAAGGCGCAAG

AAGCTGACTGGCGGGATCCCCCCGAGGGTTGCACCGCCGACCGACCTTGATCTTCTGAGAAGGGTTCGAG

TGAGAGCATGCCTGTCGGGACCCGAAAGATGGTGAACTATGCCTGAGCGGGGCGAAGCCAGAGGAAACTC

TGGTGGAGGCCCGCAGCGATACTGACGTGCAAATCGTTCGTCTGACTTGGGTATAGGGGCGAAAGACTAA

TCGAACCGTCTAGTAGCTGGTTCCCTCCGAAGTTTCCCTCAGGATAGCTGGAGCTCGGGACGAGTTCTAT

CGGGTAAAGCCAATGATTAGAGGCATCGGGGGCGCAACGCCCTCGACCTATTCTCAAACTTTAAATAGGT

AGGACGGCGCGGCTGCTTCGCTGAGCCGCGCCACGGAATCGAGAGCTCCAAGTGGGCCATTTTTGGTAAG

CAGAACTGGCGATGCGGGATGAACCGGAAGCCGGGTTACGGTGCCC

>FM895856.1 FM895856 Jatropha curcas embryo 71-95 (DAF) Jatropha curcas cDNA clone rjcpga0_002852, mRNA sequence

TGCAGGAATTCGAATTCCCTCTTTTAGGTTCCATTGAAGAAAAAAAAAAAAAAAAAAGTAATATGGATTC

TGTTGTTGCTAAAGCTCTTATTTTAGTGCAACTTTCACTATTATGCTTCCTTGCACTTAGCGATGAAGAA

ATAGGGCCAGTGCCATTGTTGCACCATCACAACAATCACCACCATAGTCTAGCTCCAACTCCAGTCGATA

ATCACCATCACCACCACCACCACGATGATCCAACTCCAACTCCAGCTCATCACCATCACCACCATCATCA

CCATGGTCCAGCCCCAGCTTATACACCACTCCATCCACCAAAGTTACACTCAAACCCACAAGTATACTCT

CCACCAACTCTAGCTCATGGTCAC

>FM895855.1 FM895855 Jatropha curcas embryo 71-95 (DAF) Jatropha curcas cDNA clone rjcpga0_002851, mRNA sequence

ATTCCTCTGATTCTCGCGGTTTTAATAGCCTGTTCGGTAACCCACGTAAGATCAGACGGTTCCGATCACC

GCTACAAGGAAGGCGACCCTGTCCCTCTCTACGCAAATAAGGTCGGCCCCTTTCACAACCCCAGTGAAAC

TTATCGCTACTTTGATCTGCCTTTTTGTGTCCCAGATCATTTGAAAGAGAAAAAAGAAGCTCTTGGAGAG

GTTTTGAATGGTGACCGTTTAGTTAGTGCTCCTTACAAACTTAACTTTAAAGATGAGAAGGTATCAGCAG

TTGTTTGTCAGAAGAAGCTTACGCAAGAGGATGTTGCTCGGTTTAGATCTGCAATTGGTAAAGATTATTA

CTTCCAAATGTACTATGATGATTTGCCCATTTGGGGCTTCATTGGAAAGGTTGACAAAGAAGGCAAGGTT

GATCCCAGTGAGTACAAGTACTTGCTTTACAAGCATATTCAATTTGATGTTCTTTACAATAAGGACCGTG

TGATTGAAGTGAGTGTTCGTATGG

>FM895854.1 FM895854 Jatropha curcas embryo 71-95 (DAF) Jatropha curcas cDNA clone rjcpga0_002850, mRNA sequence

GCTGCAGGAAATAACCACCAGGTAATGTCTACAGGCATACAGAAGGAAGCTGGTCTTAATCAAAATTCAG

CTGAGGCAGCTGGTATGCCTTTTGAGAAACTGTTAACTGAGGTCATTAAGAGAACTCAAGTACCATCTCC

ATCAAAAGAGAATAATACTAGCTCAACTGTTAAATCATGTGCAGAAGATGATGACAGTAGCGCAGATCTG

CCTCTTTCAGTTGAGCCCCTGCAATCTGTGCAGCCAGTTCTGAATAATCACCCTGCAGTTCTGTCAAGAC

CCTTGGAGAACTATAGAACTGGAAAAATGACTGAGCTGCTACAGGTTTTGCAGGAAAACATGCCCCAAAA

CCAGGTCACCGTGGACCCCAGAATGAAGGAAGATGAACTAGGCCCAGAAACCGAACATGGAGATGAAGAT

GGTGGCAATTCAATGAAACATTCACAGGCATCAAGTGTCTGACGTGATAAATGTTTCCTTGGGGAGCAAT

CTCATCGCTGCTGCTTCCTACCTAACATGTTCAGGAGCAATTTGCAGCAA

>FM895853.1 FM895853 Jatropha curcas embryo 71-95 (DAF) Jatropha curcas cDNA clone rjcpga0_002849, mRNA sequence

ATGAAAATTACTTCCAATCATCAAAAGAAGGCTACAGAAGAGACATAAAACTAACAATTAGAAGAGAAAG

ATCTTTGAAGGTCTTTGAGCAGGCCATACTACAAAAGAACAAAGAGGTCTTTCTTTTGGTTTCAAACCAC

CAAATCCATTACCTAACCTATGTGAATGCTGATAGTTAATTAGCAGATTCACTATCAGTATTATGAAACC

CACTCATTACAACTACCCTATAATTCTACCCTAAAATGGAATTTTTAACACCAAAAATTAGCAGGCACTT

GCAGACACACTAAGGAACTGCTTTATTTTCAGATGACGCAACATGCTTGGCAATTTTGGGGGAGTTATCA

ACTGCTGAATTTGATCCATTCTCTTCTAGCAGCTTCAACTCTCCGTTCATCTTCCCCACTTCAACGGATG

CTGCACCTGTTGATTCCCTCAATACACTATCCATTGATTTTTGTG

>FM895852.1 FM895852 Jatropha curcas embryo 71-95 (DAF) Jatropha curcas cDNA clone rjcpga0_002848, mRNA sequence

ATTCCTCAGTTATACCAGAAGTACAAGATCAGAGTTTGGAGATTCTGGCCTTTCCATGCAATCAATTTGG

ATCTCAGGAGCCAGGAACCAATAGAACAGATATGTGGAGTTTGCCTAGCACATCGCTTCAAGGCGTGAGT

ATCCAATATTCAGATAAGATCGATGTCAATGGANAACAATGCTGCTGCTCTTTACAAGTTCTTGAAGTCC

AGCAAAGGAGGAATTTTTGGGGATAGCATCAAGTGGAATTTCACCAAGTTTCTGGCTGATAAAGATGGCA

ATGTTGTGAACCGATATGCTCCCACTACTTCTCCTCTTAGCATCGAGAAAGATGTGAAGAAACTGCTGGG

GATTGCATGAATGCTAAGGCGGGCTATTGAATAAGTCAGGACATCAGTCTGTCTCTGTGATGAATAAGCA

GCATGAAGAGATGTAACTATATTTGTATGGCGTA

>FM895851.1 FM895851 Jatropha curcas embryo 71-95 (DAF) Jatropha curcas cDNA clone rjcpga0_002847, mRNA sequence

ATTCCCCACATGTATGTTATTAAGGATTTGGTCGTGGATATGACTAATTTCTATAATCAATATAAGAGTA

TTGAGCCTTGGCTTAAGAGAAAGAACCCGCCTCAGGTTCCTGGCAAAGAGATATTGCAGAGTAAGAAAGA

TAGGGCTAAGCTTGATGGGATGTATGAGTGTATATTGTGTGCTTGCTGCAGCACATCCTGCCCTAGCTAT

TGGTGGAATCCTGAGTCTTACTTGGGTCCAGCTGCTTTGCTCCATGCTAATCGGTGGATTAGTGATAGCC

GTGATGAATATACCAATGAGCGACTAGAGGCAATAAATGATGAGTTCAAGCTCTACCGCTGCCATACCAT

ATTGAATTGCGCACGTGCCTGCCCTAAATGCTTGAACCCTGGAAAGCAGATCATGAATATCAAACGG

>FM895850.1 FM895850 Jatropha curcas embryo 71-95 (DAF) Jatropha curcas cDNA clone rjcpga0_002846, mRNA sequence

GTAACCAATTCAAGGAATTGGAAAATGAGCTCCAATTGCAGTGTACCAGCTGGAGATTTGCGGTGGAGGC

TAACAATTTGAATCCGTGGAAAACAATTCCCCAGGATTGTGCAGAGTATGTGAGAAATTACATGATGGGA

AGAGGTTATCAAGTTGATCTCGAGAGAGTGTCGAATGAAGCTGGAGTTTATGCCAAGAGTGTCCAGTTGA

ATGAGGATGGCAAGGATGCTTGGGTGTTTGATGTTGACGAAACTTTGCTCTCCAATCTTCCTTATTATAC

TGGGCATGGTTATGGCTTGGAGATTTTTGATCCATTGGAATTTGATAAGTGGGTTGAGAAGGCTATGGCA

CCAGCTAAGAACCAAGTCTGAACCTATAAGGA

>FM895849.1 FM895849 Jatropha curcas embryo 71-95 (DAF) Jatropha curcas cDNA clone rjcpga0_002845, mRNA sequence

GCACAGATCAAATATGTTATGTTACATTACCAAAGGAAGTTAGAATCTCCAGGGTTCAATACATATTATA

TAATGGATTAAATGGTACCATTAAATATCAACTACAGATCTTTTCACAGTTCAAGATAAGCTTACGCAAC

ACAACCTAAATTATCTTTTTGTTTTTTTTCAAGGATGCAATGTGTAAATCGACTCTTCATTTGTTCATAA

CAGAGAGCATTAGACATGGGAAAACACACATCATTTGTACACAACAATGATTGACATCTAAATCTCCCAA

CCATAGACTGAGCTATCCCATGGCATACTTCTGTGTCCAGCTCCTAGCAGTTGCCTCGTACTTGGCCCTG

TCTGTCTTGTACATGTGAGCAATTTCTGGCACCAAGGGATCATCTGGGTTTGGGTCCGTCAAGAGGGAAC

ATATGGAGAGCAACACCTTAGATATTGT

>FM895848.1 FM895848 Jatropha curcas embryo 71-95 (DAF) Jatropha curcas cDNA clone rjcpga0_002844, mRNA sequence

TCTCGCTCTAGGAATGGCCGTCTTAGTCATTCTCTGAATCCGCTCTAGCATTGGCATAGCTTCAAAGTTT

GAAAAGATAAAAAAAGCCGTGTTCTTTGAGCTGTGTTTATCCAGGATGGTAGCTTTTTCTGTTCATTAAT

TAACTTTTTTTTTTGTCTGATTTTAAGCTAAAGATTTGGAATTCGAAAAGGGTCATTCTCCTATAGAAGG

AATCACTTTTATTTCTGGTTTAAAACAAGCCATTTTTTTTTTTTTTTCGCCGCTTGATTTTGATATGAAA

AAGCAGTTTTTCTCTTTTCAAATTTGCTTTTTCTAAGTACGGAAATTCAACTTAAATGGATGATTAGGGT

TTTTTTTGTTGTGTTTGAATATATTAATTGTCTATGTTTTCGAAATGTAGCTGATGGAGAACTAGTTTCA

ATGCT

>FM895847.1 FM895847 Jatropha curcas embryo 71-95 (DAF) Jatropha curcas cDNA clone rjcpga0_002843, mRNA sequence

ACCAGAAAAAGAAATGCAGGTCTTCAATTCATACAAGTAAAAAAACCAACATTAGCTTTTGTAGAAAGCA

TAAATCAGATAAAGCACAGGGCTAATCCTAAGGCAAACAAGTTCAAAGGCACAACCAAAACTCTTCGAAG

CAGAATAATTTTTCTCTAAATCGAAAAAACAAAAACTTAGCCTAGTAAAAGAACCCAAGCACTTTGCCAC

CAACATTCTTTCTTCTAGCCTCTTCATGATCCATAATGCCAGCAGAGGTTGTCAACACAATATAACCAAA

CTGTCTTGATGGAAGCAGTCGTGCAGTCCATCCTTCAATCTCCTTG

>FM895846.1 FM895846 Jatropha curcas embryo 71-95 (DAF) Jatropha curcas cDNA clone rjcpga0_002842, mRNA sequence

TGACATGTGTGCGAGTCAACGGGTGAGTAAACCCGCAAGCGCAAGGAAGCTGACTGGCGGGATCCCCCTG

AGGGTTGCACCGCCGACCGACCTTGATCTTCTGAGAAGGGTTCGAGTGAGAGCATGCCTGTCGGGACCCG

AAAGATGGTGAACTATGCCTGAGCGGGGCGAAGCCAGAGGAAACTCTGGTGGAGGCCCGCAGCGATACTG

ACGTGCAAATCGTTCGTCTGACTTGGGTATAGGGGCGAAAGACTAATCGAACCGTCTAGTAGCTGGTTCC

CTCCGAAGTTTCCCTCAGGATAGCTGGAGCTCGGGACGAGTTCTATCGGGTAAAGCCAATGATTAGAGGC

ATCGGGGGCGCAACGCCCTCGACCTATTCTCAAACT

>FM895845.1 FM895845 Jatropha curcas embryo 71-95 (DAF) Jatropha curcas cDNA clone rjcpga0_002841, mRNA sequence

ATTCGCAAAGGATTCTATCCGTCGCCCGATGGGAATTGTACTTTAANGGCGGCCCTCGCGGCTCGTCCGC

CGCGGGGGCTTGGCCAACGGCACGTGCCTCTGGGGGCCGGATGGCCCCTACTGCGGGTCGGCAAACGGGC

GACGGACACGCGCGTCGCTTCTGGCCCGGATTCTGACTTAGAGGCGTTCAGTCATAATCCAGCGCACGGT

AGCTTCGCGCCACTGGCTTTTCAACCAAGCGCGATGACCAATTGTGCGAATCAACGGTTCCTCTCGTACT

AGGTTGAATTACCATCGCGACACCATCATCAGTAGGGTAAAACTAACCTGTCTCACGACGGTCTAAACCC

AGCTCACGTTCCCTATTGGTGGGTGAACAATCCAACACTTGGTGAATTCTGCTTCACAATGATAGGAAGA

GCCGACATCGAAGGATCAAAAAGCAACGTCGCTATGAACGC

>FM895844.1 FM895844 Jatropha curcas embryo 71-95 (DAF) Jatropha curcas cDNA clone rjcpga0_002840, mRNA sequence

CTGAAGGAAAGTCAGTTGCTTTTCGATATAAGCAGAAATTCTGATGGAGAGATTTNTACAAGAAGTTCCA

ACATCAACAAAACGGGCCATTGGGGAAAAATTCTCTCAGATTCCAGATCTAATCATACAATTGAGACCTC

CAAAAATGCCAGGGTCAACCTAACTTTAACAGGCCTACTGTATTTTCTCAAATCTAAAGAAAAACAAAAG

ACCTGAATCAAAATTTCGATTTTATGTAGAAGGAAGCTATATCCCACCAGTGCTTTCCCAACCCCAAATA

GAAAGAAAAGGGGTGAAAAAAAAGAATGAGAACATTATTTTCTTTGACAATTGACAGTATATAACTACTA

ACACAGAATGTAAGCAAATCCACCAGCTTTTGTTGCAAGCCTACTGCTCAACCTTGAGCACAGGAAAAGC

AATAACATCTCTTATGCTAGG

>FM895843.1 FM895843 Jatropha curcas embryo 71-95 (DAF) Jatropha curcas cDNA clone rjcpga0_002839, mRNA sequence

GGCGTAAAGACCACAATTTCATATCCAAAGTTCAAAAACATTTCGAGTAAAGTTTAGATATACAAGTTGG

GCACAAGAATCTCTGGCTAGTTTTTGTATGCCAGTAGATTTTGTTAATAGATACAGGCACTAACTATATA

GCATCATAACCTTCAATGTTTTAACACCATCTTTGTGATTTCCATCATTCGTCAACCAGACTTGAATAGC

AGAACTGCTTTCTGGTCCAGATAAAAATAAACAAAGTGGTTTGTCTCATGGGTCACATATGAACCAAAAT

TCCGACCAACGATGCAATGCCAAGTGGGCCCATGCCTCCTATCGAACTCCTTCTTTATGTGCTCCGCAAC

ATCCTTCTCTACATTGTGCTTCTCGAACGCGGCGATGGCAATATCGACGGCCTCTTTCTGCATGTCATCT

TTC

>FM895842.1 FM895842 Jatropha curcas embryo 71-95 (DAF) Jatropha curcas cDNA clone rjcpga0_002838, mRNA sequence

TCTGACATGTGTTGCGAGTCAACGGGTGAGTAAACCCGCAAGGCGCAAGGAAGCTGACTGGCGGGATCCC

CCTNAGGGTTGCACCGCCGACCGACCTTGATCTTCTGAGAAGGGTTCGAGTGAGAGCATGCCTGTCGGGA

CCCGAAAGATGGTGAACTATGCCTGAGCGGGGCGAAGCCAGAGGAAACTCTGGTGGAGGCCCGCAGCGAT

ACTGACGTGCAAATCGTTCGTCTGACTTGGGTATAGGGGCGAAAGACTAATCGAACCGTCTAGTAGCTGG

TTCCCTCCGAAGTTTCCCTCAGGATAGCTGGAGCTCGGGACGAGTTCTATCGGGTAAAGCCAATGATTAG

AGGCATCGGGGGCGCAACGCCCTCGACCTATTCTCAAACTTTAAATAGGTAGGACGGCGCGGCTGCTTCG

CTGAGCCGCGCCACGGAATCGAGAGCTCCAAGTG

>FM895841.1 FM895841 Jatropha curcas embryo 71-95 (DAF) Jatropha curcas cDNA clone rjcpga0_002837, mRNA sequence

ATTCGTCTGACATGTGTGCGAGTCAACGGGTGAGTAAACCCGCAAGGCGCAAGGAAGCTGACTGGCGGGA

TCCCCCTNAGGGTTGCACCGCCGACCGACCTTGATCTTCTGAGAAGGGTTCGAGTGAGAGCATGCCTGTC

GGGACCCGAAAGATGGTGAACTATGCCTGAGCGGGGCGAAGCCAGAGGAAACTCTGGTGGAGGCCCGCAG

CGATACTGACGTGCAAATCGTTCGTCTGACTTGGGTATAGGGGCGAAAGACTAATCGAACCGTCTAGTAG

CTGGTTCCCTCCGAAGTTTCCCTCAGGATAGCTGGAGCTCGGGACGAGTTCTATCGGGTAAAGCCAATGA

TTAGAGGCATCGGGGGCGCAACGCCCTCGACC

>FM895840.1 FM895840 Jatropha curcas embryo 71-95 (DAF) Jatropha curcas cDNA clone rjcpga0_002836, mRNA sequence

CGAATTCATTCAGCATGCTTCTGCTGCATATAACTTCTCATTTTGCTTGCTAGTTGTAGATTTTGTCTTG

GTTTTGTTTGCATCATCTTCTTCAAGACCCATTGGTTGGTCTTCACCATCATTCATTTCTTCAAAGTTTT

CATCACCCTGGGTTGATGACCGAGTCTCCATATCTCCCTGTATCATACTTGTCATCAAAATTGATAGGAC

AACTAGGAGGTACTTCAACAGGATTGTGGTCATTAACGGACTTGAGACTACCAATAAATGAGGATTCACC

ACTGTAGACCTCATCAACATTAAATTCCTTCCCAAGCTCTGAAACAATCCTAGCTTCTGAAGGTTCCTCT

CGATTCCTAATTGGAGGCATAGTATAATACGGGATTTACCCTCATTCCAATCATGCAGAACAATTCGAGC

AGCAGCTTCAACATCCACAATA

>FM895839.1 FM895839 Jatropha curcas embryo 71-95 (DAF) Jatropha curcas cDNA clone rjcpga0_002835, mRNA sequence

ATTCCTCTCTCTCTAAAATAGCACTCTCACCGAATCTCAGAGACACTCTTTGCTTCTCTGATCCTTTTCA

TCTTGGTTTATGAGCTCAAAGGAGAGACCCACTCTTGGTGGCACGCGGATTAAGACCCGCAAACGGAACA

TAGCAGCACCGCTGGACCCTGCAGCATTTGCGGATGCAGTGGTCCAGATTTATCTGNGATAATGCTGGTG

ATTTGGAACTTATTGCCAAGAGCATTGAATCTTCAAACCTTAACTTCTCAAGATACGGTGATACCTTTTT

TGAGGTTGTTTTTACAGGAGGCCGTACACAACCTGGAACAACAAAACCTGATGAGGGGGAGCGCCATCCT

TACTCTATAATAGATTGTGAGCCTACGCGTGAAGTCATCTTACCATCTGTAATTT

>FM895838.1 FM895838 Jatropha curcas embryo 71-95 (DAF) Jatropha curcas cDNA clone rjcpga0_002834, mRNA sequence

CAGATCAAAATGCTCTTCATTCATTTCAGTTATGATGTAACCTTAATCAGTCTAATTTCAACTACTACTA

ATTCAGAGAAGATTCACCAAATAAAGGGGCTTTCATATGCTCCGCAATCAACAAATAGCTTTGAAAGCAA

GTTCCAAGCGCATCCATATGAACCCTGAATTTATGAATATAAAAGTAACTGGGAAAATTTTAAGACCCTG

>FM895837.1 FM895837 Jatropha curcas embryo 71-95 (DAF) Jatropha curcas cDNA clone rjcpga0_002833, mRNA sequence

CTCAAACTTTAAATAGGTAGGACGGCGCGGCTGCTTCGCTGAGCCGCGCCACGGAATCGAGAGCTCCAAG

TGGGCCATTTTTGGTAAGCAGAACTGGCGATGCGGGATGAACCGGAAGCCGGGTTACGGTGCCCAACTGC

GCGCTAACCTAGAACCCACAAAGGGTGTTGGTCGATTAAGACAGCAGGACGGTGGTCATGGAAGTCGAAA

TCCGCTAAGGAGTGTGTAACAACTCACCTGCCGAATCAACTAGCCCCGAAAATGGATGGCGCTTAAGCGC

GCGACCTATACCCGGCCGTCGGGGCAAGAGCCAGGCCCCGATGAGTAGGAGGGCGCGGCGGTCGCTGCAA

AACCCGGGGCGCGAGCCCGGGCGGAGCGGCCGTCGGTGCAGATCTTGGTGGTAGTAGCAAATATTCAAAT

GAGAACTTTGAAGGCCGAAGAGGGGAAAG

>FM895836.1 FM895836 Jatropha curcas embryo 71-95 (DAF) Jatropha curcas cDNA clone rjcpga0_002832, mRNA sequence

GTCTGACATGTGTGCGAGTCAACGGGTGAGTAAACCCGCAAGGCGCAAGGAAGCTGACTGGCGGGATCCC

CCCGAGGGTTGCACCGCCGACCGACCTTGATCTTCTGAGAAGGGTTCGAGTGAGAGCATGCCTGTCGGGA

CCCGAAAGATGGTGAACTATGCCTGAGCGGGGCGAAGCCAGAGGAAACTCTGGTGGAGGCCCGCAGCGAT

ACTGACGTGCAAATCGTTCGTCTGACTTGGGTATAGGGGCGAAAGACTAATCGAACCGTCTAGTAGCTGG

TTCCCTCCGAAGTTTCCCTCAGGATAGCTGGAGCTCGGGACGAGTTCTATCGGGTAAAGCCAATGATTAG

AGGCATCGGGGGCGCAACGCCCTCGACCTATTCTCAAACTTTAAATAGGTAGGACGGCGCGGCTGCTTCG

CTGAGCCGCGCCACGGAATCGAGAGCTCCAAGTGGGCCATTTTTGGTAAG

>FM895835.1 FM895835 Jatropha curcas embryo 71-95 (DAF) Jatropha curcas cDNA clone rjcpga0_002831, mRNA sequence

CGAATTCATTTGATGAACCATGCCTCCATAGCCATGAACCTCTACTATCAATTCAAGGGAAGGAACATAT

GGAATTGTGATTTTAGGAATTCTGGTCTCATTGTCCTCACTAATCCGAGTTATGGGGATTGCATCTATGC

CTAATGCTTCTTGGTACAGAAAGAACTTTTGCTCCAAATAAATAAAATTATTAGAAGAATCCTCAACCAT

TCTGTAATTTCTGGAGAAACTGAGAAATACTTAAAGTCTATTTCGACATCCACAGTAGTACAGTAGTTTA

AAACGATCAACTGATCAACAAGGAAAAGTTTTCCCGTAAAGTTCAGGTATCAATTTATGTTTTTGGACCC

TTTTATTATAATAATATACCCAATTATTACACCCCAACGGAAATTTTTCTGGAATTCATGATTTTCCTAT

GTAGTTCATTTCTCTCAAGTCCTTCAATAGTTCAATGTGGTTGAATGGTTTACA

>FM895834.1 FM895834 Jatropha curcas embryo 71-95 (DAF) Jatropha curcas cDNA clone rjcpga0_002830, mRNA sequence

ATTAAAGGATTCTATCCGTCGCCCGATGGGAATTGTACTTTAAGGCGGCCTCGCGGCTCGTCCGCCGCGG

GGGCTTGGCCAACGGCACGTGCCTCTGGGGGCCGGATGGCCCCTACTGCGGGTCGGCAAACGGGCGACGG

ACACGCGCGTCGCTTCTGGCCCGGATTCTGACTTAGAGGCGTTCAGTCATAATCCAGCGCACGGTAGCTT

CGCGCCACTGGCTTTTCAACCAAGCGCGATGACCAATTGTGCGAATCAACGGTTCCTCTCGTACTAGGTT

GAATTACCATCGCGACACCATCATCAGTAGGGTAAAACTAACCTGTCTCACGACGGTCTAAACCCAGCTC

ACGTTCCCTATTGGTGGGTGAACAATCCAACACTTGGTGAATTCTGCTTCACAATGATAGGAAGAGCCGA

CATCGAAGGATCAAAAAGCAACGTCGCTATGAACGCTTGGCTGCCACAAGC

>FM895833.1 FM895833 Jatropha curcas embryo 71-95 (DAF) Jatropha curcas cDNA clone rjcpga0_002829, mRNA sequence

CGCATATCTCGCTGCTCTTTTTGAACTCACCACTCTCTATATATCAGTTGAGATCTTTCTCTTCTCTTCT

ATTCTCTTCAAATTTCAGTTACGTCTTTTCTCATTGCCTTTCTTTCTCTCCCTCTGTTAGACGAGAAATG

GTGTATTATACTATATCAGGGAATTGGAATACATTCTCCATGTGTACCATCAGTTCACAAATCTCAGTTC

ATTGGCTTCCATGGTGATCGAAGGAGCTCTGGACTTTCCTTCTTGTTGAACAAGGAGCCATTTTCTCGGA

AGATCTTGCTGGAAAGTCCTCTTATGATTCTGACTCCTCAAATTAGCAGTCACAGCTTCTGTAAAGTCCT

TGTCCGGTGGTCATATTGATAACTCACTACTTCGAAAGATCAGTTAGAAACACTTGGTGCAGTTGCTGAA

GAATC

>FM895832.1 FM895832 Jatropha curcas embryo 71-95 (DAF) Jatropha curcas cDNA clone rjcpga0_002828, mRNA sequence

AAGATATGCAGGAAGGTGGGGAATGGGAATGGGAAGGGGAGAGGCTGTGATCTCGCATATGTAGTGTGGT

ATTATGAATTGATGAGCTCCTCTCCTTTGCTTTACCACCTAAGTGAAAGAGAGAGAGAGAGAGAGGGAGG

GTACCTCCTTGCTTTTAGAGATTGGTGGTTTACCATTATACAACTCCTCCAGTGTAATTTAGCAAAGTAC

ATAACAGGATTTTGACTAGGGGATGTATACCGGGTATAGTTGGTTGCATTTGACAATTTCAATTCTTGTC

CTTATTTTTTTCTTGTCGCCAGTTTTCTCGGGGATCGGCTTGTAAAATTATTGGTTGAATTTACGCTTCT

GGGCTTTAGGATTCATGTAAAAGAATTGTAATG

>FM895831.1 FM895831 Jatropha curcas embryo 71-95 (DAF) Jatropha curcas cDNA clone rjcpga0_002827, mRNA sequence

CAAACATGAAGTTCAACATTGCCAACCCACTACGGGATGCCAAAAGAAGCTGGAAATCGACGATGACCAG

AAACTCCGGGCTTTTTTCGACAAGAGGATCTCACANGGAGGTCAGCGCGAGATGCACNTTGGAGAGGAAT

TTAAGGGTATGTTTTCAAGATTATGGGAGGTTGTGACAAGCAAGGGTTTCCCATGAAGCAGGGAGTGTTG

ACTCCTGGCCGTGTCCGACTCTTGCTTCACAGAGGAACCCCTTGCTTTCGCGGTTATGGAAGGCGAAATG

GAGAACGCAGGAGGAAGTCTGTCCGCGGATGCATTGTCAGCCAAGACCTTTCTGTTTTGAACTTGGTAAT

TGTGAAGAAGGGAGAGCAAGATCTGCCTGGACTGACAGACACT

>FM895830.1 FM895830 Jatropha curcas embryo 71-95 (DAF) Jatropha curcas cDNA clone rjcpga0_002826, mRNA sequence

TCCGTGTTTCATAAAAAATATCGCCAGTTATGGACTAACTGAAAGTCTCCATCTCTTCTCCTTTGTCTTC

TATATATTCTTCCGTAATCATCGCCGGCGACGATTCCTGTTCGCAATGGCCTCGCTCTCCGGCCTTTGCT

CCTCTTCCCCTTCTCTAAAGCCCAAAACCCCTCTTCTTAAAACCACGCTTAATCCACAGCTCATCAAAAC

CTCACTCTCGTCTCCAACTCGGTCGTCTCGGCTTGTTCAAACTCAGGCCGTAGCTCGCGAGATCTCGTCC

AATTTGTCGAAGACAAACGATAAGTTGCTAACCAAGCCGAAGCAAGGATTGGAGAAAGATCCTAGCAGTC

TATGGAGGAGATACGTGGACTGGTTGTACCAGCACAAGGAGTTAGGGCTCTATTTGGATGTAAGTCGGGT

CGGGTTCACGGACGAGTTTGTGGCGGAGATGGAGCCCCGACTACAGAA

>FM895829.1 FM895829 Jatropha curcas embryo 71-95 (DAF) Jatropha curcas cDNA clone rjcpga0_002825, mRNA sequence

TAGAAATTTGGAGAGTCGTGGCTGTGCATTACTGGGAATTTATGATACACCAACTCTGCTTGCAAAGGAA

AGACTTTTTCTTGATCAGGGATGGCAGAGGGTTGTTGCATGGGACATGCTGAGAGTTTATAGTGATTTTA

TTGAAGCTCAAGAAAGGCGCAGGATTGAACGGCTGGAATTATTTGATGAGTTTGAAGAGTGGCACATGAT

GCAGGCAAGCTAATTCGTCATTCATGCTTCAAAAAAAAGAGCACTACTGTGTGGCTTGCGCCATAAATGA

TGCCATGGGGCTCTTTGGGAATTTCGGTTTTCCTACCCACCAGCAGCACGTGATCAGCAACCCTTCAGCA

GCATCTCCATGAAAGCCCAAGACTGCACCAGAGTGTGGCTGCTCATTGCCATGTTTGGTTAAACAAATAG

TATCAGGGCAGTGCCTATGACTGACGCGTGCCAGTTAACCAGGTTAAGAGG

>FM895828.1 FM895828 Jatropha curcas embryo 71-95 (DAF) Jatropha curcas cDNA clone rjcpga0_002824, mRNA sequence

AACTATGGATCCCGGGCTGCAGGATTGAATACCCTACACCCACCAGAGCAGAAGTAGCTGATGTTTCTGA

AGCAGTTAGGCAACGGGCTGATGCTTTGATGCTCTCTGGTGAGTCAGCCATGGGCCANGTACCCAGAGAA

GGCACTGGCTGTTCTGAGAAGTGTCAGTGTGAGAATCGAGAAATGGTGGAGAGAAGAGAAGCGTCATGAA

GTTATGGAACTCCCAGCCATAGGATCTTCATTTTCAGATTCTATTTCTGAAGAGATTTGCAACTCTGCTG

CCAAGATGGCTAACAATTTGGAGGTGGATGCACTTTTTGTCTATACTAAGAATGGTCACATGGCATCTCT

CTTGTCTCGTTGCCGGCCTGACTGCCCAATCTTTGCTTTTACAACCACGACATCAGTAAGGAGGCGCCTG

AAACTACAGTGGGGCTTTATTACTTTCCGCCTGAACTTTTCTGATGATATGGAAAGTAACCTTTATAGGA

CATTCGCCTTACTC

>FM895827.1 FM895827 Jatropha curcas embryo 71-95 (DAF) Jatropha curcas cDNA clone rjcpga0_002823, mRNA sequence

CGCCATGAAAGGAGGTAAATCAAAGTCAGACACCAAGAACGCCAAGCTTTCCGTGAACAAGAAACCCGCC

AAGGCAAGTAAGAAGGCGGGTAAGGCAGCGAAGGATCCAAATAAGCCAAAGAGGCCCGCCAGTGCTTTCT

TCGTTTTCATGGAAGAGTTCAGAGAGCAGTACAAGAAGGAACATCCTAAAAACAAATCCGTTGCTGCTGT

TGGCAAAGCTGGTGGAGATAAATGGAAGTCAATGTCAGATGCTGAGAAAGCACCTTTTGTATC

>FM895826.1 FM895826 Jatropha curcas embryo 71-95 (DAF) Jatropha curcas cDNA clone rjcpga0_002822, mRNA sequence

CGGCGTCAAACTATGGATCCCCGGGCTGCAGGATTCGAATCTCTAGCTTCACATCCAGTGTAATTAAGAC

ACACCGCCGTGGAATTTTGATCAGACGACAAAGATGCCGATCAGAAACATTGCCATAGGCCATCCTCAGG

AGGCAACTCATCCAGACGCCTTGAAAGCGGCGTTGGCTGAGTTCATCTCCACTCTTATTTTTGTCTTCGC

CGGTGAAGGCTCCGGGATGGCCTTTAATAAGCTCACTAACAACGGCGCCACCACTCCTGCCGGTCTAGTC

GCCGCATCTTTANCCCATGGTTTCGGTCTTTTTGTTGCTGTCTCAGTTGGAGCCAACATCTCCGGCGGTC

ATGTAAATCCAGCTGTTACCTTCGGTGCTTTCGTCGGT

>FM895825.1 FM895825 Jatropha curcas embryo 71-95 (DAF) Jatropha curcas cDNA clone rjcpga0_002821, mRNA sequence

GAGAAAGTGGAGATAGATAGAGAGAGGGTGGGGAATATGAAGACCAGAGAGAGATGGCTATGGCAGTGAT

TTTTTTTGGGACCAATTTCCCCCTTTCCTTTTTTCTTAAAAAAAAATTAAGTTTTGGTGAAGAATTTACC

GATAGTTCTTCCTTCTGGTGAAGAGAAATTGAAATTCCCTTCTGATAAGGTTTTTTTTTTTTTTTTTTTT

TCAATCGGCGAAACGTTTTCAGTTTTCGAAAAGGTTTTGGGATTTTCTGGCATAACGTTTCGGGGAGATG

CCTTGGGGACTATACATGATTGGATCCTCTGAGATGAGGGAAATTTCCGGTTTTGCTTAAAGGTTGTTTT

TTCATTTGGAACACATATTTGGTTATTTCATTTTTAAATTTATTGAAGACTAGGCGAAAATTGATGTTTT

TTTAATCTCGATTAGTGCGATTTCTGGGGTGGAGATTGGAAAAAGTTTTGC

>FM895824.1 FM895824 Jatropha curcas embryo 71-95 (DAF) Jatropha curcas cDNA clone rjcpga0_002820, mRNA sequence

CGCGGGTGCGGCCGCCTAAACTATGGATCCCCCGGCTGCAGGCAGGAGCCTCAAAATGGACTGTTCAGGA

GCTTGTCGAATCAGTGAAGGAGGATTTTGGGAGCATTGACATCCTTGTGCATTCACTTGCTAATGGGCCA

GAGGTTAGCAAACCTCTTCTGGAGACATCAATGTATGGGTACCTTGCAGCTATATCCGCATCAAGTTACT

CATATGTTTCCTTATTGAAGCATTTTCTTCCGATAATGAATCCTGGTGGTTCTGCGCTTTCTCTAACCTA

CATAGCCTCCGAAAGGATCATTCCAGGGTATGGTGGAGGCATGAGTTCAGCAAAGGCTGCTCTGGAGAGT

GACACTCGAGTTATGGCCTTTGAAGCTGGAAGAAAGTACAAAATCAGAGTAAACACAATATCTGCAGGCC

CATTGAGAAGCCGTGCTGCCAAGGCAATTGGCTTTATCGATATGATGATTGAGTACTCAGTGGCGAATGC

ACCTCTACAGAAGGAACTATCTGCAGATGAGGTGGGGAA

>FM895823.1 FM895823 Jatropha curcas embryo 71-95 (DAF) Jatropha curcas cDNA clone rjcpga0_002819, mRNA sequence

GGATTCTATCCGTCGCCCGATGGGAATTGTACTTTAAGGCGGCCTCGCGGCTCGTCCGCCGCGGGGGCTT

GGCCAACGGCACGTGCCTCTGGGGGCCGGATGGCCCCTACTGCGGGTCGGCAAACGGGCGACGGACACGC

GCGTCGCTTCTGGCCCGGATTCTGACTTAGAGGCGTTCAGTCATAATCCAGCGCACGGTAGCTTCGCGCC

ACTGGCTTTTCAACCAAGCGCGATGACCAATTGTGCGAATCAACGGTTCCTCTCGTACTAGGTTGAATTA

CCATCGCGACACCATCATCAGTAGGGTAAAACTAACCTGTCTCACGACGGTCTAAACCCAGCTCACGTTC

CCTATTGGTGGGTGAACAATCCAACACTTGGTGAATTCTGCTTCACAATGATAGGAAGAGCCGACATCGA

AGGATCAAAAAGCAACGTCGCTATGAACGCTTGGCTGCCACAAGCCAGTTATCCCTGTGGTAA

>FM895822.1 FM895822 Jatropha curcas embryo 71-95 (DAF) Jatropha curcas cDNA clone rjcpga0_002818, mRNA sequence

ATTCCCTGNCTGTCACCTTTGGATTGGCTCTTGNGTGGTCAAATCACCATTCTTACTGGCATCTTCTACT

GGATTGCTCAGCTTCTTGGTTCCATTGTTGCTTGCCTCCTTCTCAAAGTTGTCACCGGAGGCTTGGAAAC

ACCCACCCACAGCCTTGCAGCTGGAGTAGGAGCCATTGAAGGAGTGGTTATGGAGATTATAGTCACATTT

GCTTTGGTATACACAGTGTACGCAACAGCAGCTGATCCCAAGAAAGGATCACTAGGCACCATTGCCCCCA

TAGCCATTGGCTTCATCGTTGGTGCCAACATCTTGGCTGCAGGCCCATTTTCCGGTGGATCCATGAACCC

AGCCCGCTCTTTTGGCCCAGCTGTGGCTAGCGGTGACTTCCATGACAACTGGATCTATTGGGTTGGGCCC

CTTATTGGTGGTGGGCTAGCAGGCCTTATCTATGGAAA

>FM895821.1 FM895821 Jatropha curcas embryo 71-95 (DAF) Jatropha curcas cDNA clone rjcpga0_002817, mRNA sequence

CAAAGGATTCTATCCTGTCGCCCGATGGGAATTGTACTTTAAGGCGGCCCTCGCGGCTCGTCCGCCGCGG

GGGCTTGGCCAACGGCACGTGCCTCTGGGGGCCGGATGGCCCCTACTGCGGGTCGGCAAACGGGCGACGG

ACACGCGCGTCGCTTCTGGCCCGGATTCTGACTTAGAGGCGTTCAGTCATAATCCAGCGCACGGTAGCTT

CGCGCCACTGGCTTTTCAACCAAGCGCGATGACCAATTGTGCGAATCAACGGTTCCTCTCGTACTAGGTT

GAATTACCATCGCGACACCATCATCAGTAGGGTAAAACTAACCTGTCTCACGACGGTCTAATCCCAGCTC

ACGTTCCCTATTGGTGGGTGAACAATCCAACACTTGGTGAATTCTGCTTCACAATGATAGGAAGAGCCGA

CATCGAAGGATCAAAAAGCAACGTCGCTATGAACGCTT

>FM895820.1 FM895820 Jatropha curcas embryo 71-95 (DAF) Jatropha curcas cDNA clone rjcpga0_002815, mRNA sequence

ATTGTTCCTGNTACATCACTGTCTTGACTGTTATGGGTGTCTCTAAATCTGGCAATAAATGTGNCTACTG

TGGGTACCCAAGTATTGCTTGGGCCTTTGGTGGAATGATCTTTGCACTTGTCTACTGTACTGCTGGTATC

TCAGGAGGACACATTAACCCAGCAGTGACCTTTGGTTTGTTTCTGGCACGGAAGCTTTCTTTGACAAGGG

CTTTGTTCTACATCATCATGCAGTGCCTTGGTGCCATCTGTGGTGCTGGTGTGGTGAAAGGTTTTGAGGG

CAACCGTGTATATGAGAGTTTGGGTGGTGGAGCCAACGTTGTTGCTTCAGGCTACACTAAGGGTGACGGT

CTTGGTGCTGAGATTGTTGGCACCTTTGTTCTTGTCTACACTGTCTTCTCCGCCACTGATGCTAAGAGGA

GCGCCAGGGACTCTCATGTCCCTATTTTGGCTCCTCTTCCCATTGGGTTTGCAGTGTTCTTGGTTCACTT

GGCCACCATCCCCATTACTGGAACTGGCATTAACCCAGCCA

>FM895819.1 FM895819 Jatropha curcas embryo 71-95 (DAF) Jatropha curcas cDNA clone rjcpga0_002814, mRNA sequence

TGCAAAGGATCTATCCGTCNCCGATGGGAATTGTATTTAAGGCGGCCCTCGCGGCTCGTCCGCCGCGGGG

GCTTGGCCNAACGGCACGTGCCTCTGGGGGCCGGATGGCCCCTACTGCGGGTCGGCAAACGGGCGACGGA

CACGCGCGTCGCTTCTGGCCCGGATTCTGACTTAGAGGCGTTCAGTCATAATCCAGCGCACGGTAGCTTC

GCGCCACTGGCTTTTCAACCAAGCGCGATGACCAATTGTGCGAATCAACGGTTCCTCTCGTACTAGGTTG

AATTACCATCGCGACACCATCATCAGTAGGGTAAAACTAACCTGTCTCACGACGGTCTAAACCCAGCTCA

CGTTCCCTATTGGTGGGTGAACAATCCAACACTTGGTGAATTCTGCTTCACAATGATAGGAAGAGCCGAC

ATCGAAGGATCAAAAAGCAACGTCGCTATGAACGCTTGGCTGCCACAAGCCAGTTATCCCTGTGGTAACT

TTTCTGACACCTCTAGCTTCAAATTCCGAAGGTCTAAAGGATCGATAGGCCACGCTTTCAC

>FM895818.1 FM895818 Jatropha curcas embryo 71-95 (DAF) Jatropha curcas cDNA clone rjcpga0_002813, mRNA sequence

ATTCGTCTGACATGTGTGCGAGTCAACGGGTGAGTAAACCCGCAAGGCGCAAGGAAGCTGACTGGCGGGA

TCCCCCCGAGGGTTGCACCGCCGACCGACCTTGATCTTCTGAGAAGGGTTCGAGTGAGAGCATGCCTGTC

GGGACCCGAAAGATGGTGAACTATGCCTGAGCGGGGCGAAGCCAGAGGAAACTCTGGTGGAGGCCCGCAG

CGATACTGACGTGCAAATCGTTCGTCTGACTTGGGTATAGGGGCGAAAGACTAATCGAACCGTCTAGTTG

CTGGTTCCCTCCGAAGTTTCCCTCAGGATAGCTGGAGCTCGGGACGAGTTCTATCGGGTAAAGCCAATGA

TTAGAGGCATCGGGGGCGCAACGCCCTCGACCTATTCTCAAACTTTAAATAGGTAGGACGGCGCGGCTGC

TTCGCTGAGCCGCGCCACGGAATCGAGAGCTCCAA

>FM895817.1 FM895817 Jatropha curcas embryo 71-95 (DAF) Jatropha curcas cDNA clone rjcpga0_002812, mRNA sequence

GACGCCTCCATACGTAGATGATAGGCCTTATTGAGGTTGTTGGTTTCAGAAATGCTTGGCATGGGCTTGT

TCTGCTGGCTCCAAATGGTCATGGGACTCGTCTTNCACAGGCAAGTAGAATTCGCTTTGAGTTCCGTAAG

GGTGCAGCTGACCATACATTCATGAGAATTGATGGGGAGCCTTGGAAACAACCGCTTCCTGTTGATGATG

ACACAGTCGTCGTGGAAATTTCTAACCATGGCCAAGTAGCCATGCTTGCAACCCCATCATGCCAATCCAG

AAGTATAAATGACCCGGCTTCACCTACTAATGATCATGATGAGGACGATAATAATTCCAGTGACGAAGAC

AATGAATCTTTANAAGATGAGGAAGAGCGAAGAAAGTTTGGAGCAGCTGACACTTTTCAATTTCCTGATG

ATTTTGATATCTCTCAGCTTAGTTGAACACCAACACATTCTCCTATATTTGCTTTTTGCAGACTAGCCTT

AGATTT

>FM895816.1 FM895816 Jatropha curcas embryo 71-95 (DAF) Jatropha curcas cDNA clone rjcpga0_002811, mRNA sequence

GGCTGCAGGTGTGAAGAAGCCCACAGATATCGTCCTGGAACTGTTGCACTTCGTGAAATTCGCAAGTACC

AAAAGAGTACTGAACTCCTTATCAGGAAACTCCCATTCCAGAGGCTTGTTCGTGAAATTGCTCAGGATTT

TAAGACTGACTTGCGATTCCAGAGCCATGCGGTTTTGGCCTTACAAGAGGCAGCAGAAGCATACCTTNTT

GGATTGTTTGAGGATACTAATCTGTGTGCCATCCACGCAAAGCGTGTTACCATAATGCCCAATGACATTC

AGCTTGCCAGGAGAATCCGTGGTGAGAGGGCTTAAAAGCTCAATTTGCTACAAGGTGGTCAGCTGATTGT

TGATGATTGTGTAGCGGATGTGTTTGTTGTAGGATCTTACTCTGTCTTGTCAGTTCTTGTTTTTGAATGT

GAAAGTGGATGATCAGTAGGGTCAGACTGTATTAGGGTACTTTAAGTTGGGGATGGCTGGTACAGTGGAA

TGTTGCTATTAAACCCTGAACAACCTAACGTTGTTCGTTAAAAATGTTTTGCTGTTCATGAATTTGATG

>FM895815.1 FM895815 Jatropha curcas embryo 71-95 (DAF) Jatropha curcas cDNA clone rjcpga0_002810, mRNA sequence

GCTGCAGGAATTCGAATTCCAAAAAATCAATATTAGCATTTCCAATTTTTTTGCTCAAATTCTCTAGCAT

GCAGACAAGTCAGATTGTTCTGTGTAGGAAACAAAGCCGAAGCTAAATGGTAAAATATCAGCATGAACAA

CTATCCTAGTATTTTATATATATATATATATATTTTTTTTTAAATAACCTGGGGGATTCAAACTTTTTGA

CTTAAGATATTCTGTTACTTTTATGTGAATGTGCCTCACATTTGTGCGACAATTGAATGAATTTATGTGA

TAAGAGGTATGAATATACTGAAATTGGAAGGGAAAAATCAAACAGCCTGAAGCAGTATCTGATGAACATG

TACCAGCCAGCAATAAAGCATATGAGAAATTTGCTTCCTGCTTTCCACCCCCTCCAACAAGTGTACTCTT

TTGTTCTGTTGGTTCCCATTCACGCATTTTTCTGAATCAAACAAATAAATCATTTTATTTTTTCAGTTGA

TCTCATATTTACTTGTTCTGCTTTANCTGCTGCATTTTCAATGAATGTGAGTCTTGTTTGATTTGATTAT

TATTATTATT

>FM895814.1 FM895814 Jatropha curcas embryo 71-95 (DAF) Jatropha curcas cDNA clone rjcpga0_002809, mRNA sequence

TTGTTTGTATATGACATATTCTGGGTATTCTTCTCAGAGAGATTTTTTGGTGCCAATGTTATGGTGTCAG

TGGCAACGCAACAAGCATCAAATCCTGTTCATACAGTGGCCAATAGTTTGAGCCTCCCTGGGTTGCAATT

GATTACAAAGAAGCTTGAGTTGCCTGTGAAAATAGTCTTTCCTAGGAATTTGTTGGGTGGTGCTGTACCT

GGAGGAAATGCTGCCGATTTCATGATGCTTGGCCTCGGTGACATGGCTATTCCTGCCATGCTTCTGGCAT

TAGTCCTCTGTTTTGATCATCGGAAGAGCAGAGAGCCTGTAAGTCTCTTAGATATACATTCATCAAAGGG

GCACAAATACATATGGTATGCGCTTCCTGGATATGCCATTGGACTGCTAACTGCTTTAGCAGCTGGTGTT

TTGACCCACTCACCCCAACCTGCACTTCTTTATCTGGTGCCTTCTACTCTAGGACCTGTAATCATCATCT

CCTGGGTCAGAAGGGAACTGACTGAGTTATGGGAAGGAAG

>FM895813.1 FM895813 Jatropha curcas embryo 71-95 (DAF) Jatropha curcas cDNA clone rjcpga0_002808, mRNA sequence

GTTTACACGTCTCTTTCATATTGAAATAAATACTCTGCATCGAAGATAAATTATTACACACAATTCTAAT

GTAGCAAGTGTTTTACACAAGTCTGTTTCATTGTTTGTTTAGGACTATGACAAGATATTCAGAAAAAATT

ATTTTGTTAAATAAGGGACAACAAACAATCCAGACAGAGGAGGCCATATTCTGGTCCGAGGCCATGCAGT

GTTGCAATAAATGCAAGACCAACCGAAATGCTGATTTTGTACTTTGTTTGTCAGTAATTATTCAGTGAGG

TTGATCATTATGAATTACTTGCTCTTGCTTTTGCATATAGGTCAATGCTACCCAACTGCAGCCGTGAAGT

TGATTGAACATCTCTGACTGCCAGAAATCTGAACCTAAAAGTATTACATAGGAAACCTTCCGATCTGATT

TTGAAAGATCTCCGCTGAAAACGATTGTCAAACACCTGAGAAGTCTGTTTATCCAAGACAAGCCAACTGG

ATCCCCCAT

>FM895812.1 FM895812 Jatropha curcas embryo 71-95 (DAF) Jatropha curcas cDNA clone rjcpga0_002807, mRNA sequence

ATTCCTTCAATTCTCTCTATCTCTCTGTTTCGATCAAGCGAAGCCAAAGATGTCGTGGCAGCAGTATGTT

GATGAGCACTTGATGTGTGATATTGATGGCAACCACCTCACTTCTGCTGCTATCATCGGACAAGACGGCA

CCGTTTGGGCTCAGAGCTCTACCTTTCCTCAGTTTACAGCTGAAGAAGTAACTGGAATTATGAATGATTT

TGCTGAACCTGGAACTCTTGCACCAACTGGCCTCTTTCTTGGTGGCACAAAGTACATGGTGATTCAGGGT

GAACCAGGGGCTGTTATACGAGGAAAGAAGGGCTCTGGCGGAGTAACTATCAAGAAGACCAATCAGGCTT

TGATAATTGGCGTATATGATGAGCCTTTAACTCCTGGTCAATGCAATATGATTGTGGAAAGGCTTGGGGA

TTATCTAATTGATCAGGGTCTTTATTTCTTAGGTATGCCTTTCTATTTCTTTTATTCTTTGAGAAAAATG

CT

>FM895811.1 FM895811 Jatropha curcas embryo 71-95 (DAF) Jatropha curcas cDNA clone rjcpga0_002806, mRNA sequence

TTTTTTTTTTTTGAAGGAGAAATAGTTGTTGAATTCCATCATCTGCTTTGTTCAACTGACATAACAAACA

TGATTTGTAAATGACGAGAGCAAACCTATAATACCAGAGGAATTTAACAAACTGTTGCATAAGATCAAAC

ACGTAGAACATGTTCAACCATTTCGGTTCCCATATCACCCCATCATATGGTTGTCTCATTCAATTCTTGG

ACCATTCTTGTCAAACTAAAAATTTGATTGCCATCAAGAAGCTCCATGCTCACCTACTCAGGACAGGCTT

ACTCTTTATCTCTCCTAATATTCACAGCAAACTCATCTTTTCTTACACCAATTGCCTCAACGAGAACAAC

TTTGAAACCTTAACTAATTGCTGCAAGTTCTTAAACCCCAAAAGCCCATTACACTTCAATCCACTGCTAT

CTGATTTTTGTAAAAAAGGGTTCCCATTACATGCAGTAAAAACCTGCTCTCTTATGCATTCAGAAAGTGT

TTCTTTGGATACTTATGCTTTGTGCAGTTCCTTAAAAGCTTCATCTTCT

>FM895810.1 FM895810 Jatropha curcas embryo 71-95 (DAF) Jatropha curcas cDNA clone rjcpga0_002805, mRNA sequence

CTCGGTTCTCTCTCTGGTCCTTCTCTTATTTGGGATTCTAAATGGAATCAACAAATGGAACAGCAATTGA

AGTTCCACTCGGTCAAGTGAACGCAGAAATCGAATCGTTTTTCGAATCGGCTCCTCCTCTAAAAGACAGT

GCTGATATCAGTAAGAAATTGAAGGAGTTCATTGAACTGAATTCTGCAACGCCAGGAAATGGAAGTGCTA

GGAGAGTTGTGTGTGTGAGTTCTGGCGGCACCACAGTTCCCCTAGAACAGCGTTGTGTTCGATATATTGA

TAATTTTAGCTCAGGTCATAGAGGAGCGACGTCAACTGAATACTTTATCAATGCTGGATATGCAGTTATA

TATTTATATAGAGAGGGGAACTTTCCAGCCGTATTGTCGATCTCTTCCGGATGATCCACTGCTTGAGTGT

TTTGAATTCATTGATGAATCCGATATTCAACGTCTGTAGTTTCTATTTGAAGGAATCAGTGCTGGATCTT

CATTAGGAAATTTCAATATAGGGTGGCGATATTTCTAGAGCAAAAATGTTATAGTCAAAACA

>FM895809.1 FM895809 Jatropha curcas embryo 71-95 (DAF) Jatropha curcas cDNA clone rjcpga0_002804, mRNA sequence

CGAATTAGAGACCTATCAAATCTTCAGTTGGGAATGTTGCGGCGCAAAAGAGGCGACAGAATGCAGTGGC

TGTGGGGAAGGAAAGGAGAGAATCGTTGGTGCGGGCAAAGCGCCTTTGCAGAGTGGGAACTAGTGGTGAT

ATTGATACTCCACCTGACAGTGACATGATTATTGATGAGGAGCAATCGATTCTGGAGGCTCAAACTACTA

CAGCGGTGGACGAGTTGAAATCTGCTATTGCTTTTCAGGGAAAAGGTGCAATACAAAAAAAAGTTGGAGC

TCTTCGTGAACTAAGGCGATTGCTATCAAGATCTGAGTTCCCCCCTATCGAAGCTGCACTTAAAGCCGGA

GCAATCCCGCTACTTGTGCAGTGCCTTTCATTCGGCTCTCCTGATGAACAGTTGCTTGAGGCAGCATGGT

GCCTTACAAACATTGCAGCAGGAAAACCAGAAGAAACAAAAGCGTTACTGCCAGCATTACCATTGCTTAT

TGCACATCTTGGAGAAAAGAGTCCTTGCCTGTTGCTGAGCAGTGCGCATGGGCACTG

>FM895808.1 FM895808 Jatropha curcas embryo 71-95 (DAF) Jatropha curcas cDNA clone rjcpga0_002802, mRNA sequence

ATTCGCAAAGGATTCTATCCGTCGCCCGATGGGAATTGTACTTTAAGTGCGGCCCTCGCGGCTCGTCCGC

CGCGGGGGCTTGGCCAACGGCACGTGCCTCTGGGGGCCGGATGGCCCCTACTGCGGGTCGGCAAACGGGC

GACGGACACGCGCGTCGCTTCTGGCCCGGATTCTGACTTAGAGGCGTTCAGTCATAATCCAGCGCACGGT

AGCTTCGCGCCACTGGCTTTTCAACCAAGCGCGATGACCAATTGTGCGAATCAACGGTTCCTCTCGTACT

AGGTTGAATTACCATCGCGACACCATCATCAGTAGGGTAAAACTAACCTGTCTCACGACGGTCTAAACCC

AGCTCACGTTCCCTATTGGTGGGTGAACAATCCAACACTTGGTGAATTCTGCTTCACAATGATAGGAAGA

GCCGACATCGAAGGATCAAAAAGCAACGTCGCTATGAACGCTTGGCTGCCACAAGCCAGTTATCCCTGTG

GTAACTTTTCTGACACCTCTAGCTTCAAATTCCGAAGGTCTAAAGGA

>FM895807.1 FM895807 Jatropha curcas embryo 71-95 (DAF) Jatropha curcas cDNA clone rjcpga0_002801, mRNA sequence

ATTTGAATCAACTTTCTTTTTATTTAATTAATTTATCCATGTTTCGAGTACGAATAATTTTTTATAATGA

TTTTCTTTTGTCCTCTGCATCTATTCATACATCAAACATTCATATTTTGTCTTTTTTATTTATTCTTGCA

CTGTAATATCTAGTGATTGCTCGGGCGGAAAGAGTGAATGCCGCCTTGGAGGGTGGATGGGTGCATGAGC

TGGAGCTCGACCATGGTGATGGTGATGAGCTGGAGCTGGAGCTGGAGCTGAAGGGGGGGCCCGGTAC

>FM895806.1 FM895806 Jatropha curcas embryo 71-95 (DAF) Jatropha curcas cDNA clone rjcpga0_002796, mRNA sequence

CCAAACCAGCAAATAGCAGGGGTCCATGCTTGTAAGGTTAACACTTATCCACCATCATCTATCTTTTCAT

CACAATCCCTTCCAGAATCCCATCTGAACCAAAGTTACCAGAAAACTGCTCAGGATCAACAGGACCTGGC

AGCTGAAATGCAATAGAAAACTTTCCTGGTGGACAAAGGTTCTTTGATTGCATTTCAAAGACTTGTGAGA

ACCTGCATACAGTCCTCTCGCCGGTTGTCGTCTCACCATTTATCAGAACCCTACCACCAGCATCAACTCC

ACAGCTGAATTCCTTCTCATCTCTTTTCACCCCAGGAAGAGATATGCGAAACATATATGCATCATCGCAT

TCACCAATATCCATGAGTCCAATAATGGGTCCCACCTGTCCCATAGCAGCACTCCCAGTCAATGCAAATC

CACCTTTTCCAGCAGCCACAATTTTTTCCAAATCTTCTTTACTTGGGAAGGGAGGAAGAAATAATATTCC

GGGACTGACCTTCCCCATAGTTCTGGTGTCATCTCTGGTTGGCGGAGGAGATGTATGACTATGCTCCGGA

GGCAATGTAGT

>FM895805.1 FM895805 Jatropha curcas embryo 71-95 (DAF) Jatropha curcas cDNA clone rjcpga0_002795, mRNA sequence

AAGCTTAACTGCCTGTACATACATAGTAAAAGCAGGATAGTTATTGTGAATTTGATAGGGTTGACATCAG

AATTTCATGCTCAAAGTAGACCAGCAAATTACAAGTTATATAATGTATATAACTGGTATAAAACAAAAGA

GTTGAAATTAACAGGAACAATGAGTATAGAAAAGAGTCAGGAATCAGCAACAGCCTTCAATGATGTTATG

CATTTCTATGCTTCTTTGACTTTTTAGTTTCATGGTCATTGTTCAAGGAATACTTGTCCCTGCTTGCATT

ACCACGGTCTTGCACTTCAATTTCTCCATCAGGTTGTGTCCTCTTCTTTTTATTACTGTCAGTTATAGCA

TTTTTAACATCCCCATCCAACCAACTAATACTTCCAGTGTGAGTTGGAATCAACGATCCAGAAACATGCA

GTATTTCCACATCAAAACTCTTGACAACAAAGCGTATCACAGTTCCAACCTTTATCACATGTCGTTTCTG

AACTTTGCTAACATATAAGCCTTCACCATGTTTACGTTTATATCTAAATTCATCACGAATGTCTTCATCT

GTTATAACTCCA

>FM895804.1 FM895804 Jatropha curcas embryo 71-95 (DAF) Jatropha curcas cDNA clone rjcpga0_002794, mRNA sequence

ATTGTTGCGATTACGGGTTGGACAATTAGACATCCAACCCGTAATCGCAACGACCCAATTGCAAGAGCGG

AGCTCTACCAACTGAGCTATATCCCCCCGAGCCAAGTGGAGCATGCATGAAGGAGTCAGATGCTTCTTCT

ATTCTTTTCTTTGGCGCAGCTGGGCCATCCTGGACTTGAACCAGAGACCTCGCCCGTGAAGTAAATCATC

GCACCTACGGTCCAACTAATTGGGAGAGAATCAATAGATTCCTTTTCGGGAGCGATTCATCCTTCCCGAA

CGCAGCATACAATTCTTCGTTGTACTGCGCTCTCCAAGTGTGCTTGTTCTCCCCTTCTTCCTTACCATGG

CAAGTCTTTATGAAATAACTCCGATGAGAAGAAAAAAGAAGGCGTTAAGAGACCCTCCTGGCCCCACCTT

AGACACTCTAAGATCCTTTTTCAAACCTACTCCCATTTCGAGTCAAGAGATAGATAAATATACACATCCC

ATTGCACTGACCGGGGGCGTTCGTAGTGACTGAGGGGGTCGAATACCAAAAAGTGAGTTATTTATC

>FM895803.1 FM895803 Jatropha curcas embryo 71-95 (DAF) Jatropha curcas cDNA clone rjcpga0_002793, mRNA sequence

ATTCCAAATTCCAAAATTCTCAATTCCTTCGAAAAGCCTCCCTAAACACCTACTCTTTTGCGTTTCATGG

CCGACGAAGCCAAAGCCAAAGGCAACGCCGCATTCTCAGCCGGCGACTTTACCGGCGCCGTCAAACACTT

CACTCAAGCAATCTCACTAGCCCCTTCTAATCATGTCCTCTATTCTAACCGATCTGCTGCTTATGCCTCG

CTGCACAACTACGCTGACGCGCTAGTCGATGCAAAGAAAACCGTCGAGCTTAAGCCCGACTGGTCTAAAG

GATATTCTCGCCTGGGCGCGGCTCACCTTGGATTGCACCAAATTCAGGACGCTATCGCAGCGTATAAGAA

GGGGCTCGAGATTGATCCCAACAATGAAGCGTTGAAGTCTGGCTTGGCTGATGCTGAAGCGGCAGCTTCT

AGGTCGCGAGCCGCGCCGCCGCCTAGTCCGTTTGGGGATGCGTTCTCTGGACCTGAGATGTGGGCGAAAT

TAACGGCGGATCCGTCAACGAGGA

>FM895802.1 FM895802 Jatropha curcas embryo 71-95 (DAF) Jatropha curcas cDNA clone rjcpga0_002792, mRNA sequence

CAAAAACGCAGATTGATATAACAAACTAAAAAAGGGCAAAAAAATAAAAATAAAAAGAAAGAAAAAAGAA

AGCTTGGTACGTTCACAATTCATAGCCAAGACAAGAATACCTAAATCTCAGAATAGCAAGCAAATTAGCA

ACAAACACCTTCTCCCATTAAAAACATAGAGCGGACAAGTTCGATCCTAATTTGCAGTGTAGCAGACTGC

CTCACTGCTGTTCTTCAGTGGGTTTAGCTGCTTCTTTAATCTCATCAGCTCCATCATCCTGCATGTCTGA

GGTCCACAAAGTGAGATTGTCACGGAGAAGTTGCATGATCAAAGTGCTGTCCTTGTATGACTCCTCACCC

AGGGTATCCAACTCAGCAATTGCCTCATCAAAAGCCTGTTTAGCAAGATTGCATGCGCGATCAGGAGAAT

TCAGAATCTCATAATAAAACACAGAAAAGTTCAGAGCTAGCCCTAGACGGATTGGGTGAGTTGGAGCCAG

TTCTGCATTTGCTATATCCTGAGCAGATTTGTAAGCAGTACGGGTGCTTTCAGCAGCCTCCTTCCTCTCA

GCT

>FM895801.1 FM895801 Jatropha curcas embryo 71-95 (DAF) Jatropha curcas cDNA clone rjcpga0_002791, mRNA sequence

GTAACAACTCACCTGCCGAATCAACTAGCCCCGAAAATGGATGGCGCTTAAGCGCGCGACCTATACCCGG

CCGTCGGGGCAAGAGCCAGGCCCCGATGAGTAGGAGGGCGCGGCGGTCGCTGCAAAACCCGGGGCGCGAG

CCCGGGCGGAGCGGCCGTCGGTGCAGATCTTGGTGGTAGTAGCAAATATTCAAATGAGAACTTTGAAGGC

CGAAGAGGGGAAAGGTTCCATGTGAACGGCACTTGCACATGGGTTAGTCGATCCTAAGAGACGGGGGAAG

CCCGTCCGACAGCGCGTCCTCGCGCGAGCTTCGAAAGGGAATCGGGTTAAAATTCCCGAACCGGGACGTG

AAGGCTGACGGCGACGTTAGGGAGTCCGGAGACGTCGGCGGGGGCCTCGGGAAGAGTTATCTTTTCTGTT

TAACAGCCCGCCCACCCTGGAAACGACTCAGTCGGAGGTAGGGTCCAGCGGCTGGAAGAGCACCGCACGT

CGCGCGGTGTCCGGTGCGCCCCCGGCGGCCCATGAAAATCCGGAGGACCGAGTGCCTCCCACGCCCGGTC

GTACTCATAACCGCATCAGGTCTCCAAGGTGAACAGCCTCTGGTCGATGGAACAATGTAGGCAAGGGAAG

TCGGCAAAAT

>FM895800.1 FM895800 Jatropha curcas embryo 71-95 (DAF) Jatropha curcas cDNA clone rjcpga0_002789, mRNA sequence

TTTTTTTGTTGCCCAAATTACAGTTATAAAACACAACAATCACCAAAACAACAGTGGAGTGTCATCCCAG

TTTTTAGAGAATCCAATATCAAATAATTATCCATCTACTCAACAACGATAAGTCCATTTATCCTACTCCT

CACTAGAGAAACAGATAATTATGACCTAAAAACACTAAAAATTAATAGACTGTCAAGTATCAATAAGTGA

ATAACAGCAAACCCGATCAGTTCGTTTTTGGATCAGGCATCGGGAACAATCCAGCCCCTGCGGCTCCACG

CGGCGGTGAAAAACCTAGAAACTTCTGGAGATTTGTACGGATACTGATTGAACAAAGGAAATATAGAAAC

GCCATTGAACAATCAGTCGCATCGTCGCCTTGCAATCCTCTATGACTCATCTTCATCACGATCCCTATAG

GCTTAAACGGTAATTTTGCCACCACTTTCCCTTCGAATAGCGAATTCAATAATCCGAAAACGACAAACAG

TACCAAGGCCACAACGGCACCGGATTTGAACTTGAAGAGAGAGAGATCACGACTGGACTCTTTTAAAGAG

CTCTCGACACGGTCTATTTTCTTGGTCTTAGATTTCTTGTTGGTG

>FM895799.1 FM895799 Jatropha curcas embryo 71-95 (DAF) Jatropha curcas cDNA clone rjcpga0_002788, mRNA sequence

CACGTCCTCTCTCACCAATGGGTGAGGCCTGTTCACGGATGGATCTCACAGCAATTCATCAGATCTTGGT

TATGACACATTATAAAGATGATGAGGGAACGAATGAGTTATCTTTCCAAGAATGGACACAACAAATGAGA

GATATGCTGGAGTCAAGGAAACGAGGGGACTTTGCATTCCGTGATAAAGATTTTAAAACTGCCATTGACT

GTTATTCCCAGTTCATAGACGTAGGAACAATGGTTTCTCCAACTGTTTTTGCACGGCGCAGTCTTTGTTA

TCTTTTATGCGATCAACCAGACGCCGCCCTTCGAGATGCAATGCAAGCACAATGTGTATATCCGGAGTGG

CCAACAGCCTTTTACATGCAGTCAGTTGCCCTTGCCAAACTAGACATGCATAATGATGCCGCCGACATGT

TGAATGAAGCTGCTGCACTAGAAGAAAAAAACAACGAGGTGGGAAAGGATCATGAGACTNTACTATATCA

TCAATAAAAGATCTCGATCACAGAGTATTATTGAGGCCACGCAAGTTTGTGTAAAATTTGAGCTCTGAAT

TGATTTGTTTTATTGATAAAAGAAAATGTA

>FM895798.1 FM895798 Jatropha curcas embryo 71-95 (DAF) Jatropha curcas cDNA clone rjcpga0_002787, mRNA sequence

ATTGTGAGATATTTGGTGGAGAATCCAATAGAGATAAAGAACGAGTTATATTTTGATTACACTGAGAGTA

GGCCAGTTGCATTTGTGCCAATAATGGCATGTGGTGGCGTTGGATGTGGTGGGGATTGTGAAGAGAAGAG

TTTGGGGCCAAGGGTTATACCTCCTAATCATAAGCTGCAAGCTAATGTTCTATTAACATTGCCAGAGTCT

GCATATAACAGAAATCTTGGGATATTTCAGGTTCGAGTAGACTTTCTGTCCGCTGATGGTAAAACTCTTG

CTAGCAGAAGACAGCCATGTATGTTAAAATTCAAAAGTGAAACTCTCCATGTTCTATTAACTTTCTTTAA

GCTCGGTCCTCTGATTACTGGCTACCTGTCAGAATCCCAAACTCTGAAAGTGAAGAACAAAGGCTTTGTG

GAAGGAGATGTGCCTACTTCCTGCCTAAAAGTGATAATTGAGCAACGAGCTGAATACCGTCCTGGTGCTG

GTATCCCAGAAATATATGATGCATCCCTTGTACTTGAATCTGAACTTCCATTATTTAAAAGGATTATATG

GTGTTG

>FM895797.1 FM895797 Jatropha curcas embryo 71-95 (DAF) Jatropha curcas cDNA clone rjcpga0_002786, mRNA sequence

CTTTTTACGGTTTTATCCGGTTTGCTCCCGCTCCAATTCAGCAACCAGTCCCTTCTAATTTCTTCTTTGC

ATTTCCGCACCCAGAAACTTCTCTTTATTTTCCTTCTCCATCGATCGATGCTCTTATATTTCACTTTAGC

ACTTGAAATTAAAAACTGCAAAAAAAGATATTGTCCTATCCAATTTGTGAATTTTGTTCGTGAACTTGAC

CAGAATTTGTGAAATTAGGGTGTTTGGTTTGGGTACGTGGCTAGGTATGAAGATCGAGGAATTGGAGGAC

GTGGACTGCGATCGGGGTCAAAACGACAGTGTTCACTCGAAGCAGATGGTGAGTGTGGACGCCAAGAGAG

CGCTGGTCGGAGCCGGTGCTCGGATCCTCTTTTATCCGACCCTTTTGTATAATGTGTTTAGGAACAAAAT

CCAAGCCGAGTTCCGATGGTGGGACGAAATTGACCAGTATCTTTTGCTGGGAGCGGTTCCATTCCCCAAG

GATGTTCCACGCTTGAAGCAGCTTGGTGTTGGTGGCGTCATTACACTCAATGAACCTTATGAAACTTTGG

TTCCTTCATCCCTATATCGT

>FM895796.1 FM895796 Jatropha curcas embryo 71-95 (DAF) Jatropha curcas cDNA clone rjcpga0_002785, mRNA sequence

GGAGTCTGACATGTGTGCGAGTCAACGGGTGAGTAAACCCGCAAGGCGCAAGGAAGCTGACTGGCGGGAT

CCCCCCGAGGGTTGCACCGCCGACCGACCTTGATCTTCTGAGAAGGGTTCGAGTGAGAGCATGCCTGTCG

GGACCCGAAAGATGGTGAACTATGCCTGAGCGGGGCGAAGCCAGAGGAAACTCTGGTGGAGGCCCGCAGC

GATACTGACGTGCAAATCGTTCGTCTGACTTGGGTATAGGGGCGAAAGACTAATCGAACCGTCTAGTAGC

TGGTTCCCTCCGAAGTTTCCCTCAGGATAGCTGGAGCTCGGGACGAGTTCTATCGGGTAAAGCCAATGAT

TAGAGGCATCGGGGGCGCAACGCCCTCGACCTATTCTCAAACTTTAAATAGGTAGGACGGGCGCGGCTGC

TTCGCTGAGCCGCGCCACGGAATCGAGAGCTCCAAGTGGGCCATTTTAGGTAAGCAGAACTGGCGA

>FM895795.1 FM895795 Jatropha curcas embryo 71-95 (DAF) Jatropha curcas cDNA clone rjcpga0_002784, mRNA sequence

ATTCCACAAATTGAAAAGATCAGTGGGNGAAATTTCCGGGATACAACCATCTCAACAATTCTCTGATGAG

TTCTTTAACCCTGGATGAGCAAGATTTGTCTATAAGAAAGCTTAATGAATATCAAACACATCTTCACACC

CTTCAAAAGGAGAAGTCTGATCGTCTTCATAAGGTCTTTGAATATGTGAACGAAGTGCATACTCTCTGTG

GTGTGCTTGGGTTGGATTTTGGGCAGACGGTCAGTGATGTTCACCCAAGCTTGCATAAAGAAAACCAAGA

ACAATTGACAAATATAAGTAATAGCACTCTAGAAGGTCTAGAACAATCCATTATCAAGTTGAAGTTAGAA

AGAAAAGCACGGATCCAGAAGCTGAAAGATATTGTGGCATCATTATTTGAACTTTGGAATCTGATGGACT

CACCAAAAGAGGAGAAAAATAGCTTCTCAAGAATTACTTGTATCCTTGGATCTTCTGAATCTGAAATCAT

TGAACCAGGTGTACTCACAACAGATATAATTGAACAGGCATCAGCAGAAG

>FM895794.1 FM895794 Jatropha curcas embryo 71-95 (DAF) Jatropha curcas cDNA clone rjcpga0_002783, mRNA sequence

ATTCCAAAGGATTCTATCCGTCGCCCGATGGGAATTGTACTTTAAGGCGGCCCTCGCGGCTCGTCCGCCG

CGGGGGCTTGGCCAACGGCACGTGCCTCTGGGGGCCGGATGGCCCCTACTGCGGGTCGGCAAACGGGCGA

CGGACACGCGCGTCGCTTCTGGCCCGGATTCTGACTTAGAGGCGTTCAGTCATAATCCAGCGCACGGTAG

CTTCGCGCCACTGGCTTTTCAACCAAGCGCGATGACCAATTGTGCGAATCAACGGTTCCTCTCGTACTAG

GTTGAATTACCATCGCGACACCATCATCAGTAGGGTAAAACTAACCTGTCTCACGACGGTCTAAACCCAG

CTCACGTTCCCTATTGGTGGGTGAACAATCCAACACTTGGTGAATTCTGCTTCACAATGATAGGAAGAGC

CGACATCGAAGGATCAAAAAGCAACGTCGCTATGAACGCTTGGCTGCCACAAGCCAGTTATCCCTGTGGT

AACTTTTCTGACACCTCTAGCTTCAAATTCCGAAGGTCTAAAGGATCGATAGGCCACG

>FM895793.1 FM895793 Jatropha curcas embryo 71-95 (DAF) Jatropha curcas cDNA clone rjcpga0_002779, mRNA sequence

GTGAACTTTTTGGTTTCTTCATTATTGTTACTTAGCGCGGTTCTTGGTCCCAACATCCATGGGTTAATCT

TTCAACTTTGTGGGAGTTGAAATCAATTGTGGAGATTTGGTTATGCTCCTTTTGCAACTTTTGGGATATA

AGCTCTCGACTGATGGAGATTTCTTGAAACTTATATCCATAATCTTTCCTTTTCATGTCCTTCCTGCATG

TTTATGATCTCTGAGCATAGATGGTGTGATTTTTACAGGATTCATTGGTCGCGGAGAAGTGTGTCCCGGT

GAAGTGTAGGAATGATTTACGCTCTCTCTTTCTATGTTCGAATATTTTAAAATTCCTGAGTCACTAGCAC

TTGAGTCTGAATTTGTATGTTTGCAGGTCTCTTGGGTCTCACTTGTATTTGAAGTATGTGTATTAACATC

AATGAGGGAGTGTATTTCTTTTTGCTGCCCTGAATTCTTTTTATTTCCTCTATTGGACAGTTATCTGAGT

TCTTATTGTCTCCTTCATCAGGTCTATGACTATTGTTTTTGTTATGGAGGGAGTATGATCTTATTGTCTT

TTCATTCTGCTCTTGAACTTTTCAGACAGATCTTATAAGAAGCTGAATTGCAACTATATTATGTGCTTTC

AGGATTTTCTGAGAAATATGTGGTGGGCTGT

>FM895792.1 FM895792 Jatropha curcas embryo 71-95 (DAF) Jatropha curcas cDNA clone rjcpga0_002778, mRNA sequence

AATGCTCTGAAGAAAGAGAAACAATGAATTTCAATTCATTAGATGCTATACTAAGGAGGTGTTACAACAG

GTTAAGAAAGAAAGATTAAACATGAAGACCCACATGTTAATGAACACCGATAAACATATATGAAAAGGAA

AAAAAAGCACACAGAAAATCATACACAACATTTCTAAAGGAAACCCACACTCAGAATGTGCCAACTTGTT

TCATCAGTTGGCAAATCCCCCACAGCTGGCAGCATTGGCACTGTTCACCGCCAAGCTTCAAACAGCTCTG

TCCTAGGATTTATACAGCCTACTATACTTGTTCCTTTGGTCTTCTCTACAGTTCCTGCTGCTCCAACCAT

GACCTGCTCAGTCAACCTTTTGGACCCCTTCTTGAGAATTGAGTTACTTGATTGACCAGTTCTGTTCCTG

GCAGTTTTTACTAGGACGCCTCTCTTACTTGAAAAGCCACCCTCTAAGTTTTCATTTGTTAGTGTGTTCA

CAACTTCACAAGGACAATTATCGACCAGAGACCGATTTACTCCAGGAATGTTCTCAATAGAATTCAGGAA

TTTTTGACATTTGGTAATTGAATCATCTTGACTAGACTGAGGAGGGCTCCCTA

>FM895791.1 FM895791 Jatropha curcas embryo 71-95 (DAF) Jatropha curcas cDNA clone rjcpga0_002777, mRNA sequence

ATTCGGATTCTATCCGTCGCCCGATGGGAATATGTACTTTAAGGCGGCCCTCGCGGCTCGTCCGCCGCGG

GGGCTTGGCCAACGGCACGTGCCTCTGGGGGCCGGATGGCCCCTACTGCGGGTCGGCAAACGGGCGACGG

ACACGCGCGTCGCTTCTGGCCCGGATTCTGACTTAGAGGCGTTCAGTCATAATCCAGCGCACGGTAGCTT

CGCGCCACTGGCTTTTCAACCAAGCGCGATGACCAATTGTGCGAATCAACGGTTCCTCTCGTACTAGGTT

GAATTACCATCGCGACACCATCATCAGTAGGGTAAAACTAACCTGTCTCACGACGGTCTAATCCCAGCTC

ACGTTCCCTATTGGTGGGTGAACAATCCAACACTTGGTGAATTCTGCTTCACAATGATAGGAAGAGCCGA

CATCGAAGGATCAAAAAGCAACGTCGCTATGAACGCTTGGCTGCCACAAGCCAGTTATCCCTGTGGTAAC

TTTTCTGACACCTCTAGCTTCAAATTCCGAAGGTCTAAAGGATCGATAGGCCACGCTTTCACGGTTCGTA

TTCGTACTGGAAATCAGAATCAAACGA

>FM895790.1 FM895790 Jatropha curcas embryo 71-95 (DAF) Jatropha curcas cDNA clone rjcpga0_002776, mRNA sequence

TCTTGCCATCACACGCTCAGTCTCTTTCTCTCTGAGACAAAATCTCTTCATCTCTAAACCCCTATTTTCC

TTATCTGATCAATCGACAATCGGCGAAAATGAACGACGCAGATGTCTCCAAGCAGATCCAGCAGATGGTC

AGATTTATCCGCCAGGAAGCCGAAGAGAAAGCCAATGAGATCTCCGTTTCTGCTGAAGAAGAATTCAATA

TTGAGAAGTTGCAGCTGGTAGAGGCAGAGAAGAAGAAGATCAGGCAGGAGTATGAGCGTAAAGAGAAGCA

AGTTGAAGTTCGAAAGAAGATTGAGTACTCCATGCAGCTTAATGCTTCTCGAATTAAAGTTCTTCAAGCC

CAAGATGATGTGGTTAATGGCATGAAAGAGGCAGCAGCAAAGGATCTTCTGAATGTGAGCCGTGATCATC

ATGTGTACAAAAAGCTTCTGAAAGATCTGATAGTTCAGAGTTTGCTTAGATTGAAAGAGCCTTCTGTCTT

GTTGCGTTGCCGGAAGGACGACCTGCTTTTGGTGGAGTCTGTCCTCGATTCAGCCAGGCAGGAATATGCA

GGAAAAGTAAATGTTCATGCTCCAGAAATCATAGTTGACCATCATGTCTATCTTCCACCTGCTCCAAGCC

ATC

>FM895789.1 FM895789 Jatropha curcas embryo 71-95 (DAF) Jatropha curcas cDNA clone rjcpga0_002775, mRNA sequence

AGGAAATGATCTACCAAATTTTATTGAAATGAAACTTTATATAGGCAATGAACAACTACGGTTCAACCAG

TTGTCTTGCTCATTGTACCACTACAATTCTCTCCGTCTCCAAGGGTAGAATTTCCGTCTACAAATACCAA

AAAACAATCCTTTTCAAACAGTATTCAGGAATGGATAAACCCAAGATATCCAGAACATTTTTGTTCAGAA

TGAAATTACAGACTAGGACTCAGAATTTTACTGATATTTGCTAATTCCTCACAGAAAAGATCAAAATAAT

ACTTGGAGCTTTTGTGGCATTCCTATTTCGCCTGAGGGTGTGAAGCTATAAAGTCGACAGCAAGACTGAT

AGAACTGATCTTGTCAGCTTCATTATCTGGAATCTCAAACCCAAACTCTTCTTCAAGAGCCATCACAACC

TCCACAGTGTCCAAGCTATCTAACCCAAGATCATTCTGGAAATGGGCATTTGGTGTGACCTTTGAAGGAT

CAACCTTCTGGAAGTTCTTGACGACATTGACAACACGATCTGTGACCTCGGATTTGTCCAGGAAGGAGCC

CCTCACCTCTTCGCAGAAACGGCGCACTATCGCATT

>FM895788.1 FM895788 Jatropha curcas embryo 71-95 (DAF) Jatropha curcas cDNA clone rjcpga0_002774, mRNA sequence

ATTTCTGAGAGTTTATTTAATTTTATTATCAACAATTTTATTTGTATAGCTAGTAAGGCTATGTTGAAGA

GTATCAACTTTATATTTTTAATTGTTAAGCTATTAATTTTAATTATTAATTTTTTAAAAGATTTTACAAT

TTAGCTATGGAGAATTTTTTTGCTTCTACTATCAAATGTTTAATTTTGATTTGGTACAACTTTACAACTT

GAATTTAAGTTATACATTAATAAAGTATTAGAACATGCGATGCATATAAATATAAAATATATTAATTTAT

ATATTTAGCTATAA

>FM895787.1 FM895787 Jatropha curcas embryo 71-95 (DAF) Jatropha curcas cDNA clone rjcpga0_002773, mRNA sequence

ATTCCTTCATTTAGCATCAACTAAAATACAAAAAAGAGGTATTGTAACCAAAAAAAAAAAAAAATAGTGC

CTTGATACAATTACATTATCCATACAAATATCTTCCAAATTGTAATAATACATTATACAGGTGTTGATAT

TTCTCATGGAATGATTCATCCATGTGAGGAATCAAAATACTGAAGCGCCAGTACAGGTGCAAGGCCAATA

GACTCCATCTTCATTGGCCCATTCCATTTCAGATCCTACTCCACCGAACACAGAGGCATCAAAGCTAGTA

GTGTAAGAGCCGCTTTCACTACGGCTCGTAGGCTGGAAGAATGCAGAGATAAACCTGTTATCTTCTTCTA

GCTCAAGCAATGCTTCCAGCTTCTTGAAACAATCTTGTAGATCTACCTCTAATGGAGTATTCTCCGACGC

ATTATTGTCTAGATTAGAAACAATTGAGGAAGTTGACATATCAGTGTTCTCGCGTTGATTTTGATTTCCA

GAATCTGGAGATGTTGGTGCTAGAAAACTCCATTCACCCCCATCAGAGACCGATATATCATTTACCTTGT

TTGGATTTTGATTTTCAAAATCCCTAGTCGGTGAGCTAATTAATTTATCATTCACATAGGTTGACATAG

>FM895786.1 FM895786 Jatropha curcas embryo 71-95 (DAF) Jatropha curcas cDNA clone rjcpga0_002772, mRNA sequence

CCAAGCTTCACAGAGGCAGAAGCAGCGTCAGCCGCCATGGTTAAGTACTCAAGAGAGCCTGATAATTCCA

CCAAATCTTGCAAGGCCAGGGGCTCTGACCTTAGAGTTCATTTCAAGAACACGAGGGAGACCGCCCATGC

CTTGAGGAAGTTGCCTTTGGCTAAGGCTAAGAGGTATTTGGAGGATGTTATGGCTCACAAACAAGCTATA

CCTTTTCGACGTTTCTGTGGTGGGGTTGGACGTACTGCACAGGCAAAGAACAGGCACTCAAATGGGCAAT

GACGCTGGCCTGTTAAATCTGCCAGATTCATTCTAGATTTGCTCAAGAATGCTGAGAGTAATGCCGAGGT

CAAAGGCTTGGATGTCGATGCCCTCTACATTTCTCATATCCAAGTGAATCAGGCACAGAAGCAGAGGCGT

CGAACTTATCGCGCTCATGGAAGAATCAATCCTTACATGTCTAGCCCGTGTCATATTGAGTTGATTTTGT

CCGAGAAGGAAGAGCCAGTGAAGAAGGAGCCTGAGAACCAGCTAGCTACAAGCAAATCTAAGAAGTCTCA

AGCTCTACGAAGTGGTGCTGCCTCTTAAGTGGCCATCATATTTTACCATTTCAAGGAGCACTTTCTTATA

TCC

>FM895785.1 FM895785 Jatropha curcas embryo 71-95 (DAF) Jatropha curcas cDNA clone rjcpga0_002771, mRNA sequence

TTCGCATTTGCCCTCTAAGAAGCGTACGCATAGCATCTTTATCTGCTTCTCTTTTAATTGCCAAGGAATC

ATCTTTCAATTCCAAGCAAACTCTTTTGCGATCTCTTCATAAGTCACCTCTTCCTTTGGTTCCTCGCTCG

ATCAGAACAGGGAATTCAAGGCCTGTGCTCTATTCTTTGAGATTCGATCATACAATGGCTACCCAATCTG

AGCCTAAATCAGTCCATGACTTCACTGTTAAGGATGCTAGGGGGAACGATGTTGATCTCAGCACATACAA

GGGAAAGGTTCTCATTATTGTTAATGTTGCTTCACAATGTGGCTTGACCAATTCCAACTACACTGAACTG

ACTCAGTTATACCAGAAGTACAAGGATCAAGGTTTGGAGATTCTGGCTTTTCCATGCAATCAATTTGGAT

CTCAGGAGCCAGGGACCAATGAACAGATTGTGGAGTTTGCCTGCACTCGCTTCAAGGCTGAGTATCCAAT

ATTCGATAAGATCGATGTCAATGGAAACAATGCTGCTGCTTCTTTACCAAGTTCTTTGAAGTCCAGCAAA

GGAGGAATTTTTGGGGATAGCATCAAGTGGAATTTCCCCAGTTTCTGGCTGATAAAGATGGCAATGTTGT

GAACCGAATGCTCCACTACT

>FM895784.1 FM895784 Jatropha curcas embryo 71-95 (DAF) Jatropha curcas cDNA clone rjcpga0_002769, mRNA sequence

AGGATTCTATCCGTCGCCCGATGGGAATTGTACTTTAAGGCGGCCCTCGCGGCTCGTCCGCCGCGGGGGC

TTGGCCAACGGCACGTGCCTCTGGGGGCCGGATGGCCCCTACTGCGGGTCGGCAAACGGGCGACGGACAC

GCGCGTCGCTTCTGGCCCGGATTCTGACTTAGAGGCGTTCAGTCATAATCCAGCGCACGGTAGCTTCGCG

CCACTGGCTTTTCAACCAAGCGCGATGACCAATTGTGCGAATCAACGGTTCCTCTCGTACTAGGTTGAAT

TACCATCGCGACACCATCATCAGTAGGGTAAAACTAACCTGTCTCACGACGGTCTAACCCCAGCTCACGT

TCCCTATTGGTGGGTGAACAATCCAACACTTGGTGAATTCTGCTTCACAATGATAGGAAGAGCCGACATC

GAAGGATCAAAAAGCAACGTCGCTATGAACGCTTGGCTGCCACAAGCCAGTTATCCCTGTGGTAACTTTT

CTGACACCTCTAGCTTCAAATTCCGAAGGTCTAAAGGATCGATAGGCCACGCTTTCACGGTTCGTATTCG

TACTGGAAATCAGAATCAAACGAGCTTTTACCCTTTTGTTCCACAC

>FM895783.1 FM895783 Jatropha curcas embryo 71-95 (DAF) Jatropha curcas cDNA clone rjcpga0_002768, mRNA sequence

AAAAAAAAAAAAAAGAAAAGAACTAGACAATAAACGGGTCGGACTTTCGGCGGAGATGGATTCCGGAGCA

AAAGTAAAAAAGGGAGCTGGTGGGAGGAAAGGCGGAGGTCCGAAGAAGAAGCCTACTTCTAGGTCAGTCA

AAGCCGGTCTTCAGTTTCCTGTTGGGAGAATAGGGCGGTACTTGAAGAAGGGAAGGTATGCGCAGCGGGT

TGGATCCGGAGCTCCAGTTTACATGGCTGCTGTTCTCGAGTACCTAGCTGCTGAAGTATTGGAATTGGCT

GGTAATGCAGCTAGAGATAACAAGAAGAACAGGATAATACCAAGGCATGTATTGCTTGCAGTTAGAAATG

ATGAAGAGCTTGGAAAGTTGCTTGCAGGAGTGACCATTGCTCATGGAGGTGTTCTTCCCAATATTAATCC

TGTTCTTTTGCCCAAAAAGTCAGAGAAGGCTACTAAAGATAAGGAACCTAAATCTCCATCTAAGGCAACA

AAATCTCCAAAGAAAGCTTAAGTCATTTCTGTTGTTATTCTCTTGATCTTGTTGTATGTGATTTAGAGTA

TATTTTGTTAATGTGTAGGAAAATTGAGTTGATGGATTTTGAAAATTTGTGTAGTCCCTTTATCTTTCTG

ATAGA

>FM895782.1 FM895782 Jatropha curcas embryo 71-95 (DAF) Jatropha curcas cDNA clone rjcpga0_002765, mRNA sequence

CTGCAAGGATTCTATCCGTCGCCCGATGGGAATTGTACTTTAAGGCGGCCCTCGCGGCTCGTCCGCCGCG

GGGGCTTGGCCAACGGCACGTGCCTCTGGGGGCCGGATGGCCCCTACTGCGGGTCGGCAAACGGGCGACG

GACACGCGCGTCGCTTCTGGCCCGGATTCTGACTTAGAGGCGTTCAGTCATAATCCAGCGCACGGTAGCT

TCGCGCCACTGGCTTTTCAACCAAGCGCGATGACCAATTGTGCGAATCAACGGTTCCTCTCGTACTAGGT

TGAATTACCATCGCGACACCATCATCAGTAGGGTAAAACTAACCTGTCTCACGACGGTCTAAACCCAGCT

CACGTTCCCTATTGGTGGGTGAACAATCCAACACTTGGTGAATTCTGCTTCACAATGATAGGAAGAGCCG

ACATCGAAGGATCCAAAAGCAACGTCGCTATGAACGCTTGGCTGCCACAAGCCAGTTATCCCTGTGGTAA

CTTTTCTGACACCTCTAGCTTCAAATTCCGAAGGTCTAAAGGATCCATAGGCCACGCTTTCACGGTTCGT

ATTCGTACTGGAAATCAGAATCAAACGAGCTTTTACCCTTTTGTTCCACACGAGATTTCTGTACTCGTTG

AGCTCATCT

>FM895781.1 FM895781 Jatropha curcas embryo 71-95 (DAF) Jatropha curcas cDNA clone rjcpga0_002763, mRNA sequence

GCGAAACCACAGCCAAGGGAACGGGCTTGGCGGAATCAGCGGGGAAAGAAGACCCTGTTGAGCTTGACTC

TAGTCCGACTTTGTGAAATGACTTGAGAGGTGTAGGATAAGTGGGAGCCGAAAGGCGACGGTGAAATACC

ACTACTTTTAACGTTATTTTACTTATTCCGTGAATCGGAGGCGGGGCTATGCCCCTCTTTTTGGACCCAA

GGTCGTTTCGGCGGCCGATCCGGGCGGAAGACATTGTCAGGTGGGGAGTTTGGCTGGGGCGGCACATCTG

TTAAAAGATAACGCAGGTGTCCTAAGATGAGCTCAACGAGAACAGAAATCTCGTGTGGAACAAAAGGGTT

AAAAGCTCGTTTGATTCTGATTTCCAGTACGAATACGAACCGTGAAAGCGTTGCCTATCG

>FM895780.1 FM895780 Jatropha curcas embryo 71-95 (DAF) Jatropha curcas cDNA clone rjcpga0_002762, mRNA sequence

ATTCGCAAAGGATTCTATCCGTCGCCCGATGGGAATTGTACTTTAAGGCGGCCCTCGCGGCTCGTCCGCC

GCGGGGGCTTGGCCAACGGCACGTGCCTCTGGGGGCCGGATGGCCCCTACTGCGGGTCGGCAAACGGGCG

ACGGACACGCGCGTCGCTTCTGGCCCGGATTCTGACTTAGAGGCGTTCAGTCATAATCCAGCGCACGGTA

GCTTCGCGCCACTGGCTTTTCAACCAAGCGCGATGACCAATTGTGCGAATCAACGGTTCCTCTCGTACTA

GGTTGAATTACCATCGCGACACCATCATCAGTAGGGTAAAACTAACCTGTCTCACGACGGTCTAACCCCA

GCTCACGTTCCCTATTGGTGGGTGAACAATCCAACACTTGGTGAATTCTGCTTCACAATGATAGGAAGAG

CCGACATCGAAGGATCAAAAAGCAACGTCGCTATGAACGCTTGGCTGCCACAAGCCAGTTATCCCTGTGG

TAACTTTTCTGACACCTCTAGCTTCAAATTCCGAAGGTCTAAAGGATCGATAGGCCACGCTTTCACGGTT

CGTATTCGTACTGGAAATCAGAATCAAACGAGCTTTTACCCTTTTGTTCCACACGAGATTTCTGTTCTCG

TTGAGCTCATCT

>FM895779.1 FM895779 Jatropha curcas embryo 71-95 (DAF) Jatropha curcas cDNA clone rjcpga0_002761, mRNA sequence

TGAATATGGAGGTAGAGAAGAAGAGGAAAGCAGAGGAAACAAACCCCCTCTCTCCATTCTATCTCTAAAC

ACAACAGTAGAAGAGAGAGCAATAAATATAAATATAAATAAATAAAAATTAAGTCAATTAGGTGGGAAAA

ACCCTAATCCTTCAATCCATTTCTCAAATAAAAGTCATCTGATACACAATTTTTCAGATATAAGGCCGAT

TGGATCAGAAGAAAGAAAATTTTTTATTTTTTTATTTTGATTAGGTTGTTGAATTTGGGGATTGTGAAAT

TAGGGTTAGATCGATCTGTGCTGCATGATCAGGAAACGGTGGTTGCAAGACGAAATTAGGGTAGAGGATA

GATAGGAGAGAGAGAAGAGAAGAAGAAGAAAAGAGGAGAAAATAGAAACTATGTCTGCCATAGTGTGCGG

GAAGAGATCTTTTTTTGAAGAATTGACAGTGACCAGCCCTCCCGTTAACAAAAGAATCCGTTGTTCATCT

TCCTCTCCTGTACGTTTCTCTCCTCCAAGGTCTACCAACGCTGTTTCTGTTTCTTCCCCTGCCTCTTATC

AATCTTCTTCGCTTTTGATAGAGAAACTCATCGCTCTTTTCCCTGACATGGACAAGCAGATTATTGAGAG

AGCGCTTCAAGAATGTGGTGATGATCTGGATTC

>FM895778.1 FM895778 Jatropha curcas embryo 71-95 (DAF) Jatropha curcas cDNA clone rjcpga0_002760, mRNA sequence

GCTGCAGGAGGATCTATCCGTCGCCCGATGGGAATTGTACTTAAGGCGGCCCTCGCGGCTCGTCCGCCGC

GGGGGCTTGGCCAACGGCACGNCCTCTGGGGGCCGGATGGCCCCTACTGCGGGTCGGCAAACGGGCGACG

GACACGCGCGTCGCTTCTGGCCCGGATTCTGACTT

>FM895777.1 FM895777 Jatropha curcas embryo 71-95 (DAF) Jatropha curcas cDNA clone rjcpga0_002759, mRNA sequence

GTCTGACATGTGTGCGAGTCAACGGGTGAGTAAACCCGCAATGCGCAAGGAAGCTGACTGGCGGGATCCC

CCCGAGGGTTGCACCGCCGACCGACCTTGATCTTCTGAGAAGGGTTCGAGTGAGAGCATGCCTGTCGGGA

CCCGAGGGATGGTGAACAGTGCCTGAGCGGGGCGAAGCCAGAGGAAACTCTGGTGGAGGCCCGCAGCGAT

ACTGACGTGCAAATCGTTCGTCTGACTTGGGTATAGGGGCGAAAGACTAATCGAACCGTCTAGTAGCTGG

TTCCCTCCGAAGTTTCCCTCAGGATAGCTGGAGCTCNGGACGAGTTCTATCGGGTAAAGCCAATGATTAG

AGGCATCGGGGGCGCAACGCCCTCGACCTATTCTCAAACTTTAAAT

>FM895776.1 FM895776 Jatropha curcas embryo 71-95 (DAF) Jatropha curcas cDNA clone rjcpga0_002758, mRNA sequence

ATTCTTAAAGATTACCAAGAGAAAAAAGAAAATCTTCGTGAAACATCATGTCTTCAAAAATATTTTTCTT

TGTCGTCTTTCTCACTATGATTTCCAAAAGTTACCAAGCCTCTCCATTTGTTTCCCAATCATGTGCAAAA

ACTTCGTATAGAACCCTATGCATGTCAGTTCTAGGAACATCCAGAGCTACTAATCTGCCGAGCTTGACTA

TAGATGCACTCCACTTTGCATCATCATATGGAAGCAAAATACTCAATGTCATTTCTCAATTGCAGAAAAA

ACCTTCAAATCCGATTGTTAAGCAAGCCTTGGCACAATGTCAAGAGGTTTATAGTGATGCGATTGGTCAA

ATTAACGACTCAGTTCGTGCTGTCCAAAACAGATCTTATAACGATTTAAATACGTGGGTTAGTGCTGCTA

TGACTTATTCTCAGACGTGCGAAGAAGGTTTTACGAAAAGTGCATCTCCATTGACTACTTTCAACACACA

ATTTAACCAAGTTTGTGGTATCATATTAAAATTTTCCAATCTCTNTGGCAAACATTAATTATCCTTTTAT

GTTCATTTCTGAGTTCATTGATGTATTATTGTGAGATGG

>FM895775.1 FM895775 Jatropha curcas embryo 71-95 (DAF) Jatropha curcas cDNA clone rjcpga0_002757, mRNA sequence

CTTGCAGCAGAGTGCAGAAAAGAAGGGTTTTCACAGCCAACTCCTGCAAATACAGCTGTGCCCAACAGCT

CTCAGAAAAGAACTGCTCCTGGTCCTGTAGTGGGTTGGCCTCCAATCCGATCATTCAGGAAGAATCTTGC

AAGCAGCAGCTCTTCAAAACCATCTATTGACACACAAAATGAAAGCCCAAACAAGAAGGTTGCTGGTGAA

AAACCAATGGAAAATTGCAAGAAAGGGATGTTTGTGAAGATCAATATGGATGGTGTTCCTATTGGAAGGA

AAGTAGATCTTAAAGCCTATGACAGCTATGAGAAGCTTTCTACTGCTGTTGATGAACTATTCAGAGGCCT

ACTTGCAGCTCAAAGAGATTCCTCTGCTGGTGGAATCATGAGCAAGCAAGAGGAAGAGAAAGCAATTACG

GGTGTATTAGATGGGAGTGGGGAATATACGCTTGTTTATGAGGATAATGAAGGAGACAGGATGCTTGTTG

GGGATGTCCCATGGCACATGTTTGTGTCAACTGTGAAGAGGCTTCGCGTATTGAAGAGCTCTGAAGTTTC

TGCATTAAGCCTTGGAAAGCAGTAAGCAAGGGCAGGTACAAATGAGATGAGAGAGATGAATTTTGCTGTG

>FM895774.1 FM895774 Jatropha curcas embryo 71-95 (DAF) Jatropha curcas cDNA clone rjcpga0_002756, mRNA sequence

ATTCAGCGTACTTGCTGCTCTCTCTACTTCTTCTCTTTGTGGTTCGATCCGGGAGAAACTCAGAATCATG

TTGGTCTACCAGGATTTGCTTACCGGTGATGAGCTTCTCTCGGATTCGTTCCCTTACAAGGAAATTGAGA

ATGGCATGCTGTGGGAAGTTGAGGGAAAGTGGGTTGTTCAAGGAGCCGTTGATGTGAACATTGGTGCAAA

TCCCTCTGCTGAAGGGGCGGATGAAGATGAGGGTGTTGATGACCAGACTGTTAAAGTTGTTGACGTTGTG

GACACTTTTAGGCTCCAGGAGCAACCTGCTTTTGACAAGAAGCAGTTTGTCACATACATGAAGAGGTACA

TCAAGTTGTTGACACCCAAATTGGATGAGGAGAAGCAACAGGCTTTCAAGAAAAACATTGAGGGAGCAAC

CAAGTTCCTGCTCTCGAAGCTCAGTGACCTCCAATTTTTTGTGGGGGAGAGCATGCATGATGATGGGAGT

TTGGGGTTTGCTTACTACAAGGATGGCGCCACTGACCCAACATTTTTGTACTTCGCTTATGCTTTGAAGG

AGATCAAGTGCTAAGAATGGAGAATAGCTGGCTTCGAAGTGGAAGTTCTTGCCATATTTCTAGCTTTATG

TGTCCTTTT

>FM895773.1 FM895773 Jatropha curcas embryo 71-95 (DAF) Jatropha curcas cDNA clone rjcpga0_002755, mRNA sequence

ATGAAATTGTGAAGGAAGTTTCTTCCTACTTGAAGAAGGTGGGTTACAACCCTGACAAGATCCCATTTGT

GCCAATCTCTGGTTTTGAGGGTGATAACATGATTGAGAGGTCAACAAACCTCGATTGGTACAAGGGCCCT

ACCCTCCTTGAGGCCCTCGACTTGATCAATGAGCCAAAGAGGCCAACAGACAAGCCACTCCGTCTCCCAC

TTCAGGATGTCTACAAGATTGGTGGTATCGGAACTGTCCCTGTCGGTCGTGTTGAGACTGGTGTCTTAAA

ACCTGGTATGGTTGTCACCTTCGGACCATCTGGACTGACCACTGAAGTTAAGTCTGTTGAGATGCACCAC

GAAGCTCTCCAGGAGGCCCTTCCAGGTGACAATGTTGGCTTCAATGTGAAGAATGTTGCTGTCAAGGATT

TGAAACGTGGTTTTGTTGCCTCTAACTCCAAGGATGATCCTGCGAAGGAAGCTGCCAACTTCACCTCTCA

GGTCATCATCATGAACCACCCTGGTCAAATTGGAAACGGATATGCTCCTGTCCTTGACTG

>FM895772.1 FM895772 Jatropha curcas embryo 71-95 (DAF) Jatropha curcas cDNA clone rjcpga0_002754, mRNA sequence

GAATCAACGTTAACTTGGATCTCATAAATAGGCCTCGCTCAAGTGTTCCAAGACTGACTAGCAGGGAACT

CTACCACAAAATCGCCCGAGCAGATAAATTAGTACTGCTATGGTTGCTATGATGGAAACAAAAATCCTAA

CAGAACATTTACAGACAAATTGAACGAAAATGTATTTACAAGTGCCTTTCGATTTCTCTCCTAATCTTCC

ACTGACTGAATCTCACCCATCATGATCCGGTTACTGACAACATTAAAACCCAAGACAGCAGGGCTGACCT

TCTTGCCTTTTTTCCCTTTCTTCTTTCCTCCACCCTTGGAGGACCCATCGTTTCCCAGAGTCATATCATG

GTCGAAATCCCTATTGCTTGCATGGTCAGAATTCATGTCTCTGGCACTGAACCCAGTGGCCATCCGATCA

TTCCGACTCTGAAAGGCAATTTCGAGAACATCAGCTGGTAACAACTCTTTATAGTTCAGGAATTTGTCAA

TGAACTCGTGATCAGGATCAAATGATCCAAGATTCTCTATCAGAAGCATCTCAGCTTCCGATCTGGATTG

C

>FM895771.1 FM895771 Jatropha curcas embryo 71-95 (DAF) Jatropha curcas cDNA clone rjcpga0_002753, mRNA sequence

TTGTTTTTCTNCTGCCTATAATTACGCTCTTATTTCCTTTCTTTAGAATTATATTTTCCTTTGCCTTATT

TTATTTGTTATTCTTCTTCAAATTACTTTTAAGAGCAAAAAACAAGAAACTGATTTCTAAAGAATAAAGG

AAAACGAAGAAGAAATGGCGGATCAGCTTACCGATGATCAGATCTCCGAGTTCAAGGAAGCCTTTAGCTT

GTTCGATAAGGATGGAGACGGTTGCATTACCACAAAGGAGCTTGGGACAGTAATGAGATCTCTGGGACAG

AACCCTACAGAGGCAGAGCTACAAGACATGATTAATGAAGTTGATGCTGATGGGAATGGGACTATTGATT

TCCCAGAATTTCTAAATCTCATGGCTAGGAAGATGAAAGACACTGACTCTGAGGAGGAGCTAAAAGAGGC

ATTCAGAGTCTTTGACAAGGATCAAAATGGTTTTATTTCTGCTGCTGAGCTGCGTCATGTGATGACAAAT

CTTGGTGAGAAGCTTACTGATGAAGAAGTTGATGAGATGATAAGGGAGGCAGATGTTGATGGTGATGGCC

AGATAAATTATGAGGAATTTGTCAAAGTGATGATGGCCAAATGATCATCCAATTCCCATCAAAATGGAAA

GGAGAGGATTTTAGCAGATAAAAGAAAAGAAATC

>FM895770.1 FM895770 Jatropha curcas embryo 71-95 (DAF) Jatropha curcas cDNA clone rjcpga0_002752, mRNA sequence

AAAAAAATGGACTTTCCATATTCGTATATAAGATGGAAAAAAATTAATAATTAATAAAGAAAAAAAAATG

AAAACCATGCAATTAGATTATCTTCATCAAATAATCTAAATTTGTTTCTCACATTTGGTACAAAAAAAAA

AAAAAAAAAAAAAGAAATAGCTGAAAAATGACATGAAAATATCAATGTAGAGGGTGCCTATGGCAAGCCA

TGTTTAATGGAATTGGTGGGTCTGAAAATGAACCATCTTTAATATTTTCAAACATCCATTTGTTTGCTGC

TTGAGTGAAATGAACTCCATCCCAATTAATCCAAACTGAAGGATCTTCACATGCTTTCCCCACCAAAACT

TGTCTTCCACCTTTTGTAATATATCCTCCACATCCAATATTCTTGTTGTAGTTATACTTCCCACCATGTC

CACAACAAGCTCTCAATGACTCATTAAATCCGAACTTTCTGGCTTGACTGAAAAGACTATATTTTAGAGA

GTAAATATCACCATATGTGA

>FM895769.1 FM895769 Jatropha curcas embryo 71-95 (DAF) Jatropha curcas cDNA clone rjcpga0_002751, mRNA sequence

ATTGTAACTCCAGAAAAGGTCTTGCCTTTGCCGGCTAAAGTAGAGAAAAACGAAGATGTAGATCTGGGAT

CGACTCACCGGCTTGGACCCGATCCAGAGCTGGTTCCTACTCAGATCCGACTCGCTGAGTCTAGCAAAGC

AAATGGTTTTTACGCCGGATCGGCGTTTATTACTTCACCTCCTCCTAGTTCTCTTCCTTTGCCTGCTTTT

TTTATGAAAAAGACCGTGGGTCTTCTCGATGTGAAGAACACCGACACCGATGCTACTAGTGATTTGATCC

GGATTTTGGGCCTCAGCTTACAGTGATTGGTCCATGGACGTTTGATGGGTGGATTGTACTTGGATCTGGA

GTTGAAGAAGTTTTCTTTTGTGTTTTCCATCGCTCTTCTCTTATTTTTTCAACGGGAAGATCGAGTCGGT

TAACCCAAGCTACGCGTTGCGCGATTAAGCGCTCTAGCGGTACTT

>FM895768.1 FM895768 Jatropha curcas embryo 71-95 (DAF) Jatropha curcas cDNA clone rjcpga0_002750, mRNA sequence

AACCTAAAAAATGGCTACTGCTACTGTACTCTTCTCTACCATTTCCACCGCTCTCTTTATCCTCTTCTCC

TTTGGTCAAGCTGCGCCTCTTATTTTAACCCTAGTCAACAACTGCCCTTTCACCGTTTACCCTGCCATCC

AACCCAACGCTGGCCACCCGGTCCTCGAAAAAGGTGGCTTCCCACTCTCCACCCTAACACACCGCTCTTT

TCCCGTCCCTAACCAGCACTGGACTGGCCGGATCTGGGCTAGAACCGGATGCACTCACACCAACGGCAAG

TTCCACTGCGCCACCGGTGATTGCAACCACCAACTAGAATGCAACGGCCTCGGTGGTGCCCCTCCAGCAA

CTCTCGCTCAGTTCAGCCTTCACCATGGCCATAACGACTTCTCCTCTTACGGCGTCTCTCTCGTCGACGG

ATTTAACCTTCCGATGACCATAACTCCACATGAAGGCAAAGGTGTTTGTCCGGTGGTGGGTTGTCGAGCA

AACTTACTGACAACATGCCCTCAAAAGCTTCAGGTGAGGTATCCGGTGGGTCATGGTCCGGTGGTGGCCT

GTAAAAGTGGATGCTTGGCGTTCGGACCGACGAGCCTTGTTGCGGAATCA

>FM895767.1 FM895767 Jatropha curcas embryo 71-95 (DAF) Jatropha curcas cDNA clone rjcpga0_002749, mRNA sequence

CCGTCCCCCATTGCCCCTGGATATTTCGTAAAGTGTATTTACGAGTTATCGGTTGCTCCTCATCTTTCCC

CTTAATCTCAGCAACCCATTTGTAGTTCTTCTCAATTCCCACCTTCTTCTCCTTCTCTTCTTTAGTCTTC

TTTGCTTCTTTGATCTCCGCAGTCCACTTGTACTTCCTTTCTACCGGCCCC

>FM895766.1 FM895766 Jatropha curcas embryo 71-95 (DAF) Jatropha curcas cDNA clone rjcpga0_002748, mRNA sequence

ATTCGCAAAGGATTCTATCCGTCGCCCGATGGGAATTGTACTTTAAGGCGGCCCTCGCGGCTCGTCCGCC

GCGGGGGCTTGGCCAACGGCACGTGCCTCTGGGGGCCGGATGGCCCCTACTGCGGGTCGGCAAACGGGCG

ACGGACACGCGCGTCGCTTCTGGCCCGGATTCTGACTTAGAGGCGTTCAGTCATAATCCAGCGCACGGTA

GCTTCGCGCCACTGGCTTTTCAACCAAGCGCGATGACCAATTGTGCGAATCAACGGTTCCTCTCGTACTA

GGTTGAATTACCATCGCGACACCATCATCAGTAGGGTAAAACTAACCTGTCTCACGACGGTCTAAACCCA

GCTCACGTTCCCTATTGGTGGGTGAACAATCCAACACTTGGTGAATTCTGCTTCACAATGATAGGAAGAG

CCGACATCGAAGGATCAAAAAGCAACGTCGCTATGAACGCTTGGCTGCCACAAGCCAGTTATCCCTGTGG

TAACTTTTCTGACACCTCTAGCTTCAAATTCCGAAGGTCTAAAGGATCGATAGGCCACGCTTTCACGGTT

CGTATTCGTACTGGAAATCAGAATCAAACGAGCTTTTACCCTTTTGTTCCACACGAGATTTCTGT

>FM895765.1 FM895765 Jatropha curcas embryo 71-95 (DAF) Jatropha curcas cDNA clone rjcpga0_002747, mRNA sequence

TTTAATGTCGTCTAAGAAAAAATTTATGTTTCCAAGTGTTGCATTTTCATACATTCAAAACTCTCAAAGA

TGGGAAATGTAGACTATGCTAAATTAATCAAAACAGTAGCTAAAAGTACACTTCTTGAGATCATAGAACT

GAGATACCTATGGAAGCCAAAGAAACAGGTAAAATATGATTGGATTGAGGGAAGAATGTAAAAAAGTAAC

TTCCTGAATGCCCAATTAGAGGATTTAATATCTAGTGAGCTCGGGTCATCTTTACTTTTGGCATAGCTTT

CTTCATCTGACGGGCACGCTCTTTTGCCTCTCTCTTGCGAATAGCTTCTGCACTGAGCTTTGCTTCAGCC

TCCTCTTGCATCTTCTTCCTCTCTGCCTTCTTTCTCTGCAAGGCTTCTTGTCTTGCACCCTGAAGTTCTT

TGTATGCCTCCTGGGCAGCTTTCACTCTAGCTGCCTCTGTTTTCGATCGAGCCTGTGAACTCAACTTGTA

GCGCCCAATTAGATCAATGTAATAAGGAATAAGCGCAACCAATCTAGTCATATCAGCCATGTTATTGGCA

TTAGGAAGTGCAAACTTAAACAACAGCATCTTCTTGTGCACTCC

>FM895764.1 FM895764 Jatropha curcas embryo 71-95 (DAF) Jatropha curcas cDNA clone rjcpga0_002746, mRNA sequence

AGGATTCTATCCGTCGCCCGATGGGAATTGTACTTTAAGGCGGCCCTCGCGGCTCGTCCGCCGCGGGGGC

TTGGCCAACGGCACGTGCCTCTGGGGGCCGGATGGCCCCTACTGCGGGTCGGCAAACGGGCGACGGACAC

GCGCGTCGCTTCTGGCCCGGATTCTGACTTAGAGGCGTTCAGTCATAATCCAGCGCACGGTAGCTTCGCG

CCACTGGCTTTTCAACCAAGCGCGATGACCAATTGTGCGAATCAACGGTTCCTCTCGTACTAGGTTGAAT

TACCATCGCGACACCATCATCAGTAGGGTAAAACTAACCTGTCTCACGACGGTCTAAACCCAGCTCACGT

TCCCTATTGGTGGGTGAACAATCCAACACTTGGTGAATTCTGCTTCACAATGATAGGAAGAGCCGACATC

GAAGGATCAAAAAGCAACGTCGCTATGAACGCTTGGCTGCCACAAGCCAGTTATCCCTGTGGTAACTTTT

CTGACACCTCTAGCTTCAAATTCCGAAGGTCTAAAGGATCGATAGGCCACGCTTTCACGGTTCGTATTCG

TACTGGAAATCAGAATCAAACGAGCTTTTACCCTTTTGTTCCACACGAGATTTCTGTTCTCG

>FM895763.1 FM895763 Jatropha curcas embryo 71-95 (DAF) Jatropha curcas cDNA clone rjcpga0_002745, mRNA sequence

AATAACAAAAATTTTTTTATCAAAATATATTAATAAATACAATTAATGTTGTTAATGAAATTACATTGTA

AACAAACCCTAATTATACAGCAATTCGCCACTGCAAAATAAATACTATGAAAAGGAAAAATAACAATAAT

AGTAATGGTAAATATAGAATAGATATTAAATGCACAATTTCTTACAATGATATGTACATCAAATGTGAAA

GAATGCATAAACAGTAACACACTACAAAACAAAGTTGTAATCAAGCTCCAACCTCTTCTTTTAAGTTGGA

TTGACATCCCGCGAAACTTGACCTTGCCATCTTGAGAGTAGAAACCTTGCAAGAGGGCTTAAATTGGCAG

TCGAATTTAGTTCCTTATTTTGTTGCTTCTTCTTGTTCTCAGCCTCAGCATTTGCATTTTGTGTTTCATC

AGGAGCTCCCTTGCCCTTGAGGAACAGGCGAGCTGCTAGCTCCTGTAGATCTTTCATTGAAGATTCCTTT

GAACTTGCCAATCGCTGAAGCAAACTTCTAAGGAGGACATCAGCAGATCCAGGCTTTGTTCGCTCCATAT

T

>FM895762.1 FM895762 Jatropha curcas embryo 71-95 (DAF) Jatropha curcas cDNA clone rjcpga0_002744, mRNA sequence

ATTCATCATCTCCGTGGTGTTACTGTTCTAATGACTTTTATAATGGTTGTTGCTATCCTTACATCAATTG

CCATCGTGCGCCGCATTCTTATGGCAACATCTGTTCTCAAGGTTGCTGCAAAGGTTATAGGAGAAGTTCA

AGCACTCATAATTTTCCCAGTTATACCATATGCTGTGCTTGCAATCTTTTATATGTTCTGGTTTTCAGCT

GCTTTTCATCTCTTTAGTTCGGGTGAGATTGTCCAAAACAACTGCAACTCCAACTGTTGTGCCTATGACC

TTGGGTCAAAACGTGTTAATTGTGATCGTTGCTGTGGCTATAGCATCCATTATACTCCTCATATAGCAGT

TGCCATCTTTTTCCACTTGTTTGGGTGTTATTGGGCTACACAATTTTTTATAGCAAGCTCTGCAACTGTG

ATTGCTGGTTCTATTGCCTCCTACTACTGGGCTCGTGGTGAGACATCGGCAGAGATCCCTTTTCTTCCAG

TTTTTTCCTCTATGAAGCG

>FM895761.1 FM895761 Jatropha curcas embryo 71-95 (DAF) Jatropha curcas cDNA clone rjcpga0_002743, mRNA sequence

CTGGCGGGATCCCCCCGAGGGTTGCACCGCCGACCGACCTTGATCTTCTGAGAAGGGTTCGAGTGAGAGC

ATGCCTGTCGGGACCCGAAAGATGGTGAACTATGCCTGAGCGGGGCGAAGCCAGAGGAAACTCTGGTGGA

GGCCCGCAGCGATACTGACGTGCAAATCGTTCGTCTGACTTGGGTATAGGGGCGAAAGACTAATCGAACC

GTCTAGTAGCTGGTTCCCTCCGAAGTTTCCCTCAGGATAGCTGGAGCTCGGGACGAGTTCTATCGGGTAA

AGCCAATGATTAGAGGCATCGGGGGCGCAACGCCCTCGACCTATTCTCAAACTTTAAATAGGTAGGACGG

CGCGGCTGCTTCGCTGAGCCGCGCCACGGAATCGAGAGCTCCAAGTGGGCCATTTTTGGTAAGCAGAACT

GGCGATGCGGGATGAACCGGAAGCCGGGTTACGGTGCCCAACTGCGCGCTAACCTAGAACCCACAAAGGG

TGTAGGTCGATTAGGACAGCAGGACGGTGGTCATGGAAGTCGA

>FM895760.1 FM895760 Jatropha curcas embryo 71-95 (DAF) Jatropha curcas cDNA clone rjcpga0_002741, mRNA sequence

GCAAAGGATTCTATCCGTCGCCCGATGGGAATTGTACTTTAAGGCGGCCCTCGCGGCTCGTCCGCCGCGG

GGGCTTGGCCAACGGCACGTGCCTCTGGGGGCCGGATGGCCCCTACTGCGGGTCGGCAAACGGGCGACGG

ACACGCGCGTCGCTTCTGGCCCGGATTCTGACTTAGAGGCGTTCAGTCATAATCCAGCGCACGGTAGCTT

CGCGCCACTGGCTTTTCAACCAAGCGCGATGACCAATTGTGCGAATCAACGGTTCCTCTCGTACTAGGTT

GAATTACCATCGCGACACCATCATCAGTAGGGTAAAACTAACCTGTCTCACGACGGTCTAAACCCAGCTC

ACGTTCCCTATTGGTGGGTGAACAATCCAACACTTGGTGAATTCTGCTTCACAATGATAGGAAGAGCCGA

CATCGAAGGATCAAAAAGCAACGTCGCTATGAACGCTTGGCTGCCACAAGCCAGTTATCCCTGTGGTAAC

TTTTCTGACACCTCTAGCTTCAAATTCCGAAGGTCTAAAGGATCGATAGGCCACGCTTTCACGGTTCGTA

TTCGTACTGGAAATCAGAATCAAACGAGCTTTTACCCTTTTGTTCCACACGAGATTTCTGTTCTCGTT

>FM895759.1 FM895759 Jatropha curcas embryo 71-95 (DAF) Jatropha curcas cDNA clone rjcpga0_002740, mRNA sequence

ATTCCATCGTTCCAGGGGGTTATCTAGGGTTTCTTATCCGTTGTGTTTGCCTTCCTATCGATGGCGAAGC

CAACGCGAGGCCGCCCCTCGCCGCCCTCCGGCTCGGGCTCGGGCTCGTCTTCCCGATCACGGTCCAGGTC

ACGGTCACGCTCGTATTCCGGTTCGGATTCTCGGTCCAGCTCTCGCTCGCGGTCCTTGTCCTCCCGCTCA

CGTTCTCGATCTCGCTCCAAATCCCTCTCTTCATCCTCCTCTCCTTCTCACAGCCTCAGCTCCGGTAGCC

GTAGCCCGCCTCCTCAGAAGAAGAGTCCTACTGAAGTAGCTAAGCGAGGACGTTCTCCACCACCACAATC

TAAAAAGGCTTCTCCACCTGCAAGGAAGATTTCTCCGATTCGTGAGTCTCTCGTTCTCCATGTTGACTCA

CTCAGCCGGAATGTGAATGAAGGCCATTTGAAAGAAATATTTAGTAACTTTGGTGAAGTTGTACACGTGG

AGCTGGCAATGGACCGTACTGTTAATCTTCCGAAAGGATATGGATATGTTGAGTTTAAGACACGTGCCGA

TGCTCAAAAGGCCCTTTTATACATGGACGGGGCCCAGATTGATGGC

>FM895758.1 FM895758 Jatropha curcas embryo 71-95 (DAF) Jatropha curcas cDNA clone rjcpga0_002738, mRNA sequence

AGGCGGCCATCCCAAAACCCTCCGCTCCTCCTTCTTTCTCATCCAGATCGCCTATTGGCATTTCTAGATT

CAACCCCTCATTTTTTCTGAATCTCCAAAAGTCCATACCTCAACGCTCTCTCTACATTTCCAACTCTATA

TCTAAACCTACTACCCCTGCTCCTTCTAGCACTTCCATACCTGAAGCTTCTCCTCCTAGATTTGCCACTG

ATGAACCTCGAAAAGGAGCTGACATCCTCGTCGAAGCGCTCGAGCGCCAAGGGGTTACTGACGTGTTCGC

TTATCCTGGCGGCGCATCCATGGAGATCCACCAAGCCCTAACTCGCTCTCCAATAATACGTAATGTCCTT

CCTCGCCATGAGCAAGGTGGAGTCTTCGCTGCTGAGGGATACGCGCGCGCTTCGGGCAAGCCTGGTGTCT

GCATCGCCACCTCTGGACCTGGCGCCACCAATCTCGTTAGCGGCTTAGCTGATGCTCTCCTCGATAGTGT

CCCCATTGTGGCTATCACTGGCCAAGTCCCTCGCCGCATGATTGGTACTGATGCCTTCAGGAGACTCCTA

TGTCGAGGTAACTCGGTCGATAACTAAGCACA

>FM895757.1 FM895757 Jatropha curcas embryo 71-95 (DAF) Jatropha curcas cDNA clone rjcpga0_002737, mRNA sequence

TGCTGGAATGGTGCATGATGCCACACCAACTACAGCACGAAGAGCATGAGGGAAGATGGTGGCTATGAAG

TGATAAAGAAAGCGATTCTGAATCTATCTCTTCGCCAT

>FM895756.1 FM895756 Jatropha curcas embryo 71-95 (DAF) Jatropha curcas cDNA clone rjcpga0_002736, mRNA sequence

ATTGTAACGCTGGCCTGTGACCTAAGTGGGGACACGAAAAGCATGGAACATGGCTTCTGCCCATCTCGTC

GATCTCCACTTGGCTATTTCCTGGCTCGCCCGTTCCAAGCCGAGAGTAGAGTCAATTGACCCGGGCGAAG

GCTTGGTTCGCTTGACTCCTAAAGTAAAAAAAAAG

>FM895755.1 FM895755 Jatropha curcas embryo 71-95 (DAF) Jatropha curcas cDNA clone rjcpga0_002735, mRNA sequence

GGGGGGTAAAGAGTTCTTTAAAAAAAAAAAATAAACAATCTCAAACATGACAAAAATTCCAAACTCTCCT

AAACACAAAGGCCATCTCTGCCAGGTAAATATTTTGTACAGTCACGATCGCAAATCCACTACCTCCTCCA

TTTTTTTACACCCTTAAAACTTGTACAGTAAATTTTATCTCCTCTGCAACTGCAAGGGCATCAGTATGTT

CGCCATCTGACCCTCAGCTACCCTGAATCTAAGGTATATCCTCAAACTAACAATGTACAGTTTTAATTTA

CAACTGACAATTTTAATTTTCATTAAGCTCCTATCTATCCAAATAAATAAATGGAGCCATTGCTAAATAT

C

>FM895754.1 FM895754 Jatropha curcas embryo 71-95 (DAF) Jatropha curcas cDNA clone rjcpga0_002734, mRNA sequence

CTAGCCCCGAAAATGGATGGCGCTTAAGCGCGCGACCTATACCCGGCCGTCGGGGCAAGAGCCAGGCCCC

GATGAGTAGGAGGGCGCGGCGGTCGCTGCAAAACCCGGGGCGCGAGCCCGGGCGGAGCGGCCGTCGGTGC

AGATCTTGGTGGTAGTAGCAAATATTCAAATGAGAACTTTGAAGGCCGAAGAGGGGAAAGGTTCCATGTG

AACGGCACTTGCACATGGGTTAGTCGATCCTAAGAGACGGGGGAAGCCCGTCCGACAGCGCGTCCTCGCG

CGAGCTTCGAAAGGGAATCGGGTTAAAATTCCCGAACCGGGACGTGAAGGCTGACGGCGACGTTAGGGAG

TCCGGAGACGTCGGCGGGGGCCTCGGGAAGAGTTATCTTTTCTGTTTAACAGCCCGCCCACCCTGGAAAC

GACTCAGTCGGAGGTAGGGTCCAGCGGCTGGAAGAGCACCGCACGTCGCGCGGTGTCCGGTGCGCCCCCG

GCGGCCCATGAAAATCCGGAGGACCGAGTGCCTCCCACGCCCGGTCGTACTCATAACCGCATCAGGTCTC

CAAGGTGAACAGCCTCTGGTCGATGGAACAATGTAGGCAAGGGAAGTCGGCAAAATGGATCCGTAACCTC

GGGAAAAGGATTGGCTCTG

>FM895753.1 FM895753 Jatropha curcas embryo 71-95 (DAF) Jatropha curcas cDNA clone rjcpga0_002733, mRNA sequence

TCAAATCGGCGTTGAAAGATCAGTTCTCCTTTTTGAGTATCTAAAAATGTCTTCTTGCTGTGGTGGAAAT

TGCGGCTGCGGCTCCGGCTGCAAGTGTGGCAGCGGTTGTGGCGGATGCAAGATGTATCCTGACATGAGTT

TCTCCGAGAAAAACACCGCCGAGACTCAGGTTCTCGGAGTTGCACCGGAGAAGGCACGCTTTGTCGATGG

AGGTGAGGTCGGCGTTGGAGCTGAGAACGGATGCAAGTGCGGTGACAACTGCACCTGCAATCCTTGCAAC

TGTAAATGAGATCGGCGGCGATCAGTCTGTTTTTTAGAAGTAAAAGCAGAGATGACTCTGCTTCTCTCTT

TCTAAAAAAGGAGAAATAATTAATAGAGTCTAGATATCGTATGAGTTTGTTCGTTTGTTTCTGGTTTTGA

GCTTCAATAAAAGTAGTGGTGGCTTGTGAGCTTGTGTTTAGCAATGGCTGCCTGTTGCTTTAACGTTATA

TATTGTATAAATTAAAAACTAGCATATGAATATGTAAATGAGTACCAGGGGTGAAATAGCTTTGAAAGCT

TACTGAGAAACTCCTTTTCTATTTTCTAAA

>FM895752.1 FM895752 Jatropha curcas embryo 71-95 (DAF) Jatropha curcas cDNA clone rjcpga0_002732, mRNA sequence

GGGGGCATTCGTATTTCATAGTCAGAGGTGAAATTCTTGGATTTATGAAAGACGAACAACTGCGAAAGCA

TTTGCCAAGGATGTTTTCATTAATCAAGAACGAAAGTTGGGGGCTCGAAGACGATCAGATACCGTCCTAG

TCTCAACCATAAACGATGCCGACCAGGGATCGGCGGATGTTGCTTTTAGGACTCCGCCGGCACCTTATGA

GAAATCAAAGTCTTTGGGTTCCGGGGGGAGTATGGTCGCAAGGCTGAAACTTAAAGGAATTGACGGAAGG

GCACCACCAGGAGTGGAGCCTGCGGCTTAATTTGACTCAACACGGGGAAACTTACCAGGTCCAGACATAG

TAAGGATTGACAGACTGAGAGCTCTTTCTTGATTCTATGGGTGGTGGTGCATGGCCGTTCTTAGTTGGTG

GAGCGATTTGTCTGGTTAATTCCGTTAACGAACGA

>FM895751.1 FM895751 Jatropha curcas embryo 71-95 (DAF) Jatropha curcas cDNA clone rjcpga0_002731, mRNA sequence

CAACTCAAGTTGTCAATTTGCCATAAATTACACAGGTACAACCGTTAATAACATTAAACTGCCTACATTT

TACCTGCAATGCTAAAACTGTATTTCTAGACATGACCATATTTCAAAAAGACTAGAAACATCTCAAAACT

CGTCAAATTTTTGTATGAAAACAGAGTGAACAAGTAACTAAAACTTTTTAGATCACTCAGAAGCACAAAG

AGCATTTCATTAAAATTCTCATTCTGCTGCAGCGTCTCGGCTGTTAACCCCTTCTACACTATTCCCTGGC

AATGTATCCAGATCTATGCGAGCCTCTTTCTCATCTTGGCTGACTGAAGTATCAGAAGAATCAATGGGGT

CCTGAATAAGAGCTTCAGTTCCGTCTGTTGACGAGTTTGAATTTTCATCTGTTCCACTTGATTCATTCTT

GGTGTCTGAGTTCTCATCTTGAGTAGCATCTGCAGTCT

>FM895750.1 FM895750 Jatropha curcas embryo 71-95 (DAF) Jatropha curcas cDNA clone rjcpga0_002730, mRNA sequence

TGAATTTCCTGATGTCCGCCTCAACATCATCAGCAAGCTTGATCAAGTGAATCAGGTTATTGGAATTGAT

CTACTGTCTCAATCATTATTGCCAGCTATTGTTGAGCTTGCAGAGGATAGGCATTGGAGGGTTCGGCTTG

CAATTATAGAGTATATACCTTTATTGGCAAGTCAGTTGGGTGTTGGGTTCTTTGACGATAAACTTGGGGC

TCTGTGCATGCAGTGGTTACAGGATAAGGTTTACTCAATTCGTGATGCTGCAGCAAATAATTTAAAGCGG

CTTGCTGAAGAATTTGGTCCAGAGTGGGCAATGCAACACATAGTGCCACAGGCTCTGGAGATGATTAATA

ATCCTCATTACTTGTATCGGATGACCATCTTACGTGCTATCTCACTACTAGCTCCTGTTATGGGATCAGA

AATTACATGTTCTAAACTGCTTCCTGTGGTCATCAATGCATCAAAAGACAGGGTACCTA

>FM895749.1 FM895749 Jatropha curcas embryo 71-95 (DAF) Jatropha curcas cDNA clone rjcpga0_002729, mRNA sequence

ATTCCTTCTTTTCTCTCTCCCTTACAAGCCCTAACCCTAACTCAGACCCAGATTCGCCATGAAAGGAGGT

AAATCAAAGTCAGACACCAAGAACGCCAAGCTTTCCGTGAACAAGAAACCCGCCAAGGCAAGTAAGAAGG

CGGGTAAGGCAGCGAAGGATCCAAATAAGCCAAAGAGGCCCGCCAGTGCTTTCTTCGTTTTCATGGAAGA

GTTCAGAGAGCAGTACAAGAAGGAACATCCTAAAAACAAATCCGTTGCTGCTGTTGGCAAAGCTGGTGGA

GATAAATGGAAGTCAATGTCAGATGCTGAGAAAGCACCTTTTGTATCTAAGGCAGAAAAGAGGAAGATAG

AATATGAGAAGAAATTGAAAGCCTACAACAAGGGACA

>FM895748.1 FM895748 Jatropha curcas embryo 71-95 (DAF) Jatropha curcas cDNA clone rjcpga0_002727, mRNA sequence

GATTGAACCTCTTGCTCGCCTTTCATATAACAAAAATTAACATAATAATCTATAGTATTGCATTGATGTT

CATGACCTTAGCCTCTGACAAGCATGGAATCATGGCTTGCGAACGCTTAACAGCTTCACATCGTCAATAA

CGGGTCCACAAAGAGAAGAGAAATCATCACTTCTCATGGTGTAAAATGTGCTATAGAACATTATACGAGT

TCTGTTTGAAGCTGCAACAAATCTGAGCACTGCACGCTTGAATCCTCCTTTACCCTTCGACTCATAAGGA

ACTTTCAGTGTATCTTTTCCAGCAAAAGCCTCTACTACCATTGACCCTTCGCAAGAATTACTAGCATCGC

CTACTGCAAATGAGAGTGTATATGTTTTGCCAACAACTGTTCGAGCTACCTGTGCTATTGCACTCTCTTT

ACCTGCCACGAGTTCTACAGCTCGTCTTGCTTGCGGGACTGAGAAATGGTCCGAGTCTATGTACTTGACT

GCTTTGAGTGACTCGATCATCC

>FM895747.1 FM895747 Jatropha curcas embryo 71-95 (DAF) Jatropha curcas cDNA clone rjcpga0_002725, mRNA sequence

GAACGTTCTAGCTTCACATCCANGTGTAATTAAGACACACCGCCGTGGAATTTTGATCAGACGACAAAGA

TGCCGATCAGAAACATTGCCATAGGCCATCCTCAGGAGGCAACTCATCCAGACGCCTTGAAAGCGGCGTT

GGCTGAGTTCATCTCCACTCTTATTTTTGTCTTCGCCGGTGAAGGCTCCGGGATGGCCTTTAATAAGCTC

ACTAACAACGGCGCCACCACTCCTGCCGGTCTAGTCGCCGCATCTTTAGCCCATGGTTTCGGTCTTTTTG

TTGCTGTCTCAGTTGGAGCCAACATCTCCGGCGGTCATGTAAATCCAGCTGTTACCTTCGGTGCTTTCGT

CGGTGGAAACATCACTCTCCTCCGCGGTATTCTCTACTGGATTGCTCAGCTTCTCGGATCCACCGTAGCT

TGCTTGCTTCTCAAGTTCAGCACCGGTGGCTTGACTACGTCCGCCTTTGCTCTCTCCTCTGGTGTTGGTG

TGTGGAACGCTTTCGTTTTTGAGATTGTGATGACCTTCGGGCTTGTATACACCGTATATGCCACCGCAAT

TGACCCCAAAAAGG

>FM895746.1 FM895746 Jatropha curcas embryo 71-95 (DAF) Jatropha curcas cDNA clone rjcpga0_002724, mRNA sequence

ATTCCTCAGAACTGGTACGGACAAGGGGAATCCGACTGTTTAATTAAAACAAAGCATTGCGATGGTCCCT

GCGGATGCTCACGCAATGTGATTTCTGCCCAGTGCTCTGAATGTCAAAGTGAAGAAATTCAACCAAGCGC

GGGTAAACGGCGGGAGTAACTATGACTCTCTTAAGGTAGCCAAATGCCTCGTCATCTAATTAGTGACGCG

CATGAATGGATTAACGAGATTCCCACTGTCCCTGTCTACTATCCAGCGAAACCACAGCCAAGGGAACGGG

CTTGGCGGAATCAGCGGGGAAAGAAGACCCTGTTGAGCTTGACTCTAGTCCGACTTTGTGAAATGACTTG

AGAGGTGTAGGATAAGTGGGAGCCGAAAGGCGACGGTGAAATACCACTACTTTTAACGTTATTTTACTTA

TTCCGTGAATCGGAGGCGGGGCTATGCCCCTCTTTTTGGACCCAAGGTCGTTTCGGCGGCCGATCCGGGC

GGAAGAC

>FM895745.1 FM895745 Jatropha curcas embryo 71-95 (DAF) Jatropha curcas cDNA clone rjcpga0_002723, mRNA sequence

CAAGTCCAACCATCTACCATTTCCATTCTAATTCTATGCAGTGTACACTGCATACGTATAGAAGATATCT

TTCCAAAATTGATCTTTTTTCTGAACAATTTCCCTATTGCCTAAACTAAGTCTATTAAAAATAATAGAGC

TCGAGCCAACGTCGTATTGTTAATTAACGAATGAGATCGAAGAAGTAATTCCAGAAGGCCAGACTCGATG

TAAGCTAAAAGATTTTGATCAGTTACACCTATTCTAGCAAAGGCAGCTTCATTTTCTTGTATACAACATA

TAATTTTATCTGAACGTATACTATTGCTTCTTGGATGCTTTAGCACGCTGTCGCTTAGCCCACCTTGATC

TTGAACCGTGGCTATCCAAATTGTACCCAACTGAAGTAGGAGCATGCAATGGAGGTCTTTGATCATAGCT

TGATGATTCATTTCCATTTGACTTCCCCTTGAATGCAGGTAATTTGGCTATATGATAACCAGGAGCAGGC

TGCCGTGCGCCATGGCTCATTAGTGATTTAAGGAAATTACTTGGGCCCTGGTTCATC

>FM895744.1 FM895744 Jatropha curcas embryo 71-95 (DAF) Jatropha curcas cDNA clone rjcpga0_002722, mRNA sequence

GATAAAAATGAGCCATTTATTAAAAATTCGGATGTCTCTTTTTGTACTTCCTCAATTTTAGCATGAAGAA

AACAAAAGGAAAGGTAGAAATGTGACCTAAATTTACAACACGAACTCGTATCTTTCTACAGCTCTTTCTC

TCGTTGATAAGCCACGATTAAAACACAAACACTGGGATGAGGAAAAGAAAATTCAAATTAATCTTACACA

GCAACTGGTGTGGCTTCATCAAGAGCAAGAACACCTGGAAGCTCTTTTCCTTCTAACAACTCCAAACTGG

CACCACCACCAGTTGATATGTGGCTCATAACATCAGCAACTCCTACTTTTTCTACAGCTGCAACAGAATC

TCCACCTCCAATAATTGTTGTCACTCCCTTGCCACTGAGCTCTGCTAGCTTCTTTGCAATAGCCTCTGTC

CCAACTGCAAACTTGTCAAATTCAAACACTCCCATTGGTCCATTCCAGATGACGGTCTTGGTGGTT

>FM895743.1 FM895743 Jatropha curcas embryo 71-95 (DAF) Jatropha curcas cDNA clone rjcpga0_002720, mRNA sequence

ATTGAATGAAAGTTTGAGCTTGCCATAAATATTTAGGATGCCACTACATTGCTGTGGACAAATAGCATTT

GAGAGGTTACAGCAACATAGAATAACAGAAAAATTTGCAGGTCTAATCCAACTGCAAGAAGTGAAATGAT

GACAATGTTTGCCTAAATAAAAATGGTGACAACATCTTTGACAATATTAGAGAGGTCTTGAAAATTAATG

GTAACCATCCAGAATGCATAGAATAGTTGCATTCAATTGTTTCAATGTTTCCAAATTCATAGATTTTAAG

GTTTCCAGATCCACAGATTATCTATAACGATTAACAATACTTCCATTATATTTGGGCCCTCTAAGACAAA

GTTTTCACACCCTTGAATGAACCTGATTACCACGCAACCAAAGGTGTACTTATCTACATGTCCAATTACA

ATTGTGCTGCTGATGGATACACAATATAGCCAATATAGACTGCGAGATCCGTGCCTAACAGAAGTGAAAG

ATATGATACCCAAAACAAGAACACCATATCATGAACTTTAAAAAAA

>FM895742.1 FM895742 Jatropha curcas embryo 71-95 (DAF) Jatropha curcas cDNA clone rjcpga0_002719, mRNA sequence

TGGAGATTACGCAGCTGTCTTTTGCAGCTGATACGCCTCCTCTCCCTGTTATTGCTGCCGCTAAGGTTGC

TTCCGTCCATCTTCCCTCGCCTACTCTTGTCAACGATGTTTCCCTGCCTACTGTTGTCTTCTCCGATGGG

ACGAAATTGCAAGGTATATTTGTACTTCTTAGGTATATTGGTCGGGTTGCCAAGCTTCCCAATTTTTATG

GACAGAATGCGTACTTTTCTGGACAGATTGACGAGTGGCTAGACTATTCTCCTATTCTTTCATCCGGGTC

TGAATTCGAGAATGCGTGCGGCCATATTGACAATTATTTGGCAAAGCGTACATTTTTGGTTGGACATTCT

TTATCTATTGCGGACATTGCTATCTGGTCAGGTCTAGCAGGAACTGGGCAGAGATGGGAGAGTTTGAGGA

AGTCAAAGAAATATCCAAATCCTTGTGCCGTTGGTTCAACTCCATATCTGTTGAATATAGCGATGCC

>FM895741.1 FM895741 Jatropha curcas embryo 71-95 (DAF) Jatropha curcas cDNA clone rjcpga0_002717, mRNA sequence

GGAGTCTGACATGTGTGCGAGTCAACGGGTGAGTAAACCCGCAAGGCGCAAGGAAGCTGACTGGCGGGAT

CCCCCCGAGGGTTGCACCGCCGACCGACCTTGATCTTCTGAGAAGGGTTCGAGTGAGAGCATGCCTGTCG

GGACCCGAAAGATGGTGAACTATGCCTGAGCGGGGCGAAGCCAGAGGAAACTCTGGTGGAGGCCCGCAGC

GATACTGACGTGCAAATCGTTCGTCTGACTTGGGTATAGGGGCGAAAGACTAATCGAACCGTCTAGTAGC

TGGTTCCCTCCGAAGTTTCCCTCAGGATAGCTGGAGCTCGGGACGAGTTCTATCGGGTAAAGCCAATGAT

TAGAGGCATCGGGGGCGCAACGCCCTCGACCTATTCTCAAACTTTAAATAGGTAGGACGGCGCGGCTGCT

TCGCTGAGCCGCGCCACGGAATCGAGAGCTCCAAGTGGGCCATTTTTGGTAAGCAGAACTGGCGATGCGG

GATGAACCGGAAGCCGGGTTACGGT

>FM895740.1 FM895740 Jatropha curcas embryo 71-95 (DAF) Jatropha curcas cDNA clone rjcpga0_002716, mRNA sequence

GGAGTTATTCCATATGCTGCAACAAATTACTTTGCTTATAGAACACATTACGGGAAAGCCTATCGGAAAG

TTTTTAAGCAAGAGAAGATTGGCAACATTGAAACCCTTCTAATTGGATCAGCTGCTGGAGCTATTTCAAG

TAGTGCGACCTTCCCACTTGAGGTTGCTCG

>FM895739.1 FM895739 Jatropha curcas embryo 71-95 (DAF) Jatropha curcas cDNA clone rjcpga0_002715, mRNA sequence

CAGTTCAACAGAGTTTGGGGTGGTTATTATCCAATGAGACATACAGAAGGATCCTCATGCCAAGGATACA

CACGATAAATTTATTCCTCTGAAGTCATCTATTTCTAACCATAGATGAGAAAGGACTTGATAACAGAAAA

TTCCTCTGAAGTCATCTATTTCTAACCATAGATGAGAAAGGAGTTGATAACAGAAACAACAACCGCTCAC

ATCCAAAATTCAGTACAGGAGAAGGCTGCTTAAGTGCCCACAAGAATCTAATTCACCTCAATAGAGTAAT

TCTTCGAAAAC

>FM895738.1 FM895738 Jatropha curcas embryo 71-95 (DAF) Jatropha curcas cDNA clone rjcpga0_002714, mRNA sequence

AGGATTCTATCCGTCGCCCGATGGGAATTGTACTTTAAGGCGGCCCTCGCGGCTCGTCCGCCGCGGGGGC

TTGGCCAACGGCACGTGCCTCTGGGGGCCGGATGGCCCCTACTGCGGGTCGGCAAACGGGCGACGGACAC

GCGCGTCGCTTCTGGCCCGGATTCTGACTTAGAGGCGTTCAGTCATAATCCAGCGCACGGTAGCTTCGCG

CCACTGGCTTTTCAAGCAAGCGCGATGACCAATTGTGCGAATCAACGGTTCCTCTCGTACTAGGTTGAAT

TACCATCGCGACACCATCATCAGTAGGGTAAAACTAACCTGTCTCACGACGGTCTAAACCCAGCTCACGT

TCCCTATTGGTGGGTGAACAATCCAACACTTGGTGAATTCTGCTTCACAATGATAGGAAGAGCCGAC

>FM895737.1 FM895737 Jatropha curcas embryo 71-95 (DAF) Jatropha curcas cDNA clone rjcpga0_002713, mRNA sequence

GCTGAGATCAATCCAAAGGATCTATCCGTCCCGATGGGAATTGTACTTAAGCGCCTCGCGGCTCGTCCGC

CGCGGGGGCTTGGCCAACGGCACGTGCCTCTGGGGGCCGGATGCCCTACTGCGGGTCGGCAAACGGGCGA

CGGACACCCGTCGCTTCTGGCCCGGATTCTGACTTAGAGGCGTTCAGTCATAATCCAGCGCACGGTAGCT

TCGCGCC

>FM895736.1 FM895736 Jatropha curcas embryo 71-95 (DAF) Jatropha curcas cDNA clone rjcpga0_002712, mRNA sequence

GTTTGCTACTTTCTTAGTTCTTTCTTATTGTTTGCTTCTCAATTTTATTTTTTGGTTCTTATTAGGTACT

TTTCTCCTTGGATTCAAATGGCTACTGTTGAGGTAGTATCGGCACAAACTGCACTTTCAGAGGAAAAAGC

AGAAGAAGTGAAGGCTTCAGAGACTGTCACTCAAGTAACAGATCAGTCAGTTACTGATGAAAAACCAGAC

ACAGGAGAAAAGAAGGAAGAACAAGAAGAAGTTGCAGGAACTAAAGAAGAAGCTGTAGAACCAGAACCAG

AAGTTGCAACTGAAGTAGTGGTGGAAACTAAAGAAGTAGTAGAAGATGAGACCAAAACTGTAACAGAAGA

GCCCCCATCGGAGAAAACTGAAGAAGAAACTCCTAAGGAAACAGAACCAGAAAAAGAAGAAACTCCTAAG

GAAACAGAACCAGAAAAAGAAGAAAAGGAATCCCCAAAAGAAACAGTAACTGAGGAGACTGTTGCTGAGG

AG

>FM895735.1 FM895735 Jatropha curcas embryo 71-95 (DAF) Jatropha curcas cDNA clone rjcpga0_002711, mRNA sequence

ATTCCTCAATTTTTCTTTATTTAGGGTTTTGATATTATTCCAATAGTTTTGTTATTTTGTTGGTTCGTAT

TTCCTAGTTTCATCACGGACTATTTACTTCGGTTATTTGATAGAGCTTGATCATAAAGAATCAGAGTTTG

ATCCGTGATCGATCTGGTTCTAGATGTTTAAATCTGGCTTGGCCAACGCTGCATCTGGCATGGCAGTCCA

TGATGACTGCAAATTGAAGTTCTTGGAGCTCAAGGCGAAAAGAACTTACCGCTTCATAGTTTTTAAAATT

GAGGAGAAGCAAAAGCAAGTCATTGTGGAGAAGGTTGGTGAGCCTAACCAAAGCTATGAGGATTTTACTG

CAAGCCTTCCTGCTGATGAGTGCCGATATGCTGTTTATGATTTTGATTTTGTCACGGCAGAGAATTGCCA

GAAGAGCAGGATTTCTTCTTGCTGGTCTCTGAT

>FM895734.1 FM895734 Jatropha curcas embryo 71-95 (DAF) Jatropha curcas cDNA clone rjcpga0_002710, mRNA sequence

ATTCTTTTTTTTTGAATAAAAGGAAATAACTTAGATTACCAAAAACAAGAGTACATTTTATAATGCTGAG

ACAATTGTATTAACTTCCCAGTTTGCACCACACGATGCACAACACCAATCTGAAACCCTGAAACATATCA

CTCACAGCTTAATTATTCTCAAATCAACTCTTTCAATTTCAATACTAAACTGCATCCTAAGCCGGAAAGG

AGGAGATCCGCATCATGTGATTAGTACTATGGGGGGCTGCTGCTGTGCTCATTGCACAGAGGCTATGTGG

CAGATCAAGTCGATCACACGTGAACTGTAGCCCCATTCATTGTCATACCAAGAGACAAGTTTTCACAAAG

TTATCATTCAANTGGCAATTCCAGCCTTGGGCATCAAAGATGCTAGACCTGTTGTCACCCACAAAATCAG

TT

>FM895733.1 FM895733 Jatropha curcas embryo 71-95 (DAF) Jatropha curcas cDNA clone rjcpga0_002709, mRNA sequence

ATTCCAAAGGATTCTATCCGTCGCCCGATGGGAATTGTACTTTAAGGCGGCCCTCGCGGCTCGTCCGCCG

CGGGGGCTTAGCCAACGGCACGTGCCTCTGGGGGCCGGATGGCCCCTACTGCGGGTCGGCAAACGGGCGA

CGGACACGCGCGTCGCTTCTGGCCCGGATTCTGACTTAGAGGCGTTCAGTCATAATCCAGCGCACGGTAG

CTTCGCGCCACTGGCTTTTCAACCAAGCGCGATGACCAATTGTGCGAATCAACGGTTCCTCTCGTACTAG

GTTGAATTACCATCGCGACACCATCATCAGTAGGGTAAAACTAACCTGTCTCACGACGGTCTAAACCCAG

CTCACGTTCCCTATTGGTGGGTGAACAATCCAACACTTGGTGAATTCTGCTTCACAATGATAGGAAGAGC

CGACATCGAAGGATCAAAAAGCAA

>FM895732.1 FM895732 Jatropha curcas embryo 71-95 (DAF) Jatropha curcas cDNA clone rjcpga0_002708, mRNA sequence

ATTCGGGAAACAGCCCGGATCACCAGCTAAGGCCCCTAAATGACCGCTCAGTGATAAAGGAGGTAGGGGT

GCAGAGACAGCCAGGAGGTTTGCCTAGAAGCAGCCACCCTTGAAAGAGTGCGTAATAGCTCACTGATCGA

GCGCTCTTGCGCCGAAGATGAACGGGGCTAAGCGATCTGCCGAAGCTGTGGGATGTAAAAATGCATCGGT

AGGGGAGCGTTCCGCCTTAGAGGGAAGCACCCGCGCGAGCAGGGGTGGACGAAGCGGAAGCGAGAATGTC

GGCTTGAGTAACGCAAACATTGGTGAGAATCCAATGCCCCGAAAACCTAAGGGTTCCTCCGCAAGGTTCG

TCCGCGGAGGGTGAGTCAGGGCCTAAGATCAGGCCGAAAGGCGTAGTCG

>FM895731.1 FM895731 Jatropha curcas embryo 71-95 (DAF) Jatropha curcas cDNA clone rjcpga0_002707, mRNA sequence

TGTGAAGATAGCAATAGAGGAAAGCAGATCAGCCGGTCGGATCTGTGGGGACTTCTAATGAATACGCCGT

TTGTTTCAAGAGGCGGTTCAGTTAAAGCATCTAAGACTGATAACGACGGAGATTCAGTAAACGGTAGGAA

CTTGTTTGCGTCGGTCAAAAAACCGTTGAATTGCTTGAATGCGAAAGTGGAGGGAGGAGGAGAACCGCCA

AGTCGGAGTCGACCTGTCGTTTGACGTAATTTTATTAATTTATTGGTTTATTTATTTATTTAATTTAATA

ATTAAGCTCGATTTGAAGTGGAGGAAAGGGAAACATGGTGAATCCATTAAAAGCATGCAGTGAAGGTAGA

GGTGTCAAGTAGGAATAGAGCTGTGGAATGCTAGGCCTGGACAGAACGACAATGAAGAGAGGTGAGTCAC

ATTTTTTTGGGAAGAAATTGAGTTGAACTGATGGACCAAAACTGCACATAGGCAGAAGTCCTCGGATGCC

TCATGCTTCCAAAAAGGTGAATTGGCATTACTAGTTTGGTGAGACTTAGTTGC

>FM895730.1 FM895730 Jatropha curcas embryo 71-95 (DAF) Jatropha curcas cDNA clone rjcpga0_002706, mRNA sequence

AACAAAGCAGAAAATCCACTTAAAATTCATCAAAAATATTAACAATCAATCAATAGGCAAAACTTGAAAG

GATAACTAAAAAAAATTCAACATTTAAATTTTTCACGAGGGAGGCTAAAAAAAAAAAAAAACAAAAAAAA

GAAAAGAAGAGAAAAAAGAGGTGGGAAAAATTCACAAGATTGTGAACCTAGGTTAATAAATACTACAGCA

AGATTTCTCCTTTCCAAAAAATATTACAATGAGATTAGCGAAAACAATGTATTCTGTGCACCACTGTCAT

CAGTCAAATGTGTACGATTCCAAAGTACATAGTGATGAGTCTCAGCAGTCTCTAGTACCAATAAAAATGG

GGATGCAACAGAAAGAACGCCTGAAAGCTTCCTTCTGTTACAATCTTCAGTTCCGCCATGCCTTCATCAT

TCAACATCAATCACCTTGTAGGCAGTAAAGCTGACGGATATTGCCTGAAGCATATAACCAATCCTTGAAA

>FM895729.1 FM895729 Jatropha curcas embryo 71-95 (DAF) Jatropha curcas cDNA clone rjcpga0_002705, mRNA sequence

TTTTTTTTTGACAAGTACAAATATATAAAAAAATATTTCAAATAGGGATTATTTTAAAATTTTCATTGTA

GGAACTCCTATCTAAATTAGCAAGAATGATGACAAGGGAACTTATTATTCTCAAGACTACTCCCACCATC

TCCATGAAAAATCCATAAACTGTAGGCTTTGATTGTCATGTATAACTTAAGATGATTCTATACTTCTGAC

AAATTTCTTGGCCTGCTCTAAGGTTAGAAACATGATCACATTCCAGGATCCTAGCCGCCCAAAGTTTGGG

ATAAATCCCTTATAGAAAGCAAGAGGCCCATCATTCTTCAGGGTCTTAATGAAGCAATCAAAAGTGCTCT

TGTAAGCAGAATCCCCCATCATTCTTGACTTAACCACATCAACTGGGGAGCCAATACAAACAGCAAAGAA

ACCTGCCCCAAGACCAGAAAGAAGATGAGTGACAACATTATCTGTGAACCCAGGAATTTTCAGAATTGTC

>FM895728.1 FM895728 Jatropha curcas embryo 71-95 (DAF) Jatropha curcas cDNA clone rjcpga0_002704, mRNA sequence

GGATTCTATCCGTCGCCCGATGGGAATTGTACTTTANAGGCGGCCCTCGCGGCTCGTCCGCCGCGGGGGC

TTGGCCAACGGCACGTGCCTCTGGGGGCCGGATGGCCCCTACTGCGGGTCGGCAAACGGGCGACGGACAC

GCGCGTCGCTTCTGGCCCGGATTCTGACTTAGAGGCGTTCAGTCATAATCCAGCGCACGGTAGCTTCGCG

CCACTGGCTTTTCAACCAAGCGCGATGACCAATTGTGCGAATCAACGGTTCCTCTCGTACTAGGTTGAAT

TACCATCGCGACACCATCATCAGTAGGGTAAAACTAACCTGTCTCACACGGTCTAATCCCAGCTCACTCC

CTATTGGTGGGTGAACAAACAACACTGGTGAATTCTGCTTCCAATGATAGGAAGACCGACATCAAGGATC

AAAAGCAACG

>FM895727.1 FM895727 Jatropha curcas embryo 71-95 (DAF) Jatropha curcas cDNA clone rjcpga0_002702, mRNA sequence

CTGGAAATCAGAATCAAACGAGCTTTTACCCTTTTGTTCCACACGAGATTTCTGTTCTCGTTGAGCTCAT

CTTAGGACACCTGCGTTATCTTTTAACAGATGTGCCGCCCCAGCCAAACTCCCCACCTGACAATGTCTTC

CGCCCGGATCGGCCGCCGAAACGACCTTGGGTCCAAAAAGAGGGGCATAGCCCCGCCTCCGATTCACGGA

ATAAGTAAAATAACGTTAAAAGTAGTGGTATTTCACCGTCGCCTTTCGGCTCCCACTTATCCTACACCTC

TCAAGTCATTTCACAAAGTCGGACTAGAGTCAAGCTCAACAGGTCTTCTTTCCCCGCTGATCGACGCAAG

CCCGTTCCCTTGGCTGTGGTTTCGCTGGATAGTAGACAGGGACAGTGGGAATCTCGTTAATCCATTCATG

CGCGTCACTAATTAGATGACGAGGCATTTGGCTACCTTAAGAGAGTCATAGTTACTCCCGCCG

>FM895726.1 FM895726 Jatropha curcas embryo 71-95 (DAF) Jatropha curcas cDNA clone rjcpga0_002701, mRNA sequence

TTTTTTGACTAAGGAAAACATATATAGATTATAAAGCTAAAGACATTAATGTTTTGGGTATAAAAGAGTT

CACAAAACCTTAATACCAACATACATTGATTGATAAAATAAATAATAACCCCTAACTATCTTCACCACCA

TTCTCTCCAAACCAAGAGGACAAGGGAAAGAAAAAAACTCCAAACCAATAATAGTTCATAAAGGACTAAC

GAAAATAAATAAATTAAAAAAAAATAAATGACTCCCAAAACTTCAAAACCCCTCCCTTTCTGTTCCTCTC

CAAAGATATCAGCAAATTCTCCCTCCAAACTTCAATCGAACTCTACTGTTGCTTGCACTCTGGAGGCCTC

TCACCCTGCTGACCATCCCCAGCTTGTGTTCTTCCACCCTTCTTATGCCCGGCTGACAGCTTCTTTATGG

TCTTGCTTTCATCCTCGTCCTCATCCTCAT

>FM895725.1 FM895725 Jatropha curcas embryo 71-95 (DAF) Jatropha curcas cDNA clone rjcpga0_002696, mRNA sequence

TTTTACAAAACATTATAAATTTACTTTAAAAATGATCATCTTAGTTACATCCTAACCAAATTCTTTACCC

GTAGTTAGTAACATTTTTAACTACAGAATAATTTACAAACAAGGCCAGGTTCAAGCGTGAAAAACACAAG

CTGCAAACAAAACAGAGGTGAGTTGAAAAGCTCAACAATCTTTTTCCCTTTTGCCCAGAGCAAGCATTTG

GCATCTTTAAGCATCATTTATTGACAGCATTGAGCTGTCAAGTTAAGCACGGTCGAAGTGCCAACATCCT

CCTCTCACATCTTTTATTACAGGTAGCAGCTTTATATTGATCATGCAGCTCAATCTTCTTCTGAAGATAC

AAGCTGATGCCCCTAAATTCATGATAGTCCAGTGTTTCTTGACAACGTATCAGGAATACTGCTGCTTAAG

CGAAACTAACCTTTAAGGCATTAGCAGAAACA

>FM895724.1 FM895724 Jatropha curcas embryo 71-95 (DAF) Jatropha curcas cDNA clone rjcpga0_002695, mRNA sequence

CAAAGGATTCTATCCGTCGCCCGATGGGAATTGTACTTTAAGGCGGCCCTCGCGGCTCGTCCGCCGCGGG

GGCTTAGCCAACGGCACGTGCCTCTGGGGGCCGGATGGCCCCTACTGCGGGTCGGCAAACGGGCGACGGA

CACGCGCGTCGCTTCTGGCCCGGATTCTGACTTAGAGGCGTTCAGTCATAATCCAGCGCACGGTAGCTTC

GCGCCACTGGCTTTTCAACCAAGCGCGATGACCAATTGTGCGAATCAACGGTTCCTCTCGTACTAGGTTG

AATTACCATCGCGACACCATCATCAGTAGGGTAAAACTAACCTGTCTCACGACGGTCTAAACCCAGCTCA

CGTTCCCTATTGGTGGGTGAACAATCCAACACTTGGTGAATTCTGCTTCACAATGATAGGAAGAGCCGAC

ATCGAAGGATCAAAAAGCAACGTCGCTATGAACGCTTGGCTGCCACAAGCCAGTTATCCCTGTGGTAACT

TTTCTGACACCTCTAGCTTCAAATTCCGAAGGTCTAAAGGATCGATAGGCCACGCTTTCACGGTTCGTAT

TCGTACTGGAAATCAGAATCAAACGAGCTTTTACCCTTTTG

>FM895723.1 FM895723 Jatropha curcas embryo 71-95 (DAF) Jatropha curcas cDNA clone rjcpga0_002694, mRNA sequence

ATTCGTTGTGGTTAGGGGTGAAATGCCACTCGAACCCAGAGCTAGCCGGTTCTCCCCGAAATGCGTTGAG

GCGCAGCAGTTGACTGTACATCTAGGGATAAAGCACTGTTTCGGTGCGGGCCGCGAGAGCGGTACCAAAT

CGAGGCAAACTCTGAATACTAGATATGACCTCAAAATAACAGGGGTCAAGGTCGGCCAGTGAGACGATGG

GGGATAAGCTTCATCGTCGAGAGGGAAACAGCCCGGATCACCAGCTAAGGCCCCTAAATGACCGCTCAGT

GATAAAGGAGGTAGGGGTGCAAGACAGCCAGGAGGTTTGCCTAGAAGCAGCCACCCTTGAAAGAGTGCGT

AATAGCTCACTGATCGAGCGCTCTTGCGCCGAAGATGAACGGGGCTAAGCGATCTGCCGAAGCTGTGGGG

ATGTTAAAATGCATCGGTAGGGGAGCGTTCCGC

>FM895722.1 FM895722 Jatropha curcas embryo 71-95 (DAF) Jatropha curcas cDNA clone rjcpga0_002693, mRNA sequence

TTGATGCCAATGGTATCTTGAATGTTTCAGCAGAGGATAAGACCACTGGCCAAAAGAACAAGATCACAAT

TACAAATGACAAGGGCAGATTGTCCAAGGAAGAAATTGAGAAGATGGTCCAGGAGGCTGAGAAGTACAAG

TCCGAAGATGAGGAGCACAAGAAAAAGGTGGAGGCAAAGAATGCTTTGGAGAACTATGCATACAACATGA

GGAACACCATCAAGGATGAGAAGATCAGTTCCAAGTTGGCCGCAGCTGATAAGAAGAAGATTGAAGATGC

TATTGATCAGGCCATTCAGTGGCTAGACAGCAACCAGCTTGCAGAGGCAGATGAGTTCGAGGACAAGATG

AAAGAGCTTGAGAGCATCTGCAATCCCATCATTGCCAAGATGTATCAAGGTGCTGGTGCTGACATGGGTG

GCGGCATGGAAGATGATGTTCCTCCAGCAGGTGGCAGTGGTGCTGGNTCCAAGATCGAGGAAGTTGACTA

AGTGGCTTGTTTGATGCTTGAGTAAAGATGGTGATGAATATTTTATTTTCTTTATTGGTCAGTACAATTA

TTATTATTATTATTTTAATATT

>FM895721.1 FM895721 Jatropha curcas embryo 71-95 (DAF) Jatropha curcas cDNA clone rjcpga0_002692, mRNA sequence

TGGTGCGAGTCNAACGGGTGAGTAAACCCGCAAGGCGCAAGGAAGCTGACTGGCGGGATCCCCCCGAGGG

TTGCACCGCCGACCGACCTTGATCTTCTGAGAAGGGTTCGAGTGAGAGCATGCCTGTCGGGACCCGAAAG

ATGGTGAACTATGCCTGAGCGGGGCGAAGCCAGAGGAAACTCTGGTGGAGGCCCGCAGCGATACTGACGT

GCAAATCGTTCGTCTGACTTGGGTATAGGGGCGAAAGACTAATCGAACCGTCTAGTAGCTGGTTCCCTCC

GAAGTTTCCCTCAGGATAGCTGGAGCTCGGGACGAGTTCTATCGGGTAAAGCCAATGATTAGAGGCATCG

GGGGCGCAACGCCCTCGACCTATTCTCAAACTTTAAATAGGTAGGACGGCGCGGCTGCTTCGCTGAGCCG

CGCCACGGAATCGAGAGCTCCAAGTGGGCCATTTTTGGTAAGCAGAACTGGCGATGCGGGATGAACCGGA

AGCCG

>FM895720.1 FM895720 Jatropha curcas embryo 71-95 (DAF) Jatropha curcas cDNA clone rjcpga0_002690, mRNA sequence

CTGACATGTGTGCGAGTAACGGGTGAGTAAACCCGCAAGGCGCAAGGAAGCTGACTGGCGGGATCCCCCN

CGAGGTTGCACCGCCNGACCGACCTTGATCTTCTGAGAAGGGTTCGAGTGAGAGCATGCCTGTCGGGACC

CGAAAGATGGTGAACTATGCCTGAGCGGGGCGAAGCCAGAGGAAACTCTGGTGGAGGCCCGCAGCGATAC

TGACGTGCAAATCGTTCGTCTGACTTGGGTATAGGGGCGAAAGACTAATCGAACCGTCTAGTAGCTGGTT

CCCTCCGAAGTTTCCCTCAGGATAGCTGGAGCTCGGGACGAGTTCTATCGGGTAAAGCCAATGATTAGAG

GCATCGGGGGCGCAACGCCCTCGACCTATTCTCAAACTTTAAATAGGTAGGACGGCGCGGCTGCTTCGCT

GAGCCGCGCCACGGAATCGAGAGCTCCAAGTGGGCCATTTATGGTAAGCAGAACTGGCGATGCGGGATGA

ACCGGAAGCCGGGTTACGGTGCCCAACTGCGCGCTAACCTAGAACCCACAAAAGGTGTTGGTCGAT

>FM895719.1 FM895719 Jatropha curcas embryo 71-95 (DAF) Jatropha curcas cDNA clone rjcpga0_002689, mRNA sequence

GGAAAGCCTTTCTGTTCCATCTCTTCGCGTTTGTCTCTAATCTCTACGATTAATAACAGATACTTGTATC

TCTCTCTGCTTGTTTGTTGTTGGGATAAGCTGAGATTATTTTCAATAATGGCAACTAGGCTTATGTTTGA

GAACTCATGTGAGGTTGGAGTATTTTCCAAGCTCACCAATGCTTATTGCTTGGTTGCTATTGGAGGCTCT

GAAAGCTTCTACAGCACATTTGAGGCTGAGTTAGCAGATGTTATCCCAGTGGTTAAAACTTCAATTGCAG

GCACTAGGATTGTTGGCCGTCTTTGTGCTGGTAACAAGAATGGGCTTCTCTTGCCCCACACCACCACAGA

CCAAGAACTTCAACACTTGAGAAACAGCCTACCTGACAATGTTGTGGTTCAGCGCATTGAAGAGAAATTA

TCTGCATTGGGAAATTGTATAGCCTGCAATGACCATGTTGCTCTAACACATACAGATCTTGACGGAGAAA

CAGAGGAAATGATTGCGGATGTTCTTGGTGTANAAGTTTTTAGACAGACAGTTGCTGGTAATATTCTTGT

GGGA

>FM895718.1 FM895718 Jatropha curcas embryo 71-95 (DAF) Jatropha curcas cDNA clone rjcpga0_002688, mRNA sequence

GTCTGACATGTGTGCGAGTCAACGGGTGAGTAAACCCGCAAGGCGCAAGGAAGCTGACTGGCGGGATCCC

CCTGAGGGTTGCACCGCCGACCGACCTTGATCTTCTGAGAAGGGTTCGAGTGAGAGCATGCCTGTCGGGA

CCCGAAAGATGGTGAACTATGCCTGAGCGGGGCGAAGCCAGAGGAAACTCTGGTGGAGGCCCGCAGCGAT

ACTGACGTGCAAATCGTTCGTCTGACTTGGGTATAGGGGCGAAAGACTAATCGAACCGTCTAGTAGCTGG

TTCCCTCCGAAGTTTCCCTCAGGATAGCTGGAGCTCGGGACGAGTTCTATCGGGTAAAGCCAATGATTAG

AGGCATCGGGGGCGCAACGCCCTCGACCTATTCTCAAACTTTAAATAGGTAGGACGGCGCGGCTGCTTCG

CTGAGCCGCGCCACGGAATCGAAGAGCTCCAAGTGGGCCATTTTTGGTAAGCAGAACTGGCGATTCGGGA

TGAACCGGAAGCCG

>FM895717.1 FM895717 Jatropha curcas embryo 71-95 (DAF) Jatropha curcas cDNA clone rjcpga0_002687, mRNA sequence

TTTTTTTTTCAACTGAAAAATTACTGAAATACTGAACTATTATGCTTGTTAAATGGTACATCTTATGGAA

AATATACGGTGGTCCTGGTATTAAATGGCGCCACTTATAACTTAAAATTTACATACAAGTGTGAGATACT

AGCTGATAAAACAAACCAAGAACAAGCCCTATGATGATGCTGATGAAGCTCCTCAGATGTCCCCACGTGG

AGCATGGTGGACAGGAATCTCTCCTTGATTATCCAATGGCATGTATTGCGCCATGATAGCCCGTATCTCC

GAGTCCATGTATCTCCGAATTCTGTACTTGTAAATTGCATATCCAGCAATTCCAGCAGCAGCCAAGCCTA

GAATAATAGTCCAAATAAAGCTCCAGCTAACTTCTGCGCTTGCATCTTTACTTATGCATGTGTCATGTTC

TCGCATGTATAGTAAACCACTGCCACAGCTGCAGTCATAACTACCCCAAGTATTCTTGCATTTGCAATCT

GAACATTGGCAAGCTGTTTTGTCTTTGCATTCATCAATATCCTCACAGGAATTGACTCCATCACCTCTGA

ATCCTGGAGGACACTTGCAACCTTGTGAATCACAAGCAG

>FM895716.1 FM895716 Jatropha curcas embryo 71-95 (DAF) Jatropha curcas cDNA clone rjcpga0_002686, mRNA sequence

ATTCAGGAGGAAAAGAAGCCAGCTGCAGAAGAAACGAAAGTGGAGGAAAACAAACCAGAGGAAGCAAAGA

AACCGGAAGAAGGAGACAAAGCTGAAAAGAAAGAAGAAAAGCCAGCAGAAGAAAAGAAAGAAGAGAAAAA

ACCAGAAGATTCAAAAGAATCCAAGGATGAACCTCCTCCACCTCCACAAGAAATTGTTCTCAAGGTTTAC

ATGCACTGCGAGGGTTGTGCTCGGAAGGTTCGCAGATGCTTAAAGGGCTTTGATGGGGTTGAAGATGTAA

TTACTGATTGCAAAACTAGTAAGGTTATTGTGAAGGGAGAGAAGGCAGCCGCGGATCCATTGAAGGTTCT

AGCCAGAGTTCAGAGGAAAAGCCATAGGCAAGTCGAGCTCATCTCTCCGATCCCAAAACCGCCATCTGAA

CAGGAGGAGAAGGCGGCTGAAGAGAAAGAGAAGCCCAAGCCTGAAGAGAAAAAAGAAGAGCCTCCAGTGA

TCATAGTTGTCCTACAGGTTCACATGCATTGTGAAGCTTGTGCAATGGAAA

>FM895715.1 FM895715 Jatropha curcas embryo 71-95 (DAF) Jatropha curcas cDNA clone rjcpga0_002685, mRNA sequence

ATCCTACTTTAATTTGTTGTAAGATTTCTTATTCATTTGTTGTTTTTTATAATGCAAACAAGAGACCTAT

GAAGGATACTGAGGAGAAAAGCAGAAACAAACAAAGCATACATACTAACAGACTCGAGATTTTACAAAAT

GGTTATCTTATTACATGCCTTCCCTTCAGGAAGCAATATACAGGACTTCAGAACACAACCCATTACATAT

TCAATACATGATTCTCTTCTACTGAGTTCGTAAGACGGAGAGGAAGTGAATAAGCAGAAGCTAACCTTGC

CTCAAGCCTTGCTGTCTACTGCCATTGGCATTGGATCCTGCTTGCCAGTCCCGTTAGCTGATTTTGTTTG

CTCCAATTTACTTATCTTTGAAAATATGTTGCTATAAAATTGTGCATCCTTCTTGTTGTACTCCCCTTAC

TTTTTCCTTCAAAATTTTGTACTCCAGCTTCACATCCCGATTGTCAGGATCAATCTCGAGCGCCTTCTTG

ATGTCCATCTCTGCTAAGTCTAA

>FM895714.1 FM895714 Jatropha curcas embryo 71-95 (DAF) Jatropha curcas cDNA clone rjcpga0_002684, mRNA sequence

ATTAGCCCCGAAAATGGATGGCGCTTAAGCGCGCGACCTATACCCGGCCGTCGGGGCAAGAGCCAGGCCC

CGATGAGTAGGAGGGCGCGGCGGTCGCTGCAAAACCCGGGGCGCGAGCCCGGGCGGAGCGGCCGTCGGTG

CAGATCTTGGTGGTAGTAGCAAATATTCAAATGAGAACTTTGAAGGCCGAAGAGGGGAAAGGTTCCATGT

GAACGGCACTTGCACATGGGTTAGTCGATCCTAAGAGACGGGGGAAGCCCGTCCGACAGCGCGTCCTCGC

GCGAGCTTCGAAAGGGAATCGGGTTAAAATTCCCGAACCGGGACGTGAAGGCTGACGGCGACGTTAGGGA

GTCCGGAGACGTCGGCGGGGGCCTCGGGAAGAGTTATCTTTTCTGTTTAACAGCCCGCCCACCCTGGAAA

CGACTCAGTCGGAGGTAGGGTCCAGCGGCTGGAAGAGCACCGCACGTCGCGCGGTGTCCGATGCGCCCCC

GGCGGCCCATGAAAATCCGGAGGACCGAGTGCCTCCCACGCCCGGTCGTACTCATAACCG

>FM895713.1 FM895713 Jatropha curcas embryo 71-95 (DAF) Jatropha curcas cDNA clone rjcpga0_002683, mRNA sequence

ATCCAAAGGATCTATCCGTCGCCCGATGGGAATTGTACTTTAAGGCGGCCCTCGCGGCTCGTCCGCCGCG

GGGGCTTGGCCAACGGCACGTGCCTCTGGGGGCCGGATGGCCCCTACTGCGGGTCGGCAAACGGGCGACG

GACACGCGCGTCGCTTCTGGCCCGGATTCTGACTTAGAGGCGTTCAGTCATAATCCAGCGCACGGTAGCT

TCGCGCCACTGGCTTTTCAACCAAGCGCGATGACCAATTGTGCGAATCAACGGTTCCTCTCGTACTAGGT

TGAATTACCATCGCGACACCATCATCAGTAGGGTAAAACTAACCTGTCTCACGACGGTCTAAACCCAGCT

CACGTTCCCTATTGGTGGGTGAACAATCCAACACTTGGTGAATTCTGCTTCACAATGATAGGAAGAGCCG

ACATCGAAGGATCAAAAAGCAACGTCGCTATGAACGCTTGGCTGCCACAAGCCAGTTATCCCTGTGGT

>FM895712.1 FM895712 Jatropha curcas embryo 71-95 (DAF) Jatropha curcas cDNA clone rjcpga0_002682, mRNA sequence

ATTCCTAGCCCCGAAAATGGATGGCGCTTAAGCGCGCGACCTATACCCGGCCGTCGGGGCAAGAGCCAGG

CCCCGATGAGTAGGAGGGCGCGGCGGTCGCTGCAAAACCCGGGGCGCGAGCC

>FM895711.1 FM895711 Jatropha curcas embryo 71-95 (DAF) Jatropha curcas cDNA clone rjcpga0_002680, mRNA sequence

GATTCTATCCGTCGCCCGATGGGAATTGACTTTAAGGCGGCCCTCGCGGCTCGTCCGCCGCGGGGGCTTA

GCCAACGGCACGTGCCTCTGGGGGCCGGATGGCCCCTACTGCGGGTCGGCAAACGGGCGACGGACACGCG

CGTCGCTTCTGGCCCGGATTCTGACTTAGAGGCGTTCAGTCATAATCCAGCGCACGGTAGCTTCGCGCCA

CTGGCTTTTCAACCAAGCGCGATGACCAATTGTGCGAATCAACGGTTCCTCTCGTACTAGG

>FM895710.1 FM895710 Jatropha curcas embryo 71-95 (DAF) Jatropha curcas cDNA clone rjcpga0_002679, mRNA sequence

TTAAATAGGTAGGACGGCGCGGCTGCTTCGCTGAGCCGCGCCACGGAATCGAGAGCTCCAAGTGGGCCAT

TTGGTAAGCAGAACTGGCGATGCGGGATGAACCGGAAGCCGGGTTACGGTGCCCAACTGCGCGCTAACCT

AGAACCCACAAAGGGTGTTGGTCGATTAAGACAGCAGGACGGTGGTCATGGAAGTCGAAATCCGCTAAGG

AGTGTGTAACAACTCACCTGCCGAATCAACTAGCCCCGAAAATGGAT

>FM895709.1 FM895709 Jatropha curcas embryo 71-95 (DAF) Jatropha curcas cDNA clone rjcpga0_002677, mRNA sequence

TGGAAAGCTAACAAAGCTCAAGTCAGCAATAAAGAGATGGCCATCTTTCACTAAGCTGGGTCGTACAAAC

AGCTCCATTGCTGCTAGTACTGACGTGATCGAAGACAACATTTCAGGGAACTCCAAGCAGTTTACGTCGG

TAAATCAAGACGGCGATATCTTTTAAGCTCTGATGTCATTTGCCATCCTCTTTTTCAAGAATTGATAGAA

AGATCAGGTGGGTTTGATGACGGACAAGTAGTCATTTCTTGTGAAGTTGTGTTGTTTGAGCATTTGTTAT

GGATGCTTGAAGGCGGTGGCTCTCAGTTAGGCTCCATGGAAGAGCTTGCTGACTTTTACTACACTCGCTA

ATAACTAATTAGCTAGTATTACTGCGAGTATCCCTATACCCAAAGGGTATGTGACTATCCTCATAATTGT

TTTTTCTAGTAGAATTTGTATGGTGAGGATATTAACCCAGTAATTGATTTAATGGTGGATGTTGAATTTT

AATTAGCTTCTTTTATTATTAATATATGTATATTGGATTTGCTTCTAAT

>FM895708.1 FM895708 Jatropha curcas embryo 71-95 (DAF) Jatropha curcas cDNA clone rjcpga0_002676, mRNA sequence

GCTACCTGCTTCAGATTTCAGAGACTATTTTCTTGTGCTTGTTAAATCCTTTTTCCCCTTTTCTTTTAGT

TGTGGATTCTTTTTTGGGACTTAGGACAGGTTGGTGCCAATATTGTTGATATGACAGGTAGTTTTGGAGC

TGCAAGATGATGGAAAATTGATTACATCAGATGATCGTCCTGTGACATTTGGCTGCTTGAAATTGGATTT

TGTTCAAGAGACAGGGCTTCATTCAGGAATTCTTCTACGTTTTGCTATTAGTGAAGGAGGGTTGAGTATC

GTTGATGGAAAGCTTGAGAAAGCTGATTTACTTTTTCTTGGAAAGCGTCAATAGGGCACGTGCTTTTGCA

GACAGTTACTCCAAGGTTATATTCCAGCACATGGTTCGGCATTCTCCTTTAAAATTTGATGATCTACTAG

CATCTATTTCCTCATCAGACAATCAACAAAACAAACCTTGTGATGTTGACATGGGTGGTTGATGATGATT

TGTTAGTGATGAGATGTCTCGCTCTT

>FM895707.1 FM895707 Jatropha curcas embryo 71-95 (DAF) Jatropha curcas cDNA clone rjcpga0_002675, mRNA sequence

AGGATTCTATCCNGTCGCCCGAATGGGAATTGTACTTTAAGGCGGCCCTCGCGGCTCGTCCGCCGCGGGG

GCTTAGCCAACGGCACGTGCCTCTGGGGGCCGGATGGCCCCTACTGCGGGTCGGCAAACGGGCGACGGAC

ACGCGCGTCGCTTCTGGCCCGGATTCTGACTTAGAGGCGTTCAGTCATAATCCAGCGCACGGTAGCTTCG

CGCCACTGGCTTTTCAACCAAGCGCGATGACCAATTGTGCGAATCAACGGTTCCTCTCGTACTAGGTTGA

ATTACCATCGCGACACCATCATCAGTAGGGTAAAACTAACCTGTCTCACGACGGTCTAAACCCAGCTCAC

GTTCCCTATTGGTGGGTGAACAATCCAACACTTGGTGAATTCTGCTTCACAATGATAGGAAGAGCCGAC

>FM895706.1 FM895706 Jatropha curcas embryo 71-95 (DAF) Jatropha curcas cDNA clone rjcpga0_002674, mRNA sequence

ATTCCTTTCTCTTCAGTATATTTTCTGAACTTGGGTTCACTGTTTTGGAATATGATTAAGAACCCATGGG

TATGTAACTTATTCCATTAATTAAGTTGTGGCAC

>FM895705.1 FM895705 Jatropha curcas embryo 71-95 (DAF) Jatropha curcas cDNA clone rjcpga0_002673, mRNA sequence

GAGACCGCCCATGCCTTGNAGGAATTGCCTTTGGCTAAGGCTAAGAGGTATTTGGAGGATGTTATGGCTC

ACAAACAAGCTATACCTTTTCGACGTTTCTGTGGTGGGGTTGGACGTACTGCACAGGCAAAGAACAGGCA

CTCAAATGGGCAAGGACGCTGGCCTGTTAAATCTGCCAGATTCATTCTAGATTTGCTCAAGAATGCTGAG

AGTAATGCCGAGGTCAAAGGCTTGGATGTCGATGCCCTCTACATTTCTCATATCCAAGTGAATCAGGCAC

AGAAGCAGAGGCGTCGAACTTATCGCGCTCATGGAAGAATCAATCCTTACATGTCTAGCCCGTGTCATAT

TGAGTTGATTTTGTCCGAGAAGGAAGAGCCAGTGAAGAA

>FM895704.1 FM895704 Jatropha curcas embryo 71-95 (DAF) Jatropha curcas cDNA clone rjcpga0_002672, mRNA sequence

CGAATTGCAAAGGATTCTATCGTCGCCCGATGGGAATTGTACTTAAGGCGGCCTCGCGGCTCGTCCGCCG

CGGGGGCTTAGCCAACGGCACGTGCCTCTGGGGGCCGGATGGCCCCTACTGCGGGTCGACAAACGGGCGA

CGGACACGCGCGTCGCTTCTGGCCCGGATTCTGACTTAGAGGCGTTCAGTCATAATCCAGCGCACGGTAG

CTTCGCGCCACTGGCTTTTCAACCAAGCGCGATGACCAATTGTGCGAATCAACGGTTCCTCTCGTACTAG

GTTGAATTACCATCGCGACACCATCATC

>FM895703.1 FM895703 Jatropha curcas embryo 71-95 (DAF) Jatropha curcas cDNA clone rjcpga0_002671, mRNA sequence

CTGACATGTGTGCGAGTCAACGGGTGAGTAAACCCGCAAGGCGCAAGGAAGCTGACTGGCGGGATCCCCC

CGAGGTTGCACCGCCGACCGACCTTGATCTTCTGAGAAGGGTTCGAGTGAGAGCATGCCTGTCGGGACCC

GAAAGATGGTGAACTATGCCTGAGCGGGGCGAAGCCAGAGGAAACTCTGGTGGAGGCCCGCAGCGATACT

GACGTGCAAATCGTTCGTCTGACTTGGGTATAGGGGCGAAAGACTAATCGAACCGTCTAGTAGCTGGTTC

CCTCCGAAGTTTCCCTCAGGATAGCTGGAGCTCGGGACGAGTTCTATCGGGTAAAGCCAATGATTAGAGG

CATCGGGGGCGCAACGCCCTCGACCTATTCTCAAACTTTAAATAGGTAGGACGGCGCGGCTGCTTCGCTG

AGCCGCGCCACGGAATCGAGAGCTCCAAGTGGGCCATTTTTGGTAAGCAGAACTGGCGATGCGGGATGAA

CCGGAAGCCGGGTTACGGTGCCCAACTGCGCGCTAACCTAGAACCCACA

>FM895702.1 FM895702 Jatropha curcas embryo 71-95 (DAF) Jatropha curcas cDNA clone rjcpga0_002670, mRNA sequence

CTGATGCTGACCTCAAGGCAAAACAAGAAAGAGAATTAACAGCTGCGGCAGAAAAACTAGCGGAGTGCCA

AAGAGACTATATTTCTTCTTGGCAAGCAATTGAAAGCTTTGCGTCCTCAAACAGAAATCATGGGATCTCC

ATACAGTGAGAGGAGCCAAAGGGGTGAGGGATTTGGTGATGATGAGCCTACTACAAGTGGGATGAACTTG

CAAGATTTTGATCAGGCCGAAATGGATGCTACTGTTTCAACAAATCTGCCCAAAACAGGTGGTGAATCCC

CTACGGATTTATACAACCAATCGGACGCCGAAACAAGCCTCTCAAGATCACCAATCAGTTCAAAGCAACC

GCAACACAGATCCACCAAGTCAACTTCTTCTTCTACAACTCTTGTTGGAACACCGGAGAAACATCAAAGA

GGCTTTAG

>FM895701.1 FM895701 Jatropha curcas embryo 71-95 (DAF) Jatropha curcas cDNA clone rjcpga0_002668, mRNA sequence

CTGCAAAGGATTCTATACCGTCGCCCGATGGGAATTGTACTATTAAGGCGGCCCTCGCGGCTCGTCCGCC

GCGGGGGCTTGGCCAACGGCACGTGCCTCTGGGGGCCGGATGGCCCCTACTGCGGGTCGGCAAACGGGCG

ACGGACACGCGCGTCGCTTCTGGCCCGGATTCTGACTTAGAGGCGTTCAGTCATAATCCAGCGCACGGTA

GCTTCGCGCCACTGGCTTTTCAACCAAGCGCGATGACCAATTGTGCGAATCAACGGTTCCTCTTCGTACA

AGGTGAATTTACCATCGCAAAACCATCATCAGTAGGGTAAAACTAACCTGTCTCACGACGGTCTAAACCC

AGCTCACGTTCCCTATTGGTGGGTGAACAATCCAACACTTGGTGAATTCTGCTTCACAATGATAGGAAGA

GCCGACATC

>FM895700.1 FM895700 Jatropha curcas embryo 71-95 (DAF) Jatropha curcas cDNA clone rjcpga0_002667, mRNA sequence

AAAAAAATAAAAAAAAATAAATATTATTATAAAACCAAAATTTTTCCGCACATGTGAAAATTGACATATA

TACATATATATCACACACGCAATTCAAGAACAATGTAAATTGTAATTTTTATTGTACTGAACAAATTAAA

ATATCAATCAAATCAAATAAAAAACTAAAAAGGTCCAAAATCAAATCTATGCTGCTGCTCATCTGCAGGA

TCCGATGTCGGATCGGCGAGAGGAGTACCTGCTGGATGTGATGTAGCGGCAGGAGGGGTGACCAAGGTAT

CGTCTGCAGCATCGGGATCAAGAGCAGGAAGTGGCTGCTGCTGAGACGTCAAAACATGTCGGTCTGTAGC

AGGCGCAGGATCAGAGGAGGAAGTACCAGCACGGGCCTCACCGGACAGCCGCAGGACATGCTCTCTAAGC

TCTGCAATCTGTCTCTGCTGATCAGCAAGCTGAGCTCGTATATCATCATCATACTGCGAGTCAGTGTGCT

CCGGCTGTCGTGCTGCGCTGGCT

>FM895699.1 FM895699 Jatropha curcas embryo 71-95 (DAF) Jatropha curcas cDNA clone rjcpga0_002665, mRNA sequence

GATGATTTTCCCATGCTTGAATTTTCATTTCTTCACGTTTGAACCTGGCCATATACTTAGCTTTCTCTGC

CTCCTCCCATGCTAATGTATACAGAGCGTAGGCTTGCTTTGAGCACTCTAATTTCTTCAAAGTAACAGCG

CCGGAGGCACGACCCGGCCAGTTAAGGCCAGGAGCGCATCGCCGGCAGAAGGGACGAGCCGACAGGTGCA

CACCGCGAGGCGGACCGATCGACCCAACCCAAGGTCCAACTACGAGCTTTTTAACTGCAACAACTTAAAT

ATACGCTATTGGAGCTGGAATTACCGCGGCTGCTGGCACCAGACTTGCCCTCCAATGGATCCTCGTTAAG

GGATTTAGATTGTACTCATTCCACTTACCAGACTCT

>FM895698.1 FM895698 Jatropha curcas embryo 71-95 (DAF) Jatropha curcas cDNA clone rjcpga0_002663, mRNA sequence

ATTCCCAGAGAATAGGAGGGGCTGATCAATATAGCACATTACAGCCATGACTGACTATGTGACAGGAGCA

GGAGATGGAAATCGAAGCATTGGAAGCAATACTCATGGATGATTTCAAAGAAATTCATTCAAGCGAAAGT

GGTTTGAATACTTCAAGTCGATGCTTTCAAATAACAATATCTCCACAGGATGACGATGATGCGTATGATT

CAACAGCCATTCCAGTTCAGCTAGCTCTCATTTTCTCACACACACAGAATTATCCTGATGAGCCTCCATT

ACTAAATGTGAAAAGCATAAGAGGCATACAACCTGGTGATCTTAACATTCTGAAAGAAAAGCTTGAACAC

GAGGCATCTGAAAATCTTGGTATGGCTATGGTGTACACATTGGTTACATCGGCTAAAGAGTGGCTGTCTG

AAAAATATGGCCAAGATGCTAGTGATGAGAATATCGAAGATGAACAGGCAGCAAAAGAAGATATCATTGT

ACCACACGGAGAACCTGTTACCATTGATACATTTTTGGCATGGAGAGAAGATTTGAAGCAGAGTTGGCGC

TTGAGCGAGCCAAGCTGATGCCCGAATC

>FM895697.1 FM895697 Jatropha curcas embryo 71-95 (DAF) Jatropha curcas cDNA clone rjcpga0_002662, mRNA sequence

ATTCTTCTTTCTTTTACCCATTTCTTCGTCTTCTGTGTCTCTGAATTAAACTTTCTGTTTCTATATCCTA

TCAAAGAAACTTCAAAAATTCTTCATATAAATGGAGCCAAAAGACCCTAATCCAACAGCGGAGGATGAAA

CCCAGATCGTTGTTTCTCGGCGCATCCAGAGGTTGTCCTTTCACCTCACCCCAATACCTTGCTCTCTCTA

TGATTCCAACCAGCTTGGTATGGTGGAGTGCGCTAAAGCAGCGAAATTAGTGGTGAATACTGATAAACTG

TCGCTATACATGAGAGGGAAACATAGGGAGATACAGGAGAAGGTGTTTGCTTACTTTAATTCAAGGCCGG

AGTTGCAGACCCCAGTAAAGATAGCTAAAGATGACTATAGGGAGCTTTGTTGGAGACAGCTACTGGGTTT

GGTTAGAGAGGCTGGAATTAGGCCTTTTAGATATGTAGCAGATGATCCCTGTTAGTATTTTGCCATTCTT

GAGGCGGTTGGTAGCCTTGGATATGTCGCTTGG

>FM895696.1 FM895696 Jatropha curcas embryo 71-95 (DAF) Jatropha curcas cDNA clone rjcpga0_002661, mRNA sequence

GCAAAGGATTCTATCCGTCGCCCGATGGGAATTGTACTTTAAGGCGGCCCTCGCGGCTCGTCCGCCGCGG

GGGCTTAGCCAACGGCACGTGCCTCTGGGGGCCGGATGGCCCCT

>FM895695.1 FM895695 Jatropha curcas embryo 71-95 (DAF) Jatropha curcas cDNA clone rjcpga0_002660, mRNA sequence

CAGAAGCAGCGTCGAAGCACCGCCACTTCTTTGTAGGGAGAACCACCAGCACCGCCACAATCAACCATGA

CATTCAAGAGAAGAAACGGTGGCCGCAACAAGCACGGCCGTGGTCACGTTAAGTTCATTCGCTGTTCCAA

CTGCGGCAAATGCTGCCCTAAGGACAAAGCTATCAAGAGATTTCTTGTGAGGAACATTGTTGAGCAAGCT

GCAGTTAGGGACGTTCAGGATTCTTGTGTTTATGATGGATATGTTCTTCCTAAGCTGTATGTGAAGATGC

AATACTGTGTTTCATGTGCCATTCACTCCAGAGTTGTCAGGGTTCGCTCTCGCACTGAGCGCAGGAACCG

TGAACCACCACAGCGCTTCCTCAGGCGCAGGGATGACCTGCCAAAGCCTGGACAGCCAGGTCAGACCGGT

CAAGTAGCTCGTCCTGCAGGTGCCGGTGTTGCTGCTAGGACCTAAGTGTCGAATTCTTTCTGTTCTATGT

TTTGTTTTGAAGTTGCTCGTTAAATTTTCTAAGGAATGAAATTTGCTGTCTCAATCTTTTTAAGTTAGAC

ATTAAGGATTATATATGTTATCTTGAAAATTTTTGATCCATGAGAGTGCATTATGACATTACTTCA

>FM895694.1 FM895694 Jatropha curcas embryo 71-95 (DAF) Jatropha curcas cDNA clone rjcpga0_002659, mRNA sequence

CAAAGGATTCTATCCGTCGCCCGATGGGAATTGTACTTTAAGGCGGCCCTCGCGGCTCGTCCGCCGCGGG

GGCTTGGCCAACGGCACGTGCCTCTGGGGGCCGGATGGCCCCTACTGCGGGTCGGCAAACGGGCGACGGA

CACGCGCGTCGCTTCTGGCCCGGATTCTGACTTAAAGGCGTTCAGTCATAATCCAGCGCACGGTAGCTTC

GCGCCACTGGCTTTTCAACCAAGCGCGATGACCAATTGTGCGAATCAACGGTTCCTCTCGTACTAGGTTG

AATTACCATCGCGACACCATCATCAGTAGGGTAAAACTAACCTGTCTCACGACGGTCTAAACCCAGCTCA

CGTTCCCTATTGGTGGGTGAACAATCCAACACTTGGTGAATTCTGCTTCACAATGATAGGAAGAGCCGAC

ATCGAAGGATCAAAAAGCAACGTCGCTATGAACGCTTGGCTGCCACAAGCCAGTTATCCCTGTGGTAACT

TTTCTGACACCT

>FM895693.1 FM895693 Jatropha curcas embryo 71-95 (DAF) Jatropha curcas cDNA clone rjcpga0_002658, mRNA sequence

ATTCGTCCTCAAGTAATTCCGTTGGGTATATTACACAAAATAAACAAATCCAAAATCTGGACAGCAAAAA

TATTTAATCTAGATACAAAATTATTTATAAAAAAAAAAACTACTCAAAAGGACTCCATAATTCCATGTTA

AGAAGCTAAGTTCAGATCCTCTTCTGAATGTGCCACATTTTTTCTGGTAGATCACCCTACTGAGAATTGC

TGTCACCTTAGATAGACATGGGTACTAAGACATCGGACCTTCATCGTCCGACGAACCATAATGCTGCCTT

ACATTGAGTCCAGGAAAAACCGGTGGAATGTTTTGATTCATCGGCATAGGGCGGACTGAACTGTATATAG

TTCTCTCATTGATTTTCCCAACTTCTTTGCATACCTGATCAAGAATGGAAAGGAAATCCCTTACCACCAT

GAATATCCGGAATGGGTGAGCTTCTTCCTTCGCTGAGTTCCCATGGAAGTACTCTGTTATTTCCTTAACC

AAAGAAAGAGCAAGTTTCTCTTGTGCTTCAACCTTTACAATTTCTCCATCGGCCTTTGTTAAGAATCCAT

TCATTGATTCTGAGAACTTCCTGCTACTTTC

>FM895692.1 FM895692 Jatropha curcas embryo 71-95 (DAF) Jatropha curcas cDNA clone rjcpga0_002657, mRNA sequence

NTTTTTAAAAGAAAACCCTACTTTAATTAGCGTAATAATCAGATATATTCTGTCCAGCAAAAAACAGTAT

GTCCTGTCTAATACCTACTCAATTTATGCTAAAGCAATCTGATTCATTAATCTTACATATAATTTTTAAT

TTGATAAGAGGAGCCACATAAACTCTGCGTCGAATATCAGATCAGGATCAATCAATTTTGTCAGTTAGAG

ACCTTGAGAATCATACTCAGCCGATCGAGGCATTCAAGGATGATGTTAACCTTCTGGCAATGAAATGTTG

ATGTCAGTTCCTGATTTTCTAACATCTTGCATGGGTTGCATGGAAGTCCAGTTTTCTCTTCGGAAAGTCG

AGGAATTTTGCTGTCCATGTCCACCCATACCATCCTCTCTTTCTGAATTAGAGGCAGGCACAGGAACTGG

AACGGCTATGGTTGAGGCCGGTGTAATGGTAGTTTGTACTGAATCCATTTTTATCTTCTTTGGTTTCTGG

TCTTGCTGGCTGCCACCAGGTAGGAAACTACCTACCACAACCTGCACAGGACTAGCAGCTACTAGAAGTC

C

>FM895691.1 FM895691 Jatropha curcas embryo 71-95 (DAF) Jatropha curcas cDNA clone rjcpga0_002656, mRNA sequence

CTGTAAACTAGCGGAAAAATCTATAATGGCATCTTCTGGCTTTCAAGCTGATGTAAAAGCCCTACAAAAG

CATTTGGCAGCTAGGCACTTGATTTATCAACATCAACACAACAAGCAAATGAGCTGCCCTGCCATGGCTC

AACTACTCTCCAACACATTATACTACAAACGCTTTTTCCCTTATTATGCTTTTAATGTTCTGGGCGGTCT

TGACAGTGAAGGAAAGGGCTGTGTTTACACTTATGACGCTGTTGGTTCATACGAGAGGGTTGGATATAGT

GCCCAGGGTAGCGGTTCTACTCTTATCATGCCTTTTCTGGATAACCAACTGAAGTCACCCAGCCCGCTTT

TATTACCTGCCCAAGATGCTGTAACTCCACTTTCAGAAGTGGAAGCTGTTGATTTGGTCAAAACTGTTTT

TGCATCTGCAACTGAGAGGGATATATACACTGGAGACAAGCTTGAAATTGTTATTCTAAATGCTGATGGT

ATTCGTCGCGAGTATTTGGAACTCAGGAAAGATTAATCTGTCCAAAAAAGACTCCTTGATGGTCT

>FM895690.1 FM895690 Jatropha curcas embryo 71-95 (DAF) Jatropha curcas cDNA clone rjcpga0_002654, mRNA sequence

AAAAACTTCCCCACCGTCAGTCACTTTCTATCCTTCATTTACAAATTCTAAAATTTCTCCCCTGCAGTTC

AGATCAGTGACCAAATGCCAGAGATTCAGTTGGGTTCTCACACAATAAAATCTCATGGAGCCAAAGTTGC

CAGGGTTCATATGCACGACTGGCTGATTCTTTTGCTTCTTGTGGTGATAGATGTCGTTTTGAATGTTATA

GAACCATTTCACCGTTTTGTTGGAAGGGATATGATGACAGACCTGTCATACCCACTGAAAGACAATACTG

TTCCATTTTGGGCTGTTCCAATTGTTGGGATATTGCTGCCTTTTGTCATCATTAGCGTCTATTACTTTAT

CAGAAGGGATGTCTATGACCTGCACCATGCTATTCTAGGCCTTCTATTTTCTGTGCTTATTACTGCAGTC

ATAACCGATGCAATTAAAGATGGTGTCGGTCGTCCTAGACCTGACTTCTTTAGGCG

>FM895689.1 FM895689 Jatropha curcas embryo 71-95 (DAF) Jatropha curcas cDNA clone rjcpga0_002653, mRNA sequence

GTGCGAGTCAACGGGTGAGTAAACCCGCAAGGCGCAAGGAAGCTGACTGGCGGGATCCCCCCGAGGGTTG

CACCGCCGACCGACCTTGATCTTCTGAGAAGGGTTCGAGTGAGAGCATGCCTGTCGGGACCCGAAAGATG

GTGAACTATGCCTGAGCGGGGCGAAGCCAGAGGAAACTCTGGTGGAGGCCCGCAGCGATACTGACGTGCA

AATCGTTCGTCTGACTTGGGTATAGGGGCGAAAGACTAATCGAACCGTCTAGTAGCTGGTTCCCTCCGAA

GTTTCCCTCAGGATAGCTGGAGCTCGGGACGAGTTCTATCGGGTAAAGCCAATGATTAGAGGCATCGGGG

GCGCAACGCCCTCGACCTATTCTCAAACTTTAAATAGGTAGGACGGCGCGGCTGCTTCGCTGAGCCGCGC

CCAGGAATCGAGAGCTCCAGGTGGGCCATTTTTGGTAAGCAG

>FM895688.1 FM895688 Jatropha curcas embryo 71-95 (DAF) Jatropha curcas cDNA clone rjcpga0_002651, mRNA sequence

GAGAAAGAAGCTGCCCTTTCAGCTACTCCCTCAGATTCTCCTACCATATTTGACAAGATCATCAACAAGG

AAATACCATCAACTGTTGTGTATGAGGATGATAAGGTCTTAGCTTTTAGGGATATATCTCCTCAAGCTCC

TACGCACATTTTAATCATTCCCAAAGTCAAGGATGGCTTAACTGGACTTTCTAAGGCTGAGGAGAGGCAT

TGTGACATTCTGGGTCGCCTGCTATACACTGCCAAGCTCATTGCCAAGCAGGAGGGATTAGAAGATGGTT

TTAGGATTGTGATTAATGATGGACCAAGTGGATGTCAATCTGTGTATCACCTTCACATCCACCTCCTAGG

GGGACGTCAAATGAACTGGCCCCCTGGCTAAGATGGAGTCATCATGTGACTGGACAAATTTCAATGGTTA

TAATACATTGCCTTGTGTCTTAAATTGTACTGAACTAGTATTTCAGAAACAACTAGCCTGCAAGTTACAA

TCTTGCATGTTGTTTAAATAATGTGAAAAGTTTTTCATTGAACTGGTGTACCA

>FM895687.1 FM895687 Jatropha curcas embryo 71-95 (DAF) Jatropha curcas cDNA clone rjcpga0_002650, mRNA sequence

ATTCCTAGGAAATCAGAATCAAACGAGCTTTTACCNCTTTTGTTCCACACGAGATTTCTGTTCTCGTTGA

GCTCATCTTAGGACACCTGCGTTATCTTTTAACAGATGTGCCGCCCCAGCCAAACTCCCCACCTGACAAT

GTCTTCCGCCCGGATCGGCCGCCGAAACGACCTTGGGTCCAAAAAGAGGGGCATAGCCCCGCCTCCGATT

CACGGAATAAGTAAAATAACGTTAAAAGTAGTGGTATTTCACCGTCGCCTTTCGGCTCCCACTTATCCTA

CACCTCTCAAGTCATTTCACAAAGTCGGACTAGAGTCAAGCTCAACAGGGTCTTCTTTCCCCGCTGATTC

CGCCAAGCCCGTTCCCTTGGCTGTGGTTTCGCTGGATAGTAGACAGGGACAGTGGGAATCTCGTTAATCC

ATTCATGCGCGTCACTAATTAGATGACGAGGCATTTGGCTACCTTAAGAGAGTCATAGTTACTCCCGCCG

TTTACCCGCGCTTGGTTGAATTTCTTCCCTTTGA

>FM895686.1 FM895686 Jatropha curcas embryo 71-95 (DAF) Jatropha curcas cDNA clone rjcpga0_002649, mRNA sequence

ATTCCGCATGAATGGATTAACGAGATTCCCACTGTCCCTGTCTACTATCCAGCGAAACCACAGCCAAGGG

AACGGGCTTGGCGGAATCAGCGGGGAAAGAAGACCCTGTTGAGCTTGACTCTAGTCCGACTTTGTGAAAT

GACTTGAGAGGTGTAGGATAAGTGGGAGCCGAAAGGCGACGGTGAAATACCACTACTTTTAACGTTATTT

TACTTATTCCGTGAATCGGAGGCGGGGCTATGCCCCTCTTTTTGGACCCAAGGTCGTTTCGGCGGCCGAT

CCGGGCGGAAGACATTGTCAGGTGGGGAGTTTGGCTGGGGCGGCACATCTGTTAAAAGATAACGCAGGTG

TCCTAAGATGAGCTCAACGAGAACAGAAATCTCGTGTGGAACAAAAGGGTAAAAGCTCGTTTGATTCTGA

TTTCCAGTACGAATACGAACCGTGAAAGCGTGGCCTATCGATCCTTTAGACCTTCGGAATTTGAAGCTAG

AGGTGTCAGAAAAGTTACCACAGGGATAACTGGCTTGTGGCAGCCAAGCGTTCATAGCGACGTT

>FM895685.1 FM895685 Jatropha curcas embryo 71-95 (DAF) Jatropha curcas cDNA clone rjcpga0_002648, mRNA sequence

GCAAAGGATTCTATCCGTCGCCCGATGGGAATTGTACTTTAAGGCGGCCCTCGCGGCTCGTCCGCCGCGG

GGGCTTGGCCAACGGCACGTGCCTCTGGGGGCCGGATGGCCCCTACTGCGGGTCGGCAAACGGGCGACGG

ACACGCGCGTCGCTTCTGGCCCGGATTCTGACTTAGAGGCGTTCAGTCATAATCCAGCGCACGGTAGCTT

CGCGCCACTGGCTTTTCAACCAAGCGCGATGACCAATTGTGCGAATCAACGGTTCCTCTCGTACTAGGTT

GAATTACCATCGCGACACCATCATCAGTAGGGTAAAACTAACCTGTCTCACGACGGTCTAAACCCAGCTC

ACGTTCCCTATTGGTGGGTGAACAATCCAACACTTGGTGAATTCTGCTTCACAATGATAGGAAGAGCCGA

CATCGAAGGATCAAAAAGCAACGTCGCTATGAACGCTTGGCTGCCACAAGCCAGTTATCCCTGTGGTAAC

TTTTCTGA

>FM895684.1 FM895684 Jatropha curcas embryo 71-95 (DAF) Jatropha curcas cDNA clone rjcpga0_002647, mRNA sequence

CAACAAAATTATATTTTCTCTCGTGATCTTTTATTTCTAGACAGGAGAAGAAAGAAATGGCAACTTTCGA

GCAGGCACCACCAGGCGATGTAAAAGCCGGAGAGAAAATCTTCAAGACTAAGTGCGCGCAGTGCCACACC

GTCGAGAAGGGCGCCGGTCACAAGCAAGGACCCAATTTGAATGGCCTTTTCGGTAGGCAATCTGGTACGA

CAGCTGGGTATTCCTACTCTGCAGCTAATAAGAACATGGCTGTGATCTGGGGAGAGAATACTTTATATGA

TTACTTGCTTAATCCTAAGAAGTACATTCCTGGAACTAAGATGGTCTTCCCTGGATTGAAGAAGCCACAG

GATCGTGCAGACCTCATTGCCTATTTGAAGGAAGCAACTGCTTGATTTAGTATGGTTATTTAGCCATTGA

TTTGTGACATTAATTGATTGTCCTAAGCCATTTATTTTTCCAAAGGAAGAGAAGACTTATTCACTTCTTC

AAAATAAGCTGAATTAAAGCTCACAGATGAGTAGA

>FM895683.1 FM895683 Jatropha curcas embryo 71-95 (DAF) Jatropha curcas cDNA clone rjcpga0_002646, mRNA sequence

TTTTTAAACAAATACAATTTCTTGGTCAGATGAACACATATGCTGCTTCACTTTACATTAGGGTAAATAT

TTCCATCATAATTCTATTGTTAAAATTATTATAAAAAGAACTCGATTTTATTCTTCCATTACAGTAAACC

TCCCACTTTCATAAACATGGTACAACCAAACAGGTTCAACCAATGCTCTGGCACTTTGTAATTTCTGCTT

CGCATCTCCATCGCTTTGTTCTTCAATTTCTCTCTCAATATGAGGGTAAATGTCCGGAAGAGAGCTCAAC

ATCATATCAAGTTCACTCCAAGTTTTGTCTTCTTGGCCAACTAACTTCACACATACAAAGTGTAGGATTC

CTAATACTAATCCAAAATCCTTCCTTTCCAGGCCAACAACAGAGTAAATCAAGAAAATCACTCCGACTGA

ATCTAGTACGTTCGGATTCCTTTTCAACAAAGAAAGTATTATTTCTATAAATTTCTCGAACACAGGAAAT

TTCTCCTCCTGATTCTAAAACTTAATCCTCCTCTTTCTGGGTTAACTTCACTCATTTTGCAG

>FM895682.1 FM895682 Jatropha curcas embryo 71-95 (DAF) Jatropha curcas cDNA clone rjcpga0_002645, mRNA sequence

CTGCTTCACCTCATCCTCTCTGCAAACCAGATTATTATCCAACAGTCAAAAATGTCAGGTGAGGAGGGTG

CCGTTGCCGTTGAGACACCAGCTCAGCCACTCGGCGAGGCTATGGACTTAATGACTGCATTGCAGTTAGT

TCTAAGAAAATCATTGGCTCACGGTGGTCTTGCTCGAGGTCTTCATGAAGGCGCGAAAGTGATTGAAAAG

CATGCTGCTCAGCTTTGTGTATTGGCTGAGGACTGTAACCAGCCTGACTATGTTAAGCTTGTCAAAGGCC

TTTGTGCTGACCATGGTGTTGGCCTCTTAACTGTTCCTAGTGCTAAGACTCTTGGCGAGTGGGCTGGGTT

GTGCAAGATTGATTCTGAGGGTAAGGCCCGGAAGGTAGTTGGTTGCTCCTGCGTCGTAGTTAAGGATTAT

GGCGAGGAGACTGAAGGTCTTAATGTTGTTCAGCAACACATCAAATCTCACTGAGTAGCTGTCAAATTTG

ATTTATTGGGATCTTAATGGAAGAGTATTTGTAGAGACCTTTGTTGTTTTCATGATACCAGAAATTTTGA

GTGTTTGCTATCTGCAGTTACTTTTGATTGAGTTGTTTCTTATTAGTAGAACGTCATTCTTGATGTATGA

C

>FM895681.1 FM895681 Jatropha curcas embryo 71-95 (DAF) Jatropha curcas cDNA clone rjcpga0_002644, mRNA sequence

TTTTGTTTTGTTTTGTAATTTGCAGAAGGATGTATGGAGTGAAGAGGAGGATAAGGTACTGATACAGGTC

CATGCAGAAATAGGAAACAAATGGGCAGAAATTGCGAAGAGGTTACCAGGAAGAACTGAGAACTCAATCA

AGAACCACTGGAATGCTACAAAAAGAAAGCAATACTCAAAGAGAAAATGTCGTTCAAAGAACCCAAGAGG

CTCTCTTCTTCAAGATTATATCAAGAGCTTGAATTTAGACTCATCAGCTCCTCCACCAACTGGAATAAGA

TACCAGCCGCCGAAAAGCTCTGCTAATAGTTATGCTATGGATTATAGTACAATAACCAAAGTAGCAGCAA

ACCAGCCACAGAAGAATAATACAGTAGAGTTTTGCCAGAATAATAACGATAATATAAGGTTGGTTCCAAA

TTCTTGTGATGATTTTAATGCAGATTTTGATTTTGATGAGAAGTTTTTTGAAGAGGGTTGCTGCAGCTTT

GATTCGCTTCTTGATGAGAAAAGCTTTGAAATGGATGTAACTCCATTGATGGAATTTGAAGTGAAGAAGG

AGCTTGATTTAGTGGAGATGATTTATCACACCAATG

>FM895680.1 FM895680 Jatropha curcas embryo 71-95 (DAF) Jatropha curcas cDNA clone rjcpga0_002643, mRNA sequence

ATTGCANAAGGATTCTATCCGTCGCCCGATGGGAATTGTACTTTAAGGCGGCCCTCGCGGCTCGTCCGCC

GCGGGGGCTTGGCCAACGGCACGTGCCTCTGGGGGCCGGATGGCCCCTACTGCGGGTCGGCAAACGGGCG

ACGGACACGCGCGTCGCTTCTGGCCCGGATTCTGACTTAGAGGCGTTCAGTCATAATCCAGCGCACGGTA

GCTTCGCGCCACTGGCTTTTCAACCAAGCGCGATGACCAATTGTGCGAATCAACGGTTCCTCTCGTACTA

GGTTGAATTACCATCGCGACACCATCATCAGTAGGGTAAAACTAACCTGTCTCACGACGGTCTAAACCCA

GCTCACGTTCCCTATTGGTGGGTGAACAATCCAACACTTGGTGAATTCTGCTTCACAATGATAGGAAGAG

CCGACATCGAAGGATCAAAAAGCAACGTCGCTATGAACGCTTGGCTGCCACAAGCCAGTTATCCCTGTGG

TAACTTTTCTGACACCTCTAGCTTCAAATTCCGAAGGTCTAAAGGATCGATAGGCCACGCTTTCACGGT

>FM895679.1 FM895679 Jatropha curcas embryo 71-95 (DAF) Jatropha curcas cDNA clone rjcpga0_002642, mRNA sequence

ATTCCAGCATTTATGACTCATGGTTTGACCCAGTTGTCTCAGTTTTACCTGGGCTTATTCCTGATGGCTT

TATCTATCTTAGAGCTAGCCCTGATACTTGCCATCAGAGAATGAAGCTGCGAAGGAGAGCAGAAGAGGGC

GGTGTCTCCCTAGATTATCTCCGTGACTTGCATGAAAAGCATGAAAGCTGGCTTTTCCCTTTCCAAAGTG

GACATCATGGAGTATTATCAGTCAGTAAGCTACCAGAACAAATGGATAAATCTTTGCATCCTGACATAAG

AGATCGGGTGTTTAATTTGGAAGGTGATCATATGCATTCAAGTATTCAAAAGGTGCCTGCTTTGGTTCTT

GACTGTGAAGCCAATATTGATTTTAGCAGAGATATTGAAGCTAAGGAACACTATGCACGCCAAGTTGCGG

AATTCTTTAAATATGTGAAGAACAGGAAAGAAGTTTCATCTTCACAAGCTGGAGACAGTGGTCTTCTCGG

CAACCGACAGATCTTGCTGCCTCGTGAAGGTGGATTGTTTTTACCAAATGAAAAGCACTTCCCTCAGTCT

GCTCTCAAATCTTTGGAGTTTAGACGAGCAATGTCATTTATGTCTGGTCAGTGATTGGCTTTGCAGCCTT

CTTTTC

>FM895678.1 FM895678 Jatropha curcas embryo 71-95 (DAF) Jatropha curcas cDNA clone rjcpga0_002641, mRNA sequence

TTTTTTTAGAAAAAAGAAAGTAAATTTATTCACAAACTAAGAGAAACTCATTAACGACTCTGAGGATAAC

TAGATAAACGAAAAGGGTAAAAAAAAGCTACTACCAACAGCTATTACAGTCTGTGTTTGCAACCACGTTT

TATTCTTTACATTACTCTTCTCTAATCAAAACCCATTATCATATCATTATCAAAGCTCTTTATCAATTTT

TGCACCCTTTCAGAATTAGCCGCCTCCTTCAACTTGTCTGGTGGTGCTGCAGATGCTGAAGGCAATATAA

CTGACTCGCGTTGTGAATTAACAAAGTGCTCCAATCCTTCCTCGCTTAAGCCAAATGCAGTTGCAAGCTC

AGGGCCTCTTAAGGTAGTAAGAACTGAGTTTGAACCAGCTAAGAATTGGGGTCTATTCTTTTGAGCTGAG

GTTGTGAATCCAAAGAACTCAAAAGGAGCTGTTCTTGATGCAATTTGACAGAATGGGTGATATCTTGGCA

CCCAAAACACATCATTTTCTGCTACTTTCGCTTTCATTGCTTGTGTTCCGTTTGGATACACAATCTGTAT

TACGCCAGTTCCTCTC

>FM895677.1 FM895677 Jatropha curcas embryo 71-95 (DAF) Jatropha curcas cDNA clone rjcpga0_002640, mRNA sequence

CTGGAAATCAGAATCAAACGAGCTTTTACCCTTTTGTTCCACACGAGATTTCTGTTCTCGTTGAGCTCAT

CTTAGGACACCTGCGTTATCTTTTAACAGATGTGCCGCCCCAGCCAAACTCCCCACCTGACAATGTCTTC

CGCCCGGATCGGCCGCCGAAACGACCTTGGGTCCAAAAAGAGGGGCATAGCCCCGCCTCCGATTCACGGA

ATAAGTAAAATAACGTTAAAAGTAGTGGTATTTCACCGTCGCCTTTCGGCTCCCACTTATCCTACACCTC

TCAAGTCATTTCACAAAGTCGGACTAGAGTCAAGCTCAACAGGGTCTTCTTTCCCCGCTGATTCCGCCAA

GCCCGTTCCCTTGGCTGTGGTTTCGCTGGATAGTAGACAGGAACAGTGGGAATCTCGTTAATCCATTCAT

GCGCGTCACTAATTAGATGACGAGGCATTTGGCTACCTTAAGAGAGTCATAGTTACTCCCGCCGTTTACC

CGCGCTTGGTTGAATTTCTTCACTTTGACATTCAGAGCACTGGGCAGAAATCACATTGCGTGAGCATCCT

CA

>FM895676.1 FM895676 Jatropha curcas embryo 71-95 (DAF) Jatropha curcas cDNA clone rjcpga0_002639, mRNA sequence

ATTCGCAAAGGATTCTATCCGTCGCAGCGATGGGAATTGTACTTTAAGTGCGGCCCTCGCGGCTCGTCCG

CCGCGGGGGCTTGGCCAACGGCACGTGCCTCTGGGGGCCGGATGGCCCCTACTGCGGGTCGGCAAACGGG

CGACGGACACGCGCGTCGCTTCTGGCCCGGATTCTGACTTAGAGGCGTTCAGTCATAATCCAGCGCACGG

TAGCTTCGCGCCACTGGCTTTTCAACCAAGCGCGATGACCAATTGTGCGAATCAACGGTTCCTCTCGTAC

TAGGTTGAATTACCATCGCGACACCATCATCAGTAGGGTAAAACTAACCTGTCTCACGACGGTCTAATCC

CAGCTCACGTTCCCTATTGGTGGGTGAACAATCCAACACTTGGTGAATTCTGCTTCACAATGATAGGAAG

AGCCGACATCGAAGGATCAAAAAGCAACGTCGCT

>FM895675.1 FM895675 Jatropha curcas embryo 71-95 (DAF) Jatropha curcas cDNA clone rjcpga0_002638, mRNA sequence

CTGAGCGGGGCGAAGCCAGAGGAAACTCTGGTGGAGGCCCGCAGCGATACTGACGTGCAAATCGTTCGTC

TGACTTGGGTATAGGGGCGAAAGACTAATCGAACCGTCTAGTAGCTGGTTCCCTCCGAAGTTTCCCTCAG

GATAGCTGGAGCTCGGGACGAGTTCTATCGGGTAAAGCCAATGATTAGAGGCATCGGGGGCGCAACGCCC

TCGACCTATTCTCAAACTTTAAATAGGTAGGACGGCGCGGCTGCTTCGCTGAGCCGCGCCACGGAATCGA

GAGCTCCAAGTGGGCCATTTTTGGTAAGCAGAACTGGCGATGCGGGATGAACCGGAAGCCGGGTTACGGT

GCCCAACTGCGCGCTAACCTAGAACCCACAAAGGGTGTTGGTCGATTAAGACAGCAGGACGGTGGTCATG

GAAGTCGAAATCCGCTAAGGAGTGTGTAACAACTCACCTGCCGAATCAACTAGCCCCGAGAATGGATGGC

GCTTAAGCGCGCGAGCTATACCCGGCC

>FM895674.1 FM895674 Jatropha curcas embryo 71-95 (DAF) Jatropha curcas cDNA clone rjcpga0_002637, mRNA sequence

GGAAATCAGAATCAAACGAGCTTTTACCCTTTTGTTCCACACGAGATTTCTGTTCTCGTTGAGCTCATCT

TAGGACACCTGCGTTATCTTTTAACAGATGTGCCGCCCCAGCCAAACTCCCCACCTGACAATGTCTTCCG

CCCGGATCGGCCGCCGAAACGACCTTGGGTCCAAAAAGAGGGGCATAGCCCCGCCTCCGATTCACGGAAT

AAGTAAAATAACGTTAAAAGTAGTGGTATTTCACCGTCGCCTTTCGGCTCCCACTTATCCTACACCTCTC

AAGTCATTTCACAAAGTCGGACTAGAGTCAAGCTCAACAGGGTCTTCTTTCCCCGCTGATTCCGCCAAGC

CCGTTCCCTTGGCTGTGGTTTCGCTGGATAGTAGACAGGGACAGTGGGAATCTCGTTAATCCATTCATGC

GCGTCACTAATTAGATGACGAGGCATTTGGCTACCTTAAGAGAGTCATAGTTACTCCCGCCGTTTACCCG

CGCTTGGTTGAATTTCTTCACTTTGACATTCAGAGCACTGGGCAGAAATCACATTGCGTGAGCATCCTCA

GGGACCATCGCAATGCTTTGTTTTAATTAAACAGTCGGATTCCCCTTGTCCGTACCAGTTCTGAATCGAC

TGTTCGACGCCCGG

>FM895673.1 FM895673 Jatropha curcas embryo 71-95 (DAF) Jatropha curcas cDNA clone rjcpga0_002636, mRNA sequence

GCAAAGGATTCTATCCGTCGCCCGGTGGGAATTGTACTTTAAGGCGGCCCTCGCGGCTCGTCCGCCGCGG

GGGCTTAGCCAACGGCACGTGCCTCTGGGGGCCGGATGGCCCCTACTGCGGGTCGGCAAACGGGCGACGG

ACACGCGCGTCGCTTCTGGCCCGGATTCTGACTTAGAGGCGTTCAGTCATAATCCAGCGCACG

>FM895672.1 FM895672 Jatropha curcas embryo 71-95 (DAF) Jatropha curcas cDNA clone rjcpga0_002635, mRNA sequence

TCAATTGTAAATTCTTATTACAATAAGAACACATGCTGTGAATATACTACAGACAAATATTTTANAGCGT

TTATCATAAATAACCATAAACATAAGTACAGTTTTACTTTATCAAATAAAAGAACATATAGAATAACAGC

TATCACCCGACAAAAATTGTAGGAAAAAAAAAAAGAATTCTAATTGCCAATTCATAAACTGAGTACACCA

CTGCCTATACCCGTATAACAAGCTATGTTTCTCTTGGAGCACCGAGTGTTGCATAACTTGCAGCACTTGT

CACGTTTGATCACACAAACCAGCTTCTTTTATGTTCCAAGGTAGGTCATACCAGAGCCAGTACAGCTTGT

TAGAACATTTAGCTTTAGTCGTTTGCCTCAAATGCTTTGTCACACACAAGCGGAAATGATGACAATATAA

ACCAAGAATGTCATGATAGGACGAAACAGAGAGACAAGTTGACGGGAACCATCATTTGGTAACCATGGGT

AGGCATCCGGAGGGGGCATGACACATTGATCACGGTTAAAATAAACCCTCCTAGGGAAAGCCCATCCCTT

TTCAAAAGTGAAAGTTGATTTGTCCTTTCGGAATAGCAGCTCTGATTG

>FM895671.1 FM895671 Jatropha curcas embryo 71-95 (DAF) Jatropha curcas cDNA clone rjcpga0_002633, mRNA sequence

CATTCAAATATCTTATGAATTAGATATATAAATCAGTCAGTCCTAACAAGGGCATAACTTAAGACAAAAT

AAATAATTCTTCTACAAAGTAACATAACATAGTTCACAATACAGTTCCTATTGGATGCCAGAAATCCTTA

AGAAATGCACCAAGTTTCATATTTTCTTCTGTTTTCCTCCCAACAGGACGTAAAAATAAGTTCATTTCTA

GAATCAATTTGTCGAACGATCTGTAGAGGTCACCACAACACAAGTCAATTCGTACTGATCCATATAATAT

ATGTAAACTGACGAGTTTAGTGCTAAACAAACTCCATTTACTACAGGATTTCATTTCCAAACATGTACCC

AAATGCTGCTTCAGCTGTATTAAGTATCCACCCTTTGGCTTCCTCAAAAGTGAATATAACCAGCTATCAA

GGATTCCAAGCTGTT

>FM895670.1 FM895670 Jatropha curcas embryo 71-95 (DAF) Jatropha curcas cDNA clone rjcpga0_002632, mRNA sequence

AGGATTCTATCCGTCGCCCGATGGGAATTGTACTTTAAGGCGGCCCTCGCGGCTCGTCCGCCGCGGGGGC

TTGGCCAACGGCACGTGCCTCTGGGGGCCGGATGGCCCCTACTGCGGGTCGGCAAACGGGCGACGGACAC

GCGCGTCGCTTCTGGCCCGGATTCTGACTTAGAGGCGTTCAGTCATAATCCAGCGCACGGTAGCTTCGCG

CCACTGGCTTTTCAACCAAGCGCGATGACCAATTGTGCGAATCAACGGTTCCTCTCGTACTAGGTTGAAT

TACCATCGCGACACCATCATCAGTAGGGTAAAACTAACCTGTCTCACGACGGTCTAAACCCAGCTCACGT

TCCCTATTGGTGGGTGAACAATCCAACACTTGGTGAATTCTGCTTCACAATGATAGGAAGAGCCGACATC

GAAGGATCAAAAAGCAACGTCGCTATGAACGCTTG

>FM895669.1 FM895669 Jatropha curcas embryo 71-95 (DAF) Jatropha curcas cDNA clone rjcpga0_002631, mRNA sequence

CGCGCACAGTCTTCGTGCTCTCTCTCTTTCTTTGCGTGCTGCTTCTTGTTTTTGTTGAACCACACGCCAT

GGCTTCGATTACCGGAACTTCAATCTCCATGACCTCTCTTTCGAGGTCTCTTAGCACCCAGGCACCAAGA

AATGGTATCTCTAGTTTGAGGATTTCACTTCCCATTAGTGGAAGAAGCTTTCCTTCTCTTAGATTGCAAA

AGGGTGCATCTCGCTTCCGTGTCTCCTGTGCGGCCAAACCAGAGACGCTAAATAAAGTGTGTGAAATTGT

AAGGAAGCAGTTGGCACTAGCACCTGAAACCGCTGTTAACGGAGAGTCGAAGTTCGTTGCATTGGGAGCT

GATTCTCTTGACACGGTTGAGATTGTAATGGGACTTGAGGAGGAATTTGGAATCAGTGTAGAAGAAGAAA

CGGCACAGAGTATACTCACTGTTCAAGATGCAGCTGATCTGATTGAGAAGCTTATCGAGAAGAAAGCTGC

TTAAACTAGTTTGGAAGAGTTGAAGGCATAGTAGTTTTATATGAAGTTTAGTTCTATATTT

>FM895668.1 FM895668 Jatropha curcas embryo 71-95 (DAF) Jatropha curcas cDNA clone rjcpga0_002629, mRNA sequence

TGAATTNTCGTGTGTTAGGGGTGAAATCCAAAGATCTACGAAGGAACGCCAAAAGCGAAGGCAGCTCTCT

GGGTCCCTACCGACGCTGGGGTGCGAAAGCATGGGGAGCGAACGGGATTAGATACCCTGGTAGTCCATGC

CGTAAACGATGAGTGTTCGCCCTTGGTCTACGCGGATCAGGGGCCCAGCTAACGCGTGAAACACTCCGCC

TGGGGAGTACGGTCGCAAGACCGAAACTCAAAGGAATTGACGGGGGCCTGCACAAGCGGTGGAGCATGTG

GTTTAATTCGATACTACGCGCAAAACCTTACCAGCCCTTGACATATGAACAACAAAACCTGTCCTTAACG

GGATGGTACTGACTTTCATACAGGTGCTGCATGGCTGTCGTCAGCTCGTGTCGTGAGATGTTTGGTCAAG

TCCTATAACGAGCGAAACCCTCGTTTTGTGTTGCTGAGACATGCGCCTAAGGAGAAAGTCTTTGCAACCG

AAGTGAGCCGAGGAGCCGAGTGACGTGCCAGCGCTACTAAT

>FM895667.1 FM895667 Jatropha curcas embryo 71-95 (DAF) Jatropha curcas cDNA clone rjcpga0_002628, mRNA sequence

TGAACCAGGAACCCACTTTAAGTGCCCAGTCCAATGGGGAACTAAAGCATTCAGAGAGTGGAGCAGTTTC

AAAAGCTGGAGATGACTCAAAGGCAAGTAAGCCACTTGCCTCAGAGATTCGGAATGTAGCAAATGGGATT

GCCAGTATCTGCTAGGTTACCAAATTCCAGCTATATTATGGAAGTTTTGTTGCTTGAGAGTAAAAATGTG

TAGCTGATTGCTGTTGATGTTGTTCAAGCATTGTGCCACTCTTGTAGAGCTGAAGCTGCGAAGCCTTGAT

AACCAGAGGGATGGAAAAGAGTATAGAGATTCTTCAGTTCTGGTGAGGTTTGGTGCGCTCCTTAAAATCA

CAGTATTTCTTTATGAGTAGATCTTTCTTATAGTGGTTGGTTGGCTGCTGACAAGTTTTCATTCCCTAGA

AGTTTGGGTTTTAGTGTTGCTGTTTTATTGCTTTT

>FM895666.1 FM895666 Jatropha curcas embryo 71-95 (DAF) Jatropha curcas cDNA clone rjcpga0_002627, mRNA sequence

ATTCCGAAAATGGATGGCGCTTAAGCGCGCGACCTATACCCGGCCGTCGGGGCAAGAGCCAGGCCCCGAT

GAGTAGGAGGGCGCGGCGGTCGCTGCAAAACCCGGGGCGCGAGCCCGGGCGGAGCGGCCGTCGGTGCAGA

TCTTGGTGGTAGTAGCAAATATTCAAATGAGAACTTTGAAGGCCGAAGAGGGGAAAGGTTCCATGTGAAC

GGCACTTGCACATGGGTTAGTCGATCCTAAGAGACGGGGGAAGCCCGTCCGACAGCGCGTCCTCGCGCGA

GCTTCGAAAGGGAATCGGGTTAAAATTCCCGAACCGGGACGTGAAGGCTGACGGCGACGTTAGGGAGTCC

GGAGACGTCGGCGGGGGCCTCGGGAAGAGTTATCTTTTCTGTTTAACAGCCCGCCCACCCTGGAAACGAC

TCAGTCGGAGGTAGG

>FM895665.1 FM895665 Jatropha curcas embryo 71-95 (DAF) Jatropha curcas cDNA clone rjcpga0_002626, mRNA sequence

TTAGTTCTTTCTCCTTAACCTCATATATTTTCATTCCATTCTATATCTCCTTATTTGCTTTCCATATGGC

CAGTGTTGAGGTTCAATCAGCTAAAACAGCATTGCCAGAGAATGAGACGCAAGAAGTGACCAAGACTGAG

GAGACAACCACTGAACCTGCTGCACCATCCGCACCAGAACCAGTAGCAGAACTACCGAAGGAAGTAGTCG

CAGAGGAACCAGCGGCCAAAGAACCAGAGGTTGAAACCAAGAAAGAAGAAGAAGAGAAGAAAGAGGAACC

AGAGGCAAAAGCTGAGGATCAGCCTGAGGCTGCACCAGCAGCAGAAGAGACTCCAGATGTGAAGCCAGCA

GAGCCTGTCACTGAGGAGGAGGCCAAAGATGAGAGTACTGAGAAAGTGACAGAGGCGCCACCAGCAGCAG

ACGAAGAGAAACCAGCTACGGAAGAGAAGCCAGTTGAAAAAG

>FM895664.1 FM895664 Jatropha curcas embryo 71-95 (DAF) Jatropha curcas cDNA clone rjcpga0_002625, mRNA sequence

ATTCCTTGCCTGGATTTTCTTTTACTTCTGGTGCTAACAAAGCGATCACTGTTTGCAGGAACCTGCGCTC

ATTATTAAGTCATGCAGAGTCTCTGACTCCTAACTTTTTGAATGATGAGGATGTACAGCTTTTAAGGGTG

TTCTTTAACCAGTTACACTCACTGATTAGTCCTGCTGATTTTGAAGAAAACCAAGCTCAAGAAATCAAGT

TTGAGAGATCAATATCCTTAGACAAGTTTTCTAAACTTGACATCAATGAGCATCATCAGGAGGCTCAGAG

TACAGTAGGGTACTCACCTTTACTGAAGAAGGAAACTTCAACTCTGAACAATGAAGACAAGGGTAAAGCT

GGTGGGACTGCCTCTGCTGTTTCAAGAGAGATGGACAGGGATTTTCAGAATGTCGAAACAAGCGGTTCAG

ATACAAGTTCTACACGAGGAAAGAATTTTGTTGGTCAAATGGGGAATGGAGATTTTG

>FM895663.1 FM895663 Jatropha curcas embryo 71-95 (DAF) Jatropha curcas cDNA clone rjcpga0_002624, mRNA sequence

CAAAGGATTCTATCCGTCGCCCGATGGGAATTGTACTTTAAGGCGGCCCTCGCGGCTCGTCCGCCGCGGG

GGCTTGGCCAACGGCACGTGCCTCTGGGGGCCGGATGGCCCCTACTGCGGGTCGGCAAACGGGCGACGGA

CACGCGCGTCGCTTCTGGCCCGGATTCTGACTTAGAGGCGTTCAGTCATAATCCAGCGCACGGTAGCTTC

GCGCCACTGGCTTTTCAACCAAGCGCGATGACCAATTGTGCGAATCAACGGTTCCTCTCGTACTAGGTTG

AATTACCATCGCGACACCATCATCAGTAGGGTAAAACTAACCTGTCTCACGACGGTCTAACCCAGCTCAC

GTTCCCTATTGGTGGGTGAACAATCCAACACTTGGTGAATTCTGCTTCACAATGATAGGAAGAGCC

>FM895662.1 FM895662 Jatropha curcas embryo 71-95 (DAF) Jatropha curcas cDNA clone rjcpga0_002623, mRNA sequence

TGCAAAGGATTCTATCCGTCGCCCGATGGGAATTGTACTTTAAGGCGGCCCTCGCGGCTCGTCCGCCGCG

GGGGCTTGGCCAACGGCACGTGCCTCTGGGGGCCGGATGGCCCCTACTGCGGGTCGGCAAACGGGCGACG

GACACGCGCGTCGCTTCTGGCCCGGATTCTGACTTAGAGGCGTTCAGTCATAATCCAGCGCACGGTAGCT

TCGCGCCACTGGCTTTTCAACCAAGCGCGATGACCAATTGTGCGAATCAACGGTTCCTCTCGTACTAGGT

TGAATTACCATCGCGACACCATCATCAGTAGGGTAAAACTAACCTGTCTCACGACGGTCTAAACCCAGCT

CACGTTCCCTATTGGTGGGTGAACAATCCAACACTTGGTGAATTCTGCTTCACAATGATAGGAAGAGCCG

ACATCGAAGGATCAAAAAGCAACGTCGCTATGAACGCTTGGCTGCCACAAGCCAGTTATCCCTGTGGTAA

>FM895661.1 FM895661 Jatropha curcas embryo 71-95 (DAF) Jatropha curcas cDNA clone rjcpga0_002621, mRNA sequence

AAAGCAAAAATCGAGAAGCTCAACATTTTCACTCAGATTGTTGGGATTCCAAAACAGCAGTCTTTCCAAT

ATCCAAAATTTCAAACTCAAAGTAATAACAATTTTGTTCTCCAGACAAAACCCATAAACTAACACTTCCA

AGAACATCAATGAAATTAACGGTAACGGCGAAGTGAGAGAGTGTTATTTCTCTTCCATGTTGCTCTGCGA

GAAGGACGATTCTTGTGGTGGAGATGTCCCCTTCCACGTAGACCCCTATATTTCTTGCCAGCAGAGGTGA

GACCTCGGAGCTCCCTGTGCTTATGAACAGGGTCACATAGCCAGTTGATCCTTGGGTCATTTCGAATGGC

ATTATGTGCTACATCAACTAAGATTACCTCAAAATACTTGTAAGTAGAATCCTCATTAATCCAGTAGGAA

TTGAGAACCCTCAGACCACCCAATTTCCTACCAGCGCGC

>FM895660.1 FM895660 Jatropha curcas embryo 71-95 (DAF) Jatropha curcas cDNA clone rjcpga0_002620, mRNA sequence

GTGGTAGCAGATCGGAGATGTTAAGAAATGAGTAAATACTGCATGACAGAGAGTCCTGTGGTTTCTATGC

CAACCACAAAGCGTTTGACCAATATACGTCACGTCGTTGTTTCGTTAACCTGAGAATTACAGTTACGAAT

AAAAAATCATGCATCGAATATCACCATCCATAGCGAGACATAATTGCCACATGAGCATTGCAACGGCAGA

GAGAATGAATACCACCACCCACAATGAGAATCAATGCCAGCAATTCTGGATCTTACCTTCTCAGAACATC

ATCTCCCTCTTTGTCCAAACTTCCCCTGCCACTCGCTATCTTCATATCATAACGATCTTTCCAAAACCAG

TGGCTATCCCAACCATTCACGACAACAAGGCCGTCTTTTCTTGTCGCTATTTCATTTTGATTTACTCTTG

ATCATTTATCTACAATCTCTCGATCATATTAATTTGCTTTCCTTTAAATGCTCATATATTTATCTTCGTC

AGTGGACAGA

>FM895659.1 FM895659 Jatropha curcas embryo 71-95 (DAF) Jatropha curcas cDNA clone rjcpga0_002619, mRNA sequence

ATTCCTCACCAATCTCTCTCTCTCTCTCTCTCTCTCCGAGCGAGAATTTGGAAAAGAAGCCACCGTTGTT

CTTCGACTGGATACGTAACAAACAGGTTGATTAGGGTTTCAGGTGTATAATCATTATCTAACCATCTTGT

TTGTGAAAATCTATTGGATGGCAATTATACCTTGTGGAAGCACATCAGTTGCTCAATGGGGTATTCGACC

TCAGTTCATACTTAGATCTTATATCCAAAACAGGATAGTGACTACTCAGTATGGTCTTAATGGCAAGACA

AGCTATTTGGCAGCACCAAGTTCAAGCCTATTTTCACGTGATTCCTTTCCTGTATTATCTTTTGTTGGGC

CATCTCAAACTTCACATCGTCGAAGGGGATCACGATTTACAGTTAGAGCTGATGCTGATTACTATTCTGT

CCTTGGAGTGTCAAAAAATGCAAGTAAATCTGAAATCAAAAGTGCATACCGGAAA

>FM895658.1 FM895658 Jatropha curcas embryo 71-95 (DAF) Jatropha curcas cDNA clone rjcpga0_002618, mRNA sequence

GTTTCGGAGCCGTTTTGGCGGGGCGTACTAAACCCACTACGTACCACACCACCGGGCGGCTCGCCTGAAT

GCCGAGTCTTTCTCCGCCGCCAACTCGACGTCGTCGTCACCTGCAAGATAAGGCCAAAAACTGGACTTTA

CTAAACAAGCGAGAAAAGCCCCTTTCTCTGTTATTAGTAAAGTAAAGCGCGCTAGCTGCAATCTTTCTAA

AGCGCACACTACAAAGTGCTTCGAAAGGCGCCGGCTCCCCTCTTACTGACAGCACAGCTACGTGCTGGCA

CTCAATTAGTAGCGCTGGCCCGTCACTCGGCTCCTCGGCTCACTTCGGTTGCAAAGACTTTCTCCTTAGG

CGCATGTCTCAGCAACACAAAACCGAGGGTTTCGCTCGTTATAGGACTTGACCAAACATCTCACGACACG

AGCTGACCGACAGCCA

>FM895657.1 FM895657 Jatropha curcas embryo 71-95 (DAF) Jatropha curcas cDNA clone rjcpga0_002617, mRNA sequence

CAAATCTTCTTAAGCGATATACAAATTAGAAATGGCTCTGATTCCAAGTTTCTTCGGTAACTCACGAAGC

AGCATTTTCGATCCTTTCTCTGCTTTCGACGTTTGGGATCCATTTAAAGACTTTCCTTTCCCTTCATCTT

CTTCAATCGTCTCTCGTGAAAATTCTGCTTTTGTTAACACTCGCATAGACTGGAAAGAGACACCAGAAGC

TCATATCTTCAAGGCTGATCTTCCTGGGCTCAAGAAAGAGGAAGTGAAGGTAGAGGTTGAAGATGACAGA

GTTCTCCAAATCAGCGGAGAGAGGAATGTGGAGAAAGAAGACAAGAACGATACTTGGCGTCGGGTAGAGC

GTAGCAGTGGCAAGTTCTTGAGGAGGTTCAGGCTGCCGGAGAATGCAAAGATGGATCAAGTGAAGGCTTC

AA

>FM895656.1 FM895656 Jatropha curcas embryo 71-95 (DAF) Jatropha curcas cDNA clone rjcpga0_002616, mRNA sequence

AACTTGAAACCTAAATCGAATTTTCTGTTTGCAACAGTTTCTCTGCCGATGACGGTTCTAGTCTACAACT

AGGCCCAATTTAATCCAATCACCAGTACGCCTGACGAAAAATTATAGCACAGGCACTACTAACATGAACC

CAAAAAAGAAACCAGAAAAAATGAACCCAACACTCAAAACTCAACCTGCAATCAATGCGATAGACCAAAA

TGACAAATCACGAAAAGCCCAGTTGCTAGATGGTCTTTCGTGTCTTTGCCGGTTTATAGTATACTCTCGC

TTCATCAATAAGCATCCGCTCGGAGGAGTTAATTTTTAGTTTGCTATAAAAGGCGGCAGTAGCATACCTA

CAACGTTACAATCAAGGGAATAACAAAGAACCAACCCAAAACCAAACTCTTCAACATTATCAACACCAAA

AGCCACATATCCGTCCCCTTCTTCCGTTGTTAATAGAGAACAAAAGATCTGCTTCTTCAGATATACTGAA

CC

>FM895655.1 FM895655 Jatropha curcas embryo 71-95 (DAF) Jatropha curcas cDNA clone rjcpga0_002615, mRNA sequence

ATTCCAAAGTATTGTGGTTTTCGAGGCCAAATCCATCGGGCACTCCATTCGTTTTATCAGGAAAAAAATG

ACTACATTGGATGCAACAAGAGCAGAACTTGCTTTGTTAGTTTTGTATCTGAATAAAGCCGAGGCAAGAG

ACAAGATATGCAGGGCTATTCAATATGGTTCAAAATTCTTGAGCGACGGAAAGCCTGGTACTGCCCAGAA

TGTTGACAAATCAACCAGCTTGGCCCGCAAAGTTTTCCGACTTCTTAAGTTTGTCAATGATCTGCATGCT

CTTATTAGTCCAGTCCCTCAAGGAACTCCCCTGCCTCTTGTTTTGTTGGGAAAGTCGAAAAACGCATTAT

TGTCGACTTTCTTATTTCTTGATCAAATTGTTTGGCTTGGTAGATCGGGTATCTACAAGAACAAAGAACG

TGCTGAGCTACTTGGTAGGATCTCTCTTTACTGTTGGATGGGGTCTTCAGTCTGTACTACCTTGGTTGAG

ATTGGAAAGCTTGGAAGGCTTTCTGCATCAATCAAG

>FM895654.1 FM895654 Jatropha curcas embryo 71-95 (DAF) Jatropha curcas cDNA clone rjcpga0_002614, mRNA sequence

ATTCGTCTGACATGTGTGCGAGTCAACGGGTGAGTAAACCCGCAAGGCAAGGAAGCTGACTGGCGGGATC

CCCCCGAGGGTTGCACCGCCGACCGACCTTGATCTTCTGAGAAGGGTTCGAGTGAGAGCATGCCTGTCGG

GACCCGAAAGATGGTGAACTATGCCTGAGCGGGGCGAAGCCAGAGGAAACTCTGGTGGAGGCCCGCAGCG

ATACTGACGTGCAAATCGTTCGTCTGACTTGGGTATAGGGGCGAAAGACTAATCGAACCGTCTAGTAGCT

GGTTCCCTCCGAAGTTTCCCTCAGGATAGCTGGAGCTCGGGACGAGTTCTATCGGGTAAAGCCAATGATT

AGAGGCATCGGGGGCGCAACGCCCTCGACCTATTCTCAAACTTTAAATAGGTAGGACGGCGCGGCTGCTT

CGCTGAGCCGCGCCACGGAATCGAGAGCTCCAAGTGGGCCATTTTAGGTAAGCAGAACTGGCGATG

>FM895653.1 FM895653 Jatropha curcas embryo 71-95 (DAF) Jatropha curcas cDNA clone rjcpga0_002613, mRNA sequence

GGATTCTATCNCGTCGCCCGATGGGAATTGTACTTTAAGTGCGGCCCTCGCGGCTCGTCCGCCGCGGGGG

CTTGGCCAACGGCACGTGCCTCTGGGGGCCGGATGGCCCCTACTGCGGGTCGGCAAACGGGCGACGGACA

CGCGCGTCGCTTCTGGCCCGGATTCTGACTTAGAGGCGTTCAGTCATAATCCAGCGCACGGTAGCTTCGC

GCCACTGGCTTTTCAACCAAGCGCGATGACCAATTGTGCGAATCAACGGTTCCTCTCGTACTAGGTTGAA

TTACCATCGCGACACCATCATCAGTAGGGTAAAACTAACCTGTCTCACGACGGTCTAACCCCAGCTCACG

TTCCCTATTGGTGGGTGAACAATCCAACACTTGGTGAATTCTGCTTCACAATGATAGGAAGAGCCGACAT

CGAAGGATCAAAAAGCAACGTCGCTATGAACGCTTGGCTGCCACAAGCCAGTTATCCCTGTGGTAACTTT

TCTGACACCTCTAGCTTCAAATTCCGAAGGTCTAAAGGATCGATAGGCCACGCTTTCACGGTTCGTATAC

GTACTGGAAATCAGAATCAAACGAGCTTTTACCC

>FM895652.1 FM895652 Jatropha curcas embryo 71-95 (DAF) Jatropha curcas cDNA clone rjcpga0_002612, mRNA sequence

CAAAGGATTCTATCCGTCGCCCGATGGGAATTGTACTTTAAGGCGGCCCTCGCGGCTCGTCCGCCGCGGG

GGCTTGGCCAACGGCACGTGCCTCTGGGGGCCGGATGGCCCCTACTGCGGGTCGGCAAACGGGCGACGGA

CACGCGCGTCGCTTCTGGCCCGGATTCTGACTTAGAGGCGTTCAGTCATAATCCAGCGCACGGTAGCTTC

GCGCCACTGGCTTTTCAACCAAGCGCGATGACCAATTGTGCGAATCAACGGTTCCTCTCGTACTAGGTTG

AATTACCATCGCGACACCATCATCAGTAGGGTAAAACTAACCTGTCTCACGACGGTCTAAACCCAGCTCA

CGTTCCCTATTGGTGGGTGAACAATCCAACACTTGGTGAATTCTGCTTCACAATGATAGGAAGAGCCGAC

ATCGAAGGATCAAAAAGCAACGTCGCTATGAACGCTTGGCTGCCACAAGCCAGTTATCCCTGTGGTAACT

TTTCTGACACCTCTAGCTTCAAATTCCGAAAGTCTAAAGGATCGATAGGGCCACGCTTTCCGGTTCGTAT

TCGTACTGGAAATCAGAATCAAACGAG

>FM895651.1 FM895651 Jatropha curcas embryo 71-95 (DAF) Jatropha curcas cDNA clone rjcpga0_002611, mRNA sequence

ATTCGTCTGACATGTGTGCGAGTCAACGGGTGAGTAAACCCGCAAGGCGCAAGGAAGCTGACTGGCGGGA

TCCCCCCGAGGGTTGCACCGCCGACCGACCTTGATCTTCTGAGAAGGGTTCGAGTGAGAGCATGCCTGTC

GGGACCCGAAAGATGGTGAACTATGCCTGAGCGGGGCGAAGCCAGAGGAAACTCTGGTGGAGGCCCGCAG

CGATACTGACGTGCAAATCGTTCGTCTGACTTGGGTATAGGGGCGAAAGACTAATCGAACCGTCTAGTAG

CTGGTTCCCTCCGAAGTTTCCCTCAGGATAGCTGGAGCTCGGGACGAGTTCTATCGGGTAAAGCCAATGA

TTAGAGGCATCGGGGGCGCAACGCCCTCGACCTATTCTCAAACTTTAAATAGGTAGG

>FM895650.1 FM895650 Jatropha curcas embryo 71-95 (DAF) Jatropha curcas cDNA clone rjcpga0_002610, mRNA sequence

GTGAGACCTCATGGCCTGGCCGGAGGGATGTAGTTGCTTGTGCCTCTGTTTGTAAATCTTGGAGGGAGAT

CACAAAAGAAATTGTTAAGACACCTGAGCAATGTGGCTCGCTCACTTTTCCCATCTCGCTCAAGCAGCCT

GGTTCAAGAGATGCTCCAATTCAATGCTTTATCAGACGGGATAGGGCAACCTCAACGTATCTCTTGTATC

TTGGTTTGAGCCCTGCTCTATCTGGTGATATGAGTAAACTGTTATTTGCAGCAAAGAAGATTAGAAGGGC

AACAAGTACTGAGTTTCGAATTTCTTATGTTGCAAATGATTTTTGTCGGACTAGCAACAATCATGTTGGA

AAACTGAAGTCTAATTTCCTGGGAACAAAATTTACCGTTTACGACAGTCAACCTGCACACCCAGCTGTAC

AAGCCAATTGCAAGCCACACAGAAAACTGCATTCAATCCAGGTTCCTACATCAGTACCTGCTAAGGACTA

TAATGTGGCCACTATTTCTTATGAGCTCAATGTCCTCAGAACCAGAGGCCCGAGG

>FM895649.1 FM895649 Jatropha curcas embryo 71-95 (DAF) Jatropha curcas cDNA clone rjcpga0_002609, mRNA sequence

GAGAGAAAGAAGAAAATATAACCAAAAAGAAGATTTCAGTAACAAAATTTTAGTTTCTCTGCCATACTAG

ACATTCCAATACCTGCTCTAACAGAACTAGTGACAAAGTCTACTCAATGACAAGATTATAGCTGCTAGTA

GAAAAGAAAAGAATCAAAGAGAGGAAAAGAAGAGTAAAAAACTAGAACGACAGTGAGATTTCACTGAGAG

AAAATCATTGTTTTGGAAGGTAATAAGGGTATCCACCCATTCCCATAGGTGTTGGTGGTGAAAGACCGAG

CGGATCTTTCTTCATGGCAACTGGTGGATATGAAATTTGAGATTGAGGAGGATATGGAGCAGGTGCTGTG

GGAGGACTTGGGTAAGAGGAAGCAGGATATGATGTCATAGTTTGAGGATAAAGAGTAGCCAACAGGGGCA

GGGCCAGGATGTGCTTTTACAGGTTGAGTGTATCCAGCTGCCATTGGCAAGGGTACTGGTACCACTGGTG

GAGGCAATTGTGTTTGTGCTGTTTTGCCTTTGTTGAGTATCAGCA

>FM895648.1 FM895648 Jatropha curcas embryo 71-95 (DAF) Jatropha curcas cDNA clone rjcpga0_002608, mRNA sequence

CAAAGGATTCAATCCGATCTGCCCGATGGGAATTGTACTTGAAGGGCGGCCCTCGCGGCTCGTACGGCGA

GGGGGCTTGGCCAAACGGCACGTGCCTCTGGGGGCCGGATTGCCCCTACTGCGGGTCGGCAATACAGGCG

ACGAGAGACGCGCGTCGCTTCTGGCCCGGATTCTGACTTAGACGGAGTTCAGTAATAATACACCGCACGG

TACCTGTCCCTCCACTCGGCTTTGCAAGCTCATGCGCGATGACCAATTGTGCGAATCAACGGTTCCTCTC

GTACTACGTTGAATTACCATCGCGACCACCATCATCAGTAGGGTAAAACTAACCTGTCTCACGACGGTCT

AACCCCAGCTCAGCGTCCCTATTGGTGGGTGAACAATCCAACACTTGGTGAAATCTGCTTCACAATGATA

GGAATAACCGACATCGAAGGATCGAAAAAGCAACGTAACTATGAACGCTTGGCTGCCACAATCCAATTAT

CCCTGTGGTAACTTTTCTGACACCTCTAACTTCGAATTCCGAATGTCTAAAAGATCGATA

>FM895647.1 FM895647 Jatropha curcas embryo 71-95 (DAF) Jatropha curcas cDNA clone rjcpga0_002605, mRNA sequence

GCAAAGGATTCTATCCGTCGCCCGATGGGAATTGTACTTTAAGGCGGCCCTCGCGGCTCGTCCGCCGCGG

GGGCTTGGCCAACGGCACGTGCCTCTGGGGGCCGGATGGCCCCTACTGCGGGTCGGCAAACGGGCGACGG

ACACGCGCGTCGCTTCTGGCCCGGATTCTGACTTAGAGGCGTTCAGTCATAATCCAGCGCACGGTAGCTT

CGCGCCACTGGCTTTTCAACCAAGCGCGATGACCAATTGTGCGAATCAACGGTTCCTCTCGTACTAGGTT

GAATTACCATCGCGACACCATCATCAGTAGGGTAAAACTAACCTGTCTCACGACGGTCTAAACCCAGCTC

ACGTTCCCTATTGGTGGGTGAACAATCCAACACTTGGTGAATTCTGCTTCACAATGATAGGAAGAGCCGA

CATCGAAGGATCAAAAAGCAACGTCGCTATGAACGCTTGGCTGCCACAAGCCAGTTATCCCTGTGGTAAC

TTTTCTGACACCTCTAGCTTCAAATTCCGA

>FM895646.1 FM895646 Jatropha curcas embryo 71-95 (DAF) Jatropha curcas cDNA clone rjcpga0_002604, mRNA sequence

ATTCCACCCTTTCTCTCTCTCTCTCTCTCTCTCTCTCTCTCTATTTATCTTTTCCTTCTTTTCTCTCTCC

CTTACAAGCCCTAACCCTAACTCAGACCCAGATTCGCCATGAAAGGAGGTAAATCAAAGTCAGACACCAA

GAACGCCAAGCTTTCCGTGAACAAGAAACCCGCCAAGGCAAGTAAGAAGGCGGGTAAGGCAGCGAAGGAT

CCAAATAAGCCAAAGAGGCCCGCCAGTGCTTTCTTCGTTTTCATGGAAGAGTTCAGAGAGCAGTACAAGA

AGGAACATCCTAAAAACAAATCCGTTGCTGCTGTTGGCAAAGCTGGTGGAGATAAATGGAAGTCAATGTC

AGATGCTGAGAAAGCACCTTTTGTATCTAAGGCAGAAAAGAGGAAGATAGAATATGAGAAGAAATTGAAA

GCCTACAACAAGGGACAGGCTGAAGGCCCCAAGGAAGAAGAGGAATCTGAGAAGTCAATGTCTGAGGTGA

ATGATGAGGAAGAAGATGATGAGGAGGGACGTGGAGAGGAGGACGATGATGAGTAGGAAGAGGGGG

>FM895645.1 FM895645 Jatropha curcas embryo 71-95 (DAF) Jatropha curcas cDNA clone rjcpga0_002603, mRNA sequence

ATTATGAAAGTGGCGATTCCATATTTGTTAAGCAAATCTATGAATTTCTACAGCAGTGGTTGCTGGAACA

TCCTCAATTTTCTGAAAACTCATTTTACTTTGGTGGTGATGGATATTCTGGCAATTTGATTCCAGTGGTT

ACACAAACAGTTCTAGATGGCAATGAAGCTGGAAATATCTCTTTCTTGAATATTAAGGGTTATATGCTTG

GAAATCCTGGAGCAGATTTTAGCATTGATTTCAATGGAAGACACACTTTTGCTAACCGAATAGGATTTGT

ACCGGATGAACTCTACGAGGCTGCTGATGAGTCTTGCAGTGGAAATTTCACAAACCCACCAAATGATGTT

TGTAAGACACTTGTGGATGCTATGTCAGAGCTAACCAGGAACATTAATCCTGTACAAGTTATAGAGCCAG

CTTGTAAAAGTGTACCAAATTATCTCAAGCTGCCACAAATTGGCGAACAATCTGCTCGAAGATATCTCGA

ACAAAACCCCACTCATCCTTTATCTGATACTCCTACTTTTAAGGTGTCGTAAATATGATCTCCAGAT

>FM895644.1 FM895644 Jatropha curcas embryo 71-95 (DAF) Jatropha curcas cDNA clone rjcpga0_002602, mRNA sequence

ATTATCGGAGGCGGGGCTATGCCCCTCTTTTTGGACCCAAGGTCGTTTCGGCGGCCGATCCGGGCGGAAG

ACATTGTCAGGTGGGGAGTTTGGCTGGGGCGGCACATCTGTTAAAAGATAACGCAGGTGTCCTAAGATGA

GCTCAACGAGAACAGAAATCTCGTGTGGAACAAAAGGGTAAAAGCTCGTTTGATTCTGATTTCCAGTACG

AATACGAACCGTGAAAGCGTGGCCTATCGATCCTTTAGACCTTCGGAATTTGAAGCTAGAGGTGTCAGAA

AAGTTACCACAGGGATAACTGGCTTGTGGCAGCCAAGCGTTCATAGCGACGTTGCTTTTTGATCCTTCGA

TGTCGGCTCTTCCTATCATTGTGAAGCAGAATTCACCAAGTGTTGGATTGTTCACCCACCAATAGGGAAC

GTGAGCTGGGGTTAGACCGTCGTGAGACAGGTTAGTTTTACCCTACTGATGATGGTGTCGCGATGGTAAT

TCAACCTAGTACGAGAGGAACCGTTGATTCGCACAATTGGTCATCGCGCTTGGTTGAAAAGCCAGTGGCG

CGAAGCTACGGTGCGCTGGATTATGACTGAACGCTTCTAAGTC

>FM895643.1 FM895643 Jatropha curcas embryo 71-95 (DAF) Jatropha curcas cDNA clone rjcpga0_002601, mRNA sequence

CGAATTCGAGTACTGCAGGAATGCAAGAGAATTAGCACTGAAACTACTTGATGCAATTTCAGAAAGCTTA

GGTCTTGAAAGGGATTACATAAATAAAGCACTGGGCAAACATGCACAACACATGGCCATAAATTACTATC

CTCCATGTCCACAACCAGATCTGACTTACGGATTACCTGCCCATGCAGATCCTAATGCAATTACTATTCT

CCTTCAGGATCAGGTGCCAGGCCTTCAGGTGCTTCAGAACGGCCATTGGGTTGCTGTTAAACCTATTCCA

GATACTTTCATTGTGAACATTGGAGATCGAATGCAGGTAATTAGCAACAACCGATACAAGAGCGTGCTCC

ACCGGGCTATAGTGAACTGTGACAATGAGCGGATCTCTGTCCCAACGTTTTACTGCCCATCACCTGATGC

TGTTATGGGACCAGCCCCACTGCTGGTGGACGATGATCATCCACCGTTGCATGGGAACTTTACGTATAAT

GAATACTATCACAAGTTTTGGAACTGTGGTCTCACAACAAAGACCTGTTTGGATATGTTCAA

>FM895642.1 FM895642 Jatropha curcas embryo 71-95 (DAF) Jatropha curcas cDNA clone rjcpga0_002596, mRNA sequence

CGAGACAAACAGTGGAATTCAACTCCATTGCATTTTTATTTGATGGGCGGAGACTGCGTGGAGAGCAGAC

TCCTGATGAGCTTGAGATGGAAGATGGTGATGAAATTGATGCAATGGAGTTGAATTCCACTGATTGCCTG

TCACAATAGGCAGAGCGGAAGAAAGGCTTTTCTTTAGCTATAGGAAAGCTAGTGTATATGAAATTGAAAT

GAGTGCAACGAGACTTTAAAAG

>FM895641.1 FM895641 Jatropha curcas embryo 71-95 (DAF) Jatropha curcas cDNA clone rjcpga0_002595, mRNA sequence

GAATATTTTTTCGAAGCATTAATTATTTAATGAACGTTTACTGTTAACAAAAAAGCACTCCTCCATTGTT

ACAATTCAGAGCCATACAAATTTCTGGTCTTAAATTTCACGTTCAATTCCAAATGGGATTTTATACACTA

CCATATATGTTTCAGACTACAAGAATACATGTACAAATTATGAGGCTTGCCAATCTAAGCAAATACCAAT

TCTTGTTGCTAGTTTCCGGAAGCCAAGCAAATTCCAACATAATGGATGGGATGATGTCAGCAATCGTTCG

GATCCTCCTTGCACGCTTTTCTCGATTCAGAAGTAGCTTCAGTACCTTAATCATGAGTTGTAAAGACCAC

TTCTTCTCAAGCTGAATAAATTAGAAAAGGCACAATCAGAAAAATACTTAGCATTAATGCCAGTGTCAGC

TGGACAGAACAACTGAATTGCAAGTGAGCGGGTGCTTGCATTTGCCTTTGGAACCCAACAACCTGACCAA

AGAACTGATTTATCCAATTAAATGGTCCCCTTGCTCGAAGAATATTCTCCAGACCTTTGCTTCCAAAGAG

ACGCTTCA

>FM896628.1 FM896628 Jatropha curcas embryo 71-95 (DAF) Jatropha curcas cDNA clone rjcpga0_003892, mRNA sequence

ATTATTTCTCTTATAATCCACAGCCTTAAAGTAGAGGTACAAGTCTCACTAAAAATTTATCGCTCTCGAA

TCTCTGTGAACTCCATATCCAAAAGCGCAATGGCTCGATTAGTGTTGCTGCTCGCGCTCTGCGTGCTTCC

GGCCATCGTGAGCGCCGCCCGCCCTGCAAGAAATCCATTTGAGGTGGAAGGCCGTGTCTACTGCGACACT

TGCCGTGCTGGTTTCGAAACCCCCAAAACCACCTACATCGCTGGTGCAAAGGTAAGATTGGAATGCAAGG

ACAGGAAATCAATGGAGCTTGTATACAGCAAGGAAGGAACAACAGACTCCACAGGTACCTATAAGATCTA

TGTAGACGATGACCATGCGGATCAGCTCTGTGATGCTGTACTAGTTAGCAGCCCCAAGAAAGATTGCAGA

TAACCATCGTCAGGCCGTGATCGTGCCCGCGTTATCCTC

>FM896627.1 FM896627 Jatropha curcas embryo 71-95 (DAF) Jatropha curcas cDNA clone rjcpga0_003891, mRNA sequence

ATTCCTAAAGCCCAAAAGCATCAATCAGGCCGAATAGGCTGGCCACTAAATGCTCATTATCATTAAGTCA

GAAATTCTACAAACAGATATAATGATCCTTGCATTTGAAGTTTCATCCATCACTTTCAGTTTTAACATGT

AGCAGGAACAACAAATTCAACAAGAAACATTACACAACAAAAAAATTTTAATACAAATCTTCAAGACCCA

TATACAGATCTTTTATTTTGCAAATGAAAATTTTAGTATGCACTGGTAAGAAAGATCAACGATAAAGGCT

CTCTGATCTCAGGTTCTCTGCAATCTCAGTTTCCCATCGGTCACCATTCTCAAGTAGCTTCTCTTTGTCA

TACAAAAGTTCCTCATGAGTCTCTGTAGCAAATTCAAAGTTGGGTCCCTCAACAATTGCATCAACAGGAC

ATGCCTCTTGGCAGAAGCCACAGTAGATGCATTTTGTCATGTCACTATCATACCTAGTTGTCCTCCGGCT

CCCATCTTCACGTTCCTCGGCCT

>FM896626.1 FM896626 Jatropha curcas embryo 71-95 (DAF) Jatropha curcas cDNA clone rjcpga0_003890, mRNA sequence

TGTGAGAAGTTGAAGAAATATGCCACTGTTATTCGTGTTTTGGCCCACACTCAGATCAGGAAAATGAAGG

GATTGAAGCAGAAGAAAGCTCACCTAATGGAGATCCAGGTCAATGGTGGAACTGTTGCTCAGAAGGTGGA

TTATGCCTACGGGTTTTTTGAGAAGCAAATCCCAATTGATGCTGTCTTCCAGAAGGATGAAATGATTGAC

ATCATTGGTGTCACCAAGGGTAAAGGTTATGAAGGTGTTGTCACTCGTTGGGGTGTCACCCGTCTGCCTC

GTAAAACCCACAGGGGTCTCCGCAAGGTGGCTTGTATTGGTGCCTGGCATCCAGCTAGAGTTACATTTAC

TGTTGCCAGGACTGGTCAGAATGGATATCATCACCGTACCGAAATGAACAAGAAAGTATACAAACTTGGC

AAAGCTGGACAGGAGTCCCACACTGCTATAACTGAGTATGACAGGACTGAGAAAGGTATCA

>FM896625.1 FM896625 Jatropha curcas embryo 71-95 (DAF) Jatropha curcas cDNA clone rjcpga0_003888, mRNA sequence

ATTCCTCTACATTAACATATCTTTCTCACACTTATCATCAACAAATGGCTGAACGTTCACAGCCACACCA

AGTTCAAGTGCACCCACAACACCGCTATGAAGCTGCCTTCAAAGGTCAACAAAAAGGTCCATCAGCTCAG

AAAGTGTTAGCTGTGATCACCCTCCTGCCGGTAGGCGGTGGGCTTCTTGCACTAGCTGGTATAACCTTAG

TAGGTACCCTAATCGGCCTAGCTATTACTACCCCTCTTTTTGTCATCTTTAGCCCTGTTCTTGTCCCAGC

TGCATTAGTTATTGGCCTTTCGGTCATGGCATTTTTGGCTTCCGGGGCTATGGGGCTCACAGGGTTGTCA

TCACTCTCGTGGGTTCTGAAATACCTCCAGGAGGTCACTCGGCGAATGCCGGAGCAGCTGGATATAGCTA

AGAAGCGTATGCAAGACATGGCTGGTTTTGTGGGACAGAAGACCAAGGAAGTAGGCCAAGAAATCCAGAG

GAAAGCACATGAAGGGAAAT

>FM896624.1 FM896624 Jatropha curcas embryo 71-95 (DAF) Jatropha curcas cDNA clone rjcpga0_003887, mRNA sequence

ATTCCTATTCTCAAACTTTAAATAGGTAGGACGGCGCGGCTGCTTCGCTGAGCCGCGCCACGGAATCGAG

AGCTCCAAGTGGGCCATTTTTGGTAAGCAGAACTGGCGATGCGGGATGAACCGGAAGCCGGGTTACGGTG

CCCAACTGCGCGCTAACCTAGAACCCACAAAGGGTGTTGGTCGATTAAGACAGCAGGACGGTGGTCATGG

AAGTCGAAATCCGCTAAGGAGTGTGTAACAACTCACCTGCCGAATCAACTAGCCCCGAAAATGGATGGCG

CTTAAGCGCGCGACCTATACCCGGCCGTCGGGGCAAGAGCCAGGCCCCGATGAGTAGGAGGGCGCGGCGG

TCGCTGCAAAACCCGGGGCGCGAGCCCGGGCGGAGCGGCCGTCGGTGCAGATCTTGGTGGTAAGTAGCAA

ATATTCAAATGAGAACTTTGAAGGCCGA

>FM896623.1 FM896623 Jatropha curcas embryo 71-95 (DAF) Jatropha curcas cDNA clone rjcpga0_003886, mRNA sequence

AGTACTAATCGTATCTGCATATCAAATAAACAGAAAGTACGCAATAAGTACTCATTCTCCCACCAAAAAT

TTAAATAAGACATGTCAAATATCTGGACTACTAGATCAAAGCAAAACATACAACCGAAAAGAAATGCAAA

ATTCATCATCAGAACTTCCTTCTTGTCATAACTTCTCTCCACTACTCACTAGAAGCTCAGGACATTCTTT

GTGCGGCCTCCTTTGCCAAGACTCTCTCTTTAGCTTTGGTTTTGGCCTGAGATTTGGAGCCCATAATACC

ACCACCCCATCTCTTACGGTGCTCGTCGAACTTGTCATTAAAGTTAGCCTTGACTGCCTCCAAAATCTTG

CTAAATTCCAATTTATCTTCATTCTTAACAGATGTGAGGCATAAAGCTGCAGCTGTATTCTTGTGGACAA

TCGCTCCAAGACGTGATTTGCCCTTCACAATTGCGTAAGGGACCTCCATTTTCCGGCACAAGGCAGGTAA

CCAAACAACAAGCTCAATAGGATCGACATCATGTGCAATGACCACC

>FM896622.1 FM896622 Jatropha curcas embryo 71-95 (DAF) Jatropha curcas cDNA clone rjcpga0_003885, mRNA sequence

ATTCGTCCAAACGCTCCTCTCTTTTCTCCTTCTCCTCGCCGTCTCTACCTTCGCCGTTTCTGGCTATGAG

GACTGCGTGTATACAATATACATAAGAACCGGATCTATCATAAAAGCAGGAACCGATTCCATTATCAGCG

TCAGGCTATACGACGCCTACGGCGAGTTCATCCAGATCTCCAATCTAGAGGCCTGGGGCGGCCTCATGGG

TCCGGAATACAACTACTTCGAGAGGGGCAATCTCGACATTTTCAGCGGCAGAGGATCTTGCTTGAGCGCT

CCGGTTTGCGCTTTGAACTTGACCTCCGACGGAACCGGCTCGGTTCCAGCTGGTACTGTAACTACGTTGA

GGTCACAACCACCGGAGTTCATGAGTCTTGCTCGCAGCAGCAGTTTACGGTGGAGCAGTGGCTCTCCGAG

AAGGTATCGCCGTATCAGTTGACGGCTACCAGGAATTATTGTCCAGATCTCGCCGATGTTAGAGGCCGTG

ATCGGGTGAAATCTGCTGTACTCTGA

>FM896621.1 FM896621 Jatropha curcas embryo 71-95 (DAF) Jatropha curcas cDNA clone rjcpga0_003884, mRNA sequence

GCATCAATTTACTTTTTATTAATAAATTTATCCATACTTTGAGTACGAACAATTTTTATAATGATTTTCT

TTTGTCCTCTGTATCTATTGATACATTAAAAATTAATGTTCTGTACTTTTTATTTATTCTTGCACTATAA

TATCTAGTGAAGACTGTTCGGGAGGGTGGGAAAGAGTCAATGCTGTCTTGGAGCTGGAGCCGGATCATGT

TGATGGTGGTGATGGTGATGAGCTGGAGCTAGATCTGGACCGTCGTGGTGGTGATGATGAGCTGGAGCTG

GATCTGGACCATGATGGTGGTGGTGATGAGTCGGAGCTGAAGCTGCACCGTGGTGGTGAGAACCATGAAT

TAGAGTTGGCGGAAAGTACAACGGGGGGGGCC

>FM896620.1 FM896620 Jatropha curcas embryo 71-95 (DAF) Jatropha curcas cDNA clone rjcpga0_003883, mRNA sequence

CTCCAACAATTGATAATGATCTTTTTATGAGGATGTTAGTGCAGCCTGTGAGTTCACGAATCAACAAAGG

TGAAAAAACAATTAGGATTTCTATTGTGACATTATATTTGGGAAATGCAGATACTGTTGTGAGCTTACTG

AGTAAAGAATTGCCTGAATTAAGGTTAAAGAAAGAGAATTGCAGTGAAATGAGTTGGATTGATTCTGTTC

TTTGGTGGGCTAATTTTGACAATGGTACTTCTCCTACTGCTCTTCTTGACAGAGATCTCAATTCTGCAAA

TTTCCTGAAAAGAAAATCAGATTATGTGCAGAAACCAATTTCAAAAAATGGTTTTAATTTGATTTGTAAG

AAAATGATTGAATTGGGTAAAACTGGATTGGTTTTCAATCCTTACGGAGGCAAAATGGCTGAAATTCCAG

CCACTGATGCTCCTTTCCCTCACCGGGCCGGAAATTTGTTTAAAATTCAGTATTCAGTGAATTGGGAAGA

AGCTGGAG

>FM896619.1 FM896619 Jatropha curcas embryo 71-95 (DAF) Jatropha curcas cDNA clone rjcpga0_003882, mRNA sequence

GCAAAGGATTCTATCCGTCGCCCGATGGGAATTGTACTTTAAGGCGGCCCTCGCGGCTCGTCCGCCGCGG

GGGCTTAGCCAACGGCACGTGCCTCTGGGGGCCGGATGGCCCCTACTGCGGGTCGGCAAACGGGCGACGG

ACACGCGCGTCGCTTCTGGCCCGGATTCTGACTTAGAGGCGTTCAGTCATAATCCAGCGCACGGTAGCTT

CGCGCCACTGGCTTTTCAACCAAGCGCGATGACCAATTGTGCGAATCAACGGTTCCTCTCGTACTAGGTT

GAATTACCATCGCGACACCATCATCAGTAGGGTAAAACTAACCTGTCTCACGACGGTCTAAACCCAGCTC

ACGTTCCCTATTGGTGGGTGAACAATCCAACACTTGGTGAATTCTGCTTCACAATGATAGGAAGAGCCGA

CATCGAAGGATCAAAAAGCAACGTC

>FM896618.1 FM896618 Jatropha curcas embryo 71-95 (DAF) Jatropha curcas cDNA clone rjcpga0_003881, mRNA sequence

AATAAATTACAAAAAATTGCCCATTACATCAACAATAGTTTCAGAGCATACGTGTGATACAATACATTGT

CTTTGCACTACAAGTTTCAAAATACAAAGATTTTCTCCGCCCATGATGAACTATAACAATTTTTTTTGAC

ATACAAGGCAACCCTTCTTCCTCCAAGTTCCTTCTCCCACCAATAATAGAAAATTTAAAATTATTAACAC

ATCCAAGCACAATACATCACAGTAGAGGTCATACACATTGCATTCATGATCTCACGTCTTTCAGTACTCC

TCCCTTCTCAAGCTCACCAACTAGATCCCTCAATGAAGGAACTTTCATAGCTAAAGTTCTATACAGTCTA

GAATATCGAGCTCGGGTGCCATCTGCTGTTCTTGCTAAGGCTAGCAAGCTTAGAGCCTCAGGAAGTTGAG

AGAATACTTGTTTCATTTCCTCCAAGCTCAAGTTTTCTAATACGGGTGGTCTTGGAACAACTCTTACTAT

TTCACCGACTTTAGGTTCCTGAACTCGAGCATCTGCTTTAATGACCAA

>FM896617.1 FM896617 Jatropha curcas embryo 71-95 (DAF) Jatropha curcas cDNA clone rjcpga0_003880, mRNA sequence

ATTCCAAAGGATTCTATCCGTCGCCCGATGGGAATTGTACTTTAAGGCGGCCCTCGCGGCTCGTCCGCCG

CGGGGGCTTAGCCAACGGCACGTGCCTCTGGGGGCCGGATGGCCCCTACTGCGGGTCGGCAAACGGGCGA

CGGACACGCGCGTCGCTTCTGGCCCGGATTCTGACTTAGAGGCGTTCAGTCATAATCCAGCGCACGGTAG

CTTCGCGCCCCTGGCTTTTCAACCAAGCGCGATGACCAATTGTGCGAATCAACGGTTCCTCTCGTACTAG

GTTGAATTACCATCGCGACACCATCATCAGTAGGGGTAAACTAACCTGTCTCACGACGGTCTAAACCCAG

CTCACGTTCCCTATTGGTGGGTGAACAATCCCAACACTTGGTGAATTCTGCTTCACAATGATAGGAAGAG

CCGACATCGAAGGAATCAAAAGCAACGTTCGCTATAACGCTTGGCTGCCACAAGCCAGTTATCCCTGTGG

TAACTTTTCCTTGAACACCTCTAACTTCAAATTCC

>FM896616.1 FM896616 Jatropha curcas embryo 71-95 (DAF) Jatropha curcas cDNA clone rjcpga0_003879, mRNA sequence

GGATCTATCCGTCGCCCGGTGGGAATTGTACTNTAAGGCGGCCCTCGCGGCTCGTCCGCCGCGGGGGCTT

AGCCAACGGCACGTGCCTCTGGGGGCCGGATGGCCCCTACTGCGGGTCGGCAAACGGGCGACGGACACGC

GCGTCGCTTCTGGCCCGGATTCTGACTTAGAGGCGTTCAGTCATAATCCAGCGCACGGTAGCTTCGCGCC

ACTGGCTTTTCAACCAAGCGCGATGACCAATTGTGCGAATCAACGGTTCCTCTCGTACTAGGTTGAATTA

CCATCGCGACACCATCATCAGTAGGGTAAAACTAACCTGTCTCACGACGGTCTAAACCCAGCTCACGTTC

CCTATTGGTGGGTGAACAATCCAACACTTGGTGAATTCTGCTTCACAATGATAAGAAAAAGC

>FM896615.1 FM896615 Jatropha curcas embryo 71-95 (DAF) Jatropha curcas cDNA clone rjcpga0_003877, mRNA sequence

GTGGATCCCCCGGGCTGCAGGGTAAACCCGCAAGGCGCAAGGAAGCTGACTGGCGGGATCCCCCCGAGGG

TTGCACCGCCGACCGACCTTGATCTTCTGAGAAGGGTTCGAGTGAGAGCATGCCTGTCGGGACCCGAAAG

ATGGTGAACTATGCCTGAGCGGGGCGAAGCCAGAGGAAACTCTGGTGGAGGCCCGCAGCGATACTGACGT

GCAAATCGTTCGTCTGACTTGGGTATAGGGGCGAAAGACTAATCGAACCGTCTAGTAGCTGGTTCCCTCC

GAAGTTTCCCTCAGGATAGCTGGAGCT

>FM896614.1 FM896614 Jatropha curcas embryo 71-95 (DAF) Jatropha curcas cDNA clone rjcpga0_003875, mRNA sequence

ATTCAATCCAGCTAAAATTCCCGCCTTTTCAAGGGAGTGCCTGAGGCAGAATGTGGCAGTTGTGACAGTG

GCTTTTCCAGCAACACCACTATTGTTGGCTAGGGCACGCATATGCATATCTGCTTCTCATACCAAGGAAG

ACCTGATTAAAGCATTGGAAGTTATCAGCCAAGTTGGTGATCTTGTGGGAATAAAGTACTTCCCTGCTGA

ACCAAACAAGCACGAGGAAGATAATAAGATGGTGAAGCTGGACTGATCGACTTTCCTATTTTTCTTTAAC

TAATCAATCGTTGCAAGCAGCAGAACAGGAGAAAAGATTATTGAAGCTGAACCAAGTATGTTGCATTATA

TTCTTTTTTCCTTAAACAATTGGCAGCTGCCCTTTAGCATCACCAGTTGTGTTACAAGCCCTGCTATGTA

GTCTGTTGTATTTGCAGTGTAAGGCTTTATCATGCTTTTAAGTGGATCTGAGTTTTGCCTCTATGCTCAT

TGCTCATGCAAACACGAGCTACCGCTGAGTGCTGCTTGATTTCTTAATTTTTGGTTTATCCTTTGATTAT

TTTTTTTTTCCATGTTTTCTGATTATGTGTATATATGTAGCTGTACCCTATATTTTGTTCTGTTTGAAAT

AGTACTT

>FM896613.1 FM896613 Jatropha curcas embryo 71-95 (DAF) Jatropha curcas cDNA clone rjcpga0_003874, mRNA sequence

CGAATTCCTTCTTAGTGGGGTCCCACCCTTTTGGGGAGAGACTGAGCAGGCTATCTTTGACTCTGTTCTT

CGGGGACATATCAATTTCTCTTCTGACCCATGGCCTTCCATATCCAGCAGCGCTAAAGATCTTGTGAAGA

AGATGCTAAGAACTGATCCCAAGGATCGACTTTCTGCAGCTGAAGTCCTAAATCATCCATGGATGCGAGA

AGATGGTGATGCATCTGATAAGCCTCTTGATATTGCTGTTTTAACTAGAATGAAACAATCCAGAGCCATG

AATAAACTTAAAAAATTGGCTCTAAAGGTTATTGCTGAAAATCTTTCTGA

>FM896612.1 FM896612 Jatropha curcas embryo 71-95 (DAF) Jatropha curcas cDNA clone rjcpga0_003873, mRNA sequence

ATTCGGATTCTATCCGTCGCCCGATGGGAATTGTACTTTAAGGCGGCCCTCGCGGCTCGTCCGCCGCGGG

GGCTTAGCCAACGGCACGTGCCTCTGGGGGCCGGATGGCCCCTACTGCGGGTCGACAAACGGGCGACGGA

CACGCGCGTCGCTTCTGGCCCGGATTCTGACTTAGAGGCGTTCAGTCATAATCCAGCGCACGGTAGCTTC

GCGCCACTGGCTTTTCAACCAAGCGCGATGACCAATTGTGCGAATCAACGGTTCCTCTCGTACTAGGTTG

AATTACCATCGCGACACCATCATCAGTAGGGTAAAACTAACCTGTCTCACGACGGTCTAAACCCAGCTCA

CGTTCCCTATTGGTGGGTGAACAATCCAACACTTGGTGAATTCTGCTTCACAATGATAGGAAGAGCCGAC

ATCGAAGGATCAAAAAGCAAACGTCGCTATGAACGCTTGGCTGCCACAAGCCAGTTATCCCTGTGGTAAC

TTTTCTGACACCTCTAGCTT

>FM896611.1 FM896611 Jatropha curcas embryo 71-95 (DAF) Jatropha curcas cDNA clone rjcpga0_003872, mRNA sequence

GCCGAAAACTTCCCTAATCAACACAGGTAAAATATGAATGGATTTTACACATTGGAGAATCAATAAAATT

TGACAAAATCCCTCTCCCCACCATCTCACATTAAAATCAAATTAGAATAAGAAGAAAGCTACAATAATAC

ACGGTAGACAATGGTGTTGCACACACTTGGCCGGGACACGTGAACCAGGAAAACTGACTTCCAAGCCTAA

AGGGATAAAGACTGCACGCACCACTTGCTGTTGTGTTTGTAATTGTTTCTTGAGCCTTCTATGGCCATAA

AAGATCCCAGATAANAAGAAGGAAAAAAAGCATCAGACAATACTAGGAGGATCGCCTACCCCATTGCAAA

CAGCGCGGGGCCAACGTTGTCCTTATGCAATCCACCAAAGGTGCATGTTGAGCAGGTTC

>FM896610.1 FM896610 Jatropha curcas embryo 71-95 (DAF) Jatropha curcas cDNA clone rjcpga0_003871, mRNA sequence

ATTCCTTTCTCTTACTATGATCAGGTGCTTGACACCACAGCATTGCTTGGGGCTGTTCCACCCAGGTATG

GCTGGAACGGTGGTGAGATTGGATTCGATGTGTACTTCTCCATGGCCAGAGGAAATGCCTCTGTCCCTGC

TATGGAAATGACCAAGTGGTTCGACACCAACTACCATTTCATTGTCCCTGAATTGGGACCTGATGTCAAA

TTCTCTTATGCTTCTCACAAGGCAGTGACTGAATACAAGGAGGCCAAGGCGCTCGGAATAGATACAGTTC

CTGTCCTTGTTGGCCCTGTCTCTTACTTGTTACTTTCTAAACCTGCTAAGGGTGTGGAGAAAACCTTTTC

TCTTCTCTCCCTTCTTGGAAAGATCCTTCCAGTCTACAAGGAGGTTATTTCAGAGCTTAAAGAAGCTGGT

GCATCCTGGATTCAGTTTGACGAGCCTACGCTTGTTATGGACCTTGATTCTCACAAATTGCAAGCATTTA

CTGATGCCTACTCTGAGCTTGAGTTCACTCTATCCGGCTTGAATGTTGTGGTCGAGAACTACTTTGCTGA

TGTACCTGCTGAGGCATTCAAGACCCTCA

>FM896609.1 FM896609 Jatropha curcas embryo 71-95 (DAF) Jatropha curcas cDNA clone rjcpga0_003867, mRNA sequence

CTGGAAATCAGAATCAAACGAGCTTTTACCCTTTTGTTCCACACGAGATTTCTGTTCTCGTTGAGCTCAT

CTTAGGACACCTGCGTTATCTTTTAACAGATGTGCCGCCCCAGCCAAACTCCCCACCTGACAATGTCTTC

CGCCCGGATCGGCCGCCGAAACGACCTTGGGTCCAAAAAGAGGGGCATAGCCCCGCCTCCGATTCACGGA

ATAAGTAAAATAACGTTAAAAGTAGTGGTATTTCACCGTCGCCTTTCGGCTCCCACTTATCNCTACACCT

CTCAAGTCATTTCACAAAGTCGGACTAGAGTCAAGCTCAACAGGGTCTTCTTTCCC

>FM896608.1 FM896608 Jatropha curcas embryo 71-95 (DAF) Jatropha curcas cDNA clone rjcpga0_003866, mRNA sequence

GAATGGTGCTAATCAGAAGTTACTGTAATCAACACACAGACATTTTTTCTTCGCGTAAGAGTAAAAAGGA

GAACAAGACTTAGTTGAAACAGATGGCACTAGTTTCAGGAGGAAGATCAACTCTCAACCCAGATGCCCCA

CTCTTTATCCCTGCTGCATACCAGCAAGTAGAGGATTTCTCCCCAGAATGGTGGCAACTGGTTACAACTT

CAACATGGTATCGAGATTACTGGCTCAGTCAGCATCAAGATGAGGAAGGCTTCTATGACAATGCTGAGGA

TGACAGCTATGATGCCTGTGATGTAGCTGACTTACTTCCAGATACTTTTGATCTTGATGCTGGCGAAGAT

TTCTCCACTCTGGAAGTTCAATTTCAGGAGTTTGTGGAATCTTATCAAACTGATGTAGATAGCAGATCAC

CTCCTTCCAATGGCTTTCAAATTGAAGCTGAGGCACCCAAGAGGGATGAAACAGCTCCAGCAGCCAAGTA

TATTTAATACAAAGTGCGGCCCGCAGTATTTGCTGGAAGGGCTAACTCTCTCAAGTTGCAGTTATCCCCT

C

>FM896607.1 FM896607 Jatropha curcas embryo 71-95 (DAF) Jatropha curcas cDNA clone rjcpga0_003865, mRNA sequence

TGGTAGAAATAATCCATCCAATTAGCATAGGCTCAGCCATACAACTACTGAAATCTAACTTTAGTTAATA

TATATTTATCATCCCTCAATTAACAAGCATACATCACAATCTCTCATATTCAAATGAAAAATACACCAGA

CATTACAAAAAAGCAGCAAGCACTCCATAAACAAGTGGCTTGATCACACACCTTATATCCAGAGAAATTC

ACTGGGTACATCTGTCTTTAGAGTCAAAACATTCTCGAACTCATTCAGATGCCGATTTGTATTGAGTTTC

TTGTTCCCTTTGTCTTCTTTGTTCACGAGGAGGGCCATCTTTTCGTCTTTCATATCTTTTGCTTACATAT

TTAGAATCTCTCTGTTGCTTTGGTTGATAAGTAGGATACTTGCAAGGAATTATCTTCCCATTAATATATT

TATCACCTCCATAGTCCTTATAATTCACATCTATGTAAGAATCTGGCAAGCACCCAGAGAAACCCAGGCA

ATCCCTTGAATTTCT

>FM896606.1 FM896606 Jatropha curcas embryo 71-95 (DAF) Jatropha curcas cDNA clone rjcpga0_003864, mRNA sequence

TCTGATGTAACAACATTTTATTTCATGATTATGAATACAGCATGGTAACAGTTATACATATATCATGACA

TAAATGACTCAAATCTTCCACCTAATTTTACTTCTATCAGATGTCTATCCTAATTCCTTCAGATATGGTT

TCTCCCCCATTTTCCATTCAGTGAGATTCAGAAAATTGGACCAGCTGCCAGAATCTCCTCAGTGAGCTTG

TTGTCCTTTCCAATCTTGTGATCTGTGAATGTTACGAAGTTATTCTTGAAGAGACCAGCCAGTTTCAGCA

GTGTATCCTTGTAAGCTTTCTTGTCTGACAATGTGTTCACAGGATCCAGTATATCAGAAGGCACTCCTTC

CACCTCAGTGGGGATCTCAAGCCCACATATCTCAGTATTTATGTATTTTGCTTTACAGAGGCTGCCACAA

TGTATGGCATCAATAATTTTCCTTGTGTATGCTAACTAGATTCGG

>FM896605.1 FM896605 Jatropha curcas embryo 71-95 (DAF) Jatropha curcas cDNA clone rjcpga0_003862, mRNA sequence

CTGCAAAGGATTCTATCCGTCGCCCGATGGGAATTGTACTTTAAGGCGGCCCTCGCGGCTCGTCCGCCGC

GGGGGCTTGGCCAACGGCACGTGCCTCTGGGGGCCGGATGGCCCCTACTGCGGGTCGGCAAACGGGCGAC

GGACACGCGCGTCGCTTCTGGCCCGGATTCTGACTTAGAGGCGTTCAGTCATAATCCAGCGCACGGTAGC

TTCGCGCCACTGGCTTTTCAACCAAGCGCGATGACCAATTGTGGGAATCAACGGTTCCTCTCGTACTAGG

TTGAATTACCATCGCGACACCATCATCAGTAGGGTAAAACTAACCTGTCTCACGACGGTCTAATCCCAGC

TCACGTTCCCTATTGGTGGGTGAACAATCCAACACTTGGTGAATTCTGCTTCACAATGATAGGAAGAGCC

GACATCGAAGGATCAAAAAGCAACGTCGCTATGAACGCTTGGCTGCCACAAGCCAGTTATCCCTGTGGTA

ACTTTTCTGACACCTCTAGCTT

>FM896604.1 FM896604 Jatropha curcas embryo 71-95 (DAF) Jatropha curcas cDNA clone rjcpga0_003861, mRNA sequence

TGCAAGCTTGGCAAGAAGAAAGGCTTACCTTCTCACTAGAAATTTCTCGAATTCCAGTCCGGATGCCCTC

AGGTACTCCTCACTCACCAGCTTCTTCCGCGGCTTCGCCTCTTCGGGATCTGACGAGAACGACGTAGTCG

TAATCGGCGGCGGCCCCGGTGGCTATGTCGCCGCTATCAAGGCCGCACAGCTCGGACTCCAAACCACTTG

CATCGAGAAACGTGGAACTCTCGGTGGCACCTGCCTCAATGTTGGATGCATCCCTTCTAAGGCACTTCTT

CATTCATCCCACATGTTCCATGAAGCTCAGCATTCATTAGCAAGTCATGGTGTGAAGTTTTCATCTGTCG

AAGTCGATTTGCCTGCCATGATGGGCCCA

>FM896603.1 FM896603 Jatropha curcas embryo 71-95 (DAF) Jatropha curcas cDNA clone rjcpga0_003860, mRNA sequence

GTCCCTCTGCAGCTTTCTCTGTCTAAAAGTCACTCGTGGAGACTTGAGAGAGCCATGGCCATTGGCCAGC

CATTTCACTAAACTTTTTCCACAAACGAACTAAGCGTACTGTCTCTAATACTTCACAATACGGTGCGTGC

AGTTAGGAGGAGAGAGAGATCTGGTTTCGGTTTTCCGGCGAAATGGAGGACGAGAAAGGAGCGATAGTGC

AGAGTTTGATTGACACAGTGCACGATATAGCTTCGATCTCTGATTATAGGTGTACGGTCAAGAAACAGTA

TTGCAATTTGGCTAGGAGATTGAAGCTTTTAACGCCAATGTTCGAGGAGATTAGAGAAAGTAAGGAGCCG

ATCCCTGATGAGTCTATGAAAGCTTTAGTTTTGTTGAAAGAAGCTTTGGATTTAGCTAAGGAGTTGCTTA

GATTTGGTAGCGAAGGCAGCAAGATCTACCTGGTGTGTATGTGTTTTCTTTCTTTAGATTTTTAGTGCTT

CTGTTTGGATTTGGATCTGTTTGGCTGCTGAG

>FM896602.1 FM896602 Jatropha curcas embryo 71-95 (DAF) Jatropha curcas cDNA clone rjcpga0_003859, mRNA sequence

ATTCCTCCAAGGCGCAGATCCGAGGAGTTTGGGATTAGAAACCCTAATTTCCTCTCTCTACTTTCTAAAA

GCCTCCAATCGCTCTATTTCTCTATCAATCTCTCTCTCTCACTAGCAGTCATGGCAGGTTTGGCACCAGA

AGGATCACAGTTTGATGCTAAGCAATATGACGCCAAAATGAATGAATTGCTCTCAAGTGATGGACAAGAT

TTCTTCACCACATATGATGAGGTTTATGACAGTTTTGATGCAATGGGATTGCAAGAGAACCTTCTGAGAG

GCATCTATGCTTATGGTTTTGAGAAGCCTTCTGCTATCCAACAAAGGGGAATTGTTCCATTTTGTAAGGG

TCTTGATGTTATTCAGCAGGCCCAATCTGGAACTGGAAAAACGGCAACATTTTGCTCCGGCATTCTGCAG

CAACTTGATTATGCTACAGTTCAATGTCAAGCGTAGGTGTAGGCTCCGACTAGAGAACTGGCACAGCAGA

TTGAGAAAGT

>FM896601.1 FM896601 Jatropha curcas embryo 71-95 (DAF) Jatropha curcas cDNA clone rjcpga0_003858, mRNA sequence

ATTCCAAAGGATTCTATCCGTCGCCCGATGGGAATTGTACTTTAAGGCGGCCCTCGCGGCTCGTCCGCCG

CGGGGGCTTGGCCAACGGCACGTGCCTCTGGGGGCCGGATGGCCCCTACTGCGGGTCGGCAAACGGGCGA

CGGACACGCGCGTCGCTTCTGGCCCGGATTCTGACTTAGAGGCGTTCAGTCATAATCCAGCGCACGGTAG

CTTCGCGCCACTGGCTTTTCAACCAAGCGCGATGACCAATTGTGCGAATCAACGGTTCCTCTCGTACTAG

GTTGAATTACCATCGCGACACCATCATCAGTAGGGTAAAACTAACCTGTCTCACGACGGTCTAAACCCAG

CTCACGTTCCCTATTGGTGGGTGAACAATCCAACACTTGGTGAATTCTGCTTCACAATGATAGGAAGAGC

CGACATCGAAGGATCAAAAAGCAACGTCGCTATGAACGCTTGGCTGCCACAAGCCAGTTATCCCTGTGGT

AAC

>FM896600.1 FM896600 Jatropha curcas embryo 71-95 (DAF) Jatropha curcas cDNA clone rjcpga0_003857, mRNA sequence

CTAAAATGCCGCCCAAATTTGACCCTTCTCAGGTCGTAGATGTATTCGTTCGGGTCACCGGTGGTGAAGT

AGGTGCGGCCAGTTCTCTTGCTCCGAAAATCGGGCCTCTAGGTCTTTCCCCTAAAAAGATCGGAGAAGAC

ATTGCTAAGGAGACCGCCAAGGATTGGAAGGGACTTCGAGTTACTGTCAAGCTTACGGTGCAGAATCGTC

AGGCCAAAGTAACGGTGGTGCCATCGGCGGCCGCTTTGGTGATCAAGGCACTCAAGGAACCGGAGAGAGA

CAGGAAAAAGACTAAGAATATCAAGCACAGTGGCAACATCTCGCTCGATGATGTTATCGAGATCGCGAGG

GTGATGAGACCAAGATCCATGGCGAAGGATCTGAGCGGAACTGTTAAGGAGATATTGGGGACTTGCGTTT

CGGTTGGATGTACTGTCGATGGAAAAGACCCTAAGGATTTGCAGCAAGAGATTACTGATGGTGATGTTGA

GGTGCCGCTTGATTGAGATCGCAAGCTTTAGTGAGCTATTTCCTTTTACTTTTTCTTCTACTTCTT

>FM896599.1 FM896599 Jatropha curcas embryo 71-95 (DAF) Jatropha curcas cDNA clone rjcpga0_003855, mRNA sequence

GATCCCCCGGGCTGCAGGGGCCGGGTCGTGCCTCCGGCGCTGTTACTTTGAAGAAATTAGAGTGCTCAAA

GCAAGCCTACGCTCTGTATACATTAGCATGGGATAACATCATAGGATTTCGGTCCTATTGTGTTGGCCTT

CGGGATCGGAGTAATGATTAACAGGGACAGTCGGGGGCATTCGTATTTCATAGTCAGAGGTGAAATTCTT

GGATTTATGAAAGACGAACAACTGCGAAAGCATTTGCCAAGGATGTTTTCATTAATCAAGAACGAAAGTT

GGGGGCT

>FM896598.1 FM896598 Jatropha curcas embryo 71-95 (DAF) Jatropha curcas cDNA clone rjcpga0_003851, mRNA sequence

GTGAAAGTTTCCCTTCATACGGCTCGAATCATTCTCAGATGCCCGGAGAGGCATCCTTTCCTTGTTTGTT

GGGTTGGTTCACGCGTCACGCGCGCGCAAACGAGCACCAGCCGCAACAGCAGGGAACTATGACCAATAGA

GTCAGACACTTAGCTACATCCGCACGCAAAAGGAATGTGGTTCTGGTTGATGCTTTCTTTCCTTTATTAG

TAAAGGGGGCGCGGGACGTTCGCGGAGTCCGTGCCTTCCGTACCACCACAAGACTGGTCGTTCTTGGGCG

AATCCCGCCCCTAAACAAAACATAAGGGATAGGAGGAAGGTGGCCGGGCCTATTATATAAAATATATCAC

AAGGGGTGTAGAAAGAGAACCCGGCCACCTATTTCATTCGACGTTCGGAGGCAGGCGCCCGCCCGATTCG

ACCTACTTTTGGTTTGGA

>FM896597.1 FM896597 Jatropha curcas embryo 71-95 (DAF) Jatropha curcas cDNA clone rjcpga0_003850, mRNA sequence

AGGATTCTATCCGTCGCCCGATGGGAATTGTACTTTAAGGCGGCCCTCGCGGCTCGTCCGCCGCGGGGGC

TTGGCCAACGGCACGTGCCTCTGGGGGCCGGATGGCCCCTACTGCGGGTCGGCAAACGGGCGACGGACAC

GCGCGTCGCTTCTGGCCCGGATTCTGACTTAGAGGCGTTCAGTCATAATCCAGCGCACGGTAGCTTCGCG

CCACTGGCTTTTCAACCAAGCGCGATGACCAATTGTGGGAATCAACGGTTCCTCTCGTACTAGGTTGAAT

TACCATCGCGACACCATCATCAGTAGGGTAAAACTAACCTGTCTCACGACGGTCTAAACCCAGCTCACGT

TCCCTATTGGTGGGTGAACAATCCAACACTTGGTGAATTCTGCTTCACAATGATAGGAAGAGCCGACATC

GAAGGATCAAAAAGCAACGTCGCTATGAACGCTTGGCTGCCACAAGCCAGTTATCCCTGTGGTAACTTTT

CTGACACCTCTAGCTTCAGATTCCGAAGGTCTAAAG

>FM896596.1 FM896596 Jatropha curcas embryo 71-95 (DAF) Jatropha curcas cDNA clone rjcpga0_003849, mRNA sequence

TTTTTTTATTCCAAACACTATGTAAAAGTAAAATTAATAATTTTCAATTAATACTATTTTTCCTCGTAGG

AGGCAGCTGAACTGAAGTTCATTATCAGAGGAGAGTAATGGAGGCATACTCTTCTAAGATCAACTAATAC

TAGCACAACACAGCACAACAAGCCTGTACTAAAAGAACTCGAAGTCCAGATATTTACCACCAGACTTCAT

GTCTGTCCCCACCATCCTTTTACTGCTGCTGCCCACCCCAATTGGATCAACTGCCCCAAATTTCCCAGGC

AGCTCAGTCTCCATTGGTCTCGGTACTTCTGGAGGTGTGCTGCATCTTATTAGTGCCCAATTTACACCTT

CAAAGAATGGATGTTGCTTGATCTCAGTTGCACCCCTCTTCACTCCCAACCTGTGCTGTGGTTCCTTCAC

CAGCAAGCCTCGGATCAAGTCCCGGCTTGCATAGCTGGTTGCTGGAGAATCTGGGAATCTAAGCTGTTGT

CCCACCACATTAAACAGTGTAGCTCGATTTCCTGTGCCTTTGAATGGAGTTTACCGTACAGCAATCATGC

AA

>FM896595.1 FM896595 Jatropha curcas embryo 71-95 (DAF) Jatropha curcas cDNA clone rjcpga0_003848, mRNA sequence

CGAATTCAAGGATTCTATCCGTCGCCCGATGGGAATTGTACTTTAANGGCGGCCCTCGCGGCTCGTCCGC

CGCGGGGGCTTAGCCAACGGCACGTGCCTCTGGGGGCCGGATGGCCCCTACTGCGGGTCGACAAACGGGC

GACGGACACGCGCGTCGCTTCTGGCCCGGATTCTGACTTAGAGGCGTTCAGTCATAATCCAGCGCACGGT

AGCTTCGCGCCACTGGCTTTTCAACCAAGCGCGATGACCAATTGTGCGAATCAACGGTTCCTCTCGTACT

AGGTTGAATTACCATCGCGACACCATCATCAGTAGGGTAAAACTAACCTGTCTCACGACGGTCTAAACCC

AGCTCACGTTCCCTATTGGTGGGTGAACAATCCAACACTTGGTGAATTCTGCTTCACAATGATAGGAAGA

GCCGACATCGAAGGATCAAAAAGCAACGTCGCTATGAACGCTTGGCTGCCACAAGCCAGTTATCCCTGTG

GTAACTTTTCTGACACCTCTAGCTTCAAATTCCGAAGGTCTAAAGGATCGATAGGCCACGCTTTCACGGT

TCGTAT

>FM896594.1 FM896594 Jatropha curcas embryo 71-95 (DAF) Jatropha curcas cDNA clone rjcpga0_003847, mRNA sequence

ATTCCCAGTAGTCATATGCTTGTCTCAAGATTAAGCCATGCATGTGTAGTATGAACTAATTCAGACTGTG

AAACTGCGAATGGCTCATTAAATCAGTTATAGTTTGTTTGATGGTACCTGCTACTCGGATAACCGTAGTA

ATTCTAGAGCTAATACGTGCACCAAACCCCGACTTCTGGAAGGGATGCATTTATTAGATAAAAGGTCGAC

GCGGGCTCTGCCCGTTGCTCTGATGATTCATGATAACTCGACGGATCGCACGGCCATCGTGCCGGCGACG

CATCATTCAAATTTCTGCCCTATCAACTTTCGATGGTAGGATAGAGGCCTACCATGGTGGTGACGGGTGA

CGGAGAATTAGGGTTCGATTCCGGAGAGGGAGCCTGAGAAACGGCTACCACATCCAAGGAAGGCAGCAGG

CGCGCAAATTACCCAATCCTGACACGGGGAGGTAGTGACAATAAATAACAATACCGGGCTCAATGAGTCT

GGTAATTGGAATGAGTACAATCTAAATCCCTTAACGAGGATCCATTGGAGGGCAAGTCTGGTGCCAGCAG

CCGCGGTA

>FM896593.1 FM896593 Jatropha curcas embryo 71-95 (DAF) Jatropha curcas cDNA clone rjcpga0_003845, mRNA sequence

ATTCGAAAAGATTCAAATGGGTACCGGTTTACATTAAATCTAAAGAGATGCAATCCACATTTGCATATAA

ACAAACATAAAATTCTCACTTGCGACAAATTCTAGCAGTAGGCATTGTGACACAGACAGGACTGTACTTC

TTCTTAGCTTCTGGAGTCCCTGCCTTAGGCTCATCGTCAGCCATGGCAATTTTGGCGACGGAGAATGCAG

CAGTGGCTGCAGCTACAAATACCAAATTCCTCCTCCCATTGCTGCTCTCTTCCCTGTTCTTCATCTCCAC

ACTGACCTTGTCTCCATCTGCGGGCGTTGAGGCCTTGGCTACGATGAGCCCTCTGCGTGGAGTTGTTAAG

GGCTGTTTGGCCATGGTTGAACCGGTTGCTAGGAAGGATGCTGTCATAGTCATGGATGCCATCTCTGGAA

ATTCTGCTTACCTCTCTCTCTCTCTCTCTCACTCTGTTGAGGGAGTTTTAGTCTCAGGGGGGGCCCGGTA

C

>FM896592.1 FM896592 Jatropha curcas embryo 71-95 (DAF) Jatropha curcas cDNA clone rjcpga0_003844, mRNA sequence

AATTCAAACGAACATACATGGATAGGAGTAAAGAAGTAGGCGGAGGAGGAGGAGGAGGCCAGGGGAAGGC

GAGAAAGCTAGAAAAAATCAAAATCAAAGCGAAGCTAGAGGCGGAGTTGCTCCCAAAGGAAAATCATGCA

TAGGATATCTCTATCACTCTTCCTCTCTTAAATCCAATGGCATCAATCCTCGTTGCATCGGCATCCCTCG

TACTCTTCAACAAGTTCCCAGTAACCTTGTTGGAGTATCTAAGGCTGAAGCTTCTAAAAAGGGCGGGATT

CTTACGGATATGTATTATGGTTGTGCTGGGTACTCTATCTATGTCACTAATGACCACTCTACAGATAAGC

TACTGACAAAGCCAGCATGTTCGGGCCTTGAGTAGATAGTGGAAGGAGTTGCTAATGCTGATAGTGCATC

TGCTCCGGCTCATGTTCATAGTAAAGAAGATGGCAGAGAATTTCCTCAGCCTCGAAACAATAAACCAGGG

CATTCTGCAGGAGATGATTTTCTGAGCAGGTATACAAGGAATGCCAGCTTGGTTGCATCAGGAGTGGCGA

GA

>FM896591.1 FM896591 Jatropha curcas embryo 71-95 (DAF) Jatropha curcas cDNA clone rjcpga0_003843, mRNA sequence

TTGCTGAGAATGAGCTGGGTTAAATGCAAGCATCTCATTCTCAGCAAACCAGTTATCAATATTCCTGTTC

AACACCAACCGTCCAATTGGCTGTAGAGGGAAAATGTCTTCTGGCCAGATCTTAGTCATATCAAGTGGAT

CGAAGTCGAATCTGTCCTCATCAGCAGGATCCATTGTCTGAATATGAAGTATCCATTCAGGATAGTTACC

AGCAGCAATTGAATCGTATAAATCTTGTGTAGCGTGACTGTGGTTAGCTCCTCCAATTTTCATTGCTTCA

TCATCCGTCAAACACTTGACGCCGCAAGTGGGTTTCCAGTGAAATTTGACATAGGTCACTTTGCCCGCTT

TGTTCNAGAAACAGAAAGTGTGAACTCCAAAGCCTTCCATGTGTCTGTAATCTTGTGGAATGCCAACATC

GTCAAAGAACCCAGCGAACCGTGCTCAAACTTTCAGGGTGGTGTGATAAGAAATCGAAGATCCTCCAGTA

CTCCTGGATATGAGACTTTGGGTTTTGGCTTGAATGCCTTGGATCA

>FM896590.1 FM896590 Jatropha curcas embryo 71-95 (DAF) Jatropha curcas cDNA clone rjcpga0_003842, mRNA sequence

CTCCATTTATAGGCACTTAAATTATTGGTTCCAAAACTAACTCAGAACAATGGAAAAACATTCAACAATA

CCTTAAGAAATACAAATTGCAATTAAAAGTTATAACTTTTCTTTANTTCATTGTTTCTGCTCAAGATAGC

AGGTGAGGAATTGGCGTGTGTTGTATAGTTTCATTATCATCTGAAATTTACTCCTCCGTGGATCCAGAAG

AGTGATGAGGGAAATTTGACCACACATCAGCCCATGTCCTATGCCCATCCGTCACTGACTTCACAACTGA

TGCGGCTTCTTCCACATTAACTCGGATTTGATCCTTGCTATCCCTCTCTCTGATTGTCACAGACGATGTT

GAATCAACAGTTATTGCAAAGGGTACACCCAATTCATCTGTCCTGGCATATCGTTTGCCAATTGAAGTGC

CTGTAATATCAATCTTATGTGAGATTCCAGCAGCAGTCAATGACTTAGAAATAACCTTGGCAACCCCCTC

ATACTGCTGATTCTGAACA

>FM896589.1 FM896589 Jatropha curcas embryo 71-95 (DAF) Jatropha curcas cDNA clone rjcpga0_003841, mRNA sequence

TTTGAGAGCAGAAGAATCTCCCTTTCTCTTTCTATCTCATATCTCTCCCTCTTTATACAGTAAAATGTCT

GCTCTTGTGGGCTTCTTTGTGGGTCTTATACTGATCGGTACGGTTTACTCTGCAAGATTTGATGATCTTT

TTCAGCCCAGCTGGGCTCTCGATCACTTCATCTATGATGGAGAGCTTCTCAGTCTCAAGCTCGATAACTA

TTCTGGTGCTGGGTTTCAATCCAGAAGCAAGTACATGTTTGGGAAAGTTACTGTACAGATTAAGCTTGTA

GAAGGCGACTCTGCTGGAACTGTTACTGCTTTCTATATGTCATCGGACGGTCCTACTCACAATGAGTTTG

ATTTCGAGTTCCTGGGTAATACCACAGGTGAACCTTACCTGGTTCAAACCAATGTGTATGTTAATGGCGT

GGGTAACAGGGAGCAAAGGTTGAACCTTTGGTTTGATCCTACTAAGGATTTCCACTCATACTCTATCCTC

TGGAACCAGCACCAAGTTGTATTTCTAGTGGATGAGACTCCAATCAGATTACACACAAATATGGAGAAC

>FM896588.1 FM896588 Jatropha curcas embryo 71-95 (DAF) Jatropha curcas cDNA clone rjcpga0_003840, mRNA sequence

AGGATAGAATTAAATTAATTGAATTACATTGTATGAACAATCAAACTTCAATCAGAAAAATAAAAGAATA

CAACAGGAAACACGTATTTCACAGATAATAATGGATTCACAAGCCAAACCAACAAAACGGAGATGACAGA

TACAAACAAACTGCAGTCTATGATTGTTTATTTATCGTTAGAAGCGCAAGCACCTCCACCCAAAGCTCTC

TCCAGCCTGGTCTCAAATGGAGTAGAATGCCAATTCACTCTCTTGTCCTCCCTATACTTGCTGGTTGTGT

TTGGTATTCCTTTGAAAGAAGAAGCTGAGCCAGAATCCTTGCCAGAGCCATTGTTATTCCAATAGGTTCC

AACAGTTCCGATGATAGGCATCTCATCAGGGCTACGACTAGGTGACTTCCTTGGCGAAGAAGAAGCCGAC

AATTGTTTAAGCTCTTCCCAGGGGTTAATGCTCCTAACATAGGTCTATCTTCCAAGCTTGCAGGAGAATC

TGATCCCAATGACATGTCT

>FM896587.1 FM896587 Jatropha curcas embryo 71-95 (DAF) Jatropha curcas cDNA clone rjcpga0_003839, mRNA sequence

ATTCCGCCTTTCGGCCTGATCTTAGGCCCTGACTCACCCTCCGCGGACGAACCTTGCGGAGGAACCCTTA

GGTTTTCGGGGCATTGGATTCTCACCAATGTTTGCGTTACTCAAGCCGACATTCTCGCTTCCGCTTCGTC

CACCCCTGCTCGCGCGGGTGCTTCCCTCTAAGGCGGAACGCTCCCCTACCGATGCATTTTTACATCCCAC

AGCTTCGGCAGATCGCTTAGCCCCGTTCATCTTCGGCGCAAGAGCGCTCGATCAGTGAGCTATTACGCAC

TCTTTCAAGGGTGGCTGCTTCTAGGCAAACCTCCTGGCTGTCTCTGCACCCCTACCTCCTTTATCACTGA

GCGGTCATTTAGGGGCCTTAGCTGGTGATCCGGGCTGTTTCCCTCTCGACGATGAAGCTTATCCCCCATC

GTCTCACTGGCCGACCTTGACCCCTGTTATTTTGAGGTCATATCTAGTATTCAGAGTTTGCCTCGATTTG

GTACCGCTCTCGCGGCCCGCACCGAAACAGTGCTTTATCCCTAGATGTACAGTCAACTGCTGCGCCT

>FM896586.1 FM896586 Jatropha curcas embryo 71-95 (DAF) Jatropha curcas cDNA clone rjcpga0_003835, mRNA sequence

CTTAGAGTTTCCTTGCAGATCTGAAAATGAGCAAATCACCTCTGGAAATTGATCTCAAAGGAAAAGTGAA

AGTGAAAGAAGAACCAGTTATGTACTCGAATCAAGTGCGCATGCCTAGGTTTGGTGCTCAAAACAAATCA

AAATTTATAATCAAGACTGAGAATATGGAAGAACAAAAGCCCTCCGGTGTTGTTGATACAATGTATGTTG

TAATGAAAGAAGATTCCAAGTATCAGCGTAATTGGGTTTTTGATGGAAGATCAAAGATTGGAGCTGCGGT

TGCTAAAACATTCAATGGGAGGACATACATGGGACATGTTATAAATTATCAAGCTGCTAAGTTCAGGGTG

CTGTATGAAAACAGTTGCTTCGAGAATATTACTAGAGCTGAACTGGAAGAAATTGAAATCCCTCCTCATA

TGGTTAAAGCCTATTATGTTGAGTTAGAAGAAAGAAGGGTTGCTGCAAAGAGAGAACAAACCTCTATGGA

AAATGGACCTTCTGGTTCTGGAAGCCAGGAGCCAAAGACTAAGAAACCCAAGAAAAACAAGAATCCAACT

AACTTTGTCGCTCTGTACAACGA

>FM896585.1 FM896585 Jatropha curcas embryo 71-95 (DAF) Jatropha curcas cDNA clone rjcpga0_003834, mRNA sequence

CTCATCTCCAACCTTGAACTCAGCCAAGTACCTGTGATAATCTCCTTTCATCTTCAAATAGAAGACCTTC

GATTCACTAGCGGTCGCCGATGGTATCAGATGAGAGAAAGGCCGCTGCTGAGGATACTATGCTTGCTTAC

AAAGCCGCACAGGATATCGCACTCGCAGATCTTGCACCAACACATCCGATCAGATTAGGACTGGCACTGA

ACTTCTCTGTGTTCTACTACGAAATTCTCAATCAATCTGACAAGGCTTGTAGCATGGCTAAACAGGCATT

CGAGGAAGCAATTGCTGAGCTAGACACTTTGGGAGAGGAATCATACAAGGATAGCACTCTAATCATGCAA

CTCCTTCGGGATAACCTCACTCTTTGGACTTCAGATGTCCAGGACCAGTTAGATGAGTCATAAAAGGCAT

CATAAGCTTGGTCGGTCCTAGTCTTCTTTTGGAGGAGGTGGGCTTTGAATGTCATCCATTAGTCCTGATT

AAAAGTGTGTTTAACATGAAACTATG

>FM896584.1 FM896584 Jatropha curcas embryo 71-95 (DAF) Jatropha curcas cDNA clone rjcpga0_003833, mRNA sequence

ATTCCTCACCCTCTTCTCAACGATTCTCAGCAAAGTGTCTTTCCGTCCTCTCCCAACCAAATTCACTAAA

CCGGCCCAACCCAAACTGACCCGAAATATCGGATTAGTAGAGAGAGTAAAAGAATGGTGTCCGACTCGGA

GCTAATCGGAAGGCTAAGAGAGTTTCTCCGAAATTCGGACCTCGACACCACGACAACCGGTATCGTCCGC

CGGCAGCTAGAGGAAGATTTCAGCATTGATTTATCGGATAAGAAGGCATTTATAAGGGAACAGGTGGATT

TGTTTCTCCAAAGCCAGTTTGAAGAACAAAGCGAAAATGAAGAAGAAGGTGGGGAAGGAGGAGGAGAAGA

TGATCAGGCGGCTAATGTTAAATCAGAGGAAACCGAAGCCTCTGATTCTAGGGAGGAAGGTGATGGTAAT

GATGAAGAAGAGGAAGAAGAAGCAACGGCGAGTAACGGAAAAAATAGAGCAAACCGGAGATCTAACACAC

ATAATAATGA

>FM896583.1 FM896583 Jatropha curcas embryo 71-95 (DAF) Jatropha curcas cDNA clone rjcpga0_003832, mRNA sequence

CTACGCCTCACTGAATAACACCGTTGCGGCCTCATATCGCACTTCCTGCTCATCGCCACCGGCAACCATG

ATCATTCCGGAGAAGAACCGCCGCGAGATCTCTAAGTACCTCTTCCAAGAGGGAGTATGCTATGCAAAGA

AGGACTTCAACCTTGCAAAGCACCCACAAATTGATGTGCCGAATCTACAGGTTATTAAACTGATGCAGAG

CTTCAAGTCAAAGGAGTATGTCCGCGAGACATTTGCATGGATGCACTACTACTGGTACCTCACTAATGAT

GGTATTGAGTTTCTGCGAACTTACCTCAATTTGCCATCTGAAATTGTTCCAGCTACATTGAAGAAACAAA

GCAAACCCCTTGGTAGGCCCATGGGAGGCCCACCAGGAGATCGCCCACGTGGTCCTTCACGTTTTGATGG

AGAGAGGAGATTTGGTGGTGATCGAGATGGATACCGTGGAGGTCCACGTGGACCTGGTGGTGAGTTTGGT

GACAAGGGTGGAGCCCCTGCTGATTACAGACCTACCTACGGGGGTAGTGGTGGAAGGCCTGGCTTTGGCC

GTGGTGCTGGTGGCTTTGG

>FM896582.1 FM896582 Jatropha curcas embryo 71-95 (DAF) Jatropha curcas cDNA clone rjcpga0_003831, mRNA sequence

TTTTTTTGAACAAAAGCAACAACATGAAGTTTAAAAACACATTTACAACATTTAGAAAATAGTCTTTAAT

TTAAGACTCTACAACTTAACGAGTGCACATTTCTAGCTACCTCGAGACACCAAGTACATAAAAAGCTAGA

CTCTTACAGTTACTATTCTAGAGGCTGAAATATGTAACTGTGTTACTTTTTAACAGCCAACTTGAGTTTT

CCTCCATCCTTCTATCCCTACCACAGCAGGATGTCGCCGTTGCGTTTCCCTGACTAGCTATACACCTCTT

AATTCTAATGTTGCCCCAGTTTTTCTTGTAGCTGCAAAGGTCGAACCATAGCACAGCTACTTCACACAGA

AGCAACACCAACAGCGCTACGAGAGGTAGCACCTTGATTGGCCAAGAAGCGGACAACGACACAGGTAATA

TTATCTGCACTTCCTCTCTGGTATGCCTCTTGCAACAACCTCCTTGCTGCCTCCTCTGGGTCTGCAATTG

GTTTAGTCATTTCAACAGCTTCCTCATTAGTGACAACAT

>FM896581.1 FM896581 Jatropha curcas embryo 71-95 (DAF) Jatropha curcas cDNA clone rjcpga0_003829, mRNA sequence

TTTTTTTTTTTTGACGTCAAAATTCAATGGAACAATCAATAAAAATTCACTGTCTGATGCCAGCATATTA

ATATTAGCCGGTACAAAGTTACGTTGCTGTCAAGAGAACAAGCATAAATTTACTCTGCACAAGTCTTGTG

AAGCAAACTTTCATCAACTCAGGACCAGCTTCTACAGATATCCCTAGTGTGTTATTATCAAGGAGGGTAT

GATATTAACAGAAAATGAAAGATAAAACATTCGCAAACATTACATCAGGATATCAATCTTCTGTACAAAT

GTCGTAAAACAAACAACATTTGAACGGAGGAGCTAACAGAAATTCAAATTCCCTTCTAGTAATGTCTTTG

CCTCCACAAGAGAATTCCAAACATTTCGCAGTTCTTCTTCCATTTTTTCAGATAATAAGCATGAATTGCA

GCTTCTGGTTTCTAACAGTTTAGTCATATTCTCAGCTGCTGTTATCCTCACCATTCTTAACTCATCCAGT

CTTCTCAATTCTATCAGTATTTGTTCTTCCAATGCCTTTATCTCCTCCACTTTCAAA

>FM896580.1 FM896580 Jatropha curcas embryo 71-95 (DAF) Jatropha curcas cDNA clone rjcpga0_003828, mRNA sequence

GACAAAGGAACAATCTTCTTTTCATCATTACAGGGAAATAAATATTATCAGGGTAGGAAATCTGACGCAC

TAGCTATTATAAATCAAATCCCTTATAATACACACACTTACACTTCAATGGCTGAAGGAGGAAAACAGAA

TGAGCCCAGAACCCAAAAACAGGATTAACCTGTTCCCTCAGAAACCTGCCTGTGCCTGCCCTGTTGATCA

GAT

>FM896579.1 FM896579 Jatropha curcas embryo 71-95 (DAF) Jatropha curcas cDNA clone rjcpga0_003827, mRNA sequence

GCTTAGAAGCATGGAAATGCCATTCAGATTAAACACATAATTACAACAAGAACACCTACAACTTTTTGTG

CCCAACCATTATGAAAGAATTATATATGCAACAAAGAGGTGTTCAAATAACATCAAGTTTGAAAGAATAA

TCATTAGAACTCAATAATGTATCATCTACAAATACAGTAATGAATTATAGATTATTGACTATGAATACAG

TGACTGACAGTCATCTAAATAAAAATGAATTCGCCACCACGAACAGCATTGGCGGCACCTTTCCTCTCCT

CTTCAGCCTCTTTCTCGCACTCTTTCCAGCTCTCATATACATGATCATCAGTTTGCAGCTCATGCCGGCA

AATTGGACAAGAATTATGCTCATCCAGCCATGGCTTTAGACAAGGAGGATGGAATGTGTGCTTGCAAGGC

AACTCTTGCATCTTATCATTAACCACCAAATTCTCCTTACAAATTGCACACTCTGCATCTTGTCCAAGCC

TGGCCAAGATTTCCTCCGTGAGAGTAATGACTG

>FM896578.1 FM896578 Jatropha curcas embryo 71-95 (DAF) Jatropha curcas cDNA clone rjcpga0_003826, mRNA sequence

CTTGATATGCTTTCCAAAATCATATTTGGTAAGAAAGAATGTAATGCCAAAATCTTACAAAAGAAAAAGG

AAAAAAAAAAAAAAAAAAAAAAACAGAAAGCTGACAATCCTGATTATTTCCACGCTTACTTCCCCCTAAA

TTGTTTGCCAAAGCGACTCTGTATCCTCTTCTTCTCTTCCCGAGATCTAGCTGCCCTGGCTTTTTACGCA

GCAAAGGGCATAACCTTCTCATGTAGCTTCTGTCTATTACTTTAACCACCAGTCTTTAGGTTTTAAATAG

CAGTTCTAGCCTGGTACTACCCTGTAGCCTCTCTCCCAGCTCTCACTAATGCCAATCTTACTTCCTTG

>FM896577.1 FM896577 Jatropha curcas embryo 71-95 (DAF) Jatropha curcas cDNA clone rjcpga0_003825, mRNA sequence

CCACACGAGATTTCTGTTCTCGTTGAGCTCATCTTAGGACACCTGCGTTATCTTTTAACAGATGTGCCGC

CCCAGCCAAACTCCCCACCTGACAATGTCTTCCGCCCGGATCGGCCGCCGAAACGACCTTGGGTCCAAAA

AGAGGGGCATAGCCCCGCCTCCGATTCACGGAATAAGTAAAATAACGTTAAAAGTAGTGGTATTTCACCG

TCGCCTTTCGGCTCCCACTTATCCTACACCTCTCAAGTCATTTCACAAAGTCGGACTAGAGTCAAGCTCA

ACAGGGTCTTCTTTCCCCGCTGATTCCGCCAAGCCCGTTCCCTTGGCTGTGGTTTCGCTGGATAGTAGAC

AGGGACAGTGGGAATCTCGTTAATCCATTCATGCGCGTCACTA

>FM896576.1 FM896576 Jatropha curcas embryo 71-95 (DAF) Jatropha curcas cDNA clone rjcpga0_003823, mRNA sequence

TTTGATTGACCAATGGATTTTACGAAGGAATCAATGAAAATTTTAATTAAATCTAGAATCCAACACTTGT

TTGTTCCTACTACTACCAGAAGGAAATGTTACAGAGATAAGAACACACTTGTAGAGTTGCCCCAAGGAAC

GGGAAGTACCCTTGAATTTTGGCCTGGGTTCTTCACCACTTCATCGTTGACAGAAATACATCTGTAATAT

TTAGTTGCAGAGATTATACGAGTGCAGCAGCTTCTGTGTTTCACCCTTCATCCACAAATCAGAGACTATT

TGGCGACGGACATATTAGCTATCAATTGACTAGGGATTATTTGGCACCTTATCGAGCTGATGAAGGCAAG

ATTCAGGTTTTTCCGTTTTAATGAGCTAATAACCAACATGAACCTCCAAAGATCAGAAAAATTCCGAGCG

CCAGCCGCATTTCCCTTCATGCTTCTTCAGAATCTCCAGCATTCAATGACAGAATCTCCTACGACATCGA

GCAGATCCCAG

>FM896575.1 FM896575 Jatropha curcas embryo 71-95 (DAF) Jatropha curcas cDNA clone rjcpga0_003822, mRNA sequence

CCAGAGGAAACTCTGGTGGAGGCCGCAGCGATACTGACGTGCAAATCGTTCGTCTGACTTGGGTATAGGG

GCGAAAGACTAATCGAACCGTCTAGTAGCTGGTTCCCTCCGAAGTTTCCCTCAGGATAGCTGGAGCTCGG

GACGAGTTCTATCGGGTAAAGCCAATGATTAGAGGCATCGGG

>FM896574.1 FM896574 Jatropha curcas embryo 71-95 (DAF) Jatropha curcas cDNA clone rjcpga0_003821, mRNA sequence

ATTCCTGCTAGTCGCTCTATTTCTCCTACTGTCTCTCTTCTACTCTCTTCACTTCATTATCAATGGCTGT

CTTTTCTGATCTCCATACTGAATCAGGCCTCAAGGCTCTCGATGCCTTTCTCGCCGGAAAATCTTATATT

TCTGGGGATCATCTGTCAAAGGATGATATAAAGGTATACGCTGCCGTTGTGGGGAAGCCTGGAGATGCTT

TCCCCAATGCCAGCAAGTGGTATGCTTCTGTAGATTCACTTCTTGCCAAAAGTTTCCCTGGAAAATCTGT

TGGGGTAAGAATTGGTGGCAGAGGTGCTGATCCAGTTGAAGCTGCTGCCCCTGCTAAGGCTGATGCATCT

GCTGGAGATGACGACGATGACTTGGATCTCTTTGGTGATGAGACTGAGGAGGACAAGAAGGCAGCAGAGG

AGAGGGCAGCTAAAAAGGAATCCTCCAAGAAGAAAGAGAGTGGAAAATCTTCTGTTCTTCTGGATGTGAA

GCCTTGGGATGATGAGACAGATATGAAG

>FM896573.1 FM896573 Jatropha curcas embryo 71-95 (DAF) Jatropha curcas cDNA clone rjcpga0_003820, mRNA sequence

TTTTTTCAATAATGATTTCCGAGCAAGCAGTCTTTATGTTCCTTATCCACAAATCTCCCCCAATCCAGGT

AAAATCTCACCTAGATTTTGAAGATTTCTTTGCTGGAGGATATATCAGGCCCTCACTTTATTTAAGGATT

CCGCAAAAATTGCAAGGATGGTCTCGCAGTCCATCAGATTCAAGACCCAAATTATTAAACAATATGACGC

CCTTTGTATGTTCAACTTTCATAGCTAAGAAATTATGTTTCACAGCATCAACAGAAATTTTCTCGACAAC

AGACAAGTCAAGAAAGGGGATCATCTGAGATAAACTCTCAATTTTCATCATCTGATACACCTGTGACACC

TGCTGAAGCAGCCTTAAAGTAGCGAGCTTTTCAAGGGCAGGAACATATTGAGACAGTTGCAC

>FM895640.1 FM895640 Jatropha curcas embryo 71-95 (DAF) Jatropha curcas cDNA clone rjcpga0_002594, mRNA sequence

TTGGAATCAACCAAGGAAAGAATTGTTATGGAGAAGCACAGCCATTATCAGGCTCTACATATCCCGGTGG

TGCACCACCGGCAGTTGCAGTGGTAAACCAGGTTATAAGTACAATGAAGAAACCAATTTATCTACTAGAT

ATAACAACACTCTCACAATTAAGAAAAGATGCTCACCCTTCCACTTATAGTGGTGAAGGCTCTGGCACAG

ACTGCAGCCACTGGTGCCTTCCTGGATTACCTGATATTTGGAACCAACTCCTATATGCAGCTCTTATCAT

GTGAAGGTCAAGACTAAAAACTTACAGTTTAGTTTTTACCTTTTCACATGATTGTATATTTAGGAAGGTG

AAAGAATTTCTCATATAGTCTTGTAGGGTGGTTTAATAAGGCGGCAGAAATCAATTGCAAAGTGATTGAT

TTTCTCGATCACAATAATTGCTCTAAAAGTGAAGAATTGATAGGATTCAAAGGGTTTCTTGTGAATTAGT

GATTTATGTAGGGGAAGTAAATACTCATTGTGGCCTAAACTTAAGTCTATTCAATTATCATTGGATTTGT

ACTGGTGTTTCATTAAACTTTACATATG

>FM895639.1 FM895639 Jatropha curcas embryo 71-95 (DAF) Jatropha curcas cDNA clone rjcpga0_002593, mRNA sequence

ATTCCTACTGTGCAAGGCTTGTCTGGTACCGGTTCTTTGAGAGTTGGAGGGGAGTTTCTTGCAAGGCATT

ACCATCAACTGACAATATACATTCCACAGCCAACATGGGGAAATCATACCAAAGTCTTCACTCTTGCAGG

GTTGTCTGTAAAGACGTACCGGTACTATGATCCAGCAACACGTGGGCTGGATTTCCAAGGCTTGCTAGAG

GATCTGGGATCTGCACCAACAGGATCTGTAGTGCTTCTCCATGCATGTGCACATAACCCAACTGGCGTTG

ACCCAACCCTTGAGCAGTGGGAGCAGATCAGACAGCTGATCAGATCAAAAGCATTTTTGCCTTTCTTTGA

CAGTGCTTATCAGGGTTTTGCGAGTGGCAGCCTAGATGCAGATGCACAGCCTGTTCGGTTGTTTGTTGGA

GATGGTGGTGAATGTCTTGTAGCACAGAGTTATGCAAAAAATATGGGACTCTATGGGGAACGTGTTGGGG

CTCTCAGCATTGTCTGCAAAACAGCAGATGTGGCGAGTAG

>FM895638.1 FM895638 Jatropha curcas embryo 71-95 (DAF) Jatropha curcas cDNA clone rjcpga0_002592, mRNA sequence

GAAGATGGCAACGGCAGATTCTCCAAACAACAATATCGATCCCAATCTCGAAGAAACCAATCAAAATCAA

AACCCTAATTCTAATTCTTCAAATGAATTAATTGTTCCTGAGTCGAGCTCTCCGGCCGTCTGCCTCATCC

GCTTCGCCGGTGACTCTGCCGGCGGTGCCTTCATGGGCAGCATTTTTGGCTACGGTTCGGGATTGATTAA

GAAGCAAGGCTTTAAAGGATCCTTTGTGGAGGCTGGATCTTATGCCAAGGAGCGCCGCAGGCTCTTTTAC

AGAGCTGCCTGACTTTTGGGGCATTCTCATTTATTATTGAAGGGCTTAACAAGCAGCAACCTGCACTGGC

TCATTCATTTGCTGTGAGAAAAAAGCGCCACTACCATGATGCATCTCGTCTTGAGTACCGTAATGCTCCT

CATTCCTTTTCTCTGCCTCTTTCAGTCGCTCTACCAAATGAACTGAAAGCAGCATTCTCTTTTTTCTGCA

AGTCCTTAAAGAAACCAACTGGCAATTTTCCTGCTTCCACCTGATAAAAGATTTGGACAATGATTAATCA

ATTATGTTTG

>FM895637.1 FM895637 Jatropha curcas embryo 71-95 (DAF) Jatropha curcas cDNA clone rjcpga0_002591, mRNA sequence

CCATCTATGAATTTACCTGCGATCTTTTGATATATATATATATATATATATATATAAACCCAATGAAGAG

CAAGAAAGGAATCTGCATGCAACCATTTTATGCATGCAGATTACACATTGACTCAAATATAAGAATTAAC

AGGGGGTGAAAATTGTTACATTACAAAGAACAAAAGTGTCAGTGAGATGCTGAAGGAAGCTTCCAAAAGG

TTTTCCTTGCAACAAGAATTTTCTCTCTCGCATGTGATTCAGGTTCCTTCTCATGTGTCTCAGCTGTCCA

CCTTACAGCTCGAACTATGTAAACCACTTTGTCAATCACTTCAGGAGTATCGCGAATTATAACAGTTCCT

TCGGGACGTAATATTCTATCCATTTCCACTATCAAATCTACAAGGTTACACCTGTTCTTGCTTGAACCTG

GGCTCTTTATCAGCGATTCAATGCTGGTTACATGAATGAGATCATAAGTACGAGGATATGTTGAGAAAGG

CTCGCACCAATCATGGTAGAGTCCAATAAGGCCCCGGTCATAAA

>FM895636.1 FM895636 Jatropha curcas embryo 71-95 (DAF) Jatropha curcas cDNA clone rjcpga0_002590, mRNA sequence

TTCTCTCTCTCTCTCTCTCTCTCTCTTGGTTTGCTACTTTCTTAGTTCTTTCTTATTGTTTGCTTCTCAA

TTTTATTTTTTGGTTCTTATTAGGTACTTTTCTCCTTGGATTCAAATGGCTACTGTTGAGGTAGTATCGG

CACAAACTGCACTTTCAGAGGAAAAAGCAGAAGAAGTGAAGGCTTCAGAGACTGTCACTCAAGTAACAGA

TCAGTCAGTTACTGATGAAAAACCAGACACAGGAGAAAAGAAGGAAGAACAAGAAGAAGTTGCAGGAACT

AAAGAAGAAGCTGTAGAACCAGAACCAGAAGTTGCAACTGAAGTAGTGGTGGAAACTAAAGAAGTAGTAG

AAGATGAGACCAAAACTGTAACAGAAGAGCCCCCATCGGAGAAAACTGAAGAAGAAACTCCTAAGGAAAC

AGAACCAAGAAAAAGAAGAAACTCCTAAGGAAACAGAACCAGAAAAAGACGAACAGGAATCCCCAAAAGA

AACAGTAACTGAGGAGACTGTTGCTGAGGAGACTACTAAAGAGACCACTGAGAGTACTGAGA

>FM895635.1 FM895635 Jatropha curcas embryo 71-95 (DAF) Jatropha curcas cDNA clone rjcpga0_002589, mRNA sequence

GCAAAGGATTCTATCCGTCGCCCGATGGGAATTGTACTTTAAGGCGGCCCTCGCGGCTCGTCCGCCGCGG

GGGCTTGGCCAACGGCACGTGCCTCTGGGGGCCGGATGGCCCCTACTGCGGGTCGGCAAACGGGCGACGG

ACACGCGCGTCGCTTCTGGCCCGGATTCTGACTTAGAGGCGTTCAGTCATAATCCAGCGCACGGTAGCTT

CGCGCCACTGGCTTTTCAACCAAGCGCGATGACCAATTGTGCGAATCAACGGTTCCTCTCGTACTAGGTT

GAATTACCATCGCGACACCATCATCAGTAGGGTAAAACTAACCTGTCTCACGACGGTCTAAACCCAGCTC

ACGTTCCCTATTGGTGGGTGAACAATCCAACACTTGGTGAATTCTGCTTCACAATGATAGGAAGAGCCGA

CATCGAAGGATCAAAAAGCAACGTCGCTATGAACGCTTGGCTGCCACAAGCCAGTTATCCCTGTGGTAAC

TTTTCTGACACCTCTAGCTTCAAATTCCGAAGGTCTAAAGGATCGATAAGCCACGCTTTCACGGTTCGTA

TTCGTACTGGAAATCAGAATCA

>FM895634.1 FM895634 Jatropha curcas embryo 71-95 (DAF) Jatropha curcas cDNA clone rjcpga0_002588, mRNA sequence

ATTGCAAAGGATTCTATCCGTCGCCCGATGGGAATTGTACTTTAAGGCGGCCCTCGCGGCTCGTCCGCCG

CGGGGGCTTGGCCAACGGCACGTGCCTCTGGGGGCCGGATGGCCCCTACTGCGGGTCGGCAAACGGGCGA

CGGACACGCGCGTCGCTTCTGGCCCGGATTCTGACTTAGAGGCGTTCAGTCATAATCCAGCGCACGGTAG

CTTCGCGCCACTGGCTTTTCAACCAAGCGCGATGACCAATTGTGCGAATCAACGGTTCCTCTCGTACTAG

GTTGAATTACCATCGCGACACCATCATCAGTAGGGTAAAACTAACCTGTCTCACGACGGTCTAACCCCAG

CTCACGTTCCCTATTGGTGGGTGAACAATCCAACACTTGGTGAATTCTGCTTCACAATGATAGGAAGAGC

CGACATCGAAGGATCAAAAAGCAACGTCGCTATGAACGCTTGGCTGCCACAAGCCAGTTATCCCTGTGGT

AACTTTT

>FM895633.1 FM895633 Jatropha curcas embryo 71-95 (DAF) Jatropha curcas cDNA clone rjcpga0_002587, mRNA sequence

ATTCGTTTTTTTTGCTGATTTTATGATTCTCGTTGCTCGATGGATCTTCCTTATGGTTCAACTCCCCATT

TGTAACAAGAGTATTCTCATCTTTATCACCACTCTCGACTGAAGGCTTCGATTCAACCACTGTTAGATCA

CAATTTAATTCTGGTGTTTCATTCATTTCCAATGTATTCAGAGTGGTTTCTACATCAGGCACTTTCAACG

CTGTCAAGGTTGGCTCAGTCTTCACCACTTCAGGTAGTTTGGGTGTTGTCACAGTTGTCTCTTTCTTTAT

CTTATCCCCTGGATTGTTCATATTCTGTCCCAAATGGTGATCACCAGAAGCACCTGATACCTTTTCCATA

CTCTGTTTTGGCTTCTCAAATTCAACCTCTGACTGAGCAGTGTTTTCTACTACAGGGTTGTGAACTTTTC

TCAAATTACGTTTTACCTTTTCAAGTTCATTTTGTGGATCTTCCTGCACTGTATCAGCTGGATGATTTGA

TACCTTTCTAAGATTACGCTTGGGTTTCTCATTTTCAGTGGCTTGTACTGACGTATTATCAATATTT

>FM895632.1 FM895632 Jatropha curcas embryo 71-95 (DAF) Jatropha curcas cDNA clone rjcpga0_002586, mRNA sequence

CGAATTCATTTTGCGATGGATGTCTCAAGAATGCGGAATGTTGCTTGTTATATGAGCCACAGTTCAACTC

CAAATGTGCTGGTGCAATTTGTTCTGTATGATCATAACAATTTGATGTTCCCACATCTCATGCTTTTTGC

AATGGAGAACATTCCTCCATTGAGGGAGCTCAGCCTTGATTATGGTGTGGCTGATGAATGGACGGGGAAG

CTTTCCATTTGCAACTGAATGGGCTTGAAGAGTTGTTGGAATTTTTGTATGTAGTGTAGTAGAACATCCC

GAAGACATGATGCTATAAGGTGGAAATCTTAGTTATCTTTTGCTGGTTGAGAATCTGTCACAATGGTTTT

GCCATGATTTTCTGACCAGTGTCCTGAGTGTTTTCCGTTGGTAACTATAAATAGCCTTCACCCGTTGTAT

ACACTGCCATGGTTTATCTTTCTATTTTCTTTCGTTAATCCTAAAGTGCTTAAGGCTCAGATTTTCAGAA

TTTTTTTTTCCTGGTTGGTGTTGGGTGGAGGAGACAACTGAAACTCAAAATTTTCTGCGGTTAGTCATAT

G

>FM895631.1 FM895631 Jatropha curcas embryo 71-95 (DAF) Jatropha curcas cDNA clone rjcpga0_002585, mRNA sequence

AGGATTCTATCCGTCGCCCGATGGGAATTGTACTTTAAGGCGGCCCTCGCGGCTCGTCCGCCGCGGGGGC

TTGGCCAACGGCACGTGCCTCTGGGGGCCGGATGGCCCCTACTGCGGGTCGGCAAACGGGCGACGGACAC

GCGCGTCGCTTCTGGCCCGGATTCTGACTTAGAGGCGTTCAGTCATAATCCAGCGCACGGTAGCTTCGCG

CCACTGGCTTTTCAACCAAGCGCGATGACCAATTGTGCGAATCAACGGTTCCTCTCGTACTAGGTTGAAT

TACCATCGCGACACCATCATCAGTAGGGTAAAACTAACCTGTCTCACGACGGTCTAAACCCAGCTCACGT

TCCCTATTGGTGGGTGAACAATCCAACACTTGGTGAATTCTGCTTCACAATGATAGGAAGAGCCGACATC

GAAGGATCAAAAAGCAACGTCGCTATGAACGCTTGGCTGCCACAAGCCAGTTATCCCTGTGGTAACTTTT

CTGACACCTCTAGCTTCAAATTCCGAAG

>FM895630.1 FM895630 Jatropha curcas embryo 71-95 (DAF) Jatropha curcas cDNA clone rjcpga0_002582, mRNA sequence

TTTTTTTTTAAGGAAAACATATATAGATTATAAAGCTAAAGACATTAATGTTTTGGGTATAAAAGAGTCA

CAAAACCTTAATACCAACATACATTGATTGATAAAATAAATAATAACCCCTAACTATCTTCACCACCATT

CTCTCCAAACCAAGAGGACAAGGGAAAGAAAAAAACTCCAAACCAATAATAGTTCATAAAGGACTAACGA

AAATAAATAAATTAAAAAAAAATAAATGACTCCCAAAACTTCAAAACCCCTCCCTTTCTGTTCCTCTCCA

AAGATATCAGCAAATTCTCCCTCCAAACTTCAATCGAACTCTACTGTTGCTTGCACTCTGGAGGCCTCTC

ACCCTGCTGACCATCCCCAGCTTGTGTTCTTCCACCCTTCTTATGCCCGGCTGACAGCTTCTTAAAGGTC

TTGCTTTCATCCTCGTCCTCATCCTCATCTTCATCCTCTTCATCCTCATCATCATCATCAATATCTTCAT

CGTCGTCGACGTCATCATCATCATCATCATCTATTCCAAAGTCATCCCCCTGGATAGCCTCACCCGTAAA

CCAAGAAACAGCA

>FM895629.1 FM895629 Jatropha curcas embryo 71-95 (DAF) Jatropha curcas cDNA clone rjcpga0_002581, mRNA sequence

TTCAAGAAATCAAAATCTAGTGGATTTTTCTATCATCTTAATTTTCTGATCTCTGCAAGCAACCATGTCT

AGCACAGCCGGTCAGGTCATTCGTTGCAAAGCCGCGGTGGCATGGGAGGCAGGGAAACCACTGGTGATTG

AAGAAGTGGAGGTGGCACCACCACAAGCTAACGAAGTTCGTTTGAAGATCCTCTTCACCTCATTATGTCA

CACTGATGTTTACTTTTGGGAGGCAAAGGGACAATCGCCATTATTTCCTCGCATTTTTGGTCATGAAGCT

AGTGGAATTGTGGAGAGTGTTGGTCCTGGTGTGACTGATCTAAAACCAGGTGACCATGTCCTTCCTGTGT

TCACTGGAGAATGCAGGGAGTGCCGCCATTGTAAATCAGAAGAGAGCAACATGTGTGACCTTCTCAGAAT

CAATACCGATAGAGGTGTTATGCTTGCTGATGGCCAATCAAGGTTCTCTATCCAAGGGAAGCCCATTTAC

CACTTTCTGGGCACCTCTACTTTCAGTGAATACACTGTTGTTCATGTCGGTTGTGTTGCCAAGATCAACC

CTGATGCTCCTCTT

>FM895628.1 FM895628 Jatropha curcas embryo 71-95 (DAF) Jatropha curcas cDNA clone rjcpga0_002580, mRNA sequence

ATTCGAAAAAACCAATTTCAGTGTAGGAACTTTTTCAAGTGTAGTATTTGGACGCGAAAATGCAGTCTTG

CAGTGGAACTGCTGTTATGGGTTCTATACAACAGCCTGCTTGGACCAAAGGGTCACCCCTCCTTCAAAAA

AGGTTCGGTGTTATTGGGTTTCCACATCAGATTAAGCTCAATTCGGTGAAGCCTTGTAGATACGTCAATA

TTGAAGGGAGCTTGGCCACTGGGAGGCCACCCTCTTCTGTGTCTGTCCCTGAAGTTGGAGGTGGCAGTAG

TAGTTTTGTAGATTATGGTCTAAGTGAAGCAGATCCTGAAGTGCGTGAAATTATTGAGAAGGAGAAGAAT

AGGCAGTTCAAAAGCCTTGAATTAATTGCCTCAGAGAATTTTACATCCCGAGCAGTGATGGAAGCAGTTG

GCTCATGCCTCACAAACAAGTATTCTGAAGGACTGCCTGGTAAAAGATACTATGGTGGCAATGAGTACAT

TGATGAGCTCGAAACTCTTTGTCAAGAAAGGGCTTTGGCTGCTTTTCAATTAGATGGACAGAAGTGGGGC

GTTAACGTCCAACCATTGTCTGGTTCTCCTGCTAA

>FM895627.1 FM895627 Jatropha curcas embryo 71-95 (DAF) Jatropha curcas cDNA clone rjcpga0_002579, mRNA sequence

GAAAAACAAAAAGACGGAAGTACATTGACATATTCAAAGAGAAGATGATTAATTACACTGACCTGAACTT

ACAAACACTCCCACGGTCAAGCAAGGAAAAAAAGTACCAGCAAGACCAAAGTGGAGAGACTAACTACTCC

ATACTCATATAACCTAGAACTGACCCCCCTAATTGCAGCTCTGCAAATGCCAAAAACTTCATTGCAGAAG

CTACCTAAGACACCAAAATAAACCTGAAACAAAAATTGACAAAAAAAAAAACAAGAGTACAATAATAATT

GAAATTTAAAGAGCTGCCCAATCAATCTCCACCGAGGGGTATTTCTCCAAACCGAAATTCTCAGTCTCAT

CCCATTGTGAAGAATCAGAGAAATCCAAGAAAGAAATCTCAGACTGAGGTGAAGAAGAACCAGCCCATGA

TTCATCAGATGAAGCTGGTGCTGATGAAATCTCCAACCTATAGTCCTCA

>FM895626.1 FM895626 Jatropha curcas embryo 71-95 (DAF) Jatropha curcas cDNA clone rjcpga0_002578, mRNA sequence

TTGATGACGAAGAGCTTTTGCAATTGGTGGAATTGGAGGTGCGTGAATTGTAGTCTTCATTATGACGTTT

CCTGCGTGATGACATACCCATCATTTCTGGCTCTGCACTTTTGGCTTTGGAAGCTTTGATGGCTAATCCT

AGCATTAAGCGTGGAGATAATCAATGGGTAGACAAGATTTATGAACTCATGGATTCTGTGGATAGCTACA

TTCCAATTCCCCAAAGGCAGACTGATTTACCATTTTTACTTGCTATTGAAGATGTGTTTTCAATTACTGG

TCGTGGTACTGTGGCCACAGGCAGGGTTGAAAGAGGAACTGTTAAGGTGGGTGAAACTGTAGATATTGTG

GGTTTGAGGGAGACTAGGAATACTACTGTGACTGGAGTTGAAATGTTTCAAAAGATTTCGGATGATGCCA

TGGATGGTGATAATGTGGGATTATTGTAGAGAGGTGTCCAAAAGGCTGATAT

>FM895625.1 FM895625 Jatropha curcas embryo 71-95 (DAF) Jatropha curcas cDNA clone rjcpga0_002577, mRNA sequence

TGAAAACATATATAGATTATAAAGCTAAAGACATTAATGTTTTGGGTATAAAAGAGTTCACAAAACCTTA

ATACCAACATACATTGATTGATAAAATAAATAATAACCCCTAACTATCTTCACCACCATTCTCTCCAAAC

CAAGAGGACAAGGGAAAGAAAAAAACTCCAAACCAATAATAGTTCATAAAGGACTAACGAAAATAAATAA

ATTAAAAAAAAATAAATGACTCCCAAAACTTCAAAACCCCTCCCTTTCTGTACCTCTCCAAAGATATCAG

CAAATTCTCCCTCCAAACTTCAATCGAACTCTACTGTTGCTTGCACTCTGGAGGCCTCTCACCCTGCTGA

CCATCCCCAGCTTGTGTACTTCAAACCTTATTATACCCGGCTGACAGCTTCTTTAAAGTCTTGCTTTCAT

CCTCGTACTCATCCTCATATTCATCCTCTTCATCCTAATCATCATCATCAATATCTTCATCGTCATCGTC

GTCATCATCATCATCATCATCTATTCCAAAGTCATCCCACTGAAT

>FM895624.1 FM895624 Jatropha curcas embryo 71-95 (DAF) Jatropha curcas cDNA clone rjcpga0_002576, mRNA sequence

CCGTTGCTGAAGACATGTTTCATTGGCAAGCTACGATCATGGGTCCCCCTGACAGTCCATATGCAGGGGG

TGTTTTCCTTGTTACTATTCATTTCCCTCCGGATTATCCATTTAAGCCTCCCAAGGTGGCTTTCAGAACA

AAGGTATTCCACCCAAATATTAACAGCAACGGGAGCATTTGTCTTGACATATTGAAGGAGCAATGGAGCC

CTGCTCTAACCATATCTAAGGTGTTGCTTTCAATTTGTTCTTTGTTGACGGATCCCAATCCTGATGATCC

GTTAGTGCCAGAGATAGCACACATGTACAAGACAGACAGGAATAAGTATGAGACAACCGCTAGAAGCTGG

ACTCAAAAGTATGCAATGGGCTAAAATGGTATCTGGTGCTTGCGAGGGGAGGTCATTTTCTATTCATTTT

ATTTTTGTGTTTCAGTTGTGTATCAATGTTTGTCCTGTCTCCAATCAAAATGGGCTTAAGGAGGTCCATA

ATAACGATTTAGCTCAAGGAAAACATATATATGTTGCGTTTCAATTGATGTATGGATAAAATATTAAAGT

GGGAACTGTGCCAATGTGTCCCCAATTTAATGTCAACTTATGTACGCTCAAACTGTGAATGTGTCCCATA

TTCTGTCTGACTTTGTATCTGCTTATGAATCAAATATGACCTGT

>FM895623.1 FM895623 Jatropha curcas embryo 71-95 (DAF) Jatropha curcas cDNA clone rjcpga0_002575, mRNA sequence

TTTTTTTATATCAAAAATCAGAAAAATCATTAAACCGACTACTTTCATCATTACATAGTGTTTTCTGAAT

ACTGAAATCCTTGCCAAACTAATTTTTGCTTCAGAGGCAGGAGAATGAAACTACGGAAGCCTCACATATA

AAAATGTCATTTACACTCTTCAGTCTTCATACATTGGACGCTCCAGAACAAAGCCAAAGAAAAAAGGAAA

AAAAATTAGGAATAGCCAAGAACCCAGCCAGCAAGAGTGTAAAATCCCCTAAACTGAAGATCTAACAAAA

GTGAAATTCATAGGACACCGATCAGCCTTTCCAAACATCTGTTTAGGTTTCACGTCTGGATTGCCTTCTG

CGAGGAGCAGCAGCAGCAGCATCCTCCTTCTCAGTCTCTTCTTCCTTCTCCCCACTGCTTCCTTCTTTGT

TGGAAGTCTCGGTGGCTGGTGCAGGTTTGTCTACTTTTGCTGGCTTCTTTCCACCAGAAATTATTTTAAG

GAGAACTGTGAATATGACCACAACAATGGAGACAAGAATACCAATAGTGAGGTAAGGTTGTTTCTCTGCC

TTCTCAATGACATCAAGGATTTTGTCCTTGTGCCCACTTAAGAAAGGAATATC

>FM895622.1 FM895622 Jatropha curcas embryo 71-95 (DAF) Jatropha curcas cDNA clone rjcpga0_002574, mRNA sequence

GCTGACATGTGTGCGAGTAACGGGTGAGTAAACCCGCAAGGCGCAAGGAAGCTGACTGGCGGGATCCCCC

TGAGGGTTGCACCGCCGACCGACCTTGATCTTCTGAGAAGGGTTCGAGTGAGAGCATGCCTGTCGGGACC

CGAAAGATGGTGAACTATGCCTGAGCGGGGCGAAGCCAGAGGAAACTCTGGTGGAGGCCCGCAGCGATAC

TGACGTGCAAATCGTTCGTCTGACTTGGGTATAGGGGCGAAAGACTAATCGAACCGTCTAGTAGCTGGTT

CCCTCCGAAGTTTCCCTCAGGATAGCTGGAGCTCGGGACGAGTTCTATCGGGTAAAGCCAATGATTAGAG

GCATCGGGGGCGCAACGCCCTCGACCTATTCTCAAACTTTAAATAGGTAGGACGGCGCGGCTGCTTCGAT

GAGCCGAGCCACGGAATAGAGAGCTCACAAGTGG

>FM895621.1 FM895621 Jatropha curcas embryo 71-95 (DAF) Jatropha curcas cDNA clone rjcpga0_002573, mRNA sequence

CTAGAACCCACAAAGGGTGTTGGTCGATTAAGACAGCAGGACGGTGGTCATGGAAGTCGAAATCCGCTAA

GGAGTGTGTAACAACTCACCTGCCGAATCAACTAGCCCCGAAAATGGATGGCGCTTAAGCGCGCGACCTA

TACCCGGCCGTCGGGGCAAGAGCCAGGCCCCGATGAGTAGGAGGGCGCGGCGGTCGCTGCAAAACCCGGG

GCGCGAGCCCGGGCGGAGCGGCCGTCGGTGCAGATCTTGGTGGTAGTAGCAAATATTCAAATGAGAACTT

TGAAGGCCGAAGAGGGGAAAGGTTCCATGTGAACGGCACTTGCACATGGGTTAGTCGATCCTAAGAGACG

GGGGAAGCCCGTCCGACAGCGCGTCCTCGCGCGAGCTTCGAAAGGGAATCGGGTTAAAATTCCCGAACCG

GGACGTGAAGGCTGACGGCGACGTTAGGGAGTCCGGAGACGTCGGCGGGGGCCTCGGGAAGAGTTATCTT

TTCTGTTTAACAGCCCGCCCACCCTGGAAACGACTCAGTCGGAGGTAGGGTCCAGCGGCTGGAAGAGCAC

CGCACGTCGCGCGGTGTCCGATGCGCCCCCGGCGGCCCATGAAAATCCGGAGGACCGAG

>FM895620.1 FM895620 Jatropha curcas embryo 71-95 (DAF) Jatropha curcas cDNA clone rjcpga0_002572, mRNA sequence

ATTCCAAAGGATTCTATCCGTCGCCCGATGGGAATTGTACTTTAAGGCGGCCCTCGCGGCTCGTCCGCCG

CGGGGGCTTGGCCAACGGCACGTGCCTCTGGGGGCCGGATGGCCCCTACTGCGGGTCGGCAAACGGGCGA

CGGACACGCGCGTCGCTTCTGGCCCGGATTCTGACTTAGAGGCGTTCAGTCATAATCCAGCGCACGGTAG

CTTCGCGCCACTGGCTTTTCAACCAAGCGCGATGACCAATTGTGCGAATCAACGGTTCCTCTCGTACTAG

GTTGAATTACCATCGCGACACCATCATCAGTAGGGTAAAACTAACCTGTCTCACGACGGTCTAAACCCAG

CTCACGTTCCCTAATGGTGGGTGAACAATCCAACACTTGGTGAATTCTGCTTCACAATGATAGGAAGAGC

CGACATCGAAGGATCAAAAAGCAACGTCGCTATGAACGCTTGGCTGCCACAAGCCAGTTATCCCTGTGGT

AACTTTTCTGACACCTCTAGCTTCAAATTCCGAAGGTCTAAAGGATCGATAGGCCACGCTTTCACGGTTC

GTATACGTACTGGAAATCAGAATCAAACGAGCTT

>FM895619.1 FM895619 Jatropha curcas embryo 71-95 (DAF) Jatropha curcas cDNA clone rjcpga0_002570, mRNA sequence

ATTCATCAGTTTCATTTGAAGGAAGTAAAAGAGTTTATTTTCCTTTCAAGAGAGATAAACAGAAAGATGG

AACGCGGAGGCTTCCATGGCTATCGCAAGCTCCCTACTACAAGCTCTGGACTGAAGCTGTCCGAGATGAA

CATGAGGCTTGATGAAATCAATCGCAGCGGGGGCAGTGGCGGCAGCAACAACAACAAGAACAACACTAAC

TCTACTGGTGATGACAATGAGTGCACTGTGAGGGAACAAGACAGGTTCATGCCAATTGCCAATGTTATCC

GCATCATGCGCAAGATACTCCCTCCGCACGCCAAAATCTCAGATGACGCCAAAGAGACAATCCAAGAATG

CGTGTCTGAGTACATAAGCTTCATTACTAGCGAAGCTAATGAGCGCTGCCAACGCGAGCAGCGCAAGACT

ATAACAGCTGAGGATGTGCTTTATGCAATGAGCAAGCTAGGGTTTGATGACTACATTGAACCCTTGACTG

TGTATCTACACCGCTATCGGGAGATGGAAGGGGACCGTAGCTCGATAAGAAGTGAGCCATTGGTGA

>FM895618.1 FM895618 Jatropha curcas embryo 71-95 (DAF) Jatropha curcas cDNA clone rjcpga0_002569, mRNA sequence

ATTCCTTAAGACCTCTTTAGTATTTGTAGTCCTTGACATGGAGACTCTCTGAGGCACCCTCACCAAGAGT

AGCATTACCCTGNGTAAGTTCCAAGAGTTGCCTCTGAGTTGGCCTTAGCCCTGACCAGGAATGCAGCTTG

AGCCTTCTGCACATTCTCTGGCTTACCTGCCCAAGCCTTGAGGGTACTTTGTTGAAGTGCACGTCCAAAT

GAGAAGGAAAGGGACCATGGCTTCTTGGTCTTGAGCTTGTTCATCGCATTCAGGTTGACGGTAGCTTCCT

CCTCACTCTGCCCACCAGACAAGAACACAACAGCAGGAACAGCGGCAGGCACAGTGCATTGGAGGGCACG

GACCGTGTACTCAGCAATCACCTCTGGTGCAACCTTGGCGGATTCTGATCCTGGAGTCACCATGTTGGGT

TTCAAAAGAGTTCCCTCAAGCAAAACATGGTGGTCATTGAGAGCCTTGTAGCAAGCAGCAAGAACCCGCT

CAGTTACATCAGCGCACTTCTTAATGTCATGAGGTCCATCAACCAAAATCTCAGGCTCCACAATTGGTAC

CAAGCCATTCTCCTGGCAGATGATGGCATACCTAGCCAACCCATTCGCATTCT

>FM895617.1 FM895617 Jatropha curcas embryo 71-95 (DAF) Jatropha curcas cDNA clone rjcpga0_002568, mRNA sequence

CTTCTGCAAGTGCAATGAAAAACACTCCTCTTAAGCTCTCGTATGTTTATCAATGCATTGGTTGTGATTC

TTTTCATCTGCAGCCTATTGGCAGGACTATCTCCAAGAACAACAGTGTAAGATATCTGCCTGGGTTTGGT

CCTACTGTTCCTCAAGAGTGCAGTGACTGTGGGAAGAAATTTAACATGGGTGGACCTATATGGTCTGCAC

CGATTCATGATCAAGAATGGGTCGCTTCTATATTAGAAGGTGTAAAATCTATGAAGGACCGGTATCCTGC

TTATGATAAAATCTCTGCTGTACTGACAACAGTATCTGAGGAATTGCCTGATGTTCCTCTCTTTTGGAGT

CTGCACAACCTATGCGCAACACTGAAATGCACTGCCCCATCCGCAGTAATATTGCGTTCGGCTGTGATCA

ATGCTGGTTATCGTATTTCTGGAACACATGTCAATCCACTGGGGCTAAAATCAGATGCTCCCATGGATGT

CATTTGGGATATAATGAGATGCTGGGTCAAAAATCATCCAGTGAAAGCTCAAGCACCTGATCACTCAGGA

AGTGTGATACTTGCTAAAGAACCAGTTCTTCAAGCAAATTTTG

>FM895616.1 FM895616 Jatropha curcas embryo 71-95 (DAF) Jatropha curcas cDNA clone rjcpga0_002567, mRNA sequence

CGAGGAGTTTGGGATTAGAAACCCTAATTTCCTCTCTCTACTTTCTAAAAGCCTCCAATCGCTCTATTTC

TCTATCAATCTCTCTCTCTCACTAGCAGTCATGGCAGGTTTGGCACCAGAAGGATCACAGTTTGATGCTA

AGCAATATGACGCCAAAATGAATGAATTGCTCTCAAGTGATGGACAAGATTTCTTCACCACATATGATGA

GGTTTATGACAGTTTTGATGCAATGGGATTGCAAGAGAACCTTCTGAGAGGCATCTATGCTTATGGTTTT

GAGAAGCCTTCTGCTATCCAACAAAGGGGAATTGTTCCATTTTGTAAGGGTCTTGATGTTATTCAGCAGG

CCCAATCTGGAACTGGAAAAACGGCAACATTTTGCTCCGGCATTCTGCAGCAACTTGATTATGCTACAGT

TCAATGTCAAGCGTTGGTGTTGGCTCCGACTAGAGAACTGGCACAGCAGATTGAGAAAGTTATGCGTGCC

CTTGGTGATTATCTTGGTGTGAGGGTCATGCTTG

>FM895615.1 FM895615 Jatropha curcas embryo 71-95 (DAF) Jatropha curcas cDNA clone rjcpga0_002566, mRNA sequence

GTTCCACACGAGATTTCTGTTCTCGTTGAGCTCATCTTAGGACACCTGCGTTATCTTTTAACAGATGTGC

CGCCCCAGCCAAACTCCCCACCTGACAATGTCTTCCGCCCGGATCGGCCGCCGAAACGACCTTGGGTCCA

AAAAGAGGGGCATAGCCCCGCCTCCGATTCACGGAATAAGTAAAATAACGTTAAAAGTAGTGGTATTTCA

CCGTCGCCTTTCGGCTCCCACTTATCCTACACCTCTCAAGTCATTTCACAAAGTCGGACTAGAGTCAAGC

TCAACAGGGTCTTCTTTCCCCGCTGATTCCGCCAAGCCCGTTCCCTTGGCTGTGGTTTCGCTGGATAGTA

GACAGGGACAGTGGGAATCTCGTTAATCCATTCATGCGCGTCACTAATTAGATGACGAGGCATTTGGCTA

CCTTAAGAGAGTCATAGTTACTCCCGCCGTTTACCCGCGCTTGGTTGAATTTCTTCACTTTGACATTCAG

AGCACTGGGCAGAAATCACATTGCGTGAGCATCCGCAGGGACCATCGCAATGCTTTGTATTAATTAAACA

GTCGGATTCCCCTTATCCGTACCAGTTCTGAGTCGACTGTTCGACGCCCGGGGAAGGC

>FM895614.1 FM895614 Jatropha curcas embryo 71-95 (DAF) Jatropha curcas cDNA clone rjcpga0_002565, mRNA sequence

ATTCGGCACTTCTTTTCATGGCTCTGCTTTGCAAGAATGGCAGGAGATGGCTAACGTATTTTTTTTGCAA

TCCAAGAATACTTTCTGCTGTGGACAGATTGACAAAAGAGAAGGACAGCTATCTGCAGCAGTGTTTGGAT

GCATTTGTGCACATTGTTGCATCTACAGTACCAAGCTTACTGGACGTTATAACTGCAGATATTCAGCAGA

TGATGGGAGGAAGGCGCCATGGGCAGAGCTCTGCCATTTCCAATCGGGTTGCTTCAAAGACTAATGTTCA

TTTATTTCCTGTTGTGCTTCATCTTCTTGGGACGTCTTCTTTTAAACGCAGGTTGGTGAATCGTCAGGTG

CTACAGCAATTGACAAATTTGGTCAAGATTGTGGAGACACCATTTCAGGGAAGAGACGACTTCCAGATAA

CTCTTCTGCGAGTTCTAGAGTCTGTTGCAGAGGAATCTTCTTTAATTATTGAAAGCCCCAACATCTTTAT

AGGTGATATTCTCCCAAGTTTGGCTTTTCTATACAAGGGAAATAAAGATGGTGATGCCAGATTTTTATGC

CTGAAAATCCTGTTTGATGTGATGGTCATATTCTTGAAT

>FM895613.1 FM895613 Jatropha curcas embryo 71-95 (DAF) Jatropha curcas cDNA clone rjcpga0_002564, mRNA sequence

CTAAGATTTGAAGAAGGGATCCAAATGCTCAAGGAAGCAGGAGTGGAAGTTGATCCTTTTGGGGACTTGA

ATACAGAAGCAGAGAGAAAATTGGGCCAGCTTGTTTTAGAGAAGTATGGGACTGAGTTTTACATTCTTCA

TCACTATCCATTGGCTGTTAGGCCCTTCTACACCATGCCATGCTATGATGATGCAAAGTACAGCAACTCG

TTTGATGTCTTTATTCGAGGTGAGGAAATTATTTCGGGGGCTCAACGTATCCATGTACCAGAGTTGTTAG

AAGAACGTGCAAAGGCCTGTGGTATTGATGTCAATACAATATCAACATACATTGATGCTTTCAGGTATGG

TGCGCCTCCACACGGTGGGTTTGGGGTGGGGTTAGAGCGTGTGGTAATGTTATTCTGCGGTCTAAATAAC

ATTCGCAAAACATCACTTTTCCCTCGCGACCCTCTTAGGCTTGCTCCATGATGCGTCTCAGTCGGACTCT

GTTCTACAAAGATCAACTTTTTTCCTTCCTCTTGGAAGTTTAGCTTACATAAGTTT

>FM895612.1 FM895612 Jatropha curcas embryo 71-95 (DAF) Jatropha curcas cDNA clone rjcpga0_002563, mRNA sequence

ATTCATTCCTTCTTTCAGCCTGATAACATACAACGGAGGGAAAGGCTATAGCTCATACTATTTTACGTTA

AAGCCATGGCGCTGTATTTACTATACGAGTCGGCGTCTGGTTACAGTCTCTTTCTTGCTAACGGACTCGA

TGAAATTGGACAGAACACAGAAGCTGTTCGCAACTCCGTCGCAGATCTCAACCGCTTTGGCAAAGTCGTT

CAGTTAACTGCTTTTCTTCCTTTCGAGTCATCTTTAGATGCATTGAATCAGTGTAACTCAGTCTCTGAAG

GGCTTATGACTGAGGAATTGAGGAGCTTTTTAGAACTCAATCTTCCTAAAGTCAAGGAAGGTAAGAAGGC

AAAGTTTAGTTTAGGAGTTGCTGAGCCTAAATTGGGGTCACATATATATGAAGAGACAAAAATACCTTGC

CAAAGCAATGAATTTGTTCTTGAGCTTCTTCGTGGTGTTAGACTACATTTTGAAAGGTTCATCAATGACC

TACAGCCTGGTGACTTGGAGAAAGCCCAACTTGGTTTGGGGCACAGTTATAGCAGGGCAAAGGTCAAGTT

CAATGTCAACCGTGTTGATAATATGGTTATTCAAGCAATTTTCCTTCTTGATACTCCTTGACAAGGATAT

TAATTCCTTTTCAATGAG

>FM895611.1 FM895611 Jatropha curcas embryo 71-95 (DAF) Jatropha curcas cDNA clone rjcpga0_002562, mRNA sequence

ATTCGCAGCTCATATAGAGAAACACAATAAAAGCAGTTTCACAGGAAGAGAAAGAAGAAGAACAAGTATT

GGGAAGAATGGAAGCGCCAAAATCAATTGCTCATGAAATCGGGGGCATCCAAAACGATGCTCTTCGATTC

GGCCTTCATGGCGTCAAGAGCGACCTCGTCGGAGCTCACCCTCTCCAAACAGCCTACCAATCGTCCAGGA

AAACCCAAGAGGAGATGAATAGAAAAATCCTTGCAAACACGTATGGGTCAGCCTTCCCGTTGAAGATGGA

TCTTGATAGGCAAATTCTTTCCCGTTTTCAGAGGCCACCAGGACCAATTCCATCTTCAATGCTAGGACTT

GAAGCTCATACAGGAAGCTTGGATGATTTTGGTTTTGAGGATTATTTGAACGATCCTCGCGAATCTGAAA

CTCACCGGCCTCTCGACATGCATCATGGTATGGAAGTACAACTTGGACTTTCCAAGGGACCAGTGTATCC

CAGTTTCATATAGTGCAACCATATAGCCAATGCCTAAAGAGTTCAAAACAGTACTTCATTTTATGTCAAT

TGCTGGTGGACTTTGTTGTTGATGTTCTTAGTATTATGATCTGTTGAACTTATCATACACTCTGAGACCA

TAACAATTTTATTGTTGTCATGTTGCCAA

>FM895610.1 FM895610 Jatropha curcas embryo 71-95 (DAF) Jatropha curcas cDNA clone rjcpga0_002561, mRNA sequence

TCAAGCCTTAATATTTGTTTTCTGTTTGTCTTTCAAGTAAATCTGCAATCTCTGTTTCAAGAAAGAAGCT

AATTAATGGCGTCCAGGCAAGAGAAGGTGGACAAAGCGGAGGCAGAAGCGTGGCAAGCTGCGCGTGATCT

AAAAGATGCTAATAGAGAAAAAGAATATGAAGAAAGGGCAAAAATGGAGACAGACCTGCAACAAGAAGAA

AGACCAGGCGTTATTGGGTCTGTGCTAAAGAAAGTTGCTGATACCTATGAGCATGCCAGAGAAGCTGTTG

TAGGTAAAAGCCAAGAGGCTGCTGAGAAGGCGAAGGAAGCCAATTATAAAGCAGCTGAGAAAGCAGGAGA

GACTAAAGATTACACTGCAGAGAAGGCGGCGGAGACGGCAGACGCAGCTGCAGGAAAATGGGACGAAACA

AAAGAATCTGCAAAAGAGAAGGCGGACCAGGCAAAAGAAAAGGCCAAAGAAACGAAGGACTCGACTACGG

GAAAGTTAGGTGAGTACAAAGATTATGCAGCTGAGAAGGCGAAAGAGAGTACAGAAATGGCGAAGGGAAA

GGCTGGAGAGTACAACGATAGAGCTG

>FM895609.1 FM895609 Jatropha curcas embryo 71-95 (DAF) Jatropha curcas cDNA clone rjcpga0_002559, mRNA sequence

GTCCACTTCAAGAGCTAAATGCGGACGCTTTGCCTTCGTCATTTTCTCAACCAGAGCATTTACCCTTGTC

TTCAACTGCTGCAAATCCTTTGCTTGGAGAATCCTTTTGGCAATGCAACAGCCCTGTTCAAAGTAACCAG

TTATGGTCTCAAAATATGCAAGACCTTGGAGTCTGCGAACACATTGTTTGTCATGATGATATTAACATAC

CCGATGTTGATTTAACATTTCGGAACTTCGAAGAACTGTTTGGAGGTGAACAAGACCCTGTTAGGGCACC

GCTTGATGATAAAGATGCATCGTGCTCCTCTCTGGAGAAGGATATGTCCCTTGATACATTGCATAACAGA

AATGCGAGAAAGGGGACTTCTATTGCATCATCTGCTTACATTAGTCGGCCTGTTCACATGGACAAAGATA

AAGGTCCTTCTACCCAAGTTTACACCTTACCTGGAAGTCTGCATGCTCCTCGGACAATTCAGCCATCTTA

TTCGGCAATGTCATTCTCTGTTTCAAGGCTTAGTGCTGAAAGCAGTGCCACCGAATATATCGATAGTGGA

TTTTCGCCATATATCATAGGGGCTGAAGTTTCATGTCGTACTCCTGAAAAGGGCGCACATTCAGGAGATA

AA

>FM895608.1 FM895608 Jatropha curcas embryo 71-95 (DAF) Jatropha curcas cDNA clone rjcpga0_002558, mRNA sequence

TATGCTTGTCTCAAAGATTAAGCCATGCATGTGTAAGTATGAACTAATTCAGACTGTGAAACTGCGAATG

GCTCATTAAATCAGTTATAGTTTGTTTGATGGTACCTGCTACTCGGATAACCGTAGTAATTCTAGAGCTA

ATACGTGCACCAAACCCCGACTTCTGGAAGGGATGCATTTATTAGATAAAAGGTCGACGCGGGCTCTGCC

CGTTGCTCTGATGATTCATGATAACTCGACGGATCGCACGGCCATCGTGCCGGCGACGCATCATTCAAAT

TTCTGCCCTATCAACTTTCGATGGTAGGATAGAGGCCTACCATGGTGGTGACGGGTGACGGAGAATTAGG

GTTCGATTCCGGAGAGGGAGCCTGAGAAACGGCTACCACATCCAAGGAAGGCAGCAGGCGCGCAAATTAC

CCAATCCTGACACGGGGAGGTAGTGACAATAAATAACAAATACCGGGCTCAATGAGTCTGGTAATTGGAA

TGAGTACAATCTAAATCCCTTAACGAGGATCCATTGGAGGGCAAGTCTGGTGCCAGCAGCCGCGGTAATT

CCAGCTCCAATAGCGTATATTTA

>FM895607.1 FM895607 Jatropha curcas embryo 71-95 (DAF) Jatropha curcas cDNA clone rjcpga0_002557, mRNA sequence

CTGACATGTGTGCGAGTCAACGGGTGAGTAAACCCGCAAGGCGCAAGGAAGCTGACTGGCGGGATCCCCC

CGAGGGTTGCACCGCCGACCGACCTTGATCTTCTGAGAAGGGTTCGAGTGAGAGCATGCCTGTCGGGACC

CGAAAGATGGTGAACTATGCCTGAGCGGGGCGAAGCCAGAGGAAACTCTGGTGGAGGCCCGCAGCGATAC

TGACGTGCAAATCGTTCGTCTGACTTGGGTATAGGGGCGAAAGACTAATCGAACCGTCTAGTAGCTGGTT

CCCTCCGAAGTTTCCCTCAGGATAGCTGGAGCTCGGGACGAGTTCTATCGGGTAAAGCCAATGATTAGAG

GCATCGGGGGCGCAACGCCCTCGACCTATTCTCAAACTTTAAATAGGTAGGACGGCGCGGCTGCTTCGCT

GAGCCGCGCCACGGAATCGAGAGCTCCAAGTGGGCCATTTAAGGTAAGCAGAACTGGCGATGCGGGATGA

ACCGGAAGCCGGGTTACGGTGCCCAACTGCGCGCTAA

>FM895606.1 FM895606 Jatropha curcas embryo 71-95 (DAF) Jatropha curcas cDNA clone rjcpga0_002556, mRNA sequence

ATTCAAAGGATAATATTATTGAATGGGATAAGATTAGTTAATTAGTCTGACCAAGGTAAAATTCAGGCGT

TCCTGTAATTCTGATTTTGGGCAAGGTAAAATTCCATCTTCCCTCATCTTTAACATGCTCTGCTCTGGCG

CAGAACACCAGTGAATGGACTTAAAAGAGAGAGAGAAACAAAATACAAATGCGCAAAAGATGTAGTATCA

AGATCACTTTATGCTATCAAACAGGAACCCCTAGTTCAGAAATCTTCATTATCCGTTTGTCCAGCGCAGT

CCTGTGCTGCTTCAAACCTGATGCCATAGAAGCAACCTCAGCTCGAAGTTTCAAAGCCTCCCTACCAGAG

ATTTCTCGTAGCCCATCCATCAAATCTGCAACTTGGTTTTCAACCTTGTAAACTGATTTGGCAAGACGTT

GAATTTGACTTCCAGAATCCAAAGAGCTCAGTAAGGCCATGTTTCATATCCTTTTCAGCATAAGATGCCC

TTTCAAGTAGTTTACTGCTCTCATTCTTCATTAAATCTACAGAGAGGGTCAACTCTTTTAAATTCTTTTC

AGCTTTAAGAAATTTAGCCTCCTCACTCTGA

>FM895605.1 FM895605 Jatropha curcas embryo 71-95 (DAF) Jatropha curcas cDNA clone rjcpga0_002555, mRNA sequence

CGAATTCCACAATCAACGAGTATCAGGTTAACCGTGCGCGTTTGCACAACAAGAAAGCCGGTCTCGATTC

GCTTTGTGAAGTTGTTTGTGGGAATTTCCTTGAGATGCCGTTTCCAGACAACAGTTTCGACGGCGCGTAC

TCTATCGAAGCTACTTGTCACGCGCCGAAGCTGGAAGAGGTATACGCAGAGATCTTTCGAGTGTTGAAGC

CAGGATCTCTCTACGTGTCCTACGAATGGGTCACAACTGACAAGTACAGGGCTAGTGATCCTCAACACGT

GGAGATCATTCAAGGCATTGAAAGAGGAGATGCTTTGCCGGGGCTGAGAAGCTACGCAGACATTGCCGAG

ACGGCGAGAAAGGTAGGGTTCGAAGTAGTGAAAGAGAAGGATTTAGCAAAACCACCAGCGCAGCCATGGT

GGACTAGGCTAAAGATGGGAAGGATTTGCCTACTGGAGGAATCACATTCTCGTTACTATATTGGCTGCTT

TGGGTATCGCACCGAAGGGAACCGTGGATGTCCACGAGATGCTTTTCAGAACAGCCGATCATTTGACACA

GGGCGGCGAGACTGGAATTTTCTCTCCGAT

>FM895604.1 FM895604 Jatropha curcas embryo 71-95 (DAF) Jatropha curcas cDNA clone rjcpga0_002554, mRNA sequence

CTCAAACCCTAACAATGGCAGCGGCTCCAATCGAGTCAGTCCAGTGCTTTGGACGCAAGAAGACCGCTGT

GGCTGTCACCTACTGCAAGCGTGGAAGAGGCCTAATCAAGATCAATGGATGCCCAATCGAGCTAGTGGAG

CCTGAGATCCTCCGTTTCAAGGCATATGAGCCTATCCTTCTTCTTGGACGACACCGTTTTGCTGGCGTCG

ACATGCGCATTCGCGTCAAAGGTGGTGGTCACACCTCGCAGATCTATGCTATCCGTCAAAGTATCGCTAA

AGCTCTTGTCGCCTTCTATCAGAAGTACGTCGACGAGCAGAGCAAGAAGGAAATAAAAGATATTTTGGTC

AGGTATGATAGGACTTTGCTTGTAGCTGATCCCAGACGATGCGAGCCAAAGAAGTTCGGTGGTCGTGGTG

CTCGCGCCAGGTTCCAGAAGAGTTACCGTTGAAGTCGAGGTTATCCTTTTTTTTATGCGTCTGTTATTTT

CGAAAGTGTATTGAATTATGGATTTTGCTATTTGAGGACTAAAAATGTTAATGGATTTATGTTGAGCGGT

GTATATTTAGAGAATTTTACTCTCAATAG

>FM895603.1 FM895603 Jatropha curcas embryo 71-95 (DAF) Jatropha curcas cDNA clone rjcpga0_002553, mRNA sequence

ATTCTCGAGTTTATATTATTTATATTTCAAGTCAAGTCATCAAAATTTATAATAGTTTTGTGTGGTTATT

ATAAACCCCTCAGATATATCAGAGGAAAAGAGGACAAAAAATAATGTGTAGTTTACAAGCTTAAGCAAGA

AAAAAAATATCAAGGCTTCGATTGGTATCCCCATGGATTGTATGCTTGCCCAATTCCATTGGTCTCTGCA

CATCTCCGCTATACCATATTTCGCCTTCCAGCCAAGTTCTCTCTCAGCTTTCTCTGTGGAAGCATACACA

GCAGTGGCATCTCCAGGTCTTCTTGGACACAGATTGATGGGGATTAACTATGCCAGAAGCCTTCTCAAAT

GCAGCAACCATATCAAGCACAGACGTTCCTTGTCCCGTACCCAAGTTATAGGCTACACAACCTATATTCT

CTGTTGTAAAAAGCTTCTGAAGAGCAGGAATGTGACCAT

>FM895602.1 FM895602 Jatropha curcas embryo 71-95 (DAF) Jatropha curcas cDNA clone rjcpga0_002552, mRNA sequence

GATATAAACAAATTCATGATTGGAGTTAATTTTCTACTTCTATTTCACAATTCAAATATATTCCCTATTG

GATTTCCCTGTTCATATATGTTACAACGATTCGGAAAATCATTCCCCCAATAATATTTATTAAAAAAAAA

AAATCAAAGCTCCTCAGTGATGGCCTCTAAAGCTGGAGTCCATTGCCTGCATTTCTCCAATCGGCAACAG

GCAAAACCCATATCCAGCTCTTCCTTAACCTCACTCTCTGCCTCATTAGCCACAGCCGGTAAAACCTTAG

CGCCCCTCCGCCCTTTGCTTGACTTTTTCTTCTTAGATGCAGCAGCAGCAGACTCTATAATCCCCAGCTC

TGACTCCGAAATCTTACGGTTAAGCTTACTTAAATGAACTAGCAGGTAAAACTTTCCCGCCAAAAGCTCA

TCCTCAGCTCGCAAAGCTATTAACCGGCGGGTCCGCCGGAGTTCTTGCACAGGAGAGACGACGTGGCCGG

GTTCTTCGAGCATGAGCTCCGCTGCTTTAGCTGGGGCCTTTACTTGCCTCAAGTACCCTTGAGAATCTAT

AAGCTTAGCAGTTTTACTGGGGA

>FM895601.1 FM895601 Jatropha curcas embryo 71-95 (DAF) Jatropha curcas cDNA clone rjcpga0_002551, mRNA sequence

ATTCCCGAGGGTTGCACCGCCGACCGACCTTGATCTTCTGAGAAGGGTTCGAGTGAGAGCATGCCTGTCG

GGACCCGAAAGATGGTGAACTATGCCTGAGCGGGGCGAAGCCAGAGGAAACTCTGGTGGAGGCCCGCAGC

GATACTGACGTGCAAATCGTTCGTCTGACTTGGGTATAGGGGCGAAAGACTAATCGAACCGTCTAGTAGC

TGGTTCCCTCCGAAGTTTCCCTCAGGATAGCTGGAGCTCGGGACGAGTTCTATCGGGTAAAGCCAATGAT

TAGAGGCATCGGGGGCGCAACGCCCTCGACCTATTCTCAAACTTTAAATAGGTAGGACGGCGCGGCTGCT

TCGCTGAGCCGCGCCACGGAATCGAGAGCTCCAAGTGGGCCATTTTTGGTAAGCAGAACTGGCGATGCGG

GATGAACCGGAAGCCGGGTTACGGTTGCCCAACTGCGCGCTAACCTAG

>FM895600.1 FM895600 Jatropha curcas embryo 71-95 (DAF) Jatropha curcas cDNA clone rjcpga0_002550, mRNA sequence

TATTTTATTTTACAAGTTAAATCTAATTTTATAACTGAATCCAACATAACATCTTAAAGACACAATATTA

AGTCTAAATGGCACAACATACAACTCGCATTTTATTGGATGCAGCACACATGTTATCAAAAGATAAAACA

TATTGATTCAGCTTCAGTATCCCAACAAAGCAACAGCATTTGGAAGATTAGCATTGATTTGGTGCTTCTG

AACCAGCATGCTCTCTTTTGCGACTTGATTTGTATTTTCAAATGCTTGGCTTCACGTTCAGACGAGACGC

TAGCTTCTGACCCAGAGATCTGTCGCACTGAGTAAAGTAGTTGATACCAAATACTACAAATCTCATGTGT

GACACGAGGATCAGATAAGCAACCAACAATCCGATTGAGGAATCGCTCTTGCCTGTCAGGAGCCCAGGAT

CGATATCTCTCTCC

>FM895599.1 FM895599 Jatropha curcas embryo 71-95 (DAF) Jatropha curcas cDNA clone rjcpga0_002548, mRNA sequence

TGCTCCATCAGGTTATTCGATCAATGTTGCCACTGCTAATGCCACTCAGGTTTTATTAGCTACTGGCGGC

GGTCATTTAGTTTATCTGGAAATTGGTGATGGAACATTGACAGAGGTAAAACATGCTCAGTTGGAGTATG

AGATATCATGCCTTGACATTAATCCTATAGGTGAAAATCCCAACAATAGTCAGCTGGCTGCAGTTGGAAT

GTGGACGGATATAAGTGTCAGGATATTTTCATTGCCTGACCTAAATCTCATAACAAAGGAGCATTTGGGA

GGAGAGATAATACCTCGTTCTGTTCTTCTGTGTTCCTTTGAAGGGATATCATACTTGTTATGTGCTCTTG

GAGATGGTCATCTTTTAAACTTTCTATTGAATTTGAATACTGGGGAGTTAACAGATAGGAAAAAGGTGTC

TTTAGGCACCCAACCTATAACACTCCGAACTTTCTCATCCAAGAATTCCATTCATGTATACGCAGCATCA

GATAGGCCAACTGTCATTTACAGCAGTAACAAGAAACTACTATATAGTAATGTGAATTTGGAAGAAGTTA

GTCATATGTGTC

>FM895598.1 FM895598 Jatropha curcas embryo 71-95 (DAF) Jatropha curcas cDNA clone rjcpga0_002547, mRNA sequence

CGAATCCGTCTCTCTCGTGCAACTTTGAACCTTTTTCGTGTCTCTTTCTTTTCCTCGTGTTCGAATCAAA

GCAGAGGGTATCGTATCCTGTTCTCTTCTCTCTAGGAATCTACGCGATCTTTCCTCTCGTGGGTATCACG

AGATTCACTTCCACTATCTACCGCTCTTTCTCTCTCTAATGCATTGAACTCATTTCATTATATTCCAGTC

TCTCTTTAACCATTTAATTTGCACAAAGCAGAGAGATGGCTGGCAAGAATTTGGAGAAGATGGTATCAAT

AGATGCACAGTTGAGGCTATTGGCTCCACGGAAAGTGTCTGAGGATGACAAGTTAGTGGAGTATGATGCT

CTGCTATTGGACCGATTTCTCGATATATTGCAGGATTTGCATGGGGAGGATATTAGAGAAACGGTTCAAG

AGTGTTATGAAATTTCAGCCGAGTATGAAAGGAAGCATGATCCGCAGAAGCTGGAGGAACTTGGGAAAGT

GCTAACGAGTTTGGATCCTGAGGATTCAATTGTTGTTACAAAATCATTT

>FM895597.1 FM895597 Jatropha curcas embryo 71-95 (DAF) Jatropha curcas cDNA clone rjcpga0_002546, mRNA sequence

CTGACATGTGTGCGAGTCAACGGGTGAGTAAACCCGCAAGGCGCAAGGAAGCTGACTGGCGGGATCCCCC

CGAGGGTTGCACCGCCGACCGACCTTGATCTTCTGAGAAGGGTTCGAGTGAGAGCATGCCTGTCGGGACC

CGAAAGATGGTGAACTATGCCTGAGCGGGGCGAAGCCAGAGGAAACTCTGGTGGAGGCCCGCAGCGATAC

TGACGTGCAAATCGTTCGTCTGACTTGGGTATAGGGGCGAAAGACTAATCGAACCGTCTAGTAGCTGGTT

CCCTCCGAAGTTTCCCTCAGGATAGCTGGAGCTCGGGACGAGTTCTATCGGGTAAAGCCAATGATTAGAG

GCATCGGGGGCGCAACGCCCTCGACCTATTCTCAAACTTTAAATAGGTAGGACTGGCGCGGCTGCTTCGC

TGAGCCGCGCCACGGAATCGAGAGCTCCAAGTGGGCCATTTTTGGTAAGCAGAACTGGCGATGCGGGATG

AACCGGAAGCCGAGTTACGGTGCCCAACTGCGCGCTAA

>FM895596.1 FM895596 Jatropha curcas embryo 71-95 (DAF) Jatropha curcas cDNA clone rjcpga0_002545, mRNA sequence

CTTGGAAGCTTTCCTACTATCCTTTTATTCCCCAAACATTCTTCTAGGCCAATCAAGTACCCCTCAGAGA

AAAGGGATGTAGATTCCTTGTTAACCTTCGTGAATGCTCTCCGTTGATTGCAATGAAAGATAATTTGAGA

AACATGTGTTTGAATCACTTGAGCATAAAAGTCGGAGCTTTTCTTTCATCCTCATGAATTAAAGCCGTTC

ATTGAATCAAAGGTGAACTCCCAATGACATTTGGAATAATAGAGGCGGCTGCCAGCCTCAACCACAAGCT

AAACTTAGTTGTTTTTGCTTCTTCCAGAACTGGCAGCCGTGGCAACATAGTATAACTTTGTCTTTTGTAT

ACTGTTAGTTTATACTTTTCTTCACCCCCCTATTTTCCTTGTATAAGAGTCTGTTTCGTTTCCCCCCTCA

CAAAAGAAAAATATAAAAAATAAAAAAAATGGAAAGAAAGAAAGAAGGGAAGGAAAGAGTCTGTTTCAGG

CCAGAAATTGATTTTGGCTGAGATTGTTTGCTGATGTACTTGGAAATGG

>FM895595.1 FM895595 Jatropha curcas embryo 71-95 (DAF) Jatropha curcas cDNA clone rjcpga0_002544, mRNA sequence

GAACAAATATATTGCTTGCTATTATTGTATTAATTAATGGACAATAAGCCCAAAGGTTAATCTAAAAGGT

ATCAAAGATTCTACATCATACAAACATTTCCATGTTATGCTAAGCTTTGGCATGCTGGTTATATTTTTAA

CCAGCTTCCCAGAAGTGGATGATTAAATACACCACAGGTATATTTACAGGAGAACTTACTTTTCCAGGTT

CAGTTCATGAGAGTGTTCAACATGGATATGGGGGCAAGGTCTTTCTGGTTCTCCTCGTGTCCACAAAACG

TTGAGGTCGCCTCTTTTCTTTCCCAATGCCAAAAAGGTTCCAAGAGTGAATGGAACAATGTAAAGCAGTG

CTGGTTGGCCATGCCCATCCATCAAGTTCAGTGCCACATATGTTATGAGTAGACCTAATCCATAAGCAAT

CATTGCCCACAAGAAGTATCCAGCTCGAAGACTTTAATTTGCCAGCCAATCGTACCTAAGTGAAAATGCT

ATCAGCAATCCAGGCAAAAGAATATCGACAAATCCTATGATGCTGTAA

>FM895594.1 FM895594 Jatropha curcas embryo 71-95 (DAF) Jatropha curcas cDNA clone rjcpga0_002543, mRNA sequence

ATTCGGCGAACCCTAAGGTGTTCTTCGACATGGCCGTTGGCGGCCAGCCAGTCGGCAGAATCGTGATGGA

GCTCTACCAGGACGTCGTTCCTCGCACCGCCGAGAACTTCCGTGCTCTCTGCACTGGCGAGAAGGGGATC

GGCAAAAGCGGCAAGCCTCTCCACTACAAGGGGTCCAGCTTCCACCGTGTGATCCCCGGATTCATGTGTC

AGGGAGGTGACTTCACAGCCGGAAATGGTACCGGAGGTGAATCGATCTACGGAGCAAAGTTCGCTGATGA

GAACTTCGTCAAGAAGCACACTGGCCCTGGCATCCTTTCCATGGCTAATGCTGGTCCCGGAACTAACGGA

TCTCAGTTCTTTATTTGCGTTGCGAAGACCGAGTGGCTGGACGGAAAGCATGTCGTGTTCGGTCAGGTCG

TGGAAGGTATGGATGTTGTGAAGGCCATCGAAAAGGTCGGGTCTAGCTCCGGAAGGACCTCAAAGCCTGT

TGTGATTGCAGATTGCGGTCAACTTTCTTAAGTGACCT

>FM895593.1 FM895593 Jatropha curcas embryo 71-95 (DAF) Jatropha curcas cDNA clone rjcpga0_002542, mRNA sequence

TCCACACTCCAAAAATGGCAGTGAAGCTCTCTGTGATTCTCTGCATTGTTTCAATTCTTTCCATCTCGGG

CGCTAATGGTGCCGGCCACCACCATACAGCTCCGGCACCGTCTGTGGACTGCTCAAGTTTAGTGTTGAAC

ATGGCGGATTGCTTGTCTTTTGTTTCAAACGGCAGTACGACAACAAAACCAGAAAAAACGTGCTGCTCTG

GGTTGAAAACGGTCTTGCAAACAGATGCAGAGTGTCTTTGTGAGGCTTTTAAGAGCAGCGCTCAATTGGG

TGTCGTTTTGAATGTCACTAAGGCTTTAAGTCTTCCTACTGTCTGCAAACTCCAAGCCCCTCCTGCTTCT

AATTGTGGATTGGCTTTGGCTCCTGCTGCTGCTCCTGGTGCTCTTTCACCATCTGTTAGTGGTGCTGCTG

CCCCAACTGTGACCGCCGGAGCAACTGTGTCAGCACCAGCACCATCTCCAGGGAATGCAGGTTCTCGCGG

ATTGTCAAACTCAGTTGGCTCTCTAGCAATTGGCCTTGTAATTGCATCATTCTCTAGTTTCTGAGTAATT

ATGTCACC

>FM895592.1 FM895592 Jatropha curcas embryo 71-95 (DAF) Jatropha curcas cDNA clone rjcpga0_002541, mRNA sequence

ATTCCCTAGAACCCACAAAGGGTGTTGGTCGATTAAGACAGCAGGACGGTGGTCATGGAAGTCGAAATCC

GCTAAGGAGTGTGTAACAACTCACCTGCCGAATCAACTAGCCCCGAAAATGGATGGCGCTTAAGCGCGCG

ACCTATACCCGGCCGTCGGGGCAAGAGCCAGGCCCCGATGAGTAGGAGGGCGCGGCGGTCGCTGCAAAAC

CCGGGGCGCGAGCCCGGGCGGAGCGGCCGTCGGTGCAGATCTTGGTGGTAGTAGCAAATATTCAAATGAG

AACTTTGAAGGCCGAAGAGGGGAAAGGTTCCATGTGAACGGCACTTGCACATGGGTTAGTCGATCCTAAG

AGACGGGGGAAGCCCGTCCGACAGCGCGTCCTCGCGCGAGCTTCGAAAGGGAATCGGGTTACAATTCCCG

AACCGGGACGTGAAGGCTGACGGCGACGTTAGGGAGTCCGGAGACGTCGGCGGGGGCCTCGGGAAGAGTT

ATCTTTTCTGTTTAACAGCCCGACCACCCTGGAAACGACTCAATCGGAGGTAGGGTCCAGCGGCTGGAAG

AGCACCGCACGTCGCGCGGTGTCCGATGCG

>FM895591.1 FM895591 Jatropha curcas embryo 71-95 (DAF) Jatropha curcas cDNA clone rjcpga0_002540, mRNA sequence

GTGCGAGTCAACNGGGTGAGTAAACCCGCAAGGCGCAAGGAAGCTGACTGGCGGGATCCCCCTGAGGGTT

GCACCGCCGACCGACCTTGATCTTCTGAGAAGGGTTCGAGTGAGAGCATGCCTGTCGGGACCCGAAAGAT

GGTGAACTATGCCTGAGCGGGGCGAAGCCAGAGGAAACTCTGGTGGAGGCCCGCAGCGATACTGACGTGC

AAATCGTTCGTCTGACTTGGGTATAGGGGCGAAAGACTAATCGAACCGTCTAGTAGCTGGTTCCCTCCGA

AGTTTCCCTCAGGATAGCTGGAGCTCGGGACGAGTTCTATCGGGTAAAGCCAATGATTAGAGGCATCGGG

GGCGCAACGCCCTCGACCTATTCTCAAACTTTAAATAGGTAGGACGGCGCGGCTGCTTCGCTGA

>FM895590.1 FM895590 Jatropha curcas embryo 71-95 (DAF) Jatropha curcas cDNA clone rjcpga0_002538, mRNA sequence

CGGGGAGAGAGAGAGAGAGAGAGAGAGAGAGAGAGAGAGAGAGATCGAGGGAGGGAGAGAATGGGCAGAG

AAGTGTCAGAGAGTTGCGTTGATAGCTTACTGACGGAGATGGTGTCATCATACTGCAATAGGTTCGACGC

TAATAAACCTGAGCTCGCCGGCCGGCGGATCGAGGCTATTGGCTATCAGGTCGGCCACCAGCTAGCCGAA

CGGTATACTATG

>FM895589.1 FM895589 Jatropha curcas embryo 71-95 (DAF) Jatropha curcas cDNA clone rjcpga0_002537, mRNA sequence

CGCTTGCGCCCGTCTCTTTGATCTCATCTCATAGACAGTTTCGTTCTCCTGCTTCCTCAACTCATAGCCG

GCGCCCAGAGTCCGGTCTCCATCTCCGGTCCCTTCTTTGATTCTCATATTCGATTCCAGCTGTCAACTGA

AAATTCAATGAGTTAAACTGAGTTGTTTCATCTTTTTTTATTATTATTTTTTATTTTAGAGTCTTCTTCC

ATTTGAATTCTCAAATTTCAATTTTACTGTTTTGTTTTGATCTGAAGGCTACATGTACCAAGGAAAGGCA

AGGGAGTCAGTAGCCTTTACCTTGACTTGCCAAAGCGGCTGTAGCTATATCTATGACTGATGAGAAGTTA

ACTGGCCGGTGGTTGTATGGGATCTCTCTGAGATCTCTGTTGACATATCTGCTTACCGAGCTTCTCACAT

TTTTTTTCTTTTATTCTTGAATCGATCGACGATGAGGAGGGTGAGAGTGGGAAAATGGCATAGGATTTAG

CCTTATTGAGAGACACTCCTCTTACATTGAAAAAAGAAACATTTGTATTTGAGTCACCTTCCAGGCATTC

TGCTTTGATC

>FM895588.1 FM895588 Jatropha curcas embryo 71-95 (DAF) Jatropha curcas cDNA clone rjcpga0_002536, mRNA sequence

GTTGATGCATCAAGATCATATTTTCAGTGAAAGGGGCCAGTGCCAGAGCCAAAACTGGCTTTAGAGAAGC

AAGGCATTAATGATCCCAGATTTGGACAGCATAATGAAGACATGCAAATTGGCTATGAAGAGAAACCATC

ATTGCAAACTTTTGAAGGACTTGAGCAGAAATTCCTTGATGACATTTTGAAACTAGCCAAGGATCAAAAT

GATGCTGAGGATGCGGAAAATGCGAGACACAGAGAGAAAATAAATGCAATAAATAATCTATCAAGAACAA

CTAGTAGCACTTCGAGCCCGACATGCCAGCCGTAGAGATGAGTTCCTTCGCAAGGAGTCAAATGCACGAC

AGCATCAATATCAGCAAGCCGTGGTGGACCGATATTGCAGCAGTGGCATGGGCCCTGGTGATCCTCATGG

TTACAATGCACCCTCAGCTGGTAACAATGAAACTCACCGAGCCTATAATACTGATAATTTTGAATCCTAC

AGGGAGCGGGCTCGATTTCTTGGTGGTC

>FM895587.1 FM895587 Jatropha curcas embryo 71-95 (DAF) Jatropha curcas cDNA clone rjcpga0_002535, mRNA sequence

ATTCCAAGGAGTCTGACATGTGTGCGAGTCAACGGGTGAGTAAACCCGCAAGGCGCAAGGAAGCTGACTG

GCGGGATCCCCCCGAGGGTTGCACCGCCGACCGACCTTGATCTTCTGAGAAGGGTTCGAGTGAGAGCATG

CCTGTCGGGACCCGAAAGATGGTGAACTATGCCTGAGCGGGGCGAAGCCAGAGGAAACTCTGGTGGAGGC

CCGCAGCGATACTGACGTGCAAATCGTTCGTCTGACTTGGGTATAGGGGCGAAAGACTAATCGAACCGTC

TAGTAGCTGGTTCCCTCCGAAGTTTCCCTCAGGATAGCTGGAGCTCGGGACGAGTTCTATCGGGTAAAGC

CAATGATTAGAGGCATCGGGGGCGCAACGCCCTCGACCTATTCTCAAACTTTAAATAGGTAGGACGGCGC

GGCTGC

>FM895586.1 FM895586 Jatropha curcas embryo 71-95 (DAF) Jatropha curcas cDNA clone rjcpga0_002534, mRNA sequence

TAATAATATAAAACTTGCTTGCCATCCCCCCATTGGAAATGGGGTGATGATAGTTCTCAAGATACAGTCT

CCACATTCCCATATCTACAACTGTCATCATCATTCAAAAATAGAAGAAGAAAAAAAAAAAAATGGAAACA

TAAAAGGTTAATGGGGACCCCAACATAATTACCAACCAACAAGGCCATGTTGCTTTCATCAAAACTTCTC

ATATGTGATGGTGTTGTAGCTGCAAGAGTCTAGAGTATGCTCCGTCGGCTCGGCTAATTAATTCGGCATG

GCTGCCTTGCTCTACAATCCGCCCATCTTGAACAACTCCGATACTGTCCACCCCTCTAATGGTCGATAAC

CGGTGCGCTACCAGCACCGTGGTTCGACCTCTCATTAGCCTCTCGAGCGCTTCTTGTAGCAAACATTCAG

ATTCCGCATCAAGTGCACTGGTAGCTTCATCTAGTAAAAGTATTGCTGGGTCCTTAAGAACAGCTCTTGC

TATTGCAATTCTTTGTTTTTGTCCTCCAGAGAGCTGAAC

>FM895585.1 FM895585 Jatropha curcas embryo 71-95 (DAF) Jatropha curcas cDNA clone rjcpga0_002533, mRNA sequence

CCAGAATCGCTTCTTTTCGCTTAATAATTCCTATGGAAGAGGTAATCGGCGTTTTTCCATTTCAGCCACC

GCTGTTGTCCCTGATAAAGGGGGAACCGACACGTTCTTCGCCGAAGAAGGCGTTTCGTGGGCTTCTTTGG

GCTTATCTGATCGGCTTTGCCGTGCTATCTCTAACGCTGGCCTTGAAAGACCGTCTTTAGTTCAGGCTAA

CTCCGTACCGGCAATACTCTCGGGGAAGGATGTGGTAGTTGCAGCTGAGACTGGTAGTGGTAAAACACAT

GCCTACCTAGTTCCTTTAATTGACAAGTTATACAATCCACTGGACTATAATAGAGACTCCGTCTCTGATC

AAAGATTAAACCCGTCAGATAGACTTTCTTTGGTTCTTTGCCCAAATGTATTGTTGTGTGAACAAGTGGT

TCGAATGGCTAGTGGTCTTTGTGATGATAATGGCGAACCACTGCTTAAAGTCACTGCCATCTGTGGCCGA

CAGGGATGGCCAGTTAATCAGCCAGATATTATTGTGTCAACACCAGCTGCTCTTCTAAATAATATTGACC

CCAAAAA

>FM895584.1 FM895584 Jatropha curcas embryo 71-95 (DAF) Jatropha curcas cDNA clone rjcpga0_002532, mRNA sequence

GAGAACTAGTCTCGAGTTTTTTTAGCACGGCAAGCTTATGACCTCATAGACAGCCATATAAAACGATTGG

ATGAGGATCTAAATAACTTTGCAGAAGATTTGAAGCATGAGGGAAAAATACCAGCAGATGAACCAGCAGT

TCTTCCTCCACAACCTATATTAGTCCCTAAACTTGAAAAACGCAAGTCCTTTTATGGGACACCTCAATCA

AAAAGGAATGAGTTTAGGGACAGGGAGTGGGATCGAGAACGTGAAAGGGATTTTGAGCTCATGCCGCCTC

CAGGAATTAAGAAGGATTTTAATGTCTCTGTTGATGTTGATCAACCCATTGATCCAAATGAACCTACTTA

CTGTGTCTGCCATCAGGTGTCCTTTGGAGATATGATTGCCTGTGATAATGAAAATTGCGAAGGAGGTGAA

TGGTTCCATTATGCATGTGTAGGTCTAACGCCAGAGACGAGGTTCAGGGGAAAGTGGTATTGTCCAACCT

GCAGACAGCTACCCCATTGTCAATTATGATATATGCTTGTATGGTATCTACTGC

>FM895583.1 FM895583 Jatropha curcas embryo 71-95 (DAF) Jatropha curcas cDNA clone rjcpga0_002531, mRNA sequence

GCTGAACAAAACTAGTCGATTGAACATTAAAGTAATACAAAATGCTCCACGAGCAGAATTGACTAGAATC

TCTACTGCTAAATTCTAACAGCCAAATAAATAAAATCACATCCAAAGCTTTATTTTTCAGGACAGGAAAG

AAGCTAAAAGGGAATTAGGAAAGTTAATTTATCTATACCATATACCCATACTATGTACTACCTACTTGTA

CAAATCCGTTTCTTTCCCTTTCCATGTTATTATTCCCTGTTCTTACTCCTTACTAGCTCTGACTACAAGT

TGATTCTGTGATCTGATTATGAGCGCGATCCCTCAAGGCCCAGTTTTGCTGTGAGCCAATTACGAACCTC

GTCCATTTCTCTAGGAACTGTGTAATGCCCAAGTCCATCATATGGTTTGAATGTGAGATGTCGACAGCCT

GCTGTATTTAAGAAGTGGGCAGATCTCTCTCCATAGTTATAAGGAACTACATCATCACATGTTCCATGAC

AAAGCAAAATTGGCAATGATGCAGCACGTCTTGCAGCATCGAGTGA

>FM895582.1 FM895582 Jatropha curcas embryo 71-95 (DAF) Jatropha curcas cDNA clone rjcpga0_002530, mRNA sequence

ATTCAAAGGATTCTATCCCGTCGCCCGATGGGAATTGTACTTTAAGGCGGCCCTCGCGGCTCGTCCGCCG

CGGGGGCTTAGCCAACGGCACGTGCCTCTGGGGGCCGGATGGCCCCTACTGCGGGTCGGCAAACGGGCGA

CGGACACGCGCGTCGCTTCTGGCCCGGATTCTGACTTAGAGGCGTTCAGTCATAATCCAGCGCACGGTAG

CTTCGCGCCACTGGCTTTTCAACCAAGCGCGATGACCAATTGTGCGAATCAACGGTTCCTCTCGTACTAG

GTTGAATTACCATCGCGACACCATCATCAGTAGGGTAAAACTAACCTGTCTCACGACGGTCTAACCCCAG

CTCACGTTCCCTATTGGTGGGTGAACAATCCAACACTTGGTGAATTCTGCTTCACAATGATAGGAAGAGC

CGACATCGAAGGATCAAAAAGCAACGTCG

>FM895581.1 FM895581 Jatropha curcas embryo 71-95 (DAF) Jatropha curcas cDNA clone rjcpga0_002529, mRNA sequence

CTAATATCTGACGCGTGGTTTAAATTTGATCAAAACAGCTTGATTTAATATGAGAATGGAAGAAAAGCCG

ATGATTGGCCTTTCATGGGAGCCAAAGTTGCCGGCTTTCTCATCTTCAACCAAAGTTAAAACTAAAAGTA

ATGATCAACCGGAGGGCAGTGCACTTTGGAAATCCAATACAGAGCTTATTGATGGTCTATTTGTGCCACC

GAATGATCCTATAAAGGTTAACAAGTTACTCAGAAGCCAAGCCAAAGATTCAGCTGGAAAAGATTGGTTT

GATATGCCTGCCCCAACAATGACACCAGAGTTGAAAAAAGATCTCCAGCTACTTAAGTTGAGGAATGCTC

TTGATCCGAAGAGGCACTATAAGAAGGGTGATTCAAAAACAAAAGCACTGCCCAAGTATTTCCAGGTTGG

CACAGTTATAGAGTCGGCAACAGATTTCTTCTCAGGTAGGCTAACGAAAAAGGAGAGGAAGGCAACCCTT

GCAGAAGAGCTGCTTTCAGACCGTAACCTTGCAGCTTAC

>FM895580.1 FM895580 Jatropha curcas embryo 71-95 (DAF) Jatropha curcas cDNA clone rjcpga0_002528, mRNA sequence

GTCTGACATGTGTGCGAGTCAACGGGTGAGTAAACCCGCAAGGCGCAAGGAAGCTGACTGGCGGGATCCC

CCCGAGGGTTGCACCGCCGACCGACCTTGATCTTCTGAGAAGGGTTCGAGTGAGAGCATGCCTGTCGGGA

CCCGAAAGATGGTGAACTATGCCTGAGCGGGGCGAAGCCAGAGGAAACTCTGGTGGAGGCCCGCAGCGAT

ACTGACGTGCAAATCGTTCGTCTGACTTGGGTATAGGGGCGAAAGACTAATCGAACCGTCTAGTAGCTGG

TTCCCTCCGAAGTTTCCCTCAGGATAGCTGGAGCTCGGGACGAGTTCTATCGGGTAAAGCCAATGATTAG

AGGCATCGGGGGCGCAACGCCCTCGACCTATTCTCAAACTTTAAATAGGTAGGACGGCGCGGCTGCTTCG

CAGAGCCGCGCCACGGAATCGAAAGCTCCAAGTGGGCCAT

>FM895579.1 FM895579 Jatropha curcas embryo 71-95 (DAF) Jatropha curcas cDNA clone rjcpga0_002527, mRNA sequence

AGGATTCTATCCGTCGCCCGATGGGAATTGTACTTTAAGGCGGCCCTCGCGGCTCGTCCGCCGCGGGGGC

TTGGCCAACGGCACGTGCCTCTGGGGGCCGGATGGCCCCTACTGCGGGTCGGCAAACGGGCGACGGACAC

GCGCGTCGCTTCTGGCCCGGATTCTGACTTAGAGGCGTTCAGTCATAATCCAGCGCACGGTAGCTTCGCG

CCACTGGCTTTTCAACCAAGCGCGATGACCAATTGTGCGAATCAACGGTTCCTCTCGTACTAGGTTGAAT

TACCATCGCGACACCATCATCAGTAGGGTAAAACTAACCTGTCTCACGACGGTCTAAACCCAGCTCACGT

TCCCTATTGGTGGGTGAACAATCCAACACTTGGTGAATTCTGCTTCACAATGATAGGAAGAGCCGACATC

GAAGGATCAAAAAGCAACGTCGCTATGAACGCTTGGCTGCCACAAGCCAGTTATCCCTGTGGTAACTTTT

CTGACACCTCTAGCTTCAAATTCCGAAGGTCTAAAGGATCGATAGGCCACGCTTTCACGGTTCGTATTCG

TACTGGAAAATCAGAATCAAACGAGCTTTTACCCTTTTT

>FM895578.1 FM895578 Jatropha curcas embryo 71-95 (DAF) Jatropha curcas cDNA clone rjcpga0_002526, mRNA sequence

CTGACATGTGTGCGAGTCAACGGGTGAGTAAACCCGCAAGGCGCAAGGAAGCTGACTGGCGGGATCCCCC

CGAGGGTTGCACCGCCGACCGACCTTGATCTTCTGAGAAGGGTTCGAGTGAGAGCATGCCTGTCGGGACC

CGAAAGATGGTGAACTATGCCTGAGCGGGGCGAAGCCAGAGGAAACTCTGGTGGAGGCCCGCAGCGATAC

TGACGTGCAAATCGTTCGTCTGACTTGGGTATAGGGGCGAAAGACTAATCGAACCGTCTAGTAGCTGGTT

CCCTCCGAAGTTTCCCTCAGGATAGCTGGAGCTCGGGACGAGTTCTATCGGGTAAAGCCAATGATTAGAG

GCATCGGGGGCGCAACGCCCTCGACCTATTCTCAAACTTTAAATAGGTAGGACGGCGCGGCTGCTTCGCT

GAGCCGCGCCACGGAATCGAGAGCTCCAAGTGGGCCATTTTTGGTAAGCA

>FM895577.1 FM895577 Jatropha curcas embryo 71-95 (DAF) Jatropha curcas cDNA clone rjcpga0_002525, mRNA sequence

ATTCTCCAACTCGCGCTCGCAAACCATCCTGATATTCTTCTAGTAGACTACCAGACCACATAAAGTGGTC

AATATTATTAGTTTAGTTAGTTTTAAATTCTCCTTCAACAGATATCTTCCTCTTTCTGTCTCTCTCTGCG

ACTGCTATTTCCTGACTACTGTGACATTTTCAACTGTGATCTTTGAAACACATTCTCCTTGTGAGTGTTA

CTGCATTTGCATCCCTGAAAGTGAAACGCTTTGCTGCTTTGATTGGTCAATATCGCCATGGCGGTCTCTG

CAATTGGTTTTGAAGGTTATGAGAAGAGGCTAGAAATAACATTTTTTGAGCCTGGCATTTTCGTTGATCC

TGAAGGAAAGGGACTTCGAGCTCTCTCCAAGGCTCAGTTGGATGAGTTTCTTGGACCTGCTGAATGTACT

ATTGTTGATTCATTGAAAAATGACCATGTTGACTCTTATGTCCTTTCTGAGTCTAGCCTCTTTATATATC

CTTACAAGCTTATAATCAAAACCTGTGGGACTACAAAGCTGCTTCTTGCAATCCCACCAATTTTGAAGCT

GTCTAATGCTCTGTCCCTTAAAGTGAAATCCGTGAGGTACACTCGTGGG

>FM895576.1 FM895576 Jatropha curcas embryo 71-95 (DAF) Jatropha curcas cDNA clone rjcpga0_002524, mRNA sequence

TTTTTTTTTTTTGCAGAAAAGCATTCCTTTGAAACTTCTCATAGCATGCAATGTTTCAACATTGTAACCC

AAAAATGTATCAGAGAGTAATAGTCAGAAAGAGAAAGCGCAACATTGCAGAAAAATTGTCGTATTTTCAC

AGTACAGTCACAAAGCGATCTTGCTTCATTCTTATGAGGTTGTTTTCAATGTTCTTCAAAACTGGTTTGT

AGAGGCGTGTAAGACCATTATATGCTTGTGTGACTTCTTGTAGTTTTGCTGATAGCTGCAGGACCTCTGC

AGACAATGCTTCTGCTCTGTTCTGTTCCTTCGCAAGTTCTCCCTTGAGGTAAAAAATTTCTGTTGAACCT

GTCTCGTTTATCCCATTGCTATGTCGTTCTGCTGAAATGAACATCTCCCTTTCACCTGAAGAAAGCATAT

TGGCCTGTACTGTTCTCATCTTAACAAGAACTCCACGAAGCTCTTCATTTTGCTTAAGACAGTCTTTACA

CTGATTCTCCCATTGCTTTGCTATGCTTAT

>FM895575.1 FM895575 Jatropha curcas embryo 71-95 (DAF) Jatropha curcas cDNA clone rjcpga0_002523, mRNA sequence

CTGGAAATCAGAATCAAACGAGCTTTTACCCTTTTGTTCCACACGAGATTTCTGTTCTCGTTGAGCTCAT

CTTAGGACACCTGCGTTATCTTTTAACAGATGTGCCGCCCCAGCCAAACTCCCCACCTGACAATGTCTTC

CGCCCGGATCGGCCGCCGAAACGACCTTGGGTCCAAAAAGAGGGGCGTAGCCCCGCCTCCGATTCACGGA

ATAAGTAAAATAACGTTAAAAGTAGTGGTATTTCACCGATCGCCTTTCGGCTCCCACTTATCCTACACCT

CTCAAGTCATTTCACAAAGTCGGACTAGAGTCAAGCTCAACAGGGTCTTCTTACCCCGCTGATTCCGCCA

AGCCCGTTCCCTTGGCGGTGGTTTCGCTGGATAGTAGACAGGGACAGTGGAAATCTCGTTAATCCATTCA

TGCGCGTCACTAATTAGATGACGAGGCATTTGGCTACCTTAAGAGAGTCATAGTTACTCCCGACGTTTAC

CCGCGCTTGGTTGAATTTCTTCACTTTGACATTCAGAGCACTGGGC

>FM895574.1 FM895574 Jatropha curcas embryo 71-95 (DAF) Jatropha curcas cDNA clone rjcpga0_002521, mRNA sequence

TTAGAAGGAGCTGGGGATGTTCCTACAAACACTCATCAGCGCATAGATGATTTTGAAATTGAAAATGGAG

GATTACATTATAATTTAGCTCAATTTGAGTACGCTAATGAGTTTGAGCTTCAACCCCAACTTGAATATGA

CTCTGATGTTGAGCTTGTTCCACTATTTGATGAATGGGACCTTGATAATGCTTTTCTTGCACTCCAAGAA

ACCCTTGCCCCTGATCCTAACCCTGACCCTGACCCTGACCCTGACCCTAACTTGGTTAATGATCATGTTG

TTCCATTGGAGGAAAGGGAGGTGGTGGTTGTTGTTGAGAATGAGGAAACTCCAAATGATCTTAACATAGT

TAATGCCTATCTTGTTTCACCTGGGGAAGGTGATAACGAGATGGTGAGGGATGAGGAAGAGCTTCACTTG

TTATAACCTCAACCTTCTGTTATTTTCAGTTCTGATACAAAACCTCGTCTTCGATGGACTCCAGACCTTC

ATAATTGCAATTGTTAGAGCTGTCAGACATCTTGGTGGAC

>FM895573.1 FM895573 Jatropha curcas embryo 71-95 (DAF) Jatropha curcas cDNA clone rjcpga0_002520, mRNA sequence

TTCTTGGGTATGGGACCAAGTTGGTTTGGAATTCAGTCACATCCACATTCAAAGCACCATCAAACCTCAA

AGAGGCAGTCAATGAGGAAATCACCTGAGAGACAAGCCTGTTAAGATTTGTGTAAGTGGGTCTCTCAATG

TCAAGAGAGCGCCTGCAGATATCATAGATGGCCTCATTGTCAAGAAGCACAGCCACATCAGTGTGTTCCA

GAAGGGAGTGAGTAGAAAGGACACTGTTGTAGGGCTCAACAACAGATGTGGAGACTTGAGGAGATGGATA

GACAGTGAAACCCAACTTGGATTTCTTTCCATAATCAACAGACAAACGCTCCAAGAGAAGGGATCCAAGA

CCAGATCCAGTACCACCACCAACAGCATTAAACACAAGGAAGCCCTGGAGACCAGTGCAGTTGTCTGCAA

GCTTTCTGATACGGTCCAAGCAGAGATCAACAATTTCCTTGCCAATGGTATAATGGCCACGGGCAAAGTT

GTTGGCAGCGTCTTCCTTGCCGCTAATGAGCTGCTC

>FM895572.1 FM895572 Jatropha curcas embryo 71-95 (DAF) Jatropha curcas cDNA clone rjcpga0_002519, mRNA sequence

AAACGAAAAGCTAAAAGCACAGAATTACATAATGGAATGATAATAGCAATAATGTGTTTTCATGGCATTG

ACAAAAAGCCACATCAATCCATGAACTGTTCCAGGAATCAACTCAACACCAATAGTTAGCAGTTTCTAGT

ACATAAGATTGCATTGTAGACCATCTCCATCAGATACTGTGAAGAAGGGGGAGTGTCCAACCTGGTCCAG

CTTGAGCAAGGTTTTTCTCATGCTTATGCTCATGCCACCTCAACCATCTCCTGTTCCACTGTACTGGGCA

TAACAGGATAGTATTTGTAGTGGAGTTCTCCAACATCATCACCAGATAGCTTCTTCCATCCATTTGGTCC

AACATAATAAACGCTAGCAACTCCACCACTGGCTCCATCTCTGAATGTAGCATGATAAATAGCTCTTCTA

GCCAACTCCGCAGCTTCCTCAATTGACAAATCATACCGATAGCCACTATCAAGTACACCATAAGCATATG

GGGAACCAGAGCCAACAGAGAATCTTGTTCCTTT

>FM895571.1 FM895571 Jatropha curcas embryo 71-95 (DAF) Jatropha curcas cDNA clone rjcpga0_002517, mRNA sequence

CACTTAGGGAGATGATCCCACCTGGTGCTTTGGTTATGCTTACACCACTTGTAGCTGGAACCTTCTTCGG

AGTCGAAACTCTTTCCGGTGTTCTTGCTGGTTCACTTGTTTCTGGTGTCCAGGTTGCCATATCAGCTTCT

AACACTGGAGGGGCATGGGATAACGCCAAGAAATACATTGAGGCTGGTGCTTCTGAGCATGCTAAATCAC

TTGGTCCTAAAGGTTCTGATCCTCACAAAGCTGCTGTGATTGGCGACACGATCGGAGACCCACTAAAGGA

CACTTCTGGTCCGTCACTTAATATTTTGATCAAGCTCATGGCAGTTGAGTCCTTGGTGTTTGCTCCATTT

TTTGCTGCTCATGGAGGTCTGTTATTCAAATTGCTCTAAATGGTATCCACCGAGTAAATCGGCCAAATTA

TGTCTACTAGTAAAGCACTATCCTGTAATTCCGAGCTCTTACTGTTACAGTTTCGACATGTAAATGATAA

ACGATCAGGCTTTGCTACTGCCAATTTTACCTTACCATGAATAAATTTGTAGTTTTCATGTGTAACAAAA

AA

>FM895570.1 FM895570 Jatropha curcas embryo 71-95 (DAF) Jatropha curcas cDNA clone rjcpga0_002516, mRNA sequence

TCAAACATATATTACACTTCCCCTAAGTTAAAAATTTTTGAAAACATTCAAATGCTAGGCCAAAAAGTAC

AATGCCACTTCCCTGGAACCGAGCTGCTCTCCCTGTTATCATTCCTACTTTTACTGTAATTGAACTCTAC

TTCAATAAAATTGTGCTGCCATAAAGCAGGAAAACAAATGTGTATACATATAAGGGGACAAATGCATAAA

TCGTTTCCTCACAGGTTAGCCAAAGCTCTTCACGTTCATCAACTCTGACCC

>FM895569.1 FM895569 Jatropha curcas embryo 71-95 (DAF) Jatropha curcas cDNA clone rjcpga0_002515, mRNA sequence

AGATCACAACAATTTACTTATAATTTATCCATTCAAATCAATTAAAAAACAGAAAATTACAAAGTTAAGT

TCAATTATTAAAGAGTAAAGTACAAGTAATGACAAATCATAATACATCAGCTCACTTGCCAACCTTCCCT

TGTTATTTTAAGGTCATATCTAATATTCAAAGTTAAGTTTACATCGATTTGGCGCAGCTCTCACCTCACT

GTCAGCGTTAAACAAACCACAATCTAGTCTGTTTTATTAGACGAAGATTGCAGGTTAATTGAGGCAGAGA

AATTAGGCAGAGTCCCACAATCATAAGAATGAGCAAGATTAAAGACCTTGGGCAATAAGAACCTAAGAAA

TACAGCATCTCTTAAAAATTTCATCCACCATTGAGACCCACCTTGTTGAATCCATCCAGATAAATATGAG

CCAGTGATCAAACCAGCTACTCTCCACCTCCTTTCTCTAACATACCCATTTATGGCTCCAGCCATGTCAT

TTGAAACAAGCCTACCATTATTTCTTATGCATGATTCTCCAATGTGCCTGCCTAAAACGACAGCATCTAC

TAGTGCTGAACAAACAACTAGGCCTAAATCAGGTGTCATTGGGTGCATTGCATCACA

>FM895568.1 FM895568 Jatropha curcas embryo 71-95 (DAF) Jatropha curcas cDNA clone rjcpga0_002514, mRNA sequence

TGAAATCAGAATCAAACGAGCTTTTACCCTTTTGTACCACACGAGATTTCTGTTCTCGTTGAGCTCATCT

TAGGACACCTGCGTTATCTTTTAACAGATGTGCCGCCCCAGCCAAACTCCCCACCTGACAATGTCTTCCG

CCCGGATCGGCCGCCGAAACGACCTTGGGTCCAAAAAGAGGGGCGTAGCCCCGCCTCCGATTCACGGAAT

AAGTAAAATAACGTTAAAAGTAGTGGTATTTCACCGTCGCCTTTCGGCTCCCACTTATCCTACACCTCTC

AAGTCATTTCACAAAGTCGGACTAGAGTCAAGCTCAACAGGGTCTTCTTTCCCCGCTGATTCCGCCAAGC

CCGTTCCCTTGGCTGTGGTTTCGCTGGATAGTAGACAGGGACAGTGGAAATCTCGTTAATCCATTCATGC

GCGTCACTAATTAGATGACGAGGCATTTGGCTACCTTAAGAGAGTCATAGTTACTCCCGCCGTTTACCCG

CGCTTGGTTGAATTTCTTCACTTTGACATTCAGAGCACTGGGC

>FM895567.1 FM895567 Jatropha curcas embryo 71-95 (DAF) Jatropha curcas cDNA clone rjcpga0_002513, mRNA sequence

ACCAAACCTAAAACATAAAATTTAGGTTACCACCGAAGTCCTCCAGTACAACAATATTCTCCAATATAAC

AAAGCACATATAAGAGGTCTGCATAAGTATAACCTAAACCTCTTAGAAATATTCTTAATCATCCTTCTCT

TGCACAAACAAGTGCAAATATAACTAATCAAAAACAAAAATAA

>FM895566.1 FM895566 Jatropha curcas embryo 71-95 (DAF) Jatropha curcas cDNA clone rjcpga0_002512, mRNA sequence

TTTCTTTTTCTTTTGATATATTACAGCTCAGAATCTGAAGTTATAACCATTGATGTTAAGGCAGCCAAGG

GTTTGCATGAATCTGGCTATATCTATCTAGATGTTAGGACTGTGGAAGAATTCAAGAAAGGACATGTGCG

TGCAGAGAAGATATTTAACATTCCTTACATGTTCAACACACCAGAAGGGAGGGTAAAAAACCCTAAATTC

TTGGAGGAGGTTTCAGCTATTTGCAAAAAGGAAGATCATCTTGTTGTGGGCTGTCAGAGTGGAGTCAGAT

CTCTATATGCAACTGCTGATCTATTAACTGCTGGTTTCAAGGATGTGAGCAACATGGGAGGAGGTTATCT

GGATTGGGTGAAGAATGAATTACCAGTGGAGATGGAAGAGGAGCCAAAGATTGAGCTCTAATTTGTAAAA

ACAGAGTAAACAAAAAATATATATGTAATTTAGAAATATTATTTCCAAGTATTTCATAGAGGAAAAGAAA

TCATTTCAACATATGTAAACAGTTGTGCCCAGATTCTGTATTTAGTATTTATATTTAGAATCCTG

>FM895565.1 FM895565 Jatropha curcas embryo 71-95 (DAF) Jatropha curcas cDNA clone rjcpga0_002511, mRNA sequence

AGTTGTCAAACCAGAATTCTCTTTGGCATGGATTAAGAGTTTGATAGTTAACAAACAAGCATTAGGGTTC

AGAAACTTCTCAACACATTTGTATTCTAATACAAATCCACATGATTTACTTCCATGCCTCCATCACAATA

TCAAGTTCCCGATAGAAAATCATGTTACCACCTGCTGTATGGAAACTAGCAAACAAAATGACAGACATCC

TCCTGGCATCTATCTGCTACGCCTGGGTGACAAGTCAGCAGATCTAGACCGAGATCCAGAAGGTGACCTT

GATGCTCTACCAGGTGATTGCCTCCAAGCTCGTGCTTCCTCAGAATCATAGGCAGGTTTCTCGCCATAAC

CATTGCCATTTCCATTTAGATCATCTACACCATATCCAGGAGAATATGACCTGCGAATGTGGCCACCATC

TCGATCTGGTGGAGGGCCTCCAGGAGACCTTAGATGCTCTGCATGCCTTCTACCTGGGGAAGCAGAGTAG

TCTCTCCGTCTTGGGGCAAGAGAGTATGACCTTGAGCGGTATCGAGACCTAGAACTCATATGATGT

>FM895564.1 FM895564 Jatropha curcas embryo 71-95 (DAF) Jatropha curcas cDNA clone rjcpga0_002510, mRNA sequence

TGGAATTCCATAGTCCATTCCCCACATTCTCAATTCATTTTCTTCTCCGTTTGCCTATCTCGATTTCGCT

GAATCGCGGACTCGGAAAAGCTAATCCGTGACATTTCGCGCTATGGCACCCATTAGGGGCATTCTTTCCT

TGCAAAGAGCTGCTATGATTCGGCGTCATAGTGATAGGCTGGACATAGGATTTAGATCATTGAGCACCCA

AGCTGCAACAAATGCTGGCAGTCCACAGCCTCCCCCACCTCCCCCACCTCCGGAGAAAACCCATTTTGGT

GGCTTGAAAGACGAGGATAGGATTTTTACCAACTTGTATGGGTTGCATGACCCTTTTCTCAAAGGTGCCA

TGAAACGGGGTGACTGGTATAGAACAAAGGACTTGGTACTTAAGGGCACTGATTGGATTGTTAATGAAGT

CAAGAAATCTGGCCTCCGTGGACGTGGTGGTGCTGGTTTCCCCTCTGGTCTCAAATGGTCATTTATGCCA

AAAGTATCTGATGGCCGACCTTCCTATCTTGTGGTCAATGCTGATGAAAGTGAACCTGGAACTTGTACAG

ACAGGGAAATTATGCGCCATGATCCACACAAGCTGTAAAAGGGCTGCCTTATTGCTGGTGTGGGAATGAG

AGCCAGTGCTGCTTATATC

>FM895563.1 FM895563 Jatropha curcas embryo 71-95 (DAF) Jatropha curcas cDNA clone rjcpga0_002509, mRNA sequence

GAAATAATTTTGTCCTAGATTTATGAATTACTGGATGGTATGTCATTGATATACTTTTTTTTAATGATAC

TCTGTTGCATATTAGCATTTTTGGTTCTTTCTTTAGATTTTTTTTTCTTATGTAATTTTATCTATTTAGA

GAATCTGTGCATTATGAAATACTTCTTTTGGTCATTATGAAAGGCCATTCAGTAAGTGTTGTTTCAGTAC

TGGATCATTACATAAGCAATGAATGATTTGCATCTTGCTTGAAATTGCAGAGATTATGGACTTTGGTTAC

CCACAAAATCTGTCTCCTGAGATTTTAAAGCTTTACATCACACAAGAAGGAGTTCGCTCACCGTTTTCAT

CCAAGGTTAGATGTTTGTTTCAAGAAGAACCACAACACTTCTTTTGGACATTGATTTTCTATTTTATTTG

AATAATATCAATATGACATGACTGTAAAAGTTACTTGATTGTCTTTGTCTTGTGTTTTTGATGTCGAATA

GTCTTTGATCATGTGTTTCTTTCTCTTTTGCATTGCAAAACCAAAATAGCCTACAGATAAACCAGTACCT

AATGCA

>FM895562.1 FM895562 Jatropha curcas embryo 71-95 (DAF) Jatropha curcas cDNA clone rjcpga0_002508, mRNA sequence

ATTCCCCAACTCCGCCGCCTTATCCTACCCCTCCGGTAACACCGACACCAAAACCACCGGTGATCAAGCC

TCCGACTCTACCACCAAAGCCTCCTGTTACCCCTACACCACCGTATTATCCTACGCCGCCGGTAACACCA

GCACCGCCAACTCTACCACCAAAGCCTCCTGTTGTGAAGCCACCGCCAACTGAAACACCATGCCCTCCTC

CGCCACCTCCAGTGCCATGCCCACCTCCACCCCCAAAGCAAGAGACTTGCCCTATTGATGCTCTAAAGCT

AGGAGCATGTGTTGATGTGTTAGGTGGTTTAGTGCATATTGGGATTGGTGGTAGTGCTAAGGACGCCTGT

TGCCCAGTGCTTCAAGGGCTACTGGACTTAGATGCTGCTCTTTGTCTTTGCACTACCATTAAAGTTAAGC

TTCTGAATATAAATATTCTTTTACCCATTGCTCTAGAAGTTCTTATTGACTGTGGCAAGAACCCACCTGC

TGGATTCAAGTGTCCTGCGTAAG

>FM895561.1 FM895561 Jatropha curcas embryo 71-95 (DAF) Jatropha curcas cDNA clone rjcpga0_002507, mRNA sequence

ATTCCGGCGGTCGCTGCAAAACCCGGGGCGCGAGCCCGGGCGGAGCGGCCGTCGGTGCAGATCTTGGTGG

TAGTAGCAAATATTCAAATGAGAACTTTGAAGGCCGAAGAGGGGAAAGGTTCCATGTGAACGGCACTTGC

ACATGGGTTAGTCGATCCTAAGAGACGGGGGAAGCCCGTCCGACAGCGCGTCCTCGCGCGAGCTTCGAAA

GGGAATCGGGTTAAAATTCCCGAACCGGGACGTGAAGGCTGACGGCGACGTTAGGGAGTCCGGAGACGTC

GGCGGGGGCCTCGGGAAGAGTTATCTTTTCTGTTTAACAGCCCGCCCACCCTGGAAACGACTCAGTCGGA

GGTAGGGTCCAGCGGCTGGAAGAGCACCGCACGTCGCGCGGTGTCCGGTGCGCCCCCGGCGGCCCATGAA

AATCCGGAGGACCGAGTGCCTCCCACGCCCGGTCGTACTCATAACCGCATCAGGTCTCCATAGTGAACAG

CCTCTGGTCGATGGAACAATGTA

>FM895560.1 FM895560 Jatropha curcas embryo 71-95 (DAF) Jatropha curcas cDNA clone rjcpga0_002506, mRNA sequence

AGGATTCTATCCGTCGCCCGATGGGAATTGTACTTTAAGTGCGGCCCTCGCGGCTCGTCCGCCGCGGGGG

CTTAGCCAACGGCACGTGCCTCTGGGGGCCGGATGGCCCCTACTGCGGGTCGGCAAACGGGCGACGGACA

CGCGCGTCGCTTCTGGCCCGGATTCTGACTTAGAGGCGTTCAGTCATAATCCAGCGCACGGTAGCTTCGC

GCCACTGGCTTTTCAACCAAGCGCGATGACCAATTGTGCGAATCAACGGTTCCTCTCGTACTAGGTTGAA

TTACCATCGCGACACCATCATCAGTAGGGTAAAACTAACCTGTCTCACGACGGTCTAAACCCAGCTCACG

TTCCCTATTGGTGGGTGAACAATCCAACACTTGGTGAATTCTGCTTCACAATGATAGGAAGAGCCGACAT

CGAAGGATCAAAAAGCAACGTCGCTATGAACGCTTGGCTGCCACAAGCCAGTTATCCCAGTGGTAACTTT

TCTGACACCTCTAGCTTCAAATTCCGAAGGTCTAAAGGATCGATAGGCCACGCTT

>FM895559.1 FM895559 Jatropha curcas embryo 71-95 (DAF) Jatropha curcas cDNA clone rjcpga0_002505, mRNA sequence

ATTCCTCACACTTATCATCAACAAATGGCTGAACGTTCACAGCCACACCAAGTTCAAGTGCACCCACAAC

ACCGCTATGAAGCTGCCTTCAAAGGTCAACAAAAAGGTCCATCAGCTCAGAAAGTGTTAGCTGTGATCAC

CCTCCTGCCGGTAGGCGGTGGGCTTCTTGCACTAGCTGGTATAACCTTAGTAGGTACCCTAATCGGCCTA

GCTATTACTACCCCTCTTTTTGTCATCTTTAGCCCTGTTCTTGTCCCAGCTGCATTAGTTATTGGCCTTT

CGGTCATGGCATATTTGGCTTCCGGGGCTATGGGGCTCACAGGGTTGTCATCACTCTCGTGGGTTCTGAA

ATACCTCCAGGAGGTCACTCGGCGAATGCCGGAGCAGCTGGATATAGCTAAGAAGCGTATGCAAGACATG

GCTGGTATAGTGGGACAGAAGACCAAGGAAGTAGGCCAAGAAATCCAGAGGAAAGCACATG

>FM895558.1 FM895558 Jatropha curcas embryo 71-95 (DAF) Jatropha curcas cDNA clone rjcpga0_002504, mRNA sequence

TTCTATCCGTCGCCCGATGGGAATTGTACTTTAAGGCGGCCCTCGCGGCTCGTCCGCCGCGGGGGCTTGG

CCAACGGCACGTGCCTCTGGGGGCCGGATGGCCCCTACTGCGGGTCGGCAAACGGGCGACGGACACGCGC

GTCGCTTCTGGCCCGGATTCTGACTTAGAGGCGTTCAGTCATAATCCAGCGCACGGTAGCTTCGCGCCAC

TGGCTTTTCAACCAAGCGCGATGACCAATTGTGCGAATCAACGGTTCCTCTCGTACTAGGTTGAATTACC

ATCGCGACACCATCATCAGTAGGGTAAAACTAACCTGTCTCACGACGGTCTAACCCCAGCTCACGTTCCC

TATTGGTGGGTGAACAATCCAACACTTGGTGAATTCTGCTTCACAATGATAGGAAGAGCCGACATCGAAG

GATCACAAAGCAAAGTACGCTATGAACGCTTGGCTGCCAC

>FM895557.1 FM895557 Jatropha curcas embryo 71-95 (DAF) Jatropha curcas cDNA clone rjcpga0_002503, mRNA sequence

ATTATTAATGTGGATAGGAGTCATCCCAGCCCCTGCATTTCGTCTCCTATCCACATTAATACCATTTGAT

ACACCATTGATATGGAAATCACCAGCAATTTCCACGCAGGGAAGATTTTCCAACCTACTTTGTGTTGACA

AGCCTCTATCTTTATTGAACATGTCAGTATGCTCACCACTTTTCATGCCTATATCTCTTCCGTTTGGGTG

ATTTATGGTGGGAGGGCTATGATTAAAACTAGTTTCTGTAGATGAACTAGGCAACATATACTGTTGATCA

GGAGCAAGTTGAAGCAATGCAGCTGTTAGCCATGTTATTTTGTCATTTGACATTCTAAGTTGTTTTTCAG

CCTCCGATAAAGTTTTCAGGGCTTGGCGTAGTTTTTCCATATCTTCCTTGGACAATGGTTTTCTTCGGAA

AAACTTCCTCCTACGCCTCTCTATTGTGAAATCATAACTACCAGCT

>FM895556.1 FM895556 Jatropha curcas embryo 71-95 (DAF) Jatropha curcas cDNA clone rjcpga0_002501, mRNA sequence

CTTGCGCGCGCCGCTTTGCCATTTCCATCTGCATCGTCCAAGATCCTCTCATCCTGCGAATATAATTAAC

CATCCACGACAGTGAAGAAAGCGCAGTGATTCCAAAAGCACCGGACGTCAAAAACCCTAACACCGACAAC

CCAATGACCAACGCCGCTGGTACCAGCACCGGACTACAAATCACGAACAGCGGAGTCGCAACAGCTAGCG

CAATGAGTGTTCCAGTAAGCGTTATTCCAGCAAGGAAAAGAAGCGTCCCACTCACCGGCAAAAGAGTGAC

AACCGCTAAAATCTGTGAGGTCGAGAAGCCGCCCTTCTCCTTTAGGGCTCCTTTGTGCCCTTCTCCGCCT

GGTCGTCGCTCATACTCGGCCATGGAAGCTTAAAATAGATATCGTAATAAGCTTTTTCTTGGAAGGGAGA

AAAAGAGTAGATTTATGCAGAAAGAGATGGAGAAGAGACAGGAAAGGAGAGTGAATTTAAGGAAATGGAG

AGATGGACACGTGGAAGGAAGTGGCACGTGTTGTATATATAAGATGGTAGAGAGTTGCAATGAGGAATGT

GTCACTATATAT

>FM895555.1 FM895555 Jatropha curcas embryo 71-95 (DAF) Jatropha curcas cDNA clone rjcpga0_002496, mRNA sequence

CCACGACGAGATTTCTGTTCTCGTTGAGCTCATCTTAGGACACCTGCGTTATCTTTTAACAGATGTGCCG

CCCCAGCCAAACTCCCCACCTGACAATGTCTTCCGCCCGGATCGGCCGCCGAAACGACCTTGGGTCCAAA

AAGAGGGGCATAGCCCCGCCTCCGATTCACGGAATAAGTAAAATAACGTTAAAAGTAGTGGTATTTCACC

GATCGCCTTTCGGCTCCCACTTATCCTACACCTCTCAAGTCATTTCACAAAGTCGGACTAGAGTCAAGCT

CAACAGGGTCTTCTTTCCCCGCTGATTCCGCCAAGCCCGTTCCCTTGGCTGTGGTTTCGCTGGATAGTAG

ACAGGGACAGTGGGAATCTCGTAAATCCATTCATGCGCGTCACTACTTAGATGACGAGGCATTTGGCTAC

CTTAAGAGAGTCATAGTTACTCCCGC

>FM895554.1 FM895554 Jatropha curcas embryo 71-95 (DAF) Jatropha curcas cDNA clone rjcpga0_002495, mRNA sequence

GTAGAAATCCCCTTTTTCTATTTTGTTTAAAGAAAAGCTAAAAGCACAGAATTACATAATGGAATGATAA

TAGCAATAATGTGTTTTCATGGCATTGACAAAAAGCCACATCAATCCATGAACTGTTCCAGGAATCAACT

CAACACCAATAGTTAGCAGTTTCTAGTACATAAGATTGCATTGTAGACCATCTCCATCAGATACTGTGAA

GAAGGGGGAGTGTACAAACTAGTCCAGCTTGAGCAAGGTTTATCTCATGCTTATGCTCATGCCACCTCAA

CCATCTCCTGTTCCACTGTACTGGGCATAACAGGATAGTATTAGTAGAGGAGTTCTCCAACATCATCAAC

AGATAGCTTCTTCCATCCATTTGGTCCAACATAATAAACGCTAGCAACTCCACCACTGGCTCCAACTCTG

AATGTAGCATGATAAATAGCTCT

>FM895553.1 FM895553 Jatropha curcas embryo 71-95 (DAF) Jatropha curcas cDNA clone rjcpga0_002494, mRNA sequence

ATTCAAAGGATACTATCCGTCGCCCGATGGGAATTGTACTTTAAGGCGGCCCTCGCGGCTCGTCCGCCGC

GGGGGCTTGGCCAACGGCACGTGCCTCTGGGGGCCGGATGGCCCCTACTGCGGGTCGGCAAACGGGCGAC

GGACACGCGCGTCGCTTCTGGCCCGGATTCTGACTTAGAGGCGTTCAGTCATCATCCAGCGCACGGTAGC

TTCGCGCCACTGGCTTTTCAACCAAGCGCGATGACAAATTGTGCGAATCAACGGTTCCTCTCGTACTAGG

TTGAATTACCATCGCGACACCATCATCAGAAGGGTAAAACTAAACTGTAACACGACGGACAAACCCCAGC

TCACGTACCCTATTGGTGGGTGAACAATC

>FM895552.1 FM895552 Jatropha curcas embryo 71-95 (DAF) Jatropha curcas cDNA clone rjcpga0_002493, mRNA sequence

AATGTAAAGCACGCACAATTAAAATAGAATGATGACCAGAAATAGTATTACAGATTCTAACCAATCCAAA

ATTATAGTAAACAAGATTCTACCACATCCAAAATTCTCTTCAGAAGTTACATAACTCCCAACAAAATAAA

AAGACATTCTGCAAAATATTACTTGAAAAACCAACACTTACTACGATGTAATATACTGGGCCTTGTTGAG

GATGACACCTTCATATTTCACCTGAAACCACTTCATTGCATCCTCCTTGGTAACCCGGTGCTGAATACCG

ACCTTTGACTTGCATCTTCTGCGACGACCAACGCGATAGCCTGGACGCTCCAAAACAACATAGAAATCCA

TACCATAAATACCAGATGAAGGATCATACATGATGCCAAGATCAATGTGCTCCTGGATACCAAAATCCAA

AGCAACCAGTGTCACTG

>FM895551.1 FM895551 Jatropha curcas embryo 71-95 (DAF) Jatropha curcas cDNA clone rjcpga0_002492, mRNA sequence

AGCTTCTAACAGATTACAAGATAGATTCAACTATTCCGCGCATTAAGATTTGTATTATGTCCTCAAGTAA

TTCCGTTGGTATATTACACAAAATAAACAAATCCAAAATCTGGACAGCAAAAATATTTAATCTAGATACA

AAATTATTTATAAAAAAAAAAAAAACTACTCAAAAGGACTCCATAATTCCATGTTAAGAAGCTAAGTTCA

GATCCTCTTCTGAATGTGCCACATTTTTTCTGGTAGATCACCCTACTGAGAATTGCTGTCACCTTAGATA

GACATGGGTACTAAGACATCGGACCTTCATCGTCCGACGAACCATAATGCTGCCTTACATTGAGTCCAGG

AAAAACCGGTGGAATGTTTTGATTCATCGGCATAGGGCGGACTGAACTGTATATAGTTCTCTCATTGATT

TTCCCAACTTCTTTGCATACCTGATCAAGAATGGAAAGGAAATCCCTTACCACCATGAATATCCGGAATG

GGTGAGCTTATTCCTTCGCTG

>FM895550.1 FM895550 Jatropha curcas embryo 71-95 (DAF) Jatropha curcas cDNA clone rjcpga0_002490, mRNA sequence

TTCAAATCCAAGCCATAAGAGACTTTCTCTGTTTCATCTGTGTCTTTGTTTCGATAAAAGCCCTTCCTTT

CTTCATTAGGAATAGAAAGTCCCAGACTTTGATTTCAATCTGAGTTTCGGATCCCAAAATGGCATCTGGA

TCTTCCGGCCGGGCCAACTCGGGCTCGAAGGGATTCGATTTCGGGTCCGATGATATTCTTTGCTCTTATG

AAGATTACACTAATCAGGACTCCTCCAACGGAACTCATTCAGATCCTGTTATTGGGTCTAATTCCAGCAA

GGATTTTCACAAAGGCAGAATGGCAAGGTCTTCTGTGTTTCCTGCCAATTCCTATAGCCAGCCAGAAGAT

TCTTTCAGCCAAGATATGATATCTTTTTTTGAGAAAAGCATGAAAAAACATACAGACAACGTTATGCGCT

TTCTCGAAGGTGTCAGCTCACGGCTGTCCCAATTGGAATTATATTGCTATAACCTCGATAAGTCAATTGG

AGAAATGCGATCTGATTTAGTTCGTGACCATGGAGAAGCAGACGCAAAGCTTAAATCTCTTGGGAAACAT

ATTCAAGAGGTGCATAGGTCTGTCCAGATCCTAAGGGACAAGAAGAACTGGCTGAACCTC

>FM895549.1 FM895549 Jatropha curcas embryo 71-95 (DAF) Jatropha curcas cDNA clone rjcpga0_002489, mRNA sequence

CCCACCAGGAAACGGAAAGCATTAATTATAGTACTCGTTTACAAATACTAGTTGTTTAATTAAAACAAAT

TAGTCTCTTTAAAAGAGAATTTAAGGTTGTGAGATCAAATCAGTTGGTCTCTATCCTTTATCCTTGGCAA

GACCTCTGGACAACACTGCCTCTTCTAATCAGTTTGATTGATTCACCAATGTCATGAATTACCTGGTGAG

GATCAGGAATTGTGCTTTCATCAACCGATAGAACGATCGACATCTTGTCGGCATAACCTTTGAAAGTTAC

TCACCACAGCCTGTGGTTGATTAAAAAAACTTGGAGCTAAAAAAAGCCAGTGGATGGCCATAAAATCCAA

TATCTTCCTGAGGTCCAGCTAGGTTTGAAAA

>FM895548.1 FM895548 Jatropha curcas embryo 71-95 (DAF) Jatropha curcas cDNA clone rjcpga0_002488, mRNA sequence

ATTGTGGAGGAGGATAGCTGGAGAAAATAACCTGCCAGCCCGAAGCTTTGGAACTGTGCTGACCAGAGTA

GGAAATGGAAGGCAAAATATGGGGACGTCAGATATTGCCAGGAACGGATTGAAACAGAACAGGGCTCAAA

AGACTCCGCTTTCTATTTATAAAGACTCAAATACTGATGTCATTCTGGGGCATCAACCTGGGAAGTCAAA

AACAGATTTTAATCCTTGGAACAGTCTTGGAGCTCGTGCTGAGAGAAACAAAGAAAATAACGCAGTGCCT

ACCAAGTGGACTACCCACAAAATTCCACAGAGACCCGGACCTAGAGCCCCTGGAGCAACTGCAAGTGCCT

GCATTCAAGTATTTGTTGACGACGAATACGTGGAAACTGATAGATCACATGGTGATGGTGGCAAGCCTTC

AACTTTGCTACTCCGGCAACGGGATGACCTAGATATTCAAAAGGAGACGGAGTTATTAAGAGAGAACCCA

T

>FM895547.1 FM895547 Jatropha curcas embryo 71-95 (DAF) Jatropha curcas cDNA clone rjcpga0_002487, mRNA sequence

GATGTAAATCTAGTTTCCCGTTTCACAAGGCCCATTTGTTTCTCAAAAAGACTACTGAGGTTAAGACCCT

GAGACGTGGAGATAAGTTCAAATGCATTCATAGTCAGAGGTGCAACAGGCCCTATAGGAGCTTCTCGCCT

CTCCACAACAAGGTTTTGAGAATCCCCTGATTCATCAAAGATAGAGTTCACATCATCAAGACTGACATCA

GCTTGTTCAAAAGTGGGTGGTTTATATCCCTTTTTGAACCACTCATTTTCAATAACTTCAGCAATAGTAA

TACGGGTTGAAGGATTTGGATCCAGAATTCTTTTGATTAGTTTCTTTGCACTCGATGAGAACCACGGAGG

ACATGTGAAGTCCGCCTTAAATATCTTTTTATATAGAGCCATAAGGTTGGACTCTTCAAAAGGCAAGAAA

CCAGCCATTAAGACAAAAAGGATCACACCGCAAGACCACAAATCTGCCTTAGCTCCATCATAGCCTTTAT

TGTTGATGACCTCTGGAGCAACATAATTCGGTGTTCCACATGTGGTGTGAAGTAACCCATCTTCTCGAAC

TTGCTGAGGCAGCGCACTTAATCCAAAATCT

>FM895546.1 FM895546 Jatropha curcas embryo 71-95 (DAF) Jatropha curcas cDNA clone rjcpga0_002486, mRNA sequence

ATAAAAATTAGTTTTAGTATGGATATCATTTCCATTTATTATTTATGTAAATCCCATCTCACAATAATAC

ATCAATGAACTCAGAAATGAACATAAAAGGATAATTAATGATTTTGCCAAAGCACATTGAGAAAATTTTA

ATATGATACCACAAACTTGGTTAAATTGTGTGTTGAAAGTAGTCAATGGAGATGCACTTTTCGTAAAACC

TTCTTCGCACGTCTGAGAATAAGTCATAGCAGCACTAACCCACGTATTTAAATCGTTATAAGATCTGTTT

TGGACAGCACGAACTGAGTCGTTAATTTGACCAATCGCATCACTATAAACCTCTTGACATTGTGCCAAGG

CTTGCTTAACAATCGGATTTGAAGGTTTTATCTGCAAT

>FM895545.1 FM895545 Jatropha curcas embryo 71-95 (DAF) Jatropha curcas cDNA clone rjcpga0_002485, mRNA sequence

CCTCGAAAAAACAGAGAGAGTAACAACCCTCCTCAAATTAATATAAATGTTAAAGATTCAAATAATTTCT

GACAAGCTCCTACAGCCTACACCAAGTTACCATCTAAAACAAACTACTGATTCTTTGGAAAAAGATGAAA

TTAAAAAGAAATAAAATTTATTAAACGATCCCACAAACGAATATGCAAATTAACCCAAGACAGACTGCAC

ATTGACATTATTGTCTTGTATAAACAGTAACTATGAAGCTCATCAGCACTGAGCTAACCTACAAATCCCA

TGCCTGCAAAAAAGGCTTCCTTTAAACTGCATGGATTGGGCTGCCGTTTGAGTTCATGTTTGAATCGTTA

GAGGCTAGATTAGCATGAAAAGCAGATTGTCTTGAAGACCCTTCAAGCAATTTCAAGGAAAGAGACGAGG

GGCCAATAGAGTCTCCTAAACTTAGTTTTGACATGCCAACCAGCTCATCAACGTTGATTGGGCTTTATGA

ATGTACTGCTGTTGGCTTGAGAACCTCGTGAGTCTCCTCCTTCTTACTTGGCTCCGCATTGTATGCTGAC

CACAGTGGAAAAGAACATGGGAAGAATGGTGGGATATAAG

>FM895544.1 FM895544 Jatropha curcas embryo 71-95 (DAF) Jatropha curcas cDNA clone rjcpga0_002484, mRNA sequence

ATTCCAACGGGGTGAGTAAACCCGCAAGGCGCAAGGAAGCTGACTGGCGGGATCCCCCTGAGGGTTGCAC

CGCCGACCGACCTTGATCTTCTGAGAAGGGTTCGAGTGAGAGCATGCCTGTCGGGACCCGAAAGATGGTG

AACTATGCCTGAGCGGGGCGAAGCCAGAGGAAACTCTGGTGGAGGCCCGCAGCGATACTGACGTGCAAAT

CGTTCGTCTGACTTGGGTATAGGGGCGAAAGACTAATCGAACCGTCTAGTAGCTGGTTCCCTCCGAAGTT

TCCCTCAGGATAGCTGGAGCTCGGGACGAGTTCTATCGGGTAAAGCCAATGATTAGAGGCATCGGGGGCG

CAACGCCCTCGACCTATTCTCAAACTTTAAATAGGTAGGACGGCGCGGCTGCTTCGCTGAGCCGCACCAC

GGAATCGAGAGCTCCAAGATGGGCCATAATTGGTAAGCAGAACTGGCGA

>FM895543.1 FM895543 Jatropha curcas embryo 71-95 (DAF) Jatropha curcas cDNA clone rjcpga0_002482, mRNA sequence

ATCCTGTCCTGTCTCTCTCTCTCTCTCTCTCTCCCTTCTCTTTCTCTTTTCTCCAAAGCAAACCCTATAA

ATCCCATCTGTCTCTTTCCCTCTCTCTCTCTCTCTCTCTTCTCTCTCTAACCCATTTCCTCAATTAAAAC

CATCAGTTTTAAGAGACAATACCAGAGAGTTAGAGAGAGAAAGCTCAGGCACATGCACCTTCTCCGGATT

ACTATAAATTGGCGAAGACAAGTGCTGTAGACGATTTAGTTTTGAGGAGGAGAGACAAGATGCGCAGGTG

GCTTTGTTGTACCTGTCAAGTAGAGGAGTCGTACCCATCACGTGAGAATGAGCACCTAAGAAGCCCAAAG

CACTATCCAGATGGAAATCCTACGAAAGGCCCGAAGGTAGCAGCTCCAGTGAAATCCGAAGTGCAGAAGG

AAGCCCCTCCAATTGAAGTGCCTGCATTGTCTTTGGATGAACTGAAAGAGAAGACCGAGAATTTTGGTTC

AAAAGCATTGATTGGTGAAGGATCCTATGGAAGAGTCTATTATGCTAATTTAGAGAATGGA

>FM895542.1 FM895542 Jatropha curcas embryo 71-95 (DAF) Jatropha curcas cDNA clone rjcpga0_002481, mRNA sequence

CTGACTGGCGGGATCCCCCTGAGGGTTGCACCGCCGACCGACCTTGATCTTCTGAGAAGGGTTCGAGTGA

GAGCATGCCTGTCGGGACCCGAAAGATGGTGAACTATGCCTGAGCGGGGCGAAGCCAGAGGAAACTCTGG

TGGAGGCCCGCAGCGATACTGACGTGCAAATCGTTCGTCTGACTTGGGTATAGGGGCGAAAGACTAATCG

AACCGTCTAGTAGCTGGTTCCCTCCGAAGTTTCCCTCAGGATAGCTGGAGCTCGGGACGAGTTCTATCGG

GTAAAGCCAATGATTAGAGGCATCGGGGGCGCAACGCCCTCGACCTATTCTCAAACTTTAAATAGGTAGG

ACGGCGCGGCTGCTT

>FM895541.1 FM895541 Jatropha curcas embryo 71-95 (DAF) Jatropha curcas cDNA clone rjcpga0_002480, mRNA sequence

ACCTCCNTCTCCAGAAATGCTTTTCAAGCATGGGCTAACGTTACGCCCTTCACATTTTCTCCCTTCCATG

GCCGTGGAAATCCAGATATAACGATTGGTCTTTACAAAGGTGATCATGGAGATGGGTACCCTTTTGATGC

ATTTGGTGCCATTGCTGCTCATTCTTGGCCTCCACAGGATGGAAGGCTACATTTTAATTCGTATGGGAGA

TTTTGTATCGGTGCATTTCCAAATTTATATGATTTTGGGACAGTTGCCTCGCATGAAATAGGGCACCTTC

TTGGACTTGGACATAGCCGTGACGAAGAGGCTATTATGTATCCAGTTGCTCCTTTCGGACGGGTCAAGGG

TATACATCAATATGATATTGAAGGCGTTCGAGCTTTATATGAATTTTAATAATATTTTCTGTTTCTATTA

TTATTAAATAAGACGTGAGAAATAAATTGCCGTTGTGTAGTAATATTTTGTATTTCTATGGCTATAAAAA

GGCTATATGATGGTAAAATAAGTACTATTGATTGAATTAAAAAA

>FM895540.1 FM895540 Jatropha curcas embryo 71-95 (DAF) Jatropha curcas cDNA clone rjcpga0_002479, mRNA sequence

TTATCATCATCTTTGGTTTCTTTCTTTGATATTTTCTCTCTCTTTTTCTCTTCCTCTTCGAACTCACACA

TCTTCTTCCATGGCCGATCAGCTCACCGACGACCAGATCTCTGAGTTCAAGGAAGCCTTCAGCCTATTTG

ATAAGGACGGCGATGGCTGCATCACCACTAAGGAACTTGGGACTGTAATGCGATCATTGGGCCAGAACCC

AACTGAGGCAGAGCTCCAGGACATGATAAATGAGGTTGATGCTGATGGAAATGGTACCATTGATTTCCCA

GAGTTCCTTAATCTGATGGCCCGTAAGATGAAGGACACTGACTCTGAGGAGGAACTCAAGGAAGCTTTCC

GGGTTTTTGATAAGGATCAGAATGGCTTCATTTCTGCTGCTGAGCTGAGCCATGTCATGACAACTCTTGG

CGAGAAGCTGACAGATGAGGAGGTTGATGAGATGATTCGTGAGGCTGATGTGGACGGTGAAGGTCAGATC

>FM895539.1 FM895539 Jatropha curcas embryo 71-95 (DAF) Jatropha curcas cDNA clone rjcpga0_002478, mRNA sequence

AGGCTCTCAATTGGAAAATTATAGCCAGACTGGTAAACAATAAACTGTTTGCTCTATCTTTTCATCAAAT

GAAATAAATGGATAAACTGTTTGCTCTACCTTTTCATCAACTGAAATATCACAACACAGTCACTTCAATG

TTTTCCCATCTAATGAAATGCAGCCACCTCATCTTCGAAGTGTTTCAGTAGGCTCTCCATGCTCATTACT

GAGTGATCACCCTTGTCTCTCACTCGTACACTCACCTGTCCAGTTTTGGCTTCCTCTTCACCCACGACCA

ATATAAAGTTGTACTGTGCCAACTGAGCTTCCCGCACCTTTAATCTGAATCTTTCTGTCAGTTGTGTCAA

CATCAACATGATAACCAGCTTTATGTATCTGAGCCTGCACCTGCTCTGCATATGGCTGGGATATCTCAGA

GACAGGGCAGACAATTGCTTGACGAGGACTAAGCCAGAAAGGCCATTTCCCCTTGTAATGTACCAATAGA

ATGGCAAACATTC

>FM895538.1 FM895538 Jatropha curcas embryo 71-95 (DAF) Jatropha curcas cDNA clone rjcpga0_002477, mRNA sequence

TTTTTTTTTTCTAGAAAAAGCAATACTGCACTTCTTCACCATTTATCACCATTACGATGGATCCTCATTG

ACATCAAAATCCTCTAAAAGGGGAAAATTTTCCCCACCATTCAACAACATGACAGACAAAATTCAAGAGT

CAACATGAACTATGAACTACATATTCTTTGATTGCCAAATTATAACAAGGATATATAACGAACCAGAAAC

TATCAGAAATGGCGGACCCTTTTTGTCTTGCTTGTCTCATAGTCCATATCTTTATAATATCTTGAAAAGC

AGGCCTCCATGTGTAGCAAAGAATGGAGCGAAAACAAGCGATTCAACTGCCATAAGTTTGATAAGGATGT

TTAGTGATGGTCCAGATGTATCCTTCAATGGGTCTCCAATGGTATCCCCAATAACAGCTGCTTTGTGTGG

ATCTGAGCCTTTAGGACCAAGAGTTCTTGCATGCTCAGAGGCACCAGCCTCAATGTACTTCTTGGCATTG

TCCCAGGCGCCACCAGTGTTAGATGCAGAGATGGCAATCTGTACACCAGATACAAGTGATCCTGCTAGAA

CACCAGACAGAGTTTCGACACCGAAAAGTATTCCAACAATAAGGGGGGTGAGCATAACGAGTGCCCC

>FM895537.1 FM895537 Jatropha curcas embryo 71-95 (DAF) Jatropha curcas cDNA clone rjcpga0_002476, mRNA sequence

GAATCAACTAGCCCCGAAAATGGATGGCGCTTAAGCGCGCGACCTATACCCGGCCGTCGGGGCAAGAGCC

AGGCCCCGATGAGTAGGAGGGCGCGGCGGTCGCTGCAAAACCCGGGGCGCGAGCCCGGGCGGAGCGGCCG

TCGGTGCAGATCTTGGTGGTAGTAGCAAATATTCAAATGAGAACTTTGAAGGCCGAAGAGGGGAAAGGTT

CCATGTGAACGGCACTTGCACATGGGTTAGTCGATCCTAAGAGACGGGGGAAGCCCGTCCGACAGCGCGT

CCTCGCGCGAGCTTCGAAAGGGAATCGGGTTAAAATTCCCGAACCGGGACGTGAAGGCTGACGGCGACGT

TAGGGAGTCCGGAGACGTCGGCGGGGGCCTCGGGAAGAGTTATCTTTTCTGTTTAACAGCCCGCCCACCC

TGGAAACGACTCAGTCGGAGGTAGGGTCCAGCGGCTGGAAGAGCACCGGACGTCGCGCGGTGTCCGGTGC

GCCCCCGGCGGC

>FM895536.1 FM895536 Jatropha curcas embryo 71-95 (DAF) Jatropha curcas cDNA clone rjcpga0_002475, mRNA sequence

AGGATTCTATCCGTCGCCCGATGGGAATTGTACTTTAAGGCGGCCCTCGCGGCTCGTCCGCCGCGGGGGC

TTGGCCAACGGCACGTGCCTCTGGGGGCCGGATGGCCCCTACTGCGGGTCGGCAAACGGGCGACGGACAC

GCGCGTCGCTTCTGGCCCGGATTCTGACTTAGAGGCGTTCAGTCATAATCCAGCGCACGGTAGCTTCGCG

CCACTGGCTTTTCAACCAAGCGCGATGACCAATTGTGCGAATCAACGGTTCCTCTCGTACTAGGTTGAAT

TACCATCGCGACACCATCATCAGTAGGGTAAAACTAACCTGTCTCACGACGGTCTAATCCCAGCTCACGT

ACCCTATTGGTGGGTGAACAATCCAACACTTGGTGAATACTGCTTCACAATGATAGGAAGAGCCGACATC

GAAGGATCAAAAAGCAACGTCGCTATGAACGCTTGGCTGCCACAAGCCAGTTATACCTGTGGTAACTTTT

CTGACACCTC

>FM895535.1 FM895535 Jatropha curcas embryo 71-95 (DAF) Jatropha curcas cDNA clone rjcpga0_002474, mRNA sequence

CTTGAGCTACAAGTGCAGGAGGAGCATTTCGCTTCCCATTTAACACCTGGGCAGTAGCGAGTTACACCAT

CGAAAATGAAGTTCAATATAGCAAATCCAACGACTGGGTGCCAGAAGAAA

>FM895534.1 FM895534 Jatropha curcas embryo 71-95 (DAF) Jatropha curcas cDNA clone rjcpga0_002473, mRNA sequence

TTCTCCTTCTCTTTTCTCTCTCTCTCTCTCTCTCTCTCTCTCTCTCTATATATATATATATATATATATA

TATATATATACCAAGCAATAAGGAAGAAACAGAAACTGAGATTTGGAAGAATTCGATGATGGGCACTTTG

GGCCGCGTTTTAGCGGTTTCTTACCCTATAAAGCCTCTATGCCTGTACAGTCCTTGCTCATATCAATCTG

ATTGTAGATTATAATGCGTTAATCAAAAATTGAGCTCGAAATGGCGATCTATGGTGTCTGAGTTCGAATC

TTCTCCATTAACGCCTTCTGTTGACTCGGATACAAACGCAGCCGGTTTTTGTATTATAGAAGGGCCTGAA

ACAGTTCAGGATATCGCCAAAATGGAACTCCAAGAAATTCAAGATAATATTCGAAGTCGGCGAAACAAAA

TTTTCTTGCATATGGAGGA

>FM895533.1 FM895533 Jatropha curcas embryo 71-95 (DAF) Jatropha curcas cDNA clone rjcpga0_002471, mRNA sequence

CTAGCCCCNGAAAATGGATGGCGCTTAAGCGCGCGACCTATACCCGGCCGTCGGGGCAAGAGCCAGGCCC

CGCATGAGTAGGAGGGCGCGGCGGTCGCTGCAAAACCCGGGGCGCGAGCCCGGGCGGAGCGGCCGTCGGT

GCAGATCTTGGTGGTAGTAGCAAATATTCAAATGAGAACTTTGAAGGCCGAAGAGGGGAAAGGTTCCATG

TGAACGGCACTTGCACATGGGTTAGTCGATCCTAAGAGACGGGGGAAGCCCGTCCGACAGCGCGTCCTCG

CGCGAGCTTCGAAAGGGAATCGGGTTAAAATTCCCGAACCGGGACGTGAAGGCTGACGGCGACGTTAGGG

AGTCCGGAGACGTCGGCGGGGGCCTCGGGAAGAGTCATCTTATCTGTTTAACAGCCCGCC

>FM895532.1 FM895532 Jatropha curcas embryo 71-95 (DAF) Jatropha curcas cDNA clone rjcpga0_002470, mRNA sequence

AGAAAGCAACTGTCAACTGCCATCAAATTTGCGGGGGAAAAATAAAAAAAATCAAATACAACAGTGGCTG

ATTGGTCAGTGCATGGTTTTGTCACAACGTACAAAGCTTCTAACTCAGAGTTACAAACATCACTTTATCT

ATTTCATTGTATCTATTGTGACAATCACAAAAATGGCCATAACCTGGCCCATCTCCTAGTAACCTTTCCA

TTCTCACTTGTGGAGGTTAACAGTACCATAAACTGAGCTAAATCTCTAATCTCACCGTCCTCTTGCTTGG

AAAATTCTAAGTATCCAAGTAAACCAATGGGAACTACAACTCAATTTCCAGAAGCATGTCTTCGTTGCAC

CAACAAATCATCAGCAGAGTAATAATCGGCAATG

>FM895531.1 FM895531 Jatropha curcas embryo 71-95 (DAF) Jatropha curcas cDNA clone rjcpga0_002469, mRNA sequence

TGGAAATCAGAATCAAACGAGCTTTTACCCTTTTGTTCCACACGAGATTTCTGTTCTCGTTGAGCTCATC

TTAGGACACCTGCGTTATCTTTTAACAGATGTGCCGCCCCAGCCAAACTCCCCACCTGACAATGTCTTCC

GCCCGGATCGGCCGCCGAAACGACCTTGGGTCCAAAAAGAGGGGCATAGCCCCGCCTCCGATTCACGGAA

TAAGTAAAATAACGTTAAAAGTAGTGGTATTTCACCGTCGCCTTTCGGCTCCCACTTATCCTACACCTCT

CAAGTCATTTCACAAAGTCGGACTAGAGTCAAGCTCAACAGGGTCTTCTTTCCCCGCTGATTCCGCCAAG

CCCGTTCCCTTGGCTGTGGTTTCGCTGGATAGTAGACAGGGACAGTGGGAATCTCGTTAATCCATTCATG

CGCGTCACTAATTAGATGACGAGGCATTTGGCTAACCTTAAGAGAGTCATAGTAACTCCCGCCGTATACC

CGCGCTTGGTTGAATTTCTTCACTTAGACATTCAGAGCACTGGGCAGA

>FM895530.1 FM895530 Jatropha curcas embryo 71-95 (DAF) Jatropha curcas cDNA clone rjcpga0_002468, mRNA sequence

AAAGATAAACAAGAATTTCTCCATTCAATAAGGGGTCACAAACTTGATACATCACCAATACTAGATAATT

TACACACATGATTAACATAATTAACTTTTTCAATACCAGATTCACATGGAAGCGGAAGAGAACCCAAAAT

ACACAACAAAAGAAAAAACAAAAAAAAAAAAAACAGAACACTAAAGTAGAAAAGTGATGGTGAAATGCAA

AGCAGCATTTACGCCCTGCTCTTGAAAGGAATAGCTCTAATGACTATCTGGTGGTACAGAGCAGCACGAG

CAGCTCCAATGAAGGGCCCACCCCAAAAAACCCAGTGGTCATCCCAGGCGTGGTCTTTGTTGAAGATGAT

AGCAGCTCCAAGACTCCTGGCTGGGTTAATGCCAGTTCCAGTAATGGGGATGGTGGCCAAGTGAACCAAA

AACACTGAAACCCAATGGGAAGAGGAGCCAAATAGGGAAATGAAAGTCCCTGGCGCTCCTCTT

>FM895529.1 FM895529 Jatropha curcas embryo 71-95 (DAF) Jatropha curcas cDNA clone rjcpga0_002467, mRNA sequence

CAAAGATCCACAANTTGGCACGGAGGACTGACAAGAAGGGGCAGACTGCATTGCATATGGCTGTAAAAGG

GCAAAGCTGTGAGGTGGTAAAATTGCTTCTCGATGCAGATGCTGCTATCGTGATGCTTCCAGACAAATTT

GGTAACACAGCATTACATGTGGCAACTAGGAAGAAGCGGGTGGAGATAGTTAATGAGTTGTTATTACTTC

CCGATACCAACGTCAATGCACTGACAAGGGACCATAAGACAGCCCTTGACATTGCTGAAGAGCTTACCCT

GTCAGAAGAATCATCTGAGATTAAAGAATGCCTTTGTCGCTACGGTGCTGTTAGAGCTAATGAACTCAAC

CAACCAAGAGATGAGTTAAGGAAAACTGTAACTCAAATTAAGAAAGATGTTCACACCCAGCTTGAACAAA

CCAGGAAGACCAACAAGAATGTTCATAACATTTCTAAAGAGCTG

>FM895528.1 FM895528 Jatropha curcas embryo 71-95 (DAF) Jatropha curcas cDNA clone rjcpga0_002466, mRNA sequence

TGCAGGAATTCGAATTCCTACATTAACATATCTTTCTCACACTTATCATCAACAAATGGCTGAACGTTCA

CAGCCACACCAAGTTCAAGTGCACCCACAACACCGCTATGAAGCTGCCTTCAAAGGTCAACAAAAAGGTC

CATCAGCTCAGAAAGTGTTAGCTGTGATCACCCTCCTGCCGGTAGGCGGTGGGCTTCTTGCACTAGCTGG

TATAACCTTAGTAGGTACCCTAATCGGCCTAGCTATTACTACCCACTCTTTTTGTCATCTTTAGCCCTGT

TCTTGTCCCAGCTGCATTAGTTATTGGCCTTTCGGTCATGGCATTTTTGGCTTCCGGGGCTATGGGGCTC

ACAGGGTTGTCATCACTCTCGTGGGTTCTGAAATACCTCCAGGAGGTCACTCGGCGAATGCCGG

>FM895527.1 FM895527 Jatropha curcas embryo 71-95 (DAF) Jatropha curcas cDNA clone rjcpga0_002465, mRNA sequence

TTGGCTTTATAGGTGGAAAGAGTAGTGCCCTATTGCAGGTAAGTGGTGGCCCTTCTCCCCGTCCTTACAA

TGTTGGTTTGGCTACAGAGCCTTCTCCTCAAACTCCCATACCTAGTAAAACCCCCTATATGCCTCCTGTT

GCAGAGAAACCAGTTGTTTCAGCCGCAAGTATAAATCCTGATGAACTCCAGAGAAAAACAATTTCGCTTT

TGAAAGAATATATCAACATCCGACTTTTGGATGAAGCATTGCAATGTGTGGAGGAACTGAAGTCCCCTGC

TTACCATCCTGAAATAGTCAAGGAAGCCATCTCCCTTGGCTTGG

>FM895526.1 FM895526 Jatropha curcas embryo 71-95 (DAF) Jatropha curcas cDNA clone rjcpga0_002464, mRNA sequence

GGTTCTTTTGAGTTTATGAATTGGGTTTTCAGTTTCTGATAAGAGGCAAAACAATACAAGTTATGAGCCA

ATATCCTAATGGAGTGTACAACTAATGGAAGTGAGTACCAATCTCAGATTTCAGCGAAAGAATACAACTA

ATGACAGATATTTATGGAACAGACAGAATGTGCTATTGTAATACAAAACGTGTTGTTGAAAATATCAATG

TTCTTGAACTGGAAATCACAGGAAGAATTTTCCCCGGACAATGGTTCAAACACTTGGTGACNATTTCACA

AACTTGATAATGGTTTCCAAAGCTTGATGCGGTTCCCCTGTGCTATCAGATAACCCCACACAATTGTCAT

CTTCTCTTTGCATGCTTCATCCAAAAACATATTAGCAGCAAGGAAGCTATCTAGCCATCTTGTGGATATA

ATTGAAACTACACATCGTATACAGGCAGTCGCTCTTCGATTGTAACACGGCAGATAGGGCACTTCTTACA

CTTCT

>FM895525.1 FM895525 Jatropha curcas embryo 71-95 (DAF) Jatropha curcas cDNA clone rjcpga0_002463, mRNA sequence

GTATCGTAGCATGGCGTTGCCTCTGTTCTGTGCAAAAAGTTCCATGTCATAGTCGGATCTCACCTCATCT

AAAGTTCCAATCCAAGCCGGATGAGGCTTCTCCACCACCTGGAAATGGATGGGACTACTGCATGTTTTGG

CCTTGACCAGCTTGGATTGTGATGATAGTTCCATTAGTGATGTCTTTTTACTTTCTTCTGTTCTTGCTGT

GTGCCTTGACAATTGTTGACTGCAGCAAGTATTTTTTGACAGTACATCCTCAAATGGTGTCAAACAGTTT

ATTCGGCGTGTATCATCC

>FM895524.1 FM895524 Jatropha curcas embryo 71-95 (DAF) Jatropha curcas cDNA clone rjcpga0_002461, mRNA sequence

ATTCCAATTTGAATAGCTACCGTCTAGATAGGATTTGATTCACCTCTCATTACAGAAAATGTTTCACAGG

AATTTAAGAGAACAAGCATACTATTTTTGGCAGACTGTAATTTGTTTATAACAGCACACGATTGAAAGCA

CCAATGAACTTTTTTAGAGGACAGACACTACTCATTTGAGGAGTGTCTTGACAATAGATTTCACATTCAA

CTTTTTGAGATCTGTATAAGATTGTCCACAGCCGACGAACATTACTGGTGCGCCAGAGATGTATACCATT

GACAGCGCAGCACCAACCTTATCATCAATTGTATCAAACTTGGTAAGCAGAATGCCATCGATTAATCTTG

CATTAGGTGAAGTAGAGAGATCGGCTAGTAACTGGTTGAACTTTGATAGTTGATCAACGGCATCATATCC

AACCAATGCCTCTCAAACAAACAAG

>FM895523.1 FM895523 Jatropha curcas embryo 71-95 (DAF) Jatropha curcas cDNA clone rjcpga0_002460, mRNA sequence

ATTCTTAGAATTGGAATCGCAGGTTGCGGCGATTGGTTTCCTCTGCTTCAGCACACTCGACGAATCCGTC

TGCCTGTGACTGGGCTTTATTTGAGGCGTCTATTTCTCCTGTTCTCTTTGTTTCTATTTAGGCTTGGAGA

TGGATTCTTCTACGATGCTATATAACCAGCTCAAGGCAGCGGAGCCATTTTTCTTAATGGCAGGCCCAAT

GTGATTGAATCTGAGGAACACATTCTCAAAATGGCCAAGCACCTCAAGACCATTACAACAAAACTTGGTT

TGCCATTGGTTTTTAAGTCAAGCTTTGACAAAGCAAATAGAACATCCTCGGAGTCATTTCGAGGTCCAGG

CATGAGTGAAGGCTTGAAGATTCTTGAGAAGGTTAAAATAGCATATGACATCCCTATTATTACTGATGTG

CATGAAACTATCCAGTGTGAAGCAGTAGGTAGAGTTGCAGATATCATACAAATTCCT

>FM895522.1 FM895522 Jatropha curcas embryo 71-95 (DAF) Jatropha curcas cDNA clone rjcpga0_002459, mRNA sequence

CTTCATTTGGATTAAGCGATGGATACTGATTCTGTGTTTAGCAATATCGTTCGAGCTCCTGAAGATCCTA

TTTTAGGGGTTACTGTTGCTTATAACAAAGATACAAGCCCTAACAAGTTGAATTTGGGTGTTGGTGCTTA

TCGAACTGAGGAAGGGAAGCCTCTCGTTTTGAATGTGGTGAGGAAAGCGGAGCAGATACTAGTAAATGAC

CAGTCTCGGGTCAAAGAGTATCTTCCTATTGTTGGACTTGCTGATTTTAATAAACTGAGTGCCAAGCTTA

TCTTAGGAGCTGATAGCCCCGCTATACAAGAGAACAGAATAACTACTGTGCAAGGCTTGTCTGGTACCGG

TTCTTTGAGAGTTGGAGGGGAGTTTCTTGCAAGGCATTACCATCAACTGACAATATACATTCCACAGCCA

ACATGGGGAAATCATACCAAAGTCTTCACTCTTGCAGGGTTGTCTGTAAAGACGTACCGGTA

>FM895521.1 FM895521 Jatropha curcas embryo 71-95 (DAF) Jatropha curcas cDNA clone rjcpga0_002458, mRNA sequence

GATCTTCTGAGAAGGGTTCGAGTGAGAGCATGCCTGTCGGGACCCGAAAGATGGTGAACTATGCCTGAGC

GGGGCGAAGCCAGAGGAAACTCTGGTGGAGGCCCGCAGCGATACTGACGTGCAAATCGTTCGTCTGACTT

GGGTATAGGGGCGAAAGACTAATCGAACCGTCTAGTAGCTGGTTCCCTCCGAAGTTTCCCTCAGGATAGC

TGGAGCTCGGGACGAGTTCTATCGGGTAAAGCCAATGATTAGAGGCATCGGGGGCGCAACGCCCTCGACC

TATTCTCAAACTTTAAATAGGTAGGACGGCGCG

>FM895520.1 FM895520 Jatropha curcas embryo 71-95 (DAF) Jatropha curcas cDNA clone rjcpga0_002456, mRNA sequence

CTCTTTCTCTGCAGTCTGCACATTATTGAAGTGATATTAGGTGAATGATTTTATCATCATTAATGGCTTC

GTTTTGGTCATTCTTGACAATGTTTATTATTTTCATGGCATATTTTGCTTCACTAGCTTTTACAAGTCAT

GTAGTACATGTACAATTTTCTACTATATCAGCAGCACCAGCATTTTTACCTGAAGCTCCTCTTTCTTCCC

CACCAACTTCAAGTTTGTCACCAGACATTGAGCCTGTTTTCCCTACAACGGGAGTGGCAGTGCCTTCTCC

AGCTGAATCATCACTACCCACTATTCCTTCAAGCCCAAGCCCTCCAAATCCAGATGTTATTCATCCAGCG

CCAGGTCCTTCAATGGCATTTTCGCCGTCTGGGTCTTTACCAGCTTCTTCTGCTACTTCTGTAAACCAAG

CTGCTGTTTATTTTCTTGGTATGCTGTAGGTGGTGTTTACTTTGATGCAGCTTTCTGCTTTTTAAGGTAC

TATTACTATAGTTGGACGCTCCATTCTTGA

>FM895519.1 FM895519 Jatropha curcas embryo 71-95 (DAF) Jatropha curcas cDNA clone rjcpga0_002455, mRNA sequence

ACGAACTTGGCTATGTATTAAAACCTGCCATAAAGTTGCTTATAAAATTCTTTGTACACATCTAGCACTT

GTAAAATCATAACCTACCCATATATTTCTTTGCTATAAACAAACTACCAACAGCACCAATGTACACCAAG

ACCAAGATCAAATTCAAAGATTCCCATACGGCCACTGATCTTGAAAAGAATTCCAATACTATCCCAAAAC

TAGAACCACAATGAATCCATCTACATAGCTGACAGATCGCGGATATAGTCATCCCACATACTACTTGCAA

TAGAGTCTCGTAACTGTGAACTAAATTCAAGCTGTTCTGTCTCAAAAGCAATGTCCAGAGCCTGGTTCTC

AACTTGTAGAATTGACTGTTCCACCTCCAATGAGGGTAAAGATTCTTCCATTTCTAGTATGCTATCCTGT

TCATACATTCTAAAAATCAAATCATCTGGTTTCTCCCTACGGATGTAAATATGTATGGCACATGCTGATA

CAACCAACTTAACCTGTGTCTGCAAAGGGTAA

>FM895518.1 FM895518 Jatropha curcas embryo 71-95 (DAF) Jatropha curcas cDNA clone rjcpga0_002453, mRNA sequence

GGATTCTATCCGTCGCCCGATGGGAATTGTACTTTAAGGCGGCCCTCGCGGCTCGTCCGCCGCGGGGGCT

TGGCCAACGGCACGTGCCTCTGGGGGCCGGATGGCCCCTACTGCGGGTCGGCAAACGGGCGACGGACACG

CGCGTCGCTTCTGGCCCGGATTCTGACTTAGAGGCGTTCAGTCATAATCCAGCGCACGGTAGCTTCGCGC

CACTGGCTTTTAAACCAAGCGCGATGACCAATTGTGCGAATCAACGGTTCCTCTCGATACTAGGTTGAAT

TACCATCGCGACACCATCATCAGTAGGGTAAAACTAACCTGTCTCACGACGGTCTAAAACCCAGCTCACG

TTCCCTATAGGTGGGTGAACAATCCAACACTTGGTGAATTCTGCTTCACAAATGATAGGAAGAGCCGACC

ATCGAAGGATCAAAAAGCAACGTCGCTATGAACGCTTGGCTGCCACAAGCCAGTTATCCCTGTGGTA

>FM895517.1 FM895517 Jatropha curcas embryo 71-95 (DAF) Jatropha curcas cDNA clone rjcpga0_002452, mRNA sequence

CCAGCCTGGTTTTCGCATTGTTAAGCTGGTGAATACTTAGTATTATAGTTCTACCTCTTGTTTCTGCCAT

GACTTTAAGCATATCAATAATTTGTAAAGCAGAGGTACTATCAAGACCAGAAGTTGGCTCATCAAGAATC

AACACTTTCGGGTCATGTATAACGTCGACTCCGATTGAAACTCGTCTCCTTTCTCCGCCAGAAATACCAC

GTACTCTGTCATCGCCGGTACACACACGAGTCATGGCTACATGGTCAATGCCTAGCTCTTGGACCAAAGA

CTTCACTCTAGATCTCAGTTGAGCTTGTGGAAGACTTAAACGTAGCTTTGCACTAAACATGAGGGTTTCT

TCAACGGTAAGGAGAGGACAAAGAGTGTCTCTTTGTGTAACATAGCCTGAAATATACTGGAGCTGAGATT

TGTCAATAGGATTATGATTGACAAAAATGGAACC

>FM895516.1 FM895516 Jatropha curcas embryo 71-95 (DAF) Jatropha curcas cDNA clone rjcpga0_002451, mRNA sequence

CTTGGCCCCGGGTGCCTGAAGAAATGAGCCCTGAAGCATGGGACCTAATTGATAGGTTATTAACGGAAGA

TCCTCATCAGAGACTTGGAGCTGGAGGAGCATCTGAGGTGAAGCAGCATGTATTCTTTAAAGATATTAAC

TGGGACACGCTTGCTAGACAGAAGGCTGCATTTGTTCCCAGTTCAGAGAGTGCGCTTGACACAAGTTACT

TCACAAGTCGCTACTCATGGAATCATTCAGATGACCACGTATATCCAGCCAGTGATTTTGAGGATTCTAG

TGATGCTGACAGCTTGAGTGGCAGCAGTAGCTGCCTAAGCAATCGCCAAGATGAAGTGGGAGATGAATGT

GGGGGTCTAGCAGAGTTTGAGTCTGGCTCGTCCGTCAATTACTCATTCAGCAATTTCTCTTTCAAGAATC

TCTCACAGCTTGCATCTATCAAGTATGACCTTCTCTCCCAGGGTTGGAAGGACGATCCATCCACGAA

>FM895515.1 FM895515 Jatropha curcas embryo 71-95 (DAF) Jatropha curcas cDNA clone rjcpga0_002450, mRNA sequence

ATTCAACAACTCACCTGCCGAATCAACTAGCCCCGAAAATGGATGGCGCTTAAGCGCGCGACCTATACCC

GGCCGTCGGGGCAAGAGCCAGGCCCCGATGAGTAGGAGGGCGCGGCGGTCGCTGCAAAACCCGGGGCGCG

AGCCCGGGCGGAGCGGCCGTCGGTGCAGATCTTGGTGGTAGTAGCAAATATTCAAATGAGAACTTTGAAG

GCCGAAGAGGGGAAAGGTTCCATGTGAACGGCACTTGCACATGGGTTAGTCGATCCTAAGAGACGGGGGA

AGCCCGTCCGACAGCGCGTACTCGCGCGAGCTTCGAAAGGGAATCGGGTTAAAATTCCCGAACCGGGACG

TGAAGGCTGACGGCGACG

>FM895514.1 FM895514 Jatropha curcas embryo 71-95 (DAF) Jatropha curcas cDNA clone rjcpga0_002449, mRNA sequence

CAAAGGATTCTATCCGTCGCCCGATGGGAATTGTACTTTAAGGCGGCCCTCGCGGCTCGTCCGCCGCGGG

GGCTTAGCCAACGGCACGTGCCTCTGGGGGCCGGATGGCCCCTACTGCGGGTCGGCAAACGGGCGACGGA

CACGCGCGTCGCTTCTGGCCCGGATTCTGACTTAGAGGCGATCAGTCATAATCCAGCGCACAGTAGCTTC

GCGCCACATGGCTTTATCAACACAAGCGCGATGACCAATTGTGCGAATCAACGGTTCCTCTCGTACTAGG

TTGAATTACCATCGCGACACCATCATCAGTAGGGTAAAACTAACCTGTCTCACGACGGTCTAACCCCAGC

TCACGT

>FM895513.1 FM895513 Jatropha curcas embryo 71-95 (DAF) Jatropha curcas cDNA clone rjcpga0_002447, mRNA sequence

TTTAAATTTTCTGCTCAAATGATAAACTTGAATTACCAAAGCATGATTACAACGAAAAGATAAAGTCGAA

TATTGAAAAGCATCAGAAAATATAAACAACGCTTCCGACAAGACGAGACCAAGTAAAACGAAGACAGTAT

AATTAATACTAGGTCAGGCTGTTTATCCCGATTTGATTAGCTAAGCGCAAAGCATTTCGACTTGAGAACC

ACTGAGAGCCCAATTGGCTTCACTGACAACACGAGGCATGGTGCTTATCCTTGACTGACTATCAACCTTG

AAATTAGCTCCGCACATTACTCCTTACACTATCAATTCTTGGCATAGCCTTCATTTTGCTCTATGGATGC

TCAAAACTCATCAACCCTTGTACACTATGGAACATGCACCCTTTTGGAAATGGTGCAAATGACTTTGCAC

AGCTTCCATTAATGCCCATCCAAGATAGTCAGATATTACAACAGAACCATC

>FM895512.1 FM895512 Jatropha curcas embryo 71-95 (DAF) Jatropha curcas cDNA clone rjcpga0_002446, mRNA sequence

ATTCGAAGCGAGAGAGAGAGAGAGAGAGAGTGAGAGATAGGGTTTGAATCTAATCCTCAAACAAAGTCTC

TTCTTCGTTTACTTGGCATCTTCAGCTTTTAGCTCTAATGGCGTCCAATTCGAACAAGGATGTTCCCTCT

GATGAGAAAACGGGTTCAACAGAAAGTAAAAACCCAAAATCAGAGACCTCACCTGCGGAGTCTCAAGCAG

GACAAGGAAGATCTCCTCCTCCTGCAGGGGCTGGATTTGCCACGCCCAATCCTTTTGATTTCTCAGCCAT

GAGTGGACTGCTTAATGATCCAAGCATCAAGGACTTGGCTGAACAAATAGCAAAAGATCCTGCATTTAAC

CAAATGGCAGAACAACTCCACAAAACACTTCAGGGTGTGGGAGCTGATGAGGGGATCCCTCAATTTGATT

CGCAGCAATACTATTCCACCATGCAACAAGTTATGCAGAATCCTCAATTTATGACTATGGCTGAGCGTCT

TGGCAATGCTTTGATGCAGGATCCATCAATGTCCCAAATG

>FM895511.1 FM895511 Jatropha curcas embryo 71-95 (DAF) Jatropha curcas cDNA clone rjcpga0_002445, mRNA sequence

AGGATCTATCCGTCGCCCGATGGGAATTGTACTTTAAGGCGGCCCTCGCGGCTCGTCCGCCGCGGGGGCT

TGGCCAACGGCACGTGCCTCTGGGGGCCGGATGGCCCCTACTGCGGGTCGGCAAACGGGCGACGGACACG

CGCGTCGCTTCTGGCCCGGATTCTGACTTAGAGGCGTTCAGTCATAATCCAGCGCACGGTAGCTTCGCGC

CACTGGCTTTTCAACCAAGCGCGATGACCAATTGTGCGAATCAACGGTTCCTCTCGTACTAGGTTGAATT

ACCATCGCGACACCATCATCAGTAGGGTAAAACTAACCTGTCTCACGACGGTCTAAACCCAGCTCACGTT

CCCTATTGGTGGGTGAACAATCCAACACTTGGTGAATTCTGCTTCACAATGATAGGAAGAGCCGACATCG

AAGGATCAAAAAGCAACGTCGCTATGAACGCTTGGCTGCCACAAGCCAGTTATCCCTGTGGTAACTCTTC

TGACACCTCTAGCTTCAAATTCCGAAGGTCTAAAGGATCGATAGGGCCAGCTTTCACGGTTCGTATTCGT

ACTGGAAATCAAAATCAACGAGCTTTTACCCT

>FM895510.1 FM895510 Jatropha curcas embryo 71-95 (DAF) Jatropha curcas cDNA clone rjcpga0_002444, mRNA sequence

CTTCCAAAGCTCAAATCTTGGGATCCTATATAGGCGGGTTTCCATATCTTGCTTTAATTCTACTTCAACA

TTAAAATTTGACACTAAGAAATATGGCTACGGCCGCAACTCCTAGCACATACATGGCAACAATCAATCCT

TGCCTATCTTCTACGCATAAAGTATTTATGTCAACAACAGCTACTTTCAGTACCGAGTCCAAAGAGGCAT

CTTGGACTAGGCTTAAAAGCTCCTCTCACATCTCATCAAGGCAGCCATTCTTACAGAGTGCTACTTCAGC

GCCTGTGCGACTTAAAAGGGTTGTTACAAGAGCAATGTCTGCAGCAAATGAGAACAAGCCCCTGCCAGGA

CTGCCTGTTGATTTAAGAGGTAAGAGAGCGTTCATTGCTGGTGTAACTGATGATAATGGATATGGTAGGG

CAATTGCAAAATCCCTTGCTGCTGCAGGTGCTGAGAT

>FM895509.1 FM895509 Jatropha curcas embryo 71-95 (DAF) Jatropha curcas cDNA clone rjcpga0_002442, mRNA sequence

ATTTCAAGGCTTAAAATGCTAAGCACAACTTTAATTTCACAAAATGTCAAAATGTTGCTGATTCCGCATT

GTCATACATCAAGAATGACGTTCTACTAATAAGAAACAACTCAATCAAAAGTAACTGCAGATAGCAAACA

CTCAAAAATTCTGGTATCATGAAAACAACAAAGGTCTCTACAAATACTCTTCCATTAAGATCCCAATAAA

TCAAATTTGACAGCTACTCAGTGAGATTTGATGTGTTGCTGAACAACATTAAGACCTTCAGTCTCCTCGC

CATAATCCTTAACTACGACGCAGGAGCAACCAACTACCTTCCGGGCCTTACCCTCAGAATCAATCTTGCA

CAACCCAGCCCACTCGCCAAGAGTCTTAGCACTAGGAACAGTTAAGAGGCCAACAACCATGGTCAGCACA

AAGGCCTTTGACAAGCTTAACATAGTCAGGCTGGTTACAGTCCTCAG

>FM895508.1 FM895508 Jatropha curcas embryo 71-95 (DAF) Jatropha curcas cDNA clone rjcpga0_002440, mRNA sequence

TTTCTTTTCTTTTTGCCACTTCAACAATATTAATTTGTTGAATACTATTCTATCATACATTGTCAAAGAG

TCTCAATTTGATCACATAGCATTTTTCAGAGAATCTCCAACTCCCTGAGAAGAAGAAATATGAGAGTGCA

AAATCAGCAATAAAGATACCAACAAGTGAGATAACCACGGCTAGAAGTTGTCGATTCACCAACGCCTTTG

GCGCCTCCTGAAGTAGTAACTCCCCAAGCACAGCTCACAATTGATATGATTGCACCAAAAACTCAATGGG

GGGCCCGGTAC

>FM895507.1 FM895507 Jatropha curcas embryo 71-95 (DAF) Jatropha curcas cDNA clone rjcpga0_002439, mRNA sequence

ATTCCTGCAAAGGATTCTATCCGTCGCCCGATGGGAATTGTACTTTAAGTGCGGCCCTCGCGGCTCGTCC

GCCGCGGGGGCTTGGCCAACGGCACGTGCCTCTGGGGGCCGGATGGCCCCTACTGCGGGTCGGCAAACGG

GCGACGGACACGCGCGTCGCTTCTGGCCCGGATTCTGACTTAGAGGCGTTCAGTCATAATCCAGCGCACG

GTAGCTTCGCGCCACTGGCTTTTCAACCAAGCGCGATGACCAATTGTGCGAATCAACGGTTCCTCTCGTA

CTAGGTTGAATTACCATCGCGACACCATCATCAGTAGGGTAAAACTAACCTGTCTCACGACGGTCTAACC

CCAGCTCACGTTCCCTATTGGTGGGTGAACAATCCAACACTTGGTGAATTCTGCTTCACAATGATAGGAA

GAGCCGACATCGAAGGATCAAAAAGCAACGTCGCTATGAACGCTTGGCTGCCACAAGCCAGTTATACCTG

TGGTAAC

>FM895506.1 FM895506 Jatropha curcas embryo 71-95 (DAF) Jatropha curcas cDNA clone rjcpga0_002438, mRNA sequence

TTTTTATTTTTTCTGCAACTACAATTATTTATAGCAAATAGCCTTTAATTGTAAATAGCAACACTCTCTT

AAGTTGAAGGTTCACTTCACAGCGCCAAATTGATAAATGAACTGTTCAGACACAAATAAAGTACGATAAA

AACTAAAAACAAAATTCAGGCCTACTTCAAATATAAGGAGGAAGCAAATTCAACTGCTTCCAACGATGAA

GGCATTCTATCATGCTCTTTTAGCAAAGTCACTGTCATCATCATCCCTGCCTTCCAAAGGTTCAGCTTGA

TCATAACCTGCACCTGCATCATCAAAGCTGCTACCTCCCTGGCCAAAAAATTTATCGTCACCACCATCTT

TATTGCTGCTACCAAAGTTACCACTGCCAGCACCATTTTCACTAGTGTTTCCACCTGCAACACCAACTCC

ATAAGCTCCAAAACTCCAAACACCTCCAGAAGCTCCAGTGCTACTACTGTCAAACGTGTTTCCAGCAGAA

GTAACATCTGCATTGGCATAGTC

>FM895505.1 FM895505 Jatropha curcas embryo 71-95 (DAF) Jatropha curcas cDNA clone rjcpga0_002437, mRNA sequence

TTTTTTTTTCATCCAAACACAATCAGCCTTATGCATGAAAGAGCGTGCAGATTTACAACTTTCATGGTTT

GATGGTAAAACCAGATTCTCATTCAAGCATATAGGCAAAAATGAAACTACAAAAATATGACAAGCTATAA

TTTACAATTTCTTGCAGCCTAGCATTAATCCATAGTAATTGAGGGCTCATACCCATCATATGTCCAGATC

ATCATCTGGATCCTCGTCGGAATCTGGCTTCTCATCGTCTGAGTCCCGGAAATATATTATCTCACCCCCA

CCAGATGCTTCATTTCTTTGCAAATTCCCAGTTGAAGCAAATGTTGCCTCATTATTATTCTCTGTGAGAG

ATCTTCGACCGTCATAAATTTGTATAGGTTTACCATTTCCTTGGTGCTCAAAAGGGAGTACAACCTTAGC

CCTTTCACTCTGCTCTTTCTCTGACAGCTGTAGGCTAAACAGCACCTTAGGCAAAAGACCTTGA

>FM895504.1 FM895504 Jatropha curcas embryo 71-95 (DAF) Jatropha curcas cDNA clone rjcpga0_002436, mRNA sequence

GCCAATGATTAGAGGCATCGGGGTGCGCAACGCCCTCGACCTATTCTCAAACTTTAAATAGGTAGGACGG

CGCGGCTGCTTCGCTGAGCCGCGCACAGGAAATCGAGAGCTCCAAGTGGGCCATTTTTGGTAAGCAGAAC

TGGCGATGCGGGATGAACCGGAAGCCGGGTTGCGAGTGCCCAACTGCGCGCTAACCTAGAACCCACAAAG

GGTGTTGGTCGATTAAGACAGCAGGACGGTGGTCATGGAAGTCGAAATCCGCTAAGGAGTGTGTAACAAC

TCACCTGCCGAATCAAACTAGCCCCGAAAATGGATGGCGCTTAAGCGC

>FM895503.1 FM895503 Jatropha curcas embryo 71-95 (DAF) Jatropha curcas cDNA clone rjcpga0_002435, mRNA sequence

ATATTATCATCGTCAGCAACTAGAAGGAAGATAGATGCGTTACAGAACTTTGAGCAACAATCCCCCTAAT

AATGGAAACATTACCCAAGTAGAATTTGAAGGCAATACCAAAATGAACCTCTGCCAACAAGTAAAATACA

CTAGCTGTAACTGCCTAACAAGATTATTTCTGCAAGTTTTTCTTCTTATCATCATTTTCTTCTCATGATT

GTGATAACAGCTGAAGGCCCGCTTCCAAATCCTCCAATGTGATGGTTCGTAGTACATACAGCATCTATAC

AATCAAAATCCAGACTCAAG

>FM895502.1 FM895502 Jatropha curcas embryo 71-95 (DAF) Jatropha curcas cDNA clone rjcpga0_002433, mRNA sequence

GGATTCTATCCGTCGCCCGATGGGAATTGTACTTTAAGTGCGGCCCTCGCGGCTCGTCCGCCGCGGGGGC

TTGGCCAACGGCACGTGCCTCTGGGGGCCGGATGGCCCCTACTGCGGGTCGGCAAACGGGCGACGGACAC

GCGCGTCGCTTCTGGCCCGGATTCTGACTTAGAGGCGTTCAGTCATAATCCAGCGCACGGTAGCTTCGCG

CCACTGGCTTTTCAACCAAGCGCGATGACCAATTGTGCGAATCAACGGTTCCTCGGCGAAGATGAGCAAG

AAGTTTGATCAGAAGAAGAAAGAGGAGGAGATTGCTAGGATGCAAGAGAAGTATGGAG

>FM895501.1 FM895501 Jatropha curcas embryo 71-95 (DAF) Jatropha curcas cDNA clone rjcpga0_002432, mRNA sequence

TTTCAGAGCGCCACCTACTGTCGTCTTAGATCTTCGCATCTGCAAGTTATCCATCTAAATCCATAATGGC

GACCCAAATGAGCAAGAAGCGAAAGTTTGTTGCAGACGGTGTTTTCTTCGCCGAGCTTAACGAAGTTTTG

ACCAGAGAGCTCGCCGAGGATGGATACTCTGGTGTTGAGGTTAGGGTCACTCCTATGCGCACCGAGATCA

TCATCAGGGCCACTCGCACCCAAAACGTTCTAGGTGATAAGGGTAGGAGGATCAGAGAGCTGACCTCCGT

GGTGCAAAAGAGGTTTAAATTCCCAGAAAACAGTGTGGAGCTCTATGCTGAGAAAGTTAACAGCAGAGGC

CTTTGCGCCATTGCTCAGGCTGAGTCTCTTCGTTACAAGCTTCTCGGTGGTCTTGCTGTTCGTAGGGCCT

GCTATGGTGTGTTGAGATTCATAATGGAAAGTGGTGCTAAGGGATGTGAGGTAATTGTCAGTGGGAAACT

AAAGGCACAGCGTGCAAAATCCATGAAGTT

>FM895500.1 FM895500 Jatropha curcas embryo 71-95 (DAF) Jatropha curcas cDNA clone rjcpga0_002431, mRNA sequence

ATTCTTGTCTTTCTATCCCCAAGTATATTTGTAAGTACAAAAGATATCCTTCAGGACCACAGAAGTCCAA

ACAACTCCTAATTAAATTACCCCAATAGTTAGAAAAGTTAAAAGGGTACATCAAATGAGGAGTACAAAAA

AAAAATGTCAAGCCTAAGTTGTAATTATCTTGTATGCTAACTTTAAGGGATCAGGCATTCACTACTGTGT

CTGGAAAACAGAATTCACCATTCACCACCACAAATAATCCTTTACTAATACTGGCTCAGACCTTGTACTC

CCCGAGATGCATCCACTTTGTAGCTGACCACTTGTTTCCTCTAATTACAGGGCAACTACCATGCAAACTT

GAAGGGTCTAGTGTAGCATCAGGCCTTGTGCTCCAGAACAGCAATGCATTGCCCATCTTCGGTTTCACTG

AAAGACCCCGTTTGCCACATTCTGACAACTCATTCCACCATGGTACAGAGCTGATGTATGCCTGGGCAGC

AGGAAACACTGTCTCACCCCCTTCTTCAACATCTGACAGATACATTTAAAG

>FM895499.1 FM895499 Jatropha curcas embryo 71-95 (DAF) Jatropha curcas cDNA clone rjcpga0_002430, mRNA sequence

CGCAATCATCGCGTTTCCCTTCCCCCCACTAGTCCCCAAAATTAGCCCCTCCATTCTCTCTCAAAAACAA

CTCCGCCTCTCTACCTCTCCAAGAATCAAAAACCAGATCTAACCTCTCTCTTCCTCTGTGGATCTTTCTT

TTCTTCTTTCTTTCAACAATGGCGGTAGCACCTACGTCTCGAGAAGAGAACGTCTACATGGCCAAGCTCG

CTGAGCAGGCCGAGCGTTACGAGGAGATGGTAGAGTTTATGGAAAAGGTCTCCGCCTCAGTTGAGAACGA

AGAGCTCTCTGTCGAGGAGCGAAACCTCCTCTCCGTCGCCTACAAGAACGTGATCGGAGCTCGACGTGCC

TCGTGGCGTATTATCTCCTCTATCGAGCAGAAAGAGGAGAGCCGCGGTAACGAAGACCATGTCTCCGTGA

TCCGTGACTACAGGGCCAAGATCGAGTCGGAGCTCTCCTCGATCTGCGATGGGATCTTGAAGTTGCTCGA

CACGAGGTTGATTCCTTCTGCCTCTTCAGGAGATTCCAAGGTGTTCTATTTGACGATGAAGGGTGATTAT

CATAGGTATCTTGCCGAGTTCAAAAACTGGAGCTGAG

>FM895498.1 FM895498 Jatropha curcas embryo 71-95 (DAF) Jatropha curcas cDNA clone rjcpga0_002429, mRNA sequence

ATTCCTTTGATCTCTCTCACACACTCACTCACAAACAGATGGCGCTTAAGCTATATGGAGCGGCCATGTC

CACAAGCACCTCCCTCGTGCTAACCTGTCTGCACGAGAAAGAAGTAGATTTTGAGTTCATCCCTGTCGAT

CTTTTCGCCGGCGAACATAAACAGCCTCATTTCGGAGCCAAAAATCCCTTTGGTCAGGTTCCAGTGCTTG

AGGATGGTGATCTCACTTTATTTGAATCTAGAGCAATCACAGCATACATAACTGAAAAATACAAGGAACC

AGCGTATGATCTAACGAGGCATAAAAAACTTGAAGAAGCAGCAATGGTGAAGGTGTGGATAGAGGTAGAA

TCTCAGCGATATAACCCTGCAATAGCTCCAATAGTGTACCAACTTTATGTGGGGCCACTGAAAGGCGTTG

CACCTGACGAAGCAATCATAGACACAAACATAGAGAAGCTAAGACAGGTGCTTGACATATACGAAGAAAG

GCTGAGTTGCAGCAAATACCTTGCAGGAGATTTCTTCAGCTTAGCAGATCTTCATCATCTTCCTTACACT

TACTATCTTATGAAGACATCTGCTGCTTCTGTCATAAATGACCGTCCCCGTTGTCAGGCATGGTGGGATG

ATATC

>FM895497.1 FM895497 Jatropha curcas embryo 71-95 (DAF) Jatropha curcas cDNA clone rjcpga0_002428, mRNA sequence

CTCGAGCCGTTTTGGCGGGGCGTACTAAACCCACTACGTACCACACCACCGGGCGGCTCGCCTGAATGCC

GAGTCTTTCTCCGCCGCCAACTCGACGTCGTCGTCACCTGCAAGATAAGGCCAAAAACTGGACTTTACTA

AACAAGCGAGAAAAGCCCCTTTCTCTGTTATTAGTAAAGTAAAGCGCGCTAGCTGCAATCTTTCTAAAGC

GCACACTAGAAAGTGCTTCGAAAGGCGCCGGCTCCCCTCTTACTGACAGCACAGCTACGTGACTGGCACT

CAATTAGTAGCGCTGGCACGTCACTCGGCTCCTCGGCTCACTTCGGTTGCAAAGACTTTCTCCTTAGGCG

CATGTCTCAGCAACACAAAACGAGGGTTTCGCTCGTTATAGGACTTGACCAAACATCTCACGACACGAGC

TGACGACAGCCATGCAGCACCTGTATGAAAGTCAGTACCATCCCGTTAAGGACAGGTTTTGTTGTTCATA

TGTCAAGGGCTGGTAAGGTTTTGCGCGTAGTATCGAATTAAACCACATGCTCCACCGCTTGTGCAGGCCC

CCGTCAATTCCTTTGAG

>FM895496.1 FM895496 Jatropha curcas embryo 71-95 (DAF) Jatropha curcas cDNA clone rjcpga0_002427, mRNA sequence

AAGAAGATGAATGGAACAATTCAGAATTGATGAGATTTTTACAAGAATAAAAAGTTAAAAAAAACTTCAA

AAAGCAATCCTTAAAACTATTGCGAGAAACCCTAAAACCCCCACCCTACGATAATCAAGTTGATAAAATC

AAAATCTGCCTCCCTTTTCCTTCTCTTTCTCTCCTGTTTCTAAGCAAACAAACAAGCTAATCACGAAACC

CCTTAAGAAAACACTAAAAAATATTGCAAGCCAAAACCCAAAAATACCGAAACTATAATTACACCCTCAA

TTTACCGACAACAAAAGGCTAACAAGACCCAAAAGGAGAGAATAAAAGAAAACCTCAATCTTCCTTTTCC

CACCAGGGTAAACAGAAACAGAGAGAAAATCAAAACCCTAAAATAAAGTCCCAGAAGAAGACCCAGACTA

AGTAAAAGGATTACTTAAAAGAACACCCACCTTCTCTCTACCTTCAATGTCCGAAACATTTCCCCAAACG

AAGCCTCAATGCCCAAATCCCTACTCTCA

>FM895495.1 FM895495 Jatropha curcas embryo 71-95 (DAF) Jatropha curcas cDNA clone rjcpga0_002426, mRNA sequence

CTCTTCCATTATTGAAACCTTTACCCACCTTTATTTCAAGAATCTCTATTTTATTGGCCCAAACCAGCAC

CATTTTGCCCTTTATTGCCAATTGTTTTCCTTTCCTTCTCTCTCTTTCTTTCTTTACTTTAGCTCAATTA

TAGTGAAGATCAAATCTTTCATAAGAAAGATTTTGTGGGTGTTTCTTGGTGTATTGCAAGAAATTGAGAT

TTTCTAAGATTTATATTTTGGGTATTTCTGGGTTTACGAAAAGATATGGCGAGTGCTGCTGCTGGTATGA

TCGGAGGAAATGGGTGTGTTGGTACTTCATCTTCTTGGTTTCAGATCTAAAACAGGAGAAAGAAGAACAA

TATGGTTAAAGAAAGAGTTAAATTTTATTGTGTTTCTTCTGCTTCTTCTCCATCTTC

>FM895494.1 FM895494 Jatropha curcas embryo 71-95 (DAF) Jatropha curcas cDNA clone rjcpga0_002425, mRNA sequence

TTTTTTCATTGGAAAATCTACAATCAAATGCTTCATTGTGAAAAACATGACAAATCCTTCAGGCCAATTC

AAATGTAAGAAACAATCTACAATATTGATGATGCGTACAGTTAGATAGTACACAATGATAATTCTGTTTC

ACTCGTCCTTCAAATCCATCGATATTTGAAAAATGAACTTTCTTTTCTATTACACTTTATCCCCTCAACT

TGATGCTTTTCCAGTAACTCGCTGGGCGATCCACCCTAGACCATCATATAGAACTTCACCTGTAAGGGCA

CAGCAAGCTTGGATGTGCCAATCATGATTCTTGATGCTGTGGAGAGACAGAGCATCAGTCAGCTCAGCTG

GGGTCATGGCATCCTTAAGGTCTTGTTTATTAACAAACACCAGTATAACAGAATTTTGTAGATACTCGTG

TGCAAACAATCTGAAAAGTTCATCCTTCATAATAGAGATCCTGGCTCTATCGGTACTGTACATCACTGCA

ATGACAGCATGAGTTCCACGATAATATGTAGCCCATGATGTC

>FM895493.1 FM895493 Jatropha curcas embryo 71-95 (DAF) Jatropha curcas cDNA clone rjcpga0_002424, mRNA sequence

TCTCTCTTTTTCTCTTCCTCTTCGAACTCACACATCTTCTTCCATGGCCGATCAGCTCACCGACGACCAG

ATCTCTGAGTTCAATGAAGCCTTCAGCCTATTTGATAAGGACGGCGATGGCTGCATCACCACTAAGGAAC

TTGGGACTGTAATGCGATCATTGGGCCAGAACCCAACTGAGGCAGAGCTCCAGGACATGATAAATGAGGT

TGATGCTGATGGAAATGGTACCATTGATTTCCCAGAGTTCCTTAATCTGATGGCCGTAAGATGAAGGACA

CTGACTCTGAGGAGGAACTCAAGGAAGCTTTCCGAGTTTTTGATAAGGATCAGAATGGCTTCATTTCTGC

TGCTGAGCTGCGCCATGTCATGACAAATCTTGGCGAGAAGCTGACAGATGAGGAGGTTGATGAGATGATT

CGTGAGGCTGATGTGGACGGTGACGGTCAGATCAACTACGAGGAGTTTGTTAAAGTCATGATGGCCACGT

GAGGACCACTCAACCAAACTGAATTT

>FM895492.1 FM895492 Jatropha curcas embryo 71-95 (DAF) Jatropha curcas cDNA clone rjcpga0_002423, mRNA sequence

TTGGTCGCCTAGTTCTCCCAAAAGCATGTGCTGAAGCATATTTTCCTCCTATTTCTCAATCTGAAGGCCT

TCCTTTAAGGATTCAAGATGTGAAGGGGAGAGAATGGACATTTCAGTTCAGATTTTGGCCCAATAATAAC

AGCAGGATGTATGTCCTGGAGGGTGTAACCCCTTGCATACAGTCTATGCAATTGCAAGCGGGTGATACAA

TAACTTTTAGCCGGATAGATCCTGAGGGCAAACTTGTCATGGGATTTCGCAAGGCTACAAACAACTCTTT

AGATACACAGGATGCCCAAACATCTGCAATTCCTAATAGTGCTGCTATCACCGAAACTTCTTTTTCTGCT

GATGGGGATATTGGCTGGAACAAGAGTGAGAATCACGGAGGCAGGGTAAATGGGGATGCACTGCTGCAAT

CAACGTCAGAGAAGAAAAGAACTCGAAATATTGGACCTAAGAATAAGCGATTACTCATGAATAGTGAAGA

TGCTTTGGAGCTGCGACTTACATGGGAAGAAGCGCAGGACTTG

>FM895491.1 FM895491 Jatropha curcas embryo 71-95 (DAF) Jatropha curcas cDNA clone rjcpga0_002422, mRNA sequence

TTTTTTATATTTTTACCAAGGAAAAAGGGATATCATATCAACTCAAGTTGTCAATTTGCCATAAATTACA

CAGGTACAACCGTTAATAACATTAAACTGCCTACATTTTACCTGCAATGCTAAAACTGTATTTCTAGACA

TGACCATATTTCAAAAAGACTAGAAACATCTCAAAACTCGTCAACTTTTTGTATGAAACACAGAGTGAAC

AAGTAACTAAAACTTTTTAGATCACTCAGAAGCACAAAGAGCATTTCATTAAAATTCTCATTCTGCTGCA

GCGTCTCGGCTGTTAACCCCTTCTACACTATTCCCTGGCAATGTATCCAGATCTATGCGAGCCTCTTTCT

CATCTTGGCTGACTGAAGTATCAGAAGAATCAATGGGGTCCTGAATAAGAGCTTCAGTTCCGTCTGTTGA

CGAGTTTGAATTTTCATCTGTACCACTTGATTCATTCTTGGTGTCTG

>FM895490.1 FM895490 Jatropha curcas embryo 71-95 (DAF) Jatropha curcas cDNA clone rjcpga0_002421, mRNA sequence

TGAAAAAAATTTACTTTTCTGTGCTTGCGCTTTGATCATTTCAATATGCCTCGCCGTAGCCGCAGCTCTA

GAGGAGGAGGTTCAAGCCGGCCTGCTGCAGCTCGTTCTCCCCCAAGAAGTCGGCCTCCCAAGCCAGCTGC

TCAATCACCACCACCAGCTCCTGCAAAAGTTGCCAATTTATCCGCAATCGGAGGTTTAAGTGCTGCTATT

GCTGATGGCGTGGCTTGGGGAACTGGAACTGCTCTGGGTCGCAGGATTGCTGATGCTGTCATGGGTCCTC

CAGTGATCCGCCATGAAGCTGTTGCTTCATCTGCCCCTGCAGCTATGCCCTTGGAGAACACAATCAGTAC

AGTTGATGCATGTGATAGACAAGCCAAGGGACTCCAAGAGTGCCTGAACAACTATGGCAGTGATATT

>FM895489.1 FM895489 Jatropha curcas embryo 71-95 (DAF) Jatropha curcas cDNA clone rjcpga0_002419, mRNA sequence

TTTTTTTTAGACAAAAAGCAGACATTATCCATTGACCAAATTTACATAGTCAGAACATTGAAAAACGCTT

TCCATGAAACAAACAGACATAGCCTACATATGGTTTGAATATGCCATCTGCAAGAATCTACAGGGGAAAG

GGCAGATAATTCAAGCAATACAAGATGAAACACAGAGACGGATGGCTTCATTTAGCAAAACAGGATTCGA

TCTTATCTAAGTCTACAAAGCATTCCAATAGATGTGCATGGCTTCATCTAGCCCACTATAACTCAGGCTC

TAGCCTATCTGACTTCGGCGGATTTCCAACTACATATCCGGGCAGTTTAGCAGTTAACCGATCACAACCC

ATGATATGGGGCAATACAAAACTGCAAGCTGTCTTCAAAACTGTAAATTCACCAGCAGTGAGAGGAACAC

TGAACCAGTCGTAGGTTTAAAAAACATTGTTAGCAACGGTTAATGACAGAAAAT

>FM895488.1 FM895488 Jatropha curcas embryo 71-95 (DAF) Jatropha curcas cDNA clone rjcpga0_002418, mRNA sequence

CTTCCTATCATTGTGAATTCTGCTTCACAATGATAGGAAGAGCCGACATCGAAGGATCAAAAAGCAACGT

CGCTATGAACGCTTGGCTGCCACAAGCCAGTTATCCCTGTGGTAACTTTTCTGACACCTCTAGCTTCAAA

TTCCGAAGGTCTAAAGGATCGATAGGCCACGCTTTCACGGTTCGTATTCGTACTGGAAATCAGAATCAAA

CGAGCTTTTACCCTTTTGTTCCACACGAGATTTCTGTTCTCGTTGAGCTCATCTTAGGACACCTGCGTTA

TCTTTTAACAGATGTGCCGCCCCAGCCAAACTCCCCACCTGACAATGTCTTCCGCCCGGATCGGCCGCCG

AAACGACCTTGGGTCCAAAAAGAGGGGCATAGCCCCGCCTCCGATTCACGGAATAAGTAAAATAACGTTA

AAAGTAGTGGTATTTCACCGTCGCCTTTCGGCTCCCACTTATCCTACACCTCTCAAGTCATTTCACAAAG

TCGGACTAGAGTCAAGCTCAACAGGCTCTTCTTTCCCCGCTGATTCCGCCAAGCCCGTTCCCTTGGCTGT

GGTTTCGCTGGATAGTAGACAGGGACAGTGGG

>FM895487.1 FM895487 Jatropha curcas embryo 71-95 (DAF) Jatropha curcas cDNA clone rjcpga0_002417, mRNA sequence

ATTCCTGACAGTGACAGCCCACAGAAATGGCATGTTTACTCTGCATATGCAGATTCTGCTATCTCCCGCG

ACCTTGTTTACACTGTAGAGATGTGCATGACTGGTTTGGACCGGCAGAAGGCTGCTGTTTTCTTCAAGAC

TCGATCAGCTTCTGCAGCTGTCATGACTGTTGATTCTGGCATAAGAAAGATTCTTCCAGACTCGAAAATA

TGCGATTTTGATTTTGATCCATGTGGTTACTCAATGAATTCAATTGAAGGTTCTGCTATATCTACCATTC

ATATTACTCCAGAAGATGGTTTCAGCTATGCTAGCTTTGAAACAGTAGGATATCATCCAGAAGAAGTGAA

TCTGAACCAGTTGGTTGACAGGCTGTTGTCTTGTTTCCAGCCAAGCCAATTCTCCATTGCTGTGCATGCT

GATGTTGCTGGTAAACAACTAATGCGGACTTGTTCACTTGATGTCAAGGGATACTGTCGTGGAGAGAGTA

GCTTTGAAGAGTTTGGGAAGGGTGGTTCCACTTGTCTACCAGAAGTTCATTAGGACTGGGGACTCTGGCT

CTCCTAGGTCAACTCTGAAATGCTGCTGGAAA

>FM895486.1 FM895486 Jatropha curcas embryo 71-95 (DAF) Jatropha curcas cDNA clone rjcpga0_002416, mRNA sequence

CTTTCTCTCTCTCTCTCTCTCTCTCTCTCTCTCTATTTATCTTTTCCTTCTTTTCTCTCTCCCTTACAAG

CCCTAACCCTAACTCAGACCCAGATTCGCCATGAAAGGAGGTAAA

>FM895485.1 FM895485 Jatropha curcas embryo 71-95 (DAF) Jatropha curcas cDNA clone rjcpga0_002414, mRNA sequence

GCTGCAGGAATTCGAATTCCTTATTTATTTTTCTGAATTCTCTTTTTCTGTAGCCCCTACCCTATAATGC

GCGATGGTGTGTTTTACGCACCCCTCAATCCACCGCTTCTTCTCTCTCCCTCTCCGACACCAACCTCCGT

GAACAATTCCATTGCTGCCTCCAACCAAAACATGCTCGTCTCCGTTTTTTTGGCCCTTTTCTTGCCTTGT

ATCGGCATGAGCGCAGTTTTTCTCGTCTATATTTGTCTCCTGTGGTACGCTGCAAGTCACCAAGCCGAAA

TCCCACCGCCAGTAAAGGCGGCACCGGAGAAGGGATTATCGGCTTCTCAGCTCGAGAAATTGCCTAAATT

GACTGGGAAGGAGTTGATATTGGGGACGGAATGCGCGGTGTGTCTAGATGAGATCGAGAGCGAACAGCCG

GCTAAACTGGTTCCCGGTTGCAATCACGGTTTCCATTTGGAATGCGCTGACACTTGGCTTTGCAAGCATT

CTGTGTG

>FM895484.1 FM895484 Jatropha curcas embryo 71-95 (DAF) Jatropha curcas cDNA clone rjcpga0_002413, mRNA sequence

TTTTTATTTAAATTAAAATCAGACGACTTGTATTCCAGCCATTATTTTGTTTGTTTACAGTTACCCACTG

AGAGTGATGCATCACCTATTGCAGCTTGTCTTTCTATCCCCAAGTATATTTGTAAGTACAAAAGATATCC

TTCCGGACCACAGAAGTCCAAACAACTCCTAATTAAATTACCCCAATAGTTAGAAAAGTTAAAAGGGTAC

ATCAAATGAGGAGTAAAAAAAAAAAATGTCAAGCCTAAGTTGTAATTATCTTGTATGCTAACTTTAAGGG

ATCAGGCATTCACTACTGTGTCTGGAAAACAGAATTCACCATTCACCACCACAAATAATCCTTTACTAAT

ACTGGCTCAGACCTTGTACTCCCCGAGATGCATCCACTTTGTAGCTGACCACTTGTTTCCTCTAATTACA

GGGCAACTACCATGCAAACTTGAAGGGTCTAGTGTAGCATCAGGCCTTGTGCTCCAGAACAGCAATGCAT

TGCCCATCTTCGGTTTCACTGAAAGACCCCGTTTGCCACATTCTGACAACTCATACCAACATGGTACAGA

GC

>FM895483.1 FM895483 Jatropha curcas embryo 71-95 (DAF) Jatropha curcas cDNA clone rjcpga0_002412, mRNA sequence

CAAAGGATTCTATCCGTCGCCCGATGGGAATTGTACTTTAAGTGCGGCCCTCGCGGCTCGTCCGCCGCGG

GGGCTTGGCCAACGGCACGTGCCTCTGGGGGCCGGATGGCCCCTACTGCGGGTCGGCAAACGGGCGACGG

ACACGCGCGTCGCTTCTGGCCCGGATTCTGACTTAGAGGCGTTCAGTCATAATCCAGCGCACGGTAGCTT

CGCGCCACTGGCTTTTCAACCAAGCGCGATGACCAATTGTGCGAATCAACGGTTCCTCTCGTACTAGGTT

GAATTACCATCGCGACACCATCATCAGTAGGGTAAAACTAACCTGTCTCACGACGGTCTAAACCCAGCTC

ACGTTCCCTATTGGTGGGTGAACAATCCAACACTTGGTGAATTCTGCTTCACAATGATAGGAAGAGCCGA

CATCGAAGGATCAAAAAGCAACGTCGCTATGAACGCTTGGCTGCCACAAGCCAGTTATCCCTGTGGTAAC

TTTTCTGACACCTCTAGCTTCAAATTCCGAAGGTCTAAAGGATCGATAGGCCACGCTTTCACAGTTCGTA

TTCGTACTGGAAATCAGAATCAAACGAGCTTTTACCCTTTTGTTCCACACGAGATTTCTGTTCTCGTTGA

GCTCATCTTAGGACACCTGCGTTATCTTT

>FM895482.1 FM895482 Jatropha curcas embryo 71-95 (DAF) Jatropha curcas cDNA clone rjcpga0_002411, mRNA sequence

CAGTAAGAATCAACCCATTTCGGAGGATTACACTCCCACTGTATCTTGAAAAACTCACTTGGCACTGGCA

AGCTCGGTGCGGAGTAGCTTAGCTGCAGCAACCATGTTCTTTAGAGCAGGCTTGACCTCAGAGTACTTGC

GAGTTTTGAGACCACAGTCAGGGTTAACCCACAAGATGTTCTTCTCGAGAACCGCAAGCATCTTGTTAAT

TCTGTCAGCAATCTCCTCAGTTGAAGGTATTCTAGGAGAGTGGATATCATAAACACCAAGGCCAATTCCA

GCACCATACTTCACTCCCTCGCGGAAGACGGAGAGAAGCTTTTCATCGGATCGAGAGTTCTCAATGGTGA

TCACATCAGCATCCATATTGATGATTGAGTGAATAATGTCATTGAAGTAGGAGTAGCACATGTGAGTGTG

AATCTGGGTGGTGTCTTGTACTCCGCAAATGGTAATTCTAAAGGAGTGCACGGCCCAGTCCAAAGGTAGA

AAGCTTGC

>FM895481.1 FM895481 Jatropha curcas embryo 71-95 (DAF) Jatropha curcas cDNA clone rjcpga0_002410, mRNA sequence

GGAGTGTGTAACAACTCACCTGCCGAATCAACTAGCCCCGAAAATGGATGGCGCTTAAGCGCGCGACCTA

TACCCGGCCGTCGGGGCAAGAGCCAGGCCCCGATGAGTAGGAGGGCGCGGCGGTCGCTGCAAAACCCGGG

GCGCGAGCCCGGGCGGAGCGGCCGTCGGTGCAGATCTTGGTGGTAGTAGCAAATATTCAAATGAGAACTT

TGAAGGCCGAAGAGGGGAAAGGTTCCATGTGAACGGCACTTGCACATGGGTTAGTCGATCCTAAGAGACG

GGGGAAGCCCGTCCGACAGCGCGTCCTCGCGCGAGCTTCGAAAGGGAATCGGGTTAAAATTCCCGAACCG

GGACGTGAAGGCTGACGGCGACGTTAGGGAGTCCGGAGACGTCGGCGGGGGCCTCGGGAAGAGTTATCTT

TTCTGTTTAACAGCCCGCCCACCCTGGAAACGACTCAGTCGGAGGTAGGGTCCAGCGGCTGGAAGAGCAC

CGCACGTCGCGCGGTGTCCGGTGCGCCCCCGGCGGCCCATGAAAATCCGGAGGACCGAGTGCCTCCCACG

CCCGGTCGTACTCATAACCGCATCAGGTCTCCAAGGTGAACAGCCTCTGGTCGATGGAACAAT

>FM895480.1 FM895480 Jatropha curcas embryo 71-95 (DAF) Jatropha curcas cDNA clone rjcpga0_002409, mRNA sequence

GCTGCAGGAATTCGAATTCAAATAAACTTTTCCCATACGGTCTGCAAAATTTGTAAATTTACAATTTGAG

ATAATTTGCACCTGTGTAAACTATGAATTTCATCTCCTCAAATGGTGAAAGAACATCTGTACACTGCATT

TTATAAAAAGTTCAATGGCTTAAACATGTTGACAATGCAAATAAATTACCTTATTTCAATGTCTAATGCT

TCAATCTTGCGCTTGAGCTAATTATAATTTGAGAAAGAGGAGGCACATATCAGTAAGTAGACTTCGATAA

TCTCTGAAGTGGGGAAAAAAGGATAACATTGCATTTCCAGTAATTGCCATTCTGATAAAAGTTCAGTTGC

TGCGTCGAGCCTCTAATCTCCACCATTCAATTAATTGCATCACCACACTGATATACAGGAAGGTTAAGTC

GTAGGCCTTGTCATTGTAGATAAACCGCGTCCTACTGGTATAACCCCCGGATAGCTGTTGCCTGTTTCTT

CTTCTAAGGGAGCAGTAATCTCATCGCCCGCCTGAATGCCCGCAACCCCAATGCATGAATAGAGAGAATC

CTCATCTACCTTCAATTGTTCATATGCAAGGTATATGTCCTCAATAGTTGCAGCCTCTTACAGTGAAGTG

AGATCTTTAAT

>FM895479.1 FM895479 Jatropha curcas embryo 71-95 (DAF) Jatropha curcas cDNA clone rjcpga0_002408, mRNA sequence

ATTCTTATTTATCTTTCTCTCTACACCAAACCCACTTCACTTGTCAACAATAGCTCTTACTCACACTCCA

AAAATGGCAGTGAAGCTCTCTGTGATTCTCTGCATTGTATCAATTCTTTCCATCTCGGGCGCTAATGGTG

CCGGCCACCACCATACAGCTCCGGCACCGTCTGTGGACTGCTCAAGTTTAGTGTTGAACATGGCGGATTG

CTTGTCTTTTGTTTCAAACGGCAGTACGACAACAAAACCAGAAAAAACGTGCTGCTCTGGGTTGAAAACG

GTCTTGCAAACAGATGCAGAGTGTCTTTGTGAGGCTTTTAAGAGCAGCGCTCAATTGGGTGTCGTTTTGA

ATGTCACTAAGGCTTTAAGTCTTCCTACTGTCTGCAAACTCCAAGCCCCTCCTGCTTCTAATTGTGGATT

GGCTTTGGCTCCTGCTGCTGCTCCTGGTGCTCTTTCACCATCTGTTAGTGGTGCTGCTGCCCCAACTGTG

ACCGCCGGAGCAACTGTGTCAGCACCAGCACCATCTCCAAGGAATGCAGGTTCTCGCGGATTGTCAATCT

CAGTTGGCTCTCTAACAATTGGCCTTGTAATTGCATCATTCTCTAATTTCTGAGTAAT

>FM895478.1 FM895478 Jatropha curcas embryo 71-95 (DAF) Jatropha curcas cDNA clone rjcpga0_002407, mRNA sequence

CTGACATGTGTGCGAGTCAACGGGTGAGTAACCCGCAAGGCGCAAGGAAGCTGACTGGCGGGATCCCCCT

GAGGGTTGCACCGCCGACCGACCTTGATCTTCTGAGAAGGGTTCGAGTGAGAGCATGCCTGTCGGGACCC

GAAAGATGGTGAACTATGCCTGAGCGGGGCGAAGCCAGAGGAAACTCTGGTGGAGGCCCGCAGCGATACT

GACGTGCAAATCGTTCGTCTGACTTGGGTATAGGGGCGAAAGACTAATCGAACCGTCTAGTAGCTGGTTC

CCTCCGAAGTTTCCCTCAGGATAGCTGGAGCTCGGGACGAGTTCTATCGGGTAAAGCCAATGATTAGAGG

CATCGGGGGCGCAACGCCCTCGACCTATTCTCAAACTTTAAATAGGTAGGACGGCGCGGCTGCTTCGCTG

AGCCGCGCCACGGAATCGAGAGCTCCAAGTGGGCCATATAAGGTAAGCAGAACTGGCGATGCGGGATGAA

CCGGAGGCCGGGTTACGGTGCCCAACTGCGCG

>FM895477.1 FM895477 Jatropha curcas embryo 71-95 (DAF) Jatropha curcas cDNA clone rjcpga0_002406, mRNA sequence

TGATCTATCCGTCGCCCGATGGGAATTGTACTTTAAGGCGGCCCTCGCGGCTCGTCCGCCGCGGGGGCTT

GGCCAACGGCACGTGCCTCTGGGGGCCGGATGGCCCCTACTGCGGGTCGGCAAACGGGCGACGGACACGC

GCGTCGCTTCTGGCCCGGATTCTGACTTAGAGGCGTTCAGTCATAATCCAGCGCACGGTAGCTTCGCGCC

ACTGGCTTTTCAACCAAGCGCGATGACCAATTGTGCGAATCAACGGTTCCTCTCGTACTAGGTTGAATTA

CCATCGCGACACCATCATCAGTAGGGTAAAACTAACCTGTCTCACGACGGTCTAAACCCAGCTCACGTTC

CCTATTGGTGGGTGAACAATCCAACACTTGGTGAATTCTGCTTCACAATGATAGGAAGAGCCGACATCGA

AGGATCAAAAAGCAACGTCGCTATGAACGCTTGGCTGCCACAAGCCAGTTATCCCTGTGGTAACTTTTCT

GACACCTCTAGCTTCAAATTCCGAAGGT

>FM895476.1 FM895476 Jatropha curcas embryo 71-95 (DAF) Jatropha curcas cDNA clone rjcpga0_002404, mRNA sequence

AGGATTCTATCCGTCGCCCGATGGGAATTGTACTTTAAGGCGGCCCTCGCGGCTCGTCCGCCGCGGGGGC

TTGGCCAACGGCACGTGCCTCTGGGGGCCGGATGGCCCCTACTGCGGGTCGGCAAACGGGCGACGGACAC

GCGCGTCGCTTCTGGCCCGGATTCTGACTTAGAGGCGTTCAGTCATAATCCAGCGCACGGTAGCTTCGCG

CCACTGGCTTTTCAACCAAGCGCGATGACCAATTGTGCGAATCAACGGTTCCTCTCGTACTAGGTTGAAT

TACCATCGCGACACCATCATCAGTAGGGTAAAACTAACCTGTCTCACGACGGTCTAAACCCAGCTCACGT

TCCCTATTGGTGGGTGAACAATACAACACTTGGTGAATACAGCTTCACAATGATAGGACGAGCCGACATC

GAAGGATCAAAAAGCAACGTCGCTATGAACGCTTGGATGCCA

>FM895475.1 FM895475 Jatropha curcas embryo 71-95 (DAF) Jatropha curcas cDNA clone rjcpga0_002403, mRNA sequence

ATTCGTCTGACATGTGTGCGAGTCAACGGGTGAGTAAACCCGCAAGGCGCAAGGAAGCTGACTGGCGGGA

TCCCCCCGAGGGTTGCACCGCCGACCGACCTTGATCTTCTGAGAAGGGTTCGAGTGAGAGCATGCCTGTC

GGGACCCGAAAGATGGTGAACTATGCCTGAGCGGGGCGAAGCCAGAGGAAACTCTGGTGGAGGCCCGCAG

CGATACTGACGTGCAAATCGTTCGTCTGACTTGGGTATAGGGGCGAAAGACTAATCGAACCGTCTAGTAG

CTGGTTCCCTCCGAAGTTTCCCTCAGGATAGCTGGAGCTCGGGACGAGTTCTATCGGGTAAAGCCAATGA

TTAGAGGCATCGGGGGCGCAACGCCCTCGACCTATTCTCAAACTTTAAATAGGTAGGACGGCGCGGCTGC

TTCGCTGAGCCGCGCCACGGAATC

>FM895474.1 FM895474 Jatropha curcas embryo 71-95 (DAF) Jatropha curcas cDNA clone rjcpga0_002402, mRNA sequence

CTGGAAATCAGAATCAAACGAGTTTTACCCTTTTGTTCCACACGAGATTTCTGTTCTCGTTGAGCTCATC

TTAGGACACCTGCGTTATCTTTTAACAGATGTGCCGCCCCAGCCAAACTCCCCACCTGACAATGTCTTCC

GCCCGGATCGGACGCCGAAACGACCTTGGGTCCAAAAAGAGGGGCATAGCCCCGCCTCCGATTCACGGAA

TAAGTAAAATAACGTTAAAAGTAGTGGTATTTCACCGTCGCCTTTCGGCTCCCACTTATCCTACACCTCT

CAAGTCATTTCACAAAGTCGGACTAGAGTCAAGCTCAACAGGGTCTTCTTTCCCCGCTGATTCCGACAAG

CCCGTTCCCTTGGCTGTGGTTTCGCTGGATAGTAGACAGGGACAGTGGGAATCTCGTTCATCCATTCATG

CGCGTCACTAATTAGATGACGAGGCATTAGGCTACCTTAAGAGAGTCATAGTTACTCCCGCCGTTTACCC

GCGCTTGGTTGAATTTCTTCACTTTGACATTCAGAGCA

>FM895473.1 FM895473 Jatropha curcas embryo 71-95 (DAF) Jatropha curcas cDNA clone rjcpga0_002401, mRNA sequence

ATTCGTTCGTCTGACTTGGGTATAGGGGCGAAAGACTAATCGAACCGTCTAGTAGCTGGTTCCCTCCGAA

GTTTCCCTCAGGATAGCTGGAGCTCGGGACGAGTTCTATCGGGTAAAGCCAATGATTAGAGGCATCGGGG

GCGCAACGCCCTCGACCTATTCTCAAACTTTAAATAGGTAGGACGGCGCGGCTGCTTCGCTGAGCCGCGC

CACGGAATCGAGAGCTCCAAGTGGGCCATTTTTGGTAAGCAGAACTGGCGATGCGGGATGAACCGGAAGC

CGGGTTACGGTGCCCAACTGCGCGCTAACCTAGAACCCACAAAGGGTGTTGGTCGATTAAGACAGCAGGA

CGGTGGTCATGGAAGTCGAAATCCGCTAAGGAGTGTGTAACAACTCACCTGCCGAATCAACTAGCCCCGA

AAATGGATGGCGCTTAAGCGCGCGAACTATACCCGGCCGTCGGGGCAAGAGCCAGGCCCCGATGAGTAGG

AGGGCGCGGCGGTCGCTGCAAAACCGGGGCGCGAGCCCGGGCGGAGCGGCCGTCGGTGCAGATCTTGGTG

GTAGTAGCAAATA

>FM895472.1 FM895472 Jatropha curcas embryo 71-95 (DAF) Jatropha curcas cDNA clone rjcpga0_002396, mRNA sequence

CACTTTGACATTCAGAGCACTGGGCAGAAATCACATTGCGTGAGCATCCTCAGGGACCATCGCAATGCTT

TGTTTTAATTAAACAGTCGGATTCCCCTTGTCCGTACCAGTTCTGAGTCGACTGTTCGACGCCCGGGGAA

GGCCCCCGGAAGGGCCGTTCCCAGTCCGTCCCCCGGCCGGCACGCGGCGACCCGCTCTCGCCGCGGGAGC

AGCTCGAGCAGTCCGCCGACAGCCGACGGGTTCGGGACTGGGACCCCCGGGCCCAGCCCTCAGAGCCAAT

CCTTTTCCCGAGGTTACGGATCCATTTTGCCGACTTCCCTTGCCTACATTGTTCCATCGACCAGAGGCTG

TTCACCTTGGAGACCTGATGCGGTTATGAGTACGACCGGGCGTGGGAGGCACTCGGTCCTCCGGATTTTC

ATGGGCCGCCGGGGGCGCACCGGACACCGCGCGACGTGCGGTGCTCTTCCAGCCGCTGGACCCTACCTCC

GACTGAGTCGTTTCCAGGGTGGGCGGGCTGTTAAACAGAAAAGATAACTCTTCCCGAGGCCCCCGCCGAC

GTCTCCGGACTCCCTAACGTCGCCGTCAGCCTTCACGTCCCGGTTCGGGAAT

>FM895471.1 FM895471 Jatropha curcas embryo 71-95 (DAF) Jatropha curcas cDNA clone rjcpga0_002395, mRNA sequence

CAATGAGCAAGTGAGGGATCTCCTTGCTACTGATAGCATTAATAGAAGATTAGAAATTCGCAACAGTTCT

CAGAATGGGATCAATGTACCTGATGCAAACTTAGTACAAGTATCATCAACTGGTGATGTCCTAAATTTGA

TGAACATTGGCCAGAGGAATCGTTCAGTGAGTGCTACAGCAATGAACGACCGGAGTAGTCGATCTCATAG

CTGCCTGACAGTTCATGTTCTAGGAAGAAACCTCACGTCCGGAACTGTGCTCCGTGGTTCTATGCATCTG

GTTGATTTAGCAGGAAGTGAACGAGTCGACAAATCAGAGGTCACTGGAGATAGATTAAAGGAAGCACAGC

ATATCAACAAATCTCTTTCTGCTTTAGGAGATGTGATTTCTTCTCTTGCTCAGAAAAGTTCTCATGTTCC

ATATAGAAACAGTAAACTCACACAACTACTCCAAGATTCACTTGGAGGGCAAGCAAAGACACTTATGTTT

GTCCACATAAGTCCTGAGCATGAAGCTCTTGGAGAAACGCTTAGCACACTAAAATTTGCTGAACGGGTCG

CCACAGTTGAGCTTGGTGCTG

>FM895470.1 FM895470 Jatropha curcas embryo 71-95 (DAF) Jatropha curcas cDNA clone rjcpga0_002394, mRNA sequence

CTCAGCAGCTTCAATATCAGCAACCCTAGACATAGAATCAACACGAACACGTGCACCATAGATCTTCACT

TTATCTGTATCCATTGCAGTATTAGCTACCAAAATTTTTGCATTCTCTATGCGCTTTGGTTGTCCAACAC

CAATTTTCTTGTCAAGAATAAATCCTTCATCCAGGAAAGAATCCTTCAGTGAACCTCCAGGCTTCTTGAT

AATCTGGATTGACTCTAAGTTTGTGCTACCCTTCAGCCTCAAAACAGCATCCACAGCAAGTTTTGCAAAA

TGTTCCTTATCCTGTGATAAAATTTTGGAACTCAAAGTAGTCATTGCAATCTTCATCAAGTCCAATTTAA

ATTTCTCTTCATTCTCTTTGTTATCCACAACCTTTTGCAACAAAGCATTACGAGCACATTCTGCCGCCAT

TCGGTAGCCTGCAATAATCGTCATAGGATGAATCTTTGCTGCCACTAGCTTTTCTGCCTCCCTTAAAAGT

TCCCCAGCCAACACAACAACAGACGTTGTCCCATCACCAACTTCATCATCTTGCACTTTTGAAATATCAA

CTAGAACTTTGGCAGCTGCGTTATCAATGTGAAGGGACTTCAAAATGGTGGCCCCATCATTTGTCACAGT

AACTTCACGTC

>FM895469.1 FM895469 Jatropha curcas embryo 71-95 (DAF) Jatropha curcas cDNA clone rjcpga0_002393, mRNA sequence

ATTCCCCCTCTTTTTGGACCCAAGGTCGTTTCGGCGGCCGATCCGGGCGGAAGACATTGTCAGGTGGGGA

GTTTGGCTGGGGCGGCACATCTGTTAAAAGATAACGCAGGTGTCCTAAGATGAGCTCAACGAGAACAGAA

ATCTCGTGTGGAACAAAAGGGTAAAAGCTCGTTTGATTCTGATTTCCAGTACGAATACGAACCGTGAAAG

CGTGGCCTATCGATCCTTTAGACCTTCGGAATTTGAAGCTAGAGGTGTCAGAAAAGTTACCACAGGGATA

ACTGGCTTGTGGCAGCCAAGCGTTCATAGCGACGTTGCTTTTTGATCCTTCGATGTCGGCTCTTCCTATC

ATTGTGAAGCAGAATTCACCAAGTGTTGGATGGTTCACCCACCAATAGGGAACGTGAGCTGGGATTAGAC

CGTCGTGAGACAGGTTAGTTTTACCCTACTGATGATGGTGTCGCGATGGTAATTCAAGCTAGTACGAGAG

GAACCGTTGATTCGCACAATTGGTCATCGCGCTTGGTTGAAAAGCCAGTGGCGCGAAGCTACCGTGCGCT

GGATTATGACTGAACGCCTCT

>FM895468.1 FM895468 Jatropha curcas embryo 71-95 (DAF) Jatropha curcas cDNA clone rjcpga0_002392, mRNA sequence

GTCTGACATGTGTGCGAGTCAACGGGTGAGTAAACCCGCAAGGCGCAAGGAAGCTGACTGGCGGGATCCC

CCCGAGGGTTGCACCGCCGACCGACCTTGATCTTCTGAGAAGGGTTCGAGTGAGAGCATGCCTGTCGGGA

CCCGAAAGATGGTGAACTATGCCTGAGCGGGGCGAAGCCAGAGGAAACTCTGGTGGAGGCCCGCAGCGAT

ACTGACGTGCAAATCGTTCGTCTGACTTGGGTATAGGGGCGAAAGACTAATCGAACCGTCTAGTAGCTGG

TTCCCTCCGAAGTTTCCCTCAGGATAGCTGGAGCTCGGGACGAGTTCTATCGGGTAAAGCCAATGATTAG

AGGCATCGGGGGCGCAACGCCCTCGACCTATTCTCAAACTTTAAATAGGTAGGACGGCGCGGCTGCTTCG

CTGAGCCGCGCCACGGAATCGAGAGCTCCAAGTGGGCCATTCACGGTAAGCAGAACTGGCGATGCGGGAT

GAACCGGAAGCCGGGTTACGGTGCCCAACTGCGCG

>FM895467.1 FM895467 Jatropha curcas embryo 71-95 (DAF) Jatropha curcas cDNA clone rjcpga0_002391, mRNA sequence

CTACAGAACAAGAGGCCATTTCAGCAACTCAAGGAATCAGCAGATGATACCATACATCAAAATTCAATTA

CAGCCATAAAAAAAAGAAGCAGATAGTGATAAATTTCTCTAATTTTAATGTAAGAGTATTTGTCAAGAGT

TTAACAGCAGGCATTAGATATGCCTAAATCATAGCTCTTAATAGGTCCCTAATGCACTTCTAGGCTAGCT

TCTCTGAAATAAAAATTTGTTCACAGCGTTGTACACATAATTTTGCTTCCAATTTTTTTATCAAAAAACT

GGAAATATGTTACTCCACTGGTGGTGTATCTAAAATCCCATTACTGGATTTCCAGCAATTTAAAACCCCA

CTTCTCAACTTCTCAACATCTATTGCACCAGAGACATTTTGTCTCCCTTTCTTTAGTAGTTCAATACGAT

CTCGCAGGAGTCGATTCTGCTTTTCAGCCTTTTCCAATGTGTCCGTCAAGAAGCGGATCTCATTTTCCTT

CGCAATTGACAGCTCGCGTGCCACATCCATTATGTTGGTCTTTTTTGAAAACAATGGGTTCAAACTTTGC

TCTTCCACAAGTTGGTCCACAGTATCCAGAGTTGTCCTCACCTGTG

>FM895466.1 FM895466 Jatropha curcas embryo 71-95 (DAF) Jatropha curcas cDNA clone rjcpga0_002390, mRNA sequence

TTTTTTTTTTTAATGAGAAAAAAGAAAGTAAATTTTATTCACAAACTAAGAGAAACTCATTAACGACTCT

GAGGATAACTAGATAAACGAAAAGGGTAAAAAAAAGCTACTACCAACAGCTATTACAGTCTGTGTTTGCA

ACCACGTTTTATTCTTTACATTACTCTTCTCTAATCAAAACCCATTATCATATCATTATCAAAGCTCTTT

ATCAATTTATGCACCCTTTCAGAATTAGCCGCCTCCTTCAACTTGTCTGGTGGTGCTGCAGATGCTGAAG

GCAATATAACTGACTCGCGTTGTGAATTAACAAAGTGCTCCAATCCTTCCTCGCTTAAGCCAAATGCAGT

TGCAAGCTCAGGGCCTCTTAAGGTAGTAAGAACTGAGTTTGAACCAGCTAAGAATTGGGGTCTATTCTTT

TGAGCTGAGGTTGTGAATCCGAAGAACTCACAAGGAGCTGTTCTTGATGCAATTTGACAGAATGGGTGAT

ATCTTGGCACCCAAAACACATCATTTACTGCTACTTACGCTTACATTGCTTGTGTACC

>FM895465.1 FM895465 Jatropha curcas embryo 71-95 (DAF) Jatropha curcas cDNA clone rjcpga0_002389, mRNA sequence

TTTAGCAAATTTCACAGAGAACCCTAACAATCATGGGTTCTAAACTTGAAGCTTTGTTATTCATTTTTAT

GATTTTCATGGCAATATCATTGCCACCAATTTATGCTTGTGCACCTTGTACTCAGCCACATCCACCAGGC

CACCGTCCAACCCACCCAAAAGTTCCTCATCCTAACCCACCAAGCACCAAGCACCCACCACACCACGGCG

GCCACCCAAAACCACCTTCAACAAAAAACCCACCAGTGGTTTTGCCACCAATAGTCATTAATCCTCCTCC

TGTAGTAAACCCTCCTGTAATAATAACACCTCCTCCTGTAATAACACCGCCGCCTTCTTCAGGTTACCCA

CCATACACGCCTAGCCCTCCATCTGGTGGTGGCGGAGGCGGCGGCGGTGGCGGAGGCGGTGGAGGAGGAG

GAGGAGGAGGTGGTGGTGGTGGTGTNCCGGGGCTTAATCCTCCACCTACTACTCAACCCACTTGTCCAAT

CAACGCCCTTAAGCTAGGGGCTTGTGTTGATGTATTAGGAGGATTAGTGCATGTTGGATTAGGGAATCCA

GTTGAGAATGTTTGCTGCCCCGTAATTACAGGATTGCTAGAGCTTGAAGCAGC

>FM895464.1 FM895464 Jatropha curcas embryo 71-95 (DAF) Jatropha curcas cDNA clone rjcpga0_002388, mRNA sequence

ATTCCAAAGGATTCTATCCGTCGCCCGATGGGAATTGTACTTTANAGGCGGCCCTCGCGGCTCGTCCGCC

GCGGGGGCTTGGCCAACGGCACGTGCCTCTGGGGGCCGGATGGCCCCTACTGCGGGTCGGCAAACGGGCG

ACGGACACGCGCGTCGCTTCTGGCCCGGATTCTGACTTAGAGGCGTTCAGTCATAATCCAGCGCACGGTA

GCTTCGCGCCACTGGCTTTTCAACCAAGCGCGATGACCAATTGTGCGAATCAACGGTTCCTCTCGTACTA

GGTTGAATTACCATCGCGACACCATCATCAGTAGGGTAAAACTAACCTGTCTCACGACGGTCTAACCCCA

GCTCACGTTCCCTATTGGTGGGTGAACAATCCAACACTTGGTGAATTCTGCTTCACAATGATAGGAAGAG

CCGACATCGAAGGATCAAAAAGCAACGTCGCTATGAACGCTTGGCTGCCACAAGCCAGTTATCCCTGTGG

TAACTTTTCTGACACCTCTAGCTTCAAATTCCGAAGGTCTAAAGGATCGATAGGCCACGCTTTCACGGTT

CGTATTCGTACTGGAAATCAGAATCAAACGAGCTTT

>FM895463.1 FM895463 Jatropha curcas embryo 71-95 (DAF) Jatropha curcas cDNA clone rjcpga0_002386, mRNA sequence

TTTCAACTTCATGAAAGTGGCTACTGACCTGTCGAAATCTGGTGATGGTTCGATTGTCAATGCTGCAGTT

ATAGTGGATCCTTCAGTTCAGCAAATTATTGCAAGCGGGTGTGATCAGATCTATTCATGTCATAAAGAGA

ACCACACTGAAAACAACCACTTTAACATACCAGTTTCTCCTTTAGATCCTGATTCCAACAGTGTAGCAAG

TCATGCAACTGCAATTCCAAACAGTCTGCAATCACATGATAAACCTGAAACTTCATGTGTTGCTGTTTCT

TGTTTAAACCCTTGGCAGTGGTCTGGGCAACAATTAAATAAGCAAGGTTCTTTTAATTGGCATCCTTTAC

GACATGCTGCTATTGTTGCTATTGAATCTTCTGCAGAAAGGGATAGGCATCTCTTCCCTGTTTCTAAGGA

AAAGGCTTTTGAAGTGGAACCGATGCTGTCTTCATGTGCAGATTCTCCAGCAAAAAGACAAAAGACAAAC

ATTGCAAATGTTGAGAATGGGAAAGAAATGGATGCTCATAATGAAGCAACATCAGTGAGACCCTATCTAT

GCACTGGTTACGACATCTTTCTGGTTTGGGAGCCGTGTACCATGTGTGCAATGGCGCTTGTTCATCAAAG

AA

>FM895462.1 FM895462 Jatropha curcas embryo 71-95 (DAF) Jatropha curcas cDNA clone rjcpga0_002384, mRNA sequence

GAGAGTCTAGCTACCCTCTACGAGAAAGATTTAGAATTTGAGTTTGTTCATATCGATCTTGCCACGGGAG

AGCACAAGAGGGAGCCTTTCATTTCCCTAAATCCATTTGGTCAAGTCCCAGCTCTAGAACACGGAGATCT

CAAGCTCTTTGAATCAAGGGCAATTACCCAATACATTACCCATGAGAATCCTGACAAGGGAACCCAGCTT

CTTTGCCCAGGCAAGGTCAAGATGCCAATATTAACAGTTTGGATGGAAGTGGAGGCTCAAAAATTTGAAC

CAGCAGCCTCAAAGCTCAACTGGGAAGTGTTTTTGAAGCCAATTTTTGGTTTGACAACGGATTCAGCTGC

AGTAGAGGCGAATGAAGCTGAGCTTTCTAAGGTTCTTGATGTTTATGAGGCAAGACTAGCTCAATCTAAG

TACTTGGCTTGTGATTGCTTCACTTTGGTAGATCTACACCACTTGCCTAACTTACAGCTGCTGTTATTGA

CACAAAGCAAGAAATTGATCGAGTCAAGACCTCATGTTAGCGCATGGGCTGCTGATATTACAGCAAGACC

AGCTTGGGCTAAGGTTCTTGCTATGCAGAAGAAGTGAGAGGAGCTGGGGTGGCCTGGCTATGGTCCTTTC

TCTT

>FM895461.1 FM895461 Jatropha curcas embryo 71-95 (DAF) Jatropha curcas cDNA clone rjcpga0_002383, mRNA sequence

CACTAACAATTTTGCGTTCCACTAACCCAAGACTTGGACTAGAGGATTTATGAGTGGGAGTCAATGAGGA

ACTCCTACTCCTTTGTTTCTTATAACGCCTTGGAGTTGGAGAACGACTTTTACGACGTCTTGGTGTTGGA

GAACGACTTTTGCGTCGGCGTGGAGAAACAGAACGACTTCTCCTTCTGTATGATACAAAAGAAATTATGA

GCAACAACAGCAAGCAGAATGAATCACTAGTGTACACAATAACTTGGGAATCCTCTCACCATTTACCACA

ATTAAAATAGAAGCATATACAGTTACCTCATTCATTGTTAAGAATAACCATTGTGTGCTCCATCTTTTAT

CATATGAAGAAAGTAATATCAGAATCGAGCAACAAAAGGACACCGATCGGCACCCATCACCTGCTCATTT

CCCATCCCACTCGATGAGAAGAAACAAGTTTTTAAAATTTTCTTAGCATGAAAACTATGGCACCCTTTGC

ACTTTTCTCCCCCAAAAAATTATATTTTGTTTCCATTCAACTTCTCCATGTAAATGGCAATTCCTCAGAA

AGCAGATGCCCCCACTGAGATAGTGACATTGACTATTTGTTTACAAACATAACTATAAA

>FM895460.1 FM895460 Jatropha curcas embryo 71-95 (DAF) Jatropha curcas cDNA clone rjcpga0_002382, mRNA sequence

ATTCAAAGGGATTCTATCCGTCGCCCGATGGGAATTGTACTTTAAGGCGGGCCTCGCGGCTCGTCCGCCG

CGGGGGCTTAGCCAACGGCACGTGCCTCTGGGGGCCGGATGGCCCCTACTGCGGGTCGGCAAACGGGCGA

CGGACACGCGCGTCGCTTCTGGCCCGGATTCTGACTTAGAGGCGTTCAGTCATAATCCAGCGCACGGTAG

CTTCGCGCCACTGGCTTTTCAACCAAGCGCGATGACCAATTGTGCGAATCAACGGTTCCTCTCGTACTAG

GTTGAATTACCATCGCGACACCATCATCAGTAGGGTAAAACTAACCTGTCTCACGACGGTCTAAACCCAG

CTCACGTTCCCTATTGGTGGGTGAACAATCCAACACTTGGTGAATTCTGCTTCACAATGATAGGAAGAGC

CGACATCGAAGGATCAAAAAGCAACGTCGCTATGAACGCTTGGCTGCCACAAGCCAGTTATCCCTGTGGT

AACTTTTCTGACACCTCTAGCTTCAAATTCCGAAGGTCTAAAGGATCGATAGGCCACGCTTTCACGGTTC

GTATTCGTACTGGAAATCAGAATCAAACGAGCTTTTACCCTTTTGTTCCACACGAGATTTCTGTTCTCGT

TGAGCTCATCTTAG

>FM895459.1 FM895459 Jatropha curcas embryo 71-95 (DAF) Jatropha curcas cDNA clone rjcpga0_002381, mRNA sequence

GGAGTCTGACATGTGTGCGAGTCACGGGTGAGTAAACCCGCAAGGCGCAAGGAAGCTGACTGGCGGGATC

CCCCCGAGGGTTGCACCGCCGACCGACCTTGATCTTCTGAGAAGGGTTCGAGTGAGAGCATGCCTGTCGG

GACCCGAAAGATGGTGAACTATGCCTGAGCGGGGCGAAGCCAGAGGAAACTCTGGTGGAGGCCCGCAGCG

ATACTGACGTGCAAATCGTTCGTCTGACTTGGGTATAGGGGCGAAAGACTAATCGAACCGTCTAGTAGCT

GGTTCCCTCCGAAGTTTCCCTCAGGATAGCTGGAGCTCGGGACGAGTTCTATCGGGTAAAGCCAATGATT

AGAGGCATCGGGGGCGCAACGCCCTCGACCTATTCTCAAACTTTAAATAGGTAGGACGGCGCGGCTGCTT

CGCTGAGCCGCGCCACGGAATCGAGAGCTCCAAGTGGGCCATTTTTGGTAAGCAGAACTGGCGATGCGGG

ATGAACCGGAAGCCGGGTTACGGTGCCCAACTGCGCGCTAACCTAGAACCCACAAAGGGTGTAGGTCGAT

TAAGACAGCAGGACGGTGGTCATGGAAGTCGAAATCCG

>FM895458.1 FM895458 Jatropha curcas embryo 71-95 (DAF) Jatropha curcas cDNA clone rjcpga0_002380, mRNA sequence

CGAATTCGCAAAGGATTCTATCCGTCGCCCGATGGGAATTGTACTTTAAGGCGGCCCTCGCGGCTCGTCC

GCCGCGGGGGCTTGGCCAACGGCACGTGCCTCTGGGGGCCGGATGGCCCCTACTGCGGGTCGGCAAACGG

GCGACGGACACGCGCGTCGCTTCTGGCCCGGATTCTGACTTAGAGGCGTTCAGTCATAATCCAGCGCACG

GTAGCTTCGCGCCACTGGCTTTTCAACCAAGCGCGATGACCAATTGTGCGAATCAACGGTTCCTCTCGTA

CTAGGTTGAATTACCATCGCGACACCATCATCAGTAGGGTAAAACTAACCTGTCTCACGACGGTCTAAAC

CCAGCTCACGTTCCCTATTGGTGGGTGAACAATCCAACACTTGGTGAATTCTGCTTCACAATGATAGGAA

GAGCCGACATCGAAGGATCAAAAAGCAACGTCGCTATGAACGCTTGGCTGCCACAAGCCAGTTATCCCTG

TGGTAACTTTTCTGACACCTCTAGCTTCAAATTCCGAAGGTCTAGAGGATCGATAGGCCAC

>FM895457.1 FM895457 Jatropha curcas embryo 71-95 (DAF) Jatropha curcas cDNA clone rjcpga0_002379, mRNA sequence

CTTCCATTGTTGCAAACTTACCGCCTACAGTCTTGGTGATCCCAAGTAGAAGCACCCTTTTTAGTACTCG

TCAAAAGCTTCGCGTTTCTCCCATTAGAGAGAGACAAAGTTGTGCAGCTGTTGTTGTGAAGGCTGCTGGA

GAAAGCTCTGAGTCATCTACCTCCCTCAGCATTGTTAAGTCTGTACAGAATGTTTGGGATAAACCTGAAG

ATCGGGTGGCTCTTATTGGTTTGGGGTTTGCAGCTATAGTAGGTCTATGGGCATCAGCAAATCTTGTATC

GGCTGTTGACAAGTTGCCGGTCATCCCAAGTGCATTAGAACTAATTGGCATCCTGTACTCTTCGTGGTTT

GTATACCGGTACCTCCTATTCAAACCCGACCGGGAAGAGTTAGTCCAGATCATCAACAAAACAATATCTG

ATGTATTGGGCCAGTGAATTATGCTTCCTAATGCAGTCTGTAGTGTAATTTCAGATCATGGATCTTTTCT

ATGTTTATGCGTCAGATGCATCTGTATTAGTATATAGAAGCTATGAACATTTGATTTAAGT

>FM895456.1 FM895456 Jatropha curcas embryo 71-95 (DAF) Jatropha curcas cDNA clone rjcpga0_002378, mRNA sequence

AAATGCAAACTGCACAAACAAATCAATTACACGACCTTAGGCCATTGAACATCTCAGAAGACGAGAACAA

GCACTGGCCAAATTCTAACATCCAACGAATAAGTGCCTAGAATCATAGTACATAAATTTGATGATTCTAT

ATGTTCAATCTAAGACAATCCAAATTTTCCAACACCCTCTCACAGCATTTCAAACTGCAGGATCTTCAAG

CGTGGCCGTGGATAAATCATTTATTCGAGGATAAGCAGCTTTCTCGTCAAGTTGGTTCTGAGCCCAGATC

AGCATTTTTAACAAACTCGGAAGCTTTGGATCTTTTTCATGGCTCTGGCTAGTTAGGATGGCTGCATTCA

CTTCACTTGCTGTCTTCAGGCGCTGAGAAATATCCAGAAGTTCTCCAACAGGGCAGTTAGTGACATCTTC

AAAAGCCAGCAGGGCCACAGTCCTCTCCAACTCTTCTAAAAAGCTTTGATTTTCTTCTCCCCTGGGTGCA

AGTTCCTCCTGAGCAAACTCCAAAGCCTCTTCCACTTTTCCATTTCGAATCAATTCTATCAATCGCTGCT

GTTGGAGATGGAAAAATAG

>FM895455.1 FM895455 Jatropha curcas embryo 71-95 (DAF) Jatropha curcas cDNA clone rjcpga0_002377, mRNA sequence

TTTTTCCTGAATTCTCTTTTTCTGTAGCCCCTACCCTATAATGCGCGATGGTGTGTTTTACGCACCCCTC

AATCCACCGCTTCTTCTCTCTCCCTCTCCGACACCAACCTCCGTGAACAATTCCATTGCTGCCTCCAACC

AAAACATGCTCGTCTCCGTTTTTTTGGCCCTTTTCTTGCCTTGTATCGGCATGAGCGCAGTTTTTCTCGT

CTATATTTGTCTCCTGTGGTACGCTGCAAGTCACCAAGCCGAAATCCCACCGCCAGTAAAGGCGGCACCG

GAGAAGGGATTATCGGCTTCTCAGCTCGAGAAATTGCCTAAATTGACTGGGAAGGAGTTGATATTGGGGA

CGGAATGCGCGGTGTGTCTAGATGAGATCGAGAGCGAACAGCCGGCTAGACTGGTTCCCGGTTGCAATCA

CGGTTTCCATTTGGAATGCGCTGACACTTGGCTTTGCAAGCATTCTGTGTGTCCTGTTTGTAGGGCCAAG

CTTGATGCTCTGTTCTTCGTTGCTTCTAATGAGAATCCTTGTTAATTTTAATTTCTGAACAAGCATGATC

CATCAAACAAGAAGTTCTTCCAGCTCTGTTCTGCTATGTTGCCAGAAGATTGCCGATTGCGACTCATAAT

TACTAGG

>FM895454.1 FM895454 Jatropha curcas embryo 71-95 (DAF) Jatropha curcas cDNA clone rjcpga0_002376, mRNA sequence

ATTCCTGCCCTTTCACCGTTTACCCTGCCATCCAACCCAACGCTGGCCACCCGGTCCTCGAAAAAGGTGG

CTTCCCACTCTCCACCCTAACACACCGCTCTTTTCCCGTCCCTAACCAGCACTGGACTGGCCGGATCTGG

GCTAGAACCGGATGCACTCACACCAACGGCAAGTTCCACTGCGCCACCGGTGATTGCAACCACCAACTAG

AATGCAACGGCCTCGGTGGTGCCCCTCCAGCAACTCTCGCTCAGTTCAGCCTTCACCATGGCCATAACGA

CTTCTCCTCTTACGGCGTCTCTCTCGTCGACGGATTTAACCTTCCGATGACCATAACTCCACATGAAGGC

AAAGGTGTTTGTCCGGTGGTGGGTTGTCGAGCAAACTTACTGACAACATGCCCTCAAAAGCTTCAGGTGA

GGTATCCGGTGGGTCATGGTCCGGTGGTGGCCTGTAAAAGTGGATGCTTGGCGTTCGGAACCGACGAGCT

TTGTTGCCGGAATCATTATAACAATCCACAAACTTGCAGAGCATCGAGTTATTCAGAATTCTTCAAGCAT

GCTTGCCCTGC

>FM895453.1 FM895453 Jatropha curcas embryo 71-95 (DAF) Jatropha curcas cDNA clone rjcpga0_002375, mRNA sequence

TTGTTTTTTTCTACGAGAAATGGCCAACGCAATTCGATTCTTTACTCTAAATACCGGAGCCAAAATCCCC

TCCGTTGGCCTCGGCACTTGGCAATCTGAACCTAGCGTTGTTGGCGACGCTGTTACTGCTGCCATTAAGG

CTGGATATAGACATATTGATTGTGCTTCAGTTTATGGCAATGAGAAGGAGGTTGGTTTAGCACTGAAAAA

GCTGTTTGATGATGGCGTAGTGAAGCGTGAGGAGTTGTGGATTACCTCAAAACTCTGGTGTACTGACCAT

GATCCAGAAGATGTACCAGTGGCGTTGAATAGAACTTTGCAGGACTTGCAACTTGAATATCTAGATCTGT

ATCTGATCCATTGGCCAGCAAGCATGAAGAAGGGTTCGGTTGGGTTTAAGCCAGAGAACCTTACACGCCC

AAATATACCAAGCACATGGAAAGCAATGGAAGCACTGTATGATTCTGGTAAGGCTAGAGCTATTGGGGTT

AGTAATTTCACTGCAAAAAAGCTTGGAGATCTGCTTGAAGTTTCTAGAATTCCTCCTGCTGTTAATCAAG

TAGAGATTCACCCTGTCTGGCAGCAGCCTA

>FM895452.1 FM895452 Jatropha curcas embryo 71-95 (DAF) Jatropha curcas cDNA clone rjcpga0_002374, mRNA sequence

GGGAAAGAAGACCCTGTTGAGCTTGACTCTAGTCCGACTTTGTGAAATGACTTGAGAGGTGTAGGATAAG

TGGGAGCCGAAAGGCGACGGTGAAATACCACTACTTTTAACGTTATTTTACTTATTCCGTGAATCGGAGG

CGGGGCTATGCCCCTCTTTTTGGACCCAAGGTCGTTTCGGCGGCCGATCCGGGCGGAAGACATTGTCAGG

TGGGGAGTTTGGCTGGGGCGGCACATCTGTTAAAAGATAACGCAGGTGTCCTAAGATGAGCTCAACGAGA

ACAGAAATCTCGTGTGGAACAAAAGGGTAAAAGCTCGTTTGATTCTGATTTCCAGTACGAATACGAACCG

TGAAAGCGTGGCCTATCGATCCTTTAGACCTTCGGAATTTGAAGCTAGAGGTGTCAGAAAAGTTACCACA

GGGATAACTGGCTTGTGGCAGCCAAGCGTTCATAGCGACGTTGCTTTTTGATCCTTCGATGTCGGCTCTT

CCTATCATTGTGAAGCAGAATTCACCAAGTGTTGGATTGTTCACCCACCAATAGGGAACGTGAGCTGGGT

TTAGACCGTCGTGAGACAGGTTAGTTT

>FM895451.1 FM895451 Jatropha curcas embryo 71-95 (DAF) Jatropha curcas cDNA clone rjcpga0_002373, mRNA sequence

ATTCGCATTTGAATTCATTAAATTGTAGAAATATAAAAGATAAAACACTTCCAAGTCAACTGAACAAAAC

CGTCCAAACTCAACAACAAGCAGATATTCCTCATGTTTTTCTTATTTTGATTACCTTCCATGGATATGCT

AAAAACCTCATAAAGCAAATATAACAAAAAGACAGGTCCTAAAACTATGCCTGAGACAAAAGACAATCTA

TTATACTCCTCTGAAATTTAAAACGGAAACTTAAGACCTCTTTAGTATTTGTAGTCCTTGACATGGAGAC

TCTCTGAGGCACCCTCACCAAGAGTAGCATTACCCTGGTAAGTTCCAAGAGTTGCCTCTGAGTTGGCCTT

AGCCCTGACCAGGAATGCAGCTTGAGCCTTCTGCACATTCTCTGGCTTACCTGCCCAAGCCTTGAGGGTA

CTTTGTTGAAGTGCACGTCCAAATGAGAAGGAAAGGGACCATGGCTTCTTGGTCTTGAGCTTGTTCATCG

CATTCAGGTTGACGGTAGCTTCCTCCTCACTCTGCCCACCAGACAAGAACACAACAGCAGGAACAGCGGC

AGGCACAGTGCATTGGAGGGCACGGACCGTGTACTCAGCAATCACCTCTGGTGCAACCTTGGCGGATTCT

GATCCT

>FM895450.1 FM895450 Jatropha curcas embryo 71-95 (DAF) Jatropha curcas cDNA clone rjcpga0_002372, mRNA sequence

TGGAATCAAAAAGGTAATTGAGTATAGGTTTAACGATGATGGTAATAAGGTTAAGCTCACTACTACAACC

CGTGTTCGGAAGCTCGCTAAGGCTCGGTTGAGTAAGAGTGCTGTCGAGCGCAGAAATTGGGCTAAATTTG

GCGACGCTGTTCATGAGGATGTTGGTAGTAGGCTTACCATGGTTTCTACTGAGGAGATCTTGCTTGAAAG

ACCTAGAGCTCCAGGTACCAAAGTGGAAGAAACCAAGCTGGCAGGGGATAACTTGTCTCAATTAGGTAAA

GCTGGAGCAGTTCTCATGGTGTGCAGAACTTGTGGTAAGAAAGGTGACCACTGGACATCAAGATGCCCCT

ACAAGGATCTTGCTTCACCGTCTGAAGCCTTCATCGATAAACCACCAACATCAGATACTGCTGCTGCTGC

TGCTTCTGGACCTACAAAGGGTGCTTATGTGCCACCAAGCATGAGAGCAGGTGCAGAAAGACCTGCTGGA

TCAGATATGAGGCGCAGGAATGAAGAGAATTCAGTTCGTGTCACCAATTTATCCGAGGACACAAGGGAGC

CTGACTTGCTT

>FM895449.1 FM895449 Jatropha curcas embryo 71-95 (DAF) Jatropha curcas cDNA clone rjcpga0_002371, mRNA sequence

GCAAAGGATTCTATCCGTCGCCCGATGGGAATTGTACTTTAAGTGCGGCCCTCGCGGCTCGTCCGCCGCG

GGGGCTTGGCCAACGGCACGTGCCTCTGGGGGCCGGATGGCCCCTACTGCGGGTCGGCAAACGGGCGACG

GACACGCGCGTCGCTTCTGGCCCGGATTCTGACTTAGAGGCGTTCAGTCATAATCCAGCGCACGGTAGCT

TCGCGCCACTGGCTTTTCAACCAAGCGCGATGACCAATTGTGCGAATCAACGGTTCCTCTCGTACTAGGT

TGAATTACCATCGCGACACCATCATCAGTAGGGTAAAACTAACCTGTCTCACGACGGTCTAAACCCAGCT

CACGTTCCCTATTGGTGGGTGAACAATCCAACACTTGGTGAATTCTGCTTCACAATGATAGGAAGAGCCG

ACATCGAAGGATCAAAAAGCAACGTCGCTATGAACGCTTGGCTGCCACAAGCCAGTTATCCCTGTGGTAA

CTTTTCTGACACCTCTAGCTTCAAATTCCGAAGGTCTAAAGGATCGATAGGCCACGCTTTCACGGTTCGT

ATTCGTACTGGAAATCAGAATCAAACGAGCTTTTAC

>FM895448.1 FM895448 Jatropha curcas embryo 71-95 (DAF) Jatropha curcas cDNA clone rjcpga0_002370, mRNA sequence

ATTGGAGAAGAGAGGAAATTTAATCATCCAAAAATAATGACTTTTCCCAAAGTCAACCTAACAATCTTCC

TTTTCATTTTGGCTTTCCTATCAATAATAATCCCCTCGTTGGCCTCTCAAATTGCACCCCAAAAACCATT

AGGTATCACAGAAAAATATCGTCTACATATTGTCAACAACTTGGGCAAGAACAAGATATTACTTGTACAT

TGCAAGTCGAAAGACGATGATCTTGGTGCCCATAATATTACAGTTGGGTCTGAATTTTCTTGGAAGTTTA

GTGTGAATTTCACTTGGACAACACTTTATTGGTGCTATTTGGCTCCAGACAGTTATTCTCATGTCAATTT

CGTTATATTTGAGAAAGATGATTATATGTTGAGTAGATGTAACACTAAGGGCCTTTTCGGTTCTATTAAT

GATTGCGTTTGGATTGCGAAAGATAATGGGATTTATATGAAGGATTTCTATAGAAACATGGATGATT

>FM895447.1 FM895447 Jatropha curcas embryo 71-95 (DAF) Jatropha curcas cDNA clone rjcpga0_002369, mRNA sequence

TTGGTATTAAGAAAGCTTTGGTTTTTTACGTTGGGAAAGCTCCGAAAGGAACCAAAACGAATTGGATTAT

GCATGAATATCGTCTCTTAGAATCCTCTCGGAAAAATGGAAGCACAAAGTTGGATGATTGGGTTTTGTGT

CGGATTTATAAAAAGAATTCGGGTGCCCAAAAACCCATTTCAAATACCGTTCCAAGCAAAGAATATAGCA

ACAATGGTTCTTGTTCCTCCTCATCTTCTCATCTAGACGATGTTTTGGACTCGTTGCCGGAGATTGATGA

CCGGTTCTTTGCTATTCCGGCCACCAATTCAGCCAAACCAATGCATCATGAAGAGAAAGTCAATCTCAAT

AATTTTGGTTCGGGTAATTTTGATTGGGCCAGTCTTGCTGGATTGAACCCGGTATCTGAACTCCTTCCTA

GTGGGCCAACTCAAACGCAGGGGATGCTGAACCAGAGTTATATGAATAGCACTTGTAATGACCTTTACGT

CCCTTCCTTACCGTCTCAAAGTGTACACGTGGATTCGAAGATGCTTAACTTGGTTGAAGAGGAAGTTCAA

AGCGGTA

>FM895446.1 FM895446 Jatropha curcas embryo 71-95 (DAF) Jatropha curcas cDNA clone rjcpga0_002368, mRNA sequence

ATTCATCTAAACGTCATAATCTTCATCTCCGATTCCCGGTTCTCGTCGCAGATCTCTTTCGCTTGCAATT

CTGAAGGTATAAGGTCTGGAGGATGGCAGATAATGAAGGAGGGGAGAATGTTCCTNCGCGGGAATGAGTG

GGAAGTTGTTTCACTTACGGCATCAACATATGCTGCTGCTCCTGGTCCTAATGAAGTGGAAATGAAAGAT

GAAAACAAGGATAATGCATATGTGGGGGATGAAGCAGAAACTTCCCGTGCCTTGTTCATGTCTGGGCACT

TTGTCTTTCCACCAAGCCAGCATGAGAATTTACCACTGGAACGAGATAAAAGCGAGATTTTAAATGAGCA

AGTTGGGAAGAATGCTGCCTCTGAACTTGACGTGCAAGAAGGGGATAAATCTGGTGGGAAGGATGACGAA

ATTTGGGCACCGAAGCATGAATCTGAAGAGTTCCCTGGGATACAGATTTTTGATGAAAAGGGTAACAGAC

TACCCATCTCTGGGGCTGAATTTGAAGAAAGCATGA

>FM895445.1 FM895445 Jatropha curcas embryo 71-95 (DAF) Jatropha curcas cDNA clone rjcpga0_002367, mRNA sequence

TTTTTTTAGAGGAAAATACTTAATAGACTTTATAATATTTCTAGTAAACATCAGCCTCATCAGAAACTAA

TGTCAGACGAAAACACCCAAAAAGAAAAACACAAGCAGATTACGATTTCAACATAGCCCAAATTATTTCC

AGCACGCAGTCAAACGTGACAAAACGATCCTCTGAGCTCAAGCAATTTTAACCTCAATAGTCTTTGGCTT

TTTAGGCTCAGGCGGTGGCAATTTCTGGACAGTGACAGTCAAAACCCCATCTTGACACACAGCAGAAATG

GCATCAGTATTGGCATTCTCAGGCAGAGCAAACTTCCTCATCAATTTTCCAACTCTCCTTTCCATCCTCA

CATACTTAGCTCCTTCCTTCTCCTCCTCACGCTTTCTCTCTCCACTAATCAACAGCACATTATCATCTTC

CACCTGCACTTTGATATCTCCAGACTTCAATCCAGGCATGTCGATAATGAACACATAAGAATTCGGATAC

TCTTTGACATCGGCCGGAGTAGAAGCCATAGCCTTCGCGTCCCTCACATAAGTGCGTGTTGGCGCGTTGA

AGGATTTGTGGTCAGCATCGTCGCCAATGTCCATCATTTGCTGGAGAGTGTGGAGA

>FM895444.1 FM895444 Jatropha curcas embryo 71-95 (DAF) Jatropha curcas cDNA clone rjcpga0_002366, mRNA sequence

TGAGAGTCCATATAGGTCATTAGGTCAACAACCAAATGGCAGACCGATGGATTTGGAAGCATGGCCTGGC

ATGCAAACAGAGGAAAATGGGCATCTTCAAAGAATGGCCTCACTTCAGGCTGCTTCAATGGGCTGGCCTG

GGGTCCCTGGAGTCCCAACTACTCCTGTTGTAAAGAGAGTAGTTAGACTTGATGTGCCTGTTGACAAATA

CCCCAATTATAATTTTGTTGGCCGAATATTGGGACCACGTGGAAACTCCTTGAAAAGAGTTGAAGCCATG

ACAGAATGCAGGGTATATATAAGAGGCAAGGGATCTGTTAAGGATGCTGTAAAGGTGTTGCCTCAAATAG

ATCTTCCCCAACTTTATCTAATGGAAGAGAAACTAAAAGATAAGCCTGGTTATGAACACCTTAATGAGCC

ACTGCATGTGTTGGTGGAGGCCGAATTTCCAGAGGATATAATAAATGCTCGCTTGGATCATGCAGTAACA

ATACTAGAAAGCCTCTTGAAGCCTGTGGATGAATCATTGGATCACTATAAAAAGCAACAATTAAGAGAAT

TGGCCATGCTAAATGGTACATTAAGGGAGGAAAAGCCGAGTATGAGT

>FM895443.1 FM895443 Jatropha curcas embryo 71-95 (DAF) Jatropha curcas cDNA clone rjcpga0_002365, mRNA sequence

AGGATTCTATCCGTCGCCCGATGGGAATTGTACTTTAAGGCGGCCCTCGCGGCTCGTCCGCCGCGGGGGC

TTAGCCAACGGCACGTGCCTCTGGGGGCCGGATGGCCCCTACTGCGGGTCGACAAACGGGCGACGGACAC

GCGCGTCGCTTCTGGCCCGGATTCTGACTTAGAGGCGTTCAGTCATAATCCAGCGCACGGTAGCTTCGCG

CCACTGGCTTTTCAACCAAGCGCGATGACCAATTGTGCGAATCAACGGTTCCTCTCGTACTAGGTTGAAT

TACCATCGCGACACCATCATCAGTAGGGTAAAACTAACCTGTCTCACGACGGTCTAATCCCAGCTCACGT

TCCCTATTGGTGGGTGAACAATCCAACACTTGGTGAATTCTGCTTCACAATGATAGGAAGAGCCGACATC

GAAGGATCAAAAAGCAACGTCGCTATGAACGCTTGGCTGCCACAAGCCAGTTATCCCTGTGGTAACTTTT

CTGACACCTCTAGCTTCAAATTCCGAAGGTCTAAAGGATCGATAGGCCACGCTTTCACGGTTCGTATTCG

TACTGGAAATCAGAATCGAACGAGCTTTTACCCTTTTGTTCCACACGAGATTTCTG

>FM895442.1 FM895442 Jatropha curcas embryo 71-95 (DAF) Jatropha curcas cDNA clone rjcpga0_002364, mRNA sequence

ATTCCGCCTTCTTTCTCTATCGCCATTCATTTCTCATTAGAGAAAAAGAAGAAATGGCATCGAATATAGG

GATGATGGATAGTGCATATTTTGTTGGAAGGAATGAGATTTTAACCTGNGATCAACAATAGGCTTCAGCT

CAACCTCACTCGCATCGAAGAGGCTGCATCCGGTGCCGTGCAATGTCAGATGATGGACATGACCTATCCA

GGAGTTGTTCCCATGCACAAGGTGAATTTTGATGCCAAGAACGAATATGATATGATCCAAAATTACAAAG

TTTTAACGGAGGTGTTCCACAAGTTGAAAATTGAAAAGCATATTGAAGTTACCAGGCTTGTTTAAGGCCG

ACCTTTAGACAACTTGGAGTTCTTACAATGGTTGAAGCGATACTGTGATTCCATCAATGGTGGCATCATG

AATGAGAATTATAACCCTGTGGAACGGAGAATTAAGGGTGGAAAGGACCGT

>FM895441.1 FM895441 Jatropha curcas embryo 71-95 (DAF) Jatropha curcas cDNA clone rjcpga0_002363, mRNA sequence

ATTCCTCTGCTCTGCTTGTAGCAGAAGAACAAAAAGATAAACTCTTCTCTTTAGAAGAATCAGAAGAAAG

AAGAAAATGTTAAGTCTTGCAGTGGGTTCTTCAGCAGCAGCCACCATGGCCAAGCTTTCCACGAATTGCA

GCTATGGCAAAGCCTTATTGGCTCAAAAGCCAACCACAACCACACAATTTGGGTTCAATGGATCCCAACC

ACCTTCGCATCTCTTCATCTCCACCACCAGACCCATTTCCTCTTTTAAGACTGCTGTTGCTGCTGTCGAG

TCTAATGAGCTCGGTTCCTCTTCCCCTAATCCTCCTGACAAGAAACAACCCAACAAGTATTATTTCGTTG

TTGCAAATGCAAAATTCATGCTGGATGAAGAGGAACACTTTCAGGAACTCATGTTTGAGCGTCTTCGTTA

TTATGGAGAGCGAAACAAAGAGCAGGACTTCTGGCTTGTGATTGAGCCTAAGTTCCTTGATAAATTCCCT

AACATTACCAATAGGTTGAAGAGACCTGCTGTTTCTCTAGTCTCCACTAATGGTCCATGGATCACGTTTA

TGAAATTAAGATTGGACCGGGTTTTATCTGATAGCTACGAAGCTGACACCTT

>FM895440.1 FM895440 Jatropha curcas embryo 71-95 (DAF) Jatropha curcas cDNA clone rjcpga0_002362, mRNA sequence

ATTCCTTTTTTTTCTACAAAATCTCCTCTTTGAAGCCTCTTCTTGTACAACCTATAATCATGAGAACTTA

CAGCTTCTTACTTCCACTACTGTTTTTCACAATTATTTTAGCAGGTCCAGTTCATCTTTGTACTTGCAGG

CATCTCGACTTTGCTACCAATCAAGAACCAGATCGAACAATAAACACCATAAAGTTCCCTTTTTCCATTT

TCATATCATCGTTCTGCTGTTTCTCGCTCCCTGCAATCTCAAGATTACAAGGCCAGCCCATTATATGCTG

CATCCAATAAGCTTGTTCCAGGTGGACCAAATCCACTTCATAATTGATCTGATTTTCTACTTGTGTATTT

TGTAGGTAGGCAAAAGAAAAAGAATTCTGTTTGGTTCTTGGAAAGAATTGAAAGGAAAGAAAAGAAAGAG

ATATTCTCAAAATTGCAATTTTAGTTCTTTCCTTCATTCTTTTCAAGAACCAAATTTTTACTTCGAGTTG

TAAAAACTAAATCCATCATGGATTACTACTGTAAAGCACATTATCAATTTATATAATTACCATGATTATG

TTGCAAAAAAAAAAA

>FM895439.1 FM895439 Jatropha curcas embryo 71-95 (DAF) Jatropha curcas cDNA clone rjcpga0_002361, mRNA sequence

TTAGAATTAGGGTTAGGGTTGGTATAGTGAAAGAGGGATATCAGGCCTACAGCTATGGCCTGTTCAAGAG

GTGGAGAAATGATCACTCCACTCTCGTGGTCTACTCTTCCAGTAGATCTCTTAACTGATATAGCCAAACA

CCTCGATCCCTATTTTGATGTCCTTCATTTCCGTTCTGTTTGCCATTCATGGCGAACTTCCGTTCCTGCT

CCTAAAAAGTCTCCATCTTTGCCTTTGGAGCTTAATTTCCTCACTGACGATCCTTTCCACCCTTTTGCTA

CTTTTTCTGTCTATCGAAGGACAGTCTTTCGTCTTGAACCTGAAGGCTCTCAACATGGTAATAGCTGGTT

GATTAAGGTTCAAGAAGAGAGTGATGCAGGCAAGCTGCTTGTAACAAGTCCTTTCTCAAGATATCCCATT

TATAATTTACCTCCCACATTTCCAAGGGAGTTGAACCTGCTCAGTTTCCGTGTCTGGCCTATTGCTAGTA

CTTTCAGCCTTGAACGACATGATGAGGATGAGGAGCTCGATAGCAACGATGTTACTTATCACCCCATGGA

GATCGTTCATGGAGGAGTAGAAGAAAATGATAACGATAGTTTTATTAGCAATGACGGGGTTACCATGTCT

AGCAAGGTAACCGTTTCAGCTTCTGGAACGGATTCAG

>FM895438.1 FM895438 Jatropha curcas embryo 71-95 (DAF) Jatropha curcas cDNA clone rjcpga0_002360, mRNA sequence

AGGATTCTATCCGTCGCCCGATGGGAATTGTACTTTAAGGCGGCCCTCGCGGCTCGTCCGCCGCGGGGGC

TTGGCCAACGGCACGTGCCTCTGGGGGCCGGATGGCCCCTACTGCGGGTCGGCAAACGGGCGACGGACAC

GCGCGTCGCTTCTGGCCCGGATTCTGACTTAGAGGCGTTCAGTCATAATCCAGCGCACGGTAGCTTCGCG

CCACTGGCTTTTCAACCAAGCGCGATGACCAATTGTGCGAATCAACGGTTCCTCTCGTACTAGGTTGAAT

TACCATCGCGACACCATCATCAGTAGGGTAAAACTAACCTGTCTCACGACGGTCTAATCCCAGCTCACGT

TCCCTATTGGTGGGTGAACAATCCAACACTTGGTGAATTCTGCTTCACAATGATAGGAAGAGCCGACATC

GAAGGATCAAAAAGCAACGTCGCTATGAACGCTTGGCTGCCACAAGCCAGTTATCCCTGTGGTAACTTTT

CTGACACCTCTAGCTTCAAATTCCGAAGGTCTAAAGGATCGATAGGCCACGCTTTCACGGTTCGTATTCG

TACTGGAAATCAGAATCAAACGAGCTTTTACCCTTTTGTTCCAC

>FM895437.1 FM895437 Jatropha curcas embryo 71-95 (DAF) Jatropha curcas cDNA clone rjcpga0_002359, mRNA sequence

TTTACAATAAATTGCGGGATGGCATTCAAAATTTTACATCATATAACATATTGTACAATTTCTAAATGCA

TCATTTACAGTGATTTTTGAGAACAGTATTCCAACTATAGCTGAGTCAATGAACCTCAAAGAATAAACCT

ATAAAGTTCTCCCCTCAAATCCCTAATGAAAGAATTAAATCTAAAGGTACACTTTTTCCTGCTTAGACAT

TTTAATTGTACATTAAAAATTACAGCGTGTAAATACTATATTTTGTAACTCCAGAAACTATCATCTAGCT

AAAAGCTTAGAATTAAGTATCAATGTGCTATTGTCTTTAGCTCTTGCTTCAGGTGGTTTACACATGCAAT

CAGGCATTGGAAATGTGTAAAGTGATCCACTGGGCTTTCTCAATTCAGACCCTTTGTGATCACTGTGGCG

AAGCATATCTTTCATCTGTCGCTTGAAGACTTCCCATCTGATACTGGGTTTATCAAATAAGAGGGCCAAT

TTTCTCTTGTCAGGTTTGATCGTCTTAGCATGTCCTCCATTCAAATAACGCCTGTGACCCATCAAGAAGT

GGGG

>FM895436.1 FM895436 Jatropha curcas embryo 71-95 (DAF) Jatropha curcas cDNA clone rjcpga0_002358, mRNA sequence

ATTCCTCTCTTTTGCCATCCTTTTGTTCTACTCTCTTAAAGTGCTGTTAATTTCTCTTCTTTTGCTGCTC

AATCTTGCATAATCATGGCTGTTCTAGTGTATTTAGTGCTTTTCTTAGCACTTACTGGTTATTCAAGTGC

TACTTACTGTGTTTGTAAAGATGGATTGAGCGACGCAGCACTACAAAAGTCTCTAGATTATGCTTGCGGT

GCTGGAGCTGACTGTACTCCGATCCTTCAAAATGGTGCTTGTTTCCAACCCAACACCGTAAAAGACCACT

GCAGCTACGCTGTGAACAGCTATTTCCAAAGGAAGGGTCAAGTAACTGGAAGCTGTGATTTTGCAGGAAC

TGCTACTACTAGTGCCAGTCCCCCTTCAGGTGTTCCTTCTACTTGTGTTTATCCTGCATCTTCAAGCACT

GCCGGCACAGGCACCACAACCCCAAGCACAGGCACCGGCACAGGCACAGGCACCGGCACAGGGACAGGCA

CAGGGACAGGGACAGGGACAGGCACAGGGACTGGGACAGGCACAGGCACAAGCACAGGGACTGGGACAGG

CACAACAAGTGTGACTCCATTTGGAGGGCTGGGTCCTAC

>FM895435.1 FM895435 Jatropha curcas embryo 71-95 (DAF) Jatropha curcas cDNA clone rjcpga0_002357, mRNA sequence

CTCGATCATTATCATGGCTTCCAGAAAATTTTCCTTCTTTTCATTCACTCTTATTGTCCTAATTTTTTTC

CTTTCTTGTTCAGATTTAGCCTACTCATTTAACAAAAAATTACCACCATTTCAATTCCTCAAACGTCTTC

AAGGTTGCCACAAGGGAGATAAGATCAAAGGCATCCAAGAGCTCAAAGAGTATCTCAAATATTTTGGTTA

CTTGAGATACAAGAACAGAGCTCATGCTAATGATGATTACTTTGACGATGTTTTAGAGTCTGCCATTAAA

ATGTATCAGCAGAATTACCGTTTGAAGACTACTGGATCTTTAACCTCTGAGACAGTGTCAAAGATGATGA

TGCCTCGATGTGGGGTTGCAGACCTCACCAATGGCACCATTAAAAAAAGCTCTAACGCTTATCATACTTC

TGGGTATACCTTCTTTCCTGGGAACCCTAAATGGCCAGCTTCTAAATATGCACTCACTTACGAGCTTCGT

TATGACACCCCGCCTCACCTAATACCTCCTCTCCAGAATGCTTTTCAAGCATGGGCTAACGTTACGCCCT

TCACATTTTCTCCCTTCCATGGCCGTGGAAATCCAGATATAACGA

>FM895434.1 FM895434 Jatropha curcas embryo 71-95 (DAF) Jatropha curcas cDNA clone rjcpga0_002356, mRNA sequence

CTCTTGAACTTTGGGTCTAATGCCCTCAAAACCTCTCTGATGAACTTTGGCCTAGCCAGACACAAAGTGT

TGAAATATTCACAAAAATTATTCTGAAACAAAGTACCTGAAAGCAAGATTCTGAGATCAGTTTCCACTTT

CATCAAAACCTTCCTTAACCTTGATTTAGTACTTCTGGGGTTATGCCCTTCATCAAGTATTAGAAGTCCT

GGACTTTCACGCAACACTTTAGCCATATATTTTCTGTGAGCAAACTTTGAATCTTCTCTCATTAATGTCA

GAAATGAGGTATAACCCATGACAAGGACACTTGGTTGTGCATGCCATTTCTGAATTTTCTCCAAGCAATC

CAAAACATGCATAACATCATGGCTGGGTTTTGGAGCTCCTCGAATTGCCACTGTCTTCTGCCTGAAGACT

CTATATGTCCTTCGACCATGGATTAGATGAACCGGGATAGGAATTTGCCACTTGATAAATTCCTTGTACC

AGGTATAGAGTGTCGTATCAGGGGCAAGGACCAAAGGCCGTTTTCCAGGAAACAACTTCCGGTAGCTAAC

AAGAAAAGCAATAATGAGAAAAGTTTTCCCGCTCCCAGGAGTATGAGAGACACACAGCCACTATC

>FM895433.1 FM895433 Jatropha curcas embryo 71-95 (DAF) Jatropha curcas cDNA clone rjcpga0_002355, mRNA sequence

TGTCGATTAAGACAGCAGGACGGTGGTCATGGAAGTCGAAATCCGCTAAGGAGTGTGTAACAACTCACCT

GCCGAATCAACTAGCCCCGAAAATGGATGGCGCTTAAGCGCGCGACCTATACCCGGCCGTCGGGGCAAGA

GCCAGGCCCCGATGAGTAGGAGGGCGCGGCGGTCGCCGCAAAACCCGGGGCGCGAGCCCGGGCGGAGCGG

CCGTCGGTGCAGATCTTGGTGGTAGTAGCAAATATTCAAATGAGAACTTTGAAGGCCGAAGAGGGGAAAG

GTTCCATGTGAACGGCACTTGCACATGGGTTAGTCGATCCTAAGAGACGGGGGAAGCCCGTCCGACAGCG

CGTCCTCGCGCGAGCTTCGAAAGGGAATCGGGTTAAAATTCCCGAACCGGGACGTGAAGGCTGACGGCGA

CGTTAGGGAGTCCGGAGACGTCGGCGGGGGCCTCGGGAAGAGTTATCTTTTCTGTTTAACAGCCCGCCCA

CCCTGGAAACGTCTCAGTCGGAGGTAGGGTCCAGCGGCTGGAAGAGCACCGCACGTCGCGCGGTGTCCGA

TGCGCCCCCGGCGGCCCATGAAAATCCGGAGGACCGAGTGCCTCCCACGCCCGGTCGTACTCA

>FM895432.1 FM895432 Jatropha curcas embryo 71-95 (DAF) Jatropha curcas cDNA clone rjcpga0_002354, mRNA sequence

ACAAAACTGAAGTCAGCTCCATTTTCTGTAGAAAATTCTGTTTGGTACATCAAAATGTATTTCTATAAAA

ACCTTGAAGAACCCAAAAGAACAGAGAGAATCTAGATAGATGAACACTAAGCGTTTCAATAGCTAACAGT

CATAGCAAATTACTAGTTTGTCATAATTTTTTTTAACATGCCATCATAATTCAGGAACTTCCCTTGTCTG

TTTACTCCAAGGTTTTAGGTGGTTTTCCACCAGTCTGATCATTCTCCACCCCTCCATCAAAAGCATTGGT

TGTCAGTTTTTCTTCCTCAATGCAACTTAACAGTTTATCATAAGCATCTGCTGAGTAACAACGAGTCTCC

TTAATCATCTTCAAAAGCTCTTTTGCAGCTTCCACGTCTCCCTGACCTTTCAAATACACAAGACATGCCT

GTACATTATAAAAGCGTGGCTCTTTCCCTGGTTTACTTACTGAAACTGCTTTCTTTATTGCTTCTACTGC

CTTTGCCATCTGACCCACTCTATAATATCCAGCTGCCACACAATCCCATGCGGTCCCATTA

>FM895431.1 FM895431 Jatropha curcas embryo 71-95 (DAF) Jatropha curcas cDNA clone rjcpga0_002353, mRNA sequence

TTTTTTGTATTTTTCAACAAATTCTATTATGAAACTGGCAATTTAAGAGAAAATTTGAGCATACCAAGAT

TACCAAGTTGTCTAACAGTAGAAATGTAAGAATCAAATATAGTTAACTAGCAAATTACAAGAAATGATAG

CTACAATGGTCCACCCCATTTCTGGGTTACCAAAGTAATGCTTCATCAAAGGCCTCGATTATTTTCTGCA

TAAAGTGTATATGGTCCATAGACTGCCTGTTCCTTTCCTTATTCATTTCTGAAATTACTTTCAACGCTAA

TATCCAAGAAAGTCATCCATAAGCTCAAATTTTTACCATTGCCAAGTGCCATCTGTAAAAACATTTCCGA

GCAGCTCATCTAGAGTTCCTGACCTTAACAATGTGACAAAGTTCTCCATTCTCCAAGGTGCTTCTAGCCC

CACATCCTTCAGTATGAAACACATTTGATGTTCCTAATCAAGTAAAACCTCAAATATGAATCCTTCAAGA

TTACCAACAAGAGCCCGGGTCCCAGTAA

>FM895430.1 FM895430 Jatropha curcas embryo 71-95 (DAF) Jatropha curcas cDNA clone rjcpga0_002352, mRNA sequence

TTTACAAAAAATGTGCCTATTTCATATTTCTAGACACCAAGTTAATGGGCCAATTTAATATTTTAGGATA

CCAAGCCCCAAAATCAAACTCGTGCTTAAGTTCGTGTTAGGCTTGGAGCACACTGGTTCTTACATTCCAC

GGAATACACAGAGGCAGCAACCTACTTACAAAGAATTTACATAACCTCTCACCACTTTTCAACCTCCCCT

TTTGAAAAAAATTACAGGAAATATGAAAACATGGATACGTTTAATGCAAAATATCAAAATAGAAAAGTAA

GATAAGGAAGAAAAAATACCCCTACCCACGAAAAAACAAAATTGGCTTCCACCAATCATAAAGGCAGAAA

TTTAGAATCAAATTTCTGATATTCCAAACCATAGCATTTGCAAGTGCACCATAGTTGCATCTTGATTGTG

GAAGGTCAATGAGCCAGACCTTACCTCCCCATGCCGACAATAGATTTATCAGCCATGGGAACATTGTATT

GCTGATATACTCTTGCTCTGTTCGCTGCCCACCATTGCTCTGCTTGTTTCTCACTGAATCTCTTACGGCT

GAAACGGACACGCTTCAGATCTTTGGCACCTCCAGGCAATGGAAACAGGGTAATGTACACACCTGGTTCA

TCTTGCTCAAACCATTCATCCCC

>FM895429.1 FM895429 Jatropha curcas embryo 71-95 (DAF) Jatropha curcas cDNA clone rjcpga0_002351, mRNA sequence

AGGATTCTATCCGTCGCCCGATGGGAATTGTACTTTAAGTGCGGCCCTCGCGGCTCGTCCGCCGCGGGGG

CTTAGCCAACGGCACGTGCCTCTGGGGGCCGGATGGCCCCTACTGCGGGTCGGCAAACGGGCGACGGACA

CGCGCGTCGCTTCTGGCCCGGATTCTGACTTAGAGGCGTTCAGTCATAATCCAGCGCACGGTAGCTTCGC

GCCACTGGCTTTTCAACCAAGCGCGATGACCAATTGTGCGAATCAACGGTTCCTCTCGTACTAGGTTGAA

TTACCATCGCGACACCATCATCAGTAGGGTAAAACTAACCTGTCTCACGACGGTCTAAACCCAGCTCACG

TTCCCTATTGGTGGGTGAACAATCCAACACTTGGTGAATTCTGCTTCACAATGATAGGAAGAGCCGACAT

CGAAGGATCAAAAAGCAACGTCGCTATGAACGCTTGGCTGCCACAAGCCAGTTATCCCTGTGGTAACTTT

TCTGACACCTCTAGCTTCAAATTCCGAAGGTCTAAAGGATCGATAGGCCACGCTTTCACGGTTCGTATTC

GTACTGGAAATCAGAATCAAACGAGCTTTTACCTTTTG

>FM895428.1 FM895428 Jatropha curcas embryo 71-95 (DAF) Jatropha curcas cDNA clone rjcpga0_002350, mRNA sequence

AAAAACTATCTTNAGTATATATAACTTCAAATAACATTATAAGCTGCTTATGAATTCAGAGTTTCTTATT

TATACACACTACTCGATCCATCAATGTGAAAGCACTCTGTTTAGAAAATAGGACCAGCTGCAAGAATCTC

CTCAGTCAGACTGTTGTCTTTTCCAATCTTATAATTTGCGAACACTTCAAAATTCTTCCTGAACAGACCA

CCCAGCTTCAACAGGGTATCCTGGTAGGCTTTCTTGTCTTTCCAAGTGTTAACAGGATCCAGGATTTCTG

AAGGCACACCCTCGATCTCAGTTGGGATTTCAAGCCCAAACACTTCAGTCTTTTTGTAGTTTGCTTTCAA

GAGTCTCCCAGAATGTATGGCATCGATGATTCTTCTAGTGTATGCTAACTTCATACGGCTTCCTGAGCCA

TAGCTGCCACCTGACCAGCCGGTATTGACAAGCCAACCTGTAGCCCCATGCTTCTGCATTTTTTCAGCTA

ACATGGCTGCATACTTTGTAGGATGCAGCGTTATAAATGCTGCACCAAAGCAAGCTGAAAATGTTGCTTG

TGGCTTCCTTATTCCATCCTCTGTGCCTGCTACCAGAGCAGTATAACCACTAATGA

>FM895427.1 FM895427 Jatropha curcas embryo 71-95 (DAF) Jatropha curcas cDNA clone rjcpga0_002349, mRNA sequence

TTACTGGTAGCTATGCAGATTTCTATCTAGGGCAAACGTACACAGAGAGGATGTGGATTTAGGGGTGCAG

GCATTAGATAATTACATATATACACTTGAAAAAAGTTCAAAGCCAGCAGACATTGCTAAACCTCTAGTTG

GTTTTATCGAAATGGTTCCAGAGGCAGCAAGATTCAAATATATTGAGAAAAAAGTATTAAGTCAAATTAG

CAAAACCTTTAGGCCGGGTGGTGACATAATTAGCCTTGAGAACAACTGGGGAGACCTCTCTTATCAAATA

CAGAAATGTGTAAATGGTGTATTTCTGAAGCCAGTTCAATTACAACGTGAAAACTATACCAATATCCTAG

TGAACAATGTCACCCAAGTAGCAGGTGTCATGGGAGTCTTGTTGAATGCAGTCAATTACAAAGTCTGAAT

GGAAGAAATTATTTTCAACTACCAAAAGTGGCTGCCATGGCTTTAATCCTACTTTTGCTCTATATATAGA

GTAGCATAAATAAAGGACAACAAATTTATTATTATTGTTGCTAATGCTATATGCTATNTCCCTGTAATAT

CCTCATCTTTCCAATGTATGAATATGATGATGAATTATATATGACAAA

>FM895426.1 FM895426 Jatropha curcas embryo 71-95 (DAF) Jatropha curcas cDNA clone rjcpga0_002348, mRNA sequence

CTGCAAAGGATCTATCCGTCGCCCGATGGGAATTGTACTTTAAGTGCGGCCCTCGCGGCTCGTCCGCCGC

GGGGGCTTGGCCAACGGCACGTGCCTCTGGGGGCCGGATGGCCCCTACTGCGGGTCGGCAAACGGGCGAC

GGACACGCGCGTCGCTTCTGGCCCGGATTCTGACTTAGAGGCGTTCAGTCATAATCCAGCGCACGGTAGC

TTCGCGCCACTGGCTTTTCAACCAAGCGCGATGACCAATTGTGCGAATCAACGGTTCCTCTCGTACTAGG

TTGAATTACCATCGCGACACCATCATCAGTAGGGTAAAACTAACCTGTCTCACGACGGTCTAAACCCAGC

TCACGTTCCCTATTGGTGGGTGAACAATCCAACACTTGGTGAATTCTGCTTCACAATGATAGGAAGAGCC

GACATCGAAGGATCAAAAAGCAACGTCGCTATGAACGCTTGGCTGCCACAAGCCAGTTATCCCTGTGGTA

ACTTTTCTGACACCTCTAGCTTCAAATTCCGAAGGTCTAAAGGATCGATAGGCCACGCTTTCACGGTTCG

TA

>FM895425.1 FM895425 Jatropha curcas embryo 71-95 (DAF) Jatropha curcas cDNA clone rjcpga0_002347, mRNA sequence

GCAAAGGATTCTATCNCGTCGCCCGATGGGAATTGTACTTTAAGGCGGCCCTCGCGGCTCGTCCGCCGCG

GGGGCTTGGCCAACGGCACGTGCCTCTGGGGGCCGGATGGCCCCTACTGCGGGTCGGCAAACGGGCGACG

GACACGCGCGTCGCTTCTGGCCCGGATTCTGACTTAGAGGCGTTCAGTCATAATCCAGCGCACGGTAGCT

TCGCGCCACTGGCTTTTCAACCAAGCGCGATGACCAATTGTGCGAATCAACGGTTCCTCTCGTACTAGGT

TGAATTACCATCGCGACACCATCATCAGTAGGGTAAAACTAACCTGTCTCACGACGGTCTAACCCCAGCT

CACGTTCCCTATTGGTGGGTGAACAATCCAACACTTGGTGAATTCTGCTTCACAATGATAGGAAGAGCCG

ACATCGAAGGATCAAAAAGCAACGTCGCTATGAACGCTTGGCTGCCACAAGCCA

>FM895424.1 FM895424 Jatropha curcas embryo 71-95 (DAF) Jatropha curcas cDNA clone rjcpga0_002346, mRNA sequence

TGGTTTTTGGTGACAAGACCTGGAGATATCAGAAGTGAGAATGCTGACATGAGTAACGAGAAATCCTGTG

AAAAACACGATCGCCTGCCAGTGGAAGGCTTTCTGCGTTCAGTCAATCTACGCAGAGTGAATCGGTCCCT

AAGGAACCCCCGAAAGGGCTGCCGTCCGATGGGTACACGAAAGTGACGAAGTTGCTTTGACTACAGAACC

ATGCCTGTCTGTTGGAGCGAATTGGATGATCGGGCCGAGGACTGCCCCCTCTTCCCCTCACTCTCCTTTC

CCTAATATGAACCTTGAGTCATCAAAGCCTTTCTGACTCGGCCTGGCCCGGTCGCGAGTCTGTTTATAGT

CGCGACTGTTGTCATAGTCAACAAGGTTGAAACTTCCAGGAAAAAACTTCGAATTGGGAGGGCGATCCTC

CCGGTGAACTGACCGTACCCCAAACCGACACAGGTGAACAAGTAGAGTATACTAGGGCGCTTGAGAGAAC

CATGTCGAAGGAACTCGGCAAAATGACCCCGTAACCTCGGGAGAAGGGGTGCTCTCCTATCTTTTGATTA

GGAAAGCGGCACATACC

>FM895423.1 FM895423 Jatropha curcas embryo 71-95 (DAF) Jatropha curcas cDNA clone rjcpga0_002343, mRNA sequence

TGAAGATTTTGGTCGATGAGTGGTATAATGCTACAAAGGTTTTCAAAGGTGATGAAGGTACTCCTGACTC

TGTTAATCCATCTGTTGTTGATGATGAGGAAGAAGAAGGGCTTCCATCTCCTCCATTAGATGAAGCTTTC

TTTGCCACACAAACTACTGGAATAGAGCTCTCACAGTTCTTTGATGGCATGGATGACTTTGGAAATCCTC

GGAATTGTGGGGAATTCATCAAGAACCGTGAAAATGGAAGAAAACCATCGGTGGAGAGTCGAAATATCAC

AAAACAAAAACTGCAGACTCCTAATGAGGTAAATGTCATCGCCAAGGATAATAAGAGTCAACAAATGAGG

AGGCAAGAAGCTGTCCCGAAGCTAAATAAGCCCTCACACACTGATTCTGGTCCTGGTAGACCTCCAAGAC

AAAGTGCCGAGCAAAAAACAAAAACTGATCCAAAGATACATCGAAAAATGGATAAGGTTACCAACCTGAG

AAAGCCTCCAAGTGGTCAACAAGATAAATTGAAGTGTTCAGATGAAGTTGC

>FM895422.1 FM895422 Jatropha curcas embryo 71-95 (DAF) Jatropha curcas cDNA clone rjcpga0_002342, mRNA sequence

GTTCCACACGAGATTTCTGTTCTCGTTGAGCTCATCTTAGGACACCTGCGTTATCTTTTAACAGATGTGC

CGCCCCAGCCAAACTCCCCACCTGACAATGTCTTCCGCCCGGATCGGCCGCCGAAACGACCTTGGGTCCA

AAAAGAGGGGCATAGCCCCGCCTCCGATTCACGGAATAAGTAAAATAACGTTAAAAGTAGTGGTATTTCA

CCGTCGCCTTTCGGCTCCCACTTATCCTACACCTCTCAAGTCATTTCACAAAGTCGGACTAGAGTCAAGC

TCAACAGGGTCTTCTTTCCCCGCTGATTCCGCCAAGCCCGTTCCCTTGGCTGTGGTTTCGCTGGATAGTA

GACAGGGACAGTGGGAATCTCGTTAATCCATTCATGCGCGTCACTAATTAGATGACGAGGCATTTGGCTA

CCTTAAGAGAGTCATAGTTACTCCCGCCGTTTACCCGCGCTTGGTTGAATTTCTTCACTTTGACATTCAG

AGCACTGGGCAGAAATCACATTGCGTGAGCATC

>FM895421.1 FM895421 Jatropha curcas embryo 71-95 (DAF) Jatropha curcas cDNA clone rjcpga0_002341, mRNA sequence

TTTTTGCAATACGAAAGAAAAATCTTTTTAACTGAAAAAAAATTTTCATATTCTAAAAATGGTAGAATAA

CATCTTAAACATTTTTATGGTGTTGCCTAACTCTACAAGAGAAGCCAATAAAACCATACGGCTTAAAAAG

AGCAATCTACTAAAAGTATCCAAGGGTGCTGATCACCACAAGAAGTCTCACTCTTAAGGTGAAGCATGTC

CACCGTAGAGGGGAATGCGCCCAAGTTATAACCTACTGGGATAGAACAGACCTAGCAGCAGATTCCAAAA

TTCTCCTTGCGTCTGTGTCTGGATCTGGTAGCTCACTTGGGTTCTTTCCGTATAGGTTTGTTGTTGTCAC

AGAAGCTCCAGAAGCCAGAAGTAATCTTATAACATCCACGTGTTCACCTCTTGCTGCATGGTGAAGAGGA

GTATCACCCTCAGCATCAACTGTTTCTAACATTCTTCTGACAGTCTCTGTGCTATTGGCACTATTCAGCA

GCAACTGGACTATCTCTAGAAAACCCCCTGCACAAGCATCATGCAAAGGAATTGCCCCATCTT

>FM895420.1 FM895420 Jatropha curcas embryo 71-95 (DAF) Jatropha curcas cDNA clone rjcpga0_002340, mRNA sequence

ATTAATTTTCCATTTTATTCCAGCCGCAGCTATGATTATCATGNACATATATTCTTTATAACTTCACCAT

CACTCCATAAGTAACACAAGATCAAACCAGAAAATGACATTATCAGATGGTATGGGGACATTGTTCGTAT

GATGTGATGACTCGTCTAATCATATCCTTCGGCCCTTTGATCATTAAGCAGGAAAAACTTATGATAATTA

TACATAATGATACAGGTTAATGACTTGCAGATAGCAAACCTCATTTGAGGGACATTGATTTGTACTTCTG

CATTGTGCTTGTTATAGCTTGTTCGACAGGCAGTCTCTCCATGCTTGATGCCCCGTAAAATCCATGAACT

CCCTTTGTTCTCTTCAGTATAAATTCCGCCTCTGTAGGACCNGATATAGGACCTCCATGGCAAAGCACAA

TTACACTGGGGTTGATGTTATGTGCAGCGTCTGCAATTGCCTGCACACGAATTACGCT

>FM895419.1 FM895419 Jatropha curcas embryo 71-95 (DAF) Jatropha curcas cDNA clone rjcpga0_002338, mRNA sequence

CAAAGGATTCTATCCGTCGCCCGATGGGAATTGTACTTTAAGGCGGCCCTCGCGGCTCGTCCGCCGCGGG

GGCTTGGCCAACGGCACGTGCCTCTGGGGGCCGGATGGCCCCTACTGCGGGTCGGCAAACGGGCGACGGA

CACGCGCGTCGCTTCTGGCCCGGATTCTGACTTAGAGGCGTTCAGTCATAATCCAGCGCACGGTAGCTTC

GCGCCACTGGCTTTTCAACCAAGCGCGATGACCAATTGTGCGAATCAACGGTTCCTCTCGTACTAGGTTG

AATTACCATCGCGACACCATCATCAGTAGGGTAAAACTAACCTGTCTCACGACGGTCTAAACCCAGCTCA

CGTTCCCTATTGGTGGGTGAACAATCCAACACTTGGTGAATTCTGCTTCACAATGATAGGAAGAGCCGAC

ATCGAAGGATCAAAAAGCAACGTCGCTATGAACGCTTGGCTGCCACAAGCCAGTTATCCCTGTGGTAACT

TTTCTGACACCTCTAGCTTCAAATTCCGAAGGTCTAAAGGATCGATAGGCCACGCTTTCACGGTTCGTAT

TCGTACTGGAAATCAGAATCAAACGAGCTTTTACCCTTTTGTTCCACACGAGATTTCTGTTCTCGTTGAG

CTCATCTTAGGACACCTGCGTAATCTTTTAACAGATGTGCCGCCCCAGCCAACTCCC

>FM895418.1 FM895418 Jatropha curcas embryo 71-95 (DAF) Jatropha curcas cDNA clone rjcpga0_002337, mRNA sequence

AGGATTCTATCCGTCGCCCGATAGGAATTGTACTTTAAGGCGGCCCTCGCGGCTCGTCCGCCGCGGGGGC

TTGGCCAACGGCACGTGCCTCTGGGGGCCGGATGGCCCCTACTGCGGGTCGGCAAACGGGCGACGGACAC

GCGCGTCGCTTCTGGCCCGGATTCTGACTTAGAGGCGTTCAGTCATAATCCAGCGCACGGTAGCTTCGCG

CCACTGGCTTTTCAACCAAGCGCGATGACCAATTGTGCGAATCAACGGTTCCTCTCGTACTAGGTTGAAT

TACCATCGCGACACCATCATCAGTAGGGTAAAACTAACCTGTCTCACGAACGGTCTAACCCCAGCTCACG

TTCCCTATTGGTGGGTGAACAATCCAACACTTGGTGAATTCTGCTTCACAATGATAGGAAGAGCCGACAT

CGAAGGATCAAAAAGCAACGTCGCTATGAACGCTTG

>FM895417.1 FM895417 Jatropha curcas embryo 71-95 (DAF) Jatropha curcas cDNA clone rjcpga0_002336, mRNA sequence

GGATTCTATCCGTCGCCCGATGGGAATTGTACTTTAAGGCGGCCCTCGCGGCTCGTCCGCCGCGGGGGCT

TGGCCAACGGCACGTGCCTCTGGGGGCCGGATGGCCCCTACTGCGGGTCGGCAAACGGGCGACGGACACG

CGCGTCGCTTCTGGCCCGGATTCTGACTTAGAGGCGTTCAGTCATAATCCAGCGCACGGTAGCTTCGCGC

CACTGGCTTTTCAACCAAGCGCGATGACCAATTGTGCGAATCAACGGTTCCTCTCGTACTAGGTTGAATT

ACCATCGCGACACCATCATCAGTAGGGTAAAACTAACCTGTCTCACGACGGTCTAATCCCAGCTCACGTT

CCCTATTGGTGGGTGAACAATCCAACACTTGGTGAATTCTGCTTCACAATGATAGGAAGAGCCGACATCG

AAGGATCAAAAAGCAACGTCGCTATGAACGCTTGGCTGCCACAAGCCAGTTATCCCTGTGGTAACTTTTC

TGACACCTCTAGCTTCAAATTCCGAAGGTCTAAAGGATCGATAGGCCACGCTTTCACGGTTCGTATTCGT

ACTGGAAATCAG

>FM895416.1 FM895416 Jatropha curcas embryo 71-95 (DAF) Jatropha curcas cDNA clone rjcpga0_002334, mRNA sequence

ATTCAAATACATTGGGGAAAGTGCTAGGTTAATACGAGAAATGTTTGGATATGCTCGCGATCACCAACCT

TGTATCATTTTCATGGATGAGATTGATGCAATTGGTGGACGCCGTTTTAGTGAAGGAACAAGTGCTGATC

GTGAAATTCAAAGAACGCTGATGGAATTGCTTAATCAGCTAGATGGATTTGATCAGCTCGGGAAGGTTAA

AATGATAATGGCAACCAACAGGCCTGATGTTCTTGATCCTGCACTTCTTCGTCCGGGACGATTAGACAGA

AAGATAGAGATTCCATTGCCAAATGAACAATCAAGAATGGAAATTCTTAAAATTCATGCTGCTGGCATTG

CCAAGCATGGAGAGATTGATTATGAGGCCGTTGTAAAACTTGCAGAGGGTTTTAATGGAGCTGACCTTCG

CAATGTTTGCACGGAAGCTGGAATGTCAGCAATCCGTGCAGAACGTGATTATGTCATACATGAAGACTTC

ATGAAGGCTGTAAGGAAGCTTAATGAGGCA

>FM895415.1 FM895415 Jatropha curcas embryo 71-95 (DAF) Jatropha curcas cDNA clone rjcpga0_002333, mRNA sequence

TTTTTTTTTNTGGAAAAATATCAGATTTCATTCACATAAAAGAAAGGCCATAAACCTAAAGTACCAACTA

AACTAAAAATAAAGACAGTAGCCAATAGACAATACTACACAGTCACCACCCTTAAGCACGCTCGCCTCTA

ATTCTCCTCGCGAGCTGAATATCCTTCCTGGCTTAGATGATACGACCACTGCATGTGCTGTACAAAGCTC

TGATGCAAATGGGATTAGCGTACCTGGAAAAGCTCTTCCTGGACAGCCTATATATCCACCAGTAACAACA

GCTGGAGCTTCTTCAGAGAATGCTTCCCTTCGCTCTAAGACCAAGTGGCCAGAGCAATCGGAAGAGCTCT

TAGGGTTGATTGTGAACTCACTGAGAGCCCTTGATGGAGCTGTTCCACAACGTTGTCCTGAGCCGAGACG

AAGGCCTCAATCTGCACAGAAGATTGCTCTTGTATTGGATAAAGCACCTAGACATCTGCAGCCAGACCTA

GTAGCTTTAGTACCCAAGTTGGTTGAACATTCAGAGCATCCACTGGCAGCTTGTGCACTTCTTGACAGAC

TTAGGAAAC

>FM895414.1 FM895414 Jatropha curcas embryo 71-95 (DAF) Jatropha curcas cDNA clone rjcpga0_002332, mRNA sequence

ATTGTCTGACATGTGTGCGAGTCAACGGGTGAGTAAACCCGCAAGGCGCAAGGAAGCTGACTGGCGGGAT

CCCCCCGAGGGTTGCACCGCCGACCGACCTTGATCTTCTGAGAAGGGTTCGAGTGAGAGCATGCCTGTCG

GGACCCGAAAGATGGTGAACTATGCCTGAGCGGGGCGAAGCCAGAGGAAACTCTGGTGGAGGCCCGCAGC

GATACTGACGTGCAAATCGTTCGTCTGACTTGGGTATAGGGGCGAAAGACTAATCGAACCGTCTAGTAGC

TGGTTCCCTCCGAAGTTTCCCTCAGGATAGCTGGAGCTCGGGACGAGTTCTATCGGGTAAAGCCAATGAT

TAGAGGCATCGGGGGCGCAACGCCCTCGACCTATTCTCAAACTTTAAATAGGTAGGACGGCGCGGCTGCT

TCGCTGAGCCGCGCCACGGAATCGAGAGCTCCAAGTGGGCCATTTTTGGTAAGCAGAACTGGCGATGCGG

GATGAACCGGAAGCCGGGTTACGGTGCCCAACTGCGCGCTAACCTAGAACCCACAAAGGGTGTTGGTCGA

TTAAGACAGCAGGACGGTGGTCATGGAAGTCGAAATCCGCTAAGGAGTGTGTAACAACTCAACTGCC

>FM895413.1 FM895413 Jatropha curcas embryo 71-95 (DAF) Jatropha curcas cDNA clone rjcpga0_002331, mRNA sequence

ATTCAAAGGATTCTATCCGTCGCCCGATGGGAATTGTACTTTAAGGCGGCCCTCGCGGCGCGTCCGCACG

CGTGGGGCTTAGCCAACGGCACGTGCCTCTGGGGGCCTGGATGGCCCCTACTGCAGGTCGGCAAACGGGC

GACGGACACGCGCGTCGCTTCTGGCCCGGAGTTCTGACTTAGAGGCGTTCAGTCATAATCCAGCGCACGG

TATCTTCGCGCCACTGGCTTTTCAAGCCAAGGCGCGATGACCAATTAGTAGCGAATCAACGGTTCCTCTC

GTACTAGGTTGACATTACCGATCGCGACACCATCATCAGTAGGGTAAAACTAACCTGTCTCACGACGGTC

TAATCCCAGCTCACGTTCCCTATTTGGTGGGTGAACGATCCAACACTTGGTGAATTCTGCTTCACGATGA

TAGGAAGAGCCGACATCGAAGGATCAGACAGCAGCGTCGCTATGAACGCTTGGCTGCCACAAGACCAGTT

ATCCCTGTGGTAACTTTTCTGACACCTCTAGCTTCAAATTCCGAAGGTCTAAAGGATCGATAGGCCACGC

TTTCACG

>FM895412.1 FM895412 Jatropha curcas embryo 71-95 (DAF) Jatropha curcas cDNA clone rjcpga0_002330, mRNA sequence

CTCAAAAACAGCAGATCCTCCATTCTCTTTGCCGATCCATAATCAAAACAATGAAGGTCATCGCTGCATA

TTTGCTCGCCGTTTTGGGAGGTAACACCTCACCCTCCGCCGAGGACCTGAAGGAAATCCTCGGATCCGTT

GGAGCTGATGCTGATGATGATAAGATTCAGTTATTGTTGTCCCAAGTTGAAGGAAAGGATATCACAGAAT

TGGTTGCAGCAGGAAGGGAGAAATTGGCATCTGTGCCTTCTGGTGGTGGTGTAGCTGTTGCTGCTGCTGC

AGCACCTGCTGCTGGAGGTGGTGGTGCTGCTCCTGCTGCTGAGGCCAAAAAGGAGGAGAAGGTTGAGGAG

AAAGAGGAGTCAGATGATGATATGGGTTTCAGCCTCTTCGACTAGGAGATCTATTTGCTGGTTCCATTTG

TATCAAGGCTTTCTTTTATCTTCTAGTCTTTTAGTTAAAAAATTTACAAGTTTTTGGTCTGTAGTTGGTT

ATGATATTTAGAACTTGTTTGCGTTCTTGAATTTTGAGTTAGGTAATGGAGATTAAACCATTTCACATAT

AGTTGTTAATTTTGTGAT

>FM895411.1 FM895411 Jatropha curcas embryo 71-95 (DAF) Jatropha curcas cDNA clone rjcpga0_002329, mRNA sequence

CAAAGGATTCTATCCGTCGCCCGATGGGAATTGTACTTTAAGTGCGGCCCTCGCGGCTCGTCCGCCGCGG

GGGCTTGGCCAACGGCACGTGCCTCTGGGGGCCGGATGGCCCCTACTGCGGGTCGGCAAACGGGCGACGG

ACACGCGCGTCGCTTCTGGCCCGGATTCTGACTTAGAGGCGTTCAGTCATAATCCAGCGCACGGTAGCTT

CGCGCCACTGGCTTTTCAACCAAGCGCGATGACCAATTGTGCGAATCAACGGTTCCTCTCGTACTAGGTT

GAATTACCATCGCGACACCATCATCAGTAGGGTAAAACTAACCTGTCTCACGACGGTCTAAACCCAGCTC

ACGTTCCCTATTGGTGGGTGAACAATCCAACACTTGGTGAATTCTGCTTCACAATGATAGGAAGAGCCGA

CATCGAAGGATCAAAAAGCAACGTCGCTATGAACGCTTGGCTGCCACAAGCCAGTTATCCCTGTGGTAAC

TTTTCTGACACCTCTAGCTTCAAATTCCGAAGGTCTAAAGGATCGATAGGCCACGCTTTCACGGTTCGTA

TTCGTACTGGAAATCA

>FM895410.1 FM895410 Jatropha curcas embryo 71-95 (DAF) Jatropha curcas cDNA clone rjcpga0_002328, mRNA sequence

ATGTAGCTATCTTTCTTCTTTCTATTATGTCAGGAACTACCATACAATCAACAATTTGCATGACATCATT

TATTTTAACACTATCAGCTTACATAATTAATGCAAAATAGCATATGAACCGTGATTTTACTTCATGTCAG

CTAATTCTCTTTTCCTTCAGGCATTATCCCAAGCCGACAGCCTCACTTTGGTCGCTTTCGGTTTGGGAAC

ATAATATCATCCATGCTTTCTTTTATACGATTCCCCACTTTTTGTATGTTTCTCGCCACCCCTGCTGCAA

CCAGGCTTGCATTCCTTGTATATCTCCTCAGGAAATCATCTCCTGCAGAATCGGCTGGTTTACGTGTTTG

AGCCTGAGGACGCTCTCGGCCATCTTCTTTACCATGAACACGAGCGGGAGCAGATGCACTATCAGCAATA

TGAACTCTTTTTCTATGATCCAATAAAACTGCAGCCCTTTTGTCCGCAATAAACTCAAGGCCAACACAAA

GTGGCAGTTGAGATGTTGTCACTTGCTTATCCATAGACTGATCTTTAATGAGATATACCGAGTTCCCAGC

ACAAGCATAATAAAAATCCTTAGGACCCAATCCCCTTGCAGAAGATACAATCTCATAATCTTTG

>FM895409.1 FM895409 Jatropha curcas embryo 71-95 (DAF) Jatropha curcas cDNA clone rjcpga0_002327, mRNA sequence

TGAGTCTGACATGTGTGCGAGTCAACGGGTGAGTAAACCCGCAAGGCGCAAGGAAGCTGACTGGCGGGAT

CCCCCCGAGGTTGCACCGCCGACCGACCTTGATCTTCTGAGAAGGGTTCGAGTGAGAGCATGCCTGTCGG

GACCCGAAAGATGGTGAACTATGCCTGAGCGGGGCGAAGCCAGAGGAAACTCTGGTGGAGGCCCGCAGCG

ATACTGACGTGCAAATCGTTCGTCTGACTTGGGTATAGGGGCGAAAGACTAATCGAACCGTCTAGTAGCT

GGTTCCCTCCGAAGTTTCCCTCAGGATAGCTGGAGCTCGGGACGAGTTCTATCGGGTAAAGCCAATGATT

AGAGGCATCGGGGGCGCAACGCCCTCGACCTATTCTCAAACTTTAAATAGGTAGGACGGCGCGGCTGCTT

CGCTGAGCCGCGCCACGGAATCGAGAGCTCCAAGTGGGCCATTTTTGGTAAGCAGAACTGGCGATGCGGG

ATGAACCGGAAGCCGGGTTACGGTGCCCAACTGCGCGCTAACCTAGAACCCACAAAGGGTGTTGGTCGAT

TAAGACAGCAGGACGGTGGTCATGGA

>FM895408.1 FM895408 Jatropha curcas embryo 71-95 (DAF) Jatropha curcas cDNA clone rjcpga0_002326, mRNA sequence

CTATTGGAAAGCCATTAGCTGGATTATTGCTTATTTGTAGTTTGCCCCCAACAAGAATGAACAGAACAAA

GCCCCCAACAAGAATGAACACAACAAAGCCCCCAACAAGAATGAACACAACAAAGGTACATATACATACA

TATATGTATGTATATATGCATATACCGAAACTAACCAACTTAGTATGACCAGCACCAGAAGGCATGATCT

TCAAAACTTTCAGGATTGGAGTAATCAGCATCTTCAAGCTTTGGAAAGACATATGGAGGCAATAAGCTCT

TGCTTAAATTATCTTCTTCATCTTGCGATAAATCCGACTGATCTGGTTCAAAAACATATGATGAATCACC

AGCTTCTAAAAGTGAAGAGTGAACTCCATCAGTGTAATGTGGGCTATCTGAATCGAATATATCGCTTTTA

GCCGAGCTCATATCTTCCTGTTTGCAGGCCACAAATGAAGCTTTAGATGCTTCACCCTCAGACGTTGAGT

CACCAACTAGCGTTAAAGGTGGTTCTTGAGATGTAGTGTCTTTATCAGACAATTCTGAGTCCCCCCTCTC

TTTCACCTTCTCTGCAAGTACATTAACCTCAGCT

>FM895407.1 FM895407 Jatropha curcas embryo 71-95 (DAF) Jatropha curcas cDNA clone rjcpga0_002323, mRNA sequence

GAGAGATAGCTTGCACTTACGCTACTTTGATCCTCCACGACGACGGGATCGCCGTCACTGCGGAGAAGAT

CGCTACACTGGTGAAGGCAGCTAACGTGTCGGTTGAATCATACTGGCCAAGCCTTTTCGCTAAGTTGGCA

GAGAAGAGGAAGCTCGACGATCTTATCATGAATGTTGGTTCTGGTGGTGGCGGTGGTGCTGTTGCCGTTG

CTGCTCCCGCTGCAGGTGCTGGTGCCACCGCTGCCGCTGCTGCTCCTGCTGTTGAGGAGAAGAAGGAGGA

ACCCAAGGAAGAGAGTGATGATGACATGGGATTCAGCTTGTTTGATTAGGTGCTTTCGCCGTTTTTAGTG

TCTTTTATGTATTTTAGTTGTTTTGCGAATTAGACAAAAATTAGGATTCATCTTTTTTTGGTAATTCAAA

CGCCCATTTGGGAACTAATTCAAGCGGTTTTATTTTGGAATTGATTTTGGAGAACCAATACAAGGGGT

>FM895406.1 FM895406 Jatropha curcas embryo 71-95 (DAF) Jatropha curcas cDNA clone rjcpga0_002322, mRNA sequence

ATTCCTGGAAATCAGAATCAAACGAGCTTTTACCCTTTTGTTCCACACGAGATTTCTGTTCTCGTTGAGC

TCATCTTAGGACACCTGCGTTATCTTTTAACAGATGTGCCGCCCCAGCCAAACTCCCCACCTGACAATGT

CTTCCGCCCGGATCGGCCGCCGAAACGACCTTGGGTCCAAAAAGAGGGGCATAGCCCCGCCTCCGATTCA

CGGAATAAGTAAAATAACGTTAAAAGTAGTGGTATTTCACCGTCGCCTTTCGGCTCCCACTTATCCTACA

CCTCTCAAGTCATTTCACAAAGTCGGACTAGAGTCAAGCTCAACAGGGTCTTCTTTCCCCGCTGATTCCG

CCAAGCCCGTTCCCTTGGCTGTGGTTTCGCTGGATAGTAGACAGGGACAGTGGGAATCTCGTTAATCCAT

TCATGCGCGTCACTAATTAGATGACGAGGCATTTGGCTACCTTAAGAGAGTCCTAGTTACTCCCGCCGTT

TAACCGCGCCTGGTTGAATTTCCTCAATTTTGACTTCCAAAGCACTGGGCAGAATTCACATTGCGTGAGC

ATCCGCAGGGACCATCGCAATGCTTTGTTTTAATTAAACAGTCGGAT

>FM895405.1 FM895405 Jatropha curcas embryo 71-95 (DAF) Jatropha curcas cDNA clone rjcpga0_002321, mRNA sequence

TGGAAATCAGAATCAAACGAGCTTTTACCCTTTTGTTCCACACGAGATTTCTGTTCTCGTTGAGCTCATC

TTAGGACACCTGCGTTATCTTTTAACAGATGTGCCGCCCCAGCCAAACTCCCCACCTGACAATGTCTTCC

GCCCGGATCGGCCGCCGAAACGACCTTGGGTCCAAAAAGAGGGGCATAGCCCCGCCTCCGATTCACGGAA

TAAGTAAAATAACGTTAAAAGTAGTGGTATTTCACCGTCGCCTTTCGGCTCCCACTTATCCTACACCTCT

CAAGTCATTTCACAAAGTCGGACTAGAGTCAAGCTCAACAGGGTCTTCTTTCCCCGCTGATTCCGCCAAG

CCCGTTCCCTTGGCTGTGGTTTCGCTGGATAGTAGACAGGGACAGTGGGAATCTCGTTAATCCATTCATG

CGCGTCACTAATTAGATGACGAGGCATTTGGCTACCTTAAGAGAGTCATAGTTACTCCCGCCGTTTACCC

GCGCTTGGTTGAATTTCTTCACTTTGACATTCAGAGCACTGGGCAGAAATCACATTGCGTGAGCATCCGC

AGGGACCATCGCAATGCTTTGTTTTAATTAAACAGTCGGATTCCCCTTGTCCGTACCAGTTCTGAGTCGA

CTGTTCGACGCCCGGGGAAGGCCCCGGAATGGCCGTTCCCAGTCCGTCCCC

>FM895404.1 FM895404 Jatropha curcas embryo 71-95 (DAF) Jatropha curcas cDNA clone rjcpga0_002320, mRNA sequence

ATTCCGGCGATCGTGGACCACAGCGCGCGCCTTCGCGGCGTGCCCCGGCACCTGCGCGCTCCAGGCGTCG

GCCAGCGGGCTCCCCATTCGGCCCGTCTTGAAACACGGACCAAGGAGTCTGACATGTGTGCGAGTCAACG

GGTGAGTAAACCCGCAAGGCGCAAGGAAGCTGACTGGCGGGATCCCCCCGAGGGTTGCACCGCCGACCGA

CCTTGATCTTCTGAGAAGGGTTCGAGTGAGAGCATGCCTGTCGGGACCCGATAGATGGTGAACTATGCCT

GAGCGGGGCGAAGCCAGAGGAAACTCTGGTGGAGGCCCGCAGCGATACT

>FM895403.1 FM895403 Jatropha curcas embryo 71-95 (DAF) Jatropha curcas cDNA clone rjcpga0_002319, mRNA sequence

GAACGAAAGAAGGTAAATACTGGGATTCACCAATATTCCTGATTTGTACAAATGATTCTGAAAATCGAAT

TACATTTAAAAGAATGGAAGTTTTAACTGCCGAACAACTGATAAGAAAAAGCAAATCATAAACAAATAAT

TTTCTTATCTAAGCATCGAGAGAACTAGTTTTACATGCCACAACGAACATGGTTAACATCACAAGCCGAC

TAAAATCGATTCCCATTTAAGATGAGCAGCAACCAGACTTCTGATTGACTGGCTGTCCTCGAATTTGCAC

AGTTGGCGGCCTTGCATTGTTCATTGCTGGTTGGCTAGCCATCCTGTTCTTGATTTCAGCAGCCATAGCC

ATGAAAGCCTGCTCAACGTTTGTGGAGTTTTTAGCACTTGTTTCCATGAAAGGGATTCCAATTTCATCAG

CAAATGCCTTTGCAGTCTCATACGAGACCACTTTGTTTGCTGTGAGGTCGCACTTGTTACCAACTAGAAG

CTTGTTCACATTTTCACTTGCATAACGGTCAATTTCACTCAACCATTGTTTGACGTTGTTAAAGCTCTCT

TGAT

>FM895402.1 FM895402 Jatropha curcas embryo 71-95 (DAF) Jatropha curcas cDNA clone rjcpga0_002318, mRNA sequence

AGATTGTAATTCTTTTCAGACATATATATATATATATCCAAGAGGCACGAATAATGTTTGACTCGAAGAG

GTAAAAGCACCATGGATTATAACAACAACAAAATGCCAATACAAGGTACAATAGCTAAAAACAGGTTACC

CTCCAATCCTTGGTCCTTCACAACTATATCACTACAACAAGCTCCTAATAGTAACAATAGTAATATCAAA

CGGGTGTACACCTACAGCTCTGTCATGTTTTATTATGGTGAAACTGGAACTGGGTCAGTACCAGAAATGT

GAGAAGCCGCATCCTCTTTCGATATGGCAACATAGTTAACAGGGGCAGCTGGTCTGCTAGTACTATCTCT

CCTCCCCTCTTTAGTGCTGGACCTGCTGCCATTTTGATGAGCATTCAGGGAAAGCCGTCTGCTTGGAGTA

CCATTGGCACCTCCATTTGTACGAGGACCGACCACCTTTTTAGTACCAACTGGTCGTGCAGGGCTGGGTC

TTGAACCAAAGATAGCTTCTTGTTCTGTGCTTTGTTGCTCGTGGAACTTCTTCTGATCTCTTAGTCTTCG

CTTCTCTTCTTCCCTTTCCTGCCTGAGCATCGCATATTCATCTAACATGGCCAGGAGAGGACCACCATCA

T

>FM895401.1 FM895401 Jatropha curcas embryo 71-95 (DAF) Jatropha curcas cDNA clone rjcpga0_002317, mRNA sequence

ATTCGTCTGACATGTGTGCGAGTCAACGGGTGAGTAAACCCGCAAGGCGCAAGGAAGCTGACTGGCGGGA

TCCCCCCGAGGGTTGCACCGCCGACCGACCTTGATCTTCTGAGAAGGGTTCGAGTGAGAGCATGCCTGTC

GGGACCCGAAAGATGGTGAACTATGCCTGAGCGGGGCGAAGCCAGAGGAAACTCTGGTGGAGGCCCGCAG

CGATACTGACGTGCAAATCGTTCGTCTGACTTGGGTATAGGGGCGAAAGACTAATCGAACCGTCTAGTAG

CTGGTTCCCTCCGAAGTTTCCCTCAGGATAGCTGGAGCTCGGGACGAGTTCTATCGGGTAAAGCCAATGA

TTAGAGGCATCGGGGGCGCAACGCCCTCGACCTATTCTCAAACTTTAAATAGGTAGGACGGCGCGGCTGC

TTCGCTGAGCCGCGCCACGGAATCGAGAGCTCCAAGTGGGCCATTTTTGGTAAGCAGAACTGGCGATGCG

GGATGAACCGGAAGCCGGGTTACCGTGCCCAACTGCGCGCTAGCCTAGAACCCACAAAGGGTG

>FM895400.1 FM895400 Jatropha curcas embryo 71-95 (DAF) Jatropha curcas cDNA clone rjcpga0_002316, mRNA sequence

ATTGTACAAAGTGGTGTTAATGACAACTGAATACATAAACAAGCTAATATAATTCACACAATACAAGAAT

TTTATGACAGGATAGTACAAAATCAACAATATATCCTTAAGTGAATGTGCCATCAATCACTATGAGATTG

CAAAGTAAAACACATGCACTTATCTTTCATAGCTTCAGACTCACTTCCATACTCTTTGAATTTGTACTCA

TCCTGCTTAAAAGCAGGAGGCACCTTTCCTTCACTATAGCACTGATTACATAGACTAAAATGTGATTTCA

TCTCCTTAAAGCGGGAACCAATAATTGGGTAGCGGCAGACTGAGCAGACGTGGTTTCCATGGCGATTCCT

ATCTCCAGTTTCAAGCTTTACTAGAGTTGACATGATACCAAAACCCCAATCAGGATCATCAAACATCACA

AGAAAATCATTGAAAGATACCATTGAAGAGTCCGTTTCCCCATCAACTTCTAACATTTCACTAAGACCAT

GAGAAGGAACATAAATAGCTCTCAGCAACTTTATATAACCAACAGCATCAACCTTTTTAATCTGAGAACT

ACCTTCATTCGCAGGACGCCACAAAAGTGCATCAAAAGCTATCCGTTTCCG

>FM895399.1 FM895399 Jatropha curcas embryo 71-95 (DAF) Jatropha curcas cDNA clone rjcpga0_002315, mRNA sequence

ATTCTCAACAAGAACCACCCGCCAAAAAACTGCAATTCTCACAATAATCTTCTTTACCTTAATAACAGTA

CTCTTTGCTTCACTCTTCAGGAACTTCTCTTTTGGTTCCAAAGGAACACAAGTTGATATAACTTCCTCTG

AAACTCTAAATCCACATATTATAGTCAAGAATGCCTGTACCAGAACTCTTTACCCTTCTTTGTGCTACAC

TACCCTCTCCTTCATTTCTAATTCCAGGAATTCCACTACTTTCCACTACATTCTTGAATTAACAATGGTA

AAAGCCAAGAAATCCGTAGAGAGCACAAGATTTCACCAGATGGGCCTATTCGAATTCCAAGATTTGATTC

CTCAAGAAAAGAGAGCGCTTAAAGATTGCGTGGAGATGTTGGATCAAACTCTTTACGAACTTAGACTAGC

AATTAAATATTTGCGTTGTTTCCCTGCAGCCATTGATCATCGTCTTCATTTATTGTACGCTAATCTCAAA

ACCCTACTTAGTGCAGCCATGACAAATCAGAACACTTGCATTGATGGGTTTTCTGATTTAGATGAATCTT

CTTTTAAAAATCAGAAGGGC

>FM895398.1 FM895398 Jatropha curcas embryo 71-95 (DAF) Jatropha curcas cDNA clone rjcpga0_002314, mRNA sequence

TCTCTCTCTCTTTTCCTCTCCAAGTCCTCTCTTTCTTCGTTTCTCTCTTTGGTCTCATCGATGATGGAGA

TAGAAGCTACAAGAAGCTCTCTGTATCTGAATTTTTTTTATCTTACACTACAGCCTGTATCAGCTTTTGA

TCTTTTTGTTCTAGATCAAGTATGAGTAGAAGCTTATAGCGTTGGATCTGTTATTTTATGAGTTAAATAA

GCTGTTCTTCGCGTCAAATTCGATAATCGCTAATTGAAGCATCTGTGGATCCCTGGAGAGATTGGAAAAA

TCATCATCTGTTCCGACTGCATCTAGTATAGAAACTAAACCTAAACCAAAAACATGAATCATCTAACTGT

TGAGACTGAGGATTCTTTTTCTAGTTTACTTGAATTTGCTGCTAACAATGATGTTGAAGGCTTTAAGCAC

TTGATTGAGCGGGATGCTTCCTCTATTGATGAGGTTGGGTACTGGTATGGTCGCCAAAAGGGATCTAAGA

ATATTGTTCTTGACCACCGAACTCCACTAATGGTTGCTGCTACATATGGCAGTCTTGATGTTCTTAAGTT

TATACTCACTCACACAGATGCAGATGTTAATCTCTTATGTGGGATGGA

>FM895397.1 FM895397 Jatropha curcas embryo 71-95 (DAF) Jatropha curcas cDNA clone rjcpga0_002313, mRNA sequence

CCACAGCCACAGGAACGGGCTTGGCGGAATCAGCGGGGAAAGAAGACCCTGTTGAGCTTGACTCTAGTCC

GACTTTGTGAAATGACTTGAGAGGTGTAGGATAAGTGGGAGCCGAAAGGCGACGGTGAAATACCACTACT

TTTAACGTTATTTTACTTATTCCGTGAATCGGAGGCGGGGCTATGCCCCTCTTTTTGGACCCAAGGTCGT

TTCGGCGGCCGATCCGGGCGGAAGACATTGTCAGGTGGGGAGTTTGGCTGGGGCGGCACATCTGTTAAAA

GATAACGCAGGTGTCCTAAGATGAGCTCAACGAGAACAGAAATCTCGTGTGGAACAAAAGGGTAAAAGCT

CGTTTGATTCTGATTTCCAGTACGAATACGAACCGTGAAAGCGTGGCCTATCGATCCTTTAGACCTTCGG

AATTTGAAGCTAGAGGTGTCAGAAAAGTTACCACAGGGATAACTGGCTTGTGGCAGCCAAGCGTTCATAG

CGACGTTGCTTTTTGATCCTTCGATGTCGGCTCTTCCTATCATTGTGAAGCAGAATTCACCAAGTGTAGG

ATTGTTCACCCCACCAATAGGGAACGTGAGCTGGGGTTAGACCGTCGTGAG

>FM895396.1 FM895396 Jatropha curcas embryo 71-95 (DAF) Jatropha curcas cDNA clone rjcpga0_002312, mRNA sequence

TCCGGCTCTCGATTCGATTCCGCTTCTGCTTGGTGGGGTTCACTTCTTTTGCAGATACACAAGTTTTCCG

ATTTCCCTTATCTCAGGTTAATTGCAACACTTTTGGTAGCACACTTGTAACAAGAAAATACCTTCCCTAG

GTATGGAAACTGAGGATATGATTGGCCTCCCTGCTTCCAGCAATTCTAGAGATAGCAGTGAGAGCGGTGA

AATTCACAAGTCTGGATTTGGGTCAGGTGAAGCTGATTCTCAGCCTAGGAATTTTGAAGCTGAAGAAGAT

GTGGGTGATGGAGATAGTTTGAGGCTCAATAATGACGTTGATATTGATACAGAAAAAGGAATAAGAGGCC

CTTGATTGTTTGGCGCTTGTCGAAGACGATACTGGAGCAGAAAAAATATTAGGAGATTCTGAGGTTTTGG

ACAGCAATGAAGATGATATTGGAACAGAAGAACATGTGACAAGATTAGAGAACGAAATATTAAGTTATTG

ATGCATTGAAATATATAATTTTCAACTAACATTTTATCCATCAAAGCATAATGGATTGG

>FM895395.1 FM895395 Jatropha curcas embryo 71-95 (DAF) Jatropha curcas cDNA clone rjcpga0_002311, mRNA sequence

GTGCATAATGGAATTATTTTTAAAAGCTTGTACGAGTGTCCAATTATGTCACTGGGCAAAAAATGTCAAA

GATAATCAGAAACAGACTTGTTCTTTCTGCATACCCCGCCAACAATTATCACAAACTTTTCTGACTCGCG

GAACCCAGATAAAGCAAACTCCATCGTGCGTTACAACCAAAATGTCCTAATCACCTCTCACTCAATTATT

ATTATTATTATTATTATTATTATTATTATTATATTTTTTCTAGTCATTTCAGTTTCACAATGATACTCTG

TACTACTCGATATCTCCCACCCAAAAGATTCTCATACTCTGTCCAACCGCATTATTTTTCAAAAAAACCT

CACTAGATTGAGCAGGCCAATCACCAAGCTTTGAGAAAATTTCGAGTTATCAAAGCCGAAGGCAGGCCCA

TCAACAGGACTTTGGAGTTTGAACCACGTTCACTTTTACATGCCTTGAGCTGCGCACTAATTTATTCCAA

GTGCAGAGAGAGACTCTAGCCACACCAGCAGGCAGCTAGTGTTAATTATCCTTACAGAATAGGATATTAT

CTGATATCTGGAAAT

>FM895394.1 FM895394 Jatropha curcas embryo 71-95 (DAF) Jatropha curcas cDNA clone rjcpga0_002310, mRNA sequence

GGTTTGGTTTTTACCGACCAACTGCCGAAATGCCCTTGCTTGGGTCAGCTCTTCGGGGGTCAAGTCTTTT

GACAAGCTTTTCTGAGGCCACCTTTCTTAGTAAAGGCTTTTGTTTGTGGTCTGCGATGGGTCTAGCCCCG

TGAGCCTCTCCAAACGTTCTCAAATCGGCCTCTTTGTTGTGTTGTTACGGGCGTACGGTACTACGGCCTT

TATTATTCCACTTTCTCAAAATGTAGAGCAGCGAATCGAGAGTCTCTCGCTATGTCCCCATTCCTTTCTT

CGATTCTATGGCCGATCTTCTTTAATCCAAAGCTGGTGCAAGCAATCTCATAGGTGGATTCACGGGGAAT

AA

>FM895393.1 FM895393 Jatropha curcas embryo 71-95 (DAF) Jatropha curcas cDNA clone rjcpga0_002309, mRNA sequence

CCATTTTCTTCTTCTTTGCCGTTTCTGAGCTAGTCGTTTTCGATTCTCTCGATCTCTGTCAATGGCAAAG

GAACCAGTTCGCGTTCTCGTCACTGGGGCTGCAGGACAAATTGGATATGCGCTTGTCCCCATGATTGCTA

GGGGAGTCATGCTTGGTACTGACCAACCTGTCATCCTCCACTTGCTTGATATCCCACCTGCAGCAGAGGC

ATTGAATGGTGTGAAGATGGAGTTGGTTGATGCAGCTTTTCCTCTTCTTAAAGGTGTTGTTGCTACAACT

GACGTTGTTGAGGCTTGCACTGGGGTAAACATTGCTGTCATGGTTGGTGGCTTCCCTAGGAAAGAAGGAA

TGGAGAGAAAAGATGTGATGTCAAAAAATGTGTCAATCTACAAGTCCCAGGCTTCTGCCCTAGAGAAGCA

TGCAGCTGCAAACTGCAAGGTTCTGGTTGTCGCCAACCCGGCAAACACTAATGCATTGATTCTGAAGGAA

TTTGCACCATCCATCCCTGAGAAAAATATTACTTGCTTGACAAGACTGGATCACAATAGGGCACTTGGCC

AAATTTCAGAGAGACTGAACGTTCAAGTATCTGATGTTAAGAATGTGATCATTTGGGGGAATCATTCA

>FM895392.1 FM895392 Jatropha curcas embryo 71-95 (DAF) Jatropha curcas cDNA clone rjcpga0_002308, mRNA sequence

CGAATCCTCAGTAGGGCCAGCGAAGGAGCCTGAGAAGAAAGAAGAGCCTAAGAAGGAAGAACCCAAGAAA

GAAGAGGAAGCTAAGAAAGAGGAACCAAAGAAAGAAGAGGAAGCAAAGAAAGAGGAGCCCAAGAAAGAAG

AGGAAGCGAAGAAAGAGGAGCCCAAGAAAGAGGAAGAAAAGAAAGAAGAAGAGAAAAAGACAGAAGCAGC

TCCAGCAGCAGCACCAGTTGCAGTTGCAGCTGCACCACCACCAGATCCGGTTTTAGAGCTTGTTAAGGCT

TACAAAGCTTATAATCCTCACCTCACCACATATTATTATGTGCAAAGCATTGAAGAGAATCCAAATGCTT

GTGTTATTTGCTAAGCTGGATGGCTATATAAGTTTAAGGACAAGTATATATAGCTCTCTTTTTGCCCCAA

CTGGTGATGGGTTTTTTTCAATCTTATCAGTTGTTCAAGTGTGTTAAGAGGCCAAAACCTAAGAAGAGAA

GAACCTTGTATATAAAGTGGTGATGCCTCATTCTTCTATTCAGAAGAATAGTAATGTGTAGAGAAGAATA

AATGAAATTTTGTAACTTGTTCTTTCTTTATTTTTCACATTTTCTTTGGGGGTAGAAAAAGGCGGTGTTA

ATCCCATAATTTGGAACTTG

>FM895391.1 FM895391 Jatropha curcas embryo 71-95 (DAF) Jatropha curcas cDNA clone rjcpga0_002306, mRNA sequence

ATTCACCAGATCCCCTAACGGTGCATGCTGGGCTTAACTTGGTGGGCGCCGATTCTCGGTCTCGGCCGAT

CGAGTCGTCGTTCGTTGGGCTCGGTCTGGCTAGTCAACGTTAAGGGCGCTGGTGAGCTGTGGGCATGGTC

CCTTCCATTCCCATCGCTCGCAAGCTCCCCCGACTGCGCGTGCTGGGCTTAACTTGGTGGGCGCCGATTC

TCGGTCTCGGCCGATCGAGTCGTCGTTCGTCGGGTTTGGTCTGGCTAGTCAACGTTAGGGGCGCGGGCGA

GCGGTGGGCATGGCACTCGATGGACCCTTCCATTCCCATCGCTCGCAAGCTCCCCTGACTGCGCGTGCTG

GGCTTAACTTGGTGGGCGCCGATTCTCGGTCTCGGCCGATCGAGTCGTCGTTCGTCGGGTTTGGTCTGGC

TAGTCAACGTTAAGGGTGCGGGCGAGCGGTGGGCATGGCACTCGATGGACCCTTACATACCCATCGCTCG

CAAGCTCCCCTGACCGCGCATGCTGGGCTTAACTTGGTGGGCGCCGATTCTCGGTCTCGGCCGATCGAGT

CGTCGTTCGTCGGGTTTGGTCTGGCTAGTCAACGTTAAGGGTGCGGGCGAGCGGTGGGCATGGCACTCGA

TGGACCCTT

>FM895390.1 FM895390 Jatropha curcas embryo 71-95 (DAF) Jatropha curcas cDNA clone rjcpga0_002305, mRNA sequence

CGGAGGAGGAAACTGTTACAACTGTGGAGGATCTGGTCATTTTGCTAGGGAGTGCCCAAACAGCGGTCGC

TAAGCTAGGGCAACGAGTCGAACATGTAAAATGGTTTGACAACATTTTGCATTGTTCCATCTTTCCTGGG

TGTTTGGGTACTGGTTTAGGGTATCTGTTTGGCAAATAGGTTATGTTGGTTATCTACTATGTTGGTTATT

TTAAAGTTTCAGCGTTTTAAGGATTTGGTGATTGTGATGTCATGTTTTTGTTTGGGTTTAATGTTAGTAT

GATTAGGGTTATGAAATCTGGTCTTGAATTAGAGTGGCAGGCAGTAGAGAGAAAATTGGGAATTAATGGT

GAAAAAAAAATTACATGTTGTAATTGTAGAACTCTCACTGTTTATGGAAGATGTGTATACACAATTATTA

TATAATTAAATATACATGCTGCTGGTTTGGTTGGGTCC

>FM895389.1 FM895389 Jatropha curcas embryo 71-95 (DAF) Jatropha curcas cDNA clone rjcpga0_002304, mRNA sequence

ATTCACACACTTACCCATCACTATTGCTTCTCTTGCTCTGAAATTCTCTGATTCTAATCCATTAATACTA

GTTCTTTCAAAGATTTCTTCTTTTTTCTCTTTGCTTTTTTAATCAAGTTTTAATCTTCACGAAAATGGTG

AATGTTCTGCACTTCTTGTTTGGGGTTTTTGGAAATGCTACTGCTTTATTCCTCTTTTTGTCTCCAACGA

TTACTTTCAAGAGAATAATAAAGAGTAAATCAACAGAGCAATTTTCAGGCGTCCCATATGTGATGACTTT

GCTCAACTGTCTCCTTTCTGCCTGGTATGGCCTGCCATTTGTCTCCAAGAACAATATTTTGGTGTCAACA

ATTAACGGGACAGGATCTGTAATTGAAAGCATATACGTGTTAGTTTTCATTTTATATGCAACAAGGAAGG

AGAAGGGTAAAATCTTAGGCCTCCTTACACTTGTGCTCACCATTTTTGCTACTGTGGCCTTCGTGTCTCT

GTTTGCCCTCCACGGAAGCACCAGAAAGCTCTTCTGTGGCTTGGCTGCTACTGTCTTCTCTATCATCATG

TATGCCTCACCTCTGTCCATCATTAGGTTGGTGATTAAAACTAAGAGTGTAGAG

>FM895388.1 FM895388 Jatropha curcas embryo 71-95 (DAF) Jatropha curcas cDNA clone rjcpga0_002303, mRNA sequence

TTTTAGCAAGGTTGTCATATGTCTTTTGTTGCGGAAGACGGTTCCTTTGATGCATTATGTTAAGGCTCAT

CGGCAAATTCTTAATCAGCTGGTTGACTTGATTGGCATTACATCTCTCATGGAGGTATTAATACGGCTTA

TTGGTGCTGATGAACATATGTATTCCAACTATACGGATGCCATGCAGTGGATAGAAGAAACGGATGTGCT

GGAGATGATTGTAGACAAGTTCAGCTCTTCTGATTCTCCTGAGGTGCATGCTAATGCAGCCGAAACGCTT

TGTGCTATAACACGATTTGCTCCTCCGGGTCTTGCTGCTAAAATCTCCAGTCCGAATTTTATTGGAAGAT

TGTTTCGTCATGCTTTGGAAGAATCACGACCAAAATCTGTCCTTGTTAACTCATTGTCCATATGTATATC

CTTGTTAGACCCTAAAAGGCTAACTTTGGGGACATATCATACATATGGCCGCCAAATTAATCATGGGTCT

ACTGTGACCGCCAGTCCTGAGACAATTGAGGGCATGCTGGAGAGCTTAGGTAATCTGCTGAAGCTTTTGG

ATGTCTCATCTACAGAAAATGCCTTGTTGACCACATATGGTAAACTGCAGCCACCTCTCGGAAAGCATCG

TTTGAAG

>FM895387.1 FM895387 Jatropha curcas embryo 71-95 (DAF) Jatropha curcas cDNA clone rjcpga0_002302, mRNA sequence

ATCCAAAGGATTCTATCCGTCGCCCGATGGGAATTGTACTTTAAGGCGGCCCTCGCGGCTCGTCCGCCGC

GGGGGCTTGGCCAACGGCACGTGCCTCTGGGGGCCGGATGGCCCCTACTGCGGGTCGGCAAACGGGCGAC

GGACACGCGCGTCGCTTCTGGCCCGGATTCTGACTTAGAGGCGTTCAGTCATAATCCAGCGCACGGTAGC

TTCGCGCCACTGGCTTTTCAACCAAGCGCGATGACCAATTGTGCGAATCAACGGTTCCTCTCGTACTAGG

TTGAATTACCATCGCGACACCATCATCAGTAGGGTAAAACTAACCTGTCTCACGACGGTCTAACCCCAGC

TCACGTTCCCTATTGGTGGGTGAACAATCCAACACTTGGTGAATTCTGCTTCACAATGATAGGAAGAGCC

GACATCGAAGGATCAAAAAGCAACGTCGCTATGAACGCTTGGCTGCCACAAGCCAGTTATCCCTGTGGTA

ACTTTTCTGACACCTCTAGCTTCAAATTCCGAAGGTCTAAAGGATCGATAGGCCACGCTTTCCCGGTTCG

TATTCGTACTGGAAATCAGAATCAAACGAGCTTTTACCCTTTTGTTCCACACGAGAATTCTGTTCTCGTT

GAGCTCATC

>FM895386.1 FM895386 Jatropha curcas embryo 71-95 (DAF) Jatropha curcas cDNA clone rjcpga0_002301, mRNA sequence

GTGGTTTCATTTGGCTATGTTTTTCTGGGGGCCACGCCTTTAGTACTTTATGCAAATCCTTAATATCTTC

GAATCTCGATGTTTCTTGATCTGGGTATCAAGTGTTTCTTCTTTAGATCATTATTTTAGACTTTAAAAAT

CTTTACTTTTGTCCATCAGCAGGGAAGAATCCAACTGGTTCTTGGTCTTTTTTCAGTTTTGTTTGTTTTC

TTAACAGATTCAGAGTAATGGGTCTCTTGTTGAGGATGCAAATAAGCTTTTTCCAAAACCAGAAATATTC

TTTGTCCGTATGAGTTGTTCTTTCACGTTATTGTAAAGTTATGGGTAGAGGTAGAGGAAAGGGAAAAAAA

TTGACTGTTAGCAATCATGAGGATCCTGGAAGTGGTGAGGAAGAGAAAATTCCTGCACAAAAGAGAAGAG

GAAGGCCTCAAAAACCATTGAAGGATGATATTGATGAAGAAGAAGTTGAAAAAATAGAGGAGGAAGATGG

AGAGAATGGAAAAAC

>FM895385.1 FM895385 Jatropha curcas embryo 71-95 (DAF) Jatropha curcas cDNA clone rjcpga0_002196, mRNA sequence

TGCAGAATCGAATTCTGACATGTGTGCGAGTCAACGGGTGAGTAAACCGCAAGCGCAAGAAGCTGACTGG

CGGGATCCCCCCGATGGTTGCACCGCCGACCGACCTTAATCTTCTGAGAAGGGTTCGAGTGAGAGCATGC

CTGTCGGGACCCGAAAGATGGTGAACTATGCCTGAGCGGGGCGAAGCCAGAGGAAACTCTGGTGGAGGCC

CGCAGCGATACTGACGTGCAAATCGTTCGTCTGACTTGGGTATAGGGGCGAAAGACTAATCGAACCGTCT

AGTAGCTGGTTCCCTCCGAAGTTTCCCTCAGGATAGCTGGAGCTCGGGACGAGTTCTATCGGGTAAAGCC

AATGATTAGAGGCATCGGGGGCGCAACGCCCTCGACCTATTCTCAAACTTTAAATAGGTAGGACGGCGCG

GCTGCTTCGCTGAGCCGCGCCACGGAATCGATAGCTCCAGGTGGGCCATTTTTTGGTAAGCAGAACTGGC

GATGCGGGATG

>FM895384.1 FM895384 Jatropha curcas embryo 71-95 (DAF) Jatropha curcas cDNA clone rjcpga0_002195, mRNA sequence

ATTCCTGACATGTGTGCGAGTCAACGGGTGAGTAAACCCGCAAGGCGCAAGGAAGCTGACTGGCGGGATC

CCCCCGAGGGTTGCACCGCCGACCGACCTTGATCTTCTGAGAAGGGTTCGAGTGAGAGCATGCCTGTCGG

GACCCGAAAGATGGTGAACTATGCCTGAGCGGGGCGAAGCCAGAGGAAACTCTGGTGGAGGCCCGCAGCG

ATACTGACGTGCAAATCGTTCGTCTGACTTGGGTATAGGGGCGAAAGACTAATCGAACCGTCTAGTAGCT

GGTTCCCTCCGAAGTTTCCCTCAGGATAGCTGGAGCTCGGGACGAGTTCTATCGGGTAAAGCCAATGATT

AGAGGCATCGGGGGCGCAACGCCCTCGACCTATTCTCAAACTTTAAATAGGT

>FM895383.1 FM895383 Jatropha curcas embryo 71-95 (DAF) Jatropha curcas cDNA clone rjcpga0_002194, mRNA sequence

ATTGTCTGACATGTGTGCGAGTCAACGGGTGAGTAAACCCGCAATGCGCAAGGAAGCTGACTGGCGGGAT

CCCCCCGAGGGTTGCACCGCCGACCGACCTTGATCTTCTGAGAAGGGTTCGAGTGAGAGCATGCCTGTCG

GGACCCGAAAGATGGTGAACTATGCCTGAGCGGGGCGAAGCCAGAGGAAACTCTGGTGGAGGCCCGCAGC

GATACTGACGTGCAAATCGTTCGTCTGACTTGGGTATAGGGGCGAAAGACTAATCGAACCGTCTAGTAGC

TGGTTCCCTCCGAAGTTTCCCTCAGGATAGCTGGAGCTCGGGACGAGTTCTATCGGGTAAAGCCAATGAT

TAGAGGCATCGGGGGCGCAACGCCCTCGACCTATTCTCAAACTTTAAATAGGTAGGACGGCGCGGCTGCT

TCGCTGAGCCCGCGCCACGGAATCGAGAGCTCCAAGTGGGCCATTTTAGGTAAGCAGAACTGGCGATGCG

GGAT

>FM895382.1 FM895382 Jatropha curcas embryo 71-95 (DAF) Jatropha curcas cDNA clone rjcpga0_002193, mRNA sequence

ATATTGAAAGAACAGCAATAACCATATTGACATAACAAAGGGTTTGGAACAAATATTAGAGTTTCCCGAT

AGGTTCAGGTACGGAATGGAGCAAATAAGAAGACAAGTGCTTGCAAAACAAGTAATCCCTAAACACCCAG

GTCTAACACAAGAAGGTAACTAGGAATAAAGATACAATTTTTCATGGTCTTTTCACTCGAAGGCCCACTG

GTTCTCTCTCCGAACTTCCTCTTCTTCCTCTGGTGTGAAATCATTCTTGATGTTGAACGTTTTGCGTATC

TCCTCTGGAGTTTTGCCCTTGATCATGTCAGCCACTGTCTGACAAGTGAGATCCAGTAAACTCTTGATGT

TCAGATAATTGGCAGCCAAGATTAGATCAAAGAGGGTAGCCTGATCAACTTTGACAAAGTCCGCGTCCCA

GGTCTTAAGTTCTTCGTCGGCAGAAGAGGGGCGATCGTCGGACTTAGGCGTCTCCACATGCTTCTTACAG

TACTCAATTACCTTTAGATAAGATCTTGCTGGTGACAT

>FM895381.1 FM895381 Jatropha curcas embryo 71-95 (DAF) Jatropha curcas cDNA clone rjcpga0_002191, mRNA sequence

TTTTTTTTTTGTTAATTAAGTGGTAACAAGCTCTTATAAATCAAGAGGGCAAATGGTTAAAACAATAAAG

GAAAAGGCCATAGTTCCTTGNGTTTGTACCCTAGNTATCTGAACCAAGAACAATGTCTTATATACATTTT

GCTGACCATAAAAATACAAAAACGAATTTCCTGNGACAGAGTATGAAATATCGTACCGTGCATTACTTTG

CATTATTTTCTTAGCTCCTGAACTAGGACAGACTCGCTATTGGCCCCGAATGAAAAT

>FM895380.1 FM895380 Jatropha curcas embryo 71-95 (DAF) Jatropha curcas cDNA clone rjcpga0_002189, mRNA sequence

TTTTTTTTATCTCCAACTCAACTTAGCACTGCATTTTTCCAAATGATACATGATATCGGATTTTCCAACT

CAGCTCGAAAGAAAAAGGACCATACCAAAACTAATAAGCAGCATGTTTTTCTTGCACATGACATTTGAGC

TACAAAAATTTCTTTGGGCTTTATGCACTCATCCTTTTTATATCACATAACCCTGTATTCCTCCATCTGA

TTGCTCTTCTTTCCTTCAATCATATTCTAATTTGTACAAGAAAACAATGCTCAGTAAGACATGCTGACTG

GCTGACTGGCTGTAAAACAAAGATCCCGCAGCAATAGTACAGAAAAGTGAACTGCTAGAAAAATAAAAAC

CTACAAGCTCATGACATTCTTTTTTTCCTTTTATGTATCCATAATTAACTTCTGGGAGCTCAGTCTGCTG

CTAGTCAATTATCTGGGGAACCTCTTTCTTCCAGCATTGTTGTGCCCTTGAATATCGTCCCCTGCAGTTC

CCTTGTATATTTGGACAGTTGTCCCTCGGCTCATCTTTCTGAAGCCAAATCTTCCTTGTCCCAATTGACT

CAGCTTCCTCAGCTGCTGGTAGCACCAAGTTTACCACATTGAGGGAGCCAAGTATCTCTCAATGCATGAG

>FM895379.1 FM895379 Jatropha curcas embryo 71-95 (DAF) Jatropha curcas cDNA clone rjcpga0_002188, mRNA sequence

GACAAATAGCCCAATGCTAATATAGACACAAAATATGATAATGCACAGTCAATGTGAGCTCTGCAGGCAT

AAAAATATCCGAATCAAAATTCAAATTTACAGGTCTAGTTGTTTCTGATATGCAGTCAACAATTTGATAA

TGCTTTATGGCATTATTAACCTTTATCTATTGATTGCAATTACCTATAACTCATCGTGGATGTTTCCCTC

AGATGGCTTGGATTCTGGTTGATATTCCAGTAAGTACCTTGTAGCTAATGATGCATATAATGCTGCGCCA

TATGGAAGCACATCTTCATTGACTGTGAAATATGGTGAATGACCATTTCGAATTGGTCCTTGAGATTCAT

TTTTCATCCCAACAAAGAAGAAGTATCCAGGAATCGCTTCTGCAAAAAAGGAGAAGTCTTCTGCTCCCAT

CACTGGTTGCATTTCGATAATGTTCCTGTGCACCCAGCATGTCCGTTGCAACATTTATGAAATGCTTGTG

CAAATCTTGGTTATTTACAGTTGCTGGGTAAA

>FM895378.1 FM895378 Jatropha curcas embryo 71-95 (DAF) Jatropha curcas cDNA clone rjcpga0_002187, mRNA sequence

TATAGATCTTTATTCATCATATTAGTATGCCACCAACAGTAGAAACTTGTTAGCATGAAACAATAAAGAA

ACCTTTTTAAACAATAAAGAAACCCTTTTTAAACAATAAAGAAACCCTTTTTAAACAATAAAGGAAACCC

TTTTTTCTCCTTTTTGTCTTTTAAACTCTGATTTCATAACCTAAGCAATTGTTTTCATGGATCCACAAAT

TGGTATTTAATTACTTTCCCGCTGGTATCTCAGACAAGACTTGGGCCTCACAACTCCATAGGTTGATCCA

AAAGCATTATTGCAGGTAGAAGTGACCTCTCCTGATTCTGCAGGTCCCAATCTAATATTTCCCAAACTGA

GATTGGTACAAAGCACATTCTCACTGCAGTTCAGATCGATAACCACTTTGCTCTTCGATGTCCCAAAGAA

TCCAAAAAATTGCACATCGCTTATGTGAACTCCTGCTCTCATATTTGTTGAATGTGATTTTTTTTCACCA

TAATGTTGGTCACTAATAATCGGATTTT

>FM895377.1 FM895377 Jatropha curcas embryo 71-95 (DAF) Jatropha curcas cDNA clone rjcpga0_002186, mRNA sequence

GTAACTAATTCAACTGTATTTTCAATTTTTCATGAACTGTCGGTAACAGATATAAATTGGTCCTTCCATG

TCCAACCATACAAGTTCCACAATAGGTAATGAGCTTGAATCAAACAAGCACCAAATAATAAATAAATATA

CAAGTAAATTACATCAGAGAGTTGGAACATGCATGCTTGGCCAACGTATTTCCACCTGAGGGTATCACAT

TAAGGCAGAAAAGGATTGTGCGAGGGAGTCATCTCTTTCTGCTTTTTGTAAGCCCTGGTGGCATTCAATC

CCCCTAGTCCAACGGTTCCCAGTATAATAAAAAGGAAAGGTATGCGGATTNAAACATTATTACTTCTTCC

CCCATGAGCTCTTGGCATGCCAGTCCTCACAA

>FM895376.1 FM895376 Jatropha curcas embryo 71-95 (DAF) Jatropha curcas cDNA clone rjcpga0_002185, mRNA sequence

AACATTGGGGATGTTACTGCAAAAGGAGAATGTGTTGGCAATCTTGCAGATTTGCTTGGAACAGGCCTTA

GCATAATGATTGCAAAAAGAAACCCTAGCTTGTTTACCACATTTGCCTTCCTTTCATGCGGCTATGTTCT

CAGCTCTTACCAAGAGGTTAAATCTGTAGTGTTGCACACCTTGAACCGGGCCAGATTCAGTGTGGCAGTG

GATTCTTTTCTTAAGACAGGGCGAGTGCCCTCGTTGCAGGAGGGGAATATGAAGGAAGATATATTCAGCT

TTCCATGGTTGAAGGATAGGCCTATTGTTCTAGGACCAAGATTTAAAGAAGCATTTCAAGACCCAAGTGC

ATATCTTGCAATCCAGCCTCTATTTGAGAAAGAGAGATATATAGTGACTTACAATCCATCAAGGGATACG

ATTTATGCATTGCTCAAGGATCAAGCAAAGGCAGATGACATTCTAAAAGCAGCATTCCACGCTCATGTGC

TTTTGCATTTCATACAATCATCAAATAATGGTCATTCCTCATCACGGAAGCAACGAGAATCGGTCGTTCT

AACTATATTCTCTCGGCTAATGATTT

>FM895375.1 FM895375 Jatropha curcas embryo 71-95 (DAF) Jatropha curcas cDNA clone rjcpga0_002183, mRNA sequence

ATTCGAAAAGAATGATGATGCGCCAGAGAATAATCCTCAGTATACCACTGTTTATGTTGGCAACCTTGCT

CCCGAGGTTAGTTCTGCAGATCTTCATCAGCATTTCCATGCACTTGGTGTAGGAACTATTGAAGATGTGC

GGGTGCAACGAGATAAAGGTTTTGGTTTTGTGAGATACAGTACACATACTGAAGCAGCTCTTGCTATTCA

GATGGGAAATGCTCGTTTTCTACGTGGCAAACCTATTAAGTGCTCGTGGGGAAGTAAACCCACTCCACCA

GGAACAAGCTCTACCCCTCTTCCTCCCCCAGCTGCTGGACATATGCCTGGCCTTTCAGCTGTTGAGCTTG

CAGCCTATGAGCGGCAAATGGCATTGAGCAAAATGGCTGGTGCTCAAGCCCTCATGCTTCCACAAAGTCA

GCATGCCCTTAAGCAGGCCGTCATGGGAATGGGTGCTGTGGGATCTAGTCAAGCAATATATGACGCCGGA

TTCCCTAATGTTGCTACAAGCCAGCAGATTATGTACTATCACTAATAT

>FM895374.1 FM895374 Jatropha curcas embryo 71-95 (DAF) Jatropha curcas cDNA clone rjcpga0_002182, mRNA sequence

ACTCTTTTTCTTTAAAAAAGTGATGGTCCAATTGGACCGGCCCATCGGGTCATGCGGATCCAAGTGGCAT

CGACCTGTTATCCTTCCACTGCCAGGAGCGGGATGTGATCGCTGCTCTGTGAAACCAGCCTGACGCAGTG

CCGTCCTCTATAGGACTCGTGAGG

>FM895373.1 FM895373 Jatropha curcas embryo 71-95 (DAF) Jatropha curcas cDNA clone rjcpga0_002181, mRNA sequence

TGGCGTATAGTTTACCTCGTAAAGGAATCCTCCAGAGCGAAGCTCTCTTGAAGTATATCTANTAGAAAAA

AGTGTATATCCAAGAGAGCACGAGGAACTCAAGAATTTACGAAAAGCTACAGAGAACAAATATGGAAACC

TGAGCTTAATGTCAGTACCAGCTGATGAAGGGCAGTTCCTATCAATGCTTGTGAAGATGATGAACGCGAA

AAGGACAATAGAGATTGGTGTTTTTACTGGCTATTCTCTTCTTTCAACTGCTCTTGCCTTGCCTCATGAC

GGCCGAATAACAGCAATAGACATTGATCAAGAAGCTTATGAATTTGGATTACCATTCATTCGCAATGCAG

GCGTTGAGCACAAAATCAATTTCATTCACTCTAATGCTATTTCAGTATTAAGTGAAATGTTAAACAATGA

ACAGGAGAAAGAAGAATTTGACATGGCATTTGTAAGATGCTGATAAGCACAATTACAAAAATTACCATGA

GCAATTGCTTGAAATTAGTTAAGATTGGAGGAATAATTTGCTTACGATAACGCCTTATGGTATGGATTTG

TTGCTCA

>FM895372.1 FM895372 Jatropha curcas embryo 71-95 (DAF) Jatropha curcas cDNA clone rjcpga0_002180, mRNA sequence

TTTTTTATTTCTTTGTACTCTTTATGAAGCAAAAAGACAACCACAACCTTTACTGAAATTGAAGCAGGCT

CAATAGCCAAAGTTGAAACAATGCCGCAAAACAGGTAAAGGGAAAAAAACATTAAAAAAAAAAAAAAAAA

ATGAAGCAATGGAAAGCCTTCTTTGTCCTATCACAACTGTGAAATGACCATTATTCTTCACAACTCTTAA

AAACTTCCCAGTTGAATCTTCACATCTCAGAAACCTCCCACTTGAGTTTTATGCTTCCACTTACTCAACA

GTCAGAGGAACAGATTCAGCCAATTTCCTGTACTTGAGAGCATAGAACATCTCAAGGTAGCGCCGGCTCT

CACGGCGGTCGAGTTTAGATACATGTTGCCAGAAGCCATATACACCAGTGAGAGTCAATAGCCTTTGAGA

GTAAATTTGCCAGGTATACTTCTCTTGGATACGTTGCAGACCTCCTTGGGAGATTTTGTCCCAGTGAGAG

GGATCTACCTTGCACTTCTCGAAGAAGTCGACAAGAAGCTGAGCAGCCTGTTCGCCATGGTAAGGATCAC

TGTTGAAGCCAGAATTTCCATGCACAATGATCTCAGCA

>FM895371.1 FM895371 Jatropha curcas embryo 71-95 (DAF) Jatropha curcas cDNA clone rjcpga0_002179, mRNA sequence

ACAAACAAGACATTCTAGATCTTTATAGATTCTGAAACCATTTTGGTTTTTAACAACTCACATCATTCTA

ATGCTAAACCTGCCACAAGCTCTACAGCAAGCAAATGCCTTTGTAGAAACTAAAACAGAGTCTTATCATG

CTGAAATCATTATATCAATACTAACATCACCGCTAATAGAAACACCAAAAACAAAAACAGGTATAAATGA

AACTTAAATAGCCAAAATTATCATTCCAACTAGTCAACAATTAAGACCATAAATTGTAAGCTCAACAAGA

AGAAACTTAAAATCAAAAGCTTGGTCTACCCTCTGAGAAACAATTTCAAAATCATTCTCCACCAGCCTTC

TGGTAAACATAGGTGAGGCCGCACTTACCACAGTAGTGCCTATCAAAATGATTAGCCATAAAAGTGCCAG

CTCCACACTCCGCATTGGGACACTCCTTCCTTAACCTCTGCACTTTACCACTGTCATCGACCTTGTAGAA

CTGAAGAACAGCGAGCTTGACCTTCTTCTTCTTGTGCTTGATCTTTTTTGGCTTGGTGTAGGTCTTCTTC

TTTCTCTTC

>FM895370.1 FM895370 Jatropha curcas embryo 71-95 (DAF) Jatropha curcas cDNA clone rjcpga0_002178, mRNA sequence

CTAAATTTTCTTGTATCTTGCTACCTTAAACAACCAATTTCTCCGAAGTCAACAAAAACAAGAGCAAGAA

TCCCTTCAATTCTTACAAATTGACTACCACTATTGGCGTCAAAGCCATTGTCCAGCCTTCCCTCAGTGTG

CTCGTAAATCCGCAGCTTCATCATCTTCTTCTTCTGTTGCTGTTGAGTCTGAGAAAGACCGGCTACCAGA

CTGATATTAGAGTCACAGAGACCGTGGAGCCCAATTCAAGATTGAGGTTGACTGTAGAAGTCCCAGAAGC

TGTATGCGATGATTGTTACAGCAGTGCTCATGAATGAGTTCATGAAGCAAGCTAGGGTACCGGGTCTTCG

TCCTGGAAAGAATGTACCAGAAAGTATTCTATCTAAAGTTATGTTGGAAAGGAAAATGTACAGAAGGCTA

ACAATTGAATCTAGTAAAGAAGAGGACACTTCCACATGC

>FM895369.1 FM895369 Jatropha curcas embryo 71-95 (DAF) Jatropha curcas cDNA clone rjcpga0_002177, mRNA sequence

GAAACTGTCTTTGGACAAACTGGCCTTGAAAATGAGACAGCTCCTGCTCCTACTCCATCTTCTAGTGCCT

TTGGTGCAATGGCTAGTTTTCTTGTTCCCAAGCTTTCAGTTGGGCTGCCTTCATCTACAGATAAGTCTTC

TGGCTCTGTTGATCAATCTAAAATCTTGAAGAAAGAAAGACATGCAGTTCGTCCACCTGTGCAGCATAAT

TGGTCTCTTCCTGGAAGCAATGTACATACGAAACCACCACAGATTTTTCAGCATGAATTGCTTCAGAATT

TCGCAATTAATATGTTCTGCAAGATTCCAGTGAAGAAGGTCAAGACATATGGTGACTTGCGTAATGTTTT

GATGAAGAGAATATTTCTATCTGCCTTGCATTTTCGCATTAATACGCGATACAAGAGTTCAAATCCACCC

TTCACCTCAATTGAATTGGATCATAGCGATTCTGGAAGGGAAGGATGCACTGTAACAACACTTACAGTAA

CTGCGGAGCCCAAGAATTGGCAGGGTGCAATTCAAGTTGCTGTTCAAGAGGTCCGAAGGCTTAAAGAATT

TGGTGTTACAAACGGTGAATTAACTCG

>FM895368.1 FM895368 Jatropha curcas embryo 71-95 (DAF) Jatropha curcas cDNA clone rjcpga0_002176, mRNA sequence

AAAATGCTAAGCACAACTTTAATTTCCAAAATGTCAAAATGTTGCTGATTCCGCATTGTCATACATCAAG

AATGACGTTCTACTAATAAGAAACAACTCAATCAAAAGTAACTGCAGATAGCAAACACTCAAAAATTCTG

GTATCATGAAAACAACAAAGGTCTCTACAAATACTCTTCCATTAAGATCCCAATAAATCAAATTTGACAG

CTACTCAGTGAGATTTGATGTGTTGCTGAACAACATTAAGACCTTCAGTCTCCTCGCCATAATCCTTAAC

TACGACGCAGGAGCAACCAACTACCTTCCGGGCCTTACCCTCAGAATCAATCTTGCACAACCCAGCCCAC

TCGCCAAGAGTCTTAGCACTAGGAACAGTTAAGAGGCCAACACCATGGTCAGCACAAAGGCCTTTGACAA

GCTTAACATAGTCAGGCTGGTTACAGTCCTCAGCCAATACACAAAGCTGAGCAGCATGCTTTTCAATCAC

TTTCGCGCCTTCATGAA

>FM895367.1 FM895367 Jatropha curcas embryo 71-95 (DAF) Jatropha curcas cDNA clone rjcpga0_002175, mRNA sequence

ATTCGTAAAGCCAATGATTAGAGGCATCGGGGTGCGCAACGCCCTCGACCTATTCTCAAACTTTAAATAG

GTAGGACGGCGCGGCTGCTTCGCTGAGCCGCGCCACGGAATCGAGAGCTCCAAGTGGGCCATTTTTGGTA

AGCAGAACTGGCGATGCGGGATGAACCGGAAGCCGGGTTACGGTTGCCC

>FM895366.1 FM895366 Jatropha curcas embryo 71-95 (DAF) Jatropha curcas cDNA clone rjcpga0_002174, mRNA sequence

ATTCCTGACAATGAGTTCATTACTTTGATGCACGGGTCGGATCCGGTAAAGCTTGAGCTTAATCGGCTTG

AGAATGAAGTTAGAGATAAGGATAGAGAGTTAGGAGAAGCACAAGCTGAGATCAAAGCGCTGAGATTGTC

CGAGAGACTGAAGGAGAAGGCCGTCGAAGAGCGAACTGAGGAACTCTCAAAGGTGGAGGAAAAGCTCAAA

TTAACAGAATCCCTTCTAGAAAGCAAGAATCTTGAAATCAAGAAAATCAATGATGAGAAGAAGGCTTCCA

TGGCAGCTCAGTTTGCAGCTGAAGCAACTCTCCGAAGAGTACATGCTGCTCAGAAGGATGATGATATGCC

TCCAATTGAAGCCATTCTTGCACCCTTGGAGGCTGAGCTCAAGCTTGCTCGTCTGGAGATCACAAAGCTT

CAAGATGATAACAAAGCATTAGATCGGCTGACCAAGTCAAAAGAAGCGGCTTTACTTGAAGCTGAGAGGA

CTGTTCAAGTTGCTTTGGCTAAGGCCTCTATGGTGGATGATCTCCAAAATAAGAACCAAGAACTAATGAA

ACAGATAGAAATTTGTCAGGAAGAAAATACAATTTTAGACA

>FM895365.1 FM895365 Jatropha curcas embryo 71-95 (DAF) Jatropha curcas cDNA clone rjcpga0_002173, mRNA sequence

ATTTGAGAGGTTTTGAATCACACAATGGGGTCTTTAATGGCAGGTTGGGACTCGCCTGTCCCTGNATCCC

AAATCAGTGAAATACAAGAAGAATTGGTCATTTACTAAGGAAAATTGAAGCTTACTGGAAGTCAAAGAAG

AAGATAGAGGAGGAACATCTCAAAGCCATTTCCCGTCCATCAGGAGAAAGAATCTCAGCAGGATGATAAG

TTTTGGAATTGAAGATTCAAAGATCAAGCTCTTTGCCAGGGACTAAAACAAAGGAGGGGCTAATAGACAT

GGAAACTGAAAAAAGCTTAGAGGAATTCATAAAGAAAAATGGCTGGTGGACGAGGAGCAACTGGGCATGT

CTAAATGAACCTCCTGTGCTGGAAAACTCTTCTTACAATTATGTTCCGCAATTCCATATTGCAAATCTGG

CCACATCAAAAGACAATAATACTGGAATTAGTGCTTAATTATCCATATTGATGTTATGTTTCATGTAGGT

GTACATATAATTATATATAATATGCGGGTGATATACCTAAACAGATCCAGGTGTAGATTTAGGGTACGGT

ATGAATTGTGACGCTGTAAATATGAGTGGTTTTTGTTTATGGGTGGATTTAGATG

>FM895364.1 FM895364 Jatropha curcas embryo 71-95 (DAF) Jatropha curcas cDNA clone rjcpga0_002172, mRNA sequence

TGCAGGAATTCGAATTCCAATGGAGTCTGACATGTGTGCGAGTCAACGGGTGAGTAAACCCGCAAGGCGC

AAGGAAGCTGACTGGCGGGATCCCCCCGAGGGTTGCACCGCCGACCGACCTTGATCTTCTGAGAAGGGTT

CGAGTGAGAGCATGCCTGTCGGGACCCGAAAGATGGTGAACTATGCCTGAGCGGGGCGAAGCCAGAGGAA

ACTCTGGTGGAGGCCCGCAGCGATACTGACGTGCAAATCGTTCGTCTGACTTGGGTATAGGGGCGAAAGA

CTAATCGAACCGTCTAGTAGCTGGTTCCCTCCGAAGTTTCCCTCAGGATAGCTGGAGCTCGGGACGAGTT

CTATCGGGTAAAGCCAATGATTAGAGGCATCGGGGGCGCAACGCCCTCGACCTATTCTCAAACTTTAAAT

AGGTAGGACGGCGCGGCTGCTTCGCTGAGCCGCGCCACGGAATCGAGAGCTCCAAGTGGGCCATTTTTGG

TAAGCAGAACTGGCGATGCGGGATGAACCGGAAGCCGGGTTACGGTGCCCAACTGCGCG

>FM895363.1 FM895363 Jatropha curcas embryo 71-95 (DAF) Jatropha curcas cDNA clone rjcpga0_002171, mRNA sequence

TTTTTTTTTTACTGTAAAAACCATCTAATGTTTGTATTGAACTGATGAAATTCTTTCTCCAACAACATCC

TTTAAAAACCCCATCTTTGACCAACCAAAATCAAAAATTACAAAAGAGCGGACACAAGTTTTATTAAACA

AAGGGAAGCCTGCATCTAAACATTTGACTCAATACACAGCAGTCAAAAATCACCATCAATGTCTTCAGTG

GAGGGTGGAGGCAATGTGAGCAATCAAGTCTAGCACACGTGAACTGTAGCCCCATTCGTTGTCATACCAA

GAGACAAGTTTCACAAAGTTGTCATTCAAAGCAATTCCTGCCTTGGCATCAAAAATACTTGATCTGCTAT

CGCCCACAAAGTCAGAAGACACTACATCATCTTCAGTATAGCCCAAGATTCCCTTAAGCTTGCCCTCAGA

CTCCTCCTTGATTGCAGCTTTGATGTCCTCATATGTTGCCTTCTTTTCAAGCCTGACAGTAAGGTCAACA

ACAGAAACATCCACAGTTGGAACACGGAATGACATTCCAGTCAATTTGCCATTAAGTGCAGGAAGAACCT

>FM895362.1 FM895362 Jatropha curcas embryo 71-95 (DAF) Jatropha curcas cDNA clone rjcpga0_002170, mRNA sequence

CCGTCGCCCGAATGGGAATTGTACTTTAAGTGCGGCCCTCGCGGCTCGTCCGCCGCGGGGGCTTGGCCAA

CGGCACGTGCCTCTGGGGGCCGGATGGCCCCTACTGCGGGTCGGCAAACGGGCGACGGACACGCGCGTCG

CTTCTGGCCCGGATTCTGACTTAGAGGCGTTCAGTCATAATCCAGCGCACGGTAGCTTCGCGCCACTGGC

TTTTCAACCAAGCGCGATGACCAATTGTGCGAATCAACGGTTCCTCTCGTACTAGGTTGAATTACCATCG

CGACACCATCATCAGTAGGGTAAAACTAACCTGTCTCACGACGGTCTAACCCCAGCTCACGTTCCCTATT

GGTGGGTGAACAATCCAACACTTGGTGAATTCTGCTTCACAATGATAGGAAGAGCCGACATCGAAGGATC

AAAAAGCAACGTCGCTATGAACGCTTGGCTGCCACAAGCCAGTTATCCCTGTGGTAACTTTTCTGACACC

TCTAGCTTCAAATTCCGAAGGTCTAAAGGATCGATAGGCCACGCTTTCA

>FM895361.1 FM895361 Jatropha curcas embryo 71-95 (DAF) Jatropha curcas cDNA clone rjcpga0_002169, mRNA sequence

CGAATTCATCCTCTCCTCTGCAAAAATGCAGATCTTCGTGAAAACCCTAACGGGGAAGACGATAACCCTA

GAGGTTGAGTCTTCTGACACCATCGATAATGTCAAGGCCAAGATCCAGGACAAGGAAGGAATCCCTCCAG

ACCAGCAGCGACTGATCTTCGCCGGAAAGCAGCTGGAGGATGGCCGCACTCTTGCCGACTACAACATCCA

GAAGGAATCCACCCTCCACCTGGTGCTTCGTCTTCGTGGTGGTGCTAAGAAGAGAAAGAAGAAGACTTAC

ACCAAGCCAAAGAAGATCAAGCACAAGAAGAAGAAGGTCAAGCTTGCCGTGCTTCAGTTCTACAAGGTCG

ATGACTCCGGAAAGGTGCAGAGGCTGAGGAAGGAGTGCCCGAATGCTGAGTGTGGAGCTGGAACTTTCAT

GGCCAACCATTTTGATAGGCACTACTGCGGCAAGTGTGGCTTGACTTATGTTTACAATAAGGCTGGCGGT

GATTAGGTTATTTTTAATGCGTTTTTGTTTGGTTATATGGATTTGGTAATTCTAAGTTTGCTACTTTTGG

CTTAATTTTGTTTAGTTGAGTATGCTTCTGATGGATATTCATATACTGGTTTTATTGGGTTAGTTTCTTC

ATATACTTATCTGGATA

>FM895360.1 FM895360 Jatropha curcas embryo 71-95 (DAF) Jatropha curcas cDNA clone rjcpga0_002168, mRNA sequence

TGAAAATCCGGAGGACCGAGTGCCTCCCACGCCCGGTCGTACTCATAACCGCATCAGGTCTCCAAGGTGA

ACAGCCTCTGGTCGATGGAACAATGTAGGCAAGGGAAGTCGGCAAAATGGATCCGTAACCTCGGGAAAAG

GATTGGCTCTGAGGGCTGGGCCCGGGGGTCCCAGTCCCGAACCCGTCGGCTGTCGGCGGACTGCTCGAGC

TGCTCCCGCGGCGAGAGCGGGTCGCCGCGTGCCGGCCGGGGGACGGACTGGGAACGGCCCTTCCGGGGGC

CTTCCCCGGGCGTCGAACAGTCGACTCAGAACTGGTACGGACAAGGGGAATCCGACTGTTTAATTAAAAC

AAAGCATTGCGATGGTCCCTGAGGATGCTCACGCAATGTGATTTCTGCCCAGTGCTCTGAATGTCAAAGT

GAAGAAATTCAACCAAGCGCGGGTAAACGGCGGGAGTAACTATGACTCTCTTAAGGTAGCCAAATGCCTC

GTCATCTAATTAGTGACGCGCATGAATGGATTAACGAATTCCCACTGTCCCTGTCTACTATCCAGCGAAA

CCACAGCCAAGGGAACGGGCTTGGCGGAATCAGCGGGGAAAGAAGACCTGTTGAG

>FM895359.1 FM895359 Jatropha curcas embryo 71-95 (DAF) Jatropha curcas cDNA clone rjcpga0_002167, mRNA sequence

ATTCCTTGGAATTATGTTCTCTCTTTTGATTAGAAATAGAATAAGAATGGAAACGTGACAAGATAAGATT

ACACACTCAAAAATTACTGGGCAAAAGAACACCAGAAATAGTGCCACAGTAAATAGTCCATATACATATT

TAGTTATATACATATCGTATATTACCATAATACACGTCGAGTCATCAAAATTACACAACCAAAATCCTGC

AACCTATAACATATTATATTTTACATGGGTATCATATTATATGGTAAATTACACAAGAACCCTCTAAACA

CTGCCAGGGGGCTTGCAGTCAACCTCTCTGAGGCTAGTCCTGAGACCCTTTCCTCCACCATTAGCCAAAG

GATGTATTCCGTGATCTGGCCCTTTCATATAAGCTCTGTTCTCCCTCTCTTTCTCAAATGATACCCCATA

TCGGTTCTCCACATCTCGAGCAAGGTCATCATAAATCTGAGTATCCCTAGTCATAGCCATCATAATCTGA

GGTAAGCCAAGAGGTAAATTGTCACCTTTGTCAACTTGCCAAAAATGAATAGTCTTGCCGTAAGTTTTGC

ACACTTTCTCAAGATCCTGGTCTTGTATTGGACCAGGAATGCCAGGCATGAACAAGACACCATTTTCACC

TCATACTCGTGTGTGTGCA

>FM895358.1 FM895358 Jatropha curcas embryo 71-95 (DAF) Jatropha curcas cDNA clone rjcpga0_002166, mRNA sequence

CTGGAAATCAGAATCAAACGAGCTTTTACCCTTTTGTTCCACACGAGATTTCTGTTCTCGTTGAGCTCAT

CTTAGGACACCTGCGTTATCTTTTAACAGATGTGCCGCCCCAGCCAAACTCCCCACCTGACAATGTCTTC

CGCCCGGATCGGCCGCCGAAACGACCTTGGGTCCAAAAAGAGGGGCATAGCCCCGCCTCCGATTCACGGA

ATAAGTAAAATAACGTTAAAAGTAGTGGTATTTCACCGTCGCCTTTCGGCTCCCACTTATCCTACACCTC

TCAAGTCATTTCACAAAGTCGGACTAGAGTCAAGCTCAACAGGGTCTTCTTTCCCCGCTGATTCCGCCAA

GCCCGTTCCCTTGGCTGTGGTTTCGCTGGATAGTAGACAGGGACAGTGGGAATCTCGTTAATCCATTCAT

GCGCGTCACTAATTAGATGACGAGGCATTTGGCTACCTTAAGAGAGTCATAGTTACTCCCGCCGTTTACC

CGCGCTTGGTTGAATTTCTTCACTTTGACATTCAGAGCACTGGGCAGAAATCACATTGCGTGAGCATCCG

CAGGGACCATCGCAATGCTTGTTTTAATTAAACAGTCGGATCCCCTTGTCCGTACCAGTCTGAGTCGATG

TTCGACGCCGGGGAAGGCCCCGGAAGGCCGTCCCAGTCGTCCC

>FM895357.1 FM895357 Jatropha curcas embryo 71-95 (DAF) Jatropha curcas cDNA clone rjcpga0_002165, mRNA sequence

TGGATCCCCCGGCTGCAGGCAAAACCTTTCTGTTGAAATCTCTCCTCTATCTACTGTTCAATCATGTCTG

CTTACACGGGCAAATACGCCGATGAGCTCATCCGCAATGCTGCCTACATTGGCACCCCTGGGAAGGGTAT

TCTTGCAGCTGATGAATCTACTGGAACTATTGGCAAGCGCCTTGCCAGCATAAATGTTGAGAATGTTGAG

GAGAACAGGCGTGATCTTCGTGAGCTCCTCTTCACAACTCCTGGAGCCCTCCAATACCTCAGTGGAGTGA

TTCTCTTTGAGGAAACCCTCTACCAGAAGAGTAAATCAGGTAAACCTTTTGTTGATATTTTGAAGGAGGG

TGGTGTCCTCCCTGGTATTAAGGTCGACAACGGTACTGTTGAGCTTGCCGGCACCAATGGTGAGACTACC

ACCCACGGCTCTTGATGGCCTTGCCCAACGATGCCAGAAGTACTACGAAGCTGGTGCCCGATTTGCCAAA

TGGCGTGCTGTGCTTAAGATTGGCCCTAATGAACCATCTCAGCTTTC

>FM895356.1 FM895356 Jatropha curcas embryo 71-95 (DAF) Jatropha curcas cDNA clone rjcpga0_002164, mRNA sequence

AATTCCCCTTCTTTTCTCTATCTCTATCTCCATCTCATTCTCATCTCGTCGCAGCCATGTCGAAAAGAAA

GACTAGAGAGCCTAAGGAAGAAAATGTGACACTATGAGACCTGCTGTCAGGGGAGGGTGAGCATAGTTTT

TGGAAGTTGCCCACATCTTTGCATCTTTCAATGATACGTTCATTCACGTGACTGATCTTTCTGGAAGGGA

AACCCTGGTTCGTATAACTGGTGGGATGAAGGTGAAAGCTGACAGAGATGAGTCTTCCCCATATGCTGCC

ATGCTTGCAGCTCAGGATGTTGCACAGAGATGCAAGGAACTTGGTGTTGGTGCTCTTCATGTTAAGCTGC

GTGCTACCGGAGGGAACAAAACAAAAACACCTGGCCCCGGTGCACAGTCGGCCTTGCGTGCCCTTGCTCG

ATCTGGGATGAAAATTGGTCGTATAGAGGACGTTACACCAATTCCAACTGACAGCACTCGCAGAAAGAGT

GGTAGAAGAGGGAGAAGGCTGTGAGCTTCTTTCCTTAGTGGTCGAGGATCTTGTTTCTTATGTGGGAATT

ATCT

>FM895355.1 FM895355 Jatropha curcas embryo 71-95 (DAF) Jatropha curcas cDNA clone rjcpga0_002163, mRNA sequence

ACGAAAATATTTTTCCACATTAAATTTCACAATCATCATCACAATTTGAAATCTCACAATCATCATCACA

ATTTGAAATCTCTGGAGCAGTAACTACAAGACTAGGATGCGATCCAGAAGCATTCAGTGAAACAGGGGGA

ACATCAGACTGAGCAGATGAAACAGCTGGTAATGAATCCTTCTTATTGGCATCAGAAGCTGCATAACTAA

GATCTGCACCACTGTTATATAGGGAACTGGTCTCTGATTCACTGCAAGAAAATTCAGGAGCAGATATTCT

GTTGCAAGAAAGCAACCCAGAA

>FM895354.1 FM895354 Jatropha curcas embryo 71-95 (DAF) Jatropha curcas cDNA clone rjcpga0_002162, mRNA sequence

GCAAGTTCGGGGTAGGAGTTGGGAAGGTGGAGTGGTTAGATAATGAGGAATCATGGTTGGTGATGGGTAT

GGATGGCCAAAATCATGGCCAATTTAAAGGAGTGGTAGCATCAGACAAAAATATAGTTTCTCCAAGGTTT

AAAGATATCACAGGATGTCCACCACCACTCGATTTGAGTTGGGCTCCAGAATTGGCAGTGAAGTTCCAAG

ATATTCCTGTTAATCCGTGTTTTGCTCTTATGCTAGCATTTACCGAGCCTTTATCTCCTATACCTGTAAA

AGGCTTCTCATTCAAAAATTCTGAAGTTCTAAGCTGGGGTCACTGTGACAGCAGCAAGCCCGGTCGATCA

ACCACAAATGAACGATGGGTATTGCACTCAACAATAAAGTATGCAAAGGATATAATTGCTCAAACTGGAC

CTCAGAAGCCTTCAAGCGAAACGCTAACAAAAGTAGCTGAAGATTTGTTCCAGGAATTTGAAAGCACTGG

CCTTCACAGCTCTAGACCATTTTCAGAAAGGCTCATAAATGGGGAAGTGCTTTTCAGCTGCTAGCATA

>FM895353.1 FM895353 Jatropha curcas embryo 71-95 (DAF) Jatropha curcas cDNA clone rjcpga0_002161, mRNA sequence

CCTGTGAAAAGTGTTATTACGACGATATAATTGAAGGGATAACAAAAAATAGGTACGGGTAATCTTGCTG

GTTCACATGTCCCTGCCTGGAATTAAACTATATGGTGGCTCTTCACAACTCTGCCTCCAGAAGGACTTCT

AAACATACTTTGTAGTGGCCCTTCATGCGAATGTATCATCATGCTAATAAAAATAGATGCAAAATCCTTT

GATTATAGCAATAAGTAAAGGTTGGGATGTGCTAGGCCTATTATGGAGTGAACACGGAGGACAACACTTG

TGAAGAAAATTGGCAAAACATATTAAAAGAGAGTACGTATGGAGCATATTACTGCCCAAATACACGCTTT

GCACCGTCCACGGTGTTCCTCAGTAGCATTGCAACAGTCATTGGACCCACACCGCCAGGAACAGGAGTAA

TCCATCCGGCCACTTTACATGCTTCTTCATAATCTACATCTCCCACTAGCCTATAACCCGACTTCCTACT

TGGGTCATCTATAGCATTTGTCCCAACATCAATAACGGCAGCACCAGGTTTAATCCAGCTGCCCTTGACC

ATCTTTGCTTGCCC

>FM895352.1 FM895352 Jatropha curcas embryo 71-95 (DAF) Jatropha curcas cDNA clone rjcpga0_002160, mRNA sequence

TGCAGGAATTCGAATTCGGCATGCTCCTATCTTACAACCACATACAAACTATATTATATAGCAAAGGTAA

TCAGCTTTACATTGTAGCTATGCCCCATCAAGTCGAGTCTTTCACATATGCTCCATCTCCTCTTCCTCCT

CGTCTTCGTATTCACCCTCCTCATCAGCAGTAGCATCCTGGTACTGCTGGTACTCGGACACAAGATCATT

CATATTGCTTTCAGCTTCTGTAAACTCCATCTCATCCATTCCTTCACCAGTATACCAGTGCAAAAAGGCC

TTCCTCCTGAACATGGCAGTGAATTGCTCACTCACTCGCCTGAACATTTCTTGGATGGAGGTTGAATTTC

CAATGAAGGTGGATGCCATTGACAGCCCCCTAGGTGGAATGTCACAAACACTAGATTTCACATTGTTGGG

AATCCACTCAACAAAGTATGATGAGTTCTTGTTTTGGACATTGATCATCTGCTCATCCACTTCCTTGGTG

CTCATCTTTCCGCGGAACATGGCAGAGGCAGTGAGGTACCGACCATGCCTTGGATCAGCTGCACACATCA

TGTTCTTGGCATCCCACATTTGCTGAGTCATTCTGGGACTG

>FM895351.1 FM895351 Jatropha curcas embryo 71-95 (DAF) Jatropha curcas cDNA clone rjcpga0_002159, mRNA sequence

CTGACATGTGTGCGAGTCAACGGGTGAGTAAACCCGCAAGGCGCAAGGAAGCTGACTGGCGGGATCCCCC

CGAGGGTTGCACCGCCGACCGACCTTGATCTTCTGAGAAGGGTTCGAGTGAGAGCATGCCTGTCGGGACC

CGAAAGATGGTGAACTATGCCTGAGCGGGGCGAAGCCAGAGGAAACTCTGGTGGAGGCCCGCAGCGATAC

TGACGTGCAAATCGTTCGTCTGACTTGGGTATAGGGGCGAAAGACTAATCGAACCGTCTAGTAGCTGGTT

CCCTCCGAAGTTTCCCTCAGGATAGCTGGAGCTCGGGACGAGTTCTATCGGGTAAAGCCAATGATTAGAG

GCATCGGGGGCGCAACGCCCTCGACCTATTCTCAAACTTTAAATAGGTAGGACGGCGC

>FM895350.1 FM895350 Jatropha curcas embryo 71-95 (DAF) Jatropha curcas cDNA clone rjcpga0_002158, mRNA sequence

GAAACTGGACACAATTATTATTAAAGAAAAGTACCAAGATACACTACTATTTATTCTTCTTAATACTATT

GTTACAAGCATCTTTATAACATATAAAATTCATGAACTTGCAGATTGGAACCAACAGGCACACCTCCCCA

CTATAGACCCAACCCAAAAACATCTCCATGTTGAGTCTACCAATGCATGTCTGCCATGTGGTGAGTTCCC

ATTGGTCCAATCCAAGTAGGACCCATTAATCTAACATAACTCTCTTAAATCTCTTATTTAACCCAATAAA

AAAAAAAAGCTAATTAAAGAAAGATAGAAAATTCAAGAAACATTCAAGTTGGCATACAGCCAGAAACAAA

CAGCCTCTACACAGCACAAGTACCCATTACTCAGCTTTCGCTTCACCTTCTTCCTTAGGAGCTTCAACGG

CAGCTTCCGGTGCCGGTTCTGGCGCCGGAGTTGTTGTCTCAGTACTCTCAGTGGTCTCTTTANTAGTCTC

CTCAGCAACAGTCTCCTCAGTTACTGTTTCTTTTGGGGATTCCTTTTCTTCTTTTTCTGGTTCTGTTCCT

TAGGAGTTTCTTCTTTTCTGGTTCTGTTCCTT

>FM895349.1 FM895349 Jatropha curcas embryo 71-95 (DAF) Jatropha curcas cDNA clone rjcpga0_002157, mRNA sequence

TTCTTTTTCTTTTTCTGTCAACTTTTCTTTTAGATATGGCACTTGAAGCTGTTGTGTATCCACAAGACCC

ATTTGGTTATGTCTACAAAGATTGTTACAGTACTGGAGGAGCTTGGGTTGGCTATCCTAATGGTTTTGAT

GACTTTCAAGAAAATCAAGATATAGCTTTTCTTGGGATTCTTGACAATGGTAACAATATAGGGCAACAGA

GTTTGCAAGCTAATTGGGATTCTTCTTCATCTCCTTCTATGATTCATCGACATGCAAAAGAATGGGATCC

TAATTCTTCTTCACCTGAGACTTGCGCCGTTGATCAACCCTTTCCTCCTACTAGTGCTGGAGTTCTCCAT

CATCCATCATTGGAAACACCGGCAGCCACCACTGGCCGGCGAAAGCGAAGACGAACTAAGAGCACTTAAA

AACAAGAGGAAATTGA

>FM895348.1 FM895348 Jatropha curcas embryo 71-95 (DAF) Jatropha curcas cDNA clone rjcpga0_002156, mRNA sequence

GGCAGAAAGAAAATATGTGAGGACAAATACAAGAGAAAGGATCTTCCCTGGTTGGAAAAGGTAGAGAAAA

TATCAAATGAAGGAAACTATGCACCATTTTCGTCCCAGGCCCACAAAATTAGAATCTCTCAAATTGGAGA

AAAAAATGATGAATGAATTGAGAAAATTGCATTGTTTAATGTGAAATAGATTTCATTATTTTTCTTCCTT

TATTATTTTATTTCCTCTTCTTTCCAAACAAACAATTAGTACAAGTACTGACAAGAAAAATTAGAAAAAA

TAAAATCATTTAATAAGCATGAAATCAAACAAACCAAGATGGGGGAAAATGATTGGACGAGTTTAGAATT

AAGACCTAGTTAAGTCATGTTTCCACTAATTTGCCGCAAGAAGAAAAGTATATGCCTGTTATCGCTACCA

CTTAGGACATAATGAATGCTTTCA

>FM895347.1 FM895347 Jatropha curcas embryo 71-95 (DAF) Jatropha curcas cDNA clone rjcpga0_002155, mRNA sequence

TGCAGGAATTCGAATTCCTCTTACTCACACCCAAAAATGGCAGTGAAGCTCTCTGTGATTCTCTGCATTG

TTTCAATTCTTTCCATCTCGGGCGCTAATGGTGCCGGCCACCACCATACAGCTCCGGCACCGTCTGTGGA

CTGCTCAAGTTTAGTGTTGAACATGGCGGATTGCTTGTCTTTTGTTTCAAACGGCAGTACGACAACAAAA

CCAGAAAAAACGTGCTGCTCTGGGTTGAAAACGGTCTTGCAAACAGATGCAGAGTGTCTTTGTGAGGCTT

TTAAGAGCAGCGCTCAATTGGGTGTCGTTTTGAATGTCACTAAGGCTTTAAGTCTTCCTACTGTCTGCAA

ACTCCAAGCCCCTCCTGCTTCTAATTGTGGATTGGCTTTGGCTCCTGCTGCTGCTCCTGGTGCTCTTTCA

CCATCTGTTAGTGGTGCTGCTGCCCCAACTGTGACCGCCGGAGCAACTGTGTCAGCACCAGCACCATCTC

CAGGGAATGCAGGTTCTCGCGGATTGTCAATCTCAGTTGGCTCTCTAGCAATTGGCCTTGTAATTGCATC

ATTCTCTAGTTTCTGAGTAATATGTCACCCCTTTTGAGTCAA

>FM895346.1 FM895346 Jatropha curcas embryo 71-95 (DAF) Jatropha curcas cDNA clone rjcpga0_002154, mRNA sequence

ATTCCTCGACTTTTTACTGTTGGTCGCCTTGATGTTGCCACATCTGGATTGATTATTGTGACCAATGATG

GAGATTTTGCTCAAGCACTTGCACATCCTTCATTTAAACTATCGAAGGAATACATTGCAACAGTAGAGGG

TGAGGTAAACAAACGGCATTTGATAACCATCAGCGAAGGAACAATTGTTGAGGGTGTACACTGCACTCCA

GATTCTGTGGAGTTACTACCACGGCAGCCCGATATTTCAAGACGTCGTCTTCGTATAGTGGTTCATGAAG

GGAGGAATCATGAAGTTCGAGAACTAGTTAAGAATGCTGGACTAGAGGTTTATTCATTGAAGCGTGTGCG

AATTGGTGGTTAATAGACTTCCATCAGATCTTGGGATTGGGAAGCATGTTGAAC

>FM895345.1 FM895345 Jatropha curcas embryo 71-95 (DAF) Jatropha curcas cDNA clone rjcpga0_002153, mRNA sequence

ATTCACTACTTTTAACGTTATTTTACTTATTCCGTGAATCGGAGGCGGGGCTATGCCCCTCTTTTTGGAC

CCAAGGTCGTTTCGGCGGCCGATCCGGGCGGAAGACATTGTCAGGTGGGGAGTTTGGCTGGGGCGGCACA

TCTGTTAAAAGATAACGCAGGTGTCCTAAGATGAGCTCAACGAGAACAGAAATCTCGTGTGGAACAAAAG

GGTAAAAGCTCGTTTGATTCTGATTTCCAGTACGAATACGAACCGTGAAAGCGTGGCCTATCGATCCTTT

AAACCTTCGGAATTTGAAGCTAGAGGTGTCAGAAAAGTTACCACAGGGATAACTGGCTTGTGGCAGCCAA

GCGTTCATAGCGACGTTGCTTTTTGATCCTTCGATGTCGGCTCTTCCTATCATTGTGAAGCAGAATTCAC

CAAGTGTTGGATTGTTCACCCACCAATAGGGAACGTGAGCTGGGGTTAGACCGTCGTGAGACAGGTTAGT

TTTACCCTACTGATGATGGTGTCGCGATGGTAATTTCACCTAGTACGAGAGGAACCGTTGATTCGCACGA

TTGGTCATCGCGCTTGGTTGAAAAG

>FM895344.1 FM895344 Jatropha curcas embryo 71-95 (DAF) Jatropha curcas cDNA clone rjcpga0_002152, mRNA sequence

CGAAGGATGGGTAGATAGGTAGCGGAACCCATAACAGCAGAGGTAGAGCTGCCAATGGCGAAGCAAATGG

TGCGGTGTTTTTTACATCTTCCGCTTTCATCTAGAGCTCTTTTTTCACCTCCCTGTTTGCTTTGCCACCA

GAGCAGAGAGAACCACAGCCACCTCTACTGCTAATTCTATTAGCAACAAGAAGAAGAAGAGTAAGAAGAG

TGAGGAGAAGAAGAAAGCAGAGTTAATAGCGGTAAGGAGGGAAAAGGAGAAGAGAAGAACTCGTTCTGAT

AAAGAATTTGGTTCTTCTTCTTCATTTGATTTTCACAATAATGGAAATCATATCCCTGTTATGCTTGGTG

AAGTTGTTGACGTCTTCTCTTCATTGAATCTTCGCTCTTTTGTCGATTGTACTCTTGGCGCCGCCGGCCA

TACCTCCGCCATAATCCAGGCCCATCCTGAACTGGAAAATTATGTAGGGATGGATGTTGATACAGTTGCA

CATGCCAAGGCTCGAGCTTGCATTGATACTTTACTGCATTCTCATTCTAACTTGAAAGCACACA

>FM895343.1 FM895343 Jatropha curcas embryo 71-95 (DAF) Jatropha curcas cDNA clone rjcpga0_002151, mRNA sequence

CCGGTGAATTTGATACCGCTCGTCCTGAGAAGGTGAATCGATCGGCTCTCTCAGTGTTCAAGCCAAATAC

AAGCAAGGTAAATTACCCACTAAAGGCATGCTGAATATACCGCAGGTCTTAATTTTTGTGGTACGAACAT

GCTCGGTTGATCAGCCGGGGCGGATTCAAATCCGTCTCACTGATTCTGCTAGGCCGCAAGACGTGGTCGA

TGGTAACCAACAGGTTACCCTCGAACTTTCGAAATTGCCGGCTATAGTTAGTTTTTCGCCGAGTTATGAT

TGTCCATTAGAGACGATGGGTAAAGATGGCCTCCCTCGGTGCTTTATGATAGAAACCAGATTGTCAGGCG

TGGTAGGGCATGAAGGCACTGTTGCGCAGTGCTTCAATTACTGGGATGCAAATTTCCCCAGTAAAGGAAA

CAATTATAAATGCCACCGCACACAGGCGCAGTTCGTGATACCCTTTGATCGTCTCGTGAAATTAGATCCT

TCATGTATAGGATCTTTCATTAAGGGCGTCTCTAATCGAACAGAAGCCAGCAGTTACCTGCAATGCTCGC

GCCTACCTGATGAGGTTCCGAGTTTACATAT

>FM895342.1 FM895342 Jatropha curcas embryo 71-95 (DAF) Jatropha curcas cDNA clone rjcpga0_002150, mRNA sequence

ATTCCGAATCGGAGCGACAAGGGCTGAATCTCAGTGGATCGTGGCAGCAATGCCACTCTGCCACTTACAA

TACCCCGTCGCGTATTTAAGTCGTCTGCAAAGGATTCTATCCGTCGCCCGGTGGGAATTGTACTTTAAGG

CGGCCCTCGCGGCTCGTCCGCCGCGGGGGCTTAGCCAACGGCACGTGCCTCTGGGGGCCGGATGGCCCCT

ACTGCGGGTCGGCAAACGGGCGACGGACACGCGCGTCGCTTCTGGCCCGGATTCTGACTTAGAGGCGTTC

AGTCATAATCCAGCGCACGGTAGCTTCGCGCCACTGGCTTTTCAACCAAGCGCGATGACCAATTGTGCGA

ATCAACGGTTCCTCTCGTACTAGGTTGAATTACCATCGCGACACCATCATCAGTAGGGTAAAACTAACCT

GTCTCACGACGGTCTAAAACCAGCTCACGTTCCCTATTGGTGGGTGAACAATCCAACACTTGGTGAATTC

TGCTTCACAATGATAGGAAGAGCCGACATCGAAGGAT

>FM895341.1 FM895341 Jatropha curcas embryo 71-95 (DAF) Jatropha curcas cDNA clone rjcpga0_002149, mRNA sequence

AGGTTCTGTTCAAAAACTCGTCCGAGAACTTGTATAGAGAAGGATTTCCCGCGGCGCAGCAGTTCTTCCA

TACCAACTTAGCTGCCCGGCGCTGCTATTAGCATAACAACCGGTACACCATAGGTTGGCCCAACCCAGTC

CTCTCGTACTAGGGTTGGCTCCTCGCAGTTCTCCCTTTAACACCAACGGTAGATAGGAACCGAACTGTCT

CACGACGTTCTAAACCCAACTCACGTACCACTTGAATCGGCGAACAACCGAACCCTTGGGACCTTCTTCA

ACCCCAGGATGTGATGAGTCGACATCGAGGTGCCAAACGACTCCGTCGATAAGAGCTCTTGGGAGTCATC

AGCCTGTTATCCCCGGCGTACCTTTGATCCGTTGAGCGAGAGCCCTTCCACACGGGACTCCCGGATCACT

ATGGCCGACTTTCGTCTCTGTTCGACCAGTCGGTCTCACAGTCAGGCAGGCTTATACCATTACGCTCACG

AGCAGAATCTTAGCTTGAGCCTACCTTCGCACACCTCCGTTA

>FM895340.1 FM895340 Jatropha curcas embryo 71-95 (DAF) Jatropha curcas cDNA clone rjcpga0_002148, mRNA sequence

CGTAGAAACCTGTTAGAGCGTTTCTTGGAACCTGCCAATAATGAGTAAGCTTCAGAGTGAGACATTGAGA

GAGGCTATCTCTACTATTATGGCTGCTTCTGTGGAGAAGAAGCGTAACTTTACTGAGACAATTGAGCTCC

AGATTGGACTGAAGAACTATGATCCTCAAAAGGATAAGCGTTTCAGTGGCTCTGTGAAGTTGCCTCACAT

CCCTCGTCCTAAGATGAAGATTTGCATGCTTGGAGATGCTCAGCATGTTGAGGAGGCTGAGGCAATAGGA

CTACAATGGATGGATGTGGAAGCATTGAAGAAGCTAAACAAGAACAAAAAATTGGTTAAGAAACTTGCAA

AGCAGTACCATGCCTTCTTGGCTTCAGAATCTGTCATCAAGCAGATTCCTCGTCTTCTGGGCCCTGGTCT

CAACAAGGCAGGTAAATTTCCTACCCTTGTGACCCATCAGGAGTCCCTAGAAGTCTAAGTGAATGAAACA

AAGGCAACAGTGAAGTT

>FM895339.1 FM895339 Jatropha curcas embryo 71-95 (DAF) Jatropha curcas cDNA clone rjcpga0_002147, mRNA sequence

TTTTATAATTAAAAATATTTTATTAAATTTTATATTTTTATTGAATTAAAGCAAACATAACATCCACAGA

AGGCCGTAACAGGCCAATCAGAAAGGAAACAGAATACAACGATGATAGAGAAGCCCATTAGACTCTCTAA

GAAAATACATTCAGATGAAAGCATGAAAACAATTAAGGAAAGCAATTCCCCCCTTCAGAATCCACCACGC

AGACGCAGGACAAGGTGAAGGGTTGATTCTTTCTGGATATTGTAGTCGGCCAGAGTCCTTCCGTCCTCCA

ACTGCTTCCCAGCAAAGATCAGCCTTTGCTGATCTGGAGGAATCCCTTCCTTATCCTGGATTTTCGCCTT

CACATTATCGATTGTATCACTGCTCTCCACTTCCAGAGTGATAGTCTTCCCTGTCAGGATCTTGACGAAG

ATTTGCATTCCACCACGAAGCCTGAGCACGAGGTGGAGAGTGGATTCCTTCTGAATATTGTAATCGGCCA

AAGTACGGCCATCTTCCA

>FM895338.1 FM895338 Jatropha curcas embryo 71-95 (DAF) Jatropha curcas cDNA clone rjcpga0_002146, mRNA sequence

GCAAAGGATTCTATCCGTCGCCCGATGGGAATTGTACTTTAAGGCGGCCCTCGCGGCTCGTCCGCCGCGG

GGGCTTGGCCAACGGCACGTGCCTCTGGGGGCCGGATGGCCCCTACTGCGGGTCGGCAAACGGGCGACGG

ACACGCGCGTCGCTTCTGGCCCGGATTCTGACTTAGAGGCGTTCAGTCATAATCCAGCGCACGGTAGCTT

CGCGCCACTGGCTTTTCAACCAAGCGCGATGACCAATTGTGCGAATCAACGGTTCCTCTCGTACTAGGTT

GAATTACCATCGCGACACCATCATCAGTAGGGTAAAACTAACCTGTCTCACGACGGTCTAACCCCAGCTC

ACGTTCCCTATTGGTGGGTGAACAATCCAACACTTGGTGAATTCTGCTTCACAATGATAGGAAGAGCCGA

CATCGAAGGATCCAAAAGCAACGTCGCTATGAACGCTTGGCTGCCACAAGCCAGTTATCCCTGTGGTAAC

TTTTCTGACACCTCTAG

>FM895337.1 FM895337 Jatropha curcas embryo 71-95 (DAF) Jatropha curcas cDNA clone rjcpga0_002145, mRNA sequence

CAGAGGTTGATTTTTGCTGGCAAGCAGCTGGAAGATGGGCGCACCCTTCAGACTACAACATCCAGAAGGA

ATCCACCCTGCACTTGGTCCTTCGTCTTCGTGGTGGCATGCAAATCTTCGTGAAGACCCTCACTGGCAAG

ACTATCACATTGGAAGTCGAAAGCTCAGACACCATTGATAATGTCAAGGCAAAAATTCAGGACAAAGAGG

GGATCCCGCCAGACCAGCAGAGGCTGATCTTTGCTGGTAAGCAACTGGAGGATGGCCGCACCCTTGCTGA

CTACAACATCCAGAAGGAGTCCACCCTCCATTTGGTCCTTCGTCTTCGCGGTGGCATGCAGATATTTGTG

AAGACTCTGACAGGCAAGACAATCACGTTGGAAGTTGAAAGCTCTGATACTATTGACAATGTGAAGGCCA

AGATACAGGACAAGGAGGGCATCCCACCAGATCAGCAGAGGTTGATTTTTGCTGGAAAGCAGCTTGAAGA

TGGACGCACCCTTGCAGACTACAACATCCAGAAAGAATCCACCCTTCACTTGGTTCTACGTCTTCGTGGT

GGCATGCAGATCCTTGTGAAGACCCTTACTGGC

>FM895336.1 FM895336 Jatropha curcas embryo 71-95 (DAF) Jatropha curcas cDNA clone rjcpga0_002144, mRNA sequence

TTTTTTTTGAAATTGACAGTTCCGTACATAAGTATATGTAAGTATATATATATAAGCCCCCTTGGAATTT

TCTTGCAATATCAGTAACTTAATATTATCAGACAATATTACTTGGAAATTTATAATTTGCAATTGCAATT

TTTATCTTGTTACTCTTGACCACTGAAAAAGAAGAGAGACAAGAATGTGCCAAGTTGGACTTCAACTGCT

CTGCATTCAGATTGAATTAAATAAGCTTGAAAAAACGAGAAAAAAGAAAGCATCTATGTTAGTCCGTCCA

TCTAATTCAAATATGCCTTTTCTTACAATTGGCGACCCACTATCTTGGAATAGTAACAAAATATTACACA

ACCATGGCAGACTACACAACACTAGTTTAGCAAGTCTACATATGAATAACTAACCTTATAAAATTCCAAG

CCTTCATTTGTTAGGTTTTCATCCCCATAAAATTCCCACTTCAA

>FM895335.1 FM895335 Jatropha curcas embryo 71-95 (DAF) Jatropha curcas cDNA clone rjcpga0_002143, mRNA sequence

AAGCTGGAGCCACGCGGTGGCGGCGCTCTAGAACAGTGGTCCCCCGGCTGCAGGCACCAAACCTTTGAAG

CAATTCAAATATGGCAATTTCTCCACTCAAAACTCATACCAGATTACCATAACTGTGTAATTTAATCTCT

GAACAATCCATCTAAGTTTCCTTTTTCTTTTTGATTTCTCATTGTTCTTTGGATGTTATATATTTGATTT

AAATATAGATAGATTTGTATAAATGGAGAAGAGTTTCAGTGCCGAAGATCTGTCTACTATTGGAGGAATA

GCTACCGTTTCGCTTCTTCATTCATTCATTCCTACGCATTGGCTTCCTTTCTCCATTGTTGGCCGTGCAC

AGAAATGGACTCTCTCAAGAACCCTCCTTGTCACGGCTTTTGGGGCAGTTCTGCATGTGATATCCACTTC

ACTTCTTGGGATAACAGCAATCACCATGGCAAACACTATTGCTGGAGAAGAAACAGTGCACAAGCTTGCA

TCACTTCTTCTCATAATTCTCGGTGGTAGTTATGTATTACTTTTTCTTTCTGGGAACGGTGGTGGACACA

GTCATTCGCACA

>FM895334.1 FM895334 Jatropha curcas embryo 71-95 (DAF) Jatropha curcas cDNA clone rjcpga0_002142, mRNA sequence

TCTTCACTTTGACATTCAGAGCACTGGGCAGAAATCACATTGCGTGAGCATCCTCAGGGACCATCGCAAT

GCTTTGTTTTAATTAAACAGTCGGATTCCCCTTATCCGTACCAGTTCTGAGTCGACTGTTCGACGCCCGG

GGAAGGCCCCCGGAAGGGCCGTTCCCAGTCCGTCCCCCGGCCGGCACGCGGCGACCCGCTCTCGCCGCGG

GAGCAG

>FM895333.1 FM895333 Jatropha curcas embryo 71-95 (DAF) Jatropha curcas cDNA clone rjcpga0_002141, mRNA sequence

ATTCGGAGTGTGTAACAACTCACCTGCCGAATCAACTAGCCCCGAAAATGGATGGCGCTTAAGCGCGCGA

CCTATACCCGGCCGTCGGGGCAAGAGCCAGGCCCCGATGAGTAGGAGGGCGCGGCGGTCGCTGCAAAACC

CGGGGCGCGAGCCCGGGCGGAGCGGCCGTCGGTGCAGATCTTGGTGGTAGTAGCAAATATTCAAATGAGA

ACTTTGAAGGCCGAAGAGGGGAAAGGTTCCATGTGAACGGCACTTGCACATGGGTTAGTCGATCCTAAGA

GACGGGGGAAGCCCGTCCGACAGCGCGTCCTCGCGCGAGCTTCGAAAGGGAATCGGGTTAAAATTCCCGA

ACCGGGACGTGAAGGCTGACGGCGACGTTAGGGAGTCCGGAGACGTCGGCGGGGGCCTCGGGAAGAGTTA

TCTTTTCTGTTTAACAGCCCGCCCACCCTGGAAACGACTCAGTCGGAGGTAGGGTCCAGCGGCTGGAAGA

GCA

>FM895332.1 FM895332 Jatropha curcas embryo 71-95 (DAF) Jatropha curcas cDNA clone rjcpga0_002140, mRNA sequence

TTTTTTTTTTAGAGGATATCAAAACATAGGGAACACCAAATTCTTATATATTCTTGATTGTTTTCCTAAT

ACATTATAACTGGAAGTAAACAATGAAGAAAATGCACAAAGGTGGTGACTTTCCACACAGACACAACGAT

GAAGATGCTGCGGTGAAAACATCACTACGCGACCTTCATTTTTTACTTACAACTTCAAGCTGTTAGCAAT

CAAACTATCACAACCGTATCTATCAGATGGCATCTCTGTCCTCTGTCAGTTTAGCAAAAGTTGTAGCAAA

CCAGAATTGAAACAAACAAATGTAAAAACAAAACACCTACTCACTATATTTCTACCTCATTTCTTATAAA

ACTATTATAATCAGTACAGAAAGCCAAAAACCAAGCATGCGGACACTTCCAGTCAAAAAAATATATTTAC

TCTGTTACCAGATTATTTGCTATGCAGTCACTATTGGTCTGCTCGGTCTTTTCCCTTCCATGGCATCTTT

CTGTTTTCGACAGTTAGGGC

>FM895331.1 FM895331 Jatropha curcas embryo 71-95 (DAF) Jatropha curcas cDNA clone rjcpga0_002139, mRNA sequence

CTGACATGTGTGCGAGTCAACGGGTGAGTAAACCCGCAAGGCGCAAGGAAGCTGACTGGCGGGATCCCCC

CGAGGGTTGCACCGCCGACCGACCTTGATCTTCTGAGAAGGGTTCGAGTGAGAGCATGCCTGTCGGGACC

CGAAAGATGGTGAACTATGCCTGAGCGGGGCGAAGCCAGAGGAAACTCTGGTGGAGGCCCGCAGCGATAC

TGACGTGCAAATCGTTCGTCTGACTTGGGTATAGGGGCGAAAGACTAATCGAACCGTCTAGTAGCTGGTT

CCCTCCGAAGTTTCCCTCAGGATAGCTGGAGCTCGGGACGAGTTCTATCGGGTAAAGCCAATGATTAGAG

GCATCGGGGGCGCAACGCCCTCGACCTATTCTCAAACTTTAAATAGGTAGGACGGCGCGGCTGCTTCGCT

GAGCCGCGCCACGGAATCGAGAGCTCCAAGTGGGCCATTTTTGGTAAGCAGAACTGGCGATGCGGGATGA

ACCGGAAG

>FM895330.1 FM895330 Jatropha curcas embryo 71-95 (DAF) Jatropha curcas cDNA clone rjcpga0_002138, mRNA sequence

ATTCTATTATTTCTAATTTAATACTCTTTTTCTCCTTTATCTCTCCCCTTTCAGACATACATTTTTCATT

TTCCAACCAAGAACCTTTCCTCTTTTTTTTTTCTATTTTATGCTTCACTGTATGATGAGCTTTTGCAAGA

AAAGTGTTCACTCTTTTTTGGGTCTCACAATACCCACGGATTCTAATGATCATACTAATACTACTGTTGT

TCAAGAAAAGCCTTCCAAATGTCTTTTAACTAATACTAATGGTGGATTAGGGTTAATCTCTGCCACTGGT

GATTTTTGTAAAACACCCAATATTCTTGAATCTGCTACTGTCAAGTCTTCCTCACCGCCGATCAAGAAAG

ATCCCGGTGGGATTGGATTTCTTGATGATGTTGGTGGTGGTGTTAATGGTTTGATGTCTTGCACTGAAAG

TTTAGGATTTGAAAGTTGCGATGAAAGAAGAGTTGATGATGAGATTGACGAGTTATTATCAGCGAGAACC

GATAAGGTTAAATGGAGAAAGATTGGAGAAAGAAGAAGAGAGGTAAA

>FM895329.1 FM895329 Jatropha curcas embryo 71-95 (DAF) Jatropha curcas cDNA clone rjcpga0_002137, mRNA sequence

CTAACCTAGAACCCACAAAGGGTGTTGGTCGATTAAGACAGCAGGACGGTGGTCATGGAAGTCGAAATCC

GCTAAGGAGTGTGTAACAACTCACCTGCCGAATCAACTAGCCCCGAAAATGGATGGCGCTTAAGCGCGCG

ACCTATACCCGGCCGTCGGGGCAAGAGCCAGGCCCCGATGAGTAGGAGGGCGCGGCGGTCGCTGCAAAAC

CCGGGGCGCGAGCCCGGGCGGAGCGGCCGTCGGTGCAGATCTTGGTGGTAGTAGCAAATATTCAAATGAG

AACTTTGAAGGCCGAAGAGGGGAAAGGTTCCATGTGAACGGCACTTGCACATGGGTTAGTCGATCCTAAG

AGACGGGGGAAGCCCGTCCGACAGCGCGTCCTCGCGCGAGCTTCGAAAGGGAATCGGGTTAGAATTCCCG

AACCGGGACGTGAAGGCTGACGGCGACGTTAGGGAGTCCGGAGACGTCGGCGGGGGCCTCGG

>FM895328.1 FM895328 Jatropha curcas embryo 71-95 (DAF) Jatropha curcas cDNA clone rjcpga0_002136, mRNA sequence

ATTCAAATCCTTGTTCCGTCCGGTCCCAATACCGAGGTTTCGGATGTGACGACTTACGGCGGTGGTTCCC

AGCCGGACCTCGGTTACCTTCTGGAAGCCTCAGATGACGAGCTTGGCTTGCCGCCGACGTTCTCCGGCGA

GGAAGAAAAGGTCAGTGCTGTAGATTTGGATCCCGAATTAAGCGCATCTGGTGCGACTGGTTTCGGAAAT

ATGGCAGGCTTCGAGGATGACATACCGAGTTACGACTCGTTTGGGTTCGGACTCGGGGGTGACTCCGATA

GTAATAGTTACAACGTTAGTTATAATGACGGTGGAGATTTTGTCTCCTTAGGTGGATTGTTTGATTACGC

GGACGAGAACTATGTGCCGGCCGATATATCGGCTGTGCAGTGGCAGCCCGAGTCACTATCTGC

>FM895327.1 FM895327 Jatropha curcas embryo 71-95 (DAF) Jatropha curcas cDNA clone rjcpga0_002135, mRNA sequence

ACAGCAGGAATAAATAAGGGAAATTACCATTTATACTAACATCAGTTGTTACTGAACAAAGCTGATGATA

ATAACACATACAAAGTCATGATTTTCCGTTTGTGGTCACCTAAACATCTCAACATACAAAGGAAAACATC

ACACTACATTGATACATTATTACACTTCACCATTAGTATTAGTCTTCTTGCTAAAGTCTAAAGTCACCTG

TGTGTTTTCCTCTGACCCAGTAGAACTACTGCTCAGAAGAAAGCCCTGCTCCCATAAAACACGCGGGAAC

CACTGTTCTTTGAAGATTGAATTTTCTGATCAGAAGTTGAACTACCTGAACTGGTTGGTCCACTATCAAT

GTCCAAAGGAAGCTTCATCTTTGCCAATGACTCCGTGAATTGCTGCATGCTTTACATGTACATGTCTACT

ATGTCCTGTTGAACCACATTTGGCTCTGGTTCAATATACATAGTAATAACAGGCTTTACAA

>FM895326.1 FM895326 Jatropha curcas embryo 71-95 (DAF) Jatropha curcas cDNA clone rjcpga0_002134, mRNA sequence

CACGGATACCGGTGTTGGGCACCGGAGGAATTGTACTAAAATTCATGTTTCGATAGAGTCCTGCAAAGAG

AAAGGGTATTCAGTAGTGAATATGAGGAGCAGAGACCGGCCTAAGCTTTTATTTGATACGTTATGCGTAT

TAACTGATATGCAATATGTAGTATTCCACGCTGCAGTTAGCTCCATGGGCACTATGGCTGATCAGGAGTA

CTTTATACGACATAAAGATGGCTTTACTTTGGATACAGAAGGTGAAAGGCATAAACTGACTCAATGTTTG

ATTGCTGCGATTGAAAGAAGAGTATCCCATGGATTGAGACTAGACCTCTGCACACACAACAGAATCGGAT

TACTTTCAGACATGACACGGGCATTTCGCGAAAATGGTTTATCGATATCAAGGGCTGAGATGGGAACACA

AGGTGAGACAGCAGTAGGATCATTCTATGTAACTGATGCTTCAGGAGATGAAATTAATCCGCTGACAATA

GAAGTAGTGAGGAAAGAATAGGGGGATCTGTCTTGGTAGTTATA

>FM895325.1 FM895325 Jatropha curcas embryo 71-95 (DAF) Jatropha curcas cDNA clone rjcpga0_002133, mRNA sequence

TTCAAGTTCAAGAAACCAATATGATCACATTCTTCAGAGAATTTTATTTTTCTGTGCAAAAAATGAAGTT

TCTTAAAAATCAGAAGGTAGAAGTATGCAGCAAGGAAGAAGGTTTCGTTGGCTCTTTCTATGAAGCAACT

ATCCTCAACCCCATTTATGACAACAAACAAGTGTTCTATGAAGTTGAGTACAAGAATCTGGTAGAGGAGA

AGAACGAGAACAAGCCTCTGGTAGAGAAGGTCCGGCATGATGAAGTCCGGCCACTCCCTCCGCCAGTAGT

GAACGAGCGAGGGTATTTGGTGGATGAGTTGGTTGAGGTTTTTGATAATGATGGATGGTGGTTTGGCACG

ATTACTGGCAAAACAGGGTGCTGTTACTCTGTCTACTTTCCGACAACTGGTGACCATATGGAGTACCATG

TTTCTTGTTTGAGGCCTCATCAGGATTGGGTCCAGGATAAGTGGACTTCCTACAAGTGAAGAAGAAAAAC

ATATTTCTTCCTTTCATTTTTTGTTTTCTTCACTGTTTTAGTAACACTGTAACAGATACTAGCAAAGGAT

TGATGT

>FM895324.1 FM895324 Jatropha curcas embryo 71-95 (DAF) Jatropha curcas cDNA clone rjcpga0_002132, mRNA sequence

CTCGTTGCGGCCTCATATCGCACTTCCTGCTCATCGCCACCGGCAACCATGATCATTCCGGAGAAGAACC

GCCGCGAGATCTCTAAGTACCTCTTCCAAGAGGGAGTATGCTATGCAAAGAAGGACTTCAACCTTGCAAA

GCACCCACAAATTGATGTGCCGAATCTACAGGTTATTAAACTGATGCAGAGCTTCAAGTCAAAGGAGTAT

GTCCGCGAGACATTTGCATGGATGCACTACTACTGGTACCTCACTAATGATGGTATTGAGTTTCTGCGAA

CTTACCTCAATTTGCCATCTGAAATTGTTCCAGCTACATTGAAGAAACAAAGCAAACCCCTTGGTAGGCC

CATGGGAGGCCCACCAGGAGATCGCCCACGTGGTCCTTCACGTTTTGATGGAGAGAGGAGATTTGGTGGT

GATCGAGATGGATACCGTGGAGGTCCACGTGGACCTGGTGGTGAGTTTGGTGACCACGGTGGAGCCCCTG

CTGATTACAGACCTACCTACGGGGGTAGTGGTGGAAGGCCTGGCTTTGGCCGTGGTGCTGGTGGCTTTGG

TG

>FM895323.1 FM895323 Jatropha curcas embryo 71-95 (DAF) Jatropha curcas cDNA clone rjcpga0_002131, mRNA sequence

TGGTCTTATTCTCTTGGAATGGTCGGGCTGGGGTCAAGCGCCGGGCGGAATGGGGAATCTTCAACCCGAC

TTGTTTCGGTATCTGCCGCTGCTAGAGAGGCGATGGAGTTTGTATCGGTGTTTGAGAGAGAGTATGGTTC

TAGAAGGCCGAATTTTGTAACTGAAGGGTTCATGGATGCTCTGCAAAGGTCGAGGAATGCTTACAAGTTG

TTATTTGTGTACTTGCATTCACCAGATCACCCGGATACGCCTATGTTTTGTGAGAGGACTCTGTGTTCAG

ATGTTTTCTCCGCGTTTGTGAATGAGAACTTCGTGGCCTGGGGAGGGAGCATTAGGGCTAGCGAAGGATT

TAAGATGAGTAACAGCCTTAAGGCTTCCAGATATCCGTTTTGTGCCGTAGTCATGCCCGCCACAAATCAA

AGGATCGCGTTACTTCAACAGGTTGAGGGTCAAAAATCTCCTGAAGAAATGCTTGTGACACTACAGAGAG

TGCTTGAAGAAAGTGCACCT

>FM895322.1 FM895322 Jatropha curcas embryo 71-95 (DAF) Jatropha curcas cDNA clone rjcpga0_002130, mRNA sequence

AAACTAGTGGATCCCCGGCTGCAGAAAGGATTCTATCCGTCGCCCGATGGGAATTGTACTTTAAGGCGGC

CCTCGCGGCTCGTCCGCCGCGGGGGCTTGGCCAACGGCACGTGCCTCTGGGGGCCGGATGGCCCCTACTG

CGGGTCGGCAAACGGGCGACGGACACGCGCGTCGCTTCTGGCCCGGATTCTGACTTAGAGGCGTTCAGTC

ATAATCCAGCGCACGGTAGCTTCGCGCCACTGGCTTTTCAACCAAGCGCGATGACCAATTGTGCGAATCA

ACGGTTCCTCTCGTACTAGGTTGAATTACCATCGCGACACCATCATCAGTAGGGTAAAACTAACCTGTCT

CACGACGGTCTAATCCCAGCTCACGTTCCCTATTGGTGGGTGAACAATCCAACACTTGGTGAATTCTGCT

TCACAATGATAGGAAGAGCCGACATCGAAGGATCAAAAAGCAACGTCGCTATGAACGCTTGGCTGCCACA

AGCCAGTTATCCCTGTGGTAACTTTTCTGAC

>FM895321.1 FM895321 Jatropha curcas embryo 71-95 (DAF) Jatropha curcas cDNA clone rjcpga0_002129, mRNA sequence

AATACAAAATCACAATTTTCATTTATGCTTTTATTCTCACACCTCATCAAACCAAGATATATATGCACAC

AGTTCACTATATTTAAGAGTGAGCATCAAGGTACTATTCAATAGTCTTGCTTGCTCCACCAAAAAGGTAC

TATTTACATATAGATATGCTTGTTAACTAACTCATTAAACATATTTCTTTATTTTGGGATATAAACATAA

ATAATATTTGTAGCCTTGTCCACCGGCTGCACTTGAAAGTCGAAATGCTAACAGCTGAAAATTTCAATTT

TCAGCGTTTCAGTTATAATTTTGAACCATAAACAAGTAGTCTTGAACCGCGGCTCTAAACTAAATGCTAG

TTTTACTGTATACCCTTTGAACTAATTACTAGGAACTCAAATGTCGAACATTTGTCTCCGGAACAGCTCA

GCCAAACAGGCCCTAACATAGAGGCCATAGTCTTCTAATGCACAATGTATATTACATCGGATAGTATAAG

TGAATCTTCTCAATACTATCCAGACATGGATGATTTCCAAATCTTCCTCATTA

>FM895320.1 FM895320 Jatropha curcas embryo 71-95 (DAF) Jatropha curcas cDNA clone rjcpga0_002128, mRNA sequence

GAGAGAGCTTAGACTTCATCTGATTTTAGGGTTTCCCTTCCTATCTAATTTCTTCATCGTGAAGCCCTAA

TCCTCGGATCAGGTCTGAGCATTCTGGTTGTTAGGCTTCCTTGATAAGCTAAGTTTCATGTCTGAGCTCG

AAGCCCAGATTCCTACTACCTTTGATCCTTTTGCTGATGCAAATGCTGAAGACTCTGGTGCTGGGGCAAA

AGAGTATGTGCATATCCGTATACAGCAACGGAATGGTAGGAAAAGCTTGACTACTGTGCAGGGTTTGAAG

AAGGAATTCAGCTACAACAAGATACTCAAGGATCTCAAGAAGGAATTCTGCTGCAATGGCACTGTAGTAC

AGGACCCTGAATTAGGGCAGGTTATTCAACTTCAAGGTGATCAGCGGAAGAACGTATCTACCTTCCTTGT

CCAAGCTGGCATTGTGAAGAAGGAAAATATAAAAATCCATGGTTTCTGAGTTCCAGTAACTCAATTCTCT

GCCTGGTGCATTCCCCTCCTATGTTGGTGCGGAAATTGTTTGAACTTTAAAATCTATTTAAGCTGCTAAA

TCCCTAGTCATG

>FM895319.1 FM895319 Jatropha curcas embryo 71-95 (DAF) Jatropha curcas cDNA clone rjcpga0_002127, mRNA sequence

AAAACAAGAAAGAATGAATCACACTCGTTTGAAAAATCTTTAAAATACTATAATCCCAACACAGCGAAAC

CAATCTGCTCAGACCTAAATTTTAAAAACTATCTTAGTATATATAACTTCAAATAACATTATAAGCTGCT

TATGAATTCAGAGTTTCTTATTTATACACACTACTCGATCCATCAATGTGAAAGCACTCTGTTTAGAAAA

TAGGACCAGCTGCAAGAATCTCCTCAGTCAGACTGTTGTCTTTTCCAATCTTATAATTTGCGAACACTTC

AAAATTCTTCCTGAACAGACCACCCAGCTTCAACAGGGTATCCTGGTAGGCTTTCTTGTCTTTCCAAGTG

TTAACAGGATCCAGGATTTCTGAAGGCACACCCTCGATCTCAGTTGGGATTTCAAAGCCCAAACACTTCA

GTCTTTTTGTAGTTTGCTTTCAAGAGTCTCCCAGAATGTATGGCATCGATGATTCTTCTAGTGTATGCTA

ACTTCATACGGCTTCCTGAGCCATAGCTGCCACCTGACCAGCCGGTATTGACAAGCCAACCTGTAGCCCC

ATGCTTCTGCA

>FM895318.1 FM895318 Jatropha curcas embryo 71-95 (DAF) Jatropha curcas cDNA clone rjcpga0_002126, mRNA sequence

GAAGATTTACTTCTCTGCGGCGGGCTTGATTCTACGACAGATCATATGATAGAGTCACCCCTAAAAACGA

GCGTCGTTTGGAGAGATTCAAAACCGAAACTTTTTCAAAGTCACCACGACTGATGATCCTGTGATTCGGA

GACTTGCCAATGAAGATAAGGCTACTGTGTTTGCTACGGATACTATCCTGGCAACTTTGATGTGTGCGCC

TAGGTCGGTATATTCGTGGGATATTGTGATTCAGAGAGTTGGAAACAAGCTCTTTTTCGATAAGAGAGAT

GGTTCTCAGCTTGATTTGCTTGCAGTGCATGAGACCTCACATGAGCCATTACCAGAGGCGAAAGATGATA

TGAACTCTGCTTATTCATTGAGTGTTGAGGCAGCCTATATTAATCAGAACTTTTCACAGCAGGTTCTAAT

TAG

>FM895317.1 FM895317 Jatropha curcas embryo 71-95 (DAF) Jatropha curcas cDNA clone rjcpga0_002125, mRNA sequence

TTCAATTCTCTCTCTCTCTCTCTCTCTCTCTCTATTTATCTTTTCCTTCTTTTCTCTCTCCCTTACAAGC

CCTAACCCTAACTCAGACCCAGATTCGCCATGAAAGGATGTAAATCAAAGTCAGACACCAAGAACGCCAA

GCTTTCCGTGAACAAGAAACCCGCCAAGTCAAGTAAGAATGCGGGTAAGGCAGCGAATGATCCAAATAAG

CCAGAGAGGCCCGCCAGTGCTTTCTTCGTTTTCATGGAAGAGTTCAGAGAGCAGTACAAGAAGGAACATC

CTAAAAACAAATCCGTTGCTGCTGTTGGCAAAGCTGGTGGAGATAAATGGAAGTCAATGTCAGATGCTGA

GAAAGCACCTTTTGTATCTAAGGCAGAAAAGAGGAAGATAGAATATGAGAAGAAATTGAAAGCCTACAAC

AATGGACATGCTGAAGGCCCCAGAGAAGAAGAGGAATCTGAGAAGTCAATGTCTGAAGTGAATGATGAGG

AAGAAGATGATGAGGAGGGAAGTGGAG

>FM895316.1 FM895316 Jatropha curcas embryo 71-95 (DAF) Jatropha curcas cDNA clone rjcpga0_002124, mRNA sequence

GGAGTCTGACATGTTGTGCGAGTCAACGGGTGAGTAAACCCGCAAGGCGCAAGGAAGCTGACTGGCGGGA

TCCCCCCGAGGGTTGCACCGCCGACCGACCTTGATCTTCTGAGAAGGGTTCGAGTGAGAGCATGCCTGTC

GGGACCCGAAAGATGGTGAACTATGCCTGAGCGGGGCGAAGCCAGAGGAAACTCTGGTGGAGGCCCGCAG

CGATACTGACGTGCAAATCGTTCGTCTGACTTGGGTATAGGGGCGAAAGACTAATCGAACCGTCTAGTAG

CTGGTTCCCTCCGAAGTTTCCCTCAGGATAGCTGGAGCTCGGGACGAGTTCTATCGGGTAAAGCCAATGA

TTAGAGGCATCGGGGGCGCAACGCCCTCGACCTATTCTCAAACTTTAAATAGGTAGGA

>FM895315.1 FM895315 Jatropha curcas embryo 71-95 (DAF) Jatropha curcas cDNA clone rjcpga0_002123, mRNA sequence

TGATTCTATCCGTCGCCCGATGGGAATTGTACTTTAAGGCGGCCCTCGCGGCTCGTCCGCCGCGGGGGCT

TGGCCAACGGCACGTGCCTCTGGGGGCCGGATGGCCCCTACTGCAGGTCGGCAAACGGGCGACGGACACG

CGCGTCGCTTCTGGCCCGGATTCTGACTTAGAGGCGTTCAGTCATAATCCAGCGCACGGTAGCTTCGCGC

CACTGGCTTTTCAACCAAGCGCGATGACCAATTGTGCGAATCAACGGTTCCTCTCGTACTAGGTTGAATT

ACCATCGCGACACCATCATCAGTAGGGTAAAACTAACCTGTCTCACGACGGTCTAACCCCAGCTCACGTT

CCCTATTGGTGGGTGAACAATCCAACACTTGGTGAATTCTGCTTCACAATGATAGGAAGAGCCGACATCG

AAGGATCAAAAAGCAACGTCGCTATGAACGCTTGGCTGCCACAAGCCAGTTATCCCTGTGGTAACTTTTC

TGACACCTCTAGCTTCAAATTCCGAAGGTCTAAAGGATCGATAGGCCACGCTTTCACGGTTCGTATTCGT

ACTGGAAATCAGAATCAAACG

>FM895314.1 FM895314 Jatropha curcas embryo 71-95 (DAF) Jatropha curcas cDNA clone rjcpga0_002122, mRNA sequence

ACTGAGCTCACGCGGTGGCGGCGCTCTAGAACAGTGGATCCCCGGCTGCAGAATCGAATTCAGGATTCTA

TCCGTCGCCCGATGGGAATTGTACTTTAAGTGCGGCCCTCGCGGCTCGACCGCCGCGGGGGCTTGGCCAA

CGGCACGTGCCTCTGGGGGCCGGATGGCCCCTACTGCGGGTCGGCAAACGGGCGACGGACACGCGCGTCG

CTTCTGGCCCGGATTCTGACTTAGAGGCGTTCAGTCATAATCCAGCGCACGGTAGCTTCGCGCCACTGGC

TTTTCAACCAAGCGCGATGACCAATTGTGCGAATCAACGGTTCCTCTCGTACTAGGTTGAATTACCATCG

CGACACCATCATCAGTAGGGTAAAACTAACCTGTCTCACGACGGTCTAAACCCAGCTCACGTTCCCTATT

GGTGGGTGAACCATCCAACACTTGGTGAATTCTGCTTCACAATGATAGGAAGAGCCGACATCGAAGGATC

AAAAAGCAACGTCGCTATGAACGCTTGGCTGCCACAAGCCAGTTATCCCTGTGGTAACTTTTCTGACACC

TCTAGCTTCAAATTCCGAAGTCTAAAGGATCGAT

>FM895313.1 FM895313 Jatropha curcas embryo 71-95 (DAF) Jatropha curcas cDNA clone rjcpga0_002121, mRNA sequence

ATTCCTATTGTATCTTCTTAGTTTCGTTAAATCAAGTTGTTTATTTCAAGTGATAAGTTCAATTATGGCT

GATCAGGAGCACCATCAGAAGAGTTATGAGCCCGAGACCACTGCAGTTGAGGCCACGGATCGTGGGTTGT

TTGATTTCGGGAAGAAGAAAGAGGAAGAGAAGCCTCAAGAGGAGGTGATCGTTACAGAGTTTGAAGAGAA

GGTTCAGGTCTCTGAACCAGAGGAGAAGAAGCATGGCATCTTGGAGAAGCTTCACCGATCAGGAAGCAGC

TCTAGCTCTTCTAGCGATGAAGAGGAAGGAGAGGGAGAGGAAAAGAAGAAGAAAAAGAAAGAAAAGAAGG

GACTGAAGGAGAAGATCAAGGAGAAACTATCAGGAGAGAAAGAAG

>FM895312.1 FM895312 Jatropha curcas embryo 71-95 (DAF) Jatropha curcas cDNA clone rjcpga0_002120, mRNA sequence

GCTGCAGGCTGACATGTGTGCGAGTCAATGGGTGAGTAAACCCGCAAGGCGCAAGGAAGCTGACTGGCGG

GATCCCCCGAGGGTTGCACCGCCGACCGACCTTGATCTTCTGAGAAGGGTTCGAGTGAGAGCATGCCTGT

CGGGACCCGAAAGATTGTGAACTATGCCTGAGCGGGGCGAAGCCAGAGGAAAACTCTGGGTGGAGGCCCG

CAGCGATACTGACGTGCAAATCGTTCGTCTGACTTGGGTATTGGGGGCGAAAGACTAATCGAACCGTCTA

GTAG

>FM895311.1 FM895311 Jatropha curcas embryo 71-95 (DAF) Jatropha curcas cDNA clone rjcpga0_002119, mRNA sequence

TTTTTTTTTAGTAAGAATCAAAATCATTCCTTTCTTTCTTTCCACCCCCACAATGATATACCAAATTTTC

CCTCAATGAATGGGATCAATATATTTCCGTTACAGTATCACAATTTCATGGTCTTATGACACACTACTTC

CCTATTCTGGTACGTGCTGAGTTCTTTGTGCAAAGCTAAAGCCTCTTTTACATTTTGGTTCACCC

>FM895310.1 FM895310 Jatropha curcas embryo 71-95 (DAF) Jatropha curcas cDNA clone rjcpga0_002118, mRNA sequence

GGGATAAACAATTTCATCACTACAACAAAAACAAACTCTGCAAGAAGCAGTAAACAGTAGCATATCAAAA

CGAAAATAACACTATATATCAAAAATGAAAACGGTAAAAAGAAAAAAAAAAAAAAAAATCATCACGCTCT

AACAAGGAACTATCTTTAAAAGCAAAAAACCAATCACTCCAAATTAAAATACCGACAACACTTGCTACTG

GCCAGATTACAATAAAAAAAATCAACATAACAGATTACAATAAAATAATCAACATGACAAGCTGCACATT

TTTCTGTAGACAAAATGCAATCTATGCAATGATTCCTTGCAACTTGGCTAGAATGGCTAGCAAAGTATTC

AATCTCCATCTGATCTCATTGCAATAAGCCAATACTTCACTCATGTACTCAATGTTTTTAGCACCTAATC

TCATAAGAACCTGCTTTANTGATATAGCGGACCCACTCAACTGTATTATACCCACTCTTCTCCCATACCT

TGAGAAAGCGAACACGTAAACCAGATGCCGTAAACATGGGAACCTGGAACTCCTTCTGAATTGGTGGCC

>FM895309.1 FM895309 Jatropha curcas embryo 71-95 (DAF) Jatropha curcas cDNA clone rjcpga0_002116, mRNA sequence

GCTGCATGCCCCGAAAATGGATGCGCTTAAGCGCGCGACCTATACCCGGCCGTCGGGCAAGAGCCAGGCC

CCGAATAGTAGGAGGGCGCGGCGGTCGCTGCAAAACCCGGGGCGCGAGCCCGGGCGGAGCGGCCGTCGGT

GCAGATCTTGCGTGGTAGTAGCAAACTATTCAAATGAGAACTTTGAAGGCCGAAGAGGGGAAAGGTTCCC

TGTGAACGGCACTTGCACATCGGTTAGTCGATCCTAAGAGACGGGGGAAGCCCGTCCGACAGCGCGTCCT

CGCGCGAGCTTCGAAAGGGAATCGGGTTAAAATTCCCGAACCGGGACGTGAAGGCTGACGGCGACGTTAC

GGAGTCCGGAGACGTCGGCGGGGGCCTCGGGAAGAGTTATCTTTTCTGTTTAACAGCCCGCCCACCCTGG

AAACGACTCACTCGGAGGTAGGGTCCACCGGCTGGAAGAGCACCG

>FM895308.1 FM895308 Jatropha curcas embryo 71-95 (DAF) Jatropha curcas cDNA clone rjcpga0_002115, mRNA sequence

NTGCACAGATCAAATATGTTATGTTACATTACCAAAGGAAGTTAGAATCTCCAGGGTTCAATACATATTA

TATAATGGATTAAATGGTACCATTAAATATCAACTACAGATCTTTTCACAGTTCAAGATAAGCTTACGCA

ACACAACCTAAATTATCTTTTTGTTTTTTTTCAAGGATGCAATGTGTAAATCGACTCTTCATTTGTTCAT

AACAGAGAGCATTAGACATGGGAAAACACACATCATTTGTACACAACAATGATTGACATCTAAATCTCCC

AACCATAGACTGAGCTATCCCATGGCATACTTCTGTGTCCAGCTCCTAGCAGTTGCCTCGTACTTGGCCC

TGTCTGTCTTGTACATGTGAGCAATTTCTGGCACCANAGGATCATCTGGGTTTGGGTCCGTCAAGAGGGA

ACATATGGAGAGCAACACCTTAGATATTGTCCAGGGCTGGGCTCCACTGCTCCTTTAAAATGTCAAGGCA

AATGCTGCCATTACTGTTAATATTTGGGTGAAAAACCTTTGTCCTGAATGC

>FM895307.1 FM895307 Jatropha curcas embryo 71-95 (DAF) Jatropha curcas cDNA clone rjcpga0_002114, mRNA sequence

GAAAGGAATCTGAGATTTTATTCAAAAAGAAACCATAAATGTTTACAGGTATTATTTTCCTGTATCTGTA

ACTAAATGTGCCTTTCATGAATGGGGTATAGGATAACAATCCAACCTCCAATTATTACAATATTAATCAG

ACAATCCCATCCTAAATTCCCATTAACATGTCAATAGCGTCATTGCCATACAACAAGCTCTCCAGTGGAA

ATTTCCAAGGTTGGGATACCATGATCACGACCACGACCATGACCATGCATTGGCATCTTAGCAGTGAAGA

TGAATATTCATGTCTTATCATTCATGCTTAAGAAGGATTCAAGGATCTGGTTGCTCTACTTCTATCTTGT

TTAAGCTTAATGGTGAACTTCAAGTTGAGCCAAAAACCANCTCTTTTAAGGACGTTAGTGGCGAACTGTA

TCCATACACTACCGTGTCTC

>FM895306.1 FM895306 Jatropha curcas embryo 71-95 (DAF) Jatropha curcas cDNA clone rjcpga0_002113, mRNA sequence

CTCGTCCGCCGCGGGGTGCTTTGCCAACGGGCACGTGCCTCTTGGTGGCCGGATTGCCCCTACTGCGTGG

TCGGCAAACGGGCGACGGACACGCGCGTCGCTTCTGGCCCGGATTCTGACTTAGAGGCGTTCAGTCATAA

TCCAGCGCACGGTAGCTTCGCGCCACTGGCTTTTCAACCAAGCGCGATGACCAATTGTGCGAATCAACGG

TTCCTCTCGTACTAGGTTGAATTACCATCGCGACACCATCATCAGTAGGGTAAAACTAACCTGTCTCACG

ACGGTCTAATCCCAGCTCACGTTCCCTATTGGTGGGTGAACAATCCAACACTTGGTGAATTCTGCTTCAC

AATGATAGGAAGAGCCGACATCGAAGGATCAAAAAGCAACGTCGCTATGAACGCTTGGCTGCCACAAGCC

AGTTATCCCTGTGGTAACTTTTCTTGACACCTCTAGCTTCACATTCCGAAAGTCT

>FM895305.1 FM895305 Jatropha curcas embryo 71-95 (DAF) Jatropha curcas cDNA clone rjcpga0_002112, mRNA sequence

GCACCTACCAATCATATCCATTACATAGATTTTTTTGGAAAAATCTTTCATCTTTCTGTAAACATAACAA

TCCATTTGTCTGCTAAATTACTTTCCCTGATTGTAATTTATTTTCCGAGGATACGTATCTCAATCTGTAA

ATAATGATACACAACATTCTTGGCTAATGGCTACCATGATCTAAAATATACGCTTCCCTCCATTCAAGTT

CTTGGCTACCAGCGCAGTGACAGTCTTCTGGTCAAGTTACATTGCTCAAACACCGCCAATCCGTTTCCTT

CTACGAGCTCCTACGGGGCTTTTGCTCCAGAGGGCTTGGCCATCAATAGCAGTGAAGCTATGATCATCAA

GGCGCTTCCATGTTTTGATTGACTTAACAGCTCCCCAATCTCCTTTCTCTGACTTCTCCAAAGATGCTAC

AACCAACCTTGCCCTTCTTGACCATCCAGCCTTCCCATAGGCATGGTATCCAAACCCACCGGCATAACAA

AGGTTATACCAGTCAGTTGACC

>FM895304.1 FM895304 Jatropha curcas embryo 71-95 (DAF) Jatropha curcas cDNA clone rjcpga0_002111, mRNA sequence

ATTCTATCCGTCGCCCGATGGGAATTGTACTTTAAGTGCGGCCCTCGCGGCTCGTCCGCCGCGGGGTGCT

TGTGCCAACGGCACGTGCCTCTGGGGGCCGGATTGCCCCTACTGCGGGTCGGCAAACGGGCGACGGACAC

GCGCGTCGCTTCTGGCCCGGATTCTGACTTAGAGGCGTTCAGTCATAATCCAGCGCACGGTAGCTTCGCG

CCACTGGCTTTTCAACCAAGCGCGATGACCAATTGTGCGAATCAACGGTTCCTCTCGTACTAGGTTGAAT

TACCATCGCGACACCATCATCAGTAGGGTAAAACTAACCTGTCTCACGACGGTCTAAACCCAGCTCACGT

TCCCTATTGGTGGGTGAACAATCCAACACTTGGTGAATTCTGCTTCACAATGATAGGAAGAGCCGACATC

GAAGGATCAAAAAGCAACGTCGCTATGAACGCTTGGCTGCCACAAGC

>FM895303.1 FM895303 Jatropha curcas embryo 71-95 (DAF) Jatropha curcas cDNA clone rjcpga0_002109, mRNA sequence

ATCCAAAGGATTCTATCCGTCGCCCGATGGGAATTGNTACTTTAAGGCGGCCCTCGCGGCTCGTCCGCCG

CGGGGGCTTAGCCAACGGCACGTGCCTCTGGGGGCCGGATGGCCCCTACTGCGGGTCGGCAAACGGGCGA

CGGACACGCGCGTCGCTTCTGGCCCGGATTCTGACTTAGAGGCGTTCAGTCATAATCCAGCGCACGGTAG

CTTCGCGCCACTGGCTTTTCAACCAAGCGCGATGACCAATTGTGCGAATCAACGGTTCCTCTCGTACTAG

GTTGAATTACCATCGCGACCACATCATCAGTAGGGTAAAACTAACCTGTCTCACGACGGTCTAAACCCAG

CTCACGTTCCCTATTGGTGGGTGAACAATCCAACAATTGGTGAATTCTGCTTCACAATGATAAGAAAAGC

CGA

>FM895302.1 FM895302 Jatropha curcas embryo 71-95 (DAF) Jatropha curcas cDNA clone rjcpga0_002108, mRNA sequence

GTGGTCAGCTACAAGTGGATGCGTGTGAATGAGTATCAAGCCTGAAGTAGAGCCCTAAAGGTGATTCTCG

GTTCCCAAAATGTAGGATTAACAAGACAAAATATCTCAACTGTAATTTGCCCCTCCTGAAAGAGAGCTCC

TTGATTAACGGGCTTTGAG

>FM895245.1 FM895245 Jatropha curcas embryo 71-95 (DAF) Jatropha curcas cDNA clone rjcpga0_002038, mRNA sequence

GGTCATCCAATTGCCAGCAGTAATGATTGATATAAAATGGGAGTTTTTACATACAGCAATATCAAATTTC

AAACAAAAGGAATCTGTAATATTACTATTATCTAGACCAACTGCTGGACTTTCCACAGCAGTGATAGCAA

AATATAACCTCCCTTAGATGTACAAATCTGTATGTCTTTCTACAGAGATGCACACTCCAGCCAGAGATCG

ACATGACCTGGATTTTACTAACCATTGAATCCAAAAAAACCAAAATGCATCTGTACAACTATAAGTCAAC

ATCAGTGTAGTAAACCCGCCTCCGATTTGACCTTCGTCTCAATGGCACAAGTGCTTTTCCATACCCAGGA

AATTCCCATTTGGCGATTACTCCATCCNCAGAAGAGCTAACGATCATTGGATAGAGAGGATGCCCACTAC

AATCTCTTACAGGGCCTTCGTGATGATCAAGTCTTGCAACTTCAGCACCACTCACCAGATTCTAATATAA

ACAGGACAATCACTTGATCCGCTGTAGATATACTTCTGGACC

>FM895244.1 FM895244 Jatropha curcas embryo 71-95 (DAF) Jatropha curcas cDNA clone rjcpga0_002037, mRNA sequence

CTCAAACCCTAACAATGGCAGCGGCTCCAATCGAGTCAGTCCAGTGCTTTGGACGCAAGAAGACCGCTGT

GGCTGTCACCTACTGCAAGCGTGGAAGAGGCCTAATCAAGATCAATGGATGCCCAATCGAGCTAGTGGAG

CCTGAGATCCTCCGTTTCAAGGCATATGAGCCTATCCTTCTTCTTGGACGACACCGTTTTGCTGGCGTCG

ACATGCGCATTCGCGTCAAAGGTGGTGGTCACACCTCGCAGATCTATGCTATCCGTCAAAGTATCGCTAA

AGCTCTTGTCGCCTTCTATCAGAAGTACGTCGACGAGCAGAGCAAGAAGGAAATAAAAGATATTTTGGTC

AGGTATGATAGGACTTTGCTTGTAGCTGATCCCAGACGATGCGAGCCAAAGAAGTTCGGTGGTCGTGGTG

CTCGCGCCAGGTTCCAGAAGAGTTACCGTTGAAGTCGAGGTTATCCTTTTTTTTATGCGTCTGTTATTTT

CGAAAGTGTTTTGAATTATGGATTTTGCTATTTGAGGACTAAAAATGTTAATGGATTTTAGTTGAGCGGT

GTTTATTTAGAGATTTTTCTCTCAATAGACCCATTGCTTAGTTTTGTAGTTTGGATAATTTAATGGTTAT

ATTATAGCTAGTTGATTAAACTCAGTTATAAAA

>FM895243.1 FM895243 Jatropha curcas embryo 71-95 (DAF) Jatropha curcas cDNA clone rjcpga0_002035, mRNA sequence

ATTCAGCTTACTCTCAGTGTTTTCTTTCTCTGTCAAACTGTCAAAGATCAAATTTTTCTTGCATTTCTTC

GAAGTGGCAATGGAGGTAATTTCTCACACATCAACACTGAATCCCAACGCTCCATTGTTCGTCCCAATGG

CTTATCGTTCGGTGGAGGATTTCTCCGATCAATGGTGGTCCCTCGTCCACTCTTCTCCTTCCTTCCGCGA

TTACTGGCTTGAAGAATGCTTCACTGATCCTCAAATTGATCCTCTCCATTTCAATTTTTTCGACGAAGTC

GATGTCGATTCCCTCTTTTGCGACTGCGTTGATACCAAACGTAATAGAAAGGCGTAACTTTTATAATTTA

TTGCATTGTTTTGAATCTGCTCTGAGTCAGTGATTCAACTGAGTAACTCGGTCTTTCTTGATTTATTGCA

GAGGCGGTGGAGATGGAAGAGAAGGAGCATAGCAAGGATTTGGTTTCAATAGGAGTAATGAAGTGGCAAA

AGGGTCGAGCTCAGGTGGCGCAGGTACCCAAGTATGTAGAAAAAGCACCAAGGATTGTTAATGTGAGAGT

GAGTCCACGAACGATT

>FM895242.1 FM895242 Jatropha curcas embryo 71-95 (DAF) Jatropha curcas cDNA clone rjcpga0_002034, mRNA sequence

TCGGTTAAAAGATGTCAGGCCGTGGAAAGGGCGGAAAGGGGCTGGGCAAAGGAGGAGCAAAGCGTCATCG

GAAAGTTCTCCGTGATAACATTCAGGGAATCACAAAGCCGGCGATTCGCCGGTTGGCTCGAAGAGGTGGA

GTGAAAAGAATCAGTGGATTGATCTACGAAGAAACTCGTGGAGTACTCAAGATCTTCTTGGAGAATGTGA

TTCGTGATGCTGTGACATACACTGAGCATGCTAGGAGAAAGACGGTGACCGCCATGGATGTGGTCTATGC

TTTGAAGAGGCAAGGAAGGACTCTTTATGGATTTGGAGGGTAAGAAGGGAGTCTTGGTTTGTGGTGTTGG

AGATCGGAGAAGTCTTGCTGTCAAGAATTAGAAGTGACAATGGTGGTGGCAGTGGCGGTGGAGACGACGG

TGGTTGTTAAGGTGTTTAGGGTTAGGGTTAGGTTTTTGTAATTGTTAATTTGAATGTTGTTTCTATGTAA

TTAGATTGGTGTGTTTGGGAATCTGAGTTTATGTCAGTACTATGGAGTATCTGTCTTTGTAATTCATT

>FM895241.1 FM895241 Jatropha curcas embryo 71-95 (DAF) Jatropha curcas cDNA clone rjcpga0_002033, mRNA sequence

TTTTTTTTTTATGAAAATTATGTTTCTCAAATATAAATGTAGATCTGAATTTTTGCATCCCAAATTTACA

ACCAATCCACTTCCAGTGATAAGTTAAAAGGAGAACCCCGTACAGGGGAAAAAAAAATTGCAACATAGCT

TGGAAACATTAATCTTTTGCAACAACGTTGCCTAAGGACCATAGCAAAAGAGAATCTTCAGTCACACCAC

TCCTGAGGGAAGACGGAGAACAAGCTGTCCTCCAAAAACACTTTTAACATAATAATAACAGAAGTCTGCT

AAGATGAAAGTCTGGACAATTTCTGAGATGAGAACCATAGATGGCCATAATCCATATCCCAAGGCCACCA

GCAAGTGTCCGCGAGTATCTAAAACCTGGAGAACCCAATGGGCACAGCTCAAGAACCTTGCAACACCCAA

TGCAAATACATAATGCGCTGTAAATGGTTCAACAATCTTTGTATTCTGCATAACTCGCAACTGAGGTAGT

ACAGAGACAGCTTCCAGATATACACAAAACGCCCACAAAATCCTGTTGATTAAGTTATGAGATGTTGATG

GATGAATAAACAAGGCTAGAATAGCACAGGGCACCACCACATAATATATCGCAAAGTTATCCTTATCTTC

CATGTAACTAGAC

>FM895240.1 FM895240 Jatropha curcas embryo 71-95 (DAF) Jatropha curcas cDNA clone rjcpga0_002031, mRNA sequence

CCTGAGGGTTGCACCGCCGACCGACTTATCTTCTGAGAAGGGTTCGAGTGAGAGCATCCTGTCGGGACCC

GAAAGATGGTGAACTATGCCTGAGCGGGGCGAAGCCAGAGGAAACTCTGTGTGGAGGCCCGCAGCGATAC

TGACGTGCAAATCGTTCGTCTGACTTGGGTATAGGGGCGAAAGACTAATC

>FM895239.1 FM895239 Jatropha curcas embryo 71-95 (DAF) Jatropha curcas cDNA clone rjcpga0_002030, mRNA sequence

AATGAATAGTGACAAGTATTCAATTACACCTGCAAACCAAATAGTGTAAATGATGTATGTGCAGGCTTCA

GCTTATTTTTAGAATGCTTCATAATCATTACAAGACGAAGGCCTCAAGAATATCCATCAATACAAATCAG

AAGCTAACTAGGTTCTTCTCCTTCAAGCTTTCAA

>FM895238.1 FM895238 Jatropha curcas embryo 71-95 (DAF) Jatropha curcas cDNA clone rjcpga0_002029, mRNA sequence

TTTTTTTTTATCAAATTTTCAATTTCTACAATACAAATGAAAGATAAGATGATGAAATTATTGTCATTTC

TACAAATCCAATTTCATCCAAGAGATACAAAACAGAACTGATTATCCTGTACTACATCCACTGAGCACAA

AATGAGAAAATTGTTTCTTCTCTCTCCCACCAAACACAGCCTAATGCCCAAAAGGAACTGAAATGAAATA

GATTTAGGAGGAAGAATATGGAAGCAAAATAATATCATTACAATCTCGGTGATTTTAATAATAATAATAA

TAACAATATTCGTGATGATGATGGATAAATCTACTCGCATTTGCTTAAAGCAAATTCGTTCTGGAAAAAA

ATTTGAGAAATGGCTGCCACTGATTTCCTTTTCTAGGAAGAGATTTGTATGAAGTATGTGGGAGAGGCCA

TTTTCAAACACCAAGTTAGCTCTTCATGCTCCCAAAGAATCTTGAAAGCGGCACAGGACCATCAGCCTCT

TCATCCTCACTGCTGGAGCGCTTGGATTTCGATCTGTGCTTCTTTTCCTTCTATGTTCTCTTTGATCTGG

ACTTCGACCTTCTTGATTCTCTCTCCTCGTCATCGCTACTTGAAGAATCAGAGGATGAACCTGACGAGCT

ATAATCTGAAGATCTTCG

>FM895237.1 FM895237 Jatropha curcas embryo 71-95 (DAF) Jatropha curcas cDNA clone rjcpga0_002028, mRNA sequence

CCCGGGCTGCAGGAAAAACTTGTATCTATGGGTTTTGAGAAGACACAAGTAGAAGTCGCACTAGCAGCTG

CTGATGGGGATCTTAATGTTGCTGTTGAGATTCTCATGAGCCAACAGGGATAAACTGTGAAACAATTATC

TCATTGCAGGGATAAACTCTGCCAACCTATTTGCAGTCTATTTTGTGGATACAATGTGCATTGATATCAT

TTTTGGCACAAGTAGGTCGAAAGAAGAAAATATTCTGAGCAAAAAACGATGGCTAACTTCTGTTACAAGA

AAGCGCTAGAAGACTTATATTCTTGTAGGATATAGTTGCATGAATATTGAAATTCAATCAGAATCCGAAA

GACTGCTGTTATACCGACAATTTTTAATTCCTTTTGTATCAATTTGATAATAGAATTGTTTATCATTTGG

GCGACTTGTACAATTCTAGTATCTTTATGGCTGTATAAATGTTTTGACAAGTTGTACATTACATGTATAC

AATTCCTCG

>FM895236.1 FM895236 Jatropha curcas embryo 71-95 (DAF) Jatropha curcas cDNA clone rjcpga0_002027, mRNA sequence

GCATGTCAAATCATGTTCCAAAATAAATTACTGGAAATTTAACAACTCTGAAATCAATCAGTTCAATCTA

CAAATGAGAAGTCCGCACACAAAATTAATGTGATACCCAAACATATACTATGCCCACAGAGAGAATTACA

CATGTATCATTGATGTGACAAAAGGAAACAAAGAGTTCCTGCCACTGAGATCAACTTTAGAGACCCTTTC

ACTTTGGTCCTGCAAATTTGTTCATCATCTTAGCAATTACAGGAGCCACCTTGGGATTGGCTTGATGCTT

AGCGAGATTAGCCGGGTTCTTCATTACATCCTGAAGAGCAGCCATGATTTCAGGATCACTAAATGCAGCC

ATCAGTTCGGGGTCATTCAAGATTTTACTAAAATCCACATTTCCTGGCATCCCTCCAGGCATGGCACCTG

GGAAACCTCCAGGCATTCCACCAGGCATTCCACCAGGCATTCCTCCAGACATCCCTCCTGGCATTCCACC

AGGAAAGCCACCAGGCATTCCTCCTGGCATTCCACCAGGCATACCTCCAGGTCTTCTGCTGGAAGATGAT

TGCTCTTGCTTCTTTGCCTTCTCATATTCGGCCTGAGCTTTGGCACGTCGACGTTGT

>FM895235.1 FM895235 Jatropha curcas embryo 71-95 (DAF) Jatropha curcas cDNA clone rjcpga0_002026, mRNA sequence

ATTCCTACATTAACATATCTTTCTCACACTTATCATCAACAAATGGCTGAACGTTCACAGCCACACCAAG

TTCAAGTGCACCCACAACACCGCTATGAAGCTGCCTTCAAAGGTCAACAAAAAGGTCCATCAGCTCAGAA

AGTGTTAGCTGTGATCACCCTCCTGCCGGTAGGCGGTGGGCTTCTTGCACTAGCTGGTATAACCTTAGTA

GGTACCCTAATCGGCCTAGCTATTACTACCCCTCTTTTTGTCATCTTTAGCCCTGTTCTTGTCCCAGCTG

CATTAGTTATTGGCCTTTCGGTCATGGCATTTTTGGCTTCCGGGGCTATGGGGCTCACAGGGTTGTCATC

ACTCTCGTGGGTTCTGAAATACCTCCAGGAGGTCACTCGGCGAATGCCGGAGCAGCTGGATATAGCTAAG

AAGCGTATGCAAGACATGGCTGGTTTTGTGGGACAGAAGACCAAGGAAGTAGGCCAAGAAATCCAGAGGA

AAGCACATGAAGGGAAATAAGTAGAGAGAAATGCTGTTGGTTATTAAGGGTTGAAGTCAGAGAGCTGGGT

TTTGGTACGAGACTACTTTTGTTGTATGGGTGTTGTTTTGAGGTAGTTGAGTT

>FM895234.1 FM895234 Jatropha curcas embryo 71-95 (DAF) Jatropha curcas cDNA clone rjcpga0_002023, mRNA sequence

ATTAAATTTTATATTTTTATTGAATTAAAGCAAACATAACATCCACAGAAGGCCGTAACAGGCCAATCAG

AAAGGAAACAGAATACAACGATGATAGAGAAGCCCATTAGACTCTCTAAGAAAATACATTCAGATGAAAG

CATGAAAACAATTAAGGAAAGCAATTCCCCCCTTCAGAATCCACCACGCAGACGCAGGACAAGGTGAAGG

GTTGATTCTTTCTGGATATTGTAGTCGGCCAGAGTCCTTCCGTCCTCCAACTGCTTCCCAGCAAAGATCA

GCCTTTGCTGATCTGGAGGAATCCCTTCCTTATCCTGGATTTTCGCCTTCACATTATCGATTGTATCACT

GCTCTCCACTTCCAGAGTGATAGTCTTCCCTGTCAGGGTCTTGACGAAGATTTGCATTCCACCACGAAGC

CTGAGCACAAGGTGGAGAGTGGATTCCTTCTGAATATTGTAATCGGCCAAAGTTCGGCCATCTTCCAGTT

GCTTACCGGCGAAGATCAGCCTCTGCTGGTCTGGTGGGATGCCCTCCTTGTC

>FM895233.1 FM895233 Jatropha curcas embryo 71-95 (DAF) Jatropha curcas cDNA clone rjcpga0_002022, mRNA sequence

GTATATATGTGGTAAAGGTGGTACGTTATTCAGATTTTTAGGTGTTCTTAGATTTGCACTGAAATACTGC

TTTTGCCCACTGATTTTGATGATCATTTTATTGGAGATGGTGTAGGCTTCTTCGGGTTTTAGATAATTAA

GTTCGTGGTGGATGGCCTTTACATTTGGAGCAGGCATTTTGTTGAGATCTTCGAGGATCCTTTCATATCT

GATTCTGCTTTGACCATTTTGACTAAGAAGATTTAAGTTAGGGTCCTTTTAAGATTTGGGGAAAAGCGGA

AAAAGAAGAAGATACAAAAAAGGAAGTTTTTTCCGGATCCAGAGTATTATTATTCTACAGTTAGTAGATT

TTCTTCAAGTTATGTGATTTGGATCCTGAATCGTGATTTCTTCGGAGAACACTCTACTGTTTTTTAATTT

TTTCAAAGAAACTTTGGCTAGAAAAGATGGATTTCTTCAAAGTAAAGAAGTTCAAAAAAGCCTACAAACC

AGACCCTGAGAAGGATTTAGAGGATAAACCTGTGTCGCAGCCGGAGGAACCAAAGAATGAAAATGGCGGC

GGTGGTAATGTGAGCAACAAGTCTGCTAATGCTGATCTTGTA

>FM895232.1 FM895232 Jatropha curcas embryo 71-95 (DAF) Jatropha curcas cDNA clone rjcpga0_002021, mRNA sequence

ATTCGTCAAAAGCTGACGCTGGAGCTTCCAAAACTTACCCGCAGCAAGCCGGAACTATTAGAAAAAACGG

ATATATAGTCATTAAGAATCGCCCATGCAAGGTTGTTGAAGTTTCCACCTCCAAGACTGGAAAGCACGGT

CACGCGAAGTGCCACTTCGTTGGAATAGACATCTTCAATGGCAAAAAGCTTGAAGATATTGTCCCTTCCT

CCCATAACTGTGATGTTCCCCATGTCACTCGAACTGACTATCAGCTAATTGATATCTCTGAGGATGGATT

TGTGAGTTTGCTGACTGAAAATGGGAACACTAAGGATGATCTGAGGCTTCCAACTGATGAGAATCTTCTG

TCTCAGATTAAGGATGGGTTTGCTGAAGGAAAAGATCTTGTTGTGACTGTCATGTCTTCAATGGGAGAAG

AGCAGATCTGTGCTCTTAAGGATATTGGCCCAAAGTAATTTCTTATTATCGGCTTTAGGTGGTGGTTGCT

ATTTAAATCGACTGATTTTTGTAAAAACCATCTTTTCTTTGCTTTATATTATAGAAACTTAGAGTAGACT

AGCTCAATGCTTTGCGGATGGTATTTTATATCTGTCAAAACTGGAAACAGA

>FM895231.1 FM895231 Jatropha curcas embryo 71-95 (DAF) Jatropha curcas cDNA clone rjcpga0_002020, mRNA sequence

AGGATTCTATCCGTCGCCCGGTGGGAATTGTACTTTAAGGCGGCCCTCGCGGCTCGTCCGCCGCGGGGGC

TTAGCCAACGGCACGTGCCTCTGGGGGCCGGATGGCCCCTACTGCGGGTCGGCAAACGGGCGACGGACAC

GCGCGTCGCTTCTGGCCCGGATTCTGACTTAGAGGCGTTCAGTCATAATCCAGCGCACGGTAGCTTCGCG

CCACTGGCTTTTCAACCAAGCGCGATGACCAATTGTGCGAATCAACGGTTCCTCTCGTACTAGGTTGAAT

TACCATCGCGACACCATCATCAGTAGGGTAAAACTAACCTGTCTCACGACGGTCTAAACCCAGCTCACGT

TCCCTATTGGTGGGTGAACAATCCAACACTTGGTGAATTCTGCTTCACAATGATAGGAAGAGCCGACATC

GAAGGATCAAAAAGCAACGTCGCTATGAACGCTTGGCTGCCACAAGCCAGTTATCCCTGTGGTAACTTTT

CTGACACCTCTAGCTTCAAATTCCGAAGGTCTAAAGGATCGATAGGCCACGCTTTCACGGTTCGTATTCG

TACTGGAAATCAGAATCAAACGAGCTTTTACCCTTTTGTTCCACACGAGATTTCTGTTCTCGTTGAGCTC

ATCTTAGGACACCTGCGTTATCTTTTAACAGATGTGCCGCCCCAGCCAAACTCCCCACCTGACAATGTCT

TCCGCCCGGATCGG

>FM895230.1 FM895230 Jatropha curcas embryo 71-95 (DAF) Jatropha curcas cDNA clone rjcpga0_002019, mRNA sequence

AACAGCATGAAATATCAGTTCATGCTAAAAAGTAAATAGCAATAACATCCAAAGCAATTTTAGAAGTAAA

CAAAAAAGAGATCTCACAACACAGAATATAATTTCTGACTGGTCTACCAAATAAGATTTGAAAACAAAAG

CAATATCCAATAAACAAAAATGATAAGTCTAGGAAAAACAAAATCCATAACTGAATCCCTCTCTTTACTC

ATCATTTTTCAGATGAGGACAGCTTACATAAATAGGTTGGGCCTGGATGCCTTGTATGTTGTCCTAAGCT

TCCTGGTTGGTGGCCTAACCTTCTTGAACACCAATGGGAACTTGATCTTCGAGTTGTGGAATTGCTTGCT

GCTTTCTCTTTTGCAGAGCTTAGCTGGAATGGTGCCAGTCTTGATTATCTGAATGCATGGGAACCTTACT

CTGTGTCGGGAAGCCATCTCAGTGTACATCTGTTCCACAGCACCATTCAAAGTGGTATCTCTGTATTCCT

TGTACATGTTGTGATAGCCAGTTCGGCTTTGGTAACGCAACCAAATGCCATAAGTCTTGATCTTA

>FM895229.1 FM895229 Jatropha curcas embryo 71-95 (DAF) Jatropha curcas cDNA clone rjcpga0_002018, mRNA sequence

ATTCCTTAGAAGCACGATCTCCGAACGGCGCCAAGGAGCGAAACTAGTTGCAGCAAATAAGCCCAAGTAA

GCCACTAAATCCTCAGTACTTCGCTGCGAAATCGAGTCGTTGCATAGATATACAGGCATTGGTTTTAACA

CCGAGTTGTGTTTATTCAAGCAGAACCATGGCAGACAAAGCTGTGACGATTAGAACAAGGAAGTTTATGA

CTAACAGGCTCCTTTCAAGGAAGCAGTTCGTGATTGATGTTCTGCATCCTGGGAGGGCCAATGTCTCTAA

GGCTGAGCTGAAGGAGAAGTTGGCAAGTTTATATGAAGTGAAAGACTCAAATACAATTTTTGTATTCAAG

TTCCGTACACATTTTGGAGGTGGAAAGTCAACTGGCTTTGGACTGATATATGATTCTGTGGACAATGCTA

AGAAGTATGAGCCAAAATACAGGCTGATCAGAAATGGACTGGACACCAAGGTAGAAAAATCAAGAAAACA

AATGAAGGAAAGGAAGAACAGGTCCAAGAAAGTCCGAGGTGTAAAGAAGACTAAGGCTGGAGATGCTGCA

AAGAAGAAGTGAGATCTGAATAATAGGGAGCTCATCTTTTGTCCTAGTACACTTGGGTCCTTTAGCCTTT

>FM895228.1 FM895228 Jatropha curcas embryo 71-95 (DAF) Jatropha curcas cDNA clone rjcpga0_002017, mRNA sequence

ATTCCAAAGGATTCTATCCGTCGCCCGGTGGGAATTGTACNTTTAAGGCGGCCCTCGCGGCTCGTCCGCC

GCGGGGGCTTAGCCAACGGCACGTGCCTCTGGGGGCCGGATGGCCCCTACTGCGGGTCGGCAAACGGGCG

ACGGACACGCGCGTCGCTTCTGGCCCGGATTCTGACTTAGAGGCGTTCAGTCATAATCCAGCGCACGGTA

GCTTCGCGCCACTGGCTTTTCAACCAAGCGCGATGACCAATTGTGCGAATCAACGGTTCCTCTCGTACTA

GGTTGAATTACCATCGCGACACCATCATCAGTAGGGTAAAACTAACCTGTCTCACGACGGTCTAACCCCA

GCTCACGTTCCCTATTGGTGGGTGAACAATCCAACACTTGGTGAATTCTGCTTCACAATGATAGGAAGAG

CCGACATCGAAGGATCAAAAAGCAACGTCGCTATGAACGCTTGGCTGCCACAAGCCAGTTATCCCTGTGG

TAACTTTTCTGACACCTCTAGCTTCAAATTCCGAAGGTCTAAAGGATCGATAGGCCACGCTTTCACGGTT

CGTATTCG

>FM895227.1 FM895227 Jatropha curcas embryo 71-95 (DAF) Jatropha curcas cDNA clone rjcpga0_002015, mRNA sequence

ATTCCAAAGGATTCTATCCGTCGCCCGATGGGAATTGTACTTTAAGGCGGCCCTCGCGGCTCGTCCGCCG

CGGGGGCTTAGCCAACGGCACGTGCCTCTGGGGGCCGGATGGCCCCTACTGCGGGTCGGCAAACGGGCGA

CGGACACGCGCGTCGCTTCTGGCCCGGATTCTGACTTAGAGGCGTTCAGTCATAATCCAGCGCACGGTAG

CTTCGCGCCACTGGCTTTTCAACCAAGCGCGATGACCAATTGTGCGAATCAACGGTTCCTCTCGTACTAG

GTTGAATTACCATCGCGACACCATCATCAGTAGGGTAAAACTAACCTGTCTCACGACGGTCTAACCCCAG

CTCACGTTCCCTATTGGTGGGTGAACAATCCAACACTTGGTGAATTCTGCTTCACAATGATAGGAAGAGC

CGACATCGAAGGATCAAAAAGCAACGTCGCTATGAACGCTTGGCTGCCACAAGCCAGTTATCCCTGTGGT

AACTTTTCTGACACCTCTAGCTTCAA

>FM895226.1 FM895226 Jatropha curcas embryo 71-95 (DAF) Jatropha curcas cDNA clone rjcpga0_002014, mRNA sequence

AGGATTCTATCNCGTCGCCCGATGGGAATTGTACTTTAAGTGCGGCCCTCGCGGCTCGTCCGCCGCGGGG

GCTTGGCCAACGGCACGTGCCTCTGGGGGCCGGATGGCCCCTACTGCGGGTCGGCAAACGGGCGACGGAC

ACGCGCGTCGCTTCTGGCCCGGATTCTGACTTAGAGGCGTTCAGTCATAATCCAGCGCACGGTAGCTTCG

CGCCACTGGCTTTTCAACCAAGCGCGATGACCAATTGTGCGAATCAACGGTTCCTCTCGTACTAGGTTGA

ATTACCATCGCGACACCATCATCAGTAGGGTAAAACTAACCTGTCTCACGACGGTCTAAACCCAGCTCAC

GTTCCCTATTGGTGGGTGAACAATCCAACACTTGGTGAATTCTGCTTCACAATGATAGGAAGAGCCGACA

TCGAAGGATCAAAAAGCAACGTCGCTATGAACGCTTGGCTGCCACAAGCCAGTTATCCCTGTGGTAACTT

TTCTGACACCTCTAGCTTCAAATTCCGAAGGTCTAAAGGATCGATAGGCCACGCTTTCACGGTTCGTATT

CGTACTGGAAATCAGAATCAAACGAGCTTTT

>FM895225.1 FM895225 Jatropha curcas embryo 71-95 (DAF) Jatropha curcas cDNA clone rjcpga0_002013, mRNA sequence

AGGATTCTATCCGTCGCCCGATGGGAATTGTACTTTAAGGCGGCCCTCGCGGCTCGTCCGCCGCGGGGGC

TTGGCCAACGGCACGTGCCTCTGGGGGCCGGATGGCCCCTACTGCGGGTCGGCAAACGGGCGACGGACAC

GCGCGTCGCTTCTGGCCCGGATTCTGACTTAGAGGCGTTCAGTCATAATCCAGCGCACGGTAGCTTCGCG

CCACTGGCTTTTCAACCAAGCGCGATGACCAATTGTGCGAATCAACGGTTCCTCTCGTACTAGGTTGAAT

TACCATCGCGACACCATCATCAGTAGGGTAAAACTAACCTGTCTCACGACGGTCTAAACCCAGCTCACGT

TCCCTATTGGTGGGTGAACAATCCAACACTTGGTGAATTCTGCTTCACAATGATAGGAAGAGCCGACATC

GAAGGATCAAAAAGCAACGTCGCTATGAACGCTTGGCTGCCACAAGCCAGTTATCCCTGTGGTAACTTTT

CTGACACCTCTAGCTTCAAATTCCG

>FM895224.1 FM895224 Jatropha curcas embryo 71-95 (DAF) Jatropha curcas cDNA clone rjcpga0_002012, mRNA sequence

TATGAAGATTGTTTGGCCATGTTCTATACACGAACAAATGCATTAATATAGTGAAGGTTGATGAACAACC

AAGTTACAAGAATGAAGAAGAACAGTGTGGGCAAGGATCAACTTGCCATTAAGCAGTGAAGAAGATTTCT

AGTAACAAGTAGAGCCTTCTATTAGTATGACCCTCAACCTGGTGGAACTGGAGAAAAAAACGATATATTG

ATCCAACGGCATTTAGATCAGGAATAAGTTGAGTTGAACTAATTACCTAGACGGTTGGATATCTTTGACA

ACACAATGCATTAGCATTGGTGTTAGAAGGTAGGAAACTGAGCATCCACAAAACGGATTGCAAGTTTTAT

GACAATCATGATGTCTGGTAAAATTCATTTGTCATCTCTTTTGGAGTTTTGAAAGGAGTTAGTTTAAACA

ACTGAATAATGTGCTATCTTTACGGGCCATTCCTATCTTCGAACAGCATAATCTCAGGCCTGCAATAAAT

AGGTTCATTTCCTGGCGAT

>FM895223.1 FM895223 Jatropha curcas embryo 71-95 (DAF) Jatropha curcas cDNA clone rjcpga0_002011, mRNA sequence

GTCTGACATGTGTGCGAGTCAACGGGTGAGTAAACCCGCAAGGCGCAAGGAAGCTGACTGGCGGGATCCC

CCCGAGGGTTGCACCGCCGACCGACCTTGATCTTCTGAGAAGGGTTCGAGTGAGAGCATGCCTGTCGGGA

CCCGAAAGATGGTGAACTATGCCTGAGCGGGGCGAAGCCAGAGGAAACTCTGGTGGAGGCCCGCAGCGAT

ACTGACGTGCAAATCGTTCGTCTGACTTGGGTATAGGGGCGAAAGACTAATCGAACCGTCTAGTAGCTGG

TTCCCTCCGAAGTTTCCCTCAGGATAGCTGGAGCTCGGGACGAGTTCTATCGGGTAAAGCCAATGATTAG

AGGCATCGGGGGCGCAACGCCCTCGACCTATTCTCAAACTTTAAATAGGTAGGACGGCGCGGCTGCTTCG

CTGAGCCGCGCCACGGAATCGAGAGCTCCAAGTGGGCCATTTTTGGTAAGCAGAACTGGCGATGCGGGAT

GAACCGGAAGCCGGGTTACGGTGCCCAACTGCGCGCTAACCT

>FM895222.1 FM895222 Jatropha curcas embryo 71-95 (DAF) Jatropha curcas cDNA clone rjcpga0_002010, mRNA sequence

TTATGGTTCACAAGCCTGAAGAAGGATTCTTCTCTTGGATCTTGGCAATCTTGCTTTCACCTCTGCTTTG

GATATTTTCAACATTAATTGAGTTCTATTTGAAGTGGACACAACCATTGAAGAAATATAATATGGTCCCA

TCTCATAGTTTCATCAAACAGATTTCTTCGTGCTCGCTCTCATCTCTACCAGCTAATTTTTATGGCAATG

TCAAAGAAGGAAGCCTTATCCCAAAGAAATCAGAAAACCTCAGCTTCTGCAAAAATGGTTTGATCATAGA

AGGTGAGGAGAATCCAATGGAAACTGAAATTGTTATCTTTGCAACGGGATACAAAAGTGAAGAAAAGCTC

AAGAACATTTTCAAATCCAGCTACTTTCAAAAATGCATTATTGGGTCATCAGCTCCTTTATACAGAGATT

GCATTCATCCTCGAATTCCACAGCTAGCTATACTTGGATATGCATACAGTCATTCAGATTTATATGCTGC

GGAAATGAGAAGCAAGTGGGTGGCACATTTTCTTGCAGGAAAATTCACATTACCAACCATAAATGAAATG

GAAGCTCAAGTGACA

>FM895221.1 FM895221 Jatropha curcas embryo 71-95 (DAF) Jatropha curcas cDNA clone rjcpga0_002009, mRNA sequence

AGGATTCTATCCGTCGCCCGATGGGAATTGTACTTTAAGCGCGGCCCTCGCGGCTCGTCCGCCGCGGGGG

CTTAGCCAACGGCACGTGCCTCTGGGGGCCGGATGGCCCCTACTGCGGGTCGGCAAACGGGCGACGGACA

CGCGCGTCGCTTCTGGCCCGGATTCTGACTTAGAGGCGTTCAGTCATAATCCAGCGCACGGTAGCTTCGC

GCCACTGGCTTTTCAACCAAGCGCGATGACCAATTGTGCGAATCAACGGTTCCTCTCGTACTAGGTTGAA

TTACCATCGCGACACCATCATCAGTAGGGTAAAACTAACCTGTCTCACGACGGTCTAACCCCAGCTCACG

TTCCCTATTGGTGGGTGAACAATCCAACACTTGGTGAATTCTGCTTCACAATGATAGGAAGAGCCGACAT

CGAAGGATCAAAAAGCAACGTCGCTATGAACGCTTGGCTGCCACAAGCCAGTTATCCCTGTGGTAACTTT

TCTGACACCTCT

>FM895220.1 FM895220 Jatropha curcas embryo 71-95 (DAF) Jatropha curcas cDNA clone rjcpga0_002008, mRNA sequence

TGGAGCCAAAACTCGTTCAATTAAGTCTAACTTGACTCATTGTGCTTATTGTTTCAACTCGTCGCAACCA

TGAGCCGATTCCGGAGAATCGAGATTCTCGAGTCTCACTGCCCCTCGCTCTTTGTTAGAGAAGCTGTTTT

AGCTCCCAAGGCTCTAGCTTTTCCCTCATTCCTAGTAGAAGAGGAACACGATGATCTCAGTTTTGCTCTT

GATCTTTTAAACCCTAATCCCACTCCCTTTGAGCTCTTCGACCCAGTGTCGGATCTGATCCAAATCGAAA

AAATGCCATCGTTTTACTCTTACAAGAGGGTCCAGCGACGACTGGGGACCGAGTTGTTCTTAGAGACCTT

ATGTGATCGAGTCAGCGCTCTCGAGTCTAGGTTTGACCGCTTGCTCAGCTCAGAGATCCGCGGTGGAGAC

CGGAAGTACCCATGGACAGCAGAGATCAAGGGGCCGGTAGAAAGGAAGTACAAGTGGACTGCGGAGATC

>FM895219.1 FM895219 Jatropha curcas embryo 71-95 (DAF) Jatropha curcas cDNA clone rjcpga0_002007, mRNA sequence

TTTTTTGGCAAATTGGTACTCAGATTCATCTATAAAACTATAAAATTAAACTCAAAGTTTAGCCTACATG

TATGTAAAATTCATCCAGCGAAAGAAAAGCCTAACCTAATTACTATTAAAATGTACAAGCTGACATTTAA

ATAGCAACTAGACTTCGTTCATTCCAGGTTGTTGAGGTTTATCAAGGGCAATGATTGGCTGGACAGGAGG

AGCGTTGCTTGTTGTCCCATTCTCAATAGGAGAAGACACAGTCCATTTACCACCAGATTCAAGAGGCTCA

AAGACCATAACATTCCCATCCGCTAGCCCTATAGCGAACTGGTTAGCTTCTTGAGGATGTGCTGCTATCA

CAAGTGGGTATACTGTAGACCTGATATCTGGTGGAAGGTATGCACAAGGATTAATTTGACATTGAACTTC

AAGATTTGATGCGCTAAATATGCGTAGAATTCCATCCAGGAAGCTACTGTATACTAGCTGGCTATCGCAT

GAGAAAGTTGCGTGGGAGATTGGTGCTGCAAATTCTCCAATTGTCCACTGCT

>FM895218.1 FM895218 Jatropha curcas embryo 71-95 (DAF) Jatropha curcas cDNA clone rjcpga0_002006, mRNA sequence

ATTCGGATTCTATCCGTCGCCCGATGGGAATTGTACTTTAAGGCGGCCCTCGCGGCTCGTCCGCCGCGGG

GGCTTGGCCAACGGCACGTGCCTCTGGGGGCCGGATGGCCCCTACTGCGGGTCGGCAAACGGGCGACGGA

CACGCGCGTCGCTTCTGGCCCGGATTCTGACTTAGAGGCGTTCAGTCATAATCCAGCGCACGGTAGCTTC

GCGCCACTGGCTTTTCAACCAAGCGCGATGACCAATTGTGCGAATCAACGGTTCCTCTCGTACTAGGTTG

AATTACCATCGCGACACCATCATCAGTAGGGTAAAACTAACCTGTCTCACGACGGTCTAAACCCAGCTCA

CGTTCCCTATTGGTGGGTGAACAATCCAACACTTGGTGAATTCTGCTTCACAATGATAGGAAGAGCCGAC

ATCCGAAGGATCAAAAAGCAACGTCGCTATGAACGCTTGGCTGCCACAAGCCAGTTATCCCTGTGGTAAC

TTTTCTGACACCTCTAGCTTCAAATTCCGAAGGTCTAAAGGATCGAT

>FM895217.1 FM895217 Jatropha curcas embryo 71-95 (DAF) Jatropha curcas cDNA clone rjcpga0_002005, mRNA sequence

CTGTCGGGACCCGAAAGATGGTGAACTATGCCTGAGCGGGGCGAAGCCAGAGGAAACTCTGGTGGAGGCC

CGCAGCGATACTGACGTGCAAATCGTTCGTCTGACTTGGGTATAGGGGCGAAAGACTAATCGAACCGTCT

AGTAGCTGGTTCCCTCCGAAGTTTCCCTCAGGATAGCTGGAGCTCGGGACGAGTTCTATCGGGTAAAGCC

AATGATTAGAGGCATCGGGGGCGCAACGCCCTCGACCTATTCTCAAACTTTAAATAGGTAGGACGGCGCG

GCTGCTTCGCTGAGCCGCGCCACGGAATCGAGAGCTCCAAGTGGGCCATTTTTGGTAAGCAGAACTGGCG

ATGCGGGATGAACCGGAAGCCGGGTTACGGTGCCCAACTGCGCGCTAACCTAGAACCCACAAAGGGTGTT

GGTCGATTAAGACAGCAGGACGGTGGTCATGGAAGTCGAAATCCGCTAAGGAGTGTGTAACAACTCACCT

GCCGAATCAACTAGCCCCGAAAATGGATGGCGCTTAAGCGCGCGACCTATACCCGGCCGTCGGGGCAAGA

GCCAGGC

>FM895216.1 FM895216 Jatropha curcas embryo 71-95 (DAF) Jatropha curcas cDNA clone rjcpga0_002004, mRNA sequence

ATTGGGAAAGGTTCCATGTGAACGGCACTTGCACATGGGTTAGTCGATCCTAAGAGACGGGGGAAGCCCG

TCCGACAGCGCGTCCTCGCGCGAGCTTCGAAAGGGAATCGGGTTAAAATTCCCGAACCGGGACGTGAAGG

CTGACGGCGACGTTAGGGAGTCCGGAGACGTCGGCGGGGGCCTCGGGAAGAGTTATCTTTTCTGTTTAAC

AGCCCGCCCACCCTGGAAACGACTCAGTCGGAGGTAGGGTCCAGCGGCTGGAAGAGCACCGCACGTCGCG

CGGTGTCCGATGCGCCCCCGGCGGCCCATGAAAATCCGGAGGACCGAGTGCCTCCCACGCCCGGTCGTAC

TCATAACCGCATCAGGTCTCCGAGGTGAACAGCCTCTGGTCGATGGAACAATGTAGGCAAGGGAAGTCGG

CAAAATGGATCCGTAACCTCGGGAAAAGGATTGGCTCTGAGGGCTGGGCCCGGGGGTCCCAGTCCCGAAC

CCGTCGGCTGTCGGCGGACTGCTCGAGCTGCTCCCGCGGCGAGAGCGGGTCGCCGCGTGCCGGCCGGGGG

ACAGACTGGGAACGGCCCTTCCGGTGGCCTTCCCCGGGCGTCGAACAGTC

>FM895215.1 FM895215 Jatropha curcas embryo 71-95 (DAF) Jatropha curcas cDNA clone rjcpga0_002003, mRNA sequence

GCAAAGGATTCTATCCGTCGCCCGATGGGAATTGTACTTTAAGGCGGCCCTCGCGGCTCGTCCGCCGCGG

GGGCTTGGCCAACGGCACGTGCCTCTGGGGGCCGGATGGCCCCTACTGCGGGTCGGCAAACGGGCGACGG

ACACGCGCGTCGCTTCTGGCCCGGATTCTGACTTAGAGGCGTTCAGTCATAATCCAGCGCACGGTAGCTT

CGCGCCACTGGCTTTTCAACCAAGCGCGATGACCAATTGTGCGAATCAACGGTTCCTCTCGTACTAGGTT

GAATTACCATCGCGACACCATCATCAGTAGGGTAAAACTAACCTGTCTCACGACGGTCTAAACCCAGCTC

ACGTTCCCTATTGGTGGGTGAACAATCCAACACTTGGTGAATTCTGCTTCACAATGATAGGAAGAGCCGA

CATCGAAGGATCAAAAAGCAACGTCGCTATGAACGCTTGGCTGCCACAAGCCAGTTATCCCTGTGGTAAC

TTTTCTGACACCTCTAGCTTCAAATTCCGAAGGTCTAAAGGATC

>FM895214.1 FM895214 Jatropha curcas embryo 71-95 (DAF) Jatropha curcas cDNA clone rjcpga0_002002, mRNA sequence

ATTCCAAACACAAGTTCAGCGCTCTAGTTTTTCCGGATCTGCGAATATGCAGAGTGCAGCCTTCACCCTC

TCTTCTTCTCCTTCATTTCCATCTTTCAAGGCCACGGCGGCCTCTGAATAACCCTACTTTCGCTCCTAGA

TTTGATCCTATTCGTGCCTCTTCCAAACGACAGGATCTAGACACCTCAACTAATGTCGTTTTCCCCCGCC

GATCATGGTCTTTCTCTTCTTCTCCCTCTCTTTTCAGGCCATGGAATCCTCTGCCTCCTCTGGTTTCTGA

ACCTAAAACGGATGGGTTTGAGGTTAGAGCAACGCCTGTGCCCGAGAGTGCCGGTGAGGGCGAGAAGTCG

AATAGCTTAATGAAGACTTTGGAGCTTGGATTGTTGTTTGGCCTTTGGTATCTTTTCAATATATACTTCA

ATATCTACAACAAGCAGGTTCTGAAGGTATTTCCAAACCCAGTAACTGTCACTGTAGGTCAGTTTGCTGT

TGGGACAGTGTTAGTCATTCTGATGTGGACTTTCAATCTTTACAAAAGGCC

>FM895213.1 FM895213 Jatropha curcas embryo 71-95 (DAF) Jatropha curcas cDNA clone rjcpga0_002001, mRNA sequence

TCAAACCAATTCGGTTCCCTCAATGACTTAGCCCACGAGCTCGCATCTCTCCAGGACCTTGCCAACCGCG

GCAGCTGGCGCTCGATCCTCGACAAGGTCGCTCGTGCTCGCTCTCTGGCTCTCCTCACCACTCCTCACGA

TCACCTCACTTACCTGGCCTACAACGTCCTCGCTTTCGTAAAACTACGCCGCTTCAAAGACGCTCTCACG

GAACTTGATTCCGTCGACGATTTCGATAGCCACCATTACCGTTACGAAACTTATCCAAAAATCTACCCTC

ATCGGCACGGTTCTATGGTTCCTTTCTCTCTCCGATGGCTCCACGCTGTGATCCCCTTAAAATTAGGTAA

TCGTCAAGAAGGATTAGATCGGTTTTATATTTTGCTCGATTTTGTTCGCGGAAAACTGAAGGAAAAACAA

GGTTTTGATTTTTCAAGTAAAATTTGGAGAAATAGAGAGGTTTTTGTTATGAATTGGATAATTAGTGAGC

ATTTGAAGAATAAAGAGTTCACTATCTGTTTGGATTTGATTAAAGATTTGGTTTCCCGTGGTAACTTTGA

TCCAGTTTTGTTGTCAAAATTAGGTTGTATACAGATGCAAATTGGGGATTTAGAAGGAGCAAAGGGTTCA

TTCGAGAAGGTAGAGAAATTGTTGGAGGAG

>FM895212.1 FM895212 Jatropha curcas embryo 71-95 (DAF) Jatropha curcas cDNA clone rjcpga0_001996, mRNA sequence

TTTTTTTTTGAATTTCTCAATCAAGGTATTCACTATCATTTAAAAAAAAAAAAGAATTTTAATTTTCTTG

GAGAACAATCAAATAATCTTTTCCAAAAACTATAAAAAAATCCAGTAAATCCAAGTATTTCACTATGTGC

ATGTAATTCTTTGAGTAAATAAAATAAAAAAAAAAGAATGTCTATATTCAAGAAAATTTTTAATAATATA

GAACTCGACCTATGATGTGATTTCGCATGGTTGACCAACAGCAGTCAACATCACCTCCCACTGGAATCCG

AATTCTCTGACCCAGGATCCGAACCCGATGACAAAGGCCCAGCATCGGCAGAATCTATAAAATTATTAGT

AATAATCCAACCGAAACCAGTCGTCGGCCTTCTCTGGCGCTGCCCATCTTCACTACCGACACCATCATCC

TCCACAGGCATCCCAAACCGACAAATCGGGCACGACCCATGGATACCCAACCACTTTTCTATACAACCGG

AATGAAATCCGTGTTTACACGGCATCTCCCGAATCTCGCCGCCGATACTCATCTCGTCCAAACAAATTGG

GCAGTCCTCAACGCGATCTTCAGTCACTATTATTCTTGGCATCGCGTCTATCGACTCTTTCG

>FM895211.1 FM895211 Jatropha curcas embryo 71-95 (DAF) Jatropha curcas cDNA clone rjcpga0_001995, mRNA sequence

ACTAGTGGATCCCCGGGCTGCAGCAAAGGATTCTATCCGTCGCCCGATGGGAATTGTACTTTAAGGCGGC

CCTCGCGGCTCGTCCGCCGCGGGGGCTTGGCCAACGGCACGTGCCTCTGGGGGCCGGATGGCCCCTACTG

CGGGTCGGCAAACGGGCGACGGACACGCGCGTCGCTTCTGGCCCGGATTCTGACTTAGAGGCGTTCAGTC

ATAATCCAGCGCACGGTAGCTTCGCGCCACTGGCTTTTCAACCAAGCGCGATGACCAATTGTGCGAATCA

ACGGTTCCTCTCGTACTAGGTTGAATTACCATCGCGACACCATCATCAGTAGGGTAAAACTAACCTGTCT

CACGACGGTCTAAACCCAGCTCACGTTCCCTATTGGTGGGTGAACAATCCAACACTTGGTGAATTCTGCT

TCACAATGATAGGAAGAGCCGACATCGAAGGATCAAAAAGCAACGTCGCTATGAACGCTTGGCTGCCACA

AGCCAGTTATCCCTGTGGTAACTTTTCTGACACCTCTAGCTTCAAATTCCGAAGGTCTAAAGGATCGATA

GGCCACGCTTTCACGGTTCG

>FM895210.1 FM895210 Jatropha curcas embryo 71-95 (DAF) Jatropha curcas cDNA clone rjcpga0_001994, mRNA sequence

ATTCCAAATATGAGTGAAGCATATAACAATTTCACACAACCTTCAACAGGCTATAAATTGCCAACATATA

TCACACCTTCTGCAACCCATTGTAGTGCAAAAATGTCTACTCCATACATGAAGCTGCTGCTTTACTTCTT

CATTGCTTTGACGGTTTTTGTCATCCCATCCCTCTCCCATGATCAACCCAAAAAACCTAAGTTCAATGGA

TGAAAATAATAACGATCATCATCAGTGTGCC

>FM895209.1 FM895209 Jatropha curcas embryo 71-95 (DAF) Jatropha curcas cDNA clone rjcpga0_001993, mRNA sequence

TTTTATTTTTATTTGTGAGCCCAAAATGAATTCATATCTACTCCATTAAGAGAGGACTAATGCAAGTACA

CCAAAATACCAAATTCAAGCAAAATTAAGTTCCTCATATTATATAATCCTCTGCTTGACTCAAAAGGGTG

ACATAATTACTCAGAAACTAGAGAATGATGCAATTACAAGGCCAATTGCTAGAGAGCCAACTGAGATTGA

CAATCCGCGAGAACCTGCATTCCCTGGAGATGGTGCTGGTGCTGACACAGTTGCTCCGGCGGTCACAGTT

GGGGCAGCAGCACCACTAACAGATGGTGAAAGAGCACCAGGAGCAGCAGCAGGAGCCAAAGCCAATCCAC

AATTAGAAGCAGGAGGGGCTTGGAGTTTGCAGACAGTAGGAAGACTTAAAGCCTTAGTGACATTCAAAAC

GACACCCAATTGAGCGCTGCTCTTAAAAGCCTCACAAAGACGCTCTGCATCTGTATGCAAGACCGT

>FM895208.1 FM895208 Jatropha curcas embryo 71-95 (DAF) Jatropha curcas cDNA clone rjcpga0_001992, mRNA sequence

AGGATTCTATCCGTCGCCCGATGGGAATTGTACNTTTAAGGCGGCCCTCGCGGCTCGTCCGCCGCGGGGG

CTTAGCCAACGGCACGTGCCTCTGGGGGCCGGATGGCCCCTACTGCGGGTCGGCAAACGGGCGACGGACA

CGCGCGTCGCTTCTGGCCCGGATTCTGACTTAGAGGCGTTCAGTCATAATCCAGCGCACGGTAGCTTCGC

GCCACTGGCTTTTCAACCAAGCGCGATGACCAATTGTGCGAATCAACGGTTCCTCTCGTACTAGGTTGAA

TTACCATCGCGACACCATCATCAGTAGGGTAAAACTAACCTGTCTCACGACGGTCTAAACCCAGCTCACG

TTCCCTATTGGTGGGTGAACAATCCAACACTTGGTGAATTCTGCTTCACAATGATAGGAAGAGCCGACAT

CGAAGGATCAAAAAGCAACGTCGCTATGAACGCTTGGCTGCCACAAGCCAGTTATCCCTGTGGTAACTTT

TCTGACACCTCTAGCTTCAAATTCCGAAGGTCTAAAGGATCGATAGGCCACGCTTTCACGGTTCGTATTC

GTACTGGAAATCAGAATCAAACGAGCTTTTACCCTTTTG

>FM895207.1 FM895207 Jatropha curcas embryo 71-95 (DAF) Jatropha curcas cDNA clone rjcpga0_001990, mRNA sequence

GAATTCCTCTCTCCAATTCACATTCATCGACGTCAATGTTCAGTGTTCGACCCGGGCCCCACCATAGGAG

GGCCCACTCCGAGATGAGCTTCCGATTGCCGGACGACATGACTATGATGATGATGGGTCTGTCGCCTTCT

GATCCGATCAATGGCGGTGGAGGTGGAAATGAAGGTGGCGGATCTTCTGCTTGTAGTCTCGAGGAGATCG

GGTCAGAGGACGACCTCTTTTCTACCTATATTGACGTCGATAAGCTTAACGGAGATGGGCGCAACTCTAC

GGATCAGAGTAATAACAATAACAACAACAACAACAACGGTGAAGGTAATGGAGATAAAGGAGGAGCTTCT

ACTTCAACGTCGAGGCCAAAGCACAGGCATAGCAACTCCGTTGACGGAAGCGTTTATGGAGACGTAATGG

AGGCTACCAAAGCTATGCCTCCTGATAAGTTACAAGAGCTTTGGAGCATTGATCCAAAAAGAGCAAAAAG

GATACTAGCTAATCGGCAGTCTGCTGCTCGTTCTAAAGAGAGGAAGGCCCGATATATTCTAGAGCTTGAG

>FM895206.1 FM895206 Jatropha curcas embryo 71-95 (DAF) Jatropha curcas cDNA clone rjcpga0_001988, mRNA sequence

CTGTAAATGATAATAATTAGAGTAAAGAAAAACTTACTGTATCTTTTTCAATTCCAATTGCTATTAACTC

CCATGCAAATATGCATAAAAGGGGAAAATCATACCATCTAATGCCCAATAAAAAGAAAACAAAAAAAATG

CAAATTGTATCTTTAAATGTTTCTTTACATCTCAGGCAATTAAGCTCCCAAGAGAGAAGCTCTTCTATTT

TCTAGAGTAAGAAAGAAGAAAGAAGACGATGGCCAAGGAAGTAAGTGAAGAAACGCAAACTACCCATGCA

AAGGATTATGTAGATCCACCACCTGCGCCACTGATCGACATGGCTGAAATCAAGCTCTGGTCATTACTAC

AGAGCTTTAATAGCTGAGTATCATAGCCACTCTCCTTATCCTTAACATAACTGTAGCCACTGTAATTGGC

TACAAGAAACAAACTGATCCATGTGGTGGAGTAGGTCTATAGGGCATTGCATGGGCCTTTGGTGGCATGA

TCTTAAT

>FM895205.1 FM895205 Jatropha curcas embryo 71-95 (DAF) Jatropha curcas cDNA clone rjcpga0_001987, mRNA sequence

CAAAATTCCGTCAGTTCTGCAAGCCTGGTTTGGAAGGGAAGAATTGTATTGCTGATGAACATTATTTGCC

AACCTTTTTCAATATTGTTGATCCTGGTGGTATTGCAAATTGGTCAATAACACATGTTGATTGGTCTGAG

AGGAAGTGGCATCCTAAGTCTTATAGGGCTCAGGATGTTACTTTCGAGCTTCTGAAGAATATTACGTCAA

TTGACTTGAGCATACATGTGACAAGTGATGAAAAGAAGGAAGTGCTAATACAGCCATGCTTATGGAATGG

TATCCAACGGCCATGTTACTTATTCGCTAGGAAATTCTATCCAGAAGCTATTGATAACCTGCTGCAGCTA

TTCTCCAATTATTCAACAATTTGAGTGAGATAATTTTGACTCTAAATTCTTTGATTAAATCAACAGTTCA

TCCCCACCATCACATGTCATCTTAGTTTCTCATATATCCTAAAAGTTTGTAAATGCTGCGTGCAAATGCC

CCCTGGCACTGTTCTCTTGTGCATGGGCCGCTCGGAGGGGTCTTAGAGTGACTTAGCTGAGCTGAAGGCT

AACAATTCGGGGTGGATAATATATTGTCCATTTTTCTCCCTTCCATTTTGGGCTCTTGATGGGT

>FM895204.1 FM895204 Jatropha curcas embryo 71-95 (DAF) Jatropha curcas cDNA clone rjcpga0_001986, mRNA sequence

ATTCCTCAGCATTTCTAACCAAAGTGTCAAGGTCCACTTGCGTTCTTTATCTTTTTGCGCAAGTTTTTTA

TCCCTAATAGGTGTGCGTTTTTTGTGGCAACTCTACGCTGCTAATATTATGGGATCTAACGTAAGAACTA

TCTTCTCTGGAACTTCACATACAACTGGTACTGTTGCCAAGCACGGCACTGGAGGGGATTCTGTCGATGA

GGCTTATAAAGCCTTATTTAACTCTGAAGAGAATCGCCTGGCTTGGGCTCGTGAGTCGAAATTGGGATGC

GCTAATTATCTTAAGTGCTCCAAGCCGGTGAATTTGATACCGCTCGTCCCTGAGAAGGTGAATCGATCGG

CTCTCTCAGTGTTCAAGGCCAAATACAAGCAAGGTAAATTACCCACTAAAGGCATGCTGAATATACCGCA

GGTCTTAATTTTTGTGGTACGAACATGCTCGGTTGATCAGCCGGGGCGGATTCAAATCCGTCTCACTGAT

TCTGCTAGGCCGCAAGACGTGGTCGATGGTAACCAACAGGTTACCCTCGAACTTTCGAAATTGCCGGCTA

TAGTTAGTTTTTCGCCGAGTTATGATTGTCCATTAGAGACGATGGGTAAAGATGGCCTCCCTCGGTGCTT

TATGATAGA

>FM895203.1 FM895203 Jatropha curcas embryo 71-95 (DAF) Jatropha curcas cDNA clone rjcpga0_001985, mRNA sequence

ATTCCTGACATGTGTGCGAGTCAACGGGTGAGTAAACCCGCAAGGCGCAAGGAAGCTGACTGGCGGGATC

CCCCCGAGGGTTGCACCGCCGACCGACCTTGATCTTCTGAGAAGGGTTCGAGTGAGAGCATGCCTGTCGG

GACCCGAAAGATGGTGAACTATGCCTGAGCGGGGCGAAGCCAGAGGAAACTCTGGTGGAGGCCCGCAGCG

ATACTGACGTGCAAATCGTTCGTCTGACTTGGGTATAGGGGCGAAAGACTAATCGAACCGTCTAGTAGCT

GGTTCCCTCCGAAGTTTCCCTCAGGATAGCTGGAGCTTGGGACGAGTTCTATCGGGTAAAGCCAATGATT

AGAGGCATCGGGGGCGCAACGCCCTCGACCTATTCTCAAACTTTAAATAGGTAGGACGGCGCGGCTGCTT

CGCTGAGCCGCGCCACGGAATCGAGAGCTCCAAGTGGGCCATTTNTGGTAAGCAAACTGGCGATGCGGGA

TGAACCGGAAGCCGGGTTACGGTGCCCAACTGCGCGCTAACCTAAAACCCACAAGGGTGTTGGTCGATTA

AGAACAGCGGAACGTGGTCAT

>FM895202.1 FM895202 Jatropha curcas embryo 71-95 (DAF) Jatropha curcas cDNA clone rjcpga0_001984, mRNA sequence

ATTCGCTAGATACTATTAAAACAGGTTTAAAATCACCTCAATTACCTATCCCGGTTAATCAGGTTGTGCA

ACTCCTTGAAGCGGGTTCATTATGCTCTCAATTAAATATGCTTGTCACTTCACGAGATTTGTTCGCTGTG

ACTGGAGTTGAACAATTTCAAAACCAAAGAACCACTACCAATTTTGGAGGGGCTCATGTTTCTGGTTGCC

CCTTGATACTCGTACAGCCAAGTTCCGCTATCGATATGCAAACTGGGCTGGAAGATGCAATCTGTTATGT

TGAAGCTAATCGATTCAACGGACGTGATGCCAACCATTGATTATTATTTTAGAGCTGCACGTCCCAACCA

TTGATTATTATTTTAGAGCTGATCGAGAAACAGAAAGGGGACTCGACACATAAAACAAGGCTGAAGTCGT

TTACCTTTGGAGCCTGATAATGCGCTCTGCATATATTTGTCTAATCTTGTATCATAGTTTATTCATATTT

CATGTGGTGTGGAAAAGCCGTGGGAGGAAAGTGATTTGTAGTTCAATGTGCAATTCTTGCTCTAAAATTA

TAGACACCGGTCCTTGTAAAGTTCTTCCCTACAAAGA

>FM895201.1 FM895201 Jatropha curcas embryo 71-95 (DAF) Jatropha curcas cDNA clone rjcpga0_001983, mRNA sequence

AAGAACCACAGTATGATCTTGCAGGCAATTGTGGATTCTGAGATGAGGTTTCTGGACATAGTCACTGGAT

GGCCTGGAAAAATGGAAGATAGGGTAGTGTTTCAAAGCTCAAACTTCTATAAACTGTGTGAAAGTGGCGA

AAGGTTGAATGGGAAAAATTTACAGCTTTCAGACGGATCAGAAATAAGAGAATATATAATAGGGGATTCA

GGCTTTCCACTGCTTCCCTATCTTATTGTTCCTTATGAAGGGAAAGAACTATCAGAAACGAAAGCTGAGT

TCAACAAGCGACATTTTGCAACTCAGATGGTGGCCCAAAGAGCACTGGCAAGGCTCCAGGAGATGTGGAG

GATCATCCATGGAGTAATGTGGAGACCTGACAAGCATAGGTTGCCAAGAATAATTCTAGTCTGCTGTTTG

CTCCATAACATTATCCTTGATATGGA

>FM895200.1 FM895200 Jatropha curcas embryo 71-95 (DAF) Jatropha curcas cDNA clone rjcpga0_001982, mRNA sequence

ATTCCAAATAGGTCCAATATATTAAAAAATGCACAAACGCATCCTTACATCTAAAATTACTCAAATTTTC

GTCAAATGGCAAATAAAATGACCATACTAACCTTTATAATATACAAAATTTTCTTTACAATTAATTACTT

CTATTCCTCCATCTCTTTCCTTGAATCCTTCACAGAAACAGAAAACGGTCATCTCTCTCTTTAATGCCGT

GATCTGCACTTTCTGAACTCTCTCATTTTCACAAATCTGAACCATAAATTACGCATGGAGCAATCTATGT

GAGAGCAAGGCCTTCAAAAATGAAATTACGAACAAATAAATGAATTTAAGCATAGGCTAATTTCCTTTCC

CTTTCTCATGGTAGAGATGCCATTATTGCTATTTCATTATTTTTTTTTCCTTTTCCTATCACCGGCTCAT

GTACGGCTAGCAGCGACCTCCTCCCTTTGCCTCACCGCCGTTCTCTCTCTCCATTTCTTTTCCGATTGGA

GCAATGACAGGGTGTGAGCTGCCTTCGTTTTCCGAATGGAGTGGCTCCACCTCAGTCTCCTTCAATA

>FM895199.1 FM895199 Jatropha curcas embryo 71-95 (DAF) Jatropha curcas cDNA clone rjcpga0_001981, mRNA sequence

CAAATGGCTACTGTTGAGGTAGTATCGGCACAAACTGCACTTTCAGAGGAAAAAGCAGAAGAAGTGAAGG

CTTCAGAGACTGTCACTCAAGTAACAGATCAGTCAGTTACTGATGAAAAACCAGACACAGGAGAAAAGAA

GGAAGAACAAGAAGAAGTTGCAGGAACTAAAGAAGAAGCTGTAGAACCAGAACCAGAAGTTGCAACTGAA

GTAGTGGTGGAAACTAAAGAAGTAGTAGAAGATGAGACCAAAACTGTAACAGAAGAGCCCCCATCGGAGA

AAACTGAAGAAGAAACTCCTAAGGAAACAGAACCAGAAAAAGAAGAAACTCCTAAGGAAACAGAACCAGA

AAAAGAAGAAAAGGAATCCCCAAAAGAAACAGTAACTGAGGAGACTGTTGCTGAGGAGACTACTAAAGAG

ACCACTGAGAGTACTGAGACAACAACTCCGGCGCCAGAACCGGCACCGGAAGCTGCCGTTGAAGCTCCTA

AGGAAGAAGGTGAAGCGAAAGCTGAGTAATGGGTACTTGTGCTGTGTAGAGGCTGTTTGTTTCTGGCTGT

ATGCCAACTTGAATGTTTCTTGAATTTTCTATCTTTCTTTAATTAGCT

>FM895198.1 FM895198 Jatropha curcas embryo 71-95 (DAF) Jatropha curcas cDNA clone rjcpga0_001980, mRNA sequence

CCAGGATCTCCCTTCTGTACAAGAAAATCACATTTCTGAGCAACCAATTGCTTTATCTGAGGAAGCCAAT

GGGGAGGAAGTCTATGACCCATCTGAGAATGGGGATGGTTCAATTGAAGAAGAGGAACAACCAGTGCCTG

AAGTTGTCGATGAAATTCCTGATGATGCTCAGATGGTTACTGAGTCACAGATAGTTGCTGAATCTAGTTC

TAAGATTGAAGAAGTGCCAAAGAAGTCGTATGCCTCCATTGTGAAAGTTATGAAGGAGAATGCTGCGCCG

TTTTCAAGCCCTGCCCCTTCTCCTCTCAGATCTACACCAAAGAGTCAAGAACAAGTAATTGCTGCAGTTA

TTCCTACTGCGGCATCTGAGACACATGTCTCCACTTCAAATGCTACTGAAAATGGGAATGCCCAGGAGGG

TGAAGCTGAGGGTCCTTCAATCTATGTAAAAGGTCTACCTTTGGATGCTACACATGCTCTACTTGATAGT

GAGTTTAAGAAGTTTGGACCTATTAGGACTGGTGGGATTCAAGTTAGGTGTCACAAGGGGTTTTGCTTTG

GCTTTGTGGAA

>FM895197.1 FM895197 Jatropha curcas embryo 71-95 (DAF) Jatropha curcas cDNA clone rjcpga0_001979, mRNA sequence

TATCCGTCGCCCGATGGGAATTGTACTTTAAGGCGGCCCTCGCGGCTCGTCCGCCGCGGGGGCTTGGCCA

ACGGCACGTGCCTCTGGGGGCCGGATGGCCCCTACTGCGGGTCGGCAAACGGGCGACGGACACGCGCGTC

GCTTCTGGCCCGGATTCTGACTTAGAGGCGTTCAGTCATAATCCAGCGCACGGTAGCTTCGCGCCACTGG

CTTTTCAACCAAGCGCGATGACCAATTGTGCGAATCAACGGTTCCTCTCGTACTAGGTTGAATTACCATC

GCGACACCATCATCAGTAGGGTAAAACTAACCTGTCTCACGACGGTCTAAACCCAGCTCACGTTCCCTAT

TGGTGGGTGAACAATCCAACACTTGGTGAATTCTGCTTCACAATGATAGGAAGAGCCGACATCGAAGGAT

CAAAAAGCAACGTCGCTATGAACGCTTGGCTGCCACAAGCCAGTTATCCCTGTGGTAACTTTTCTGACAC

CTCTAGCTTCAAATTCCGAAGGTCTAAAGGATCGATAGGCCACGCTTTCACGGTTCGTATTCGTACTGGA

AATCAGAATCAAACGAGCTTTTACCCTTTTGTTCCACACGAGATTTCTG

>FM895196.1 FM895196 Jatropha curcas embryo 71-95 (DAF) Jatropha curcas cDNA clone rjcpga0_001978, mRNA sequence

AGGATTCTATCCGTCGCCCGATGGGAATTGTACTTTAAGGCGGCCTCGCGGCTCGTCCGCCGCGGGGGCT

TGGCCAACGGCACGTGCCTCTGGGGGCCGGATGGCCCCTACTGCGGGTCGGCAAACGGGCGACGGACACG

CGCGTCGCTTCTGGCCCGGATTCTGACTTAGAGGCGTTCAGTCATAATCCAGCGCACGGTAGCTTCGCGC

CACTGGCTTTTCAACCAAGCGCGATGACCAATTGTGCGAATCAACGGTTCCTCTCGTACTAGGTTGAATT

ACCATCGCGACACCATCATCAGTAGGGTAAAACTAACCTGTCTCACGACGGTCTAAACCCAGCTCACGTT

CCCTATTGGTGGGTGAACAATCCAACACTTGGTGAATTCTGCTTCACAATGATAGGAAGAGCCGACATCG

AAGGATCAAAAAGCAACGTCGCTATGAACGCTTGGCTGCCACAAGCCAGTTATCCCTGTGGTAACTTTTC

TGACACCTCTAGCTTCAAATTCCGAAGGTCTAAAGGATCGATAGGCCACGCTTTCACGGTTCGTATTCGT

ACTGGAAATCAAATCAAACGAGCTTTTACCCTTTTGTTCCACACGAAATTTCTGTTCTCGTTGAGCTCAT

C

>FM895195.1 FM895195 Jatropha curcas embryo 71-95 (DAF) Jatropha curcas cDNA clone rjcpga0_001977, mRNA sequence

GTCGTCTGCAAAGGATTCTATCCGTCGCTTCTGGCCCGGATTCTGACTTAGAGGCGTTCAGTCATAATCC

AGCGCACGGTAGCTTCGCGCCACTGGCTTTTCAACCAAGCGCGATGACCAATTGTGCGAATCAACGGTTC

CTCTCGTACTAGGTTGAATTACCATCGCGACACCATCATCAGTAGGGTAAAACTAACCTGTCTCACGACG

GTCTAAACCCAGCTCACGTTCCCTATTGGTGGGTGAACAATCCAACACTTGGTGAATTCTGCTTCACAAT

GATAGGAAGAGCCGACATCGAAGGATCAAAAAGCAACGTCGCTATGAACGCTTGGCTGCCACAAGCCAGT

TATCCCTGTGGTAACTTTTCTGACACCTCTAGCTTCAAATTCCGAAGGTCTAAAGGATCGATAGGCCACG

CTTTCACGGTTCGTATTCGTACTGGAAATCAGAATCAAACGAGCTTTTACCCTTTTGTTCCACACGAGAT

TTCTGTTCTCGTTGAGCTCATCTTAGGACACCTGCGTTATCTTTTAACAGATGTGCCGCCCCAGCCAAAC

TCCCCACCTGACAATGTCTTCCGCCCGGATCGGCCGCCGAAACGACCTTGGGTCCAGAAAGAGGGGCATA

GCCCCGCCTCCGATTCACGGAAATAAGTAAAATAACGGTTAAAGTAGTGGTAT

>FM895194.1 FM895194 Jatropha curcas embryo 71-95 (DAF) Jatropha curcas cDNA clone rjcpga0_001976, mRNA sequence

GAGCCTTCAAAATTTATGAAAAATTATTTAATCATTGCTCAAAAAAACAACAAATGTTAGTTCAAAGCTA

ACCTAAGGCAGTCCAGTTCAGGTGTCAAGATTCAACTTAAAATACCGTATTACAACAGAAAAACAACTGA

GTTAAGAATCAAGACATATCTTCCCCCTAAAAAGAAAATTCTTCAGGTAACAGTTCCTTGCCTCTCGGTT

CGATTTTGCGACTCTTGTGCAGATATACTGTAAGAAGAATCATGATGAATTTACTCCCAGCTGTTTTTAA

TCCTCAAATTGATGCATTGCTTTCATGTTATCTACGCGGTAGCAAAATTGAATTGTGACGGTGATTATCA

ACTGAATTATGCTGAACCTGCAGTAACCTTCTTCCGGACAACCCTTTTTCTTCGTGTCTTTGGAGCTGAT

GCTCCCCCGTCAACAGAATCAACGCTCCCTTGTTTGATGCCAAGAAGCCCTAGCCCAGAAACAAAGGTTT

TCTTTGATATAAACCATTTTGAAGATGATGTANCAACATCTTCAACTGGTCTATTGTAAGCCTTCAAAAG

GGTATCCGACTGGGCC

>FM895193.1 FM895193 Jatropha curcas embryo 71-95 (DAF) Jatropha curcas cDNA clone rjcpga0_001975, mRNA sequence

GCAATAAAGAGGCCAAACATATCCATACTATATAGCAAGCACATGCAATCACAATTCCGAGTTCATTGTT

ATTTAAACAGGGTAGAGAAGGACTTTTCAAGTTCTGCTGCCAAACTAGGAAATTAATCAGCCGTCCAACT

CTGCTCAACTATTTCCCGTACTCTCCTATTGTATTCCCTCTTGTTCTCACTAAACATCCGTGCAGCTTCT

GAATTTGCAGGAGAATTTGGATTTGGATCACAGAGCAATGACTGGATTGATGTTAGTATGGCTGCCACAT

CATAAATAGGACTCCACTGATTCTGTAGGATGTCCAAACAAATGCTTCCATCTGCATAAATATTTGGATG

GAACATTCTAGAAACAAAACACACTGTTGGGGGCTTATTAGGATAATCTTCTGTAAACTGAAGTGTCAAT

TTGAACGTACCTCCATCCCATGGTGTGTCATCAGGCCCAAATATAACAGCATTCCATAGCATAATATTAT

TCTCTTGAGGAGCACCACTAATCCCAGCAGGAGGATCCTGTTGCAGCCTCTTAAAATCCCTCATCAGTCT

CTTCCTTGATGGGGTAGTCATCCT

>FM895192.1 FM895192 Jatropha curcas embryo 71-95 (DAF) Jatropha curcas cDNA clone rjcpga0_001974, mRNA sequence

GGATTCTATCCGTCGCCCGATGGGAATTGTACTTTAAGGCGGCCCTCGCGGCTCGTCCGCCGCGGGGGCT

TGGCCAACGGCACGTGCCTCTGGGGGCCGGATGGCCCCTACTGCGGGTCGGCAAACGGGCGACGGACACG

CGCGTCGCTTCTGGCCCGGATTCTGACTTAGAGGCGTTCAGTCATAATCCAGCGCACGGTAGCTTCGCGC

CACTGGCTTTTCAACCAAGCGCGATGACCAATTGTGCGAATCAACGGTTCCTCTCGTACTAGGTTGAATT

ACCATCGCGACACCATCATCAGTAGGGTAAAACTAACCTGTCTCACGACGGTCTAAACCCAGCTCACGTT

CCCTATTGGTGGGTGAACAATCCAACACTTGGTGAATTCTGCTTCACAATGATAGGAAGAGCCGACATCG

AAGGATCAAAAAGCAACGTCGCTATGAACGCTTGGCTGCCACAAGCCAGTTATCCCTGTGGTAACTTTTC

TGACACCTCTAGCTTCAAATTCCGAAGGTCTAAAGGATCGATAGGCCACGCTTTCACGGTTCGTATTCGT

ACTGGAAATCAGAATCAAACGAGCTTTTACCCTTTTGTTCCACACGAGATTTCTGTTCTCGTTGAGCTCA

TCTTAGGA

>FM895191.1 FM895191 Jatropha curcas embryo 71-95 (DAF) Jatropha curcas cDNA clone rjcpga0_001972, mRNA sequence

CGTGTCTACTCTTTCAGTGGATTGATTCCATTGCCGTGGACATGAAAGAAAAGAAATTAACGGTGATCGG

CACAGTTGATCCTATAACTGTAGTGAGCAAATTGAGAAAGTATTTTCAAACAGATATAATCTCAGTAGGG

CCAGCGAAGGAGCCTGAGAAGAAAGAAGAGCCTAAGAAGGAAGAACCCAAGAAAGAAGAGGAAGCTAAGA

AAGAGGAACCAAAGAAAGAAGAGGAAGCAAAGAAAGAGGAGCCCAAGAAAGAAGAGGAAGCGAAGAAAGA

TGGAGCCCAAGAAAGAGGAAGAGAAGAAAGAAGAAGAGAAAAAGACAGAAGCAGCTCCAGCAGCAGCACC

AGTTGCAGTTGCAGCTGCACCACCACCAGATCCGGTTTTAGAGCTTGTTAAGGCTTACAAAGCTTATAAT

CCTCACCTCACCACATATTATTATGTGCAAAGCATTGAAGAGAATCCAAATGCTTGTGTTATTTGCTAAG

CTGGATGGCTATATAAGTTTAAGGACAAGTATATATAGCTCTCTTTTTGCCCCAACTGGTGATGGGTTAT

ATTCAATCTTATCAGTTGTTCAAGTGTGTCAAGAGGCCAAAACCTAAGAAGAGAAGAACCTTGTATATCA

AGTGGTGATG

>FM895190.1 FM895190 Jatropha curcas embryo 71-95 (DAF) Jatropha curcas cDNA clone rjcpga0_001971, mRNA sequence

ATTAAATTTTATAGTTTTTATTGAATTAAAGCAAACATAACATCCACAGAAGGCCGTAACAGGCCAATCA

GAAAGGAAACAGAATACAACGATGATAGAGAAGCCCATTAGACTCTCTAAGAAAATACATTCAGATGAAA

GCATGAAAACAATTAAGGAAAGCAATTCCCCCCTTCAGAATCCACCACGCAGACGCAGGACAAGGTGAAG

GGTTGATTCTTTCTGGATATTGTAGTCGGCCAGAGTCCTTCCGTCCTCCAACTGCTTCCCAGCAAAGATC

AGCCTTTGCTGATCTGGAGGAATCCCTTCCTTATCCTGGATTTTCGCCTTCACATTATCGATTGTATCAC

TGCTCTCCACTTCCAGAGTGATAGTCTTCCCTGTCAGGGTCTTGACGAAGATTTGCATTCCACCACGAAG

CCTGAGCCCAAGGTGGAGAGTGGATTCCTTCTGAATATTGTAATCGGCCAAAGTTCGGCCATCTTCCAGT

TGCTTACCGGCGAAGATCAGCCTCTGCTGGTCTGGTGGGATGCCCTCCTTGTCTTGAATTCTTGCCTTCC

CGTTATCAATAGTGTCACTGCTCTCAACCTCCAGGGTGATGGTCTTTC

>FM895189.1 FM895189 Jatropha curcas embryo 71-95 (DAF) Jatropha curcas cDNA clone rjcpga0_001970, mRNA sequence

GAAAATTAGATACTGCATACCGTGAAGCTAAGGAAAAGCAAGCAACCATGCCCGGCTGTTTCTGTTCTTT

TCGGTAAATGCTAGTGCTCAGTTCTGTGGAGTGGCTGAAATGGTTGGACCTGTTGATTTTGACAAGAGTG

TGGATTATTGGCAGCAAGATAAGTGGAGTGGGCAGTTCCCTGTTAAGTGGCATATCATTACAGATGTTCC

AAACAGTCAGTTTCGTCATATTGTACTGGAAAATAATGACAACAAGCCTGTGACTAACAGTCGAGACACA

CGAGAGGTGAAATTGGAACAGGGTGTTGAGATGTTGAACATTTTTAAGAACTATGAAAGTAATATGTCAC

TCCTGGATGACTTTTGATTTTTATGAAGACAGACAGAAAGCCATGCAAGAGCGGAAGGCCAGACAGCTGC

AAGCAGGCCTGATGGCTGGTGGTGTAGTTGGAGAAAATGAGCAGAAAAATGCAGTTACATTACCCAGCAA

TTTGATCAGGCAGATGTCCAAAAGTTTTGCTCAAGTTGTCCGCTTAGAGGAGTCTAATAAAGAAGTCCCT

GTTGTAGATAAAGCAAGCTCCGTCTCAGATAGTTCTACCAGAGTAAAGGTGGAAGATGCTATCACAGGTG

TCTCCCCTGCAAAGACCAGTTAGGAAGACATTTAATAGTG

>FM895188.1 FM895188 Jatropha curcas embryo 71-95 (DAF) Jatropha curcas cDNA clone rjcpga0_001969, mRNA sequence

CGGGCTGCAGGAATTCGAATTCCAAAGGATTCTATCCGTCGCCCGATGGGAATTGTACTTTAAGGCGGCC

CTCGCGGCTCGTCCGCCGCGGGGGCTTGGCCAACGGCACGTGCCTCTGGGGGCCGGATGGCCCCTACTGC

GGGTCGGCAAACGGGCGACGGACACGCGCGTCGCTTCTGGCCCGGATTCTGACTTAGAGGCGTTCAGTCA

TAATCCAGCGCACGGTAGCTTCGCGCCACTGGCTTTTCAACCAAGCGCGATGACCAATTGTGCGAATCAA

CGGTTCCTCTCGTACTAGGTTGAATTACCATCGCGACACCATCATCAGTAGGGTAAAACTAACCTGTCTC

ACGACGGTCTAATCCCAGCTCACGTTCCCTATTGGTGGGTGAACAATCCAACACTTGGTGAATTCTGCTT

CACAATGATAGGAAGAGCCGACATCGAAGGATCAAAAAGCAACGTCGCTATGAACGCTTGGCTGCCACAA

GCCAGTTATCCCTGTGGTAACTTTTCTGACACCTCTAGCTTCAAATTCCGAAGGTCTAAAGGATCGATAG

GCCACGCTTTCACGGTTCGTATTCGTACTGGAAATCAGAATCAAACGAGCTTTTACCCTTTTGTTCCACA

CGAGATTTCTGTTCTCGTTGAGCTCATCTTAGGACACCTGCGTTA

>FM895187.1 FM895187 Jatropha curcas embryo 71-95 (DAF) Jatropha curcas cDNA clone rjcpga0_001968, mRNA sequence

CTCATCGATTGAAGCCTGGAGAACGAATCCTGGACGTTGGATGCGGCGTCGGGGGCCCCATGCGCGCCAT

CGCCGCTCACTCGCGCGCTAATGTTGTAGGCATCACAATCAACGAGTATCAGGTTAACCGTGCGCGTTTG

CACAACAAGAAAGCCGGTCTCGATTCGCTTTGTGAAGTTGTTTGTGGGAATTTCCTTGAGATGCCGTTTC

CAGACAACAGTTTCGACGGCGCGTACTCTATCGAAGCTACTTGTCACGCGCCGAAGCTGGAAGAGGTATA

CGCAGAGATCTTTCGAGTGTTGAAGCCAGGATCTCTCTACGTGTCCTACGAATGGGTCACAACTGACAAG

TACAGGGCTAGTGATCCTCAACACGTGGAGATCATTCAAGGCATTGAAAGAGGAGATGCTTTGCCGGGGC

TGAGAAGCTACGCAGACATTGCCGAGACGGCGAGAAAGGTAGGGTTCGAAGTAGTGAAAGAGAAGGATTT

AGCAAAACCACCAGCGCAGCCATGGTGGACTAGGCTAAAGATGCCCTGGATTGCCTACTGGAGGAATCAC

ATTCTCGTTACTATATTGGCTGCTTTGGGTAT

>FM895186.1 FM895186 Jatropha curcas embryo 71-95 (DAF) Jatropha curcas cDNA clone rjcpga0_001967, mRNA sequence

AGACAAGAAACCTTAACTTATTCCTTTGTCAACACCTTTTACAGATTCAACAAGAATCCTTAAAAGAAAA

AACCTCCAATGCTAAGAACACGGGAAGAAACTTGGAGAGCAGTATACATAAATCTGTAAAATGTTCCCTA

ACTAACGAAACAGGGAAGGAAAGAGAAGACAAAAGACAAGTGTCTCCAATGAAGTTAGCATCAGATGCTC

TGAGCAACCCTAATATACACACGCCAAAGAGCAAGAAGACAACAGTGCCGCCACCACCACTACAACAAGC

ATAATTACTATTATCATAACAAGAAGAATAGAAAACACTGTCTAATGGCATGCTGATTGCTGTGCATTGA

AAAACTCTTGAGAGCTGGAACCATATGCAAAGTCTGTGGACTTGCAACTTTATTCTCAATCCTTTTACAC

TTCCTCCCTGTCTCGGCTGCTACTCTTCAAGATATTGTACTTGCAAAGTGGGCAGGTAGCATTAATATAC

AGCCACTTATCTACACAGGCACAATGGAAGTGGTGACCAC

>FM895185.1 FM895185 Jatropha curcas embryo 71-95 (DAF) Jatropha curcas cDNA clone rjcpga0_001966, mRNA sequence

ATTCCGTGATGTTACCGATGCCGGCATGTCAGCTTTTGCCAAGAATTGCAGAGGTTTGAAGAAGCTCTCG

TGTGGATCGTGCACGTTTGGAGCCAAAGGTATGAATGCCGTCCTCGATAACTGTGCATCGCTTGAAGAGT

TGTCAGTGAAGCGTCTTCGCGGAATCACCGATGGAGCCGCCGCTGAGCCTATTGGACCAGGTTTGGCGGC

ATCTTCGCTCAAAACAATATGCTTAAAGGAGCTTTATAATGGACAGTGTTTTGGTCCGCTTATAATTGGG

TCAAAGAATCTCAGAACTTTGAAACTTTTCAGATGCTCTGGTGATTGGGACAAGCTTCTTCAAGTGATTG

CGGATCGGGTCACGGGAATGCTGGAGATCCATCTCGAGAGAATCCAGGTAAGTGATGTTGGCCTCGCGGC

AATCTCTAATTGTTTGGATCTGGAGATATTGCACCTTGTCAAGACCCCCGAGTGTACCAATTTAGGCCTA

GTTTCCGTAGCAGAGCGTTGTAGGCTTCTAAGAAAGCTTCATATTGATGGATGGAAGGCCAATCGAATAG

GCGATGAGGGACTAATAGCAGTTGCGAAAAATTGCCCTAATTTGCAAGAACTGGTCCTTATTGGAGTCAA

TCCTA

>FM895184.1 FM895184 Jatropha curcas embryo 71-95 (DAF) Jatropha curcas cDNA clone rjcpga0_001965, mRNA sequence

TTTTTTTTAGAAAAAAATAAATAAATAAAAACTCATAAAAAAGGTCACATCTGCGCAGCAAATATAGTAT

TGTGATTTATCATCATTAAGTTCTGATGAACATTGTCTATCCAAAAATACTACCTTGCAAGAAATAGGTA

ATACAGATTCCAGCAAAGATATCAAGCATCAAAAGAATCAAATCATAGACCAGCTCAAAGAATCTTCTTT

CTTGGCACTTTCACTGGATGTAGGATGAGAAGCAAGCAACAATTAGATGTATAAATAACCTTGATTCTAC

ACAACCATGGATTTATCCTTGGTTTCTGCTGAATCTTGAGCATCCAAAATGGTATTATTCCCATTCAATA

GTTGATGGAGGCTTTGATGTAATGTCTTGAACAACTCGGTTTACGCCCCTTACACCGTTACAAATTTTTC

GGGCCACGTCATTAAGGAACTTGTGTTCAAAATAGTACCAATCAGCAGTCATTCCATCTTGACTTGTAAC

AGCTCTTAAAGCAACAA

>FM895183.1 FM895183 Jatropha curcas embryo 71-95 (DAF) Jatropha curcas cDNA clone rjcpga0_001964, mRNA sequence

CCCTGACAATCCCTGGTCATTCCATTTATCTGATCTGCAGCTGGGGACGGAAGCAATGTACACACAATTG

TCGAAAGATGTCGTACCTGAAGGAGATCGTCCTGTTCTCACCTGCCTACGCATAGGTTACTATAACAACC

ACATCTGAACGGTGCCTGTATAG

>FM895182.1 FM895182 Jatropha curcas embryo 71-95 (DAF) Jatropha curcas cDNA clone rjcpga0_001963, mRNA sequence

CTGCGATCTCAGATCTAAACGGCACCGTAATCAAATCGACTCGTCAAACATGTCTTCTGTATTCAGCGGC

GATGAAACTGCTCCTTTCTTTGGCTTTCTCGGCGCCGCTGCTGCCCTCGTCTTCTCCTGTATGGGAGCTG

CATATGGAACAGCCAAGAGTGGCGTTGGTGTGGCATCAATGGGAGTTATGAGGCCTGAGCTTGTGATGAA

GTCTATTGTACCAGTTGTTATGGCTGGTGTGTTGGGTATATATGGCTTGATTATTGCTGTTATTATCAGT

ACTGGAATTAACCCCAAGGCTAAGTCATATTACCTTTTTGATGGGTATGCACATCTCTCATCTGGTCTTG

CCTGTGGCCTTGCTGGGCTTTCTGCTGGAATGGCCATTGGAATTGTTGGTGATGCAGGTGTTAGAGCTAA

TGCACAACAGCCCAAACTTTTTGTTGGAATGATCCTCATCCTCATCTTTGCTGAAGCATTGGCATTGTAT

GGGCTTATTGTTGGGATTATCTTGTCTTCGCGAGCTGGCCAGTCTAGGGCAGAATGAGCCAGGAGATTAG

TACCAATTGTTATTCTTTAATGACTTAGCGGTTTGACTGC

>FM895181.1 FM895181 Jatropha curcas embryo 71-95 (DAF) Jatropha curcas cDNA clone rjcpga0_001962, mRNA sequence

ATTCCAACTTTAGATAATGGAGATTTCTTGGATGATTAGGGCATTTCTGATTCCGGATCATACTGATGAA

TCATATTTGTTTATAACCTTGAAGTTCATTGAATGGTGCATTACAGGTCATCCAGATGAACATACAAGAA

GATATACGCAAACAAATTGTTCAGGAGCTGAAAATAAACCAAGCCTCGCAGTGTTCTCATGTTGTTGTTT

GCTACCACTCATTCTATCACAATGGAGCTATTTCTCTTGTGTTGGAATATATGGACCGTGGATCTCTAGC

GGATGTGATTAGACAAGTTAAAACCATTCTTGAGCCTTATCTAGCTGTCGTGTGTTTTACAGGGTCTTGT

TTACCTGCACCACGAACGGCATGTCATACATAGGGACATTAAGCCATCTAATCTATTGGTAAACCAGAAG

GGAGAAGTGAAGATCACTGATTTTGGAGTGAGTGCAATGCTAGCTAGTTCTATGGGCCANAGGGACACAT

TTGTTGGAACTTACAATTACATGTCGGTATGTCTTGGTTTTATTTCTGTGCTAGCCCAAGATAGCGAAAA

CTATTAACTTGACCAGTTCATTAATTATGATAACCCCTCACC

>FM895180.1 FM895180 Jatropha curcas embryo 71-95 (DAF) Jatropha curcas cDNA clone rjcpga0_001961, mRNA sequence

TTTTTTACAAATACAAGTTAAATCTAATTTTATAACTGAATCCAACATAACATCTTAAAGACACAATATT

AAGTCTAAATGGACAACATACAACTCGCATTTTATTGGATGAGAACATGTTATCAAAAGATAAAACATAT

TGATTAGCTTAGTATCCCAACAAAGCAACAGCATTTGGAAGATTAGCATTGATTTGGTGCTTCTGAACCA

GCATGCTCTCTTTTGCGACTTGATTTGTATTTTCAAATGCTTGGCTTCACGTTCAGACGAGACGCTAGCT

TCTGACCCAGAGATCTGTCGCACTGAGTAAAGTAGTTGATCCAAATACTACAAATCTCATGTGTGACACG

AGGATCAGATAAGCAACCAACAATCCGATTGAGGAATCGCTCTTGCCTGTCAGGAGCCCAGGATCGATAT

CTCTCTCCAGGTTGCTTGAAATTGTTCTCCTTTGGTATCACACACTTCTCACGCTTTCCACTGCATATAG

TGGCCGGAAAGGGGAACGCCTCAGAATGGCGAACCGGATCGTATCTGGACGGGAAGTAATTGACCTCCTC

ATCCCTATGCATGAAATTCATGAAACCTTCATGGTGATTGTTATGATGAGCACACTTGGGAGCATTACAG

GGAGCTGTAGATAGT

>FM895179.1 FM895179 Jatropha curcas embryo 71-95 (DAF) Jatropha curcas cDNA clone rjcpga0_001960, mRNA sequence

ATTCGTTAAATTACTGGGTTCTTATTCAAAGAGAGGTTTGCATTTGCTAATTTATGAATATATGGATAAA

GGATCCCTTGAGCAAGCTTTATTCGGTCCTCATTTTTCACTGCCGCTTAATTGGGAAATTAGATACCGTA

TTTGCCAACAAATAGCCCAAGGTTTAGAATATATACATAACCAGGAGCCCCCCATAATCCACAGAAGTAT

CAACGCCAGTAATATTCTGCTTTATAGAGATTATAATGCTAAAATCTCAGACTTTGGATTGGCAAATCTG

TATGAGGAGAATGATCCATTCACGTTCATTAGAGCAGGAAGGACACTAAGGTACATGGCACCTGAGTATG

CTGTTGGAAAAGAAGTAACAGTGAAATCTGATGTGTATAGTTTTGGTATTCTGCTCCTTGAAATCCTTAG

TGGGATAAAGATGGACACAAATTTGGGCGATCGTGGTAATAGTATTTATCTTTTAGACAAGGCTTTTGAA

TGTCACAAGCAAAAAAACTACGAGGAACTAGTCGATGAAATATTAAAGGAGTATCGATTATGGAAAGTA

>FM895178.1 FM895178 Jatropha curcas embryo 71-95 (DAF) Jatropha curcas cDNA clone rjcpga0_001959, mRNA sequence

CTCATCGAAGATCCATGTATGAGTAGAGATTTTCTATTTGATATAATAAGCAATCTTAACAAAGTTGATG

TTCATACATCAACCACTGAATTACAAAACTGGAATGTATAATCAGTTGATTGCTTTTATTTTAACTGAAG

AATTTTACAGGCATCTGTCACTTTGAAGCATTAGGGGGAAGCTGCAACCAAATCTAGAAGACCCAAGCTG

ATTGATCTTCCACTGTTGATCCTTCCAACCATGTTTGATGAAGGTGCATAAAAGTTGCTTCCACAGATTT

CTTTATATTTATTATAGCAGTGTTCACACAACCCTCCCGCTTATCACCAGATGATTCCCACTGATTGAAC

TTTACTAGCCATGCATCTGAACCAATTTTTGTAGGTATGACCTGATCAACCCAAATCCGAAATGGCTTTC

CCTTTTTCTCGCTGTAGTGACTTCTCAGTCCATTTACAGCATCATGAAGAGTTAGCTCAGCACCAGAAGG

ATGGATAAGGATACCAGATGGATCACAATCAGCTTTCACGCTTGCCAAGTACATCTCACAATTCTCAATT

TCTGCGCGCCTCCATTTCTCGAGGGGGGGCCCGGTAC

>FM895177.1 FM895177 Jatropha curcas embryo 71-95 (DAF) Jatropha curcas cDNA clone rjcpga0_001958, mRNA sequence

GCATCAAATAAATAAAATATATTGCAATTTTATCTAGATGTATGGCTTGTACAAAAACCTAAAGCAAGAA

ATTCTGTGAAAATAAAGTTTCATTAAATAATTGTTGGATTATCAAATACTGTTAAACAATAAAAAGTGCT

TCAAAAGGATGATATCCAACTAGTAATGGTTGAAAAACAATCACCTCAAACGAAGATCATGGAACCCGAT

GTGAAGAGCTAAAAGAAACAAGAAAAGAGTTTCTACTACCCTCACATAAAGGATTTGCCTTCATAGATAA

AGTCCTTCTGGCACCCCTTCTTTCTTTTTGAACATCACTTTCTCCTTTGCTTTCTTGAAGTCTGCATGAG

TCACCTTCATGCGCCGCTCTCTTAAAGCTAGCAAGCCAGCTTCTGTACATATAGCTTTGATATCAGCCCC

AGAGAATTCATCCTTGGTCATTACAAACTCTTCTAAGTTAACATCATCTGCCAATGTCATCCTCGATGTA

TGTATCTGGAATATGCGCCTTCTGGTTTTGATATCTGGAAGGGGAAATTCAATCTTTCTGTCTATTCTTC

CAAGGCGCAGCAAAGCAGGATCAAGACTTTCTATTCTGTATGTTGCAAGAATTACTTTTACATCTCCCCT

GGAATCAAAACCATCCAACTG

>FM895176.1 FM895176 Jatropha curcas embryo 71-95 (DAF) Jatropha curcas cDNA clone rjcpga0_001957, mRNA sequence

TTTAAAACAAAATTTAGTATCACTTGACAAAGGGTATCAAAAAGTCAAAAGAACTAGCACGACGCCATCG

TCACAGATAAGAAAAATAAAGCTCATTAAATAAAAATCAGGAAATAAACGGCATGGGCTTTTAGTCTAAG

CTACTGCTACTGGTACTGGGACCTCGATCTCGGTCGCCACAACAGGTACCCGGGCGTACTCCTCTTCCTC

CTTGGGGGGATGGATCGTGACCAAATCTGGTAGAGGAGTTGTTGGGCCCTGCTTCCCTTTAGGATCCCAA

TCAAGCATTATCTTGACCTTGATACCAAGAACCCCCTGTCGTAGGAGGACATGTCTCACAGCAGAATCAA

TATAATCCTTTACTGGTTGGCCAGATGAAATCATGTATCCATCCTTGAACTTCATGGATTTTGCACGCTG

TGCCCTTAGTTTCCCACTGACAATTACCTCACATCCCTTAGCACCACTTTCCATTATGAATCTCAACACA

CCATAGCAGGCCCTACGAACAGCAAGACCACCGAGAAGCTTGTAACGAAGAGACTCAGCCTGAGCAATGG

CGCAAAGGCCTCTGCTGTTAACTTTCTCAGCATAGAGCTCCACACTGTTTTCTGGGAATTTAACCTCTTT

TGCACCACGGAGGTCAGCTCTCTGAT

>FM895175.1 FM895175 Jatropha curcas embryo 71-95 (DAF) Jatropha curcas cDNA clone rjcpga0_001956, mRNA sequence

ATTCGTCTGACATGTGTGCGAGTCAACGGGTGAGTAAACCCGCAAGGCGCAAGGAAGCTGACTGGCGGGA

TCCCCCTGAGGGTTGCACCGCCGACCGACCTTGATCTTCTGAGAAGGGTTCGAGTGAGAGCATGCCTGTC

GGGACCCGAAAGATGGTGAACTATGCCTGAGCGGGGCGAAGCCAGAGGAAACTCTGGTGGAGGCCCGCAG

CGATACTGACGTGCAAATCGTTCGTCTGACTTGGGTATAGGGGCGAAAGACTAATCGAACCGTCTAGTAG

CTGGTTCCCTCCGAAGTTTCCCTCAGGATAGCTGGAGCTCGGGACGAGTTCTATCGGGTAAAGCCAATGA

TTAGAGGCATCGGGGGCGCAACGCCCTCGACCTATTCTCAAACTTTAAATAGGTAGGACGGCGCGGCTGC

TTCGCTGAGCCGCGCCACGGAATCGAGAGCTCCAAGTGGGCCATTTTTGGTAAGCAGAACTGGCGATGCG

GGATGAACCGGAAGCCGGGTTACGGTGCCCAACTGCGCGCTAACCTAGAACCCACAAAGGGTGTTGGTCG

ATTAAGA

>FM895174.1 FM895174 Jatropha curcas embryo 71-95 (DAF) Jatropha curcas cDNA clone rjcpga0_001955, mRNA sequence

GTTTTTCTCTACATTAACATATCTTTCTCACACTTATCATCAACAAATGGCTGAACGTTCACAGCCACAC

CAAGTTCAAGTGCACCCACAACACCGCTATGAAGCTGCCTTCAAAGGTCAACAAAAAGGTCCATCAGCTC

AGAAAGTGTTAGCTGTGATCACCCTCCTGCCGGTAGGCGGTGGGCTTCTTGCACTAGCTGGTATAACCTT

AGTAGGTACCCTAATCGGCCTAGCTATTACTACCCCTCTTTTTGTCATCTTTAGCCCTGTTCTTGTCCCA

GCTGCATTAGTTATTGGCCTTTCGGTCATGGCATTTTTGGCTTCCGGGGCTATGGGGCTCACAGGGTTGT

CATCACTCTCGTGGGTTCTGAAATACCTCCAGGAGGTCACTCGGCGAATGCCGGAGCAGCTGGATATAGC

TAAGAAGCGTATGCAAGACATGGCTGGTTTTGTGGGACAGAAGACCAAGGAAGTAGGCCAAGAAATCCAG

AGGAAAGCACATGAAGGGAAATAAGTAGAGAGAAATGCTGTTGGTTATTAAGGGTTGAAGTCAGAGAGCT

GGGTTTTGGTACGAGACTACTTTTGTAGTATGGGTGTAGTATTGAGGTAG

>FM895173.1 FM895173 Jatropha curcas embryo 71-95 (DAF) Jatropha curcas cDNA clone rjcpga0_001954, mRNA sequence

ATTCCATGCCCCTGTTGTCTCTCCCATGCATGCTCCAGCTCATGCACCCGTTCACCCACCAAAGCCGCAC

CCACACCCACACCCATCGGTTCAGCCTCCCATGCACTCTCCAAAACCACACCTACCGGTTAAACCACCAG

TTCACCCTCCCGTACACTCTGCACCACCAAAGCCATTACCACATCCACACCCACACCCACCAGTTAAACC

ACCAGCTGAGGCACCGATCTACCACTTCGAAAGAAGCTTTGTTTCCGTGCAAGGGGTTGTTTATTGCAAG

TCATGCAAATATTCTGGAGTTGAAACTCTCTTGGGAGCTTCACCACTTTCTGGTGCAACAGTAAAACTGC

AGTGCAATAATACAAAATACCCACTAGAAGCCAAGGCTACAACGGACAAAAATGGTTACTTCTTCCTTGA

AGCACCAAAGAGTATCACCAACTTCGGGGCCCACAAATGCAAGGTGTCATTAGTCTCTGCACCAAACACC

GCTTGCAGCAAGCCAACCGACCTCCACGGCGGAGTTAGTGGTGGCTTGTTGAGGCCGGAGAAGAAATATA

TTTCAAATAAGCTTCCGTTTGTTCTGTTTACAGTTGGACCATTTGCCTTTGAACCCAAGTGCCCTCGTTA

AATTAATGAATAAC

>FM895172.1 FM895172 Jatropha curcas embryo 71-95 (DAF) Jatropha curcas cDNA clone rjcpga0_001953, mRNA sequence

CCACTACACCCACTCCACCAGATTGCAGAAACACCGACACACAAACTCCTTTTGAAGCAATGGCTAAAAG

AAGAGGAGCTAATCCTAAACAGAGTTGCTCTTAAAGAAACCCAAATTGATTCAGTACGTAAAGAAATAAC

ACAGCTTTATATCTTCTTCTTCCTCTTCCATTCCATTTCCTTGCTTCTTCTCTTCAATGCCTCTTCTAGA

GACCCACCTGGAAGTGGGTCCTACTGTAAGAGATCATGGATCCCATCATTGTGTTCTCTTTTGTGTTCTT

TGGGGATAATCTGGGCAGTTCGATATAAAACAGATGTAGAATGCCATATGGAGAAGTTGTTGGAGAGAGA

GAAAGAAGATGGGAAGTTATTGGCCAAGTGTGTAGAAGAGTTGAAGAAGAAAGGAATAGAATTTGATCTT

TTAAAGGAAGTTGATGCGCTTAGAAGAGCTAAGAGTTTGCGCGTGGAGACGAAGGTGGTAAGGAAATGGT

CTGCTAGGGATTTTGTTACTCTGTTTTTCTTTACCGTGTCATGTTTAGTTCTGGGATTGACCAGGATCAT

TTTGTGCAGTTAGGTTGGGTTATGGGGTTACGGGATTTTGAATATGATGGGCAAAATTCTTTGTTGCTGC

TATTTGTCTT

>FM895171.1 FM895171 Jatropha curcas embryo 71-95 (DAF) Jatropha curcas cDNA clone rjcpga0_001952, mRNA sequence

AAAAACTATCTTAGTATATATAACTTCAAATAACATTATAAGCTGCTTATGAATTCAGAGTTTCTTATTT

ATACACACTACTCGATCCATCAATGTGAAAGCACTCTGTTTAGAAAATAGGACCAGCTGCAAGAATCTCC

TCAGTCAGACTGTTGTCTTTTCCAATCTTATAATTTGCGAACACTTCAAAATTCTTCCTGAACAGACCAC

CCAGCTTCAACAGGGTATCCTGGTAGGCTTTCTTGTCTTTCCAAGTGTTAACAGGATCCAGGATTTCTGA

AGGCACACCCTCGATCTCAGTTGGGATTTCAAGCCCAAACACTTCAGTCTTTTTGTAGTTTGCTTTCAAG

AGTCTCCCAGAATGTATGGCATCGATGATTCTTCTAGTGTATGCTAACTTCATACGGCTTCCTGAGCCAT

AGCTGCCACCTGACCAGCCGGTATTGACAAGCCAACCTGTAGCCCCATGCTTCTGCATTTTTTCAGCTAA

CATGGCTGCATACTTTGTAGGATGCAGCGTTATAAATGCTGCACCAAAGCAAGCTGAAAATGTTGCTTGT

GGCTCCTTAATTCCATCCTCTGTGCCTGCTACCAGAGCAGTATAACCACTAATGAAGTGATACATGGTCT

GTGCCAAGCTCA

>FM895170.1 FM895170 Jatropha curcas embryo 71-95 (DAF) Jatropha curcas cDNA clone rjcpga0_001951, mRNA sequence

AAACAAATTAGACAACAAAAATCATTTATCCAAAATAAAAATAATGTAGTTGTATTCTAAGTTACTTGTT

TGATGTAAATCCTACATGGCAAAAATATTCTACCAAAAGTAATGAAAATGTCGGAAGAAGTATTTTAACC

ATGAGGGGCAAAAATCCCCTATTACATAATCTTCACACTAAGAATGAAAGATTCAATGTGATTAAGCTTT

TCCAAGACAGATTCAGTACACATCATGTATATAGTAACCAACAACAAACAAAAGCATCCCCTCAAATTGT

GAAAGAATCAAGAGAGCACCACCAATCAAAGTGCAGTTTATGTGCTAGAGAAATTTCATTTTTCCCTTCA

CCGAAGACTTTGCCAGCTCTATGCGACACAATGCTTTCTCTTTTCCCATCAATCCAGAACATGATCCAGT

GGCCCCTAAAGTAACAGCAAAATATAGGTATATGGTGCATAGGAAAGCCAAAAGAACCAAAGCTGTTAAG

AGAAAACGGTGCCTTTGAATGAGTGTTGACAAACTACCCAACTCATCCCGTGGGAATTGTTTCCGGAACG

ACGGTGATCTACGATGAGGAGAAGATAAAATGTTGTCGAGTACCAATAGCCTTTAAACTGAAAC

>FM895169.1 FM895169 Jatropha curcas embryo 71-95 (DAF) Jatropha curcas cDNA clone rjcpga0_001950, mRNA sequence

ATTCGTTCTGTTCAAAAACCTCGTCCGAGAACTTGTATAGAGAAGGATTTCCCGCGGCGCAGCAGTTCTT

CCATACCAACTTAGCTGCCCGGCGCTGCTATTAGCATAACAACCGGTACACCATAGGTTGGCCCAACCCA

GTCCTCTCGTACTAGGGTTGGCTCCTCGCAGTTCTCCCTTTAACACCAACGGTAGATAGGAACCGAACTG

TCTCACGACGTTCTAAACCCAACTCACGTACCACTTGAATCGGCGAACAACCGAACCCTTGGGACCTTCT

TCAACCCCAGGATGTGATGAGTCGACATCGAGGTGCCAAACGACTCCGTCGATAAGAGCTCTTGGGAGTC

ATCAGCCTGTTATCCCCGGCGTACCTTTGATCCGTTGAGCGAGAGCCCTTCCACACGGGACTCCCGGATC

ACTATGGCCGACTTTCGTCTCTGTTCGACCAGTCGGTCTCACAGTCAGGCAGGCTTATACCATTACGCTC

ACGAGCAGAATCTTAGCTTGAGCCTACCTTCGCCAAAGGATTCTATCCGTCGCCCGATGGGAATTGTACT

TTAGGGCGGCCCTCGCGGCTCGTCCGCCGCGGGGGCTTGGCCAAC

>FM895168.1 FM895168 Jatropha curcas embryo 71-95 (DAF) Jatropha curcas cDNA clone rjcpga0_001949, mRNA sequence

TCAAAGGATCTATCCGTCGCCCGATGGGAATTGTACTTTAAGCGCGGCCCTCGCGGCTCGTCCGCCGCGG

GGGCTTGGCCAACGGCACGTGCCTCTGGGGGCCGGATGGCCCCTACTGCGGGTCGGCAAACGGGCGACGG

ACACGCGCGTCGCTTCTGGCCCGGATTCTGACTTAGAGGCGTTCAGTCATAATCCAGCGCACGGTAGCTT

CGCGCCACTGGCTTTTCAACCAAGCGCGATGACCAATTGTGCGAATCAACGGTTCCTCTCGTACTAGGTT

GAATTACCATCGCGACACCATCATCAGTAGGGTAAAACTAACCTGTCTCACGACGGTCTAATCCCAGCTC

ACGTTCCCTATTGGTGGGTGAACAATCCAACACTTGGTGAATTCTGCTTCACAATGATAGGAAGAGCCGA

CATCGAAGGATCGAAAAGCAACGTCGCTATGAACGCTTGGCTGCCACAAGCCAGTTATCCCTGTGGTAAC

TTATCTGACACCTCTAGCTTCAAATTCCGAAGGTCTAAAGGATCGATAGGCCACGCTTTCACGGTTCGTA

TTCGTACTGGAAATCAGAATCAAACGAGCTTTTACCCTTTTGTTCCACACGAGATTTCTGTTCTCGTTG

>FM895167.1 FM895167 Jatropha curcas embryo 71-95 (DAF) Jatropha curcas cDNA clone rjcpga0_001948, mRNA sequence

CAAGCAAAATTCAAATCCATCCTTTGTTCATTAAGAAGCACTGGAAAACTGATAAGAAAGGAAGTATGCT

TTCCTCAAATCAGATGCCAACCATCTTTTATGAGCACCTCACAATTCCACTCCCTCTATTTAAATGACAT

TATCAGGTTACTGAAAAACAAGGGAAACCAGCACCTGTAATACTACTGCTAGTTTTTGTATGGTTTTCAA

TATATCAGAAGGAAAAAAAAAAAAAATGTATAATTAACCTGTGTAGACTGTTAAGTTACAAGTGTAAATT

AAAGGGCCTTCAATGGCTTGAAGTTATTCTTGGTGGTCTGTGCCCTTCTTGTCTTCTGAATTTAGTGGGG

AACCCTTCCTTTCCAGTATACGCTTGCATAACAAGTCTAAATCCTCTGTCCTGCACTTTATCTCCTTTGA

CCACAATAACTGGGTTGGCATTCTTTGGTAAGGAATCTTGTAGTCGAGTTCTCTCTCTTAACCACTGTTC

TGCTTTATTGCACTCATT

>FM895166.1 FM895166 Jatropha curcas embryo 71-95 (DAF) Jatropha curcas cDNA clone rjcpga0_001947, mRNA sequence

CTGACATGTGTGCGAGTCAACGGGTGAGTAAACCCGCAAGGCGCAAGGAAGCTGACTGGCGGGATCCCCC

CGAGGGTTGCACCGCCGACCGACCTTGATCTTCTGAGAAGGGTTCGAGTGAGAGCATGCCTGTCGGGACC

CGAAAGATGGTGAACTATGCCTGAGCGGGGCGAAGCCAGAGGAAACTCTGGTGGAGGCCCGCAGCGATAC

TGACGTGCAAATCGTTCGTCTGACTTGGGTATAGGGGCGAAAGACTAATCGAACCGTCTAGTTGCTGGTT

CCCTCCGAAGTTTCCCTCAGGATAGCTGGAGCTCGGGACGAGTTCTATCGGGTAAAGCCAATGATTAGAG

GCATCGGGGGCGCAACGCCCTCGACCTATTCTCAAACTTTAAATAGGTAGGACGGCGCGGCTGCTTCGCT

GAGCCGCGCCACGGAATCGAGAGCTCCAAGTGGGCCATTTTTGGTAAGCAGAACTGGCGATGCGGGATGA

ACCGGAAGCCGGGTTACGGTGCCCAACTGCGCGCTAACCTAGAACCCACAAAGGGTGTTGGTCGATTAAG

ACAGCAGGACGGTGGTCATGGAAGTCGAAATCCGC

>FM895165.1 FM895165 Jatropha curcas embryo 71-95 (DAF) Jatropha curcas cDNA clone rjcpga0_001946, mRNA sequence

TGCTGACATGTGTGCGAGTCAACGGGTGAGTAAACCCGCAAGGCGCAAGGGAAGCTGACTGGCGGGATCC

CCCCGAGGGTTGCACCGCCGACCGACCTTGATCTTCTGAGAAGGGTTCGAGTGAGAGCATGCCTGTCGGG

ACCCGAAAGATGGTGAACTATGCCTGAGCGGGGCGAAGCCAGAGGAAACTCTGGTGGAGGCCCGCAGCGA

TACTGACGTGCAAATCGTTCGTCTGACTTGGGTATAGGGGCGAAAGACTAATCGAACCGTCTAGTAGCTG

GTTCCCTCCGAAGTTTCCCTCAGGATAGCTGGAGCTCGGGACGAGTTCTATCGGGTAAAGCCAATGATTA

GAGGCATCGGGGGCGCAACGCCCTCGACCTATTCTCAAACTTTAAATAGGTAGGACGGCGCGGCTGCTTC

GCTGAGCCGCGCCACGGAATCGAGAGCTCCAAGTGGGCCATTTATGGTAAGCAGAACTGGCGATGCGGGA

TGAACCGGAAGCCGGGTTACGGTGCCCAACTGCGCGCTAACCTAGAACCCACAAAGGGTGTTGGTCGATT

AAGACAGCAGGACGGTGGTCATGGAAGTCGAAATCCGCTAAGGAGTGTGTAACAACTCCACTGCCGAATC

AACTAGCC

>FM895164.1 FM895164 Jatropha curcas embryo 71-95 (DAF) Jatropha curcas cDNA clone rjcpga0_001945, mRNA sequence

ATCCTNTCCTTTCTTTTCTTTTGTTCCTTTCCTTTCTTTCTTTCTTCCTTTCCTCTTCATTGCGCCTGCG

TTTTTGGCGAGACAGCAGCAGATTGCGCGACACCTCTAGTCGATCGGGCCTCCTCGCGCGCCACCTCGCT

GCAGCTTGACGTCGCAGCAGCTCTGCCAGTAGCTCCGCCCTCTGTTACGCCAGTTGCAGCTGCACACAGT

TCCAGCGCAACACCAGACCGCGCCACCGCTTGCTCCACCACCAGATCGCGCCATCGCCTAGCCCTGCCGC

CACTCCAGCGTGCCTCCGACCACCAGGAACGAGCCTGTCCAGCTTCTTCGCGTCATCTCCGCCTGCCTTT

GCCATTGTCAGTCGGACCCGGCCACTTCCGGCGCGCGTAGCTCCGATCCAGCGCGAGCAAACTCCCGATC

TAGCAACAAATTCGAGAAAAGATTATATTTTGGGATCGCCCGGGTCCATCTAGTGCTCCTCCAGGACAGA

ATCAAAGCTAGAACTTCGCTCTCCACCACTGTACTAGGAAGCAGCTTATTCGACTGAAGCTCTGCAATCT

TCTCCGTAGAGTTACAATGTAAACTTTATATTCATGTTATTTAATTTTGGATGTATAAATTATTCTGTTA

TCAG

>FM895163.1 FM895163 Jatropha curcas embryo 71-95 (DAF) Jatropha curcas cDNA clone rjcpga0_001943, mRNA sequence

CGTACTCTTATTTGCTTCAATTTGCACTCTTGCTTTTGCATTCGATAATAGTCCTCTTCAAGATTTTTGT

GTGGCAGATACAACTGGTCAAGTAAGAGTGAATGGCTTTCCATGCAAGGACCCGAAGCAAGTTCAAGCAA

ATGATTTCTTCTTTACTGGGCTTCATTTACCTACCAACACATCGAACCCATCTGGTTTAGGGTCTAACAT

AATCACAGCGACTCGATTACCAGGTCTAAACACTCTTGGGATCTCTTTGGCTCGTGTAGATTTCGCACCA

TCGGGTGTTAGCTCTCCACACATACATCCAAGGGCTACTGAAGTTGTGACCATTTTGGAGGGTACCCTAG

AAATGGGATTTGTGACTTCTCTTCCAGACAACCGTCTGATCTCAAAGGTCCTTAATAAGGGTGATGTGTT

CGTCGTCCCTCCTGGTCTTATCCATTATGCAAGAAACCCTGGAAAGAGTAATACTGCTTGTATTGTTGCT

TTTAGTAGCCAAAGCCCAGGGTTCCTTCTCATCCCCCAGGCAGTGTTTGGGTCAAACCCTAATATTTCTA

GTGATATTCTTGCCAAGTCTTTTCAAGTTGACGAGAACATTATTTCTCAGATTAAAGCCAAGTTTTAGGA

AAATTAGCTAGAGTGGCTATGAATTGTC

>FM895162.1 FM895162 Jatropha curcas embryo 71-95 (DAF) Jatropha curcas cDNA clone rjcpga0_001942, mRNA sequence

ATTCGTTAGAAAGTTTTTTCATTTACGTCCCTATAGTGATAGCCATCATAAGCTGCTTCAGCGACAACCA

CCATGTGTGGCGGTGCTATCATCTCCGACTTTATACCTCCCGCGGTCGCTGGGCGATCTTCTCGGCGGTT

GACAGCTGATTTTCTTTGGCCTGATCTAAAGAAACCCATTGGAAAGCAGTACTCAAAACCTGTTGTTGAT

CTTGACAATGATTTCGAGGCTGATTTTCAGGAGTTCAAAGATGAATCTGATGCCGATGAGGAAGATGATG

TCATGCTTGATGTCAAGCCTTTTGCTTTTTCTGCTACTGCCTCTCCTCCTGCTCGCAATCGCTCTTCCTC

TCGTGGATCTGCAGCTGTCAAATCTGTGGAATTCAATGGGCAGGCAGAAAAATCTGCAAAAAGAAAGAGA

AAGAACCAGTATAGAGGAATCCGGCAGCGCCCATGGGGAAAATGGGCTGCTGAGATTCGTGATCCCCGGA

AAGGGGTGCGTGTCTGGCTAGGAACATTCAATACTGCAGAAGAAGCTGCAAGAGCATATGACGCGGAGGC

ACGTAGAATTCGTGGCAAGAAAGCTAAAGTTAACTTCCCCGAGGAAGCTCCGCATGCTTCACCAAAACGT

CCATC

>FM895161.1 FM895161 Jatropha curcas embryo 71-95 (DAF) Jatropha curcas cDNA clone rjcpga0_001940, mRNA sequence

AGGATTCTATCCGTCGCCCGATGGGAATTGTACTTTAAGGCGGCCCTCGCGGCTCGTCCGCCGCGGGGGC

TTGGCCAACGGCACGTGCCTCTGGGGGCCGGATGGCCCCTACTGCGGGTCGGCAAACGGGCGACGGACAC

GCGCGTCGCTTCTGGCCCGGATTCTGACTTAGAGGCGTTCAGTCATAATCCAGCGCACGGTAGCTTCGCG

CCACTGGCTTTTCAACCAAGCGCGATGACCAATTGTGCGAATCAACGGTTCCTCTCGTACTAGGTTGAAT

TACCATCGCGACACCATCATCAGTAGGGTAAAACTAACCTGTCTCACGACGGTCTAAACCCAGCTCACGT

TCCCTATTGGTGGGTGAACAATCCAACACTTGGTGAATTCTGCTTCACAATGATAGGAAGAGCCGACATC

GAAGGATCAAAAAGCAACGTCGCTATGAACGCTTGGCTGCCACAAGCCAGTTATCCCTGTGGTAACTTTT

CTGACACCTCTAGCTTCAAATTCCGAAGGTCTAAAGGATCGATAGGCCACGCTTTCACGGTTCGTATTCG

>FM895160.1 FM895160 Jatropha curcas embryo 71-95 (DAF) Jatropha curcas cDNA clone rjcpga0_001939, mRNA sequence

GTAATTTCCGTGAGAACTTAAAAAAGAATAAATATCTGTACTTTACCACTGCACTAAGTACAGTTAGCTC

CAAACTAGGAATTAGGAGATTGTACCTCCAAAACACAATATTTTCAACAAATGACTCATACACTCCACAA

GAGAGATGTTACCCTCTTCGCAACACCTCAAGCTTGGAGTTCTGATCCCAGGGTTTCAATCTTCAAAAGT

TGCTCCTCGTGTTGCAAACACATAAAATAAAGCTGTTAGCAATTATAAGCCAGAATCATGATAGCAAACA

AGTCCATTTTCACATGAATTTGGACTTCCTATCTGCAGGCTTAAGTATTTGGAAAGAGCACATTAAGGTC

TCATCAACACTCATCATCGCACCAGCATTATCAAATTCACCGCAATAATTAGGAGCAGAAAATATTGTAA

CAAGCTGCCTGTCTGCAAAGAATTCATACCCATCCTCCACAACCTGATGGGCACGACAAACAATGTCCAT

ATCATTATTTGTAAGGAATTCTGACACCTTATCTGGCCCAAAGGTGTATGAGACCCCTCGGTCATTCATA

CCCCAGCCTTTAGCATCCTACTAGGATCAGACCAAGTAAATCACGAGCAACCCAG

>FM895159.1 FM895159 Jatropha curcas embryo 71-95 (DAF) Jatropha curcas cDNA clone rjcpga0_001938, mRNA sequence

CTTGCACACACACTCACACACCCAAACCTAACAGAGCGCTCTCTGATTCAGGAAAATGGGTCGCGTACGT

ACAAAGACAGTGAAGAAATCCTCTCGCCAAGTGATAGAGCGCTACTACTCGCGCATGACACTTGATTTTC

ATACCAACAAGAAGATTCTTGAAGAGGTGGCTATCATCCCTTCAAAACGTCTCCGAAATAAGATTGCAGG

GTTTTCTACCCATCTAATGAAACGGATCCAAAAGGGTCCGGTTCGCGGGATCTCACTCAAGCTTCAAGAG

GAAGAGCGCGAGCGGCGCATGGACTTTGTACCTGATGTCTCTGCCATCAAGACTGATCAGATTGAGGTCG

ATAAGGAGACTATTGACATGCTTGCTGCTTTGGGTATGACTGATATTCCTGGGATTCTCCAAGTCGAACC

GGCTGCAGCGCCTGCGACCCAGTTCGTGTTTGGGAGAGGTGGTGCTGGTGGCAGACCTGGTGGAAGGTTT

TAAAAACTTATTAGAAAAATTTTTGTTTTTATTTCGTTTTATGTTGTTTTTGTTTGACTGGATTTTTAAT

CATGTTGGAAATATTTTGTTGCTATGCCATCTATAAGTATTGTGATGATTTAGTTAAATGCTT

>FM895158.1 FM895158 Jatropha curcas embryo 71-95 (DAF) Jatropha curcas cDNA clone rjcpga0_001937, mRNA sequence

GATTAATGAAAACATCCTTGGCAAATGCTTTCGCAGTTGTTCGTCTTTCATAAATCCAAGAATTTCACCT

CTGACTATGAAATACGAATGCCCCCGACTGTCCCTGTTAATCATTACTCCGATCCCGAAGGCCAACACAA

TAGGACCGAAATCCTATGATGTTATCCCATGCTAATGTATACAGAGCGTAGGCTTGCTTTGAGCACTCTA

ATTTCTTCAAAGTAACAGCGCCGGAGGCACGACCCGGCCAGTTAAGGCCAGGAGCGCATCGCCGGCAGAA

GGGACGAGCCGACAGGTGCACACCGCGAGGCGGACCGATCGACCCAACCCAAGGTCCAACTACGAGCTTT

TTAACTGCAACAACTTAAATATACGCTATTGGAGCTGGAATTACCCGCGGCTGCTGGCACCAGACTTGCC

CTCCAATGGATCCTCGTTAAGGGATTTAGATTGTACTCATTCCAATTACCAGACTCATTGAGCCCGGTAT

TGTTATTTATTGTCACTACCTCCCCGTGTCAGGATTGGGTAATTTGCGCGCCTGCTGCCTTCCTTGGATG

TGGTAGCCGTTTCTCAGGCTCCCTCTCCGGAATCGAACCCCTATTCTCCGTCACCCGTTCCCACCATGGT

A

>FM895157.1 FM895157 Jatropha curcas embryo 71-95 (DAF) Jatropha curcas cDNA clone rjcpga0_001936, mRNA sequence

ATTCGGGTGGATGCATCTGATTTGGAATCATAGGAGGTAATAGGCCAGTTGTTTATTCATCAAAGAACGG

TCATGCTAGTTTCCCTCGCCCTGGCACTTACCTTCAAGGGTCAACAAAGCTTGGAATTGGAGTGAGGAAT

GATGCTGCTAGAAGCAAATATTATGTGGATTCAAGCCAAATGTATCAGCTTGTTGCTGCAGAGTATCTTG

GCGATGGAGTTGTCACAGAACCATTTTGGTTGCAGTTTATGAGAGAATGGGGTCCAACCATTTTGTATGA

CTCACGATCCGAAGTAGATAAGATTATTAATCTCCTCCCGTTCTTTGTTCGGTTTTCGGTAGAGAACATC

TTTGACCTTTTCCCAACAGAGCTTTATGGTGAGGAGGGGCCAACTGGACCGAAGGAAAAACGAAACTGGT

TGGGAGATGAAATATCTGGTTGAATTTGCTGCATGTGAATTCTCATTGCATATTATTGTCTCACTTTCTT

TACTGCCTGGTTATGTTGATAGAGAGTTCAGCGAAAACACAGGTATCTGCAGTTGAAATGTCAACTGTAG

CATGCTCTTCTTGTTGTACATGCAA

>FM895156.1 FM895156 Jatropha curcas embryo 71-95 (DAF) Jatropha curcas cDNA clone rjcpga0_001935, mRNA sequence

CACTTACCCATCACTATTGCTTCTCTTGCTCTGAAATTCTCTGATTCTAATCCATTAATACTAGTTCTTT

CAAAGATTTCTTCTTTTTTCTCTTTGCTTTTTTAATCAAGTTTTAATCTTCACGAAAATGGTGAATGTTC

TGCACTTCTTGTTTGGGGTTTTTGGAAATGCTACTGCTTTATTCCTCTTTTTGTCTCCAACGATTACTTT

CAAGAGAATAATAAAGAGTAAATCAACAGAGCAATTTTCAGGCGTCCCATATGTGATGACTTTGCTCAAC

TGTCTCCTTTCTGCCTGGTATGGCCTGCCATTTGTCTCCAAGAACAATATTTAGGTGTCAACAATTAACG

GGACAGGATCTGTAATTGAAAGCATATACGTGTTAGTTTTCATTTTATATGCAACAAGGAAGGAGAAGGG

TAAAATCTTAGGCCTCCTTACACTTGTGCTCACCATTTTTGCTACTGTGGCCTTCGTGTCTCTGTTTGCC

CTCCACGGAAGCACCAGAAAGCTCTTCTGTGGCTTGGCTGCTACTGTCTTCTCTATCATCATGTATGCCT

CACCTCTGTCCATCATTAGGTTGGTGATTAAAACT

>FM895155.1 FM895155 Jatropha curcas embryo 71-95 (DAF) Jatropha curcas cDNA clone rjcpga0_001934, mRNA sequence

GAACCGGAAGCCGGGTTACGGTGCCCAACTGCGCGCTAACCTAGAACCCACAAAGGGTGTTGGTCGATTA

AGACAGCAGGACGGTGGTCATGGAAGTCGAAATCCGCTAAGGAGTGTGTAACAACTCACCTGCCGAATCA

ACTAGCCCCGAAAATGGATGGCGCTTAAGCGCGCGACCTATACCCGGCCGTCGGGGCAAGAGCCAGGCCC

CGATGAGTAGGAGGGCGCGGCGGTCGCTGCAAAACCCGGGGCGCGAGCCCGGGCGGAGCGGCCGTCGGTG

CAGATCTTGGTGGTAGTAGCAAATATTCAAATGAGAACTTTGAAGGCCGAAGAGGGGAAAGGTTCCATGT

GAACGGCACTTGCACATGGGTTAGTCGATCCTAAGAGACGGGGGAAGCCCGTCCGACAGCGCGTCCTCGC

GCGAGCTTCGAAAGGGAATCGGGTTAAAATTCCCGAACCGGGACGTGAAGGCTGACGGCGACGTTAGGGA

GTCCGGAGACGTCGGCGGGGGCCTCGGGAAGAGTTATCTTTTCTGTTTAACAGCCCGCCCACCCTGGAAA

CGACTCAGTCGGAGGTAGGGTCCAGCGGCTGGAAGAGCACCGCACGTCGCGCGGTGTCCGATGCGCCCCC

GGCGGCCCATGAAAATCCGGAGGACCGAGTGCCTCCCACGCCCGGTCGTACTCAT

>FM895154.1 FM895154 Jatropha curcas embryo 71-95 (DAF) Jatropha curcas cDNA clone rjcpga0_001933, mRNA sequence

ATTCATATAGTAATATATATTGCAAAAGTTTATGGCGCTAATGACCCAAAACCACCATATAACAATCAGA

AACATAATAACTTTTAAGAACTTTAAGTTACAAAACTGCCAGATAACTGAAACAGAAACCAAAAACAACA

GCAAGACACAGGTTAAAATCAAAATATAACTAAACTAATAACTGTAACTTCTTCATTCACCCGATGCTTT

ACTTTTTCTTTGCTGCGGATTTGGTGACCTTAGCACCACTTGGGTCCTTCTTCTCAACACTCTTGATGAC

ACCAACAGCAACTGTCTGGCGCATGTCCCTCACAGCAAAACGACCCAGAGGAGGGTACTCAGAAAAGGTC

TCCACAACCATGGGCTTGGTGGGAATCATCTTAACAAATCCAGCATCACCATTCTTCAAGAATTTTGGCT

CCTTCTCAAGCTCTTTACCAGATCGCCTATCAATCTTGGTTAGGATCTCAGCAAACTTAACAGCAATGTG

GGAGGTGTGGCAGTCAAGGACTGGAGCATATCCATTTCCAATCTGACCAGGGTGGTTCATGATGATGACC

TGAGAGGTGAAGTTGGCAGCTTCCTTCGCAGGATCATCCTTGGAGTTAGAGGCAACAAAACCA

>FM895153.1 FM895153 Jatropha curcas embryo 71-95 (DAF) Jatropha curcas cDNA clone rjcpga0_001932, mRNA sequence

ATTCTCCTCTTGCCATCACACGCTCAGTCTCTTTCTCTCTGAGACAAAATCTCTTCATCTCTAAACCCCT

ATTTTCCTTATCTGATCAATCGACAATCGGCGAAAATGAACGACGCAGATGTCTCCAAGCAGATCCAGCA

GATGGTCAGATTTATCCGCCAGGAAGCCGAAGAGAAAGCCAATGAGATCTCCGTTTCTGCTGAAGAAGAA

TTCAATATTGAGAAGTTGCAGCTGGTAGAGGCAGAGAAGAAGAAGATCAGGCAGGAGTATGAGCGTAAAG

AGAAGCAAGTTGAAGTTCGAAAGAAGATTGAGTACTCCATGCAGCTTAATGCTTCTCGAATTAAAGTTCT

TCAAGCCCAAGATGATGTGGTTAATGGCATGAAAGAGGCAGCAGCAAAGGATCTTCTGAATGTGAGCCGT

GATCATCATGTGTACAAAAAGCTTCTGAAAGATCTGATAGTTCAGAGTTTGCTTAGATTGAAAGAGCCTT

CTGTCTTGTTGCGTTGCCGGAAGGACGACCTGCTTTTGGTGGAGTCTGTCCTCGATTCAGCCAGGCAGGA

ATATGCAGGAAAAGTAAATGTTCATGCTCCAGAAATCATAGTTGACCATCATGTCTATCTTCCACCTGCT

CCAAGCCATCATAATGCCCATGGTCCTTACTGTTCTGGTGGTGTGGTGCTGGCTTCTCG

>FM895152.1 FM895152 Jatropha curcas embryo 71-95 (DAF) Jatropha curcas cDNA clone rjcpga0_001931, mRNA sequence

ACAAAGGATCTATCCGTCGCCCGATGGGAATTGTACTTTAAGGCGGCCCTCGCGGCTCGTCCGCCGCGGG

GGCTTAGCCAACGGCACGTGCCTCTGGGGGCCGGATGGCCCCTACTGCGGGTCGGCAAACGGGCGACAGA

CACGCGCGTCGCTTCTGGCCCGGATTCTGACTTAAAGAGCGTTCAGTCATAATCCAGCGCACGGTAGCTT

CGCGCCACTGGCTTTTCAACCAAGCGCGATGACCAATTGTGCGAATCAACGGTTCCTCTCGTACTAGGTT

GAATTACCATCGCGACACCATCATCAGTAGGGTAAAACTAACCTGTCTCACGACGGTCTAAACCCANCTC

ACGTTCCCTATTGGTGGGTGAACAATCCAACACTTGGTGAATTCTGCTTCACAATGATAGGAAGAGCCGA

CATCGAAGGATCAAAAAGCAACGTCGCTATGAACGCTTGGCTGCCCCAAGCCAGTTATCCCTGTGGTAAC

TTTTCTGACACCTCTAGCTTCCAATTCCGAAGGTCTAAAGGATCGATAAGCCACGCTTTCACGGTTCGTA

TTCGTACTGGAAATCAGAATCAGACGAGCTTTTAGCCTTTTGTTCCGCACGAGATTTCTGTTCTCGTTGA

GCTCATC

>FM895151.1 FM895151 Jatropha curcas embryo 71-95 (DAF) Jatropha curcas cDNA clone rjcpga0_001930, mRNA sequence

CAACGGAGGGAAAGGCTATAGCTCATACTATTTTACGTTTAAAAGCCATGGGCGCTGTATTTACTATACG

AGTCGGCGTCTGGTTACAGTCTCTTTCTTGCTAACGGACTCGATGAAATTGGACAGAACACAGAAGCTGT

TCGCAACTCCGTCGCAGATCTCAACCGCTTTGGCAAAGTCGTTCAGTTAACTGCTTTTCTTCCTTTCGAG

TCATCTTTAGATGCATTGAATCAGTGTAACTCAGTCTCTGAAGGGCTTATGACTGAGGAATTGAGGAGCT

TTTTAGAACTCAATCTTCCTAAAGTCAAGGAAGGTAAGAAGGCAAAGTTTAGTTTAGGAGTTGCTGAGCC

TAAATTGGGGTCACATATATATGAAGAGACAAAAATACCTTGCCAAAGCAATGAATTTGTTCTTGAGCTT

CTTCGTGGTGTTAGACTACATTTTGAAAGGTTCATCAAGGACCTAAAGCCTGGTGACTTGGAGAAAGCCC

AACTTGGTTTGGGGCACAGTTATAGCAGGGCAAAGGTCAAGTTCAATGTCAACCGTGTTGATAATATGGT

TATTCAAGCAATTTTCCTTCTTGATACTCTTGACAAGGATATTAATTCCTT

>FM895150.1 FM895150 Jatropha curcas embryo 71-95 (DAF) Jatropha curcas cDNA clone rjcpga0_001929, mRNA sequence

CTGCAAAGGATTCTATCCGTCGCCCGATGGGAATTGTACTTTAAGGCGGCCCTCGCGGCTCGTCCGCCGC

GGGGGCTTGGCCAACGGCACGTGCCTCTGGGGGCCGGATGGCCCCTACTGCGGGTCGGCAAACGGGCGAC

GGACACGCGCGTCGCTTCTGGCCCGGATTCTGACTTAGAGGCGTTCAGTCATAATCCAGCGCACGGTAGC

TTCGCGCCACTGGCTTTTCAACCAAGCGCGATGACCAATTGTGCGAATCAACGGTTCCTCTCGTACTAGG

TTGAATTACCATCGCGACACCATCATCAGTAGGGTAAAACTAACCTGTCTCACGACGGTCTAAACCCAGC

TCACGTTCCCTATTGGTGGGTGAACAATCCAACACTTGGTGAATTCTGCTTCACAATGATAGGAAGAGCC

GACATCGAAGGATCAAAAAGCAACGTCGCTATGAACGCTTGGCTGCCACAAGCCAGTTATCCCTGTGGTA

ACTTTTCTGACACCTCTAGCTTCAAATTCCGAAGGTCTAAAGGATCGATAGGCCACGCTTTCACGGTTCG

TATTCGTACTGGAAATCAGAATCAAACGAGCTTTTACCCTTTTGTTCCACACGAGATTTCTGTTCTCGTT

GAGCTCATCTTAGGACACCTGCGTTATCTTTTAACAGATGTGCCGCCCCAGCCAACTCCCCACCTGACAA

TGTCTTCCGCCGGATCGGCCGC

>FM895149.1 FM895149 Jatropha curcas embryo 71-95 (DAF) Jatropha curcas cDNA clone rjcpga0_001928, mRNA sequence

GAATTCCTTACCATGGTTCAACTTTGATATCAGCCAGTCTTTCTGGACTTCCAGCATTGCACCAAAAGTT

TGATCTTCCCGCTCCATTTGTCTTGCACACTGACTGCCCTCATTACTGGCGTTATCATCTTCCAGGTGAG

ACAGAGGAAGAATTCTCAAGCAGATTAGCTAAGAATTTGGAGGATCTCATCCTGAAAGAGGGACCAGAGA

CGATTGCTGCATTTATTGCGGAGCCTGTCATGGGGGCAGGAGGTGTGATACCTCCACCTGCATCTTATTT

CGAAAAGGTGCAAGCTGTTGTAAAAAAATATGACATTCTTTTTATAGCAGATGAGGTAATTTGTGCTTTT

GGAAGGCTTGGAACAATGTTTGGGTGTGACAAATACAACATTAAACCAGACCTTGTATCTGTAGCAAAGG

CGCTTTCTTCTGCATATTTGCCAATTGGAGGTGTTATGGTTAGCCCAGAAGTTTCAGATGTTATACACTC

ACAAAGCAACAAACTCGGCTCTTTTTCTCATGGATTCACTTATTCTGGCCACCCTGTATCATGTGCGGTT

GCCATTGAAGCACTCAAGATATACAAGGAGAGAAACATTCCTGAGCAAGTAAGTAAAATTGCCCCAAAAT

TCCAAGATGGTTTAAAAGCATTCTCCGACAGTCCCATCATAGGAGAGATTCGGGGAACT

>FM895148.1 FM895148 Jatropha curcas embryo 71-95 (DAF) Jatropha curcas cDNA clone rjcpga0_001927, mRNA sequence

CGTTTGAACCCTTACTAGATAACTGAAAAAAGAGCTATAATGTTTTCCATCCAAGGCAGTACCACCAAAA

CTATCCAAAATACCCAACCACTAAAACAGTACCGCAATTATAACATATTATAATAATAAAGTTAAAAAAA

ACAAAAAACAAAAACAAAAAATAAAAGAGGAACCACCGAAGATAATGTTAAAAAGTTCTTATCAGATAAC

TTGCTTAATCAACTTCCTCCATCTTGGATCCTTCAGCATCAACATCAGCATCCTCAAGTGCAGGCATGTC

AGCATCTCCTTCACCAGCATCCTCATCAATGCTCAGTCCAAGTTTCAACATCCTATGGATTCTGTTGCCG

AAAGTGTTGGGATCATCAAGGCTGAACCCTGAGGTAAGAAGAGCAGTCTCAAATAGCAGCAGGACAAGAT

CCTTCACTGACTTATCGTTCTTGTCAGCATCAGCTCTCTTCCTTGACTCCTCCATGATAGGATACTCAGG

GTTGATCTCCATTGTCTTCTTGCTTGACATGTATCCAGCCATGCTATTATCCCTCAAAGCTTGAGCACTC

AATAA

>FM895147.1 FM895147 Jatropha curcas embryo 71-95 (DAF) Jatropha curcas cDNA clone rjcpga0_001926, mRNA sequence

CTGGAAATCAGAATCAAACGAGCTTTTACCCTTTTGTCCACACGAGATTTCTGTTCTCGTTGAGCTCATC

TTAGGACACCTGCGTTATCTTTTAACAGATGTGCCGCCCCAGCCAAACTCCCCACCTGACAATGTCTTCC

GCCCGGCATCGGTCGCCGAAACGACCTTGGGTCCAAAAAGAGGGGCATAGCCCCGCCTCCGATTCACGGA

ATAAGTAAAATAACGTTAAAAGTAGTGGTATTTCACCGTCGCCTTTCGGCTCCCACTTATCCTACACCTC

TCAAGTCATTTCACAAAGTCGGACTAGAGTCAAGCTCAACAGGGTCTTCTTTCCCCGCTGATTCCGCCAA

GCCCGTTCCCTTGGCTGTGGTTTCGCTGGATAGTAGACAGGAACAGTGGGAATCTCGTTAATCCATTCAT

GCGCGTCACTAATTAGATGACGAGGCATTTGGCTACCTTAAGAGAGTCATAGTTACTCCCGCCGTTTACC

CGCGCTTGGTTGAATTTCTTCACTTTGACATTCAGAGCACTGGGCAGAAATCACATTGCGTGAGCATCCT

CAGGGACCATCGCAATGCTTTGTTTTAATTAAACAGTCGGATTCCCCTTGTACGTACCAGTTCTGAGTCG

ACTGTTCGAC

>FM895146.1 FM895146 Jatropha curcas embryo 71-95 (DAF) Jatropha curcas cDNA clone rjcpga0_001925, mRNA sequence

CGAATCAACTAGCCCCGAAAATGGATGGCGCTTAAGCGCGCGACCTATACCCGGCCGTCGGGGCAAGAGC

CAGGCCCCGATGAGTAGGAGGGCGCGGCGGTCGCTGCAAAACCCGGGGCGCGAGCCCGGGCGGAGCGGCC

GTCGGTGCAGATCTTGGTGGTAGTAGCAAATATTCAAATGAGAACTTTGAAGGCCGAAGAGGGGAAAGGT

TCCATGTGAACGGCACTTGCACATGGGTTAGTCGATCCTAAGAGACGGGGGAAGCCCGTCCGACAGCGCG

TCCTCGCGCGAGCTTCGAAAGGGAATCGGGTTAAAATTCCCGAACCGGGACGTGAAGGCTGACGGCGACG

TTAGGGAGTCCGGAGACGTCGGCGGGGGCCTCGGGAAGAGTTATCTTTTCTGTTTAACAGCCCGCCCACC

CTGGAAACGACTCAGTCGGAGGTAGGGTCCAGCGGCTGGAAGAGCACCGCACGTCGCGCGGTGTCCGGTG

CGCCCCCGGCGGCCCATGAAAATCCGGAGGACCGAGTGCCTCCCACGCCCGGTCGTACTCATAACCGCAT

CAGGTCTCCAAAGTGAACAGCCTCTGGTCGATGGAACAATGTAGGCAACGGAAGTCGGCAAAATGGATCC

GTAACCTCGGGAAAAGGATTGGCTCTG

>FM895145.1 FM895145 Jatropha curcas embryo 71-95 (DAF) Jatropha curcas cDNA clone rjcpga0_001924, mRNA sequence

ATTCGGAAAGCTTTTGAAAGAGTCAGATAAAGAAGTTGGGCATAAGAAGACGATAACATCACTTACAAAA

TCTTCTGATGGTTCACACTTTGTTACTGGTTCTCTAGATAAATCTGCAAAGCTATGGGACATCAGAACGT

TGACTCTTATCAAGACCTATGTGACAGAACGTCCAGTCAATGCTGTTACTATGTCTCCACTTCTTGATCA

TGTTGTGCTTGGAGGTGGTCAGGATGCTTCAGCTGTGACTACCACTGATCACCGTGCTGGGAAATTCGAA

GCCAAATTCTATGACAAGATTCTTCAAGAAGAAATTGGAGGCGTGAAAGGTCATTTTGGACCTATAAATG

CTTTGGCTTTCAACCCTGATGGGAAAAGCTTCTCAAGCGGAGGTGAAGATGGGTACGTGAGGTTACATCA

TTTCGATCCAGATTACTTCAACATTAAGATTTAAATTTGGCAGACATGTGTATTAGATAACTATGTATCA

GAAGGAGGAAAAGAATCAACTTTGGAGAGAGATGGAAAGGGATTTTGGTTTTTGTATGTTGAAGGAGCTT

AGTGTGACAAAATACTTTGTAACACCAATTGTTTAGAGAGTCCTTATTTTAGTACAATACTTAGATAATG

AAGCTAATTGTTTTTTTACTTTTTTAGACCGAGTATATTTGAATCTACAACTTTA

>FM895144.1 FM895144 Jatropha curcas embryo 71-95 (DAF) Jatropha curcas cDNA clone rjcpga0_001923, mRNA sequence

GGGCAAAGGATTCTATCCGTCGCCCGATGGGAATTGTACTTTAAGGCGGCCCTCGCGGCTCGTCCGCCGC

GGGGGCTTGGCCAACGGCACGTGCCTCTGGGGGCCGGATGGCCCCTACTGCGGGTCGGCAAACGGGCGAC

GGACACGCGCGTCGCTTCTGGCCCGGATTCTGACTTAGAGGCGTTCAGTCATAATCCAGCGCACGGTAGC

TTCGCGCCACTGGCTTTTCAACCAAGCGCGATGACCAATTGTGCGAATCAACGGTTCCTCTCGTACTAGG

TTGAATTACCATCGCGACACCATCATCAGTAGGGTAAAACTAACCTGTCTCACGACGGTCTAACCCCAGC

TCACGTTCCCTATTGGTGGGTGAACAATCCAACACTTGGTGAATTCTGCTTCACAATGATAGGAAGAGCC

GACATCGAAGGATCAAAAAGCAACGTCGCTATGAACGCTTGGCTGCCACAAGCCAGTTATCCCTGTGGTA

ACTTTTCTGACACCTCTAGCTTCAAATTCCGAAGGTCTAAAGGATCGATAGGCCACGCTTTCACGGTTCG

TATTCGTACTGGAAATCAGAATCAAACGAGCTTTTACCCTTTTGTTCCACACGAGATTTCTGTTCTCGTT

GAGCTCATCTT

>FM895143.1 FM895143 Jatropha curcas embryo 71-95 (DAF) Jatropha curcas cDNA clone rjcpga0_001922, mRNA sequence

AAGGATTCTATCCGTCGCCCGATGGGAATGTACTTTAAGGCGGCCCTCGCGGCTCGTCCGCCGCGGGGGC

TTGGCCAACGGCACGTGCCTCTGGGGGCCGGATGGCCCCTACTGCGGGTCGGCAAACGGGCGACGGACAC

GCGCGTCGCTTCTGGCCCGGATTCTGACTTAGAGGCGTTCAGTCATAATCCAGCGCACGGTAGCTTCGCG

CCACTGGCTTTTCAACCAAGCGCGATGACCAATTGTGCGAATCAACGGTTCCTCTCGTACTAGGTTGAAT

TACCATCGCGACACCATCATCAGTAGGGTAAAACTAACCTGTCTCACGACGGTCTAATCCCAGCTCACGT

TCCCTATTGGTGGGTGAACAATCCAACACTTGGTGAATTCTGCTTCACAATGATAGGAAGAGCCGACATC

GAAGGATCAAAAAGCAACGTCGCTATGAACGCTGTGGCTGCCACAAGCCAGTTATCCCTGTGGTAACTTT

TCTGACACCTCTAGCTTCAAATTCCGAAGGTCTAAAGGATCGATAGGCCACGCTTTCACGGTTCGTATTC

GTACTGGAGATCAGAATCAAACGAGCTTATACCCTTTTGTTCCACACGAGATTTCTGTACTCGTTGAGCT

CATCT

>FM895142.1 FM895142 Jatropha curcas embryo 71-95 (DAF) Jatropha curcas cDNA clone rjcpga0_001921, mRNA sequence

ATTCCGCTCTCTCAATTTCTGCGGGAGAGCACTTCTTCTGCTGGCGAGTCTCCTCCTCTAATAGTCGTTT

TCTCTGTTATTATTTTTAAGCTTCTCTTCCAAATTTTATCATGGGTAAGGAGAAGGTTCACATTAACATT

GTGGTCATTGGCCATGTCGACTCTGGCAAGTCAACAACCACCGGTCATTTAATCTACAAGCTTGGAGGTA

TTGACAAGCGTGTGATTGAGAGATTTGAGAAGGAAGCTGCTGAAATGAACAAAAGGTCATTCAAGTATGC

TTGGGTCTTGGACAAGCTTAAGGCTGAACGTGAGCGTGGTATCACCATTGATATTGCTCTTTGGAAGTTT

GAGACCACTAAATATTACTGCACTGTCATTGATGCTCCTGGACATCGCGACTTCATCAAGAACATGATCA

CTGGTACCTCACAGGCTGACTGTGCTGTGCTGATTATTGACTCTACCACTGGTGGTTTTGAAGCTGGTAT

TTCCAAGGATGGTCAGACCCGTGAACATGCTCTGCTTGCTTTCACTCTTGGTGTGAAGCAGATGATCTGT

TGCTGCAACAAGATGGATGCCACCACCCCCAAGTACTCTAAGGCTAGATACGATGAAATTGTGAAGGAAG

TTTCTTCCTACTT

>FM895141.1 FM895141 Jatropha curcas embryo 71-95 (DAF) Jatropha curcas cDNA clone rjcpga0_001920, mRNA sequence

TTTTTTTTAAAGCAAACAACAAACTTTCCTTGTGTTCATTAAAAAACACACACAAGTTGAATACATAATT

TCTACAGTTTACATGCCACAAAGTTTGAAGTTTTGAACAAAAACAAAACAACCCCTAAACAATGGCTACC

TTGTCATATTCTTCTACTTCCCCACAAGTTTCATCCTCATCTAATTTCCCTTTTTTTTAAAAAAAAAAAA

ACAGGGGGAAAAAAAGACATTTGGAACTTGAAAGCAGAACCTATCCTTCTCAAGTGCCTCCAATCATCAA

AAATAAAAAGAAACAAGAAAAGGAAAGAATTTATAGGAAAATAAATGACAAGAATAAGACAAGTAGGCTA

CTTGAGCCATACATTTAAACATTCTAAATACTATTAGTATCCTTAAAAAAAGAAAAATACAATCATCCAT

ATAAGAATGTATTATGAATGAGCATTATCATCATTTTCGTCATCATCATCAATTCCATCAAAGAATGGAT

CTTTGAGGAGATCTGCAGCAAAAGGTCTTGCTCTAGGCTGAGCAAGGCATCTCTCAATGAATGCCCTAAC

TTCAGGGTCTCTGACCTTGCTTAAGGCCTGAGGCCTAAATCCAGATGAA

>FM895140.1 FM895140 Jatropha curcas embryo 71-95 (DAF) Jatropha curcas cDNA clone rjcpga0_001919, mRNA sequence

ATTCCTTTCATCTACAAAGGTCCTAAAACCCTAGCCCCTCCCCTTAAGCACATTTGCCTCGCAGGAGTCG

CGCGCCGAGCAGCAAAAGGAGGAACGACAATGGGTATCGATTTGAAGGCCGGAGGCAAGAGCAAGAAGAC

TAAGCGCACTGCGCCTAAGTCTGATGATATCTACCTCAAGCTACTTGTCAAGTTGTATCGATTTCTGGTG

AGGAGAACTGGTAGTAAATTCAATGCGGTGATTCTTAAGAGGCTCTTCATGAGCAAGATTAACAAGCCAC

CTCTCTCCCTCTCTCGTTTGATTAGTTTCACAAAGGGCAAAGAGGATAAGATTGCCTGTGGTTGTTGGGA

ACTGTAACTGATGATATTCGTGTGTATGAAGTCCCTGCTTTGAAGGTTACTGCACTGAAGTTCACTGAGA

CAGCAACGGCCACGAATTGAGAAGGCAGGTGGCGAGTGCTTGACCTTTGATCAACTTGCTCTTAGAGCAC

CTCTTGGTCAGAATACGGTTCTCCTAAGAGGTCCTAAGAATGCTCGTGAGGCAGTTAAGCACTTTGGACC

AGCTCCTGGTGTACCACACAGCCATACCAAACCCTATG

>FM895139.1 FM895139 Jatropha curcas embryo 71-95 (DAF) Jatropha curcas cDNA clone rjcpga0_001918, mRNA sequence

CTTTGTCCTTCCTCTTTCCCGCTCTCTCAAGGGTTAGGGTTTCTCTTTTTCCAAAATTTCAATCACAGTA

ACAATGTCGACATCCAAGAAAGTCACTCTCAAATCGTCCGACGGCGAGACTTTTGAGGTGGAGGAAGCGG

TGGCGGTAGAGTCGCAGACTATAAAACACATGATCGAGGATGACTGCGCCGACACTGAGATACCGTTGCC

TAACGTGACCAGCAAGATCTTGGCTAAGGTCATCGAATACTGCAAGAAGCATGTCGAGGCCGCCACTGCT

GATAAGGACAAGGATAAGTCCTCCTCTGATGAGGCTCTCACGACTTGGGACGCCGATTTTGTCAAGGTTG

ACCAGAATACCCTTTTTGATCTCATCCTGGCTGCTAACTATTTGAACATCAAGAGTTTACTAGATTTGAC

TTGCCAGACTGTGGCTGACATGATCAAAGGGAAGACTCCGGAGGAGATTCGCAAGACATTCAACATTAAG

AATGATTTCACCCCAGAGGAAGAAGAGGAGGTTCGAAGGGAGAATCAGTGGGCATTTGAATGATGAATGT

ACAATGTCCTTGCAATGTGCATATGATGGTATGATGATAGGAATGTCGATATACGATAGGATGCTGTAAA

TGTTCTGTGTCTCTTAATGTGGAGGATGAT

>FM895138.1 FM895138 Jatropha curcas embryo 71-95 (DAF) Jatropha curcas cDNA clone rjcpga0_001917, mRNA sequence

GAAGAATCTTAGTGTTTTCTTCTTCGCTCTCCTCCTCCTCGCTTCCCCTCTCCTTCAAGTCGCTAGGTGT

CAGTCAGATTCTGATGTGGAAGTCACAGACGCTGCTGAAGAAGTCAGTGATCTTGGGATTGTTGGTGAGG

ATGCTCAGGATTTTGGTGATGGGACTTTTAGCCCGGCTCCTGGAGTAGAGACTGTTTGTGTTTTCCCCAA

AAACAGTGCCCGTGTGGTGCCAGCTGGGGAAGAGGCTGAACTACTTGTTGGAATTAAGAATGATGGGGAG

TCGAGCATAAATATCATTGCAGTTAAGGCCAGTGTTCACCTTCCTTTTGATCATCGTCTGCTGGTTCAGA

ATCTCACTGCTCAGGCCTTCAACAATGCAACTGTTCCTCCCTCAACACAGGCGACTTTCCCATACGTTTT

TGCTGTCAGCAAGTACTTGCAGCCTGGCAACTTTGATCTTGTAGGATCCATTATTTATGAGATAGACCAG

CAAGGATATCAAAGCACCTTCTACAATGGTACTATTGAAGTTGTTGAGTCTGGTGGCTTCCTCAGCATCG

AGTCAGTTTTTCTGGTTACCCTTGGAATTGCCCTTCTTGTACTCTTTGGTTTGTGGATTCATGGTCAAGT

ACAAAACCTTTCCAAGAAAACAAGAGGGCTCCAAAGTGGAAGTTGAACTGGGACTAGGATG

>FM895137.1 FM895137 Jatropha curcas embryo 71-95 (DAF) Jatropha curcas cDNA clone rjcpga0_001916, mRNA sequence

CTCTCTTATACCTAAATTTAAAGTTAGAGTTTCAGAAATATGGCTACGAAGAACACATCAACAGTTGCTC

TCTTTCTTGTATTTAACCTTTTGTTCTTTACCCTAGCCAACGCATGTGGTGGGGGCTGCCCTACTCCTAG

GCCACCAAGACCAAAGCCAACTCCAAAGCCAACACCAACTCCGTCACCTTCAGGCGGAAAATGTCCTAGA

GATGCACTTAAACTAGGCGTATGTGCTAAGGTGCTCGGTTCATTGCTTAATATCACCATTGGCGATCCTC

CTGTCAAACCTTGCTGCTCTCTTCTAGAGGGTCTTGTTGATCTTGAAGCTGCTGTTTGCCTTTGCACTGC

CATTAAAGCTAATATTTTGGGTATCAACCTCAATGTTCCACTTTCTTTGAGTTTGCTTCTAAATGTTTGT

AGCAAAAAGGTTCCTTCTGATTTCCAATGCGCCTAAACATATATAGACATTTAACTATATATCTATATAT

GGGTTTTGAGTTTTACGTGTTGTTCGATCAAGTTTAGGAGTTCGCTAAACTTATGTTTGTATTTTAAGGT

TGTAATCGTTTGTTTTGTTTAATAGCTTGTTAAAATGTTTGTAATTTCTGTTTATTGGGGGGTTAATCGA

AACAAATAAGTGTTGTAT

>FM895136.1 FM895136 Jatropha curcas embryo 71-95 (DAF) Jatropha curcas cDNA clone rjcpga0_001915, mRNA sequence

GTAACAACTCACCTGCCGAATCAACTAGCCCCGAAAATGGATGGCGCTTAAGCGCGCGACCTATACCCGG

CCGTCGGGGCAAGAGCCAGGCCCCGATGAGTAGGAGGGCGCGGCGGTCGCTGCAAAACCCGGGGCGCGAG

CCCGGGCGGAGCGGCCGTCGGTGCAGATCTTGGTGGTAGTAGCAAATATTCAAATGAGAACTTTGAAGGC

CGAAGAGGGGAAAGGTTCCATGTGAACGGCACTTGCACATGGGTTAGTCGATCCTAAGAGACGGGGGAAG

CCCGTCCGACAGCGCGTCCTCGCGCGAGCTTCGAAAGGGAATCGGGTTAAAATTCCCGAACCGGGACGTG

AAGGCTGACGGCGACGTTAGGGAGTCCGGAGACGTCGGCGGGGGCCTCGGGAAGAGTTATCTTTTCTGTT

TAACAGCCCGCCCACCCTGGAAACGACTCAGTCGGAGGTAGGGTCCAGCGGCTGGAAGAGCACCGCACGT

CGCGCGGTGTCCGGTGCGCCCCCGGCGGCCCATGAAAATCCGGAGGACCGAGTGCCTCCCACGCCCGGTC

GTACTCATAACCGCATCAGGTCTCCAAGGTGAACAGCCTCTGGTCGATGGAACAATGT

>FM895135.1 FM895135 Jatropha curcas embryo 71-95 (DAF) Jatropha curcas cDNA clone rjcpga0_001914, mRNA sequence

ATTCATTAATCCTTTGTTTAACAATTATTGAGGTTTACAAATATACAATTTTTTTTCTAATTAGGAAAGA

GATAAACATACCTTACTAGGATGCCTAGATCCCATCATTGGCTTAAAACCATAACCACGATGGTTTTTCT

TTGAAAGATTGACCAACATTACATTATATATAGAAGACTATTCTGCTGAGCTTCTGTTACAAAGTTTGCT

TCTGCTTGACAAAAATTTGTTGAGCATAATATAAGTAGGTTACCTTTACACCATCAAGTCTATTCATTCC

TAAATGACCTTCCAATGTTGCTTAACTATAATTATGTGATTGGGAATAGTTAAATTGCCTTCTCAAATTT

GGGAGAAGTGGTCTTGATGGGGGCCCAGAGATCTGCACCATTGCTGCGCGCAACTCCGAGGGTGCACGAG

ATTGGTACAAGACATAAACCTCTGCTTCTCAGACTGTATTGCTCCAATTCCTGGCAGCTGAGCTGGAGTA

ACTTGGA

>FM895134.1 FM895134 Jatropha curcas embryo 71-95 (DAF) Jatropha curcas cDNA clone rjcpga0_001913, mRNA sequence

ATTCGCCAATTTTGCCCAGCAACTCTCTCTTAAAGGGTTTGCTGGAGACACAGCCAAGGCTCATGCAAAG

ACTAGCATGATAGCTGGTGAAGGAGTGAGCAATATCCGAACTGTTGCAGCTTTTAATGCCCAAGATAAAA

TCCTCTCTCTATTCTGCCATGAACTCCGTGTCCCACAGCTCCGTAGCCTACGCAAGAGCCAAACCTCTGG

CCTTCTCTTTGGCCTCTCTCAGCTTGCTCTCTATGCCTCTGAGGCTCTCATCCTTTGGTACGGTGCTCAT

CTTGTGAGCAAAGGTGCCTCCACATTTTCAAAGGTTATTAAGGTCTTTGTAGTCCTGGTGATCACAGCTA

ATTCAGTTGCAGAAACTGTTAGTCTTGCTCCAGAGATCATCAGGGGCGGTGAAGCTGTTGGTTCTGTGTT

TTCCATTTTAGACCGTTCTACTCGAATTGACCCGGATGATCCTGAGGCTGAGCCTGTAGAATCAGTACGT

GGAGAGATTGAGCTTAGACATGTTGATTTTGCATACCCTTCAAGACCTGATGTTCCAGTGTTTAAAGACC

TTAACCTTAGAATCCGAGCTGGTCAAAGCCAAGCCCTTGTTGG

>FM895133.1 FM895133 Jatropha curcas embryo 71-95 (DAF) Jatropha curcas cDNA clone rjcpga0_001912, mRNA sequence

GGATCTATCCGTCGCCCGATGGGAATTGTACTTTAAGGCGGCCTCGCGGCTCGTCCGCCGCGGGGGCTTG

GCCAACGGCACGTGCCTCTGGGGGCCGGATGGCCCCTACTGCGGGTCGGCAAACGGGCGACGGACACGCG

CGTCGCTTCTGGCCCGGATTCTGACTTAGAGGCGTTCAGTCATAATCCAGCGCACGGTAGCTTCGCGCCA

CTGGCTTTTCAACCAAGCGCGATGACCAATTGTGCGAATCAACGGTTCCTCTCGTACTAGGTTGAATTAC

CATCGCGACACCATCATCAGTAGGGTAAAACTAACCTGTCTCACGACGGTCTAACCCCAGCTCACGTTCC

CTATTGGTGGGTGAACAATCCAACACTTGGTGAATTCTGCTTCACAATGATAGGAAGAGCCGACATCGAA

GGATCAAAAAGCAACGTCGCTATGAACGCTTGGCTGCCACAAGCCAGTTATCCCTGTGGTAACTTTTCTG

ACACCTCTAGCTTCAAATTCCGAAGGTCTAAAGGATCGATAGGCCACGCTTTCACGGTTCGTATTCGTAC

TGGAAATCAGAATCAAACGAGCTTTTACCCTTTTGTTCCACACGAGATTTCTGTTCT

>FM895132.1 FM895132 Jatropha curcas embryo 71-95 (DAF) Jatropha curcas cDNA clone rjcpga0_001911, mRNA sequence

ATTCCTCCCCGATACAGCTTTTCAAAAAGAAAAATCTCGCAGACTTCATACAACAGAAAGCTCTGAGAAT

TAGATCTCTCTCATCTAAAGCAAAAATCCATTATAATAGAGAATGAGCTATTATAACCAACAACAGCCTC

CTGTTGGTGTTCCTCCTCCTCAAGGTTATCCACCTGAAGGATATCCAAAAGACGCTTATCCACCACCCGG

ATATCCCCCACAAGGATATCCTCAGGGTTATCCTCCTCAGGGATATCCTCCTCAGGGTTATCCTCCTCCT

CCTCCTGCTTATGCCCCTCAGTATGGTCAACCACCTCCTCAACAACAAAGCAGCGGCCCTGGTTGCTTGG

AAGGATGTTTGGCTGCTCTTTGCTGTTGCTGCCTGCTAGATGCTTGCTTTTAATAATGAGGAGAAAGCTT

TAAGAGTGAGATTCAACAGAAAATTGTGATTTGAAATTGAATCT

>FM895131.1 FM895131 Jatropha curcas embryo 71-95 (DAF) Jatropha curcas cDNA clone rjcpga0_001909, mRNA sequence

GGAAGCGCAAGAGGCAGCAACTACCTCAGTCCGCCACCATGGGTCGCATGCACAGTCGAGGTAAGGGTAT

TTCAGCTTCCGCTTTGCCTTATAAGAGGACACCGCCCAGTTGGCTGAAGATATCTGCTCAGGATGTCGAG

GATAACATCTGCAAATTTGCAAAGAAAGGCTTGACTCCATCTCAAATTGGTGTCATCCTTCGTGATTCTC

ATGGTATTGCCCAAGTGAGGAGTGTTACTGGTAACAAGATTTTGCGGATATTGAAGGCACATGGGCTTGC

TCCTGAAATACCTGAAGATTTGTACCACCTCATCAAGAAAGCAGTTGCCATTAGGAAACATTTGGAGAGG

AACAGGAAGGATAAAGATTCCAAATTTAGGTTGATTTTGGTTGAGAGCAGGATCCACCGCCTAGCTCGCT

ATTACAAGAAGACAAAGAAGCTTCCACCAGTATGGAAATATGAATCAACTACTGCCAGCACCCTTGTGGC

ATAGTCAAGTATTTGCTTCTGGTTACTTGCCTGAAGTTGCTCCTGCATAAAGCTGGTCTCGGTTTTCCAA

TGTCAGTTGTGATTTCTTTTTAGTTAAGGACTTGTTCGCAGGTGTTTTAAGCAAAGATTCGAAGAGGTGA

CAAAAGGTCTTCATTTTATGTTTCAGATTGGTCTATCTT

>FM895130.1 FM895130 Jatropha curcas embryo 71-95 (DAF) Jatropha curcas cDNA clone rjcpga0_001907, mRNA sequence

AAAATCAAAACCCCCCTTATAGTATCAGAGTTTATTTCTTTATTGCACTACTGAAAAGATAAATTTTCTC

TCTTGTAATTTTACAATGATTACAGGAAATAAATCAGCCTCATTGGTGATTAAACGAAATACATTATCCT

CCCCCCTATTGCGTCTGACAATAAATATGGGGGAACAGTCTTCTTCCCAAACCCAAAATATGAATACAAG

ACGTATTGGTTCAATGTGTTAGGAAATTAAATTAACTAACTCAGGTACATTCTCTTTTAACTTATAGAGT

TTGTTCTTTCTAAACTAGCTCAGCTGCTCAAGACATTCTCCTTTTCTGTATCTCTCCTACTTCAACAGAT

ATTATATACTTTGGAGGTTAGACTTAGGCCTTACCAGGGTGGGTGGTCAAGCTCTGGCGTCTCTGGTGGC

ATTGGTTTCCAATCGGGTTTCTCATCCCAGTACATGTCATAATATACGTGTCCTGGGGTGTGATTTTCCA

CGCAGCACCATATACCAACATACCGCTTCACACGATTCCTAAATTTTTCTTCTCTTGCCTTGTCGGTGAA

ACCTAGGAATCCATCTTGCATTGAAG

>FM895129.1 FM895129 Jatropha curcas embryo 71-95 (DAF) Jatropha curcas cDNA clone rjcpga0_001906, mRNA sequence

ATTCGAAAGCTGGAAGTTATCGAAATAAACATTGTATAGCTCTTCTAACCACGGTCACCGACCGATCAAG

AAATGGCAGGAGTATCGTTGAAGTGCGGGGACTGTGGTGCTCTCCTGAAGTCCGTTCAGGAGGCTCAAGA

GCACGCCGAGCTGACTTCTCACTCCAATTTCTCCGAGTCGACGGAGGCTGTGCTCAATCTCGTTTGCACT

ATCTGTAACAAACCTTGCCGATCCAAAACAGAAAGTGATCTGCACACAAAGAGAACTGGGCATACAGAGT

TTGTAGATAAGACTTCTGAGGCAGCAAAACCAATCAGTCTAGAGGTTCCTAAGGCACCAATGGATGAGGA

TGAGCCCGTCGACGCCGGCCCTAGTGACCAGTCTGCAGAAATGGTAGCTCCAGAGGTTGACCAAAAGCTC

CTTGAGGAACTAGAAGGAATGGGTTTCCCAAAAGCACGGGCAACCCGGGCACTTCATTACTCTGGGAATG

CCAGCATTGAAGCTGCTGTAAACTGGATAGTTGAGCATGAGAATGACCCAGACATAGATGACATGCCCAT

GGGGCCTGCTAACAAAAACGTTGAGGATCCT

>FM895128.1 FM895128 Jatropha curcas embryo 71-95 (DAF) Jatropha curcas cDNA clone rjcpga0_001905, mRNA sequence

TCGAGTTCTTCCTTATCAAGTTCCTCTGCAGGGCTGGATTGATCATCATCAGCTTCGTGTCCTAACAAAT

TTCCCGTGTCCTCATCCTTCGGCTGGTATTGTTCAGCTGAGACTTCCGCCTGGACTGACACTGAAGGAAT

TCTATGAACAGGAACCTGGAAAGACGCATAGTTTCCAGCAGCCGCAGCTACTTCCACTGGAGCTTTCATC

TCCGGTGTGGTGGTGAAGTAATCAGAAGCCATAATCTTGTTCAGCCTCTCCCTCAGTTCCGCATAAGAAG

CGTTAGCATTGGGCTCAATTGGCTGGTCGGAATTAGCAAGCCACAACTTGGCACGCTCGATGCACCGTTG

CAACGCATTCCTATGAGATAAGTTGGAATCAACAGGGCGCGAAATCAACAACCCGCCAAGCTTCGAAATC

ATGTCCAAATCCCTTTCCCCAAGCAGATCCGTAGCATCATCAGTTACATAATCATAAGTCAAGCAGCAAC

CTCTCTCGTGCGTACGAGTCAACATCGTGGCAGTGAAGTCATTCTGTGACTTCACGTCGAACAACGAACC

GAAATACAATAATTCAACAGATCCTCCACCATAGAACCACCGT

>FM895127.1 FM895127 Jatropha curcas embryo 71-95 (DAF) Jatropha curcas cDNA clone rjcpga0_001904, mRNA sequence

CTGCTTCTACTTCTTCTTCTTCTTCTACTACCTCTAAACTTGGCTTTTTCTTTTTCCAACAAAAACCAGC

ATTAATTTGCTTTTTCCTCGCTCAATATGTCCATTCTTGAGGCAAGATTTCATGATTGAGCACACCCTCT

GCACGTATATTTCTCTTCTGCTGCGCCAAAAGTGCATTCCTTCTTGCCATAAATGCATCATAGTCAGAGG

CCAAAGAACTATTGAAGCAAGGCTGTGTCTGGTTATGAGTGCGGCCCTTAATGTTCACATCTTCAAAGTT

CAAATTTAGAGCTTGAACAACCTTTGCTGGTAGCAGAACTGTAGAGCAAGCAGATTTTTTCTTCGACTCA

GTTGGGTTTCCGTATCTGCGAGGCAAGAAAACACCAGTCCCTGCACATTCTTTTTTAACACCAGATCCAC

CAAGAAAAAACTGCTCTCATGCCGGAATTAGTACCGGATTGTTGCTGTTGTTGTTGATGGTGGTTCTGCT

TTTGAAGAGAAGGCCAAGCAGACTGAGGTACAGCTACTGGATGCGCGCTTCTTCCATGTTCGCCAGCAAC

TCCTCTGTTTCTGTTCTGCATCTGCTGTTGCTGGTGGTGGTGCT

>FM895126.1 FM895126 Jatropha curcas embryo 71-95 (DAF) Jatropha curcas cDNA clone rjcpga0_001903, mRNA sequence

AAATTTTAATATTCTTCAAATAAGCACAAATGCATAAAAGTTCTAATTAAACATCATCCGAATTCTCCCA

AATAAACATAAACATCATTCAAACGCCATCCAATTTCTCCAAATAAACATAAACATCATCTAAATTACAA

TTTAGATGAGGAAGTACAAACAACAACTTCAACATCTTTATACAATTCCTCCGGCTCTAAGTTAAGCTCC

ATAGTTTCTCTTGTAAGTTCTTCCAACTCTGCTTTCTTTGCATCTCCTGGATCAGGGGCTCTGTTGCTAT

TGCCAGATGCATTGGGTGGTAAAGAATTCGGCATAGGATTTTCAGACTTGGACTCCATTGTAAGCATCTT

CAAAGAAATTTTCCGTAGATAATCCGGCTGGCTTGCAGCAGCAGTATAAATCTTTTCCTCAAACCGCACT

GCAATTTTCTTGAGTTCTTCCAATCCCTCTTGACTAGAAAATGGAAGATGTCTCTTTAATGTCTCCGTTA

TCTTGTAGACAATTCGCTGCCGCGAATCAGGCTGCAACTGAGCTCCTCCATCACCAGCATTCCATTGTAG

GTTCTGCACCTTGGC

>FM895125.1 FM895125 Jatropha curcas embryo 71-95 (DAF) Jatropha curcas cDNA clone rjcpga0_001902, mRNA sequence

AGATCATCGCGGAGTACATATGGATCGGTGGATCTGGCATGGACTTGAGGAGCAAAGCAAGGACCCTCTC

TGGACCAGTCAGTGATCCTTCAAAGCTTCCAAAGTGGAACTACGATGGTTCCAGCACAGGCCAGGCTCCT

GGAGAAGATAGTGAAGTGATCCTCTATCCTCAAGCTATTTTTAGGGATCCATTTAGAAGGGGCAACAACA

TCCTTGTCATTTGTGATGCTTACACCCCAGCCGGTGAGCCAATTCCAACAAACAAGAGACATGCCGCCGC

TAAGATTTTCAGCCATCCTGATGTGGTTGCTGAAGTGCCTTGGTATGGCATTGAGCAAGAA

>FM895124.1 FM895124 Jatropha curcas embryo 71-95 (DAF) Jatropha curcas cDNA clone rjcpga0_001696, mRNA sequence

ATTCGCAAAGGATCTATCCGTCGCCCGATGGGAATTGTACTTTAAGGCGGCCCTCGCGGCTCGGTCCGCC

GCGGGGGCTTAGCCAACGGCACGTGCCTCTGGGGGCCGGATGGCCCCTACTGCGGGTCGGCAAACGGGCG

ACGGACACGCGCGTCGCTTCTGGCCCGGATTCTGACTTAGAGGCGTTCAGTCATAATCCAGCGCACGGTA

GCTTCGCGCCACTGGCTTTTCAA

>FM895123.1 FM895123 Jatropha curcas embryo 71-95 (DAF) Jatropha curcas cDNA clone rjcpga0_001695, mRNA sequence

ATTCCTTTGTTTGAGATAGAACCTGATAATCCTGCCAACTATGTCTTATTGGCTAGAATTTATGCCAGCA

TGGGGAGACACGGGGAAGCTGAGAGAATGAGAAGAGAAATGAAAGAGAAGGGAGTGAAGGTAGCTCCTGG

CGGTAGCTGGGTGACATAAAGACTTGCTGATAGACTTATGACATGATTATCTGCCAACTGAAAGTTGCTT

TAGAAGGCTAAACAGGTGTAGCAGATCTCTCTTTAATGCTCGGTCAAAGCTCCATCTTTTATGAGCAAAC

AAGAAGTGGTAAAAGGTAGCCCTTAAAGGATGCCAATTCAAAGATGGAGGCTTGCACTTTTTGTGAGGTT

GAACTGTTGCATTAACAACTTTGAAGTCAAGCCTGCATAATAAAAGAGGAGGTGAAATGATGGAGAAGGT

AAGTTTGGGATTGTCCTTGTTTGAATTTGATGGGTATTACTTGTGCTGGTAAAACCGTTCCAATCAATGT

GCTAACTGGCAGGAAAACTGATGGATATGTTGGGGGTAGCATC

>FM895122.1 FM895122 Jatropha curcas embryo 71-95 (DAF) Jatropha curcas cDNA clone rjcpga0_001694, mRNA sequence

TAGAATGGAATTTAATTCACTGTTTGGCACTGCGAATCAAACCCTAAATGATCAAGTCACAGACACTAAC

ACTAACCAAAAAGAAAGAAACTAACACAAAAACTAAGGACAAATGAATTTACCCCATAAGATTAACCTTA

TTGGTGGAGTAGTACAATGTTTCAAAGAGGAGGACACACAAAACCAGGAGGTGGATTCTTTCCACAAGTT

ATTAAAACTTGAAGAGCAAGAGGAATGAAAATGTTGATATTAAGAAGCTTAAGCCTTATAGTTGTGCATA

GACAAACTG

>FM895121.1 FM895121 Jatropha curcas embryo 71-95 (DAF) Jatropha curcas cDNA clone rjcpga0_001693, mRNA sequence

CCCCTCCAAAATTCGCTCTCATTATTTCTCTCAAAACCTCAAGTAACCATTACTCACCATTTCGGTACCT

CTCGTTAGAAAGTTTTTTCATTTACGTCCCTATAGTGATAGCCATCATAAGCTGCTTCAGCGACAACCAC

CATGTGTGGCGGTGCTATCATCTCCGACTTTATACCTCCCGCGGTCGCTGGGCGATCTTCTCGGCGGTTG

ACAGCTGATTTTCTTTGGCCTGATCTAAAGAAACCCATTGGAAAGCAGTACTCAAAACCTGTTGTTGATC

TTGACAATGATTTCGAGGCTGATTTTCAGGAGTTCAAAGATGAATCTGATGCCGATGAGGAAGATGATGT

CATGCTTGATGTCAAGCCTTTTGCTTTTTCTGCTACTGCCTCTCCTCCTGCTCGCAATCGCTCTTCCTCT

CGTGGATCTGCAGCTGTCAAATCTGTGGAATTCAATGGGCAGGCAGAAAAATCTGCAAAAAGAAA

>FM895120.1 FM895120 Jatropha curcas embryo 71-95 (DAF) Jatropha curcas cDNA clone rjcpga0_001692, mRNA sequence

ATCCACCGATGTGGTGATTACAGGTAATAATGGCACAATCGATGGACAAGGAGCAACTNTGGTGGAAGAA

GTTCCATAAGGGTCAACTGAAGATTACCAGACCATATCTCATTGAGTTTTTATACTCTAATCAGATTCAA

ATATCCCATATCACTTTAATCAACTCTCCTTCTTGGAATGTTCATCCCACATATTCCAGTAATGTGCTGA

TCCAACGGTTGACTATTCTTGCACCAGTTGACT

>FM895119.1 FM895119 Jatropha curcas embryo 71-95 (DAF) Jatropha curcas cDNA clone rjcpga0_001691, mRNA sequence

TGAATTCTAAGGTACNTGGGAATTCATCATTCGAAGGGAAGTAGACTACTCAAGAATTTCACATTTCATT

TAAGTCGTAATTGTGAGAATTGAATAAGTTGTGATAATTAAATAAAGAATTCAGAAATTCTAAAAAAAAA

TGGAAAAGAAGAATGAATCAATGAAAGCTTGCTTGGTATTCATCGGGAACTTGAGTAAGGAGTAGCTCTT

TTGGGGGTTTTTCTCGATTTTGAAAGCAGGAACGAACCCCCTTTTTTTATTTGGTATAACTACTGGAGCC

GGATGAGAGGAAACTTTCACGTCCGATTTTGAAGGGGGGAGACCCTATAGGATCCTATCCCAATTTTTCT

TTTGCTAGGCCCATAGCTAAAAAACCCACTTTTTTACGATTACGAGGTTCATTCGAATATGAAATCCAAT

CTTGGAAATACAGTATTCCACTTTTTTTTACTACCCAAGGCTTCGATACATTTCGAAATCGAGAAATTT

>FM895118.1 FM895118 Jatropha curcas embryo 71-95 (DAF) Jatropha curcas cDNA clone rjcpga0_001690, mRNA sequence

GTGGGCCTGCTGTAAAGGAGTTGATACCCAAGCTTGCTATTGTCAATAAACACCTTTCCTCCAAATTGGG

GGTAGTGATACCTGATATTGATCCTAGGAATCTGGTTGCTGGCATGATTGTCCTTAAGGGTCTCGGAGGC

TTCCTCTTTGTATTTGGCACACCGTTTGGTGCTTATCTACTGCTTCTGCACTTGGCCGTTAGCTCTCCAC

TTCTGTACGACTTCTATAATTATGGTCGGGATCAGTCTGAATACTTCATTCTCTTAAATGAATTCTTGCA

GAGTGTTGCACTTTTTGGGGCATTGCTCTTCTTCATAGGAATGAAAAACTTGATTCCAAAGAGACAAATC

AAGAAGAAAGCCCCAAAGGCAAAAGTGGGCTAATTGTGTCAAAAGATGCCATATAAAAAAATGGATGATT

CTTGCAGCTGCAATTTTACTGGGTGTCAGATTAGCTGCACATATCATTTTAATGGATTCTTTTTGAGCTC

TAA

>FM895117.1 FM895117 Jatropha curcas embryo 71-95 (DAF) Jatropha curcas cDNA clone rjcpga0_001689, mRNA sequence

GAAGAGGTAACAAGTCTACAGCCAAAGATCCAATGGATTTAAAGTGCAGGGGAGAAAATGTAGCTTCAAC

AGAGTTTTACCATAATATGCCAAAAAAACACATTTAACCAAATTCAAGAATCAAAATTTTAAATAGTATC

CTATCTGCTGCTGAAATCTTAGAACTCTAGAAACAGGGACAAAAACTCTCAACAGATGTTCAAGCCTCTG

TTATAGGATACTCAAAAACCACATCTTTCCCAGTAAGCTTCCTGTAAACTCCAGCAAATGTCTCCAACTT

GTACTCAGTGTTGTTGCGCTCTCTGGGATCCAAGAAAATCTTGATAATTTTGGACCCATCGATTCTGTAT

CTGATGCGTTTTCCAACAATCTCAGCGGGATACACTACATCCTCAAGCATGGCATCATGCACAGATGTAA

GGGTTCGGGAACGAGGG

>FM895116.1 FM895116 Jatropha curcas embryo 71-95 (DAF) Jatropha curcas cDNA clone rjcpga0_001688, mRNA sequence

ATAAAATAGTAGAATTCAACTTGATAGCAAGACAAACTTACTAAAACAAAAGTCTGAAATTTAAAAGCAA

CAATGGCCAGNGGAACACATAACAGGCCATTTCAAAAAGAGTTCAACTTATTCAAATCCATTTAACAATG

CCCACAACCATACAACAGTGTGACCCATGAAAAACAGTCTGCAAGACCCATAGTTTAAAAGTGAAACGCG

TTAGCACAACATTAGAAGCCTCCACGGAGACGGAGGACAAGATGCAGAGTGGACTCCTTCTGGATGTTGT

AATCTGCCAAAGTTCGGCCATCTTCCAGCTGCTTCCCTGCAAAGATGAGCCTCTGCTGGTCCGGGGGAAT

TCCCTCCTTGTCCTGGATCTTAGCCTTCACATTGTCAATGGTATCAGAGCTCTCCACCTCCAGGGTGATG

GTCTTCCCTGTCAAAGTCTTCACAAAAATCTGCATACCGCCTCTGAGACGAAGCACCAAGTGGAGAGTGG

ACTCCTTCTGGATGTTGTAGTCGGCA

>FM895115.1 FM895115 Jatropha curcas embryo 71-95 (DAF) Jatropha curcas cDNA clone rjcpga0_001687, mRNA sequence

ACACAAATCACAACCNTTGGATGATGATGATGGAAAAAGATCAACGATCACAAATTAAACAAAAGATAAA

TAAAAATACCCTCAAAAGATAAAATAAATACACCAAAGAAGTTAAAAAAAGCCGAGCCACCCGATCATTC

ATCCCCAACCACAACCGCCGATCGCCGGTTTTTAGTAGTCAGCGGTAGGAAGTTGCTCG

>FM895114.1 FM895114 Jatropha curcas embryo 71-95 (DAF) Jatropha curcas cDNA clone rjcpga0_001686, mRNA sequence

ATTCGATTCTATCCGTCGCCCGATGGGAATTGTACTTTAAGGCGGCCCTCGCGGCTCGTCCGCCGCGGGG

GCTTGGCCAACGGCACGTGCCTCTGGGGGCCGGATGGCCCCTACTGCGGGTCGGCAAACGGGCGACGGAC

ACGCGCGTCGCTTCTGGCCCGGATTCTGACTTAGAGGCGTTCAGTCATAATCCAGCGCACGGTAGCTTCG

CGCCACTGGCTTTTCAACCAAGCGCGATGACCAATTGTGCGAATCAACGGTTCCTCTCGTACTAGGTTGA

ATTACCATCGCGACACCATCATCAGTAGGGTAAAACTAACCTGTCTCACGACGGTCTAATCCCAGCTCAC

GTTCCCTATTGGTGGGTGAACAATCCAACACTTGGTGAATTCTGCTTCACAATGATAGGAAGAGCCGACA

TCGAAGGATCAAAAAGCAACGTCGCTATGAACGCTTGGCTGCCACAAGCCAGTTA

>FM895113.1 FM895113 Jatropha curcas embryo 71-95 (DAF) Jatropha curcas cDNA clone rjcpga0_001685, mRNA sequence

ATGACAGTGCATATGGCTCGATCACTTATTGCTGCTCGACATGCAGGCGAGAGACTCCTTAGGTGTTGGG

GAGGTGGGACTGGTTGGGCTGTGGAGGATGCAAAAGACAAGATATTGAAGTTATTAGAGGAGTATGAAAG

TGGGGGGGTTGTCAGTGAGGCTTGCCAGTGTATCCGTGATCTTGGGATGCCCTTCTTTAATCATGAGGTG

GTGAAGAAGGCATTAGTTATGGCCATGGAGAAGAAGAACGATAGGATACTTGATCTGCTACAGGAGTGTT

TCAATGAAGGTCTGATTACTATCAATCAGATGACCAAAGGCTTTACCCGCATCAAGGATGGGCTTGATGA

TCTCGCTCTTGACATTCCAAATGCAAAGGAGAAATTCAGTTTCTATGTGGACTATGCCCAGAAGAAAGGG

TGGCTCCAAGCATCTTTCGGGTTGTCTGTTGCAGATGCAACTTCAACCTTAGCAAAAGCTACTTGAGATG

AACATATGTCCGTTAGTTTCTCTCGTGTCTG

>FM895112.1 FM895112 Jatropha curcas embryo 71-95 (DAF) Jatropha curcas cDNA clone rjcpga0_001684, mRNA sequence

CTAACTATAATAAGTCTCTTGAGCTGCTCAATGAAGCTATGAATAAGGGGGACTCTGCTTCTGTTGTCAA

GTTGCAGAGCGCCATCAAGTTCAATGGTGGAGGTCATATCAACCACTCCATTTCTGGAAGAATCTCGCCC

CTGTTCGTGAAGGAGGTGGTGAACCTCCGCATGGTTCCCTTGGTTGGGCGATTGATGAAGATTTNGGTTC

TTTGGAAAAGTTGATCCAGAAAATCAGCACAGAAGGTGCTGCTGTCCAAGGATCTGGATGGGTGTGGCTT

GGTTTGGACAAAGAAGCAAAGAAACTTGTAGTTGAGACAACTGCAAACCAGGACCCATTGGTAACTAAAG

GACCAACTTTAGTTCCTTTGCTTGGCATTGATGTTTGGGAGCATGCATACTACTTACAGTACAAGAATGT

CAGACCTGATTATCTAAAGAACATATGGAAGGTAATAAAT

>FM895111.1 FM895111 Jatropha curcas embryo 71-95 (DAF) Jatropha curcas cDNA clone rjcpga0_001683, mRNA sequence

CGAATCAATGGATGGCGCTTAAGCGCGCGACCTATACCCGGCCGTCGGGGCAAGAGCCAGGCCCCGATGA

GTAGGAGGGCGCGGCGGTCGCTGCAAAACCCGGGGCGCGAGCCCGGGCGGAGCGGCCGTCGGTGCAGATC

TTGGTGGTAGTAGCAAATATTCAAATGAGAACTTTGAAGGCCGAAGAGGGGAAAGGTTCCATGTGAACGG

CACTTGCACATGGGTTAGTCGATCCTAAGAGACGGGGGAAGCCCGTCCGACAGCGCGTCCTCGCGCGAGC

TTCGAAAGGGAATCGGGTTAGAATTCCCGAACCGGGACGTGAAGGCTGACGGCG

>FM895110.1 FM895110 Jatropha curcas embryo 71-95 (DAF) Jatropha curcas cDNA clone rjcpga0_001682, mRNA sequence

GGCAGGATGGTAATTTGAGTAAAATTCCGAGTCGACCGTTGAAAAACAACTCAAGAATTCACGCAGCCAC

TGCGCGGATTATTCGCAAATCTGAACATTTAGGCGTTTGAAACAGGTTCATATGAAGAACCACATGTAAA

CAATCCGCCAAAAGAGGAAAAAATAATTTAACGTATGGCATGTCAACTTAAAAAAAAAAATTTCAGGGTG

TCAACCATGCGCATGACAAAAAAGGAAGGGGGAAAGCAAATTTGCACGCGCTGGGCACCAACACCGGCAC

TGGCACCCTCTTCTTCATTTCCTTTCAAGGACGCACTAAAACTTGGTAAATAGGAACAGTGCTTTGCTTT

TGAATCAAAATCCCTACTTAGTGTAATGGTAACCAAACTATACAGTTGTGTTTATATCTGAGGTAATAGG

TCTTTGCTAAAATTGAAAACTTTGCTCGTAGGATACACTCTCCAAGAGACCTAAAATCCAGATAACCCAG

AAAAATGC

>FM895109.1 FM895109 Jatropha curcas embryo 71-95 (DAF) Jatropha curcas cDNA clone rjcpga0_001681, mRNA sequence

AGGATTCTATCCGTCGCCCGATGGGAATTGTACTTTAAGGCGGCCCTCGCGGCTCGTCCGCCGCGGGGGC

TTGGCCAACGGCACGTGCCTCTGGGGGCCGGATGGCCCCTACTGCGGGTCGGCAAACGGGCGACGGACAC

GCGCGTCGCTTCTGGCCCGGATTCTGACTTAGAGGCGTTCAGTCATAATCCAGCGCACGGTAGCTTCGCG

CCACTGGCTTTTCAACCAAGCGCGATGACCAATTGTGCGAATCAACGGTTCCTCTCGTACTAGGTTGAAT

TACCATCGCGACACCATCATCAGTAGGGTAAAACTAACCTGTCTCACGACGGTCTAAACCCAGCTCACGT

TCCCTATTGGTGGGTGAACAATCCAACACTTGGTGAATTCTGCTTCACAATG

>FM895108.1 FM895108 Jatropha curcas embryo 71-95 (DAF) Jatropha curcas cDNA clone rjcpga0_001680, mRNA sequence

ATTCGAGATTTCCACTGTCCCTGTCTACTATCCAGCGAAACCACAGCCAAGGGAACGGGCTTGGCGGAAT

CAGCGGGGAAAGAAGACCCTGTTGAGCTTGACTCTAGTCCGACTTTGTGAAATGACTTGAGAGGTGTAGG

ATAAGTGGGAGCCGAAAGGCGACGGTGAAATACCACTACTTTTAACGTTATTTTACTTATTCCGTGAATC

GGAGGCGGGGCTACGCCCCTCTTTTTGGACCCAAGGTCGTTTCGGCGGCCGATCCGGGCGGAAGACATTG

TCAGGTGGGGAGTTTGGCTGGGGCGGCACATCTGTT

>FM895107.1 FM895107 Jatropha curcas embryo 71-95 (DAF) Jatropha curcas cDNA clone rjcpga0_001677, mRNA sequence

GTGGATCCCCCGGCTGCAGGCAAAGGATTCATCCGTCCCCGATTGGGAATTGTACTTTAAGGCGGCCCTC

GACGGCTCGTCCGCCGCGGGGGCTTGGCCAACGGCACTTCCTCTGGGGGCCTGATGGCCCCTACTGCGGG

TCGTGCAAACGGGCGACGG

>FM895106.1 FM895106 Jatropha curcas embryo 71-95 (DAF) Jatropha curcas cDNA clone rjcpga0_001675, mRNA sequence

GGGCCAAATCGAATTTTAGCAATTATGTAAGTAAAGCAATGATATACTCACATATAAGTAAAGCACGCAC

AATTAAAATAGAATGATGACCAGAAATAGTATTTCAGATTCTAACCAATCCAAAATTATAGTAAACAAGA

TTCTACCAAATCCAAAATTCTCTTCAGAAGTTACATAACTCCCAACAAAATAAAAAGACATTCTGCAAAA

TATTACTTGAAAAACCAACACTTTCTACGATGTAATATTCTGGGCCTTGTTGAGGATGACACCTTCATAT

TTCACCTGAAACCACTTCATTGCATCCTCCTTGGTAACCCGGTGCTGAATACCGACCTTTGACTTGCATC

TTCTGCGACGACCAACGCGATAGCCTGGACGCTCCAAAACAACATAGAAATCCATACCATAAATACCAGT

TGAAGGATCATACTTGATGCCAAGAT

>FM895105.1 FM895105 Jatropha curcas embryo 71-95 (DAF) Jatropha curcas cDNA clone rjcpga0_001674, mRNA sequence

TTTGCTGATTTGCCTGAGATAAAAATGTGCATGCTTGGGAGTCACCAACTACAAAATGCAGCTACTGCAG

CATGTGCAGCACTATGCCTCCGCAATCAAGGCTGGGGCATTTCAGATCGATCTATTAGGGTTGGTATTGA

AAACACATGCCTACCTGGAAGAAGTCAATTTCTTGCATCCAAAGAAGCTGAGGCATTGGGACTACCTGGA

GCCACAGTACTGCTTGATGGAGCTCACACTAGAGAATCTGCGAAAGCTTTGGTGGATACAATACAGATGA

CATTTCCAAAGGCAACATTGGCCCTTGTGGTTGCAATGGCTAGTGACAAAGACCATTTGGCTTTTGCTAG

AGAATTTTTTCTAGGTAAAAATTTAGGGGCTGTCTTCCTAACAGAAGCTGACATTGCTGGAGGCAAATCC

AGAACAACGACAGC

>FM895104.1 FM895104 Jatropha curcas embryo 71-95 (DAF) Jatropha curcas cDNA clone rjcpga0_001673, mRNA sequence

GCAAAGGATTCTATCCGTCGCCCGATGGGAATTGTACTTTAAGGCGGCCCTCGCGGCTCGTCCGCCGCGG

GGGCTTGGCCAACGGCACGTGCCTCTGGGGGCCGGATGGCCCCTACTGCGGGTCGGCAAACGGGCGACGG

ACACGCGCGTCGCTTCTGGCCCGGATTCTGACTTAGAGGCGTTCAGTCATAATCCAGCGCACGGTAGCTT

CGCGCCACTGGCTTTTCAACCAAGGCGCGATGACCAATTGTGCGAATCAACGGTTCCTCTCGTACTAGGT

TGAATTACCATCGCGACACCATCATCAGTAGGGTAAAACTAACCTGTCTCACGACGGTCTAAACCCAGCT

CACGTTCCCTATTGGTGGGTGAACAATCCAACACTTGGTGAATTCTGCTTCACAATGATAGGAAGAGCCG

ACATCGAAG

>FM895103.1 FM895103 Jatropha curcas embryo 71-95 (DAF) Jatropha curcas cDNA clone rjcpga0_001672, mRNA sequence

ATTCCCTAATTAAGCTTTGCTTTTCCGATTCCTTCTGTTAGCGTCAAAATGGCGGATTCTCCTGGCAAAA

GGAATTCACGATCTCTTTCCCCTTGGAGAGAACAATCAAGGTCTAGGTCAAGATCCAGGTCAAGGGCAAG

GTCCAGGTCCAGATCTAGATCTTTATCCCGGCCAAGGCATAGATCACGGTCCCGAAGTCGTGGAAGATCA

AGATCCAGAAGTCGTGGCAGACCTGATGCCATAAATCCTGGAAACACACTGTATGTCACTGGTCTATCTA

CAAGGGGTCACAGAAAGGGACTTAGAAGAACATTTCTCTAAGGAGGGAAAGGTTGCTTCATGCCTTTCTT

GTTGTGGAGCC

>FM895102.1 FM895102 Jatropha curcas embryo 71-95 (DAF) Jatropha curcas cDNA clone rjcpga0_001671, mRNA sequence

TTTTTTGTTTGTTTCGGAGCCGTTTTGGCGGGGCGTACTAAACCCACTACGTACCACACCACCGGGCGGC

TCGCCTGAATGCCGAGTCTTTCTCCGCCGCCAACTCGACGTCGTCGTCACCTGCAAGATAAGGCCAAAAA

CTGGACTTTACTAAACAAGCGAGAAAAGCCCCTTTCTCTGTTATTAGTAAAGTAAAGCGCGCTAGCTGCA

ATCTTTCTAAAGCGCACACTAGAAAGTGCTTCGAAAGGCGCCGGCTCCCCTCTTACTGACAGCACAGCTA

CGTGCTGGCACTCAATTAGTAGCGCTGGCACGTCACTCGGCTCCTCGGCTCACTTCGGTTGCAAAGACTT

TCTCCTTAGGCGCATGTCTCAGCAACACAAAACGAGGGTTTCGCTCGTTATAGGACTTGACCAAACATCT

CACGACAC

>FM895101.1 FM895101 Jatropha curcas embryo 71-95 (DAF) Jatropha curcas cDNA clone rjcpga0_001670, mRNA sequence

ATTCGCAAAGGATTCTATCCGTCGCCCGATGGGAATTGTACTTTAAGGCGGCCCTCGCGGCTCGTCCGCC

GCGGGGGCTTGGCCAACGGCACGTGCCTCTGGGGGCCGGATGGCCCCTACTGCGGGTCGGCAAACGGGCG

ACGGACACGCGCGTCGCTTCTGGCCCGGATTCTGACTTAGAGGCGTTCAGTCATAATCCAGCGCACGGTA

GCTTCGCGCCACTGGCTTTTCAACCAAGCGCGATGACCAATTGTGCGAATCAACGGTTCCTCTCGTACTA

GGTTGAATTACCATCGCGACACCATCATCAGTAGGGTAAAACTAACCTGTCTCACGACGGTCTAAACCCA

GCTCACGTTCCCTATTGGTGGGTGAACAATCCAACACTTGGTGAATTCTGCTTCACAATGATAGGAAGAG

CCGACATCGAAGGATCAAAAAGCAACGTCGCTATGAACGCTTGGCTGCCACAAGCCAGTTATCCCTGTGG

TAACTTTTCTGACACCTCTAGCTTCAAATTCCGAA

>FM895100.1 FM895100 Jatropha curcas embryo 71-95 (DAF) Jatropha curcas cDNA clone rjcpga0_001669, mRNA sequence

AGGATCTATCCGTCGCCCGATGGGAATTGTACTTTAAGGCGGCCCTCGCGGCTCGTCCGCCGCGGGGGCT

TGGCCAACGGCACGTGCCTCTGGGGGCCGGATGGCCCCTACTGCGGGTCGGCAAACGGGCGACGGACACG

CGCGTCGCTTCTGGCCCGGATTCTGACTTAGAGGCGTTCAGTCATAATCCAGCGCACGGTAGCTTCGCGC

CACTGGCTTTTCAACCAAGCGCGATGACCAATTGTGCGAATCA

>FM895099.1 FM895099 Jatropha curcas embryo 71-95 (DAF) Jatropha curcas cDNA clone rjcpga0_001668, mRNA sequence

AATAAGAAGCTTTCACAAGCAAAAATAATACAAAACTTCGTTCTCATTACAACACAATCGCATTTGGAAA

CCCTCACAACAATACAGGCCATCTAGGAACAAACACCATGAAAAAACAATCCAGAAAGTTTCCAACAGTT

GGTTATACACAAGGAATAACGTGAGCATAACCGACAGCATGAATATCATGATCAGATATTGACAGAAAAT

TGAATCAAGGGAAAAAGAAATGAAGTCACCCTCAGCCATCCTAGGAAAGTAAAATGGAAATCGAAACCTG

ATTTGGCAATAAGATTATTAACCGCCAGAGTGAGAGACTGGAGTAGTTTCAAAGTGGCCATCCTTGAATA

AATGGATATACTGTTGTGGCAATGCATGTTCCACCCAATCACCATCAGGCTGTGCACCTGGTACCAGAAC

GTTGACCTCACTT

>FM895098.1 FM895098 Jatropha curcas embryo 71-95 (DAF) Jatropha curcas cDNA clone rjcpga0_001667, mRNA sequence

TTGATGTTGCAGAGACGCAAACATCTGATGTGCAAAAAACTCCCAGTCTTGAAAAATCCTTCAGAATTTG

ATGTTTCCAACTCTGACATGGAAATTTCATCACAACTGGATAGGCAGATGCCATGACATTTCTTTCTAAG

ATTGGGATTCAAGCAGTCTCATCAGGATACTGTCAGACCAGGTTTTGGTTCCTCATCCAATCTGGTTGAT

TATGGCTAGGAAGTTGATCAGATCGTGCAATGTCTCCATGGTTATAATTTGCCATAATTGTGGTAGAAAC

CTGTTGTGTTGCAGGTCATTTGCTTAAGGTATCTCCAGTTCTCAATGGCAACTTGCAGTAAAAAAAATTC

ATGGAATATATAATATATATATTTGAGCATGAGCTTCGAAGAACATGGAAACAAG

>FM895097.1 FM895097 Jatropha curcas embryo 71-95 (DAF) Jatropha curcas cDNA clone rjcpga0_001665, mRNA sequence

GGCTCACATATAAGTAAAGCACGCACAATTAAAATAGAATGATGACCAGAAATAGTATTTCAGATTCTAA

CCAAGTCCAAAATTATAGTAAACAAGATTCTACCAAATCCAAAATTCTCTTCAGAAGTTACATAACTCCC

AACAAAATAAAAAGACATTCTGCAAAATATTACTTGAAAAACCAACACTTTCTACGATGTAATATTCTGG

GCCTTGTTGAGGATGACACCTTCATATTTCACCTGAAACCACTTCATTGCATCCTCCTTGGTAACCCGGT

GCTGAATACCGACCTTTGACTTGCATCTTCTGCGACGACCAACGCGATAGCCTGGACGCTCCAAAACAAC

ATAGAAATCCATACCATAAATACCAGTTGAAGGATCATACTTGATGCCAAGATCAATGTGCTCCTGGATT

CCAAATCCAAAGCAACCAGTGTCACTGAAGTTTCTTCTCAACAGCTCGTATTCCTTCACTTTCAAACCAC

TTTC

>FM895096.1 FM895096 Jatropha curcas embryo 71-95 (DAF) Jatropha curcas cDNA clone rjcpga0_001664, mRNA sequence

CTGGACTCGGTGCTGCACTGCCTCCCAACACAGATGCTCTTGGGCAACTTCTGTCTGAGTATGCTAAGCG

GGTCTACACTTCCCAATTGCAACATTTAAAGGATATTGCTGGCACCTTAGCTACTGAGGAGGCAGAAGAT

GCTACACAAGTAGCAAAACTGCGTTCAGCTCTTGAGTCTGTTGATCATAAAAGAAGAAAGATTTTGCAAC

AATTGAGGGGCGATGTAGCCATGTTAACGTTGGAGGATGGAGGTTCACCTATTCATAATCCTTCTACTGC

TGCTGAGGATGCACGATTAGCATCTTTAATTTCTCTGGATGGCATACTGAAGCAAGTGAAGGATATATTG

AGACAATCCTCAGTAGATGTCTTGAGTAAAAGTAAGAAGAAATCAATGC

>FM895095.1 FM895095 Jatropha curcas embryo 71-95 (DAF) Jatropha curcas cDNA clone rjcpga0_001663, mRNA sequence

CAACTTCACTGCTACTTCAACAGCCGACTCGGACTGCTTCCTTGCATAATCTCTCAGTCAGATCTCTCAT

CTCTCTCAATCTCCACTTCCCAATTACTCTTCCAACAGGGTTCCTGAAAAAATGGCCGATTCAAAACCAG

GATTGAGGAAGCCTGTATTCACCAAGGTTGACCAGCTACGCCCAGGCACCAGTGGGCATACTCTCACTGT

GAAAGTTGTAAGTACAAAGATGGTCTTGCAGAAAGGTCGTGCTGATGGTCCTCAAATACGCCAAATGCGA

ATTGCTGAATGTTTGGTAGGAGATGAGACAGGAATGATTATCTTCACAGCTAGAAATGATCAAGTGGACT

TGGTGAAAGAAGGAACTACTATAATCCTTCGCAATGCAAAAATCGACATGTTTCAAGGATCAATGAGGCT

TGCTGTTGACAAGTGGGGACGTGTTGAAGTAGCTGAACCTGCCAGTTTCACTGTGAAGGAAGACAACAAC

CTATCCCTGATTGAGTATGAACTCGTGAATGTAGTTGAAGAGTGACTTGTCTGTGGTGCG

>FM895094.1 FM895094 Jatropha curcas embryo 71-95 (DAF) Jatropha curcas cDNA clone rjcpga0_001662, mRNA sequence

ATTCAAAGAATCAAATACAGATATAGGAGCCATGGATAGAGGTTTTAAGGAAGTTGTGGTATTGATGATA

TTGATAGCAGCGAGTCTTATGGTTATGGTGGCTGAATCAAATGAAGCCTCTCCACCCCCAGATTCCGACT

ACGATGATCCACCTGCACCAAGCCATGATTGGAAACACTCTCTTTGTTCTTGTAGCGTAGAATGTGCAGA

GGACTGTCATCCAACTGCGCATCACTTCCCTGTCCTTTGTATGAGTAAATGTGCGCTAAAATGCCTGAAA

AAGTCTCTACCTCCCTATTATTTCGTTTCCAACTGTGCAAATTCCACCACCTCTAATGATAAAAAACAAT

TGGAAAGCTACAGGGCCTTGGAACAAGTTGGGAGATTTGTAGATTCCTGTTACAAGAACTGCAAGGAGCT

GATAAATTAGCAGTTGATCCTGATGATAAAATGTGTAATAATGGTTCAACATCTTTTGTTTGATACTT

>FM895093.1 FM895093 Jatropha curcas embryo 71-95 (DAF) Jatropha curcas cDNA clone rjcpga0_001659, mRNA sequence

ATTCCTCTTTCTCTCTCTGTCAAATCTATTTTCATTTCACCAGCACCACCACCTCTTCCTCCGCCTCCGC

CATAATCAAAAGCTAAGGAGGCGATTCCATATTTTCTCTCTTCTTATCATGGCGGAAAAGGGCCAACCAT

TACCAAAGTTTGGTGAATGGGATGTCAATGACCCAGCATCAGCTGAGGGATTCACAGTCATCTTCAACAA

GGCTCGCAATGAGAAAAAGACAGGTGGAAAGCCTGACTCGCCAGCAAGAGATAACTCCTCATTCAAGCCT

GGCAGTAACACTCTTGGAAAACCTCAATCTAAAAAATGGTTTTGCTGCATACAAGCTGCTCCTGCAGAAT

AAAAAGGGTGGAAGAACCTTGTAAGAGCCACAATGGGAGAGTATATCTATCTACGCCAGGCCCTAGCATT

ATATAATCCAGGAGACTGGAGCTGTATTCAGAAGCTATGATGGGTCCTACTGGAATCTCTTTATTGTGGC

TTAAAACTTTGTGAAAAAGTGGGCTTGGGGAAGAAATGTGATGAAAGAACAATGTGGTT

>FM895092.1 FM895092 Jatropha curcas embryo 71-95 (DAF) Jatropha curcas cDNA clone rjcpga0_001658, mRNA sequence

TTTTTTTTTTGAACAAAAACCAAATGTTTCTTCTGACTTGTGTTCTTTAATCACTCGTTCAAAAATACAT

ATATCCATGTACAACTCATAAAGTCATAACCTTAAAACCCCCTACCTTGATTACAATCAATTAATAACTC

AAAAAGAAAAAATTTAAATTAAAAACACATCCATTTCCTATCTTAAATAATAAAAAAATAATTACTAACT

TTACAACGATCAGAAATAGGACGAGTTTGGGGAATGAAGATCTCTACCCGCCGCAACAACTCGGTACCGT

GACCGGCTTAGTCCACCAGATTCCAATGCCCCTTCCCAATCAGTCTCTTCATCTTCCTCACCTCTTCCTC

TCATCTTCTGTAAAATCTTCTTTGCTTTCTCCTTCGCTCTCTGGCTCCCTCTCTCTTCTATTTCCCGCAA

CGCCTCTACTGCTCTTGCCTCCTTAGCCAGCCCTTTAAATCTTAAGCTCCCATGACTCAATGCAAATAAT

GCCGCTACACAATTCTCCCGGATCGCTTCTGAGTCAGCCTGCTCATTTTCCTCCTTCAGCATTCCCA

>FM895091.1 FM895091 Jatropha curcas embryo 71-95 (DAF) Jatropha curcas cDNA clone rjcpga0_001657, mRNA sequence

ATTTGGAATGATATTTTCATATTATTGATATCCAACATTCTAATCAAATTAGTAAGTTAAACATCGTAAA

CCTAGTTGAAGAGGAAGCACCTCCCAATCTTTGTTTTTACCCACCAAAAATCCCTAAAGTGTTCAAGTGT

AGTCAAGTACAAAGAACATTTTCATGTGTCCATTTTCAGTTGTAACACTATAAGAATGGTGTATAAACCT

GTCCGCGATCACCAGTATATACTATGTTGCAAGAATGACCTTGTTGAATTGGCTGTCCTCCCTGAGATTT

TTCTCCCTGAAATCAGGAACTTCTTCAAACAGTAAAACAATTCTCACTTGGTTGCAAATGTTTAAGATTT

CCACTTTCC

>FM895090.1 FM895090 Jatropha curcas embryo 71-95 (DAF) Jatropha curcas cDNA clone rjcpga0_001656, mRNA sequence

AGGATTCTATCCGTCGCCCGATGGGAATTGTACTTTAAGGCGGCCCTCGCGGCTCGTCCGCCGCGGGGGC

TTGGCCAACGGCACGTGCCTCTGGGGGCCGGATGGCCCCTACTGCGGGTCGGCAAACGGGCGACGGACAC

GCGCGTCGCTTCTGGCCCGGATTCTGACTTAGAGGCGTTCAGTCATAATCCAGCGCACGGTAGCTTCGCG

CCACTGGCTTTTCAACCAAGCGCGATGACCAATTGTGCGAATCAACGGTTCCTCTCGTACTAGGTTGAAT

TACCATCGCGACACCATCATCAGTAGGGTAAAACTAACCTGTCTCACGACGGTCTAATCCCAGCTCACG

>FM895089.1 FM895089 Jatropha curcas embryo 71-95 (DAF) Jatropha curcas cDNA clone rjcpga0_001655, mRNA sequence

CTGGCAAGTTGGAAGTTCAATATTTCCACCACCATGCAACCTTCGCCACGGCTGTTGCATTGAGCAAATC

TCCTGCTGTTGATTTTTCAGCCACCATTGGTAC

>FM895088.1 FM895088 Jatropha curcas embryo 71-95 (DAF) Jatropha curcas cDNA clone rjcpga0_001654, mRNA sequence

GTAGAAACTATCCCCTTTCTATTAGGTAAATCTCCATACTGATGATTCGTAACACCAGAAGCATAGTAAA

ACAGTATGCAGTGTTCTGGATTGATTTTATGTCTTTCTCCTACTCATATAACAATAATGAAAAGAAAAAG

AAATTAAAAGAAGCAAAGATTAAAATTAGTTCTTAAGATTCCCATGCCTATAAAGATTTAAGCAGTATGC

CTATAAGATCCATTTTTTACCTATTTGAACAACACAACTTCATAGGCCTACAGATAAATGGTGAGAGAGT

AATCCCCAGCAAAATCCTCAACTGAAACTTGGTAAATACTGCAGAAGTTGAAGTGCCTAATGGAAAAAAA

AAAATATGACAAGATTTAAGAACATTCCTGGAAGTTACCAGATATACTTCCGAATGGCTTTGACTTATTC

CATTACAACTTTGGCCCCGACTGCTTTCATCTTCTCAATTATTTTCTCAGCTTCTTCCTTTGACACTCCT

TGCTTTCAG

>FM895087.1 FM895087 Jatropha curcas embryo 71-95 (DAF) Jatropha curcas cDNA clone rjcpga0_001653, mRNA sequence

CTGCAAAGGATTCTATCCGTCGCCCGATGGGAATTGTACTTTAAGGCGGCCCTCGCGGCTCGTCCGCCGC

GGGGGCTTAGCCAACGGCACGTGCCTCTGGGGGCCGGATGGCCCCTACTGCGGGTCGGCAAACGGGCGAC

GGACACGCGCGTCGCTTCTGGCCCGGATTCTGACTTAGAGGCGTTCAGTCATAATCCAGCGCACGGTAGC

TTCGCGCCACTGGCTTTTCAACCAAGCGCGATGACCAATTGTGCGAATCAACGGTTCCTCTCGTACTAGG

TTGAATTACCATCGCGACACCATCATCAGTAGGGTAAAACTAACCTGTCTCACGACGGTCTAAACCCAGC

TCACGTTCCCTATTGGTGGGTGAACAATCCAACACTTGGTGAATTCTGCTTCACAATGATAGGAAGAGCC

GA

>FM895086.1 FM895086 Jatropha curcas embryo 71-95 (DAF) Jatropha curcas cDNA clone rjcpga0_001652, mRNA sequence

TTCTCAATCCCAGTCGCTTAGGGTTCTTAGTCTCGTCGCTGATTTGGCTTACGGTTTTGATGATGGACTT

GGGCTTCGTTTGAATTCCTTCGAGCTTCCTGCACCTTGGTTCATCTACTCTTTTATGGGCGTGGGCATTA

TATTGTGTTGCATTACTCTCATTGGTTGCATTGCAGCTGAGGCTATTAATGGCTGTTGCCTGTGCTTTTA

CACCATACTCAAAATCTTATTCATTCTGATAGAAGCAGCTTTAGTGGCCTTCATAGCAATTGATCATCGT

TGGCAAAAGGATATTCCTTTTGATCCAACTGGAGAACTTCAAACCCTTAAGTCTTTTATTGAAGACAATG

TTGATATATGCAAATGGGTTGGCATTA

>FM895085.1 FM895085 Jatropha curcas embryo 71-95 (DAF) Jatropha curcas cDNA clone rjcpga0_001651, mRNA sequence

ATTCCCCAAGGTCGTTTCGGCGGCCGATCCGGGCGGAAGACATTGTCAGGTGGGGAGTTTGGCTGGGGCG

GCACATCTGTTAAAAGATAACGCAGGTGTCCTAAGATGAGCTCAACGAGAACAGAAATCTCGTGTGGAAC

AAAAGGGTAAAAGCTCGTTTGATTCTGATTTCCAGTACGAATACGAACCGTGAAAGCGTGGCCTATCGAT

CGCTTTAGACCTTCGGAATTTGAAGCTAGAGGTGTCAGAAAAGTTACCACAGGGATAACTGGCTTGTGGC

AGCCAAGCGTTCATAGCGACGTTGCTTTTTGATCCTTCGATGTCGGCTCTTCCTATCATTGTGAAGCAGA

ATTCACCAAGTGTTGGATTGTTCACCCACCAATAGGGAACGTGAGCTGGGTTTAGACCGTCGTGAGACAG

GTTAGTTTTACCCTACTGATGATGGTGTCGCGATGGTAATTCAACCTAGTACGAGAGGAACCGTTGATTC

CCAC

>FM895084.1 FM895084 Jatropha curcas embryo 71-95 (DAF) Jatropha curcas cDNA clone rjcpga0_001650, mRNA sequence

TCAAAGGATTCTATCCGTCGCCCGATGGGAATTGTACTTTAAGGCGGCCCTCGCGGCTCGTCCGCCGCGG

GGGCTTGGCCAACGGCACGTGCCTCTGGGGGCCGGATGGCCCCTACTGCGGGTCGGCAAACGGGCGACGG

ACACGCGCGTCGCTTCTGGCCCGGATTCTGACTTAGAGGCGTTCAGTCATAATCCAGCGCACGGTAGCTT

CGCGCCACTGGCTTTTCAACCAAGCGCGATGACCAATTGTGCGAATCAACGGTTCCTCTCGTACTAGGTT

GAATTACCATCGCGACACCATCATCAGTAGGGTAAAACTAACCTGTCTCACGACGGTCTAAACCCAGCTC

ACGTTCCCTATTGGTGGGTGAACAATCCAACACTTGGTGAATTCTGCTTCACAATGATAGGAAGAGCCGA

CATCGAAGGATCAAAAAGCAACGTCGCTATGAACGCTTGGCTGCCACAAGCCANTTATCCCTGTGGTAAC

TTTTCTGACACCTCTAGCTTCAAATTCCGAAGGTCTAAAGGATCGATAGGCCACGCTTTCACGGTTCGTA

TTCG

>FM895083.1 FM895083 Jatropha curcas embryo 71-95 (DAF) Jatropha curcas cDNA clone rjcpga0_001649, mRNA sequence

ATTGCAAAGGATTCTATCCGTCGCCCGATGGGAATTGTACTTTAAGGCGGCCCTCGCGGCTCGTCCGCCG

CGGGGGCTTGGCCAACGGCACGTGCCTCTGGGGGCCGGATGGCCCCTACTGCGGGTCGGCAAACGGGCGA

CGGACACGCGCGTCGCTTCTGGCCCGGATTCTGACTTAGAGGCGTTCAGTCATAATCCAGCGCACGGTAG

CTTCGCGCCACTGGCTTTTCAACCAAGCGCGATGACCAATTGTGCGAATCAACGGTTCCTCTCGTACTAG

GTTGAATTACCATCGCGACACCATCATCAGTAGGGTAAAACTAACCTGTCTCACGACGGTCTAATCCCAG

CTCACGTTCCCTATTGGTGGGTGAACAATCCAACACTTGGTGAATTCTGCTTCACAATGATAGGAAGAGC

CGACATCGAAGGATCAAAAAGCAACGTCGCTATGAACGCTTGGCTGCCACAAGCCAGTTATCCCTGTGGT

AACTTTTCTGACACCTCTAGCTTCAAATTCCGA

>FM895082.1 FM895082 Jatropha curcas embryo 71-95 (DAF) Jatropha curcas cDNA clone rjcpga0_001648, mRNA sequence

GCAAAGGATTCTAATCCGTCGCCGATGGGAATTGTACTTTAAGGCGGCCCTCGCGGCTCGTCCGCCGCGG

GGGCTTGGCCAACGGCACGTGCCTCTGGGGGCCGGATGGCCCCTACTGCGGGTCGGCAAACGGGCGACGG

ACACGCGCGTCGCTTCTGGCCCGGATTCTGACTTAGAGGCGTTCAGTCATAATCCAGCGCACGGTAGCTT

CGCGCCACTGGCTTTTCAACCAAGCGCGATGACCAATTGTGCGAATCAACGGTTCCTCTCGTACTAGGTT

GAATTACCATCGCGACACCATCATCAGTAGGGTAAAACTAACCTGTCTCACGACGGTCTAAACCCAGCTC

ACGTTCCCTATTGGTGGGTGAACAATCCAACACTTGGTGAATTCTGCTTCACAATGATA

>FM895081.1 FM895081 Jatropha curcas embryo 71-95 (DAF) Jatropha curcas cDNA clone rjcpga0_001647, mRNA sequence

ATCGCCACAAGCCCCAATATTATACTATTTGAAACAGCATACAATGTTCTAGACAGAAAGTAGAAACTTA

AAGTAAAACAAGAAAATAATAAATAAAAACAGAACCTGGAGCTGCAGATAGATACTAAAACATTTCTGCA

TTTCCTCGAATTCCATTTCATGAATGCAACTCAATAACATTAAGGATCCTAGACAAAAACGATAAATTGT

ACTGAACAAAGAGTCAAGACAATGCCTCTTACAGATTTCTTTATGTCAATTTTTGGCCTTAACAGAATTA

TTTTCCTTGATTTTAGCACGGTTGTTTAGCTTGACAAAGCGTTTGAGCTCTTCATAGGAGAGAGTTCTAA

CATTTTTCCTTTTTAATTCACTCAGTGCAGTACGTTTATCGAGCAGGTGCCACAACCAATCGGGGTATTC

AGAGTCAGGCAAGACCTTGGGATCTGCTCCATCCTTAAGAATATTGGCACCAACTACAGTGGTAGACTTA

ACTTCTTTGCTCAG

>FM895080.1 FM895080 Jatropha curcas embryo 71-95 (DAF) Jatropha curcas cDNA clone rjcpga0_001646, mRNA sequence

ATTCCCTTNATCGAATGTGTAGGATAGGTGGGAGGTGGTGACACACAACGACCAATCCTGAAAGACCACT

CTTTCGTCTAAGGATGCCTAACCGCCGCACCGAAAAATCGGGGGGGGCGGGACACTGCGAGGTGGGTAGT

TTATCTGGGGCGGATGCCTCCTAAAGAGTAACGGAGGTGTGCGAAGGTAGGCTCAAGCTAAGATTCTGCT

CGTGAGCGTAATGGTATAAGCCTGCCTGACTGTGAGACCGACTGGTCGAACAGAGACGAAAGTCGGCCAT

AGTGATCCGGGAGTCCCGTGTGGAAGGGCTCTCGCTCAACGGATCAAAGGTACGCCGGGGATAACAGGCT

GATGACTC

>FM895079.1 FM895079 Jatropha curcas embryo 71-95 (DAF) Jatropha curcas cDNA clone rjcpga0_001643, mRNA sequence

TTTTTTTTACAAAGTGATGGCAATCCCATCAGAAAAAGTACTATTTCAAACAGAACAAAAATATAGGGTA

CAGCTACATATATACACATAATCAGAAAACATGGAAAAAAAAAATAATCAAAGGATAAACCAAAAATTAA

GAAATCAAGCAGCACTCAGCGGTAGCTCGTGTTGCATGAGCAATGAGCATAGAGGCAAAACTCAGATCCA

CTTAAAAGCATGATAAAGCCTTACACTGCAAATACAACAGACTACATAGCAGGGCTTGTAACACAACTGG

TGATGCTAAAGGGCAGCTGCCAATTGTTTAAGGAAAAAAGAATATAATGCAACATACTTGGTTCAGCTTC

AATAATCTTTTCTCCTGTTCTGCTGCTTGGCAACGATTGATTAGTTAAAGAAAAATAGGAAAGTCGATCA

GTCCAGCT

>FM895078.1 FM895078 Jatropha curcas embryo 71-95 (DAF) Jatropha curcas cDNA clone rjcpga0_001642, mRNA sequence

ATTCATCATTTCTAGGGTTTTAACAGCTCTGCGATTAACTGCATACCTAAATTCTAATTTCCTGTGTCTC

TTTTCGAATACTCGTGGCCGAGGAATCTCGTTAAGGTTGTTGGTGCTTGTTTTGTTTGTGACAATGGCTC

GGGTTTACGTGGGGAATTTGGAGCCGAGAGTCTCTGAGAGGGATCTTGAAGATGAATTCAGAATGTATGG

CGTTCTTCGGAGGTAATTTTCTGATCTCATTTTCTTATCAGGTCAAATTTGTTGGTGCCAAATGCTTATT

ATTGTCTCCTTTTCGTTCTTTGAATTTTCCAGAAGATATTGGTAAATGATTAGCAAATAAATAAGTTTAG

CATAGGGCATTTTTCTACTTTCATGGCAATTT

>FM895077.1 FM895077 Jatropha curcas embryo 71-95 (DAF) Jatropha curcas cDNA clone rjcpga0_001641, mRNA sequence

ATTCCAAAGGATTCTATCCNGTCGCCCGATGGGAATTGTACTTTAAGGCGGCCCTCGCGGCTCGTCCGCC

CCGCGGGGGCTTGGCCAACGGCACGTGCCTCTGGGGGCCGGATGGCCCCTTNACTGCGGGTCGGCAAACG

GGGNCGACGGACACGCGCGTCGCCCTTCTGGCCCGGATTCTGACTTAGAGGCGTTCAGTCATAATCCAGC

GCACGGTAGCTTCGCGCCACTGGCTTTTCAACCAAGCGCGATGACCAATTGTGCGAATCAACGGTTCCTC

TCGTACTAGGTTGAATTACCATCGCGACACCATCATCAGTAGGGTAAAACTAACCTGTCTCACGACGGTC

TAATCCCAGCTCACGTTCCCTATTGGTGGGTGAACAATCCAACACTTGGTGA

>FM895076.1 FM895076 Jatropha curcas embryo 71-95 (DAF) Jatropha curcas cDNA clone rjcpga0_001640, mRNA sequence

TTTTTTTTATTGGCAGAAATAAAATAAATCATTTAAAAATTATCCAGTTGAAACCTAGGAATCAAAAGGA

AGGCAAAATCCAACAGCGAAAGGCAAATGCGTAGGGACACGTTAAAACTCCATAAATTATCCTTATCCGA

GGGCCCTAAGAAGTTCATTATACTTCTTTGTGTCTATACTTTCCGGACCTGAATATTGTGAGTAGATTGA

AACCTCCAGATTGGCTGTCTGCCACTCTGAAGAAGTGCAACTTCCTCTCCTTCCTTCACCATTCCTTGAT

TCTTGAGCACTGACAAAGCATTTGCAAAGGTTTGTTCAGCATCGTCTGAAAACTCCATGTATATGGGGCA

AACTCCTTGGTATAAAGCAAGTCTCTGTTGTATTTCCTTCTCATTTGTGAAGGCAAAAATAGTGCCAGTA

GGTCGATAATGACTCAGCAAAATAGCCATGAAGCCA

>FM895075.1 FM895075 Jatropha curcas embryo 71-95 (DAF) Jatropha curcas cDNA clone rjcpga0_001639, mRNA sequence

CTGACATGTGTGCGAGTCAACGGGTGAGTAAACCCGCAAGGCGCAAGGAAGCTGACTGGCGGGATCCCCC

TGAGGGTTGCACCGCCGACCGACCTTGATCTTCTGAGAAGGGTTCGAGTGAGAGCATGCCTGTCGGGACC

CGAAAGATGGTGAACTATGCCTGAGCGGGGCGAAGCCAGAGGAAACTCTGGTGGAGGCCCGCAGCGATAC

TGACGTGCAAATCGTTCGTCTGACTTGGGTATAGGGGCGAAAGACTAATCGAACCGTCTAGTAGCTGGTT

CCCTCCGAAGTTTCCCTCAGGATAGCTGGAGCTCGGGACGAGTTCTATCGGGTAAAGCCAATGATTAGAG

GCATCGGGGGCGCAACGCC

>FM895074.1 FM895074 Jatropha curcas embryo 71-95 (DAF) Jatropha curcas cDNA clone rjcpga0_001638, mRNA sequence

TTTTCTTCTTGGATTGTTTAGTTGAACCTTAATAACAAAAACAAAGATGGTCATCAACAAAATCCTTCGA

TCCACTCTCAAAAACCAGCTTGGTTCACACAGCAAACATGTTGCAGCTTCTGGCAGTTCACAGAAGCATC

TTGCTTCGGCTCCATGCCTGGCCAAATTGTATGGCACAGAGGCATCTTTGCAAAAGGATGACTCAACGAC

TGATTCGAGGGATGGTAGAGGTTATAAGGGGCATGATATGTTGGCACCTTTCACTGCTGGGTGGCAGAGT

ACTGATTTGCATCCTCTCGTCATAGATAAGTCTGAGGGTAGCTATGTTTATGACATTAATGGGAAGAAGT

ATCTTGATTCTCTTGCTGGTCTTTGGTGCACTGCTTTGGGGGGAAGTGAACC

>FM895073.1 FM895073 Jatropha curcas embryo 71-95 (DAF) Jatropha curcas cDNA clone rjcpga0_001635, mRNA sequence

GCAATCAAACCAGTCAACTATTTTTTATATTGAAGCACAAAAGTTTTATAACAATTCAATGCAATTCGGT

AGACAATTTCTATTTTACACGAAGCTTAAAGACACGCAACAGGGTAAAGTCACATTAAAGCAGAGACAAA

GAAACCCCAATAATCATACAATTGGACAGGACGCCAAAGGTACAGGGTAAAAGTTGGTAATCTCACAGGA

TGGTTGATTTTTAATGGCCGTGCTACTGTCACATGCCTCCAGAGCTTCATATAGATTCGGACAGAACTAA

GTTTAATCAGCTGTCCAGCTCTGCTCCACAATTTCCCTAACTCTTCGGTTGTACTCGCGCTTGTTTTCAC

TAAACATCCGAGCAGCTTCTGAATTTGCTGGAGAATTGGGGTTGGGGTCACAGAGCAATGACTGTATAGA

GGTAAGTATAGCTGCCACATCATATATAGGACTCCACTGAT

>FM895072.1 FM895072 Jatropha curcas embryo 71-95 (DAF) Jatropha curcas cDNA clone rjcpga0_001634, mRNA sequence

ATTCCCTCTGGTCGATGGAACAATGTAGGCAAGGGAAGTCGGCAAAATGGATCCGTAACCTCGGGAAAAG

GATTGGCTCTGAGGGCTGGGCCCGGGGGTCCCAGTCCCGAACCCGTCGGCTGTCGGCGGACTGCTCGAGC

TGCTCCCGCGGCGAGAGCGGGTCGCCGCGTGCCGGCCGGGGGACGGACTGGGAACGGCCCTTCCGGGGGC

CTTCCCCGGGCGTCGAACAGTCGACTCAGAACTGGTACGGACCAGGGGAATCCGACTGTTTAATTAAAAC

AAAGCATTGCGATGGTCCTGCGGATGCTCACGCAATGTGATTTCTGCCCAGTGCTCTGAATGTCAAAGTG

AAGAAATTCAACCAAGCGC

>FM895071.1 FM895071 Jatropha curcas embryo 71-95 (DAF) Jatropha curcas cDNA clone rjcpga0_001633, mRNA sequence

AACGTTTAACTTCAGNTGTCCTATGGTTTCGTTCACATTAGTTTCTGAGCAACTAGAAACTATATATGTT

GATCAACTTGTTCCATACCTTTCTTTACAAATAATGCATGAGATATGCTGATATTAAGACAAAAAGAAAC

ACAAATAAAAGAGTAAATAAAATATCAATGAGCCAGAACAATAATTTCCATTTNCTTTGCAATATCCTCC

AGAGGACGCCTCTTTATCCAGGTCTTAGGGGCTGTCAGACTACCAACTTGGACCTTCTTCATGCTCAGTG

CATTCTCGTTAATAGCCCATGAGTCAAAAACTTTTGGGGTGTTCCTTGAATAAAGCCAGTCAAGTTCCTC

AATGGACTTGCCTTTTTAGCTGATTGAAACATGCCTTGGCAGCTTTTACCATCTCCAGCAGTG

>FM895070.1 FM895070 Jatropha curcas embryo 71-95 (DAF) Jatropha curcas cDNA clone rjcpga0_001630, mRNA sequence

CTTTCTTCGAGTTGAAGGGAAGCTCTGTGGCTTGCGTTAGCGAGACTATCACTGGCTTGCCTTATTGGTT

GCAGTAGCAGCTGTTAAGCTCCAGTCAAAAGCACCACCACCGGCGCAATCAACCATGACATTCAAGAGAA

GAAATGGCGGCCGCAACAAGCATGGTCGTGGTCATGTCAAGTTCATTCGCTGCTCTAACTGTGGCAAATG

CTGCCCTAAGGATAAAGCCATCAACAGATTTCTTGTGAGGAACATTGTTGAGCAAGCTGCAGTTAGGGAT

GTCCAGGATTCTTGTGTTTATGATGGATATGTTCTTCCTAAGCTGTATGTGAAGATGCAGTACTGTGTTT

CATGTGCTATTCACTCCAGAGTTGTTAGGGTTCGTTCTCGCACTGCGCGAAGGAACCGTGAGCCACCACA

GCGCTTTCTCAGGCGCAGGGATGACCTGCCAAAGCCTGCACAGCCAGGTCAGCCTGG

>FM895069.1 FM895069 Jatropha curcas embryo 71-95 (DAF) Jatropha curcas cDNA clone rjcpga0_001629, mRNA sequence

TCGAAATCCGCTAAGGAGTGTGTAACAACTCACCTGCCGAATCAACTAGCCCCGAAAATGGATGGCGCTT

AAGCGCGCGACCTATACCCGGCCGTCGGGGCAAGAGCCAGGCCCCGATGAGTAGGAGGGCGCGGCGGTCG

CTGCAAAACCCGGGGCGCGAGCCCGGGCGGAGCGGCCGTCGGTGCAGATCTTGGTGGTAGTAGCAAATAT

TCAAATGAGAACTTTGAAGGCCGAAGAGGGGAAAGGTTCCATGTGAACGGCACTTGCACATGGGTTAGTC

GATCCTAAGAGACGGGGGAAGCCCGTCCGACAGCGCGTCCTCGCGCGAGCTTCGAAAGGGAATCGGGTTA

AAATTCCCGAACCGGGACGTGAAGGCTGACGGCGACGTTAGGGAGTCCGGAGACGT

>FM895068.1 FM895068 Jatropha curcas embryo 71-95 (DAF) Jatropha curcas cDNA clone rjcpga0_001628, mRNA sequence

CTAGTGGATCCCCGGGCTGCAGGCAAAGGATTCTATCCGTCGCCCGATGGGAATTGTACTTTAAGGCGGC

CCTCGCGGCTCGTCCGCCGCGGGGGCTTGGCCAACGGCACGTGCCTCTGGGGGCCGGATGGCCCCTACTG

CGGGTCGGC

>FM895067.1 FM895067 Jatropha curcas embryo 71-95 (DAF) Jatropha curcas cDNA clone rjcpga0_001627, mRNA sequence

AATCGAATTCGAGAGATAGAGGTAGTGTTTGGCAGGAGAGAGTGTTAGAATAGAGGCGAAAGCTCGCACT

CTTTCTTGCTTTTCAGGAACAAAATGGTGGGTCTTGATCTCAGGCAAGAACTGAAGATTCTTATAACGA

>FM895066.1 FM895066 Jatropha curcas embryo 71-95 (DAF) Jatropha curcas cDNA clone rjcpga0_001622, mRNA sequence

CTTATCTTCTTTCGCTGCTCTTGTCCTTCTTCCTAAATCATCTAAATACGACAAATCTAATGAAAGAACT

TTTAGAGGAAGGGATGGAGTGATTTTACTTTTTAATGCAGCAGATCCTGTT

>FM895065.1 FM895065 Jatropha curcas embryo 71-95 (DAF) Jatropha curcas cDNA clone rjcpga0_001621, mRNA sequence

TATCCGTCGCCCGATGGGAATTGTACTTTAAGGCGGCCCTCGCGGCTCGTCCGCCGCGGGGGCTTGGCCA

ACGGCACGTGCCTCTGGGGGCCGGATGGCCCCTACTGCGGGTCGGCAAACGGGCGACGGACACCGCGTCC

TTCTGGCCCGGATTCTGACTTAAGGCGTTCAGTCATAATCCAGCGCACGGTACTTC

>FM895064.1 FM895064 Jatropha curcas embryo 71-95 (DAF) Jatropha curcas cDNA clone rjcpga0_001619, mRNA sequence

ATTCCCAAAAGATATCAAACATGACCAAAGATTTGATTTTGATCGTGTTCCTGCAGTTGTTGAGCTATGC

AGGCAAGCAGGTGCTGAGGTATCTTCGGAAATTGTCATCGGTGATTCTGATGAAGCTCAGTCTTTGTCAC

CTTCGGATCTTGTGTTTGTAGCTAATAAAACTTTGAGAACCTGGGAAAACGGTAGATCCGGGGTTGCGAA

ATTGGCGGTTGGTTAATCCTTCAAAAGTTGGCAAACATGGTTCAGAAGTTCATGTAGGGCCATCCGGACA

TAAGGCTCGCCTTTGTGGGATATTTAAGTATGAGAGTTGGCGTGGAACACACTTCTGGAAGAAGGCAGGG

GTAGATGATTTGGTGCCTCCTAAGATTGTGTGGCGGAGACGGCCACAAGATCCTCCAGTTCTTTTGGATG

AATGGCGGAATTTTTATGGACATGCACCAGCTGTCGTGGATCTGTTCACAAAGGCTGGTGCTATTGCACC

TACAAAATATTACTGCATGATGAAAGTTCAG

>FM895063.1 FM895063 Jatropha curcas embryo 71-95 (DAF) Jatropha curcas cDNA clone rjcpga0_001616, mRNA sequence

GCTAACTGGGGCTTTTAATGTCACTAGTCATTTCACTCTTTATCTCCAAAAGAATGCACTTCTTCTTGCT

TCTCAGGATGAAGCAGAATGGCCCGTTCTCGCTCCATTGCCTTCTTATGGGAGAGGAAGAGATAAACCTG

GTGGAAGGTTTAGCAGTCTCATTTTTGGTACACACCTCACCGATGTGGTGATTACAGGTAATAATGGCAC

AATCGATGGACAAGGAGCAACTTGGTGGAAGAAGTTCCATAAGGGTCAACTGAAGATTACCAGACCATAT

CTCATTGAGTTTTTATACTCTAATCAGATTCAAATATCCCATATCACTTTAATCAACTCTCCTTCTTGGA

ATGTTCATCCCACATATTCCAGTAATGTGCTGATCCAACGGTTGACTATTCTTGCACCAGTTGACTCTCC

TAACACAGATGGGATTAATCCAGACTCGTGCACGAATACTCGAATCGAAGATTGTTATATTGTGTCAGGA

GATGATTGTATTGCAGTTAAAAGTGGGTGGGATCAGTATGGAATCAAA

>FM895062.1 FM895062 Jatropha curcas embryo 71-95 (DAF) Jatropha curcas cDNA clone rjcpga0_001615, mRNA sequence

ATTCTCTTTTCCCTAATAAGAAGCCATACATACAATAGATACCTTCTCCAAAGTAACNAAAGTAAAGGGA

AAGAAGAGAGACAGAGAGAGAGAAGAAAGAAAATGCCAGGCGATAGAAAGATCGGTGTGGCCATGGATCG

TTCGCCCAGCAGCAAGAAAGCTCTAAAATGGGCCATCGATAACTTGGCTGATAGAAAAGATACTCTTTAC

CTAATCCATGTCAATCCAAATTCTCTTGACGAGTCTCACCAACTCTGGGCCAAGTCTGGTTCTCCTCTTA

TTCCATTGGCTCAGTTTAGAGAGCCGGAGACTCTGAAAAGTTATGATGTGAAGATTGATATCGATGTTCT

TGATATGCTCGACACTATTTCCAGGCAGAAAGAGGTAAATGTAATTACAAAATTGTACTGGGGAGGAGAC

GCCAGAGATAAGCTGCTGGATGCAATTGATGATCTGAAACTCGACTCTTTGGTTATGGGGAGCAGAGGAC

TTGGCACTGTTAAAAGGATACTGCTGGGAAGCGTAAGCACATTTGTGATGACACATGCAACATGCCCTGT

GACGGTTGTGAAGGATCTAG

>FM895061.1 FM895061 Jatropha curcas embryo 71-95 (DAF) Jatropha curcas cDNA clone rjcpga0_001614, mRNA sequence

CAATTAAACTCACTCCCGATAAAGATCGAGAAATTCTTATATGGATTTAATCGACTGTGTACTGGCCAAC

ATGGATCTGCCGCCGCATTCTCGCTCTCTAACGTATCTAAGATATCTAAGCCAACACCAGCTCTTAATAT

TCAAATTTATTTTCTACCAACTTGAACTAACCGAACTTCTATTGTATTCTGATTAAATATATGTTCCATC

TTCTGGAAAAGCCTCGCTTCAATTCGCAATCCAAAGTCATGGATTAAAGAGTAAAGATTCTCAGGCAACA

AAATTGGCTAGCTGCTTCTGCTCACAAACACTAAACTGTAGAGTTAACCATTACAATGACACGAGCACAA

AGCCTATTAAAAAGATTTCATTATACAATACACTTCAAAGCCATGACTTGCAAACAAGTGCAAAGACCGA

TGTTTGAACCAGGCATATGCTGGTATGCTGTCCCTCAGATTCCAGGAAGGCAACATAATTAACCAATTGA

GATGTTCTACTAATCGCAATATATTGGAGTGAGGACAG

>FM895060.1 FM895060 Jatropha curcas embryo 71-95 (DAF) Jatropha curcas cDNA clone rjcpga0_001612, mRNA sequence

CTGGAAATCAGAATCAAACGAGCTTTTACCCTTTTGTTCCACACGAGATTTCTGTTCTCGTTGAGCTCAT

CTTAGGACACCTGCGTTATCTTTTAACAGATGTGCCGCCCCAGCCAAACTCCCCACCTGACAATGTCTTC

CGCCCGGATCGGCCGCCGAAACGACCTTGGGTCCAAAAAGAGGGGCATAGCCCCGCCTCCGATTCACGGA

ATAAGTAAAATAACGTTAAAAGTAGTGGTATTTCACCGTCGCCTTTCGGCTCCCACTTATCCTACACCTC

TCAAGTCATTTCACAAAGTCGGACTAGAGTCAAGCTCAACAGGGTCTTCTTTCCCCGCTGATTCCGCCAA

GCCCGTTCCCTTGGCTGTGGTTTCGCTGGATAGTAGACAGGGACAGTGGGAATCTCGTTAATCCATTCAT

GCGCGTCACTAATTAGATGACGAGGCATTTGGCTACCTTAAGAGAGTCATAGTTACTCCCGCCGTTTACC

CGCGCTTGGTTGAATTTCTTCACTTTGACATTCAGAGCACTGGGCAGAAATCACATTGCGTGAGCATCCG

CAGGGACCATCGCAATGCTGTGTTTTAATTAAACAGTCGGATTCCCCTTGTCCGTA

>FM895059.1 FM895059 Jatropha curcas embryo 71-95 (DAF) Jatropha curcas cDNA clone rjcpga0_001611, mRNA sequence

AGGATTCTATCCGTCGCCCGATGGGAATTGTACTTTAAGGCGGCCCTCGCGGCTCGTCCGCCGCGGGGGC

TTGGCCAACGGCACGTGCCTCTGGGGGCCGGATGGCCCCTACTGCGGGTCGGCAAACGGGCGACGGACAC

GCGCGTCGCTTCTGGCCCGGATTCTGACTTAGAGGCGTTCAGTCATAATCCAGCGCACGGTAGCTTCGCG

CCACTGGCTTTTCAACCAAGCGCGATGACCAATTGTGCGAATCAACGGTTCCTCTCGTACTAGGTTGAAT

TACCATCGCGACACCATCATCAGTAGGGTAAAACTAACCTGTCTCACGACGGTCTAAACCCAGCTCACGT

TCCCTAATGGTGGGTGAACAATCCAACACTTGGTGAATTCTGCTTCACAATGATAGGAAGAGCCGACATC

GAAGGATCAAAAAGCAACGTCGCTATGAACGCTTGGCTGCCACAAGCCAGTTATCCCTGTGGTAACTTTT

CTGACACCTCTAGCTTCAAATTCCGAAGGTCTAAAGGATCGATAGGCCACGCTTTCACGGTTCGTATTCG

TACTGGAAATCAGAATCAAACGAGCTTTTACCCTTTTGTTCC

>FM895058.1 FM895058 Jatropha curcas embryo 71-95 (DAF) Jatropha curcas cDNA clone rjcpga0_001610, mRNA sequence

TTTTTTTTTTACTCAGACATCCAGATAATTATATTAAGAAACTAAACAAAAAAAAACCAGTATCAATATC

CATCATACTCAACTAATCAAAATTATGCAAAAAGTAGCAAACTTAGAATTACCAAATCCATATCACGAAA

CAAAAACGCATTAACAATAACCTAATCACCGCCAGCCTTATTGTAAACATAAGTCAAGCCGCACTTGCCG

CAGTAGTGCCTATCAAAATGGTTGGCCATGAAAGTTCCAGCTCCACACTCAGCATTGGGGCACTCCTTCC

TCAGCCTCTGCACCTTTCCGGAGTCATCGACCTTGTAGAACTGAAGCACGGCAAGTTTGACCTTCTTCTT

CTTGTGCTTGATCTTCTTTGGCTTGGTGTAAGTCTTCTTCTTTCTCTTCTTAGCACCACCACGAAGACGA

AGCACCAGGTGGAGAGTGGATTCCTTCTGGATATTGTAGTCGGCAAGAGTGCGGCCATCCTCTAGCTGCT

TTCCGGCGAAGATCAGTCGCTGCTGGTCTGGAGGGATGCCTTCCTTGTCCTGGATCTTGGCCTTGACATT

ATCGATAGTATCAGAAGACTCAACCTCTAGGGTTATCGTCTTCCCGGTTAGGGTTTTCACGAAGATCTGC

ATT

>FM895057.1 FM895057 Jatropha curcas embryo 71-95 (DAF) Jatropha curcas cDNA clone rjcpga0_001609, mRNA sequence

GCAAAGGATTCTATCCGGTCGCCCGATGGGAATTGTACTTTAAGGCGGCCCTCGCGGCTCGTCCGCCGCG

GGGGCTTGGCCAACGGCACGTGCCTCTGGGGGCCGGATGGCCCCTACTGCGGGTCGGCAAACGGGCGACG

GACACGCGCGTCGCTTCTGGCCCGGATTCTGACTTAGAGGCGTTCAGTCATAATCCAGCGCACGGTAGCT

TCGCGCCACTGGCTTTTCAACCAAGCGCGATGACCAATTGTGCGAATCAACGGTTCCTCTCGTACTAGGT

TGAATTACCATCGCGACACCATCATCAGTAGGGTAAAACTAACCTGTCTCACGACGGTCTAAACCCAGCT

CACGTTCCCTATTGGTGGGTGAACAATCCAACACTTGGTGAATTCTGCTTCACAATGATAGGAAGAGCCG

ACATCGAAGGATCAAAAAGCAACGTCGCTATGAACGCTTGGCTGCCACAAGCCAGTTATCCCTGTGGTAA

CTTTTCTGACACCTCTAGCTTCAAATTCCGAAGGTCTAAAGGATCGATAGGCCACGCTTTCACGGTTCGT

ATTCGTACTGGAAATCAGAATCAAACGAGCTTTTACCCTTTTGTTCCACACGAGATTTCTGTTCTCGTT

>FM895056.1 FM895056 Jatropha curcas embryo 71-95 (DAF) Jatropha curcas cDNA clone rjcpga0_001608, mRNA sequence

ATTCCACAAGGATTCTATCCGTCGCCCGATGGGAATTGTACTTTAAGGCGGCCTCGCGGCTCGTCCGCCG

CGGGGGCTTGGCCAACGGCACGTGCCTCTGGGGGCCGGATGGCCCTACTGCGGGTCGGCAAACGGGCGAC

GGACACGCGCGTCGCTTCTGGCCCGGATTCTGACTTAGAGGCGTTCAGTCATAATCCAGCGCACGGTAGC

TTCGCGCCCCTGGCTTTTCAACCAAGCGCGATGACCAATTGTGCGAATCAACGGTTCCTCTCGTACTAGG

TTGAATTACCATCGCGACACCATCATCAGTAGGGTAAAACTAACCTGTCTCACGACGGTCTAAACCCAGC

TCACGTTCCCTATTGGTGGGTGAACAATCCAACACTTGGTGAATTCTGCTTCACAATGATAGGAAGAGCC

GACATCGAAGGATCAAAAAGCAACGTCGCTATGAACGCTTGGCTGCCACAAGCCAGTTATCCCTGTGGTA

ACTTTTCTGACACCTCTAGCTTCAAATTCCGAAGGTCTAAAGGATCGATAGGCCACGCTTTCACGGTTCG

TATTCGTACTGGAAATCAGAATCAAACGAGCTTTTACCCTTTTGTTCCACACGAGATTTCTGTTCTCGTT

GAGCTCATCTTAGGACACCTGCGTTATCTTTAACAGATGTGCCGCCCCAGCCAAATCCCCACCTGACAAT

GTCTTCCGCCGGATCGGCGCCGAACGACCTGGGT

>FM895055.1 FM895055 Jatropha curcas embryo 71-95 (DAF) Jatropha curcas cDNA clone rjcpga0_001606, mRNA sequence

CGAATTCGAGAGACTGTGGCTGTGGATATCTCTTTTGTATTCAAGAAGGACTCCAACTTCCACGAATGCC

TGGAAGCAAACTCACCTGGTCCTACAGAAGAGGCAGTTCTTACTGGCCCGTTTGACCATTTGAGATAGGC

CTTCCTGATGGATTGATAGAGACAGGAACGACAACTGGATGGATGAAAGAGGAAGGACTGCTTTGATAGA

GCAAGGAACGATCCATTAAGCAACAGGGAAAGAGCACGGGATGGAACAGAACTAGCTTAAGCTTAGGTCC

TTTCCCCGGATGGCTTTCCTACTTGAATGCTGGCTTTCCTAAGATCCCTATTACCTTTTTGCCCACTTGC

TTAACTTAGTGCTTGGCCAAAGGAAGAAAGAGAAAGGAAAATGATAGTAGGCCTACATACACAGGGGAAG

AAAGCATAACAGGAGAAGAAAAAGATAGAGCTGGCTTAAAAGCGAATTATTTACACACCGGTAAAACTTA

CTTTCCGGC

>FM895054.1 FM895054 Jatropha curcas embryo 71-95 (DAF) Jatropha curcas cDNA clone rjcpga0_001605, mRNA sequence

GTTCTCCAATCTACATGCTGCCTATTTGACATTCCATTCGAGCTGCTTCAGGCAAATTACAACAATAATA

AGATGCAAGCTACCAGAAAATGAAATGCAAAATCTTCAATTCATAACAAGTTAAAAAAAAGCAGCACTAG

CTTTTGTAGAAAGCATAAATCAGACGAAGCACATGGCTAATCCTATGGCAAACAAGTTCAAAGGCACAAC

CAAAACTCTCGGAAGCAGAATAATTTTTCTCTAAAGTGAAAAAAACAAAAGCTGAGCCTAGTAAAAGAAT

CCAAGCACTTTGCCACCAACATTCTTTCTTCTCGCCTCTTCATGATCCATAATGCCAGCAGAGGTAGTCA

ACACAATATAACCAAACTGTCTTGATGGAAGAAGCCTTGCAGTCCATCCTTCAATTTCTTTGACACCGAT

ATCAAAACGAGGGCTGATAACTCCACACTTGTTCCACCTCCCATTCAATTCAACCACAATTTTACCAGCT

CTAGGATCATCAACATACTCAAACTCGCCAATGTATCCATGCTTTTGCATCACCAGCAGAAATTTGATG

>FM895053.1 FM895053 Jatropha curcas embryo 71-95 (DAF) Jatropha curcas cDNA clone rjcpga0_001604, mRNA sequence

AGGATTCTATCCGTCGCCCGATGGGAATTGTACTTTAAGGCGGCCCTCGCGGCTCGTCCGCCGCGGGGGC

TTAGCCAACGGCACGTGCCTCTGGGGGCCGGATGGCCCCTACTGCGGGTCGGCAAACGGGCGACGGACAC

GCGCGTCGCTTCTGGCCCGGATTCTGACTTAGAGGCGTTCAGTCATAATCCAGCGCACGGTAGCTTCGCG

CCACTGGCTTTTCAACCAAGCGCGATGACCAATTGTGCGAATCAACGGTTCCTCTCGTACTAGGTTGAAT

TACCATCGCGACACCATCATCAGTAGGGTAAAACTAACCTGTCTCACGACGGTCTAACCCCAGCTCACGT

TCCCTATTGGTGGGTGAACAATCCAACAC

>FM895052.1 FM895052 Jatropha curcas embryo 71-95 (DAF) Jatropha curcas cDNA clone rjcpga0_001603, mRNA sequence

CTATCCGTCGCCCGATGGGAATTGTACTTTAAGGCGGCCCTCGCGGCTCGTCCGCCGCGGGGGCTTGGCC

AACGGCACGTGCCTCTGGGGGCCGGATGGCCCCTACTGCGGGTCGGCAAACGGGCGACGGACACGCGCGT

CGCTTCTGGCCCGGATTCTGACTTAGAGGCGTTCAGTCATAATCCAGCGCACGGTAGCTTCGCGCCACTG

GCTTTTCAACCAAGCGCGATGACCAATTGTGCGAATCAACGGTTCCTCTCGTACTAGGTTGAATTACCAT

CGCGACACCATCATCAGTAGGGTACAACTAACCTGTCTCACGACGGTCTAAACCCAGCTCACGTTCCCTA

TTGGTGGGTGAAACAATCCAACACTTGGTGAATTCTGCTTCACAATGATAGGAAGACGCCGACATCGAAG

GA

>FM895051.1 FM895051 Jatropha curcas embryo 71-95 (DAF) Jatropha curcas cDNA clone rjcpga0_001602, mRNA sequence

TCAACTAGCCCCGAAAATGGATGGCGCTTAAGCGCGCGACCTATACCCGGCCGTCGGGGCAAGAGCCAGG

CCCCGATGAGTAGGAGGGCGCGGCGGTCGCTGCAAAACCCGGGGCGCGAGCCCGGGCGGAGCGGCCGTCG

GTGCAGATCTTGGTGGTAGTAGCAAATATTCAAATGAGAACTTTGAAGGCCGAAGAGGGGAAAGGTTCCA

TGTGAACGGCACTTGCACATGGGTTAGTCGATCCTAAGAGACGGGGGAAGCCCGTCCGACAGCGCGTTCT

CGCGCGAGCTTCGAAAGGGAATCGGGTTAAAATTCCCGAACCGGGACGTGAAGGCTGACGGCGACGTTAG

GGAGTCCGGAGACGTCGGCGGGGGCCTCGGGAAGAGTTATCTTTTCTGTTTAACAGCCCGCCCACCCTGG

AAACGACTCAGTCGGAGGTAGGGTCCAGCGGCTGGAAGAGCACCGCACGTCGCGCGGTGTCCGGTGCGCC

CCCGGCGGCCCATGAAAATCCGGAGGACCGAGTGCCTCCCACGCCCGGTCGTACTCATA

>FM895050.1 FM895050 Jatropha curcas embryo 71-95 (DAF) Jatropha curcas cDNA clone rjcpga0_001601, mRNA sequence

CATCTAAAATTATAAACTGCATTGTAAAAAACTATACGGTGCAAACAAATAATACAAATCAGAATACAGA

ACATAGCTAAATTCTGCCTCCCAAAGTAAAAAAATGAATAAAATATAAGAGTCAATTTGATGTGAAACTA

ATATCTACTCCACATTCCTTTCATGTAATTCTCGCGCAAATCAAAGAATCGAATTAAGAATTCCCAATAT

GTCCTGTTTTTCCAGTAGGCCACAAACTAGATATTCAAGCAAGAGTATCACTTAAGGGCATAGATCTTGA

AACGGTTGCTCCCTCAGTATCAGGTATTTGTGCTGGTGGTACTTCAGTTGGGGTTGATGTTGTATCGCTC

GTGGACACTGGTGGGATACTTTCTCCTGATGCAACAAGATCTGGGTTGCTTCCTGAAGATGGGACAGGAG

CATTATCACCAACAGAAACAGGAGGTTCACCTGCTTGAGTTTGCAGTTTAGCACTTTCTACTTCTGGGGT

AGAACTAGGAGGTGCATTTTCTGGTGTTGTTGCAGCAGTAGTGTTAGGAATAGATGTAGATACTGGTTCG

GTTGTAGCAACTGGGGC

>FM895049.1 FM895049 Jatropha curcas embryo 71-95 (DAF) Jatropha curcas cDNA clone rjcpga0_001395, mRNA sequence

ATTCTTTCTCTCAAAACCTCAAGTAACCATTACTCACCATTTCGGTACCTCTCGTTAGAAAGTTTTTTCA

TTTACGTCCCTATAGTGATAGCCATCATAAGCTGCTTCAGCGACAACCACCATGTGTGGCGGTGCTATCA

TCTCCGACTTTATACCTCCCGCGGTCGCTGGGCGATCTTCTCGGCGGTTGACAGCTGATTTTCTTTGGCC

TGATCTAAAGAAACCCATTGGAAAGCAGTACTCAAAACCTGTTGTTGATCTTGACAATGATTTCGAGGCT

GATTTTCAGGAGTTCAAAGATGAATCTGATGCCGATGAGGAAGATGATGTCATGCTTGATGTCAAGCCTT

TTGCTTTTTCTGCTACTGCCTCTCCTCCTGCTCGCAATCGCTCTTCCTCTCGTGGATCTGCAGCTGTCAA

ATCTGTGGAATTCAATGGGCAGGCAGAAAAATCTGCAAAAAGAAAGAGAAAGAACCAGTATAGAGGAATC

CGGCAGCGCCCATGGGGAAAATGGGCTGCTGAGATT

>FM895048.1 FM895048 Jatropha curcas embryo 71-95 (DAF) Jatropha curcas cDNA clone rjcpga0_001394, mRNA sequence

ATTCCGAAGCCGTCGAGATCCGAAACATCAGATGATCCTGTGATTCTGCTCACCAAAGATTTGCGAGTGA

GACACGAATTTCTATTTTAGATTTCTTCTCGATGGATTTTAGCCAGCCTGCAGGGGAAAACTATGCTAAT

CCAAGGACATGCTTCTTTCATGTGCTCTTTAAGGCTGCAGCCCTAGCATTCTACATACTCTCAGCCCTGT

TTGTTGATAGTTTTGTCATCATCTTTGTGGTTACTGTTGTTCTTGCTGCCCTTGATTTTTGGGTAGTGAA

GAATGTTAGCGGCCGGATCTTAGTAGGGCTTAGGTGGTGGAATGAAATAAATGAGGATGGTGAGAGCGTG

TGGAAATTTGAATGTCTTGATCAAGAGTCATTAGCTCGAATGAACAAGAAGGATTCATGGCTATTTTGGT

GGACATTATACCTTACTGCAGCTGATTGGATCGTCCTTGGAATATTTTCAATCATAAGGTTCCAGGTTGA

TTATGTCCTGGTAATCGGAGTTTGTTTGAGCCTTAGCATTGCAAATATTATTGGGTTCACCAAATGCCGC

AAAGATGCCAAGAAGCAGATTC

>FM895047.1 FM895047 Jatropha curcas embryo 71-95 (DAF) Jatropha curcas cDNA clone rjcpga0_001392, mRNA sequence

ATTCGGATTCTATCCGTCGCCCGATGGGAATTGTACTTTAAGGCGGCCCTCGCGGCTCGTCCGCCGCGGG

GGCTTAGCCAACGGCACGTGCCTCTGGGGGCCGGATGGCCCCTACTGCGGGTCGGCAAACGGGCGACGGA

CACGCGCGTCGCTTCTGGCCCGGATTCTGACTTAGAGGCGTTCAGTCATAATCCAGCGCACGGTAGCTTC

GCGCCACTGGCTTTTCAACCAAGCGCGATGACCAATTGTGCGAATCA

>FM895046.1 FM895046 Jatropha curcas embryo 71-95 (DAF) Jatropha curcas cDNA clone rjcpga0_001390, mRNA sequence

CAAATGGCTACTGTTGAGGTAGTATCGGCACAAACTGCACTTTCAGAGGAAAAAGCAGAAGAAGTGAAGG

CTTCAGAGACTGTCACTCAAGTAACAGATCAGTCAGTTACTGATGAAAAACCAGACACAGGAGAAAAGAA

GGAAGAACAAGAAGAAGTTGCAGGAACTAAAGAAGAAGCTGTAGAACCAGAACCAGAAGTTGCAACTGAA

GTAGTGGTGGAAACTAAAGAAGTAGTAGAAGATGAGACCAAAACTGTAACAGAAGAGCCCCCATCGGAGA

AAACTGAAGAAGAAACTCCTAAGGAAACAGAACCAGAAAAAGAAGAAACTCCTAAGGAAACAGAACCAGA

AAAAGAAGAAAAGGAATCCCCAAAAGAAACAGTAACTGAGGAGACTGTTGCTGAGGAGACTACTAAAGAG

ACCACTGAGAGTACTGAGACAACAACTCCGGCGCCAGAACCGGCACCGGAAGCTGC

>FM895045.1 FM895045 Jatropha curcas embryo 71-95 (DAF) Jatropha curcas cDNA clone rjcpga0_001389, mRNA sequence

CGAATCCGAAAACCCTAGAGGTGCTTCTCGAAGCTTGTCTTCATATTAGAAAAATTCGATTTATTTCCAT

CGTCAACCTTTCTGCAGTTCCGTTTATATTATTGTATCGAAGGCTTAAAAGAGTTTGATAAATCACGTCG

ATTATATTCAGCTATGGCAGCTAAGATTCTTGCCAACTTAATTGTAATGGGATCTGGCATTTTGGCTCGG

GCTTTTGTTCAAGCCTACCGTCAAGCACTTGCAAATGCCTCAAAATCTGGTGTTGCCCAAGAGACAATTC

AAAACACTATCCGTAAGGGAAGCAAAGTCATGACAGAACAAGAGGCACGACAGATTCTTGGTGTAACTGA

GGAAACTGCTTGGGAGGAGATAGTAAAGAAATATGACACTTTATTTGAGAGAAATGCCAAGAATGGAAGC

TTTTACCTTCAGTCAAAGGTTCACAGGGCTAAGGAATGTTTAGAGGCTGTACATCGAGGAAAAGGCGAGG

GTACACCTAGCTGAGAATTTTTTTAAGGCCCTTCTGTTGCACATACTGACCTCCTGTATTATTATT

>FM895044.1 FM895044 Jatropha curcas embryo 71-95 (DAF) Jatropha curcas cDNA clone rjcpga0_001387, mRNA sequence

TGGAAATCAGAATCAAACGAGCTTTTACCCTTTTGTTCCACACGAGATTTCTGTTCTCGTTGAGCTCATC

TTAGACACCTGCGTTATCTTTTAACAGATGTGCCGCCCCAGCCAAACTCCCCACCTGACAATGTCTTCCG

CCCGGATCGGCCGCCGAAACGACCTTGGGTCCAAAAAGAGGGGCATAGCCCCGCCTCCGATTCACGGAAT

AAGTAAAATAACGTTAAAAGTAGTGGTATTTCACCGTCGCCTTTCGGCTCCCACTTATCCTACACCTCTC

AAGTCATTTCACAAAGTCGGACTAGAGTCAAGCTCAACAGGGTCTTCTTTCCCCGCTGATTCCGCCAAGC

CCGTTCCCTTGGCTGTGGTTTCGCTGGATAGTAGACAGGGACAGTGGGAATCTCGTTAATCCATTCATGC

GCGTCACTAATTAGATGACGAGGCATTTGGCTACCTTAAGAGAGTCATAGTTACTCCCGCCGTTTACCCG

CGCTTGGTTGAATTTCTTCACTTTGACATTCAGAGCACTGGGCAGAAATCACATTGC

>FM895043.1 FM895043 Jatropha curcas embryo 71-95 (DAF) Jatropha curcas cDNA clone rjcpga0_001386, mRNA sequence

AGGTTTTTAATTAAAACCCATCGAATTGATCAATTTCCCACCAACCTTAGCCCATGGATTCGTTAGAGGA

CGGCTGTTGCTGGGATTTTCTTGATTATAACTTTATCGACGAAACCGCAACGTCTTCTGATTTCTTATGG

CATAATAATCAAAGTGGCGACAGCGCCGGTGGCGGCGTGGATATTGATTTTTCTTCTTCTGGTACTGTAT

CTGAAGCAAAACAATTCTCGCGCAAGAGGGGACGCAGCGATTCTTGCAGCAAACCGGTGACCAAAGCTTG

CCGAGAAAAGTTACGGAGGGAAAGATTAAATGACAGGTTTCATGATTTGAGCTCTGTTTTAGAACCTGGC

AGACCCGCGAGAACTGACAAACCAGCCATACTTGATGACGCTATTAGAGTTCTGAATCAACTGAAAACTG

AAGCTCAGGAGCTCAAAGAAACAAATCAAAAGCTTCTAGAAGAAATAACAAGTCTGAAGGCTGACAAGAA

TGAACTTCGTGAAGAGAAACT

>FM895042.1 FM895042 Jatropha curcas embryo 71-95 (DAF) Jatropha curcas cDNA clone rjcpga0_001385, mRNA sequence

CAAGGATTCTATCCGTCGCCCGATGGGAATTGTACTTTAAGGCGGCCCTCGCGGCTCGTCCGCCGCGGGG

GCTTGGCCAACGGCACGTGCCTCTGGGGGCCGGATTGCCCCTACTGCGGGTCGGCAAACGGGCGACGGAC

ACGCGCGTCGCTTCTGGCCCGGATTCTGACTTAGAGGCGTTCAGTCATAATCCAGCGCACGGTAGCTTCG

CGCCACTGGCTTTT

>FM895041.1 FM895041 Jatropha curcas embryo 71-95 (DAF) Jatropha curcas cDNA clone rjcpga0_001384, mRNA sequence

ATTATTCCTCTTTCTTCCCCAACAATTTAGGGTTCCACAAAAATCACTCTCCGATTCGTGCGATAATAAT

CTCTCCAATCCCTACAAAACCATGAAGCAGCATTCCAAGAAGAAGAAGAAGCAACAGCCGGCACGCGCCT

CCAATCCCTCCGGCAACCACAAGGGGCAACAAGATGAACAGCAAGAAACCAAAGCTGCGTTGGCGTTAAT

GGAAGCTTTTGATTCGATCTCATTAGAAGAAGTTTCCTCGGCTTGTAGAGAAGCCGACGGTGATATCTAC

AAAGCGGCTGGGATATTATCCAACTTGAGCGACAGCGCGGACGATCCGTCCACGAGTGGGTTATCGAGTT

CTGACTTGACTTCAACTTCGGGTTTTGGTTCGAGTTCTAGTAGTACCACTTCGCGTTCGGGCTCGTCGGA

GGGGTACTTGGAGGCTGATTTGGTAAAAAGAAAGGGCTTTCGAGGTAACAATAAGCAGAAGAGAGCCGTG

GCTGTTACGG

>FM895040.1 FM895040 Jatropha curcas embryo 71-95 (DAF) Jatropha curcas cDNA clone rjcpga0_001383, mRNA sequence

ATTCTTCAGGGAACTATCAGTGACCTCGACTTCAACAGAGCCAGAGACTGAAAAACATAAAGAAACAGTA

GTTCCGGTGATCTCAACTCCTAGAGAGTCAGGTACAGTGCTTGAACCATCATCAACCCAAGAATCTGTGG

AGCCAGTGAAGCAAGAACGCATTGGTGAAACAATATCAGAGACTGAAAAACCAAAGGAATCCTTAAAACA

AGAACCGTTATCAACTGAAAAACTTTTGGAGCCAGAGAAACAAGAGACTGAAAAACTTGTGGAGCCAGCA

AAACAAGAACCGTTAGTGACTGAAAACTCTGCAGAGGTTTTAGTCAAAAAATCCGAAATCCCAGTTGAAC

CAACTCCATCTTGGGCTGTTGATGCTGCTTCTATAAAGGTAGAGAAGGTAATTGAAGCCATACCAGAAAC

TAAGTGAGGACATTAGCCAAGAATATGATTTTATGAAAATCTTTCTTAGATTGATAATAGGCAACCAAAA

ACCATTTACTTTATTTGGGTTTTGTGAGTTTGTTTCATATAAATTTTCTTCTTTTCGTTTGGGTTTGATC

GGTTTTGTGTCCTTGAGCATTG

>FM895039.1 FM895039 Jatropha curcas embryo 71-95 (DAF) Jatropha curcas cDNA clone rjcpga0_001382, mRNA sequence

CTTTGCTAAAGGACTCGTTGAAAGATCTCTGCTTTCGCTTCTAAGCCAAAATCCTAGATCTGCTCTCTCT

TGTGTTTTTGGTTCAGTTGCAAGAATGGAGACCTTCCTATTCACTTCCGAATCTGTTAACGAGGGCCATC

CCGACAAGCTTTGCGATCAGGTTTCAGATGCCATCCTCGATGCTTGCCTTGAGCAAGATCCTGACAGCAA

GGTTGCTTGCGAGACTTGCACCAAGACCAACATGGTCATGGTCTTTGGTGAGATCACAACCAAAGCCAAT

GTAGACTATGAAAAGATTGTTCGTGACACCTGCCGTTCAATTGGATTTGTTTCTGATGATGTTGGTCTTG

ATGCTGACAAATGCAAGGTCCTAGTTTACATTGAGCAGCAGAGCCCTGATATTGCTCAGGGTGTCCATGG

CCATCTTACCAAG

>FM895038.1 FM895038 Jatropha curcas embryo 71-95 (DAF) Jatropha curcas cDNA clone rjcpga0_001380, mRNA sequence

CATCAAAAACCAAAAATGTATCAAAATTCAATGCCAAATATAGGTTAGCTGAAAAGTTATATTCCCAGCA

GCGCACATATGAAACAACACCAGCATCAACTAGTTGCAAAATCAATCCGAAAAATAAAACAAAATTGACC

TTTTAGCAAACAGATTTCATTTCTATGCTAATCAACCTGATCAAGCTCGCATCACCTGAGACACCTAGAA

TATAAGAAATTGACTACTTAAATAAGGTCATCTAAAACAGAAACGATCAGATGACAAGAAACAGTCATTT

TTCAACTCTGCTCTTCAGCATCTGCCATTTCCCTCTCTTCAGCAGCAGCAGCCATCAATTCATCGAAATT

TTCAGTTTTCCCGAGCAATATCTCAATCTTAGCTTTCTGTACAGGCCGCCCTCTTGCTTCATCTCTCATA

TCAACTGTTGAAGTCATGATCTTCTTTTCAATAGCCAATCCATTGTTTTTCAGGATTTCTGCAACAGTGA

CAACAGTGGCTATAGCCATTCCAAGTGCAGACAGTTCCACCTCGTTGTGTTGCTGCA

>FM895037.1 FM895037 Jatropha curcas embryo 71-95 (DAF) Jatropha curcas cDNA clone rjcpga0_001379, mRNA sequence

TTGAAGTCAGGTGGTTTTCGTGTGACTGGGAGGATATCTACTCAGTGTTCCTTGTTGGATCCTATCAGTG

GAGAGCTAACAGTAGAAAGATCTGCAGTTCCCATTTCCTCCATTGATATTCACTTGCTTCGGGTGGAGTC

AATTCTTTATGGAGAGAAAATTGTGACTGAAACCTCTTTGATTCAGACAACACAGATAGCAGATGGAGAT

GTCTCCCATAATATGACTTTGCCCATTTACGTTATCATTCCCCGTCTTTTGACTTGTCCAACTGTCTTGG

CTGGTCCATTTTCAATTGAGTTCAAGGTGTCCATTGTTATTAGCTTTCAGTCAGAACTGTCCAAATTGCA

TAAAAAATCTGATCCCCGAAATCCAAGACTCTGGCTGGCAATGGAAACTTTACCCCTTGAACTTGTCCGA

ACAAGTTTGCAGTTAGAAGAGAAAATAGGAAGAACAAGCAAGGAAAACTAGTTAGCGCTATGATATCTAC

CCTCGCCAAATTAGGTAAAGTAGAGCTTGCATAGATCGTGTTTGAGACCCCTGT

>FM895036.1 FM895036 Jatropha curcas embryo 71-95 (DAF) Jatropha curcas cDNA clone rjcpga0_001378, mRNA sequence

AACACCTAGTACGAATATGACACTACTATCAAAAAACCTTTAGTGGTGAGTATTTCACATCTCTCAACCA

CCTGTCTATGCAGCAACAGATGGCTTAGAAGCAGCAGAGAGCCTTGATCTCTTCTTTGCCAAGCTTTCAC

TACGGCGTCTCGCTGTCCTTCATCTAGTGGCAAGAAGTTTCTGATATTCAGCTGCCTCTGCCTTGGCCTT

TGCAATTCTCTTCTTTTAGTCTGCAATCCTAGCACGTTTCCGCTGAAGTGTCAATGGAGTCACAAGTCTT

TGTATCTTTGGAGCTTTACTGACTTTCTTCCCAGATTTTGTTGTAAAGGTTCTACGGTATGTGTTGACAT

ATTTGCGGACATCATCTTCCTTGGAAAGGTTAAAAAGCTTCCGGATCTTGGATGCCCTCTTTGGACCTCT

CATCCTAGGTTTTTCAGTGTCAGTCAAACCGGGCAACTCATTTTCACCCTTCTTCACTATAACCAAGTTC

AAAACAGAAAGGTCCTGACT

>FM895035.1 FM895035 Jatropha curcas embryo 71-95 (DAF) Jatropha curcas cDNA clone rjcpga0_001377, mRNA sequence

TTTGACGAGCCTACGCTTGTTATGGACCTTGATTCTCACAAATTGCAAGCATTTACTGATGCCTACTCTG

AGCTTGAGTCAACTCTATCCGGCTTGAATGTTGTTGTCGAGACCTACTTTGCTGATGTACCTGCTGAGGC

ATTCAAGACCCTCACATCTTTGAAGGGTGTCACTGGATATGGTTTTGATTTGGTTCGTGGAACTAAAACT

CTTG

>FM895034.1 FM895034 Jatropha curcas embryo 71-95 (DAF) Jatropha curcas cDNA clone rjcpga0_001376, mRNA sequence

CAGACTGCTTAGTTAGACTTGTAAAAATTTCTAATCACTCTCTCTCTCTCTCTCTCTCTCTCTCTCTATT

TCTTTAAAGATAGAGAGGATAGAGAGAGAGAAGGACAGAGACAGAGATTTCTAGTCCTATTATATCTCTC

CACCATTCGGACACTTCTTCATAGCCCCTCTCTTCCCCTCTCAAGATTTTCACTTTTCTCTGGTTGGTTT

GATAAGAAAAGATTGAGAAATGGGTTTGTGGGCTTATTTTATTTTCACTATTATTTCTCATTTCTTGGTT

CTTCCTTATACAATAAAAGCTACTAGTAGCTGCGATTTCTTTACTGGTACTTGGGTCATTGACAGTTCAT

ACCCTCTTTACAACTCCTCAATCTGTCCTTTCATTGAGAGAGAATTCAGCTGCGAAAAGAATGGCCGTTC

CGATTTATTATACACCAAGTACAAATGGCAGCCATTTGGTTGCGATTTACAGAGGTTGGACGGTGTGGAT

TTCTTGGAAAGATTTAGAGGGAAAAGCATAATGTACGTTGGTGATTCCCTAAGTCGGA

>FM895033.1 FM895033 Jatropha curcas embryo 71-95 (DAF) Jatropha curcas cDNA clone rjcpga0_001375, mRNA sequence

AGAATTAGATGTATAACTCTAAGAAATATATATAAGATATTTGTTATACATATTTTAGTGATAAGNTTAG

AATCTCTCCCTTGGATTCCTTCTGAGAACATATTTGAAAAAGAAGACTTTTACAACAAGCTTAAAGAAGA

CTGCTGATTCTTTGAGCTATAAAAATCTGGATTTTGGACAAAGATGGAATAGTTAGCTGAATACAAGGAA

GTGGACAAGCAGAGAAGAAAAAGGCTCCC

>FM895032.1 FM895032 Jatropha curcas embryo 71-95 (DAF) Jatropha curcas cDNA clone rjcpga0_001374, mRNA sequence

ATTGCAAAGGATTCTATCCGTCGCCCGGTGGGAATTGTACTTTAAGGCGGCCCTCGCGGCTCGTCCGCCG

CGGGGGCTTAGCCAACGGCACGTGCCTCTGGGGGCCGGATGGCCCCTACTGCGGGTCGGCAAACGGGCGA

CGGACACGCGCGTCGCTTCTGGCCCGGATTCTGACTTAGAGGCGTTCAGTCATAATCCAGCGCACGGTAG

CTTCGCGCCACTGGCTTTTCAACCAAGCGCGATGACCAATTGTGCGAATCAACGGTTCCTCTCGTACTAG

GTTGAATTACCATCGCGACACCATCATCAGTAGGGTAAAACTAACCTGTCTCACGACGGTCTAAACCCAG

CTCACGTTCCCTATTGGTGGGTGAACAATCCAACACTTGGTGAATTCTGCTTCACAATGATAGGAAGAGC

CGACATCGAAGGATCAAAAAGCAACGTCGCTATGAACGCTTGGCTGCCACAAGCCAGTTATCCCTGTGGT

AACTTTTCTGACACCTCTAGCTTCAAATTCCGAAGGTCTAAAGGATCGATAGGCCACGCTTTCACGGTTC

GTATTCGTACTGGAAATCAGAATCAAACGAGCTT

>FM895031.1 FM895031 Jatropha curcas embryo 71-95 (DAF) Jatropha curcas cDNA clone rjcpga0_001373, mRNA sequence

AAGGATTCTATCCGTCGCCCGATGGGAATTGTACTTTAAGGCGGCCCTCGCGGCTCGTCCGCCGCGGGGG

CTTGGCCAACGGCACGTGCCTCTGGGGCCGGATGGCCCCTACTGCGGGTCGGCAAACGGGCGACGGACAC

GCG

>FM895030.1 FM895030 Jatropha curcas embryo 71-95 (DAF) Jatropha curcas cDNA clone rjcpga0_001372, mRNA sequence

CTTGCTTCTCCATGCATACAAAGAGAAATCTCACATGTGTAGATGTGGCATCTATTATGGATGGTAAAGG

AGTGAGTCCTGAAAATACAGATCACTCGGCCTGTGTAATGCCACCTCCAACAAAACGTTCTAAACTCATA

TCAAGTCAGCACCTATCATTATCAAATCATCCAGAAGCAGTTTCTGGCCTAACAAAAATTACAAGCTCTC

AGGATACTTTTACCTGTAGGGATTTTCACTTGCAGTCAACTTCGGGGAGTGACATGCCAAATGATTGTAT

TGTCTCTAAGTTCCCTGCGCGAGCTCATGAGCATTGCCTGCTTTCAAAAGATATTCCAGTTCCCCTTGCT

ACAAAAATTTATTACTCTCAGAGGAATAATCGCCTTCGGTTGGCAATTGCTCATATGTACCATGAAGCAT

CAGCTAAGGGACGATTCAGTAATGTCATGAGTCCAGAAGTATCAGAGGAAACCATAATTCAATTACCGGC

TTCATCTGATAGAGGTTGTTCACGTAA

>FM895029.1 FM895029 Jatropha curcas embryo 71-95 (DAF) Jatropha curcas cDNA clone rjcpga0_001371, mRNA sequence

CGGCGCCTAAACTATGGATCCCCCGGCTGCAGGAAAGTTTTGATGATCAACAGGATCTGGTCATGGATAT

TTTATGTGGCAAAGATATTGCTTCTGAGATAGACCCAAGAAAATGTGAAACTCATTCTAACATCCAAAAC

ATGTTAAGCAACCAGACAAAAGGCCAAGAAGGCAAAACAGATGGAATGCAGAATCTGGTGAATAGTGAAC

CTGTGGCCTCTAGTACAGGAAAAGAGAGAAAGAAAATATCAGACAAGGGAATGGAGTTTGATCCAATCAG

ACAGCACAGACACTTTTGCCCCTGGATTGTATCAACAAGTAGTGGAGCAGCTGGTTGGCAACAAACACTA

TCTGCTTTACTTCGACAAAAAGAGTATTCTAGTCCTGCAAAATCTCCTCCATCCACCTCCATCATCAAGG

TGGATGATCCGATCACTTCAGTAAGAAAGCTCTTTATGTCTCCATCCGCCAAAAGGATGAAACCCTCCAG

CGGGTCTAGTTGAAGCGTGCAGCTGATTAATTTTGCAGGTGTCAATATGCACCCAGTTAACATGTATTTT

ATGATACTGCTCCCCTTTGTATATTATAGATGCAATGATGTATTCAAA

>FM895028.1 FM895028 Jatropha curcas embryo 71-95 (DAF) Jatropha curcas cDNA clone rjcpga0_001368, mRNA sequence

TCAAAGCTAGTTAGTCCAGTTCCCTTATTAAGCTAAATTTTTTTCGAAGATGGCCAAAATTGCCTTTGGT

CGTTTTGATGATTCTTTTAGTTTAGGCTCTTTTAAAGCTTATCTTGCAGAATTTATCTCAACTCTGCTCT

TCGTTTTTGCTGGTGTTGGTTCTGCCATAGCTTACAATAAATTGACAGGAAATGCAGCTCTTGATCCTGC

TGGTTTAGTAGCAATTGCTATTTGCCATGGATTTGCTCTCTTTGTTGCTGTCGCCGTCGGTGCCAACATC

TCCGGTGGCCATGTTAATCCTGCTGTCACCTTTGGATTGGCTCTTGGTGGTCAAATCACCATTCTTACTG

GCATCTTCTACTGGATTGCTCAGCTTCTTGGTTCCATTGTTGCTTGCCTCCTTCTCAAAGTTGTCACCGG

AGGCTTGGAAACACCCACCCACAGCCTTGCAGCTGGAGTAGGAGCCATTGAAGGAGTGGTT

>FM895027.1 FM895027 Jatropha curcas embryo 71-95 (DAF) Jatropha curcas cDNA clone rjcpga0_001365, mRNA sequence

GTAACAGCAGATATTGAAAATTGCCTTTATAAAACAGTGAGACAAAAGAATAACAAATACATTAGTTCAC

CTGAACAGAAAATAACGAACAAGAAGCTAAAAATCTTGTGCAATCATATCTTACTAATTCATAAATTATC

CAGGAAAAAAAATGGCCTTCACAATCCTTATTAACTTCTGACATACAGGAACAAGATTACATGGGAAAAT

TTGGTTACCTCTTTCACCAAGGGAGAAATAAGTTACCTCTTTCTTTGTAGTAGACTCCTTACACGCTGCA

TAACACGAACAAACTCAAAAAACAATGAAAAGTCGAAAGCAGATGTTGAAATACTCTCTGGTCCAATTTT

AGGACTTTTTGCAGAAAATGTGCATGGACTCTGACAGATGATGAAGTCAAAACCACCAAACTTCTCTATC

AGTCTGTCAATCTTACTACTCGTTAGCTTTTGAATGTCTTCTATCTGTACTAGTTCCCCAGTTTGTTCAC

TACTGTGCCACCACCTTCTTAGGATCCTCTGCTTTGTTTCAGAAGTTTCCACTGAGACAACACCCTTCAT

ATGGATCCCGAGGGCGGTGCAAAGCAACTTCTGCC

>FM895026.1 FM895026 Jatropha curcas embryo 71-95 (DAF) Jatropha curcas cDNA clone rjcpga0_001361, mRNA sequence

ATTTAGAGACTTGTTTACCGTTTGAGGGTTGAGAATCGAATTGAAAGATGGCGTTGGAAAAGGCTAAGGA

GATTGTTTCTTCCAATTCCGTCGTCGTTTTTAGCAAAACTACTTGCCCTTTTTGCACAACCGTGAAGAAG

CTTTTTAATCAATTGGGAGCAGCTTTCAAGGCCATCGAATTGGATACTGAGAGTGATGGTAAAGAGATAC

AATCAGCTTTAGCAGAGTGGACTGGACAACGCACTGTGCCTAACGTTTTCATTGGGGGCAAGCACATTGG

TGGCTGTGATGCAACTACAGGCTTGCACGGTGAAGGAAAGCTGGTTCCTCTGCTTACTGAAGCTGGAGCT

CTTGCTGCTGCTGTTGCCAAGGCTTAAGCTATAAAGGAGTTGGACTACCTTGTTAATAAAAAATAACTAT

GTTTGTGTTTATTTTATGTTGGTAAAACTTTAAA

>FM895025.1 FM895025 Jatropha curcas embryo 71-95 (DAF) Jatropha curcas cDNA clone rjcpga0_001360, mRNA sequence

GGAAAGGATTCTATCCGTCGCCCGATGGGAATTGTACTTTAAGGCGGCCCTCGCGGCTCGTCCGCCGCGG

GGGCTTAGCCAACGGCACGTGCCTCTGGGGGCCGGATGGCCCCTACTGCGGGTCGGCAAACGGGCGACGG

ACACGCGCGTCGCTTCTGGCCCGGATTCTGACTTAGAGGCGTTCAGTCATAATCC

>FM895024.1 FM895024 Jatropha curcas embryo 71-95 (DAF) Jatropha curcas cDNA clone rjcpga0_001357, mRNA sequence

ATTCCCTTGATCTTCTGAGAAGGGTTCGAGTGAGAGCATGCCTGTCGGGACCCGAAAGATGGTGAACTAT

GCCTGAGCGGGGCGAAGCCAGAGGAAACTCTGGTGGAGGCCCGCAGCGATACTGACGTGCAAATCGTTCG

TCTGACTTGGGTATAGGGGCGAAAGACTAATCGAACCGTCTAGTAGCTGGTTCCCTCCGAAGTTTCCCTC

AGGATAGCTGGAGCTCGGGACGAGTTCTATCGGGTAAAGCCAATGATTAGAGGCATCGGGGGCGCAACGC

CCTCGACCTATTCTCAAACTTTAAATAGGTAGGAC

>FM895023.1 FM895023 Jatropha curcas embryo 71-95 (DAF) Jatropha curcas cDNA clone rjcpga0_001356, mRNA sequence

GAGGACAAACTCGATAATATTTTATTTTATTCTATTTGGCTTAAAAATCTTGCCCCTTTACAAATAACCA

AAAGCAAATCTCCATACTGGAAATCAGGAAGACATATATCATATTCTATTTTATTCCATTTTTTCTTGGT

CAACTTAATCTAGGCTGAAACCGGAAGACAGAAATCATATTCTTATAGTCTTGGATATGAACCAGGACAT

ATTTTATCCTTGGGTAAATCTAAACGACATTATGATTAATAAGGTCGTCTCCCAATATAATTACCAGGAG

GATCATAATTGCATCCAATGAAGGTTCCTCCATTAGTGCAGGTAACCTTAGCACACCCTAGGCGAACCGA

TTCGCGCCAGACTACTTGAGTATAGTGGCCACAAACCTCGCCAGGAACACAAGAATTAGAATTATAGTCG

TAATAAGTTTTCTCATCAACCCACA

>FM895022.1 FM895022 Jatropha curcas embryo 71-95 (DAF) Jatropha curcas cDNA clone rjcpga0_001355, mRNA sequence

CAAACTACTACAGCCTTATCAAACACAGTGAAGGAAGCTTTCAAGAGGAGCACAAGAAATCTACAAACAT

TGCACAGCAACAGGCCAACTAGTTCAAATCTGCTCCATATGTCTCAATACAACAACAAAATGTCTGGACA

CCAACTAAACCCAAACCCGAAAAAAGGGACAATCTTTTACAAACTCATGTAGTCACAAGCACGCCTCAGA

AGGGACAGCAGCCCAGCATCGAGTCTCTTCAATCTTCATTAATCATTTGCACAACTTGAGCCACTCCTGC

TGCTATTTCCTTCTGAATGACAGCATCAGGCATATCAAATGTCCGTCTTTTCATTGAAAATAATTTCCCA

GGAGGCTGAGGGTCCACCACATCAAGCATTGCCTCCAGCTCATCTACCATTGACCTTTTTGCACCTCTCA

CCATCAGATCAGCTCCCTCAATGGCATCAACTGTGAGGAGCAGGAC

>FM895021.1 FM895021 Jatropha curcas embryo 71-95 (DAF) Jatropha curcas cDNA clone rjcpga0_001354, mRNA sequence

ATTCCATCACTATTGCTTCTCTTGCTCTGAAATTCTCTGATTCTAATCCATTAATACTAGTTCTTTCAAA

GATTTCTTCTTTTTTCTCTTTGCTTTTTTAATCAAGTTTTAATCTTCACGAAAATGGTGAATGTTCTGCA

CTTCTTGTTTGGGGTTTTTGGAAATGCTACTGCTTTATTCCTCTTTTTGTCTCCAACGATTACTTTCAAG

AGAATAATAAAGAGTAAATCAACAGAGCAATTTTCAGGCGTCCCATATGTGATGACTTTGCTCAACTGTC

TCCTTTCTGCCTGGTATGGC

>FM895020.1 FM895020 Jatropha curcas embryo 71-95 (DAF) Jatropha curcas cDNA clone rjcpga0_001353, mRNA sequence

ACAACTCAATAATCTGTCTCAAACTTTTAGTCCAAAATTACCACCACACTTTACTATGTTGAATTTGATG

GTAGCTTCAATTATTTATAGTTTACCAGAAGAAGAAGAAGAAGAAGAAAGAGGAGGTGCAAGTGACACAG

AATTAGCAAAAGCTTAAGGAGTAAATATGAAGAAGCTATACAAGGATTTACAAATGTGCTAAATACAATT

TATGAACCCCATCTCTAACCTAATCTCAACCTCGCCAACCAACTCCAAGTTGGATTCTTCAGAAAAGAAA

AGCACTGATATTTTTCTAATTTGGCCTAAATATTTGCCAAATCAATTTCAGTGACCTTATGCAATGCAAC

GGAAACAGAGAGGAAACTACTCATCAAGATCTCTCTTTTTTTCTTTTTCTGGGTTTGGGATTGATACTAC

TAACCCAAACTCGTTGGGAGATGGAGATCTCAGACCATTCTTGTGCCTATCTAAGATGAGAATCATAAGT

GTCAAAAAGGGCATCTGGAACAAT

>FM895019.1 FM895019 Jatropha curcas embryo 71-95 (DAF) Jatropha curcas cDNA clone rjcpga0_001352, mRNA sequence

AAACTGTTTCAACTGTCGCCGTCGGATCTCTCTCTCCTTCACCATCACCGTCTGTTCTATCTTCTCTTAC

CTCTCTCTCTCTCTCTCTCTCTCTCTGTGTCTGTGTCTCTGCTGCTCACTAAACACTGCTTCCATGATTG

GTCGCGGTAAGCGACTGCACTCGATTAGTCGATCTGAAGTTGAAGTTGCGATCCGATGGAACCGTTGTCT

TTGCCGTTTTCTTGAATCTCCGGCTTGTTAAAGCGATGGAAGAGGAGAGTCAGGACAGCAAACTCAATAG

AATGAGTTGCAGCAGTGGCATGCCCGATGTCGTTTTAGAATGTGTGATGCCGTACATAGATGACCCACGA

GACCGCGACGCTGTTTCACTGGTTTGTCGTCGCTGGTATGAGCTTGACGCCTTAACGCGAAAGCATATAA

CGATCGCTTTATGCTATACC

>FM895018.1 FM895018 Jatropha curcas embryo 71-95 (DAF) Jatropha curcas cDNA clone rjcpga0_001351, mRNA sequence

TATTTCCAGTGTATATCCATGTAATACAACAAGGTCAGAGTCAATCAATTATTGGAGCTTCTTTTGTGTA

ACAGAAAAAAAAAACATGCATCATCATGATTGTCATTTGTTTGAATTCAATCATACAACGCATCCACATT

ACACTCCCCAAGGAAAACTTTGATACTGAGCGGTGAACCACAACTACCTTCAGCTATAATTGCTCTGTCA

TTGGCGGCCATGTGTGCAATAATCTTGTATATCATTTCAACATCTTCTTCTGCATGGCAACGTTCAGCTA

CTTCTTGAAGTGTCAATGGTTCAACAGGCTCTTTACAGCTTGCCTCATTAAGTACTGCCAAAACCCGCTT

CTGAAGGGCTAAAACTTCCCCTGCTGCCTTTTTCCCAGCTTCCACACCAGGTTGATGATAAGCATTGATA

TTAACTAGTGAGGCATATATCCCAACTGCTCGTTCATAAAGTGCAACAAGAGCCCCCACGGTCCT

>FM895017.1 FM895017 Jatropha curcas embryo 71-95 (DAF) Jatropha curcas cDNA clone rjcpga0_001350, mRNA sequence

ATTATTATTTATAATTTATATATATTTAAATCAACTAGCTATAATATACCCATTAAATTATCCAAACAAC

AAAACTAAGCAATGGGTCTATTGAGAGAAAAATCTCTAAATAAACACCGCTCAACAAAAATCCATTAACA

TTTTTAGTCCTCAAATAGCAAAATCCATAATTCAAAACACTTTCGAAAATAACAGACGCATAAAAAAAAA

GGATAACCTCGACTTCAACGGTAACTCTTCTGGAACCTGGCGCGAGCACCACGACCACCGAACTTCTTTG

GCTCGCATCGTCTGGGATCAGCTACAAGCAAAGTCCTATCATACCTGACCAAAATATCTTTAATTTCCTT

CTTGCTCTGCTCGTCGACGTACTTCTGATAGAAGGCGACAAGAGCTTTAGCGATACTTTGACGGATAGCA

TAAATCTGCGAGGTGTGACCACCAC

>FM895016.1 FM895016 Jatropha curcas embryo 71-95 (DAF) Jatropha curcas cDNA clone rjcpga0_001346, mRNA sequence

CAAGGATTCTATCCGTCGCCCGATGGGAATTGTACTTTAAGGCGGCCTCGCGGCTCGTCCGCCGCGGGGG

CTTGGCCAACGGCACGTGCCTCTGGGGGCCGGATGGCCCCTACTGCGGGTCG

>FM895015.1 FM895015 Jatropha curcas embryo 71-95 (DAF) Jatropha curcas cDNA clone rjcpga0_001345, mRNA sequence

ATAGCAATCTACAGCTATCACTGATGTTAAGGAATAAACAATGACACTAAAGAGTTTAGACAGCCAAATC

TCCTTTACAGTGTACTAATGGACAAATATACATCAGACGCTCTTAACATTTTCCTGCGTCAATTCCTATC

TGAAACCAGAAGGAAATAATACATTTGCACTTCAATGAATACAAATCATTCAATTGCACACGTTCATAAT

GATTAAAACCACTAACCTTTACTTTGAAGTTGTTCTTCATGCTCACAGATTAACGCGGGACAATTACACT

AGTCACCGCCGCCAATGCTCTTCTCTATGTTAGACTCATCATCCTCGTGGTCATCCTTCTCTGCAGGCAC

TCTGTCCTGCACAATCTCATTCCCAAAACCATCATCCTTGGACAACTCGTATTGTGCAACCACTTTGTCC

ACAACTACATTCCTCTCTTCACCCTTTGCTTCATCCTCTTTCACAACCAGTTTATCTTTAACTACAATCT

CACTCGCTCCATTTTTTATCTCGTCCACATCCTTCAGTTCTCCATGATTAGGCGCAACCTCTTCCATACA

AACATCATGGCTCTCCTTAGCACAATTGTTA

>FM895014.1 FM895014 Jatropha curcas embryo 71-95 (DAF) Jatropha curcas cDNA clone rjcpga0_001344, mRNA sequence

GTAGAGGTACAATCTCACTAAAAATTTATCGCTCTCGAATCTCTGTGAACTCCAAATCCAAAAGCGCAAT

GGCTCGATTAGTGTTGCTGCTCGCGCTCTGCGTGCTTCCGGCCATCGTGAGCGCCGCCCGCCCTGCAAGA

AATCCATTTGAGGTGGAAGGCCGTGTCTACTGCGACACTTGCCGTGCTGGTTTCGAAACCCCCAAAACCA

CCTACATCGCTGGTGCAAAGGTAAGATTGGAATGCAAGGACAGGAAATCAAT

>FM895013.1 FM895013 Jatropha curcas embryo 71-95 (DAF) Jatropha curcas cDNA clone rjcpga0_001343, mRNA sequence

TCTGATAGAAAATCTTAAGGCTCCCAGCTGCCGCCATGTCTGATGAAGAGCATCATTTTGAGTCTAAGGC

TGACGCTGGGGCTTCGAAGACTTACCCTCAACAAGCTGGAACTATAAGAAAGAACGGTTACATCGTCATT

AAAGCCCGTCCATGCAAGGTTGTTGAGGTCTCAACATCCAAAACTGGCAAGCATGGTCATGCCAAATGTC

ACTTTGTTGGAATTGACATCTTCAATGGCAAGAAACTTGAAGATATTGTTCCTTCTTCCCACAATTGTGA

TGTTCCCCATGTTACACGTACTGACTACCAGCTGATTGATATCTCTGAGGACGGATTTGTGAGTCTGCTG

ACTGAGAATGGCAATACCAAGGATGACCTGAGGCTTCCAACCG

>FM895012.1 FM895012 Jatropha curcas embryo 71-95 (DAF) Jatropha curcas cDNA clone rjcpga0_001342, mRNA sequence

CCTAAAAACACCAGTTAAGCTCTTTTTCTGCTAATTTCAGCTCCCTAATCTTTATAATTAAGCTAAAACC

CAAGTTTCTGTAGATTAAATCTCACTGTAAAATTTATCTTCTAGCTCCTGCTTAGCTCCTCAAAACCCAG

AAATTTCTTTCTTTTTTTTCGTTTTTGTGTGTGTTTTTTAAGATTACTATTTTTTTTTCTTTACTCTGTC

TTCTGTGTTCTAAATTTTAAGCTTGTTTTACTAACAGTGCTTGGATTTTTCTGTTATTTTTTTTTCTCAA

AAATTGAACTTTGTTTTAGATGATTTAAGCTGAAAACAAAATGGTGTTTTAGTAACAGTTTAGAAAATAA

AATCTATAGGGTTTAAGCATGCAAGCAAGAGAAACTGTTAGTAGTAGCAGTGGAGGTGGGGTTACAGTGG

TGGGATCCGATGTTCCTTCCGACTACCAAATCGCTCCAAGGTCGGCTGACAACCCTAGCCCTGCCACTGG

ATCAGCTCCAAC

>FM895011.1 FM895011 Jatropha curcas embryo 71-95 (DAF) Jatropha curcas cDNA clone rjcpga0_001341, mRNA sequence

ATTCCTGCTCTCTCTACTTCTTCTCTTTGTGGTTCGATCCGGGAGAAACTCAGAATCATGTTGGTCTACC

AGGATTTGCTTACCGGTGATGAGCTTCTCTCGGATTCGTTCCCTTACAAGGAAATTGAGAATGGCATGCT

GTGGGAAGTTGAGGGAAAGTGGGTTGTTCAAGGAGCCGTTGATGTGAACATTGGTGCAAATCCCTCTGCT

GAAGGGGCGGATGAAGATGAGGGTGTTGATGACCAGACTGTTAAAGTTGTTGACGTTGTGGACACTTTTA

GGCTCCAGGAGCAACCTGCTTTTGACAAGAAGCAGTTT

>FM895010.1 FM895010 Jatropha curcas embryo 71-95 (DAF) Jatropha curcas cDNA clone rjcpga0_001340, mRNA sequence

TGACTTCAGAGTTCCAACTACTCTTCTCTCTAATGGCATTCTTGTGTCATTTCCAGCATTTCTAACGCCT

TATCAGAGCCTTAGAACCTTCTTCTCCAATGAGCCGCTCAATCATGGCTCCTTGCAAGCTCAATTCCACT

GATTCTTATGGAAATGGAGATGTGAGTTTTTCTTGTTCACAGTTAAATAATTCACTTCCTATCTTACATT

CTGAGAAGGCAGCACAGAATGGAAATGGAGATGTGAGTTTTTCATGTTCACAGCTAGATAATTCACTTTC

TGAGAAGGAAGTGCAGGAGCTTCTTCAACAGACTCCTATTCAGGGAACTGATGAACATCTTATAGAGTTC

TCAGAGGCACTAAGAACTGTTGCAAAGACATTGAGACGAGTGGCTGAAGGAAAAGCTTTTGCTCAAGCTG

AGGCTGCTGAATGGAAGCGTAGATATGAATTGGAGAGGGAAAGAAATCAGCGGCTGCAGCGTAAAGAACA

TTTACCCAGGGAGTGCAATGGTGATTGTAACAAAGAGAGGACATTGAACTCAAAAAACCTGTTAGTTTCG

AGTAATGGCACAAATGGACAATCTGAA

>FM895009.1 FM895009 Jatropha curcas embryo 71-95 (DAF) Jatropha curcas cDNA clone rjcpga0_001338, mRNA sequence

TAGTGGATCCCCCGAGGCTGCAGGCAAGCTTTGTTCTCTCCACTCTTCCTTTATCTTTCTTTCTATTTGC

CAAATTCATCGACCACCATCAGCACCACCACCGGAGCTGCCATCGCCATGTGCGATCCGCCGTCGTTTGA

CTGAGCTTTTCTCATTGATAGCAATTTTTGCTTTCCTTCCCTGAATTAAGTGCGGTGTCGTATATTTGTA

TCCGAAAGTAATGGCGATTGAAGAAGACTTAGAGAGTTGTGGAAGCAGAGCAGTGGAGTCGTATTCACAG

GCGAATCCACGACACCATATGCAAAAGCTGGAGGTGTATAACGAAGTGCTTAGGCGAATTCAAGATTTTA

ATTATGAGGAAGCGAATCTTCCTGGCTTCGATGATCAAGCTTTGGCTCCATTTCAATCGATTACCTGCAA

GATATGCATTGGATGTGAATGTGGAGAGAGCAGAAGATGTGCTTACCCATAAGAGATTGCTGCAGTTGGC

AGAAGATCCTGCTAATCGACCAGCATTTGAAGTTCGTCTTGTCCA

>FM895008.1 FM895008 Jatropha curcas embryo 71-95 (DAF) Jatropha curcas cDNA clone rjcpga0_001337, mRNA sequence

TTGACACTGTTTTGCAACCCTTAATCCAAAGGCTTCCACAACTAAAGGTTGTGATGGAGCATATCACTAC

AATGGATGCTGTTAGGTTTGTCGAGTCTTGTGATAATGGATCTGTGGCAGCAACTGTTACTCCTCAGCAT

CTTGTTCTGAATAGAAATGCTCTATTCCAAGGAGGACTGCAACCACACAGTTATTGCCTTCCAGTACTCA

AAAGAGAAACCCACAGACAAGCAATTGTTTCAGCTGTTACTAGTGGAAGTAAAAGATTTTTTCTTGGGAC

CGATAGTGCTCCTCATGAAAGAAAGAGAAAGGAATGTCCTTGTGGATGTGCTGGAATTTATAATGCCCCT

GTTGCCTTGTCACTATATACCAAGGTTTTTGAAGAGGCTGGCGCACTTGATAAGTTAGAGGCATTTACAA

GCTTTAACGGACCAGACTTTTACGGACTTCCAAGGAACATATCAAAGATCAAGTTGACCAAGGCCCAGTG

GAAGGTGCCAGAGTCTTTCTCATTTCCGTTCGGAGATATCGTTC

>FM895007.1 FM895007 Jatropha curcas embryo 71-95 (DAF) Jatropha curcas cDNA clone rjcpga0_001336, mRNA sequence

CCACACGAGATTTCTGTTCTCGTTGAGCTCATCTTAGGACACCTGCGTTATCTTTTAACAGATGTGCCGC

CCCAGCCAAACTCCCCACCTGACAATGTCTTCCGCCCGGATCGGCCGCCGAAACGACCTTGGGTCCAAAA

AGAGGGGCATAGCCCCGCCTCCGATTCACGGAATAAGTAAAATAACGTTAAAAGTAGTGGTATTTCACCG

TCGCCTTTCGGCTCCCACTTATCCTACACCTCTCAAGTCATTTCACAAAGTCGGACTAGAGTCAAGCTCA

ACAGGGTCTTCTTTCCCCGCTGATTCCGCCAAGCCCGTTCCCTTGGCTGTGGTTTCGCTGGATAGTAGAC

AGGGACAGTGGGAATCTC

>FM895006.1 FM895006 Jatropha curcas embryo 71-95 (DAF) Jatropha curcas cDNA clone rjcpga0_001335, mRNA sequence

CGTTGAAAAATTTGGAACACTGGATATTCTGGATCAACAATGCTGGGTTTATCAGTCCACCCTGTTCAGA

CATACGAAATGTAAATTTATCACATTTCGAACAGGTGTTTGATGTAAATGTGAATGGTGCATTTATTGGA

ATGAAGCATGCACCACGGATTATGATCCCGCTAAATAACGGTTCGATTGTTTCTATCTGCAGTGTCAGCG

GTGTAATATGAGGCCTGAGTCCCCATGCATATACAGGCTCCAAGCATGCTCTTTTGGGGCTGACCAGAAA

TGTTGCAGCTGAACTACGAAAATATGGGATACGTGTCAACTGTGTTTCTCCTTATGCAGTTCTAACCGAC

CTGTGCCTTAGCTCACTTGCCCGAGGACGACGAGAACAGAAGATGCAATGGCAGGTTTCCGAGCCTTTGC

TGCAAAGAATGCTAATTTGCCAGGGGTGGAATTGACGCCTAATGATGTGGCTAATGTTGTACTCTTCTTG

GCAAGTGAAGAAGCAAGGTACATAACTGGGGCTAATCTAATGGTCGAC

>FM895005.1 FM895005 Jatropha curcas embryo 71-95 (DAF) Jatropha curcas cDNA clone rjcpga0_001334, mRNA sequence

TTTTTTGATCAGTAGATGGAAATTTCTTATTAGAGAAGGAGAAATTATACAATAGTTTCATATTTATGTC

AAATTATTTCTCAAGTGCCATACTTTTTTTATTATTTATCATAAGGTTCACAACAATAGAAACTACTCTT

CCATGTTAATAATGCACCTCAAGCCTTCCCCTTTCAGCATCAAGTCAAAAGCCTTATTGATGTCAGAAAA

AGGAACCCTATGAGTTATAAACTTGTCCAATTCCAGTTTCTTGTTCATATACATGTCAACAACAGATGGG

AGATCGGTTCGCGGTTTATAGTTCCCAAAGAAAGTTCCTTTTAGAGTTCTTTCATTAAGCAAATTGATTG

GCTTGGTCATAAAAACTGCTTCTTTGTTCGGTACCCCGACGAGCACAGCCACCCGCCATCCATCATGAAC

ACATTCAAAAGCTGCAACCATGGCGTCAATGTGCCCTGTGCACTCCCCACTCCTGTCTACTCCACCAATT

GTAATCTACTGCAATTATCTGGTTGAACTGGACGGTCATGATCCTTCGGGT

>FM895004.1 FM895004 Jatropha curcas embryo 71-95 (DAF) Jatropha curcas cDNA clone rjcpga0_001332, mRNA sequence

ATTCAACAGAATACGATACACAGGAACAACTACCAGACCCATTTTCTCTCTCTTTTTCCCTTATAACCCA

TTGTTTTCTTGTTTTTCTTCGTCAGACAAGCATGGTGGCTCTTGAAATAGGTCTATCAAAAGCCATGAAC

GCAAGAATCATCGGCTCCGGTAGCGAAGTCCTGGTTCTTGCACATGGGTATGGAGGAGATCAGAGTGCTT

GGGATAAAATAGTTCCAAACTTGTCTATGCATTTCAGGATTCTGGTTTTTGACTGGATATTTTCTAGTGC

TGTTACAGAAAATCAAGAAGGAGAAGGGCTTTTTGACCCTGAAAAGTACTCTTCTTTTGATGCTTTTGCT

GACGACTTAATTTCTCTTATAGAAGAGATGAACCTTAAATCACCACTAGTTTTTGTTGGTCATTCCATGT

CTGGTATGATTGGTTGCATTGCTTCCATTAAGAGGCCTGACCTTTTTCAGAGATTGATACTTATTGCAGC

TTCTCCCAGGTACATAAATGCAGATAATTATGAAGGAGGGTTCAAGAGCT

>FM895003.1 FM895003 Jatropha curcas embryo 71-95 (DAF) Jatropha curcas cDNA clone rjcpga0_001331, mRNA sequence

AGGATTCTATCCGTCGCCCGGTGGGAATTGTACTTTAAGGCGGCCCTCGCGGCTCGTCCGCCGCGGGGGC

TTAGCCAACGGCACGTGCCTCTGGGGGCCGGATGGCCCCTACTGCGGGTCGGCAAACGGGCGACGGACAC

GCGCGTCGCTTCTGGCCCGGATTCTGACTTAGAGGCGTTCAGTCATAATCCAGCGCACGGTAGCTTCGCG

CCACTGGCTTTTCAACCAAGCGCGATGACCAATTGTGCGAATCAACGGTTCCTCTCGTACTAGGTTGAAT

TACCATCGCGACACCATCATCAGTAGGGTAAAACTAACCTGTCTCACGACGGTCTAATCCCAGCTCACGT

TCCCTATTGGTGGGTGAACAATCCAACACTTGGTGAATTCTGCTTCACAATGATAGGAAGAGCCGACATC

GAAGGATCAAAAAGCAACGTCGCTATGAACGCTTGGCTGCCACAAGCCAGTTATCC

>FM895002.1 FM895002 Jatropha curcas embryo 71-95 (DAF) Jatropha curcas cDNA clone rjcpga0_001330, mRNA sequence

GGTGCCATCAAATAATCCAAGAAAATTCATTTGGCTTTCAACAATTAAACAGAAGTTCTATAATTTCACA

CTTTCACAAGTACCTTAATCATTATTTGTCCGTTTCAATTAGTTTTCACATAAAAATAGTCATCTCAAAC

TGAAAAAATACACATCCAACATATATTTTGAACAAAAATGAGAAGCATATTTATTTTCGATGATCCATTC

AGGCGCAAAGAGGTGAGATGTGAGAAAGGGGACCCCAGAGTATGATTTACATCTCCAACAACAAACACAG

ACGATTCAGGGAATAGTGACAACTTCTTCTCACTAATTTGGCAACATCTTTCTTTCTCTGAAATGTGTTG

AGAAATTAACTCTCAGCAGTGGATCTTTTCTTGGTGGTGTAAGGGTAGTATTCCTTGCCATTGTCTATTC

TCGTGGTCTGACATCCAGCATCAATAACTGCAGAAGTCACTGCCAGTGCTGCTCCAGCAGAGATAGCTGT

TGAGATGCTCCTCCCTTTGAGCCCCAGAATTGAAGCCCCT

>FM895001.1 FM895001 Jatropha curcas embryo 71-95 (DAF) Jatropha curcas cDNA clone rjcpga0_001329, mRNA sequence

CTATGGATCCCCCGGGCTGCAGGTAAAAGAGAGATCGGAAATTTCTGTGAGCTAAAACCATGGCTACCAT

TGTTGTTCCTGCCAATGTTTCTTATGTTGAAGATGCTGAGACTCTCA

>FM895000.1 FM895000 Jatropha curcas embryo 71-95 (DAF) Jatropha curcas cDNA clone rjcpga0_001328, mRNA sequence

TTTATTTCCTTAAATGCATAAAAAATCGAAGGGGAAATGAAAAAGAGAACCATCTCTCCTGCGACGCATA

GCTTCTTTGTCTCTTAAATTTTGTGTTTCTGTAGCAGAAAAATTGTCATACATTCTTAATTTAGGTAATT

TATATAGGAATAGCCATTGAGGCAACCATGCGAATTTAAGTTATTCAAGATGCTTACAAACCCTAATTCT

GGATTAGGGTTATAAGCCAATGGATAGAGTGATAACTTACATATACATTGGCTAAATACAATGTAAAAGA

GCCTGAGGAATAAGAGTTTTACTCCCCAAAAAAAAAGGCAATATTGTAGCGATCTGTTCACTGGTCCTGG

CTCAGCAGATTGGTGAAGAAAGGCACTGAGAATGGAAAACAGGCATATATAGCAGGAAACAAGAAGAAAA

GAGGATCCTATACTGCACCAGCCAGCTCAGATGTTAGTTTTTTTGCTCTGTTTTTACCCGTTTATGGTTC

GTTAGTTGTTCAAGCCCTGCTGTGTAACCCTATTAGTTTGGTGAA

>FM894999.1 FM894999 Jatropha curcas embryo 71-95 (DAF) Jatropha curcas cDNA clone rjcpga0_001327, mRNA sequence

ATTCCTTTTGACTGCCCTTGCTGTTTTTGGACATGGTCCAACACTAGATGAAGCAAGTAGGAGATTTCAT

GCTTTTGTAGAAGACAGAAATACTCTACTCCTTCCACCTGATATAAGAAAGGCAGCATATGTGGCAGTAA

TGCAGAGAGTTAGTGCATCAAATAGATCAGATTATGAATCTCTTCTAAGAGTCTATAGAGAGACTGATCT

AAGCCAAGAGAAAACACGCATTCTGGGTTCTTTGGCATCTTGTCCGGATCCCAGCATAGTTCTTGAAGTT

CTCAACTTTGTGTTGTCCTCTGAGGTTCGCAGTCAAGATGCTG

>FM894998.1 FM894998 Jatropha curcas embryo 71-95 (DAF) Jatropha curcas cDNA clone rjcpga0_001324, mRNA sequence

ATTCCAAAGGATTCTATCCGTCGCCCGATGGGAATTGTACTTTAAGGCGGCCCTCGCGGCTCGTCCGCCG

CGGGGGCTTGGCCAACGGCACGTGCCTCTGGGGGCCGGATGGCCCCTACTGCGGGTCGGCAAACGGGCGA

CGGACACGCGCGTCGCTTCTGGCCCGGATTCTGACTTAGAGGCGTTCAGTCATAATCCAGCGCACGGTAG

CTTCGCGCCACTGGCTTTTCAACCAAGCGCGATGACCAATTGTGCGAATCAACGGTTCCTCTCGTACTAG

GTTGAATTACCATCGCGACACCATCATCAGTAGGGTAAAACTAACCTGTCTCACGACGGTCTAAACCCAG

CTCACGTTCCCTATTGGTGGGTGAACAATCCAACACTTGGTGAATTCTGCTTCACAATGATAGGAAGAGC

CGACATCGAAGGATCAAAAAGCAACGTCGCTATGAACGCTTGGCTGCCACAAGCCAGTTATCCCTGTGGT

AACTTTTCTGACACCTCTAGCTTCAAATTCCGAAG

>FM894997.1 FM894997 Jatropha curcas embryo 71-95 (DAF) Jatropha curcas cDNA clone rjcpga0_001323, mRNA sequence

ATTCCTCAAAACCCTAAACCAGCAAAACCTGTTCTTTGACTCTGACGCCGCCACTCTTTCCTCTCAACGT

GCAGTACTACGAATTCGTTTCTTCGATAAAGATTCTAGCTGCGTTATCTCGCTAAAGGCCAGGCCTTTAC

TAATCAATGGTATAAGTCGCTTTGAGGAAGATGAAGAGGAAATTGATCCGTTAATTGGTCGTGAATGCCT

GGCCGATCCATTAAAGTTAAGTTCGATCGAATCGAGGGTTTTGAGGAGGTGTAGAGATGAGTTTGGAGTT

TTGGGTGAAATGAGTTTTGTGTGTTTGGGTGGATTTGAGAATGTTAGAGAAGTTTATGAATGGAGAGGAT

TGAAACTAGAGGTGGATGAGACTAAGTATAGCTTTGGGGTTTGTTATGAGGTGGAGTGTGAGACTGCTGA

TCCTGAAGAGGCTAAGAGAGAGCTGGAAGGGTTTTTAAAGGAGAATGGGATTGATTATAAGTATTCAGAG

ATGTCAAAGTTTGCAGTTTTTCGAT

>FM894996.1 FM894996 Jatropha curcas embryo 71-95 (DAF) Jatropha curcas cDNA clone rjcpga0_001321, mRNA sequence

CCTTTTGATGCTAACCCTTGTACAATATTATTAAACATTACAATAACATTGTTGAGTAAAAAAAGAAAAT

ATAAAAGAAAAGGAAGCTGCTTGCTAATTTGACATTGTTTACAATTTCAATGCTGCCAAGTTTAGAGTCC

CAGAAGGCTTTCAACATGGCAACATATGTGATTCATTTTATGCAGGCAGCGTCCCGACAAGGACGATAAC

GAAATCAGCAAAGTCAGAATAATTAAAATTATAAAAATGAGTCTTCGACTGGAAAAACCAAGCACACATT

TTGGTGACTCCTCTGAACTTGGTAATTTAGTTTTCTTTTTCTGTGAAGGATACTTGTTGATGTTGCCTTC

AGATGCCTCAGGTATTTGCGTCAACTTAATTCTTCTGTTTGCTGTCCCTTGGATAGGAATCGACTGCAAA

GGAGGTGCACCTGAAACAACTAATTCCATAGGCTGTTGTTGAGCTTGTGTCTGAACCTCAGGCTTATTGG

CAAGCGTTGGTAGGAGCTCAGGAATGGCAGTCAACGGAGTTTCGTCTAGGTTAACTATATCATCATTTAG

TATATCAGCA

>FM894995.1 FM894995 Jatropha curcas embryo 71-95 (DAF) Jatropha curcas cDNA clone rjcpga0_001319, mRNA sequence

CTCTTCTGTCTCAGCTTTTAACACATAGATACACAGTTGTCAGTGATAAACGAGAATCTGAACTAGATAA

AAGTTGAAAACTCTAGCCGTGTAATCGATTATTAGAGACCAGAACAGGTAAGCAAACAACAAATTTAAAG

AGGGATATGGGATCTGGAGCTGGAAGCTTCTTGAAGGTGCTTCTTAAGAACTTCGATGTTCTTGCTGGGC

CTGTGGTTAGTCTTGTTTATCCTCTATATGCCTCAATTAGGGCAATTGAAACCAAGTCTCCCATTGATGA

TCAGCAATGGCTTACTTACTGGGTCCTCTATTCAATGATTACGCTCTTTGAGCTCACTTTTGCTAAAGTT

ATTGAATGGATACCAATCTGGTCATATGCAAAGCTTATCATGACATGCTGGTTAGTGATCCCATACTTCA

GTGGCGCTGCTTATGTTTATGAGCACTTTGTGAGACCTCTCTTTGTTAACCCAAAACAAACCGTTAATAT

CTGGTACGTC

>FM894994.1 FM894994 Jatropha curcas embryo 71-95 (DAF) Jatropha curcas cDNA clone rjcpga0_001318, mRNA sequence

AAAAACGCAGATTGATATAACAAACTAAAAAAGGGCAAAAAAATAAAAATAAAAAGAAAGAAAAAAGAAA

GCTTGGTACGTTCACAATTCATAGCCAAGACAAGAATACCTAAATCTCAGAATAGCAAGCAAATTAGCAA

CAAACACCTTCTCCCATTAAAAACATAGAGCGGACAAGTTCGATCCTAATTTGCAGTGTAGCAGACTGCC

TCACTGCTGTTCTTCAGTGGGTTTAGCTGCTTCTTTAATCTCATCAGCTCCATCATCCTGCATGTCTGAG

GTCCACAAAGTGAGATTGTCACGGAGAAGTTGCATGATCAAAGTGCTGTCCTTGTATGACTCCTCACCCA

GGGTATCCAACTCAGCAATTGCCTCATCAAAAGCCTGTTTAGCAAGATTGCATGCGCGATCAGGAGAATT

CAGAATCTCATAATAAAACACAGAAAAGTTCAGAGCTAGCCCTAGACGGATTGGGTGAGTTGGAGCCAGT

TCTGCATTTGCTATATCCTGAGCAGAT

>FM894993.1 FM894993 Jatropha curcas embryo 71-95 (DAF) Jatropha curcas cDNA clone rjcpga0_001317, mRNA sequence

AAGTAATAAAAAATCAAATATTATGGTATTATTAGCTAGCAAGAAAAATAGTTTCCTGTTGGAGTTTACA

AGACAATAATAATACATGAACAGTCCTAATTTATTGTCAAATGTCAACTGAATGCCCACCAAGAAGCCAA

TTTTTCTGCTCTCTCACTTCCATTACAGGGACCCTCCACAGTTGCCATATTTCAGGAGAACCAAGGGCGC

TTCCCTNATCGAAAAACTGCAAACTTTGACATCTCTGAATCCTTATAATCGAATCCCATTCTCCTTTAAA

AACCCTTCCAGCTCTATCTTAGCCTCTTCAGGATCAGCAGTCTCCCACTCCACCTCATAACAAACCCCAA

AGCTATACTTAGTCTCATCCAACTCTAGTTTCAATCCTCTCCATTCATAAACTTCTCTAACATTCTCAAA

TCCACCCAAACACACAAAAATCATTTCACCCAAAACTCCAAAATCGTCTCTACACCTCCTCAAAACCCTC

GATTCGATCGAACTTAACTTT

>FM894992.1 FM894992 Jatropha curcas embryo 71-95 (DAF) Jatropha curcas cDNA clone rjcpga0_001316, mRNA sequence

CGAATTCGTTGTTCGTATCCAGAGGCAAAGGAGGATTCTGAAGCAACGCCTGAAGGTTCCTCCTGCCGTG

AATCAGTTTACAAAGACACTTGACAAGAATCTTGCAACCCAACTCTTTAAGTTGTTGCTCAAATATAGAC

CTGAGGATAAGGCAGCAAAGAAGGAAAGGCTTCTTCAGAGAGCACAAGCTGAAGCTGAGGGGAAGACTGT

GGAATCAAAGAAGCCCATTGTTGTTAAATATGGGCTTAACCATGTCACTTACCTCATTGAGCAGAACAAG

GCTCAATTGGTGGTCATTGCACATGATGTCGATCCTATTGAGCTTGTTGTTTGGTTACCTGCCTTGTGCC

GGAAAATGGAGGTCCCTTACGCAATTGTGAAGGGCAAATCACGTCTTGGAGCGATTGTCCACAAGAAAAC

AGCTGCAGCTTTATGCCTCACATCTGTTAAGAATGAAGATAAATTGGAATTTAGCAAGATTTTGGAGGCA

GT

>FM894991.1 FM894991 Jatropha curcas embryo 71-95 (DAF) Jatropha curcas cDNA clone rjcpga0_001315, mRNA sequence

AAACCAAACAATACAAATACAAGTTAAATCTAATTTTATAACTGAATCCAACATAACATCTTAAAGACAC

AATATTAAGTCTAAATGGACAACATACAACTCGCATTTTATTGGATGAGAACATGTTATCAAAAGATAAA

ACATATTGATTAGCTTAGTATCCCAACAAAGCAACAGCATTTGGAAGATTAGCATTGATTTGGTGCTTCT

GAACCAGCATGCTCTCTTTTGCGACTTGATTTGTATTTTCAAATGCTTGGCTTCACGTTCAGACGAGACG

CTAGCTTCTAACCCAGACATCTGTCGCACTGACTAAAGTAGTTGATCCAAATACTACAAATCTCATGTGT

GACACGACGATCAGATAAGCAACCAACAATCCGATTGAGGAATCGCTCTTGCCTGTCAGGAGCCCAGGAT

CGATATCTCTCTCCAGGTTGCTTGAAATTGTTCTCCTTTGGTATCACACACTTCTCACGCTTTCCACTGC

ATATAGTGGCCGGAAAGGGGAACGCCTCAGAATGGCGAA

>FM894990.1 FM894990 Jatropha curcas embryo 71-95 (DAF) Jatropha curcas cDNA clone rjcpga0_001313, mRNA sequence

CGAATCCTCTAAAGCTCTATACCATTTTTCACTACTCAAACTCTCACTCTATTTACTGACTGATAATGTT

GTTTTTTATTTCTGGGTGGGATTATGTGAGTGCAGTTAATTCTTCCGGCGAGATTTGGCCGGAGAATGGC

ATATATGTGCGCAGACAGCGGAAATCTAATGGCAATAGCCCAACAAGTGATCAAGCAAAAGCAGCAGCAA

GAGCAACAACAGCAACACCATCACCACCAGCAACAACTTTTAGGCCTAAATTCTTTCCCTTTGAATCCTT

GGCCTGCTTCAATTTCTGCAAATCCTAATCTTGGTTATGCTGGGCTTACCGGTAGTGGTTTCACAGAGCC

GTTTCAGGTTTCCGGCGGCGGAGATACCGGAGAACCGGGGTTTCAGTTTCCACAGATGGAGCATCACTCT

AGTGG

>FM894989.1 FM894989 Jatropha curcas embryo 71-95 (DAF) Jatropha curcas cDNA clone rjcpga0_001312, mRNA sequence

CCGGAGCAGAAGGAATTTAATGAATATATGGAAATCATTTTTCTGTAATTACAAAATTTCTCCTCTTGGG

CCTTAAAATAGAACCCATTAGATGAGAGTAAAGACGAGTTCAGCATAATATCCCATCAATACAACGAGCA

TCACAATTCACTAGTGTTCAATATCGCAAAGTTGAGTGCAACTGTAAAATCCGCCTGATATTAAACACTA

AAGCCTATCAGCAAAACCTCTTGCAGTGGCAATTATTGAGAAGAGAACTACTAAATCAACCAGATTAGGT

GCAAGCAACTTAATGTTGCTTCACTAAGAACTCGACTGCAACTTCAGGTTGAATCTTACATTATTTAGTT

TGAGCACCAGACCCTGTCCTCTCGTGCAATCGACAAATCAGATTAGACTTGGACATCTGGGGTTCTCTTT

CTAGCACGGCAATTACATCCTTGACAGAAATGGTGCGTGCTACACTAATTTGCATTACTTGGTTCTTCCC

CAAATTCCTGGCAGCCCTTGATGC

>FM894988.1 FM894988 Jatropha curcas embryo 71-95 (DAF) Jatropha curcas cDNA clone rjcpga0_001310, mRNA sequence

ATTCTTCTGGTTTGTGTAGATAATCATATACGCTTCCACCAGACATAAATTCTGTTACAATGCACAAACT

CGGAGGGTATTTTTAAGCTTCCATCCCTGCTTAAAGTAGCAATAGACGTTTCCAAAGGAATGAACTACTT

GCACCAAAATAATATAATCCACAGGGATTTGAAGGCTGCAAACCTTCTCATGGATGAAAATGAAGTGGTT

AAGGTTGCTGATTTTGGGGTTGCGAGAGTAAAGGCTCAATCTGGAGTTATGACAGCTGAAACTGGGACCT

ATAGATGGATGGCTCCTGAGGTCATAGAACACAAACCATATGACCACAAGGCTGATGTTTTTAGTTTCGG

GATTGTGCTGTGGGAGTTGTTAACTGGGAAGCTTCCATACGAGTATTTAACCCCATTACAAGCAGCTGTT

GGTGTGGTCCAAAAGGGACTACGACCTACAATTCCAAAGAACACTAATTCAAAACTGGCTGAGCTGCTTG

AGAGATGTTGGCAGCAAGATCCAGCACTACGACCTGATTTTGCCGAGATTATAGAGATCTTGCGGCAAAT

AATGAAAGAGGTTGGAGATGAAAGCGATGGTCGCAAGGACAAATCATCTGGAGGATTCCTCTCTGTCCTT

AG

>FM894987.1 FM894987 Jatropha curcas embryo 71-95 (DAF) Jatropha curcas cDNA clone rjcpga0_001308, mRNA sequence

CAAAGGATTCTATCCGTCGCCCGATGGGAATTGTACTTTAAGGCGGCCCTCGCGGCTCGTCCGCCGCGGG

GGCTTGGCCAACGGCACGTGCCTCTGGGGGCCGGATGGCCCCTACTGCGGGTCGGCAAACGGGCGACGGA

CACGCGCGTCGCTTCTGGCCCGGATTCTGACTTAGAGGCGTTCAGTCATAATCCAGCGCACGGTAGCTTC

GCGCCACTGGCTTTTCAACCAAGCGCGATGACCAATTGTGCGAATCAACGGTTCCTCTCGTACTAGGTTG

AATTACCATCGCGACACCATCATCAGTAGGGTAAAACTAACCTGTCTCACGACGGTCTAACCCCAGCTCA

CGTTCCCTATTGGTGGGTGAACAATCCAACACTTGGTGAATTCTGCTTCACAATGATAGGAAGAGCCGAC

ATCGAAGGATCAAAAAGCAACGTCGCTATGAACGCTTGGCTGCCACAAGCCAGTTATCCCTGTGGTAACT

TTT

>FM894986.1 FM894986 Jatropha curcas embryo 71-95 (DAF) Jatropha curcas cDNA clone rjcpga0_001305, mRNA sequence

CAGCCTTTGACTGTTCGTTTTGCGGATCCCAAAAAACGCAGGACTGGAGAATTTAGGGGAAATTATGGAT

TTGGTGGACAAAACTTTGGTCCATGTTCCCAGGAACCAATGATGAGGCCAGCACCCAATTTTGGTGACTC

CATAGGAG

>FM894985.1 FM894985 Jatropha curcas embryo 71-95 (DAF) Jatropha curcas cDNA clone rjcpga0_001304, mRNA sequence

TTTTTTTGATCATGGTCTTTCATTAATTAATTAATTTTTGGACTTCAAACAGACAATGAACAATAGGAAG

AAAGAGCAAAAATGACAGCAGTATACATATATATATATTACAAAATACAAATTATGACAGCAGAATTCGT

CGTCCACGAGAACTTGCTATCTGCAGCCTTTCAGCAGATAGGAAGAGGGGCTAGAGAAGCTCATAGCAGA

TGAAGATTTCCCTCTTTCTGTTCCCTTCCCAGGTGTCTCACATTCACTGAAGTCTAGGGTACGGCATTCC

GTCAAAGCCAA

>FM894984.1 FM894984 Jatropha curcas embryo 71-95 (DAF) Jatropha curcas cDNA clone rjcpga0_001303, mRNA sequence

ATTCGTGAAAGGGCCTGTATTGGTGGAAGACACGTGTCTCTGCTACAATGCTCTAAAGGGACTTCCAGGG

NCCTTACATGTAAGTTTGTATTCTCTTTTTTATTCCATTCGCGAAGTAAATTCTGTCTTTCTTAAGTTGT

GTTCAGGTCCCTTTTTGATTCTAATGTTTTGCATAGAACGTTGATTGT

>FM894983.1 FM894983 Jatropha curcas embryo 71-95 (DAF) Jatropha curcas cDNA clone rjcpga0_001302, mRNA sequence

AGGATCTATCCGTCGCCCGATGGGAATTGTACTTTAAGGCGGCCCTCGCGGCTCGTCCGCCGCGGGGGCT

TGGCCAACGGCACGTGCCTCTGGGGGCCGGATGGCCCCTACTGCGGGTCGGCAAACGGGCGACGGACACG

CGCGTCGCTTCTGGCCCGGATTCTGACTTAGAGGCGTTCAGTCATAATCCAGCGCACGGTAGCTTCGCGC

CACTGGCTTTTCAACCAAGCGCGATGACCAATTGTGCGAATCAACGGTTCCTCTCGTACTAGGTTGAATT

ACCATCGCGACACCATCATCAGTAGGGTAAAACTAACCTGTCTCACGACGGTCTAACCCCAGCTCACGTT

CCCTATTGGTGGGTGAACAATCCAACACTTGGTGAATTCTGCTTCACAATGATAGGAAGAGCCGACATCG

AAGGATCAAAAAGCAACGTCGCTATGAACGCTTGGCTGCCACAAGCCAGTTATCCCTGTGGTAACTTTTC

TGA

>FM894982.1 FM894982 Jatropha curcas embryo 71-95 (DAF) Jatropha curcas cDNA clone rjcpga0_001301, mRNA sequence

GAAGAGAACATTGCATACTAGGAGAATCCATTAATAAAAGATCATAACCCTCTCAATCACATATGGAGGT

TTAAATTTAAACTCCATCCCTAATCTAATTTAAAAATTAAAAAAAAGCAATAACCCTCTCAATTACTTCA

TCCTCACCATCAAGAAGTAATTAACTATTGTAAAATAAATATTATTATCATCCATAAAAATACTTAATAA

TTATAAGAGTGCACTTTAGAGATCTAAAATGCCATTAACAAATAGAGAATCAAACGTCTCCATTTCATAC

TTCTGCAAAACTAAGCCAATCTCAACACCACCACTTTCATCTTTGCTCTCTACCATAGAAATTACACCAC

TTCGATCTATAGATACTATCTCCACCTTTTTTGGCCTCCCCCATCCAAAATCTATATCGTAAAGCTTGAA

CTTAGGTGAACCAGCAACTCCAATCGCTTCTAATTTACTCAACTCCATAGGCTTCAAAGAGTTTGCAAGC

TTAGCTCCCTTAAGAGTTCCTTTCTCTAACCCTTGTATTTTATCGTCGAGCTTCTTAACAATAAAGTTAA

TCCCATATCTCTATCAAAAGAGGTCCTGCTTCTAATAGTATAACATTTCCGATAA

>FM894981.1 FM894981 Jatropha curcas embryo 71-95 (DAF) Jatropha curcas cDNA clone rjcpga0_000896, mRNA sequence

CTGACATGTGTGCGAGTCAACGGGTGAGTAAACCCGCAAGGCGCAAGGAAGCTGACTGGCGGGATCCCCC

CGAGGGTTGCACCGCCGACCGACCTTGATCTTCTGAGAAGGGTTCGAGTGAGAGCATGCCTGTCGGGACC

CGAAAGATGGTGAACTATGCCTGAGCGGGGCGAAGCCAGAGGAAACTCTGGTGGAGGCCCGCAGCGATAC

TGACGTGCAAATCGTTCGTCTGACTTGGGTATAGGGGCGAAAGACTAATCGAACCGTCTAGTAGCTGGTT

CCCTCCGAAGTTTCCCTCAGGATAGCTGGAGCTCGGGACGAGTTCTATCGGGTAAAGCCAATGATTAGAG

GCATCGGGGGCGCAACGCCCTCGACCTATTCTCAAACTTTAAATAGGTAGGACGGCGCGGCTGCTTCGCT

GAGCCGCGCCACGGAATCGAGAGCTCCAAGTGGGCCATTTTTGGTAAGCAGAACTGGCGATGCGGGATGA

ACCGGAAGCCGGGTTACGGTGCCCAACTGCGCGCTAACCTAGAACCCACAAAGGGTGTTGGTCGATTAAG

ACAGCAGGACGGTGGTCATGGAA

>FM894980.1 FM894980 Jatropha curcas embryo 71-95 (DAF) Jatropha curcas cDNA clone rjcpga0_000895, mRNA sequence

TTTTTTTTTAAGAAGGCCCGTCATATTATCATCGTCAGCAACTAGAAGGAAGATAGATGCGTTTACAGAA

CTTTGAGCAACAATCCCCCTAATAATGGAAACATTACCCAATTGAATTTGAAGGCAATACCAAAATGAAC

CTCTGCCAACAATAAAATACACTAGCTGTAACTGCCTAACAAGATTATTTCTGCAAGTTTTTCTTCTTAT

CATCATTTTCTTCTCATGATTGTGATAACAGCTGAAGGCCCGCTTCCAAATCCTCCAATGTGATGGTTCG

TAGTTCATCAGCATCTATACAATCAAAATCCAGCCTCAAGTCTAAGTTCTCTCTAGCATTTACTAGCATT

GTATCTACTAAACCTCCATTCATCTCCGTGCGTTGCTTTTCTGTTGTTCCTTTCTCTATCAATCTCGCAA

TGGAATCTATACTGCAGTTAGAATGTAAATTAAATCCATACAACAAACCATCTTCTTCTTGATTTTGCAT

CTTGATATGGACTATCTTAGCTAAATCTTCAGAAGTGAAGTCATCAGAATGGAAAAACTTTGTAACCCTT

CTACAGAAGCCTTCAT

>FM894979.1 FM894979 Jatropha curcas embryo 71-95 (DAF) Jatropha curcas cDNA clone rjcpga0_000894, mRNA sequence

GAAAAGACTCATGATGAACAATTAGTTGGAGATGTCCATGCACCCCAGCTGGCCTTAAGGCATATGTGAC

ATCATATACAGCAAAGTCATTGTCTGTCTGCCGGGAAGCATGTGGAGGACATGGGTATGCTGCTGTCAAT

CGGTTTGGTACCTTGAGAAATGACCATGACATTTTTCAGACATTTGAAGGGGATAACACAGTGCTGCTAC

AACAGGTAGCAGGTGATCTCTTAAAGCAATACAAGGAGAAGTTCCAAGGTGGGACATTGGCAGTGACATG

GAACTATCTGAGAGAATCAATGAATACATATCTATCTCAACCAAATCCAGTAACTGCACGATGGGAGGGT

GAAGATCACTTGAGAGACCCTAAATTCCAGTTGGATGCCTTCAGATACCGAACATCTCGACTGCTTCAAA

GTGTTGCTGTCCGACTTCGCAAGCACTCTAAAAGTCTTGGTAGCTTTGGTGCTTGGAATCGGTGCTTGAA

TCACCTTCTCACACTTGCAGAATCTCACGTTGAATCCGTGATCCTTGCAAAATTTATTGAAGCTGTGCAG

AATTGTCCTGATGCAAGCTCTCGAGCTGCTTTAAAACTTGTCTGTGATCTTTATGCCTTGGACCGGATCT

GGAATGA

>FM894978.1 FM894978 Jatropha curcas embryo 71-95 (DAF) Jatropha curcas cDNA clone rjcpga0_000893, mRNA sequence

TGCTTGCCTCCTTCTCAAAGTTGTCACCGGAGGCTTGGAAACACCCACCCACAGCCTTGCAGCTGGAGTA

GGAGCCATTGAAGGAGTGGTTATGGAGATTATAGTCACATTTGCTTTGGTATACACAGTGTACGCAACAG

CAGCTGATCCCAAGAAAGGATCACTAGGCACCATTGCCCCCATAGCCATTGGCTTCATCGTTGGTGCCAA

CATCTTGGCTGCAGGCCCATTTTCCGGTGGATCCATGAACCCAGCCCGCTCTTTTGGCCCAGCTGTGGCT

AGCGGTGACTTCCATGACAACTGGATCTATTGGGTTGGGCCCCTTATTGGTGGTGGGCTAGCAGGCCTTA

TCTATGGAAACTTGTACATTAATAACGATCATGCCCCATTGTCCAATGATTTCTAAGTTATTGTGTTTCT

AATCCATTTTTATCTGTGTTTTGAAATAAAAGAAAGGGAAGCAGCTTATGTTATGCTTTCCTTTCTTTTT

CTTCTCTTTTCTTTTCTTTTTCGTCTTTTTGTTTTAATATTTCCTTTTTAGTTCATTTAAATTATCTTTG

TTGTTGTACATCTTTGTTGTGATAAG

>FM894977.1 FM894977 Jatropha curcas embryo 71-95 (DAF) Jatropha curcas cDNA clone rjcpga0_000892, mRNA sequence

CAAAGGATTCTATCCGTCGCCCGATGGGAATTGTACTTTAAGGCGGCCCTCGCGGCTCGTCCGCCGCGGG

GGCTTGGCCAACGGCACGTGCCTCTGGGGGCCGGATGGCCCCTACTGCGGGTCGGCAAACGGGCGACGGA

CACGCGCGTCGCTTCTGGCCCGGATTCTGACTTAGAGGCGTTCAGTCATAATCCAGCGCACGGTAGCTTC

GCGCCACTGGCTTTTCAACCAAGCGCGATGACCAATTGTGCGAATCAACGGTTCCTCTCGTACTAGGTTG

AATTACCATCGCGACACCATCATCAGTAGGGTAAAACTAACCTGTCTCACGACGGTCTAAACCCAGCTCA

CGTTCCCTATTGGTGGGTGAACAATCCAACACTTGGTGAATTCTGCTTCACAATGATAGGAAGAGCCGAC

ATCGAAGGATCAAAAAGCAACGTCGCTATGAACGCTTGGCTGCCACAAGCCAGTTATCCCTGTGGTAACT

TTTCTGACACCTCTAGCTTCAAATTCCGAAGGTCTAAAGGATCGATAGGCCACGCTTTCACGGTTCGTAT

TCGTACTGGAAATCAGAATCAAACGAGCTTTACCCT

>FM894976.1 FM894976 Jatropha curcas embryo 71-95 (DAF) Jatropha curcas cDNA clone rjcpga0_000891, mRNA sequence

CGAATTCGGTGGTTTCAGTAATTGCTTATTGCTTTCAAATCGCAGCGAAGAAGAAGAGGAGGAATTTCCA

ATCGTCTTCACTCCTGTCGGTAATACTCTAATGGCTGCGATCTTAGCTCGCAAGTCTATCTCCGCGCTTC

GCGCTCGTCGGCTTCTTGTATCTGGACAACTATTTCCGGGCTCACACCAGCATGCACTACAATCTAGTGC

ACACTCATTTTCTACCAAGAAAGAGGATGAAGAAAGAGAGCAGCTAGCAAAGGAGATCTCCAAGGACTGG

AGTTCTGTTTTTGAGCGAAGCATAAACACACTATTTCTTACTGAAATGGTTCGTGGTTTGAACTTAACGT

TGAAGTATTTCTTCGAAAGGAAAGTTACTATCAATTACCCATTTGAGAAGGGTCCTTTAAGCCCCCGGTT

TCGTGGTGAGCATGCGCTCCGACGATATCCAACTGGAGAGGAACGTTGCATTGCCTGCAAACTCTGTGAA

GCTATCTGTCCTGCACAAGCAATCACAATAGAGGCCGAGGAACGTGAAGATGGGAGCCGGAGGACAACTA

GGTATGATATTGACATG

>FM894975.1 FM894975 Jatropha curcas embryo 71-95 (DAF) Jatropha curcas cDNA clone rjcpga0_000890, mRNA sequence

AGGATTCTATCCGTCGCCCGATGGGAATTGTACTTTAAGTGCGGCCCTCGCGGCTCGTCCGCCGCGGGGG

CTTAGCCAACGGCACGTGCCTCTGGGGGCCGGATGGCCCCTACTGCGGGTCGGCAAACGGGCGACGGACA

CGCGCGTCGCTTCTGGCCCGGATTCTGACTTAGAGGCGTTCAGTCATAATCCAGCGCACGGTAGCTTCGC

GCCACTGGCTTTTCAACCAAGCGCGATGACCAATTGTGCGAATCAACGGTTCCTCTCGTACTAGGTTGAA

TTACCATCGCGACACCATCATCAGTAGGGTAAAACTAACCTGTCTCACGACGGTCTAAACCCAGCTCACG

TTCCCTATTGGTGGGTGAACAATCCAACACTTGGTGAATTCTGCTTCACAATGATAGGAAGAGCCGACAT

CGAAGGATCAAAAAGCAACGTCGCTATGAACGCTTGGCTGCCACAAGCCAGTTATCCCTGTGGTAACTTT

TCTGACACCTCTAGCTTCAAATTCCGAAGGTCTAAAGGATCGATAGGCCACGCTTTCACGGTTCG

>FM894974.1 FM894974 Jatropha curcas embryo 71-95 (DAF) Jatropha curcas cDNA clone rjcpga0_000889, mRNA sequence

AATCAAGATCTATCCGTCGCCCGATGGAATTGTACTTTAAGGCGGCCCTCGCGGCTCGTCCGCCGCGGGG

GCTTGGCCAACGGCACGTGCCTCTGGGGGCCGGATGGCCCCTACTGCGGGTCGGCAAACGGGCGACGGAC

ACGCGCGTCGCTTCTGGCCCGGATTCTGACTTAGAGGCGTTCAGTCATAATCCAGCGCACGGTAGCTTCG

CGCCACTGGCTTTTCAACCAAGCGCGATGACCAATTGTGCGAATCAACGGTTCCTCTCGTACTAGGTTGA

ATTACCATCGCGACACCATCATCAGTAGGGTAAAACTAACCTGTCTCACGACGGTCTAAACCCAGCTCAC

GTTCCCTATTGGTGGGTGAACAATCCAACACTTGGTGAATTCTGCTTCACAATGATAGGAAGAGCCGACA

TCGAAGGATCAAAACGCAACGTCGCTATGAACGCTTGGCTGCCACAAGCCAGTTATCCCTGTGGTAACTT

TTCTGACACCTCTAGCTTCAAATTCCGAAGGTCTAAAGGATCGATAGGCCACGCTTTCACGGTTCGTATT

CGTACTGGAAATCAGAATCAAACGAGCTTTTACCCTTTTGTTCCACACGAGATT

>FM894973.1 FM894973 Jatropha curcas embryo 71-95 (DAF) Jatropha curcas cDNA clone rjcpga0_000888, mRNA sequence

AGAATCATCGNGTGAATTTACATACACCACATAATTATATTCCAACAACCAACCCACCGCCGGCCGGCCG

GCCGGACAAGATCAAACCCCTAAATCCCAAATCATACAGCAAACATAAAATCAACCTTTCTTTTCGTCTA

AAACTCATATTTTTTCAAGATTCTCTGCCTTGACAAGAGGATTGGCCTTAGCAATCTGCTCCTTGCCCAT

AGCCTTGAAATCAAAGGTACAATTATGCCGTTCCGGATACCTGTGCGTCCCGCAAAACATCATCTCGCAC

CTGCACTTAAATCCCGTGAGCCCCACGCGCTTCCTGCACGTCGAGCACCTGTTTGGCCGCACGACCGGTG

TCTCGTCCTTATCTTCCACCGCCGCCGCTGATGGCTGATTACTGCTTACTTCAGGCAGATCGACGGTCAC

CGGAGACGAAATAGAAATAAAAGCAGAAGCAGTCGATGATGCGGAAGAAGACGATGACGGCGATGAGGCG

GAGGTTAGGGTTTGGTTCCAGACAAGCTTGGCGTTAGAGGACTGCTGCTCTTTGAGCTGAAGATCGCGAT

AACATTTGGAGCAAAGATTTTG

>FM894972.1 FM894972 Jatropha curcas embryo 71-95 (DAF) Jatropha curcas cDNA clone rjcpga0_000887, mRNA sequence

TCTGACATGTGTGCGAGTCAACGGGTGAGTAAACCCGCAAGGCGCAAGGAAGCTGACTGGCGGGATCCCC

CCGAGGGTTGCACCGCCGACCGACCTTGATCTTCTGAGAAGGGTTCGAGTGAGAGCATGCCTGTCGGGAC

CCGAAAGATGGTGAACTATGCCTGAGCGGGGCGAAGCCAGAGGAAACTCTGGTGGAGGCCCGCAGCGATA

CTGACGTGCAAATCGTTCGTCTGACTTGGGTATAGGGGCGAAAGACTAATCGAACCGTCTAGTAGCTGGT

TCCCTCCGAAGTTTCCCTCAGGATAGCTGGAGCTCGGGACGAGTTCTATCGGGTAAAGCCAATGATTAGA

GGCATCGGGGGCGCAACGCCCTCGACCTATTCTCAAACTTTAAATAGGTAGGACGGGCGCGGGCTGCTTC

GCTGANGCCGCGCCACGGAATCGAGAGCTCCAAGTGGGCCATTTTTGGTAAGCAGAACTGGCGATGCGGG

ATGAACCGGAAGCCGGGTTACGGTGCCCAACTGCGCGCTAACCTAGAACCCACAAAGGGTGTTGGTCGAT

TAAGACAGCAGGACGGT

>FM894971.1 FM894971 Jatropha curcas embryo 71-95 (DAF) Jatropha curcas cDNA clone rjcpga0_000886, mRNA sequence

CTGAAGACCTTTTTGGGTATGATCCATAGGCTGATGAAGAAGTCTGTACAAGTAGGCTTAAGCAAAAGAG

ACAAGTTTTCAGTATTCAGACATGGAAATAGTACGTACATTTCTCTATCGGCATGTTATTTAACAAAGGA

GATCAATTTAAGGATGCAGTAACACAATGGGTTATAGAAAATAATAGAGATGTAAAATTCATAAGGAATA

CCAAAACATAATTTAGAGCTAAGTGTGTGAAAAAAAAAATTGTCCTTAGACCATATATGCGTCTAAGGAA

AAAAAAAAAGAATGATATTCAAGTGAAAATCCCTTATTGATAAACACACATGTTCTATTTCATATAAAAA

TAGGAGGGTTACTGCTAAGTGGTTAGCTAAAAAGGTTTTACTTAAGTATAAATCTATCGAGTCCATGAAA

TTAGTTGAACTTAAGGCACAGTTAAAAGAGGATATCAGTGTAGAAATTAAGTTGTGCAGATTGAGAAGAG

TAAAGTTACTATATCTGTCTACAATGTTGGTGATACTGAGGGTGAATATGCCAAATTGCAGGACTGTCTA

GGTGAAGTGAAAAGATCAAATCCCTTAAGTACAATTGAGATGAAGGTTCAGAGGCTTATATTTGATGAGA

TGGTTG

>FM894970.1 FM894970 Jatropha curcas embryo 71-95 (DAF) Jatropha curcas cDNA clone rjcpga0_000885, mRNA sequence

TTCCTTGAGATGCCGTTTCCAGACAACAGTTTCGACGGCGCGTACTCTATCGAAGCTACTTGTCACGCGC

CGAAGCTGGAAGAGGTATACGCAGAGATCTTTCGAGTGTTGAAGCCAGGATCTCTCTACGTGTCCTACGA

ATGGGTCACAACTGACAAGTACAGGGCTAGTGATCCTCAACACGTGGAGATCATTCAAGGCATTGAACGA

GGAGATGCTTTGCCGGGGCTGAGAAGCTACGCAGACATTGCCGAGACGGCGAGAAAGGTAGGGTTCGAAG

TAGTGAAAGAGAAGGATTTAGCAAAACCACCAGCGCAGCCATGGTGGACTAGGCTAAAGATGGGAAGGAT

TGCCTACTGGAGGAATCACATTCTCGTTACTATATTGGCTGCTTTGGGTATCGCACCGAAGGGAACCGTG

GATGTCCACGAGATGCTTTTCAAAACAGCCGATCATTTGACACAGGGCGGCGAGACTGGAATTTTCTCTC

CGATGCATATGGTTCTCTGCAGAAAACCTGAGAGCCCTTCAAATTCTTAACCCCCCAAAACCAAAAAAGA

CTGAGGTAACACCTGGTTAATTATGTTGCAGTAACTCTGTTCTTCCCTTTCTTTCCCTTGTGACCTTAGT

TTTCCT

>FM894969.1 FM894969 Jatropha curcas embryo 71-95 (DAF) Jatropha curcas cDNA clone rjcpga0_000884, mRNA sequence

GAAAACCTCAGCATCCTCAAGCAGGACAGGACGGCCGGAGTTCACACAGAAAATGGCCAACCTCAGGACG

GCTATAGACTCAGCGTTCTGGGATCAGCCCGTTTCTTCTCCAATAACCCTAGAAGGTTGTGCCCGTTCCA

TCCCCGGCGAGCCATTCCCTCTCGACGCCACTCGAGCTAGCCGAGCCCTTAGGATTCAACAGCTTTCCCT

CTTAGGAAATGGCTTTCCTCTCGGTATCATTCCCTCTTATTCTCCTCCTTCTCACAAGGACCTCGGTTCT

TTCTCTCTCCAGTCTGTCTTGCTTAAGCTTGCTACTCCTGACTGTTGGCTTGCATTAGTCGGGCAGCTTC

GACCAAAGAAATTGATTTCTTCTATCAAAGCAGAATTCGCTAATGCTGAGGAACTTGAATTCTCGGTATT

CAGAGATGCGGCCAGACATATTCTGGACAAGTCTCTTTATTCTCTCGGTTTATCCGCACAGTTTTCTCCG

ACTCCCTCAACTTCTGTGTTGCTGACCACAGAACGACATGGTGAGAAGAAGAGACCTCGCTAC

>FM894968.1 FM894968 Jatropha curcas embryo 71-95 (DAF) Jatropha curcas cDNA clone rjcpga0_000883, mRNA sequence

AGTTTTTCTCTTTTAGGTCTCTCCCTAGAGACTTAGTATAACAAAGTTATACTATCGATACTCGGTATCA

TGCTGCTTCATGAAACAGCTACCTTGCACCCGCGGGAGAGCTGCGGGATGAGTATCTAGATTCTTTGTAA

AGAATCTGCGTGTAAACCACGACTATCTGTTATAGAACAGATTCACCTATTGGCTGCAGGGGCGGTTTAC

AGCCGCAACTGTTCAGTCCAGTCTGAATAGCCACAACCCTACATTGGCTAAAGACCGGTAGTTTAACGTC

TTCGGACGAAACGTAATCAGATACTGAAGGCATTATCATATTTGGGAGCTTCATGCTCAACCTCTAAATG

CACAACAATATCAGTAGCAGACAGCGCCGCGTCGCTATAGAGATAAACTTTAAGTTTATTTAACTCTTCC

AATGTGGTACCACGGTAGTGTGATTTCAAATCTACTACGTGACCCAATGGATCCCCAGAAGGGAGTTTAG

CTATATGCGTAGCGCTCTGAAACGCCTGTATATCATCCGAAG

>FM894967.1 FM894967 Jatropha curcas embryo 71-95 (DAF) Jatropha curcas cDNA clone rjcpga0_000882, mRNA sequence

GAATTAAAGCAAACATAACATCCACAGAAGGCCGTAACAGGCCAATCAGAAAGGAAACAGAATACAACGA

TGATAGAGAAGCCCATTAGACTCTCTAAGAAAATACATTCAGATGAAAGCATGAAAACAATTAAGGAAGG

AATTCCCCCAGATCAGCAGAGGCTGATCTTCGCCGGAAAACAGCTAGAGGACGGTCGTACCTTGGCCGAT

TACAACATTCAGAAGGAGTCCACCCTTCACCTCGTGCTCCGCCTCCGTGGAGGGATGCAGATTTTCGTTA

AGACCTTGACTGGGAAGACCATCAC

>FM894966.1 FM894966 Jatropha curcas embryo 71-95 (DAF) Jatropha curcas cDNA clone rjcpga0_000881, mRNA sequence

TTGGGGGTTCAAAGCGCTCTCTCTTTCTCTCTCTCTGTCTCTGGGGTTACTCACTGGTCAAGTTCCAAGT

CAACTCCCTTTCTCTTTCTCTTGCTCTTGTATACCTTAACAAGATGCTCTGATATCTTATTTATCTGTCT

ATTCAGCCTTAGTTTCCACTAACTTGTTAATTTGTCAAGTAATTACATAATTAATGGAGAACTTGGCCAG

TTTGGAACCATTCCTCTTCAGAACTTTTCAGGACTCATGGATTTCTGAAGCCTATGCTCGCGACACCGAA

ACTCTAACTAAAGCCCTCCAGAAATCACTCTTTTCCGATAACAACCTTATCTGCTCCGATACACAACACC

CTCCTACCACTCCTACCGCTTCTAACGTTTCGGGGTCGGACCCTGAAACGGTTGCTCCTCCCAAGCGTCA

GCGAAACGGGATCCCTGCTACTTCTGGCAAAGTCTCGAAACGCAAGTCTCGCGCGTCCAAGCGTTCTCAG

ACTACGTTTATAACTGCGGATCCGGCGAATTTCAGGCAGATGGTGCAACAGGTTACTGGTGTGAGATTCG

GTAACGCCCAGTTGCCACCTGTCCCTGTGCTCAAGCCTGAGCCACAG

>FM894965.1 FM894965 Jatropha curcas embryo 71-95 (DAF) Jatropha curcas cDNA clone rjcpga0_000880, mRNA sequence

CGAATTAAAAGAACCAGCCAAGGTGGCGTTAACTTCAAAGTTCTTGGAGCTTACATTTAATAACATCATG

AGGATGATCGCTGGAAAACGTTACTATGGCAAAAATGTTATGGATCAAGGAGGAGAATTCTTGCAGGATA

TCATAAAAGAAATGGAGGCACTTCGTGGAAGTTCAAATCTAAATGATTACTTCCCTGTGCTGCAATGGGT

GGATTACCAAGGGGTGGAGAAAAGGATGCAGAAATTAATGAAGAAGATGGACAACTTCTTACAGCAGCTC

ATTGATGAGCACATGAAAACAAGGGGTGACTCATTTCCACCAGTGAATCTGTCTGCTGCAAGTAACCAAA

AGAAGCACACAACTTTAATAGATGTTATGTTATCTTTGAAAGAAACAGAACCAGAATTCTACACAGATCA

AACCATCAAAGGTGTTATTTTGTCAAC

>FM894964.1 FM894964 Jatropha curcas embryo 71-95 (DAF) Jatropha curcas cDNA clone rjcpga0_000879, mRNA sequence

ATTCCCGAAGTTTCCCTCAGGATAGCTGGAGCTCGGGACGAGTTCTATCGGGTAAAGCCAATGATTAGAG

GCATCGGGGGCGCAACGCCCTCGACCTATTCTCAAACTTTAAATAGGTAGGACGGCGCGGCTGCTTCGCT

GAGCCGCGCCACGGAATCGAGAGCTCCAAGTGGGCCATTTTTGGTAAGCAGAACTGGCGATGCGGGATGA

ACCGGAAGCCGGGTTACGGTG

>FM894963.1 FM894963 Jatropha curcas embryo 71-95 (DAF) Jatropha curcas cDNA clone rjcpga0_000878, mRNA sequence

CAAAGGATTCTATCCGTCGCCCGATGGGAATTGTACTTTAAGGCGGCCCTCGCGGCTCGTCCGCCGCGGG

GGCTTAGCCAACGGCACGTGCCTCTGGGGGCCGGATGGCCCCTACTGCGGGTCGGCAAACGGGCGACGGA

CACGCGCGTCGCTTCTGGCCCGGATTCTGACTTAGAGGCGTTCAGTCATAATCCAGCGCACGGTAGCTTC

GCGCCACTGGCTTTTCAACCAAGCGCGATGACCAATTGTGCGAATCAACGGTTCCTCTCGTACTAGGTTG

AATTACCATCGCGACACCATCATCAGTAGGGTAAAACTAACCTGTCTCACGACGGTCTAAACCCAGCTCA

CGTTCCCTATTGGTGGGTGAACAATCCAACACTTGGTGAATTCTGCTTCACAATGATAGGAAGAGCCGAC

ATCGAAGGATCAAAAAGCAACGTCGCTATGAACGCTTGGCTGCCACAAGCCAGTTAT

>FM894962.1 FM894962 Jatropha curcas embryo 71-95 (DAF) Jatropha curcas cDNA clone rjcpga0_000877, mRNA sequence

TGAAGAACAAGAAGAAGTTGCAGGAACTAAAGAAGAAGCTGTAGAACCAGAACCAGAAGTTGCAACTGAA

GTAGTGGTGGAAACTAAAGAAGTAGTAGAAGATGAGACCAAAACTGTAACAGAAGAGCCCCCATCGGAGA

AAACTGAAGAAGAAACTCCTAAGGAAACAGAACCAGAAAAAGAAGAAACTCCTAAGGAAACAGAACCAGA

AAAAGAAGAAAAGGAATCCCCAAAAGAAACAGTAACTGAGGAGACTGTTGCTGAGGAGACTACTAAAGAG

ACCACTGAGAGTACTGAGACAACAACTCCGGCGCCAGAACCGGCACCGGAAGCTGCCGTTGAAGCTCCTA

AGGAAGAAGGTGAAGCGAAAGCTGAGTAATGGGTACTTGTGCTGTGTAGAGGCTGTTTGTTTCTGGCTGT

ATGCCAACTTGAATGTTTCTTGAATTTTCTATCTTTCTTTAATTAGCTTTTTTTTTTTATTGGGTTAAAT

AAGAGATTTAAGAGAG

>FM894961.1 FM894961 Jatropha curcas embryo 71-95 (DAF) Jatropha curcas cDNA clone rjcpga0_000875, mRNA sequence

GGAGTTTGAAATGGCATTGCAAGATCGGGTTATGGAGGAAACCAAAGACAAGAAAAATGCTGTTGAAGCG

TATGTCTATGATATGAGAAACAAGCTTAGCGACAAATATCAGGAGTTTGTCACTGATCCAGAGAGGGAAG

ATTTCACAGCCAAACTGCAGGAAACAGAAGATTGGTTGTATGAAGATGGTGAGGACGAAACTAAAGGTGT

TTACATAGCCAAACTTGAAGAGCTCAAAAAGCAAGGTGATCCCATTGAGGAGCGCTACAAGGAGCACATG

GAAAGGGGATCTGTAATTGATCAACTTGGTTATTGTATTAATAGTTACAGAGAGGCAGCTGTGTCGAGTG

ATCCTAAATTTGATCACATTGACTTAGCTGAAAAGCAGAAGGTATTGAATGAGTGTGTAGAAGCAGAATC

CTGGTTAAGAGAGAA

>FM894960.1 FM894960 Jatropha curcas embryo 71-95 (DAF) Jatropha curcas cDNA clone rjcpga0_000874, mRNA sequence

TTTTTTTTTAATAGATATTAACTTTATTGTCCATATATTATAATAATAGAGTAAAAGCTGCGACAGGATG

AACCCATCACAGCAATTTCAAACACTGACATCTAGAGTGATGAAACTTGGACACAACAAGAGAATAAAAG

AAGTTCAGCTTATTCATGGCCACTCATTTACGACCATTGGACGCACACAGACATCAACAAATTTAAATAA

ACAATCACCCAAGATATTATAGATATAAGTAACTGCTTATAAACCAAACAGCAAACAGATTAGAAACCAC

CACGGAGTCGGAGGACAAGGTGAAGAGTGGACTCTTTCTGGATATTATAGTCTGCAAGGGTTCGTCCATC

TTCAAGTTGTTTACCAGCAAAAATCAATCGTTGTTGGTCCGGGGGAATACCCTCCTTGTCCTGGATTTTA

GCCTTCACATTGTCAATTGTGTCAGAGCTCTCAACCTCGAGGGTAATGGTTTTCCCTGTCAG

>FM894959.1 FM894959 Jatropha curcas embryo 71-95 (DAF) Jatropha curcas cDNA clone rjcpga0_000873, mRNA sequence

ATTCAACAATTTGATTGCAAAAAGATAAAACCCAATAATTATGACTGGGTCCCATTCTTATCATAACAAC

AGAGCTTCAGCTCGAGCTTGATAATGGGATGACAAAGGGTCTCTTCACAGTTAATTAGAGAACTCTAGAG

AACTGGAGTTCAAGCTTATAGAACACAAAATTTAGTATGCAAATCCGGATTATTGAACGGAGAAATTTCA

ATACTAATGGCTTAAAAAAAGTCTTAAGATAAGACTAAGATCGTGTTCGTAATGACTTTTTAAGTATTTT

GTACTGTATTCGCTGTGAAAAGCTTTATCTTTATACTATTCATCTACAATTTTTGACATAAAAAAGTGTT

CTTGACCACTTTAAATCTTGCATCTTTTGTAAGTTTAGCACATGTTCACATCTTAGATTGAAAGAAATAA

GTTTTATCTAATAATATAATTAACCCATTTTTTCAATGCAGATTGTGATGAGTAGTTTGAGAGGAG

>FM894958.1 FM894958 Jatropha curcas embryo 71-95 (DAF) Jatropha curcas cDNA clone rjcpga0_000872, mRNA sequence

TTGGAAATTCGGTGGCCTCTACCCGCTTTCTATTGCAAGATGAATTCAACATTCAGACCACACAATGTGA

TAATTGCATTATTGGTTTTATGTTCTGTCTCCAACAAATTGCATGTATATTCTCCATTGTGGCAATGATT

GTTGGGAGTGATGAAATTTCAGAGGCTTCTCAGTTACTTTCATGTTTGGCTGATATGGTGTATTGCACGG

TTTGCGCCTGTATGCAGACTCAACACAAGATTGAAATGGACAAACGAGATGGCAAATTCGGGCCACAAGT

GATGGCTGTGCCCCCTGTTCAGCAGATGTCACGCATTGATCAGCCAGTTCCTCCCAATGTTGGATATGGA

CAGCCCTACGGTTATCCACAAGCTCAAGGCTATCCTGCGGCAGGTTATCCTCCGCCTGCCAATTACCCAC

CGCCTGGTTACCCACCTTCTGGCTA

>FM894957.1 FM894957 Jatropha curcas embryo 71-95 (DAF) Jatropha curcas cDNA clone rjcpga0_000871, mRNA sequence

CCTTCATCGTGCTTCTTGAAACCCTAGATTTCGCCTTCCTACATCTAGTCCAAAATGAAGCTTGTCAGGT

TTTTGATGAAGTTGAACAACGAGACTGTATCAATTGAGCTTAAGAACGGAACCGTTGTTCACGGCACTAT

TACAGGTGTGGATATTAGTATGAATACTCATCTGAAGACTGTTAAACTAACACTTAAGGGGAAAAATCCA

GTAACTTTGGATCATCTAAGTGTGAGGGGTAACAATATTCGCTATTATATCCTTCCCGACAGCTTGAATC

TTGAGACTTTGCTGGTTGAAGAGACACCTAGGGTCAAACCTAAGAAGCCAACTGCTGGGAGGCCTGTGGG

ACGTGGTCGGGGCCGTGGACGTGGTCGTGGACGTGGTAGAGGCCGCTAGATCTGCTGCACGTATTTCAAT

TTTTGTGGCATCTGGTTATGTAAAACGCCTATGTTGTAAGGAGAATTTGTT

>FM894956.1 FM894956 Jatropha curcas embryo 71-95 (DAF) Jatropha curcas cDNA clone rjcpga0_000870, mRNA sequence

AGGATTCTATCCGTCGCCCGATGGGAATTGTACTTTAAGGCGGCCCTCGCGGCTCGTCCGCCGCGGGGGC

TTGGCCAACGGCACGTGCCTCTGGGGGCCGGATGGCCCCTACTGCGGGTCGGCAAACGGGCGACGGACAC

GCGCGTCGCTTCTGGCCCGGATTCTGACTTAGAGGCGTTCAGTCATAATCCAGCGCACGGTAGCTTCGCG

CCACTGGCTTTTCAACCAAGCGCGATGACCAATTGTGCGAATCAACGGTTCCTCTCGTACTAGGTTGAAT

TACCATCGCGACACCATCATCAGTAGGGTAAAACTAACCTGTCTCACGACGGTCTAAACCCAGCTCACGT

TCCCTATTGGTGGGTGAACAATCCAACACTTGGTGAATTCTGCTTCACAATGATAGGAAGAGCCGACATC

GAAGGATCAAAAAGCAACGTCGCTATGAACGCTTGGCTGCCACAAGCCAGTTATCCCTGTGGTAACTTT

>FM894955.1 FM894955 Jatropha curcas embryo 71-95 (DAF) Jatropha curcas cDNA clone rjcpga0_000869, mRNA sequence

GCATTTCTAACCACAGAGTCAAGGTCTACTTGCGTCTTTATCTTTTTGCGCAAGTTTTTTATCCCTAATA

GGTGTGCGTTTTTTGTGGCAACTCTACGCTGCTAATATTATGGGATCTAACGTAAAGACTATTTTCTCTG

GAACTTCACATACAACTGGTACAGTTGCCAAGCATGGCACTGGAGGGGATTCTGTTGATGAGGCTTATAA

AGCCCTATTGCGAACATGCTCGGTTGATCAGCCGGGGCGGATTCAAATCCGCCTCACTGATTCTGCTAGG

CCGCAAGACGTGGTCGATGGTAACCAACAGGTTACCCTCGAACTTTCGAAATTGCCGGCTATAGTTAGTT

TTTCGCCGAGTTATGATTGCCCATTGGAGACGATGGGTAAAGATGGCCCCCCTCGGTGCTTTATGATAGA

AACCAGATTGTCGGGTGTGGTAGGGCATGATG

>FM894954.1 FM894954 Jatropha curcas embryo 71-95 (DAF) Jatropha curcas cDNA clone rjcpga0_000868, mRNA sequence

GCAAAGGATTCTATCCGTCGCCCGGTGGGAATTGTACTTTAAGGCGGCCCTCGCGGCTCGTCCGCCGCGG

GGGCTTAGCCAACGGCACGTGCCTCTGGGGGCCGGATGGCCCCTACTGCGGGTCGGCAAACGGGCGACGG

ACACGCGCGTCGCTTCTGGCCCGGATTCTGACTTAGAGGCGTTCAGTCATAATCCAGCGCACGGTAGCTT

CGCGCCACTGGCTTTTCAACCAAGCGCGATGACCAATTGTGCGAATCAACGGTTCCTCTCGTACTAGGTT

GAATTACCATCGCGACACCATCATCAGTAGGGTAAAACTAACCTGTCTCACGACGGTCTAACCCCAGCTC

ACGTTCCCTATTGGTGGGTGAACAATCCAACACTTGGTGAATTCTGCTTCACAATGATAGGAAGAGCCGA

CATCGAAGGATCAAAAAGCAACGTCGCTATGAACGCTTGGCTG

>FM894953.1 FM894953 Jatropha curcas embryo 71-95 (DAF) Jatropha curcas cDNA clone rjcpga0_000867, mRNA sequence

GAATATTTGCTACTACCACCAAGATCTGCACCGACGGCCGCTCCGCCCGGGCTCGCGCCCCGGGTTTTGC

AGCGACCGCCGCGCCCTCCTACTCATCGGGGCCTGGCTCTTGCCCCGACGGCCGGGTATAGGTCGCGCGC

TTAAGCGCCATCCATTTTCGGGGCTAGTTGATTCGGCAGGTGAGTTGTTACACACTCCTTAGCGGATTTC

GACTTCCATGACCACCGTCCTGCTGTCTTAATCGACCAACACCCTTTGTGGGTTCTAGGTTAGCGCGCAG

TTGGGCACCGTAACCCGGCTTCCGGTTCATCCCGCATCGCCAGTTCTGCTTACCAAAAATGGCCCACTTG

GAGCTCTCGATTCCGTGGCGCGGCTCAGCGAAGCAGCCGCGCCGTCCTACCTATTTAAAGTTTGAGAATA

GGTCGAGGGCGTTGCGCCCCCGATGCCTCTAATCATTGGCTTTACCCGATAGAACTCGT

>FM894952.1 FM894952 Jatropha curcas embryo 71-95 (DAF) Jatropha curcas cDNA clone rjcpga0_000864, mRNA sequence

TGTCGTACTCATAACCGCATAGGTCTCCAAGGTGAACAGCCTCTGGTCGATGGAACAATGTAGCAAGGGA

AGTCGGCAAAATGGATCCGTAACCTCGGGATCGAGGATTGGCTCTGAGGGCTGGGCCCGGGGGTCCCAGT

CCCGAACCCGTCGGCTGTCGGCGGACTGCTCGAGCTGCTCCCGCGGCGAGAGCGGGTCGCCGCGTGCCGG

CCGGGGGACGGACTGGGAACGGCCCTTCCGGGGGCCTTCCCCGGGCGTCGAACAGTCGACTCAGAACTGG

TACGGACAAGGGGAATCCGACTGTTTAATTAAAACAAAGCATTGCGATGGTCCCTGCGGATGCTCACGCA

ATGTGATTTCTGCCCAGTGCTCTGAATGTCAAAGTGAAGAAATTCAACCAAGCGCGGGTAAACGGCGGGA

GTAACTATGACTCTCTTAAGGTAGCCAAATGCCTCGTCATCTAATTAGTGACGCGCATGAATGGATTAAC

GAGATTTCCACTGTCCCTGTCTACTATCCAGCGAAACCACAGCCAAGGGAACGGGCTTGGCGGAATCAGC

GGGGAAAGAAGACCCTGTTGAGCTTGACTCTA

>FM894951.1 FM894951 Jatropha curcas embryo 71-95 (DAF) Jatropha curcas cDNA clone rjcpga0_000863, mRNA sequence

GATAAACACGATAATGGATTATCATTAATTGTCAAAACAAGTGATCCAATTCAGCACATTTATGTCTTCC

ATACACAGATGCCAGATAACTTGCTAAACTTTAGAAACACTTTTGGAGAGAAATGCCAATGGAAACTAAC

AGTAAAATGGAACAATTTCACCATTTTTTCAGAAGTAGATTTCCCCGTCGAGTATTGCAGCCGCAAGCCT

CTAACAACTCAGTATAGAATATGAATCTCTCATCTCTTTGCCTTGACAATCTTGAAATATCAATCAAGTA

TCCACAAATCTCATGCACTTAAGTGAGCAGCATTTAGGGGTATCGATGATTATGCTTCATCAAGTTTCAG

ACGAGGAATAATATTCATGGATTGGAGTTCCTGAAGTACAAGCTTGCAAGCATATGGCAGTTTCATAGTA

GAACTATTATCCCCAT

>FM894950.1 FM894950 Jatropha curcas embryo 71-95 (DAF) Jatropha curcas cDNA clone rjcpga0_000862, mRNA sequence

AGGATTCTATCCGTCGCCCGATGGGAATTGTACNTTTAAGGCGGCCCTCGCGGCTCGTCCGCCGCGGGGG

CTTGGCCAACGGCACGTGCCTCTGGGGGCCGGATGGCCCCTACTGCGGGTCGGCAAACGGGCGACGGACA

CGCGCGTCGCTTCTGGCCCGGATTCTGACTTAGAGGCGTTCAGTCATAATCCAGCGCACGGTAGCTTCGC

GCCACTGGCTTTTCAACCAAGCGCGATGACCAATTGTGCGAATCAACGGTTCCTCTCGTACTAGGTTGAA

TTACCATCGCGACACCATCATCAGTAGGGTAAAACTAACCTGTCTCACGACGGTCTAAACCCAGCTCACG

TTCCCTATTGGTGGGTGAACAATCCAACACTTGGTGAATTCTGCTTCACAATGATAGGAAGAGCCGACAT

CGAAGGATCAAAAAGCAACGTCGCTATGAACGCTTGGCTGCCACAAGCCAGTTATCCCTGTGGTAACTTT

TCTGACACCTCTAGCTTCAAATTCCGAAGGTCTAAAGGATCGATAGGCCACGCTTTCACGGTTCGTATTC

GTACTGGAAATCAGAAATCAACGAGCTTTTACCCCTTTTGTTCCACACGAAATTTCTGTTCTCGTTGAAG

CTCATCTT

>FM894949.1 FM894949 Jatropha curcas embryo 71-95 (DAF) Jatropha curcas cDNA clone rjcpga0_000861, mRNA sequence

ATTCTTTTTTTTTACAGAAAATTTCCCTTCCACCACATTTTGATTAAATAAACTGCATTTAAGGTATCCA

ACAACCAATTCCTCAAGCCATAAAACCCAAGAAGAAATTAATTCAAATACCTTCCTTCAACAAGATGCAA

ATAGCTGTGTTATATATATATGCAAAAAGAATCGCCCACCATCGTGAGCCATACCAGAGTGCAACTTCTG

GATTTGTTTTTCTGGCCCCTTCGGCGTTGCTCAACTCCAGTATCAACAACTTCTAAACCAGAAGAAACAA

GCATTGCGACTAACGTATCGGGTGGTATTGTGCTGATATTCTCTTGGGAGGCAACTTTCGCAAGATGTAG

AGCACTAGAGCTGAAGGTAGAATCTCAACCAACATGTAATATATCAAGTTCAAAACCGGATGATCCAAAA

CATCAAGTGACGCATCTGTATCAAAAGCAGAAAGAAGAACCACAAAGCATCTAATCAGGAAGCATGTAAA

GCATATAGCCGTAACTGATCCAACCTCATGGAGCTTCTTTCTTCTCCCTTTAGACTCAATAGGGAAGCGT

CTCAGCATGAAAAATAATCGTCCTCCATATAACAAGAAACCTAATGCAGCTATAACTGAGACCACTCCAA

TAAATATCTTCCCAATGAATTCCACAATGCTGTTGTCATCTACCCA

>FM894948.1 FM894948 Jatropha curcas embryo 71-95 (DAF) Jatropha curcas cDNA clone rjcpga0_000859, mRNA sequence

CTTGTCTCAAAGATTAAGCCATGCATGTGTAAGTATGAACTAATTCAGACTGTGAAACTGCGAATGGCTC

ATTAAATCAGTTATAGTTTGTTTGATGGTACCTGCTACTCGGATAACCGTAGTAATTCTAGAGCTAATAC

GTGCACCAAACCCCGACTTCTGGAAGGGATGCATTTATTAGATAAAAGGTCGACGCGGGCTCTGCCCGTT

GCTCTGATGATTCATGATAACTCGACGGATCGCACGGCCATCGTGCCGGCGACGCATCATTCAAATTTCT

GCCCTATCAACTTTCGATGGTAGGATAGAGGCCTACCATGGTGGTGACGGGTGACGGAGAATTAGGGTTC

GATTCCGGAGAGGGAGCCTGAGAAACGGCTACCACATCCAAGGAAGGCAGCAGGCGCGCAAATTACCCAA

TCCTGACACGGGGAGGTAGTGACAATAAATAACAATACCGGGCTCAATGAGTCTGGTAATTGGAATGAGT

ACAATCTAAATCCCTTAACGAGGATCCATTGGAGGGCAAGTCTGGTGCCAGCAGCCGCGGTAATTCCAGC

TCCAATAGCGTATATTTAAGTTGTTGCAGTTAAAAAGCTCGTA

>FM894947.1 FM894947 Jatropha curcas embryo 71-95 (DAF) Jatropha curcas cDNA clone rjcpga0_000858, mRNA sequence

GGATTCTATCCGTCGCCCGATGGGAATTGTACTTTAAGGCGGCCCTCGCGGCTCGTCCGCCGCGGGGGCT

TGGCCAACGGCACGTGCCTCTGGGGGCCGGATGGCCCCTACTGCGGGTCGGCAAACGGGCGACGGACACG

CGCGTCGCTTCTGGCCCGGATTCTGACTTAGAGGCGTTCAGTCATAATCCAGCGCACGGTAGCTTCGCGC

CACTGGCTTTTCAACCAAGCGCGATGACCAATTGTGCGAATCAACGGTTCCTCTCGTACTAGGTTGAATT

ACCATCGCGACACCATCATCAGTAGGGTAAAACTAACCTGTCTCACGACGGTCTAATCCCCGCTCACGTT

CCCTATTGGTGGGTGAACAATCCAACACTTGGTGAATTCTGCTTCACAATGATAGGAAGAGCCGACATCG

AAGGATCAAAAAGCAACGTCGCTATGAACGCTTGGCTGCCACAAGCCAGTTATCCCTGTGGTAACTTTTC

TGACACCTCTAGCTTCAAATTCCGAAGGTCT

>FM894946.1 FM894946 Jatropha curcas embryo 71-95 (DAF) Jatropha curcas cDNA clone rjcpga0_000857, mRNA sequence

TGCGCGCTAACCTAGAACCCACAAAGGGTGTTGGTCGATTAAGACAGCAGGACGGTGGTCATGGAAGTCG

AAATCCGCTAAGGAGTGTGTAACAACTCACCTGCCGAATCAACTAGCCCCGAAAATGGATGGCGCTTAAG

CGCGCGACCTATACCCGGCCGTCGGGGCAAGAGCCAGGCCCCGATGAGTAGGAGGGCGCGGCGGTCGCTG

CAAAACCCGGGGCGCGAGCCCGGGCGGAGCGGCCGTCGGTGCAGATCTTGGTGGTAGTAGCAAATATTCA

AATGAGAACTTTGAAGGCCGAAGAGGGGAAAGGTTCCATGTGAACGGCACTTGCACATGGGTTAGTCGAT

CCTAAGAGACGGGGGAAGCCCGTCCGACAGCGCGTCCTCGCGCGAGCTTCGAAAGGGAATCGGGTTAAAA

TTCCCGAACCGGGACGTGAAGGCTGACGGCGACGTTAGGGAGTCCGGAGACGTCGGCGGGGGCCTCGGGA

AGAGTTATCTTTTCTGT

>FM894945.1 FM894945 Jatropha curcas embryo 71-95 (DAF) Jatropha curcas cDNA clone rjcpga0_000856, mRNA sequence

TGGAAATCAGAATCAAACGAGCTTTACCCTTTTGTTCCACACGAGATTTCTGTTCTCGTTGAGCTCATCT

TAGGACACCTGCGTTATCTTTTAACAGATGTGCCGCCCCAGCCAAACTCCCCACCTGACAATGTCTTCCG

CCCGGATCGGCCGCCGAAACGACCTTGGGTCCAAAAAGAGGGGCATAGCCCCGCCTCCGATTCACGGAAT

AAGTAAAATAACGTTAAAAGTAGTGGTATTTCACCGTCGCCTTTCGGCTCCCACTTATCCTACACCTCTC

AAGTCATTTCACAAAGTCGGACTAGAGTCAAGCTCAACAGGGTCTTCTTTCCCCGCTGATTCCGCCAAGC

CCGTTCCCTTGGCTGTGGTTTCGCTGGATAGTAGACAGGGACAGTGGGAATCTCGTTAATCCATTCATGC

GCGTCACTAATTAGATGACGAGGCATNTGGCTACCTTAAGAGAGTCATAGTTACTCCCGCCGTTTACCCG

CGCTTGGTTGAATTTCTTCACTTTGACATTCAGAGCACTGGGCAGAAATCACATTGCGTGAGCATCCTCA

GGGACCATCGCAATGCTTTGTTTTAATTAAACAGTCGG

>FM894944.1 FM894944 Jatropha curcas embryo 71-95 (DAF) Jatropha curcas cDNA clone rjcpga0_000855, mRNA sequence

ACTGTAAAAACCATCTAATGTTTGTATTGAACTGATGAAATTCTTTCTCCAACAACATCCTTTAAAAACC

CCATCTTTGACCAACCAAAATCAAAAATTACAAAAGAGCGGACACAAGTTTTATTAAACAAAGGGAAGCC

TGCATCTAAACATTTGACTCAATACACAGCAGTCAAAAATCACCATCAATGTCTTCAGTGGAGGGTGGAG

GCAATGTGAGCAATCAAGTCTAGCACACGTGAACTGTAGCCCCATTCGTTGTCATACCAAGAGACAAGTT

TCACAAAGTTGTCATTCAAAGCAATTCCTGCCTTGGCATCAAAAATACTTGATCTGCTATCGCCCACAAA

GTCAGAAGACACTACATCATCTTCAGTATAGCCCAAGATTCCCTTAAGCTTGCCCTCAGACTCCTCCTTG

ATTGCAGCTTTGATGTCCTCATATGTTGCCTTCTTTTCAAGCCTGACAGTAAGGTCCACAACAGAAACAT

CCACAGTTGGAACACGGAATGACATTCCAGTCAATTTGCCATTAAGTGCAGGAAGAACCTTTTCCACAGC

CTTAGCAGCTCCAGTGCTGCTAGGAATGATGTTGAAGG

>FM894943.1 FM894943 Jatropha curcas embryo 71-95 (DAF) Jatropha curcas cDNA clone rjcpga0_000854, mRNA sequence

GCTGCAGGAATTCGAATTCAGGATTCTATCCGTCGCCCGATGGGAATTGTACTTTAAGGCGGCCCTCGCG

GCTCGTCCGCCGCGGGGGCTTAGCCAACGGCACGTGCCTCTGGGGGCCGGATGGCCCCTACTGCGGGTCG

GCAAACGGGCGACGGACACGCGCGTCGCTTCTGGCCCGGATTCTGACTTAAGGCGTTCAGTCATAATCCA

GCGCACGGTAGCTTCGCGCCACTGGCTTTTCAACCAAGCGCGATGACCAATTGTGCGAATCAACGGTTCC

TCTCGTACTAGGTTGAATTACCATCGCGACACCATCATCAGTAGGGTAAAACTAACCTGTCTCACGACGG

TCTAAACCCAGCTCACGTTCCCTATTGGTGGGTGAACAATCCAACACTTGGTGAATTCTGCTTCACAATG

ATAGGAAGAGCCGACATCGAAGGATCAAAAAGCAACGTGGCTATGAACGCTTGGCTGCCACAAGCCAGTT

ATCCCTGTGGTAACTTTTCTGACACCTCTAGCTTCAAATTCCGAAGGTCTAAAGGATCGATAGGCCACGC

TTTCACGGTTCGTATTCGTACTGGAAACTCAGAATCAACGAGCTTTTACCCCTTTTGTTCCCACGAGATT

TCTGTTCTCGTTGAGCTCATCTTAAGACA

>FM894942.1 FM894942 Jatropha curcas embryo 71-95 (DAF) Jatropha curcas cDNA clone rjcpga0_000853, mRNA sequence

AAAACAGTACCGCAATTATAACATATTATAATAATAAAGTTAAAAAAAACAAAAAACAAAAACAAAAAAT

AAAAGAGGAACCACCGAAGATAATGTTAAAAAGTTCTTATCAGATAACTTGCTTAATCAACTTCCTCCAT

CTTGGATCCTTCAGCATCAACATCAGCATCCTCAAGTGCAGGCATGTCAGCATCTCCTTCACCAGCATCC

TCATCAATGCTCAGTCCAAGTTTCAACATCCTATGGATTCTGTTGCCGAAAGTGTTGGGATCATCAAGGC

TGAACCCTGAGGTAAGAAGAGCAGTCTCAAATAGCAGCAGGACAAGATCCTTCACTGACTTATCGTTCTT

GTCAGCATCAGCTCTCTTCCTTAACTCCTCCATGATAGGATTCTCAGGGTTGATCTCCATTGTCTTCTTG

CTTGACATGTATCCAGCCATGCTATTATCCCTCAAAGCTTGAGCTCTCATAATTCTTTCCATGTTAGCAG

TCCACCCATATTCCCCTGTGACNCAACAGCAAGGAGAATCAACCACGCGGTCTGAAACTACAACCTTCTC

TACCCTGTCTCCCAGTACATCCTTGA

>FM894941.1 FM894941 Jatropha curcas embryo 71-95 (DAF) Jatropha curcas cDNA clone rjcpga0_000852, mRNA sequence

ATTCAGGCAATGCACCAAAATTTACTGTTTCAAAATTTGCAATTACATCTGTAAAGTATTTCAAGGCGGA

TCGTCCATCCATTGCTAGATGTGGTTCCTTTATGAATTTCTATTCCTAAGAAACTCTGAAGCCTAACAGC

CACCACTAGGGAAAAGTCTAAAACAGAAGTGAGAAAGAATTACAGGGTGATTCCTCTAGATTCAGCTTCA

AGATACTTGCAGATCCTCTCCATAACCTCTCTACCTGTTCGCACTATTCTGCAAGAACTTCTCAACACAT

CGTTGTTCTACCTTTTCTGCCATTGATGCTAGTGCCGATAATGGTTTGATCTGTATACTAGTCTCCTGCC

GACAAATTGTCCATTCGCTTGGATTATCAGGGTGAGGATCATAGCGGATCTTCTCCTCAACTTCTATGAA

CTTCTGAAGACTAATGTTGCGGCTGGTGAGCTGCATGGAGTGTGTTTCGGCATCAACAACTGTTGATTCA

ATACAGTGGCAAATCTCCTGGCCTATAAATTTGCGGATGAACCATGGTCCCGGAGCATGCACAGTAACAG

CCCGAGTGGTGTAAAGCTTCCCGAATTCCGAA

>FM894940.1 FM894940 Jatropha curcas embryo 71-95 (DAF) Jatropha curcas cDNA clone rjcpga0_000851, mRNA sequence

TTCTAGTGTTGCTAAATACACACGAACACTTACCAATGTGGTTGGTGCGGGGACTTATAAGGCTTCAATT

TCAGGTCAGCTTACGGGAGTTAAAATTTCAGTTAATCCTCAAGTGTTGAACTTTAGCCAAGCAAAGGAGA

AGAAAACTTTTACTGTTACTGTAACTGGAAGTTCATTGCCAATTAATACTAACGCGTTTGCTCGTTTAGA

GTGGAGTGATGGGAAACATGTTGTTGGTAGTCCAATAGCAGTTAGCTGGACTTAGCCCAAGAAGAAGAAG

CATATGAAATAGGAGAAGTTGGGTTTTTAATTTGCTCCTTCTCGAAGAGAGGAGTGGATTAGATAAGTTG

TTAGTTTTAAAATTCTATGAATGTCATATTAATTTGTTTATATCCAGGAAATAATGGAAGGCTGGATTAG

TGTTTGATGTTGTTCTCTGAGAAAGAAAACAAGAAAAACCATGAAAAAGAAAAGGCAATGTGTGGAGATG

TTTTCTTTCTTAGTGTACAGTTTTATTTACATTTTAGTATTTGTATTGCTATGCTTGATTAGTAGATTTC

TCAAGATTTCAAAAAAAAA

>FM894939.1 FM894939 Jatropha curcas embryo 71-95 (DAF) Jatropha curcas cDNA clone rjcpga0_000850, mRNA sequence

TTCAGGCCAGCTTCACGGTTCGTATTCGTACTGGAAATCAGAATCAAACGAGCTTTTACCCTTTTGTTCC

ACACGAGATTTCTGTTCTCGTTGAGCTCATCTTAGGACACCTGCGTTATCTTTTAACAGATGTGCCGCCC

CAGCCAAACTCCCCACCTGACAATGTCTTCCGCCCGGATCGGCCGCCGAAACGACCTTGGGTCCAAAAAG

AGGGGCATAGCCCCGCCTCCGATTCACGGAATAAGTAAAATAACGTTAAAAGTAGTGGTATTTCACCGTC

GCCTTTCGGCTCCCACTTATCCTACACCTCTCAAGTCATTTCACAAAGTCGGACTAGAGTCAAGCTCAAC

AGGGTCTTCTTTCCCCGCTGATTCCGCCAAGCCCGTTCCCTTGGCTGTGGTTTCGCTGGATAGTAGACAG

GGACAGTGGGAATCTCGTTAATCCATTCATGCGCGTCACTAATTAGATGACGAGGCATTTGGCTACCTTA

AGAGAGTCATAGTTACTCCCGCCGTTTACCCGCGCTTG

>FM894938.1 FM894938 Jatropha curcas embryo 71-95 (DAF) Jatropha curcas cDNA clone rjcpga0_000849, mRNA sequence

AGCATCAACATGTGGCCTTCAAAACATTGTTATTCAAATATATTATGAGTAAAGCTTTCAGTTAGTTCCT

CAAATGTATGGCTACGAATAAAATCAAGCAATCTAATGACACCTTTCTCTCTGATTCAATTATGGTTTTC

ACTACTACACTGTAAGGCTTAAACAGAGCATGTATCTGGAAATCCTAATAAGACACAAATCCTAAAATAG

AAAGGACTCGGCATTTTGAATTCCATTATTTACTCATTTTTCTGTCTGATTCTCTATTTCCATACTACGT

TCACCAACATCACCAGTGTCAATATATTCATCATCATCGTCGTCGTCATCTTCGTCTTCGTAGTCCCCGC

TGGTGTCATCATCAGACAAGCTAATTCGTGAAAATTTGCTGAGGTCGCCTCTAATATCATCATCACCCAG

AAACTGAAACCCAAATTTAGTATCCCGGTGTTTAACTAGAAGCCACCGCAATAGGCGATCTTCCTTGTAT

AGGTAATGCAGCTCCTTGTTGATGTAGGGTGTGGCCATCATTGTCATCTGCATTAATTGGCCCTGATAAT

ATCTTCCATCAAGCTTCTTAATA

>FM894937.1 FM894937 Jatropha curcas embryo 71-95 (DAF) Jatropha curcas cDNA clone rjcpga0_000848, mRNA sequence

CGAATTCGGTTGCTCTGCATTGAAGAAGATAATCAAAATAGAAGTATGAACAAGACAAATAAGAAGAAGA

ATCCAACTAAGAAAAGAAAGGTGAACTCTGAGCAGGAGGTTACAACTCTNTGGGGCAGAAGACAGCTTGC

AACAAATGGACAAATTAAACTCAAGAAGTGTAACCCTAGATGGCTATTATGGGGCACAACAAAGTGTGCC

AGGAATGGTGCAACTGAACTTAATGGCACCAACACGGGATAACTACTATGGGAACCAACAGACTATCCAG

GGGCTGGGGCAGTTAAACTCAATAGCACCTAGCCACGATGGTTACTACAATGCCCAGCAAAGCATGCATG

GGCTGGGACAGATGGATTTCTTCCGCGCACAAGCTGGTTTCACTTATGGGATCCGGGATGACCCAAATGT

TAGAACAGCACCGCTGCATGATAATGCATCCAGACATGCATGAAGATCACCTTTCTCGCTCTCAAGGTGA

GGAGAAAGAGTACTTAT

>FM894935.1 FM894935 Jatropha curcas embryo 71-95 (DAF) Jatropha curcas cDNA clone rjcpga0_000844, mRNA sequence

ATTCCTTTTTCTTCATCATTCTCTTTATCATTTCAAAGAAAGGGTTTTAGGAGATTGCAAGTAGTTTCAG

CGTCAATGGCGGATTCGCGTGCAAGCACTGTTCTTGTCACTGGAGCTGGTGGCAGAACCGGGTCAATAGT

TTATAAGAAATTAAAGGAGAGATCTGACCAGTATGTTGCAAGAGGCTTGGTCAGAACTCAAGAAAGCAAA

GAAAAAATTGGTGGAGCAGAGGATGTATTTGTTGGGGATATAAGGCAACCAGAAAGCATCAATCCTGCTA

TCCAAGGAATTGATGCTCTCATAATTCTTACGAGTGCGGTGCCAAAGATGAAACCTGGGTTTGATCCATC

TAAAGGTGAAAGGCCTGAGTTTTGTTTTGAAGATGGAGCATATCCTGAGCAGGTTGATTGGATTGGTCAG

AAAAATCAAATAGATGCTGCTAAGGCTGCAGGGGTGAAGCAAATTGTGTTGGTTGGATCTATGGGTGGAA

CAAACCCTAATCATCCCCTGAACAGTTTGGGCAATGGAAATATATTGGTTTGGAAGAGAAAGGCTGAACA

GTATCTCGCGGGACTCTGGCATTCCATACACAATTATAATGGCTGGTGGCCTGCAAGACAAAGATGG

>FM894934.1 FM894934 Jatropha curcas embryo 71-95 (DAF) Jatropha curcas cDNA clone rjcpga0_000843, mRNA sequence

ATTCGCAAAGGATTCTATCGTCGCCCGATGGGAATTGTACTTTAAGGCGGCCCTCGCGGCTCGTCCGCCG

CGGGGGCTTGGCCAACGGCACGTGCCTCTGGGGGCCGGATGGCCCCTACTGCGGGTCGGCAAACGGGCGA

CGGACACGCGCGTCGCTTCTGGCCCGGATTCTGACTTAGAGGCGTTCAGTCATAATCCAGCGCACGGTAG

CTTCGCGCCACTGGCTTTTCAACCAAGCGCGATGACCAATTGTGCGAATCAACGGTTCCTCTCGTACTAG

GTTGAATTACCATCGCGACACCATCATCAGTAGGGTAAAACTAACCTGTCTCACGACGGTCTAACCCCAG

CTCACGTTCCCTATTGGTGGGTGAACAATCCAACACTTGGTGAATTCTGCTTCACAATGATAGGAAGAGC

CGACATCGAAGGATCACAAAGCAACGTCGCTATGAACGCTTGGCTGCCACAAGCCAGTTATCCCTGTGG

>FM894933.1 FM894933 Jatropha curcas embryo 71-95 (DAF) Jatropha curcas cDNA clone rjcpga0_000842, mRNA sequence

AGGATTCTATCCGTCGCCCGATGGGAATATGTACTTTAAGGCGGCCCTCGCGGCTCGTCCGCCGCGGGGG

CTTGGCCAACGGCACGTGCCTCTGGGGGCCGGATGGCCCCTACTGCGGGTCGGCAAACGGGCGACGGACA

CGCGCGTCGCTTCTGGCCCGGATTCTGACTTAGAGGCGTTCAGTCATAATCCAGCGCACGGTAGCTTCGC

GCCACTGGCTTTTCAACCAAGCGCGATGACCAATTGTGCGAATCAACGGTTCCTCTCGTACTAGGTTGAA

TTACCATCGCGACACCATCATCAGTAGGGTAAAACTAACCTGTCTCACGACGGTCTAACCCCAGCTCACG

TTCCCTATTGGTGGGTGAACAATCCAACACTTGGTGAATTCTGCTTCACAATGATAGGAAGAGCCGACAT

CGAAGGATCAAAAAGCAACGTCGCTATGAACGCTTGGCTGCCACAAGCCAGTTATCCCTGTGGTAACTTT

TCTGACACCTCTAGCTTCAAATTCCGAAGGTCTAAAGGATCGATAGGCCACGCTTTCACGGTTCGTATTC

GTACTGGAAATCAGAATCAAACGAGCTTTTACCCTTTTGTTCCACACGAGATTTCTGTTCT

>FM894932.1 FM894932 Jatropha curcas embryo 71-95 (DAF) Jatropha curcas cDNA clone rjcpga0_000841, mRNA sequence

GGGTTTTTTTTTTATGCCCATGACAACTTGAAAGAATTGAAACGGAAGAACATAATTTGTACACTCTTAA

AAACTCATAATTTATATTAAGATACAGCTTTACAAGAAGATATTCTAGATTTGCTGTCAAAATCCAAGTA

AATTATGGAGGTGTTATCCTTTGTCCGCAGTGTTCTAGCTTCACTCAATAAAAAATTAGCAACCTTCTCA

GCAGAATTCTCTCCATCCGAAAGGTATTTCTCCTTTGTCTGAAGCACAAGTTGAACTGCCTTTTTAACAC

CAATGACATCCCAAAAGCCATCACTTGCCAACAGTGCGAAGGTGCTGCTTGCTCGATCTATATGCACAGC

TTTGCTTATGTAAGGTTCTGAACTGAATCGGGCATCCTGCTGTTTCAAAAATTTGTCTCCAAGCATCCGA

GCGAGGTTCAAGCCACAAAGACGTGTTTCCCCATCTTTCAATGGTTCTCCTGTTTCATTGATTCGAAGCC

TTTCAGAACAACTAGTTATTCTATGGTCTTCCGTCATCTTAATCTGCTTCCCATCAACATTCATGACACA

AGCTGAATCCCCAACATTGGCACACTGGAGCAAAGAGATTTTCGTCATCA

>FM894931.1 FM894931 Jatropha curcas embryo 71-95 (DAF) Jatropha curcas cDNA clone rjcpga0_000840, mRNA sequence

CAAGAAGAATGAATGATAGTTTCCATTATCAATCAATGGGCCTTGTATCAATGCTGCTCTGCAGCTGACT

CTTCATATTCCTATTCTCTTTTCCAGCTATCACCCAAAGGCAGCCCCTCTCTACTTTACATGCTTTCTTC

AAGTAAAATTCAGACCACATATATATTCTTATATATGGAAAAGAAAAGTAAACAAAAGCAAACCAAAAAA

TAAAACGAAAATAAAAGCAAAAACAAGCAAACGATAGGAGATTTTTTTGGGGCAAAGAAGCTGCAACGGG

CAAATGCGAAGTTTAGTCTTGAGGCTTCTCCCATTTGAGGGGCTTCACCACCTCCCATGTGAAGTCAGCG

TCATCCCTTCCAAAGTGACCATAGGCAGCTGTCTTCAAGAACCTACCATTGCCGCCTCTCTTGAGATCTA

AGTTAATAGCAATCATACCAGGCCTGAAGTCGAAGTTTACTTTTACAATCTTGAGAATCTCCTTGTCTGG

GATCTTTCCGGTGCCATAGGAATCAACAAACACGGATAATGGTTCTGGCACGCCAATAGCATAAGACACT

TGAACAATGCATCT

>FM894930.1 FM894930 Jatropha curcas embryo 71-95 (DAF) Jatropha curcas cDNA clone rjcpga0_000839, mRNA sequence

ATTCCTGACGGCGACGTTAGGGAGTCCGGAGACGTCGGCGGGGGCCTCGGGAAGAGTTATCTTTTCTGTT

TAACAGCCCGCCCACCCTGGAAACGACTCAGTCGGAGGTAGGGTCCAGCGGCTGGAAGAGCACCGCACGT

CGCGCGGTGTCCGGTGCGCCCCCGGCGGCCCATGAAAATCCGGAGGACCGAGTGCCTCCCACGCCCGGTC

GTACTCATAACCGCATCAGGTCTCCAAGGTGAACAGCCTCTGGTCGATGGAACAATGTAGGCAAGGGAAG

TCGGCAAAATGGATCCGTAACCTCGGGAAAAGGATTGGCTCTGAGGGCTGGGCCCGGGGGTCCCAGTCCC

GAACCCGTCGGCTGTCGGCGGACTGCTCGAGCTGCTCCCGCGGCGAGAGCGGGTCGCCGCGTGCCGGCCG

GGGGACGGACTGGGAACGGCCCTTCCGGGGGCCTTCCCCGGGCGTCGAACAGTCGACTCAGAACTGGTAC

GGACAATGGGAATCCGACTGTTTAATTAAA

>FM894929.1 FM894929 Jatropha curcas embryo 71-95 (DAF) Jatropha curcas cDNA clone rjcpga0_000838, mRNA sequence

GTCTTCAATAACCTCTCCCCAGTCACCACCTTACCATTTTTCCATCTTTAAGCTAAGAAAGCACCTGATC

AATTATATTTTTGATATTCACTTATACCAAATAAGTGATCTTTGGAATAAAAATTAAATTGAAATGAAAA

AAAAAAAACCTTTGAATATCACCTGTAAAAATTTCCACTAAATTTTTCTATAAGAATAATATTGAAAATA

AAAAAAATCTATTTGACGGTGTTCGAAAATGGAGGGCCTTCAAAGCGAATATAATCGTACATAATCCCTT

GGAAAGGGCTTTGATCGGCTGTTTGTGTCAAAAAGATTGTGTTAATCCCTTCAACAAGGTGAGCTCCTGG

TACATCTACATTGAATAGCCTGTACAGCCCATGAATTCCATGTCTCGCTATTGTATTGTCCTTCCCAATT

CTTCCAGTTGAAAATAAAGGAGAACTTGCATTTACATCATTAATCCGAACCTGTAAATTGGCATTATTTG

CTGTCGCAAGTGCCAAACGTAGTTTATATATCCCACTTCGATCAACTGCTTCTTGCTTGAACTTAATTTG

CCATGTAGTTCCTTCATATGTGTCATTTTCCTTCTTCCTTGTTACTTGTGCAAAAAA

>FM894928.1 FM894928 Jatropha curcas embryo 71-95 (DAF) Jatropha curcas cDNA clone rjcpga0_000837, mRNA sequence

ATTCCAAGCACAACAATGAGGCTTGACATGGAAGCGGTGCCAAAGGACAAAGCCACTCCAGTCAGCTTAG

CCCAGCATACTTACTGGAACTTAGCTGGCCACAACTCAGGAAGTATACTTGACCATTCAATTCAAATATG

GGGATCTCATATTACACCTGTTGATGAGAATATTGTACCTACTGGTGAAATAATGCCAGTTAAAGGCACC

CCTTTTGATTTCACCTCTGAGAAGAAGATTGGTGCTTCAATCGATGAAGTAGGTTTAGGGTATGATCACA

ACTACGTGCTCGACTGCGGGGAAGAGAAAAATGGTTTAAAACATGCTGGGAAAGTTAAAGATCCATCAAG

CTTGAGGGTCCCTTAACTTGTGG

>FM894927.1 FM894927 Jatropha curcas embryo 71-95 (DAF) Jatropha curcas cDNA clone rjcpga0_000836, mRNA sequence

ATTCCAATCATGGGTTCTAAACTTGAAGCTTTGTTATTCATTTTTATGATTTTCATGGCAATATCATTGC

CACCAATTTATGCTTGTGCACCTTGTACTCAGCCACATCCACCAGGCCACCGTCCAACCCACCCAAAAGT

TCCTCATCCTAACCCACCAAGCACCAAGCACCCACCACACCACGGCGGCCACCCAAAACCACCTTCAACA

AAAAACCCACCAGTGGTTTTGCCACCAATAGTCATTAATCCTCCTCCTGTAGTAAACCCTCCTGTAATAA

TAACACCTCCTCCTGTAATAACACCGCCGCCTTCTTCAGGTTACCCACCATACACGCCTAGCCCTCCATC

TGGTGGTGGCGGAGGCGGCGGCGGTGGCGGAGGCGGTGGAGGAGGAGGAGGAGGAGGTGGTGGTGGTGGT

GTTCCGGGGCTTAATCCTCCACCTACTACTCAACCCACTTGTCCAATCAACGCCCTTAAGCTAGGGGCTT

GTGTTGATGTATTACGAGGATTAGTGCATGTTGGATTAGGGAATCCAGTTGAGAATGTATGCTGCCCCGT

AATTAAAGGATTGCT

>FM894926.1 FM894926 Jatropha curcas embryo 71-95 (DAF) Jatropha curcas cDNA clone rjcpga0_000835, mRNA sequence

ATTGTTTGGAGGAGAGTTTCAGTCTGGACACACAGCAGATGTACTGAGCATCTCAATCAATGGGTCAAGC

TCAAGAATGTTCGTATCTGGCTCCTGTGACTCAACCGCCCGATTGTGGGATACTCGCGTTGCTAGTCGAG

CTGTGCGTACTTTTCATGGGCATGAGGGAGATGTTAATGCTGTGAAGTTCTTTCCAGATGGCAATAGATT

TGGAACTGGATCTGACGATGGAACTTGTAGATTGTTTGACATCAGAACTGGGCATCAACTCCAAGTATAC

TATCAACAGCATGGTGACAATGAAGTCCCTCATGTGACCTCTATTGCATTCTCCATATCAGGAAGACTTC

TCTTTGCTGGATATTCAAATGGTGATTGCTATGTTTGGGATACTTTATTAGCGCAGGTTGGTTTAAACTT

GGGATCTCTGCAGAATTCGCATGAGGGTCGGATAAGCTGTTTGGGCTTGTCAGCAGATGGGAGTGCCTTG

TGTACAGGAAGTTATGATACAAACCTAAAGATTTGGGCGTTCGGAGGCCATAGGAAGGTGATTTGAGAAT

GGAAATCAAAGAAAAAAGTTACCGTGTGGTCCT

>FM894925.1 FM894925 Jatropha curcas embryo 71-95 (DAF) Jatropha curcas cDNA clone rjcpga0_000834, mRNA sequence

AGGTTTTTATCTCGAAATGGAGAACCAAGCCTTGCTTCAACAAATTGCCGCCATGAGGAAATCTTTCTTT

GATGAGGGAACTCTCGACTCGTTTTTTGTTCAACTGGAACAACTCCAAGGCCCTGATGATCCAAATTTCG

TGGAGGAAATACTGACTTTGTTCTTCTCAGACTCCATGGATATGCTAGCTACTGTAGAGGAGAAACTGAC

GAAGAGCCCTGTCGATTTCACTGGAATTAACAAGATACTTCATCAGCTGAAGGTCAACAGCGCGAGCATT

GGAGCTAAAAAGGTTAAGGACAAAGTGATTGCAATGAGGAACAATAGCAAGGAACATAATGTGGAAGGTA

TCAGAGTCTCCCTGCAAGAGCTGAGGATGGAATATGAGACCCTGAAAGGGAAGTTTGAGCCCTATTTTGA

GCTGCTGAAACAAGCTGGCCCTCAAGAGGGAGAAGCACTGCCTTCACTGTACGGCGGTCGTTTCTAGAAG

GCGACAAAATGGCTTGGATGGTGGCCAACAATTGCTCTATCTGCAAATTAATCCACCATTTCTATGTTT

>FM894924.1 FM894924 Jatropha curcas embryo 71-95 (DAF) Jatropha curcas cDNA clone rjcpga0_000833, mRNA sequence

ATTCTCTTAACCAGCCTCAATAGCCGGTCCCTATGGTTCTTGGTGCTGACACTTTCATCAGGCATGTGGT

GAAGCACAAATGCAAAATTCACAGCTAGAGCCTGTCTCCAAAAGTGGTGACCCTTGTTGAGCAGGAATCT

AACACAAATACTGCTGCATTCTTCCCTCGGTTCCTTGAGACATTAGATTATTATATGGCTATGTTTGAAT

CAATTGATGTAACGCTCCCAAGAGAGCATAAGGAGAGGATCAATGTTGAGCAGCACTGCCTGGCAAGGGA

TGTTGTTAACATAATAGCATGTGAAGAAACTGAGAGAGTGGAACGACATGAACTTCTTGGGAAGTGGCGG

TCACGGTTCAGAATGGCAGGTTTTGCTCCTTACCCTTTAAGCTCCTTGGTAAATGCCACCATTAAAACTC

TTCTAGAGAATTATTGTGATAAATATAGGCTTCAAGAGAGACATGGAGCTCTATATCTTGGTTGGATGAA

CAGAGAGCTGGTGGCATCTTGTGCATGGAAATGAAAGCCGAGTATAATTTACTCTTACTTCAGCATGTGT

GCAGCCAGCTGAGGCAGAACTTTAGTACTTCAATATTTTCCTTTTTCACCCTTTTGCCTC

>FM894923.1 FM894923 Jatropha curcas embryo 71-95 (DAF) Jatropha curcas cDNA clone rjcpga0_000832, mRNA sequence

GCTGCAGGTTTTTTTTAAGGATCGACGAATCAAATGATATTTTTGTTCCAATTTTTGCCTCTTCTTCTCC

CGCTGAATCAAACTTTTCCTTGCCATAATATTTCACTTCCTATTAGTATCGCGGATACAAGTCGGATCCT

AGATGTAGAAATATAAAAAATCGCCTTATCCAGCGAAAAAAATGATATTTTCGCGGATACAGCACAAAAA

GAATTAACCAAATTTACCTGATGTAGAGGCAATCAAGAAAGCCGCATAAGTGAAGATATAACCTACAGAA

AAGTGGGCTAATCCAACCAATCTTGCTTGCACAATGGAAAGAGCTACTGGTTTATCTCTCCATCGAATCA

AATTAGCCAAAGGCGTACGTTCATGAGCCCACGCTAAAGTTTCAATCAATTCCTGCCAATATCCACGCCA

AAAAAAAAAAA

>FM894922.1 FM894922 Jatropha curcas embryo 71-95 (DAF) Jatropha curcas cDNA clone rjcpga0_000831, mRNA sequence

ATTCAAAGGATTCTATCCGTCGCCCGATCCCGCCCCCCGTCCCCCCCCTGCGGCCCTCGCGGCTCGTCCG

CCGCGGGGGCTTGGCCAACGGCACGTGCCTCTGGGGGCCGGATGGCCCCTACTGCGGGTCGGCAAACGGG

CGACGGACACGCGCGTCGCTTCTGGCCCGGATTCTGACTTAGAGGCGTTCAGTCATAATCCAGCGCACGG

TAGCTTCGCGCCACTGGCTTTTCAACCAAGCGCGATGACCAATTGTGCGAATCAACGGTTCCTCTCGTAC

TAGGTTGAATTACCATCGCGACACCATCATCAGTAGGGTAAAACTAACCTGTCTCACGACGGTCTAAACC

CAGCTCACGTTCCCTATTGGTGGGTGAACAATCCAACACTTGGTGAATTCTGCTTCACAATGATAGGAAG

AGCCGACATCGAAGGATCAAAAAGCAACGTCGCTATGAACGCTTGGCTGCCACAAGCCAGTTATCCCTGT

GGTAACTTTTCTGACACCTCTAGCTTCAAATTCCGAAGGTCTAAAGGATCGATAGGCCACGCTTTCACGG

TTCGTATTCGTACTGGAAATCAGAATCAAACGAGCTTTTACCCTTTTGTTCCACACGAGATTTCTGTTCT

CGTTGAGCTCATCTTAGGACACCTGCG

>FM894921.1 FM894921 Jatropha curcas embryo 71-95 (DAF) Jatropha curcas cDNA clone rjcpga0_000830, mRNA sequence

CATGGTTCGATCCTTCCCANGTAAAACGCGGCGTGTTCGAATTCTGATCGCTTTTACGCGAAGAAAGGGG

GAACCACCCTCTAAGCCTAAGTATTCCTCAATGACCGATAGCGTACAAGTACCGTGAGGGAAAGGTGAAA

>FM894920.1 FM894920 Jatropha curcas embryo 71-95 (DAF) Jatropha curcas cDNA clone rjcpga0_000829, mRNA sequence

GGAAATTTGTCTGGACAACGTCAAGGGCAACCTGTCTTTATCACCAGATTGGAAGGTTATAGGAATGCTT

CAGCTTCTGAATCGCTTGCAGCAAACTGGCCACCAACAATGCAGATAGTTCGGCTCATTTCTCAGGACCA

CATGAATAACAAGCAGTATGTTGGGAAGGCAGATTTTCTAGTATTTCGGGCAATGAATCAGCATGGATTT

CTTGGCCAACTGCAAGAAAAGAAGCTTTGTGCAGTCATCCAGTTGCCATCACAGACGTTGCTGCTTTCTG

TTTCGGACAAAGCTTGCCGCTTGATAGGGATGCTTTTTCCTGGGGATATGGTTGTGTTTAAGCCTCAAAT

ACCCAGTCAGCAGCAGCAACAGCAACAAATGCAACAACAGCAGCAACAGCAACAAATGCAACAACAGCAT

CACCAGCAGATGCAATCGCAGCAGCATCAACAGCTACAACAACAACAGCACCCGCAGCTTCAACAGCAGC

AGCAACTTCCGCAGCTGCAGCAGCAGCAGCAGCTTCCACAAATGCAACATCAGCAGCAGCATCCCAGTCA

GTTGCAGCAACAGCAACAACTCTCTCAGCTTCAACAACAGCAGCAACTTCAGCAGCAGCAACAGCAGCAG

CTTC

>FM894919.1 FM894919 Jatropha curcas embryo 71-95 (DAF) Jatropha curcas cDNA clone rjcpga0_000828, mRNA sequence

ATTCCCGAAAGATGGTGAACTATGCCTGAGCGGGGCGAAGCCAGAGGAAACTCTGGTGGAGGCCCGCAGC

GATACTGACGTGCAAATCGTTCGTCTGACTTGGGTATAGGGGCGAAAGACTAATCGAACCGTCTAGTAGC

TGGTTCCCTCCGAAGTTTCCCTCAGGATAGCTGGAGCTCGGGACGAGTTCTATCGGGTAAAGCCAATGAT

TAGAGGCATCGGGGGCGCAACGCCCTCGACCTATTCTCAAACTTTAAATAGGTAGGACGGCGCGGCTGCT

TCGCTGAGCCGCGCCACGGAATCGAGAGCTCCAAGTGGGCCATTTTTGGTAAGCAGAACTGGCGATGCGG

GATGAACCGGAAGCCGGGTTACGGTGCCCAACTGCGCGCTAACCTAGAACCCACAAAGGGTGTTGGTCGA

TTAAGACAGCAGGACGGTGGTCATGGAAGTCGAAATCCGCTAAGGAGTGTGTAACAACTCACCTGCCGAA

TCAACTAGCCCCGAAAATGGATGGCGCTTAAGCGCGCGACCTATAACCCGGCCGTCGGGGCAAGAGCCAG

GCCCCGATGAGTAGGAGGGCGCGGCGG

>FM894918.1 FM894918 Jatropha curcas embryo 71-95 (DAF) Jatropha curcas cDNA clone rjcpga0_000827, mRNA sequence

GCTGCAGGAAGGATTCTATCCGTCGCCCGAATGGGAATTGTACTTTAAGGCGGCCCTCGCGGCTCGTCCG

CCGCGGGGGCTTGGCCAACGGCACGTGCCTCTGGGGGCCGGATGGCCCCTACTGCGGGTCGGCAAACGGG

CGACGGACACGCGCGTCGCTTCTGGCCCGGATTCTGACTTAGAGGCGTTCAGTCATAATCCAGCGCACGG

TAGCTTCGCGCCACTGGCTTTTCAACCAAGCGCGATGACCAATTGTGCGAATCAACGGTTCCTCTCGTAC

TAGGTTGAATTACCATCGCGACACCATCATCAGTAGGGTAAAACTAACCTGTCTCACGACGGTCTAAACC

CAGCTCACGTTCCCTATTGGTGGGTGAACAATCCAACACTTGGTGAATTCTGCTTCACAATGATAGGAAG

AGCCGACATCGAAGGATCAAAAAGCAACGTCGCTATGAACGCTTGGCTGCCACAAGCCAGTTATCCCTGT

GGTAACTTTTCTGACACCTCTAGCTTCAAATTCCGAAGGTCTAAAGGATCGATAGGCCACGCTTT

>FM894917.1 FM894917 Jatropha curcas embryo 71-95 (DAF) Jatropha curcas cDNA clone rjcpga0_000826, mRNA sequence

TTTTTTATTTTGTTCAAGTTCATTCCATAACTTGTAAAATATAAACCAAACTATGTTTTTTTTTTTTTTT

TTTTCCAAACCAGCAAATAGCAGGGGTCCATGCTTGTAAGGTTAACACTTATCCACCATCATCTATCTTT

TCATCACAATCCCTTCCAGAATCCCATCTGAACCAAAGTTACCAGAAAACTGCTCAGGATCAACAGGACC

TGGCAGCTGAAATGCAATAGAAAACTTTCCTGGTGGACAAAGGTTCTTTGATTGCATTTCAAAGACTTGT

GAGAACCTGCATACAGTCCTCTCGCCGGTTGTCGTCTCACCATTTATCAAAACCCTACCACCAGCATCAA

CTCCACAGCTGAATTCCTTCTCATCTCTTTTCACCCCAGGAAGAGATATGCGAAACATATATGCATCATC

GCATTCACCAATATCCATGAGTCCAATAATGGGTCCCACCTTCCCCATAGTTCTGGTGTCATCTCTGGTT

GGCGGAGGAGATGTATGACTATGCTCCGGAGGCAATGTAGTACATGGCACAATGCTAAGAGGCTCCACAA

AATGGACATGCTGATCCTCAGGTTGCATGGTGCTAGCA

>FM894916.1 FM894916 Jatropha curcas embryo 71-95 (DAF) Jatropha curcas cDNA clone rjcpga0_000825, mRNA sequence

ATTCGCAAAGGATTCTATCCGTCGCCCGATGGGAATTGTACTTTAAGGCGGCCCTCGCGGCTCGTCCGCC

GCGGGGGCTTGGCCAACGGCACGTGCCTCTGGGGGCCGGATGGCCCCTACTGCGGGTCGGCAAACGGGCG

ACGGACACGCGCGTCGCTTCTGGCCCGGATTCTGACTTAGAGGCGTTCAGTCATAATCCAGCGCACGGTA

GCTTCGCGCCACTGGCTTTTCAACCAAGCGCGATGACCAATTGTGCGAATCAACGGTTCCTCTCGTACTA

GGTTGAATTACCATCGCGACACCATCATCAGTAGGGTAAAACTAACCTGTCTCACGACGGTCTAATCCCA

GCTCACGTTCCCTATTGGTGGGTGAACAATCCAACACTTGGTGAATTCTGCTTCACAATGATAGGAAGAG

CCGACATCGAAGGATCAAAAAGCAACGTCGCTATGAACGCTTGGCTGCCACAAGCCAGTTATCCCTGTGG

TAACTTTTCTGACACCTCTAGCTTCAAATTCCGAAGGTCTAAAGGATCGATAGGCCACGCTTTCACGGTT

CGTATTCGTACTGGAAATCAGAATC

>FM894915.1 FM894915 Jatropha curcas embryo 71-95 (DAF) Jatropha curcas cDNA clone rjcpga0_000824, mRNA sequence

GGATTCTATCCGTCGCCCGATGGGAATTGTACTTTAAGGCGGCCCTCGCGGCTCGTCCGCCGCGGGGGCT

TGGCCAACGGCACGTGCCTCTGGGGGCCGGATGGCCCCTACTGCGGGTCGGCAAACGGGCGACGGACACG

CGCGTCGCTTCTGGCCCGGATTCTGACTTAGAGGCGTTCAGTCATAATCCAGCGCACGGTAGCTTCGCGC

CACTGGCTTTTCAACCAAGCGCGATGACCAATTGTGCGAATCAACGGTTCCTCTCGTACTAGGTTGAATT

ACCATCGCGACACCATCATCAGTAGGGTAAAACTAACCTGTCTCACGACGGTCTAATCCCAGCTCACGTT

CCCTATTGGTGGGTGAACAATCCAACACTTGGTGAATTCTGCTTCACAATGATAGGAAGAGCCGACATCG

AAGGATCAAAAAGCAACGTCGCTATGAACGCTTGGCTGCCACAAGCCAGTTATCCCTGTGGTAACTTTTC

TGACACCTCTAGCTTCAAATTCCGAAGGTCTAAAGGATCGATAGGCCACGCTTTCACGGTTCGTATTCGT

ACTGGAAATCAGA

>FM894914.1 FM894914 Jatropha curcas embryo 71-95 (DAF) Jatropha curcas cDNA clone rjcpga0_000823, mRNA sequence

ATGTTTGTTTCGGAGCCGTTTTGGCGGGGCGTACTAAACCCACTACGTACCACACCACCGGGCGGCTCGC

CTGAATGCCGAGTCTTTCTCCGCCGCCAACTCGACGTCGTCGTCACCTGCAAGATAAGGCCAAAAACTGG

ACTTTACTAAACAAGCGAGAAAAGCCCCTTTCTCTGTTATTAGTAAAGTAAAGCGCGCTAGCTGCAATCT

TTCTAAAGCGCACACTAGAAAGTGCTTCGAAAGGCGCCGGCTCCCCTCTTACTGACAGCACAGCTACGTG

CTGGCACTCAATTAGTAGCGCTGGCACGTCACTCGGCTCCTCGGCTCACTTCGGTTGCAAAGACTTTCTC

CTTAGGCGCATGTCTCAGCAACACAAAACGAGGGTTTCGCTCGTTATAGGACTTGACCAAACATCTCACG

ACACGAGCTGACGACAGCCATGCAGCACCTGTATGAAAGTCAGTACCATCCCGTTAAGGACAGGTTTTGT

TGTTCATATGTCAAGGGCTGGTAAGGTTTTGCGCGTAGTATCGAATTAAACCACATGCTCCACCGCTTGT

GCAGGCCCCCGTCAATTCCTTTGAGTTTCGGTCTTGCGACCGTACTCCCCAGGCGGAGTGT

>FM894913.1 FM894913 Jatropha curcas embryo 71-95 (DAF) Jatropha curcas cDNA clone rjcpga0_000822, mRNA sequence

ATTCCGAAGCTTTCTTTCTCTCTTTTCTCTCCACAGCACAACCAAACCAGTCACAGAGACATGGTTCTTG

GAGCTAACGGCGTCGTTTGGATGGGAGGAAAAGAGAGGAGCAGTGGAGGAAGAGAAGAAGATGTTGTTTC

TTCTGGACTAGAACCAAGTAAGGATAATGAAGTAAGCGTAAAGAAAAAAATTGGGTATCTGGTTCTCTCT

CTACTTTTAATCATGCTTGAAACTGATTGGTATATCAATTCTGATATAAACCCGATCTCCTCTCCAAAAC

CACCATGAAATCAGAGACCTTGCTTTTTGCTCTAACCCAACACCAGAAAACCTTCTTTTACAGCCTATGG

ACTCTTCCTCTTCTTGTTCTCCTTCACAAGCATTTTCTCTCGATCCTTCACAATCACACCATCCTTTTTT

GCCCTCTAAA

>FM894912.1 FM894912 Jatropha curcas embryo 71-95 (DAF) Jatropha curcas cDNA clone rjcpga0_000821, mRNA sequence

AAGGATTCGTGTTAGAGGAGGCAATGTTAAGTGGAGAGCCCTGAGATTGGACACAGGAAATTATTCCTGG

GGTAGTGAGGCTGTTACCCGCAAGACTCGTATCCTTGATGTGGTTTATAATGCCTCCAACAACGAACTTG

TTAGGACCCAAACTTTGGTGAAGAGTGCTATTGTTCAGGTTGATGCTGCTCCATTTAAGCAATGGTATCT

CCAGCACTATGGTGTTGACATAGGCAGGAAGAAGAAGACTGTTGCTAAGAAGGAAACTACTGAGGAAGGG

GATGCTGCTGCTCCTGCCACGGCAGAGGAAGCTAAGAAGAGCAACCATGTACAGAGGAAGCTTGAGAAAC

GCCAGAAGGATCGTAAGCTTGACCCTCACATTGAAGAGCAATTTGGAGCTGGAAGATTGTTGGCCTGCAT

CTCATCTCGACCTGGTCAATGTGGCAGAGCTGATGGGTACATATTGGAGGGTAAGGAACTTGAGTTCTAT

ATGAAGAAGATCCAAAAGAAGAAGGGCAANGGTGCTGCCTAAATGTTTTAACTGTCAAGATTTTGCTGCC

GACTGTCTGGTTTATTTTAGTTTAGGTTTCTTGAGTTTTAAAAATACGATTTGATACCTTAATGGTAGAA

TGCTA

>FM894911.1 FM894911 Jatropha curcas embryo 71-95 (DAF) Jatropha curcas cDNA clone rjcpga0_000820, mRNA sequence

AGGAATTCTATCCGTCGCCCGAATGGGAATTGTACTTTAAGTGCGGCCCTCGCGGCTCGTCCGCCGCGGG

GGCTTGGCCAACGGCACGTGCCTCTGGGGGCCGGATGGCCCCTACTGCGGGTCGGCAAACGGGCGACGGA

CACGCGCGTCGCTTCTGGCCCGGATTCTGACTTAGAGGCGTTCAGTCATAATCCAGCGCACGGTAGCTTC

GCGCCACTGGCTTTTCAACCAAGCGCGATGACCAATTGTGCGAATCAACGGTTCCTCTCGTACTAGGTTG

AATTACCATCGCGACACCATCATCAGTAGGGTAAAACTAACCTGTCTCACGACGGTCTAAACCCAGCTCA

CGTTCCCTATTGGTGGGTGAACAATCCAACACTTGGTGAATTCTGCTTCACAATGATAGGAAGAGCCGAC

ATCGAAGGATCAAAAAGCAACGTCGCTATGAACGCTTGGCTGCCACAAGCCAGTTATCCCTGTGGTAACT

TTTCTGACACCTCTAGCTTCAAATTCCGAAGGTCTAAAGGATCGATAGGCCACGCTTTCACGGTTCGTAT

TCGTACTGGAAATCAGAATCAAACGAGCTTTTACCCTTTTGTTCCACACGAGATTTCTGTTC

>FM894910.1 FM894910 Jatropha curcas embryo 71-95 (DAF) Jatropha curcas cDNA clone rjcpga0_000819, mRNA sequence

CAAAACCAAACCATTAACCTGATAGTTTTTAAAACTGGCAATCAAAACATAGTGCAAGTAAAATCCAATT

ACATTTTCTTCTCTTCTTCCAGAGGGGCGGTAAATAAACAATTAACAAAGAGGCACCAAGTGATGTGATG

GGATTTGCAGCAATGAGAAGACGAGATACAAAATGGTCCTATTGGTGCTTGGGCCTCTTTATGGAATCAA

TTACAATTCTCCGGCTCGGGTGCACAATCTTACAGTTGTTGCTCATACCTGGCTCTGAAGGCAAAATGTA

CAAGCAGCCTCAGCTCCTGCTTCTAAGGAAACACCAAAAGCAGAGCATAATTAATGGCATTTATACATAT

TCTTGTGCTTCTTCAAATTGTCTACAGCCTCTCTTTCAATGCAAATTGCAGCAGCCACTGTCACCACTTC

ATTATTCATGGACAATAGACCAGGCCACCTTATGCCAAGTATCTGAACAAGTTAGGATTATCTTTGTCCC

TTTCGAGATAATCTCGTGTAATCAGATCTTCAATCCGCTTTTTAATTGCCTTGAAATCAGGCTTGAACAT

GCGACCCAACTGCTCAACACACTCCATAACCAGCTGTTGGTGACCCAAAACTTTCCGGCTCTT

>FM894909.1 FM894909 Jatropha curcas embryo 71-95 (DAF) Jatropha curcas cDNA clone rjcpga0_000818, mRNA sequence

CCGCGGTGGCGGCGCTCTAAACTATGGATCCCCGGCTGCAGAATCGAATCCCATCTCTCTTTCAATGGCA

GCCGCTGCCGGTTCATCAATCTCCATGCAGTCTCGTCCTAGACTGGCTGCAACCAGAATCTCTAGCCTCA

AATCTTTTTCGTTTCCAAGCCAGAGAAGTTTCCTGTCTTTCAGAGTGCAGCCAGTTCCAGCATCTCGCCT

TCGGATTTCTTGTGCTGCCAAACCAGAAACAGTAGAAAAGGTGTGTCAAATAGTGAAGAAGCAGCTGGCA

TTAGCCCCAGAAGCTGTTGTTACCGGCGAGTCAAAATTTACAACACTAGGAGCAGATTCTCTTGATACAG

TTGAGATTGTGATGGGACTGGAGGAGGAATTCGATATCAGTGTGGAAGAGGAGAGTGCCCAGAGCATCAC

AACCGTTCAGGAAGCTGCTGATCTTATTGAAAAACTTGTTGAGAAGAAGGACGCTTAGAAGATCAGTCTA

GGAAATCCTTCAATTCCCTTTCCCCNCTGGCGCCCCCCCCCCCCCCCTCAACATAATTGCAGTTATGCTT

TGTGTTAGCCCGCCATATGATTGTGCCAGGCAATCTTGTTGTGAACTATGTTTTTGTGGATTTCATCTAG

AAAAACACTCTAAAATTTCCTAATTTATGCAATTATGACGTGTATTTTCTTT

>FM894908.1 FM894908 Jatropha curcas embryo 71-95 (DAF) Jatropha curcas cDNA clone rjcpga0_000817, mRNA sequence

ATTCCCCNGGGTTTAAAACCTGTCCGTCACAACTATAGTGTCTCAGTGGGCCAGAGTGTGTTTCGTCCTG

GGGAAGGTCACCCACGCGCTAAATGATGATACATTACTTCAAGCATTGATGGATAGTAGATACCCAACTG

AGGGACTGACAAATTATGATGAATGGACTATTGATTTGAGGAAACTGAATATGGGGACAGCCTTTGCCCA

AGGTGCTTTTGGAAAGTTATATAGAGGAACATATAATGGAGAGGATGTCGCAATAAAGATACTGGAGAGG

CCAGAGAATAGCCCGGAGAAGGCCCAACTGATGGAGCAGCAATTTCAGCAGGAAGTCATGATGCTTGCAA

CACTAAAGCACCCTAACATTGTGCGCTTTATTGGCGCATGTCGTAAGCCCATGGTTTGGTGTATTGTGAC

AGAATATGCAAAGGGAGGGTCAGTTCGGCAATCTCTGAATAAGAGGCATAGTCGAGCGGTACCACTGAAA

TTGGCAGTTAAGCAGGCATTAGATGTCGCAAGGGGAATGGCATACGTACCTGGGCTTGGGTTTATACACC

GGGAT

>FM894907.1 FM894907 Jatropha curcas embryo 71-95 (DAF) Jatropha curcas cDNA clone rjcpga0_000816, mRNA sequence

CTCAAATTGAAACTGCTGCTAGACCATATGCCTTCATTAAATTTGATACATTCATACAGAAAACAAAGAA

ATTGTACCAGGACACCCGTACTCAGCGGAATATTGCAAAGTTGAATGATGAACTTTATGAAGTGCACCAA

ATAATGACCCGCAATGTGCAGGAAGTACTTGGCGTTGGTGAAAAACTAGACCAGGTCAGTGAAATGTCCA

GCAGATTAACATCAGAATCTCGCATATATGCTGACAAGGCAAGAGATTTGAATCGGCAGGCCTTGATTAG

AAAGTGGGCTCCTGTTGCTATCGTGTTAGGAATTGTCTTCCTCCTTTTTTGGGTTAAGAGAAAGCTCTGG

TGATCTAAATGCCTAAGAATTGTTGCATCATTTTACTGGGTTTCTCTTTGTTGCTGCCATTTACTTGCGT

TCATCGGGGATACGTACTAAAGGGAACTTTTGAGTGGGACCTAGATGCACAGGAAGACCTAAGTCATGTT

TCAAGCTGGACTGTAGATTTTGTTCGTGGGAAAGTGCCTTACGACGACCATGTGGAAATGTGAAAGAGAG

ACCC

>FM894906.1 FM894906 Jatropha curcas embryo 71-95 (DAF) Jatropha curcas cDNA clone rjcpga0_000815, mRNA sequence

CTGGCGGGATCCCCCTGAGGGTTGCACCGCCGACCGACCTTGATCTTCTGAGAAGGGTTCGAGTGAGAGC

ATGCCTGTCGGGACCCGAAAGATGGTGAACTATGCCTGAGCGGGGCGAAGCCAGAGGAAACTCTGGTGGA

GGCCCGCAGCGATACTGACGTGCAAATCGTTCGTCTGACTTGGGTATAGGGGCGAAAGACTAATCGAACC

GTCTAGTAGCTGGTTCCCTCCGAAGTTTCCCTCAGGATAGCTGGAGCTCGGGACGAGTTCTATCGGGTAA

AGCC

>FM894905.1 FM894905 Jatropha curcas embryo 71-95 (DAF) Jatropha curcas cDNA clone rjcpga0_000814, mRNA sequence

AAAAATATAAAAAAAAGGACCAGGACCTTTTCCAAATTCCATCAGCTGTCTCAATAATGCTCAGTAGGCG

CGAGTACGCTAGTCAAAAAATATAAATTGTATCAACAATTAAGCATTTAAACTTAAGCAGTCTCACTAGC

GTCTCTTATTGGGTTCCAGAAACAGGCAAGTTCAGTAACACTGCCAGTGAGGCACCCCTGATGTACATCA

AATACCTATCTGTAGTATATTTGCAAAAAAAAAAAAAAAAAAAAAAAAATCTAAAAATCCGACGACTTTG

GGGTGCTGTATACTCTCATTCAACCGCAGTAAATATGAACAACTCTTCACAAAAACACGGGACCCATCAC

TACCGTGATCGTGGCTAACATTTTTATGAGGACATGAAGCGAAGGTCCAGCTGTATCTTTGAACGGGTCT

CCTACAGTATCTCCTGTAACTGCTGCTTTATGGCACTCGCTCCC

>FM894904.1 FM894904 Jatropha curcas embryo 71-95 (DAF) Jatropha curcas cDNA clone rjcpga0_000813, mRNA sequence

TTCACATCCGTAAACTTCCAGCTTCTTCTGCTGCACAAGCAAATGCTTTATGGTTGGCTAAATGTGAGGA

CTCATCAAAACCAAATATATCAGAGTCTGCCAAAGATATTTCTAGCACTGGAGATGATAGCATGGATGTT

GTTGATGATAATGATGATAATAAATCAGAGAGCCATAGTTGGAACGGTAGGCTGCACCACAAGGAAGGAG

AATTTGATGTATAGTGGTACTTACTAAACTGATTCTGATATATAATGCCCATGAATTTTGCAGATACAAA

TATACGTGGGAAATCAGATTGTAAAGAATAATGAAGTTGACAAACATAGGATTTTAAGTTTGGAGTTTAG

TTTATGTTTTTTTTTTTTCTCGAACTAAGAGCTAATGTTATATATACATCAGAGAGGAGGACATAGGGAT

TTTGCCGATACTACTGATGCGGATTTAATTTTCTGATCTCCAGCTTGAGTTCTGTCTCTTCCTGTATCTG

TTATTTGTTGTTCATTTGCAGTTTATGTAATGCATATTGATGGGTTGGTACTTGAC

>FM894903.1 FM894903 Jatropha curcas embryo 71-95 (DAF) Jatropha curcas cDNA clone rjcpga0_000812, mRNA sequence

TTNGGACTTCAAATCCTTGTCTATATATATGAATATTCATATCCCCAATAACAACAATGTATAGCTCTAT

TGTTGCAATCAAAGCTAACAAAGTTTACATATACTATAAATAAATTACTCATAAGCTTAGCACTATAGAG

AGTTGCAAAAATCTTGGATAATGCACAAAAACCACTAAGAGAATCCCAAGAACTCTCTTTTCTATCTTCC

TAACAAGGACAAAAAGAACAACTCCAATAATATTATATCTCTTCACCAAAATTCTATAATCCCTCAATTC

CTAACTGCAAAAATTATCATAGGCATGTAAAATTTAAAGTTTCCTCAACAAAAGCAAGAGCCTCAAGTTC

AATATTAGGAAGATCCATTACCTCATCTATAGCAAAATCTACCATCTCTCCACTTTCTGCCACAGAAATA

TCTTGTGTATAAAAAAAGTTACCAAAATCGTTCTCAAACAGCGAGCAGTAATCAGAACCAAGCAGTTCTT

GACTAGCAGAAGGGGAAAGCACAGGCTCTTCCCATAAATCTGAAATCTGTAACTCTTGAAATTTATCCTC

CACATTGCTTGAACTTTCAAGCCCATG

>FM894902.1 FM894902 Jatropha curcas embryo 71-95 (DAF) Jatropha curcas cDNA clone rjcpga0_000810, mRNA sequence

CTGACATGTGTGCGAGTCAACGGGTGAGTAAACCCGCAAGGCGCAAGGAAGCTGACTGGCGGGATCCCCC

CGAGGGTTGCACCGCCGACCGACCTTGATCTTCTGAGAAGGGTTCGAGTGAGAGCATGCCTGTCGGGAAC

CCGAAAGATGGTGAACCCAGCCTGAGCGGGGCGAAGCCAGAGGAAACTCTGGTGGAGGCCCGCAGCGATA

CTGACGTGCAAATCGTTCGTCTGACTTGGGTATAGGGGCGAAAGACTAATCGAACCGTCTAGTAGCTGGT

TCCCTCCGAAGTTTCCCTCAGGATAGCTGGAGCTCGGGACGAGTTCTATCGGGTAAAGCCAATGATTAGA

GGCATCGGGGGCGCAACGCCCTCGACCTATTCTCAAACTTTAAATAGGTAGGA

>FM894901.1 FM894901 Jatropha curcas embryo 71-95 (DAF) Jatropha curcas cDNA clone rjcpga0_000809, mRNA sequence

CTCCTCCACAGAAAATTCTGTAGCTCCCTCTCCTTCCTTTCGCCGTCCTCTTCTTTCAACTCCTCAGCGG

AAGGCGGAGAGATTAGATTCCTAATCGCCGCCACTTGTCAGGGAAGTCACAATGGTTGATCAGGCTCGAA

ACCCGTCTCTCATGCAGAAGGTAGCTGGCCAGCATCTTCATTCCGGCATTTCTCAGGACATCCAGAGGCC

TGCTCTTTACCAAAGGCGTGCATATGGAAACTACTCGAATGCTGCATTCCAATATCCGTTGGCACAGTCA

TACACTGCCTCTACTGACATTTCTATTATTCCATCAACTGCATCTTCTGTTTGCTTACATGCCCCTTCTG

AAAAAGGATTTTCCAGCTTTGCTATTGATTTCCTAATGGGTGGAGTCTCAGCTGCCGTATCAAAAACTGC

TGCAGCTCCAATTGAGCGTGTTAAGCTCTTGATTCAAAATCAGGATGAGATGATTAAAAGTGGTAGACTC

TCAGAACCATACAAGGGTATTGGTGATTGC

>FM894900.1 FM894900 Jatropha curcas embryo 71-95 (DAF) Jatropha curcas cDNA clone rjcpga0_000796, mRNA sequence

CTGAAACGCCGAAATATAGTTTTCATTAATGCATAACAACTATTCCAAACCATGTTTAATTATATGCTAC

ATGTCTAGATCAACACTATGCTTTTTAATAGAAGACAAAGCACGTTAATCTCTAAGAGCTCTTATGTTGC

AGAGCTCATCTTTGCTTATCCAAGAAAAAGAAAAAGAAATTCAAATTTAATTCATCCCATAAGTGCACTT

TTGGAGCTTTACCGAGTCAATCGGTATATTCTCAGGTGGAAAGTCGCACTCTGTCAGTTTCTCCGGCCCT

TGGTCAGGCAACAAATCACACGGTCGAGGACTAACCCCACTTGTAATCCCTGCAATCTCAGTGCATGTCC

ATGGTACCTTCTTGGCCTTAGCTGCCATTCCAATTGTTACATTAGAAATGCAAATTTCCTTAAATGGGTC

GCCGGAGATCCCTTCCAACCTTGCAGCCATGGTTACATTATCAGCAACCATGTCCCTGTAATTAATCCCT

TGAATAACCGGCAATGCATTTGGGTCATAGTTTTTATCAGCATGTGACCCATAATTTCCGGTCATCCAAA

AGACCCATTTCATACTATGCATAGTCATTCTCTTTACATATATGTCTTTAACAAATCCTCCTCTGCCTAC

GGCAGTTTTGATCCTGAACCCTGA

>FM894899.1 FM894899 Jatropha curcas embryo 71-95 (DAF) Jatropha curcas cDNA clone rjcpga0_000795, mRNA sequence

TCTGACATGTGTGCGAGTCAACGGGTGAGTAAACCCGCAAGGCGCAAGGAAGCTGACTGGCGGGATCCCC

CTGAGGGTTGCACCGCCGACCGACCTTGATCTTCTGAGAAGGGTTCGAGTGAGAGCATGCCTGTCGGGAC

CCGAAAGATGGTGAACTATGCCTGAGCGGGGCGAAGCCAGAGGAAACTCTGGTGGAGGCCCGCAGCGATA

CTGACGTGCAAATCGTTCGTCTGACTTGGGTATAGGGGCGAAAGACTAATCGAACCGTCTAGTAGCTGGT

TCCCTCCGAAGTTTCCCTCAGGATAGCTGGAGCTCGGGACGAGTTCTATCGGGTAAAGCCAATGATTAGA

GGCATCGGGGGCGCAACGCCCTCGACCTATTCTCAAACTTTAAATAGGTAGGACGGCGCGGCTGCTTCGC

TGAGCCGCGCCACGGAATCGAGAGCTCCAAGTGGGCCATTTTTGGTAAGCAGAACTGGCGATGCGGGATG

AACCGGAAGCCGGGTTACGGTGCCCAACTGCGCGCTAACCTAGAACCCACAAAGGGTGTTGGTCGATTAA

GACA

>FM894898.1 FM894898 Jatropha curcas embryo 71-95 (DAF) Jatropha curcas cDNA clone rjcpga0_000794, mRNA sequence

GTCTGACATGTGTGCGAGTCAACGGGTGAGTAAACCCGCAAGGCGCAAGGAAGCTGACTGGCGGGATCCC

CCCGAGGGTTGCACCGCCGACCGACCTTGATCTTCTGAGAAGGGTTCGAGTGAGAGCATGCCTGTCGGGA

CCCGAAAGATGGTGAACTATGCCTGAGCGGGGCGAAGCCAGAGGAAACTCTGGTGGAGGCCCGCAGCGAT

ACTGACGTGCAAATCGTTCGTCTGACTTGGGTATAGGGGCGAAAGACTAATCGAACCGTCTAGTAGCTGG

TTCCCTCCGAAGTTTCCCTCAGGATAGCTGGAGCTCGGGACGAGTTCTATCGGGTAAAGCCAATGATTAG

AGGCATCGGGGGCGCAACGCCCTCGACCTATTCTCAAACTTTAAATAGGTAGGACGGCGCGGCTGCTTCG

CTGAGCCGCGCCACGGAATCGAGAGCTCCAAGTGGGCCATTTTTGGTAAGCAGAACTGGCGATGCGGGAT

GAACCGGAAGCCGGGTTACGGTGCCCAACTGCGCGCTAAACCTAGAACCCACAAAGGGTGTTGGTCGATT

A

>FM894897.1 FM894897 Jatropha curcas embryo 71-95 (DAF) Jatropha curcas cDNA clone rjcpga0_000793, mRNA sequence

CCGCATCAGGTCTCCAATGTGAACAGCCTCTGGTCGATGGAACAATGTANGCAATGGAAGTCGGCAAAAT

GGATCCGTAACCTCGGGAAAAGGATTGGCTCTGAGGGCTGGGCCCGGGGGTCCCAGTCCCGAACCCGTCG

GCTGTCGGCGGACTGCTCGAGCTGCTCCCGCGGCGAGAGCGGGTCGCCGCGTGCCGGCCGGGGGACGGAC

TGGGAACGGCCCTTCCGGGGGCCTTCCCCGGGCGTCGAACAGTCGACTCAGAACTGGTACGGACAAGGGG

AATCCGACTGTTTAATTAAAACAAAGCATTGCGATGGTCCCTGAGGATGCTCACGCAATGTGATTTCTGC

CCAGTGCTCTGAATGTCAAAGTGAAGAAATTCAACCAAGCGCGGGTAAACGGCGGGAGTAACTATGACTC

TCTTAAGGTAGCCAAATGCCTCGTCATCTAATTAGTGACGCGCATGAATGGATTAACGAGATTCCCACTG

TCCCTGTCTACTATCCAGCGAAACCACAGCC

>FM894896.1 FM894896 Jatropha curcas embryo 71-95 (DAF) Jatropha curcas cDNA clone rjcpga0_000792, mRNA sequence

ATACGCAAAGGATTCTATCCGTCGCCCGATGGGAATTGTACTTTAAGGCGGCCCATCGCGGCTCGTCCGC

CGAGGGGGCTTAGCCAACGGCACGTGCCTCATGAGGGGCCGGATGGCCCCTACTGCGGGTCGGCAAACCG

GCGACTGACACGCGCGTCGCTTCTGGCCCGGATTCTGACTAACAGGCGTTCAGTCATAATCCACCGCACG

GTAGCTTCGCGCCAGTGGCTTTACAACCAAGCGCGATGACCAATTGTGCAGAATCGGCGGATTCCTCTCG

TACTGAGTTGATATTACCATCGCTGACACCATCATCAGTAGGGTAAAACTAACCTGTCTCACGACGGTCA

TATCCCAGCTCACGTACCCATATAGGTGGGTGAACAATCCAACACTTGGTGAATTCTGCTT

>FM894895.1 FM894895 Jatropha curcas embryo 71-95 (DAF) Jatropha curcas cDNA clone rjcpga0_000790, mRNA sequence

TTTTTTTTTTAAGCACTATATAAGAAATATATATTATATATAGGAATAAGCTAAAAAAAAAAAAAACATG

CACTACCAGATCTTCCTCGAAATAACAACAAAATCCTAGAGCTTTGAATGTCTTATAACAAAGACACCAA

ATTAGGCAAAAGATGAAACAAGATAAGAACATAAAACAAACAGAAAGATACTAAGGCAGTCATTTTTGGA

CCCTTTTAACTTCTCATTCACACAACCAACATCCCTTCCCTTCTTCCTCTTGCTCCTGCTCCTGCTGCTT

GTCAAGTCAATACCATTTCTTCTCTCTGTTTCTTTTCCTTTTTCTAACAAAGACCACCTTGTAATAGAAA

GAAGCACGAAAAAAACCTTTCCATCAAAGGCGTACTAATTAATTAATTAATAATTCATTAATTATTCAGA

CCATTAGATAATTACTACACCAGTGGATTGGTCGTCGTGGACGATCCATAAGCGTTGATCATTCCAGTCC

ATGATCCCAGATTGTTTAGATACCCATATGTCTCTTTTCCATTATCTGAGCAACCCTGGTCTTGCCATTC

AAGGGACAAAAGCTTTGCATTAGGCTTCACATCTTCATTTCCTTGATGATCAT

>FM894894.1 FM894894 Jatropha curcas embryo 71-95 (DAF) Jatropha curcas cDNA clone rjcpga0_000789, mRNA sequence

ATTCAACATAATTGCTTGCAAATTGTACAGGCCTGGATCTGTTGGATTTGTCTCCAAATCTGGTGGCATG

TCAAATGAGCTATACAATACAATTGCCCGTGTAACAGATGGCATATATGAAGGTATTGCAATTGGAGGAG

ATGTCTTTCCAGGCTCCACTCTTTCTGATCATGTTTTGCGGTTCAACAACATCCCACAGGTTAAGATGAT

GGTTGTCCTTGGAGAACTTGGTGGACGAGATGAGTATTCTCTTGTTGAAGCCCTCAAACAGGGAAAGGTG

ACCAAACCAGTAGTTGCTTGGGTCAGTGGAACTTGTGCTCGACTCTTCAAGTCAGAAGTACAATTTGGTC

ATGCTGGTGCTAAAAGTGGCAGTGAACTGGAGTCTGCTCAAGCAAAAAATCAAGCATTAAAGGATGCTGG

AGCTGTTGTTCCCACCTCATATGAAGCTTTTGAGACTGCAATTAAGGAAACATTTCAGAAGCTGGTTGAA

GATGGTAAGATTACACCT

>FM894893.1 FM894893 Jatropha curcas embryo 71-95 (DAF) Jatropha curcas cDNA clone rjcpga0_000788, mRNA sequence

TTTTTTTTTTTGAAAAAACCAAATTAATATCCAATGGTTAGCCACTTAAAGATTAACCAGTTCGGTTAGA

AAAAATTGCCAGAAACCTGTTTATCGAACATCGATTACCAAACAAGCAATAGAATCTTATCAACTCAAGG

CCGTAGTATTGCTGCAACGACCGACAGGCATTGATGGATCCCGAGAAATGTTCAGAGGCAATCAAAACAA

GTAATTGAAGCTTAACCCGTCAATTATTATTGAATTAATATTTACAAGTTGAAGGGTTTGTGTGATTTTG

AGACGTCACAACTAATTGGAACATTGGGTGCGTATGTACAAAGTCAGCATGGCTCCATTTCAGGTAAAAC

TTTAAACATTTCAGTTGCTGATATCGAAATATCTAGGATCGTCCGGGAAGTGATATTCCACATTCAACTT

GCCCTCATCAGCATCTGAATGGCGATGAGGGAACACAACTCTCAGGTCATTGTATAAATAGAACCTGCGG

TCCCCTTCCGCATCTAAATGTGTATTTTGTTTTAGAGAAGTGGGATCAGATTTGCATCTCTGAACTGACC

TAGAAGACTTTTTAGGGGAAGGGCATAGAAAACGGAGATGCAGGGCA

>FM894892.1 FM894892 Jatropha curcas embryo 71-95 (DAF) Jatropha curcas cDNA clone rjcpga0_000785, mRNA sequence

GAAACGACTCAGTCGGAGGTAGGGTCCAGCGGCTGGAAGAGCACCGCACGTCGCGCGGTGTCCGATGCGC

CCCCGGCGGCCCATGAAAATCCGGAGGACCGAGTGCCTCCCAC

>FM894891.1 FM894891 Jatropha curcas embryo 71-95 (DAF) Jatropha curcas cDNA clone rjcpga0_000784, mRNA sequence

ATTCAAAGGATTCTATCCGTCGCCCGATGGGAATTGTACTTTAAGGCGGCCCTCGCGGCTCGTCCGCCGC

GGGGGCTTGGCCAACGGCACGTGCCTCTGGGGGCCGGATGGCCCCTACTGCGGGTCGGCAAACGGGCGAC

GGACACGCGCGTCGCTTCTGGCCCGGATTCTGACTTAGAGGCGTTCAGTCATAATCCAGCGCACGGTAGC

TTCGCGCCACTGGCTTTTCAACCAAGCGCGATGACCAATTGTGCGAATCAACGGTTCCTCTCGTACTAGG

TTGAATTACCATCGCGACACCATCATCAGTAGGGTAAAACTAACCTGTCTCACGACGGTCTAAACCCAGC

TCACGTTCCCTATTGGTGGGTGAACAATCCAACACTTGGTGAATTCTGCTTCACAATGATAGGAAGAGCC

GACATCGAAGGATCAAAAAGCAACGTCGCTATGAACGCTTGGCTGCCACAAGCCAGTTATCCCTGTGGTA

ACTTTTCTGACACCTCTAGCTTCAAATTCCGAAGGTCTAAAGGATCGATAGGCCACGCTTTCACGGTTCG

TATTCGTACTGGAAATCAGAATCAAACGAGCTTTTACCCTTTTGTTCCACACGAGATTTCTGTTCTCGTT

GAGCTCATCTTAGGAC

>FM894890.1 FM894890 Jatropha curcas embryo 71-95 (DAF) Jatropha curcas cDNA clone rjcpga0_000783, mRNA sequence

CGAAATCCTCTCTTTCTCTGCTTCTGCATCCGCAACCATGGTGAGAGTTAGTGTTTTGAATGATGCTTTG

AAGAGCATGTACAATGCTGAAAAGCGTGGGAAGCGCCAGGTTATGATCAGGCCTTCCTCAAAAGTGATCA

TCAAATTTCTGCTGGTGATGCAAAAGCATGGATACATTGGCGAGTTTGAGTATGTTGATGATCATAGAGC

TGGTAAAATTGTGGTTGAATTGAATGGGAGGTTGAACAAGTGTGGAGTTATCAGCCCTCGTTTTGATATC

GGTGTCAAAGAAATTGAAGGATGGACTGCAAGGCTTCTTCCATCAAGACAGTTTGGTTATATTGTGTTGA

CTACCTCTGCTGGCATTATGGATCATGAAGAGGCGAGAAGAAAGAATGTTGGTGGCAAAGTGCTTGGATT

CTTTTACTAGGCTCAGCTTTTGTTTTTTTCACTTTAGAGAAAAATTATTCTGCTTCCGAGAGTTTTGGTT

GTGCCTTTGAACTTGTTTGCCATAGGATTAGCCATGTGCTTCGTCTGATTTATGCTTTCTACAAAAGCTA

GTGCTGCTTTTTTTTAACTTGTTATGAATTGA

>FM894889.1 FM894889 Jatropha curcas embryo 71-95 (DAF) Jatropha curcas cDNA clone rjcpga0_000782, mRNA sequence

CTGACATGTGTGCGAGTCAACGGGGTGAGTAAACCCGCAAGGCGCAAGGAAGCTGACTGGCGGGATCCCC

CCGAGGGTTGCACCGCCGACCGACCTTGATCTTCTGAGAAGGGTTCGAGTGAGAGCATGCCTGTCGGGAC

CCGAAAGATGGTGAACTATGCCTGAGCGGGGCGAAGCCAGAGGAAACTCTGGTGGAGGCCCGCAGCGATA

CTGACGTGCAAATCGTTCGTCTGACTTGGGTATAGGGGCGAAAGACTAATCGAACCGTCTAGTAGCTGGT

TCCCTCCGAAGTTTCCCTCAGGATAGCTGGAGCTCGGGACGAGTTCTATCGGGTAAAGCCAATGATTAGA

GGCATCGGGGGCGCAACGCCCTCGACCTATTCTCAAACTTTAAATAGGTAGGAACGGCGCGGCTGCTTCG

CTGAGCCGCGCCACG

>FM894888.1 FM894888 Jatropha curcas embryo 71-95 (DAF) Jatropha curcas cDNA clone rjcpga0_000781, mRNA sequence

CTGCAATTACATATTCCCGTAATCTTTATCCTATGTATACATAACATACAACCAAGTAAAATTTGCAGCA

CATAACTTTAATACACAAGAGATCGGAGAAGGGGAGAGTTGAAGAAAAAGAGCCCCACAACTGAACACTC

CTGATCTTATAGAGTATTTAAGCTGCTTTTCAAAATTAAAAAGCCAAATATTTACCCAGTTGAGGCAGAA

TGACGACCCACGCCATGAGCTGTGAGGAGTTGACATCACAGCCTTGCTTGATCTAGACTGCTCATTTAGT

TCCAATTGGTTTGTTGGATAATCTGCGCTTGTAACGTTTAAGCACAGAAGCCAAATTTTCTTTTCCCTTT

GGAAAAGCTACATCTCCAGCAGCAGTGCCTGATCGACCCTTCCGACAAGATATGGCAATCTCTACATCCT

TTATCAAGTCAAGAACCATCGCTCCAGTTGTGCATTTCCCACTTCCTGCTCCTGATTTCACAGCAGGGGG

CAACATTCCATTTGTAACAGGTGATCTTGCCTTGC

>FM894887.1 FM894887 Jatropha curcas embryo 71-95 (DAF) Jatropha curcas cDNA clone rjcpga0_000780, mRNA sequence

CGAATTCCAAAGGATTCTATCCGTCGCCCGATGGGAATTGTACTTTAAGGCGGCCCTCGCGGCTCGTCCG

CCGCGGGGGCTTAGCCAACGGCACGTGCCTCTGGGGGCCGGATGGCCCCTACTGCGGGTCGACAAACGGG

CGACGGACACGCGCGTCGCTTCTGGCCCGGATTCTGACTTAGAGGCGTTCAGTCATAATCCAGCGCACGG

TAGCTTCGCGCCACTGGCTTTTCAACCAAGCGCGATGACCAATTGTGCGAATCAACGGTTCCTCTCGTAC

TAGGTTGAATTACCATCGCGACACCATCATCAGTAGGGTAAAACTAACCTGTCTCACGACGGTCTAATCC

CAGCTCACGTTCCCTATTGGTGGGTGAACAATCCAACACTTGGTGAATTCTGCTTCACAATGATAGGAAG

AGCCGACATCGAAGGATCAAAAAGCAACGTCGCTATGAACGCTTGGCTGCCACAAGCCAGTTATCCCTGT

GGTAACTTTTCTGACACCTCTAGCTTCAAATTCCGAAAGTCTAAAGGATCGATAGGCCACGCTTTCACGG

TTCGTATTCGTACTGGAAAT

>FM894886.1 FM894886 Jatropha curcas embryo 71-95 (DAF) Jatropha curcas cDNA clone rjcpga0_000779, mRNA sequence

GCAGAAAAACCAAGGCGATCTTATTGTTCAACTGTTTTAGATACAATCTCTCCTGTCATAATCAGAGAAT

GGACCTCTTTTTCCATAACCAGAGAATTCAACTAGGCAATCCTAAAGCAGCATCTTGCTGCCAAGCTTCC

AGATTTAGGAAGTGTCAACAGGTGTGTCAGGATGTGCACCCCATTCAGTCCAGGATCCATCATAAACTGC

AACATCTTGCTTCCCAAGTCGATGCAGACCCAAAGCAAGAATGCAAGCAGTTACGCCAGTCCCACAAGAA

GTAACAATAGAGCTTTCCAAGGAGATGCCTTCTTGATCAAACCGTTTCTTAAGCTCATCCGCTGGTAAGA

GTGTCTGTGAAGCATCCAACATTTGGGGAAAAGGAATGCACTTGCTCCCAGGTATGTGACCACTTTTTAT

TCCCTTTCGAGGTTCTGGTGCAGCTCCATCAAACCTGGCCTTTGAACGGGCATCAATATGTTGGTGCATC

TTGTCCTCAATGTTTTTCTTAACCTGCTCAAGGGTCCAAACAAGATGCGGCTGAAATTT

>FM894885.1 FM894885 Jatropha curcas embryo 71-95 (DAF) Jatropha curcas cDNA clone rjcpga0_000778, mRNA sequence

TTTTTTTTATTTTAAATAACCAACATACATTGATTGATAAAATAAATAATAACCCCTAACTATCTTCACC

ACCATTCTCTCCAAACCAAGAGGACAAGGGAAAGAAAAAAACTCCAAACCAATAATAGTTCATAAAGGAC

TAACGAAAATAAATAAATTAAAAAAAAATAAATGACTCCCAAAACTTCAAAACCCCTCCCTTTCTGTTCC

TCTCCAAAGATATCAGCAAATTCTCCCTCCAAACTTCAATCGAACTCTACTGTTGCTTGCACTCTGGAGG

CCTCTCACCCTGCTGACCATCCCCAGCTTGTGTTCTTCCACCCTTCATATGCCCGGCTGACAGCTTCTTA

TAGGTCTTGCTTTCATCCTCGTCCTCATCCTCATCTTCATCCTCTTCATCCTCATCATCATCATCAATAT

CTTCATCGTCGTCGTCGTCATCATCATCATCATCATCTATTCCAAGGTCATCCCCCTGGATAGCCTCACC

CGTAAACCAAGACACAGCATGAGGGATGATCTTGTCTCGAATAGTTGACCCAACGTCATAATCTTGTTCC

ATTTGATTTTGGAGCTCCTCCGCAGTATCTTCATCAATATCTTCATCATCCTCAGGGACTTGAGGTGGAT

TGAAGAAGTTGAAAAAA

>FM894884.1 FM894884 Jatropha curcas embryo 71-95 (DAF) Jatropha curcas cDNA clone rjcpga0_000777, mRNA sequence

ATTCCGAAGTTTCCCTCAGGATAGCTGGAGCTCGGGACGAGTTCTATCGGGTAAAGCCAATGATTAGAGG

CATCGGGGGCGCAACGCCCTCGACCTATTCTCAAACTTTAAATAGGTAGGACGGCGCGGCTGCTTCGCTG

AGCCGCGCCACGGAATCGAGAGCTCCAAGTGGGCCATTTTTGGTAAGCAGAACTGGCGATGCGGGATGAA

CCGGAAGCCGGGTTACGGTGCCCAACTGCGCGCTAACCTAGAACCCACAAAGGGTGTTGGTCGATTAAGA

CAGCAGGACGGTGGTCATGGAA

>FM894883.1 FM894883 Jatropha curcas embryo 71-95 (DAF) Jatropha curcas cDNA clone rjcpga0_000776, mRNA sequence

ATTCCCAGCTTCACGGTTCGTATTCGTACTGGAAATCAGAATCAAACGAGCTTTTACCCNTTTTGTTCCA

CACGAGATTTCTGTTCTCGTTGAGCTCATCTTAGGACACCTGCGTTATCTTTTAACAGATGTGCCGCCCC

AGCCAAACTCCCCACCTGACAATGTCTTCCGCCCGGATCGGCCGCCGAAACGACCTTGGGTCCAAAAAGA

GGGGCATAGCCCCGCCTCCGATTCACGGAATAAGTAAAATAACGTTAAAAGTAGTGGTATTTCACCGTCG

CCTTTCGGCTCCCACTTATCCTACACCTCTCAAGTCATTTCACAAAGTCGGACTAGAGTCAAGCTCAACA

GGGTCTTCTTTCCCCGCTGATTCCGCCAAGCCCGTTCCCTTGGCTGTGGTTTCGCTGGATAGTAGACAGG

GACAGTGGGAATCTCGTTAATCCATTCATGCGCGTCACTAATTAGATGACGAGGCATTTGGCTACCTTAA

GAGAGTCATAGTTACTCCCGCCGTTTACCCGCGCTTGGTTGAATTTCTTCAC

>FM894882.1 FM894882 Jatropha curcas embryo 71-95 (DAF) Jatropha curcas cDNA clone rjcpga0_000775, mRNA sequence

AGTTTGTCTCCAAGAACAATATTTTGGTGTCAACAATTAACGGGACAGGATCTGTAATTGAAAGCATATA

CGTGTTAGTTTTCATTTTATATGCAACAAGGAAGGAGAAGGGTAAAATCTTAGGCCTCCTTACACTTGTG

CTCACCATTTTTGCTACTGTGGCCTTCGTGTCTCTGTTTGCCCTCCACGGAAGCACCAGAAAGCTCTTCT

GTGGCTTGGCTGCTACTGTCTTCTCTATCATCATGTATGCCTCACCTCTGTCCATCATTAGGTTGGTGAT

TAAAACTAAGAGTGTAGAGTTTATGCCATTCTTCTTGTCACTATTTGTGTTCCTATGCGGCACTTCCTGG

TTCGTCTATGGTCTACTCGGCCATGATCCTTTTGTTGCTGTCCCAAATGGGTTCGGATGTGGTCTAGGTA

CAGTTCAGCTAATCTTATACTTCATCTACAGAAATGGCAAAGGAGTGGACGATAGTGATGATGTCAAAAA

ACCCACATCACAGTCTGTTGAAATGGGTGT

>FM894881.1 FM894881 Jatropha curcas embryo 71-95 (DAF) Jatropha curcas cDNA clone rjcpga0_000774, mRNA sequence

ATTCATATCAAATCGGCGTTGAAAGATCAGTTCTCCTTTTTGAGTATCTAAAAATGTCTTCTTGCTGTGG

TGGAAATTGCGGCTGCGGCTCCGGCTGCAAGTGTGGCAGCGGTTGTGGCGGATGCAAGATGTATCCTGAC

ATGAGTTTCTCCGAGAAAAACACCGCCGAGACTCAGGTTCTCGGAGTTGCACCGGAGAAGGCACGCTTTG

TCGATGGAGGTGAGGTCGGCGTTGGAGCTGAGAACGGATGCAAGTGCGGTGACAACTGCACCTGCAATCC

TTGCAACTGTAAATGAGATCGGCGGCGATCAGTCTGTTTTTTAGAAGTAAAAGCAGAGATGACTCTGCTT

CTCTCTTTCTAAAAAAGGAGAAATAATTAATAGAGTCTAGATATCGTATGAGTTTGTTCGTTTGTTTCTG

GTTTTGAGCTTCAATAAAAGTAGTGGTGGCTTGTGAGCTTGTGTTTAGCAATGGCTGCCTGTTGCTTTAA

CGTTATATATTGTATAAATTAAAAACTAGCATATGA

>FM894880.1 FM894880 Jatropha curcas embryo 71-95 (DAF) Jatropha curcas cDNA clone rjcpga0_000772, mRNA sequence

TTTTTTATTTTTGTAAAAACCATCTAATGTTTGTATTGAACTGATGAAATTCTTTCTCCAACAACATCCT

TTAAAAACCCCATCTTTGACCAACCAAAATCAAAAATTACAAAAGAGCGGACACAAGTTTTATTAAACAA

AGGGAAGCCTGCATCTAAACATTTGACTCAATACACAGCAGTCAAAAATCACCATCAATGTCTTCAGTGG

AGGGTGGAGGCAATGTGAGCAATCAAGTCTAGCACACGTGAACTGTAGCCCCATTCGTTGTCATACCAAG

AGACAAGTTTCACAAAGTTGTCATTCAAAGCAATTCCTGCCTTGGCATCAAAAATACTTGATCTGCTATC

GCCCACAAAGTCAGAAGACACTACATCATCTTCAGTATAGCCCAAGATTCCCTTAAGCTTGCCCTCAGAC

TCCTCCTTGATTGCAGCTTTGATGTCCTCATATGTTGCCTTCTTTTCAAGCCTGACAGTAAGGTCAACAA

CAGAAACATCCACAGTTGGAACACGGAATGACATTCCAGTCAATTTGCCATTAAGTGCAGGAAGAACCTT

TCCAACAGCCTTA

>FM894879.1 FM894879 Jatropha curcas embryo 71-95 (DAF) Jatropha curcas cDNA clone rjcpga0_000771, mRNA sequence

ATTCCAAAGGATTCTATCCGTCGCCCGATGGGAATTGTACTTTAAGGCGGCCCTCGCGGCTCGTCCGCCG

CGGGGGCTTGGCCAACGGCACGTGCCTCTGGGGGCCGGATGGCCCCTACTGCGGGTCGGCAAACGGGCGA

CGGACACGCGCGTCGCTTCTGGCCCGGATTCTGACTTAGAGGCGTTCAGTCATAATCCAGCGCACGGTAG

CTTCGCGCCACTGGCTTTTCAACTAAGCGCGATGACCAATTGTGCGAATCAACGGTTCCT

>FM894878.1 FM894878 Jatropha curcas embryo 71-95 (DAF) Jatropha curcas cDNA clone rjcpga0_000770, mRNA sequence

AGGATTCTATCCGTCGCCCGATGGGAATTGTACTTTAAGGCGGCCCTCGCGGCTCGTCCGCCGCGGGGGC

TTGGCCAACGGCACGTGCCTCTGGGGGCCGGATGGCCCCTACTGCGGGTCGGCAAACGGGCGACGGACAC

GCGCGTCGCTTCTGGCCCGGATTCTGACTTAGAGGCGTTCAGTCATAATCCAGCGCACGGTAGCTTCGCG

CCACTGGCTTTTCAACCAAGCGCGATGACCAATTGTGCGAATCAACGGTTCCTCTCGTACTAGGTTGAAT

TACCATCGCGACACCATCATCAGTAGGGTAAAACTAACCTGTCTCACGACGGTCTAAACCCAGCTCACGT

TCCCTATTGGTGGGTGAACAATCCAACACTTGGTGAATTCTGCTTCACAATGATAGGAAGAGCCGACATC

GAAGGATCAAAAAGCAACGTCGCTATGAACGCTTGGCTGCCACAAGCCAGTTATCCCTGTGGTAACTTTT

CTGACACCTCTAGCTTCAAATTCCGAAGGTCTAAAGGATCGATAGGCCACGCTTACACGGTTCGTATTCG

TACTGGAAATCAGAATCAAACGAGCTTTAACC

>FM894877.1 FM894877 Jatropha curcas embryo 71-95 (DAF) Jatropha curcas cDNA clone rjcpga0_000769, mRNA sequence

AGGATTCTATCNCGTCGCCCGAATGGGAATTGTACTTTAAGTGCGGCCCTCGCGGCTCGTCCGCCGCGGG

GGCTTAGCCAACGGCACGTGCCTCTGGGGGCCGGATGGCCCCTAGTGCGGGTCGGCAAACGGGCGACGGA

CACGCGCGTCGCTTCTGGCCCGGATTCTGACTTAGAGGCGTTCAGTCATAATCCAGCGCACGGTAGCTTC

GCGCCACTGGCTTTTCAACCAAGCGCGATGACCAATTGTGCGAATCAACGGTTCCTCTCGTACTAGGTTG

AATTACCATCGCGACACCATCATCAGTAGGGTAAAACTAACCTGTCTCACGACGGTCTAAACCCAGCTCA

CGTTCCCTATTGGTGGGTGAACAATCCAACACTTGGTGAATTCTGCTTCACAATGATAGGAAGAGCCGAC

ATCGAAGGATCAAAAAGCAACGTCGCTATGAACGCTTGGCTGCCACAAGCCAGTTATCCCTGTGGTAACT

TTTCTGACACCTCTAGCTTCAAATTCCGAAGGTCTAAAGGATC

>FM894876.1 FM894876 Jatropha curcas embryo 71-95 (DAF) Jatropha curcas cDNA clone rjcpga0_000768, mRNA sequence

AGGATCTATCCGTCGCCCGATGGGAATTGTACTTTAAGGCGGCCCTCGCGGCTCGTCCGCCGCGGGGGCT

TGGCCAACGGCACGTGCCTCTGGGGGCCGGATGGCCCCTACTGCGGGTCGGCAAACGGGCGACGGACACG

CGCGTCGCTTCTGGCCCGGATTCTGACTTAGAGGCGTTCAGTCATAATCCAGCGCACGGTAGCTTCGCGC

CACTGGCTTTTCAACCAAGCGCGATGACCAATTGTGCGAATCAACGGTTCCTCTCGTACTAGGTTGAATT

ACCATCGCGACACCATCATCAGTAGGGTAAAACTAACCTGTCTCACGACGGTCTAATCCCAGCTCACGTT

CCCTATTGGTGGGTGAACAATCCAACACTTGGTGAATTCTGCTTCACAATGATAGGAAGAGCCGACATCG

AAGGATCAAAAAGCAACGTCGCTATGAACGCTTGGCTGCCACAAGCCAGTTATCCCTGTGGTAACTTTTC

TGACACCTCTAGCTTCAAATTCCGAAGGTCTAAAGGATCGATAGGCCACGCTTTCACGGTTCGTATTCGT

ACTGGAAATC

>FM894875.1 FM894875 Jatropha curcas embryo 71-95 (DAF) Jatropha curcas cDNA clone rjcpga0_000767, mRNA sequence

CGAATTCCAAAGGATTCTATCCGTCGCCCGATGGGAATTGTACTTTAAGGCGGCCCTCGCGGCTCGTCCG

CCGCGGGGGCTTGGCCAACGGCACGTGCCTCTGGGGGCCGGATGGCCCCTACTGCGGGTCGGCAAACGGG

CGACGGACACGCGCGTCGCTTCTGGCCCGGATTCTGACTTAGAGGCGTTCAGTCATAATCCAGCGCACGG

TAGCTTCGCGCCACTGGCTTTTCAACCAAGCGCGATGACCAATTGTGCGAATCAACGGTTCCTCTCGTAC

TAGGTTGAATTACCATCGCGACACCATCATCAGTAGGGTAAAACTAACCTGTCTCACGACGGTCTAAACC

CAGCTCACGTTCCCTATTGGTGGGTGAACAATCCAACACTTGGTGAATTCTGCTTCACAATGATAGGAAG

AGCCGACATCGAAGGATCAAAAAGCAACGTCGCTATGAACGCTTGGCTGCCACAAGCCAGTTATCCCTGT

GGTAACTTTTCTGACACCTCTAGCTTCAAATTCCGAAGGTCTAAAGGATCGATAGGCCACGCTTTCAC

>FM894874.1 FM894874 Jatropha curcas embryo 71-95 (DAF) Jatropha curcas cDNA clone rjcpga0_000766, mRNA sequence

ATTCATAATCAATTTTGCTTTGAACGAGCCAAGTGAGGCAATAATCGGCGAAGAATGGCGCATTCATTAG

TATCCTCATCGGCCTCTTCCCTCAGATTCAGCTCTCTCGTCTTTTCTCCAAACCCTAGCACTACTTCCGA

TTGCCAGAAGCTTTCTTTACCTTTTGAGCCTCTTCGTTCCAGGAAAGTGGGAAAGTTGAGTAGTTTTAGA

AAGAATTTTCAGCCATCTCCAACAAAGGCTGTTTATTCTGGCGAGTTTTGGGCACCTCAGAGAACTTCTC

GAAAGGGGATTTGGTCTATAAGGGATGATTTGGAAATCCCATCTTCACCTTATTTCCCTGCATATGCCAA

TGGACAAGGTCAAGGACAAGGGCCACCTCCAATGGTGCATGAGAGGTTCCAGAGTGTTGTTAGTCAACTT

TTTCAACATAGAATTATACGCTGTGGTGGAGCTGTTGATGATGACATGGCAAATATTATTGTTGCTCAAC

TTCTTTATCTTGATGCAGTTGATCCTAATAAGGATATTGTAATGTATGTAAATTCTCCA

>FM894873.1 FM894873 Jatropha curcas embryo 71-95 (DAF) Jatropha curcas cDNA clone rjcpga0_000765, mRNA sequence

CTTTTTCTCCTCTTAAACTCCTCTTCCTTTCCCTCTCTTCCCCTCATTCTCTCTTTCTCTTGCTTCTGCA

GCTTTCTGTTTACTACAGAAACAGAAAAGAGAGAGAGATAAGTTAGCGCAACTCCAATGGCGACTGCTTT

CTCTGCTACCAAGTTTACCCAGCCTTTGCCCCTCAATACCACCTCCTCCAGATCTCATGAAAAGCACACT

CTTTTTGATCCTCTTAAAACTACTGCTTCTTTTCTCGGATCCACCCGCAAACTTCGTTTCAATTCTGTTC

CTAAATTCAATCAAATCAATTCCCACCGTCGATCTGCTATTGTTGCTGTATCTGAAGCCGTTAAGGAGAA

GAAGCTCAAATCAACCTCCAATCTGTTGATCACAAAAGAAGAAGGCTTAGAGCTATATGAGGATATGGTG

TTGGGCAGATACTTTGAGGACATGTGTGCCCAGATGTATTACAGAGGAAAAATGTTTGGTTTTGTTCATC

TCTATAATGGCCAGGAGGCTGTGTCCACTGGGTTCATCAAGCTTTTGAAAAAGGAGGATTCTGTTGTCAG

CACTTATCGTG

>FM894872.1 FM894872 Jatropha curcas embryo 71-95 (DAF) Jatropha curcas cDNA clone rjcpga0_000764, mRNA sequence

TTTTTTTTTTTTAGACCAGCAATTACAGTTATATAATGTATATAACTGGTATAAAACAAAAGAGTTGAAA

TTAACAGGAACAATGAGTATAGAAAAGAGTCAGGAATCAGCAACAGCCTTCAATGATGTTATGCATTTCT

ATGCTTCTTTGACTTTTTAGTTTCATGGTCATTGTTCAAGGAATACTTGTCCCTGCTTGCATTACCACGG

TCTTGCACTTCAATTTCTCCATCAGGTTGTGTCCTCTTCTTTTTATTACTGTCAGTTATAGCATTTTTAA

CATCCCCATCCAACCAACTAATACTTCCAGTGTGAGTTGGAATCAACGATCCAGAAACATGCAGTATTTC

CACATCAAAACTCTTGACAACAAAGCGTATCACAGTTCCAACCTTTATCACATGTCGTTTCTGAACTTTG

CTAACATATAAGCCTTCACCATGTTTACGTTTATATCTAAATTCATCACGAATGTCTTCATCTGTTATAA

CTCCAGAAGAAAACCCAAGAACAATACAATGAATGTATTCTAGTGTAAGTTTCACAACCTTTCCCTCTAA

AAGCATATCTGGCTTTGGAGAGAAAAGCAAT

>FM894871.1 FM894871 Jatropha curcas embryo 71-95 (DAF) Jatropha curcas cDNA clone rjcpga0_000763, mRNA sequence

ATTCGCAAAGGATTCTATCCGTCGCCCGATGGGAATTGTACTTTANAGGCGGCCCTCGCGGCTCGTCCGC

CGCGGGGGCTTGGCCAACGGCACGTGCCTCTGGGGGCCGGATGGCCCCTACTGCGGGTCGGCAAACGGGC

GACGGACACGCGCGTCGCTTCTGGCCCGGATTCTGACTTAGAGGCGTTCAGTCATAATCCAGCGCACGGT

AGCTTCGCGCCACTGGCTTTTCAACCAAGCGCGATGACCAATTGTGCGAATCAACGGTTCCTCTCGTACT

AGGTTGAATTACCATCGCGACACCATCATCAGTAGGGTAAAACTAACCTGTCTCACGACGGTCTAACCCC

AGCTCACGTTCCCTATTGGTGGGTGAACAATCCAACACTTGGTGAATTCTGCTTCACAATGATAGGAAGA

GCCGACATCGAAGGATCAAAAAGCAACGTCGCTATGAACGCTTGGCTGCCACAAGCCAGTTATCCCTGTG

GTAACTTTTCTGACACCTCTAGCTTCAAATTCCGAAGGTCTAAAGGATCGATAGGCCACGCTTT

>FM894870.1 FM894870 Jatropha curcas embryo 71-95 (DAF) Jatropha curcas cDNA clone rjcpga0_000762, mRNA sequence

CAAGGATTCTATCCGTCGCCCGATGGGAATTGTACTTTAAGGCGGCCCTCGCGGCTCGTCCGCCGCGGGG

GCTTGGCCAACGGCACGTGCCTCTGGGGGCCGGATGGCCCCTACTGCGGGTCGGCAAACGGGCGACGGAC

ACGCGCGTCGCTTCTGGCCCGGATTCTGACTTAGAGGCGTTCAGTCATAATCCAGCGCACGGTAGCTTCG

CGCCACTGGCTTTTCAACCAAGCGCGATGACCAATTGTGCGAATCAACGGTTCCTCTCGTACTAGGTTGA

ATTACCATCGCGACACCATCATCAGTAGGGTAAAACTAACCTGTCTCACGACGGTCTAATCCCAGCTCAC

GTTCCCTATTGGTGGGTGAACAATCCAACACTTGGTGAATTCTGCTTCACAATGATAGGAAGAGCCGACA

TCGAAGGATCAAAAAGCAACGTCGCTATGAACGCTTGGCTGCCACAAGCCAGTTATCCCTGTGGTAACTT

TTCTGACACCTCTAGCTTCAAATTCCGAAGGTCTAAAGGATCGATAGGCCACGCTTTCACGGTTCGTATT

C

>FM894869.1 FM894869 Jatropha curcas embryo 71-95 (DAF) Jatropha curcas cDNA clone rjcpga0_000760, mRNA sequence

CAAGCTGTGAACTATACCCCCACAAACCATCATATAAATTGTACGTAAATATCACACTTCAAGGGTATAC

AACCCCACGAATCCACAAATATATAACGACACGACTTAGAGAAGCAAATTCTAGGTTCAAATTTTTATAA

ACATTACATTTAATGGACCAGATAATATGGAATCTCCATCACATTGTAGGTATCCTACATTCAATTTGAT

CGGCTTCTAATAACATCAAGACCCATTTCTGTAGGATCAGTTGCTTGCAACTCTACCTGAATACCGTCCA

ACTCTCTTTTGAACCTGTCCTTTGAGCTAGCATAAATCATCTTGCTTCTCACCTTAGATGTATCAGGAGA

CCATGCAATAAAAACAATCCTACTCTTCTGGACATTCTCCTCTGTTACAAAATCAAAATCATAGACAGCA

TAACGGCACTCATCAGCAGGAAGGCTTGCTGTGAAATCCTCATAACTGTTAGCTGGTTCGCCAAGCTTCT

CCAAAAACAACTGCTTCTGCTTCTCCTCAATCTTGAAG

>FM894868.1 FM894868 Jatropha curcas embryo 71-95 (DAF) Jatropha curcas cDNA clone rjcpga0_000759, mRNA sequence

ATTCTGCATCCAAAGGACCTGTAATTCTTGTTATAATGTCTGGAGGTGGTATGGATGTATCTTTTGCCAA

ATCTAATGACAAGATCACAAGCATCCTATGGGTTGGTTACCCCGGTGAAGCTGGTGGAGCTGCCATTGCT

GATATTATTTTTGGATATTATAATCCAAGTGGAAGACTGCCAATGACATGGTATCCGCAATCATATGTGG

ACAAAGTGCCAATGACGAATATGAATATGAGACCAGACCCTTCCAGTGGTTATCCCGGTCGATCCTATAG

GTTCTACACTGGAGAAACTGTTTATTCATTTGGAGATGGGCTGAGCTATTCAAAATTCGAGCAACAACTA

GTTCAAGCCCCTAAATTAGTAACTGTTCCCTTAGAGGAAGGACATGCTGGTCAATCTACAAAATGCATAT

CAGTTGATGCTGTAGACCGTACTTGTCAAAATTTAGCTTTTGATATACATCTGAAAGTTAAAAACATGGG

AAGAATTAGAGGAAGCCATACAGTGTTCTTGTTCTCTACACCTCCA

>FM894867.1 FM894867 Jatropha curcas embryo 71-95 (DAF) Jatropha curcas cDNA clone rjcpga0_000758, mRNA sequence

TTTTTTTTTACAATCAACAACCAATCCAGTAAAGCCTTAAAATAATTCATCCACTACAAAAATGCGCTTC

TGTAAAGCTGCAAAGATAATCTAACTATAGATTCAATGTAGGATTAACCATATAAAGGCCCGTTTTCCAA

TTAAATACAATTTCATCCCTTTTTCCCATGAATGTAGTTGTCTCGCATTGGCTTTCTGGTGTTTGTTGTC

ACAACCGACTTGTGAACAGGAATATGCTAAAGCTTGTCATTTCTTACTTAATACGCCAGAGGTAGAAATG

AGGCGGAGTGCTTGATTGGAGTTATTCAAAACTGAAGATGATCTTGAAACCTGCATTTTCTTTTTCCTCA

GAAATTCCAAGGAGTTCTGTAAGCATGGAGGAGGTGACAAATCCTTCAAAATAACAGTTTCCATCCCAGA

AGTATAAGGCTTCTTCTTCCTTGCAGAAAGCTTTGTCTCAACCTTCTCATCTTCGGAGTCTGATAAGAAA

ACTTTCTTCTCACGAGCTGCAAGCAGTTGTACAATGTCATTTGGAAGCATATCCCCAGCACCCAAAGGCT

CCTTATCCATTTCAGTTTCCATTACATCTTGTACACTTTCATCTACTA

>FM894866.1 FM894866 Jatropha curcas embryo 71-95 (DAF) Jatropha curcas cDNA clone rjcpga0_000757, mRNA sequence

ATTCCAAAGGATTCTATCCGTCGCCCGATGGGAATTGTACTTTAAGGCGGCCCTCGCGGCTCGTCCGCCG

CGGGGGCTTGGCCAACGGCACGTGCCTCTGGGGGCCGGATGGCCCCTACTGCGGGTCGGCAAACGGGCGA

CGGACACGCGCGTCGCTTCTGGCCCGGATTCTGACTTAGAGGCGTTCAGTCATAATCCAGCGCACGGTAG

CTTCGCGCCACTGGCTTTTCAACCAAGCGCGATGACCAATTGTGCGAATCAACGGTTCCTCTCGTACTAG

GTTGAATTACCATCGCGACACCATCATCAGTAGGGTAAAACTAACCTGTCTCACGACGGTCTAATCCCAG

CTCACGTTCCCTATTGGTGGGTGAACAATCCAACACTTGGTGAATTCTGCTTCACAATGATAGGAAGAGC

CGACATCGAAGGATCAAAAAGCAACGTCGCTATGAACGCTTGGCTGCCACAAGCCAGTTATCCCTGTGGT

AACTTTTCTGACACCTCTAGCTTCAAATTCCGAAGGTCTAAAGGATCGATAGGCCACGCTTTCAC

>FM894865.1 FM894865 Jatropha curcas embryo 71-95 (DAF) Jatropha curcas cDNA clone rjcpga0_000756, mRNA sequence

CAAAGGATTCTATCCGTCGCCCGATGGGAATTGTACTTTAAGGCGGCCCTCGCGGCTCGTCCGCCGCGGG

GGCTTAGCCAACGGCACGTGCCTCTGGGGGCCGGATGGCCCCTACTGCGGGTCGGCAAACGGGCGACGGA

CACGCGCGTCGCTTCTGGCCCGGATTCTGACTTAGAGGCGTTCAGTCATAATCCAGCGCACGGTAGCTTC

GCGCCACTGGCTTTTCAACCAAGCGCGATGACCAATTGTGCGAATCAACGGTTCCTCTCGTACTAGGTTG

AATTACCATCTGCGACNNACCATCATCAGTAGGGGGTAAAACTAACCTGGTCTCACNGACGGTCTAATCC

CAGCTCACGTTCCCTATTGGTGGGTGAACAATCCAACACTTGGTGAATTCTGCTTCACAATGATAGGAAG

AGCCGACATCGAAGGATCAAAAAGCAACGTCGCT

>FM894864.1 FM894864 Jatropha curcas embryo 71-95 (DAF) Jatropha curcas cDNA clone rjcpga0_000755, mRNA sequence

CGAATCAAAGGATTCTATCCGTCGCCCGATGGGAATTGTACTTTAAGGCGGCCCTCGCGGCTCGTCCGCC

GCGGGGGCTTGGCCAACGGCACGTGCCTCTGGGGGCCGGATGGCCCCTACTGCGGGTCGGCAAACGGGCG

ACGGACACGCGCGTCGCTTCTGGCCCGGATTCTGACTTAGAGGCGTTCAGTCATAATCCAGCGCACGGTA

GCTTCGCGCCACTGGCTTTTCAACCAAGCGCGATGACCAATTGTGCGAATCAACGGTTCCTCTCGTACTA

GGTTGAATTACCATCGCGACACCATCATCAGTAGGGTAAAACTAACCTGTCTCACGACGGTCTAAACCCA

GCTCACGTTCCCTATTGGTGGGTGAACAATCCAACACTTGGTGAATTCTGCTTCACAATGATAGGAAGAG

CCGACATCGAAGGATCAAAAAGCAACGTCGCTATGAACGCTTGGCTGCCACAAGCCAGTTATCCCTGTGG

TAACTCTTCTGACACCTCTAGCTTCAAATTCCGAAGGTCTAAAGGATCGATAGGCCACGCTTTCACGGTT

CGTATTCGTACTG

>FM894863.1 FM894863 Jatropha curcas embryo 71-95 (DAF) Jatropha curcas cDNA clone rjcpga0_000754, mRNA sequence

GGATCCCCCGGCTGCAGGGAAGATTTCTGGAATGAGGCAATTAAACTTGCAGACCTGCACCATCCAAATG

TGGTTGCTTTCTATGGTGTTGTGCTTGATGGTCCTGGACGTTCTGTGGCAACTGTTACAGAATACATGGT

TAATGGTTCTTTAAGAAATGCTTTGCAGAAGAATGAGAGGAGCCTTGACAAGCGTAAGCGTCTCCTGATT

GCAATGGATGTGGCTTTTGGGATGGAATATTTGCATGGAAAGAACATAGTACACTTCGATTTGAAGAGTG

ACAATTTACTTGTCAATCTTCGAGATCCTCACCGCCCAATATGCAAGGTTGGTGATTTGGGCTTGTCCAA

GGTGAAACGTCAAACACTTATCTCAGGTGGTGTTCGGGGAACACTTCCATGGATGGCACCAGAACTTCTT

AATGGCAGCAGTAGTCTTGTTTCTGAGAAGGTTGATGTGTTCTCATTTGGTGTTGTGCTGTGGGAACTTC

TAACTGGAGAAGAGCCATATGCAGACTTGCACTACGGGGCTATTATAGGCGGTATTGTGAGCAACACATT

GCGTACACCAGTGCCTGAATCTTGCG

>FM894862.1 FM894862 Jatropha curcas embryo 71-95 (DAF) Jatropha curcas cDNA clone rjcpga0_000753, mRNA sequence

TTCTGACATGTGTGCGAGTCAACGGGTGAGTAAACCCGCAAGGCGCAAGGAAGCTGACTGGCGGGATCCC

CCCGAGGGTTGCACCGCCGACCGACCTTGATCTTCTGAGAAGGGTTCGAGTGAGAGCATGCCTGTCGGGA

CCCGAAAGATGGTGAACTATGCCTGAGCGGGGCGAAGCCAGAGGAAACTCTGGTGGAGGCCCGCAGCGAT

ACTGACGTGCAAATCGTTCGTCTGACTTGGGTATAGGGGCGAAAGACTAATCGAACCGTCTAGTAGCTGG

TTCCCTCCGAAGTTTCCCTCAGGATAGCTGGAGCTCGGGACGAGTTCTATCGGGTAAAGCCAATGATTAG

AGGCATCGGGGGCGCAACGCCCTCGACCTATTCTCAAACTTTAAATAGGTAGGACGGCGCGGCTGCTTCG

CTGAGCCGCGCCACGGAATCGAGAGCTCCAAGTGGGCCAT

>FM894861.1 FM894861 Jatropha curcas embryo 71-95 (DAF) Jatropha curcas cDNA clone rjcpga0_000751, mRNA sequence

ATTCGTTCTGTTCAAAAACCTCGTCCGAGAACTTGTATAGAGAAGGATTTCCCGCGGCGCAGCAGTTCTT

CCATACCAACTTAGCTGCCCGGCGCTGCTATTAGCATAACAACCGGTACACCATAGGTTGGCCCAACCCA

GTCCTCTCGTACTAGGGTTGGCTCCTCGCAGTTCTCCCTTTAACACCAACGGTAGATAGGAACCGAACTG

TCTCACGACGTTCTAAACCCAACTCACGTACCACTTGAATCGGCGAACAACCGAACCCTTGGGACCTTCT

TCAACCCCAGGATGTGATGAGTCGACATCGAGGTGCCAAACGACTCCGTCGATAAGAGCTCTTGGGAGTC

ATCAGCCTGTTATCCCCGGCGTACCTTTGATCCGTTGAGCGAGAGCCCTTCCACACGGGACTCCCGGATC

ACTATGGCCGACTTTCGTCTCTGTTCGACCAGTCGGTCTCACAGTCAGGCAGGCTTATACCATTACGCTC

ACGAGCAGAATCTTAGCTTGAGCCTACCTTCGCACACCTCCGTTACTCTTTAGGAGGCATCCGCCCC

>FM894860.1 FM894860 Jatropha curcas embryo 71-95 (DAF) Jatropha curcas cDNA clone rjcpga0_000750, mRNA sequence

CTAGTGGATCCCCCGGCTGCATGCTGCAAAGGATTCTATCCGTCGCCCGATGGGAATTGTACTTTAACGG

CGGCCCTCGCGGCTCGTCCGCCGCGGGGGCTTGCCAACGGCACGTGCCTCTGGGGGCCGGATGGCCCCTA

CTGCGGGTCGGCAAACGGGCGACGGACACGCGCGTCGCTTCTGGCCCGGATTCTGACTTAGAGGCGTTCA

GTCATAATCCAGCGCACGGTAGCTTCGCGCCACTGGCTTTTCAACCAAGCGCGATGACCAATTGTGCGAA

TCAACGGTTCCTCTCGTACTAGGTTGAATTACCATCGCGACACCATCATCAGTAGGGTAAAACTAACCTG

TCTCACGACGGTCTAAACCCAGCTCACGTTCCCTATTGGTGGGTGAACAATCCAACACTTGGTGAATTCT

GCTTCACAATGATAGGAAGAGCCGACATCGAAGGATCAAAAAGCAACGTCGCTATGAACGCTTGGCTGCC

ACAAGCCAGTTATCCCTGTGGTAACTTTTCTGACACCTCTAGCTTCAAATTCCGAAGGTCTAAAGGATCG

ATAGGCCACGCTTTCACGGTTCGTATTCGTACTGGAAATCAGAATCAAACGAGCTTTTACCCTTTTGTTC

CACACGAGATTTC

>FM894859.1 FM894859 Jatropha curcas embryo 71-95 (DAF) Jatropha curcas cDNA clone rjcpga0_000749, mRNA sequence

TTTGTTGTTAGTTTCAAAGTCTCGTCTAATGCTGAAGGCTTTTCGATTGGAAGTGATATTAAGCATGCTG

TTTCTGGTATTACTACATCTTCACAAGGTGCTCTGCGAAATTTGCCGATGGAGTCCTCTACAGTTGATCT

GAATCAGCCAATAGAATCCACTGCAGTTGATTTGAATCCAACTATGGAATCAGCCAATGGAAGTGAGAGT

CAACCAGTTTCAGAATGTGGGGGATCTCTGGGAAAAGCATCAAGTCTTGGTTCAAGCAACACAAGGACTC

CTAAATCACCTTGGATGCCTTTCCCTATGTTGTTTGCTGCAATCTCAAACAAAATTCCTCGTAAAGAAAT

GGAACTCGTAACTACAAATTATGAACTTTTCCAGGCCAAAAAGATAAGCAGGGGAGATTTTATAAAAAGT

TTGAGGTTGATAGTTGGAGATGCTTTGCTGAAGTCAACAATAACAAGTCTTCAGTGCAAGGTACCATTCA

AGTGTGTGGTGGAATCACCTAAGCCAACTGTAGAAGGGTCCAACGGTCCTTGATCTATCAAAGCAGCATA

TTTCGTTGGATG

>FM894858.1 FM894858 Jatropha curcas embryo 71-95 (DAF) Jatropha curcas cDNA clone rjcpga0_000748, mRNA sequence

CCGAAGTTTCCCTCAGGATAGCTGGAGCTCGGGACGAGTTCTATCGGGTAAAGCCAATGATTAGAGGCAT

CGGGGGCGCAACGCCCTCGACCTATTCTCAAACTTTAAATAGGTAGGACGGCGCGGCTGCTTC

>FM894857.1 FM894857 Jatropha curcas embryo 71-95 (DAF) Jatropha curcas cDNA clone rjcpga0_000747, mRNA sequence

TGCAGAGCTTATGAGACTGCACAATCTCTCTGGCCCTCCAAGATTTCTCTTCACAATTAAAGAGGAAACA

AAGGAAGATTTAGAATCTGATGATGGGAAATCAAGAGGTGATAGAAGCAGAAAAGCTTCAAGAACAAGAA

GCTTAAGTGATCTTATTTTAAGTGTTGATACCCCCTTTTTAACCCCTTTAGCTTCTCCTAAATTAAAGTC

CCCACCTTTAAACGCTTTAGATTATTATAATCACCATGGATTCAACCCTCTCTTTGAATCATCATCAATG

GATGCAGAAACTCACAGGCTTAGATCTTCTCCACCTCCAAAATTCAAATTCTTAAGAGATGCTGAAGATA

AACTTTATAAAAGATTAATGGAAGAAAGAGCTTTGAAAAATACAAATGTTTCTGTTCAAGATTCAAGAAA

TCAAGATTCAAGAATCTTATTAGCAGAGGAAGCAGAGGGGTCTTTTTTGGGCTTTATTGTTAGTAGAAAC

AAAGAAAGAGATGTTTTTAAACATCATCATCTTCCACAATACCCTTCTAGTTCATCTCAGGTACTTCCAT

TGGCTTCTTCCCCTACGGCATTCAGTCCATTAGACAAGAAGCCCTGATGTAAAATAATTT

>FM894856.1 FM894856 Jatropha curcas embryo 71-95 (DAF) Jatropha curcas cDNA clone rjcpga0_000746, mRNA sequence

GAGAAACAGAAATTAAAATTAAGCCAAACTTTGTTCACATTCCAACAATGTTCAGCAAATGTTAAAACTC

AATTCTACCTTGGTTTAAGCATATAAAAGGAGGCTCAGATTCGCCTTTTTCCAAACAGAAAAGTGAACAG

GAAAACCTCACCCTTTGCTTGAAATATTTTCCGGAAAACAAACAGAGCCCAAACAGAGAAATGCACAGAA

ACGGAGGTTCAGATCAGATATGCATTTGAATCAAACAAGAAAGTAACACCACCACATCATGGCTGCAGCC

AAGAGCTACCAGGAAAAGGAGGGAAACCCACATTCCAGCGACTCCAAAGGCTACCCCGCCAGAAGAATGA

TGTTTATATTCTAGTCTGATTCTATAACACTGATATATCATCATAGCCTCATTACTTTACCTTCATAAAC

CTTCCATCGTATCATCTCATCCTTGGACAAAGTACAAAGTTAGGGGTGAATTTATGTATTCGGCCTCTCT

GTACATACGAGGGAAGATGGCTGTTGGCAAAATAAGAAGACAAATCACGGGCTCCACTGCTTATCAAAGA

GGTGAACTCAA

>FM894855.1 FM894855 Jatropha curcas embryo 71-95 (DAF) Jatropha curcas cDNA clone rjcpga0_000745, mRNA sequence

TGGCCTCTTCAAGCTGTTTGACAAGAACAGAAAAGAGAGGAAAGGAAAATGGGGAATTGTCAAGCCATAG

ATGCAGCAACGCTAGTGATACAACACCCAAGTGGTAAAGTTGACAAATTTTATTGGCCTGTTAGTGCAGC

TGAGATTATGAAGATGAACCCTGAGTCACTATGTTGCTCTTCTTCTCTCCACCACCTTATATCCAACTAC

TAAAAATGCTGAATGTACTAACACCACCACCACCGTCGTCGTCACCCCCACCACCACCACCACCAATAAT

AATAATAGCTCTGTGCGGATAACAAGAATTAAGCTTCTCAAGCCTACAGATACTCTTCTTCTTGGTCATG

TTTATAGACTCATAACTACTCAAGAGGTAATGAAGGGTCTGTGGGAAAAAAAACAAGCACAAA

>FM894854.1 FM894854 Jatropha curcas embryo 71-95 (DAF) Jatropha curcas cDNA clone rjcpga0_000743, mRNA sequence

ATTCGCAAAGGATTCTATCCGTCGCCCGATGGGAATTGTACTTTAAGGCGGCCCTCGCGGCTCGTCCGCC

GCGGGGGCTTGGCCAACGGCACGTGCCTCTGGGGGCCGGATGGCCCCTACTGCGGGTCGGCAAACGGGCG

ACGGACACGCGCGTCGCTTCTGGCCCGGATTCTGACTTAGAGGCGTTCAGTCATAATCCAGCGCACGGTA

GCTTCGCGCCACTGGCTTTTCAACCAAGCGCGATGACCAATTGTGCGAATCAACGGTTCCTCTCGTACTA

GGTTGAATTACCATCGCGACACCATCATCAGTAGGGTAAAACTAACCTGTCTCACGACGGTCTAATCCCA

GCTCACGTTCCCTATTGGTGGGTGAACAATCCAACACTTGGTGAATTCTGCTTCACAATGATAGGAAGAG

CCGACATCGAAGGATCAAAAAGCAACGTCGCTATGAACGCTTGGCTGCCACAAGCCAGTTATCCCTGTGG

TAACTTTTCTGACACCTCT

>FM894853.1 FM894853 Jatropha curcas embryo 71-95 (DAF) Jatropha curcas cDNA clone rjcpga0_000742, mRNA sequence

TTTTTTTTTTACAAATTTGTAGGCTCATCGCCAATCAAATAACAAAAAAAGGAGAAAAAACAAACATGAC

AAAGATAACAGGTTTACATCAGACAAGAACTCAACTCAGATCATACTTCCAATCCCAGGTCAAATACAAA

AATTAAATATTTTACCACAACTACGGCAACTCCCAAAAGAACTGCAGTCTTTCCATTTATCTTGTTTTTA

CTTTTTTTTCAACATTCAATCACCAGAGCTTAGATCCATTCCTTCAAGTTTCAACTCATTCTCCGATAAC

ACCGAGTAAAAAATTGAAAGAAAAATAAAAAAATTGAAAATGGAGCACAGTGTTTCTTTTCCTAATATAT

ATGAACAAGTCCAAACTACTAAAAAACCAATTTAGTCAGTGAAGAGTCTCTACTGATTCCGTGATCTCAT

GTTTGTTTCGAATTTGCTGCTCCATTAGATTGTTGCCTTTGAATGCTATTTCGCTTGCACCAGCATAGAT

ACTGAGGGATTGGCCCATTGTCTCCAATAACCCAACTGTGTTTAGCAGCAGAATCTCATGTTATCCTTTG

CTTTGGATCTT

>FM894852.1 FM894852 Jatropha curcas embryo 71-95 (DAF) Jatropha curcas cDNA clone rjcpga0_000741, mRNA sequence

TGAGTCTGACATGTGTAGCGAGTCAACGGGTGAGTAAACCCGCAAGGCGCAAGGAAGCTGACTGGCGGGA

TCCCCCCGAGGGTTGCACCGCCGACCGACCTTGATCTTCTGAGAAGGGTTCGAGTGAGAGCATGCCTGTC

GGGACCCGAAAGATGGTGAACTATGCCTGAGCGGGGCGAAGCCAGAGGAAACTCTGGTGGAGGCCCGCAG

CGATACTGACGTGCAAATCGTTCGTCTGACTTGGGTATAGGGGCGAAAGACTAATCGAACCGTCTAGTAG

CTGGTTCCCTCCGAAGTTTCCCTCAGGATAGCTGGAGCTCGGGACGAGTTCTATCGGGTAAAGCCAATGA

TTAGAGGCATCGGGGGCGCAACGCCCTCGACCTATTCTCAAACTTTAAATAGGTAGGACGGCGCGGCTGA

TTCGCTAAGCCGCGCCAAGGAATCGAGAGCTCCAAGTGGGCCATAGAAGGTAAGCAGAACTGGCGATGCG

GGATG

>FM894851.1 FM894851 Jatropha curcas embryo 71-95 (DAF) Jatropha curcas cDNA clone rjcpga0_000740, mRNA sequence

GAAAGACCTTAGAGAGCTCTACTCTGAAGTGGAAGTCAAGGTCGCAGATCCTGTTGTTTCATTTTGTGAA

ACTGTGGTGGAGTCTTCATCAATGAAATGTTTTGCTGAAACACCCAACAAGAAGAATAAAATAACCATGA

TTGCAGAGCCATTGGAGAAAGGGCTGGCAGAAGACATTGAAAATGGTGTTGTTAGTATTGATTGGAACCG

CAAAGCTCTTGGTGACTTCTTCAAGACAAAATATGACTGGGATTTGCTAGCAGCACGATCCATTTGGGCA

TTCGGCCCTGACAAGCAGGGACCTAACATTTTATTGGATGACACACTTCCAACTGAAGTTGACAAAGGAT

TGCTGGGTGCTGTCAAGGATTCCATAGTTCAAGGTTTTCAGTGGGGTGCACGAGAAGGTCCATTGTGTGA

TGAACCTATCAGAAATGTCAAGTTCAAAATAGTTGATGCTAGAATTGCACCAAAACCACTGCATAGAGGA

TCTGGTCAGATTATTCCCACAGCTAGACGTGTCGCCTATTCGGCTTTTCTGATGGCAACCCCACGA

>FM894850.1 FM894850 Jatropha curcas embryo 71-95 (DAF) Jatropha curcas cDNA clone rjcpga0_000739, mRNA sequence

GACAAAGCAATTAAGCTCACAAGAGAGAAGCTCTTCTATTTTCTAGAGTAAGAAAGAAGAAAGAAGACGA

TGGCCAAGGAAGTAAGTGAAGAAACGCAAACTACCCATGCAAAGGATTATGTTGATCCACCACCTGCGCC

ACTGATCGACATGGCTGAAATCAAGCTCTGGTCTTTCTACAGAGCTTTAATAGCTGAGTTCATAGCCACT

CTCCTTTTCCTTTACATAACTGTAGCCACTGTAATTGGCTACAAGAAACAAACTGATCCATGTGGTGGAG

TTGGTCTTTTGGGCATTGCATGGGCCTTTGGTGGCATGATCTTTATCCTTGTTTACTGCACTGCCGGTAT

ATCTGGTGGTCATATTAACCCAGCAGTGACCTTTGGGCTGTTTTTAGCAAGAAAGGTGTCACTAATTCGA

GCCTTGGCTTACATGGTGGCTCAGTGTTTAGGTGCCATCTGTGGTGTAGGGTTGGTCAAAGCTTTTATGA

A

>FM894849.1 FM894849 Jatropha curcas embryo 71-95 (DAF) Jatropha curcas cDNA clone rjcpga0_000737, mRNA sequence

CGAATTCGAATAAATCAGCTTCCTTTTATCGGCAATTATTTCATCACATTAAAATGTATACAGTAACCTA

AAAAGAAAAGATGTGGCTTAGTTAGACAAGCTTTCTAAAAAGAAACACAGATGCTAATCTTGCATTTGAA

CTAGTTATGCAGAAATCATGTCAGATCGCCCTCCTGTCATCCTTTCAGCTTTCTCTCCTCGCTCACCAGT

GCGCACTCTTATTATATCTGAAACAGGAACCAAGAAAATCTTGCCATCTCCAATCTCTCCTGTTCTTCCC

TCCTCAATGATCTTTTCTATCACGGGTTCAACCTGGTCTTTGCTCACCACAATCTCCATCTTAACTTTAG

CAACAAACTTGTCTTCAGAAAATTCTGAGCCACCATGCCTCTCTGTTGAACCTCCTTGAGCGCCAAAACC

TCGAACATCAGAAACAGTAACACCTCGGATACCAATTTTCAGCAGAGCCGACGAAACTCGCGTGACCCGC

CAGGGCCTGAGAATTGCTTCCACTTTGTAGAATTTAGCATCCGGTATATACTCAGGCGAGCTTTGAGCTC

TTACTACAGGGACAATGGGTGC

>FM894848.1 FM894848 Jatropha curcas embryo 71-95 (DAF) Jatropha curcas cDNA clone rjcpga0_000735, mRNA sequence

ATTGCAAGAGGTGACCTCACAACAGGCTGTGGAAAGTTATTACACTTGGATTCAGATATGCTTTCACAAG

CCTAATTATGAAAAATTAAAGTCCATAGGTCTGTTAGTGAACCCATGCGCAGGTCATTGGAGCAATCAAA

TGGAAAATAAATGAAAGTTCTTTGTCAAAAGTATAAAAAAACAATAAAAGAAGCAGGGCCTACCAGAAAT

GCAACTGAGAGAACTCTTTGGTGCTTCTGTAATCCAGAGAGTTCAATTGCTTCCCCTGAGCTCAAATTCT

TTGCTTTTATCTTCACTGCTCTTCTTCGCAATCCATATAGAGATTCAGAAAAAGAAGCATCTGAATAGGA

GTAATATACCAAAGAAATAAGTGCCAACCAGCAGCTATTGTTCAAAAGTAGTCAATAGATGTAAAACCAC

CATAAGCATTACTATATTAATGTTTTAATCAAAACAACATTAAGACTGCAAAACAATGACAAGAAGATTT

AATGTTATTATTAAATAAAAAGGAGGAAGGATACCTGTTATCC

>FM894847.1 FM894847 Jatropha curcas embryo 71-95 (DAF) Jatropha curcas cDNA clone rjcpga0_000734, mRNA sequence

TTGCCAGGAAGTATAAGTTGCACTGGATAGTGATGCATGGGAATTTGGAGGACAAGGAAGGGTCAGCCAT

GAGGCAGAACATTTTACATCTCCTGAAGGGATACCTGGAGTGGCTGAAACAAATTTCAACAATCGTCCGA

ACTCCTTCAAAGTACTTTCCCCAGCTCGCACATGTGTGGCTTATTACAGAGTTGACGAAAGCCAAGAAAT

TAACAATGGAGGCAATCTGGGCGCTGTTGATGAGACATTGACAGCTGATGCTGTAGAGAAGCTAGAGGTT

TCTGAAGAAGTTGTCAAGCAGAACTTAGCCACAGATACAATGGAAAAGAAGGACAATTTGGAAGAAGTAA

ACACCAAGAAATTAGCTGCAGATACTGCGGTGAACCAGGAGGATATTAAAGAACCTGCTGATTCGACGAT

GAATGGTGATGCTGGTCCAACTATGGCAGGGAATGTAGAAGCAGAAGAGATTGAGGGAGGGAAAT

>FM894846.1 FM894846 Jatropha curcas embryo 71-95 (DAF) Jatropha curcas cDNA clone rjcpga0_000733, mRNA sequence

ATTCCTGCTCTCTCTACTTCTTCTCTTTGTGGTTCGATCCGGGAGAAACTCAGAATCATGTTGGTCTACC

AGGATTTGCTTACCGGTGATGAGCTTCTCTCGGATTCGTTCCCTTACAAGGAAATTGAGAATGGCATGCT

GTGGGAAGTTGAGGGAAAGTGGGTTGTTCAAGGAGCCGTTGATGTGAACATTGGTGCAAATCCCTCTGCT

GAAGGGGCGGATGAAGATGAGGGTGTTGATGACCAGACTGTTAAAGTTGTTGACGTTGTGGACACTTTTA

GGCTCCAGGAGCAACCTGCTTTTGACAAGAAGCAGTTTGTCACATACATGAAGAGGTACATCAAGTTGTT

GACACCCAAATTGGATGAGGAGAAGCAACAGGCTTTCAAGAAAAACATTGAGGGAGCAACCAAGTTCCTG

CTCTCGAAGCTCAGTGACCTCCAATTTTTTGTGGGGGAGAGCATGCATGATGATGGGAGTTTGGTGTTTG

CTTACTACAAGGATGGCGCCACTGACC

>FM894845.1 FM894845 Jatropha curcas embryo 71-95 (DAF) Jatropha curcas cDNA clone rjcpga0_000732, mRNA sequence

TTGGGAAGGTCAGAGAGATCCATTCTATATATGCAAAGAGATCGAGCTCTCTCTGCTAAGAACACAAGTA

AAAACACTTAAGACAGCAAACAAATTCAGTATCATTACCGAATTATTTTCTTCTGTTCCTTAAAAACATC

AATCTCGAAAGTTGTTTCTGAAGTTTTTAAGATTTGAATCGAATGGCACCTGCGGCGACTCAAGAAGATG

ATGAGATAATCAAGGACGAGAAGAATCCTCGTCCTCTTGATGAAGACGATATTGCCCTCCTCAAGACCTA

TGGTTTAGGTCCATATTCAAACAGCATTAAGAAAGAGGAGAAGGAAATCAAGGAACTGGCTAAGAAGATC

AATGACCTCTGTGGTATCAAGGAATCGGACACTGGGTTAGCTGCACCAAGTCAGTGGGATCTTGTTTCTG

ATAAGCAAATGATGCAGGAGGAGCAACCTCTTCAAGTTGCAAGGTGTACTAAAATCATTAATCCAAATAC

GGATGATGCCAAGTATGTCAT

>FM894844.1 FM894844 Jatropha curcas embryo 71-95 (DAF) Jatropha curcas cDNA clone rjcpga0_000730, mRNA sequence

ATTCCAAAGACTACTCAGACTGTTAGTGATTACAGTTGCAATGGACGTCACGTGTTTACACATACTAGAG

AGCTTGAGCGATCACTTGTTGGTTCAATTCTACAAAGTATGTGGGGAGTGTCACCAACCCATTTGTTATG

GAGTTCAAGGCATAACAATACTTTGGTTGATTATACTTGGAGTGTTGGGCAGACTCCATTTGGGCCTTTC

TCGGAGACTTCGTCACTGTCTTTTGTGCAGAGGGATGCCGCTCGGAGAAATGTTATATTGACGTCAATGA

ATTACAGTATAACTAGCGCTATTGATGTTCTTGAGTCTATAGTAGGACATGGTGGTGACAGGAATCTCCT

TAAACAGAATCAGCATGCTGAGTTCATACAGAGATGGAATCTGTTTAAGTACAAGCTGGACAAAGCAGTT

TCTGCAATGTCTCATTTTGATTTTGAGATGGCTTTATATTACTTGAGGTCTTCAGACCATGATTTATATG

CCATTCATTCTCTTGTTTACCATGCTTCCCAG

>FM894843.1 FM894843 Jatropha curcas embryo 71-95 (DAF) Jatropha curcas cDNA clone rjcpga0_000729, mRNA sequence

CAATTTTTCTACAAATGTCATATTTTGATTTCCCAGTGTTTCTTTACAAAAAGAAAAGAAAATTATTACA

CAGGCAAGCAAGAATCATGAATTAATACAATCAGACAGACAAACTTAACCTCACTTCAGGTTGAGACTTC

AGGACTCTCCCAGGTACACTTTGATTAACAGGGGGTCAGACATGCAAAACCTCTTTTCACATTAGCTAAA

TCTGATTGAACTCATTCTACAACGACTGGCGAAATTGTATCACGCATGGAAACCTTCTCAATGTAGATAT

AAACGCAAGGTTATCTTCAACAAGGCACTACTACATGTCTATATTAGCATCATGAAGGCGAACGAGGCAG

CAAACAGCACATAAACTATCAAATCTTCACTATACAAGCCACTTTGAGCTGATGCGGCTGCAGGAAGTGC

TGGAATTTACACAGTTTAGTTGCATGATGATGCCGGTAATTGAGCTGAAGAGGGCCAAGGGGCAGGAATA

TTGTCATTCAGATCACAAATGAAGCCAATCCCCGAAGACTTGTCTAGTGTTGCATCAGAAGGCAAGCTTG

GCAGAAGGCATCCAGACTCTTGCTTTGCAACTTGGAGTG

>FM894842.1 FM894842 Jatropha curcas embryo 71-95 (DAF) Jatropha curcas cDNA clone rjcpga0_000728, mRNA sequence

GATATAAAAAAGCTCATCATACAGGAAAAAGAAAAAAAAGTGTATCTGACTCATCCATGCAATGAATGGT

CAGTCAAATAAGATTTTGATAACACAGATTTAAAGAAAGAAAGCTTTACAGAAAGCATAATCCCATCAAA

GAAACAGTTAAAACTGCAGGGCTCCCTCTAAGTGTATAGCCTCCGCAGTTTACTATAACCTCCCAACAAT

CTCTTCAATCGAATAAGATGCCTATTGGAACTTCAGAACCAAACTTTCTGATATCCTCAAAACATTTACG

GCCCTCGGCACCTGCGGAAACAAAGACAACCTTGTGGCTGATCACCTGGGATCATACCTCCACCCTTGGA

TGTGTCAATTGCCTCTATCATGGACACAACCTCTTCCATCTCTGGCCGCCTGTCAGGGTTGGCGTCCCAG

CATCGCTTCATTACATTTGCAAGGGAGCTTGGGCAACATCGAGGTATTTCTGGCCGCAGATTCTGTCGGA

CAACAGCAGATGTAACTTCTGAGAAGCTGAGGTCTGGATATGGCATGTCACAGCAGTATATCTCCCACAG

GCAGATACCAAAACTGTAAACATCGCACTTTCTATTATATGGGCTGCCATTGAGAACCTCTGGTGCCATG

TAT

>FM894841.1 FM894841 Jatropha curcas embryo 71-95 (DAF) Jatropha curcas cDNA clone rjcpga0_000727, mRNA sequence

CGTAAAATACAAAGGTATATTTATATATTTTGGCCATAAAGTCGTAATATATAGTTAGTTTCAAACTCAG

CAATCAGCGTCACCTTCTCCGTTGTTGAACAATCGAGCTTTTCCATTATAGCAACACTCAAGTTAGCAAA

CCAAAAACAAGTTTTACTGATATCAACAGACAATTGAGTTGAACAATCCCTGATACAATAATACAACAAA

CTTTACAGGGGAGATTTCACCCCGAACTTGAATCTGTTCCAACTCTTTAACAATTGTAATAGGCAATAGG

CAGTGAAGGTGCACATTTCCTACACTATCTTATTTTACAGGCACTAAGGGAGTTTTTTCTAATCTACTCT

TGCCATCCACAATGCATAATACATCATTCTTTACAGAAATTCTCTCTTCAGTAAACTCCTCCGCTACAGC

ACAAAAATAAGTACTCTTTCTCCTCACCTTGAGAGCGAGAAAGGTGATCTTCATGCATGTCTGGATGCAT

TATCATGCAGCGGTGCTGTTCTACCATTTGGGTCATCCCGGATCCCATAAGTGAAACCAGCTTGTGCGCG

GAAGAAACCATCTGTCCCAGCACATGCATGCTTTGCTGGGCATTGTAG

>FM894840.1 FM894840 Jatropha curcas embryo 71-95 (DAF) Jatropha curcas cDNA clone rjcpga0_000726, mRNA sequence

ATTTTTGGGGTTAATTTTTCCTTTATTATTTAAGAAACAAAAAGTTGCAAGATTACAAGATGCTACACAT

AAGATGAAATGAAACCCAGAAAAACAAACTAGCAAATGAAAGTTTGGGTCTTATGTACAACAGTCAACAA

CTACAAATTAAGTAAATAATCTGAGTTATTCTCAAACTCACTGACTCACATACTCTACCTCGGCTGCTGA

ATCGTTCGTGGACTCACTCTCACACTAACAATCCTTGGTGCTTTTTCTACATACTTGGGTACCTGCGCCA

CCTGAGCTCGACCCTTTTGCCACTTCATTACTCCTATTGAAACCAAATCCTTGCTATGCTCCTTCTCTTC

CATCTCCACCGCCTGTTTGGTATCAACGCAGTCGCAAAAGAGGGAATCGAAATCGACTTCGTCGAAAAAA

TTGAAATGGAGAGGATCAATTTGAGGATCAGTGAAGCATTCTTCAAGCCAGTAATCGCGGAAGGAAGGAG

AAGAGTGGACGAGGGACCACCATTGATCGGAGAAATCCTCCACCGAACGATTAGCCATTGGGACGAACAA

TGGAACGTTGGGATTCCGTG

>FM894839.1 FM894839 Jatropha curcas embryo 71-95 (DAF) Jatropha curcas cDNA clone rjcpga0_000725, mRNA sequence

TCGTGTTAATGATGATGAGACATNCCCATCATTGAAGCCAAGATGACTGATGAGCCAGGAGAGGATCCAT

TTGCCAAGAGAAAGACTGAGAAGAAGAACAGAGTTGAAAAACAACAGAAGAATCGGCTGCAGAACTTGAA

ACAAGCTGCAAAAGTTGGTGCTTTGCCAAGCCATGTTCAGCTAGCTGCCACGGCTTTGCCCATAACAGGA

ACTCATGCTGCAACCAAGAAGGGTTCCAAAGAATAACCTAGGAAAGTTACCGGGACTACCGGCAACTGCA

ACAGCCAGTGGTGGGAAGTTTGACAAGAAGTTGCCAAGAGAAAAGCCTGCCAAAAAGCAAGGAAAGAATC

GCAAGTTTCTACCAGTCGTGGAAGGTTCAGGAATTGGCAGACAAGAGAAGGAGCAAACTGACAAGGTTCT

AAGCAAAATAATTTCACAGAATTCACATGAGATACTTAACATTGATAAGGCTGTTTCAAAATATACAGTC

AAGAAAGAGAGGAATTGGAAGAATCAGCAAGGCAAATCTTCAACAACTCCAAGCAAGTTGAAAGCAAAAG

GCAAACCTATGAAAGTGA

>FM894838.1 FM894838 Jatropha curcas embryo 71-95 (DAF) Jatropha curcas cDNA clone rjcpga0_000724, mRNA sequence

CAAGGATCTATCCGTCGCCCGATGGGAATTGTACTTTAAGGCGGCCCTCGCGGCTCGTCCGCCGCGGGGG

CTTAGCCAACGGCACGTGCCTCTGGGGGCCGGATGGCCCCTACTGCGGGTCGACAAACGGGCGACGGACA

CGCGCGTCGCTTCTGGCCCGGATTCTGACTTAGAGGCGTTCAGTCATAATCCAGCGCACGGTAGCTTCGC

GCCACTGGCTTTTCAACCAAGCGCGATGACCAATTGTGCGAATCAACGGTTCCTCTCGTACTAGGTTGAA

TTACCATCGCGACACCATCATCAGTAGGGTAAAACTAACCTGTCTCACGACGGTCTAAACCCAGCTCACG

TTCCCTATTGGTGGGTGAACAATCCAACACTTGGTGAATTCTGCTTCACAATGATAGGAAGAGCCGACAT

CGAAGGATCAAAAAGCAACGTCGCTATGAACGCTTGGCTGCCACAAGCCAGTTATCCCTGTGGTAACTTT

TCTGACACCTCTAGCTTCAGATTCCGAAGGTCTAAAGGATCGATAGGCCACGCTTT

>FM894837.1 FM894837 Jatropha curcas embryo 71-95 (DAF) Jatropha curcas cDNA clone rjcpga0_000723, mRNA sequence

TTTTTTTTTTGAATCACACTCGTTTGAAAAATCTTTAACATACTATAATCCCAACACAGCGAAACCAATC

TGCTCAGACCTAAATTTTAAAAACTATCTTAGTATATATAACTTCAAATAACATTATAAGCTGCTTATGA

ATTCAGAGTTTCTTATTTATACACACTACTCGATCCATCAATGTGAAAGCCCTCTGTTTAGAAAATAGGA

CCAGCTGCAAGAATCTCCTCAGTCAGACTGTTGTCTATTCCAATCTTATAATATGCGAACACTTCAAAAT

TCTTCCTGAACAGACCACCCAGCTTCAACAGGGTATCCTGGTAGGCTTTCTTGTCTTTCCAAGTGTTAAC

AGGATCCAGGATTTCTGAAGGCACACCCTCGATCTCAGTTGGGATTTCAAGCCCAAACACTTCAGTCTTT

ATGTAGTTTGCTTTCAAGAGTCTCCCAGAATGTATGGCATAGATGATTCTTCTAATGTATGCTAACTTCA

TACGGATTCCTG

>FM894836.1 FM894836 Jatropha curcas embryo 71-95 (DAF) Jatropha curcas cDNA clone rjcpga0_000722, mRNA sequence

GTGGATCCCCCGGGCTGCAGGCAACGCCCTCGACCTATTCTCAAACTTTAAATAGGTAGGACGGCGCGGC

TGCTTCGCTGAGCCGCGCCACGGAATCGAGAGCTCCAAGTGGGCCATTTTTGGTAAGCAGAACTGGCGAT

GCG

>FM894835.1 FM894835 Jatropha curcas embryo 71-95 (DAF) Jatropha curcas cDNA clone rjcpga0_000721, mRNA sequence

ATTCAATTAATTAGAAATCGAAAGAGAAACCCATCGTTCAACACAAGGTTCTTAGGCGGTTCCAGACTTC

CAGTCAGTCAGTCGGGCGGGAATGGGCGGTTCTGCATCTGTCCCTGAGAAATCAATCCATGAATTCACTG

TCAAGGATGCGCGAGGCCAGGAAGTCGACCTCAGCGTCTACAAAGGGAAGGTTCTCCTTGTCGTCAATGT

CGCCTCTAAATGTGGATTTACGGATTCAAATTACACCCAGTTGACTGAGCTTTATAAAAAGTACAAGGAC

CAAGGTCTTGAAATCTTGGCTTTTCCATGCAATCAATTTCTAAAACAAGAGCCTGGGTCAAGTGAAAATG

CGCAAGAGTTTGCTTGTACACGATACAAGGCTGAATACCCAATCTTCCAAAAGGTTCGTGTCAATGGGCC

GAATACAGCACCTGTCTACAAATTCCTAAAGGCGAGTAAATTTGGCTTTATGGGATCTAGCATAAAGTGG

AATTTTACGAAGTTAATAATCAACAGGGAAGGGCAAGTCATCAATCGCTATGGCCCAAACAACAATCCTT

TGTCTTAAGAGGATCATATCAAGAAAGCATTGGAAATTAAGT

>FM894834.1 FM894834 Jatropha curcas embryo 71-95 (DAF) Jatropha curcas cDNA clone rjcpga0_000720, mRNA sequence

ATTGTCTATAGCATATGTGAGGTACAAAGGGGAAAACTGTAGTGAAAATCTGCATGGCAAAATCTTCTAG

CCAATACTTTGGGCGTGAACCAAAAATGGAGGAAGGTAATGCTCCAAAATAGCCAAGAGCTCTCTGCCCT

GACTAACCCCTTCTAAGGACCCACCTCCCAGCCGTCTCACAGCTTCAACAAGCTGTCCCCTTAACCTTTG

TGAAAATAAACAACCACCTGCTGCAACACACTCCCGATAGAGTGGAAGATAAGCAATTGCCACCCTGAGA

GGGCCAGGCAGATCTCTTATGCACATCGGAGAGCTACCCCAATTCAAAATCATCATTAGATTCATATAAT

GTTCTTGCCCTTGCTTTAATCCATCCAAGAAAGCTGCCTCCGACCAGTGCTCCCGAAGAAAAGCCCTTAG

CTCAGGGGCAACAGCCTCATCATCGGACCTAGCAATGCTGTCTGCAACAGCATAAGTAGCAACAGGGTCG

CCTAGGAAATCAGAACGGAGGAGAAACGCCACACCATCAAGTGACCCACCACTTCGCTCACCAGCAGCCT

TG

>FM894833.1 FM894833 Jatropha curcas embryo 71-95 (DAF) Jatropha curcas cDNA clone rjcpga0_000719, mRNA sequence

ATTATTATTATATAATATTAGCTTTTAATTTTTTATTTATTATTAGCTTTTTTTTAATTTTTAAGAGATC

AGAGCGAGAGGACTCACCGACCACCTGGCAATGGTTGCAGTAGGCGGATCTGGCGGTTCTAGTTACGTCT

TCTTCGACTATATCAAGTAAAACCACGACCGGCGAGAGATCTGGACCGTCCACCAGATCTCCGACACGAA

ACACGATCTGCCACATAAGCAGGCTTGGAAAAAGTGGAGGGGGGAAAGAAACGCGTGCATGGCGAGATAG

AAACTGTTTAATGTTATCCCGGAATGGACTCGACGGCAACTGTTCCTCCGCCACTCCGGTCGGAAACGTG

TAGAAGTCGTAGAGATTGGCAGTGACTCTGTTTTTCCTGGGTCTTTTTAGGGGCCTGTCGTTCACTACCA

TTGTCTATAAGTTAAAACGACGTTACTTTTCGAATTTGAACGTCGCGTGTAGCGTATGTATATATAGAGA

GGGGTTGAAGAGGGCGAGAGGGA

>FM894832.1 FM894832 Jatropha curcas embryo 71-95 (DAF) Jatropha curcas cDNA clone rjcpga0_000718, mRNA sequence

TATCCGTCGCCCGATGGGAATTGTACTTTAAGGCGGCCCTCGCGGCTCGTCCGCCGCGGGGGCTTAGCCA

ACGGCACGTGCCTCTGGGGGCCGGATGGCCCCTACTGCGGGTCGGCAAACGGGCGACGGACACGCGCGTC

GCTTCTGGCCCGGATTCTGACTTAGAGGCGTTCAGTCATAATCCAGCGCACGGTAGCTTCGCGCCACTGG

CTTTTCAACCAAGCGCGATGACCAATTGTGCGAATCAACGGTTCCTCTCGTACTAGGTTGAATTACCATC

GCGACACCATCATCAGTAGGGTAAAACTAACCTGTCTCACGACGGTCTAAACCCAGCTCACGTTCCCTAT

TGGTGGGTGAACAATCCAACACTTGGTGAATTCTGCTTCACAATGATAGGAAGAGCCGACATCGAAGGAT

CAAAAAGCAACGTCGCTATGAACGCTTGGCTGCCACAAGCCAGTTATCCCTGTGGTAACTTTTCTGAC

>FM894831.1 FM894831 Jatropha curcas embryo 71-95 (DAF) Jatropha curcas cDNA clone rjcpga0_000717, mRNA sequence

ATTCCAGGTTTTTTCTCCTATGCCAGCTAATCCTCCGGAGGATCAGGATCAGGTAGAGGATAAATCAGAT

ACTAGTTCACTTATTAATCAACTTGGTCGCGAAGTGTCAATAAGTTGTTTCCTTCACTGCTCAAGGTCTG

ATTATGGCACAATTGCTTCATTGAATAAAAGCTTTCAGTCGCTAGTCCGGAGTGGTCTGTTGTATAAACT

GAGGCGGGAAATGGGCATCATTGAGCATTGGGTTTACTTCTCCTGCAATCTTCTTGAATGGGAGGCTTTT

GATCCTATTCGATGCCGATGGATGCATTTGCCAAGAATGAATTCAAATGAATGCTTCATGTGTTCAGACA

AGGAATCTTTGGCAGTTGGTACAGAACTGCTTGTATTTGGAAAGGAAATAGAATCCCATGTTATTTATAA

ATATAGCATTTTAACTAACATGTGGACATCCGGCATGACGATGAACATGCCCCGTTGCTTGTAAGGGTCC

GCCAGTCTGGGGGAAATTGCA

>FM894830.1 FM894830 Jatropha curcas embryo 71-95 (DAF) Jatropha curcas cDNA clone rjcpga0_000716, mRNA sequence

CAGAATCAAACGAGCTTTTACCCTTTTGTTCCACACGAGATTTCTGTTCTCGTTGAGCTCATCTTAGGAC

ACCTGCGTTATCTTTTAACAGATGTGCCGCCCCAGCCAAACTCCCCACCTGACAATGTCTTCCGCCCGGA

TCGGCCGCCGAAACGACCTTGGGTCCAAAAAGAGGGGCATAGCCCCGCCTCCGATTCACGGAATAAGTAA

AATAACGTTAAAAGTAGTGGTATTTCACCGTCGCCTTTCGGCTCCCACTTATCCTACACCTCTCAAGTCA

TTTCACAAAGTCGGACTAGAGTCAAGCTCAACAGGGTCTTCTTTCCCCGCTGATTCCGCCAAGCCCGTTC

CCTTGGCTGTGGTTTCGCTGGATAGTAGACAGGGACAGTGGGAATCTCGTTAATCCATTCATGCGCGTCA

CTAATTAGATGACGAGGCATTTGGCTACCTTAAGAGAGTCATAGTTACTCCCGCCGTTTACCCGCGCTTG

GTTGAATTTCTTCACTTTGACATTCAGAGCACTGGGCAGAAATCACATTGCGTGAGCATCCTCAGGGACC

ATCGCAATGCTTTGTTTTAATTAAACAGTCGGATTCCCCTTGTCCGT

>FM894829.1 FM894829 Jatropha curcas embryo 71-95 (DAF) Jatropha curcas cDNA clone rjcpga0_000715, mRNA sequence

CGGGATGAACCGGAAGCCGGGTTACGGTGCCCAACTGCGCGCTAACCTAGAACCCACAAAGGGTGTTGGT

CGATTAAGACAGCAGGACGGTGGTCATGGAAGTCGAAATCCGCTAAGGAGTGTGTAACAACTCACCTGCC

GAATCAACTAGCCCCGAAAATGGATGGCGCTTAAGCGCGCGACCTATACCCGGCCGTCGGGGCAAGAGCC

AGGCCCCGATGAGTAGGAGGGCGCGGCGGTCGCTGCAAAACCCGGGGCGCGAGCCCGGGCGGAGCGGCCG

TCGGTGCAGATCTTGGTGGTAGTAGCAAATATTCAAATGAGAACTTTGAAGGCCGAAGAGGGGAAAGGTT

CCATGTGAACGGCACTTGCACATGGGTTAGTCGATCCTAAGAGACGGGGGAAGCCCGTCCGACAGCGCGT

CCTCGCGCGAGCTTCGAAAGGGAATCGGGTTAAAATTCCCGAACCGGGACGTGAAGGCTGACGGCGACGT

TAGGGAGTCCGGAGACGTCGGCGGGGGCCTCGGGAAGAGTTATCTTTTCTGTTTAACAGCCCGCCCACCC

TGGAAACGACTCAGTCGGAGGTAGGGTCCAGCGGCTGGAAGAGCACCGCACGTCGCGCGGTGTCCGGTGC

GCCCCCGGCGGCCCATGACAATCCGGAGGACCGAGTGCCTCCCACGCCCGGTCGTACTCATA

>FM894828.1 FM894828 Jatropha curcas embryo 71-95 (DAF) Jatropha curcas cDNA clone rjcpga0_000714, mRNA sequence

TCTATCCGTCGCCCGATGGGAATTGTACTTTAAGGCGGCCCTCGCGGCTCGTCCGCCGCGGGGGCTTGGC

CAACGGCACGTGCCTCTGGGGGCCGGATGGCCCCTACTGCGGGTCGGCAAACGGGCGACGGACACGCGCG

TCGCTTCTGGCCCGGATTCTGACTTAGAGGCGTTCAGTCATAATCCAGCGCACGGTAGCTTCGCGCCACT

GGCTTTTCAACCAAGCGCGATGACCAATTGTGCGAATCAACGGTTCCTCTCGTACTAGGTTGAATTACCA

TCGCGACACCATCATCAGTAGGGTAAAACTAACCTGTCTCACGACGGTCTAACCCCAGCTCACGTTCCCT

ATTGGTGGGTGAACAATCCAACACTTGGTGAATTCTGCTTCACAATGATAGGAAGAGCCGACATCGAAGG

ATCAAAAAGCAACGTCGCTATGAACGCTTGGCTGCCACAAGCCAGTTATCCCTGTGGTAACTTTTCTGAC

ACCTCTAGCTTCAAATTCCGAAGGTCTAAAGGATCGATAGGCCACGCTTTCACGGTTCGTATTCGTACTG

GAAATCAGAATC

>FM894827.1 FM894827 Jatropha curcas embryo 71-95 (DAF) Jatropha curcas cDNA clone rjcpga0_000713, mRNA sequence

CTTTACGAATCGGAGCGACAAAGGGCTGAATCTCAGTGGATCGTGGCAGCAAGGCCACTCTGCCACTTAC

AATACCCCGTCGCGTATTTAAGTCGTCTGCAAAGGATTCTATCCGTCGCCCGATGGGAATTGTACTTTAA

GGCGGCCCTCGCGGCTCGTCCGCCGCGGGGGCTTGGCCAACGGCACGTGCCTCTGGGGGCCGGATGGCCC

CTACTGCGGGTCGGCAAACGGGCGACGGACACGCGCGTCGCTTCTGGCCCGGATTCTGACTTAGAGGCGT

TCAGTCATAATCCAGCGCACGGTAGCTTCGCGCCACTGGCTTTTCAACCAAGCGCGATGACCAATTGTGC

GAATCAACGGT

>FM894826.1 FM894826 Jatropha curcas embryo 71-95 (DAF) Jatropha curcas cDNA clone rjcpga0_000712, mRNA sequence

CCTCCTTAACAATAACATATATATAATCACAGTAGTAATTACTAATTAAATATTTAATTTACACATAGTT

TCAAAAATTACACAAAATGAAGATCACTTAACCAATTCAGAAATCCAATCAAGATGAGCCAATTCACCTT

CCGATTCCTCAATTCCTCCGCCGCCTTTCTTCTCTTCATCCACAATATTCCAATTCCTCAAACTATTCAG

CAACTCATCCATCCTAATACCCGATGACGTCGAACCCTCCTCACTCCCTCCGTTCATTCTCGCCCTGTAT

ACAAAAGGACACGCATACTTCTTCTCCGATCGGAGCTGCTCCGGCGTGTGAGCAAAGAAACAAACCTTAC

GCTGGCAAAAACGCCCCGCATTGCATGCACGTGTACGGTAACGTGCAGGGTGCAGCCAGTACTCAAAAAC

GCCATGTGCGTATTCGCACGTGTCACCTTTATGACATTTCCCGTTTCGAAACCCAGGGCAAGCGATGGCT

ACGTAAGGAACCTTTCGCGGGTCTCGACGAGTCACTTTCTCTCCGCGATGGGCGTAGGGGCACTCCGTCC

AGTCGTGGCTGCGAGTCCTGGTGCACCGTTTGATCTTGTAAGCGTACATGCGGAACTCATCGGAGCCG

>FM894825.1 FM894825 Jatropha curcas embryo 71-95 (DAF) Jatropha curcas cDNA clone rjcpga0_000711, mRNA sequence

TGCGGCCCTCGCGGCTCGTCCGCCGCGGGGGCTTGGCCAACGGCACGTGCCTCTGGGGGCCGGATGGCCC

CTACTGCGGGTCGGCAAACGGGCGACGGACACGCGCGTCGCTTCTGGCCCGGATTCTGACTTAGAGGCGT

TCAGTCATAATCCAGCGCACGGTAGCTTCGCGCCACTGGCTTTTCAACCAAGCGCGATGACCAATTGTGC

GAATCAACGGTTCCTCTCGTACTAGGTTGAATTACCATCGCGACACCATCATCAGTAGGGTAAAACTAAC

CTGTCTCACGACGGTCTAAACCCAGCTCACGTTCCCTATTGGTGGGTGAACAATCCAACACTTGGTGAAT

TCTGCTTCACAATGATAGGAAGAGCCGACATCGAAGGATCAAAAAGCAACGTCGCTATGAACGCTTGGCT

GCCACAAGCCAGTTATCCCTGTGGTAACTTTTCTGACACCTCTAGCTTCAAATTCCGAAGGTCTAAAGGA

TCGATAGGCCACGCTTTCACGGTTCGTATTCGTACTGGAAATCACGAATCAACGAGCTTTTACCCTTTTG

TTCCACACGAGATTTCTGTACTCGTTGAGCTCATC

>FM894824.1 FM894824 Jatropha curcas embryo 71-95 (DAF) Jatropha curcas cDNA clone rjcpga0_000710, mRNA sequence

GAGGCCCTTCCAGGTGACAATGTTGGCTTCAATGTGAAGAATGTTGCTGTCAAGGATTTGAAACGTGGTT

TTGTTGCCTCTAACTCCAAGGATGATCCTGCGAAGGAAGCTGCCAACTTCACCTCTCAGGTCATCATCAT

GAACCACCCTGGTCAAATTGGAAACGGATATGCTCCTGTCCTTGACTGCCACACCTCCCACATTGCTGTT

AAGTTTGCTGAGATCCTAACCAAGATTGATAGGCGATCTGGTAAAGAGCTGGAGAAGGAGCCAAAATTCT

TGAAGAATGGTGATGCTGGATTTGTGAAGATGATTCCCACCAAGCCCATGGTTGTGGAGACCTTTTCTGA

GTACCCTCCTCTGGGTCGTTTTGCTGTGAGGGACATGCGCCAGACAGTTGCTGTTGGTGTCATCAAGAGT

GTTGAGAAGAAGGATCCATCTGGTGCAAAGGTCACCAAATCCGCAGCAAAGAAAAAGTAAAGCATTGGGT

GAATGAAGTTACAGTTAGTTTAGTTATGTTTTGTTTTAAGTTATGTCTTGCTGTTGTTTTTGGTTTCAGT

TTCAGTTATCTG

>FM894823.1 FM894823 Jatropha curcas embryo 71-95 (DAF) Jatropha curcas cDNA clone rjcpga0_000709, mRNA sequence

CTTAAACCATTACCATCATTACCGTTCGTCTCCTGTCACGGCCAAGCCAAACCTGGTTTATTTCTCAGCT

ATTCTCTGTCTATTTATTCCTCTAGCTAGAGGGTTTGATTGAGCAAAAGAACGACTTACCGTTTATCTCA

CCACTGATAACACCATGGCAAGACCTTTGTCAAATATGCTCTTGAAAGGCATTGCAGGTCTCTCTGCATC

GCGGTCTAGCAGAATTGTGTCCGGATCCTTTTACTACAATGGAATGCGCTACTCTACTACTGTGCCCAAT

GATCCTGACACACATGAAGATTTTCAACCAAATATTAAGACTGAGAGTTCTGGAGTATCTTTGAATGATA

TTGTTGAACAGGATGTCAAGGACAATCCTGTGATGATTTACATGAAAGGGGTGCCTGATTATCCTCAATG

TGGATTCAGTGCCCTGGCAGTACGGGTATTAAAACAATACAATGTTCCTTTGAGTG

>FM894822.1 FM894822 Jatropha curcas embryo 71-95 (DAF) Jatropha curcas cDNA clone rjcpga0_000696, mRNA sequence

ATTCCAAAGGATTCTATCCGTCGCCCGATGGGAATTGTACTTTAAGGCGGCCCTCGCGGCTCGTCCGCCG

CGGGGGCTTGGCCAACGGCACGTGCCTCTGGGGGCCGGATGGCCCCTACTGCGGGTCGGCAAACGGGCGA

CGGACACGCGCGTCGCTTCTGGCCCGGATTCTGACTTAGAGGCGTTCAGTCATAATCCAGCGCACGGTAG

CTTCGCGCCACTGGCTTTTCAACCAAGCGCGATGACCAATTGTGCGAATCAACGGTTCCTCTCGTACTAG

GTTGAATTACCATCGCGACACCATCATCAGTAGGGTAAAACTAACCTGTCTCACGACGGTCTAACCCCAG

CTCACGTTCCCTATTGGTGGGTGAACAATCCAACACTTGGTGAATTCTGCTTCACAATGATAGGAAGAGC

CGACATCGAAGGATCAAAAAGCAACGTCGCTATGAACGCTTGGCTGCCACAAGCCAGTTATCCCTGTGGT

AACTTTTCTGACACCTCTAGCTTCAAATTCCGAAGGTCTAAAGGATCGATAGGCCACGCTTTCACGGTTC

GTATTCGTACTGGAAAT

>FM894821.1 FM894821 Jatropha curcas embryo 71-95 (DAF) Jatropha curcas cDNA clone rjcpga0_000695, mRNA sequence

CCCGCGGCTGCAGGAATTCGAATTCCAAAGGATTCTATCCGTCGCCCGGTGGGAATTGTACTTTAAGGCG

GCCCTCGCGGCTCGTCCGCCGCGGGGGCTTAGCCAACGGCACGTGCCTCTGGGGGCCGGATGGCCCCTAC

TGCGGGTCGGCAAACGGGCGACGGACACGCGCGTCGCTTCTGGCCCGGATTCTGACTTAGAGGCGTTCAG

TCATAATCCAGCGCACGGTAGCTTCGCGCCACTGGCTTTTCAACCAAGCGCGATGACCAATTGTGCGAAT

CAACGGTTCCTCTCGTACTAGGTTGAATTACCATCGCGACACCATCATCAGTAGGGTAAAACTAACCTGT

CTCACGAAGGTCTAATCCCAGCTCACGTTCCCTATTGGTGGGGTGAACAATCCAACAC

>FM894820.1 FM894820 Jatropha curcas embryo 71-95 (DAF) Jatropha curcas cDNA clone rjcpga0_000694, mRNA sequence

CTTTAGCTTTCCATTGAAGAAAAAAATAATATGGGTTTTGCTATAGCAAAAACTCTTATTTTAATGCAAC

TTTCACTGTTATGCTTCATTGCGCTTAGCGATGAAGAAATAGAACCAGTGTCAGTATTACACCATCACAA

CAATCAACACCGATAGATCTAGCTCCTACTCCAGATCGATGATCACCATCACCACCACCATCACCTTGGT

CCAGCTCCAGCTCCAGCTCACCACCACCACCACCACCACCATGGTCCAACTCCAGCTCATGCACCAATCC

ATCCACCAAAACTGCATCCACACCCACCTGTATTACTTTCCACCAACTCTAGCTCATGGTTACCACCACC

ATGGTCCAACGTCAGCTCCGACTCATCACCACCACCAACACCAATAGTCCAGATTCAGCTCCAGCTCCAG

CTCCAGCTCATCACCATCACCATGGTCGAGCTCCAGCTCATGCACCCATCCACCCTCCAAAGCGGCATTC

ACTCTTTCCGACCGAGCAATCACTAGATATTACAGTGCAAGAAT

>FM894819.1 FM894819 Jatropha curcas embryo 71-95 (DAF) Jatropha curcas cDNA clone rjcpga0_000693, mRNA sequence

CTGCCAGTAGTCATATGCTTGTCTCAAGATTAAGCCATGCATGTGTAAGTATGAACTAATTCAGACTGTG

AAACTGCGAATGGCTCATTAAATCAGTTATAGTTTGTTTGATGGTACCTGCTACTCGGATAACCGTAGTA

ATTCTAGAGCTAATACGTGCACCAAACCCCGACTTCTGGAAGGGATGCATTTATTAGATAAAAGGTCGAC

GCGGGCTCTGCCCGTTGCTCTGATGATTCATGATAACTCGACGGATCGCACGGCCATCGTGCCGGCGACG

CATCATTCAAATTTCTGCCCTATCAACTTTCGATGGTAGGATAGAGGCCTACCATGGTGGTGACGGGTGA

CGGAGAATTAGGGTTCGATTCCGGAGAGGGAGCCTGAGAAACGGCTACCACATCCAAGGAAGGCAGCAGG

CGCGCAAATTACCCAATCCTGACACGGGGAGGTAGTGACAATAAATAACAATACCGGGCTCAATGAGTCT

GGTAATTGGAATGAGTACAATCTAAATCCCTTAACGAGGATCCATTGGAGGGCAAGTCTGGTGCCAGCAG

CCGCGGTAATTCCAGCTCCAATAGCG

>FM894818.1 FM894818 Jatropha curcas embryo 71-95 (DAF) Jatropha curcas cDNA clone rjcpga0_000692, mRNA sequence

ATCCAAAGGATTCTATCCGCGCCCGATGGGAATTGTACTTTAAGGCGGCCCTCGCGGCTCGTCCGCCGCG

GGGGCTTGGCCAACGGCACGTGCCTCTGGGGGCCGGATGGCCCCTACTGCGGGTCGGCAAACGGGCGACG

GACACGCGCGTCGCTTCTGGCCCGGATTCTGACTTAGAGGCGTTCAGTCATAATCCAGCGCACGGTAGCT

TCGCGCCACTGGCTTTTCAACCAAGCGCGATGACCAATTGTGCGAATCAACGGTTCCTCTCGTACTAGGT

TGAATTACCATCGCGACACCATCATCAGTAGGGTAAAACTAACCTGTCTCACGACGGTCTAATCCCAGCT

CACGTTCCCTATTGGTGGGTGAACAATCCAACACTTGGTGAATTCTGCTTCACAATGATAGGAAGAGCCG

ACATCGAAGGATCAAAAAGCAACGTCGCTATGAACGCTTGG

>FM894817.1 FM894817 Jatropha curcas embryo 71-95 (DAF) Jatropha curcas cDNA clone rjcpga0_000690, mRNA sequence

TACAAATCCCCTAATCGCCACTCGCAAAAAGGCTTGAACTTTGAAGGCACTTTAAGATAATTTCTATATT

CTATGTAACCCTTCAAGGCTTCAACAAGAAAATTAGAACCGAAATAGCCTTTCTCTTCCTTGTTCGCTCA

GTGTAGCACCCCGCAATTATTGGCCAGATTTCTGTCTCTCTGTTGGGAGCTGGACTTGAATCTTTGCTTC

GCTTTGACTGAGGAGAGGACTTTCACTGAACGGAGTTTTTGGTAGAGAAAGAGCAATGTCACTCAGACCC

AGTTCGCGTACTGAGTCGCGAAAGAAATCGTACAAGAGTGGCGTCGATGGAGACGAGGCTCGCCGAAGAA

GAGAGGACAATTTGGTTGAGATCAGGAAGAGCAAGCGAGAGGACAGTCTTCTTAAGAAAAGGAGAGAAGG

GCTTCTTCTTCAATCTCAGAATTCAAATGACCTTAATGCTGGGATCGAGAAA

>FM894816.1 FM894816 Jatropha curcas embryo 71-95 (DAF) Jatropha curcas cDNA clone rjcpga0_000689, mRNA sequence

CAATAAGCTTAAGATTTAAAACTTAGCCTCCACTTTATCTGAATATCTGAATTACAAAATAATCAAACCT

AGTTTAAAATCCCTTTACTTGGGCTTCGAATCGTCCTTGGGCTTTCAAGCCGGACTTGTTGTGGTCCCAA

AAGAACGAAAAGTCAATTTTAAAATATTCAGTCAATTCTTCGACCTATCATAAATGCATTTATTATCACA

AACATTACTGAGTTTCTCGAAAACACTGATATTACAATTTGAAAAAATTATTACTCTTATCCTCCAAACG

CCATTATTTTGTTTTTACATTCTAACCATTCCCAAAGCCTTACTTCAAAATTGGAGTTTATCGTCTAATC

TGATACATACGTGATATTCTCATCCGAGAAGATAATAATTGCATGGACGACTCTCCACGACCCGAACACA

GGCCATTTATGCATTTAGCCACGAGAAGACGCTGGGGGATGATGGCAGCCTCGCCGTAAGAACCTCTACT

CCAGTTTCTGTCACCAAAAGAGTATGCTCAAATTGA

>FM894815.1 FM894815 Jatropha curcas embryo 71-95 (DAF) Jatropha curcas cDNA clone rjcpga0_000688, mRNA sequence

AGAGGAAATACTTAATAGACTTTATAATATTTCTAGTAAACATCAGCCTCATCAGAAACTAATGTCAGAC

GAAAACACCCAAAAAGAAAAACACAAGCAGATTACGATTTCAACATAGCCCAAATTATTTCCAGCACGCA

GTCAAACGTGACAAAACGATCCTCTGAGCTCAAGCAATTTTAACCTCAATAGTCTTTGGCTTTTTAGGCT

CAGGCGGTGGCAATTTCTGGACAGTGACAGTCAAAACCCCATCTTGACACACAGCAGAAATGGCATCAGT

ATTGGCATTCTCAGGCAGAGCAAACTTCCTCATCAATTTTCCAACTCTCCTTTCCATCCTCACATACTTA

GCTCCTTCCTTCTCCTCCTCACGCTTATCTCTCTCCACTAATCAACAGCACATTATCATCTTCCACCTGC

ACTTTGATATCTCCAGACTTCAATCCAGGCATGTCGATAATGAACACATAAGAATTCGGATACTCTTAGA

CATCGGCCGGAGTAGAAGCCATAGCCTTCGCGTCCCTCACATAAGTGCGTG

>FM894814.1 FM894814 Jatropha curcas embryo 71-95 (DAF) Jatropha curcas cDNA clone rjcpga0_000687, mRNA sequence

ACAATAGAAGAAATCCCCATAAAAGAAAAATAAAGAATGATATACATTTTTAATTTAAGGCTTACAAACA

AATTATCAAGCTTTTGCCTTTAGCAGATGGCTTTGCTTAAAATAAACTCACAATTTCTCCCTCTCCGGCA

TATATAATTTCCTGATTTCAAATTGCTAGAGCTAACAGCCAAACAAAACACCAACTCTAACTCGATTACA

GAATAATTATTAATTTAAGTAAACCTTGCACTGTTCCTTCTTCCCCACCACTTCTTTTTCACCCTGTCCA

GGGCTCCTTCTTCATTTCCTAGTTTATTTAAAATTACCTTTGGTTCCCGTAGTCTAGCTCCATAATCCTT

C

>FM894813.1 FM894813 Jatropha curcas embryo 71-95 (DAF) Jatropha curcas cDNA clone rjcpga0_000686, mRNA sequence

CTTGATGAATGCCGTAAATGGAATCTGGTCTGGGTTGGCAGGAATAAAGTTGCTCCACTTGAACCGGATG

AAGTAGAGATGCTTTTGGGTTTCCCAAGGAACCACACAAGGGGAGGTGGATTAAGTAGGACTGACAGATA

TAAATCACTCGGTAACTCATTCCAGGTCGACACTGTTGCCTACCATCTTTCAGTTTTGAAAGACATGTTC

CCTGGAGGTATCAATGTTCTCTCTCTTTTTTCGGGGATTGGTGGTGCAGAAGTTGCTCTTCACCGGCTTG

GTATTCGTCTGA

>FM894812.1 FM894812 Jatropha curcas embryo 71-95 (DAF) Jatropha curcas cDNA clone rjcpga0_000685, mRNA sequence

ATTCGATAGTGATTGCAATAAGTCCTGACCATACAAAAACAATGGTCGGTGTTCTTCCTCGTCTTCCCAT

TAGACCTTTGGCAAACGGGTAGAGATGAGCCAAAACCCAGAAACTGAAGAAAACTCCACCTATTAAACGG

CTCCATTGCGGTAAAACACTGTATAATTGTTCGGGCTAAACCCAAACAGCTATTAGCAATTTAAGTTGGT

>FM894811.1 FM894811 Jatropha curcas embryo 71-95 (DAF) Jatropha curcas cDNA clone rjcpga0_000684, mRNA sequence

GAAAACAGAATTTCTGTTGCCTCTTTACCACAACTTCTGAACCAAACATGTCCTAGATGTCCACAAGTCC

TCATAATGCCCTACGCTACTTGGGCCAAAAACGGAAAGAAATTTCTTGATCTGCTAATAAACCATTGATT

CAGCAAGTTGCGTTGCATAATTTGTGTTGGAGAAAGCTTACAATTCGTTGCACAAACATGCACTAAGCAG

ACAAGTATAAATTTTCTACCAAGTTTGTTTGACAACATTACATGACGACTCCCATAAAGAGCATCAAAAA

CTGATCTGACTAGAGCAGAGTGTTTAGACAACAATCAAGAAGCCTAAACAATAAACCTTTACCAGAGAAC

CTGCTGCAAATAAGCTGATGTACACTTCAATGGCTGAAGGAGGAAAACAGAATGAGCCCAGAACCCAAAA

ACAGGATTAACCTGTTCCCTCAGAAACCTGCCTGTGCCTGCCCTGTTGATCAGATAGATAATACAAGAGA

CCCAGGGCCAACAGGCCAAACAATTTCCTATATCATAGCAATCAGGTCCCAACTCTTT

>FM894810.1 FM894810 Jatropha curcas embryo 71-95 (DAF) Jatropha curcas cDNA clone rjcpga0_000683, mRNA sequence

CCATTGCTACATTCCGAATTCTGAGGCAAGTGGGATGCCAGTATCTGCTAGGTTACCAAATTCCAGCTAT

ATTATGGAAGTTTTGTTGCTTGAGAGTAAAAATGTGTAGCTGATTGCTGTTGATGTTGTTCAAGCATTGT

GCCACTCTTGTAGAGCTGAAGCTGCGAAGCCTTGATAACCAGAGGGATGGAAAAGAGTATAGAGATTCTT

CAGTTCTGGTGAGGTTTGGTGCGCTCCTTAAAATCACAGTATTTCTTTATGAGTAGATCTTTCTTATAGT

GGTTGGTTGGCTGCTGACAAGTTTTCATTCCCTAGAAGTTTGGGTTTTAGTGTTGCTGTTTTATTGCTTT

TGTATTCTTATATGATGTGGTTTCTAGCTTTTTGCTTTTCTTTATAGTTTCTATTTTACCTGCATGGCCG

TTTTGTGCCTTCTGACAGTCCAATAGTTAACAGGGTTCTGTTTATTGGCATATGTAGAGAGCTATGAGCA

AAAGTGGAAAAAGCTTTTAAAATACATAGTTGACTAAAAATAGCTTTTCGTTTTAAC

>FM894809.1 FM894809 Jatropha curcas embryo 71-95 (DAF) Jatropha curcas cDNA clone rjcpga0_000682, mRNA sequence

CATAATCCTACAAACAAATGAAGGACAAAAATATCTGAAGCAACACTTGGAACCTGATCATATTGCTCCT

TGGGTGAAGGAATACAAGGAAGGCAAGGTGCTACCATTCAAGAAATCTGAACCTATCCCGGAAGTTAACA

ATGAGCCTGTGAAAGTTGTGGTGGCTGATAGTCTCCAGGACATTGTTTTCAACTCTGGAAAAAACGTTCT

TCTAGAGTTTTATGCTCCATGGTGTGGACACTGTAAAAAATTGGCTCCAATCTTGGATGAAGTTGCCGTC

TCATACCAAAATGATCCTGATGTAGTCATTGCAAAGCTTGATGCAACTGCAAATGATATCCCGAGTGATA

CCTTTGATGTCAAGGGATATCCCACAGTATACTTCAGGTCTTCAAGTGGGAATTTGGTGCAGTATGATGG

GGATAGGACGAAAGAAGATATTATTGATTTCATCGAAAAGAATCGGGACCAAGATAAAGTTCCCCAGCAA

CAAGATAAAGAAACCCTGCAAGAATCAGAAGAGACAAAGGATGAGCTCTAAATCTTGTATCAATTATGAG

CTTAGTGTACTACTAAAAGTTATGTCAGAAATCA

>FM894808.1 FM894808 Jatropha curcas embryo 71-95 (DAF) Jatropha curcas cDNA clone rjcpga0_000681, mRNA sequence

GCCACGCGGGGCGGCGCCTAGAACTAGTGGATCCCGGGCTGCAGGTTTATTTGGAATCAAATCATGAACT

ATATATAACAAAGAGATGGCAAAGAAATCCAACTTCAACTTAATTAATACTAAAAGAGAAAGCGGTAATT

AAATTTACAAACTCTCCTTTTAGGTCCTTAAGTTATTGAGGCATATATATATATAGATAGATATAATCAA

ATTTAGAGTTCATCTCTGCGAGAATCGGGTGATTTCTTGTTGTCTGAGCTATTCTTGATAGGGTAAGAGG

CCTCCAAAGTTATACCACACAGTCCCTCTTCTGCATCAATCCCTCTTTCCATTCTTATGTAACCATTCTC

TCCCCAATCACTTCCCCATGAATTCTTCACTATCCAATACTTTGTGCCATCTAGTGTTGTGCCATATCCC

ACAAGTGCTACCCCATGGTTTAGCTCTGTCCCGCAATCTCCAGTGAATACTCCCTCATAATAGAATTGCA

TATCTTTTCCGCCTGCATCTATTGCAATAGATACTGGTTGGTTAGCAACAGCTTTCATCAATGCATTCTC

GTCGTTTTCTGGCACCATTTCGTATCCATCAATTTGCACAACAGGAGCAT

>FM894807.1 FM894807 Jatropha curcas embryo 71-95 (DAF) Jatropha curcas cDNA clone rjcpga0_000680, mRNA sequence

TTTTTTTTGACTAAGGAAAACATATATAGATTATAAAGCTAAAGACATTAATGTTTTGGGTATAAAAGAG

TTCACAAAACCTTAATACCAACATACATTGATTGATAAAATAAATAATAACCCCTAACTATCTTCACCAC

CGATTCTCTCCAAACCAAGAGGACAAGGGAAAGAAAAAAACTCCAAACCAATAATAGTTCATAAAGGACT

AACGAAAATAAATAAATTAAAAAAAAATAAATGACTCCCAAAACTTCAAAACCCCTCCCTTTCTGTTCCT

CTCCAAAGATATCAGCAAATTCTCCCTCCAAACTTCAATCGAACTCTACTGTTGCTTGCACTCTGGAGGC

CTCTCACCCTGCTGACCATCCCCAGCTTGTGTACTTCCACCCTTCTTATGCCCGGCTGACAGCTTCTTAA

AGGTCTTGCTTTCATCCTCGTCCTCATCCTCATCTTCATCCTCTTCATCCTCATCATCATCATCAATATC

TTCATCGTCGTCGTAATCATCATCAATCATCATCATCTA

>FM894806.1 FM894806 Jatropha curcas embryo 71-95 (DAF) Jatropha curcas cDNA clone rjcpga0_000679, mRNA sequence

CAAAGGATCTATCCGTCGCCCGATGGGAATTGTACTTTAAGGCGGCCCTCGCGGCTCGTCCGCCGCGGGG

GCTTGGCCAACGGCACGTGCCTCTGGGGGCCGGATGGCCCCTACTGCGGGTCGGCAAACGGGCGACGGAC

ACGCGCGTCGCTTCTGGCCCGGATTCTGACTTAGAGGCGTTCAGTCATAATCCAGCGCACGGTAGCTTCG

CGCCACTGGCTTTTCAACCAAGCGCGATGACCAATTGTGCGAATCAACGGTTCCTCTCGTACTAGGTTGA

ATTACCATCGCGACACCATCATCAGTAGGGTAAAACTAACCTGTCTCACGACGGTCTAACCCCAGCTCAC

GTTCCCTATTGGTGGGTGAACAATCCAACACTTGGTGAATTCTGCTTCACAATGATAGGAAGAGCCGACA

TCGAAGGATCAAAAAGCAACGTCGCTATGAACGCTTGGGCTGCCACAAGCCAGTTATCCCTG

>FM894805.1 FM894805 Jatropha curcas embryo 71-95 (DAF) Jatropha curcas cDNA clone rjcpga0_000678, mRNA sequence

CTAAGGAGTGTGTAACAACTCACCTGCCGAATCAACTAGCCCCGAAAATGGATGGCGCTTAAGCGCGCGA

CCTATACCCGGCCGTCGGGGCAAGAGCCAGGCCCCGATGAGTAGGAGGGCGCGGCGGTCGCTGCAAAACC

CGGGGCGCGAGCCCGGGCGGAGCGGCCGTCGGTGCAGATCTTGGTGGTAGTAGCAAATATTCAAATGAGA

ACTTTGAAGGCCGAAGAGGGGAAAGGTTCCATGTGAACGGCACTTGCACATGGGTTAGTCGATCCTAAGA

GACGGGGGAAGCCCGTCCGACAGCGCGTCCTCGCGCGAGCTTCGAAAGGGAATCGGGTTAAAATTCCCGA

ACCGGGACGTGAAGGCTGACGGCGACGTTAGGGAGTCCGGAGACGTCGGCGGGGGCCTCGGGAAGAGTTA

TCTTTTCTGTTTAACAGCCCGCCCACCCTGGAAACGACTCAGTCGGAGGTAGGGTCCAGCGGCTGGAAGA

GCACCGCACGTCGCGCGGTGTCCGGTGCGCCCCCGGCGGCCCATGAAAATCCGGAGGACCGAGTGC

>FM894804.1 FM894804 Jatropha curcas embryo 71-95 (DAF) Jatropha curcas cDNA clone rjcpga0_000677, mRNA sequence

ATTCAGATTATAAAGCTAAAGACATTAATGTTTTGGGTATAAAAGAGTTCACAAAACCTTAATACCAACA

TACATTGATTGATAAAATAAATAATAACCCCTAACTATATTCACCACCATTCTCTCCAAACCAAGACGAC

AAGGGAAAGAAAAAAACTCCAAACCAATAATAGTTCATAAAGGACTAACGAAAATAAATAAATTAAAAAA

AAATAAATGACTCCCAAAACTTCAAAACCCCTCCACTTTCTGTTCCTCTCCAAAGATATCAGCAAAATTC

TCCCTCCCAACTTCATTCGAACTCTACTGTTGCTTGCACTCTGGAAGCCTCTCACCCTGCTGACCATCCC

CAGC

>FM894803.1 FM894803 Jatropha curcas embryo 71-95 (DAF) Jatropha curcas cDNA clone rjcpga0_000676, mRNA sequence

CGAATTCAAAAAAGAGTAATTTTTATAAAAATTAAGCAATGTAAATTCCACAGGTTAGCTCTGATGTTAT

ATCAGTCTAAACAATAGCATCCAAACAAATAAAATAGAAAATCATTTCTAAGTCATACTTTGAATAGAAG

CTTCTGTTTTCCTCTCACCTAGGAGAGCTGCTCGTATCTATCTCCTGTCATGTTTCATGCTTGCATATTC

AATTATATATGATCATATCAATCCCCGCGGCAGGCCCATCTCTGCCAACGATCCTGCTCTGAATATGGAT

CCAGAATACAATTCCTGGATTCTACTACCATCGTGTCTCTCAAAAGAACGGCTCCAAAATGTGCTGCAGA

CTGCCTTCAAGGCAATAAAAGTACACTCGTAGCTATATTCACATTGTGGAATCATCTGCACTCCATAACT

TCTTTCTCACAATCAAAACTCGTTCTTCACCTGAGGAGAGAGCATCACTCCTTGGTTC

>FM894802.1 FM894802 Jatropha curcas embryo 71-95 (DAF) Jatropha curcas cDNA clone rjcpga0_000674, mRNA sequence

GGCGCACGGTTGTTACTCTAAACCCTTAAGGGTTGGTCGTATAAACGACAAAAGCTCATACAGTCGTTAT

ACTTTTTATTTTTTATTTTTTACATAAAATACAAAATAAACGACATTCCAAAGGGTGCAGAAGTTGTTTA

GTGCCAATAAATATCTCCTTCAACTCTTCATCTCACGCGCCTCTTCTTACGAGTTTGGTCATCCTTACGA

TGGCATTTTAGCGATTTCCTTATCCAAGCATGAAATTGTAATCATCAAGAGGGAGGCAGCCATTCTGTTT

CTGGAACTTCATCTTCATCTTCCTCCAAGATGTAAACCAAGGCGCGTTTTCTTGCAGCAAAAATGCAGGC

TACACCTCTGGATGCACTGACAGCCAAGGGAGCAATTACAGAATGAGGAATATAGCGAACCTTTTCATTC

TCCATTTGCAGCTGCACGACAGCATCCCTAAGTTGATGCGATTTCCACATCTTCAAAGAAGTCGACCTTG

AAATAGATATAAATGGAAGGTCATTAGCTTGCAAAATCATCA

>FM894801.1 FM894801 Jatropha curcas embryo 71-95 (DAF) Jatropha curcas cDNA clone rjcpga0_000673, mRNA sequence

CCCTTTCAATATGCTGAAAACAGCAATGATAGATTACTTTGAACACAAACCGGGATTTCAACTAGTTGAT

GAATTGTATGGCTTCAATCTTTGCTTTGAAAGGCCAGACTCTGCAGGGTTCGGACAGCTTCCGAGAATCA

CATTTCATTTTGAAGGGGGTGATATTGAAGTGAGGCCAGAGAATCTGTATGTGCTTGGTGAACATGGCCG

GTTTTATAAATTTTTTTGCTTGGCGATGTACAAAGCAACAAATGGATTGACCATTATCGGAGCACGCCAA

CAAGCAGACCAGCGTGTGATTTATGACACTCAACATATGCAATTGCATTTAGGTCCTGAGGATTGTGCTC

ATAATGCTTGAAGTTTGCCTTGTTTATCTTTGAATTTATAATAAATATGATCCATCCACCCGTTAAT

>FM894800.1 FM894800 Jatropha curcas embryo 71-95 (DAF) Jatropha curcas cDNA clone rjcpga0_000672, mRNA sequence

CGAATTTTCATTTCGTGAAAGATCAGAAAATGTTGACCTNTGTAGTGGAGATTTCTCTACAGCCTTGGAA

TGTCTTTAAGCCAGATGGGGTCATTTTATTCTCAGATATTCTTACCCCACTTTCTGGGATGAATATACCT

TTTGACATTGTAAAAGGTAAGGGGCCTGTTATATTCAATCCTTTACGCACTGCTGCTGATGTTGATCGAG

TGAGAGAGTTTGTGCCTGAAGAGTCAGTTCCATATGTTGGAGAATCATTGACAATCCTGCGAAAAGAGGT

AGATAATAAAGCTGCAGTGCTTGGTTTTGTTGGTGCTCCTTTCACCCTGGCATCATATGTTGTTGAAGGC

GGTTCATCGAAGCACTTCTCAAAAATAAAGAGAATGGCTTTCTCTCAGCCCGAGGTTCTTCATGCATTGC

TTCAGAAATTTGCAACCTCCATGGCAAAGTACATTCAATATCAAGCTGACAATGGGGCACAAGCTGTTCA

GATCTTTGACTCATGGGCTACAGAGCTTAGTCCTGTCGATTTTGAAGTGTTCAGTCTTCCATACCTGAAG

CAGATTGTGGATGCTGTTAACAAGACGCAT

>FM894799.1 FM894799 Jatropha curcas embryo 71-95 (DAF) Jatropha curcas cDNA clone rjcpga0_000671, mRNA sequence

CTAAAAGTAGGCTTTCATTAAAAAAATGAGACAGATTAATATGCTATTCAACGGAAAAGGCTACAAATGA

TCAAACGACCAACACGTTTCCCAAAATTGAAATGAGGTCAGAAAAAAGATAAAACCACCATAACTGATAT

TCCTGGAGAGCTTTCCTTCACTTGTTGCGAAGGGAAATATAGTAGTTGAGGTTCCAGTGAACTGTGCCAC

AGTTCTCAAAGATATTGGTAGAACACCTAACAGAATCTAGAGAAGGTGGATGGTGAAAATGATATATAGT

CCGCCTCAAGCAATCTACAAATTTCTCTCCCCTACTGTTGGAAGAGAAAAATAATGTTGGTGTAAGGCTA

AAACAAGAATATTGTAATGTACCTACACTAGGAACCAAATTCATGGCTTAAGATCATCTGACATTGCAGA

AGGTATGCAATGTGTAGTAAGAACATCTACAGTAGCTGCCTTGGCTGTCTTTGTCCTG

>FM894798.1 FM894798 Jatropha curcas embryo 71-95 (DAF) Jatropha curcas cDNA clone rjcpga0_000670, mRNA sequence

CTGCGAAGCAGCAACCAGTTACTTCTCTCTTCTCTCCCACTGCTGCCGGAGCTTTGATTTGCAAAGATGT

ACACTACTTGGAAGAAGATCCGAAAAGACAAGGATGCTGAACCAACTGAATTTGAGGAGTCAGTTGCTCA

GGCCCTGTTTGATCTCGAAAATACCAACCCAGAACTGAAAAGTGATTTAAAAGATCTCTTCATAAATCAG

GCAGTTCAAATGGATGTTTCTGGAAATCGGAAAGCTGTTGTTATTTATGTTCCCTACAGATTGAGGAAGG

CCTACCGCAAGATTCATCTTCGTCTTGTTAGAGAGTTGGAGAAAAAGTTCAGCGGAAAGGACGTTGTCCT

CATTGCCACCCGGAGGATCGTGCGCCCACCGAAGAAGGGTTCTGCTGTTCAGCGCCCTCGTTCCCGAACC

CTTACATCTGTGCATGATGCAATGCTTGAGGATGTAGTGTATCCCGCTGAGATTGTTGGAAAACGCATCA

GAT

>FM894797.1 FM894797 Jatropha curcas embryo 71-95 (DAF) Jatropha curcas cDNA clone rjcpga0_000669, mRNA sequence

AACATCACACACCGTACCAGAGACGCAGACGAACCCGCCACCGCCACCGCGAATAGAGATATGAAAACTA

GGCAAGACAAAAGAATGGCTATGGCCAAACGCGGTATAAGATCACTAGCCATTGCGGTTGCGCTTCCTCT

CTCTTTGACACTATTTAACTTCTATTTCTTCGGCTCTAGTAATGGATATGGGATCGCATCAAAACCCTCA

TGGTTTCCTCCTATATGGGCTCTACATTTCTTCTCCTTCGTTTCGACTTTCTTGATGGGTCTGTCCGCTT

GGCTCGTTTGGGCTGAAGGAGGGTTTCATAAAAAACCAAGGGCTATTTCTCTTTACTTGGCTCAGTTAGG

TTTGGGTTTGGCTTGGGACCCGATTGTGTTCCGGATGGGAGCTACTTGGGTCGGGTTGCTTGCTTGCTTG

GCTATGTTTGGAGCCATTGTTGGGTGTTCACGCCAGTTCAAAGAGGTGAATCCCATTGCTGGA

>FM894796.1 FM894796 Jatropha curcas embryo 71-95 (DAF) Jatropha curcas cDNA clone rjcpga0_000668, mRNA sequence

GCAAAGGATTCTATCCGTCGCCCGATGGGAATTGTACTTTAAGGCGGCCCTCGCGGCTCGTCCGCCGCGG

GGGCTTGGCCAACGGCACGTGCCTCTGGGGGCCGGATGGCCCCTACTGCGGGTCGGCAAACGGGCGACGG

ACACGCGCGTCGCTTCTGGCCCGGATTCTGACTTAGAGGCGTTCAGTCATAATCCAGCGCACGGTAGCTT

CGCGCCACTGGCTTTTCAACCAAGCGCGATGACCAATTGTGCGAATCAACGGTTCCTCTCGTACTAGGTT

GAATTACCATCGCGACACCATCATCAGTAGGGTAAAACTAACCTGTCTCACGACGGTCTAACCCCAGCTC

ACGTTCCCTATTGGTGGGTGAACAATCCAACACTTGGTGAATTCTGCTTCACAATGATAGGAAGAGCCGA

CATCGAAGGATCAAAAAGCAACGTCGCTATGAACGCTTGGCTGCCACAAGCCAGTTATCCCTGTGGTAAC

TTTTCTGACACCTCTAG

>FM894795.1 FM894795 Jatropha curcas embryo 71-95 (DAF) Jatropha curcas cDNA clone rjcpga0_000665, mRNA sequence

GATCCCCGGGCTGCATGTCGTCTGACTTGGGTATAGGGGCGAAAGACTAATCGAACCGTCTAGTAGCTGG

TTCCCTCCGAAGTTTCCCTCAGGATAGCTGGAGCTCGGGACGAGTTCTATCGGGTAAAGCCAATGATTAG

AGG

>FM894794.1 FM894794 Jatropha curcas embryo 71-95 (DAF) Jatropha curcas cDNA clone rjcpga0_000664, mRNA sequence

TACAATTTGTACACTATTATCACCAAAATGCAGTAGCTGCCCGCTTAACGAAGAAGCCGAATGCGGGACA

AAACAACTGAAAAACCACAATCTGTGGACTAGGGTTGTCACCCAAGTGGAACTGCATATGAATCCCTATG

GTCAATGATACACATATTTCAGTTCATGGGATCCACAATAAGACCCTTTCCTCCACTTACTTCTACATCA

TGCTGGAAAACAAAGTCTGGAATGGTCAACTGCTCCCGAGGAACATGCCTGAAC

>FM894793.1 FM894793 Jatropha curcas embryo 71-95 (DAF) Jatropha curcas cDNA clone rjcpga0_000663, mRNA sequence

ATTTTTCATCTAAAATTATAGACTGCATTGTAAAAAACTATACGGTGCAAACAAATAATACAAATCAGAA

TACAGAACATAGCTAAATTCTGCCTCCCAAAGTAAAAAAATGAATAAAATATAAGAGTCAATTTGATGTG

AAACTAATATCTACTCCACATTCCTTTCATGTAATTCTCCGCAAATCAAAGAATCAATTAAGAATTCCCA

ATATGTCCTGTTTTTCCAGTAGGCCACAAACTAGATATTCAAGCAAGAGTATCACTTAAGGGCATAGATC

TTGAAACGGTTGCTCCCTCAGTATCAGGTATTAGTGCTGGTGGTACTTCAGTTGGGGTTGATGTTGTATC

GCTCGTGGACACTGGTGGGATACTTTCTCCTGATGCAACAAGATCTGGGTTGCTTCCTGAAGATGGGACA

GGAGCATTATCACCAACAGAAACAGG

>FM894792.1 FM894792 Jatropha curcas embryo 71-95 (DAF) Jatropha curcas cDNA clone rjcpga0_000662, mRNA sequence

ATTCGGATGTTTTATTGAAGTTACAATAGTAAGGAGCTCGAAAAAGAGCATCCATTGCCACACAACTACG

GATAAGCTATGATACAGTTCATATATGTTACAATTCTGCTTTCTCGAAACCGAAATACTGCAATAATATT

CACAAAGTCTAGCAGAAATAACTTCCCATTACACAGACCAAAGTCTAATTTTGTGAAAATAACTTGGGCG

GAAACAATCAGATCATAAATCTCATACATCCACCGGATATACTCATATCAGCTATGCACGGAAGCAACTC

AAGAAAAACTTTCAACGTATTGCAGCTTATTTATCAATCCGTTTCATGGTCAGGAGCAGGTGAGTACCAA

GCTGGAAATCTTCCTTTTGTTCGAAGTAATCTAGTATACTGTGAATCTGGCATTCTTGCTCTCTCTTCCT

CTTACAGCTCCTCCTTCGATTTTGGCAATCCCTCTGGTATTGGACATGATCTGTCTCTGCCTGCTCCATG

GTCCTTGGTGCCATGAAACCAAAACCAT

>FM894791.1 FM894791 Jatropha curcas embryo 71-95 (DAF) Jatropha curcas cDNA clone rjcpga0_000660, mRNA sequence

CAGGAAATAACTGATAACATATATCAGATAGCATTAAAAATCTACTGCAAGCACAGGAAATGAAGTTCTC

ATCTCTCTAATAAAAAAACTCTAATCAAGTCGAGCATTTCAATTTTACTAGCAATTCCAACAAAACAAAA

ATAACAGCAATCCAAGAAGAAAAGGTGATCTTTCAAGTAAAGACACTTCTATAGGTAACTGGAATGCTAC

ATCTTGTAAAAATCTCAAAACTCCAAATAAGCTGCTCCTTAAAAGACGCGGACTGGGTTACCCATTGTAG

TCTTGATGTTTAGGCACTTCACATTTTGCCAGTTCTTCTTCAACAAGGAAACAAGGAAATTGACGCTCAT

TTGCACATTTTGGAAGACTTGCTTCTCCTCCATAGCACAGTTGCCAACAGCAACTCCCATGCAGAGCACT

TTCTTCAATTGGAACTTCACTGTTGCCTTTGTTTCATTCACTTTAGACTCTAGGGACTCCTGATGGGTCA

CAAGGGTAGGAAATTTACCTGCCTTGTTGAGACCAGGGCCCAGAAGACGAACATCTGCTTGATGACAGAT

TCTGAAG

>FM894790.1 FM894790 Jatropha curcas embryo 71-95 (DAF) Jatropha curcas cDNA clone rjcpga0_000659, mRNA sequence

TTTTTGGAGAGAATAAAAAATTAGTACTTACGAGGGGAAATAAAGCATGAAATATCAGTTCATGCTAAAA

AGTAAATAGCAATAACATCCAAAGCAATTTAGAAGCTAAACAAAAAAGAGATCTCA

>FM894789.1 FM894789 Jatropha curcas embryo 71-95 (DAF) Jatropha curcas cDNA clone rjcpga0_000658, mRNA sequence

CAAATTGCTACTCCTCTTCAACTAATTCAATCTTCCCTTGAGAAGAAAAGGATGAGGCTGAAGGAAATGT

GTAAAAAAAGTAAAGGTTGGAAGAAGAAATCATGGCCCCAAGTGTATGAAGACGTGGAGCTGTTGTTTGG

GCTAATAGACATAAAAATCTTATCCAGTGTTCTTAGGATGGTGAGGATCAGTAAGGAGCAGTTAATTTGG

TGTGAAGAAAAGATGAAAAAACTACATATATCAGATGGGAAATTTGGAAGAGATCCCTCACCTATCCTGT

TCCCTTGTTGATAGCTGTGAAAAAGGATGGATGAAGAAGG

>FM894788.1 FM894788 Jatropha curcas embryo 71-95 (DAF) Jatropha curcas cDNA clone rjcpga0_000657, mRNA sequence

AGGATTCTATCCGTCGCCCGATGGGAATTGTACTTTAAGGCGGCCCTCGCGGCTCGTCCGCCGCGGGGGC

TTGGCCAACGGCACGTGCCTCTGGGGGCCGGATGGCCCCTACTGCGGGTCGGCAAACGGGCGACGGACAC

GCGCGTCGCTTCTGGCCCGGATTCTGACTTAGAGGCGTTCAGTCATAATCCAGCGCACGGTAGCTTCGCG

CCACTGGCTTTTCAACCAAGCGCGATGACCAATTGTGCGAATCAACGGTTCCTCTCGTACTAGGTTGAAT

TACCATCGCGACACCATCATCAGTAGGGTAAAACTAACCTGTCTCACGACGGTCTAAACCCAGCTCA

>FM894787.1 FM894787 Jatropha curcas embryo 71-95 (DAF) Jatropha curcas cDNA clone rjcpga0_000656, mRNA sequence

CTACTTTGATAGGTTTATTTCAACTTTAAAGTTTCCAAGAGATAAGCCTGGATGGGCCAACTTGCTGCAG

TCGCTTGTTTGTCTCTGGCTGCTAAAGTGGAGGAAACCCAAGTGCCTCTTCTTCTGGACTTGCAAGTGGA

GGAATCAAAATATGTTTTTGAAGCTAAGACTATGAAGAGAATGGAGCTTCTGGTGCTCTCTACTCTTCAA

TGGAGGATGAATCCTGTGACTCCAATTTCTTTCTTTGATCACATTATAAGGAGGCTTGGTCTAAAGAACC

ACTTACATTGGGAATTTTTGAGGAGGTGTGAGCGTTTACTTCTCTCTGTCATCGCTGATTCAAGATTCAC

GAGTTATCTTCCTTCTATATTTGCTACTGCAATTATGTTGCATGTTATTAAGGAGGTTGAGGCATGTAAT

CAAGTGGAATACCAGAATCAGCTTATGGCTGTGATCAAAATTAGTGAGGACAAAGTCAAAGAGTGTTACA

AGCACATTTTGGAGCTATCAGGCAGCCAGAATTCAAGTAGTAAGCGCAAACATTCATGGATA

>FM894786.1 FM894786 Jatropha curcas embryo 71-95 (DAF) Jatropha curcas cDNA clone rjcpga0_000655, mRNA sequence

CCACTGCTAGGGGCTGCTTGGCCTGTCATAGTGTAAGGGTGCTAAGATTTTCTCAAGTCTCCTGCAATGG

CTGCTTTTAGGAATTTCTTGAAGAAAAAAGCTTTAACATGCAACGAGCTTCCACTGCGGAGATTGTTCTC

CGGCGAAGCAGCTGTTGATTCTTCTTCTTTTGCTCAAAGAGTCAGGGATCTGCCCAAAAAACTTCCTGGA

ACTAAAATCAAGACTGAAGTTTCTCAACTCATTGGGAGAACTCCCCTGGTATATCTTAACAGAGTGACTG

ATGGATGTGGAGCATATATTGCTGTCAAGCAAGAGATGATGCAACCAACTGCCAGTATCAAAGACAGACC

AGCCTTTTCAATGATCAATGATGCAGAACAGAAGAATTTAATCACTCCTGGAAAGACTACCTTGATAGAG

CCAACATCTGGTAATATGGGTATTAGTATGGCATTTATGGCAGCCATGAAAGGGTACAAGATGGTTTTAA

CTATGCCCTCGTACAC

>FM894785.1 FM894785 Jatropha curcas embryo 71-95 (DAF) Jatropha curcas cDNA clone rjcpga0_000654, mRNA sequence

CTTCTTTCTTTTAATAGATTAAATCAGAAAAAGAAACAAAAAAATTAAGGATGCAGGCGATTTCACCTCC

CTCCCCTCTCAAGTCATTAATACCGACTCGCCGCCGACTCGCCCAAGTTTACTCCCACCAAACTCGTCGT

CCATTCCAATCTATAGGCCGGACTCGGTTAACTTACCCAACG

>FM894784.1 FM894784 Jatropha curcas embryo 71-95 (DAF) Jatropha curcas cDNA clone rjcpga0_000653, mRNA sequence

AAAGAGAAACCAAGTACTAGCAGAATTCTGATTAAATTAAATCTAGTTCTACAAGCATTGGAAATTACAC

TGTTGCGGCAGTAAGAACAAAAATCATTACTTTCATTTCCGTACAATAAGTTACCAAAGATGTAAGATTC

TGTACTATCATCTGCTCTACAATAACCATAGTTGGTCAATGGATGACAGGCTTCAGAGGATGAGCTCTTA

GATTTGACAAGTCAAGATGTGAATCCAGCTCCTCATCCAGTTCTCCTACAATGCTTATGTTGTCACCCCG

TATTATGTACAAACCCAGAAACAAAGTTGTTGAACACCTTCCTTA

>FM894783.1 FM894783 Jatropha curcas embryo 71-95 (DAF) Jatropha curcas cDNA clone rjcpga0_000652, mRNA sequence

ATTCCAAAGGATTCTATCCGTCGCCCGATGGGAATTGTACTTTAAGGCGGCCCTCGCGGCTCGTCCGCCG

CGGGGGCTTGGCCAACGGCACGTGCCTCTGGGGGCCGGATGGCCCCTACTGCGGGTCGGCAAACGGGCGA

CGGACACGCGCGTCGCTTCTGGCCCGGATTCTGACTTAGAGGCGTTCAGTCATAATCCAGCGCACGGTAG

CTTCGCGCCACTGGCTTTTCAACCAAGCGCGATGACCAATTGTGCGAATCAACGGTTCCTCTCGTACTAG

GTTGAATTACCATCGCGACACCATCATCAGTAGGGTAAAACTAACCTGTCTCACGACGGTCTAATCCCAG

CTCACGTTCCCTATTGGTGGGTGAACAATCCAACACTTGGTGAATTCTGCTTCACAATGATAGGAAGAGC

CGACATCGAAGGATCAAAAAGCAACGTCGCTATGAACGCTTGGCTGCCACAAGCCAGTTATCCCTGTGGT

AACTTTTCTGACACCTCTAGCTTCAAATTCCGAAGGTCTAAAGGATCGATAGGCCACGCTTTCACGGTTC

GTATT

>FM894782.1 FM894782 Jatropha curcas embryo 71-95 (DAF) Jatropha curcas cDNA clone rjcpga0_000651, mRNA sequence

CCCGAGACATATATATATTACTTTGATTATATGAATATTTTTATATTCACATGCCTCCACCATCCTTTCT

TACTTCACTTATCAATGCTAATAAATTATCCATTAGAATTGGTCTTCCCACATGAACAACTTGCAGGATT

TAATTTCTTCCCAAAAGCACATGATGGCTAGTCATTACAACAAGCAGAAAAAGGAAACCCACAGTAATTG

GAGCAAATATTCATGGTTCAGATATAATATCTAGCAGATTAAAAGAGAACCCCGACATGGTTCAAGTGAA

TCTCGCAGTTCAGTTGATTAACCTCCGAGCAGGCTATCATCTACCGCTCTACCATGATCCTTCAGCACCA

ACTGTTGGATTCTGGAATGCAGTGAATCCCCAGAAGGTCTTCAGTGCATATGGGGGTGAAATTGTTACAC

CTTCCTCCGTGATTTTAGCTATCTGAAGCTTGTTTACAGCAGATCTATCACGATAT

>FM894781.1 FM894781 Jatropha curcas embryo 71-95 (DAF) Jatropha curcas cDNA clone rjcpga0_000650, mRNA sequence

CAAAGGATTCTATCCGTCGCCCGATGGGAATTGTACTTTAAGGCGGCCCTCGCGGCTCGTCCGCCGCGGG

GGCTTGGCCAACGGCACGTGCCTCTGGGGGCCGGATGGCCCCTACTGCGGGTCGGCAAACGGGCGACGGA

CACGCGCGTCGCTTCTGGCCCGGATTCTGACTTAGAGGCGTTCAGTCATAATCCAGCGCACGGTAGCTTC

GCGCCACTGGCTTTTCAACCAAGCGCGATGACCAATTGTGCGAATCAACGGTTCCTCTCGTACTAGGTTG

AATTACCATCGCGACACCATCATCAGTAGGGTAAAACTAACCTGTCTCACGACGGTCTAATCCCAGCTCA

CGTTCCCTATTGGTGGGTGAACAATCCAACACTTGGTGAATTCTGCTTCACAATGATAGGAAGAGCCGAC

ATCGAAGGATCAAAAAGCAAACGTCGCTATGAACGCTTGGCTGCCACAAGCCAGTTATCCCT

>FM894780.1 FM894780 Jatropha curcas embryo 71-95 (DAF) Jatropha curcas cDNA clone rjcpga0_000649, mRNA sequence

TTTGCGTGCTAACAAGCTCGAAGCGCCGAACCAGTTCACTGCTTAATTTGTCCAAAGCATGGCTGCCCAA

ATCGAGTAAGAAGCGTAAGTTTGTTGCAGATGGGGTTTTCTACGCAGAGCTAAATGAGGTGCTGACTAGA

GAGCTGGCCGAGGATGGCTACTCTGGTGTTGAAGTTAGAGTTACTCCTATGCGTACTGAGATTATCATTA

GGGCTACTCGTACCCAGAATGTTCTTGGTGAGAAAGGAAGAAGGATCAGGGAGCTTACATCTGTTGTTCA

GAAACGATTCAAGTTCCCAGAGAACAGTGTGGAACTGTATGCTGAGAAAGTCAACAATAGGGGTCTTTGT

GCTATTGCACAGGCTGAATCTCTTAGATACAAGCTCCTTGGAGGCCTTGCTGTTCGGAGGGCTTGCTATG

GTGTCTTGAGATTTATTATGGAGAGTGGGGCAAAAGGATGTGAGGTTATTGTTAGGTGGAAAGC

>FM894779.1 FM894779 Jatropha curcas embryo 71-95 (DAF) Jatropha curcas cDNA clone rjcpga0_000648, mRNA sequence

TTTTTTTCAAAAACAACCTACTTGATCATATAGCAAATTAACAACTTAGCAAACAGGCAAGTTTTAGTCT

AAACAAGTACCATCAGAATATCACTGAAACTAAACAGTGAACTGAAAAAAACTTGCCACACCCTAACATA

AGCATGAAATTCCACATAAAATACATAAACATATTTGCTGCTAAGGAAGATTAGTACTTTGTTCTTCCAT

CACCTTCTGTTATCACATCTTTAAAAGCTCCACCACTTGGGATCATGGGCAAAACATGCTCTTGATGGGG

CACAATCACATCCAACAAATATGGTCCCGGAGTATCTAACATTGTTTTGATTGCTTCTCTAAGATCCTTT

ATCCTTGTCACACGGGCAGCAGGTATTCCACAAGCTTCAGCAAACTTCAACATATAGGGGAAAATCTCAG

TCTCTCTTGATGGATCCCCTAAATAAGTATGAGCTCTATTTGCCTTGTAAAACCGATCCTCCCATTGCA

>FM894778.1 FM894778 Jatropha curcas embryo 71-95 (DAF) Jatropha curcas cDNA clone rjcpga0_000647, mRNA sequence

ATCCTTTCTCCANGTTCTAAAGCTGCTTTTCCTTCTCCTTTTCTCTCCAGTCTTTTTCACTTCCATTTTA

GCTCTTAACACATCTTCTACAACTTCTCTACAGACCCATGATTTCTCTTCCCTTAGAAATTTCTGCCAAA

GCAGACCATACCCAGATGCTTGTTTTGATTCATTAAAACTCACCATTTCTATAAATATCAGTCCAAACAT

TCTTACTTATCTTCTCCATACTCTCCAAACTGCAATCTCTGAAGCAGGAAAATAGACCAGTCTATTCTCA

ACAGCTGGGACGTCTAATATTATTGAAAAACAAAAAGGAGCTATTCAAGATTGCAAAGAATTACATCAAA

TCACTTTGCTTTCACTGCAAAAATCAGTTTCAAGAATCCAAACACGTGATTCGCGTAAACTAGCTGATGC

AAAAGCTTATCTTAGTGCAGCACTTACAAACAAGAACACTTGTATAGAAGGATTAGATTCTGCAACTGGT

CCATTAAAGCCTGTGATAGTAAATTCTTTGATTAGTACTTACGAACACGTACACAATTCT

>FM894777.1 FM894777 Jatropha curcas embryo 71-95 (DAF) Jatropha curcas cDNA clone rjcpga0_000646, mRNA sequence

ATTCCAATTACAAAAAGTGGTTCTGTCAGAAGTGGAAGGAAGTTTCGATGGGTAAAGCTTGAGGATTCAG

TGGACACAAAGCTGTTGGGATACCCACAGGCTTTGACTAAATTCTGTTACTTTATTATGGATGCCTTGAG

AGAGAAAGGAGCTAGAGCAAAGCCCTTGGTTTGTGCATGCTTATCACAAGAACCAAACAAGATGTTGATT

GTTGGGGTTTGTGGAAAGCCACGACTTGGTGCAGTTCAAGGAAATGCATTTGGAGTTGCATTTAGAAATG

CAGCGGAGGCAGATTGGAGCAGAGTTCTTCCATGAGTTGTTTGAATCTTCATGGATTGTTTTAGACAAAG

GTGCAGTCAATAATTTTATGATCAGATTAACTGAGAAGCTCTGATGTAGAACTTAGTATTCTAACTAGAT

AATCTCTCATTCTTCTTTA

>FM894776.1 FM894776 Jatropha curcas embryo 71-95 (DAF) Jatropha curcas cDNA clone rjcpga0_000644, mRNA sequence

AGGATTCTATCCGTCGCCCGATGGGAATTGTACTTTAAGGCGGCCCTCGCGGCTCGTCCGCCGCGGGGGC

TTGGCCAACGGCACGTGCCTCTGGGGGCCGGATGGCCCCTACTGCGGGTCGGCAAACGGGCGACGGACAC

GCGCGTCGCTTCTGGCCCGGATTCTGACTTAGAGGCGTTCAGTCATAATCCAGCGCACGGTAGCTTCGCG

CCACTGGCTTTTCAACCAAGCGCGATGACCAATTGTGCGAATCAACGGTTCCTCTCGTACTAGGTTGAAT

TACCATCGCGACACCATCATCAGTAGGGTAAAACTAACCTGTCTCACGACGGTCTAAACCCAGCTCACGT

TCCCTATTGGTGGGTGAACAATCCAACACTTGGTGAATTCTGCTTCACAATGATAGGAAGAGCCGACATC

GAAGG

>FM894775.1 FM894775 Jatropha curcas embryo 71-95 (DAF) Jatropha curcas cDNA clone rjcpga0_000643, mRNA sequence

ATTCGCCAGTAGTCATATGCTTGTCTCAAAGATTAAGCCATGCATGTGTAAGTATGAACTAATTCAGACT

GTGAAACTGCGAATGGCTCATTAAATCAGTTATAGTTTGTTTGATGGTACCTGCTACTCGGATAACCGTA

GTAATTCTAGAGCTAATACGTGCACCAAACCCCGACTTCTGGAAGGGATGCATTTATTAGATAAAAGGTC

GACGCGGGCTCTGCCCGTTGCTCTGATGATTCA

>FM894774.1 FM894774 Jatropha curcas embryo 71-95 (DAF) Jatropha curcas cDNA clone rjcpga0_000642, mRNA sequence

ATTAAATTTTATATTTTTATTGAATTAAAGCAAACATAACATCCACAGAAGGCCGTAACAGGCCAATCAG

AAAGGAAACAGAATACAACGATGATAGAGAAGCCCATTAGACTCTCTAAGAAAATACATTCAGATGAAAG

CATGAAAACAATTAAGGAAAGCAATTCCCCCCTTCAGAATCCACCACGCAGACGCAGGACAAGGTGAAGG

GTTGATTCTTTCTGGATATTGTAGTCGGCCAGAGTCCTTCCGTCCTCCAACTGCTTCCCAGCAAAGATCA

GCCTTTGCTGATCTGGAGGAATCCCTTCCTTATCCTGGATTTTCGCCTTCACATTATCGATTGTATCACT

GCTCTCCACTTCCAGAGTGATAGTCTTCCCTGTCAGGGTCTTGACGAAGATTTGCATTCCACCACGAAGC

CTGAGCACAAGGTGGAGAGTGGATTCCTTCTGAATATTGTAATCGGCCAAAGTTCGGCCATCTTCCAGTT

GCTTACCGGCGAAGATCAGCCTCTGCTGGTCTGGTGGGATGCCCTCCTTGTCTTGAATTTTAGCCTTCAC

GTAATCAATAGTGTCACTGCTC

>FM894773.1 FM894773 Jatropha curcas embryo 71-95 (DAF) Jatropha curcas cDNA clone rjcpga0_000641, mRNA sequence

GAAAAACTAGCATTATTTGGCTACTCATATGCTCCTATTTGGAGTAATAATGCTCAAAACACGGAGCAAC

ATTGAAAAGGTTACAGAAGCTTTATAGAGAAATCAAGTAAGATCCCCAGCTCTGGTTTTTCTTTCCTGCT

TAAATAAAAAAGTAGGACAACCAACATTTTCCCTTATTTGCTATGACTAGGCTTCCATGAAAACCAACTT

ATGCTCCATACTTTGGGGCCTTCCAAAGCTTGAATGCAACTTCATGCATAAATCTATCGGCTGGTATTAC

AAGATTTCCACCATGCTCTTTAAAATCTTTAAAAGAGAATGTCTCAGCCATATCCATGCGTGTCACAGCA

TACTGCCACTGAGCAACCAACATGGGACAGTGGTGCCTAACATCATTTCGTGGCTTTTCTCCTCTCATTG

TGTAACTTTGACGAGGGAAGAGCATGGAAAGATCAGAGTTCCTTTCTTCTTTAGAAACCAGAACTGATAA

ATTTGTAGAGTCCATTTCTCTGATATTTCCATAGCTCATAACAAGAGACAAGTGCAGGCGGGAACCAATT

TCTGAACCAGGTTCTGCTGCGCGCATCACAC

>FM894772.1 FM894772 Jatropha curcas embryo 71-95 (DAF) Jatropha curcas cDNA clone rjcpga0_000640, mRNA sequence

ATTCATTTGGAATCCCTTCCGATCAAAATTGGCGGCTGCCATTCTCGGTGGTGTGGATAACATATGGATT

AAACCTGGTGCGAAGGTTCTCTACCTTGGAGCGGCTTCGGGCACTACCGTCTCCCATGTGTCTGACCTAG

TTGGCCCTACAGGAGTAGTCTATGCAGTCGAATTTTCTCACAGAAGTGGTAGAGATTTGGTTAACATGGC

AAAAAAGCGCACAAATGTTATACCTATCATTGAAGATGCTAGACATCCAGCAAAGTATCGAATGCTTGTT

GGCATGGTTGATGTGATATTTTCTGATGTTGCTCAACCTGATCAGGCAAGGATTTTAGCTCTGAATGCTT

CATACTTCCTGAAAGCTGGTGGCCATTTTGTAATTTCAATAAAGGCTAATTGCATCGACTCTACTGTACC

TGCTGAGGCCGTTTTTGAAAGTGAAGTGAAGAAGATGACACAGGAGCAGTTCAAGCCTTCCGAGCAGGTG

ACCCTCGAGCCATTTGAGCGAGACCATGCTTGTGTTATTGGTGGGTATCGTATGCCAAAGAAACAGAAAG

TTGCTGCATAGCAAGATAGGTTACTGATCGAAAGCTGTTCGTACTTGTTATGAAT

>FM894771.1 FM894771 Jatropha curcas embryo 71-95 (DAF) Jatropha curcas cDNA clone rjcpga0_000639, mRNA sequence

ATTCCAAAGGATTCTATCCGTCGCCCGATGGGGAATTGTACTTTAATGGCGGCCCTCGCGGCTCGTCCGC

CGCGGGGGCTTGCCAACGGCACGTGCCTCTGGGGGCCGGATGGCCCCTACTGCGGGTCGGCAAACGGGCG

ACGGACACGCGCGTCGCTTCTGGCCCGGATTCTGACTTAGAGGCGTTCAGTCATAATCCAGCGCACGGTA

GCTTCGCGCCACTGGCTTTTCAACCAAGCGCGATGACCAATTGTGCGAATCAACGGTTCCTCTCGTACTA

GGTTGAATTACCATCGCGACACCATCATCAGTAGGGTAAAACTAACCTGTCTCACGACGGTCTAACCCCA

GCTCACGTTCCCTATTGGTGGGTGAACAATCCAACACTTGGTGAATTCTGCTTCACAATGATAGGAAGAG

CCGACATCGAAGGATCAAAAAGCAACGTCGCTATGAACGCTTGGCTGCCACAAGCCAGTTATCCCTGTGG

TAACTTTTCTGACACCTCTAGCTTCAAATTCCGAAGGTCTAAAGGATCGATAGGCCACGCTTTCACGGTT

CGTATTCGTACTGGAAATCAGAATCAAACGAGCTTTTACCCTTTTGTTCCACACG

>FM894770.1 FM894770 Jatropha curcas embryo 71-95 (DAF) Jatropha curcas cDNA clone rjcpga0_000638, mRNA sequence

CTAGTGGATCCCCCGGGGCTGCAGGGTCTGACATGTGTGCGAGTCAACGGGTGAGTAAACCCGCAAGGCG

CAAGGAAGCTGACTGGCGGGATCCCCCCGAGGGTTGCACCGCCGACCGACCTTGATCTTCTGAGAAGGGT

TCGAGTGAGAGCATGCCTGTCGGGACCCGAAAGATGGTGAACTATGCCTGAGCGGGGCGAAGCCAGAGGA

AACTCTGGTGGAGGCCCGCAGCGATACTGACGTGCAAATCGTTCGTCTGACTTGGGTATAGGGGCGAAAG

ACTAATCGAACCGTCTAGTAGCTGGTTCCCTCCGAAGTTTCCCTCAGGATAGCTGGAGCTCGGGACGAGT

TCTATCGGGTAAAGCCAATGATTAGAGGCATCGGGGGCGCAACGCCCTCGACCTATTCTCAAACTTTAAA

TAGGTAGGACGGCGCGGCTGCTACGCTGAGCCGCGCCACGGAATCGAGAGCTCCAAGTGGGCCA

>FM894769.1 FM894769 Jatropha curcas embryo 71-95 (DAF) Jatropha curcas cDNA clone rjcpga0_000637, mRNA sequence

GTTTATATTTATTTTACCTCAGTTCATTATGGGCGTGCTTTATAAGACATTTCTGAATAGCCAAAAGTGC

GTCGCATACGACAACATAAGACATTCTCACCTATTTCAGAGTTCAATCAGCAAACTACGAGGCTCCAATA

CTTTTTTAAGTCTCAAGCTATGAGAAAAAAACCAAAATAATATTCAAGAATTCAAGCCCCTTGCTGACTA

GAAATATCTGTAAGATGCCAAGCTTGTTAGCTTCAGAACGAGTCATTGACGGCCATATGAAACACTCCTG

TCATCACCATATGGATGAGCTGTATCACTACCGTATGCTGGACTAACGGTATTACCATATGCAGGATCAG

TGTTGCTCCCATACACAGGGCTCTTGTCGCTCTCATATGCAGGAGTTGTAGCATTCCCACGTACAGAATT

TGCATAGCCCCAGTTTGCGGGACCAGTTGTATTCCCATATACAGAAGTTGTGTTACTCCCATATGCAGGA

CCTGTGGAATGCCCATATGCAGGACCCATGTAGCTACCGTGTGCAGGACCCAT

>FM894768.1 FM894768 Jatropha curcas embryo 71-95 (DAF) Jatropha curcas cDNA clone rjcpga0_000636, mRNA sequence

TTTTTTATCTTTTTGTCAAAGAGCTGAACAATAAAAATAGATGGCGAGTGCCTAATCGAATTGATCGGGT

CATGTAGGAACAAGGTTCAAGTCTACCGGTCTGTTAGGATGCCTCAGCTGCATACATCACTGCACTTCCA

CTTGACACCTATCGTAATGATAAACGGCTCGTCTCGCCGTGACCTTCTCTTGAATTCTCAAAACTTCTGT

CACTCCATCCCCGCAGGGGCAGAGAACCCGTCGCTGTCTCGGCTGTGCTACCGAAGGTTCTGGGGAAGTC

GGAATAGGAGAGCGCTCATCTTGGGGTGGGCTTCCTACTTAGATGCTTTCAGCAGTTATCCGCTCCGCAC

TTGGCTACCCAGCGTTTACCGTGGGCACGATAACTGGTACACCAGAGGTGCGTACTTCCCGGTCCTCTCG

TACTAGGGAAAGGTCCTCTCAATGCTCTAACGCCCACACCGGATATGGACCGAACTGTCTCACGACGTTC

TGAACCCAGCTCACGTACCGATTTAATGGGCGAACAGGCCAACCCTTGGAACATACTACAGACCCAGGTG

GCGAAGAGCCGACATCGAAGTGCCAACCTTTCCGTCGATGTGAGCTCTTGGGGAAG

>FM894767.1 FM894767 Jatropha curcas embryo 71-95 (DAF) Jatropha curcas cDNA clone rjcpga0_000634, mRNA sequence

CCTAACCCTAACTGCCTGCATCTCAATCTTCGTCTGCATCTCTTTGCACGCCCACTCACACACCCGAACC

CACACGCAGAAATCCTCTCTGATTCAGGAAAATGGGTCGCGTCCGTACGAAGACAGTGAAGAAATCCTCT

CGCCAAGTGATAGAGCGCTACTACTCGCGCATGACTCTTGATTTTCACACCAACAAGAAGATTCTTGAAG

AGGTCGCCATCATCCCTTCAAAGCGTCTCCGCAACAAGATTGCGGGGTTTTCTACCCATCTCATGAAACG

GATCCAAAAGGGTCCGGTTCGCGGGATCTCACTCAAGCTTCAAGAGGAAGAGCGCGAGCGCCGCATGGAC

TTTGTACCTGATGTCTCTGCCATCAAGACTGATCAGATTGAGGTCGATAAGGAGACTATTGACATGCTTG

CTGCTTTGGGCATGAGTGATATTCCTGGGATTCTCCAAGTCGAACCGGCTGCTGCGCCTGCGACCCAGTT

CGTGTATGGGAGAGGTGGTGCTGGTGGCAGACCTGGTGGAAGGTTTTAAAAACTTTTTAGGAAAGTCTTG

TATATATATCGTTATATGTAGTGTTTCTCGAC

>FM894766.1 FM894766 Jatropha curcas embryo 71-95 (DAF) Jatropha curcas cDNA clone rjcpga0_000633, mRNA sequence

GAAATCAGAATCAAACGAGCTTTTACCCTTTGTTCCACACGAGATTTCTGTTCTCGTTGAGCTCATCTTA

GGACACCTGCGTTATCTTTTAACAGATGTGCCGCCCCAGCCAAACTCCCCACCTGACAATGTCTTCCGCC

CGGATCGGCCGCCGAAACGACCTTGGGTCCAAAAAGAGGGGCATAGCCCCGCCTCCGATTCACGGAATAA

GTAAAATAACGTTAAAAGTAGTGGTATTTCACCGTCGCCTTTCGGCTCCCACTTATCCTACACCTCTCAA

GTCATTTCACAAAGTCGGACTAGAGTCAAGCTCAACAGGGTCTTCTTTCCCCGCTGATTCCGCCAAGCCC

GTTCCCTTGGCTGTGGTTTCGCTGGATAGTAGACAGGGACAGTGGGAATCTCGTTAATCCATTCATGCGC

GTCACTAATTAGATGACGAGGCATTTGGCTACCTTAAGAGAGTCATAGTTACTCCCGCCGTTTACCCGCG

CTTGGTTGAATTTCTTCACTTTGACATTCAGAGCACTGGGCAGAAATCACATTGCGTGAGCATCCTCAGG

GACCATCGCAATGCTTAGTTTTAATTAAACAGTCGGATTCCCCTTGTCCGTACCAGTTCTGAGTCGA

>FM894765.1 FM894765 Jatropha curcas embryo 71-95 (DAF) Jatropha curcas cDNA clone rjcpga0_000632, mRNA sequence

AAAGCACAGGAAGCATCCGGGGTGGTCGCGGTAACGCCGGAGGCATGCACCACCACAGAATTCTCTTTGA

CAAGTACCATCCGGGGTATTTTGGTAAGGTTGGTATACGCTACTTCCACAAGCTCCGCAACACGTTCTAC

TGTCCTATCGTCAACATAGACAAGCTCTGGTCCTTGATTCCTCCCGAGGCGACGGCAACAGCTTCTAAGG

ATAATGTGCCCTTGCTCGATGTTACC

>FM894764.1 FM894764 Jatropha curcas embryo 71-95 (DAF) Jatropha curcas cDNA clone rjcpga0_000631, mRNA sequence

CGATTAAGACAGCAGGACGGTGGTCATGGAAGTCGAAATCCGCTAAGGAGTGTGTAACAACTCACCTGCC

GAATCAACTAGCCCCGAAAATGGATGGCGCTTAAGCGCGCGACCTATACCCGGCCGTCGGGGCAAGAGCC

AGGCCCCGATGAGTAGGAGGGCGCGGCGGTCGCTGCAAAACCCGGGGCGCGAGCCCGGGCGGAGCGGCCG

TCGGTGCAGATCTTGGTGGTAGTAGCAAATATTCAAATGAGAACTTTGAAGGCCGAAGAGGGGAAAGGTT

CCATGTGAACGGCACTTGCACATGGGTTAGTCGATCCTAAGAGACGGGGGAAGCCCGTCCGACAGCGCGT

CCTCGCGCGAGCTTCGAAAGGGAATCGGGTTAAAATTCCCGAACCGGGACGTGAAGGCTGACGGCGACGT

TAGGGAGTCCG

>FM894763.1 FM894763 Jatropha curcas embryo 71-95 (DAF) Jatropha curcas cDNA clone rjcpga0_000630, mRNA sequence

ATTCTTAGGGATAAAGTGACAGAATATGTCAAGGAACGAATTTCAGCATTAAGGATAGCTAATCCAGGTC

AATATCAAAATGTCTCAGTGGTTCGAACCAATTCTATGAAATACATACCAAATTACTTTGGGAAAGGACA

GCTTACGAAGATGTTTTCCTGTCCCGGATCCCCACTTCAAGGAGAAGAATCATCGTCGAAGGGTTATTAG

TCCGCATTTGCTAGATGAGTATGCCTATGTTCTTGAGACTGGCGGGATTGTTTATACAATTACCGATGTG

GAAGAATTGGGTGAGTGGATGAAGGCTTGTTTGGAGAATCACCCTATGTTTGAAGCCCTCACAGAGGAGG

AGCTTGGAGTAGATCCTGTTGTAGAGCTCCTGAGCACAGCAACTGAAGAAGGACAAAAGGTGGCTAGGAA

TAGGGGACAGACTTTTCAAGCAGTCTATAGACGTATTGCACCATCTCTATGATACATAAGAAACTTAGAC

ATACTTAAACAGATGGCTTAAACTAATT

>FM894762.1 FM894762 Jatropha curcas embryo 71-95 (DAF) Jatropha curcas cDNA clone rjcpga0_000628, mRNA sequence

CTAGTGGATCCCCCGCGGCTGCAGGTTTTTTTTTACACGAAAATAGATCCACTTTCTAAAATAGAAATTT

AAGGAAAACTGAAATAAACAGTAAAGAAAGATGGGCGAAATTGAAGAAAAATACTACAAAATAAGTTCCT

AACTTTCATCATCACATTTAAGAATGTGAGATCTANCTCCGCTAATTCTTCAAGGACCTCCTCTTCATCC

CTGTAAACAAAATCAATCATCCTCCATATCTGAAGAGTACCACCTCCTGAAGTGCTTTCACAATCATCAG

AAACACTGACAATTGTCCAGGGATCAGAGGCATTCCAATGGAAGTCAACAACCTTATCCCTATGCCCAGC

ATGCCGGAAACATAATCCTGCAGGAGCATTTGGCAATTTCAACCCAACAGAATCTTGCTTCTTACCAACC

TTCTCATAATCCCATATATATAGAATAGCCCTC

>FM894761.1 FM894761 Jatropha curcas embryo 71-95 (DAF) Jatropha curcas cDNA clone rjcpga0_000627, mRNA sequence

TCACTTTTCTTTCCACTATTGGATTACGCTTCCGTCTTTTCTTTCAACGAACCTCTCAACTTAGCCTACT

CACGTACTAACATACCATGAATTTGGGGTTTTCTCTTCGGATTCGATCTATATTGTGAGTGCTTCTTGTC

ATGATGTCTAGACCTATGCTACTTGTGTTTCTATTACTTGTACTCATAATCACCTCTCAGTTTGAGTGGA

GACAACAACTTGTAAATGACATTGATGCCACCCCTGCTGTTACCCAGAAGCAGCAACACGTATCANAGAG

GGAAGAAGCTGTCAAGGAGAAGATTATCTTATCAAAAGAGAGGAATATTTTTAGACTTAATGAGCTTGTC

CGGAGTCTTCAACAGCACCTATTGCAGTGCAGGTGCAATAATGATACTAGAAATGGCACTGTTGATTTGA

CAGAGCGTGTAATTGAGCTTGAACG

>FM894760.1 FM894760 Jatropha curcas embryo 71-95 (DAF) Jatropha curcas cDNA clone rjcpga0_000626, mRNA sequence

CTAAGGAGTGTGTACAACTCACCTGCCGAATCAACTAGCCCCGAAAATGGATGGCGCTTAAGCGCGCGAC

CTATACCCGGCCGTCGGGGCAAGAGCCAGGCCCCGATGAGTAGGAGGGCGCGGCGGTCGCTGCAAAACCC

GGGGCGCGAGCCCGGGCGGAGCGGCCGTCGGTGCAGATCTTGGTGGTAGTAGCAAATATTCAAATGAGAA

CTTTGAAGGCCGAAGAGGGGAAAGGTTCCATGTGAACGGCACTTGCACATGGGTTAGTCGATCCTAAGAG

ACGGGGGAAGCCCGTCCGACAGCGCGTCCTCGCGCGAGCTTCGAAAGGGAATCGGGTTAAAATTCCCGAA

CCGGGACGTGAAGGCTGACGGCGACGTTAGGGAGTCCGGAGACGTCGGCGGGGGCCTCGGGAAGAGTTAT

CTTTTCTGTTTAACAGCCCG

>FM894759.1 FM894759 Jatropha curcas embryo 71-95 (DAF) Jatropha curcas cDNA clone rjcpga0_000625, mRNA sequence

TTTAACATCAAGTTTGAAAGAATAATCATTAGAACTCAATAATGTATCATCTACAAATACAGTAATGAAT

TATAGATTATTGACTATGAATACAGTGACTGACAGTCATCTAAATAAAAATGAATTCGCCACCACGAACA

GCATTGGCGGCACCTTTCCTCTCCTCTTCAGCCTCTTTCTCGCACTCTTTCCAGCTCTCATATACATGAT

CATCAGTTTGCAGCTCATGCCGGCAAATTGGACAAGAATTATGCTCATCCAGCCATGGCTTTAGACAAGG

AGGATGGAATGTGTGCTTGCAAGGCAACTCTTGCATCTTATCATTAACCACCAAATTCTCCTTACAAATT

GCACACTCTGCATCTTGTCCAAGCCTGGCCAAGATTTCCTCCGTGAGAGTAATGACTGGAAGCTTTGCAA

CAACCTCTTTACTGGCGGGAGGCGCTTAAGGAACTCTAGATTCATTGTCTGGAACCATAATCTCAAGTAT

AATGTCATCA

>FM894758.1 FM894758 Jatropha curcas embryo 71-95 (DAF) Jatropha curcas cDNA clone rjcpga0_000624, mRNA sequence

TTTTTTTTTTAAAAAGAGAAACTAACAAGCCAACTACTAAACGGGAAACAGCGCAAACAAGTAGATAGTA

ATGAAAAGTGCCACAAAATAATGCAAAACAAGAAAAATAGACAACCAACCAAAGCTAATAACACCAAATG

AAGATGTCCTAAAGGGAAATAGAATTCTCATCAAACTTATCTGCCCTGTTTCCCTTTTTCTTTTTATGCT

TTTAAAATTCAGTTTGGTTGAGTGGTCCTCACTTGGCCATCATGACTTTAACAAACTCCTCGTAGTTGAT

CTGACCGTCACCGTCCACATCAGCCTCACGAATCATCTCATCAACCTCCTCATCTGTCAGCTTCTCGCCA

AGATTTGTCATGACATGGCGCAGCTCAGCAGCAGAAATGAAGCCATTCTGATCCTTATCAAAAACCCGGA

AAGCTTCCTTGAGTTCCTCCTCAGAGTCAGTGTCCTTCATCTTACGGGCCATCAGATTAAGGAACTCTGG

GAAATCAATGGTACCATTACCATCAGCATCAACCTCATATATCATGTCCT

>FM894757.1 FM894757 Jatropha curcas embryo 71-95 (DAF) Jatropha curcas cDNA clone rjcpga0_000623, mRNA sequence

ATTCAATGTTATATAGAGGGTTAACCTTTTATAATATATTACTTTTAACCTCTAAAATAACTAGTTCAAC

ACTATGTCACTTAATATTATAAAAAATAGCGTCAAAACGCTAAAAGTTGTAAACATGACCTGAGACTTGT

TATTGTTAATAGTTTTTTGCACATCAATTTCCTATGATAAATGCCAATAATTTCTTCTTTTTGTAGGTGT

TTCAAAGGGAAAGATTAAACGGGCATTACCATTCAAGTGCATTTGTTGTTAGCAACACACTTGCATCAGC

ACCTTACTTGCTTCTAATATCCATAATTCCAGGAGCAATTACATACTATCTTGTCGGTCTCCAACAAGAA

AGTGCACACTTCATCTATTTTGCTTTAACACTTATTGCTTGTATGATGTTAGTAGAAGGCCTAATGCTAA

TTGTGGCAAGCATTGTTCCAAATATCCTCATGGGTTTAATCACCGGCGCCGGAATTCAAGGCCTAATGAT

GCTAAGTGGTGGA

>FM894756.1 FM894756 Jatropha curcas embryo 71-95 (DAF) Jatropha curcas cDNA clone rjcpga0_000622, mRNA sequence

TGGAGATTCGCAAATGGAATGTTTGCTTTGTTCTTTCATTTGGACTTCTGCTGACTGCACTAAGAAGCAG

AAAAGCAAGGTCTTGGCGCTATGGTTCTGGATCTCTTCGAGGCTTTATAGCAGATTATGGTGTACCATTG

ATGGTGCTGGTTTGGACAGCTGTTTCCTATATTCCATCAAAAAGTATTCCAAAAGGAATTCCAAGGCGTC

TTTTTAGTCCAAATCCATGGTCAACAGGCGCATATGAAAACTGGACTGTCATGAAGGATATGCTAAACGT

ACCAGTTCTCTATATAGTTGGAGCTTTCATTCCGGCAACAATGATTGCAGTGCTCTATTATTTTGACCAC

AGTGTAGCGTCACAGCTTGCTCAACAGAAAGAATTTAACTTGAGAAAGCCTCCTTCTTTCCACTATGATT

TACTTCTTTTAGGATTCATGGTCATCTTATGTGGTCTTATTGGGATCCCTCCATCAAATGGTGTCATTCC

ACAGTCTCCCATGCATACTA

>FM894755.1 FM894755 Jatropha curcas embryo 71-95 (DAF) Jatropha curcas cDNA clone rjcpga0_000621, mRNA sequence

CTAGTGGATCCCCGGGCTGCAGGCTGACATGTGTGCGAGTAACGGGTGAGTAACCCGCAAGGCGCAAGGA

AGCTGACTGGCGGGATCCCCCCGAGGGTTGCACCGCCGACCGACCTTGATCTTCTGAGAAGGGTTCGAGT

GAGAGCATGCCTGTCGGGACCCGAAAGATGGTGAACTATGCCTGAGCGGGGCGAAGCCAGAGGAAACTCT

GGTGGAGGCCCGCAGCGATACTGACGTGCAAATCGTTCGTCTGACTTGGGTATAGGGGCGAAAGACTAAT

CGAACCGTCTAGTAGCTGGTTCCCTCCGAAGTTTCCCTCAGGATAGCTGGAGCTCGGGACGAGTTC

>FM894754.1 FM894754 Jatropha curcas embryo 71-95 (DAF) Jatropha curcas cDNA clone rjcpga0_000620, mRNA sequence

TTAAAACCAAAAAGTATGGAAACATCAAATTCATCAACAAACAAAGTAAGACTTGATTACAGAGACCTTC

AACACATGACAAGAAAATTATTTTAGACAGCTCTCAAAAGAGGGAAGACCCGTTAACAAATTACATAGAA

GAAACAAAAAGATTAAGCGAAAAAAGAAATACACCAGACAAGCAATGGCCTTCCATGTTAATTAAAGGAC

TCATATATTTTTATATATATATCAAAGCCATTTTATGTTCTCTGCATCAACCACCAAAACCCACTTCTCT

TTTAAGTCTGTTGACCTGTTACATGCTGTACAAACTGCTCAGCTCTGTCCTTCATCTCTCTTGCCTTTCC

CGCCAACTTTAAGCGTGCCTGGTCCAGACTTTCTGCTCCTGGTGGATGCTTTCCAGTAACATACCTATAA

ATCCAGGACAAGACAGAAATGGCAGCGACGCCAAAACCGACTGAGGCTAAAAAACCTGTGATTATCAAAC

AAACTGTGATAACCGCAGGAACAAGAACCGGACTGAATATGACC

>FM894753.1 FM894753 Jatropha curcas embryo 71-95 (DAF) Jatropha curcas cDNA clone rjcpga0_000619, mRNA sequence

ATTCAACCATTGCAGCAGCTGCTTATTTGAGGTTAGCAGGCAGGCCACCGGTTCTCCCTTCAAGCAACCT

TTCTTATGCGGAGAACTTCTTGTACATGCTAGACTCATTAGGTAATCGATCTTATAAACCTAATCCTCGA

CTTGCACGAGTTCTAGATGTTCTCTTCATTTTGCACGCAGAACATGAAATGAATTGCTCTACTGCTGCTG

CCCGGCATCTTGCGTCCAGTGGTGTTGATGTATACACTGCTCTTGCTGGGGCTGTTGGAGCATTGTATGG

TCCTCTTCATGGTGGAGCAAATGAGGCTGTGCTTAAGATGCTGAGTGAGATTGGAACTGTTGAAAACATT

CCAGATTTCATTGAGGGCGTAAAAAGCAGGAAGCGAAAGATGTCAGGTTTTGGGCATCGAGTATACAAAA

ACTATGATCCCAGAGCAAAAGTCATTAGGAAACTAGCTGAGGAAGTGTTCTCCATTGTTGGTCGAGATCC

ACTTATTGAGGTAGCAGTTGCTTTGGAAA

>FM894752.1 FM894752 Jatropha curcas embryo 71-95 (DAF) Jatropha curcas cDNA clone rjcpga0_000618, mRNA sequence

CGAATCTGTTCATTTTTTCAATTCTCACCAATCTAGAGAACAGAAACAGAAGATGGAAAAATGTTGGGAA

AAAAAAAAAAGAAAGAAAGAAATCACAGACCAAAAATACTAAGTTGCCAAAATGGCTAACACTAGAATCA

GTAGCATATAAGTATTTTGCTTATATTTTGTTGAAGGCAACAATGTCACAGCTCTGAATATATTCGTTGC

AAGGCTCAACTGTCAGATGTTCCTCTATGAGAGAATCAACGGAAACAAGGTCATCAACAATGGTGAGCAT

GATCTGCAATTTCTTGATGCCATAACCAACTGGAACAAGTTTTGATGCTCCCCACAGTAGACCAGGCATC

TCTATACTTCGAACTGCCTCCTCTAACTTCTTCATATCTGTCTCATCATCCCAAGGCTTCACATCCAGAA

GAACAGAAGATTTTCCACTCTCTTTCTTCTTGGAGGATTCCT

>FM894751.1 FM894751 Jatropha curcas embryo 71-95 (DAF) Jatropha curcas cDNA clone rjcpga0_000617, mRNA sequence

ATTCCGAACGTCTTCTTCAACCGATCTGTCATTTTGTTTCATATTTTCTCGCACTTCCTCGGTCGTATTC

TCGTTCTCTCCTTCTCTAATCAATGGACGCGTTCGCGTCGTTCTTCGACTCCCAATCGGCTTCAAGGAGT

AGCTGGACCTACGATTCGCTGAAGAACTTCCGCCAGATCTCTCCTGTCGTCCAAACTCACCTTAAGCAGG

TCTATTTGACCTTATGTTGCGCACTGGTTGCATCGGCTGCTGGGGCTTACTTGCACATTCTGTGGAATAT

TGGTGGTCTTCTGACAACTTTTGCGTGCTTGGGATGTATGGCTTGGCTACTTTCTGTACCTCCTTATGAA

GAGCAAAAGAGAGTCTCTCTTTTGATGGCAGCTGCACTTCTTGAAGGGGCTTCAGTTGGTCCTTTGATTG

ATTTGGCCATTGAGATCGATCCAAGTGTTCTGATCACAGCATTTGTGGGAACTTCGGTGGCCTTTGGTTG

TTTCTCAGCGGCAGCCACGTTGGCAAGGCGTAGAGAATACCTATATCTTGGTGGCTTGCTTTCATCTGGC

TTGTCCATCCTTCTCTGGTTGCAGTTTGCCTCCTCCATCTTTGGGGGAT

>FM894750.1 FM894750 Jatropha curcas embryo 71-95 (DAF) Jatropha curcas cDNA clone rjcpga0_000616, mRNA sequence

ACACCAGATGAAGGAGCATTTGAATTCATTAAATAGTAGAAATATAAAAGATAAAACACTTCCAAGTCAA

CTGAACAAAACCGTCCAAACTCAACAACAAGCAGATATTCCTCATGTTTTTCTTATTTTGATTACCTTCC

ATGGATATGCTAAAAACCTCATAAAGCAAATATAACAAAAAGACAGGTCCTAAAACTATGCCTGAGACAA

AAGACAATCTATTATACTCCTCTGAAATTTAAAACGGAAACTTAAGACCTCTTTAGTATTTGTAGTCCTT

GACATGGAGACTCTCTGAGGCACCCTCACCAAGAGTAGCATTACCCTGGTAAGTACCAAGAGTTGCCTCT

GAGTTGGCCTTAGCCCTGACCAGGAATGCAGCTTGAGCCTTCTGCACATTCTCTGGCTTACCTGCCCAAG

CCTTGAGGGTACTTTGTTGAAGTGCACGTACAAATGAGAAGGAAAGGGACCATGGCTTCTTGGTCTTGAG

CTTGTTCATCGCATTCAGGTTGAC

>FM894749.1 FM894749 Jatropha curcas embryo 71-95 (DAF) Jatropha curcas cDNA clone rjcpga0_000615, mRNA sequence

ATTCGCAAAGGATTCTATCCGTCGCCCGATGGGAATTGTACTTTAAGGCGGCCCTCGCGGCTCGTCCGCC

GCGGGGGCTTGGCCAACGGCACGTGCCTCTGGGGGCCGGATGGCCCCTACTGCGGGTCGGCAAACGGGCG

ACGGACACGCGCGTCGCTTCTGGCCCGGATTCTGACTTAGAGGCGTTCAGTCATAATCCAGCGCACGGTA

GCTTCGCGCCACTGGCTTTTCAACCAAGCGCGATGACCAATTGTGCGAATCAACGGTTCCTCTCGTACTA

GGTTGAATTACCATCGCGACACCATCATCAGTAGGGTAAAACTAACCTGTCTCACGACGGTCTAACCCCA

GCTCACGTTCCCTATTGGTGGGTGAACAATCCAACACTTGGTGAATTCTGCTTCACAATGATAGGAAGAG

CCGACATCGAAGGATCAAA

>FM894748.1 FM894748 Jatropha curcas embryo 71-95 (DAF) Jatropha curcas cDNA clone rjcpga0_000614, mRNA sequence

AGGATTCTATCCGTCGCCCGGTGGGAATTGTACTTTAAGGCGGCCCTCGCGGCTCGTCCGCCGCGGGGGC

TTAGCCAACGGCACGTGCCTCTGGGGGCCGGATGGCCCCTACTGCGGGTCGGCAAACGGGCGACGGACAC

GCGCGTCGCTTCTGGCCCGGATTCTGACTTAGAGGCGTTCAGTCATAATCCAGCGCACGGTAGCTTCGCG

CCACTGGCTTTTCAACCAAGCGCGATGACCAATTGTGCGAATCAACGGTTCCTCTCGTACTAGGTTGAAT

TACCATCGCGACACCATCATCAGTAGGGTAAAACTAACCTGTCTCACGACGGTCTAATCCCAGCTCACGT

TCCCTATTGGTGGGTGAACAATCCAACACTTGGTGAATTCTGCTTCACAATGATAGGAAGAGCCGACATC

GAAGGATCAAAAAGCCACGTCGCTATGAACGCTTGGGCTG

>FM894747.1 FM894747 Jatropha curcas embryo 71-95 (DAF) Jatropha curcas cDNA clone rjcpga0_000613, mRNA sequence

GATTAATGAAAACATCCTTGGCAAATGCTTTCGCAGTTGTTCGTCTTTCATAAATCCAAGAATTTCACCT

CTGACTATGAAATACGAATGCCCCCGACTGTCCCTGTTAATCATTACTCCGATCCCGAAGGCCAACACAA

TAGGACCGAAATCCTATGATGTTATCCCATGCTAATGTATACAGAGCGTAGGCTTGCTTTGAGCACTCTA

ATTTCTTCAAAGTAACAGCGCCGGAGGCACGACCCGGCCAGTTAAGGCCAGGAGCGCATCGCCGGCAGAA

GGGACGAGCCGACAGGTGCACACCGCGAGGCGGACCGATCGACCCAACCCAAGGTCCAACTACGAGCTTT

TTAACTGCAACAACTTAAATATACGCTATTGGAGCTGGAATTACCGCGGCTGCTGGCACCAGACTTGCCC

TCCAATGGATCCTCGTTAAGGGATTTAGATTGTACTCATTCCAATTACCAGACTCATTGAGCCCGGTATT

GTTATTTATT

>FM894746.1 FM894746 Jatropha curcas embryo 71-95 (DAF) Jatropha curcas cDNA clone rjcpga0_000612, mRNA sequence

ATTCCAAAGGATTCTATCCGTCGCCCGATGGGAATTGTACTTTAAGGCGGCCCTCGCGGCTCGTCCGCCG

CGGGGGCTTAGCCAACGGCACGTGCCTCTGGGGGCCGGATGGCCCCTACTGCGGGTCGGCAAACGGGCGA

CGGACACGCGCGTCGCTTCTGGCCCGGATTCTGACTTAGAGGCGTTCAGTCATAATCCAGCGCACGGTAG

CTTCGCGCCACTGGCTTTTCAACCAAGCGCGATGACCAATTGTGCGAATCAACGGTTCCTCTCGTACTAG

GTTGAATTACCATCGCGACACCATCATCAGTAGGGTAAAACTAACCTGTCTCACGACGGTCTAACCCAGC

TCACGTTCCCTATTGGTGGGTGAACAATCCAACACTTGGTGAATTCTGCTTCACAATGATAGGAAGAGCC

GACATCGAAGGATCAAAAAGCAACGTCGCTATGAACGCTTGGCTGCCACAAGCCAGTTATCCCTGTGGTA

ACTTTTCTGACACCTCTAGCTTCAA

>FM894745.1 FM894745 Jatropha curcas embryo 71-95 (DAF) Jatropha curcas cDNA clone rjcpga0_000611, mRNA sequence

TTACATTTTGACACAACCTACAGGTCTAATAGGTATCGCTTGCCATTTGCACCGTTCACAGAGGGTAAAC

CATCATGGACACCCTGTTTTGTTTGGTTGTGCTTTCCTAATAAATGAAACTGAAGCCTCATTTGTCTGGC

TGTTCAATACATGGCTTACTGCAATGTCTGGTCGTCATCCAGTGTCAATCACCACCGATCATGATGCAGT

GATAAGATCGGCCATCATGCAGGTTTTCCCAGAAACTCGCCATCGCTTTTGCAAATGGCATATCTTTAAG

AAATGCCAAGAGAAGTTGTCCCATGTGTTTCTGAAACACCCAAATTTTGAGGCAGAGTTCCATAAATGTG

TTAACTTGACTGAGTCAATTGAAGAATTCGAATCTTGCTGGTTGTCACTTGTTGACAGATATGACCTCCG

GGATCATGAGTGGCTTCAAACAATATATTCTGCTCGTAGGCAGTGGGTCCCAGTATATTTGCGGGATACT

ATTTTTGCCGAGATGTCCATAAC

>FM894744.1 FM894744 Jatropha curcas embryo 71-95 (DAF) Jatropha curcas cDNA clone rjcpga0_000610, mRNA sequence

GCCAGTAGTCATATGCTTGTCTCAAAGATTAAGCCATGCATGTGTAAGTATGAACTAATTCAGACTGTGA

AACTGCGAATGGCTCATTAAATCAGTTATAGTTTGTTTGATGGTACCTGCTACTCGGATAACCGTAGTAA

TTCTAGAGCTAATACGTGCACCAAACCCCGACTTCTGGAAGGGATGCATTTATTAGATAAAAGGTCGACG

CGGGCTCTGCCCGTTGCTCTGATGATTCATGATAACTCGACGGATCGCACGGCCATCGTGCCGGCGACGC

ATCATTCAAATTTCTGCCCTATCAACTTTCGATGGTAGGATAGAGGCCTACCATGGTGGTGACGGGTGAC

GGAGAATTAGGGTTCGATTCCGGAGAGGGAGCCTGAGAAACGGCTACCACATCCAAGGAAGGCAGCAGGC

GCGCAAATTACCCAATCCTGACACGGGGAGGTAGTGACAATAAATAACAATACCGGGCTCAATGAGTCTG

GTAATTGGAATGAGTACAATCTAAATCCCTTAACGAGGATCC

>FM894743.1 FM894743 Jatropha curcas embryo 71-95 (DAF) Jatropha curcas cDNA clone rjcpga0_000609, mRNA sequence

CAATGGAAGAAAGTCAGGTTGTTAAGCTTATGACGGTGGTTATGACTCTGTTCTTGCTTTCGGCAGTTTC

AGAGCAGGCTTCAGAGGAGGCTCAAGGTGTTGATTGCATCAGAGGATGCGACGATCGGTGTGGTCCTTAT

GGAAGTAGATATGTCAACTGTTGTATCAGAATTTGTCAAAAACATGAGCGTACTGAAGATTGTAAATTTT

CTATATGTGGCAATTTTGGTGATGTCTCAGAGGATGCTAAAGATGGTGGTTGCGCCAGAAAATGCATGCT

AAAATGTGCGGCTCTTAGAAGGGCAGCGCCCTTTTGTGTAAGCTTGTGTCTAAAAGATAGTGAGAAACCT

CCTCTAGAGGGCATTTACGAGGAACATGGCTCCATCCGAAATTGTGAACATTCCAAATGTGACGAATTCT

GGTAATG

>FM894742.1 FM894742 Jatropha curcas embryo 71-95 (DAF) Jatropha curcas cDNA clone rjcpga0_000596, mRNA sequence

CTCATTATTATATGAGGAATTACTTACTCTTGTGTTTTACCCTGTGATACTTCATAGAAACAAAATATGA

ATAAATTTCCCCCTCCTTTGAGATGTGGGATATATACCATAAGTAACAGCCCAATGTTCTA

>FM894741.1 FM894741 Jatropha curcas embryo 71-95 (DAF) Jatropha curcas cDNA clone rjcpga0_000595, mRNA sequence

TATTATTGAGCATATTGTTGAGCTGATGCACCGTGAAAAAATCAAGCCTGATGGGGCAACCTGCAATTTT

GTGTTCTGTACATACGTGGAGTGTGATTTTCATAACACAGCGATAGAAGCATTGCAAGTTTTGAGTTTGC

GTATGATTTGTGAAGATGACAATTCTTTTCAAGAAAAGAAAGAAGAATTCGAGAATAATTTCATCCTTTC

TGACGACCAAGATGTTGAGTCACGAATACTTGAGATGTTTGGAGGTCATCAGGAGACTACTGCTGTTGCC

CTCTTGCATTTAAGATGGTCTGCCATTCTTAGGTTCCCAATTTGTTGGTCACCAAATGAAACTCTATGGG

CAAGGAGACTTTCAGTTAACTATGATGCTAGAAAGAGTATTGTGTGATAGATATTTGTTGGTTATCGTTG

ATGAAGTTACATTATAAGAAAGGGGGACAAAGATTTGCCTGAAGGTCCTGGATTCGAGCCCAGGACTTGC

GGTTTGCCTCA

>FM894740.1 FM894740 Jatropha curcas embryo 71-95 (DAF) Jatropha curcas cDNA clone rjcpga0_000594, mRNA sequence

ATTCCACATTGAATATTCTTATTCCAAACCCATGGGAAGCAAGTGATTTAGTTAAGATACTAGGCGTTCA

CCGAGTCACACCATTAGCTAGCTCACCCAAATCCACTACCGAGTCATCTCTT

>FM894739.1 FM894739 Jatropha curcas embryo 71-95 (DAF) Jatropha curcas cDNA clone rjcpga0_000592, mRNA sequence

TTGAATTTTCTTATTCCAAACCCATGGAAGCAAGTGATTTAGTTAAGATACTAGGCGTTCACCGAGTCAC

ACCATTAGCTAGCTCACCCAAATCCACTACCGAGTCATCTCTTGCTCTTACACATTTTGATATGATTTGC

CTCAAATTTTATCCAGTTGAGCAAATCTTTTTCTATGAGTTCAACAATTTAACTTTGTCATTCTTCAACT

CAGTGATCATCCCTAAACTCAAATCATCACTCTCACTTGCTCTCCTCCATTTCCTTCCTCTAGCAGGAAA

CATCAAATGGCCTTCTAATGCTACCAAACCATGCATCTTATACACCGTAAATGATGGCATTTTGGTTAGG

ATTGCAGAATCAGCTGCAAACTTCACCCATCTTTCGAGCAACCAAATGAAGGAAGCAATTGAGTCACGTC

TTNATATACCCGGAATTGAATATATCTGACTCAACAGCTGCCATTATAGCTATTCAAATAACTTTATTTC

CATATCAAGGGTTTTCCA

>FM894738.1 FM894738 Jatropha curcas embryo 71-95 (DAF) Jatropha curcas cDNA clone rjcpga0_000590, mRNA sequence

ATTCAGAAAAGTTACCACAGGGATAACTGGCTTGTGGCAGCCAAGCGTTCATAGCGACGTTGCTTTTTGA

TCCTTCGATGTCGGCTCTTCCTATCATTGTGAAGCAGAATTCACCAAGTGTTGGATTGTTCACCCACCAA

TAGGGAACGTGAGCTGGGTTTAGACCGTCGTGAGACAGGTTAGTTTTACCCTACTGATGATGGTGTCGCG

ATGGTAATTCAACCTAGTACGAGAGGAACCGTTGATTCGCACAATTGGTCATCGCGCTTGGTTGAAAAGC

CAGTGGCGCGAAGCTACCGTGCGCTGGATTATGACTGAACGCCTCTAAGTCAGAATCCGGGCCAGAAGCG

ACGCGCGTGTCCGTCGCCCGTTTGCCGACCCGCAGTAGGGGCCATCCGGCCCCCAGAGGCACGTGCCGTT

GGCCAAGCCCCCGCGGCGGACGAGCCGCGAGGGCCGCCTTAAAGTACAATTCCCATCGGGCGACGGATAG

AATCCGGGGGGGCCCGGTAC

>FM894737.1 FM894737 Jatropha curcas embryo 71-95 (DAF) Jatropha curcas cDNA clone rjcpga0_000589, mRNA sequence

CGGTAAGACGTGAAAACACCCGATCCCATTCCGACCTCGATATGTGGAATCGTCTTGCGCCATATGTACT

GAGATTGTTCGGGAGACATGGTCCAAGCCCGGT

>FM894736.1 FM894736 Jatropha curcas embryo 71-95 (DAF) Jatropha curcas cDNA clone rjcpga0_000588, mRNA sequence

TTTTCTTCCTAATTTTGAGGTATATTAGCACCAAAATGGATAGAAAATCTGTACACGAACAAAATAGATG

CATTTCTAAGGTAAAAGTACAAACATTCTATGCTGTCAAGAAACAAGTAGGGTCGAAAAGTAGCAAAACC

CACTCAAAATCGGAAAAGCCGATGAGTTCTCAGTGTGAACTGGAAGCCTGCTTTCTTAGTTGATCTACTA

TACAACCTTACTTCCACAAAGAAAAAGATAACATGGAAACCACAGTTGCTTATGCAACTAAAGTTTGAGA

GCCTCTCTGAACCCAATGCTGAAACTATATTTTAGGCTCGTCCTACCATGACCTGCCACATTCCCTTTCT

GCATCATAGCCAAAAA

>FM890742.1 FM890742 Jatropha curcas embryo 56-70 (DAF) Jatropha curcas cDNA clone rjcaeb1_000629, mRNA sequence

ATTCCAATGCCACGGGACGTCGTGGGAGAAACGTGTGCTGCACGTGAGCATGGAGGCACAGGAAGAAAGG

AAGAGGTGGTGGTTCTTGAAGAAACACCAGCAGGAACCGTGGCATCCACCCTGAAAGAGTCTGATCAGAT

TAGTGCTCAAACTTTCAACGATGTTGGACGGTTAGATGATGAAGGTGTTACTCATCTAAAGGTCGTCCAC

CGTTCAGACAATAAGTGATAACGTCCACCGAATTCACGGTGTACTTCCTACTTCTTTTACTTTTTCACTT

TTACACCACCATGTTCATTAGTAGCTTTTTGGGCCTTCTTGTTGTGCTTTTTTTTTGTTTTGAGTAACTA

TAAACAAAGCTTTTGTGGGCTTGTTTGCCCTTCTGGATCTTATGTGCCTAATCTTATATGGATAAGATTG

GAAGGTTATCATCTCTGTGAGTTTGTATTGTAATTTAATGGTTGTGATGTGAGGTTTAACAAGCGGACGT

TGAACTATTTAACGCTCAGTAATTGCCCAT

>FM890741.1 FM890741 Jatropha curcas embryo 56-70 (DAF) Jatropha curcas cDNA clone rjcaeb1_000628, mRNA sequence

ATTCAAAATGGGCTCCAAAATTCTTCCTCAGGCGCTAGTTGTGATCCCAAGAAACGCTACTCAGTTTCCT

CCTTCAAGGAAATTAGGCTTTTCTGCAACAGTCTATCGCGGTTCCTCTCTCTGCAATTCAACTTCTAGGG

TTTCGGTTTCTCAGCTTTCGACAAACTCAATTGGTTCAAATCTGGACGCAATCATCTCCGCTTTTGCGAG

CCAGAGGTTCGTTGCGAGAGCGGAATCAGAGGAGTCTGGAGGCGGTGAAGTTGCGGATAATAGCGAAAAT

GTTGAATTAGAAGAACAGGAAGATAGTGAAAATGCTGAAGTAGAAGAACAGGAAGAGGAAAAACAACGTG

ATGAGGTGAAGGCCGAAGAAAAAGTGAAAGAACCGAGAAAGCCAAGAATCAAGCTTGGGGAGATAATGGG

GATATTGAC

>FM890740.1 FM890740 Jatropha curcas embryo 56-70 (DAF) Jatropha curcas cDNA clone rjcaeb1_000626, mRNA sequence

GGCTGCAGGAATTCGAATTCTGAATAGCCAAGTGAGTGATTGAATTTGAATAGCCAAGTGAGTGAAAATG

GAGTCGCTGAAGGAAACCGCAGCTAACGTTGGAGCCTCTGCTAAGGCTGGCATGGAAAAAACCAGAGCAG

AGGCGCAAGAGAAGGTGGAGAGAATGTCAACACGTGATCCAGTGCAGAAGGAAATGGCGAGAGAGAAGAA

AGAAGAGAGGAAAACGGAGGCAGAATTGCGGAAACTAGAAGCGCGCGAGGATAACGCAGCGGCGAGGCAG

GCAGCCAAGGCTGGTGGCCATGTACAGTACACAACTGCTGGTCCGGGCGCTCAAGGCACAACCGAAACCT

ATACCCATTCAACAACTGGGACAACCGGTTACCCTACAGGGACCCAGCAAATGTCGGCCATGCCTGGACA

C

>FM890739.1 FM890739 Jatropha curcas embryo 56-70 (DAF) Jatropha curcas cDNA clone rjcaeb1_000625, mRNA sequence

GGTTTAAAAAAAAAGGGCGTGCTTAATTCAAGGGGGAAATAAGGATAAAAGTTTTAACANGTCAACCTCA

AAACTCGAAAGCACTCTTCCATACTATATTTGCCCAGATTTTTACCANAGAAATATGATCCAAGACCTCT

ATAAATTACGACCCCAAACTGACCAAAAGAAATACCAAAGTCATCCACCAGACCCAACACAAACATAAAC

TGTGTGAATATACCAACAATCCCAATCTGTGGGAAAAATAACATCCACTAACAACTCAAAACCAAGACTA

TGCTGAAGGCATGCTGCATCAGAAAATAACAATATCCTTCATTTTCATGCTGATTATGCAGCATTGTCCT

CCTTTTTGGCCTCTTCTTTTGCTTTCTCACCTTCAGCATCACTGCACTCCTTCGCTTCAACAGGTACCTG

CTTCTTCTCTTTTGCCTCACCTTCAACATCTTTAACACTCAAGTTCTCA

>FM890738.1 FM890738 Jatropha curcas embryo 56-70 (DAF) Jatropha curcas cDNA clone rjcaeb1_000623, mRNA sequence

ATTCATAAGCTCTGACTATGTTGGAATCAAGCAGTTAGTGTTCGGTGGATCGGAATTTGAGAATTGGAAG

GAGAACTGGACATCAGAAGACGCAGGTTGCAGTGTCCAGAAGATTTAGAGTTGTCTTTGGCGTCCAATTT

TTCGTCTTTGAATTCTATGTATATGTTGAACAAGAGGAGATAAAACAAAACAATAATACTCAATTTAGTT

CAGGATGTGTAATTGGTAGGAAATTAGGAAAAAGCACTCCATTGGTGGCTCATGAGGCATACATATCATG

CCTTTGCATTTAAACACCATTCCTGGTTTCAAT

>FM890737.1 FM890737 Jatropha curcas embryo 56-70 (DAF) Jatropha curcas cDNA clone rjcaeb1_000621, mRNA sequence

ACAGTAAATCCCAAATTTTGTTATTACATTTCTCCAAACAGAAGACAGAAGTCCTTCTTAAACACACAAA

CCAAAGCTCCAACACACTTGGGGATGCAAGCTCAACCAGCCACACATCACCCTATTACAAACTGATCGAT

AAAAAGCAAATCAGCCACACAACATAACAAATAAGGGCATTGCGTGCGTATAATACATTATTAAGTAGAG

TATTAAAGAACACCTAGTGGTGCTCGTGGTGGTGGGAGCCAGGAAGCTTCTCCTTAATCTTATCCATTAT

TCCTTTCTTCTCATGATCATGGCCATACTCAGCTGAATGATAACCACCTGGAGTTGTAGCTGAGGTTGCT

TGAGTTCTTTCTTCCTTATGATGACCTGGTAGCTTCTCTTTTATCTTTTCTTTAAGCCCTTNCTTCCTCC

TTCCACCATGTCCATCATCTTCCGAGGAGCTAGAGCTGGAGGAGCCAGAACGGTGAAGCTGAACAGACTC

TCCGTAGTGTT

>FM890736.1 FM890736 Jatropha curcas embryo 56-70 (DAF) Jatropha curcas cDNA clone rjcaeb1_000618, mRNA sequence

ATTCAGAAGACAATATGGAAAAAGATCAATGCAATGCGCAAAATAGTAGGATATACAACTGCACGCATGG

GACGTGTATCGAGAGCTTAAGCTCTCTATGTTTTCACTGGCGTTGAGCCACCTGCTTCATTCAAGGAAAC

ATCTGATCTGGTTGAAGTGAATGATAAGCTTAGGTTTCTGATGTCTATTGTTGGAGTAAAATAGACACTT

GGTTTTCATATAGGACTTGATTTCAAATCTTTTAGCAGTATCTTCTTCCTAATAATTAATGTGAATACTG

TTGTGTTTTTGCAATTCTGTATGCATTTGGACATGATGATTATTACTGGTGCATTGTGGAGTGGCATTTA

TAATAGTTGACAAGTTCCTG

>FM890735.1 FM890735 Jatropha curcas embryo 56-70 (DAF) Jatropha curcas cDNA clone rjcaeb1_000617, mRNA sequence

AACCTCAAAACTCGAAAGCAGTCTTCCATACTATATTTGCCCAGATTTTACCAAGAAATATGATCCAAGA

CCTCTATAAATTACGACCCCAAACTGACCAAAAGAAATACCAAAGTCATCCACCAGACCCAACACAAACA

TAAACTGTGTGAATATACCAACAATCCCAATCTGTGGGAAAAATAACATCCACTAACAACTCAAAACCAA

GACTATGCTGAAGGCATGCTGCATCAGAAAATAACAATATCCTTCATTTTCATGCTGATTATGCAGCATT

GTCCTCCTTTTTGGCCTCTTCTTTTGCTTTCTCACCTTCAGCATCACTGCACTCCTTCGCTTCAACAGGT

ACCTGCTTCTTCTCTTTAGCCTCACCTTCAACATCTTTAACACTCAAGTTCTCAAGT

>FM890734.1 FM890734 Jatropha curcas embryo 56-70 (DAF) Jatropha curcas cDNA clone rjcaeb1_000612, mRNA sequence

GAATACAATTATATTATAGAATATATTGATTAGGGAATACTTAATTACGAATTAGGATGATTACATTACT

TTCATCACTTATTTTCATAATATATTACATACTTCATTAGCGTGAGATCATAACTCCCCTCGTAATACTA

CTTACTTTCACTATCTACACACCCAAAAAGGCTACTTCATTATTTACTCCTCGGCATATCTTCCAGACTC

GGTCCTGGAAATAGTCAAGGTGGTCTCTTGTCTTCCAAACTTAATCCTTCTTGCCTCCTCTGCTGATACC

CTAAATGCATTTGCAATAACCGCCAACGGCATTGCTCTAACGGCGGAGGAGCGACCTGCAAGATCATTGG

TCATCGCGTTATCGCTTGTCTTGAAGGTTACATATTCGAATCTTTCACTTTCTGCTCGTTTCACCACTAC

GAAGTTTTGTGGCACCGTCAAAACCTGTCCTTTGCTTACACTTCCGTCGAAGACGGTGTTGCCGTTTTCA

TCCGTCACCTGAACCCGCGCTTGTCCTTTGAGTGCGTAAATTACGCTGTGAGAGTTTATGTGCCAGTGGG

GTAATCTCATGGCATCA

>FM890733.1 FM890733 Jatropha curcas embryo 56-70 (DAF) Jatropha curcas cDNA clone rjcaeb1_000609, mRNA sequence

GGCCACGAAATAGTCCATCCATATATAAGAAAAATTTTACATACCAAACATACATTATTATCTCCGCTAC

TAACAAAGAGAGGAAGTGCTTGACATCTACGCAAAAACTATACAATAGTATTTCTTAGATAAGAGTGTAT

CATATCGTCCGGCTGAGATATATTCTAGTCGACATAGGTGAAACGTAGATATTCCTTCTTGGAAGGAAGG

TCAACAGTCTTGTATCCCTGTGGAAACATCTTCTTGGCCTCTTCATTCGACACATTAGAGGATATTACCA

CCGGATCTCCTGGTTTCCAATCCACAGGAGTAGCAATCTTGTGCTTTTCTGCCCCTCTGCAGGGAATCCA

GCACCCGGATCACTTCATCCCATGTTCCCTTCAGTACTTGCTGGATAAAGGAAAGCTCA

>FM890732.1 FM890732 Jatropha curcas embryo 56-70 (DAF) Jatropha curcas cDNA clone rjcaeb1_000605, mRNA sequence

ATTCCACAAGAAGACAAAACTAAACCTGGAAAATTCACAAATGCACAAAAACCTCAAGCTTCGCTATCGT

TACTCTCCCATACGATAGTCAGGTAATTGAAGAGAGTGTATTTCCATTGAGACCAGAAATCAATCATTGC

CATGGCAACCTCTCTCCTATCGCAACTCTCAGCCCCAACGCCAGAGCTAGCGAGTCCCTCTTCTGGGCTC

CCAAAATCCTCTTTCCTCCCCAATACAAAGCTCTTCTTCTTAACCAATTCAAGAAAACAGAGACTTTTTC

AACAGAGATGCTTCAGAGCCCCTTCTGCATCTGGGAAATCTCTCAACCACATACCCAATCAGTTCAGACA

AGAGAATCTCAAGGATGGATTGATGGAAAATTACAAGAACGTACCGCGTCATCTTTATGGTCTCACACCT

TCACAGCTAGACATGTTCATGACTGAAGATAATCCAGTTCGTCGACAGTCAGAAAGTGTTACAGAGGAAA

GTATCTCATCTAGAAGTAACTATTTGGAACATGGGGGAATGTACAGTGTATCAGGCATGATGGATAG

>FM890731.1 FM890731 Jatropha curcas embryo 56-70 (DAF) Jatropha curcas cDNA clone rjcaeb1_000603, mRNA sequence

GCTGCAGGAATTCGAATTCGGCCAGAGCGGGAGGTGGATGAATGGAATGGAAGAAGCCATGTGCACAATG

AAAGACTATACATAACCTTGAGTCTCGTAGGGAAGCTGATTTTTACACAAAGGAAGCTGGTAGAATCCAC

CTTGCAAATCAACGAAAGCTCCCCATTCTCAGTTTCATGGACATGAGCGCTGAGCGAGGCGTTCTGCTGC

CGAGGGCAATGCACAGCCCACACTGGTCCATAAACGATCAGAGAATAGTGTGTGCTCTTCGCGGCGACAT

GCACATACAGATTGTGGATGACAGTGGAAACACAGTAATGGACGAAAGGGTGAAAGAAGGAGACATGTTC

GTGATCCCTCAATTCTATGCAGCACTTGGAAGGGCAGGAGAAAATGGGTTCGAGTACGTAACATTTAAAA

CAAGTAGCCAGCCTATCAAGAAGCCATTGGCGGGGTTATACTCAGTGATGAGAGCCATGCCTCTTGATGT

TCTGATGACCTCTTACAAATGTCTCCAGCGAAGCTCATCAGTGA

>FM890730.1 FM890730 Jatropha curcas embryo 56-70 (DAF) Jatropha curcas cDNA clone rjcaeb1_000602, mRNA sequence

TGCAGGAATTCGAATTGCTGGAGCTAAATTCTATGACCCGAGAGCTCCAACTGAAGCACAGCAATGCCAT

CGCCAGAGAATTACCACAACCCAACCTTCTCATCGGATTGACTCAGAGGGTGGGGAAACGGAGATTTGGG

ATCCATCGGACGATCAGTTTCAGTGTGCCGGTGTTGCCGCTTATAGAGATAGAATCCAGCCCAGCTCTCT

CTCCTTGCCCAAG

>FM890729.1 FM890729 Jatropha curcas embryo 56-70 (DAF) Jatropha curcas cDNA clone rjcaeb0_000695, mRNA sequence

CGAATTCATCGGATTGACTCAGAGGGTGGGGAAACGGAGATTTGGGATCCATCGGAGGATCAGTTTCAGT

GTGCCGGTGTTGCCGCTTATAGAGATAGAATCCAGCCCAACTCTCTCTCCTTGCCCAAGTTTTTACCTGC

TCCTCGCTTGGTTTATGTCATCCAAGGTAGAGGAGTGCTTGGACTTAGCTACCCTAGCGGATGCCCCCAA

ACATTCCACGCAGAGCAACAATCGAGAGGAGGAGAAAGCCAAACAGGAGCAAGACACGACCCACACCAGA

AAATTCGCAGGATTCGTCGTGGAGACATCATTGCCATACCACATGGCGCAGTTGATTGGTGCTACAATGA

TGGTAACGAGGAGCTTGTAGCCGTCACTGTTGTCCATCTTAACAACAGGCACAACCAGCTCGACCAGTAC

TTGAGGGCATTTTTGCTCGGAGGCGGCCAATCCCGACAAAGAGAGAGAGAGAGTCCTGAAAGACCCCTTC

GATCTATACGTGCACGAAAATCTGAGAAAATCAGAGAAACTTTCCAGAATGTTTTCCGGTCGTTGGATGA

GCAGGCACTGGCCGGAGTCTTTAA

>FM890728.1 FM890728 Jatropha curcas embryo 56-70 (DAF) Jatropha curcas cDNA clone rjcaeb0_000694, mRNA sequence

ATTCCTGCCTTGCGAGCTTCTATGTCACTCCNGGTTGCAACTATTGCTGAGATTGCTTCCTATAATATGT

GCAGATGGGGAACCATCTGGCCGGAACATGAGGTACATGCTTGCATCTGTAATACTTCGTCTGCTAGGTC

ATCGGGTTGTGCATGAGGATGCAGACCTATCTCTCTCTCCTATGCAAAGTTCTCAATCCAAGATGGAACT

GGAGTCAACATCAGAAGCTGCTTCTGGAGATTTATCTGGTGAATCTCTATTTGATCGGTTATTATTAGTC

TTGCATGGTCTCTTGAGTAATAGCCAGCCAAGTTGGTTGAAATCAAGGGCAGCTTCCAAGTTAATGAATG

AATTTTCAAAGGATTCTGCTGGACTTGATCGTGACGTGGTGGAGAGTTTGCAGAATGACTTGGATCACAT

GCAGCTGCCTGGTTCAATCCGGTGCCGCATCCAAGCTGCAATGCCAATACTTCTTCCTTCTGTACGATGG

TCCATATCTTGCCAGTTACCTTCTGTTCCTATTGCTTCTGTTGCTTCACTTCAACCTAGTTCCACAATAT

CTGGGTTACACACTGGAAA

>FM890727.1 FM890727 Jatropha curcas embryo 56-70 (DAF) Jatropha curcas cDNA clone rjcaeb0_000692, mRNA sequence

ATTNCGAGAGAGAGAGCAAAAGATTAATTATATTGGTGGAGAAATTTGTTAGCAAAAAAGGGCAAAAATG

TCTTCAAATCAATCAAGACAAGAGCTTGATGAAAGAGCAAGGCGTGGAGAGACTGTTGTCCCTGGAGGTA

CTGGGGGCAAGAGCCTTGAAGCTCAAGAGCATCTTGCTGAAGGGAGAAGCAGAGGAGGACAGACCAGGAA

GGAGCAGCTGGGGACAGAAGGATATCAGGAGATGGGCCGGAACGGTGGGCTGAGCACGACGGACGAGTCT

GGTGGAGAAAGAGCTGAAAAGGAGGGCATCACTATCGATGAGTCGAAGTTCACGACTGGGAGCTCGTGTG

ATGATTAAAACTATAAATGGT

>FM890726.1 FM890726 Jatropha curcas embryo 56-70 (DAF) Jatropha curcas cDNA clone rjcaeb0_000688, mRNA sequence

ATTCCTTCATNGCAGCAGAATCAATGTCAGAAGCTAGAGTTGCTGCTGAAAATGGAGATCTAGCCCGTGC

AGTCTCCGTATTGGATAGCTGTTACAAGTCATTGTCAGAAACTCCATCTGCACAGGCTGGTGACAGATTG

TGTGTTGCATTATGTGCTGAGTTAAAGGAGATGCAAGAAAGGATGGCAAGCCGACAAGTCTATGAGTCAT

CTGGAAGAGCATATGTTCTATCAGGTTTGAGCTCACACTCATGGCAGAGAGCAACTGCAAGAGGTGATTC

CACTAATAGTACGAGCCTTCTACAAGCTTACCAAACTCCATCCATGGTTGAGATGGTAACCAAGTCTCAG

ACAATGTTGTTAGGGAATCCATCATCTCGCTCATCTCATCGGAAACTCCGACAAGCTCTATCATTTCCGG

CTGCTAGACCGCAACCGAGGTAACTGGCAGCAATCTCGCCAAAGTTTCTCCTCACCCTACATTCATCTAT

CAACATCCAAGAACCTTTGTCTACTTAAATTTTGTTTTTAATTATTATTATTATTATTATTATTT

>FM890725.1 FM890725 Jatropha curcas embryo 56-70 (DAF) Jatropha curcas cDNA clone rjcaeb0_000687, mRNA sequence

ATTCAAAGAAATGAAGACTTTCACTTTCTTATTTGTTTTAGCTCTTTTACTTGTTTCAGGATCATTATCT

ACTTCCACCCGACCCCAACCCCAACCCAATTCATCCAGAGGCAAACCTGCTACTGTCCCTAGAACCAGAG

GTGGTGGAAATGATAATCCTGGAATGGGCGGATACTTTGGGCCCGGAGCCGGGTTTGGAATACCGGGATT

TGGAAAAGGTTGGGGAAATGGGATTGTTGGTGGCGGGTATGGAGCTGGTTATGGAGGTCCAAATGGAGGG

TACTCAAAAGGTGGTATTATAAGGCCTACTGTGGTGTGCAAGGAGAGAGGTCCTTGTTACAAGAAGAAGC

TGACTTGTCCTGCAAAGTGTTTCACCTCCTATAGCCGGTCAGGGAAGGGATATGGAGCTGGCGGTGGTGG

GGGTGGCTGCACCATCGACTGTAAGAAAAAGTGTACTGCTTATTGTTAGATGATTGATTGTGGTAACCGG

TAGCCGGTAATAGTTTCTGCTATATTTTATACACCTATTACTAGTA

>FM890724.1 FM890724 Jatropha curcas embryo 56-70 (DAF) Jatropha curcas cDNA clone rjcaeb0_000685, mRNA sequence

ATTTATTGAATACAATTATATTTATAGAATATATTGATTAGGGAATACTTAATTACTAATTAGGATGATT

ACATTACTTTCATCACTTATTTTCATAATATATTACATACTTCATTAGCGTGAGATCATAACTCCCCTCG

TAATACTACTTACTTTCACTATCTACACACCCAAAAAGGCTACTTCATTATTTACTCCTCGGCATATCTT

CCAGACTCGGTCCTGGAAATAGTCAAAGTGGTCTCTTGTCTTCCAAACTTAATCCTTCTTGCCTCCTCTG

CTGATACCCTAAATGCATTATGCAATAACCGACAACGGCATTGCTCTAACGGCGGAGGAGCGACCTGCAA

GATCATTGGTCATCGCGTTATCGCTTGTCTTGAAGGTTACATATTCGAATCTTTCAATTTCATGCTCG

>FM890723.1 FM890723 Jatropha curcas embryo 56-70 (DAF) Jatropha curcas cDNA clone rjcaeb0_000680, mRNA sequence

ATTTTATCTTGAAATGGATACCTACAGGTATCATGGTCACCATGTCTGATCCTGGTAGCACCTACCGTAC

TCGTGACGAGATCAGTGGGTCAACGGACGTGATCCAATTGCAAAGAGTAAGGAAGGTGATATTAGCTCAT

GATCTAGCTACTGAGAAAGAGCTAAAGGATATTGAGAAGGAAATAAGCAAAAGAAGTAGACGAAGCCATT

GCTCAAGCCAAGGAAAGTCCAATGCCAGAACCATCAGAACTTTTCTCAAATGTGTATGTGAAAGGGCTTG

GAACTGAGGCATTTGGGGCAGATAGGAAAGAAGTCAGAGTTGTGCTTCCATAAACAAACATATGCGGAAA

AGTATCCAACAACAACCCCACTCTCAAAAAAAACTTTACCCAGCAGAAGTTATAGAAAAGTGGTAACAAA

TAAATGTTAGCGAAGATGGGTATCTTTTTCCAGCAATCATTTTGTTTCTCACATATTTAAGTATAGAATA

CTGATTCTTTCAAAGTTGTGGGGGTGAGAATTGTATCTTTTTTGTCTGTGTGGTTCTCTAATAGGTTCTG

TGATATTCAATTGAATAGGGTTCTAATGTGCGTGAGGAAAAAA

>FM890722.1 FM890722 Jatropha curcas embryo 56-70 (DAF) Jatropha curcas cDNA clone rjcaeb0_000679, mRNA sequence

ATTGTTCTATCCCAACAATCATTCAGTGCATACTCTTTTGACCACACAGCAGTTTCAACAGCTTCAAATT

CATTCTCACGAAACAACAGCATCGAGTTCCAGCAGCATCAACTGCATCAGCTTCAGCAGCAACAATTGCA

GCAGCAGGATCAACAGCAGCAAGTTGGAGATTTGAAGATGAGAGGAAATATGTCTTCTCAAAACCTGAAA

GATAATGCATCGGATGTTAGCTCTACTGCAACCAATGACTGAGGGATTTGGCCACTGGGCCGTGTTGTAC

AAGGGTGGCAGCTTTTATGTACAATCCTAGTTGCAGTCTGTTGTCAGATAGGATGTCCTCACTCTCTCTC

TCTCTCTCTCTCCGAAACCTCATTTTATTAATTTTTTCCCCCAGTATGTTTAAGAACATTTATATTATAT

ATTTATTTTAGGCAATTCTATATTCTAAGTTCAGTCTGAAAAAAAAAAGGGGGGGCCCGGTACCCC

>FM890721.1 FM890721 Jatropha curcas embryo 56-70 (DAF) Jatropha curcas cDNA clone rjcaeb0_000678, mRNA sequence

TTTCTCTTTCTCTCAACCCTAACCCCCCTTAGCTAAAACCATGGCGGAAAGAGGAGCTGGAGGAGAAAGA

TGTGCCTTTCGCCGATGCTTCAGCGGTGCCCCTCGATGGTGACCGCGGAGGAAGAGGCCGACGTCGTGCT

CGCCGTGATGATGAGGAGAAGTGGGTGCCAGTTACTAAACTCGGACGTTTGGTTAAAGAAGGCAAGATCA

GAAGCTTCGAGCAGATATACCTTCATTCTCTCCCAATCAAAGAGCACCCGATCGTCGACACTCTCATCGG

TCCATCCCTCGAGGATGAGGTCATGAAGATCACTCACGTGCAGAAACAAACCCGTGCGGGT

>FM890720.1 FM890720 Jatropha curcas embryo 56-70 (DAF) Jatropha curcas cDNA clone rjcaeb0_000674, mRNA sequence

ATTGAACCATTACTGAAGTTGAAGCATTTGCTCTAATGGCTGATGACTTGAAATTTGTTGCTTCTCAATT

CCGGCGGCTTCACAGTAAGCAACTCCGCCACACTTTCAGGTTTTACTCGCAACAATGGAGGACATGGGCA

GCCTGCTTTATACAAGCTGCATGGCGTCGATATAATAAAAAGAAGCTGGAAGAATCTCTGCAACAAGAGG

AGGATAGGTTGCAAGATGCATTGGCCAAGACAACTGGGAACTCACCGAGTTTAGGTGCCACCATATATGC

CTCACGATTTGCTGCTAATGCACTTCGTGCTCTACGGCGTAGTGGTACTCGCAAGACAAGGTTGTTGGAG

AGGGTTCCGCCCATTTTGCTTCAGAAGCCTGCAGAACCTGATTTTACAACTGGAGAAAGATAGATGTAGA

AGACTATTTGCTATGATATTTTTTATGTTACATTGTAAATGTACAAAAATGAAATTAGAACTACTGTGGC

CAATGAAGAAATGAACATAGGGGGG

>FM890719.1 FM890719 Jatropha curcas embryo 56-70 (DAF) Jatropha curcas cDNA clone rjcaeb0_000672, mRNA sequence

ATTCCATTCGTCTTCAAAGTTTATAATTCACGCATTATCGTCGATTTTGGAGCAACGGAAGATGTAAAGA

TAGGTCATGCAGTGTGCGCCATTGATCAAGTTTCTGCAAAAGGAACTCATTTGGAAGATGGAAGAGAAAT

AATGCAGGTACTCTCCGAGGAGAAAAATTTTCCCTTGACTATCAAATTTGCCAAGCCGGTCCCCACTTCC

AACGATCGTCTACATTTGGCCGGATTGTGGCACGGTTTGCATCGTTTGTCTCGACACTTGAGTCCATTGT

CTGAAACGTCAACTAACGACTTTTCCGATTCGCAAGAAAAACGAGCTGCTGCGGTTTTCAACTCGGGCAT

CCAGAGTCTAGAATGCGCATCCTGTCGTATTCACTGCTTGGAATCGCGTGTTGGTGTGAAATTTTTACTG

ATTACTGATGCTAAATTGCCGTCAGCCTCGCGTGAAGCACTCAAGCGAATTTACGAAGCGTATACAGATT

ATGTCCTGAAAAACCCCTTCTACGCGCCAAATCAACTGTTCAACTACGATT

>FM890718.1 FM890718 Jatropha curcas embryo 56-70 (DAF) Jatropha curcas cDNA clone rjcaeb0_000670, mRNA sequence

GTTGAAATAAACCACTCTCTCTCACCGTACCAACAAAATCTGCTTCATCTGAATCACGAGAGGGAAGGAG

AGTGAAAGCCAAAATGGGTTATGTAATTGTGGTTTCATTGCCATTGATATTATTCATCTTAATAGTGGCA

CTTGCTTGTTACCTTGTGGGTCGAAATTTAAGCAGAAGAGAAGCAGCTCGATTGCCTCAGTATTATGGCC

CCCCTGCACCTCCTCTTCGACCTCAATCTTCACCGGGGAACACACCATCTCAATCTAAATTAGTATTGTA

TTTCTATAAACATGTTCCTTTATCCTTTTTAGTATTACTTTCTGTTACCAAATGATTATGTTTTGCATTT

GCTTCCACTGTAAAATGTGTTTCTTTCTGTAACTTCCATGATCGATTGTTGTGCAAAG

>FM890717.1 FM890717 Jatropha curcas embryo 56-70 (DAF) Jatropha curcas cDNA clone rjcaeb0_000669, mRNA sequence

ATTCGAAAGAAAAAATGGAGAAGCTTACACCTCAGCCGCTCTACTCTGCATTCTGCTAGTGCACATAGCC

AATGCAGTCTCCGGCTATGTGACAACCACCATCACCACCGTTGAAATCGACGATGATTCTTCTAACACTA

GTCAGCGAAAATGCAGGAGGGAAGCTGAAAGGGCAGATTTGAGCTCCTGTGAGAGTTATATGAGATCGTC

AAGAAGGCCGTCAGAAGAGATGCTAGCTTTGCGAGGAATTGAAAACCAGCAACAACGGGATCTTCGCCAA

TGTTGTAACGAATTAAGGCAGCTCAGTCATGATTGTCCATGTGAAGGAATAAAGTATGTAT

>FM890716.1 FM890716 Jatropha curcas embryo 56-70 (DAF) Jatropha curcas cDNA clone rjcaeb0_000668, mRNA sequence

ATTCCCACGGGGACTGCCCTCCTGCCATGAGAGCCTTAATTGAGCAATGCTGGTCCTTGCAACCTTGAAA

AGAGGCCCGAATTTTGGCAAATCGTGAAGGTATTAGAGGAATTTGAGTCTTCGCTTGCCCGTGATGGGAC

TCTAAATTTGGTACAAAACCCAACTTTCCAAGATCACAAGAAAGGTCTTCTTCATTGGATTCAAAAGCTT

GGTCCCGTGCACCCTAGTGGCTCCCCCACCCCTACCCCTAAACCTAAATTCACATGAGTTTCTCTTATGG

CTTTTGTAGTGCACCAGGTTACTTGTAATTCAAAGGTTTGTTTCGTAAGCCTCGTAATTTGTACTCTTCT

ATGTCACATTCATGTGACAGTGAATTAATAGATTGGTAGGCATCCATTGAGGTGAAAAAGATGTCTTCCC

TTCACAGAAAANAACAAAAAA

>FM890715.1 FM890715 Jatropha curcas embryo 56-70 (DAF) Jatropha curcas cDNA clone rjcaeb0_000667, mRNA sequence

CCCGCAAGATATAATTTACATTAATGATAGTGCAAAGAACCAAACAAGGTCTTTATCATGCCCACTTATA

AAGGCTTTATTTTTACACCTCTCTATTACTCTCCTACATAGCCACTAGCTAAGCGTTTTTTCTCTCCTTA

TTACAATTACATAGCTGCCTTTTTAGTCCCTTCAATTGCGAGAAGAAGGCCTAGTCCCAGCGACTGAACA

GAGTCACTTCTTGTCTATTCTCTTTCAGTCTCCTCGCGTCCTCCCTTGAAACCTGGAATACGTTTGCCAA

CACCTCTTCTGGAATTGATCTGATTGCCGAGACCCGTCCTGCTAGTTGGTCAATCTTGGCATTGTCATTA

GTCTTGAAGGATACCCATTCAAGA

>FM890714.1 FM890714 Jatropha curcas embryo 56-70 (DAF) Jatropha curcas cDNA clone rjcaeb0_000664, mRNA sequence

ATTCGATTTANTCCCGGTAAAGGCATCAGATTTATTCGTTCTGATTCTCAGGTCTTCCTGTTTGCTAATT

CAAAATGCAAGAGGTACTTTCACAACCGTTTGAAGCCATCAAAGCTTACATGGACGGCCATGTATAGGAA

GCAGCATAAGAAGGATATTGCTCAAGAGGCTGTAAAGAAGAGGAGACGTGCCACCAAGAAGCCTTACTCC

AGGTCTATTGTGGGTGCCACTTTGGAGGTTATCCAGAAGAGAAGAACTGAGAAGCCAGAAGTTCGTGATG

CTGCAAGGGAAGCTGCCCTTCGCGAAATCAAGGAAAGGATCAAGAAAACCAAAGACGAAAAGAGGGCAAA

GAAGGCAGAAGTGACAGCCAAGGCACAGAAGACACAAGGCAAAGGAAATGCTCCCAAGACTGCAGGAAAG

GGTCCCAAACTTGGAGGTGGTGGTGGAAAGCGCTGAGCATATAGTCTATTATCTGTACTGTGCATTTCAT

CACTAGTTACAGTCTATTATCTATCCTTTCAAGTTGGAGTTATAGGGATTTTATGTGTACTTTTAGGCTT

AATAGACTTTGATTGGCC

>FM890713.1 FM890713 Jatropha curcas embryo 56-70 (DAF) Jatropha curcas cDNA clone rjcaeb0_000663, mRNA sequence

ATTCCACTACTTGTAATTAGAAGTTGGAGCCAACTGTAGCTGTAAAGTTGCTCTTCCTGAGCAGAGGACT

CTAATATGAGCTCTTCATCTACTCAACTTTCTGCCTTGAGGGGTATGGGCATTTATGAGCCATTCCACCA

GATTAGCTCATGGGGAGACACTTTCAGAGGCAATGATAACCTAAATATTGGTCCATCCACAATCATGCAG

GTGGATGATGGGCTAAACAAGGGTGAATATGTTTCTCAAGAATCAATGGAACCTTCTAGAAGTGATCAAG

AAGCAAACAGACCTGCTGATAAGGTGCAGAGACGTTTGGCACAAAATCGTGAAGCTGCTCGCAAAAGTCG

CATGCGGAAAAAGGCCTACGTTCAACAACTAGAATCTAGCCGCGTGAAGTTGGTGCAGCTGGAGCAGGAA

CTTGAGAGAGCTCGTCACCAGGGTTTGTTTGTGGGCAGCGTACCAGATGCCACTCATTTATTGCCTGGAA

ATATAAATTAAGGAATTACTACATTTGAAATAGAGTATGGACACTGGGTTGAGGAGCAACATAAACCTGT

TTCTGAACTTTGGAATGCATTACACGCTCACATCACCGATATAGAGCTTCGGATACTTGTGGA

>FM890712.1 FM890712 Jatropha curcas embryo 56-70 (DAF) Jatropha curcas cDNA clone rjcaeb0_000657, mRNA sequence

ATATCCGCTTACTATGCTTCTCTTCACTCGAGACGATGGTAACTTACAAGTTTCACCAATACCAGGTTGT

TGGAAGAGGGTTGCCCACTGAGAATGATGAGCATCCAAAAATTATCGGATGAAGCTGTGGGCCACTAATG

AGGTTAGGGCCAAGTCCAAGTACTGGTACTTCTTGAGGAAGCTGAAAAAGGTCAAGAAGAGCAATGGCCA

AGTACTTGCTATTAACGAGATCTTTGAGAAGAACCCAACTAAGATCAAGAATTATGGCATATGGTTGCGT

AACCAAAGCCGAACTGGCTATCACAACATGTACAAGGAATACAGAGATACCAATTTGAATGGTGCTGTGG

AACAGATGTACAACTGAGATGGCTACCC

>FM890711.1 FM890711 Jatropha curcas embryo 56-70 (DAF) Jatropha curcas cDNA clone rjcaeb0_000655, mRNA sequence

AATTCCTCTTCTAGTTCTCTCCGGTTTGACCCTTGCTGGTACGGTCATTGCACTGACCGTAGCCACTCCA

TTGCTGGTCATATTCAGTCCGGTTCTTGTTCCTGCGGTTATCACAGTTTGTTTGATAATCACAGGGTTTT

TAGCCTCAGGCGGTTTTGGCGTCGCTGCCATTTCTGTCTTGTCCTGGATTTATAGGTATGTTACTGGAAA

GCATCCACCAGGAGCAGAAAGTCTGGACCAGGCACGCTTAAAGTTGGCGGGAAAGGCAAGAGAGATGAAG

GACAGAGCTGAGCAGTTTGGACAGCATGTAACAGGTCAACAGACTTAAAAGAGAAGTGGGTTTTGGTGGT

TGATGCAGAGAACATAAAATGGCTTTGATATATATATAAAAATATATGAGTCCTTTAATTAACATGGAAG

GCCATTGCTTGTCTGGTGTATTTCTTTTTTCGCTTAAATCTTTTTGTTTCTTCTATGTAATTTGTTAACG

GGTCTTCCCTCTTTTGAGAGCTGTCTAAAATAATTTTCTT

>FM890710.1 FM890710 Jatropha curcas embryo 56-70 (DAF) Jatropha curcas cDNA clone rjcaeb0_000654, mRNA sequence

ATTCTATCATATCAAATCGGCGTTGAAAGATCAGTTCTCCTTTTTGAGTATCTAAAAATGTCTTCTTGCT

GTGGTGGAAATTGCGGCTGCGGCTCCGGCTGCAAGTGTGGCAGCGGTTGTGGCGGATGCAAGATGTATCC

TGACATGAGTTTCTCCGAGAAAAACACCGCCGAGACTCAGGTTCTCGGAGTTGCACCGGAGAAGGCACGC

TTTGTCGATGGAGGTGAGGTCGGCGTTGGAGCTGAGAACGGATGCAAGTGCGGTGACAACTGCACCTGCA

ATCCTTGCAACTGTAAATGAGATCGGCGGCGATCAGTCTGTTTTTTAGAAGTAAAAGCAGAGATGACTCT

GCTTCTCTCTTTCTAAAAAAGGAGAAATAATTAATAGAGTCTAGATATCGTATGAGTTTGTTCGTTTGTT

TCTGGTTTTGAGCTTCAATAAAAGTAGTGGTGGCTTGTGAGCTTGTGTTTAGCAATGGCTGCCTG

>FM890709.1 FM890709 Jatropha curcas embryo 56-70 (DAF) Jatropha curcas cDNA clone rjcaeb0_000653, mRNA sequence

ATAAATAAAATATCTACTAAAAGATACCTTTTATTCAACTGGAATTGCTTAACAAGAAAAAGACACCAAA

CAGAAAACATAGATATATTACATAACAGGAGCTAAAGCACATGCCATGCCCTACACACGTGGGTTATGGC

CAGTGGTGTCTCCGGGCAACCCAGATGGGCGAGTCCTGGTCAAACCCGTTGGGTCCACCTCTCCACCGTA

TGGCTGCCCTGTGCCGTGTCCAGGCATGGCCGACATTTGCTGGGTCCCTGTAGGGTAACCGGTTGTCCCA

GTTGTTGAATGGGTATAGGTTTCGGTTGTGCCTTGAGCGCCCGGACCAGCAGTTGTGTACTGTACATGGC

CACCAGCCTTGGCTGCCTGCCGGGGGGGCCCGGTAC

>FM890708.1 FM890708 Jatropha curcas embryo 56-70 (DAF) Jatropha curcas cDNA clone rjcaeb0_000648, mRNA sequence

ATTCCAATGGCTAACTCTTCTTTTCTCTCTCTCGGTCTCTGCTTTCTCGTTCTTTTCCAAACCTGCTTTG

GTCAATTAGAACAATTAACAACTGAGAATCGCCGGCCCTGGCAATTCCGTGACCAGCGCTCTCAACAGGA

TGCGTGTAGACTCGAACGCATTAACGCTGTGGAGCCTTCCCGTAGAATTCAATCAGAGGCCGGTGTAACT

GACATCTGGGACGAAAACGACGAGCAGTTCCGATGCGCTGGTGTCGTAGCCATGCGTCATACAATTCAAG

AGAGAGGCCTCTTGTTGCCCCTATATGCCAATGGTCCTAAACTCATTTACGTGCTCCAAGGTAGAGGTAT

CCACGGAGCCGTATTTCCAGGTTGTCCTGAGACATTCCAGTCACCATCAGAACCACAGGAACGGTACGGA

TTCGGTGCAGGAGAAAGAGAGAGTCGGAGATTTGATCAACACCAGAAAGTTCGTTCACTCCGACAAGGTG

ATGTTATTGCTTTGCCAGCTGGAGTAGCTCAGTGGATTTACAACAGTGGCCGGACTCCTCTTGTTCTTGT

CCAAATTATTGAT

>FM890707.1 FM890707 Jatropha curcas embryo 56-70 (DAF) Jatropha curcas cDNA clone rjcaeb0_000647, mRNA sequence

TTTAAGCAAAAATGGCAAAACTTGCAATCTCAACATCTCTAATCTGCGTTCTTCTAGCGATCATAGCCAA

TGCAGTCTCCGGCTATCTGACAGCCACCGCCATCACAGTTGAAATGGAAGATGCTTATCGAGTTCAGGGA

ATATGCAAGAAGGAAGCTGAAAAAAAAGATTTGAGCTCTTGTGAGAATTACGTAAGACTGTCAAGAACAA

GGTCCGAAGAGACGCCAGCTATAAGAAGAATTGGAAACCGGCAACAACAGGTTCCTGGGCAATGTTGTAA

CCAAGCATGGAAACTAAGTGGCTTTTGTCGATGTCAATCAATAAGGTATCTACCAGAGAAGCAAGGATAC

GTTGGAAGCCAAGAATATGATGAGGCAATGCGTAGGGCTGACGATATAATTAGTGTTTGCTTTGACAATA

TCTGCCCTCGTTAAAATGCTATTGCTACCGAAAATAAAATTTCACTCGCTTGAATGTGAGCGAGAGTTAC

GTTTAATAATAATCCATAAACAATATGAGTTTCGGAGTAAGTTTGTAATGTTGCTTCAAGTTTTCTACAT

GTCCTGTAATAAAAGCTAATACACTGTTGAGTTATTGAACTG

>FM890706.1 FM890706 Jatropha curcas embryo 56-70 (DAF) Jatropha curcas cDNA clone rjcaeb0_000646, mRNA sequence

ATTCGAGAAAGCTGGGTCCTAGTCGTCGTTGGTGGCCGTCAGCCTTCACGTCCCGGTTCGGGAATTTTAA

CCCGATTCCCTTTCGAAGCTCGCGCGAGGACGCGCTGTCGGACGGGCTTCCCCCGTCTCTTAGGATCGAC

TAACCCATGTGCAAGTGCCGTTCACATGGAACCTTTCCCCTCTTCGGCCTTCAAAGTTCTCATTTGAATA

TTTGCTACTACCACCAAGATCTGCACCGACGGCCGCTCCGCCCGGGCTCGCGCCCCGGGTTTTGCAGCGA

CCGCCGCGCCCTCCTACTCATCGGGGCCTGGCTCTTGCCCCGACGGCCGGGTATAGGTCGCGCGCTTAAG

CGCCATCCATTTTCGGGGCTAGTTGATTCGGCAGGTGAGTTGTTACACACTCCTTAGCGGATTTCGACTT

CCATGACCACCGTCCTGCTGTCTTAATCGACCAACACCCTTTGTGGGTTCTAGGTTAGCGCGCAGTTGGG

CACCGTAAACCGGCTTCCGGTTCATCCCGCATCGCCAGTTCTGCTTACCAAAAATGGCCCACTTGGAGCT

CTCGATTCCGT

>FM890705.1 FM890705 Jatropha curcas embryo 56-70 (DAF) Jatropha curcas cDNA clone rjcaeb0_000645, mRNA sequence

ATTCCTTCATTCTGCTTGGAGGCGCCTCTCTTTCTCTGCTTTCTGCATCCGCAACCATGGTGAGAGTTAG

TGTTTTGAATGATGCTTTGAAGAGCATGTACAATGCTGAAAAGCGTGGGAAGCGACAGGTTATGATCAGG

CCTTCCTCAAAAGTGATCATCAAATTTCTGCTGGTGATGCAAAAGCATGGATACATTGGCGAGTTTGAGT

ATGTTGATGATCATAGAGCTGGTAAAATTGTGGTTGAATTGAATGGGAGGTTGAACAAGTGTGGAGTTAA

TCCGCCCTCGTTTTGATATCGGTGTCAAAGAAATTGAAAGATGGACTGCCC

>FM890704.1 FM890704 Jatropha curcas embryo 56-70 (DAF) Jatropha curcas cDNA clone rjcaeb0_000643, mRNA sequence

ATTCCACACTGGTCCATAAACGATCAGAGAATAGTGTGTGCTCTTCGCGGCGACATGCACATACAGATTG

TGGATGACAGTGGAAACACAGTAATGGACGAAAGGGTGAAAGAAGGAGACATGTTCGTGATCCCTCAATT

CTATGCAGCACTTGGAAGGGCAGGAGAAAATGGGTTCGAGTACGTAACATTTAAAACAAGTAGCCAGCCT

ATCAAGAGCCCATTGGCGGGTTATACATCAGTGATGAGAGCCATGCCTCTTGATGTTCTGATGAACTCTT

ACAGAATGTCTCCAAGCGAAGCTCATCAAGTGAAGAGGAACAGGGATCCAAAGTCCATGTTGTTTACTCC

CAACAGCAGGAGACCATAAGTAAGTTAATACTAAAATAGCATTGTGATTGGTGCTTCTACTAACCA

>FM890703.1 FM890703 Jatropha curcas embryo 56-70 (DAF) Jatropha curcas cDNA clone rjcaeb0_000642, mRNA sequence

GATAAGTGTAACAGAGCAAAAGATTAATTATATTGGTGGAGAAATTTGTTAGCAAAAAAGGGCAAAAATG

TCTTCAAATCAATCAAGACAAGAGCTTGATGAAAGAGCAAGGCGTGGAGAGACTGTTGTCCCTGGAGGTA

CTGGGGGCAAGAGCCTTGAAGCTCAAGAGCATCTTGCTGAAGGGAGAAGCAGAGGAGGACAGACAAGGAA

GGAGCAGCTGGGGACAGAAGGATATCAGGAGATGGGCCGGAAAGGTGGGCTGAGCACGACGGACGAGTCT

GGTGGAGAAAGAGCTGAGAAGGAGGGCATCACTATCGATGAGTCGAAGTTCAGGACTGGGAGCTAGTGTG

ATGATTAAAACTATAAATGGTATTTAGTAGTAATAATAATAATAATG

>FM890702.1 FM890702 Jatropha curcas embryo 56-70 (DAF) Jatropha curcas cDNA clone rjcaeb0_000640, mRNA sequence

TTTTTTTTTTAGCAGAAACATGCTGTGCTAAGCAACACCATTATATACAAAAACATTATTAATTTGTAAT

TACATCACATTAGGAAGTTCAAATACACATCTTCCTCGTTCTTCTGCAAAAGGTAATTAAGGGCTAACCA

AACTTAAAAGAGAAATAAAGTAACATGTATACATGTCGCGTAGCTCAATGCCGCTTAGGATCAATCCCAA

CAGACTTGAGAGTCTCGGCGTGTGGCTTGTAAGCAAATGCATCTCTATGAATATTAGCATCAGGCAAGCT

TTGAAGTCCACTATCAAACTCTTGAACATGCGCTACTTTCCTAAAGAAAGACTTCTTCAGAAATTTATCA

GCTTCATCCACAACACTATCAGGGTCTTCCACCTCCGGTATCGTCTCCCTTATCTTCTTCTTCACGCGCA

CATTCGGCGAGTGCAATATCTGATGCTTTGCAGTGTACAAGACCCAGAGAGACCGATAAAGCAATCAGTC

CTATCGCCACATAAACAGGCACATACTCTCCTTTTATAGTCTTCTTCGTATGCTTTACTTGGTGATGAGA

>FM890701.1 FM890701 Jatropha curcas embryo 56-70 (DAF) Jatropha curcas cDNA clone rjcaeb0_000638, mRNA sequence

ATTTATTATATTATTTTTCAAACATCAAATAAGCATGGCCAATTTTCATCATAGTGATGCAAACACAGAA

AACTACTACTAGTTACTCATTTACATGTACTTGTTACCTTTACATAGGCTTTCGATCGATAGCTTAGACT

TTGCACGCAACCAACAAAAAAATACAAACAAAAAGCAACAAAACGCAACATACATCCAAATTTCGCAAAA

CGCTCACCCTAGTACTGTATTTAAGTCAAACCCATAACTCCAGAAGCTGAAGCGTATAAAAGCTTTATCT

ATTAGTAGTAAAAGACCATATTTGCACTTCTAGCCAGTGATCTCAAAAGGCTTAATCTGAGGCTGCTTCT

ATTGTACTTAAGGCACAA

>FM890700.1 FM890700 Jatropha curcas embryo 56-70 (DAF) Jatropha curcas cDNA clone rjcaeb0_000637, mRNA sequence

AAATAAAATATCTACTAAAAGATACCTTTTATTCAACTGGAATTGCTTAACAAGAAAAAGACACCAAACA

GAAAACATAGATATATTACATAACAGGAGCTAAAGCACATGCCATGCCCTACACACGTGGGTTATGGCCA

GTGGTGTCTCCGGGCAACCCAGATGGGCGAGTCCTGGTCAAACCCGTTGGGTCCACCTCTCCACCGTATG

GCTGCCCTGTGCCGTGTCCAGGCATGGCCGACATTTGCTGGGTCCCTGTAGGGTAACCGGTTGTCCCAGT

TGTTGAATGGGTATAGGTTTCGGTTGTGCCTTGAGCGCCCGGACCAGCAGTTGTGTACTGTACATGGCCA

CCAGCCTTGGCTGCCTGCCTCGCACGCTGCGTTATCCTCGCGCGCTTCTAGTTTCCGCAATTCTGCCTCC

GTTTTCCTCTCTTCTTACTTCTCTCTCGCCATTTCCTTCTGCACTGGATCACGTGTTGACAT

>FM890699.1 FM890699 Jatropha curcas embryo 56-70 (DAF) Jatropha curcas cDNA clone rjcaeb0_000633, mRNA sequence

ATTCAAAGATTAATTATATTGGTGGAGAAATTTGTTAGCAAAAAAGGGCAAAAATGTCTTCAAATCAATC

AAGACAAGAGCTTGATGAAAGAGCAAGGCGTGGAGAGACTGTTGTCCCTGGAGGTACTGGGGGCAAGAGC

CTTGAAGCTCAAGAGCATCTTGCTGAAGGGAGAAGCAGAGGAGGACAGACAAGGAAGGAGCAGCTGGGGA

CAGAAGGATATCAGGAGATGGGCCGGAAAGGTGGGCTGAGCACGACGGACGAGTCTGGTGGAGAAAGAGC

TGAGAAGGAGGGCATCACTATCGATGAGTCGAAGTTCAGGACTGGGAGCTAGTGTGATGATTAAAACTAT

AAATGGTA

>FM890698.1 FM890698 Jatropha curcas embryo 56-70 (DAF) Jatropha curcas cDNA clone rjcaeb0_000631, mRNA sequence

ACTGGAGCTCACGCGGGGCGGCGCTCTAAACTAGTGATCCCCCGGCTGCAGAATTCGAATTCCGGCAATT

GCGGACTCTGGAACTTCCTTGTTGGCAGGTCCAACGACTGTGGTTACCATGATAAATCAAGCTATTGGTG

CTACTGGAGTTGTTAGCCAGAAGTGCAAAGCTGTTGTTGCTCAATATGGACAAACCATAATGGATTTGCT

TCTCATGGAGGTTCAGCCAAAGAAAATCTGCTCACAAGTTGGATTGTGCACATTTGATGGGACACATGGT

GTTAGCATGGGCATTCAGAGTGTGGTTGATGAGAGAAATGATAAGTCATCATGGTATTGCTCGTGATGCT

ATGTGCTCA

>FM890697.1 FM890697 Jatropha curcas embryo 56-70 (DAF) Jatropha curcas cDNA clone rjcaeb0_000624, mRNA sequence

ATTCCGGTTAGATGATGAAGGTGTTACTCATCTAAAGGTCGTCCACCGTTCAGACAATAAGTGATAACGT

CCACCGAATTCACGGTGTACTTCCTACTTCTTTTACTTTTTCACTTTTACACCACCATGTTCATTAGTAG

CTTTTTGGGCCTTCTTGTTGTGCTTTTTTTTTGTTTTGAGTAACTATAAACAAAGCTTTTGTGGGCTTGT

TTGCCCTTCTGGATCTTATGTGCCTAATCTTATATGGATAAGATTGGAAGGTTATCATC

>FM890696.1 FM890696 Jatropha curcas embryo 56-70 (DAF) Jatropha curcas cDNA clone rjcaeb0_000622, mRNA sequence

ATTCCGAAGCTACTCTCGCTACGATCGACCAAATCTAAGGCTTTCAGATCGAAAAATGGCATTGATTCCA

AGCTTTTTCGGTAACTCACGAAGCAACATCTTCGATCCTTTCTCTGCTCTCGACGTTTGGGATCCATTCA

GAGACTTTTCTTTCCCTTCATCTTCTTCCCTCGTCTCTCGTGAAAATTCTGCTTTTATTAACACTCGCAT

TGACTGGAGAGAGACACCAGAGGCTCATATATTCAAGGCTGATCGCCCTGGGCTCAACAAAGAGGAAGTG

AAAGTAGAAATTGAAGATGACAGAGTGCTTCCAATCAGCGGGGAGAGAAATGTGGAGAAGGAAGACAAGA

ACGACACGTGGCATCACGTGGAGCGTAGCAGTGGAAAGTTCATGAGGAGGTTCAGGGCTGCGGAGAATGC

CAAGATGGATCAAGTAAAGGCTTAAATGGAAAAACGGAGTACTTACTGTCACTGTTCCTAAAGAG

>FM890695.1 FM890695 Jatropha curcas embryo 56-70 (DAF) Jatropha curcas cDNA clone rjcaeb0_000620, mRNA sequence

ATTCGGATGCAAGATTTAAGAAGACTGAGATTTGCCAAACGTGTAGTAAGTTGAAAAATGTTTGTCAAGT

CTGTCTTTTGGATCTTGAATATGGATTGCCGGTTCAGGTTCGAGATACTGCTCTCCATATCAGTTCCAAT

GATTCCATTCCAAAGAGTGATGTCAACAGGGAGTACTTTGCTGAGGAGCATGACCGAAGGGCTAGAGCTG

GTATAGATTATGAATCTTCATATGGGAAGGTGCGGCCAAATGACACTATTCTGAAGCTTCAAAGGACGAC

ACCGTACTATAAAAGAAATCGAGCACATGTTTGTAGTTTCTATGTTCGAGGTGAATGCACACGAGGTGCT

GAGTGCCCTTATAGGCATGAGATGCCAATAACTGGGGAGTTGTCACAACAGAATATAAAGGATCGCTATT

ATGGAGTAAATGATCCAGTGGCTTTGAAGCTGCTTAACAAGGCTGGTGAAATGCC

>FM890694.1 FM890694 Jatropha curcas embryo 56-70 (DAF) Jatropha curcas cDNA clone rjcaeb0_000619, mRNA sequence

AGACGTAACGATATATATTGAACACTGACTTTTTATACGTACATGAACACAAGTGGATTAATTAAACATA

AAGGACGATCAAAGTCCAAAGTCCAAAGTCCAAACCAGACCCAGATACATCCCCGCCAAAAGGGGATATG

TAAATGACACAAGATAATTCTTTTAGCATTACATGTACATATATTATTACATTGTTGTTGCTTAAACCCG

AGTCCCTGTCACTGCTGTGCCACCCTTTGGAATTGGTTCCCCACCAGCTCCTACAGCGTATTCGGTGTCC

CTGTAACCAGGGCCATACCTGCTGCTAAATACCCCAGCATGAAGCAACAGGACATACAAGAGTTGGGTAA

ATGTCAAGATTATCATGAATGCCTCTAATACCCTCAGTCTCCATCCTCTGTGCCCTCCTAGGTTTATTTC

CTTGCATGCCAACCCAAAAGCTAGGGCAGTGATGGCCCAAGTCACAAGTGAAGAAGAGCCTGCAAAGGCT

AGACTGTCATTTCTCCAGCTCCTGATATGAAGGCCACCTGCCAACTTGGATACTATACCA

>FM890693.1 FM890693 Jatropha curcas embryo 56-70 (DAF) Jatropha curcas cDNA clone rjcaeb0_000616, mRNA sequence

AAAAGAAAAACTTACATTTTATTAACGAAAACTCAGATTACATAATAAAAACAGCAACTCCATAAGGAAA

CAAGCTCACAACAACAAACCCATTACACACATCTATTTATTTTATTGGATGGATGGGTACCATTTAGACT

TAGACACACTCAAACATAACTAAAAGTTTCAACCAGAAATCTCAATAGCCTTAACGTCCGGTTTCTTGAC

TTCCACTTTAGGCACTGTCACAGTCAAGACTCCATTTTCCATGCTAGCCTTAACCTCATTAACCTTTGCA

TTCTCTGGTAGCCTAAACCTTCTCAAGAACTTACCACTTGACCTTTCAACTCTATGCCACTTATCATTCT

TTTCTTCTTACTCTTTGCTTCTCTCTCCGCTTATCTGCAAGACCCTACCTTCCTCTATCTCCACTTTCAC

TTCCTCTTTCTTAAGCCCTGGAAGATCAGCCTTGAATACATGAGCTTCTGGTGT

>FM890692.1 FM890692 Jatropha curcas embryo 56-70 (DAF) Jatropha curcas cDNA clone rjcaeb0_000615, mRNA sequence

TCGTTGCTATTCCCACCAAGAAGCGCAGCAAGAAGAAAGACGAGAAAGAGAGGAAGAGACCCAGAAACAA

TTAGAGGGAACAGGTTACAGACCAGGTGGCGGCCAAGGAAGATTCGTTAATGGTGTGGAAGAAACTTTCT

GCACTGCTAGACTCGTACACAACATCAATGACCCGTCACGTGCCGATGTATTCAACCCACGTGCTGGCCG

TCTCACCAACATCAACGGCCAAGACCTCCCCATCCTCAGTTATATCCAGCTAAGCGTTCAGAAAGGTGTC

CTCTACAGAAATGCTCTAATGTCACCACACTGGAACGTCGATGCCCATAGCATAAACTACTTCACAAGAG

GAAATGGCCATATCCAGATCGTAGATGATAATGGAGAGACAGTATTTGATGGAGAAGTTCAAGCAGGACA

AATATTAACATCTCCACAAAACTTTGTGGTAGTAAAGAG

>FM890691.1 FM890691 Jatropha curcas embryo 56-70 (DAF) Jatropha curcas cDNA clone rjcaeb0_000611, mRNA sequence

ATTCATCAAAGGCATAGATACCATTTTTGTTAGATAATCCCATGAATCTATTTCTAAGCTCTTGTTCTTG

AGTCTTGTACACCTATATATAATTTCTTTGTCAAAGCTCCTTTATAGTGCCAGGTTTTTGGCTCCTTTAA

CAGGATTTAGCCTCATCTTGATCATATTCAAAAGCAATTAACCTCACAAGATCACATATCAAGAAAAGAG

GGAAAACATCTGGATCTCGCTTATTTGTCTCCTCTGTGATTGATAAAGAATTGACCCTTTTACTTCCAAT

TTGATCCGAAATATGGGTCTCTCAAATCTTCCAGCTCCATCTGAAGGAGTACTATGTGTGCTTTTGGTAA

ACACTGCCTTATCAATCTCCCTCTTCAAAGGGTTGGTCAGGACGATTCTTCACATTGTTGGCATCCGTTT

ATCATGGTCCTCATCATCCTCCTCATCAACCCCATCCTTGGATTACACAGAAGAGCCCAGTGAATCATTT

GAATTTCGTTTGCCTCCACCACAGACTTACATTCAGGAGTTCCGGAGCAGGACTCCAACAACGCGATTTG

ATGCAGTATGTAGTTGT

>FM890690.1 FM890690 Jatropha curcas embryo 56-70 (DAF) Jatropha curcas cDNA clone rjcaeb0_000610, mRNA sequence

GAGCTCACGCGTGGCGGCGCCTAACTATGATCCCGGGCTGCATGTAAGAATTCGTGCAGGCACTCCTCTC

TATTTGATTAACAGAGACGAAAAAGAGAAACTTTACGTTGTCAG

>FM890689.1 FM890689 Jatropha curcas embryo 56-70 (DAF) Jatropha curcas cDNA clone rjcaeb0_000608, mRNA sequence

ATTCCGCGTCGATGACAAGATCGTGAAGGCTCAGATTTGGGATACCGCCGGCCAAGAAAGGTACCGGGCG

ATCACGAGTGCATACTACAGAGGAGCTGTTGGTGCATTGCTAGTTTATGATGTAACAAGACATGTGACTT

TTGAGAATGTGGAACGATGGTTGAAGGAGCTACGAGGTCACACAGATGCTAATATAGTGATTATGCTTGT

GGGTAACAAGGCAGATCTACGTCACTTGCGTGCTGTTTCTACTGAGGATGCCAAGGCCTTTGCAGAGAGG

GAGAAAACATTCTTTATGGAAACATCTGCCCTTGAGTCTATGAATGTTGAGAATGCTTTCACCGAAGTGC

TGACTCAAATATATCATGTGGTGAGCCGGAAAGCACTTGATATAGGGGATGATC

>FM890688.1 FM890688 Jatropha curcas embryo 56-70 (DAF) Jatropha curcas cDNA clone rjcaeb0_000606, mRNA sequence

AAATCCCAAATTTTGTTATTACATTTCTCCAAACAGAAGACAGAAGTCCTTCTTAAACACACAAACCAAA

GCTCCAACACACTTGGGGATGCAAGCTCAACCAGCCACACATCACCCTATTACAAACTGATCGATAAAAA

GCAAATCAGCCACACAACATAACAAATAAGGGCATTGCGTGCGTATAATACATTATTAAGTAGAGTATTA

AAGAACACCTAGTGGTGCTCGTGGTGGTGGGAGCCAGGAAGCTTCTCCTTAATCTTATCCATTATTCCTT

TCTTCTCATGATCATGGCCATACTCAGCTGAATGATAACCACCTGGAGTTGTAGCTGAGGTTGCTTGAGT

TCTTTCTTCCTTATGATGACCTGGTAGCTTCTCTTTTATCTTTTCGGGGGGGCCCGGTAC

>FM890687.1 FM890687 Jatropha curcas embryo 56-70 (DAF) Jatropha curcas cDNA clone rjcaeb0_000604, mRNA sequence

ATGGAAACATCAAATTCATCAACAAACAAAGTAAGACTTGATTACAGAGACCTTCAACACATGACAAGAA

AATTATTTTAGACAGCTCTCAAAAGAGGGAAGACCCGTTAACAAATTACATAGAAGAAACAAAAAGATTT

AAGCGAAAAAAGAAATACACCAGACAAGCAATGGCCTTCCATGTTAATTAAAGGACTCATATATTTTTAT

ATATATATCAAAGCCATTTTATGTTCTCTGCATCAACCACCAAAACCCACTTCTCTTTTAAGTCTGTTGA

CCTGTTACATGCTGTCCAAACTGCTCAGCTCTGTCCTTCATCTCTCTTGCCTTTCCCGCCAACTTTAAGC

GTGCCTGGTCCAGACTTTCTGCTCCTGGTGGATGCTTTCCAGTAACATACCTATAAATCCAGGACAAGAC

AGAAATGGCAGCGACGCCAAAACCGACTGAGGCTAAAAACCCTGTGATTATCAAACAAACTGTGATAA

>FM890686.1 FM890686 Jatropha curcas embryo 56-70 (DAF) Jatropha curcas cDNA clone rjcaeb0_000596, mRNA sequence

ATTCGGGTGTTTTGCCAGGGCATGTACCCACAATTGCTGAACTGAAACCTGGTGTTTTATCTGTGCATGA

AGGAAATGACACGACTAAATATTTTCTCAGCAGTGGCTTTGCTTTTATCCATGCGAACTCTGTCGCTGAT

ATAATTGCTATTGAAGCTGTGCCAATTGATCAGATTGACCCAAGCTTAGTTCAGAAGGGGCTTGCAGACT

TCACTCAGAAGGTCAACTCAGCTACGACTGACTTGGAGAAAGCCGAGGCTCAGATTGGTGTCGATGTTCA

CAGTGCTCTCAACTCAGCTCTTACAGGCTAATGGTCTCTGCATACCGCCTCTAGTTTTTCTTACCTTCCC

TTTTTGTGCTTCTTTTGCTGTTTAATCTTTTTCCCTCATAATCTGTATAACCACATAGGCCTGTGAAGAT

TTCTCTTTGCCGTGATTCCAGAAAGGTAAATAAAGTCTTCAGGAACATGAAACACAATGCATCCACATTT

TAAGGCACCTCTTAGCTTGGAATGTTGCTTTTTCCAAGTGTTGATTTGTTAGAGAATGTTAAAGCCTGGT

ATAATGGAAATGACCCAAATTTCTCT

>FM890685.1 FM890685 Jatropha curcas embryo 56-70 (DAF) Jatropha curcas cDNA clone rjcaeb0_000595, mRNA sequence

CTTGTACAAGTACAATGTTTTTCAGATCGCATTTATTGTTTTAGCGGGTTTGACTTTCGTTTATTATGCT

GCCTTTGGATGGAGAAGAAAAAAGCCAAGCGGCAGATTTCAGCTTTCCTCCTAAGGCAGGATTTTGACTG

GAGTGCACGCACCCACTTGGAGCCCTCGGAGTTGCCAGTTGTATCATCCTTGGGGGAGTG

>FM890684.1 FM890684 Jatropha curcas embryo 56-70 (DAF) Jatropha curcas cDNA clone rjcaeb0_000594, mRNA sequence

ATTCAAAAGACATTAAGCAGGCTGGAAGGGATACAAAAAGCATGCCAGGAGTCTGGGCTTCTAAACAAAA

GGAAATCATACATTAATAGGAGTAAGCCAAGATACAATTCTGCACTGATTGTCCTACTTTTTGTAATACT

GTCCATCGTTTTCTCTTATCTTTCTGCAAATCGACCAAGTATCTAGAGATTGTGACTCTGTTCAACTTTG

TTCATGACTTGCTCCATCGAACTTCGTCTTTTACAATGTTTGCACAGAATATTGTAATGCCAGAAGTCTG

TTCCCTGATATTTAAACAAAAAGGCCTATAAGTAAAGTTTGCACCCCCTTAACCCTGAAATCCCATTTAG

TTAATTTATTGCTTGATTGTTGTTACTCTACTCTTAATAGTGGTGGTGGAGCAATAATATTAAGATTGTA

AAGTGC

>FM890683.1 FM890683 Jatropha curcas embryo 56-70 (DAF) Jatropha curcas cDNA clone rjcaeb0_000593, mRNA sequence

ACTGGGCAAAAGAACACCAGAAATAGTGCCACAGTAAATAGTCCATATACATATTTAGTTATATACATAT

CGTATATTACCATAATACACGTCGAGTCATCAAAATTACACAACCAAAATCCTGCAACCTATAACATATT

ATATTTTACATGGGTATCATATTATATGGTAAATTACACAAGAACCCGTCTAAACACTGCCAGGGGGCTT

GCAGTCAACCTCTCTGAGGCTAGTCCTGAGACCCTTTCCTCCACCATTAGCCAAAGGATGTATTCCGTGA

TCTGGCCCTTTCATATAAGCTCTGTTCTCCCTCTCTTTCTCAAATGATACCCCATATCGGTTCTCCACAT

CTCGAGCAAGGTCATCATAAATCTGAGTATCCCTAGTCATAGCCATCATAATCTGAGGTAAGCCAAGAGG

TAAATAGTCACCTATTGTCAACTTGCCAAAAATGAATAGTCTTGCCGTAAGTTTTGCACACTTGCTCAAG

ATCCTGTCTTTGTATTGGACCAGGAATTCAAGGCATGAAC

>FM890682.1 FM890682 Jatropha curcas embryo 56-70 (DAF) Jatropha curcas cDNA clone rjcaeb0_000592, mRNA sequence

ATTCTAACGAAAGTGAAAGTTTTACTGTAAGAAATACTAAAAGCTCTATCTTTCTTTCGTGTTAGGCTTT

GAATTAAAAGGACAAAAGATGGCAACTGTGGGCAGGAACATAGCAGCTCCATTGCTGTTTCTTAACTTAA

TCATGTATGCTATTGCTTTAGGTTTTGCCAGTTGGTGCCTTAATAAGTATATCAATGGACAAACCTACCA

TCCAAGTTTTGGAGGGAATGGAGCAACAGCTTTCTTTTTGACTTTTGCTATATTAGCTTCTATTGTTGGT

ATAGTATCCAAGTTGGCAGGTGGCCTTCATATCAGGAGCTGGAGAAATGACAGTCTAGCCTCTGCAGGCT

CTTCTTCACTTGTGACTTGGGCCATCACTGCCCTAGCTTTTGGGTTGGCATGCAAGGAAATAAACCTAGG

AGGACACAGAGGATGGAGACTGAGGGTATTAGAGGCATTCATGATAATCTTGACATTTACCCAACTCTTG

TATGTCCTGTTGCTTCATGCTGGGGTATTTAGCAGCAGGTATGGCCCTGGTTACAGGGACACCGAATACG

CTGTAGGAGCTGGTGGGGAACCAATTCC

>FM890681.1 FM890681 Jatropha curcas embryo 56-70 (DAF) Jatropha curcas cDNA clone rjcaeb0_000591, mRNA sequence

ATTCCTAACAAAGACGGAGACAGAGAGAACAGAGTCTCAAAGACCTCATCTTCGGTCTCAGAGACATAAA

GAAAAAGGATGGCTTCAGATCCCAAAATCCACAAATTTGAAGACGTTGCAGTGCACAACAAAACAAAGGA

TTGTTGGCTTATTATCTCTGGCAAGGTGTATGATGTCACTTCATTCATGGATGATCATCCTGGAGGAGAT

GAAGTCTTGCTGTCATCAACTGGAAAAGATGCAACAAATGATTTTGAAGATGTGGGTCACAGTGACTCTG

CTAGAGATATGATGGAGAAGTACTACATTGGTGAAATAGATACATCAACGATTCCTAAAAAGCGAGCTCC

GGTTCCTCCCAGGCTAGCTGCGCACAATCAAGATAAGAGCTCAGAGCTTTTCATTAAGATCTTGCAATTT

CTTGTGCCTCTCTTGATCTTGGGTTTAGCCTTTGCGGTCCGACATTACACCAAGAAAGATTAGGCTAATT

ACAGATCGTTAATACCATTTATAAGGGTTAATTATGGAGTTGCATTTTAGAACG

>FM890680.1 FM890680 Jatropha curcas embryo 56-70 (DAF) Jatropha curcas cDNA clone rjcaeb0_000590, mRNA sequence

TTTTTTATTTTTTAAAAAAAGGGTTAAATAGCAACATTTAATTCTAAACATAAAAAGCAAAGCAAATTAC

TCAGACATCCAGATAATTATATTAAGAAACTAAACAAAAAAAAACCAGTATCAATATCCATCATACTCAA

CTAATCAAAATTATGCAAAAAGTAGCAAACTTAGAATTACCAAATCCATATCACGAAACAAAAACGCATT

AACAATAACCTAATCACCGCCAGCCTTATTGTAAACATAAGTCAAGCCGCACTTGCCGCAGTAGTGCCTA

TCAAAATGGTTGGCCATGAAAGTTCCAGCTCCACACTCAGCATTGGGGCACTCCTTCCTCAGCCTCTGCA

CCTTACCGGAGTCATCGACCTTGTAGAACTGAAGCACGGCAAGTTTGACCTTCTTCTTCTTGTGCTTGAT

CTTCTTTGGCTTGGTGTAAGTCTTCTTCTTTCTCTTCTTAGCACCACCACGAAGACGAAGCAACAGGTGG

AAAGTGGATTCCTTCTGGATATTGTAGT

>FM890679.1 FM890679 Jatropha curcas embryo 56-70 (DAF) Jatropha curcas cDNA clone rjcaeb0_000589, mRNA sequence

ATTCTGACAACTGTAGGAGATGGCCCTAATGGAAGGAAAGTAGAGGGGTTTCTTTACAAGTATAGCGAAG

GTCAAGTGAACATAGTATGCATTTGTCATGGCAGCTTCTTCTCTCCGGAAGAATTCGTAAGGCATGCAGG

CTGTGAAGATGTCGCCAACCCCATGAAGCGTATCACAGTCTCCTCTTCTTTTTCCTTTTAGTAGCCCCAA

TTGGATCGGGAACAATGACATACTTTGGAATTAAGTACGTGATAGGGATTCTGACCCTCCTCTTCTCTAC

CATAAATACCCTTAGTTTGTTATAAAATATCATATTTGCCTTTGCTTTGAACTTTGTATGTCATTATGAA

TGTTTTTTATACAATAAAGACCTTATATTGATTGTTGTTTGTTG

>FM890678.1 FM890678 Jatropha curcas embryo 56-70 (DAF) Jatropha curcas cDNA clone rjcaeb0_000588, mRNA sequence

ATTCCGAACCCCTATGAGAAAGTGAAAGGAGGGAGACTCACCTTCAAGGGAGGAAGCGTAGCCACCAGTA

GCAAAGGCATTGACAAGAAGAAGAAGAAGAACAAGAACAAGATTAAAAAGGTTGCTGATGATGAAATCGA

CGCCGCCGCGGTAGAGGTGAATAATAGTGAAACTGGCGGTGGAGGTGATATTTACACAATCGACGCTGCC

AAGAAGCTCCAGTACGAGCAGCTTTTTCCGGTGGAGGCGAAGAAGTTCTGTTATGAGGAAAAGTCCAAAT

TCAATTCGGTAGAGGATGCTCTCGATGATCGCGTCAAGAAGAAAGCCGATCGCTACTGTAAATGAGCGAT

CATTCAGGTCCGCTTCTTTCTTAGGGTTATGTTATATACATCTTGTACTTCTGATTGTAATCAAATTAAT

TAGGGTTTATGATTAGCTATGTTCTTTTGTTTAAGATTACCTAACAAATTAGAGAATATTTTTGAATTAC

ATTGTATAACCCTAAGTAATGCATTGATTTGAAACTTTCTAGTAAAATGCTAATTTAATT

>FM890677.1 FM890677 Jatropha curcas embryo 56-70 (DAF) Jatropha curcas cDNA clone rjcaeb0_000587, mRNA sequence

ACTAGGAAATATGAATTACATACAATATAGTTCACGATACTTGTACATCTCACAAAAGATCATCCCGACA

AGCATAACAATGTCATGTATTAGTTGCAACAATTAGTGGTATTTCCGGGTCGAGTACCAAGTCACATTAC

AGCTTAAAGTATTTTAACTTCACATGCGAACCCACCTACATTTAACTCCAGTAATCCCAAAAAAAGCTGT

GTCCAATACACTACATGTCCCAAACTTTCCATCAGAAAGCAACT

>FM890676.1 FM890676 Jatropha curcas embryo 56-70 (DAF) Jatropha curcas cDNA clone rjcaeb0_000586, mRNA sequence

GAAAAAAAGATACAGCAATGCCGTGATGAAAACAGCATCAATATCAAAATTTGGAACATCAGTTCAATTA

CTTTGTGACAGAAAACAAGATAGTGTTTAACTTAATAACAATATAATCAAGATGACAACCCACTCCTAAA

TTTGTCGATTTACACTTTATTAAGACATTATAAAACCAAGTCTTGCTCAATTGAAGATACAAGAAACGAT

TTGCCACACTGCAAGATACAGCAATTCAACTTCTACACTCAATTCACATCATTGTAATCCTTCGTCCCAA

AGTTCTTGCACCATAGCTGCGATGGAATCGGGCGAACTGGATGATGTGGTCGTTG

>FM890675.1 FM890675 Jatropha curcas embryo 56-70 (DAF) Jatropha curcas cDNA clone rjcaeb0_000584, mRNA sequence

ATTCAGAAGGCCTCTAGCTGCGACTGAAAAAATGGCGACTGCTAGAACTGTGAAGGACGTGTCTCCCCAC

GAGTTCGTCAAGGCTTATGCTTCCCATCTCAAGAGATCCGGCAAGATTGAGTTGCCTCCCTGGACTGATA

TTGTGAAAACTGGAACACTCAAGGAGCTTGCACCTTATGATCCAGACTGGTACTACATTAGAGCCGCCTC

CATGGCAAGGAAGATTTACTTGAGGGGTGGCCTTGGTGTGGGTGCTTTCCGTAGAATCTATGGTGGGAGC

AAGAGAAATGGAAGTCGTCCACCTCATTTCGGCAAGAGCAGTGGTTCAATTGCTCGTCACATTCTTCAAC

AATTGCAGACAATGAACATTATCGATGTTGACCCAAAGGGTGGGAGGAGAATCACTTCTAGCGGTCAACG

GGATCTTGACCAAGTGGCTGGACGGATTGTGGTTGCTGCATGATGAACCAATGGACCGTATAG

>FM890674.1 FM890674 Jatropha curcas embryo 56-70 (DAF) Jatropha curcas cDNA clone rjcaeb0_000583, mRNA sequence

AAAATAGAATGATGACCAGAAATAGTATTTCAGATTCTAACCAATCCAAAATTATAGTAAACAAGATTCT

ACCAAATCCAAAATTCTCTTCAGAAGTTACATAACTCCCAACAAAATAAAAAGACATTCTGCAAAATATT

ACTTGAAAAACCAACACTTTCTACGATGTAATATTCTGGGCCTTGTTGAGGATGACACCTTCATATTTCA

CCTGAAACCACTTCATTGCATCCTCCTTGGTAACCCGGTGCTGAATACCGACCTTTGACTTGCATCTTCT

GCGACGACCAACGCGATAGCCTGGACGCTCCAAAACAACATAGAAATCCATACCATAAATACCAGTTGAA

GGATCATACTTGATGCCAAGATCAATGTGCTCCTGGATTCCAAATCCAAAGCAACCAGTGTCACTGAAGT

TTCTTCTCAACAGCTCGTATTCCTTCACTTTCAAACCACTTTCAAGTAGCTGCATAGCTTTGTCCCCCCT

CACGGTCACATAGCAAGCGATCTTTTCATTACGCCTGATACCAAAAGATCGAACAGTGTACCTAGCCTTA

GAGAAGACAGGAGTCTGACCGCTAAGTTGATCGAG

>FM890673.1 FM890673 Jatropha curcas embryo 56-70 (DAF) Jatropha curcas cDNA clone rjcaeb0_000582, mRNA sequence

GCACTAAACCTGTAAATCTTGATAGAAAGCACAACAAAAATGAATGCAGAATACTTGTTTTGTTTATCAA

GTACAAAAACTTATTTTGTTTTAATACTCTCTTCTAATTTTGAGAAACTCTCTTTAAGGGTTTTTGTTTA

GTCCAAAGATCTATGACTGTCCTAAATGTACCAAGTTCAATAAACTTCAGAAATCCTAACTCAATTGCAC

TGTAAAAATTTTAAAAATGATTGGTTGGACTTGGAAAGGATGTGAGACACCAAAACCAGCCAGAAACTAC

AACACAGCTCAGGTATCTTTTATTCCATAGCTTAAGTCAACTGAAAATCCCCATCTGCGAGGAAAACTAT

AATCCCACGTTTTTCTCCAGCTTTTGGATCTGGTTCAGGAATATCCAGGTCATCATGACATGGGGAGCAA

TTCTAACACAATACACGGGGAATCCTGTGTAGAATTTGAATGGTCCTCCTGCCTTGAGAGTCTTCATGGC

ACAGTCTAACGAGCCAGTGTATGGATACTTTCCCTGGGCATCAGGTTGCATTTTCTGAATTTGAGTTTTA

AGGGGGGGCCCGGTAC

>FM890672.1 FM890672 Jatropha curcas embryo 56-70 (DAF) Jatropha curcas cDNA clone rjcaeb0_000581, mRNA sequence

ATTCGCCAAGTGAGTGATTGAATTTGAATAGCCAAGTGAGTGAAAATGGAGTCGCTGAAGGAAACCGCAG

CTAACGTTGGAGCCTCTGCTAAGGCTGGCATGGAAAAAACCAGAGCAGAGGCGCAAGAGAAGGTGGAGAG

AATGTCAACACGTGATCCAGTGCAGAAGGAAATGGCGAGAGAGAAGAAAGAAGAGAGGAAAACG

>FM890671.1 FM890671 Jatropha curcas embryo 56-70 (DAF) Jatropha curcas cDNA clone rjcaeb0_000579, mRNA sequence

AAGCATAAATGAAACAGCTTTCAAAATGTGATATATCCACCCAGTTTATAACGTTAACAGAAAATACAAA

TCATCGAATCCAGTCACCGCTATCATAAATCTCTCATTTCCTATGGCATGCCTAACAAAATCTTCTAACT

TCGATCCTCCCGATATCTCTCAACAAAAATTTAATTCCATTTACTTCCCACCATGTTTGGCCTTAGAGTG

AGACTGCAGAGCACCATCCGAGCCAAATGACCTGTCGCAAGATTTGCAAGAAAATTGACCTCCAGATTTC

GGTGACTTCGCATTGCTGCCGACTGCAGCAGTTTTTGCAGCACCCTTAGCTGGATGGGGAGTTGCAGTAT

GGCCGCCCTTCTTACCGTCAGTCTTCTGAGGAGTAACTGATTTTATCTTCTTCGAGGGGACAGGTGTCTT

TGTTGCAGAGTCATTGGGCCTCTTTTTGCCTTTTACAGCCTTCTTGGGGTCTCCTCATCTTCATCTCAAC

TCATCCTCTCTCTCATCTGAATCA

>FM890670.1 FM890670 Jatropha curcas embryo 56-70 (DAF) Jatropha curcas cDNA clone rjcaeb0_000578, mRNA sequence

ATTCCATTAACATATCTTTCTCACACTTATCATCAACAAATGGCTGAACGTTCACAGCCACACCAAGTTC

AAGTGCACCCACAACACCGCTATGAAGCTGCCTTCAAAGGTCAACAAAAAGGTCCATCAGCTCAGAAAGT

GTTAGCTGTGATCACCCTCCTGCCGGTAGGCGGTGGGCTTCTTGCACTAGCTGGTATAACCTTAGTAGGT

ACCCTAATCGGCCTAGCTATTACTACCCCTCTTTTTGTCATCTTTAGCCCTGTTCTTGTCCCAGCTGCAT

TAGTTATTGGCCTTTCGGTCATGGCATTTTTGGCTTCCGGGGCTATGGGGCTCACAGGGTTGTCATCACT

CTCGTGGGTTCTGAAATACCTCCAGGAGGTCACTCGGCGAATGCCGGAGCAGCTGGATATAGCTAAGAAG

CGTATGCAAGACATGGCTGGTTTTGTGGG

>FM890669.1 FM890669 Jatropha curcas embryo 56-70 (DAF) Jatropha curcas cDNA clone rjcaeb0_000577, mRNA sequence

ATTGAAGAAGGTCACGCTTGCCGTGCTTCAGTTCTACAAGGTCGATGACTCCGGAAAGGTGCAGAGGCTG

AGGAAGGAGTGCCCGAATGCTGAGTGTGGAGCTGGAACTTTCATGGCCAACCATTTTGATAGGCACTACT

GCGGCAAGTGTGGCTTGACTTATGTTTACAATAAGGCTGGCGGTGATTAGGTTATTTTTAATGCGTTTAA

GTAAGGTTATATGGATTTGGTAATTCTAAGTTTGCTACTTTTGGCTTAATTTTGTTTAGTTGAGTATGCT

TCTGATGGATATTCATATACTGGTTTTATAGGTTTAGTTTCTTAATATACTTATCTGGATAGCTTATATC

TAAAGGGGGGGCCCGGTACCCAATTCGACCTATAGTGAGT

>FM890668.1 FM890668 Jatropha curcas embryo 56-70 (DAF) Jatropha curcas cDNA clone rjcaeb0_000576, mRNA sequence

CGAATCCAATTGCTCTCTCTCTCTCTCTCTCTCTCTCTCTCATAAAGATAAAGAAAATTTAAGCAAAGAA

AAGGATATTTTATTTTCCTTTGTGAGTTTTTCTTGGAATTAGGAGGTGGAAAAATTAAAATCACTAGATG

GGTCTTTCAAGCTTTCCTAGTGCAGCAGAAGGAGTATTACCAATTTTGGTAATGAACACAGTTCTATCAG

TAGCTTTACTGAAGAATATGGTAAGGTCTTTGCTTCAAGTTGTGGGTGCTAATTGGATCCCACAAGATTA

TGAACAAGACCCAGATGAGTTCCCTCGAGAAAATGCAAGAATGAGAACAATTTCAATTACCCAGTTCAAA

TCTTTGACTCCCCACAGTAATGGTGGCGGCAGCTGCAGCGGCGGCAGCCGCGGTGCTATAGTGGAATGCT

GTGTGTGTTTATGTGGGTTTGAAGCTGAAGAGGAGGTGAGTGAGTTG

>FM890667.1 FM890667 Jatropha curcas embryo 56-70 (DAF) Jatropha curcas cDNA clone rjcaeb0_000574, mRNA sequence

CGAATTCCAACCAGCTCGACCAGTACTTGAGGGCATTTTTGCTCGGAGGCGGCCAATCCCGACAAAGAGA

GAGAGAGAGTCCTGAAAGACCCCTTCGATCTATACGTGCACGAAAATCTGAGAAAATAAGAGAAACTTTC

CAGAATGTTTTCCGGTCGTTGGATGAGCAGGCACTGGCCGAGTCCTTTAATGTTCCAACAGAAATTGTAA

GGAGAATGCAGGCAGGAAACAACAGAGGAACAATAATTAGGTGCGACAAAGAAATGCGCACGATCGTGCC

TGATGAACGAGAAGAAGAAGAGTTCACGTACAGCAGACCCGGCCAGAGCGGGA

>FM890666.1 FM890666 Jatropha curcas embryo 56-70 (DAF) Jatropha curcas cDNA clone rjcaeb0_000573, mRNA sequence

ATTCTTCGTCTTCGCGGGTGGCATGCAGATATTTGTGAAGACTCTGACAGGGAAGACAATCACGTTGGAA

GTTGAAAGCTCTGATACTATTGACAATGTGAAGGCCAAGATACAGGACAAGGAGGGCATCCCACCAGATC

AGCAGAGGTTGATTTTTGCTGGAAAGCAGCTTGAAGATGGACGCACCCTTGCAGACTACAACATCCAGAA

AGAATCCACCCTTCACTTGGTTCTACGTCTTCGTGGTGGCATGCAGATCTTTGTGAAGACCCTTACTGGC

AAGACCATTACCTTGGAGGTTGAGAGTTCTGACACTATTGATAATGTTAAGGCTAAAATCCAAGACAAGG

AAGGCATCCCACCTGATCAGCAGAGATTGATTTTTGCTGGGAAGCAGCTTGAGGATGGGAGGACCCTTGC

CGATTACAATATCCAGAAGGAGTCTACCTTGCACCTTGTCCTGC

>FM890665.1 FM890665 Jatropha curcas embryo 56-70 (DAF) Jatropha curcas cDNA clone rjcaeb0_000572, mRNA sequence

CGTAATTTACGCACTCAAAGGACAAGCGCGGGTTCAGGTGACGGATGAAAACGGCAACACCGTCTTCGAC

GGAAGTGTAAGCAAAGGACAGGTTTTGACGGTGCCACAAAACTTCGTAGTGGTGAAACGAGCAGAAAGTG

AAAGATTCGAATATGTAACCTTCAAGACAAGCGATAACGCGATGACCAATGATCTTGCAGGTCGCTCCTC

CGCCGTTAGAGCAATGCCGTTGGCGGTTATTGCAAATGCATTTAGGGTATCAGCAGAGGAGGCAAGAAGG

ATTAAGTTTGGAAGACAAGAGACCACCTTGACTATTTCCAGGACCGAGTCTGGAAGATATGCCGAGGAGT

AAATAATGAAGTAGCCTTTTTGGGTGTGTAGATAGTGAAAGTAAGTAGTATTACGAGGGGAGTTATGATC

TCACGCTAATGAAGTATGTAATATATTATGAAAATAAGTGATGAAAGTAATGTAATCATCCTAATTAGTA

ATTAAGTATTCCCTAATC

>FM890664.1 FM890664 Jatropha curcas embryo 56-70 (DAF) Jatropha curcas cDNA clone rjcaeb0_000571, mRNA sequence

ATTCTAATAACTATATTTCTGCTTCCCCATTAACATTAAGTGTGGTCTGGAAAGAAAAATATGCTACCAA

AAAATTTTGCTTTCCTTTTATCACTTTGGCTTTCAGTCTTTGAGTATGCTGAATTGAGAAAACTTTCCAG

TGATGTTTTGCCTTCACCAAGTACGTTTTGTCAATATCCGTGCCATCCTCTTGCTCCTCCACCACCACTT

TCTGAGTATCCAATATATGGAGCACCGNCACCACCACTTTCATATGGAAGTCCACCATCTCAAGTCAACT

GCACACAAAATCCAGTTCAGTGCTGTCCTTATTCCCCTCCAGCTCCTTATGCTTTTGGATATCAGCCCCC

TCCTTATCCTTTAGGATATCAGCCCATTAGCTATACTGCTTCCCTTTCCTCTTTTTATTACATTTCAGTT

ATGTTTTCGCTACTCTCAATTGCTGTTCTGCTCTAGGTTCATATACTATA

>FM890663.1 FM890663 Jatropha curcas embryo 56-70 (DAF) Jatropha curcas cDNA clone rjcaeb0_000570, mRNA sequence

GAAAAATCCAAGAGCATACTTTATATTATCATAAAATATCCAGAATAATTCAAAACCAAAGCAAAATCCC

TAGAACAATTGTTTAAAGACCTAGACATATAAAGCAACAATTTAAATAGACAAAATATTGATCAACATAG

CCGTCCTCAGCGTAATAGGAAATAACTAGAGTATCCAAGCTGCAGCAAAACATTTCACTTAAGAGGAATG

AACCTAGACGAGTGGGTGGCACCAATACCAGGTCTACCATGTTTCACAGGCTTGTATGAGATTGAGAACT

CAGCAAGATAATGCCCGATCATCTCAGGCTTGATTTCAACTTGGTTAAAGGTCTTCCCATTGTACACACC

AATAATGCTTCCAATCATTTCGGGAACAATAATCATATTGCGGAGGTGGGTTCGAACTGGTTCTGGTGGG

GGGGCCCGGTAC

>FM890662.1 FM890662 Jatropha curcas embryo 56-70 (DAF) Jatropha curcas cDNA clone rjcaeb0_000569, mRNA sequence

ACCAACTTAGGCTGGTAAATTTTAGAATACACGCCCAATAGAAGATTGAATTATTTGTCAAACAAGAAAC

TAATAACCAAAGAAGGAGGAAAACATGTCTAGTCAATAATTAACCAAAATAAGGCATGAAGCACCAAATA

TAACAGTACAGAGATGGAAAAGGACACATAAAGCTAGAAATATGGCAAGAACTTCCACTTCGAAGCCAGC

TATTCTCCATTCTTAGCACTTGATCTCCTTCAAAGCATAAGCGAAGTACAAAAATGTAGGGTCAGTGGCG

CCATCCTTGTAGTAAGCAAACACCAAACTCCCATCATCATGCATGCTCTCCCCCACAAAAAATTGGAGGT

CACTGAGCTTCGAGAGCAGGAACTTGGTTGCTCCCTCAATGTTTTTCTTGAAAGCCTGTTGCTTCTCCTC

ATCCAATTAGGGTGTCAACAACTTGATGTACCTCTTCATGTATGTGACAAACTGCTTCTTGTCAAAAGCA

GGTTGCTCCTGGAACCTAAAAGTGTCCACAACGTCAACAACTTTAACAGTCTGGTCAT

>FM890661.1 FM890661 Jatropha curcas embryo 56-70 (DAF) Jatropha curcas cDNA clone rjcaeb0_000568, mRNA sequence

ATTCCCTGTTTCTGAGAGTTGCTGCCTTGAGGCTAAGAAAATAGTGCAAGATACAAGCTGTCTTTGTGCT

ATTGTACTTTCAAACACCGCTAAGGCTGCTGGGATGATCCCTGAAGTTGCCATTACCATTCCCAAGAGAT

GCAACATTGCTGACCGTCCTGTCGGTCACCAGTGTGGAGATTATACACTTCCATGAAGTTATAACAAGCA

TTCTGAACATGAAGTACTGGGAAGATAGCAGCCAACCACTTGTTCAATAGCAACTTGGTTGCCCTACGTA

CTAGTCTTACTCTTCTGAATAAAAAAACTAGCACTTAGCTAGCTCTGGTTGTACGTGTATCCTGAATAAT

ATGTTCAGAATTTCAAATC

>FM890660.1 FM890660 Jatropha curcas embryo 56-70 (DAF) Jatropha curcas cDNA clone rjcaeb0_000567, mRNA sequence

ATTCGAGAGAGAGAGAGACTTGGAGAAAAAGCTGTCAAGGAAGAAGAAAATGAGGACAAGACGATGTAGA

ACAGCAATATCCACACCTTCCAGCGCAACAATGTCAAGTAAGCCTTCCCCAAGGAAGAGATCTGTTGGTG

TGGCAATATAATATCTATATGTATTGTAAATAACAGCTTACCACATCTTCTGTTTGAGGTGATGAGTGCA

TTACAATAAAATCTGTTAGATATGTATTCTTGTGATAAAAAAA

>FM890659.1 FM890659 Jatropha curcas embryo 56-70 (DAF) Jatropha curcas cDNA clone rjcaeb0_000566, mRNA sequence

ATTCTGCTTCAGCGTCCTCTGATTCAGCTCTCAGCACAAATGGTGACACGGTGGTGAAAGCAAGTCAAAA

TAGGTGCGCCACTTGCAGGAAACGCGTTGGTCTAACAGGATTCAGTTGTCGTTGCGGGAATCTTTTCTGT

TCAGTTCATCGCTACTCAGACAAACATGGTTGCACCTATGATTATCGAACTGCTGGACAAAATGCTATTG

CCAAGGCCAACCCCATTGTGAAAGCTAAAAAGCTTGATAACATCTGACCCTCATCAAACTTCTGCTGAAG

GTATCACATCTATGTTCTGGGATGCCACATGCAAGGCTTTTATCTCGTTGTTCTTGTTATTTAGATGTCG

TGTTGCTCAAAAGGTTGAAATAACATGTGAACTGGCATCTTTCCAGTTACT

>FM890658.1 FM890658 Jatropha curcas embryo 56-70 (DAF) Jatropha curcas cDNA clone rjcaeb0_000565, mRNA sequence

TGAAAGAAGGAGACATGTTCGTGATCCCTCAATTCTATGCAGCACTTGGAAGGGCAGGAGAAAATGGGTT

CGAGTACGTAACATTTAAAACAAGTAGCCAGCCTATCAAGAGCCCATTGGCGGGTTATACATCAGTGATG

AGAGCCATGCCTCTTGATGTTCTGATGAACTCTTACAGAATGTCTCCAAGCGAAGCTCATCAAGTGAAGA

GGAACAGGGATCCACAGTCCATGTTGTTTACTCCCACCAGCAGGAGACCATAAGTAAGTTACTACTAAAA

TAGCATTGTGATTGGTGCTTCTACTAGCCAAGGCCGCAACTAAGAGATCCTTCTTGTAAATTTAGAGAAG

GGATCCGTCGTATATTGTAGTATAGAGAGTTGTAGAGTGTTAAAAATGTAGAACTTCAACTTCTGCAGAA

ATCAATAAAATGTGGCCTTCTTTTGTATCTAATTAATAAAATATAAATTCCATCGGCTTAACAGAGG

>FM890657.1 FM890657 Jatropha curcas embryo 56-70 (DAF) Jatropha curcas cDNA clone rjcaeb0_000564, mRNA sequence

GGCACATACAAAGATAATACATTGAAAATCAACAATGGAACCCAAAAGACCAAAAAGAGAAATACAAATG

CAACAAAGAAGATCCCCCATAAACCAACTCAACTACCTCAAAACAACACCCATACAACAAAAGTAGTCTC

GTACCAAAACCCAGCTCTCTGACTTCAACCCTTAATAACCAACAGCATTTCTCTCTACTTATTTCCCTTC

ATGTGCTTTCCTCTGGATTTCTTGGCCTACTTCCTTGGTCTTCTGTCCCACAAAACCAGCCATGTCTTGC

ATACGCTTCTTAGCTATATCCAGCTGGGGGGGCCCGGTAC

>FM890656.1 FM890656 Jatropha curcas embryo 56-70 (DAF) Jatropha curcas cDNA clone rjcaeb0_000563, mRNA sequence

CCGGGCTGCAGGAATTCGAATTCCTTCAAGCAAAACCCTAACAACGACGCCGATCAGGATTCAGGCGCAA

CAAAGAAGAAGATTCCCTGAGTTCTGCGATCAATTAGAACCGATTAAAATAAAGTATTAGATTTTCAATT

GAAAAATGATGAATTTTACAACAATGTTTTGCAAACGTCTAAACGTGAAGGAGCTTGTCACCAATGTTCC

TGTATACGAAAGCATTAGAGATAGCTCTGCTGGTGGATTAAGCTTGATTTTAAGGCGTTGGGCTACCAAG

AAGACAGCCGGATCCACCAAGAATGGCCGAGATTCGAAACCTAAGAACCTTGGAGTGAAAAAATTTGGCG

GCGAGAGGGTGATACCTGGAAATATCATAGTTAGGCAGCGGGGAACTCGATTTCATCCTGGAAACTATGT

CGGGATGGGGAAAGATCACACCCTTTATGCCCTGAAGGAAGGAAATGTCAAGTTTGAGACTCACAAACTT

AGTGGACGCAAGTGGGTGCATGTTGAACCCAAAGATGGA

>FM890655.1 FM890655 Jatropha curcas embryo 56-70 (DAF) Jatropha curcas cDNA clone rjcaeb0_000562, mRNA sequence

ATTCATCCAATATCACTGATCGTAAACTCCATTGAATTTACTCTGTGCTTCGATCGCGTCAATGGCGGAG

CACCCACAGTCGCAGCATGTTGGCCAACAGACACGATCCCACCAAGTAGTGAAAGCAGCCACTGCAGTCA

CTGCCGGTGGATCTCTTCTAATTCTCTCCGGTTTGAACCTTGCTGGTAAGGTCATTGCACTGACCGTAGC

CACTCCATTGCTGGTCATATTC

>FM890654.1 FM890654 Jatropha curcas embryo 56-70 (DAF) Jatropha curcas cDNA clone rjcaeb0_000561, mRNA sequence

GCTAAGATAATCACCACATCTTCTCGAATTGATCATTTCCCGCAGCTCACAGTTCTACAGTACTCTAATC

ATGCAAACGAAAATAAAATAATTATGCATAACATGTTTGTCCTAAGAAGGCTACCTCATAATCTAAGTAG

TTCCATAAAAAGTTAGAGAACAAGTCACAGGAAATAACGTATTTGAGGAGCCTCTACTATTTTGTCTATC

TCACTTCTTCCTCTTCTTTGGGGGCTGGAGGGCTTCCTCGTCTTCATCCTCCTCATCTTCATTCTCTACC

TCCTCAACTTCCTCCTCATCTTCTTCCCCACCATCGTCGTCGTCGTCATCATCATCGTCGTCATCGTCAT

CATCTTGATCCTCACCATCTTCTTCATCATCATCATCCTCTTCCTCTTCTCCATTCTCTTCTGCTC

>FM890653.1 FM890653 Jatropha curcas embryo 56-70 (DAF) Jatropha curcas cDNA clone rjcaeb0_000560, mRNA sequence

ATTCCGCAGTAGGGGCCATCCGGCCCCCAGAGGCACGTGCCGTTGGCCAAGCCCCCGCGGCGGACGAGCC

GCGAGGGCCGCCTTAAAGTACAATTCCCATCGGGCGACGGATAGAATCCTTTGCAGACGACTTAAATACG

CGACGGGGTATTGTAAGTGGCAGAGTGGCCTTGCTGCCACGATCCACTGAGATTCAGCCCTTTGTCGCTC

CGATTCGTAAAAT

>FM890652.1 FM890652 Jatropha curcas embryo 56-70 (DAF) Jatropha curcas cDNA clone rjcaeb0_000559, mRNA sequence

ATTCCATCTCTCAGTGCACACAGAGAAAACGAAGAGTAAGCTATATTAATCATATCATCCAAAATGGGAG

GAGGACCAGAGCATGGACACGGAGCTGATGGCGCCCATGGGGACTTCAGGGGGAAAGTGTGGAGCATGAG

TGGAGGTCCATACTGCAGGCCCAAGCATTGGCGCCGTAACACTGCCATCGCTATGGCTAGCGTTTTCCTG

ATCTGCATCCCTATCGCCATGAAATCTGCAGAGCTCGAGCAACGACCACATCATCCAGTTCGCCCGATTC

CATCGCAGCTATGGTGCAAGAACTTCGGGACGAAGGATTACAATGATGTGAATTGAGTGTAGAAGTTGAA

TTGCTGTATCTTGCAATGTGGCAAATCGTNTCTTGTATCTTCAATTGAGCAAGACTTGGTTTTATAATGT

CTAAATAAAGTTTAAATCGACAAATTTAAAAA

>FM890651.1 FM890651 Jatropha curcas embryo 56-70 (DAF) Jatropha curcas cDNA clone rjcaeb0_000558, mRNA sequence

TATTAAGATTAAGATGGCATTGCGAAGGTTGGGTTCGCGTGGATCGGTCGGAATACTTTCTAATATCGGT

CAATCTCTTCTACAGAATTTATCGTTCCGTTGCCAAAGGGTGCAATGCCTTCGAATTGGTAATGCTGAAA

TCCCAAATGTGAAAAACGGTTGAGGTATGCCCTTCAGCATATTCAGGGAATTGGCCGCCAAAGAGCTCAC

AAAATCCTTGTCGACCTAAGCCTAGAGAACAAGCCTGCCAAGGACTTAACTGGATTTGAACTTCACTCTC

TTAGAGAGGAAGTCTCCAAGTACTTGATCGGAGATGATTTAAGGCGACATGTTAAAGCTGATATAGAGAG

ATTGGTAGGTATTCAGTGCCACAGAGGATTTAAATTCATGTTTGGCATTGATGGCCATGGC

>FM890650.1 FM890650 Jatropha curcas embryo 56-70 (DAF) Jatropha curcas cDNA clone rjcaeb0_000557, mRNA sequence

GTGATCGATAAAGTGTAACAGAGCAAAAGATTAATTATATTGGTGGAGAAATTTGTTAGCAAAAAAGGGC

AAAAATGTCTTCAAATCAATCAAGACAAGAGCTTGATGAAAGAGCAAGGCGTGGAGAGACTGTTGTCCCT

GGAGGTACTGGGGGCAAGAGCCTTGAAGCTCAAGAGCATCTTGCTGAAGGGAGAAGCAGAGGAGGACAGA

CAAGGAAGGAGCAGCTGGGGACAGAAGGATATCAGGAGATGGGCCGGAAAGGTGGGCTGAGCACGACGGA

CGAGTCTGGTGGAGAAAGAGCTGAGAAGGAGGGCATCACTATCGATGAGTCGAAGTTCAGGACTGGGAGC

TAGTGTGATGATTAAAACTATAAATGGTATTTTGTAGTAATAATAATAATAATGTTGGTAATAAAGTATT

AGCTTAGCAGTGTACTTTAGTTTAGTAATGTAGTTTACATGTGGGGTTAAATGACTAGGGGTTAGCTAGT

ATGTGTGTT

>FM890649.1 FM890649 Jatropha curcas embryo 56-70 (DAF) Jatropha curcas cDNA clone rjcaeb0_000556, mRNA sequence

GTGGCAGAGACTGGATGGCCTTATAAGGGAGATGACAATGAAGTAGGACCAAGTATTGAGAATGCTAAGG

CTTATAATGGTAATTTAATTGCACACCTTAGATCAATGGTGGGTACTCCATTGATGCCTGGGAAATCAGT

AGATACATATCTGTTTGCTTTATATGATGAAGACTTGAAACCTGGACCTGGTTCAGAGCGAGCATTTGGA

CTTATCAAGCCTGATCTTACCATGATTTATGATATTGGACTCTCCAAGAGTAATCAGGCGCCTACTACGC

CAAAAACTCCTTCGGTGAGTCCATCACAAACTCAAAACCAAGCAACATGGTGTACGCCCAAGGCTGCTGT

CTCAGATGCCAAATCGCAAGCAAATCTGGATTATGCTTATGAAAATGGGAAGGA

>FM890648.1 FM890648 Jatropha curcas embryo 56-70 (DAF) Jatropha curcas cDNA clone rjcaeb0_000555, mRNA sequence

TTTTATTTTATTTTAAAACTGCTAAGCACAACTTTAATTTCACAAAATGTCAAAATGTTGCTGATTCCGC

ATTGTCATACATCAAGAATGACGTTCTACTAATAAGAAACAACTCAATCAAAAGTAACTGCAGATAGCAA

ACACTCAAAAATTCTGGTATCATGAAAACAACAAAGGTCTCTACAAATACTCTTCCATTAAGATCCCAAT

AAATCAAATTTGACAGCTACTCAGTGAGATTTGATGTGTTGCTGAACAACATTAAGACCTTCAGTCTCCT

CGCCATAATCCTTAACTACGACGCAGGAGCAACCAACTACCTTCCGGGCCTTACCCTCAGAATCAATCTT

GCACAACCCAGCCCACTCGCCAAGAGTCTTAGCACTAGGAACAGTTAAGAGGCCAACACCATGGTCAGCA

CAAAGGCCTTTGACAAGCTTAACATAGTCAGGCTGGTTACAGTCCTCAGCCAATACACAAAGCTGAGCAG

CATGCTTTTCAATCACTTTCGCGCCTTCATGAAGACCTCGAGCAAGACCAACGTGA

>FM890647.1 FM890647 Jatropha curcas embryo 56-70 (DAF) Jatropha curcas cDNA clone rjcaeb0_000554, mRNA sequence

ATTCGAGAAGTACAAGTCCGAAGATGAGGAGCACAAGAAAAAGGTGGAGGCAAAGAATGCTTTGGAGAAC

TATGCATACAACATGAGGAACACCATCAAGGATGAGAAGATCAGTTCCAAGTTGGCCGAAGCTGATAAGA

AGAAGATTGAAGATGCTATTGATCAGGCCATTCAGTGGCTAGACAGCAACCAGCTTGCAGAGGCAGATGA

GTTCGAGGACAAGATGAAAGAGCTTGAGAGCATCTGCAATCCCATCATTGCCAAGATGTATCAAGGTGCT

GGTGCTGACATGGGTGGCGGCATGGAAGATGATG

>FM890646.1 FM890646 Jatropha curcas embryo 56-70 (DAF) Jatropha curcas cDNA clone rjcaeb0_000553, mRNA sequence

CAGCTCAACAAGAAAGCAACACGATTTCATATCTACAGAGCTCAATCTACATATGAAAGTAAGTCTGAAT

CCAAATCAAGAAAGCAAAACATATATAGGTATTTGTACAGAATTTTAAACCATTGTTACGCTACAACTTG

ACCATATGCGACTAGTTTAAACGTTCTTCTATGTTTCAAAAGAAAAGATGTCAAAGAGAGAGAGAAAAAG

CCACGGAAGCATATCCTTTTGCACATTGTTGCGACCAAGATTTTATCATTTCTTGCGAGACAGTGCAGCA

GGCAATTCCACCAGCTATGAGTCCCCCAGCAGTAGCAGTGGATG

>FM890645.1 FM890645 Jatropha curcas embryo 56-70 (DAF) Jatropha curcas cDNA clone rjcaeb0_000552, mRNA sequence

ATTCTGGTTATTGGGTTGCCTCTACTTAAGCTCCTCAGTTCCATACTCAGTGTTGTCGTTTGTGTTCTAC

ACATTTATTTGATCGTGTATGGCTGGAATTCCAATATGCGTCCAATGCGGCACCACTAGCAACCCTTGCC

GGTGCAAGGTGGTTGGTCCAACTCTGGGGTTCTTGGCGTTTGCCGTGGCGGCGGTGGTAGAGTGGCCGGT

GGGAGCTTTTGTTTACCTCTTTAAACACAGGAAAGGTCGCAGGATCATGGCACATCCAGCGACTGTCGTT

TACCCATCAGTCACCAATTCAATTCCCGTTTAATTAATAGATTTGATGCTTTTGTTTTGTTTTCAGGTTC

TTTGTGTTCCTTTTATGTAATGGCCACTGTGTTTCTTATATATGCTATAATAAAATAATAGTGTGGCTTT

GTGTACGCTATTTAGTATAC

>FM890644.1 FM890644 Jatropha curcas embryo 56-70 (DAF) Jatropha curcas cDNA clone rjcaeb0_000551, mRNA sequence

AGACGTAACGATATATATTGAACACTGACTTTTTATACGTACATGAACACAAGTGGATTAATTAAACATA

AAGGACGATCAAAGTCCAAAGTCCAAAGTCCAAACCAAACCCAGATACATCCCCGCCAAAAGGGGATATG

TAAATGACACAAGATAATTCTTTTAGCATTACAGTTACATATATTATTACATTGTTGTTGCTTAAACCCG

AGTCCCTGTCACTGCTGTGCCACCCTTTGGAATTGGTTCCCCACCAGCTCCTACAGCGTATTCGGTGTCC

CTGTAACCAGGGCCATACCTGCTGCTAAATACCCCAGAATGAAGCAACAGGACATACAAGAGTTGGGTAA

ATGTCNAGATTATCATGAATGCCTCTAATACCCTCAGTCTCCATCCTCTGTGCCCTCCTAGGTTTATTTC

CTTGCATGCCAACCCAAAAGCTAGGGCAGTGATGGCCCAAGTCACAAGTGAA

>FM890643.1 FM890643 Jatropha curcas embryo 56-70 (DAF) Jatropha curcas cDNA clone rjcaeb0_000550, mRNA sequence

TTTTTTTTGACATATACAGAACTGGTTTTTTTATTGTTAAAAAACCTCACAAATACATTAACATATACCT

ATACATTGGAAGGAAACAGAAACCAGAGCGAGCAAACCACACATAATCTCATTTCATACACATCAACGAC

AAAGCAATTTATTGTTTTTATATCTTACAGAAGATTGAAACTCATGCTCATGCACCGCTAATCTCAATAG

ACTTGACTTGAGGCTTCTTCTCCTCTGCCTTTGGAATAGTAACAGTAAGTACTCCATTCTCCATTGATGC

CTTAACTTCAACCTTTGCATTTTCCGAGTAACCAAAACCTTCTCAAGAACTTACCTCTGCTTCTCTCAAC

GCGGTGCCATTTCTCGTTCTTCTCTTCCTTATCAACATTTCTCTCTCCGGTAATCTG

>FM890642.1 FM890642 Jatropha curcas embryo 56-70 (DAF) Jatropha curcas cDNA clone rjcaeb0_000548, mRNA sequence

ATTCCTCAAAACCTTCAAAAGAAATGAAGACTTTCACTTTCTTATTTGTTTTAGCTCTTTTACTTGTTTC

AGGATCATTATCTACTTCCACCCGACCCCAACCCCAACCCAATTCATCCAGAGGCAAACCTGCTACTGTC

CCTAGAACCAGAGGTGGTGGAAATGATAATCCTGGAATGGGCGGATACTTTGGGCCCGGAGCCGGGTTTG

GAATACCGGGATTTGGAAAAGGTTGGGGAAATGGGATTGTTGGTGGCGGGTATGGAGCTGGTTATGGAGG

TCCAAATGGAGGGTACTCAAAAGGTGGTATTATAAGGCCTACTGTGGTGTGCAAGGAGAGAGGTCCTTGT

TACAAGAAGAAGCTGACTTGTCCTGCAAAGTGTTTCACCTCCTACAGCCGGTCAGGGAAGGGATATGGAG

CTGGCGGTGGTGGGGGTGGCTGCACCATCGACTGTAAGAAAAAGTGTACTGCTTATTGTAAGATGATTGA

TTGTGGTAGCCGGTAGCCGGTAATAG

>FM890641.1 FM890641 Jatropha curcas embryo 56-70 (DAF) Jatropha curcas cDNA clone rjcaeb0_000547, mRNA sequence

GAAAATACTCTTTCCAACCCTTCAACTTCAAATCAAGTATCCAGCTAGAATGGCTTTAAGCTTAGGAAGG

AGGAGCAACATCTTTGATCCGTTCTCACTAGATATTTGGGACCCATTTGAGGGCTTACCTTTCTATAGCG

GAACAGTGGCCAATGTTCCGTCCACTCAACACGAAACAGCCGCCATTGCCAACGCACGAGTGGACTGGAG

GGAGACTCCGGAGGCTCACATATTTGCTGTTGATCTTCCTGGGTTGAAAAAAGAAGAGGTAAAAGTGGAG

GTAGAAGATGGCAGAGTTTTGCAGATAAGTGGGGAGAGAAGCAAAGAACAAGAAGAGAAGAACGATAAGT

GGCATCGCGTAGAAAGAAGCACTGGTAAGTTCTTGAGGAGGTTTAGGTTGCCGGAGAACACCATGATGGA

TCNACGTCAGGCCACTATGGAGAACGGTGTGCTTAAAGTGGTTGTGCCTCAAAAACAAGAGACGCAGCCT

CAAATTCAGCCTATTGAGATCACTGGCTCAAAATGCCAATATGGTCTTCTACTACTAATAGATCAAGCTT

TAACGCTGCCGCTTCTGGAGTAATGGGTATGACTAACATACAGT

>FM890640.1 FM890640 Jatropha curcas embryo 56-70 (DAF) Jatropha curcas cDNA clone rjcaeb0_000546, mRNA sequence

ATTCTTGAATAGCCAAGTGAGTGAAATGGAGTCGCTGAAGGAAACCGCAGCTAACGTTGGAGCCTCTGCT

AAGGCTGGCATGGAAAAAACCAGAGCAGAGGCGCAAGAGAAGGTGGAGAGAATGTCAACACGTGATCCAG

TGCAGAAGGAAATGGCGAGAGAGAAGAAAGAAGAGAGGAAAACGGAGGCAGAATTGCGGAAACTAGAAGC

GCGCGAGGATAACGCAGCGGCGAGGCAGGCAGCCAAGGCTGGTGGCCATGTACAGTACACAACTGCTGGT

CCGGGCGCTCAAGGCACAACCGAAACCTATACCCATTCAACAACTGGGACAACCGGTTACCCTACAGGGA

CCCAGCAAATGTCGGCCATGCCTGGACACGGCACAGGGCAGCCATACGGTGGAGAGGTGGACCCAACGGG

TTTGACCAGGACTCGCCCATCTGGGTTGCCCGGAGACACCACTGGCCATAACCCACGTGTGTAGGGCATG

GCATGTGCTTTAGCTCCTGTTATGTAATATATCT

>FM890639.1 FM890639 Jatropha curcas embryo 56-70 (DAF) Jatropha curcas cDNA clone rjcaeb0_000544, mRNA sequence

ATTCGATCTTTGATCTTCTCCAAAAACAATCAAGAGAATGTCTCTGATTCCAAGCACCCTCTTCGGTGGC

CGGAGAACCAACATTTTCGATCCCTTCTCTCTCGATATCTGGGATCCATTTCAGGACTTCCCTTTCACAA

GCACTGCTCTTTCTGCTCCTCGTTCTGAGTTATTGAACGAAACGGCAGCGTTTGCCAACACCCGCATAGA

CTGGAAAGAAACACCAGAAGCTCATGTATTCAAGGCTGATCTTCCAGGGCTAAAGAAAGAGGAAGTGAAA

GTGGAGATAGAGGAAGGTAGGGTCTTGCAGATAAGCGGAGAGAGAAGCAAAGAGAAAGAAGAAAAGAATG

ATAAGTGGCATAGAGTTGAAAGGTCAAGTGGTAAGTTCTTGAGAAGGTTTAGGCTACCAGAGAATGCAAA

GGTTAATGAGGTTAAGGCTAGCATGGAAAATGGAGTCTTGACTGTGACAGTGCCTAAAGTGGAAGTCAAG

AAACCGGACGTTAAGGCTATTGAGATTTCTGGTTGAAACTTTTAGTTATGTTTGAGTGTGTCTAAGTCTC

AATGGTACCCATCCATCCAATAAAATAAATAGATGTGTGTAATGGGTTTGTTGTTGTGAGCTTGTTCCTT

TATGG

>FM890638.1 FM890638 Jatropha curcas embryo 56-70 (DAF) Jatropha curcas cDNA clone rjcaeb0_000543, mRNA sequence

CAGACAACGCAAATATGGAATTATTATCAAATCTGCAAATCAGCCAACATGCCAAAAGCTCGACGAAAAA

TATATCTTGTTCTACCAAATATGTTCAAAAACCTATCAAACACACTACAAGTTCCAACTTCCAAGTTCCA

ACAACCTACTTAAGCCTAGAATAACCAAATATTGGATGGTCTAAATCTGCTTACCAAGAAATAAAATGGT

GTCTAGAGCTGCTTAACCATAAATATAACTAACTCTTGAAATTGCAAAACCACTTGATGGGCATGTCTTA

GTTCACCAAAAACATTCTTTAGGAAACCAGGCCATCATTCAGGGATAGTGCATGCAAGCTGATCGGCAGG

GTTGGCTGCCTGCTGCTGAACAGCAACATTTCTAAGGACTTCCATGGCCTCTGCAACTTTAGCTTTTAAA

GCCTCTGGTGATTCAAGCAGGATGTAAACTTCAGTTTGATCAATCTCCAGAAGCATGCCTGTAACCTTGG

CTGCTGAATCATGCTCCAGTTGATCCACCAATGGGTACAGACTTTC

>FM890637.1 FM890637 Jatropha curcas embryo 56-70 (DAF) Jatropha curcas cDNA clone rjcaeb0_000542, mRNA sequence

ATTCCGGAGGCAGAATTGCGGAAACTAGAAGCGCGCGAGGATAACGCAGCGGCGAGGCAGGCAGCCAAGG

CTGGTGGCCATGTACAGTACACAACTGCTGGTCCGGGCGCTCAAGGCACAACCGAAACCTATACCCATTC

AACAACTGGGACAACCGGTTACCCTACAGGGACCCAGCAAATGTCGGCCATGCCTGGACACGGCACAGGG

CAGCCATACGGTGGAGAGGTGGACCCAACGGGTTTGACCAGGACTCGCCCATCTGGGTTGCCCGGAGACA

CCACTGGCCATAACCCACGTGTGTAGGGCATGGCATGTGCTTTAGCTCCTGTTATGTAATATATCTATGT

TTTCTGTTTGGTGTCTTTTTCTTGTTAAGCAATTCCAGTTGAATAAAAGGTATCTTTTAGTAGATATTTT

ATTT

>FM890636.1 FM890636 Jatropha curcas embryo 56-70 (DAF) Jatropha curcas cDNA clone rjcaeb0_000541, mRNA sequence

ATTCAAACAGGTATATTTATGATCATTTATTTGTTGCCCACACATTTGAATGTTTGTTGTATTAACTGCC

GTATCAATACAAAACCTGAAAGGTAAGATAAATATCTAAACATAGGGTTACAGCTCTGGTAACTGAATTG

CGAAACTTTCTGAACTAATGTAAATGTTTGTTTCACTAATTTTTTGGACTGAACTGGATAACAAAATTAA

CTGACATTTTTTCAGTTAACCGGTGGTTTCAGTTCAGTCTGCCAGTTTATTGTCGTCATTTTTCAGCCAA

ATGACACCTTTTGAATTTTCTGTTCACCTCTTACTGTGTTAAAGCATGTCAGAGTCAAGCACAGCTAGCA

CTCAAATCATTCAGAGACAAAATTAAACCACAAATGTCCAACTAGGCTATAAATGAATGGAGGAGGTGAA

TGCATCTGAGGAAAAAAAAGGGGGGGCCCGGTAC

>FM890635.1 FM890635 Jatropha curcas embryo 56-70 (DAF) Jatropha curcas cDNA clone rjcaeb0_000540, mRNA sequence

CGAATTCCACCGTTGATATTGAAGAGTTTAGAACTGAAGTCAGGAAAATATTGCTTGCGATTGCTGATGG

GTTAGGATCCAGTCCCATCCAGATGGCTCTTGAAGATGATGATCAGAGTTTTCTCAAGCAGGCTGCCGAT

CTCGAAGCCTCTAAGCTTCAGTCTTCTGTTTCTTAACTAATTTAGATCCGTTTTCTATCTTGCCTGTCAA

ATCTTTTTATCTTTACATCTTATAAGGCTTTTGTTTGTTCCTACCTTCTTTATCTGTTTTTCAATATTTC

AATAAATTTTGGCAATTTAATCTCTTCTCAGAGCTTGATTAAAAAAAAAGGGGGGGCCCGGTAC

>FM890634.1 FM890634 Jatropha curcas embryo 56-70 (DAF) Jatropha curcas cDNA clone rjcaeb0_000539, mRNA sequence

ATTCATTCAAGCTATGAGTCATCGTGAAGAAGGTGGCGGCGTTTGGCCTTTCGGAACTGAATCCATCGGT

CCAATTAGTATCCTGAAGAACCCTGTTTTTAGGAATAACTATGGTCAACTCTTTGAAGTTGGCTCTAGAG

ATTTCAAGCAACAGCTCCGAGACCTTGACCTTTCAGTCTCCGTTGCCAACATCTCTCGTGGAGCAATGTC

TGGGCCCTATTACAACTCCAGAGTAACCAAGATATCAGTGGTTTTGGATGGCGAAGGCTACTTTGAAATG

GCTTGTCCTCACCTTAGCGGCTCGCGGGGAGACACGACGGGTGAAAGTCAGAAGACTTACCAGAAAGTAA

GTTCACGGTTGAAACGTGGAACAGCAGTGGTCGTCCCTGCAGGCCATCCGGCGGCT

>FM890633.1 FM890633 Jatropha curcas embryo 56-70 (DAF) Jatropha curcas cDNA clone rjcaeb0_000538, mRNA sequence

ATTCCAGAGCAAAAGATTAATTATATTGGTGGAGAAATTTGTTAGCAAAAAAGGGCAAAAATGTCTTCAA

ATCAATCAAGACAAGAGCTTGATGAAAGAGCAAGGCGTGGAGAGACTGTTGTCCCTGGAGGTACTGGGGG

CAAGAGCCTTGAAGCTCAAGAGCATCTTGCTGAAGGGAGAAGCAGAGGAGGACAGACAAGGAAGGAGCAG

CTGGGGACAGAAGGATATCAGGAGATGGGCCGGAAAGGTGGGCTGAGCACGACGGACGAGTCTGGTGGAG

AAAGAGCTGAGAAGGAGGGCATCACTATCGATGAGTCGAAGTTCAGGACTGGGAGCTAGTGTGATGATTA

ANAACTATAAATGGTATTTTGTAGTAATAATAATAATAATGTTGGTAATAAAGTATTAGCTTAGCAGTGT

ACTTTAGTTTAGTAATGTAGTTTACATGTGGGTT

>FM890632.1 FM890632 Jatropha curcas embryo 56-70 (DAF) Jatropha curcas cDNA clone rjcaeb0_000537, mRNA sequence

ATTCCAAAAGATAAGGCAAAGAAGACATCACAGCATCTCAAGGATAACGTGGCTGGTGCCGGTGGTGAAT

TGCCGGACGAAGAGCTCTGATTTGTTTCTTTGGCTGTTCTGTTGTCGTTTTGGCGTCTATTTTACATATC

TGGTGTCCTGTCTTGTATTTTGTTGGGCTTGAATGAAGTAGAAATGAAGTATAGTTGGGATGAGTGACGA

CAGAGCCCATGTGAGGCCTGTATGAGCCCATATGTATATTTTCTACATGGATGGATTAGTCAACGAAGAA

AATGTTCCTGTAAAAAAAAAAAAAAAA

>FM890631.1 FM890631 Jatropha curcas embryo 56-70 (DAF) Jatropha curcas cDNA clone rjcaeb0_000535, mRNA sequence

ATTCCTAAATCCTGATACTTGATAACACAGGCACACGGGCACGGTGGTTACCAAATAGAAAATGAATATT

AGTAAGAAACTGGAGATAATACTCCCATTTCTCCTTACAAGTAGGGATTCATTACAATATTAGTTCTTAA

AACCTTCAAATATCAAATCGGCGTTGAAAGATCAGTTCTCCTTTTTGAGTATCTAAAAATGTCTTCTTGC

TGTGGTGGAAATTGCGGCTGCGGCTCCGGCTGCAAGTGTGGCAGCGGTTGTGGCGGATGCAAGATGTATC

CTGACATGAGTTTCTCCGAGAAAAACACCGCCGAGACTCAGGTTCTCGGAGTTGCACCGGAGAAGGCACG

CTTTGTCGATGGAGGTGAGGTCGGCGTTGGAGCTGAGAACGGATGCAAGTGCGGTGACAACTGCACCTGC

AATCCTTGCAACTGTAAATGAGATCGGCGGCGATCAGT

>FM890630.1 FM890630 Jatropha curcas embryo 56-70 (DAF) Jatropha curcas cDNA clone rjcaeb0_000534, mRNA sequence

ATTCTTTTTTTTTAACATTGAAAATTTGTTTATTCAAATGCTTGATATAACAAACTGACAATACCACCTC

AGGAAACAGAAGATCAAACAATACAGAAAACACTGACATCCAAACGCTAGTAGATACATGAACAGAGGAA

GGTGACTCATTAAACCTCTCGTTAGCCACACCTCAGTAACTAAGCTTCTTAACCCTCATGCTCATAATTA

TCTTCCATGCCCTCCTCATACTCAACGTCCTCCTCAGCAGTTGCATCCTGGTATTGTTGGTACTCTGCCA

CCAAATCATTCATGTTGCTCTCTGCTTCAGTGAATTCCATTTCGTCCATCCCTTCCCCAGTATACCAGTG

CAAGAAGGCCTTGCGACGGAACATGGCTGTGAATTGCTCACTTACCCTCCTGAACATCTCCTGGATTGAT

GTGGAGTTCCCCACGAAGGTTGATGCCATCCTCAAACCCTTTGGAGGAATATCGCACACGCTGGACTTCA

CATTGTTAGGAATCCACTCCACAAAATAAGAGGAATTTTTGGGGGGGCCCGGTAC

>FM890629.1 FM890629 Jatropha curcas embryo 56-70 (DAF) Jatropha curcas cDNA clone rjcaeb0_000533, mRNA sequence

ATTCTTTTAGATTCCAGACACTGCATTTTCGACAAGATTTAAGTACACGATCATCATAGGAAGGGGCGAG

CAAAACAAAAGGAAAAAACCAGGATGAGTAACGAAACATACAAATAGAACAGTCTACATAATTACAGCCT

CCGGTGTGACCGAGGTCACTAGAGTAATTTGGAAACGGCTCATATCCCAACTACCTTTTACCTTTGGCGG

CGAGTCACCGTGTTACCAGATCGCCCTGGTTTGGGAACTTTGGGATTCTTCAACAAAGCCAAATATTCAT

TTTCAATGACGGGAGTATCATCTGAATCAACCGATTTCGAGGGTTTCTGAGCCACATGTGTACGACCAGC

CTTTGAAGAACACCAATGCCACAGGCCGACCAGCACTGCGATGCTCACGGCGATCACCAGCATAACGAGG

AANCACATCTGTGTGTCTATGCCTTCTTCCACTTCATACAGATTTATAGTCTGGTTGAAAAGACGATGTG

CGTAGTACACCCCATTTTCGTCATGATAATGAGCCACGATGGACAGATCGAACGGTCTTCCCACAAGTTC

AAGAGCTGGCCGAAATTGTAGTA

>FM890628.1 FM890628 Jatropha curcas embryo 56-70 (DAF) Jatropha curcas cDNA clone rjcaeb0_000532, mRNA sequence

ATTTTTAGAGGAAAATACTTAATAGACTTTATAATATTTCTAGTAAACATCAGCCTCATCAGAAACTAAT

GTCAGACGAAAACACCCAAAAAGAAAAACACAAGCAGATTACGATTTCAACATAGCCCAAATTATTTCCA

GCACGCAGTCAAACGTGACAAAACGATCCTCTGAGCTCAAGCAATTTTAACCATCAATAGTCTTTGGCTT

TTTAGGCTCA

>FM890627.1 FM890627 Jatropha curcas embryo 56-70 (DAF) Jatropha curcas cDNA clone rjcaeb0_000529, mRNA sequence

ATTCGCAGGAAACTTTTGTGGAGGAAGAAGAAAGAAAGAAAGAAAGAAAAGGGAGATCTATCAAATTCGT

AACAGAGAAGAAGCGAAAAATGGCGTGGATATGGTTAGAGGCAGCTCTACCACTTGGAATCATAGCAGGA

ATGCTTTGTGTTATGGGTAATGCTCAATATTACATCCACAAAGCCGCTCATGGCCGGCCAAAGCACATCG

GAAATGATATGTGGGATGTGGCCATGGAAAGAAGGGACAAGAAGCTCGTGGAGAATCTCTCTGCCCCTTC

TACTAATTAGGGAAGCTTTCTGAACAACTCCTGAAGAAGGAATAAAGTGCTCCTCTGATACTGCATTCAT

GTTAAAGAAGTCTGATGAGCAAAATTACTTTGTATGTGAATTTGCATGAACTTTTGAAGAAAATGTAGAT

TGTGCTTGGTGATT

>FM890626.1 FM890626 Jatropha curcas embryo 56-70 (DAF) Jatropha curcas cDNA clone rjcaeb0_000528, mRNA sequence

ATCCAAATATCACTGATCGTAAACCTCCATTGAATTACTCTGTGCTTCGATCGCGTCAATGGCGGAGCAC

CCACAGTCGCAGCATGTTGGCCAACAGCCACGATCCC

>FM890625.1 FM890625 Jatropha curcas embryo 56-70 (DAF) Jatropha curcas cDNA clone rjcaeb0_000527, mRNA sequence

ATTCTCATCTCGTCTCGTCTCGTCTGGTCTATTCTCGTCTCCTATTCCCAGTAGAATACACCTTAGTACC

TGCAAAACTTAAACCTAAATCACTTTCTTTTTTCCCTTTCTATTGTTATTCAATTTCCAATATTCAAACC

CTAACAGATCGGTTCTTCATCGTCCGTACTCCGAATTAATCAATCATGCCTTCCCGTCGAAGAACTCTCT

TGAAGGTCATAATCCTCGGCGATAGCGGGGTCGGGAAGACCTCTTTGATGAATCAATATGTGAATAAGAA

GTTTAGCAATCAATATAAGGCAACAATCGGAGCTGATTTCTTGACTAAGGAAGTGCAGTTTGAAGATAGG

CTCTTCACTTTACAGATCTGGGATACTGCTGGCCAGGAAAGATTCCAAAGTCTTGGCGTTGCTTTCTATC

GTGGTGCTGATTGTTGTGTTCTTGTATATGATG

>FM890624.1 FM890624 Jatropha curcas embryo 56-70 (DAF) Jatropha curcas cDNA clone rjcaeb0_000526, mRNA sequence

ATTCGGGAATACTGGATTTGCAGCTCCAACAGGGGCATATGGCATGCCTGGTAACCTGAAAAATAGTTGG

GGTAGCCAAGTTCCTTCTAATTATGCTGCTTCAGGTTATGGTGCAAATGCAAGTTATGGGGCCACAACTC

CATGGAGTGCATCTGGTGGTGGCAATTCTGCTTCAGCTAATATGGGCCAATCCCCAGGTGGAACTCCTGG

GTATGGTGGCTATGGTTATGGCAATTATGGTGGAAATGATGGTCCTTATTCTGCTGGATATGGAACAGCC

GGTGGGCGCACTGGAAATGCCCAAAATAACAGTGCTGGCACCGGAGCTGCTGGGGGAGAACAACAGGGGA

GTGGAGGTGGCTACCCAAGTGGTGGCTATGGTG

>FM890623.1 FM890623 Jatropha curcas embryo 56-70 (DAF) Jatropha curcas cDNA clone rjcaeb0_000525, mRNA sequence

AGGATCAAGAAATAAAATTTATTTCAGTAAACAGAAACATACAGCCAACCAAAGCTATGGGCAAACAAAC

AAGTCTTATTACATATTTATGAAATCCATCATCAAACTCAAACTATATTAGCTTATATTATTTACCAAAC

AACAACTACTGCTAGACAACCTGATCATGTCTTTGCAACTTCCTGGGCCTTCTGGCCCACCTCCCTAGCC

TTCTGGCCCAATTGACCCGTCGTTTCTTGCGCGCGCCGCTTTGCCATTTCCATCTGCATCGTCCAAGATC

CTCTCATCCTGCGAATATAATTAACCATCCACGACAGTGAAGAAAGCGCAGTGATTCCAAAGCACGGACG

TCAAAAACCTAACACG

>FM890622.1 FM890622 Jatropha curcas embryo 56-70 (DAF) Jatropha curcas cDNA clone rjcaeb0_000524, mRNA sequence

ATTCCCTCTCCCTCTCTCTTTCTCTCTTCACTCGCAGATTGAGTTGCATCAATGGCCTTTGTCAAGGCTC

AGAAGAGCAAGGCTTACTTTAAGCGGTTTCAGGTCAAGTTTAAGAGAAGAAGAGCGGGAAAGACTGACTA

TAGAGCTAGGATTCGCCTGATCAATCAGGACAAGAACAAGTACAACACTCCTAAATATCGCTTTGTTGTG

CGATTTTCCAACAAGGACATTGTTGCACAAATCGTATCAGCTAGTATTGCTGGCGATTTGGTCCTAGCTT

CTGCCTATTCTCATGAGCTTCCTCATTATGGACTTGAAGTGGGTCTTACAAACTATGCAGCAGCTTATTG

CACTGGACTTCTTTTGGCCCGTCGTACCTTGAAAATGCTTGAAATGGATGATGAATATCAGGGCAATGTG

GAGGCCACTGGAGAAGACTTCTCAGTTGAACCAACAGACAACAGGAGGCCATTCCGTGCTTTACTT

>FM890621.1 FM890621 Jatropha curcas embryo 56-70 (DAF) Jatropha curcas cDNA clone rjcaeb0_000523, mRNA sequence

ATTCCGTACTTGCTGCTCTCTCTACTTCTTCTCTTTGTGGTTCGAATCCGGGAGAAACTCAGAATCATGT

TGGTCTACCAGGATTTGCTTACCGGTGATGAGCTTCTCTCGGATTCGTTCCCTTACAAGGAAATTGAGAA

TGGCATGCTGTGGGAAGTTGAGGGAAAGTGGGTTGTTCAAGGAGCCGTTGATGTGAACATTGGTGCAAAT

CCCTCTGCTGAAGGGGCGGATG

>FM890620.1 FM890620 Jatropha curcas embryo 56-70 (DAF) Jatropha curcas cDNA clone rjcaeb0_000522, mRNA sequence

ATTCCATCACTATCGATGAGTCGAAGTTCAGGACTGGGAGCTAGTGTGATGATTAAAACTATAAATGGTA

TTTTGTAGTAATAATAATAATAATGTTGGTAATAAAGTATTAGCTTAGCAGTGTACTTTAATTTAGTAAT

GTAGTTTACATGTGGGTTAAAATGACTAGGGTTTAGCTAGTATGTGTGTTGTAACTTTGAGAGGGGTTTA

TGGTCGTTCCCTTTCTGGGAGGACTGCTAATACTTTCTCCCTATTTGTAAAATAACCTGCAGTATATATA

ATTACCTGTTGGTT

>FM890619.1 FM890619 Jatropha curcas embryo 56-70 (DAF) Jatropha curcas cDNA clone rjcaeb0_000521, mRNA sequence

ATTCAAACCAAGAAGGAAGAATGCAAGGAAGTACACAGGCGAAGGTCAAGGGTGCTCAAAAGAATATATC

GCCGCCATTAGAATGGTACAACGTACCAGAAGGAACAAAGAGCCTGGCTTTGGTGGTGCAAGACATAGAT

GCACCTGACCCAGATGGTCCCATTGTTCCTTGGACCCATTGGGTTGTGGTTAATATACCACCTACGTTGA

ATAGAATACCAGAAGGGTTTTCAGGGAAAGAAGAGGAAATAGGTGGTGAATATGCTGGAATCAAAGAAGG

AAATAATGACTGGAAAGTTCCAGGTTGGCGCGGCCCCAAGCTTCCTAATCATGGACATAGGTTTGAGTTC

AAGCTTTTTGCCTTGGATGAGGAATTGAATCTGGGAAATAAGGTAACAAAAGAGAAGCTGTTGGAA

>FM890618.1 FM890618 Jatropha curcas embryo 56-70 (DAF) Jatropha curcas cDNA clone rjcaeb0_000520, mRNA sequence

CGAATCCATATTCCTGTGATGTTGGCTATGAACGTTTTCTTGGACCTGAGGTTTTCTTTAATCCAGAGAT

ATATAGCAGTGACTTCACCACTCCATTGCCAGCTGTAATAGACAAGTGTATTCAATCTGCGCCAATTGAT

ACAAGGAGGGCATTATATAAGAATATAGTGTTGTCTGGGGGATCAACCATGTTCAAAGACTTTGGCAGAA

GGTTGCAACGGGATCTGAAGAAGATTGTGGATTCACGGGTTCTTGCATCTGAAGCTAGACTGGATGGGGA

AGTGAAATTACAACCAGTTGAAGTAAATGTGGT

>FM890617.1 FM890617 Jatropha curcas embryo 56-70 (DAF) Jatropha curcas cDNA clone rjcaeb0_000519, mRNA sequence

GAGTGGAAAGGCCGATGGCGAGTTGTTATATTACATAACCCATAAGTTAGATTTACAGTAAATTGATTTT

GCAATAAAGGAGGATTCCTGGAAAACTACAACTCTTGTTGCACATGTTCATTGTCCTTGGAGGGAGTAGT

AACATTACCCTTCACCTTCTCACTTCGAAGCTGCTCAATAACTTCTTCCAAAGGAGGGTCTCCAGGCCTA

CGGTAGACTTTATCCCCGATCTTTATCTCATAAGATTCAGGTTCATTGAATATGAATTCCTTCAACTCCT

TCGTATGTCGGGCTTTTTCCAGGTTGAACATGATTGTGCTGCTATCGACACCTGTGAACTTCACCCCAAT

TGCACCAGTTCTTAGAACTTTGGTCCATTTCATAGC

>FM890616.1 FM890616 Jatropha curcas embryo 56-70 (DAF) Jatropha curcas cDNA clone rjcaeb0_000518, mRNA sequence

ATTAGAACTTATCAAACAATACTGCTGGTTTCTTATATCCTTCAAATCAATTAGGTCAAAAAACTTCAGT

GCTCAGATTCAAGCAGGTCACAACTGCGTCGAGTAATCCTGAGAATCCACACGACATGGATAAGGCTCAG

AGCCCAAATGAGAAATCCAAAGAAGCAATAGAACATGGGGATGTAATGTCCCATTCATTTGGACAAGCGT

ATGCCACGAGATCTGATGAAGAAGGATTTGGTGGAATTTATGGAGGAAATCAATCATGTGATAACGATGA

TGATAATAATGGCAAGAAAATCCATGAGAATCACCCCGATTATGACAGGAGCC

>FM890615.1 FM890615 Jatropha curcas embryo 56-70 (DAF) Jatropha curcas cDNA clone rjcaeb0_000517, mRNA sequence

ATTCGATATGGTGATTGAGGCCGTCATTGAGAATATTTCTTTGAAGCAACAAATTTTTTCTGATCTTGAA

AAATATTGCCCACCGAATTGCATACTTGCAAGCAACACCTCAACAATTGATTTGAACTTGATAGGCCAGA

ACACCAGGTCTCAGGATCGGATCATTGGAGCCCATTTCTTTAGTCCGGCTCATGTTATGCCACTTTTGGA

GATTGTCCGTACCAATCAAACATCTCCTCAAGTAATTGTGGACTTACTAGATGTTGGAAAGAAGATAAGG

AAGACTCCCGTTGTGGTTGGAAATTGCACAGGATTTGCTGTCAACAGGATGTTTTTTCCTTACACACAAG

CTGGTCTTTTGCTAGTTGAACGTGGGACGGACCTTTATCGGATTGATAGGGCAATCACCAAATTTGGAAT

GCCAATGGG

>FM890614.1 FM890614 Jatropha curcas embryo 56-70 (DAF) Jatropha curcas cDNA clone rjcaeb0_000516, mRNA sequence

ATTCCATATTTCTAGCTTTATGTGTCCTTTCCATCTCTGTACTGTTATATTTGGTGCTTCATGCCTTATT

TTGGTTAATTATTGACTAGACATGTTTTCCTCCTTCTTTGGTTATTAGTTTCTTGTTTGACAAATAATTC

AATCTTCTATTGGGCGTGTATTCTAAAATTTACCAGCCTAAGTTGGTAATTGTTTGATGTTACAACTTCT

TTGTTCCCTTTGTATTTGTGGAAGGCTTGTCAAAATAGATATGTATTACTGGCTCT

>FM890613.1 FM890613 Jatropha curcas embryo 56-70 (DAF) Jatropha curcas cDNA clone rjcaeb0_000514, mRNA sequence

ATTCAACATCTCTCGTGGAGCAATGTCTGGGCCCTATTACAACTCCAGAGTAACCAAGATATCAGTGGTT

TTGGATTGGCGAAGGCTACTTTGAAATGGCTTGTCCTCACCTTAGCGGCTCGCGGGGAGACACGACGGGT

GAAAGTCAGAAGACTTACCAGAAAGTAAGTTCACGGTTGAAACGTGGAACAGCAGTGGTCGTCCCTGCAG

GCCATCCGGCGGCTTTTGTAGCTTCAAGAAACAATAATCTGGAAGTAGTCTGCTTTGAGATTAATGAAGA

AGGCAATACTAGGCATCTACTTGCAGGGAAAAATAATATTGTAAATAAGATGGAGAAGCAAGCCAAAGAG

TTAGCCTTCGGTGTAAGTGAAAGAGAAGTGGATCAGGCTTTTGGAAGACAAAACGAGGAGTGGTTCTTTC

CAGGGCCAACGAGAGGTTCCTTCGAGGGTCATGCTGTGGCATGAGAAGCTAATAACGTGATGGGCAGTGT

TTCGGCTTTTTGGGGTGACTTGTGACATCGACCTTGAGGGTTTACTAATGTAAATAGTTGCTGTGTATGG

GTTGAATAAATAATG

>FM890612.1 FM890612 Jatropha curcas embryo 56-70 (DAF) Jatropha curcas cDNA clone rjcaeb0_000513, mRNA sequence

TGCAGGAATTCGAATTCTTTTTTTTTGACTAGTAAACAATATGACAATAATTTATAGAAAGGTAAAAAGG

GTATATAGTACAAAAACCCATCAAGCAAAACTTTCCAAGAGGACCCAAAAAATAAAAATAAAAAGTAAAT

AAGAACTAGACAATCCCTTCAGATTGCAATTGAGAAGGCTGATATGAGATAAACTGTCAGATGTCTGAAA

ATATTAAATTAAGACACTAAACAGGATGGAAAACAACACGTTAAATAGTACTTGCAAAATAAGAAACCCT

TTCTCAACAAGCACCAAAAATTCGCATCACAGAAGATCAGCAACATTCGACGGCAGTTCCTCAATAACCA

CGTTGTAGAACTTCTGAATGTCAAACAGCATTCTCTCATCATCCCTTGTCACGAAGTTAATGGCAACACC

CTTTCTTCCAAACCGTCCACCTCTTCCGATTCGATGAAGGTAGTTTTCAGGTTGAGTCGGCAGATCATAA

TTTATCACAAGGGAGACTTGCTGCACATCAATACCGCGGGCCAAGAGATCAGTGGTGATGAGAACTCGAG

AAGAGCCAGAACGGAATT

>FM890611.1 FM890611 Jatropha curcas embryo 56-70 (DAF) Jatropha curcas cDNA clone rjcaeb0_000512, mRNA sequence

ATTCCATTAACAATGGCCATAACTAACTCTTTCCTTCTTTCACTGTGTCTAATTGTTATCTTCACTGGCT

CTATAGCAAGTCAGCAACTTCAGCAGCAAAACCAGTGTCAACTCGATAGGCTCGACGCCCTCGAGCCAGA

TAATAGTATTCAAAGCGAAGCTGGTGTGGTCGAGTCTTGGAACCCTAACCAAGACCAGTTCCAATGTGCT

GGTGTGGCTATTGTTAGGCTCACCATTCAACCCAAGGGCCTTCTGTTGCCATCTTATAGCAACGCTCCTC

AACTCGTTTACATTGTCCAAGGTACGGGTATGACAGGAGCCCTGTTTCCTGGATGCGCTGAAACATATCA

GGAGTCTCAAGGATCAGGTGGTAGCAGTAGGGTTGAAGCCCAGCACCAGAAGATTCGTCGTTTCCGTCCA

GGTGATATCATTGCTTTGCCTGCCGGAGTTGCCCATTGGTGTTACAACGACGGCAATGAGCCTGTTGTCG

CCGTTTCTGTTCTTGACACCGCCAACAGCGCCAACCAGCTTGACATGAACCCTAGACATTTCAATCTAGC

TGGTAACCCAGAAGATGAATTCCCGCAACTGCATGA

>FM890610.1 FM890610 Jatropha curcas embryo 56-70 (DAF) Jatropha curcas cDNA clone rjcaeb0_000510, mRNA sequence

ATTCAAACATCAAATAAGCATTCCAATTTTCATCATAGTGATGCAAACACAGAAAACTACTACTAGTTAC

TCATTTACATGTACTTGTTACCTTTACATAGGCTTTCGATCGATAGCTTAGACTTTGCACGCAACCAACA

AAAAAATACAAACAAAAAGCACCAAAACGCAACATACATCCAAATTTCGCAAAACGCTCACCCCTAGTAC

TGTATTTTAGTCATACCCATAACTCCAGAAGCTGCAGCGTTAAAAGCTTTATCTATTAGTAGTAAAAGAC

CATATTTGCACTTCTAGCCAGTGATCTCAGGGGGGGCCCGGTAC

>FM890609.1 FM890609 Jatropha curcas embryo 56-70 (DAF) Jatropha curcas cDNA clone rjcaeb0_000509, mRNA sequence

ATTCATCANGCTGGTTATCATTAAGAAGGCCTTTCCGCTTATCACAAAGTGGCCTTCAAAGTACAAGTGT

AAAGCTTTATATCATAATGTTCCACAGCAAAGCCCATGACAGATCTGTAGCTTTATTATAACAAATCTAT

AACTCTATCATATAGTCCTATAACAGATATTTCACATCAGGAACACTAAAATTTCTAAAATCAGT

>FM890608.1 FM890608 Jatropha curcas embryo 56-70 (DAF) Jatropha curcas cDNA clone rjcaeb0_000508, mRNA sequence

AAACCATTGGGACTCACCCCCCAAAATCAATATGAACAACATTTTTCCCAGTTCCCATTACACATGAACA

ACACTAAAGCTACTGGCACAGAGACAACTTTCTCTTTTGGGGTTATTCCATGCCGACAAGCAGTAACTAC

ATTAAACACCTGATGCTAATTCACATAAAAAAAATCAAAGATGATGAGTCATTTGCCGGAATTGCAAATA

ACAGCACGCAATCTTAAGAAAGTTTTGCAGTTCAATTTTAGTTCACGAGCATGTGATCAAAACCCCCACC

AGCCTGAAGCCCTGGAACCCGATTCACTCCTACATTGTGAAGCAACTGCTGCAGCCATCGCCTAGTTGTT

GGATACGAAGGCAACTACTGAAGGTGGCATCTCGTAGCATGTCTGGCAGGCAACTTCTTAATGCGTCACA

AGCCCTAGGATTATCTTGACAGTCGAAGATAACATCGAATAATATGTT

>FM890607.1 FM890607 Jatropha curcas embryo 56-70 (DAF) Jatropha curcas cDNA clone rjcaeb0_000507, mRNA sequence

GCCGGCGCCCCTGTCTATCTTGCGGCCGTTCTCGAGTATCTTGCCGCTGAGGTACTTGAATTAGCTGGAA

ATGCAGCCCGTGACAACAAGAAGACTCGAATTGTTCCACGACACATCCAATTAGCAGTGAGGAATGACGA

GGAACTAAGCAAGCTTCTTGGTGATGTTACTATTGCTAATGGAGGTGTGATGCCAAATATCCACAACCTT

CTTCTGCCAAAGAAGACTGGTACCTCAGGCTCGAAGGCCTCTGCTGATGATGATTCTTAAATTCAACAAG

AAAGGGTTCAAAAGGAAATATTCTTAGAAAGTCTTTGCGTTAATGTGATACCACTATGTTTGTTCAGTAG

ATTTTTATGTTAGGAGTTTGGTTCTTTTAATGCTTGTGAAGTAGTTTCTGTTACAGAGTTAGGTTTTTCA

GTTAGGCGATTGTGAATATAATCGATTATCTTGTAGTTGCTTAAATAGGAATGATTTGTGGAAAGATTT

>FM890606.1 FM890606 Jatropha curcas embryo 56-70 (DAF) Jatropha curcas cDNA clone rjcaeb0_000505, mRNA sequence

ATTCGCAAATTGATTGATGGTCGGATGGCTGTCTGTAATCTAGCATGTGAAGGGTTAAGTGGGGTGAGTA

CTACGCCTGATTTAGCCCAGAGAAAACTCTATATTGGAAGTTTGTCACCAGAGATCACAAGTGAATTGCT

GCTCAACTTTTTTGGAAGGCATGGTGAGATTGAAGAAGGTTCAGTTGCTTATGACAAAGATACCAGGTGA

ATCGCGTGGGTTTGGTTTTGTTACATACAAGACAGTGGAGGCTGCAAAGAAGGCCATAGATGATCCACAG

AAGACACTTGGGGGGAGAACTATTGTTGTGAAGCTTGCTGATACTCACAAAGGCAAAACAGCACAAACAC

AATTGCCTCCACCAGTGGTACCAGTACCCTTGCCAATGGCAGCTGGATACACTCAACCTGTAAAAGCACA

TCCTGGCCCTGCCCCTGTTGGCTACTCTTATCCTCAAACTATGACATCATATCCTGCTTCCTCTTACCCA

AGTCCTCCCA

>FM890605.1 FM890605 Jatropha curcas embryo 56-70 (DAF) Jatropha curcas cDNA clone rjcaeb0_000504, mRNA sequence

ATTCCGACGTCTTCTCTAAAACCCTAGCCTTCTCCCTTTCTCTCTGTCATCCTCTATGGAATTCTCGGGG

TGTTGAAGTCAAGGCTGGAGAGCCTCTCAAAGTAAAACCCGAATCTGATAGTATATTACATCTTTCTCAG

GCGGCTCTAGGTGAGTCAAAGAAAGAAAAAGGAAATGAATCAGTTCCCCTATTTGTGAAGGTTGATGACA

AGAAGCTTGTACTGGGTACTCTTTCTACCGAAAACATACCTCAGATTTCGCTTGATTTGGTCTTCGAGAA

AGAGGTGGAACTATCTCACAACTGGAAGAATGGCAGCGTTTACTTCTGTGGTTATCAAACCCTTATGCCA

GATGAAGAAGATATGTATTCTGATGAGGACTCAGAAGAGGACAAGCCACTTCCATTGGTGAATGCAGACA

ATGGAAAGCCACATGTAGAGCCTGTAAAGGCTATTGCTGGCAAGCCTAAGCCTGATTCTCCCGCAAAAAC

AAAAGCCTAAGCCTGCAGAGCCAAGCAAAGGACAGAAAGCAGAGGATGAATCTGATGAAGATGAATCAGA

TGATGATGATTC

>FM890604.1 FM890604 Jatropha curcas embryo 56-70 (DAF) Jatropha curcas cDNA clone rjcaeb0_000503, mRNA sequence

TGGAAACACACTAAACCAAGAAACTTTCTCTCACCGATCTTTGATCTTCTCCAAAAACCAATCAAGAGAA

TGTCTCTGATTCCAAGCACCCTCTTCGGTGGCCGGAGAACCAACATTTTCGATCCCTTCTCTCTCGATAT

CTGGGATCCATTTCAGGACTTCCCTTTCACAAGCACTGCTCTTTCTGCTCCTCGTTCTGAGTTATTGAAC

GAAACGGCAGCGTTTGCCAACACCCGCATAGACTGGAAAGAAACACCAGAAGCTCATGTATTCAAGGCTG

ATCTTCCAGGGCTAAAGAAAGAGGAAGTGAAAGTGGAGATAGAGGAAGGTAGGGTCTTGCAGATAAGCGG

AGAGAGAAGCAAAGAGAAAGAAGAAAAGAATGATAAGTGGCATAGAGTTGAAAGGTCAAGTGGTAAGTTC

TTGAGAA

>FM890603.1 FM890603 Jatropha curcas embryo 56-70 (DAF) Jatropha curcas cDNA clone rjcaeb0_000501, mRNA sequence

ATTCGGAAGAGGTAACTTTGCAGAACGTTTTTGATGCTCTTGACAAGGTGTCAAAGGGGTATTGATGGTT

GGCAAAATACGATATCCAATTTCTATTAAGTGAAAGGTATTATCATTAAATGCTTTAGTATTACCATGTA

ATTACCGAAGCTGTAATGCAGAATTAATGCTTTACGGGATCTTAAAGAACATATTGGAACATTTTTTAGA

ATTCTGCTGTTGGAGCCTGAAGAATATCAGTATCCAATGGAATGGTTTAGCTGCCCTGTAAATATGTACC

GTTTGAACCTCCCAACCAGACTGTAAAGGACCTTTAATTGAATTAACAAGGGATAAACAGCGGCGGGGGG

GCCCGGTAC

>FM890602.1 FM890602 Jatropha curcas embryo 56-70 (DAF) Jatropha curcas cDNA clone rjcaeb0_000496, mRNA sequence

AGCAACATTGTGTTCTCCATGCAGTTCTCAATGATTTTCTTATAACCCTCAAAGCCAAGCCTTATAAACT

GATAATATTGTGCAATTATTTGACTAGAGCCTTTTGAGAAGTTGAGTGTGAAAGTGGGTTGATCAGAGCC

AAGGTAGTTAATGTGAAAGATAAGCTCCTCTGGCAAGTCTTCCTTAGTCCTCCAAACAGCCCAACCAACA

CCAGCATAAACGAGGCCATACTTGTGACCGCTGACATTAATGCTCTTCACCAATGGCAGACGAAAATCCC

ATTCAAGATCTGGGTAAAGGAATGGAGCAATAAACCCTCCACTGGCTGCATCCACGTGAATGGGGGTGTC

CCAACCAGTCTCCTCATTCTTTTTGATCAGAAGTTCATTAAGAAGCTTCACATCTTCGAACTCCCCAGTA

AGTGTTGATCCCAAAATGGCTGCAACGCAAATTGTATTCTCATCCAACATCTCAACAGCT

>FM890601.1 FM890601 Jatropha curcas embryo 56-70 (DAF) Jatropha curcas cDNA clone rjcaeb0_000495, mRNA sequence

ATTCCTGAAGAACAAGTTTGGGTTCGATGATGCTTTCAATTACAAGGAAGAGCCAGATTTAGATGCAGCT

CTTAAAAGGTATTTTCCTAGAAGGCATAGATATTTACTTCGAAAATGTTGGTGGGAAAATGCTTGATGCA

GTGCTCCTCAACATGAGGATTCGTGGTCGAATTGCTGTTTGTGGAATGATCTCTCAGTACAATCTTGAAA

ATCCCGAAGGTGTACACAACTTAGCATCTATTATTGCGAAACGAATTCGCATGGAAGGATTCCTGGCTGG

TGATTTTTACCACCTTTATCCCAAATTTCTAGAAATGGTTATCCCTTACATCAAACAAGGCAAGATTGTA

TATGTAGAAGATGTAGCTGAAGGACTTGAGAGTGGACCTACATCTCTAATAGGACTATTCACTGGCCGGA

ATGTTGGAAAACAGTTGGTTGTTGTTGCTCGTGAATGAAAATCATATGATTATAGTCTACTTTTTAGTCT

TTAAA

>FM890600.1 FM890600 Jatropha curcas embryo 56-70 (DAF) Jatropha curcas cDNA clone rjcaeb0_000494, mRNA sequence

ATTGTTTGAATCAACTAGCTATAATATAACCATTAAATTATCCAAACAACAAAACTAGGCAATGGGTGTA

CTAAGAGAAAAACAACAAAAATCCATCACATTTTTAGTCCTCAAATAGCAAAATCCATAATTCAAAACAC

TTTGGAAAATAACAGAAACATAAAAAAGCATAATACCGAATTCAACGGTAACTCTTCTGGAACCTGGCGC

GAGCACCACGACCACCGAACTTCTTTGGCTCGCATCGCCTGGGATCAGCTACAAGCAAAGTCCTATCATA

CCTGACCAAAATATCTTTGATTTCCTTCTTGCTCTGCTCGTCAACGTACTTCTGATAGAAGGCGACAAGA

GCTTTAGCGATACTTTGACGGATAGCATAGATCTGCGAGGTGTGACCACCACCTTTGACGCGAATGCGCA

TGTCGACACCGGCAAAACGATGTCGTCCAAGAAGAAGGATTGGCTCGTAAGCC

>FM890599.1 FM890599 Jatropha curcas embryo 56-70 (DAF) Jatropha curcas cDNA clone rjcaeb0_000493, mRNA sequence

GTTTTTGAAAAATCCAAGAGCATACTTTATATTATCATAAAATATCCAGAATAATTCAAAACCAAAGCAA

AATCCCTAGAACAATTGTTTAAAGACCTAGACATATAAAGCAACAATTTAAATAGACAAAATATTGATCA

ACATAGCCGTCCTCATGTAATAGGAAATAACTAGAGTATCCAAGCTGCAGCAAAACATTTCACTTAAGAG

GAATGAACCTAGACGAGTGGGTGGCACCAATACCAGGTCTACCATGTTTCACAGGCTTGTATGAGATTGA

GAACTCAGCAAGATAATGCCCGATCATCTCAGGCTTGATTTCAACTTGGTTAAAGGTCTTCCCATTGTAC

ACACCAATAATGCTTCCAATCATTTCGGGAACAATAATCATATTGCGGAGGTGGGTTCGAACTGGTTCTG

GTTTCTCACCAGGTGGTGCTTCCCTTTTCGCCTTGCGCAGCTTCTTGATAAGAGCCATTGGCTTCCTCTT

CAGACCTCTCTGAAACCTTCTGCGAGCACGGGCAGGAAAGAGTTTGACAAGCTCATCAGTGGTCATG

>FM890598.1 FM890598 Jatropha curcas embryo 56-70 (DAF) Jatropha curcas cDNA clone rjcaeb0_000492, mRNA sequence

AGATAACAAAATTGTACAGCCAACTTATTTATCGAAAGACAGACAAGCCTGAGTATGACTCCAATCGACA

CCGCATCCCTCAACTGCCCAAAAATTGTAAAGCACTTCAAGGAAATGGTGGAATCGGAAATTATGAACAT

AAAAAAAAAGACCACCTTATTGCCCTTTCCTCGGCCATTAAAGAGGAGGGAAAAAATCATATTGTGTCTC

CCTATGACCTATGACCTAAAGCCTTATAATGTATACCCTGCAAATGAGTCTAAGCAGATTTTGTGACCTT

ATGGATAGCGTTCGCCAAATATCCCACATTCCCCGTAGTAATGCCAGCCATACTGATTCGACCATTACGG

GTCATATAAATATGAAATTCTTTTGTNCAACGATCCACTTGTACGGGTGTCATACCACTATAGCAAAACA

TGCCGATCTGATTGGTGATGTGCTTCCATGAACAAGGTGACCCCACCTGTTCAAGGGTTTCTCGTAGAGC

CATTCGCATGCCAATGATACGATCCAGCCATTGTC

>FM890597.1 FM890597 Jatropha curcas embryo 56-70 (DAF) Jatropha curcas cDNA clone rjcaeb0_000491, mRNA sequence

GGCTTCCATAAAGTACAGTAGCATTCAGTAACGAATACATATTAATTAAACACGTATTTTACGTAATTAA

GACCATTTTTAGAACATGTATATAGCGGATTCTACTTCCAAAAACACCAAAACCAAAAAACAAGCAACCA

CCTATACATCCTGATATCCACACTGACACTATCCATATTGGTTTTTTACTTTATAAACACAAAATCTGCA

TATATATGATCATGGAAAAGGTGCCCTCAATTTCATTTCAGTCCTTTCAATGTCAATGGCAAAACCTTTC

CCATTCTGCTTCCAGTAATCTGCCTGAGGATTGATCCAATCTGGGCTCTGCCAATCTCAATCCTCGAGTT

CTTTATAGCATCTGTCAATATATTATACTTGTAATCCCTTTACTTCATCAGGGCTGGAGAAACCATGCCC

AA

>FM890596.1 FM890596 Jatropha curcas embryo 56-70 (DAF) Jatropha curcas cDNA clone rjcaeb0_000490, mRNA sequence

ATTCCTAGCGGCCTAATCCTGTTCGAGCTTATTTCTCCGGTTAGGGTTTTCTGATTCCAATCAACTACCA

GCAACCATGGCGGATGTGGAGACAGAGGTGGCTGCAGGACAGCCAAAGAAGAGAACGTTCAAGAAGTTCA

GCTTTAGGGGAGTGGATTTGGATGCTCTCCTTGACATGACCACTGATGAGCTTGTCAAACTCTTTCCTGC

CCGTGCTCGCAGAAGGTTTCAGAGAGGTCTGAAGAGGAAGCCAATGGCTCTTATCAAGAAGCTGCGCAAG

GCGAAAAGGGAAGCACCACCTGGTGAGACACCAGAACCAGTTCGAACCCACCTCCGCAATATGATTATTG

TTCCCGAAATGATTGGAAGCATTATTGGTGTGTACAATGGGAAGACCTTTAACCAAGTTGAAATCAAGGC

CTG

>FM890595.1 FM890595 Jatropha curcas embryo 56-70 (DAF) Jatropha curcas cDNA clone rjcaeb0_000489, mRNA sequence

TTTGGGGCTGCAAAGACTGTGGTAAAGTCAAAGCAGTCGGTGCCTACACTTTGAACACCGCCAGTGCCGT

GACTGTGAGGAGCACAATAAGAAGGCTTAGAGAACAAACCGAACTGACTACCATTGAACCTATCTGCTGC

TGTTGGTTATTGCTGTATCTTCTTATAATGACTTTTGAGGATTTAATTTNAATTTTTCTTACAAGTTAAA

GTTCTTCCGTGTAGTGCACTTGTGTTTTGAAGCTTAGGTTATTAAGGCTTTAACTCTATCGTTTGCTTGG

TTTTTCCTCGTCTCCAACTGTTTTCCTGTTGTGGATGTTCTATATTATAAGCTGGTATTTGAAGATAGAC

TTGGTTTGTATGAAAAGGGGGGCCCGGTACCC

>FM890594.1 FM890594 Jatropha curcas embryo 56-70 (DAF) Jatropha curcas cDNA clone rjcaeb0_000486, mRNA sequence

ATTCCAATTGCCGTCATTTTACGGCATGAAATCCAGGCCGTTGAAACCACTATGAGGCCAACCAACCAAT

CTAATAAAACTCTAAAATTACGTTTATATCCTAGAGCTGGTATTGGTGAACAAAATCCAGAAGAGTCTTA

TTTTCCACTTACATCCATCTCCATTAAAGAGTCTGCTGATCAATCGACGTTAACAGAAGAAAATGGCATC

CCATGCTTTCGCAACAGCTGTAAGTGCTCCAACGAGAGAAACGTTGATCTTTTCTTCGTCTTTCTCGCGA

CGATCCGACGGTTTCTGCAACCTTTTCCCCAATTCGTTAGCCTTAAAAGGAAGTAGAAGTAAAGCGGGTT

TGAATCTGAGGGTGAAAGCGAGGGGAGGAACACCGAAGTTCAAAGGGACGCAGATGAGGGAAAAGCAGCT

GACGGAGATGATAGAGCAGAAGGTGATAGAAGCGAAGGAGGTATGCGAAGGAGACAGAACCTCAGACGAG

TGCAAAGTGGCGTGGGATGAGGTAGAGGAGGTGAGCGAGGCCAAAGCCAATTTCAGGCTCAAGTTGGAGA

AACGGGATCCTCTCGAGT

>FM890593.1 FM890593 Jatropha curcas embryo 56-70 (DAF) Jatropha curcas cDNA clone rjcaeb0_000485, mRNA sequence

GAATCACACTCGTTTGAAAAATCTTTAAAATACTATAATCCCAACACAGCGAAACCAATCTGCTCAGACC

TAAATTTTAAAAACTATCTTAGTATATATAACTTCAAATAACATTATAAGCTGCTTATGAATTCAGAGTT

TCTTATTTATACACACTACTCGATCCATCAATGTGAAAGCACTCTGTTTAGAAAATAGGACCAGCTGCAA

GAATCTCCTCAGTCAGACTGTTGTCTTTTCCAATCTTATAATTTGCGAACACTTCAAAATTCTTCCTGAA

CAGACCACCCAGCTTCAACAGGGTATCCTGGTAGGCTTTCTTGTCTTTCCAAGTGTTAACAGGATCCAGG

ATTTCTGAAGGCACACCCTCGATCTCAGTTGGGATTTCAAGCCCAAACACTTCAGTCTTTTGGTAGTTTG

CTTTCAAGAGTCTCCCAGAATGTATGGCATCGATGATTCTTCTAGTGTATGCTAACTTCATACGGCTTCC

TGAGCCATAGCTGCCACCTGACCAGCCGGTATT

>FM890592.1 FM890592 Jatropha curcas embryo 56-70 (DAF) Jatropha curcas cDNA clone rjcaeb0_000484, mRNA sequence

NTCGAATTCAATACTTGGTGGGTCCCATGAACAAGGAGTATCTGATAAAGAGCAGAAAGCATGTGAGTGC

TTGGTGGGATAATATTAGCAACAGGCCATCTTGGAAGAAGGTCCTCCAACTCTAGAATCCATATATGATA

AATCTGTTATCTTTTGAATAATTTTTACTAGTTAATGTTTGGTTTTATGTTTTGTTCTTTTATATCTACA

ATCTGGCTTTGGGCAAATGCTGGATGAACTTTTATCGCCTTTTTTTTCTAAAAAATCAAGGTTATGTTCT

TGTTCTAGCTGTAGTAGAAATGGAAGTATTTGCAATTACAAAGGGGGGGCCCGGTAC

>FM890591.1 FM890591 Jatropha curcas embryo 56-70 (DAF) Jatropha curcas cDNA clone rjcaeb0_000483, mRNA sequence

GGCCAAATAAAATTTACAGTTCTTTGTTAGACTCAAAATTTTACATGGCAAAATATCAGCCTCAGGTATA

CAACCAACTGTGAAATCTTTGTGAAAAAAATACAAAATTTAAGATTTAATTAAAAGCACAGATATACAGA

ATATAATATAATATTACAACACACAACAGTAATCTATATGCAAAGTAATCTCTGTTTAAGCATACCGCAA

ATCCACTTCTGGAGTTATATATGGAGGCTCCAAATTATGTTTTGAAGTTCTCTTTGCTATTCTTAATTTC

TTCCTTCTCAAGGTATCACTTACCGGCATTGGAGACCAAAGCAACACAACTTATTGTTTTTCTTCCGACT

CGATTGTTTCTTTTCTTGCAATCTATTTTCATGGATGCCATTTACCTCAATGTTCATTTGCTTATTCTCC

TGCGACATTACGTCTATATTTGGTAGGTCTGCGATCCCATCTTCGGAAGAACCCAAGACTTGGTTGCTTT

CACTAGGAATTG

>FM890590.1 FM890590 Jatropha curcas embryo 56-70 (DAF) Jatropha curcas cDNA clone rjcaeb0_000482, mRNA sequence

GCAAACTAAATGTAAACATCCCATAAAATTCTAACAAAATCAAATAAAACAGATGGTTTGGGTTTACACA

GTTGTTTATCAAACTCAAATATTCTTTACAGAAAAAGGCCAATGGCAAAATATAGTAACTAACTAATTAC

CATGAAATCTAACAAACACACATACTAAAATTCAATTAAGCACGTTCCCCGCGAAATCCGCCTAGCCAGC

TGAATATCNCTTGGGCATAATTGTAACCACTCTTAGCATGAATGGCGCAGAGATTGGTGTCCTCAAACAG

ACCCACCAGATAGGCCTCCGCTGCCTCCTGAAGTGCAAGAACCGCGTGACTCTGGGAACCTCAAGTCCGT

CTTGAAATCCTGTGCGATTTCACGAACAAGTCGCTGAAAGGGTAACTTGCGTATCAAAAGCTCAGTGCTC

TTCTGGTACTTGCGAATTTCACGGGGGGGCCCGGTAC

>FM890589.1 FM890589 Jatropha curcas embryo 56-70 (DAF) Jatropha curcas cDNA clone rjcaeb0_000481, mRNA sequence

ATTCGAGAACTACCAATGTCAACCCCAAAACACAAAAGCTTCATACCCCCAATAATGGCACCGAGGAAAC

GAGTTTCAGTAGAACCAGTGACATATAAACACATAAATGACCCAATTTCACCTGGTATGCCTATAGTTCC

TGTGCCAAATCCTCTGAAAACGTTCGACAGACCAAAGAGCGCAAGGATTATTATTCCATCGAGAAAAATT

AATGAAGAACTCCTTAATTTGATTGAGAATCAGGGAAAACCTTCGCATGTTGATGGAAGAAATGGGGTGG

GATACCTTAGCAAGGTTTGTAGTTAGGGTGGTGCTGGAGGTTTTAGTTACATGATTAGCGGCGGATTTTG

GAAATCCTCCGATGATGGTTTTACCCCCTAGACCTCCACCTAACCCCCTATGAGAGTTGCCCGTTCCTGT

CTACAGTTAGTGCTTTTTGGTAAAGGTGGGAA

>FM890588.1 FM890588 Jatropha curcas embryo 56-70 (DAF) Jatropha curcas cDNA clone rjcaeb0_000480, mRNA sequence

ATTATTCACTCTACATATTCACATCCAAGTGAACCAAGCACAAAAACAACGTCGCCGTACTTATAGGGCA

CATGGAAGGATAAATCCTTACATGTCCTCACCATGCCACATTGAGTTGATTTTGTCAGAAAAAGAAGAGC

CTGTTAAGAAAGAGCCTGAGACTCATCTGGCTCCTAGGAAACCAAAGGCCCAAGCTCTACGCAGTGGGGC

TTCCTCCTGAAGGCAAGCAGTAAACAGGTCCTGAAAATTGGCTTTATCTGTTTCTTCTCTGCTGGAATGC

TGTCTCATCAGTGACCTCTGCGTTTGTTTTCTGAGATGTTTGTCCTAGATCTTATACTCATTTAGACTCT

TACTGTAAACTGATCCCCCTACTCTGCAGTGGATGGGCCAAAGATTGGGATATGAAAGCTTTCTCTAAAA

TTTTGAT

>FM890587.1 FM890587 Jatropha curcas embryo 56-70 (DAF) Jatropha curcas cDNA clone rjcaeb0_000479, mRNA sequence

ATTCCTTTATATCAAGCCCAGATCAGGTGACAGTTGGCCGTAAGCCAGTTACTGGTCTTAGACTTAGCCT

AGAAGGCACCAAGCAAAACAGGCTCTCCATTCATTTGCAACATCTGGTGTCTCTTCCGAAAATTCTTCAT

CCACATTGGGATGCACATATAGCCATAGGAGCACCCAAGTGGAAAGGTCCTGAAGAGCAAGACAGCCGTT

GGTTTGAGCCTATCAAGTGGAAGAGCTTCTCTCATGTAAGCACTGCACCAATAGAGTACACTGATACTTC

CATCGGAGACCTCTCTGGTGTTCACATTGTCACTGGTGCTCAGCTTGGCGTTTGGGATTTTGGAGCCAAA

AATGTGTTGCACCTCAAACTCCTCTTCTCCAAAGTCCCAGGCTGTACAATCAGGCGGTCTGTATGGGACC

ATAGCCCCTCCAGTCCTTCACAGCAGCCTAACGGGGCATCATCATCAATGTTCAATGAAAAAGCCTCCAG

TGAT

>FM890586.1 FM890586 Jatropha curcas embryo 56-70 (DAF) Jatropha curcas cDNA clone rjcaeb0_000478, mRNA sequence

AACAGAAAACAAACTTAGCTTGATGTGCTCATATCTTTTGTGCATTATTATACATAACTCTGATCACTCA

CACACCAAGTGAGTGAAATTTTTATTTAACTGAAGCACATCCACGTTATAGCTAGCAGATTAGGAACGAA

TATCGCAGCTTGTACGCAGGCCACAAGCAGAAGGTATACTTCTAGCCCTACGCACTGCCTCCAAACGCTC

TCGCTGTCCTTGCTCCAGCTGCTGCTCTAATACATACTTTATTCCTTCACATGGACAATCATGACTTAGC

TGCCTTAATTCGTTACAACATTGGCGAAGATCCCGTTGTTGCTGGTTTTCAATTCCTCGCAAAGCTAGCA

TCTCTTCTGACGGCCTTCTTGACGATCTCATATAACTCTCACAGGAGCTCAAATCTGCCCTTTCAGCTTC

CCTCCTGCATTTTCGCTGACTAGTGTTAGAAGAATCATCGTCGAATTTCAACGGTGGTGATGGT

>FM890585.1 FM890585 Jatropha curcas embryo 56-70 (DAF) Jatropha curcas cDNA clone rjcaeb0_000475, mRNA sequence

ATTCCAAATATCACTGATCGTAAACCTCCATTGAATTTACTCTGTGCTTCGATCGCGTCAATGGCGGAGC

ACCCACAGTCGCAGCATGTTGGCCAACAGCCACGATCCCACCAAGTAGTGAAGGCAGCCACTGCAGTCAC

TGCCGGTGGATCTCTTCTAGTTCTCTCCGGTTTGACCCTTGCTGGTACGGTCATTGCACTGACCGTAGCC

ACTCCATTGCTGGTCATATTCAGTCCGGTTCTTGTTCCTGCGGTTATCACAGTTTGTTTGATAATCACAG

GGTTTTTAGCCTCAGGCGGTTTTGGCGTCGCTGCCATTTCTGTCTTGTCCTGGATTTATAGGTATGTTAC

TGGAAAGCATCCACCAGGAGCAGAAAGTCTGGACCAGGCACGCTTAAAGTTGGCGGGAAAGGCAAGAGAG

ATGAAGGACAGAGCTGAGCAGTTTGGACAGCATGTAACAGGTCAACAGACTTAAAAGAGAAGTGGGTTTT

GGTGGTTGATGCAGAGAACATAAAATGGCTTTGATATATATATAA

>FM890584.1 FM890584 Jatropha curcas embryo 56-70 (DAF) Jatropha curcas cDNA clone rjcaeb0_000473, mRNA sequence

TTTCTGAATTCTGTCCGAAACAAGAGATTAGGCTTGCACTGGCAATTCACTGAAATTCCATATTACATCA

AAGCAAAAGTTATAACAGATTGATGCTAGTCCGCTCACAAATTGCC

>FM890583.1 FM890583 Jatropha curcas embryo 56-70 (DAF) Jatropha curcas cDNA clone rjcaeb0_000472, mRNA sequence

ATTCCTGGTAATATCAGCTGAGCCGATCTCGAACCCACATCATTATCAACCGTACGGGGAAATCTACACG

ATTAAATTAGACGGCTCTGATTTGAAGAGGTTGACACATAATTCGTTTGAAGATGGGACCCCAGCATGGG

GTCCTATATATATTGAGCCAAACGACGTGGAGTGGCCAAACGAGAGACCACAGTGCCAGTTTGAGGACTG

TCATTGGCTTAATGGAATGCCAAGCCGAGATGTTGGGGTTTTGAGTAAGAATCAATGCAAAGCATAGTTT

ATATTTATTTTCTGCACTGTAAATGAGAGAAAGATCATATAAAGGCATAATGACTGTATATTTTATGTGG

TTTTCGTGGAAAGATTGTAATTAAAAAATACTATGGG

>FM890582.1 FM890582 Jatropha curcas embryo 56-70 (DAF) Jatropha curcas cDNA clone rjcaeb0_000471, mRNA sequence

ATTCCAATGCCTCATTATCATGCAATGGAGGCCACTAAAGCAATCAAGCCTATATTGGGCGAGTATTATC

AGTTTGATGGCACTCCGATTCTTATGGCGCTCTGGAGGGAGGCCAAGGAGTGCCTGTTTGTCGAGCCAGA

AGAGGGAGGTCCCAACCGAGGAGTTCTCTGGTATGGAAATAAGTATTAAGAGCTATAATATGTGGTTTGG

CCTTCAGAGAAGTTAATTAGAATAACTATGCAGAGAGTGTGAATGTAGTATAGATGTTAGTAATGGAGAA

GAAAAACTATATGCGTAGTAGCTTTCTTTACGGGAAAGTGGTGTATGAGTTATGTCTGTATATGGAACTC

TAC

>FM890581.1 FM890581 Jatropha curcas embryo 56-70 (DAF) Jatropha curcas cDNA clone rjcaeb0_000470, mRNA sequence

GTAATCAAAGGAACAAGCTTTAGAAATATTAATAATTACAGAAATTAAACCAGATAAAGCATTAACAATC

CACGCATATAGATATTATCATTTCAGTATATCAAGGAGTTAGACGTGTGCCTACATCTGATTGAGAAGGA

AGTTGCCTTCATTGTTTTTGTGCACCTCAACCTTGTACTTCTCTTCATTGCTTCCCCTCTTCACCTTAAG

GAGCATATCAAACTTTGCAAAATCATCCACTACCTCAGCCTTCGCATGAACAATCTCTTGAAGCTCATAA

GGGAACAATGAATTGGACCTCTGCTGAATGGTCTTCACAGCATGATTTGCAGCATCTTGCACTGCAGGAT

CATGTGCTGGCACTTCTCTCCATCCAGGCACATGCCCATCTCTTTTGACACCAAGATCTGAAGAAGGTGA

AGGATGAAGCACCACCATCAGGTCCATCAACATCCCCAGCATGCTTGAACTCCTGTAATTCTTTGAAGTT

T

>FM890580.1 FM890580 Jatropha curcas embryo 56-70 (DAF) Jatropha curcas cDNA clone rjcaeb0_000469, mRNA sequence

ACAAGTTAAGTTCAATTATTAAAGAGTAAAGTACAAGTAATGACAAATCATAATACATCAGCTCACTTGC

CAACCTTCCCTTGTTATTTTAAGGTCATATCTAATATTCAAAGTTAAGTTTACATCGATTTGGCGCAGCT

CTCACCTCACTGTCAGCGTTAAACAAACCACAATCTAGTCTGTTTTATTAGACGAAGATTGCAGGTTAAT

TGAGGCAGAGAAATTAGGCAGAGTCCCACAATCATAAGAATGAGCAAGATTAAAGACCTTGGGCAATAAG

AACCTAAGAAATACAGCATCTCTTAAAAATTTCATCCACCATTGAGACCCACCTTGTTGAATCCATCCAG

ATAAATATGAGCCAGTGATCAAACCAGCTACTCTCCACCTCCTTTCTCTAACATACCCATTTATGGCTCC

AGCCATGTCATTTGAAACAAGCCTACCATTATTTCTTATGCATGATTCTCCAATGTGCCTGCCTAAAACG

ACAGCATCTTCTAGTGCTGAACAACCACCTTGGCCT

>FM890579.1 FM890579 Jatropha curcas embryo 56-70 (DAF) Jatropha curcas cDNA clone rjcaeb0_000468, mRNA sequence

AACAGAAAACAAACTTAGCTTGATGTGCTCATTCTTTTGTGCATTATTATACATAACTCTGATCACGTCA

CACACCAAGTGAGTGAAATTTTTATTTAACTGAAGCACATCCACGTTATAGCTAGCAGATTAGGAACGAA

TATCGCAGCTTGTACGCAGGCCACAAGCAGAAGGTATACTTCTAGCCCTACGCACTGCCTCCAAACGCTC

TCGCTGTCCTTGCTCCAGCTGCTGCTCTAATACATACTTTATTCCTTCACATGGACAATCATGACTTAGC

TGCCTTAATTCGTTACAACATTGGCGAAGATCCCGTTGTTGCTGGTTTTGGGGGGGCCCGGTAC

>FM890578.1 FM890578 Jatropha curcas embryo 56-70 (DAF) Jatropha curcas cDNA clone rjcaeb0_000466, mRNA sequence

GGATCGAAATATCAAAGTAAAGACAAGATTTTAAAGACAAGATTTTAAACATCGGGTAAGAGTAAGAGCT

CCAAACAACAGACATTGTCTACATCAAAGCATCTTGATCATACAGTTATTTCATGCCAAACCCAAGATAT

AGATTCCACTGAAGAACTCAAGTCCATCCCTAGCCTCTATCGAACCAAACCAAGTAAATAAGTTAATAAA

ACAGTTTTCAACACATTCCACCATAAACATCACCCGATTAGTGAACAACCAGTATCAACCCATGGCATAC

TTCTGTGTCCAGCTGCGTGCAGTTGCTTCATACTTTGCTCGATCAGTCTTATACATATGGGCAATCTCTG

GGACAAGTGGGTCATCGGGATTTGGATCAGTCAGCAATGAGCAAATAGATAGCAATACCTTAGAAATGGT

GAGAGCAGGGCTCCATTGTTCTTT

>FM890577.1 FM890577 Jatropha curcas embryo 56-70 (DAF) Jatropha curcas cDNA clone rjcaeb0_000465, mRNA sequence

GAATACAATTATATTTATAGAATATATTGATTAGGGAATACTTAATTACTAATTAGGATGATTACATTAC

TTTCATCACTTATTTTCATAATATATTACATACTTCATTAGCGTGAGATCATAACTCCCCTCGTAATACT

ACTTACTTTCACTATCTACACACCCAAAAAGGCTACTTCATTATTTACTCCTCGGCATATCTTCCAGACT

CGGTCCTGGAAATAGTCAAGGTGGTCTCTTGTCTTCCAAACGGGGGGGCCCGGTAC

>FM890576.1 FM890576 Jatropha curcas embryo 56-70 (DAF) Jatropha curcas cDNA clone rjcaeb0_000464, mRNA sequence

AGTTGTCAAATGGACAAAAAGAATCCCGATTGAGATTAAATAAGGTAACAAAATATTGTTCATCCACAAT

TCTAAAAAACAATAGTAACTCCTCAAAACTCGAAAGCATTCAACACATTTAAACTTAGCTTATATAGAAC

AACTAAAAAGCAGATCCTCCAAGCTATCAATAAGCCCAATCTGCTATCTACCGGTCTTGTGATACTTATA

TGTTGCTAGGGTACATGAAGACTCTAACCCTTGCACCCATTGATTTCGGAGGCAGATTGGACTTGAACTT

AGCTCGTACAACACCACTGTTACCATGAGGCCTAGTGACCTTTCCCCAAATGCAGCGATAGTGTGATCCA

TTCTTCTTTACCTTGGCCTTGTAGATATAAGCCATGCGCTTACCAGCATACCAGGCAACCTCCTCCTTGG

TGTTCACTCCCTCAATCTGGATTAATGATGTATTTGGGTACTGGTTTGACTTGGAACTCTTGTATCCAAT

GACGGTCCCTCTG

>FM890575.1 FM890575 Jatropha curcas embryo 56-70 (DAF) Jatropha curcas cDNA clone rjcaeb0_000463, mRNA sequence

ATTCATTTTTCTGTTTTGGGAGAAGGATATTATCAGAAACCCTAGAACCATGGGAGTATTCACATTTGTT

TGCCGTCAATCCGGTGGCGAGTGGTCCGCTAAGCAGATCGCTGGTGGTGATCTCGAGGCTTCCGCTTCTT

CCACCTATGATCTGCAGAGAAAGCTTGTACAGACCGCTCTCTCTTCCGACTCCTCCGGCGGTGTCCAGTC

TTCTTTCTCTACAATCACCCCTTCTTCGGCCGTTTTTCAGGTGATAATTGGTGGTGGCGGCGGCGGAGCT

TTTATTGGTGGAGGAGCTGCTGCTGCAGCTCCAGCTGGAGGCGCAGCACCAGCAGCTGAAGCACCTGCTG

CTGAGGAAAAGAAGAAGGAAGAACCAGCGGAAGAGAGTGATGATGATATGGGATTCTCTCTC

>FM890574.1 FM890574 Jatropha curcas embryo 56-70 (DAF) Jatropha curcas cDNA clone rjcaeb0_000462, mRNA sequence

AACCAACAGGTAATTATATATACTGCAGGTTATTTTACAAATAGGGAGAAAGTATTAGCAGTCCTCCCAG

AAAGGGAACGACCATAAACCCCTCTCAAAGTTACAACACACATACTAGCTAAACCCTAGTCATTTTAACC

CACATGTAAACTACATTACTAAACTAAAGTACAGCTGCTAAGCTAATACTTTATTACCAACATTATTATT

ATTATTACTAGAAAATACCATTTAGTAGGTTTTAAGTCATCACACGTAGCTCCCAGTCCTGACACTTCGA

CTCATCGATAGTGATGCCCTCCTTCTCAGCTCTTTCTCCACCAGACTCGTCCGTCGTGCTCAGCCCACCT

TTCCGGCCCATCTCCTGATATCCT

>FM890573.1 FM890573 Jatropha curcas embryo 56-70 (DAF) Jatropha curcas cDNA clone rjcaeb0_000461, mRNA sequence

ATTCCATTAACGTATCTTTCTCAGACTTGTCATCAACAAATGGCTGAACGTTCGCAGCCAGGCCAAGTTC

AAGTGCACCCACAACACCGCTATGAAGCTGCCGTTCAAAGGTCAACAAAAAGGTCCATCAGCTCAGAAAG

TGTTAGCTGTGATCACCCTCCTGC

>FM890572.1 FM890572 Jatropha curcas embryo 56-70 (DAF) Jatropha curcas cDNA clone rjcaeb0_000460, mRNA sequence

AGGAAAGCAAACAAATCCATTTCCACCTTCGTCAAAACAAAACAAATACTAGATCCCGAAAAATCAAACA

AAACCGTTAAAAGAAAAAGATAGCGTGCTTAAAGTCTTAAGAACTATGGGGTAATTAACATGAATTGTTC

ATCCCTATATTTTCTCATATTTGAGAACTCAGTCTCATTTGTCTGCGTCAAGCTTCAACCCTCTCCACTT

CTTCAATCAGGACTTTTGTAGGTTTTCCCAACAGATCAGTGTACTCCTGGAAAGGAGACTTTGTAAAGCG

GGTTTCCCTCCAGAAGTCAGGAGTCAAGAACCCGTAGGTCCTAAGAAGGCAATCAAAGGTGGCCTTCACA

AAGTTTCCAAGTGTCTTAGTAGAGCCCCTGGATGAGGTGAACACATCCTCAATTCCAGCAAACTGCAAAA

CCTTCTTAGGGACTCGAG

>FM890571.1 FM890571 Jatropha curcas embryo 56-70 (DAF) Jatropha curcas cDNA clone rjcaeb0_000459, mRNA sequence

AAATAAAATATCTACTAAAAGATACCTTTTATTCAACTGGAATTGCTTAACAAGAAAAAGACACCAAACA

GAAAACATAGATATATTACATAACAGGAGCTAAAGCACATGCCATGCCCTACACACGTGGGTTATGGCCA

GTGGTGTCTCCGGGCAACCCAGATGGGCGAGTCCTGGTCAAACCCGTTGGGTCCACCTCTCCACCGTATG

GCTGCCCTGTGCCGTGTCCAGGCATGGCCGACATTTGCTGGGTCCCTGTAGGGTAACCGGTTGTCCCAGT

TGTTGAATGGGTATAGGTTTCGGTTGTGCCTTGAGCGCCCGGACCAGCAGTTGTGTACTGTACATGGCCA

CCAGCCTTGGCTGCCTGCCTCGCCGCTGCGTTATCCTCGCGCGCTTCTAGTTTCCGCAATTCTGCCTCCG

GTTTCCTCTCTTCTTTCTTCTCTCTCGCCATTTCCTTCTGCACTGGATCACGTGTTGACATTCTCTCCAC

CTTCTCTTGCGCCTCTGCTCTGG

>FM890570.1 FM890570 Jatropha curcas embryo 56-70 (DAF) Jatropha curcas cDNA clone rjcaeb0_000458, mRNA sequence

ATTNCGAATTCATTTGATGACGTGTTTGAGACTTGGATTGCAGATAGTAAGTTTAAACTATGGCGTTGTT

GCCTCTGTAACAAATTACCTAAAATAGTGCAGAAGAAAAACCCTAGCAATTGGACCAGTGAAGGAGCTCC

GATCAAGAATACTATTGTGCACTCATCTTTCAACTTAAGGCTTGAGAGGCAAACAATGGTAAACGCGCAG

CAAACAATGGTAAATGCGCAGTGAACTGTGGGGATATATATAGGGAGAGACGAGAGAGAATTTATTTAAT

TTTTTTTTTCCAAATTTTACAGCAAAATAGGGATTATGCAAAGTTTATTTATCTTTCAGTTTTGAGAAAA

ATAAAAGTCTCCCTTACTTTCACCGTTTTGATGCTTTATCTATATTTTTGTTCTTTTTCATATTTGAGTT

CTGGAAATTATTATAAGTTTTGGGGCTGGTCTGAATTTCTGGAAGATCTCTAGCCTATTAACATCAACTT

CCTTACAGATTATCATGTTGTACTTCATGTGTTGAAGAGCTTTG

>FM890569.1 FM890569 Jatropha curcas embryo 56-70 (DAF) Jatropha curcas cDNA clone rjcaeb0_000457, mRNA sequence

ATTCTTTGCCCTCTAAGAAGCGTACGCATAGCATCTTTATCTGCTTCTCTTTTAATTGCCAAGGGAATCA

TCTTTCAATTCCAAGCAAACTCTTTTGCGATCTCTTCATAAGTCACCTCTTCCTTTGGTTCCTCGCTCGA

TCAGAACAGGGAATTCAAGGCCTGTGCTCTATTCTTTGAGATTCGATCATACAATGGCTACCCAATCTGA

GCCTAAATCAGTCCATGACTTCACTGTTAAGGATGCTAGGGGGAACGATGTTGATCTCAGCACATACAAG

GGAAAGGTTCTCATTATTGTTAATGTTGCTTCACAATGTGGCTTGACCAATTCCAACTACACTGAACTGA

CTCAGTTATACCAGAAGTACAAGGATCAAAGTTTGGAGATTCTGGCTTTTCCATGCAATCAATTTGGATC

TCAGGAGCCAGGGACCAATGAACAGATTGTGGAGTTTGCCTGCACTCGCTTCAAGGCTGA

>FM890568.1 FM890568 Jatropha curcas embryo 56-70 (DAF) Jatropha curcas cDNA clone rjcaeb0_000456, mRNA sequence

TTTTTTATTTATTTATTTTTCATTCAAGCTTTCAAGTTTTCATTCATTTCTTTCCTTCCCCAAAAATTTC

AGCTCTCATCCATCTAACAAATCTGATTTCAGATGCGTTATTAGACAGTAACAAGAACTAGTGTAAACAA

CCTTGTCATTTAGACAACAAGTTTCAAAGCTTCATTTAAGTCTGCGATAAGAAATGGATTTCAAAACTGA

AAAAGGTGCAGGCGGCGGATCACCGAACATTCACCAGCTCAACATCAAACACAAGCCACGAATTTGGCGG

TATAGCCTTGCCAGCTCCCCTATCTCCATAACCCATTGATGGTGGAATCGTGAGTCTA

>FM890567.1 FM890567 Jatropha curcas embryo 56-70 (DAF) Jatropha curcas cDNA clone rjcaeb0_000454, mRNA sequence

ATTCCTTCTGCTAGTCATGACAAGATGGTCAAATTCTGGAAGTGATTTCATGCATCTCTTGGATCTGCCT

CGCATTCCCAGGTATTCTGACAGATGCCTTTGCAGCTGTTTGTTTATATTGGCTAATTGTTACAGCCTTT

TTAGTCAAATGAGCTAATAGCTTTTGTCCATTTTACACTCCACACTAACCCAGTGTCTGTAGTAACCTCA

AGATTTTAATTTAACTGAAGATCTCTGGAAAGACAAGTTTAAGTTTGACATTGATATGTAGATTTACAGT

TGGGGAAACCGACTAAAGTGAAAGTTGTCGCTGTAAATGCTACATACATTACCTATTGCATAGTAAATGA

ATGCAATGATTATATATATATATATATATATATATATATATATATATATATATATATATATATATATATA

TATATA

>FM890566.1 FM890566 Jatropha curcas embryo 56-70 (DAF) Jatropha curcas cDNA clone rjcaeb0_000453, mRNA sequence

ATTCCAGAAAGGATGGTGTCTACATGTACTTTGAAGATAATGCTGGTGTTATTGTGAACCCCAAAGGTGA

AATGAAAGGATCTGCAATTACTGGTCCAATTGGAAAGGAATGCGCTGATCTGTGGCCAAGGATTGCAAGT

GCAGCTAATGCTATAGTATAAGAGTTTGCTACTTTCTTTCCCCACTGGGTTGGGAGCTATATTGTCACAG

TTTTATTCCTTGAGAGTGTAGGATGTTCCTTTGAACTTCAGCAAATTTTGTTTGTGGATTATGCATTTGG

TTAATGACATGGAATTTATGCAGGAAATGTCAATTTTATTGGTTTTGTTTCTGAAAAAAAAAAAAAAAAA

AAACTCGAGTCTCGAGNTTTTTATATTTGTTTTGGAGAAAAATCTTAGAAAAATTTGTAAATACACTATC

TTGCTTTTGCTTCTCTGTTTTGGCTAACTTCTGATCAACTTATGGTGTAAAGTAAATTATATGCACTCGC

ATG

>FM890565.1 FM890565 Jatropha curcas embryo 56-70 (DAF) Jatropha curcas cDNA clone rjcaeb0_000452, mRNA sequence

AGAGCCAGTAAAACATATCTATTTTGACAAGCCTTCCACAAATACAAAGGGAACAAAGAAGTTGTAACAT

CAAACAATTACCAACTTAGGCTGGTAAATTTTAGAATACACGCCCAATAGAAGATTGAATTATTTGTCAA

ACAAGAAACTAATAACCAAAGAAGGAGGAAAACATGTCTAGTCAATAATTAACCAAAATAAGGCATGAAG

CACCAAATATAACAGTACAGAGATGGAAAAGGACACATAAAGCTAGAAATATGGCAAGAACTTCCACTTC

GAAGCCAGCTATTCTCCATTCTTAGCACTTGATCTCCTTCAAAGCATAAGCGAAGTACAAAAATGTTGGG

TCAGTGGCGCCATCCTTGTAGTAAGCAAACACCAAACTCCCATCATCATGCATGCTCTCCCCCACAAAAA

ATTGGAGGTCACTGAGCTTCGAGAGCAGGAACTTGGTTGCTCCCTCAATGTTTTTCTTGAAAGCCTGTTG

CTTCTCCTCATCCAATTTGGGTGTCAACAACTTGAT

>FM890564.1 FM890564 Jatropha curcas embryo 56-70 (DAF) Jatropha curcas cDNA clone rjcaeb0_000450, mRNA sequence

GATCCAAAACACAGTTGTGTGGCAATATTCAAGAATTTGTTGGCGGCATACTTTGCAAGTATCTGTGAAG

CATTACCGACCAAATATACGCGCATATTTTTTTATTGGATATCGTAAATT

>FM890563.1 FM890563 Jatropha curcas embryo 56-70 (DAF) Jatropha curcas cDNA clone rjcaeb0_000449, mRNA sequence

AGAAAAGATAATCAGGAATTATTATATTTTACATTATTAAAATGAAACAGTAATAATAAACATTCTTTCA

GTACATACTTTCTCACACACATATCATTATATATATATATATATATATATATATATATATATATACATAT

ATATATAGAGATAACATCATTATAAGCTGAAACTTAACCAGAGATCTCAATAGCCTTAACATCAGGTTTC

TTGACTTCCTCTTTAGGAACAGTGACAGTAAGTACTCCGTTTTCCATTGAAGCCTTTACTTGATCCATCT

TAGCATTCTCCGGCAGCCTGAACCTCCTCATGAACTTGCCACTGCTACGCTCCACGCGATGCCACGTGTC

GTTCTTGTCTTCCTTCTCCACATATCTCTCCCCGCTGATTTGAAGCACTCTGTCATCTTCAAATTTCTAC

>FM890562.1 FM890562 Jatropha curcas embryo 56-70 (DAF) Jatropha curcas cDNA clone rjcaeb0_000448, mRNA sequence

ATTCGCTCCTTCCGCGGTCCTCTGCTTCAATCAGAGGAGGTGGTACTCTGGAACTGGCAGCACATAGTTC

AAGAGGTTATAAATACCCAATGCTTAACCTCATTTTCTTCATTTGTATTAAGATTCTTTTTTTCTTTTTC

CGAAGAGGAGAGTTTAATGAAAGAATTCTTTTTTCCCTTTTCTTTTCTTTTTCTTTTTTTTTCCCTTCTT

TTTCCTACTGTATAGCCACGCTCCTCGGAAACTTACTTGAAGAAGTCATAAAGAGAACTCCATAGATAGT

AGAGAACTGATGCCTGGACAGAGAGATGCCTCAGGGTATTGGTTGTCGTCTGGTTATTGGCATTTTATAC

TTGATGAGCTATTCCTCATTAGAGCTATGGTTTTACTCTACTGCCTCAAGAATTATATAAATGGATGTTG

CCATTGCTATTGATATGAAATTTAACCAATGAAATATTTTACAGTTTCAGTTC

>FM890561.1 FM890561 Jatropha curcas embryo 56-70 (DAF) Jatropha curcas cDNA clone rjcaeb0_000447, mRNA sequence

GAAGAGGTAACAAGTTCTACAGCCAAAGATCCAATGGATTTAAAGTGCAGGGAGAAAATGTAGCTTCAAC

AGAGTTTTACCATAATATGCCAAAAAAACACATTTAACCAAATTCAAGAATCAAAATTTTAAATAGTATC

CTATCTGCTGCTGAAATCTTAGAACTCTAGAAACAGGGACAAAAACTCTCAACAGATGTTCAAGCCTCTG

TTATAGGATACTCAAAAACCACATCTTTCCCAGTAAGCTTCCTGTAAACTCCAGCAAATGTCTCCAACTT

GTACTCAGTGTTGTTGCGCTCTTTGGGAT

>FM890560.1 FM890560 Jatropha curcas embryo 56-70 (DAF) Jatropha curcas cDNA clone rjcaeb0_000446, mRNA sequence

ATCCGAATTGATGGCGTCCAAGTGAATCAGTTGCAGTCTAAGGCATGTCAGGCATCGAAGTTTGTTGCCA

AGCACGGAACTGCCTACTACAAGCAATTGTTAGAGCAGAATAAGCAATATATTCAGGAGCCTGCTACTGT

TGAGAAGTGCAATTTGTTGTCAAAGCAATTGTTCTACACTCGTCTTGCTAGTATTCCTAATCGATATGAA

TCATTCTGGAAGGAACTTGATTATGTGAAGAATTTATGGAAGAACAGGCAGGAGCTGAAGGTTGAAGATG

CTGGTATTGCTGCTTAGTTTGGTTTGGAGTGTTTTACTTGGTTTTGTGCTGGTGAGATTGTGGGAAGAGG

ATTTACTATCACTGGCTACTATCCTTGAGGAGAACAGCTTCACAGAATGTGTTCCCGAGGATTCTGCTGT

CATTTCGTGATTGATCACAGGCACTGTACTTGCAAATTTTCGTTTTTTCTGCCAAATTTTTGAAGCTGAG

AAATCAGTGGGCTTCTGTGATTGCCTCAATAATTTTACAATGAAGCCGCATAGGATGTTATTATGCTTCC

ATTAACT

>FM890559.1 FM890559 Jatropha curcas embryo 56-70 (DAF) Jatropha curcas cDNA clone rjcaeb0_000445, mRNA sequence

ATTCCAAACAGGAAAAGCTATGGCCTTCTCCAAGCTTCTGATTGCTTCACTTGTCATCTCTCTTCTTCTT

CTTCTCCATTTTGCTGAGGCTGATCATCAAACGGTCAAGTCAAAACTAGACGCAACTAATTCTCCCGCTG

AGGAAATTGATTGCGGTAGCGCTTGCACAGCCAGGTGTCAGTTATCGTCAAGGCCAAACCTATGCAAGAG

GGCATGCGGGACATGCTGTGCCCGTTGCAAGTGTGTTCCGCCGGGAACTGCCGGTAACTATGAGGCCTGC

CCCTGTTACGCCAGCTTGACTACCCATGGTGGCCGACGCAAGTGTCCTTGAACCCTTTTGCTTTAAGACG

CAAATTATCTCTTTCACACGCACATATATGTATGGGGTGATCACTTATATTTTCTTATTTACTTCCTCAG

TTCATGGAACTTTGGTTCTGTATCTCTTTCTGTTTCTATATGGAATAAATAATTGCTAAT

>FM890558.1 FM890558 Jatropha curcas embryo 56-70 (DAF) Jatropha curcas cDNA clone rjcaeb0_000444, mRNA sequence

ATTCCTCATCTGGTAGCGAGATCTTGCGTAAATCACCGAGATCTAGCTTGTCTAGATCTCCTATTCGAGA

TCCGTTACGCCAGACGAATCAAAAGGTGCAGACGTCGACAGATCGGGAAAAATCAGACGCAGCGGTGGCT

AGGAGGGAGGTGAACCGATGCTCGGGATGTAGAAGGAAGGTTGGGTTGACCGGATTCCGATGCCGGTGTG

GAGAGCTTTTCTGTTGGGAACACCGGTATTCTGACCGTCACGACTGCAGTTACGACTATAAAGCCGCTGG

CAGGGAGGCGATCACTAGAGAAAACCCCGTGGTCAAAGCGGCGAAGATTGTTAGAGTTTGATAAATGGGA

AAGAACGTCAAAATTTGAACAGAGAACTTCAAAACTTCACGATGATCGATGCTCTCGATCTGATCATTCC

TCCTTTTTCCTCTATAGAGAATGGCTTGAGATAGATTTT

>FM890557.1 FM890557 Jatropha curcas embryo 56-70 (DAF) Jatropha curcas cDNA clone rjcaeb0_000443, mRNA sequence

CGATTCAATTAATCGGGATTGTGAATTTTAGAAGCCGAGAGAATTATGGATTACAGTCTTTTGGTTGGCC

TTCACTTCCGAGATGATAACACTTATGACAAAATGGGTTTGTCACCGTTTGTTTTGCGCTCTGGAAGTAA

GGATTCCTATCAAAATGAGAAGTTCATGCGTGGCTGCCGCTTTCTCGAGGCAGAGCTACAAGACAGAGAT

CGGATCTTATCTGGCCGGAAACCTCTGATAAGGCTGGGAGCAAACATGCCAGCAAGAGCAGAACGAATGG

CAAGAAGGAGTGATTTTGATCAGTACACCCCTGGTGGGATTAGCCATTTAACCCCCTCAAGAAATGGTGA

GATCTATGAGGTGGTCCTTTATTTTGGGATCATTGATATCTTGCAAGACTACGATATT

>FM890556.1 FM890556 Jatropha curcas embryo 56-70 (DAF) Jatropha curcas cDNA clone rjcaeb0_000442, mRNA sequence

ATTCTGGAGACACAACTCAGGATGGCGGAAACCCAATGGACCTGTGGAGCTTTGATGACCTCGCTGGCAT

GGTTGGGGGTGTTTATTGATCAAAATCTTCATTACTCATTAGCTTCTGTAAATAAGTCTACATGTTAGGA

TGATTCTGTGTCTGATATGTAGTACAAGCAATCTGGACTTTGCTTAATCCTCTGTGGAGTAGGTGTTCTG

AGGAGCTTTTGGTGCAAGAGTAGTACATAGGTATTTAGGTTCCCATTTACATTTTGAGACATTGGACTTG

GCTTGTTGAATTTGATGCTTGTATTTGGCAATGCCAGTGTGATTTGTGTTGAAAACTAAGAAACTCATAT

>FM890555.1 FM890555 Jatropha curcas embryo 56-70 (DAF) Jatropha curcas cDNA clone rjcaeb0_000441, mRNA sequence

GAAAAATCCAAGAGCATACTTTATATTATAATAAAATATCCAGAATAATTCAAAACCAAAGCAAAATCCC

TAGAACAATTGTTTAAAGACCTAAACATATAAAGCAACAATTTAAATAGACAAAATATTGATCAACATAG

CCGTCCTCATGTAATAGGAAATAACTAGAGTATCCAAGCTGCAGCAAAACATTTCACTTAAGAGGAATGA

ACCTAGACGAGTGGGTGGCACCAAATACCCGGTCTACCATGTTTCACAGGCCTGTATGAGATTGAAAACT

CAGCAAGAATAATGCCCGATCATCTCAGGCTTGATTCAACTTGGTTAAAGGTCTTCCCATTGTACACACC

AATAATGCTTCCAATCATTTCGGGAACAATAATCATATTGCGGAGGTGGGTTCGAACTGGTTCTGGTTTC

TCACCAGGTGGTGCTTCCCT

>FM890554.1 FM890554 Jatropha curcas embryo 56-70 (DAF) Jatropha curcas cDNA clone rjcaeb0_000440, mRNA sequence

AACAAGAAAAGCCCCGTGGAGGATTCTGTTATTTGCTTTGTCGGATTCCGTTTACATAGAGCATTCAGCA

CACACTTTGACAAGAAACAGTACAGGCCTGAGATGAGAGATATAATAAGCTTTTCAGATAGGCATGGATA

AAAGATGCATTAAGCTTTTAATTTCTCATTATGGTTTTAACAAAATGCACATATAATACTACATGAACAA

CGCAGACACAGACGAAACTAGCAGAGTAAGCACAACTCTTATTTTCTCTGAAACAGGTGACTCATGATTA

TCACATAAGAAGATCTACAGCTCTTGTTTGGCCCCACTATCTGTGAGCATGGCATTTTCTTCTCCAGCAA

TGCTAACGACCCTTGGACCTTTGATCTTATCAGGAGACAACTTGGAGAAAGATAAAGTAAGCACACCATT

TTCAAGCTTAGCCTTAACAGAGTCCAAATCCACATTGTCTGGCAACTTAAACTGTCTCCAAAACTTGCCA

TATGATCTTTCTACCCTATGCCAATGATCCCCTTTCTTCTCTTCCTCTCT

>FM890553.1 FM890553 Jatropha curcas embryo 56-70 (DAF) Jatropha curcas cDNA clone rjcaeb0_000439, mRNA sequence

CGAATTCTTCTTCTGCTGTAGAAGGAACTACTTTCTTCAGGAATATATGATTCCTAGGGGTTCAGCCTCA

TTCTGTCTTTATGACTCCCGTGGTTTGTCTGATGACTTCTCTGAGAACGTTGAAATGCTAAAGCAATGGA

TCACGAAAGGTGTCCGTCATGGAGAACTCATTA

>FM890552.1 FM890552 Jatropha curcas embryo 56-70 (DAF) Jatropha curcas cDNA clone rjcaeb0_000437, mRNA sequence

TTTTATTTTTATTTATTTACCTCAAGGTATGAGTGGCAGAGTGGCCGGTGGGAGCTTTGGTTTACCTCTT

TAAACACAGGAAAGGTCGCAGGATCATGGCACATCCAGCGACTGTCGTTTACCCATCAGTCACCAATTCA

ATTCCCGTTTAATTAATAGATTTGATGCTTTGGTTTGGTTTTCAGGTTCTTTGTGTTCCTTTTATGTAAT

GGCCACTGTGTTTCTTATATATGCTATAATAAAATCAATAGTGTGGCTTGGTGTACGCT

>FM890551.1 FM890551 Jatropha curcas embryo 56-70 (DAF) Jatropha curcas cDNA clone rjcaeb0_000436, mRNA sequence

ATTCGAATACACAACAGCTATTGACATCTGGTCTGCTGGCTGCGTTCTTGCTGAACTACTGCTTGGACAG

CCTTTATTTCCTGGGGAAAGTGGAGTTGACCAGCTTGTTGAGATCATCAAGGTTTTGGGGACTCCAACAA

GGGAGGAAATTAAATGCATGAACCCAAACTATACAGAGTACAAATTTCCTCAGATTAAAGCTCACCCATG

GCACAAGATATTCCACAAGCGTATGCCTCCAGAAGCTGTTGATCTCTTTTCAAGACTACTGCAATACTCT

CCTAACCTAAGATGCTCTGCTCTGGATGCCTTGACTCATCCCTTTTTTGATGAGTTGCGTGACCCAAACA

CACGCCTGCCAAATGGACGTTTCCTTCCACCATTGTTCAACTTTAAATCTCAT

>FM890550.1 FM890550 Jatropha curcas embryo 56-70 (DAF) Jatropha curcas cDNA clone rjcaeb0_000435, mRNA sequence

AAATTTAACAGCTCTTGTGAAACATAATATTCAATGGCTCTAGTAAAACAAAATTTAGTATCACTTGACA

AAGGGTATCAAAAAGTCAAAAGAACTAGCACGACGCCATCGTCACAGATAAGAAAAATAAAGCTCATTAA

ATAAAAATCAGGAAATAAACGGCATGGGCTTTTAGTCTAAGCTACTGCTACTGGTACTGGGACCTCGATC

TCGGTCGCCACAACAGGTACCCGGGCGTACTCCTCTTCCTCCTTGGGGGGATGGATCGTGACCAAATCTG

GTAGAGGAGTTGTTGGGCCCTGCTTCCCCAGAGTTTTTTTTTTATATTTTTTTTTCAACTGAAAAATTAC

TGAAATACTGAACTATTATGCTTGTTAAATGGTACATCTTATGGAAAATATACGGGTGGTCCTGGCTATT

AAATGGCGCCACTTATACTTAAA

>FM890549.1 FM890549 Jatropha curcas embryo 56-70 (DAF) Jatropha curcas cDNA clone rjcaeb0_000434, mRNA sequence

TTCCTGAGGAGGCATACTCNGATTCGAAGCCATGGTCTCTTGAGAATACATTTACCAAACCCTAGTTAGA

TTTTCGCCTGCAACTACTCCATTCCTTATCTCAAGTGCTTGTTGTCCTTTGCTGGATGTGAATACACACT

TTGAATGTCAGCTCGTCAATTGATGTTTGATGGCATATTGATAATCGATCCCTCGACCAAGGTTACTTTG

TTCAAGTCATGTGCAGTCTCTTGTTTTAATAAGTTTATCTCAGTTGCTAGTGTCTTTATAAATATTAATA

ATGCTTGAAGCTCTTGTTACTTTGTTTATAAAGTAACAAAGGTTTATAAGGGTTACTTTGTTCAAGTCAT

GTGGAGAGTCTCTTGTTTTGTTAAGTTTATCTTAGTTGGGAGTGTCTTTATAAATATTAGTAATACTTGA

AGCTCTTGTAGCGGTCGAGGTTCTTGTGTGTCCTTGGTGTGTAAGGTTGCTTAGGGCAGTTCTGTGTCTT

GGGGTGCCTCAGATCAGCAGGCTTTGGCAGTGGCATTATGGGTGATAACTT

>FM890548.1 FM890548 Jatropha curcas embryo 56-70 (DAF) Jatropha curcas cDNA clone rjcaeb0_000433, mRNA sequence

ATTCCGGAGCACGACTTCCGCCAAAACCGAATGCCACCACCATCATCCGCCGCCACGCGTTCCGACCATG

ATGACGACGATCATGCTCCCTCGTCCTCCGATCCACCTGTCAAAATCTCTGATTCCAAGCTCGATAACGA

CAAACAACACGAGGTCGAAGTTGAAGAAGTAGAGGAACAAGAAGAAGACGTTAATGATAATGACGATGGC

AGCGAAGAAGAAGAAGCAGAAGAAGAAGAGTACGAAGAGATTGAAGT

>FM890547.1 FM890547 Jatropha curcas embryo 56-70 (DAF) Jatropha curcas cDNA clone rjcaeb0_000431, mRNA sequence

ATTCCAAAAAGGTCCATCAGCTCAGAAAGTGTTAGCTGTGATCACCCTCCTGCCGGTAGGCGGTGGGCTT

CTTGCACTAGCTGGTATAACCTTAGTAGGTACCCTAATCGGCCTAGCTATTACTACCCCTCTTTTTGTCA

TCTTTAGCCCATGTTCTTGTCCCAGCTGCATTAGTTATTGGCCTTTCGGTCATGGCATTTTTGGCTTCCG

GGGCTATGGGGCTCACAGGGTTGTCATCACTCTCGTGGGTTCTGAAATACCTCCAGGAGGTCACTCGGCG

AATGCCGGAGCAGCTGGATATAGCTAAGAAGCGTATGCAAGACATGGCTGGTTTTGTGGGACAGAAGACC

AAGGAAGTAGGCCAAGAAATCCAGAGGAAAGCACATGAAGGGAAATAAGTAGAGAGAAATGCTGTTGGTT

ATTAAGGGTTGAAGTCAGAGAGCTGGGTTTTGGTACGAGACTACTTTTGTTGTATGGGTGTTGTTTTGAG

GTAGTTGAGTTGGTTTATGGGGGATCTTCTTTGTTGCATTTGTATTTCTCTTTTAGGTCTTTTGGGTT

>FM890546.1 FM890546 Jatropha curcas embryo 56-70 (DAF) Jatropha curcas cDNA clone rjcaeb0_000430, mRNA sequence

CTCTGTGCTTCGATCGCGTCAATGGCGGAGCACCCACAGTCGCAGCATGTTGGCCAACAGCCACGATCCC

ACCAAGTAGTGAAGGCAGCCACTGCAGTCACTGCCGGTGGATCTCTTCTAGTTCTCTCCGGTTTGACCCT

TGCTGGTACGGTCATTGCACTGACCGTAGCCACTCCATTGCTGGTCATATTCAGTCCGGTTCTTGTTCCT

GCGGTTATCACAGTTTGTTTGATAATCACAGGGTTTTTAGCCTCAGGCGGTTTTGGCGTCGCTGCCATTT

CTGTCTTGTCCTGGATTTATAGGTATGTTACTGGAAAGCATCCACCAGGAGCAGAAAGTCTGGACCAGGC

ACGCTTAAAGTTGGCGGGAAAGGCAAGAGAGATGAAGGACAGAGCTGAGCAGTTTGGACAGCATGTAACA

GGTCAACAGACTTAAAAGAGAAGTGGGTTTTGGTGGTTGATGCAGAGAACATAAAATGGCTTTGATATAT

ATATAAAAATAT

>FM890545.1 FM890545 Jatropha curcas embryo 56-70 (DAF) Jatropha curcas cDNA clone rjcaeb0_000427, mRNA sequence

GTAAAGACTTGATTACAGAGACCTTCAACACATGACAAGAAAATTATTTTAGACAGCTCTCAAAAGAGGG

AAGACCCGTTAACAAATTACATAGAAGAAACAAAAAGATTTAAGCGAAAAAAGAAATACACCAGACAAGC

AATGGCCTTCCATGTTAATTAAAGGACTCATATATTTTTATATATATATCAAAGCCATTTTATGTTCTCT

GCATCAACCACCAAAACCCACTTCTCTTTTAAGTCTGTTGACCTGTTACATGCTGTCCAAACTGCTCAGC

>FM890544.1 FM890544 Jatropha curcas embryo 56-70 (DAF) Jatropha curcas cDNA clone rjcaeb0_000426, mRNA sequence

AAATAAAATATCTACTAAAAGATACCTTTTATTCAACTGGAATTGCTTAACAAGAAAAAGACACCAAACA

GAAAACATAGATATATTACATAACAGGAGCTAAAGCACATGCCATGCCCTACACACGTGGGTTATGGCCA

GTGGTGTCTCCGGGCAACCCAGATGGGCGAGTCCTGGTCAAACCCGTTGGGTC

>FM890543.1 FM890543 Jatropha curcas embryo 56-70 (DAF) Jatropha curcas cDNA clone rjcaeb0_000424, mRNA sequence

ATTCCTCTACATTAACATATCTTTCTCACACTTATCATCAACAAATGGCTGAACGTTCACAGCCACACCA

AGTTCAAGTGCACCCACAACACCGCTATGAAGCTGCCTTCAAAGGTCAACAAAAAGGTCCATCAGCTCAG

AAAGTGTTAGCTGTGATCACCCTCCTGCCGGTATGCGGTGGGCTTCTTGCAC

>FM890542.1 FM890542 Jatropha curcas embryo 56-70 (DAF) Jatropha curcas cDNA clone rjcaeb0_000422, mRNA sequence

AACAATTAAAAGAAAAACTTACATTTTATTAACGAAAACTCAGATTACATAATAAAAACAGCAACTCCAT

AAGGAAACAAGCTCACAACAACAAACCCATTACACACATCTATTTATTTTATTGGATGGATGGGTACCAT

TTAGACTTAGACACACTCAAACATAACTAAAAGTTTCAACCAGAAATCTCAATAGCCTTAACGTCCGGTT

TCTTGACTTCCACTTTAGGCACTGTCACAGTCAAGACTCCATTTTCCATGCTAGCCTTAACCTCATTAAC

CTTTGCATTCTCTGGTAGCCTAAACCTTCTCAAG

>FM890541.1 FM890541 Jatropha curcas embryo 56-70 (DAF) Jatropha curcas cDNA clone rjcaeb0_000421, mRNA sequence

ATTCCAGGCCTGGTAATTTTCGATAGCAATCCATTGCTGGAACAATGACTGGATTAGCTCATGGCATATG

GTACAGGCAAGAACGTGCGAGCTACATATGGAATTATTTGTGAATCTGTGAAATACTTTAAAGCAAAAGG

TTATGTACAAAAATTCTTTTCATTTTTTTCTTTCTGATACATTATCTTCAGAAATTAATGGGTGTTGGCT

GGGACTGCACATAAAAGAAAAATGTAAATTTATTGCTTGAAATGATAACTTCATTGCGCTT

>FM890540.1 FM890540 Jatropha curcas embryo 56-70 (DAF) Jatropha curcas cDNA clone rjcaeb0_000420, mRNA sequence

ATTCGCTGATAAGAAGAAGATTGAAGATGCTATTGATCAGGCCATTCAGTGGCTAGACAGCAACCAGCTT

GCAGAGGCAGATGAGTTCGAGGACAAGATGAAAGAGCTTGAGAGCATCTGCAATCCCATCATTGCCAAGA

TGTATCAAGGTGCTGGTGCTGACATGGGTGGCGGCATGGAAGATGATGTTCCTCCAGCAGGTGGCAGTGG

TGCTGGTCCCAAGATCGAGGAAGTTGACTAAGTGGCTTGTTTGATGCTTGAGTAAAGATGGTGATGAATA

TTTTATTTTCTTTATTGGTCAGTACAATTATTATTATTATTATTTTAATATTCTACTTAGACATTATGTA

AACCATCTGTC

>FM890539.1 FM890539 Jatropha curcas embryo 56-70 (DAF) Jatropha curcas cDNA clone rjcaeb0_000419, mRNA sequence

CGAATCTGAAGATCAGAGTCCTTTACGAGGCATCAGCTGGGAGTTGCTTGAGGCTGCTCTTAAAACCGAA

AGGAGGAGGTTAGAGCATATATTCAGGCAAAAGCAAGGAGCTATTGTAAAAGCCTCCAGGGAGCAAATTC

AAGCTATGAGTCATCGTGAAGAAGGTGGCGGCGTTTGGCCTTTCGGAACTGAATC

>FM890538.1 FM890538 Jatropha curcas embryo 56-70 (DAF) Jatropha curcas cDNA clone rjcaeb0_000418, mRNA sequence

TGCAGGAATTCGAATTCTTTTTTTTGTAATCAGGAATTATTATATTTTACATTATTAAAATGAAACAGTA

ATAATAAACATTCTTTCAGTACATACTTTCTCACACACATATCATTATATATATATATATATATATATAT

ATATATATATATATATATATATATATTGAGATAACATCATTATAAGCTGAAACTTAACCAGAGATCTCAA

TAGCCTTAACATCAGGTTTCTTGACTTCCTCTTTAGGAACAGTGACAGTAAGTACTCCGTTTTCCATTGA

AGCCTTTACTTGATCCATCTTTGCATTCTCCGGCAGCCTGAACCTCCTCATGAACTTGCCACTGCTACGC

TCCACGCGATGCCACGTGTCGTTCTTGTCTTCCTTCTCCACATTTCTCTCCCC

>FM890537.1 FM890537 Jatropha curcas embryo 56-70 (DAF) Jatropha curcas cDNA clone rjcaeb0_000417, mRNA sequence

TGAACACTTCCAATGGCTACAACTAACTTGCTTACTGTCTCTTCTTTCTCTGCTTGCTTTTGCTGCTGGA

GCTAAATTCTATGACCCGAGAGCTCCAACTGAAGCACAGCAATGCCATCTCCAGAGAATTACCACAACCC

AACCTTCTCATCGGATTGACTCAGAGGGTGGGGAAAA

>FM890536.1 FM890536 Jatropha curcas embryo 56-70 (DAF) Jatropha curcas cDNA clone rjcaeb0_000416, mRNA sequence

ATTCGTTTACCCGTCCTGGTAAACTCAGTGATCGGTTTCCTCAGCCATATGCAAATGAACAAGCAGCTAG

ATTTGCTAATGGAGGGGCCTATCCTCCTGATCTAAGTCTTATTACGAAAGCTCGTCACAATGGTCAGAAC

TATGTTTTCGCTCTCTTAACAGGATACCGTGACCCTCCTGCTGGTGTTTCTATCAGGGAGGGATTGCATT

ACAATCCTTACTTTCCTGGAGGAGCTATTGCCATGCCTAAAATGTTGAATGACGGTGCTGTTGAGTATGA

AGATGGTACGCCTGCAACAGAAGCACAGATGGGAAAAGATGTTGTATCATTTTTATCATGGGCTGCAGAA

CCAGAAATGGAAGAAAGGAAATTGATGGGTTTTAAGTGGATATTTGTACTCTCACTGGCATTACTTCAGG

CAGCGTATTACCGGCGCTTGAAGTGGTCAGTTCTCAAGTCACGCAGGCTTGTTCTCGATGTTGTCAACTA

GCTTCCAACCAGTTTATCTCACTGTAATGCTTATGGGAGGACTTGGATTGG

>FM890535.1 FM890535 Jatropha curcas embryo 56-70 (DAF) Jatropha curcas cDNA clone rjcaeb0_000414, mRNA sequence

CCGGGCTGCAGGAATTCGAATTCCATATCTCTAAAGATCTCTCGCGAAAACACTAGGAAAAATGGATGTC

AGGTTCGTAGGTCTCGATTCACCACTTCTCCACACTCTCCAGCAAATGATGGACATTGGCGACGATGCTG

ACCACAAATCCTTCAACGCGCCAACACGCACTTATGTGAGGGACGCGAAGGCTATGGCTTCTACTCCGGC

CGA

>FM890534.1 FM890534 Jatropha curcas embryo 56-70 (DAF) Jatropha curcas cDNA clone rjcaeb0_000412, mRNA sequence

ATTCTGATAATTCCTTTTGTTTATTTTGCATCTCTGCTTAAGATGACTACCNGAAAAAGAAGCTGAAGTT

GCTTCCACCGTTGAACAGCCACCGCCTGCGACAGAGGAGGCTAAGCCGGCGGAGACTAAACCTGTGAAGG

AGAAGAAAACCAGAGCTCCAAGAGAGAAGAAGCCTAAACAGCCTAAAACTGCTTCTCATCCTCCATATTT

TCAGATGATCAAAGAAGCTCTGCTGGCTTTAAACGAAAAGAGTGGATCGAGCCCATACGCCATAGCTAAA

TACATGGAAGAAAAGCACAAAGCAGTATTACCAGCAAATTTCAAGAAAATCTTACCTTTACAGTTAAAAA

ACTCGGCTGCTCGAGGAAAGCTGACAAAGATCAGAGCTTCTTATAAATTATCAGCAGGCAAGAAAGAGAA

AGACACAACCACCAAAGAAGTTTCAAAGAGTACTAAAACTACTAACAAAGAAAACAACAAACCCAAAACA

GCTACGACAAGAAAGACAAGGTCTGTTAACAAAACTGTCGAAGCTGCAAAGAAGCCTGCGAAGAGAGCTG

TGGGAGCTAAGAAGACACAGAAATCAACTCCTGCTAAACCTAAACAGCCCAAGTCAGTTAAATCTCCTAC

TGCTAAAAGAAC

>FM890533.1 FM890533 Jatropha curcas embryo 56-70 (DAF) Jatropha curcas cDNA clone rjcaeb0_000411, mRNA sequence

AACCAAAAGTGACCGTTTTTTCATTTTGCAAGTTCACAAACCAACTACAATTCTACGGAGTGGCAACTAA

ACATAAAGCCAAAAAAGAAGACCCAAAAACCATGGGATGCCACTTCCTGTCAGTGGGGAATACAACAGGT

TACACACTGATGCCCAGAAAACAAACCCCGACACCAACCAAAAACAACCAACATACCGCAGTTACTACTA

CCATATGTACAAACCTACTCGCAACTAGTTCAAAAAGAGGAACTACTAGTTAAAACACTGATATGAAGAA

CCTAACTAACGGGTTTTATACTTGGACTCATCAATTTCAATCCCCTCCTCAGCAGCACGTTCTTCACCAG

ACTTGTCCATGGTGCTAAGTCCGCCTTTTCGGCCCATCTCCTGATACCCTTCATGCCCAATCTGCTCCCT

TCTTGTCTCACCTCCACGGTGACCAAGTTCCTTGTACCCCTCAGTTCCCAGCTGCTCCCTCCTTTGTCTG

CCCTCCACGGCTC

>FM890532.1 FM890532 Jatropha curcas embryo 56-70 (DAF) Jatropha curcas cDNA clone rjcaeb0_000410, mRNA sequence

ATTCCTTTGAATACTTATCATTCATCAACAATGGCAGGTCTTAAGTTACTTAGCATTTTGGTTCTCTGCA

TGTTTGTCGCTGCGCCTATAACAGAGGCCATAACATGTGGACAAGTGAGCGGTGCCCTGGCTCCATGCAT

TAACTACCTGAGAACAGCAGGTGCCAAAGCTCCACCGCCGAATTGCTGCAATGGAGTGAGAAGCATCAAC

TCTGCTGCAAAATCGACCCCTGACCGCCAACAAGCATGTCAGTGCTTGAAACAAGCAGCAGCAAGCGTCT

CTGGTATCATCCCTACGAACGCTGAGGCTCTTCCTGGTGCTTGCAGCGTCAACATTCCTTACAAGATCAG

CCTCTCCACTAACTGCAACAATGTCAGGTGAGTTATACATGGAGCAGAGTTCGATTTTCAGGCCTGGAGA

TCAGGTAGCGTAAGAAGAATAAGAGGGAGAGAGAGGATAATGGTGTCTCCACTTTAGCGAGTCTCCGTTT

CTCTTTAATTACTACTGTTTTCTCT

>FM890531.1 FM890531 Jatropha curcas embryo 56-70 (DAF) Jatropha curcas cDNA clone rjcaeb0_000409, mRNA sequence

ATTCAATCCATCGGTCCAATTAGTATCCTGAAGAACCCTGTTTTTAGGAATAACTATGGTCAACTCTTTG

AAGTTGGCTCTAGAGATTTCAAGCAACAGCTCCGAGACCTTGACCTTTCAGTCTCCGTTGCCAACATCTC

TCGTGGAGCAATGTCTGGGCCCTATTACAACTCCAGAGTAACCAAGATATCAGTGGTTTTGGATGGCGAA

GGCTACTTTGAAATGGCTTGTCCTCACCTTAGCGGCTCGCGGGGAGACACGACGGGTGAAAGTCAGAAGA

CTTACCAGAAAGTAAGTTCACGGTTGAAACGTGGAACAGCAGTGGTCGTCCCTGCAGGCCATCCGGCGGC

TTTTGTAGCTTCAAGAAACAATAATCTGGAAGTAGTCTGCTTTGAGATTAATGAAGAAGGCAATACTAGG

CATCTACTTGCAGGGAAAAATAATATTGTAAATAAG

>FM890530.1 FM890530 Jatropha curcas embryo 56-70 (DAF) Jatropha curcas cDNA clone rjcaeb0_000407, mRNA sequence

ATTCCGATGAGTTCGTCCTAGCGAGCTCATATTTTGGAGGGAGAACCTACTGCTTGCTACAATTTCATTG

TAGTTTTGATATCCAGACGGGATTCTCATGCGGAATGATTTAGAAAGTGCATGGGGTTCACATCAACAGG

TTGCTTGTCAAGGGGCGAGTGATGAAATAGGCGTTACCACTATTCAGTCGGGAGTGGGGTTCTGATATCA

CCATTTTCAGCAAAAACAATTGAAGAAATTAGGCCCAGGAGAATTCAGAAGGCCTCGTCAGCAAAACAAA

TTCCAAAAGCACCAAGTCTGTAATTTTGATTTCAGAAGGCTAAACATCTTGTGATCGATGAGTTTCTCAT

TTATATCATATGTCAAAAACAGGTGGTGGTTTTCTTGGTTTGTAAATTATTTATGATCAAAGATTTATGA

ATATGCTAGAAGGATGTGAAATTAATTTATGGTTGGTTAGATTAT

>FM890529.1 FM890529 Jatropha curcas embryo 56-70 (DAF) Jatropha curcas cDNA clone rjcaeb0_000406, mRNA sequence

CGAATTCACTTTCAAAACCAATATGGCGCAGTTAACTATACGGACGAATACGGCAATCCAGTAGACGAAT

ACGGTCGCGCAATACGCCAAACAGACGAAGTTATGGTAATCCAATCCCTCAAACTGGTACTACCACTGCA

TACGGTGGCGCACCAGAGACTGGTACTACTGGCCTTGGCATGGGACACCGCCAAGAACACTACGGAGAGT

CTGGTCAGCTTCACCGTTCTGGCTCCTCC

>FM890528.1 FM890528 Jatropha curcas embryo 56-70 (DAF) Jatropha curcas cDNA clone rjcaeb0_000405, mRNA sequence

ATTCTTTTTTTTTAGCTAATCTGTAAAATAACTAACTTGCCAATATGATTTCTGAACTACAAAGGCATCA

CGTGGCCAATCATATAACCCCTGAAGTTTGAACTTAACCTCACTGAAACTTTAAATCTTAGATCATGAAT

TCGTAAATACATGAAAGAAAAAATAACATTCCACAACATTTGCACATCTGGCGCCCCTTTATCCCCTATA

AAGCAAGTAATTTGTTCGATTCACAAGAATCTAGCACTCCTGCCTGCAAGCTATTAATTTGAGAAAAATC

AGACTGGCCTTCGCCATGCAAATGATGATACAAGTCCTTCTGTCCCTTCATTAAACCCCAATGTTGATGT

GACCATCATAATCCATGAATCAATGGCCTTTTCAAAATGGTTCCACTAGTATGGCCTGTAGCGCATAGGT

CGTCTGAATCTTGGAACCCTCCCATAACTGTATGGAGGATAGAATGGGGCAGCAGGCATAAACGGCCTTC

GGGATCTGAACCCAAAATAAGGGTTTGGACGCCTTCCTCGATACTGCTTCATTCCAGGAATGTGGGTTCG

CTTCGCAGAGA

>FM890527.1 FM890527 Jatropha curcas embryo 56-70 (DAF) Jatropha curcas cDNA clone rjcaeb0_000404, mRNA sequence

GACCAGAAATAGTATTTCAGATTCTAACCAATCCAAAATTATAGTAAACAAGATTCTACCAAATCCAAAA

TTCTCTTCAGAAGTTACATAACTCCCAACAAAATAAAAAGACATTCTGCAAAATATTACTTGAAAAACCA

ACACTTTCTACGATGTAATATTCTGGGCCTTGTTGAGGATGACACCTTCATATTTCACCTGAAACCACTT

CATTGCATCCTCCTTGGTAACCCGGTGCTGAATACCGACCTTTGACTTGCATCTTCTGCGACGACCAACG

CGATAGCCTGGACGCTCCAAAACAACATAAGAAATCCATACCATAAATACCAGTTGAAGGATCATACTTG

ATGCCAAGATCAATGTGCTCCTGGATTCCAAATCCAAAGCAACCAGTGTCACTGAAGTTTCTTGGGGGGG

CCCCGGTAC

>FM890526.1 FM890526 Jatropha curcas embryo 56-70 (DAF) Jatropha curcas cDNA clone rjcaeb0_000403, mRNA sequence

ATTCACTTAGCCTTCAATGTCTCTGGACGACCTTATAATCGGTTTGCTTGTCACTTTGATCCTTCTGGAC

CTATATATTCTGATAGAGAAAGGCTTCAGATTTACATTTCTGACATTGGGATTTTCGCTGCAACTTATGT

GCTCTATCAGATTGCCATGGCAAAAGGGTTAGCTTGGCTGATATCTATTTATGGGATACCATTGCTTATT

GTTAATGCTTTTCTTGTGACAATCACATATTTGCAGCACACTCACCCTGCATTGCCACACTATGACTCGT

CCGAATGGGATTGGCTCCGGGGAGCTTTGTCGACAGTGGATAGAGATTATGGGGTGTTGAATAAGGTTTT

CCAGTAATATTAACAAGACACTCATGTAAACCACCATCTCTTCTCTACAATGCCTCATTATCATGCCATG

GAGG

>FM890525.1 FM890525 Jatropha curcas embryo 56-70 (DAF) Jatropha curcas cDNA clone rjcaeb0_000402, mRNA sequence

ATTCCAAGTTTTCTCCACCTACTCAGACAACCAACCTGGTGTTTTGATCCAGGTCTACGAGGGTGAGAGA

ACCAGAACAAGAGACAACAACTTGCTCGGCAAATTTGAGCTCTCCGGCATTCCTCCAGCACCAAGAGGGG

TTCCTCAGATCACTGTTTGCTTTGATATTGATGCCAATGGTATCTTGAATGTTTCAGCAGAGGATAAGAC

CACTGGCCAAAAGAACAAGATCACAATTACAAATGACAAGGGCAGATTGTCCAAGGAAGAAATTGAGAAG

ATGGTCCAGGAGGCTGAGAAGTACAAGTCCGAAGATGAGGAGCACAAGAAAAAGGTGGAGGCAAAGAATG

CTTTGGAGAACTATGCATACAACATGAGGAACACCATCAAGGATGAGAAGATCAGTTCCAAGTTGGCCGC

AGCTGATAAGAAGAAGATTGAAGATGCTATTGATCAGGCCATTCAGTGGCTAGACAGCAACCAGCTTGCA

GAGGCAGATGAGTTCGAGGACAAGATGAAAGAGCTTGAGAGCATCTGCAATCCCATCATTGCCAAGATGT

A

>FM890524.1 FM890524 Jatropha curcas embryo 56-70 (DAF) Jatropha curcas cDNA clone rjcaeb0_000401, mRNA sequence

ATCGATTGGTGCTACAATGATGGTAACGAGGAGCTTGTAGCCGTCACTGTTGTCCATCTTAACAACAGGC

ACAACCAGCTCGACCAGTACTTGAGGGCATTTTTGCTCGGAGGCGGCCAATCCCGACAAAGAGAGAGAGA

GAGTCCTGAAAGACCCCTTCGATCTATACG

>FM890523.1 FM890523 Jatropha curcas embryo 56-70 (DAF) Jatropha curcas cDNA clone rjcaeb0_000196, mRNA sequence

ATTCTGAGACTATTGACTCTCTTGCTGAAGCATTGAATGAATGGGATGGTGGTTTGGTTCTTGTTAGCCA

TGACTTCAGGCTTATAAACCAGGTAGCAGAAGAGATATGGGTTTGTGAAAATCAAGCCGTTACCCGATGG

GAGGGTGATATTATGGACTTCAAAGAGCACCTCAAGAGAAAGGCTGGCTTATCTGATTGAGTCGGATTAT

ATTAATAGAAGTGAAGAATATGATCCTACTATCATCATGCCCTAATCTATCGTTGAGAATCACAGCACAC

ACAGGTTATGGTTGTGGATCACAACATGGGCAGGTGAGTGCCGCCTAAGGATGGTGTTTGACCAAGGTGA

ATTACGGTGAAAGTTAAGCTAGAGAGCAGAGAGGTAACAAAAGGCTTGGTTATTGTTTTGAGCTGAAATG

TGCCTTTCCTGTGCTTAGTGACCAGGTGATTATTT

>FM890522.1 FM890522 Jatropha curcas embryo 56-70 (DAF) Jatropha curcas cDNA clone rjcaeb0_000194, mRNA sequence

GCTGCAGGATTCGAATTCGAAGCTGTGAAACACTTTGGCCCAGCTCCTGGTGTGCCACACAGCCATACCA

AACCATATGTTCGATCAAAGGGTAGGAAGTTTGAGAGAGCTAGAGGAAGGAGGAACAGCAGGGGATTTAG

GGTTTGACGTGTTTTTAGTTCTAAATTGTGAGGCACTTGTTTTACTTTTCCCAAATTTTGTTAGACTTTA

GGTTCCTTTGGAACTTGTTTATGTTATTCTTCTCGGTTATCAATTATCAGTCTGAATTGTTTT

>FM890521.1 FM890521 Jatropha curcas embryo 56-70 (DAF) Jatropha curcas cDNA clone rjcaeb0_000193, mRNA sequence

ATTCCGCTTTCCTGTTTGCTTCAGAGGGGAGTAATTTCTGAGTGACAGCCCTTTAAAGACCTTTTCAATC

GCCACGAGTCACCGGTTTTTAAAGATTATCTCCTCTGAAAGCAGGAAATTCAACTTATTTAAATAATGGA

GCACAATGAGACAGGATGCCAAGTTCCACTTGGGGCTCCTAAACTTTGTGCCAATGGTTGTGGATTCTTT

GGAACTGCAGCGACCATGAACTTGTGCTCTAAGTGCCACAAAGACTTCATAATGAAGCAAGAACAAACCA

AACTTGCTTCATCGTCCCTTGAGAGTATTGTGAACGGTAGCTCAAGCAACATCAGGAATGAGCCTGCTGT

TGCTATTGATCTGCAAACTTATTCAGCAGAGACTATGGTCAGTTCTGCTTCAGCGTCCTCTGATTCAGCT

CTCAGCACAAATGGTGACACGGTGGTGAAAGCAAGTCAAAATAGGTGCGCCACTTGCAGGAAACGCGTTG

GTCTAACAGGATTCAGTTGTCGTTGCGGGAATCTTTTCTGTTCAGTTCATCGCTACTCAGACAAACA

>FM890520.1 FM890520 Jatropha curcas embryo 56-70 (DAF) Jatropha curcas cDNA clone rjcaeb0_000192, mRNA sequence

ATTGAAGGTAATAAATTGGAAGTATGCAAGTGAAGTGTATGCAAATGAATGCCCTTCAGCTTAGAGCGCT

CACCACTCTTATGATTGAAATAAAATGATGTGTTAATGTATTATAAAATATTAAGGGCCCCTTCTCTCCA

CCGAGCTGTTCACAGGTTGTCTGCACTAGTGTGTGCTTTTGTTCATTTCTTCTTTGTCAGTCCTATCATG

GAACATATTGCTCTTGATGTCCTGTTATATGGATCTTCTAATAACAACAATTTGGGCACTAGATTTTATT

CAAGGGGCTTGAAATGATCATAATTCCAAAGCCTAATTAGGCAAGTTCCCTGTGATTATATATGAAAATT

AGTTTGTTAATGCGTC

>FM890519.1 FM890519 Jatropha curcas embryo 56-70 (DAF) Jatropha curcas cDNA clone rjcaeb0_000191, mRNA sequence

TTGATTCGAATTCCCGCTTGTGAACCGCCTTGAGCCAGATCATGTAGCAAAGGTGACTGGAATGTTGTTG

GAGATGGATCAAACAGAGGTCCTACATCTCATCGAGTCTCCAGATGCTCTGAAAAACAAGGTGGCAGAAG

CAATGCAAGTCCTCCGAGAAGCTAGTGCTTCTAGTGTTGGTGATCAGCTTGGTTCGCTGGGACTGAATGA

CTGAAGTGCATAATTCATGGTTATTTATTTTTTGGTCGTCTGTTAGCCCTGTTTTTTTATAGGGCTTCAT

GAGCAGTCATTATTTTGGGGCTATTCTGCTTAATTAGTTTTCTAGTATTTAGTATTGAACAGTGTCTTTT

ATATGTTAGGATTTTGGCTCTATTCTTCTTTTCCCTTTTTATTCTTTTAGATTTATTTGAC

>FM890518.1 FM890518 Jatropha curcas embryo 56-70 (DAF) Jatropha curcas cDNA clone rjcaeb0_000189, mRNA sequence

ATTCGCCAAGTGAGTGATTGAATTTGAATAGCCAAGTGAGTGAAAATGGAGTCGCTGAAGGAAACCGCAG

CTAACGTTGGAGCCTCTGCTAAGGCTGGCATGGAAAAAACCAGAGCAGAGGCGNCAAGAGAAGGTGGNAG

AGAATGTCAACACGTGATCCAGTGCAGAAGGAAATGGCGAGAGAGAAGAAAGAAGAGAGGAAAACGGAGG

CAGAATTGCGGAAACTAGAAGCGCGCGAGAGATAACGCAGCGGCGAGGCAGGCAGCCAAGGCTGGTGGCC

ATGTACAGTACACAACTGCTGGTCCGGGCGCTCAAGGCACAACCGAAACCTATACCCATTCAACAACTGG

GACAACCGGTTACCCTACAGGGACCCAGCAAATGTCGGCCATGCCTGGACACGGCACAGGGCAGCCATAC

GGTGGAGAGGTGGACCCAACGGGTTT

>FM890517.1 FM890517 Jatropha curcas embryo 56-70 (DAF) Jatropha curcas cDNA clone rjcaeb0_000188, mRNA sequence

ATTCCACAGTCGCAGCATGTTGGCCAACAGCCACGATCCCACCAAGTAGTGAAGGCAGCCACTGCAGTCA

CTGCCGGTGGATCTCTTCTAGTTCTCTCCGGTTTGACCCTTGCTGGTACGGTCATTGCACTGACCGTAGC

CACTCCATTGCTGGTCATATTCAGTCCGGTTCTTGTTCCTGCGGTTATCACAGTTTGTTTGATAATCACA

GGGTTTTTAGCCTCAGGCGGTTTTGGCGTCGCTGCCATTTCTGTCTTGTCCTGGATTTATAGGTATGTTA

CTGGAAAGCATCCACCAGGAGCAGAAAGTCTGGACCAGGCACGCTTAAAGTTGGCGGGAAAGGCAAGAGA

GATGAAGGACAGAGCTGAGCAGTTTGGACAGCATGTAACAGGTCAACAGACTTAAAAGAGAAGTGGGTTT

TGGTGGTTGATGCAGAGAACATAAAATGGCTTTGA

>FM890516.1 FM890516 Jatropha curcas embryo 56-70 (DAF) Jatropha curcas cDNA clone rjcaeb0_000187, mRNA sequence

ATTCCAGCAATGAAGTTCAATATCGCAAATCCAACTACTGGATGCCAAAAGAAGCTCGAGATCGATGACG

ATCAGAAACTTCGAGCATTTTTTGATAAGAGGATCTCACAGGAGGTCAGTGGAGATGCGTTGGGTGAGGA

GTTCAAAGGGTATGTTTTCAAAATCATGGGAGGGTGTGACAAACAAGGATTTCCCATGAAGCAGGGAGTT

CTCACTCCTGGTCGTGTCCGTTTGTTGCTACACAGAGGCACCCCATGTTTTCGTGGATATGGAAGACGGA

ATGGTGAGCGCAGGAGAAAGTCTGTTCGCGGGTGTATTGTTAGTCAGGACCTTTCTGTTTTGAACTTGGT

TATAGTGAAGAAGGGTGAAAATGATTTGCCCGGTTTGACTGACACTGAAAAACCTAGGATGAGAGGTCCA

AAGAGGGCATCCAAGATCCGGAAGCTTTTTAACCTTTCCAA

>FM890515.1 FM890515 Jatropha curcas embryo 56-70 (DAF) Jatropha curcas cDNA clone rjcaeb0_000186, mRNA sequence

TCGTGGAACAGCAGTGGTCGTCCCTGCAGGCCATCCGGCGGCTTTTGTAGCTTCAAGAAACAATAATCTG

GAAGTAGTCTGCTTTGAGATTAATGAAGAAGGCAATACTAGGCATCTACTTGCAGGGAAAAATAATATTG

TAAATAAGATGGAGAAGCAAGCCAAAGAGTTAGCCTTCGGTGTAAGTGAAAGAGAAGTGGATCAGGCTTT

TGGAAGACAAAACGAGGAGTGGTTCTTTCCAGGGCCAAGGAGAGGTTCCTTCGAGGGTCATGCTGTGGCA

TGAGAAGCTAATAACGTGATGGCAAGTGTTTCGGCTTTTTGGGGTGACTGTGACATCGACCTTGAGGGTT

TACTAATGTAAATAGTGCTGTGTATGGTGAATAAATAAT

>FM890514.1 FM890514 Jatropha curcas embryo 56-70 (DAF) Jatropha curcas cDNA clone rjcaeb0_000185, mRNA sequence

ATTCCATTTATATGGGGGAGGATTTGCTGAGTTTGTTGTGGCTCAAGGAGAGCTTGACAACTGAAAGGCC

ACCAGAAGTTTCAGCCGCAGAAGGTGCAGGATTGCTAGTTGCTGGTCTCACAGCTCACCAGGCTGTCACT

CAAGCTGCTGGGATCAACCTTGATAAAACCGGCGATCCAAAAAACATTCTGATCACTGCTGCTTCTGGCG

GTGTGGGTCATTATGCAGTTCAACTAGCAAAACTCGGAAACACGAATGTAACAGCCACTTGTGGTGCACG

TAACATTGAATTTGTGAAAAGCTTAGGAGCTGATGAGGTTCTTGATTACAGGACCCCAGATGGGGAAGCA

CTTAAGAGCCCATCTGGTCGGAAATACGATGCTGTGATACACTGTGCAACAGGTATTCCTTGGAAGACAT

TTGAGCCTAATTTGAGCGAAAATGGGAAGGTTATAGATATAACCCCAAGTCCTAGTGCAATGTGGACTTT

TGCTCTAAAGAAGGTTACATTCTCTAAAAAGCAGTTAGTGCCTTTGCTTTTGAGTCCCAAGGTTGAGAAC

ATGAACTATCTTATTAAACTGGTGAAGA

>FM890513.1 FM890513 Jatropha curcas embryo 56-70 (DAF) Jatropha curcas cDNA clone rjcaeb0_000184, mRNA sequence

ATTCCTCCTTCTTCCTTTGCGATAATGGTGAGTCGCAGTAGCCCAAATCCCTAATCTCATCTCCTCTTCT

TGCTTTTGATTTGCTAACTTTCTTGCCGTTTTTGTTGAGTTGTATCCTTGAATACGTTTATGGAATCTGA

AAGTGAAGATAGCGCTAGGTCAGTGAACTCCATTGTTGCTTGATCTAGACTGAGATGTCGGCGTAATCTT

CCCGTCCAAATAATGACATCGTGGAGTAGAACAAATCTAAGTAGACAATTCTGTCGATGCCCATATTGGA

TAAAGAAGGTAAAACAATAGGAATTGCTTCCTATTTTCTCTTTTCACTAGTACTTTGGTCTACACATACC

AATGTCAATTTCATGTTAATTGTTTCGGGAAGGGGACCCTAAAAATTATGACTTCTTTAGGTGGATGGAC

AGTGAACTTCCACCCAGGAGCATGGAGATTGTAAACAACATGTTTGCAAGAATTGATGGGTTGAAAACTA

TTGCGCAA

>FM890512.1 FM890512 Jatropha curcas embryo 56-70 (DAF) Jatropha curcas cDNA clone rjcaeb0_000183, mRNA sequence

TGGGAACTAATTATTAATTAAAACCAAAAAGTATGGAAACATCAAATTCATCAACAAACAAAGTAAGACT

TGATTACAGAGACCTTCAACAACATGACAAGAAAATTATTTTAGACAGCTCTCAAAAGAGGGAAAGACCG

TTAACAAATT

>FM890511.1 FM890511 Jatropha curcas embryo 56-70 (DAF) Jatropha curcas cDNA clone rjcaeb0_000182, mRNA sequence

ATATAAAGTGTAACAGAGCAAAAGATTAATTATATTGGTGGAGAAATTTGTTAGCAAAAAAGGGCAAAAA

TGTCTTCAAATCAATCAAGACAAGAGCTTGATGAAAGAGCAAGGCGTGGAGAGACTGTTGTCCCTGGAGG

TACTGGGGGCAAGAGCCTTGAAGCTCAAGAGCATCTTGCTGAAGGGAGAAGCAGAGGAGGACAGACAAGG

AAGGAGCAGCTGGGGACAGAAGGATATCAGGAGATGGGCCGGAAAGGTGGGCTGAGCACGACGGACGAGT

CTGGTGGAGAAAGAGCTGAGAAGGAGGGCATCACTATCGATGAGTCGAAGTTCAGGACTGGGAGCTAGTG

TGATGATTAAAACTATAAATGGTATTTTGTAGTAATAATAATAATAATGTTGGTAATAAAGTATTAGCTT

AGCAGTGTACTTTAGTTTAGTAATGTAGTTTACATGTGGGT

>FM890510.1 FM890510 Jatropha curcas embryo 56-70 (DAF) Jatropha curcas cDNA clone rjcaeb0_000181, mRNA sequence

CGAATTCCCCGCGGTTCGATTGATGTAGTTGCTATGCTTAGTGTGCGACTCGTTGGGTTTTTATTTTAGG

GTCAGGTTTAAAAAAAAAGGACTGACCTGTACTATGAACTAAAAATATAAGTAAGTATGCAACTAATAAC

CAGCCCAGCGCTTTGTAGTATCTCGATCCAAAGAAATTGTCAAGACATGATATATTAGCCATATCATTGT

AGCCATTGAAATTCTTTTTGCACAAACAAAAGAAAAACCAATCTGATTATGAGTTGTAAATCAAAATATA

AAATTAGATAGATAAGTGGAAGGGTGAAAGAAAGAGAGAAAGAGAGAAAAAGAATATCCAATGATATATG

ATTCCTAATATGTACAGGTCTATGAAGTCATCACAGTAAAAATTATAAACAATTAATGTAATAAAGCAGC

AATACCAATTGATTTATAATTAAA

>FM890509.1 FM890509 Jatropha curcas embryo 56-70 (DAF) Jatropha curcas cDNA clone rjcaeb0_000180, mRNA sequence

ATTCGAATTCAAAATAAGTCTTTGGGAATGGAGACAGAAGAAGGTGCAGTGTTAATGGAGCAGAAGCAGG

AGCAGAGGCAGAAGAAGAAGGTGATGGTGGCACTTGATGAGAGTGATACGAGCTTCTATGCTCTAAAATG

GACCCTTGATAACCTTATAATCAAAGGTGTTAGTCAAGATTCAGATATGGTTACACTAGTTCATGTCCAA

CATTCTTTCCAGACTAGTATCGTCCCTCTTGGTCCTGCTGGAGCAGCTCTTTACACACCGTCTTCAATAG

CGGATTCTGTGAAGAAAGCTCAGCAAGAAAATTCTGCAGCAATAGTCTCCCGAGCATTAAAGATGTGCGA

TGAAAAAATGGTGAAAGCAGAAAGTCTAATTCTTGAAGGGGATGCGAAGGACATGATTTGTAAAGCTACA

GAGCAAATGCAGGTTGATCTC

>FM890508.1 FM890508 Jatropha curcas embryo 56-70 (DAF) Jatropha curcas cDNA clone rjcaeb0_000179, mRNA sequence

CGTTCCCNAAACACAGTTTTTTCCATAACAGAATTATAAATAAATAGTCTACACAAGGTTATCCGAGAAT

AACAAGGGATTTAACAAAAAAAAAAAAAAAAAATGAAAACTATGGGGAATGAAACTGTAAATAGTTTTGG

AAACATCCAAAGGTGTTTAGAAGCATACTCTAACCAGTAGATTCAACTTCTTCACACCAAGTTACAAAAA

TATAAATAAACAATAAAAGGCTACTTAATGACAAGTCTTATAAGTGAAGCCACATCTCATGTAAAATGTT

CTTGAGATGGGCCCGATTCCCAAACCCTCAGACCCACGGCTAAACTACAGTGATCAGCACTCAACAATGG

ATGACCTTCCACAAAGCAAGAACAACTACTTTGGGCCAATGTCCTTAAGAGCACAAATCTGCGGGGGGGC

CCGGTAC

>FM890507.1 FM890507 Jatropha curcas embryo 56-70 (DAF) Jatropha curcas cDNA clone rjcaeb0_000178, mRNA sequence

GGTATAATCTGGTATTTATTTCCACAATGTAACAAATGTTACAAAGCTACAAAATGTACTTAGTATATAT

CTTCTCCGAATACATTAATCGAAAGGGAAATGGAAGGGAAAAAATAGGGAAAACCCTACACCAACCCTAT

ATATATATGTTTGAGTTTTGAAATCAAACAAAAGGATTACAGTTATTCTAGTAAAAAAACTAAAATAAAT

GGGCTTACATCTTTAATTTACTACATAAATTGCAATGATTCCCTTCTAAGTTGGCTTCATTTGTAGCGGT

TGCTCTCCTTGCTTGCCGAGAGAACCAGTTCCTACTGCAGAGGTTGCAGAGGTATTCAGTCCACTGGATT

CTGGGTGATTGATTCAGGAGACTTCTTGCATAGGCTGTATGGTGCTGCACTCATCACCCTCTCCTTGCGC

TTCTCCAAAAACCGAGCTAAGGACGCCTTTCGAGCTTGGGGCACAGATGGTATCATGGTTGTTGCAGCAA

CAGATCCCATTGTGCTTGCAATTCTTGGAGACTCCAGTTTACTAACAGGAGTTGT

>FM890506.1 FM890506 Jatropha curcas embryo 56-70 (DAF) Jatropha curcas cDNA clone rjcaeb0_000177, mRNA sequence

ATTCTAACAATGGCCAAACTAACTCTTTCCTTCTTTCACTGTGTCTAATTGTTATCTTCACTGGCTCTAT

AGCAAGTCAGCAACTTCAGCAGCAAAACCAGTGTCAACTCGATAGGCTCGACGCCCTCGAGCCAGATAAT

AGTATTCAAAGCGAAGCTGGTGTGGTCGAGTCTTGGAACCCTAACCAAGACCAGTTCCAATGTGCTGGTG

TGGCTATTGTTAGGCTCACCATTCAACCCAAGGGCCTTCTGTTGCCATCTTATAGCAACGCTCCTCAACT

CGTTTACATTGTCCAAGGTACGGGTATGACAGGAGCCCTGTTTCCTGGATGCGCTGAAACATATCAGGAG

TCTCAAGGATCAGGTGGTAGCAGTAGGGTTGAAGCCCAGCACCAGAAGATTCGTCGTTTCCGTCCAGGTG

ATATCATTGCTTTGCCTGCCGGAGTTGCCCATTGGTGTTAC

>FM890505.1 FM890505 Jatropha curcas embryo 56-70 (DAF) Jatropha curcas cDNA clone rjcaeb0_000175, mRNA sequence

ATTCCTTCAACAACAGGAGAATCACTGAGAAGATATCAACTGATAAATACAAGAATTATGCAGTGGTTGC

TGGTATAGTTACTAGTTTTGGTGCTCTGGGATGGTATCTGAATTCTCGAGGGAAGAAAACTGAAGAGGTT

CAAGATTGAGACCACTGTGATAGAGCGTGAAGAGGCATCTTGAGACAAATGGTGGCAGTTAGCAATAAAC

AGGATTTTGGGCATCAACTCTATTTTAACTTTTAGAGCTACGCTTGGCTGGATTTTATTTACTTCTGCAT

TTGAAAATGGCTTTTGGATGGGGGGGATGTCCTCAATGCTTTTACCCTTAACATCAAAGGGGTTTTT

>FM890504.1 FM890504 Jatropha curcas embryo 56-70 (DAF) Jatropha curcas cDNA clone rjcaeb0_000174, mRNA sequence

ATTCGAGATGGGCCGGAAAGGTGGGCTGAGCACGACGGACGAGTCTGGTGGAGAAAGAGCTGAGAAGGAG

GGCATCACTATCGATGAGTCGAAGTTCAGGACTGGGAGCTAGTGTGATGATTAAAACTATAAATGGTATT

TTGTAGTAATAATAATAATAATGTTGGTAATAAAGTATTAGCTTAGCAGTGTACTTAGTTTATTAATGTA

GTTTACATGTGGGTTAAAATGACTAGGGTTTAGCTAGTATGTGTGTTGTAACTTTGAGAGGGGTTTATGG

TCGTTCCCTTTCTGGGAGGACTGCTAATACTTTCTCCCTATTTGTAAAATAACCTGCAGTATATATAATT

ACCTGTTGGTT

>FM890503.1 FM890503 Jatropha curcas embryo 56-70 (DAF) Jatropha curcas cDNA clone rjcaeb0_000173, mRNA sequence

ATTCCACATTTGTTTGCCGTCAATCCGGTGGCGAGTGGTCCGCTAAGCAGATCGCTGGTGGTGATCTCGA

GGCTTCCGCTTCTTCCACCTATGATCTGCAGAGAAAGCTTGTACAGACCGCTCTCTCTTCCGACTCCTCC

GGCGGTGTCCAGTCTTCTTTCTCTACAATCACCCCTTCTTCGGCCGTTTTTCAGGTGATAATTGGTGGTG

GCGGCGGCGGAGCTTTTATTGGTGGAGGAGCTGCTGCTGCAGCTCCAGCTGGAGGCGCAGCACCAGCAGC

TGAAGCACCTGCTGCTGAGGAAAAGAAGAAGGAAGAACCAGCGGAAGAGAGTGATGATGATATGGGATTC

TCTCTCTTTGATTAGAGAATATGCTAAGTTACTCTTTTTTTATGTGCTTTATTTTGTCTCTCTGTTTTGT

TTTGCTGGAGTTGGTGGATTGGATTTAGAT

>FM890502.1 FM890502 Jatropha curcas embryo 56-70 (DAF) Jatropha curcas cDNA clone rjcaeb0_000172, mRNA sequence

ATTCCGAAGACCTAGAAAGAAATTGAAATGTCGGGAGTGTCCAATCAAGAGGAGGACAAGAAACCCACCG

ATCAATCGGCTCACATCAATCTCAAAGTCAAAGGCCAGGATGGCAATGAAGTCTTCTTCAGGATTAAGAG

AAGCACTCAACTGAAGAAGCTCATGAATGCTTACTGTGATCGACAGTCAGTGGAGTTCAACTCTATTGCA

TTTTTATTTGATGGTCGCCGGCTTCGTGGAGAGCAGACACCAGATGAGTTGGAAATGGAGGATGGGGATG

AGATAGACGCAATGCTGCACCAAACAGGTGGCACTATTGCCTAATGTAATCGCTCGATGAGTTTGTTAAT

TTGGTAGGAGCAAATTATTTAGTATTAAGTGGATTAGGATCACTGGAAAACAATGGCTCTTCAGTTACAC

ATGCAGCCTATATTTTCTGAACTTTGTTGGCAAAATAGCTAAGATAT

>FM890501.1 FM890501 Jatropha curcas embryo 56-70 (DAF) Jatropha curcas cDNA clone rjcaeb0_000171, mRNA sequence

ATTCCAACTACGCAGTTGACCCAGCATCACTTCCTGAGGGTGTGGAAGTGACTCATATCAATCTGAATGA

TGGAAGCTGTGCTGGTCTTGCTTATCCTCCACTAAACATCATGTCACTTCAATACCACCCTGAGGCATCC

CCTGGCCCTCACGATTCAGATATGGTGTTTGGGGAATTTGTAGATCTAATGAAGCAAGCGAAGCAAAAGG

CTTGAGATGAGATAGTTTTGCTTGAAGTGAGCTTTGCCGTATTTTCATGCTGTCGATATTGCTAATCCAT

GCAAGATAAAATTAGTCATGGGCATCTTAGCATCAGTTGCTGGAGGTGTCTGTTAAATAGTTGAGATGGT

TTATAAATTCGTGTCGTTCTCTGAAGTTATGCAGTTTTGCTTAACCGCTGATGATATGTGACTACTATGG

ATTGGTTGGATTCCATTTGTAGGTTATCATGAATTTGCATTCAGTAAAAAA

>FM890500.1 FM890500 Jatropha curcas embryo 56-70 (DAF) Jatropha curcas cDNA clone rjcaeb0_000169, mRNA sequence

CCCCGGGTCTGCAGGATTCGAATTCTTTTATTTTTAACTAGAATTTATCTCTTTATTAATTATTAAACCA

GAAGCCACTATAGTCCGCAGCAGTGATCTTTCTCTTTTCTTAAAGACTAATTTACACATGTACCTTCACC

TTTCATTATTTATTCACCATACACAGCACTATTTACATTAGTAAACCCTCAAGGTCGATGTCACAGTCAC

CCCAAAAAGCCGAAACACTTGCCATCACGTTATTAGCTTCTCATGCCACAGCATGACCCTCGAAGGAACC

TCTCCTTGGCCCTGGAAAGAACCACTCCTCGTTTTGTCTTCCAAAAGCCTGATCCACTTCTCTTTCACTT

ACACCGAAGGCTAACTCTTTGGCTTGCTTCGGGGGGGCCCGGTAC

>FM890499.1 FM890499 Jatropha curcas embryo 56-70 (DAF) Jatropha curcas cDNA clone rjcaeb0_000168, mRNA sequence

TGCAAATCTAATTACCTCAACAGCCCTACTCTGCGTTCTTCTAGTGATCATAGCCAATGCAGTCACTGGC

TACAGAACTATCATCACCACCGTTGAAGTCGACGATTCTTCCAATGTTCGGGATATATGCAAGAAGGAAG

CTGAAAGAAGAGATTTGAGTTCCTGTGAGAATTATATAACACAGAGAAGAGGAAGGTCAGAAGATATGTT

AGCTATGCCAGGAATTGAAAACCCTCGACAGCAGGTTCCTAGACAATGCTGTAACCAAGCAAAGGAACTA

AGTGCTATTTGTCGATGTGAATCAATACATTATCTGTTAGAGAAGCAGCTGGAGGAAGGAGAAGTTGGAA

GCGAAGATGAGGCAAGGCGCAGGACTAAAAATATAATTGATGTTTGCTTTGGGTCTATATGCCCTCGTTA

AAATGCTAGCTGCAGGTGAATAAAACTTCATTCACTTGTATGTGAGTGAGAGTTATATATAATGATGTAT

AAGAAGTAAGAGCATCAGAATAAAAAAGGGGGGGCCCGGTAC

>FM890498.1 FM890498 Jatropha curcas embryo 56-70 (DAF) Jatropha curcas cDNA clone rjcaeb0_000167, mRNA sequence

GCAATATAACGAAATTTGGGTAACAAAATCACATAATTCTAATTAACAAAAACAAAAAAGGATAATATGC

TCAACATTGTCTGAGTCTAAATATCAAAAATTCAACAGTATGCCAGTACCCAGAAAAACAAGAGACATAA

TCCAAAACAACCAATATAAGACTTCACCTACATAACTATTAAAAACTTGTTATCCTGTCACACCTTACTT

CTCAGATGAGTTGGGTCCTCTCTTCTTGCCGAAACCCACAACTGCGGTGACGAAACGTCGGTTGTATTGC

ATCCTCTTGTGGGCGCGGCCACGTGGCTTCTTCTTCTTGTCCTGCTTTGCCACTTTCGGAGTTTGGCCTC

TAACCTTACCAGCACGAGCCAAAGATCCATGAACCTTACCCATGGCGGCTTCTTGAGCTTTTTCTGCTGA

GTGGGGGAGCTGTTGCTCTGAA

>FM890497.1 FM890497 Jatropha curcas embryo 56-70 (DAF) Jatropha curcas cDNA clone rjcaeb0_000166, mRNA sequence

ATTCCCCCTGGCTTCCTCCTAACCCTAACTCAGACCCAGATTCGCCATGAAAGGAGGTAAATCAAAGTCA

GACACCAAGAACGCCAAGCTTTCCGTGAACAAGAAACCCGCCAAGGCAAGTAAGAAGGCGGGTAAGGCAG

CGAAGGATCCAAATAAGCCAAAGAGGCCCGCCAGTGCTTTCTTCGTTTTCATGGAAGAGTTCAGAGAGCA

GTACAAGAAGGAACATCCTAAAAACAAATCCGTTGCTGCTGTTGGCAAAGCTGGTGGAGATAAATGGAAG

TCAATGTCAGATGCTGAGAAAGCACCTTTTGTATCTAAGGCAGAAAAGAGGAAGATAGAATATGAGAAGA

AATTGAAAGCCTACAACAAGGGACAGGCTGAAGGCCCCAAGGAAGAAGAGGAATCTGAGAAGTCAATGTC

TGAGGTGAATGATGAGGAAGAAGATGATGAGGAGGGAAGTGGAGAGGAGGACGAT

>FM890496.1 FM890496 Jatropha curcas embryo 56-70 (DAF) Jatropha curcas cDNA clone rjcaeb0_000165, mRNA sequence

ATTCCTTCCTTGCTGGTAACCCACATCGAAAAGTACAGAGCCAGAGAGGTGAGTCCGAAAGAAGGCATGA

GTGGTCCAGAGGCGAAAGCCTAAGGAGTCATCAAAGAAGCGGCAATGTGTTTTCTGGCTTGGACGAGCAA

CTACTAGCTGAAGCTTTCAATGTCAACCGGGATATTGCAAGAAGACTAAAAGGAGAAAATGATAACAGAG

GGATTATTGTTAGTGTGGAGGGTGAGCTNTGAGTTTACTTTAGCCCACGAAGGAACCANAGAAGAAGAAA

AACGAGAGAGACAATTCGTTAATGGCATAGAAGAAACTTTCTGCACAGCCAGACTCAAG

>FM890495.1 FM890495 Jatropha curcas embryo 56-70 (DAF) Jatropha curcas cDNA clone rjcaeb0_000164, mRNA sequence

ATTCCTCAGCTTTCATTGGCACTGCTGCGCAGATATACAAAAAAGAACCCTAAAAATGGCGCCTCAGTTT

CTTAAGCTTCCCATCATCTCTTGCTATTGTAGACATTGCAATATTATCAATCACAGCTTCTCGCTATTAT

CCAAATCCTACCCTTCTAATCTTACCGTTAGATGGGTCAAACTGGAAACGCCTTGTTCTTTCAGGAGTTT

TGGTGTTGCTAAGGCAGGAAAGATTGCAATACCTAAAAAAAAAAAGAAGACTGGATGAAATATGTCTTGA

AAGGTTCCAGCAATACAGCCGAACATTCATACAGTCATGGATTTTACAAGGCAAAGTATTTGTGGATGGA

AAGATGGTGAACAAAGCAGGCACACCTGTCTCCGACAAAGCTGTTGTACAAATAATGGCTGAAATTCCAA

AATATGTATGTAGAGCCGGATATAGGTTAGAAGGCGCCATCGAACAATTGGGGGT

>FM890494.1 FM890494 Jatropha curcas embryo 56-70 (DAF) Jatropha curcas cDNA clone rjcaeb0_000163, mRNA sequence

ATTCCGAGGAATAAAGAAATTTCAAGCGAGAAGAATGGCTACAGAGCAAGAAAGGGCTGAACTTGATGCT

AGAGCAAGGCAAGGAGAGACTGTTGTACCTGGTGGAACTGGTGGCAAGAGCCTTGAAGCCCAGGAACATC

TAGCTGAAGGGCGGAGCCGTGGAGGGCAGACAAGGAGGGAGCAGCTGGGAACTGAGGGGTACAAGGAACT

TGGTCACCGTGGAGGTGAGACAAGAAGGGAGCAGATTGGGCATGAAGGGTATCAGGAGATGGGCCGAAAA

GGCGGACTTAGCACCATGGACAAGTCTGGTGAAGAACGTGCTGCTGAGGAGGGGATTGAAATTGATGAGT

CCAAGTATAAAACCCGTTAGTTAGGTTCTTCATATCAGTGTTTTAACTAGTAGTTCCTCTTTTTGAAC

>FM890493.1 FM890493 Jatropha curcas embryo 56-70 (DAF) Jatropha curcas cDNA clone rjcaeb0_000162, mRNA sequence

ATTCTTTATTTCTTCTTCTTCGTGCGGAAGGCAAAAACATAAGACAGAGGACAAAAAAAACAAAGAAAAG

ATTTATGTCTAAATCAATCATGACTAATAGTACTATTCCAGAGAAAAAACAGCTGAGGAAGCCTGCTCAG

GCTAGTTCAAGGAAAGGGTGCATGAGAGGCAAAGGAGGCCCAGAAAATGCTCTCTGCACTTACAAAGGTG

TTCGCCAAAGGACTTGGGGAAAATGGGTTGCTGAAATTCGAGAACCCAATCGCGGTGCCCGTCTTTGGCT

CGGTACTTTTGACACCTCTCACGAAGCTGCAATGGCTTACGATGCTGCTGCTCGTAAACTATATGGTCCC

GAAGCCAAGCTCAACCTACCGGAGTTGCACCC

>FM890492.1 FM890492 Jatropha curcas embryo 56-70 (DAF) Jatropha curcas cDNA clone rjcaeb0_000161, mRNA sequence

ATTCCCTCTCTCGGCCATCAATACTTGTGAGGCCTTGGACGCCATATTTTCTGTACAACCCCATCAAGAG

AAACAAGGGAAGTAGCGCGGAGAAAGAGAATGAAGGGCTTGCAATAGATCAGGAGAGAGCTTCTTCAACA

GCTGATGAGTTCAAAAGAGTTGCAAAGGAGAAGGAAAAGGCTGAAGAGGCAACGCGTGTCGCAGAAGATC

AAGGCATTACTAGTCAAACTGGTGAAAAGACAATCGACGGCGCAGAAGAGGCTTCTGGAGATTCTAATAT

CGAATCTGTTAAAAACAGGTATAAGAAGCATGAACTGGGTGCTAATTCTGGTAGAACAGGTTGATTAATT

AGTGA

>FM890491.1 FM890491 Jatropha curcas embryo 56-70 (DAF) Jatropha curcas cDNA clone rjcaeb0_000160, mRNA sequence

ATTCCTTCAACTTCAAATCAAGTATCCAGCTAGAATGGCTTTAAGCTTAGGAAGGAGGAGCAACATCTTT

GATCCGTTCTCACTAGATATTTGGGACCCATTTGAGGGCTTACCTTTCTATAGCGGAACAGTGGCCAATG

TTCCGTCCACTCAACACGAAACAGCCGCCATTGCCAACGCACGAGTGGACTGGAGGGAGACTCCGGAGGC

TCACATATTTGCTGTTGATCTTCCTGGGTTGAAAAAAGAAGAGGTAAAAGTGGAGGTAGAAGATGGCAGA

GTTTTGCAGATAAGTGGGGAGAGAAGCAAAGAACAAGAAGAGAAGAACGATAAGTGGCATCGCGTAGAAA

GAAGCACTGGTAAGTTCTTGAGGAGGTTTAGGTTGCCGGAGAACACAATGATGGATCAAGTCAAGGCCAC

TATGGAGAACGGTGTGCTTAAAGTGGTTGTGCCTAAAGAACAAGAGAAGCAGCCTCAGATTAAGCCTATT

GAGATCACTGGCTAGAAGTGCAAATATGGTCTTTTACT

>FM890490.1 FM890490 Jatropha curcas embryo 56-70 (DAF) Jatropha curcas cDNA clone rjcaeb0_000159, mRNA sequence

ATTCCTTCAAATCAAGTATCCAGCTAGAATGGCTTTAAGCTTAGGAAGGAGGAGCAACATCTTTGATCCG

TTCTCACTAGATATTTGGGACCCATTTGAGGGCTTACCTTTCTATAGCGGAACAGTGGCCAATGTTCCGT

CCACTCAACACGAAACAGCCGCCATTGCCAACGCACGAGTGGACTGGAGGGAGACTCCGGAGGCTCACAT

ATTTGCTGTTGATCTTCCTGGGTTGAAAAAAGAAGAGGTAAAAGTGGAGGTAGAAGATGGCAGAGTTTTG

CAGATAAGTGGGGAGAGAAGCAAAGAACAAGAAGAGAAGAACGATAAGTGGCATCGCGTAGAAAGAAGCA

CTGGTAAGTTCTTGAGGAGGTTTAGGTTGCCGGAGAACACAATGATGGATCAAGTCAAGGCCACTATGGA

GAACGGTGTGCTTAAAGTGGTTGTGCCTAAAGAACAAGAGAAGCAGCCTCAGATTAAGCCTATTGAGATC

ACTGGCTAGAAGTGCAAATATGGTCTTTTACTACT

>FM890489.1 FM890489 Jatropha curcas embryo 56-70 (DAF) Jatropha curcas cDNA clone rjcaeb0_000158, mRNA sequence

ATTCCAAGTTCCTGCTCTCGAAGCTCAGTGACCTCCAATTTTTTGTGGGGGAGAGCATGCATGATGATGG

GAGTTTGGTGTTTGCTTACTACAAGGATGGCGCCACTGACCCAACATTTTTGTACTTCGCTTATGCTTTG

AAGGAGATCAAGTGCTAAGAATGGAGAATAGCTGGCTTCGAAGTGGAAGTTCTTGCCATATTTCTAGCTT

TATGTGTCCTTTTCCATCTCTGTACTGTTATATTTGGTGCTTCATGCCTTATTTTGGTTAATTATTGACT

AGACATGTTTTCCTCCTTCTTTGGTTATTAGTTTCTTGTTTGACAAATAATTCAATCTTCTATTGGGCGT

GTATTCTAAAATTTACCAGCCTAAGTTGGTAATTGTTTGATGTTACAACTTCTTTGTTCCCTTTGTATTT

GTGGAAGGCTTGTCAAAATAGATATGTTTTACTGGCTCTAATCATTAGCAATTTGGCAACATTCTATTAT

TATATACTTTAAGGTGGTGCAGGCAAGCAAGCATGT

>FM890488.1 FM890488 Jatropha curcas embryo 56-70 (DAF) Jatropha curcas cDNA clone rjcaeb0_000156, mRNA sequence

GGAATTGGCAAACATATTCCACACACTAAGTATAATGACCAGCTACACGTGAAAATCAGGAATCAGGAAA

CACCAGCTAACTTTATCAATAAGGCATTTAGATAATCATTTGCAAACGCCATTAGAACCAAGGTTTTACA

GAGGAAATAAGGGTTTCTTCCCCTACTTTTCCTCGAGCAGCACAAGCAAAATTGCAAAAATGTCCGCGTG

TAATAAGTGAATGCCATAAGAGCAAGGTAGGAGTAACATACAGCAAAAGGTTCAACTTCCTCCCATATTT

CATGAAGATCTATCTCCTGCAAAGGTTTGAGACCAAGGCGAGAAAGTCCAGAATGGCTAAATTTCTCAAA

GAGTAAATATTTGTCCGCAGAAGCATTTCCTTCACATCTCATAATCCCATTGCAAGACAAGGTATTTATG

CTTGACTCCGCT

>FM890487.1 FM890487 Jatropha curcas embryo 56-70 (DAF) Jatropha curcas cDNA clone rjcaeb0_000155, mRNA sequence

GAAATTTCAAGCGAGAAGAATGGCTACAGAGCAAGAAAGGGCTGAACTTGATGCTAGAGCAAGGCAAGGA

GAGACTGTTGTACCTGGTGGAACTGGTGGCAAGAGCCTTGAAGCCCAGGAACATCTAGCTGAAGGGCGGA

GCCGTGGAGGGCAGACAAGGAGGGAGCAGCTGGGAACTGAGGGGTACAAGGAACTTGGTCACCGTGGAGG

TGAGACAAGAAGGGAGCAGATTGGGCATGAAGGGTATCAGGAGATGGGCCGAAAAGGCGGACTTAGCACC

ATGGACAAGTCTGGTGAAGAACGTGCTGCTGAGGAGGGGATTGAAATTGATGAGTCCAAGTATAAAACCC

GTTAGTTAGGTTCTGCATATCAGTGTTTTAACTAGTAGTTCCTCTTTTTGAACTAGTTGCGAGTAGGTTT

GTACATATGGTAGTAGTAACTGCGGTATGTTGGTTGTTTTTGGATTGGTGTCGGGGTTTGTATTCTGGGC

ATCAGTGTGTAACCTGTTGTATTCCCCACTGAC

>FM890486.1 FM890486 Jatropha curcas embryo 56-70 (DAF) Jatropha curcas cDNA clone rjcaeb0_000154, mRNA sequence

GCATGTGGATCATGCTGGGCTTTTAGTACCACTGGAGCGATTGAAGGAGCTAATTTTATAGCAACAGGAA

AGCTTCTTAATCTTAGTGAGCAACAATTGGTGGACTGCGATAATAAGTGTGACATGGTGGACAAGACTGC

ATGCGATAGTGGTTGCGGGGGAGGTCTTATGACGAATGCTTATAAATATTTGATGGAGGCAGGAGGGTTA

GAAGAAGAGAGTTCATATCCATATACAGGAAAACGCGGTGAATGCAAGTTTGATAAGGAGAAAATAGCAG

TCAGAGTTGCTAATTTCACCAATATCGCTATTAATGAGGATCAAATTGCAGCTCATTTAGTTCACTATGG

ACCTTTGGCAGTTGGATTGAATGCGATTTATATGCAAACTTACATTGGAGGAGTGTCATGTCCACTTATT

TGCGGCCAGAAATGGATCAACCATGGAGTTTTGCTTGTTGGGTATGGAGCTAAAGGGTTCTCCATTCTTA

GATTAAGTAACAAGACGTATTGGATCATCAAGAACT

>FM890485.1 FM890485 Jatropha curcas embryo 56-70 (DAF) Jatropha curcas cDNA clone rjcaeb0_000153, mRNA sequence

TTCTCTCACTCCTTCCTCCTCTCTCTCTCGCCCTCGATTTTTTTTCACTTAAACGGCAAGCAAGTAAAAA

ACAAAAAAAATCCAAGGGATGTCGAGTACAACCGCTGCTACCGATCCAGAGCACAACAACAAAGACAGAG

AAGAGGAAGAGAACGCTTCTGCCGTCGACGATGAGGACACCGGAGCTCAGGTTGCTCCCATCGTCAAACT

CGAAGAGGTCGCTGTTTCCACTGGCGAGGAAGATGAGGATCCTATCCTTGATCTGAAAGCGAAGCTCTAT

CGATTTGATAAGGACGGAAACCAGTGGAAGGAGAGAGGAGCTGGCAATGTGAAGCTGTTGAAGCACAAGG

AGTCTGGCAAGGTTCGCCTTGTCATGCGCCAATCTAAGACTCTCAAGATCTGCGCTAATCACCTAGTTCT

ACCATCAATGAATGTCCAAGAGCATCATGGAAATGATAAATCATGTGTGTGGCATGCCACTGATTTTGCT

GATGGAG

>FM890484.1 FM890484 Jatropha curcas embryo 56-70 (DAF) Jatropha curcas cDNA clone rjcaeb0_000152, mRNA sequence

AACCAAGCAAACGATAGAGTTAAAGCCTTAATAACCTAAGCTTCAAAACACAAGTGCACTACACGGAAGA

ACTTTAACTTGTAAGAAAAATTAAATTAAAATCCTCAAAAAAGTCAATTACTAAGAAGATAACAATGCAA

ATAACCAAAACAGCAGCAGATAGGTTCAATGGTAGCTCAGCTCTCGGTTTGTTATCTAAGCCTTCTTATT

GTGCTCCTCACAGTCACGGCACTGGCGGTGTTCAAAGTGTAGGCACCGCCTGCTTTGACTTTACCACAGT

CTTTGCAGCCCCAAATTCCCACAGCCTTTCTCTTCACAGCGTACTTCCCACAGAATTCACAGAAGTATTT

GCTGTGCTGACTGACCTCCATCTTCTTAATCTGTTTACGCAGACTTGCACCATACCTGGTACCATATTTT

CCAACAATACCAGCCTTCTTTGTTCGCTTTGTCATGGTTGTTGGCTTGAAGGACCTC

>FM890483.1 FM890483 Jatropha curcas embryo 56-70 (DAF) Jatropha curcas cDNA clone rjcaeb0_000151, mRNA sequence

ATTCCACAAATTAGCCCACTAGCAGGACGGACTCGGCAATTAGGGCTCTGCCAGAGGGCAGTGGTTGCAA

ACGCATTCCAGATTTCCAGAGAAGATGCCAGGAGGCTTAAGGAGAATAGACAGGAAGTCACTGTGTTTAG

CCCTGGGTCTAGATCTCGTAATTAATATTGCAACTATGCAATTATACCAAGAGGATGTATAATAAACTGG

TTTCTACATAGTTTAGATTTGGAGAAGAATAAGCCTATTTTTATAGGCAGTTTTAGTTCCTTGCAATAAT

ATAAAAATGTAGATCTGTATATGCCTTATGTTATGAGATAATGAAAGTTTGTGGAGCTAAAAAAAAAAAA

>FM890482.1 FM890482 Jatropha curcas embryo 56-70 (DAF) Jatropha curcas cDNA clone rjcaeb0_000150, mRNA sequence

ATTCCTTATCATCAACAAATGGCTGAACGTTCACAGCCACACCAAGTTCAAGTGCACCCACAACACCGCT

ATGAAGCTGCCTTCAAAGGTCAACAAAAAGGTCCATCAGCTCAGAAAGTGTTAGCTGTGATCACCCTCCT

GCCGGTAGGCGGTGGGCTTCTTGCACTAGCTGGTATAACCTTAGTAGGTACCCTAATCGGCCTAGCTATT

ACTACCCCTCTTTTTGTCATCTTTAGCCCTGTTCTTGTCCCAGCTGCATTAGTTATTGGCCTTTCGGTCA

TGGCATTTTTGGCTTCCGGGGCTATGGGGCTCACAGGGTTGTCATCACTCTCGTGGGTTCTGAAATACCT

CCAGGAGGTCACTCGGCGAATGCCGGAGCAGCTGGATATAGCTAAGAAGCGTATGCAAGACATGGCTGGT

TTTGTGGGACAGAAGACCAAGGAAGTAGGCCAAGAAATCCAGAGGAAAGCACATGAAGGGAAATAAGTAG

AGAGAAATGCTGTTGGTTATTAAGGGTTGAAGTCA

>FM890481.1 FM890481 Jatropha curcas embryo 56-70 (DAF) Jatropha curcas cDNA clone rjcaeb0_000149, mRNA sequence

CCCTAACTAACAAAAGTTAAGTTGAACAAGTCTAACTAACTGTCAAGCCTTTCAAAAAAGTATAGATACC

CCATATCTTTGGGAGGGTTTACAGAAGCCCCGACCTTGTTGTCGTTGAAAATAGTCCATCTGCCATCTTT

AAATACATGCGCAACATAATGCCCGCATTGCGTAGAGGTTCCAATGTGGCTCACTATTCCGAAAAGCCTG

TATCTCCCTCCTCCATCAGGCAGTCCAGCATCATCCGGGTTAGGTAAGTTACTAGATGTGGTGGCATCCA

TATCAGATGAGACAGAAGCATCGGGATTGCTGAATATCCAATCTGTTGCTTTCTCGGGGGGGCCCGGTAC

>FM890480.1 FM890480 Jatropha curcas embryo 56-70 (DAF) Jatropha curcas cDNA clone rjcaeb0_000148, mRNA sequence

ATTCGTGGCTTCCAGTTTATATTGACCCACTTAGATGGAAGGCAACTGCTCATAAAATCTCAACCANGGG

GAAGTAGTGAAGCCTGATCAATTCAAGGCCATAAATGATGAAGGGATGCCGATGTATCAGAGGCCGTTCA

TGAGGGGGAAGTTGTACATTCATTTCACTGTAGATTTCCCTGACTCTCTACCTCCAGATCAGTGCAAAGC

CCTAGAGGCAGTGCTTCCCTCAAGATCGTCGGTGCAGCTCTCTGACATGGAGCTGGATGAATGTGAGGAG

ACAACTTTACATGATGTTAACTTTGAAGAGGAGATGCGGAGGAAACAACAACATGCTGCAGAGGCATATG

ATGAAGATGAAGATATGCATGGTGGTTCCCAGAGGGTACAATGTGCTCAGCAATAAATTAACGTTAAGGA

GATTATCTGAATCAAGGCTGAGAAATCAAGACTCATAGTATTTGAGTATAAGATTCTCAAACGCTCTTTG

CCTTGTGCTATAGGAT

>FM890479.1 FM890479 Jatropha curcas embryo 56-70 (DAF) Jatropha curcas cDNA clone rjcaeb0_000147, mRNA sequence

TGCAGGCGGGCCGCGAGTAGCTGCAGAAGGATTGATCCATTCGAGAATCTATTTCTCAGGGGATCGAATA

GCTTCTGCAGTATCTGCTTACTCTGTACATATATCTACCAATAAAATGACTCGCTCTCTTTCCACCGCTA

AATTTCTGGTTGCTTCAATTGTTGAGGATCTACCACTTTCTATCTTCCGGAGAGGCTATGCGGGTGCAGC

ACATGGGTCTGAGTCGGCTAGCTTTGGTAGGCGAGGGTCACGGACTAGTATTGTGGGGAAAGTAGAAGAA

AGAGCTGCGACGAAAGAAGATGATTCAGGATCATCTACTGCGTGGGCACCAGACCCCATCACGGGGTACT

ACAGGCCAGCTAATTCTGCGGCTGAGATCGACCCGGCTGAGCTCCGGGAAATGCTATTGCACAAACGGGT

TAGGCCACGGTAGAAAGTCTGAGATTCAGATGCTGAAGTGGTGGCGTTCCCTTTTAGATGTGTGTCTTAC

ACCAGCTTATAGTAGTACTG

>FM890478.1 FM890478 Jatropha curcas embryo 56-70 (DAF) Jatropha curcas cDNA clone rjcaeb0_000146, mRNA sequence

CGAATTCGATTAACAGAGACGAAAAAGAGAAACTTTACGTTGTCAGCGTCATGGTACCCGTCAATATTCC

GGGCGAGTTCGAGATATTCAGTGCGTCTGGTACTGAAGATTCAGAGTCCTTTTACGAGGCATTCAGCTGG

GAGTTGCTTGAGGCTGCTCTTAAAACCGAAAGGAGGAGGTTAGAGCATATATTCAGGCAAAAGCAAGGAG

CTATTGTAAAAGCCTCCAGGGAGCAAATTCAAGCTATGAGTCATCGTGAAGAAGGTGGCGGCGTTTGGCC

TTTCGGAACTGAATCCATCGGTCCAATTAGTATCCTGAAGAACCCTGTTTTTAGGAATAACTATGGTCAA

CTCTTTGAAGTTGGCTCTAGAGATTTCAAGCAACAGCTCCGAGACCTTGACCTTTCAGTCTCCGTTGCCA

ACATCTCTCGTGGAGCAATGTCTGGGCCCTATTACAACTCCAGAGTAACCAAGATATCAGTGGTTTTGGA

TGGCGAAGGCTACTTTGAAATGGCTTGTCCTCACCTTAGCGGCTCGCGGGGAGACACGACGGGTGAAAGT

CAGAAGACTTACCAGAAAGT

>FM890477.1 FM890477 Jatropha curcas embryo 56-70 (DAF) Jatropha curcas cDNA clone rjcaeb0_000145, mRNA sequence

ATTCCTCTTTCCAACCCTTCAACTTCAAATCAAGTATCCAGCTAGAATGGCTTTAAGCTTAGGAAGGAGG

AGCAACATCTTTGATCCGTTCTCACTAGATATTTGGGACCCATTTGAGGGCTTACCTTTCTATAGCGGAA

CAGTGGCCAATGTTCCGTCCACTCAACACGAAACAGCCGCCATTGCCAACGCACGAGTGGACTGGAGGGA

GACTCCGGAGGCTCACATATTTGCTGTTGATCTTCCTGGGTTGAAAAAAGAAGAGGTAAAAGTGGAGGTA

GAAGATGGCAGAGTTTTGCAGATAAGTGGGGAGAGAAGCAAAGAACAAGAAGAGAAGGACGATAAGTGGC

ATCGCGTAGAAAGAAGCACTGGTAAGTTCTTGAGGAGGTTTAGGTTGCCGGAGAACACAATGATGGATCA

AGTCAAGGCCACTATGGAGAACGGTGTGCTTAAAGTGGTTGTGCCTAAAGAACAAGAGAAGCAGCCTCAG

ATTAAGCCTATTGAGATCACTGGCT

>FM890476.1 FM890476 Jatropha curcas embryo 56-70 (DAF) Jatropha curcas cDNA clone rjcaeb0_000143, mRNA sequence

GCAGTACAAAGTACATAAACTAATTCTGAAAGTTATTGGGCACAAGTTAACATCACATTACAAATCCAGA

TACATTATTGAACTGATTTTTAACTATAATGAACACAACAGTTACATATGCATGGCCAACACACTAAGCG

GAAGCCGCTGCTGCAGCTTCTTCCAGCAACAGTTTAACCTTCGTGATATAGTCACTCATTGCTTCTTCCT

TAGATTTGCCTTCAACAGCCTTCCATGCATCCCACTTTGCTCTGTCCCTCATGTAGAACATTCCAGGACG

GCTGGTGTACACAGGTCCAACAGTGGCTTGTATAAAAAGCCCATACAAAATAAGTTTGTTCTCATTTGTT

GTGTTTTCCGGAAGGGTCTTAGCTTTCTCAGCATACTCCTCAAATTCCTCCTTCAAACGGGGGGGCCCGG

TAC

>FM890475.1 FM890475 Jatropha curcas embryo 56-70 (DAF) Jatropha curcas cDNA clone rjcaeb0_000142, mRNA sequence

ATTCCGATAAAGTGTAACAGAGCAAAAGATTAATTATATTGGTGGAGAAATTTGTTAGCAAAAAAGGGCA

AAAATGTCTTCAAATCAATCAAGACAAGAGCTTGATGAAAGAGCAAGGCGTGGAGAGACTGTTGTCCCTG

GAGGTACTGGGGGCAAGAGCCTTGAAGCTCAAGAGCATCTTGCTGAAGGGAGAAGCAGAGGAGGACAGAC

AAGGAAGGAGCAGCTGGGGACAGAAGGATATCAGGAGATGGGCCGGAAAGGTGGGCTGAGCACGACGGAC

GAGTCTGGTGGAGAAAGAGCTGAGAAGGAGGGCATCACTATCGATGAGTCGAAGTTCAGGACTGGGAGCT

AGTGTGATGATTAAAACTATAAATGGTATTTTGTAGTAATAATAATAATAATGTTGGTAATAAAGTATTA

GCTTAGCAGTGTACTTTAGTTTAGTAATGTAGTTTACATGTGGGTTAAAATGACTAGGGTTTAGCTAGTA

TGTGTGTTGTAACTTTGAGAGGGGTTTATGGTCGTTCCCTTTCTGGGAGGACTGCTAATACTTTCTCCCT

ATTTGTAAAATAACCTGCAGTATATATAA

>FM890474.1 FM890474 Jatropha curcas embryo 56-70 (DAF) Jatropha curcas cDNA clone rjcaeb0_000141, mRNA sequence

AAAAAAGGACACCTGTTTCATTTTTCATTAATAAGCACATTTCCTAACCAGGTAATACAAAGTTACAATA

GAAACACTTCATAGCAAGCTGCTTTTTAAGCACACTTAGACCCACTATTTTATTTATGGCTTTCCCTGTT

TATAAGATGCTACATAAACTCTCATCTTAGAGAAAGCCCAAGACCCCAATACACACATAAAATTTATTAG

CCATAGGTAGCAGTACTAGTAGCTAATACCTAGTGTCTGTGTCCCTATAAGTTGTGGTCTGCTGATGGCC

AGAACGATCCTTAACATGATCTTCTTCTTCTTCATGGTGAGCCATACCAAAAGCGCTTTTGACAGTATCA

GCAGTTTTTTGAGCTGCATGCTTCATTTTCTCCCCAGTTTCTTCCAGAACTCCCTTTGTCTTCTCCT

>FM890473.1 FM890473 Jatropha curcas embryo 56-70 (DAF) Jatropha curcas cDNA clone rjcaeb0_000140, mRNA sequence

ATTCGTGATCGATAAAGTGTAACAGAGCAAAAGATTAATTATATTGGTGGAGAAATTTGTTAGCAAAAAA

GGGCAAAAATGTCTTCAAATCAATCAAGACAAGAGCTTGATGAAAGAGCAAGGCGTGGAGAGACTGTTGT

CCCTGGAGGTACTGGGGGCAAGAGCCTTGAAGCTCAAGAGCATCTTGCTGAAGGGAGAAGCAGAGGAGGA

CAGACAAGGAAGGAGCAGCTGGGGACAGAAGGATATCAGGAGATGGGCCGGAAAGGTGGGCTGAGCACGA

CGGACGAGTCTGGTGGAGAAAGAGCTGAGAAGGAGGGCATCACTATCGATGAGTCGAAGTTCAGGACTGG

GAGCTAGTGTGATGATTAAAACTATAAATGGTATTTTGTAGTAATAATAATAATAATGTTGGTAATAAAG

TATTAGCTTAGCAGTGTTATTTAGTTTAGTAATGTAGTTTACATGTGGGTTAAAATGACTAGGGTTTAGC

TAGTATGTGTGTAGTAACTCTGAGAGGGGTTTATGGTCGTTCCCTTTCTGGGAGGACTGCTAATACTTTC

TCCCTATTTGTAAAAT

>FM890472.1 FM890472 Jatropha curcas embryo 56-70 (DAF) Jatropha curcas cDNA clone rjcaeb0_000139, mRNA sequence

ATTCGCTGCTTTGCCAAAGGCTCCATCATCCCAATATGTGAATGTGTCCCGGTGGTACAAGCACATTGAT

GCACTCCTAAGAATTTCTGGTGTATCTGGAGAAGGGTCAGGAGTTACTGTTCAGGGATTTTGCACCCGTC

ACAGAGGAGGCAATTGCAACTCCTCCAGCTGCTGATTCAAAGGCTGCTGCCGAGGATGATGATGATGATG

TGGATCTGTTTGGTGAGGAGACTGAGGAGGAAAAGAAGGCCGCAGAAGAACGTGCAGCTGCTGTCAAGGC

ATCTGCAAAAAAGAAAGAGTCTGGTCAGTCATCCGTTCTATTGGACGTCAAGCCATGGGATGATGAGACT

GACATGAAAAAGCTTGAGGAAGCAGTTAGAAGCATTCAGATGGAAGGATTGCTTTGGGGAGCATCCAAGC

TTGTACCGGTTGGATATGGTATAAAGAAACTGCAGATTATGCTTACTATTGTGGATGACCTAGTGTCTGT

CGACAGTCTAGTTGAGGAACATCTGACTG

>FM890471.1 FM890471 Jatropha curcas embryo 56-70 (DAF) Jatropha curcas cDNA clone rjcaeb0_000138, mRNA sequence

ATTCCTTTAACTGCATCCTATGAAGGGTTAGATGCCTCAAGTTATGATGCTCTTGTTATCCCAGGAGGCC

GGGCTCCAGAATACTTGGCCCTAGATGAGACGGTGATTGCATTGGTGAAAGAATTCATGGGATCTAAGAA

GCCTGTGGCATCCATTTGCCATGGGCAGCAGATCTTAGCTGCTGCTGGTGTTCTGAAGGGAAGAAAATGT

ACTGCATATCCTACGGTGAAGCTTAATGTTGTCTTGTCCGGGGCAACATGGCTAGAACCTGATCCGATAG

ATCGATGCTACACTGATGGAAATTTGGTTACTGGAGCAGCATGGCCAGGGCATCCACAGTTCATCTCTCA

ATTGATGGCATTGCTAGGTATCCGGGTCTCATTCTAGTTGCTGTCACTATGCTTGTAGATTTCATCGTAT

GCCTTCTTATATCAGATAGCGTGTTAATATAACTCATGCTTTCTTGTATCCAATTGCATGTTGAATATCC

AGCATATCACCTCCTAAAAGCATGCGTGTGTCAAAACTAAAAAATAACAAAA

>FM890470.1 FM890470 Jatropha curcas embryo 56-70 (DAF) Jatropha curcas cDNA clone rjcaeb0_000137, mRNA sequence

ATTCCCAAGATCCTTTGGATGAGGAGAATGCGTGTCCTGAGGCGTCTGCTGCGCAAATACAGGGAATCCA

AAAAAATTGACAAGCACATGTATCATGACATGTACATGAAGGTAAAGGGTAATGTATTCAAGAATAAACG

TGTGCTGATGGAAAGTATCCATAAATCAAAGGCTGAAAAGGCAAGAGAGAAGACTTTATCTGACCAATTT

GAGGCTAAGCGTGCAAAGAACAAGGCAAGCAGGGAGAGGAAGATAGCCAGAAGAGAGGAGCGTTTGGCTC

AGGGGCCTGGAGAGAAGGCACCACAAGCACCTGCCCCACAGCAGACCGAGGGAGCAAAGAAATCCAAGAA

GTGAATGTAAGACTGATGAATGCAGGAGGTTGATACCTGAGTTATCTTTATGTCTGGTCTAGTAGGAGTT

TGTCTCTTAAAGTTACTATGTTTTCATTTTCTGGGATATTTCTTTGAAATATAGAATTTTTGAATTCCAT

GAGCC

>FM890469.1 FM890469 Jatropha curcas embryo 56-70 (DAF) Jatropha curcas cDNA clone rjcaeb0_000136, mRNA sequence

ATTCAAACAATCCTCCTAATGCAGAAGGCAATGATTTAGAGGTGCGTGCAGCTACAAAACGTGAAATGCC

TGTGGAGCTTTTGCGCGCATGCCTTCCCCATACTGATGAACGCCAGATGGAGCTTTTAAGACACTTTGGA

CAGCAACGAAATTCTGTTACTGGGTTGAATGCACATTCAGGGAATAGTGGTCAGATGAACTCTGAATTGG

TGAGGAGTTGAAGAACGAACTAAAGATATCCAGAATGCCGTAGCTCTTCTCTGTGCATCCCACTTGATAC

TTTGGCGGAGAAACTGCTTGGTATTGCAGGTGGGCAAAAAGCGTAAGGTTACTGCTTAATTATCTTTCCT

TGAAAGTGTGTATTATGATGAAAATTATTCCTGGGAGTTGGCGACTAGAAGTTTGTTTGTTTATGAGGGG

GCTTGTTTAGTCGCAACTGCAGGAAAGTTACAAGCACAGGGGATAGAATGTACAGGGTGGTCTTAGAGAA

CCATCAAATCCCTCTTTTCATCAGCCATATCAGTTTGAAGACTTTTCTGATTCAAAAATGGCATGATGTG

C

>FM890468.1 FM890468 Jatropha curcas embryo 56-70 (DAF) Jatropha curcas cDNA clone rjcaeb0_000135, mRNA sequence

ATTCAACAATAATGTCCTTTATCTTGTTAGCCCCTGTGGCTGTCTTCATGGAAGGGGTCAAGTTTACTCC

TGCATATCTCCAATCTGCTGGCTTGAATGTTAAACAGGTGTACACAAGGTCTCTTATTGCTGCTCTCTGC

TTCCATGCCTATCAACAGGTTTCATACATGATATTGCAGAGGGTATCACCTGTTACTCACTCTGTAGGCA

ATTGTGTGAAGCGTGTGGTGGTCATTGTCAGCTCCGTTCTCTTCTTCAGAACGCCTGTGTCACCTATAAA

CTCTCTAGGCACTGGGATTGCCCTTGCTGGAGTTTTTCTGTATTCAAGAGTGAAGCGCATTAAGCCAAAG

CCGAAGACAGCTTGAAGTTAGGCAGAGCATTTTTGCATTTAAATGTGGTTATTAGACATAGGTTGGACAA

CTTCTTTGTAGCTGAGGCCAGAATGTCGATTATGCTTTTGTTTTCTT

>FM890467.1 FM890467 Jatropha curcas embryo 56-70 (DAF) Jatropha curcas cDNA clone rjcaeb0_000134, mRNA sequence

ATTCCACAAAATGGCTCACTTTCAAAACCAATATGGCGCAGTTAACTATACGGACGAATACGGCAATCCA

GTAGACGAATACGGTCGCGCAATACGCCAAACAGACGAGTATGGTAATCCAATCCCTCAAACTGGTACTA

CCACTGCATACGGTGGCGCACCAGAGACTGGTACTACTGGCCTTGGCATGGGACACCGCCAAGAACACTA

CGGAGAGTCTGGTCAGCTTCACCGTTCTGGCTCCTCCAGCTCTAGCTCCGTAAGTTAAAATATTACTCTG

AAAATTTATCATGCGTAATTTTTTTTTAATTAACACTTTCATCTTGCTATTTTTAAAAAATAAATTGCAA

TCACGTCTTTTACTTTTGCTATAAGCTTACCATCAGGTTCTTTCGTTTCGTTAAAAAAGTTAGAAACATG

TTTGTTTATATGGAAAACCTAATCTGCAGTCTGAAGATGATGGACATGGTGGAAGGAGGAAGAAAGGGCT

AACAGA

>FM890466.1 FM890466 Jatropha curcas embryo 56-70 (DAF) Jatropha curcas cDNA clone rjcaeb0_000133, mRNA sequence

ATTGCAATATATTCGTTGATTTGGATTTCCAGGTTACGGAAGCGAAGATGATATGGGTTGATAGGCTGGG

TTTCGACATGCGACTCTACTGCCCTCAGAAAGGTGTTTTTGATGTCCGAATTCCTTTCCCAAGAGAAGTG

ACTGATGTAAAGGGTGCAAAGTCCTCATTCAATGGTATGTCACAACTTGCTTGGGAAGTGGAAAAAAACT

ATAATATGCCTGATTTTAAGAGGGTGAAACAGTTGAAAAGGATAACATATAGGGGATTGTAAAATAGGAT

TTTTGTCTCAGGAGTTTTTTGTGAAATAATGTGTTAGGAGTGAGATGGAAATAATTTCTTCTTCAGACAA

ACAAATCTGATGCTTTTGTGGGTTTAATTTTTCTCACATGCGGTTTAATGATTGAGTTCTAATGAGCTTA

CTATAATCTCAATTTCTAGTTCTTTATTTTGTTCATTGGATGATCTACTCAGCATAAAAGTATTAGTTTC

TGTGGGCTTCTCTAAGCTACTAAACAAGGAATTGGTGGTGCTTGTAC

>FM890465.1 FM890465 Jatropha curcas embryo 56-70 (DAF) Jatropha curcas cDNA clone rjcaeb0_000132, mRNA sequence

CCCGGGCTGCAGGGAATCGAATTCGAATCCTCTTCCAAACAAAGGGAGCATGACTCTAGCGGCCTTGATC

TTTCTGGTTTGGCCAGAGATGTTTAAATCAGTATGTAAAAACACGAATTTCTTAAGTTGGGATTTGCTGT

TCTAATCTTTTTTTCGCATTATTATTAGCAAAATATAGACTCTTCAAATACATTTTTACTGAAATTGAGA

GCTAGTTCTTGAAAAAAAAAAAAAAAAAAAAAAAAAAAAAAAAAAAAAACCAATCAAGAGAATGTCTCTG

ATTCCAAGCACCCTCTTCGGTGGCCGGAGAACCAACATTTTCGATCCCTTCTCTCTCGATATCTGGGATC

CATTTCAGGACTTCCCTTTCACAAGCACTGCTCTTTCTGCTCCTCGTTCTGAGTTATTGAACGAAACGGC

AGCGTTTGCCAACACCCGCATAGACTGGAAAGAAACACCAGAAGCTCATGTA

>FM890464.1 FM890464 Jatropha curcas embryo 56-70 (DAF) Jatropha curcas cDNA clone rjcaeb0_000131, mRNA sequence

ATAGAAGACTTACCAGAAAAGTAAGTTCACGGGTTGAAACGTGGCAACAGCAGTGGTCGTCCCTGCAGGC

CATCCGGCGGCTTTTGTAGCTTCAAGAAACAATAATCTGGAAGTAGTCTGCTTTGAGATTAATGAAGAAG

GCAATACTAGGCATCTACTTGCAGGGAAAAATAATATTGTAAATAAGATGGAGAAGCAAGCCAAAGAGTT

AGCCTTCGGTGTAAGTGAAAGATCGAAGTGGATCAGGCTTTTGGAAGACAAAACGAGGAGTGGTTCTTTC

CAGGGCCAAGGAGAGGTTCCTTCGAGGGTCATGCTGTGGCATGAGAAGCTAATAACGTGATGGCAAGTGT

TTCGGCTTTTTGGGGTGACTGTGACATCGACCTTGAGGGTTTACTA

>FM890463.1 FM890463 Jatropha curcas embryo 56-70 (DAF) Jatropha curcas cDNA clone rjcaeb0_000130, mRNA sequence

ATTCCACGCTCCAAATCGTAGATGAGAATGGAGATGCCGTGTTCGATGGACAAGTTCAACAAGGTCAAAT

ATTAACAGCTCCACAGAACTTTGTGGTTTTGAAGAGGGCAAGCAATGAAGGGCTAGAGTGGGTATCCTTC

AAGACTAACGAAAATGCACAAATTAGCCCACTAGCAGGACGGACCTCGGCAATTAGGGCTCTGCCAGAGG

CAGTGGTTGCAAACGCATTCCAGATTTCCAGAGAAGATGCCAGGAGGCTTAAGGAGAATAGACAGGAAGT

CACTGTGTTTAGCCCTGGGTCTAGAATCTCNGTAATTAATATTGCAACTATGCAATTATACCAAGAGGAT

GTATAATAAACTGGTTTCTACATAGTTTAGATTTGGAGAAGAATAAGCCTATTTTTATAGGCAGTTTTAG

TTCCTGGCCAATAAATATAAAAATGTAGATTCTGGTATATGGCCTTTATGTTATGAGATAATGAAAGTTT

GTGGAGCTATAAGTTATAGT

>FM890462.1 FM890462 Jatropha curcas embryo 56-70 (DAF) Jatropha curcas cDNA clone rjcaeb0_000129, mRNA sequence

AGAAGGCAAACGGCAAATACGATAAATCCACAACTGTGACCAATGCAACATGAGGAGTAACAATCTAATA

ATAATATTACATTCGATACATGATATGAAGATGTGAATACCTCGTATGCATACAACTGTAATGATTCCAT

GATGAGTTGAAGCGTGCAAAATTGGGAAAGCCTTCCATTGATGTAGCAAAGTTGCAACAGTTAATTGGCA

GCTCAAAATCTACCTAGAAGAATTCTTCCTTGCAGTGTATGCGCGAAAAGCTGCCACTCCGACAACTGTG

AAAGCAGCCCCAACTGACATGAATAGCAGAGAGTGACTGAAAAGCTGCCGAAAGTACTTGCGCTTCCTCC

CAGCCTCAGTTTCAGGAATATT

>FM890461.1 FM890461 Jatropha curcas embryo 56-70 (DAF) Jatropha curcas cDNA clone rjcaeb0_000128, mRNA sequence

CGTTGATGGGCGAGCATCTATTTCTTTGTCTAGTTATAGAACAAAAAAAAAAACAAACAAACAAACCAAC

CATCGCTGACTTTATTATTTCTTGCCTACTCGTACCATTGCGTCTACTAACTGGGCCAGGGCAAAACGAG

AAGACCCTCCTTCGCTCGCTGCCTCCGCCGGCAGCCTCTTTCATGGCAATAACTCGCTTTTGAATCGTTT

GCCCTTTCTCCGAGTTCATCAACTCGGTTAGCCGCTGCTCTAACTCCTCCCCACTCACAAAACCATCTTC

AGGCTGATTCACCGGCAAAGCCATTTTCATCTCTTCGACCAAGAAAACACTGTTCATTTTTTGCTCCGCA

TAAAGAGGCCATGCCAACATAGGCACGCCGGCACAAAGCGATTCGAGAATCGAGTTCCACCCGCAGTGAG

TCACAAACAGACCCACCGAGTCATGATTAAGTAGAGAGAGAGAGAGAGAGAATAGTCTCAGGGGGGGCCG

GTACCCATTCG

>FM890460.1 FM890460 Jatropha curcas embryo 56-70 (DAF) Jatropha curcas cDNA clone rjcaeb0_000127, mRNA sequence

ATTTATATATATATATATATTAACCCTTGTAACCGAAGCTTGAGTCCTGCATTCTGGAAATTGCCTTCTC

AAATTGTGACCATTTCTGCTCCAAATTGCCAATATACCCAATAGACATCCTAATTAACCCAGGCGAAATC

CCAGCTAGTTTCTTTTCCTCAGGACTCATTTCACTGCTGGTGCTACTCCCAGAAACTGACATTAACGTCT

CGTAGTATCCCAAACTAACTGCCATAAGCCCAAACTGGGTGTAGTTCTGCAAGTGGTTCATCAACCGGTT

AGCCCTTTCTTCAGTTTCCATGTCAAGGCAAACAAGTGCACTGAAACCATAATCTTTATTAGCCATGGAT

TTCAAGAGCTGATGTTGTGGATGCTCTTCAAGACCTGGGTATATGACTTTGAGTCCCAGTTTCTTCATTC

TGGTGGCATATTCCATTGCTCTGTGACAATGTTCTCTCATTCTCAATCCCAAGTGAGGAAGCCTTCCTGA

TATTCAAAGC

>FM890459.1 FM890459 Jatropha curcas embryo 56-70 (DAF) Jatropha curcas cDNA clone rjcaeb0_000126, mRNA sequence

AACTAGAATTTATCTCTTTATTAATTATTAAACCAGAAGCCACTATAGTCCGCAGCAGTGATCTTTCTCT

TTTCTTAAAGACTAATTTACACATGTACCTTCACCTTTCATTATTTATTCACCATACACAGCACTATTTA

CATTAGTAAACCCTCAAGGTCGATGTCACAGTCACCCCAAAAAGCCGAAACACTTGCCATCACGTTATTA

GCTTCTCATGCCACAGCATGACCCTCGAAGGAACCTCTCCTTGGCCCTGGAAAGAACCACTCCTCGTTTT

GTCTTCCAAAAGCCTGATCCACTTCTCTTTCACTTACACCGAAGGCTAACTCTTTGGCTTGCTTCTCCAT

CTTATTTACAATATTATTTTTCCCTGCAAGTAGATGCCTAGTATTGCCTTCTTCATTAATCTCAAAGCAG

ACTACTTCCAGATTATTGTTTCTTGAA

>FM890458.1 FM890458 Jatropha curcas embryo 56-70 (DAF) Jatropha curcas cDNA clone rjcaeb0_000124, mRNA sequence

ATTCCCAATTCCTCGCTCGCTCAACAAACTCCTTTCACCGCCGCACTAAACTGCTACTACATATATTCCC

CATCCACACCCTCTATCTCTCAACAAATTCAAAAATTTTCTCAAACTTTCTCTCCGTCCAAACAGTCCCC

ACTCATAATCATGTTTCGTCTAAGCAACAATTTGGTAGGGATTCTCAACTTTGTCACTTTCCTTCTCTCA

ATTCCGATCCTCGGCGGTGGGATCTGGCTTCGGAATCATGGATCCTCTGAATGCGAGAAGTTTCTGGACA

CGCCAGTTATAGTCCTCGGCGTGTTCCTGATGGTGGTTTCTCTGGCCGGACTGATCGGCGCGTGTTGTCG

CGTGTCGTGGTTACTCTGGTTTTATCTCCTCGTCATGTTCCTTCTTATTGTGTTACTCTTCTGTAAACGA

TCTTAGCTTATGTAGTGACTAACAAAGGGGCTGGAAAGGT

>FM890457.1 FM890457 Jatropha curcas embryo 56-70 (DAF) Jatropha curcas cDNA clone rjcaeb0_000123, mRNA sequence

CGAATCCAATGCCGTTGGCGGTTATTGCAAATGCATTTAGGGTATCAGCAGAGGAGGCAAGAAGGATTAA

GTTTGGAAGACAAGAGACCACCTTGACTATTTCCAGGACCGAGTCTGGAAGATATGCCGAGGAGTAAATA

ATGAAGTAGCCTTTTTGGGTGTGTAGATAGTGAAAGTAAGTAGTATTACGAGGGGAGTTATGATCTCACG

CTAATGAAGTATGTAATATATTATGAAAATAAGTGATGAAAGTAATGTAATCATCCTAATTAGTAATTAA

GTATTCCCTAATCAAGGGGGGGCCCGGTAC

>FM890456.1 FM890456 Jatropha curcas embryo 56-70 (DAF) Jatropha curcas cDNA clone rjcaeb0_000122, mRNA sequence

CGAATTCGAATTCTCTGGATCCGAAGAGGCACTATAAGAAGGGTGATTCAAAAACAAAAGCACTGCCCAA

GTATTTCCAGGTTGGCACAGTTATAGAGTCGGCAACAGATTTCTTCTCAGGTAGGCTAACGAAAAAGGAG

AGGAAGGCAACCCTTGCAGAAGAGCTGCTTTCAGACCGTAACCTTGCAGCTTACAGGAAGCGTAAGGTTA

GAGAGATAGAAGAGCAAAGTCGGCCAGCTGGAAATGAAAAGTGGAAGATAAAGGGTCGACAATCTAGGAA

GAGAGCCAAAGAAAGAAGGCATTAATAAAACCATTTTTAGAGAACTCGAAAAGTAAGGCACTGGTATTTC

TTGCTGACATTTTGTGTCAAAGGTTATACAAGAGCGATATTTGTACCATCCGAGGAAATTTTTCTTTAAT

TGCTTTAATTACATATTTATGTATGTGTTTTAAAAAAAAAGGGGGGGCCCGGTAC

>FM890455.1 FM890455 Jatropha curcas embryo 56-70 (DAF) Jatropha curcas cDNA clone rjcaeb0_000121, mRNA sequence

ATTCGTCTGACATGTGTGCGAGTCAACGGGTGAGTAAACCCGCAAGGCGCAAGGAAGCTGACTGGCGGGA

TCCCCCCGAGGGTTGCACCGCCGACCTACCTTGATCTTCTGAGAAGGGTTCGAGTGAGAGCATGCCTGTC

GGGACCCGAAAGATGGTGAACTATGCCTGAGCGGGGCGAAGCCAGAGGAAACTCTGGTGGAGGCCC

>FM890454.1 FM890454 Jatropha curcas embryo 56-70 (DAF) Jatropha curcas cDNA clone rjcaeb0_000120, mRNA sequence

CTGAAAAACTGCATTGCAATAGAATTATCAAGTTTGACAGAAACAACCAATAATTTAGCCACCTGATGAT

ATGTGACCCAATCTGACTACATTAATTAAATGTTCCAAGAACTCATTAACATAAAACAAGATTAAAAGAT

AAAAACCATGGAGCCACCAGAACCAAGTTAATTAGTAAGCTCCATTATAGCACTAACAATGTCTCCATTG

TGTGTCCTGAGAGCCTTAACTGCCTTGCTCCTTGACACTCCTGCTTGTGTCATAACTAGTTCAATGTCTC

GAGGCTCAACTCC

>FM890453.1 FM890453 Jatropha curcas embryo 56-70 (DAF) Jatropha curcas cDNA clone rjcaeb0_000118, mRNA sequence

ATTCGAATTCCAAATATCACTGATCGTAAACCTCCATTGAATTTACTCTGTGCTTCGATCGCGTCAATGG

CGGAGCACCCACAGTCGCAGCATGTTGGCCAACAGCCACGATCCCACCAAGTAGTGAAGGCAGCCACTGC

AGTCACTGCCGGTGGATCTCTTCTAGTTCTCTCCGGTTTGACCCTTGCTGGTACGGTCATTGCACTGACC

GTAGCCACTCCATTGCTGGTCATATTCAGTCCGGTTCTTGTTCCTGCGGTTATCACAGTTTGTTTGATAA

TCACAGGGTTTTTAGCCTCAGGCGGTTTTGGCGTCGCTGCCATTTCTGTC

>FM890452.1 FM890452 Jatropha curcas embryo 56-70 (DAF) Jatropha curcas cDNA clone rjcaeb0_000117, mRNA sequence

ATTCCTTGATGAAAGAGCAAGGCGTGGAGAGACTGTTGTCCCTGGAGGTACTGGGGGCAAGAGCCTTTAA

GCTCAAGAGCATCTTGCTGAAGGGAGAAGCAGAGGAGGACAGACA

>FM890451.1 FM890451 Jatropha curcas embryo 56-70 (DAF) Jatropha curcas cDNA clone rjcaeb0_000116, mRNA sequence

TTATTTTGTTTTTTGGTTTTTGTTGTCTTTTAGTTAGTTGTCTTAGCCATCTTTGTTACCATACTCTTAG

AAGTGGCTATCATTGAGAGGGGGATGTAAGTTTGGGTGGGGCTGCAGGAATAGAGGTGTACAATGTTGTT

AGGTTTTGGTGAGAAAGTTCTAACAAATTAGGAATTAATTCATGCTCGCTCTTCTTTTTTATCCGTTTTT

GTTCTTTTTAGTGCAACTTTATTCGGTGTTGTTGGGTTGTGGAATATGTAGCAGGAGAGCGTAGAGTTTC

AAATCCGCTGTATCCATGTTAATCTCTAGGCATGTTCTTTTCAACCCAGTTTGTCGGGGGTATATTGTCG

AGGCATCTTGTCCTGCCATATCTCTGGCTTATCTTCTTCCCCTTTGAAAGGAGCTTTTAAGGCTTTTGTA

ATATTTCAGTGGACAATGAAATGGGGGGGCCCGGTAC

>FM890450.1 FM890450 Jatropha curcas embryo 56-70 (DAF) Jatropha curcas cDNA clone rjcaeb0_000115, mRNA sequence

ATTCCGGGCTTGGCGGAATCAGCGGGGAAAGAAGACCCTGTTGAGCTTGACTCTAGTCCGACTTTGTGAA

ATGACTTGAGAGGTGTAGGATAAGTGGGAGCCGAAAGGCGACGGTGAAATACCACTACTTTTAACGTTAT

TTTACTTATTCCGTGAATCGGAGGCGGGGCTATGCCCCTCTTTTTGGACCCAAGGTCGTTTCGGCGGCCG

ATCCGGGCGGAAGACATTGTCAGGTGGGGAGTTTGGCTGGGGCGGCACATCTGTTAAAAGATAACGCAGG

TGTCCTAAGATGAGCTCAACGAGAACAGAAATCTCGTGTGGAACAAAAGGGTAAAAGCTCGTTTGATTCT

GATTTCCAGTACACGGAACCGGCCGTTAGGGGGGGCCCGGTAC

>FM890449.1 FM890449 Jatropha curcas embryo 56-70 (DAF) Jatropha curcas cDNA clone rjcaeb0_000114, mRNA sequence

ATTCCATNGATTGTCCATGTGAAGGAATAAAGTATGTATTAGAGCAGCAGCTGGAGCAAGGACAGCGAGA

GCGTTTGGAGGCAGTGCGTAGGGCTAGAAGTATACCTTCTGCTTGTGGCCTGCGTACAAGCTGCGATATT

CGTTCCTAATCTGCTAGCTATAACGTGGATGTGCTTCAGTTAAATAAAAATTTCACTCACTTGGTGTGTG

AGTGATCAGAGTTATGTATAATAATGCACAAAAGAATGAGCACATCAAGCTAAGTTTGTTTTCTGTTAAA

AAAGGGGGGGCCCGGTAC

>FM890448.1 FM890448 Jatropha curcas embryo 56-70 (DAF) Jatropha curcas cDNA clone rjcaeb0_000113, mRNA sequence

AACTAGAATTTATCTCTTTATTAATTATTAAACCAGAAGCCACTATAGTCCGCAGCAGTGATCTTTCTCT

TTTCTTAAAGACTAATTTACACATGTACCTTCACCTTTCATTATTTATTCACCATACACAGCACTATTTA

CATTAGTAAACCCTCAAGGTCGATGTCACAGTCACCCCAAAAAGCCGAAACACTTGCCATCACGTTATTA

GCTTCTCATGCCACAGCATGACCCTCGAAGGAACCTCTCCTTGGCCCTGGAAAGAACCACTCCTCGTTTT

GTCTTCCAAAAGCCTGATCCACTTCTCTTTCACTTACACCGAAGGCTAACTCTTTGGCTTGCTTCTCCAT

CTTATTTACAATATTATTTTTCCCTGCAAGTAGATGCCTAGTATTGCCTTCTTCATTAATCTCAAAGCAG

ACTA

>FM890447.1 FM890447 Jatropha curcas embryo 56-70 (DAF) Jatropha curcas cDNA clone rjcaeb0_000112, mRNA sequence

ATTATCTTTAGCCCTGTTCTTGTCCCAGCTGCATTAGTTATTGGCCTTTCGGTCATGGCATTTTTGGCTT

CCGGGGCTATGGGGCTCACAGGGTTGTCATCACTCTCGTGGGTTCTGAAATACCTCCAGGAGGTCACTCG

GCGAATGCCGGAGCAGCTGGATATAGCTAAGAAGCGTATGCAAGACATGGCTGGTTTTGTGGGACAGAAG

ACCAAGGAAGTAGGCCAAGAAATCCAGAGGAAAGCACATGAAGGGAAATAAGTAGAGAGAAATGCTGTTG

GTTATTAAGGGTTGAAGTCAGAGAGCTGGGTTTTGGTACGAGACTACTTTTGTTGTATGGGTGTTGTTTT

GAGGTAGTTGAGTTGGTTTATGGGGGATCTTCTTTGTTGCATTTGTATTTCTCTTTTTGGTCTTTTGGGT

TCCATTGTTGATTTTCAATGTATTATCTTTGTATGTGCCATCTGTGATTCTTCTCACACATCTCATCCTC

GCATGCTTGTCTTATATTAGGAGTCTACGGTCCAGGTTTTCCTTTCACTTCAGATGAA

>FM890446.1 FM890446 Jatropha curcas embryo 56-70 (DAF) Jatropha curcas cDNA clone rjcaeb0_000111, mRNA sequence

ATCACGGAATTGTTCTGAGAGGAACTGGCGTAATACAGATTGTGTATCCAAACGGAACACAAGCAATGAA

AGCGAAAGTAGCAGAAAATGATGTGTTTTGGGTGCCAAGATATCACCCATTCTGTCAAATTGCATCAAGA

ACAGCTCCTTTTGAGATTCTTTGGATTCACAACCTCAGCTCAAAAGAATAGACCCCAATTCTTAGCTGGT

TCAAACTCAGTTCTTACTACCTTAAGAGGCCCTGATGCTTGCAACTGCATTTGGCTTAAGCGAGGAAGGA

TTGGAGCACTTTGTTAATTCACAACCCGAGTCAGTTATATTGCCTTCAGCATCTGCAGCACCACCAGACA

AGTTGAAGGAGGCGGCTAATTCTGAAAGGGTGCAAAAATTGATAAAGAGCTTTGATAATGATATGATAAT

GGGTTTTGATTAGAGAAGAGTAATGTAAAGAATAA

>FM890445.1 FM890445 Jatropha curcas embryo 56-70 (DAF) Jatropha curcas cDNA clone rjcaeb0_000110, mRNA sequence

GCATTTGAATTCATTAAATTGTAGAAATATAAAAGATAAAACACTTCCAAGTCAACTGAACAAAACCGTC

CAAACTCAACAACAAGCAGATATTCCTCATGTTTTTCTTATTTTGATTACCTTCCATGGATATGCTAAAA

ACCTCATAAAGCAAATATAACAAAAAGACAGGTCCTAAAACTATGCCTGAGACAAAAGACAATCTATTAT

ACTCCTCTGAAATTTAAAACGGAAACTTAAGACCTCTTTAGTATTTGTAGTCCTTGACATGGAGACTCTC

TGAGGCACCCTCACCAAGAGTAGCATTACCCTGGTAAGTTCCAAGAGTTGCCTCTGAGTTGGCCTTAGCC

CTGACCAGGAATGCAGCTTGAGCCTTCTGCACATTCTCTGGCTTACCTGCCCAAGCCTTGAGGGTACTTT

GTTGAAGTGCACGTCCAAATGAGAAGGAAAGGGACCATGGCTTCTTG

>FM890444.1 FM890444 Jatropha curcas embryo 56-70 (DAF) Jatropha curcas cDNA clone rjcaeb0_000108, mRNA sequence

CGGCACGAGGTCAAAGAGGAGTAGATGTGTAGAAAAGAGTTTGGTTTTGGACAGTTACACTTGAGTTGTC

TATACAGCTTAATCTACTTGTAGAGTTGTATAAATAGGCAACATAGTGTAGATATTTAAACACATGAGTT

TTGGAATCGAGACTGATTTCACCATTGACCTTAATCTGATTAGTTCACTTTCTTTAAGTCTTTGTTTTCT

CATCTATAATTGAGATAGTAATAAACCCTCAAAGGATTATTACAAAATTATAGATAATAAAGTGTTTAAA

CATAGTACTTGGCATATAGAACGAAGTAAATTTTAGTTACTAATATATATGACAGTAAAACTTCACACTC

AAATATGTGGAGTTAAGATGAAAAGATGACCATTAAAAATACATTTCACAGTACATTTTCTTATACTTGA

ACTACTAATCTAAAATAGCTGTCATTTTTGGACAGTCTTTTAAAAATATGATTTATTAATTTCTTTAGCT

CTTTTTTGA

>FM890443.1 FM890443 Jatropha curcas embryo 56-70 (DAF) Jatropha curcas cDNA clone rjcaeb0_000107, mRNA sequence

CGGCACGAGGTCAAAGAGGAGTAGATGTGTAGAAAAGAGTTTGGTTTTGGACAGTTACACTTGAGTTGTC

TATACAGCTTAATCTACTTGTAGAGTTGTATAAATAGGCAACATAGTGTAGATATTTAAACACATGAGTT

TTGGAATCGAGACTGATTTCACCATTGACCTTAATCTGATTAGTTCACTTTCTTTAAGTCTTTGTTTTCT

CATCTATAATTGAGATAGTAATAAACCCTCAAAGGATTATTACAAAATTATAGATAATAAAGTGTTTAAA

CATAGTACTTGGCATATAGAACGAAGTAAATTTTAGTTACTAATATATATGACAGTAAAACTTCACACTC

AAATATGTGGAGTTAAGATGAAAAGATGACCATTAAAAATACATTTCACAGTACATTTTCTTATACTTGA

ACTACTAATCTAAAATAGCTGTCATTTTTGGACAGTCTTTTAAAAATATGATTTATTAATTTCTTTAGCT

CTTTTTTGACAGATGTGTGACTTGCTCTAAATCATCTGTTTTGAATAGAAAAC

>FM890442.1 FM890442 Jatropha curcas embryo 56-70 (DAF) Jatropha curcas cDNA clone rjcaeb0_000105, mRNA sequence

GCACGAGGTCAAAGAGGAGTAGATGTGTAGAAAAGAGTTTGGTTTTGGACAGTTACACTTGAAGTTGTCT

ATACAGCTTAATCTACTTGTAGAGTTGTATAAATAGGCAACATAGTGTAGATATTTAAACACATGAGTTT

TGGAATCGAGACTGATTTCACCATTGACCTTAATCTGATTAGTTCACTTTCTTTAAGTCTTTGTTTTCTC

ATCTATAATTGAGATAGTAATAAACCCTCAAAGGATTATTACAAAATTATAGATAATAAAGTGTTTAAAC

ATAGTACTTGGCATATAGAACGAAGTAAATTTTAGTTACTTAATATATATGACAGTAAAAACTTCACACT

CCAATATGTGGAGTTAAGATGAAAAGATGACCATTAAAAATACATTTTCACAGTACATTTTCTTATACTT

GAACTACTAAT

>FM890441.1 FM890441 Jatropha curcas embryo 56-70 (DAF) Jatropha curcas cDNA clone rjcaeb0_000104, mRNA sequence

GCACGAGGTCAAAGAGGAGTAGATGTGTAGAAAAGAGTTTGGTTTTGGACAGTTACACTTGAGTTGTCTA

TACAGCTTAATCTACTTGTAGAGTTGTATAAATAGGCAACATAGTGTAGATATTTAAACACATGAGTTTT

GGAATCGAGACTGATTTCACCATTGACCTTAATCTGATTAGTTCACTTTCTTTAAGTCTTTGTTTTCTCA

TCTATAATTGAGATAGTAATAAACCCTCAAAGGATTATTACAAAATTATAGATAATAAAGTGTTTAAACA

TAGTACTTGGCATATAGAACGAAGTAAATTTTAGTTACTAATATATATGACAGTAAAACTTCACACTCAA

ATATGT

>FM890440.1 FM890440 Jatropha curcas embryo 56-70 (DAF) Jatropha curcas cDNA clone rjcaeb0_000103, mRNA sequence

CGGCACGAGGTCAAAGAGGAGTAGATGTGTAGAAAAGAGTTTGGTTTTGGACAGTTACACTTGAGTTGTC

TATACAGCTTAATCTACTTGTAGAGTTGTATAAATAGGCAACATAGTGTAGATATTTAAACACATGAGTT

TTGGAATCGAGACTGATTTCACCATTGACCTTAATCTGATTAGTTCACTTTCTTTAAGTCTTTGTTTTCT

CATCTATAATTGAGATAGTAATAAACCCTCAAAGGATTATTACAAAATTATAGATAATAAAGTGTTTAAA

CATAGTACTTGGCATATAGAACGAAGTAAATTTTAGTTACTAATATATATGACAGTAAAACTTCACACTC

AAATATGTGGAGTTAAGATGAAAAGATGACCATTAAAAATACATTTCACAGTACATTTTCTTATACTTGA

ACTACTAATCTAAAATAGCTGTCATTTTTGGACAGTCTTTTAAAAATATGATTTATTAATTTCTTTAGCT

CTTTTTTGACAGATGTGTGACTTGCTCTAAATCATCTGTTTTGAATAGAAAAC

>FM890439.1 FM890439 Jatropha curcas embryo 56-70 (DAF) Jatropha curcas cDNA clone rjcaeb0_000102, mRNA sequence

GCACGAGGTCAAAGAGGAGTAGATGTGTAGAAAAGAGTTTGGTTTTGGACAGTTACACTTGAGTTGTCTA

TACAGCTTAATCTACTTGTAGAGTTGTATAAATAGGCAACATAGTGTAGATATTTAAACACATGAGTTTT

GGAATCGAGACTGATTTCACCATTGACCTTAATCTGATTAGTTCACTTTCTTTAAGTCTTTGTTTTCTCA

TCTATAATTGAGATAGTAATAAACCCTCAAAGGATTATTACAAAATTATAGATAATAAAGTGTTTAAACA

TAGTACTTGGCATATAGAACGAAGTAAATTTTAGTTACTAATATATATGACAGTAAAACTTCACACTCAA

ATATGTGGAGTTAAGATGAAAAGATGACCATTAAAAATACATTTCACAGTACATTTTCTTATACTTGAAC

TACTAATCTAAAATA

>FM890438.1 FM890438 Jatropha curcas embryo 56-70 (DAF) Jatropha curcas cDNA clone rjcaeb0_000101, mRNA sequence

CGGCACGAGGTCAAAGAGGAGTAGATGTGTAGAAAAGAGTTTGGTTTTGGACAGTTACACTTGAGTTGTC

TATACAGCTTAATCTACTTGTAGAGTTGTATAAATAGGCAACATAGTGTAGATATTTAAACACATGAGTT

TTGGAATCGAGACTGATTTCACCATTGACCTTAATCTGATTAGTTCACTTTCTTTAAGTCTTTGTTTTCT

CATCTATAATTGAGATAGTAATAAACCCTCAAAGGATTATTACAAAATTATAGATAATAAAGTGTTTAAA

CATAGTACTTGGCATATAGAACGAAGTAAATTTTAGTTACTAATATATATGACAGTAAAACTTCACACTC

AAATATGTGGAGTTAAGATGAAAAGATGACCATTAAAAATACATTTCACAGTACATTTTCTTATACTTGA

ACTACTAATCTAAAATAGCTGTCATTTTTGGACAGTCTTTTAAAAATATGATTTATTAATTTCTTTAGCT

CTTTTTTGACA

>FM892681.1 FM892681 Jatropha curcas embryo 56-70 (DAF) Jatropha curcas cDNA clone rjcaeb0_003290, mRNA sequence

GAAATCAAAATTGATATTCTTATCTATTAATCCAAAGTCAAGCCAAACATACCCGTAGAAAATCCACAAG

AAATAATGAGAAAAAAGAAAGTAAATTTTATTCACAAACTAAGAGAAACTCATTAACGACTCTGAGGATA

ACTAGATAAACGAAAAGGGTAAAAAAAAGCTACTACCAACAGCTATTACAGTCTGTGTTTCGAACCACGT

TTTATTCTTTACATTACTCTTCTCTAATCAAAACCCATTATCATATCATTATCAAAGCTCTTTATCAATT

TTTGCACCCTTTCAGAATTAGCCGCCTCCTTCAACTTGTCTGGTGGTGCTGCCGATGCTGAAGGC

>FM892680.1 FM892680 Jatropha curcas embryo 56-70 (DAF) Jatropha curcas cDNA clone rjcaeb0_003288, mRNA sequence

TTGGCGTCTTCCTTAAGTTGGTTGCAGGCGGAGTTTTGGATCTGTTTGCTTCTGACCATTTTGGGATATA

TTCCTGGGATTATCTATGCTGTCTATGCAATCACCAAGTGATCTTCCATCTACGGTGGAAAGCTACATAA

CCATTTGATTTGATGGAGGGGTCTGTTTTGGTGAAGTCAGATGTATCATTCTAATTGTGTGTGTGTGTTT

ACGTGTTTGTCTTCTATGAAGATTTCCATTTCATTTTTTAATTTTCTGTAGTTAAAAAAAAATTAGCCAT

TGAGTTGGGTTTGTGAGTAGAAACAATGGCTTGTCAATTATCATTTTAGTTTATCTTTGAACTTGAAATT

TGTTTTAATTTTAATGTGATTGTATAATTATTGTCTACCTATTTATCTTCTTGATTGTTCATTATTATTA

TGAAGCACAAACAGCATATCCCCTGAGGATTCTGAATAAAAAAGGGGGGGCCCGGTAC

>FM892282.1 FM892282 Jatropha curcas embryo 56-70 (DAF) Jatropha curcas cDNA clone rjcaeb0_002803, mRNA sequence

ATTCCGCAAGGGCGAGCTCGGGCGCTTCGGCCCCCGCGCGAGCCCAAAGGCCGAGCCGGGTGCTGCTGGG

AAATGCCTCGGCCCTTCCCGCACGCTCGACCCGGTCCACAAACCAACCCCGGCGCAGGACGCGCCAAGGA

AACACGAAACGAGAAGAGCACGCGCCCGTCGGCCCCGGAAACGGGCAGCCTCAGGGATGCGCTGTCCTCT

ATCTA

>FM892281.1 FM892281 Jatropha curcas embryo 56-70 (DAF) Jatropha curcas cDNA clone rjcaeb0_002802, mRNA sequence

ATTCCTTTCTGCAGAGTTAGATCATAAGAAGAAGGACTACAAGTTCGGGTTTGGTTTGACAGTGGGTTAA

AATTAAAATTGAAGATCCAAACATTTGTATCATCAAAATCAAATTTTACATAAGAAGTTGTCGGTGATAG

CTACTGAGGAAGGTGAACTTTGCGTTTGGTGCAAACGTCGAGGGATCTTTTTGTACTGTTTTGAGAGAAC

ATTATTTGCGGGGGAGTATCAGCCTGCTGCAGACAGTTTTTATTAGCTTTTGAGTATTATTCATTGAATT

GGTATTTGTCATTTCATTTTCATTCTAGAGCTGTACAATTTTTATGTTAATGAGAAGTAGGGATGAAATG

ATTGCTATAATAAAGCTTTCCTTGAATAATTTTC

>FM892280.1 FM892280 Jatropha curcas embryo 56-70 (DAF) Jatropha curcas cDNA clone rjcaeb0_002801, mRNA sequence

GGGAGAGAAAAGATACTTCGCTCTCATTGTTAAAGAGAATGCTATAACATGCAAAACAGGTTACAGGTAC

ATGTAGACTGAACATAAAACTCGGTGCCATTTCTATGTCCGCTTCGAGAAGACACTTGTTATAAGTACAT

ACGCAAATACACTCCTACACACAAGGGGAAGTAACAGAACAAAACAAAGCAAACATCATATACAAACAAA

CACCATTCATATTAGTAACTCTTCATTAGTGTATCAGTAGTTAATGGTCTCATTAAGCCTAGCAGCTGTC

GTCATTGACACCGCAACACCACCTGGATGCGTGTTTAAATTCGGATTGTTCCTCAACTCTGCACTAACCA

CCCCTTCAGCATCTGCCCTTGTCACGACCTTATCCGCCGCCAATTTCGAAGTTGCACCCGGGGGGGGCCC

GGTAC

>FM892279.1 FM892279 Jatropha curcas embryo 56-70 (DAF) Jatropha curcas cDNA clone rjcaeb1_002796, mRNA sequence

AAAAAGGTAATATAGCAGCAGTTTTATCAGTCAAGACTACAGCTAATTTGGCTACAATTTCAGTTTGAGT

GGTACAAAGTTAGAACCAAAAATGACTAAATAGTACTTGTGAACTAGAAATTAATGAACAGAAGCAATGG

CAGCATCTAACTTCAAAAACCAATGCTGCACAAAACTAAGAAATGTTCTAAGGACTGCATAAATTCCTAA

GGTCATCTTCCTTTGCATACCAGCTTGGTATAATCCTTGAAGCATAAGGATACAAATCCTTATAAGAATT

TCTTATAGTCCCTTCCGCCACTCCGGTAGCCAGCGATATATCTTTTAGAAGTTTCTTGTCGTCGGAGAGT

TGAGTTATCATGTAAATTATTGCTGCTGCTATCGATATTGGGCTTCGCCTTATATCAAGCTCTTCAGATT

TCTGCACAGATTCTGAGCTGCTTTACTGTGATGTCATCCCAAGATGAGACAAAACTTTCAGAAAGTTCAG

ATGATAGTTCCATTTCATTGAT

>FM892278.1 FM892278 Jatropha curcas embryo 56-70 (DAF) Jatropha curcas cDNA clone rjcaeb1_002793, mRNA sequence

ATTCGTTTGTTCTCCAAAAGTGATGGAAGGAAGATATCTGGTTCAGAAGATATCAAGTCGGATAAAAAGA

GCTCAAGAATTGTTAAGGGAAGTGGAAACAAAAATGCCTTCACCTGAGCTAACAGGTATATTAGGGTTTA

ATCTTGATGATCATGATTTTGTTAAAGGGCTTGAGGAAATGATGGAAGAATGGGCACCAGCAAGATCAAA

GAGACTTCCCATATTTGAACAGAGTCTCTTCATTTAGAGATCAATTAGCATGCTAGTTCTTTCTTTTTCT

GTGGGTTTGTGAGATTGGAGTTGGGTTTGTTTTTATAATTTATCATTGTTTATAAGGGTAGTGTTGTAAT

TATATATATTTACAAAATTAGAATTGTTGTTTGGAAGAACAACAAAT

>FM892277.1 FM892277 Jatropha curcas embryo 56-70 (DAF) Jatropha curcas cDNA clone rjcaeb1_002790, mRNA sequence

CGAATTCGGAAAAAATACGGANGCTCCGAGAGAGATACAGAAAGAAGAATCCTGAAGAAATTTCGGCTAA

ACCTTCATCGTTGAGCCCTTATTCTCTCTTTTCGTTTCTCTCTTTAGCGCTACCTCCGATTTCTTCAACA

TTCAGCTTTCTTCGTCGGCCAGATTGGTGCATATCTGCCATTACTCCCTCGCCAGTTCTTAAACTCGGTC

TTCGATCTTCTTGAGTTATACCAAAATGGTGTTCTGGGTTTTTGGCTATGGTTCGTTGGTGTGGAACCCG

GGATTTGAATATGATGAGAAAGTGATAGGCTTTATCAAGGATTACAGACGAGTATTTGATCTTGCATGCA

TTGATCACAGAGGTNACACCTGAACGTTCCTGCGAGAACTTGCACATTTTGGAAAATGTTGAAGGATCTG

TTTGCTGGGGTGCTGCTTACTGTGTTCGTGGAGGACCTGAAAGGGGAAAAGACTAACAAATGGAAAGTAT

TTGGAACGGAGAGAATGTGAATATGATAAGAAAACCCTTGTAGAATTTTTAACAAGGGAAGGGGATCCTC

GCGACCTGTTTACGGAGTCTATTTTCC

>FM892276.1 FM892276 Jatropha curcas embryo 56-70 (DAF) Jatropha curcas cDNA clone rjcaeb1_002783, mRNA sequence

ATTCCTCCCACTTCAGGATGTCTACAAGATTGGTGGTATCGGAACTGTCCCTGTCGGTCGTGTTGAGACT

GGTGTCTTAAAACCTGGTATGGTTGTCACCTTCGGACCATCTGGACTGACCACTGAAGTTAAGTCTGTTG

AGATGCACCACGAAGCTCTCCAGGAGGCCCTTCCAGGTGACAATGTTGGCTTCAATGTGAAGAATGTTGC

TGTCAAGGATTTGAAACGTGGTTTTGTTGCCTCGGGCTCCAAGGATGATCCTGCGAAGGAAGCTGCCAAC

TTCACCTCTCAGGTCATCATCATGAACCACCCTGGTCAGATTGGAAATGGATATGCTCCAGTCCTTGACT

GCCACACCTCCCACATTGCTGTTAAGTTTGCTGAGATCCTAACCAAGATTGATAGCGATCTGGTAAAGAG

CTTGAAAGGAGCCAAAATTCTTGAACAATGGTGATGCTGGATTTGTAAGATGATTCCCACCAAGCCCATG

TTGTGGAGACTTTCTGAGTACCCTACTCTGGTCGTTTGCTGTGAGGGACATGCGCCAGACTGTCGCTGTG

GTGTCTCAGAGTG

>FM892275.1 FM892275 Jatropha curcas embryo 56-70 (DAF) Jatropha curcas cDNA clone rjcaeb1_002782, mRNA sequence

ATTCCAAAGCAATTCCCTCCATTGTTACAAATCTTCTACCTTTCAGCTTTCGATCTCTGCCGAGATTCTT

CTTTTCCAATTCCTCTCTAAAGATCATGAAGCTCGATACCAGTGGTCTTGAAT

>FM892274.1 FM892274 Jatropha curcas embryo 56-70 (DAF) Jatropha curcas cDNA clone rjcaeb1_002780, mRNA sequence

AGAGTAGACAAAAAAGAGACAGCAGACAATGTTATAGCTGGAAAGAGGAAATATTTACCTCACAGACAAA

AAAAACTAATAAACGAGTAATACATTCAAGGACACGCTAAGCTTGTACATATGACGATTTTTTTACTGGC

TCTAAGATATTAATCTGAGATGGTTTGCAGTATCTGCACAAGGTGAATACCATTTCTCAATTTATTACAG

AAGTAACAATTTGCTCATATCAGCTTTACTTCACATATAGCAGCCACCATTGCTTTACATTCTAATACAT

CTTAGACTCGCTACCCATGTTCATTTTAGCACAATATTCGAACAAACTATCGATCAAACAGCGCCGAACA

GCTTCTTTAGATAGAAAGCCTTCTTC

>FM892273.1 FM892273 Jatropha curcas embryo 56-70 (DAF) Jatropha curcas cDNA clone rjcaeb1_002778, mRNA sequence

CGAATTCTCGAGTTTCAGAATGTGGGGGATCTCTGGGAAAAGCATCAGTCTTGGTTCAGGCGACACAAGG

ACTCCTAAATCACCTTGGATGCCTTTCCCTATGTTGTTTGCTGCAATCTCAAACAAAATTCCTCGTAAAG

AAATGGAACTCGTAACTACAAATTATGAACTTTTCCAGGCCAAAAAGATAAGCAGGGGAGATTTTATAAA

AAGTTTGAGGTTGATAGTTGGAGATGCTTTGCTGAAGTCAACAATAACAAGTCTTCAGTGCAAGGTACCA

TTCAAGTGTGTGGTGGAATCACCTAAGCCAACTGTAGAAGGGTCCAACGGTCCTTGATCTATCAAAGCAG

CATATTTCGTTGGATGTTTAGGTAAAACTCGTTCTGTACTTGCGTGCTTGAATGCGTCGACATGATGTTG

TGAATAATATGTTGGATATGTTTAGGTTTAGGTTTAGTTATATCACAACCTGCTAGTTTCTTTTTTCTTT

TTTCTTTCCCTGTTGCGAC

>FM892272.1 FM892272 Jatropha curcas embryo 56-70 (DAF) Jatropha curcas cDNA clone rjcaeb1_002777, mRNA sequence

ATTCGACAGGTTAGTTTTACCCTACTGATGATGGTGTCGCGATGGTAATTCAACCTAGTACGAGAGGAAC

CGTTGATTCGCACAATTGGTCATCGCGCTTGGTTGAAAAGCCAGTGGCGCGAAGCTACCGTGCGCTGGAT

TATGACTGAACGCCTCTAAGTCAGAATCCGGGCCAGAAGCGACGCGCGTGTCCGTCGCCCGTTTGCCGAC

CCGCAGTATGGGCCATCCGGCCCCCAGAGGCACGTGCCGTTGGCCAAGCCCCCGCGGCGGACGAGCCGCG

AGGGCCGCCTTAAAGTACAATTCCCATCGGGCGACGGATAGAATCCTTTGCAGGGGGGGGCCCGGTAC

>FM892271.1 FM892271 Jatropha curcas embryo 56-70 (DAF) Jatropha curcas cDNA clone rjcaeb1_002775, mRNA sequence

GTTAAATCTAATTTAACTAATATTTTCAACAGCAATAACTGGATCATCTCACAAGCATGAGATCCATCCA

GGTGATTTATGCTGCTTCCACTTTTGTATTGCAACCATAAGCATGAGATCCATCACATTCCAAGTAGCTT

TCAGGAACTTGTCAACTATTATAAATGCCACTCCACAATGCACCAGTACTAATCATCATGTGCAAATGCA

TACAGAATTGCAAAAACACAACAGTATTCACATTAATTATTAGGAAGAAGATACTGCTAAAAGATTTGAA

ATCAAGTCCTATATGAAAACCAAGTGTCTATTTTACTCCAACAATAGACATCAGAAACCTAAGCTTATCA

TTCACTTCA

>FM892270.1 FM892270 Jatropha curcas embryo 56-70 (DAF) Jatropha curcas cDNA clone rjcaeb1_002774, mRNA sequence

ATTCGGAAAGAGAGCGGGCGCGAGTACTGCAGAAGGATTGATCCATTCGAGAATCTATTTCTCAGGGGAT

CGAATAGCTTCTGCAGTATCTGCTTACTCTGTACATATATCTACCAATAAAATGACTCGCTCTC

>FM892269.1 FM892269 Jatropha curcas embryo 56-70 (DAF) Jatropha curcas cDNA clone rjcaeb1_002773, mRNA sequence

ATTCATCAGCATTTGAAGATGCTTCACTCGGTAAAAGTTTTGATGAAGAGGGAACAGATGTGGATGATGA

GTATGAAGATGATGAATATTATGATAGCGACTCATTTGATGAGTTAGATGCTTCAGTACCTCTGGGATCA

TTACCTGTTGATCGGATAGTCAAAAAACTCGATCAAGAATGAAATGCTAGATGAACAGGTTAGTAATGCT

TAAACCTTTCGCATTCCAGAGGCTGGCTATGCAAGTTGCTCGAAAGTAATGAAGGTGTCTGCAACTCATT

GTCTCTAGCAAAGGTTACCGAAGGTGTACATTTGTAGCAGTAGAAGGGTCATTGGTGAATTTTCCCTGTG

CATTTAAGTATACTTTTACTCACAATGATGTTTATAAGTCCCATCCAGTATAAGCAATAAAGTGAATATA

TC

>FM892268.1 FM892268 Jatropha curcas embryo 56-70 (DAF) Jatropha curcas cDNA clone rjcaeb1_002772, mRNA sequence

ATTCCTCTTTTCTCTGTCTTCTTTTGTGATCGGAGAGTCAGGTTTTTGCGGCGACAAAATGGCCAGACTC

GGTGGAGTTCGCGAAGTAAGCGCCAACAGCGTCGAGATCGACGGTCTGGCTCGTT

>FM892267.1 FM892267 Jatropha curcas embryo 56-70 (DAF) Jatropha curcas cDNA clone rjcaeb1_002771, mRNA sequence

ATTCATCAAGCCTTAATATTTGTTTTCTGTTTGTCTTTCAAGTAAATCTGCAATCTCTGGTTCAAGAAAG

AAGCTAATTAATGGCGTCCAGGCAAGAGAAGGTGGACAAAGCGGAGGCAGAAGCGTGGCAAGCTGCGCGT

GATCTAAAAGATGCTAATAGAGAAAAAGAATATGAAGAAAGGGCAAAAATGGAGACAGACCTGCAACAAG

AAGAAAGACCAGGCGTTATTGGGTCTGTGCTAAAGAAAGTTGCTGATACCTATGAGCATGCCAGAGAAGC

TGTTGTAGGTAAAAGCCAAGAGGCTGCTGAGAAGGCGAAGGAAGCCAATTATAAAGCAGCTGAGAAAGCA

GGAGAGACTANAGATTACACTGCAGAGAAGGCGGCGGAGACGGCAGACGCAGCTGCAGGAAAATGGGACG

AAACAAAAGAATCTGCAAAAGAGAAGGCGG

>FM892266.1 FM892266 Jatropha curcas embryo 56-70 (DAF) Jatropha curcas cDNA clone rjcaeb1_002770, mRNA sequence

ATTCCCACCACAACAGAAGCGGCAACAGCAGAAGAGGCAGCCATGGTTCTCAAGACAGAGCTATGTCGTT

TTAGTGGGGCTAAGATTTATCCCGGTAAAGGCATCAGATTTATTCGTTCTGATTCTCAGGTCTTCCTGTT

TGCTAATTCAAAATGCAAGAGGTACTTTCACAACCGTTTGAAGCCATCAAAGCTTACATGGACGGCCATG

TATAGGAAGCAGCATAAGAAGGATATTGCTCAAGAGGCTGTAAAGAAGAGGA

>FM892265.1 FM892265 Jatropha curcas embryo 56-70 (DAF) Jatropha curcas cDNA clone rjcaeb1_002769, mRNA sequence

ATTCATCAATCAAGACAAGAGCTTGATGAAAGAGCAAGGCGTGGAGAGACTGTTGTCCCTGGAGGTACTG

GGGGCAAGAGCCTTGAAGCTCAAGAGCATCTTGCTGAAGGGAGAAGCAGAGGAGGACAGACAAGAAGAGC

ACTGGGGACAGAAGGATATCAGGAGATGGGCCGGAAAGGTGGGCTGAGCACGACGGACGAGTCTGGTGGA

GAAAGAGCTGAGAAGGAGGGCATCACTATCGATGAGTCGAAGTTCAGGACTGGGAGCTAGTGTGATGATT

AAAACTATAAATGGTATTTTGTAGTAATAATAATAATAATGTTGGTAATAAAGTATTAGCTTAGCAGTGT

ACTTTAGTTTAGTAATGTAGTTTACATGTGGGTTAAAATGACTAGGGTTTAGCTAGTATGTGTGTTGTAA

CTTTGAGAGGGGTTTATGGTCGTTCCCTTTCTGGG

>FM892264.1 FM892264 Jatropha curcas embryo 56-70 (DAF) Jatropha curcas cDNA clone rjcaeb1_002768, mRNA sequence

CAAAGCTGATGATAACAAAATCTAATGACCATGTACATAGTAAAACAGGTAACACCCAATTTTACATTGA

AAACAATAGAAGAAAGACATTATGATTTATGGGATTCACTCGATTGGAATCATCACCTCATTAGTCCTAC

AAGGAGGTAAAGTCCAACGTGGATTATACCTTGCCAGCACAAATTCCCCAATTACCGTATACCCATCTCT

CTCCAAGCTTTACTTC

>FM892263.1 FM892263 Jatropha curcas embryo 56-70 (DAF) Jatropha curcas cDNA clone rjcaeb1_002767, mRNA sequence

ATTCCAAAATAGNTAAATAATATCAAAACATCCTTCAATGTCAAAAAACACAAATTAGATAAAAGCACAG

GTTTCTCTGTTTTATAAAATTTAACAAAAAAAAGAAGTAGAAGAAAAAGAAAAAGGAAATAGCTCACTAA

AGCTTGCGATCTCAATCAAGCGGCACCTCAACATCACCATCAGTAATCTCTTGCTGCAAATCCTTAGGGT

CTTTTCCATCGACAGTACATCCAACCGAAACGCAAGTCCCCAATATCTCCTTAACAGTTCCGCTCAGATC

CTTCGCCATGGATCTTGGTCTCATCACCCTCGCGATCTCGATAACATCATCGAGCGAGATGTTGCCACTG

TGCTTGATATTCTTAGTCTTTTTCCTGTCTCTCTCCGGTTCCTTGAGTGCCTTGATCACCAAAGCGGCCG

CCGATGGCACCACCGTTACTTTGGCCTGACGATTCTGCACCGTAAGCTTGACAGTAACTCGAAGTCCCTT

CCAATCCTTGGCGGTCTCCTTAGCAATGTCTTCTCCGATCTTTTTAGGGGAAAGACCTAGAGGCCCGATT

TTCGGAGCAAGAGAACTGGCCGCACCTACTTCACCACCCGGTGACCGAACGAATACATCTACGACCTGAG

AAGGGTCAATGGGGGGGCCCGGTACCCATTCGCCTATAGTGAGTCGATTAC

>FM892262.1 FM892262 Jatropha curcas embryo 56-70 (DAF) Jatropha curcas cDNA clone rjcaeb1_002766, mRNA sequence

ATTCCCAATTTCTTGGTTTCTTGCTTGTTATCGCTGTTTCTCTCTCAATTTACCCGACTTTTACTTACTT

AGAGGGGTCCCTTCTTTTCTCTATGTTCTTGCTCAGGAACCGAAATTTGGGGATTTCTAATTGAAACAAA

GAACCAAGAAAAGAAAGGAAAACAAAAATGAGTGCTTCTAGGTTCATTAAATGCGTCACAGTTGGTGATG

GTGCTGTTGGCAAGACTTGCATGCTCATCTCCTACACCAGCAACACTTTTCCTACTGACTATGTGCCTAC

TGTGTTTGACAACTTCAGTGCGAATGTGGTTGTGGATGGGAGCACAGTCAATCTAGGGTTGTGGGATACT

GCTGGCCAGGAAGATTATAATAGATTAAGACCTTTGAGCTATCGTGGGGCTGATGTTTTCTTGCTTGCTT

TCTCTCTCATTAGCAAGGCCAGTTATGAAAATGTTGCAAAGAAGTGGATTCCTGAATTGAGGCATTATGC

ACCTGGAGTACCAATTATCCTTGTTGGGACTAAGCTTGATCTCCGAGATGATAAGCAATTCTTCATAGAT

CACCCTGGT

>FM892261.1 FM892261 Jatropha curcas embryo 56-70 (DAF) Jatropha curcas cDNA clone rjcaeb1_002765, mRNA sequence

ATTTATGGAAACATCAAGAATTCATCAACAAACAAAGTAAGACTTGATTACAGAGACCTTCAACACATGA

CAAGAAAATTATTTTAGACAGCTCTCAAAAGAGGGAAGACCCGTTAACAAATTACATAGAAGAAACAAAA

AGATTTAAGCGAAAAAAGAAATACACCAGACAAGCAATGGCCTTCCATGTTAATTAAAGGACTCATATAT

TTTTATATATATATCAAAGCCATTTTATGTTCTCTGCATCAACCACCAAAACCCACTTCTCTTTTAAGTC

TGTTGACCTGTTACATGCTGTCCAAACTGCTCAGCTCTGTCCTTCATCTCTCTTGCCTTTCCCGCCAACT

TTAAGCGTGCCTGGTCCAGACTTTCTGCTCCTGGTGGATGCTTTCCAGTAACATACCTATAAATCCAGGA

CAAGACAGAAATGGCAGCGACGCCAAAACCGGCTGAGGGCTAAAACCTGTGATTATCAAACAAACTGTGA

TAACCGCAGGAACCAAGAACCGGACTG

>FM892260.1 FM892260 Jatropha curcas embryo 56-70 (DAF) Jatropha curcas cDNA clone rjcaeb1_002763, mRNA sequence

ATTCCCTTTTCCTTCCGTTGTTTCTCCAAACACTTCCCTATCATTTCCTTCCTCGCTTTCTCTCCTCACA

GACGCCATTAAAAATGAGAAACCTTACCTCGATTCTTTACTACACAAAACTGGAGCGATTCTCTTTAGAG

GCTTTCCCGTAAACACCGCCTCGGACTTTAACGACGTCGTGGAAGCATTCGGTTTCGAGGAGTTACCTTA

CGTTGGTGGCGCCGCTCCTCGGACTAATGTCGTCGGCCGTGTCTTCACTGCCAATGAGTCCCCACCTGAT

CAGAAAATACCTTTCCACCATGAAATGGCTCAGGTTCCTGAGTTTCCATCCAAGTTGTTCTTCTTCTGTG

AAATTGAACCAGGGAGTGGAGGAGAAACTCCGTTAGTTCTAAAGCCATATAGTATATGAAAGAATGAAAG

AAAAGTATCCAGATTTTGTTGAGAGATTAGAAGAGCATGGTTTGATCTATATTCGAGTATTGGGTGAAGA

TGATGATCCTTCATCTCCAATTGGCCG

>FM892259.1 FM892259 Jatropha curcas embryo 56-70 (DAF) Jatropha curcas cDNA clone rjcaeb1_002761, mRNA sequence

CTTTTTCAAATATTTTTCATATGCCAAACGAAAATAGTAACAGAACAAGTCCATGCTGAAAGTCAATGGA

AAAGAAAAAATTAAGGCAACGAAGACAAACTATTGATGATCTCGAGGCATCATTAAACGAACAAACAAAC

AAATTGATGGTGATGATCAAACTGCTAGAAAATGTCGAGAATCTCGAGGATTAATCGATTACAAAGGGGG

CAGGACCCTCGATTCAGCCAAACCTCTCTCGAACACACCCTGCAATATGTATGCCCACATGGGATAAACG

CCGCGCCCTTTTTCGGGGGGGCCCGGTAC

>FM892258.1 FM892258 Jatropha curcas embryo 56-70 (DAF) Jatropha curcas cDNA clone rjcaeb1_002759, mRNA sequence

GAGAAGGGTACAACTTTTATTTATCCAAACAAAGACGACATTTAACACCTATATAAGCTATAGAAGGAGA

CACCATTAAACCAGCAACACTCTAGATATATAACAAAAAGACAAAAAGAACTTTTGACTTTCTTCACAGC

TATCTCCATCAACCATTCACAATAACGCCTCCATTGGGGTGAAGAACTTGGCCAGTGATATGGGAGGAGC

AATGGTTACAAGCAAGAAACACATAAGAGGGTGCAACCTCACTTGGCTGGCCAGCTCTCTGCATAGGCGT

CTGTATCCCAAACTTAGCCACCTCCTCTTCACTGAAGAGAAGAGGGTATCCATAGGAGTCCACATAGGTC

CAGGAG

>FM892257.1 FM892257 Jatropha curcas embryo 56-70 (DAF) Jatropha curcas cDNA clone rjcaeb1_002757, mRNA sequence

ATTCCTCTTGTTTTGTTAAGTTTATCTTAGTTGGGAGTGTCTTTATAAATATTAGTAGTACTTGAAGCTC

TTGTAGCGGTCGAGGTTCTTGTGTGTCCTTGGTGTGTAAGGTTGCTTAGGGCAGTTCTGTGTCTTGGGGT

GCCTCAGATCAGCAGGCTTTGGCAGTGGCATTATGGGTGATAACTTTGTTTTGTATTCTAAGCCAGGCTT

GATTTTTCAGGTGCGCTTTGATTATGAATTGTTCTCTGCAACTGCTTCAATAAAATGTAATATTTCCCTT

TCTGTTGTGTAAAAAAAAAGGGGGGGCCCGGTAC

>FM892256.1 FM892256 Jatropha curcas embryo 56-70 (DAF) Jatropha curcas cDNA clone rjcaeb1_002756, mRNA sequence

ATTAGAAAGCAATTTATAAATTAGGAAGTAAGCTTGTGGACAGTGTCTATATTACACAAATTTAGCCAAT

AATCTTATTTCATTAGAAAAATAATCACACTAAAATTCTCCTCAAGTTGAACTTACAACCTCAAATTTAA

AACAATCATCCACTAATTAAACTAATTGAAAAAGCATATTAATAAATAAATATCTGATCTAAATTCCCTC

CTGTTGAGATACTATCACATTTCCCTTGTTAGCAGTACTAATATTCTTCAATTTCAATACAGAATTAAAC

CATACATCATCTTCTACCATATTAAAATTTGAAGAAAAATAATAACAATTGCTTACTTGTATAAAGCACA

GGCTTATTGGATTAATCATCGAGCATCTTTCGAACGCGTTGTTTGATATAATCGCTAACGGGCTTTAGCT

CGATCTTGTTGAGGGGCTATATTGACCAGTGACAGGTGAGCTCCATAAGCAATCATTGAAGCTCCGATGA

TAAAAAAAGCGACTGTTATGGGTAATTGTCTTCGCGTCGGTGGATA

>FM892255.1 FM892255 Jatropha curcas embryo 56-70 (DAF) Jatropha curcas cDNA clone rjcaeb1_002755, mRNA sequence

AAAAAAGGACACCTGTTTCATTTTTCATTAATAAGCACATTTCCTAACCAGGTAATACAAAGTTACAATA

GAAATCGCTTCATAGCAAGCTGCTTTTTAAGCACACTTAGACCCACTATTTTATTTATGGCTTTCCCTGT

TTATAAGATGCTACATAAACTCTCATCTTAGAGAAAGCCCAAGACCCCAATACACACATAAAATTTATTA

GCCATAGGTAGCAGTACTAGTAGCTAATACCTAGTGTCTGTGTCCCTATAAGTTGTGGTCTGCTGATGGC

CAGAACGATCCTTAACATGATCTTCTTCTTCTTCATGGTGAGCCATACCAAAAGCGCTTTTGACAGTATC

AGCAGTTTTTTGAGCTGCATGCTTCATTTTCTCCCCAGTTTCTTCCAGAACTCCCTTTGTCTTCTCCTTC

CCTTCATGAGCCTTCTCCTTGGTTGCCTCTGCAGTCTCAGATGTCTTTTCTCTTGTCTCATGGGCTTTTT

CTC

>FM892254.1 FM892254 Jatropha curcas embryo 56-70 (DAF) Jatropha curcas cDNA clone rjcaeb1_002754, mRNA sequence

ATTCATAACACTATCAAACCTAAACCAAATACCATCCTTCAATCAAATCAGATAGATAAGAGATGGAGAA

TAAGAACAACAACAGCAGCAGCGGCTTCATCTCATGCGATAAGCTTGATCGAGTGGCCAATTGGGTGGGA

AGCAACGTGGCCTCCGCATTCTTCGCCTCCCTTGAACGGTGCTCCTGCATCAACCTCAACACCGCCGACC

TCGAAGACGAAGACGATGAAGGGGCCAAGGATCGACCTCTTATGCTCTGCAAGCCCACTATCCGCGACAA

TCCTGAAGACTATCTCGCCGCCGCCCAGGCCGGCACCACGGCTGCCCATTAGTCCGCCTTCTAATGATGA

TAATTAATGATTATCTATCAAATAGGCTTCGTGTCCTCTTCTTTTTTTGGTGGTGCTTTTATATATGCAA

GTCGAGTTATCGCAAGAATGTTTCTTGGATTGTATTAAGTAGTTATGAACTAAACAATTATTCAACCGCT

TTTGTAGAAATTTGAGGCTTGTAATGTCAATATTTCTCTCAGATCTATTGTGATTCGTGTTATTTCTCAA

GAATTTATGTCTGGTTGCATTTGGTTCCAAAAAAAA

>FM892253.1 FM892253 Jatropha curcas embryo 56-70 (DAF) Jatropha curcas cDNA clone rjcaeb1_002753, mRNA sequence

ATTCGGCCGAGTCACCATCAACGCCTGAGTCCAATGCCACGTCTTTCGGTTCAGATTATGCTGAGATTAT

TATATTACGCCATGGTGAAACTCTGTGGAATGTTGATGGAAGAATTCAGGGACATGTTGATGTTGAATTG

AATGAGGCTGGGAGACAGCAAGCAGCTTTGGTGGCTAATCGACTTTCCAGAGAGCCTAATATCTCAGCTA

TATATTCTTCTGACTTGAAGCGAGCACTTGAAACAGCAGAGACTATTGCTGCAAGATGCGGCGGTCTACA

GGTCATTAAAGACACAGACTTACGGGAGAGACACTTAGGAGATCTTCAAGGCCTTGTATTTAGTGAAGCT

GCCAAAATTAATCCTGAGGCTTTCCGGGCTCTTTTATCCCACAAAACTAGTCAAGATATCCCAGGTGGTG

GAGAGAGTCTGGATCAACTTTATCAACGTTGTACATCTTGTTTGCAAAGAATTGCTAGGAAGCATATAGG

AGAGCGAGTGATTGTGGTTACTCATGGGGGGGTCATTAGTACTTTGTACGAAAATGCTCGCCCCAATAAC

AAGGCTCCAAGAAAGGTATTGAACACATCTATTCATATTTTCCTCATACCTGATGAGAAAAATTGGACCA

TAAAAACTGAGGGTGATGTTAGTCATCTTAACCAAACAGA

>FM892252.1 FM892252 Jatropha curcas embryo 56-70 (DAF) Jatropha curcas cDNA clone rjcaeb1_002752, mRNA sequence

CTCCAAAGTTTAGCATTTATTTAAAGATCCAAAAGTTGAAGCAGGTCTGTAATAGAATTATACTACATTG

ATAAAGTAAACTAAATTGCGAAACAAAGTTCACCCCACACATGCATCCGAGTAAACATCACTCACTCTTG

GCATCTCCATAAGTAATTGCTTCCTGGATTTCTAATCATGTTCCTAAATCTTAATATTACTCCTTGACTT

CAGCATTAGTGTGTTCCACAGCTCCATCAGCTGATGGACCATCTTCTGCAGGCTTAGCAACATCAGCTTC

TTTAGCTTCTGCAGGTTCACCAGCGGAAGCAGCTTCATCAGTTACTGGAACAGGCTTCTTGATAGTTCCC

ACCTTAACATACTTGCTCATGAACTTATACTCCCAATCCTGCAAGGCCTCCAATTCGAATGGACCAAGAC

CAGAGATATCAACAGTCAAATCTTATACTTCAAAGGACATCTTTGCAAGAGCTCTGATGGCGTCCTTTCC

GGCAAATAATGCATAAGGGCCACCAAGTCCATAAAACATCCTGCTTTGAGAG

>FM892251.1 FM892251 Jatropha curcas embryo 56-70 (DAF) Jatropha curcas cDNA clone rjcaeb1_002751, mRNA sequence

ATCCCGAGTCTGAAGTCTCGGAACAAGATACTTCAAGAAATTTTGAAGAAGCCAAACATGCATACCCAGC

TTCCATTAACGAAATTGACCAAATAACCACACCAATGTATGTGCAAGTTGAGCAGGATCTTGTATCTGGT

GGTGTGAACGGGATTCAGAATTCCAAGCTCCTAAATCAAGAGAAGTTGCTGAACAGTGACATGAAGCATT

TAGCTCTTATCAATGATCATTTTAGAGCTGAAAGTTTGAAATCTAGCATGGACCACTTAAGTAATGAGTT

GGAGAGGATGAAAAATGAGAATTCATTTCTGGAAGATGATCATGATTTTCAGCAAAAATTTCCTGCTCTA

CAAAGAGAACTTGTGCAGTTGGAAAAGGTCAATGAAGAACTAGGAAGCATGTTCCCCTTGTTTAATGAAA

TTTCAGGGAGTGGAAATGCTTTAGAAAGGGTACTTGCATTAGAAATTGAGCTTGCTGAAGCTCTGCAGGC

AAAGAAGAGATCCAGCATCAATTTTCAGA

>FM892250.1 FM892250 Jatropha curcas embryo 56-70 (DAF) Jatropha curcas cDNA clone rjcaeb1_002750, mRNA sequence

AAACAAAAGCTTCGATGTTTGTTACGAATATGTTGCTGAGCAAGGGAGGGAGCTCTACGAGCTGGTGGCA

CATCTGCCCGACAAAGCGAACAACTATTTGACTCCTGCATTATCTGTTTTAGAAAAGAACACTTGAACAT

AACACATTCACTAAAAAACACAGTGGATCGAGAGATTTTGGAAGAAAGCACAACGAAATAGAAGAAATCT

CCTGTGATGTCTTTAAATAAAACATAGTTTTGTTCTTTTTCTATATATTGTGAAATTTGTGGTCCACGGT

TGTCATACTGGACTCTTAGTGTTTTCCTTTTTGTATATTCAGGTGTAGATAACATGTGTATGTACTGACT

GCATATTGCAGTACCACTAACAGAGGTGAGAGGATGTCCAGAACTGGAAATTGTGTGTAGTGAAAACTTA

GAAGGCCAAAATTTAGAATTTGTTTGGGTATAAATGTATTTCCATAAAGTTTAATTAAAC

>FM892249.1 FM892249 Jatropha curcas embryo 56-70 (DAF) Jatropha curcas cDNA clone rjcaeb1_002749, mRNA sequence

ATTCATCTCCATTTGCAGGACGATATTGTTCCAGATATTGCACACGAGTTCACTGAGAATGGCTTTTTTA

ACAGTGACCTTGACGATAACATTGGTTATGGTTGGAAGGCATGACTAACATGGTATGGCCTAGTCATTCC

CTGTCATTGAGTGAATGTTTGGCCATAGATATTTGTACAGAGTATTAAATAGCTGCTTTGCCCCACCCTC

TCCTTGCTTTGCTAATTTTCGAATTTGTTGTGACATCATGGTGGCACTTGTATTTCTATAGGCTAAAGTT

AGCTTTTTTTCTCTCTCTCTCTCTCTCTCTCTCTCTCTCTCTGTTTTTGTTTCTTTCTTCTCACCTCTTC

TCTGAGAGTTTCTGTCAAGTCTTGCAAGTGTAATCTATTGGAGACTAAAACTTGGACAGTTAATATGATA

TTTTACCGAGTTTTGC

>FM892248.1 FM892248 Jatropha curcas embryo 56-70 (DAF) Jatropha curcas cDNA clone rjcaeb1_002748, mRNA sequence

ATTCCTTATAGTTACAGAGCCGGTGAGGCTACAGGCCGAGCCGAGGAGAAGGGGAACAGAATGGGCGATA

CGGTGAAAGAGAAGGCTGGAGAAGCGAAAGACTGGACAGGAGAAAAAGCCCATGAGACAAGAGAAAAGAC

ATCTGAGACTGCAGAGGCAACCAAGGAGAAGGCTCATGAAGGGAAGGAGAAGACAAAGGGAGTTCTGGAA

GAAACTGGGGAGAAAATGAAGCATGCAGCTCAAAAAACTGCTGATACTGTCAAAAGCGCTTTTGGTATGG

CTCACCATGAAGAAGAAGAAGATCATGTTAAGGATCGTTCTGGCCATCAGCAGACCACAACTTATAGGGA

CACAGACACTAGGTATTAGCTACTAGTACTGCTACCTATGGCTAATAAATTTTATGTGTGTATTGGGGTC

TTGGGCT

>FM892247.1 FM892247 Jatropha curcas embryo 56-70 (DAF) Jatropha curcas cDNA clone rjcaeb1_002747, mRNA sequence

ATTCATCGCGTCAATGGCGGAGCACCCACAGTCGCAGCATGTTGGCCAACAGCCACGATCCCACCAAGTA

GTGAAGGCAGCCACTGCAGTCACTGCCGGTGGATCTCTTCTAGTTCTCTCCGGTTTGACCCTTGCTGGTA

CGGTCATTGCACTGACCGTAGCCACTCCATTGCTGGTCATATTCAGTCCGGTTCTTGTTCCTGCGGTTAT

CACAGTTTGTTTGATAATCACAGGGTTTTTAGCCTCAGGCGGTTTTGGCGTCGCTGCCATTTCTGTCTTG

TCCTGGATTTATAGGTATGTTACTGGAAAGCATCCACCAGGAGCAGAAAGTCTGGACCAGGCACGCTTAA

AGTTGGCGGGAAAGGCAAGAGAGATGAAGGACAGAGCTGAGCAGTTTGGACAGCATGTAACAGGTCAACA

GACTTAAAAGAGAAGTGGGTCTTGGTGGTTGATGCAGAGAACATAACATGGCTTTGATATATATATAAAA

ATATATGAGTCCTTTAATTAACATGGAAGGCCATTGC

>FM892246.1 FM892246 Jatropha curcas embryo 56-70 (DAF) Jatropha curcas cDNA clone rjcaeb1_002746, mRNA sequence

ATTCCTCTACATTAACATATCTTTCTCACACTTATCATCAACAAATGGCTGAACGTTCACAGCCACACCA

AGTTCAAGTGCACCCACAACACCGCTATGAAGCTGCCTTCAAAGGTCAACAAAAAGGTCCATCAGCTCAG

AAAGTGTTAGCTGTGATCACCCTCCTGCCGGTAGGCGGTGGGCTTCTTGCACTAGCTGGTATAACCTTAG

TAGGTACCCTAATCGGCCTAGCTATTACTACCCCTCTTTTTGTCATCTTTAGCCCTGTTCTTGTCCCAGC

TGCATTAGTTATTGGCCTTTCGGTCATGGCATTTTTGGCTTCCGGGGCTATGGGGCTCACAGGGTTGTCA

TCACTCTCGTGGGTTCTGAAATACCTCCAGGAGGTCACTCGGCGAATGCCGGAGCAGCTGGATATAGCTA

AGAAGCGTATGCAAGACATGGCTGGTTTAGTGGGACAGAAGACCCAGGAAGTAGGCCAAGAAATCCAGAG

GAAAGCAC

>FM892245.1 FM892245 Jatropha curcas embryo 56-70 (DAF) Jatropha curcas cDNA clone rjcaeb1_002745, mRNA sequence

ATTCACTTTCTTTTGGATGGCAACAAGGTCGGATGTATATGATGATGACGATTTTAGTGAAATTTACAAG

GAATACACTGGTCCTCCGGGATCTAATGTTAACAATACACAAGATGGATCAAAAGAAAATAAACGATCCC

ATGCAGGCTCTGATGAGGAAGAGGAAACTCGGGACCCAAATGCTGTGCCGACTGACTTCACAAGCCGAGA

AGCTAAGGTTTGGGAAGCTAAATCGAAAGCTACAGAGCGGAATTGGAAGAAAAGAAAAGAAGAAGAAATG

ATTTGTAAAATATGTGGAGAATCAGGTCATTTTACTCAGGTATTGTTTTCTCGCTTCAGCAATACCCTAC

TAGCTTTTTATGTATAGCTGAATATTGAAAATTACTGTTACACTTAGTCTCCAAGCTGTCTTACACACAA

GGATGATGCAATTCTGTAATTCTGTATGTACTAAGAGTGACATTATTTTTAAGCTCTTATTTTTTAGCCA

TATG

>FM892244.1 FM892244 Jatropha curcas embryo 56-70 (DAF) Jatropha curcas cDNA clone rjcaeb1_002744, mRNA sequence

ATTCGTTGGTGGCCGTCAGCCTTCACGTCCCGGTTCGGGAATTTTAACCCGATTCCCTTTCGAAGCTCGC

GCGAGGACGCGCTGTCGGACGGGCTTCCCCCGTCTCTTAGGATCGACTAACCCATGTGCAAGTGCCGTTC

ACATGGAACCTTTCCCCTCTTCGGCCTTCAAAGTTCTCATTTGAATATTTGCTACTACCACCAAGATCTG

CACCGACGGCCGCTCCGCCCGGGCTCGCGCCCCGGGTTTTGCAGCGACCGCCGCGCCCTCCTACTCATCG

GGGCCTGGCTCTTGCCCCGACGGCCGGGTATAGGTCGCGCGCTTAAGCGCCATCCATTTTCGGGGCTAGT

TGATTCGGCAGGTGAGTTGTTACACACTCCTTAGCGGATTTCGACTTCCATGACCACCGTCCTGCTGTCT

TAATCGACCAACACCCTTTGTGGGTTCTAGGTTAGCGCGCAGTTGGGCACCGTAACCCGGCTTCCGGTTC

ATCCCGCATCGCCAGTTCTGCTTACCAAAAATGGCCCACTT

>FM892243.1 FM892243 Jatropha curcas embryo 56-70 (DAF) Jatropha curcas cDNA clone rjcaeb1_002743, mRNA sequence

ACAAAAAGAAAATGATCCATTGAAACTAGAAACACAAATTACAGACTCTTGCTAGCTATATCCTGAAATA

TATTAGGATTAAGGAGTACTACGAGTTAACTTAAACAAGAGTTTCAAGAGCAATTAAGATTTGTCGAGAT

GATTAGAATTTACAGTCTTTTCCACCTCTTCCCTCTTTTTCTCTTTTTCCTCTTTCCCTTTACTTATCTC

CTTCTCTGCTTCCTCCTTGTTGATGGTTTCAGTGACAGGTCGCTTGGTTCCATGTACAGCAGCTCTGTTG

GAAGCTTTTGTAGTGATGAGTCTCTTCATTAAATCATCTCTATTTTTAACCGTATGATCAGCAATGTTTT

GAGTCCTCCACAGCCAACCCACCACCAGTCTTCCCTCCATTTTTATCAATCTTTATCTTGCGAGAGATAC

AGCAATGGAGGGCAAGGAAGAAGATGTTAGACTAGGAGCAAACAAGTTTACAGAAAGGCAACCCATAGGG

ACTGCAGCTCAAACAGACAA

>FM892242.1 FM892242 Jatropha curcas embryo 56-70 (DAF) Jatropha curcas cDNA clone rjcaeb1_002742, mRNA sequence

ATTCGCTTCAAGCTGCCGCAAGGAACCTTTATGTCATTTTCCCACATGAAGTGTTCTGACTCATCTGTTT

TCTTCCTCCCTAACCCTTTAACGCCGATCTTTTTCTCTTCAAAGGATTTCGTATCCTCTGGCAGGCTCGA

AATCTCAGTTGCTTGATCTCCAAGAGAAGCAACGGCTAGAACCAGGAATCCTTCTGGTCTATCTATAGCA

GTAAAACCATATCTAGCTGCTTCTGCAGCAGCATCAGAACAAACAATTGCCTTGCCAAACATATAACCCG

GTACCGGAAGAGAACAAGCAGCTGGTAAGAAGCCCTTCTGCAAATGCCTTAACAAGTTCGAACTTCGGGT

ACCACACCACAACAAGACTTTGTTCGGCAACTTCTTGATTTCATCTAAAGAAGGCCAAGCGCTTGATTCA

ACGGCAAAAATATTCTCAACTGATACTCCATAATCAATATCTCCAACTTTGATTGGTTCATAAGTTTTTT

CCAAGTATTTGACAATCATTTTATATTCATCAGAATCTTTCTCCAGTGGAGACATTGAGCATCCTA

>FM892241.1 FM892241 Jatropha curcas embryo 56-70 (DAF) Jatropha curcas cDNA clone rjcaeb1_002741, mRNA sequence

TTTCTACAACTAATTAATTTCTTTTTTAAGAAAGGAGAAGGACAAAATAAGAGGGCCAAGAAGAAGAGAG

AGGAGATGTCCAGTGTTGTCCGCAAGATGAGGGGTGCTGGAGGTGGTGAAAAGAAGAAGTGAGCAGACCT

CAATGATTTTAGACAGACAGATCTCAATCTGTCTGTTAGACATTATAATAGCTAGAGGCGACTTCCATGT

TTTTTTAGACGTTTGAGATTTTGATCAGAACCTTGTTTTGTTTTAATTAGAAGCAAATGATGTCCTTTTA

GTTTCCCTT

>FM892240.1 FM892240 Jatropha curcas embryo 56-70 (DAF) Jatropha curcas cDNA clone rjcaeb1_002740, mRNA sequence

ATTCGTCGAAGTTCAGGACTGGGAGCTAGTGTGATGATTAAAACTATAAAGGGTATTTTGTAGTAATAAT

AATAATAATGTTGGTAATAAAGTATTAGCTTAGCAGTGTACTTTAGTCTAGTAATGTAGTTTACATGTGG

GTTAAAATGACTAGGGTTTAGCTAGTATGTGTGTTGTAACTTTGAGAGGGGTTCATGGTCGTTCCCTTTC

TGGGAGGACTGCTAATACTTTCTCCCTATTTGTAAAATAACCTGCAGTATATATAC

>FM892239.1 FM892239 Jatropha curcas embryo 56-70 (DAF) Jatropha curcas cDNA clone rjcaeb1_002739, mRNA sequence

ATTCGAGGAAGGTAGGGTCTTGCAGATAAGCGGAGAGAGAAGCAAAGAGAAAGAAGAAAAGAATGATAAG

TGGCATAGAGTTGAAAGGTCAAGTGGTAAGTTCTTGAGAAGGTTTAGGCTACCAGAGAATGCAAAGGTTA

ATGAGGTTAAGGCTAGCATGGAAAATGGAGTCTTGACTGTGACAGTGCCTAAAGTGGAAGTCAAGAAACC

GGACGTGGAGGCTATTGAGATTTCTGGTTGAAACTTTTAGTTCATGTTTGAGTGTGTCTAAGTCTAAATG

GTACCCATCCATCCAATAAAATAAATAGATGTGTGTCATGGGTTTGTCGTAGTGAGCTTGTATCCTTATG

GAGTTGCTG

>FM892238.1 FM892238 Jatropha curcas embryo 56-70 (DAF) Jatropha curcas cDNA clone rjcaeb1_002738, mRNA sequence

ATCCTTCTCTTCTCTTCTCTTCTCTATAAATAACTAAGCTCCATGGCAGCACTGGTGGCTGGTGAGCGGT

GACAGCCAGAGAACGCACAGAAGCGGATCTCTGCAATTTTATAATTCTTCATATTAGGTCTCTGTGTTTG

TAGTCTTAGGATATGATAACTCTTTTGCAATATTTTGAAGCCTAATATTGCAATTCTTTGATCTCTTCCG

AAATCGGAGATAGATCAAGGAATCTATCGTTAGGCAATAGTAATTGTTTTATTTTGTATGCATGAAATTT

TTCAATTTCATGTATACTCTGCCTTTAGCTCCGATTTTCCTCTCCTTTCTTTAGGAATTATCGGTGACAA

NGTAGATACAATTG

>FM892237.1 FM892237 Jatropha curcas embryo 56-70 (DAF) Jatropha curcas cDNA clone rjcaeb1_002737, mRNA sequence

GAGCCTCAGAGAGGCGGCCCTGCCTCAGTCATGCAATCTGCGGCAAACGTCAACGTTAGGACTGGTGCCG

TCGAGCGGGATGATGTGAGTGATGTTGTTAGAGAACAGGGCATTAACGTTGCTGAAATAGATATCGGCGG

CACTCGCGTTATCACAGAGAAAGTCGGTGGAGAGGTTGTGGGACAATATGTTCAGCCAAGAGTTCCAGCG

ACATATCCAATGCCAGGTATGGATATTACAATGGGTGAAGCTTTAGAGGCAACTGCTTATTCTGCCGCAG

GAGACAAACCAATTGATCAAAGCGACGCCGCTGCTATAAAAGCTGCAGAGGTGAGAGCTCTTCGCAGTAC

TCAAACCCCGGCGGGTGGAATAGGCGCAGAAGCACAGTCGGCAGCTGATCGTAATACCAGAGTTATGCTC

GACGAAGATAAGACTACCCTCTCTGATGTTTTAGCGGACGCGACTGCCAAGTTGCCTAGAGACAAGACGG

TAACTCGGGATGATGCTG

>FM892236.1 FM892236 Jatropha curcas embryo 56-70 (DAF) Jatropha curcas cDNA clone rjcaeb1_002736, mRNA sequence

TCAATTCATGGTATTCAAATCGAGTATATTGACATTTCACCATTGCCTATGCTCAACACCGATCTTGAAG

GTCCCGACGGTTCTTTCCCGCCGGTCGTTGTAGCTTTCCGGAAAAAGATTCGGCAAGCTGATAGTGTCCT

TTTCGCCTCCCCTGAGTATAATTTCTCAGTCACTGCACCTTTGAAAAATGCAATTGACTGGGCATCTAGA

CCACCAAACTGTTGGGCTGATAAAGCTGCTGCCATTGTAAGTGCTGGTGGAAGTTTTGGCGGTGGGCGAT

CACAGTACCATCTTCGCCAGATTGGAGTTTATCTCGACCTTCATTTTATCAACAAACCTGAGTTTTTCTT

GAATGCATTTGCACCTCCAGCGAAATTTGATGCTGATGGCAACTTGATTGATCCAGAAGCAAAGGAGAGA

ATAAAGGATCTTCTTTTAAGCTTGCAAGCATTTACTTTGCGACTCAAAGGAAAGTGCTAATTGTATATTC

ATTGGTTTATTGTTTGATCCATTTGAAATTGATAATCACTTTGAAA

>FM892235.1 FM892235 Jatropha curcas embryo 56-70 (DAF) Jatropha curcas cDNA clone rjcaeb1_002735, mRNA sequence

GAATTCGACGAAGGGTACAACTTTTATTTATCCAAACAAACACGACATTTAACACCTATATAAGCTATAG

AAGGAGACACCATTAAACCAGCAACACTCTAGATATATAACAAAAAGACAAAAAGAACTTTTGACTTTCT

TCACAGCTATCTCCATCAACCATTCACAATAACGCCTCCATTGGGGTGAAGAACTTGGCCAGTGATATAA

GAGGAGCAATGGTTACAAGCAAGAAACACATAAGAGGGTGCAACCTCACTTGGCTGGCCAGCTCTCTGCA

TAGGCGTTTGTTTCCCAAAGTTAGCCACCTCCTCTTCACTGAAAGAAGAGGGTATCAATGGAGTCCAAAT

AGGTCCAGGAGCAACACCATTGACTCGTATGCCTTTTTTCACTAGCTGAAGTGCTAGTCCTCTAGTGAAA

GCCACTATTGCCCCTTTAGTTGCTGTGTAATCAAGCAGTTTCTTATTTCCCTTGTATGCATTAACTGATG

TCGTGTCGATTATAGAGCTGCCCTCTTTCATGTGCTTCAGAC

>FM892234.1 FM892234 Jatropha curcas embryo 56-70 (DAF) Jatropha curcas cDNA clone rjcaeb1_002734, mRNA sequence

CCTTCAATGCTCCTCCATAAACCCAAAAAGTGTCCCACGCAAAACAGAAACAAATAAAGCCACGAATGTG

AAGTAGAAATTAGTGAGAATTGCTAAAACTCAATCAAAAACCCAACAAATATAACTGCAATATCAAAAAA

TGAATTTCAAACACGCAAAAAGCCACTACAAAATCTAAGATATACAGTACAAAATACAGGCAGAGGATCG

CCAGAGCTACATTAGCAATTCATCCGGAGGTAGTATTTTGCTCATCAATATTAACTTTAGCATTTGAAGT

CCCTTGGGCATTTGCAGCCTCCGGTGGAGGACCCAACCCCAATAACAAAGGAGTTTGTTTCTTTTCAGCA

GAACTGGAATTTTTATTAGAATCCGGATTTGCAGCCCAAGCTCGAAGAGGAGGTTCAAAATGCTTGCTGG

CAACATCAGGTGGTAAGGAATCTTCTCCGGGTAGAACAAGCAGTC

>FM892233.1 FM892233 Jatropha curcas embryo 56-70 (DAF) Jatropha curcas cDNA clone rjcaeb1_002733, mRNA sequence

ACGAATTAAAATTAACAAAAGTTAAAGGTAATACCTCAACACCCACTACAAAGAAGTGCATCAACATACC

AACAATTGGTGGTTCAGCCACATTGGAGAAGACAAGATCACAAATTGTTAGAGCTTGTTACATACGACAC

TACAACAGTGATGTCATCAAGTTTACCACCATAGTAGCTGAACCCAGCGCGTTAAGCAGCAGTAGAAAAA

GGTGTCTGTCGGTTCTCATCCAGTGCTCGTTGACTTGTCATAGCTGCAATTTTCTGAGCTGTTACTTGAG

GCCCTAAGCCAGCTCTTAGGGCATCAACAACAGCAGTGGTAACCTAGTTATACAAGTTGTCGAATAATCC

ATCTGTTCCGGCAATAATTACGTCACCCGGAGAAATAGGAACTATGAATACCTGGCCAGAGCTTGGTAGA

TCACCCTCATTGCCACTCTCTAGTTGGTATGGAAAATTAAAGCAATGCTGCTGTGCTGGGGATTGGAACA

CTGTGCATCCATCTCTAA

>FM892232.1 FM892232 Jatropha curcas embryo 56-70 (DAF) Jatropha curcas cDNA clone rjcaeb1_002732, mRNA sequence

ATTCAATACACTCTCTCTTATACCTAAATTTAAAGTTAGAGTTTCAGAAATATGGCTACGAAGAACACAT

CAACAGTTGCTCTCTTTCTTGTATTTAACCTTTTGTTCTTTACCCTAGCCAACGCATGTGGTGGGGGCTG

CCCTACTCCTAGGCCACCAAGACCAAAGCCAACTCCAAAGCCAACACCAACTCCGTCACCTTCAGGCGGA

AAATGTCCTAGAGATGCACTTAAACTAGGCGTATGTGCTAAGGTGCTCGGTTCATTGCTTAATATCACCA

TTGGCGATCCTCCTGTCAAACCTTGCTGCTCTCTTCTAGAGGGTCTTGTTGATCTTGAAGCTGCTGTTTG

CCTTTGCACTGCCATTAAAGCTAATATTTTGGGTATCAACCTCAATGTTCCACTTTCTTTGAGTTTGCTT

CTAAATGTATGTAGCACAAAGGTTCCTTCTGATTTCCAATGCGCCTAAACATATATAGACATTTAACTAT

ATATCTATATATGGGT

>FM892231.1 FM892231 Jatropha curcas embryo 56-70 (DAF) Jatropha curcas cDNA clone rjcaeb1_002729, mRNA sequence

ATTCGATTACCTAAAAAATGGCTACTGCTACTGTACTCTTCTCTACCATTTCCACCGCGCTCTTTATCCT

CTTCTCCTTTGGTCAAGCTGCGCCTCTTATTTTAACCCTAGTCAACAACTGCCCTTTCACCGTTTACCCT

GCCATCCAACCCAACGCTGGCCACCCGGTCCTCGAAAAAGGTGGCTTCCCACTCTCCACCCTAACACACC

GCTCTTTTCCCGTCCCTAACCAGCACTGGACTGGCCGGATCTGGGCTAGAACCGGATGCACTCACACCAA

CGGCAAGTTCCACTGCGCCACCGGTGATTGCAACCACCAACTAGAATGCAACGGCCTCGGTGGTGCCCCT

CCAGCAACTCTCGCTCAGTTCAGCCTTCACCATGGCCATAACGACTTCTCCTCTTACGGCGTCTCTCTCG

TCGACGGATTTAACCTTCCGATGACCATAACTCCACATGAAGGCAAAGGTGTTTGTCCGGTG

>FM892230.1 FM892230 Jatropha curcas embryo 56-70 (DAF) Jatropha curcas cDNA clone rjcaeb1_002728, mRNA sequence

ACTAAAAGATACCTTTTATTCAACTGGAATTGCTTAAGCAAGAAAAAGACACCAAACAGAAAACATAGAT

ATATTACATAACAGGAGCTAAAGCACATGCCATGCCCTACACACGTGGGTTATGGCCAGTGGTGTCTCCG

GGCAACCCAGATGGGCGAGTCCTGGTCAAACCCGTTGGGTCCACCTCTCCACCGTATGGCTGCCCTGTGC

CGTGTCCAGGCATGGCCGACATTTGCTGGGTCCCTGTAGGGTAACCGGTTGTCCCAGTTGTTGAATGGGT

ATAGGTTTCGGTTGTGCCTTGAGCGCCCGGACCAGCAGTTGTGTACTGTACATGGCCACCAGCCTTGGCT

GCCTGCCTCGCCGCTGCGTTATCCTCGCGCGCTTCTAGTTTCCGCAATTCTGCCTCCGTTTTCCTCTCTT

CTTTCTTCTCTCTCGCCATTTCCTTCTGCACTGGATCACGTGTTGACATTCTCTCCACCTTCTCTTGCGC

CTCTGCTCTGGTTTTTTCCATGCCAGCCTTAGCAGAGGCTCCAACGTTAGCT

>FM892229.1 FM892229 Jatropha curcas embryo 56-70 (DAF) Jatropha curcas cDNA clone rjcaeb1_002727, mRNA sequence

ATTCCAAAAAACTCATTCAAGGAGAAAAAAGAAGAAAGCTTGTCTTTGAAGACTTATCCATCATTAACAA

TGGCAGGTCTTAAGTTAATTAGCATTTTGGTTCTCTGCATGTTTGTCGCTGCGCCTATAACAGAGGCCAT

AACGTGTGGACAAGTGACCAGTGCCCTGGCTCCATGCATTACTTACCTGAGAACAAGAGGTGCCAAAACT

CCACCGCCAAATTGCTGCAACGGAGTGAGAAGCATCAACTCCGCCGCAAAATCCACCCCTGACCGCCAAC

AAGCATGTCAGTGCTTGAAACAAGCAGCAGGAAGCGTCTCTGGCATCATCCCCGGGAACGCTGAGGCTCT

GCCTGGTGCTTGCAAAGTTAACATTCCTTACAAGATCAGCCTCTCCACTAATTGCAACAATGTCAGGTAA

GGTTTACATATCGAGCAGCAATGGAT

>FM892228.1 FM892228 Jatropha curcas embryo 56-70 (DAF) Jatropha curcas cDNA clone rjcaeb1_002726, mRNA sequence

ATTCGAGAGAACTAGTCTCCAAGAAAGCTGGGTCCTAGTCGTAGCGGGAGTAGAGCAGCTCGAGAAGGGA

AAGGAAAGCAAGACCCTGATTGTGGTTGATTTTACTGCTACCTGGTGTGGGCCTTGTCGTTTCATCACTC

CAATTCTGCAAGATTTGGTTAAGAAGATGCCTCATGTCACGTTCTTGAAGGTGGACGTGGATGAATTGAG

GACTGTTGCTGAAGATTGGGCTGTTGAGGCAATGCCTACTTTTATGTTCTTGAAAGAAGGGAAAATCGTG

GACAAGGTTGTTGGTGCCAAGAAAGAGGAACTGCAGATGACAATTGTAAAGCATGCCACAGAGGTTGCAG

CAGCTTAATGGATGAAGAACACTAGGACTTCCATGTCCACTAATGGCAGCTTAGTTAACTGTCTTACAAA

TATTTGTATTATCTGGAACCAATATGTTTATCTTATTATGTGGTCATGCGAGGCCACCACCGTTTGTGTT

TAATACTTTCTATATATTAGATCTTTCTATGTTTGCTT

>FM892227.1 FM892227 Jatropha curcas embryo 56-70 (DAF) Jatropha curcas cDNA clone rjcaeb1_002725, mRNA sequence

ATTCCCACCTTATTCCACAACAGATTTCCGATAACCTCCATTGCTAAATCTCTCTCTTTTTGCTTCTCTC

CTTTCAAGAAAATCGTCTCCTTCTTTTGTAAAAAATAAAAAGA

>FM892226.1 FM892226 Jatropha curcas embryo 56-70 (DAF) Jatropha curcas cDNA clone rjcaeb1_002723, mRNA sequence

ATTCCCCCACAAAACCCTTACAATGGCGTCCTCAGCGAAGAAGCGACCGGCTATAAGCAAGCGGCAACGA

AGGAATAGTCGCCAGGGTGACACGCACAGTTTCAGAGTCGGCGGTATTGAACACGGCGAAACAAGCAGCC

AACGACACGGCCTACGTGGCGAAGAGACTGGCTAAGAGCACGGGAAAGGCGGCGTGGATCGCGGGAACCA

CGTTCCTTATCCTGGTAGTCCCACTCATCATCGAGATGGACCGCGAGCAGCAGTTCAATGAGCTCGAGTT

GCAGCAGCAGAGCCTGCTTGGAGCC

>FM892225.1 FM892225 Jatropha curcas embryo 56-70 (DAF) Jatropha curcas cDNA clone rjcaeb1_002722, mRNA sequence

ACTAAAAGATACCTTTTATTCAACTGGAATTGCTTAACAAGAAAAAGACACCAAACAGAAAACATAGATA

TATTACATAACAGGAGCTAAAGCACATGCCATGCCCTACACACGTGGGTTATGGCCAGTGGTGTCTCCGG

GCAACCCAGATGGGCGAGTCCTGGTCAAACCCGTTGGGTCCACCTCTCCACCGTATGGCTGCCCTGTGCC

GTGTCCAGGCATGGCCGACATTTGCTGGGTCCCTGTAGGGTAACCGGTTGTCCCAGTTGTTGAATGGGTA

TAGGTTTCGGTTGTGCCTTGAGCGCCCGGACCAGCAGTTGTGTACTGTACATGGCCACCAGCCTTGGCTG

CCTGCCTCGCCGCTGCGTTATCCTCGCGCGCTTCTAGTTTCCGCAATTCTGCCTCCGTTTTCCTCTCTTC

TTTCTTCTCTCTCGCCATTTCCTTCTGCACTGGATCACGTGTTGACATTCTCTCCACCTTCTCTTGCGCC

TCTGCTCTGGTAAAATCCATGCCAGCCTTAGC

>FM892224.1 FM892224 Jatropha curcas embryo 56-70 (DAF) Jatropha curcas cDNA clone rjcaeb1_002721, mRNA sequence

ATTGGAATAAGAAATTTCAAGCGAGAAGAATGGCTACAGAGCAAGAAAGGGCTGAACTTGATGCTAGAGC

AAGGCAAGGAGAGACTGTTGTACCTGGTGGAACTGGTGGCAAGAGCCTTGAAGCCCAGGAACATCTAGCT

GAAGGGCGGAGCCGTGGAGGGCAGACAAGGAGGGAGCAGCTGGGAACTGAGGGGTACAAGGAACTTGGTC

ACCGTGGAGGTGAGACAAGAAGGGAGCAGATTGGGCATGAAGGGTATCAGGAGATGGGCCGAAAAGGCGG

ACTTAGCACCATGGACAAGTCTGGTGAAGAACGTGCTGCTGAGGAGGGGATTGAAATTGATGAGTCCAAG

TATAAAACCCGTTAGTTAGGTTCTTCATATCAGTGTTTTAACTAGTAGTTCCTCTTTTTGAACTAGTTGC

GAGTAGGTTTGTACATATGGTAGTAGTAACTGCGGTATGTTGGTTGTTTTTGGTTGGTGTCGGGGTTTGT

TTTCTGGGCATCAGTGTGTAACCTGTTGTATTCCCCACTGACAGGAAGTGGCATCCCATGGTT

>FM892223.1 FM892223 Jatropha curcas embryo 56-70 (DAF) Jatropha curcas cDNA clone rjcaeb1_002720, mRNA sequence

GATTACGGACAAAATTTATTGGAAAGAGCAAAAGCAAATTCTTAGCAGTGGATCATTTGAAAGCAAATAT

GTGAACTCAGACCAAAAACTTTAAAAAATGTTTGAGGCACCAGGAAAAGCACTGTAACACAAACATATTC

TACTAGTTATCCCTCATCAGAGATGATATACAAGAATTTTCTAAATTATGGGACTGGCTAAGTGAAAAAC

TGGCAGCCTTGGAAACTTAGATTCAACCCGCGAAAACTTTCTTCACAAATCCTTGCAAAGAATTCCCCAT

TCCAGCGCCAACAGCTTCAGCCAAATACTTTGCCTCTTGAATTGACTTCTTGAAGTCGAAGGCATCTTTC

ACTCGATCATCCAAGAATGCCCATGTATCACGAAAATCTGGGGAACTATCAGTCAGCATGTAGATC

>FM892222.1 FM892222 Jatropha curcas embryo 56-70 (DAF) Jatropha curcas cDNA clone rjcaeb1_002717, mRNA sequence

ATTCGCTGTTTCTACTTATCCGCTCTCTGCCTCCAAGAAGTATCAGGTAGGGTTTTAACGAGCTTCTCTT

GCGGCAACCATGGCGGACAAGGCGGTCACTATACGTACCAGGAAGTTTATGACCAATCGCCTCCTCTCCA

GGAAGCAATTCGTTATTGATGTTCTTCACCCAGGAAGGCCCAATGTCTCCAAGGCAGAGTTGAAGGAGAA

GCTAGCAAGCTTGTATGAGGTGAAGGATCCCAATACAATTTTTGTATTCAAGTTCAGGACTCATTTTGGA

GGTGGGAAATCCACAGGCTTTGGTTTGATCTATGATTCAGTTGACAATGCAAAGAAGTATGAGCCCAAGT

ACAGACTCATCAGGAATGGGCTTGATACTAAAGTAGAGAAGTCAAGGAAACAAATGAAGGAAAGAAAGAA

CAGGTCCAAGAAGGTTCGCGGAGTAAGAAGACCAGCTGGTATGCTGCCAAAAGAAGTG

>FM892221.1 FM892221 Jatropha curcas embryo 56-70 (DAF) Jatropha curcas cDNA clone rjcaeb1_002715, mRNA sequence

ATTCAGCTCAATGGATTTACAATAATGGCCGGTCTCCTCTTGTTCTTGTCCAAAGTCAGTTGACACTAGC

AATCCTGCCAACCAGCTAGATCAAAGTCACAGATTGTTCTTCCTTGCTGGTAACCCACATCGAAAAGTAC

AGAGCCAGAGAGGTGAGTCCGAAAGAAGGCATGAGTGGTCCAGAGGCGAAAGCCTAAGGAGTCATCAAAG

AAGCGGCAATGTGTTTTCTGGCTTGGACGAGCAACTACTAGCTGAAGCTTTCAATGTCAACCGGGATATT

GCAAGAAGACTAAAAGGAGAAAATGATAACAGAGGGATTATTGTTAGTGTGGAGGGTGAGCTTGAGTACT

TTAGCCCACGAAGGAACCAAGAAGAAGAAAAACGAGAGAGACAATTCGTTAATGGCATAGAAGAAACTTT

CTGCACAGCCAGACTCACGCACAATATCAACGACCCATCACGTGCCGATATCTATAATCCACGTGGTGGC

CCATGTCACTAATTGTCATAGCCACAACTTCCCGATCCTCAGACACCTCCGACTCAGCGTTCAGAAAGGT

GTCTCTTTAGGAATGCTATATTTGCACCAGACTGGAACATC

>FM892220.1 FM892220 Jatropha curcas embryo 56-70 (DAF) Jatropha curcas cDNA clone rjcaeb1_002713, mRNA sequence

GCCAACTTCACCTCTCAGGTCTTCATCATGAACCACCCTGGTCAAATTGGAAACGGATATGCTCCTGTCC

TTGACTGCCACACCTCCCACATTGCTGTTAAGTTTGCTGAGATCCTAACCAAGATTGATAGGCGATCTGG

TAAAGAGCTGGAGAAGGAGCCAAAATTCTTGAAGAATGGTGATGCTGGATTTGTGAAGATGATTCCCACC

AAGCCCATGGTTGTGGAGACCTTTTCTGAGTACCCTCCTCTGGGTCGTTTTGCTGTGAGGGACATGCGCC

AGACAGTTGCTGTTGGTGTCATCAAGAGTGTTGAGAAGAAGGATCCATCTGGTGCAAAGGTCACCAAATC

CGCAGCAAAGAAAAAGTAAAGCATTGGGTGAATGAAGTTACAGTTAGTTTAGTTATGTTTTGTTTTAAGT

TATGTCTTGCTGTTGTTTTTGGTTTCAGTTTCAGTTATCTGGCAGTTTTGTGACTTAGGTTCTTAAGTTA

TTGTCTTTTGCATCCTCTTCTGATTGTTATATGTTGTTATTGGATTTTAGAGCCACAAACTTCAATTTAT

CATACTATCTTCATTCCATTT

>FM892219.1 FM892219 Jatropha curcas embryo 56-70 (DAF) Jatropha curcas cDNA clone rjcaeb1_002711, mRNA sequence

ATTGTTTACTTTTAGTATTGATCTAAGCGGACCTGAAAATTTTATTTTCGGGTTTGTCTTCTCTATTTTG

TTGCAAAGTTCGGTTAATCACTGCTAACTTGGGGTAAGTTTATGAATCCCCGAGTCAATTTCCAAGACAG

AATATATGCACTATAACCTCAAGAATACCATGGGAAAAAAAAAAAAAAAAGAGGTAGGCTAAACTTGGAT

GTTCTGACCATTGCAATTATCTTGGGTGTATCATACAAAGAGGAGAATATTATGAGGAAATCCATGTGAA

TGGAGAAGGGGGATAATACTTTAATGTACAGAAGTCCTTGAAGGACTGAAAGAATACAATTCCCCCCTTA

AAACTTGTCTAATTATGAAAGTGAACACAGAAAAGCAA

>FM892218.1 FM892218 Jatropha curcas embryo 56-70 (DAF) Jatropha curcas cDNA clone rjcaeb1_002710, mRNA sequence

ATTCGGTATATTCTGGAGGGTAAGGAGCTGGAGTTCTACATGAAGAAGATCCAGAGGAAGAAGGGCAAGG

GAGCTGGGGCTGCCTAAAATTTTGCAATCCAAACTTTCTATTCAGATCAGACTTGTCTCCCCCCTCTTTC

TTTTCCTTGTGTCTGGAGATTGTATTTTTTATGAGATGAGAGATTAAACTTGTAGCAGCAACTATTATTA

TCGTTGAATTCCCAATTAGAAACCTAATATTGTCTTTTATTTAACAGCAGCATAAGATGATCTTTTGATT

TTGT

>FM892217.1 FM892217 Jatropha curcas embryo 56-70 (DAF) Jatropha curcas cDNA clone rjcaeb1_002709, mRNA sequence

CCAGCATTAATGTGATACTTCCATATAAACTTTATCAAACCAGTAGGAATATCAATAGCTGTTAGAGTTT

CTCTTCGAACTATAGAAAAACCGAATACATCTACTAGTTTGCAGTGCAACAAAGACATTCTCCATTAACA

GAGACAAACACATTCTCTATCCTCCATCAACAAAATCAACATCTTCATCTGACCTCAGCGAGCTTCATAC

TGAACAATGTCTACGGGTGCGTTTTTTGTAGAGAATACGGTAACCGCACTCTCGGCATTGGATAACATCT

CCTGGCTTTAGAGTATTTTCCATTCCACAATCGGCTCTGGTTGAGGATCCATTGGTCTGAGCTCGTTCTA

GGGTTTTGGGGGGGCCCGGTAC

>FM892216.1 FM892216 Jatropha curcas embryo 56-70 (DAF) Jatropha curcas cDNA clone rjcaeb1_002708, mRNA sequence

AAAATTGAAGCCGTGAATCGTGAAAGGAAATATCATCAGCAAAATACTGCATACGAGATGAGTGCTTTGT

CCATGCAATGGAAGGAGCTTTGTCAGAAAAATATAGAAATCCAAGCGGCGTGTACCAAAATAGAAATGCA

GATCGAAGAATTGAAGAAAGAAGCTGCAGAAAGAGGCTGGAATTTGGAAGCAAACATAGAGAATGGTTCT

GTTATTACATAGAAAACAATAAGGCTTTCTATGCAGATTTGAAAAATAGGAATTCCATTCAGAAAAATAT

GAAAATACATTTCTATGGTTTCTGGCGGCTCATTGCTCTGGATTTTGCAGACTCATGTTGTGGGTTCTCG

CGCGGAAAAACCTTTGTTTCTAGTTTTGTCCTGCTTCTGTTTTTAAGATCAATTATTGTTTAGGCTTAAT

ATTAATGTTGTATTTGATGAGACTTGTTCTGGTTGTATATCTTATGATGAAGAGAATGGACTTATTCGTT

CAGCT

>FM892215.1 FM892215 Jatropha curcas embryo 56-70 (DAF) Jatropha curcas cDNA clone rjcaeb1_002707, mRNA sequence

ATTCTGCCAACTTCACCTCTCAGGTCATCATCATGAACCACCCTGGTCAAATTGGAAACGGATATGCTCC

TGTCCTTGACTGCCACACCTCCCACATTGCTGTTAAGTTTGCTGAGATCCTAACCAAGATTGATAGGCGA

TCTGGTAAAGAGCTGGAGAAGGAGCCAAAATTCTTGAAGAATGGTGATGCTGGATTTGTGAAGATGATTC

CCACCAAGCCCATGGTTGTGGAGACCTTTTCTGAGTACCCTCCTCTGGGTCGTTTTGCTGTGAGGGACAT

GCGCCAGACAGTTGCTGTTGGTGTCATCAAGAGTGTTGAGAAGAAGGATCCATCTGGTGCAAAGGTCACC

AAATCCGCAGCAAAGAAAAAGTAAAGCATTGGGTGAATCGAAGTTACAGTTAGTTTAGTTATGTATTGTT

TTAAGTTATGTCTTGCTGTTGTCTTTGGTTTCAGTTTCAGTTATCTGGCAGTTTTGTGACTTAGGTTCTT

AAGTTATTGTCTTCTGCTTCCTCTTCTGATTGTAATATGTTGTTTTT

>FM892214.1 FM892214 Jatropha curcas embryo 56-70 (DAF) Jatropha curcas cDNA clone rjcaeb1_002706, mRNA sequence

ATTCACTATTTAGAAGCAGTACAGGAGATCTTGGTTGTGCTCTCGCCGGTGCCGGAGATGGATGGTAAAG

GGAAAGAGAAAAGGAGGAGGATTGACAAAGAATCCGATGAAGCTAAGAGTAAAAAGGTTAGACATGGAGA

TAATAAGGAGGAGGCGACGGACGAGGAAGTTGAGGAATTCTTTGCGATTCTAAGGAGTATGCAAGTAGCT

GTCAAGTACTTCGAGAAAGGAAAACGAGAAGGCTGGCGTGCTGCCGTCGAAGCTGAAGTGGTTGCGGTGG

TTGGTGGTTCCAAAGAAGATGAAGACGATGATCATCAGATTAAAAAGAAGAAAGGATCGCCAGCAAAGAT

AAATCCGATTGTGGAGGAGGAGGAGATGGCGGTTTTGGATTTGAACGCCTTGCCGGCGGTGGAAAGTAGT

GAGATATGTATAACTGAATCGCCTGATCAAACGTGGTTGAGATCAAGGAAGCTGCCGTCGCGTGCGGTAG

TGGAGGAGCAGCTCGTGAG

>FM892213.1 FM892213 Jatropha curcas embryo 56-70 (DAF) Jatropha curcas cDNA clone rjcaeb1_002705, mRNA sequence

ATTCGTGATGATACGGCTTAATAGCAGAGGATTGAGTTCCCAAGCTGCCTTGGCAGAGCGGCTACAGCCT

TGTGCAACGATTGAAGTTGAAGGTTTTCTAGTAATTGCTTTCAGCTTGTTCTCTGTAATGTACAGATATT

AATTATAGTGTCATCATCAATACATGAGGTTCTCTCTTTCCTTCGGTACTTTACTTGAGATTGCCTCTTG

AGGATTTTATGCCGGATGTATCACATATCTATCTCTGATTGATATTTGGTAATTTTATAT

>FM892212.1 FM892212 Jatropha curcas embryo 56-70 (DAF) Jatropha curcas cDNA clone rjcaeb1_002702, mRNA sequence

TCGAGAGAGAGAATAGTCGTCGTTGGTGGCCGCAGCTAACGTTGGAGCCTCTGCTAAGGCTGGCATGGAA

AAAACCAGAGCAGAGGCGCAAGAGAAGGTGGAGAGAATGTCAACACGTGATCCAGTGCAGAAGGAAATGG

CGAGAGAGAAGAAAGAAGAGAGGAAAACGGAGGCAGAATTGCGGAAACTAGAAGCGCGCGAGGATAACGC

AGCGGCGAGGCAGGCAGCCGGGGGCTGGTGGCCATGTACAGTACACAACTGCTGGTCCGGGCGCTCAAGG

CACAACCGAAACCTATACCCATTCAACAACTGGGACAACCGGTTACCCTACAGGGACCCAGCAAATGTCG

GCCATGCCTGGACACGGCACAGGGCAGCCATACGGTGGAGAGGTGGACCCAACGGGTTTGACCAGGACTC

GCCCATCTGGGTTGCCCGGAGACA

>FM892211.1 FM892211 Jatropha curcas embryo 56-70 (DAF) Jatropha curcas cDNA clone rjcaeb1_002701, mRNA sequence

AATTCCTCTTTTGCCGGTGAGTGGGACGCCNTTCTTTTCCTTGCTGGAATAACGCTTACTGGAGCACTCA

TTGCGCTAGCTGTTGCGACTCCGCTGTTCGTGATTTGTAGTCCGGTGCTGGTACCAGCGGCGTTGGTCAT

TGGGTTGTCGGTGTTAGGGTTTTTGACGTCCGGTGCTTTTGGAATCACTGCGCTTTCTTCACTGTCGTGG

ATGGTTAATTATATTCGCAGGATGAGAGGATCTTGGACGATGCAGATGGAAATGGCAAAGCGGCGCGCGC

AAGAAACGACGGGTCAATTGGGCCAGAAGGCTAGGGAGGTGGGCCAGAAGGCCCAGGAAGTTGCAAAGAC

ATGATCAGGTTGTCTAGCAGTAGTTGTTGTTTGGTAAATAATATAAGCTAATATAGTTTGAGTTTGATGA

TGGATTTCATAAAT

>FM892210.1 FM892210 Jatropha curcas embryo 56-70 (DAF) Jatropha curcas cDNA clone rjcaeb0_002794, mRNA sequence

ATTCAGAAAAACGCATCTCACTCTCCCCCTCACTCTGCAACTCCAGCCATGAGTCTTTTTGGACTGGGCA

GAAACCAAAGGACATTCCGGCCCAAAAAGAGTGCACCATCTGGGAGTAAGGGTGCACAACTTAGAAAGCA

CATTGATGCCACCTTAGGTAGTGGAAACCTGAGGGAAGCTGTAAGACTTCCTCCTGGGGAGGATCTGAAT

GAGTGGCTTGCTGTCAACACTGTGGATTTCTTCAACCAGGTGAATCTTCTCTATGGTACCCTCACAGAGT

TCTGTACTCCAGAGAACTGTTCTACAATGTCTGCAGGCCCCAAGTATGAATATAGATGGGCAGATGGAGT

GCAAATCAAGAAACCTATTGAAGTTTCTGCTCCAAAATATGTTGAATATTTAATGGACTGGATTGAAGCT

CAGCTTGATGATGAATCCATATTTCCTCAAAAGCTTGGTGCGCCATTTCCCCTCAATTTCAAGGAGGTTG

TTAAGACAATATTTAAACGTCTATTCCGTGTATATGCACATATTTATCACTCTCACTTTCAGAAAATTGT

GAGCCTCAA

>FM892209.1 FM892209 Jatropha curcas embryo 56-70 (DAF) Jatropha curcas cDNA clone rjcaeb0_002792, mRNA sequence

ATTCGTCTCCCTTGNTGATAAATTATGATCTGCCGACTCAACCTGAAAACTACCTTCAGCGAATCGGAAG

AGGTGGACGGTTTGGAAGAAAGGGTGTTGCCATTAACTTCGTGACAAGGGATGATGAGAGAATGCTGTTT

GACATTCAGAAGTTCTACAACGTGGTTATTGAGGAACTGCCGTCGAATGTTGCTGATCTTCTGTGATGCG

AATTTTTGGTGCTTGTTGAGAAAGGGTTTCTTATTTTGCAAGTACTATTTAACGTGTTGTTTTCCATCCT

GTTTAGTGTCTTAATTTAATATTTTCAGACATCTGACAGTTTATCTCATATCAGCCTTCTCAATTGCAAT

CTGAAGGGATTGTCTAGTTCTTATTTACTTTTTATTTTTATTTTTTGGGTCCTCTTGGAAAGTTTTGCTT

GATGGGTTTTTGTACTATATACCCTTTTTACCTTTCTATAAATTATTGTCATATTGTTTACTAGTC

>FM892208.1 FM892208 Jatropha curcas embryo 56-70 (DAF) Jatropha curcas cDNA clone rjcaeb0_002791, mRNA sequence

ATTCCAAGAAGCTGTGCAAAAATCTGAAGAGTTCGATATAAGGAGAAGTCCTATATCAATCGCAGCAGCA

GTCATTTATATCATTTCTCAGCTTTCAGATGATAAGAAGCCTCTCCGAGAAATTTCACAGGCTACTGGAG

TTGCAGAAGGGACAATTAGAAATTCCTACAAGGACCTCTATCCCCATGTTTCGAAGATAATACCAGGCTG

GTATGCCAAAGAAGAGGATCTCAAGAATCTTTGCAGCCCTTAAATTATAGAGAAAGACAATAAAGGGAAT

ACCGACACGGACACATTTGAAATGGTTTGGAGCCTTCCATCTTGAATTGTATTGGTAACTGCAGAGACAA

AAAGAAGAAAAAAAAAAGGAAAATTTGTGCAAGGCCCTTTATTTTCATTTTCTTTCGGCATTCAATTACA

CTGCTACCTTTAGTTACGAGGGAGGAAAGCAAAAATATTTTCAGCCATTCAATTCTAACTGGCGCTTAAC

AACGCAGCAGCTACATTTTCACGTTTGAGTGGATATTGATGTGTATTATGCAAGTTGCCAAATCTCACTT

TTTAAACATTTCAATTTACAAACTT

>FM892207.1 FM892207 Jatropha curcas embryo 56-70 (DAF) Jatropha curcas cDNA clone rjcaeb0_002789, mRNA sequence

ATTCCATTTGAAGAGTAAGAATAGCCAACAACCAAACAAGGATCCATCTTAGATGGGATTGTCACGTGTA

GAGTGAGATCGGTTTTTGTCTAATCAAGGATCAACGGTCCCTATTCAGTCGTCGCCACCAAAACAACTTC

ACTGCTACTTCAACAGCCGACTCGGACTGCTTCCTTGCATAATCTCTCAGTCAGATCTCTCATCTCTCTC

AATCTCCACTTCCCAATTACTCTTCCAACAGGGTTCCTGAAAAAATGGCCGATTCAAAACCAGGATTGAG

GAAGCCTGTATTCACCAAGGTTGACCAGCTACGCCCAGGCACCAGTGGGCATACTCTCACTGTGAAAGTT

GTAAGTACAAAGATGGTCTTGCAGAAAGGTCGTGCTGATGGTCCTCAAATACGCCAAATGCGAATTGCTG

AATGTTTGGTAGGAGATGAGACAGGAATGATTATCTTCACAGCTAGAAATGATCAAGTGGACTTGGTGAA

AGAAGGAACTACTATAATCCTTCGCAATGCAAAAATCGACATGNTTAAAGGATCAATGAGGCTTGCTGTT

GACAAGTGGGGACGTGTTGAAGTAGCTGA

>FM892206.1 FM892206 Jatropha curcas embryo 56-70 (DAF) Jatropha curcas cDNA clone rjcaeb0_002787, mRNA sequence

ATTCCTCAAGAGCATCTTGCTGAAGGGAGAAGCAGAGGAGGACAGACAAGGAAGGAGCAGCTGGGGACAG

AAGGATATCAGGAGATGGGCCGGAAAGGTGGGCTGAGCACGACGGACGAGTCTGGTGGAGAAAGAGCTGA

GAAGGAGGGCATCACTATCGATGAGTCGAAGTTCAGGACTGGGAGCTAGTGTGATGATTAAAACTATAAA

TGGTATTTTGTAGTAATAATAATAATAATGTTGGTAATAAAGTATTAGCTTAGCAGTGTACTTTAGTTTA

GTAATGTAGTTTACATGTGGGTTAAAATGACTAGGGTTTAGCTAGTATGTGTGTTGTAACTTTGAGAGGG

GTTTATGGTCGTTCCCTTTCTGGGAGGACTGCTAATACTTTCTCCCTATTTGTAAAATAACCTGCAGTAT

ATATAATTACCTGTTGGTT

>FM892205.1 FM892205 Jatropha curcas embryo 56-70 (DAF) Jatropha curcas cDNA clone rjcaeb0_002784, mRNA sequence

CAAAAGGAACAAGCTTTAGAAATATTAATAATTACAGAAATTAAACCAGATAAAGCATTAACAATCCACG

CATATAGATATTATCATTTCAGTATATCAAGGAGTTAGACGTGTGCCTCCATCTGATTGAGAAGGAAGTT

GCCTTCATTGTTTTTGTGCACCTCAACCTTGTACTTCTCTTCATTGCTTCCCCTCTTCACCTTAAGGAGC

ATATCAAACTTTGCAAAATCATCCACTACCTCAGCCTTCGCATGAACAATCTCTTGAAGCTCATAAGGGA

ACAATGAATTGGACCTCTGCTGAATGGTCTTCACAGCATGATTTGCAGCATCTTGCACTGCAGGATCATG

TGCTGGCACTTCTCTCCATCCAGGCCCATGCCCATCTCTTTTGACACCAAGATCTGAAGAGGTGAAGGAT

GAAGCACCACCATCAGGTCCATCAACATCCCCAGCATGCTTGAACTCCTGTAATCTTGAAGTTTAG

>FM892204.1 FM892204 Jatropha curcas embryo 56-70 (DAF) Jatropha curcas cDNA clone rjcaeb0_002781, mRNA sequence

TTTTTTTTATCCAACAGGTAATAATATAGACTGCAGGTGATTTAACAAATAGGGATGAAAGTATTAGCAG

TCCTCCTCAGAAAGGGAACGACCATAAACCCCTCTCAAAGATTACGACACAGCATACTAGCTAAACCCTA

GTCATTTGAACCCACATGTAAACTACATTACTAAACTAAAGTACACTGCTTAAGCTAATACTTGATAACC

AACATTATTATTATTATTACTACAAAATACCAGTTATAGTTTTAATCATCACACTAGCTCCGCAGGTACG

TAGAACTTCGACTCATCGATAGTGATGCCCGTCCTTCTCAGCTCTTTCTCCACCAGACTCGTCCGTCGTG

CTCAGCCCACCTTTCCGGCCCATCTCCTGATATCCTTCTGTCCCCAGCTGCTCCTTCCTTGTCTGTCCTC

CTCTGCTTCTCCCTTCAGCAAGATGCTCTTGAGCTTCAAGGCTCGGGGGGGCCCGGTA

>FM892203.1 FM892203 Jatropha curcas embryo 56-70 (DAF) Jatropha curcas cDNA clone rjcaeb0_002760, mRNA sequence

CTCGCAAACAATAGCTCATAACTGAAATTTTTTCTACAATACAAATTTCTAGGTATCAATATGCATGCTT

GAGGAGCTGTTTCGACCGAGCAGCTAAAACATGATCTTTTTGCAACACTGCTTTTCATAATTTAACAAAT

ATACTTGAATCTCTAATTTCCTTCACTGTCGCAAAGCTTCTGTATGGTAACTGTGAAAGAGCCTAATTTA

TCTACAAAATGGGATTTGATCATTATCTCACAGGTAACAGATTTGGACCATTATTTCGGGTTCAAGATTC

TAATTGTACCATTGACACGCTTGGTACAAATGATGCCTCCTCCA

>FM892202.1 FM892202 Jatropha curcas embryo 56-70 (DAF) Jatropha curcas cDNA clone rjcaeb0_002758, mRNA sequence

ATTCCTCCCTTAGCAGATCGGACTATCTCAAGATATAACTATGTCGGTCGGAGAAATTGCTTGCTCTTAC

GCTGTTATGATTCTATATGATGATGGTATTCCCATCACTGCAGAGAAAATTGCCCAATTGGTCAAAGCAG

CCAATGTGCAAGTTGAATCTTACTGGCCTAGCTTGTTTGCTAAGCTAGCCGAGAAGCGTAACATTGAGGA

TCTTATCACAAATGTTGGGTCTGGTGGTGGTGGTGGTGCCCCAGTTGCTGTTGCAGCTCCAGCTGGAGGT

GCTGCTGCTGGTGGAGCTGCTGCCCCTCCTCCTGCTGAAGAGAAGAAGAAGGAAGAGCCACAAGAAGAGA

GTGATGACGACATGGGATTTAGCTTGTTTGATAAGGAGCTCCTCTCTGTATGCCAGAGTCCTACGTTTGT

CTTAGTAGTTCATTAGTTCATTTTGGATCTTTTTGCTGTTTCGTTTCATTATAAATTTCGTG

>FM892201.1 FM892201 Jatropha curcas embryo 56-70 (DAF) Jatropha curcas cDNA clone rjcaeb0_002731, mRNA sequence

ATTCGTTGCAGTACTTAAGCAGCAATATGGCTTGAGCAAGACTGCAAATGAAGTAATCATTGTTATTGAA

GCTTACAGAACCCTCAGAGATCGAGGGCCTTATCCTGCTGATCAGGTTGTCAAAGATATTGAAGGGAAAT

TTGCTTTCATTTTATATGATAGCACTTCAAAAGCCACATTTATAGCTGCTGATGCTGATGGAAGTGTACC

CTTCTTCTGGGGAACCGATTGTGAAGGTCATCTTGTTCTTTCAGATGATGTTCAGATTTTGAAGCAGGGC

TGTGGGAAATCATTTGCACCATTCCCTAAAGGATGCTTCTTCACAACTTCTGGAGGCTTGAGGAGCTTTG

AGCACCCGTTGAATGAGTTGAAGCCAATTCCAAGAGTTGATAGTTCAGGTCAGGTTTGTGGTGCGAATTT

CATGGTGGATGAAGGGGTTAAGAAGGGAGGTGGCATGCCAAGAGTTGGAAGTGCTTATGACTGGTCCTCT

AACTACTAATGAGAAGTTCTTATCAGCAGGCTCGTGTTATTAACGAGCCCGATTGGCTTTTACTATAGCT

TTTGCTGATCTTACATTCCCCATG

>FM892200.1 FM892200 Jatropha curcas embryo 56-70 (DAF) Jatropha curcas cDNA clone rjcaeb0_002730, mRNA sequence

ATTCCCAGCTTCCTGCTTCTCTGCAGCAATTGCAAAAACAAGGTTTTCCTAGCCAACGGCTATCAATCAT

GATTATTTCAGAGAAGAACAGGAGGGAGATCTCCAAGTATCTCTTTCAAGAGGGAGTTTGCTACGCAAAG

A

>FM892199.1 FM892199 Jatropha curcas embryo 56-70 (DAF) Jatropha curcas cDNA clone rjcaeb0_002719, mRNA sequence

AAGAAATTCTTCATACCAACTATTGCATTTATGGTTAAAATGAATACAACCAACGATGACTATCGCATAC

TTTTTTCTTTAGGCTCTGTTTAATTACTGATTAAAAAAGGCTCAGCAACATTTGTAATTTATTATATGAG

CACCTTATAAATCTCCGATTGAGAGAAAAATGAAACAAAGGTACAATCTCAGTTGGTCCAACTCCAATAT

GTGCGTCTAGTTATGGCAGCTCTGGTCCCCATGGCCAGTGTTCTTATCATCCCCAGCTTCCATTTGTTGT

TCTCATCCTCAGAAGATGTATGGTTATTCCCACCATCAACTGCATCACAATCGTCTGCAGGAGCATAAGC

TGATGGGGGGGCCCGGTAC

>FM892198.1 FM892198 Jatropha curcas embryo 56-70 (DAF) Jatropha curcas cDNA clone rjcaeb0_002716, mRNA sequence

ATTCTTTTTAAATTGACAAATATAGCTTTAAATAAAAGATTACCAACATGAATTCACATCTCTCAAAATT

GAGGTCTTCAACTAAAGCTATGCCCCCAAAAAACATAAAACCAAAAGAGAAAAACAAGTTTCATCACGCA

ACAAATGTTGATAATCCTTCAAAACTAACTCATGTTTATAGTAAATGTCCAATACATAGCGTAAAATTAA

AAAACCTTACTCCCAGTTACCACCAGCATCTCCAACAGCAGCAGTTGCAATTTGAGGTGGGGGTGGTGCA

GCATCCCAGTCCCCAGCTGGAGCTTGCTCTGCACTCCAGTTGGCTGCAGGAATAGCTGGAATTGGCGCTT

GAGTTGCATCATTGGACCATCCAGCATCAGCACCAGGGGTATTCCAGTTATCAGCACCACCACCAATCGT

ATAATCAGCACCAGGAAGGCCATATTCAATGGGAGCAACAACGTCCTCCTCTTCCTGTTGCTTAGTTTCC

TCAGGTTCCCTGTAGAAAAATAAGTCCACCATGACCTCCCACTTGTGACCAGGAAGAATAGTGCCTCGCA

TCTGAAGAACCATCCTAGCAAGGAGCCAAAAGAGGCATCCAATGCTGTGCTTCCCCTTGTTATTGGCAGG

AATACCAATATCCACATAGCGCA

>FM892197.1 FM892197 Jatropha curcas embryo 56-70 (DAF) Jatropha curcas cDNA clone rjcaeb0_002714, mRNA sequence

CTATATTTTATATTGAATACAATTATATTTATAGAATATATTGATTAGGGAATACTTAATTACTAATTAG

GATGATTACATTACTTTCATCACTTATTTTCATAATATATTACATACTTCATTAGCGTGAGATCATAACT

CCCCTCGTAATACTACTTACTTTCACTATCTACACACCCAAAAAGGCTACTTCATTATTTACTCCTCGGC

ATATCTTCCAGACTCGGTCCTGGAAATAGTCAAGGTGGTCTCTTGTCTTCCAAACTTAATCCTTCTTGCC

TCCTCTGCTGATACCCTAAATGCATTTGCAATAACCGCCAACGGCATTGCTCTAACGGCGGAGGAGCGAC

CTGCAAGATCATTGGTCATCGCGTTATCGCTTGTCTTGAAGGTTACATATTCGAATCTTTCACTTTCTGC

TCGTTTCACCACTACGAAGTTTTGTGGCACCGTCAAAACCTGTCCTTTGCTTACACTTCCGTCGAAGACG

GTGTTGCCGTTTTCATCCGTCACCTGAACCCGCGCTTGTCCTTTGAGTGCGTTAATTACGCTGTGAGAGT

TTCTGGGGGGGGCCCGGTAC

>FM892196.1 FM892196 Jatropha curcas embryo 56-70 (DAF) Jatropha curcas cDNA clone rjcaeb0_002712, mRNA sequence

CGAATTCCTTCATTGTTAGCTCATGTGCTTGGTTTCCCGACTCAAAGCGACTTGTGTGTGGCAGTTCCGA

CCCTGAGAAGGGCATTTGCATGTGGGACTCTGATGGGAACGAGATAAAAGCATGGAGGGGCACGAGGATG

CCTAAGGTTTTGGATCTTGCTGTGACACCAGATGGGGAACATCTCATCAGTGTATTTTCTGATAAGGACA

TTCGGATATTCAATTTGGTGACAAATGCTGAAAGAGTCATCTCTGAGGAGCACCCAATCACCTCCCTGTC

AGTTTCCATTGATAGCAAGTACTTTATAGTCAATCTTAACAGCCAAGAGATTCATTTGTGGGATGTGGCT

GGAAAACAGAAGGCATTAAAATATATGGGCCACAAACAGCACAAGTATGTCATACGTTCCTGCTTTGGTG

GATTGAATAGCTCATTTATTGCCAGTGGCAGTGAGAATTCACAGGTGTACATTTGGAACAGGCGAATACC

TATGCCGATCGAGGTCTTATCTGGCCATTTAATGACGGTGAACTGTGTGAGCTGGAACCCTAGAA

>FM892195.1 FM892195 Jatropha curcas embryo 56-70 (DAF) Jatropha curcas cDNA clone rjcaeb0_002704, mRNA sequence

ATTCCCTTCTCAATAATATTATGCTTTATGCAATTGTACTTGGTATTGGACAGATCACCATATTCTAAGT

ACTCTTATTATTTTGTGCAGGAACAAGAAGCTTTGTCTGCTTTGAAGGAGGGGCAAGTAATAGGCATCCA

TTCTGTTAGTGAGCTGGAAACCAAATTGAATGCTGCTTCAAGAACATCTCGCCTTGCAGTCTTGTACTTT

ACAGCAACATGGTGTGGGCCTTGTCGTTTCATTTCTCCTCTTTTTACAAATTTAGCTGCAAAGTACCTCA

AAACTGTTTTTTTGAAAGTTGATATCGATGAGGCAAGGGATGTTGCTGCAAGATGGAATATTAGCAGTGT

TCCTACCTTTTTCTTCATAAAGAATGGAGAGGAGATTGACAAGGTAGTGGGGGCTGATAAAACTGGACTT

GAAAGGAAAATTGAACAATATGCAAGCTAGTCTGGAATTAATCTTTTGTAAGAACCTCAAAACTGTTTGT

TTCTTTGGTCTGGTAGCTTTCTTCGATCAAACTCATCACTAGTATTGGGTATGGGATAAACTTGTAA

>FM892194.1 FM892194 Jatropha curcas embryo 56-70 (DAF) Jatropha curcas cDNA clone rjcaeb0_002696, mRNA sequence

ATTGCACATTCAATCTAGGGAATTGAAGCGGCAATCTTTGCTTTCTGGTTATGAAGTTTCTTCAGGTAAT

GCAAATGCTGTAAAGAAGTTAGCCGACTCACCAGTGAAACGTCAGGCCATGACACCTCAAGAAAAAATTG

AAAAATTAAGAAAACGGCAGCAAATGCAGGCGATGCTTGCTATTCAGAAACAGCAGCAGCAGTTTGGTCA

CCAAGTTTCCTGTTGTGATCATTCTATCACTCAAAAACATTCCCAAGAAAATCAAACCCAGCATGTTGAG

GGAGCTGACCTAGAGGTGGAGGATTTGACCACTTTTCCTGCTTTCGATCCAAACTCGCCTGTTGAACAAG

ATGATTCCAATACAATCTCTCTGGCAGTTAATGATTACTCAGCAGAGGATACTGTGCTCTACCGACTTCA

GGATATTATTGCAAAGCTGGATGTCAGAATTAGACTTTGCATACGGGATAGCTTGTTCCGACTGGCTCAA

AGCGCAATGCAAAGGCATTATGCTAGTGATACTAGCAGTACCAACAATAGTAGCAGGGATGAGCAGGTTG

TTGCAAAAGAGGAAACCAGTGGTCATAACAGGAATTCTAAGATACCTGAAGCCGAAACGGAGACCAATCC

CTTAGAT

>FM892193.1 FM892193 Jatropha curcas embryo 56-70 (DAF) Jatropha curcas cDNA clone rjcaeb0_002695, mRNA sequence

TGGCCGTTCCCCCAGCTGCTTATTTATCAGAGAAGTACAACCATGTTGTTAAGGACTTAAGCCAGAAAGG

GTATACGGTTTTTGGTTATCTGCCTTTGGTTCCAATTGATGAGATAGCCAAGGTATTTAAACAAGATGAA

GCAGAGAAGAAAGGAAATGCAACTGGACATAAATCTGATTCATCAGATTCAGATTAGAAGATTGCATTTT

AAATCTGATATTGTTTGCTTATTCTAGTGTAGTGGTTTCTGAAAATATAGTAGGACTTGTGGGTCTGAGA

AGAAATGCTAAGAGATGGCAAGTGTGTAAATCTCTTACTTGTGATTATGAATGTTGGATGGTTTGTAAAT

TCTGCCTTGTACTTTATGGATATTTGTCCTTGATGGATACTTCTTGAAGTTTCTTATC

>FM892192.1 FM892192 Jatropha curcas embryo 56-70 (DAF) Jatropha curcas cDNA clone rjcaeb0_002694, mRNA sequence

ATTCCGTTAATGGTGTGGAAGAAACTTTCTGCACTGCTAGACTCGTACACAACATCAATGACCCGTCACG

TGCCGATGTATTCAACCCACGTGCTGGCCGTCTCACCAACATCAACGGCCAAGACCTCCCCATCCTCAGT

TATATCCAGCTAAGCGTTCAGAAAGGTGTCCTCTACAGAAATGCTCTAATGTCACCACACTGGAACGTCG

ATGCCCATAGCATAAACTACTTCACAAGAGGAAATGGCCATATCCAGATCGTAGATGATAATGGAGAGAC

AGTATTTGATGGAGAAGTTCAAGCAGGACAAATATTAACATCTCCACAAAACTTTGTGGTAGTAAAGAGG

GCAGGAAGCGAAGGTCTTGAATGGGTATCCTTCAAGACTAATGACAATGCCAAGATTGACCAACTAGCAG

GACGTGTCTCGGCAATCAGATCAATTCCAGAAGAGGTGTTGGCAAACGTATTCCAGGTTTCAAGGGAGGA

CGCGAGGAGACTGAAAGAGAATAGACAAGAAGTGACTCTGTTCAGCCCTGGGACTAGGCCTTCTTCTCGC

AATTGAAGGGACTAAAAAGGCAGCTA

>FM892191.1 FM892191 Jatropha curcas embryo 56-70 (DAF) Jatropha curcas cDNA clone rjcaeb0_002693, mRNA sequence

ATTCCCTTCAAACAACAAAAATGGCTTCGGAGAAGAAGCTCTCAAACCCAATGAGAGAGATTAAGGTACA

GAAGCTTGTACTAAATATATCCGTCGGAGAAAGCGGTGATAGGCTCACCTCG

>FM892190.1 FM892190 Jatropha curcas embryo 56-70 (DAF) Jatropha curcas cDNA clone rjcaeb0_002691, mRNA sequence

CGAATTCCTTAATAAGTATATCAATGGACAAACCTACCATCCAAGTTTTGGAGGGAATGGAGCAACAGCT

TTCTTTTTGACTTTTGCTATATTAGCTTCTATTGTTGGTATAGTATCCAAGTTGGCAGGTGGCCTTCATA

TCAGGAGCTGGAGAAATGACAGTCTAGCCTCTGCAGGCTCTTCTTCACTTGTGACTTGGGCCATCACTGC

CCTAGCTTTTGGGTTGGCATGCAAGGAAATAAACCTAGGAGGGCACAGAGGATGGAGACTGAGGGTATTA

GAGGCATTCATGATAATCTTGACATTTACCCAACTCTTGTATGTCCTGTTGCTTCATGCTGGGGTATTTA

GCAGCAGGTATGGCCCTGGTTACAGGGACACCGAATACGCTGTAGGAGCTGGTGGGGAACCAATTCCAAA

GGGTGGCACAGCAGTGACAGGGACTCGGGTTTAAGCAACAACAATGTAATAATATATGTACATGTAATGC

TAAAAGAATTATCTTGTGTCATTTACATATCCCCTTTTGGCGGGGATGTATCTGGGTCT

>FM892189.1 FM892189 Jatropha curcas embryo 56-70 (DAF) Jatropha curcas cDNA clone rjcaeb0_002689, mRNA sequence

TGCAGGAATTCGAATTCGGATGTGGTGGATTTGAGGAGGCGGGCCGGTGGAGGAGAAAGCGATGATGTTA

AGAAGAGAAAGGATTAGTTCAAAGTATAATTTTAAGAAGTTGGTTAATTGGTTCTTTTTGTCGAGTTTAA

TTAAATTAGAGACTATGGCCAAATAAAATAAATGGAATGCATGGTTTATTATCTTGTAGCTCTCTTATAA

TCTTGTTTCTTAAGAAAAATCATGGATATATGAAGTTTTCAATT

>FM892188.1 FM892188 Jatropha curcas embryo 56-70 (DAF) Jatropha curcas cDNA clone rjcaeb0_002688, mRNA sequence

ATTCGGAAACAATGACGGAATGAATCTCTCAGCACCACAAACTTTCCGTTCAAAGGAAATCTCCAAAAGC

AATGTTGTTGATGACATGGTATCTAGTAATGGCATCTTGTATGAACCTGGTGAACATCCTGACCATGTTG

TGGTCATTAAGTATGTGCCATATGTGGGGGATAGTAAGAGAGCTATGGATGAGTATACTTCAGAGATTTT

CATGGGTGGCAAAAACACCATAGTCCTGCACAACACATGTGAGGACTCTCTTTTGGCTGCACCAATCATC

CTGGATTTGGTCCTTCTTGCTGAGCTGAGCACCAGGATCCAGCTTAAAGCTGAAGGGGAGGGTAAGTTCC

ATTCCTTCCACCCTGTGGCTACCATTCTCAGTTACCTCACCAAGGCTCCTCTTGTTCCACCAGGCAACCC

AGTGGTGAATGCACTGTCAAAGCAGCGAGCAATGCTTGAGAACATACTGAGGGCTTGTGTTGGGCTGGCT

CCAGAGAATAACATGATCTTGGAGTACAAATGAGAGAGAAGAGGCCATACTAGGGAGACAAGAAAAATAG

TGGAAGAAGATCGGTCCGTTCCAGT

>FM892187.1 FM892187 Jatropha curcas embryo 56-70 (DAF) Jatropha curcas cDNA clone rjcaeb0_002685, mRNA sequence

ATTCCTAAGCTTGCTTTACTGTCTCTTCTTTCTCTGCTTGCTTTTGCTGCTGGAGCTAAATTCTATGACC

CGAGAGCTCCAACTGAAGCACAGCAATGCCATCTCCAGAGAATTACCACAACCCAACCTTCTCATCGGAT

TGACTCAGAGGGTGGGGAAACGGAGATTTGGGATCCATCGGAGGATCAGTTTCAGTGTGCCGGTGTTGCC

GCTTATAGAGATAGAATCCAGCCCAACTCTCTCTCCTTGCCCAAGTTTTTACCTGCTCCTCGCTTGGTTT

TATGTCATTCCAAAGTAGAGGAGTGCTTGGACTTAGCTACCCTAGCGGATGCCCCCAAACATTCCACGCC

AG

>FM892186.1 FM892186 Jatropha curcas embryo 56-70 (DAF) Jatropha curcas cDNA clone rjcaeb0_002684, mRNA sequence

ATTCGGCATCTAATGATCAGTCTTATAGTTACAGAGCCGGTGAGGCTACAGGCCGAGCCGAGGAGAAGGG

GAACAGAATGGGCGATACGGTGAAAGAGAAGGCTGGAGAAGCGAAAGACTGGACAGGAGAAAAAGCCCAT

GAGACAAGAGAAAAGACATCTGAGACTGCAGAGGCAACCAAGGAGAAGGCTCATGAAGGGAAGGAGAAGA

CAAAGGGAGTTCTGGAAGAAACTGGGGAGAAAATGAAGCATGCAGCTCAAAAAACTGCTGATACTGTCAA

AAGCGCTTTTGGTATGGCTCACCATGAAGAAGAAGAAGATCATGTTAAGGATCGTTCTGGCCATCAGCAG

ACCACAACTTATAGGGACACAGACACTAGGTATTAGCTACTAGTACTGCTACCTATGGCTAATAAATTTT

ATGTGTGTATTGGGGTCTTGGGCTTTCTCTAAGATGAGAGTTTATGTAGCATCTTTATAAACCAGGGAAA

GCCATAAATAAAATAGTGGGTCTAGGTGTGCTTAAAAAGCAGCTTGCTATTAAGTGTTTCTATTG

>FM892185.1 FM892185 Jatropha curcas embryo 56-70 (DAF) Jatropha curcas cDNA clone rjcaeb0_002683, mRNA sequence

ATTGAATAAAGAAATTTCAAGCGAGAAGAATGGCTACAGAGCAAGAAAGGGCTGAACTTGATGCTAGAGC

AAGGCAAGGAGAGACTGTTGTACCTGGTGGAACTGGTGGCAAGAGCCTTGAAGCCCAGGAACATCTAGCT

GAAGGGCGGAGCCCACAGGGCTAGGAGGAAGCGGGGGAGGAAGTTTAAGGGGGGGCCCGGTAC

>FM892184.1 FM892184 Jatropha curcas embryo 56-70 (DAF) Jatropha curcas cDNA clone rjcaeb0_002682, mRNA sequence

GATAACCTTCCAATCTTATCCATATAAGATTAGGCACATAAGATCCAGAAGGGCAAACAAGCCCACAAAA

GCTTTGTTTATAGTTACTCAAAACAAAAAAAAAGCACAACAAGAAGGCCCAAAAAGCTACTAATGAACAT

GGTGGTGTAAAAGTGAAAAAGTAAAAGAAGTAGGAAGTACACCGTGAATTCGGTGGACGTTATCACTTAT

TGTCTGAACGGTGGACGACCTTTAGATGAGTAACACCTTCATCATCTAACCGTCCAACATCGTTGAAAGT

TTGAGCACTAATCTGATCAGACTCTTTCAGGGTGGATGCCACGGTTCCTGCTGGTGTTTCTTCAAGAACC

ACCACCTCTTCCTTTCTTCCTGTGCCTCCATGCTCACGTGCAGCACACGTTTCTCCCACGACGTCCCGTG

GCATTGTAAGGGGGGGCCCGGTAC

>FM892183.1 FM892183 Jatropha curcas embryo 56-70 (DAF) Jatropha curcas cDNA clone rjcaeb0_002681, mRNA sequence

ATTCCAAATATCACTGATCGTAAACCTCCATTGAATTACTCTGTGCTTCGATCGCGTCAATGGCGGAGCA

CCCACAGTCGCAGCATGTTGGCCAACAGCCACGATCCCACCAAGTAGTGAAGGCAGCCACTGCAGTCACT

GCCGGTGGATCTCTTCTAGTTCTCTCCGGTTTGACCCTTGCTGGTACGGTCATTGCACTGACCGTAGCCA

CTCCATTGCTGGTCATATTCAGTCCGGTTCTTGTTCCTGCGGTTATCACAGTTTGTTTGATAATCACAGG

GTTTTTAGCCTCAGGCGGTTTTGGCGTCGCTGCCATTTCTGTCTTGTCCTGGATTTATAGGTATGTTACT

GGAAAGCATCCACCAGGAGCAGAAAGTCTGGACCAGGCACGCTTAAAGTTGGCGGGAAAGGCAAGAGAGA

TGAAGGACAGAGCTGAGCAGTTTGGACAGCATGTAACAGGTCAACAGACTTAAAAGAGAAGTGGGTTTGG

GTGGTTGATGCANAGAACATAAAATGGCTTTGATATATATATAAAAATATATGAGTCCTTTAATTAACAT

GGAAGGC

>FM892182.1 FM892182 Jatropha curcas embryo 56-70 (DAF) Jatropha curcas cDNA clone rjcaeb0_002679, mRNA sequence

ATTCGTCATGGAAGTCGAAATCCGCTAAGGAGTGTGTAACAACTCACCTGCCGAATCACCTAGCCCCGAA

AATGGATGGCGCTTACGCGCGCGACCTATACCCGGCCGTCGGGGCAAGAGCCAGGCCCCGATGAGTAGGA

GGGCGCGGCGGTCGCTGCAAAACCCGGGGCGCGAGCCCGGGCGGAGCGGCCGTCGGTGCAGATCTTGGTG

GTAGTAGCACATATTCAAATGAGACCTTTGAAGGCCGAAGAGGGGAAAGGTTCCATGTGAACGGCACTTG

CACATGGGTTAGTCGATCCTAAGAGACGGGGGAAGCCCGACCGACAGCGCGTCCTACGCGCGAGCTTCGA

AAGGG

>FM892181.1 FM892181 Jatropha curcas embryo 56-70 (DAF) Jatropha curcas cDNA clone rjcaeb0_002678, mRNA sequence

ATTCGATAAAGTGTAACAGAGCAAAGATTAATTATATTGGTGGAGAAATTTGTTAGCAAAAAAGGGCAAA

AATGTCTTCAAATCAATCAAGACAAGAGCTTGATGAAAGAGCAAGGCGTGGAGAGACTGTTGTCCCTGGA

GGTACTGGGGGCAAGAGCCTTGAAGCTCAAGAGCATCTTGCTGAAGGGAGAAGCAGAGGAGGACAGACAA

GGAAGGAGCAGCTGGGGACAGAAGGATATCAGGAGATGGGCCGGAAAGGTGGGCTGAGCACGACGGACGA

GTCTGGTGGAGAAAGAGCTGAGAAGGAGGGCATCACTATCGATGAGTCGAAGTTCAGGACTGGGAGCTAG

TGTGATGATTAAAACTATAAATGGTATTTTGTAGTAATAATAATAATAATGTTGGTAATAAAGTATTAGC

TTAGCAGTGTACTTTAGTTTAGTAATGTAGTTTACATGTGGGTTAAAATGACTAGGGTTTAGCTAGTATG

TGTGTAGTAACTTTGAGAGGGGTAATGGTCGTTCCCTTTCTGGGAGGACTG

>FM892180.1 FM892180 Jatropha curcas embryo 56-70 (DAF) Jatropha curcas cDNA clone rjcaeb0_002677, mRNA sequence

AATTTTTCAAAAAAAAAAAAAAAGGATTAAGACTGATGGATAGATTCACCTAATAACTAAAATGAAAGCA

ACTTGCAAATGTACTGAATTTCCCGCGTTCGCTACATCACACTCCCGGTTCACAACACTACATAACTACA

TATTTGCTTCTATGGCTGCTATGTTTCCAGAAAAGTATTACAGTAAATCCCACATTTAGTTGTTACATTT

CTCAAAACAGAAGACAGAAGTCCTTCTTAAACCCACAAACCAAAGCTCCAACACACTTGGGGATGCAAGC

TCAACCAGCCACACATCACCCTATTACAAACTGATCGATAAAAAGCAAATCNGCCACACAACATAACAAA

TAAGGGCATTGCGTGCGTATAATACATTATTAAGTAGAGTATTAACGAACACCTAGTGGTGCTCGTGGTG

GTGGGAG

>FM892179.1 FM892179 Jatropha curcas embryo 56-70 (DAF) Jatropha curcas cDNA clone rjcaeb0_002676, mRNA sequence

ATTCCCTTTCTCTCTCTCGCACATGGATAGTGTTTCTTCTTTACAATGGCTGCTTCTTATTTTCATATAT

TTCAGTTCCATAATTTCGACACTCGCTCAGTTGCCTGGCACTTGGGAGCTGTTGGTCCCTAATGCCGGCA

TAGCCTCTATGCACACAGCAGTTACCCGCTTTAACACCGTTGTTCTTCTCGACCGGACTAACATTGGCCC

ATCTCGGAAAATGCTGCCCAAAGGCCATTGTCGCTTTGACAAAAACGATGCCATCTAGAAGCGCGATTGC

TATGCACATTCCGTTCTCTTGGATCTTCAAACCAAACAAATTCGACCTTTAATGATCCTCACCGACACTT

GGTGCTCATCCGGTCAGTTTCTCCCTGACGGTACTCTCTTACACACCGGGGGTGACTTGGACGGATTTAC

AAAGATCCGGAAGTTCGAACCTTGTGACCCAAACGGGTTGTGTGATTGGGTGGAGCTTGATGATGTGAAA

TTGAGTGACGGAAGATGGTATGCTACGAATCCAGTATTACCGGACGGTTCGGTGAATATTGTTGG

>FM892178.1 FM892178 Jatropha curcas embryo 56-70 (DAF) Jatropha curcas cDNA clone rjcaeb0_002675, mRNA sequence

AGCACAAATCANGTAGGTTCTCACTCATTTATTTATAAACAAAAGAAATCATACTGATATAACCGCAACA

GTGTAACCTGAATTCATGTCAAATATGAATTCGGCTCTCCCATGTCTCTCTCTTCTATAGCACAGTAACT

AAAGTAATAACTCTTTTCAAACAGTAGTAGAAGCATGAAAATGTAAGTACTATGGGTGTGTGAAAAGCAT

TATCTTTGCAAGTTCCAGTGCTTTCGGTCGTCCAAACAGCTTCTCAACAATTGCCAACGCAAACTCCATT

GAAGTTCCAGGCCCTCTACTAGTAATGAGGTTGCCATCAACCACAACCCTGTTCTCGGTTTCACTTCGAT

CTGACAGCTTCTCGCACAGCGGAGGAAAAGCTGTGGCTTTTTTACCCTTAAGCAAGCCATGAGGCTCCAG

GACTAAAGCTGGAGATGCACATATTGCTCCATATGGTCTATTTGATTCCCTCTGTTACTTTAGCATATTA

ACCAGATTTTCTGATTTTGCAAATGCTTGGGCACCACCCAGCCCA

>FM892177.1 FM892177 Jatropha curcas embryo 56-70 (DAF) Jatropha curcas cDNA clone rjcaeb0_002674, mRNA sequence

TTTATTTTTATTTTAATTTAATATTTTCAGTTGCTTTTTCTTTTTCCGTCTTCCATAAAACACTGCTTCG

AAAGGTGTTTTTATTTACTTCCATGGTTGTAATACTACAGAAGGGAGGAAACACCATAAAGTAGCTCTTT

GGAAGTTGGGAAATTTTATTTAATATATACTCTGAACATGAGCGAAGCAAACCAAGGGAATAATCCTGAT

GCATCAGCGATTGTTCCTGTGAAGCGAAAGCGTGGTCGTCCTCGCAAATATCCAAGACCGGATCCTAATC

ATGGGGGCAATGCTCATGCTTCTAGGAATCAGAATCCAAATCATGGGGGCAGCTCACGTGTTCCACCTGG

ATTTGTCAGGGTTAACGGGAATCAGCCCCGTCAAGAAGACCCAGTAATCGATGCAAATGATGTCATGGTG

GGTCAGGTAGTCCATGGCGTCATTGAAGCATGATTTGATGCTGGATATTTACTCTCTGTCAGGGTTGGCA

ACTCAGAAAACACTTTGCG

>FM892176.1 FM892176 Jatropha curcas embryo 56-70 (DAF) Jatropha curcas cDNA clone rjcaeb0_002673, mRNA sequence

ATTCCATACTTCGATCGACTCCTCTCTTCCCAGCAAGTCTGTTTCTCCCATCAGTTTCGGCGACAAAGCT

TGCCTGACGAAAACGCCATGAGCGACGGTGGCGAAGCCACTATCAGGGTCATTTCCGACGAAACAAGGCT

CACCCAATCCAAATTGATCACGGATCCGGAGTCCCCATGAGATTCCGAGTGTTTTGGTTTATATGTTGTA

AAATTCTGGACTAAAATCACTTTTCCGACTGATCCCTTTTTTTTTAAAATAAAATGAAATAAAACAAAAT

GATCCTTTTTTTTTTAAAAAAAAAAGGGGGGGCCCGGTAC

>FM892175.1 FM892175 Jatropha curcas embryo 56-70 (DAF) Jatropha curcas cDNA clone rjcaeb0_002672, mRNA sequence

ATTCGTACGTATAGCAGCTGCTGGAAAAGTAACCAAGTTCTGCCGCATTTTAAATCCAGAACTTGCCATT

CAGCATATTCTCCCTTGTGTGAAGGAATTGTCATCAGATTCTTCCCAGCATGTGCGGTCCGCTTTGGCTT

CAGTTATTATGGGAATGGCTCCTGTCCTGGGGAAGGATGCTACAATTGAGCAGCTTCTTCCAATATTTCT

TTCACTGTTGAAGGATGAATTTCCTGATGTCCGCCTCAACATCATCAGCAAGCTTGATCAAGTGAATCAG

GTTATTGGAATTGATCTACTGTCTCAATCATTATTGCCAGCTATTGTTGAGCTTGCAGAGGATAGGCATT

GGAGGGTTCGGCTTGCAATTATAGAGTATATACCTTTATTGGCAAGTCAGTTGGGTGTTGGGTTCTTTGA

CGATAAACTTGGGGCTCTGTGCATGCAGTGGTTACAGGATAAGGTTTACTCAATTCGTGATGCTGCAGCA

AATAATTTAAAGCGGCTTGCTGAAGAATTTGGTCCAGAGTGGGCAATGCAACACATAGTGCCACAGGCTC

TGGAGATGATTAATAATCCTCATTACTTGTATCGGATGACCATCTTACGT

>FM892174.1 FM892174 Jatropha curcas embryo 56-70 (DAF) Jatropha curcas cDNA clone rjcaeb0_002671, mRNA sequence

TTTTTTTTTTAAAAAAGGAACTGTTCCTTCGATCATTAATAAGCACATTTCCTAACCAGGTAATACAAAG

TTACAATAGAAACACTTCATAGCAAGCTGCTTTTTAAGCACACTTAGACCCACTATTTTATTTATGGCTT

TCCCTGTTTATAAGATGCTACATAAACTCTCATCTTAGAGAAAGCCCAAGACCCCCATACACACATAAAA

TTTATTAGCCATAGGTAGCAGTACTAGTAGCTAATACCTAGTGTCTGTGTCCCTATAAGTTGTGGTCTGC

TGATGGCCAGAACGATCCTTAACATGATCTTCTTCTTCTTCATGGTGAGCCATACCAAAAGCGCTTTTGA

CAGTATCAGCAGTTTTTTGAGCTGCATGCTTCATTTTCTCCCCAGTTTCTTCCAGAACTCCCTTTGTCTT

CTCCTTCCCTTCATGAGCCTTCTCCTTGGTTGCCTCTGCAGTCTCAGATGTCTTNACTCTTGTCTCATGG

GCTTTTACTCCTGTCCAGTCTTTCGCTTCTCCAGCCTTCTCTTTCACGGGGGGGCCCGGTAC

>FM892173.1 FM892173 Jatropha curcas embryo 56-70 (DAF) Jatropha curcas cDNA clone rjcaeb0_002670, mRNA sequence

ATTCCANCTGTACTTAATTCTGTCTTCTTCGAGTAACCGTTAAAGAAGTATCTTGTTCCTTTCGGTCTTC

TTTCCAACATGGATTCTCAAATAGAACGCCCACAAGGTTATGGTTACGACCAAGAACCCCGGAATGCTGC

ATTCCACGCTGAAGGGGAAGACGACCATCACAATGAGAAGAAGTCCGTTTTGAAGAAGGTGAAGGCAAAA

GCCAAGAAAATAAAAGACACGCTAAAATTACATGGCCACGGTCAAGGTTATGAACAGGATCATTTGCAGC

ATGAAGGTCATCATGTGCCTGATGATCACGACCTAGAGGAGGAAGATGATGAGGAAGAGGATGAAGAAAT

GGTTATGGACCCTGAAACTCATGGAGGATCAGCATTCGAGTCCGCAGTTATTAGGAGCTCTTTGCCTTTG

CAAGTGGAGAAGTTAGGTGATTCAGTAGAAACAAGAGATTCTAATAAAGCCAAAGTCACTGATTCAGTAC

GTAGCCTCTTTCCTATTCAAGAAGAAGATCAAGGACGTAAACTCGCTGTTGGGAAGACAAGTGATGTTAA

GGAAGTGCCTCATGCTCTACTAAATAACCTGCATCTATTACACCTGGAATTGTG

>FM892172.1 FM892172 Jatropha curcas embryo 56-70 (DAF) Jatropha curcas cDNA clone rjcaeb0_002669, mRNA sequence

CGACTCGAATTCCAGAAAACAAACTTAGCTTGATGTGCTCATTCTTTTGTGCATTATTATACATAACTCT

GATCACTCACACACCAAGTGAGTGAAATTTTTATTTAACTGAAGCACATCCACGTTATAGCTAGCAGATT

AGGAACGAATATCGCAGCTTGTACGCAGGCCACAAGCAGAAGGTATACTTCTAGCCCTACGCACTGCCTC

CAAACGCTCTCGCTGTCCTTGCTCCAGCTGCTGCTCTAATACATACTTTATTCCTTCACATGGACAATCA

TGACTTAGCTGCCTTAATTCGTTACAACATTGGCGAAGATCCCGTTGTTGCTGGTTTTCAATTCCTCGCA

AAGCTAGCATCTCTTCTGACGGCCTTCTTGACGATCTCATATAACTCTCACAGGAGCTCAAATCTGCCCT

TTCAGCTTCCCTCCTGCATTTTCGCTGACTAGTGTTAGAAGAATCATCGTCGATTTCAACGGTGGTGATG

GTGGTTGTCACATAGCCGGAGACTGCATTGGCTATGTGCACTAGCAGAATGCAGAGTAGAGCGGCTGAGG

TTGTAAGCTTCTCCATTTTTTCGGGGGGGCCC

>FM892171.1 FM892171 Jatropha curcas embryo 56-70 (DAF) Jatropha curcas cDNA clone rjcaeb0_002668, mRNA sequence

ATTCCTCACACTTATCATCAACAAATGGCTGAACGTTCACAGCCACACCAAGTTCAAGTGCACCCACAAC

ACCGCTATGAAGCTGCCTTCAAAGGTCAACAAAAAGGTCCATCAGCTCAGAAAGTGTTAGCTGTGATCAC

CCTCCTGCCGGTAGGCGGTGGGCTTCTTGCACTAGCTGGTATAACCTTAGTAGGTACCCTAATCGGCCTA

GCTATTACTACCCCTCTTTTTGTCATCTTTAGCCCTGTTCTTGTCCCAGCTGCATTAGTTATTGGCCTTT

CGGTCATGGCATTTTTGGCTTCCGGGGCTATGGGGCTCACAGGGTTGTCATCACTCTCGTGGGTTCTGAA

ATACCTCCAGGAGGTCACTCGGCGAATGCCGGAGCAGCTGGATATAGCTAAGAAGCGTATGCAAGACATG

GCTGGTTTTGTGGGACAGAAGACCAAGGAAGTAGGCCAAGAAATCCAGAGGAAAGCACATGAAGGGAAAT

AAGTAGAGAGAAATGCTGTTGGTTATTAAGGGTTGAAGTCAGAGAGCTGGGTTTTGGTACGAGACTACTT

TTGTTGTATGGGTG

>FM892170.1 FM892170 Jatropha curcas embryo 56-70 (DAF) Jatropha curcas cDNA clone rjcaeb0_002667, mRNA sequence

ATTCATCTTTCTCACACTTATCATCAACAAATGGCTGAACGTTCACAGCCACACCAAGTTCAAGTGCACC

CACAACACCGCTATGAAGCTGCCTTCAAAGGTCAACAAAAAGGTCCATCAGCTCAGAAAGTGTTAGCTGT

GATCACCCTCCTGCCGGTATGCGGTGGGCTTCTTGCACTAGCTGGTATAACCTTAGTAGGTACCCTAATC

GGCCTAGCTATTACTACCCCTCTTTTTGTCATCTTTAGCCCTGTTCTTGTCCCAGCTGCATTAGTTATTG

GCCTTTCGGTCATGGCATTTTTGGCTTCCGGGGCTATGGGGCTCACAGGGGTTGTCATCACTCTCGTGGG

TTCTGAAATACCTCCAGGAGGTCACTCGGCGAATGCCGGAGCAGCTGGATATAGCTAAGAAGCGTATGCA

AGACATGGCTGGTTTTGTGGGACAGAAGACCAATGAAGTAGGCCAAGAAATCCAGAGGAAAGCACATGAA

TGGAAATAAGTAGAGAGAAATGCTGTTGG

>FM892169.1 FM892169 Jatropha curcas embryo 56-70 (DAF) Jatropha curcas cDNA clone rjcaeb0_002666, mRNA sequence

ATTCTACATTTTCCAATTCCCAATCACCAAGACCATCATCGTCCTCTTCTCCGTCGCTATCTTTTAATTA

ATATTCTTCATCATGCTTCTGATCCAAGTGTAAATTCTCCTTTTCTGTCTTCTCAACATCAAAGTCTTTC

CTTACAGCCTTCCCAAACTTCTTCCAGCCGTTCCGGACTTCCATCTGCGGCTTTCCTCGGATCTTGCGCC

GCCCGCAAAATCCTTCGCCGGCCATGTAACGATAAGTGTTATTCAGCTTCCTATTCTACTCCAAGTGCAG

AATCTCATAATTTCACTGTTGATAGCGTCTTGGGACTTAGCAATGTCGAGTTCTTTATGAAGGAGGGACT

CAAGATCAGTGTTGGAGATTGTGATGTTTTCAAGCCACCCCAGGAGGACTCCCCACCTTTCGTTGGAATA

ATTCGATCGCTGATTGTCGGTGAAGTGCTCAAGTTGTGTGTGAATTGGTTATATCGACCTGCTGAACTGA

AGCTTCGCAAAGGCATCCCTTTGGAAGCTGCACCAAATGAAGTATTCTATTCCTTTCATAAGGATGAGAT

TCTTGCTGCATCATTACTCCAT

>FM892168.1 FM892168 Jatropha curcas embryo 56-70 (DAF) Jatropha curcas cDNA clone rjcaeb0_002665, mRNA sequence

ATTCGAATTCGGAAGATGGCAACGGCAGATTCTCCAAACAACAATATCGATCCCAATCTCGAAGAAACCA

ATCAAAATCAAAACCCTAATTCTAATTCTTCAAATGAATTAATTGTTCCTGAGTCGAGCTCTCCGGCCGT

CTGCCTCATCCGCTTCGCCGGTGACTCTGCCGGCGGTGCCTTCATGGGCAGCATTTTTGGCTACGGTTCG

GGATTGATTAAGAAGCAAGGCTTTAAAGGATCCTTTGTGGAGGCTGGATCTTATGCCAAGACATTTGCAG

TCCTATCTGGAGTGCACAGTTTGGTTGTCTGCTTTTTGAAGAAGCTTCGAGGGAAGGATGACGTCATCAA

TGCTGGAGTGGCTGGATGTTGCACTGGTCTTGCTTTAAGTTTTCCAGGAGCGCCGCAGGCTCTTTTACAG

AGCTGCCTGACTTTTGGGGCATTCTCATTTATTATTGAAGGGCTTAACAAGCAGCAACCTGCACTGGCTC

ATTCATTTGCTGTGAGAAAAAAGCGCCACTACCATGATGCATCTCGTCTTGAGTACCGTAATGCTCCTCA

TTCCTTTTCTCTGCCTCTTTCAGTCGCTCTACCAAATGAACTGAAAGCAGCATTCTCTTTAAACTGCAAG

TCCTTAAAGAAA

>FM892167.1 FM892167 Jatropha curcas embryo 56-70 (DAF) Jatropha curcas cDNA clone rjcaeb0_002663, mRNA sequence

AAACATAAAATACATGTCTTCTAGAAATAGGATATTTGATCTTCGTCTGCTATAATGTTACAACATGTTG

TGATACAGGAATAGAGTAATCAGACCACCCATGGAGGCTTGGACAAACATCAAAAACCATGTCTCTAAAT

CTGCTTTTACATAATAACAAGGTGATACAACAAAATCACGGAAACAGAAAGCGAACCACATAGCATAGAT

AGAGGGGACAAACACCATAGCTTCCATGTCAAGGCATTGCAGACAAACATTTGCCCTACATTTCATTTTG

AATCATAAGCCAAGAAAAGGACATTGCTTTCTAGCATGCTCTGGTATCAATTTAGCCACAGTCTCAGCTG

CTACGCCCTTCAATTCATGAGATTTACAGTTGAACCATGGTGGAAGGAAACGTTCCATTTTGGCAGGCGT

GT

>FM892166.1 FM892166 Jatropha curcas embryo 56-70 (DAF) Jatropha curcas cDNA clone rjcaeb0_002662, mRNA sequence

ATTCCTCTCTTCTAGGAGGTGTTTTGGAGCAAAAATTTTCTTCTCATTTTAAATACTGCAACAACTTTTT

TCTTTGAAGGGAATTTTTCTTCTGTTTTGAAGTACTGCAACAAAGATGGGACGCGAATGGAAGAAGGAGA

TGGAGCAAAACCTGAAACCTCTCACTCCTTCTCTGCTCCCATCGCGGCCGTCTACTACCGGATTCCTCCA

CGCCGGCGTCATGAAATTGATTTTGTTCGGTTCTTTGGCGGCCGGTATTATTGTCAAGCTACTTCCCTAC

GAGTACACTGACCGCGGCGATCCAGTTCCAACCGGCATATTCAAGGGGCTTCCGGTGACAACGTTTCATG

TTTTTGTAATCTCGATCATTTTTTCCTTCGCCGGGGCGTTTAGCTCGTTGCTGACTAGCGGAAGACCAAA

AAATGCGAATTATGAAAAATTCTGCCGGTATTATTCGCTTGTGTTTTCGGCTTTGGCCATAGCAATTCTT

GTGCGGGCTGCGATAAGGTTGACTTTTCGATGGGCGCCGATTGTTGATGACTAAGGACAGATCCGAGTAT

TAAAGAATCAGGTGACTATGTAAG

>FM892165.1 FM892165 Jatropha curcas embryo 56-70 (DAF) Jatropha curcas cDNA clone rjcaeb0_002661, mRNA sequence

TGAGGGAGAGTTTTATGTGATAGACACAGAGAAATTAGAAAATGTGTTGTCTTTGTTACAGAGCCGCAGT

GATGGTTCCTTAGAGTTTGAGCCTTGATAGTATGGATTTGGATTTACATGAGGAGTTTATAGTGAAAGTA

CTGGAAACTCCTCTTGTTTCGGGCGATAATTTGATTAAGTTTTTCAGGTGGGCAATTGAGAAGCCAGAAA

TTAGTGTGACTAACCGTGTAGTTGATTTGCTTGTTCAATCTATCTGTAGTGACCTTAGGAAGAAAAACGC

ATATGCATTGTGGGATTTGGTCCAGGATGTTGCTCAGAAGGATGCTGCTGTTCTGAATACTGTGATTCTT

AATCAGCTGATAATTTTGTTTTCAAAGTTGGGTAAAGGGAAAGCAGCTTATGAGGTTTTCAACAAGTTTG

TGGATTTTGGGTATGTCCCGGATTCAGATACTTATTATTATACNATTTGACGCCCTTTGTAGGCGATCTT

TTCTCGACTGGGCTTGGTCAGTTTGTGAGAAGATGCTTAATGCTGAAACTTTGCCTG

>FM892164.1 FM892164 Jatropha curcas embryo 56-70 (DAF) Jatropha curcas cDNA clone rjcaeb0_002660, mRNA sequence

TTTTTTATTTTTTACGAATCGGACCGACAAAGGGCTGAATCTCAGTGGATCGTGGCAGCAAGGCCACTCT

GCCACTTACAATACCCCGTCGCGTATTTAAGTCGTCTGCAAAGGATTCTATCCGTCGCCCGATGGGAATT

GTACTTTAAGGCGGCCCTCGCGGCTCGTCCGCCGCGGGGGCTTAGCCAACGGCACGTGCCTCTGGGGGCC

GGATGGCCCCTACTGCGGGTCGGCAAACGGGCGACGGACACGCGCGTCGCTTCTGGCCCGGATGGGGGGC

CCGGTAC

>FM892163.1 FM892163 Jatropha curcas embryo 56-70 (DAF) Jatropha curcas cDNA clone rjcaeb0_002659, mRNA sequence

ATACCGAATTCCAAGCCAATTCACATTCAATTTTAGTATCCAAAATACACAGCTTCAAATCCCTTGCCTT

CTTGTATTTCAGATTGGTAGGTTTGCTTCTGTTCACTTTTTCTCAGTAACCAAGCAGGAAAAGAAAATTA

AAAACTAAACTGAAATTAAAAAAGAAAAAACTAAATGGAGTCTTCAAGCTGATCCAGCTTCTCTTTCTTC

TTCAATTCACTCTCATATTTTTTCAATCTCTCATTCATCTTCTCCTTGTAGCCGACTTCGGTGCGGTGCA

TAACGTAGAAGGCGAGAATGCCACCCAGAATCGTGCCAGTGATGATCCGTATATAACCCCACTTCACCGC

GTCTAGCACCATAATTTCCCTTTCTCGGGGGGGCCCGGTAC

>FM892162.1 FM892162 Jatropha curcas embryo 56-70 (DAF) Jatropha curcas cDNA clone rjcaeb0_002658, mRNA sequence

ATTTCAGCAATGTCATAGATTTCCGGCGATGGAAGACACCGGAATATTTGCAAGTAACCTGATGGATGTA

ACAGGATGGGACGCGTTGCACACTCCATGGTGCATGTAAATTTCCATGTGTCCATGCGTTTGAAGTTGAT

GAAAAAAGAAAGCTACGATGTAGCCACATAGGGTTTTCTGGTCTTTTATCGTGGCTCTTGTTCATTGTAC

TCTCCCCACCCCCCTCATATACCCATGAGTCACGTGCAGGTATCCATGAATCACGTGCTTCTACTTTGTG

TGATACACCCTGATACTGGTCATGTATGTAACATAATAGACCTAGTAACAGTTTGATATATATTATACTA

CTAAAAGATTTT

>FM892161.1 FM892161 Jatropha curcas embryo 56-70 (DAF) Jatropha curcas cDNA clone rjcaeb0_002657, mRNA sequence

TCTGTCTATATTCGAAAGGTCAAGATCTTGAAGGCACCCAAATTTGACCTTGGAAAGTTGATGGAGGTTC

ACGGTGACTATTCGGCCGATGATATTGGTGTGAAGTTGGACAGGCCGGCTGATGAAACAATTGCTGAGGC

AGAGACTGAAGTTGTCGGTGCAGCCTAAGGAGTATTTTAGTTTGTTATAATCCTCTTGTTTTCAGACTTG

TGAACATTTAGTACTTGGAAATTTTGCATTTTTGGATATTAAGAGGTGCTGAGCTAAGGTCTGTATATGA

CCAATATGTGTGGATTCTTTAGATATTAATAGCCATTAGCTTTTTCCTTATGT

>FM892160.1 FM892160 Jatropha curcas embryo 56-70 (DAF) Jatropha curcas cDNA clone rjcaeb0_002656, mRNA sequence

ATTAGAGGAACTCTGAGAAGTCAATGTCTGAGGTGAATGATGAGGAAGAAGATGATGAGGAGGGAAGTGG

AGAGGAGGACGATGATGAGTAGGAAGAGGGGGAGAGGAGTAGCATAGTCATTTAGGCTGTGACAAATGTT

TGAACAACTCATGTTAATGGGGATATTTGCTTGTTAATTTTTTTCTTGCTTTTGTCTTGTAAGAGAAGGA

TGTCTCTGCTCCCTCTTATTTTGTCCTTCTCCTTTCTTAAAAAAGAAATTAATAGTTGTAGAAAGGAGAT

TGCTTTTGCGTACCTTGCACCAAAATTAATGATTTAGCTAAGTGGTTTTCTTTTGTAGAGAAAGTTCTCT

GTGATGAACTATGATGGTTGTTTGTCTGACTTCGCA

>FM892159.1 FM892159 Jatropha curcas embryo 56-70 (DAF) Jatropha curcas cDNA clone rjcaeb0_002655, mRNA sequence

ATGCACAGCCCACACTGGTCCATAAACGATCAGAGAATAGTGTGTGCTCTTCGCGGCGACATGCACATAC

AGATTGTGGATGACAGTGGAAACACAGTAATGGACGAAAGGGTGAAAGAAGGAGACATGTTCGTGATCCC

TCAATTCTATGCAGCACTTGGAAGGGCAGGAGAAAATGGGTTCGAGTACGTAACATTTAAAACAAGTAGC

CAGCCTATCAAGAGCCCATTGGCGGGTTATACATCAGTGATGAGAGCCGTGCCTCTTGATGTTCTGATGA

ACTCTTACAGAATGTCTCCAAGCGAAGCTCATCAAGTGAAGAGGAACAGGGATCCACAGTCCATGTTGTT

TACTCCCACCAGCAGGAGACCATAAGTAAGTTACTACTAAAATAGCATTGTGATTGGTGCTTCTACTATC

CAAGGCCGCAACTAGGAGATCCTTCTTGTAAATTTAGAGAAGGGATCCGTCGAATATAGTAGTATAGAGA

G

>FM892158.1 FM892158 Jatropha curcas embryo 56-70 (DAF) Jatropha curcas cDNA clone rjcaeb0_002654, mRNA sequence

ATTCCGATTTCTTCTCTATCGGCATCAACAATTCCTTCTTTGAAGACACCGCAGTTACAGTCAAGTACCA

CCAAATTAACTTCCTTATCTTCTCTGCAATTCCACTCTCATCTTCAAAGGCTTCAATTTGGGAATGGAGG

AATCACTTCTTCTCCTTCCAGGCCTCGAATTTTGCCTCTGGTTGCAGCAAAGAAGCAAACATTTTCCTCC

TTCGATGAATTGCTGGAAAATGCTGACAAACCGGTGCGGTCCCTGTCAAATTATGGGTCCAATTCTGAAT

GAAGTCAGTACTGTTCTGAAAGACACTATCCAGGTGGTCAAAATAGATACCGAGAAGTACCCTACCATTG

CTGACACATACAGAATAGAAGCATTGCCTACATTTCTCATATTTAAAGATGGGAAACCTTATGATCGCTT

TGAGGGTGCTTTTTCTAAAGATAAATTCATTCAACGCATTGAAATTTCACTGCAGGTGAACCAGTAGTTA

CGGTATTAACGAACAATGTGATA

>FM892157.1 FM892157 Jatropha curcas embryo 56-70 (DAF) Jatropha curcas cDNA clone rjcaeb0_002653, mRNA sequence

AACTAGAGTTTAATCAACTAGCTATAATATAACCATTAAATTATCCAAACAACAAAACTAAGCAATGGGT

CTATTGAGAGAAAAATCTCTAAATAAACACCGCTCAACAAAAATCCATTAACATTTTTAGTCCTCAAATA

GCAAAATCCATAATTCAAAACACTTTCGAAAATAACAGACGCATAAAAAAAAGGATAACCTCGACTTCAA

CGGTAACTCTTCTGGAACCTGGCGCGAGCACCAAGACCACCGAACTTCTTTGGCTCGCATCGTCTGGGAT

CAGCTACAAGCAAAGTCCTATCATACCTGACCAAAATATCTTTTATTTCCTTCTTGCTCTGCTCGTCGAC

GTACTTCGGGGGGGCCCGGTAC

>FM892156.1 FM892156 Jatropha curcas embryo 56-70 (DAF) Jatropha curcas cDNA clone rjcaeb0_002651, mRNA sequence

AACTGATAACATATATCAGATAGCATTAAAAATCTACTGCAAGCACAGGAAATGAAGTTCTCATCTCTCT

AATAAAAAAACTCTAATCAAGTCGAGCATTTCAATTTTACTAGCAATTCCAACAAAACAAAAATAACAGC

AATCCAAGAAGAAAAGGTGATCTTTCAAGTAAAGACACTTCTATAGGTAACTGGAATGCTACATCTTGTA

AAAATCTCAGAACTCCAAATAAGCTGCTCCTTAAAAGACGCGGACTGGGTTACCCATTGTAGTCTTGATG

TTTAGGCACTTCACATTTTGCCAGTTCTTCTTCAACAAGGAAACAAGGAAATTGACGCTCATTTGCACAT

TTTGGAAGACTTGCTTCTCCTCCATAGCACAGTTGCCAACAGCAACTCCCATGCAGAGCACTTTCTTCAA

TTGGAACTTCACTGTTGCCTTTGTTTCATTCACTTTAAACTCTAGGGAATCCTGATGGGTCACAAAGGT

>FM892155.1 FM892155 Jatropha curcas embryo 56-70 (DAF) Jatropha curcas cDNA clone rjcaeb0_002650, mRNA sequence

ATTGGCNTACAAAAGAACTCTTTTGCTCCTCTGCTTTGAGAAACTGCAATGAGAATCCCATCTTGATCTT

GACACACGGCGACTTATTAACAAGTGAGGAGAGAATTGAAGGCAGGCTAAAGATATGTGAAAGTTTAGGC

ATATCAGAGACAAATGGGATATACGATGTCGTTTGCCTTACAGAATATGGATTTTTAGCAGAAGAATCTG

ACCCTGTTAGTGCTTACAATGTAGCTGAAGCAGTATATAGGGCATTGCTCATTTCTGACAGGGAACACTT

GCCAAAGAAAACGTTGCAAGACTGGACGCTCTTCATATTATCATGGCTAATGTCTCTCATGGCTATTTTT

TTCGCTTTTCTTGCTCAAATTTGCTCTGCACTTGGACATAGCAACAAGCTGAAGCGTTACAATTTAAAGA

GAAGGAGATTAGGAAATACATACATATATATTTTCTGTATATTATATTGTTATGTGTAGTTTAGCTTATA

ATGACAATTTCAGTAAGGCTAACTGAATATCTGTTTAATCT

>FM892154.1 FM892154 Jatropha curcas embryo 56-70 (DAF) Jatropha curcas cDNA clone rjcaeb0_002649, mRNA sequence

ATTCAATTATATTATAGAATATATTGATTAGGGAATACTTAATTACTAATTAGGATGATTACATATACTT

TCATCACTTATTTTCATAATATATTACATACTTCATTAGCGTGAGATCATAACTCACCCTCGTAATACTA

CTTACTTTCACTATCTACACACCCAAAAAGGCTACTTCATTATTTACTCCTCGGCATATCTTCCAGACTC

GGTCCTGGAAATAGTCAAGGTGGTCTCTTGTCTTCCAAACTTAATCCTTCTTGCCTCCTCTGCTGATACC

CTAAATGCATTTGCCATAACCGCCAACGGCATAGCTCTAAACGGCGGAAGAGCGACCTGCAAGATCATTG

GTCGTCGCGTTATCGCTTGTCTTGAAGGTTACATATTCGAATCTTTCACTTACTGCTCGTTT

>FM892153.1 FM892153 Jatropha curcas embryo 56-70 (DAF) Jatropha curcas cDNA clone rjcaeb0_002648, mRNA sequence

AAAGCTTAAATCAAGTTATTTATAAATCTAGAATCCCAAAACAAGCTTGGTACTTATAATTTGAAGCTTA

CAAAAACACTACCATAAGGGCATCAAAACAAATCTTCCGCTTGCACTGCTACATAAGTAACACATAAATG

GCAAAAATGCATGAAGACCTTTAAGCAAATCCAGAAGAACTAGTTTGAGCTGCCCCACCATAACCACCAC

CACCACCACGACCAAAGCCAGGCCTACCACCTGAGCCCCTAAACGATGGCTGGAAGTCCGCTGGAGCACC

TCCCTTTTCACCACCAAAATCACCCCCTCGAGGGCCTCCACGGTACCCATCACGATCACCAAATCTTGGG

CGGTCTCCATCAAAGCGAGGTGGGCCTCGGGGGCGATCACCAGGTGGGCCACCCATTGGCCGACCAGTGG

GCCTTGCAGATTTCTTCAATGTGGCAGGAACAATTTCTGATGGAAGATTAAGGTAAGTCCT

>FM892152.1 FM892152 Jatropha curcas embryo 56-70 (DAF) Jatropha curcas cDNA clone rjcaeb0_002646, mRNA sequence

ATTCCTTCAACTTCAAATCAAGTATCCAGCTAGAATGGCTTTAAGCTTAGGAAGGAGGAGCAACATCTTT

GATCCGTTCTCACTAGATATTTGGGACCCATTTGAGGGCTTACCTTTCTATAGCGGAACAGTGGCCAATG

TTCCGTCCACTCAACACGAAACAGCCGCCATTGCCAACGCACGAGTGGACTGGAGGGAGACTCCGGAGGC

TCACATATTTGCTGTTGATCTTCCTGGTGTTGAAAAAAGAAGAGGTAAAAGTGGAGGTAGAAGATGGCAG

AGTTTTGCAGATAAGTGGGGAGAGAAGCAAAGAACAAGAAGAGAAGAACGATAAGTGGCATCGCGTAGAA

AGAAGCACTGGTAAGTTCTTGAGGACGGTCTACGTTGCCGGAGAACACAATGATGGATCAAGTCAATGCC

ACTATGGCGAACGGTGTGCTTAAAGTGGTTGTGCC

>FM892151.1 FM892151 Jatropha curcas embryo 56-70 (DAF) Jatropha curcas cDNA clone rjcaeb0_002644, mRNA sequence

ATTAAATTAATATGACATTCATAGAATTTAAAACTAACAACTTATCTAATCCACTCCTCTCTTCGAGAAG

GAGCAAATTAAAAACCCAACTTCTCCTATTTCATATGCTTCTTCTTCTTGGGCTAAGTCCAGCTAACTGC

TATTGGACTACCAACAACATGTTTCCCATCACTCCACTCTAAACGAGCAAACGCGTTAGTATTAATTGGC

AATGAACTTCCAGTTACAGTAACAGTAAAAGTTTTCTTCTCCTTTGCTTGGCTAAAGTTCAACACTTGAG

GATTAACTGAAATTTTAACTCCCGTAAGCTGACCTGAAATTGAAGCCTTATAAGTCCCCGCACCAACCAC

ATTGGTAAGTGTTCGTGTGTATTTAGCAACACTAGAACCACCATTGATCATTTGTGTAGTATCAAAGTTA

ACAGCAAAAGAAGGATAGTTAAAATCATTCAAACTATACTTCTTATTCGAATCGCAACTGAATCTTCTTC

TTGCAAGGCTAGTGATTTGTGATGCTGTGTAATTAACGCGCAGCAGAAACTTAAGTAGTCCTCGGATGTT

AGATCGTAAACGAGCCCCGGATCAAGGGCTGATACTGGAT

>FM892150.1 FM892150 Jatropha curcas embryo 56-70 (DAF) Jatropha curcas cDNA clone rjcaeb0_002643, mRNA sequence

ATTCGTTACTATTAATCTGGTTGATGACNCACAAGATACCCACATAATGCTGTACCGCCAAATATTGCTC

CTTTCTTGAATATTGAGCCTAGTACAGGGAATTACTATCCCACCATATTCTTCAACGAGTTTTGGTTGCT

TAGAGATAAGTTGATCCCAGTGAACGAGACAGCGACTGAGTTGCCACTTAATCTGGAGGTGGGACCTATA

AGCATGACAAAATGGCAACTATTCCTGCAGATTGATCAGTCATTCCAGATTCATCGTAATTATGGAAGCA

TGCTTGAAGGCGAGGCCGATGAACTGAAGAGGGTCTTCTTGGAAGGAAATCCTTACCTCTTGGGGATTAC

AATGGTTGTTTCATTACTTCATTCAGTTTTTGACTTCTTGGCATTCAAAAATGATATCCAATTTTGGAAC

AAAAATAAGTCTATGGAGGGGCTGTCTGCAAAGTCTGTTGTTGTGAGCTTTATATGTCAGCTCATTGTCT

ATCTTTATCTGCTTGACAACGACACTTCATGGATGATACTTGCAAGTTCTGGAATTGGTTGCTGTATTGA

GTTCTG

>FM892149.1 FM892149 Jatropha curcas embryo 56-70 (DAF) Jatropha curcas cDNA clone rjcaeb0_002642, mRNA sequence

ACAAAGATTAGCTCTTATTATTTATGGAGATTATTATACATAACTCTCACTCACATTCAAGTGAGTGAAA

TTTTATTTTTCTGCAGAATATATATATTAATATATCCATGTTGTAGGTAGCATTTTAACGAGGGCAGGCC

GAGCCAAAGCAAACATCGATTATATTGTAAGACCTACTCTCTGCCTCTTCATATTCTTCGCTTCCAACAA

ATCCTTGCTTCTCTGGTAGATACAATATGGATTCACATCGACAAATGGCACGTAGCTTCCTTGCTTGGTC

ACAACATTGTCTAGGAACGTTATGGTGCCGGTTTTCGATTCCTTTCATAGCTAACAGAGCTTCTGACCTT

GTTCTCGACTGTCTTATATACCTCTCACAAGAGCTCAAATTTGTTCTTTCAGCTTCTCTCTTGCACATTT

CCCGAACTTCATAAGCATCGTCCATTTCAACGGTGGTGGTGGTGGTGGTGGTTGTCATATGGCCGG

>FM892148.1 FM892148 Jatropha curcas embryo 56-70 (DAF) Jatropha curcas cDNA clone rjcaeb0_002641, mRNA sequence

TCCTCATTCCATCAACCGAAATCCTCTTAATATTACTGAACTTCCTACGGAGTGTCAAAACGTTGACGAA

TTTCAAAGAGATGACTTACTTGTGTGCAAGACCGAGACAGTGGATGATGATCCTCCTTGCGTGCTTAACA

CAGAAGCATCATTTCCTTTACTCCAATATAAAAGGAGAAAGCACAATAATGATACAGTATCTAATCATTC

AAATGCTTCGGACAAGTCAACCAGGACAACTCAAGATCCTTGCTCATCTACAACATCAACAGCAGCAACA

ACAACAACTACAGACACAACGATGACAACAGCTGCAGCAACCACTACTGCAATCTCTGCATTATAGGAGT

TTTCTCTAATGGAATCAATTGACCCCAAAGTAAATCGTCATGCTCCTCCTCCTAGCCTCGGTACTATTAG

TCTTGATTCGAAAGTGCCTCCTAAACTGC

>FM892147.1 FM892147 Jatropha curcas embryo 56-70 (DAF) Jatropha curcas cDNA clone rjcaeb0_002640, mRNA sequence

TCGATTAATTATATTGGTGGAGAAATTTGTTAGCAAAAAAGGGCAAAAATGTCTTCAAATCAATCAAGAC

AAGAGCTTGATGAAAGAGCAAGGCGTGGAGAGACTGTTGTCCCTGGAGGTACTGGGGGCAAGAGCCTTGA

AGCTCAAGAGCATCTTGCTGAAGGGAGAAGCAGAGGAGGACAGACAAGGAAGGAGCAGCTGGGGACAGAA

GGATATCAGGAGATGGGCCGGAAAGGTGGGCTGAGCACGACGGACGAGTCTGGTGGAGAAAGAGCTGAGA

AGGAGGGCATCACTATCGATGAGTCGAAGTTCAGGACTGGGAGCTAGTGTGATGATTAAAACTATAAATG

GTATTTTGTAGTAATAATAATAATAATGTTGGTAATAAAGTATTAGCTTAGCAGTGTACTTTAGTTTAGT

AATGTAGTTTACATGTGGGTTAAAATGACTAGGGTTTAGCTAGTATGTGTGTTGTAACTTTGAGAGGGGT

TTATGGTCGTTCCCTTTCTGGGAGGACTGCTAATACTTTCTCCCTATTTGTAAAATAACCTGCAGTATAT

ATAATTACCTGTTGGTTAAAAAAAAAGGGGGGGCCCGGTACCCAATTCGCCCT

>FM892146.1 FM892146 Jatropha curcas embryo 56-70 (DAF) Jatropha curcas cDNA clone rjcaeb0_002639, mRNA sequence

ATTCCAGGTTTCTTCAGGTTTACGTCTCAAAGTAACAAAAATGGCTTTCTGTAACAAACTTGGGAGCCTG

GTGAGACAAAGTATCTCTCAGAATGGGCAAGTTCCAGTGAATTCAATGCTGAATTCTATTCGGTGCATGT

CGTCTTCCAAACTTTTCATAGGAGGCCTTTCATATGGAACTGATGACCAGTCTCTGAGAGATGCCTTTTC

TAGTTTCGGTGAAGTCGTTACTGCAAGAGTTGTCACTGATAGAGATACTGGGAGGTCACGGGGATTTGGG

TTTGTTGACTTCTCCAGCGAGGAGTCTGCTAGCTCGGCCTTATCAGCCATGGATGGCCAGGAATTACAAG

GACGAAGTGTTCGCGTCAGTTATGCTCAGGAAAGAACTGGTCCTCCAAGACATAATTATAGTAATTTTCG

TGGGAATGGAAATTTTAACAATGAAGGCTTCTAAGCGGTTATCCCAATCTATGGGCTTGTTTCTATAGCT

AGTTGTTAGTTGTTTGGAACGAATGGGCATTTATTTGATTATGATCGATACTAAACAACTTTTGTATTAG

GTTTCAACTAATTGTGCTGGTTATAACCTATAATATATTTGCTGGGTAAGTT

>FM892145.1 FM892145 Jatropha curcas embryo 56-70 (DAF) Jatropha curcas cDNA clone rjcaeb0_002638, mRNA sequence

ATTCGAAATGCGCACGATCGTGCCTGATGAACGAGAAGAAGAAGAGTTCACGTACAGCAGACCCGGCCAG

AGCGGGAGGTGGATGAATGGAATGGAAGAAGCCATGTGCACAATGAAAACTATACATAACCTTGAGTCTC

GTAGGGAAGCTGATTTTTACACAAAGGAAGCTGGTAGAATCCACCTTGCAAATCAACGAAAGCTCCCCAT

TCTCAGTTTCATGGACATGAGCGCTGAGCGAGGCGTTCTGCTGCCGAGGGCAATGCACAGCCCACACTGG

TCCATAAACGATCAGAGAATAGTGTGTGCTCTTCGCGGCGACATGCACATACAGATTGTGGATGACAGTG

GAAACACAGTAATGGACGAAAGGGTGAAAGAAGGAGACATGTTCGTGATCCCTCAATTCTATGCAGCACT

TGGAAGGGCAGGAGAAAATGGGTTCGAGTACGTAACATTTAAAACAAGTAGCCAGCCTATCAAGAGCCCA

TTGGCGGGTTATACATCAGTGATGAGAGCCATGCCTCTTGATGTTCTGATGAACTCTTACAGAATGTCTC

CAAGCGAAGCTCATCAAGTGAAGAGG

>FM892144.1 FM892144 Jatropha curcas embryo 56-70 (DAF) Jatropha curcas cDNA clone rjcaeb0_002637, mRNA sequence

GAAGAGCCCGCGTTCTACAGAAATTCACTAAAGATCGGAGCTTCTACGTGGAATTAAGAATTATCGTGTA

GGAATTCTTGAAGCTAATCCTCAAACATTTGTATCTCAGGACCATTGGGATGCTGATGGAGTTCTCTATG

TGGCTAGAGGACGAGGAACAATATCTATGATTATTGAGGAAAAGAGACAGAGCTTTAACGTCCAGCGTGG

AGATGTGCTAAGAATTCGTGCAGGAACTCCTCTCTATTTGATTAACAGAGACGAAAAAGAGAAACTTTAC

GTTGTCAGCGTCATGGTACCCGTCAATATTCCGGGCGAGTTCGAGATATTCAGTGCGTCTGGTACTGAAG

ATTCAGAGTCCTTTTACGAGGCATTCAGCTGGGAGTTGCTTGAGGCTGCTCTTAAAACCGAAAGGATGAG

GTTAGAGCATATATTCAGGCAAAAGCAAGGAGCTATTGTAAAAGCCTCCAGGGAGCAAATTCAAGCTATG

AGTCATCGTGAAGAAGGTGGCGGCGTTTGGCCTTTCGGAACTGAATCCATCGGTCCAATTAGTATCCTGA

AGAACCCTGTATTTAGGAATAACTATGGTCAACTCTTTGAAGTTGGCTCTAGAGATTTCAAGCAACAGCT

CCGAGACCTTGACCTTTCAGTCTCCGTTGCCAACATCT

>FM892143.1 FM892143 Jatropha curcas embryo 56-70 (DAF) Jatropha curcas cDNA clone rjcaeb0_002635, mRNA sequence

CAATTCGATCATAAAGTGTAACAAGCAAAAGATTATTATATGGTGGAAAATTTGTTACAAAAAGGGCAAT

GTCTTAATCTCGACAACGATGAAAGAGCAGCGTGGAAGACTGTGTCCCTGAGTACTGGGGCAACTGAACT

CAGACATCTTGCTGAAGGAAAGCAAGAGACAACAGAAGACATGGGGACAAAGATTAGAATGGGCCGGAAG

TGGGCTGACCACGACGATTGGTGAAAAGAGCTGAGAAGGAGGGCATCACTATCGATGAGTCGAAGTTCAG

GACTGGGAGCTAGTGTGATGATTAAAACTATAAATGGTATTTTGTAGT

>FM892142.1 FM892142 Jatropha curcas embryo 56-70 (DAF) Jatropha curcas cDNA clone rjcaeb0_002634, mRNA sequence

ATTGTCTCGTGGGGGGTTTGGGACAGATTCAGATAGAGAGAGAATGGAAGCCGTAGGTGTAGTTGGTAGT

GGTGGACTATGCGTCCCTGCGTCATCCATTAATCGCTCATCAACCCACACAACTTACAGCTTATCCTTCG

TCCCCGGCAATAAGCTTGGCCTACGCAGTAGTCACCTAGCAGCATTCCATTCACCATCCCATCTTTTCTC

TTATTCCCCTTCACGTCCTTATAATGCTAAGCATCGCAAGCCTACCAAAACCCATATCTTTCTTCCTCAT

TTGGTTGCTTCCCTGGAACAGGTTGAAGAGACATATATCATGGTCAAGCCTGACGGCGTTCAACGTGGCC

TTGTTGGAGAAATAATCTCAAGATTTGAGAAGAAAGGATTTAAACTTACTGGCTTGAAGCTTTTCCAATG

CCCTAAGGAGTTGGCAGAGGAACATTATAAGGATCTCAAGGCTAGAACCTTTCTTCCCTAAGTCGATCAA

TTACATAACTTCAGGTCCAGTTGTGTGTATGGC

>FM892141.1 FM892141 Jatropha curcas embryo 56-70 (DAF) Jatropha curcas cDNA clone rjcaeb0_002633, mRNA sequence

AACCAACAGGTAATTATATATACTGCAGGTTATTTACAAATAGGGAGAAAGTATTAGCAGTCCTCCCAGA

AAGGGAACGACCATAAACCCCTCTCAAAGTTACAACACACATACTAGCTAAACCCTAGTCATTTTAACCC

ACATGTAAACTACATTACTAAACTAAAGTACACTGCTAAGCTAATACTTTATTACCAACATTATTATTAT

TATTACTACAAAATACCATTTATAGTTTTAATCATCACACTAGCTCCCAGTCCTGAACTTCGACTCATCG

ATAGTGATGCCCTCCTTCTCAGCTCTTTCTCCACCAGACTCGTCCGTCGTGCTCAGCCCACCTTTCCGGC

CCATCTCCTGATATCCTTCTGTCCCCAGCTGCTCCTTCCTTGTCTGTCCTCCTCTGCTTCTCCGGGGGGG

CCCGGTAAC

>FM892140.1 FM892140 Jatropha curcas embryo 56-70 (DAF) Jatropha curcas cDNA clone rjcaeb0_002632, mRNA sequence

AGACAAATTATCATTATGACAACGAAGATAAACCATATCAATTATTCCTAGACTAACAAAATATTTACCA

TGCTAGAACCAGATTTTGATGAACCTATCACTTGAAATGAATTACACCATAACATTTTCACAAGTTAAAT

AACCAACAATTAAAATATCAAATACAGAGCCAAATTAATCTATTCATGCTTCTTCCCCTTAGAGCCTTCC

TTTGGGAATCTTACTAAGGAAACCGGAATCCAGCTTACGGTAGTGACCTTGCAGCTGTTCAAGTACCTTG

TACACAAAGTCATCTACTTGGTCTTCATACCTCTCGTATAGGACTGGCAATGTGTGTGCAGCTACAAAAC

CAATATACATAACAGTTAGAAAATTGCACCAGCTCCCAATGACAGCAGCAGCCCACAAACTAGCTATAAC

CAAAAGAAATTGTTTCGAATTTCCTCCAGATGATACATCCTGAAGAAACCGCAAAGCACGGTTAAGCTCA

AGCCCAATTGAACTGCCAATACTGACG

>FM892139.1 FM892139 Jatropha curcas embryo 56-70 (DAF) Jatropha curcas cDNA clone rjcaeb0_002631, mRNA sequence

ATTCGAGGAGCATCACTTCGAGTCAAAAGCTGACGCTGGAGCTTCCAAAACTTACCCGCAGCAAGCCGGA

ACTATTAGAAAAAACGGATATATAGTCATTAAGAATCGCCCATGCAAGGTTGTTGAAGTTTCCACCTCCA

AGACTGGAAAGCACGGTCACGCGAAGTGCCACTTCGTTGGAATAGACATCTTCAATGGCAAAAAGCTTGA

AGATATTGTCCCTTCCTCCCATAACTGTGATGTTCCCCATGTCACTCGAACTGACTATCAGCTAATTGAT

ATCTCTGAGGATGGATTTGTGAGTTTGCTGACTGAAAATGGGAACACTAAGGATGATCTGAGGCTTCCAA

CTGATGAGAATCTTCTGTCTCAGATTAAGGATGGGTTTGCTGAAGGAAAAGATCTTGTTGTGACTGTCAT

GTCTTCAATGGGAGAAGAGCAGATCTGTGCTCTTAAGGATATTGGCCCAAAGTAATTTCTTATTATCGGC

TTTAAGTGGTGGTTGCTATTTACATCGACTGATTTTTGTAAAAAACCATCTT

>FM892138.1 FM892138 Jatropha curcas embryo 56-70 (DAF) Jatropha curcas cDNA clone rjcaeb0_002628, mRNA sequence

CCGGGTTGCAGGATTCGAATTCAATTTTCAATCTTTGTTTCAATGGCGACCCCTAAGGTGTTCTTCGACA

TGGCCGTTGGCGGCCAGCCAGTCGGCAGAATCGTGATGGAGCTCTACCAGGACGTCGTTCCTCGCACCGC

CGAGAACTTCCGTGCTCTCTGCACTGGCGAGAAGGGGATCGGCAAAAGCGGCAAGCCTCTCCACTACAAG

GGGTCCAGCTTCCACCGTGTGATCCCCGGATTCATGTGTCAGGGAGGTGACTTCACAGCCGGAAATGGTA

CCGGAGGTGAATCGATCTACGGAGCAAAGTTCGCTGATGAGAACTTCGTCAAGAAGCACACTGGCCCTGG

CATCCTTTCCATGGCTAATGCTGGTCCCGGAACTAACGGATCTCAGTTCTTTATTTGCGTTGCGAAGACC

GAGTGGCTGGACGGAAAGCATGTCGTGTTCGGTCAGGTCGTGGAAGGTATGGATGTTGTGAAGGCCATCG

AAAAGGTCGGGTCTAGCTCCGGAAGGACCTCAAAGCCTGTTGTGATTGCAGATTGCGGTCACCTTTCTTT

AAGTGACCTAGGATCTCAGTCTTATAAGTTGCTGAGGCGGCGGAGGTGTCCTTTATATGTGATGATGTGT

G

>FM892137.1 FM892137 Jatropha curcas embryo 56-70 (DAF) Jatropha curcas cDNA clone rjcaeb0_002626, mRNA sequence

TCTTGGTGACTCCCGTGCTGTTTTGGGTAGAGCTGTAAAGGCAACTGGGGAAGTTTTGTCCATTCAGCTC

TCAGCAGAGCACAATGTTTGTATAGAGTCTGTGAGACAGGAGATGCAATCTTTGCATCCTGATGATTCAC

AGATTGTTGTTTTAAAACACAATGTATGGCGTGTAAAAGGCCTCATACAGGTTTCTAGATCTATTGGTGA

TGTCTATTTGAAGAAGGCTGAGTTCAACAGGGAGCCCTTATATACCAAGTTCCGACTACGTGAACCTTTC

AAAAAGCCAATATTAAGTGCAGAACCATCAATTTCAATGCACCAGCTCCAGCCACATGATCAATTTATTA

TATTTGCATCTGATGGCCTTTGGGAGCACCTTAGCAATCAAGAAGCAGTGGATATAGTACAACATCATCC

ACGCAGTGGAAGTGCTAGGAGACTGGTAAAAGCTGCCTTGCAAGAGGCAGCAAAGAAGAGAGAAATGAGG

TATTCAGACTTGAAGAAGATTGATCGCGGTGTTCGACGGCATTTCCACGATGATATCACCGTGATAGTCC

TGTTTCTTGAACTCAAATCTTGTATCAACGGCTAGTTCAGTCAAGTGCTCGAATATATCTGTGAGAGGGG

GTGGAATCAGCATACCCTCAAA

>FM892136.1 FM892136 Jatropha curcas embryo 56-70 (DAF) Jatropha curcas cDNA clone rjcaeb0_002625, mRNA sequence

ACCACAATCACCTCTTAATGCTTGTTATTCAAAGTTGCTGAAGTATAGCAATTAGCAGCCAACCAAGCAC

AAGCAACAGTTAAAAAAGAAGTCACATTGAAATTATCAGAAGATTATAATGAAACTAGACTAAACATCAA

AAAGTGCAAAAGAACAAAACTGCAAAATATATGACAGTGACTTTGAAAAGTTTTTTTTTGCTAAAATGGT

GAAATTAATTGCACAAATCCATGGATGTAAAGTTTGACACCATTCAAAAAGCCGAGCACATTTCCTTCTA

ATGTATCTACTCAATGATGGACTGAATGACACCAGCTCCAACCGTCTTCCCTCCTTCTCTGATGGCAAAC

CTCATCCCTTGCTCACAAGCCACAGGCACAATAAGTTCCACAACCATCTTCACACGATCACCGGGCATAA

CCATCTTTGACGGGGGGGCCCGGTAC

>FM892135.1 FM892135 Jatropha curcas embryo 56-70 (DAF) Jatropha curcas cDNA clone rjcaeb0_002624, mRNA sequence

AACCAACAGGTAATTATATATACTGCAGGTTATTTGACAAATAGGGAGAAAGTATTAGCAGTCCTCCCAG

AAAGGGAACGACCATAAACCCCTCTCAAAGTTACAACACACATACTAGCTAAACCCTAGTCATTTTAACC

CACATGTAAACTACATTACTAAACTAAAGTACACTGCTAAGCTAATACTTTATTACCAACATTATTATTA

TTATTACTACAAAATACCATTGATAGTTTTAATCATCACACTAGCTCCCAGTCCTGAACTTCGACTCATC

GATAGTGATGCCCTCCTTCTCAGCTCTTTCTCCACCAGACTCGTCCGTCGTGCTCAGCCCACCTTTCCGG

CCCATCTCCTGATATCCTTCTGTCCCCAGCTGCTCCTTCCTTGTCTGTCCTCCTCTGCTTCTCCCTTCAG

GCAGATGCTCTTGAGCTTCAAGGCTCTTGCCCCCAGTACCTCCAGGGACAACAGTCTCTCCACGCCTTGC

TCTNTCATCAAGCTCTTGTCTTGATTGATTTGAAGGGGGGGGCCGGTAC

>FM892134.1 FM892134 Jatropha curcas embryo 56-70 (DAF) Jatropha curcas cDNA clone rjcaeb0_002623, mRNA sequence

ATTCCAACAAGATCCTCCGTATTCTTAAAGCTCATGGGTTGGCTCCGGAGATTCCAGAAGATTTGTACCA

TTTGATCAAGAAGGCAGTTGCGATTCGCAAGCATTTGGAGAGGAACAGGAAGGATAAGGATTCCAAGTTC

AGGTTGATTTTGGTGGAAAGTAGGATCCACAGGCTCGCTCGCTACTACAAGAAAACCAAGAAGCTCCCTC

CTGTTTGGAAATACGAGTCAACTACAGCAAGCACCCTGGTTGCTTAATGAAGGCGTGGCTACCTACTAGC

TAGATGAGCAATTCTCTCATACTAGTCATATCGGATCTTTGTACTGAAATTGAACTCATTTACCTGAATT

TTAGAGCAGACAGAAAACAATTTCTTGCATTTTTTAAAAATTTATTTTAGTTTTTAAAGGGAAAAAAAAG

GGGGGGCCCGGTAC

>FM892133.1 FM892133 Jatropha curcas embryo 56-70 (DAF) Jatropha curcas cDNA clone rjcaeb0_002620, mRNA sequence

CGAATCCACAGAGAGAGAGAGTGAGTGAAATGCCGGCCGGTCATGTCTTCGCTCCGAACTCGTGATTTGT

TCTCAGCCTTTCGGAAGAAGGTTACTCCCATTGACCACTTATCTTAGACCTACAGATCGTAATATTGATA

TCAAGTTAACGCCCGCCACAAGGTTCCTCACAATTCTACCCATGGCCGTACCGGTCGTGTATGGAACGTT

ACCAAACGCGC

>FM892132.1 FM892132 Jatropha curcas embryo 56-70 (DAF) Jatropha curcas cDNA clone rjcaeb0_002618, mRNA sequence

ATTCCACTAAACCAAGAAACTTTCTCTCACCGATCTTTGATCTTCTCCAAAAACCAATCAAGAGAATGTC

TCTGATTCCAAGCACCCTCTTCGGTGGCCGGAGAACCAACATTTTCGATCCCTTCTCTCTCGATATCTGG

GATCCATTTCAGGACTTCCCTTTCACAAGCACTGCTCTTTCTGCTCCTCGTTCTGAGTTATTGAACGAAA

CGGCAGCGTTTGCCAACACCCGCATAGACTGGAAAGAAACACCAGAAGCTCATGTATTCAATGCTGATCT

TCCAGTGCTAAAGAAAGAGGAAGTGAAAGTGGAGATAGAGGAAGGTAGGGTCTTGCAGATAAGCGGAGAG

AGAAGCAAAGAGAAAGAAGAAAAGAATGATAAGTGGCATAGAGTTGAAAGGTCCAGTGGTAAGTTCTTGA

GAAGGTTTAGGCTACCAGAGAATGCAAAGGTTAATGAGGTTAAGGCTAGCATGGAAAATGGAGTCTTGAC

TGTGACAGTGCCTAAAGTGGAAGTCACGAAACCGGACGTTAAGGCTATTGAGATTTCTGGTTGAAACTTT

TAGTTATGTTTGAGTGTGTC

>FM892131.1 FM892131 Jatropha curcas embryo 56-70 (DAF) Jatropha curcas cDNA clone rjcaeb0_002617, mRNA sequence

ATTCCACTAAACCAAGAAACTTTCTCTCACCGATCTTTGATCTTCTCCAAAAACCAATCAAGAGAATGTC

TCTGATTCCAAGCACCCTCTTCGGTGGCCGGAGAACCAACATTTTCGATCCCTTCTCTCTCGATATCTGG

GATCCATTTCAGGACTTCCCTTTCACAAGCACTGCTCTTTCTGCTCCTCGTTCTGAGTTATTGAACGAAA

CGGCAGCGTTTGCCAACACCCGCATAGACTGGAAAGAAACACCAGAAGCTCATGTATTCAAGGCTGATCT

TCCAGGGCTAAAGAAAGAGGAAGTGAAAGTGGAGATAGAGGAAGGTAGGGTCTTGCAGATAAGCGGAGAG

AGAAGCAAAGAGAAAGAAGAAAAGAATGATAAGTGGCATAGAGTTGAAAGGTCAAGTGGTAAGTTCTTGA

GAAGGTTTAGGCTACCAGAGAATGCAAAGGTTAATGAGGTTAAGGCTAGCATGGAAAATGGAGTCTTGAC

TGTGACAGTGCCTAAAGTGGAAGTCAAGAAACCGGACGTTAAGGCTATTGAGATTTCTGGTTGAAACTTT

TAGTTATGTTTGAGTGTGTCTAAGTCTAAATGGTACCCATCCATCCCATTAACATAAATAGATGTGTGTA

>FM892130.1 FM892130 Jatropha curcas embryo 56-70 (DAF) Jatropha curcas cDNA clone rjcaeb0_002616, mRNA sequence

ATTCCTTTCATTTTTGTCTGCCGGAGAACCTTGGGTTGGAAAGTAAAAAGCTTTTTGGTCTCTGTAGCGA

CCATATGAGTTTTCAATGGAGAAAATGGACATTTCGAGTTTAGTTTTATTGTTTTTCTTTTGGAATTCGA

ACTTCTCGTTTGCATATGCTGTGATTTCACTTCGAAATCCAATTTCCTCTTCTGGTTAATCTTGAACTCT

TAT

>FM892129.1 FM892129 Jatropha curcas embryo 56-70 (DAF) Jatropha curcas cDNA clone rjcaeb0_002615, mRNA sequence

ATTCCGAAGCTACTCTCGCTACGATCGACCAAATCTAAGGCTTTCAGATCGAAAAATGGCATTGATTCCA

AGCTTTTTCGGTAACTCACGAAGCAACATCTTCGATCCTTTCTCTGCTCTCGACGTTTGGGATCCATTCA

GAGACTTTTCTTTCCCTTCATCTTCTTCCCTCGTCTCTCGTGAAAATTCTGCTTTTATTAACACTCGCAT

TGACTGGAGAGAGACACCAGAGGCTCATATATTCAAGGCTGATCTCCCTGGGCTCAAAAAAGAGGAAGTG

AAAGTAGAAATTGAAGATGACAGAGTGCTTCAAATCAGCGGGGAGAGAAATGTGGAGAAGGAAGACAAGA

ACGACACGTGGCATCGCGTGGAGCGTAGCAGTGGCAAGTTCATGAGGAGGTTCAGGCTGCCGGAGAATGC

AAAGATGGATCAAGTAAAGGCTTCAATGGAAAACGGAGTACTTACTGTCACTGTTCCTAA

>FM892128.1 FM892128 Jatropha curcas embryo 56-70 (DAF) Jatropha curcas cDNA clone rjcaeb0_002614, mRNA sequence

ATTCAGGAGAGGTTCCTTCGAGGGTCATGCTGTGGCATGAGAAGCTAATAACGTGATGGCAAGTGTTTCG

GCTTTTTGGGGTGACTGTGACATCGACCTTGAGGGTTTACTAATGTAAATAGTGCTGTGTATGGTGAATA

AATAATGAAAGGTGAAGGTACATGTGTAAATTAGTCTTTAAGAAAAGAGAAAGATCACTGCTGCGGACTA

TAGTGGCTTCTGGTTTAATAATTAATAAAGAGATAAATTCTAGTT

>FM892127.1 FM892127 Jatropha curcas embryo 56-70 (DAF) Jatropha curcas cDNA clone rjcaeb0_002612, mRNA sequence

ATTCGAAGAAACACCGAGCAGAATAAAAGATGGAGGGCGAGAAGAAGACAAATTCGGGACCAGAATTGAG

ACGCTTAGGGTTCGTAAGGATTGCTGCTATTCAGACTTTGGTGTATGTTTCGAATTTATACAATTACGCC

AAGCAGAACTCTGGGCCTTTGAGATCTACGGTTGGTACTGTGGAAGGTGCTGTTACCGCCGTAGTTGGCC

CAGTCTACCGGAAATTCAAGAACGTGCCTGATGATCTTCTTGTGTTTCTTGATAAGAAGGTAGATGAAGG

CACACAAAAGTTCGATAAGCATGCTCCACCAGCTGCTTAGCGAGCTGTGAGTCAAGCCCAG

>FM892126.1 FM892126 Jatropha curcas embryo 56-70 (DAF) Jatropha curcas cDNA clone rjcaeb0_002611, mRNA sequence

GGACTATAACTTATAGCTCCACAAACTTTCATTATCTCATAACATAAGGCATATACAGATCTACATTTTT

ATATTATTGCAAGGAACTAAAACTGCCTATAAAAATAGGCTTATTCTTCTCCAAATCTAAACTATGTAGA

AACCAGTTTATTATACATCCTCTTGGTATAATTGCATAGTTGCAATATTAATTACGAGATCTAGACCCAG

GGCTAAACACAGTGACTTCCTGTCTATTCTCCTTAAGCCTCCTGGCATCTTCTCTGGAAATCTGGAATGC

GTTTGCAACCACTGCCTCTGGCAGAGCCCTAATTGCCGAGGTCCGTCCTGCTAGTGGGCTAATTTGTGCA

TTTTCGTTAGTCTTGAAGGATACCCACTCTAGCCCTTCATTGCTTGCCCTCTTCAAAACCACAAAGTTCT

GTGGAGCTGTTAATATTTGACCTTGTTGAACTTGTCCATCGAACACTGCATCTCCATTCTCATCTACGAT

TTGGGGGGGGCCCGGTAC

>FM892125.1 FM892125 Jatropha curcas embryo 56-70 (DAF) Jatropha curcas cDNA clone rjcaeb0_002609, mRNA sequence

ATTCTGCAAATTAAATAGAGTATTATTCCACAGTGAAATTAGCACTACAATACATACATGCACAGACACA

TTTATAGAAAATCAGGGAAGAGCTTAAGATGCTTGAGTAGCCTCTTGAGCCTTGTGTTGCATATACTGTC

CATACTCTTTAGCTTTTTCTTTAATATCATGAGCCTTTCTCGCCAGTTGTCTCTCTCGGGGGGGCCCGGT

AC

>FM892124.1 FM892124 Jatropha curcas embryo 56-70 (DAF) Jatropha curcas cDNA clone rjcaeb0_002608, mRNA sequence

ATTCATGCAAGGAAGGACGCTGGCTGAAGAAAACATGAGAGGTATCTGCTTTGAAGTCTGTGATGTGGTG

CTCCACACTGATGCTATTCATAGAGGTGGTGGTCAGATTATTCCAACTGCTAGGAGGGTTATCTACGCAT

CACAGCTTACAGCCAAGCCCAGGCTTATGGAACCTGTTTACTTGGTGGAGATCCAAGCCCCAGAGCAGGC

ACTTGGAGGTATCTACAGTGTTCTTAACCAGAAACGTGGGCATGTCTTTGAGGAGTTACAGAGGCCTGGT

ACCCCATTGTACAACATCAAGGCATACCTACCGGTTATTGAGTCCTTTGGATTCTCAAGCACTTTGAGGG

CTGCCACATCAGGACAGGCCTTCCCACAATCCGTGTTTGATCACTGGGAGATGATGTCTTCTGATCCAAT

GGAAGCTGGTACACAAGCATCANCTCTCGTGGCAGAGATTCGCAAGAGAAAGGGTTTGAAGGAGCAGATG

ACTCCTCTATCTGAGTACGAGGACAGGCTGTAATCCTTTGTTGAGTGAGTGTGAAGATGATAAGACAATT

TTTTTTGCTTACTTTTTTGTACTCAAGTTATTTTTTTAT

>FM892123.1 FM892123 Jatropha curcas embryo 56-70 (DAF) Jatropha curcas cDNA clone rjcaeb0_002607, mRNA sequence

CGAATTCTGATCTTCTGAGAAGGGTTCGAGTGAGAGCATGCCTGTCGGGACCCGAAAGATGGTGAACTAT

GCCTGAGCGGGGCGAAGCCAGAGGAAACTCTGGTGGAGGCCCGCAGCGATACTGACGTGCAAATCGTTCG

TCTGACTTGGGTATAGGGGCGAAAGACTAATCGAACCGTCTAGTAGCTGGTTCCCTCCGAAGTTTCCCTC

AGGATAGCTGGAGCTCGGGACGAGTTCTATCGGGTAAAGCCAATGATTAGAGGCATCGGGGGCGCAACGC

CCTCGACCTATTCTCAAACTTTAAATAGGTAGGACGGCGCGGCTGCTTCGCTGAGCCGCGCCACGGAATC

GAGAGCTCCAAGTGGGCCATTTTAGGTAAGCAGAACTGGCGATGCGGGATGAACCGGAAGCCGGGTTACA

GTGCCCAACTGCGCGCTAACCTAGAACCCACAAAGGGTGTTGGTCGAATAAGACAGCAGGACAGTGGTCA

TGGAAGTCGAAATCC

>FM892122.1 FM892122 Jatropha curcas embryo 56-70 (DAF) Jatropha curcas cDNA clone rjcaeb0_002606, mRNA sequence

ATTCCCGCTTCCGCTTTTTCTTGCCCACTCTGCATGGAAACCCATAAATTCTTGTTCCTACTCTCCAACT

ATGCTCTGTTTCTTGTTCTTTTACTTTCTTTTCTAGATTTACAAAGTGGCTCTGCCTCTCGGTTGAACCC

ATTTGAACCTGGAATTCTTTTACCGACCGAAGAAGATGCCTCAGAAGTTGATGATGATCCAATTGGCACC

AGATGGG

>FM892121.1 FM892121 Jatropha curcas embryo 56-70 (DAF) Jatropha curcas cDNA clone rjcaeb0_002605, mRNA sequence

ATTCTTGCAAAAAGAGCTCTTCTTTGACTCTCTATCCATTGCAACTGCATCACCATTCTGCTCATTTCTC

TTCTCAAGTCTTTCATGCGCAGTATCCATCAGATAATTTCCTCTAGACTTGGAACGGGATTTAACCTTCT

TCCTTTGAGAGTTTTGTAGAATCCGATGAAACTAACATGGATGCATTTGATGTAGACGGCCCAACCTCTG

AAGAAAAGCAATTACTCTCCAGCAACTGTTCTTTAATAAGGTATTGGGTCTCTAAAGTGCAACGCTTGCT

CTTTTTATGTTTTATCTTTTTCCGTAGCCCCAAAGATGCTCTAAAGAATTTTTGTAGATGCATGTTTGGA

ATCCCGTGCTTGCAAAAGTTTCTTTTTTAACTTCTGTTGAGGTGAGGAATCAGGAACTTTCTTCTGTAGA

GATTCATTTGGTACGACTGATGCTGATGATGACTGAACGGGGGGGCCCGGTAC

>FM892120.1 FM892120 Jatropha curcas embryo 56-70 (DAF) Jatropha curcas cDNA clone rjcaeb0_002604, mRNA sequence

AATTTTATTTGTCTTGGAATTATGTTCTCTCTTTTGATTAGAAATAGAATAAGAATGGAAACGTGACAAG

ATAAGATTACACACTCAAAAATTACTGGGCAAAAGAACACCAGAAATAGTGCCACAGTAAATAGTCCATA

TACATATTTAGTTATATACATATCGTATATTACCATAATACACGTCGAGTCATCAAAATTACACAACCAA

AATCCTGCAACCTATAACATATTATATTTTACATGGGTATCATATTATATGGTAAATTGGGGGGGCCCGG

TAC

>FM892119.1 FM892119 Jatropha curcas embryo 56-70 (DAF) Jatropha curcas cDNA clone rjcaeb0_002603, mRNA sequence

ATTCAGAAATTGAGTGGGAGGCGAGCACATCATCTTTCCGGTGCGACCCATACAAACCACCACTATCCAA

CTACAGTAGATCCTGTAGTTGGAACCCATGCACATCAGCCTGTAGGAACCACAGGTACCATGGCTGGAAC

AACTGTTCCTACTTACCCTTTAGGAGGCCATCCTCCAGGTTATAAGTACGTCTAGGCATAATTTGTATCT

TTTTTTTTATATCCTTTTCGTATGTGTCTGGTGTGTAGATGCTTGTGTTCTCTAATGAGTTCAATGCGTG

AATAAGGAGCAAGAAAGACTTCAAAGCTTTCAAATACTAAAAAAAAAGGGGGGGCCCGGTAC

>FM892118.1 FM892118 Jatropha curcas embryo 56-70 (DAF) Jatropha curcas cDNA clone rjcaeb0_002602, mRNA sequence

ATTCGGAGACTGAAAGGTCAAGGTCTCCGTTGCCAACATCTCTCGTGGAGCAATGTCTGGGCCCTATTAC

AACTCCAGAGTAACCAAGATATCAGTGGTTTTGGATGGCGAAGGCTACTTTGAAATGGCTTGTCCTCACC

TTAGCGGCTCGCGGGGAGACACGACGGGTGAAAGTCAGAAGACTTACCAGAAAGTAAGTTCACGGTTGAA

ACGTGGAACAGCAGTGGTCGTCCCTGCAGGCCATCCGGCGGCTTTTGTAGCTTCAAGAAACAATAATCTG

GAAGTAGTCTGCTTTGAGATTAATGAAGAAGGCAATACTAGGCATCTACTTGCAGGGAAAAATAATATTG

TAAATAAGATGGAGAAGCAAGCCAAAGAGTTAGCCTTCGGTGTAAGTGAAAGAGAAGTGGATCAGGCTTT

TGGAAGACAAAACGAGGAGTGGTTCTTTCCAGTGCCAAGGAGAGGTTCCTTCGAGGGTCATGCTGTGGCA

TGAGAAGCTAATAACGTGATGGCAAGTGTATCGGCTATATGGGGTTACTGTGACATCGACCTTGAGGGTT

TACTAATGTCAATAGTGCTGTGTATGGTGAATAAATAATGAAAGGTGAAGGTAC

>FM892117.1 FM892117 Jatropha curcas embryo 56-70 (DAF) Jatropha curcas cDNA clone rjcaeb0_002601, mRNA sequence

ATTCATTGAATTGGCAGATGAAGTGAGGAAGGTACTTGTTGCGCTTAATTCCTATGTTCCAGCCCTTAAA

CTACGTCCACTCACGGAAGCTGTGGCGCTGGATTCTTAATATTGAAGACAGATAACTGAAGGAAAGGGGG

GCATAAACCAGAGCTCCACTAACTTGATTACACTCCTGGAGAATCCTCTCTGTTTTTTCGTTTGTTACAC

CCTGCCAAAAGATTATTATTATTATTATTATTTTTTTGTCGTATGTTCCCGTCATTGCTTCTTAGTTATT

AGCATCCACTTGTCTGTATAGGCTCATAGCATTGCTGTGTCTCTAACATTAACCATAGTGTTTGAGCAAA

AGAGATGTAACTGGCCAACGGCCGCCCGCTATAAACAATGATAATTTTTATTTTTGAGAACAGTGATAAG

TGATAACTGGTGATTCCTT

>FM892116.1 FM892116 Jatropha curcas embryo 56-70 (DAF) Jatropha curcas cDNA clone rjcaeb0_002596, mRNA sequence

AAGGGAACTCAGGTTTAGAAACTGGTATTACATTTGGCAAAAAAAAAACTAGATCTTTATGCAGCAACAT

GCCAGCAACTCACACTAAACAAGTCATATAATAAGTTCATAGCACAACTTATCTCAAGGTTCGGCAAATT

ATCGCGAGTAGTCACAATACTTAAACAAAACTCAAAACTAAAAATCTTAAATGTCATTGTAAAAGCTTTA

GACTCTAATGATACAACTTCATCAGTCAAAGCCTCCCATGGACCAGCTGCTAACAATCCAAGAGGCCCAT

CACACCGAATTGCAATCCGTAATATTTCATAT

>FM892115.1 FM892115 Jatropha curcas embryo 56-70 (DAF) Jatropha curcas cDNA clone rjcaeb0_002593, mRNA sequence

ATTCCACATTCTCATTGATGGAGAACCCGCAACTGGTTGCCTTGTGGATTTCGGTCTTTATTTTTACCAC

AACTATGCTGCATTTCGCAGGACACAAGGTGCAGGCTATGGTCCTTTCTTCTATCTCCCTAAAATGGAGC

ACTCAAGGGAAGCTAAGATATGGAACTGTGTGTTTGAGAGAGCTGAGAAGATGGCAGGAATAGAAAGAGG

AAGTATTAGAGCTACTGTTCTAATTGAAACACTTCCAGCTGTTTTTCAAATGAATGAAATCCTCTATGAA

TTAAGAGATCACTCTGTTGGCTTGAATTGTGGAAGATGGGATTATATTTTCAGCTATGTCAAGACATTCC

AGGCTCACCTGGATCGCCTGTTACCCGATAGGGTTCAAGTTGGCATGACCCAACATTTCATGAAGAGTTA

CTCTGATCTCCTCATTTGGACATGCCACAGGCGTGGTGTGCATGCCATGGGTGGCATGGCAGCACAAATT

CCAATTAGAGATGATAAAGAAGCAAATGAAGCAGCACTAGAATTGGT

>FM892114.1 FM892114 Jatropha curcas embryo 56-70 (DAF) Jatropha curcas cDNA clone rjcaeb0_002592, mRNA sequence

TTTTTTTTTTTGTCATTTAACATTATAATACATATATCAGGAATAAGAGAAGAAGAAAAAAAACCTAGTA

TATACTACAATACAGCACATGATTGTTTTTCTAAAGAAAAACAGGTTTAAGTGGAAGAACTACTATAAGT

TTATACAAAAAAGAGCCATGGACTACTTAGCTTTCTTATCTGTTGGTTGGGAT

>FM892113.1 FM892113 Jatropha curcas embryo 56-70 (DAF) Jatropha curcas cDNA clone rjcaeb0_002591, mRNA sequence

AGCCATCAAGAAGTCGAATCCATTCAACAAAAACCAGATTAAAGTCTGATCATACATCAATATGCACAAA

CGAAAGGGAGAGAGAACTAACTCTAGAAGACTAGACTAGACCCCACACTACATATTTTCAAATTCACAAA

ACACCTCGCGCCACACCAGATAGCATGTCGTTTTAACCACCTGTCCATATGATCATATAACATCACTTCC

CAACCTTCCCTCTGAGCTTCACACCAAGCACTTTCTCCATCTTCGCCAAAATAGCCTGGTTAGGAACCGC

CTTCCCGTTCTCATACTCCTGAACCACTTTAGGCTGCTCGTTGATTAACTTAGCCAGCTCTGTTTGGCTC

ATCTACTTCTCTAGCCGTGCCTTCTGGATAGCTTGCCTTACCTCCGGTGACACCCTGTCCAGCGCTGCGG

GTTCCGTTCCTTCGTCCAACTTTCTAGCCGTCACAACCGGGGCTGCTTACTTGTTAGAG

>FM892112.1 FM892112 Jatropha curcas embryo 56-70 (DAF) Jatropha curcas cDNA clone rjcaeb0_002590, mRNA sequence

CTACTAAAAGATACCTTTTATTCAACTGGAATTGCTTAACAAGAAAAAGACACCAAACAGAAAACATAGA

TATATTACATAACAGGAGCTAAAGCACATGCCATGCCCTACACACGTGGGTTATGGCCAGTGGTGTCTCC

GGGCAACCCAGATGGGCGAGTCCTGGTCAAACCCGTTGGGTCCACCTCTCCACCGTATGGCTGCCCTGTG

CCGTGTCCAGGCATGGCCGACATTTGCTGGGTCCCTGTAGGGTAACCGGTTGTCCCAGTTGTTGAATGGG

TATAGGTTTCGGTTGTGCCTTGAGCGCCCGGGGGGGCCCGGTAC

>FM892111.1 FM892111 Jatropha curcas embryo 56-70 (DAF) Jatropha curcas cDNA clone rjcaeb0_002589, mRNA sequence

AAAACTAATAAAAGCATGCCAATTAGCATTCCAAAAGTAAACGACTAGAGGCAGAGGGTCAACTTGAAAA

TGAACTAACTGTGCCGTCTAGCCTCTTGCAAACAGTTACATATTAGCTTGTAGTAGACATAAGACTACAA

GATGACACACATAAGTTCTATAAATAACAAATCACAAGATATACCAGAAACCATAGATTCATGCTCCAGA

TGAATAAAATGACTTTATGGAAAAACTCAGAAACCGATATCACAATTGTCCACGTTCACCATCCCACAAA

CTATAAACATTTCACTCCAGTAAAGAAGAGTCTGAATGGAGGAACTCCTTGCTCAAAGGCATACGGTTCA

CATTCAGTTGGCACGACTTCTAAAGACATCGAGCCCCATCTCAGTAGGGTCGGTTGCCTGCAATTCTACC

TGAATTCCATCAAGCTCTCTCTTAAATCTGTCTTTCGAGCTAGCATAAATCATCTTGCTTCTGACTCTTG

ATGCATCAGGAGACCATGCAATGAAGAAAATCCTGCTCTTCTGGCAATTCTCTGCC

>FM892110.1 FM892110 Jatropha curcas embryo 56-70 (DAF) Jatropha curcas cDNA clone rjcaeb0_002588, mRNA sequence

ATTGAGAGAGAGAACTAGTTCTCTCTCTCTGAAAAGGTTGGAAGATCTGGCCAAAGAAGGTGATAGCCCT

GTAGATTCGTCCCATGGTTCGATCCTTCCCAGTAAAACGCGGCGTGTTCGAATTCTGATCGCTTTACGCG

AGAAAGGGGGACCACCCTCTAAGCCTAAGTATTCCTCAATGACCGATAGCGTACAAGTACCGTGAGGGAA

AGGTGAAAAGAACCCTATTTAGGGAGTGCAATAGAGAACCTGAGATCCGATGCGAACAATCAGTCGAAGG

AGCGGAGCCTTTACTTAA

>FM892109.1 FM892109 Jatropha curcas embryo 56-70 (DAF) Jatropha curcas cDNA clone rjcaeb0_002586, mRNA sequence

ATTCTTGAAAGGATCGGATTTTTTGTAAGAGCAAGGACCGAAGTTGTCGATCCTCCACTGCGCTAGTCTT

GAGATGGTCTCCACCCTTGAATCCGCCATAGCTGCGTGTCTTTCTATCTTGTTGGCTTTGAGTGAGGTGT

TTGAAAGAGAGTCAGGAGAGACACGACAGGAGACTTATTGTTTAAGGTGTCGTTGCTAGATGACGGAGGG

CTTAGTAAGATTTTAAATTGCAGCTAAAGTAAACATTTTCTTCTCTCGTTGAGAGTTTGAGAGACATAAA

TCCAAACATAAAAGGAACTTTGGTTGGTAGATGTGACTTTACTGTTTAAGCACTTTTTAGGGAAAAAAAT

TCAAAAGGAAATCAAAGCTTGTTGCTTCACTTCAAGGGTAATCTACTATTTAGTATATAGTCGCTAAATT

TTCATTAAATTTATATAACATATATGCATTATTTGTTAAAATCACTAATTCATTCATAA

>FM892108.1 FM892108 Jatropha curcas embryo 56-70 (DAF) Jatropha curcas cDNA clone rjcaeb0_002585, mRNA sequence

ATTCCGCTGCCATTTCTGTCTTGTCCTGGATTTATAGGTATGTTACTGGAAAGCATCCACCAGGAGCAGA

AAGTCTGGACCAGGCACGCTTAAAGTTGGCGGGAAAGGCAAGAGAGATGAAGGACAGAGCTGAGCAGTTT

GGACAGCATGTAACAGGTCAACAGACTTAAAAGAGAAGTGGGTATAGGTGGTTGATGCAGAGAACATAAA

ATGGCTTTGATATATATATAAAAATATATGAGTCCTTTAATTAACATGGAAGGCCATTGCTTGTCTGGTG

TATTTCTTTTTACGCTTAAATCTTTTTGTTTCTTCTATGTAATTTGTTAACGGGTCTTCCCTCTTTTGAG

AGCTGTCTAAAATAATTTACTTGTCATGTGTTGAAGGTCTCTGTAATCAAGTCTTACTT

>FM892107.1 FM892107 Jatropha curcas embryo 56-70 (DAF) Jatropha curcas cDNA clone rjcaeb0_002584, mRNA sequence

AGAAAAAGCAGCGGTCATTTTTATAATTTGATTCAGGTCCAACACAGTAAAACTAAAATAAAGCACAAAC

CAGAACAACAATCCACATAATACACAATCTGAAAGGGTTTAGGGAACGTGTATGTCTTTAGCAAAGTGAT

TTTATACATTTATCCGAGTGCTCATCTTATTAATACTTCAACTCATTACACCTTCCTAGAGGACTCAGGT

TTTAAGTTCGACTGAATGATTCTGCAAATTAAGGTATCTTAGCTTAAAGATGACCTTGCTTCTCAGTTCA

TTTCACAGTATGAAGCAATATGAGAGATCATTCGCGAGGTGATGAGTTCTTTATAATCAATGCATAGGCA

TCACTATATATGGCCACCAAGTAAGAGAATACAGCAAGGACAATCATGAAAATACACAAGATCTTGTCCT

TCTTTGTAGCTATATCATGACGATTCCTAAGAGTGATGGAAGCAGGAAAAATGAATCCAAGGCAAACC

>FM892106.1 FM892106 Jatropha curcas embryo 56-70 (DAF) Jatropha curcas cDNA clone rjcaeb0_002583, mRNA sequence

ATTCGTCGAAGTTCAGGACTGGGAGCTAGTGTGATGATTAAAACTATAAATGGTATTTTGTAGTAATAAT

AATAATAATGTTGGTAATAAAGTATTAGCTTAGCAGTGTACTTTAGTTTAGTAATGTAGTTTACATGTGG

GTTAAAATGACTAGGGTTTAGCTAGTATGTGTGTAGTAACTTTGAGAGGGGTTTATGGTCGTTCCCTTTC

TGGGAGGACTGCTAATACTTTCTCCCTATTTGTAAAATAACCTGCAGTATATATAATTACCTGTTGGTT

>FM892105.1 FM892105 Jatropha curcas embryo 56-70 (DAF) Jatropha curcas cDNA clone rjcaeb0_002582, mRNA sequence

GGGAGAGAAAAGATTACTTCGTCTCATTGTTAAAGAGAATGCTATAACATGCAAAACAGGTTACAGGTAC

ATGTAGACTGAACATAAAACTCGGTGCCATTTCTATGTCCGCTTCGAGAAGACACTTGTTATAAGTACAT

ACGCAAATACACTCCTACACACAAGGGGAAGTAACAGAACAAAACAAAGCAAACATCATATACAAACAAA

CACCATTCATATTAGTAACTCTTCATTAGTGTATCAGTAGTTAATGGTCTCATTAAGCCTAGCAGCTGTC

GTCATTGACACCGCAACACCACCTGGATGCGTGTTTAAATTCGGATTGTTCCTCAACTCTGCACTAACCA

CCCCTTCAGCATCTGCCCTTGTCACGACCTTATCCGACGCCAATTTCGAAGTTGCACCCGATAGAACGTC

GGCGAGTTTAATCTTTTCCTCAGCGCGATTCGTAGCGGCATTGTAAGATGCGGCAGATTGAGCTGTGGCT

GCGATTCCACCTGGTACAATAACATTGCTGCCAGTTGCTCTTACTTCTGCCTCTTG

>FM892104.1 FM892104 Jatropha curcas embryo 56-70 (DAF) Jatropha curcas cDNA clone rjcaeb0_002581, mRNA sequence

TTTTTTCTTCTTTTAGCAAAATCATATAGCAATCTACAAAATGTTCCACATGCATATATAACGTTAACAA

TTCAAATTTAGGAGCATGGTCATATCAATCCTTCTTTTTTAATCTCATCTTCGCTTCTGCATCTCCACTA

TC

>FM892103.1 FM892103 Jatropha curcas embryo 56-70 (DAF) Jatropha curcas cDNA clone rjcaeb0_002580, mRNA sequence

ATTCGGTCTGAAGAGACGTCAGCTAAGCAAAGAATTGGAAACCGGCACCATGTTCCTACGCAATGTTGCG

ACCAAGCATGGAAACTACATCCTGTCTGTCAATGTGAAGCAATAATGTATCTAGCACAGAAGCAAGGACA

TATTGGAAGCCAAGAATATGATGAGGCAGAACGTAGGGCTTTCAATATAATTGATGTTTGCTTAGGCTCG

GTTTGCCCTCGTTAAAATGCTGCAGGTAAATAAAATTTCACTCGCTTGAGTGTGAGTGAGACAGAGTTAT

GTGTAATAATCTATAAAAAACTAA

>FM892102.1 FM892102 Jatropha curcas embryo 56-70 (DAF) Jatropha curcas cDNA clone rjcaeb0_002579, mRNA sequence

ATTCAGAGTTTGTGTTTGCGTTCACAAATGGCTCACTTTCAAAACCAATATGGCGCAGTTAACTATACGG

ACGAATACGGCAATCCAGTAGACGAATACGGTCGCGCAATACGCCAAACAGACGAGTATGGTAATCCAAT

CCCTCAAACTGGTACTACCACTGCATACGGTGGCGCACCAGAGACTGGTACTACTGGCCTTGGCATGGGA

CACCGCCAAGAACACTACGGAGAGTCTGGTCAGCTTCACCGTTCTGGCTCCTCCAGCTCTAGCTCCTCTG

AAGATGATGGACATGGTGGAAGGAGGAAGAAAGGGCTAAAAGAAACGATAAAAGAGAAGCTACCAGGTCA

TCATAAGGAAGAAAGAACTCAAGCAACCTCAACTACAACTCCAGGTGGTTATCATTCAGCTGACTATGGA

CATGATCATGAG

>FM892101.1 FM892101 Jatropha curcas embryo 56-70 (DAF) Jatropha curcas cDNA clone rjcaeb0_002578, mRNA sequence

ATTCNAGATTTCAAGCAACAGCTCCGAGACCTTGACCTTTCAGTCTCCGTTGCCAACATCTCTCGTGGAG

CAATGTCTGGGCCCTATTACAACTCCAGAGTAACCAAGATATCAGTGGTTTTGGATGGCGAAGGCTACTT

TGAAATGGCTTGTCCTCACCTTAGCGGCTCGCGGGGAGACACGACGGGTGAAAGTCAGAAGACTTACCAG

AAAGTAAGTTCACGGTTGAAACGTGGAACAGCAGTGGTCGTCCCTGCAGGCCATCCGGCGGCTTTTGTAG

CTTCAAGAAACAATAATCTGGAAGTAGTCTGCTTTGAGATTAATGAAGAAGGCAATACTAGGCATCTACT

TGCAGGGAAAAATAATATTGTAAATAAGATGGAGAAGCAAGCCAAAGAGTTAGCCTTCGGTGTAAGTGAA

AGAGAAGTGGAT

>FM892100.1 FM892100 Jatropha curcas embryo 56-70 (DAF) Jatropha curcas cDNA clone rjcaeb0_002577, mRNA sequence

ATTCATCAAATATCACTGATCGTAAACCTCCATTGAATTTACTCTGTGCTTCGATCGCGTCAATGGCGGA

GCACCCACAGTCGCAGCATGTTGGCCAACAGCCACGATCCCACCAAGTAGTGAAGGCAGCCACTGCAGTC

ACTGCCGGTGGATCTCTTCTAGTTCTCTCCGGTTTGACCCTTGCTGGTACGGTCATTGCACTGACCGTAG

CCACTCCATTGCTGGTCATATTCAGTCCGGTTCTTGTTCCTGCGGTTATCACAGTTTGTTTGATAATCAC

AGGGTTTTTAGCCTCAGGCGGTTTTGGCGTCGCTGCCATTTCTGTCTTGTCCTGGATTTATAGGTATGTT

ACTGGAAAGCATCCACCAGGAGCAGAAAGTCTGGACCAGGCACGCTTAAAGTAGGCGGGAAAGGCAAGAG

AGATGAAGGACAGAGCTGAGCAGTTTGGACAGCATGTAACAGGTCAACAGACTTAA

>FM892099.1 FM892099 Jatropha curcas embryo 56-70 (DAF) Jatropha curcas cDNA clone rjcaeb0_002576, mRNA sequence

ATTCGATAAAGTGTAACAGAGCAAAAGATTAATTATATTGGTGGAGAAATTTGTTAGCAAAAAAGGGCAA

AAATGTCTTCAAATCAATCAAGACAAGAGCTTGATGAAAGAGCAAGGCGTGGAGAGACTGTTGTCCCTGG

AGGTACTGGGGGCAAGAGCCTTGAAGCTCAAGAGCATCTTGCTGAAGGGAGAAGCAGAGGAGGACAGACA

AGGAAGGAGCAGCTGGGGACAGAAGGATATCAGGAGATGGGCCGGAAAGGTGGGCTGAGCACGACGGACG

AGTCTGGTGGAGAAAGAGCTGAGAAGGAGGGCATCACTATCGATGAGTCGAAGTTCAGGACTGGGAGCTA

GTGTGATGATTAAAACTATAAATGGTATTTTGTAGTAATAAT

>FM892098.1 FM892098 Jatropha curcas embryo 56-70 (DAF) Jatropha curcas cDNA clone rjcaeb0_002575, mRNA sequence

ATTCCTTTCTCCAAGTTGGGAGAACTGATGAGGGGACATGTTACCTTGAGGGATGCTGTTGACTGGGACT

ATAACCTACCATGATACGGCTAGAAACAGAGTTTACAAGCTTAATGATCAACTAGCTAAGCTCTTTGTCC

GTCCAACGAGGCTGGCATCTACCTGACTCTCACATTCTCAATTGATGGCTAACCCGCAACTGGTTGCCTT

GTGGA

>FM892097.1 FM892097 Jatropha curcas embryo 56-70 (DAF) Jatropha curcas cDNA clone rjcaeb0_002574, mRNA sequence

ATTCATCAAATCGGCGTTGAAAGATCAGTTCTCCTTTTTGAGTATCTAAAAATGTCTTCTTGCTGTGGTG

GAAATTGCGGCTGCGGCTCCGGCTGCAAGTGTGGCAGCGGTTGTGGCGGATGCAAGATGTATCCTGACAT

GAGTTTCTCCGAGAAAAACACCGCCGAGACTCAGGTTCTCGGAGTTGCACCGGAGAAGGCACGCTTTGTC

GATGGAGGTGAGGTCGGCGTTGGAGCTGAGAACGGATGCAAGTGCGGTGACAACTGCACCTGCAATCCTT

GCAACTGTAAATGAGATCGGCGGCGATCAGTCTGTTTTTTAGAAGTAAAAGCAGAGATGACTCTGCTTCT

CTCTTTCTAAAAAAGGAGAAATAATTAATAGAGTCTAGATATCGTATGAGTTTGTTCGTATGTATCTGGT

AAAGAGCTTCAATAAAAGTAGTGGTGGCTTGTGAGCTTGT

>FM892096.1 FM892096 Jatropha curcas embryo 56-70 (DAF) Jatropha curcas cDNA clone rjcaeb0_002573, mRNA sequence

AAGCACATGGGTTCTGAGCTGGCACAGAGGGTTGGCATTAATGAAATTGCAGAAAGCATGATGGCTTTTA

ACACCAATTACAAAGACACTGGCCTATTTGGTGTTTACGCTGTTGCCAAGCCGGATGGCTTGGATGATTT

AGCTTGGGCAATTATGCATGAGACAGCCAAGTTATGTTATCGGGTTTCTGACGCTGATGTGACAAGGGCT

TGTAATCAGTTGAAATCTTCATTGCTACTTCACATAGATGGAACCAGCCCTGTAGCTGAAGATATTGGAC

GACAGTTGCTTACTTATGGTCGGAGAATTCCATTCGCTGAATTGTTTGCTAGGATTGATGCTGTTGATGC

AAGTACTATTAAACGTGTTGCAAACAGATTTATTCATGACAAGGATGTTGCAATTGCTGCCCGGGTCCTA

TCCAGGGCTTGCCTGATTACAACTGGTTCAGACGCAGGACCTACATGAACCGCTACTAAAGTGTTGTGCT

TTTTATTTGTTACATTACTGCATGGATACATGCATTTTGTTGTTTTTACAGCACCAAC

>FM892095.1 FM892095 Jatropha curcas embryo 56-70 (DAF) Jatropha curcas cDNA clone rjcaeb0_002572, mRNA sequence

CTAAAAACCCCCTCACAAAATGAAGATTACAGGGCAAAAAACCATCAGTTCCAACTGCAAGCAAAACCCA

ATATCACATTTCTATTGGAAAGCCATTANGATGGAATTTATATGCGTTATATTGTAGATTTGCCCCCAAC

AAGAATGAACAGAACAAAGCCCCCAACAAGAATGAACACAACAAAGCCCCCAACAAGAATGAACACAACA

AAGGTACATATACATACATATATGTATGTATATATGCATATACCGAAACTAACCAACTTAGTATGACCAG

CACCAGAAGGCATGATCTTCAAAACTTTCAGGATTGGAGTAATCAGCATCTTCAAGCTTTGGAAAGACAT

ATGGAGGCAATAAGCTCTTGCTTAAATTATCTTCTTCATCTTGCGATAAATCCGACTGATCTGGTTCAAA

AACATATGATGAATCACCAGCTTCTAAAAAGTGA

>FM892094.1 FM892094 Jatropha curcas embryo 56-70 (DAF) Jatropha curcas cDNA clone rjcaeb0_002571, mRNA sequence

CATTCCGACCAAGGGGTTTCGTTTTTGGCTATGGACTGCATTTGCTGGAAAACTACCCNCTTGGAGAATG

TGAAACATAATAAGAGCTTATTCATGAGGTTTCAAGGAGTTAATGCCGGAGCGAGATGATCTCACCGGAT

GGTGCAGACGAGATTGCGAACCATTGAAGATGTATTTCCAACACCAGTTGGACAAATTGATTAATCTAGC

AATGCTTACTGAAAGATCTCCATTCTATTTTAAAAATGAGTTTGCCTGTATTTTAAAATGTAATTCTCAT

TATAGCTTATGTATTTCTCCATTTTGTTTTGAAGTTACTACTATTTATTTCAATTATATCTATCTAACTA

GGATTTATTCTTGTGGTAACTTAACAACAATGGAATTCATAATAAAAACGAAAATAACTTATAGTTTCAT

AAAAC

>FM892093.1 FM892093 Jatropha curcas embryo 56-70 (DAF) Jatropha curcas cDNA clone rjcaeb0_002570, mRNA sequence

ATTCGAGAGAGAACTAGTCTCGAGTTTTATTATATTTTATTACATAATGTCTAAGTAGAATATTAAAATA

ATAATAATAATAATAGTACTGACCAATAAAGAAAATAAAATATTCATCACCATCTTTACTCAAGCATCAA

ACAAGCCACTTAGTCAACTACCTCGATCTTGGGACCAGCACCACTGCCACCTGCTGGAGGAACATCATCT

TCCATGCCGCCACCCATGTCAGCACCAGCACCTTGATACATCTTGGCAATGATGGGATTGCAGATGCTCT

CAAGCTCTTTCATACTTGTCCTCGAACTCAATCTGACTCTGCAAGCTGGTTGCTGTATAGCCACTGAATG

GCCTGATCCATAACATCTACAATCTTCTTCTTATCAGCTGCGGGGGGGGCCCGGTAC

>FM892092.1 FM892092 Jatropha curcas embryo 56-70 (DAF) Jatropha curcas cDNA clone rjcaeb0_002569, mRNA sequence

ATTCAGAAATTTCAAGCGAGAAGAATGGCTACAGAGCAAGAAAGGGCTGAACTTGATGCTAAAGCAAGGC

AAGGAGAGACTGTTGTACCTGGTGGAACTGGTGGCAAGAGCCTTGAAGCCCAGGAACATCTAGCTGAAGG

GCGGAGCCGTGGAGGGCAGACAAGGAGGGAGCAGCTGGGAACTGAGGGGTACAAGGAACTTGGTCACCGT

GGAGGTGAGACAAGAAGGGAGCAGATTGGGCATGAAGGGTATCAGGAGATGGGCCGAAAAGGCGGACTTA

GCACCATGGACAAGTCTGGTGAAGAACGTGCTGCTGAGGAGGGGATTGAAATTGATGAGTCCAAGTATAA

AACCCGTTAGTTAGGTTCTTCATATCAGTGTTTTAACTAGTAGTTCCTCTTTTTGAACTAGTTGCGAGTA

GGTTTGTACATATGGTAGTAGTAACTGCGGTATGTTGGTTGTTTATGGTTGGTGTCGGGGTTTGTTTTCT

GGGCATCAGTGTGTAACCTGT

>FM892091.1 FM892091 Jatropha curcas embryo 56-70 (DAF) Jatropha curcas cDNA clone rjcaeb0_002568, mRNA sequence

AGAGTAATATTGATTATTGCAAGTAATTAAGCATACATGGCTTTATCTTGTCATCATTATCAGAAAGGCA

CACCAAATATAATTAAAGCTGGAACTTTTAAGTTAAATATATCTCAGGAATTGATCGCAAATTGAACATG

GAACAAGGTCGAATGTTGCATGAGCAGACTGCACTTATTCCTCTTCCTCTGCCTCTTCCAGCCAATTAAC

AAAGGGCTCAAGGGCCTTCACAAAGGTTTGCCTGCCCTTAGGGTTTGTTCCCTTGCGGAACCAGTGAAGA

ATGGTGTCTTCTGCAAGGACATCCTGGTCATAGAGAGACCTCACTATCTCAGGGAACAGCTTCATCAGCT

TAGCATCCTCATAGCACTGCATTTGAACCTTGTACAAGAGTTCCAGTTCAAGTTTTCCATTGGTGCAGAA

GGTATTTACCAGTTCCGCCCATGTTTTCACCTGGCGCAATGCTGCATTCGCATTCTGCTGCTGATTCTTT

CCAGACCACTGAACAGCGTCCATCAATACATCCCATAAAACCCGCACCACCTCATTATCTGGGAATTTAG

CATCGTTAACCCGTTGC

>FM892090.1 FM892090 Jatropha curcas embryo 56-70 (DAF) Jatropha curcas cDNA clone rjcaeb0_002567, mRNA sequence

GGCCTATAATTAACTTTTATGAGAAAGAAGCAAATAAGTAATGACATTTCCAACTGTTAGTTCTATTGGA

TTAACTTCTCACATTCATTTCCTAAACCACTGCCCTTCCCCCTTTTTCACTTTGATATCGTGTTATTCTT

TTTTTTCCCCAGTGTTCCAATAGTCCTACCAGTCAAAATGCCAACAAATAACTCAAAAAATAAAAAAGGG

CATCCCATCACATTGTGATGCCCCGCCCTCCTTTCCCCGCAAATGAGATAAACAAATGCCTAAATTGGCA

TTAAGGAACCTGCAACCATGAATTTTTATGTGTGGCTGAACCATGGTCCAACTGCACCATTCACGTGCCC

ATTGCCATGGATTCCGACACCATTTGGAATTAGTGAAGGAGGCAATCCAACGGGGGGGCCCGGTAC

>FM892089.1 FM892089 Jatropha curcas embryo 56-70 (DAF) Jatropha curcas cDNA clone rjcaeb0_002566, mRNA sequence

TTGTGGCTACCAAGGATGGCCGTATTGGCATGAAGAACCAGATTCAGGTAAATGTTACAGCAGATCATCG

TGTAATCTATGGTGCTGATTTGTCTTCCTTCTTGCAAACTTTGGCAAAGATTATTGAGGATCCCAAAGAT

CTTACCTATTAGGCAACATCAAATCAGGCCTGATTTTGCATCTGTTACATTTCCTTAACCTTGTTGTATG

TTGCCAGAGCTCATTTTTGTCGCCATCAATCCTTGATATCGTGGAAGAAACTTGTGCAGAAAAAATTTAG

GAGCTCAACCACTCTGTTCAGAGTATACAATGAACGGGAGCAGAATAGTGAAGAGCAATGTTTTTTTGTG

TATCAAACATTACAATTTTTGAACTTTTTAATGATACCATAAAAACTGTCCATTACCTGGA

>FM892088.1 FM892088 Jatropha curcas embryo 56-70 (DAF) Jatropha curcas cDNA clone rjcaeb0_002565, mRNA sequence

ATTCGTCTTGACTGTTATGGGTGTCTCTAAATCTGGCAATAAATGTGCTACTGTGGGTACCCAAGGTATT

GCTTGGGCCTTTGGTGGAATGATCTTTGCACTTGTCTACTGTACTGCTGGTATCTCAGGAGGACACATTA

ACCCAGCAGTGACCTTTGGTTTGTTTCTGGCACGGAAGCTTTCTTTGACAAGGGCTTTGTTCTACATCAT

CATGCAGTGCCTTGGTGCCATCTGTGGTGCTGGTGTGGTGAAAGGTTTTGAGGGCAACCGTGTATATGAG

AGTTTGGGTGGTGGAGCCAACGTTGTTGCTTCAGGCTACACTAAGGGTGACGGTCTTGGTGCTGAGATTG

TTGGCACCTTTGTTCTTGTCTACACTGTCTTCTCCGCCACTGATGCTAAGAGGAGCGCCAGGGACTCTCA

TGTCCCTATTTTGGCTCCTCTTCCCATTGGGTTT

>FM892087.1 FM892087 Jatropha curcas embryo 56-70 (DAF) Jatropha curcas cDNA clone rjcaeb0_002563, mRNA sequence

ATTCGAATTCGGAACGTTTAGGAATCATTGCCCCTCTAGCCATCGGTTTAATAGTTGGGGCTAACATTTT

AGTGGGAGGTCCTTTTGATGGTGCGGCAATGAATCCAGCAAGAGCATTTGGGCCTGCGCTGGTCGGATGG

AGATGGAGTAACCACTGGATTTACTGGGATTGGTCCTTTCATTGG

>FM892086.1 FM892086 Jatropha curcas embryo 56-70 (DAF) Jatropha curcas cDNA clone rjcaeb0_002562, mRNA sequence

ATTATCTTTTCCATTCTTTTCTCTCTCCCTTACAAGCCCTAACCCTAACTCAGACCCAGTAAGTTCCGTC

GATTTTTTAACGATTTTGGCTGTTCGATCGATTTAGACCGTTTATTAGCTGATCT

>FM892085.1 FM892085 Jatropha curcas embryo 56-70 (DAF) Jatropha curcas cDNA clone rjcaeb0_002553, mRNA sequence

CTGCAGGATTCGAATTCTATCTTTACCCTTTGCTCTGTAGAGAACGCCATCCGTGGAAAAATGGCGACCG

CCAAGACTGTAAAGGACGTGTCTCCTCATGAGTTCGTCAAAGCTTACTCTGCCCATCTTAAGCGATCCGG

CAAGATCGAGTTGCCTCCATGGACTGATATTGTGAAGACAGGAACACTCAAGGAGCTTGCACCTTATGAC

CCAGATTGGTACTTTGTTAGAGCTGCCTCCATGGCAGGAAGATTATATGAGGGGAGGCCTTGGTGTGGGT

GCTTTCCGCAGATCTATGGTGGAAGCAA

>FM892084.1 FM892084 Jatropha curcas embryo 56-70 (DAF) Jatropha curcas cDNA clone rjcaeb0_002552, mRNA sequence

ATTCATGTTACTGGAAAGCATCACCAGGAGCAGAAAGTCTGGACCAGGCACGCTTAAAGTTGGCGGGAAA

GGCAAGAGAGATGAAGGACAGAGCTGAGCAGTTTGGACAGCATGTAACAGGTCAACAGACTTAAAAGAGA

AGTGGGTTTTGGTGGTTGATGCAGAGAACATAAAATGGCTTGATATTATATACAAATATATGAGTCCTTT

ATTACATGG

>FM893873.1 FM893873 Jatropha curcas embryo 56-70 (DAF) Jatropha curcas cDNA clone rjcaeb0_004808, mRNA sequence

ATTCGGTCTTCCATGTCTGTTTAATTATTGAAGTAAAATGCTCTTCTTCAAACACATAAGGGTTCTTTTC

TTCTCCCCAAACTGTGTTCCCCTTCGTCCCTTCCAAACTTCTGTTGGCACCGGAAGCGACAAAGCGCTCT

CTCTTCTCCCTTTTGTCTCTCGCATTGGCTTTGACACTGTCCTACCCTCCTTTCGGCTTCTTCTTCTTCT

TCTCGTCCTTCTTCTCCATGCTCTCGCCCCTTCTTCTCTTTGTAATAATCCTCGCACTGCTGGATGCATC

TTTTCTTCTCGCCCTCATCATAGTGGCGTTGGACTCTGCATTGGTGTTGGCACTGTTCTAGCTCTGGGTC

TCTTTTAGCTAGAGCCAAGCCAGAACAAAGAACCAAAAGGGAAACGAGTAAAACAGATAAAACAAAAACT

TCATTCTCATAGCCATTGTATGGGGGGGCCCGGTACC

>FM893872.1 FM893872 Jatropha curcas embryo 56-70 (DAF) Jatropha curcas cDNA clone rjcaeb0_004807, mRNA sequence

ATTCGATTGGGACTGAGCAGTATATTGACCGAATAACATCAGAGCTTTACTATTTTAGAATAGAAAAGGC

TAAAAATGGGAAGGGAGAGAGAACCCCAAAAGCTGCAGCAACAGCAGCAAGTCTCCTACACTGTCGAGCA

GCTCGTAGCCGTCAATCCCTACAATCCAGATATCCTCCCCGATCTCGAAAACTATGTTAATGAACAGGTT

TCATCACAAACCTACAGTCTGGATGCGAATCTATGCCTTCTTCGCCTATATCAGTTTGAGCCAGAGAGAA

TGAGCACCCAAATTGTTGCTCGCATCTTGGTTAAGGCATTGATGGCAATGCCAGCTCCAGATTTTAGCCT

TTGCCTGTTTTAGATTCCTGAACGAGTGCAAATGGAGGAACAGTTCAAGACATTGATTGTTCTCTCACAT

TATCTGGAGACGGGAAGGTTCCGCCACTTCTGGGATGAAGCAAGCCAGAGTCGCCACATTGTTGAATGCT

GTGCCGGTTTTGAGCAAGCAATCCAAGCATATGCAATTCATCTGCTTTCTCTGACTTATCAAAAGGTTCC

C

>FM893871.1 FM893871 Jatropha curcas embryo 56-70 (DAF) Jatropha curcas cDNA clone rjcaeb0_004806, mRNA sequence

CGCTGCGATCATACAATAGGAGCATTAAAAACAATATCCACACCCCACATCAGGAAAGGGTTGCAAATCC

CGAGTTAATAGAAGCACAAGTGCAACTAGAAAGGGTAAGAAAATAAATGAAGGAAACAGAATATCCACAG

GATCCACAAATTGACAATTAATTGCTCAAGCCAATTTGCCAATATTTAATTCTAGGTTT

>FM893870.1 FM893870 Jatropha curcas embryo 56-70 (DAF) Jatropha curcas cDNA clone rjcaeb0_004804, mRNA sequence

ATTCCAAAACTTCGATGAAGGTTGCCATTATCAAGAATCCATCATTCAAAAGTGTTGACGGTGAAAGATG

CTAGATGGGATGTTGATGATTTACATTAAAAAGTTAACTATTTTTGTTTGTGCTGTTAATTAGAAGGTTT

ATTTTGAAGTTTTAGTTCATTATATGTTAATGTTTGTAGCTCTGTTCAGATCTATTAATTGCTTATTATC

>FM893869.1 FM893869 Jatropha curcas embryo 56-70 (DAF) Jatropha curcas cDNA clone rjcaeb0_004803, mRNA sequence

ATAAATAAAATATCTACTAAAAGATACCCATTCAACAACTGGGACAACCGGTTACCCTACAGGGACCCAG

CAAATGTCGGCCATGCCTGGACACGGCACAGGGCAGCCATACGGTGGAGAGGTGGACCCAACGGGTTTGA

CCAGGACTCGCCCATCTGGGTTGCCCGGAGACACCACTGGCCATAACCCACGTGTGTAGGGCATGGCATG

TGCTTTAGCTCCTGTTATGTAATATATCTATGTTTTCTGTTTGGTGTCTTTTTCTTGTTAAGCAATTCCA

GTTGAATAAAAGGTATCTTTTAGTAGATATTTTATTTAT

>FM893868.1 FM893868 Jatropha curcas embryo 56-70 (DAF) Jatropha curcas cDNA clone rjcaeb0_004802, mRNA sequence

AAAAGAAGTTTCTGCAGCTATAATTCCAAGTAAATCTAGCACCCGGTTAGAGTGAGATACACCCTTCCAA

GAATTACAGACATAAGATTCAACAAAGCCAACTCTAGTTCACAAGCACATCAAAAAGATAATCCAAGACA

AAACTTGACAACCAAATGGGTTTATAATTGCAAGATACCAAAACCAATCTATTTAGCAAAAATTGCCCTA

CACCTTGATAGCATACTTTCTCATGGGGAAGTACATTTCTCTCTTCTTCTCACGTTCTGTCTTCAACGAT

TGCTGGTGTTTGGTAAGCCTCCTGCGGATGGCTCTTGTCTTCTTCGGACGCAGATCAAGTGGCAAGAACT

TCTTGTTCTTGTATTGCG

>FM893867.1 FM893867 Jatropha curcas embryo 56-70 (DAF) Jatropha curcas cDNA clone rjcaeb0_004801, mRNA sequence

ATTCGGAATCCTCATAAGTTGATCTCATAATACAAACATAAAACAGTACAAGAAGAACACATTCGACCAA

ATTGAAGTAAAATGTCCGGTGTATCTCTGGCTACAGCTGCTAGAAGTGAACTTGATGGTACTACAACTAA

ACCTGCAAATCCCAAAGAAAAGCAACAGCAGGCGATGATGGGGGGTATAATGGGATCGTTGCGAGTAATA

GAACTCCAACTAGTTGCTTTCATAATGGTTTTCTCAGCTAGTGGCCTTGTTCCACTTCTTGATTTGGTGT

TCCCAGCTCTTGCTTCCACTTACATTTTAATCCTAGCTCGATGGGCCTTTCCTACACATGGCAGAGCCAA

TACAGGCTCTCAAGAGATTTTTCAAGGAACCAAATTCTTTAGGTTTTATGTTATTGTGGGAACATCAATA

GGCCTGTTCTTGCCACTGGCATATGTATTAGGAGGGTTTGCTAGAGGCGATGGTTATGCTGTTCGGGTCA

GCGACACCTCACTTGTTCTTGCTATCATTTCAGATACTTACTGA

>FM893866.1 FM893866 Jatropha curcas embryo 56-70 (DAF) Jatropha curcas cDNA clone rjcaeb0_004796, mRNA sequence

ATTCAGATTATTGGAGTTATGCAATTGGAAGTAACAATATGGACTTCTCTAAATTCAAATCCTTGAGAAC

TGGTCGTGGTCCACTTTTGGATGGATGGCAGGATAGGTGCAATCCCGTTATGACAGCATACAAGTTGGTA

ACAGTAGATGCACCATACTGGGGCTTTGGTTATCGACTTGAACAGGCTTTATTAGCGGGGGAAAGGGCTC

TTTTCTTAGAGAGTCATCGGAATTGTTTTGTTTGGATTGATGAATGGTGTGAGATGACAATGCAACAATT

ACGTGAACTTGAACGACAGAGCGATTCATCATTAAATAAGAAAATTGGGATGCCAACAGCAATGGCAACA

GCAGAAGAATTCGACCAGAGATTGTTGCTTGACAATGAAGATGCTAACAGCAAGAGGATTCAAGTTGCTG

TTGATACTTAGGAAACAAAATTGGATGAAGTCTCCATATTGGAATTGTATTGTAATGTTATTAGAATTAT

TTAATTTG

>FM893865.1 FM893865 Jatropha curcas embryo 56-70 (DAF) Jatropha curcas cDNA clone rjcaeb0_004795, mRNA sequence

ATTCCACTGATTCTAAGCTTTCTTTTTTTCATCAACACCATGTCTAGCACCTGCGGCAACTGCGACTGCG

CTGACAAGTCTCAGTGCGTGAAAAAGGGCAGCAGCTACACTGCTGACATCGTTGAGACTGAGAAGAGCTT

TGTCTCCACCATTGTCATGGATGTTCCGGCCGGCGCTGAGAACGACGGCAAGTGCAAGTGTGGTCCAAGC

TGCACCTGTGTGGACTGCGGTTGTGGTCATTGAAGCATATAATAAAAATGAAAGAGTGGTGTGTGAGAGT

TTACTAATAATGTAATTGTGCTAAATAATTTGTATTTCCTGTTAAAATACATGTGGGTGTGAAGTGAAAT

GTGTGAAAGAGCCATGTCTCTGTTTCCTTGCATACCTTGTGTAATGGCCATATCTATGGCTTCTTGTGTG

GTTTAATGAATTACTCTTAGTAT

>FM893864.1 FM893864 Jatropha curcas embryo 56-70 (DAF) Jatropha curcas cDNA clone rjcaeb0_004794, mRNA sequence

ATTCCTTCTCTCAACCATATCTCGNAGAGAATCATCGGATATTAGGCGCCTTGCTAACTTCTACAAAGAG

ATCTTTGGGTTTGAGGAGATTGAGAGTCCAGATTTCGGAGAATTCAAAGTTATATGGCTTAATCTCCCTC

AAGCATTCTCTCTCCACCTAATAGAGAGAAGCTCTATCACGAAGCTTCCGGAAGGCCCGTACAGCGCCAC

TTCACCGGTTCTTGATCCTAGCCATCTCCCCAGAGGCCATCATATCTGCTTCTCCGTTTCCAATTTCGAT

TCCTTTGTTCAAACTCTCAAGGATAAGGGAATTGAAACATTTCAAAGGTCTGTTCCTGGTCGACCAATTA

GGCAGGTCTTCTTCTTTGATCCTGATGGTAATGGATTGGAGGTTGCAAGTCGTGATGGATAAAATTGCAC

TCCACTCAGGAATGTGGTTAATGTATAATGGAGTAGAAGAGGGAAAGATAAATAAACAAGAGTAATAATT

TGTACTGAGATTTCCAAATGTGCTGTGAAATTTTGTTTTAAAAAAAAAGGGGGGGCCCGGTACCAATTTC

G

>FM893863.1 FM893863 Jatropha curcas embryo 56-70 (DAF) Jatropha curcas cDNA clone rjcaeb0_004793, mRNA sequence

ATTCGTCTTCTTGCTGTGGTGGAAATTGCGGCTGCGGCTCCGGCTGCAAGTGTGGCAGCGGTTGTGGCGG

ATGCAAGATGTATCCTGACATGAGTTTCTCCGAGAAAAACACCGCCGAGACTCAGGTTCTCGGAGTTGCA

CCGGAGAAGGCACGCTTTGTCGATGGAGGTGAGGTCGGCGTTGGAGCTGAGAACGGATGCAAGTGCGGTG

ACAACTGCACCTGCAATCCTTGCAACTGTAAATGAGATCGGCGGCGATCAGTCTGTTTTTTAGAAGTAAA

AGCAGAGATGACTCTGCTTCTCTCTTTCTAAAAAAGGAGAAATAATTAATAGAGTCTAGATATCGTATGA

GTTTGTTCGTTTGTTTCTGGTTTTGAGCTTCAATAAAAGTAGTGGTGGCTTGTGAGCTTGT

>FM893862.1 FM893862 Jatropha curcas embryo 56-70 (DAF) Jatropha curcas cDNA clone rjcaeb0_004792, mRNA sequence

ATTCATTTCTTTTCTCTCTCTCATTTCTAAAGGGGAAAAAATGCAAGCAGCAGCTGCTGTAGCAGCCATG

GCTGCTCATACAATCCTTGCAGCCTCTCCATCCTCTCATCCTCTCCTCTATCCATTCCCAAACCCTATCC

TTTCCCACTCTTCACCTCTGCATTCCTCATTCCATGGTGTTTCTCTCAAACTTCCTCGTCAATCGCTACC

TCTTTCTCTTACCACCGCCGCCGCCCCAAAAAAACCCCTTGCTGTCGTTGCTGCTACCAAAAAGGCCGTC

GCTGTCCTTAAGGGTACCTCCAATGTTGAAGGCGTTGTCACTTTGACCCAGGAAGACGATGGTCCCACAA

CAGTGAATGTTCGTGTCACTGGCCTTACTCCAGGGCCTCATGGATTCCAACTACATGAGTATGGTGACAC

AACAAATGGATGCATTTCTACAGGAGCTCATAACAATCCGAACAACAAGACACATGGTGCTCCTG

>FM893861.1 FM893861 Jatropha curcas embryo 56-70 (DAF) Jatropha curcas cDNA clone rjcaeb0_004791, mRNA sequence

ATTCCNTGAGTCAGGAACGAGTGTGAAACGAGACGGGTTATCACTCGGAGAAATCGTTTACGAGGTGGGT

CACTCATGTTAGGTTATCTGAAAGATCCGGCCAGCACATCAAAGTGTATGAAAGAGAACGGCTGGTTTTA

CACCGGCGACGTCGCTGTAATGCATCCAGATGGTTATGTAGAAATTAAAGATAGATCCAAGGATGTTATT

ATTAGCGGCGGAGAGAATTTAAGCAGCGTTGAAGTTGAGTCAGTTTTATATACGAACCCCTTAATCAACG

AAGCAGCAGTTGTGGCTCGACCGGACGAGTATTGGGGCGAGACGCCATGTGCGTTTGTTAGTTTGAAGAA

AGGCGAGTCAATCATAACAGAAAAGGATATAATTGATTATTGCAGAGCTAAATTGCCACATTACATGGTG

CCAAAAACTGTGGTGATTAAAGACGAATTGCCAAAAACGTCGACGGGTAAGATTCAGAAGTTTGTGCTTA

GAGAAATGGCTAAGGCAATGGGGTCGTCAAAAGTGAGTCGCATGTGACTCAATTT

>FM893860.1 FM893860 Jatropha curcas embryo 56-70 (DAF) Jatropha curcas cDNA clone rjcaeb0_004790, mRNA sequence

TGAAAATGGAGTCGCTGAAGGAAACCGCAGCTAACGTTGGAGCCTCTGCTAAGGCTGGCATGGAAAAAAC

CAGAGCAGAGGCGCAAGAGAAGGTGGAGAGAATGTCAACACGTGATCCAGTGCAGAAGGAAATGGCGAGA

GAGAAGAAAGAAGAGAGGAAAACGGAGGCAGAATTGCGGAAACTAGAAGCGCGCGAGGATAACGCAGCGT

CGAGGCAGGCAGCCAAGGCTGGTGGCCATGTACAGTACACAACTGCTGGTCCGGGCGCTCAAGGCACAAC

CGAAACCTATACCCATTCAACAACTGGGACAACCGGTTACCCTACAGGGACCCAGCAAATGTCGGCCATG

CCTGGACACGGCACAGGGCAGCCATACGGTGGAGAGGTGGACCCAACGGGTTTGACCAGGACTCGCCCAT

CT

>FM893859.1 FM893859 Jatropha curcas embryo 56-70 (DAF) Jatropha curcas cDNA clone rjcaeb0_004788, mRNA sequence

CGGAATCCACTGGAACATCAATGCGCATAGCATATATTACTTTACAAGAGGAAATGGTCACGTCCAAATC

GTAGATGAGAATGGAGATGCAGTGTTCGATGGACAAGTTCAACAAGGTCAAATATTAACAGCTCCACAGA

ACTTTGTGGTTTTGAAGAGGGCAAGCAATGAAGGGCTAGAGTGGGTATCCTTCAAGACTAACGAAAATGC

ACAAATTAGCCCACTAGCAGGACGGACCTCGGCAATTAGGGCTCTGCCAGAGGCAGTGGTTGCAAACGCA

TTCCAGATTTCCAGAGAAGATGCCAGGAGGCTTAAGGAGAATAGACAGGAAGTCACTGTGTTTAGCCCTG

GGTCTAGATCTCGTAATTAATATTGCAACTATGCAATTATACCAAGAGGATGTATAATAAACTGGTTTCT

ACATAGTTTAGATTTGGAGAAGAATAAGCCTATAATTATAGGCAGAATTAGTTCCTTGCAATAATATAAA

AATGTAGATCTGT

>FM893858.1 FM893858 Jatropha curcas embryo 56-70 (DAF) Jatropha curcas cDNA clone rjcaeb0_004787, mRNA sequence

TCGAGAGAGAGAGAGAGTGGCTTCTTTGGATTGTTGTAGATATTCCAGAAGGATCCGATGCTACCAAAGG

GCATGAGTTGGTGTCGTACATGGGACCTCAGCCTCCAACCGGCATTCACCGTTATGTTTTTGCGCTGTTC

AAGCAGAAAGGTGCATTGATGGGAAGGATTCAACCTCCAGATGGTCGTGGCAACTTCAACACCCGCCACT

TTGCTGCCCAAAGTGGACTTGGGCTTCCGGTTGCTGCTGTGTATTTTAACTCGCAGAAGGAACCGGCAGT

TAAAAAACGTTAATTCTGTTTAGTTCTTTTTTTTTTTTTTTTAACACGGGATAGCAACATTGGGATTACG

GTTATATGTTTACGCTGCGTCCTGTATTTGCTAGTTTTGTGCTGTTAATGCTTGTATTGTTCTGTTTTAC

TTTGCTTTTTGTAAAAAAAAGGGGGGGCCCGGTAC

>FM893857.1 FM893857 Jatropha curcas embryo 56-70 (DAF) Jatropha curcas cDNA clone rjcaeb0_004786, mRNA sequence

ATCGGTCTCCTCTTGTTCTTGTCCAAATCATTGACACTAGCAATCCTGCCAACCAGCTAGATCAAAGTCA

CAGATTGTTCTTCCTTGCTGGTAACCCACATCGAAAAGTACAGAGCCAGAGAGGTGAGTCCGAAAGAAGG

CATGAGTGGTCCAGAGGCGAAAGCCTAAGGAGTCATCAAAGAAGCGGCAATGTGTTTTCTGGCTTGGACG

AGCAACTACTAGCTGAAGCTTTCAATGTCAACCGGGATATTGCAAGAAGACTAAAAGGAGAAAATGATAA

CAGAGGGATTATTGTTAGTGTGGAGGGTGAGCTTGAGTACTTTAGCCCACGAAGGAACCAAGAAGAAGAA

AAACGAGAGAGACAATTCGTTAATGGCATAGAAGAAACTTTCTGCACAGCCAGACTCAAGCACAATATCA

ACGACCCATCACGTGCCGATATCTATAATCCACGTGGTGGCCATGTCACTAATGTCAATAGCCACAACTT

CCCGATCC

>FM893856.1 FM893856 Jatropha curcas embryo 56-70 (DAF) Jatropha curcas cDNA clone rjcaeb0_004785, mRNA sequence

ATTGGACAACCGATAGCGTGATGGGACTTCCCAAAGCTCTTACTGAAAAACTTATGAAGGAGGCTCTCTA

GCCTAGTGCAGCTATTATCTTTTCCATTCTTTTGGTATTTCCCGACTGACATCCTTTGCTTTCCTTTACT

GTTATGCTTCCTCTGAACATTGCTTGATTTGAAATGGTTAAACAAATTATTGGTTATCGTTCCCCATCAG

CTGATAGAGTAGGCACTGATTTTCGTTTATCAGGGATGACTAGTTACTTAAATGTTGAGCTGTGAACAAA

ATGCTATTTGCCAAAGTTGCCCTTTTGTTAAAGCTACTTGATAGGAATATATAGTAGACTGCATATTTTT

ATGTTACTGGGCATGATATGAGTAGAGAAAATTTTATTT

>FM893855.1 FM893855 Jatropha curcas embryo 56-70 (DAF) Jatropha curcas cDNA clone rjcaeb0_004784, mRNA sequence

AAGGTTAAAAAAATATATATAATAAAATAAAGGAAAGGAAAAAGAAACATCAAGTTCAAGATTCTACATC

TTTCCATACATCATCGTTATGCAAATATAAGTGAAATCAATCCCATCCCACTGACAGAAAACTAAAATGG

TCTAAAATCCTATAGCACAAGGCAAAGAGCGTTTGAGAATCTTATACTCAAATACTATGAGTCTTGATTT

CTCAGCCTTGATTCAGATAATCTCCTTAACGTTAATTTATTGCTGAGCACATTGTACCCTCTGGGAACCA

CCATGCATATCTTCATCTTCATCATATGCCTCTGCAGCATGTTGTTGTTTGGGGGGGCCCGGTAC

>FM893854.1 FM893854 Jatropha curcas embryo 56-70 (DAF) Jatropha curcas cDNA clone rjcaeb0_004783, mRNA sequence

TCCAAAACCCCCACCATAATCATGCAAGAACAGAAATTGGATATCAACACCTCCCCCTCCCCCTCCGCTG

ACAGTGGGCGCGTTAAGAGCTTCCCGGCTGAAAATATAAGGCTGAGAGATAAGAAAGTGAGAGACCTCAT

GA

>FM893853.1 FM893853 Jatropha curcas embryo 56-70 (DAF) Jatropha curcas cDNA clone rjcaeb0_004782, mRNA sequence

ATTCCATGCCCCTTATAAGTGATCTCATGCGTGTGTTGGTGGGAGAGAGAGCATAGAAGAGAATTAAGAT

AAAGAATTGAGTGGAACATTTGTGCGGTGAAGAACTATACCTTTTACTGATGCTTGTTATTGTTCGGCAC

TGTTTCATTTTTCGTTTTTAACCCGAGTTTTCAAGACGAGTCACTTTAACTGATCAGTGATTATTGCAGC

GGTATACTAGTTAAAAAACTCCAGATGGAACTCACTACT

>FM893852.1 FM893852 Jatropha curcas embryo 56-70 (DAF) Jatropha curcas cDNA clone rjcaeb0_004781, mRNA sequence

TCTTTTTTTTTAACGCAAAACAGTTTTATTCATGCAACGAATGTAATGTGATGAATGTGGTATCACCATT

CAATCAACGATTTTTATTCTTCCTCAAACGCACGTTTCTAGCACAGTCTGATTTTCGCGCATATATCCTG

CGATTTTTGCTTCTGTATGAAATCGAAGACAATATCGATTCCCTTGCCAACCAACGCTTCGCACAAACCC

TTGACAGGACCAAACGCTGCACAAACGCCTTTCATCAAAACTTCAACCTCGTTCCTGACAGCGTCAATTT

GCAACAGTTTTTTCAATTGATCCAAGGCTACAGTGCATACTTCACACGGACTAACATCTGCATCTGGTTC

CTGGAATTGCACCTCAGCGTTGGCCAAAGCCACAGTTGCCAGAACCAACAAAATCAGTGGATTCATTTTG

GGGGGGCCCGGTAC

>FM893851.1 FM893851 Jatropha curcas embryo 56-70 (DAF) Jatropha curcas cDNA clone rjcaeb0_004780, mRNA sequence

CAAATAAAACCCTTCCATAGAATATCAACAATGTATGAAAAATCATACCCAACCTTCAAGACAAAGAACA

AAAGCATCATTTGCTGGCGTTGAAGTCATGATTCTTGACATAGATTTATTAGGCACACACACAAAAGAAA

AAAAAAGGTAATACCAACTCCTTAATGCATAAATTTGATTTTAAAACTATGCATTAGGACTTAGCATTTG

GTCGAGTTTCGTCCTATTTCCTTCTCCTTCACTTCACTTCCCTGGCTCCTGTCATAATCGGGGTGATTCT

AATGGATTTTCTTGCCATTATTATCGTCATCGTTATCACATGATTGATTTCCTCCATCCATTCCACCAAA

TCCTTCTT

>FM893850.1 FM893850 Jatropha curcas embryo 56-70 (DAF) Jatropha curcas cDNA clone rjcaeb0_004779, mRNA sequence

ATTCGTGAAAGAGTGGAAGCAGCAGGCGGAAGAGTCATAGACTGGAACGGTAGCCGCATCCTAGGCGTAC

TCGGAACTTCAAGATCAATAGGAGATCTGTATCTGAAGCCTTTTGTGACATCAGAACCAGAGGTTACAGT

AACTGAGCGAAGTATATCAGACCAATTTATCGTGATAGGAACAGATGGGTTATGGGATGTAGTTTCCAAC

GAGGTTGCTTGTGAAGTTGTGAAGAAATGTTTTGATGGGCATATTAGAAACAAGAGATTCTTGGATGAGT

TTAATGGAACAAGGGCTGCAGAGGCAGCAGCGATTCTAGCCGAATTGGCGATGGCTCGAGGAAGCAAAGA

TAATATCAGCGTCATAGTAGTTCAACTGGAAAGCCCCAAATAGCAGCTGCCGGCCCGCCCGCCCGCCTCT

TTCGTTACGCCAATTTTGTTTATATGTATTTTACCTTTGGGCTCTGTGTGTATTATCGTTGCGTTTCATT

TTCTAAATTGTGGTTAAAGTCGGCTGGCAGCCTGTGTGTGGTATCTACAAGATTCAAATGC

>FM893849.1 FM893849 Jatropha curcas embryo 56-70 (DAF) Jatropha curcas cDNA clone rjcaeb0_004778, mRNA sequence

ATTCCTCCCTATGCTCTGGATCAGGCCATTCGGCGGCGTTTTGACAAGAGAATATACATTCCTCTCCCAG

ATTTGAAGGCAAGGCAACATATGTTTAAGGTGCATCTTGGAGATACTCCCCACAACTTAACTGAAAGTGA

TTTTGAGCACTTGGCTCAGGAAACAGAAGGATTTTCAGGTTCAGATATTTCTGTTTGTGTTAAAGATGTA

CTCTTTGAACCTGTTCGTAAAACCCGAGATGCTAAATATTTTATGGAGACTTCTGATGGCATGTGGCTGC

CTTGTGAACCAACCCAACAAGGAGCTATCAAGATCACACTAGAGGAACTTGATGAACAAGGCCTTGCTTC

AAAGATCCTTCCGCCGCCGATAACAAGAGCAGATTTCGATAAAGTGCTTGCAAGGCAGAAGCCAACAGTG

AGTAAAGCTGATCTTGAAGTACATGAAAGATTCACAAAGGAGTTTGGGGAAGAGGGCTGATAACTTTTGG

CAGAAGAT

>FM893848.1 FM893848 Jatropha curcas embryo 56-70 (DAF) Jatropha curcas cDNA clone rjcaeb0_004777, mRNA sequence

ATTCCGAAATCCTTTGAAGAGAAAAAGATCGGCGTTAAAGGGTTAGGGAGGAAGAAAACAGATGAGTCAG

AACACTTCATGTGGGAAAATGACATAAAGGTTCCTTGCGGCAGCTTGAAGCTATCAGAGCATAAAGATAG

TCCTCTGGAATATAATGAGTATGCTGTCTACGATCCAAAACAGACATGCATAAGGTTCCTGGTGGGAGTC

AAGTACGAGGAGAAGGATGTGATTATGGACACTGCAGAACCATGAGTTGAAGAAAGGGGACTAACAAAGT

TCTTTTTGTGTATTCCTAGCTTTTTGCCATTCTCTTGTTCTGGGGACAGAGGAACATAGACAGGGGACTT

TTTTTTTTTTTTGCTAAGGTTCTAAGTTTTTAACATCTAATGCTCTCAAGTATATGTTGATGGATTTTGT

TTGTATATAACATAATCATCACCGTGGCT

>FM893847.1 FM893847 Jatropha curcas embryo 56-70 (DAF) Jatropha curcas cDNA clone rjcaeb0_004776, mRNA sequence

ATTCGGAAGCTTATAATAATGTAAGCATATTATATCTACTTGCTGTCATATCACAAATCGAGATTAACTT

AATGATATGTAATGTTTGATGCTGTTTCTCTATAGTTTAAAGTATCTGTTTCTCTATAGACCAATGTTTA

GATGAAACTGCAATTTTTCATGCACATTTTTCATGTATATTGTTTTTGCTCCATTTAGCTTTCGTGTGGT

GGCCTCAAGGCCAAAATTAGAGAAAGGAATAACCTTTCTATGACTGCGTGGCATGTATTAAACGATTGAA

TAAACTGGTTATGATTGAGATTAAATTAGTTTTGTCGT

>FM893846.1 FM893846 Jatropha curcas embryo 56-70 (DAF) Jatropha curcas cDNA clone rjcaeb0_004775, mRNA sequence

TCGAAAGGTTGAAATCAGGAGCGGGGAATCAGCGGTTCTGCAAGACAAGAGAGGCTGTTGCTGTGGATCT

GCTTGAAAGCCGTTTCCCCTTTTTGACCGCTATTTACGTCCGATCGTGATTTTCCCTTTCAAACCCTACT

CTTCAACTTCACTTGCAGGTCC

>FM893845.1 FM893845 Jatropha curcas embryo 56-70 (DAF) Jatropha curcas cDNA clone rjcaeb0_004774, mRNA sequence

ATTCTCTCTACCTAAGCATTCTCTACCTTCTCTCCTTCCTACTCAGAAACCCAGACACTCTCCTTCTCAA

AATCTCCCAATTTTCTCCAAAGCTTCACAATCCCAATTTTATGGCCTCAAGCTCTCTAATTTATCTTCTC

TCACAATTCCATCTTCTTCTTCTTTGAAGACTACCATTTTTGCTAAGGTGAATAAAGGACAGGTGCCTCC

GTCTTTCACATTGAAAGATCAAGAGGGGAAAAATGTGAGCCTTTCTAAATTCAAAGGGAAGCCTGTAGTT

GTTTATTTCTACCCAGCTGATGAGACCCCTGGCTGCACCAAACAGGCCTGTGCTTTCAGGGATTCTTATG

AGAAGTTTAAGAAAGCAGGAGCAGAGGTTATTGGAATCAGTGGTGATGATACATCGTCGCACAATGCTTT

TGCGAAAAAATATAGACTTCCATTTACATTGCTGAGTGACGAAGGAAATAAAGTGAGAAAAGAGTGGGGC

ATTCCAGGAGATTT

>FM893844.1 FM893844 Jatropha curcas embryo 56-70 (DAF) Jatropha curcas cDNA clone rjcaeb0_004773, mRNA sequence

ATTCCGCGATATTTGCAGCGCTGGAGTGGAAACAAACGACACAGTAAAGGCATAAGGAGGAGTTGCATCG

TCGAAGGTTTTCCGTCTGCTTTCTTGATAAATCCTCTTGGGTGCCGCGATCCAAACGTATAGGTAGCTAC

GTATCTATACGCATATGAATCTCCGTATTATCAGTTGATTTTGCATATTTTGTGATTGACATTTGAAGTT

AGGGTTTCTGGGATTGATTTACTTGAATCGGACATTTTGAATCCACAATCAATACAGAGGTAGAATTAGG

AGTCGATCTACTATGAGTTGGATTTAAGTGTTTATTCAAAGCAGAAGTTCACCTATATCATACTTTTCTA

TTAAAATAGCTTTTATAATTGGACAAGGAAGACCACTTTCTTAATATCGGATGTTGAAGTAGATGTTTCT

GTTCATAGATGGCGCTGCAAGAAAAATTAGATAGATTCAAGAAGCAGCAAGAGAAGTGCCAAACGACTCT

TACCAGCATTGCGTCCAGGCCTTCTAAACCCGCTGCAGCTCAAAAACATGTACCTTTAGCTGGCCCAACA

AATCCAAGGACTCCAGGTCCACCTGGTCAATTTTCAAATGACACGGAGAGACTCAACACATCAACAGCAT

TCGAAAAGCGCTG

>FM893843.1 FM893843 Jatropha curcas embryo 56-70 (DAF) Jatropha curcas cDNA clone rjcaeb0_004772, mRNA sequence

ATTCAGAAACTCCGAGCAATCTGAAGACGGAACAGTGAAGTTCTTCCCAAGTTTGGTTGCAAGGCAAATA

ACTGAAGGCGGTGATATCCTTACACTAGTGGATCCTAGACTAGGTGGCAATGCTGATTTGGATGAGCTAA

CAAGACTCTGCAAGGTCGCATGCTGGTGCATCCAAGATGATGAAACCCAAAGGCCATCAATGGGACAAGT

GGTTCAGATTCTTGAGGGAGTCTTGACTGTGAATCTGCCTCCAATTCCAAGATCTCTCCAGGTTTTAGTT

GATGAACAGGAACACATAATTTTCTTCACAGAATCATCTTCTGGCCAGAGTTCGCAGTCGCAATCACAGA

CACAAAGCAACAACTCAATTGCTTCTTCGCAGGCCAAGAGCAGCACATCATCTATTACTT

>FM893842.1 FM893842 Jatropha curcas embryo 56-70 (DAF) Jatropha curcas cDNA clone rjcaeb0_004771, mRNA sequence

TCCAGGTTCGTAGGTCTCGATTCACCACTTCTCCACACTCTCCAGCAAATGATGGACATTGGCGACGATG

CTGACCACAAATCCTTCAACGCGCCAACACGCACTTATGTGAGGGACGCGAAGGCTATGGCTTCTACTCC

GGCCGATGTCAAAGAGTATCCGAATTCTTATGTGTTCATTATCGACATGCCTGGATTGAAGTCTGGAGAT

ATCAAAGTGCAGGTGGAAGATGATAATGTGCTGTTGATTAGTGGAGAGAGAAAGCGTGAGGAGGAGAAGG

AAGGAGCTAAGTATGTGAGGATGGAAAGGAGAGTTGGAAAATTGATGAGGAAGTTTGCTCTGCCTGAGAA

TGCCAATACTGATGCCATTTCTGCTGTGTGTCAAGATGGGGTTTTGACTGTCACTGTCCAGAAATTGCCA

CCGCCTGAGCCTAAAAAGCCAAAGACTATTGAGGTTAAAATTGCTTGAGCTCAGAGGATCGTTTTGTCAC

GTTTGACTGCGTGCTGGAAAT

>FM893841.1 FM893841 Jatropha curcas embryo 56-70 (DAF) Jatropha curcas cDNA clone rjcaeb0_004770, mRNA sequence

ATTCAGAAAAAATGGAGAAGCTTACAACCTCAGCCGCTCTACTCTGCATTCTTGCTAGTGCACATAGCCA

ATGCAGTCTCCGGCTATGTGACAACCACCATCACCACCGTTGAAATCGACGATGATTCTTCTAACACTAG

TCAGCGAAAATGCAGGAGGGAAGCTGAAAGGGCAGATTTGAGCTCCTGTGAGAGTTATATGAGATCGTCA

AGAAGGCCGTCAGAAGAGATGCTAGCTTTGCGAGGAATTGAAAACCAGCAACAACGGGATCTTCGCCAAT

GTTGTAACGAATTAAGGCAGCTAAGTCATGATTGTCCCATGTGAAGGAATAAAGTATGTATTTAGAGCAG

CAGCTGGAGCAAGGACAGCGAGAGCGTTTGGAGGCAGTGCGTAAGGGCTAGAAGTATACCTTCTGCTTGT

GGCCTGCGTACAAGCTGCGATATTCGTCCCTATTCTGTTAGCTATAACGTGGATGTGCTTCAGTTAAATA

AAATTTCACTCCACTTGGTGTGTGAGTGA

>FM893840.1 FM893840 Jatropha curcas embryo 56-70 (DAF) Jatropha curcas cDNA clone rjcaeb0_004769, mRNA sequence

ATTCCTCTTCTAATACCCAATATCTCAAACTCAATCACTACATTTGGAGTTTGAAGGAGAATGGCCAAGA

GCAGCTTTAAGCTAGAACATGATTTTGAGAAGAGGCGTGCTGAGGCTATGAGAATTAGGGAGAAATATCC

AGATAGAATTCCGGTAATTGTTGAGAAGGCTGAGAGAAGTGACATTCCTAACATTGATAAGAAGAAATAC

TTAGTCCCAGCTGACCTGACAGTTGGTCAGTTTGTTTATGTGATCCGGAAGAGAATTACACTGAGTGCAG

AAAAGGCAATCTTCATATTTGTGGACAATGTCCTCCCTCCAACGGGGGCAGTGATGTCTACTATCTATGA

TGAAAAGAAGGATGAAGATGGATTTTTGTACGTCACATACAGTGGAGAGAACACTTTCGGGTGGCAGATG

ATACTGTAGATAAAGTTCTTTCCTTGTATTCCATTCTTTGTGTTGCTGTCCAGTATGTCTCTCATCATAA

TGACTTGGCAAAATCTTTGCTAGTGACTAGTCTCATTTAGTTGAAAGGCTATGATATCAAGCCTAATTA

>FM893839.1 FM893839 Jatropha curcas embryo 56-70 (DAF) Jatropha curcas cDNA clone rjcaeb0_004768, mRNA sequence

ATTCAGAACCAGTTCGAACCCACCTCCGCAATATGATTATTGTTCCCGAAATGATTGGAAGCATTATTGG

TGTGTACAATGGGAAGACCTTTAACCAAGTTGAAATCAAGCCTGAGATGATCGGGCATTATCTTGCTGAG

TTCTCAATCTCATACAAGCCTGTGAAACATGGTAGACCTGGTATTGGTGCCACCCACTCGTCTAGGTTCA

TTCCTCTTAAGTGAAATGTTTTGCTGCAGCTTGGATACTCTAGTTATTTCCTATTACATGAGGACGGCTA

TGTTGATCAATATTTTGTCTATTTAAATTGTTGCTTTATATGTCTAGGTCTTTAAACAATTGTTCTAGGG

ATTTAGCTTTGGTTTTGAATTATTCTGGATATTTTATGATAATATAAAGTATGCTCTTGGATTTTACAAT

TTT

>FM893838.1 FM893838 Jatropha curcas embryo 56-70 (DAF) Jatropha curcas cDNA clone rjcaeb0_004767, mRNA sequence

ATTCCAAAATGAGTCCTGAAGAAGTGAAGGCAGCAGGAGTAGAGGGCTCCAAAAGGCCACCAGGCCATAA

CCCAGGAGGGGTCTTGCACCAGAGGCGCAATTTGCCTTTCAGCAAAACCACCATTACAGTAGCTGGCTTC

TTTATCACTGCTGCTATTGGATACATGGTCTTGTATGCCAAGAAAAAGCCTGAAGCAAGCGCTAGAGATG

TTGCCAAAGTTGCCACTGGTACTGCTGACCCTAGAGATACTCATCCTAGGAAGTAAATATAACTGTAATT

TTGTGTATTTTCTGTTCTGTTCGTTATCTAATATATGCATTACTTTTAGTGTTT

>FM893837.1 FM893837 Jatropha curcas embryo 56-70 (DAF) Jatropha curcas cDNA clone rjcaeb0_004766, mRNA sequence

TCCTTGAGAAGGTTTAGGCTACCAGAGAATGCAAAGGTTAATGAGGTTAAGGCTAGCATGGAAAATGGAG

TCTTGACTGTGACAGTGCCTAAAGTGGAAGTCAAGAAACCGGACGTTCAGGCTATTGAGATTTCTGGTTG

AAACTTTTAGTTATGTTTGAGTGTGTCTAAGTCTAAATGGTACCCATCCATCCAATAAAATAAATAGATG

TGTGTAATGGG

>FM893836.1 FM893836 Jatropha curcas embryo 56-70 (DAF) Jatropha curcas cDNA clone rjcaeb0_004765, mRNA sequence

ATTCTGATTATTTCAGAGAAGAACAGGAGGGAGATCTCCAAGTATCTCTTTCAAGAGGGAGTTTGCTACG

CAAAGAAGGATTTCAACCTTGCAAAACACCCAAATATCGATGTCCCAAATCTGCAGGTTATTAAGCTTAT

GCAGAGTTTCAAGTCCAAGGAGTATGTTCGTGAGACATTTGCATGGATGCACTACTACTGGTACCTCACA

AATGATGGGATTGAGTTTTTGAGGACTTACCTTAATCTTCCATCAGAAATTGTTCCTGCTACATTGAAGA

AATCTGCAAGGCCCACTGGTCGGCCAATGGGTGGCCCACCTGGTGATCGCCCCCGAGGCCCACCTCGCTT

TGATGGAGACCGCCCAAGATTTGGTGATCGTGATGGGTACCGTGGAGGCCCTCGAGGGGGTGATTTTGGT

GGTGAAAAGGGAGGTGCTCCAGCGGACTTCCAGCCATCGTTTAGGGGCTCAGGTGGTAGGCCTGGCT

>FM893835.1 FM893835 Jatropha curcas embryo 56-70 (DAF) Jatropha curcas cDNA clone rjcaeb0_004764, mRNA sequence

GGATCCCCGGGCTGCAGGAATCGAATCCGAGATGGCAAATCGGGCCACAAGTGATGGCTGTGCCCCTGTT

CAGCAGATGTCACGCATTGATCAGCCAGTTCCTCCCAATGTTGGATATGGACAGCCCTACGGTTATCCAC

AAGCTCAATGCTATCCTGCGGCAGGTTATCCTCCGCCTGCCAATTACCCACCGCCTGGTTACCCACCTTC

TGGCTACCCACCTTCTGGCTATTCCAGGTAGATTGGTGACTTTAATGAATGTTGGTATGCCGTTCCCAAG

GAGTGTTACCGTGGTTAATATTGATT

>FM893834.1 FM893834 Jatropha curcas embryo 56-70 (DAF) Jatropha curcas cDNA clone rjcaeb0_004763, mRNA sequence

ATTCCGGCTTTTGCCGCGTTTTCTCAGTTGCCAAACTTTATGACTTCTCGAGAGCTTGTTCTTGTCTCGA

CAGCAACGATCTTCGTTGCATTAACATCTGCACTTGTGGTTCGTTCCTTGTTCTTCAACCACAAAAAGCG

GTTTGTCCCGATCGATCCATCTGGAAATGGTGTCATTTCAAAGAAACGCTCGTCTCAGAGCCCGTTTGAT

CCTTCCAAACGGAAAGAATATTTGTCGTGGGACGATTATTTTATGGCTATTGCGTTTCTATCAGCTGAAA

GATCAAAAGATCCTAACAGGCAGGTTGGTGCATGCTTAGTTAGTCAAAATGGCGTAATTCTTGGCATTGG

CTATAATGGATTTCCCAGAGGTTGCTCAGATGACAAACTTCCTTGGGCAAAGAAATCTAAAACTGGGAAT

CCTCTGGAGACAAAGTATCCTTATGTTTGTCATGCTGAAGTTAATGCTATCCTGAACAGAAACCATGCTT

CTGCTGCCGGGCAGAAGCTTTACGTGACCATGTTCCCCTGCAATGAATGTG

>FM893833.1 FM893833 Jatropha curcas embryo 56-70 (DAF) Jatropha curcas cDNA clone rjcaeb0_004761, mRNA sequence

ATTCGAAGGACATTGCATTTATGATGGTGGCTATTGTAAGCTTCATCTATCATACTCTCGTCATACTGAT

CTCAATGTAAAGGCCTACAGTGAGAAAAGTAGAGATTACACCATCCCGGATGCAAGTTTGCTTGCAGCAG

GACAGATTTCTGGTGTCCCTGCTGCTCCGACAGCGTGGCAGAATCCTCAGGCTGCTCCAATTTACCCTCC

AAACAACTATGCCGCTCCCCCTCAAGTTCCTGTAGGGCAGGTGCCAGCTTGGGATCCAACGGTTCGGGCC

AGTGGACCAGCTTATGCATCAGTTCCAGGCACATACCCTGGTCAAACATATTCATCACCACCGGTACTGG

CATATGCCTCTGCAGCAATGCCTGTTGGATCCTCTCCACTTTCACAAGGCAGTCCGATTTCTCCTGGAGT

GGCTTCCATGCCAATGAATCAGACAGGGGTTAATGTGCGACCT

>FM893832.1 FM893832 Jatropha curcas embryo 56-70 (DAF) Jatropha curcas cDNA clone rjcaeb0_004760, mRNA sequence

ATTCGTTAGTTGATAAGGCACGGGCTAAGCAGCTGCAACCTCATGAATACAATACAGGTACTTTTACACT

CTCTAACCTTGGAATGTTTGGGGTGGATCGGTTTGATGCCATTCTGCCCCCTGGAACTGGTGCAATCATG

GCTGTTGGAGCATCCCAGCCTACTGTCGTGGCTACTAAGGACGGCCGTATTGGCATGAAGAACCAGATGC

AGGTAAATGTTACAGCAGATCATCGTGTAATCTATGGTGCTGATTTGGCTTCTTTCTTGCAAACTTTGGC

AAAGATCATTGAGGACCCCAAAGATCTTACCTTGTAGTCAACATCAAAATCAAGTCTTGTCTTTTGTGTC

TGTCACATTTCCTTAACCTTGTTGTATATTCCCAAAGCTGATTTCCCTCATCATCAATCCTTGATATTTA

TGGAAGACTTGCGCAGAAAAATGGT

>FM893831.1 FM893831 Jatropha curcas embryo 56-70 (DAF) Jatropha curcas cDNA clone rjcaeb0_004759, mRNA sequence

ATTCCATCAGTGATGAGAGCCATGCCTCTTGATGTTCTNGATGAACTCTTACAGAATGTCTCCAAGCGAA

GCTCATCAAGTGAAGAGGAACAGGGATCCACAGTCCATGTTGTTTACTCCCACCAGCAGGAGACCATAAG

TAAGTTACTACTAAAATAGCATTGTGATTGGTGCTTCTACTAGCCAAGGCCGCAACTAAGAGATCCTTCT

TGTAAATTTAGAGAAGGGATCCGTCGTATATTGTAGTATAGAGAGTTGTAGAGTGTTAAAAATGTAGAAC

TTCAACTTCTGCAGAAATCAATAAAATGTGGCCTTCTTTTGTTTCT

>FM893830.1 FM893830 Jatropha curcas embryo 56-70 (DAF) Jatropha curcas cDNA clone rjcaeb0_004758, mRNA sequence

CGAATCCTTTCTTCTTTCCTCCCTTGTTTGTCTTCTTAGTCGTTATACTCGGTCATATCATTAATAATCG

ATTCATTCCAAATCACTATTTCTGCTCTCTTTCTCTCTCCTCAAACTAATTCCACACTTCATTTTGGACA

TATACTGGTTTATTTCTAATGAATTGACTCTTTTCTACGCTATTCTAACGCGAATCCATGATTTATTCTT

GCTTGGCACTCTGACCTTGATCTCCCATCTGATTGTTTTCCACATACCCACTTAAACACGTCTTTCGATG

AAAGTAACGTCAGTTAGTAATAAACAGATACACACATGCCACGGGGCTTTCATTTCCAATGAGTCTTTGT

TTGATCTCGGTCTCGAATCTGTTTTATTTATGCTTCTGGG

>FM893829.1 FM893829 Jatropha curcas embryo 56-70 (DAF) Jatropha curcas cDNA clone rjcaeb0_004757, mRNA sequence

ATTCGCTTCTCTCCCCACTTATCTGCAAAACTCTGCCATCTTCTACCTCCACTTTTACCTCTTCTTTTTT

CAACCCAGGAAGATCAACAGCAAATATGTGAGCCTCCGGAGTCTCCCTCCAGTCCACTCGTGCGTTGGCA

ATGGCGGCTGTTTCGTGTTGAGTGGACGGAACATTGGCCACTGTTCCGCTATAGAAAGGTAAGCCCTCAA

ATGGGTCCCAAATATCTAGTGAGAACGGATCAAAGATGTTGCTCCTCCTTCCTAAGCTTAAAGCCATTCT

AGCTGGATACTTGATTTGAAGTTGAAGGGTTGGAAAGAGTATTTTCGGGGGGGCCCGGTAC

>FM893828.1 FM893828 Jatropha curcas embryo 56-70 (DAF) Jatropha curcas cDNA clone rjcaeb0_004756, mRNA sequence

ATTCGGACTTCTTGAATATGTATTTAAGGATATCTACAAGCCACTGCCTCTGGTTTACAATCTTGTTCTG

GCAATGTTGTGGCGTCATCCGGAGAATGTGGAGCTTGACAAGGTCAAAGTCGTTCACTACTGTGCAGCGG

GCTCTAAGCCATGGAGATACACTGGCAAAGAGGAGAACATGCAGAGAGAAGACATAAAGATGCTGGTGAA

GAAATGGTGGGACATTTATAATGATGAATCACTCGACTATAAGAAGGTTCCAGCTGCGGATGGTGATGCT

GAGCCAGTGAACTTGCAGCCATTCATTGCAGCTCTTTCGGAAGCTGGTGCTGTTCAATACGTGACGGCC

>FM893827.1 FM893827 Jatropha curcas embryo 56-70 (DAF) Jatropha curcas cDNA clone rjcaeb0_004755, mRNA sequence

ATAAAAATCTTCAAATTGGTGTTTATTTATGTACAATTCGAATCTTGTGGATGTAAAAAAATTAATTATT

CATGTTACACGCTAATTACATATTATTTGAGTTTGGAGAGAAGGCGAATTACTACACAAACAACATATAT

TTTACTTCATAATATTATAAACAGGAGAAGATATGACAAGTTATTTTTCAATCAAATTGTCGTCTCCAAG

ATCTTTTCTTTACTGCAGGAATTGGAGCTTCTCCATTTCCAGGAAATTCCCATTTCACAATATCCCCATC

CCATGAAGAGCTGACGAGCATTGGATAATACGGGTGCCAACTACAATCCCTTACAGGTGATTTATGGTGT

TTGAGTACTGCAACTTGGTCTCCCGTAACCAAGTCATATACATAAATGCAAGAGTCATGAGAGCCAGTGT

ATATGTACTTTTGGCCAGTACTATACGCGGGGGAAAAGTAGCAGCGACTGAGAGTACGCAAGACTGAATG

ACCTCTATNTGTAGCCACAGACTGATCACCTGGGTGTTTCAAATCTCTTGCCTGTGGAGGATAGTCCATC

CATCTGTAGTCCCATTCATA

>FM893826.1 FM893826 Jatropha curcas embryo 56-70 (DAF) Jatropha curcas cDNA clone rjcaeb0_004754, mRNA sequence

GCTATCAAAGATAAAAGCATTCATAAAAATCATAAAAGCACTATATATAACAACTTCATAATACAATCAC

CACCCGAGGCTCAAAATATCAAGAAGCAAAACTTTAAGTTATCTGGCCCCATTAGATAATTCTATGCATA

CCCAGCCATCCCGACATTCGAACTAGTACGATTTGCCTTGCTGGCTGCCTGAGGGGCAGTAGCCTTGGAG

AAATACATGTAGTAGTATACATTAAGAATCATTGTTGCAACTACAAACACAGCTCCAGCAACAAAAACAC

CCTTACGCAATGTTTCACATGAGAAATTTTGAGCATATATCATTCCCCTGTACTTGGTGTGGTATGCATT

TTTTGTTGCACCTGCAAGTAGACATGCTTCTGCAACCAGAAAAGTCAACCATGATGAGACAAAATATATT

ATAGACCATGCTCGATTTCCACCTGGAGCTAATGGTCTTCCAAAACACATGCACTTGGTTACACCCATTA

GCAGTGATTCACTCGAGAGA

>FM893825.1 FM893825 Jatropha curcas embryo 56-70 (DAF) Jatropha curcas cDNA clone rjcaeb0_004753, mRNA sequence

AACCAACAGGTAATTATATATACTGCAGGTTATTTTACAAATAGGGAGAAAGTATTAGCAGTCCTCCCAC

AAAGGGAACGACCATAAACCCCTCTCAAAGTTACAACACACATACTAGCTAAACCCTAGTCATTTTAACC

CACATGTAAACTACATTACTAAACTAAAGTACACTGCTAAGCTAATACTTTATTACCAACATTATTATTA

TTATTACTACAAAATACCATTTATAGTTTTAATCATCACACTAGCTCCCAGTCCTGAACTTCGACTCATC

GATAGTGATGCCCTCCTTCTCAGCTCTTTCTCCACCAGACTCGTCCGTCGTGCTCAGCCCACCTTTCCGG

CCCATCTCCTGATATCCTTCTGTCCCCAGCTGCTCCTTCCTTGTCTGTCCTCCTCTGCTTCTCCCTTCAG

CAAGATGCTCTTGAGCTTCAAGGCTCTTGCCCCCAGTACCTCCAGGGACAACAGTCTCTCCACGCCTTGC

TCTTTCATCAAGCTCTTGTCTTGATTGATTTGAAGACATTTTTGCCCTTTTTAGCTAACAAATTTCTCCA

CCAATATAATTAATCTTTTGCTCTGTTACGGGGGGGCCCGGTACCCAATTCGCCC

>FM893824.1 FM893824 Jatropha curcas embryo 56-70 (DAF) Jatropha curcas cDNA clone rjcaeb0_004752, mRNA sequence

TTAACAACAAACCACCTTTTATGCATCAACTGCATGTCAAAATAAAAAGAGAGCACGTAAAACAAAATGG

CCAGTAGCGGGCTAAGAAAGATGAAAAATCTCAATATCCTCCAAAACTTAAAAAAAAAAAAACATTTCTC

TCTTACTCACGTTTACTGTTTCAGTCAAAAGACAAAATATGTTGGTGCTAGCTATGTTATTGAGAAGGTA

AAAAG

>FM893823.1 FM893823 Jatropha curcas embryo 56-70 (DAF) Jatropha curcas cDNA clone rjcaeb0_004751, mRNA sequence

GATATATTAACCATTTAATAGCAATATCATAATTGAAGTTCCCCTTCCAAAATGACACAAAAGGCTCCTT

AAAGACCAACAAAGCAAAAGTTCAAGACAATACATAACAAGAACAGGAAATCAAATCCCACATGACAGGG

TTTGAGAAGGTACCCACGGCTTCTTTTATTTACAGCAAACATGGATTATTACACATAATTCAACTGATTA

ACCATAAAAGGCCAAGACTAATAACTGCTTAGTAGGAGCCTTTTCCCTTCTGGTTTCTCATTAGCAGTTG

CATGGATCACAGCTGCAGTTTGATCCACACTTGCAGCCATGACCACCCTCAGCTCCAAAGCTCATCTCAG

AACTCTCATTCAACTTCTTGGAGGGTGCAACTCCTGCAATGAGGGTCTCAGTTCTGGTGCTCTCTGCAAT

GTCAGGGTACATGCCACATCCGTTGCCGATGCCGCAGATGCAGCCAGAGCCGCATCTGCAGGCAGAA

>FM893822.1 FM893822 Jatropha curcas embryo 56-70 (DAF) Jatropha curcas cDNA clone rjcaeb0_004750, mRNA sequence

ATTCGTACTTTTTCAGCAGCTATGTCATTCAGGGGACTAAGCCGGCCTAATGCAACATCAGGGATGGGTG

TTGCTGATCACAGCATAAACACATTCTTGGAACTGCAGAGGAAGAAAGTCACCGCTATGTGGTATTTAGA

ATTGATGAAAAAAAGAAGGAAGTCGTGGTTGAGAAGACTGGTGGCCCTGCTGAGAGCTACGAGGATTTCG

CTGCATCTTTGCCTGAGAATGACTGCAGATATGCTGTATATGACTTTGATTTTGTGACTTCTGAGAATTG

TCAGAAGAGCAAGATATTTTTCATTGCATGGTCTCCTTCAACCTCTCGAATCCGTGCCAAGATGCTCTAC

GCAACTTCTAAAGACAGGTTCAGACGAGAGCTGGATGGTATCCACTATGAGATTCAGGCCACTGACCCTA

CTGAGATGGATCTGGAAGTGATCAGAGAACGCGCTAATTAAACAGTCCCCGCAAACCTAAGGTTTCTTCC

TCAAAGGAGACAAATCTGTTATGTATATGCCAATTTATGAATAATGAATCTTGCTACACTTAAGTCAATT

ACAGAATCCTTTCCAGCA

>FM893821.1 FM893821 Jatropha curcas embryo 56-70 (DAF) Jatropha curcas cDNA clone rjcaeb0_004749, mRNA sequence

ATTCGAGGAATGGCTGGAAAATAAGGAAAAATCACTGCTTATTTCTCAGTCATGCTCCAGTGAATTGGAG

AGCGAAATAATGCCAAGTGAAGTCAGAACTTGCGGAGAGGCCCTTGCTCTACTTTTTTCAACACTGGAAA

TATTATTGGCACCAGATTCAAATTCAAGAAAGCAGATAGAGAGAATGTCACACCAGATAAGAGAATATGA

AGTTGTTTTGAAGCAGCTTAAGGAAGATTTGCGAGTGGAAAAATTGAAGGCAAAGGAAGAAGCTGAGGAT

CTTGCACAAGAAATGGCTGAGCTGAGGTACCAAATGACATGTTTGCTCCAAGAAGAGTCCAAGCGCCGCG

CTTCCATTGAACAAGTATCATTACAGAGAATAGCAGAATTAGAGGCGCAGATTCAAAAAGAACAGAAGAA

CTCATTTCATGGAATCAGGCATCTCCATGAAGCATAGGAATCAGCTCTATCTAGAACTATGGGTTTCAAT

TAATTGGAAATTGTGATTGAGGAGACTTGATACATTCCAGGAGATGGCAACATGTATGGATCTGCAGGAC

ATCGGTGTCTGGAGATGGTGCAGCACTTTATGGGGCAACTATAGAGTGCAG

>FM893820.1 FM893820 Jatropha curcas embryo 56-70 (DAF) Jatropha curcas cDNA clone rjcaeb0_004747, mRNA sequence

ATTCCTTCTGAACATTTTCGAAATCAGGTTAGTTCACATCCGGAATTTACAAAGATGAAGACTATATTGT

CATCGGAGACTATGGATATACCCGATGGCGTTAAAATCAAAGTTAACGCCAAGGTTATAGAGGTAGAAGG

GCCTAGAGGCAAGCTCACTCGCAACTTCAAGCACTTGAATCTTGATTTTCAGCTCATCAAAGATGAGGCC

ACTGGGCAACGGAAACTCAAGATCGAAGCTTGGTTTGGATCCAGGAAGACCAGCGCCTCCATTCGCACCG

CCCTCAGCCATGTTGAGAATTTGATCACAGGTGTCACCAAGGGCTATCGCTATAAGATGCGATTTGTCTA

TGCTCACTTCCCTATCAACGCCTCCATTACCAATTCCAACACAGCTATTGAGATCCGCAACTTCCTTGGC

GAGAAGAAGGTCAGGAAAGTTGACATGCTGGAAGGTGTCACTGTTGTTCGGTCAGAGAAGGTTAAGGATG

AGTAGGTATTGGATGGCAATGACATAGAGCTCGTGTCACGTTCTTGTGCCCTCATTAATCAGGAATGCCA

TGTGAAGAAGAAAGATATTAGGAAATTCCTTGATGGCATCTAG

>FM893819.1 FM893819 Jatropha curcas embryo 56-70 (DAF) Jatropha curcas cDNA clone rjcaeb0_004746, mRNA sequence

TTTTTTTAACTAGAATTTATCTCTTTATTAATTATTAAACCAGAAGCCACTATAGTCCGCAGCAGTGATC

TTTCTCTTTTCTTAAAGACTAATTTACACATGTACCTTCACCTTTCATTATTTATTCACCATACACAGCA

CTATTTACATTAGTAAACCCTCAAGGTCGATGTCACAGTCACCCCAAAAAGCCGAAACACTTGCCATCAC

GTTATTAGCTTCTCATGCCACAGCATGACCCTCGAAGGAACCTCTCCTTGGCCCTGGAAAGAACCACTCC

TCGTTTTGTCTTCCAAAAGCCTGATCCACTTCTCTTTCACTTACACCGAAGGCTAACTCTTTGGCTTGCT

TCTCCATCTTATTTACAATATTATTTTTCCCTGCAAGTAGATGCCTAGTATTGCCTTCTTCATTAATCTC

AAAGCAGACTACTTCCAGATTATTGTTTCTTGAAGCTACAAAAGCCGCCGGATGGCCTGCAGGGACGACC

ACTGCTGTTCCACGTTTCAACCGTGAACTTACTTTCTGGTAAGTCTTCTGACTTTCACCCGTCGTGTCTC

CCCGCGAGCCGCTAAGGTGAGGACAAGCCATTTCAAAGTAGCCTTCGCCATCCAAACCACTGATATCTTG

GTTACTCTGGAGTTGTAATAG

>FM893818.1 FM893818 Jatropha curcas embryo 56-70 (DAF) Jatropha curcas cDNA clone rjcaeb0_004744, mRNA sequence

ATTCCCTGNTCTTTCAGAGTGCAGCCAGTTCCAGCATCTCGCCTTCGGATTTCTTGTGCTGCCAAACCAG

AAACAGTAGAAAAGGTGTGTCAAATAGTGAAGAAGCAGCTGGCATTAGCCCCAGAAGCTGTTGTTACCGG

CGAGTCAAAATTTACAACACTAGGAGCAGATTCTCTTGATACAGTTGAGATTGTGATGGGACTGGAGGAG

GAATTCGATATCAGTGTGGAAGAGGAGAGTGCCCACAGCACCCCCACCGCTCAGGAAGCTGCTGATCTTA

TTGAAAACCTTGTTGAGAAGAAGGACGCTTAGAAGATCACCCTCACGAAATCCTTCAATTCCTTTCCCCC

TGGCGCCCCCCCCACCCCCCTCCACATAATTGCAG

>FM893817.1 FM893817 Jatropha curcas embryo 56-70 (DAF) Jatropha curcas cDNA clone rjcaeb0_004743, mRNA sequence

ATTCCTTGAAGGATCCTCATAAGTTGATCTCATAATACAAACATAAAACAGTACAAGAAGAACACATTCG

ACCAAATTGAAGTAAAATGTCCGGTGTATCTCTGGCTACAGCTGCTAGAAGTGAACTTGATGGTACTACA

ACTAAACCTGCAAATCCCAAAGAAAAGCAACAGCAGGCGATGATGGGGGGTATAATGGGATCGTTGCGAG

TAATAGAACTCCAACTAGTTGCTTTCATAATGGTTTTCTCAGCTAGTGGCCTTGTTCCACTTCTTGATTT

GGTGTTCCCAGCTCTTGCTTCCACTTACATTTTAATCCTAGCTCGATGGGCCTTTCCTACACATGGCAGA

GCCAATACAGGCTCTCAAGAGATTTTTCAAGGAACCAAATTCTTTAGGTTTTATGTTATTGTGGGAACAT

CAATAGGCCTGTTCTTGCCACTGGCATATGTATTAGGAGGGTTTGCTAGAGGCGATGGTTATGCTGTTCG

GTCAGCGACACCTCACTTGTTCTTGCTATCATTTCAGATACTTACTGAGAATATAATAAGTGGGTTGTCA

TTGTTCTCGCCTCCCGTGAGAGCATTAGTGCCATTGCTTTATACTGTGAGGAGGA

>FM893816.1 FM893816 Jatropha curcas embryo 56-70 (DAF) Jatropha curcas cDNA clone rjcaeb0_004742, mRNA sequence

GGACTATAACTTATAGCTCCACAAACTTTCATTATCTCATAACATAAGGCATATACAGATCTACATTTTT

ATATTATTGCAAGGAACTAAAACTGCCTATAAAAATAGGCTTATTCTTCTCCAAATCTAAACTATGTAGA

AACCAGTTTATTATACATCCTCTTGGTATAATTGCATAGTTGCAATATTAATTACGAGATCTAGACCCAG

GGCTAAACACAGTGACTTCCTGTCTATTCTCCTTAAGCCTCCTGGCATCTTCTCTGGAAATCTGGAATGC

GTTTGCAACCACTGCCTCTGGCAGAGCCCTAATTGCCGAGGTCCGTCCTGCTAGTGGGCTAATTTGTGCA

TTTTCGTTAGTCTTGAAGGATACCCACTCTAACCCTTCATTGCTTGCCCTCTTCAAAACCACAAAGTTCT

GTGGAGCTGTTAATATTTGACCTTGTTGAACTTGTCCAT

>FM893815.1 FM893815 Jatropha curcas embryo 56-70 (DAF) Jatropha curcas cDNA clone rjcaeb0_004741, mRNA sequence

GAGATTAAGATATGATTGAATTCAACAATGAAAACTAATACAAACGGTAGACCAAACGATAAATCTGGCA

CGGAACATTGTACGAATCTACGATTTTATGCAAGTTCATGCTTGCTTCTTACATGACTTAGTAGAGATCA

CTCTCCTTGTGAGTACGAATAACAGAGTCTGAAGTTGGATATGAGACACAGGTAAGTACATATCCTTTCT

CCATTTGCTCTTCGCTCAGGAATGACCCATCTGATTGGTCTACAGATCCAGAAACCAGCTGACCAGCACA

AGTAGAGCAGGCCCCTGACCCTACATGAGTAAGGCAGTTCTATTCCTGCATTTTCAGGGGGGGCCCGGTA

C

>FM893814.1 FM893814 Jatropha curcas embryo 56-70 (DAF) Jatropha curcas cDNA clone rjcaeb0_004740, mRNA sequence

CCATTTTACGAAGCTGGCTCCTGAATATTGGAGAAATCTCGACCCTTATTGGAAAGGCACTGAGCCTCTC

ATGCACCGGTACTTCCATCAAGATGGAAGCCAAACTGCTGATCCTGGTGACAGAGTATGGGGCGGTCACG

AATGCTGTTTTTCAGTTGTAACTAGTTTTATTGCTGATGGAACAATCAGGGAGCACTATGTTAGAATAAA

CCGATGGCCCCGGATGTCAGTTTCTAGGAAACAGGACTGGAGCTGGGAAATGTCCAACCACCTCTTCTGT

TACTCCAGTGTTCCTGATGCCTACAAGAAAGGTGGAACTGGGCCAGTCTTTTAGTTTAGGTAAATATTGT

ATTATTGTTGTATATTTCTCGCTTTAGAATGTGTNCTTTCTGCTAGCATACGTATATGCAGTAATTGGGA

GTGTGCAAGCT

>FM893813.1 FM893813 Jatropha curcas embryo 56-70 (DAF) Jatropha curcas cDNA clone rjcaeb0_004739, mRNA sequence

ATTCGTTATCTTCGCCGCAGGATTATTAAGAAGTTTTCCCAGTTCCATAATTGTTTTGCTATGTAATTAT

ATGTATAAGAAGCTATGTATAACAAACAGTTAGTGACTCCGCAGTTGTAGAATTTACATACGCCTAGTCG

TGTAGTAATATAGATGAAGTAATTCTGTTAGCCCATATTAAAAAATGTGTTATATTGTAAATGGGGCTCG

AACTGTGAAATGTAGAAGATAAACTTGGGCCGAGAAATAATCAAATATTGAAGTTTATTTTTT

>FM893812.1 FM893812 Jatropha curcas embryo 56-70 (DAF) Jatropha curcas cDNA clone rjcaeb0_004738, mRNA sequence

ATTCCTTGCACATTGGGAAAACAAGGATGATGATATACAAGTGCATAGGTACCTTCCAAAAGGCGTATCA

TACTACGAAATGATGAGAAAAAGCAAGTTCTGCCTCTTGTCCTAGGTGGTTACGAAATTTGCAAGTCCTA

AAGTCGTTGAGGCCTTTCTTCCGGGTTTGTGTGCCAGTGCTAATTTCAGACCACTATGTACCGCCTTTCA

GCGATGTTCTGAATTGGAAGTCATTCTCCGTTGAGGTTCCGGTGAGCAAAATCCCCAACCTGAAGAGAAT

CTTGACAAGCATATCTCCAAGACAATACATTAGAATGCAAAGAAGAGTACAACAAATAAGGAGGCATTTT

GAGGTAAATTCTCCGCCTAAGCGGTACGATGTTTTTCATATGATTCTTCACTCTATTTGGCTTCGAAGAT

TAAATGTTAGGATCCATAATGATCAAGTAGCCATTACCAGTTGATTGAACAGCATATCTTGTTTAATAGG

TAATTTAGTTTAGGGAAGTACTTCAGGAATACGTGTGTGT

>FM893811.1 FM893811 Jatropha curcas embryo 56-70 (DAF) Jatropha curcas cDNA clone rjcaeb0_004737, mRNA sequence

ATTATCAATCGTAACACCATTTGTAGTAAATGCGAAGGTCGTGGTGGTAAAAGCGGAGCTGTACAGACAT

GTCGAACTTGTCGCGGAACAGGTATTCAAACGCATATCAGACACCTGTCTCCTGGTTTTGTCCAGCAAAT

TCAGAGTACATGCTCCACATGCAAAGGGGAGCGAGAGATCATTGACCCTAAAGATTGTTGCAGGACTTGC

GAGGGGAAGAAAGTAGTCAGTGAAAAGAAGTTAATCGAGGTTCCCATTGCCATTGGAATGGCTGATGGAG

AAACGATTAAGTTTGCCGGTGAAGGGGATCGGGAACCTGGTATTGAACCGGGAGATGTGATCGTTGTTAT

TGACGAAATGCCTCACAGCAGATTCGTCCGTCGCCGAATGGATCTTATTCACACTGTTACCCTTACCCTC

AGCGAAGCGCTATGTGGGTTTACCAGGACGGTCAATACTTTGGACGATCGCACGTTACTCTTGCGCTCCA

AACCAGGGGAGGTTTTCACTGGGTCGGAGTTTCGAGCCATTGAA

>FM893810.1 FM893810 Jatropha curcas embryo 56-70 (DAF) Jatropha curcas cDNA clone rjcaeb0_004736, mRNA sequence

ACTTTATAGAATATATTGATTAGGGAATACTTAATTACTAATTAGGATGATCACATCACTTTCATCACTT

ATTTTCATAATATATTACATACTTCATTAGCGTGAGATCATAACTCCCCTCGATAATACTACTTACTTTC

ACTATCTACACACCCAAAAAGGCTACTTCATTATTTACTCCTCGGCATATCTTCCAGACTCGGTCCTGGA

AATAGTCAAGGTGGTCTCTTGTCTTCCAAACTTAATCCTTCTTGCCTCCTCTGCTGATACCCTAAATGCA

TTTGCAATAACCGCCAACGGCATTGCTCTAACGGCGGAGGAGCGACCTGCAAGATCATTGGTCATCGCGT

TATCGCTTGTCTTGAAGGTTACATATTCGAATCTTTCACTTTCTGCTCGTTTCACCACTACGAAGTTTTG

TGGCACCGTCAAAACCTGTCCTTTGCTTACACTTCCGTCGAAGACGGTGTTGCCGTTTACATCCGTCACC

TGAAACCGCGCTTGTCCTTTGAGTGCGTAAATTACGCTGTGAG

>FM893809.1 FM893809 Jatropha curcas embryo 56-70 (DAF) Jatropha curcas cDNA clone rjcaeb0_004735, mRNA sequence

ATTCAATTTAACGAAAGTGAAAGTTTTACTGTAAGAAATACTAAAAGCTCTATCTTTCTTTCGTGTTAGG

CTTTGAATTAAAAGGACAAAAGATGGCAACTGTGGGCAGGAACATAGCAGCTCCATTGCTGTTTCTTAAC

TTAATCATGTATGCTATTGCTTTAGGTTTTGCCAGTTGGTGCCTTAATAAGTATATCAATGGACAAACCT

ACCATCCAAGTTTTGGAGGGAATGGAGCAACAGCTTTCTTTTTGACTTTTGCTATATTAGCTTCTATTGT

TGGTATAGTATCCAAGTTGGCAGGTGGCCTTCATATCAGGAGCTGGAGAAATGACAGTCTAGCCTCTGCA

GGCTCTTCTTCACTTGTGACTTGGGCCATCACTGCCCTAGCTTTTGGGTTGGCATGCAAGGAAATAAACC

TAGGAGGGCACAGAGGATGGAGACTGAGGGTATTAGAGGCATTCATGATAATCTTGACATTTACCCAACT

CTTGTATGTCCTGTTGCTTCATGCTGGGGTAATTAGCAGCAGGTATGGCCCTGGTTACAGGGACACCGAA

TACGCTGTAGGAG

>FM893808.1 FM893808 Jatropha curcas embryo 56-70 (DAF) Jatropha curcas cDNA clone rjcaeb0_004734, mRNA sequence

CACGTTTTTATTACAAGACATTAGGAAAGCTTGAAGCAACGTTACAAGAAGCAGTTTCGAACAAACTTAT

TCTGATGCTCTTACTTCTTATACATCATTATATATAACTCTCACTCACATACAAGTGAATGAAGTTTTAT

TCACCTGCAGCTAGCATTTTAACGAGGGCATATAGACCCAAAGCAAACATCAATTATATTTTTAGTCCTG

CGCCTTGCCTCATCTTCGCTTCCAACTTCTCCTTCCTCCAGCTGCTTCTCTAACAGATAATGTATTGATT

CACATCGACAAATAGCACTTAGTTCCTTTGCTTGGTTACAGCATTGTCTAGGAACCTGCTGTCGAGGGTT

TTCAATTCCTGGCCTAGCTAACATATCTTCTGACCTTCCTC

>FM893807.1 FM893807 Jatropha curcas embryo 56-70 (DAF) Jatropha curcas cDNA clone rjcaeb0_004733, mRNA sequence

TTTTTAAACATAAGGACTAAAATTGAGTCCTTTCTATTATTAGGGTGTGGGAAAATAAAACCACCATACT

ATATCTTTGACAAATACATTTCAAATAAATACCTATAGGTATAGGACAAAGGGAAAAAGCAAATTGGTTG

CAAGAAATGAGGGCACGTCTTGCCTCACATTATGCAGCAATTTGGCTGGGACTCGAGCATCTCTGGAGAT

ATTTGGCCAAGATTAGCAAAGTCAATATCAGGAGCAAGCTCTTGCACAATATCATTTGGAGGTTGGCCAC

ATTCTTGCATTTTCTGCATGAGATCAACAATCTTTGTGAAATTATTAGGTTCATTCTCATAAACTTTGTT

AAGTTTGTTGATGAGTTCATATTGGTGAGAATAACGCTCGTATTCATCTTTGGTTAAACTGGTTTTGTGT

TCTTCCAACCACTTTGGATATCTTGCTCCAATTTCCTTCATAGGTTCCTGAAGAATCTCCTTAGACAAAA

GTTGTTGCATCATCGTCTCCACGATAGATTCCATGTCCTGAGAGCCAGCAAGCTCCTCAAATTGCTTAAA

CCAATCCTCCAGAAATGCATCCTTACCAAAATCATCTGGTTTTGGACTTGTGACAGACTCCAAGCCCTTG

>FM893806.1 FM893806 Jatropha curcas embryo 56-70 (DAF) Jatropha curcas cDNA clone rjcaeb0_004732, mRNA sequence

AAGAGCTTGATGAAAGAGCAGGCGTGGAGAGATGTTGTCCCTGGAGGTACTGGGGGCAAGAGCCTTGAAG

CTCAAGAGCATCTTGCTGAAGGGAGAAGCAGAGGAGGACAGACAAGGAAGGAGCAGCTGGGGACAGAAGG

ATATCCGGAGATGGGCCGGAAAGGTGGGCTGAGCACGACGGACGAGTATGGTGGAGAAAGA

>FM893805.1 FM893805 Jatropha curcas embryo 56-70 (DAF) Jatropha curcas cDNA clone rjcaeb0_004731, mRNA sequence

AAATATAACGAAAGCATAAACATATAAACGAAAATTGATGCTACATAGTGCCAATATTTTAAACTTCTTC

CATCTAAGATATGAAGTAACATAGTCTAAATCAACAAAAGACCACACCAAATCAACAAAACTATTCTGCT

CAAACAACAGAATGTCAGAACCTCTGGATCTCATTTCTTGGATTTCTTAGACCCCTCAGTTGGTTGAGAT

GCAGCAGCAGGCGGAGCAGCTACAGGTTTCTCTCCCCCAGGACCCTGGGCCAATCGTTCCTCCCTCCTCG

CAAATTTCCTTTCCCTGCTTGCCTTGTTCTTTGCCCTCTTAGCCTCAAACTGATCAGACAGAGTCTTCTC

TCTAGCTTTCTCAGCCTTTGACTTGTGAATACTTTCCATAAGAACACGCTTGTTCTTAAAGACATTACCC

TTCACCTTCATGTACATGTCATGGTACATGTGCTTGTCAATCTTCTTTGATTCTCTATACTTGCGGAGCA

GACGCCTGAGAACACGCATCCTTCTCATCCAGAGAATCTTTGTTGGCAGCCTAGCCTCTCTTGTACCCTT

GCGCTTACCATATCCAGAGTGTCGCCCTTTTCTTTTGGCTTTCTTCATTCAACGTGCCCGAGATCG

>FM893804.1 FM893804 Jatropha curcas embryo 56-70 (DAF) Jatropha curcas cDNA clone rjcaeb0_004730, mRNA sequence

ATTCAGTCAATCATCCNTCTGGCACCTCACCAAGGAAGAGAAAACCTACGGTGTCCCCCGCCGCCGCCTC

ACCGGTCATTCTCACTTTGTCCAGGATGTTGTTCTGTCTTCGGATGGTCAGTTTGCTCTTTCCGGCTCCT

GGGACGGCGAACTCCGTCTTTGGGATCTTGCTGCTGGCGTTTCTGCTCGTCGTTTTGTAGGCCATACTAA

GGATGTACTATCTGTTGCATTCTCGATTGATAATCGTCAAATCGTATCGGCCTCTCGTGACCGTACGATT

AAGTTGTGGAATACTCTTGGAGAGTGCAAGTACACAATTCAGGATGGTGATGCTCATAGTGATTGGGTTA

GCTGTGTTCGTTTCAGCCCTAATACCCATCAGCCTACTATTGTTTCTGCTTCATGGGACCGAACTGTGAA

GGTTTGGAATTTGACTAACTGTAAGCTGCGATGCACTTTGGCAGGGCCTAATGGTTATGTTAACACTGTT

GCTGTGTCGCCTGATGGGTCTTTGTGCGCAAGTGGTGGGAAAGATGGAGTGATTTTGCTCTGGGATTTGG

CAGAGGGAAAGAGGCTTTACCATCTTGATGCTGGTGCAGTCATCCATGCTCTGT

>FM893803.1 FM893803 Jatropha curcas embryo 56-70 (DAF) Jatropha curcas cDNA clone rjcaeb0_004729, mRNA sequence

ATTCTTCATTCTGCTTGGAGGGAGGCGCCTCTCTTTCTCTGCTTTCTGCATCCGCAACCATGGTGAGAGT

TAGTGTTTTGAATGATGCTTTGAAGAGCATGTACAATGCTGAAAAGCGTGGGAAGCGCCAGGTTATGATC

AGGCCTTCCTCAAAAGTGATCATCAAATTTCTGCTGGTGATGCAAAAGCATGGATACATTGGCGAGTTTG

AGTATGTTGATGATCATAGAGCTGGTAAAATTGTGGTTGAATTGAATGGGAGGTTGAACAAGTGTGGAGT

TATCAGCCCTCGTTTTGATATCGGTGTCAAAGAAATTGAAGGATGGACTGCAAGGCTTCTTCCATCAAGA

CAGTTTGGTTATATTGTGTTGACTACCTCTGCTGGCATTATGGATCATGAAGAGGCGAGAAGAAAGAATG

TTGGTGGCAAAGTGCTTGGATTCTTTTACTAGGCTCAGCTTTAGTATTTTTCACTTTAGAGAAAAATTAT

TCTGCTTCCGAGAGTTTAGGTTGTGCCTTTGAACTTGTTTGCCATAGGATTAGCCATGTGCTTCGTCT

>FM893802.1 FM893802 Jatropha curcas embryo 56-70 (DAF) Jatropha curcas cDNA clone rjcaeb0_004728, mRNA sequence

ATTCCCATAGAGATATACCCCACTGGCACATAAACTCTCACAGCGTAATTTACGCACTCAAAGGACAAGC

GCGGGTTCAGGTGACGGATGAAAACGGCAACACCGTCTTCGACGGAAGTGTAAGCAAAGGACAGGTTTTG

ACGGTGCCACAAAACTTCGTAGTGGTGAAACGAGCAGAAAGTGAAAGATTCGAATATGTAACCTTCAAGA

CAAGCGATAACGCGATGACCAATGATCTTGCAGGTCGCTCCTCCGCCGTTAGAGCAATGCCGTTGGCGGT

TATTGCAAATGCATTTAGGGTATCAGCAGAGGAGGCAAGAAGGATTAAGTTTGGAAGACAAGAGACCACC

TTGACTATTTCCAGGACCGAGTCTGGAAGATATGCCGAGGAGTAAATAATGAAGTAGCCTTTTTGGGTGT

GTAGATAGTGAAAGTAAGTAGTATTACGAGGGGAGTTATGATCTCACGCTAATGAAGTATGTAATATATT

ATGAACATAAGTGATGAAAGTAATGTAATCATCCTAATTAGTAATTAAGTATTCCCTACTCAATATATTC

TATAAATATAATTGTA

>FM893801.1 FM893801 Jatropha curcas embryo 56-70 (DAF) Jatropha curcas cDNA clone rjcaeb0_004727, mRNA sequence

GATAATGGATAGTTTTCTTCATTGATATGATAAGTCATAACCAAGACTACAGGAGTCTTTAAGACAATAA

GAGTGGTATGACGCAACTGGCAACATTTAAAAACTTCTAGTACAGTACTACTATATGCTGGTGTAAGAAA

CACACTAAAAAGTGAACGCCACCACTTCAGCATCTGAATCTCAGACTTCTACCGTGGCCTAACCCGGTTG

TGCAATAGCATTTCCCGGAGCTCAGCCGGGTCGATCTCAGCCGCAGAATTAGCTGGCCTGTAGTACCCCG

TGATGGGGTCTGGTGCCCACGCAGTAGATGATCCTGAATCATCTTCTT

>FM893800.1 FM893800 Jatropha curcas embryo 56-70 (DAF) Jatropha curcas cDNA clone rjcaeb0_004726, mRNA sequence

ATTCCAGAAAGCTGGGTCGTGTTAGCACCGTTAATAGCCATAACCTCCCTATTCTTCGGTGGCTACAACT

CAGCGCTTCCTATGGCGTTCTCCGCAATGATGCCATGAGATTACCCCACTGGCACATAAACTCTCACAGC

GTAATTTACGCACTCAAAGGACAAGCGCGGGTTCAGGTGACGGATGAAAACGGCAACACCGTCTTCGACG

GAAGTGTAAGCAAAGGACAGGTTTTGACGGTGCCACAAAACTTCGTAGTGGTGAAACGAGCAGAAAGTGA

AAGATTCGAATATGTAACCTTCAAGACAAGCGATAACGCGATGACCAATGATCTTGCAGGTCGCTCCTCC

GCCGTTAGAGCAATGCCGTTGGCGGTTATTGCAAATGCATTTAGGGTATCAGCAGAGGAGGCAAGAAGGA

TTAAGTTTGGAAGACAAGAGACCACCTTGACTATTTCCAGGACCGAGTCTGGAAGATATGCCGAGGAGTA

AATAATGAAGTAGCCTTTTTGGGTGTGTAGATTGTGAAAGTAAGTAGTATTACGAGGGGAGTTATGATCT

CAC

>FM893799.1 FM893799 Jatropha curcas embryo 56-70 (DAF) Jatropha curcas cDNA clone rjcaeb0_004725, mRNA sequence

CCCGCAAGATATAATTTACATTAATGATAGTGCAAAGAACCAAACAAGGTCTTTATCATGCCCACTTATA

AAGGCTTTATTTTTACACCTCTCTATTACTCTCCTACATAGCCACTAGCTAAGCGTTTTTTCTCTCCTTA

TTACAATTACATAGCTGCCTTTTTAGTCCCTTCAATTGCGAGAAGAAGGCCTAGTCCCAGGGCTGAACAG

AGTCACTTCTTGTCTATTCTCTTTCAGTCTCCTCGCGTCCTCCCTTGAAACCTGGAATACGTTTGCCAAC

ACCTCTTCTGGAATTGATCTGATTGCCGAGACGGGGGGGCCCGGTAC

>FM893798.1 FM893798 Jatropha curcas embryo 56-70 (DAF) Jatropha curcas cDNA clone rjcaeb0_004724, mRNA sequence

ATTCCCTCCATCGAGTCTTTTTTACACTCTCACACTCCTGATCAGTCTCGCCACTTCTTCTCCCTCACCT

TTCCGACTCTAATTTGCAAGCTCTACGGCTTCGGTGATGCCTCATCTCCCCCAAATGGACCGCACTTCGC

TTCATCCAATGTTGGCTGGATCGATATTATTCTCCAATCGGATGACGCCGATCTTGCTTCTAAAGTCTTC

AACCTCCTCTCACCAAATGGTATGGTTTTTCAGTCAATTTTCGCCGTTGATCGTCAGTCCCTTGTCAAAT

ACGTGTTTCCAATCGAGCGGTTGCCTGAGTGGGCCCGGTTCATGCTATCAAGCGAGAGAGATTGTCAGGT

CTTGAACAATCTTTGCCCTCTCTTTAGAGATAAAGTCAAGGAGGATTCACTTAAAGGGTCTTTGTATTAT

CAAAGTACAATTAAACGTTTATGAGTATTTCATGTATAGGTTTGCTTTATAATCCTATCTGCAAAGGGAA

TTGGCGAAACATTTAA

>FM893797.1 FM893797 Jatropha curcas embryo 56-70 (DAF) Jatropha curcas cDNA clone rjcaeb0_004723, mRNA sequence

TTTATTTTATTTTTCTATCGAGATGTTGGGAGTGTTTAGCAGCTCTATAGTATCTCCGCCCGACGAGCTG

GTGGCGGCAGGAAGCCGGACGCCATCTCCGAAGACAACGGCGGATGCGCTGGTGAAGCGATTCATCGACA

CCAATCCCTCCGCCGTATCCTTACAGATCGGAGACAACGCGCAGTTGGCTTACACCCATCACAGCGAGTC

CTTGCTGCATCCCAGATCATTCGCTGTTAAGGATGATGTCTTCTGCCTGTTTGAGGGTGCACTGGACAAC

TTGGGAAGTCTGAGGCAACAATATGGGCTTGCAAAATCGGCAAATGAGGTGATTTTGGTAATTGAGGCAT

ACAAGGCTCTTCGTGACAGGGCACCTTACCCTGCAAACCACGTTGTTGGTCATCTTAGTGGGAGCTTTGC

TTTCATTGTCTTTGACAATTCCTACCTCTACCTTGTTTGTGGCTTCTGATCAATTTGGAAAAGTTCCTCT

ATATTGGGGAATCACTGCTGATGGTTTCGTGGCCTTTGCCGATAATATTGAATTGCTTAAAGGTGCTTGT

GGCCAGTACCTGCCCTCTTTCCCTGAAAGGGTGCTCTACTCCACAGAG

>FM893796.1 FM893796 Jatropha curcas embryo 56-70 (DAF) Jatropha curcas cDNA clone rjcaeb0_004722, mRNA sequence

ATTCCATGAACTTGTGCTCTAAGTGCCACAAAGACTTCATAATGAAGCAAGAACAAACCAAACTTGCTTC

ATCGTCCCTTGAGAGTATTGTGAACGGTAGCTCAAGCAACATCAGGAATGAGCCTGCTGTTGCTATTGAT

CTGCAAACTTATTCAGCAGAGACTATGGTCAGTTCTGCTTCAGCGTCCTCTGATTCAGCTCTCAGCACAA

ATGGTGACACGGTGGTGAAAGCAAGTCAAAATAGGTGCGCCACTTGCAGGAAACGCGTTGGTCTAACAGG

ATTCAGTTGTCGTTGCGGGAATCTTTTCTGTTCAGTTCATCGCTACTCAGACAAACATGGTTGCACCTAT

GATTATCGAACTGCTGGACAAAATGCTATTGCCAAGGACAAACCCATTGTGAAAGCTAAAAAGCTTGATA

ACATCTGACACCTCATCCAACTTCTGCTGAAGGTATCACAT

>FM893795.1 FM893795 Jatropha curcas embryo 56-70 (DAF) Jatropha curcas cDNA clone rjcaeb0_004721, mRNA sequence

GCTGCAGGAATTCGAATTCCGAGTTTTTTGGCACAGCCGCCTCTGCTCGTACTGGACCAAACATGAAGTT

CAACATTGCCAACCCCACTACGGGATGCCAAAAGAAGCTGGAAATCGACGATGACCAGAAACTCCGGGCT

TTTTTCGACAAGAGGATCTCACAGGAGGTCAGCGGAGATGCACTTGGAGAGGAATTTAAGGGTTATGTTT

TCAAGATTATGGGAGGTTGTGACAAGCAAGGGTTTCCCATGAAGCAGGGAGTGTTGACTCCTGGCCGTGT

CCGACTCTTGCTTCACAGAGGAACCCCTTGCTTTCGCGGTTATGGAAGGCGAAATGGAGAACGCAGGAGG

AAGTCTGTCCGCGGATGCATTGTCAGCCAAGACCTTTCTGTTTTGAACTTGGTAATTGTGAAGAAGGGAG

AGCAAGATCTGCCTGGACTGACAGACACTGAGAAACCCAGAATGAGAGGACCTAAGAGGGCTTCAAAGAT

CCGCAAGCTCTTTAATCTCTCCAAAGAGGATGATGTTCGGAAGTAACGTAAATACTT

>FM893794.1 FM893794 Jatropha curcas embryo 56-70 (DAF) Jatropha curcas cDNA clone rjcaeb0_004720, mRNA sequence

GGTGGCTACATTCATGGTGTCTTGCGTAGCATGCACTGAAATGTGCCAAATATAAGAGTGAGACATATGG

TTAATAACTAGGCTTGATCTTTGGTCAATGCATATAGGAGCTGATGTCAGTATCTTTTATTTTTACTACT

TGGTATGTAAAATTTGTGTTCTACAATTAGGATGGAACTTTTTATGGATGAATCAAGCAGCTAAAGATTC

TATT

>FM893793.1 FM893793 Jatropha curcas embryo 56-70 (DAF) Jatropha curcas cDNA clone rjcaeb0_004719, mRNA sequence

CTTCGTTGACTAATCCATCCATGTAGAAAATATACATATGGGCTCATACAGGCCTCACATGGGCTCTGTC

GTCACTCATCCCAACTATACTTCATTTCTACTTCATTCAAGCCCAACAAAATACAAGACAGGACACCAGA

TATGTAAAATAGACGCCAAAACGACAACAGAACAGCCAAAGAAACAAATCAGAGCTCTTCGTCCGGCAAT

TCACCACCGGCACCAGCCACGTTATCCTTGAGATGCTGTGATGTCTTCTTTGCCTTATCTTTTGCCTCTT

CGGACTTGTGGTATGCTCTCTCTTTCGCAGCTCCAACTGTCTCTCTGGCCTTGTCCTTTGCTTCCTCAGC

CTTCTCAAAGGCCTTGTCCTTTGCTTCCTCAGCCTTCTCAAAGGCCGTGTTCTTTGCCTCCTCACCTTTC

TCCTTTGCTGCTTCTGTAGCATCGCTGGTCTGGCCTTCAGCCGCACTGAGCTTCTCCTTAGCCGCATTCG

TCATTTCAGAGGCCTTATCTTTCATTTCGTTGGCCTTCTGC

>FM893792.1 FM893792 Jatropha curcas embryo 56-70 (DAF) Jatropha curcas cDNA clone rjcaeb0_004717, mRNA sequence

GAACAGGGAAAACATAGTCATATATTTTATTAAGCGAATGAAAGGTAGGTCAAGTTTTCTTAGACAAAAC

CATACAACTGAAAATAAAACACATCAAACGACATGTGCCAAGGTGAAACCATAGCCATCATTATTTTGAA

ACGGACTCACAGCTCAATCCTCCTTATATCCGACAAGTGCCAGCAGCATTTTTTCATAGTCCCCATGGGT

ATCCTTGATAATGGCCCGATCAAGAGGAACACTGTTCCTTTGCTGGTACTCATCTTTTATAACCTTCAAA

TCTACCTCTGCTCTTGTAGAAACAACTCTTGTCAGAGCTCCTTCATCTGTTCCTGTTCTGTTGATAGCCA

GACGAAGAACCTTCTCAAAATGGGGGGGCCCGGTAC

>FM893791.1 FM893791 Jatropha curcas embryo 56-70 (DAF) Jatropha curcas cDNA clone rjcaeb0_004716, mRNA sequence

GGGCTGCAGGAATTCGAATTCACAAAGTCTCCAAAGAAAGCTTAGGCCATTCCAGTTATTATTCTCTTAT

GCTCTTGTTGTATGTAGTTTTGCCATATTTTGTTAATTTGTAGGACGAAGTTGATGGATTTTGATAAAAT

TGTGTAGTCCCTTGATCTTTGTGATACTATGTTAGGATTGACAT

>FM893790.1 FM893790 Jatropha curcas embryo 56-70 (DAF) Jatropha curcas cDNA clone rjcaeb0_004715, mRNA sequence

ATTCGCGATCTTGTTCNCGATTTCAAGCTAAAAATGTCGCTAACTCCTTTCTTCGGTAACAGGAGGACCA

ACATCTTTGATCCTTTCTCTCTTGACATTTGGGATCCGTTCAAGGATTTCCCTTTTCCTTCTTCTTCTTC

CTCTTCTTCGCTTTTCCCTCGTGAAACCTCTGCTTTTGTTAGTACTCGCGTAGATTGGAAGGAAACACCA

GAAGCTCATGTTTTCAAGGCTGATCTTCCTGGGATCAAAAAGGAAGAAGTGAAAGTAGAGATTGAAGACG

ATAGAGTGCTGCAAATTAGTGGAGAGAAGCATATGGAGAAGGAAGACAAGAATGACACTTGGCATCGTGT

GGAGCGTAGCAGCGGCAAGTTCTCTAGGAGATTCAGGTTGCCGGAGAATGCAAAGATAGATCAGGTTAAG

GCTTCAATGGAAAATGGGGTTCTTAC

>FM893789.1 FM893789 Jatropha curcas embryo 56-70 (DAF) Jatropha curcas cDNA clone rjcaeb0_004714, mRNA sequence

GCAATTGAGAAGGCTGATATGAGATAAACTGTCAGATGTCTGAAAATATTAAATTAAGACACTAAACAGG

ATGGAAAACAACACGTTAAATAGTACTTGCAAAATAAGAAACCCTTTCTCAACAAGCACCAAAAATTCGC

ATCACAGAAGATCAGCAACATTCGACGGCAGTTCCTCAATAACCACGTTGTAGAACTTCTGAATGTCAAA

CAGCATTCTCTCATCATCCCTTGTCACGAAGTTAATGGCAACACCCTTTCTTCCAAACCGTCCACCTCTT

CCGATTCGATGAAGGTAGTTTCCAGGTTGAGTCGGCAGATCATAATTTATCACAAGGGAGACTTGCTGCA

CATCAATACCGCGGGCCAAGAGATCAGTGGTGATGAGAACTCGAGAAGAGCCAGAACGGAATTCCCGCAT

AATGATATCACGGGTGTACAGGTCCATATCCCCATGGG

>FM893788.1 FM893788 Jatropha curcas embryo 56-70 (DAF) Jatropha curcas cDNA clone rjcaeb0_004713, mRNA sequence

ATTCTTTTTTTTGAAACCATAATATTCAATGGCTCTAGTAAAACAAAATTTAGTATCACTTGACAAAGGG

TATCAAAAAGTCAAAAGAACTAGCACGACGCCATCGTCACAGATAAGAAAAATAAAGCTCATTAAATAAA

AATCAGGAAATAAACGGCATGGGCTTTTAGTCTAAGCTACTGCTACTGGTACTGGGACCTCGATCTCGGT

CGCCACAACAGGTACCCGGGCGTACTCCTCTTCCTCCTTGGGGGGATGGATCGTGACCAAATCAGGGGGG

GGCCCGGTAC

>FM893787.1 FM893787 Jatropha curcas embryo 56-70 (DAF) Jatropha curcas cDNA clone rjcaeb0_004712, mRNA sequence

GATCCCCGGGCTGCATGAATTCTAATTGAATGGTGTTTGTTTGTATATGATGTTTGCTTTGTTTTGTTCT

GTTACTTCCCCTTGTGTGTAGTGCAGAGTTGAGGAACA

>FM893786.1 FM893786 Jatropha curcas embryo 56-70 (DAF) Jatropha curcas cDNA clone rjcaeb0_004710, mRNA sequence

TGCAGGACTAAACCAACGAAACTTTCTCTCACCGATCTTTGATCTTCTCCAAAAACCAATCACGAGAATG

TCTCTGATTCCAAGCACCCTCTTCGGTGGCCGGAGAACCAACATTTTCGATCCCTTCTCTCTCGATATCT

GGGATCCATTTCAGGGAACTTCCCTTTCACAAGCACTGCTCTTTCTGCTCCTCGTTCTGAGTTATTGAAC

GAAACGGCAGCGTTTGCCAACACCCGCATAGACTGGAAAGAAACACCAGAAGCTCATGTATTCAAGGCTG

ATCTTCCAGGCCTAAAGAAAGAGGAAGTGAAAGTGGAGATAGAGGAAGGTAGGGTCTTGCAGATAAGCGG

AGAGAGAAGCAAAGAGAAAGAAGAAAAGAATGATAAGTGGCATAGAGTTGAAAGGTCAAGTGGTAAGTTC

TTGAAAAGGTTTAGGCTAC

>FM893785.1 FM893785 Jatropha curcas embryo 56-70 (DAF) Jatropha curcas cDNA clone rjcaeb0_004709, mRNA sequence

TCGAAAGTAGTTTGAAACTTGGTATGATAAAAGATGTCGCGAAAAGGTTTGATGGAGCAAGACTTGAGCA

AATTGGATGTGACAAAATTACATCCACTTTCTCCTGAAGTTATATCTCGCCAAGCAACTATAAATATAGG

CACCATAGGTCATGTGGCACATGGGAAGTCAACAGTTGTGAAAGCAATTTCTGGTGTTCAGACTGTTCGT

TTTAAGAATGAGTTGGAGCGCAACATTACAATTAAGCTTGGATATGCAAATGCTAAGATATACAAGTGTG

AAGATGAACGGTGTCCCCGTCCTATGTGCTACAAGGCATATGGAAGTGGAAAGGAAGATAATCCCATGTG

TGATGTACCTGGATTTGAAAATTGTAAAATGAAATTGCTGAGACACGTCTCTTTTGTTGATTGCCCGGGT

CATGATATTCTCATGGCTACGATGCTTAATGGAGCAGCAATTATGGATGGTGCGTTACTTCTCATAGCTG

CAAATGAAAGCTGTCCCCAAACACAGACTTCTGAGCATCTTGCTGCTGTTGACATTATGCGTCTTCAGCA

TATCAT

>FM893784.1 FM893784 Jatropha curcas embryo 56-70 (DAF) Jatropha curcas cDNA clone rjcaeb0_004707, mRNA sequence

GAGAAGAAATTGATGAGAACTCTACTGTCTACATAGATGCAGCGCCAAGCGGGAACGGCTTGAGTTATCA

GGTAGAGAAAAATGGTGGCCTTGTAATGCAGCCACAGGCCAGAAGGCATGAAGTCTAATCAGTTACCAAG

TGTCCCAAGAAATGATGCAGCTCAAGCAGTAAAGAAGATGAAGATCGAAGAAATTGTTGACAATGATGAA

GATGAAATGGTTGAGTAAGTGTTCATATGGTCGGTGGGGATCCATTGCAGATGTATTAGCATGTTTTGGG

TATACATTTAGTTGTTTTGAAGTGTAAAATGGGGCAAGTAAAGAGGTGGTTGATTGGCTATCCTCTTGTT

GCATCTTCTATTATCAGAAAATAACTTCTGG

>FM893783.1 FM893783 Jatropha curcas embryo 56-70 (DAF) Jatropha curcas cDNA clone rjcaeb0_004704, mRNA sequence

CACAGCTTGTGGAACGAGAAAGAGAACGATTTGGACTGTTAGCAAAAAATGGGGAAACCATTGGGGACAA

CGGGAGAGTCTTTAAGCGAAGGGACGAATGGAGGAAGCATCCGATGCTCACCAATCAGTTCCGCCACGCA

ACTCCTGGTCTTGGCATCGCCATTGTCGCCTTCGGCATCTACCTTGTTGGAGAACAAGTCTACAATAAGC

TTTATGCTCTTTCGCCTACCCACCACGCCTCTTCGTCTTCTCACTCTCACTGATTTTTATCCATCAGTGT

TATTTTGCGCTTTTCAGATGGACAACTGGTGGAATGTTCAAGCTTGGACTCTCCATTATACTTGCCTTCA

TAGTAACTTGGATCAATAAGTTCTCATGTGAAATTGAGACTTTTAACTCTTATAGTTTCTTTGGTGTGTT

TGGATATGGGACATGCATTTGAGATGTTTTATAAAATGTATTAAGTTGCTCTC

>FM893782.1 FM893782 Jatropha curcas embryo 56-70 (DAF) Jatropha curcas cDNA clone rjcaeb0_004703, mRNA sequence

CGAATCCTTGCTGGTACGGTCATTGCACTGACCGTAGCCACTCCATTGTTGGTCATATTCAGTCCGGTTC

TTGTTCCTGCGGTTATCACAGTTTGTTTGATAATCACAGGGTTTTTAGCCTCAGGCGGTTTTGGCGTCGC

TGCCATTTCTGTCTTGTCCTGGATTTATAGGTATGTTACTGGAAAGCATCCACCAGGAGCAGAAAGTCTG

GACCAGGCACGCTTAAAGTTGGCGGGAAAGGCAAGAGAGATGAAGGACAGAGCTGAGCAGTTTGGACAGC

ATGTAACAGGTCAACAGACTTAAAAGAGAAGTGGGTTTTGGTGGTTGATGCAGAGAACATAAAATGGCTT

TGATATATATATAAAAATATATGAGTCCTTTAATTAACATGGAAGGCCATTGCTTGTCTGGTGTCTTTCT

TTTTTCGCTTCACTCTTTTCGTTCCTTCTATGTCATTTGTTCACGGGTCTTCCCTCTTTTG

>FM893781.1 FM893781 Jatropha curcas embryo 56-70 (DAF) Jatropha curcas cDNA clone rjcaeb0_004702, mRNA sequence

TTAAGTGGGAATGGTTCTGCAAAATTGGGTACAATTGCAACTGTAGTTGCTGCTGCCAATTCAACAGCTC

TCGAATGCTCTAAAGAAATTGAAGCTGCAATGCAGATTTCTTTAAGAAATGCTTTGGGAATGGTGAATAA

TAGAATAACTGATGGTCCAATGGATGATCTGGCAATCATGAAGGAGACACTAAAAGTTAAGGATGATGAA

CTGAAGAATTTGGCTCGGGACCTTCGTGCTCGTGATTCAACAATAAGGGACATAGCAGAGAAACTATCAG

AGACTGCTTAAGCTGCTGAGGCTGCAGCATCTGCAGCGCATACAATGGACGAACAAAGGAGACTTGCTTG

TGCTGAAATAGAACGGTTGTCAAAAGCTTCACAGAAGCAGCTGGAGTCATCCATGTTGAAGCTGAAAGAA

TTTGAAGAAAAAGTTATGACTCTCATCAAAGAAAAAGACGAACTGATCAAGCAGCGAGATTCTGCACATC

AGGAGGCACATTTATGGCGTTCTGAGCTTTGCAAAAGCTAAAGAGCGTGTTGTGATATTGGA

>FM893780.1 FM893780 Jatropha curcas embryo 56-70 (DAF) Jatropha curcas cDNA clone rjcaeb0_004701, mRNA sequence

TCCAAAGATGATGATCTGGAGGAACCTCCGTACACATTCTAATCCAACCGTCCAAATCCCTCCCTGGGAC

CCTTTTTTCGACGAGCCAAACATCACCGTTACCTTCTCCATTCGCTAACTGCGGAAATACTAACTCAGCC

TTTTCTCGACTCCTTAACCGCGCTCCACCGTTACCTTCCGTCAAATGAGCCTGATTCGTTGAGCGATGAC

TTGGACTTTGCTGTGGATGCTTTCTCGTGCGATCATTTCCGCATGTATGAGTTCAAGGTGAGGCGGTGCG

CACGTGGGAGGTCACACGACTGGACGGAATGTCCATACGCACATCCTGTAGAGAAGGCTCGCCGGACGTG

ACCCACGCCGGTATCATTACTCTGGTACGGCCTGCCCCGACTTTCGCAAAGGTGGGTGCAAGAAAGGTGA

CTCGTGTGAGTTTGCTCATGG

>FM893779.1 FM893779 Jatropha curcas embryo 56-70 (DAF) Jatropha curcas cDNA clone rjcaeb0_004696, mRNA sequence

TGAGCCTTAGCTCTTAGGAAGCAGAGGAGGAAATACTCTTTTTCTCTTTTATTTTCTCCTCTCTTCAATC

TTTACCCCTACGAAATAAACTACGGCTAAGGTTCCGTGTTCTTCCTTCAATAAAGATGTAATCTTTTTCT

GTGTTTGATATAGCATTTATATATATACATATATATATATAGATGGGCTTCTGTTTGATCTCTGTTTTAG

GTTCTGGGTTAGTTTAATTTGTCGACAAGAAAAAATCTTTGTGGTTTTTGGTTTTGGTGTGGGTTTTGAT

TATTTGAGGAGAAGAAATGGCTAATGGAAAAGATGAAAGCAAAAGCAAAGACTTCTATCAAGTACTTGGG

TTGAATAAGGAATGCACAGCAACAGAGCTGAAGAATGCTTATAGGAAACTTGCACTGAGATGGCACCCAG

ATCGCTGTTCAGCTTCCGGAAATTCAAAGTTTGTAGAAGAAGCAAAACAAAATTTTCAGGCAATANCACA

GGCCTACTCCGTTCTATCTGACGCCAACAAGAGGTTTCTGTACGATGTAGGTGCTTATGACAGTGATGAC

GACAAAAATGGGCTGGGTGATTTTCTGAATGAAATGGCGTTATGATGAGCCAAA

>FM893778.1 FM893778 Jatropha curcas embryo 56-70 (DAF) Jatropha curcas cDNA clone rjcaeb0_004695, mRNA sequence

ATTCGGACAGCCGATGTCCGCGGAACAACACATGCTGGACAAGGGAGCACAGATGTTGCAGTCATTGAAA

CCAATAAAACAAATGAGCCAGCATGTTTGTACCTTTGCTAGTTATAGCCATGACATGACTCGTCAGATCG

AAATTCACCACTATGTCACCAGACTCAACCAGGATTTCCTTCAGTGCGCTGTATACGATTCCGATGACAA

GAATGGCCGTCTCATCGGAGTGGAATACATAGTTTCAGATCGGATATTTGAATCTTTGCCACCTGAAGAA

CAGAAGCTCTGGCATTCCCATGCTTACGAGGTCAAATCAGGTCTGTTGGATCATCCCAGAGTTCCAGAAA

TGGTGGTGAAACCGGAACTGGAAAATTTAGCCAAAACGTATGGCAAGTTTTGGTGCACATGGCAAGTGGA

TAGAGGCGATAGGCTTCCATTAGGAGCGCCTGCATTGATGATGTCACCACAGGCAGTGAACTTGGGCATG

GTTTCTCCAGCCCTGATTGAGAAAAGGGATTACAAGTATAATATATTGACAGATGCTATAAAGAACTCGA

GGATTGAGATTGCAGAGCCAGAGTGGATCAATCCTCAGGCAGATTACTGGAAGCAGAATGGGAAAGGTTT

TGCCATTGACATTGAAAGGACTGAAATGAATTGAGGGCACCTTTCCATGATCATATATATGCAGA

>FM893777.1 FM893777 Jatropha curcas embryo 56-70 (DAF) Jatropha curcas cDNA clone rjcaeb0_004694, mRNA sequence

ATTCGAATTCGCTCGATGCAGCATCCATGATGCAGAACAGAGATCAAGTGGAAACGGCACAAAATGCCCA

ATTTTCCTCTTTTTTTCGGTTAATGGAAGTGGGCAATTTGTTGGGGCAGCTGAGATGGTTGGGAAGGTGG

ATTTTGATAAAGACATGGACTTTTGGCAACTTGACAAGTGGAATGGTTTCTTCCCAGTCAAATGGCATGT

CGTAAAAGACATTCCTAACAATCAGCTGCGCCATATCATTCTCGAAAATAATGATAGAAGGCCTGTAACT

TTTAGTAGGGATACCCAAGAGATTGAACTTAAGCAAGGTTTGGAAATGCTGAAAATTTTTAAGAGCTATT

CAGCTAAATCGTCGTTGTTGGATGACTTTAATTTTTATGAAATTCGAGAGAAGTCCCTTAATGTGAAAAA

GAGCAGTAAGCCTGCAACTCTGCGAATGGAAATACACAACAATGATGATTTCCCAAAGCAATCAAAACAG

GAGAAAGGACATTTGAAGATGACTTGAGGACTAAGAAAACGACTAATCCTTCATCTCTCATCCATCTAAC

TAAAAATCTCTCTCTCCACAGCTACAATCCGAAGAGTAACAATTCTATAAAGAAGCCCATAGAAAATTCA

GTCCCTGCACCATAAGCGTTTTTACTGTGCTTTTTAGTTCTTCCAATTCA

>FM893776.1 FM893776 Jatropha curcas embryo 56-70 (DAF) Jatropha curcas cDNA clone rjcaeb0_004693, mRNA sequence

ATTGAGAAAGCTGGGTCCTAGTCGTCTTGATGTCGCACACGTGAGGCTGGAATCCAGGACATTCATCACA

AAATTAGGCCAGAAGAAGTTGAGCTTGTTCGCCGTGATTATACAGCAAATGAAGGTTGGGAAACATTTCT

GTCATATGAGGATACGCGGCAGGACATCCTTGTTGGCTTGTTGCGATTGCGAAAGTGTGGTCGCAATGTT

ACTTGCCCAGAGCTCATGCAGAAATGCTCTATTGTTCGTGAGCTCCATGTTTATGGAACTGCTGTTCCTG

TTCATGGGCGTGAGGCAGACAAGTTGCAACATCAGGGTTATGGTACATTGTTAATGGAAGAAGCAGAGCG

GATTGCTCGCAATGAGCATAGATCAACTAAAATAGCAGTCATTTCTGGTGTCGGAACGCGGTAATTATTA

TAGGAAATAGGGCTATGAG

>FM893775.1 FM893775 Jatropha curcas embryo 56-70 (DAF) Jatropha curcas cDNA clone rjcaeb0_004692, mRNA sequence

ATTCCACCTGACAGGAAAGACCGGTTTATACGACGTTGGGTTGAGGCCTTGTCTGACCCAAGAGTCACCT

ATGAAATTCGCAGCATTTGGATCTCATACTGGTCTCAGGCTGACAAATCTTTGGGTCAGAAAATTGCCTC

TCACCTCAACATGAGGCCAACCATGTGATGATGTTTCTGTTTCTGGTGCTGAGCAGAAGCAATGTCTCAT

ATACTTAAAATAGTGATTCCATCAAATTCTTATTGTACCTTATATATGATATGTGATTAAGAAAAAAACA

ATGCAGTTTTGTGACACATACTGAAAAGAGTCTTTGTAATAAAGATGTTCTAATTAT

>FM893774.1 FM893774 Jatropha curcas embryo 56-70 (DAF) Jatropha curcas cDNA clone rjcaeb0_004691, mRNA sequence

ATTGTTGTATTATCGTTTTTTACACCCTGCCAAAAGATTATTATTATTATTATTATTTTTATTTCGTATG

TTCCCGTCATTGCTTCTTAGTTATTAGCATCCACTTGTCTGTTTAGGCTCATAGCATTGCTGTGTCTCTA

ACATTAACCATAGTGTAAGAGCAAAAGAGATGTAACTGGCCAAAGGACGCCCGCTATAAACAATGATAAT

ATAAATAATAGAG

>FM893773.1 FM893773 Jatropha curcas embryo 56-70 (DAF) Jatropha curcas cDNA clone rjcaeb0_004690, mRNA sequence

GACTCGGAGCATTCAAATGACAATGCTCGTAATATATAACCCCATCTCACACCGCTTTATTCAATTTAAA

AAGCAGAAAAGAAAACAACATATAATAAAAATAATAACTATTCAACTTCCTCGTCCTTCTCATCCTCACT

CCAGCAACATTTCAGGATTGACCTTGGAGATGAACAGCCTTTGGTTTTCACAAAACTCTGATAGATGATC

GATCCACCCTTCCCAAGAACTTCATAGTTACTGCCTCCGCAGGAATATCCCTTCACATCCAGAGACGACA

TAAGTCCAAGTTCATCATGCACAAAGTCAGAATGCAATGCAATGGAGAACTCGGCCGGTTCAAAGCAAGC

CGACACCCTTTCGAGCAGCAGAGTTAATTTCACTTGTTCAAGATTGTAACCAACAGCTTCAAAGCTTGCA

TAGCTAAATCCGTCTTCCGGAGTAACATGGATTGTACAAATTGCACCCCCTTCGATGGAA

>FM893772.1 FM893772 Jatropha curcas embryo 56-70 (DAF) Jatropha curcas cDNA clone rjcaeb0_004689, mRNA sequence

ATTCCCAAGTTGCNTTGTGGTCTGCACAATCGACGTAATTAAATAAGTGACTGATCGATCTGCTCCTCCA

AGCATAGAGCGCTAAGAAAAAATGGCGACCGGCACAGCCAATCCTGCACTGGATCCGGATCTCGACAAAC

CCGATAACCCGTCTCACGAATTCGCTCAGTTCGGAGCAGGCTGCTTCTGGGGAGTGGAGCTGGCTTTTCA

ACGAGTGGTTGGGGTGGTGAAGACGGAGGTCGGCTACTCTCAGGGTCATGTGGATAATCCCAATTACAAA

CTGGTTTGTACCGGCACCACTAACCACGTGGAGGTGGTGCGCGTCCAATTCGACCCGGAACTTTGCCCAT

ATACCAATCTCCTCTCTCTTTTCTGGTCTCGCCACGATCCTACAACCCTAAATCGCCAGGGCGGTGATGT

GGGTGCGCAATACAGATCAGGAATATACTATTACAATGAAACCCAAGCAAAGTTAGCCCAAGAATCAAAG

GAAGCTAAGCAGCTGGAACTTAAAGATAAGAAGATTGTGACTGAAATTCTCCAGCAAAAAGGTTTACAGA

GCAGAAGAGTATCATCAGCAATATCTTGAAAAAGAGGAGGTGAGGGATTGAAGCAATCTGCCT

>FM893771.1 FM893771 Jatropha curcas embryo 56-70 (DAF) Jatropha curcas cDNA clone rjcaeb0_004687, mRNA sequence

ATTCCACTTACATCCTTCAAATTGTCTAGATCATAAGCCGGAGTGGGTTATTTATAATGAATATGTCCTA

ACAAGTCGGAATTTTATTCGCTACAGTGACTGATATTCGTAGGTGAATGGTTAGTAGATGTTGCACCGCA

TTACTATGATTTAGCCAACTTCCCTAATTGTGAGGCCAAGCGAGTTCTCGAGAGACTTTACAAGAAGCGG

GAGAGGGAGAAGGAAGAGAGCAAGAATAAAAAATGAAGTGTGAACATGTCCAAATATTTTGATTTATTCA

ATTTTCTTCACCTGATAGTGTGACATTGGTGCTCCTTAAAGTGCTATTGTATGACAACATCCTCCCACTG

TGCCGCCATCATCCCCTTTCCCCCTGGGTGCTTTATGCCCTTCTCCGGTATCAACCGGTCAAGCTCCATC

CATTTCTTGAAATTATAGGTTTAGAAATCAGCATATTCTATTGTACCCTTCGCATTCACATTTATTTAAT

AGATGTGGCTATATGGCGTAGGTTGCTATTAATGTTTAGTTGAACATCGGAAAAA

>FM893770.1 FM893770 Jatropha curcas embryo 56-70 (DAF) Jatropha curcas cDNA clone rjcaeb0_004686, mRNA sequence

ATTCCGCGAATGGAAGAAGGAGATGGAGCAAAACCTGAAACCTCTCACTCCTTCTCTGCTCCCATCGCGG

CCGTCTACTACCGGATTCCTCCACGCCGGCGTCATGAAATTGATTTTGTTCGGTTCTTTGGCGGCCGGTA

TTATTGTCAAGCTACTTCCCTACGAGTACACTGACCGCGGCGATCCAGTTCCAACCGGCATATTCAAGGG

GCTTCCGGTGACAACGTTTCATGTTTTTGTAATCTCGATCATTTTTTCCTTCGCCGGGGCGTTTAGCTCG

TTGCTGACTAGCGGAAGACCAAAAAATGCGAATTATGAAAAATTCTGCCGGTATTATTCGCTTGTGTTTT

CGGCTTTGGCCATAGCAATTCTTGTGCGGGCTGCGATAAGGTTGACTTTTCGATGGGCGCCGATTGTTGA

TGACTAAGGACAGATCCGAGTATTAAAGAATCAGGTGACTATGTAAGAGATAGCTATGGCGTTTGCTGTG

CGTTGCTTTCTCTGATCTGCCTGGCAATGTTAAAGATGACAAACTCAATCCATGTAGGAAATAGCTATGG

CTATCGCTGTGTGTTTCTTCATCTGATCTGCTTGGGCATTGGTGATCAAATCAATCAATAAACTTGCGCT

TTTCGTAAAAAAAAAAGGGGGGGCCCGGTAC

>FM893769.1 FM893769 Jatropha curcas embryo 56-70 (DAF) Jatropha curcas cDNA clone rjcaeb0_004683, mRNA sequence

TCGTAGCTTTCGATCTTTCACAACATCAATAAGGAAAAATGGCATCGGCGATTGCACTAAGGAGAACAGC

ATCTTCTACTCTGATCTCGAAGCTCATAAACCCTGTTCGCTCTGCTGCCGTGGCTCCCTCTGTATCTCGT

TTCTTCAGCACCGAAACCCAGGTCACAAACATCGGAGGTGATGATCTTGATACCGTCGACGTGGATCGCC

GCTCCACAGGTCGCAGCGTCTCTCGCCGCCGCGATATGTCTCCTGGTTTCTTCCCAGATATAGGTGGACC

CATTTTCGACAACAAGAACTCTAAGCCAAGT

>FM893768.1 FM893768 Jatropha curcas embryo 56-70 (DAF) Jatropha curcas cDNA clone rjcaeb0_004682, mRNA sequence

AAACCAAAAGTGACCGTTTTTTCATTTTGCAAGTCACAAACCAACTACAATTCTACGGAGTGGCAACTAA

ACATAAAGCCAAAAAAGAAGACCCAAAAACCATGGGATGCCACTTCCTGTCAGTGGGGAATACAACAGGT

TACACACTGATGCCCAGAAAACAAACCCCGACACCAACCAAAAACAACCAACATACCGCAGTTACTACTA

CCATATGTACAAACCTACTCGCAACTAGTTCAAAAAGAGGAACTACTAGTTAAAACACTGATATGAAGAA

CCTAACTAACGGGTTTTATACTTGGACTCATCAATTTCAATCCCCTCCTCAGCAGCACGTTCTTC

>FM893767.1 FM893767 Jatropha curcas embryo 56-70 (DAF) Jatropha curcas cDNA clone rjcaeb0_004681, mRNA sequence

TCGACTGGAGGAAGGTGCTGAGATAGTAAGGGGAAGGACTGCGTGGAGGCCAAAGTATCGCAGCAACTTT

GGAATTATGGGTCAGATTCCAGTTGAAAGTGCCTAAGGATCCAATACCCCTCTGTTCTTTGTCGACTGAT

GTGGCATTAAGTCTCTTTATGTCACACAACGCCTGTGTAGAATCTTCCTTTGTTCAGTTCTTGGAATTAA

TAGATGTTCCTTCTTATATATATTATATTTATTTATTATATATATTTTTCTGATTTCTTGAAAAAAAGGA

AGTTGTAAGCTTTTGTAATTACTAATTAGTTCTTGCT

>FM893766.1 FM893766 Jatropha curcas embryo 56-70 (DAF) Jatropha curcas cDNA clone rjcaeb0_004680, mRNA sequence

ATTCCCTAAAGCTATTCTCTTTTTCCTCTTTTTCGCTTTCTCTTCAACGTTCTCTCAAGAACATGACGCC

GCCACCTGGACCCTACTCCGGCCTCAGCACCTTGGCCCTGGTGGCTCGTGTGTCAGCTTTCTCTGCGGGT

CTTGTGTATGGGAACTTGAAGCTCAAATATCTCCGGGCAAAGGCAAAATCTCAGAAGAAAGCTGAAGCTA

AAGCCCAAGCAAAAGCGGCCCATCACTGAAGAGAGATTTGTTATTGAAAGTTGGTAGACTTGAGTTTTGC

TGGCATAGGAATAATGCATAGCAGCAGAATTGTACCAGATTCTTGTTTTGATGTAAAGCTTTTCTAAACC

ATGGTACACTGGTTATAATACTTTCCAAGATTTTTTTTTTTTTTTTAAGGTTCTTAACCTGGTTTTATAA

GAATTGTTTATTTGGTCTGCCTTGGCCACCATGAAGGATATCTTATATTGCGTACCTGACAATGTGTAGG

TGATGGAAGATTAGATCTTCCACTGATTGAAATAATAAATATATGCAACTTTTTGTCT

>FM893765.1 FM893765 Jatropha curcas embryo 56-70 (DAF) Jatropha curcas cDNA clone rjcaeb0_004679, mRNA sequence

ATTGGTTGTCACTCTTTTGCCGGTGAGTGGGACGCTTCTTTTCCTTGCTGGAATAACGCTTACTGGAACA

CTCATTGCGCTAGCTGTTGCGACTCCGCTGTTCGTGATTTGTAGTCCGGTGCTGGTACCAGCGGCGTTGG

TCATTGGGTTGTCGGTGTTAGGGTTTTTGACGTCCGGTGCTTTTGGAATCACTGCGCTTTCTTCACTGTC

GTGGATGGTTAATTATATTCGCAGGATGAGAGGATCTTGGACGATGCAGATGGAAATGGCAAAGCGGCGC

GCGCAAGAAACGACGGGTCAATTGGGCCAGAAGGCTAGGGAGGTGGGCCAGAAGGCCCAGGAAGTTGCAA

AGACATGATCAGGTTGTCTAGCAGTAGTTGTTGTTTGGTAAATAATATAAGCTAATATAGTTTGAGTTTG

ATGATGGATTTCATAAATATGTAATAAGACTTGTTTGTTTGCCCATAGCTTTGGTTGGCTGTATGTTTCT

GTTTACTGAAATAAATTTTATTTCTTGATCCTAAAAAAAAAGGGGGGGCCCGGTAC

>FM893764.1 FM893764 Jatropha curcas embryo 56-70 (DAF) Jatropha curcas cDNA clone rjcaeb0_004678, mRNA sequence

ATTCCTGAGAAGCTGTCAGTTCTTGCTGAAAGTAATGAGAGGGCGATAGAGGCAAGAGTCGGTGGTGGTG

GTGGTTTGGATCTGCCTGTGAGGCGGAAAGAGGGCCAGGATTATGCTAGCATGGCTGCTGCAGGTGGCAA

GTGGCAAGACTCTTACACTCCGGGAAGGCAAGGTTCAGGGCGTTCAGGATATAATGTTGGTGGTGGCAGA

CCACCAGCACTAGGCCAAGCTACTGGTGGTGGCTATTCAAGAGGTCAGTCACGAACAGGTGGATACTCAG

GCGGGTCAAGGTATCTAGATGGTGCATATGGTGGTTCAGGAAGGACTTCTGCAAGGGGATCTCAGTTGGA

TGGTTCAAATCAAATGGTTAGTCTCAACAGGGGGGTTCGTGCATAGAGGAATAGTAAGAGATGCAATCAA

TTTTCTTACGAGTAGACAAGTCTTTTCCACCTGTTCCTGAATTTTGTTTGTTGTTTTTGTGCCCTTTAAT

TTCATTTCTATTATAATCTGAATTGGGACTAAAAAAATAGACGTGTAAGTTATATTTTC

>FM893763.1 FM893763 Jatropha curcas embryo 56-70 (DAF) Jatropha curcas cDNA clone rjcaeb0_004676, mRNA sequence

AATTCGAATTCGTCTGCGTAAACAGATTAAGAAGATGGAGGTCAGTCAGCACAGCAAATACTTCTGTGAA

TTCTGTGGGAAGTACGCTGTGAAGAGAAAGGCTGTGGGAATTTGGGGCTGCAAAGACTGTGGTACAGTCT

AAGCAGGCGGTGCCTACACTTTGAACACCGCCAGTGCCAGTGACTGTGAAGAGCACAATTACGAAGGCTT

AGAGAACATACCGAGAGCTGAGCTACCATTGAACCTATCTGCTGCTGTATAGGTTATTTGCATTGTTATC

TTCTTAGTAATTGACTTGTTTGAGGATTTTAATTTAATTTTTCTTACAAGTTAAAGTTCTTACGTGTAGT

GCACTTGTGTTTTGAAGCTTACGTTATTAAGGCTTTAACTCTATCGTATGCTTGGTTTATCCT

>FM893762.1 FM893762 Jatropha curcas embryo 56-70 (DAF) Jatropha curcas cDNA clone rjcaeb0_004675, mRNA sequence

AGCAGAAAACAGGTTTAGATTCAAAATTAACCAACACTCAACCCAAAACAAAATAATACCAATAAGGTTC

ATCAGCCTAATGCAAAGAGTATAGGCAATAGCAAAATAAACATAATGTTTACTCATCCTCCTGGCTCCGC

AACCTAGCCAGTTTGTTGGTAACAACAACATCCAACTGAGCTGCACGCTCAACAATCTCCTTTCTCTTTC

TGGTGGATACATCATGGGCAATCTCAGCACAGTAAGTCCTGTTGTGCATCATCAGAAGTTC

>FM893761.1 FM893761 Jatropha curcas embryo 56-70 (DAF) Jatropha curcas cDNA clone rjcaeb0_004674, mRNA sequence

TCGAATTCATTCTGTGTCTTTCTTTGTATCTCCCGTCGCTTTCGTTTTTGACCGTCGCTTTCGTTTTTGA

CCNGTCGCCTCCGGCAGCAGTCGTGCTCTTCAAGCGTACAAGAGCCACATTCTTGTTCATGTGCGGAGTC

GAATGGTGTCTTTTGTGCGGAAGAAGATTATGTTGATTGTTAATCCTATGCAATCTTGGTTCTTGAAAAG

TGCAGATACAGCTAGAA

>FM893760.1 FM893760 Jatropha curcas embryo 56-70 (DAF) Jatropha curcas cDNA clone rjcaeb0_004672, mRNA sequence

ATTCATAAAGTGTAACAGAGCAAAAGATTAATTATATTGGTGGAGAAATTTGTTAGCAAAAAAGGGCAAA

AATGTCTTCAAATCAATCAAGACAAGAGCTTGATGAAAGAGCAAGGCGTGGAGAGACTGTTGTCCCTGGA

GGTACTGGGGGCAAGAGCCTTGAAGCTCAAGAGCATCTTGCTGAAGGGAGAAGCAGAGGAGGACAGACAA

GGAAGGAGCAGCTGGGGACAGAAGGATATCAGGAGATGGGCCGGAAAGGTGGGCTGAGCACGACGGACGA

GTCTGGTGGAGAAAGAGCTGAGAAGGAGGGCATCACTATCGATGAGTCGAAGTTCAGGACTGGGAGCTAG

TGTGATGATTAAAACTATAAATGGTATTTTGTAGTAATAATAATAATAATGTTGGTAATAAAGTATTAGC

TTAGCAGTGTACTTTAGTTTAGTAATGTAGTTTACATGTGGGTTAAAATGACTAGGGTTTAGCTAGTATG

TGTGTTGTAACTTTGAGAGGGGTTTATGGTCGTTCCCTTTCTGGGAGGACTGCTAATACTTTCTCCCTAT

TTGTAAAATAACCTGCAGTATATATAATTACCTGTTGGTTAA

>FM893759.1 FM893759 Jatropha curcas embryo 56-70 (DAF) Jatropha curcas cDNA clone rjcaeb0_004671, mRNA sequence

ATTCATCAAAAACAACAGCACTATAAAAACTACCAACAGCAACAACAGAAACCACAGCTTCCTGCAGCGG

AGGATGTCAAGACTATATGGATTGGTGACTTGCTTAATTGGATGGATGAGCCTTATCTACATAATTGTTT

CTCTCATACAGGCGAGGTTTCCACCGTCAAAGTTATACGCAATAAGCAAACTGGTCAGTCAGAAGGGTAT

GGATTCGTTGAGTTCCATTCACATGCGACAGCTGAGAAAGTCTTGCAGAACTATAATGGCTCTGTGATGC

CAAACACAGAGCAGCCTTTCCGCCTCAATTGGGCAAGCTTTGCGGGTGATAGGAGAGCAGATTCTGGTTC

TGATCTATCAATATTTGTTGGAGATTTGGCTACAGACGTGACTGATTCTATGTTGCAGGAAACCTTTGCC

AGTAAATACTCTTCTGTCAAGGGAGCAAAAGTTGTCACTGATCTAAATACTGGCCGCTCAAAAGGTTATG

GCTTTGTTAGGTTTGGTGATGAAAATGAGAGGTCAAGGGCCATGTCAGAGATGAATGGTGTTTATTGCTC

TAGTAGGCCAATGCGCATAGGTGTTGCAACTCCCAAGAAGTCATCTGCATATCAACAACAGTACTCTTCA

CAAGCTTTGGTGTTAGCTGGTGGGCATGCTCCAA

>FM893758.1 FM893758 Jatropha curcas embryo 56-70 (DAF) Jatropha curcas cDNA clone rjcaeb0_004670, mRNA sequence

TGCGGTGATCGTCCTTCAAGGCACGCTACGCCGGTCGCAAGGCGGTAATCATCAAGTCCTTTGACGAAGG

CACACGCGACCGCCCTTACGGTCATTGTTTGGTTGCAGGGATAAAAAAGTACCCGAGCAAGGTTATCAAG

AAGGACTCAGCAAAGAAGACTGCCAAGAAATCCCGCGTCAAGTGCTTCATCAAGCTTGTCAACTACCAGC

ATTTGATGCCCACTCGCTACACTCTTGACGTCGACCTTAAGGACGTTGTCACCGTCGACTCTCTGCAGAG

CAAGGAGAAGAAGGTGGCGGCTGCTAAGGAGACTAAGGCGAGGTTCGAGGAGCGGTTCAGGACTGGAAAG

AATAGGTGGTTCTTTACCAAGCTCAGGTTTTGAGGATTCATTTCTTA

>FM893757.1 FM893757 Jatropha curcas embryo 56-70 (DAF) Jatropha curcas cDNA clone rjcaeb0_004668, mRNA sequence

ATTCCTTCTTTTTAAAGGTTTCCCTCTCTTTTAGTTCTATTTTAAGATCAAGAATGGCTTTGGCACGACT

AGCCTTGAGGAACTCACTGTCCACAAGGGTTAGTTCTTCTTCTTCTTCTTCTTCTTCTTTAGTGGGTAGT

GTTGGTGATGTGAAAGGGCAAATTTGGAACAGTGAATTGTTGAAGAGGTTTATGGTAACTGGTGCTGATA

AGGTAGCTGGTGAGACCTCAGATGGCAAAGAGGTTGCTGTGAGTGATAGTAAGAAGCCTTCTAAGCTTTT

TTCTAAGAGGAAAGGCAGGAGGGGCCTTTGGAGGGGCAACGGACAAGAATTTGTTCCTCAACTTTTTGGA

TTAGGAAATGCACTATTGCAAGCAAGTGAAAACATGAACAGACTAATCCAAAACCTAAATTTGACACCAA

CAAACCTGATGGGTCGTTTCAAAGAGAAAGACCAGTGCTACAAAGTAAGATATGAAGTGCCTGGACTTTC

CAAAGAAGACCTGAAAATCACCATTGACGATGGAGTTTAAACAATAAAAGGAGAGCATACAGAAGAGGAA

GAAGAAGGATCAGATGATG

>FM893756.1 FM893756 Jatropha curcas embryo 56-70 (DAF) Jatropha curcas cDNA clone rjcaeb0_004667, mRNA sequence

ATTCCTTCTTTCCTCGCTTATTCTTCAATGGACCTGAACTTGGGACCTGGAACTCATAAGTGGCTCTATT

TTATTGTTTAAGCAATGTCCAACTTCTACATTATCGAAAATATGCTATCTTTGTAATCTGGATAACAGTA

TGTGAGATGAATTGCTGATTT

>FM893755.1 FM893755 Jatropha curcas embryo 56-70 (DAF) Jatropha curcas cDNA clone rjcaeb0_004666, mRNA sequence

ATTGAGATTTCAAGCAACAGCTCCGAGACTTGACCTTTCAGTCTCCGTTGCCAACATCTCTCGTGGAGCA

ATGTCTGGGCCCTATTACAACTCCAGAGTAACCAAGATATCAGTGGTTTTGGATGGCGAAGGCTACTTTG

AAATGGCTTGTCCTCACCTTAGCGGCTCGCGGGGAGACACGACGGGTGAAAGTCAGAAGACTTACCAGAA

AGTAAGTTCACGGTTGAAACGTGGAACAGCAGTGGTCGTCCCTGCAGGCCATCCGGCGGCTTTTGTAGCT

TCAAGAAACAATAATCTGGAAGTAGTCTGCTTTGAGATTAATGAAGAAGGCAATACTAGGCATCTACTTG

CAGGGAAAAATAATATTGTAAATAAGATGGAGAAGCAAGCCAAAGAGTTAGCCTTCGGTGTAAGTGAAAG

AGAAGTGGATCAGGCTTTTGGAAG

>FM893754.1 FM893754 Jatropha curcas embryo 56-70 (DAF) Jatropha curcas cDNA clone rjcaeb0_004665, mRNA sequence

AATTTGGGTTCTGGAACATTGAATAGAAGTCGACATGTTTCATCAAATAATTTTGGCTCTCTAGCTGAAG

ATGCAAAAGCAAGAGCCCTGGTCAGAAGCATGGGTGAGATATTGTTCCTATGTGGAAGTAATAAGCGGGC

TGTAATTGTTACTTTGAATGTCATTGGCTGTGACACTGTGGGCTCTACAAAGGATGAGATTATTGCAAAA

GCACTCGAGGGCCTTTCAATTGAATCAGCTTCTACTCTGCAGAAAATTTTAAGAATTGAGACATACACAT

CCCAACCAAGTGCATTGCAGAGGCTCCAGGACATGATTCCTGTCTTCCAATCTCGAATGGGGGCACTTCT

ATTCCTTATTTCCGCATTGCTGTCCAGAGGATTGGATTCTGTTCAAGCTGACAGGGATGATCCCAGCCTT

CCTTTAGTCACTGCACCCTTTGGGCATGCCTCACAGGAAATTGTGAATCTACTGCTCTGTGGACAGGCTG

TTCCTAATGTGTATGATGGTAGAATGGAT

>FM893753.1 FM893753 Jatropha curcas embryo 56-70 (DAF) Jatropha curcas cDNA clone rjcaeb0_004664, mRNA sequence

ATTCGGAAGTAGTCTGCTTTGAGATTAATGAAGAAGGCAATACTAGGCATCTACTTGCAGGGAAAAATAA

TATTGTAAATAAGATGGAGAAGCAAGCCAAAGAGTTAGCCTTCGGTGTAAGTGAAAGAGAAGTGGATCAG

GCTTTTGGAAGACAAAACGAGGAGTGGTTCTTTCCAGGGCCAAGGAGAGGTTCCTTCGAGGGTCATGCTG

TGGCATGAGAAGCTAATAACGTGATGGCAAGTGTTTCGGCTTTTTGGGGTGACTGTGACATCGACCTTGA

GGGTTTACTAATGTAAATAGTGCTGTGTATGGTGAATAAATAATGAAAGGTGAAGGTACATGTGTAAATT

AGTCTTTAAGAAAAGAGAAAGATCACTGCTGCGGACTATAGTGGCTTCTGGTTTAATAATTAATAA

>FM893752.1 FM893752 Jatropha curcas embryo 56-70 (DAF) Jatropha curcas cDNA clone rjcaeb0_004663, mRNA sequence

ATTATCAAATATCACTGATCGTAAACCTCCATTGAATTTACTCTGTGCTTCGATCGCGTCAATGGCGGAG

CACCCACAGTCGCAGCATGTTGGCCAACAGCCACGATCCCACCAAGTAGTGAAGGCAGCCACTGCAGTCA

CTGCCGGTGGATCTCTTCTAGTTCTCTCCGGTTTGACCCTTGCTGGTACGGTCATTGCACTGACCGTAGC

CACTCCATTGCTGGTCATATTCAGTCCGGTTCTTGTTCCTGCGGTTATCACAGTTTGTTTGATAATCACA

GGGTTTTTAGCCTCAGGCGGTTTTGGCGTCGCTGCCATTTCTGTCTTGTCCTGGATTTATAGGTATGTTA

CTGGAAAGCATCCACCAGGAGCAGAAAGTCTGGACCAGGCACGCTTAAAGTTGGCGGGAAAGGCAAGAGA

GATGAAGGACAGAGCTGAGCAGTTTGGACAGCATGTAACAGGTCAACAGACTTAAAAGAGAAGTGGGTTT

TGGTGGTTGATGCAGAGAACAT

>FM893751.1 FM893751 Jatropha curcas embryo 56-70 (DAF) Jatropha curcas cDNA clone rjcaeb0_004662, mRNA sequence

GGGCTGCAGGCTCTCTCTCTCTCTCTCTCTCTCTCTCTCTCTCTCTCTCTCTCTCTCTCTCTCTCTCTCT

CTCTCTCTCTCTCTCTCTCGTCTTGTACTATGCCTTTTCACTAGACTCCTGAAAACTGTGGCTTGAAATT

TGCCAGAACGCTTGCGGATAGGTTTGTTTACGGTGACAACCCACTTACCAGGATGAGTTCTGTGGCTTAT

CTTGCTAGTTATTTGGCTCGTGCAAAGTTCCTGTCTTCTGCTTATGTTGCTAACACGTTGACGAGGCTGG

TGGATTGGTGTTTGGAATACAGTAAAACCCAAGATGGTCACATGAATCCAAAAGCACATCGAGTTATCTA

TTCTGGATGTCAGGCCATCATGTATGTGCTTTGCTTCCGTATGAGGTCGATAATGGATGTTCCTGCGCTC

CAATCCCATCTTCTCCTCAAACCTATGGAGCTCATCTTGA

>FM893750.1 FM893750 Jatropha curcas embryo 56-70 (DAF) Jatropha curcas cDNA clone rjcaeb0_004661, mRNA sequence

ATTCGAGAACAATGGCATGTCAGCTTCAGTTGGTAAGGACAGTACAGATTGTCAAGATACCATACCAAAT

GGGATCGATTATTTTGAGTATATGACCTTAAATTTGACAGAAACAAAGTTAAAAGAATACCATTATGAAT

CTGAAGTTATAGATAACACAAAAGATGAAGTCACATTGTTAAGACGGCCGCGGAGAGGTCAGGCCCGGAG

AGGAAGGCAGCGGAAGGACTTTCCGAGAGATCTACTTCCCAGCCTTTCCTCTCTCTCCACGAATGATGTG

GCTAATGA

>FM893749.1 FM893749 Jatropha curcas embryo 56-70 (DAF) Jatropha curcas cDNA clone rjcaeb0_004660, mRNA sequence

AATTCGAATTCCATGTTCCTTCCAGTACTTGCTGGATAAAGGAAGCTCAGCTTGATCTTCTTATCAGCGC

CCACAATATGCAGAGCTCGGGAGGGAAGACTG

>FM893748.1 FM893748 Jatropha curcas embryo 56-70 (DAF) Jatropha curcas cDNA clone rjcaeb0_004659, mRNA sequence

ATTCATCATGACAAGGCAAAATGAGGGTCCAATCGTATATTTGGACGATTCTCGAGCACCGAAAGCAAGC

GTATGGAAAAAGTCATTCAAATGAAGGAGCAAGACAGACTTCAGCACCTAAAGAGAATGATGAAGTCCAA

CAAGAACCAATGGAAGAAGAGGAAGAAGAGGAGGAAACAACATGGTCATGGAAGAAACTCATGAGTTCTG

TCTTCGGGCAGGAAAATGAGACAAACAAGCGTGAGAGGACCGG

>FM893747.1 FM893747 Jatropha curcas embryo 56-70 (DAF) Jatropha curcas cDNA clone rjcaeb0_004658, mRNA sequence

CTACTAAAAGATACCTTTTATTCAACTGGAATTGCTTAACAAGAAAAAGACACCAAACAGAAAACATAGA

TATATTACATAACAGGAGCTAAAGCACATGCCATGCCCTACACACGTGGGTTATGGCCAGTGGTGTCTCC

GGGCAACCCAGATGGGCGAGTCCTGGTCAAACCCGTTGGGTCCACCTCTCCACCGTATGGCT

>FM893746.1 FM893746 Jatropha curcas embryo 56-70 (DAF) Jatropha curcas cDNA clone rjcaeb0_004657, mRNA sequence

AATCGAATTCCACTTGCAGACTACAATATCCAAAAGGAATCCACACTCCATTTGGTGCTGCGATTGAGGG

GTGGTATGCAGATATTTGTCAAGACTCTCACTGGCAAGACTATCACTTTGGAAGTTGAGAGTTCGGATAC

CATCGATAATGTCAAGGCCAAAATACAAGACAAGGAGGGTATTCCACCAGATCAGCAAAGATTGATTTTT

GCTGGGAAACAGCTAGAGGATGGTCGCACACTTGCTGACTATAATATTCAGAAAGAGTCTACGCTTCACT

TGGTGCTCCGACTCCGTGGTGGTATGCAAATCTTTGTGAAAACCTTAACTGGCAAGACCATCACCTTGGA

GGTTGAGAGCTCTGACACCATTGACAATGTGAAAGCCAAGATCCAAGACAAGGAGGGTATTCCCCCAGAC

CAGCAGAGGCTTATCTTTGCTGGTAAGCAGCTCGAGGATGGGCGCACCCTTGCTGACTACAACATTCAGA

AGGAGTCCACCTTGCATTTGGTGCTTCGTCTCCGTGGTGGCATGCAGATTTTTGTGAAGACCCTTACT

>FM893745.1 FM893745 Jatropha curcas embryo 56-70 (DAF) Jatropha curcas cDNA clone rjcaeb0_004656, mRNA sequence

ATTCCTCTCTCTCTCTCTCTCTCTCTATTTATCTTTTCCTTCTTTTCTCTCTCCCTTACAAGCCCTAACC

CTAACTCAGACCCAGATTCGCCATGAAAGGAGGTAAATCAAAGTCAGACACCAAGAACGCCAAGCTTTCC

GTGAACAAGAAACCCGCCAAGGCAAGTAAGAAGGCGGGTAAGGCAGCGAAGGATCCAAATAAGCCAAAGA

GGCCCGCCAGTGCTTTCTTCGTTTTCATGGAAGAGTTCAGAGAGCAGTACAAGAAGGAACATCCTAAAAA

CAAATCCGTTGCTGCTGTTGGCAAAGCTGGTGGAGATAAATGGAAGTCAATTACAGATGCTGAGAAAG

>FM893744.1 FM893744 Jatropha curcas embryo 56-70 (DAF) Jatropha curcas cDNA clone rjcaeb0_004653, mRNA sequence

ATTCAAACATCAAATAAGCATTCCAATTTTCATCATAGTGATGCAAACACAGAAAACTACTACTAGTTAC

TCATTTACATGTACTTGTTACCTTTACATAGGCTTTCGATCGATAGCTTAGACTTTGCACGCAACCAACA

AAAAAATACAAACAAAAAGCACCAAAACGCAACATACATCCAAATTTCGCAAAACGCTCACCCCTAGTAC

TGTATTTTAGTCATACCCATAACTCCAGAAGCTGCAGCGTTAAAAGCTTTATCTATTAGTAGTAAAAGAC

CATATTTGCACTTCTAGCCAGTGATCTCAATAGGCTTAATCTGAGGCTGCTTCTCTTGTTCTTTAGGCAC

AACCACTTTAAGCACACCGTTCTCCATAGTGGCCTTGACTTGATCCATCATTGTGTTCTCCGGCAACCTA

AACCTCCTCAAGAACTTACCAGTGCTTCTTTCTACGCGATGCCACTTATCGTTCTTCTCTTCTTGTTCTT

TGCTTCTCTCCCCACTTATCTGCAAAACTCTGCCATCTTCTACCTCCACTTTTACCTCTTCTTTTTTCAA

CCCAGGAAGATCAACAGCAAATATGTGAGCCTCCGGAGTCTCCCTTCCAGTCCACTCGTGCGTAGGCCAT

GGCGGCTG

>FM893743.1 FM893743 Jatropha curcas embryo 56-70 (DAF) Jatropha curcas cDNA clone rjcaeb0_004652, mRNA sequence

TCCCTTCTGTTGCCATCTTATAGCAACGCTCCTCAACTCGTTTACATTGTCCAAGGTACGGGTATGACAG

GAGCCCTGTTTCCTGGATGCGCTGAAACATATCAGGAGTCTCAAGGATCAGGTGGTAGCAGTAGGGTTGA

AGCCCAGCACCAGAAGATTCGTCGTTTCCGTCCCGGTGATATCATTGCTTTGCCTGCCGGAGTTGCCCAT

TGGTGTTACAACGACGGCAATGAGCCTGTTGTCGCCGTTTCTGTTCTTGACACCGCCAACAGCGCCAACC

AGCTTGACATGAACCCTAGACATTTCAATCTAGCTGGTAACCCAGAAGATGAATTCCCGCAACTGCATGA

GGGTCGCCGTGAGGGTGGCAGCCAGGAAAAATATCCACGGGGCCGCCTTTCCTCGAGAGAACGTGGCTCT

TGTAACAATCTTTTTTGCGGAATGGACTCGAGATTTGTTGCTGAAGCTTTCAATATCGACGAATCGTTGG

CTAGGAAACTCCAAAGCGAGAATGACAACAGGGGCAATATTATCAGTGTTGAAGATGGTGAGCTTCCAGT

GGATAGACCAACAAGAACTCAACAAGAGGCGCAACAGC

>FM893742.1 FM893742 Jatropha curcas embryo 56-70 (DAF) Jatropha curcas cDNA clone rjcaeb0_004651, mRNA sequence

ATTCGATTAATTATATTGGGTGGAGAAATTTGTTAGCAAAAAAGGGCAAAAATGTCTTCAAATCAATCAA

GACAAGAGCTTGATGAAAGAGCAAGGCGTGGAGAGACTGTTGTCCCTGGAGGTACTGGGGGCAAGAGCCT

TGAAGCTCAAGAGCATCTTGCTGAAGGGAGAAGCAGAGGAGGACAGACAAGGAAGGAGCAGCTGGGGACA

GAAGGATATCAGGAGATGGGCCGGAAAGGTGGGCTGAGCACGACGGACGAGTCTGGTGGAGAAAGAGCTG

AGAAGGAGGGCATCACTATCGATGAGTCGAAGTTCAGGACTTCGGGAGCTAGTGTGATGATTAAAACTAT

AAATGGTATTTTGTAGTAATAATAATAATAATGTTGGTAATAAAGTATTAGCTTAGCAGTGTACTTTAGT

TTAGTAATGTAGTTTACATGTGGGTTACAATGACTAGGGTTTAGCTAGTATGTGTGTCGT

>FM893741.1 FM893741 Jatropha curcas embryo 56-70 (DAF) Jatropha curcas cDNA clone rjcaeb0_004650, mRNA sequence

ATTCCGATAAAGTGTAACAGAGCAAAAGATTAATTATATTGGTGGAGAAATTTGTTAGCAAAAAAGGGCA

AAAATGTCTTCAAATCAATCAAGACAAGAGCTTGATGAAAGAGCAAGGCGTGGAGAGACTGTTGTCCCTG

GAGGTACTGGGGGCAAGAGCCTTGAAGCTCAAGAGCATCTTGCTGAAGGGAGAAGCAGAGGAGGACAGAC

AAGGAAGGAGCAGCTGGGGACAGAAGGATATCAGGAGATGGGCCGGAAAGGTGGGCTGAGCACGACGGAC

GAGTCTGGTGGAGAAAGAGCTGAGAAGGAGGGCATCACTATCGATGAGTCGAAGTTCAAGACTGGGAGCT

AGTGTGATGATTAAAACTATAAATGGTATTTTGTAGTAATAATTATAATAATGTTGGTAATAAAGTATTA

GCTTAGCAGTGTACATTAGTTTAGTAATGTAG

>FM893740.1 FM893740 Jatropha curcas embryo 56-70 (DAF) Jatropha curcas cDNA clone rjcaeb0_004649, mRNA sequence

ATTCCAAGGATCTCCTTCAAACAACAAAAATGGCTTCGGAGAAGAAGCTCTCAAACCCAATGAGAGAGAT

TAAGGTACAGAAGCTTGTACTAAATATATCCGTCGGAGAAAGCGGTGATAGGCTCACTCGTGCTGCTAAG

GTGCTCGAGCAACTTAGCGGTCAGACTCCTGTCTTCTCTAAGGCTAGGTACACTGTTCGATCTTTTGGTA

TCAGGCGTAATGAAAAGATTGCTTGCTATGTGACCGTGAGGGGGGACAAAGCTATGCAGCTACTTGAAAG

TGGTTTGAAAGTGAAGGAATACGAGCTGTTGAGAAGAAACTTCAGTGACACTGGTTGCTTTGGATTTGGA

ATCCAGGAGCACATTGATCTTGGCATCAAGTATGATCCTTCAACTGGTATTTATGGTATGGATTTCTATG

TTGTTTTGGAGCGTCCAGGCTATCGCGTTGGTCGTCGCAGAAGATGCAAGTCCAAGGTCGGTATTCAGCA

CCGGGTTACCAAGGAGGATGCAATGAAGTGGTTTCAGGTGAAATATGAAGGTGTCATCCTCAACAAGGCC

CAGAATATTACATCGTAGAAAGTGTTGGTTTTTCAAGTAAATATTTGC

>FM893739.1 FM893739 Jatropha curcas embryo 56-70 (DAF) Jatropha curcas cDNA clone rjcaeb0_004647, mRNA sequence

GCAGAAAAAGGTAAATAGGTTCTAAAAATTTCACAAACAGTACTAAAATATAAAAAGCCCACAAAAGTCA

AACCTCAGACCAAAATCTGCTATGGACAAAAAGATGATCGTAGTACCTTTTCTTCAAGTCCAACCAGCCT

AAAAATTCCTCTAAAGGTGATAATTATGTAACCAACCTTTTGTTCTAATTCTTCTTATGCCTTCTTGTCC

TTCACTTCAATTTTGCCTTC

>FM893738.1 FM893738 Jatropha curcas embryo 56-70 (DAF) Jatropha curcas cDNA clone rjcaeb0_004646, mRNA sequence

ATTCCTCTGCATTCTGCTAGTGCACATAGCCAATGCAGTCTCCGGCTATGTGACAACCACCATCACCACC

GTTGAAATCGACGATGATTCTTCTAACACTAGTCAGCGAAAATGCAGGAGGGAAGCTGAAAGGGCAGATT

TGAGCTCCTGTGAGAGTTATATGAGATCGTCAAGAAGGCCGTCAGAAGAGATGCTAGCTTTGCGAGGAAT

TGAAAACCAGCAACAACGGGATCTTCGCCAATGTTGTAACGAATTAAGGCAGCTAAGTCATGATTGTCCA

TGTGAAGGAATAAAGTATGTATTAGAGCAGAGCTGGAGCAAGACGCGAGAGCGTTGGAGGCAGTGCGTAG

GCTAGAAGTATCCTTCTGCTTGTGGCCTGCGTACAAGCTG

>FM893737.1 FM893737 Jatropha curcas embryo 56-70 (DAF) Jatropha curcas cDNA clone rjcaeb0_004645, mRNA sequence

ATTCCTTCCATCTTTCATCTCAGTCACTCCTCCCTCTCTCCAGCTTGTCGATTTACAGGTAAGCAATGGC

GACAGCTGTAGTAGCACCGCCGCCGGCAACCCCAGAACCTACACAGCCTTACACTGAAGTGAAGCTCTTT

AATCGATGGACTTTTGAGGAAGTTCAGGTTAACGACATTTCCTTGTGCGACTACATTGGTGTGCAACCAG

CCAAGCATGCCACCTATGTTCCACACACTGCTGGAAGGTACTCTGTCAAGCGTTTCAGGAAGGCCCAGTG

CCCAATTGTTGAGAGGCTTACAAATTCATTGATGATGCACGGTAGAAACAATGGAAAGAAATTGATGGCA

GTCAGGATTGTGAAACATGCTATGGAGATCATTCATCTTCTGACCGATCAAAACCCAATCCAAGTTATTG

TTGATGCTGTGATTACAGTGGTCCCGGA

>FM893736.1 FM893736 Jatropha curcas embryo 56-70 (DAF) Jatropha curcas cDNA clone rjcaeb0_004644, mRNA sequence

TCCAGAAAAATCGTTAACGCTCCCTGTGTGAGATTCTCAGTTTTGAAAAGGGAAACAGAATGGCTGAAGA

AGGTCAAGTTATTGCCTGCCACACCGTTGAGGCCTGGAACGAGCAGCTCGAGAAGGGAAAGGAAAGCAAG

ACCCTGATTGTGGTTGATTTTACTGCTACCTGGTGTGGGCCTTGTCGTTTCATCACTCCAATTCTGCAAG

ATTTGGCTAAGAAGATGCCTCATGTCACGTTCTTGAAGGTGGACGTGGATGAATTGAGGACTGTTGCTGA

AGATTGGGCTGTTGAGGCAATGCCTACTTTTATGTTCTTGAAAGAAGGGAAAATCGTGGAC

>FM893735.1 FM893735 Jatropha curcas embryo 56-70 (DAF) Jatropha curcas cDNA clone rjcaeb0_004643, mRNA sequence

GGCTGCAGGAATTCGAATTCCACACTTGGAGCGGTCGTCAGAAAAGAAAATTATAAAAAACACACCGGAA

AATGAGAAAAGATTGAATCTTTGGGTCGGAGATATGGAGAGCAAAAATAACAAAAGAAGCGAAATCGGCA

AACCGATGATTGGATTGATCTGTGGTAGTTTGGTTTATTACCACTGTGCCTATCGGAATGCCAGCCTTCT

GTCTTTGGTTTCCGATGTACTCATCGTCCTCCTCTGCTCCCTTGCCATTCTCGGCCTTCTCTTCCGCCAG

ATGAACATCT

>FM893734.1 FM893734 Jatropha curcas embryo 56-70 (DAF) Jatropha curcas cDNA clone rjcaeb0_004642, mRNA sequence

CGGGAAGTCAGATGATGTCAATAACATCATGGCTTTATATATAGACTCGGATTGGGCTAGTGATGTTAAA

GATGCTATGGCAAGAGGAATGGAGTATGGAAGAACCAGAATTAGTAATGCTAATGAAGAAGAAGCCAAAC

GAGAAACAGAGACGCTTGAAAATATTCCTGTACAGAACGGAGGTGATGCCATAGAGGCTTTAGAAGGACC

GATGGGATATGGATCATGCGACGAGATGGATGACTTTTATCAATCTATAAATAAAGTCGGGGAGGCATAT

AGGTCATCCAAAGAGACAATGTCTGGTGAGGCAAAAGCTAAGTACGAATCCGCCAANGAGAGGATGTCTA

GGCACTGGTGCTGTGGAGACGAATGAGAAAGAGCAGCAGAGGTTGAGTATACTACCACTTTTACATACTA

TCTT

>FM893733.1 FM893733 Jatropha curcas embryo 56-70 (DAF) Jatropha curcas cDNA clone rjcaeb0_004641, mRNA sequence

ATTCCTTTGATCTTAGATTACATTTGTAAGATAGATAATGATGAATGCCTCTCAGAAGAGCTTTTGAATA

AACCAATTCATCTGTCTCTGCCAAAACTTACATTTGTTGAAATAAGAGGTTGTGAAGAAGTAGAGAGGAT

TGCATTCTTCTGGCACTTTTATTTGATCATGGAGTAGTTCTGGAAAAGGTTATTTTGTTTCCCCCGAGTG

CGGATGCTTCCCGTAAAGTAGTGATTCTGCAAAGAACAGGCCCTTAGCCATCCATTGCCCGAGGATGGTG

AAGTTTCTAATGGTTTTAACATTATTAAGGATAGAGTAACTAGATTTGCAGTGCTCCTCCTCATGAGAAA

CTTTTTGTTTCAAACCCAAGAGCAAGATTTGGCTGCCTCAGTATCTTCGACAGGCTCATCAAATCAACAA

TACAGATGTGGCTATTGAGTAGAAGGT

>FM893732.1 FM893732 Jatropha curcas embryo 56-70 (DAF) Jatropha curcas cDNA clone rjcaeb0_004640, mRNA sequence

ATTCCACACAATGAGCATATGCAGCTTTAGCTTCTGACGCTGCCAAAAACTTTTCTTTATGGCTTGTGCC

ACTTGAGGTTCTTTCTCTAATTGATCACTTCTTTTTATGTTGCTCCAGGCTACGTGTGTTCGTTGTGTTG

TTGATAGTAGTACCACGCCTATGAAGACCTTAGGTTTCTTTGTTAATTTTAATTTTGCACATGAACATAG

AATGTGAATTTGTATGTTTGGTTTGCCGTTGCTCATATTTATATATTGTTAAATATAAAGACTAGAAGAT

GAGAAATTTGATGTAAAAAAAAAGGGGGGGCCCGGTAC

>FM893731.1 FM893731 Jatropha curcas embryo 56-70 (DAF) Jatropha curcas cDNA clone rjcaeb0_004639, mRNA sequence

ATTCGAGTTGAAGGAATCTTGCTATGTCCCGAGCTATAGGTGACAAATATTTAAAGCCAGTTGTGACAGC

AGAGCCTGAGATTACTTTCACAAAAAGAGAAGCTGAAGACGAGTGCTTAATTCTTGCTAGCGATGGGTTA

TGGGATGTTTTGTCAAGTGACTTGGCTTGTGAGGTAGCTTGTAAGTGTCTACGAGAAGGAAATCCTCCTA

CTTTTGCTGCAAATGCTGGGTCCCAGATAGAAGATGAAAGGATTATGGCATCATATCCATCTCGAAGCAC

ATTGGCTGCCCAGTTGCTCACTCGCCTTGCTTTAGGACGAAGAAGCTCTGACAACATCAGTGTGCATTGT

TGTGATCTTAAGAGGAGCTGAGGACTAAAACAAGTCTGGGATGAGATCGGATGATAGATTCATGTCAACT

CAAGTGTGTATAAAACAGCATGCTAATAGGTTTATTGGAGTAGCAAA

>FM893730.1 FM893730 Jatropha curcas embryo 56-70 (DAF) Jatropha curcas cDNA clone rjcaeb0_004638, mRNA sequence

GGCTGCAGGAATTCGAATTCAGAATATGATGAGGCAATGCGTAGGGCTGACGATATAATTAGTGTTTGCT

TTGACAATATCTGCCCTCGTTAAAATGCTATTGCTACCGAAAATAAAATTTCACTCGCTTGAATGTGAGC

GAGAGTTACGTTTAATAATAATCCATAAACAATATGAGTTTCGGAGTAAGTTTGTAATGTTGCTTCAAGT

TTTCTTCATGTCCTGTAATAAAAGCTAATCAACTGTTGAGTTATTG

>FM893729.1 FM893729 Jatropha curcas embryo 56-70 (DAF) Jatropha curcas cDNA clone rjcaeb0_004637, mRNA sequence

ATTCCAACTAATCCCCACTGTTTATTGGCTTCCTTCTCCCATTTCACCACCGATGCCACTCATCACAACC

AGCAATCGCATTTGCCGACGCAGTATGCGGGCCTAGAGCCCACAAAGTCCACCGAGAAGCCGAGGGTGGT

GGTATTAGGATCTGGGTGGGCTGGCTGCCGCCTCATGAAAGGCATAGATACCGAGTTGTACGATGTCGTC

TGCGTGTCGCCCCGCAATCACATGGTGTTCACTCCTTTACTGGCTTCTACTTGCGTTGGCACCCTCGAGT

TCAGGTCCGTGGCCGAGCCAATTGCGCGCATCCAGCCTGCAATTTCTAAGGAGCCCGGCTCCTATTACTT

TCTTGCCAGCTGTACCGCTCTTGACACTCGTAACCATGTGGTGCATTGCGAGACTGTTATG

>FM893728.1 FM893728 Jatropha curcas embryo 56-70 (DAF) Jatropha curcas cDNA clone rjcaeb0_004636, mRNA sequence

ACTAAAAGATACCTTTTATTCAACTGGAATTGCTTAACAAGAAAAAGACACCAAACAGAAAACATAGATA

TATTACATAACAGGAGCTAAAGCACATGCCATGCCCTACACACGTGGGTTATGGCCAGTGGTGTCTCCGG

GCAACCCAGATGGGCGAGTCCTGGTCAAACCCGTTGGGTCCACCTCTCCACCGTATGGCTGCCCTGTGCC

GTGTCCAGGCATGGCCGACATTTGCTGGGTCCCTGTAGGGTAACCGGTTGTCCCAGTTGTTGAATGGGTA

TAGGTTTCGGTTGTGCCTTGAGCGCCCGGACCAGCAGTTGTGTACTGTACATGGCCCCACCAGCCTTGGC

TGCCTGCCTCGCCGCTGCGTTATCCTCGCGCGCTTCTAGTTTCCGCAATTCTGCCTCCGTTTTCCTCTCT

TCTTTCTTCTCTCTCGCCATTTCCTTCTGCACTGGATCACGTGTTGACATTCTCTCCACCTTCTCTTGCG

CCTCTGCTCTGGTT

>FM893727.1 FM893727 Jatropha curcas embryo 56-70 (DAF) Jatropha curcas cDNA clone rjcaeb0_004634, mRNA sequence

GATCTCTACCTAATGAGATTTATTAAACTGAAAAATTTCATGAAATCTTTAATTTTACACTCCACAATTT

CATTATACATATTGTTATACAATTTATATAATATAAA

>FM893726.1 FM893726 Jatropha curcas embryo 56-70 (DAF) Jatropha curcas cDNA clone rjcaeb0_004633, mRNA sequence

ATTCTGCTTTACCTGCACCACCAATGCCAGGTATGGGGGGACCAGCACCAATGGGCGGAGGTTTTGCGGC

ACCACCACCAATGGGTGGAATGGGAATGCCTCCAATGCCTCCTTTTGGAATGCCACCTATGGGCAGCTAT

TGATACATGCAAGGCGACCCTTTTTGTAGATTTTAGCTGTGAATCCAAGTTGGTTACAAAGGGCTTTTCC

ATTTGGTGGCGTTTACTGGGCTTTGGCGCCTTTAAATTTCCTTTTGTGTTTGTTTTGGTGGGATGACGGT

GTAGTTGAATAATGCCATTTTTCATTTTCAAATCTTCATTTAGGTGTGGATATGTGTTGTAATATTCTTT

TGGTAGGAGAGGCATTTTTTGGCTGCTCTTAGATGGCAGGCCGAGGATTTCAT

>FM893725.1 FM893725 Jatropha curcas embryo 56-70 (DAF) Jatropha curcas cDNA clone rjcaeb0_004632, mRNA sequence

GATTAATGAAAACATCCTTGGCAAATGCTTTCGCAGTTGTTCGTCTTTCATAAATCCAAGAATTTCACCT

CTGACTATGAAATACGAATGCCCCCGACTGTCCCTGTTAATCATTACTCCGATCCCGAAGGCCAACACAA

TAGGACCGAAATCCTATGATGTTATCCCATGCTAATGTATACAGAGCGTAGGCTTGCTTTGAGCACTCTA

ATTTCTTCAAAGTAACAGCGCCGGAGGCACGACCCGGCCAGTTAAGGCCAGGAGCGCATCGCCGGCAGAA

GGGACGAGCCGACAGGTGCACACCGCGAGGCGGACCGATCGACCCAACCCAAGGTCCAACTACGAGCTTT

TTAACTGCAACAACTTAAATATACGCTATTGGAGCTGGAATTACCGCGGCTGCTGGCACCAGACTTGCCC

TCCAATGGATCCTCGTTAAGGGATTTAGATTGTACTCATTCCAATTACCAGACTCATTGAGCCCGGTATT

GTTATTTATTGTCACTACCTCCCCGTGTCAGGATTGGGTAATTTGCGCGCCTGCTGCCTTCCTTGGATGT

GGTAGCCGTTTCTCAGGCTCCCTCTCCGGAATCGAACCCTAATTC

>FM893724.1 FM893724 Jatropha curcas embryo 56-70 (DAF) Jatropha curcas cDNA clone rjcaeb0_004631, mRNA sequence

CAATCTACAAATCAAAGAAATAACTATAATCTTCTGAAGATACATATGATAAAATTCAGCTCATCTATTT

GAGGGGTACAACTCATATATGTGCGAGCAACGCAGGTCAGTTCTCTGGAGACATGCATTGCAATTTGCAA

AACAAAATAAACAGTACATCCGCCAATGCCATTATATACACTGTCTATCTTCAAATTTTATAACTGAACA

ACTGGTATGTGCTAAAATTGAAATTCTTGCCGCTGGAAATCTCCACATCCCGACTTTTCTAAAGATAAAA

GAAAATCCAGAGTTCATTGCAAAACAAATATATCTGATGGATGTCACTGATGCTAAAGTCCCAAACTAGT

TTATGGCTTCTGCATTTCCTCTGCGGATAAAGTGCTGTTCAAGGGATTTGGAGCAACATCTGATTTCAAA

GGCTTAGTGTCGCCAGCTTGATTGTTGACGGGGCTCTGTTCTATAAGCTCGTTTGACTTTTGATTTAACT

TGAATGGCCTTTCAAAATCTTTAGC

>FM893723.1 FM893723 Jatropha curcas embryo 56-70 (DAF) Jatropha curcas cDNA clone rjcaeb0_004630, mRNA sequence

ATTAGATATTAACTTTATTGTCCATATATTATAATAATAGAGTAAAAGCTGCGACAGGATGAACCCATCA

CAGCAATTTCAAACACTGACATCTAGAGTGATGAAACTTGGACACAACAAGAGAATAAAAGAAGTTCAGC

TTATTCATGGCCACTCATTTACGACCATTGGACGCACACAGACATCAACAAATTTAAATAAACAATCACC

CAAGATATTATAGATATAAGTAACTGCTTATAAACCAAACAGCAAACAGATTAGAAACCACCACGGAGTC

GGAGGACAAGGTGAAGAGTGGACTCTTTCTGGATATTATAGTCTGCAAGGGTTCGTCCATCTTCAAGTTG

TTTACCAGCAAAAATCAATCGTTGTTGGTCCGGGGGAATACCCTCCTTGTCCTGGATTTTAGCCTTCACA

TTGTCAATTGTGTCAGAGCTCTCAACCTCGAGGGTAATGGTTTTCCCTGTCAGGGTTTTGACNAAAATCT

GCATTCCACCACGCAGACGAAGCACCAAATGCAGAGTAGATTCTTTCTGAATATTATAATCTGCAAGAGT

CCTTCCATCTTCAAGCTGCTTCCCAGCAAAGATCAACCTCTGCTGGTCTGGTGGGATCCCCTCCTTATCT

T

>FM893722.1 FM893722 Jatropha curcas embryo 56-70 (DAF) Jatropha curcas cDNA clone rjcaeb0_004629, mRNA sequence

CGAATTCATCAAGGATGAGAAGATCAGTTCCAAGTTGGCCGCAGCTGATAAGAAGAAGATTGAAGATGCT

ATTGATCAGGCCATTCAGTGGCTAGACAGCAACCAGCTTGCAGAGGCAGATGAGTTCGAGGACAAGATGA

AAGAGCTTGAGAGCATCTGCAATCCCATCATTGCCAAGATGTATCAAGGTGCTGGTGCTGACATGGGTGG

CGGCATGGAAGATGATGTTCCTCCAGCAGGTGGCAGTGGTGCTGGTCCCAAGATCGAGGAAGTTGACTAA

GTGGCTTGTTTGATGCTTGAGTAAAGATGGTGATGAATATTTTATTTTCTTTATTGGTCAGTACAATTAT

TATTATTATTATTTTAATATTCTACTTAGACATTATGTAAACCATCTGTCCGATTTTTACTTTCAATATC

TTGGCTGCAATA

>FM893721.1 FM893721 Jatropha curcas embryo 56-70 (DAF) Jatropha curcas cDNA clone rjcaeb0_004627, mRNA sequence

ATTCAGAACTGCAAGTATTAGTTCACGAAATGCACCAAGCCCATCTGTTCAACGAACTTCACGAAAGGTA

TAGAGCAAAAATGGAGTTTTGAAGATCGAGACAAGAGAGAGAGTTCCAGAAACTTCAAGATTACATGCAA

ACGTGGTACTTCTCTCCATCACTCTACCATTTACCTCTTTTCGGAACTAGAAAACGTTTTCAACGATAGA

AATTATGTTAAAGAGGATGTATCATATTAAGTGGAGAGGGGTTGGTTCTCTATGGAGGATGGCTCTTAGT

GTGGTGGTCAT

>FM893720.1 FM893720 Jatropha curcas embryo 56-70 (DAF) Jatropha curcas cDNA clone rjcaeb0_004626, mRNA sequence

ATTCAGAAGACTATTACCCTACCTCCACAGCGCCGAGGCTGCCATCTTATTACTCCCAAGATTTTGAATG

AAANTGGTCCAGACTTATCAGAATTCAAGTGCGGCCTCGCTCATCTCTTCTTACACCACACAAGCGCCTC

TCTTACTATCAACGAGAATTATGACTCTGATGTTCGTGATGACACCGAGACTTTCCTCAATAAGATAGTT

CCAGAGGGGAGATCTGCTCCTTGGAAGCATACACTGGAAGGCCCAGATGACATGCCAGCACACATCAAGT

CATCAATGTTTGGATGTGAACTCACGATACCAATCACAAATGGAAAGCTTAATATGGGCACTTGGCAGGG

GATATGGCTGTGTGAACATCGTGATCACCCTACTGCACGCAGAGTTGTGGTTACCCTTAACGGAATATAA

GTACATGATATTCAGAGACATTCCGTGTCTTTTCTTCAGAGATATACGGTTATAAGTAGGCGGTTAGTTT

CATGAAAATATATTGTCTGTAATCTCTGCAACTCTGTTTACTTATTCATGGATTAGTACTCTGGCGAGTG

GCTTGTTAGAACTAAGAATTGCTGAATAACGTGGAGACCATGGCATGA

>FM893719.1 FM893719 Jatropha curcas embryo 56-70 (DAF) Jatropha curcas cDNA clone rjcaeb0_004625, mRNA sequence

ATTCCAGAAAGGATATCAGGAGATGGGCCGGAAAGGTGGGCTGAGCACGACGGACGAGTCTGGTGGAGAA

AGAGCTGAGAAGGAGGGCATCACTATCGATGAGTCGAAGTTCAGGACTGGGAGCTAGTGTGATGATTAAA

ACTATAAATGGTATTTTGTAGTAATAATAATAATAATGTTGGTAATAAAGTATTAGCTTAGCAGTGTACT

TTAGTTTAGTAATGTAGTTTACATGTGGGTTAAAATGACTAGGGTTTAGCTAGTATGTGTGTTGTAACTT

TGAGAGGGGTTTATGGTCGTTCCCTTTCTGGGAGGACTGCTAATACTTTCTCCCTATTTGTAAAATAACC

TGCAGTATATATAATTACCTGTTGGTT

>FM893718.1 FM893718 Jatropha curcas embryo 56-70 (DAF) Jatropha curcas cDNA clone rjcaeb0_004624, mRNA sequence

TCGATGCACCACGAAGCTCTCCAGGAGGCCCTTCCAGGTGACAATGTTGGCTTCAATGTGAAGAATGTTG

CTGTCAAGGATTTGAAACGTGGTTTTGTTGCCTCTAACTCCAAGGATGATCCTGCGAAGGAAGCTGCCAA

CTTCACCTCTCAGGTCATCATCATGAACCACCCTGGTCAAATTGGAAACGGATATGCTCCTGTCCTTGAC

TGCCACACCTCCCACATTGCTGTTAAGTTTGCTGAGATCCTAACCAAGATTGATAGGCGATCTGGTAAAG

AGCTGGAGAAGGAGCCAAAATTCTTGAAGAATGGTGATGCTGGATTTGTGAAGATGATTCCCACCAAGCC

CATGGTTGTGGAGACCTTTTCTGAGTACCCTCCTCTGGGTCGTTTTGCTGTGAGGGACATGCGCCAGACA

GTTGCTGTTGGTGTCATCAAGAGTGTTGAGAAGAAGGATCCATCTGGTGCAAAGGTCACCAAATCCGCAG

CAAAGAAAAAGTAAAGCATTGGGTGAATGAAGTTACAGTTAGTTTAGTTATGTTTTGTTTTAAGTTATGT

CTTGCTGTTGTTTTTGGTTTCAGTTTCAGTTATCTGGCAGTTTTGTGACT

>FM893717.1 FM893717 Jatropha curcas embryo 56-70 (DAF) Jatropha curcas cDNA clone rjcaeb0_004623, mRNA sequence

ATTCAGAGAGAGAGAGAGAGAGTTGATGGAGAAAGAATGGCAAAGAGTAAGGATGATATCAAGTATGGGA

CAGCACAGGCAAAGCTATCAGAAGATGAAAGTCTAAGGATGAGGTATAAGCATGGGACTCCGCTTGAAGG

AGGCAAGATTGCTGATTCTGAGACTGTTGATCTCTTCTCTAGTGCTCATAATATTCCCAAATCATCGGAC

TCCGCCACTTCCACTGATCAATCTCAGAGCCACCGCCCCTCGAATGCCGCCGGAGGAAATGCTACCAATA

ACAATCCCACCACTACCAATGATTGAGAGTTTTATTTTGTTTCATGTAATCTGAGAGCGTGTCGTAGAAA

AAAGAAGTGTTAGAATGTTTCTTCTGCTGCTCTCCAGAAACTATATGTGTAATAATAATTTAGTTTTTTT

TTTCCTAAGATAGCAATATATAAAAATTATTAGATAGTTTTGTAATAATTCCTGG

>FM893716.1 FM893716 Jatropha curcas embryo 56-70 (DAF) Jatropha curcas cDNA clone rjcaeb0_004621, mRNA sequence

ATTCCATATATTACTTTACAAGAGGAAATGGTCACGTCCAAATCGTAGATGAGAATGGAGATGCAGTGTT

CGATGGACAAGTTCAACAAGGTCAAATATTAACAGCTCCACAGAACTTTGTGGTTTTGAAGAGGGCAAGC

AATGAAGGGCTAGAGTGGGTATCCTTCAAGACTAACGAAAATGCACAAATTAGCCCACTAGCAGGACGGA

CCTCGGCAATTAGGGCTCTGCCAGAGGCAGTGGTTGCAAACGCATTCCAGATTTCCAGAGAAGATGCCAG

GAGGCTTAAGGAGAATAGACAGGAAGTCACTGTGTTTAGCCCTGGGTCTAGATCTCGTAATTAATATTGC

AACTATGCAATTATACCAAGAGGATGTATAATAAACTGGTTTCTACATAGTTTAGATTTGGAGAAGAATA

AGCCTATTTTTATAGGCAGTTTTAGTTCCTTGCAATAATATAAAAATGTAGATCTGTATATGCCTTATGT

TATGAGATAATGAAAGTTAGTGGAGCAAA

>FM893715.1 FM893715 Jatropha curcas embryo 56-70 (DAF) Jatropha curcas cDNA clone rjcaeb0_004620, mRNA sequence

ATTCGTCTTGTTGAAAACCAGATTTCAATTTCTCATTAACTAAGCTTCTTTTCTCATTTCTCAAGTTCTA

AACAATGGCGTACTCGGTTGCTCTTAAAAGGCTTGTCTCTTCAAACCTCATTCCCTCTTCTCTCCGATTG

ATCCGCCCAGTAGCCACGACCCCAACGGGCTCTCGCCTTTTCAACACCAATGCTCTCCGCGAGTTCGACA

ATGATGACAACGAGCGTGGTAGTGAAATTGATCGCCGTTCGGGTCGCTCTCTGTCCCGCCATCGAGATGA

TTTCTTCTCAGATGTGGTGGACCCATTCTTGCCAAGCAGGAGCCTGAGCCAAGTGCTCAACATGGTGGAC

CAATTCATGGACAATCCTTTATTCTCTTCCTCCAGAGGAATTGGATCCGGACTTCGCAGAGGCTTCGACG

TTCGTGAGACCGATAATGCTCTGAATCTGCGGATTGACATGCCAGGACTAGGCAAAGAAGATGTGAAGGT

CTCCGTGGAACAGAACACTCTAATCATCAAAGGCGAGGGAGAGAAGGAATCAGATGAGGAAGAGAGCGGC

AGGAGGTACACCAGTAGGATTGATCTACCGGAGAAATTGTATAAGACTGATGAGATCAAAGCGG

>FM893714.1 FM893714 Jatropha curcas embryo 56-70 (DAF) Jatropha curcas cDNA clone rjcaeb0_004619, mRNA sequence

AACTAGAATTTATCTCTTTATTAATTATTAAACCAGAAGCCACTATAGTCCGCAGCAGTGATCTTTCTCT

TTTCTTAAAGACTAATTTACACATGTACCTTCACCTTTCATTATTTATTCACCATACACAGCACTATTTA

CATTAGTAAACCCTCAAGGTCGATGTCACAGTCACCCCAAAAAGCCGAAACACTTGCCATCACGTTATTA

GCTTCTCATGCCACAGCATGACCCTCGAAGGAACCTCTCCTTGGCCCTGGAAAGAACCACTCCTCGTTTT

GTCTTCCAAAAGCCTGATCCACTTCTCTTTCACTTACACCGAAGGCTAACTCTTTGGCTTGCTTCTCCAT

CTTATTTACAATATTATTTTTCCCTGCAAGTAGATGCCTAGTATTGCCTTCTTCATTAATCTCAAAGCAG

ACTACTTCCAGATTATTGTTTCTTGAAGCTACAAAAGCCGCCGGATGGCCTGCAGGGACGACCACTGCTG

TTCCACGTTTCAACCGTGAACTTACTTTCTGGTAAGTCTTCTGACTTTCACCCGTCGTGTCTCCCCGCGA

GCCGCTAAGGTGAGGACAAGCCATTTCAAAGTAGCCTTCGCCATCCCAAACCACTGATATCTTGGTTACT

CTGGA

>FM893713.1 FM893713 Jatropha curcas embryo 56-70 (DAF) Jatropha curcas cDNA clone rjcaeb0_004618, mRNA sequence

ATTCCGTTGCATGATAATGGCTTCTGAAAAAGCATACCGGGTGTTGCTCTGCTTGGGGTTCGCGATGCTG

TTGATAGTTGTTGTTGTCGGTAGCCTAGAACATATGCCTTCCACGAGCGAAAAAAGCGAGGACTCGGAAG

CAATTAAGGACAGGGCAAAAGATGCCAAACGAACATCGGAGTCGTGGACAGAATGGGCGAAGGACAAAAT

CTCCGAGACTGTCGGACGAAAACAAGATGAAGCCAAGGAGGCTGCTAAGAAAGCGTCTGACTCTACCGCT

GAAACTGCTCAGAGGACCAAACACAAAGCTTCTGAAAAAGCTGAAGAGCTGAAGGATGCTGCAACGCAGA

AGGCCAACGAAATGAAAGATAAGGCCTCTGAAATGACGAATGCGGCTAAGGAGAAGCTCAGTGCGGCTGA

AGGCCAGACCAGCGATGCTACAGAAGCAGCAAAGGAGAAAGGTGAGGAGGCAAA

>FM893712.1 FM893712 Jatropha curcas embryo 56-70 (DAF) Jatropha curcas cDNA clone rjcaeb0_004617, mRNA sequence

ATTCCCAAACATAAACAGTAATGGAAGCATTTGTCTTGATATCCTCAAAGAACAATGGAGCCCTGCTCTC

ACCATTTCTAAGGTATTGCTATCTATTTGCTCATTGCTGACTGATCCAAATCCCGATGACCCACTTGTCC

CAGAGATTGCCCATATGTATAAGACTGATCGAGCAAAGTATGAAGCAACTGCACGCAGCTGGACACAGAA

GTATGCCATGGGTTGATACTGGTTGTTCACTAATCGGGTGATGTTTATGGTGGAATGTGTTGAAAACTGT

TTTATTAACTTATTTACTTGGTTNGGTTCGATAGAGGCTAGGGATGGACTTGAGTTCTTCAGTGGAATCT

ATATCTTGGGTTTGGCATGAAATAACTGTATGATCAAGATGCTTTGATGTAGACAATGTCTGTAGTTTGG

>FM893711.1 FM893711 Jatropha curcas embryo 56-70 (DAF) Jatropha curcas cDNA clone rjcaeb0_004616, mRNA sequence

ATTCAATAGTGCTGTGTATGGTGAATAAATAATGAAAGGTGAAGGTACATGTGTAAATTAGTCTTTAAGA

AAAGAGAAAGATCACTGCTGCGGACTATAGTGGCTTCTGGTTTAATAATTAATAAAGAGATAAATTCTAG

TT

>FM893710.1 FM893710 Jatropha curcas embryo 56-70 (DAF) Jatropha curcas cDNA clone rjcaeb0_004615, mRNA sequence

CGAATTCCATCAATCAAATCTTCTTAAGTGATATACAAATTAGAAATGGCTCTGATTCCAAGTTTCTTCG

GTAACTCGCGAAGCAGCATTTTCGATCCTTTCTCTGCTTTTGACGTTTGGGATCCATTCAAAGATTTTCC

TTTTCCTTCATCTTCTTCAATCGTCTCTCGTGAAAATTCTGCTTTTGTGAACACTCGCATAGACTGGAAA

GAGACACCGGAAGCTCATGTCTTCAAGGCTGATCTTCCTGGGCTCAAGAAAGACGAAGTGAAGGTAGAGG

TTGAAGATGACAGAGTTCTCCAAATCAGCGGAGAGAGGAATGTGGAGAAAGAAGACAAGAACGATACTTG

GCATCGGATAGAGCGTAGCAGTGGCAAGTTCTTGAGGAGGTTCAGGCTGCCGGAGAATGCAAATATGGAT

GAAGTGAAGGCTTCAATGGAAAATGGAGTTCTTACTGTGACTGTTCCTAAGGAGGAAATCCAGAAACGTG

ATGTGAAGGCTATTGAGATTTCTGGCTGAGTTTTACAAATTTGAT

>FM893709.1 FM893709 Jatropha curcas embryo 56-70 (DAF) Jatropha curcas cDNA clone rjcaeb0_004614, mRNA sequence

CTACGCCGCCGGGTAACACCAGCACCGCCAACTCTACCACCAAAGCCTCCTGTTGTGAAGCCACCGCCAA

CTGAAACACCATGCCCTCCTCCGCCACCTCCAGTGCCATGCCCACCTCCACCCCCAAAGCAAGAGACTTG

CCCTATTGATGCTCTAAAGCTAGGAGCATGTGTTGATGTGTTAGGTGGTTTAGTGCATATTGGGATTGGT

GGTAGTGCTAAGGACGCCTGTTGCCCAGTGCTTCAAGGGCTACTGGACTTAGATGCTGCTCTTTGTCTTT

GCACTACCATTAAAGTTAAGCTTCTGAATATAAATATTCTTTTACCCATTGCTCTTGAGGTTCTTATTGA

CTGTGGCAAGAACCCACCTGCTGGATTCAAGTGTCCTGCGTAAGCTACACTGATCAAATCTTGAGGATTC

TGCTATAGCACTATATATTTGCTCCTTTGTTTTGACAAGTTACAAGCAATATGCTGATGCATGGGATTTC

TGTATCGCTAAAGAAGCTGTATTTCTTAGTTTTAGTATTTTTCAGAGAATAACTTGTGAAAGTCTTGTGT

TTTATTTCTTGTTTTTCCCTTTTGTTCTTACTGCCTTACAATGGAAATATACATTTGAGGACTTGCCCGC

CATTGCCAGGAGGCTTAAGGAGAATAGACGGGAAGTCACTGTGTTTACCCTG

>FM893708.1 FM893708 Jatropha curcas embryo 56-70 (DAF) Jatropha curcas cDNA clone rjcaeb0_004613, mRNA sequence

TTCGAATGTGACGGATGACCATTTGAGAGAGCTTTTTGGCCAATATGGCCAATTATTACATGTAAAAATA

CCTGCTGGCAAGCGATGTGGATTCGTTCAGTTTGCTGACAGGAGCTGTGCAGAGGAAGCGTTACGGATGT

TAAATGGAGCTCAGTTGAGTGGACAAAATATTCGGCTTTCATGGGGACGCAGTCCTTCAAACAAGCAGCC

ACAGCCAGATGCTAGTCAATATGCTGGATATTATGGATATGCACAAGGATATGAAAACTATGGATATGCC

CCTGCTCCCCAAGATCCTAACATGTACTATGGAGGCTATCCTGGGTATGGCAATTATCAGCAGCCCCAGC

AGCAGCAGCAGCAGCAGCAACAAGTGGGATATAGTTGAGAAGTTTTGACCTAATTATGCAATCATTTATT

TCAGTTGTGAGATGGACTGGCGTTGGTTCTATTTATGTGGCTTTCATGGTAAACAAATTCTGGCTGTGTT

GTTTCATTGCTAATAGCATATGCACTGTAGAAACCTCAGTTCTACTAAATTTTGCACAATTACTATCTTA

ATGTGCCAATTACGTTGTTTTGTTTCTAT

>FM893707.1 FM893707 Jatropha curcas embryo 56-70 (DAF) Jatropha curcas cDNA clone rjcaeb0_004611, mRNA sequence

ATTCCTTGTGCGACTGAGACAAAGAGAGAGCTTAGAGACGAAGGGGACGAATGGCAGCAGAAGATGATGA

ATTTTACCTTCGATATTATGTTGGCCACAAGGGTAAATTCGGTCACGAGTTCCTGGAGTTCGAGTTCCGG

CCAGATGGCAAGCTCCGATACGCTAACAACTCCAACTACAAGAACGACACCATGATCCGGAAGGAAGTCT

TCCTCACCCCCGCCGTTCTTAAGGAATGCCGCCGCATCATCGCCGAGAGCGAGATCATGAAGGAAGATGA

TGCCAACTGGCCAGAACCAGACAGGGTTGGGAGACAAGAGCTCGAGATTGTAATGGGAAACGAGCATATC

TCCTTCACTACCTCAAAGATTGGGTCACTTGTTGATGTCCAGAGCAGTAAGGATCCTGAAGGGCTTCGTA

TCTTCTATTACCTTGTTCAGGACTTGAAGTGCTTTGTTTTCTCCCTTATCTCACTCCATTTCAAGATCAA

GCCTATCTAGAGAACATTATGCCTGGCTTTCCATTTGTATTGAGGAAGT

>FM893706.1 FM893706 Jatropha curcas embryo 56-70 (DAF) Jatropha curcas cDNA clone rjcaeb0_004610, mRNA sequence

CGAATTCGGTGGACCCAACGGGTTTGACCAGGACTCGCCCATCTGGGTTGCCCGGAGACACCACTGGCCA

TAACCCACGTGTGTAGGGCATGGCATGTGCTTTAGCTCCTGTTATGTAATATATCTATGTTTTCTGTTTG

GTGTCTTTTTCTTGTTAAGCAATTCCAGTTGAATAAAAGGTATCTTTTAGTAGATATTTTATTTAAAAAA

AAAGGGGGGGCCCGGTAC

>FM893705.1 FM893705 Jatropha curcas embryo 56-70 (DAF) Jatropha curcas cDNA clone rjcaeb0_004609, mRNA sequence

ATTCAGAAAGGAAAGGAGAAAAAGCTTCGAGCAAGCGGAAACGATGTCGGGCAGAGGAAAGGGAGGCAAG

GGATTGGGAAAGGGAGGAGCCAAGAGGCACAGGAAGGTCTTAAGAGATAACATACAAGGCATCACCAAAC

CTGCAATTCGCCGATTGGCTAGACGTGGTGGCGTTAAGCGTATCTCCGGTCTAATTTACGAGGAAACCAG

AGGTGTTTTGAAGATTTTCCTCGAGAATGTTATTCGAGATGCTGTTACCTATACAGAGCACGCCCGCCGC

AAGACTGTTACTGCAATGGATGTTGTCTATGCCTTGAAGAGGCAGGGCCGTACTCTCTATGGTTTTGGTG

GTTAGGGATTTTAGGATGTATGGATGGATCTAGAATGATTAGGGGTTGGGTCTAGGGTTTCTTTTTGTAA

TTTGGGGCCTCATTTTGGTTTATAGTTTTTTGGAGTTTGTATCTGTGCAAAGTAGTAATGAAATGAATGG

ATTGCATTTAGTTAGTTCTGTTGCTGGATATGAAATTTAGCCAAATTGGGAAAAAAAAAAAAAAAAAACT

CGAGACTAGGGCAGAGTGGCCTTGCTGCCACGATCCACTGAGATTCAGCCCTTTGTCGCTCCGATTCGTC

CCT

>FM893704.1 FM893704 Jatropha curcas embryo 56-70 (DAF) Jatropha curcas cDNA clone rjcaeb0_004608, mRNA sequence

CGAATCCTGGGATCATTTCAGGACTTCCCTTTCACAAGCACTGCTCTTTCTGCTCCTCGTTCTGAGTTAT

TGAACGAAACGGCAGCGTTTGCCAACACCCGCATAGACTGGAAAGAAACACCAGAAGCTCATGTATTCAA

GGCTGATCTTCCAGNGCTAAAGAAAGAGGAAGTGAAAGTGGAGATAGAGGAAGGTAGGGTCTTGCAGATA

AGCGGAGAGAGAAGCAAAGAGAAAGAAGAAAAGAATGATAAGTGGCATAGAGTTGAAAGGTCAAGTGGTA

AGTTCTTGAGAAGGTTTAGGCTACCAGAGAATGCAAAGGTTAATGAGGTTAAGGCTAGCATGGAAAATGG

AGTCTTGACTGTGACAGTGCCTAAAGTGGAAGTCAAGAAACCGGACGTTAAGGCTATTGAGATTTCTGGT

TGAAACTTTTAGTTATGTTTGAGTGTGTCTAAGTCTAAATGGTACCCATCCATCGNATAAAATAAATAGA

TGTGTGTAATGGGTTTGTTGTTGTGAGCTTG

>FM893703.1 FM893703 Jatropha curcas embryo 56-70 (DAF) Jatropha curcas cDNA clone rjcaeb0_004607, mRNA sequence

CAAATTAATTTAGTAATTATTTCATCACCTAGAATTACACATTTTTCATGTCAAAGGAAACCAAAACCAA

AACAACACAAATATACAAATTAAACCTTCAAGTGAGGATCAACCTCAATTCTTGCCACTCCCTCTTTTGC

CTCTTTAGCCACCTCCACCTCTCCAACAACCAATTCACCCTCTTCTCCTCTCAGTAGCTTCTCCTCCTTT

TCCTCATCCACATAGTTCGGATCCTTCTCATCAATCGCCGGCGGCGCCGCGTCCAGCTCGCTCTCCGCCA

TGTCCTCAGGTCCTTCCCACGTGTACTTTCCACCGTGTCCTGACTTGGGCGGCGGTTTCCGTATCCCACC

GATCCCGTCAACCGGATGCCTGTCCATGCGGTGAACACCCTTGTCCATGAGCTTCTTTTCGACGTACTTG

ATAGTGTCTTTGTTGTGCGTATGTAGCTCTGTCTTTTCCACTCTTTTCCTTCCTTCTTCGTCATGGGACA

CTGCCCTTATCACCACCTTATCCTTCTTCTCGTCAATGGGTTTCATTTTCTCTAGTTCTTGGGGGGGGCC

CGGTAC

>FM893702.1 FM893702 Jatropha curcas embryo 56-70 (DAF) Jatropha curcas cDNA clone rjcaeb0_004606, mRNA sequence

TTTCTAAGCAGAGAGCGAGAATGGCGGGGAGGTGGTCCACATCAGGATTCAGGTGGCTGGGGCTAGACTT

AGCTTACTCTTCCCTCCGGTCTTCGATCTTCCGGTGGCCGGCGCAATTAATGTCGTCCTCGTACTTATCG

CCGAGGTGGACTCCGATAGGAGTACTAAATAGGGACCCGCAGAGGTTCTTAAGGTGGCCGGAGCTGAATT

TCTCAATAATCGACGATGTCATGTGGAGCTTGATTACTGCGTTTGAGTCTGTGGC

>FM893701.1 FM893701 Jatropha curcas embryo 56-70 (DAF) Jatropha curcas cDNA clone rjcaeb0_004605, mRNA sequence

TAAATCCAACTACGATGATGAAGATTGGAGATGATATTTGTAGTAAGTATGTTTTAGACTCGCTACCTCT

TCTATCTCTCTCTCTATGATAAATTTCTCTTCAGTTGTAAGTCTTGTGCTAGCTGAACAGGTTCCTGCAT

GTCTGTGGCTAGTTTTCTTTGTCATCACTTTAGTTTGTAGGTAACACCTTTTCAATCAACGTTGGAAGGA

TTCTGCAGAAGCCTTTTCGGTT

>FM893700.1 FM893700 Jatropha curcas embryo 56-70 (DAF) Jatropha curcas cDNA clone rjcaeb0_004604, mRNA sequence

ATTCGTAACTTCGAAGGGTCATCAGCTCGTGGGTATGAAGAAAACTTTAGTTTTTTACAGAGGAAAACCT

CCACATGGCACTAGAACTGATTGGATTATGCATGAATATCGCCTTATGACCACTGAAACCAATTCCCCAC

AAAAGAAGAACATAATTCAGAGTCCTTCGATGCCAATGGAAAGTTGGGTTCTTTGCCGAATATTTTTGAA

GAAAAGAAGCACAAAAAATGAAGAGGAAAATGTCCAATTTGGAAATGAAAATAAAGTGCGCAAGCTGAGA

AATACAAAGCCTGTGTTCTATGATTTCTTGACAAAGGAAAGAACTGATTTGAATCTTGCTCCTTCTTCAT

CATCTTCAGGATCAAGTGGAATTACTGAAGTTTCCTCAAATGAATTAGATGATCATGAAGAAAGTAGTAG

TTGCAATAGTTTTCCTTTTTTCAGAAGAAAAGCATGACTAGAATCTAAAATATCTTCTTCCTTAGTTTTT

AGTATTTTTTAGTTCTTCCCCTCTCTTCAAGTTCCTGTATCAGTAATTTGCTTCTGGTTATCAAATCAGA

ATAACTCATCTAAGTTATCT

>FM893699.1 FM893699 Jatropha curcas embryo 56-70 (DAF) Jatropha curcas cDNA clone rjcaeb0_004603, mRNA sequence

CGAATTGGACTTAAGCAGGTTGTTCTGTTAACGGATGGAATGGACACTCGGCCATATAGACTTAACTGGC

CAACTTCATCTATAATATTTGGGATGTCTCCAGAAAGGATATTTAAGAAATCAGCTGAAAGGCTTAAAGG

TGTTGGAGCTAAGATTCCTAGAAATTGTGTGTTTCTTCATGTCCCACTGGAGTCCTCTAATATACAAGAG

AATCTGCAAGCTAAGGGCTTCAATGGCAATCAGCCAAGCATATGGGCCATGCAGGGACTGTCAATTATGA

ATTTGTCCAGTTTTGAAGAAATTTTGTTCATTGTTAGTAGTCTGGCTATGAATGGATGTCTGTTCTTGGG

AGAACTGCCTGCGTGGTTGGCGGAAACTGAAATTGGGTGTAAGTCCAGCACGGAGAAATGGATGGACAAA

CTTTTCATGAGCAATGGTTTTCGGGTGGAGATGATTAAATATGATGAGGTTGCAAGGAGTTTAGGCAAGG

AATTAGCACCAGGAGATTACAAGAATATACTTTTTGTTGCAGAACAGTTACGATTTTCAGATGATCAGAT

GGAAACATGGAGGAGAGAATACCACAGGATAGAGGAGGAAGGAGATGAAGAACGGTCTGAGGAGCTCTAA

ATATACAGAGGGATCTAAAGACATGGGTGGCATCCTCTCTCTTTTGGTAGTTCCACTTTGTTTCTAAGCA

ACCAAATGGAATGTGTATATATATAAAAATATTGCATGTATCTTATCGAGGGGCGCCAA

>FM893698.1 FM893698 Jatropha curcas embryo 56-70 (DAF) Jatropha curcas cDNA clone rjcaeb0_004602, mRNA sequence

AACGCAAAACAGTTTTATTCATGCAACGAATGTAAGTGTGATAATGTGGTATCACCATTCAATCAACGAT

TTTTATTCTTCCTCAAACGCACTTTTCTAGCACAGTCTGATTTTCGCGCATATATCCTGCGATTTTTGCT

TCTGTATGAAATCGAAGACAATATCGGATTCCCTTGCCAACCAACGCTTCGCACAAACCCTTGACAGGAC

CAAACGCTGCACAAACGCCTTTCATCAAAACTTCAACCTCGTTCCTGACAGCGTCAATTTGCAACAGTTT

TTTCAATTGATCCAAGGCTACAGTGCAATACTTCACACGGACTAACATCTGCATCTGGTTCCTGGAATTG

CACCTCAGCGTTGGCCAAAGCCACAGTTGCCAAAACCAACAAAATCAGTGGATTTCATTTTTGGGGGGGC

CCGGTAC

>FM893697.1 FM893697 Jatropha curcas embryo 56-70 (DAF) Jatropha curcas cDNA clone rjcaeb0_004601, mRNA sequence

TTGCAAGAAAGTGGTTGATGAAGTGGTAAATGCATATGGGCGGATTGATATTTTGGTTAACAATGCAGCT

GAGCAGTACAAAAGTAGCTCTGTTGAGGAAATCGATGAGGAGAGGCTCGAAAGGGTGTTCAGAACTAACA

TCTTCTCCTACTTCTTCACAACCAGACATTGTCTGAAGCACATGAAAGAGGGCAGCTCTATAATCAACAC

GACATCAGTTAATGCATACAAGGGAAATAAGAAACTGCTTGATTACACAGCAACTAAAGGGGCAATAGTG

GCTTTCACTAGAGGACTAGCACTTCAGCTAGTGAAAAAAGGCATACGAGTCAATGGTGTTGCTCCTGGAC

CTATTTGGACTCCATTGATACCCTCTTCTTTCAGTGAAGAGGAGGTGGCTAACTTTGGGAAACAAACGCC

TATGCAGAGAGCTGGCCAGCCAAGTGAGGTTGCACCCTCTTATGTGTTTCTTGCTTGTAACCATTGCTCC

TCTTATATCACTGGCCAAGTTCTTCACCCCAATGGAGGCGTTATTGTGAATGGTTGATGGAGATAGCTGT

GAAGAAAGTCAAAAGTTCTTTTTGTCTTTTTGTTATATATCTAGAGTGTTGC

>FM893696.1 FM893696 Jatropha curcas embryo 56-70 (DAF) Jatropha curcas cDNA clone rjcaeb0_004596, mRNA sequence

ATTCCGGGCAAAGCCATTTGAAGACATTCAAGATGCAAATCTATTGTGAAAACCCTTAACTCGGCTAATA

CAATCACCCTTGAATGTGGAAAGCTCCGACTACCATTGACAATGTCAAAGCAAAGATCCAGTACAAAGAG

GGAATCCCCCGTACCAGGATAAGCTTATCTTCGCTGGTAAGCAGCTCGAGGACGGGCGTACACTTGCAGA

CTACTATATCCAATAGGAATCCACTCTCCATTTGGTGCTGCGATTGATCGGGTGGTATGCGCCATATTCG

TCAAGACTCTCGCTGGCTAGCACTATCACTTTGGAAGTTGA

>FM893695.1 FM893695 Jatropha curcas embryo 56-70 (DAF) Jatropha curcas cDNA clone rjcaeb0_004595, mRNA sequence

ATTCCTGTGATCGAATTCCAAGCCCACTGGGGCAGTCAGCTGTGGACTGCGGAAGTCTCTCTTCAATGCC

TATTATTTCCTTCACAATAGCAGGAAAGCTTTTTGAACTCACCCCAGAGGAGTACATTCTCAAGGTGGGC

GAGGGTTCGGCAGCTCAGTGCATTAGCGGGTTTACTGGTTTGGATGTGCCTCCTCCTCGTGGACCTCTTT

GGATCTTGGGAGATATTTTCATGGGTCGCTACCACACTGTGTTCGATTTTGGCAATAACAGAGTTGGATT

TGCAGAAGCAGCGTAGAGGTTTCCCTTCCACACAATTCCGACTCCTGTGTACTACCAGCATATGTAATAT

ATGTGCCTTTCAAAAATGGTGTAGTTCCTGGAAACTTTATAGTGGTGTTGGGCCTGGCGTAAGAGGCGAA

TGTTTGCTTGTAAATATTTTTTATGTCTTGTGTGGATAAGCGGATTTTAGTTCAAGACAATGATGCGCTT

CACCTCTTGGTTATGGGTACTTTATCTACTGAGCTTAAATTGTGAGCCGGTGACGACTAAGTAGGGTCTC

TACTGTGT

>FM893694.1 FM893694 Jatropha curcas embryo 56-70 (DAF) Jatropha curcas cDNA clone rjcaeb0_004594, mRNA sequence

AAATGCAATTTAAAACATTATGTATAGCATAAACAACCTTATGAGCTAAGACAAACTTACTTTAAAAATG

TTAATATCACCAAGTTACATTGATTGCAAAATTTAAATTATCAAAAGGTGGTAAGCAAACTAGATGATTC

TCACTCCTAACAAGACAAAAAATTCCCAGGTTCAGCTCATTCATCTTCCTGGCTGCGCAACCTAGCCAGC

TTGTACGTAACAACAACATCAAGTTGAGCAGCTCGCTCCACAATCTCTTCCCTCTTCCTTGTTGACACGT

CATGTGCAATCTCAGCACAGTAGGTTCTGTTATGCATCATCAACAGCTCAAGTTCCCCAACATTGTGTAC

AACAAATTTCTTGAAGCCATTTGGAAGATAATGGCGAGTCTTTATGTCTGAGCCATACCCAATATTGGGC

ATCAAAGTACATCCTTTGAACTTCCTTCGGACTCTAGAATCAATACCTTTTGGTCTACGCCAGTTTTCCT

T

>FM893693.1 FM893693 Jatropha curcas embryo 56-70 (DAF) Jatropha curcas cDNA clone rjcaeb0_004593, mRNA sequence

ATTCCAAGAGACGAGCTGAAGTTGCAAGGCTTCGGGAGCTTCACACACTCAAAGGACATGTCGAGTCAGT

GGTGAAACTGAAGGGTTTGGACATTGACACAATCCAGCAACATTATACAGTGTAAAAATCCTTAAGCCTG

CAATAACATTGCTTGAGAAGGAAAGGGCTGCTTTTCAGTGACGAACCGAAGATCAAGAAAGCTAAAAAGG

CCAAAATAGTTTAAAGCTTTAGATAGGGAGAGAATGTCTATGCTATAGTTCTCCTAATCTATTTTTCTTT

ACCTTTCTTTTGTACTTCAACTGCTGGAAGAAAGGCTTCTGTTTACCAACTTCTTGCTCTGTAATGTTAC

CTTGTCACAGTTTTTGAGTTTTAATTTTACTTCTCTTCTGCCT

>FM893692.1 FM893692 Jatropha curcas embryo 56-70 (DAF) Jatropha curcas cDNA clone rjcaeb0_004592, mRNA sequence

ATTCGGATTATCAAGAAGAGGATCCATGTTAGAATTGAGCATTTGCAACCTTCGAGGTGTACCGAGGAAT

TCCGCCTAAGGAAAAAGAAGAACGATGAGCTGAAGGCAGAGGCAAAGGCTAAAGGTGAGGTCATTAGCAC

AAAGAGGCAGCCAGAAGGACCAAAACCCGGATTCAAGGTCGAGGGAGCAACATTGGAAACTGTTACACCC

ATTCCATATGATGTTGTTAATGATCTCAAGGGTGGTTATTAGAGCTACAAGTTTTAGGTTTTGTTCATGC

TGTTTGGTTTGTGATGCAAACTTTATTGTTGACTGGATTAGCTTATTCATATGTCTTTTTAAGTACTTGA

AGTATTGAAGAGGTTCATCCGATATAATGAACTATGCTTTATTTCACTTCCTCTTCA

>FM893691.1 FM893691 Jatropha curcas embryo 56-70 (DAF) Jatropha curcas cDNA clone rjcaeb0_004591, mRNA sequence

AATCGAATTCCACAGATTAAAGCCTTGGCAGGAGCTGCTATTGGAGGAGAAGACATACTGCAAAATATGA

TGCAATATCTATTACCAAACACAGAGCGCCTTTTCTTGAGTCATGGCATCAATGAGATCAAAACAATGAG

TTTGCTTCGAGTTGCAACCCTAGATGCAATCCATAGGCTTTCCTCTCTGACAAGTGTTCAGATTGATGTT

GATTTGGGAAATGGTACAAAAATATGTAAAATCGTTACTCAAGAGGAATTCGAGGAAGTGAACCAGAAGC

TATTTGAAAAGTGTGAAAGCCTCATAAAACAATGCTTAAATGACTCAAAGGTAGATATTGAGGACTTGAG

TGATGTAATACTGGTAGGCGGATGCTCATACATACCAAAGATAAGAAATCTTGTAAAGAGTATATGTAAA

ANAGAGGAACTCTACGAAGGAATGAACCCATTGGAAGCTCCAGTTTGTGGGGCTGCCCTGGAAGGTGCAG

TTGCTTTCGGAATCAGTGATCCTTTTGGAAGTTTGGATCTGTTGACTATCCCGGCTACCCC

>FM893690.1 FM893690 Jatropha curcas embryo 56-70 (DAF) Jatropha curcas cDNA clone rjcaeb0_004590, mRNA sequence

ATTCCTTTCTCCAAATCCATCACCATGGGTGTAGCCAACAACATAACAGCAGTTCTTAATTTCGTAGCCT

TTCTATGCTCAATTCCCATAATAGCTTCCGGTATCTGGCTTGCCTCCAAACCGGACAACGGGTGCATCCA

TCTCTTCCGCTGGCCGGTAGTCCTTCTCGGAATCCTCGTCCTCCTGGTCTCGCTCGCCGGCTTCGTCGGC

GCCTACTGGTATAAGGAGACACTCCTTGGTTTCTATCTCTGCTGCATGGCCATTCTCATCGGACTTCTCT

TAATTCTACTCGTTTTCGCTTTTGTTGTCACGCGCCCGGATGGCGGGTATGATGTTCCCGGGAGAGGTTA

TAAAGAATATAGGCTTGAAGGATTTTCCTCTTGGCTGAGAAATCACGTCGTGAATTCAAAGAATTGGGTT

AAAATTAGGAATTGTTTGGCTGAGAGCGATGTCTGTGCTAAATTAAGTCAGAATTATATCACTGCTGATC

AGTTCTTCACCGCCCATATCTCTCCTCTTCAGTCAGGGTG

>FM893689.1 FM893689 Jatropha curcas embryo 56-70 (DAF) Jatropha curcas cDNA clone rjcaeb0_004588, mRNA sequence

ATTCCATACTTCGTGGATCTGTTTGTTAGAGCGTCAAATACACCAGCCATAAAGATGTACGAGAAGCTCG

GGTATGTAATCTATAGACGAGTGCTCCGATATTACTCAGNGGGAGGAAGATGGCTTAGACATGAGGAAAG

CTTTATCGCGGGATGTTGAAAAGAAGTCTATTATTCCACTCAAACGGCCAGTTACTCCTGATGAACTGGA

ATATGATTAGATCGGAAAGCTGAATAATTTTGTGATTCTTGAATGAAAGAGTAAGTACCTTGTATCAGTT

GCACATTGTTGAGCAGGACGATATGTTGAGCGTGAGAATATCAAAGAGAAAGGTGCACATTGTTATTGGT

TTTCCCATCTTTTGAGTTTTGAATGCTGTATGGCCTCTGTACTGAGTAAGAATTAAGTTAGATTTTATAT

ACTATAAATTAAGTATAAAACTTTAAATTGAAAATCTCATGTTCAATTTATTCTTTGTTATCTATATATT

TCAACAATTAAAAAAAACCACAAGGGGGGGCCCGGTAC

>FM893688.1 FM893688 Jatropha curcas embryo 56-70 (DAF) Jatropha curcas cDNA clone rjcaeb0_004587, mRNA sequence

ATTAATTAGAAACAAAAGAAGGCCACATTTTATTGATTTCTGCAGAAGTTGAAGTTCTACATTTTTAGCA

CTCTACAACTCTCTATACTACAATATACGACGGATCCCTTCTCTAAATTTACAAGAAGGATCTCTTAGTA

GCGGCCTTGGCTAGTAGAAGCACCAATCACAATGCTATTTTAGTAGTAACTTACTTATGGTCTCCTGCTG

GTGGGAGTAAACAACATGGACTGTGGATCCCTGTTCCTCTTCACTTGATGAGCTTCGCTTGGAGACATTC

TGTAAGAGTTCATCAGAACATCAAGAGGCATGGCTCTCATCACTGATGTATAACCCGCCAATGGGCTCTT

GATAGGCTGGCTACTTGTTTAAAATGTTACGTACTCGAACCCATTTTCTCCTGCCCTTCCAAGTGCTGCA

TAGACTTGAGGGATCACGAACATGTCTCCTTCTATCACGGGGGGGC

>FM893687.1 FM893687 Jatropha curcas embryo 56-70 (DAF) Jatropha curcas cDNA clone rjcaeb0_004586, mRNA sequence

ATTCCTACGATCTCTCTATTTCTGCGAGAGAGCACTTCTTCGGCTAGCGAATCTCCTCCTCTAAGAGTCG

TTTTCTCTGGTTATTTTTAAGCTTCTCTCCCAACTTTTATCATGGGTAAGGAGAAGGTTCACATTAACAT

CGTGGTCATTGGCCATGTCGACTCTGGCAAGTCAACAACCACTGGTCATTTAATCTACAAGCTTGGAGGT

ATTGACAAGCGTGTGATTGAGAGATTTGAGAAGGAAGCTGCTGAAATGAACAAAAGGTCCTTCAAGTATG

CTTGGGTCTTGGACAAGCTTAAGGCTGAACGTGAGCGTGGTATCACCATTGATATTGCTCTTTGGAAGTT

TGAGACCACTAAATATTACTGCACTGTCATTGATGCTCCTGGACATCGGGACTTTATCAAGAACATGATC

ACTGGTACCTCACAGGCTGACTGTGCTGTGCTCATTATTGACTCTACCACTGGTGGTTTTGAAGCTGGTA

TTTCCAAGGATGGTCAGACCCGTGAGCATGCTCTGCTTGCTTTCACTCTTGGTGTGAAGCAGATGATCTG

TTGCTGCAACAAGATGGATGGCACAACCCCCAAGTACTCTAAGGGCTAAATACGATGAAATTGTGAAGGA

AGTTTCTTCCCTACT

>FM893686.1 FM893686 Jatropha curcas embryo 56-70 (DAF) Jatropha curcas cDNA clone rjcaeb0_004585, mRNA sequence

GGGCTGCAGGAATTCGAATTAGAAGATGCCAGGAGGCTTAAGGAGAATAGACAGGAAGTCACTGTGTTTA

GCCCTGGGTCTAGATCTCGTAATTAATATTGCAACTATGCAATTATACCAAGAGGATGTATAATAAACTG

GTTTCTACATAGTTTAGATTTGGAGAAGAATAAGCCTATTTTTATAGGCAGTTTTAGTTCCTTGCAATAA

TATAAAAATGTAGATCTGTATATGCCTTATGTTATGAGATAATGAAAGTTTGTGGAGCTATAAGTTATAG

TCC

>FM893685.1 FM893685 Jatropha curcas embryo 56-70 (DAF) Jatropha curcas cDNA clone rjcaeb0_004584, mRNA sequence

ATTCCACAAAATGGCTCACTTTCAAAACCAATATGGCGCAGTTAACTATACGGACGAATACGGCAATCCA

GTAGACGAATACGGTCGCGCCCTACGCCACACAGACGAGTATGGTAATCCAATCCCTCAAACTGGTACTA

CCACTGCATACGGTGGCGCACCAGAGACTGGTACTACTGGCCTTGGCATGGGACACCGCCAAGAACACTA

CGGAGAGTCTGGTCAGCTTCACCGTTCTGGCTCCTCCAGCTCTAGCTCCTCTGAAGATGATGGACATGGT

GGAAGGAGGAAGAAAGGGCTAAAAGAAAAGATAAAAGAGAAGCTACCAGGTCATCATAAGGAAGACAGAA

CTCAAGCAACCTCAGCTACAACTCCAGGTGGTTATCATTCAGCTGAGTATGGCCATGATCATGAGAAGAA

AGGAATAATGGATAAGATTAAGGAGAAGCTTCCTGGCT

>FM893684.1 FM893684 Jatropha curcas embryo 56-70 (DAF) Jatropha curcas cDNA clone rjcaeb0_004583, mRNA sequence

ATTCCGTACTTGCTGCTCTCTCTACTTCTTCTCTTTGTGGTTCGATCCGGGAGAAACTCAGAATCATGTT

GGTCTACCAGGATTTGCTTACCGGTGATGAGCTTCTCTCGGATTCGTTCCCTTACAAGGAAATTGAGAAT

GGCATGCTGTGGGAAGTTGAGGGAAAGTGGGTTGTTCAAGGAGCCGTTGATGTGAACATTGGTGCAAATC

CCTCTGCTGAAGGGGCGGATGAAGATGAGGGTGTTGATGACCAGACTGTTAAAGTTGTTGACGTTGTGGA

CACTTTTAGGCTCCAGGAGCAACCTGCTTTTGA

>FM893683.1 FM893683 Jatropha curcas embryo 56-70 (DAF) Jatropha curcas cDNA clone rjcaeb0_004582, mRNA sequence

ATTCCTNGATCTTTTAATGAGAGTTGCAAGAAAATTTTTGAATAAAGATGATAAGTCCATGAAACAGTTA

GAGTTGAATGTGCAACCCTCTTATGTTTTCTTCCCTATTGGTTCACAGGGAAATATTACAAGTTACTTCA

CTACACCTACAGCAGAGAATGAGAAGGGTAGACAAGATGCTATTTTCAAGAAGATATTGAGCGAGTCATT

GACCTTTCACTTTTGGAGCAGCTTGACATCAGCACTCATTCCAGAACCAGGGAGCCTTGTGGCTCGGCTT

ATTGACCGCACCTGTATCCATTGCTTTGATATATTGTGATTTTTACAGTACTTGTCGGTTCTGTAAAAAT

AATCATCAAGTTGCTGAAATTAAAGCAGCAATCTGATTATCAAAGCACATCAGGTTCCTGCTAGAGCTCA

ACAAAATGTCCAAGATTGAGCTTTTATAGATATTGGAGCTTCGAATTCATGATGGAATTATGAAGAATGC

CTGTTCCTGTAAGTGTACAGTTCTCTATCC

>FM893682.1 FM893682 Jatropha curcas embryo 56-70 (DAF) Jatropha curcas cDNA clone rjcaeb0_004581, mRNA sequence

ATTCAGAAAGGTGGCGGCGTTTGGCCTTTCGGAACTGAATCCATCGGTCCAATTAGTATCCTGAAGAACC

CTGTTTTTAGGAATAACTATGGTCAACTCTTTGAAGTTGGCTCTAGAGATTTCAAGCAACAGCTCCGAGA

CCTTGACCTTTCAGTCTCCGTTGCCAACATCTCTCGTGGAGCAATGTCTGGGCCCTATTACAACTCCAGA

GTAACCAAGATATCAGTGGTTTTGGATGGCGAAGGCTACTTTGAAATGGCTTGTCCTCACCTTAGCGGCT

CGCGGGGAGACACGACGGGTGAAAGTCAGAAGACTTACCAGAAAGTAAGTTCACGGTTGAAACGTGGAAC

AGCAGTGGTCGTCCCTGCAGGCCATCCGGCGGCTTTTGTAGCTTCAAGAAACAATAATCTGGAAGTAGTC

TGCTTTGAGATTAATGAAGAAGGCAATACTAGGCATCTACTTGCAGGGAAAAATAATATTGTAAATAAGA

TGGAGAAGCAAGCCAAAGAGTTAGCCTTCGGTGTAAGTGAAAGAGAAGTGGATCAGGCTTTTGGAAGACA

AAACGAGGAG

>FM893681.1 FM893681 Jatropha curcas embryo 56-70 (DAF) Jatropha curcas cDNA clone rjcaeb0_004580, mRNA sequence

ATTCGTTACTTCTCTCTTCTCTCCCACTGCTGCCGGAGCTTTGATTTGCAAAGATGTACACTACTTGGAA

GAAGATCCGAAAAGACAAGGATGCTGAACCAACTGAATTTGAGGAGTCAGTTGCTCAGGCCCTGTTTGAT

CTCGAAAATACCAACCCAGAACTGAAAAGTGATTTAAAAGATCTCTTCATAAATCAGGCAGTTCAAATGG

ATGTTTCTGGAAATCGGAAAGCTGTTGTTATTTATGTTCCCTACAGATTGAGGAAGGCCTACCGCAAGAT

TCATCTTCGTCTTGTTAGAGAGTTGGAGAAAAAGTTCAGCGGAAAGGACGTTGTCCTCATTGCCACCCGG

AGGATCGTGCGCCCACCGAAGAAGGGTTCTGCTGTTCAGCGCCCTCGTTCCCGAACCCTTACATCTGTGC

ATGATGCCATGCTTGAGGATGTAGTGTATCCCGCTGAGATTGTTGGAAAACGCATCAGATACAGAATCGA

TGGGTCCAA

>FM893680.1 FM893680 Jatropha curcas embryo 56-70 (DAF) Jatropha curcas cDNA clone rjcaeb0_004579, mRNA sequence

ATTCCTTCGTGTGAATCATGCAGTCTTGGCTGTCGGTTATGGAACACAGGGTGGTACTGACTATTGGATT

GTGAAAAATAGTTGGGGATCGTCCTGGGGTGAGCGTGGTTACATTCGAATGGTTAGGAACCGAGGTAACA

TGTGTGGAATTGCTTCGCTGGCCAGTCTCCCGATGGTGGCACGATTTCCGTGATACGTTTCTGTCATTAT

GAAAACGCACTGAACAATAAATTTCATTCTGCTTTGCTTAAAAAAAAAAAAAAAAAAAAAAAAAAAAAAA

AAAAAAAAAAAAAAAAAAAAAACACAAAAAACTACTAGTTAAAACACTGATATGAAAGAACCTAACTAAC

GGGTTTTATACTGGGACTCATCAATTTCATTCCCCTCCTCAGCAGCACGTACTTCACCAGACTTGTCCAT

GGTGCTAAGT

>FM893679.1 FM893679 Jatropha curcas embryo 56-70 (DAF) Jatropha curcas cDNA clone rjcaeb0_004578, mRNA sequence

GGGCTGCAGGAATTCGAATTCTAACAGAGCAAAAGATTAATTATATTGGTGGAGAAATTTGTTAGCAAAA

AAGGGCAAAAATGTCTTCAAATCAATCAAGACAAGAGCTTGATGAAAGAGCAAGGCGTGGAGAGACTGTT

GTCCCTGGAGGTACTGGGGGCAAGAGCCTTGAAGCTCAAGAGCATCTTGCTGAAGGGAGAAGCAGAGGAG

GACAGACAAGGAAGGAGCAGCTGGGGACAGAAGGATATCAGGAGATGGGCCGGAAAGGTGGGCTGATCAC

GACGGACGAGTCTGGTGGAGAAAGAGCTGAGAAGGAGGGCATCACTATCGATGAGTCGAAGTTCAGGACT

GGGAGCTAGTGTGATGATTAAAACTATAAATGGTATTTTGTAGTAATAATAATAATAATGTTGGT

>FM893678.1 FM893678 Jatropha curcas embryo 56-70 (DAF) Jatropha curcas cDNA clone rjcaeb0_004577, mRNA sequence

ATTCGAAATTCAAGATAATTATCCTCTCCTAGCCAAAAAAATGTCTCTGATCCGATCTTTCTTCGGCAAC

CCACTGTCTACTGATCTATGGGCTCCATCTGCTTCGAATAATGAGATTTCCGCAATCGCTAATGTACAAG

TTGATTGGAAGGAAACACCAGAAGCCCATATATTCAAAGCTGATCTTCCAGGATTAAAGAAAGAGGAAGT

GAAAGTTGAAATTGAAGAAGGAAGAGTTCTTCAGATTAGCGGAGAGAGAAATGTTGATAAGGAAGAGAAG

AACGAGAAATGGCACCGCGTTGAGAGAAGCAGAGGTAAGTTCTTGAGAAGGTTTTGGTTACCGGAAAATG

CAAAGGTTGGTGAAGTTAAGGCATCAATGGAGAATGGAGTACTTACTGTTACTATTCCAAAGGCAGAGGA

GAAGAAGCCTCAAGTCAAGTCTATTGAGATTAGCGGTGCATGAGCATGAGTTTCAATCTTCTTTAAGATA

TAAAAACAATAAATTGCTTTGTCGTTGATGTGTATGAAATGAGATTATGTGTGGTTTGCTCGCTCTGGTT

TCTGTTACCTTCCAATGTATAGGTATATGTTAATGTA

>FM893677.1 FM893677 Jatropha curcas embryo 56-70 (DAF) Jatropha curcas cDNA clone rjcaeb0_004575, mRNA sequence

ATTATCAGAGTCAANGCTTCTTCTGGTTCTCTATTCTTTCAATTGTTGCAATTAGCAGTTTCAGTGGGCT

ATCTTTCTAGGAAGCTAAAATGAGTTTCACTGGTCCCTCCGTGGGTTCTGGTGGTAAAACTGCTAGAAGG

GCATTGGAGTTTGGAAGGACATATGTGGTTAGGCCCAAAGGTAAACACCAAGCTACCGTTATATGGCTAC

ATGGTCT

>FM893676.1 FM893676 Jatropha curcas embryo 56-70 (DAF) Jatropha curcas cDNA clone rjcaeb0_004574, mRNA sequence

GAACAGAAGAAGTGTTTTCCTCCATGGTATTGAAAATTATATCAAAAATCAGAAAAATCATTAAACCGAC

TACTTTCATCATTACATAGTGTTTTCTGAATACTGAAATCCTTGCCAAACTAATTTTTGCTTCAGAGGCA

GGAGAATGAAACTACGGAAGCCTCACATATAAAAATGTCATTTACACTCTTCAGTCTTCATACATTGGAC

GCTCCAGAACAAAGCCAAAGAAAAAAGGAAAAAA

>FM893675.1 FM893675 Jatropha curcas embryo 56-70 (DAF) Jatropha curcas cDNA clone rjcaeb0_004573, mRNA sequence

ATTCCCTACCATCCAAGTTTTGGAGGGAATGGAGCAACAGCTTTCTTTTTGACTTTTGCTATATTAGCTT

CTATTGTTGGTATAGTATCCAAGTTGGCAGGTGGCCTTCATATCAGGAGCTGGAGAAATGACAGTCTAGC

CTCTGCAGGCTCTTCTTCACTTGTGACTTGGGCCATCACTGCCCTAGCTTTTGGGTTGGCATGCAAGGAA

ATAAACCTAGGAGGGCACAGAGGATGGAGACTGAGGGTATTAGAGGCATTCATGATAATCTTGACATTTA

CCCAACTCTTGTATGTCCTGTTGCTTCATGCTGGGGTATTT

>FM891089.1 FM891089 Jatropha curcas embryo 56-70 (DAF) Jatropha curcas cDNA clone rjcaeb0_001088, mRNA sequence

AAACTAGTGGATCCCGGCTGCAGGAATCGAATCCAGAATGCAAATTTATGATAAACTACAAATGGAATCC

AACCAATCCATAATAGTCACATATCATCAGCGGTTAACCAAAACTGCATAACTTAAGAGAACGACACGAA

TTTATAAACCATCTCAACTATTTAACAGACACCTCCAGCAACTGATGCCAAGATGCCCATGACTAACTTT

ATCTTGCATGTTTTAGCAATATCGACAGCATGAAAATACCGGCAAAGCTCACTTCAAGCAAAACTATCTC

ATCTCAAGCCTTTTGCTTCGCTTGCTTCATTAGATCTACAAATTCCCCAAACGCAGTATCTGAATCGTGA

GGGCCAGGGGATGCCTCAGGGTGGTATTGAAGTGACATGATGTTTAGTGCCGGATAAGCAGGCGGGGCCC

GGTAC

>FM894116.1 FM894116 Jatropha curcas embryo 56-70 (DAF) Jatropha curcas cDNA clone rjcaeb0_005096, mRNA sequence

AGAAATCAGTCCTGTACTTTCGATCTTTCAAAACATCAATAAGGAAAAATGGCATCGGCGATTGCACTAA

GGAGAACAGCATCTTCTACTCTGATCTCGAAGCTCATAAACCCTGTTCGCTCTGCTGCCGTGGCTCCCTC

TGTATCTCGTTTCTTCAGCACCGAAACCCAGGTCACAAACATCGGAGGTGATGATCTTGATACCGTCGAC

GTGGATCGCCGCTCCACAGGTCGCAGCGTCTCTCGCCGCCGCGATATGTCTCCTGGTTTCTTCCCAGATT

TGGTGGACCCATTTTCGCCAACAAGAACTCTAAGCCAAGTCTTCAATTTAATGGACCAGTTAATGGATTT

TCCCCTGTCACGCGGCATAGGAGCTGGCGGTGTTCCACGGCGAGGGTGGGATGTGAAAGAGGATAATGAT

GCTCTTTTACTACGATTCGACATGCCTGGACTACGCCAAGAAGATGTGAAAGTTTGCGTAGAGCAAAACA

CTTTGATTATAAAAGGCGAAGGACCTAAAGAAAATGAAGAAGAAGAAGAAGAAGAAGGAGAAAGTGGAAG

GAGATA

>FM894115.1 FM894115 Jatropha curcas embryo 56-70 (DAF) Jatropha curcas cDNA clone rjcaeb0_005095, mRNA sequence

ATTTTTGGTGATATATCCACCCAGTTTATAACGTTAACAGAAAATACAAATCATCGAATCCAGTCACCGC

TATCATAAATCTCTCATTTCCTATGGCATGCCTAACAAAATCTTCTAACTTCGATCCTCCCGATATCTCT

CAACAAAAATTTAATTCCATTTACTTCCCACCATGTTTGGCATTAGAGTGAGACTGCAGAGCACCATCCG

AGCCAAATGACCTGTCGCAAGATTTGCAAGAAAATTGACCTCCAGATTTCGGTGACTTCGCATTGCTGCC

GACTGCAGCAGTTTTTGCAGCACCCTTAGCTGGATGGGGAGTTGCAGTATGGCCGCCCTTCTTACCGTCA

GTCTTCTGAGGAGTAACTGATTTTATCTTCTTCGAGGGGACAGGTGTCTTAGTTGCAGAGTCATTGGGCC

TCTTTTTGCCTTTTACAGCCTTCTATGGGGTCTCCTCATCTTCACTCTCAGAATCATCCTCATCCTCATC

ATCTGAATCAACAGAC

>FM894114.1 FM894114 Jatropha curcas embryo 56-70 (DAF) Jatropha curcas cDNA clone rjcaeb0_005094, mRNA sequence

ATTCCGGAAAGGTGGGCTGAGCACGACGGACGAGTCTGGTGGAGAAAGAGCTGAGAAGGAGGGCATCACT

ATCGATGAGTCGAAGTTCAGGACTGGGAGCTAGTGTGATGATTAAAACTATAAATGGTATTTTGTAGTAA

TAATAATAATAATGTTGGTAATAAAGTATTAGCTTAGCAGTGTACTTTAGTTTAGTAATGTAGTTTACAT

GTGGGTTAAAATGACTAGGGTTTAGCTAGTATGTGTGTTGTAACTTTGAGAGGGGTTTATGGTCGTTCCC

TTTCTGGGAGGACTGCTAATACTTTCTCCCTATTTGTAAAATAACCTGCAGTATATATAATTACCTGTTG

GTT

>FM894113.1 FM894113 Jatropha curcas embryo 56-70 (DAF) Jatropha curcas cDNA clone rjcaeb0_005093, mRNA sequence

AAGGAATAGAGATGTTCATATGATAGAATACAAAAAAGGCCCTCTGCTACATTATTGTTATACAAGGCAT

GTGGAGAACAATTAGATTCAAATGATAATTGTTGGGTGAAAAAAATCATGTCATAAATAGATTCATTGAG

CACTAATTAAAATCAAATATTGATGACATTTAAGTCGTGTTGGATGTGTGGAGAATAGATTGCTACTGCT

ACTGTTTCACCATGAAAGAGCTTTTACAACTGCACCCACCAACTGCGCTTGGATTTATAGTCACTACAAA

AGCAGAACGAATCAGCTCCTCAACATAGTCAATAGTTGCCCCTTTCACAAAATCATATGAAATATTGTCA

ACTACCAATTTTATTCCTTCTCTCTCAAAAACTCTGTCATCCAGATTGATTTCATTGTCTAAGTCGAATA

CATACTGGAAACCAGAACATCCACCAGTTTCCAAACCCAAGCGAAGCAGTATAACATCTGCTGATCCTTC

ACTGGCTTGCAGCTCCTTCATCCTCC

>FM894112.1 FM894112 Jatropha curcas embryo 56-70 (DAF) Jatropha curcas cDNA clone rjcaeb0_005092, mRNA sequence

TCCACCTGACAGGAAAGACCGGTTTATACGACGTTGGGTTGAGGCCTTGTCTGACCCAAGAGTCACCTAT

GAAATTCGCAGCATTTGGATCTCATACTGGTCTCAGGCTGACAAATCTTTGGGTCAGAAAATTGCCTCTC

ACCTCAACATGAGGCCAACCATGTGATGATGTTTCTGTTTCTGGTGCTGAGCAGAAGCAATGTCTCATAT

ACTTAAAATAGTGATTCCATCAAATTCTTATTGTACCTTATATATGATATGTGATTAAGAAAAAAACAAT

GCAGTTTTGTGACACATACTGAAAAGAGTCTTTGTAATAAAGATGTTCTAATTAT

>FM894111.1 FM894111 Jatropha curcas embryo 56-70 (DAF) Jatropha curcas cDNA clone rjcaeb0_005091, mRNA sequence

ATTCCCTCAAACTGGTACTACCACTGCATACGGTGGCGCACCAGAGACTGGTACTACTGGCCTTGGCATG

GGACACCGCCAAGAACACTACGGAGAGTCTGGTCAGCTTCACCGTTCTGGCTCCTCCAGCTCTAGCTCCT

CTGAAGATGATGGACATGGTGGAAGGAGGAAGAAAGGGCTAAAAGAAAAGATAAAAGAGAAGCTACCAGG

TCATCATAAGGAAGAAAGAACTCAAGCAACCTCAGCTACAACTTCCAGGTGGTTATTCATTCAGTGGAGT

ATGGCCATGATCATGAGAAGAAAGGAATAATGGATAAGATTAAGGAGAAGCTTCCTGGCTCCCACCACCA

CGAGCACCACTAGGTGTTCTTTAATACTCTACTTAATAATGCATTATACGCACGCAATGCCCTTATTTGT

TATGTTGTGTGGCTGATTTGATTTTTATCGATCAGTTTGGTATAGGGTGATGTGTGGCTGGTTG

>FM894110.1 FM894110 Jatropha curcas embryo 56-70 (DAF) Jatropha curcas cDNA clone rjcaeb0_005090, mRNA sequence

ATTCTAGGGCTTCTCTGCATTTGCTGCAATGGGTAAGGGAACAGGGAGTTTTGGTAAGAGGAGGAACAAG

ACCCACACCCTCTGTGTCAGGTGTGGTCGCCGCAGCTTCCACCTCCAGAAGAGTCGTTGTTCAGCCTGTG

CTTTCCCTGCTGCCCGCAAGAGGAAATACAACTGGAGTGTGAAGGCCATCAGAAGAAAGACTACTGGAAC

TGGAAGGATGAGGTATCTCCGCCATGTACCTCGCAGGTTCAA

>FM894109.1 FM894109 Jatropha curcas embryo 56-70 (DAF) Jatropha curcas cDNA clone rjcaeb0_005089, mRNA sequence

ATTCGCTTGACTTAGACATGTCTGCTCTACAATATATGATCAAGGAATATCCGGGGAATGAGGAAGAAAA

AATGAGAGCAGCAATTGGGAAGTTTGGGCTGACTGGCAAAGCCCAGGTGATGCCCATGAAGAATCTCTCA

GATGGGCAGAGGAGCAGGGTGATATTTGCTTGGTTAGCTTACAGGCAACCCCAATTGCTGCTGTTAGATG

AGCCAACTAACCATTTAGATATTGAGACTATTGACTCTCTTGCTGAAGCATTGAATGAATGGGATGGTGG

TTTGGTTCTTGTTAGCCATGACTTCAGGCTTATAAACCAGGTAGCAGAAGAGATATGGGTTTGTGAAAAT

CAAGCCGTTACCCGATGGGAGGGTGATATTATGGACTTCAAAGAGCACCTCAAGAGAAAGGCTGGCTTAT

CTGATTGAGTCGGATTATATTACTAGAAGTGAAGAATATGATCCTACTATCATCATGCCCTAATCTATCG

TTGAGAATCACAGCACACACAG

>FM894108.1 FM894108 Jatropha curcas embryo 56-70 (DAF) Jatropha curcas cDNA clone rjcaeb0_005088, mRNA sequence

ATTCCCAACCCTTGGAACATACTACAGCCCCAGGTGGCGAAGAGCCGACATCGAGGTGCCAAACCTTCCC

GTCGATGTGAGCTCTTGGGGAAGATCAGCCTGTTATCCCTAGAGTAACTTTTATCCGTTGAGCGACGGCC

CTTCCACTCGGCACCGTCGGATCACTAAGGCCAACTTTCGTCCCTGCTCGACGGGTGGGTCTTGCAGTCA

AGCTCCCTTTTGCCTTTGCACTCGAGGGCCAATCTCCGTCCGGCCCGAGGAAACCTTTGCACGCCTCCGT

TACCTTTTGGGAGGCCTACGCCCCATAGAAACTGTCTACCTGAGACTGTCCCTTGGCCCGTAGGTCCTGA

CACAAGGTTAGAATTCTAGCTCTTCCAGAGTGGTATCTCACTGATGGCTCGGACCCCCCCGGAAGGGGGC

CTTCTTCGCCTTCCACCTAAGCTGCGCAGGAAAGGCCCAAAGCCAATCCCAGGGAACAGTGAAGCTTCAT

AGGGTCTTTCTGTCCAGGTGCAGGTAGTCCGCATCTTCACAGACATGTCTATTTCACCGAGCCTCTCTCC

GAGACAGTGCCCAGATCGTTA

>FM894107.1 FM894107 Jatropha curcas embryo 56-70 (DAF) Jatropha curcas cDNA clone rjcaeb0_005087, mRNA sequence

ATTCCACTATCATATCAAATCGGCGTTGAAAGATCAGTTCTCCTTTTTGAGTATCTAAAAATGTCTTCTT

GCTGTGGTGGAAATTGCGGCTGCGGCTCCGGCTGCAAGTGTGGCAGCGGTTGTGGCGGATGCAAGATGTA

TCCTGACATGAGTTTCTCCGAGAAAAACACCGCCGAGACTCAGGTTCTCGGAGTTGCACCGGAGAAGGCA

CGCTTTGTCGATGGAGGTGAGGTCGGCGTTGGAGCTGAGAACGGATGCAAGTGCGGTGACAACTGCACCT

GCAATCCTTGCAACTGTAAATGAGATCGGCGGCGATCAGTCTGTTTTTTAGAAGTAAAAGCAGAGATGAC

TCTGCTTCTCTCTTTCTAAAAAAGGAGAAATAATTAATAGAGTCTAGATATCGTATGAGTTTGTTCGTTT

GTTTCTGGTTTTGAGCTTCAATAAAAGTAGTGGTGGCTTGTGAGCTTGTGTTTAGCAATGGCTGCCTGTT

GCTTTAACGTTATATATTGTATAAATTAAAAACTAGCATATGAATATGTAAATGAGTA

>FM894106.1 FM894106 Jatropha curcas embryo 56-70 (DAF) Jatropha curcas cDNA clone rjcaeb0_005086, mRNA sequence

ATTCCTTCCTCCGCCTCCGCCATAATCAAAAGCTAAGGAGGCGATTCCATATTTTCTCTCTTCTTATCAT

GGCGGAAAAGGGCCAACCATTACCAAAGTTTGGTGAATGGGATGTCAATGACCCAGCATCAGCTGAGGGA

TTCACAGTCATCTTCAACAAGGCTCGCAATGAGAAAAAGACAGGTGGAAAGCCTGACTCGCCAGCAAGAG

ATAACTCCTCATTCAAGCCTGGCAGTAACACTCTTGGAAAACCTCAATCTAAAAAATGGTTTTGCTGCAT

ACAAGCTGCTCCTGCAGAATAAAAAGGGTGGAAGAACCTTGTAAGAGCCACAATGGGAGAGTATATCTAT

CTACGCCAGGCCCTAGCATTATATAATCCAGGAGACTGGAGCTGTATTCAGAAGCTATGATGGGTCCTAC

TGGAATCTCTTTATTGTGGCTTAAAACTTTGTGAAAAAGTGGGCTTGGGGAAGAAATGTGATGAAAGAAC

AATGTGGTTTTCTGTTCATATAATACTGTTGGGTTATTTTTGTTATTTTTTTTTTTTCTTTAA

>FM894105.1 FM894105 Jatropha curcas embryo 56-70 (DAF) Jatropha curcas cDNA clone rjcaeb0_005085, mRNA sequence

CTACTAAAAGATACCTTTTATTCAACTGGAATTGCTTAACAAGAAAAAGACACCAAACAGAAAACATAGA

TATATTACATAACAGGAGCTAAAGCACATGCCATGCCCTACACACGTGGGTTATGGCCAGTGGTGTCTCC

GGGCAACCCAGATGGGCGAGTCCTGGTCAAACCCGTTGGGTCCACCTCTCCACCGTATGGCTGCCCTGTG

CCGTGTCCAGGCATGGCCGACATTTGCTGGGTCCCTGTAGGGTAACCGGTTGTCCCAGTTGTTGAATGGG

TATAGGTTTCGGTTGTGCCTTGAGCGCCCGGACCAGCAGTTGTGTACTGTACATGGCCACCAGCCTTGGC

TGCCTGCCTCGCCGCTGCGTTATCCTCGCGCGCTTCTAGTTTCCGCAATTCTGCCTCCGTTTTCCTCTCT

TCTTTCTTCTCTCTCGCCATTTCCTTCTGCACTGGATCACGTGTT

>FM894104.1 FM894104 Jatropha curcas embryo 56-70 (DAF) Jatropha curcas cDNA clone rjcaeb0_005083, mRNA sequence

AACAAAAGATAATAGCAGATAACTTTATTTATTAACTGAAATAAACTTTCGAAAAAAAGTACAGCATCTT

ACAATTGATGATAACTTACTATACAGGGAAGACTAGCTTTTAGTATACGGGGATTTCACATATTTTACAG

ATAAAGCCGCAAAGACCTTATGTAGCTTGAAACTGGAAAGAGCCAAGTGTTTGAACAATCCTTAAAGACA

GAGAAGCTACGTATATAGCTAAAAGTCCTGATCCCAACACCCCATCAAGCTTCATGTTTCTACATGGTAA

GATCACAAGTGCCCATAGCAAGCCAGCCAACAGAAACCCTAATGTCTCGAACAGATATGGATCTGCTGGT

ACTACAATAGGAGATGGGTATTTATACCAGCATGAACTAACAAGAGATAATCCCAAACCAAACAGGATAT

TGAAAATAGGACCTGCATAA

>FM894103.1 FM894103 Jatropha curcas embryo 56-70 (DAF) Jatropha curcas cDNA clone rjcaeb0_005082, mRNA sequence

GGAGAACGGTGTGCTTAAAGTGGTTGTGCCTAAAGAACAAGAGAAGCAGCCTCAGATTAAGCCTATTGAG

ATCACTGGCTAGAAGTGCAAATATGGTCTTTTACTACTAATAGATAAAGCTTTTAACGCTGCAGCTTCTG

GAGTTATGGGTATGACTAAAATACAGTACTAGGGGTGAGCGTTTTGCGAAATTTGGATGTATGTTGCGTT

TTGGTGCTTTTTGTTTGTATTTTTTTGTTGGTTGCGTGCAAAGTCTAAGCTATCGATCGAAAGCCTATGT

AAAGGTAACAAGTACATGTAAATGAGTAACTAGTAGTAGTTTTCTGTGTTTGCATCACTATGATGAAAAT

TGGAATGCTTATTTGATGTTTGAATAAAAAAAAAGGGGGGGCCCGGTAC

>FM894102.1 FM894102 Jatropha curcas embryo 56-70 (DAF) Jatropha curcas cDNA clone rjcaeb0_005079, mRNA sequence

ATTCCCTTTTCCTGTCCTGTTTTCCTGGCCAAATTGCCATTTTTCCCGGTGAATGAATGCTCTAAACTCT

AGAGCTCGTGCTAGACGAACACATTGTTGGCCTCTGTTCGCCCAATAAAAGCCGGACTAAGAGAAACCAT

GAATAAGTAGTGTGATACCGTAACATCACATTTTCCGGTGATTCCCGCTGTCACCGGCGAAGACAATCAT

CAATGAGGCTTTTGATGACGCGCCATCACCTCACGAATGATTTCCTTCCATCAATTCTGGCGATGAAGCT

TGGATGGCAAAACCACCAAGAATGGCGGTGGTACAACGACTATCGGACCTATTTCAGCAAATTGAAGCGG

TGACTCAAAAAATTGACATCAGAATTGGG

>FM894101.1 FM894101 Jatropha curcas embryo 56-70 (DAF) Jatropha curcas cDNA clone rjcaeb0_005078, mRNA sequence

ATTCGAGAGAGAACTATATAACATATCTTCTGACCTTCCTCTTCTCTGTGTTATATAATTCTCACAGGAA

CTCAAATCTCTTCTTTCAGCTTCCTTCTTGCATATATCCCGAACATTGGAAGAATCGTCGACTTCAACGG

TGGTGATGATAGTTCTGTAGCCAGTGACTGCATTGGCTATGATCACTAGAAGAACGCAGAGTAGGGCTGT

TGAGGTAATTAGATTTGCCATTTTTGGGGGGGGCCCGGTAC

>FM894100.1 FM894100 Jatropha curcas embryo 56-70 (DAF) Jatropha curcas cDNA clone rjcaeb0_005077, mRNA sequence

ATTCGGACGAAAGGGTGAAAGAAGGAGACATGTTCGTGATCCCTCAATTCTATGCAGCACTTGTGAAGGG

CAGGAGAAAATGGGTTCGAGTACGTAACATTTAAAACAAGTAGCCAGCCTATCAAGAGCCCATTGGCGGG

TTATACATCAGTGATGAGAGCCATGCCTCTTGATGTTCTGATGAACTCTTACAGAATGTCTCCAAGCGAA

GCTCATCAAGTGAAGAGGAACAGGGATCCACAGTCCATGTTGTTTACTCCCACCAGCAGGAGACCATAAG

TAAGTTACTACTAAAATAGCATTGTGATTGGTGCTTCTACTAGCCAAGGCCGCAACTAAGAGATCCTTCT

TGTAAATTTAGAGAAGGGATCCGTCGTATATTGTAGTATAGAGAGTTGTAGAGTGTTAAAAATGTAGAAC

TTCAACTTCTGCAGAAATCAATAAAATGTGGCCTTCTTTTGTTTCTAATTAATAAAATATAAATTCCATC

GGCTTTACAGTGGAAATTCACTACAAAAAAAAGGGGGGGCCCGGTACCCAATTCGCCCTAT

>FM894099.1 FM894099 Jatropha curcas embryo 56-70 (DAF) Jatropha curcas cDNA clone rjcaeb0_005076, mRNA sequence

ATTCCGAGCTTTGAGCAAAAATGGCAAAGCTAATTACCTCAACTGCTCTAATCTGCATTCTTCTAGTGAT

CATAGCCAATGCAGTCTCCGGCCATATGACAACCACCACCACCACCACCACCGTTGAAATGGACGATGCT

TATGAAGTTCGGGAAATGTGCAAGAGAGAAGCTGAAAGAACAAATTTGAGCTCTTGTGAGAGGTATATAA

GACAGTCGAGAACAATGTCAGAAAGCTCTGTTAGCTATGAAAGGAATCGAAAACCGGCACCATAACGTTC

CTAGACAATGTTGTGACCAAGCAAGGAAGCTACTTGCCATTTGTCGAT

>FM894098.1 FM894098 Jatropha curcas embryo 56-70 (DAF) Jatropha curcas cDNA clone rjcaeb0_005074, mRNA sequence

AACTAAGAATTTATCTCTTTATTAATTATTAAACCAGAAGCCACTATAGTCCGCAGCAGTGATCTTTCTC

TTTTCTTAAAGACTAATTTACACATGTACCTTCACCTTTCATTATTTATTCACCATACACAGCACTATTT

ACATTAGTAAACCCTCAAGGTCGATGTCACAGTCACCCCAAAAAGCCGAAACACTTGCCATCACGTTATT

AGCTTCTCATGCCACAGCATGACCCTCGAAGGAACCTCTCCTTGGCCCTGGAAAGAACCACTCCTCGTTT

TGTCTTCCAAAAGCCTGATCCACTTCTCTTTCACTTACACCGAAGGCTAACTCTTTGGCTTGCTTCTCCA

TCTTATTTACAATATTATTTTTCCCTGCAAGTAGATGCCTAGTATTGCCTTCTTCATTAATCTCAAAGCA

GACTACTTCCAGATTATTGTTTCTTGAAGCTACAAAAGCCGCCGGATGGCCTGCAGGGACGACCACTGCT

GTTCCACGTTTCAACCGTGAACTTACTTTCTGGTAAGTCTTCTGACTTTCACCCGTCGTGTATCCCCGCG

AGCCGCTAAGGTGAGGACAAGCCATTTCAAAGTAGCCTTCGCCATCCAAAACCACTGATATCTTGGTTAC

TCTGGAGTTGTAA

>FM894097.1 FM894097 Jatropha curcas embryo 56-70 (DAF) Jatropha curcas cDNA clone rjcaeb0_005073, mRNA sequence

GGCCAAATATTTTAAAAATTACTAATTAATAATACCGAAGCATTTAACTAAAATCATCACAATTACTTAT

AGATTGCATAGCAACAAAATATTTCCAACATGATTAAAAATCCAGTCAAACAAAAACAACATAAAACGAA

ATAAAAACAAAAATTTTTCTAATAAGTTTTTAAAACCTTCCACCAGGTCTGCCACCAGCACCACCTCTCC

CAAACACGAACTGGGTCGCAGGCGCTGCAGCCGGTTCGACTTGGAGAATCCCAGGAATATCAGTCATACC

CAAAGCAGCAAGCATGTCAATAGTCTCCTTATCGACCTCAATCTGATCAGTCTTGATGGCAGAGACATCA

GGTACAAAGTCCATGCGCCGCTCGCGCTCTTCCTCTTGAAGCTTGAGTGAGATCCCGCGAACCGGACCCT

TTTGGATCCGTTTCATTAGATGGGTAGAAAACCCTGCAATCTTATTTCGGAGACGTTTTGAAGGGATGAT

AGCCACCTCTTCAAGAATCTTCTTGTTGGTATGAAAATCAAGTGTCATGCGCGAGTAGTAGCGCTCTATC

ACTTGGCGAGAGGATTTCTTCACTGTCTTTGTACGTACGCGAACCATTTTCCTGAATCAG

>FM894096.1 FM894096 Jatropha curcas embryo 56-70 (DAF) Jatropha curcas cDNA clone rjcaeb0_005072, mRNA sequence

ATTCTCTCGATATCAACCCGGGTTAGGGCTAAGGAGCTCTCTAACATGCGTGCTGCTGGCAAAGATGAGA

ATTTGAAACTATCTCCACCTCGATTGATGACTCTTGAAGAAGCCATTGGCTATGTTGCTTCCGATGAGCT

TATTGAGGTTACGCCTAAGGCCATTCGTTTGAGAAAAAGATACCTGGATGTCAATAAGCGCAAAGCCATG

AGTAAGAGGCCGAAGGAATGAAATCAATCGAACACAACTCTGTAACTTATACCTCTCAATTCTTGAATTC

TGCGGAGCAGTTAACCTAAATATAGGTATATTTTACACAATACTGAAGAAAGTATTCTTTGTCAAATTCA

ATTAAACAATAATGCACAATAAAACTTATCCTCATTGATATGTAAACCCAATATTGATTTTTCATGTTCA

GCAACTCAGAAAGCCATTCTACTGGCTACATAGCTTACTAAAAAAAAAAGGGGGGGCCCGGTAC

>FM894095.1 FM894095 Jatropha curcas embryo 56-70 (DAF) Jatropha curcas cDNA clone rjcaeb0_005071, mRNA sequence

ATTCATTCGAATATGTAACCTTCAAGACAAGCGATAACGCGATGACCAATGATCTTGCAGGTCGCTCCTC

CGCCGTTAGAGCAATGCCGTTGGCGGTTATTGCAAATGCATTTAGGGTATCAGCAGAGGAGGCAAGAAGG

ATTAAGTTTGGAAGACAAGAGACCACCTTGACTATTTCCAGGACCGAGTCTGGAAGATATGCCGAGGAGT

AAATAATGAAGTAGCCTTTTTGGGTGTGTAGATAGTGAAAGTAAGTAGTATTACGAGGGGAGTTATGATC

TCACGCTAATGAAGTATGTAATATATTATGAAAATAAGTGATGAAAGTAATGTAATCATCCTAATTAGTA

ATTAAGTATTCCCTAATCAATATATTCTATAAATATAATTGTATTC

>FM894094.1 FM894094 Jatropha curcas embryo 56-70 (DAF) Jatropha curcas cDNA clone rjcaeb0_005070, mRNA sequence

GGAAAAGTAAAATGTATAATATATATAAAAACAATCTGTTGTGAATCTATGAGAGCAACCTACATTGTGA

ACACATAACCAGATGAACTGTCTACAATATAGTTAAATACCTTTTCAACTCAGCATTAAATGACAAAGAT

TCGAAAAACCAGCCTCTCTATAGCAAGTCTACTGATTCTTCTTTAGTGTAGAAGTTGAGGATCGACGATC

TTGGTTAGAAGTTCTGATAATAAAAAGCAAAGTTTCTACAAGCATTCCACAAACTAGACCAAGAATCCCT

CCAGCAGCACTCATTCCAAGATTGTGGCCAAACAATGCCCTAAATGTTGCATATCCCACCAAGTAGCCAG

TAAACATGGTTAGAGCAACATGTAAACCAAAACCAAGCTGATCCTTGTAAGAGGAGAAAGGCTCAGTCTC

GTCGTTCCTTGGTGTAATATCCATAACAAGCTTCTGGTATGCATTCCTCTCTGCCAATTCCTCAAGTTTC

CTCAGTCTTGCCTTTAACGCTTCACTCTTTTCTCTAGGTTTAGGACTAGTAAAGAGGAATTCAGATCCTG

AGAAGAGGCTGGCCAAATCAGGTCGGTTGGAAGGCTGGCATGCTATCCATAAGCTACGTAGAGACTTGTA

GTGTATATTATTTTGAGTGAGGAGAGTGGAAGCAAGGGCTCGAAGGTTCTG

>FM894093.1 FM894093 Jatropha curcas embryo 56-70 (DAF) Jatropha curcas cDNA clone rjcaeb0_005068, mRNA sequence

CTACTAAAAGATACCTTTTATTCAACTGGAATTGCTTAACAAGAAAAAGACACCAAACAGAAAACATAGA

TATATTACATAACAGGAGCTAAAGCACATGCCATGCCCTACACACGTGGGTTATGGCCAGTGGTGTCTCC

GGGCAACCCAGATGGGCGAGTCCTGGTCAAACCCGTTGGGTCCACCTCTCCACCGTATGGCTGCCCTGTG

CCGTGTCCAGGCATGGCCGACATTTGCTGGGTCCCTGTAGGGTAACCGGTTGTCCCAGTTGTTGAATGGG

TATAGGTTTCGGTTGTGCCTTGAGCGCCCGGACCAGCAGTTGTGTACTGTACATGGCCACCAGCCTTGGC

TGCCTGCCTCGCCGCTGCGTTATCCTCGCGCGCTTCTAGTTTCCGCAATTCTGCCTCCGTTTTCCTCTCT

TCTTTCTTCTCTCTCGCCATTTCCTTCTGCACTGGATCACGTGTTGACATTCTCTCCACCTTCTCTTGCG

CCTCTGCTCTGGTTTTTTCCATGCCAGCCTTAGCAGAGGCTCCAACGTTAGCTGCGGTTTCCTTCAGCGA

CTCCATTTTCACTCACTTGGCTATTCAAATTCAAGGGGGGGCCCGGTAC

>FM894092.1 FM894092 Jatropha curcas embryo 56-70 (DAF) Jatropha curcas cDNA clone rjcaeb0_005067, mRNA sequence

ATTCCATTTCCAAACCCTCCCTTTCTTTCTCATCTCCTCGGTCTATAAAACTCCCCTCTATTCATCCACA

CTCAACCTTCAAACCGCTCACCTCCAACGCGCGTTCTGTCCTCGTTCCACGCTCCACCGCCACTCCCTCT

TCCACCACTTCCACAATCGCAACCACTTTTCATGGACTCTGCTACGTTGTTGGAGACAACATCGACACCG

ACCAAATAATTCCCGCCGAGTATCTAACCCTTGTACCTTCAAACCCAGCCGAATACGAAAAGCTTGGCTC

TTATGCCTTAATTGGCCTTCCAGCCTCTTATGAAACCAGGTTCATTGAACCNGGGTGAAACGAAGACCAA

ATACTCAATAGTGATTGGAGGCGATAATTTTGGATGCGGATCATCGCGTGAGCACGCGCCGGTGGCTCTT

GAGCTGCGGCTGGCGTTGCGGCTGTGGTTGCGGAATCTTATGCTCGGATCTTCTTCAGGAACTCGGTGGC

CACCGGAAAAATTATCCTCTGGAATCAGAGGTTAGGATTTGTGAGGAATGC

>FM894091.1 FM894091 Jatropha curcas embryo 56-70 (DAF) Jatropha curcas cDNA clone rjcaeb0_005066, mRNA sequence

GGACTATAACTTATAGCTCCACAAACTTTCATTATCTCATAACATAAGGCATATACAGATCTACATTTTT

ATATTATTGCAAGGAACTAAAACTGCCTATAAAAATAGGCTTATTCTTCTCCAAATCTAAACTATGTAGA

AACCAGTTTATTATACATCCTCTTGGTATAATTGCATAGTTGCAATATTAATTACGAGATCTAGACCCAG

TGCTAAACACAGTGACTTCCTGTCTATTCTCCAGAGAGAGAGAGAGAGAGAACTAGTCTCGAGGGGGGGG

CCCGGTAC

>FM894090.1 FM894090 Jatropha curcas embryo 56-70 (DAF) Jatropha curcas cDNA clone rjcaeb0_005065, mRNA sequence

ATTCTGCTTACCGGTGATGAGCTTCTCTCGGATTCGTTCCCTTACAAGGAAATTGAGAATGGCATGCTGT

GGGAAGTTGAGGGAAAGTGGGTTGTTCAAGGAGCCGTTGATGTGAACATTGGTGCAAATCCCTCTGCTGA

AGGGGCGGATGAAGATGAGGGTGTTGATGACCAGACTGTTAAAGTTGTTGACGTTGTGGACACTTTTAGG

CTCCAGGAGCAACCTGCTTTTGACAAGAAGCAGTTTGTCACATACATGAAGAGGTACATCAAGTTGTTGA

CACCCAAATTGGATGAGGAGAAGCAACAGGCTTTCAAGAAAAACATTGAGGGAGCAACCAAGTTCCTGCT

CTCGAAGCTCAGTGAACTCCCAATTTTTGTGGGGGAGAGCATGCATGATGATGGGAGTTTGGT

>FM894089.1 FM894089 Jatropha curcas embryo 56-70 (DAF) Jatropha curcas cDNA clone rjcaeb0_005064, mRNA sequence

AACCAAAAAGTGACCGTTTTTTCATTTTGCAAGTCACAAACCAACTACAATTCTACGGAGTGGCAACTAA

ACATAAAGCCAAAAAAGAAGACCCAAAAACCATGGGATGCCACTTCCTGTCAGTGGGGAATACAACAGGT

TACACACTGATGCCCAGAAAACAAACCCCGACACCAACCAAAAACAACCAACATACCGCAGTTACTACTA

CCATATGTACAAACCTACTCGCAACTAGTTCAAAAAGAGGAACTACTAGTTAAAACACTGATATGAAGAA

CCTAACTAACGGGTTTTATACTTGGACTCATCAATTTCAATCCCCTCCTCAGCAGCACGTTCTTCACCAG

ACTTGTCCATGGTGCTAAGTCCGCCTTTTCGGCCCATCTCCTGATACCCTTCATGCCCAATCTGCTCCCT

TCTTGTCTCACCTCCACGGTGACCAAGTTCCTTGTACCCCTCAGTTCCCAGCTGCTCCCTCCTTGTCTGC

CCTCCACGGCTCCGCCCTTCAGCTAGATGTTCCTGGGCTTCAAGGCTCTTGCCACCAGTTCAACAGGTAC

AACAGTCTCTCCTTGCCTCGCTCTAGCATCAAGTTCAGCCCTTTCTTGCTCTGTAGCCATTC

>FM894088.1 FM894088 Jatropha curcas embryo 56-70 (DAF) Jatropha curcas cDNA clone rjcaeb0_005063, mRNA sequence

ATTCCTCTTTTTCTCCCTTCCAAGAAAAAGCTTATTACGATATCTATTTTAAGCTTCCATGGCCGAGTAT

GAGCGACGACCAGGCGGAGAAGGGCACAAAGGAGCCTTAAAGGAGAAGGGCGGCTTCTCGACCTCACAGA

TTTTAGCGGTTGTCACTCTTTTGCCGGTGAGTGGGACGCTTCTTTTCCTTGCTGGAATAACGCTTACTGG

AACACTCATTGCGCTAGCTGTTGCGACTCCGCTGTTCGTGATTTGTAGTCCGGTGCTGGTACCAGCGGCG

TTGGTCATTGGGTTGTCGGTGTTAGGGTTTTTGACGTCCGGTGCTTTTGGAATCACTGCGCTTTCTTCAC

TGTCGTGGATGGTTAATTATATTCGCAGGATGAGAGGATCTTGGACGATGCAGATGGAAATGGCAAAGCG

GCGCGCGCAAGAAACGACGGGTCAATTGGGCCAGAAGGCTAGGGAGGTGGGCCAGAAGGCCCAGGAAGTT

TGCAAAGACATGATCAGGTTGTCTAGCAGTAGTTGTTGTTTGGTAAATAATATAAGCTAATATAGTTTGA

GTTTGATGATG

>FM894087.1 FM894087 Jatropha curcas embryo 56-70 (DAF) Jatropha curcas cDNA clone rjcaeb0_005062, mRNA sequence

ATTCCAATATTGGACAAATCTTGACGAACCTGACCAAATTCGCCATCGATTCTACCCTCGACCACTCTCT

CAAAGGCGTTAATGGCCCAAAGAAACTATACAGGATCGTGCAAGAGAGATTAAAGAATCCACCCACTTCT

CTTTCCTTAAAAAACTGTAAGAAACCTGAAGATGTTAAATTATTGGAAGATATGCAGATAAAAGAAGAAG

AAACAGAAGGCATGGTGAAGTTGAAGAAGCAAAATGATACTTCAGCTAAATTTGTAAGGGGAAAAGAGGG

TCTGAAGAAGGAACCAAATGAAGGCAACAGGAGCTCGGATGTTTTGGAACCTGAAAAGAATAGAATTTTT

ATTCGATCTCGGCTGTAAAATAGGGCTAATTGTAATAATCATAACGCCTGAATCTGCAAAAGATTAAAAT

AACTGTATTGTATTTCTCAGGATTTTCTTAGACAAATTCGCATATTAGTGTCCATATTATATGAACTTTG

TAGCTTTTACTGTGTTTTGTCTGTCAGATTTTCCAGTGAAATGAGTTATCTTTTGATCATTTTGTAT

>FM894086.1 FM894086 Jatropha curcas embryo 56-70 (DAF) Jatropha curcas cDNA clone rjcaeb0_005061, mRNA sequence

AATTCGTTGGATGATATTGGCATTGACCTACCAAAAGCACCTCATAATTTTGGTGAAATTATTGGGAAAC

TGGTTTTGGCTGGGGCATTGGACTTTAAAGTGGTGAGAGAGGTCCTTAAGAAGATGGAAGATGATATGTA

TCAAAAAGCTGTACTTGATGCAGCGATAAGGACCATTGGTTGCAGTCCAAATGGGCAATGCTTATTAGAT

TCGCAGGCATCTGATATTGAGGCTTGCCAGAGTTTGCTTTAGTAGGCGTGGCATGCAATTGTGTGAGTAC

TTGAGCGTCTGTATCCACAAGAACTTTACGTGCTTTGTTTCGAATCATAAATGCCCCGTCCCGAAACCTT

TATGAAGGAGAGCAATTTGTACTAATGTGATAAGATGAA

>FM894085.1 FM894085 Jatropha curcas embryo 56-70 (DAF) Jatropha curcas cDNA clone rjcaeb0_005060, mRNA sequence

ATTCAGAAGAAGAACAAGAACAAGAAGAGATGGGAGTGGTGGTAATAGATGGATCCACGGTCCGTAACTT

TGTGGAGAATGAAGCCCACTTCAAGAAGAGCGTGGAAGATACGTTCGCCAAGCTGGACCTTAACAACGAC

GGGGTGTTGTCCCGCTCTGAGCTCCGACGAGCCTTCGAGTCGCTGCGTTTGGTGGAAACCCACTTCGGAG

TGGACGTCACCGCCACACCAGAGCAGCTGACTCAGTTGTACGACTCTATCTTTGAGTCTTTTGATTGTGA

>FM894084.1 FM894084 Jatropha curcas embryo 56-70 (DAF) Jatropha curcas cDNA clone rjcaeb0_005059, mRNA sequence

ATTCCATATATTTCCCATACAAGAAAAGATTTGCCATCAAACACACACGTATACACATACTTAATCACAC

CTATCTATCAACTCCCATTTCTTCAAGAAATGGACAAAGAAGCCATCGTTCCTCTACCTCACCGGGGATC

TTCGTGCCTCTCGGCCGGTTGCATGATGTCTCCTTCATGCTTCCCGGTGTACGACGGAATGGAGTACCAG

CTCGTTCAATATAACAGTAACAACAGTAGCAGCATCAAGAGAAGCCGGAGATGGCGATACTTGATAAGAA

GGCTAGTAAGAGAAGGAAAAACCAGCATATATGGATCAAAGCCTCTATCTTTTCATTATGACGCTGTTAG

TTACTCACAAAACTTCGATGAAGGTTGCCATTATCAAGAATCCATCATTCAAAAGTGTTGACGGTGAAAG

ATGCTAGATGGGATGTTGATGATTAACATTAAAAAG

>FM894083.1 FM894083 Jatropha curcas embryo 56-70 (DAF) Jatropha curcas cDNA clone rjcaeb0_005057, mRNA sequence

ACTAAATAGCGTACACAAAGCCACACTATTATTTTATTATAGCATATATAAGAAACACAGTGGCCATTAC

ATAAAAGGAACACAAAGAACCTGAAAACAAAACAAAAGCATCAAATCTATTAATTAAACGGGAATTGAAT

TGGTGACTGATGGGTAAACGACAGTCGCTGGATGTGCCATGATCCTGCGACCTTTCCTGTGTTTAAAGAG

GTAAACAAAAGCTCCCACCGGCCACTCTACCACCGCCGCCACGGCAAACGCCAAGAACCCCAGAGTTGGA

CCAACCACCTTGCACCGGCAAGGGTTGCTAGTGGTGCCGCATTGGACGCATATTGGAATTCCAGCGGGGG

GGCCCGGTAC

>FM894082.1 FM894082 Jatropha curcas embryo 56-70 (DAF) Jatropha curcas cDNA clone rjcaeb0_005056, mRNA sequence

CGAATTCGGTTCCTGAGGTTTTAGTTGATGAAGACTTAGATGTGAATGACTCTAAGTTTTACCTTGAATT

GGAGGATTTGACAGGAAAGCCAAGAAGTTGGGATGGATATGTAACTGAGTTGGTACAGCAAGCGGGTTGT

ATTTAGTTCTTGATTGCACTGGATGTGAATTGTCAGGCAGGTGGCCCTTCCATTTGAGCTGCTCCTAGCC

CGCATTGGAAATGCTAGTGTTTGACAATGGTCATACATGTTCATATTTCCTGAGTCGTTTTTTTCTTTTT

CTTTTTCTTTTTATTCTGTCACCAATGATGAATCATCTAGACTGTAAATATTCCCACACGCAGTAAATAC

CATAGTTTTATCATCCTGCATCAAAAGTTTTAAACTTTGTAATATCAGTAGTCAGACTTTGGGTATCAAA

CTACATAATAGTATGGAATAGCACTTGAGGTTT

>FM894081.1 FM894081 Jatropha curcas embryo 56-70 (DAF) Jatropha curcas cDNA clone rjcaeb0_005055, mRNA sequence

GGAAAAAGAAACAAATATTGCTACTAGATGTCAACAACAATGGAAATATATGAATTATTCTGAAGCTACA

GGAAAAAAATTGCAATGTTTGGACGAAAAATGACAAACACGGTTAAACAGGTACAGCCGCTAAAGGAGGC

TTAAAAAAATTCAGAAGACAAAGGAGCACTATCAATTGTGTATTCCGGTACTTTGTTGCAATCAAAAACT

CAAGAGTTACGCCATCACCTATGCCCTGTTTGCAATATTGTTTGAGAGCCAGTCCAGTCCCTCGTACAAC

CCTTCACCAGAAGTGGCACA

>FM894080.1 FM894080 Jatropha curcas embryo 56-70 (DAF) Jatropha curcas cDNA clone rjcaeb0_005054, mRNA sequence

TCCCAGAGGCAGTGGTTGCAAACGCATTCCAGATTTCCAGAGAAGATGCCAGGAGGCTTAAGGAGAATAG

ACAGGAAGTCACTGTGTTTAGCCCTGGGTCTAGATCTCGTAATTAATATTGCAACTATGCAATTATACCA

AGAGGATGTATAATAAACTGGTTTCTACATAGTTTAGATTTGGAGAAGAATAAGCCTATTTTTATAGGCA

GTTTTAGTTCCTTGCAATAATATAAAAATGTAGATCTGT

>FM894079.1 FM894079 Jatropha curcas embryo 56-70 (DAF) Jatropha curcas cDNA clone rjcaeb0_005053, mRNA sequence

ATTCAATAAATCGAAAGAGAATTGTCCTCATGGCTTTTCCAAGTGTAAAAACGTTTCGTATCCGATTCGT

AGGTCATGGAGCGAAGGTTGCTTGGCTTTAGTTGCAGCCGCTTCCTGCCATTCCTCAGCGTCTTCTTCAC

CGTCTTTGCAAATCGCAAGGTCGTCGACGCAGTTAGGATTGTGCAACGATCTAGACGAGGTTGATGATGG

TGATCAAGGTTCTTCTACAACTTCTTCAACGCCGTTATCGTCGTCAAGGGTTTGATGAGTTAACAATTTC

TCGATCGCCGTCGTTTTTCCGTTTGTTTCATGAATAATTAATGTATGTAATGTGAGACCGAAAAGTTCGT

GAAACGTTCTGATCTTTGATTCAAGTCAAATCGGATCGGGTTTTGAATCTATAAATTTGTCATGAAAACG

GGCTTGCCAGCT

>FM894078.1 FM894078 Jatropha curcas embryo 56-70 (DAF) Jatropha curcas cDNA clone rjcaeb0_005051, mRNA sequence

AAAAATACAAACATATAGGGATATGATACAAGTACTTCAGTTTGGATCGAAATATCAAAGTAAAGACAAG

ATTTTAAAGACAAGATTTTAAACATCGGTAAGAGTAAGAGCTCCAAACAACAGACATTGTCTACATCAAA

GCATCTTGATCATACAGTTATTTCATGCCAAACCCAAGATATAGATTCCACTGAAGAACTCAAGTCCATC

CCTAGCCTCTATCGAACCAAACCAAGTAAATAAGTTAATAAAACAGTTTTCAACACATTCCACCATAAAC

ATCACCCGATTAGTGAACAACCAGTATCAACCCATGGCATACTTCTGTGTCCAGCTGCGTGCAGTTGCTT

CATACTTTGCTCGATCAGTCTTATACATATGGGCAATCTCTGGGACAAGTGGGTCATCGGGATTTGGATC

AGTCAGCAATGAGCAAATAGATAGCAATACCTTAGAAATGGTGAGAGCAGGGCTCCATTGTTCTTTGAGG

ATATCAAGACAAATGCTTCCATTACTGTTTATGTTTGGATGAAACACCTTGGTCCTAAATGCAACCTTCG

GAGGCTTGAAAGGATAATCTGG

>FM894077.1 FM894077 Jatropha curcas embryo 56-70 (DAF) Jatropha curcas cDNA clone rjcaeb0_005050, mRNA sequence

ATTCCCTTCACAATAGCAGGAAAGCTTTTTGAACTCACCCCAGAGGAGTACATTCTCAAGGTGGGCGAGG

GTTCGGCAGCTCAGTGCATTAGCGGGTTTACTGGTTTGGATGTGCCTCCTCCTCGTGGACCTCTTTGGAT

CTTGGGAGATATTTTCATGGGTCGCTACCACACTGTGTTCGATTTTGGCAATAACAGAGTTGGATTTGCA

GAAGCAGCGTAGAGGTTTCCCTTCCACACAATTCCGACTCCTGTGTACTACCAGCATATGTAATATATGT

GCCTTTCAAAAATGGTGTAGTTCCTGGAAACTTTATAGTGGTGTTGGGCCTGGCGTAAGAGGCGAATGTT

TGCTTGTAAATATCTTTTATGTCTTGTGTGGA

>FM894076.1 FM894076 Jatropha curcas embryo 56-70 (DAF) Jatropha curcas cDNA clone rjcaeb0_005049, mRNA sequence

ATTCCGAATTCCTTCAAATCAAGTATCCAGCTAGAATGGCTTTAAGCTTAGGAAGGAGGAGCAACATCTT

TGATCCGTTCTCACTAGATATTTGGGACCCATTTGAGGGCTTACCTTTCTATAGCGGAACAGTGGCCAAT

GTTCCGTCCACTCAACACGAAACAGCCGCCATTGCCAACGCACGAGTGGACTGGAGGGAGACTCCGGAGG

CTCACATATTTGCTGTTGATCTTCCTGGGTTGAAAAAAGAAGAGGTAAAAGTGGAGGTAGAAGATGGCAG

AGTTTTGCAGATAAGTGGGGAGAGAAGCAAAGAACAAGAAGAGAAGAACGATAAGTGGCATCGCGTAGAA

AGAAGCACTGGTAAGTTCTTGAGGAGGTTTAGGTTGCCGGAGAACACAATGATGGATCAAGTCAAGGCCA

CTATGGAGAACGGTGTGCTTAAAGTGGTTGTGCCTAAAGAACAAGAGAAGCAGCCTCAGATTAAGCCTAT

TGAGATCACTGGCTAGAAGTGCAAATTTGGTCTTTTACTACTAATAGATAAAGCTTTTAACGCTGCAGCT

T

>FM894075.1 FM894075 Jatropha curcas embryo 56-70 (DAF) Jatropha curcas cDNA clone rjcaeb0_005048, mRNA sequence

CGTCTCATTGTTAAAGAGAATGCTATAACATGCAAAACAGGTTACAGGTACATGTAGACTGAACATAAAA

CTCGGTGCCATTTCTATGTCCGCTTCGAGAAGACACTTGTTATAAGTACATACGCAAATACACTCCTACA

CACAAGGGGAAGTAACAGAACAAAACAAAGCAAACATCATATACAAACAAACACCATTCATATTAGTAAC

TCTTCATTAGTGTATCAGTAGTTAATGGTCTCATTAAGCCTAGCAGCTGTCGTCATTGACACCGCAACAC

CACCTGGATGCGTGTTTAAATTCGGATTGTTCCTCAACTCTGCACTAACCACCCCTTCAGCATCTGCCCT

TGTCACGACCTTATCCGCCGCCAATTTCGAAGTTGCACCCGATAGAACGTCGGCGAGTTTAATCTTTTCC

TCAGCGCGATTCGTAGCGGCATTGTAAGATGCGGCAGATTGAGCTGTGGCTGCGATTCCACCTGGTACAA

TAACATTGCTGCCAGTTGCTCTTACTTCTGCCTCTTGAATTGCGGCAGCATCGCTCTGGTCCACTGCCTT

GTCACCAGCTGTTTGTGCTGAGGCTTCCAGCGCTTCTCCTATAGTAATCGCA

>FM894074.1 FM894074 Jatropha curcas embryo 56-70 (DAF) Jatropha curcas cDNA clone rjcaeb0_005047, mRNA sequence

AAAGCGTTAAGAGTGGAGGACTAGCATGTCAAAAGACTACAGATACACAATCCTCACCAGAAGCACACAA

CATTACAACACTACATACAACATTACAAGCGCGTATTGTTGGACAGGTAGAGCAGATTCTATAAAAAGCC

TCACCATAAGTACACAACATTAACATTATACAATCAAGCTGAAGTGACTTAATATTTCAAACAGACCTGT

CAATCTCTAGATGCTCAGCACACTCCAACTCTCTTGCCTTCTACAAGCTTTTTCCAATACTGGGTGACTT

CTTCTTGCATCTCCCTCTCACTCTTTTTAACTATTTTCTTCCCTTCACTGTCCAGCAATGTCTTATTAAC

AATGGCAGTTTGGCTGGAAATATGCACAGGAAGTAAGTCCATTTCCTTCAGAACTTGTCCAATGTGAGAT

ACAAGTCTTTCTGCCAAGCTGCGGCTCAAATCCTCCCGGACCACCACCCGAAGTACAGCAATGTGCTCAG

CATCTGCAGGCATTGTATAGGCTGGAAGGGGGGGCCCGGTAC

>FM894073.1 FM894073 Jatropha curcas embryo 56-70 (DAF) Jatropha curcas cDNA clone rjcaeb0_005046, mRNA sequence

GTTGTAACCAAGCATGGAAACTAAGTGGCTTTTGTCGATGTCAATCAATAAGGTATCTACCAGAGAAGCA

AGGATACGTTGGAAGCCAAGAATATGATGAGGCAATGCGTAGGGCTGACGATATAATTAGTGTTTGCTTT

GACAATATCTGCCCTCGTTAAAATGCTATTGCTACCGAAAATAAAATTTCACTCGCTTGAATGTGAGCGA

GAGTTACGTTTAATAATAATCCATAAACAATATGAGTTTCGGAGTAAGTTTGTAATGTTGCTTCAAGTTT

TCTTCATGTCCTGTAATAAAAGCTAATCAACTGTTGAGTTATTGAAAAAA

>FM894072.1 FM894072 Jatropha curcas embryo 56-70 (DAF) Jatropha curcas cDNA clone rjcaeb0_005045, mRNA sequence

ATTCCTCAGCAAAAAATATCAACAGCATCAAGAAGAGGAAGAAGTGGGTGTTTTTGAAGAAAAATGTGTA

ATCCAATCATACCTTTGGTTAGCCATGGGGTCTTCGAGCGAAGATGGTTTGTTTTCCAAGCAGTAGAGAC

CATGACTGAACATGCAAGGTTAGAGACAGCTGCTGTTGCTGGTGGTGGTGGTGGTGCTGCTGCTGCTGCT

GGAGGTGGCTCGATAAAGAGCTGTATTTGTTCCCCGACAAGACACGCAGGATCATTCAGGTGCAGATATC

ACCATGGTGATTATGAATGGGGTCGTAGAATAACAAAGAAGTGAGTACACTTGTAAGTTTGTAATTAAAG

TTCAAAGGCTTAATGCAAATGTATAAACTACATAACAAAAATGGGTTTTAATCC

>FM894071.1 FM894071 Jatropha curcas embryo 56-70 (DAF) Jatropha curcas cDNA clone rjcaeb0_005044, mRNA sequence

TCACTATCGATGAGTCGAAGTTCAGGACTGGGAGCTAGTGTGATGATTAAAACTATAAATGGTATTTTGT

AGTAATAATAATAATAATGTTGGTAATAAAGTATTAGCTTAGCAGTGTACTTTAGTTTAGTAATGTAGTT

TACATGTGGGTTAAAATGACTAGGGTTTAGCTAGTATGTGTGTTGTAACTTTGAGAGGGGTTTATGGTCG

TTCCCTTTCTGGGAGGACTGCTAATACTTTCTCCCTATTTGTAAAATAACCTGCAGTATATATAATTACC

TGTTGGTTAAAAAAAAGGGGGGGCCCGGTAC

>FM894070.1 FM894070 Jatropha curcas embryo 56-70 (DAF) Jatropha curcas cDNA clone rjcaeb0_005043, mRNA sequence

GTACGACATCATGTATTCAAAGGAATATAAAGAACACACGGGCGTATGCGTCACAATCGGACGAACGAAC

GAGACGCTCGGAACGCGACCCTAACGAACAGCAGCGACGGCGAAGCAGTACGTATCCGCATGCACTCCAG

GATTAACAGTATTAAATGCAAAACGAAGCCTGCTACTCCGCTCGGCATGGAAATTGCGACACCTTTTTAC

GGACGCCCATAGGAATACCTTGCAGGAACTGGTGAGCGACTATCACCACCGTAGTCGTAATAGCCGTAAT

CATAGCCCGAATCGTAATTGCTCGGGGAACTAGTCTCGAGTTTTT

>FM894069.1 FM894069 Jatropha curcas embryo 56-70 (DAF) Jatropha curcas cDNA clone rjcaeb0_005042, mRNA sequence

TTTATTTTTATCGGTACTCATTTATTCTAACTCATCAGGGGATCTTTTTCTCCGCTTCTTACTGTACCTT

GGAGCAATCAACGCTAGGGCTAATTTTGTAGGGAATGCTAACACCACATTTGCCAGGAAGACCAGCTGCA

AGATTTAAGTCAATACCAGGAATGGAGTTTGAAGCACTTTTCAAACATTTGCAAGCCTGTTGGCGGTCAG

CAGTGGTCTTAGCCGCCGCATTAAGGCTCCTAACGCCGCTGCAGCAGGCTGGAGGAGGCGCAGCTACGCC

TTTCCTCAGATAGGGAATACATGCTGATACTGAACTTGCCACCTGCCCACATGTAATTGCAGCTTCGGCC

AGAGGTGCACCCACCACCATGCACATCACCAACACACAAGCTAACTTAGTAGCCATTGAGCAAGCCATTA

TATAGGGGGGGCCCGGTACCCA

>FM894068.1 FM894068 Jatropha curcas embryo 56-70 (DAF) Jatropha curcas cDNA clone rjcaeb0_005041, mRNA sequence

ATTCCCATGTCTGAGGTTGAGGCGGCAAAGACGAGCGTGGACAGGGTGTTGAAGCGGCGGAAAGTGTCGG

GGAAACGGTTTCTCGAGCACAAGAAGTTCCTACAGCGAGTGAACCGTCGCCTAAAAGCTAAGGGGGAGCC

ACGCGCCAAGGGTCGCAAACCAACTGGAAAGTCGGGAGTCCAAGCAGCCGAGCCGAAACGCAGATTTCCT

CGCAGTTATGTCACTCAACAAGGCAAGACAAAACTGAGACTCAGGAAGACTAAGTGTTTCGCAGACCATG

TGCGACGTTTTCGTCCAAGCTTGATCCCAGGCACGGTTCTCATTTTGCTGGCCGGTCCGCACAAAGGGAA

ACGTGTTGTCCTCTTGAACACCTTGCGCAGTGGGCTGCTGCTTGTTACTGGCCCATTCAAGTATAATGGC

GTACCAATGCGTCGTGTCAACCAGCGGTATGTTATTGCCACATCTACGCACATTGAC

>FM894067.1 FM894067 Jatropha curcas embryo 56-70 (DAF) Jatropha curcas cDNA clone rjcaeb0_005040, mRNA sequence

ATTCCGAAAAATGGCATTGATTCCAAGCTTTTTCGGTAACTCACGAAGCAACATCTTCGATCCTTTCTCT

GCTCTCGACGTTTGGGATCCATTCACAGACTTTTCTTTCCCTTCATCTTCTTCCCTCGTCTCTCGTGAAA

ATTCTGCTTTTATTAACACTCGCATTGACTGGAGAGAGACACCAGAGGCTCATATTCAAGGCTGATCTCC

CTGGGCTCAAAAAAGAGGAAGTGAAAGTAGAAATTGAAGATGACAGAGTGCTTCAAATCAGCGGGGAGAG

AAATGTGGAGAAGGAAGACAAGAACGACACGTGGCATCGCGTGGAGCGTAGCAGTGGCAAGTTCATGAGG

AGGTTCAGGCTGCCGGAGAATGCACAGATGGATCAAGTAAAGGCTTCAATGGAAAACGGAGTACTTACTG

TCACTGTTCCTAAAGAGGAAGTCAAGAAACCTGATGTTAAGGCTATTGAGATCTCTGGTTAAGTTTCAGC

TTATAATGATGTTATCTCAATATATATATATATATATATATCTATATATATATATATATATATA

>FM894066.1 FM894066 Jatropha curcas embryo 56-70 (DAF) Jatropha curcas cDNA clone rjcaeb0_005039, mRNA sequence

CGGAAGAATGGCTACAGAGCAAGAAAGGGCTGAGCTTGATGCTAGAGCAAGGCAAGGAGAGACTGTTGTA

CCTGGTGGAACTGGTGGCAAGAGCCTTGAAGCCCAGGAACATCTAGCTGAAGGGCGGAGCCGTGGAGGGC

AGACAAGGAGGGAGCAGCTGGGAACTGAGGGGTACAAGGAACTTGGTCACCGTGGAGGTGAGACAAGAAG

GGAGCAGATTGGGCATGAAGGGTATCAGGAGATGGGCCGAAAAGGCGGACTTAGCACCATGGACAAGTCT

GGTGAAGAACGTGCTGCTGAGGAGGGGATTGAAATTGATGAGTCCAAGTATAAAACCCGTTAGTTAGGTT

CTTCATATCAGTGTTTTAACTAGTAGTTCCTCTTTTTGAACTAGTTGCGAGTAGGTTTGTACATATGGTA

GTAGTAACTGCGGTATGTTGGTTGTTTTTGGTTGGTGTCGGGGTTTGTTTTCTGGGCATCAGTGTGTAAC

CTGTTGTATTCCCCACTGACAGGAAGTGGCATCCCATGGTTTTTGGGTCTTCTTTTTTGGCTTTATGTTT

AGTTGCCACTCCGTAGAATTGTAGTTGGTTTGTGAACTTGCAAATGACAAACGGTCACTT

>FM894065.1 FM894065 Jatropha curcas embryo 56-70 (DAF) Jatropha curcas cDNA clone rjcaeb0_005038, mRNA sequence

ATTCCGGCGATGTGTTTAATGTCGGAGGCGATGTCGCCTCCCAGCCGATTGCACCAGTAGACGCTGCTAA

CATGCAGTCTGCTGAGAGCCAAGTCCTTGGAGAGCCTCAGAGAGGCGGCCCTGCCTCAGTCATGCAATCT

GCGGCAAACGTCAACGTTAGGACTGGTGCCGTCGAGCGGGATGATGTGAGTGATGTTGTTAGAGAACAGG

GCATTAACGTTGCTGAAATAGATATCGGCGGCACTCGCGTTATCACAGAGAAAGTCGGTGGAGAGGTTGT

GGGACAATATGTTCAGCCAAGAGTTCCAGCGACATATCCAATGCCAGGTATGGATATTACAATGGGTGAA

GCTTTAGAGGCAACTGCTTATTCTGCCGCAGGAGACAAACCAATTGATCAAAGCGACGCCGCTGCTATAA

AAGCTGCAGAGGTGAGAGCTCTTCGCAGTACTCAAACCCCGGCGGGTGGAATAGGCGCAGAAGCACAGTC

GGCAGCTGATCGTAATACCAGAGTTATGCTCGACGAAGATAAGACTACCCTCTCTGATGTTTTAGCGGAC

GCGACTGCCAAGTTGCCTAGAGACAAGACGGTAACTCGGGATGA

>FM894064.1 FM894064 Jatropha curcas embryo 56-70 (DAF) Jatropha curcas cDNA clone rjcaeb0_005037, mRNA sequence

AATGAATAAGGTACTCTAATGATAGAACAAGAAACAGACTGTTTCCAAGCAATACCATGAGAAGACACGT

TTACAATAGGAATAACTAAATTCGGAACCAGGGAATATAAAAACATGACATGAAATGCCCTATGAATCCC

ACAAATATTTTTTTAAAAATAAAATAAAAAAAGAAGAAGAAGACGACTGATTATGTAACTGACTACAACA

CTTCAAGATGTAGCAACCCACAGCAGTGAAGACCACACAGATGACAAATTGGTCTATCATACTTCTTTAA

ATCCTTACCAAGTCACAAGTTAAAGGAAGGCATCCTTCTCGAGCAACTTCAGTACTTCATGCTGGTTGTT

TAGCTTGGCAACATCAATGGGTGTCTTGCCATCCAGGTTCTGAAGAGTAACAGCAGCTCCATTCTCTAGC

AGTAGGGCCACACACTCTTTCCTCCCATAACCAGCTGCATAATGAAGCGCAGTGTTCTTATTTTTATCCA

GTGCATCAACAGTTGCTCCCGCCTCAACAAGGATTTGAGCACACTTCACCTCTCCATACCCACAAGCCAA

AATGCACGCAGTTCTTCCTTCTGAATCTTCTTCGTTCTTGTCAGCACCAGATG

>FM894063.1 FM894063 Jatropha curcas embryo 56-70 (DAF) Jatropha curcas cDNA clone rjcaeb0_005036, mRNA sequence

TGAGAATCGGAGACTCGAACACGAAGATGGCGAGATCGAGGTTGCTTGCTCTAGTTCTGCTATCGACTCT

TGTTCTATGTGGAACCCTAACTGTTACTCAGGCGAAGAAGTCTAAGGAAGATTTGAAAGAGATAACCCAT

AAAGTTTACTTTGATGTTGAGATTGCTGGAAAACCTGCAGGCAGAATCGTCATGGGTCTCTTTGGGAAGG

CTGTTCCTAAAACTGCAGAAAATTTCCGAGCTTTGTGCACAGGGGAAAAAGGTGTTGGGAAGAGTGGAAA

ACCCCTTCACTACAAAGGAAGTGTATTCCACAGGATTATTCCCAGCTTCATGCTCCAAGGAGGTGATTTT

ACACTAGGTGACGGAAGAGGAGGAGAATCAATTTATGGTGAGAAGTTCGCTGATGAGAATTTCAAGTTGA

AGCATACGGGACCCGGGCTTTTGTCTATGGCAAATGCTGGTCCTGACACAAACGGTTCACAATTCTTCAT

CACAACTGTGACAACTAACTGGTTGGATGGAAGACATGTTGTATTTGGAAAGGTGATTTCTGGAATGGAC

GCTGTTTATAAAAT

>FM894062.1 FM894062 Jatropha curcas embryo 56-70 (DAF) Jatropha curcas cDNA clone rjcaeb0_005034, mRNA sequence

TTTTTTTTAGAGAGAAAAAAAAAAAACCTCTATTCCTTTTCATCCTCAATCAAATTCATTTGATTAATAA

ATACAACAATGCGCATAAACTAAATGAGGCGCAGACTCTGACTGAAAAATTTGTAAATAAAATGTTACAA

TTTTAACTGAAGGGTTAAGGCCTTAAGGGTGTAGATCGGTAAGGAGATCAGTAAGGAAAACCAATGTTCT

ATGCTCAATTTAAGCTAGTCATAATCTAATGATTGTCAATATATATTCTTCTCCACTGCTAACTGTTTCC

CTCATCAACACGAGACTGAACAACTGAGGATTGGTTGCTTTGTTCGAGGTTAGCTACTGCATCTGGTCCG

TACATTCGTACCAACTCTTCCTGAAAGGGGGGGCCCGGTAC

>FM894061.1 FM894061 Jatropha curcas embryo 56-70 (DAF) Jatropha curcas cDNA clone rjcaeb0_005033, mRNA sequence

ATTCGTTATTGGGTCTGTCCTGCAACGACGTTTTGTCCCATGTTGAGTGGGTTAAGGACATTGAAGCATA

CAGCCCCNGGAAGCAAGGTAACATATCCAATTATTGCAGATCCTAACCGTCAGGTCCTGAATCAACTAAA

CATGGTGGATCCAACTGAGAAAGATTCCTCAGGGAACAGTCTTCCCTCCCGAGCTCTGCATATTGTGGGC

GCTGATAAGAAGATCAAGCTGAGCTTCCTTTATCCAGCAAGTACTGGAAGGAACATGGATGAAGTGATCC

GGGTGCTGGATTCCCTGCAGAGGGCAGAAAAGCACAAGATTGCTACTCCTGTGGATTGGAAACCAGGAGA

TCCGGTGGTAATATCCTCTAATGTGTCGAATGAAGAGGCCAAGAAGATGTTTCCACAGGGATACAAGACT

GTTGACCTTCCTTCCAAGAAGGAATATCTACGTTTCACCTATGTCGACTAGAATATATCTCAGCCGGACG

ATATGATACACTCTTATCTAAGAAATACTATTGTATAGTTTTTGCGTAGATGTCAAGCACTTCCTCTCTT

TGTTAGTAGC

>FM894060.1 FM894060 Jatropha curcas embryo 56-70 (DAF) Jatropha curcas cDNA clone rjcaeb0_005032, mRNA sequence

AAATCAAACAACTGGTGCTGGCACCATTCATTAAGCTAGCGTAACAACTGCTGCTGCTGCCATACTTAGA

CCAAACACATTTCGACATTTAACATTTAGAACAAAGGAGACAAGCTAGTAACTGGAAAGATGCCAGTTCA

CATGTTATTTCAACCTTTTGAGCAACACGACATCTAAATAACAAGAACAACGAGATAAAAGCCTTGCATG

TGGCATCCCAGAACATAGATGTGATACCTTCAGCAGAAGTTTGATGAGGGTCAGATGTTATCAAGCTTTT

TAGCTTTCACAATGGGGTTGGCCTTGGCAATAGCATTTTGTCCAGCAGTTCGATAATCATAGGTGCAACC

ATGTTTGTCTGAGTAGCGATGAACTGAACAGAAAAGATTCCCGCAACGACAACTGAATCCTGTTAGACCA

ACGCGTTTCCTGCAAGTGGCGCACCTATTTTGACTTGCTTTCACCACCGTGTCACCATTTGTGCTGAGAG

CTGAATCAGAGGACGCTGAAGCAGAACTGACCATAGTCTCTGCTGAATAAGTTTGCAGATCAATAGCAAC

AGCAGGCTCATTCCTGATGTTGCTTGAGCTACCGTTCACAATACTCTCAAGGGAC

>FM894059.1 FM894059 Jatropha curcas embryo 56-70 (DAF) Jatropha curcas cDNA clone rjcaeb0_005031, mRNA sequence

GAACTAAGAAGCGTCAAGGCACTTTTCATAATCATCAAACGAAACACCTCAAGGTATGAAAATTCAAATA

AAAACCTTAAACATTTCATCTTGCTAACAAAACTCCATGATTTTAAGTAACATGGACCTAAGCTGCAACA

GATGGCTTAGATGCAGCAGAGAGCCTGGATCTTTTCTTGGCTAAGCTCTCACTTCGTCGTTCTCTCTGCT

CCTTCAATCTTGTGGCAAGAAGCTTCTGGTACTCAGCGGCCTCTGACTTGGCCTTAGCAATTCTTTTCTT

CTTGTCAGCAATTCTTGCTCGCTTTCTTTGCAAGGTCAATGGAGTAACCAACCTCTGAATTTTGGGAGCT

TTACTAACCTTCTTACCAGATTTAGTTGTGAATGATCTGCGGTAAGTATTTACGTACTTCCGAACATCAT

CCTCTTTTGAGAGATTAAAGAGCTTGCGGATCTTTGAAGCCCTCTTAGGTCCTCTCATTCTTGGTTTCTC

AGTGTCTGTCAGTCCAGGCAGATCTTGCTCTCCCTTCTTCACAATTACCAAGTTCAAAAAGAAAGGTCTG

GTGACATGCATCCGGGACAGA

>FM894058.1 FM894058 Jatropha curcas embryo 56-70 (DAF) Jatropha curcas cDNA clone rjcaeb0_005030, mRNA sequence

ATTCCTCTAATCTCCTCAACCTCATGCAACAATCCTCTGCTTCTCAGGCCCAACTAATGAAGCTCCCAAA

AAACCTCCTAGCAAAAGCCTCCACAATGAAGAACACTGGGCAAGCTTTAGAGCAGATGCCTCAAGTGATT

TCATCCCTGGATGCACATATGGAAAATGGCTTGGAGAGTGTACCTCATTTGGGAACTGTAATTCAGTTGC

TCAAAAAAAAAAAAAAAAAAAAAAAAAAAAAAAAAAAAAAAAAAAAAAAAAAAAAAAAAAAAAAA

>FM894057.1 FM894057 Jatropha curcas embryo 56-70 (DAF) Jatropha curcas cDNA clone rjcaeb0_005029, mRNA sequence

ATTCCTGGCCAATGGCAGCCTTTGAACCGAGGTTATAGTGCCTAAATTAGTCACATGGCAGGAAATGTGC

GAGTAGTTATTCATAGTGTTTCACAAAAATCACTACTGAGTTTAGAAATTTTAGTGTTCCTGATGTGAAA

TATCTGTTATAGGACTATATGATAGAGTTATAGATTTGTTATAATAAAGCTACAGATCTGTCATGGGCTT

TGCTGTGGAACATTATGATATAAAGCTTTACACTTGTACTTTGAAGGCCACTTTGTGATAAGCGGAAAGG

CCTTCTTAATGATAACAAGCTGATCTTTAATGT

>FM894056.1 FM894056 Jatropha curcas embryo 56-70 (DAF) Jatropha curcas cDNA clone rjcaeb0_005028, mRNA sequence

ATTCCCACATTATTGCAACGACTTCTCCTCCATAAACTCGAATTTCTTGTGGTTTCTGCCTCCAAAGTTT

CCATACACAATCTTTGTCTTGTTTGTGTTTGCTTTTCAAAGATGAGCGACCCCAAGTATGCTTATCCTTA

CCCTGCTCAAGGTTATTATCAAGGACCTCCAGTCATGGCACCCCCACAATATCAATATGCTCCTCCACCC

AGAAGAGAGCCTGGTTTTCTTGAAGGATGCCTTGCAGCTTTATGTTGCTGTTGCCTTATTGATGAGTGTT

GCTGTGACCCATCCATCATCTTTATTAGCTGATACAAAATCTGTAATTTCTTCCACAAATGATTCTACTT

GGTGTGATATAAATATAGTGTATGATGGGTGGTATTATTTGTCCTGGAACATTTCTATCTAATTCTCTTA

TCATTCTAATGTTCAAGTCCTTTTACTATGGATCTCCTTTTGATCTTTGTTTGTGTACTGAACGTTACAA

CAATTTATATCCTTTTCCATTTCCCTTTAAAAAAGGGGGGGCCCGGTAC

>FM894055.1 FM894055 Jatropha curcas embryo 56-70 (DAF) Jatropha curcas cDNA clone rjcaeb0_005027, mRNA sequence

TTCGATCGCGTAATGGCGGAGCACCCACAGTCGCAGCATGTTGGCCAACAGCCACGATCCCACCAAGTAG

TGAAGGCAGCCACTGCAGTCACTGCCGGTGGATCTCTTCTAGTTCTCTCCGGTTTGACCCTTGCTGGTAC

GGTCATTGCACTGACCGTAGCCACTCCATTGCTGGTCATATTCAGTCCGGTTCTTGTTCCTGCGGTTATC

AACAGTTTGTATGATAATCACAGGGTTTTTAGCCTCAGGCGGTTTAGGCGTCGCTGCCATTTCTGTCTTG

TCCT

>FM894054.1 FM894054 Jatropha curcas embryo 56-70 (DAF) Jatropha curcas cDNA clone rjcaeb0_005026, mRNA sequence

ATTCCGCAAGAGAAGGTGGAGAGATGTCAACACGTGATCCAGTGCAGAAGGAAATGGCGAGAGAGAAGAA

AGAAGAGAGGAAAACGGAGGCAGAATTGCGGAAACTAGAAGCGCGCGAGGATAACGCAGCGGCGAGGCAG

GCAGCCAAGGCTGGTGGCCATGTACAGTACACAACTGCTGGTCCGGGCGCTCAAGGCACAACCGAAACCT

ATACCCATTCAACAACTGGGACAACCGGTTACCCTACAGGGACCCAGCAAATGTCGGCCATGCCTGGACA

CGGCACAGGGCAGCCATACGGTGGAGAGGTGGACCCAACGGGTTTGACCAGGACTCGCCCATCTGGGTTG

CCCGGAGACACCACTGGCCATAACCCACGTGTGTAGGGCATGGCATGTGCTTTAGCTCCTGTTATGTAAT

ATATCTATGTTTTCTGTTTGGTGTCTTTTTCTTGTTAAGCAATTCCAGTTGAATAAAAGGTATCTTTTAG

TAGATATTTTATTTAT

>FM894053.1 FM894053 Jatropha curcas embryo 56-70 (DAF) Jatropha curcas cDNA clone rjcaeb0_005025, mRNA sequence

AACAGAAAACAAACTTAGCTTGATGTGCTCATTCTTTTGTGCATTATTATACATAACTCTGATCACTCAC

ACACCAAGTGAGTGAAATTTTTATTTAACTGAAGCACATCCACGTTATAGCTAGCAGATTAGGAACGAAT

ATCGCAGCTTGTACGCAGGCCACAAGCAGAAGGTATACTTCTAGCCCTACGCACTGCCTCCAAACGCTCT

CGCTGTCCTTGCTCCAGCTGCTGCTCTAATACATACTTTATTCCTTCACATGGACAATCATGACTTAGCT

GCCTTAATTCGTTACAACATTGGCGAAGATCCCGTGTGCTGGTTTCAATTCCTCGCAAAGCTAGCATCTC

TTCTGACGGCCTTCTTGACGATCTCATATAACTCTCACAGGAGCTCAAATCTGCCCTTTCAGCTTCCCTC

CTGCATTTTCGCTGACTAGTGTAGAAGAATCATCGTCGATTCAACGGTGGTGATGGTGGTTGTCACATAC

CGGAGACTGATTGGCTATGTGCACTAGCAGAATGCAGA

>FM894052.1 FM894052 Jatropha curcas embryo 56-70 (DAF) Jatropha curcas cDNA clone rjcaeb0_005024, mRNA sequence

TGCGCATTCGCGTCAAAGGTGGTGGTCACACCTCGCAGATCTATGCTATCCGTCAAAGTATCGCTAAAGC

TCTTGTCGCCTTCTATCAGAAGTACGTCGACGAGCAGAGCAAGAAGGAAATAAAAGATATTTTGGTCAGG

TATGATAGGACTTTGCTTGTAGCTGATCCCAGACGATGCGAGCCAAAGAAGTTCGGTGGTCGTGGTGCTC

GCGCCAGGTTCCAGAAGAGTTACCGTTGAAGTCGAGGTTATCCTTTTTTTTATGCGTCTGTTATTTTCGA

AAGTGTTTTGAATTATGGATTTTGCTATTTGAGGACTAAAAATGTTAATGGATTTTTGTTGAGCGGTGTT

TATTTAGAGATTTTTCTCTCAATAGACCCATTGCTTAGTTTTGTTGTTTGGATAATTTAATGGTTATATT

ATAGCTAGTTGATTAAACTC

>FM894051.1 FM894051 Jatropha curcas embryo 56-70 (DAF) Jatropha curcas cDNA clone rjcaeb0_005023, mRNA sequence

ATTCCAACATTCAGAAAGGAGTCCACCTTNCATTTGGTGCTTCGTCTCCGTGGTGGCATGCAGATTTTTG

TGAAGACCCTTACTGGAAAGACAATCACCTTGGAGGTAGAGAGCTCTGACACAATTGATAATGTCAAGGC

CAAGATCCAAGATAAGGAGGGGATCCCACCAGACCAGCAGAGGTTGATCTTTGCTGGGAAGCAGCTTGAA

GATGGAAGGACTCTTGCAGATTATAATATTCAGAAAGAATCTACTCTGCATTTGGTGCTTCGTCTGCGTG

GTGGAATGCAGATTTTTGTCAAAACCCTGACAGGGAAAACCATTACCCTCGAGGTTGAGAGCTCTGACAC

AATTGACAATGTGAAGGCTAAAATCCAGGACAAGGAGGGTATTCCCCCGGACCAACAACGATTGATTTTT

GCTGGTAAACAACTTGAAGATGGACGAACCCTTGCAGACTATAATATCCAGAAAGAGTCCACTCTTCACC

TTGTCCTCCGACTCCGTGGTGGTTTCTAATCTGTTTGCTGTTTGGTTTATAAGCAGTTACTTATATCTAT

AATATCTGGGTGATGTTATTTAAATTGTGAGTCTGGTGCGTCCAGGTCGTA

>FM894050.1 FM894050 Jatropha curcas embryo 56-70 (DAF) Jatropha curcas cDNA clone rjcaeb0_005022, mRNA sequence

ATTCCTCTCTCTCTCTCTCTCTCTCTCTATTTATCTTTTCCTTCTTTTCTCTCTCCCTTACAAGCCCTAA

CCCTAACTCAGACCCAGATTCGCCATGAAAGGAGGTAAATCAAAGTCAGACACCAAGAACGCCAAGCTTT

CCGTGAACAAGAAACCCGCCAAGGCAAGTAAGAAGGCGGGTAAGGCAGCGAAGGATCCAAATAAGCCAAA

GAGGCCCGCCAGTGCTTTCTTCGTTTTCATGGAAGAGTTCAGAGAGCAGTACAAGAAGGAACATCCTAAA

AACAAATCCGTTGCTGCTGTTGGCAAAGCTGGTGGAGATAAATGGAAGTCAATGTCAGATGCTGAGAAAG

CACCTTTTGTATCTAAGGCAGAAAAGAGGAAGATAGAATATGAGAAGAAATTGAAAGCCTACAACAAGGG

ACAGGCTGAAGGCCCCAAGGAAGAAGAGGAATCTGAGAAGTCAATGTCTGAGGTGAATGATGAGGAAGAA

GATGATGAGGAGGGAAGTGGAGAGGAGGACGATGATGAGTAGGAAGAGGGGGAGAGGA

>FM894049.1 FM894049 Jatropha curcas embryo 56-70 (DAF) Jatropha curcas cDNA clone rjcaeb0_005021, mRNA sequence

ATTCATCCATTTGTGAAAATTTATGTAAGTGAGCCAAATAAACATTCAGGAATCCTGCATTGTGAGGTCA

GGGCATGAAAATACAATTGCTTAAGAAAAAGAACAAAATTACAAAGTTTAGTCCTATGCCCACCTGGATG

GCTTACGATTCCATCAGAAAGAACATGATTTGTTAGGAATAACCAGCTTCAGCTTTAGACCCATATACTG

TAAAGCTTCATTCATGGTCCTCTTCTTCTTCTTTTCAAAATCTACACCGCTCCAAAGTGTTAATTTTACA

CGTTTTTATACATTTTGTGGTCAAGCAGCAGTTATCTCCTCCACATTGCTGATTTCGTCATCAAATTGTG

CCTTTGGCTCCAAATCTATGAATTGGTTGGACTTACTTGCTAGATGTGAGATGCTAACCCTCCCCTGGCG

CTTAATATAGTCAGCAACAGCTTTCATCTCTTCCTGTGAGATGTATATATACTTTCCTCTATCGTCCATC

ACACCAGAAAGTCTCCCCATGCTTTCTAGGGAGGTTATCCGATTAATGCATTCCTGAGTCCTCAGCTTGA

ATTCCGCAGCAAGATCTTCTAAGGGAACACATTTGTGTTTCTTTATGTATTCCACAAACTCGGATAACAA

ATCCT

>FM894048.1 FM894048 Jatropha curcas embryo 56-70 (DAF) Jatropha curcas cDNA clone rjcaeb0_005020, mRNA sequence

GGGTAACAAAATCACATAATTCTAATTAACAAAAACAAAAAAGGATAATATGCTCAACATTGTCTGAGTC

TAAATATCAAAAATTCAACAGTATGCCAGTACCCAGAAAAACAAGAGACATAATCCAAAACAACCAATAT

AAGACTTCACCTACATAACTATTAAAAACTTGTTATCCTGTCACACCTTACTTCTCAGATGAGTTGGGTC

CTCTCTTCTTGCCGAAACCCACAACTGCGGTGACGAAACGTCGGTTGTATTGCATCCTCTTGTGGGCGCG

GCCACGTGGCTTCTTCTTCTTGTCCTGCTTTGCCACTTTCGGAGTTTGGCCTCTAACCTTACCAGCACGA

GCCAAAGATCCATGAACCTTACCCATGGCGGCTTCTTGAGCTTTTTCTGCTGAGTGGGGGAGCTGTTGCT

CTGAAGGGGGGGCCCGGTAC

>FM894047.1 FM894047 Jatropha curcas embryo 56-70 (DAF) Jatropha curcas cDNA clone rjcaeb0_005019, mRNA sequence

GAAAAGGAACAAGTCCTCCTAATGGTCTCATATTTTTTTTCTCCCTTCCAATAAAAACAAACAGTTCATT

TATTTCCATAAACATCACAAACAAGACTATTTCAGACCAACATTGACTATTTGGATTCTTGACAATGTTT

GATTACATAGCATTAGGTAATATAAGAGCTTTAAACACACAAACAACTAGAGAAATAGCTACTTGAACAA

ACTTAGAGAACCTTTAGGATTTCATCAACACCGGAGACAGTGAACTCTCCACCTTCTTCAAGATTTGCCG

CCTGCACCTTCAGATTATCAACTAAAAGAGCAAATCTCCTTGATCGAGTGCCAAGTCCCTTCTCTGTCAA

GTCAAGCTGAAGCCGAGAGAGAACTAGTTCTCTCTCGGGGGGCCCGGTAC

>FM894046.1 FM894046 Jatropha curcas embryo 56-70 (DAF) Jatropha curcas cDNA clone rjcaeb0_005018, mRNA sequence

ATTCCTTCATATCTCTAAAGATCTCTCGCGAAAACACTAGGAAAAATGGATGTCAGGTTCGTAGGTCTCG

ATTCACCACTTCTCCACACTCTCCAGCAAATGATGGACATTGGCGACGATGCTGACCACAAATCCTTCAA

CGCGCCAACACGCACTTATGTGAGGGACGCGAAGGCTATGGCTTCTACTCCGGCCGATGTCAAAGAGTAT

CCGAATTCTTATGTGTTCATTATCGACATGCCTGGATTGAAGTCTGGAGATATCAAAGTGCAGGTGGAAG

ATGATAATGTGCTGTTGATTAGTGGAGAGAGAAAGCGTGAGGAGGAGAAGGAAGGAGCTAAGTATGTGAG

GATGGAAAGGAGAGTTGGAAAATTGATGAGGAAGTTTGCTCTGCCTGAGAATGCCAATACTGATGCCATT

TCTGCTGTGTGTCAAGATGGGGTTTTGACTGTCACTGTCCAGAAATTGCCACCGCCTGAGCCTAAAAAGC

CAAAGACTATTGAGGTTAAAATTGCTTGAGCTCAGAGGATCGTTTTGTCACGTTTGACTGCGTGCTGGAA

ATAATTTGGGCTATGTTGAAATCGTAATCTGCTTGTGTTTTTCTT

>FM894045.1 FM894045 Jatropha curcas embryo 56-70 (DAF) Jatropha curcas cDNA clone rjcaeb0_005017, mRNA sequence

ATTCCTGCATCATTAGGAATTAGCAGTACCAAATTGATTGGGGTTGATCACAACTCCATAAACCGGTCGT

CTCCCGCGTCGGAGATTTCTGTCAGAAGGTGGTGCTGCAGAAATCAATGGCTGAGCATCAGGAGGCACAG

CCATTTCAGCAGAATGACCAGAAATGCAGAGAGAACCAGAATCATCTTGCTCCCAGTATACTTCAACCAT

GTTACCTCTAGGATTCTCTGGAGATTCTGTTCCTGGAATCCCAACAAATCTTGCTCCTACAGGTATCTGC

ATATCAATTGAAGCAACAAAAGGAAAAAAAAAAAAAAAAATGAATGAATGATTTTATTTTTTCCTTTTTA

TGAGTTAGAATACAATAATTCCATATAAATTGCGCTTACCTCAACCGAGACTCCAGATGTTAAGGACAAA

GTAACCAATCTCCCTTCGGCTGCAGCACTTTCTAGCTCTGCTTGCTTGGCAATATTTAAGAAAACTTCTT

CGAGCGTGGTGAGGCCAATTTGGATGTCTGCTA

>FM894044.1 FM894044 Jatropha curcas embryo 56-70 (DAF) Jatropha curcas cDNA clone rjcaeb0_005016, mRNA sequence

CGAATTCCCTCTCTTTCTATCTTCTTTTTCTTTCTTTCGCTCAATTTCTCTCAAATTACCTATTTCATTT

TGATATTCGTTCCAATAATTGTCAGGCTACCTTTCATATCATAACGATGGATAACAACAACAACCCCGAC

ACCAATGCCAACGCCAATGCCATCGTATCGATGGATAACCTGAACCAGGTCGCCACCTGGGTTAGCACCA

CCGTCATCTCCGCCTTCTTTTCGTCTCTGGAGCGTTTTTCCTGCGTCAATATCGCTACCTTTGATCCCGA

CGAGGATGATTTTGAAGATGAGGAGAAGGACCGCCCTCTCGCTCTCACCAACAACCCCCACCAAAGCGAA

GACGACGACGTCGCCAACCTTCCAGTTTGATTACCCGATGATAAATTTCATTGGTAATTTTAGTATCGCA

TGCCTGCATATCCCCTCTCCAAAACCCCCCGATCCCCCAAGTTTTAAATTTTATCTTCTGTTTGTGGATT

TTATGGGTTTTATACATACAAACCGTAATTCTGTGATTATATGATGATTTTCAATCTTATCAATGTCGAA

TTCGTATCAAAAAAGGGGGGGCCCGGTAC

>FM894043.1 FM894043 Jatropha curcas embryo 56-70 (DAF) Jatropha curcas cDNA clone rjcaeb0_005015, mRNA sequence

ATTCCCCAAATTCATTTCACTCTTCTTATGTAAAATCAACCATGGCTAAGCTCACCATCCTCTTAGCCTC

CTTCATAGCCCTCCTCTTCCTCGTCGACGCCTCCATCTACCGAACCACCCTCATCACCGACGAAAACGAC

GATAACCAATTCCCGCCATACGACCGCCCAACAAGCTGTCAACAGCAAATCCAATCACAGCAAAGGTTAA

GAGCATGCCAGCAGTATCTCCGGCAACAAACTGGCTCCCCCTGGGGCCGTCGTTTCGACAACGAGGAAAA

TCAGATGGAGCGAGAAGAGTGCTGCAGACAGCTTCAAAGAATGGAAGCTCGGTGCCGGTGCGACGGTCTC

CGACAGGCTATTGATCAGCTACAGAGGCGTGGAATCCTTGGGGGCCAGGATGTCCGTGAGGCGTACATGT

TAGCTAGGCAATTGCCGTCGGAATGCGGCGTTTCACCAAGGCAATGCCATTTCGGGACACGGTGGTGGTT

CTAAAAGAGTGGTGGACGTAAGGTGGCAAGGCGTAGGATAAATGCTGTACTGGTAGAAGAATAAAAGTCA

CAGCTCTGGTGGTTTTGCTAGAGATTTGAGTGATTTTGTTATTAGTGAAAATAAAAAATTATAAGC

>FM894042.1 FM894042 Jatropha curcas embryo 56-70 (DAF) Jatropha curcas cDNA clone rjcaeb0_005014, mRNA sequence

CAAAGACTCAAAATGGTGCTACTTCTGCATTTCATATATCAAGACAAAATGTTCAACCAAAATAATGCGA

CACAACTGATTTGCTAGCAACTGCATCTAGCAAACACTCAAAAACCCCACAGGAGGGTATCATGAAAACA

TTAAAAGAAGGTCTACGTATAAACTAAAATTTAGATCCCAATAAATCAAATACACTGTTGCTCAGTGAGA

CTTGATGTGTTGCTGAACAACGTTGGGGGGGCCCGGTAC

>FM894041.1 FM894041 Jatropha curcas embryo 56-70 (DAF) Jatropha curcas cDNA clone rjcaeb0_005013, mRNA sequence

ATTCGTTCTATGTCAATGTTGAGAAAGAAGAATGGAAACTTGAAACACTCTGCGATCTGTATGAGACCCT

AGCCATCACCCAGAGTGTCATTTTTGTCAACACACGGCGCAAGGTGGACTGGCTCACTGACAAGATGCGT

GGCCGTGACCATACAGTCTCGGCCACCCATGGGGATATGGACCAGAACACCCGTGATATCATTATGCGGG

AATTCCGTTCTGGCTCTTCTCGAGTTCTCATCACCACTGATCTCTTGGCCCGCGGTATTGATGTGCAGCA

AGTCTCCCTTGTGATAAATTATGATCTGCCGACTCAACCTGAAAACTACCTTCATCGAATCGGAAGAGGT

GGACGGTTTGGAAGAAAGGGTGTTGCCATTAACTTCGTGACAAGGGATGATGAGAGAATGCTGTTTGACA

TTCAGAAGTTCTACAACGTGGTTATTGAGGAACTGCCGTCGAATGTTGCTGATCTTCTGTGATGCGAATT

TTTGGTGCTTGTTGAGAAAGGGTTTCTTATTTTGCAAGTACTATTTAACGTGTTGTNTTCCATCCTGTTT

AGTGTCTTAATTTAATATTTTCAGACATCTGACAGTTTATCTCATATCAGCCTTCTCAATTGCAATCTGA

AGGGATTGTCTAGTTCTTATTTACTTTTTATTTTTATTTTTTGGGTCCTCTTGGAAAG

>FM894040.1 FM894040 Jatropha curcas embryo 56-70 (DAF) Jatropha curcas cDNA clone rjcaeb0_005012, mRNA sequence

CAGGAAATATCAGTCTCTTTTTGCTGCTTTTTTGTCCCTTCAATAGGAGAATCAGCAGTTCTAATATACT

CTGCCTCGGTTCCCCTTTCAACATGCATCACTTTAACATGGCTTCCACGATATATAAGGCCTTAACTAAT

TATGCATTGCTGAAAATACAGTGATGAGGGTAGACAAAAAGGATTTTGACTTGCAAAAATATTTTCACAT

GGTATGCTATGATCAACCAACATATCCACATAAATCTTAAAGCTAAACCTATGGAGAGCAAACTTACTAC

TATTCCATCACTTGAAAATAATCACAAAAACTCATGACGTGATTATTCCATTGAAATCATCTTCATTTAT

CCTCCCCATAGAGTTCTTCCATTGTATCCTTCAAGCGTTCAACGTCAGCACCAACAATTTCAGCTGCCTT

TTTACCATTTTCGAAGAAATGGAGAGTTGGCACAGAAATAATAGATAATCTACTCAATAAGCTCTCAAGA

CCTTCCTGGTCTATATCAATCTTAAACGTTGTAACATTAGGGTATTTCTCACTTTGCTCTGCAAGTATAG

GAGATATGAACTTGCAGGGTCCACACCACTTGGCAGTAAAATAGAA

>FM894039.1 FM894039 Jatropha curcas embryo 56-70 (DAF) Jatropha curcas cDNA clone rjcaeb0_005011, mRNA sequence

CTGAAATGAAAATAGCATTATAATCAGCCAATTGGTTAGAAAAGTTCAGAACATACCAAGTAAGACAACA

TCACTATAGACCTTAATACTAGAACAAACATAAAGGATTAATCCCAAAATACTACAACTGACATTCATAA

ACATAAATAACCGAGTACAAAAACAAAAAGGCAGCAAAAACATTGTCGTATCATCTTCACACTCACTCAA

CAAATGATTACAGCTTGTCCTCGAACTCAGATAGCGGAGTCATCTGCTCCTTCAAACCCTTTCTCTTGCG

AATCTCCGCCACGAGAGTTGATGCTTGTGTACCAGCCTCCATTGGATCAGAAGACATCATCTCCCAATGA

TCAAACACACATTGTGGGAAGGCCTGTCCTGATGTGGCAGCCCTCAAAGTGCTTGAGAATCCAAAGGACT

CAATAACCGGTAGGTATGCCTTGATGTTGTACAATGGGGTACCAGGCCTCTGTAACTCCTCAAAGACATG

CCCACGTTTTTGGTTAAGAACACTGTAGATACCTCCAAGTGCCTGCTCTGGGGCTTGGATCTCCACCAAG

TAAACAGGTTCCAGAAGCCTTGGCTTGGCTGTAAGCTGTGATGCGTAGATAACCCT

>FM894038.1 FM894038 Jatropha curcas embryo 56-70 (DAF) Jatropha curcas cDNA clone rjcaeb0_005009, mRNA sequence

CGCATTCCAGATTTCCAGAGAAGATGCCAGGAGGCTTAAGGAGAATAGACAGGAAGTCACTGTGTTTAGC

CCTGGGTCTAGATCTCGTAATTAATATTGCAACTATGCAATTATACCAAGAGGATGTATAATAAACTGGT

TTCTACATAGTTTAGATTTGGAGAAGAATAAGCCTATTTTTATAGGCAGTTTTAGTTCCTTACAATAATA

TAAAAATGTAGATCTGTATATGCCTTATGTTATGAGATAATGAAAGTTTGTGGAGCTATAAGTTATAGTC

C

>FM894037.1 FM894037 Jatropha curcas embryo 56-70 (DAF) Jatropha curcas cDNA clone rjcaeb0_005008, mRNA sequence

ATTCCACCAGACCCCATCACGGGGTACTACAGGCCAGCTAATTCTGCGGCTGAGATCGACCCGGCTGAGC

TCCGGGAAATGCTATTGCACAACCGGGTTAGGCCACGGTAGAAGTCTGAGATTCAGATGCTGAAGTGGTG

GCGTTCACTTTTTAGTGTGTTTCTTACACCAGCATATAGTAGTACTGTACTAGAAGTTTTTAAATGTTGC

CAGTTGCGTCATACCACTCTTATTGTCTTAAAGACTCCTGTAGTCTTGGTTATGACTTATCATATCAATG

AAGAAAACTATCCATTATCAAAATTTAAAAAAAAAAAAAAAAAAAAAAA

>FM894036.1 FM894036 Jatropha curcas embryo 56-70 (DAF) Jatropha curcas cDNA clone rjcaeb0_005007, mRNA sequence

ATTCGATTAATTATATTGGGTGGAGAAATTTGTTAGCAAAAAAGGGCAAAAATGTCTTCAAATCAATCAA

GACAAGAGCTTGATGAAAGAGCAAGGCGTGGAGAGACTGTTGTCCCTGGAGGTACTGGGGGCAAGAGCCT

TGAAGCTCAAGTCACCTCTTGCTGAACGGAGAAGCCAGAGGAGGACAGACAAGGAAGGAGCAGCTGGGGA

CAGAAGGATATCCGCGAGATGGGCCGGAGAGGTGGGCTGAGCACGACGGACGAGTCTGGTGGAGAAAGAG

CTGAGAAGGAGGGCATCACTATCGATGAGTCGAAGTTCAGGACTGGGAGCTAGTGTGATGATTAAAAATA

TAAATGGTATTTTGTAGTAGCTAATAATAATAATGTTGGTAATAAAGTATTAGCTTAGCAGTGTACTTTA

GTTTAGTAATGTAGTTTACATGTGGGTTAAAATGACTAGGGTTTAGCTAGTATGTGTGTTGTAACTTTGA

GAGGGGTTTATGGTCGTTCCCTTTCTGGCAGGACTGCTAATACTTTCCCCCTATTTGTAAAATAACCTGC

AGTATA

>FM894035.1 FM894035 Jatropha curcas embryo 56-70 (DAF) Jatropha curcas cDNA clone rjcaeb0_005005, mRNA sequence

ATTCCCTTGGGATGATGAGACAGATATGAAGAAGTTAGAGGAGGCAGTTCGAAGTATAGAGATGCCTGGT

CTACTGTGGGGAGCATCAAAACTTGTTCCAGTTGGTTATGGCATCAAGAAATTGCAGATCATGCTCACCA

TTGTTGATGACCTTGTTTCCGTTGATTCTCTCATAGAGGAACATCTGACAGTTGAGCCTTGCAACGAATA

TATTCAGAGCTGTGACATTGTTGCCTTCAACAAAATATAAGCAAAATACTTATATGCTACTGATTCTAGT

GTTAGCCATTTTGGCAACTTAGTATTTTTGGTCTGTGATTTCTTTCTTTCTTTTTTTTTTTTCCCAACAT

TTTTCCATCTTCTGTTTCTGTTCTCTAGATTGGTGAGAATTGAAAAAATGAACAATTAAAAAAAAAGGGG

GGGCCCGGTAC

>FM894034.1 FM894034 Jatropha curcas embryo 56-70 (DAF) Jatropha curcas cDNA clone rjcaeb0_005004, mRNA sequence

AACTAGAATTTATCTCTTTATTAATTATTAAACCAGAAGCCACTATAGTCCGCAGCAGTGATCTTTCTCT

TTTCTTAAAGACTAATTTACACATGTACCTTCACCTTTCATTATTTATTCACCATACACAGCACTATTTA

CATTAGTAAACCCTCAAGGTCGATGTCACAGTCACCCCAAAAAGCCGAAACACTTGCCATCACGTTATTA

GCTTCTCATGCCACAGCATGACCCTCGAAGGAACCTCTCCTTGGCCCTGGAAAGAACCACTCCTCGTTTT

GTCTTCCAAAAGCCTGATCCACTTCTCTTTCACTTACACCGAAGGCTAACTCTTTGGCTTGCTTCTCCAT

CTTATTTACAATATTATTTTTCCCTGCAAGTAGATGCCTAGTATTGCCTTCTTCATTAATCTCAAAGCAG

ACTACTTCCAGATTATTGTTTCTTGAAGCTACAAAAGCCGCCGGATGGCCTGCAGGGACGACCACTGCTG

TTCCACGTTTCAACCGTGAACTTACTTTCTGGTAAGTCTTCTGACTTTCACCCGTCGTGTCTCCCCGCGA

GCCGCTAAGGTGAGGACAAGCCATTTCAAAGTAGCCTT

>FM894033.1 FM894033 Jatropha curcas embryo 56-70 (DAF) Jatropha curcas cDNA clone rjcaeb0_005003, mRNA sequence

ATTCTCTTCTCCACNCATTTTCCGATCGCCTGAACTCAACTCCGGCCGACTCAACCACCCTTCCCTTGCT

AGCAGTTCCTCCAGTCACCCGCTTTCCCCCTATTTTCAGTCAGTCTTAGCGCAAAACGACAGAGAAAGAG

GAGCTGTGTTAACTATCGATTCAAGCTCTTGCATTTCCGGTCACCTGAGCTCGAGTTGTGCCAACTAGAC

CTTCTCCTTGCCACTGTCGCTCCTCCACCGCTCTCTCCTTCTCCGTTTCCGGTTCCGGCTATAGTGCCGC

GATCGCAGGTGAAGTTGCGATTATGTTTTTCCGATCAAACAATTTCACTTCCATTCATTTAGCTTTCCGG

TGAAACCAGCCAAGGACCGGCTGGATTACACTCCCTCCTCACCTAATGCAGTTCTCCAGCCACCAACCTG

TTCATTTCCGGCCAAGCTAGCGTCGACTAGAATAGATTTTGAGCAAAGGAGCTGCTGCCGGTCATTTTAA

TCGGAAAATTACGAATTCCAGCATGTTTTGCAGCCAGGTTAGCCATATTCCCACTCTCTTAGTTTGGATT

AATGAGTTCTTGATCTTAGTTGAAGGATTTGAAGTATAGATTTAGTTACTGCAGATTAACTTAGAGAATT

TCGCTAAAATTATTATTAGAAGTTATAATCTGAGGTTTAAATAA

>FM894032.1 FM894032 Jatropha curcas embryo 56-70 (DAF) Jatropha curcas cDNA clone rjcaeb0_005002, mRNA sequence

TAGCATTCGTGCATGCAAATGCACGTAATGTACCTTCTTCTGATCATGCAGGTCTGATCAGTGACCAACA

GAATTTATTCATCCTTGTCCACCAAAGGAAACTACTGTTGACGATAACCTACCAAATCAGCAAGTATTTG

TGCATGCGAGTGCTACTGCACCAGTAATGGCCGAGTCACCCGCAAGCACTGGCCTTAAAGACAAAAAGAA

TTTCATCTATGGTGGTGTAGGTGGCTTTGCCGGAATGGGTGGTTATGCCGGCGTTGTTGGTGGATTTCCG

GTTCTAGGTGGAGTCGGTGGAATAGGGAAGTATGGTGGAATTGGAGGAATTGGTGGCATATGTGGCTATG

GTGGTCTTGGTGGTACAGGAGGAGGTCTTGGTGGTATGGGTGGTTTAGGTAGGTGGTGCTGCTGGTGGTG

CT

>FM894031.1 FM894031 Jatropha curcas embryo 56-70 (DAF) Jatropha curcas cDNA clone rjcaeb0_004996, mRNA sequence

CAGTTTTTATTACAAGACATTAGGAAAGCTTGAAGCAACGTTACAAGAAGCAGTTTCGAACAAACTTATT

CTGATGCTCTTACTTCTTATACATCATTATATATAACTCTCACTCACATACAAGTGAATGAAGTTTTATT

CACCTGCAGCTAGCATTTTAACGAGGGCATATAGACCCAAAGCAAACATCAATTATATTTTTAGTCCTGC

GCCTTGCCTCATCTTCGCTTCCAACTTCTCCTTCCTCCAGCTGCTTCTCTAACAGATAATGTATTGATTC

ACATCGACAAATAGCACTTAGTTCCTTTGCTTGGTTACAGCATTGTCTAGGAACCTGCTGTCGAGGGTTT

TCAATTCCTGGCATAGCTAACATATCTTCTGACCTTCCTCTTCTCTGTGTTATATAATTCTCACAGGAAC

TCAAATCTCTTCTTTCAGCTTCCTTCTGCTATATCCCGAACTTGGAAGATCGCGACTTCACGGT

>FM894030.1 FM894030 Jatropha curcas embryo 56-70 (DAF) Jatropha curcas cDNA clone rjcaeb0_004994, mRNA sequence

ATTCAGAGAAGGTTGAGGCAAAACTGAAGGAGCTCAAGGATGCAATTGCCGATGGATCAACACAAGCAAT

GAAAGATGCCATGGCTGCCCTAAATCAAGAAGTAATGCAGCTTGGCCAGTCTCTTTACAACCAACCAGGT

GCAGGTGGTGCTGGCCCTGCTCCTGGCAGTGAAGCTGGGCCTGGACCTTCAGATTCATCAAGCAAAGGAC

CTGATGGCGATGTTATTGACGCAGATTTCACTGACAGCAAGTGAGATGGCAAGATAGTTGGCTAGTTTTT

GTGCCCCTTGCTGCTGATGTGTATAAGAAATTTGATTTTGCCTGGTCTGAGGGTTACTGCATTTCTAGAC

AACTTTTGCTCTTTGTGGAGTGATAAGGAAATCGTTTTTAGGAATATAGAGAACCAAGCAGCAAACAGCA

AGCAACCCACAATAGTTAAAGAAAAGAACTTTGGGTGTTAAAGCCAGTGCACTTATTTATAGTGGTGTTT

AGATTTAGTATGGCGGATTGTTCTTTATTGAATTACGCATTGATTACTGAATCCCTAATGTCATTATTAT

ATTTCACTGATTA

>FM894029.1 FM894029 Jatropha curcas embryo 56-70 (DAF) Jatropha curcas cDNA clone rjcaeb0_004993, mRNA sequence

GGAGAAACTAAGATAGTTTAAAACAGTTTACCCAAGTTCTACAGATAACTTCCATACAAACAATTAAAAG

CAGCAAATTAGACCTCTCCCTTTTGGTCTGCTCATAAGAGGCAGAGATAAACACTTTCATCATTCATACA

TATATATATATATTTTAACTTAAAGACCAAAGCCACAGGGAAAGAAGCCCTCCCTATCACACATACAAAG

CAGCATAGTTAACCCATAGCATACTTCTGGGTCCAGCTCCTTGCTGTTGCCTCGTATTTGCTCCTATCAG

TCTTATACATGTGGGCAATTTCTGGCACCAACGGATCGTCAGGATTTGGGTCCGTCAATAGAGAACAAAT

TGAAAGCAACACCTTGGAAATGGTAAGGGCAGGGCTCCACTGCTCCTTCAAAATATCAAGGCAAATGCTA

CCATTGTGTT

>FM894028.1 FM894028 Jatropha curcas embryo 56-70 (DAF) Jatropha curcas cDNA clone rjcaeb0_004992, mRNA sequence

GCAGGAAACCAAGTAAAAGTCTATTTACATAGAGATTCAAAGTTTATACATTCTCGAAAGGACACGACAA

AAAGTGTATCACTTTGGCCACCATCATTCGTAACTAGCAAAACAGGGGAACGGTTCATATAGCAAGCGAC

AGTGCAGCAATTATATAAAACGAAGGCAACTTCTCTATAAAAAGAAAGAAAAAGAAAACAGAACGTGAAT

GGTATTATGGGTTCTGATTAAGCCTAGCAGCTGCAGCCACAGAAGCAGCAACTCCACCAGGCGTAGTCCT

CATATTAGGTTTGTTTCTTATTTCCGCTCCAATCACACCTTCAGACATCATCCCGAGTTACCGTCTTGTC

TCTAGGCAACTTGGCAGTCGCGTCCGCTAAAACATCAGAGAGGGTAGTCTTATCTTCGTCGAGCATAACT

CTGGTATTACGATCAGCTGCCGACTGTGCTTCTGCGCCTATTCCACCGCCGGGG

>FM894027.1 FM894027 Jatropha curcas embryo 56-70 (DAF) Jatropha curcas cDNA clone rjcaeb0_004991, mRNA sequence

ATTCCATACTCGTACTGGTACGTAGTTTATGATCGTTGCATGATAATGGCTTCTGAAAAAGCATACCGGG

TGTTGCTCTGCTTGGGGTTCGCGATGCTGTTGATAGTTGTTGTTGTCGGTAGCCTAGAACATATGCCTTC

CACGAGCGAAAAAAGCGAGGACTCGGAAGCAATTAAGGACAGGGCAAAAGATGCCAAACGAACATCGGAG

TCGTGGACAGAATGGGCGAAGGACAAAATCTCCGAGACTGTCGGACGAAAACAAGATGAAGCCAAGGAGG

CTGCTAAGAAAGCGTCTGACTCTACCGCTGAAACTGCTCAGAGGACCAAACACAAAGCTTCTGAAAAAGC

TGAAGAGCTGAAGGATGCTGCAACGCAGAAGGCCAACGAAATGAAAGATAAGGCCTCTGAAATGACGAAT

GCGGCTAAGGAGAAGCTCAGTGCGGCTGAAGGCCAGACCAGCGATGCTACAGAAGCAGCAAAGGAGACAG

GTGAGGAGGCAA

>FM894026.1 FM894026 Jatropha curcas embryo 56-70 (DAF) Jatropha curcas cDNA clone rjcaeb0_004990, mRNA sequence

GAAATGGAGATCGGTTTGGTGGGCGTAACAGGCATGCTGGTAGTCGTGGAGGTCCAGGAACTGATCGATA

CAATCGCGATCGCACTGGACCATATGAACGCCGTGGTTCAGGAGGTTTTCGTTCTGGATAGAACTTCTGC

TGATTGTGGTATGCCACCTATTACTGAACTCTACATGTCTTGGTCAGTCAGCAGAATGTGTTTGTGTCCT

CATGCAGCTGGGGAAGTGGAAACATCGGTGCTACTATCTGTGAATTGTTTAAGGTTTTTGTTTTAGTTTT

TTCAAACTTTATAATGGATGTGTTATCTTCGTTGTTCTCTTGCTGGATCAATCAGTTTTATCTAGCCCTA

AACTCGGCACAAATACTTAAGCTGTTTTACTTCCTAAAATGCTGACTTCCATGTTTTGATC

>FM894025.1 FM894025 Jatropha curcas embryo 56-70 (DAF) Jatropha curcas cDNA clone rjcaeb0_004989, mRNA sequence

ATTCCTGACCCAACATTTTTGTACTTCGCTTATGCTTTGAAGGAGATCAAGTGCTAAGAATGGAGAATAG

CTGGCTTCGAAGTGGAAGTTCTTGCCATATTTCTAGCTTTATGTGTCCTTTTCCATCTCTGTACTGTTAT

ATTTGGTGCTTCATGCCTTATTTTGGTTAATTATTGACTAGACATGTTTTCCTCCTTCTTTGGTTATTAG

TTTCTTNTTTGAC

>FM894024.1 FM894024 Jatropha curcas embryo 56-70 (DAF) Jatropha curcas cDNA clone rjcaeb0_004988, mRNA sequence

GAATTGATTAAGGTAAATGTGGAAGGTACTACTAAAGTAACACAAGCTGTTTTGCCCGGGATGTTGAAGA

GGAAGAAAGGTGCTATAGTTAATATTGGTTCTGGTGCAGCTATTGTTATTCCTTCTGACCCTCTCTATGC

AGTTTATGCTGCTACTAAAGCGTATATAGACCAATTCTCTAGGTGCCTTTATGTTGAATACAAGAAGAGT

GGGATTGATGTGCAGTGTCAGGTTCCGCTCTATGTGGCCACAAAGATGGCATCAATAAAGAGATCTTCTT

TCTTCGTTCCATCAACAGATGGTTATGCCCGAGCAGGTTTGCGTTGGATAGGCTATGAACCTCGCTGCAC

ACCTTACTGGCCTCATTCCTTTATTTGGGGTTTGTTACATGCATTGCCAGAATCTGCTGTTGATACATGG

CGATTCCGCTTTTGCCTTGGCATTCGAAAGAGAGGACACGAAAGATCTAGGAA

>FM894023.1 FM894023 Jatropha curcas embryo 56-70 (DAF) Jatropha curcas cDNA clone rjcaeb0_004987, mRNA sequence

AGAAACAAGAGAACTGAGGGTTGGAATAGCTCTTTGATCATAATCTCACAAAATGATGTTAATATAGGAA

ATTGCACAACTTATAAACACAACAAGTCTAATTGGCTATAGTGTTCACATCACCACATCACTGAAAATGC

AGAGAAAAACCTCTAGAAACTCTCAGGTTTTGAAGGGAAATCTCAGGTACCCCTATGGCTCATGTTCCAG

AACTCAACTTCATGTTCAAGAACATGCAGTAAAGTTACTTCAGCTTTTGCAAATCACTTCCAGCTTGAAA

GCCTTCTCAAGGTATCGATTTGCAATTTCCTTCAAAGAACGACAATATTCCCCAAAACCTTCATTGCCCC

ATCTTCGGCAAGTTTCCTGTAATTCTGGTGGGGTTTTGCTGCCATCTTCCAAGCAGTGAGCAAAGCTCTC

TTGGTATGTAGCTTCGATGGCCCATAAGGCTGTAAGGGCCATGCATATCACTTCTCGTGACATCAACCTC

CAAAATCTCATAATTGGTTTGCCTTA

>FM894022.1 FM894022 Jatropha curcas embryo 56-70 (DAF) Jatropha curcas cDNA clone rjcaeb0_004986, mRNA sequence

ATAATTAACACACACATCGCTACCATTAAAAAGGTTAGTGATTACAAACAAGCCAGTTGAGAAAAGCATT

AAATGAGATTGGGGTCATCATATCAGTGGTTACCATCTTCAACTTCCAAGTAGTGTCATGTCTATCGGTA

GTAAGCCTGCTGATAAGCTGGTGCGAAACCATTGCCATAACCTCCATATGCAGGAGAATTCAAAGGAGCT

CCCCGATATAAACCTGCCATGGGGGGAGG

>FM894021.1 FM894021 Jatropha curcas embryo 56-70 (DAF) Jatropha curcas cDNA clone rjcaeb0_004985, mRNA sequence

ATTCTCTNGAACATTTTCGAAATCAGGTTAGTTCACATCNCGGAATTTACAAAGATGAAGACTATATTGT

CATCGGAGACTATGGATATACCCGATGGCGTTAAAATCAAAGTTAACGCCAAGGTTATAGAGGTAGAAGG

GCCTAGAGGCAAGCTCACTCGCAACTTCAAGCACTTGAATCTTGATTTTCAGCTCATCAAAGATGAGGCC

ACTGGGCAACGGAAACTCAAGATCGAAGCTTGGTTTGGATCCAGGAAGACCAGCGCCTCCATTCGCACCG

CCCTCAGCCATGTTGAGAATTTGATCACAGGTGTCACCAAGGGCTATCGCTATAAGATGCGATTTGTCTA

TGCTCACTTCCCTATCAACGCCTCCATTACCAATTCCAACACAGCTATTGAGATCCGCAACTTCCTTGGC

GAGAAGAAGGTCGGAAAGTTGACTGCTGGAGGTTCCTGTTGTCGT

>FM894020.1 FM894020 Jatropha curcas embryo 56-70 (DAF) Jatropha curcas cDNA clone rjcaeb0_004984, mRNA sequence

AAGTATTAAGTGTAACACTTGATATAGATCTCTCTCTCTCTCTCTCTCTCTCTCTCTCTCTCTATATATA

TATATATATATATATAACTCACAAAATACTTAGTAGGAGTATATAGAGTCGACTTGAAAGAGCCTGTTCT

TCATTTTCAGTCTTTAACAGCTACAGGTAGGACACGTGCAACCAACGCCACACTTGCAATTCGCCCTACC

AGCCGCAATAGTCTCTCTACCTTTCGGACATGTGCACGGATTGCAACCGCAATGCTCGCCGCACGAGCAC

CGGCTGTGTTCATATCCCGTTCCCATAGCGCCACCACCGCTTGCCGTCTCACTTACTGTTCTGCACCTGC

ATGCTGTGCCGACGGGGCATGGAACTGGGCATCCGCACCGGTCAGTGCAGGCAAATACGCTCGTTCGGGG

GGGCCCGGTAC

>FM894019.1 FM894019 Jatropha curcas embryo 56-70 (DAF) Jatropha curcas cDNA clone rjcaeb0_004983, mRNA sequence

CAACTGAAATGAAACAGTTCAAACTCTTTAACACTCAAATATCCGATCTTAAAACAGAAAATAATCATCA

TAAGCAAAAATATCAGGGGCATAACAATCTTAATAGACTAATTTACTAGTACTGACACAAAATTTGATAC

TAAAAAAACATAATTATGCCTTCAGACGTCCAAAGAACTTCTGTTTCTCCTGTGTTGTCTGGAAGCGGCC

ATGTCCAAACTTTGAAGATGTATCAATAAACTTGAGCTTAATCTCCTCCAGAGCCAACCGAGATGTCTGG

GTAAGCAGAGACTGGCGGAGAGTAACAACCCTCTTTTTAGTTCCAACACAGCATCCCTTGATCATTATGT

AGTCATCCTTCACCTCACCATAGTGAGGGAAACCACCAATTGGATTGATACCTTACTCAGTCCTGTCATA

CTCAGTTATAGCAGTGTGGGA

>FM894018.1 FM894018 Jatropha curcas embryo 56-70 (DAF) Jatropha curcas cDNA clone rjcaeb0_004982, mRNA sequence

TCTTTGATCTTCTCCAAAAACCAATCAAGAGAATGTCTCTGATTCCAAGCACCCTCTTCGGTGGCCGGAG

AACCAACATTTTCGATCCCTTCTCTCTCGATATCTGGGATCCATTTCAGGACTTCCCTTTCACAAGCACT

GCTCTTTCTGCTCCTCGTTCTGAGTTATTGAACGAAACGGCAGCGTTTGCCAACACCCGCATAGACTGGA

AAGAAACACCAGAAGCTCATGTATTCAAGGCTGATCTTCCAGGGCTAAAGAAAGAGGAAGTGAAAGTGGA

GATAGAGGAAGGTAGGGTCTTGCAGATAAGCGGAGAGAGAAGCAAAGAGAACGAAGAAAAGAATGATCAG

TGGCATAGAGTAGAAAGGTCAAGTGGTAAGTTC

>FM894017.1 FM894017 Jatropha curcas embryo 56-70 (DAF) Jatropha curcas cDNA clone rjcaeb0_004981, mRNA sequence

TTCAATTCCGAGTTTTGTATTTTAATAATTGTGAGATAAGCTAGGACCGGCCTTGATTTGTTTATTTATT

TCTTTATGTTCCAACATATGAAGTGGTTCAAAGC

>FM894016.1 FM894016 Jatropha curcas embryo 56-70 (DAF) Jatropha curcas cDNA clone rjcaeb0_004980, mRNA sequence

AACCCAACAGGTAATTATATATACTGCAGGTTATTTTACAAATAGGGAGAAAGTATTAGCAGTCCTCCCA

GAAAGGGAACGACCATAAACCCCTCTCAAAGTTACAACACACATACTAGCTAAACCCTAGTCATTTTAAC

CCACATGTAAACTACATTACTAAACTAAAGTACACTGCTAAGCTAATACTTTATTACCAACATTATTATT

ATTATTACTACAAAATACCATTTATAGTTTTAATCATCACACTAGCTCCCAGTCCTGAACTTCGACTCAT

CGATAGTGATGCCCTCCTTCTCAGCTCTTTCTCCACCAGACTCGTCCGTCGTGCTCAGCCCACCTTTCCG

GCCCATCTCCTGATATCCTTCTGTCCCCAGCTGCTCCTTCCTTGTCTGTCCTCCTCTGCTTCTCCCTTCA

GCAAGATGCTCTTGAGCTTCAAGGCTCTTGCCCCCAGTACCTCCAGGGACAACAGTCTCTCCACGCCTTG

CTCTTTCATCAAGCTCTTGTCTTGATTGATTTGAAGACATTTTGCCCTTTTTTGCTAACAAATTTCTCCA

CCAATATAATTAATCTTTGCTCTGTTACACTTTATCGATGGGGGGC

>FM894015.1 FM894015 Jatropha curcas embryo 56-70 (DAF) Jatropha curcas cDNA clone rjcaeb0_004979, mRNA sequence

ATGTGTAACAGAGCAAAGATAATTATATTGGTGGAGAAATTTGTTAGCAAAAAAGGGCAAAAATGTCTTC

AAATCAATCAAGACAAGAGCTTGATGAAAGAGCAAGGCGTGGAGAGACTGTTGTCCCTGGAGGTACTGGG

GGCAAGAGCCTTGAAGCTCAAGAGCATCTTGCTGAAGGGAGAAGCAGAGGAGGACAGACAAGGAAGGAGC

AGCTGGGGACAGAAGGATATCAGGAGATGGGCCGGAAAGGTGGGCTGAGCACGACGGACGAGTCTGGTGG

AGAAAGAGCTGAGAAGGAGGGCATCACTATCGATGAGTCGAAGTTCAGGACTGGGAGCTAGTGTGATGAT

TAAAACTATAAATGGTATTTTGTAGTAATAATAATAATAATGTTGGTAATAAAGTATTAGCTTAGCAGTG

TACTTTAGTTTAGTAATGTAGTTTACATGTGGGTTAAAATGACTAGGGTTTAGCTAGTATGTGTGTTGTA

ACTTTGAGAGGGGTATATGGTCGTTCCCTTTCTGGGAGCGACTGCTAATACTTTCTCCCTATT

>FM894014.1 FM894014 Jatropha curcas embryo 56-70 (DAF) Jatropha curcas cDNA clone rjcaeb0_004978, mRNA sequence

TCCATCATTTACTGCTGTGCTCTAACGTTCTTCTATCCTCCAATTCTCTCCTCTACCGCCGCTTTCATCT

TGCAGAAGATTCAATCAATCTTTTCTCGCACTTCAGTTGGCAGAGCTAGCTATAAACACTCGACAGCAGG

AAGAAACTTGGGAAGAGATTGGTGTAATTTCATTTCTCAACGCTTTGTATCGAGAAAGTCATGGTGAGTC

CATATGGGCAAATGGGCAACCAGGATTTGGGTCCGCCATGGTTAAAACCAATGCTTCGAGCGAGCTATTT

CACCCCCTGTTCAATTCATGGGGATTCCAATAAAAGTGAATGCAATATGTTCTGTTTGGACTGTATGGGT

AATGCCCTTTGTTCTTATTGTTTGGTCAATCACAAAGATCACCGAGTTGTTCAGATTAGGCGATCTTCAT

ACCATAACGTGGTGAGGGTTAATTGAAATACAGAAGTACATAGATATATCGTGCGTCCAGACTTACATTA

TCCATAGCGCCAAGATTGTATTCCTATACGAGCGGCCACAGACAAGGCCTG

>FM894013.1 FM894013 Jatropha curcas embryo 56-70 (DAF) Jatropha curcas cDNA clone rjcaeb0_004977, mRNA sequence

CGTTGACTAATCCATCCATGTAGAAAATATACATATGGGCTCATACAGGCCTCACATGGGCTCTGTCGTC

ACTCATCCCAACTATACTTCATTTCTACTTCATTCAAGCCCAACAAAATACAAGACAGGACACCAGATAT

GTAAAATAGACGCCAAAACGACAACAGAACAGCCAAAGAAACAAATCAGAGCTCTTCGTCCGGCAATTCA

CCACCGGCACCAGCCACGTTATCCTTGAGATGCTGTGATGTCTTCTTTGCCTTATCTTTTGCCTCTTCGG

ACTTGTGGTATGCTCTCTCTTTCGCAGCTCCAACTGTCTCTCTGGCCTTGTCCTTTGCTTCCTCAGCCTT

CTCAAAGGCCTTGTCCTTTGCTTCCTCAGCCTTCTCAAAGGCCGTGTTCTTTGCCTCCTCACCTTTCTCC

TTTGCCTGGTTCTGTAGCATCGCTGGTCTGGCCTTCAGCCGCACTGAGC

>FM894012.1 FM894012 Jatropha curcas embryo 56-70 (DAF) Jatropha curcas cDNA clone rjcaeb0_004974, mRNA sequence

ATTCCTCCTCTCCTCTGCAAAAATGCAGATCTTCGTGAAAACCCTAACCGGGAAGACGATAACCCTAGAG

GTTGAGTCTTCTGATACTATCGATAATGTCAAGGCCAAGATCCAGGACAAGGAAGGCATCCCTCCAGACC

AGCAGCGACTGATCTTCGCCGGAAAGCAGCTAGAGGATGGCCGCACTCTTGCCGACTACAATATCCAGAA

GGAATCCACTCTCCACCTGGTGCTTCG

>FM894011.1 FM894011 Jatropha curcas embryo 56-70 (DAF) Jatropha curcas cDNA clone rjcaeb0_004973, mRNA sequence

AGTTCGGTCATGTTGCTGCATATATGAAGACATTAATGGAGGATGAACCAGAGAAATACCAGACTCACTT

CAGTGAATATGTCAAGAGGGGAATTGAGCCAGACAACTTGGAGGCATTATACAAGAAGGTTCATGCTGCA

ATTCGTGCTGATCCTACTCTGAAGAAAATAGAGAAGCAACCACCCAAGGAACCCAAGAGGTACAAT

>FM894010.1 FM894010 Jatropha curcas embryo 56-70 (DAF) Jatropha curcas cDNA clone rjcaeb0_004972, mRNA sequence

ATTCCAGAAGCCGCTTCCATCTTTCATCTCAGTCACTCCTCCCTCTCTCCAGCTTGTCGATTTACAGGTA

AGCAATGGCGACAGCTGTAGTAGCACCGCCGCCGGCAACCCCAGAACCTACACAGCCTTACACTGAAGTG

AAGCTCTTTAATCGATGGACTTTTGAGGAAGTTCAGGTTAACGACATTTCCTTGTGCGACTACATTGGTG

TGCAACCAGCCAAGCATGCCACCTATGTTCCACACACTGGCTGGAAGGTACTCTGTCAAGCCGTT

>FM894009.1 FM894009 Jatropha curcas embryo 56-70 (DAF) Jatropha curcas cDNA clone rjcaeb0_004971, mRNA sequence

ATTCCTCTNGAAGAAAGTCGAAACAACTGACTCGAAAGTCGAATTTGATTCTAATCAAAAGCCCTAGTAA

AAATGGGTCAGATCCAGTATTCTGAGAAGTATTTCGATGACACTTTCGAGTACAGGCATGTGGTGCTCCC

TCCTGAAGTCGCCAAACTTGTGCCCAAAAATCGGCTTCTCTCTGAAAATGAATGGCGTGCCATTGGTGTC

CAACAGAGCCGTGGTTGGGTGCACTATGCAATTCATCGCCCTGAGCCACACATCATGCTCTTCAGGAGGC

CACTCAACTATCAGCAGCAGCAGGAGATTCAAGCTCAGCAAAATATGCTTG

>FM894008.1 FM894008 Jatropha curcas embryo 56-70 (DAF) Jatropha curcas cDNA clone rjcaeb0_004970, mRNA sequence

ATTCCTCTCTTCATTCTGCTTGGAGGCGCCTCTCTTTCTCTGCTTTCTGCATCCGCAACCATGGTGAGAG

TTAGTGTTTTGAATGATGCTTTGAAGAGCATGTACAATGCTGAAAAGCGTGGGAAGCGACAGGTTATGAT

CAGGCCTTCCTCAAAAGTGATCATCAAATTTCTGCTGGTGATGCAAAAGCATGGATACATTGGCGAGTTT

GAGTATGTTGATGATCATAGAGCTGGTAAAATTGTGGTTGAATTGAATGGGAGGTTGAACAAGTGTGGAG

TTATCAGCCCTCGTTTTGATATCGGTGTCAAGGAGATTGAAGGATGGACTGCACGACTGCTTCCATCAAG

ACAGTTTGGTTATATTGTGTTGACGCTGATCTGCTGGCTGTTTAGGACCAAAAAGGGGGGGCCCGGTAC

>FM894007.1 FM894007 Jatropha curcas embryo 56-70 (DAF) Jatropha curcas cDNA clone rjcaeb0_004969, mRNA sequence

ATTCCGCCAATGTTGTAACGAATTAAGGCAGCTAAGTCATGATTGTCCATGTGAAGGAATAAAGTATGTA

TTAGAGCAGCAGCTGGAGCAAGGACAGCGAGAGCGTTTGGAGGCAGTGCGTAGGGCTAGAAGTATACCTT

CTGCTTGTGGCCTGCGTACAAGCTGCGATATTCGTTCCTAATCTGCTAGCTATAACGTGGATGTGCTTCA

GTTAAATAAAAATTTCACTCACTTGGTGTGTGAGTGATCAGAGTTATGTATAATAATGCACAAAAGAATG

AGCACATCAAGCTAAGTTTGTTTTCTGTTATCTAAAAAAAAAAGGGGGGGCCCGGTAC

>FM894006.1 FM894006 Jatropha curcas embryo 56-70 (DAF) Jatropha curcas cDNA clone rjcaeb0_004968, mRNA sequence

CAGACATTGTCTACATCAAAGCATCTTGATCATACAGTTATTTCATGCCAAACCCAAGATATAGATTCCA

CTGAAGAACTCAAGTCCATCCCTAGCCTCTATCGAACCAAACCAAGTAAATAAGTTAATAAAACAGTTTT

CAACACATTCCACCATAAACATCACCCGATTAGGGGGGGCCCGGTAC

>FM894005.1 FM894005 Jatropha curcas embryo 56-70 (DAF) Jatropha curcas cDNA clone rjcaeb0_004966, mRNA sequence

GCTGCAGGAATTCGAATTCCCAAAAACCAATCAAGAGAATGTCTCTGATTCCAAGCACCCTCTTCGGTGG

CCGGAGAACCAACATTTTCGATCCCTTCTCTCTCGATATCTGGGATCCATTTCAGGACTTCCCTTTCACA

AGCACTGCTCTTTCTGCTCCTCGTTCTGAGTTATTGAACGAAACGGCAGCGTTTGCCAACACCCGCATAG

ACTGGAAAGAAACACCAGAAGCTCATGTATTCAAGGCTGATCTTCCAGGGCTAAAGAAAGAGGAAGTGAA

AGTGGAGATAGAGGAAGGTAGGGTCTTGCAGATAAGCGGAGAGAGAAGCAAAGAGAATGAAGAAAAGAAT

GATAAGT

>FM894004.1 FM894004 Jatropha curcas embryo 56-70 (DAF) Jatropha curcas cDNA clone rjcaeb0_004965, mRNA sequence

AGGAGAAACTAAGATAGTTTAAAACAGTTTACCCAAGTTCTACAGATAACTTCCATACAAACAATTAAAA

GCAGCAAATTAGACCTCTCCCTTTTGGTCTGCTCATAAGAGGCAGAGATAAACACTTTCATCATTCATAC

ATATATATATATATTTTAACTTAAAGACCAAAGCCACAGGGAAAGAAGCCCTCCCTATCACACATACAAC

GCAGCATAGTTAACCCATAGCATACTTCTGGGTC

>FM894003.1 FM894003 Jatropha curcas embryo 56-70 (DAF) Jatropha curcas cDNA clone rjcaeb0_004964, mRNA sequence

ATTCCAACTCCANGGTGGTTATCATTCAGCTGAGTATGGCCATGATCATGAGAAGAAAGGAATAATGGAT

AAGATTAAGGAGAAGCTTCCTGGCTCCCACCACCACGAGCACCACTAGGTGTTCTTTAATACTCTACTTA

ATAATGTATTATACGCACGCAATGCCCTTATTTGTTATGTTGTGTGGCTGATTTGCTTTTTATCGATCAG

TTTGTAATAGGGTGATGTGTGGCTGGTTGAGCTTGCATCCCCAAGTGTGTTGGAGCTTTGGTTTGTGTGT

TTAAGAAGGACTTCTGTCTTCTGTTTGGAGAAATGTAATAACAAAATTTGGGATTTACTGT

>FM894002.1 FM894002 Jatropha curcas embryo 56-70 (DAF) Jatropha curcas cDNA clone rjcaeb0_004963, mRNA sequence

AGTTCTAAGAGATGGAAAAACAAGCTACAGAAAGTCGTGAAGAGCAGGAAAAGAAAGAGAAATTAGAGGG

TCTTCCAGTTGAGGACAGCCCTTATCTGCAATACAAGGACTTGGAAGATTACAAGCTAAAGGGCTATGGA

ACTCAAGGCCATCTCCAGCCAAAGCCTGGCCGCGGAGCCGGCGCCACCGATGCACCCACTCTTTCTGGTG

GCTCTGTCCCTCGAGATGCTGATGTTAGTACCATTGATGCTATAACTAGTCGTCCCATGAAGCCTTAAAT

GCTTATTCCTCAAAATTAAAAAAGAGAACCACCTTAGTTTTGTTTAGTTTNATTATTTTTGTGGGATGAA

TATATAGATGGGGATTAAATAAAGTTGGGTAATTAAACTTTCAAGCTTTCTTTTTTCAAATTAAAAAAAG

ATAATAAATAACAGTGATTAAGGAAAATGTGACCTGTGTATATTTGTATGTTCTTTCTATAATTGATGTG

TTTAATTATATATCTTCTGTTCATGAAAC

>FM894001.1 FM894001 Jatropha curcas embryo 56-70 (DAF) Jatropha curcas cDNA clone rjcaeb0_004962, mRNA sequence

CGCGAGCTAGTCTGTCAGAATGGAAGGAAGTGGCAACACTGTACCCAGTATTGTTGTTTACGTCACTGTG

CCTAATAGAGAAGCTGGAAAGAATTTGGCTCAAAGCATTGTCAAAGAGAAATTAGCAGCATGTGTGAACA

TAGTCCCAGGTATTGAGTCAGTATATGAGTGGAAAGGAGAGATCCAGACAGATTCAGAGGAACTGCTTAT

AATTAAGACTAGGCAATCTCTGTTGGAAGCATTGACAGAGCATGTCAAGGCAAATCATGAATACGAGGTA

CCGGAAGTAATCTCTTTGCCGATTACTGGAGGCAGCATTCCATACCTGGAATGGCTAAAGAACAGCACAA

GGGATTGACTTCTCAAGTTTACTGCAAAAATTTGATTGCTAAAACTTTGCTCTTAATGAAAATGGCGATC

ATCACTTACATGGTCTCCAGCTCTATGGTTGCTTGTAAATTGGCTTTTTATGCTTTGAAACCTATATGTA

CATGCTCTTGTTTTGTCGGTTGTCTTTTAGTCGAATAAATATATTTATATATATATATATATATATATAT

ATATA

>FM894000.1 FM894000 Jatropha curcas embryo 56-70 (DAF) Jatropha curcas cDNA clone rjcaeb0_004960, mRNA sequence

ATTGCTTCGGCTACTGCTCCTCTCCTCTGCAAAAATGCAGATCTTCGTGAAAACCCTAACCGGGAAGACG

ATAACCCTAGAGGTTGAGTCTTCTGATACTATCGATAATGTCAAGGCCAAGATCCAGGACAAGGAAGGCA

TCCCTCCAGACCAGCAGCGACTGATCTTCGCCGGAAAGCAG

>FM893999.1 FM893999 Jatropha curcas embryo 56-70 (DAF) Jatropha curcas cDNA clone rjcaeb0_004959, mRNA sequence

ATCTTTGTCTGTATCACCATCTTTCTTTTCTTTATATTTTCCCTCATTATTTGATTTCTTGTCTCGAGTT

TTCTTGATACTTCACTTGATTCCTTGCTACACGAGCTCCTTCGCTATTATCCTCCATTCTTAATGATCTT

CTATCCTCAATAGCTTGCAAAACATTTATGAGTTCTAAGAGAGTTATGCTTGAAAGATCTCCAGAATCTT

CTACAGGAGGTATATATTCAAATATATCAGGAATGCTAAACCAAGTCTTTCTTCACTGTTATATAATTTA

CCTCTTCATCCATC

>FM893998.1 FM893998 Jatropha curcas embryo 56-70 (DAF) Jatropha curcas cDNA clone rjcaeb0_004958, mRNA sequence

ATTCCTGCTCCTCTCCTCTGCAAAAATGCAGATCTTCGTGAAAACCCTAACCGGGAAGACGATAACCCTA

GAGGTTGAGTCTTCTGATACTATCGATAATGTCAAGGCCAAGATCCAGGACAAGGAAGGCATCCCTCCAG

ACCAGCAGCGACTGATCTTCGCCGGAAAGCAGCTAGAGGATGGCCGCACTCTTGCCGACTACAATATCCA

GAAGGAATCCACTCTCCACCTGGTGCTTCGTCTTCGTGGTGGTGCTAAGAAGAGAAAGAAGAAGACTTAC

ACCAAGCCAAAGAAGATCAAGCACAAGAAGAAG

>FM893997.1 FM893997 Jatropha curcas embryo 56-70 (DAF) Jatropha curcas cDNA clone rjcaeb0_004957, mRNA sequence

CTTCAATGGTCCTTGTTGGATGTCCTATGTGCCTCATGTATGTTATGCTGTCTGAAAATGACCCTAAATG

CCCCAAGTGCAAGAGTACTGTGTTGCTCGATTTTCTCCATGACAATACTACCCAAACAAGGAACAATTGA

AACAAAGAAAAACGAAAAAAGGAAAAACAGAGTAGGGTTCCTTGTTCTGATTGTGCAACCATTAGGATTA

ATAGGCTCTCTATATCTTGCTTCTTTTATTATTGTATCTAAAATAATGCTATCTGGGCTCCATTATCTTT

TTTATGGGTGGAGG

>FM893996.1 FM893996 Jatropha curcas embryo 56-70 (DAF) Jatropha curcas cDNA clone rjcaeb0_004956, mRNA sequence

ATTCATCACTGATCGTAAACCTCCATTGAATTTACTCTGTGCTTCGATCGCGTCAATGGCGGAGCACCCA

CAGTCGCAGCATGTTGGCCAACAGCCACGATCCCACCAAGTAGTGAAGGCAGCCACTGCAGTCACTGCCG

GTGGATCTCTTCTAGTTCTCTCCGGTTTGACCCTTGCTGGTACGGTCATTGCACTGACCGTAGCCACTCC

ATTGCTGGTCATATTCAGTCCGGTTCTTGTTCCTGCGGTTATCACAGTTTGTTTGATAATCACAGGGTTT

TTAGCCTCAGGCGGTTTTGGCGTCGCTGCCATTTCTGTCTTGTCCTGGATTTATAGGTATGTTACTGGAA

AGCATCCACCAGGAGCAGAAAGTCTGGACCAGGCACGCTTAAAGTTGGCGGGAAAGGCAAGAGAGATGAA

GGACAGAGCTGAGCAGTTTGGACAGCATGTAACAGGTCAACAGACTTAAAAGAGAAGTGGGTTTTGGTGG

TTGATGCAGAGAACATAAAATGGCTTTGATATATATATAAAAATATATGAGTCCTTTAATTAACATGGAA

TGCCATTGCTTGTCTGGTGTA

>FM893995.1 FM893995 Jatropha curcas embryo 56-70 (DAF) Jatropha curcas cDNA clone rjcaeb0_004955, mRNA sequence

GATCAACAATAGAAATATAATTATTTTTCAAGAACAAAACTTTTTACAATGTTCGCATATAGTTTCAAGA

GTAATCAACTATAGAATCACAACAACATCAAATTCAGATGCTCGCTTCTCCAACTATAATACGTCAACAA

GGAAAAGGTACAAATGGAAACAAGCACAAGAATTACCAAAATATGTATAGCTACGTCTTCTTCTTGCGAA

ATACATACACATCAGTCTCTTCATATGCATAATCTGGATGCAAATCCTCATGCGGAACCTTCTCGATCTC

AAAAACATCTCCACAAATTTCCCAAAACTTCTTATCCGCCTCCGGCGACCTCACTTGATACCCTAACAAA

ATGACTCCATCATCCGCCACCAGCGCTTGCATAGCTCCTACGAGGTGCCCTACGGACTCCTCAATATAAA

CTACATCCGCTGCAATCACAATATCAAACGGCGGATTCAAGGCCTTGATTTGATCCTGATTGTTCCAATA

GAGGATCGAGGTCTTCAACATCTTTCCTAGGACTTGTATATTCCGCTTGAGGTTGTGCTTCAATGCTGGC

ATGACCGGAGCAATGTCGGTGAGGATAATAT

>FM893994.1 FM893994 Jatropha curcas embryo 56-70 (DAF) Jatropha curcas cDNA clone rjcaeb0_004954, mRNA sequence

ATTAAAGATGATATCACAAGAGAGGAGGCAGTTCAGCTTGCACTTAAGGTGCTGAGCAAGACAATGGACA

GTACAAGTCTCACTTCTGATAAGCTCGAACTAGCTGAGGTCTTTCTCCTGCCCACTGGAGAAGTTAAGTA

CCAGGTTCGTTCACCAGACTCTCTGACTAGGTTGTTAGTGAAGTCTGGAGTTACCCAACCTCCTGCTGAG

GCTGCTTAGGTAAGTTTCAGCAGTAATTTGGTAGGTGTATGTCGTTTTGAGTACTTATGCAACTTTTTCT

CAAAAGACGATCTTGCTTTGGTTGGACAAATTCAATATTGAACTCTCATGTTTGATCTGGGGTATAAGGC

TGATGATATCTAAGATCTGATTGAATTTCTGTG

>FM893993.1 FM893993 Jatropha curcas embryo 56-70 (DAF) Jatropha curcas cDNA clone rjcaeb0_004953, mRNA sequence

ATTCCGCCGTGCCTCGAGAGAACGTGGCTCTTGTAACAATCTTTTTTGCGGAATGGACTCGAGATTTGTT

GCTGAAGCTTTCAATATCGACGAATCGTTGGCTAGGAAACTCCAAAGCGAGAATGACAACAGGGGCAATA

TTATCAGTGTTGAAGATGGTGAGCTTCAAGTGGTTAGACCACCAAGAACTCAACAAGAGGCGCAACAGCA

AGAAGAGAGAGAAGGATGCCCCTCCGGACGCGACAATGGTCTTGAAGAGACCATTTGCAGCATGAGAATT

GGAGAGAACATTGCTGATCCCTCGCGTGCTGACGTTTATGTACCAGAAGCTGGTCGTGTTAAGCACCGTT

AATAGCCATAACCTCCCTATTCTTCGGTGGCTACAACTCAGCGCTTCCTATGGCGTTCTCCGCAATGATG

CCATGAGATTACCCCACTGGCACATAAACTCTCACAGCGTAATTTACGCACTCAAAGGACAAGCGCGGGT

TCAGGTGACGGATGAAAACGGCAACACCGTCTTCAACGGAAGTGTAAGCAAAGGACAGGTTTTGACGGTG

CCACAAAACTTCGTAGTGGTGAAAACGAGCAGAAAGTG

>FM893992.1 FM893992 Jatropha curcas embryo 56-70 (DAF) Jatropha curcas cDNA clone rjcaeb0_004952, mRNA sequence

GGGGAGAGAAAAGATTACTTCGTCTCATTGTTAAAGAGAATGCTATAACATGCAAAACAGGTTACAGGTA

CATGTAGACTGAACATAAAACTCGGTGCCATTTCTATGTCCGCTTCGAGAAGACACTTGTTATAAGTACA

TACGCAAATACACTCCTACACACAAGGGGAAGTAACAGAACAAAACAAAGCAAACATCATATACAAACAA

ACACCATTCATATTAGTAACTCTTCATTAGTGTATCAGTAGTTAATGGTCTCATTAAGCCTAGCAGCTGT

CGTCATTGACACCGCAACACCACCTGGATGCGTGTTTAAATTCGGATTGTTCCTCAACTCTGCACTAACC

ACCCCTTCAGCGGGGGGGCCCGGTAC

>FM893991.1 FM893991 Jatropha curcas embryo 56-70 (DAF) Jatropha curcas cDNA clone rjcaeb0_004951, mRNA sequence

ATTCCGTTCCAAAGATTGTACACAAAAAAGATGGAACCAGAGCTGAATGGGAGTATCCATTTGCTGTGGC

TGGTATAAACATTTCCTTCATGTTGGTACAAATGTTGGATCTTCAAACTGGGAAGCCTTCCACTCGAGCA

GGAATCCGATTCTTGGAATTTCTTGCAGAGGATGAAATGGCATTTGACAATCTTTATTGTGTAGCATTTC

ACATGATGGATGCTCAATGGCTTGCAAAGCGTGCTTCATACATGCAATTTAACGATGTTTTGAAGTCTAC

AAGAATGCAGCTAGAACGTGAGCTTGCCCTTGAAGATGTCCATAGTGTCAAAGATATACCACCATACAAT

CTGTTGACTGGGTAAAACTATCTTCAAATGTAAGAAGAGAATGTACCAAGTATATACTCTATTTAGTGTT

GTTTACATCAAAGTTGAATTACAAATC

>FM893990.1 FM893990 Jatropha curcas embryo 56-70 (DAF) Jatropha curcas cDNA clone rjcaeb0_004949, mRNA sequence

ATTCCTCTCATTTCCTTTCCCTCGCTACTTTTAAAACACAGTCTCTCTTGCTTGTCTCTTTAATCTTTTC

TCTCCTTTGTTTTTACTGCTGGACATGGCTACTTTTGGTGCTGCAGCGGCTGCAAATCCAAACCCCAACA

AATCTTTTGAGGTGGGGGGGGCCCGGTAC

>FM893989.1 FM893989 Jatropha curcas embryo 56-70 (DAF) Jatropha curcas cDNA clone rjcaeb0_004946, mRNA sequence

ATTCTAACACTTGCAGCCACCAAGCAAAAGCCCTAGGAAGGAGTCTTAAGATATGGCGACAGTTCCAGGG

CAATTGATATGGGAGCTTGTGAAGAAGAACAACTAATTTTTGGTGAAACACGATTAGGAAGAAGAACAGC

TAACCTCCAGTTCAGCAAAGAGCCAACAATCTCTACAATCTCAACTCCTACAAGCACTCTGGGTTGGCCA

ATAAGAAAAAAGT

>FM893988.1 FM893988 Jatropha curcas embryo 56-70 (DAF) Jatropha curcas cDNA clone rjcaeb0_004944, mRNA sequence

ATTCGTGATTGAATTTTGAATAGCCAAGTGAGTGAAAATGGAGTCGCTGAAGGAAACCGCAGCTAACGTT

GGAGCCTCTGCTAAGGCTGGCATGGAAAAAACCAGAGCAGAGGCGCAAGAGAAGGTGGAGAGAATGTCAA

CACGTGATCCAGTGCAGAAGGAAATGGCGAGAGAGAAGAAAGAAGAGAGGAAAACGGAGGCAGAATTGCG

GAAACTAGAAGCGCGCGAGGATAACGCAGCGGCGAGGCAGGCAAGCCAAGGCTGGTGGCCATGTACACTA

CACCACTGCTGGTCCGGGCGCTCAAGGCACAACCGAATAATATACCCATTCAACAACAGGGACAACCGGT

TACCCTACAGGGACCCAACCCAATGTCGG

>FM893987.1 FM893987 Jatropha curcas embryo 56-70 (DAF) Jatropha curcas cDNA clone rjcaeb0_004943, mRNA sequence

ATTCCTGTGATTTCACAGAAGCAGAAGGCTGCATTGAGGGACGCATACAAGAACAAGAAGTTCTTGCCAC

TTGATCTGCGTCCGAAGAAGACAAGAGCCATCCGCAGGAGGCTTACCAAACACCAGCAATCGTTGAAGAC

AGAACGTGAGAAGAAGAGAGACAATGTACTTCCCCATGAGAAAGTATGCTATCAAGGTGTAGGGCAATTT

TTGCTAAATAGATTGGTTTTGGTATCTTGCAATTATAAACCCATTTGGTTGTCAAGTTTTGTCTTGGATT

ATCTTTTTGATGTGCTTGTGAACTACAGTTGGCTTTGTTGAATCTTATGTCTGTAATCTTGGAAGGGTGT

ATCTCACTCTAACCGGGTGCTAGATTTACTTGG

>FM893986.1 FM893986 Jatropha curcas embryo 56-70 (DAF) Jatropha curcas cDNA clone rjcaeb0_004942, mRNA sequence

CATTTGAATATTTGCTACTACCACCAAGATCTGCACCGACGGCCGCTCCGCCCGGGCTCGCGCCCCGGGT

TTTGCAGCGACCGCCGCGCCCTCCTACTCATCGGGGCCTGGCTCTTGCCCCGACGGCCGGGTATAGGTCG

CGCGCTTAAGCGCCATCCATTTTCGGGGCTAGTTGATTCGGCAGGTGAGTTGTTACACACTCCTTAGCGG

ATTTCGACTTCCATGACCACCGTCCTGCTGTCTTAATCGACCAACACCCTTTGTGGGTTCTAGGTTAGCG

CGCAGTTGGGCACCGTAACCCGGCTTCCGGTTCATCCCGCATCGCCAGTTCTGCTTACCAAAAATGGCCC

ACTTGGAGCTCTCGATTCC

>FM893985.1 FM893985 Jatropha curcas embryo 56-70 (DAF) Jatropha curcas cDNA clone rjcaeb0_004941, mRNA sequence

ATTGATCTCTCGCGAAAACACTAGGAAAAATGGATGTCAGGTTCGTAGGTCTCGATTCACCACTTCTCCA

TACTCTCCAGCAAATGATGGACATTGGCGACGATGCTGACCACAAATCCTTCAACGCGCCAACACGCACT

TATGTGAGGGACGCGAAGGCTATGGCTTCTACTCCGGCCGATGTCAAAGAGTATCCGAATTCTTATGTGT

TCATTATCGACATGCCTGGATTGAAGTCTGGAGATATCAAAGTGCAGGTGGAAGATGATAATGTGCTGTT

GATTAGTGGAGAGAGAAAGCGTGAGGAGGAGAAGGAAGGAGCTAAGTAATGTGAGGATGGAAAGGAGAGT

TGGAAAATTGATGAGGAAGTTTGCTCTGCCTGAGAATGCCAATACTGATGCCATTTCTGCTGTGTGTCAA

GATGGGGTTTTGACTGTCACTGTCCAAAAATTGCCACCGCCTGAGCCTAACAAGCCAAAGACTA

>FM893984.1 FM893984 Jatropha curcas embryo 56-70 (DAF) Jatropha curcas cDNA clone rjcaeb0_004940, mRNA sequence

ATTCCTTTAACAGGAGTCATAGTTTTCACTTCCACTCCNGGATAAAATATCAAACAAGTACTACTTGGGA

CCTGCTCCCTTGGAGGAAATGGCTAGCCAAATTGCAACGGCCCATGGACCTTGTGGGAATAACAGAGACT

ACCTATTCCTGTTAGAAAAAGCCATGTTTGATATTGGTCATGAAGATGACATGGTGATTGAATTGGCAAA

TGAAGTGAGGAAGGTACTTGTTGCGCTTAATTCCTATGTTCCAGCCCTTAAACTACGTCCACTCACGGAA

GCTGTGGCGCTGGATTCTTAATATTGAAGACAGATAACTGAAGGAAAGGGGGGCATAAACCAGAGCTCCA

CTAACTTGATTACACTCCTGGAGAATCCTCTCTGTTTTTTCGTTTTTTACACCCTGCCAAAAGATTATTA

TTATTATTATTATTTTTTTTTCGTAAGAAAAAAGGGGGGGCCCGGTAC

>FM893983.1 FM893983 Jatropha curcas embryo 56-70 (DAF) Jatropha curcas cDNA clone rjcaeb0_004939, mRNA sequence

ATTCAATCAGTGGATTGATCTACGAAGAAACTCGTGGAGTACTCAAGATCTTCTTGGAGAATGTGATTCG

TGATGCTGTGACATACACTGAGCATGCTAGGAGAAAGACGGTGACCGCCATGGATGTGGTCTATGCTTTG

AAGAGGCAAGGAAGGACTCTTTATGGATTTGGAGGGTAAGAAGGGAGTC

>FM893982.1 FM893982 Jatropha curcas embryo 56-70 (DAF) Jatropha curcas cDNA clone rjcaeb0_004938, mRNA sequence

ATTCGGTCACTGCTACTGCCAGTCGTCTAGAGGTGATGGCTCCAAATACCGGCCTCTTTGTGGGATTGAA

CAAAGGTCACATAGTAACCAAGAAAGAATTAGCTCCTCGTCCTTCTAACCGCAAAGGGAAAACAAGCAAA

CGGGTTCACTTTGTGAGAAATTTGATAAGGGAAGTTGCTGGTTTTGCACCATATGAGAAGCGGATCACTG

AACTTCTGAAAGTTGGGAAGGATAAGCGGGCCTTGAAGGTGGCTAAAAGAAAGTTGGGGACTCACAAGAG

GGCCAAGAAGAAGAGAGAGGAGATGTCCAGTGTTGTCCGCAAGATGAGGGGTGCTGGAGGTGGTGAAAAG

AAGAAGTTGAGCAGACCTCAGTGATTTTAGACAGACAGATCTCAATCTGTCTGTTAGACATTATAATAGC

TAGAGGCGACTTCCATGTTTTTTTAGACGTTTGAGATTTTGATCAGAACCCTGTTTTGTTTTAATTAG

>FM893981.1 FM893981 Jatropha curcas embryo 56-70 (DAF) Jatropha curcas cDNA clone rjcaeb0_004937, mRNA sequence

ATTCGATTTTCTTTATCCATTCTTAAAGAAGCTTTCAAATATCAATTGCAAAGGAAAATGGCATCTAATG

ATCAGTCTTATAGTTACAGAGCCGGTGAGGCTACAGGCCGAGCCGAGGAGAAGGGGAACAGAATGGGCGA

TACGGTGAAAGAGAAGGCTGGAGAAGCGAAAGACTGGACAGGAGAAAAAGCCCATGAGACAAGAGAAAAG

ACATCTGAGACTGCAGAGGCAACCAAAGAGAAGGCTCATGAAGGGAAGGAGAAGACAAAGGGAGTTCTGG

AAGAAACTGGGGAGAAAATGAAGCATGCAGCTCA

>FM893980.1 FM893980 Jatropha curcas embryo 56-70 (DAF) Jatropha curcas cDNA clone rjcaeb0_004936, mRNA sequence

ATTGCAAAGGATTCTATCCGTCGCCCGATGGGAATTGTACTTTAAGGCGGCCCTCGCGGCTCGTCCGCCG

CGGGGGCTTGGCCAACGGCACGTGCCTCTGGGGGCCGGATGGCCCCTACTGCGGGTCGGCAAACGGGCGA

CGGACACGCGCGTCGCTTCTGGCCCGGATTCTGACTTAGAGGCGTTCAGTCATAATCCAGCGCACGGTAG

CTTCGCGCCACTGGCTTTTCAACCAAGCGCGATGACCAATTGTGCGAATCAACGGTTCCTCTCGTACTAG

GTTGAATTACCATCGCGACACCATCATCAGTAGGGTAAAACTAACCTGTCTCACGACGGTCTAATCCCAG

CTCACGTTCCCTATTGGTGGGTGAACAGGGGGGGCCCGGTACCG

>FM893979.1 FM893979 Jatropha curcas embryo 56-70 (DAF) Jatropha curcas cDNA clone rjcaeb0_004934, mRNA sequence

CAGTTTTTATTACAAGACATTAGGAAAGCTTGAAGCAACGTTACAAGAAGCAGTTTCGAACAAACTTATT

CTGATGCTCTTACTTCTTATACATCATTATATATAACTCTCACTCACATACAAGTGAATGAAGTTTTATT

CACCTGCAGCTAGCATTTTAACGAGGGCATATAGACCCAAAGCAAACATCAATTATATTTTTAGTCCTGC

GCCTTGCCTCATCTTCGCTTCCAACTTCTCCTTCCTCCAGCTGCTTCTCTAACAGATAATGTATTGATTC

ACATCGACAAATAGCACTTAGTTCCTTTGCTTGGTTACAGCATTGTCTAGGAACCTGCTGTCGAGGGTTT

TCAATTCCTGGCATAGCTAACATATCTTCTGACCTTCCTCTTCTCTGTGTTATATAATTCTCACAGGAAC

TCAAATCTCTTCTTTCAGCTTCCTTCTGGCATATATCCCGAACATGGGAAGAATCGTCGACTTCAACGGT

GGTGATGATAGTTCTGGTAGCCGTGACTGCATTGGCTATGATCACTAGAAGAACGCAGAGTAGGGCTGTT

GAGGTATTAGATTGGCGGGGGGGCCCGG

>FM893978.1 FM893978 Jatropha curcas embryo 56-70 (DAF) Jatropha curcas cDNA clone rjcaeb0_004933, mRNA sequence

CTTTTTGGGCACATACAAAGATAATACATTGAAAATCAACCATGGAACCCAAAAGACCAAAAAGAGAACT

ACAAATGCAACAAAGAAGATCCCCCATAAACCAACTCAACTACCTCAAAACAACACCCATACAACAAAAG

TTAGTCTCGTTACCAAAACCCAGCTCTCTGACTTCAACCCTTAATAACCAACAGCATTTCTCTCTACTTA

TTTCCCTTCATGTGCTTTCCCTCTGGATATCTTGGCCTACTTCCTT

>FM893977.1 FM893977 Jatropha curcas embryo 56-70 (DAF) Jatropha curcas cDNA clone rjcaeb0_004932, mRNA sequence

ATTGCAGCTGTTATTGAGGAGGAAGGATCACAGTTGCTTCTGCTGAATGCCATCGAGTCTTTCTTTTGTA

AGGCAAGCCCTGACGTAGTAAAAGAAGTAGCGTTGATTTTGAAAGTGTTATATGACAATGATGTTCTGGA

AGAAGAGTTTATAATGGAGTGGTACCAGAAGGGTGTGAATGGTGGCAACAAAAACTCGCCAGTTTGGAAG

AGTGCGAAGCCTTTTGTTGAGTGGCTTGAGAATGCTGAATCTGAAACTGAAGAGGAGTAATGCCTGATGG

CGCGCTTCTTCTCTATTTGGGTGCAGAACTATCTTTCTTTCTTTGGAATAAAAGAGTTTGATTTTTTTTT

GAGTCTAGTCTTTCGAGTCATGTAATACGGGCGTACAATTCCTTGCATCTGTGTCGCTCGATGATTTTAT

GCTTTTGGCTGGCTTCTTCGTCTTTTCTGGTTGTCTGATATTGTCACAGTGTATTATCTGGATTTTGGAC

TTTTATGATCATTACTAGTTTGTGTTTGGTC

>FM893976.1 FM893976 Jatropha curcas embryo 56-70 (DAF) Jatropha curcas cDNA clone rjcaeb0_004931, mRNA sequence

ATGAGCCGCTTCCATCTTTCATCTCAGTCACTCCTCCCTCTCTCCAGCTTGTCGATTTACAGGTAAGCAA

TGGCGACAGCTGTAGTAGCACCGCCGCCGGCAACCCCAGAACCTACACAGCCTTACACTGAAGTGAAGCT

CTTTAATCGATGGACTTTTGAGGAAGTTCAGGTTAACGACATTTCCTTGTGCGACTACATTGGTGTGCAA

CCAGCCAAGCATGCCACCTATGTTCCACACACTGCTGGAAGGTACTCTGTCAAGCGTTTCAGGAAGGCCC

AGTGCCCAATTGTTGAGAGGCTTACAAATTCATTGATGATGCACGGTAAGACACAATGGAAAGACATTGA

TGGCAGTCAGGATTGTGAAACATGCTATGGAGATCATTCATCTTCTGACCGATCAAAACCCAATCCAAGT

TATTGTTGATGCTGTGATTAACAGTGGTCCCA

>FM893975.1 FM893975 Jatropha curcas embryo 56-70 (DAF) Jatropha curcas cDNA clone rjcaeb0_004930, mRNA sequence

GGCACATACAAAGATAATACATTGAAAATCAACAATGGAACCCAAAAGACCAAAAAGAGAAATACAAATG

CAACAAAGAAGATCCCCCATAAACCAACTCAACTACCTCAAAACAACACCCATACAACAAAAGTAGTCTC

GTACCAAAACCCAGCTCTCTGACTTCAACCCTTAATAACCAACAGCATTTCTCTCTACTTATTTCCCTTC

ATGTGCTTTCCTCTGGATTTCTTGGCCTACTTCCTTGGTCTTCTGTCCCACAAAACCAGCCATGTCTTGC

ATACGCTTCTTAGCTATATCCAGCTGCTCCGGCATTCGCCGAGTGACCTCCTGGAGGTATTTCAGAACCC

ACGAGAGTGATGACAACCCTGTGAGCCCCATAGCCCCGGAAGCCAAAAATGCCATGACCGAAAGGCCAAT

AACTAATGCAGCTGGGACAAGAACAG

>FM893974.1 FM893974 Jatropha curcas embryo 56-70 (DAF) Jatropha curcas cDNA clone rjcaeb0_004929, mRNA sequence

ATTCCCAGTTTATTCAATGCTAGGCATAATGAGTATAAGGACCTCAAAAAATTCTCTTTTCGTCCTATCC

TATGAACTTTAAGGTGTATGAAGTTTCATATTTGATTCTTTAAGCAGAAGCGGGGCGGTGGAGATTCCAT

TTAACTTAGGTTAATTTAAGCCAAAAGCAGACCTACGTCAGGATAACCTTTCTTTGAAACACTTTGGTAG

TGCTCTCGGATCGGAATCCATAAATCAGAGCACATGGAGCCATCTTCTTATCTTTCTGCCAAGAGAATTA

TGGTAGACTAACTGATTATTTATATCAGTTAATGAACGAGCCCAATGCAAAAAAAATGCATGTTGGGTCT

TTGAAAAAGTTCGAATCATTTTGATAATAATCAGTTTGATCTG

>FM893973.1 FM893973 Jatropha curcas embryo 56-70 (DAF) Jatropha curcas cDNA clone rjcaeb0_004928, mRNA sequence

ATTCGGAATGCCAGCGGAAAAAGGCACCATGAAGAGAAAGGTCTCACTAGGTCAAATGCTCAGTAAACCA

AATGGTGATGTGCCCTCAAATCGTAAGGTCGAGAAGGAAAGCAGTCACAAGCAGTTCTCTGGTAAGAGGA

AGAAGTTTGGTTTTGCTAAATTCTCTTTACTCTTAACAAAGGAGAGTAAGAAAAAGAAGAAGCCAACTCT

AAACTTGAGGTAATTTGCCCGCCAATATTTCCAATCAGCTAACTTCTAATTTCCCTCCCTTCCTGTGGTG

GACTCTTTTATATTCCTGTTTTGCAGTGTTCTAGTTCCCGTGTAGTTGTTTCTATTAACTATATATGTAT

CGTATCATTGGTTGTTGTAAAGATGATATTTATGATATTTCCCATATTTCCTATATCCTTTTCAATGCT

>FM893972.1 FM893972 Jatropha curcas embryo 56-70 (DAF) Jatropha curcas cDNA clone rjcaeb0_004927, mRNA sequence

ATTCCGTAGATATATTAATGAATATGTCATTTGCCTTGGTTGCAAAAGTCCAGACACTATACTTTCGAAG

GAGAATCGTCTATTTTTTCTCAGATGTGAAAAGTGTGGTTCTGGACGATCAGTTGCCCCCATCAAAGCTG

GTTTTGTTGCTCGTGTTGGCCGCAGAAATGCAGGGACTTGAGTCTTCTAAAGCTAAGTTTGTTGCATATG

TTACCAGTAGAAATATACTGGGACATGAACCTTTCAAAGGTGAGCTGTGCAGATGAGAAGTTTTGGACCT

TGTTTTACCGTACCTACTCTTTACTCTTTACTGTTCACTTTGATGTTTACCAAAGTTAAACAATACTTTG

TCTCGAGTTACAATTCTACTGGCATTTAATGTCTTGAAGGAAACAGTATTGATTTTGGTTTATTGATCAT

TATCTCTACTGCAATAATATATGC

>FM893971.1 FM893971 Jatropha curcas embryo 56-70 (DAF) Jatropha curcas cDNA clone rjcaeb0_004926, mRNA sequence

AAGACAAGCATGCGAGGATGAGATGTGTGAGAAGAATCACAGATGGCACATACAAAGATAATACATTGAA

AATCAACAATGGAACCCAAAAGACCAAAAAGAGAAATACAAATGCAACAAAAGAAGATCCCCCATAAACC

AACTCAACTACCTCAAAACAACACCCATACAACAAAAGTAGTCTCGTACCAAAACCCAGCTCTCTGACTT

CAACCCTTAATAACCAACAGCATTTCTCTCTACTTATTTCCCTTCATGTGCTTTCCTCTGGATTTCTTGG

CCTACTTCCTTGGTCTTCTGTCCCACAAAACCAGCCATGTCTTGCATACGCTTCTTAGCTATATCCAGCT

GCTCCGGCATTCGCCGAGTGACCTCCTGGAGGTATTTCAGAACCCACGAGAGTGATGACAACCCTGTGAG

CCCCATAGCCCCGGAAGCCAAAAATGCCATGACCGAAAGGCCAATAACTAATGCAGCTGGGACAAGAACA

GGGCTAAAGATGACAAAAAGAGGGGTAGTAATAGCTAGGCCGATTAGGGTACCTATTAAGGTTATACAGC

TAGTGCAAGAAGC

>FM893970.1 FM893970 Jatropha curcas embryo 56-70 (DAF) Jatropha curcas cDNA clone rjcaeb0_004925, mRNA sequence

ATTCGACAACCCTCTAGCCACCCCTGCCGCCGCCTTCCTCCTCCTTTCTTCTTCATCTTCTTTCTTTCTT

TCTTTTCTTTTATTTCTTCTCCTTCCTTCTTCCCTGCGTCTGTCTGCGTGTGACTGGCGAGAGACAGAGC

AGGTCCGGCGCGCGCCACCCAGCCCTCGCGCACCACCTCGCCGCCACTCCACGCCGCAGCAGCTTCGCCG

TCGCCGCTCTTCTCCGTTCGACGCTAGCAAGATCGACAGTCGCAAAGGACTGTGGCCTCACCAGATCGAG

TCGCGCCACTGCCTCCATCGCAGCTGCGCCGCCAGCCGCGCCTCCGCCCTCTGCCAGACCACCCAGCCAC

CGCTGCAGCTCGCTGTCGCAGAAGCTCCACCAGATCGCACCACCGCTGCAGCTCAGGCCGTTTTCACATC

GGTCTCAGGTGGTCCAGCCACTCCGACCCGACTCCGGCCATTCATTTACAGTCAGATCCGGCCACCGACG

TTCACTGCTCTCCGATCCGGCACACGTAAAATCCCGATCTAGCAAGAACAATATATTTTTGGGAGTAGCC

TCTTTGGGCGGACCTGATGATCTAGATTTGATTGTTGCCCGTAGGGTGGCCTTCTGTTGTGTGCGCCTGA

TGATC

>FM893969.1 FM893969 Jatropha curcas embryo 56-70 (DAF) Jatropha curcas cDNA clone rjcaeb0_004924, mRNA sequence

TTTTTTTTTAGCTCCACAAACTTTCATTATCTCATAACATAAGGCATATACAGATCTACATTTTTATATT

ATTGCAAGGAACTAAAACTGCCTATAAAAATAGGCTTATTCTTCTCCAAATCTAAACTATGTAGAAACCA

GTTTATTATACATCCTCTTGGTATAATTGCATAGTTGCAATATTAATTACGAGATCTAGACCCAGGGCTA

AACACAGTGACTTCCTGTCTATTCTCCTTAAGCCTCCTGGCATCTTCTCTGGAAATCTGGAATGCGTTTG

CAACCACTGCCTCTGGCAGAGCCCTAATTGCCGAGGTCCGTCCTGCTAGTGGGCTAATTTGTGCATTTTC

GTTAGTCTTGAAGGATACCCACTCTAGCCCTTCATTGCTTGCCCTCTTCAAAACCACAAAGTTCTGTGGA

GCTGTTAATATTTGACCTTGTTGAACTTGTCCATCGAACACTGCATCTCCATTCTCATCTACGATTTGGA

CGTGACCATTTCCTCTTGTAAAGTAATATATGCTATGCGCATTGATGTTCCAGTGTGGTGCAAATATAGC

ATTCCTAAAGAGGACACCTTTCTGAACGCTGAGTCGGAGGTGTCTGAGGATCGGGAAGTTGTGGCTATTG

ACATTAGTGACATGGCCACACGTGGATTATAGATTTCGGCACGTGATGGGGGGGCCCGGTACCAATTCGC

CTAT

>FM893968.1 FM893968 Jatropha curcas embryo 56-70 (DAF) Jatropha curcas cDNA clone rjcaeb0_004923, mRNA sequence

ATTCTTGCTGCTCTCTCTACTTCTTCTCTTTGTGGTTCGATCCGGGAGAAACTCAGAATCATGTTGGTCT

ACCAGGATTTGCTTACCGGTGATGAGCTTCTCTCGGATTCGTTCCCTTACAAGGAAATTGAGAATGGCAT

GCTGTGGGAAGTTGAGGGAAAGTGGGTTGTTCAAGGAGCCGTTGATGTGAACATTGGTGCAAATCCCTCT

GCTGAAGGGGCGGATGAAGATGAGGGTGTTGATGACCAGACTGTTAAAGTTGTTGACGTTGTGGACACTT

TTAGGCTCCAGGAGCAACCTGCTTTTGACAAGAAGCAGTTTGTCACATACATGAAGAGGTACATCAAGTT

GTTGACACCCAAATTGGATGAGGAGAAGCAACAGGCTTTCAAGAAAAACATTGAGGGAGCAACCAAGTTC

CTGCTCTCGAAGCTCAGTGACCTCCAATTTTTTGTGGGGGAGAGCATGCATGATGATGGGAGTTTGGTGT

TTGCTTACTACAAGGATGGCGCCACTGACCCAACATTTTTGTACTTCGCTTATGCTTTGAAGGAGATCAA

GTGCTAAGAATGGAGAATAGCTGGCTTCGAAGTGGAAGTTCTTGCCATATTTCTAGCTTTATGTGTCCTT

TTCCATCTCTGTACTGTTATATTTGGTGCTTCCATGCCTTATTTTGGTT

>FM893967.1 FM893967 Jatropha curcas embryo 56-70 (DAF) Jatropha curcas cDNA clone rjcaeb0_004922, mRNA sequence

ATTCAAAACTACTAACAAAGAAAACAACAAACCCAAAACAGCTACGACAAGAAAGACAAGGTCTGTTAAC

AAAACTGTCGAAGCTGCAAAGAAGCCTGCAAAGAGAGCTGTTGGAGCTAAGAAGACAAAGAAATCAACTC

CTGCTAAACCTAAACAGCCCAAGTCAATTAAATCTCCTACTGCTAAAAGAACCAAGAAGGCTGCTGTTGC

TACTGCTTCTTCTTGAGCGTAGAAAATGGATAAAGGATGGTAATTGTATTAGGATTAGCGATAATAATAT

TATGTTGATGTAAATAAGAGAGAGTGTGTGTCTGTTTGTTTATGTTTATGTATGCCCTGTTTTGCACATA

ATGGGTATTGTTTTGTTTTGTGATCTCTGAAAAATTAGGGCTGATGGAACTCTGTTTTGTGCTGTGATGT

TAGCTCCTGCTTTTTTATATAATATAAATCTGAATTTTTAGAGCTG

>FM893966.1 FM893966 Jatropha curcas embryo 56-70 (DAF) Jatropha curcas cDNA clone rjcaeb0_004921, mRNA sequence

ATTCCATGGAGTCAATAGCTCATAGATTTCTTTCAGCAGCTTTGAAGCTAGATAGGAAACCATCCAAATG

TGTGGTGTTCGAGGATGATCCCAGAGGCATAACTGCTGCTCACAACTGTACTATGATGGCTGTGGCATTG

ATTGGTGCTCATCCCGCGTATGAATTAGTGCAGGCTGATCTCGCTGTTGCTAGCTTCAATGAACTCTCAG

TGATAAACCTTAGGAGGTTATTTGCTAATAATGGTTCTGGATTTATGGACCGACAAAAGCAGATTATAGA

GAAATCTCCCCCAAAAAGAAAGCTAACAATTGACACCATTTTCTGATTTCTTTACATCTTGCCCCTCTAT

TTCTTTATTTTATCCTAGCATCTTCTTGTTCTTTTTTTTTTTTTTTTCCCATTCCCATCCACTCCCCTGA

TAGAATATTCATTTGTTATTTACATTCTTTATGTGAATGTACAATAATTTAGAGGTTTTGTAGCTTCCTA

TGTGATCAACATGTTGGAATATTATTATCCAATTGTCAAAGTTTAGCACTCAATGTATAGTTGAGTCAAA

GTTGAGTTGCAATGTAATGCATTCCTTCTAAGCTGTATACTGTTGGGAGTAGGTGAAAAATTGGGACGGA

TTGGGTATCAACCATTTTGAACTGCAG

>FM893965.1 FM893965 Jatropha curcas embryo 56-70 (DAF) Jatropha curcas cDNA clone rjcaeb0_004920, mRNA sequence

ATCAACATAATGGATTTCTTCTATTGAATTAAGGTATAGTACATACTGAAAATGTTTCGATCAAAGGAAA

ACAACATTGGCAAAACTTTATCAAAATAGAATTAAAGTAAGAAAATAAAAGTAAAATGTAACAGCTGAGA

CTCGACCTCAAATGATTATGAAAAATGTATGCCAGCCATATTGAACCGAGAAAGCAGGTATAAGAACTCC

TCTTGTGTGAGTTTAGAGGACCTCTTCTCATAAAAGTTTCTCTCTTTCAACACAGCTAAAACTTTTTCTT

TGAACTCAGACACCTCCACATCAGTATCACCATCCTCCACCTCCATTTCATCATCTGTTCCGTTATCCAA

GTCCATGCTATGATCTTCCTTATGATCCCCTAGTCCAGAAATATCCATCTGGGCATCAGTACCTTCCAAG

GAACCATTTTGAGAAGTCTGTATTGCCTGTAGGGTCTTGTAGTTCTTTTCCAACATAGACAAGACATATT

TCGGGGGGGCCCGGTAN

>FM893964.1 FM893964 Jatropha curcas embryo 56-70 (DAF) Jatropha curcas cDNA clone rjcaeb0_004919, mRNA sequence

ATTGAGAGAGAGTTGATGGAGAAAGAATGGCAAAGAGTAAGGATGATATCAAGTATGGGACAGCACAGTG

CAAAGCTATCAGAAGATGAAAGTCTAAGGATGAGGTATAAGCATGGGACTCCGCTTGAAGGAGGCAAGAT

TGCTGATTCTGAGACTGTTGATCTCTTCTCTAGTGCTCATAATATTCCCAAATCATCGGACTCCGCCACT

TCCACTGATCAATCTCAGAGCCACCGCCCCTCGAATGCCGCCGGAGGAAATGCTACCAATAACAATCCCA

CCACTACCAATGATTGAGAGTTTTATTTTGTTTCATGTAATCTGAGAGCGTGTCGTAGAAAAAAGAAGTG

TTAGAATGTTTCTTCTGCTGCTCTCCAGAAACTATATGTGTAATAATAATTTAGTTTTTTTTTTCCTAAG

ATAGCAATATATAAAAATTATTAGATAGTTTTGTAATAATTCCTGGAAAAAAAAAAAGCATGAGACGACC

AGTCCGAAGGATCGTCTTTAAAAATAAAGCAACCAGACACGTTC

>FM893963.1 FM893963 Jatropha curcas embryo 56-70 (DAF) Jatropha curcas cDNA clone rjcaeb0_004918, mRNA sequence

ATTCTATTAATTTTTTGAAATCAACTGCATCTGAGAATTAATGAAGTAATATAAAGACTAATACACACTG

AATTCCAAGGCATTGGGTACGATAAAGAACTTTGTGTGCACTTAGGAAGGGGGGAAAAAAAAGAGCTGTC

ATCTAATAAAAAACAATGTAAACAATTAGGAGGCCTAATACACAAGCCACAAATCTTGAAAGTAGCCCAG

AGTGTAGAAAATGTCCACTCAAGCAAATGCGCGGTCTGCCTGCAAAGCATGTAATAAGGTCGTATTTCTC

CTGACCTCCCCATTACAACTCTCCCACAATATGCGGTCACAATGCTTCAGCAAATTTTACAAGAAATGCA

CCCAAGGACTCCTTCATCATAAGAAATAAGGAACTCCAGTCCTAGGTCCTCTCATCTGGTTGTTCTCCTC

TGTTTCAGTGAAAAATTTCACTTCTCTTTCCATGTTCTCATTGAAGCTCAATATCGAAGCTACATTCCCG

CAACGATAACAGTAATTTGGTGCAGACCAGACAGTTACTAAGCCTTTGTCTTGAAACATGTACTTAAGAC

CTTCTTGTACCAGTTGGTGTGCTCGGCAAACCAGATCAAGATTGTTAATGTGGTTGAACTCAGAAGTCAC

CC

>FM893962.1 FM893962 Jatropha curcas embryo 56-70 (DAF) Jatropha curcas cDNA clone rjcaeb0_004917, mRNA sequence

ACTAATTAGGATGATTACATTACTTTCATCAATTCTATACATAATCTCATTACATACTTCATTAGCGTGA

GATCTTAACTCCCCTCGTAATACTACTTACTTTCACTATCTACACACCCAAAAAGGCTACTTCATTATTT

ACTCCTCGGCATATCTTCCAGACTCGGTCCTGGAAATAGTCAAGGTGGTCTCTTGTCTTCCAAACTTAGT

CCTTCTTGCCTCCTCTGCTGATACCCTAAATGCATTTGCAATAACCGCCAACGGCATTGCTCTAACGGCG

GAGGAGCGACCTGCAAGATCATTGGTCATCGCGTTATCGCTTGTCTTGAAGGTTACATATTCGAATCTTT

CACTTTCTGCTCGTTTCACCACTACGAAGTTTTGTGGCACCGTCAAAACCTGTCCTTTGCTTACACTTCC

GTCGAAGACGGTGTTGCCGTTTTCATCCGTCACCTGAACCCGCGCTTGTCCTTTGAGTGCGTAAATTACG

GGGGGGCCCGGTACCCAATT

>FM893961.1 FM893961 Jatropha curcas embryo 56-70 (DAF) Jatropha curcas cDNA clone rjcaeb0_004916, mRNA sequence

AAGACTAAACCAAAATTTTATTCTCTNGATTACAGTCTCAGGTTCCACTTAACTAAAACATAAAATCCTT

TTTTTCAGCTATTTTTATTATTAATTTATTAGTACAGAAAAACTATGATAATAAAAAATTAAAAACCTCA

TTGATCACTGGATCTTAACCTCATGAACATCCCTTCTCTCTTCCTCCTTCACTTTAGGCACCACAACCTT

CAAAACACCATTCTTCATTTCAGCTTTAATGTCCTGAAGTTTATACAGATTTGGTGGCAAGTCCAGTCTG

CTAGAATATCTCCTTCCACTTTCTCCTTCTTCTTCTTCTTCTTCTTCATTTTCTTTAGGTCCTTCGCCTT

TTATAATCAAAGTGTTTTGCTCTACGCAAACTTTCACATCTTCTTTGCCTAGTCCAGGCATGTCGAATCG

TAGTAAAAGAGCATCATTATCCTCTTTCACATCCCACCCTCGCCGTGGAACACCGCCAGCTCCTATGCCG

CGTGACAGGGGAAAATCCATTAACTGGTCCATTAAATTGAAGACTTGGCTTAGAG

>FM893960.1 FM893960 Jatropha curcas embryo 56-70 (DAF) Jatropha curcas cDNA clone rjcaeb0_004915, mRNA sequence

ATTAGAGATGCTAGCTTTGCGAGGAATTGAAAACCAGCAACAACGGGATCTTCGCCAATGTTGTAACGAA

TTAAGGCAGCTAAGTCATGATTGTCCATGTGAAGGAATAAAGTATGTATTAGAGCAGCAGCTGGAGCAAG

GACAGCGAGAGCGTTTGGAGGCAGTGCGTAGGGCTAGAAGTATACCTTCTGCTTGTGGCCTGCGTACAAG

CTGCGATATTCGTTCCTAATCTGCTAGCTATAACGTGGATGTGCTTCAGTTAAATAAAAATTTCACTCAC

TTGGTGTGTGAGTGATCAGAGTTATGTATAATAATGCACAAAAGAATGAGCACATCAAGCTAAGTTTGTT

TTCTGTT

>FM893959.1 FM893959 Jatropha curcas embryo 56-70 (DAF) Jatropha curcas cDNA clone rjcaeb0_004914, mRNA sequence

ATTCGCTCAAACTGGAGCTGATAGGGAAGTTCAAAGAATCTTAATGGAACTTCTAAATCAGATGGATGGG

TTTGATCAGACAGTGAACGTTAAGGTCATAATGGCAACAAATCGCGCAGACACCTTGGACCCCGCACTCC

TTCGTCCTGGAAGGCTTGACAGAAAAATTGAATTTCCATTGCCTGATAGACGGCAAAAGAGGCTTGTTTT

TCAGGTTTGCACATCTAAAATGAATCTCAGTGATGAGGTAGACTTGGAAGATTATGTTTCTAGGCCAGAT

AAAATTAGTGCTGCTGAGATTGCAGCTATTTGTCAGGAAGCAGGCATGCATGCGGTGAGGAAAAATAGGT

ATGTGATACTGCCAAAAGACTTTGAGAAAGGTTATCGAACCAACGTGAAGAAGTCGGACACGGACTTTGA

ATTCTACAAATGAAGAGAGCCAAACACGAGTATTGCTAAGACTGGACTGTGCTTTTGAGGGATTGATAGC

TTATGTCAGCTAATACTAATTGCTGAAAGATTCCCATTTGCATGATGCTATATGG

>FM893958.1 FM893958 Jatropha curcas embryo 56-70 (DAF) Jatropha curcas cDNA clone rjcaeb0_004913, mRNA sequence

ATTCCAGACAAGGAAGGAGCAGCTGGGGACAGAAGGATATCAGGAGATGGGCCGGAAAGGTGGGCTGAGC

ACGACGGACGAGTCTGGTGGAGAAAGAGCTGAGAAGGAGGGCATCACTATCGATGAGTCGAAGTTCAGGA

CTGGGAGCTAGTGTGATGATTAAAACTATAAATGGTATTTTGTAGTAATAATAATAATAATGTTGGTAAT

AAAGTATTAGCTTAGCAGTGTACTTTAGTTTAGTAATGTAGTTTACATGTGGGTTAAAATGACTAGGGTT

TAGCTACTATGTGTGTTGTAACTTTGAGAGGGGTTTATGGTCGTTCCCTTTCTGGGAGGACTGCTAATAC

TTTCTCCCTATTTGTAAAATAACCTGCAGTATATATAATTACCTGTTGGTTAAAAAAAAAAA

>FM893957.1 FM893957 Jatropha curcas embryo 56-70 (DAF) Jatropha curcas cDNA clone rjcaeb0_004912, mRNA sequence

ATTCGTTTGTAACATGAGGAAGTGCAATACACCGAAGCCAGGATCTCAGTGCTGCAAAGTCAGAGAAAAG

TTCCAAACAAAAAATGCCTGATGGAAGCTGGAAGTGTGAGAAATGTAACAATATAAACTATCCATTCAGG

ACCAAGTGCAATAGACAGAATTGTGGAGCTGAGAAACCTAATGAGTCAAAGAAGTCCCCTTCACCGGTGG

CAGAGGAGAATGATCAGTGAGTACTAGGACATTTTTGAGGTTTGAGAAGGTCCAGAGTTGTCGTCCAGCA

TTGCTCAATATGGGTGTCTGAAAGTCAGTCGTGTCATCCTCATGTTGCAGCGGTTGTCAAGTTACCTTCT

CATGATGTCTGTCAAATTGTTGTATTACTTGCAATGATGTTAACGGTCTTTATGAATCCTTGATGCATTA

TGTCTCTGCCAGCTTGATCTGGCAAGATTACAGGTTGGTTCTGGTTTCTCTTAGTTAGGCTTATGTAATA

TAACGGTATCAACTCATCTAATATAGTGGGTATGCTACCGTGAACTTAGAAT

>FM893956.1 FM893956 Jatropha curcas embryo 56-70 (DAF) Jatropha curcas cDNA clone rjcaeb0_004910, mRNA sequence

ATTCCTTTCCCTTTTTAATTCAAATTTCACAGATTCTTTGCTTTCCTTCAAATCTCTTCAATTCCATATT

TTCCTTTCCTTTTCTCTCTTTTTTCTCTTAAATCTCTCTTAATTACCTCAGATTCCTACCCAAATTCCTT

TCTCCCTCCCTCTAAAGCTCCTGTTTTTCAGAATCATTGTCAAAGTTCTTCCCTTTCTACGGTTTTTAAC

GTGGGAGATATGAATTGAAGCTGAATTGTGATTGATTAGGAGGTTTTTGGAGCTGAATTTAGGGTTTTTG

TTTGTTTTCATTGTATTTGATACCTTTGGTAAATAACAGAAGGTGAAATGGAGAAGTACGAGCTGGTGAA

AGATATAGGATCTGGGAATTTTGGAGTGGCTAGGCTCATGAGGAACAAGGAGACCAAAGAGCTTGTTGCT

ATGAAGTTACATCGAGCGCGGGCACGAGATTGATGAAGATGTGGCCAGGGAGA

>FM893955.1 FM893955 Jatropha curcas embryo 56-70 (DAF) Jatropha curcas cDNA clone rjcaeb0_004909, mRNA sequence

ATTCGTCTATCTTAAGCCATGGCAGATCAGTTCAGGTTAGTTTCACCNGGATATAAACCAAGAAGGAAGA

ATGCCAAGGGAAGTACACAGGCGAAGGTCAAGGTGCTCAAAAGAATATATCGCCGCCATTAGAATGGTAC

AACGTACCAGAAGGAACAAAGAGCCTGGCTTTGGTGGTGCAAGACATAGATGCACCTGACCCAGATGGTC

CCATTGTTCCTTGGACCCATTGGGTTGTGGTTAATATACCACCTACGTTGAATAGAATACCAGAAGGGTT

TTCAGGGAAAGAAGAGGAAATAGGTGGTGAATATGCTGGAATCAAAGAAGGAAATAATGACTGGAAAGTT

CCAGGTTGGCGCGGCCCCAAGCTTCCTAATCATGGACATAGGTTTGAGTTCAAGCTTTTTGCCTTGGATG

AGGAATTGAATCTGGGAAATAAGGTAACAAAAGAGAAGCTGTTGGAAGCAATTGAAGGGCATGTTCTAGG

AGAAGCAGTGCTGATGTGCAAGTTTTGAACTTT

>FM893954.1 FM893954 Jatropha curcas embryo 56-70 (DAF) Jatropha curcas cDNA clone rjcaeb0_004908, mRNA sequence

ATTCCTACTTAAGCTCTCAGTTCCATACTCAGTGTTGTCGTTTGTGTTCTACACATTTATTTGATCGTGT

ATGGCTGGAATTCCAATATGCGTCCAATGCGGCACCACTAGCAACCCTTGCCGGTGCAAGGTGGTTGGTC

CAACTCTGGGGTTCTTGGCGTTTGCCGTGGCGGCGGTGGTAGAGTGGCCGGTGGGAGCTTTTGTTTACCT

CTTTAAACACAGGAAAGGTCGCAGGATCATGGCACATCCAGCGACTGTCGTTTACCCATCAGTCACCAAT

TCAATTCCCGTTTAATTAATAGATNTGATGCTTTTGTTTTGTTTTCAGGTTCTTTGTGTTCCTTTTATGT

AATGGCCACTGTGTTTCTTATATATGCTATAATAAAATAATAGTGTGGCTTTGTGT

>FM893953.1 FM893953 Jatropha curcas embryo 56-70 (DAF) Jatropha curcas cDNA clone rjcaeb0_004907, mRNA sequence

ATTCCCATAATCAAAACAATGAAGGTCATCGCTGCATATTTGCTCGCCGTTTTGGGAGGTAACACCTCAC

CCTCCGCCGAGGACCTGAAGGAAATCCTCGGATCCGTTGGAGCTGATGCTGATGATGATAAGATTCAGTT

ATTGTTGTCCCAAGTTGAAGGAAAGGATATCACAGAATTGGTTGCAGCAGGAAGGGAGAAATTGGCATCT

GTGCCTTCTGGTGGTGGTGTAGCTGTTGCTGCTGCTGCAGCACCTGCTGCTGGAGGTGGTGGTGCTGCTC

CTGCTGCTGAGGCCAAAAAGGAGGAGAAGGTTGAGGAGAAAGAGGAGTCAGATGATGATATGGGTTTCAG

CCTCTTCGACTAGGAGATCTATTTGCTGGTTCCATTTGTATCAAGGCTTTCTTTTATCTTCTAGTCTTTT

AGTTAAAAAATTTACAAGTTTTTGGTCTGTAGTTGGTTATGATATATAGAACTTGTTTGCGTTCTTGAAT

TTTGAGTTAGGTAATGGAGATTAAACCATTTCACATATAGTTG

>FM893952.1 FM893952 Jatropha curcas embryo 56-70 (DAF) Jatropha curcas cDNA clone rjcaeb0_004906, mRNA sequence

ATTCCAAAAGGCATAACAATGGCCATAACTAACTCTTTCCTTCTTTCACTGTGTCTAATTGTTATCTTCA

CTGGCTCTATAGCAAGTCAGCAACTTCAGCAGCAAAACCAGTGTCAACTCGATAGGCTCGACGCCCTCGA

GCCAGATAATAGTATTCAAAGCGAAGCTGGTGTGGTCGAGTCTTGGAACCCTAACCAAGACCAGTTCCAA

TGTGCTGGTGTGGCTATTGTTAGGCTCACCATTCAACCCAAGGGCCTTCTGTTGCCATCTTATAGCAACG

CTCCTCAACTCGTTTACATTGTCCAAGGTACGGGTATGACAGGAGCCCTGTTTCCTGGATGCGCTGAAAC

ATATCAGGAGTCTCAAGGATCAGGTGGTAGCAGTAGGGTTGAAGCCCAGCACCAGAAGATTCGTCGTTTC

CGTCCAGGTGATATCATTGCTTTGCCTGCCGGAGTTGCCCATTGGTGTTACAACGACGGCAATGAGCCTG

TTGTCGCCGTTTCTGTTCTTGACACCGCCAACAGCGCCAACCAGCTTGACATGAACCCTAGACATTTCAA

TCTAGCTGGTAACCCAGAAGATGAATTCCCCGCACTGCATGAGGGTCGCCGTGAGGGTGGCAGCCAGGAA

>FM893951.1 FM893951 Jatropha curcas embryo 56-70 (DAF) Jatropha curcas cDNA clone rjcaeb0_004905, mRNA sequence

ATTCCTCCGAAGTTTCCCTCAGGATAGCTGGAGCTCGGGACGAGTTCTATCGGGTAAAGCCAATGATTAG

AGGCATCGGGGGCGCAACGCCCTCGACCTATTCTCAAACTTTAAATAGGTAGGACGGCGCGGCTGCTTCG

CTGAGCCGCGCCACGGAATCGAGAGCTCCAAGTGGGCCATTTTTGGTAAGCAGAACTGGCGATGCGGGAT

GAACCGGAAGCCGGGTTACGGTGCCCAACTGCGCGCTAACCTACAACCCACAAAGGGTGTTGGTCGATTA

CGACAGCAAGACGGTGGTCATGGAAGTCGAAATC

>FM893950.1 FM893950 Jatropha curcas embryo 56-70 (DAF) Jatropha curcas cDNA clone rjcaeb0_004904, mRNA sequence

ATTCCTTTCTCACACTTATCATCAACAAATGGCTGAACGTTCACAGCCACACCAAGTTCAAGTGCACCCA

CAACACCGCTATGAAGCTGCCTTCAAAGGTCAACAAAAAGGTCCATCAGCTCAGAAAGTGTTAGCTGTGA

TCACCCTCCTGCCGGTAGGCGGTGGGCTTCTTGCACTAGCTGGTATAACCTTAGTAGGTACCCTAATCGG

CCTAGCTATTACTACCCCTCTTTTTGTCATCTTTAGCCCTGTTCTTGTCCCAGCTGCATTAGTTATTGGC

CTTTCGGTCATGGCATTTTTGGCTTCCGGGGCTATGGGGCTCACAGGGTTGTCATCACTCTCGTGGGTTC

TGAAATACCTCCAGGAGGTCACTCGGCGAATGCCGGAGCAGCTGGATATAGCTAAGAAGCGTA

>FM893949.1 FM893949 Jatropha curcas embryo 56-70 (DAF) Jatropha curcas cDNA clone rjcaeb0_004903, mRNA sequence

ATTCCGTTTTGATGAGGCAGTGGCACTGTTTATGGAAATGCAAATCAGAAGAGTTAAACCTGACAAATTC

ATAATGGTTTCTCTTCTTACATGTTGTGCTCAAACAGGAGCATTAGAGCAAGGTAAATGGATTCATGAAT

TCATAGATGAGAACAGTATAACAGTAGATGCAGTTGTTGGAACTGCTCTAATAGAAATGTATGCAAAGTG

TGGGTGCATAGAAAAAGCTTTGGAGATTCTCTATGGGTTAATAGAAAAAGATACAGCCTCATGGACTTCA

ACTATATGCCGGCTTGCCGTGAATGGAAAGACCAAAAATGTCACTCGATTTGTTTT

>FM893948.1 FM893948 Jatropha curcas embryo 56-70 (DAF) Jatropha curcas cDNA clone rjcaeb0_004896, mRNA sequence

GCTGCAGGAATTCGAATTCCACTTCTCCTTTCTATAGCAATGGCTAACTCTTCTTTTCTCTCTCTCGGTC

TCTGCTTTCTCGTTCTTTTCCAAACCTGCTTTGGTCAATTAGAACAATTAACAACTGAGAATCGCCGGCC

CTGGCAATTCCGTGACCAGCGCTCTCAACAGGATGCGTGTAGACTCGAACGCATTAACGCTGTGGAGCCT

TCCCGTAGAATTCAATCAGAGGCCGGTGTAACTGACATCTGGGAAAAACGACGAGCAGTTCCGATGCGCT

GGTGTCGTAGCCATGCGTCATACAATTCAAGAGAGAGGCCTCTTGTTGCCCCTATATGCCAATGGTCCTA

AACTCATTTACGTGCTCCAAGGTAGAGGTATCCACGGAGCCGTATTTCCAGGTTGTCCTGAGACATTCCA

GTCACCATCAGAACCACAGGAACGGTACGGATTCGGTGCAGGAGAAAGAGAGAGTCGGAGATTTGATCAA

CACCAGAAAGTTCGTTCACTCCGACAAGGTGATGTTATTGCTTTGCCAGCTGGAGTAGCTCAGTGGATTT

ACAACAGTGGCCGGACTCCTCTTGTTCTTGTCCAAATTATTGATATGGAATGCTGCA

>FM893947.1 FM893947 Jatropha curcas embryo 56-70 (DAF) Jatropha curcas cDNA clone rjcaeb0_004895, mRNA sequence

ATAAATAAAATATCTACTAAAAGATACCTTTTATTCAACTGGAATTGCTTAACAAGAAAAAGACACCAAA

CAGAAAACATAGATATATTACATAACAGGAGCTAAAGCACATGCCATGCCCTACACACGTGGGTTATGGC

CAGTGGTGTCTCCGGGCAACCCAGATGGGCGAGTCCTGGTCAAACCCGTTGGGTCCACCTCTCCACCGTA

TGGCTGCCCTGTGCCGTGTCCAGGCATGGCCGACATTTGCTGGGTCCCTGTAGGGTAACCGGTTGTCCCA

GTTGTTGAATGGGTATAGGTTTCGGTTGTGCCTTGAGCGCCCGGACCAGCAGTTGTGTACTGTACATGGC

CACCAGCCTTGGCTGCCTGCCTCGCCGCTGCGTTATCCTCGCGCGCTTCTAGTTTCCGCAATTCTGCCTC

CGTTTTCCTCTCTTCTTTCTTCTCTCTCGCCATTTCCTTCTGCACTGGATCACGTGTTGACATTCGGGGG

GGCCCGGTAC

>FM893946.1 FM893946 Jatropha curcas embryo 56-70 (DAF) Jatropha curcas cDNA clone rjcaeb0_004894, mRNA sequence

GTTTAATCAAAACTTGATATTTATTTCCTCATTATATCCTACATTACATAGCAAGTTCTTAGGACTCCAT

AAATAGAGAAGGTGATACTTGCCCAGTATGCCAGCGATAAAACTTATCCGTCATCTTTCTCCCTGTAACG

CATACATCCTTCGCATTAAGAACAATGCACATATCCCCATCGTCACGATATGGAGCATATGTAGGCTTAT

CTTTGCCTTGAACTACTGTTGATATTTGAGATGCTAATCTTCCAAGGACCTGGCCTTTAGCATCAAATAC

TCTCCAACGCAAGCCTTCAAGATTGATACGTCTGAGGCCAGCAATCGCTTTCTTCACGTTGCCATTGAAA

GAGCTAGCTGCTTGGCTAGCCATGTTTCCCTGTACGATGAGTATTCTCGTTTATCACCCACAAATCATTC

ACCTTGAGTAGCAGTAGGGTTTGTTGGGGGGGCCCGGTAC

>FM893945.1 FM893945 Jatropha curcas embryo 56-70 (DAF) Jatropha curcas cDNA clone rjcaeb0_004893, mRNA sequence

ATTCCACACTACACATTTGCCAAGGTTTTGGTCTTTTGTGTTTTGCCTCCATAGCTGCCAAGAACCATGG

ATTTTGTAAGTCTAATCTTGGATTTCGCCACTAGCTTCTGCAATTGCACCGCCAGGCATACAAATTATAT

CCGTAAGCTTAAAAAGAATCTTTATTCTTTGAGTAACGCAATGAAGAACTTAAGAACCTTAATGCAGATG

TCAAGAGAAGGATCCAACTTGCTGAGCAGCAACACTTGTTCTGTACAAACCTAGTGAAAAGCTGGGTAGA

GAGAGTAAAAGTTATGGAGGAGGTGGTTAATGAAATTCTGCAAAAAGGGGATCTTGAGATGCAGAACAAA

TGTTTTAGAAGTCGATGTCCTACGAATTGGCGATCCTGCTACGAACTTGGAAAGATGGTAATCCAGAAGC

TCAATTCTGTCAGGGAACTAGTGAATAAAGGCAATTTAGATATAGTTGCTTAAAGCTAGACTCAACAT

>FM893944.1 FM893944 Jatropha curcas embryo 56-70 (DAF) Jatropha curcas cDNA clone rjcaeb0_004892, mRNA sequence

GCAAAAGATTATTATATTGGTGGAGAAATTTGTTAGCAAAAAAGGGCAAAAATGTCTTCAAATCAATCAA

GACAAGAGCTTGATGAAAGAGCAAGGCGTGGAGAGACTGTTGTCCCTNGGAGGTACTGGTGGGCAAGAGC

CTTGACAGCTCAAGAAGCATCTTGACTGAAGGGGAGAAGCAGAGGGAGGACAGACAAGGGAAGGAGCAGC

TGGGGCACAGAAGGATATCAGGAGATGGGCCGGAAAGGTGGGCTGAGCACGACGGACGAGTCTGGTGGAG

AAAGAGCTGAGAAGGAGGGCATCACTATCGATGAGTCGAAGTTCAAGACTGGGAGCTAGTGTGATGATTA

AAACTATAAATGGTATTTTGTAGTAATAATAATAATAATGTTGGTAATAAAGTATTAGCTTAGCAGATGT

ACTTTAGTTTAGTAGTGTAGTTTACATGTGGGTTAAAATGACTAGGG

>FM893943.1 FM893943 Jatropha curcas embryo 56-70 (DAF) Jatropha curcas cDNA clone rjcaeb0_004891, mRNA sequence

CCAAAAAGCATAAACTTGCAAAATTTATTCATCTAAACTAGTTTACATGAACATTGCTTAATTAAAGGAT

GACTAGGTTAGGTTTTCACCCCTTTGTTCCATCTCTATCTTGAAGCTCTCTGTGCGCTCCAGCCATTTCT

CATTCTCAAGATGCCTTTCATAGAAGTTGACCTCTCCACCTGATTTCACGGAAACTGCAGAGGTGCTTCT

CGTTGCATATNCGTCCCAATGGAGTGTCTGTGTCAACAAATATGGAACTTAACTGATGTTCAAATTCAGC

AGGATAAATTTTGGGTAACATGTTTTCCTCATCTTTGATAGTG

>FM893942.1 FM893942 Jatropha curcas embryo 56-70 (DAF) Jatropha curcas cDNA clone rjcaeb0_004890, mRNA sequence

ATTCATTCCGTCCAAGGCTAAATCAGGCGAGAGACCGATAGCGAACAAGTACGCGAGGGAAAGATGAAAA

GGACTTTGACAAGAGAGTCAAAGAGTGCTTGAAATTGTCGGGAGGGAAGCGGATGGGGGCCGGCGATGCG

CCCCGGTCGGATGTGGAACGGCGACAAGCCGGTCCGCCGATCGGCTCGGGGCGCGGACCGACGCGGATCG

CTGC

>FM893941.1 FM893941 Jatropha curcas embryo 56-70 (DAF) Jatropha curcas cDNA clone rjcaeb0_004889, mRNA sequence

ATTCCAAAAAAGCAGTTGCTCAACACAATCAAACACACCCAATTCTCTTTGAATCCAATTCAAGAAAGCA

AGATAAAGAAAGAAAAATGCCAAGTGAAGGCACAGCTACTTGCATAGATATCCTCTTGGCTGTTATCTTG

CCTCCTCTTGGCGTCTTCCTTAAGTTTGGTTGCAAGGCGGAGTTTTGGATCTGTTTGCTTCTGACCATTT

TGGGATATATTCCTGGGATTATCTATGCTGTCTATGCAATCACCAAGTGATCTTCCATCTACGGTGGAAA

GCTACATAACCATTTGATTTGATGGAGGGGTCTGTTTTGGTGAAGTCAGATGTATCATTCTAATTGTGTG

TGTGTGTTTACGTGTTTGTCTTCTATGAAGATTTCCATTTCATTTTTTAATTTTCTGTAGTTAAAAAAAA

ATTAGCCATTGAGTTGGGTTTGTGAGTAGAAACAATGGCTTGTCAATTATCATTTTAGTTTATCTTTGAA

CTTGAAATTTGTTTTAATTTTAAATGTGATTGTAT

>FM893940.1 FM893940 Jatropha curcas embryo 56-70 (DAF) Jatropha curcas cDNA clone rjcaeb0_004888, mRNA sequence

ATTCCAACCAAGCAAAAGCCCTAGGAAGGAGTCTTAAGATATGGCGACAGTTCCAGGGCAATTGATATGG

GAGATTGTGAAGAAGAACAACTCATTTTTGGTGAAACAGTTTGGAAGAGGAACAGCTAGCCTCCAGTTCA

GCAAAGAGCCTAACAATCTCTACAATCTCAACTCCTACAAGCACTCTGGGTTGGCCAATAAGAAAACAGT

CACCATTCAGCCTGGGAAAGATCAATCTGTTGTTCTTGCTACTACTAAGACTAAGAAACAGAATAAGCCT

TCAGCTTTGCTTCACAAGTCTGTCATGAAGAAAGAGTTCCGTCGGATGGCGAAGGCTGTGGAAAACCAGG

TTGGAGATAACTATTACAGGCCTGACTTGAAGAAAGCAGCTCTTGCAAGGTTGAGTGTTGTCCACAGGAG

CCTGAAGGTCGCCAAATCTGGTGTCAAGAAGAGGAATAGACAAGTTCAGAAGAGTAGGAAGTGAATAGTG

AGGTGAAAGGGAAAGATATGCGCCCCTCCCCCCTTTCTCTATTTTGTGAGACCCCCANAATTTCTGAACC

CAGTTTCTAGTTTATTTGGATATCGTTTTATGGCTAGGAATT

>FM893939.1 FM893939 Jatropha curcas embryo 56-70 (DAF) Jatropha curcas cDNA clone rjcaeb0_004887, mRNA sequence

ATTCACGAGTTTTACCAGAAGGTGCTGCTGAATTGTGTGGCATACAACCTTCAGAAGTGGCATGGTTAGA

AGAAACTCCGCCTCCAGAAAACTTTGTGGTGCAGAGGGGTCTATATAAAGGCCCAAATATTAGGCTTTAA

TAAAGGGTTTTTCCCCCCTCCCCTTTTGGATAGGATGTAAAGTACCATTAATCAAATCAAATGAACAAAT

GGATAACCCTGGAATTGATTGCTGGAAAGTTAGCTGTCAACTGACATGGCAAGTAATAATTAGTCCTTGG

CTTTGTAAAAAGTCTGTCCCTAAAACCCTGCCACTCTGCATCGAAGAGCTCATCTTGTTCATTGCTTATT

CAGGTGTCTAATTCAGTCATGTTTATGACAAGATTTATTGGGAGAACCCTCTTTTCTGCTGCTAAATCTG

AAACCTCTGCTGCTGCTGCTGCTACGATTCAGTTTGCACGTAACCCTCTCGAGGAGTTCTTTGAAGCAGA

TAGAAGCCAGGATGAGGACAACAAAATTTTCTATGGTCGAAGTTGGAAAGCTTCTGAACTACGCCTGAAG

TCTTGGG

>FM893938.1 FM893938 Jatropha curcas embryo 56-70 (DAF) Jatropha curcas cDNA clone rjcaeb0_004886, mRNA sequence

ATCTGCTCGAGAGGAAGTACCTGGAAGGCGTGGATATCCTGGGTACATGTATACTGATTTGGCGACTATA

TATGAGCGGGCTGGACGTATTGAGGGGCGAAAAGGCTCCATCACACAGATTCCAATTTTAACTATGCCTA

ATGATGATATTACACATCCCACCCCAGATCTTACGGGATATATTACAGAGGGACAGATATATATTGACAG

GCAGCTTCACAATAGACAGATATATCCTCCAATCAATGTCCTCCCATCTCTCTCTCGTCTGATGAAGAGT

GCCATTGGTGAGGGGATGACTAGGAGGGACCATGCTGATGTGTCTAACCAGCTTTATGCAAATTATGCAA

TTGGGAAGGACGTCCAGGCAATGAAAGCTGTGGTTGGAGAAGAAGCTCTTTCCTCGGAAGACCTGCTATA

TTTGGAGTTCTTGGACACATTTGAGCGAAAATTTGTGACTCAAGGCGCTTATGACACTCGCAACATCTTC

CAATCACTCGATTTAGCTTGGACTCTGCTCCGCATCTTTCCTCGTGAGCTACTCCACCGAATACCTGCTA

AGACACTT

>FM893937.1 FM893937 Jatropha curcas embryo 56-70 (DAF) Jatropha curcas cDNA clone rjcaeb0_004885, mRNA sequence

CAATGCCAAAACAAGCACAAATCAGTAGGCTCCTTAAAAAACAACAAAGCAAAAGTTCAAGACAATACAT

AAAACAAGAAACAACAGGAAATCAAATCCCACATGACAGGGTTTGAGAAGGTACCCATGTGCTTCTTTTA

TTTACACCAAACATGGATTATTACACATAATTCAACTGATTAACCATAAAAGGCCAAGACTAATAACTGC

TTTGTTGGAGCCTTTTCCCTTCTGGTTTCTCATTAGCAGTTGCATGGGTCACAGTTGCAGTTTGATCCAC

ACTTGCAGCCATGACCACCCTCAGATCCAAAGCTCATATCAGAACTCTCATTCAAATTCTTGGAAGGTGC

AACTCCTGCAATGAGGGT

>FM893936.1 FM893936 Jatropha curcas embryo 56-70 (DAF) Jatropha curcas cDNA clone rjcaeb0_004884, mRNA sequence

CGAATTGTTCCATATTATGATTATGCACTTGATCTGATATTGGATGTTGAATCCTCTCATGGGCGATATG

TTTACTGAAGAACAGAATGAGCTGGTGGAATCTGCAGCAGAGATGCTGTATGGTTTAATCCATGTGCGAT

ATATACTGACTACCAATGGAATGGCTGCGATGTTAGAGAAGTACAAAAATTATGACTTTGGGAGATGCCC

AAGAGTTTACTGCTGTGGACAGCCCTGCCTTCCGGTTGGTCAATCAGACATACCTCGTTCAAGCACTGTT

AAAATCTACTGTCCAAAGTGTGAGGATATATATTACCCTAGATCAAAATATCAATGCAACATTGATGGAG

CTTATTTTGGAACGACGTTTCCTCACCTATTTCTGATGACGTATGGGCACCTGAAGCCACAAAAGGCAGC

ACAGAGCTACGTACCTAG

>FM893935.1 FM893935 Jatropha curcas embryo 56-70 (DAF) Jatropha curcas cDNA clone rjcaeb0_004881, mRNA sequence

GCAAGTAACATATAAGTGCCCACAAAAAGTAATACAATTTCAATATTAAACTCTTTAATACATGAATTCT

TTATTAAAATATTCCTGCCTAAAAAGTCTATCCCACAGGATATATCCACTCTGACTTCCTCAATCTACTG

TTGCATGCTGCTGCACAGATCCAATGGCTCTTTAAGTACCGTGAGACAGCTCGCAAAAAGCCCGCAAGTT

AGCATTGAGACGTGATGTGAGGAGATTTGTGCCGGCCATGCTATGCAGAGGCAAGAATGATTGCAAACAT

CCCAATTCTCCCAAGTTCCTGGAAGGACCAAAGGAGACGCGGCGGGACTGAAACCGAAGAGAG

>FM893934.1 FM893934 Jatropha curcas embryo 56-70 (DAF) Jatropha curcas cDNA clone rjcaeb0_004880, mRNA sequence

ATTCTGTAAATAGTGCTGTGTATGTGTGAATAAATAATGAAAGGTGAAGGTACATGTGTAAATTAGTCTT

TAAGAAAAGAGAAAGATCACTGCTGCGTGACTATAGATGGCTTCTGTGTTTAAGTAATTAATAAATGAGA

TAAATTCTAGCTTAAAC

>FM893933.1 FM893933 Jatropha curcas embryo 56-70 (DAF) Jatropha curcas cDNA clone rjcaeb0_004878, mRNA sequence

ATTCCAACATCTCTCNGTGGAGCAATGTCTGGGCCCTATTACAACTCCAGAGTAACCAAGATATCAGTGG

TTTTGGATGGCGAAGGCTACTTTGAAATGGCTTGTCCTCACCTTAGCGGCTCGCGGGGAGACACGACGGG

TGAAAGTCATAAGACTTACCAGAAAGTAAGTTCACGGTTGAAACGTGGAACAGCAGTGGTCGTCCCTGCA

AGCCATCCGGAGGCTTTTGTAGCTTCCAGACACAATAATCTGGAAGTAGTCTGCTTTGAGATTAATGAAG

AAGGCAATACTAGGAATCTACTTGCAGGGAAAAATAATATTGTAAATAAGATGGATAAGACAGCCAAAGA

G

>FM893932.1 FM893932 Jatropha curcas embryo 56-70 (DAF) Jatropha curcas cDNA clone rjcaeb0_004877, mRNA sequence

TCCGACGAAGCTACTCTCGCTACGATCGACCAAATCTAAGGCTTTCAGATCGAAAAATGGCATTGATTCC

AAGCTTTTTCGGTAACTCACGAAGCAACATCTTCGATCCTTTCTCTGCTCTCGACGTTTGGGATCCATTC

AGAGACTTTTCTTTCCCTTCATCTTCTTCCCTCGTCTCTCGTGAAAATTCTGCTTTTATTAACACTCGCA

TTGACTGGAGAGAGACACCAGAGGCTCATATATTCAAGGCTGATCTCCCTGGGCTCAAAAAAGAGGAAGT

GAAAGTAGAAATTGAAGATGACAGAGTGCTTCAAATCAGCGGGGAGAGAAATGTGGAGAAGGAAGACAAG

AACGACACGTGGCATCGCGTGGAGCGTAGCAGTGGCAAGTTCATGAGGAGGTTCAGGCTGCCGGAGAATG

CAAAGATGGATCAAGTAACGGCTTCAATGGAAAACGGAGTACTTACTGTCACTGTACCTAACGAGGAAGT

CAAGAAACCTGATGTTAAGGCT

>FM893931.1 FM893931 Jatropha curcas embryo 56-70 (DAF) Jatropha curcas cDNA clone rjcaeb0_004874, mRNA sequence

GACTAGTTCTCTCTCTCTCTCTCTCTGCTATTGGAAATGGAGGAAGTGGTGGCCGTGCACGAGCTGCATA

TATGGGCCATCACAGTGGGCAAAGTCCTCTTAGCTTGTCATGTAAAAATCAGACCAGAAGCGAATGCAGA

CATGGTTCTGGACAATGTGATAGACTACATCAGAAGGGAATAC

>FM893930.1 FM893930 Jatropha curcas embryo 56-70 (DAF) Jatropha curcas cDNA clone rjcaeb0_004873, mRNA sequence

TACAAGTTCAACAAATACTGACTGTGCTGCCTGGAACAATGATCCGAATATTCTGTGCTTCAATTGCAAT

TCATGCAAGGCTGGATTGCTAGACAATATAAAGAGAGATTGGAAGAAGGTTGCTGTTGTGAACATTATCT

TCCTTATCTTCCTCATCATTGTCTACTCTGTTGGATGCTGTGCTTTCAGGAACAACAGAAGGGACAATGC

TTATTCTGGTGGTGGATGGAATAAGCACCCTTGAGACTTTCAGATTGGAGGGTGTTATA

>FM893929.1 FM893929 Jatropha curcas embryo 56-70 (DAF) Jatropha curcas cDNA clone rjcaeb0_004872, mRNA sequence

ATGTTCGATCNCGGGAGAAACTCAGAATCATGTTGGTCTACCAGGATTTGCTTACCGGTGATGAGCTTCT

CTCGGATTCGTTCCCTTACAAGGAAATTGAGAATGGCATGCTGTGGGAAGTTGAGGGAAAGTGGGTTGTT

CAAGGAGCCGTTGATGTGAACATTGGTGCAAATCCCTCTGCTGAAGGGGCGGATGAAGATGAGGGTGTTG

ATGACCAGACTGTTAAAGTTGTTGACGTTGTGGACACTTTTAGGCTCCAGGAGCAACCTGCTTTTGACAA

GAAGCAGTTTGTCACATACATGAAGAGGTACATCAAGTTGTTGACACCCAAATTGGATGAGGAGAAGCAA

CAGGCTTTCAAGAAAAACATTGAGGGAGCAACCAAGTTCCTGCTCTCGAAGCTCAGTGACCTCCAGATTT

TTGTGGGGGAGAGCATGCATGATGATGGGAAGTTGGGTGTATGCTTACTACCAGGATGGCGCCACTGAAC

CAACATTTTTGGTACTTCGCTTATGC

>FM893928.1 FM893928 Jatropha curcas embryo 56-70 (DAF) Jatropha curcas cDNA clone rjcaeb0_004871, mRNA sequence

ATTCCCAGAAGCTGGTCGTGTTAGCACCGTTAATAGCCATAACCTCCCTATTCTTCGGTGGCTACAACTC

AGCGCTTCCTATGGCGTTCTCCGCAATGATGCCATGAGATTACCCCACTGGCACATAAACTCTCACAGCG

TAATTTACGCACTCAAAGGACAAGCGCGGGTTCAGGTGACGGATGAAAACGGCAACACCGTCTTCGACGG

AAGTGTAAGCAAAGGACAGGTTTTGACGGTGCCACAAAACTTCGTAGTGGTGAAACGAGCAGAAAGTGAA

AGATTCGAATATGTAACCTTCAAGACAAGCGATAACGCGATGACCAATGATCTTGCAGGTCGCTCCTCCG

CCGTTAGAGCAATGCCGTTGGCGGTTATTGCAAATGCATTTAGGGTATCAGCAGAGGAGGCAAGAAGGAT

TAAGTTTGGAAGACAAGAGACCACCTTGACTATTTCCAGGACCGAGTCTGGAAGATATGCCGAGGAGTAA

ATAATGAAGTAGCCTTTTTGGGTGTGTAGATAGTGAAAGTAAGTAGTATTACGAGGGGAGTTATGATCTC

ACGCTAATGAAGTATGTATTATATTATG

>FM893927.1 FM893927 Jatropha curcas embryo 56-70 (DAF) Jatropha curcas cDNA clone rjcaeb0_004869, mRNA sequence

TTTTAGGATAAAAGATGCATTAAGCTTTTAATTTCTCATTATGGTTTTAACAAAATGCACATATAATACT

ACATGAACAACGCAGACACAGACGAAACTAGCAGAGTAAGCACAACTCTTATTTTCTCTGAAACAGGTGA

CTCATGATTATCACATAAGAAGATCTACAGCTCTTGTTTGGCCCCACTATCTGTGAGCATGGCATTTTCT

TCTCCAGCAATGCTAACGACCCTTGGACCTTTGATCTTATCAGGAGACAACTTGGAGAAAGATAAAGTAA

GCACACCATTTTCAAGCTTAGCCTTAACAGAGTCCAAATCCACATTGTCTGGCAACTTAAACTGTCTCCA

AAACTTGCCATATGATCTTTCTACCCTATGCCAATGATCCCCTTTCTTCTCTTCCTCTCTTTTCCTTTCT

CCACTCACTCTGAGTACCCTATTCTCTTCCACTTCAATCTTCAGCTCTTCCTTCTTCAAACCAGGAACAT

CTAACATGATAAGATGACCCTCAGGTGTTTCTTTCCAGTCTACTCTGGCCGGAGAGAGTGCCACACTATC

ATCTCTCTCAATCTCAAGGGGGGGGCCGGTAACCAATTCGCCCTAT

>FM893926.1 FM893926 Jatropha curcas embryo 56-70 (DAF) Jatropha curcas cDNA clone rjcaeb0_004866, mRNA sequence

ATTCATCAAGGAATATCCGGGGAATGAGGAAGAAAAAATGAGAGCAGCAATTGGGAAGTTTGGGCTGACT

GGCAAAGCCCAGGTGATGCCCATGAAGAATCTCTCAGATGGGCAGAGGAGCAGGGTGATATTTGCTTGGT

TAGCTTACAGGCACCCCCAATTGCTGCTGTTAGATGAGCCAACTAACCATTTAGATATTGAGACTATTGA

CTCTCTTGCTGACAGCATTGAATGAATGGGATGGTGGTTTGGTTCTTGTTAGCCATGACTTCAGGCTTAT

AAACCAGGTAGCAGAAGAGATATGGGTATGTGAAAATCAAGCCGTTACACGATGGGAGGGTGATA

>FM893925.1 FM893925 Jatropha curcas embryo 56-70 (DAF) Jatropha curcas cDNA clone rjcaeb0_004865, mRNA sequence

ATTCATCAAATATCACTGATCGTAAACCTCCATTGAATTTACTCTGTGCTTCGATCGCGTCAATGGCGGA

GCACCCACAGTCGCAGCATGTTGGCCAACAGCCACGATCCCACCAAGTAGTGAAGGCAGCCACTGCAGTC

ACTGCCGGTGGATCTCTTCTAGTTCTCTCCGGTTTGACCCTTGCTGGTACGGTCATTGCACTGACCGTAG

CCACTCCATTGCTGGTCATATTCAGTCCGGTTCTTGTTCCTGCGGTTATCACAGTTTGTTTGATAATCAC

AGGGTTTTTAGCCTCAGGCGGTTTTGGCGTCGCTGCCATTTCTGTCTTGTCCTGGATTTATAGGTATGTT

ACTGGAAAGCATTCCACCAGGAGCAGAAAGTCTGGACCAGGCACGCTT

>FM893924.1 FM893924 Jatropha curcas embryo 56-70 (DAF) Jatropha curcas cDNA clone rjcaeb0_004864, mRNA sequence

ATAAAATATTTAAATAATTAATTTAGATTTAATATAAACATTACGAAAACAAAATATTGAATACAATTAT

ATTTATAGAATATATTGATTAGGGAATACTTAATTACTAATTAGGATGATTACATTACTTTCATCACTTA

TTTTCATAATATATTACATACTTCATTAGCGTGAGATCATAACTCCCCTCGTAATACTACTTACTTTCAC

TATCTACACACCCAAAAAGGCTACTTCATTATTTACTCCTCGGCATATCTTCCAGACTCGGTCCTGGAAA

TAGTCAAGGTGGTCTCTTGTCTTCCAAACTTAATCCTTCTTGCCTCCTCTGCTGATACCCTAAATGCATT

TGCAAGAGCCCGCGTTCTACAGAAATTCACTAAAAGATCGGAGCTTCTACGTGGAATTAAGAATTATCGT

GTAGGAATTCTTGAAGCTAATCCTCAAACATTTGTATCTCAGGGCCATTGGGATGCTGATGGAGTTCTCT

ATGTGGCTAGAGGACGAGGACCAATATCTATGATTATTGAGGAAAAGAGACAGAGCTTTAACGTCCAGCG

TGGAGATGTGCTAAGAATTCGTGCAAGCACTCCTCTCTATTTGATTACCAGAGAC

>FM893923.1 FM893923 Jatropha curcas embryo 56-70 (DAF) Jatropha curcas cDNA clone rjcaeb0_004863, mRNA sequence

CGAGGAGTGGTTCTTTCCAGGGCCAAGGAGAGGTTCCTTCGAGGGTCATGCTGTGGCATGAGAAGCTAAT

AACGTGATGGCAAGTGTTTCGGCTTTTTGGGGTGACTGTGACATCGACCTTGAGGGTTTACTAATGTAAA

TAGTGCTGTGTATGGTGAATAAATAATGAAAGGTGAAGGTACATGTGTAAATTAGTCTTTAAGAAAAGAG

AAAGATCACTGCTGCGGACTATAGTGGCTTCTGGTTTAATAATTAATAAAGAGATAAATTCTAGTTAAAA

AAAAAAAAAAAAAAACTGACGCTTAAACTCTCAAGTCTCTGCTTCAAAACTCAGCAGAAATGGCTCTACC

GAATCAGCAGACTGTAGATTATCCAAGCTTCAAACTTGTTATTGTCGGCGATGGTGGTACAGGGAAAACT

ACATTCGTGAAAAGGCATCTTACAGGCGAGTTCGAGAAGAAATATGAGCCAACCATTGGCGTGGAAGTTC

ATCCCTTGGACTTCTTTACCAACTGTGGGAAAATTAGATTCTACTGTTGGGACACAGCTGGACAAGAGAA

GTTTGGTGGCCTTAGGGATGGATACTATATTCATGGCCAATGTGCTATTATTATGTTTGATGTTACTGCT

CGGTTGACTTACAAGAATGTGCCCACATGGCATCGGGACCTTTGCCGGGTTTGTGAAAATATTCCTATCG

TCCTCTGT

>FM893922.1 FM893922 Jatropha curcas embryo 56-70 (DAF) Jatropha curcas cDNA clone rjcaeb0_004862, mRNA sequence

GAATTCATCAAATATCACTGATCGTAAACCTCCATTGAATTTACTCTGTGCTTCGATCGCGTCAATGGCG

GAGCACCCACAGTCGCAGCATGTTGGCCAACAGCCACGATCCCACCAAGTAGTGAAGGCAGCCACTGCAG

TCACTGCCGGTGGATCTCTTCTAGTTCTCTCCGGTTTGACCCTTGCTGGTACGGTCATTGCACTGACCGT

AGCCACTCCATTGCTGGTCATATTCAGTCCGGTTCTTGTTCCTGCGGTTATCACAGTTTGTTTGATAATC

ACAGGGTTTTTAGCCTCAGGCGGTTTTGGCGTCGCTGCCATTTCTGTCTTGTCCTGGATTTATAGGTATG

TTACTGGAAAGCATCCACCAGGAGCAGAAAGTCTGGACCAGGCACGCTTAAAGTTGGCGGGAAAGGCAAG

AGAGATGAAGGACAGAGCTGAGCAGTTTGGACAGCATGTAACAGGTCAACAGACTTAAAAGAGAAGTGGG

TTAAGGTGGTTGATGCAGAGAACATAAAATGGCTTTGATATATATATAAAAATATATGAGTCCTTTAATT

ACCATGGAAGGCCATTGCTTGTCTGGTGTA

>FM893921.1 FM893921 Jatropha curcas embryo 56-70 (DAF) Jatropha curcas cDNA clone rjcaeb0_004861, mRNA sequence

ATTATAGGATAGATGATCTTATTCTGAATAAAGAGGAAATATATGAGATATGATTCTCTCTCCTCTGATC

AGTACATATATGGAATATAACCGACTAACAATGCCTAGTGCAGAAGCAGCGGCGGCGCAGCCCTCGGCAG

TCTCCTCCGGTGAAGCCTTCCGTTTTGCAAACGTTAGCACAGTTAGTCTCACTTACGCATGTTCCCTTGA

ACTTATGGCTTTGAGACTCGCATGTCCTCGCCTCCGACATCTTTGACCC

>FM893920.1 FM893920 Jatropha curcas embryo 56-70 (DAF) Jatropha curcas cDNA clone rjcaeb0_004860, mRNA sequence

AATTCATTAGGTGCGACAAAGAAATGCGCACGATCGTGCCTGATGAACGAGAAGAAGAAGAGTTCACGTA

CAGCAGACCCGGCCAGAGCGGGAGGTGGATGAATGGAATGGAAGAAGCCATGTGCACAATGAAAACTATA

CATAACCTTGAGTCTCGTAGGGAAGCTGATTTTTACACAAANGGAAGCTGGTACAATCCACCTTGCAAAT

CAACGAAAGCTCCCCATTCTCAGTTTCATGGACATGAGCGCTGAGCGAGGCGTCCTGCTGCCGAGGGCAA

TGCACAGCCCACACTGGTCCATAAACGATCAC

>FM893919.1 FM893919 Jatropha curcas embryo 56-70 (DAF) Jatropha curcas cDNA clone rjcaeb0_004859, mRNA sequence

ATCAGAAACGTAGATAGATATCATTAATAACACAGACAGTACAATAGACCCAAAGACGCAAACACCAAAT

AGTAAAAATTACAACAAAAACATACAGAGAAAACAGTAGTAATTAAAGAGAAACGGAGACTCGCTAAAGT

GGAGACACCATTATCCTCTCTCTCCCTCTTATTCTTCTTACGCTACCTGATCTCCAGGCCGGACAATCGA

ACTCTGCTCCATGTATAACTCACCTGACATTGTTGCAGTTAGTGGAGAGGCTGATCTTGTAAGGAATGTT

GACGCTGCAAGCACCAGGAAGAGCCTCAGCGTTCGTAGGGATGATACCAGAGACGCTTGCTGCTGCTTGT

TCCAAGCACTGACATGCTTGTTGGCG

>FM894735.1 FM894735 Jatropha curcas embryo 71-95 (DAF) Jatropha curcas cDNA clone rjcpga0_000584, mRNA sequence

GGAAATCAGAATCAAACGAGCTTTNTACCCTTTTGTTCCACACGAGATTTCTGTTCTCGTTGAGCTCATC

TTAGGACACCTGCGTTATCTTTTAACAGATGTGCCGCCCCAGCCAAACTCCCCACCTGACAATGTCTTCC

GCCCGGATCGGCCGCCGAAACGACCTTGGGTCCAAAAAGAGGGGCATAGCCCCGCCTCCGATTCACGGAA

TAAGTAAAATAACGTTAAAAGTAGTGGTATTTCACCGTCGCCTTTCGGCTCCCACTTATCCTACACCTCT

CAAGTCATTTCACAAAGTCGGACTAGAGTCAAGCTCAACAGGGTCTTCTTTCCCCGCTGATTCCGCCAAG

CCCGTTCCCTTGGCTGTGGTTTCGCTGGATAGTAGACAGGGACAGTGGGAATCTCGTTAATCCATTCATG

CGCGTCACTAATTAGATGACGAGGCATTTGGC

>FM894734.1 FM894734 Jatropha curcas embryo 71-95 (DAF) Jatropha curcas cDNA clone rjcpga0_000583, mRNA sequence

TTTTTTTTTATGAAAATTCAAAATATGCTTCCTTGATCCAAGTTTTTTGAGAACACTTCCTAAATCCAAA

GCATATTGAATAATTACGAGTTCTTTAAGTAAAGCAAGTAAACACATGGTCTTAAAGATGACAAGGCACA

AATGGGCTACCCCCAGTTCCATTCGAGGCTGCACTTCTACGGTGACTCACTGCCTCTAGGGGAGCGGCGG

TTGTCCTCGTAATCATCATCAACAGGGCTCCTATCATCCCTGGGACTGGGGCTGCGGCTGCGGCCATTAG

TTTGAGCTGGACTTTCATACCTCCCATCTGCAGGGCTCTGTCTTTCAGGGCTTCTGCTCCTCCTCCTTGG

GCTACCACTATATGATCTATCTTGCTGATCATCCACTTTCCGAACTTTGGGAGAAGGGCGTGGGCTACCA

TTATCAGGCGTTGGGCTGCGCTTCCTGCTCTTGGAGGGGAGACCATTCTTTGGCTCATGGCTTGGGGACC

TGTCTTCAATATCAACACTTCGGTCTCTCT

>FM894733.1 FM894733 Jatropha curcas embryo 71-95 (DAF) Jatropha curcas cDNA clone rjcpga0_000581, mRNA sequence

ATGCAAGGATTCTATCCGTCGCCCGATGGGAATTGTACTTTAAGGCGGCCCTCGCGGCTCGTCCGCCGCG

GGGGCTTAGCCAACGGCACGTGCCTCTGGGGGCCGGATGGCCCCTACTGCGGGTCGGCAAACGGGCGACG

GACACGCGCGTCGCTTCTGGCCCG

>FM894732.1 FM894732 Jatropha curcas embryo 71-95 (DAF) Jatropha curcas cDNA clone rjcpga0_000579, mRNA sequence

GGCGCAGCTGGGCCATCCTGGACTTGAACCAGAGACCTCGCCCGTGAAGTAAATCATCGCACCTACGGTC

CAACTAATTGGGAGAGAATCAATAGATTCCTTTTCGGGAGCGATTCATCCTTCCCGAACGCAGCATACAA

TTCTTCGTTGTACTGCGCTCTCCAAGTGTGCTTGTTCTCCCCTTCTTCCTTACCATGGCAAGTCTTTTTG

AAATAACTCCGATGAGAAGAAAAAAGAAGGCGTTAAGAGACCCTCCTGGCCCCACCTTAGACACTCTAAG

ATCCTTTTTCAAACCTACTCCCATTTCGAGTCAAGAGATAGATAAATAGACACATCCCATTGCACTGACC

GGGGGCGTTCGTAGTGACTGAGGGGGTCGAAGACCAAGAAGTGAGTTATTTATCAGCCAAGCATTCTTCT

TACGGCTAGATCCAATCTCCTGGTCCCTGCGGAAAGGAAAAAGAATTTCACCTCCTTCCTTTCGCCTTTC

GGGAAGGGAGGGTTAGGAAAATCCTATTGATTGCAGCTTTCTCCAGACCTCCGGGAAAAGCATGAAAAAA

AAAGGCTCGAATGGTACGATCCCTCCGTCGCCCCAGAATGAAAGGGGCGATCTCGTAGTTCT

>FM894731.1 FM894731 Jatropha curcas embryo 71-95 (DAF) Jatropha curcas cDNA clone rjcpga0_000578, mRNA sequence

TGCCAGTAGTCATATGCTTGTCTCAAAGATTAAGCCATGCATGTGTAAGTATGAACTAATTCAGACTGTG

AAACTGCGAATGGCTCATTAAATCAGTTATAGTTTGTTTGATGGTACCTGCTACTCGGATAACCGTAGTA

ATTCTAGAGCTAATACGTGCACCAAACCCCGACTTCTGGAAGGGATGCATTTATTAGATAAAAGGTCGAC

GCGGGCTCTGCCCGTTGCTCTGATGATTCATGATAACTCGACGGATCGCACGGCCATCGTGCCGGCGACG

CATCATTCAAATTTCTGCCCTATCAACTTTCGATGGTAGGATAGAGGCCTACCATGGTGGTGACGGGTGA

CGGAGAATTAGGGTTCGATTCCGGAGAGGGAGCCTGAGAAACGGCTACCACATCCAATGAATGCAGCAGG

CGCGCAAATTACCCAATCCTGACACGGGGAGGTAGTGACAATAAATAACAATACCGGGCTCAATGAGTCT

GGTAATTGCAATGAGTACAATCTAAATCCCTTAACGATGATCCATTGGAGGGCAAGTCTGGTGCCAGCAG

CCGCGGTAATTCCAGCTCCAATAGCGTATATTTAGTTTGTTGCAGTTAAAAAGCTCGTA

>FM894730.1 FM894730 Jatropha curcas embryo 71-95 (DAF) Jatropha curcas cDNA clone rjcpga0_000577, mRNA sequence

ACACCAGATGAAGGAGCATTTGAATTCATTAAATTGTAGAAATATAAAAGATAAAACACTTCCAAGTCAA

CTGAACAAAACCGTCCAAACTCAACAACAAGCAGATATTCCTCATGTTTTTCTTATTTTGATTACCTTCC

ATGGATATGCTAAAAACCTCATAAAGCAAATATAACAAAAAGACAGGTCCTAAAACTATGCCTGAGACAA

AAGACAATCTATTATACTCCTCTGAAATTTAAAACGGAAACTTAAGACCTCTTTAGTATTTGTAGTCCTT

GACATGGAGACTCTCTGAGGCACCCTCACCAAGAGTAGCATTACCCTGGTAAGTTCCAAGAGTTGCCTCT

GAGTTGGCCTTAGCCCTGACCAGGAATGCAGCTTGAGCCTTCTGCACATTCTCTGGCTTACCTGCCCAAG

CCTTGAGGGTACTTTGTTGAAGTGCACGTCCAAATGAGAAGGAAAGGGACCATGGCTTCTTGGTCTTGAG

CTTGTTCATCGCATTCAGGTTGACGGTAGCTTCCTCCTCACTCTGCCCACCAGACAAGAACACAACAGCA

GGAACAGCGGCAGGCACAGTGCATTGGAGGGCACGGAC

>FM894729.1 FM894729 Jatropha curcas embryo 71-95 (DAF) Jatropha curcas cDNA clone rjcpga0_000575, mRNA sequence

ATTCCAAAGGATTCTATCCGTCGCCCGATGGGAATTGTACTTTAAGGCGGCCCTCGCGGCTCGTCCGCCG

CGGGGGCTTGGCCAACGGCACGTGCCTCTGGGGGCCGGATGGCCCCTACTGCGGGTCGGCAAACGGGCGA

CGGACACGCGCGTCGCTTCTGGCCCGGATTCTGACTTAGAGGCGTTCAGTCATAATCCAGCGCACGGTAG

CTTCGCGCCACTGGCTTTTCAACCAAGCGCGATGACCAATTGTGCGAATCAACGGTTCCTCTCGTACTAG

GTTGAATTACCATCGCGACACCATCATCAGTAGGGTAAAACTAACCTGTCTCACGACGGTCTAAACCCAG

CTCACGTTCCCTATTGGTGGGTGAACAATCCAACACTTGGTGAATTCTGCTTCACAATGATAGGAAGAGC

CGACATCGAAGGATCAAAAAGCAACGTCGCTATGAACGCTTGGCTGCCACAAGCCAGTTATCCCTGTGGT

AACTTTTCTGACACCTCTAGCTTCAAATTCCGAAGGTCTAAAGGATCGATAGGCCACGCTTTCACGGTTC

GTATTCGTACTGGAAATCAGAATCAAACGAGCTTTTACCCTTTTGTTCCACACGAGATTTCT

>FM894728.1 FM894728 Jatropha curcas embryo 71-95 (DAF) Jatropha curcas cDNA clone rjcpga0_000574, mRNA sequence

TTTTTTTTTTGAGACACAAATTTTAACATTTATTATATTATTTTAGGAAAAAATCGCTTACACCGAGTAC

AATGCCCCCACTATTCTATAACAAGTTTTACTTGTTTCATTGAACTACGGAAGATGAAAGAGAAGGTGAA

ATACAAAGAGACGACCACCAAAATTTTTAACATGAACTGAATCAAACATCATCTATCCAAAATAAATGTA

TTTACTAATTACCAGTGTCTCTGTTATAAAACTTAAACCAAATTGGGTCCAACTTAACCGAGTTACAGGT

TCCCCATCCTAACACCAGCGTCTCTGTTATATGAAGGCTGACCGTCATTTGCCCTGGAATGGCTTACATA

GTAAAGCTCAGACACTATGCCGGTAAGGACGACAAATGCAGCTCCAGCTCCGAAGACTCCCTTTCTCAGT

TGTCGGCATGACGGGGAATTTTCTGACAAGTAGTACTTGGTGTGGTAGGCATTTCTAACAGAACCCGCTA

ACAAGCAGACCTCAGCAATGAAAAAGAATACCCAGCAGGTGATGAATAGAACAATTGCCCATGTCCTAGA

CCCGCTAGGCCTCATGGCTTTCCCACAGCACAAGCATCGACTTGCAATCATTATTAGGATTTGACTAGCC

ATTAGAGATAATAAAGCACCAC

>FM894727.1 FM894727 Jatropha curcas embryo 71-95 (DAF) Jatropha curcas cDNA clone rjcpga0_000572, mRNA sequence

GGATTCTATACCGTCGCCCGATGGGAATTGTACTTTAAGGCGGCCCTCGCGGCTCGTCCGCCGCGGGGGC

TTGGCCAACGGCACGTGCCTCTGGGGGCCGGATGGCCCCTACTGCGGGTCGGCAAACGGGCGACGGACAC

GCGCGTCGCTTCTGGCCCGGATTCTGACTTAGAGGCGTTCAGTCATAATCCAGCGCACGGTAGCTTCGCG

CCACTGGCTTTTCAACCAAGCGCGATGACCAATTGTGCGAATCAACGGTTCCTCTCGTACTAGGTTGAAT

TACCATCGCGACACCATCATCAGTAGGGTAAAACTAACCTGTCTCACGACGGTCTAAACCCAGCTCACGT

TCCCTATTGGTGGGTGAACAATCCAACACTTGGTGAATTCTGCTTCACAATGATAGGAAGAGCCGACATC

GAAGGATCAAAAAGCAACGTCGCTATGAACGCTTGGCTGCCACAAGCCAGTTATCCCTGTGGTAACTTTT

CTGACACCTCTAGCTTCAAATTCCGAAGGTCTAAAGGATCGATAGGCCACGCTTTCACGGTTCGTATTCG

TACTGGAAATC

>FM894726.1 FM894726 Jatropha curcas embryo 71-95 (DAF) Jatropha curcas cDNA clone rjcpga0_000570, mRNA sequence

CCAAAACAACCATTTAGTAATCAACAATGGCAAAAGTTACTCTTCTTTCGCTACTTATCCCTATTCTCTA

CATTTTAGATACAGCAAGATCTGCAGCCACTGCAAGATCCACTGCCGCTTCCTCAAATTTCATTAAGGCC

TCCTGTAGAACCACTACCTACCCTGTATTATGTGTAGAATCTCTCTCAGCATACGCTCCTTCAATTCAGC

AGAACCCACGCCAATTGACCGAGGCTGCCTTGTCCGTGAGCGTAGCCAGTGCTCAGGCCACCAGGACATT

CGTGTACAAGCTCACAAAGTTCAAGGGAGTTAAGCCCAGAGAAAGGGAAGCCATTAACGACTGTTTAGAG

GAGATTGATGATACTGTTGATAGACTTAGCAAGTCTTTTAAAGAATTAAAGAGCGCTAAAGGTAAGGACT

TTCAATGGCACATGAGCAATGTTGAAACTTGGGTTAGTGCTGCTTTGACTGATGAGAACACTTGCGTTGA

TGGCTTTGCCGGTAAGGCCTTGGATGGGAAGCTGAAGGCTTCTATTAAGGCAAGGATTGTTACTGTTGCT

CAGGTCACTAGC

>FM894725.1 FM894725 Jatropha curcas embryo 71-95 (DAF) Jatropha curcas cDNA clone rjcpga0_000569, mRNA sequence

ATTCGTCTATGAGTCATCTGGAAGAGCATATGTTCTATCAGGTTTGAGCTCACACTCATGGCAGAGAGCA

ACTGCAAGAGGTGATTCCACTAATAGTACGAGCCTTCTACAAGCTTACCAAACTCCATCCATGGTTGATA

TGGTAACCAAGTCTCAGACAATGTTGTTAGGGAATCCATCATCTCGCTCATCTCATCGGAAACTCCGACA

AGCTCTATCATTTCCGGCTGCTAGACCGCAACCGAGGTAACTGGCAGCAATCTCGCCAAAGTTTCTCCTC

ACCCTACATTCATCTATCAACATCCAAGAACCTTTGTCTACTTAAATTTTGTTTTTAATTATTATTATTA

TTATTATTATTTTACTTTTGTTGTTTGGGAATTGGGTAAATTGGAAAGAGAGGGAGTAGGTTTTTTATAT

TATGGGTATATTGTCCTTATTTTGTAAATGTAAATGAACAAGAATTTGGGGGATAGCTAAGAGATGAGTT

GTTCAGAGGAAAATGGTGAAAGGTAAAGGCGGGGTTAGGATATTTAACAGTCTCATTATGGGGGTGATGT

AAA

>FM894724.1 FM894724 Jatropha curcas embryo 71-95 (DAF) Jatropha curcas cDNA clone rjcpga0_000568, mRNA sequence

GTGGATCCCCGGGCTGCAGGCGACAAGATAGCAAAGAATGTTGATATCCTTACAAAAGGAGACAACAATG

ATGTAGATGATAGGTCTTTATATGCTTATGGTCAGTATTGGTTGAAGCGGCAACATATCATGGGGAGAGC

TGTTGGATTCTTGCCTTATGTTGGTTGGGTGACAATTATCATGACTGAGAAGCCTTTAATCAAGTACATA

CTCATAAGCATTTTGGGTTTGCTAGTCATTACCTCAAAGGATTAGGTAAACAACGTTTTGAAGACTAATT

TAGTACTGGGAGCAAAGAAAATTAGATATGAGATCTCATCATGCGATTTTGTGCTTGTAGTTGATGAAAT

ATATTTGTTCCTTGAGAAGCTATTATGTGAATGATACT

>FM894723.1 FM894723 Jatropha curcas embryo 71-95 (DAF) Jatropha curcas cDNA clone rjcpga0_000567, mRNA sequence

ATTCAGAAAATATGACGACAAGTACCATTTTGCAAGTCAGTTTACAGCTGATCTTATTGCAATGAACAAT

GCAGATTTTATTATCACTAGTACGTACCAGGAGATTGCAGGAAGCAAGACTAATGTTGGACAGTATGAGA

GCCATACTGCTTTTACTCTTCCTGGGCTGTATCGAGTTGTTCATGGTATTGACGTTTTTGATCCCAAGTT

TAATATAGTCTCTCCGGGAGCAGATATGTGCATATATTTTCCATACTCCGAAAGGGAAAGAAGACTTACT

GCTTTACATGGTTCAATTGAAGAACTCTTATATGATCCTGAGCAGAATGAGGAGCATGTAGGTTTTTTAA

CAGACCAATCAAAGCCCATCATCTTTTCCATGGCAAGACTTGATAGGGTGAAGAACCTGACAGGACTTGT

TGAGTGCTATGGTAGAAGCAGCCAGCTAAGAGAACTTGTGAATCTTGTTGTGGTTGGTGGGTACATAAAT

GTGAAGCAATCCAGGGATAGAGAAGAAATGGCAGAGATTGAAAAGATGCATGCCCTCATCAAGCAGTATG

ATCTGCATGGCCAGTTTCGCTGGATAGCTGCCCAAATGAACCGTG

>FM894722.1 FM894722 Jatropha curcas embryo 71-95 (DAF) Jatropha curcas cDNA clone rjcpga0_000566, mRNA sequence

CTGCAAGGATTCTATCCGTCGCCCGATGGGAATTGTACTTTAAGTGCGGCCCTCGCGGCTCGTCCGCCGC

GGGGGCTTGGCCAACGGCACGTGCCTCTGGGGGCCGGATGGCCCCTACTGCGGGTCGGCAAACGGGCGAC

GGACACGCGCGTCGCTTCTGGCCCGGATTCTGACTTAGAGGCGTTCAGTCATAATCCAGCGCACGGTAGC

TTCGCGCCACTGGCTTTTCAACCAAGCGCGATGACCAATTGTGCGAATCAACGGTTCCTCTCGTACTAGG

TTGAATTACCATCGCGACACCATCATCAGTAGGGTAAAACTAACCTGTCTCACGACGGTCTAAACCCAGC

TCACGTTCCCTATTGGTGGGTGAACAATCCAACACTTGGTGAATTCTGCTTCACAATGATAGGAAGAGCC

GACATCGAAGGATCCAAAAGCAACGTCGCTATGAACGCTTGGCTGCCACAAGCCAGTTATCCCTGTGGTA

ACTTTTCTGACACCTCTAGCTTCAAATTCCGAAAGTCT

>FM894721.1 FM894721 Jatropha curcas embryo 71-95 (DAF) Jatropha curcas cDNA clone rjcpga0_000565, mRNA sequence

TCTCACTCCTTCCTCCTCTCTCTCTCGCCCTCGATTTTTTTTCACTTAAACGGCAAGCAAGTAAAAAACA

AAAAAAATCCAAGGATGTCGAGTACAACCGCTGCTACCGATCCAGAGCACAACAACAAAGACAGAGAAGA

GGAAGAGAACGCTTCTGCCGTCGACGATGAGGACACCGGAGCTCAGGTTGCTCCCATCGTCAAACTCGAA

GAGGTCGCTGTTTCCACTGGCGAGGAAGATGAGGATCCTATCCTTGATCTGAAAGCGAAGCTCTATCGAT

TTGATAAGGACGGAAACCAGTGGAAGGAGAGAGGAGCTGGCAATGTGAAGCTGTTGAAGCACAAGGAGTC

TGGCAAGGTTCGCCTTGTCATGCGCCAATCTAAGACTCTCAAGATCTGCGCTAATCACCTAGTTCTACCA

TCAATGAATGTCCAAGAGCATCATGGAAATGATAAATCATGTGTGTGGCATGCCACTGATTTTGCTGATG

GAGAATTGAAGGATGAGCTCTTCTGCATCAGATTTGCTTCAGTTGAGAATTGC

>FM894720.1 FM894720 Jatropha curcas embryo 71-95 (DAF) Jatropha curcas cDNA clone rjcpga0_000564, mRNA sequence

CCATCTGCTTCTTGGGTAAAGAAGATAGTCCAACCACTGTAACTGGAAGCATATCTGGCCTTAAGCCAGG

ACTTCATGGTTTCCATGTTCATGCCCTTGGAGACACAACAAATGGTTGCATGTC

>FM894719.1 FM894719 Jatropha curcas embryo 71-95 (DAF) Jatropha curcas cDNA clone rjcpga0_000563, mRNA sequence

GCAACAAGAAAATACACTATCATCAACATTCATGTGTCTTAAGCATTCTACAAGGGAAATACGCCATACA

AATATAGTTACATCTCTTCATGCTGCTTAATCATCACAGAGACAGACTGATGTCCTGACTTATTCAATAG

CCCGCCTTAGCATTCATGCAATCCCCAGCAGTTTCTTCACATCTTTCTCGATGCTAAGAGGAGAAGTAGT

GGGAGCATATCGGTTCCAACATTGCCATCTTTATCAGCCAAACTTGTGAAATCCACTTATCTATCCCCAA

AAATCCTCCTTGCTGACTTCAAGAACT

>FM894718.1 FM894718 Jatropha curcas embryo 71-95 (DAF) Jatropha curcas cDNA clone rjcpga0_000559, mRNA sequence

CCACACGAGATTTCTGTTCTCGTTGAGCTCATCTTAGGACACCTGTGTATCTTTAACAGAGTGTGCCGCC

CCAGCCAAACTCCCCACCTGACAATGTCTTCCGCCCGGATCGGCCGCCGAAAGACTTGGTCCAAGAGGGG

CTAGCCCCGCCTCCGATTACGGAATAGT

>FM894717.1 FM894717 Jatropha curcas embryo 71-95 (DAF) Jatropha curcas cDNA clone rjcpga0_000557, mRNA sequence

ATTCCGATAGAGCTATGCTCCGCTACAAAGACGAAGACCGAAATATCGGGTAATGGAGCTAGCATTGGTA

TCTAGAACTGCTAATCATGCAGCGACGACGTCGTCGATAAGCGGGAGAATTGGCGGTTCATCGAGAATCA

GTGAATTTCCGGCAAGAAAGAGAAGACTTAGCTTCAGTGATGGAGACAATAATAAAGTCCAAACACGGTG

CGCTAAAGCCTCTACGGAGAGGAGCAGTGAAGGGATAGAAGAAAGAAAAAGCACGCCCGGAGGAGGGTTC

AGTGGAAGTGCAATGGAAGTGACCA

>FM894716.1 FM894716 Jatropha curcas embryo 71-95 (DAF) Jatropha curcas cDNA clone rjcpga0_000554, mRNA sequence

ATTCCCCATGTAGCCAATCCTGCTGCTAATTTGAACATTGGAGTTATTAATCCAGCTGCCTCTTTATATC

AAACTATTGATCCTAGATTGGCTCAGCCAATGCTGGGAGTGGGATCCATTATCTACCCTCAACGACCTGG

ACAGACAGAATGCGATTTCTACATGAAGACTGGTGAATGCAAGTTTGGAGAAAGGTGCAAGTTCCATCAC

CCTATTAATCGGTTCTGCAC

>FM894715.1 FM894715 Jatropha curcas embryo 71-95 (DAF) Jatropha curcas cDNA clone rjcpga0_000553, mRNA sequence

GCAAAGGATTCTATCCGTCGCCCNGATGGGAATTGTACTTTAAGGCGGCCCTCGCGGCTCGTCCGCCGCG

GGGGCTTGGCCAACGGCACGTGCCTCTGGGGGCCGGATGGCCCCTACTGCGGGTCGG

>FM894714.1 FM894714 Jatropha curcas embryo 71-95 (DAF) Jatropha curcas cDNA clone rjcpga0_000552, mRNA sequence

CTACCTTCCTCTTCCTCCCTTTTCCTAGCCTCTTCTTCCTTCCTTTTCCTCTCCTCTTCTTCTCTCTTTC

TAGCCTCCTCTTCCTCCCTTTCTCTTTCTCTTTCCATTTCTTCCTCCATCTTCCTTTCCTCTTCTTCTGC

ACAAGAAATGCACTCCAATATGATTTCTTCCTCTTGGGGATTCAAGAGCTTATCAACTGTGGTATTGGGA

CATTGAATGATAAGCCAGATCTCTTAGTTCAAGTTTGGAAGAAGATCTCTTCCCTGCTAGACATTGAGGG

TCATATAACTTGTTGATGTGC

>FM894713.1 FM894713 Jatropha curcas embryo 71-95 (DAF) Jatropha curcas cDNA clone rjcpga0_000551, mRNA sequence

ATTCAAAGTTCTATCCGTCGCCCGATGGGAATTGTACTTTAAGGCGGCCTCGCGGCTCGTCCGCCGCGGG

GGCTTGGCCAACGGCACGTGCCTCTGGGGGCCGGATGGCCCCTACTGCGGGTCGGCAAA

>FM894712.1 FM894712 Jatropha curcas embryo 71-95 (DAF) Jatropha curcas cDNA clone rjcpga0_000549, mRNA sequence

GCTGCAGAATCGAATTCAATCTGTGTTCTTTGTTGTCTTATAAGCTAGGAATTACGCAGTGCAACATTGC

GCATGCATTGGAGAAAATAAAGTATCCGGATTCTGATATATACTGGAGAAAATATGACGACAAGTACCAT

TTTGCAAGTCAGTTTACAGCTGATCTTATTGCAATGAACAATGCAGATTTTATTATCACTAGTACGTACC

AGGAGATTGCAGGAAGCAAGACTAATGTTGGACAGTATGAGAGCCATACTGCTTTTACTCTTCCT

>FM894711.1 FM894711 Jatropha curcas embryo 71-95 (DAF) Jatropha curcas cDNA clone rjcpga0_000548, mRNA sequence

ATTTTGAGAATGGGGTGTACTACTACTCCTCCCAATCTGTTTTTGTGTCAAAGACTTCGCTGTCTCTCCA

TTTCTTTCCTTTTTCTCTTGATTTCAAGCTCTACCCATCTCAGATTCAAGGCAGAAGGTCGAGCTAATCC

AAAATCAGCTGGCGTTTCAGGGACAGAAAATGAAGAGAAGATGATAATGAGATCCCAGATAGGGTCAAGG

CCTCCAAGATGTGAGAGAAGGTGTAGTTCTTGTGGACATTGTGAGGCAATTCAAGTGCCTACAAATCCCC

AAGTAAATCATGGCAATAGAAACTACTCATCTGTCTCAAATATTGCTTATGCAAGAGGTGATGGTGCTTC

CAACTACAAGCCTATGAGTTGGAAATGCAAATGTGGCGATTTAATTTTCAACCCGTGACCATTTTTCCA

>FM894710.1 FM894710 Jatropha curcas embryo 71-95 (DAF) Jatropha curcas cDNA clone rjcpga0_000547, mRNA sequence

AGGATTCTATCCGTCGCCCGATGGGAATTGTACTTTAAGGCGGCCCTCGCGGCTCGTCCGCCGCGGGGGC

TTGGCCAACGGCACGTGCCTCTGGGGGCCGGATGGCCCCTACTGCGGGTCGGCAAACGGGCGACGGACAC

GCGCGTCGCTTCTGGCCCGGATTCTGACTTAGAGGCGTTCAGTCATAATCCAGCGCACGGTAGCTTCGCG

CCACTGGCTTTTCAACCAAGCGCGATGACCAATTGTGCGAATCAACAGTTCCTCTCGTACTAGGTTGAAT

TACCATCGCGACACCATCATCAGTAGGGTAAAACTAACCTGTCTCACGACGGTCTAATCCCAGCTCACGT

TCCCTATTGGTGGGTGAACAATCCAACACTTGGTGAAT

>FM894709.1 FM894709 Jatropha curcas embryo 71-95 (DAF) Jatropha curcas cDNA clone rjcpga0_000546, mRNA sequence

TGATTGAATTTGTTGCAAGGCTGCAAAATCTTGTGGATGCATTCGAAGAATTAAGTGAGAAAGCAAATTT

TAAAGAACCAGAGGAGCTGATAGCAGAAGCAGTGATAGAACCTACCGGGTTCTGGAAGAGATTGCTCAGA

GGTTTGAAGTTGCGGAACTAATTAAATTCTGAACATGATTAAAGATCTTGAAGCGCAGAAAACTCTGAAA

AGGATCAATTCCTTGATTAGAATGACATTTTACTGATACTTGTATATTCTAGGTTCTGAAATACAATAAT

TTTTTTGGCTTCGACTTGATGGAACATCACAATGACCAGTGCCAGAGTTTGATTCCACGGGGTGCTCAAG

GGATGTCGATATTTAGGATGATATTGCATGAGCGCTCAAGGTGTTCGGTTCAGATCATCTCCAGATAAAG

AAGGATAGGCAGTAATTTT

>FM894708.1 FM894708 Jatropha curcas embryo 71-95 (DAF) Jatropha curcas cDNA clone rjcpga0_000545, mRNA sequence

ATTCCTCTTTCTTTTCCTCCTCCTCCTCCATCCTCCTCCACCGCCACCATTATCCTTCCTTCGCCGCCTC

CTTACCATTTCTACTCTTCAATTTTCACCAACGCTGTTTCCTTCATCCTAGATCGTACCCATGTCCGCGA

CTGCTGCTGGATCTCCGTCCCACCAGGATTCTACTCTCCTCCCATCCAATCCGATACCGGCGCTTCCAGC

GCCACCGCAGCCCTCTACCAACCCGCGCCGTCTCCCTCCGCCGTGTTGGTCCCACGACGAGACCGTCGCC

CTGATCGATGCTTACCGCGACAAATGGTATACCCTCCGCCGTGGAAACCTCAAGGCTAACCATTGGCAAG

AAGTCGCGGATGCCGTGGCTCGTCGATGCCCGACGGCATCCCCGCCAAAGACCGCTGTGCAGTGCCGTCA

TAAGATGGAGAAACTCCGCAAACGCTACCGCACTGAGATCCAGCGGGCTCGATCCATGCCTGTTTCAAGA

TTCTCTTCTTCTTG

>FM894707.1 FM894707 Jatropha curcas embryo 71-95 (DAF) Jatropha curcas cDNA clone rjcpga0_000544, mRNA sequence

CTCAACTTTAAATAGGTAGGACGGCGCGGCTGCTTCGCTGAGCCGCGCCACGGAATCGAGAGCTCCAAGT

GGGCCATTTTTGGTAAGCAGAACTGGCGATGCGGGATGAACCGGAAGCCGGGTTACGGTGCCCAACTGCG

CGCTAACCTAGAACCCACAAAGGGTGTTGGTCGATTAAGACAGCAGGACGGTGGTCATGGAAGTCGAAAT

CCGCTAAGGAGTGTGTAACAACTCACCTGCCGAATCAACTAGCCCCGAAAATGGATGGCGCTTAAGCGCG

CGACCTATACCCGGCCGTCGGGGCCAGAGCCAGGCCCCGATGAGTAGGAGGGCGCGGCGGTCGCTGCAAA

ACCCGGGGCGCGAGCCCGGGCGGAGCGGCCGTCGGTGCAGATCTTGGTGGTAGTAGCAAATATTCAAATG

AGAACTTTGAAGGCCGAAGAGGGGAAAGGTTC

>FM894706.1 FM894706 Jatropha curcas embryo 71-95 (DAF) Jatropha curcas cDNA clone rjcpga0_000541, mRNA sequence

CTGAAAGGAAGATCCAACGCCTACGCCTCCCCATCTTTCTTATTTATTTTTCCTGAATTCTCTTTTTCTG

TAGCCCCTACCCTATAATGCGCGATGGTGTGTTTTACGCACCCCTCAATCCACCGCTTCTTCTCTCTCCC

TCTCCGACACCAACCTCCGTGAACAATTCCATTGCTGCCTCCAACCAAAACATGCTCGTCTCCGTTTTTT

TGGCCCTTTTCTTGCCTTGTATCGGCATGAGCGCAGTTTTTCTCGTCTATATTTGTCTCCTGTGGTACGC

TGCAAGTCACCAAGCCGAAATCCCACCGCCAGTAAAGGCGGCACCGGAGAAGGGATTATCGGCTTCTCAG

CTCGAGAAATTGCCTAAATTGACTGGGAAGGAGTTGATATTGGGGACGGAATGCGCGGTGTGTCTAG

>FM894705.1 FM894705 Jatropha curcas embryo 71-95 (DAF) Jatropha curcas cDNA clone rjcpga0_000540, mRNA sequence

ATCCCCCAAATTCATTTCACTCTTCTTATGTAAAATCAACCATGGCTAAGCTCACCATCCTCTTAGCCTC

CTTCATAGCCCTCCTCTTCCTCGTCGACGCCTCCATCTACCGAACCACCCTCATCACCGACGAAAACGAC

GATAACCATCTCCCGCCATACGACCGCCCAACAAGCTGTCAACAGCAAATCCAATCACAGCAAAGGTTAA

GAGCATGCCAGCAGTATCTCCGGCAACAAACTGGCTCCCCCTGGGGCCGTCGTTTCGACAACGAGGAAAA

TCAGATGGAGCGAGAAGACTGCTGCAGACAGCTTCAAAGAATGGAAGCTCGGTGCCGGTGCGACGGTCTC

CGACAGGCTATTGATCAGCTACAGAGGCGTGGAATCCTTGGGGGCCAGGATGTCCGTGAG

>FM894704.1 FM894704 Jatropha curcas embryo 71-95 (DAF) Jatropha curcas cDNA clone rjcpga0_000538, mRNA sequence

AACAAAAAATTGGAAGTGATTTTGATATGGAAAATTCATACACTTGTGACATGCAAGCTACATTGTTTTG

ATAACAACAATTATTCTAAATATCCCAATTTGTAACTAAAATATCAGTCTCTTTACTATCCATTATCTTG

AATAGCTGGAATATATTCTTAACTTGAGCGTAAGGTCTCTGCATCCATTCGTTCGAGGATTCTTCGTGCT

TCTTCCTCTGTTGCTGGAACCACATTGCGTAACCTAAATCCATTCTTCATGGCTTTTGCTTCTGGACGTA

CGGTGAAAGTGAAATAAAATTCATTTGACACCTCCCTTGATCTCAGCTCAGGCCTGGTAACTTGAGCAAC

GACTTCCATGTTAATCAAAGGCTGCTCAGGGTTTTCGAGTTCTGTGTAAAGAACACATGATTTCAAACGC

AGGAAAT

>FM894703.1 FM894703 Jatropha curcas embryo 71-95 (DAF) Jatropha curcas cDNA clone rjcpga0_000537, mRNA sequence

GTGATTATTACACTCTTAAACTCTCGCGACTCTCCCAAGTTAGAACCCCTAATCTGCAAAAACGTTAGCT

TTGATTCTTCACTCTCGAGAGAAAGAAACAGAGTGATTAATGGAGAGTAACTCAGATGGGAACGAGGATA

CGGCGGTCTCCACCATCAAAAGCCTACGTTCTCAGCTCGAATCTCGAATAGAAACTCAGCAAAGGACCCA

ACTTGACCTCTTAGCTTCTCTCCAATCTCTAGTACCTAATATCGTCACTTCTATTGACCTCTCTCTTCAA

ATTGTGTCTTCCTTCAATCACAAACCGTTTACCCCTACGCCCTCTCTCCCTAACCCAATAAAGAAAAC

>FM894702.1 FM894702 Jatropha curcas embryo 71-95 (DAF) Jatropha curcas cDNA clone rjcpga0_000536, mRNA sequence

GAAGTGCTGTCCTAGATGATTATTTCGGCAAACTTGGTGGGGGCAGTATGGCTTATGTAATGGGTGGGTC

TGACAGTGACAGCCCACAGAAATGGCATGTTTACTCTGCATATGCAGATTCTGCTATCTCCCGCGACCTT

GTTTACACTGTAGAGATGTGCATGACTGGTTTGGACCGGCAGAAGGCTGCTGTTTTCTTCAAGACTCGAT

CAGCTTCTGCAGCTGTCATGACTGTTGATTCTGGCATAAGAAAGATTCTTCCAGACTCGAAAATATGCGA

TTTTGATTTTGATCCATGTGGTTACTCAATGAATTCAATTGAAGGTTCTGCTATATCTACCATTCATATT

ACTCCAGAAGATGGTTTCAGCTATGCTAGCTTTGAAA

>FM894701.1 FM894701 Jatropha curcas embryo 71-95 (DAF) Jatropha curcas cDNA clone rjcpga0_000535, mRNA sequence

ATTCTCGATCTCCGACCACCACTCGACCAAAACGGAGTGGCGATCGGTTCGTCCGACGCCAGTGGTCTCA

ATCCCAGCCTCCCTTCACGTTGAACGGACCTGCAGCGGCGCGGATGGAGCTTTCTCCCACGCGACTGTCT

CCTCTTCCATCTTTGGCGATCCACACCATTTCCGGCGGCCCCAGCGGAGGAAGCGGGTATTAATCGACTC

TCTACCACGCTGTGAGTGAATTTCCTCTAACAATTTGTGGATTTATCGATGTTTTGAGGAGCAACCTTGC

GTGGAGGAGATGCAGCGGCTGTCGAGCCCGATCCGGCCAATCAAGGACCGACCTCACTAAATTAACGCCA

AAAACGGACTCCTTGGTGAATTTTGAGTTGAATGATGTGATCGTTGCAAAT

>FM894700.1 FM894700 Jatropha curcas embryo 71-95 (DAF) Jatropha curcas cDNA clone rjcpga0_000533, mRNA sequence

CTGCAAAGGATTCTATCCGTCGCCCGGTGGGAATTGTACTTTAAGGCGGCCCTCGCGGCTCGTCCGCCGC

GGGGGCTTAGCCAACGGCACGTGCCTCTGGGGGCCGGATGGCCCCTACTGCGGGTCGGCAAACGGGCGAC

GGACACGCGCGTCGCTT

>FM894699.1 FM894699 Jatropha curcas embryo 71-95 (DAF) Jatropha curcas cDNA clone rjcpga0_000532, mRNA sequence

ATAATACAGAAAGCCAAACACTGCCAGAAAAACAAAAATGTCAGATGAAGAACATCACTTCGATTCCAAA

GCTGATGCTGGAGCATCAAAGACTTTCCCTCAGCAAGCTGGTACTATCCGCAAAAATGGCTACATAGTTA

TCAAGAATCGTCCCTGCAAGGTTGTGGAGGTCTCTACCTCAAAGACAGGGAAACATGGACACGCCAAGTG

CCACTTTGTGGGAATTGATATATTCAATGGAAAAAAGCTTGAAGATATTGTTCCTTCATCCCACAATTGT

GATGTTCCTCATGTGAATCGTACTGATTATCAGCTGATTGATATCTCTGAAGATGGCTTTGTGAGTCTTT

TGACTGAAAATGGAAACACCAAAGATGATCTGAGGCTTCCCACCGATGAAAATCTGCTTAGCCAGATTAA

AGATGGGTTTGCTGAGGGGAAGGACCTTGTGGTGACTGTTATGTCTGCAATGGGAGAGGAGCAGATCTGT

GCTCTTAAGGATATAGGTCCTAAGAATTTAAAATGATGCTATAGCATTAGCCATAGCTGATGGTG

>FM894698.1 FM894698 Jatropha curcas embryo 71-95 (DAF) Jatropha curcas cDNA clone rjcpga0_000531, mRNA sequence

CAACGCCCTCGACCTATTCTCAAACTTTAAATAGGTAGGACGGCGCGGCTGCTTCGCTGAGCCGCGCCAC

GGAATCGAGAGCTCCAAGTGGGCCATTTTTGGTAAGCAGAACTGGCGATGCGGGATGAACCGGAAGCCGG

GTTACGGTGCCCAACTGCGCGCTAACCTAGAACCCACAAAGGGTGTTGGTCGATTAAGACAGCAGGACGG

T

>FM894697.1 FM894697 Jatropha curcas embryo 71-95 (DAF) Jatropha curcas cDNA clone rjcpga0_000530, mRNA sequence

ATTCTCGTCCAAAACCGCTTAGTGTTGAAGAAGAACAATTATGCGGGTGTACTATGAATATAAGCTTCGA

GAAGTGTGTAATGCCTTCTACTTTCCACATAAGATTCAGGCAACAGCACTCATATACTTTAAAAGGTTTT

ATCTTCAATGGTCGGTCATGGAGCATGATCCAAAAAATATAATGTTAACCTGTATATATTCAGCTTGCAA

GATAGAGGAAAATCATGTGTCGGCAGAGGAGCTTGGTAAGGGGATTTCACAGGATCATCATATGATTCTC

AATTATGAGATGATAATTCTTCAGAGCTTGGAATTTGATCTTATTGTCTATGCACCATACCGTTCAGTTG

AGGGTTTTATCAATGACATGGAGGAATTCTGCCATGCGACAGATGACCAAATTCAAATGCTGAAGGAG

>FM894696.1 FM894696 Jatropha curcas embryo 71-95 (DAF) Jatropha curcas cDNA clone rjcpga0_000529, mRNA sequence

ATACAAATAGACGAGTCTTCCATTCAAGGCGATGAAAGAAGAATCAGTACAGAATATGGATCCATCAGCA

AGAAATTTAAAGCAAAGGATTCTATCCGTCGCCCGATGGGAATTGTACTTTAAGGCGGCCCTCGCGGCTC

GTCCGCCGCGGGGGCTTAGCCAACGGCACGTGCCTCTGGGGGCCGGATGGCCCCTACTGCGGGTCGACAA

ACGGGCGACGGACACGCGCGTCGCTTCTGGCCCGGATTCTGACTTAGAGGCGTTCAGTCATAATCCAGCG

CACGGTAGCTTCGCGCCACTGGCTTTTCAACCAAGCGCGATGACCAATTGTGCGAATCAACGGTTCCTCT

CGTACTAGGTTGAATTACCATCGCGACACCATCATCAGTAGGGTAAAACTAACCTGTCTCACGACGGTCT

AACCCCAGCTCACGTTCCCTATTGGTGGGTGAACAATCCAACACTTGGTGAATTCTGCTTCACA

>FM894695.1 FM894695 Jatropha curcas embryo 71-95 (DAF) Jatropha curcas cDNA clone rjcpga0_000528, mRNA sequence

GGATCCCCGGGCTGCAGGAGAAGCTTTGCAAAGATGGATAAGGAGGTAGAGGAGTGGTGCAACGACGTAG

ATAAGACCGCTAACTGTCGTTGCGAGCTTCAGACTCCGCAGTGCGATGCCGTTGGATCAACGGCTGTTGT

TGCAG

>FM894694.1 FM894694 Jatropha curcas embryo 71-95 (DAF) Jatropha curcas cDNA clone rjcpga0_000527, mRNA sequence

TATCCGTCGCCCGATGGGAATTGTACTTTAAGTGCGGCCCTCGCGGCTCGTCCGCCGCGGGGGCTTGGCC

AACGGCACGTGCCTCTGGGGGCCGGATGGCCCCTACTGCGGGTCGGCAAACGGGCGACGGACACGCGCGT

CGCTTCTGGCCCGGATTCTGACTTAGAGGCGTTCAGTCATAATCCAGCGCACGGTAGCTTCGCGCCACTG

GCTTTTCAACCAAGCGCGATGACCAATTGTGCGAATCAACGGTTCCTCTCGTACTAGGTTGAATTACCAT

CGCGACACCATCATCAGTAGGGTAAAACTAACCTGTCTCACGACGGTCTAATCCCAGCTCACGTTCCCTA

TTGGTGGGTGAACAATCCAACACTTGGTGAATTCTGCTTCACAATGATAGGAAGAGCCGACATCGAAGGA

TCAAAAAGCAACGTCGCTATGAACGCTTGGCTGCCACAAGCCAGTTATCCCTGTGGTAACTTTTCTGACA

CCTCTAGCTTCAAATTCCGAAGGTCT

>FM894693.1 FM894693 Jatropha curcas embryo 71-95 (DAF) Jatropha curcas cDNA clone rjcpga0_000525, mRNA sequence

GGGAAGGAGATATTTTCAATCTATTTATTAATTTTCAGTATTGAAGGCTAATGAATGGAACTTTATGATA

ATAAGGGTGGGTGCTTGACATTGACTATCTTTACCAGTCATGTTATAAGCAGTAAAGGAGTTAGGAATTA

AAGTTAAAGGATTTAAAAATTTTCAAGAATTAAAAGATGACGAAAATTGAAAAATTTATTATTTTAAAGA

GTAAAATGAAAAATTTTAAAATTTTATGAGATAATTCTTAAAAAAAAAATTTAATTAGGGGTTGGTGCGG

AGGCCGCCACCCCTAGATCTCCTAGCTCCATTAGTGGTTGTAATGTAAATAAAAATTAATGTAAGTTAAA

GTGATTAATTTTTTACTTTTTATAAAAATTATAATCAATTTAGTAATTAATTAAATATTATGCATATAAA

GTGGCAGCCGTGCTCCCCCTAACCCAAAATT

>FM894692.1 FM894692 Jatropha curcas embryo 71-95 (DAF) Jatropha curcas cDNA clone rjcpga0_000524, mRNA sequence

CACACGAGATTTCTGTTCTCGTTGAGCTCATCTTAGGACACCTGCGTTATCTTTTAACAGATGTGCCGCC

CCAGCCAAACTCCCCACCTGACAATGTCTTCCGCCCGGATCGGCCGCCGAAACGACCTTGGGTCCAAAAA

GAGGGGCATAGCCCCGCCTCCGATTCACGGAATAAGTAAAATAACGTTAAAAGTAGTGGTATTTCACCGT

CGCCTTTCGGCTCCCACTTATCCTACACCTCTCAAGTCATTTCACAAAGTCGGACTAGAGTCAAGCTCAA

CAGGGTCTTCTTTCCCCGCTGATTCCGCCAAGCCCGTTCCCTTGGCTGTGGTTTCGCTGGATAGTAGACA

GGGACAGTGGGAATCTCGTTAATCCATTCATGCGCGTCACTAATTAGATGACGAGGCATTTGGCTACCTT

AAGAGAGTCATAGTTACTCCCGCCGTTTACCCGCGCTTGGTTGAATTTCTTCACTTTGACATTCAGAGCA

CTGGGCAGAAATCACATTGCGTGAGCATCCGCAGGGACCATCGCAATGCTTTGTTTTAATTAAACAGTCG

GATTCCCCTTGTCCGTACCAGTTCTGAGTCG

>FM894691.1 FM894691 Jatropha curcas embryo 71-95 (DAF) Jatropha curcas cDNA clone rjcpga0_000523, mRNA sequence

CTGATTTTAAAACTGGCTCATATTTGCTACTTTTTCCTCAATCAGCTTTAAGGTGACAAAAGTGAAAGAA

AAGCTGGTCCAACAGATTTCCTTCCGACTTTTTGAACAAAAGTCTCCTATTAGCAATAACAATGCCAACT

AGTAGTGTCTTTATCTCCATATAAACATGTTGGTTAAGTTGTCTGAAAACTGAAAATTGTTATCTTGTTT

CTATATAGATTAAAGATGGGTTTGCTGAGGGGAAGGACCTTGTGGTGACTGTTATGTCTGCAATGGGAGA

GGAGCAGATCTGTGCTCTTAAGGATATTGGTCCTAAGAATTAAAAATGATGCTATAAGCATTAGCCATAG

CTGATGGTGAAACAATCTATTTAAGTTTGCTGATAGAGAATTTTATCTACTATGAATATGTACAACAAAC

ATTTATTAAGATGGTATTTAAGCTTTGTAATCCAGACTCATAAAGCTTGT

>FM894690.1 FM894690 Jatropha curcas embryo 71-95 (DAF) Jatropha curcas cDNA clone rjcpga0_000521, mRNA sequence

TGAAAATGGGCCCACCAAAAATGCTAGTACATATAAAAGTAAAGTTGATGTTTCTGAAGTGAAGAATGGA

ACTTTGATGAAAGACATTCCGCTTGATCAGGTTTCAGATTGTTCATTGTATAGGGGAAACAAGATGGAGA

AGACCGAGAATGATAATCAGATGCTCAAGCTATGGGAATCTGCTGAGCAAGACTATAGCCTTAATCCAAT

GTCCAGTGCTATACAAAAGCAGGCAGCTTCACAGTTGGAGAATGTTAATGCCCCTCATCAGTTCAAAGAT

GCCAACCATAAGAGTCGAAATCCTCCTTTGGAGTTACAAGAAGAGAGGGAAGTGGGCATTGACAGGCTAG

AGGTGTCTACAAGCATGAATAAAGAGCCAAATCAAGGAGGGAACAGAGGAAAGATCCTGGAGAGACTTGC

TTCTGATGCACAGAAATTAGTATCTCTTCAAACAGCTGTGGCAGATTTGAAGGAAGAAAATGG

>FM894689.1 FM894689 Jatropha curcas embryo 71-95 (DAF) Jatropha curcas cDNA clone rjcpga0_000520, mRNA sequence

CGAATTCCTAAAACATGGAACACCGATCACCAAATGCTTCGAGAGGGATTAGAGTTCAAGCTCCACTAGT

TGATTCTGTATCTTGTTATTGCAAAGTAGATTCTGGATTAAAAACAGTTGCTGAAGCCAGGAAATTTGTC

CCTGGATCAAAACTTTGTATCCAGCCTGACATCAATCCTAATGCACATAAGAGTAAAAATTTGCGGAGGG

AGAGAACCCGAGTGCAGGCCCCACTACTGCCTGGACTTCCTGATGATCTTGCTATTGCCTGTCTGATTCG

AGTTCCCCGAGCTGAACATCGAAAACTCCGTCTAGTTTGTAAGAGATGGTACCATCTTCTGGCTGGAAAT

TTTTTCTATTCTCTTAGGAAAAGTCTTGGAATGGCAGAGGAGTGGGTTTATGTCATCAAGCGAGATCGGG

ATGGGAAAATCTCATGGAATGCTTTTGACCCTGTCTATCAGCTCTGGCAGCCACTTCCACCAGTTCCCAT

GGAATATTCCGAGGCTCTTGGTTTTGGCTGTGCTGTT

>FM894688.1 FM894688 Jatropha curcas embryo 71-95 (DAF) Jatropha curcas cDNA clone rjcpga0_000519, mRNA sequence

ATTGACATGTGTGCGAGTCAACGGGTGAGTAAACCCGCAAGGCGCAAGGAAGCTGACTGGCGGGATCCCC

CCGAGGGTTGCACCGCCGACCGACCTTGATCTTCTGAGAAGGGTTCGAGTGAGAGCATGCCTGTCGGGAC

CCGAAAGATGGTGAACTATGCCTGAGCGGGGCGAAGCCAGAGGAAACTCTGGTGGAGGCCCGCAGCGATA

CTGACGTGCAAATCGTTCGTCTGACTTGGGTATAGGGGCGAAAGACTAATCGAACCGTCTAGTAGCTGGT

TCCCTCCGAAGTTTCCCTCAGGATAGCTGGAGCTCGGGACGAGTTCTATCGGGTAAAGCCAATGATTAGA

GGCATCGGGGTCGCAACGCCCTCGACCTATTCTCAAACTTTAAATAGGTAGGACGGCGCGGCTGCTTCGC

TGAGCCGCGCCACGGAATCGAGAGCTCCAAGTGGGCCATTTTTGGTAAGCAGAACTGGCGATGCGGGATG

AACCGGAAGCCGGGTTACGGTGCCC

>FM894687.1 FM894687 Jatropha curcas embryo 71-95 (DAF) Jatropha curcas cDNA clone rjcpga0_000518, mRNA sequence

CGAATTCCACCGTGTCTTTGGTTCTCCTCCATTTTGTTTCAGCGTCGCGACTAAGAGAGGGAGTTGCGAT

TTCGATCGACAGCTCGCTCCTCTCTCCTCGTCTTTCCGGCGAATCCAGCACGATCGATCGACTCCAGCTT

CCTTCTCGTTGACGGCGAAGCTCCGATCTCTCTCTCCCTTCGTTTCCGGCTGGCACAACAAATGTAAGGA

GCTGAGGTTGCGTTCCGATCGAGACAGTAGCGCAACCAGCCCTGTTCCTTCGTTTACCTGTTCCTTCGTT

TTTTCAGCGACGCTTGTCCTTAGCCGGCGGTTATGATCTGACCTTCTCATTTGCAGCTTCTTCAGCCATT

GTTGCCTTCACCTTCGATCGAGCTAGCGTCGACAGAAATGGTTTTGCGCAAGCTGTTTCCGGTCACTCTA

ACAGAAAAATTTCAGTCTTCAGTGCGTTTTTCAGCTGGAAGCAGATATTTCTTTGTCTTAGTTAAAGCTC

TGTTTCCTTCACCGTAAAGCTAGAAAAGTAATTTCTTGATTCCAGTAATTATTTACATTATGAGCCTTAT

GT

>FM894686.1 FM894686 Jatropha curcas embryo 71-95 (DAF) Jatropha curcas cDNA clone rjcpga0_000517, mRNA sequence

AAAAACAGTATATAAAAAATAAATATTCATTTATTCTATTTACAGAAATGTCCTCCTGCTATAACCTGTC

ACCAAAAGCATAGCATAGCAATGTTATCCCATCATATCAACTTGCTCATCACTTTCCCTATAAAAATTAC

TACGGCAAATAGTAAAAAAAAAAAAAAAATTCAAGTAAAAAGAATAGTATAAATAATACAAAGTAATTGC

TTTTAATTTGGCTTTATGCATTCTTCTGTATGGCATGGGGAGCATTTTTTTTTTTTCCTTCTAACCCCGA

AATCTCTCATAGGACTTCAATACCTGCTATGTCCATTCATTTCCTTGCTGCATCAGATGATGTGTTATCC

AAGGAGGTGAACATGAAATTCCCAGAAAATAAGGCTTCTTTGTCTGTTAAAGAAAGACAATTGACTTCGC

GGCGTACATATCTTGCCATTGCCTGGACCGCCGGTAATGCAGTAGCATCTGGCTCTGATTTACCATCATC

AGAGAATACATTTCTCCATACCACCTTCACGAGCTCATTGGATTTAACCTCTGGAAGTAGAACAGAATCA

TAAGCAACAATATTTCCATA

>FM894685.1 FM894685 Jatropha curcas embryo 71-95 (DAF) Jatropha curcas cDNA clone rjcpga0_000516, mRNA sequence

CATCGAATCCAAAAGACTGTTGATGGGCCATCAAGCAAGGATTGGAGAGGTGGAAGAGCTGCTTCCTTCA

ACATCATTCCTAGCAGCACAGGAGCTGCTAAGGCTGTTGGGAAGGTGCTACCTGCTCTGAACGGCAAGCT

TACTGGAATGGCTTTCCGTGTTCCAACTGTTGATGTCTCTGTGGTTCATCTCACTGTTAGGCTAGAGAAG

GCTGCTACATATGAACAAATTAAAGCTGCTATCAAAGAGGAGTCTGAGGGAAAGCTGAAGGGAATATTGG

GCTATGTCGAGGAAGATCTGGTTTCCTCTGACTTCATTGGTGACAACAGGTCAAGTATTTTTGATGCCAA

AGCTGGAATTGCTTTGAATGATAAGTATGTGAAGCTTGTTTCTTGGTACGACAATGAGGTTGGTTACAGC

ACCCGGGTGGTTGACTTGATCTGCCATATGGCATCTCATCATTAAGGCTGCAGCAATATGTTATGAGGCT

TCCTCCAACATGTCTTGGTGGTGTTTATGT

>FM894684.1 FM894684 Jatropha curcas embryo 71-95 (DAF) Jatropha curcas cDNA clone rjcpga0_000515, mRNA sequence

AATATAATTTCCATAAGTTTTATAAACGCAAACTGACACTAGATAAAAATTCTCATACAATAACTATTAA

CATTAATGTTCTCAGGGGGAAAAAACCCTTCAAATTTTGAAGCAAGAAAAATAAAATCAAATGAAAGAAA

AGAAAAGAAAAGAAAAAAGCAGCACAATCACTCTCCAGCTACAAGCATCCTACTAAAAGGAGACATTGGT

TGAACCACAAAAGATTGCGAGGAAAGTAATGACTTGAGCACCAAAGTGTCTGCCCACCACATCCAGAGGG

ACCATTACAGTTGAGCCTCTTAATTTCGGCTTTAACATACATGAGTAGCAATAGATGGAAAGCTATTTGA

TACATCAAGATCAGATATACCCTCCTTGAATGACCCCATCCGACACTTTTTCACATCGACCATACTTGAG

TTCATGACACCTTGACACGAAAAAAGCCAACTAAATGCAAAAAAGAACTCGCCATCAAAGCACCTGTAGA

ACTTAGAAGCACTTCCTGTGGACAATGGATGGACCAGACTCATCATACTCGCCCTTGGAAATCCACATCT

GTTGGAAGGTGCTA

>FM894683.1 FM894683 Jatropha curcas embryo 71-95 (DAF) Jatropha curcas cDNA clone rjcpga0_000514, mRNA sequence

AGGATTCTATCCGTCGCCCGATGGGAATTGTACTTTAAGGCGGCCCTCGCGGCTCGTCCGCCGCGGGGGC

TTGGCCAACGGCACGTGCCTCTGGGGGCCGGATGGCCCCTACTGCGGGTCGGCAAACGGGCGACGGACAC

GCGCGTCGCTTCTGGCCCGGATTCTGACTTAGAGGCGTTCAGTCATAATCCAGCGCACGGTAGCTTCGCG

CCACTGGCTTTTCAACCAAGCGCGATGACCAATTGTGCGAATCAACGGTTCCTCTCGTACTAGGTTGAAT

TACCATCGCGACACCATCATCAGTAGGGTAAAACTAACCTGTCTCACGACGGTCTAAACCCAGCTCACGT

TCCCTATTGGTGGGTGAACAATCCAACACTTGGTGAATTCTGCTTCACAATGATAGGAAGAGCCGACATC

>FM894682.1 FM894682 Jatropha curcas embryo 71-95 (DAF) Jatropha curcas cDNA clone rjcpga0_000513, mRNA sequence

ATTCCAAAGGATTCTATCCGTCGCCCGATGGGAATTGTACTTTAAGGCGGCCCTCGCGGCTCGTCCGCCG

CGGGGGCTTGGCCAACGGCACGTGCCTCTGGGGGCCGGATGGCCCCTACTGCGGGTCGGCAAACGGGCGA

CGGACACGCGCGTCGCTTCTGGCCCGGATTCTGACTTAGAGGCGTTCAGTCATAATCCAGCGCACGGTAG

CTTCGCGCCACTGGCTTTTCAACCAAGCGCGATGACCAATTGTGCGAATCAACGGTTCCTCTCGTACTAG

GTTGAATTACCATCGCGACACCATCATCAGTAGGGTAAAACTAACCTGTCTCACGACGGTCTAAACCCAG

CTCACGTTCCCTATTGGTGGGTGAACAATCCAACACTTGGTGAATTCTGCTTCACAATGATAGGAAGAGC

CGACATCGAAGGATCAAAAAGCAACGTCGCTATGAACGCTTGGCTGCCACAAGCCAGTTATCCCTGTGGT

AACTTTTCTGACACCTCTAGCTTCAAATTCCGAAGGTCTAAAGGATCGATAGGCCACGCTTTCACGGTTC

GTATTCGTACTGGAAATCAGAATCAAACGAGCTTTTACCCTTTTGTTCCACACGAGATATCTGTTCTCGT

TGAGCTCATC

>FM894681.1 FM894681 Jatropha curcas embryo 71-95 (DAF) Jatropha curcas cDNA clone rjcpga0_000512, mRNA sequence

ATTGGCCATTTTTGGTAAGCAGAACTGGCGATGCGGGATGAACCGGAAGCCGGGTTACGGTGCCCAACTG

CGCGCTAACCTAGAACCCACAAAGGGTGTTGGTCGATTAAGACAGCAGGACGGTGGTCATGGAAGTCGAA

ATCCGCTAAGGAGTGTGTAACAACTCACCTGCCGAATCAACTAGCCCCGAAAATGGATGGCGCTTAAGCG

CGCGACCTATACCCGGCCGTCGGGGCAAGAGCCAGGCCCCGATGAGTAGGAGGGCGCGGCGGTCGCTGCA

AAACCCGGGGCGCGAGCCCGGGCGGAGCGGCCGTCGGTGCAGATCTTGGTGGTAGTAGCAAATATTCAAA

TGAGAACTTTGAAGGCCGAA

>FM894680.1 FM894680 Jatropha curcas embryo 71-95 (DAF) Jatropha curcas cDNA clone rjcpga0_000509, mRNA sequence

AGCCTAAGAACCACAAAAGCAATCTCATTTCAAGAGCCTGAAATTCAGATGCATCACTTTGTTCCAAACT

CATATATGATCAAAAAATAGATAAACTGTCGACACGACACTCAGGTAAAAATAACACCATCATCGTTAGC

AGCTGTCAAGACATCTTCTAGACACATATTATAGGAATGGGCAAATTTATTAAAATAGCTTAGTTCAGAA

CAAGAATAACAAAGTCAAGTATTTCCATCTCAACTACAGGCTAACATCTTCCACCTTTCAATTATGTTTG

TACTACATTTATGCCATGCCTCCGCTTGTAGCAGTCCTGATTATCTGGAATAGAGATGATCCAATGACCA

CAAATATGAAAAATCCAAGCAGAACAGGGCCAACAGGATAGTCCTTTGCCTTTTTAGTAGTGGTTTCAGG

TACAGCTCCTCTCTTGGTAATGTTTTTCTCAAACTTCTCAACTTTCCTATCTGCAAGGCGCCTTGATGTA

GTCATGGTTACGGGGAGATCTGATAGCCGGGAACGATTGGAGCGATGAAGCTTACAGAGAAACTACAGGT

GATTTCGTCTCCTCCCTTCTCCGGCGAGCTCTTTCTCTCTCCCCTTTCCCTCTC

>FM894679.1 FM894679 Jatropha curcas embryo 71-95 (DAF) Jatropha curcas cDNA clone rjcpga0_000496, mRNA sequence

TTTTTTTTTATGATAAAAATAAAATGAGCATAATTCAAGGACAATATCTCTTCACCTCTTTAAAAAATAA

ACATCCACAGTAGATTAAAATCACAAGTGACTTCAAACATTACCTCATTGAACAAAATTCATACTAACTC

ATTATCTTTCTACAAAATTCAAAAATCCAGGATAAAACCACATGCAAAAACTACAAAATGATGTCACAAC

TAGATTCCATCCAAGTAAAAGTAGTGAACATAACCAATCTATTAAAGCACCACCACCAAACTGGATATGC

AGTTGACAGGAAACACTCTACAGTCTTCTTCCCCTTCTACCACTCTTTCTGCGAGTGCTGTCAGTGGGAA

TTGGTGTCACGTCCTCAATTCGACCAATTTTCATTCCAGAACGAGCAAGTGCCCTAAGAGCAGACTGAGC

ACCTGGACCAGGTGTCTTAGTTTTATTCCCTCCAGTAGCACGAAGCTTTATATGAAGAGCAGTAATGCCA

AGCTCCTTGCATCTTGCTGCAACATCTTGTGCTGCAAGCATAGCAGCATATGGTGAAGATTCAT

>FM894678.1 FM894678 Jatropha curcas embryo 71-95 (DAF) Jatropha curcas cDNA clone rjcpga0_000495, mRNA sequence

ATTCGTCTGACATGTGTGCGAGTCAACGGGTGAGTAAACCCGCAAGGCGCAAGGAAGCTGACTGGCGGGA

TCCCCCTGAGGGTTGCACCGCCGACCGACCTTGATCTTCTGAGAAGGGTTCGAGTGAGAGCATGCCTGTC

GGGACCCGAAAGATGGTGAACTATGCCTGAGCGGGGCGAAGCCAGAGGAAACTCTGGTGGAGGCCCGCAG

CGATACTGACGTGCAAATCGTTCGTCTGACTTGGGTATAGGGGCGAAAGACTAATCGAACCGTCTAGTAG

CTGGTTCCCTCCGAAGTTTCCCTCAGGATAGCTGGAGCTCGGGACGAGTTCTATCGGGTAAAGCCAATGA

TTAGAGGCATCGGGGGCGCAACGCCCTCGACCTATTCTCAAACTTTAAATAGGTAGGACGGCGCGGCTGC

TTCGCTGAGCCGCGCCACGGAATCGAGAGCTCCAAGTGGGCCATTTTTGGTAAGCAGAACTGGCGATGCG

GG

>FM894677.1 FM894677 Jatropha curcas embryo 71-95 (DAF) Jatropha curcas cDNA clone rjcpga0_000494, mRNA sequence

ATTCCAAAATCTCCGATTTGACAAGGTCATCAGTTGAAGCAGCGGCAAAAACCAAATCCCCTCTCTTTGC

TTTGCATTCTGCGCAGTTGAGGAGAAAGAGGAATCAATGGGGTCTGCAATTCGTGAGTGGGTCGGGTTCC

AGCAGTTCCCTGCTGCTACGCAAAGCAAATTGGTTGAATTGTTTGGCCAATTAAAGGAAGAGGGTGTTAG

TACTTTGACCATTCTTGTTATGGGCCAAGGCGGAGTTGGAAAATCTTCCACTGTCAATTCTCTCATTGGG

GAACGAGTGGTTAACGTGAATTCCTTCTCGGCAGAGATATCAAGACCTGTAATGGTTTCACGAACTCGGG

CGGGATTCACATTAAACATTATAGACACTCC

>FM894676.1 FM894676 Jatropha curcas embryo 71-95 (DAF) Jatropha curcas cDNA clone rjcpga0_000493, mRNA sequence

GTACTAAACAATGACTAAATAAAAACAGATCCAGAAATGGTACATGTAAAAATCAAAACGCATCCCCTTG

TCAATATGCATCAGAATCTGTTTAAGGAAGATTTACGAAAAGGTCACTCTGATCAGATGCAAAGTTGCAA

ACTCAAATGGCAATTTTGCTCATATAAAAGGAAACTGCCAAATTGTTCATCAATCAGATGGCAGCTGTTT

GTACTCTTCCAGAAGCTTCGGGATGTCATCCATTCGTAGCTGAGAAGCATTTTTCATGTGATAAAACTGA

TAAGGCATATTTCCCAGGAAAACCAGATTCCCTTGAATGCAAATCAGATCCGCCTTTTGCTAAATGAT

>FM894675.1 FM894675 Jatropha curcas embryo 71-95 (DAF) Jatropha curcas cDNA clone rjcpga0_000492, mRNA sequence

GACGATTGCGTGATTTTTGGCTCCCATGTTCGACTGAACCCGTTTTCCCTTAGCTGATGCAAACATGGAT

TCTACTTTAAACGCCTTAACTGCTGCCGGTTTTGGTGCTGCTGGACAACGTTGCATGGCACTGAGTACAG

TAGTTTTTGTTGGGGACTCAAAATCATGGGAGAGCAAACTTGTGGAACGTGCAAAAGCTCTTAAAGTAAA

TGCTGGAACAGAGCCTGATGCGGACCTGGGTCCAGTCATTAGTAAACAGGCCAAGGAACGAATACATAGA

CTAATTCAAAGTGGTGTTGAAAATGGTGCCAGACTACTGCTTGATGGAAGAAATATAGTGGTTCCGGGAT

ATGAGCAANGTAATTTTATTGGTCCAACCATCTTATCAGGAGTCACAGCTGATATGGAGTGCTACAAGGA

GGAAATCTTTGGGCCAGTTCTCCTATGCATGGAGGCTGACAGCTTTGAAGAAGCCATCAACATTGTTAAC

AGAAACAAATATGGCAATGG

>FM894674.1 FM894674 Jatropha curcas embryo 71-95 (DAF) Jatropha curcas cDNA clone rjcpga0_000491, mRNA sequence

AGGATTCTATCCGTCGCCCGGTGGGAATTGTACTTTAAGGCGGCCCTCGCGGCTCGTCCGCCGCGGGGGC

TTAGCCAACGGCACGTGCCTCTGGGGGCCGGATGGCCCCTACTGCGGGTCGGCAAACGGGCGACGGACAC

GCGCGTCGCTTCTGGCCCGGATTCTGACTTAGAGGCGTTCAGTCATAATCCAGCGCACGGTAGCTTCGCG

CCACTGGCTTTTCAACCAAGCGCGATGACCAATTGTGCGAATCAACGGTTCCTCTCGTACTAGGTTGAAT

TACCATCGCGACACCATCATCAGTAGGGTAAAACTAACCTGTCTCACGACGGTCTAAACCCAGCTCACGT

TCCCTATTGGTGGGTGAACAATCCAACACTTGGTGAATTCTGCTTCACAATGATAGGAAGAGCCGACATC

GAAGGATCAGAAAGCAACGTCGCTATGAACGCTTGGCTGCCACAAGCCAGTTAT

>FM894673.1 FM894673 Jatropha curcas embryo 71-95 (DAF) Jatropha curcas cDNA clone rjcpga0_000490, mRNA sequence

ATTAATGGCTTCAATGCAGGAACCATGCCTTCTTGAAAATGGCAACGTTAAATTATTAAGCAAGGAGATG

CGACACGGAAGAACTGCTCATAACATGTCTTCCTCTTCTCTGCGCAAAAAATCTGATCTGACCCTTGTTT

CGAAACTCCGCTGTGGGTTGCTTAGAAATTTGTTAACAAATCTTCAGGAAGTCATTTTAGGGACTAAGCT

TTCTGTTCTTTTCCCCGCCATTGCACTCGCCATTGCTGCTAAGTGCTATGGCTTCGGAAGACCGTGGGTT

TTTGCTCTCAGTTTGCTTGGACTAACCCCGCTAGCCGAACGAGTCAGCTTTCTCACTGAGCAAATTGCTT

ATTATACCGGTCCAACAGTGGGAGGACTCCTAAATGCAACTTGTGGCAATGCAACTGAACTGATAATAGC

TATTTTTGCACTAAGCCAACAAAAGATAGAGGTGGTAAAGTATTCCCTCTTGGGTTCCGTACTGTCA

>FM894672.1 FM894672 Jatropha curcas embryo 71-95 (DAF) Jatropha curcas cDNA clone rjcpga0_000489, mRNA sequence

ACTAGTGGATCCCCGGGCTTGCATGCGCCATCAATGCTTTGTCGGACCTCGTTTTCCTTCTTCTCCATCC

ACACTGTCTAGCCTTTCTTCTTCTCTTCTTCGAATCAGATTTTGGTTTGGAGGGTCTGGGAATTAAGGTT

TTGGCAATATGACGATGAATGAGCGGAAGACGATTGACCTAGAGCAAGGATGGGAGTTTATGCCAACAGG

GATTACGAAGCTGAAGAACATCCTGGAAGGATTGCCTGAGCCACAGTTCAGCTCCGAGGACTACATGATG

CTCTATACTACCATCTACAACATGTGTACGCAGAAGCCTCCTCATGATTACTCCCAACAACTGTATGACA

AGTACCGTGAGTCTTTCGAAGAATACATTACTTCAACGGATTTGCCATCCTTGAGAGAAAAACATGATGA

GTTTATGTTGAGAAGAGCTTGTGA

>FM894671.1 FM894671 Jatropha curcas embryo 71-95 (DAF) Jatropha curcas cDNA clone rjcpga0_000487, mRNA sequence

CTGGAAATCAGAATCAAACGAGCTTTTACCCTTTTGTTCCACACGAGATTTCTGTTCTCGTTGAGCTCAT

CTTAGGACACCTGCGTTATCTTTTAACAGATGTGCCGCCCCAGCCAAACTCCCCACCTGACAATGTCTTC

CGCCCGGATCGGCCGCCGAAACGACCTTGGGTCCAAAAAGAGGGGCATAGCCCCGCCTCCGATTCACGGA

ATAAGTAAAATAACGTTAAAAGTAGTGGTATTTCACCGTCGCCTTTCGGCTCCCACTTATCCTACACCTC

TCAAGTCATTTCACAAAGTCGGACTAGAGTCAAGCTCAACAGGGTCTTCTTTCCCCGCTGATTCCGCCAA

GCCCGTTCCCTTGGCTGTGGTTTCGCTGGATAGTAGACAGGGACAGTGGGAATCTCGTTAATCCATTCAT

GCGCGTCACTAATTAGATGA

>FM894670.1 FM894670 Jatropha curcas embryo 71-95 (DAF) Jatropha curcas cDNA clone rjcpga0_000486, mRNA sequence

GAGAACTAGTTCGAGGTATAGAGATGCCTGGTCTACTGTGGGGAGCATCAAAACTTGTTCCAGTTGGTTA

TGGCATCAAGAAATTGCAGATCATGCTCACCATTGTTGATGACCTTGTTTCCGTTGATTCTCTCATAGAG

GAACATCTGACAGTTGAGCCTTGCAACGAATATATTCAGAGCTGTGACATTGTTGCCTTCAACAAAATAT

AAGCAAAATACTTATATGCTACTGATTCTAGTGTTAGCCATTTTGGCAACTTAGTATTTTTGGTCTGTGA

TTTCTTTCTTTCTTTTTTTTTTTTCCCAACATTTTTCCATCTTCTGTTTCTGTTCTCTAGATTGGTGAGA

ATTGAAAAAATGAACAATTAAAAACCCGTTCTGT

>FM894669.1 FM894669 Jatropha curcas embryo 71-95 (DAF) Jatropha curcas cDNA clone rjcpga0_000484, mRNA sequence

CGAATCCTGAGTCTGATAAGCCATCATGTTTCTGTTAGATTGGTTTTACGGAGTTCTTGCATCTCTTGGA

TTATGGCAAAAGGAGGCCAAGATTCTCTTCTTGGGCCTTGATAATGCCGGCAAGACCACTCTCCTTCACA

TGTTGAAAGATGAGAGACTGGTACAGCACCAGCCGACCCAGCACCCAACATCGGAGGAGTTGAGCATAGG

CAAAATCAAGTTCAAAGCCTTCGATTTGGGTGGCCATCAGATCGCTCGTCGTGTCTGGAAAGATTACTAT

GCTAAGGTGGATGCAGTGGTTTACCTGGTGGATGCATATGACAAGGAGAGGTTTGCAGAATCGAAAAAGG

AATTGGACGCCCTTCTTTCGGATGAGGCCCTTGCCAATGTTCCTTTTCTCATTCTTGGCAACAAGATTGA

CATTCCGTATGCAGCTTCTGAGGATGAGCTTCGTTATCATCTAGGACTTACCAATTTCACAACTGGCAAT

GGGAAGGTGAATCTTACAGACTCCAATGTTCGCCCCCTCGA

>FM896528.1 FM896528 Jatropha curcas embryo 71-95 (DAF) Jatropha curcas cDNA clone rjcpga0_003761, mRNA sequence

GCAGCCTTTGTCGCAAGCTGCTCGTAAGTCTGCCCCAACCACAGGTGGTGTGAAGAAGCCCCACAGATAT

CGTCCTGGAACTGTTGCACTTCGTGAAATTCGCAAGTACCAAAAGAGTACTGAACTCCTTATCAGGAAAC

TCCCATTCCAGAGGCTTGTTCGTGAAATTGCTCAGGATTTTAAGACTGACTTGCGATTCCAGAGCCATGC

GGTTTTGGCCTTACAAGAGGCAGCAGAAGCATACCTTGTTGGATTGTTTGAGGATACTAATCTGTGTGCC

ATCCACGCAAAGCGTGTTACCATAATGCCCAAGGACATTCAGCTTGCCAGGAGAATCCGTGGTGAGAGGG

CTTAAAAGCTCAATTTGCTACAAGGTGGTCAGCTGATTGTTGATGATTGTGTAGCGGATGTGTATGTTGT

AGGATCTTACTCTGTCTTGTCAGTTCTTGTTTTTGAATGTGAAAGTGGATGATCAGTAGGGTCAGACTGT

ATTACGGTACTTTAAGTTGAGGGATGGCTGGTACAGTGGAATGTTGCTATTAAAC

>FM896527.1 FM896527 Jatropha curcas embryo 71-95 (DAF) Jatropha curcas cDNA clone rjcpga0_003760, mRNA sequence

ACAAAGATGAAGGAACTTACATGGCATGCTTAGCATGCGCAATTGAAGACAGACTTTCCAAAGCACTTGG

CTTTAGTTGCAATCTTTCTGACCCCCATTTCATGAAGAGAGAATCTTGTGCTAGGAGACAGGAGAAGTCT

GTCCATTATGTTGATCTTTGGAGCCTCAAGAATTGCCTTAGGAAGTGATAATGCCCATTGCATGCTCCAA

ATTGCTAGTGGTCTCATATAAATGAGGGAACGGAAGTGCCCATCAATCGTCCAGCCCTCTGGTGTCTGGA

ACCAGTATCCATATCCCTCCTCTGACCAACCTGCAAGGAAAATGCCCTCAGCAGTAGTGAATGCCTCATC

CTCCATTCCAGCAAGAATCATGTTAGCAGCCACAGCATAGGTGACACCAGTCCATATTTCTCGTGACTGC

ATACAAGTATCATCCACCTTTCCATTGGGATGCATTCCATTTACAGCACCCATCTTACCTCCTTTAACTT

TCATTACATTAAAATCATAAATTTTCTGAAGAGCACTTCTAATTTTGGAATCATCAAAAAGCGGTGGCAA

GCCTGAAGAT

>FM896526.1 FM896526 Jatropha curcas embryo 71-95 (DAF) Jatropha curcas cDNA clone rjcpga0_003759, mRNA sequence

GGATTCTATCCGTCGCCCGATGGGAATTGTACTTTAAGGCGGCCCTCGCGGCTCGTCCGCCGCGGGGGCT

TGGCCAACGGCACGTGCCTCTGGGGGCCGGATGGCCCCTACTGCGGGTCGGCAAACGGGCGACGGACACG

CGCGTCGCTTCTGGCCCGGATTCTGACTTAGAGGCGTTCAGTCATAATCCAGCGCACGGTAGCTTCGCGC

CACTGGCTTTTCAACCAAGCGCGATGACCAATTGTGCGAATCAACGGTTCCTCTCGTACTAGGTTGAATT

ACCATCGCGACACCATCATCAGTAGGGTAAAACTAACCTGTCTCACGACGGTCTAAACCCAGCTCACGTT

CCCTATTGGTGGGTGAACAATCCAACACTTGGTGAATTCTGCTTCACAATGATAGGAAGAGCCGACATCG

AAGGATCAAAAAGCAACGTCGCTATGAACGCTTGGCTGCCACAAGCCAGTTATCCCCTGTGGTAACTTTT

CTTGACACCTCTAGCTTCAAATTCCGAAGGTCTAAAGGATCGATAAGGCCACGCTTTCACGGTTC

>FM896525.1 FM896525 Jatropha curcas embryo 71-95 (DAF) Jatropha curcas cDNA clone rjcpga0_003758, mRNA sequence

CAACTAGCCCCGAAAATGGAGCGCTTAGCGCGCGACCTATACCCGGCCGTCGGGGCAAGAGCCAGGCCCC

GATGAGTAGGAGGGCGCGGCGGTCGCTGCAAAACCCGGGGCGCGAGCCCGGGCGGAGCGGCCGTCGGTGC

AGATCTTGGTGGTAGTAGCAAATATTCAAATGAGAACTTTGAAGGCCGAAGAGGGGAAAGGTTCCATGTG

AACGGCACTTGCACATGGGTTAGTCGATCCTAAGAGACGGGGGAAGCCCGTCCGACAGCGCGTCCTCGCG

CGAGCTTCGAAAGGGAATCGGGTTAAAATTCCCGAACCGGGACGTGAAGGCTGACGGCGACGTTAGGGAG

TCCGGAGACGTCGGCGGGGGCCTCGGGAAGAGTTATCTTTTCTGTTTAACAGCCCGCCCACCCTGGAAAC

GACTCAGTCGGAGGTAGGGTCCAGCGGCTGGAAGAGCACCGCACGTCGCGCGGTGTCCGGTGCGCCCCCG

GCGGCCCATGAAAATCCGG

>FM896524.1 FM896524 Jatropha curcas embryo 71-95 (DAF) Jatropha curcas cDNA clone rjcpga0_003757, mRNA sequence

GCGAAGCAGCAACCAGTTACTTCTCTCTTCTCTCCCACTGCTGCCGGAGCTTTGATTTGCAAAGATGTAC

ACTACTTGGAAGAAGATCCGAAAAGACAAGGATGCTGAACCAACTGAATTTGAGGAGTCAGTTGCTCAGG

CCCTGTTTGATCTCGAAAATACCAACCCAGAACTGAAAAGTGATTTAAAAGATCTCTTCATAAATCAGGC

AGTTCAAATGGATGTTTCTGGAAATCGGAAAGCTGTTGTTATTTATGTTCCCTACAGATTGAGGAAGGCC

TACCGCAAGATTCATCTTCGTCTTGTTAGAGAGTTGGAGAAAAAGTTCAGCGGAAAGGACGTTGTCCTCA

TTGCCACCCGGAGGATCGTGCGCCCACCGAAGAAGGGTTCTGCTGTTCAGCGCCCTCGTTCCCGAACCCT

TACATCTGTGCATGATGCCATGCTTGAGGATGTAGTGTATCCCGCTGAGATTGTTGGAAAACGCATCAGA

TACAGAATCGATGGGTCCAAAATTATCAAGATTTTCTTGGATCCCAAAGAGCGCAACAACACTGAGTACA

AGTTGGAGACATTTGCTGGAGTTTACAGGAAG

>FM896523.1 FM896523 Jatropha curcas embryo 71-95 (DAF) Jatropha curcas cDNA clone rjcpga0_003756, mRNA sequence

GAGAAAAGCTCAAAATCTTCTTTCGTCTTTCCTCCGATACGTCCTGTTGGTGACTTCAAATGGCGGGCAG

TCGATGGCTTAGGCCTGAGGTCTATCCTCTTTTTGCGGCCGTCGGAGTTGCCGTCGGGATCTGCGGGTTC

CAGCTGGTCCGCAACATCTGCATCAACCCTGAAGTCAGGGTTAGCAAGCAGACCAGGGCTGCCGGAGTTT

TGGACAACTTTGAGGAGGGAGAAAAATACTCTCAACATTTTCTACGGAAATATGTTCGCAACAGGGCCCC

TGAAATAATGCCATCCCTCAACAGCTTCTTCACAGACCCCAAATGAAAAGTGATGACAGGCGCTTGATGT

TCTAAACTATAAAGAAAATTTGAGATACAGTTCATTGTGGCTGTTTTTGTGACAGAAGTTCGTGTAACAT

CTTGGGTTGAACGAGTGTTTTTGAACTGAAAATAAACTCTGGATGGATTTATGATAAGCTCAAGCTTTGT

CGAACCTTGAATTAATTGAGACAGCATCATTGAATCCTTTTTTCTTTTTATATGTTTCGATGTTGTATGA

AAGCTGCAACAT

>FM896522.1 FM896522 Jatropha curcas embryo 71-95 (DAF) Jatropha curcas cDNA clone rjcpga0_003754, mRNA sequence

ATTTTGTACTGAAACAAGGGTTAATCATCATTCAGTATATAGTTTAGAGACATTAAAAAATGATGGATCC

CCAGTGGCCTAAATTACAATTAAAACCTGACACTCATTCTAAAAAAATCCAAAAAATAAAATGTCATCAA

CAACAATCGCTATTAAAAAAAAAAATCTTGAGCTCTGCAAAGCTAATCCAAACTATGCATTGCAAGTTTC

CCCCATCCTTTAACATCCATCACACCTACAACAGGCCAATCTCCCACTGCCAATAACAGATGACTCTCAA

GTCATATCCAACCACCCGGAGTTTAGACGAAAATGAAAAATGAGGAACAGAGTACCATCAGCAGAGAAAT

ATACAGTTCCAGCTTACTAATAAGAATAGAGATAGCTGCAGAATTATCAGTCTTCTGCAAGGAACGAAGG

GCGCCACAATATCTAACATTATCAGGCGGACCCATCTCTGTTTCACCATTTTCCTCAGGATTGATGATTC

TCATGTATGTTTCATGGCCTAGATACCATCGATCAACGTTCTCAACTCTGAGATTCCATACTCCCGACAT

TGTC

>FM896521.1 FM896521 Jatropha curcas embryo 71-95 (DAF) Jatropha curcas cDNA clone rjcpga0_003753, mRNA sequence

CTGGCAATCCCATAAAAATCAAATTCCCAGATGTGAAATCAGCTCCAGATGGGTATGTTGCTAAATGTGT

GTGGTTTGATTTACAAGGCTCACCAAATTTTAGCAATGTAGCATCTGGAAATGAATGTTCAACAATTGAC

CAAGGGCATTTTTCTATAGTTGTTGAATATAATGCCCCTTCTCCAGCACCAGTTTTTCCTACTCCATCTG

GTGAAGCTCCAAATGTTCCAGGACGAGAAGGACATAAAAAGAATAATTCTAAAGTGTGGATAATTGTTGG

TTCTGTGCTGGGTGGCCTATTGTTGTTGGTGCTATTGTCATTTCTAGTGCTGTGGGTGCATAAGTTGAAG

GAAAGGAAGAAACTGCAAGAGATGGAGAGGGCTGCAGATGTGGGAGAAGCCCTGCAAATGACATCAGTTG

GGGAAACAAAAGCACCTGCAGCAATGGTTACTCGAACGCCACCAACCCTTGAGAATGACTATGTGCCATA

ACTCTCACCATTTGTACAAGCTTCCTTTACCTTG

>FM896520.1 FM896520 Jatropha curcas embryo 71-95 (DAF) Jatropha curcas cDNA clone rjcpga0_003752, mRNA sequence

ATTCCATCAAGGCTATCTCAGAAGGTTTATGGTGGCTTCTGGCATTTTAATCTTGGACATTGCTGGAATA

CAGTTTGCATCGCTTTGTTTTCCACATCAAAACGAAGACTTATTGGTGGAACACCTTACACTACCTTATT

CACGGCTGTCATCACAAGCACCCTATGGATGGTTTGCGGCTTGTCTTTCCTCCAGCTGCAACAGCTGTTC

TTTGTGTGCCATTCTGGAACTTGGTTCAACTTTTCGCTACTCCCTCAACTGCTCCTGCGTTATTTGGAGG

TGGTTTGAAGGGTTATGTGATGTACGACTGTACTCATTATTACTTGCATCATGGTCAGCCATCAAGTGGA

GTACCAAAGGATCTCAAGAAATACCACTTGAATCATCACTTCAGAATCCAAAATATGGGTTTTGGAATCA

CTTCAGCCCTGTGGGACAGGGTGTTTGGAACACTTCCTCCATCAAAAGT

>FM896519.1 FM896519 Jatropha curcas embryo 71-95 (DAF) Jatropha curcas cDNA clone rjcpga0_003751, mRNA sequence

GTCTGACATGTGTGCGAGTCAACGGGTGAGTAAACCCGCAAGGCGCAAGGAAGCTGACTGGCGGGATCCC

CCCGAGGGTTGCACCGCCGACCGACCTTGATCTTCTGAGAAGGGTTCGAGTGAGAGCATGCCTGTCGGGA

CCCGAAAGATGGTGAACTATGCCTGAGCGGGGCGAAGCCAGAGGAAACTCTGGTGGAGGCCCGCAGCGAT

ACTGACGTGCAAATCGTTCGTCTGACTTGGGTATAGGGGCGAAAGACTAATCGAACCGTCTAGTAGCTGG

TTCCCTCCGAAGTTTCCCTCAGGATAGCTGGAGCTCGGGACGAGTTCTATCGGGTAAAGCCAATGATTAG

AGGCATCGGGGGCGCAACGCCCTCGACCTATTCTCAAACTTTAAATAGGTAGGACGGCGCGGCTGCTT

>FM896518.1 FM896518 Jatropha curcas embryo 71-95 (DAF) Jatropha curcas cDNA clone rjcpga0_003749, mRNA sequence

CTCAAGCTCCAACCAGCAGCTGTATTAGCGGCGTGCAGCCTCGGAGGTCCAACACGCTAAAGCTCTAATG

GGGATCGATTTGAAAGCAGGAGGTAAGAGCAAAAAGACCAAGCGCACAGCTCCTAAATCTGATGATATCT

ATCTCAAGCTACTTGTTAAGCTATACCGATTTCTGGTAAGGAGAACCGGTAGCAAGTTCAATGCGGTGAT

ATTGAAGAGGCTGTTCATGAGCAAGGTCAACAAAACCCCGCTCTCTCTCTCTAGGCTCATCAGGTTCATG

AAGGGAAAGGAGAGTAAGATTGCAGTGGTTGTGGGCACAGTTACCGATGACATTAGGGTTTATGAGGTTC

CGGCATTGAAGGTTACTGCCTTGAGGTTTACAGAGACAGCAAGAGCCAGAATCGAGAAGGCTGGGGGAGA

GTGTTTGACATTTGACCAGCTTGCTCTGAGAGCTCCTTTGGGGCAGAACACAGTTCTCTTAAGAGGTCCT

AAGAATGCTCGTGAAGCTGTGAAACACTTTGGCCCAGCTCCTGGTGTGCCACACAGCCATACCAAACCAT

ATGTTCGATCAAAGGGTAGGAAG

>FM896517.1 FM896517 Jatropha curcas embryo 71-95 (DAF) Jatropha curcas cDNA clone rjcpga0_003748, mRNA sequence

TTTTTTTTTTGAAATGGAGAAACCATTACAATTAGAAGCAACCATCTAGTATACAATAAGTGGACATTAA

CAAGTGCCCTTAAAACCATTAAATTTACAATGCTTCTAATCACATTGGAAAATACATAGGAATTTTCATG

TGGTCATCTATGTTCTCTTCTTTTCTACCTCTCTCCCCAGTCTATCGAGTCACTTTTCATGCTCTGCTCT

GAACTTACCTGGTCGATTTCTCAAGGATAGAAGTTATTTCGTGTTCTCTGAAATTACTTGATAGATCAAG

TAATTTCTCTTAGGGTAGTAGAAGGAGAAGAAGTCCCTTTGAAGATAAGGAAGTTGAGGGTCTCCAATGT

GAAGTAGATGACAGTCCCTTTAGTATGAGTAAAGAAACACCCAAAATGGGGGGGCCCGGTAC

>FM896516.1 FM896516 Jatropha curcas embryo 71-95 (DAF) Jatropha curcas cDNA clone rjcpga0_003747, mRNA sequence

GTTTTGGGATTGGTGAACCGAGCCGATCGGTAGTGTCGTTCCTCTTCTTGTGTTTAGGCTTCATCATACG

AATTTCTTAGTTGTTCGGTCTTATCTCTGCCTTTGCGATCTGCGATCTGTATTAGAATGTCATATTTTGT

TATCTTTGTTTCGTTTCTTTGATCTAGTTTTCATATCATCTTATATGCGTCATGATATCGGCTTCTGTAT

GATTTGGTTTATGGATTTCAGTTTTGAAATTAGGTGATTGAATTGGCTCGGGCGTAGTTGATGCGAAGGC

AACAGTTGGTTTGACTGATCCTTTAGATACTAGCTTTATGTTGAAGATTTGTCTGCCTGAGGCTTTGTTT

CTTTTTTGATTTATTGGATCGTCAATTTGATGGTAGAATACCTGCTATGGTTGTATATTGGTGTTAGGTT

TTGAATAAGCGGAAACGAAGCAGGTTTATGAAGTCCAAGTTACGGAATCAGGGTC

>FM896515.1 FM896515 Jatropha curcas embryo 71-95 (DAF) Jatropha curcas cDNA clone rjcpga0_003746, mRNA sequence

CTGCTGCATGGACCGAATTGGGGTCAAAACTTGCATCCACGTTACATTTGAAACCTGTCAAAAGTGGATG

CCATCTGTTTTGCATATTACTCTCCCATGGTACCAACTGATATTCCTCTATCGAGTTCGCAATTCGCCAC

GCATTAAGAGAATACTGTACATGTCTCACAATAGCCGCTGAATCACGTACTTGCTGCTGCCAAATCAACT

TGTTTCTAGCATTCCATAAATCCCAAGCCGCAACACTAGCCTCCCTGTTTTTGCATCATCCATCCGTTGC

ATTAAGAATTCAAACCATTTATTTAAGCCCGAAACATTACCCCACCCTCCTATAGAAGTAGATAACCACG

CATCTTTTGCAAATGGACAATACAGTAAACAATGGGCAACTGTTTCATCCTCCTGATGACATCGAGGACA

AAGTATAGGTACCTCAATTCGTCTTCTTTGCAAAACTGAAAAAACAGGAAGAATGTCTAACAAAGCTCTC

CACATAAAGTTTAGAACTTTAGGTGGAATTTTTAACTGCCCAAGCTTGCGCCAAATTCCTTACTGAAGTC

>FM896514.1 FM896514 Jatropha curcas embryo 71-95 (DAF) Jatropha curcas cDNA clone rjcpga0_003743, mRNA sequence

TGTGTGCGAGTCAACGGGTGAGTAAACCCGCAAGGCGCAAGGAAGCTGACTGGCGGGATCCCCCCGAGGG

TTGCACCGCCGACCGACCTTGATCTTCTGAGAAGGGTTCGAGTGAGAGCATGCCTGTCGGGACCCGAAAG

ATGGTGAACTATGCCTGAGCGGGGCGAAGCCAGAGGAAACTCTGGTGGAGGCCCGCAGCGATACTGACGT

GCAAATCGTTCGTCTGACTTGGGTATAGGGGCGAAAGACTAATCGAACCGTCTAGTAGCTGGTTCCCTCC

GAAGTTTCCCTCAGGATAGCTGGAGCTCGGGACGAGTTCTATCGGGTAAAGCCAATGATTAGAGGCATCG

GGGGCGCAACGCCCTCGACCTATTCTCAAACTTTAAATAGGTAGGACGGGCGCGGCTGCTTCGCTGAGCC

GCGCCACGGAATCGAGAGCTCCAAGTGGGCCATT

>FM896513.1 FM896513 Jatropha curcas embryo 71-95 (DAF) Jatropha curcas cDNA clone rjcpga0_003742, mRNA sequence

ATTCTGAGGTTTTTGCTATTGAGAATAGGTCTCCAGCTGAAGGAGCTTATACTGAAGGACTCCTTAATGC

TAAGCTGACCCAGGATGGTTGTATAGTTTGCCCGACGACTGATAGCACATTTGATCTTCGAACTGGAGCT

ATCAAAGAATGGTATCCAAAAAATCCTGTGCTGAGAGTTCTTACGCCTGCTTTGAGAACACTCTACGTAT

TTCCTGTAAAAACTGATGAAGAAAATATTTACATCAGCTTGAGAGGAGGTAAAAAAGCTGATGCATCAGC

CGAAATAGTGTTTAGTGGGAAGGCTCAACCTGGTTTGACTGCAAGTGATGTCAATGTGGATGAGGTGAGA

ATGGTAATTGACGAGGATTCAGAGGCATTTGGCTTCACCGGCAAGAATGAATTGATAAATGGGAAAGCAG

CAGTAATTGGCTTCTTGTTGTTATTAGATTTTGAGCTCTTGACTGGGAAAGGTCTCCTCAAGGGA

>FM896512.1 FM896512 Jatropha curcas embryo 71-95 (DAF) Jatropha curcas cDNA clone rjcpga0_003741, mRNA sequence

CGAATTCCAAAGGATTCTATCCGTCGCCCGATGGGAATTGTACTTTAAGGCGGCCCTCGCGGCTCGTCCG

CCGCGGGGGCTTGGCCAACGGCACGTGCCTCTGGGGGCCGGATGGCCCCTACTGCGGGTCGGCAAACGGG

CGACGGACACGCGCGTCGCTTCTGGCCCGGATTCTGACTTAGAGGCGTTCAGTCATAATCCAGCGCACGG

TAGCTTCGCGCCACTGGCTTTTCAACCAAGCGCGATGACCAATTGTGCGAATCAACGGTTCCTCTCGTAC

TAGGTTGAATTACCATCGCGACACCATCATCAGTAGGGTAAAACTAACCTGTCTCACGACGGTCTAAACC

CAGCTCACGTTCCCTATTGGTGGGTGAACAATCCAACACTTGGTGAATTCTGCTTCACAATGATAGGAAG

AGCCGACATCGAAGGATCAAAAAGCAACGTCGCTATGAACGCTTGGCTGCCACAAGCCAGTTATCCCTGT

GGTAACTTTTCTGACACCTCTAGCTTCAAA

>FM896511.1 FM896511 Jatropha curcas embryo 71-95 (DAF) Jatropha curcas cDNA clone rjcpga0_003740, mRNA sequence

TTCANAATGTGATGGTTTGTGAGCGTGGAACTATGTTTGGCTATAGTGATTTAATTGTTGATCCGCGTAA

TTTGGAGTGGATGAGGGATGCTGATTGTCCTGTTGTGGCTGATGTGACACATTCACTACAACAGCCTGCT

GGGAAAAAGTTGGATGGTGGTGGTGTTGCCAGTGGAGGTCTTCGTGAATTGATACCATGTATTGCAAGGA

CAGCAGTTGCTGTTGGGGTAGATGGAATTTTTATGGAGGTGCATGATGATCCTCTGAGTGCTCCTGTGGA

TGGTCCAACACAATGGCCTCTACGCCATTTGGAGGAATTACTTGAAGAGCTCATGGCAATAGCTAGGGCA

AGTAAGGGGAAGATAAGTATGGACATTGATCTCACACCATTTCGTGATTAGAAAAGCTTGAGAGCCTCTG

TTGAGTTATTGTCTGTGGATTCAGTCTAGCTTTACTACGAGATGACAAGAATGGCAAGTATTTGTNCACT

TCTGCTATTAAATGCTATGTCGTTTAATATTTCCAACTTGAATT

>FM896510.1 FM896510 Jatropha curcas embryo 71-95 (DAF) Jatropha curcas cDNA clone rjcpga0_003738, mRNA sequence

ATTCAAAGGATTCTATCCGTCGCCCGATGGGAATTGTACTTTAAGGCGGCCCTCGCGGCTCGTCCGCCGC

GGGGGCTTGGCCAACGGCACGTGCCTCTGGGGGCCGGATGGCCCCTACTGCGGGTCGGCAAACGGGCGAC

GGACACGCGCGTCGCTTCTGGCCCGGATTCTGACTTAGAGGCGTTCAGTCATAATCCAGCGCACGGTAGC

TTCGCGCCACTGGCTTTTCAACCAAGCGCGATGACCAATTGTGCGAATCAACGGTTCCTCTCGTACTAGG

TTGAATTACCATCGCGACACCATCATCAGTAGGGTAAAACTAACCTGTCTCACGACGGTCTAAACCCAGC

TCACGTTCCCTATTGGTGGGTGAACAATCCAACACTTGGTGAATTCTGCTTCACAATGATAGGAAGAGCC

GACATCGAAGGATCAAAAAGCAACGTCGCTATGAACGCTTGGCTGCCACAAGCCAGTTATCCCTGTGGTA

ACTTTTCTGACACCTCTAGCTTCAAATTCCGAAGGTCTAAAGGATCGATAGGCCACGCTTTCACGGTTCG

TATTCGTACTGGAAATCAGAATCAAACGAGCTTTTACCCTTTTGTTCCACA

>FM896509.1 FM896509 Jatropha curcas embryo 71-95 (DAF) Jatropha curcas cDNA clone rjcpga0_003737, mRNA sequence

GTGGGCCATTTTTGGTAAGCAGAACTGGCGATGCGGGATAAACCGGAAGCCGGGTTACGGTGCCCAACTG

CGCGCTAACCTAGAACCCACAAAGGGTGTTGGTCGATTAAGACAGCAGGACGGTGGTCATGGAAGTCGAA

ATCCGCTAAGGAGTGTGTAACAACTCACCTGCCGAATCAACTAGCCCCGAAAATGGATGGCGCTTAAGCG

CGCGACCTATACCCGGCCGTCGGGGCAAGAGCCAGGCCCCGATGAGTAGGAGGGCGCGGCGGTCGCTGCA

AAACCCGGGGCGCGAGCCCGGGCGGAGCGGCCGTCGGTGCAGATCTTGGTGGTAGTAGCAAATATTCAAA

TGAGAACTTTGAAGGCCGAAGAGGGGAAAGGTTCCATGTGAACGGCACTTGCACATGGGTTAGTCGATCC

TAAGAGACGGGGGAAGCCCGTCCGACAGCGCGTCCTCGCGCGAGCTTCGAAAGGGAATCGGGTTAAAATT

CCCGAACCGGGACGT

>FM896508.1 FM896508 Jatropha curcas embryo 71-95 (DAF) Jatropha curcas cDNA clone rjcpga0_003736, mRNA sequence

GAACCCACAAAGGGTGTTGGTCGATTAAGACAGCAGGACGGTGGTCATGGAAGTCGAAATCCGCTAAGGA

GTGTGTAACAACTCACCTGCCGAATCAACTAGCCCCGAAAATGGATGGCGCTTAAGCGCGCGACCTATAC

CCGGCCGTCGGGGCAAGAGCCAGGCCCCGATGAGTAGGAGGGCGCGGCGGTCGCTGCAAAACCCGGGGCG

CGAGCCCGGGCGGAGCGGCCGTCGGTGCAGATCTTGGTGGTAGTAGCAAATATTCAAATGAGAACTTTGA

AGGCCGAAGAGGGGAAAGGTTCCATGTGAACGGCACTTGCACATGGGTTAGTCGATCCTAAGAGACGGGG

GAAGCCCGTCCGACAGCGCGTCCTCGCGCGAGCTTCGAAAGGGAATCGGGTTAAAATTCCCGAACCGGGA

CGTGAAGGCTGACGGCGACGTTAGGGAGTCCGGAGACGTCGGCGGGGGCCTCGGGAAGAGTTATCTTTTC

TGTTTAACAGCCCGCCCACCCTGGAAACGACTCAGTCGGAGGTAGGGTCCAGCGGCTGGAAGAGCACCGC

ACGTCGCGCGGT

>FM896507.1 FM896507 Jatropha curcas embryo 71-95 (DAF) Jatropha curcas cDNA clone rjcpga0_003734, mRNA sequence

ATTCGCAAAGGATTCTATCCGTCGCCCGATGGGAATTGTACTTTAAGGCGGCCCTCGCGGCTCGTCCGCC

GCGGGGGCTTGGCCAACGGCACGTGCCTCTAGGGGCCGGATGGCCCCTACTGCGGGTCGGCAAACGGGCG

ACGGACACGCGCGTCGCTTCTGGCCCGGATTCTGACTTAGAGGCGTTCAGTCATAATCCAGCGCACGGTA

GCTTCGCGCCACTGGCTTTTCAACCAAGCGCGATGACCAATTGTGCGAATCAACGGTTCCTCTCGTACTA

GGTTGAATTACCATCGCGACACCATCATCAGTAGGGTAAAACTAACCTGTCTCACACGGTCTAAACCCAG

CTCACGTTCCCTATTGGTGGGTGAACAATCCAACACTTGGTGAATTCTGCTTCACAATGATA

>FM896506.1 FM896506 Jatropha curcas embryo 71-95 (DAF) Jatropha curcas cDNA clone rjcpga0_003733, mRNA sequence

TTTTTTCTTTTTTCCATTGAAGTGCATGAATTGATTTTCTGATTTTTCACGTACAACGACAAAAGTACGC

GTACGCAATAAAATCAAACGGAAGAATTGTTAACGTTTTTAGCTCAGGCAAAAAAGTGAAGCCATTTGTA

CAAATTTTACTTGAAAGGACAAAGTCACCAAAAAATGAGATCATATCACTCTTCTGCCGCAGGAATAGCA

CTCAAGTCCAAACCCTCTGCTGGTAGATCAGAAGTCAGCGGACCCAATATTCCATCTGAACTGAGCGTTA

GTCCACTCTCAGGCTGGAATCTCAGTATGTCAGCACGAGCCATAGCTTCTGCTTGAGCAATTCTCTGCCT

GAATGACTGAGCCATCTCCTCTGCCTTCTCAAAAACAAGCTTAGGATTGCGAATCATATCGCCAGGAGTA

GGTTCTAACTTTTAGGTAGAAAGACTTACTCTACCTCTCTCACGGTCATGGCTTAGTATCATGACCTTGA

GAGTAT

>FM896505.1 FM896505 Jatropha curcas embryo 71-95 (DAF) Jatropha curcas cDNA clone rjcpga0_003732, mRNA sequence

GACTAAGGAAAACATATATAGATTATAAAGCTAAAGACATTAATGTTTTGGGTATAAAAGAGTTCACAAA

ACCTTAATACCAACATACATTGATTGATAAAATAAATAATAACCCCTAACTATCTTCACCACCATTCTCT

CCAAACCAAGAGGACAAGGGAAAGAAAAAAACTCCAAACCAATAATAGTTCATAAAGGACTAACGAAAAT

AAATAAATTAAAAAAAAATAAATGACTCCCAAAACTTCAAAACCCCTCCCTTTCTGTTCCTCTCCAAAGA

TATCAGCAAATTCTCCCTCCAAACTTCAATCGAACTCTACTGTTGCTTGCACTCTGGAGGCCTCTCACCC

TGCTGACCATCCCCAGCTTGTGTTCTTCCACCCTTCTTATGCCCGGCTGACAGCTTCTTTAAGGTCTTGC

TTTCATCCTCGTCCTCATCCTCATCTTCATCCTCTTCATCCTCATCATCATCATCAATATCTTCATCGTC

GTCGTCGTCATCATCATCATCATCATCTATTCCAAGGTCATCCC

>FM896504.1 FM896504 Jatropha curcas embryo 71-95 (DAF) Jatropha curcas cDNA clone rjcpga0_003731, mRNA sequence

TTTTGAGAAACAGAAATTAAATTAAGCCAAACTTTGTTCACATTCCAACAATGTTCAGCAAATGTTAAAA

CTCAATTCTACCTTGGTTTAAGCATATAAAAGGAGGCTCAGATTCGCCTTTTTCCAAACAGAAAAGTGAA

CAGGAAAACCTCACCCTTTGCTTGAAATATTTTCCGGAAAACAAACAGAGCCCAAACAGAGAAATGCACA

GAAACGGAGGTTCAGATCAGATATGCATTTGAATCAAACAAGAAAGTAACACCACCACATCATGGCTGCA

GCCAAGAGCTACCAGGAAAAGGAGGGAAACCCACATTCCAGCGACTCCAAAGGCTACCCCGCCAGAAGAA

TGATGTTTATATTCTAGTCTGATTCTATAACACTGATATATCATCATAGCCTCATTACTTTACCTTCATA

AACCTTCCATCATATCATCTCATCCTTGGTCAAAGTACAAAGTTAGGGGTGAATTTATGTATTCGGCCTC

TCTGTACATACGAGGGAAGATGGCTGTTGGCAAATTAGAAGACAAATCACGGGCTCCCCTGCTTATCAAA

GAGGTGAACTCAACCCCCCTGAAACCTTCCCCTCAG
[truncated: 30,655,851 more chars]
